# A gene network switch enhances the oxidative capacity of ovine skeletal muscle during late fetal development

**DOI:** 10.1186/1471-2164-11-378

**Published:** 2010-06-15

**Authors:** Keren Byrne, Tony Vuocolo, Cedric Gondro, Jason D White, Noelle E Cockett, Tracy Hadfield, Christopher A Bidwell, Jolena N Waddell, Ross L Tellam

**Affiliations:** 1 CSIRO Livestock Industries, Queensland Bioscience Precinct, 306 Carmody Rd., St. Lucia, 4067, Queensland, Australia; 2 Department of Animal Science, University of New England, Armidale, 2351, NSW, Australia; 3 Department of Veterinary Science, The University of Melbourne, Melbourne, 3010, VIC, Australia; 4 Department of Animal, Dairy and Veterinary Science, Utah State University, Logan, Utah, 84322-4815, USA; 5 Department of Animal Sciences, Purdue University, West Lafayette, Indiana, 47907-20242, USA

## Abstract

**Background:**

The developmental transition between the late fetus and a newborn animal is associated with profound changes in skeletal muscle function as it adapts to the new physiological demands of locomotion and postural support against gravity. The mechanisms underpinning this adaption process are unclear but are likely to be initiated by changes in hormone levels. We tested the hypothesis that this developmental transition is associated with large coordinated changes in the transcription of skeletal muscle genes.

**Results:**

Using an ovine model, transcriptional profiling was performed on *Longissimus dorsi *skeletal muscle taken at three fetal developmental time points (80, 100 and 120 d of fetal development) and two postnatal time points, one approximately 3 days postpartum and a second at 3 months of age. The developmental time course was dominated by large changes in expression of 2,471 genes during the interval between late fetal development (120 d fetal development) and 1-3 days postpartum. Analysis of the functions of genes that were uniquely up-regulated in this interval showed strong enrichment for oxidative metabolism and the tricarboxylic acid cycle indicating enhanced mitochondrial activity. Histological examination of tissues from these developmental time points directly confirmed a marked increase in mitochondrial activity between the late fetal and early postnatal samples. The promoters of genes that were up-regulated during this fetal to neonatal transition were enriched for estrogen receptor 1 and estrogen related receptor alpha cis-regulatory motifs. The genes down-regulated during this interval highlighted de-emphasis of an array of functions including Wnt signaling, cell adhesion and differentiation. There were also changes in gene expression prior to this late fetal - postnatal transition and between the two postnatal time points. The former genes were enriched for functions involving the extracellular matrix and immune response while the latter principally involved functions associated with transcriptional regulation of metabolic processes.

**Conclusions:**

It is concluded that during late skeletal muscle development there are substantial and coordinated changes in the transcription of a large number of genes many of which are probably triggered by increased estrogen levels. These changes probably underpin the adaption of muscle to new physiological demands in the postnatal environment.

## Background

The transition from the mammalian fetal environment to that enveloping a newborn animal is profound and associated with major physiological changes. Skeletal muscle in particular, must rapidly adapt to meet the demands of locomotion and to provide postural support against gravity in the newborn animal. These skeletal muscle adaptations are of even greater importance in newborn ruminants, which stand, walk and run within one to two hours of birth - a physiological trait that is required to avoid predation and allow movement with the mother as she feeds on different pastures and supplies nourishment to the newborn animal.

Individual skeletal muscles are adapted to specific functions. Muscles undergoing slow but continuous contractions such as various postural muscles, are characterized by predominance of slow twitch oxidative fibres (type 1 fibres), while muscles requiring rapid contraction generating substantial force such as some locomotory muscles, have a greater proportion of fast twitch glycolytic fibres (type IIb fibres). Fibre types intermediate between these two exist and most muscles contain a mixture of fibre types. The developmental program that produces the differing functional outcomes in specific adult skeletal muscles is unclear but it is known to involve the programming of fetal muscles [1-3].

Skeletal muscle development in sheep is characterized by the sequential formation of primary, secondary and tertiary myofibres beginning approximately 32, 38 and 62-76 days of fetal life, respectively [1,3,4]. Parturition is at 147 days from conception. The primary fibres are composed of multinucleated myotubes, which are derived from the fusion of committed myoblasts present in the embryonic dermamyotome. Secondary and tertiary myotubes, which surround the primary myotubes, are formed from myoblasts associated with primary myotubes. The formation of secondary myotubes typically occurs after innervation. The primary myotubes have a greater chance of becoming type I fibres while the secondary and tertiary myotubes have a greater propensity of forming type IIb fibres in the adult. The wave of tertiary myotube formation is complex and initially consists of different muscle groups that populate the spaces between secondary fibers and along the borders of fascicles using the secondary fibers as scaffolds. The different muscle developmental stages are characterized by progressive changes in the expression of embryonic, neonatal and adult myosin heavy chain genes [4].

The molecular events controlling the preparation of fetal skeletal muscle during its late developmental phase for the demands of the post-partum environment are unknown. *Longissimus dorsi *(LD) skeletal muscle, which has both locomotory and postural roles, is one of the largest muscles of the back spanning the entire thoracic and lumbar regions, and contains a mixture of fibre types in the adult state [5]. We have chosen ovine LD skeletal muscle to examine transcriptional changes occurring during development from stages representing the beginning of tertiary myotube formation (80 days of fetal development) through to birth and to a young immature lamb of 3 months of age. The objective of this research was to define the hierarchical transcriptional changes associated with skeletal muscle development and to interpret this information in the context of progressive changes in biological functions. We hypothesize that there is a major developmental switch in gene expression occurring during late fetal development which is associated with tertiary myotube formation and changes in the oxidative status of muscle in preparation for the new physiological demands on the muscle in the postnatal environment.

## Results

### Experimental model

**Figure 1:**
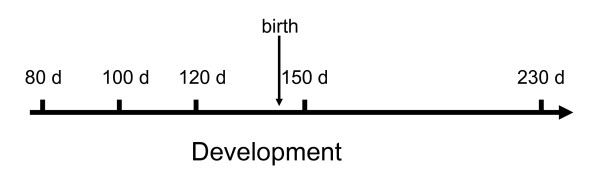
**Experimental design**. LD skeletal muscle samples were taken from three animals at each of five developmental stages i.e. 80, 100, and 120 days of fetal development, 150 days of development (i.e. 1-3 days post- partum) and 230 days of development (i.e. 83 days post- partum). Birth was at approximately 147 days of development.

Gene expression was measured using microarray analysis of samples representing various stages in late fetal and postnatal development of ovine LD skeletal muscle. Figure 1 shows the experimental model, which included three fetal and two postnatal samples (n = 3 for each developmental stage). Birth was at approximately 147 days of development. The fetal samples corresponded with the period of formation of tertiary myotubes [1,3,4].

### Global gene expression across LD skeletal muscle development

**Figure 2:**
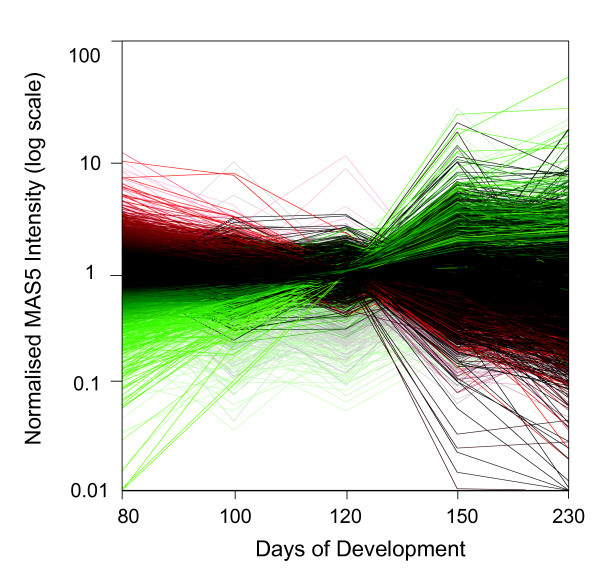
**Visual representation of the changes in global gene expression in ovine skeletal muscle during late fetal and postnatal development**. Affymetrix MAS5 data were globally scaled to 200 on each microarray and normalized across all microarrays to enable visualization of the expression of all genes. Each analysis time point consisted of triplicate samples, and the mean for each developmental stage is presented. The genes are differentially colored according to their normalized expression intensity relative to the global gene intensity at 80 days of fetal development. Genes with mean intensities less than the global average at 80 days are colored green, while those with mean intensities greater than the global average at 80 days are colored red. Gene intensities that were not different from the 80 day fetal global mean are colored black. Genes represented by unsaturated colors were of low confidence according to a defined set of criteria. The ordinate shows the logarithm of the MAS5 intensity data while the abscissa corresponds to the five developmental stages i.e. 80, 100 and 120 days of fetal development, 150 days of development (i.e. 1-3 days after birth) and 230 days of development (young lamb).

Figure 2 shows a visual representation of the expression of all genes expressed in at least one stage across the skeletal muscle developmental time course. The mean MAS5 intensity data at each developmental stage (n = 3) were used. Genes were differentially coloured relative to the global mean at 80 days of development. The predominant feature of the diagram is a major switch in gene expression occurring between the last fetal developmental time point (120 d) and the stage 1-3 d after birth (150 d). This major switch in gene expression involved 2,471 significantly differentially expressed genes and dominated less apparent trends occurring between the other developmental stages. The range in gene expression levels was substantially restricted in the fetal 120 d sample compared with all other samples. Moreover, the range in gene expression intensities at 80 days of fetal development was not as great as in the postnatal 230 d sample.

The gene expression data were subjected to statistical analysis to identify genes that were significantly differentially expressed between any two adjacent developmental time points. Three data processing methods were employed and each was subjected to independent statistical analysis. Genes that were significantly differentially expressed between any two adjacent developmental time points, as measured by the convergence of the three analysis methods, are listed in Additional File 1. This conservative approach using multiple methods provided increased robustness in the identification of differentially expressed genes. A total of 2,798 unique genes were differentially expressed at some stage during the total developmental period. These represented 17.9% (2,798/15,642) of the total number of genes that were expressed at any stage during development. This result indicated that skeletal muscle development during the late fetal stages and postnatal period was associated with substantial transcriptional changes.

The differentially expressed genes were clustered into co-expression patterns. Table 1 summarizes the number of genes in each of the major clusters (n ≥ 50 genes) using specific terminology to reflect the direction of the expression changes in the four contrasts between adjacent developmental stages i.e. U, up; D, down; F, flat. For example, the 'FFUF' cluster contains genes that were only differentially expressed between 120 days of fetal development and the 150 d sample taken 1-3 days after birth. Details of the differentially expressed genes present in the major clusters are listed in Additional File 1. As the three microarray analysis methodologies produced unrelated measures of signal intensity, only MAS5.0 signal data were used to define the gene expression clusters. However, the significantly differentially expressed genes were defined from the convergence of the three microarray analysis methodologies. 77 clusters were identified using this approach but only 12 clusters containing 50 or more genes were subject to more detailed analysis. None of the remaining 65 smaller clusters was over-represented in any gene ontology terms, pathways or key words.

**Table 1:** Summary of clusters of differentially expressed genes during the development of LD skeletal muscle

Cluster^1^	Number of genes^2^	Biological description of the cluster
FFFU	545	Change in postnatal development
FFFD	612	Change in postnatal development
FFUF	787	Developmental switch between late fetal development and birth
FFDF	1392	Developmental switch between late fetal development and birth
FFUD	150	Transient change around birth
FFDU	440	Transient change around birth
FFDD	96	Continuation of the developmental switch between late fetal development and birth into the young lamb
FUFF	113	Fetal changes in preparation for the major developmental switch between late fetal development and birth.
FDFF	104	Fetal changes in preparation for the major developmental switch between late fetal development and birth.
UFFF	221	Fetal changes in preparation for the major developmental switch between late fetal development and birth.
DFFF	109	Fetal changes in preparation for the major developmental switch between late fetal development and birth.
DUFF	79	Transient early fetal change

^1^D (Down), significantly decreased expression; U (Up), significantly increased expression; F (Flat), no significant change. Each symbol (D, U, F) represents the direction of change in gene expression between any two adjacent developmental time points. Each cluster is represented by these symbols across four developmental intervals. Only clusters containing more than 50 genes are shown.

^2^Number of significantly differentially expressed genes in each cluster.

As anticipated from the visual representation of global gene expression shown in figure 2, the largest clusters, containing 787 (FFUF) and 1392 genes (FFDF), reflected changes between the samples taken at 120 days of fetal development and the samples taken 1-3 days after birth (150 days of development). These two clusters combined represented 77.9% of the genes that were differentially expressed at any stage throughout the developmental profile. This result reinforced the visual representation shown in figure 2 that there was a major developmental switch in gene expression between late fetal development and birth. Four clusters, FUFF, FDFF, UFFF and DFFF containing 113, 104, 221 and 109 genes, respectively, reflected changes in gene expression that were occurring during fetal development prior to the much larger transcriptional changes occurring between late fetal development (120 d) and the samples taken just after birth (150 d). Two clusters, FFUD (150 genes) and FFDU (440 genes), represented transient changes in gene expression coincident with, or in preparation for, the birth process. There was also a substantial number of genes associated with postnatal muscle development (i.e. FFFU (545 genes) and FFFD (612 genes)).

**Figure 3:**
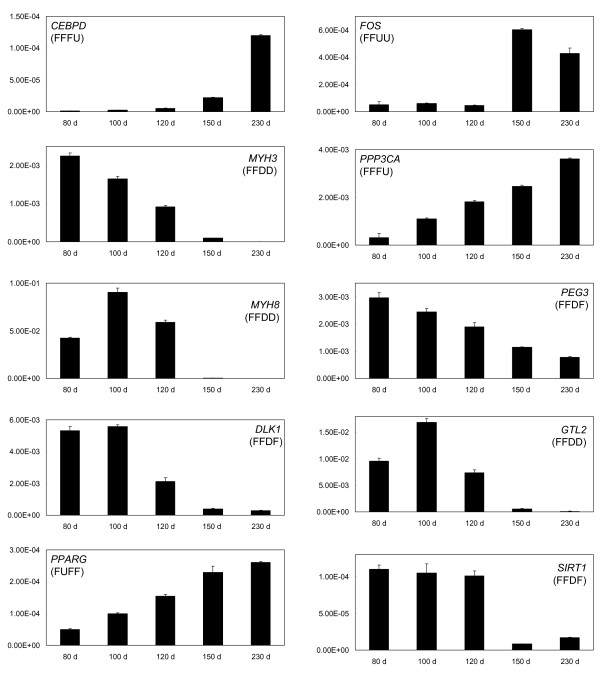
**qRT-PCR expression patterns for selected genes from the microarray gene expression clusters**. The mRNA expression patterns of selected genes from the major gene expression clusters were also measured by qRT-PCR. The expression levels are expressed as mean normalised expression (MNE) values relative to the reference gene, 18S RNA. The corresponding gene expression cluster derived from microarray data is shown in brackets. Fetal samples at 80, 100 and 120 days of development and postnatal samples at 150 d and 230 d of development are shown. The result for each gene shows the mean (n = 3) while the error bar denotes 1 S.D.

**Figure 4:**
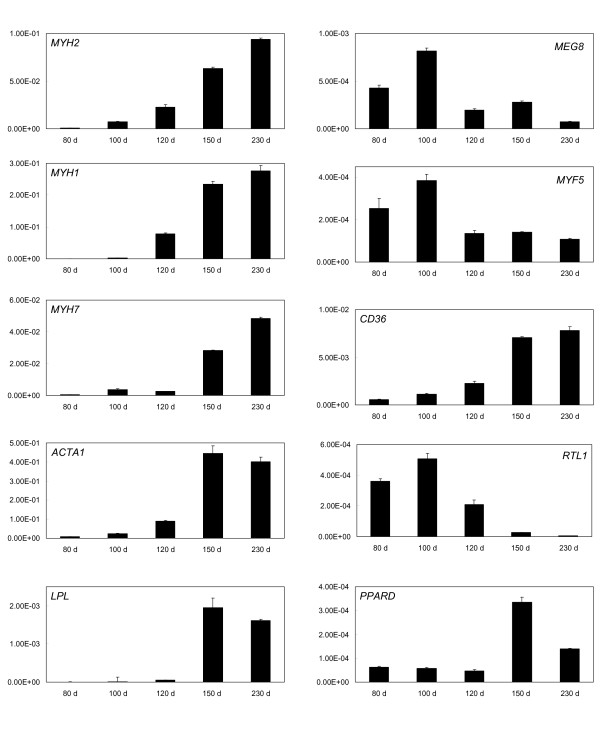
**qRT-PCR expression patterns for selected genes not represented or not reporting on the microarray**. The mRNA expression patterns of genes that were not represented or not reporting on the microarray were measured by qRT-PCR. The expression levels were expressed as mean normalised expression (MNE) values relative to 18S RNA. Fetal samples at 80, 100 and 120 days of development and postnatal samples at 150 d and 230 d of development are shown. The result for each gene shows the mean (n = 3), while the error bar denotes 1 S.D.

Figure 3 shows qRT-PCR expression patterns for selected genes representative of some of the clusters identified by microarray analysis. Typically, the qRT-PCR results showed good agreement with the major trends identified by microarray analysis e.g. *SIRT1 *(see also [6,7]). Precise replication of the cluster designation did not always occur e.g. *MYH8 *and *FOS*. This may result from differences between the sensitivities and dose-response curves for data derived from microarrays and qRT-PCR as well as potentially confounding issues associated with differential splice variants. However, it is clear that the dramatic changes in gene expression in the predominant FFUF and FFDF microarray clusters were replicated by the qRT-PCR analysis for many of the tested genes. In addition, qRT-PCR was used to analyse expression patterns of a number of genes that were not represented on the microarray, not reporting on the microarray due to cross-species analysis issues, or not providing a unique signal that discriminated between gene family members (Figure 4). This analysis reinforced the observations shown in figures 2 and 3 that many genes dramatically changed their expression patterns between late fetal development and 1-3 days post-partum e.g. lipoprotein lipase (*LPL*) and *CD36 *were strongly up-regulated during this transition while the non-coding RNA, *MEG8*, the imprinted retrotransposon-like gene, *RTL1 *[8], and the promyogenic transcription factor, *MYF5*, were correspondingly strongly down-regulated. *PPARD*, a muscle specific ligand-activated nuclear receptor, also showed strongly increased postnatal expression, although this was greater immediately after birth (150 d) than at 230 d of development.

Of particular note are the skeletal muscle structural proteins α-actin (*ACTA1*) and a number of myosin heavy chain genes, *MYH1, MYH2, MYH3*, *MYH7*, and *MYH8*. Fetal and embryonic myosin heavy chain genes (*MYH8 *and *MYH3*, respectively) reflected their names in that both were strongly down-regulated during the fetal to new-born transition between 120 d and 150 d of development, and both were present in the FFDD cluster. In terms of absolute expression, *MYH8 *was generally more prevalent than *MYH3*. Three 'adult' myosin heavy chain genes, *MYH7, MYH2 *and *MYH1*, showed a reciprocal pattern to the *MYH8 *and *MYH3 *pair, in that the former group was strongly up-regulated in the postnatal samples. These three genes characterize type I (slow oxidative), type IIa (fast oxidative/glycolytic) and type IIx (intermediate between types IIa and IIb (fast glycolytic)) skeletal muscle fibres, respectively. Concomitant with these changes is a strong postnatal increase in α-actin expression.

Gene ontology (GO) analyses were performed using the DAVID functional analysis tool to identify GO terms as well as pathways and keywords that were significantly over-represented (p < 0.05; Benjamini correction for multiple testing) in specific gene expression clusters [9,10]. DAVID was then used to group significantly enriched terms into *functional annotation clusters *(hereafter referred to as *Functional Groups*) (summarized in Additional file 2 with the full analysis listed in Additional file 3). This process provided a higher level perspective of the enriched functions associated with each gene expression cluster.

The dominant FFUF and FFDF clusters, representing genes that were differentially expressed between late fetal development and birth, were characterized by highly significant changes in many GO terms, however there were marked differences between these two clusters. The FFUF cluster contained 15 functional groups. Functional Groups 1-7 were directly associated with mitochondrial structure or function and all were highly significant. For example, Functional Group 1 in the FFUF cluster contained 39 terms which were largely associated with mitochondrial structure, while Functional Group 3 was enriched for terms associated with the TCA (tricarboxylic) cycle and Function Group 7 was enriched for terms associated with oxidative metabolism. Thus, there were strong indications that the transition between late fetal development and birth corresponded with enhanced mitochondrial function, which could be due to enhanced mitochondrial number, size or activity. The genes *TOMM70A*, *TOMM40 *and *TOMM34 *were present in this cluster. They encode structural proteins located in the outer mitochondrial membrane and are involved in the translocation of proteins into the mitochondria. These three mitochondrial genes were up-regulated 2.1, 2.34 and 2.56 fold, respectively in the contrast between the fetal 120 d and postnatal 150 d samples, thereby suggesting an increased size and/or number of mitochondria. The remaining functional groups primarily related to aspects of metabolism and in several cases indirectly reflected mitochondrial function (e.g. Functional Groups 8 and 13). Functional Group 15 represented terms linked with amino acid metabolism, suggesting an emphasis on protein synthesis. Three Kegg pathways were over-represented in the FFUF cluster: *Oxidative metabolism *(Functional Group 1); *Citrate cycle (TCA cycle) *(Functional Group 3) and *Phenylalanine, tyrosine and tryptophan biosynthesis *(Functional Group 15). The first two pathways related to mitochondrial function and together suggested that energy production and conversion were the major themes of this cluster. Although the FFUF and FFDF gene expression clusters were associated with several GO terms linked to skeletal muscle structure and function, these were not significant after correction for multiple testing. The under-representation of skeletal muscle genes on the microarray and in some instances its inability to discriminate between gene family members may have contributed to this outcome.

The FFDF cluster by contrast, was characterized by 22 functional groups but none was related to mitochondrial function. There was also greater diversity in the functional groups associated with the FFDF cluster. Several functional groups involved aspects of development, morphogenesis and differentiation (Functional Groups 3, 4, 12, 17, 21, 22) suggesting that these processes were concluding during the fetal to postpartum transition. Functional Groups 3 and 4 contained terms linked to neurogenesis, which may correspond with completion of the muscle innervation process that began with secondary myotube formation [11]. The FFDF cluster was also associated with several functional groups suggesting completion of tissue remodeling as these groups contained terms associated with the extracellular matrix, cytoskeleton and transmembrane receptors (Functional Groups 2, 5-8, 10, 11, 14, 15 and 22). (The related FFDU cluster contained a similar functional group encompassing aspects of cell adhesion.) Other distinctive functional groups contained terms associated with cell signaling (Functional Groups 1, 6, 9, 13, 18, 19 and 23) and transcriptional regulation (Functional Group 16). Indeed, postnatal down-regulation of the expression of a number of promyogenic transcription factors (*PAX7*, *MYF5*, *MYOD*, *MYOG *and *MEF2A*) in similar samples has previously been reported [12]. Two Kegg pathways were associated with the FFDF cluster: *Cell adhesion molecules *and *Wnt signaling pathway*. Although the FFUF cluster showed strong evidence for enhanced mitochondrial oxidative activity, the FFDF cluster showed no corresponding enrichment for glycolytic metabolism. Thus, the acquisition of enhanced oxidative capacity between the 120 d fetal time point and the postpartum sample (150 d) required continued maintenance of glycolytic function.

The FFFU cluster contained four functional groups, all of which were related to transcriptional regulation, while three of the four also contained terms relating to transcriptional control of metabolic processes. The FFUD cluster, representing transient changes around birth, contained two functional groups involving aspects of nucleotide binding and amino acid metabolism as well as one Kegg pathway (*Glycine, serine and threonine metabolism*). Immune response was a major theme in one of the two functional groups in the FUFF cluster. The second functional group in this cluster broadly related to extracellular glycoproteins. The UFFF cluster (up-regulated between 80 and 100 d of fetal development) was the only other cluster containing significant functional groups. Three of the four functional groups in this cluster involved aspects of cell adhesion indicating that this theme temporally preceded the tissue remodeling theme present in the FFDF cluster. Two Kegg pathways were present in the UFFF cluster: *Cell adhesion molecules *and *Cytokine-cytokine receptor interaction*. The former is also present in the FFDF cluster while the latter overlaps with immune response terms enriched in the closely related FUFF cluster.

### Changes in LD skeletal muscle oxidative activity during development

**Figure 5:**
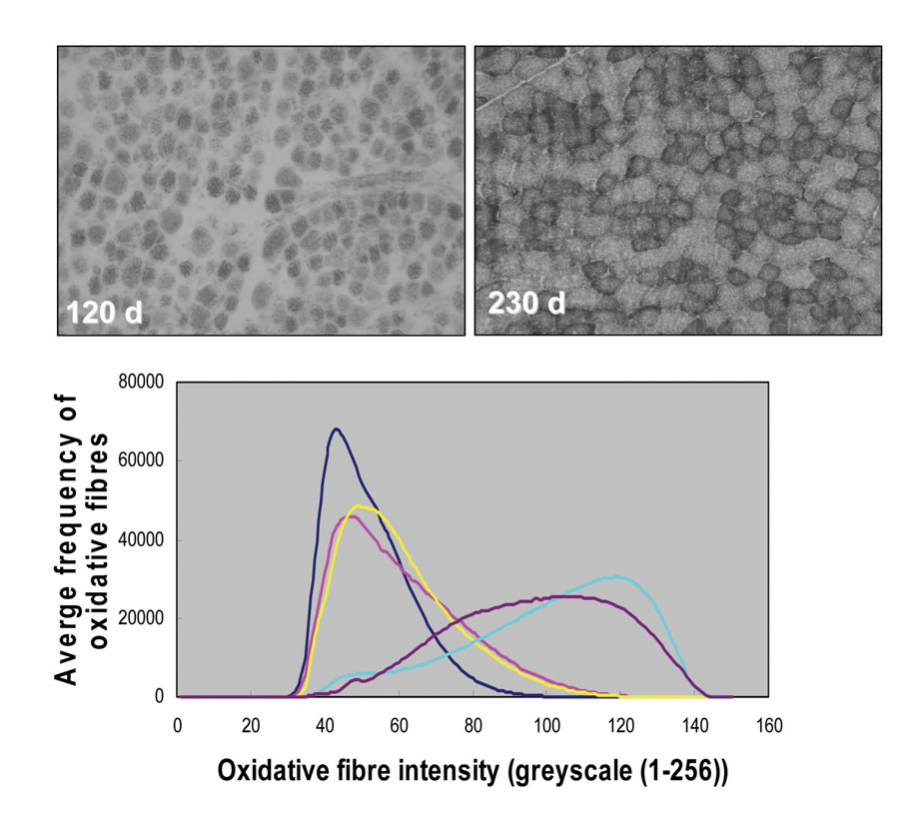
**Muscle fibre type histochemistry showing the frequency of oxidative fibres in samples across the developmental period**. NADH-Tr histochemistry was used to identify oxidative fibres in sections of LD muscles from animals sampled at 80, 100, 120, 150 and 230 days of development (n = 3 at each developmental stage). (a) Examples of histochemical staining patterns in a 120 d fetal sample (left panel) and a sample taken at 230 d of development (young immature lamb) (right panel). (b) A series of random grey-scale images was collected from each section and converted to pixel grayscale intensity values. The average frequency across the images from each sample was calculated and the mean for the three animals at each developmental time was then graphed. Greater pixel intensity corresponded with increased staining and therefore higher oxidative activity. The developmental times are differentially colored: 80 d (black); 120 d (yellow); 100 d (red); 150 d (blue), and; 230 d (purple).

The GO, keyword and pathway analyses for the genes in the FFUF cluster strongly suggested that there was a large change in the oxidative capacity of LD skeletal muscle during the late fetal to postpartum transition. This prediction was experimentally examined using sections of muscle samples stained with nicotinamide adenine dinucleotide-nitro-blue tetrazolium reductase (NADH-TR), a stain for oxidative fibres [13]. Examples of the staining patterns in the 120 d fetal sample and a sample at 230 d (young immature lamb) are shown in figure 5(a). The latter sample has larger fibres and a greater proportion of darkly stained oxidative fibres. Figure 5(b) shows the mean greyscale frequency distribution (n = 3) for each of the developmental samples. A larger greyscale value indicates stronger staining intensity and therefore higher oxidative capacity. There was a marked difference between the fetal and postnatal samples, which was consistent with the gene expression analysis. Compared with the two postnatal samples, the three fetal samples were characterised by relatively low greyscale values, indicative of less abundant oxidative fibre contents. Moreover, the fetal frequency peaks were relatively sharp, which infers that the fetal muscles at these three time points were relatively homogeneous in their oxidative capacities compared with the postnatal samples. The higher intensity edges of the fetal 100 d and 120 d peaks showed extensions toward higher oxidative capacity compared with the fetal 80 d sample. Thus, fetal development showed progressive increase in muscle oxidative capacity. The postpartum (150 d) and young immature lamb (230 d) peaks were very different from the fetal samples in that the frequency profiles of the former two samples were markedly shifted toward higher staining intensities and the peaks were relatively broad and asymmetrical. These results are consistent with a gain in overall oxidative capacity and greater heterogeneity in muscle fibre oxidative status in the postnatal samples. The 150 d sample showed a greater shift toward higher greyscale values compared with the 230 d postnatal sample and hence there presumably was a greater proportion of oxidative fibres at birth relative to the young lamb (230 d) sample.

### Identification of conserved cis-regulatory motifs in gene expression clusters

Each gene expression cluster was examined for enrichment of conserved cis-acting regulatory motifs using the Molecular Signatures Database (MSigDB) [14]. This database included conserved gene-associated anonymous motifs in the human, mouse, rat and dog genomes [15] and also conserved gene-associated transcription factor binding sites as well as conserved miRNA recognition sites. The conserved anonymous motifs and transcription factor binding sites were restricted to a sequence window corresponding to 2 kb up-stream and 2 kb down-stream of the transcription start site while the conserved miRNA recognition sites were restricted to the 3' untranslated region of genes. Only the FFUF and FFDF clusters contained significantly over-represented cis-regulatory motifs after Bonferroni correction for multiple testing (Tables 2 and 3). In the FFUF cluster five motifs were over-represented. Two of these conserved motifs matched binding sites for the transcription factors estrogen-related receptor alpha (ESRRA) and estrogen receptor 1 (ESR1). Two related motifs corresponded with SP1 (splicing factor 1) recognition sites, while the last motif corresponded with MLLT7 (mixed-lineage leukemia translocated 7/FOXO4; Forkhead 4 transcription factor) sites. No anonymous conserved motifs or miRNA motifs were identified in this cluster. The FFDF cluster was enriched for 19 motifs, only one of which (MLLT7) was also identified in the FFUF cluster. Strikingly, 14 of the motifs in this cluster were conserved miRNA recognition sites. There were also two SOX9 motifs, one anonymous motif and an LEF1 (lymphoid enhancer-binding factor 1) motif.

**Table 2:** Enrichment of conserved cis-regulatory motifs for genes in the FFUF cluster^1^

Gene motif (number of genes)	Description	Genes in overlap	p-value^2^
TGACCTY_V$ERR1_Q2 (1081)	Genes with promoter regions [-2 kb, 2 kb] around transcription start site containing the motif TGACCTY which matches annotation for ESRRA: estrogen-related receptor alpha	76	1.08E-10
TGACCTTG_V$SF1_Q6 (258)	Genes with promoter regions [-2 kb, 2 kb] around transcription start site containing the motif TGRCCTTG which matches annotation for SF1: splicing factor 1	26	2.91E-7
V$SF1_Q6 (264)	Genes with promoter regions [-2 kb, 2 kb] around transcription start site containing the motif TGRCCTTG which matches annotation for SF1: splicing factor 1	25	1.49E-6
V$ER_Q6_02 (261)	Genes with promoter regions [-2 kb, 2 kb] around transcription start site containing the motif NAGGTCANNNY which matches annotation for ESR1: estrogen receptor 1	22	3.48E-5
TTGTTT_V$FOXO4_01 (2149)	Genes with promoter regions [-2 kb, 2 kb] around transcription start site containing the motif TTGTTT which matches annotation for MLLT7: myeloid/lymphoid or mixed-lineage leukemia (trithorax homolog, Drosophila); translocated to, 7	43	4.31E-5

^1^The FFUF gene expression cluster was examined for enrichment of conserved cis-acting regulatory motifs by using the Molecular Signatures Database (MSigDB) and its associated analysis procedures [14]. The database contained motifs conserved in the human, mouse rat and dog genomes. The conserved transcription factor binding sites and anonymous conserved motifs were restricted to a sequence window corresponding to ± 2 kb of the transcription start site while the conserved miRNA recognition sites were restricted to the 3' untranslated region of the genes in this cluster.

^2^P-values are uncorrected for multiple testing and were calculated as described [14]. Only motifs with p-values < 0.05 after Bonferonni correction for multiple testing are shown.

**Table 3:** Enrichment of conserved cis-regulatory motifs for genes in the FFDF cluster^1^

Gene motif (number of genes)	Description	Genes in overlap	p-value^2^
CTTTGT_V$LEF1_Q (22036)	Genes with promoter regions [-2 kb, 2 kb] around transcription start site containing the motif CTTTGT which matches annotation for LEF1: lymphoid enhancer-binding factor 1	185	7.12E-11
TTTGTAG,MIR-520D (337)	Targets of MicroRNA TTTGTAG, MIR-520D	48	5.19E-09
GTACTGT,MIR-101 (257)	Targets of MicroRNA GTACTGT, MIR-101	38	7.22E-08
GTGCCAA,MIR-96 (304)	Targets of MicroRNA GTGCCAA,MIR-96	42	1.04E-07
ACCAAAG,MIR-9 (501)	Targets of MicroRNA ACCAAAG, MIR-9	59	1.05E-07
AACTTT_UNKNOWN (1963)	Genes with promoter regions [-2 kb, 2 kb] around transcription start site containing motif AACTTT. Motif does not match any known transcription factor	166	1.06E-07
CTTTGTA,MIR-52 (437)	Targets of MicroRNA CTTTGTA, MIR-524	52	4.17E-07
ACACTGG,MIR-199A,MIR-199B (160)	Targets of MicroRNA ACACTGG, MIR-199A, MIR-199B	26	1.29E-06
CATTGTYY_V$SOX9_B1 (370)	Genes with promoter regions [-2 kb, 2 kb] around transcription start site containing the motif CATTGTYY which matches annotation for SOX9: SRY (sex determining region Y)-box 9 (campomelic dysplasia, autosomal sex-reversal)	45	1.32E-06
CAGTATT,MIR-200B,MIR-200C,MIR-429 (470)	Targets of MicroRNA CAGTATT, MIR-200B, MIR-200C, MIR-429	53	1.60E-06
TGCCTTA,MIR-124A (556)	Targets of MicroRNA TGCCTTA, MIR-124A	59	2.91E-06
ATACTGT,MIR-144 (199)	Targets of MicroRNA ATACTGT, MIR-144	29	3.05E-06
ATATGCA,MIR-448 (212)	Targets of MicroRNA ATATGCA, MIR-448	30	3.77E-06
TGTTTAC,MIR-30A-5P,MIR-30C,MIR-30D,MIR-30B,MIR-30E-5P (582)	Targets of MicroRNA TGTTTAC, MIR-30A-5P, MIR-30C, MIR-30D, MIR-30B, MIR-30E-5P	60	5.75E-06
V$SOX9_B1 (241)	Genes with promoter regions [-2 kb, 2 kb] around transcription start site containing the motif NNNNAACAATRGNN which matches annotation for SOX9: SRY (sex determining region Y)-box 9 (campomelic dysplasia, autosomal sex-reversal)	32	6.91E-06
GTGCAAT,MIR-25,MIR-32,MIR-92,MIR-363,MIR-367 (311)	Targets of MicroRNA GTGCAAT, MIR-25, MIR-32, MIR-92, MIR-363, MIR-367	38	7.23E-06
TTGTTT_V$FOXO4_01 (2149)	Genes with promoter regions [-2 kb, 2 kb] around transcription start site containing the motif TTGTTT which matches annotation for MLLT7: myeloid/lymphoid or mixed-lineage leukemia (trithorax homolog, Drosophila); translocated to, 7	168	8.87E-06
TGAGATT,MIR-216 (107)	Targets of MicroRNA TGAGATT, MIR-216	19	8.87E-06
GCACCTT,MIR-18A,MIR-18B (119)	Targets of MicroRNA GCACCTT, MIR-18A, MIR-18B	20	1.20E-05

^1^The FFDF gene expression cluster was examined for enrichment of conserved cis-acting regulatory motifs using the Molecular Signatures Database (MSigDB) [14]. The conserved transcription factor binding sites and anonymous conserved motifs were restricted to a sequence window corresponding to ± 2 kb of the transcription start site while the conserved miRNA recognition sites were restricted to the 3' untranslated regions of the genes in this cluster.

^2^P-values are uncorrected for multiple testing and were calculated as described [14]. Only motifs with p-values < 0.05 after Bonferonni correction for multiple testing are shown.

## Discussion

LD skeletal muscle development from the late fetal state to postnatal life was associated with a remarkable transition in the gene expression program, which probably underpinned major metabolic and structural changes. This transition is clearly preparing skeletal muscle for the immediate postnatal environment where the muscle contributes to locomotion and the maintenance of posture in response to gravity. The LD muscle is a large epaxial muscle lying along the thoracic and lumbar regions of the back and is involved in both of these functions. Strikingly, the range in gene expression levels is highly constrained in the sample taken just prior to birth (fetal 120 d) possibly indicating that transcriptional activity is minimized at this time in anticipation of the energy demands of birth.

Enhanced oxidative metabolism in postnatal LD skeletal muscle was strongly evident in the gene expression program and histological sections of the muscle. This is consistent with increased requirement for skeletal muscle fibre types that are fatigue resistant in the context of postnatal movement and support against gravity. Skeletal muscle oxidative fibres (type I and type IIa fibres) are known to have these attributes and are characterized by high mitochondrial content and a rich capillary supply [16]. Indeed, adult ruminant LD muscle contains a strong contribution from fast twitch oxidative fibres (type IIa fibres) [17]. Also consistent with this conclusion was the major transition in myosin heavy chain gene expression during the fetal to neonatal transition with strong postnatal up-regulation of *MYH7*, *MYH2 *and *MYH1*, which characterize type I slow twitch oxidative fibres, type IIa fast twitch oxidative fibres, and the predominant type IIx fast twitch oxidative-glycolytic fibres, respectively, and down-regulation of the fetal and embryonic myosin heavy chain genes (*MYH8 *and *MYH3*, respectively). Moreover, the postpartum change in the gene expression program in the FFUF cluster was strongly associated with many GO terms related to mitochondrial structure and function, including the TCA cycle, as well as enrichment of Kegg pathways for *oxidative phosphorylation *and *citrate (TCA) cycle*. The postpartum period involves separation of the newborn from the placental oxygen supply, the onset of lung activity and changes in the oxygen carrying capacity of blood. These major physiological events directly parallel the increased capacity for skeletal muscle oxidative metabolism suggested by the gene expression program during this time interval.

The late fetal to postnatal developmental transition in some species has been associated with major changes in muscle fibre type diameter and also expansion of the skeletal muscle in the axial direction, which was associated with the formation of new sarcomeres [18]. These changes are thought to occur in the absence of formation of new fibres. The histological staining for oxidative capacity indicated that there was greater diversity of muscle fibre types in the postnatal samples. Hence, the increased oxidative status of LD muscle during this period was most likely the result of enhanced metabolic capability, presumably the consequence of increased mitochondrial activity and/or number within type I, type IIa and to a lesser extent type IIx muscle fibres. The developmental transition between 120 d (late fetal developmental) and 150 d (3 d post-partum) needs to be further investigated at finer temporal resolution to ascertain whether the observed major gene expression changes occurred prior to birth, at birth or within the 1-3 days after birth. It is noteworthy that the large changes in gene expression between late fetal development (120 d) and the postnatal samples were often preceded by qualitatively similar, though much smaller changes, in the earlier developmental intervals suggesting that these changes were in preparation for the birth process.

The gene expression clusters revealed many additional system-wide changes within LD skeletal muscle during the fetal to neonatal transition. In the FFDF cluster there was evidence for de-emphasis of restructuring of myofibres as reflected in the enriched Kegg pathways: *Cell adhesion molecules, ECM-receptor interaction, Focal adhesion, Adherins junction*, and *Tight junction*. These could reflect the finalization of muscle hypertrophy responses or fibre type changes [11]. The FFDF cluster also contained many GO terms relating to development as might be expected from the nature of the developmental time-course being examined. The *Wnt *pathway was over-represented in this cluster suggesting that its role is diminished in postpartum skeletal muscle. This evolutionary conserved pathway is involved intercellular communication underpinning many aspects of embryonic development including body axis specification, morphogenic signaling and axon guidance [19]. In skeletal muscle the *Wnt *pathway is also involved in signaling satellite cell proliferation during muscle cell regeneration [20,21]. Thus, the down-regulation of genes involved in this pathway within the FFDF cluster suggests the completion of muscle developmental processes dependent on satellite cell formation. There were also multiple GO terms relating to neuron development in this cluster. These could reflect the completion of the innervation process during fetal secondary and tertiary myotube formation as the obligatory precursor to muscle use in the postnatal environment.

The actual birth process was anticipated to be associated with transient increased synthesis of stress related genes. However, this was not evident in either the FFUD or FFDU gene expression clusters. One possibility is that the samples taken at 150 d corresponded to a period of 1-3 days post- partum and hence there may have been sufficient time for the muscle to recover from the stress of birth. Functional Group 12 of the FFUF cluster and Functional Group 2 of the related FUFF cluster contained stress response terms. In the former instance, there was up-regulation of stress responsive genes during the transition between late fetal development (120 d) and 1-3 days postpartum but these remained up-regulated even at 230 days of development (i.e. 83 days post-partum). Hence, it is unlikely that the enhanced expression of these genes was in response to the birth process itself. An alternative possibility is that these stress related genes present in the FFUF and FUFF clusters were coordinately up-regulated in parallel with the increased muscle oxidative capacity. The stress response genes may encode proteins that protect muscle cells from damage caused by the side effects of high levels of oxidative metabolism. The extremes of both hypoxia and hyperoxia are also known to cause transcriptional changes and therefore it is probable that enhanced oxidative metabolism during development directly regulated the transcription of some of the genes during this period [22,23].

There were also changes in the gene expression program between the two postnatal samples. These related to increased transcription of genes involved in transcriptional regulation of metabolic processes and therefore may indicate emphasis during this period on the adaption of skeletal muscle to the changing metabolic requirements of the immature lamb. The weaning age for lambs is 2-3 months. The metabolic processes changing between the new-born lamb (150 d) and the immature lamb (230 d i.e. 83 d postpartum) may have corresponded with adaptation to the nutritional transition between diets of milk and forage, and the associated establishment of rumen functions.

One caveat on the gene function analyses is that highly conserved bovine genes, especially those contributing to conserved metabolic functions and cellular structures, are more likely to have been annotated and have attributed Gene Ontology terms. Hence, there may be some bias in these analyses. The histological data provided independent confirmation of the increased emphasis on oxidative metabolism in the postnatal samples thereby providing confidence in the analysis strategy.

The mechanisms underpinning the large changes in gene expression during the fetal to postnatal transition are unclear but were likely triggered by hormonal changes. These mechanisms orchestrate coordinate changes in the expression of a large number of genes within the genome in a progressive hierarchical manner that has exquisite reproducibility, accuracy and robustness. One possibility is that hormonally triggered transcription factor cascades are involved, which ultimately result in coordinated changes in the transcription of a large number of target genes. This process enables cooperativity in the production of specialized functional units within a cell such as muscle fibre structures, mitochondria and metabolic circuits. Moreover, the process results in a high level of system control by hierarchical amplification of a small initial input signal. Consistent with this concept, analysis of cis-regulatory motifs associated with genes from the FFUF cluster identified over-representation of the estrogen receptor 1 (ESR1) and the estrogen related receptor alpha (ESRRA) motifs. The ovine placenta produces increasing amounts of estrogen during pregnancy with a substantial peak just prior to birth [24]. Thus, estrogen levels may be a primary means of coordinately altering the expression of a large number of genes during the fetal to newborn transition. To coordinate the gene expression changes it is speculated that a threshold level of estrogen is required to activate the estrogen receptors. Notably, the *ESR1 *gene was present in the UUFF gene expression cluster and thus it was up-regulated early in the developmental period perhaps in preparation for its enhanced functional role in changing the expression of genes later in development just prior to birth in response to estrogen levels. The SP1 and MLLT7 (FOXO4) motifs enriched in this cluster are also interesting as the FOXO4 protein not only has characteristic DNA binding capability that regulates gene expression but it also directly binds SP1, which probably has a co-activator role [25]. Moreover, motifs for these two transcription factors co-occur with estrogen receptor motifs in the promoters of genes whose transcription is subject to control by estrogen [26]. In addition, both FOXO4 and SP1 have been strongly implicated in the regulation of gene transcription initiated by insulin in skeletal muscle [27]. Thus, it is likely that these three transcription factors act in concert with the estrogen receptors to help orchestrate large estrogen-induced gene expression changes in the FFUF cluster.

The situation in the FFDF cluster is not as clear as it is dominated by miRNA motifs thereby suggesting post-transcriptional regulatory mechanisms. The potential roles of miRNA in this cluster require further investigation to ascertain whether they are spatially and temporally expressed with their putative target transcripts and whether the miRNA affect the transcription levels of their target genes. The over-representation of the Wnt signalling pathway in the FFDF gene expression cluster and the enrichment of the LEF1 transcription factor binding site motif in these genes is consistent with previous studies indicating that LEF1 is a down-stream target in the Wnt pathway [28]. This suggests that decreased Wnt signalling led to reduced LEF1 mediated gene transcription during this developmental period. SOX9 motifs were also over-represented in this cluster. Consistent with this, SOX9 has been implicated as a negative regulator of expression of sarcoglycan, a skeletal muscle structural protein [29]. Collectively, there is strong evidence for the involvement of these transcription factors in regulating muscle development.

## Conclusions

In conclusion, this study has demonstrated that there is a major change in the gene expression program in skeletal muscle during the fetal to postnatal developmental transition. Enhanced mitochondrial function during the transition from the late fetal state to the postpartum state is probably regulated by estrogen acting via a number of transcription factors. The enhanced oxidative capacity of the muscle reflects new functional demands on the muscle and the radically different nature of the primary energy and oxygen supplies in the newborn animal.

## Methods

### Biological samples

Sheep used in this experiment were bred from a research flock of Dorset/Suffolk/Rambouillet cross-bred sheep raised at Utah State University and cared for and euthanased for sample collection in accordance with the animal ethics guidelines of Utah State University (Utah, USA). *Longissimus dorsi *(LD) skeletal muscle samples were taken from fetal lambs at 80 days (three males), 100 days (two males and one female) and 120 days (two males and one female) of gestation, newborn lambs 1 to 3 days postpartum (i.e. 150 days of development; three males) and young immature lambs at 12 weeks of age (230 days of development; three males). LD muscle was dissected from each animal at a pre-determined site within 15 minutes of euthanasia, snap frozen under liquid nitrogen and stored at -80°C.

### Muscle fibre characterisation

LD muscle samples were mounted onto cork blocks using Tragacanth Gum (5% w/v) in a transverse orientation and snap frozen in liquid nitrogen quenched isopentane. Frozen blocks were stored at -80°C until required for analysis. Serial 8 μm sections were collected onto room temperature slides. The orientation of each block was confirmed as transverse by hematoxylin and eosin staining. Slides were stored at -20°C until required for staining. LD muscle sections were stained with nicotinamide adenine dinucleotide-nitro-blue tetrazolium reductase (NADH-TR), a stain for oxidative fibres, to screen for changes in oxidative capacity in the muscle samples as a function of development [13]. The sections were incubated in NADH-TR (1:1) solution in 0.05 M Tris-HCl pH 7.6 for 30 min at 37°C, followed by three water washes and three exchanges with increasing (30, 60, and 90% vol/vol) and then decreasing acetone solutions (30 s each). The slides were rinsed with water and mounted with polyvinyl acetate (PVA) aqueous mounting media. The staining highlights oxidative fibres, which primarily includes both slow oxidative (type I fibres) and fast oxidative/glycolytic fibres (type IIa fibres). Digital images were collected using an Olympus BX60 and a SPOT RT CCD camera. A series random grey-scale images was collected from each section at 400× magnification. Collection proceeded until ten images were collected with transverse fibres filling the entire image area. All images were collected in 8-bit grey-scales and saved in Tiff file format using SPOT Advanced software (Diagnostic Instruments, Sterling Heights, MI, USA). Pixel intensity histograms were generated using Image Pro Plus Version 4.1 (Media Cybernetics, Bethesda, MD, USA). For each image the frequency of each pixel (0-256) was collected. The average frequency across the images from each sample was calculated and the mean for the three animals at each developmental time point was then graphed. Greater pixel intensity corresponded with increased staining and therefore higher oxidative activity.

### RNA extraction

Total RNA was extracted from 4 g of LD muscle by pulverisation under liquid nitrogen followed by extraction using Trizol reagent (Invitrogen). Samples from three animals were extracted at each of the five developmental time points i.e. 80, 100, and 120 days of fetal development, 150 days of development (i.e. 1-3 days post-partum) and 230 days of development (i.e. 83 days post-partum). The extracted RNA was treated with DNase1 (Ambion), purified using an RNeasy Mini Kit (Qiagen) and finally subjected to on-column DNase1 treatment [7]. This process ensured removal of any traces of genomic DNA contamination. The RNA was quantified spectrophotometrically and its integrity validated by the OD_260_/OD_280 _absorption ratio (> 1.8) and by visualization on an agarose gel.

### Microarray transcript profiling

Gene expression analyses were performed using RNA isolated from ovine LD muscle samples at each developmental stage. Samples from three animals at each of the five developmental stages were accessed (Figure 1). Different animals were used at each developmental stage. The Bovine Genome Array Affymetrix GeneChip, (Affymetrix, Santa Clara, CA), containing 24,072 probe sets was employed, as previously described [6,7]. The target labelling, hybridisations, fluidics and chip scanning were performed by the Australian Genome Research Facility Ltd (The Walter and Eliza Hall Institute of Medical Research, Melbourne, Australia). All microarray images and quality control measurements were within recommended limits.

#### Microarray data processing

Previous studies indicated that the bovine Affymetrix microarray could be used for analysis of ovine gene expression although there was some minor data loss due to species specific sequence differences in the probe sets [6,7]. At present, there are several probe set algorithms available for analysis of microarray data. All of the algorithms, which include MAS5 (Microarray Suite), RMA (Robust Multichip Average), GC-RMA (Robust Multichip Average with adjustment for GC content of probes) and PLIER (Probe Logarithmic Intensity Error), have strengths and weaknesses, and none was designed to take advantage of the unique strengths afforded by a time series [30]. Consequently, the image data were independently pre-processed by using three probe set algorithms, i.e. RMA, GC-RMA and MAS5. Each dataset was independently analyzed by employing the statistical procedures described below to identify probe sets that were significantly differentially expressed between any two adjacent developmental times. An intersection list between the three analysis algorithms was then generated. This conservative strategy selected probe sets that were differentially expressed between two adjacent developmental times with the highest level of confidence.

MAS5 data were background corrected for both PM (Perfect Match) and MM (MisMatch) probe sets [31]. The lowest 2% of probe intensities were used as background for each of 16 grids on each microarray. Each probe was then adjusted based upon a weighted (distance between the location of the probe and the centroid) average of the background for each grid. Signal intensities were normalized for each microarray using a scaled normalization, where all microarrays were scaled to the same mean value (200). The signal for a specific probe set was calculated from the weighted average of all probe signals in the probe set using One-step Tukey's Biweight estimates and summarized as log_2 _scaled averages. RMA data were background corrected using convolution, followed by quantile normalization and median polish summarization [32]. In this case, MM intensities were not used. The GCRMA data were background corrected using an affinity measure model based on probe sequences and MM intensities, followed by quantile normalization and median polish summarization [33]. The data discussed in this publication have been deposited in NCBI's Gene Expression Omnibus [34] [GEO accession: GSE20112].

#### Statistical analysis of microarray data

Differential expression was tested on log_2 _expression intensities obtained using the MAS5, RMA and GCRMA methods. P-values for each method were calculated by the moderated F-statistic which stabilizes the gene-specific variance using an empirical Bayes method to shrink the probe-wise sample variances toward a common value and to augment the degrees of freedom for the individual variances [35]. Significance after correction for false discovery rates (FDR) using the Benjamini Hochberg method for a targeted p-value of 0.01, was calculated for each summarization set. Log_2 _fold changes based on the raw data and not the fitted intensities were calculated. Comparisons were made between adjacent developmental time points. The convergence in percentage of the gene across the three analysis methods was calculated. A value of 100 indicated that the gene was detected as significantly differentially expressed for all three summarizations. The identified genes were then filtered for genes with a convergence greater than 50% and called as *Present *by MAS5 in at least half of the samples. Annotation of the differentially expressed probe sets was previously described [7] and since then has been augmented by multifaceted searches of several publically available resources based on the Btau 4.0 bovine genome assembly [36]. The term 'probe set' has been replaced by 'gene' in subsequent text.

#### Visual representation of global gene expression profiles

For the visual representation of global gene expression profiles, the MAS5 signal intensities were normalized for each chip using a distribution of all genes around the 50^th ^percentile and scaled to 200. The signal intensities for each microarray were then loaded into the microarray analysis and visualization program, GeneSpring GX7.3 (Silicon Genetics, Redwood City, CA). Data were normalized using the default normalization method recommended by the GeneSpring software. Each analysis time point consisted of triplicate samples, and the mean for each probe set was used in the analysis. Genes that were called *Present *or *Marginal *in at least one developmental stage were used. The software differentially colored genes according to their normalized expression intensity relative to the global gene intensity at 80 days of fetal development. Thus, genes that had mean intensities less than the global average at 80 days of development were colored green, while those that had mean intensities greater than the global average at 80 days of fetal development were colored red. Gene intensities that were not different from the 80 day fetal global mean were colored black, while genes represented by unsaturated colors were of low confidence according to a defined set of GeneSpring criteria (i.e. *trustworthiness*)

### Clustering of differentially expressed genes

The genes that were differentially expressed between each pair of adjacent developmental time points were used for cluster analysis across the complete developmental time course. Clusters of the differentially expressed genes were defined by a four letter combination of the following categories across the whole developmental time course: Flat (F, no significant change); Up (U, significantly up-regulated), and; Down (D, significantly down-regulated). Each category (i.e. F, U, D) defined the result of the analysis between two adjacent developmental times. The differentially expressed genes were identified by the summation of the three independent methodologies, but only normalized MAS5 intensity data for these genes were used to define clusters (e.g. FFUF). The clustering analysis using the Flat, Up, Down categories across the five development times points (i.e. 4 developmental intervals) had potential to generate 81 clusters. However, only major clusters containing 50 or more genes are reported. None of the unreported clusters contained significantly enriched gene ontology terms, keywords or pathways.

### Functional annotation of gene expression clusters for gene ontology terms, keywords, and pathways

To identify the higher level biological themes present in the data, the genes within each cluster were analysed by using the Database For Annotation, Visualization And Integrated Discovery (DAVID) [9,10] with a background containing all genes present on the bovine Affymetrix microarray. Probe sets without annotation were removed from the analysis. DAVID provided statistical methods for identification of enriched biological terms within data sets. Statistically over-represented Gene Ontology (GO) terms, keywords and pathways were identified by selecting those with a Benjamini adjusted p-value < 0.05. It should be noted that the correction for multiple testing using the Benjamini adjusted p-values is a conservative approach as many GO terms are not independent. Therefore, *Functional Annotation Clustering *of genes present in each gene expression cluster was also performed using the DAVID analysis system. This higher level process displays similar functional annotations together based on overlaps of genes associated with each function term and therefore gives a clearer overview of gene function information associated with large datasets. The geometric mean of the Benjamini-corrected p-values for terms in each functional cluster was calculated. Values less than or equal to 0.05 were considered significant.

### Identification of conserved cis-regulatory motifs

The Molecular Signatures Database (MSigDB) [37] was used to identify cis-regulatory motifs that were enriched in the gene expression clusters. The database contains gene-associated motifs that are conserved across the human, mouse, rat and dog genomes, including conserved transcription factor binding sites, conserved anonymous motifs and conserved recognition sites for miRNA [14]. The latter were restricted to the 3' untranslated regions of genes while the former two were restricted to regions 2 kb upstream to 2 kb down stream of the translational start site. The analysis yielded a p-value associated with motif enrichment and the number of genes in the cluster compared with the number genes containing the motif. Only motifs with p-values less than 0.05 after Bonferonni correction for multiple testing are reported. It is assumed that the identified motifs are also conserved in the ovine genome.

### Quantitative real time reverse transcription PCR

Quantitative real-time reverse transcription PCR (qRT-PCR) using the Sybr Green based fluorescent detection system and the ABI Prism 7900 Sequence Detection System (PE Applied Biosystems, Forster City, CA) was used to measure mRNA abundance [7,38,39]. A constant amount of cDNA, corresponding to 10 ng of reverse transcribed RNA derived from each skeletal muscle sample was used for qRT-PCR measurements. Primer pairs are listed in Additional file 4 and are derived from publically available bovine genome sequence information supplemented with bovine ESTs [36]. Four technical replicates and three biological replicates were performed for each gene investigated. This process allowed quantification of the target gene relative to a constant reference gene in each sample using threshold cycle (Ct) data. *18S ribosomal RNA (18S rRNA*; Genbank accession AY779625) was used as the reference gene following demonstration that its expression was relatively constant in all samples. Control experiments were performed in the absence of reverse transcriptase. qRT-PCR data analyses used the Relative Expression Software Tool (REST) and were expressed as Mean Normalized Expression (MNE) relative to the reference gene [40].

## Authors' contributions

RT conceived and designed the experiments, analysed the results and wrote the manuscript. KB and TV performed the gene expression analyses, CG performed the statistical analyses and JW performed the histochemical analyses of muscle sections. CB and JF-W helped conceive the experimental design and contributed to interpretation of data. NC and TH provided samples from animals. All authors read and approved the final manuscript.

## Supplementary Material

Additional File 1 **Gene expression data**. This file contains links to the gene expression data. The genes that are significantly differentially expressed between adjacent development times are listed as Experiment 1 (80 d vs 100 d), Experiment 2 (100 d vs 120 d), Experiment 3 (120 d vs 150 d) and Experiment 4 (150 d vs 230 d) in the section entitled *Open microarray analysis report for developmental expression*. Each experiment contains an MA plot, Volcano plot and Heatmap for each of the three microarray processing programs MAS5, RMA and GCRMA. For each of these processing programs there is a list of significantly differentially expressed probe sets, and for each of these there is an FDR corrected probability, fold change, percentage of microarrays with a MAS5 *Present *call, and a convergence percentage for all three microarray processing programs. The file also contains annotation of the probe sets. In a second section entitled *Time series analysis of developmental expression*, there is a list of genes in each gene expression cluster and diagrams showing MAS5 gene expression values for these genes. Click here for file

Additional File 2 **Functional clustering of genes present in each expression cluster**. The file contains a summary table listing functional groups and functional terms significantly associated with each gene expression cluster. Click here for file

Additional File 3 **Functional clustering of genes present in each expression cluster**. The file contains complete details of the functional groups and functional terms significantly associated with each gene expression cluster. Each functional group was associated with an enrichment score. Each enriched term was listed with the gene count, percentage of genes associated with this term, p-value, a list of genes, fold enrichment, Bonnferroni-corrected p-value, Benjamini-corrected p-value and an FDR (percent of false predictions expected). Click here for file

Additional File 4 **Primer sequences used for quantitative RT-PCR analyses**. Primer sequences used for quantitative RT-PCR analyses. Click here for file
