# Supplementary material for: A gene network switch enhances the oxidative capacity of ovine skeletal muscle during late fetal development
Source: BMC Genomics. 2010 Jun 15;11:378. doi: 10.1186/1471-2164-11-378 (PMC2894804; doi:10.1186/1471-2164-11-378)
Supplement: Additional File 1 — Gene expression data. This file contains links to the gene expression data. The genes that are significantly differentially expressed between adjacent development times are listed as Experiment 1 (80 d vs 100 d), Experiment 2 (100 d vs 120 d), Experiment 3 (120 d vs 150 d) and Experiment 4 (150 d vs 230 d) in the section entitled Open microarray analysis report for developmental expression. Each experiment contains an MA plot, Volcano plot and Heatmap for each of the three microarray processing programs MAS5, RMA and GCRMA. For each of these processing programs there is a list of significantly differentially expressed probe sets, and for each of these there is an FDR corrected probability, fold change, percentage of microarrays with a MAS5 Present call, and a convergence percentage for all three microarray processing programs. The file also contains annotation of the probe sets. In a second section entitled Time series analysis of developmental expression, there is a list of genes in each gene expression cluster and diagrams showing MAS5 gene expression values for these genes. [file 1471-2164-11-378-S1.ZIP › Custom/CustomResults.html]

## DevelopmentalExperiment - Time series analysis of differentially expressed genes

The different patterns of expression for the probes are shown. F - flat (no change in expression), U - up (up regulation) and D - down (down regulation). NOTE: The profiles shown are for MAS results. Unannotated probes were removed from the analysis but not from the summary, thus the numbers might differ. Patterns with no link in the summary table have no annotated genes (or are not DE - first row, all flat). Not all patterns are necessarily in the summary table since some might not have any representatives. Probes are shown in their hierachical clustering order. The number of DE genes is based on fold change, meaning that the genes are not necessarily differentially expressed from a stastical standpoint - the number of DE genes tends to be overinflated.

Summary of Expression Patterns


# Summary of Expression Patterns


|  |  |  |  |  |  |  |
| --- | --- | --- | --- | --- | --- | --- |
| **pattern** | **mas** | **rma** | **gcrma** | **maspercent** | **rmapercent** | **gcrmapercent** |
| FFFF | 9995 | 14025 | 12557 | 63.898 | 89.662 | 80.278 |
| FFFU | 545 | 101 | 211 | 3.4842 | 0.6457 | 1.3489 |
| FFFD | 612 | 188 | 323 | 3.9125 | 1.2019 | 2.065 |
| FFUF | 787 | 383 | 590 | 5.0313 | 2.4485 | 3.7719 |
| FFUU | 26 | 11 | 27 | 0.1662 | 0.0703 | 0.1726 |
| FFUD | 150 | 63 | 175 | 0.959 | 0.4028 | 1.1188 |
| FFDF | 1392 | 668 | 1128 | 8.8991 | 4.2706 | 7.2114 |
| FFDU | 440 | 13 | 68 | 2.8129 | 0.0831 | 0.4347 |
| FFDD | 96 | 25 | 75 | 0.6137 | 0.1598 | 0.4795 |
| FUFF | 113 | 15 | 22 | 0.7224 | 0.0959 | 0.1406 |
| FUFU | 25 | 2 | 13 | 0.1598 | 0.0128 | 0.0831 |
| FUFD | 16 | 0 | 4 | 0.1023 | 0 | 0.0256 |
| FUUF | 32 | 11 | 31 | 0.2046 | 0.0703 | 0.1982 |
| FUUU | 5 | 0 | 5 | 0.032 | 0 | 0.032 |
| FUUD | 12 | 1 | 4 | 0.0767 | 0.0064 | 0.0256 |
| FUDF | 18 | 0 | 5 | 0.1151 | 0 | 0.032 |
| FUDU | 18 | 0 | 1 | 0.1151 | 0 | 0.0064 |
| FUDD | 2 | 0 | 0 | 0.0128 | 0 | 0 |
| FDFF | 104 | 9 | 34 | 0.6649 | 0.0575 | 0.2174 |
| FDFU | 22 | 0 | 0 | 0.1406 | 0 | 0 |
| FDFD | 30 | 0 | 12 | 0.1918 | 0 | 0.0767 |
| FDUF | 99 | 0 | 0 | 0.6329 | 0 | 0 |
| FDUU | 16 | 0 | 0 | 0.1023 | 0 | 0 |
| FDUD | 35 | 2 | 4 | 0.2238 | 0.0128 | 0.0256 |
| FDDF | 36 | 11 | 18 | 0.2301 | 0.0703 | 0.1151 |
| FDDU | 8 | 0 | 0 | 0.0511 | 0 | 0 |
| FDDD | 11 | 3 | 14 | 0.0703 | 0.0192 | 0.0895 |
| UFFF | 221 | 23 | 48 | 1.4129 | 0.147 | 0.3069 |
| UFFU | 49 | 2 | 10 | 0.3133 | 0.0128 | 0.0639 |
| UFFD | 28 | 2 | 5 | 0.179 | 0.0128 | 0.032 |
| UFUF | 37 | 1 | 3 | 0.2365 | 0.0064 | 0.0192 |
| UFUU | 9 | 0 | 0 | 0.0575 | 0 | 0 |
| UFUD | 14 | 1 | 2 | 0.0895 | 0.0064 | 0.0128 |
| UFDF | 43 | 5 | 16 | 0.2749 | 0.032 | 0.1023 |
| UFDU | 33 | 0 | 3 | 0.211 | 0 | 0.0192 |
| UFDD | 2 | 0 | 0 | 0.0128 | 0 | 0 |
| UUFF | 19 | 13 | 21 | 0.1215 | 0.0831 | 0.1343 |
| UUFU | 7 | 0 | 1 | 0.0448 | 0 | 0.0064 |
| UUFD | 3 | 0 | 2 | 0.0192 | 0 | 0.0128 |
| UUUF | 14 | 7 | 16 | 0.0895 | 0.0448 | 0.1023 |
| UUUU | 2 | 1 | 4 | 0.0128 | 0.0064 | 0.0256 |
| UUUD | 1 | 0 | 2 | 0.0064 | 0 | 0.0128 |
| UUDF | 2 | 0 | 2 | 0.0128 | 0 | 0.0128 |
| UUDU | 4 | 0 | 2 | 0.0256 | 0 | 0.0128 |
| UDFF | 18 | 2 | 4 | 0.1151 | 0.0128 | 0.0256 |
| UDFU | 13 | 0 | 1 | 0.0831 | 0 | 0.0064 |
| UDFD | 1 | 0 | 0 | 0.0064 | 0 | 0 |
| UDUF | 25 | 0 | 0 | 0.1598 | 0 | 0 |
| UDUU | 5 | 0 | 0 | 0.032 | 0 | 0 |
| UDUD | 6 | 0 | 0 | 0.0384 | 0 | 0 |
| UDDF | 4 | 0 | 1 | 0.0256 | 0 | 0.0064 |
| UDDU | 3 | 0 | 0 | 0.0192 | 0 | 0 |
| DFFF | 109 | 29 | 97 | 0.6968 | 0.1854 | 0.6201 |
| DFFU | 27 | 2 | 1 | 0.1726 | 0.0128 | 0.0064 |
| DFFD | 21 | 1 | 9 | 0.1343 | 0.0064 | 0.0575 |
| DFUF | 34 | 1 | 6 | 0.2174 | 0.0064 | 0.0384 |
| DFUU | 4 | 0 | 1 | 0.0256 | 0 | 0.0064 |
| DFUD | 16 | 3 | 3 | 0.1023 | 0.0192 | 0.0192 |
| DFDF | 24 | 5 | 21 | 0.1534 | 0.032 | 0.1343 |
| DFDU | 16 | 0 | 1 | 0.1023 | 0 | 0.0064 |
| DFDD | 2 | 0 | 4 | 0.0128 | 0 | 0.0256 |
| DUFF | 79 | 1 | 2 | 0.5051 | 0.0064 | 0.0128 |
| DUFU | 11 | 1 | 0 | 0.0703 | 0.0064 | 0 |
| DUFD | 12 | 0 | 0 | 0.0767 | 0 | 0 |
| DUUF | 12 | 0 | 1 | 0.0767 | 0 | 0.0064 |
| DUUU | 3 | 0 | 1 | 0.0192 | 0 | 0.0064 |
| DUUD | 4 | 0 | 0 | 0.0256 | 0 | 0 |
| DUDF | 14 | 3 | 4 | 0.0895 | 0.0192 | 0.0256 |
| DUDU | 13 | 0 | 0 | 0.0831 | 0 | 0 |
| DUDD | 3 | 0 | 0 | 0.0192 | 0 | 0 |
| DDFF | 7 | 2 | 10 | 0.0448 | 0.0128 | 0.0639 |
| DDFU | 2 | 0 | 0 | 0.0128 | 0 | 0 |
| DDFD | 3 | 1 | 3 | 0.0192 | 0.0064 | 0.0192 |
| DDUF | 5 | 0 | 0 | 0.032 | 0 | 0 |
| DDUD | 4 | 0 | 3 | 0.0256 | 0 | 0.0192 |
| DDDF | 7 | 4 | 6 | 0.0448 | 0.0256 | 0.0384 |
| DDDD | 2 | 1 | 5 | 0.0128 | 0.0064 | 0.032 |

### FFFU genes: 365

FFFU Cluster

Time course of FFFU genes

Link to DE genes in NCBI

| Gene Symbol | Gene Symbol | Gene Symbol | Gene Symbol | Gene Symbol | Gene Symbol | Gene Symbol | Gene Symbol | Gene Symbol | Gene Symbol | Gene Symbol | Gene Symbol | Gene Symbol |
| --- | --- | --- | --- | --- | --- | --- | --- | --- | --- | --- | --- | --- |
 TP53INP1 | SEC61A2 | FCHSD1 | SLC11A2 | PIP3-E | MFNG | ELMO2 | ARRB1 | THAP7 | NPHP4 | ITGA10 | PPP3CC | SART1 || THAP1 | TCP11L2 | TOR1AIP1 | OSR2 | MPEG1 | CCR1 | C14orf125 | ZNF609 | PRKCA | FMO5 | PER2 | FOXO1A | ACSS1 |
| MANBA | COMMD8 | TEAD1 | CHD4 | ASCIZ | ACP5 | ABCC1 | MAFF | CHMP4A | MINA | ADRM1 | PPP1R3E | PHACS |
| ADRA2B | LRRC20 | SLC1A3 | PDRG1 | GDF9 | MGAT5B | PAX8 | FADD | API5 | BDA20 | LGP1 | CG018 | SLC24A1 |
| MGC13024 | ARHGAP30 | SLC35F2 | GPR116 | CRIM1 | SEC22L3 | XPR1 | ACVR2B | KIAA0146 | FBXL4 | SLC16A6 | LIPG | LPIN1 |
| CREM | ODF1 | AK3L1 | SORBS1 | ARID5B | AMPD3 | AKR1C1 | CREBL2 | TIMP4 | FLJ20366 | FLJ11000 | GPD2 | FLJ16636 |
| CREG1 | CLDN14 | CC2D1B | CXCL3 | GNA12 | SLC8A1 | PKHD1 | PPP1R3D | CD83 | RABL5 | U1SNRNPBP | KRT4 | STAT3 |
| SH3YL1 | FECH | EIF2S1 | MAP1D | PRP2 | TBX4 | NALP5 | ETF1 | RGS12 | SH3BGRL2 | KIAA1604 | WDR32 | KIAA0746 |
| DELGEF | 76P | RAB3GAP2 | CTSB | RCBTB1 | PCAF | HAND1 | KPNA1 | SLC37A4 | LTBP2 | TFB1M | ZNFN1A3 | CH25H |
| DUSP14 | PRPF38B | FLJ13096 | BRRN1 | MLKL | GNPAT | GABRB3 | DEDD2 | KBTBD6 | DCP2 | MARCH9 | EIF2S2 | TMEM16B |
| DLX5 | ABT1 | SFRS2IP | UQCRB | ITGA5 | MAP3K2 | CLASP2 | RPA1 | ZA20D2 | FUNDC1 | ACSL6 | C6orf97 | PNRC1 |
| LPHN3 | PDZRN4 | FABP2 | MYBPC1 | CD8A | CHD2 | C13orf1 | IL1B | CD3G | UBE2D4 | TOMM40L | TRIM5 | RBM21 |
| HINT3 | CD47 | WISP2 | B2M | CHRM4 | ZNF435 | ANK3 | SMURF2 | TXNDC10 | MTMR9 | TNFSF13B | HSD17B6 | C14orf103 |
| UBD | KIAA1217 | PAG12 | FLJ10292 | BACE1 | ARRDC2 | LLGLH | GABRA1 | TRPC4AP | SAMSN1 | AGMAT | C7orf24 | BANK1 |
| EIF2AK4 | EFNA5 | PRP9 | NEDD4 | P2RY10 | GUCA1B | NARG1L | CARKL | F3 | SLC9A7 | CL-46 | HAO2 | C6orf163 |
| ECHDC3 | MBD5 | FARP1 | MYCBP2 | INHBA | SLITRK4 | ATAD1 | HRMT1L6 | HAO1 | EIF1AX | IFI44 | DKFZp434H2226 | STATH |
| UCKL1 | STAT5B | CRNKL1 | DIAPH1 | CBX7 | CA3 | AGTPBP1 | FDFT1 | KLF11 | MYLK2 | THADA | MGC4399 | FHOD1 |
| MITF | CTNNAL1 | CIB2 | ADHFE1 | PDE4C | TGIF | DKFZp313G1735 | STXBP1 | VPS41 | MMP11 | CDGAP | SCYL3 | PCDHAC1 |
| ZNF12 | RPIA | KLF15 | TTC17 | SNTB1 | STX12 | CDKN2C | MYNN | ASS | FBXL20 | C1orf112 | KBTBD9 | PKNOX1 |
| C5orf15 | ALDH3A2 | SYF2 | CUGBP2 | ALDH6A1 | ProSAPiP2 | NUP50 | ABCC5 | SLC25A37 | FRAT2 | GRB10 | FYCO1 | SRCAP |
| CD3E | DERL1 | FUNDC2 | TRAF3IP2 | MUCDHL | FAM63A | CTSS | TLE1 | PCBP3 | MDFI | C1orf21 | TAF3 | MGC127677 |
| JUN | CLIC2 | KIAA0828 | STARD10 | AOX1 | RASD1 | PFKFB3 | FBXO32 | PRKCQ | BoLA-DRB3 | KLF2 | THBD | DUSP7 |
| NXN | CPEB2 | REV3L | NDUFB2 | BNIP3 | SESN1 | NFKBIA | MYEOV2 | DUSP3 | C6orf106 | GKAP1 | IVNS1ABP | PFKFB4 |
| ABHD4 | DUSP26 | EPHX1 | ASB11 | CEBPD | CORO6 | EPAS1 | EIF2C4 | MGC29814 | ASB2 | MGC12966 | ZFP36 | PPP1R15A |
| CUL3 | FABP5 | NDRG1 | CWF19L2 | C10orf26 | SDC4 | IBRDC3 | HBP1 | NFE2L1 | ProSAPiP1 | CD82 | CYR61 | SELENBP1 |
| SPOCK2 | MLLT7 | UBE2G1 | TSC22D3 | PFKM | MTUS1 | BCL6 | RGC32 | MID1IP1 | NEK7 | ASB5 | SET7 | UBC |
| ID1 | KLF4 | FLJ13910 | CHURC1 | MYF6 | FEM1A | TPT1 | DMN | TXNIP | HSPA1B | ANXA11 | GSTM3 | DNTTIP1 |
| CAB39 | RHOBTB1 | MXI1 | ST3GAL6 | KBTBD2 | TAGLN | TACC2 | MYOZ3 | PTPRB | TFDP2 | NUMA1 | GLUL | DDIT4 |
| ATF4 |

### FFFD genes: 450

FFFD Cluster

Time course of FFFD genes

Link to DE genes in NCBI

| Gene Symbol | Gene Symbol | Gene Symbol | Gene Symbol | Gene Symbol | Gene Symbol | Gene Symbol | Gene Symbol | Gene Symbol | Gene Symbol | Gene Symbol | Gene Symbol | Gene Symbol | Gene Symbol | Gene Symbol |
| --- | --- | --- | --- | --- | --- | --- | --- | --- | --- | --- | --- | --- | --- | --- |
 SLC39A7 | DRB1 | SEMA6C | RCN2 | PLOD2 | COPZ2 | SLC35E3 | BRWD3 | ILF3 | LEO1 | TIMM9 | NUCB2 | ELOVL1 | FASN | CCNJ || PIGW | PPRC1 | SCML1 | PPARGC1A | GPRASP1 | ABHD14A | CD248 | PHKG2 | CAPN1 | MAP1B | JAG2 | DLG5 | ARF5 | GPR89 | SPINK2 |
| CHRND | SPPL2B | GNAS | EHD4 | TXLNA | CWF19L1 | COMMD5 | MGC11256 | C1QTNF5 | GAS7 | RASA3 | C10orf33 | VARSL | SPRY4 | H2AFJ |
| NAGK | MGC39633 | PPM2C | CANX | NCF4 | MUS81 | MGC35097 | RDH10 | C9orf102 | MGC5987 | HLF | CYB5R4 | KPNA2 | LIG1 | CDCA8 |
| CSNK1G3 | AACS | KIAA0433 | SLC38A1 | GMPPB | PRAF1 | COL13A1 | CCNB1 | PGM3 | HIATL1 | GALM | ENY2 | TTYH3 | MYRIP | ENAH |
| CHST3 | FLJ23825 | EPPB9 | PVRL2 | CALML4 | PCNT1 | CDK8 | RET | FLJ22624 | PLOD3 | SOCS1 | C20orf20 | SLC35D1 | UBE2J1 | MGC20781 |
| UCK1 | FES | C6orf115 | KIAA0528 | KIFC1 | ITGA10 | RNF24 | MAGEH1 | ETV1 | DPP4 | FUT4 | TNPO2 | MGC5306 | RGS16 | TGFBR2 |
| PEX12 | CREB5 | AKAP8 | GGPS1 | EYA2 | TRIP13 | PTK7 | CRYBB2 | RIOK1 | MAP3K13 | GTPBP3 | DKFZp547K054 | POLL | FLJ20674 | ACAD9 |
| MEF2C | C13orf3 | DKFZP564O0823 | ITGAV | ZFP2 | PHACS | TRAF1 | INHA | CKLFSF3 | RWDD2 | DOCK10 | NUDCD1 | GPI7 | TSP-NY | CACNA2D1 |
| RINT-1 | KCTD9 | TRPM4 | MORC4 | ODZ3 | RAD51AP1 | PRC1 | KIF20A | FAM64A | SPAG5 | LEF1 | CACNA1H | MXD3 | CENPA | MTPN |
| ADCK5 | TSPAN9 | LMNB2 | SLC2A1 | AMPD2 | SRPX2 | C9orf81 | RGS10 | FGD1 | FGF11 | MYH10 | IGSF1 | HBB | PCTK1 | FLJ14466 |
| KIAA0007 | CACNG6 | PTS | TPCN1 | FADS1 | SLC25A1 | PRO1855 | TSSC1 | EPHX2 | GDAP1 | BRWD1 | COPG2 | NID2 | FLJ14054 | NCK1 |
| RCN3 | SEC24D | HKE2 | AP1B1 | NSDHL | GALNTL1 | KIAA0657 | GPRC5B | FLJ20920 | ADD3 | FBXW7 | IDH1 | CSRP2 | ELN | PYCR2 |
| B3GAT3 | CHKB | RXRG | RAG1AP1 | MGST1 | DNAJB1 | GUK1 | FLJ22318 | LRRN1 | GSPT2 | ACLY | P4HA2 | FGFRL1 | RTN3 | MYOM3 |
| TTF2 | MAN2A1 | SLIT3 | NUDT14 | SLC25A5 | CITED1 | KIAA0101 | GALT | MDS032 | C14orf169 | EBNA1BP2 | SMAD9 | TKT | DCTN5 | ATP1A1 |
| KCTD2 | TRAF5 | PHCA | FKBP11 | JMJD3 | PYGO2 | HRB | DNM1L | NUDT22 | CHP | PX19 | XTP3TPA | LMNA | AMD1 | HEBP2 |
| GLCE | TMEM45A | C11orf31 | C21orf7 | INSIG1 | BRI3BP | ALG14 | CCDC28B | AFURS1 | PDGFRL | JARID2 | PCOLCE | CREB3L1 | STEAP1 | SDC2 |
| PRKRA | B4GALT1 | MAGEF1 | CDC42EP2 | TCEAL4 | PSMD10 | BF | GLT28D1 | FEM1C | PTPLA | CD79B | FLJ22531 | P2RX7 | MCTP2 | KIAA0376 |
| VAPB | ADAM1 | RAD9B | FLJ20422 | MIER1 | TTBK2 | FGFR1OP2 | GDAP2 | STAM | ELOVL6 | CBLN4 | FAM36A | 5HTR2A | XPO4 | DSC2 |
| PLEK | GSTK1 | MYOD1 | WDR76 | MYEF2 | Ells1 | ZNF165 | C10orf89 | MTHFD2 | SUGT1 | SLC10A1 | CDKL2 | EMAPL | RHBDL2 | FBXL12 |
| MEIS2 | HSDL2 | FLNB | MGC128730 | MELK | CNKSR3 | KIAA1524 | RARRES1 | CDH1 | NCOA7 | RASA2 | SSX2IP | KIAA0090 | DST | ANLN |
| LY6G5B | FZD3 | REPS2 | PDZK6 | NFAT5 | IQGAP2 | KIAA1199 | COASY | C13orf10 | ZFP276 | C9orf80 | PHTF1 | IQGAP3 | POLQ | ZNF609 |
| CBS | CTNNAL1 | RYR3 | ZBTB8 | SPATA13 | SLC35A5 | GOLGB1 | ASPH | TMEM67 | PRKCD | RRM2 | PTDSR | MGC3731 | BCAT1 | AFF2 |
| ITGA9 | JARID1B | MAP4K2 | ATAD2 | FLJ20160 | AASDHPPT | FAM46A | ARMC2 | ZADH1 | PLCB1 | RNF7 | LCMT2 | SMPD3 | BAG5 | PPARA |
| TFPI | SSR1 | MGC15912 | SCML4 | PIG-Y | SNX10 | MAP3K8 | PSCD4 | C3orf1 | KCNT2 | KCNB2 | GNAO1 | FLJ13611 | KCNQ3 | LGR4 |
| YT521 | YAP1 | CTSL | CNTNAP4 | FANCL | MGC90512 | KIAA0913 | MGC26963 | SOST | ETV6 | NME1 | EIF4A1 | POLE4 | CKAP4 | RBM3 |
| ARL1 | FKBP2 | AZI2 | SEC14L1 | EMP1 | NANS | ARMET | SOCS2 | SGK | HSPB3 | ACO1 | PALMD | HSF2 | TIMM8A | PGAM1 |
| NOL5A | POLR2G | MGC52010 | SMARCA4 | HMGCS1 | DKFZp762C186 | PLAGL1 | CLCN5 | DMPK | SLC25A24 | RBM9 | EPLIN | FMOD | TNMD | COL2A1 |
| CCT7 | AKR1B1 | ITGB1BP2 | HIF1A | C5orf13 | S100A2 | CNIH | FBL | COX7A2 | ENO1 | SLC38A2 | FLJ37562 | COL5A2 | NGFRAP1 | CAPN6 |
| SERPINH1 | S100A11 | SLC25A6 | PPIB | LGALS1 | SSR4 | HSPA5 | AUTS2 | COL1A1 | COL1A2 | RPS6 | ACTC | RPL29 | K-ALPHA-1 | TUBB |
|

### FFUF genes: 568

FFUF Cluster

Time course of FFUF genes

Link to DE genes in NCBI

| Gene Symbol | Gene Symbol | Gene Symbol | Gene Symbol | Gene Symbol | Gene Symbol | Gene Symbol | Gene Symbol | Gene Symbol | Gene Symbol | Gene Symbol | Gene Symbol | Gene Symbol | Gene Symbol | Gene Symbol | Gene Symbol | Gene Symbol | Gene Symbol | Gene Symbol |
| --- | --- | --- | --- | --- | --- | --- | --- | --- | --- | --- | --- | --- | --- | --- | --- | --- | --- | --- |
 CLPP | MGC128406 | S100A16 | NDUFA11 | SAMM50 | MRPL2 | MRPL21 | IPO13 | GYS1 | KAT3 | LRPPRC | SLC35B1 | MRPL43 | IDH3A | ACSL4 | TOMM70A | ICT1 | C14orf153 | MRPL13 || DERL2 | PSMC4 | HIP2 | AARS | C2orf33 | RRAGD | PPA1 | TUFM | PSME3 | QIL1 | DNASE1L1 | MRPL37 | NDUFC1 | NDUFS6 | BZW2 | MRPL35 | TBRG4 | MTCH2 | ATP6V1F |
| RHOQ | C16orf33 | CAMK2A | ETFB | BLOC1S2 | CFL2 | CDC34 | CALM3 | FLJ42461 | ZFP91 | LDB3 | BRP44L | PREB | MAP2K6 | ASB12 | SLC25A34 | NDUFA10 | CUTC | MRPS15 |
| MLF1 | NNT | DLAT | TA-PP2C | GOT2 | FLJ32452 | HIRIP5 | FH | CHCHD3 | UBADC1 | PDHX | IDH3G | ECH1 | GYG | HADHB | POLDIP2 | STARS | ADSL | MGC4825 |
| DKFZP564J0123 | UCHL3 | AK2 | UBL4A | GSPT1 | OAT | PSMD14 | TMOD1 | MAP2K3 | FKBP4 | DNAJA2 | DNAJC7 | PSMA1 | CTGF | GPX4 | GAMT | WDR1 | C1QBP | UCRC |
| FLJ20643 | OPTN | GPX1 | KIAA1434 | PSMB5 | DULLARD | COX4NB | G3BP2 | HDGF | ACAA2 | PSMC5 | MRPL51 | PMPCB | NUTF2 | MGC128110 | CACNB1 | SLMAP | PSMD8 | HADHA |
| PTP4A2 | MAPK6 | KPNA3 | CSDA | NDUFA13 | NDUFA3 | NDUFB7 | NDUFS8 | SLC25A12 | DUSP13 | NDUFV1 | SLC25A11 | SUCLA2 | BCKDHB | CGI-69 | FLJ10803 | DBI | MGC3196 | TEAD4 |
| AVEN | HCCS | ARPC5L | PLAA | TAF9 | KPNA6 | TIMM13 | C6orf93 | ETF1 | UXT | C10orf9 | HSPA4 | FDPS | PCCB | SLC38A2 | QKI | CPT1B | PPP6C | NOLA2 |
| DNAJB5 | GADD45GIP1 | COQ6 | PFDN1 | PRDX5 | MRPL12 | HMBS | PURB | AMACR | SAP18 | PDE4DIP | FLJ13220 | C7orf25 | GK001 | JUN | FBXW5 | C18orf55 | PEX7 | COX10 |
| C13orf1 | SAR1B | NDUFB1 | TRAP1 | MRPS14 | XK | HMGA1 | FBXO9 | RUVBL1 | OSGEPL1 | HN1 | ZC3HDC5 | PDHA1 | RABGGTA | BCL2L13 | CRAT | NUDT8 | NDUFA7 | RNF34 |
| LRRC28 | ENDOG | SLC16A3 | DHDH | KIAA0828 | PRDX3 | PHKB | HIF1AN | ADSSL1 | GLRX2 | KIAA0664 | CLIC5 | MRPL38 | MPI | MCEE | LIAS | PBEF1 | ZADH1 | ALS2CR2 |
| ALAD | DNPEP | S100A14 | ATPAF1 | OGDH | ETFDH | PLA2G7 | CS | ZNF650 | HSPB6 | SMYD2 | NDUFV3 | SITPEC | HOMER2 | MGC15416 | CA14 | CYCS | ASCC2 | MRPS18A |
| SLC35A4 | GRHPR | TIGD2 | COMTD1 | LYPLA1 | AKT1S1 | RG9MTD1 | MRPS22 | TOMM40 | MRPS9 | BXDC1 | FLJ31795 | MRPL28 | SYNGR1 | MGC11324 | TPD52 | FDX1 | CYB5R1 | CUTL1 |
| THAP7 | WDR18 | FLAD1 | TRPT1 | GHITM | 15E1.2 | KNS2 | SOD2 | KIAA1881 | MRPS36 | CUGBP1 | ARIH2 | MGC128026 | IBRDC2 | QP-C | ABCD1 | PITRM1 | LETM1 | C15orf24 |
| TXNRD1 | MRPS28 | C1orf163 | POP5 | MON1A | ACTR3B | KPNA1 | FLJ21415 | MRPS35 | C17orf37 | MTERFD1 | 2-PDE | IARS | TRMU | C6orf79 | C20orf7 | C6orf203 | MRPS11 | CCDC6 |
| SLC3A2 | SPECC1 | DNAJA3 | PLEKHB1 | NSUN4 | HINT3 | KS | CLCN4 | THY28 | C1orf121 | NOL3 | BAG2 | MRPL44 | USP38 | PDF | CAMK2D | TMPIT | C1orf26 | ABCB6 |
| DKFZp564I122 | RSN | FAHD1 | NR4A1 | VLDLR | NQO1 | TTC4 | VARS | PDPK1 | HOMER1 | CPT1A | MARS | TNPO1 | DHX33 | SYNJ2BP | SMARCA5 | KIAA0427 | FGF6 | UMPS |
| HSPH1 | APRT | MRPS25 | ME3 | TRAPPC6B | SLC43A2 | C4orf14 | MRPL32 | HCLS1 | DKFZp761P1121 | KIF1C | RAB3A | FHOD3 | KIAA0372 | ZNF652 | PAPD5 | MAP4 | CLN3 | ZNF639 |
| ZFP106 | KIAA0859 | ECG2 | WWP1 | CA2 | MLYCD | PPM1J | SCN4B | TGM1 | RPL7L1 | UGT3A1 | C19orf2 | MTERFD2 | MRPL14 | PIK3R1 | PCK1 | CXADR | GOT1 | DLD |
| IPO7 | FLJ22405 | COX6B2 | USP7 | MTRF1 | MAP1D | CBR1 | FLJ13639 | ITPK1 | C6orf69 | DKFZP434C171 | LIN7B | YARS | TFDP2 | EGLN3 | PACSIN1 | ABCF2 | SAA3 | TIMP4 |
| PTD004 | TNFSF5IP1 | VCP | FLJ39378 | PARK7 | SUGT1 | FLII | MGC126931 | C2orf4 | PRKAR2A | MSRB3 | NDUFA6 | COX6B | ATP5D | NDUFC2 | TIMM8B | NDUFB11 | SUCLG1 | PCMT1 |
| C10orf70 | PDHB | COPS5 | FKBP3 | IDH3B | NDUFS7 | PFKM | MGC133598 | NDUFS3 | NDUFA5 | NDUFS1 | NDUFB9 | BRP44 | FABP3 | ACO2 | ATP5I | NDUFAB1 | VDAC1 | PDLIM7 |
| UQCRC2 | SDHA | SDHC | NDUFB4 | ACSL1 | FGFR3 | MDH1 | VDAC2 | PSMA7 | SNX3 | ATPIF1 | PABPC4 | HSPB8 | CRYAB | MGC126935 | HSPC138 | PSMB6 | MYOZ2 | NDUFV2 |
| ATP5G1 | GPI | NDUFS2 | UQCRC1 | NDUFB10 | NDUFB6 | UQCR | NDUFA9 | SDHB | ATP5B | ATP5F1 | ATP2A2 | COX5B | MDH2 | AK1 | DAP13 | ATP5H | NDUFB3 | NDUFB8 |
| COX7C | UQCRFS1 | ATP5J | COX7B | CYC1 | PYGM | NDUFA4 | ATP5C1 | COX5A | SLC25A3 | LDHA | SLC25A4 | FHL1 | UQCRB | ATP5G3 | SMPX | ATP5E | GDNF | CHORDC1 |
| TM4SF20 | GSR | PPAN | C19orf21 | KCNIP2 | RABGEF1 | MARK3 | TTLL7 | ZYG11B | PARVB | FLJ20628 | LIPE | MASP1 | SERPINA11 | SARS | C1QB | WASL | TAF6 | PDE8B |
| ATPBD1B | SELM | FLJ14775 | CA12 | SSH2 | ONECUT2 | LRMP | TAT | LOXL4 | HSPA9B | MMP16 | C6orf107 | TBXAS1 | ALG8 | PAG21 | DKFZP564I122 | ZMYM1 | DKFZP434F0318 | C13orf24 |
| IRAK3 | TAF1 | ZNF394 | C6orf32 | TOMM34 | TMEM68 | RAB28 | SMC4L1 | ANKK1 | THBS1 | KRT15 | CRYBA1 | MUSTN1 | TNFSF10 | HNRPLL | LYZ | ACSL3 | ATP1A1 | HFE |
| LBP | SLC15A2 | ZNF568 | FBP2 | PSTPIP1 | RSRC1 | FLJ38819 | ARL6IP4 | NUP98 | PON1 | FLJ23191 | ANKRD10 | TSKS | COMMD2 | SLC1A1 | SYCP3 | DSP | SFTPB | SATB1 |
| NTS | SIX3 | RPS27A | WDR72 | WBP4 | DAB2 | CSNK1G1 | FMO5 | CLGN | UNQ9217 | GPR101 | GYS2 | RAB19B | IL1F6 | CLCA3 | C18orf17 | LRRC19 |

### FFUU genes: 18

FFUU Cluster

Time course of FFUU genes

Link to DE genes in NCBI

| Gene Symbol |
| --- |
 MGC12966 || LRRN6A |
| RNPC1 |
| MAPRE2 |
| GPX3 |
| PDK4 |
| CEBPB |
| ADORA1 |
| FOS |
| PDE8A |
| PACRG |
| MB |
| HLA-DQB1 |
| FKBP5 |
| C1orf51 |
| PFKFB1 |
| RGS13 |
| TPP2 |
|

### FFUD genes: 102

FFUD Cluster

Time course of FFUD genes

Link to DE genes in NCBI

| Gene Symbol | Gene Symbol | Gene Symbol | Gene Symbol |
| --- | --- | --- | --- |
 IGJ | XPNPEP1 | IMP3 | SCD || RWDD4A | IFRD1 | DUSP10 | BCKDK |
| MRPS31 | DDT | C3F | IMMT |
| PDE4DIP | DEXI | ZNF45 | CHCHD4 |
| DLGAP4 | HDDC3 | FKSG24 | VARS |
| MRPS34 | AK1 | MTP18 | DUSP23 |
| KIAA1394 | ABHD11 | UCK2 | SMARCA4 |
| PSPH | PSAT1 | ANKRD1 | FABP4 |
| C6orf136 | TXNL5 | DNAJC7 | MRP63 |
| GARS | FBXO9 | C2orf7 | DNAJA4 |
| CYP2D6 | USP2 | DNAJB5 | SARS |
| TNFRSF12A | ALAS1 | HAGH | HINT2 |
| FLII | HDAC1 | ARL1 | ODC1 |
| NAT5 | FCGRT | IDH2 | ITGB1BP3 |
| ASF1B | EXOSC4 | ACY1 | CYP51A1 |
| PCK2 | PNCK | OLFM1 | MGC16471 |
| HSPH1 | SORD | NDRD | MECR |
| CRTAC1 | CIAPIN1 | P2RX5 | ACAD10 |
| GK | ARHGEF19 | FCGBP | UCK1 |
| IQSEC1 | COL11A2 | CTPS | CHI3L1 |
| BAG5 | SPTBN2 | SLC13A4 | TAF2 |
| VEZATIN | PSMC5 | CXorf45 | MOSC2 |
| RPS6KA6 | EIF2AK2 | ABHD3 | CENTA1 |
| PRDM2 | TTYH1 | ARID3B | PADI3 |
| DIO1 | CXCL5 | BEX1 | RAB3A |
| MUSK | CD28 |

### FFDF genes: 1026

FFDF Cluster

Time course of FFDF genes

Link to DE genes in NCBI

| Gene Symbol | Gene Symbol | Gene Symbol | Gene Symbol | Gene Symbol | Gene Symbol | Gene Symbol | Gene Symbol | Gene Symbol | Gene Symbol | Gene Symbol | Gene Symbol | Gene Symbol | Gene Symbol | Gene Symbol | Gene Symbol | Gene Symbol | Gene Symbol | Gene Symbol | Gene Symbol | Gene Symbol | Gene Symbol | Gene Symbol | Gene Symbol | Gene Symbol | Gene Symbol | Gene Symbol | Gene Symbol | Gene Symbol | Gene Symbol | Gene Symbol | Gene Symbol | Gene Symbol | Gene Symbol | Gene Symbol |
| --- | --- | --- | --- | --- | --- | --- | --- | --- | --- | --- | --- | --- | --- | --- | --- | --- | --- | --- | --- | --- | --- | --- | --- | --- | --- | --- | --- | --- | --- | --- | --- | --- | --- | --- |
 ADRBK2 | NCAM1 | PIGV | SYNGR1 | ZNF653 | C6orf134 | NR3C2 | KIAA1754L | ARL7 | TSPAN15 | FGF2 | ADAM33 | ZNF397 | LZTFL1 | ITGA8 | CBFA2T2 | SHC2 | TCP11L1 | DCTD | PHF11 | PMF1 | TNFRSF25 | RAB3D | AKAP12 | CNOT8 | Cep192 | MDC1 | FLJ44216 | ZIM2 | WDSUB1 | RAI14 | ZNF292 | CNNM4 | SLC16A2 | C1orf96 || SH3BP5L | BCL10 | CRK7 | ZNF518 | DDHD1 | PTPRF | PLEKHA5 | ST8SIA4 | PLSCR4 | IL17RD | CDC14B | SPA17 | IQGAP2 | PTPRK | FLJ13710 | HOXB8 | TTC3 | EDN3 | SPATA6 | LFNG | IRF3 | SOX9 | MCM7 | NSUN6 | PPM1K | STX6 | THBS2 | ANTXR2 | DNAL4 | AGPAT1 | SLC7A6OS | VCAM1 | CENTB1 | FLJ11127 | SRGAP2 |
| POLR3B | ZNF470 | CPNE2 | PIGS | TMEM51 | C15orf29 | PTHR1 | DNAJC18 | HNRPLL | KLHL11 | HPS5 | DDX26B | CTTNBP2NL | MYO1B | P15RS | HYPC | AVO3 | DKFZp762K222 | RBBP6 | ZCCHC8 | CREB5 | FLJ10808 | VPS13B | CNIH | XRCC4 | POFUT2 | ARL10A | LHFPL3 | HIP1 | STAC | CLK1 | ITPR2 | PDE7B | CDGAP | VAV3 |
| PARP12 | TBC1D14 | VANGL1 | RFX2 | BOMB | COL4A5 | GATA6 | RAMP1 | TSPYL1 | PPIG | PHF16 | DAF | CDC2L5 | MICAL1 | GPATC1 | ROBO1 | PLP1 | JAM2 | FOXF1 | RASIP1 | WARS2 | CCPG1 | CLDN1 | COVA1 | CG018 | CXorf45 | SEMA6D | SMARCAD1 | SLC22A17 | DDAH1 | STIM2 | MIA | MYO9A | NME3 | KIAA1244 |
| DAB2 | WDR17 | NCOA1 | DDEFL1 | MYO6 | ZNF532 | AHCTF1 | OTUD5 | KREMEN1 | ARHGEF3 | RCBTB1 | HSPA5BP1 | BNIP3L | FGFR4 | SRISNF2L | BRD3 | TRPC1 | NCOA7 | RCBTB2 | CD44 | ZCCHC3 | IGSF3 | FN3KRP | CARHSP1 | MAGI3 | MECP2 | CNTNAP1 | SORBS2 | NAV3 | KIAA0433 | SFPQ | PHLDB2 | WDR71 | GDF8 | EIF2S3 |
| ZNF207 | BCAR3 | CDCA7 | KIAA1731 | SUFU | PPP1R16B | NOS2A | PCDH12 | C1orf115 | HOXB6 | ALDH9A1 | PELI2 | LMO2 | KIAA1522 | CDH5 | KIAA1462 | EDG2 | SPSB3 | THOC2 | CBX6 | TPX2 | DNMT1 | EVI1 | FAM76B | NEO1 | ABCG2 | FGL1 | KIAA1632 | ST13 | SCPEP1 | MORC3 | ZRANB1 | ICK | FLJ10970 | KCNMB4 |
| FLJ20097 | SLC16A13 | CKLFSF8 | IMPA2 | TGFB1 | FAM63A | CD47 | KIAA1109 | DKFZP566M1046 | GLS | CCNL1 | TM2D2 | C16orf28 | SLC7A7 | ATXN3 | F2R | SLC35A5 | TJP2 | ACD | FAM40B | FLJ21742 | ICF45 | C6orf130 | RNUXA | C6orf192 | TUBGCP5 | DNAJC5 | PDE4DIP | TGFB3 | FKBP9 | WNT11 | SRGAP1 | ISLR | HDGF2 | CD3Z |
| C20orf14 | BCL9L | C8orf76 | ADCK5 | HDAC9 | SPATA7 | MGC14289 | YES1 | PKD2 | NRP2 | ZNF261 | PTK7 | MMP16 | HPGD | PAFAH1B3 | STARD13 | ABI2 | PTPRR | WDR10 | ANKRD44 | NTRK2 | PGCP | SEMA6A | FLJ20130 | C8orf70 | RASGRP2 | EZH2 | RACGAP1 | NES | SDC3 | EPB41L4A | EVC | PRDM11 | PBX3 | SFRS14 |
| GPC6 | CKIP-1 | NOV | ADD1 | LCAT | CUGBP2 | MGC16028 | RAB31 | ALDH1A3 | PDGFC | EMP3 | FAM80B | PURG | SMO | CAMTA1 | MGC128808 | TGIF | LEPREL1 | TIA1 | COL27A1 | FSCN1 | ADAM12 | MGC13204 | TOP2A | HMGB3 | CSPG6 | FAM48A | ARHGAP12 | ELK4 | DKFZP564J0863 | C10orf45 | EBF | FAM21C | GATAD2B | DNCI2 |
| MEOX2 | BAZ2B | RGL1 | SEPT8 | PRPF3 | SPTLC2 | SEC63 | FANCG | C13orf23 | RAB3IP | FLJ10707 | FAM43A | ADAR | SHC1 | SDCCAG33 | IGF1R | FLJ10652 | BRD1 | STAG1 | DMTF1 | WHSC1L1 | AEBP1 | FRS2 | FALZ | FOXP1 | IGSF4 | MCCC1 | FCHSD2 | FLJ11305 | SPTLC1 | JMJD1A | COL14A1 | CHRDL1 | FLJ10287 | AXL |
| MCP | PRKAG2 | IHPK1 | VEGFC | SNIP1 | STK24 | CHD1L | STK4 | GOLGA5 | DOCK6 | CD34 | JUP | TCN2 | GRK5 | KIAA1972 | IHPK2 | CXCL12 | PSEN1 | KIAA0892 | AGRN | CPNE1 | NRM | BTBD3 | KIAA2002 | PHF10 | CYP20A1 | WEE1 | LRRN5 | PTGIS | OACT2 | CLIPR-59 | MEN1 | SNAI2 | RHOJ | MTSS1 |
| P2RY5 | ARL3 | KIAA1627 | PIP5K3 | EVL | CDH11 | DOCK7 | EFHD2 | IGF2R | NPR2 | COLEC12 | DCBLD2 | LBH | VIL2 | FN5 | GNAI1 | TCF7L2 | C10orf6 | KIAA0323 | DZIP1 | SCRIB | TULP4 | HNRPH3 | UGCG | ZSWIM6 | RIT1 | PELI1 | COG3 | KIAA0738 | APOA1 | LRIG3 | PI15 | ZNF187 | CTDSPL | KLHDC5 |
| EML4 | USP46 | NPC2 | TIE1 | FGD5 | FLJ25476 | ACVR1 | AKT3 | HBXAP | SLB | CXorf39 | NR2F1 | FBXO25 | JAM3 | NTN4 | C20orf47 | INPP5F | BRAF | MDFIC | ST6GALNAC6 | CYGB | CTDSPL2 | SCUBE2 | DKFZp761N1114 | STX12 | NUMB | BBC3 | TNK2 | ATXN7 | C10orf10 | ESAM | NDRG2 | RPS27L | POGZ | ZNF184 |
| GCL | DNASE2 | NDST2 | PPP1R9B | RBM5 | SBNO1 | SR140 | SIRT1 | CDC73 | NMI | CLDN11 | S100A13 | ADAMTSL3 | FLI1 | TSRC1 | SHANK3 | TEK | GUCY1A3 | DOCK9 | ADAM10 | CMKOR1 | F11R | S100B | RASGRP3 | TCTE1L | MDK | GDI1 | KIAA0826 | MAGI1 | TNFRSF21 | MYO1C | LRP1 | PLK2 | KIAA1915 | PSCD2 |
| MXRA8 | FLJ20436 | TIMP2 | GSTA4 | TNS3 | MSH6 | SACS | ARHGAP29 | FLJ23342 | FLJ21616 | NCK2 | ABI3BP | SMAD2 | FLJ31951 | ING4 | CRIM1 | GAB1 | ITPR1 | SPPL3 | FNBP1L | NRAS | RNF2 | APP | MYH10 | SMARCA3 | S100PBPR | HIVEP1 | MTMR12 | CBLB | PLEKHA2 | MYOM3 | CCDC23 | CHD1 | KIF2 | AP3S1 |
| ZFHX4 | SFRP1 | SEPT11 | SFRS12 | KIAA1223 | LHFPL2 | NOTCH2 | C5orf5 | PRCP | MMD | SGCE | SPINT2 | WSB1 | MARCKS | TTC14 | GLT8D2 | WDR44 | FST | LXN | DACT1 | BACH2 | QSCN6 | C1orf63 | SNRK | MLLT3 | NCALD | PVRL3 | PTPRD | DKFZp762A217 | CLCN5 | FLJ12505 | EPB41L3 | DAP | LRRC17 | FAT4 |
| FZD8 | CPE | OLFML3 | PTGFRN | MAGED4 | FAP | GPR27 | ZNF608 | KIAA0515 | TNIK | APLN | FAT | SH3MD1 | MEGF10 | GULP1 | BASP1 | FER1L3 | SDC2 | DBN1 | CKS2 | WDFY3 | FAM62B | GOPC | MED12 | PDCD4 | FLJ20032 | RNF19 | FTO | PCF11 | SLC30A1 | EMP2 | ABLIM1 | KIAA0355 | H3F3B | CDC2L6 |
| CSNK2A2 | C14orf43 | KIAA0460 | IRF2 | SUV420H1 | ZF | FLJ23861 | HDGFRP3 | LDB2 | C1orf119 | RAB6IP1 | BTBD7 | WWTR1 | ZNF436 | KCNJ8 | ABI1 | PAN3 | NOTCH3 | PIK3C2B | ZZZ3 | CD302 | TPM1 | KLF6 | DGKH | ZNF704 | UTRN | TLE1 | FZD4 | RNF38 | RNF103 | GNG11 | KITLG | PLP2 | FRZB | CYYR1 |
| LAPTM4B | CAV2 | RYBP | PORIMIN | SNX4 | MBTPS1 | HNRPA0 | PTOV1 | SFRS5 | RAP2C | FUBP1 | FLJ35954 | CKLFSF3 | C1QDC1 | VAT1 | ARHGEF2 | ZNF262 | CASP6 | KDR | PEG3 | TRIO | ANTXR1 | ANKS1 | DDAH2 | SESN3 | FN1 | CLIC1 | PLXND1 | RAPH1 | C1orf93 | MATN2 | MARCKSL1 | ASPN | MXRA7 | VGLL4 |
| FBLN5 | LHFP | MYO10 | CREB3L2 | PHC3 | C1orf24 | PITX2 | PDZK3 | TCF4 | IFIT5 | C14orf32 | OGN | KIAA1546 | CYFIP1 | JMJD1C | TRA2A | YT521 | TGFBR2 | GABARAPL1 | PECAM1 | KIAA1429 | MLL3 | DAAM1 | PAM | RAMP2 | PSIP1 | TGFBR3 | ETS1 | PBXIP1 | TSPAN6 | PCGF5 | PCTK2 | GNB1 | HNRPH1 | DYRK2 |
| CREBBP | DLC1 | IGFBP6 | PPAP2B | XPO1 | CTDSP2 | LAMB1 | FMR1 | CLEC3B | RNASE4 | CITED2 | URB | TJP1 | CAV1 | MAGED1 | COL5A1 | SPRY1 | TUBB2 | GPC3 | SOX4 | SPG21 | LAMA4 | IGF1 | HMGB2 | ATP6V1G1 | NOPE | HTRA1 | YWHAQ | DNAPTP6 | LASP1 | COL5A2 | FSTL1 | STMN1 | SDPR | SPARCL1 |
| FBN1 | CDKN1C | DLK1 | EPB41L5 | FLJ10094 | MGC15619 | MCFP | GPM6A | ZNF26 | EDNRA | NBLA04196 | PTPRE | CBR3 | KIAA1553 | REM1 | KIAA1447 | D2S448 | CBX8 | EPM2AIP1 | RALGDS | MBP | FLJ10979 | KIAA1107 | AGPAT4 | ADRA2A | ROR2 | TAC1 | EFNA5 | HMMR | B3GNT5 | TUFT1 | GNG2 | MLL5 | CENPC1 | PLEKHF1 |
| TMEM14A | RSBN1 | EDN1 | LECT1 | GNB2 | ARL4 | CENTB2 | VEZATIN | PRICKLE1 | NOTCH4 | AIM1 | SDCCAG3 | DKFZP564D166 | CSPG5 | VTI1A | MOCOS | POLR3GL | MLSTD2 | PARP9 | GNGT2 | WNT2B | KIAA0649 | SEC22L3 | GOLGA3 | TMEM27 | EIF2AK4 | FLJ23518 | MYEF2 | TIAM1 | ALCAM | C14orf37 | FXR1 | NRBP2 | KIT | GEFT |
| PTGS1 | MPZL1 | CYLN2 | MTA2 | PIK3R4 | RASL11B | DCAMKL1 | SPFH1 | SLC26A2 | CHRNG | TANC | AFAP | TRIB2 | MUM1 | AMIGO2 | C8orf41 | AASDHPPT | NF1 | NKD2 | SLC41A1 | S100A8 | KIF26A | IMPG2 | FLJ20125 | CDCA5 | ZNF567 | RNASE6 | FREM1 | HMCN1 | RP2 | TCEAL1 | KIAA0980 | URP2 | SEMA5A | TTC19 |
| YAP1 | DNAJC1 | MED19 | PTPDC1 | SLITRK2 | IGSF2 | MGC133870 | DDX46 | BMP7 | CCHCR1 | PLEKHG2 | ECT2 | HSPC196 | SPINK5 | RUTBC1 | MAMDC2 | CXXC1 | SERPING1 | CPT1C | FBXW8 | GPNMB | CCNC | ECE1 | C1orf156 | C10orf35 | SNX7 | FZD1 | JARID1A | ANKIB1 | WBP1 | USP49 | C6orf60 | LUZP1 | YEATS2 | PAQR8 |
| E2F2 | MAN1A1 | KIAA0040 | ALPK1 | SGOL2 | RHPN2 | MARVELD2 | TMEM44 | TMEM16A | DDX42 | ALS2CR19 | TSPAN5 | ATR | NELF | H2AFY2 | C3orf21 | MAP1A | TROAP | IGSF10 | PLSCR3 | SGNE1 | KIAA1913 | TSPYL2 | CSPG4 | CENTB5 | TMEFF1 | TTYH2 | MGC21644 | EPHA4 | C14orf139 | BUB1B | PRICKLE2 | ASPM | EIF5A2 | CD109 |
| FLJ10719 | FLJ30046 | FBXW9 | OACT1 | MFAP3 | BRRN1 | HNRPD | KIAA1727 | ELAVL1 | MGC4707 | SMC4L1 | JM11 | PEX10 | C20orf67 | MGC16943 | SCDR10 | GCNT2 | RetSat | GJB1 | COG6 | RBBP9 | PKD1L2 | TFPI2 | RPGR | COL6A3 | C6orf111 | SFRS11 | C8orf42 | CNGA2 | MGC11335 | ADAMTS6 | CKAP2 | FLJ12975 | SPG3A | SORCS1 |
| DTX1 | LYST | DLEU2 | MBNL3 | NEDD9 | CCNB1 | PHLDA1 | TSPAN33 | DPY19L3 | C14orf118 | P2RY14 | GABRG2 | RERG | COL12A1 | WDTC2 | CSPP1 | RGNEF | FLJ14166 | MCMDC1 | DKK1 | LIX1 | SPTBN1 | SMC2L1 | FLJ32745 | ZNF482 | TSPAN18 | C6orf155 | WHSC1 | B4GALT4 | CCND1 | C9orf39 | MUTYH | CACNB2 | C13orf25 | CSRP2BP |
| STXBP5 | MARCH9 | FLJ40852 | RAP1GDS1 | MGC126930 | ZNF22 | KHDRBS1 | C18orf54 | PACS2 | ZBTB8 | GALNT13 | SLITRK6 | SFXN2 | DKFZP586H2123 | EFNB3 | ST3GAL5 | FZD10 | HNRPA2B1 | GPBP1 | CNTN1 | MMRP19 | C9orf4 | CDKL4 | FGF9 | FLJ20105 | PRKCB1 | ELF1 | IMPG1 | RBPMS | SSTR1 | ZBTB37 | TOR1AIP1 | FBXO47 | ZNF624 | CCDC18 |
| LRRN1 | ZIC3 | ANK1 | SDCCAG10 | MYBL1 | SRI | KIAA0960 | C1orf85 | TCF7 | COL24A1 | EPB41 |

### FFDU genes: 305

FFDU Cluster

Time course of FFDU genes

Link to DE genes in NCBI

| Gene Symbol | Gene Symbol | Gene Symbol | Gene Symbol | Gene Symbol | Gene Symbol | Gene Symbol | Gene Symbol | Gene Symbol | Gene Symbol | Gene Symbol |
| --- | --- | --- | --- | --- | --- | --- | --- | --- | --- | --- |
 TLR1 | ZNF297B | ZNF263 | FOXP2 | NR4A2 | RCOR1 | OSR2 | C1GALT1 | SLC25A29 | ANKRD29 | C10orf72 || C1orf96 | MLLT10 | STXBP6 | NCAM1 | FLJ23322 | PSD4 | SERPINA1 | STEAP2 | C13orf10 | XPA | C3 |
| ARHGAP9 | SLC16A14 | CXCR3 | PAMCI | TLR3 | PGM2L1 | FLJ43654 | MTHFR | FAM43A | TMEM24 | C13orf1 |
| SLC38A1 | NY-SAR-48 | CRYGS | NEBL | RRAS2 | PHF2 | EPHA4 | COL11A1 | ZNF278 | PRR3 | CYYR1 |
| GNA13 | ST6GALNAC2 | MAP3K12 | ZWILCH | ABCA8 | MMP24 | KIAA0984 | TBC1D17 | PTGS1 | FANCM | AEBP2 |
| SLC24A6 | ZC3HAV1 | ZC3H6 | DKFZp434C0328 | DOC1 | CSF3 | CTTNBP2NL | PDE4D | SSBP4 | TCF7 | HMOX1 |
| SNAI1 | WDR21A | S100PBPR | ZDHHC23 | ANGPT4 | TRIM38 | ENPP6 | DZIP3 | INHBA | FAM20A | KCNA3 |
| ZCSL3 | RPS6KC1 | RC74 | ANKRD12 | NUDT11 | C10orf83 | AMIGO2 | KRT10 | NAP1L1 | NCOA6IP | HOXB5 |
| FMO5 | RAP2A | C1orf165 | ITGA2 | KIAA0753 | RAP1GDS1 | JAM2 | GLT25D2 | FLJ21816 | ABCA1 | NFATC4 |
| LGI1 | FOXO3A | TCN2 | DDEF1 | PRKCB1 | C3orf19 | GSTK1 | Ells1 | ICA1 | UNC84A | CABLES1 |
| MXD1 | UBE2G2 | KCNMB4 | MXI1 | ANKRD42 | CALCOCO1 | SEPN1 | PCGF3 | MGC45840 | DAF | DATF1 |
| PMM1 | PAWR | AXUD1 | PYGO1 | TIMELESS | WAS | BBS2 | PTX3 | ZNF605 | NDE1 | SEMA5B |
| GABRE | FLJ90709 | HHIP | DMBT1 | MGC11335 | GPATC3 | MRE11A | CHST13 | STYX | NFKBIE | PRRT1 |
| SOX12 | CACNA1G | DAB1 | BOLA-DQA2 | FBXO32 | RAP140 | BCL10 | NUP160 | LCP2 | AQP11 | TIMM9 |
| IPMK | KIAA1005 | HECW1 | MGC17301 | EVI2B | ZNF365 | HIST1H2BC | FLJ21159 | HS3ST5 | DLG2 | FAM84A |
| CASC3 | CALCRL | DQX1 | BOLL | OCLN | HRASLS3 | CD3D | C20orf117 | GLTSCR2 | BVd1.18 | IFI16 |
| ARAF | DSU | OSRF | MGC39715 | EVA1 | HSPC117 | DAPK1 | LCK | CCL3L1 | PPP2R1B | SLC16A12 |
| SE57-1 | POLI | LMO3 | CHST7 | PRKCH | OVOL1 | GPR88 | BHLHB9 | ZNF317 | HHEX | IGSF4D |
| SMARCC2 | MGC133938 | RELA | MTMR9 | FLJ14001 | RBPMS | RIOK3 | E2F8 | PHLDA1 | IQCA | SELE |
| FKRP | FLJ13941 | LUZP1 | SDFR2 | HEATR1 | GTF2H3 | RNU47 | AP1GBP1 | MGC133720 | DPY19L3 | C20orf23 |
| GMFG | PB1 | CCDC14 | SHANK2 | ARMC8 | IFI44 | NPAL1 | C10orf45 | CELSR1 | SOS1 | KIAA0373 |
| PDE10A | CD96 | PVRL3 | DCAMKL1 | AFF1 | MCTS1 | ACOX1 | SLC25A21 | C9orf46 | HABP2 | TBX3 |
| C1orf55 | EPS8 | HDAC4 | LTBP4 | ZFYVE1 | CNNM3 | RNF103 | FBXL20 | TJP2 | C1orf71 | OXR1 |
| ADAM9 | MEIS1 | SEMA6D | CRAMP1L | AQP4 | NAGA | KLF6 | CXCL14 | HSPA2 | MR-1 | RNF144 |
| KIAA0556 | CSPP1 | HRMT1L1 | DNCL1 | GRB10 | KIAA0831 | DUSP19 | MAML3 | CYP2U1 | MDS1 | VPS4B |
| AGTRL1 | TPX2 | ERBB2 | ZNF470 | NCALD | EHMT2 | MGEA5 | TRIM45 | MGC127324 | FLJ30092 | PXMP2 |
| TDE1 | LAMP2 | EZH1 | PCMTD2 | LKAP | XAB2 | HBP1 | UQCRB | WDFY1 | DUSP26 | RC3H1 |
| CCPG1 | BTC | YPEL3 | ARID5B | ZFP36L2 | IRF2BP2 | GSN | BTG1 |

### FFDD genes: 76

FFDD Cluster

Time course of FFDD genes

Link to DE genes in NCBI

| Gene Symbol | Gene Symbol | Gene Symbol |
| --- | --- | --- |
 UBE2T | RBM15 | LATS1 || SLC4A2 | TCF19 | LIP8 |
| CDC42BPB | HS2ST1 | PVRL1 |
| PLVAP | EDNRA | NUP107 |
| IMP-3 | SRGAP3 | MOV10 |
| ZNF275 | ROBO1 | KIAA1223 |
| CXCR4 | PPM1K | SERTAD4 |
| MGC33887 | PPP2R2B | CYP39A1 |
| IRS1 | C16orf34 | HPGD |
| DACT1 | MYO9A | TSPYL5 |
| ARMCX1 | NUDT2 | NRK |
| TOP2A | GPC4 | MEST |
| CDH11 | FAM46A | GNG2 |
| TM4SF18 | IFITM3 | RNASE1 |
| PIGC | ENC1 | ADAMTS4 |
| PAK3 | SBBI54 | PKD1L2 |
| MARVELD1 | CDKN3 | CBX2 |
| PTGIS | CCND1 | KNTC2 |
| HOXD3 | NRIP3 | GSTK1 |
| FLJ40311 | TGIF | C18orf24 |
| KIAA0409 | SN | PXMP2 |
| RAD54B | WDR33 | FPGT |
| ASPN | GPX7 | HBG |
| ECRG4 | TGFBI | ITGA7 |
| MYH8 | MYH3 | IGF2 |
| GTL2 |

### FUFF genes: 70

FUFF Cluster

Time course of FUFF genes

Link to DE genes in NCBI

| Gene Symbol | Gene Symbol | Gene Symbol |
| --- | --- | --- |
 AKT2 | UBE2E2 | PITX1 || CEBPG | SLC1A3 | SH3GL2 |
| NFATC1 | ANKRD11 | CREB3L3 |
| PIGH | KIAA0182 | FLJ20489 |
| IGFBP5 | CRADD | AK3 |
| DSG1 | MTUS1 | ADSSL1 |
| F10 | RNF31 | FLJ22662 |
| ROCK2 | AQP7 | PPARG |
| OGT | CRISPLD2 | MCF2L |
| VEGF | RRAD | C3 |
| MUSTN1 | C1orf21 | AQP4 |
| CAMK2D | ACAT1 | BOLA-DQA |
| MMP13 | HPSE | IMPAD1 |
| BTBD15 | SOS2 | ZNF569 |
| ENPEP | C1orf82 | EGFR |
| TNFSF13 | C14orf35 | C4A |
| NPR3 | EFCAB1 | CA6 |
| IGSF6 | TSHR | TECTB |
| EHF | ERO1L | UCK1 |
| AVPR1B | CD47 | PAG16 |
| NCRMS | TUBGCP3 | SLC39A12 |
| MGC4266 | THAP6 | ADAMDEC1 |
| CAMTA1 | CADPS | TRIM9 |
| FBXL5 |

### FUFU genes: 15

FUFU Cluster

Time course of FUFU genes

Link to DE genes in NCBI

| Gene Symbol |
| --- |
 SOX15 || MIOX |
| DAG1 |
| LRRC2 |
| S100A1 |
| PRSS23 |
| CRSP3 |
| FLJ14054 |
| C18orf25 |
| PTPRC |
| ZFY |
| KIAA0408 |
| CCDC21 |
| VLDLR |
| MN1 |
|

### FUFD genes: 10

FUFD Cluster

Time course of FUFD genes

Link to DE genes in NCBI

| Gene Symbol |
| --- |
 NUDT13 || SHH |
| KIAA1117 |
| PPP1R3B |
| ELN |
| NOL8 |
| LRP2 |
| CHL1 |
| PIGX |
| QPCT |
|

### FUUF genes: 25

FUUF Cluster

Time course of FUUF genes

Link to DE genes in NCBI

| Gene Symbol |
| --- |
 ATP2A2 || TA-PP2C |
| SLC25A12 |
| PLN |
| TFRC |
| UCP3 |
| PADI2 |
| CAMK2A |
| P2RY1 |
| ALDH1A1 |
| NBLA03831 |
| TRIM7 |
| RORC |
| CCRK |
| MAPT |
| NOL8 |
| ACADVL |
| NEB |
| CABC1 |
| PHKA1 |
| GPD1 |
| PPP1R1A |
| ATP2A1 |
| TPI1 |
| PGK1 |
|

### FUUU genes: 3

FUUU Cluster

Time course of FUUU genes

Link to DE genes in NCBI

| Gene Symbol |
| --- |
 MGC13275 || ZBTB16 |
| PFKFB4 |
|

### FUUD genes: 7

FUUD Cluster

Time course of FUUD genes

Link to DE genes in NCBI

| Gene Symbol |
| --- |
 PTPLA || ANKRD1 |
| CHAD |
| SLC27A6 |
| SLC26A6 |
| FLJ20323 |
| OGG1 |
|

### FUDF genes: 11

FUDF Cluster

Time course of FUDF genes

Link to DE genes in NCBI

| Gene Symbol |
| --- |
 GPNMB || RB1CC1 |
| ERBP |
| ATXN1 |
| C14orf24 |
| EDNRB |
| ST3GAL5 |
| TESK2 |
| PCDH19 |
| MORC3 |
| EIF2C3 |
|

### FUDU genes: 8

FUDU Cluster

Time course of FUDU genes

Link to DE genes in NCBI

| Gene Symbol |
| --- |
 FLT3LG || MGC127492 |
| LAMA3 |
| MTMR11 |
| DNAH1 |
| MOXD1 |
| CETN3 |
| ZA20D2 |
|

### FDFF genes: 81

FDFF Cluster

Time course of FDFF genes

Link to DE genes in NCBI

| Gene Symbol | Gene Symbol | Gene Symbol |
| --- | --- | --- |
 PIP5K2A | KIAA0339 | C2 || PLXNB1 | C10orf4 | EPB41L1 |
| BYSL | TRIM25 | DAB1 |
| SEPT3 | MMP3 | TIFA |
| ITGA4 | VSNL1 | PPP1R11 |
| SLC11A2 | TMPO | ZFHX1B |
| DKFZp762E1312 | CCNE2 | CDCA8 |
| ESPL1 | UNC119 | SRPX2 |
| CAMK1 | FRMPD1 | TSPAN12 |
| SHRM | CKAP2 | GPSM1 |
| UHRF1 | CAND1 | ABCC8 |
| FBXO30 | ACOX1 | PAG1 |
| STAM2 | CDO1 | DCAMKL1 |
| RBP1 | CYP4V2 | TMEM39A |
| PDE3B | PAPOLA | PTPN2 |
| DOCK5 | HSD11B2 | MAGI3 |
| FLJ20297 | TLK2 | KCNK7 |
| NKX2-2 | FLJ13111 | FLJ12886 |
| NRG2 | PLD1 | CYLC1 |
| NAPB | PHLPPL | OGT |
| FLJ31978 | RPE65 | MGC16943 |
| WDR20 | B3GALT1 | LIG4 |
| CEP4 | GTDC1 | LY6D |
| UFM1 | CCNB1 | RAD54B |
| INPP4B | CLYBL | RAB35 |
| VTI1A | FGFR2 | KIAA0701 |
| STARD3NL | PPM1E | BCAT1 |
|

### FDFU genes: 12

FDFU Cluster

Time course of FDFU genes

Link to DE genes in NCBI

| Gene Symbol |
| --- |
 FLJ10081 || ABAT |
| ENTPD5 |
| CACNA1A |
| FASLG |
| TSEN2 |
| NUP43 |
| RBM15 |
| ALDOB |
| GSK3B |
| LHFPL1 |
| GNTIVH |
|

### FDFD genes: 23

FDFD Cluster

Time course of FDFD genes

Link to DE genes in NCBI

| Gene Symbol |
| --- |
 CHEK1 || MAP1B |
| FLJ11029 |
| WBSCR17 |
| DKFZP564O0823 |
| TMED1 |
| ACOT7 |
| PLXNA1 |
| CALD1 |
| TS |
| COL22A1 |
| BIRC5 |
| SLC25A15 |
| CDC2 |
| COL18A1 |
| RBM3 |
| LIG1 |
| TRIM36 |
| MGC133728 |
| CCNA2 |
| KHDRBS1 |
| DLGAP4 |
| FLJ32810 |
|

### FDUF genes: 62

FDUF Cluster

Time course of FDUF genes

Link to DE genes in NCBI

| Gene Symbol | Gene Symbol | Gene Symbol |
| --- | --- | --- |
 TMEM19 | SP1 | FLJ20699 || ABL1 | IGSF1 | RAD54L |
| PPRC1 | ODC1 | IKIP |
| KIF21A | ESR2 | KIAA1272 |
| KTI12 | GNL3 | SLC27A4 |
| BRI3BP | N4BP3 | SETDB2 |
| TRAF3IP3 | KLHL22 | FCGR2B |
| KIAA0103 | FLJ39822 | PGBD5 |
| C14orf108 | HTATIP2 | PLCB3 |
| FBXO40 | DIRAS3 | ZBTB10 |
| PLD1 | SFRS14 | RNF32 |
| ST6GALNAC3 | EPHA5 | PALM2-AKAP2 |
| NUP155 | N4BP2 | ABI1 |
| TMEM2 | ANPEP | GATAD1 |
| NMES1 | CYFIP2 | AMPD3 |
| NFE2L2 | ASCIZ | PRIM1 |
| WHSC1 | ZNF599 | MGC134214 |
| ZC3H12B | P53CSV | DAPP1 |
| TRAM1L1 | KIAA1212 | PHIP |
| ZNF529 | DAO | ANGPTL3 |
| MS4A1 | HSA9761 |

### FDUU genes: 9

FDUU Cluster

Time course of FDUU genes

Link to DE genes in NCBI

| Gene Symbol |
| --- |
 MGC13017 || CXCL10 |
| RNF40 |
| DTNBP1 |
| IRF5 |
| FLJ13868 |
| RALA |
| C1orf75 |
| PLD5 |
|

### FDUD genes: 26

FDUD Cluster

Time course of FDUD genes

Link to DE genes in NCBI

| Gene Symbol |
| --- |
 ZNFN1A3 || TMEM7 |
| MGC3731 |
| LARP2 |
| SVH |
| CARS |
| KIAA0040 |
| MPHOSPH9 |
| BAX |
| ADAM12 |
| C9orf48 |
| MOCS3 |
| NMNAT2 |
| PHGDH |
| CSDC2 |
| RFC5 |
| SMURF1 |
| LECT2 |
| TRIM9 |
| HOOK1 |
| ANKRD42 |
| SEPT11 |
| DHX32 |
| CDKN1A |
| SQLE |
| TRA1 |
|

### FDDF genes: 30

FDDF Cluster

Time course of FDDF genes

Link to DE genes in NCBI

| Gene Symbol |
| --- |
 CSPG2 || CHAF1A |
| MYO9A |
| SYTL2 |
| CCNB2 |
| KIAA1602 |
| LMNB1 |
| KLHL13 |
| MGC16121 |
| KIAA1914 |
| ABAT |
| MID1 |
| CTNND2 |
| SPAG5 |
| SLC39A6 |
| ODZ3 |
| RICS |
| PRICKLE1 |
| MFHAS1 |
| NUDT10 |
| GJA7 |
| BCAT1 |
| NUDT6 |
| KIAA1005 |
| ERBB3 |
| FLJ12505 |
| COL21A1 |
| SEPT9 |
| MAGED2 |
| NES |
|

### FDDU genes: 6

FDDU Cluster

Time course of FDDU genes

Link to DE genes in NCBI

| Gene Symbol |
| --- |
 FABP7 || GPR126 |
| DNER |
| SRD5A2L |
| SLC16A9 |
| KIAA1012 |
|

### FDDD genes: 8

FDDD Cluster

Time course of FDDD genes

Link to DE genes in NCBI

| Gene Symbol |
| --- |
 PDLIM4 || NUSAP1 |
| SPP1 |
| MGC16121 |
| BCAT1 |
| IGFBP2 |
| CIT |
| CAPN6 |
|

### UFFF genes: 149

UFFF Cluster

Time course of UFFF genes

Link to DE genes in NCBI

| Gene Symbol | Gene Symbol | Gene Symbol | Gene Symbol | Gene Symbol |
| --- | --- | --- | --- | --- |
 MX1 | KIAA0040 | HIP1R | TSPAN13 | SLC6A1 || PRKCH | RSAD2 | IL15 | SCARF1 | LYN |
| TNS4 | SPSB2 | FLJ90586 | CDH1 | C14orf120 |
| GPR103 | SORL1 | DNMT3B | MGC14141 | GBP5 |
| BIRC4BP | CLDN1 | ECM2 | KIAA1838 | SAMD9 |
| PRELP | CKMT1B | GMFG | IL2RG | USP53 |
| MYO5C | ATP6V1B2 | LGALS3BP | C17orf28 | PITRM1 |
| SLC22A5 | C10orf78 | FLJ90805 | GRSF1 | FGFR1 |
| MGC10993 | GATA2 | IFIH1 | PERP | KIAA0527 |
| RUNX2 | RNPEP | GPM6B | CCR7 | TRIB3 |
| SEMA4B | AHR | AQP3 | ZNF507 | HEG |
| CD6 | GIT2 | FGL2 | RGS5 | TESK2 |
| PAG11 | RAB15 | KIAA1627 | ATP13A4 | EPB49 |
| SPG3A | RNF39 | GCLC | ADPGK | CORO1A |
| F2RL2 | IL4R | NOSTRIN | JARID2 | PAG5 |
| CPLX2 | GPR55 | VPS26 | FH | DKFZp762A217 |
| PKIB | FLJ39155 | OFD1 | C6orf57 | SSNA1 |
| SLC2A4 | SMURF1 | MGC21382 | ATP6V1D | SEC63D1 |
| DSC2 | TRRAP | RHBG | CCKBR | HDAC2 |
| CDH2 | TTLL7 | CD86 | PRPF39 | WDFY2 |
| KIAA1737 | WDR69 | PRSS22 | FES | KLHL5 |
| DKFZP566N034 | BVd1.19 | CTSC | AGMAT | HTR1F |
| ACVR2A | FUT1 | PCNT1 | SLC30A4 | PJA2 |
| CYP1B1 | DENND1A | EAF2 | KPNA4 | PRLR |
| PPP1R3C | RAFTLIN | BHLHB2 | GENX-3414 | ANXA4 |
| DKFZP586A0522 | SQSTM1 | DNAJC5 | AMOT | NT5C3 |
| CHRNE | SERF2 | CXADR | ELTD1 | BVES |
| TRIM24 | PARP14 | NCOR1 | LRP11 | RASIP1 |
| PCOLCE2 | ZBTB20 | ALDH5A1 | ATP1A2 | S100A4 |
| FLJ20152 | DDIT4L | MGP | LNPEP |

### UFFU genes: 29

UFFU Cluster

Time course of UFFU genes

Link to DE genes in NCBI

| Gene Symbol |
| --- |
 IFIH1 || MRPS10 |
| AHNAK |
| TCEB1 |
| FVT1 |
| C9orf42 |
| CAPSL |
| DEPC-1 |
| PSMD1 |
| SCNN1A |
| C6orf70 |
| MGC127133 |
| OSRF |
| CX36 |
| FLJ20054 |
| HLA-DRA |
| MAOB |
| ADSSL1 |
| TMEM8 |
| CA4 |
| TMEM16A |
| VPS13B |
| MTAC2D1 |
| NR1D1 |
| PTGER4 |
| PSMB9 |
| MYH11 |
| RGS5 |
| ALDH2 |
|

### UFFD genes: 18

UFFD Cluster

Time course of UFFD genes

Link to DE genes in NCBI

| Gene Symbol |
| --- |
 CATHL1 || VAMP4 |
| VprBP |
| RNF185 |
| MCP |
| RGN |
| CSGlcA-T |
| KIAA0971 |
| C16orf50 |
| C10orf89 |
| GPR174 |
| ZNF533 |
| SCIN |
| CCL21 |
| IBRDC2 |
| GATM |
| PDE4B |
| NOL9 |
|

### UFUF genes: 21

UFUF Cluster

Time course of UFUF genes

Link to DE genes in NCBI

| Gene Symbol |
| --- |
 DCI || TMEM38B |
| MGC128473 |
| CLIC5 |
| COX6A1 |
| L2HGDH |
| TRIM7 |
| FAM51A1 |
| RGS20 |
| CAMKK2 |
| C20orf9 |
| DNASE1L3 |
| RDH8 |
| FLJ10652 |
| GPT2 |
| ORF1-FL49 |
| SYNPO |
| SNTA1 |
| GPT |
| SCN1B |
| TNFRSF9 |
|

### UFUU genes: 5

UFUU Cluster

Time course of UFUU genes

Link to DE genes in NCBI

| Gene Symbol |
| --- |
 CYP4B1 || MGC128590 |
| BOLA-DQA1 |
| PDK4 |
| C20orf35 |
|

### UFUD genes: 8

UFUD Cluster

Time course of UFUD genes

Link to DE genes in NCBI

| Gene Symbol |
| --- |
 HLF || CST6 |
| ZNF385 |
| KCNAB1 |
| CD80 |
| OLFM1 |
| KIF21A |
| FABP4 |
|

### UFDF genes: 33

UFDF Cluster

Time course of UFDF genes

Link to DE genes in NCBI

| Gene Symbol | Gene Symbol |
| --- | --- |
 LRRFIP1 | C9orf26 || NR5A2 | IL15 |
| CHST7 | CAMP |
| DHX30 | MYH8 |
| DHRS7 | SOX18 |
| FAH | TUB |
| RGS16 | LYPD1 |
| CLCN2 | TNS |
| C18orf43 | COBLL1 |
| FN3K | OSBP2 |
| IGF1R | AOX2 |
| KIF27 | ZNF235 |
| NT5C2L1 | ARHGAP27 |
| ETS1 | ASL |
| NR3C2 | ELK3 |
| PODXL | PLCD1 |
| COL15A1 |

### UFDU genes: 17

UFDU Cluster

Time course of UFDU genes

Link to DE genes in NCBI

| Gene Symbol |
| --- |
 RASGEF1B || CG018 |
| NEDD4 |
| ITGAX |
| LRCH2 |
| HCLS1 |
| SREBF2 |
| RAB35 |
| PNKP |
| CHML |
| TBDN100 |
| SH2D1A |
| LASS6 |
| GPR155 |
| CTSB |
| KIAA1639 |
| ALS2CL |
|

### UFDD genes: 1

Time course of UFDD genes

Link to DE genes in NCBI

| Gene Symbol |
| --- |
 ACHE ||

### UUFF genes: 12

UUFF Cluster

Time course of UUFF genes

Link to DE genes in NCBI

| Gene Symbol |
| --- |
 MYH1 || ACTN3 |
| ATP1B1 |
| PPP1R3C |
| BHLHB2 |
| RPL3L |
| ESR1 |
| S100G |
| GIPC2 |
| KIAA0391 |
| HSD11B1 |
| AFM |
|

### UUFU genes: 3

UUFU Cluster

Time course of UUFU genes

Link to DE genes in NCBI

| Gene Symbol |
| --- |
 TCAP || DDO |
| BTNL9 |
|

### UUFD genes: 3

UUFD Cluster

Time course of UUFD genes

Link to DE genes in NCBI

| Gene Symbol |
| --- |
 IER3 || PEBP4 |
| APOC2 |
|

### UUUF genes: 10

UUUF Cluster

Time course of UUUF genes

Link to DE genes in NCBI

| Gene Symbol |
| --- |
 KLF9 || ART3 |
| ANKRD23 |
| PLN |
| CKMT2 |
| TMOD4 |
| COX7A1 |
| ATP2A1 |
| PON3 |
| AGXT2L1 |
|

### UUUU genes: 2

UUUU Cluster

Time course of UUUU genes

Link to DE genes in NCBI

| Gene Symbol |
| --- |
 LRRC2 || BoLA |
|

### UUUD genes: 1

Time course of UUUD genes

Link to DE genes in NCBI

| Gene Symbol |
| --- |
 TMEM52 ||

### UUDF genes: 1

Time course of UUDF genes

Link to DE genes in NCBI

| Gene Symbol |
| --- |
 NAB1 ||

### UUDU genes: 2

UUDU Cluster

Time course of UUDU genes

Link to DE genes in NCBI

| Gene Symbol |
| --- |
 FGL2 || FLJ20628 |
|

### UDFF genes: 11

UDFF Cluster

Time course of UDFF genes

Link to DE genes in NCBI

| Gene Symbol |
| --- |
 TMC4 || SSFA2 |
| TSCOT |
| KDELR2 |
| 6-16 |
| ISG15 |
| SLC9A4 |
| MMP1 |
| IFITM5 |
| DSG2 |
| FLJ23235 |
|

### UDFU genes: 11

UDFU Cluster

Time course of UDFU genes

Link to DE genes in NCBI

| Gene Symbol |
| --- |
 TLN2 || ULK3 |
| ST7L |
| TRPV6 |
| CAST1 |
| WBP4 |
| GLS |
| SLC36A4 |
| ACTN4 |
| ZBTB24 |
| A |
|

### UDFD genes: 1

Time course of UDFD genes

Link to DE genes in NCBI

| Gene Symbol |
| --- |
 ANKRD26 ||

### UDUF genes: 15

UDUF Cluster

Time course of UDUF genes

Link to DE genes in NCBI

| Gene Symbol |
| --- |
 ISG20 || FLJ21415 |
| DMBT1 |
| CART1 |
| CENPC1 |
| PDE4D |
| ACRP30 |
| FBXO21 |
| PPP3CB |
| CLCA3 |
| KLRA1 |
| VCL |
| TMEM56 |
| GPT2 |
| CRP |
|

### UDUU genes: 2

UDUU Cluster

Time course of UDUU genes

Link to DE genes in NCBI

| Gene Symbol |
| --- |
 SDS || FBXL14 |
|

### UDUD genes: 4

UDUD Cluster

Time course of UDUD genes

Link to DE genes in NCBI

| Gene Symbol |
| --- |
 PTK9 || ALG3 |
| SLC10A1 |
| FLJ11806 |
|

### UDDF genes: 3

UDDF Cluster

Time course of UDDF genes

Link to DE genes in NCBI

| Gene Symbol |
| --- |
 PITPNM3 || SOSTDC1 |
| GALK1 |
|

### UDDU genes: 2

UDDU Cluster

Time course of UDDU genes

Link to DE genes in NCBI

| Gene Symbol |
| --- |
 KRT19 || C6orf155 |
|

### DFFF genes: 83

DFFF Cluster

Time course of DFFF genes

Link to DE genes in NCBI

| Gene Symbol | Gene Symbol | Gene Symbol |
| --- | --- | --- |
 LATS1 | FLJ14803 | KIAA0467 || CLOCK | HYPE | AGC1 |
| YEATS2 | CHST3 | EEDA |
| COL11A1 | RP2 | ZCCHC12 |
| ALDH1A2 | SEPT11 | MYO5B |
| D4S234E | TAF1B | TAF6L |
| PAG6 | PIP3-E | ENTPD3 |
| SLC6A6 | ACACA | CA5A |
| ROPN1L | YIPF5 | PIP5K3 |
| BHLHB9 | KCNE2 | STK4 |
| TM9SF4 | PGBD2 | NFATC2 |
| DCAMKL1 | TAF1 | NEF3 |
| DSPG3 | CUTL2 | NRXN3 |
| CNN1 | FLJ37562 | RHOU |
| TM7SF2 | ADAM12 | ODZ3 |
| FZD9 | CDC2 | KIAA0265 |
| KBTBD8 | PIK3R1 | PRKG1 |
| PHF3 | DDX55 | CGI-121 |
| HYPB | USP7 | CHD4 |
| GOLGB1 | MATN1 | FN1 |
| HMGA2 | ASB1 | GSTA2 |
| TBDN100 | GABRB3 | DIP |
| EXPH5 | NOL1 | CHDH |
| PRICKLE1 | KIAA1596 | USP37 |
| AMY2B | SEH1L | COQ6 |
| CLDN16 | SEC23IP | grp78 |
| CCND2 | TAGLN3 | FRMD3 |
| ACTA2 | HSPCA |

### DFFU genes: 16

DFFU Cluster

Time course of DFFU genes

Link to DE genes in NCBI

| Gene Symbol |
| --- |
 ARMC8 || SEMA4A |
| FLT1 |
| KIR2DL1 |
| ITGB3 |
| MAEL |
| ONECUT2 |
| MUC19 |
| GPR139 |
| STARD4 |
| TUBG2 |
| PMAIP1 |
| KIAA1189 |
| RNF12 |
| FRMD3 |
| RGMB |
|

### DFFD genes: 17

DFFD Cluster

Time course of DFFD genes

Link to DE genes in NCBI

| Gene Symbol |
| --- |
 PITPNB || ASPSCR1 |
| COL12A1 |
| DPP4 |
| TEX14 |
| FLJ20485 |
| IMP-3 |
| MLLT11 |
| PCYT2 |
| UCHL1 |
| TNNT2 |
| ARPC4 |
| SOCS4 |
| SDHD |
| EPOR |
| HGF |
| RANBP1 |
|

### DFUF genes: 20

DFUF Cluster

Time course of DFUF genes

Link to DE genes in NCBI

| Gene Symbol |
| --- |
 KPNA4 || PRDX6 |
| ATF3 |
| ME1 |
| MGC128711 |
| FOS |
| NCL |
| C8B |
| RBM25 |
| SLC6A15 |
| KCNJ1 |
| GNB2L1 |
| MGC20781 |
| LY9 |
| C20orf26 |
| ITGA4 |
| TOMM34 |
| PRG4 |
| ZFHX1B |
| PRSS7 |
|

### DFUU genes: 3

DFUU Cluster

Time course of DFUU genes

Link to DE genes in NCBI

| Gene Symbol |
| --- |
 GPX2 || ANGPTL4 |
| C4orf17 |
|

### DFUD genes: 10

DFUD Cluster

Time course of DFUD genes

Link to DE genes in NCBI

| Gene Symbol |
| --- |
 HAPLN1 || LRFN5 |
| PRKAA2 |
| MBP |
| PDXK |
| HP |
| BAI3 |
| F13B |
| SNX13 |
| MLLT11 |
|

### DFDF genes: 22

DFDF Cluster

Time course of DFDF genes

Link to DE genes in NCBI

| Gene Symbol |
| --- |
 EPHA7 || NRXN1 |
| UST |
| AGTR2 |
| CHRM2 |
| B3GALT2 |
| ALAS2 |
| ZNF79 |
| ACOT4 |
| GAS7 |
| NHS |
| CLDN23 |
| TRPC6 |
| HMMR |
| MGC9564 |
| GOLGB1 |
| GPR37 |
| XIST |
| LDLRAD3 |
| DCAMKL1 |
| COL16A1 |
| PTN |
|

### DFDU genes: 10

DFDU Cluster

Time course of DFDU genes

Link to DE genes in NCBI

| Gene Symbol |
| --- |
 GABRA3 || C2GNT3 |
| KCNN2 |
| GPR23 |
| WNK3 |
| CCDC4 |
| DPP10 |
| HAS2 |
| KIDINS220 |
| COL24A1 |
|

### DFDD genes: 2

DFDD Cluster

Time course of DFDD genes

Link to DE genes in NCBI

| Gene Symbol |
| --- |
 RICS || TOX |
|

### DUFF genes: 45

DUFF Cluster

Time course of DUFF genes

Link to DE genes in NCBI

| Gene Symbol | Gene Symbol |
| --- | --- |
 FLOT2 | SLC9A2 || ABHD5 | GFOD1 |
| BTBD15 | LATS1 |
| NAG | OPN1LW |
| C8A | ATP13A4 |
| IL17RD | CDMP-2 |
| TM9SF4 | ANPEP |
| TGOLN2 | KIT |
| FSD1L | SLC25A32 |
| C5orf3 | ZNF31 |
| RPE | C6orf105 |
| STX6 | SPEN |
| ARHGEF10 | SATB1 |
| TRPC3 | ADAMTS5 |
| MAN1A1 | CYP3A4 |
| FGB | BACH2 |
| KIAA0586 | GNB4 |
| C1orf27 | GNGT1 |
| G6PC | PHF10 |
| PAK7 | FLJ35880 |
| OPTN | ATG4A |
| HOXA10 | LRRC41 |
| HOXA9 |

### DUFU genes: 4

DUFU Cluster

Time course of DUFU genes

Link to DE genes in NCBI

| Gene Symbol |
| --- |
 MAOB || C1orf138 |
| LRRN6C |
| KIAA1961 |
|

### DUFD genes: 7

DUFD Cluster

Time course of DUFD genes

Link to DE genes in NCBI

| Gene Symbol |
| --- |
 CLCA2 || SULT2A1 |
| NOX4 |
| SUCLA2 |
| PPAPDC2 |
| SMEK2 |
| LZTR1 |
|

### DUUF genes: 9

DUUF Cluster

Time course of DUUF genes

Link to DE genes in NCBI

| Gene Symbol |
| --- |
 DLD || MAT1A |
| CXADR |
| UCP1 |
| CNKSR1 |
| F2RL2 |
| ANGPTL3 |
| FLJ20507 |
| ANKMY2 |
|

### DUUD genes: 2

DUUD Cluster

Time course of DUUD genes

Link to DE genes in NCBI

| Gene Symbol |
| --- |
 SLC7A11 || CWF19L2 |
|

### DUDF genes: 10

DUDF Cluster

Time course of DUDF genes

Link to DE genes in NCBI

| Gene Symbol |
| --- |
 SLC2A3 || RNF24 |
| RetSat |
| CABP5 |
| TKDP2 |
| SLC17A6 |
| MGC33887 |
| YIPF5 |
| NUP88 |
| XIST |
|

### DUDU genes: 6

DUDU Cluster

Time course of DUDU genes

Link to DE genes in NCBI

| Gene Symbol |
| --- |
 RECQL || RABGAP1 |
| ORF1 |
| PDCD4 |
| IMPG2 |
| ARHGAP12 |
|

### DUDD genes: 3

DUDD Cluster

Time course of DUDD genes

Link to DE genes in NCBI

| Gene Symbol |
| --- |
 PCDH19 || DCUN1D2 |
| XIST |
|

### DDFF genes: 3

DDFF Cluster

Time course of DDFF genes

Link to DE genes in NCBI

| Gene Symbol |
| --- |
 PIK3R2 || HES6 |
| ERBB3 |
|

### DDFU genes: 2

DDFU Cluster

Time course of DDFU genes

Link to DE genes in NCBI

| Gene Symbol |
| --- |
 HAL || MCTP1 |
|

### DDFD genes: 3

DDFD Cluster

Time course of DDFD genes

Link to DE genes in NCBI

| Gene Symbol |
| --- |
 MASK || NNAT |
| DCX |
|

### DDUF genes: 2

DDUF Cluster

Time course of DDUF genes

Link to DE genes in NCBI

| Gene Symbol |
| --- |
 COTL1 || NRG1 |
|

### DDUD genes: 4

DDUD Cluster

Time course of DDUD genes

Link to DE genes in NCBI

| Gene Symbol |
| --- |
 NNAT || CRABP2 |
| RAB9B |
| IPMK |
|

### DDDF genes: 7

DDDF Cluster

Time course of DDDF genes

Link to DE genes in NCBI

| Gene Symbol |
| --- |
 DPYSL5 || DBN1 |
| RYR3 |
| CKB |
| CRABP1 |
| LPHN3 |
| POSTN |
|

### DDDD genes: 2

DDDD Cluster

Time course of DDDD genes

Link to DE genes in NCBI

| Gene Symbol |
| --- |
 PRSS35 || BCAT1 |
|
