# Supplementary material for: A gene network switch enhances the oxidative capacity of ovine skeletal muscle during late fetal development
Source: BMC Genomics. 2010 Jun 15;11:378. doi: 10.1186/1471-2164-11-378 (PMC2894804; doi:10.1186/1471-2164-11-378)
Supplement: Additional File 2 — Functional clustering of genes present in each expression cluster. The file contains a summary table listing functional groups and functional terms significantly associated with each gene expression cluster. [file 1471-2164-11-378-S2.PDF]

Additional File 2. Functional clustering of genes present in each expression cluster[1].

Clusters[2]	Biological description of the cluster	Category	Term	Geometric Mean of P-values for each functional group
**FFFU**	Change in postnatal development	**Functional Group 1**		0.008
		SP_PIR_KEYWORDS	dna-binding	
		INTERPRO	IPR001092:Basic helix-loop-helix dimerisation region bHLH	
		SMART	SM00353:HLH	
		**Functional Group 2**		0.025
		GOTERM_BP_ALL	GO:0031323~regulation of cellular metabolic process	
		GOTERM_BP_ALL	GO:0019222~regulation of metabolic process	
		GOTERM_BP_ALL	GO:0050794~regulation of cellular process	
		GOTERM_BP_ALL	GO:0010468~regulation of gene expression	
		SP_PIR_KEYWORDS	dna-binding	
		GOTERM_BP_ALL	GO:0019219~regulation of nucleobase, nucleoside, nucleotide and nucleic acid metabolic process	
		GOTERM_BP_ALL	GO:0065007~biological regulation	
		GOTERM_MF_ALL	GO:0030528~transcription regulator activity	
		GOTERM_BP_ALL	GO:0006357~regulation of transcription from RNA polymerase II promoter	
		GOTERM_MF_ALL	GO:0003677~DNA binding	
		GOTERM_BP_ALL	GO:0045449~regulation of transcription	
		GOTERM_MF_ALL	GO:0003700~transcription factor activity	
		GOTERM_BP_ALL	GO:0006366~transcription from RNA polymerase II promoter	
		GOTERM_BP_ALL	GO:0050789~regulation of biological process	
		GOTERM_BP_ALL	GO:0006350~transcription	
		GOTERM_BP_ALL	GO:0006355~regulation of transcription, DNA-dependent	
		GOTERM_BP_ALL	GO:0006351~transcription, DNA-dependent	
		GOTERM_BP_ALL	GO:0032774~RNA biosynthetic process	
		GOTERM_CC_ALL	GO:0005634~nucleus	
		GOTERM_MF_ALL	GO:0046983~protein dimerization activity	
		GOTERM_MF_ALL	GO:0003676~nucleic acid binding	
		SP_PIR_KEYWORDS	activator	
		GOTERM_MF_ALL	GO:0043565~sequence-specific DNA binding	

		GOTERM_BP_ALL	GO:0016070~RNA metabolic process	
		SP_PIR_KEYWORDS	Transcription regulation	
		GOTERM_BP_ALL	GO:0010467~gene expression	
		SP_PIR_KEYWORDS	Transcription	
		SP_PIR_KEYWORDS	nucleus	
		GOTERM_BP_ALL	GO:0006139~nucleobase, nucleoside, nucleotide and nucleic acid metabolic process	
		GOTERM_BP_ALL	GO:0043283~biopolymer metabolic process	
		GOTERM_BP_ALL	GO:0044237~cellular metabolic process	
		GOTERM_BP_ALL	GO:0043170~macromolecule metabolic process	
		GOTERM_BP_ALL	GO:0044238~primary metabolic process	
		**Functional Group 3**		0.026
		GOTERM_BP_ALL	GO:0031325~positive regulation of cellular metabolic process	
		GOTERM_BP_ALL	GO:0009893~positive regulation of metabolic process	
		GOTERM_BP_ALL	GO:0006357~regulation of transcription from RNA polymerase II promoter	
		GOTERM_BP_ALL	GO:0006366~transcription from RNA polymerase II promoter	
		GOTERM_BP_ALL	GO:0048522~positive regulation of cellular process	
		GOTERM_BP_ALL	GO:0045941~positive regulation of transcription	
		GOTERM_BP_ALL	GO:0045944~positive regulation of transcription from RNA polymerase II promoter	
		GOTERM_BP_ALL	GO:0045935~positive regulation of nucleobase, nucleoside, nucleotide and nucleic acid metabolic process	
		GOTERM_BP_ALL	GO:0048518~positive regulation of biological process	
		GOTERM_BP_ALL	GO:0045893~positive regulation of transcription, DNA-dependent	
		GOTERM_BP_ALL	GO:0048523~negative regulation of cellular process	
		GOTERM_BP_ALL	GO:0031324~negative regulation of cellular metabolic process	
		GOTERM_BP_ALL	GO:0048519~negative regulation of biological process	
		GOTERM_BP_ALL	GO:0009892~negative regulation of metabolic process	
		**Functional Group 4**		0.034
		GOTERM_BP_ALL	GO:0006357~regulation of transcription from RNA polymerase II promoter	
		GOTERM_BP_ALL	GO:0006366~transcription from RNA polymerase II promoter	
		GOTERM_BP_ALL	GO:0009653~anatomical structure morphogenesis	
		GOTERM_BP_ALL	GO:0040008~regulation of growth	
		GOTERM_BP_ALL	GO:0040007~growth	
**FFFD**	Change in post-natal development			N.S.
**FFUF**	Developmental	**Functional Group 1**		6.47E-24

switch between late fetal development and birth	GOTERM_CC_ALL	GO:0005739~mitochondrion
	SP_PIR_KEYWORDS	Mitochondrion
	GOTERM_CC_ALL	GO:0044429~mitochondrial part
	KEGG_PATHWAY	Oxidative phosphorylation
	SP_PIR_KEYWORDS	transit peptide
	GOTERM_CC_ALL	GO:0005740~mitochondrial envelope
	GOTERM_CC_ALL	GO:0031966~mitochondrial membrane
	GOTERM_CC_ALL	GO:0005743~mitochondrial inner membrane
	GOTERM_CC_ALL	GO:0019866~organelle inner membrane
	GOTERM_CC_ALL	GO:0031967~organelle envelope
	GOTERM_CC_ALL	GO:0031975~envelope
	GOTERM_BP_ALL	GO:0006091~generation of precursor metabolites and energy
	GOTERM_MF_ALL	GO:0016655~oxidoreductase activity, acting on NADH/ NADPH, quinone or similar compound as acceptor
	GOTERM_MF_ALL	GO:0016651~oxidoreductase activity, acting on NADH or NADPH
	SP_PIR_KEYWORDS	oxidoreductase
	GOTERM_MF_ALL	GO:0050136~NADH dehydrogenase (quinone) activity
	GOTERM_MF_ALL	GO:0003954~NADH dehydrogenase activity
	GOTERM_MF_ALL	GO:0008137~NADH dehydrogenase (ubiquinone) activity
	GOTERM_CC_ALL	GO:0044444~cytoplasmic part
	SP_PIR_KEYWORDS	ubiquinone
	GOTERM_CC_ALL	GO:0005737~cytoplasm
	GOTERM_MF_ALL	GO:0009055~electron carrier activity
	GOTERM_MF_ALL	GO:0016491~oxidoreductase activity
	SP_PIR_KEYWORDS	NAD
	GOTERM_BP_ALL	GO:0006118~electron transport
	GOTERM_CC_ALL	GO:0031090~organelle membrane
	GOTERM_CC_ALL	GO:0043231~intracellular membrane-bound organelle
	GOTERM_CC_ALL	GO:0043227~membrane-bound organelle
	GOTERM_CC_ALL	GO:0044446~intracellular organelle part
	GOTERM_CC_ALL	GO:0044422~organelle part
	SP_PIR_KEYWORDS	Direct protein sequencing
	GOTERM_CC_ALL	GO:0044424~intracellular part
	GOTERM_CC_ALL	GO:0043229~intracellular organelle
	GOTERM_CC_ALL	GO:0043226~organelle
	GOTERM_CC_ALL	GO:0005622~intracellular
	SP_PIR_KEYWORDS	membrane
	GOTERM_CC_ALL	GO:0044464~cell part
	GOTERM_CC_ALL	GO:0005623~cell
	GOTERM_CC_ALL	GO:0016020~membrane

**Functional Group 2**		2.25E-7
GOTERM_CC_ALL	GO:0031980~mitochondrial lumen	
GOTERM_CC_ALL	GO:0005759~mitochondrial matrix	
GOTERM_CC_ALL	GO:0031974~membrane-enclosed lumen	
GOTERM_CC_ALL	GO:0043233~organelle lumen	
**Functional Group 3**		1.74E-6
GOTERM_BP_ALL	GO:0006732~coenzyme metabolic process	
GOTERM_BP_ALL	GO:0045333~cellular respiration	
GOTERM_BP_ALL	GO:0046356~acetyl-CoA catabolic process	
GOTERM_BP_ALL	GO:0009060~aerobic respiration	
GOTERM_BP_ALL	GO:0006099~tricarboxylic acid cycle	
GOTERM_BP_ALL	GO:0006084~acetyl-CoA metabolic process	
GOTERM_BP_ALL	GO:0009109~coenzyme catabolic process	
GOTERM_BP_ALL	GO:0051187~cofactor catabolic process	
KEGG_PATHWAY	Citrate cycle (TCA cycle)	
GOTERM_BP_ALL	GO:0044248~cellular catabolic process	
GOTERM_BP_ALL	GO:0044262~cellular carbohydrate metabolic process	
GOTERM_BP_ALL	GO:0009056~catabolic process	
GOTERM_BP_ALL	GO:0005975~carbohydrate metabolic process	
SP_PIR_KEYWORDS	Tricarboxylic acid cycle	
**Functional Group 4**		7.56E-6
GOTERM_CC_ALL	GO:0044455~mitochondrial membrane part	
GOTERM_CC_ALL	GO:0005746~mitochondrial respiratory chain	
SP_PIR_KEYWORDS	electron transport	
GOTERM_MF_ALL	GO:0008121~ubiquinol-cytochrome-c reductase activity	
GOTERM_MF_ALL	GO:0016681~oxidoreductase activity, acting on diphenols as donors, cytochrome as acceptor	
GOTERM_MF_ALL	GO:0016679~oxidoreductase activity, acting on diphenols and related substances as donors	
**Functional Group 5**		
GOTERM_CC_ALL	GO:0044455~mitochondrial membrane part	1.23E-5
GOTERM_CC_ALL	GO:0005746~mitochondrial respiratory chain	
GOTERM_CC_ALL	GO:0005747~mitochondrial respiratory chain complex I	
GOTERM_CC_ALL	GO:0045271~respiratory chain complex I	
GOTERM_CC_ALL	GO:0030964~NADH dehydrogenase complex (quinone)	
**Functional Group 6**		2.01E-5

SP_PIR_KEYWORDS	respiratory chain	
SP_PIR_KEYWORDS	electron transfer	
SP_PIR_KEYWORDS	mitochondrial inner membrane	
GOTERM_MF_ALL	GO:0016676~oxidoreductase activity, acting on heme group of donors, oxygen as acceptor	
GOTERM_MF_ALL	GO:0016675~oxidoreductase activity, acting on heme group of donors	
GOTERM_MF_ALL	GO:0004129~cytochrome-c oxidase activity	
GOTERM_MF_ALL	GO:0015002~heme-copper terminal oxidase activity	
**Functional Group 7**		3.35E-5
SP_PIR_KEYWORDS	oxidative phosphorylation	
GOTERM_BP_ALL	GO:0006119~oxidative phosphorylation	
SP_PIR_KEYWORDS	membrane-associated complex	
GOTERM_MF_ALL	GO:0015077~monovalent inorganic cation transmembrane transporter activity	
GOTERM_BP_ALL	GO:0051186~cofactor metabolic process	
GOTERM_MF_ALL	GO:0015078~hydrogen ion transmembrane transporter activity	
GOTERM_MF_ALL	GO:0022890~inorganic cation transmembrane transporter activity	
SP_PIR_KEYWORDS	respiratory chain	
GOTERM_BP_ALL	GO:0006732~coenzyme metabolic process	
GOTERM_BP_ALL	GO:0006120~mitochondrial electron transport, NADH to ubiquinone	
GOTERM_BP_ALL	GO:0042773~ATP synthesis coupled electron transport	
GOTERM_BP_ALL	GO:0042775~organelle ATP synthesis coupled electron transport	
GOTERM_BP_ALL	GO:0046034~ATP metabolic process	
GOTERM_MF_ALL	GO:0008324~cation transmembrane transporter activity	
GOTERM_CC_ALL	GO:0045259~proton-transporting ATP synthase complex	
GOTERM_BP_ALL	GO:0051188~cofactor biosynthetic process	
GOTERM_MF_ALL	GO:0015075~ion transmembrane transporter activity	
GOTERM_BP_ALL	GO:0009144~purine nucleoside triphosphate metabolic process	
GOTERM_BP_ALL	GO:0009199~ribonucleoside triphosphate metabolic process	
GOTERM_BP_ALL	GO:0009205~purine ribonucleoside triphosphate metabolic process	
GOTERM_BP_ALL	GO:0009141~nucleoside triphosphate metabolic process	
GOTERM_MF_ALL	GO:0022857~transmembrane transporter activity	
GOTERM_MF_ALL	GO:0046933~hydrogen ion transporting ATP synthase activity, rotational mechanism	
GOTERM_BP_ALL	GO:0015986~ATP synthesis coupled proton transport	
GOTERM_MF_ALL	GO:0022891~substrate-specific transmembrane transporter activity	
GOTERM_BP_ALL	GO:0009150~purine ribonucleotide metabolic process	
GOTERM_BP_ALL	GO:0009108~coenzyme biosynthetic process	
GOTERM_MF_ALL	GO:0046961~hydrogen ion transporting ATPase activity, rotational mechanism	
GOTERM_BP_ALL	GO:0006754~ATP biosynthetic process	
GOTERM_BP_ALL	GO:0006753~nucleoside phosphate metabolic process	

	GOTERM_BP_ALL	GO:0009259~ribonucleotide metabolic process
	GOTERM_BP_ALL	GO:0006163~purine nucleotide metabolic process
	GOTERM_BP_ALL	GO:0006818~hydrogen transport
	GOTERM_BP_ALL	GO:0015992~proton transport
	SP_PIR_KEYWORDS	transport
	GOTERM_MF_ALL	GO:0019829~cation-transporting ATPase activity
	GOTERM_MF_ALL	GO:0042625~ATPase activity, coupled to transmembrane movement of ions
	GOTERM_CC_ALL	GO:0045263~proton-transporting ATP synthase complex, coupling factor F(o)
	GOTERM_CC_ALL	GO:0033177~proton-transporting two-sector ATPase complex, proton-transporting domain
	GOTERM_MF_ALL	GO:0022892~substrate-specific transporter activity
	SP_PIR_KEYWORDS	CF(0)
	GOTERM_BP_ALL	GO:0009142~nucleoside triphosphate biosynthetic process
	GOTERM_BP_ALL	GO:0009201~ribonucleoside triphosphate biosynthetic process
	GOTERM_BP_ALL	GO:0009206~purine ribonucleoside triphosphate biosynthetic process
	GOTERM_BP_ALL	GO:0009145~purine nucleoside triphosphate biosynthetic process
	GOTERM_CC_ALL	GO:0016469~proton-transporting two-sector ATPase complex
	SP_PIR_KEYWORDS	hydrogen ion transport
	GOTERM_BP_ALL	GO:0016310~phosphorylation
	GOTERM_MF_ALL	GO:0043492~ATPase activity, coupled to movement of substances
	GOTERM_MF_ALL	GO:0042626~ATPase activity, coupled to transmembrane movement of substances
	GOTERM_MF_ALL	GO:0016820~hydrolase activity, acting on acid anhydrides, catalyzing transmembrane movement
	GOTERM_BP_ALL	GO:0055086~nucleobase, nucleoside and nucleotide metabolic process
	GOTERM_BP_ALL	GO:0009152~purine ribonucleotide biosynthetic process
	GOTERM_BP_ALL	GO:0009260~ribonucleotide biosynthetic process
	GOTERM_MF_ALL	GO:0015405~P-P-bond-hydrolysis-driven transmembrane transporter activity
	GOTERM_MF_ALL	GO:0015399~primary active transmembrane transporter activity
	GOTERM_MF_ALL	GO:0005215~transporter activity
	GOTERM_BP_ALL	GO:0006164~purine nucleotide biosynthetic process
	GOTERM_BP_ALL	GO:0009117~nucleotide metabolic process
	SP_PIR_KEYWORDS	ATP biosynthesis
	GOTERM_BP_ALL	GO:0006793~phosphorus metabolic process
	GOTERM_BP_ALL	GO:0006796~phosphate metabolic process
	GOTERM_BP_ALL	GO:0009165~nucleotide biosynthetic process
	GOTERM_MF_ALL	GO:0022804~active transmembrane transporter activity
	GOTERM_BP_ALL	GO:0015672~monovalent inorganic cation transport
	GOTERM_MF_ALL	GO:0017111~nucleoside-triphosphatase activity
	GOTERM_MF_ALL	GO:0016887~ATPase activity
	GOTERM_MF_ALL	GO:0016462~pyrophosphatase activity
	GOTERM_MF_ALL	GO:0016818~hydrolase activity, acting on acid anhydrides, in phosphorus-containing anhydrides

GOTERM_MF_ALL	GO:0016817~hydrolase activity, acting on acid anhydrides	
GOTERM_MF_ALL	GO:0042623~ATPase activity, coupled	
GOTERM_BP_ALL	GO:0006810~transport	
GOTERM_BP_ALL	GO:0006812~cation transport	
GOTERM_BP_ALL	GO:0051234~establishment of localization	
SP_PIR_KEYWORDS	ion transport	
GOTERM_BP_ALL	GO:0051179~localization	
GOTERM_BP_ALL	GO:0006811~ion transport	
SP_PIR_KEYWORDS	ATP synthesis	
GOTERM_MF_ALL	GO:0016787~hydrolase activity	
SP_PIR_KEYWORDS	hydrolase	
GOTERM_BP_ALL	GO:0006139~nucleobase, nucleoside, nucleotide and nucleic acid metabolic process	
GOTERM_CC_ALL	GO:0044425~membrane part	
**Functional Group 8**		4.72E-5
SP_PIR_KEYWORDS	iron-sulfur	
GOTERM_MF_ALL	GO:0051540~metal cluster binding	
GOTERM_MF_ALL	GO:0051536~iron-sulfur cluster binding	
SP_PIR_KEYWORDS	iron-sulfur protein	
SP_PIR_KEYWORDS	4Fe-4S	
GOTERM_MF_ALL	GO:0051539~4 iron, 4 sulfur cluster binding	
SP_PIR_KEYWORDS	metalloprotein	
SP_PIR_KEYWORDS	iron	
SP_PIR_KEYWORDS	2Fe-2S	
GOTERM_MF_ALL	GO:0051537~2 iron, 2 sulfur cluster binding	
GOTERM_MF_ALL	GO:0005506~iron ion binding	
INTERPRO	IPR001041:Ferredoxin	
SP_PIR_KEYWORDS	metal-binding	
GOTERM_MF_ALL	GO:0046914~transition metal ion binding	
**Functional Group 9**		7.80E-4
GOTERM_BP_ALL	GO:0008152~metabolic process	
GOTERM_BP_ALL	GO:0044237~cellular metabolic process	
GOTERM_BP_ALL	GO:0044238~primary metabolic process	
GOTERM_BP_ALL	GO:0043170~macromolecule metabolic process	
GOTERM_BP_ALL	GO:0009987~cellular process	
**Functional Group 10**		0.011
GOTERM_BP_ALL	GO:0009058~biosynthetic process	

	GOTERM_BP_ALL	GO:0044249~cellular biosynthetic process	
	GOTERM_BP_ALL	GO:0009059~macromolecule biosynthetic process	
	**Functional Group 11**		0.015
	INTERPRO	IPR001431:Peptidase M16, zinc-binding site	
	INTERPRO	IPR007863:Peptidase M16, C-terminal	
	INTERPRO	IPR011765:Peptidase M16, N-terminal	
	INTERPRO	IPR011237:Peptidase M16, core	
	PIR_SUPERFAMILY	PIRSF001210:mitochondrial processing peptidase alpha chain	
	GOTERM_MF_ALL	GO:0004222~metalloendopeptidase activity	
	**Functional Group 12**		0.015
	GOTERM_BP_ALL	GO:0006979~response to oxidative stress	
	GOTERM_BP_ALL	GO:0042221~response to chemical stimulus	
	GOTERM_BP_ALL	GO:0006950~response to stress	
	GOTERM_BP_ALL	GO:0050896~response to stimulus	
	**Functional Group 13**		0.024
	SP_PIR_KEYWORDS	ATP biosynthesis	
	GOTERM_CC_ALL	GO:0045261~proton-transporting ATP synthase complex, catalytic core F(1)	
	SP_PIR_KEYWORDS	cf(1)	
	GOTERM_CC_ALL	GO:0033178~proton-transporting two-sector ATPase complex, catalytic domain	
	SP_PIR_KEYWORDS	ATP synthesis	
	**Functional Group 14**		0.030
	INTERPRO	IPR012335:Thioredoxin fold	
	GOTERM_MF_ALL	GO:0016209~antioxidant activity	
	SP_PIR_KEYWORDS	Peroxidase	
	GOTERM_MF_ALL	GO:0016684~oxidoreductase activity, acting on peroxide as acceptor	
	GOTERM_MF_ALL	GO:0004601~peroxidase activity	
	**Functional Group 15**		0.030
	GOTERM_BP_ALL	GO:0006082~organic acid metabolic process	
	GOTERM_BP_ALL	GO:0019752~carboxylic acid metabolic process	
	KEGG_PATHWAY	Phenylalanine, tyrosine and tryptophan biosynthesis	
	INTERPRO	IPR004838:Aminotransferases, class-I, pyridoxal-phosphate-binding site	
	SP_PIR_KEYWORDS	aminotransferase	
	GOTERM_MF_ALL	GO:0008483~transaminase activity	
	GOTERM_MF_ALL	GO:0030170~pyridoxal phosphate binding	

		INTERPRO	IPR015421:Pyridoxal phosphate-dependent transferase, major region, subdomain 1	
		INTERPRO	IPR004839:Aminotransferase, class I and II	
		GOTERM_MF_ALL	GO:0016769~transferase activity, transferring nitrogenous groups	
		SP_PIR_KEYWORDS	pyridoxal phosphate	
		GOTERM_MF_ALL	GO:0019842~vitamin binding	
		GOTERM_BP_ALL	GO:0006520~amino acid metabolic process	
		GOTERM_BP_ALL	GO:0006519~amino acid and derivative metabolic process	
		GOTERM_BP_ALL	GO:0009308~amine metabolic process	
		GOTERM_BP_ALL	GO:0006807~nitrogen compound metabolic process	
**FFDF**	Developmental switch between late fetal development and birth	**Functional Group 1**		2.26E-6
		GOTERM_BP_ALL	GO:0007154~cell communication	
		GOTERM_BP_ALL	GO:0007165~signal transduction	
		GOTERM_BP_ALL	GO:0007166~cell surface receptor linked signal transduction	
		GOTERM_BP_ALL	GO:0007242~intracellular signaling cascade	
		**Functional Group 2**		5.96E-6
		GOTERM_BP_ALL	GO:0007155~cell adhesion	
		GOTERM_BP_ALL	GO:0022610~biological adhesion	
		SP_PIR_KEYWORDS	cell adhesion	
		**Functional Group 3**		0.001
		GOTERM_BP_ALL	GO:0032502~developmental process	
		GOTERM_BP_ALL	GO:0007399~nervous system development	
		GOTERM_BP_ALL	GO:0009653~anatomical structure morphogenesis	
		GOTERM_BP_ALL	GO:0007275~multicellular organismal development	
		GOTERM_BP_ALL	GO:0032501~multicellular organismal process	
		GOTERM_BP_ALL	GO:0022008~neurogenesis	
		GOTERM_BP_ALL	GO:0048856~anatomical structure development	
		GOTERM_BP_ALL	GO:0030154~cell differentiation	
		GOTERM_BP_ALL	GO:0048869~cellular developmental process	
		GOTERM_BP_ALL	GO:0009887~organ morphogenesis	
		GOTERM_BP_ALL	GO:0048731~system development	
		GOTERM_BP_ALL	GO:0048468~cell development	
		GOTERM_BP_ALL	GO:0048513~organ development	
		**Functional Group 4**		0.002
		GOTERM_BP_ALL	GO:0007399~nervous system development	
		GOTERM_BP_ALL	GO:0022008~neurogenesis	

GOTERM_BP_ALL	GO:0016477~cell migration	
GOTERM_BP_ALL	GO:0051674~localization of cell	
GOTERM_BP_ALL	GO:0006928~cell motility	
GOTERM_BP_ALL	GO:0048699~generation of neurons	
**Functional Group 5**		0.003
INTERPRO	IPR000299:Band 4.1, N-terminal	
INTERPRO	IPR011993:Pleckstrin homology-type	
SMART	SM00295:B41	
INTERPRO	IPR000798:Ezrin/radixin/moesin ERM	
INTERPRO	IPR014352:FERM-type 3-helical bundle	
UP_SEQ_FEATURE	domain:FERM	
**Functional Group 6**		0.008
SP_PIR_KEYWORDS	membrane	
GOTERM_CC_ALL	GO:0005886~plasma membrane	
SP_PIR_KEYWORDS	transmembrane	
UP_SEQ_FEATURE	topological domain:Cytoplasmic	
GOTERM_CC_ALL	GO:0044425~membrane part	
GOTERM_CC_ALL	GO:0016020~membrane	
GOTERM_CC_ALL	GO:0016021~integral to membrane	
GOTERM_CC_ALL	GO:0031224~intrinsic to membrane	
UP_SEQ_FEATURE	topological domain:Extracellular	
UP_SEQ_FEATURE	transmembrane region	
**Functional Group 7**		0.010
GOTERM_MF_ALL	GO:0004714~transmembrane receptor protein tyrosine kinase activity	
UP_SEQ_FEATURE	domain:Ig-like C2-type 2	
UP_SEQ_FEATURE	domain:Ig-like C2-type 1	
UP_SEQ_FEATURE	domain:Fibronectin type-III 1	
UP_SEQ_FEATURE	domain:Fibronectin type-III 2	
GOTERM_MF_ALL	GO:0004713~protein-tyrosine kinase activity	
INTERPRO	IPR003961:Fibronectin, type III	
SMART	SM00060:FN3	
INTERPRO	IPR008957:Fibronectin, type III-like fold	
UP_SEQ_FEATURE	domain:Fibronectin type-III 3	
GOTERM_BP_ALL	GO:0007169~transmembrane receptor protein tyrosine kinase signaling pathway	
**Functional Group 8**		0.010

SP_PIR_KEYWORDS	glycoprotein	
SP_PIR_KEYWORDS	signal	
UP_SEQ_FEATURE	glycosylation site:N-linked (GlcNAc...)	
UP_SEQ_FEATURE	signal peptide	
UP_SEQ_FEATURE	disulfide bond	
**Functional Group 9**		0.015
GOTERM_MF_ALL	GO:0060089~molecular transducer activity	
GOTERM_MF_ALL	GO:0004871~signal transducer activity	
GOTERM_MF_ALL	GO:0004872~receptor activity	
GOTERM_MF_ALL	GO:0004888~transmembrane receptor activity	
SP_PIR_KEYWORDS	receptor	
**Functional Group 10**		0.016
INTERPRO	IPR011993:Pleckstrin homology-type	
INTERPRO	IPR001849:Pleckstrin-like	
SMART	SM00233:PH	
**Functional Group 11**		0.018
GOTERM_MF_ALL	GO:0008092~cytoskeletal protein binding	
GOTERM_MF_ALL	GO:0003779~actin binding	
SP_PIR_KEYWORDS	actin-binding	
SP_PIR_KEYWORDS	cytoskeleton	
**Functional Group 12**		0.019
GOTERM_BP_ALL	GO:0022402~cell cycle process	
GOTERM_BP_ALL	GO:0007049~cell cycle	
GOTERM_BP_ALL	GO:0000278~mitotic cell cycle	
GOTERM_BP_ALL	GO:0000074~regulation of progression through cell cycle	
GOTERM_BP_ALL	GO:0051726~regulation of cell cycle	
GOTERM_BP_ALL	GO:0022403~cell cycle phase	
**Functional Group 13**		0.020
GOTERM_CC_ALL	GO:0019898~extrinsic to membrane	
GOTERM_CC_ALL	GO:0005834~heterotrimeric G-protein complex	
GOTERM_CC_ALL	GO:0019897~extrinsic to plasma membrane	
INTERPRO	IPR001770:G-protein, gamma subunit	
SP_PIR_KEYWORDS	transducer	
UP_SEQ_FEATURE	propeptide:Removed in mature form	

SP_PIR_KEYWORDS	prenylation	
**Functional Group 14**		0.021
UP_SEQ_FEATURE	domain:Ig-like C2-type 3	
UP_SEQ_FEATURE	domain:Ig-like C2-type 2	
UP_SEQ_FEATURE	domain:Ig-like C2-type 1	
SP_PIR_KEYWORDS	immunoglobulin domain	
UP_SEQ_FEATURE	domain:Ig-like C2-type 4	
UP_SEQ_FEATURE	domain:Ig-like C2-type 5	
KEGG_PATHWAY	bta04514:Cell adhesion molecules (CAMs)	
INTERPRO	IPR013098:Immunoglobulin I-set	
INTERPRO	IPR013151:Immunoglobulin	
SMART	SM00409:IG	
INTERPRO	IPR003599:Immunoglobulin subtype	
INTERPRO	IPR003598:Immunoglobulin subtype 2	
INTERPRO	IPR007110:Immunoglobulin-like	
INTERPRO	IPR013783:Immunoglobulin-like fold	
SMART	SM00408:IGc2	
**Functional Group 15**		0.022
GOTERM_CC_ALL	GO:0005578~proteinaceous extracellular matrix	
GOTERM_CC_ALL	GO:0031012~extracellular matrix	
GOTERM_CC_ALL	GO:0005576~extracellular region	
GOTERM_CC_ALL	GO:0044421~extracellular region part	
SP_PIR_KEYWORDS	extracellular matrix	
**Functional Group 16**		0.034
GOTERM_BP_ALL	GO:0065007~biological regulation	
GOTERM_BP_ALL	GO:0050789~regulation of biological process	
GOTERM_BP_ALL	GO:0050794~regulation of cellular process	
GOTERM_BP_ALL	GO:0019219~regulation of nucleobase, nucleoside, nucleotide and nucleic acid metabolic process	
GOTERM_BP_ALL	GO:0031323~regulation of cellular metabolic process	
GOTERM_BP_ALL	GO:0045449~regulation of transcription	
GOTERM_BP_ALL	GO:0019222~regulation of metabolic process	
GOTERM_BP_ALL	GO:0006350~transcription	
GOTERM_BP_ALL	GO:0006355~regulation of transcription, DNA-dependent	
GOTERM_BP_ALL	GO:0032774~RNA biosynthetic process	
GOTERM_BP_ALL	GO:0006351~transcription, DNA-dependent	
GOTERM_BP_ALL	GO:0010468~regulation of gene expression	

GOTERM_BP_ALL	GO:0016070~RNA metabolic process	
GOTERM_BP_ALL	GO:0006139~nucleobase, nucleoside, nucleotide and nucleic acid metabolic process	
GOTERM_BP_ALL	GO:0043283~biopolymer metabolic process	
GOTERM_MF_ALL	GO:0003677~DNA binding	
GOTERM_BP_ALL	GO:0010467~gene expression	
GOTERM_MF_ALL	GO:0030528~transcription regulator activity	
GOTERM_MF_ALL	GO:0003700~transcription factor activity	
GOTERM_CC_ALL	GO:0005634~nucleus	
GOTERM_MF_ALL	GO:0043565~sequence-specific DNA binding	
GOTERM_MF_ALL	GO:0003676~nucleic acid binding	
**Functional Group 17**		0.039
GOTERM_BP_ALL	GO:0007389~pattern specification process	
GOTERM_BP_ALL	GO:0009887~organ morphogenesis	
GOTERM_BP_ALL	GO:0014032~neural crest cell development	
GOTERM_BP_ALL	GO:0014033~neural crest cell differentiation	
GOTERM_BP_ALL	GO:0001763~morphogenesis of a branching structure	
GOTERM_BP_ALL	GO:0048754~branching morphogenesis of a tube	
GOTERM_BP_ALL	GO:0009792~embryonic development ending in birth or egg hatching	
GOTERM_BP_ALL	GO:0043009~chordate embryonic development	
GOTERM_BP_ALL	GO:0009880~embryonic pattern specification	
GOTERM_BP_ALL	GO:0001569~patterning of blood vessels	
GOTERM_BP_ALL	GO:0048762~mesenchymal cell differentiation	
GOTERM_BP_ALL	GO:0014031~mesenchymal cell development	
GOTERM_BP_ALL	GO:0035295~tube development	
GOTERM_BP_ALL	GO:0035239~tube morphogenesis	
GOTERM_BP_ALL	GO:0001568~blood vessel development	
GOTERM_BP_ALL	GO:0001944~vasculature development	
GOTERM_BP_ALL	GO:0001701~in utero embryonic development	
GOTERM_BP_ALL	GO:0045934~negative regulation of nucleobase, nucleoside, nucleotide and nucleic acid metabolic process	
GOTERM_BP_ALL	GO:0048514~blood vessel morphogenesis	
GOTERM_BP_ALL	GO:0001525~angiogenesis	
GOTERM_BP_ALL	GO:0009790~embryonic development	
GOTERM_BP_ALL	GO:0048646~anatomical structure formation	
GOTERM_BP_ALL	GO:0007507~heart development	
GOTERM_BP_ALL	GO:0009892~negative regulation of metabolic process	
GOTERM_BP_ALL	GO:0031324~negative regulation of cellular metabolic process	
GOTERM_BP_ALL	GO:0009889~regulation of biosynthetic process	
GOTERM_BP_ALL	GO:0008284~positive regulation of cell proliferation	

GOTERM_BP_ALL	GO:0016481~negative regulation of transcription	
GOTERM_BP_ALL	GO:0046903~secretion	
**Functional Group 18**		0.039
SP_PIR_KEYWORDS	Wnt signaling pathway	
KEGG_PATHWAY	Wnt signaling pathway	
GOTERM_BP_ALL	GO:0016055~Wnt receptor signaling pathway	
**Functional Group 19**		0.046
GOTERM_MF_ALL	GO:0004714~transmembrane receptor protein tyrosine kinase activity	
GOTERM_MF_ALL	GO:0004713~protein-tyrosine kinase activity	
UP_SEQ_FEATURE	binding site:ATP	
INTERPRO	IPR001245:Tyrosine protein kinase	
INTERPRO	IPR008266:Tyrosine protein kinase, active site	
SMART	SM00219:TyrKc	
SP_PIR_KEYWORDS	tyrosine-protein kinase	
**Functional Group 20**		0.049
INTERPRO	IPR003645:Follistatin-like, N-terminal	
SMART	SM00274:FOLN	
INTERPRO	IPR002350:Proteinase inhibitor I1, Kazal	
SMART	SM00280:KAZAL	
**Functional Group 21**		0.050
GOTERM_BP_ALL	GO:0009653~anatomical structure morphogenesis	
GOTERM_BP_ALL	GO:0032989~cellular structure morphogenesis	
GOTERM_BP_ALL	GO:0000902~cell morphogenesis	
GOTERM_BP_ALL	GO:0016049~cell growth	
GOTERM_BP_ALL	GO:0008361~regulation of cell size	
GOTERM_BP_ALL	GO:0040007~growth	
GOTERM_BP_ALL	GO:0001558~regulation of cell growth	
GOTERM_BP_ALL	GO:0040008~regulation of growth	
GOTERM_BP_ALL	GO:0016043~cellular component organization and biogenesis	
**Functional Group 22**		0.050
SP_PIR_KEYWORDS	cell shape	
GOTERM_BP_ALL	GO:0050793~regulation of developmental process	
GOTERM_BP_ALL	GO:0022603~regulation of anatomical structure morphogenesis	
GOTERM_BP_ALL	GO:0008360~regulation of cell shape	

		GOTERM_BP_ALL	GO:0022604~regulation of cell morphogenesis	
**FFUD**	Transient change in response to birth	**Functional Group 1**		0.014
		GOTERM_MF_ALL	GO:0032553~ribonucleotide binding	
		GOTERM_MF_ALL	GO:0032555~purine ribonucleotide binding	
		GOTERM_MF_ALL	GO:0017076~purine nucleotide binding	
		GOTERM_MF_ALL	GO:0000166~nucleotide binding	
		GOTERM_MF_ALL	GO:0005524~ATP binding	
		GOTERM_MF_ALL	GO:0032559~adenyl ribonucleotide binding	
		GOTERM_MF_ALL	GO:0030554~adenyl nucleotide binding	
		SP_PIR_KEYWORDS	nucleotide-binding	
		**Functional Group 2**		0.024
		GOTERM_BP_ALL	GO:0006082~organic acid metabolic process	
		GOTERM_BP_ALL	GO:0019752~carboxylic acid metabolic process	
		KEGG_PATHWAY	Glycine, serine and threonine metabolism	
		GOTERM_BP_ALL	GO:0006519~amino acid and derivative metabolic process	
		GOTERM_BP_ALL	GO:0009308~amine metabolic process	
		GOTERM_BP_ALL	GO:0006807~nitrogen compound metabolic process	
		GOTERM_BP_ALL	GO:0006520~amino acid metabolic process	
		GOTERM_BP_ALL	GO:0032787~monocarboxylic acid metabolic process	
**FFDU**	Transient change in response to birth	**Functional Group 1**		0.036
		GOTERM_BP_ALL	GO:0022610~biological adhesion	
		GOTERM_BP_ALL	GO:0007155~cell adhesion	
		SP_PIR_KEYWORDS	cell adhesion	
**FFDD**	Continuation of the developmental switch between late fetal development and birth into the young lamb			N.S.
**FUFF**	Fetal changes in preparation for the major	**Functional Group 1**		0.007
		SP_PIR_KEYWORDS	glycoprotein	
		SP_PIR_KEYWORDS	signal	

	developmental switch between late fetal development and birth.	UP_SEQ_FEATURE	glycosylation site:N-linked (GlcNAc...)	
		UP_SEQ_FEATURE	signal peptide	
		UP_SEQ_FEATURE	disulfide bond	
		**Functional Group 2**		0.034
		GOTERM_BP_ALL	GO:0002449~lymphocyte mediated immunity	
		GOTERM_BP_ALL	GO:0002443~leukocyte mediated immunity	
		GOTERM_BP_ALL	GO:0045087~innate immune response	
		GOTERM_BP_ALL	GO:0002252~immune effector process	
		GOTERM_BP_ALL	GO:0048518~positive regulation of biological process	
		GOTERM_BP_ALL	GO:0002376~immune system process	
		GOTERM_BP_ALL	GO:0006955~immune response	
		GOTERM_BP_ALL	GO:0006950~response to stress	
		GOTERM_BP_ALL	GO:0050896~response to stimulus	
		GOTERM_BP_ALL	GO:0032501~multicellular organismal process	
		GOTERM_BP_ALL	GO:0006952~defense response	
**FDFF**	Fetal changes in preparation for the major developmental switch between late fetal development and birth.			N.S.
**UFFF**	Fetal changes in preparation for the major developmental switch between late fetal development and birth.	**Functional Group 1**		0.003
		GOTERM_MF_ALL	GO:0004872~receptor activity	
		GOTERM_MF_ALL	GO:0060089~molecular transducer activity	
		GOTERM_MF_ALL	GO:0004871~signal transducer activity	
		GOTERM_MF_ALL	GO:0004888~transmembrane receptor activity	
		SP_PIR_KEYWORDS	receptor	
		**Functional Group 2**		0.021
		SP_PIR_KEYWORDS	cell junction	
		GOTERM_CC_ALL	GO:0030054~cell junction	
		GOTERM_CC_ALL	GO:0044459~plasma membrane part	
		GOTERM_CC_ALL	GO:0005886~plasma membrane	
		UP_SEQ_FEATURE	topological domain:Extracellular	

GOTERM_CC_ALL	GO:0016020~membrane	
SP_PIR_KEYWORDS	glycoprotein	
UP_SEQ_FEATURE	topological domain:Cytoplasmic	
SP_PIR_KEYWORDS	membrane	
GOTERM_CC_ALL	GO:0044425~membrane part	
SP_PIR_KEYWORDS	transmembrane	
GOTERM_CC_ALL	GO:0016021~integral to membrane	
GOTERM_BP_ALL	GO:0022610~biological adhesion	
GOTERM_BP_ALL	GO:0007155~cell adhesion	
GOTERM_CC_ALL	GO:0031224~intrinsic to membrane	
UP_SEQ_FEATURE	transmembrane region	
UP_SEQ_FEATURE	glycosylation site:N-linked (GlcNAc...)	
UP_SEQ_FEATURE	disulfide bond	
**Functional Group 3**		0.023
SP_PIR_KEYWORDS	cell junction	
SP_PIR_KEYWORDS	calcium binding	
GOTERM_CC_ALL	GO:0030054~cell junction	
INTERPRO	IPR014868:Cadherin prodomain like	
PIR_SUPERFAMILY	PIRSF002504:cadherin	
GOTERM_CC_ALL	GO:0044459~plasma membrane part	
INTERPRO	IPR000233:Cadherin cytoplasmic region	
GOTERM_BP_ALL	GO:0016337~cell-cell adhesion	
INTERPRO	IPR002126:Cadherin	
GOTERM_BP_ALL	GO:0007156~homophilic cell adhesion	
KEGG_PATHWAY	Cell adhesion molecules (CAMs)	
SMART	SM00112:CA	
SP_PIR_KEYWORDS	cell adhesion	
GOTERM_BP_ALL	GO:0022610~biological adhesion	
GOTERM_BP_ALL	GO:0007155~cell adhesion	
GOTERM_MF_ALL	GO:0005509~calcium ion binding	
SP_PIR_KEYWORDS	calcium	
SP_PIR_KEYWORDS	duplication	
SP_PIR_KEYWORDS	Cleavage on pair of basic residues	
GOTERM_MF_ALL	GO:0043167~ion binding	
GOTERM_MF_ALL	GO:0046872~metal ion binding	
GOTERM_MF_ALL	GO:0043169~cation binding	
**Functional Group 4**		0.028

		GOTERM_MF_ALL	GO:0004888~transmembrane receptor activity	
		KEGG_PATHWAY	bta04060:Cytokine-cytokine receptor interaction	
		INTERPRO	IPR003961:Fibronectin, type III	
		SMART	SM00060:FN3	
		INTERPRO	IPR008957:Fibronectin, type III-like fold	
		KEGG_PATHWAY	Jak-STAT signaling pathway	
**DFFF**	Fetal changes in preparation for the major developmental switch between late fetal development and birth.			N.S.
**DUFF**	Transient fetal change			N.S.

[1]Functional annotation clustering of genes present in each expression cluster was performed using DAVID {Dennis, 2003 #707; Huang da, 2009 #708}. The geometric mean of the p-values for terms in each functional cluster is listed. Functional annotation clustering displays similar annotations together based on overlaps of genes associated with each term to give a higher order perspective of the information. [2]D (Down), significantly decreased expression; U (Up), significantly increased expression; F (Flat), no significant change. Each symbol represents the direction of change in gene expression between any two adjacent developmental time points. Each cluster is represented by these symbols across four developmental intervals. Only clusters containing 50 or more genes are shown . [3]N.S., not significant.
