# Supplementary material for: A gene network switch enhances the oxidative capacity of ovine skeletal muscle during late fetal development
Source: BMC Genomics. 2010 Jun 15;11:378. doi: 10.1186/1471-2164-11-378 (PMC2894804; doi:10.1186/1471-2164-11-378)
Supplement: Additional File 4 — Primer sequences used for quantitative RT-PCR analyses. Primer sequences used for quantitative RT-PCR analyses. [file 1471-2164-11-378-S4.PDF]

Additional File 4. Primer sequences used for quantitative RT-PCR analyses.

Gene-name (accession number)	Position	Forward (5′ to 3′)	Reverse (5′ to 3′)	Amplicon size (bp)
18S rRNA (DQ222453.1)	1580-1731	gtaacccgttgaaccccatt	ccatccaatcggtagtagcg	152
DLK1 (AF354168) (C2 variant)[2]	59000-59012, 61033-61247	tgcgtggtgaatggctc	ggctgcaggtcttgtcca	229
MYF5 (NM_174116)[1]	265-459	tggctgctttcggggctcac	ggttgaccttcttcaggcgtctcc	195
GTL2 (AF354168)	148045-157841	gatcgtgaacgcctggac	aggattccagatgatggcttc	141
RTL1 (AF354168)	190327-190034	cttccactctccctactgcct	gcatccacaggttcccac	294
MEG8 (AF354168)	200893-201457	cccagggagtgtgaggctcttct	ggacccacggctgacctgtt	100
FOS (NM_182786)[1]	729 - 854	tcatcctagcggctcaccgacc	cctcagattcaggggtggcagc	126
MYH1 (AB059399)[1]	5727-5904	ggaggaacaatccaatgtcaac	gtcactttttagcatttggatgagtta	178
MYH7 (AB059400)[1]	5583-5832	aagaacctgctgcggctg	ccaagatgtggcacggct	250
MYH2 (AB059398)[1]	5683 -5855	gaggaacaatccaatacaaatctatct	cccatagcatcaggacacga	173
MYH8 (XM_865879)[1]	5661 - 5905	atcgcaagaatgtactcaggttg	caggtgtgttcactctgcact	245
MYH3 (NM_001101835)[1]	5850 - 6010	tctcaccaagttccgaaaagc	gtcgcagaggaggggct	161
CEBPD (AJ_276820)	1013 - 1180	acgcgagcgcaacaacatcg	tgcttgaagaagcgccgcag	168
PPARG (NM_181024)[1]	786 - 1010	atgtctcataatgccatcaggtt	gataacaaacggtgatttgtctgtc	225
PPARD (NM_001083636)[1]	1064 - 1225	ccagctttggcgacctctt	ttcggaggctgcgcaggaa	162
PPP3CA (NM 174787)[1]	1489 - 1658	cagcggcgtgctttctggag	tggcatctctgcgaggtggc	170
PEG3 (AY427787)[1]	8501 - 8656	ttgcctttaaagagtgggttgt	actggtcacttaaggcaggaag	156
SIRT1 (XM_864818.2)[1]	1395 - 1618	gcctcacctgcattttgatgtagag	ttctttctggtgaacttgagccttc	224
ACTA1 (NM_174225)[1]	1078 - 1345	agcaccatgaagatcaagatcat	cgctgatggttggagagc	268
LPL (NM_001075120)[1]	1318 - 1616	taccctgcctgaagtttccac	cccagtttcagccagactttc	299
CD36 (NM_174010)[1]	1364 - 1560	tggtgtgctagacattggcaaatg	tgttgacctgcagccgttttgc	197

[1] Derived from bovine sequence.
[2] Primers traverse mRNA splice sites.
