# Supplementary material for: A gene network switch enhances the oxidative capacity of ovine skeletal muscle during late fetal development
Source: BMC Genomics. 2010 Jun 15;11:378. doi: 10.1186/1471-2164-11-378 (PMC2894804; doi:10.1186/1471-2164-11-378)
Supplement: Additional File 1 — Gene expression data. This file contains links to the gene expression data. The genes that are significantly differentially expressed between adjacent development times are listed as Experiment 1 (80 d vs 100 d), Experiment 2 (100 d vs 120 d), Experiment 3 (120 d vs 150 d) and Experiment 4 (150 d vs 230 d) in the section entitled Open microarray analysis report for developmental expression. Each experiment contains an MA plot, Volcano plot and Heatmap for each of the three microarray processing programs MAS5, RMA and GCRMA. For each of these processing programs there is a list of significantly differentially expressed probe sets, and for each of these there is an FDR corrected probability, fold change, percentage of microarrays with a MAS5 Present call, and a convergence percentage for all three microarray processing programs. The file also contains annotation of the probe sets. In a second section entitled Time series analysis of developmental expression, there is a list of genes in each gene expression cluster and diagrams showing MAS5 gene expression values for these genes. [file 1471-2164-11-378-S1.ZIP › Report/AdditionalQCs.html]

Percent of calls detected as present in each array using the MAS5 method with tau=0.015, alpha1=0.04 and alpha2=0.06.Experiment mean: 42.79%. Per slide:

| Reference | CEL File | Percent Present |
| --- | --- | --- |
| d80\_1 | af1c1228-5-219-161007.CEL | 44.67 % |
| d80\_2 | af1c1228-6-232-161007.CEL | 49.85 % |
| d80\_3 | af1c1228-7-244-161007.CEL | 47.18 % |
| d100\_1 | af1c1228-2-204-161007.CEL | 44.31 % |
| d100\_2 | af1c1228-3-205-161007.CEL | 48.3 % |
| d100\_3 | af1c1228-9-288-161007.CEL | 48.57 % |
| d120\_1 | af1c1228-1-196-161007.CEL | 43.12 % |
| d120\_2 | af1c1228-4-215-161007.CEL | 44.93 % |
| d120\_3 | af1c1228-8-261-161007.CEL | 49.3 % |
| d150\_1 | af1c1228-12-4555-161007.CEL | 37.33 % |
| d150\_2 | af1c1228-14-4576-161007.CEL | 40.81 % |
| d150\_3 | af1c1228-15-4580-161007.CEL | 38.62 % |
| d230\_1 | af1c1228-10-4531-161007.CEL | 35.7 % |
| d230\_2 | af1c1228-11-4544-161007.CEL | 36.57 % |
| d230\_3 | af1c1228-13-4572-161007.CEL | 32.61 % |

BioB, BioC, BioD and Cre controls. All should be detected as present. Arrays marked as absent or marginal for:

BioB: None

BioC: None

BioD: None

Cre: None

Percentage of probes for which the MM intensity is greater than the PM intensity. Experiment mean: 28.7%. Mean per slide:

| Reference | CEL File | Percent MM>PM |
| --- | --- | --- |
| d80\_1 | af1c1228-5-219-161007.CEL | 28.17 % |
| d80\_2 | af1c1228-6-232-161007.CEL | 26.11 % |
| d80\_3 | af1c1228-7-244-161007.CEL | 27.12 % |
| d100\_1 | af1c1228-2-204-161007.CEL | 28.14 % |
| d100\_2 | af1c1228-3-205-161007.CEL | 26.8 % |
| d100\_3 | af1c1228-9-288-161007.CEL | 26.84 % |
| d120\_1 | af1c1228-1-196-161007.CEL | 28.64 % |
| d120\_2 | af1c1228-4-215-161007.CEL | 27.87 % |
| d120\_3 | af1c1228-8-261-161007.CEL | 26.18 % |
| d150\_1 | af1c1228-12-4555-161007.CEL | 30.5 % |
| d150\_2 | af1c1228-14-4576-161007.CEL | 29.34 % |
| d150\_3 | af1c1228-15-4580-161007.CEL | 29.7 % |
| d230\_1 | af1c1228-10-4531-161007.CEL | 31.43 % |
| d230\_2 | af1c1228-11-4544-161007.CEL | 31.02 % |
| d230\_3 | af1c1228-13-4572-161007.CEL | 32.64 % |
