# Supplementary material for: A gene network switch enhances the oxidative capacity of ovine skeletal muscle during late fetal development
Source: BMC Genomics. 2010 Jun 15;11:378. doi: 10.1186/1471-2164-11-378 (PMC2894804; doi:10.1186/1471-2164-11-378)
Supplement: Additional File 1 — Gene expression data. This file contains links to the gene expression data. The genes that are significantly differentially expressed between adjacent development times are listed as Experiment 1 (80 d vs 100 d), Experiment 2 (100 d vs 120 d), Experiment 3 (120 d vs 150 d) and Experiment 4 (150 d vs 230 d) in the section entitled Open microarray analysis report for developmental expression. Each experiment contains an MA plot, Volcano plot and Heatmap for each of the three microarray processing programs MAS5, RMA and GCRMA. For each of these processing programs there is a list of significantly differentially expressed probe sets, and for each of these there is an FDR corrected probability, fold change, percentage of microarrays with a MAS5 Present call, and a convergence percentage for all three microarray processing programs. The file also contains annotation of the probe sets. In a second section entitled Time series analysis of developmental expression, there is a list of genes in each gene expression cluster and diagrams showing MAS5 gene expression values for these genes. [file 1471-2164-11-378-S1.ZIP › Report/AnnotationExp1.html]

## Annotation of differentially expressed genes for experiment 1.

The links connect to external resources. The first column is the probe ID and links to the Affymetrix annotation site. The second column links to Entrez. The third column shows how many times the Entrez gene ID was detected as significantly differentially expressed and how many probes target the gene on the chip - this provides an additional indication of reliability of differential expression. The fourth column is the RefSeq annotation (there can be more than one link). The fifth column is the RefSeq annotation provided by SheepGenomics (it should be more reliable than the original Affymetrix annotation). The sixth column is the gene ontology annotation. The seventh column links to Pubmed. The eighth column links to the Gene Symbol in Entrez. The last column links to SheepGenomics. Note: the GO and Pubmed annotation were derived from the EntrezGene ID, the gene symbols are based on the human annotation.

|  |  |  |  |  |  |  |  |  |  |  |  |  |  |  |  |  |  |  |  |  |  |  |  |  |  |  |  |  |  |  |  |  |  |  |  |  |  |  |  |  |  |  |  |  |  |  |  |  |  |  |  |  |  |  |  |  |  |  |  |  |  |  |  |  |  |  |  |  |  |  |  |  |  |  |  |  |  |  |  |  |  |  |  |  |  |  |  |  |  |  |  |  |  |  |  |  |  |  |  |  |  |  |  |  |  |  |  |  |  |  |  |  |  |  |  |  |  |  |  |  |  |  |  |  |  |  |  |  |  |  |  |  |  |  |  |  |  |  |  |  |  |  |  |  |  |  |  |  |  |  |  |  |  |  |  |  |  |  |  |  |  |  |  |  |  |  |  |  |  |  |  |  |  |  |  |  |  |  |  |  |  |  |  |  |  |  |  |  |  |  |  |  |  |  |  |  |  |  |  |  |  |  |  |  |  |  |  |  |  |  |  |  |  |  |  |  |  |  |  |  |  |  |  |  |  |  |  |  |  |  |  |  |  |  |  |  |  |  |  |  |  |  |  |  |  |  |  |  |  |  |  |  |  |  |  |  |  |  |  |  |  |  |  |  |  |  |  |  |  |  |  |  |  |  |  |  |  |  |  |  |  |  |  |  |  |  |  |  |  |  |  |  |  |  |  |  |  |  |  |  |  |  |  |  |  |  |  |  |  |  |  |  |  |  |  |  |  |  |  |  |  |  |  |  |  |  |  |  |  |  |  |  |  |  |  |  |  |  |  |  |  |  |  |  |  |  |  |  |  |  |  |  |  |  |  |  |  |  |  |  |  |  |  |  |  |  |  |  |  |  |  |  |  |  |  |  |  |  |  |  |  |  |  |  |  |  |  |  |  |  |  |  |  |  |  |  |  |  |  |  |  |  |  |  |  |  |  |  |  |  |  |  |  |  |  |  |  |  |  |  |  |  |  |  |  |  |  |  |  |  |  |  |  |  |  |  |  |  |  |  |  |  |  |  |  |  |  |  |  |  |  |  |  |  |  |  |  |  |  |  |  |  |  |  |  |  |  |  |  |  |  |  |  |  |  |  |  |  |  |  |  |  |  |  |  |  |  |  |  |  |  |  |  |  |  |  |  |  |  |  |  |  |  |  |  |  |  |  |  |  |  |  |  |  |  |  |  |  |  |  |  |  |  |  |  |  |  |  |  |  |  |  |  |  |  |  |  |  |  |  |  |  |  |  |  |  |  |  |  |  |  |  |  |  |  |  |  |  |  |  |  |  |  |  |  |  |  |  |  |  |  |  |  |  |  |  |  |  |  |  |  |  |  |  |  |  |  |  |  |  |  |  |  |
| --- | --- | --- | --- | --- | --- | --- | --- | --- | --- | --- | --- | --- | --- | --- | --- | --- | --- | --- | --- | --- | --- | --- | --- | --- | --- | --- | --- | --- | --- | --- | --- | --- | --- | --- | --- | --- | --- | --- | --- | --- | --- | --- | --- | --- | --- | --- | --- | --- | --- | --- | --- | --- | --- | --- | --- | --- | --- | --- | --- | --- | --- | --- | --- | --- | --- | --- | --- | --- | --- | --- | --- | --- | --- | --- | --- | --- | --- | --- | --- | --- | --- | --- | --- | --- | --- | --- | --- | --- | --- | --- | --- | --- | --- | --- | --- | --- | --- | --- | --- | --- | --- | --- | --- | --- | --- | --- | --- | --- | --- | --- | --- | --- | --- | --- | --- | --- | --- | --- | --- | --- | --- | --- | --- | --- | --- | --- | --- | --- | --- | --- | --- | --- | --- | --- | --- | --- | --- | --- | --- | --- | --- | --- | --- | --- | --- | --- | --- | --- | --- | --- | --- | --- | --- | --- | --- | --- | --- | --- | --- | --- | --- | --- | --- | --- | --- | --- | --- | --- | --- | --- | --- | --- | --- | --- | --- | --- | --- | --- | --- | --- | --- | --- | --- | --- | --- | --- | --- | --- | --- | --- | --- | --- | --- | --- | --- | --- | --- | --- | --- | --- | --- | --- | --- | --- | --- | --- | --- | --- | --- | --- | --- | --- | --- | --- | --- | --- | --- | --- | --- | --- | --- | --- | --- | --- | --- | --- | --- | --- | --- | --- | --- | --- | --- | --- | --- | --- | --- | --- | --- | --- | --- | --- | --- | --- | --- | --- | --- | --- | --- | --- | --- | --- | --- | --- | --- | --- | --- | --- | --- | --- | --- | --- | --- | --- | --- | --- | --- | --- | --- | --- | --- | --- | --- | --- | --- | --- | --- | --- | --- | --- | --- | --- | --- | --- | --- | --- | --- | --- | --- | --- | --- | --- | --- | --- | --- | --- | --- | --- | --- | --- | --- | --- | --- | --- | --- | --- | --- | --- | --- | --- | --- | --- | --- | --- | --- | --- | --- | --- | --- | --- | --- | --- | --- | --- | --- | --- | --- | --- | --- | --- | --- | --- | --- | --- | --- | --- | --- | --- | --- | --- | --- | --- | --- | --- | --- | --- | --- | --- | --- | --- | --- | --- | --- | --- | --- | --- | --- | --- | --- | --- | --- | --- | --- | --- | --- | --- | --- | --- | --- | --- | --- | --- | --- | --- | --- | --- | --- | --- | --- | --- | --- | --- | --- | --- | --- | --- | --- | --- | --- | --- | --- | --- | --- | --- | --- | --- | --- | --- | --- | --- | --- | --- | --- | --- | --- | --- | --- | --- | --- | --- | --- | --- | --- | --- | --- | --- | --- | --- | --- | --- | --- | --- | --- | --- | --- | --- | --- | --- | --- | --- | --- | --- | --- | --- | --- | --- | --- | --- | --- | --- | --- | --- | --- | --- | --- | --- | --- | --- | --- | --- | --- | --- | --- | --- | --- | --- | --- | --- | --- | --- | --- | --- | --- | --- | --- | --- | --- | --- | --- | --- | --- | --- | --- | --- | --- | --- | --- | --- | --- | --- | --- | --- | --- | --- | --- | --- | --- | --- | --- | --- | --- | --- | --- | --- | --- | --- | --- | --- | --- | --- | --- | --- | --- | --- | --- | --- | --- | --- | --- | --- | --- | --- | --- | --- | --- | --- | --- | --- | --- | --- | --- | --- | --- | --- | --- | --- | --- | --- | --- | --- | --- | --- | --- | --- | --- | --- | --- | --- | --- | --- | --- | --- | --- | --- | --- | --- | --- | --- | --- | --- | --- | --- | --- | --- | --- | --- | --- | --- | --- | --- | --- | --- | --- | --- | --- | --- | --- | --- | --- | --- | --- | --- | --- | --- | --- | --- | --- | --- | --- | --- | --- | --- | --- | --- | --- | --- | --- | --- | --- | --- | --- | --- | --- |
| AFFY Probe | Entrez Gene | Dif. Expressed | RefSeq | SG RefSeq | Gene Ontology | Pubmed | Gene Symbol | SheepGenomics\|  \|  \|  \|  \|  \|  \|  \|  \|  \| \| --- \| --- \| --- \| --- \| --- \| --- \| --- \| --- \| --- \| \| Bt.12300.1.S1\_at \| 281337 \| 3/6 \| NM\_174117 \| NM\_174117 \| GO:0000166  GO:0003774  GO:0003779  GO:0005516  GO:0005524  GO:0005863  GO:0006941  GO:0016459 \| 9880677 \| MYH1 \| Bt.12300.1.S1\_at \| \| Bt.15968.1.S1\_at \| 539139 \| 1/1 \| XM\_873620 \|  \|  \|  \| KLF9 \| Bt.15968.1.S1\_at \| \| Bt.21761.1.S1\_a\_at \| 505645 \| 1/1 \| NM\_001075215 \| NM\_001075215 \|  \|  \| TMOD4 \| Bt.21761.1.S1\_a\_at \| \| Bt.29854.1.S1\_x\_at \| 281337 \| 3/6 \| NM\_174117 \|  \| GO:0000166  GO:0003774  GO:0003779  GO:0005516  GO:0005524  GO:0005863  GO:0006941  GO:0016459 \| 9880677 \|  \| Bt.29854.1.S1\_x\_at \| \| Bt.25658.1.A1\_at \| 618440 \| 1/1 \| NM\_001035501 \|  \|  \| 12477932 \| RPL3L \| Bt.25658.1.A1\_at \| \| Bt.29697.1.S1\_at \| 407147 \| 1/2 \| NM\_001040496 \|  \| GO:0003956  GO:0006471  GO:0016740 \|  \| ART3 \| Bt.29697.1.S1\_at \| \| Bt.25689.1.S1\_at \|  \|  \|  \| XM\_001252094 \|  \|  \|  \| Bt.25689.1.S1\_at \| \| Bt.6802.1.S1\_at \|  \|  \|  \|  \|  \|  \| RGS5 \| Bt.6802.1.S1\_at \| \| Bt.3804.1.S1\_at \| 513618 \| 1/1 \| NM\_001075629 \| NM\_001075629 \|  \|  \| PCOLCE2 \| Bt.3804.1.S1\_at \| \| Bt.27281.1.S1\_at \|  \|  \|  \|  \|  \|  \| ZBTB20 \| Bt.27281.1.S1\_at \| \| Bt.26688.2.S1\_a\_at \|  \|  \|  \|  \|  \|  \| FRMD3 \| Bt.26688.2.S1\_a\_at \| \| Bt.28208.1.S1\_at \| 514066 \| 1/1 \| NM\_001046164 \| NM\_001046164 \| GO:0005975  GO:0006094  GO:0016787  GO:0042132  GO:0042578 \|  \|  \| Bt.28208.1.S1\_at \| \| Bt.5129.2.A1\_at \|  \|  \|  \|  \|  \|  \| NNAT \| Bt.5129.2.A1\_at \| \| Bt.20512.2.S1\_at \| 537532 \| 1/1 \|  \|  \|  \|  \| COL15A1 \| Bt.20512.2.S1\_at \| \| Bt.20512.1.S1\_at \|  \|  \|  \|  \|  \|  \| COL15A1 \| Bt.20512.1.S1\_at \| \| Bt.2023.1.S1\_at \| 525407 \| 1/1 \| XR\_027887 \| XR\_027887 \|  \|  \|  \| Bt.2023.1.S1\_at \| \| Bt.9773.1.A1\_at \|  \|  \|  \|  \|  \|  \| ZBTB20 \| Bt.9773.1.A1\_at \| \| Bt.7084.1.S1\_at \| 539375 \| 1/1 \| NM\_001076157 \| NM\_001076157 \| GO:0003779  GO:0005509 \|  \| ACTN3 \| Bt.7084.1.S1\_at \| \| Bt.10278.1.S1\_at \| 777789 \| 1/1 \| NM\_001078164  XM\_001251769 \|  \|  \|  \| ORF1-FL49 \| Bt.10278.1.S1\_at \| \| Bt.7575.1.A1\_at \|  \|  \|  \| XM\_870732 \|  \|  \| GPT2 \| Bt.7575.1.A1\_at \| \| Bt.1390.1.A1\_at \|  \|  \|  \|  \|  \|  \| KBTBD8 \| Bt.1390.1.A1\_at \| \| Bt.3980.1.S1\_at \| 281478 \| 1/1 \| NM\_174177 \|  \|  \| 1651929  1847925  2153078  7891673  8119933  8780652  15686479 \| SCIN \| Bt.3980.1.S1\_at \| \| Bt.5848.1.S1\_at \| 518117 \| 1/2 \| NM\_001075767 \|  \| GO:0000166  GO:0000287  GO:0005388  GO:0005509  GO:0005515  GO:0005524  GO:0006812  GO:0006816  GO:0008152  GO:0015992  GO:0016020  GO:0016021  GO:0016529  GO:0016787  GO:0016820  GO:0031448 \|  \| ATP2A1 \| Bt.5848.1.S1\_at \| \| Bt.9012.1.S1\_at \| 508247 \| 1/1 \| XM\_585000 \| XM\_585000 \|  \|  \| KIAA1639 \| Bt.9012.1.S1\_at \| \| Bt.12825.1.S1\_at \| 515610 \| 1/1 \| NM\_001034502 \| NM\_001034502 \| GO:0000166  GO:0001725  GO:0005198  GO:0005515  GO:0005515  GO:0005524  GO:0005524  GO:0005625  GO:0005829  GO:0005856  GO:0005865  GO:0005884  GO:0017022  GO:0030240  GO:0043531  GO:0048741 \| 499690  12477932  12658628 \| ACTA2 \| Bt.12825.1.S1\_at \| \| Bt.76.1.S1\_at \| 338086 \| 1/1 \| NM\_176674 \| NM\_176674 \| GO:0004129  GO:0005739  GO:0005746  GO:0006118  GO:0009055  GO:0016020  GO:0016021 \| 1316159  1717471  3006725  8638158  10089392  10771420 \| COX7A1 \| Bt.76.1.S1\_at \| \| Bt.3595.1.S1\_at \| 282660 \| 1/1 \| NM\_174707 \|  \| GO:0001503  GO:0005509  GO:0005576  GO:0007275  GO:0030154  GO:0030500  GO:0051216 \| 1939157  3387234  3877721  8061611  12477932  12658628  14587031  15456771  15982861 \| MGP \| Bt.3595.1.S1\_at \| \| Bt.2911.1.A1\_at \| 540068 \| 1/2 \| NM\_001015672 \|  \|  \| 11282978  12477932 \| FLJ20152 \| Bt.2911.1.A1\_at \| \| Bt.23123.1.S1\_at \| 506945 \| 1/2 \| NM\_001024929 \| NM\_001024929 \|  \| 11282978 \| BHLHB2 \| Bt.23123.1.S1\_at \| \| Bt.9202.1.S1\_at \| 511711 \| 1/2 \| NM\_001046097 \|  \| GO:0005102  GO:0007165 \|  \| FGL2 \| Bt.9202.1.S1\_at \| \| Bt.4071.1.S1\_at \| 616916 \| 1/2 \| XM\_869056 \|  \|  \|  \| C2orf23 \| Bt.4071.1.S1\_at \| \| Bt.22169.1.S1\_at \| 540303 \| 1/1 \| NM\_001034702 \| NM\_001034702 \| GO:0000015  GO:0000287  GO:0004634  GO:0006096  GO:0016829 \| 12477932 \| ENO3 \| Bt.22169.1.S1\_at \| \| Bt.13980.2.A1\_a\_at \| 538944 \| 2/2 \| NM\_001034656 \|  \| GO:0000166  GO:0004111  GO:0005524  GO:0005739 \| 12477932 \|  \| Bt.13980.2.A1\_a\_at \| \| Bt.4417.1.S1\_at \| 281062 \| 1/1 \| XM\_001250829 \| XM\_001250829 \|  \| 2390969 \|  \| Bt.4417.1.S1\_at \| \| Bt.8736.2.S1\_at \|  \|  \|  \| XM\_598835 \|  \|  \| C1orf9 \| Bt.8736.2.S1\_at \| \| Bt.14676.1.S1\_at \| 540789 \| 1/1 \| XM\_001251104  XM\_613140 \| XM\_001251104 \| GO:0003950  GO:0005634  GO:0006471 \|  \| PARP14 \| Bt.14676.1.S1\_at \| \| Bt.12300.2.S1\_at \| 281337 \| 3/6 \| NM\_174117 \|  \| GO:0000166  GO:0003774  GO:0003779  GO:0005516  GO:0005524  GO:0005863  GO:0006941  GO:0016459 \| 9880677 \|  \| Bt.12300.2.S1\_at \| \| Bt.6630.1.S1\_at \| 534583 \| 1/1 \| NM\_001046379 \| NM\_001046379 \| GO:0003779  GO:0005516  GO:0031032 \|  \| CNN1 \| Bt.6630.1.S1\_at \| \| Bt.20822.1.A1\_at \|  \|  \|  \|  \|  \|  \|  \| Bt.20822.1.A1\_at \| \| Bt.22379.1.S1\_at \| 536925 \| 1/1 \| NM\_001046419 \| NM\_001046419 \| GO:0000166  GO:0005524  GO:0005739  GO:0006457  GO:0016301  GO:0016740 \| 11282978  16305752 \| CABC1 \| Bt.22379.1.S1\_at \| \| Bt.23217.1.S1\_at \| 515067 \| 1/1 \| NM\_001038111 \| NM\_001038111 \| GO:0004082  GO:0004083  GO:0004619  GO:0006096  GO:0008152  GO:0016787  GO:0016853 \|  \| PGAM2 \| Bt.23217.1.S1\_at \| \| Bt.5306.1.S1\_at \| 513257 \| 1/2 \| NM\_001014915 \| NM\_001014915 \| GO:0005515 \| 12477932 \| TCAP \| Bt.5306.1.S1\_at \| \| Bt.5129.1.S1\_a\_at \| 353114 \| 1/2 \| NM\_178323 \| NM\_178323 \|  \| 14668210 \| NNAT \| Bt.5129.1.S1\_a\_at \| \| Bt.23106.1.S1\_at \| 613381 \| 1/1 \| NM\_001034734 \|  \| GO:0005634 \| 12477932 \| HN1 \| Bt.23106.1.S1\_at \| \| Bt.11199.1.S1\_at \| 281939 \| 1/1 \| NM\_174397 \| NM\_174397 \| GO:0005198  GO:0005634 \|  \| MYOZ1 \| Bt.11199.1.S1\_at \| \| Bt.923.2.S1\_at \| 507116 \| 1/2 \| XM\_583674 \| NM\_001080234 \|  \|  \| ZCCHC3 \| Bt.923.2.S1\_at \| \| Bt.9996.1.S1\_at \| 533726 \| 1/1 \| XR\_027855 \| XR\_027855 \|  \|  \| DHCR24 \| Bt.9996.1.S1\_at \| \| Bt.13871.1.S1\_at \| 538856 \| 1/2 \|  \|  \|  \|  \| BCL6B \| Bt.13871.1.S1\_at \| \| Bt.25404.2.S1\_at \| 531254 \| 1/2 \| XM\_609742 \|  \|  \|  \| COL24A1 \| Bt.25404.2.S1\_at \| \| Bt.10349.1.S1\_at \| 532626 \| 1/1 \| NM\_001075969 \|  \| GO:0001584  GO:0004872  GO:0007186  GO:0016021 \|  \| GPR37 \| Bt.10349.1.S1\_at \| \| Bt.4437.1.S1\_at \| 282182 \| 1/2 \| NM\_174518 \| NM\_174518 \| GO:0005634  GO:0030528  GO:0045449 \| 12477932 \| CITED1 \| Bt.4437.1.S1\_at \| \| Bt.28193.1.S1\_at \|  \|  \|  \|  \|  \|  \| RICS \| Bt.28193.1.S1\_at \| \| Bt.3452.1.S1\_at \| 510718 \| 1/1 \| XM\_587895 \|  \| GO:0003735  GO:0005622  GO:0005840  GO:0006412 \|  \|  \| Bt.3452.1.S1\_at \| \| Bt.3879.1.S1\_at \| 286816 \| 1/2 \| NM\_174771 \| NM\_174771 \|  \| 2584239  3122824  6615417  7748902  10066775 \| TNNT2 \| Bt.3879.1.S1\_at \| \| Bt.20552.1.S1\_at \| 509593 \| 1/3 \| XR\_028343 \|  \|  \|  \|  \| Bt.20552.1.S1\_at \| \| Bt.17278.3.S1\_a\_at \| 505926 \| 1/4 \| XM\_582290 \| XM\_582290 \|  \|  \| BCAT1 \| Bt.17278.3.S1\_a\_at \| \| Bt.13980.1.A1\_at \| 538944 \| 2/2 \| NM\_001034656 \|  \| GO:0000166  GO:0004111  GO:0005524  GO:0005739 \| 12477932 \| CKMT2 \| Bt.13980.1.A1\_at \| \| Bt.28008.1.S1\_s\_at \| 404177 \| 1/1 \| NM\_205792  NM\_205814 \|  \| GO:0005216  GO:0005516  GO:0006813  GO:0016020  GO:0016021  GO:0016286 \| 17062575 \| KCNN2 \| Bt.28008.1.S1\_s\_at \| \| Bt.26077.1.A1\_at \|  \|  \|  \| XM\_593525 \|  \|  \| NRXN3 \| Bt.26077.1.A1\_at \| \| Bt.22856.1.S1\_at \| 281347 \| 1/1 \| XM\_582135 \| XM\_001252036 \|  \|  \| NEF3 \| Bt.22856.1.S1\_at \| \| Bt.10855.1.S1\_at \| 513055  616414 \| 1/1 \| NM\_001075596  XM\_868432 \| NM\_001075596 \|  \|  \|  \| Bt.10855.1.S1\_at \| \| Bt.20316.1.S1\_at \|  \|  \|  \| XM\_001249467 \|  \|  \| ANKRD23 \| Bt.20316.1.S1\_at \| \| Bt.14398.1.S1\_at \| 513254 \| 1/2 \| NM\_001034440  XM\_001250504 \| NM\_001034440 \|  \| 12477932 \| PEBP4 \| Bt.14398.1.S1\_at \| \| Bt.27202.1.A1\_at \| 523184 \| 1/1 \| NM\_001046265 \| NM\_001046265 \|  \|  \| SOSTDC1 \| Bt.27202.1.A1\_at \| \| Bt.5084.1.S1\_at \| 516210 \| 1/1 \| NM\_001015613 \| NM\_001015613 \| GO:0000166  GO:0004111  GO:0005524 \| 11003702  11282978  12477932  16305752 \| CKB \| Bt.5084.1.S1\_at \| |
