# Supplementary material for: A gene network switch enhances the oxidative capacity of ovine skeletal muscle during late fetal development
Source: BMC Genomics. 2010 Jun 15;11:378. doi: 10.1186/1471-2164-11-378 (PMC2894804; doi:10.1186/1471-2164-11-378)
Supplement: Additional File 1 — Gene expression data. This file contains links to the gene expression data. The genes that are significantly differentially expressed between adjacent development times are listed as Experiment 1 (80 d vs 100 d), Experiment 2 (100 d vs 120 d), Experiment 3 (120 d vs 150 d) and Experiment 4 (150 d vs 230 d) in the section entitled Open microarray analysis report for developmental expression. Each experiment contains an MA plot, Volcano plot and Heatmap for each of the three microarray processing programs MAS5, RMA and GCRMA. For each of these processing programs there is a list of significantly differentially expressed probe sets, and for each of these there is an FDR corrected probability, fold change, percentage of microarrays with a MAS5 Present call, and a convergence percentage for all three microarray processing programs. The file also contains annotation of the probe sets. In a second section entitled Time series analysis of developmental expression, there is a list of genes in each gene expression cluster and diagrams showing MAS5 gene expression values for these genes. [file 1471-2164-11-378-S1.ZIP › Report/AnnotationExp2.html]

## Annotation of differentially expressed genes for experiment 2.

The links connect to external resources. The first column is the probe ID and links to the Affymetrix annotation site. The second column links to Entrez. The third column shows how many times the Entrez gene ID was detected as significantly differentially expressed and how many probes target the gene on the chip - this provides an additional indication of reliability of differential expression. The fourth column is the RefSeq annotation (there can be more than one link). The fifth column is the RefSeq annotation provided by SheepGenomics (it should be more reliable than the original Affymetrix annotation). The sixth column is the gene ontology annotation. The seventh column links to Pubmed. The eighth column links to the Gene Symbol in Entrez. The last column links to SheepGenomics. Note: the GO and Pubmed annotation were derived from the EntrezGene ID, the gene symbols are based on the human annotation.

|  |  |  |  |  |  |  |  |  |  |  |  |  |  |  |  |  |  |  |  |  |  |  |  |  |  |  |  |  |  |  |  |  |  |  |  |  |  |  |  |  |  |  |  |  |  |  |  |  |  |  |  |  |  |  |  |  |  |  |  |  |  |  |  |  |  |  |  |  |  |  |  |  |  |  |  |  |  |  |  |  |  |  |  |  |  |  |  |  |  |  |  |  |  |  |  |  |  |  |  |  |  |  |  |  |  |  |  |  |  |  |  |  |  |  |  |  |  |  |  |  |  |  |  |  |  |  |  |  |  |  |  |  |  |  |  |  |  |  |  |  |  |  |  |  |  |  |  |  |  |  |  |  |  |  |  |  |  |  |  |  |  |  |  |  |  |  |  |  |  |  |  |  |  |  |  |  |  |  |  |  |  |  |  |  |  |  |  |  |  |  |  |  |  |  |  |  |  |  |  |  |  |  |  |  |  |  |  |  |  |  |  |  |  |  |  |  |  |  |  |  |  |  |  |  |  |  |  |  |  |  |  |  |  |  |  |  |  |  |  |  |  |  |  |  |  |  |  |  |  |  |  |  |  |  |  |  |  |  |  |  |  |  |  |  |  |  |  |  |  |  |  |  |  |  |  |  |  |  |  |  |  |  |  |  |  |  |  |  |  |  |  |  |  |  |  |  |  |  |  |  |  |  |  |  |  |  |  |  |  |  |  |  |  |  |  |  |  |  |  |  |  |  |  |  |  |  |  |  |  |  |  |  |  |  |  |  |  |  |  |  |  |  |  |  |  |  |  |  |  |  |  |  |  |  |  |  |  |  |  |  |  |  |  |  |  |  |  |  |  |  |  |  |  |  |  |  |  |  |  |  |  |  |  |  |  |  |  |  |  |  |  |  |  |  |  |
| --- | --- | --- | --- | --- | --- | --- | --- | --- | --- | --- | --- | --- | --- | --- | --- | --- | --- | --- | --- | --- | --- | --- | --- | --- | --- | --- | --- | --- | --- | --- | --- | --- | --- | --- | --- | --- | --- | --- | --- | --- | --- | --- | --- | --- | --- | --- | --- | --- | --- | --- | --- | --- | --- | --- | --- | --- | --- | --- | --- | --- | --- | --- | --- | --- | --- | --- | --- | --- | --- | --- | --- | --- | --- | --- | --- | --- | --- | --- | --- | --- | --- | --- | --- | --- | --- | --- | --- | --- | --- | --- | --- | --- | --- | --- | --- | --- | --- | --- | --- | --- | --- | --- | --- | --- | --- | --- | --- | --- | --- | --- | --- | --- | --- | --- | --- | --- | --- | --- | --- | --- | --- | --- | --- | --- | --- | --- | --- | --- | --- | --- | --- | --- | --- | --- | --- | --- | --- | --- | --- | --- | --- | --- | --- | --- | --- | --- | --- | --- | --- | --- | --- | --- | --- | --- | --- | --- | --- | --- | --- | --- | --- | --- | --- | --- | --- | --- | --- | --- | --- | --- | --- | --- | --- | --- | --- | --- | --- | --- | --- | --- | --- | --- | --- | --- | --- | --- | --- | --- | --- | --- | --- | --- | --- | --- | --- | --- | --- | --- | --- | --- | --- | --- | --- | --- | --- | --- | --- | --- | --- | --- | --- | --- | --- | --- | --- | --- | --- | --- | --- | --- | --- | --- | --- | --- | --- | --- | --- | --- | --- | --- | --- | --- | --- | --- | --- | --- | --- | --- | --- | --- | --- | --- | --- | --- | --- | --- | --- | --- | --- | --- | --- | --- | --- | --- | --- | --- | --- | --- | --- | --- | --- | --- | --- | --- | --- | --- | --- | --- | --- | --- | --- | --- | --- | --- | --- | --- | --- | --- | --- | --- | --- | --- | --- | --- | --- | --- | --- | --- | --- | --- | --- | --- | --- | --- | --- | --- | --- | --- | --- | --- | --- | --- | --- | --- | --- | --- | --- | --- | --- | --- | --- | --- | --- | --- | --- | --- | --- | --- | --- | --- | --- | --- | --- | --- | --- | --- | --- | --- | --- | --- | --- | --- | --- | --- | --- | --- | --- | --- | --- | --- | --- | --- | --- | --- | --- | --- | --- | --- | --- | --- | --- | --- | --- | --- | --- | --- | --- | --- | --- | --- | --- | --- | --- | --- | --- | --- | --- | --- | --- | --- | --- | --- | --- | --- | --- | --- | --- | --- | --- | --- | --- | --- | --- | --- | --- | --- | --- | --- | --- | --- | --- | --- | --- | --- | --- |
| AFFY Probe | Entrez Gene | Dif. Expressed | RefSeq | SG RefSeq | Gene Ontology | Pubmed | Gene Symbol | SheepGenomics\|  \|  \|  \|  \|  \|  \|  \|  \|  \| \| --- \| --- \| --- \| --- \| --- \| --- \| --- \| --- \| --- \| \| Bt.29854.1.S1\_x\_at \| 281337 \| 4/6 \| NM\_174117 \|  \| GO:0000166  GO:0003774  GO:0003779  GO:0005516  GO:0005524  GO:0005863  GO:0006941  GO:0016459 \| 9880677 \|  \| Bt.29854.1.S1\_x\_at \| \| Bt.15968.1.S1\_at \| 539139 \| 1/1 \| XM\_873620 \|  \|  \|  \| KLF9 \| Bt.15968.1.S1\_at \| \| Bt.11622.1.A1\_at \|  \|  \|  \| XM\_001250326 \|  \|  \|  \| Bt.11622.1.A1\_at \| \| Bt.12300.1.S1\_at \| 281337 \| 4/6 \| NM\_174117 \| NM\_174117 \| GO:0000166  GO:0003774  GO:0003779  GO:0005516  GO:0005524  GO:0005863  GO:0006941  GO:0016459 \| 9880677 \| MYH1 \| Bt.12300.1.S1\_at \| \| Bt.13980.2.A1\_a\_at \| 538944 \| 2/2 \| NM\_001034656 \|  \| GO:0000166  GO:0004111  GO:0005524  GO:0005739 \| 12477932 \|  \| Bt.13980.2.A1\_a\_at \| \| Bt.22379.1.S1\_at \| 536925 \| 1/1 \| NM\_001046419 \| NM\_001046419 \| GO:0000166  GO:0005524  GO:0005739  GO:0006457  GO:0016301  GO:0016740 \| 11282978  16305752 \| CABC1 \| Bt.22379.1.S1\_at \| \| Bt.29854.1.S1\_at \| 281337 \| 4/6 \| NM\_174117 \|  \| GO:0000166  GO:0003774  GO:0003779  GO:0005516  GO:0005524  GO:0005863  GO:0006941  GO:0016459 \| 9880677 \|  \| Bt.29854.1.S1\_at \| \| Bt.5848.2.S1\_at \| 518117 \| 1/2 \| NM\_001075767 \|  \| GO:0000166  GO:0000287  GO:0005388  GO:0005509  GO:0005515  GO:0005524  GO:0006812  GO:0006816  GO:0008152  GO:0015992  GO:0016020  GO:0016021  GO:0016529  GO:0016787  GO:0016820  GO:0031448 \|  \| ATP2A1 \| Bt.5848.2.S1\_at \| \| Bt.19517.1.S1\_at \|  \|  \|  \| XR\_028216 \|  \|  \| ERBB3 \| Bt.19517.1.S1\_at \| \| Bt.76.1.S1\_at \| 338086 \| 1/1 \| NM\_176674 \| NM\_176674 \| GO:0004129  GO:0005739  GO:0005746  GO:0006118  GO:0009055  GO:0016020  GO:0016021 \| 1316159  1717471  3006725  8638158  10089392  10771420 \| COX7A1 \| Bt.76.1.S1\_at \| \| Bt.29854.1.A1\_at \| 281337 \| 4/6 \| NM\_174117 \|  \| GO:0000166  GO:0003774  GO:0003779  GO:0005516  GO:0005524  GO:0005863  GO:0006941  GO:0016459 \| 9880677 \|  \| Bt.29854.1.A1\_at \| \| Bt.29697.1.S1\_at \| 407147 \| 1/2 \| NM\_001040496 \|  \| GO:0003956  GO:0006471  GO:0016740 \|  \| ART3 \| Bt.29697.1.S1\_at \| \| Bt.7394.1.A1\_at \| 615191 \| 1/2 \| XM\_866930 \|  \| GO:0005515  GO:0005524  GO:0016311  GO:0016791  GO:0044267 \|  \| PTPLA \| Bt.7394.1.A1\_at \| \| Bt.2129.2.S1\_at \|  \|  \|  \| XM\_001253937 \|  \|  \| CSPG2 \| Bt.2129.2.S1\_at \| \| Bt.20316.1.S1\_at \|  \|  \|  \| XM\_001249467 \|  \|  \| ANKRD23 \| Bt.20316.1.S1\_at \| \| Bt.25689.1.S1\_at \|  \|  \|  \| XM\_001252094 \|  \|  \|  \| Bt.25689.1.S1\_at \| \| Bt.13980.1.A1\_at \| 538944 \| 2/2 \| NM\_001034656 \|  \| GO:0000166  GO:0004111  GO:0005524  GO:0005739 \| 12477932 \| CKMT2 \| Bt.13980.1.A1\_at \| \| Bt.4584.1.S1\_at \|  \|  \|  \|  \|  \|  \| C1orf21 \| Bt.4584.1.S1\_at \| \| Bt.25282.1.A1\_at \| 536187 \| 1/2 \| XM\_871851 \| XM\_871851 \|  \|  \| C1QDC1 \| Bt.25282.1.A1\_at \| \| Bt.25658.1.A1\_at \| 618440 \| 1/1 \| NM\_001035501 \|  \|  \| 12477932 \| RPL3L \| Bt.25658.1.A1\_at \| \| Bt.6993.1.S1\_at \|  \|  \|  \| NM\_001015656 \|  \|  \| ATP1B1 \| Bt.6993.1.S1\_at \| \| Bt.10278.1.S1\_at \| 777789 \| 1/1 \| NM\_001078164  XM\_001251769 \|  \|  \|  \| ORF1-FL49 \| Bt.10278.1.S1\_at \| \| Bt.4138.2.S1\_at \| 281572 \| 1/2 \| NM\_174216 \|  \| GO:0000074  GO:0001525  GO:0007275  GO:0008083  GO:0008201  GO:0008283  GO:0016020  GO:0030154 \| 2479986  2610687  2735925  12126787  12213803  12448348  12649282  12777375  12779248  14551058  15075216  15456771  15501243  15501249  15616224  15637071  15649872  16257644  16899768  17043385  17178724 \| VEGF \| Bt.4138.2.S1\_at \| \| Bt.17613.1.A1\_at \| 530667 \| 1/1 \|  \|  \|  \|  \| PHKA1 \| Bt.17613.1.A1\_at \| \| Bt.28162.1.S1\_at \|  \|  \|  \|  \|  \|  \| PLN \| Bt.28162.1.S1\_at \| \| Bt.5078.1.S1\_at \| 514372 \| 1/1 \| NM\_001075665 \| NM\_001075665 \|  \|  \| MAGED2 \| Bt.5078.1.S1\_at \| \| Bt.25086.1.S1\_at \| 536607 \| 1/1 \| NM\_001034635 \| NM\_001034635 \| GO:0003779  GO:0005509  GO:0005925  GO:0006936  GO:0030018  GO:0030035  GO:0030175  GO:0030375  GO:0042802  GO:0046983  GO:0048041  GO:0051289  GO:0051370  GO:0051374 \| 12477932 \| ACTN2 \| Bt.25086.1.S1\_at \| \| Bt.25052.1.S1\_at \|  \|  \|  \| XM\_586655 \|  \|  \| CAPN6 \| Bt.25052.1.S1\_at \| \| Bt.2660.1.S1\_at \| 615387 \| 1/1 \| NM\_001076369 \| NM\_001076369 \|  \|  \| CDK2AP1 \| Bt.2660.1.S1\_at \| \| Bt.22169.1.S1\_at \| 540303 \| 1/1 \| NM\_001034702 \| NM\_001034702 \| GO:0000015  GO:0000287  GO:0004634  GO:0006096  GO:0016829 \| 12477932 \| ENO3 \| Bt.22169.1.S1\_at \| \| Bt.13128.1.S1\_at \| 286840 \| 1/1 \| NM\_001040483 \|  \| GO:0005739  GO:0005743  GO:0006118  GO:0009055  GO:0016020  GO:0051536 \| 16751257 \| SDHB \| Bt.13128.1.S1\_at \| \| Bt.16843.1.A1\_at \| 615846 \| 1/1 \| XM\_867767 \|  \|  \|  \|  \| Bt.16843.1.A1\_at \| \| Bt.21731.1.S1\_at \| 505406 \| 1/2 \| NM\_001045917 \|  \|  \|  \| DBN1 \| Bt.21731.1.S1\_at \| \| Bt.28208.1.S1\_at \| 514066 \| 1/1 \| NM\_001046164 \| NM\_001046164 \| GO:0005975  GO:0006094  GO:0016787  GO:0042132  GO:0042578 \|  \|  \| Bt.28208.1.S1\_at \| \| Bt.22174.1.S1\_at \| 523661 \| 1/1 \| XM\_601963 \| XM\_601963 \|  \|  \| CTNND2 \| Bt.22174.1.S1\_at \| \| Bt.2129.1.S1\_at \|  \|  \|  \|  \|  \|  \| CSPG2 \| Bt.2129.1.S1\_at \| \| Bt.5002.1.S1\_at \| 525042 \| 1/1 \| NM\_001035354 \|  \|  \| 11282978  16305752 \| GPD1 \| Bt.5002.1.S1\_at \| \| Bt.8319.3.S1\_at \| 515016 \| 1/4 \| NM\_001075692 \| NM\_001075692 \|  \|  \| MTUS1 \| Bt.8319.3.S1\_at \| \| Bt.2892.1.S1\_at \| 777787 \| 1/1 \| NM\_001078162  XM\_001249630 \| NM\_001078162 \| GO:0006810  GO:0008289 \|  \| FABP7 \| Bt.2892.1.S1\_at \| \| Bt.17278.3.S1\_a\_at \| 505926 \| 1/4 \| XM\_582290 \| XM\_582290 \|  \|  \| BCAT1 \| Bt.17278.3.S1\_a\_at \| \| Bt.5129.2.A1\_at \|  \|  \|  \|  \|  \|  \| NNAT \| Bt.5129.2.A1\_at \| \| Bt.27202.1.A1\_at \| 523184 \| 1/1 \| NM\_001046265 \| NM\_001046265 \|  \|  \| SOSTDC1 \| Bt.27202.1.A1\_at \| \| Bt.5129.1.S1\_a\_at \| 353114 \| 1/2 \| NM\_178323 \| NM\_178323 \|  \| 14668210 \| NNAT \| Bt.5129.1.S1\_a\_at \| |
