# Supplementary material for: A gene network switch enhances the oxidative capacity of ovine skeletal muscle during late fetal development
Source: BMC Genomics. 2010 Jun 15;11:378. doi: 10.1186/1471-2164-11-378 (PMC2894804; doi:10.1186/1471-2164-11-378)
Supplement: Additional File 1 — Gene expression data. This file contains links to the gene expression data. The genes that are significantly differentially expressed between adjacent development times are listed as Experiment 1 (80 d vs 100 d), Experiment 2 (100 d vs 120 d), Experiment 3 (120 d vs 150 d) and Experiment 4 (150 d vs 230 d) in the section entitled Open microarray analysis report for developmental expression. Each experiment contains an MA plot, Volcano plot and Heatmap for each of the three microarray processing programs MAS5, RMA and GCRMA. For each of these processing programs there is a list of significantly differentially expressed probe sets, and for each of these there is an FDR corrected probability, fold change, percentage of microarrays with a MAS5 Present call, and a convergence percentage for all three microarray processing programs. The file also contains annotation of the probe sets. In a second section entitled Time series analysis of developmental expression, there is a list of genes in each gene expression cluster and diagrams showing MAS5 gene expression values for these genes. [file 1471-2164-11-378-S1.ZIP › Report/AnnotationExp3.html]

## Annotation of differentially expressed genes for experiment 3.

The links connect to external resources. The first column is the probe ID and links to the Affymetrix annotation site. The second column links to Entrez. The third column shows how many times the Entrez gene ID was detected as significantly differentially expressed and how many probes target the gene on the chip - this provides an additional indication of reliability of differential expression. The fourth column is the RefSeq annotation (there can be more than one link). The fifth column is the RefSeq annotation provided by SheepGenomics (it should be more reliable than the original Affymetrix annotation). The sixth column is the gene ontology annotation. The seventh column links to Pubmed. The eighth column links to the Gene Symbol in Entrez. The last column links to SheepGenomics. Note: the GO and Pubmed annotation were derived from the EntrezGene ID, the gene symbols are based on the human annotation.

|  |  |  |  |  |  |  |  |  |  |  |  |  |  |  |  |  |  |  |  |  |  |  |  |  |  |  |  |  |  |  |  |  |  |  |  |  |  |  |  |  |  |  |  |  |  |  |  |  |  |  |  |  |  |  |  |  |  |  |  |  |  |  |  |  |  |  |  |  |  |  |  |  |  |  |  |  |  |  |  |  |  |  |  |  |  |  |  |  |  |  |  |  |  |  |  |  |  |  |  |  |  |  |  |  |  |  |  |  |  |  |  |  |  |  |  |  |  |  |  |  |  |  |  |  |  |  |  |  |  |  |  |  |  |  |  |  |  |  |  |  |  |  |  |  |  |  |  |  |  |  |  |  |  |  |  |  |  |  |  |  |  |  |  |  |  |  |  |  |  |  |  |  |  |  |  |  |  |  |  |  |  |  |  |  |  |  |  |  |  |  |  |  |  |  |  |  |  |  |  |  |  |  |  |  |  |  |  |  |  |  |  |  |  |  |  |  |  |  |  |  |  |  |  |  |  |  |  |  |  |  |  |  |  |  |  |  |  |  |  |  |  |  |  |  |  |  |  |  |  |  |  |  |  |  |  |  |  |  |  |  |  |  |  |  |  |  |  |  |  |  |  |  |  |  |  |  |  |  |  |  |  |  |  |  |  |  |  |  |  |  |  |  |  |  |  |  |  |  |  |  |  |  |  |  |  |  |  |  |  |  |  |  |  |  |  |  |  |  |  |  |  |  |  |  |  |  |  |  |  |  |  |  |  |  |  |  |  |  |  |  |  |  |  |  |  |  |  |  |  |  |  |  |  |  |  |  |  |  |  |  |  |  |  |  |  |  |  |  |  |  |  |  |  |  |  |  |  |  |  |  |  |  |  |  |  |  |  |  |  |  |  |  |  |  |  |  |  |  |  |  |  |  |  |  |  |  |  |  |  |  |  |  |  |  |  |  |  |  |  |  |  |  |  |  |  |  |  |  |  |  |  |  |  |  |  |  |  |  |  |  |  |  |  |  |  |  |  |  |  |  |  |  |  |  |  |  |  |  |  |  |  |  |  |  |  |  |  |  |  |  |  |  |  |  |  |  |  |  |  |  |  |  |  |  |  |  |  |  |  |  |  |  |  |  |  |  |  |  |  |  |  |  |  |  |  |  |  |  |  |  |  |  |  |  |  |  |  |  |  |  |  |  |  |  |  |  |  |  |  |  |  |  |  |  |  |  |  |  |  |  |  |  |  |  |  |  |  |  |  |  |  |  |  |  |  |  |  |  |  |  |  |  |  |  |  |  |  |  |  |  |  |  |  |  |  |  |  |  |  |  |  |  |  |  |  |  |  |  |  |  |  |  |  |  |  |  |  |  |  |  |  |  |  |  |  |  |  |  |  |  |  |  |  |  |  |  |  |  |  |  |  |  |  |  |  |  |  |  |  |  |  |  |  |  |  |  |  |  |  |  |  |  |  |  |  |  |  |  |  |  |  |  |  |  |  |  |  |  |  |  |  |  |  |  |  |  |  |  |  |  |  |  |  |  |  |  |  |  |  |  |  |  |  |  |  |  |  |  |  |  |  |  |  |  |  |  |  |  |  |  |  |  |  |  |  |  |  |  |  |  |  |  |  |  |  |  |  |  |  |  |  |  |  |  |  |  |  |  |  |  |  |  |  |  |  |  |  |  |  |  |  |  |  |  |  |  |  |  |  |  |  |  |  |  |  |  |  |  |  |  |  |  |  |  |  |  |  |  |  |  |  |  |  |  |  |  |  |  |  |  |  |  |  |  |  |  |  |  |  |  |  |  |  |  |  |  |  |  |  |  |  |  |  |  |  |  |  |  |  |  |  |  |  |  |  |  |  |  |  |  |  |  |  |  |  |  |  |  |  |  |  |  |  |  |  |  |  |  |  |  |  |  |  |  |  |  |  |  |  |  |  |  |  |  |  |  |  |  |  |  |  |  |  |  |  |  |  |  |  |  |  |  |  |  |  |  |  |  |  |  |  |  |  |  |  |  |  |  |  |  |  |  |  |  |  |  |  |  |  |  |  |  |  |  |  |  |  |  |  |  |  |  |  |  |  |  |  |  |  |  |  |  |  |  |  |  |  |  |  |  |  |  |  |  |  |  |  |  |  |  |  |  |  |  |  |  |  |  |  |  |  |  |  |  |  |  |  |  |  |  |  |  |  |  |  |  |  |  |  |  |  |  |  |  |  |  |  |  |  |  |  |  |  |  |  |  |  |  |  |  |  |  |  |  |  |  |  |  |  |  |  |  |  |  |  |  |  |  |  |  |  |  |  |  |  |  |  |  |  |  |  |  |  |  |  |  |  |  |  |  |  |  |  |  |  |  |  |  |  |  |  |  |  |  |  |  |  |  |  |  |  |  |  |  |  |  |  |  |  |  |  |  |  |  |  |  |  |  |  |  |  |  |  |  |  |  |  |  |  |  |  |  |  |  |  |  |  |  |  |  |  |  |  |  |  |  |  |  |  |  |  |  |  |  |  |  |  |  |  |  |  |  |  |  |  |  |  |  |  |  |  |  |  |  |  |  |  |  |  |  |  |  |  |  |  |  |  |  |  |  |  |  |  |  |  |  |  |  |  |  |  |  |  |  |  |  |  |  |  |  |  |  |  |  |  |  |  |  |  |  |  |  |  |  |  |  |  |  |  |  |  |  |  |  |  |  |  |  |  |  |  |  |  |  |  |  |  |  |  |  |  |  |  |  |  |  |  |  |  |  |  |  |  |  |  |  |  |  |  |  |  |  |  |  |  |  |  |  |  |  |  |  |  |  |  |  |  |  |  |  |  |  |  |  |  |  |  |  |  |  |  |  |  |  |  |  |  |  |  |  |  |  |  |  |  |  |  |  |  |  |  |  |  |  |  |  |  |  |  |  |  |  |  |  |  |  |  |  |  |  |  |  |  |  |  |  |  |  |  |  |  |  |  |  |  |  |  |  |  |  |  |  |  |  |  |  |  |  |  |  |  |  |  |  |  |  |  |  |  |  |  |  |  |  |  |  |  |  |  |  |  |  |  |  |  |  |  |  |  |  |  |  |  |  |  |  |  |  |  |  |  |  |  |  |  |  |  |  |  |  |  |  |  |  |  |  |  |  |  |  |  |  |  |  |  |  |  |  |  |  |  |  |  |  |  |  |  |  |  |  |  |  |  |  |  |  |  |  |  |  |  |  |  |  |  |  |  |  |  |  |  |  |  |  |  |  |  |  |  |  |  |  |  |  |  |  |  |  |  |  |  |  |  |  |  |  |  |  |  |  |  |  |  |  |  |  |  |  |  |  |  |  |  |  |  |  |  |  |  |  |  |  |  |  |  |  |  |  |  |  |  |  |  |  |  |  |  |  |  |  |  |  |  |  |  |  |  |  |  |  |  |  |  |  |  |  |  |  |  |  |  |  |  |  |  |  |  |  |  |  |  |  |  |  |  |  |  |  |  |  |  |  |  |  |  |  |  |  |  |  |  |  |  |  |  |  |  |  |  |  |  |  |  |  |  |  |  |  |  |  |  |  |  |  |  |  |  |  |  |  |  |  |  |  |  |  |  |  |  |  |  |  |  |  |  |  |  |  |  |  |  |  |  |  |  |  |  |  |  |  |  |  |  |  |  |  |  |  |  |  |  |  |  |  |  |  |  |  |  |  |  |  |  |  |  |  |  |  |  |  |  |  |  |  |  |  |  |  |  |  |  |  |  |  |  |  |  |  |  |  |  |  |  |  |  |  |  |  |  |  |  |  |  |  |  |  |  |  |  |  |  |  |  |  |  |  |  |  |  |  |  |  |  |  |  |  |  |  |  |  |  |  |  |  |  |  |  |  |  |  |  |  |  |  |  |  |  |  |  |  |  |  |  |  |  |  |  |  |  |  |  |  |  |  |  |  |  |  |  |  |  |  |  |  |  |  |  |  |  |  |  |  |  |  |  |  |  |  |  |  |  |  |  |  |  |  |  |  |  |  |  |  |  |  |  |  |  |  |  |  |  |  |  |  |  |  |  |  |  |  |  |  |  |  |  |  |  |  |  |  |  |  |  |  |  |  |  |  |  |  |  |  |  |  |  |  |  |  |  |  |  |  |  |  |  |  |  |  |  |  |  |  |  |  |  |  |  |  |  |  |  |  |  |  |  |  |  |  |  |  |  |  |  |  |  |  |  |  |  |  |  |  |  |  |  |  |  |  |  |  |  |  |  |  |  |  |  |  |  |  |  |  |  |  |  |  |  |  |  |  |  |  |  |  |  |  |  |  |  |  |  |  |  |  |  |  |  |  |  |  |  |  |  |  |  |  |  |  |  |  |  |  |  |  |  |  |  |  |  |  |  |  |  |  |  |  |  |  |  |  |  |  |  |  |  |  |  |  |  |  |  |  |  |  |  |  |  |  |  |  |  |  |  |  |  |  |  |  |  |  |  |  |  |  |  |  |  |  |  |  |  |  |  |  |  |  |  |  |  |  |  |  |  |  |  |  |  |  |  |  |  |  |  |  |  |  |  |  |  |  |  |  |  |  |  |  |  |  |  |  |  |  |  |  |  |  |  |  |  |  |  |  |  |  |  |  |  |  |  |  |  |  |  |  |  |  |  |  |  |  |  |  |  |  |  |  |  |  |  |  |  |  |  |  |  |  |  |  |  |  |  |  |  |  |  |  |  |  |  |  |  |  |  |  |  |  |  |  |  |  |  |  |  |  |  |  |  |  |  |  |  |  |  |  |  |  |  |  |  |  |  |  |  |  |  |  |  |  |  |  |  |  |  |  |  |  |  |  |  |  |  |  |  |  |  |  |  |  |  |  |  |  |  |  |  |  |  |  |  |  |  |  |  |  |  |  |  |  |  |  |  |  |  |  |  |  |  |  |  |  |  |  |  |  |  |  |  |  |  |  |  |  |  |  |  |  |  |  |  |  |  |  |  |  |  |  |  |  |  |  |  |  |  |  |  |  |  |  |  |  |  |  |  |  |  |  |  |  |  |  |  |  |  |  |  |  |  |  |  |  |  |  |  |  |  |  |  |  |  |  |  |  |  |  |  |  |  |  |  |  |  |  |  |  |  |  |  |  |  |  |  |  |  |  |  |  |  |  |  |  |  |  |  |  |  |  |  |  |  |  |  |  |  |  |  |  |  |  |  |  |  |  |  |  |  |  |  |  |  |  |  |  |  |  |  |  |  |  |  |  |  |  |  |  |  |  |  |  |  |  |  |  |  |  |  |  |  |  |  |  |  |  |  |  |  |  |  |  |  |  |  |  |  |  |  |  |  |  |  |  |  |  |  |  |  |  |  |  |  |  |  |  |  |  |  |  |  |  |  |  |  |  |  |  |  |  |  |  |  |  |  |  |  |  |  |  |  |  |  |  |  |  |  |  |  |  |  |  |  |  |  |  |  |  |  |  |  |  |  |  |  |  |  |  |  |  |  |  |  |  |  |  |  |  |  |  |  |  |  |  |  |  |  |  |  |  |  |  |  |  |  |  |  |  |  |  |  |  |  |  |  |  |  |  |  |  |  |  |  |  |  |  |  |  |  |  |  |  |  |  |  |  |  |  |  |  |  |  |  |  |  |  |  |  |  |  |  |  |  |  |  |  |  |  |  |  |  |  |  |  |  |  |  |  |  |  |  |  |  |  |  |  |  |  |  |  |  |  |  |  |  |  |  |  |  |  |  |  |  |  |  |  |  |  |  |  |  |  |  |  |  |  |  |  |  |  |  |  |  |  |  |  |  |  |  |  |  |  |  |  |  |  |  |  |  |  |  |  |  |  |  |  |  |  |  |  |  |  |  |  |  |  |  |  |  |  |  |  |  |  |  |  |  |  |  |  |  |  |  |  |  |  |  |  |  |  |  |  |  |  |  |  |  |  |  |  |  |  |  |  |  |  |  |  |  |  |  |  |  |  |  |  |  |  |  |  |  |  |  |  |  |  |  |  |  |  |  |  |  |  |  |  |  |  |  |  |  |  |  |  |  |  |  |  |  |  |  |  |  |  |  |  |  |  |  |  |  |  |  |  |  |  |  |  |  |  |  |  |  |  |  |  |  |  |  |  |  |  |  |  |  |  |  |  |  |  |  |  |  |  |  |  |  |  |  |  |  |  |  |  |  |  |  |  |  |  |  |  |  |  |  |  |  |  |  |  |  |  |  |  |  |  |  |  |  |  |  |  |  |  |  |  |  |  |  |  |  |  |  |  |  |  |  |  |  |  |  |  |  |  |  |  |  |  |  |  |  |  |  |  |  |  |  |  |  |  |  |  |  |  |  |  |  |  |  |  |  |  |  |  |  |  |  |  |  |  |  |  |  |  |  |  |  |  |  |  |  |  |  |  |  |  |  |  |  |  |  |  |  |  |  |  |  |  |  |  |  |  |  |  |  |  |  |  |  |  |  |  |  |  |  |  |  |  |  |  |  |  |  |  |  |  |  |  |  |  |  |  |  |  |  |  |  |  |  |  |  |  |  |  |  |  |  |  |  |  |  |  |  |  |  |  |  |  |  |  |  |  |  |  |  |  |  |  |  |  |  |  |  |  |  |  |  |  |  |  |  |  |  |  |  |  |  |  |  |  |  |  |  |  |  |  |  |  |  |  |  |  |  |  |  |  |  |  |  |  |  |  |  |  |  |  |  |  |  |  |  |  |  |  |  |  |  |  |  |  |  |  |  |  |  |  |  |  |  |  |  |  |  |  |  |  |  |  |  |  |  |  |  |  |  |  |  |  |  |  |  |  |  |  |  |  |  |  |  |  |  |  |  |  |  |  |  |  |  |  |  |  |  |  |  |  |  |  |  |  |  |  |  |  |  |  |  |  |  |  |  |  |  |  |  |  |  |  |  |  |  |  |  |  |  |  |  |  |  |  |  |  |  |  |  |  |  |  |  |  |  |  |  |  |  |  |  |  |  |  |  |  |  |  |  |  |  |  |  |  |  |  |  |  |  |  |  |  |  |  |  |  |  |  |  |  |  |  |  |  |  |  |  |  |  |  |  |  |  |  |  |  |  |  |  |  |  |  |  |  |  |  |  |  |  |  |  |  |  |  |  |  |  |  |  |  |  |  |  |  |  |  |  |  |  |  |  |  |  |  |  |  |  |  |  |  |  |  |  |  |  |  |  |  |  |  |  |  |  |  |  |  |  |  |  |  |  |  |  |  |  |  |  |  |  |  |  |  |  |  |  |  |  |  |  |  |  |  |  |  |  |  |  |  |  |  |  |  |  |  |  |  |  |  |  |  |  |  |  |  |  |  |  |  |  |  |  |  |  |  |  |  |  |  |  |  |  |  |  |  |  |  |  |  |  |  |  |  |  |  |  |  |  |  |  |  |  |  |  |  |  |  |  |  |  |  |  |  |  |  |  |  |  |  |  |  |  |  |  |  |  |  |  |  |  |  |  |  |  |  |  |  |  |  |  |  |  |  |  |  |  |  |  |  |  |  |  |  |  |  |  |  |  |  |  |  |  |  |  |  |  |  |  |  |  |  |  |  |  |  |  |  |  |  |  |  |  |  |  |  |  |  |  |  |  |  |  |  |  |  |  |  |  |  |  |  |  |  |  |  |  |  |  |  |  |  |  |  |  |  |  |  |  |  |  |  |  |  |  |  |  |  |  |  |  |  |  |  |  |  |  |  |  |  |  |  |  |  |  |  |  |  |  |  |  |  |  |  |  |  |  |  |  |  |  |  |  |  |  |  |  |  |  |  |  |  |  |  |  |  |  |  |  |  |  |  |  |  |  |  |  |  |  |  |  |  |  |  |  |  |  |  |  |  |  |  |  |  |  |  |  |  |  |  |  |  |  |  |  |  |  |  |  |  |  |  |  |  |  |  |  |  |  |  |  |  |  |  |  |  |  |  |  |  |  |  |  |  |  |  |  |  |  |  |  |  |  |  |  |  |  |  |  |  |  |  |  |  |  |  |  |  |  |  |  |  |  |  |  |  |  |  |  |  |  |  |  |  |  |  |  |  |  |  |  |  |  |  |  |  |  |  |  |  |  |  |  |  |  |  |  |  |  |  |  |  |  |  |  |  |  |  |  |  |  |  |  |  |  |  |  |  |  |  |  |  |  |  |  |  |  |  |  |  |  |  |  |  |  |  |  |  |  |  |  |  |  |  |  |  |  |  |  |  |  |  |  |  |  |  |  |  |  |  |  |  |  |  |  |  |  |  |  |  |  |  |  |  |  |  |  |  |  |  |  |  |  |  |  |  |  |  |  |  |  |  |  |  |  |  |  |  |  |  |  |  |  |  |  |  |  |  |  |  |  |  |  |  |  |  |  |  |  |  |  |  |  |  |  |  |  |  |  |  |  |  |  |  |  |  |  |  |  |  |  |  |  |  |  |  |  |  |  |  |  |  |  |  |  |  |  |  |  |  |  |  |  |  |  |  |  |  |  |  |  |  |  |  |  |  |  |  |  |  |  |  |  |  |  |  |  |  |  |  |  |  |  |  |  |  |  |  |  |  |  |  |  |  |  |  |  |  |  |  |  |  |  |  |  |  |  |  |  |  |  |  |  |  |  |  |  |  |  |  |  |  |  |  |  |  |  |  |  |  |  |  |  |  |  |  |  |  |  |  |  |  |  |  |  |  |  |  |  |  |  |  |  |  |  |  |  |  |  |  |  |  |  |  |  |  |  |  |  |  |  |  |  |  |  |  |  |  |  |  |  |  |  |  |  |  |  |  |  |  |  |  |  |  |  |  |  |  |  |  |  |  |  |  |  |  |  |  |  |  |  |  |  |  |  |  |  |  |  |  |  |  |  |  |  |  |  |  |  |  |  |  |  |  |  |  |  |  |  |  |  |  |  |  |  |  |  |  |  |  |  |  |  |  |  |  |  |  |  |  |  |  |  |  |  |  |  |  |  |  |  |  |  |  |  |  |  |  |  |  |  |  |  |  |  |  |  |  |  |  |  |  |  |  |  |  |  |  |  |  |  |  |  |  |  |  |  |  |  |  |  |  |  |  |  |  |  |  |  |  |  |  |  |  |  |  |  |  |  |  |  |  |  |  |  |  |  |  |  |  |  |  |  |  |  |  |  |  |  |  |  |  |  |  |  |  |  |  |  |  |  |  |  |  |  |  |  |  |  |  |  |  |  |  |  |  |  |  |  |  |  |  |  |  |  |  |  |  |  |  |  |  |  |  |  |  |  |  |  |  |  |  |  |  |  |  |  |  |  |  |  |  |  |  |  |  |  |  |  |  |  |  |  |  |  |  |  |  |  |  |  |  |  |  |  |  |  |  |  |  |  |  |  |  |  |  |  |  |  |  |  |  |  |  |  |  |  |  |  |  |  |  |  |  |  |  |  |  |  |  |  |  |  |  |  |  |  |  |  |  |  |  |  |  |  |  |  |  |  |  |  |  |  |  |  |  |  |  |  |  |  |  |  |  |  |  |  |  |  |  |  |  |  |  |  |  |  |  |  |  |  |  |  |  |  |  |  |  |  |  |  |  |  |  |  |  |  |  |  |  |  |  |  |  |  |  |  |  |  |  |  |  |  |  |  |  |  |  |  |  |  |  |  |  |  |  |  |  |  |  |  |  |  |  |  |  |  |  |  |  |  |  |  |  |  |  |  |  |  |  |  |  |  |  |  |  |  |  |  |  |  |  |  |  |  |  |  |  |  |  |  |  |  |  |  |  |  |  |  |  |  |  |  |  |  |  |  |  |  |  |  |  |  |  |  |  |  |  |  |  |  |  |  |  |  |  |  |  |  |  |  |  |  |  |  |  |  |  |  |  |  |  |  |  |  |  |  |  |  |  |  |  |  |  |  |  |  |  |  |  |  |  |  |  |  |  |  |  |  |  |  |  |  |  |  |  |  |  |  |  |  |  |  |  |  |  |  |  |  |  |  |  |  |  |  |  |  |  |  |  |  |  |  |  |  |  |  |  |  |  |  |  |  |  |  |  |  |  |  |  |  |  |  |  |  |  |  |  |  |  |  |  |  |  |  |  |  |  |  |  |  |  |  |  |  |  |  |  |  |  |  |  |  |  |  |  |  |  |  |  |  |  |  |  |  |  |  |  |  |  |  |  |  |  |  |  |  |  |  |  |  |  |  |  |  |  |  |  |  |  |  |  |  |  |  |  |  |  |  |  |  |  |  |  |  |  |  |  |  |  |  |  |  |  |  |  |  |  |  |  |  |  |  |  |  |  |  |  |  |  |  |  |  |  |  |  |  |  |  |  |  |  |  |  |  |  |  |  |  |  |  |  |  |  |  |  |  |  |  |  |  |  |  |  |  |  |  |  |  |  |  |  |  |  |  |  |  |  |  |  |  |  |  |  |  |  |  |  |  |  |  |  |  |  |  |  |  |  |  |  |  |  |  |  |  |  |  |  |  |  |  |  |  |  |  |  |  |  |  |  |  |  |  |  |  |  |  |  |  |  |  |  |  |  |  |  |  |  |  |  |  |  |  |  |  |  |  |  |  |  |  |  |  |  |  |  |  |  |  |  |  |  |  |  |  |  |  |  |  |  |  |  |  |  |  |  |  |  |  |  |  |  |  |  |  |  |  |  |  |  |  |  |  |  |  |  |  |  |  |  |  |  |  |  |  |  |  |  |  |  |  |  |  |  |  |  |  |  |  |  |  |  |  |  |  |  |  |  |  |  |  |  |  |  |  |  |  |  |  |  |  |  |  |  |  |  |  |  |  |  |  |  |  |  |  |  |  |  |  |  |  |  |  |  |  |  |  |  |  |  |  |  |  |  |  |  |  |  |  |  |  |  |  |  |  |  |  |  |  |  |  |  |  |  |  |  |  |  |  |  |  |  |  |  |  |  |  |  |  |  |  |  |  |  |  |  |  |  |  |  |  |  |  |  |  |  |  |  |  |  |  |  |  |  |  |  |  |  |  |  |  |  |  |  |  |  |  |  |  |  |  |  |  |  |  |  |  |  |  |  |  |  |  |  |  |  |  |  |  |  |  |  |  |  |  |  |  |  |  |  |  |  |  |  |  |  |  |  |  |  |  |  |  |  |  |  |  |  |  |  |  |  |  |  |  |  |  |  |  |  |  |  |  |  |  |  |  |  |  |  |  |  |  |  |  |  |  |  |  |  |  |  |  |  |  |  |  |  |  |  |  |  |  |  |  |  |  |  |  |  |  |  |  |  |  |  |  |  |  |  |  |  |  |  |  |  |  |  |  |  |  |  |  |  |  |  |  |  |  |  |  |  |  |  |  |  |  |  |  |  |  |  |  |  |  |  |  |  |  |  |  |  |  |  |  |  |  |  |  |  |  |  |  |  |  |  |  |  |  |  |  |  |  |  |  |  |  |  |  |  |  |  |  |  |  |  |  |  |  |  |  |  |  |  |  |  |  |  |  |  |  |  |  |  |  |  |  |  |  |  |  |  |  |  |  |  |  |  |  |  |  |  |  |  |  |  |  |  |  |  |  |  |  |  |  |  |  |  |  |  |  |  |  |  |  |  |  |  |  |  |  |  |  |  |  |  |  |  |  |  |  |  |  |  |  |  |  |  |  |  |  |  |  |  |  |  |  |  |  |  |  |  |  |  |  |  |  |  |  |  |  |  |  |  |  |  |  |  |  |  |  |  |  |  |  |  |  |  |  |  |  |  |  |  |  |  |  |  |  |  |  |  |  |  |  |  |  |  |  |  |  |  |  |  |  |  |  |  |  |  |  |  |  |  |  |  |  |  |  |  |  |  |  |  |  |  |  |  |  |  |  |  |  |  |  |  |  |  |  |  |  |  |  |  |  |  |  |  |  |  |  |  |  |  |  |  |  |  |  |  |  |  |  |  |  |  |  |  |  |  |  |  |  |  |  |  |  |  |  |  |  |  |  |  |  |  |  |  |  |  |  |  |  |  |  |  |  |  |  |  |  |  |  |  |  |  |  |  |  |  |  |  |  |  |  |  |  |  |  |  |  |  |  |  |  |  |  |  |  |  |  |  |  |  |  |  |  |  |  |  |  |  |  |  |  |  |  |  |  |  |  |  |  |  |  |  |  |  |  |  |  |  |  |  |  |  |  |  |  |  |  |  |  |  |  |  |  |  |  |  |  |  |  |  |  |  |  |  |  |  |  |  |  |  |  |  |  |  |  |  |  |  |  |  |  |  |  |  |  |  |  |  |  |  |  |  |  |  |  |  |  |  |  |  |  |  |  |  |  |  |  |  |  |  |  |  |  |  |  |  |  |  |  |  |  |  |  |  |  |  |  |  |  |  |  |  |  |  |  |  |  |  |  |  |  |  |  |  |  |  |  |  |  |  |  |  |  |  |  |  |  |  |  |  |  |  |  |  |  |  |  |  |  |  |  |  |  |  |  |  |  |  |  |  |  |  |  |  |  |  |  |  |  |  |  |  |  |  |  |  |  |  |  |  |  |  |  |  |  |  |  |  |  |  |  |  |  |  |  |  |  |  |  |  |  |  |  |  |  |  |  |  |  |  |  |  |  |  |  |  |  |  |  |  |  |  |  |  |  |  |  |  |  |  |  |  |  |  |  |  |  |  |  |  |  |  |  |  |  |  |  |  |  |  |  |  |  |  |  |  |  |  |  |  |  |  |  |  |  |  |  |  |  |  |  |  |  |  |  |  |  |  |  |  |  |  |  |  |  |  |  |  |  |  |  |  |  |  |  |  |  |  |  |  |  |  |  |  |  |  |  |  |  |  |  |  |  |  |  |  |  |  |  |  |  |  |  |  |  |  |  |  |  |  |  |  |  |  |  |  |  |  |  |  |  |  |  |  |  |  |  |  |  |  |  |  |  |  |  |  |  |  |  |  |  |  |  |  |  |  |  |  |  |  |  |  |  |  |  |  |  |  |  |  |  |  |  |  |  |  |  |  |  |  |  |  |  |  |  |  |  |  |  |  |  |  |  |  |  |  |  |  |  |  |  |  |  |  |  |  |  |  |  |  |  |  |  |  |  |  |  |  |  |  |  |  |  |  |  |  |  |  |  |  |  |  |  |  |  |  |  |  |  |  |  |  |  |  |  |  |  |  |  |  |  |  |  |  |  |  |  |  |  |  |  |  |  |  |  |  |  |  |  |  |  |  |  |  |  |  |  |  |  |  |  |  |  |  |  |  |  |  |  |  |  |  |  |  |  |  |  |  |  |  |  |  |  |  |  |  |  |  |  |  |  |  |  |  |  |  |  |  |  |  |  |  |  |  |  |  |  |  |  |  |  |  |  |  |  |  |  |  |  |  |  |  |  |  |  |  |  |  |  |  |  |  |  |  |  |  |  |  |  |  |  |  |  |  |  |  |  |  |  |  |  |  |  |  |  |  |  |  |  |  |  |  |  |  |  |  |  |  |  |  |  |  |  |  |  |  |  |  |  |  |  |  |  |  |  |  |  |  |  |  |  |  |  |  |  |  |  |  |  |  |  |  |  |  |  |  |  |  |  |  |  |  |  |  |  |  |  |  |  |  |  |  |  |  |  |  |  |  |  |  |  |  |  |  |  |  |  |  |  |  |  |  |  |  |  |  |  |  |  |  |  |  |  |  |  |  |  |  |  |  |  |  |  |  |  |  |  |  |  |  |  |  |  |  |  |  |  |  |  |  |  |  |  |  |  |  |  |  |  |  |  |  |  |  |  |  |  |  |  |  |  |  |  |  |  |  |  |  |  |  |  |  |  |  |  |  |  |  |  |  |  |  |  |  |  |  |  |  |  |  |  |  |  |  |  |  |  |  |  |  |  |  |  |  |  |  |  |  |  |  |  |  |  |  |  |  |  |  |  |  |  |  |  |  |  |  |  |  |  |  |  |  |  |  |  |  |  |  |  |  |  |  |  |  |  |  |  |  |  |  |  |  |  |  |  |  |  |  |  |  |  |  |  |  |  |  |  |  |  |  |  |  |  |  |  |  |  |  |  |  |  |  |  |  |  |  |  |  |  |  |  |  |  |  |  |  |  |  |  |  |  |  |  |  |  |  |  |  |  |  |  |  |  |  |  |  |  |  |  |  |  |  |  |  |  |  |  |  |  |  |  |  |  |  |  |  |  |  |  |  |  |  |  |  |  |  |  |  |  |  |  |  |  |  |  |  |  |  |  |  |  |  |  |  |  |  |  |  |  |  |  |  |  |  |  |  |  |  |  |  |  |  |  |  |  |  |  |  |  |  |  |  |  |  |  |  |  |  |  |  |  |  |  |  |  |  |  |  |  |  |  |  |  |  |  |  |  |  |  |  |  |  |  |  |  |  |  |  |  |  |  |  |  |  |  |  |  |  |  |  |  |  |  |  |  |  |  |  |  |  |  |  |  |  |  |  |  |  |  |  |  |  |  |  |  |  |  |  |  |  |  |  |  |  |  |  |  |  |  |  |  |  |  |  |  |  |  |  |  |  |  |  |  |  |  |  |  |  |  |  |  |  |  |  |  |  |  |  |  |  |  |  |  |  |  |  |  |  |  |  |  |  |  |  |  |  |  |  |  |  |  |  |  |  |  |  |  |  |  |  |  |  |  |  |  |  |  |  |  |  |  |  |  |  |  |  |  |  |  |  |  |  |  |  |  |  |  |  |  |  |  |  |  |  |  |  |  |  |  |  |  |  |  |  |  |  |  |  |  |  |  |  |  |  |  |  |  |  |  |  |  |  |  |  |  |  |  |  |  |  |  |  |  |  |  |  |  |  |  |  |  |  |  |  |  |  |  |  |  |  |  |  |  |  |  |  |  |  |  |  |  |  |  |  |  |  |  |  |  |  |  |  |  |  |  |  |  |  |  |  |  |  |  |  |  |  |  |  |  |  |  |  |  |  |  |  |  |  |  |  |  |  |  |  |  |  |  |  |  |  |  |  |  |  |  |  |  |  |  |  |  |  |  |  |  |  |  |  |  |  |  |  |  |  |  |  |  |  |  |  |  |  |  |  |  |  |  |  |  |  |  |  |  |  |  |  |  |  |  |  |  |  |  |  |  |  |  |  |  |  |  |  |  |  |  |  |  |  |  |  |  |  |  |  |  |  |  |  |  |  |  |  |  |  |  |  |  |  |  |  |  |  |  |  |  |  |  |  |  |  |  |  |  |  |  |  |  |  |  |  |  |  |  |  |  |  |  |  |  |  |  |  |  |  |  |  |  |  |  |  |  |  |  |  |  |  |  |  |  |  |  |  |  |  |  |  |  |  |  |  |  |  |  |  |  |  |  |  |  |  |  |  |  |  |  |  |  |  |  |  |  |  |  |  |  |  |  |  |  |  |  |  |  |  |  |  |  |  |  |  |  |  |  |  |  |  |  |  |  |  |  |  |  |  |  |  |  |  |  |  |  |  |  |  |  |  |  |  |  |  |  |  |  |  |  |  |  |  |  |  |  |  |  |  |  |  |  |  |  |  |  |  |  |  |  |  |  |  |  |  |  |  |  |  |  |  |  |  |  |  |  |  |  |  |  |  |  |  |  |  |  |  |  |  |  |  |  |  |  |  |  |  |  |  |  |  |  |  |  |  |  |  |  |  |  |  |  |  |  |  |  |  |  |  |  |  |  |  |  |  |  |  |  |  |  |  |  |  |  |  |  |  |  |  |  |  |  |  |  |  |  |  |  |  |  |  |  |  |  |  |  |  |  |  |  |  |  |  |  |  |  |  |  |  |  |  |  |  |  |  |  |  |  |  |  |  |  |  |  |  |  |  |  |  |  |  |  |  |  |  |  |  |  |  |  |  |  |  |  |  |  |  |  |  |  |  |  |  |  |  |  |  |  |  |  |  |  |  |  |  |  |  |  |  |  |  |  |  |  |  |  |  |  |  |  |  |  |  |  |  |  |  |  |  |  |  |  |  |  |  |  |  |  |  |  |  |  |  |  |  |  |  |  |  |  |  |  |  |  |  |  |  |  |  |  |  |  |  |  |  |  |  |  |  |  |  |  |  |  |  |  |  |  |  |  |  |  |  |  |  |  |  |  |  |  |  |  |  |  |  |  |  |  |  |  |  |  |  |  |  |  |  |  |  |  |  |  |  |  |  |  |  |  |  |  |  |  |  |  |  |  |  |  |  |  |  |  |  |  |  |  |  |  |  |  |  |  |  |  |  |  |  |  |  |  |  |  |  |  |  |  |  |  |  |  |  |  |  |  |  |  |  |  |  |  |  |  |  |  |  |  |  |  |  |  |  |  |  |  |  |  |  |  |  |  |  |  |  |  |  |  |  |  |  |  |  |  |  |  |  |  |  |  |  |  |  |  |  |  |  |  |  |  |  |  |  |  |  |  |  |  |  |  |  |  |  |  |  |  |  |  |  |  |  |  |  |  |  |  |  |  |  |  |  |  |  |  |  |  |  |  |  |  |  |  |  |  |  |  |  |  |  |  |  |  |  |  |  |  |  |  |  |  |  |  |  |  |  |  |  |  |  |  |  |  |  |  |  |  |  |  |  |  |  |  |  |  |  |  |  |  |  |  |  |  |  |  |  |  |  |  |  |  |  |  |  |  |  |  |  |  |  |  |  |  |  |  |  |  |  |  |  |  |  |  |  |  |  |  |  |  |  |  |  |  |  |  |  |  |  |  |  |  |  |  |  |  |  |  |  |  |  |  |  |  |  |  |  |  |  |  |  |  |  |  |  |  |  |  |  |  |  |  |  |  |  |  |  |  |  |  |  |  |  |  |  |  |  |  |  |  |  |  |  |  |  |  |  |  |  |  |  |  |  |  |  |  |  |  |  |  |  |  |  |  |  |  |  |  |  |  |  |  |  |  |  |  |  |  |  |  |  |  |  |  |  |  |  |  |  |  |  |  |  |  |  |  |  |  |  |  |  |  |  |  |  |  |  |  |  |  |  |  |  |  |  |  |  |  |  |  |  |  |  |  |  |  |  |  |  |  |  |  |  |  |  |  |  |  |  |  |  |  |  |  |  |  |  |  |  |  |  |  |  |  |  |  |  |  |  |  |  |  |  |  |  |  |  |  |  |  |  |  |  |  |  |  |  |  |  |  |  |  |  |  |  |  |  |  |  |  |  |  |  |  |  |  |  |  |  |  |  |  |  |  |  |  |  |  |  |  |  |  |  |  |  |  |  |  |  |  |  |  |  |  |  |  |  |  |  |  |  |  |  |  |  |  |  |  |  |  |  |  |  |  |  |  |  |  |  |  |  |  |  |  |  |  |  |  |  |  |  |  |  |  |  |  |  |  |  |  |  |  |  |  |  |  |  |  |  |  |  |  |  |  |  |  |  |  |  |  |  |  |  |  |  |  |  |  |  |  |  |  |  |  |  |  |  |  |  |  |  |  |  |  |  |  |  |  |  |  |  |  |  |  |  |  |  |  |  |  |  |  |  |  |  |  |  |  |  |  |  |  |  |  |  |  |  |  |  |  |  |  |  |  |  |  |  |  |  |  |  |  |  |  |  |  |  |  |  |  |  |  |  |  |  |  |  |  |  |  |  |  |  |  |  |  |  |  |  |  |  |  |  |  |  |  |  |  |  |  |  |  |  |  |  |  |  |  |  |  |  |  |  |  |  |  |  |  |  |  |  |  |  |  |  |  |  |  |  |  |  |  |  |  |  |  |  |  |  |  |  |  |  |  |  |  |  |  |  |  |  |  |  |  |  |  |  |  |  |  |  |  |  |  |  |  |  |  |  |  |  |  |  |  |  |  |  |  |  |  |  |  |  |  |  |  |  |  |  |  |  |  |  |  |  |  |  |  |  |  |  |  |  |  |  |  |  |  |  |  |  |  |  |  |  |  |  |  |  |  |  |  |  |  |  |  |  |  |  |  |  |  |  |  |  |  |  |  |  |  |  |  |  |  |  |  |  |  |  |  |  |  |  |  |  |  |  |  |  |  |  |  |  |  |  |  |  |  |  |  |  |  |  |  |  |  |  |  |  |  |  |  |  |  |  |  |  |  |  |  |  |  |  |  |  |  |  |  |  |  |  |  |  |  |  |  |  |  |  |  |  |  |  |  |  |  |  |  |  |  |  |  |  |  |  |  |  |  |  |  |  |  |  |  |  |  |  |  |  |  |  |  |  |  |  |  |  |  |  |  |  |  |  |  |  |  |  |  |  |  |  |  |  |  |  |  |  |  |  |  |  |  |  |  |  |  |  |  |  |  |  |  |  |  |  |  |  |  |  |  |  |  |  |  |  |  |  |  |  |  |  |  |  |  |  |  |  |  |  |  |  |  |  |  |  |  |  |  |  |  |  |  |  |  |  |  |  |  |  |  |  |  |  |  |  |  |  |  |  |  |  |  |  |  |  |  |  |  |  |  |  |  |  |  |  |  |  |  |  |  |  |  |  |  |  |  |  |  |  |  |  |  |  |  |  |  |  |  |  |  |  |  |  |  |  |  |  |  |  |  |  |  |  |  |  |  |  |  |  |  |  |  |  |  |  |  |  |  |  |  |  |  |  |  |  |  |  |  |  |  |  |  |  |  |  |  |  |  |  |  |  |  |  |  |  |  |  |  |  |  |  |  |  |  |  |  |  |  |  |  |  |  |  |  |  |  |  |  |  |  |  |  |  |  |  |  |  |  |  |  |  |  |  |  |  |  |  |  |  |  |  |  |  |  |  |  |  |  |  |  |  |  |  |  |  |  |  |  |  |  |  |  |  |  |  |  |  |  |  |  |  |  |  |  |  |  |  |  |  |  |  |  |  |  |  |  |  |  |  |  |  |  |  |  |  |  |  |  |  |  |  |  |  |  |  |  |  |  |  |  |  |  |  |  |  |  |  |  |  |  |  |  |  |  |  |  |  |  |  |  |  |  |  |  |  |  |  |  |  |  |  |  |  |  |  |  |  |  |  |  |  |  |  |  |  |  |  |  |  |  |  |  |  |  |  |  |  |  |  |  |  |  |  |  |  |  |  |  |  |  |  |  |  |  |  |  |  |  |  |  |  |  |  |  |  |  |  |  |  |  |  |  |  |  |  |  |  |  |  |  |  |  |  |  |  |  |  |  |  |  |  |  |  |  |  |  |  |  |  |  |  |  |  |  |  |  |  |  |  |  |  |  |  |  |  |  |  |  |  |  |  |  |  |  |  |  |  |  |  |  |  |  |  |  |  |  |  |  |  |  |  |  |  |  |  |  |  |  |  |  |  |  |  |  |  |  |  |  |  |  |  |  |  |  |  |  |  |  |  |  |  |  |  |  |  |  |  |  |  |  |  |  |  |  |  |  |  |  |  |  |  |  |  |  |  |  |  |  |  |  |  |  |  |  |  |  |  |  |  |  |  |  |  |  |  |  |  |  |  |  |  |  |  |  |  |  |  |  |  |  |  |  |  |  |  |  |  |  |  |  |  |  |  |  |  |  |  |  |  |  |  |  |  |  |  |  |  |  |  |  |  |  |  |  |  |  |  |  |  |  |  |  |  |  |  |  |  |  |  |  |  |  |  |  |  |  |  |  |  |  |  |  |  |  |  |  |  |  |  |  |  |  |  |  |  |  |  |  |  |  |  |  |  |  |  |  |  |  |  |  |  |  |  |  |  |  |  |  |  |  |  |  |  |  |  |  |  |  |  |  |  |  |  |  |  |  |  |  |  |  |  |  |  |  |  |  |  |  |  |  |  |  |  |  |  |  |  |  |  |  |  |  |  |  |  |  |  |  |  |  |  |  |  |  |  |  |  |  |  |  |  |  |  |  |  |  |  |  |  |  |  |  |  |  |  |  |  |  |  |  |  |  |  |  |  |  |  |  |  |  |  |  |  |  |  |  |  |  |  |  |  |  |  |  |  |  |  |  |  |  |  |  |  |  |  |  |  |  |  |  |  |  |  |  |  |  |  |  |  |  |  |  |  |  |  |  |  |  |  |  |  |  |  |  |  |  |  |  |  |  |  |  |  |  |  |  |  |  |  |  |  |  |  |  |  |  |  |  |  |  |  |  |  |  |  |  |  |  |  |  |  |  |  |  |  |  |  |  |  |  |  |  |  |  |  |  |  |  |  |  |  |  |  |  |  |  |  |  |  |  |  |  |  |  |  |  |  |  |  |  |  |  |  |  |  |  |  |  |  |  |  |  |  |  |  |  |  |  |  |  |  |  |  |  |  |  |  |  |  |  |  |  |  |  |  |  |  |  |  |  |  |  |  |  |  |  |  |  |  |  |  |  |  |  |  |  |  |  |  |  |  |  |  |  |  |  |  |  |  |  |  |  |  |  |  |  |  |  |  |  |  |  |  |  |  |  |  |  |  |  |  |  |  |  |  |  |  |  |  |  |  |  |  |  |  |  |  |  |  |  |  |  |  |  |  |  |  |  |  |  |  |  |  |  |  |  |  |  |  |  |  |  |  |  |  |  |  |  |  |  |  |  |  |  |  |  |  |  |  |  |  |  |  |  |  |  |  |  |  |  |  |  |  |  |  |  |  |  |  |  |  |  |  |  |  |  |  |  |  |  |  |  |  |  |  |  |  |  |  |  |  |  |  |  |  |  |  |  |  |  |  |  |  |  |  |  |  |  |  |  |  |  |  |  |  |  |  |  |  |  |  |  |  |  |  |  |  |  |  |  |  |  |  |  |  |  |  |  |  |  |  |  |  |  |  |  |  |  |  |  |  |  |  |  |  |  |  |  |  |  |  |  |  |  |  |  |  |  |  |  |  |  |  |  |  |  |  |  |  |  |  |  |  |  |  |  |  |  |  |  |  |  |  |  |  |  |  |  |  |  |  |  |  |  |  |  |  |  |  |  |  |  |  |  |  |  |  |  |  |  |  |  |  |  |  |  |  |  |  |  |  |  |  |  |  |  |  |  |  |  |  |  |  |  |  |  |  |  |  |  |  |  |  |  |  |  |  |  |  |  |  |  |  |  |  |  |  |  |  |  |  |  |  |  |  |  |  |  |  |  |  |  |  |  |  |  |  |  |  |  |  |  |  |  |  |  |  |  |  |  |  |  |  |  |  |  |  |  |  |  |  |  |  |  |  |  |  |  |  |  |  |  |  |  |  |  |  |  |  |  |  |  |  |  |  |  |  |  |  |  |  |  |  |  |  |  |  |  |  |  |  |  |  |  |  |  |  |  |  |  |  |  |  |  |  |  |  |  |  |  |  |  |  |  |  |  |  |  |  |  |  |  |  |  |  |  |  |  |  |  |  |  |  |  |  |  |  |  |  |  |  |  |  |  |  |  |  |  |  |  |  |  |  |  |  |  |  |  |  |  |  |  |  |  |  |  |  |  |  |  |  |  |  |  |  |  |  |  |  |  |  |  |  |  |  |  |  |  |  |  |  |  |  |  |  |  |  |  |  |  |  |  |  |  |  |  |  |  |  |  |  |  |  |  |  |  |  |  |  |  |  |  |  |  |  |  |  |  |  |  |  |  |  |  |  |  |  |  |  |  |  |  |  |  |  |  |  |  |  |  |  |  |  |  |  |  |  |  |  |  |  |  |  |  |  |  |  |  |  |  |  |  |  |  |  |  |  |  |  |  |  |  |  |  |  |  |  |  |  |  |  |  |  |  |  |  |  |  |  |  |  |  |  |  |  |  |  |  |  |  |  |  |  |  |  |  |  |  |  |  |  |  |  |  |  |  |  |  |  |  |  |  |  |  |  |  |  |  |  |  |  |  |  |  |  |  |  |  |  |  |  |  |  |  |  |  |  |  |  |  |  |  |  |  |  |  |  |  |  |  |  |  |  |  |  |  |  |  |  |  |  |  |  |  |  |  |  |  |  |  |  |  |  |  |  |  |  |  |  |  |  |  |  |  |  |  |  |  |  |  |  |  |  |  |  |  |  |  |  |  |  |  |  |  |  |  |  |  |  |  |  |  |  |  |  |  |  |  |  |  |  |  |  |  |  |  |  |  |  |  |  |  |  |  |  |  |  |  |  |  |  |  |  |  |  |  |  |  |  |  |  |  |  |  |  |  |  |  |  |  |  |  |  |  |  |  |  |  |  |  |  |  |  |  |  |  |  |  |  |  |  |  |  |  |  |  |  |  |  |  |  |  |  |  |  |  |  |  |  |  |  |  |  |  |  |  |  |  |  |  |  |  |  |  |  |  |  |  |  |  |  |  |  |  |  |  |  |  |  |  |  |  |  |  |  |  |  |  |  |  |  |  |  |  |  |  |  |  |  |  |  |  |  |  |  |  |  |  |  |  |  |  |  |  |  |  |  |  |  |  |  |  |  |  |  |  |  |  |  |  |  |  |  |  |  |  |  |  |  |  |  |  |  |  |  |  |  |  |  |  |  |  |  |  |  |  |  |  |  |  |  |  |  |  |  |  |  |  |  |  |  |  |  |  |  |  |  |  |  |  |  |  |  |  |  |  |  |  |  |  |  |  |  |  |  |  |  |  |  |  |  |  |  |  |  |  |  |  |  |  |  |  |  |  |  |  |  |  |  |  |  |  |  |  |  |  |  |  |  |  |  |  |  |  |  |  |  |  |  |  |  |  |  |  |  |  |  |  |  |  |  |  |  |  |  |  |  |  |  |  |  |  |  |  |  |  |  |  |  |  |  |  |  |  |  |  |  |  |  |  |  |  |  |  |  |  |  |  |  |  |  |  |  |  |  |  |  |  |  |  |  |  |  |  |  |  |  |  |  |  |  |  |  |  |  |  |  |  |  |  |  |  |  |  |  |  |  |  |  |  |  |  |  |  |  |  |  |  |  |  |  |  |  |  |  |  |  |  |  |  |  |  |  |  |  |  |  |  |  |  |  |  |  |  |  |  |  |  |  |  |  |  |  |  |  |  |  |  |  |  |  |  |  |  |  |  |  |  |  |  |  |  |  |  |  |  |  |  |  |  |  |  |  |  |  |  |  |  |  |  |  |  |  |  |  |  |  |  |  |  |  |  |  |  |  |  |  |  |  |  |  |  |  |  |  |  |  |  |  |  |  |  |  |  |  |  |  |  |  |  |  |  |  |  |  |  |  |  |  |  |  |  |  |  |  |  |  |  |  |  |  |  |  |  |  |  |  |  |  |  |  |  |  |  |  |  |  |  |  |  |  |  |  |  |  |  |  |  |  |  |  |  |  |  |  |  |  |  |  |  |  |  |  |  |  |  |  |  |  |  |  |  |  |  |  |  |  |  |  |  |  |  |  |  |  |  |  |  |  |  |  |  |  |  |  |  |  |  |  |  |  |  |  |  |  |  |  |  |  |  |  |  |  |  |  |  |  |  |  |  |  |  |  |  |  |  |  |  |  |  |  |  |  |  |  |  |  |  |  |  |  |  |  |  |  |  |  |  |  |  |  |  |  |  |  |  |  |  |  |  |  |  |  |  |  |  |  |  |  |  |  |  |  |  |  |  |  |  |  |  |  |  |  |  |  |  |  |  |  |  |  |  |  |  |  |  |  |  |  |  |  |  |  |  |  |  |  |  |  |  |  |  |  |  |  |  |  |  |  |  |  |  |  |  |  |  |  |  |  |  |  |  |  |  |  |  |  |  |  |  |  |  |  |  |  |  |  |  |  |  |  |  |  |  |  |  |  |  |  |  |  |  |  |  |  |  |  |  |  |  |  |  |  |  |  |  |  |  |  |  |  |  |  |  |  |  |  |  |  |  |  |  |  |  |  |  |  |  |  |  |  |  |  |  |  |  |  |  |  |  |  |  |  |  |  |  |  |  |  |  |  |  |  |  |  |  |  |  |  |  |  |  |  |  |  |  |  |  |  |  |  |  |  |  |  |  |  |  |  |  |  |  |  |  |  |  |  |  |  |  |  |  |  |  |  |  |  |  |  |  |  |  |  |  |  |  |  |  |  |  |  |  |  |  |  |  |  |  |  |  |  |  |  |  |  |  |  |  |  |  |  |  |  |  |  |  |  |  |  |  |  |  |  |  |  |  |  |  |  |  |  |  |  |  |  |  |  |  |  |  |  |  |  |  |  |  |  |  |  |  |  |  |  |  |  |  |  |  |  |  |  |  |  |  |  |  |  |  |  |  |  |  |  |  |  |  |  |  |  |  |  |  |  |  |  |  |  |  |  |  |  |  |  |  |  |  |  |  |  |  |  |  |  |  |  |  |  |  |  |  |  |  |  |  |  |  |  |  |  |  |  |  |  |  |  |  |  |  |  |  |  |  |  |  |  |  |  |  |  |  |  |  |  |  |  |  |  |  |  |  |  |  |  |  |  |  |  |  |  |  |  |  |  |  |  |  |  |  |  |  |  |  |  |  |  |  |  |  |  |  |  |  |  |  |  |  |  |  |  |  |  |  |  |  |  |  |  |  |  |  |  |  |  |  |  |  |  |  |  |  |  |  |  |  |  |  |  |  |  |  |  |  |  |  |  |  |  |  |  |  |  |  |  |  |  |  |  |  |  |  |  |  |  |  |  |  |  |  |  |  |  |  |  |  |  |  |  |  |  |  |  |  |  |  |  |  |  |  |  |  |  |  |  |  |  |  |  |  |  |  |  |  |  |  |  |  |  |  |  |  |  |  |  |  |  |  |  |  |  |  |  |  |  |  |  |  |  |  |  |  |  |  |  |  |  |  |  |  |  |  |  |  |  |  |  |  |  |  |  |  |  |  |  |  |  |  |  |  |  |  |  |  |  |  |  |  |  |  |  |  |  |  |  |  |  |  |  |  |  |  |  |  |  |  |  |  |  |  |  |  |  |  |  |  |  |  |  |  |  |  |  |  |  |  |  |  |  |  |  |  |  |  |  |  |  |  |  |  |  |  |  |  |  |  |  |  |  |  |  |  |  |  |  |  |  |  |  |  |  |  |  |  |  |  |  |  |  |  |  |  |  |  |  |  |  |  |  |  |  |  |  |  |  |  |  |  |  |  |  |  |  |  |  |  |  |  |  |  |  |  |  |  |  |  |  |  |  |  |  |  |  |  |  |  |  |  |  |  |  |  |  |  |  |  |  |  |  |  |  |  |  |  |  |  |  |  |  |  |  |  |  |  |  |  |  |  |  |  |  |  |  |  |  |  |  |  |  |  |  |  |  |  |  |  |  |  |  |  |  |  |  |  |  |  |  |  |  |  |  |  |  |  |  |  |  |  |  |  |  |  |  |  |  |  |  |  |  |  |  |  |  |  |  |  |  |  |  |  |  |  |  |  |  |  |  |  |  |  |  |  |  |  |  |  |  |  |  |  |  |  |  |  |  |  |  |  |  |  |  |  |  |  |  |  |  |  |  |  |  |  |  |  |  |  |  |  |  |  |  |  |  |  |  |  |  |  |  |  |  |  |  |  |  |  |  |  |  |  |  |  |  |  |  |  |  |  |  |  |  |  |  |  |  |  |  |  |  |  |  |  |  |  |  |  |  |  |  |  |  |  |  |  |  |  |  |  |  |  |  |  |  |  |  |  |  |  |  |  |  |  |  |  |  |  |  |  |  |  |  |  |  |  |  |  |  |  |  |  |  |  |  |  |  |  |  |  |  |  |  |  |  |  |  |  |  |  |  |  |  |  |  |  |  |  |  |  |  |  |  |  |  |  |  |  |  |  |  |  |  |  |  |  |  |  |  |  |  |  |  |  |  |  |  |  |  |  |  |  |  |  |  |  |  |  |  |  |  |  |  |  |  |  |  |  |  |  |  |  |  |  |  |  |  |  |  |  |  |  |  |  |  |  |  |  |  |  |  |  |  |  |  |  |  |  |  |  |  |  |  |  |  |  |  |  |  |  |  |  |  |  |  |  |  |  |  |  |  |  |  |  |  |  |  |  |  |  |  |  |  |  |  |  |  |  |  |  |  |  |  |  |  |  |  |  |  |  |  |  |  |  |  |  |  |  |  |  |  |  |  |  |  |  |  |  |  |  |  |  |  |  |  |  |  |  |  |  |  |  |  |  |  |  |  |  |  |  |  |  |  |  |  |  |  |  |  |  |  |  |  |  |  |  |  |  |  |  |  |  |  |  |  |  |  |  |  |  |  |  |  |  |  |  |  |  |  |  |  |  |  |  |  |  |  |  |  |  |  |  |  |  |  |  |  |  |  |  |  |  |  |  |  |  |  |  |  |  |  |  |  |  |  |  |  |  |  |  |  |  |  |  |  |  |  |  |  |  |  |  |  |  |  |  |  |  |  |  |  |  |  |  |  |  |  |  |  |  |  |  |  |  |  |  |  |  |  |  |  |  |  |  |  |  |  |  |  |  |  |  |  |  |  |  |  |  |  |  |  |  |  |  |  |  |  |  |  |  |  |  |  |  |  |  |  |  |  |  |  |  |  |  |  |  |  |  |  |  |  |  |  |  |  |  |  |  |  |  |  |  |  |  |  |  |  |  |  |  |  |  |  |  |  |  |  |  |  |  |  |  |  |  |  |  |  |  |  |  |  |  |  |  |  |  |  |  |  |  |  |  |  |  |  |  |  |  |  |  |  |  |  |  |  |  |  |  |  |  |  |  |  |  |  |  |  |  |  |  |  |  |  |  |  |  |  |  |  |  |  |  |  |  |  |  |  |  |  |  |  |  |  |  |  |  |  |  |  |  |  |  |  |  |  |  |  |  |  |  |  |  |  |  |  |  |  |  |  |  |  |  |  |  |  |  |  |  |  |  |  |  |  |  |  |  |  |  |  |  |  |  |  |  |  |  |  |  |  |  |  |  |  |  |  |  |  |  |  |  |  |  |  |  |  |  |  |  |  |  |  |  |  |  |  |  |  |  |  |  |  |  |  |  |  |  |  |  |  |  |  |  |  |  |  |  |  |  |  |  |  |  |  |  |  |  |  |  |  |  |  |  |  |  |  |  |  |  |  |  |  |  |  |  |  |  |  |  |  |  |  |  |  |  |  |  |  |  |  |  |  |  |  |  |  |  |  |  |  |  |  |  |  |  |  |  |  |  |  |  |  |  |  |  |  |  |  |  |  |  |  |  |  |  |  |  |  |  |  |  |  |  |  |  |  |  |  |  |  |  |  |  |  |  |  |  |  |  |  |  |  |  |  |  |  |  |  |  |  |  |  |  |  |  |  |  |  |  |  |  |  |  |  |  |  |  |  |  |  |  |  |  |  |  |  |  |  |  |  |  |  |  |  |  |  |  |  |  |  |  |  |  |  |  |  |  |  |  |  |  |  |  |  |  |  |  |  |  |  |  |  |  |  |  |  |  |  |  |  |  |  |  |  |  |  |  |  |  |  |  |  |  |  |  |  |  |  |  |  |  |  |  |  |  |  |  |  |  |  |  |  |  |  |  |  |  |  |  |  |  |  |  |  |  |  |  |  |  |  |  |  |  |  |  |  |  |  |  |  |  |  |  |  |  |  |  |  |  |  |  |  |  |  |  |  |  |  |  |  |  |  |  |  |  |  |  |  |  |  |  |  |  |  |  |  |  |  |  |  |  |  |  |  |  |  |  |  |  |  |  |  |  |  |  |  |  |  |  |  |  |  |  |  |  |  |  |  |  |  |  |  |  |  |  |  |  |  |  |  |  |  |  |  |  |  |  |  |  |  |  |  |  |  |  |  |  |  |  |  |  |  |  |  |  |  |  |  |  |  |  |  |  |  |  |  |  |  |  |  |  |  |  |  |  |  |  |  |  |  |  |  |  |  |  |  |  |  |  |  |  |  |  |  |  |  |  |  |  |  |  |  |  |  |  |  |  |  |  |  |  |  |  |  |  |  |  |  |  |  |  |  |  |  |  |  |  |  |  |  |  |  |  |  |  |  |  |  |  |  |  |  |  |  |  |  |  |  |  |  |  |  |  |  |  |  |  |  |  |  |  |  |  |  |  |  |  |  |  |  |  |  |  |  |  |  |  |  |  |  |  |  |  |  |  |  |  |  |  |  |  |  |  |  |  |  |  |  |  |  |  |  |  |  |  |  |  |  |  |  |  |  |  |  |  |  |  |  |  |  |  |  |  |  |  |  |  |  |  |  |  |  |  |  |  |  |  |  |  |  |  |  |  |  |  |  |  |  |  |  |  |  |  |  |  |  |  |  |  |  |  |  |  |  |  |  |  |  |  |  |  |  |  |  |  |  |  |  |  |  |  |  |  |  |  |  |  |  |  |  |  |  |  |  |  |  |  |  |  |  |  |  |  |  |  |  |  |  |  |  |  |  |  |  |  |  |  |  |  |  |  |  |  |  |  |  |  |  |  |  |  |  |  |  |  |  |  |  |  |  |  |  |  |  |  |  |  |  |  |  |  |  |  |  |  |  |  |  |  |  |  |  |  |  |  |  |  |  |  |  |  |  |  |  |  |  |  |  |  |  |  |  |  |  |  |  |  |  |  |  |  |  |  |  |  |  |  |  |  |  |  |  |  |  |  |  |  |  |  |  |  |  |  |  |  |  |  |  |  |  |  |  |  |  |  |  |  |  |  |  |  |  |  |  |  |  |  |  |  |  |  |  |  |  |  |  |  |  |  |  |  |  |  |  |  |  |  |  |  |  |  |  |  |  |  |  |  |  |  |  |  |  |  |  |  |  |  |  |  |  |  |  |  |  |  |  |  |  |  |  |  |  |  |  |  |  |  |  |  |  |  |  |  |  |  |  |  |  |  |  |  |  |  |  |  |  |  |  |  |  |  |  |  |  |  |  |  |  |  |  |  |  |  |  |  |  |  |  |  |  |  |  |  |  |  |  |  |  |  |  |  |  |  |  |  |  |  |  |  |  |  |  |  |  |  |  |  |  |  |  |  |  |  |  |  |  |  |  |  |  |  |  |  |  |  |  |  |  |  |  |  |  |  |  |  |  |  |  |  |  |  |  |  |  |  |  |  |  |  |  |  |  |  |  |  |  |  |  |  |  |  |  |  |  |  |  |  |  |  |  |  |  |  |  |  |  |  |  |  |  |  |  |  |  |  |  |  |  |  |  |  |  |  |  |  |  |  |  |  |  |  |  |  |  |  |  |  |  |  |  |  |  |  |  |  |  |  |  |  |  |  |  |  |  |  |  |  |  |  |  |  |  |  |  |  |  |  |  |  |  |  |  |  |  |  |  |  |  |  |  |  |  |  |  |  |  |  |  |  |  |  |  |  |  |  |  |  |  |  |  |  |  |  |  |  |  |  |  |  |  |  |  |  |  |  |  |  |  |  |  |  |  |  |  |  |  |  |  |  |  |  |  |  |  |  |  |  |  |  |  |  |  |  |  |  |  |  |  |  |  |  |  |  |  |  |  |  |  |  |  |  |  |  |  |  |  |  |  |  |  |  |  |  |  |  |  |  |  |  |  |  |  |  |  |  |  |  |  |  |  |  |  |  |  |  |  |  |  |  |  |  |  |  |  |  |  |  |  |  |  |  |  |  |  |  |  |  |  |  |  |  |  |  |  |  |  |  |  |  |  |  |  |  |  |  |  |  |  |  |  |  |  |  |  |  |  |  |  |  |  |  |  |  |  |  |  |  |  |  |  |  |  |  |  |  |  |  |  |  |  |  |  |  |  |  |  |  |  |  |  |  |  |  |  |  |  |  |  |  |  |  |  |  |  |  |  |  |  |  |  |  |  |  |  |  |  |  |  |  |  |  |  |  |  |  |  |  |  |  |  |  |  |  |  |  |  |  |  |  |  |  |  |  |  |  |  |  |  |  |  |  |  |  |  |  |  |  |  |  |  |  |  |  |  |  |  |  |  |  |  |  |  |  |  |  |  |  |  |  |  |  |  |  |  |  |  |  |  |  |  |  |  |  |  |  |  |  |  |  |  |  |  |  |  |  |  |  |  |  |  |  |  |  |  |  |  |  |  |  |  |  |  |  |  |  |  |  |  |  |  |  |  |  |  |  |  |  |  |  |  |  |  |  |  |  |  |  |  |  |  |  |  |  |  |  |  |  |  |  |  |  |  |  |  |  |  |  |  |  |  |  |  |  |  |  |  |  |  |  |  |  |  |  |  |  |  |  |  |  |  |  |  |  |  |  |  |  |  |  |  |  |  |  |  |  |  |  |  |  |  |  |  |  |  |  |  |  |  |  |  |  |  |  |  |  |  |  |  |  |  |  |  |  |  |  |  |  |  |  |  |  |  |  |  |  |  |  |  |  |  |  |  |  |  |  |  |  |  |  |  |  |  |  |  |  |  |  |  |  |  |  |  |  |  |  |  |  |  |  |  |  |  |  |  |  |  |  |  |  |  |  |  |  |  |  |  |  |  |  |  |  |  |  |  |  |  |  |  |  |  |  |  |  |  |  |  |  |  |  |  |  |  |  |  |  |  |  |  |  |  |  |  |  |  |  |  |  |  |  |  |  |  |  |  |  |  |  |  |  |  |  |  |  |  |  |  |  |  |  |  |  |  |  |  |  |  |  |  |  |  |  |  |  |  |  |  |  |  |  |  |  |  |  |  |  |  |  |  |  |  |  |  |  |  |  |  |  |  |  |  |  |  |  |  |  |  |  |  |  |  |  |  |  |  |  |  |  |  |  |  |  |  |  |  |  |  |  |  |  |  |  |  |  |  |  |  |  |  |  |  |  |  |  |  |  |  |  |  |  |  |  |  |  |  |  |  |  |  |  |  |  |  |  |  |  |  |  |  |  |  |  |  |  |  |  |  |  |  |  |  |  |  |  |  |  |  |  |  |  |  |  |  |  |  |  |  |  |  |  |  |  |  |  |  |  |  |  |  |  |  |  |  |  |  |  |  |  |  |  |  |  |  |  |  |  |  |  |  |  |  |  |  |  |  |  |  |  |  |  |  |  |  |  |  |  |  |  |  |  |  |  |  |  |  |  |  |  |  |  |  |  |  |  |  |  |  |  |  |  |  |  |  |  |  |  |  |  |  |  |  |  |  |  |  |  |  |  |  |  |  |  |  |  |  |  |  |  |  |  |  |  |  |  |  |  |  |  |  |  |  |  |  |  |  |  |  |  |  |  |  |  |  |  |  |  |  |  |  |  |  |  |  |  |  |  |  |  |  |  |  |  |  |  |  |  |  |  |  |  |  |  |  |  |  |  |  |  |  |  |  |  |  |  |  |  |  |  |  |  |  |  |  |  |  |  |  |  |  |  |  |  |  |  |  |  |  |  |  |  |  |  |  |  |  |  |  |  |  |  |  |  |  |  |  |  |  |  |  |  |  |  |  |  |  |  |  |  |  |  |  |  |  |  |  |  |  |  |  |  |  |  |  |  |  |  |  |  |  |  |  |  |  |  |  |  |  |  |  |  |  |  |  |  |  |  |  |  |  |  |  |  |  |  |  |  |  |  |  |  |  |  |  |  |  |  |  |  |  |  |  |  |  |  |  |  |  |  |  |  |  |  |  |  |  |  |  |  |  |  |  |  |  |  |  |  |  |  |  |  |  |  |  |  |  |  |  |  |  |  |  |  |  |  |  |  |  |  |  |  |  |  |  |  |  |  |  |  |  |  |  |  |  |  |  |  |  |  |  |  |  |  |  |  |  |  |  |  |  |  |  |  |  |  |  |  |  |  |  |  |  |  |  |  |  |  |  |  |  |  |  |  |  |  |  |  |  |  |  |  |  |  |  |  |  |  |  |  |  |  |  |  |  |  |  |  |  |  |  |  |  |  |  |  |  |  |  |  |  |  |  |  |  |  |  |  |  |  |  |  |  |  |  |  |  |  |  |  |  |  |  |  |  |  |  |  |  |  |  |  |  |  |  |  |  |  |  |  |  |  |  |  |  |  |  |  |  |  |  |  |  |  |  |  |  |  |  |  |  |  |  |  |  |  |  |  |  |  |  |  |  |  |  |  |  |  |  |  |  |  |  |  |  |  |  |  |  |  |  |  |  |  |  |  |  |  |  |  |  |  |  |  |  |  |  |  |  |  |  |  |  |  |  |  |  |  |  |  |  |  |  |  |  |  |  |  |  |  |  |  |  |  |  |  |  |  |  |  |  |  |  |  |  |  |  |  |  |  |  |  |  |  |  |  |  |  |  |  |  |  |  |  |  |  |  |  |  |  |  |  |  |  |  |  |  |  |  |  |  |  |  |  |  |  |  |  |  |  |  |  |  |  |  |  |  |  |  |  |  |  |  |  |  |  |  |  |  |  |  |  |  |  |  |  |  |  |  |  |  |  |  |  |  |  |  |  |  |  |  |  |  |  |  |  |  |  |  |  |  |  |  |  |  |  |  |  |  |  |  |  |  |  |  |  |  |  |  |  |  |  |  |  |  |  |  |  |  |  |  |  |  |  |  |  |  |  |  |  |  |  |  |  |  |  |  |  |  |  |  |  |  |  |  |  |  |  |  |  |  |  |  |  |  |  |  |  |  |  |  |  |  |  |  |  |  |  |  |  |  |  |  |  |  |  |  |  |  |  |  |  |  |  |  |  |  |  |  |  |  |  |  |  |  |  |  |  |  |  |  |  |  |  |  |  |  |  |  |  |  |  |  |  |  |  |  |  |  |  |  |  |  |  |  |  |  |  |  |  |  |  |  |  |  |  |  |  |  |  |  |  |  |  |  |  |  |  |  |  |  |  |  |  |  |  |  |  |  |  |  |  |  |  |  |  |  |  |  |  |  |  |  |  |  |  |  |  |  |  |  |  |  |  |  |  |  |  |  |  |  |  |  |  |  |  |  |  |  |  |  |  |  |  |  |  |  |  |  |  |  |  |  |  |  |  |  |  |  |  |  |  |  |  |  |  |  |  |  |  |  |  |  |  |  |  |  |  |  |  |  |  |  |  |  |  |  |  |  |  |  |  |  |  |  |  |  |  |  |  |  |  |  |  |  |  |  |  |  |  |  |  |  |  |  |  |  |  |  |  |  |  |  |  |  |  |  |  |  |  |  |  |  |  |  |  |  |  |  |  |  |  |  |  |  |  |  |  |  |  |  |  |  |  |  |  |  |  |  |  |  |  |  |  |  |  |  |  |  |  |  |  |  |  |  |  |  |  |  |  |  |  |  |  |  |  |  |  |  |  |  |  |  |  |  |  |  |  |  |  |  |  |  |  |  |  |  |  |  |  |  |  |  |  |  |  |  |  |  |  |  |  |  |  |  |  |  |  |  |  |  |  |  |  |  |  |  |  |  |  |  |  |  |  |  |  |  |  |  |  |  |  |  |  |  |  |  |  |  |  |  |  |  |  |  |  |  |  |  |  |  |  |  |  |  |  |  |  |  |  |  |  |  |  |  |  |  |  |  |  |  |  |  |  |  |  |  |  |  |  |  |  |  |  |  |  |  |  |  |  |  |  |  |  |  |  |  |  |  |  |  |  |  |  |  |  |  |  |  |  |  |  |  |  |  |  |  |  |  |  |  |  |  |  |  |  |  |  |  |  |  |  |  |  |  |  |  |  |  |  |  |  |  |  |  |  |  |  |  |  |  |  |  |  |  |  |  |  |  |  |  |  |  |  |  |  |  |  |  |  |  |  |  |  |  |  |  |  |  |  |  |  |  |  |  |  |  |  |  |  |  |  |  |  |  |  |  |  |  |  |  |  |  |  |  |  |  |  |  |  |  |  |  |  |  |  |  |  |  |  |  |  |  |  |  |  |  |  |  |  |  |  |  |  |  |  |  |  |  |  |  |  |  |  |  |  |  |  |  |  |  |  |  |  |  |  |  |  |  |  |  |  |  |  |  |  |  |  |  |  |  |  |  |  |  |  |  |  |  |  |  |  |  |  |  |  |  |  |  |  |  |  |  |  |  |  |  |  |  |  |  |  |  |  |  |  |  |  |  |  |  |  |  |  |  |  |  |  |  |  |  |  |  |  |  |  |  |  |  |  |  |  |  |  |  |  |  |  |  |  |  |  |  |  |  |  |  |  |  |  |  |  |  |  |  |  |  |  |  |  |  |  |  |  |  |  |  |  |  |  |  |  |  |  |  |  |  |  |  |  |  |  |  |  |  |  |  |  |  |  |  |  |  |  |  |  |  |  |  |  |  |  |  |  |  |  |  |  |  |  |  |  |  |  |  |  |  |  |  |  |  |  |  |  |  |  |  |  |  |  |  |  |  |  |  |  |  |  |  |  |  |  |  |  |  |  |  |  |  |  |  |  |  |  |  |  |  |  |  |  |  |  |  |  |  |  |  |  |  |  |  |  |  |  |  |  |  |  |  |  |  |  |  |  |  |  |  |  |  |  |  |  |  |  |  |  |  |  |  |  |  |  |  |  |  |  |  |  |  |  |  |  |  |  |  |  |  |  |  |  |  |  |  |  |  |  |  |  |  |  |  |  |  |  |  |  |  |  |  |  |  |  |  |  |  |  |  |  |  |  |  |  |  |  |  |  |  |  |  |  |  |  |  |  |  |  |  |  |  |  |  |  |  |  |  |  |  |  |  |  |  |  |  |  |  |  |  |  |  |  |  |  |  |  |  |  |  |  |  |  |  |  |  |  |  |  |  |  |  |  |  |  |  |  |  |  |  |  |  |  |  |  |  |  |  |  |  |  |  |  |  |  |  |  |  |  |  |  |  |  |  |  |  |  |  |  |  |  |  |  |  |  |  |  |  |  |  |  |  |  |  |  |  |  |  |  |  |  |  |  |  |  |  |  |  |  |  |  |  |  |  |  |  |  |  |  |  |  |  |  |  |  |  |  |  |  |  |  |  |  |  |  |  |  |  |  |  |  |  |  |  |  |  |  |  |  |  |  |  |  |  |  |  |  |  |  |  |  |  |  |  |  |  |  |  |  |  |  |  |  |  |  |  |  |  |  |  |  |  |  |  |  |  |  |  |  |  |  |  |  |  |  |  |  |  |  |  |  |  |  |  |  |  |  |  |  |  |  |  |  |  |  |  |  |  |  |  |  |  |  |  |  |  |  |  |  |  |  |  |  |  |  |  |  |  |  |  |  |  |  |  |  |  |  |  |  |  |  |  |  |  |  |  |  |  |  |  |  |  |  |  |  |  |  |  |  |  |  |  |  |  |  |  |  |  |  |  |  |  |  |  |  |  |  |  |  |  |  |  |  |  |  |  |  |  |  |  |  |  |  |  |  |  |  |  |  |  |  |  |  |  |  |  |  |  |  |  |  |  |  |  |  |  |  |  |  |  |  |  |  |  |  |  |  |  |  |  |  |  |  |  |  |  |  |  |  |  |  |  |  |  |  |  |  |  |  |  |  |  |  |  |  |  |  |  |  |  |  |  |  |  |  |  |  |  |  |  |  |  |  |  |  |  |  |  |  |  |  |  |  |  |  |  |  |  |  |  |  |  |  |  |  |  |  |  |  |  |  |  |  |  |  |  |  |  |  |  |  |  |  |  |  |  |  |  |  |  |  |  |  |  |  |  |  |  |  |  |  |  |  |  |  |  |  |  |  |  |  |  |  |  |  |  |  |  |  |  |  |  |  |  |  |  |  |  |  |  |  |  |  |  |  |  |  |  |  |  |  |  |  |  |  |  |  |  |  |  |  |  |  |  |  |  |  |  |  |  |  |  |  |  |  |  |  |  |  |  |  |  |  |  |  |  |  |  |  |  |  |  |  |  |  |  |  |  |  |  |  |  |  |  |  |  |  |  |  |  |  |  |  |  |  |  |  |  |  |  |  |  |  |  |  |  |  |  |  |  |  |  |  |  |  |  |  |  |  |  |  |  |  |  |  |  |  |  |  |  |  |  |  |  |  |  |  |  |  |  |  |  |  |  |  |  |  |  |  |  |  |  |  |  |  |  |  |  |  |  |  |  |  |  |  |  |  |  |  |  |  |  |  |  |  |  |  |  |  |  |  |  |  |  |  |  |  |  |  |  |  |  |  |  |  |  |  |  |  |  |  |  |  |  |  |  |  |  |  |  |  |  |  |  |  |  |  |  |  |  |  |  |  |  |  |  |  |  |  |  |  |  |  |  |  |  |  |  |  |  |  |  |  |  |  |  |  |  |  |  |  |  |  |  |  |  |  |  |  |  |  |  |  |  |  |  |  |  |  |  |  |  |  |  |  |  |  |  |  |  |  |  |  |  |  |  |  |  |  |  |  |  |  |  |  |  |  |  |  |  |  |  |  |  |  |  |  |  |  |  |  |  |  |  |  |  |  |  |  |  |  |  |  |  |  |  |  |  |  |  |  |  |  |  |  |  |  |  |  |  |  |  |  |  |  |  |  |  |  |  |  |  |  |  |  |  |  |  |  |  |  |  |  |  |  |  |  |  |  |  |  |  |  |  |  |  |  |  |  |  |  |  |  |  |  |  |  |  |  |  |  |  |  |  |  |  |  |  |  |  |  |  |  |  |  |  |  |  |  |  |  |  |  |  |  |  |  |  |  |  |  |  |  |  |  |  |  |  |  |  |  |  |  |  |  |  |  |  |  |  |  |  |  |  |  |  |  |  |  |  |  |  |  |  |  |  |  |  |  |  |  |  |  |  |  |  |  |  |  |  |  |  |  |  |  |  |  |  |  |  |  |  |  |  |  |  |  |  |  |  |  |  |  |  |  |  |  |  |  |  |  |  |  |  |  |  |  |  |  |  |  |  |  |  |  |  |  |  |  |  |  |  |  |  |  |  |  |  |  |  |  |  |  |  |  |  |  |  |  |  |  |  |  |  |  |  |  |  |  |  |  |  |  |  |  |  |  |  |  |  |  |  |  |  |  |  |  |  |  |  |  |  |  |  |  |  |  |  |  |  |  |  |  |  |  |  |  |  |  |  |  |  |  |  |  |  |  |  |  |  |  |  |  |  |  |  |  |  |  |  |  |  |  |  |  |  |  |  |  |  |  |  |  |  |  |  |  |  |  |  |  |  |  |  |  |  |  |  |  |  |  |  |  |  |  |  |  |  |  |  |  |  |  |  |  |  |  |  |  |  |  |  |  |  |  |  |  |  |  |  |  |  |  |  |  |  |  |  |  |  |  |  |  |  |  |  |  |  |  |  |  |  |  |  |  |  |  |  |  |  |  |  |  |  |  |  |  |  |  |  |  |  |  |  |  |  |  |  |  |  |  |  |  |  |  |  |  |  |  |  |  |  |  |  |  |  |  |  |  |  |  |  |  |  |  |  |  |  |  |  |  |  |  |  |  |  |  |  |  |  |  |  |  |  |  |  |  |  |  |  |  |  |  |  |  |  |  |  |  |  |  |  |  |  |  |  |  |  |  |  |  |  |  |  |  |  |  |  |  |  |  |  |  |  |  |  |  |  |  |  |  |  |  |  |  |  |  |  |  |  |  |  |  |  |  |  |  |  |  |  |  |  |  |  |  |  |  |  |  |  |  |  |  |  |  |  |  |  |  |  |  |  |  |  |  |  |  |  |  |  |  |  |  |  |  |  |  |  |  |  |  |  |  |  |  |  |  |  |  |  |  |  |  |  |  |  |  |  |  |  |  |  |  |  |  |  |  |  |  |  |  |  |  |  |  |  |  |  |  |  |  |  |  |  |  |  |  |  |  |  |  |  |  |  |  |  |  |  |  |  |  |  |  |  |  |  |  |  |  |  |  |  |  |  |  |  |  |  |  |  |  |  |  |  |  |  |  |  |  |  |  |  |  |  |  |  |  |  |  |  |  |  |  |  |  |  |  |  |  |  |  |  |  |  |  |  |  |  |  |  |  |  |  |  |  |  |  |  |  |  |  |  |  |  |  |  |  |  |  |  |  |  |  |  |  |  |  |  |  |  |  |  |  |  |  |  |  |  |  |  |  |  |  |  |  |  |  |  |  |  |  |  |  |  |  |  |  |  |  |  |  |  |  |  |  |  |  |  |  |  |  |  |  |  |  |  |  |  |  |  |  |  |  |  |  |  |  |  |  |  |  |  |  |  |  |  |  |  |  |  |  |  |  |  |  |  |  |  |  |  |  |  |  |  |  |  |  |  |  |  |  |  |  |  |  |  |  |  |  |  |  |  |  |  |  |  |  |  |  |  |  |  |  |  |  |  |  |  |  |  |  |  |  |  |  |  |  |  |  |  |  |  |  |  |  |  |  |  |  |  |  |  |  |  |  |  |  |  |  |  |  |  |  |  |  |  |  |  |  |  |  |  |  |  |  |  |  |  |  |  |  |  |  |  |  |  |  |  |  |  |  |  |  |  |  |  |  |  |  |  |  |  |  |  |  |  |  |  |  |  |  |  |  |  |  |  |  |  |  |  |  |  |  |  |  |  |  |  |  |  |  |  |  |  |  |  |  |  |  |  |  |  |  |  |  |  |  |  |  |  |  |  |  |  |  |  |  |  |  |  |  |  |  |  |  |  |  |  |  |  |  |  |  |  |  |  |  |  |  |  |  |  |  |  |  |  |  |  |  |  |  |  |  |  |  |  |  |  |  |  |  |  |  |  |  |  |  |  |  |  |  |  |  |  |  |  |  |  |  |  |  |  |  |  |  |  |  |  |  |  |  |  |  |  |  |  |  |  |  |  |  |  |  |  |  |  |  |  |  |  |  |  |  |  |  |  |  |  |  |  |  |  |  |  |  |  |  |  |  |  |  |  |  |  |  |  |  |  |  |  |  |  |  |  |  |  |  |  |  |  |  |  |  |  |  |  |  |  |  |  |  |  |  |  |  |  |  |  |  |  |  |  |  |  |  |  |  |  |  |  |  |  |  |  |  |  |  |  |  |  |  |  |  |  |  |  |  |  |  |  |  |  |  |  |  |  |  |  |  |  |  |  |  |  |  |  |  |  |  |  |  |  |  |  |  |  |  |  |  |  |  |  |  |  |  |  |  |  |  |  |  |  |  |  |  |  |  |  |  |  |  |  |  |  |  |  |  |  |  |  |  |  |  |  |  |  |  |  |  |  |  |  |  |  |  |  |  |  |  |  |  |  |  |  |  |  |  |  |  |  |  |  |  |  |  |  |  |  |  |  |  |  |  |  |  |  |  |  |  |  |  |  |  |  |  |  |  |  |  |  |  |  |  |  |  |  |  |  |  |  |  |  |  |  |  |  |  |  |  |  |  |  |  |  |  |  |  |  |  |  |  |  |  |  |  |  |  |  |  |  |  |  |  |  |  |  |  |  |  |  |  |  |  |  |  |  |  |  |  |  |  |  |  |  |  |  |  |  |  |  |  |  |  |  |  |  |  |  |  |  |  |  |  |  |  |  |  |  |  |  |  |  |  |  |  |  |  |  |  |  |  |  |  |  |  |  |  |  |  |  |  |  |  |  |  |  |  |  |  |  |  |  |  |  |  |  |  |  |  |  |  |  |  |  |  |  |  |  |  |  |  |  |  |  |  |  |  |  |  |  |  |  |  |  |  |  |  |  |  |  |  |  |  |  |  |  |  |  |  |  |  |  |  |  |  |  |  |  |  |  |  |  |  |  |  |  |  |  |  |  |  |  |  |  |  |  |  |  |  |  |  |  |  |  |  |  |  |  |  |  |  |  |  |  |  |  |  |  |  |  |  |  |  |  |  |  |  |  |  |  |  |  |  |  |  |  |  |  |  |  |  |  |  |  |  |  |  |  |  |  |  |  |  |  |  |  |  |  |  |  |  |  |  |  |  |  |  |  |  |  |  |  |  |  |  |  |  |  |  |  |  |  |  |  |  |  |  |  |  |  |  |  |  |  |  |  |  |  |  |  |  |  |  |  |  |  |  |  |  |  |  |  |  |  |  |  |  |  |  |  |  |  |  |  |  |  |  |  |  |  |  |  |  |  |  |  |  |  |  |  |  |  |  |  |  |  |  |  |  |  |  |  |  |  |  |  |  |  |  |  |  |  |  |  |  |  |  |  |  |  |  |  |  |  |  |  |  |  |  |  |  |  |  |  |  |  |  |  |  |  |  |  |  |  |  |  |  |  |  |  |  |  |  |  |  |  |  |  |  |  |  |  |  |  |  |  |  |  |  |  |  |  |  |  |  |  |  |  |  |  |  |  |  |  |  |  |  |  |  |  |  |  |  |  |  |  |  |  |  |  |  |  |  |  |  |  |  |  |  |  |  |  |  |  |  |  |  |  |  |  |  |  |  |  |  |  |  |  |  |  |  |  |  |  |  |  |  |  |  |  |  |  |  |  |  |  |  |  |  |  |  |  |  |  |  |  |  |  |  |  |  |  |  |  |  |  |  |  |  |  |  |  |  |  |  |  |  |  |  |  |  |  |  |  |  |  |  |  |  |  |  |  |  |  |  |  |  |  |  |  |  |  |  |  |  |  |  |  |  |  |  |  |  |  |  |  |  |  |  |  |  |  |  |  |  |  |  |  |  |  |  |  |  |  |  |  |  |  |  |  |  |  |  |  |  |  |  |  |  |  |  |  |  |  |  |  |  |  |  |  |  |  |  |  |  |  |  |  |  |  |  |  |  |  |  |  |  |  |  |  |  |  |  |  |  |  |  |  |  |  |  |  |  |  |  |  |  |  |  |  |  |  |  |  |  |  |  |  |  |  |  |  |  |  |  |  |  |  |  |  |  |  |  |  |  |  |  |  |  |  |  |  |  |  |  |  |  |  |  |  |  |  |  |  |  |  |  |  |  |  |  |  |  |  |  |  |  |  |  |  |  |  |  |  |  |  |  |  |  |  |  |  |  |  |  |  |  |  |  |  |  |  |  |  |  |  |  |  |  |  |  |  |  |  |  |  |  |  |  |  |  |  |  |  |  |  |  |  |  |  |  |  |  |  |  |  |  |  |  |  |  |  |  |  |  |  |  |  |  |  |  |  |  |  |  |  |  |  |  |  |  |  |  |  |  |  |  |  |  |  |  |  |  |  |  |  |  |  |  |  |  |  |  |  |  |  |  |  |  |  |  |  |  |  |  |  |  |  |  |  |  |  |  |  |  |  |  |  |  |  |  |  |  |  |  |  |  |  |  |  |  |  |  |  |  |  |  |  |  |  |  |  |  |  |  |  |  |  |  |  |  |  |  |  |  |  |  |  |  |  |  |  |  |  |  |  |  |  |  |  |  |  |  |  |  |  |  |  |  |  |  |  |  |  |  |  |  |  |  |  |  |  |  |  |  |  |  |  |  |  |  |  |  |  |  |  |  |  |  |  |  |  |  |  |  |  |  |  |  |  |  |  |  |  |  |  |  |  |  |  |  |  |  |  |  |  |  |  |  |  |  |  |  |  |  |  |  |  |  |  |  |  |  |  |  |  |  |  |  |  |  |  |  |  |  |  |  |  |  |  |  |  |  |  |  |  |  |  |  |  |  |  |  |  |  |  |  |  |  |  |  |  |  |  |  |  |  |  |  |  |  |  |  |  |  |  |  |  |  |  |  |  |  |  |  |  |  |  |  |  |  |  |  |  |  |  |  |  |  |  |  |  |  |  |  |  |  |  |  |  |  |  |  |  |  |  |  |  |  |  |  |  |  |  |  |  |  |  |  |  |  |  |  |  |  |  |  |  |  |  |  |  |  |  |  |  |  |  |  |  |  |  |  |  |  |  |  |  |  |  |  |  |  |  |  |  |  |  |  |  |  |  |  |  |  |  |  |  |  |  |  |  |  |  |  |  |  |  |  |  |  |  |  |  |  |  |  |  |  |  |  |  |  |  |  |  |  |  |  |  |  |  |  |  |  |  |  |  |  |  |  |  |  |  |  |  |  |  |  |  |  |  |  |  |  |  |  |  |  |  |  |  |  |  |  |  |  |  |  |  |  |  |  |  |  |  |  |  |  |  |  |  |  |  |  |  |  |  |  |  |  |  |  |  |  |  |  |  |  |  |  |  |  |  |  |  |  |  |  |  |  |  |  |  |  |  |  |  |  |  |  |  |  |  |  |  |  |  |  |  |  |  |  |  |  |  |  |  |  |  |  |  |  |  |  |  |  |  |  |  |  |  |  |  |  |  |  |  |  |  |  |  |  |  |  |  |  |  |  |  |  |  |  |  |  |  |  |  |  |  |  |  |  |  |  |  |  |  |  |  |  |  |  |  |  |  |  |  |  |  |  |  |  |  |  |  |  |  |  |  |  |  |  |  |  |  |  |  |  |  |  |  |  |  |  |  |  |  |  |  |  |  |  |  |  |  |  |  |  |  |  |  |  |  |  |  |  |  |  |  |  |  |  |  |  |  |  |  |  |  |  |  |  |  |  |  |  |  |  |  |  |  |  |  |  |  |  |  |  |  |  |  |  |  |  |  |  |  |  |  |  |  |  |  |  |  |  |  |  |  |  |  |  |  |  |  |  |  |  |  |  |  |  |  |  |  |  |  |  |  |  |  |  |  |  |  |  |  |  |  |  |  |  |  |  |  |  |  |  |  |  |  |  |  |  |  |  |  |  |  |  |  |  |  |  |  |  |  |  |  |  |  |  |  |  |  |  |  |  |  |  |  |  |  |  |  |  |  |  |  |  |  |  |  |  |  |  |  |  |  |  |  |  |  |  |  |  |  |  |  |  |  |  |  |  |  |  |  |  |  |  |  |  |  |  |  |  |  |  |  |  |  |  |  |  |  |  |  |  |  |  |  |  |  |  |  |  |  |  |  |  |  |  |  |  |  |  |  |  |  |  |  |  |  |  |  |  |  |  |  |  |  |  |  |  |  |  |  |  |  |  |  |  |  |  |  |  |  |  |  |  |  |  |  |  |  |  |  |  |  |  |  |  |  |  |  |  |  |  |  |  |  |  |  |  |  |  |  |  |  |  |  |  |  |  |  |  |  |  |  |  |  |  |  |  |  |  |  |  |  |  |  |  |  |  |  |  |  |  |  |  |  |  |  |  |  |  |  |  |  |  |  |  |  |  |  |  |  |  |  |  |  |  |  |  |  |  |  |  |  |  |  |  |  |  |  |  |  |  |  |  |  |  |  |  |  |  |  |  |  |  |  |  |  |  |  |  |  |  |  |  |  |  |  |  |  |  |  |  |  |  |  |  |  |  |  |  |  |  |  |  |  |  |  |  |  |  |  |  |  |  |  |  |  |  |  |  |  |  |  |  |  |  |  |  |  |  |  |  |  |  |  |  |  |  |  |  |  |  |  |  |  |  |  |  |  |  |  |  |  |  |  |  |  |  |  |  |  |  |  |  |  |  |  |  |  |  |  |  |  |  |  |  |  |  |  |  |  |  |  |  |  |  |  |  |  |  |  |  |  |  |  |  |  |  |  |  |  |  |  |  |  |  |  |  |  |  |  |  |  |  |  |  |  |  |  |  |  |  |  |  |  |  |  |  |  |  |  |  |  |  |  |  |  |  |  |  |  |  |  |  |  |  |  |  |  |  |  |  |  |  |  |  |  |  |  |  |  |  |  |  |  |  |  |  |  |  |  |  |  |  |  |  |  |  |  |  |  |  |  |  |  |  |  |  |  |  |  |  |  |  |  |  |  |  |  |  |  |  |  |  |  |  |  |  |  |  |  |  |  |  |  |  |  |  |  |  |  |  |  |  |  |  |  |  |  |  |  |  |  |  |  |  |  |  |  |  |  |  |  |  |  |  |  |  |  |  |  |  |  |  |  |  |  |  |  |  |  |  |  |  |  |  |  |  |  |  |  |  |  |  |  |  |  |  |  |  |  |  |  |  |  |  |  |  |  |  |  |  |  |  |  |  |  |  |  |  |  |  |  |  |  |  |  |  |  |  |  |  |  |  |  |  |  |  |  |  |  |  |  |  |  |  |  |  |  |  |  |  |  |  |  |  |  |  |  |  |  |  |  |  |  |  |  |  |  |  |  |  |  |  |  |  |  |  |  |  |  |  |  |  |  |  |  |  |  |  |  |  |  |  |  |  |  |  |  |  |  |  |  |  |  |  |  |  |  |  |  |  |  |  |  |  |  |  |  |  |  |  |  |  |  |  |  |  |  |  |  |  |  |  |  |  |  |  |  |  |  |  |  |  |  |  |  |  |  |  |  |  |  |  |  |  |  |  |  |  |  |  |  |  |  |  |  |  |  |  |  |  |  |  |  |  |  |  |  |  |  |  |  |  |  |  |  |  |  |  |  |  |  |  |  |  |  |  |  |  |  |  |  |  |  |  |  |  |  |  |  |  |  |  |  |  |  |  |  |  |  |  |  |  |  |  |  |  |  |  |  |  |  |  |  |  |  |  |  |  |  |  |  |  |  |  |  |  |  |  |  |  |  |  |  |  |  |  |  |  |  |  |  |  |  |  |  |  |  |  |  |  |  |  |  |  |  |  |  |  |  |  |  |  |  |  |  |  |  |  |  |  |  |  |  |  |  |  |  |  |  |  |  |  |  |  |  |  |  |  |  |  |  |  |  |  |  |  |  |  |  |  |  |  |  |  |  |  |  |  |  |  |  |  |  |  |  |  |  |  |  |  |  |  |  |  |  |  |  |  |  |  |  |  |  |  |  |  |  |  |  |  |  |  |  |  |  |  |  |  |  |  |  |  |  |  |  |  |  |  |  |  |  |  |  |  |  |  |  |  |  |  |  |  |  |  |  |  |  |  |  |  |  |  |  |  |  |  |  |  |  |  |  |  |  |  |  |  |  |  |  |  |  |  |  |  |  |  |  |  |  |  |  |  |  |  |  |  |  |  |  |  |  |  |  |  |  |  |  |  |  |  |  |  |  |  |  |  |  |  |  |  |  |  |  |  |  |  |  |  |  |  |  |  |  |  |  |  |  |  |  |  |  |  |  |  |  |  |  |  |  |  |  |  |  |  |  |  |  |  |  |  |  |  |  |  |  |  |  |  |  |  |  |  |  |  |  |  |  |  |  |  |  |  |  |  |  |  |  |  |  |  |  |  |  |  |  |  |  |  |  |  |  |  |  |  |  |  |  |  |  |  |  |  |  |  |  |  |  |  |  |  |  |  |  |  |  |  |  |  |  |  |  |  |  |  |  |  |  |  |  |  |  |  |  |  |  |  |  |  |  |  |  |  |  |  |  |  |  |  |  |  |  |  |  |  |  |  |  |  |  |  |  |  |  |  |  |  |  |  |  |  |  |  |  |  |  |  |  |  |  |  |  |  |  |  |  |  |  |  |  |  |  |  |  |  |  |  |  |  |  |  |  |  |  |  |  |  |  |  |  |  |  |  |  |  |  |  |  |  |  |  |  |  |  |  |  |  |  |  |  |  |  |  |  |  |  |  |  |  |  |  |  |  |  |  |  |  |  |  |  |  |  |  |  |  |  |  |  |  |  |  |  |  |  |  |  |  |  |  |  |  |  |  |  |  |  |  |  |  |  |  |  |  |  |  |  |  |  |  |  |  |  |  |  |  |  |  |  |  |  |  |  |  |  |  |  |  |  |  |  |  |  |  |  |  |  |  |  |  |  |  |  |  |  |  |  |  |  |  |  |  |  |  |  |  |  |  |  |  |  |  |  |  |  |  |  |  |  |  |  |  |  |  |  |  |  |  |  |  |  |  |  |  |  |  |  |  |  |  |  |  |  |  |  |  |  |  |  |  |  |  |  |  |  |  |  |  |  |  |  |  |  |  |  |  |  |  |  |  |  |  |  |  |  |  |  |  |  |  |  |  |  |  |  |  |  |  |  |  |  |  |  |  |  |  |  |  |  |  |  |  |  |  |  |  |  |  |  |  |  |  |  |  |  |  |  |  |  |  |  |  |  |  |  |  |  |  |  |  |  |  |  |  |  |  |  |  |  |  |  |  |  |  |  |  |  |  |  |  |  |  |  |  |  |  |  |  |  |  |  |  |  |  |  |  |  |  |  |  |  |  |  |  |  |  |  |  |  |  |  |  |  |  |  |  |  |  |  |  |  |  |  |  |  |  |  |  |  |  |  |  |  |  |  |  |  |  |  |  |  |  |  |  |  |  |  |  |  |  |  |  |  |  |  |  |  |  |  |  |  |  |  |  |  |  |  |  |  |  |  |  |  |  |  |  |  |  |  |  |  |  |  |  |  |  |  |  |  |  |  |  |  |  |  |  |  |  |  |  |  |  |  |  |  |  |  |  |  |  |  |  |  |  |  |  |  |  |  |  |  |  |  |  |  |  |  |  |  |  |  |  |  |  |  |  |  |  |  |  |  |  |  |  |  |  |  |  |  |  |  |  |  |  |  |  |  |  |  |  |  |  |  |  |  |  |  |  |  |  |  |  |  |  |  |  |  |  |  |  |  |  |  |  |  |  |  |  |  |  |  |  |  |  |  |  |  |  |  |  |  |  |  |  |  |  |  |  |  |  |  |  |  |  |  |  |  |  |  |  |  |  |  |  |  |  |  |  |  |  |  |  |  |  |  |  |  |  |  |  |  |  |  |  |  |  |  |  |  |  |  |  |  |  |  |  |  |  |  |  |  |  |  |  |  |  |  |  |  |  |  |  |  |  |  |  |  |  |  |  |  |  |  |  |  |  |  |  |  |  |  |  |  |  |  |  |  |  |  |  |  |  |  |  |  |  |  |  |  |  |  |  |  |  |  |  |  |  |  |  |  |  |  |  |  |  |  |  |  |  |  |  |  |  |  |  |  |  |  |  |  |  |  |  |  |  |  |  |  |  |  |  |  |  |  |  |  |  |  |  |  |  |  |  |  |  |  |  |  |  |  |  |  |  |  |  |  |  |  |  |  |  |  |  |  |  |  |  |  |  |  |  |  |  |  |  |  |  |  |  |  |  |  |  |  |  |  |  |  |  |  |  |  |  |  |  |  |  |  |  |  |  |  |  |  |  |  |  |  |  |  |  |  |  |  |  |  |  |  |  |  |  |  |  |  |  |  |  |  |  |  |  |  |  |  |  |  |  |  |  |  |  |  |  |  |  |  |  |  |  |  |  |  |  |  |  |  |  |  |  |  |  |  |  |  |  |  |  |  |  |  |  |  |  |  |  |  |  |  |  |  |  |  |  |  |  |  |  |  |  |  |  |  |  |  |  |  |  |  |  |  |  |  |  |  |  |  |  |  |  |  |  |  |  |  |  |  |  |  |  |  |  |  |  |  |  |  |  |  |  |  |  |  |  |  |  |  |  |  |  |  |  |  |  |  |  |  |  |  |  |  |  |  |  |  |  |  |  |  |  |  |  |  |  |  |  |  |  |  |  |  |  |  |  |  |  |  |  |  |  |  |  |  |  |  |  |  |  |  |  |  |  |  |  |  |  |  |  |  |  |  |  |  |  |  |  |  |  |  |  |  |  |  |  |  |  |  |  |  |  |  |  |  |  |  |  |  |  |  |  |  |  |  |  |  |  |  |  |  |  |  |  |  |  |  |  |  |  |  |  |  |  |  |  |  |  |  |  |  |  |  |  |  |  |  |  |  |  |  |  |  |  |  |  |  |  |  |  |  |  |  |  |  |  |  |  |  |  |  |  |  |  |  |  |  |  |  |  |  |  |  |  |  |  |  |  |  |  |  |  |  |  |  |  |  |  |  |  |  |  |  |  |  |  |  |  |  |  |  |  |  |  |  |  |  |  |  |  |  |  |  |  |  |  |  |  |  |  |  |  |  |  |  |  |  |  |  |  |  |  |  |  |  |  |  |  |  |  |  |  |  |  |  |  |  |  |  |  |  |  |  |  |  |  |  |  |  |  |  |  |  |  |  |  |  |  |  |  |  |  |  |  |  |  |  |  |  |  |  |  |  |  |  |  |  |  |  |  |  |  |  |  |  |  |  |  |  |  |  |  |  |  |  |  |  |  |  |  |  |  |  |  |  |  |  |  |  |  |  |  |  |  |  |  |  |  |  |  |  |  |  |  |  |  |  |  |  |  |  |  |  |  |  |  |  |  |  |  |  |  |  |  |  |  |  |  |  |  |  |  |  |  |  |  |  |  |  |  |  |  |  |  |  |  |  |  |  |  |  |  |  |  |  |  |  |  |  |  |  |  |  |  |  |  |  |  |  |  |  |  |  |  |  |  |  |  |  |  |  |  |  |  |  |  |  |  |  |  |  |  |  |  |  |  |  |  |  |  |  |  |  |  |  |  |  |  |  |  |  |  |  |  |  |  |  |  |  |  |  |  |  |  |  |  |  |  |  |  |  |  |  |  |  |  |  |  |  |  |  |  |  |  |  |  |  |  |  |  |  |  |  |  |  |  |  |  |  |  |  |  |  |  |  |  |  |  |  |  |  |  |  |  |  |  |  |  |  |  |  |  |  |  |  |  |  |  |  |  |  |  |  |  |  |  |  |  |  |  |  |  |  |  |  |  |  |  |  |  |  |  |  |  |  |  |  |  |  |  |  |  |  |  |  |  |  |  |  |  |  |  |  |  |  |  |  |  |  |  |  |  |  |  |  |  |  |  |  |  |  |  |  |  |  |  |  |  |  |  |  |  |  |  |  |  |  |  |  |  |  |  |  |  |  |  |  |  |  |  |  |  |  |  |  |  |  |  |  |  |  |  |  |  |  |  |  |  |  |  |  |  |  |  |  |  |  |  |  |  |  |  |  |  |  |  |  |  |  |  |  |  |  |  |  |  |  |  |  |  |  |  |  |  |  |  |  |  |  |  |  |  |  |  |  |  |  |  |  |  |  |  |  |  |  |  |  |  |  |  |  |  |  |  |  |  |  |  |  |  |  |  |  |  |  |  |  |  |  |  |  |  |  |  |  |  |  |  |  |  |  |  |  |  |  |  |  |  |  |  |  |  |  |  |  |  |  |  |  |  |  |  |  |  |  |  |  |  |  |  |  |  |  |  |  |  |  |  |  |  |  |  |  |  |  |  |  |  |  |  |  |  |  |  |  |  |  |  |  |  |  |  |  |  |  |  |  |  |  |  |  |  |  |  |  |  |  |  |  |  |  |  |  |  |  |  |  |  |  |  |  |  |  |  |  |  |  |  |  |  |  |  |  |  |  |  |  |  |  |  |  |  |  |  |  |  |  |  |  |  |  |  |  |  |  |  |  |  |  |  |  |  |  |  |  |  |  |  |  |  |  |  |  |  |  |  |  |  |  |  |  |  |  |  |  |  |  |  |  |  |  |  |  |  |  |  |  |  |  |  |  |  |  |  |  |  |  |  |  |  |  |  |  |  |  |  |  |  |  |  |  |  |  |  |  |  |  |  |  |  |  |  |  |  |  |  |  |  |  |  |  |  |  |  |  |  |  |  |  |  |  |  |  |  |  |  |  |  |  |  |  |  |  |  |  |  |  |  |  |  |  |  |  |  |  |  |  |  |  |  |  |  |  |  |  |  |  |  |  |  |  |  |  |  |  |  |  |  |  |  |  |  |  |  |  |  |  |  |  |  |  |  |  |  |  |  |  |  |  |  |  |  |  |  |  |  |  |  |  |  |  |  |  |  |  |  |  |  |  |  |  |  |  |  |  |  |  |  |  |  |  |  |  |  |  |  |  |  |  |  |  |  |  |  |  |  |  |  |  |  |  |  |  |  |  |  |  |  |  |  |  |  |  |  |  |  |  |  |  |  |  |  |  |  |  |  |  |  |  |  |  |  |  |  |  |  |  |  |  |  |  |  |  |  |  |  |  |  |  |  |  |  |  |  |  |  |  |  |  |  |  |  |  |  |  |  |  |  |  |  |  |  |  |  |  |  |  |  |  |  |  |  |  |  |  |  |  |  |  |  |  |  |  |  |  |  |  |  |  |  |  |  |  |  |  |  |  |  |  |  |  |  |  |  |  |  |  |  |  |  |  |  |  |  |  |  |  |  |  |  |  |  |  |  |  |  |  |  |  |  |  |  |  |  |  |  |  |  |  |  |  |  |  |  |  |  |  |  |  |  |  |  |  |  |  |  |  |  |  |  |  |  |  |  |  |  |  |  |  |  |  |  |  |  |  |  |  |  |  |  |  |  |  |  |  |  |  |  |  |  |  |  |  |  |  |  |  |  |  |  |  |  |  |  |  |  |  |  |  |  |  |  |  |  |  |  |  |  |  |  |  |  |  |  |  |  |  |  |  |  |  |  |  |  |  |  |  |  |  |  |  |  |  |  |  |  |  |  |  |  |  |  |  |  |  |  |  |  |  |  |  |  |  |  |  |  |  |  |  |  |  |  |  |  |  |  |  |  |  |  |  |  |  |  |  |  |  |  |  |  |  |  |  |  |  |  |  |  |  |  |  |  |  |  |  |  |  |  |  |  |  |  |  |  |  |  |  |  |  |  |  |  |  |  |  |  |  |  |  |  |  |  |  |  |  |  |  |  |  |  |  |  |  |  |  |  |  |  |  |  |  |  |  |  |  |  |  |  |  |  |  |  |  |  |  |  |  |  |  |  |  |  |  |  |  |  |  |  |  |  |  |  |  |  |  |  |  |  |  |  |  |  |  |  |  |  |  |  |  |  |  |  |  |  |  |  |  |  |  |  |  |  |  |  |  |  |  |  |  |  |  |  |  |  |  |  |  |  |  |  |  |  |  |  |  |  |  |  |  |  |  |  |  |  |  |  |  |  |  |  |  |  |  |  |  |  |  |  |  |  |  |  |  |  |  |  |  |  |  |  |  |  |  |  |  |  |  |  |  |  |  |  |  |  |  |  |  |  |
| --- | --- | --- | --- | --- | --- | --- | --- | --- | --- | --- | --- | --- | --- | --- | --- | --- | --- | --- | --- | --- | --- | --- | --- | --- | --- | --- | --- | --- | --- | --- | --- | --- | --- | --- | --- | --- | --- | --- | --- | --- | --- | --- | --- | --- | --- | --- | --- | --- | --- | --- | --- | --- | --- | --- | --- | --- | --- | --- | --- | --- | --- | --- | --- | --- | --- | --- | --- | --- | --- | --- | --- | --- | --- | --- | --- | --- | --- | --- | --- | --- | --- | --- | --- | --- | --- | --- | --- | --- | --- | --- | --- | --- | --- | --- | --- | --- | --- | --- | --- | --- | --- | --- | --- | --- | --- | --- | --- | --- | --- | --- | --- | --- | --- | --- | --- | --- | --- | --- | --- | --- | --- | --- | --- | --- | --- | --- | --- | --- | --- | --- | --- | --- | --- | --- | --- | --- | --- | --- | --- | --- | --- | --- | --- | --- | --- | --- | --- | --- | --- | --- | --- | --- | --- | --- | --- | --- | --- | --- | --- | --- | --- | --- | --- | --- | --- | --- | --- | --- | --- | --- | --- | --- | --- | --- | --- | --- | --- | --- | --- | --- | --- | --- | --- | --- | --- | --- | --- | --- | --- | --- | --- | --- | --- | --- | --- | --- | --- | --- | --- | --- | --- | --- | --- | --- | --- | --- | --- | --- | --- | --- | --- | --- | --- | --- | --- | --- | --- | --- | --- | --- | --- | --- | --- | --- | --- | --- | --- | --- | --- | --- | --- | --- | --- | --- | --- | --- | --- | --- | --- | --- | --- | --- | --- | --- | --- | --- | --- | --- | --- | --- | --- | --- | --- | --- | --- | --- | --- | --- | --- | --- | --- | --- | --- | --- | --- | --- | --- | --- | --- | --- | --- | --- | --- | --- | --- | --- | --- | --- | --- | --- | --- | --- | --- | --- | --- | --- | --- | --- | --- | --- | --- | --- | --- | --- | --- | --- | --- | --- | --- | --- | --- | --- | --- | --- | --- | --- | --- | --- | --- | --- | --- | --- | --- | --- | --- | --- | --- | --- | --- | --- | --- | --- | --- | --- | --- | --- | --- | --- | --- | --- | --- | --- | --- | --- | --- | --- | --- | --- | --- | --- | --- | --- | --- | --- | --- | --- | --- | --- | --- | --- | --- | --- | --- | --- | --- | --- | --- | --- | --- | --- | --- | --- | --- | --- | --- | --- | --- | --- | --- | --- | --- | --- | --- | --- | --- | --- | --- | --- | --- | --- | --- | --- | --- | --- | --- | --- | --- | --- | --- | --- | --- | --- | --- | --- | --- | --- | --- | --- | --- | --- | --- | --- | --- | --- | --- | --- | --- | --- | --- | --- | --- | --- | --- | --- | --- | --- | --- | --- | --- | --- | --- | --- | --- | --- | --- | --- | --- | --- | --- | --- | --- | --- | --- | --- | --- | --- | --- | --- | --- | --- | --- | --- | --- | --- | --- | --- | --- | --- | --- | --- | --- | --- | --- | --- | --- | --- | --- | --- | --- | --- | --- | --- | --- | --- | --- | --- | --- | --- | --- | --- | --- | --- | --- | --- | --- | --- | --- | --- | --- | --- | --- | --- | --- | --- | --- | --- | --- | --- | --- | --- | --- | --- | --- | --- | --- | --- | --- | --- | --- | --- | --- | --- | --- | --- | --- | --- | --- | --- | --- | --- | --- | --- | --- | --- | --- | --- | --- | --- | --- | --- | --- | --- | --- | --- | --- | --- | --- | --- | --- | --- | --- | --- | --- | --- | --- | --- | --- | --- | --- | --- | --- | --- | --- | --- | --- | --- | --- | --- | --- | --- | --- | --- | --- | --- | --- | --- | --- | --- | --- | --- | --- | --- | --- | --- | --- | --- | --- | --- | --- | --- | --- | --- | --- | --- | --- | --- | --- | --- | --- | --- | --- | --- | --- | --- | --- | --- | --- | --- | --- | --- | --- | --- | --- | --- | --- | --- | --- | --- | --- | --- | --- | --- | --- | --- | --- | --- | --- | --- | --- | --- | --- | --- | --- | --- | --- | --- | --- | --- | --- | --- | --- | --- | --- | --- | --- | --- | --- | --- | --- | --- | --- | --- | --- | --- | --- | --- | --- | --- | --- | --- | --- | --- | --- | --- | --- | --- | --- | --- | --- | --- | --- | --- | --- | --- | --- | --- | --- | --- | --- | --- | --- | --- | --- | --- | --- | --- | --- | --- | --- | --- | --- | --- | --- | --- | --- | --- | --- | --- | --- | --- | --- | --- | --- | --- | --- | --- | --- | --- | --- | --- | --- | --- | --- | --- | --- | --- | --- | --- | --- | --- | --- | --- | --- | --- | --- | --- | --- | --- | --- | --- | --- | --- | --- | --- | --- | --- | --- | --- | --- | --- | --- | --- | --- | --- | --- | --- | --- | --- | --- | --- | --- | --- | --- | --- | --- | --- | --- | --- | --- | --- | --- | --- | --- | --- | --- | --- | --- | --- | --- | --- | --- | --- | --- | --- | --- | --- | --- | --- | --- | --- | --- | --- | --- | --- | --- | --- | --- | --- | --- | --- | --- | --- | --- | --- | --- | --- | --- | --- | --- | --- | --- | --- | --- | --- | --- | --- | --- | --- | --- | --- | --- | --- | --- | --- | --- | --- | --- | --- | --- | --- | --- | --- | --- | --- | --- | --- | --- | --- | --- | --- | --- | --- | --- | --- | --- | --- | --- | --- | --- | --- | --- | --- | --- | --- | --- | --- | --- | --- | --- | --- | --- | --- | --- | --- | --- | --- | --- | --- | --- | --- | --- | --- | --- | --- | --- | --- | --- | --- | --- | --- | --- | --- | --- | --- | --- | --- | --- | --- | --- | --- | --- | --- | --- | --- | --- | --- | --- | --- | --- | --- | --- | --- | --- | --- | --- | --- | --- | --- | --- | --- | --- | --- | --- | --- | --- | --- | --- | --- | --- | --- | --- | --- | --- | --- | --- | --- | --- | --- | --- | --- | --- | --- | --- | --- | --- | --- | --- | --- | --- | --- | --- | --- | --- | --- | --- | --- | --- | --- | --- | --- | --- | --- | --- | --- | --- | --- | --- | --- | --- | --- | --- | --- | --- | --- | --- | --- | --- | --- | --- | --- | --- | --- | --- | --- | --- | --- | --- | --- | --- | --- | --- | --- | --- | --- | --- | --- | --- | --- | --- | --- | --- | --- | --- | --- | --- | --- | --- | --- | --- | --- | --- | --- | --- | --- | --- | --- | --- | --- | --- | --- | --- | --- | --- | --- | --- | --- | --- | --- | --- | --- | --- | --- | --- | --- | --- | --- | --- | --- | --- | --- | --- | --- | --- | --- | --- | --- | --- | --- | --- | --- | --- | --- | --- | --- | --- | --- | --- | --- | --- | --- | --- | --- | --- | --- | --- | --- | --- | --- | --- | --- | --- | --- | --- | --- | --- | --- | --- | --- | --- | --- | --- | --- | --- | --- | --- | --- | --- | --- | --- | --- | --- | --- | --- | --- | --- | --- | --- | --- | --- | --- | --- | --- | --- | --- | --- | --- | --- | --- | --- | --- | --- | --- | --- | --- | --- | --- | --- | --- | --- | --- | --- | --- | --- | --- | --- | --- | --- | --- | --- | --- | --- | --- | --- | --- | --- | --- | --- | --- | --- | --- | --- | --- | --- | --- | --- | --- | --- | --- | --- | --- | --- | --- | --- | --- | --- | --- | --- | --- | --- | --- | --- | --- | --- | --- | --- | --- | --- | --- | --- | --- | --- | --- | --- | --- | --- | --- | --- | --- | --- | --- | --- | --- | --- | --- | --- | --- | --- | --- | --- | --- | --- | --- | --- | --- | --- | --- | --- | --- | --- | --- | --- | --- | --- | --- | --- | --- | --- | --- | --- | --- | --- | --- | --- | --- | --- | --- | --- | --- | --- | --- | --- | --- | --- | --- | --- | --- | --- | --- | --- | --- | --- | --- | --- | --- | --- | --- | --- | --- | --- | --- | --- | --- | --- | --- | --- | --- | --- | --- | --- | --- | --- | --- | --- | --- | --- | --- | --- | --- | --- | --- | --- | --- | --- | --- | --- | --- | --- | --- | --- | --- | --- | --- | --- | --- | --- | --- | --- | --- | --- | --- | --- | --- | --- | --- | --- | --- | --- | --- | --- | --- | --- | --- | --- | --- | --- | --- | --- | --- | --- | --- | --- | --- | --- | --- | --- | --- | --- | --- | --- | --- | --- | --- | --- | --- | --- | --- | --- | --- | --- | --- | --- | --- | --- | --- | --- | --- | --- | --- | --- | --- | --- | --- | --- | --- | --- | --- | --- | --- | --- | --- | --- | --- | --- | --- | --- | --- | --- | --- | --- | --- | --- | --- | --- | --- | --- | --- | --- | --- | --- | --- | --- | --- | --- | --- | --- | --- | --- | --- | --- | --- | --- | --- | --- | --- | --- | --- | --- | --- | --- | --- | --- | --- | --- | --- | --- | --- | --- | --- | --- | --- | --- | --- | --- | --- | --- | --- | --- | --- | --- | --- | --- | --- | --- | --- | --- | --- | --- | --- | --- | --- | --- | --- | --- | --- | --- | --- | --- | --- | --- | --- | --- | --- | --- | --- | --- | --- | --- | --- | --- | --- | --- | --- | --- | --- | --- | --- | --- | --- | --- | --- | --- | --- | --- | --- | --- | --- | --- | --- | --- | --- | --- | --- | --- | --- | --- | --- | --- | --- | --- | --- | --- | --- | --- | --- | --- | --- | --- | --- | --- | --- | --- | --- | --- | --- | --- | --- | --- | --- | --- | --- | --- | --- | --- | --- | --- | --- | --- | --- | --- | --- | --- | --- | --- | --- | --- | --- | --- | --- | --- | --- | --- | --- | --- | --- | --- | --- | --- | --- | --- | --- | --- | --- | --- | --- | --- | --- | --- | --- | --- | --- | --- | --- | --- | --- | --- | --- | --- | --- | --- | --- | --- | --- | --- | --- | --- | --- | --- | --- | --- | --- | --- | --- | --- | --- | --- | --- | --- | --- | --- | --- | --- | --- | --- | --- | --- | --- | --- | --- | --- | --- | --- | --- | --- | --- | --- | --- | --- | --- | --- | --- | --- | --- | --- | --- | --- | --- | --- | --- | --- | --- | --- | --- | --- | --- | --- | --- | --- | --- | --- | --- | --- | --- | --- | --- | --- | --- | --- | --- | --- | --- | --- | --- | --- | --- | --- | --- | --- | --- | --- | --- | --- | --- | --- | --- | --- | --- | --- | --- | --- | --- | --- | --- | --- | --- | --- | --- | --- | --- | --- | --- | --- | --- | --- | --- | --- | --- | --- | --- | --- | --- | --- | --- | --- | --- | --- | --- | --- | --- | --- | --- | --- | --- | --- | --- | --- | --- | --- | --- | --- | --- | --- | --- | --- | --- | --- | --- | --- | --- | --- | --- | --- | --- | --- | --- | --- | --- | --- | --- | --- | --- | --- | --- | --- | --- | --- | --- | --- | --- | --- | --- | --- | --- | --- | --- | --- | --- | --- | --- | --- | --- | --- | --- | --- | --- | --- | --- | --- | --- | --- | --- | --- | --- | --- | --- | --- | --- | --- | --- | --- | --- | --- | --- | --- | --- | --- | --- | --- | --- | --- | --- | --- | --- | --- | --- | --- | --- | --- | --- | --- | --- | --- | --- | --- | --- | --- | --- | --- | --- | --- | --- | --- | --- | --- | --- | --- | --- | --- | --- | --- | --- | --- | --- | --- | --- | --- | --- | --- | --- | --- | --- | --- | --- | --- | --- | --- | --- | --- | --- | --- | --- | --- | --- | --- | --- | --- | --- | --- | --- | --- | --- | --- | --- | --- | --- | --- | --- | --- | --- | --- | --- | --- | --- | --- | --- | --- | --- | --- | --- | --- | --- | --- | --- | --- | --- | --- | --- | --- | --- | --- | --- | --- | --- | --- | --- | --- | --- | --- | --- | --- | --- | --- | --- | --- | --- | --- | --- | --- | --- | --- | --- | --- | --- | --- | --- | --- | --- | --- | --- | --- | --- | --- | --- | --- | --- | --- | --- | --- | --- | --- | --- | --- | --- | --- | --- | --- | --- | --- | --- | --- | --- | --- | --- | --- | --- | --- | --- | --- | --- | --- | --- | --- | --- | --- | --- | --- | --- | --- | --- | --- | --- | --- | --- | --- | --- | --- | --- | --- | --- | --- | --- | --- | --- | --- | --- | --- | --- | --- | --- | --- | --- | --- | --- | --- | --- | --- | --- | --- | --- | --- | --- | --- | --- | --- | --- | --- | --- | --- | --- | --- | --- | --- | --- | --- | --- | --- | --- | --- | --- | --- | --- | --- | --- | --- | --- | --- | --- | --- | --- | --- | --- | --- | --- | --- | --- | --- | --- | --- | --- | --- | --- | --- | --- | --- | --- | --- | --- | --- | --- | --- | --- | --- | --- | --- | --- | --- | --- | --- | --- | --- | --- | --- | --- | --- | --- | --- | --- | --- | --- | --- | --- | --- | --- | --- | --- | --- | --- | --- | --- | --- | --- | --- | --- | --- | --- | --- | --- | --- | --- | --- | --- | --- | --- | --- | --- | --- | --- | --- | --- | --- | --- | --- | --- | --- | --- | --- | --- | --- | --- | --- | --- | --- | --- | --- | --- | --- | --- | --- | --- | --- | --- | --- | --- | --- | --- | --- | --- | --- | --- | --- | --- | --- | --- | --- | --- | --- | --- | --- | --- | --- | --- | --- | --- | --- | --- | --- | --- | --- | --- | --- | --- | --- | --- | --- | --- | --- | --- | --- | --- | --- | --- | --- | --- | --- | --- | --- | --- | --- | --- | --- | --- | --- | --- | --- | --- | --- | --- | --- | --- | --- | --- | --- | --- | --- | --- | --- | --- | --- | --- | --- | --- | --- | --- | --- | --- | --- | --- | --- | --- | --- | --- | --- | --- | --- | --- | --- | --- | --- | --- | --- | --- | --- | --- | --- | --- | --- | --- | --- | --- | --- | --- | --- | --- | --- | --- | --- | --- | --- | --- | --- | --- | --- | --- | --- | --- | --- | --- | --- | --- | --- | --- | --- | --- | --- | --- | --- | --- | --- | --- | --- | --- | --- | --- | --- | --- | --- | --- | --- | --- | --- | --- | --- | --- | --- | --- | --- | --- | --- | --- | --- | --- | --- | --- | --- | --- | --- | --- | --- | --- | --- | --- | --- | --- | --- | --- | --- | --- | --- | --- | --- | --- | --- | --- | --- | --- | --- | --- | --- | --- | --- | --- | --- | --- | --- | --- | --- | --- | --- | --- | --- | --- | --- | --- | --- | --- | --- | --- | --- | --- | --- | --- | --- | --- | --- | --- | --- | --- | --- | --- | --- | --- | --- | --- | --- | --- | --- | --- | --- | --- | --- | --- | --- | --- | --- | --- | --- | --- | --- | --- | --- | --- | --- | --- | --- | --- | --- | --- | --- | --- | --- | --- | --- | --- | --- | --- | --- | --- | --- | --- | --- | --- | --- | --- | --- | --- | --- | --- | --- | --- | --- | --- | --- | --- | --- | --- | --- | --- | --- | --- | --- | --- | --- | --- | --- | --- | --- | --- | --- | --- | --- | --- | --- | --- | --- | --- | --- | --- | --- | --- | --- | --- | --- | --- | --- | --- | --- | --- | --- | --- | --- | --- | --- | --- | --- | --- | --- | --- | --- | --- | --- | --- | --- | --- | --- | --- | --- | --- | --- | --- | --- | --- | --- | --- | --- | --- | --- | --- | --- | --- | --- | --- | --- | --- | --- | --- | --- | --- | --- | --- | --- | --- | --- | --- | --- | --- | --- | --- | --- | --- | --- | --- | --- | --- | --- | --- | --- | --- | --- | --- | --- | --- | --- | --- | --- | --- | --- | --- | --- | --- | --- | --- | --- | --- | --- | --- | --- | --- | --- | --- | --- | --- | --- | --- | --- | --- | --- | --- | --- | --- | --- | --- | --- | --- | --- | --- | --- | --- | --- | --- | --- | --- | --- | --- | --- | --- | --- | --- | --- | --- | --- | --- | --- | --- | --- | --- | --- | --- | --- | --- | --- | --- | --- | --- | --- | --- | --- | --- | --- | --- | --- | --- | --- | --- | --- | --- | --- | --- | --- | --- | --- | --- | --- | --- | --- | --- | --- | --- | --- | --- | --- | --- | --- | --- | --- | --- | --- | --- | --- | --- | --- | --- | --- | --- | --- | --- | --- | --- | --- | --- | --- | --- | --- | --- | --- | --- | --- | --- | --- | --- | --- | --- | --- | --- | --- | --- | --- | --- | --- | --- | --- | --- | --- | --- | --- | --- | --- | --- | --- | --- | --- | --- | --- | --- | --- | --- | --- | --- | --- | --- | --- | --- | --- | --- | --- | --- | --- | --- | --- | --- | --- | --- | --- | --- | --- | --- | --- | --- | --- | --- | --- | --- | --- | --- | --- | --- | --- | --- | --- | --- | --- | --- | --- | --- | --- | --- | --- | --- | --- | --- | --- | --- | --- | --- | --- | --- | --- | --- | --- | --- | --- | --- | --- | --- | --- | --- | --- | --- | --- | --- | --- | --- | --- | --- | --- | --- | --- | --- | --- | --- | --- | --- | --- | --- | --- | --- | --- | --- | --- | --- | --- | --- | --- | --- | --- | --- | --- | --- | --- | --- | --- | --- | --- | --- | --- | --- | --- | --- | --- | --- | --- | --- | --- | --- | --- | --- | --- | --- | --- | --- | --- | --- | --- | --- | --- | --- | --- | --- | --- | --- | --- | --- | --- | --- | --- | --- | --- | --- | --- | --- | --- | --- | --- | --- | --- | --- | --- | --- | --- | --- | --- | --- | --- | --- | --- | --- | --- | --- | --- | --- | --- | --- | --- | --- | --- | --- | --- | --- | --- | --- | --- | --- | --- | --- | --- | --- | --- | --- | --- | --- | --- | --- | --- | --- | --- | --- | --- | --- | --- | --- | --- | --- | --- | --- | --- | --- | --- | --- | --- | --- | --- | --- | --- | --- | --- | --- | --- | --- | --- | --- | --- | --- | --- | --- | --- | --- | --- | --- | --- | --- | --- | --- | --- | --- | --- | --- | --- | --- | --- | --- | --- | --- | --- | --- | --- | --- | --- | --- | --- | --- | --- | --- | --- | --- | --- | --- | --- | --- | --- | --- | --- | --- | --- | --- | --- | --- | --- | --- | --- | --- | --- | --- | --- | --- | --- | --- | --- | --- | --- | --- | --- | --- | --- | --- | --- | --- | --- | --- | --- | --- | --- | --- | --- | --- | --- | --- | --- | --- | --- | --- | --- | --- | --- | --- | --- | --- | --- | --- | --- | --- | --- | --- | --- | --- | --- | --- | --- | --- | --- | --- | --- | --- | --- | --- | --- | --- | --- | --- | --- | --- | --- | --- | --- | --- | --- | --- | --- | --- | --- | --- | --- | --- | --- | --- | --- | --- | --- | --- | --- | --- | --- | --- | --- | --- | --- | --- | --- | --- | --- | --- | --- | --- | --- | --- | --- | --- | --- | --- | --- | --- | --- | --- | --- | --- | --- | --- | --- | --- | --- | --- | --- | --- | --- | --- | --- | --- | --- | --- | --- | --- | --- | --- | --- | --- | --- | --- | --- | --- | --- | --- | --- | --- | --- | --- | --- | --- | --- | --- | --- | --- | --- | --- | --- | --- | --- | --- | --- | --- | --- | --- | --- | --- | --- | --- | --- | --- | --- | --- | --- | --- | --- | --- | --- | --- | --- | --- | --- | --- | --- | --- | --- | --- | --- | --- | --- | --- | --- | --- | --- | --- | --- | --- | --- | --- | --- | --- | --- | --- | --- | --- | --- | --- | --- | --- | --- | --- | --- | --- | --- | --- | --- | --- | --- | --- | --- | --- | --- | --- | --- | --- | --- | --- | --- | --- | --- | --- | --- | --- | --- | --- | --- | --- | --- | --- | --- | --- | --- | --- | --- | --- | --- | --- | --- | --- | --- | --- | --- | --- | --- | --- | --- | --- | --- | --- | --- | --- | --- | --- | --- | --- | --- | --- | --- | --- | --- | --- | --- | --- | --- | --- | --- | --- | --- | --- | --- | --- | --- | --- | --- | --- | --- | --- | --- | --- | --- | --- | --- | --- | --- | --- | --- | --- | --- | --- | --- | --- | --- | --- | --- | --- | --- | --- | --- | --- | --- | --- | --- | --- | --- | --- | --- | --- | --- | --- | --- | --- | --- | --- | --- | --- | --- | --- | --- | --- | --- | --- | --- | --- | --- | --- | --- | --- | --- | --- | --- | --- | --- | --- | --- | --- | --- | --- | --- | --- | --- | --- | --- | --- | --- | --- | --- | --- | --- | --- | --- | --- | --- | --- | --- | --- | --- | --- | --- | --- | --- | --- | --- | --- | --- | --- | --- | --- | --- | --- | --- | --- | --- | --- | --- | --- | --- | --- | --- | --- | --- | --- | --- | --- | --- | --- | --- | --- | --- | --- | --- | --- | --- | --- | --- | --- | --- | --- | --- | --- | --- | --- | --- | --- | --- | --- | --- | --- | --- | --- | --- | --- | --- | --- | --- | --- | --- | --- | --- | --- | --- | --- | --- | --- | --- | --- | --- | --- | --- | --- | --- | --- | --- | --- | --- | --- | --- | --- | --- | --- | --- | --- | --- | --- | --- | --- | --- | --- | --- | --- | --- | --- | --- | --- | --- | --- | --- | --- | --- | --- | --- | --- | --- | --- | --- | --- | --- | --- | --- | --- | --- | --- | --- | --- | --- | --- | --- | --- | --- | --- | --- | --- | --- | --- | --- | --- | --- | --- | --- | --- | --- | --- | --- | --- | --- | --- | --- | --- | --- | --- | --- | --- | --- | --- | --- | --- | --- | --- | --- | --- | --- | --- | --- | --- | --- | --- | --- | --- | --- | --- | --- | --- | --- | --- | --- | --- | --- | --- | --- | --- | --- | --- | --- | --- | --- | --- | --- | --- | --- | --- | --- | --- | --- | --- | --- | --- | --- | --- | --- | --- | --- | --- | --- | --- | --- | --- | --- | --- | --- | --- | --- | --- | --- | --- | --- | --- | --- | --- | --- | --- | --- | --- | --- | --- | --- | --- | --- | --- | --- | --- | --- | --- | --- | --- | --- | --- | --- | --- | --- | --- | --- | --- | --- | --- | --- | --- | --- | --- | --- | --- | --- | --- | --- | --- | --- | --- | --- | --- | --- | --- | --- | --- | --- | --- | --- | --- | --- | --- | --- | --- | --- | --- | --- | --- | --- | --- | --- | --- | --- | --- | --- | --- | --- | --- | --- | --- | --- | --- | --- | --- | --- | --- | --- | --- | --- | --- | --- | --- | --- | --- | --- | --- | --- | --- | --- | --- | --- | --- | --- | --- | --- | --- | --- | --- | --- | --- | --- | --- | --- | --- | --- | --- | --- | --- | --- | --- | --- | --- | --- | --- | --- | --- | --- | --- | --- | --- | --- | --- | --- | --- | --- | --- | --- | --- | --- | --- | --- | --- | --- | --- | --- | --- | --- | --- | --- | --- | --- | --- | --- | --- | --- | --- | --- | --- | --- | --- | --- | --- | --- | --- | --- | --- | --- | --- | --- | --- | --- | --- | --- | --- | --- | --- | --- | --- | --- | --- | --- | --- | --- | --- | --- | --- | --- | --- | --- | --- | --- | --- | --- | --- | --- | --- | --- | --- | --- | --- | --- | --- | --- | --- | --- | --- | --- | --- | --- | --- | --- | --- | --- | --- | --- | --- | --- | --- | --- | --- | --- | --- | --- | --- | --- | --- | --- | --- | --- | --- | --- | --- | --- | --- | --- | --- | --- | --- | --- | --- | --- | --- | --- | --- | --- | --- | --- | --- | --- | --- | --- | --- | --- | --- | --- | --- | --- | --- | --- | --- | --- | --- | --- | --- | --- | --- | --- | --- | --- | --- | --- | --- | --- | --- | --- | --- | --- | --- | --- | --- | --- | --- | --- | --- | --- | --- | --- | --- | --- | --- | --- | --- | --- | --- | --- | --- | --- | --- | --- | --- | --- | --- | --- | --- | --- | --- | --- | --- | --- | --- | --- | --- | --- | --- | --- | --- | --- | --- | --- | --- | --- | --- | --- | --- | --- | --- | --- | --- | --- | --- | --- | --- | --- | --- | --- | --- | --- | --- | --- | --- | --- | --- | --- | --- | --- | --- | --- | --- | --- | --- | --- | --- | --- | --- | --- | --- | --- | --- | --- | --- | --- | --- | --- | --- | --- | --- | --- | --- | --- | --- | --- | --- | --- | --- | --- | --- | --- | --- | --- | --- | --- | --- | --- | --- | --- | --- | --- | --- | --- | --- | --- | --- | --- | --- | --- | --- | --- | --- | --- | --- | --- | --- | --- | --- | --- | --- | --- | --- | --- | --- | --- | --- | --- | --- | --- | --- | --- | --- | --- | --- | --- | --- | --- | --- | --- | --- | --- | --- | --- | --- | --- | --- | --- | --- | --- | --- | --- | --- | --- | --- | --- | --- | --- | --- | --- | --- | --- | --- | --- | --- | --- | --- | --- | --- | --- | --- | --- | --- | --- | --- | --- | --- | --- | --- | --- | --- | --- | --- | --- | --- | --- | --- | --- | --- | --- | --- | --- | --- | --- | --- | --- | --- | --- | --- | --- | --- | --- | --- | --- | --- | --- | --- | --- | --- | --- | --- | --- | --- | --- | --- | --- | --- | --- | --- | --- | --- | --- | --- | --- | --- | --- | --- | --- | --- | --- | --- | --- | --- | --- | --- | --- | --- | --- | --- | --- | --- | --- | --- | --- | --- | --- | --- | --- | --- | --- | --- | --- | --- | --- | --- | --- | --- | --- | --- | --- | --- | --- | --- | --- | --- | --- | --- | --- | --- | --- | --- | --- | --- | --- | --- | --- | --- | --- | --- | --- | --- | --- | --- | --- | --- | --- | --- | --- | --- | --- | --- | --- | --- | --- | --- | --- | --- | --- | --- | --- | --- | --- | --- | --- | --- | --- | --- | --- | --- | --- | --- | --- | --- | --- | --- | --- | --- | --- | --- | --- | --- | --- | --- | --- | --- | --- | --- | --- | --- | --- | --- | --- | --- | --- | --- | --- | --- | --- | --- | --- | --- | --- | --- | --- | --- | --- | --- | --- | --- | --- | --- | --- | --- | --- | --- | --- | --- | --- | --- | --- | --- | --- | --- | --- | --- | --- | --- | --- | --- | --- | --- | --- | --- | --- | --- | --- | --- | --- | --- | --- | --- | --- | --- | --- | --- | --- | --- | --- | --- | --- | --- | --- | --- | --- | --- | --- | --- | --- | --- | --- | --- | --- | --- | --- | --- | --- | --- | --- | --- | --- | --- | --- | --- | --- | --- | --- | --- | --- | --- | --- | --- | --- | --- | --- | --- | --- | --- | --- | --- | --- | --- | --- | --- | --- | --- | --- | --- | --- | --- | --- | --- | --- | --- | --- | --- | --- | --- | --- | --- | --- | --- | --- | --- | --- | --- | --- | --- | --- | --- | --- | --- | --- | --- | --- | --- | --- | --- | --- | --- | --- | --- | --- | --- | --- | --- | --- | --- | --- | --- | --- | --- | --- | --- | --- | --- | --- | --- | --- | --- | --- | --- | --- | --- | --- | --- | --- | --- | --- | --- | --- | --- | --- | --- | --- | --- | --- | --- | --- | --- | --- | --- | --- | --- | --- | --- | --- | --- | --- | --- | --- | --- | --- | --- | --- | --- | --- | --- | --- | --- | --- | --- | --- | --- | --- | --- | --- | --- | --- | --- | --- | --- | --- | --- | --- | --- | --- | --- | --- | --- | --- | --- | --- | --- | --- | --- | --- | --- | --- | --- | --- | --- | --- | --- | --- | --- | --- | --- | --- | --- | --- | --- | --- | --- | --- | --- | --- | --- | --- | --- | --- | --- | --- | --- | --- | --- | --- | --- | --- | --- | --- | --- | --- | --- | --- | --- | --- | --- | --- | --- | --- | --- | --- | --- | --- | --- | --- | --- | --- | --- | --- | --- | --- | --- | --- | --- | --- | --- | --- | --- | --- | --- | --- | --- | --- | --- | --- | --- | --- | --- | --- | --- | --- | --- | --- | --- | --- | --- | --- | --- | --- | --- | --- | --- | --- | --- | --- | --- | --- | --- | --- | --- | --- | --- | --- | --- | --- | --- | --- | --- | --- | --- | --- | --- | --- | --- | --- | --- | --- | --- | --- | --- | --- | --- | --- | --- | --- | --- | --- | --- | --- | --- | --- | --- | --- | --- | --- | --- | --- | --- | --- | --- | --- | --- | --- | --- | --- | --- | --- | --- | --- | --- | --- | --- | --- | --- | --- | --- | --- | --- | --- | --- | --- | --- | --- | --- | --- | --- | --- | --- | --- | --- | --- | --- | --- | --- | --- | --- | --- | --- | --- | --- | --- | --- | --- | --- | --- | --- | --- | --- | --- | --- | --- | --- | --- | --- | --- | --- | --- | --- | --- | --- | --- | --- | --- | --- | --- | --- | --- | --- | --- | --- | --- | --- | --- | --- | --- | --- | --- | --- | --- | --- | --- | --- | --- | --- | --- | --- | --- | --- | --- | --- | --- | --- | --- | --- | --- | --- | --- | --- | --- | --- | --- | --- | --- | --- | --- | --- | --- | --- | --- | --- | --- | --- | --- | --- | --- | --- | --- | --- | --- | --- | --- | --- | --- | --- | --- | --- | --- | --- | --- | --- | --- | --- | --- | --- | --- | --- | --- | --- | --- | --- | --- | --- | --- | --- | --- | --- | --- | --- | --- | --- | --- | --- | --- | --- | --- | --- | --- | --- | --- | --- | --- | --- | --- | --- | --- | --- | --- | --- | --- | --- | --- | --- | --- | --- | --- | --- | --- | --- | --- | --- | --- | --- | --- | --- | --- | --- | --- | --- | --- | --- | --- | --- | --- | --- | --- | --- | --- | --- | --- | --- | --- | --- | --- | --- | --- | --- | --- | --- | --- | --- | --- | --- | --- | --- | --- | --- | --- | --- | --- | --- | --- | --- | --- | --- | --- | --- | --- | --- | --- | --- | --- | --- | --- | --- | --- | --- | --- | --- | --- | --- | --- | --- | --- | --- | --- | --- | --- | --- | --- | --- | --- | --- | --- | --- | --- | --- | --- | --- | --- | --- | --- | --- | --- | --- | --- | --- | --- | --- | --- | --- | --- | --- | --- | --- | --- | --- | --- | --- | --- | --- | --- | --- | --- | --- | --- | --- | --- | --- | --- | --- | --- | --- | --- | --- | --- | --- | --- | --- | --- | --- | --- | --- | --- | --- | --- | --- | --- | --- | --- | --- | --- | --- | --- | --- | --- | --- | --- | --- | --- | --- | --- | --- | --- | --- | --- | --- | --- | --- | --- | --- | --- | --- | --- | --- | --- | --- | --- | --- | --- | --- | --- | --- | --- | --- | --- | --- | --- | --- | --- | --- | --- | --- | --- | --- | --- | --- | --- | --- | --- | --- | --- | --- | --- | --- | --- | --- | --- | --- | --- | --- | --- | --- | --- | --- | --- | --- | --- | --- | --- | --- | --- | --- | --- | --- | --- | --- | --- | --- | --- | --- | --- | --- | --- | --- | --- | --- | --- | --- | --- | --- | --- | --- | --- | --- | --- | --- | --- | --- | --- | --- | --- | --- | --- | --- | --- | --- | --- | --- | --- | --- | --- | --- | --- | --- | --- | --- | --- | --- | --- | --- | --- | --- | --- | --- | --- | --- | --- | --- | --- | --- | --- | --- | --- | --- | --- | --- | --- | --- | --- | --- | --- | --- | --- | --- | --- | --- | --- | --- | --- | --- | --- | --- | --- | --- | --- | --- | --- | --- | --- | --- | --- | --- | --- | --- | --- | --- | --- | --- | --- | --- | --- | --- | --- | --- | --- | --- | --- | --- | --- | --- | --- | --- | --- | --- | --- | --- | --- | --- | --- | --- | --- | --- | --- | --- | --- | --- | --- | --- | --- | --- | --- | --- | --- | --- | --- | --- | --- | --- | --- | --- | --- | --- | --- | --- | --- | --- | --- | --- | --- | --- | --- | --- | --- | --- | --- | --- | --- | --- | --- | --- | --- | --- | --- | --- | --- | --- | --- | --- | --- | --- | --- | --- | --- | --- | --- | --- | --- | --- | --- | --- | --- | --- | --- | --- | --- | --- | --- | --- | --- | --- | --- | --- | --- | --- | --- | --- | --- | --- | --- | --- | --- | --- | --- | --- | --- | --- | --- | --- | --- | --- | --- | --- | --- | --- | --- | --- | --- | --- | --- | --- | --- | --- | --- | --- | --- | --- | --- | --- | --- | --- | --- | --- | --- | --- | --- | --- | --- | --- | --- | --- | --- | --- | --- | --- | --- | --- | --- | --- | --- | --- | --- | --- | --- | --- | --- | --- | --- | --- | --- | --- | --- | --- | --- | --- | --- | --- | --- | --- | --- | --- | --- | --- | --- | --- | --- | --- | --- | --- | --- | --- | --- | --- | --- | --- | --- | --- | --- | --- | --- | --- | --- | --- | --- | --- | --- | --- | --- | --- | --- | --- | --- | --- | --- | --- | --- | --- | --- | --- | --- | --- | --- | --- | --- | --- | --- | --- | --- | --- | --- | --- | --- | --- | --- | --- | --- | --- | --- | --- | --- | --- | --- | --- | --- | --- | --- | --- | --- | --- | --- | --- | --- | --- | --- | --- | --- | --- | --- | --- | --- | --- | --- | --- | --- | --- | --- | --- | --- | --- | --- | --- | --- | --- | --- | --- | --- | --- | --- | --- | --- | --- | --- | --- | --- | --- | --- | --- | --- | --- | --- | --- | --- | --- | --- | --- | --- | --- | --- | --- | --- | --- | --- | --- | --- | --- | --- | --- | --- | --- | --- | --- | --- | --- | --- | --- | --- | --- | --- | --- | --- | --- | --- | --- | --- | --- | --- | --- | --- | --- | --- | --- | --- | --- | --- | --- | --- | --- | --- | --- | --- | --- | --- | --- | --- | --- | --- | --- | --- | --- | --- | --- | --- | --- | --- | --- | --- | --- | --- | --- | --- | --- | --- | --- | --- | --- | --- | --- | --- | --- | --- | --- | --- | --- | --- | --- | --- | --- | --- | --- | --- | --- | --- | --- | --- | --- | --- | --- | --- | --- | --- | --- | --- | --- | --- | --- | --- | --- | --- | --- | --- | --- | --- | --- | --- | --- | --- | --- | --- | --- | --- | --- | --- | --- | --- | --- | --- | --- | --- | --- | --- | --- | --- | --- | --- | --- | --- | --- | --- | --- | --- | --- | --- | --- | --- | --- | --- | --- | --- | --- | --- | --- | --- | --- | --- | --- | --- | --- | --- | --- | --- | --- | --- | --- | --- | --- | --- | --- | --- | --- | --- | --- | --- | --- | --- | --- | --- | --- | --- | --- | --- | --- | --- | --- | --- | --- | --- | --- | --- | --- | --- | --- | --- | --- | --- | --- | --- | --- | --- | --- | --- | --- | --- | --- | --- | --- | --- | --- | --- | --- | --- | --- | --- | --- | --- | --- | --- | --- | --- | --- | --- | --- | --- | --- | --- | --- | --- | --- | --- | --- | --- | --- | --- | --- | --- | --- | --- | --- | --- | --- | --- | --- | --- | --- | --- | --- | --- | --- | --- | --- | --- | --- | --- | --- | --- | --- | --- | --- | --- | --- | --- | --- | --- | --- | --- | --- | --- | --- | --- | --- | --- | --- | --- | --- | --- | --- | --- | --- | --- | --- | --- | --- | --- | --- | --- | --- | --- | --- | --- | --- | --- | --- | --- | --- | --- | --- | --- | --- | --- | --- | --- | --- | --- | --- | --- | --- | --- | --- | --- | --- | --- | --- | --- | --- | --- | --- | --- | --- | --- | --- | --- | --- | --- | --- | --- | --- | --- | --- | --- | --- | --- | --- | --- | --- | --- | --- | --- | --- | --- | --- | --- | --- | --- | --- | --- | --- | --- | --- | --- | --- | --- | --- | --- | --- | --- | --- | --- | --- | --- | --- | --- | --- | --- | --- | --- | --- | --- | --- | --- | --- | --- | --- | --- | --- | --- | --- | --- | --- | --- | --- | --- | --- | --- | --- | --- | --- | --- | --- | --- | --- | --- | --- | --- | --- | --- | --- | --- | --- | --- | --- | --- | --- | --- | --- | --- | --- | --- | --- | --- | --- | --- | --- | --- | --- | --- | --- | --- | --- | --- | --- | --- | --- | --- | --- | --- | --- | --- | --- | --- | --- | --- | --- | --- | --- | --- | --- | --- | --- | --- | --- | --- | --- | --- | --- | --- | --- | --- | --- | --- | --- | --- | --- | --- | --- | --- | --- | --- | --- | --- | --- | --- | --- | --- | --- | --- | --- | --- | --- | --- | --- | --- | --- | --- | --- | --- | --- | --- | --- | --- | --- | --- | --- | --- | --- | --- | --- | --- | --- | --- | --- | --- | --- | --- | --- | --- | --- | --- | --- | --- | --- | --- | --- | --- | --- | --- | --- | --- | --- | --- | --- | --- | --- | --- | --- | --- | --- | --- | --- | --- | --- | --- | --- | --- | --- | --- | --- | --- | --- | --- | --- | --- | --- | --- | --- | --- | --- | --- | --- | --- | --- | --- | --- | --- | --- | --- | --- | --- | --- | --- | --- | --- | --- | --- | --- | --- | --- | --- | --- | --- | --- | --- | --- | --- | --- | --- | --- | --- | --- | --- | --- | --- | --- | --- | --- | --- | --- | --- | --- | --- | --- | --- | --- | --- | --- | --- | --- | --- | --- | --- | --- | --- | --- | --- | --- | --- | --- | --- | --- | --- | --- | --- | --- | --- | --- | --- | --- | --- | --- | --- | --- | --- | --- | --- | --- | --- | --- | --- | --- | --- | --- | --- | --- | --- | --- | --- | --- | --- | --- | --- | --- | --- | --- | --- | --- | --- | --- | --- | --- | --- | --- | --- | --- | --- | --- | --- | --- | --- | --- | --- | --- | --- | --- | --- | --- | --- | --- | --- | --- | --- | --- | --- | --- | --- | --- | --- | --- | --- | --- | --- | --- | --- | --- | --- | --- | --- | --- | --- | --- | --- | --- | --- | --- | --- | --- | --- | --- | --- | --- | --- | --- | --- | --- | --- | --- | --- | --- | --- | --- | --- | --- | --- | --- | --- | --- | --- | --- | --- | --- | --- | --- | --- | --- | --- | --- | --- | --- | --- | --- | --- | --- | --- | --- | --- | --- | --- | --- | --- | --- | --- | --- | --- | --- | --- | --- | --- | --- | --- | --- | --- | --- | --- | --- | --- | --- | --- | --- | --- | --- | --- | --- | --- | --- | --- | --- | --- | --- | --- | --- | --- | --- | --- | --- | --- | --- | --- | --- | --- | --- | --- | --- | --- | --- | --- | --- | --- | --- | --- | --- | --- | --- | --- | --- | --- | --- | --- | --- | --- | --- | --- | --- | --- | --- | --- | --- | --- | --- | --- | --- | --- | --- | --- | --- | --- | --- | --- | --- | --- | --- | --- | --- | --- | --- | --- | --- | --- | --- | --- | --- | --- | --- | --- | --- | --- | --- | --- | --- | --- | --- | --- | --- | --- | --- | --- | --- | --- | --- | --- | --- | --- | --- | --- | --- | --- | --- | --- | --- | --- | --- | --- | --- | --- | --- | --- | --- | --- | --- | --- | --- | --- | --- | --- | --- | --- | --- | --- | --- | --- | --- | --- | --- | --- | --- | --- | --- | --- | --- | --- | --- | --- | --- | --- | --- | --- | --- | --- | --- | --- | --- | --- | --- | --- | --- | --- | --- | --- | --- | --- | --- | --- | --- | --- | --- | --- | --- | --- | --- | --- | --- | --- | --- | --- | --- | --- | --- | --- | --- | --- | --- | --- | --- | --- | --- | --- | --- | --- | --- | --- | --- | --- | --- | --- | --- | --- | --- | --- | --- | --- | --- | --- | --- | --- | --- | --- | --- | --- | --- | --- | --- | --- | --- | --- | --- | --- | --- | --- | --- | --- | --- | --- | --- | --- | --- | --- | --- | --- | --- | --- | --- | --- | --- | --- | --- | --- | --- | --- | --- | --- | --- | --- | --- | --- | --- | --- | --- | --- | --- | --- | --- | --- | --- | --- | --- | --- | --- | --- | --- | --- | --- | --- | --- | --- | --- | --- | --- | --- | --- | --- | --- | --- | --- | --- | --- | --- | --- | --- | --- | --- | --- | --- | --- | --- | --- | --- | --- | --- | --- | --- | --- | --- | --- | --- | --- | --- | --- | --- | --- | --- | --- | --- | --- | --- | --- | --- | --- | --- | --- | --- | --- | --- | --- | --- | --- | --- | --- | --- | --- | --- | --- | --- | --- | --- | --- | --- | --- | --- | --- | --- | --- | --- | --- | --- | --- | --- | --- | --- | --- | --- | --- | --- | --- | --- | --- | --- | --- | --- | --- | --- | --- | --- | --- | --- | --- | --- | --- | --- | --- | --- | --- | --- | --- | --- | --- | --- | --- | --- | --- | --- | --- | --- | --- | --- | --- | --- | --- | --- | --- | --- | --- | --- | --- | --- | --- | --- | --- | --- | --- | --- | --- | --- | --- | --- | --- | --- | --- | --- | --- | --- | --- | --- | --- | --- | --- | --- | --- | --- | --- | --- | --- | --- | --- | --- | --- | --- | --- | --- | --- | --- | --- | --- | --- | --- | --- | --- | --- | --- | --- | --- | --- | --- | --- | --- | --- | --- | --- | --- | --- | --- | --- | --- | --- | --- | --- | --- | --- | --- | --- | --- | --- | --- | --- | --- | --- | --- | --- | --- | --- | --- | --- | --- | --- | --- | --- | --- | --- | --- | --- | --- | --- | --- | --- | --- | --- | --- | --- | --- | --- | --- | --- | --- | --- | --- | --- | --- | --- | --- | --- | --- | --- | --- | --- | --- | --- | --- | --- | --- | --- | --- | --- | --- | --- | --- | --- | --- | --- | --- | --- | --- | --- | --- | --- | --- | --- | --- | --- | --- | --- | --- | --- | --- | --- | --- | --- | --- | --- | --- | --- | --- | --- | --- | --- | --- | --- | --- | --- | --- | --- | --- | --- | --- | --- | --- | --- | --- | --- | --- | --- | --- | --- | --- | --- | --- | --- | --- | --- | --- | --- | --- | --- | --- | --- | --- | --- | --- | --- | --- | --- | --- | --- | --- | --- | --- | --- | --- | --- | --- | --- | --- | --- | --- | --- | --- | --- | --- | --- | --- | --- | --- | --- | --- | --- | --- | --- | --- | --- | --- | --- | --- | --- | --- | --- | --- | --- | --- | --- | --- | --- | --- | --- | --- | --- | --- | --- | --- | --- | --- | --- | --- | --- | --- | --- | --- | --- | --- | --- | --- | --- | --- | --- | --- | --- | --- | --- | --- | --- | --- | --- | --- | --- | --- | --- | --- | --- | --- | --- | --- | --- | --- | --- | --- | --- | --- | --- | --- | --- | --- | --- | --- | --- | --- | --- | --- | --- | --- | --- | --- | --- | --- | --- | --- | --- | --- | --- | --- | --- | --- | --- | --- | --- | --- | --- | --- | --- | --- | --- | --- | --- | --- | --- | --- | --- | --- | --- | --- | --- | --- | --- | --- | --- | --- | --- | --- | --- | --- | --- | --- | --- | --- | --- | --- | --- | --- | --- | --- | --- | --- | --- | --- | --- | --- | --- | --- | --- | --- | --- | --- | --- | --- | --- | --- | --- | --- | --- | --- | --- | --- | --- | --- | --- | --- | --- | --- | --- | --- | --- | --- | --- | --- | --- | --- | --- | --- | --- | --- | --- | --- | --- | --- | --- | --- | --- | --- | --- | --- | --- | --- | --- | --- | --- | --- | --- | --- | --- | --- | --- | --- | --- | --- | --- | --- | --- | --- | --- | --- | --- | --- | --- | --- | --- | --- | --- | --- | --- | --- | --- | --- | --- | --- | --- | --- | --- | --- | --- | --- | --- | --- | --- | --- | --- | --- | --- | --- | --- | --- | --- | --- | --- | --- | --- | --- | --- | --- | --- | --- | --- | --- | --- | --- | --- | --- | --- | --- | --- | --- | --- | --- | --- | --- | --- | --- | --- | --- | --- | --- | --- | --- | --- | --- | --- | --- | --- | --- | --- | --- | --- | --- | --- | --- | --- | --- | --- | --- | --- | --- | --- | --- | --- | --- | --- | --- | --- | --- | --- | --- | --- | --- | --- | --- | --- | --- | --- | --- | --- | --- | --- | --- | --- | --- | --- | --- | --- | --- | --- | --- | --- | --- | --- | --- | --- | --- | --- | --- | --- | --- | --- | --- | --- | --- | --- | --- | --- | --- | --- | --- | --- | --- | --- | --- | --- | --- | --- | --- | --- | --- | --- | --- | --- | --- | --- | --- | --- | --- | --- | --- | --- | --- | --- | --- | --- | --- | --- | --- | --- | --- | --- | --- | --- | --- | --- | --- | --- | --- | --- | --- | --- | --- | --- | --- | --- | --- | --- | --- | --- | --- | --- | --- | --- | --- | --- | --- | --- | --- | --- | --- | --- | --- | --- | --- | --- | --- | --- | --- | --- | --- | --- | --- | --- | --- | --- | --- | --- | --- | --- | --- | --- | --- | --- | --- | --- | --- | --- | --- | --- | --- | --- | --- | --- | --- | --- | --- | --- | --- | --- | --- | --- | --- | --- | --- | --- | --- | --- | --- | --- | --- | --- | --- | --- | --- | --- | --- | --- | --- | --- | --- | --- | --- | --- | --- | --- | --- | --- | --- | --- | --- | --- | --- | --- | --- | --- | --- | --- | --- | --- | --- | --- | --- | --- | --- | --- | --- | --- | --- | --- | --- | --- | --- | --- | --- | --- | --- | --- | --- | --- | --- | --- | --- | --- | --- | --- | --- | --- | --- | --- | --- | --- | --- | --- | --- | --- | --- | --- | --- | --- | --- | --- | --- | --- | --- | --- | --- | --- | --- | --- | --- | --- | --- | --- | --- | --- | --- | --- | --- | --- | --- | --- | --- | --- | --- | --- | --- | --- | --- | --- | --- | --- | --- | --- | --- | --- | --- | --- | --- | --- | --- | --- | --- | --- | --- | --- | --- | --- | --- | --- | --- | --- | --- | --- | --- | --- | --- | --- | --- | --- | --- | --- | --- | --- | --- | --- | --- | --- | --- | --- | --- | --- | --- | --- | --- | --- | --- | --- | --- | --- | --- | --- | --- | --- | --- | --- | --- | --- | --- | --- | --- | --- | --- | --- | --- | --- | --- | --- | --- | --- | --- | --- | --- | --- | --- | --- | --- | --- | --- | --- | --- | --- | --- | --- | --- | --- | --- | --- | --- | --- | --- | --- | --- | --- | --- | --- | --- | --- | --- | --- | --- | --- | --- | --- | --- | --- | --- | --- | --- | --- | --- | --- | --- | --- | --- | --- | --- | --- | --- | --- | --- | --- | --- | --- | --- | --- | --- | --- | --- | --- | --- | --- | --- | --- | --- | --- | --- | --- | --- | --- | --- | --- | --- | --- | --- | --- | --- | --- | --- | --- | --- | --- | --- | --- | --- | --- | --- | --- | --- | --- | --- | --- | --- | --- | --- | --- | --- | --- | --- | --- | --- | --- | --- | --- | --- | --- | --- | --- | --- | --- | --- | --- | --- | --- | --- | --- | --- | --- | --- | --- | --- | --- | --- | --- | --- | --- | --- | --- | --- | --- | --- | --- | --- | --- | --- | --- | --- | --- | --- | --- | --- | --- | --- | --- | --- | --- | --- | --- | --- | --- | --- | --- | --- | --- | --- | --- | --- | --- | --- | --- | --- | --- | --- | --- | --- | --- | --- | --- | --- | --- | --- | --- | --- | --- | --- | --- | --- | --- | --- | --- | --- | --- | --- | --- | --- | --- | --- | --- | --- | --- | --- | --- | --- | --- | --- | --- | --- | --- | --- | --- | --- | --- | --- | --- | --- | --- | --- | --- | --- | --- | --- | --- | --- | --- | --- | --- | --- | --- | --- | --- | --- | --- | --- | --- | --- | --- | --- | --- | --- | --- | --- | --- | --- | --- | --- | --- | --- | --- | --- | --- | --- | --- | --- | --- | --- | --- | --- | --- | --- | --- | --- | --- | --- | --- | --- | --- | --- | --- | --- | --- | --- | --- | --- | --- | --- | --- | --- | --- | --- | --- | --- | --- | --- | --- | --- | --- | --- | --- | --- | --- | --- | --- | --- | --- | --- | --- | --- | --- | --- | --- | --- | --- | --- | --- | --- | --- | --- | --- | --- | --- | --- | --- | --- | --- | --- | --- | --- | --- | --- | --- | --- | --- | --- | --- | --- | --- | --- | --- | --- | --- | --- | --- | --- | --- | --- | --- | --- | --- | --- | --- | --- | --- | --- | --- | --- | --- | --- | --- | --- | --- | --- | --- | --- | --- | --- | --- | --- | --- | --- | --- | --- | --- | --- | --- | --- | --- | --- | --- | --- | --- | --- | --- | --- | --- | --- | --- | --- | --- | --- | --- | --- | --- | --- | --- | --- | --- | --- | --- | --- | --- | --- | --- | --- | --- | --- | --- | --- | --- | --- | --- | --- | --- | --- | --- | --- | --- | --- | --- | --- | --- | --- | --- | --- | --- | --- | --- | --- | --- | --- | --- | --- | --- | --- | --- | --- | --- | --- | --- | --- | --- | --- | --- | --- | --- | --- | --- | --- | --- | --- | --- | --- | --- | --- | --- | --- | --- | --- | --- | --- | --- | --- | --- | --- | --- | --- | --- | --- | --- | --- | --- | --- | --- | --- | --- | --- | --- | --- | --- | --- | --- | --- | --- | --- | --- | --- | --- | --- | --- | --- | --- | --- | --- | --- | --- | --- | --- | --- | --- | --- | --- | --- | --- | --- | --- | --- | --- | --- | --- | --- | --- | --- | --- | --- | --- | --- | --- | --- | --- | --- | --- | --- | --- | --- | --- | --- | --- | --- | --- | --- | --- | --- | --- | --- | --- | --- | --- | --- | --- | --- | --- | --- | --- | --- | --- | --- | --- | --- | --- | --- | --- | --- | --- | --- | --- | --- | --- | --- | --- | --- | --- | --- | --- | --- | --- | --- | --- | --- | --- | --- | --- | --- | --- | --- | --- | --- | --- | --- | --- | --- | --- | --- | --- | --- | --- | --- | --- | --- | --- | --- | --- | --- | --- | --- | --- | --- | --- | --- | --- | --- | --- | --- | --- | --- | --- | --- | --- | --- | --- | --- | --- | --- | --- | --- | --- | --- | --- | --- | --- | --- | --- | --- | --- | --- | --- | --- | --- | --- | --- | --- | --- | --- | --- | --- | --- | --- | --- | --- | --- | --- | --- | --- | --- | --- | --- | --- | --- | --- | --- | --- | --- | --- | --- | --- | --- | --- | --- | --- | --- | --- | --- | --- | --- | --- | --- | --- | --- | --- | --- | --- | --- | --- | --- | --- | --- | --- | --- | --- | --- | --- | --- | --- | --- | --- | --- | --- | --- | --- | --- | --- | --- | --- | --- | --- | --- | --- | --- | --- | --- | --- | --- | --- | --- | --- | --- | --- | --- | --- | --- | --- | --- | --- | --- | --- | --- | --- | --- | --- | --- | --- | --- | --- | --- | --- | --- | --- | --- | --- | --- | --- | --- | --- | --- | --- | --- | --- | --- | --- | --- | --- | --- | --- | --- | --- | --- | --- | --- | --- | --- | --- | --- | --- | --- | --- | --- | --- | --- | --- | --- | --- | --- | --- | --- | --- | --- | --- | --- | --- | --- | --- | --- | --- | --- | --- | --- | --- | --- | --- | --- | --- | --- | --- | --- | --- | --- | --- | --- | --- | --- | --- | --- | --- | --- | --- | --- | --- | --- | --- | --- | --- | --- | --- | --- | --- | --- | --- | --- | --- | --- | --- | --- | --- | --- | --- | --- | --- | --- | --- | --- | --- | --- | --- | --- | --- | --- | --- | --- | --- | --- | --- | --- | --- | --- | --- | --- | --- | --- | --- | --- | --- | --- | --- | --- | --- | --- | --- | --- | --- | --- | --- | --- | --- | --- | --- | --- | --- | --- | --- | --- | --- | --- | --- | --- | --- | --- | --- | --- | --- | --- | --- | --- | --- | --- | --- | --- | --- | --- | --- | --- | --- | --- | --- | --- | --- | --- | --- | --- | --- | --- | --- | --- | --- | --- | --- | --- | --- | --- | --- | --- | --- | --- | --- | --- | --- | --- | --- | --- | --- | --- | --- | --- | --- | --- | --- | --- | --- | --- | --- | --- | --- | --- | --- | --- | --- | --- | --- | --- | --- | --- | --- | --- | --- | --- | --- | --- | --- | --- | --- | --- | --- | --- | --- | --- | --- | --- | --- | --- | --- | --- | --- | --- | --- | --- | --- | --- | --- | --- | --- | --- | --- | --- | --- | --- | --- | --- | --- | --- | --- | --- | --- | --- | --- | --- | --- | --- | --- | --- | --- | --- | --- | --- | --- | --- | --- | --- | --- | --- | --- | --- | --- | --- | --- | --- | --- | --- | --- | --- | --- | --- | --- | --- | --- | --- | --- | --- | --- | --- | --- | --- | --- | --- | --- | --- | --- | --- | --- | --- | --- | --- | --- | --- | --- | --- | --- | --- | --- | --- | --- | --- | --- | --- | --- | --- | --- | --- | --- | --- | --- | --- | --- | --- | --- | --- | --- | --- | --- | --- | --- | --- | --- | --- | --- | --- | --- | --- | --- | --- | --- | --- | --- | --- | --- | --- | --- | --- | --- | --- | --- | --- | --- | --- | --- | --- | --- | --- | --- | --- | --- | --- | --- | --- | --- | --- | --- | --- | --- | --- | --- | --- | --- | --- | --- | --- | --- | --- | --- | --- | --- | --- | --- | --- | --- | --- | --- | --- | --- | --- | --- | --- | --- | --- | --- | --- | --- | --- | --- | --- | --- | --- | --- | --- | --- | --- | --- | --- | --- | --- | --- | --- | --- | --- | --- | --- | --- | --- | --- | --- | --- | --- | --- | --- | --- | --- | --- | --- | --- | --- | --- | --- | --- | --- | --- | --- | --- | --- | --- | --- | --- | --- | --- | --- | --- | --- | --- | --- | --- | --- | --- | --- | --- | --- | --- | --- | --- | --- | --- | --- | --- | --- | --- | --- | --- | --- | --- | --- | --- | --- | --- | --- | --- | --- | --- | --- | --- | --- | --- | --- | --- | --- | --- | --- | --- | --- | --- | --- | --- | --- | --- | --- | --- | --- | --- | --- | --- | --- | --- | --- | --- | --- | --- | --- | --- | --- | --- | --- | --- | --- | --- | --- | --- | --- | --- | --- | --- | --- | --- | --- | --- | --- | --- | --- | --- | --- | --- | --- | --- | --- | --- | --- | --- | --- | --- | --- | --- | --- | --- | --- | --- | --- | --- | --- | --- | --- | --- | --- | --- | --- | --- | --- | --- | --- | --- | --- | --- | --- | --- | --- | --- | --- | --- | --- | --- | --- | --- | --- | --- | --- | --- | --- | --- | --- | --- | --- | --- | --- | --- | --- | --- | --- | --- | --- | --- | --- | --- | --- | --- | --- | --- | --- | --- | --- | --- | --- | --- | --- | --- | --- | --- | --- | --- | --- | --- | --- | --- | --- | --- | --- | --- | --- | --- | --- | --- | --- | --- | --- | --- | --- | --- | --- | --- | --- | --- | --- | --- | --- | --- | --- | --- | --- | --- | --- | --- | --- | --- | --- | --- | --- | --- | --- | --- | --- | --- | --- | --- | --- | --- | --- | --- | --- | --- | --- | --- | --- | --- | --- | --- | --- | --- | --- | --- | --- | --- | --- | --- | --- | --- | --- | --- | --- | --- | --- | --- | --- | --- | --- | --- | --- | --- | --- | --- | --- | --- | --- | --- | --- | --- | --- | --- | --- | --- | --- | --- | --- | --- | --- | --- | --- | --- | --- | --- | --- | --- | --- | --- | --- | --- | --- | --- | --- | --- | --- | --- | --- | --- | --- | --- | --- | --- | --- | --- | --- | --- | --- | --- | --- | --- | --- | --- | --- | --- | --- | --- | --- | --- | --- | --- | --- | --- | --- | --- | --- | --- | --- | --- | --- | --- | --- | --- | --- | --- | --- | --- | --- | --- | --- | --- | --- | --- | --- | --- | --- | --- | --- | --- | --- | --- | --- | --- | --- | --- | --- | --- | --- | --- | --- | --- | --- | --- | --- | --- | --- | --- | --- | --- | --- | --- | --- | --- | --- | --- | --- | --- | --- | --- | --- | --- | --- | --- | --- | --- | --- | --- | --- | --- | --- | --- | --- | --- | --- | --- | --- | --- | --- | --- | --- | --- | --- | --- | --- | --- | --- | --- | --- | --- | --- | --- | --- | --- | --- | --- | --- | --- | --- | --- | --- | --- | --- | --- | --- | --- | --- | --- | --- | --- | --- | --- | --- | --- | --- | --- | --- | --- | --- | --- | --- | --- | --- | --- | --- | --- | --- | --- | --- | --- | --- | --- | --- | --- | --- | --- | --- | --- | --- | --- | --- | --- | --- | --- | --- | --- | --- | --- | --- | --- | --- | --- | --- | --- | --- | --- | --- | --- | --- | --- | --- | --- | --- | --- | --- | --- | --- | --- | --- | --- | --- | --- | --- | --- | --- | --- | --- | --- | --- | --- | --- | --- | --- | --- | --- | --- | --- | --- | --- | --- | --- | --- | --- | --- | --- | --- | --- | --- | --- | --- | --- | --- | --- | --- | --- | --- | --- | --- | --- | --- | --- | --- | --- | --- | --- | --- | --- | --- | --- | --- | --- | --- | --- | --- | --- | --- | --- | --- | --- | --- | --- | --- | --- | --- | --- | --- | --- | --- | --- | --- | --- | --- | --- | --- | --- | --- | --- | --- | --- | --- | --- | --- | --- | --- | --- | --- | --- | --- | --- | --- | --- | --- | --- | --- | --- | --- | --- | --- | --- | --- | --- | --- | --- | --- | --- | --- | --- | --- | --- | --- | --- | --- | --- | --- | --- | --- | --- | --- | --- | --- | --- | --- | --- | --- | --- | --- | --- | --- | --- | --- | --- | --- | --- | --- | --- | --- | --- | --- | --- | --- | --- | --- | --- | --- | --- | --- | --- | --- | --- | --- | --- | --- | --- | --- | --- | --- | --- | --- | --- | --- | --- | --- | --- | --- | --- | --- | --- | --- | --- | --- | --- | --- | --- | --- | --- | --- | --- | --- | --- | --- | --- | --- | --- | --- | --- | --- | --- | --- | --- | --- | --- | --- | --- | --- | --- | --- | --- | --- | --- | --- | --- | --- | --- | --- | --- | --- | --- | --- | --- | --- | --- | --- | --- | --- | --- | --- | --- | --- | --- | --- | --- | --- | --- | --- | --- | --- | --- | --- | --- | --- | --- | --- | --- | --- | --- | --- | --- | --- | --- | --- | --- | --- | --- | --- | --- | --- | --- | --- | --- | --- | --- | --- | --- | --- | --- | --- | --- | --- | --- | --- | --- | --- | --- | --- | --- | --- | --- | --- | --- | --- | --- | --- | --- | --- | --- | --- | --- | --- | --- | --- | --- | --- | --- | --- | --- | --- | --- | --- | --- | --- | --- | --- | --- | --- | --- | --- | --- | --- | --- | --- | --- | --- | --- | --- | --- | --- | --- | --- | --- | --- | --- | --- | --- | --- | --- | --- | --- | --- | --- | --- | --- | --- | --- | --- | --- | --- | --- | --- | --- | --- | --- | --- | --- | --- | --- | --- | --- | --- | --- | --- | --- | --- | --- | --- | --- | --- | --- | --- | --- | --- | --- | --- | --- | --- | --- | --- | --- | --- | --- | --- | --- | --- | --- | --- | --- | --- | --- | --- | --- | --- | --- | --- | --- | --- | --- | --- | --- | --- | --- | --- | --- | --- | --- | --- | --- | --- | --- | --- | --- | --- | --- | --- | --- | --- | --- | --- | --- | --- | --- | --- | --- | --- | --- | --- | --- | --- | --- | --- | --- | --- | --- | --- | --- | --- | --- | --- | --- | --- | --- | --- | --- | --- | --- | --- | --- | --- | --- | --- | --- | --- | --- | --- | --- | --- | --- | --- | --- | --- | --- | --- | --- | --- | --- | --- | --- | --- | --- | --- | --- | --- | --- | --- | --- | --- | --- | --- | --- | --- | --- | --- | --- | --- | --- | --- | --- | --- | --- | --- | --- | --- | --- | --- | --- | --- | --- | --- | --- | --- | --- | --- | --- | --- | --- | --- | --- | --- | --- | --- | --- | --- | --- | --- | --- | --- | --- | --- | --- | --- | --- | --- | --- | --- | --- | --- | --- | --- | --- | --- | --- | --- | --- | --- | --- | --- | --- | --- | --- | --- | --- | --- | --- | --- | --- | --- | --- | --- | --- | --- | --- | --- | --- | --- | --- | --- | --- | --- | --- | --- | --- | --- | --- | --- | --- | --- | --- | --- | --- | --- | --- | --- | --- | --- | --- | --- | --- | --- | --- | --- | --- | --- | --- | --- | --- | --- | --- | --- | --- | --- | --- | --- | --- | --- | --- | --- | --- | --- | --- | --- | --- | --- | --- | --- | --- | --- | --- | --- | --- | --- | --- | --- | --- | --- | --- | --- | --- | --- | --- | --- | --- | --- | --- | --- | --- | --- | --- | --- | --- | --- | --- | --- | --- | --- | --- | --- | --- | --- | --- | --- | --- | --- | --- | --- | --- | --- | --- | --- | --- | --- | --- | --- | --- | --- | --- | --- | --- | --- | --- | --- | --- | --- | --- | --- | --- | --- | --- | --- | --- | --- | --- | --- | --- | --- | --- | --- | --- | --- | --- | --- | --- | --- | --- | --- | --- | --- | --- | --- | --- | --- | --- | --- | --- | --- | --- | --- | --- | --- | --- | --- | --- | --- | --- | --- | --- | --- | --- | --- | --- | --- | --- | --- | --- | --- | --- | --- | --- | --- | --- | --- | --- | --- | --- | --- | --- | --- | --- | --- | --- | --- | --- | --- | --- | --- | --- | --- | --- | --- | --- | --- | --- | --- | --- | --- | --- | --- | --- | --- | --- | --- | --- | --- | --- | --- | --- | --- | --- | --- | --- | --- | --- | --- | --- | --- | --- | --- | --- | --- | --- | --- | --- | --- | --- | --- | --- | --- | --- | --- | --- | --- | --- | --- | --- | --- | --- | --- | --- | --- | --- | --- | --- | --- | --- | --- | --- | --- | --- | --- | --- | --- | --- | --- | --- | --- | --- | --- | --- | --- | --- | --- | --- | --- | --- | --- | --- | --- | --- | --- | --- | --- | --- | --- | --- | --- | --- | --- | --- | --- | --- | --- | --- | --- | --- | --- | --- | --- | --- | --- | --- | --- | --- | --- | --- | --- | --- | --- | --- | --- | --- | --- | --- | --- | --- | --- | --- | --- | --- | --- | --- | --- | --- | --- | --- | --- | --- | --- | --- | --- | --- | --- | --- | --- | --- | --- | --- | --- | --- | --- | --- | --- | --- | --- | --- | --- | --- | --- | --- | --- | --- | --- | --- | --- | --- | --- | --- | --- | --- | --- | --- | --- | --- | --- | --- | --- | --- | --- | --- | --- | --- | --- | --- | --- | --- | --- | --- | --- | --- | --- | --- | --- | --- | --- | --- | --- | --- | --- | --- | --- | --- | --- | --- | --- | --- | --- | --- | --- | --- | --- | --- | --- | --- | --- | --- | --- | --- | --- | --- | --- | --- | --- | --- | --- | --- | --- | --- | --- | --- | --- | --- | --- | --- | --- | --- | --- | --- | --- | --- | --- | --- | --- | --- | --- | --- | --- | --- | --- | --- | --- | --- | --- | --- | --- | --- | --- | --- | --- | --- | --- | --- | --- | --- | --- | --- | --- | --- | --- | --- | --- | --- | --- | --- | --- | --- | --- | --- | --- | --- | --- | --- | --- | --- | --- | --- | --- | --- | --- | --- | --- | --- | --- | --- | --- | --- | --- | --- | --- | --- | --- | --- | --- | --- | --- | --- | --- | --- | --- | --- | --- | --- | --- | --- | --- | --- | --- | --- | --- | --- | --- | --- | --- | --- | --- | --- | --- | --- | --- | --- | --- | --- | --- | --- | --- | --- | --- | --- | --- | --- | --- | --- | --- | --- | --- | --- | --- | --- | --- | --- | --- | --- | --- | --- | --- | --- | --- | --- | --- | --- | --- | --- | --- | --- | --- | --- | --- | --- | --- | --- | --- | --- | --- | --- | --- | --- | --- | --- | --- | --- | --- | --- | --- | --- | --- | --- | --- | --- | --- | --- | --- | --- | --- | --- | --- | --- | --- | --- | --- | --- | --- | --- | --- | --- | --- | --- | --- | --- | --- | --- | --- | --- | --- | --- | --- | --- | --- | --- | --- | --- | --- | --- | --- | --- | --- | --- | --- | --- | --- | --- | --- | --- | --- | --- | --- | --- | --- | --- | --- | --- | --- | --- | --- | --- | --- | --- | --- | --- | --- | --- | --- | --- | --- | --- | --- | --- | --- | --- | --- | --- | --- | --- | --- | --- | --- | --- | --- | --- | --- | --- | --- | --- | --- | --- | --- | --- | --- | --- | --- | --- | --- | --- | --- | --- | --- | --- | --- | --- | --- | --- | --- | --- | --- | --- | --- | --- | --- | --- | --- | --- | --- | --- | --- | --- | --- | --- | --- | --- | --- | --- | --- | --- | --- | --- | --- | --- | --- | --- | --- | --- | --- | --- | --- | --- | --- | --- | --- | --- | --- | --- | --- | --- | --- | --- | --- | --- | --- | --- | --- | --- | --- | --- | --- | --- | --- | --- | --- | --- | --- | --- | --- | --- | --- | --- | --- | --- | --- | --- | --- | --- | --- | --- | --- | --- | --- | --- | --- | --- | --- | --- | --- | --- | --- | --- | --- | --- | --- | --- | --- | --- | --- | --- | --- | --- | --- | --- | --- | --- | --- | --- | --- | --- | --- | --- | --- | --- | --- | --- | --- | --- | --- | --- | --- | --- | --- | --- | --- | --- | --- | --- | --- | --- | --- | --- | --- | --- | --- | --- | --- | --- | --- | --- | --- | --- | --- | --- | --- | --- | --- | --- | --- | --- | --- | --- | --- | --- | --- | --- | --- | --- | --- | --- | --- | --- | --- | --- | --- | --- | --- | --- | --- | --- | --- | --- | --- | --- | --- | --- | --- | --- | --- | --- | --- | --- | --- | --- | --- | --- | --- | --- | --- | --- | --- | --- | --- | --- | --- | --- | --- | --- | --- | --- | --- | --- | --- | --- | --- | --- | --- | --- | --- | --- | --- | --- | --- | --- | --- | --- | --- | --- | --- | --- | --- | --- | --- | --- | --- | --- | --- | --- | --- | --- | --- | --- | --- | --- | --- | --- | --- | --- | --- | --- | --- | --- | --- | --- | --- | --- | --- | --- | --- | --- | --- | --- | --- | --- | --- | --- | --- | --- | --- | --- | --- | --- | --- | --- | --- | --- | --- | --- | --- | --- | --- | --- | --- | --- | --- | --- | --- | --- | --- | --- | --- | --- | --- | --- | --- | --- | --- | --- | --- | --- | --- | --- | --- | --- | --- | --- | --- | --- | --- | --- | --- | --- | --- | --- | --- | --- | --- | --- | --- | --- | --- | --- | --- | --- | --- | --- | --- | --- | --- | --- | --- | --- | --- | --- | --- | --- | --- | --- | --- | --- | --- | --- | --- | --- | --- | --- | --- | --- | --- | --- | --- | --- | --- | --- | --- | --- | --- | --- | --- | --- | --- | --- | --- | --- | --- | --- | --- | --- | --- | --- | --- | --- | --- | --- | --- | --- | --- | --- | --- | --- | --- | --- | --- | --- | --- | --- | --- | --- | --- | --- | --- | --- | --- | --- | --- | --- | --- | --- | --- | --- | --- | --- | --- | --- | --- | --- | --- | --- | --- | --- | --- | --- | --- | --- | --- | --- | --- | --- | --- | --- | --- | --- | --- | --- | --- | --- | --- | --- | --- | --- | --- | --- | --- | --- | --- | --- | --- | --- | --- | --- | --- | --- | --- | --- | --- | --- | --- | --- | --- | --- | --- | --- | --- | --- | --- | --- | --- | --- | --- | --- | --- | --- | --- | --- | --- | --- | --- | --- | --- | --- | --- | --- | --- | --- | --- | --- | --- | --- | --- | --- | --- | --- | --- | --- | --- | --- | --- | --- | --- | --- | --- | --- | --- | --- | --- | --- | --- | --- | --- | --- | --- | --- | --- | --- | --- | --- | --- | --- | --- | --- | --- | --- | --- | --- | --- | --- | --- | --- | --- | --- | --- | --- | --- | --- | --- | --- | --- | --- | --- | --- | --- | --- | --- | --- | --- | --- | --- | --- | --- | --- | --- | --- | --- | --- | --- | --- | --- | --- | --- | --- | --- | --- | --- | --- | --- | --- | --- | --- | --- | --- | --- | --- | --- | --- | --- | --- | --- | --- | --- | --- | --- | --- | --- | --- | --- | --- | --- | --- | --- | --- | --- | --- | --- | --- | --- | --- | --- | --- | --- | --- | --- | --- | --- | --- | --- | --- | --- | --- | --- | --- | --- | --- | --- | --- | --- | --- | --- | --- | --- | --- | --- | --- | --- | --- | --- | --- | --- | --- | --- | --- | --- | --- | --- | --- | --- | --- | --- | --- | --- | --- | --- | --- | --- | --- | --- | --- | --- | --- | --- | --- | --- | --- | --- | --- | --- | --- | --- | --- | --- | --- | --- | --- | --- | --- | --- | --- | --- | --- | --- | --- | --- | --- | --- | --- | --- | --- | --- | --- | --- | --- | --- | --- | --- | --- | --- | --- | --- | --- | --- | --- | --- | --- | --- | --- | --- | --- | --- | --- | --- | --- | --- | --- | --- | --- | --- | --- | --- | --- | --- | --- | --- | --- | --- | --- | --- | --- | --- | --- | --- | --- | --- | --- | --- | --- | --- | --- | --- | --- | --- | --- | --- | --- | --- | --- | --- | --- | --- | --- | --- | --- | --- | --- | --- | --- | --- | --- | --- | --- | --- | --- | --- | --- | --- | --- | --- | --- | --- | --- | --- | --- | --- | --- | --- | --- | --- | --- | --- | --- | --- | --- | --- | --- | --- | --- | --- | --- | --- | --- | --- | --- | --- | --- | --- | --- | --- | --- | --- | --- | --- | --- | --- | --- | --- | --- | --- | --- | --- | --- | --- | --- | --- | --- | --- | --- | --- | --- | --- | --- | --- | --- | --- | --- | --- | --- | --- | --- | --- | --- | --- | --- | --- | --- | --- | --- | --- | --- | --- | --- | --- | --- | --- | --- | --- | --- | --- | --- | --- | --- | --- | --- | --- | --- | --- | --- | --- | --- | --- | --- | --- | --- | --- | --- | --- | --- | --- | --- | --- | --- | --- | --- | --- | --- | --- | --- | --- | --- | --- | --- | --- | --- | --- | --- | --- | --- | --- | --- | --- | --- | --- | --- | --- | --- | --- | --- | --- | --- | --- | --- | --- | --- | --- | --- | --- | --- | --- | --- | --- | --- | --- | --- | --- | --- | --- | --- | --- | --- | --- | --- | --- | --- | --- | --- | --- | --- | --- | --- | --- | --- | --- | --- | --- | --- | --- | --- | --- | --- | --- | --- | --- | --- | --- | --- | --- | --- | --- | --- | --- | --- | --- | --- | --- | --- | --- | --- | --- | --- | --- | --- | --- | --- | --- | --- | --- | --- | --- | --- | --- | --- | --- | --- | --- | --- | --- | --- | --- | --- | --- | --- | --- | --- | --- | --- | --- | --- | --- | --- | --- | --- | --- | --- | --- | --- | --- | --- | --- | --- | --- | --- | --- | --- | --- | --- | --- | --- | --- | --- | --- | --- | --- | --- | --- | --- | --- | --- | --- | --- | --- | --- | --- | --- | --- | --- | --- | --- | --- | --- | --- | --- | --- | --- | --- | --- | --- | --- | --- | --- | --- | --- | --- | --- | --- | --- | --- | --- | --- | --- | --- | --- | --- | --- | --- | --- | --- | --- | --- | --- | --- | --- | --- | --- | --- | --- | --- | --- | --- | --- | --- | --- | --- | --- | --- | --- | --- | --- | --- | --- | --- | --- | --- | --- | --- | --- | --- | --- | --- | --- | --- | --- | --- | --- | --- | --- | --- | --- | --- | --- | --- | --- | --- | --- | --- | --- | --- | --- | --- | --- | --- | --- | --- | --- | --- | --- | --- | --- | --- | --- | --- | --- | --- | --- | --- | --- | --- | --- | --- | --- | --- | --- | --- | --- | --- | --- | --- | --- | --- | --- | --- | --- | --- | --- | --- | --- | --- | --- | --- | --- | --- | --- | --- | --- | --- | --- | --- | --- | --- | --- | --- | --- | --- | --- | --- | --- | --- | --- | --- | --- | --- | --- | --- | --- | --- | --- | --- | --- | --- | --- | --- | --- | --- | --- | --- | --- | --- | --- | --- | --- | --- | --- | --- | --- | --- | --- | --- | --- | --- | --- | --- | --- | --- | --- | --- | --- | --- | --- | --- | --- | --- | --- | --- | --- | --- | --- | --- | --- | --- | --- | --- | --- | --- | --- | --- | --- | --- | --- | --- | --- | --- | --- | --- | --- | --- | --- | --- | --- | --- | --- | --- | --- | --- | --- | --- | --- | --- | --- | --- | --- | --- | --- | --- | --- | --- | --- | --- | --- | --- | --- | --- | --- | --- | --- | --- | --- | --- | --- | --- | --- | --- | --- | --- | --- | --- | --- | --- | --- | --- | --- | --- | --- | --- | --- | --- | --- | --- | --- | --- | --- | --- | --- | --- | --- | --- | --- | --- | --- | --- | --- | --- | --- | --- | --- | --- | --- | --- | --- | --- | --- | --- | --- | --- | --- | --- | --- | --- | --- | --- | --- | --- | --- | --- | --- | --- | --- | --- | --- | --- | --- | --- | --- | --- | --- | --- | --- | --- | --- | --- | --- | --- | --- | --- | --- | --- | --- | --- | --- | --- | --- | --- | --- | --- | --- | --- | --- | --- | --- | --- | --- | --- | --- | --- | --- | --- | --- | --- | --- | --- | --- | --- | --- | --- | --- | --- | --- | --- | --- | --- | --- | --- | --- | --- | --- | --- | --- | --- | --- | --- | --- | --- | --- | --- | --- | --- | --- | --- | --- | --- | --- | --- | --- | --- | --- | --- | --- | --- | --- | --- | --- | --- | --- | --- | --- | --- | --- | --- | --- | --- | --- | --- | --- | --- | --- | --- | --- | --- | --- | --- | --- | --- | --- | --- | --- | --- | --- | --- | --- | --- | --- | --- | --- | --- | --- | --- | --- | --- | --- | --- | --- | --- | --- | --- | --- | --- | --- | --- | --- | --- | --- | --- | --- | --- | --- | --- | --- | --- | --- | --- | --- | --- | --- | --- | --- | --- | --- | --- | --- | --- | --- | --- | --- | --- | --- | --- | --- | --- | --- | --- | --- | --- | --- | --- | --- | --- | --- | --- | --- | --- | --- | --- | --- | --- | --- | --- | --- | --- | --- | --- | --- | --- | --- | --- | --- | --- | --- | --- | --- | --- | --- | --- | --- | --- | --- | --- | --- | --- | --- | --- | --- | --- | --- | --- | --- | --- | --- | --- | --- | --- | --- | --- | --- | --- | --- | --- | --- | --- | --- | --- | --- | --- | --- | --- | --- | --- | --- | --- | --- | --- | --- | --- | --- | --- | --- | --- | --- | --- | --- | --- | --- | --- | --- | --- | --- | --- | --- | --- | --- | --- | --- | --- | --- | --- | --- | --- | --- | --- | --- | --- | --- | --- | --- | --- | --- | --- | --- | --- | --- | --- | --- | --- | --- | --- | --- | --- | --- | --- | --- | --- | --- | --- | --- | --- | --- | --- | --- | --- | --- | --- | --- | --- | --- | --- | --- | --- | --- | --- | --- | --- | --- | --- | --- | --- | --- | --- | --- | --- | --- | --- | --- | --- | --- | --- | --- | --- | --- | --- | --- | --- | --- | --- | --- | --- | --- | --- | --- | --- | --- | --- | --- | --- | --- | --- | --- | --- | --- | --- | --- | --- | --- | --- | --- | --- | --- | --- | --- | --- | --- | --- | --- | --- | --- | --- | --- | --- | --- | --- | --- | --- | --- | --- | --- | --- | --- | --- | --- | --- | --- | --- | --- | --- | --- | --- | --- | --- | --- | --- | --- | --- | --- | --- | --- | --- | --- | --- | --- | --- | --- | --- | --- | --- | --- | --- | --- | --- | --- | --- | --- | --- | --- | --- | --- | --- | --- | --- | --- | --- | --- | --- | --- | --- | --- | --- | --- | --- | --- | --- | --- | --- | --- | --- | --- | --- | --- | --- | --- | --- | --- | --- | --- | --- | --- | --- | --- | --- | --- | --- | --- | --- | --- | --- | --- | --- | --- | --- | --- | --- | --- | --- | --- | --- | --- | --- | --- | --- | --- | --- | --- | --- | --- | --- | --- | --- | --- | --- | --- | --- | --- | --- | --- | --- | --- | --- | --- | --- | --- | --- | --- | --- | --- | --- | --- | --- | --- | --- | --- | --- | --- | --- | --- | --- | --- | --- | --- | --- | --- | --- | --- | --- | --- | --- | --- | --- | --- | --- | --- | --- | --- | --- | --- | --- | --- | --- | --- | --- | --- | --- | --- | --- | --- | --- | --- | --- | --- | --- | --- | --- | --- | --- | --- | --- | --- | --- | --- | --- | --- | --- | --- | --- | --- | --- | --- | --- | --- | --- | --- | --- | --- | --- | --- | --- | --- | --- | --- | --- | --- | --- | --- | --- | --- | --- | --- | --- | --- | --- | --- | --- | --- | --- | --- | --- | --- | --- | --- | --- | --- | --- | --- | --- | --- | --- | --- | --- | --- | --- | --- | --- | --- | --- | --- | --- | --- | --- | --- | --- | --- | --- | --- | --- | --- | --- | --- | --- | --- | --- | --- | --- | --- | --- | --- | --- | --- | --- | --- | --- | --- | --- | --- | --- | --- | --- | --- | --- | --- | --- | --- | --- | --- | --- | --- | --- | --- | --- | --- | --- | --- | --- | --- | --- | --- | --- | --- | --- | --- | --- | --- | --- | --- | --- | --- | --- | --- | --- | --- | --- | --- | --- | --- | --- | --- | --- | --- | --- | --- | --- | --- | --- | --- | --- | --- | --- | --- | --- | --- | --- | --- | --- | --- | --- | --- | --- | --- | --- | --- | --- | --- | --- | --- | --- | --- | --- | --- | --- | --- | --- | --- | --- | --- | --- | --- | --- | --- | --- | --- | --- | --- | --- | --- | --- | --- | --- | --- | --- | --- | --- | --- | --- | --- | --- | --- | --- | --- | --- | --- | --- | --- | --- | --- | --- | --- | --- | --- | --- | --- | --- | --- | --- | --- | --- | --- | --- | --- | --- | --- | --- | --- | --- | --- | --- | --- | --- | --- | --- | --- | --- | --- | --- | --- | --- | --- | --- | --- | --- | --- | --- | --- | --- | --- | --- | --- | --- | --- | --- | --- | --- | --- | --- | --- | --- | --- | --- | --- | --- | --- | --- | --- | --- | --- | --- | --- | --- | --- | --- | --- | --- | --- | --- | --- | --- | --- | --- | --- | --- | --- | --- | --- | --- | --- | --- | --- | --- | --- | --- | --- | --- | --- | --- | --- | --- | --- | --- | --- | --- | --- | --- | --- | --- | --- | --- | --- | --- | --- | --- | --- | --- | --- | --- | --- | --- | --- | --- | --- | --- | --- | --- | --- | --- | --- | --- | --- | --- | --- | --- | --- | --- | --- | --- | --- | --- | --- | --- | --- | --- | --- | --- | --- | --- | --- | --- | --- | --- | --- | --- | --- | --- | --- | --- | --- | --- | --- | --- | --- | --- | --- | --- | --- | --- | --- | --- | --- | --- | --- | --- | --- | --- | --- | --- | --- | --- | --- | --- | --- | --- | --- | --- | --- | --- | --- | --- | --- | --- | --- | --- | --- | --- | --- | --- | --- | --- | --- | --- | --- | --- | --- | --- | --- | --- | --- | --- | --- | --- | --- | --- | --- | --- | --- | --- | --- | --- | --- | --- | --- | --- | --- | --- | --- | --- | --- | --- | --- | --- | --- | --- | --- | --- | --- | --- | --- | --- | --- | --- | --- | --- | --- | --- | --- | --- | --- | --- | --- | --- | --- | --- | --- | --- | --- | --- | --- | --- | --- | --- | --- | --- | --- | --- | --- | --- | --- | --- | --- | --- | --- | --- | --- | --- | --- | --- | --- | --- | --- | --- | --- | --- | --- | --- | --- | --- | --- | --- | --- | --- | --- | --- | --- | --- | --- | --- | --- | --- | --- | --- | --- | --- | --- | --- | --- | --- | --- | --- | --- | --- | --- | --- | --- | --- | --- | --- | --- | --- | --- | --- | --- | --- | --- | --- | --- | --- | --- | --- | --- | --- | --- | --- | --- | --- | --- | --- | --- | --- | --- | --- | --- | --- | --- | --- | --- | --- | --- | --- | --- | --- | --- | --- | --- | --- | --- | --- | --- | --- | --- | --- | --- | --- | --- | --- | --- | --- | --- | --- | --- | --- | --- | --- | --- | --- | --- | --- | --- | --- | --- | --- | --- | --- | --- | --- | --- | --- | --- | --- | --- | --- | --- | --- | --- | --- | --- | --- | --- | --- | --- | --- | --- | --- | --- | --- | --- | --- | --- | --- | --- | --- | --- | --- | --- | --- | --- | --- | --- | --- | --- | --- | --- | --- | --- | --- | --- | --- | --- | --- | --- | --- | --- | --- | --- | --- | --- | --- | --- | --- | --- | --- | --- | --- | --- | --- | --- | --- | --- | --- | --- | --- | --- | --- | --- | --- | --- | --- | --- | --- | --- | --- | --- | --- | --- | --- | --- | --- | --- | --- | --- | --- | --- | --- | --- | --- | --- | --- | --- | --- | --- | --- | --- | --- | --- | --- | --- | --- | --- | --- | --- | --- | --- | --- | --- | --- | --- | --- | --- | --- | --- | --- | --- | --- | --- | --- | --- | --- | --- | --- | --- | --- | --- | --- | --- | --- | --- | --- | --- | --- | --- | --- | --- | --- | --- | --- | --- | --- | --- | --- | --- | --- | --- | --- | --- | --- | --- | --- | --- | --- | --- | --- | --- | --- | --- | --- | --- | --- | --- | --- | --- | --- | --- | --- | --- | --- | --- | --- | --- | --- | --- | --- | --- | --- | --- | --- | --- | --- | --- | --- | --- | --- | --- | --- | --- | --- | --- | --- | --- | --- | --- | --- | --- | --- | --- | --- | --- | --- | --- | --- | --- | --- | --- | --- | --- | --- | --- | --- | --- | --- | --- | --- | --- | --- | --- | --- | --- | --- | --- | --- | --- | --- | --- | --- | --- | --- | --- | --- | --- | --- | --- | --- | --- | --- | --- | --- | --- | --- | --- | --- | --- | --- | --- | --- | --- | --- | --- | --- | --- | --- | --- | --- | --- | --- | --- | --- | --- | --- | --- | --- | --- | --- | --- | --- | --- | --- | --- | --- | --- | --- | --- | --- | --- | --- | --- | --- | --- | --- | --- | --- | --- | --- | --- | --- | --- | --- | --- | --- | --- | --- | --- | --- | --- | --- | --- | --- | --- | --- | --- | --- | --- | --- | --- | --- | --- | --- | --- | --- | --- | --- | --- | --- | --- | --- | --- | --- | --- | --- | --- | --- | --- | --- | --- | --- | --- | --- | --- | --- | --- | --- | --- | --- | --- | --- | --- | --- | --- | --- | --- | --- | --- | --- | --- | --- | --- | --- | --- | --- | --- | --- | --- | --- | --- | --- | --- | --- | --- | --- | --- | --- | --- | --- | --- | --- | --- | --- | --- | --- | --- | --- | --- | --- | --- | --- | --- | --- | --- | --- | --- | --- | --- | --- | --- | --- | --- | --- | --- | --- | --- | --- | --- | --- | --- | --- | --- | --- | --- | --- | --- | --- | --- | --- | --- | --- | --- | --- | --- | --- | --- | --- | --- | --- | --- | --- | --- | --- | --- | --- | --- | --- | --- | --- | --- | --- | --- | --- | --- | --- | --- | --- | --- | --- | --- | --- | --- | --- | --- | --- | --- | --- | --- | --- | --- | --- | --- | --- | --- | --- | --- | --- | --- | --- | --- | --- | --- | --- | --- | --- | --- | --- | --- | --- | --- | --- | --- | --- | --- | --- | --- | --- | --- | --- | --- | --- | --- | --- | --- | --- | --- | --- | --- | --- | --- | --- | --- | --- | --- | --- | --- | --- | --- | --- | --- | --- | --- | --- | --- | --- | --- | --- | --- | --- | --- | --- | --- | --- | --- | --- | --- | --- | --- | --- | --- | --- | --- | --- | --- | --- | --- | --- | --- | --- | --- | --- | --- | --- | --- | --- | --- | --- | --- | --- | --- | --- | --- | --- | --- | --- | --- | --- | --- | --- | --- | --- | --- | --- | --- | --- | --- | --- | --- | --- | --- | --- | --- | --- | --- | --- | --- | --- | --- | --- | --- | --- | --- | --- | --- | --- | --- | --- | --- | --- | --- | --- | --- | --- | --- | --- | --- | --- | --- | --- | --- | --- | --- | --- | --- | --- | --- | --- | --- | --- | --- | --- | --- | --- | --- | --- | --- | --- | --- | --- | --- | --- | --- | --- | --- | --- | --- | --- | --- | --- | --- | --- | --- | --- | --- | --- | --- | --- | --- | --- | --- | --- | --- | --- | --- | --- | --- | --- | --- | --- | --- | --- | --- | --- | --- | --- | --- | --- | --- | --- | --- | --- | --- | --- | --- | --- | --- | --- | --- | --- | --- | --- | --- | --- | --- | --- | --- | --- | --- | --- | --- | --- | --- | --- | --- | --- | --- | --- | --- | --- | --- | --- | --- | --- | --- | --- | --- | --- | --- | --- | --- | --- | --- | --- | --- | --- | --- | --- | --- | --- | --- | --- | --- | --- | --- | --- | --- | --- | --- | --- | --- | --- | --- | --- | --- | --- | --- | --- | --- | --- | --- | --- | --- | --- | --- | --- | --- | --- | --- | --- | --- | --- | --- | --- | --- | --- | --- | --- | --- | --- | --- | --- | --- | --- | --- | --- | --- | --- | --- | --- | --- | --- | --- | --- | --- | --- | --- | --- | --- | --- | --- | --- | --- | --- | --- | --- | --- | --- | --- | --- | --- | --- | --- | --- | --- | --- | --- | --- | --- | --- | --- | --- | --- | --- | --- | --- | --- | --- | --- | --- | --- | --- | --- | --- | --- | --- | --- | --- | --- | --- | --- | --- | --- | --- | --- | --- | --- | --- | --- | --- | --- | --- | --- | --- | --- | --- | --- | --- | --- | --- | --- | --- | --- | --- | --- | --- | --- | --- | --- | --- | --- | --- | --- | --- | --- | --- | --- | --- | --- | --- | --- | --- | --- | --- | --- | --- | --- | --- | --- | --- | --- | --- | --- | --- | --- | --- | --- | --- | --- | --- | --- | --- | --- | --- | --- | --- | --- | --- | --- | --- | --- | --- | --- | --- | --- | --- | --- | --- | --- | --- | --- | --- | --- | --- | --- | --- | --- | --- | --- | --- | --- | --- | --- | --- | --- | --- | --- | --- | --- | --- | --- | --- | --- | --- | --- | --- | --- | --- | --- | --- | --- | --- | --- | --- | --- | --- | --- | --- | --- | --- | --- | --- | --- | --- | --- | --- | --- | --- | --- | --- | --- | --- | --- | --- | --- | --- | --- | --- | --- | --- | --- | --- | --- | --- | --- | --- | --- | --- | --- | --- | --- | --- | --- | --- | --- | --- | --- | --- | --- | --- | --- | --- | --- | --- | --- | --- | --- | --- | --- | --- | --- | --- | --- | --- | --- | --- | --- | --- | --- | --- | --- | --- | --- | --- | --- | --- | --- | --- | --- | --- | --- | --- | --- | --- | --- | --- | --- | --- | --- | --- | --- | --- | --- | --- | --- | --- | --- | --- | --- | --- | --- | --- | --- | --- | --- | --- | --- | --- | --- | --- | --- | --- | --- | --- | --- | --- | --- | --- | --- | --- | --- | --- | --- | --- | --- | --- | --- | --- | --- | --- | --- | --- | --- | --- | --- | --- | --- | --- | --- | --- | --- | --- | --- | --- | --- | --- | --- | --- | --- | --- | --- | --- | --- | --- | --- | --- | --- | --- | --- | --- | --- | --- | --- | --- | --- | --- | --- | --- | --- | --- | --- | --- | --- | --- | --- | --- | --- | --- | --- | --- | --- | --- | --- | --- | --- | --- | --- | --- | --- | --- | --- | --- | --- | --- | --- | --- | --- | --- | --- | --- | --- | --- | --- | --- | --- | --- | --- | --- | --- | --- | --- | --- | --- | --- | --- | --- | --- | --- | --- | --- | --- | --- | --- | --- | --- | --- | --- | --- | --- | --- | --- | --- | --- | --- | --- | --- | --- | --- | --- | --- | --- | --- | --- | --- | --- | --- | --- | --- | --- | --- | --- | --- | --- | --- | --- | --- | --- | --- | --- | --- | --- | --- | --- | --- | --- | --- | --- | --- | --- | --- | --- | --- | --- | --- | --- | --- | --- | --- | --- | --- | --- | --- | --- | --- | --- | --- | --- | --- | --- | --- | --- | --- | --- | --- | --- | --- | --- | --- | --- | --- | --- | --- | --- | --- | --- | --- | --- | --- | --- | --- | --- | --- | --- | --- | --- | --- | --- | --- | --- | --- | --- | --- | --- | --- | --- | --- | --- | --- | --- | --- | --- | --- | --- | --- | --- | --- | --- | --- | --- | --- | --- | --- | --- | --- | --- | --- | --- | --- | --- | --- | --- | --- | --- | --- | --- | --- | --- | --- | --- | --- | --- | --- | --- | --- | --- | --- | --- | --- | --- | --- | --- | --- | --- | --- | --- | --- | --- | --- | --- | --- | --- | --- | --- | --- | --- | --- | --- | --- | --- | --- | --- | --- | --- | --- | --- | --- | --- | --- | --- | --- | --- | --- | --- | --- | --- | --- | --- | --- | --- | --- | --- | --- | --- | --- | --- | --- | --- | --- | --- | --- | --- | --- | --- | --- | --- | --- | --- | --- | --- | --- | --- | --- | --- | --- | --- | --- | --- | --- | --- | --- | --- | --- | --- | --- | --- | --- | --- | --- | --- | --- | --- | --- | --- | --- | --- | --- | --- | --- | --- | --- | --- | --- | --- | --- | --- | --- | --- | --- | --- | --- | --- | --- | --- | --- | --- | --- | --- | --- | --- | --- | --- | --- | --- | --- | --- | --- | --- | --- | --- | --- | --- | --- | --- | --- | --- | --- | --- | --- | --- | --- | --- | --- | --- | --- | --- | --- | --- | --- | --- | --- | --- | --- | --- | --- | --- | --- | --- | --- | --- | --- | --- | --- | --- | --- | --- | --- | --- | --- | --- | --- | --- | --- | --- | --- | --- | --- | --- | --- | --- | --- | --- | --- | --- | --- | --- | --- | --- | --- | --- | --- | --- | --- | --- | --- | --- | --- | --- | --- | --- | --- | --- | --- | --- | --- | --- | --- | --- | --- | --- | --- | --- | --- | --- | --- | --- | --- | --- | --- | --- | --- | --- | --- | --- | --- | --- | --- | --- | --- | --- | --- | --- | --- | --- | --- | --- | --- | --- | --- | --- | --- | --- | --- | --- | --- | --- | --- | --- | --- | --- | --- | --- | --- | --- | --- | --- | --- | --- | --- | --- | --- | --- | --- | --- | --- | --- | --- | --- | --- | --- | --- | --- | --- | --- | --- | --- | --- | --- | --- | --- | --- | --- | --- | --- | --- | --- | --- | --- | --- | --- | --- | --- | --- | --- | --- | --- | --- | --- | --- | --- | --- | --- | --- | --- | --- | --- | --- | --- | --- | --- | --- | --- | --- | --- | --- | --- | --- | --- | --- | --- | --- | --- | --- | --- | --- | --- | --- | --- | --- | --- | --- | --- | --- | --- | --- | --- | --- | --- | --- | --- | --- | --- | --- | --- | --- | --- | --- | --- | --- | --- | --- | --- | --- | --- | --- | --- | --- | --- | --- | --- | --- | --- | --- | --- | --- | --- | --- | --- | --- | --- | --- | --- | --- | --- | --- | --- | --- | --- | --- | --- | --- | --- | --- | --- | --- | --- | --- | --- | --- | --- | --- | --- | --- | --- | --- | --- | --- | --- | --- | --- | --- | --- | --- | --- | --- | --- | --- | --- | --- | --- | --- | --- | --- | --- | --- | --- | --- | --- | --- | --- | --- | --- | --- | --- | --- | --- | --- | --- | --- | --- | --- | --- | --- | --- | --- | --- | --- | --- | --- | --- | --- | --- | --- | --- | --- | --- | --- | --- | --- | --- | --- | --- | --- | --- | --- | --- | --- | --- | --- | --- | --- | --- | --- | --- | --- | --- | --- | --- | --- | --- | --- | --- | --- | --- | --- | --- | --- | --- | --- | --- | --- | --- | --- | --- | --- | --- | --- | --- | --- | --- | --- | --- | --- | --- | --- | --- | --- | --- | --- | --- | --- | --- | --- | --- | --- | --- | --- | --- | --- | --- | --- | --- | --- | --- | --- | --- | --- | --- | --- | --- | --- | --- | --- | --- | --- | --- | --- | --- | --- | --- | --- | --- | --- | --- | --- | --- | --- | --- | --- | --- | --- | --- | --- | --- | --- | --- | --- | --- | --- | --- | --- | --- | --- | --- | --- | --- | --- | --- | --- | --- | --- | --- | --- | --- | --- | --- | --- | --- | --- | --- | --- | --- | --- | --- | --- | --- | --- | --- | --- | --- | --- | --- | --- | --- | --- | --- | --- | --- | --- | --- | --- | --- | --- | --- | --- | --- | --- | --- | --- | --- | --- | --- | --- | --- | --- | --- | --- | --- | --- | --- | --- | --- | --- | --- | --- | --- | --- | --- | --- | --- | --- | --- | --- | --- | --- | --- | --- | --- | --- | --- | --- | --- | --- | --- | --- | --- | --- | --- | --- | --- | --- | --- | --- | --- | --- | --- | --- | --- | --- | --- | --- | --- | --- | --- | --- | --- | --- | --- | --- | --- | --- | --- | --- | --- | --- | --- | --- | --- | --- | --- | --- | --- | --- | --- | --- | --- | --- | --- | --- | --- | --- | --- | --- | --- | --- | --- | --- | --- | --- | --- | --- | --- | --- | --- | --- | --- | --- | --- | --- | --- | --- | --- | --- | --- | --- | --- | --- | --- | --- | --- | --- | --- | --- | --- | --- | --- | --- | --- | --- | --- | --- | --- | --- | --- | --- | --- | --- | --- | --- | --- | --- | --- | --- | --- | --- | --- | --- | --- | --- | --- | --- | --- | --- | --- | --- | --- | --- | --- | --- | --- | --- | --- | --- | --- | --- | --- | --- | --- | --- | --- | --- | --- | --- | --- | --- | --- | --- | --- | --- | --- | --- | --- | --- | --- | --- | --- | --- | --- | --- | --- | --- | --- | --- | --- | --- | --- | --- | --- | --- | --- | --- | --- | --- | --- | --- | --- | --- | --- | --- | --- | --- | --- | --- | --- | --- | --- | --- | --- | --- | --- | --- | --- | --- | --- | --- | --- | --- | --- | --- | --- | --- | --- | --- | --- | --- | --- | --- | --- | --- | --- | --- | --- | --- | --- | --- | --- | --- | --- | --- | --- | --- | --- | --- | --- | --- | --- | --- | --- | --- | --- | --- | --- | --- | --- | --- | --- | --- | --- | --- | --- | --- | --- | --- | --- | --- | --- | --- | --- | --- | --- | --- | --- | --- | --- | --- | --- | --- | --- | --- | --- | --- | --- | --- | --- | --- | --- | --- | --- | --- | --- | --- | --- | --- | --- | --- | --- | --- | --- | --- | --- | --- | --- | --- | --- | --- | --- | --- | --- | --- | --- | --- | --- | --- | --- | --- | --- | --- | --- | --- | --- | --- | --- | --- | --- | --- | --- | --- | --- | --- | --- | --- | --- | --- | --- | --- | --- | --- | --- | --- | --- | --- | --- | --- | --- | --- | --- | --- | --- | --- | --- | --- | --- | --- | --- | --- | --- | --- | --- | --- | --- | --- | --- | --- | --- | --- | --- | --- | --- | --- | --- | --- | --- | --- | --- | --- | --- | --- | --- | --- | --- | --- | --- | --- | --- | --- | --- | --- | --- | --- | --- | --- | --- | --- | --- | --- | --- | --- | --- | --- | --- | --- | --- | --- | --- | --- | --- | --- | --- | --- | --- | --- | --- | --- | --- | --- | --- | --- | --- | --- | --- | --- | --- | --- | --- | --- | --- | --- | --- | --- | --- | --- | --- | --- | --- | --- | --- | --- | --- | --- | --- | --- | --- | --- | --- | --- | --- | --- | --- | --- | --- | --- | --- | --- | --- | --- | --- | --- | --- | --- | --- | --- | --- | --- | --- | --- | --- | --- | --- | --- | --- | --- | --- | --- | --- | --- | --- | --- | --- | --- | --- | --- | --- | --- | --- | --- | --- | --- | --- | --- | --- | --- | --- | --- | --- | --- | --- | --- | --- | --- | --- | --- | --- | --- | --- | --- | --- | --- | --- | --- | --- | --- | --- | --- | --- | --- | --- | --- | --- | --- | --- | --- | --- | --- | --- | --- | --- | --- | --- | --- | --- | --- | --- | --- | --- | --- | --- | --- | --- | --- | --- | --- | --- | --- | --- | --- | --- | --- | --- | --- | --- | --- | --- | --- | --- | --- | --- | --- | --- | --- | --- | --- | --- | --- | --- | --- | --- | --- | --- | --- | --- | --- | --- | --- | --- | --- | --- | --- | --- | --- | --- | --- | --- | --- | --- | --- | --- | --- | --- | --- | --- | --- | --- | --- | --- | --- | --- | --- | --- | --- | --- | --- | --- | --- | --- | --- | --- | --- | --- | --- | --- | --- | --- | --- | --- | --- | --- | --- | --- | --- | --- | --- | --- | --- | --- | --- | --- | --- | --- | --- | --- | --- | --- | --- | --- | --- | --- | --- | --- | --- | --- | --- | --- | --- | --- | --- | --- | --- | --- | --- | --- | --- | --- | --- | --- | --- | --- | --- | --- | --- | --- | --- | --- | --- | --- | --- | --- | --- | --- | --- | --- | --- | --- | --- | --- | --- | --- | --- | --- | --- | --- | --- | --- | --- | --- | --- | --- | --- | --- | --- | --- | --- | --- | --- | --- | --- | --- | --- | --- | --- | --- | --- | --- | --- | --- | --- | --- | --- | --- | --- | --- | --- | --- | --- | --- | --- | --- | --- | --- | --- | --- | --- | --- | --- | --- | --- | --- | --- | --- | --- | --- | --- | --- | --- | --- | --- | --- | --- | --- | --- | --- | --- | --- | --- | --- | --- | --- | --- | --- | --- | --- | --- | --- | --- | --- | --- | --- | --- | --- | --- | --- | --- | --- | --- | --- | --- | --- | --- | --- | --- | --- | --- | --- | --- | --- | --- | --- | --- | --- | --- | --- | --- | --- | --- | --- | --- | --- | --- | --- | --- | --- | --- | --- | --- | --- | --- | --- | --- | --- | --- | --- | --- | --- | --- | --- | --- | --- | --- | --- | --- | --- | --- | --- | --- | --- | --- | --- | --- | --- | --- | --- | --- | --- | --- | --- | --- | --- | --- | --- | --- | --- | --- | --- | --- | --- | --- | --- | --- | --- | --- | --- | --- | --- | --- | --- | --- | --- | --- | --- | --- | --- | --- | --- | --- | --- | --- | --- | --- | --- | --- | --- | --- | --- | --- | --- | --- | --- | --- | --- | --- | --- | --- | --- | --- | --- | --- | --- | --- | --- | --- | --- | --- | --- | --- | --- | --- | --- | --- | --- | --- | --- | --- | --- | --- | --- | --- | --- | --- | --- | --- | --- | --- | --- | --- | --- | --- | --- | --- | --- | --- | --- | --- | --- | --- | --- | --- | --- | --- | --- | --- | --- | --- | --- | --- | --- | --- | --- | --- | --- | --- | --- | --- | --- | --- | --- | --- | --- | --- | --- | --- | --- | --- | --- | --- | --- | --- | --- | --- | --- | --- | --- | --- | --- | --- | --- | --- | --- | --- | --- | --- | --- | --- | --- | --- | --- | --- | --- | --- | --- | --- | --- | --- | --- | --- | --- | --- | --- | --- | --- | --- | --- | --- | --- | --- | --- | --- | --- | --- | --- | --- | --- | --- | --- | --- | --- | --- | --- | --- | --- | --- | --- | --- | --- | --- | --- | --- | --- | --- | --- | --- | --- | --- | --- | --- | --- | --- | --- | --- | --- | --- | --- | --- | --- | --- | --- | --- | --- | --- | --- | --- | --- | --- | --- | --- | --- | --- | --- | --- | --- | --- | --- | --- | --- | --- | --- | --- | --- | --- | --- | --- | --- | --- | --- | --- | --- | --- | --- | --- | --- | --- | --- | --- | --- | --- | --- | --- | --- | --- | --- | --- | --- | --- | --- | --- | --- | --- | --- | --- | --- | --- | --- | --- | --- | --- | --- | --- | --- | --- | --- | --- | --- | --- | --- | --- | --- | --- | --- | --- | --- | --- | --- | --- | --- | --- | --- | --- | --- | --- | --- | --- | --- | --- | --- | --- | --- | --- | --- | --- | --- | --- | --- | --- | --- | --- | --- | --- | --- | --- | --- | --- | --- | --- | --- | --- | --- | --- | --- | --- | --- | --- | --- | --- | --- | --- | --- | --- | --- | --- | --- | --- | --- | --- | --- | --- | --- | --- | --- | --- | --- | --- | --- | --- | --- | --- | --- | --- | --- | --- | --- | --- | --- | --- | --- | --- | --- | --- | --- | --- | --- | --- | --- | --- | --- | --- | --- | --- | --- | --- | --- | --- | --- | --- | --- | --- | --- | --- | --- | --- | --- | --- | --- | --- | --- | --- | --- | --- | --- | --- | --- | --- | --- | --- | --- | --- | --- | --- | --- | --- | --- | --- | --- | --- | --- | --- | --- | --- | --- | --- | --- | --- | --- | --- | --- | --- | --- | --- | --- | --- | --- | --- | --- | --- | --- | --- | --- | --- | --- | --- | --- | --- | --- | --- | --- | --- | --- | --- | --- | --- | --- | --- | --- | --- | --- | --- | --- | --- | --- | --- | --- | --- | --- | --- | --- | --- | --- | --- | --- | --- | --- | --- | --- | --- | --- | --- | --- | --- | --- | --- | --- | --- | --- | --- | --- | --- | --- | --- | --- | --- | --- | --- | --- | --- | --- | --- | --- | --- | --- | --- | --- | --- | --- | --- | --- | --- | --- | --- | --- | --- | --- | --- | --- | --- | --- | --- | --- | --- | --- | --- | --- | --- | --- | --- | --- | --- | --- | --- | --- | --- | --- | --- | --- | --- | --- | --- | --- | --- | --- | --- | --- | --- | --- | --- | --- | --- | --- | --- | --- | --- | --- | --- | --- | --- | --- | --- | --- | --- | --- | --- | --- | --- | --- | --- | --- | --- | --- | --- | --- | --- | --- | --- | --- | --- | --- | --- | --- | --- | --- | --- | --- | --- | --- | --- | --- | --- | --- | --- | --- | --- | --- | --- | --- | --- | --- | --- | --- | --- | --- | --- | --- | --- | --- | --- | --- | --- | --- | --- | --- | --- | --- | --- | --- | --- | --- | --- | --- | --- | --- | --- | --- | --- | --- | --- | --- | --- | --- | --- | --- | --- | --- | --- | --- | --- | --- | --- | --- | --- | --- | --- | --- | --- | --- | --- | --- | --- | --- | --- | --- | --- | --- | --- | --- | --- | --- | --- | --- | --- | --- | --- | --- | --- | --- | --- | --- | --- | --- | --- | --- | --- | --- | --- | --- | --- | --- | --- | --- | --- | --- | --- | --- | --- | --- | --- | --- | --- | --- | --- | --- | --- | --- | --- | --- | --- | --- | --- | --- | --- | --- | --- | --- | --- | --- | --- | --- | --- | --- | --- | --- | --- | --- | --- | --- | --- | --- | --- | --- | --- | --- | --- | --- | --- | --- | --- | --- | --- | --- | --- | --- | --- | --- | --- | --- | --- | --- | --- | --- | --- | --- | --- | --- | --- | --- | --- | --- | --- | --- | --- | --- | --- | --- | --- | --- | --- | --- | --- | --- | --- | --- | --- | --- | --- | --- | --- | --- | --- | --- | --- | --- | --- | --- | --- | --- | --- | --- | --- | --- | --- | --- | --- | --- | --- | --- | --- | --- | --- | --- | --- | --- | --- | --- | --- | --- | --- | --- | --- | --- | --- | --- | --- | --- | --- | --- | --- | --- | --- | --- | --- | --- | --- | --- | --- | --- | --- | --- | --- | --- | --- | --- | --- | --- | --- | --- | --- | --- | --- | --- | --- | --- | --- | --- | --- | --- | --- | --- | --- | --- | --- | --- | --- | --- | --- | --- | --- | --- | --- | --- | --- | --- | --- | --- | --- | --- | --- | --- | --- | --- | --- | --- | --- | --- | --- | --- | --- | --- | --- | --- | --- | --- | --- | --- | --- | --- | --- | --- | --- | --- | --- | --- | --- | --- | --- | --- | --- | --- | --- | --- | --- | --- | --- | --- | --- | --- | --- | --- | --- | --- | --- | --- | --- | --- | --- | --- | --- | --- | --- | --- | --- | --- | --- | --- | --- | --- | --- | --- | --- | --- | --- | --- | --- | --- | --- | --- | --- | --- | --- | --- | --- | --- | --- | --- | --- | --- | --- | --- | --- | --- | --- | --- | --- | --- | --- | --- | --- | --- | --- | --- | --- | --- | --- | --- | --- | --- | --- | --- | --- | --- | --- | --- | --- | --- | --- | --- | --- | --- | --- | --- | --- | --- | --- | --- | --- | --- | --- | --- | --- | --- | --- | --- | --- | --- | --- | --- | --- | --- | --- | --- | --- | --- | --- | --- | --- | --- | --- | --- | --- | --- | --- | --- | --- | --- | --- | --- | --- | --- | --- | --- | --- | --- | --- | --- | --- | --- | --- | --- | --- | --- | --- | --- | --- | --- | --- | --- | --- | --- | --- | --- | --- | --- | --- | --- | --- | --- | --- | --- | --- | --- | --- | --- | --- | --- | --- | --- | --- | --- | --- | --- | --- | --- | --- | --- | --- | --- | --- | --- | --- | --- | --- | --- | --- | --- | --- | --- | --- | --- | --- | --- | --- | --- | --- | --- | --- | --- | --- | --- | --- | --- | --- | --- | --- | --- | --- | --- | --- | --- | --- | --- | --- | --- | --- | --- | --- | --- | --- | --- | --- | --- | --- | --- | --- | --- | --- | --- | --- | --- | --- | --- | --- | --- | --- | --- | --- | --- | --- | --- | --- | --- | --- | --- | --- | --- | --- | --- | --- | --- | --- | --- | --- | --- | --- | --- | --- | --- | --- | --- | --- | --- | --- | --- | --- | --- | --- | --- | --- | --- | --- | --- | --- | --- | --- | --- | --- | --- | --- | --- | --- | --- | --- | --- | --- | --- | --- | --- | --- | --- | --- | --- | --- | --- | --- | --- | --- | --- | --- | --- | --- | --- | --- | --- | --- | --- | --- | --- | --- | --- | --- | --- | --- | --- | --- | --- | --- | --- | --- | --- | --- | --- | --- | --- | --- | --- | --- | --- | --- | --- | --- | --- | --- | --- | --- | --- | --- | --- | --- | --- | --- | --- | --- | --- | --- | --- | --- | --- | --- | --- | --- | --- | --- | --- | --- | --- | --- | --- | --- | --- | --- | --- | --- | --- | --- | --- | --- | --- | --- | --- | --- | --- | --- | --- | --- | --- | --- | --- | --- | --- | --- | --- | --- | --- | --- | --- | --- | --- | --- | --- | --- | --- | --- | --- | --- | --- | --- | --- | --- | --- | --- | --- | --- | --- | --- | --- | --- | --- | --- | --- | --- | --- | --- | --- | --- | --- | --- | --- | --- | --- | --- | --- | --- | --- | --- | --- | --- | --- | --- | --- | --- | --- | --- | --- | --- | --- | --- | --- | --- | --- | --- | --- | --- | --- | --- | --- | --- | --- | --- | --- | --- | --- | --- | --- | --- | --- | --- | --- | --- | --- | --- | --- | --- | --- | --- | --- | --- | --- | --- | --- | --- | --- | --- | --- | --- | --- | --- | --- | --- | --- | --- | --- | --- | --- | --- | --- | --- | --- | --- | --- | --- | --- | --- | --- | --- | --- | --- | --- | --- | --- | --- | --- | --- | --- | --- | --- | --- | --- | --- | --- | --- | --- | --- | --- | --- | --- | --- | --- | --- | --- | --- | --- | --- | --- | --- | --- | --- | --- | --- | --- | --- | --- | --- | --- | --- | --- | --- | --- | --- | --- | --- | --- | --- | --- | --- | --- | --- | --- | --- | --- | --- | --- | --- | --- | --- | --- | --- | --- | --- | --- | --- | --- | --- | --- | --- | --- | --- | --- | --- | --- | --- | --- | --- | --- | --- | --- | --- | --- | --- | --- | --- | --- | --- | --- | --- | --- | --- | --- | --- | --- | --- | --- | --- | --- | --- | --- | --- | --- | --- | --- | --- | --- | --- | --- | --- | --- | --- | --- | --- | --- | --- | --- | --- | --- | --- | --- | --- | --- | --- | --- | --- | --- | --- | --- | --- | --- | --- | --- | --- | --- | --- | --- | --- | --- | --- | --- | --- | --- | --- | --- | --- | --- | --- | --- | --- | --- | --- | --- | --- | --- | --- | --- | --- | --- | --- | --- | --- | --- | --- | --- | --- | --- | --- | --- | --- | --- | --- | --- | --- | --- | --- | --- | --- | --- | --- | --- | --- | --- | --- | --- | --- | --- | --- | --- | --- | --- | --- | --- | --- | --- | --- | --- | --- | --- | --- | --- | --- | --- | --- | --- | --- | --- | --- | --- | --- | --- | --- | --- | --- | --- | --- | --- | --- | --- | --- | --- | --- | --- | --- | --- | --- | --- | --- | --- | --- | --- | --- | --- | --- | --- | --- | --- | --- | --- | --- | --- | --- | --- | --- | --- | --- | --- | --- | --- | --- | --- | --- | --- | --- | --- | --- | --- | --- | --- | --- | --- | --- | --- | --- | --- | --- | --- | --- | --- | --- | --- | --- | --- | --- | --- | --- | --- | --- | --- | --- | --- | --- | --- | --- | --- | --- | --- | --- | --- | --- | --- | --- | --- | --- | --- | --- | --- | --- | --- | --- | --- | --- | --- | --- | --- | --- | --- | --- | --- | --- | --- | --- | --- | --- | --- | --- | --- | --- | --- | --- | --- | --- | --- | --- | --- | --- | --- | --- | --- | --- | --- | --- | --- | --- | --- | --- | --- | --- | --- | --- | --- | --- | --- | --- | --- | --- | --- | --- | --- | --- | --- | --- | --- | --- | --- | --- | --- | --- | --- | --- | --- | --- | --- | --- | --- | --- | --- | --- | --- | --- | --- | --- | --- | --- | --- | --- | --- | --- | --- | --- | --- | --- | --- | --- | --- | --- | --- | --- | --- | --- | --- | --- | --- | --- | --- | --- | --- | --- | --- | --- | --- | --- | --- | --- | --- | --- | --- | --- | --- | --- | --- | --- | --- | --- | --- | --- | --- | --- | --- | --- | --- | --- | --- | --- | --- | --- | --- | --- | --- | --- | --- | --- | --- | --- | --- | --- | --- | --- | --- | --- | --- | --- | --- | --- | --- | --- | --- | --- | --- | --- | --- | --- | --- | --- | --- | --- | --- | --- | --- | --- | --- | --- | --- | --- | --- | --- | --- | --- | --- | --- | --- | --- | --- | --- | --- | --- | --- | --- | --- | --- | --- | --- | --- | --- | --- | --- | --- | --- | --- | --- | --- | --- | --- | --- | --- | --- | --- | --- | --- | --- | --- | --- | --- | --- | --- | --- | --- | --- | --- | --- | --- | --- | --- | --- | --- | --- | --- | --- | --- | --- | --- | --- | --- | --- | --- | --- | --- | --- | --- | --- | --- | --- | --- | --- | --- | --- | --- | --- | --- | --- | --- | --- | --- | --- | --- | --- | --- | --- | --- | --- | --- | --- | --- | --- | --- | --- | --- | --- | --- | --- | --- | --- | --- | --- | --- | --- | --- | --- | --- | --- | --- | --- | --- | --- | --- | --- | --- | --- | --- | --- | --- | --- | --- | --- | --- | --- | --- | --- | --- | --- | --- | --- | --- | --- | --- | --- | --- | --- | --- | --- | --- | --- | --- | --- | --- | --- | --- | --- | --- | --- | --- | --- | --- | --- | --- | --- | --- | --- | --- | --- | --- | --- | --- | --- | --- | --- | --- | --- | --- | --- | --- | --- | --- | --- | --- | --- | --- | --- | --- | --- | --- | --- | --- | --- | --- | --- | --- | --- | --- | --- | --- | --- | --- | --- | --- | --- | --- | --- | --- | --- | --- | --- | --- | --- | --- | --- | --- | --- | --- | --- | --- | --- | --- | --- | --- | --- | --- | --- | --- | --- | --- | --- | --- | --- | --- | --- | --- | --- | --- | --- | --- | --- | --- | --- | --- | --- | --- | --- | --- | --- | --- | --- | --- | --- | --- | --- | --- | --- | --- | --- | --- | --- | --- | --- | --- | --- | --- | --- | --- | --- | --- | --- | --- | --- | --- | --- | --- | --- | --- | --- | --- | --- | --- | --- | --- | --- | --- | --- | --- | --- | --- | --- | --- | --- | --- | --- | --- | --- | --- | --- | --- | --- | --- | --- | --- | --- | --- | --- | --- | --- | --- | --- | --- | --- | --- | --- | --- | --- | --- | --- | --- | --- | --- | --- | --- | --- | --- | --- | --- | --- | --- | --- | --- | --- | --- | --- | --- | --- | --- | --- | --- | --- | --- | --- | --- | --- | --- | --- | --- | --- | --- | --- | --- | --- | --- | --- | --- | --- | --- | --- | --- | --- | --- | --- | --- | --- | --- | --- | --- | --- | --- | --- | --- | --- | --- | --- | --- | --- | --- | --- | --- | --- | --- | --- | --- | --- | --- | --- | --- | --- | --- | --- | --- | --- | --- | --- | --- | --- | --- | --- | --- | --- | --- | --- | --- | --- | --- | --- | --- | --- | --- | --- | --- | --- | --- | --- | --- | --- | --- | --- | --- | --- | --- | --- | --- | --- | --- | --- | --- | --- | --- | --- | --- | --- | --- | --- | --- | --- | --- | --- | --- | --- | --- | --- | --- | --- | --- | --- | --- | --- | --- | --- | --- | --- | --- | --- | --- | --- | --- | --- | --- | --- | --- | --- | --- | --- | --- | --- | --- | --- | --- | --- | --- | --- | --- | --- | --- | --- | --- | --- | --- | --- | --- | --- | --- | --- | --- | --- | --- | --- | --- | --- | --- | --- | --- | --- | --- | --- | --- | --- | --- | --- | --- | --- | --- | --- | --- | --- | --- | --- | --- | --- | --- | --- | --- | --- | --- | --- | --- | --- | --- | --- | --- | --- | --- | --- | --- | --- | --- | --- | --- | --- | --- | --- | --- | --- | --- | --- | --- | --- | --- | --- | --- | --- | --- | --- | --- | --- | --- | --- | --- | --- | --- | --- | --- | --- | --- | --- | --- | --- | --- | --- | --- | --- | --- | --- | --- | --- | --- | --- | --- | --- | --- | --- | --- | --- | --- | --- | --- | --- | --- | --- | --- | --- | --- | --- | --- | --- | --- | --- | --- | --- | --- | --- | --- | --- | --- | --- | --- | --- | --- | --- | --- | --- | --- | --- | --- | --- | --- | --- | --- | --- | --- | --- | --- | --- | --- | --- | --- | --- | --- | --- | --- | --- | --- | --- | --- | --- | --- | --- | --- | --- | --- | --- | --- | --- | --- | --- | --- | --- | --- | --- | --- | --- | --- | --- | --- | --- | --- | --- | --- | --- | --- | --- | --- | --- | --- | --- | --- | --- | --- | --- | --- | --- | --- | --- | --- | --- | --- | --- | --- | --- | --- | --- | --- | --- | --- | --- | --- | --- | --- | --- | --- | --- | --- | --- | --- | --- | --- | --- | --- | --- | --- | --- | --- | --- | --- | --- | --- | --- | --- | --- | --- | --- | --- | --- | --- | --- | --- | --- | --- | --- | --- | --- | --- | --- | --- | --- | --- | --- | --- | --- | --- | --- | --- | --- | --- | --- | --- | --- | --- | --- | --- | --- | --- | --- | --- | --- | --- | --- | --- | --- | --- | --- | --- | --- | --- | --- | --- | --- | --- | --- | --- | --- | --- | --- | --- | --- | --- | --- | --- | --- | --- | --- | --- | --- | --- | --- | --- | --- | --- | --- | --- | --- | --- | --- | --- | --- | --- | --- | --- | --- | --- | --- | --- | --- | --- | --- | --- | --- | --- | --- | --- | --- | --- | --- | --- | --- | --- | --- | --- | --- | --- | --- | --- | --- | --- | --- | --- | --- | --- | --- | --- | --- | --- | --- | --- | --- | --- | --- | --- | --- | --- | --- | --- | --- | --- | --- | --- | --- | --- | --- | --- | --- | --- | --- | --- | --- | --- | --- | --- | --- | --- | --- | --- | --- | --- | --- | --- | --- | --- | --- | --- | --- | --- | --- | --- | --- | --- | --- | --- | --- | --- | --- | --- | --- | --- | --- | --- | --- | --- | --- | --- | --- | --- | --- | --- | --- | --- | --- | --- | --- | --- | --- | --- | --- | --- | --- | --- | --- | --- | --- | --- | --- | --- | --- | --- | --- | --- | --- | --- | --- | --- | --- | --- | --- | --- | --- | --- | --- | --- | --- | --- | --- | --- | --- | --- | --- | --- | --- | --- | --- | --- | --- | --- | --- | --- | --- | --- | --- | --- | --- | --- | --- | --- | --- | --- | --- | --- | --- | --- | --- | --- | --- | --- | --- | --- | --- | --- | --- | --- | --- | --- | --- | --- | --- | --- | --- | --- | --- | --- | --- | --- | --- | --- | --- | --- | --- | --- | --- | --- | --- | --- | --- | --- | --- | --- | --- | --- | --- | --- | --- | --- | --- | --- | --- | --- | --- | --- | --- | --- | --- | --- | --- | --- | --- | --- | --- | --- | --- | --- | --- | --- | --- | --- | --- | --- | --- | --- | --- | --- | --- | --- | --- | --- | --- | --- | --- | --- | --- | --- | --- | --- | --- | --- | --- | --- | --- | --- | --- | --- | --- | --- | --- | --- | --- | --- | --- | --- | --- | --- | --- | --- | --- | --- | --- | --- | --- | --- | --- | --- | --- | --- | --- | --- | --- | --- | --- | --- | --- | --- | --- | --- | --- | --- | --- | --- | --- | --- | --- | --- | --- | --- | --- | --- | --- | --- | --- | --- | --- | --- | --- | --- | --- | --- | --- | --- | --- | --- | --- | --- | --- | --- | --- | --- | --- | --- | --- | --- | --- | --- | --- | --- | --- | --- | --- | --- | --- | --- | --- | --- | --- | --- | --- | --- | --- | --- | --- | --- | --- | --- | --- | --- | --- | --- | --- | --- | --- | --- | --- | --- | --- | --- | --- | --- | --- | --- | --- | --- | --- | --- | --- | --- | --- | --- | --- | --- | --- | --- | --- | --- | --- | --- | --- | --- | --- | --- | --- | --- | --- | --- | --- | --- | --- | --- | --- | --- | --- | --- | --- | --- | --- | --- | --- | --- | --- | --- | --- | --- | --- | --- | --- | --- | --- | --- | --- | --- | --- | --- | --- | --- | --- | --- | --- | --- | --- | --- | --- | --- | --- | --- | --- | --- | --- | --- | --- | --- | --- | --- | --- | --- | --- | --- | --- | --- | --- | --- | --- | --- | --- | --- | --- | --- | --- | --- | --- | --- | --- | --- | --- | --- | --- | --- | --- | --- | --- | --- | --- | --- | --- | --- | --- | --- | --- | --- | --- | --- | --- | --- | --- | --- | --- | --- | --- | --- | --- | --- | --- | --- | --- | --- | --- | --- | --- | --- | --- | --- | --- | --- | --- | --- | --- | --- | --- | --- | --- | --- | --- | --- | --- | --- | --- | --- | --- | --- | --- | --- | --- | --- | --- | --- | --- | --- | --- | --- | --- | --- | --- | --- | --- | --- | --- | --- | --- | --- | --- | --- | --- | --- | --- | --- | --- | --- | --- | --- | --- | --- | --- | --- | --- | --- | --- | --- | --- | --- | --- | --- | --- | --- | --- | --- | --- | --- | --- | --- | --- | --- | --- | --- | --- | --- | --- | --- | --- | --- | --- | --- | --- | --- | --- | --- | --- | --- | --- | --- | --- | --- | --- | --- | --- | --- | --- | --- | --- | --- | --- | --- | --- | --- | --- | --- | --- | --- | --- | --- | --- | --- | --- | --- | --- | --- | --- | --- | --- | --- | --- | --- | --- | --- | --- | --- | --- | --- | --- | --- | --- | --- | --- | --- | --- | --- | --- | --- | --- | --- | --- | --- | --- | --- | --- | --- | --- | --- | --- | --- | --- | --- | --- | --- | --- | --- | --- | --- | --- | --- | --- | --- | --- | --- | --- | --- | --- | --- | --- | --- | --- | --- | --- | --- | --- | --- | --- | --- | --- | --- | --- | --- | --- | --- | --- | --- | --- | --- | --- | --- | --- | --- | --- | --- | --- | --- | --- | --- | --- | --- | --- | --- | --- | --- | --- | --- | --- | --- | --- | --- | --- | --- | --- | --- | --- | --- | --- | --- | --- | --- | --- | --- | --- | --- | --- | --- | --- | --- | --- | --- | --- | --- | --- | --- | --- | --- | --- | --- | --- | --- | --- | --- | --- | --- | --- | --- | --- | --- | --- | --- | --- | --- | --- | --- | --- | --- | --- | --- | --- | --- | --- | --- | --- | --- | --- | --- | --- | --- | --- | --- | --- | --- | --- | --- | --- | --- | --- | --- | --- | --- | --- | --- | --- | --- | --- | --- | --- | --- | --- | --- | --- | --- | --- | --- | --- | --- | --- | --- | --- | --- | --- | --- | --- | --- | --- | --- | --- | --- | --- | --- | --- | --- | --- | --- | --- | --- | --- | --- | --- | --- | --- | --- | --- | --- | --- | --- | --- | --- | --- | --- | --- | --- | --- | --- | --- | --- | --- | --- | --- | --- | --- | --- | --- | --- | --- | --- | --- | --- | --- | --- | --- | --- | --- | --- | --- | --- | --- | --- | --- | --- | --- | --- | --- | --- | --- | --- | --- | --- | --- | --- | --- | --- | --- | --- | --- | --- | --- | --- | --- | --- | --- | --- | --- | --- | --- | --- | --- | --- | --- | --- | --- | --- | --- | --- | --- | --- | --- | --- | --- | --- | --- | --- | --- | --- | --- | --- | --- | --- | --- | --- | --- | --- | --- | --- | --- | --- | --- | --- | --- | --- | --- | --- | --- | --- | --- | --- | --- | --- | --- | --- | --- | --- | --- | --- | --- | --- | --- | --- | --- | --- | --- | --- | --- | --- | --- | --- | --- | --- | --- | --- | --- | --- | --- | --- | --- | --- | --- | --- | --- | --- | --- | --- | --- | --- | --- | --- | --- | --- | --- | --- | --- | --- | --- | --- | --- | --- | --- | --- | --- | --- | --- | --- | --- | --- | --- | --- | --- | --- | --- | --- | --- | --- | --- | --- | --- | --- | --- | --- | --- | --- | --- | --- | --- | --- | --- | --- | --- | --- | --- | --- | --- | --- | --- | --- | --- | --- | --- | --- | --- | --- | --- | --- | --- | --- | --- | --- | --- | --- | --- | --- | --- | --- | --- | --- | --- | --- | --- | --- | --- | --- | --- | --- | --- | --- | --- | --- | --- | --- | --- | --- | --- | --- | --- | --- | --- | --- | --- | --- | --- | --- | --- | --- | --- | --- | --- | --- | --- | --- | --- | --- | --- | --- | --- | --- | --- | --- | --- | --- | --- | --- | --- | --- | --- | --- | --- | --- | --- | --- | --- | --- | --- | --- | --- | --- | --- | --- | --- | --- | --- | --- | --- | --- | --- | --- | --- | --- | --- | --- | --- | --- | --- | --- | --- | --- | --- | --- | --- | --- | --- | --- | --- | --- | --- | --- | --- | --- | --- | --- | --- | --- | --- | --- | --- | --- | --- | --- | --- | --- | --- | --- | --- | --- | --- | --- | --- | --- | --- | --- | --- | --- | --- | --- | --- | --- | --- | --- | --- | --- | --- | --- | --- | --- | --- | --- | --- | --- | --- | --- | --- | --- | --- | --- | --- | --- | --- | --- | --- | --- | --- | --- | --- | --- | --- | --- | --- | --- | --- | --- | --- | --- | --- | --- | --- | --- | --- | --- | --- | --- | --- | --- | --- | --- | --- | --- | --- | --- | --- | --- | --- | --- | --- | --- | --- | --- | --- | --- | --- | --- | --- | --- | --- | --- | --- | --- | --- | --- | --- | --- | --- | --- | --- | --- | --- | --- | --- | --- | --- | --- | --- | --- | --- | --- | --- | --- | --- | --- | --- | --- | --- | --- | --- | --- | --- | --- | --- | --- | --- | --- | --- | --- | --- | --- | --- | --- | --- | --- | --- | --- | --- | --- | --- | --- | --- | --- | --- | --- | --- | --- | --- | --- | --- | --- | --- | --- | --- | --- | --- | --- | --- | --- | --- | --- | --- | --- | --- | --- | --- | --- | --- | --- | --- | --- | --- | --- | --- | --- | --- | --- | --- | --- | --- | --- | --- | --- | --- | --- | --- | --- | --- | --- | --- | --- | --- | --- | --- | --- | --- | --- | --- | --- | --- | --- | --- | --- | --- | --- | --- | --- | --- | --- | --- | --- | --- | --- | --- | --- | --- | --- | --- | --- | --- | --- | --- | --- | --- | --- | --- | --- | --- | --- | --- | --- | --- | --- | --- | --- | --- | --- | --- | --- | --- | --- | --- | --- | --- | --- | --- | --- | --- | --- | --- | --- | --- | --- | --- | --- | --- | --- | --- | --- | --- | --- | --- | --- | --- | --- | --- | --- | --- | --- | --- | --- | --- | --- | --- | --- | --- | --- | --- | --- | --- | --- | --- | --- | --- | --- | --- | --- | --- | --- | --- | --- | --- | --- | --- | --- | --- | --- | --- | --- | --- | --- | --- | --- | --- | --- | --- | --- | --- | --- | --- | --- | --- | --- | --- | --- | --- | --- | --- | --- | --- | --- | --- | --- | --- | --- | --- | --- | --- | --- | --- | --- | --- | --- | --- | --- | --- | --- | --- | --- | --- | --- | --- | --- | --- | --- | --- | --- | --- | --- | --- | --- | --- | --- | --- | --- | --- | --- | --- | --- | --- | --- | --- | --- | --- | --- | --- | --- | --- | --- | --- | --- | --- | --- | --- | --- | --- | --- | --- | --- | --- | --- | --- | --- | --- | --- | --- | --- | --- | --- | --- | --- | --- | --- | --- | --- | --- | --- | --- | --- | --- | --- | --- | --- | --- | --- | --- | --- | --- | --- | --- | --- | --- | --- | --- | --- | --- | --- | --- | --- | --- | --- | --- | --- | --- | --- | --- | --- | --- | --- | --- | --- | --- | --- | --- | --- | --- | --- | --- | --- | --- | --- | --- | --- | --- | --- | --- | --- | --- | --- | --- | --- | --- | --- | --- | --- | --- | --- | --- | --- | --- | --- | --- | --- | --- | --- | --- | --- | --- | --- | --- | --- | --- | --- | --- | --- | --- | --- | --- | --- | --- | --- | --- | --- | --- | --- | --- | --- | --- | --- | --- | --- | --- | --- | --- | --- | --- | --- | --- | --- | --- | --- | --- | --- | --- | --- | --- | --- | --- | --- | --- | --- | --- | --- | --- | --- | --- | --- | --- | --- | --- | --- | --- | --- | --- | --- | --- | --- | --- | --- | --- | --- | --- | --- | --- | --- | --- | --- | --- | --- | --- | --- | --- | --- | --- | --- | --- | --- | --- | --- | --- | --- | --- | --- | --- | --- | --- | --- | --- | --- | --- | --- | --- | --- | --- | --- | --- | --- | --- | --- | --- | --- | --- | --- | --- | --- | --- | --- | --- | --- | --- | --- | --- | --- | --- | --- | --- | --- | --- | --- | --- | --- | --- | --- | --- | --- | --- | --- | --- | --- | --- | --- | --- | --- | --- | --- | --- | --- | --- | --- | --- | --- | --- | --- | --- | --- | --- | --- | --- | --- | --- | --- | --- | --- | --- | --- | --- | --- | --- | --- | --- | --- | --- | --- | --- | --- | --- | --- | --- | --- | --- | --- | --- | --- | --- | --- | --- | --- | --- | --- | --- | --- | --- | --- | --- | --- | --- | --- | --- | --- | --- | --- | --- | --- | --- | --- | --- | --- | --- | --- | --- | --- | --- | --- | --- | --- | --- | --- | --- | --- | --- | --- | --- | --- | --- | --- | --- | --- | --- | --- | --- | --- | --- | --- | --- | --- | --- | --- | --- | --- | --- | --- | --- | --- | --- | --- | --- | --- | --- | --- | --- | --- | --- | --- | --- | --- | --- | --- | --- | --- | --- | --- | --- | --- | --- | --- | --- | --- | --- | --- | --- | --- | --- | --- | --- | --- | --- | --- | --- | --- | --- | --- | --- | --- | --- | --- | --- | --- | --- | --- | --- | --- | --- | --- | --- | --- | --- | --- | --- | --- | --- | --- | --- | --- | --- | --- | --- | --- | --- | --- | --- | --- | --- | --- | --- | --- | --- | --- | --- | --- | --- | --- | --- | --- | --- | --- | --- | --- | --- | --- | --- | --- | --- | --- | --- | --- | --- | --- | --- | --- | --- | --- | --- | --- | --- | --- | --- | --- | --- | --- | --- | --- | --- | --- | --- | --- | --- | --- | --- | --- | --- | --- | --- | --- | --- | --- | --- | --- | --- | --- | --- | --- | --- | --- | --- | --- | --- | --- | --- | --- | --- | --- | --- | --- | --- | --- | --- | --- | --- | --- | --- | --- | --- | --- | --- | --- | --- | --- | --- | --- | --- | --- | --- | --- | --- | --- | --- | --- | --- | --- | --- | --- | --- | --- | --- | --- | --- | --- | --- | --- | --- | --- | --- | --- | --- | --- | --- | --- | --- | --- | --- | --- | --- | --- | --- | --- | --- | --- | --- | --- | --- | --- | --- | --- | --- | --- | --- | --- | --- | --- | --- | --- | --- | --- | --- | --- | --- | --- | --- | --- | --- | --- | --- | --- | --- | --- | --- | --- | --- | --- | --- | --- | --- | --- | --- | --- | --- | --- | --- | --- | --- | --- | --- | --- | --- | --- | --- | --- | --- | --- | --- | --- | --- | --- | --- | --- | --- | --- | --- | --- | --- | --- | --- | --- | --- | --- | --- | --- | --- | --- | --- | --- | --- | --- | --- | --- | --- | --- | --- | --- | --- | --- | --- | --- | --- | --- | --- | --- | --- | --- | --- | --- | --- | --- | --- | --- | --- | --- | --- | --- | --- | --- | --- | --- | --- | --- | --- | --- | --- | --- | --- | --- | --- | --- | --- | --- | --- | --- | --- | --- | --- | --- | --- | --- | --- | --- | --- | --- | --- | --- | --- | --- | --- | --- | --- | --- | --- | --- | --- | --- | --- | --- | --- | --- | --- | --- | --- | --- | --- | --- | --- | --- | --- | --- | --- | --- | --- | --- | --- | --- | --- | --- | --- | --- | --- | --- | --- | --- | --- | --- | --- | --- | --- | --- | --- | --- | --- | --- | --- | --- | --- | --- | --- | --- | --- | --- | --- | --- | --- | --- | --- | --- | --- | --- | --- | --- | --- | --- | --- | --- | --- | --- | --- | --- | --- | --- | --- | --- | --- | --- | --- | --- | --- | --- | --- | --- | --- | --- | --- | --- | --- | --- | --- | --- | --- | --- | --- | --- | --- | --- | --- | --- | --- | --- | --- | --- | --- | --- | --- | --- | --- | --- | --- | --- | --- | --- | --- | --- | --- | --- | --- | --- | --- | --- | --- | --- | --- | --- | --- | --- | --- | --- | --- | --- | --- | --- | --- | --- | --- | --- | --- | --- | --- | --- | --- | --- | --- | --- | --- | --- | --- | --- | --- | --- | --- | --- | --- | --- | --- | --- | --- | --- | --- | --- | --- | --- | --- | --- | --- | --- | --- | --- | --- | --- | --- | --- | --- | --- | --- | --- | --- | --- | --- | --- | --- | --- | --- | --- | --- | --- | --- | --- | --- | --- | --- | --- | --- | --- | --- | --- | --- | --- | --- | --- | --- | --- | --- | --- | --- | --- | --- | --- | --- | --- | --- | --- | --- | --- | --- | --- | --- | --- | --- | --- | --- | --- | --- | --- | --- | --- | --- | --- | --- | --- | --- | --- | --- | --- | --- | --- | --- | --- | --- | --- | --- | --- | --- | --- | --- | --- | --- | --- | --- | --- | --- | --- | --- | --- | --- | --- | --- | --- | --- | --- | --- | --- | --- | --- | --- | --- | --- | --- | --- | --- | --- | --- | --- | --- | --- | --- | --- | --- | --- | --- | --- | --- | --- | --- | --- | --- | --- | --- | --- | --- | --- | --- | --- | --- | --- | --- | --- | --- | --- | --- | --- | --- | --- | --- | --- | --- | --- | --- | --- | --- | --- | --- | --- | --- | --- | --- | --- | --- | --- | --- | --- | --- | --- | --- | --- | --- | --- | --- | --- | --- | --- | --- | --- | --- | --- | --- | --- | --- | --- | --- | --- | --- | --- | --- | --- | --- | --- | --- | --- | --- | --- | --- | --- | --- | --- | --- | --- | --- | --- | --- | --- | --- | --- | --- | --- | --- | --- | --- | --- | --- | --- | --- | --- | --- | --- | --- | --- | --- | --- | --- | --- | --- | --- | --- | --- | --- | --- | --- | --- | --- | --- | --- | --- | --- | --- | --- | --- | --- | --- | --- | --- | --- | --- | --- | --- | --- | --- | --- | --- | --- | --- | --- | --- | --- | --- | --- | --- | --- | --- | --- | --- | --- | --- | --- | --- | --- | --- | --- | --- | --- | --- | --- | --- | --- | --- | --- | --- | --- | --- | --- | --- | --- | --- | --- | --- | --- | --- | --- | --- | --- | --- | --- | --- | --- | --- | --- | --- | --- | --- | --- | --- | --- | --- | --- | --- | --- | --- | --- | --- | --- | --- | --- | --- | --- | --- | --- | --- | --- | --- | --- | --- | --- | --- | --- | --- | --- | --- | --- | --- | --- | --- | --- | --- | --- | --- | --- | --- | --- | --- | --- | --- | --- | --- | --- | --- | --- | --- | --- | --- | --- | --- | --- | --- | --- | --- | --- | --- | --- | --- | --- | --- | --- | --- | --- | --- | --- | --- | --- | --- | --- | --- | --- | --- | --- | --- | --- | --- | --- | --- | --- | --- | --- | --- | --- | --- | --- | --- | --- | --- | --- | --- | --- | --- | --- | --- | --- | --- | --- | --- | --- | --- | --- | --- | --- | --- | --- | --- | --- | --- | --- | --- | --- | --- | --- | --- | --- | --- | --- | --- | --- | --- | --- | --- | --- | --- | --- | --- | --- | --- | --- | --- | --- | --- | --- | --- | --- | --- | --- | --- | --- | --- | --- | --- | --- | --- | --- | --- | --- | --- | --- | --- | --- | --- | --- | --- | --- | --- | --- | --- | --- | --- | --- | --- | --- | --- | --- | --- | --- | --- | --- | --- | --- | --- | --- | --- | --- | --- | --- | --- | --- | --- | --- | --- | --- | --- | --- | --- | --- | --- | --- | --- | --- | --- | --- | --- | --- | --- | --- | --- | --- | --- | --- | --- | --- | --- | --- | --- | --- | --- | --- | --- | --- | --- | --- | --- | --- | --- | --- | --- | --- | --- | --- | --- | --- | --- | --- | --- | --- | --- | --- | --- | --- | --- | --- | --- | --- | --- | --- | --- | --- | --- | --- | --- | --- | --- | --- | --- | --- | --- | --- | --- | --- | --- | --- | --- | --- | --- | --- | --- | --- | --- | --- | --- | --- | --- | --- | --- | --- | --- | --- | --- | --- | --- | --- | --- | --- | --- | --- | --- | --- | --- | --- | --- | --- | --- | --- | --- | --- | --- | --- | --- | --- | --- | --- | --- | --- | --- | --- | --- | --- | --- | --- | --- | --- | --- | --- | --- | --- | --- | --- | --- | --- | --- | --- | --- | --- | --- | --- | --- | --- | --- | --- | --- | --- | --- | --- | --- | --- | --- | --- | --- | --- | --- | --- | --- | --- | --- | --- | --- | --- | --- | --- | --- | --- | --- | --- | --- | --- | --- | --- | --- | --- | --- | --- | --- | --- | --- | --- | --- | --- | --- | --- | --- | --- | --- | --- | --- | --- | --- | --- | --- | --- | --- | --- | --- | --- | --- | --- | --- | --- | --- | --- | --- | --- | --- | --- | --- | --- | --- | --- | --- | --- | --- | --- | --- | --- | --- | --- | --- | --- | --- | --- | --- | --- | --- | --- | --- | --- | --- | --- | --- | --- | --- | --- | --- | --- | --- | --- | --- | --- | --- | --- | --- | --- | --- | --- | --- | --- | --- | --- | --- | --- | --- | --- | --- | --- | --- | --- | --- | --- | --- | --- | --- | --- | --- | --- | --- | --- | --- | --- | --- | --- | --- | --- | --- | --- | --- | --- | --- | --- | --- | --- | --- | --- | --- | --- | --- | --- | --- | --- | --- | --- | --- | --- | --- | --- | --- | --- | --- | --- | --- | --- | --- | --- | --- | --- | --- | --- | --- | --- | --- | --- | --- | --- | --- | --- | --- | --- | --- | --- | --- | --- | --- | --- | --- | --- | --- | --- | --- | --- | --- | --- | --- | --- | --- | --- | --- | --- | --- | --- | --- | --- | --- | --- | --- | --- | --- | --- | --- | --- | --- | --- | --- | --- | --- | --- | --- | --- | --- | --- | --- | --- | --- | --- | --- | --- | --- | --- | --- | --- | --- | --- | --- | --- | --- | --- | --- | --- | --- | --- | --- | --- | --- | --- | --- | --- | --- | --- | --- | --- | --- | --- | --- | --- | --- | --- | --- | --- | --- | --- | --- | --- | --- | --- | --- | --- | --- | --- | --- | --- | --- | --- | --- | --- | --- | --- | --- | --- | --- | --- | --- | --- | --- | --- | --- | --- | --- | --- | --- | --- | --- | --- | --- | --- | --- | --- | --- | --- | --- | --- | --- | --- | --- | --- | --- | --- | --- | --- | --- | --- | --- | --- | --- | --- | --- | --- | --- | --- | --- | --- | --- | --- | --- | --- | --- | --- | --- | --- | --- | --- | --- | --- | --- | --- | --- | --- | --- | --- | --- | --- | --- | --- | --- | --- | --- | --- | --- | --- | --- | --- | --- | --- | --- | --- | --- | --- | --- | --- | --- | --- | --- | --- | --- | --- | --- | --- | --- | --- | --- | --- | --- | --- | --- | --- | --- | --- | --- | --- | --- | --- | --- | --- | --- | --- | --- | --- | --- | --- | --- | --- | --- | --- | --- | --- | --- | --- | --- | --- | --- | --- | --- | --- | --- | --- | --- | --- | --- | --- | --- | --- | --- | --- | --- | --- | --- | --- | --- | --- | --- | --- | --- | --- | --- | --- | --- | --- | --- | --- | --- | --- | --- | --- | --- | --- | --- | --- | --- | --- | --- | --- | --- | --- | --- | --- | --- | --- | --- | --- | --- | --- | --- | --- | --- | --- | --- | --- | --- | --- | --- | --- | --- | --- | --- | --- | --- | --- | --- | --- | --- | --- | --- | --- | --- | --- | --- | --- | --- | --- | --- | --- | --- | --- | --- | --- | --- | --- | --- | --- | --- | --- | --- | --- | --- | --- | --- | --- | --- | --- | --- | --- | --- | --- | --- | --- | --- | --- | --- | --- | --- | --- | --- | --- | --- | --- | --- | --- | --- | --- | --- | --- | --- | --- | --- | --- | --- | --- | --- | --- | --- | --- | --- | --- | --- | --- | --- | --- | --- | --- | --- | --- | --- | --- | --- | --- | --- | --- | --- | --- | --- | --- | --- | --- | --- | --- | --- | --- | --- | --- | --- | --- | --- | --- | --- | --- | --- | --- | --- | --- | --- | --- | --- | --- | --- | --- | --- | --- | --- | --- | --- | --- | --- | --- | --- | --- | --- | --- | --- | --- | --- | --- | --- | --- | --- | --- | --- | --- | --- | --- | --- | --- | --- | --- | --- | --- | --- | --- | --- | --- | --- | --- | --- | --- | --- | --- | --- | --- | --- | --- | --- | --- | --- | --- | --- | --- | --- | --- | --- | --- | --- | --- | --- | --- | --- | --- | --- | --- | --- | --- | --- | --- | --- | --- | --- | --- | --- | --- | --- | --- | --- | --- | --- | --- | --- | --- | --- | --- | --- | --- | --- | --- | --- | --- | --- | --- | --- | --- | --- | --- | --- | --- | --- | --- | --- | --- | --- | --- | --- | --- | --- | --- | --- | --- | --- | --- | --- | --- | --- | --- | --- | --- | --- | --- | --- | --- | --- | --- | --- | --- | --- | --- | --- | --- | --- | --- | --- | --- | --- | --- | --- | --- | --- | --- | --- | --- | --- | --- | --- | --- | --- | --- | --- | --- | --- | --- | --- | --- | --- | --- | --- | --- | --- | --- | --- | --- | --- | --- | --- | --- | --- | --- | --- | --- | --- | --- | --- | --- | --- | --- | --- | --- | --- | --- | --- | --- | --- | --- | --- | --- | --- | --- | --- | --- | --- | --- | --- | --- | --- | --- | --- | --- | --- | --- | --- | --- | --- | --- | --- | --- | --- | --- | --- | --- | --- | --- | --- | --- | --- | --- | --- | --- | --- | --- | --- | --- | --- | --- | --- | --- | --- | --- | --- | --- | --- | --- | --- | --- | --- | --- | --- | --- | --- | --- | --- | --- | --- | --- | --- | --- | --- | --- | --- | --- | --- | --- | --- | --- | --- | --- | --- | --- | --- | --- | --- | --- | --- | --- | --- | --- | --- | --- | --- | --- | --- | --- | --- | --- | --- | --- | --- | --- | --- | --- | --- | --- | --- | --- | --- | --- | --- | --- | --- | --- | --- | --- | --- | --- | --- | --- | --- | --- | --- | --- | --- | --- | --- | --- | --- | --- | --- | --- | --- | --- | --- | --- | --- | --- | --- | --- | --- | --- | --- | --- | --- | --- | --- | --- | --- | --- | --- | --- | --- | --- | --- | --- | --- | --- | --- | --- | --- | --- | --- | --- | --- | --- | --- | --- | --- | --- | --- | --- | --- | --- | --- | --- | --- | --- | --- | --- | --- | --- | --- | --- | --- | --- | --- | --- | --- | --- | --- | --- | --- | --- | --- | --- | --- | --- | --- | --- | --- | --- | --- | --- | --- | --- | --- | --- | --- | --- | --- | --- | --- | --- | --- | --- | --- | --- | --- | --- | --- | --- | --- | --- | --- | --- | --- | --- | --- | --- | --- | --- | --- | --- | --- | --- | --- | --- | --- | --- | --- | --- | --- | --- | --- | --- | --- | --- | --- | --- | --- | --- | --- | --- | --- | --- | --- | --- | --- | --- | --- | --- | --- | --- | --- | --- | --- | --- | --- | --- | --- | --- | --- | --- | --- | --- | --- | --- | --- | --- | --- | --- | --- | --- | --- | --- | --- | --- | --- | --- | --- | --- | --- | --- | --- | --- | --- | --- | --- | --- | --- | --- | --- | --- | --- | --- | --- | --- | --- | --- | --- | --- | --- | --- | --- | --- | --- | --- | --- | --- | --- | --- | --- | --- | --- | --- | --- | --- | --- | --- | --- | --- | --- | --- | --- | --- | --- | --- | --- | --- | --- | --- | --- | --- | --- | --- | --- | --- | --- | --- | --- | --- | --- | --- | --- | --- | --- | --- | --- | --- | --- | --- | --- | --- | --- | --- | --- | --- | --- | --- | --- | --- | --- | --- | --- | --- | --- | --- | --- | --- | --- | --- | --- | --- | --- | --- | --- | --- | --- | --- | --- | --- | --- | --- | --- | --- | --- | --- | --- | --- | --- | --- | --- | --- | --- | --- | --- | --- | --- | --- | --- | --- | --- | --- | --- | --- | --- | --- | --- | --- | --- | --- | --- | --- | --- | --- | --- | --- | --- | --- | --- | --- | --- | --- | --- | --- | --- | --- | --- | --- | --- | --- | --- | --- | --- | --- | --- | --- | --- | --- | --- | --- | --- | --- | --- | --- | --- | --- | --- | --- | --- | --- | --- | --- | --- | --- | --- | --- | --- | --- | --- | --- | --- | --- | --- | --- | --- | --- | --- | --- | --- | --- | --- | --- | --- | --- | --- | --- | --- | --- | --- | --- | --- | --- | --- | --- | --- | --- | --- | --- | --- | --- | --- | --- | --- | --- | --- | --- | --- | --- | --- | --- | --- | --- | --- | --- | --- | --- | --- | --- | --- | --- | --- | --- | --- | --- | --- | --- | --- | --- | --- | --- | --- | --- | --- | --- | --- | --- | --- | --- | --- | --- | --- | --- | --- | --- | --- | --- | --- | --- | --- | --- | --- | --- | --- | --- | --- | --- | --- | --- | --- | --- | --- | --- | --- | --- | --- | --- | --- | --- | --- | --- | --- | --- | --- | --- | --- | --- | --- | --- | --- | --- | --- | --- | --- | --- | --- | --- | --- | --- | --- | --- | --- | --- | --- | --- | --- | --- | --- | --- | --- | --- | --- | --- | --- | --- | --- | --- | --- | --- | --- | --- | --- | --- | --- | --- | --- | --- | --- | --- | --- | --- | --- | --- | --- | --- | --- | --- | --- | --- | --- | --- | --- | --- | --- | --- | --- | --- | --- | --- | --- | --- | --- | --- | --- | --- | --- | --- | --- | --- | --- | --- | --- | --- | --- | --- | --- | --- | --- | --- | --- | --- | --- | --- | --- | --- | --- | --- | --- | --- | --- | --- | --- | --- | --- | --- | --- | --- | --- | --- | --- | --- | --- | --- | --- | --- | --- | --- | --- | --- | --- | --- | --- | --- | --- | --- | --- | --- | --- | --- | --- | --- | --- | --- | --- | --- | --- | --- | --- | --- | --- | --- | --- | --- | --- | --- | --- | --- | --- | --- | --- | --- | --- | --- | --- | --- | --- | --- | --- | --- | --- | --- | --- | --- | --- | --- | --- | --- | --- | --- | --- | --- | --- | --- | --- | --- | --- | --- | --- | --- | --- | --- | --- | --- | --- | --- | --- | --- | --- | --- | --- | --- | --- | --- | --- | --- | --- | --- | --- | --- | --- | --- | --- | --- | --- | --- | --- | --- | --- | --- | --- | --- | --- | --- | --- | --- | --- | --- | --- | --- | --- | --- | --- | --- | --- | --- | --- | --- | --- | --- | --- | --- | --- | --- | --- | --- | --- | --- | --- | --- | --- | --- | --- | --- | --- | --- | --- | --- | --- | --- | --- | --- | --- | --- | --- | --- | --- | --- | --- | --- | --- | --- | --- | --- | --- | --- | --- | --- | --- | --- | --- | --- | --- | --- | --- | --- | --- | --- | --- | --- | --- | --- | --- | --- | --- | --- | --- | --- | --- | --- | --- | --- | --- | --- | --- | --- | --- | --- | --- | --- | --- | --- | --- | --- | --- | --- | --- | --- | --- | --- | --- | --- | --- | --- | --- | --- | --- | --- | --- | --- | --- | --- | --- | --- | --- | --- | --- | --- | --- | --- | --- | --- | --- | --- | --- | --- | --- | --- | --- | --- | --- | --- | --- | --- | --- | --- | --- | --- | --- | --- | --- | --- | --- | --- | --- | --- | --- | --- | --- | --- | --- | --- | --- | --- | --- | --- | --- | --- | --- | --- | --- | --- | --- | --- | --- | --- | --- | --- | --- | --- | --- | --- | --- | --- | --- | --- | --- | --- | --- | --- | --- | --- | --- | --- | --- | --- | --- | --- | --- | --- | --- | --- | --- | --- | --- | --- | --- | --- | --- | --- | --- | --- | --- | --- | --- | --- | --- | --- | --- | --- | --- | --- | --- | --- | --- | --- | --- | --- | --- | --- | --- | --- | --- | --- | --- | --- | --- | --- | --- | --- | --- | --- | --- | --- | --- | --- | --- | --- | --- | --- | --- | --- | --- | --- | --- | --- | --- | --- | --- | --- | --- | --- | --- | --- | --- | --- | --- | --- | --- | --- | --- | --- | --- | --- | --- | --- | --- | --- | --- | --- | --- | --- | --- | --- | --- | --- | --- | --- | --- | --- | --- | --- | --- | --- | --- | --- | --- | --- | --- | --- | --- | --- | --- | --- | --- | --- | --- | --- | --- | --- | --- | --- | --- | --- | --- | --- | --- | --- | --- | --- | --- | --- | --- | --- | --- | --- | --- | --- | --- | --- | --- | --- | --- | --- | --- | --- | --- | --- | --- | --- | --- | --- | --- | --- | --- | --- | --- | --- | --- | --- | --- | --- | --- | --- | --- | --- | --- | --- | --- | --- | --- | --- | --- |
| AFFY Probe | Entrez Gene | Dif. Expressed | RefSeq | SG RefSeq | Gene Ontology | Pubmed | Gene Symbol | SheepGenomics\|  \|  \|  \|  \|  \|  \|  \|  \|  \| \| --- \| --- \| --- \| --- \| --- \| --- \| --- \| --- \| --- \| \| Bt.13980.1.A1\_at \| 538944 \| 2/2 \| NM\_001034656 \|  \| GO:0000166  GO:0004111  GO:0005524  GO:0005739 \| 12477932 \| CKMT2 \| Bt.13980.1.A1\_at \| \| Bt.3960.1.S1\_a\_at \| 282119 \| 2/2 \| NM\_174485 \| NM\_174485 \| GO:0005515  GO:0005741  GO:0006820  GO:0006915  GO:0008308  GO:0015288  GO:0016020  GO:0016021  GO:0019867 \| 7507248  7685355  12477932 \| VDAC1 \| Bt.3960.1.S1\_a\_at \| \| Bt.13980.2.A1\_a\_at \| 538944 \| 2/2 \| NM\_001034656 \|  \| GO:0000166  GO:0004111  GO:0005524  GO:0005739 \| 12477932 \|  \| Bt.13980.2.A1\_a\_at \| \| Bt.1316.1.S1\_at \| 286886 \| 2/2 \| NM\_174806 \|  \| GO:0004069  GO:0004069  GO:0005739  GO:0005739  GO:0005743  GO:0006520  GO:0009058  GO:0009986  GO:0030170 \| 446759  965157  7641080  12140684  12477932  12658628  16305752 \| GOT2 \| Bt.1316.1.S1\_at \| \| Bt.23502.1.S1\_at \| 504230 \| 1/1 \| XM\_580314 \| XM\_580314 \|  \|  \| RAMP2 \| Bt.23502.1.S1\_at \| \| Bt.23662.2.S1\_at \| 506764 \| 2/2 \| NM\_001014858 \|  \|  \| 11282978  16305752 \| RNF34 \| Bt.23662.2.S1\_at \| \| Bt.22664.1.A1\_at \| 506574 \| 1/1 \| XM\_583040 \| XM\_583040 \|  \|  \| LRIG3 \| Bt.22664.1.A1\_at \| \| Bt.24931.1.A1\_at \| 529762 \| 1/2 \| NM\_001037608 \|  \| GO:0005739  GO:0016020  GO:0016021 \|  \| MGC133598 \| Bt.24931.1.A1\_at \| \| Bt.7034.1.S1\_at \| 780788 \| 1/1 \| NM\_001079643 \|  \| GO:0004849  GO:0005524  GO:0007229 \|  \| ITGB1BP3 \| Bt.7034.1.S1\_at \| \| Bt.2894.1.S1\_at \| 507245 \| 1/1 \| NM\_001034286 \| NM\_001034286 \|  \| 12477932 \| SITPEC \| Bt.2894.1.S1\_at \| \| Bt.16843.1.A1\_at \| 615846 \| 1/1 \| XM\_867767 \|  \|  \|  \|  \| Bt.16843.1.A1\_at \| \| Bt.11178.1.S1\_at \| 615239 \| 1/1 \| NM\_001035463 \| NM\_001035463 \| GO:0005578 \| 12477932 \| GPC3 \| Bt.11178.1.S1\_at \| \| Bt.27381.1.S1\_at \| 538821 \| 1/2 \| NM\_001076130 \|  \|  \|  \| FLJ32452 \| Bt.27381.1.S1\_at \| \| Bt.1153.1.S1\_at \| 287327 \| 1/2 \| NM\_174819 \| NM\_174819 \| GO:0003954  GO:0005739  GO:0005747  GO:0006120  GO:0006800  GO:0006917  GO:0008137  GO:0030308  GO:0031966 \| 1899621  1907966  12381726 \| NDUFS3 \| Bt.1153.1.S1\_at \| \| Bt.27421.1.S1\_at \| 505940 \| 2/3 \| XM\_001251347  XM\_582310 \| XM\_001251347 \|  \|  \| ARHGEF2 \| Bt.27421.1.S1\_at \| \| Bt.3865.3.S1\_a\_at \|  \|  \|  \| NM\_001079511 \|  \|  \| MGC15416 \| Bt.3865.3.S1\_a\_at \| \| Bt.21766.3.S1\_at \| 533379 \| 1/1 \| NM\_001038158 \| NM\_001038158 \| GO:0002318  GO:0003677  GO:0005515  GO:0005634  GO:0005737  GO:0006350  GO:0007050  GO:0007275  GO:0030154 \|  \| MLF1 \| Bt.21766.3.S1\_at \| \| Bt.22519.1.S1\_at \| 616718 \| 1/1 \| XM\_868814 \|  \|  \|  \| BRP44 \| Bt.22519.1.S1\_at \| \| Bt.848.1.S1\_at \| 282290 \| 1/1 \| NM\_174565 \| XM\_001250334 \| GO:0003954  GO:0005506  GO:0005739  GO:0006118  GO:0008137  GO:0016491  GO:0046872  GO:0051287  GO:0051537 \| 2500970  2598272  6861757  12477932  16150735 \| NDUFV2 \| Bt.848.1.S1\_at \| \| Bt.7056.5.A1\_x\_at \| 511735 \| 3/6 \| NM\_001014902 \|  \| GO:0005344  GO:0005506  GO:0005833  GO:0006810  GO:0015671  GO:0019825  GO:0020037  GO:0046872 \| 5958205  6322113 \|  \| Bt.7056.5.A1\_x\_at \| \| Bt.11236.1.S1\_at \| 538173 \| 1/1 \| NM\_001038172 \| NM\_001038172 \| GO:0000287  GO:0008152  GO:0016787  GO:0016791 \|  \|  \| Bt.11236.1.S1\_at \| \| Bt.12510.1.S1\_a\_at \| 509824 \| 4/6 \| XM\_872590 \| XM\_872590 \|  \|  \|  \| Bt.12510.1.S1\_a\_at \| \| Bt.2696.1.A1\_at \| 535363 \| 2/2 \| XM\_615431 \| XM\_001256656 \| GO:0005509  GO:0007156  GO:0016020 \|  \| CDH11 \| Bt.2696.1.A1\_at \| \| Bt.11770.1.S1\_a\_at \|  \|  \|  \|  \|  \|  \|  \| Bt.11770.1.S1\_a\_at \| \| Bt.5002.1.S1\_at \| 525042 \| 1/1 \| NM\_001035354 \|  \|  \| 11282978  16305752 \| GPD1 \| Bt.5002.1.S1\_at \| \| Bt.17613.1.A1\_at \| 530667 \| 1/1 \|  \|  \|  \|  \| PHKA1 \| Bt.17613.1.A1\_at \| \| Bt.11794.1.S1\_at \| 768057 \| 1/1 \| NM\_001077114 \|  \|  \|  \|  \| Bt.11794.1.S1\_at \| \| Bt.4770.1.S1\_at \| 506724 \| 2/2 \| XM\_583210 \|  \|  \|  \| PDCD4 \| Bt.4770.1.S1\_at \| \| Bt.6576.1.S1\_at \| 506428 \| 2/2 \|  \| XR\_027451 \|  \|  \|  \| Bt.6576.1.S1\_at \| \| Bt.4777.1.S1\_at \| 288380 \| 1/2 \| NM\_174820 \|  \| GO:0003954  GO:0005506  GO:0005515  GO:0005739  GO:0005739  GO:0005747  GO:0005758  GO:0006800  GO:0006915  GO:0008137  GO:0016020  GO:0042773  GO:0045333  GO:0046034  GO:0046872  GO:0051537  GO:0051539  GO:0051881 \| 2514801 \| NDUFS1 \| Bt.4777.1.S1\_at \| \| Bt.9222.1.S1\_at \|  \|  \|  \|  \|  \|  \| PRKAR2A \| Bt.9222.1.S1\_at \| \| Bt.20229.1.S1\_at \| 767855 \| 1/1 \| NM\_001076967 \| NM\_001076967 \| GO:0004672  GO:0005524  GO:0006915 \|  \| TBRG4 \| Bt.20229.1.S1\_at \| \| Bt.4704.1.S1\_at \| 327714 \| 2/2 \| NM\_175829 \| NM\_175829 \| GO:0003954  GO:0005739  GO:0005743  GO:0006118  GO:0008137 \| 1518044  1907966  12477932 \| NDUFA5 \| Bt.4704.1.S1\_at \| \| Bt.21896.1.S1\_at \| 533456 \| 1/1 \| XM\_612868 \|  \| GO:0005737  GO:0006817 \|  \| COL21A1 \| Bt.21896.1.S1\_at \| \| Bt.4072.1.S1\_at \| 287014 \| 1/1 \| NM\_174808 \| NM\_174808 \| GO:0003954  GO:0005506  GO:0005739  GO:0005743  GO:0006120  GO:0008137  GO:0010181  GO:0046872  GO:0051287  GO:0051539 \| 1900194  2034666 \| NDUFV1 \| Bt.4072.1.S1\_at \| \| Bt.1316.1.S2\_at \| 286886 \| 2/2 \| NM\_174806 \| NM\_174806 \| GO:0004069  GO:0004069  GO:0005739  GO:0005739  GO:0005743  GO:0006520  GO:0009058  GO:0009986  GO:0030170 \| 446759  965157  7641080  12140684  12477932  12658628  16305752 \| GOT2 \| Bt.1316.1.S2\_at \| \| Bt.3117.1.S1\_at \| 327660 \| 1/1 \| NM\_175783 \| NM\_175783 \| GO:0003954  GO:0005739  GO:0008137  GO:0016491 \| 1518044  12477932 \| NDUFB9 \| Bt.3117.1.S1\_at \| \| Bt.27814.1.S1\_at \| 506724 \| 2/2 \| XM\_583210 \|  \|  \|  \| PDCD4 \| Bt.27814.1.S1\_at \| \| Bt.836.1.S1\_at \| 509273 \| 1/1 \| NM\_001075400 \| XM\_001253360 \|  \|  \| FAHD1 \| Bt.836.1.S1\_at \| \| Bt.4758.1.S1\_at \| 281758 \| 1/1 \| NM\_174313 \|  \| GO:0006810  GO:0008289 \| 1483473  2070787  2335570  2398054  3409882  3667628  7601110  8312397  8354262  9490052  12140684  12477932  14687081 \| FABP3 \| Bt.4758.1.S1\_at \| \| Bt.18753.1.A1\_at \| 532751 \| 1/1 \| XM\_876527 \| XM\_876527 \|  \|  \| PTPRD \| Bt.18753.1.A1\_at \| \| Bt.5120.1.S1\_at \| 280878 \| 2/2 \| NM\_173943 \| NM\_173943 \|  \| 2005110  3196335  3277960  3325062  3426633  8011636  10581554 \| NNT \| Bt.5120.1.S1\_at \| \| Bt.17963.1.A1\_at \| 517345 \| 1/1 \| NM\_001046229 \|  \| GO:0000166  GO:0005525  GO:0005622  GO:0005794  GO:0007264 \|  \| ARL1 \| Bt.17963.1.A1\_at \| \| Bt.13330.2.A1\_at \| 507367 \| 3/3 \| XM\_583960 \|  \|  \|  \| PDK4 \| Bt.13330.2.A1\_at \| \| Bt.3247.1.S1\_at \|  \|  \|  \|  \|  \|  \| MATN2 \| Bt.3247.1.S1\_at \| \| Bt.3960.2.S1\_at \| 282119 \| 2/2 \| NM\_174485 \|  \| GO:0005515  GO:0005741  GO:0006820  GO:0006915  GO:0008308  GO:0015288  GO:0016020  GO:0016021  GO:0019867 \| 7507248  7685355  12477932 \| VDAC1 \| Bt.3960.2.S1\_at \| \| Bt.1793.1.A1\_at \| 512666 \| 2/2 \| XM\_590221 \|  \|  \|  \| AVEN \| Bt.1793.1.A1\_at \| \| Bt.18572.1.A1\_at \|  \|  \|  \| XR\_027658 \|  \|  \| PURG \| Bt.18572.1.A1\_at \| \| Bt.3873.1.S1\_at \| 510127 \| 1/1 \| XM\_587231 \| XM\_587231 \|  \|  \| MRPL37 \| Bt.3873.1.S1\_at \| \| Bt.6391.1.A1\_at \| 617129 \| 1/1 \| XM\_869323 \| XM\_869323 \|  \|  \| C6orf79 \| Bt.6391.1.A1\_at \| \| Bt.18752.1.S1\_at \| 615229 \| 1/1 \| NM\_001076364 \| NM\_001076364 \| GO:0005634  GO:0008270 \|  \| SMYD2 \| Bt.18752.1.S1\_at \| \| Bt.12510.1.S1\_at \| 509824 \| 4/6 \| XM\_872590 \|  \|  \|  \| GTL2 \| Bt.12510.1.S1\_at \| \| Bt.19508.1.A1\_at \| 540179 \| 1/1 \| XM\_592079 \| XM\_592079 \|  \|  \| GPC4 \| Bt.19508.1.A1\_at \| \| Bt.4473.1.S1\_at \| 338060 \| 1/1 \| NM\_176655 \|  \| GO:0003954  GO:0005524  GO:0005739  GO:0006139  GO:0008137  GO:0016491  GO:0016773 \| 1832859  12477932  15848193 \| NDUFA10 \| Bt.4473.1.S1\_at \| \| Bt.946.2.A1\_a\_at \| 280716 \| 1/3 \| NM\_173889 \| NM\_173889 \| GO:0000166  GO:0004017  GO:0005524  GO:0005739  GO:0005739  GO:0005743  GO:0005743  GO:0006139  GO:0016301  GO:0016740 \| 2227435  3002789  3040716  8868479  11282978  16305752 \| AK2 \| Bt.946.2.A1\_a\_at \| \| Bt.11516.1.S1\_at \| 407109 \| 3/4 \| XM\_581602 \| XM\_581602 \| GO:0008152  GO:0016624 \|  \| PDHA1 \| Bt.11516.1.S1\_at \| \| Bt.5395.1.S1\_a\_at \| 282662 \| 1/1 \| NM\_181035 \| NM\_181035 \| GO:0005509  GO:0005529  GO:0005540  GO:0005578  GO:0007155 \| 1720020  9624174 \| CSPG2 \| Bt.5395.1.S1\_a\_at \| \| Bt.19517.1.S1\_at \|  \|  \|  \| XR\_028216 \|  \|  \| ERBB3 \| Bt.19517.1.S1\_at \| \| Bt.22082.1.S1\_at \| 511291 \| 1/1 \| NM\_001075507 \| NM\_001075507 \| GO:0008654  GO:0016020  GO:0016740  GO:0016780 \|  \|  \| Bt.22082.1.S1\_at \| \| Bt.11449.1.S1\_at \| 525256 \| 1/1 \| NM\_001076875 \| NM\_001076875 \|  \|  \| PREB \| Bt.11449.1.S1\_at \| \| Bt.1403.1.S1\_at \| 280878 \| 2/2 \| NM\_173943 \|  \|  \| 2005110  3196335  3277960  3325062  3426633  8011636  10581554 \| NNT \| Bt.1403.1.S1\_at \| \| Bt.16082.1.S1\_at \| 518321 \| 1/1 \| NM\_001034527 \| NM\_001034527 \| GO:0005515  GO:0005739  GO:0005759 \| 12477932 \| C1QBP \| Bt.16082.1.S1\_at \| \| Bt.13313.1.S1\_at \| 525346 \| 4/4 \| XM\_867432 \| XM\_867432 \|  \|  \| NCOA1 \| Bt.13313.1.S1\_at \| \| Bt.21372.3.A1\_at \| 513054 \| 3/6 \| NM\_001075595 \|  \|  \|  \| WHSC1L1 \| Bt.21372.3.A1\_at \| \| Bt.27926.1.S1\_at \| 520170 \| 1/1 \| NM\_001040541 \| NM\_001040541 \| GO:0004221  GO:0005622  GO:0006511  GO:0006512  GO:0008234 \|  \| UCHL3 \| Bt.27926.1.S1\_at \| \| Bt.21.1.S1\_at \| 338046 \| 1/1 \| NM\_176642 \| NM\_176642 \| GO:0003954  GO:0005739  GO:0005743  GO:0006120  GO:0008137  GO:0016491 \| 1426273  12477932 \| NDUFC2 \| Bt.21.1.S1\_at \| \| Bt.7215.1.S1\_at \| 282130 \| 1/1 \| NM\_174494 \|  \| GO:0003995  GO:0005739  GO:0006118  GO:0006629  GO:0006631  GO:0008152  GO:0050660 \| 12140684  12477932  16305752 \| ACADVL \| Bt.7215.1.S1\_at \| \| Bt.12694.1.S1\_at \| 537922 \| 1/2 \| NM\_001076911 \|  \| GO:0004872  GO:0005515  GO:0007165 \|  \| TNFRSF21 \| Bt.12694.1.S1\_at \| \| Bt.5732.1.S1\_at \|  \|  \|  \|  \|  \|  \| SESN3 \| Bt.5732.1.S1\_at \| \| Bt.5519.2.S1\_at \| 512562 \| 1/1 \| NM\_001046125 \| NM\_001046125 \|  \|  \| MAGED1 \| Bt.5519.2.S1\_at \| \| Bt.18479.1.A1\_at \| 509992 \| 1/1 \| XM\_874011 \| XM\_874011 \|  \|  \| ZNF608 \| Bt.18479.1.A1\_at \| \| Bt.5483.1.S1\_at \| 287027 \| 1/1 \| NM\_174818 \|  \| GO:0003954  GO:0005506  GO:0005739  GO:0006118  GO:0006979  GO:0008137  GO:0046872  GO:0051539 \| 1901022  12857734 \| NDUFS8 \| Bt.5483.1.S1\_at \| \| Bt.99.1.S1\_at \| 327706 \| 1/1 \| NM\_175822 \| NM\_175822 \| GO:0003954  GO:0005739  GO:0005743  GO:0006118  GO:0006979  GO:0008137  GO:0016491 \| 1518044  12477932  12658628  12857734 \| NDUFB4 \| Bt.99.1.S1\_at \| \| Bt.378.1.S1\_at \| 338040 \| 1/2 \| NM\_176639 \| NM\_176639 \| GO:0005739  GO:0006811  GO:0015986  GO:0015992  GO:0016469  GO:0045263  GO:0046872  GO:0046933  GO:0046961 \| 1827992  12477932 \| ATP5I \| Bt.378.1.S1\_at \| \| Bt.9556.1.S1\_at \| 404180 \| 2/3 \| XM\_591933 \|  \| GO:0003824  GO:0016020  GO:0016021 \| 14668210 \| MEST \| Bt.9556.1.S1\_at \| \| Bt.4262.1.S1\_a\_at \| 327717 \| 1/1 \| NM\_175830 \| NM\_175830 \| GO:0003954  GO:0005739  GO:0008137  GO:0016491 \| 1778979  1902801  9344673  12477932 \| NDUFV3 \| Bt.4262.1.S1\_a\_at \| \| Bt.27215.1.S1\_at \|  \|  \|  \|  \|  \|  \| C2orf4 \| Bt.27215.1.S1\_at \| \| Bt.5210.1.S1\_at \| 280976 \| 1/1 \| NM\_173977 \| NM\_173977 \|  \| 1547214  2511202  3372519  9195992  12477932 \| ACO2 \| Bt.5210.1.S1\_at \| \| Bt.27193.1.A1\_at \| 539374 \| 1/2 \| XM\_586734 \| XM\_586734 \|  \|  \| FAM43A \| Bt.27193.1.A1\_at \| \| Bt.1758.1.S1\_at \| 512161 \| 1/1 \| XM\_877857 \| XM\_877857 \| GO:0000785  GO:0003677  GO:0005634 \| 330164  793593 \|  \| Bt.1758.1.S1\_at \| \| Bt.27318.1.S1\_at \|  \|  \|  \| XR\_028639 \|  \|  \|  \| Bt.27318.1.S1\_at \| \| Bt.3448.2.S1\_at \| 538094 \| 2/3 \| XM\_605323 \| XM\_605323 \| GO:0004674  GO:0004707  GO:0005524  GO:0006468 \|  \| MAPK6 \| Bt.3448.2.S1\_at \| \| Bt.10013.1.S1\_at \| 512771 \| 1/3 \| NM\_001034428 \| NM\_001034428 \| GO:0000050  GO:0004056  GO:0008652  GO:0016829  GO:0042450 \| 12477932 \| ASL \| Bt.10013.1.S1\_at \| \| Bt.298.1.S1\_at \| 281742 \| 1/1 \| NM\_174306 \|  \| GO:0003954  GO:0005739  GO:0006118  GO:0006979  GO:0008137  GO:0016020 \| 9827566  12477932  12857734 \| DAP13 \| Bt.298.1.S1\_at \| \| Bt.23164.1.S1\_at \| 282393 \| 1/2 \| NM\_174629 \| NM\_174629 \| GO:0005739  GO:0005739  GO:0005746  GO:0006118  GO:0006810  GO:0008121  GO:0016020  GO:0016491 \| 1712295  2848575  8592474  9204897  9651245  12477932 \| UQCRC1 \| Bt.23164.1.S1\_at \| \| Bt.20899.1.A1\_at \|  \|  \|  \|  \|  \|  \|  \| Bt.20899.1.A1\_at \| \| Bt.13313.2.S1\_at \| 525346 \| 4/4 \| XM\_867432 \| XM\_867432 \|  \|  \| NCOA1 \| Bt.13313.2.S1\_at \| \| Bt.216.1.S1\_at \| 286825 \| 1/1 \| NM\_174774 \|  \|  \| 10077598 \| WDR44 \| Bt.216.1.S1\_at \| \| Bt.28704.1.S1\_at \| 539290 \| 1/1 \| XM\_586210 \| NM\_001081732 \|  \|  \| PLSCR4 \| Bt.28704.1.S1\_at \| \| Bt.28975.1.S1\_at \| 538841 \| 1/1 \| XM\_583205 \|  \| GO:0008270 \|  \| ZSWIM6 \| Bt.28975.1.S1\_at \| \| Bt.20141.1.S1\_at \| 509274 \| 1/1 \| NM\_001035274 \| NM\_001035274 \|  \| 12477932 \| HAGH \| Bt.20141.1.S1\_at \| \| Bt.3487.1.S1\_at \| 281543 \| 1/1 \| NM\_001013589 \| NM\_001013589 \|  \| 10790536  12140684  12477932  16305752 \| TPI1 \| Bt.3487.1.S1\_at \| \| Bt.1242.1.S1\_at \| 338053 \| 2/2 \| NM\_176649 \|  \| GO:0005739  GO:0006811  GO:0008289  GO:0015986  GO:0015992  GO:0016020  GO:0016469  GO:0045263  GO:0046933  GO:0046961 \| 1827992  1829867  2868890  12477932 \| ATP5G1 \| Bt.1242.1.S1\_at \| \| Bt.27039.1.A1\_at \|  \|  \|  \|  \|  \|  \|  \| Bt.27039.1.A1\_at \| \| Bt.20505.1.S1\_at \| 532427 \| 1/1 \| XM\_610945 \|  \|  \|  \| TIGD2 \| Bt.20505.1.S1\_at \| \| Bt.9811.1.S1\_at \| 533993 \| 1/1 \| NM\_001046365 \|  \|  \|  \| TNFSF5IP1 \| Bt.9811.1.S1\_at \| \| Bt.22379.1.S1\_at \| 536925 \| 1/1 \| NM\_001046419 \| NM\_001046419 \| GO:0000166  GO:0005524  GO:0005739  GO:0006457  GO:0016301  GO:0016740 \| 11282978  16305752 \| CABC1 \| Bt.22379.1.S1\_at \| \| Bt.23098.1.S1\_at \|  \|  \|  \|  \|  \|  \| QSCN6 \| Bt.23098.1.S1\_at \| \| Bt.1035.1.S1\_a\_at \| 338062 \| 1/1 \| NM\_176657 \|  \| GO:0002474  GO:0004872  GO:0006955  GO:0016020  GO:0019882  GO:0042612 \| 10657638  12477932  12514749  16095719 \| FCGRT \| Bt.1035.1.S1\_a\_at \| \| Bt.24211.2.S1\_at \| 507990 \| 2/2 \| NM\_001034309 \|  \|  \| 12477932 \| ASPN \| Bt.24211.2.S1\_at \| \| Bt.27091.2.S1\_at \| 540085 \| 2/2 \| XM\_001249410  XM\_591382 \|  \|  \|  \| GAB1 \| Bt.27091.2.S1\_at \| \| Bt.5571.1.S1\_at \| 767982 \| 1/1 \| BC103275 \|  \| GO:0016020  GO:0016021 \|  \|  \| Bt.5571.1.S1\_at \| \| Bt.22510.1.S1\_at \| 511492 \| 1/1 \| XM\_588837 \| XM\_588837 \|  \|  \| C2orf7 \| Bt.22510.1.S1\_at \| \| Bt.20984.1.S1\_at \| 767938 \| 2/2 \| NM\_001077011 \| NM\_001077011 \|  \|  \|  \| Bt.20984.1.S1\_at \| \| Bt.10278.1.S1\_at \| 777789 \| 1/1 \| NM\_001078164  XM\_001251769 \|  \|  \|  \| ORF1-FL49 \| Bt.10278.1.S1\_at \| \| Bt.5922.1.S1\_at \| 510811 \| 1/1 \| NM\_001075467 \| NM\_001075467 \|  \|  \| HOMER2 \| Bt.5922.1.S1\_at \| \| Bt.136.1.S1\_at \| 281563 \| 1/1 \| NM\_174210 \|  \|  \| 10582284  16775060 \| UCP3 \| Bt.136.1.S1\_at \| \| Bt.19266.1.A1\_at \|  \|  \|  \|  \|  \|  \|  \| Bt.19266.1.A1\_at \| \| Bt.1408.1.S1\_at \| 282420 \| 1/2 \| NM\_174636 \| NM\_174636 \| GO:0000074  GO:0000166  GO:0004674  GO:0004682  GO:0005515  GO:0005515  GO:0005524  GO:0006468  GO:0006468  GO:0016055  GO:0016740 \| 2159007  10782213 \| CSNK2A2 \| Bt.1408.1.S1\_at \| \| Bt.76.1.S1\_at \| 338086 \| 1/1 \| NM\_176674 \| NM\_176674 \| GO:0004129  GO:0005739  GO:0005746  GO:0006118  GO:0009055  GO:0016020  GO:0016021 \| 1316159  1717471  3006725  8638158  10089392  10771420 \| COX7A1 \| Bt.76.1.S1\_at \| \| Bt.1808.1.S1\_at \| 512500 \| 1/1 \| NM\_001038090 \| NM\_001038090 \| GO:0005506  GO:0005739  GO:0005746  GO:0006118  GO:0006810  GO:0009055  GO:0016020  GO:0016021  GO:0020037  GO:0046872 \| 6286615  9204897  9651245  12435729  14667821  16924113 \| CYC1 \| Bt.1808.1.S1\_at \| \| Bt.1129.2.S1\_at \| 280682 \| 2/2 \| NM\_001044721 \| NM\_001044721 \| GO:0004108  GO:0005737  GO:0005739  GO:0006099  GO:0016740 \| 16751257 \| CS \| Bt.1129.2.S1\_at \| \| Bt.75.1.S1\_at \| 338087 \| 1/1 \| NM\_176675 \| NM\_176675 \| GO:0004129  GO:0005739  GO:0006118  GO:0016491 \| 229067  2548168  2844245  6262305  8638158  10089392  10771420  12477932  12658628 \| COX6B \| Bt.75.1.S1\_at \| \| Bt.13324.2.S1\_a\_at \| 281235  613338 \| 1/2 \| NM\_181012  XM\_864062  XM\_872571 \|  \|  \|  \| IDH3B \| Bt.13324.2.S1\_a\_at \| \| Bt.20512.2.S1\_at \| 537532 \| 1/1 \|  \|  \|  \|  \| COL15A1 \| Bt.20512.2.S1\_at \| \| Bt.1280.2.S1\_a\_at \| 282523 \| 1/2 \| NM\_174671 \|  \| GO:0005215  GO:0005488  GO:0005739  GO:0005743  GO:0006810  GO:0016020  GO:0016021 \| 1457818  2271695  11282978  16305752 \| SLC25A11 \| Bt.1280.2.S1\_a\_at \| \| Bt.12358.1.S1\_at \| 506115 \| 2/3 \| NM\_001045935 \|  \|  \|  \| CTDSP2 \| Bt.12358.1.S1\_at \| \| Bt.12453.1.A1\_a\_at \|  \|  \|  \|  \|  \|  \| SOX4 \| Bt.12453.1.A1\_a\_at \| \| Bt.6494.1.S1\_at \| 404161 \| 1/1 \| NM\_001033620 \|  \| GO:0003954  GO:0005739  GO:0008137  GO:0016020  GO:0016021  GO:0016491 \| 12381726  12477932  15056672 \| NDUFB11 \| Bt.6494.1.S1\_at \| \| Bt.1582.1.S1\_at \| 615447 \| 1/1 \| NM\_001076376 \|  \| GO:0000166  GO:0000287  GO:0004550  GO:0005524  GO:0006183  GO:0006228  GO:0006241  GO:0009117  GO:0016020  GO:0016301  GO:0016740 \|  \|  \| Bt.1582.1.S1\_at \| \| Bt.20295.1.A1\_at \|  \|  \|  \|  \|  \|  \| FST \| Bt.20295.1.A1\_at \| \| Bt.5123.3.S1\_a\_at \| 514446 \| 3/4 \| XM\_592304 \| XM\_592304 \| GO:0003779  GO:0005515 \|  \| FLII \| Bt.5123.3.S1\_a\_at \| \| Bt.4475.1.S1\_at \| 327697 \| 1/1 \| NM\_001075137 \| NM\_001075137 \| GO:0003954  GO:0005506  GO:0005739  GO:0005747  GO:0006118  GO:0006979  GO:0006979  GO:0008137  GO:0046872  GO:0051287  GO:0051539 \| 2498081  12857734 \| NDUFS2 \| Bt.4475.1.S1\_at \| \| Bt.21383.1.S1\_at \| 614973 \| 2/2 \| XM\_878838 \| XM\_878838 \|  \|  \|  \| Bt.21383.1.S1\_at \| \| Bt.8549.2.S1\_at \| 511765 \| 1/1 \| NM\_001046099 \| NM\_001046099 \|  \|  \|  \| Bt.8549.2.S1\_at \| \| Bt.321.1.S1\_at \| 327702 \| 1/2 \| NM\_001040488 \|  \| GO:0000036  GO:0005504  GO:0005739  GO:0005759  GO:0006120  GO:0006633  GO:0006633  GO:0008137  GO:0016491  GO:0016491  GO:0031177  GO:0031966  GO:0048037 \| 1907568  16109413 \| NDUFAB1 \| Bt.321.1.S1\_at \| \| Bt.1861.1.S1\_at \|  \|  \|  \|  \|  \|  \| PAN3 \| Bt.1861.1.S1\_at \| \| Bt.893.1.S1\_at \| 327665 \| 1/1 \| NM\_175787 \| NM\_175787 \| GO:0003954  GO:0005739  GO:0008137  GO:0016491 \| 1518044  12477932 \| NDUFB6 \| Bt.893.1.S1\_at \| \| Bt.15941.1.S1\_at \| 515358 \| 1/2 \| XM\_593366  XM\_874685 \| XM\_593366 \| GO:0003723 \|  \|  \| Bt.15941.1.S1\_at \| \| Bt.1059.2.S1\_at \| 540568 \| 3/4 \| XM\_612129 \| XM\_612129 \| GO:0003824  GO:0005524  GO:0006810  GO:0006812  GO:0008152  GO:0015662  GO:0016020 \|  \| ATP2A2 \| Bt.1059.2.S1\_at \| \| Bt.20308.1.S1\_at \|  \|  \|  \|  \|  \|  \| BACH2 \| Bt.20308.1.S1\_at \| \| Bt.23497.1.S1\_at \| 618352 \| 1/1 \| NM\_001076501 \| NM\_001076501 \|  \|  \|  \| Bt.23497.1.S1\_at \| \| Bt.8964.2.S1\_a\_at \| 767949 \| 1/1 \| XM\_001252861 \|  \| GO:0004864  GO:0007165 \|  \| PPP1R1A \| Bt.8964.2.S1\_a\_at \| \| Bt.25555.1.S1\_at \| 532910 \| 1/1 \| NM\_001038156 \| NM\_001038156 \| GO:0005515 \|  \| LRRC28 \| Bt.25555.1.S1\_at \| \| Bt.21218.1.A1\_at \| 515614 \| 1/1 \| NM\_001046207 \| NM\_001046207 \| GO:0004418  GO:0006783  GO:0016740 \|  \| HMBS \| Bt.21218.1.A1\_at \| \| Bt.26992.1.A1\_at \| 535192 \| 2/3 \| NM\_001035410 \|  \| GO:0004806  GO:0005319  GO:0008201  GO:0016042  GO:0016787 \| 12477932 \| ADAM10 \| Bt.26992.1.A1\_at \| \| Bt.13505.1.S1\_at \| 280808 \| 1/1 \| NM\_001040471 \|  \|  \| 11376940 \| GPI \| Bt.13505.1.S1\_at \| \| Bt.7856.1.S1\_at \| 505005 \| 1/1 \| NM\_001045906 \| NM\_001045906 \|  \|  \| BCKDK \| Bt.7856.1.S1\_at \| \| Bt.24824.1.S1\_at \| 523294 \| 1/1 \| NM\_001046266 \|  \|  \|  \| GLT8D2 \| Bt.24824.1.S1\_at \| \| Bt.8062.1.S1\_at \| 282015 \| 1/2 \| NM\_174441 \|  \| GO:0000166  GO:0000502  GO:0005524  GO:0005634  GO:0005737  GO:0005829  GO:0008134  GO:0016787  GO:0016887  GO:0030163  GO:0031531  GO:0043069  GO:0043161  GO:0043193 \| 9701609 \| PSMC5 \| Bt.8062.1.S1\_at \| \| Bt.315.1.S1\_at \| 338079 \| 1/1 \| NM\_001038022 \| NM\_001038022 \| GO:0003954  GO:0005506  GO:0005739  GO:0005743  GO:0006120  GO:0008137  GO:0046872  GO:0051539 \| 1577158 \| NDUFS7 \| Bt.315.1.S1\_at \| \| Bt.1129.1.S1\_at \| 280682 \| 2/2 \| NM\_001044721 \| NM\_001044721 \| GO:0004108  GO:0005737  GO:0005739  GO:0006099  GO:0016740 \| 16751257 \| CS \| Bt.1129.1.S1\_at \| \| Bt.27908.1.S1\_at \| 513847 \| 1/1 \| NM\_001075639 \| NM\_001075639 \| GO:0005319  GO:0006869  GO:0016020  GO:0016021 \|  \| MGC4825 \| Bt.27908.1.S1\_at \| \| Bt.13240.1.S1\_at \|  \|  \|  \| XM\_001254885 \|  \|  \| CKLFSF3 \| Bt.13240.1.S1\_at \| \| Bt.16003.1.S1\_at \| 327664 \| 1/1 \| NM\_175786 \|  \| GO:0000166  GO:0005975  GO:0005980  GO:0008184  GO:0016757  GO:0030170 \| 8845714 \| PYGM \| Bt.16003.1.S1\_at \| \| Bt.6683.1.S1\_at \| 517402 \| 1/1 \| NM\_001075751 \|  \| GO:0005515  GO:0008152  GO:0008415 \|  \| PDHX \| Bt.6683.1.S1\_at \| \| Bt.5344.1.S1\_at \| 281556 \| 1/1 \| NM\_174207 \|  \|  \| 8547323  10715211  12445479  15557323  17130126 \| TUFM \| Bt.5344.1.S1\_at \| \| Bt.27543.1.A1\_at \|  \|  \|  \|  \|  \|  \| FAT4 \| Bt.27543.1.A1\_at \| \| Bt.17079.1.S1\_a\_at \|  \|  \|  \|  \|  \|  \| VGLL4 \| Bt.17079.1.S1\_a\_at \| \| Bt.1696.1.S1\_at \| 618777 \| 1/1 \| NM\_001046623 \| NM\_001046623 \|  \| 11282978  16305752 \| SAMM50 \| Bt.1696.1.S1\_at \| \| Bt.371.1.S1\_at \| 282478 \| 1/1 \| NM\_174658 \| NM\_174658 \| GO:0005215  GO:0005488  GO:0005739  GO:0005743  GO:0006810  GO:0006839  GO:0016020  GO:0016021 \| 2540808  3017341  7076130  12477932  14603310 \| SLC25A4 \| Bt.371.1.S1\_at \| \| Bt.5520.1.S1\_at \| 327669 \| 1/1 \| NM\_175790 \|  \| GO:0000287  GO:0004450  GO:0004450  GO:0005739  GO:0005739  GO:0005743  GO:0005743  GO:0006097  GO:0006099  GO:0008152  GO:0016491  GO:0016616  GO:0030145 \| 8318002  12477932 \| IDH2 \| Bt.5520.1.S1\_at \| \| Bt.25087.3.S1\_at \| 521244 \| 3/3 \|  \|  \|  \|  \| RHOQ \| Bt.25087.3.S1\_at \| \| Bt.4606.1.S1\_at \| 505884 \| 3/5 \| NM\_001035271 \|  \|  \| 12477932 \| KLF6 \| Bt.4606.1.S1\_at \| \| Bt.11791.1.A1\_at \|  \|  \|  \|  \|  \|  \| TA-PP2C \| Bt.11791.1.A1\_at \| \| Bt.4804.2.A1\_at \|  \|  \|  \| XM\_001252310 \|  \|  \| CDKN1C \| Bt.4804.2.A1\_at \| \| Bt.22518.1.S1\_a\_at \| 540962 \| 1/1 \| XM\_613885  XM\_882913 \| XM\_613885 \|  \|  \| QIL1 \| Bt.22518.1.S1\_a\_at \| \| Bt.13330.1.S1\_at \| 507367 \| 3/3 \| XM\_583960 \| XM\_583960 \|  \|  \| PDK4 \| Bt.13330.1.S1\_at \| \| Bt.23127.1.S1\_at \| 534927 \| 1/1 \| NM\_001038164 \| NM\_001038164 \| GO:0004180  GO:0004188  GO:0005764  GO:0006508  GO:0008236 \|  \| PRCP \| Bt.23127.1.S1\_at \| \| Bt.7915.1.S1\_at \| 281306 \| 1/1 \| NM\_001013587 \|  \| GO:0004459  GO:0005739  GO:0006096  GO:0006099  GO:0006108  GO:0016491  GO:0030060 \| 12140684  16751257 \| MDH2 \| Bt.7915.1.S1\_at \| \| Bt.13943.1.A1\_at \| 540526 \| 1/1 \| XM\_611908 \| XM\_611908 \|  \|  \| MSH6 \| Bt.13943.1.A1\_at \| \| Bt.22843.1.S1\_at \| 614033 \| 1/1 \| NM\_001034746 \| NM\_001034746 \|  \| 12477932 \| MGC126935 \| Bt.22843.1.S1\_at \| \| Bt.23603.3.S1\_at \| 511798 \| 3/3 \| NM\_001034412 \| NM\_001034412 \| GO:0006512 \| 12477932 \| FBXO9 \| Bt.23603.3.S1\_at \| \| Bt.7394.1.A1\_at \| 615191 \| 2/2 \| XM\_866930 \|  \| GO:0005515  GO:0005524  GO:0016311  GO:0016791  GO:0044267 \|  \| PTPLA \| Bt.7394.1.A1\_at \| \| Bt.26702.1.S1\_a\_at \| 511801 \| 1/2 \| NM\_001075539 \| NM\_001075539 \| GO:0006464  GO:0006512  GO:0016874  GO:0019787 \|  \|  \| Bt.26702.1.S1\_a\_at \| \| Bt.9949.3.S1\_a\_at \| 533851 \| 2/3 \| NM\_001017947 \|  \| GO:0001503  GO:0005515  GO:0005856  GO:0007275  GO:0008270  GO:0030154  GO:0046872 \| 11282978  16305752 \| PDLIM7 \| Bt.9949.3.S1\_a\_at \| \| Bt.18990.1.S1\_at \| 519213 \| 1/1 \| XM\_597427 \|  \|  \|  \| PIGV \| Bt.18990.1.S1\_at \| \| Bt.29931.1.S1\_at \| 514928 \| 1/2 \| XM\_001253800  XM\_592847 \| XM\_001253800 \| GO:0003777  GO:0005524  GO:0005875  GO:0007018 \|  \|  \| Bt.29931.1.S1\_at \| \| Bt.5042.1.S1\_at \| 282394 \| 1/1 \| NM\_174630 \|  \| GO:0004222  GO:0005739  GO:0005746  GO:0006118  GO:0006508  GO:0006810  GO:0008121  GO:0016020  GO:0016491 \| 1712295  2841307  2848575  9204897  9651245  12140684  12477932  12658628  16305752 \| UQCRC2 \| Bt.5042.1.S1\_at \| \| Bt.19355.1.A1\_at \|  \|  \|  \| XM\_594211 \|  \|  \| FLJ20130 \| Bt.19355.1.A1\_at \| \| Bt.5537.1.S1\_at \| 514801 \| 1/3 \| XR\_028679 \|  \|  \|  \| PSME3 \| Bt.5537.1.S1\_at \| \| Bt.5246.1.S1\_at \| 510949 \| 1/2 \| XM\_868352  XM\_881618 \| XM\_868352 \| GO:0003824  GO:0004018  GO:0009152 \|  \| ADSL \| Bt.5246.1.S1\_at \| \| Bt.5848.2.S1\_at \| 518117 \| 2/2 \| NM\_001075767 \|  \| GO:0000166  GO:0000287  GO:0005388  GO:0005509  GO:0005515  GO:0005524  GO:0006812  GO:0006816  GO:0008152  GO:0015992  GO:0016020  GO:0016021  GO:0016529  GO:0016787  GO:0016820  GO:0031448 \|  \| ATP2A1 \| Bt.5848.2.S1\_at \| \| Bt.27152.1.A1\_at \|  \|  \|  \|  \|  \|  \| PI15 \| Bt.27152.1.A1\_at \| \| Bt.28133.1.S1\_at \|  \|  \|  \|  \|  \|  \| MLL3 \| Bt.28133.1.S1\_at \| \| Bt.17968.1.S1\_at \| 512891 \| 1/1 \| NM\_001035111 \| NM\_001035111 \| GO:0005829  GO:0005838  GO:0006508 \| 12477932 \| PSMD8 \| Bt.17968.1.S1\_at \| \| Bt.16815.1.S1\_at \| 513526 \| 1/1 \| XM\_591219 \| XM\_591219 \|  \| 12477932 \| C16orf33 \| Bt.16815.1.S1\_at \| \| Bt.9730.1.S1\_at \| 507179 \| 1/1 \| XM\_583747 \| XM\_583747 \|  \|  \| COPS5 \| Bt.9730.1.S1\_at \| \| Bt.26975.1.A1\_a\_at \| 539164 \| 1/1 \| XM\_585339 \| XM\_585339 \|  \|  \| SLB \| Bt.26975.1.A1\_a\_at \| \| Bt.5123.2.S1\_at \| 514446 \| 3/4 \| XM\_592304 \| XM\_592304 \| GO:0003779  GO:0005515 \|  \| FLII \| Bt.5123.2.S1\_at \| \| Bt.2049.1.S1\_at \| 510634 \| 1/1 \| XM\_587797 \| XM\_587797 \|  \|  \| ZC3HDC5 \| Bt.2049.1.S1\_at \| \| Bt.5089.1.S2\_at \| 617051 \| 3/5 \| XM\_883110 \|  \|  \|  \|  \| Bt.5089.1.S2\_at \| \| Bt.3856.1.S1\_at \| 353217 \| 1/1 \| NM\_181338 \| NM\_181338 \| GO:0005351  GO:0005783  GO:0006810  GO:0016020  GO:0016021 \| 12770764 \| SLC35B1 \| Bt.3856.1.S1\_at \| \| Bt.16900.1.A1\_at \|  \|  \|  \| XM\_001250788 \|  \|  \| KIAA1429 \| Bt.16900.1.A1\_at \| \| Bt.265.1.S1\_at \| 287020 \| 1/1 \| NM\_174813 \| NM\_174813 \| GO:0005506  GO:0005739  GO:0005739  GO:0005746  GO:0006118  GO:0006810  GO:0008121  GO:0016020  GO:0016021  GO:0016491  GO:0045285  GO:0046872  GO:0051537 \| 2157409  2996928  3036596  8386158  8736555  9204897  9651245  15766251  15865449  15878858 \| UQCRFS1 \| Bt.265.1.S1\_at \| \| Bt.11492.1.A1\_at \| 538561 \| 1/2 \| XR\_027849 \| XR\_027849 \|  \|  \| MGEA5 \| Bt.11492.1.A1\_at \| \| Bt.20601.1.A1\_at \| 407101 \| 2/4 \| XM\_873993 \|  \| GO:0005515 \|  \| TJP2 \| Bt.20601.1.A1\_at \| \| Bt.5447.1.S1\_at \| 281357 \| 1/1 \| NM\_174126 \|  \| GO:0004383  GO:0004383  GO:0004672  GO:0004872  GO:0005524  GO:0006182  GO:0006182  GO:0006468  GO:0007242  GO:0008528  GO:0016020  GO:0016021  GO:0016941  GO:0016941  GO:0042802  GO:0042802 \| 7845391  8103329  16818377 \| NPR2 \| Bt.5447.1.S1\_at \| \| Bt.22122.2.A1\_at \|  \|  \|  \|  \|  \|  \| ABLIM1 \| Bt.22122.2.A1\_at \| \| Bt.1568.1.S1\_at \| 287012 \| 1/1 \| NM\_001034046 \| XM\_001249385 \| GO:0004129  GO:0005739  GO:0005740  GO:0006118  GO:0008270  GO:0016020  GO:0016491  GO:0046872 \| 2840351  2844245  6292069  8638158  10089392  10771420  12477932 \| COX5B \| Bt.1568.1.S1\_at \| \| Bt.11659.1.S1\_at \| 615666 \| 1/2 \| NM\_001034787 \|  \| GO:0003735  GO:0005739  GO:0005761  GO:0006412  GO:0030529 \| 11402041  12477932 \| MRP63 \| Bt.11659.1.S1\_at \| \| Bt.23361.1.S1\_at \|  \|  \|  \| NM\_001034797 \|  \|  \| UQCRB \| Bt.23361.1.S1\_at \| \| Bt.28187.1.S1\_at \| 540322 \| 1/1 \| XM\_593176 \| XM\_593176 \|  \|  \| WEE1 \| Bt.28187.1.S1\_at \| \| Bt.21533.1.S1\_at \| 511059 \| 1/1 \| XM\_588315 \| XM\_588315 \| GO:0015986  GO:0016469  GO:0030246  GO:0046933  GO:0046961 \|  \| KIAA0826 \| Bt.21533.1.S1\_at \| \| Bt.19167.1.A1\_at \| 532631 \| 1/1 \| XM\_611767 \|  \|  \|  \| ANKS1 \| Bt.19167.1.A1\_at \| \| Bt.9826.1.S1\_at \| 444878 \| 1/1 \| NM\_001002891 \|  \| GO:0004129  GO:0004129  GO:0005506  GO:0005739  GO:0005739  GO:0005743  GO:0005743  GO:0006118  GO:0016020  GO:0016491  GO:0046872 \| 220224  2844245  8638158  10089392  10771420 \| COX5A \| Bt.9826.1.S1\_at \| \| Bt.4864.2.S1\_at \| 532483 \| 2/3 \| NM\_001035389 \|  \| GO:0005515  GO:0005634  GO:0007049  GO:0008270  GO:0045786  GO:0046872 \| 12477932 \| MGC127324 \| Bt.4864.2.S1\_at \| \| Bt.2726.1.S1\_at \| 538778 \| 1/2 \| XR\_028578 \| XR\_028578 \|  \|  \| DACT1 \| Bt.2726.1.S1\_at \| \| Bt.12303.1.S1\_at \| 280890 \| 1/1 \| NM\_173948 \| NM\_173948 \|  \| 2059626  3153462 \| PAM \| Bt.12303.1.S1\_at \| \| Bt.3015.1.A1\_at \|  \|  \|  \|  \|  \|  \| LHFPL2 \| Bt.3015.1.A1\_at \| \| Bt.9047.1.S1\_at \| 615999 \| 1/1 \| XM\_867947 \|  \|  \|  \| DDT \| Bt.9047.1.S1\_at \| \| Bt.8950.1.S1\_at \| 338084 \| 1/1 \| NM\_176672 \|  \| GO:0003954  GO:0005515  GO:0005524  GO:0005634  GO:0005654  GO:0005737  GO:0005739  GO:0005747  GO:0006606  GO:0006800  GO:0006915  GO:0008137  GO:0008624  GO:0016020  GO:0016021  GO:0016491  GO:0017148  GO:0030262  GO:0030308  GO:0045892 \| 11522775  12477932 \| NDUFA13 \| Bt.8950.1.S1\_at \| \| Bt.11855.1.S1\_at \| 510727 \| 1/1 \| NM\_001038074 \| NM\_001038074 \|  \|  \| COX4NB \| Bt.11855.1.S1\_at \| \| Bt.2973.2.S1\_a\_at \| 613610 \| 1/3 \| NM\_001035435 \|  \| GO:0004739  GO:0005739  GO:0006096  GO:0016491 \| 2829898  12140684  16305752 \| PDHB \| Bt.2973.2.S1\_a\_at \| \| Bt.2129.2.S1\_at \|  \|  \|  \| XM\_001253937 \|  \|  \| CSPG2 \| Bt.2129.2.S1\_at \| \| Bt.5290.1.S1\_at \| 281729 \| 1/1 \| NM\_174295 \| NM\_174295 \| GO:0000166  GO:0000922  GO:0003700  GO:0003777  GO:0005515  GO:0005524  GO:0005634  GO:0005694  GO:0005737  GO:0006259  GO:0006281  GO:0006355  GO:0007049  GO:0007052  GO:0007062  GO:0007067  GO:0007126  GO:0007165  GO:0008278  GO:0016020  GO:0016363  GO:0016887  GO:0043565  GO:0045502  GO:0046982  GO:0051276  GO:0051301 \| 10072753 \| CSPG6 \| Bt.5290.1.S1\_at \| \| Bt.25099.1.A1\_at \| 533630 \| 2/2 \| NM\_001046355 \| NM\_001046355 \| GO:0000287  GO:0004647  GO:0006564  GO:0008152  GO:0008652  GO:0016787 \|  \| PSPH \| Bt.25099.1.A1\_at \| \| Bt.5183.1.S1\_at \| 777775 \| 2/2 \| NM\_001078158  XM\_001251388 \| NM\_001078158 \| GO:0000166  GO:0003924  GO:0005198  GO:0005525  GO:0005874  GO:0007018  GO:0043234  GO:0051258 \| 9571153  12140684  16305752 \|  \| Bt.5183.1.S1\_at \| \| Bt.7415.1.S1\_at \| 506485 \| 1/1 \| NM\_001075265 \| NM\_001075265 \|  \| 11282978  16305752 \| PBXIP1 \| Bt.7415.1.S1\_at \| \| Bt.3101.1.A1\_at \| 539379 \| 1/1 \| XM\_586763 \| XM\_586763 \|  \|  \| STARS \| Bt.3101.1.A1\_at \| \| Bt.10154.1.S1\_at \| 531444 \| 1/1 \| NM\_001034057 \| NM\_001034057 \| GO:0005739  GO:0008270  GO:0046872 \| 12477932  14570702 \| C10orf70 \| Bt.10154.1.S1\_at \| \| Bt.9825.1.S1\_at \| 540278 \| 1/2 \| XM\_592911 \|  \| GO:0005739  GO:0030529 \| 11282978  12477932  16305752 \| MRPL35 \| Bt.9825.1.S1\_at \| \| Bt.20307.1.S1\_at \| 506882 \| 1/1 \| NM\_001045952 \| NM\_001045952 \|  \|  \| DNPEP \| Bt.20307.1.S1\_at \| \| Bt.9434.1.S1\_at \| 613652 \| 1/1 \| NM\_001076281 \|  \|  \|  \| UBADC1 \| Bt.9434.1.S1\_at \| \| Bt.11791.2.S1\_at \|  \|  \|  \|  \|  \|  \| TA-PP2C \| Bt.11791.2.S1\_at \| \| Bt.1294.1.S1\_at \| 513397 \| 1/1 \| XM\_867796 \| NM\_001080270 \|  \|  \| DNAJA3 \| Bt.1294.1.S1\_at \| \| Bt.22842.2.S1\_at \| 510698 \| 1/1 \| XM\_587873 \|  \|  \|  \| VAT1 \| Bt.22842.2.S1\_at \| \| Bt.14143.1.S1\_at \| 280753 \| 1/1 \| NM\_173903 \|  \|  \| 2396993  3020433 \| CPE \| Bt.14143.1.S1\_at \| \| Bt.25051.1.A1\_at \| 512635 \| 1/2 \| XM\_590182 \|  \|  \|  \| TTC14 \| Bt.25051.1.A1\_at \| \| Bt.5750.1.S1\_at \| 508465 \| 1/1 \| NM\_001035059 \|  \|  \| 12477932 \| DERL2 \| Bt.5750.1.S1\_at \| \| Bt.6063.1.S1\_at \| 517535 \| 1/1 \| NM\_001034524 \| XR\_028101 \| GO:0005524  GO:0006457  GO:0051082 \| 12140684  12477932  16305752 \|  \| Bt.6063.1.S1\_at \| \| Bt.9586.1.A1\_at \| 510568 \| 1/1 \| XM\_587722 \| XM\_587722 \|  \|  \| XPO1 \| Bt.9586.1.A1\_at \| \| Bt.12211.1.S1\_at \|  \|  \|  \|  \|  \|  \|  \| Bt.12211.1.S1\_at \| \| Bt.24265.1.S1\_a\_at \| 508712 \| 1/1 \| NM\_001075370 \| NM\_001075370 \|  \|  \| KAT3 \| Bt.24265.1.S1\_a\_at \| \| Bt.27091.1.S1\_at \| 540085 \| 2/2 \| XM\_001249410  XM\_591382 \| XM\_001249410 \|  \|  \| GAB1 \| Bt.27091.1.S1\_at \| \| Bt.20281.2.S1\_a\_at \| 534402 \| 1/2 \| NM\_001076903 \| NM\_001076903 \| GO:0005975  GO:0016868 \|  \|  \| Bt.20281.2.S1\_a\_at \| \| Bt.28559.1.S1\_a\_at \| 515176 \| 1/1 \| NM\_001038634 \| NM\_001038634 \| GO:0004519  GO:0004536  GO:0005783  GO:0006308  GO:0016787 \| 11282978  16107205  16305752 \| DNASE1L1 \| Bt.28559.1.S1\_a\_at \| \| Bt.23569.1.S1\_at \| 522006 \| 1/1 \| NM\_001035342 \|  \|  \| 11282978  12477932  16305752 \| ACAA2 \| Bt.23569.1.S1\_at \| \| Bt.23396.1.S1\_at \| 507834 \| 2/4 \| XR\_028758 \| XR\_028758 \|  \| 15772903 \|  \| Bt.23396.1.S1\_at \| \| Bt.4651.1.S1\_at \| 534126 \| 1/1 \| NM\_001046370 \| NM\_001046370 \|  \|  \| FAM48A \| Bt.4651.1.S1\_at \| \| Bt.8504.1.A1\_at \| 507482 \| 1/1 \| XM\_584093 \| XM\_584093 \|  \|  \| FMR1 \| Bt.8504.1.A1\_at \| \| Bt.22528.1.A1\_at \| 614183 \| 2/2 \| XM\_865921 \| NM\_001081618 \|  \|  \|  \| Bt.22528.1.A1\_at \| \| Bt.12423.1.S1\_at \|  \|  \|  \| XR\_028841 \|  \|  \| FAM62B \| Bt.12423.1.S1\_at \| \| Bt.27939.2.A1\_a\_at \| 525346 \| 4/4 \| XM\_867432 \|  \|  \|  \| NCOA1 \| Bt.27939.2.A1\_a\_at \| \| Bt.8679.1.A1\_at \| 512723 \| 2/2 \| XM\_590291 \|  \|  \|  \| DLAT \| Bt.8679.1.A1\_at \| \| Bt.884.1.S1\_at \| 513597 \| 1/1 \| XM\_873525 \|  \|  \|  \| FER1L3 \| Bt.884.1.S1\_at \| \| Bt.14229.1.S1\_at \| 538970 \| 1/1 \| NM\_001076140 \|  \|  \|  \| C7orf25 \| Bt.14229.1.S1\_at \| \| Bt.4099.1.S1\_at \| 282120 \| 1/1 \| NM\_174486 \| NM\_174486 \| GO:0005739  GO:0005741  GO:0005743  GO:0006820  GO:0008308  GO:0015288  GO:0016020  GO:0016021  GO:0019867 \| 11311949  12140684  12477932  14739283  16305752 \| VDAC2 \| Bt.4099.1.S1\_at \| \| Bt.9878.1.A1\_at \| 530506 \| 1/1 \| XM\_001251199  XM\_608980 \|  \| GO:0003677  GO:0004386  GO:0004522  GO:0005524 \|  \| SMARCAD1 \| Bt.9878.1.A1\_at \| \| Bt.1059.3.S1\_a\_at \| 540568 \| 3/4 \| XM\_612129 \| XM\_612129 \| GO:0003824  GO:0005524  GO:0006810  GO:0006812  GO:0008152  GO:0015662  GO:0016020 \|  \| ATP2A2 \| Bt.1059.3.S1\_a\_at \| \| Bt.5676.1.S1\_at \| 533208 \| 1/2 \| NM\_001075984 \| NM\_001075984 \| GO:0005739  GO:0006744  GO:0008168  GO:0016740 \|  \|  \| Bt.5676.1.S1\_at \| \| Bt.20025.1.S1\_at \| 618609 \| 1/1 \| NM\_001076514 \|  \|  \| 11551941 \| MRPL21 \| Bt.20025.1.S1\_at \| \| Bt.5966.1.A1\_at \| 505968 \| 1/1 \| NM\_001075235 \| NM\_001075235 \|  \|  \|  \| Bt.5966.1.A1\_at \| \| Bt.7175.1.S1\_at \| 523399 \| 1/1 \| XM\_866991 \| XM\_866991 \|  \|  \| IPO13 \| Bt.7175.1.S1\_at \| \| Bt.5029.1.S1\_at \| 282710 \| 1/1 \| NM\_174724 \| NM\_174724 \|  \| 2890767  11282978  12477932  16305752 \| ATP5H \| Bt.5029.1.S1\_at \| \| Bt.24404.1.A1\_at \| 532616 \| 1/1 \| XM\_865681 \| XM\_865681 \| GO:0003779  GO:0005198  GO:0005488  GO:0005737  GO:0005856  GO:0016020  GO:0030866 \|  \| EPB41L3 \| Bt.24404.1.A1\_at \| \| Bt.10942.1.S1\_at \| 539829 \| 1/2 \| NM\_001076919 \| NM\_001076919 \|  \|  \| NRM \| Bt.10942.1.S1\_at \| \| Bt.27412.1.A1\_at \|  \|  \|  \|  \|  \|  \| AKT3 \| Bt.27412.1.A1\_at \| \| Bt.20984.1.S1\_a\_at \| 767938 \| 2/2 \| NM\_001077011 \|  \|  \|  \|  \| Bt.20984.1.S1\_a\_at \| \| Bt.21766.2.S1\_at \|  \|  \|  \|  \|  \|  \| MLF1 \| Bt.21766.2.S1\_at \| \| Bt.26453.1.S1\_at \|  \|  \|  \|  \|  \|  \| OACT2 \| Bt.26453.1.S1\_at \| \| Bt.8650.1.A1\_at \| 541299 \| 1/1 \| XM\_875156 \| XM\_875156 \|  \|  \| STIM2 \| Bt.8650.1.A1\_at \| \| Bt.21721.1.A1\_at \| 522980 \| 1/1 \| NM\_001046263 \| NM\_001046263 \|  \|  \| USP2 \| Bt.21721.1.A1\_at \| \| Bt.4977.1.S2\_at \| 281849 \| 1/2 \| NM\_174352 \|  \| GO:0004872  GO:0005215  GO:0005515  GO:0005764  GO:0006810  GO:0016020  GO:0016021 \| 1332979  2545438  2951738  2963004  11900967  12127995  12658628  12665524  15085180  15169779  15252023  17150312 \| IGF2R \| Bt.4977.1.S2\_at \| \| Bt.21698.1.S1\_a\_at \| 614435 \| 2/2 \| XM\_001254288  XM\_865931 \|  \|  \|  \| PTP4A2 \| Bt.21698.1.S1\_a\_at \| \| Bt.56.1.S1\_at \| 327691 \| 1/1 \| NM\_175810 \| NM\_175810 \| GO:0003954  GO:0005739  GO:0008137  GO:0016491 \| 1518044  1907966 \| NDUFS6 \| Bt.56.1.S1\_at \| \| Bt.28269.1.S1\_at \| 540619 \| 1/1 \| XM\_612355 \| XM\_612355 \|  \|  \| RHOJ \| Bt.28269.1.S1\_at \| \| Bt.29746.1.S1\_at \| 407109 \| 3/4 \| XM\_581602 \| XM\_581602 \| GO:0008152  GO:0016624 \|  \| PDHA1 \| Bt.29746.1.S1\_at \| \| Bt.615.1.S1\_at \| 513155 \| 1/2 \| NM\_001075601 \|  \| GO:0016021  GO:0019835 \|  \| MMD \| Bt.615.1.S1\_at \| \| Bt.5495.1.S1\_at \| 326579 \| 1/1 \| XM\_584557 \| XM\_584557 \| GO:0003743  GO:0006446 \| 12658628 \| BZW2 \| Bt.5495.1.S1\_at \| \| Bt.23438.1.S1\_at \| 510080 \| 4/5 \| NM\_001076826 \| NM\_001076826 \|  \|  \| ANTXR2 \| Bt.23438.1.S1\_at \| \| Bt.3922.1.S1\_a\_at \| 617005 \| 1/1 \| NM\_001035489 \|  \|  \| 11551941  12477932 \| MRPL38 \| Bt.3922.1.S1\_a\_at \| \| Bt.5183.2.S1\_at \| 777775 \| 2/2 \| NM\_001078158  XM\_001251388 \| NM\_001078158 \| GO:0000166  GO:0003924  GO:0005198  GO:0005525  GO:0005874  GO:0007018  GO:0043234  GO:0051258 \| 9571153  12140684  16305752 \|  \| Bt.5183.2.S1\_at \| \| Bt.3149.1.S1\_at \|  \|  \|  \|  \|  \|  \|  \| Bt.3149.1.S1\_at \| \| Bt.20411.1.S1\_at \| 539332 \| 1/1 \| NM\_001076154 \| NM\_001076154 \| GO:0003779  GO:0005515  GO:0005622  GO:0005634  GO:0005856 \|  \| CFL2 \| Bt.20411.1.S1\_at \| \| Bt.405.1.S1\_at \| 327681 \| 1/1 \| NM\_175801 \|  \| GO:0000122  GO:0005515  GO:0007276  GO:0007389  GO:0008585  GO:0030509  GO:0042475  GO:0045596 \| 8011323 \| FST \| Bt.405.1.S1\_at \| \| Bt.6465.1.S1\_at \| 510767 \| 1/1 \| NM\_001046061 \|  \| GO:0005506  GO:0005739  GO:0005746  GO:0006118  GO:0006810  GO:0009055  GO:0020037  GO:0046872 \| 5933874 \| CYCS \| Bt.6465.1.S1\_at \| \| Bt.24875.1.S1\_at \| 617922 \| 1/1 \| NM\_001076948 \|  \|  \|  \|  \| Bt.24875.1.S1\_at \| \| Bt.4565.1.S1\_at \| 520631 \| 1/1 \| NM\_001034538 \| NM\_001034538 \| GO:0003677  GO:0005622  GO:0005634  GO:0006350  GO:0006355  GO:0007275  GO:0008270  GO:0009314  GO:0046872 \| 10895307  12477932 \| SNAI2 \| Bt.4565.1.S1\_at \| \| Bt.10189.1.S1\_at \|  \|  \|  \|  \|  \|  \| ZFP91 \| Bt.10189.1.S1\_at \| \| Bt.2587.1.S1\_at \| 520260 \| 2/2 \| NM\_001075803 \| NM\_001075803 \| GO:0003824  GO:0004333  GO:0006106  GO:0045239 \|  \| FH \| Bt.2587.1.S1\_at \| \| Bt.547.1.S1\_at \| 286885 \| 1/1 \| NM\_174805 \|  \| GO:0005739  GO:0005746  GO:0006118  GO:0006810  GO:0008121  GO:0016020  GO:0016491 \| 2164842  3009231  7592738  9204897  9651245  12477932 \| QP-C \| Bt.547.1.S1\_at \| \| Bt.24096.1.A1\_at \|  \|  \|  \|  \|  \|  \|  \| Bt.24096.1.A1\_at \| \| Bt.8679.2.S1\_at \| 512723 \| 2/2 \| XM\_590291 \| XM\_590291 \|  \|  \| DLAT \| Bt.8679.2.S1\_at \| \| Bt.4694.1.S1\_at \| 521747 \| 1/1 \| NM\_001015628 \|  \|  \| 12140684  12477932  16305752 \| EIF3S2 \| Bt.4694.1.S1\_at \| \| Bt.20321.1.S1\_at \|  \|  \|  \|  \|  \|  \|  \| Bt.20321.1.S1\_at \| \| Bt.12728.1.A1\_a\_at \|  \|  \|  \|  \|  \|  \| ZNF704 \| Bt.12728.1.A1\_a\_at \| \| Bt.8618.1.S1\_at \| 505129 \| 2/5 \| XM\_001252366  XM\_001252396  XM\_001252429  XM\_581371 \| NM\_001080921 \|  \|  \|  \| Bt.8618.1.S1\_at \| \| Bt.28226.3.A1\_s\_at \| 617051 \| 3/5 \| XM\_883110 \| XM\_883110 \|  \|  \| H3F3B \| Bt.28226.3.A1\_s\_at \| \| Bt.4880.1.S1\_at \| 282477 \| 1/1 \| NM\_174657 \| NM\_174657 \| GO:0005488  GO:0005739  GO:0005739  GO:0005743  GO:0005743  GO:0006810  GO:0015293  GO:0016020  GO:0016021 \| 2036364  3038521  4066697  8144629 \| SLC25A3 \| Bt.4880.1.S1\_at \| \| Bt.3428.1.A1\_s\_at \|  \|  \|  \|  \|  \|  \| SACS \| Bt.3428.1.A1\_s\_at \| \| Bt.15664.1.S1\_at \| 509966  541081 \| 1/1 \| NM\_001046045  XM\_614469 \| XM\_001254886 \|  \|  \| PTD004 \| Bt.15664.1.S1\_at \| \| Bt.23576.1.A1\_at \| 539655 \| 1/1 \| XM\_588580 \|  \| GO:0004674  GO:0005524  GO:0006468 \|  \| PCTK2 \| Bt.23576.1.A1\_at \| \| Bt.28238.1.A1\_at \|  \|  \|  \|  \|  \|  \| PPM1K \| Bt.28238.1.A1\_at \| \| Bt.22270.1.S1\_at \| 514325 \| 1/1 \| XM\_592158 \|  \|  \|  \|  \| Bt.22270.1.S1\_at \| \| Bt.20064.2.S1\_at \| 516106 \| 1/1 \| XM\_594245 \| XM\_594245 \| GO:0003676  GO:0003700  GO:0005622  GO:0005634  GO:0006355  GO:0008270  GO:0043565 \|  \| SDCCAG33 \| Bt.20064.2.S1\_at \| \| Bt.23308.1.S1\_at \| 535746 \| 1/5 \| XM\_615857 \|  \|  \|  \| SEPT11 \| Bt.23308.1.S1\_at \| \| Bt.10965.1.S1\_at \|  \|  \|  \|  \|  \|  \| MSRB3 \| Bt.10965.1.S1\_at \| \| Bt.20937.1.A1\_at \|  \|  \|  \|  \|  \|  \| ANKRD44 \| Bt.20937.1.A1\_at \| \| Bt.8279.3.S1\_a\_at \| 511968 \| 1/3 \| NM\_001046105 \|  \| GO:0005634 \|  \| DKFZP564J0123 \| Bt.8279.3.S1\_a\_at \| \| Bt.7459.1.S1\_at \|  \|  \|  \| XM\_877179 \|  \|  \| ETS1 \| Bt.7459.1.S1\_at \| \| Bt.2138.1.S1\_at \| 282277 \| 1/1 \| NM\_174563 \| NM\_174563 \| GO:0005739  GO:0005762  GO:0030529 \|  \| MRPL43 \| Bt.2138.1.S1\_at \| \| Bt.19934.1.S1\_at \| 515509 \| 1/1 \| NM\_001046203 \|  \| GO:0005488 \|  \| SUGT1 \| Bt.19934.1.S1\_at \| \| Bt.15870.1.S1\_at \| 528165 \| 1/1 \| XM\_606577 \| XM\_606577 \| GO:0005744  GO:0006886  GO:0008152  GO:0015450  GO:0016740 \|  \|  \| Bt.15870.1.S1\_at \| \| Bt.25185.1.S1\_at \|  \|  \|  \|  \|  \|  \|  \| Bt.25185.1.S1\_at \| \| Bt.9756.1.S1\_at \| 530865 \| 1/1 \| NM\_001017944 \| NM\_001017944 \| GO:0003824  GO:0008152  GO:0009107  GO:0016992  GO:0051536 \| 11282978 \| LIAS \| Bt.9756.1.S1\_at \| \| Bt.23357.1.S1\_at \| 614145 \| 1/1 \| NM\_001076313 \|  \| GO:0000166  GO:0004449  GO:0005524  GO:0005739  GO:0006099  GO:0008152  GO:0016491  GO:0016616 \| 11282978  16305752 \| IDH3G \| Bt.23357.1.S1\_at \| \| Bt.64.1.S1\_at \| 338065 \| 1/1 \| NM\_176660 \| NM\_176660 \| GO:0003954  GO:0005739  GO:0005743  GO:0006118  GO:0008137  GO:0016491 \| 1518044  12477932 \| NDUFB7 \| Bt.64.1.S1\_at \| \| Bt.19409.1.A1\_s\_at \|  \|  \|  \|  \|  \|  \| MDFIC \| Bt.19409.1.A1\_s\_at \| \| Bt.21731.1.S1\_at \| 505406 \| 1/2 \| NM\_001045917 \|  \|  \|  \| DBN1 \| Bt.21731.1.S1\_at \| \| Bt.2347.1.S1\_at \| 506544 \| 2/3 \| NM\_001075268 \|  \|  \| 8001974 \| PFKM \| Bt.2347.1.S1\_at \| \| Bt.2711.1.S1\_at \| 509983 \| 1/1 \| NM\_001035082 \|  \|  \| 12140684  16305752 \| SUCLG1 \| Bt.2711.1.S1\_at \| \| Bt.6546.1.A1\_at \|  \|  \|  \|  \|  \|  \| HBXAP \| Bt.6546.1.A1\_at \| \| Bt.77.1.S1\_at \| 327718 \| 1/1 \| NM\_175831 \| NM\_175831 \| GO:0004129  GO:0005739  GO:0006118  GO:0016020  GO:0016021  GO:0016491 \| 213363  1655579  2554257  2844245  8638158  9092564  10089392  10771420  12477932 \| COX7C \| Bt.77.1.S1\_at \| \| Bt.5164.1.S1\_at \| 509027 \| 1/1 \| XM\_585907 \| XM\_585907 \|  \|  \| CA14 \| Bt.5164.1.S1\_at \| \| Bt.29873.1.S1\_at \| 404180 \| 2/3 \| XM\_591933 \|  \| GO:0003824  GO:0016020  GO:0016021 \| 14668210 \| MEST \| Bt.29873.1.S1\_at \| \| Bt.12722.1.A1\_at \| 616089 \| 1/1 \| NM\_001040590 \|  \| GO:0003676  GO:0004519  GO:0004522  GO:0016787 \| 12140684 \| RNASE4 \| Bt.12722.1.A1\_at \| \| Bt.12872.1.S1\_at \| 513420 \| 1/1 \| XM\_591093 \| XM\_591093 \| GO:0005515 \|  \| BTBD3 \| Bt.12872.1.S1\_at \| \| Bt.22461.1.S1\_at \|  \|  \|  \|  \|  \|  \| CUGBP2 \| Bt.22461.1.S1\_at \| \| Bt.20426.1.S1\_at \| 613426 \| 1/3 \| XM\_873696 \| XM\_873696 \| GO:0016020  GO:0016021 \|  \| C1orf119 \| Bt.20426.1.S1\_at \| \| Bt.23335.1.S1\_at \|  \|  \|  \|  \|  \|  \| PORIMIN \| Bt.23335.1.S1\_at \| \| Bt.26628.1.S1\_at \| 515181 \| 1/1 \| NM\_001075700 \|  \| GO:0000166  GO:0005525  GO:0005622  GO:0007264  GO:0016020 \|  \| RAP2C \| Bt.26628.1.S1\_at \| \| Bt.13289.2.S1\_at \|  \|  \|  \|  \|  \|  \| ARID5B \| Bt.13289.2.S1\_at \| \| Bt.7395.1.S1\_at \| 616066 \| 1/2 \| NM\_001078058  XM\_001249618 \| XM\_001249618 \| GO:0006915 \| 16305752 \| DAP \| Bt.7395.1.S1\_at \| \| Bt.5040.1.S1\_at \| 281570 \| 1/1 \| NM\_174215 \|  \| GO:0005739  GO:0005746  GO:0006122  GO:0006810  GO:0008121  GO:0009055  GO:0016020  GO:0016021  GO:0031966  GO:0045285 \| 2995132  9161705  9204897  9651245  12477932 \| UQCR \| Bt.5040.1.S1\_at \| \| Bt.11059.1.S1\_at \| 534216 \| 2/6 \| NM\_001046374 \| NM\_001046374 \| GO:0000166  GO:0003723  GO:0005622  GO:0005634  GO:0007049  GO:0008270  GO:0045786  GO:0046872 \|  \| RBM5 \| Bt.11059.1.S1\_at \| \| Bt.13289.1.S1\_at \|  \|  \|  \|  \|  \|  \| ARID5B \| Bt.13289.1.S1\_at \| \| Bt.12412.1.S1\_at \| 515320 \| 1/2 \| NM\_001046196 \|  \| GO:0003677  GO:0005634  GO:0006350  GO:0006355  GO:0016055 \|  \| HBP1 \| Bt.12412.1.S1\_at \| \| Bt.11055.1.S1\_at \| 532333 \| 1/1 \| XM\_610845 \|  \|  \|  \| SDPR \| Bt.11055.1.S1\_at \| \| Bt.3126.1.A1\_at \| 407209 \| 2/2 \| NM\_001075145 \| NM\_001075145 \| GO:0005509  GO:0005515  GO:0005856  GO:0007156  GO:0016012  GO:0016020  GO:0016021 \| 14668210 \| SGCE \| Bt.3126.1.A1\_at \| \| Bt.4431.1.S1\_a\_at \| 327675 \| 1/2 \| NM\_175796 \|  \| GO:0000166  GO:0005515  GO:0005515  GO:0005524  GO:0005739  GO:0005743  GO:0006811  GO:0008553  GO:0015986  GO:0015992  GO:0016787  GO:0017111  GO:0045261  GO:0046872  GO:0046933  GO:0046961 \| 1827992  1833193  2458129  2864455  2896550  2898218  6223927  6298222  7397110  8065448  8790345  9687365  16751257 \| ATP5B \| Bt.4431.1.S1\_a\_at \| \| Bt.6832.1.S1\_at \| 505019 \| 1/3 \| XM\_871967 \|  \|  \|  \| SR140 \| Bt.6832.1.S1\_at \| \| Bt.10969.1.S1\_at \|  \|  \|  \|  \|  \|  \|  \| Bt.10969.1.S1\_at \| \| Bt.13588.1.A1\_at \| 533044 \| 2/2 \| XM\_612314 \| XM\_612314 \| GO:0003824  GO:0006564  GO:0008152  GO:0008483  GO:0030170 \|  \|  \| Bt.13588.1.A1\_at \| \| Bt.24376.1.S1\_at \| 523498 \| 1/2 \| XM\_601798 \|  \|  \|  \| MTSS1 \| Bt.24376.1.S1\_at \| \| Bt.12613.1.S1\_at \| 505884 \| 3/5 \| NM\_001035271 \|  \|  \| 12477932 \| KLF6 \| Bt.12613.1.S1\_at \| \| Bt.12453.1.A1\_at \|  \|  \|  \|  \|  \|  \| SOX4 \| Bt.12453.1.A1\_at \| \| Bt.28143.1.S1\_at \|  \|  \|  \|  \|  \|  \| CDC2L6 \| Bt.28143.1.S1\_at \| \| Bt.28618.1.S1\_at \| 508556 \| 1/2 \| XM\_585347 \|  \|  \|  \| FLJ35954 \| Bt.28618.1.S1\_at \| \| Bt.10543.1.S1\_at \|  \|  \|  \|  \|  \|  \|  \| Bt.10543.1.S1\_at \| \| Bt.27901.1.S1\_at \|  \|  \|  \| XR\_027926 \|  \|  \| MAGI1 \| Bt.27901.1.S1\_at \| \| Bt.11990.1.S1\_at \|  \|  \|  \|  \|  \|  \| C10orf45 \| Bt.11990.1.S1\_at \| \| Bt.4540.1.S1\_at \| 327668 \| 1/2 \| NM\_001075136 \| NM\_001075136 \| GO:0005515  GO:0005739  GO:0006811  GO:0015986  GO:0015992  GO:0016020  GO:0016469  GO:0016787  GO:0045261  GO:0046872  GO:0046933  GO:0046961 \| 1827992  2526651  2864455  7397110  8065448  8391483  8790345  9687365 \| ATP5C1 \| Bt.4540.1.S1\_at \| \| Bt.67.1.S1\_at \| 282289 \| 1/1 \| NM\_174564 \|  \| GO:0003954  GO:0005739  GO:0008137  GO:0016491 \| 1518044  12477932 \| NDUFC1 \| Bt.67.1.S1\_at \| \| Bt.4864.3.S1\_a\_at \| 532483 \| 2/3 \| NM\_001035389 \| NM\_001035389 \| GO:0005515  GO:0005634  GO:0007049  GO:0008270  GO:0045786  GO:0046872 \| 12477932 \| ING4 \| Bt.4864.3.S1\_a\_at \| \| Bt.832.1.S1\_at \| 281811 \| 1/1 \| NM\_174336 \|  \| GO:0003988  GO:0005739  GO:0006629  GO:0006631  GO:0008415  GO:0016740 \| 10417336  12140684  12477932  16305752  17143551 \| HADHB \| Bt.832.1.S1\_at \| \| Bt.3854.1.S1\_at \|  \|  \|  \|  \|  \|  \| SH3MD1 \| Bt.3854.1.S1\_at \| \| Bt.14095.2.S1\_at \|  \|  \|  \|  \|  \|  \|  \| Bt.14095.2.S1\_at \| \| Bt.21766.1.A1\_at \|  \|  \|  \|  \|  \|  \| MLF1 \| Bt.21766.1.A1\_at \| \| Bt.9857.1.S1\_at \| 616477 \| 1/1 \| NM\_001035475 \|  \|  \| 12140684  12477932  16305752 \| CHCHD3 \| Bt.9857.1.S1\_at \| \| Bt.12377.1.S1\_at \| 534293 \| 1/2 \| XM\_614012 \|  \|  \|  \| MCM3AP \| Bt.12377.1.S1\_at \| \| Bt.2988.1.S1\_at \| 511812 \| 1/1 \| NM\_001024523 \| NM\_001024523 \| GO:0004871  GO:0005834  GO:0007165  GO:0007186  GO:0016020 \| 12140684  12477932  16305752 \| GNG11 \| Bt.2988.1.S1\_at \| \| Bt.20512.1.S1\_at \|  \|  \|  \|  \|  \|  \| COL15A1 \| Bt.20512.1.S1\_at \| \| Bt.22130.1.S1\_at \| 515091 \| 1/1 \| NM\_001075698 \|  \| GO:0005488 \|  \| IFIT5 \| Bt.22130.1.S1\_at \| \| Bt.21009.1.S1\_at \| 532887 \| 2/3 \| XM\_612103 \|  \|  \|  \| PLEKHA5 \| Bt.21009.1.S1\_at \| \| Bt.23148.1.S1\_at \|  \|  \|  \|  \|  \|  \| COL5A1 \| Bt.23148.1.S1\_at \| \| Bt.4898.1.S1\_at \| 286842 \| 1/1 \| NM\_174780 \| NM\_174780 \| GO:0016020 \| 9310187  9749536 \| BASP1 \| Bt.4898.1.S1\_at \| \| Bt.9708.1.S1\_at \|  \|  \|  \|  \|  \|  \| CUTC \| Bt.9708.1.S1\_at \| \| Bt.14116.1.A1\_at \| 539299 \| 1/1 \| XM\_864909 \|  \|  \|  \| DNAPTP6 \| Bt.14116.1.A1\_at \| \| Bt.4775.1.S1\_at \| 525716 \| 1/1 \| NM\_001034555 \| NM\_001034555 \|  \| 710399  2039467  3722149  4026304  6615778  7250124  8805590  11264299  12477932  14661952 \| S100B \| Bt.4775.1.S1\_at \| \| Bt.5345.1.S1\_at \| 535182 \| 1/1 \| NM\_001034628 \| NM\_001034628 \| GO:0004459  GO:0006096  GO:0006099  GO:0006108  GO:0016491  GO:0030060 \| 12477932 \| MDH1 \| Bt.5345.1.S1\_at \| \| Bt.15968.1.S1\_at \| 539139 \| 1/1 \| XM\_873620 \|  \|  \|  \| KLF9 \| Bt.15968.1.S1\_at \| \| Bt.11196.1.S1\_at \| 281816 \| 1/1 \| NM\_174340 \|  \| GO:0005739  GO:0016787 \|  \| HINT2 \| Bt.11196.1.S1\_at \| \| Bt.9731.1.S1\_at \| 513762 \| 1/2 \| NM\_001040523 \|  \| GO:0006118  GO:0006810  GO:0009055  GO:0015035  GO:0045454 \|  \| GLRX2 \| Bt.9731.1.S1\_at \| \| Bt.27657.1.A1\_at \| 338053 \| 2/2 \| NM\_176649 \|  \| GO:0005739  GO:0006811  GO:0008289  GO:0015986  GO:0015992  GO:0016020  GO:0016469  GO:0045263  GO:0046933  GO:0046961 \| 1827992  1829867  2868890  12477932 \|  \| Bt.27657.1.A1\_at \| \| Bt.20494.1.S1\_at \| 616217 \| 1/2 \| XM\_868187 \| XM\_868187 \| GO:0005622  GO:0008270 \|  \| RYBP \| Bt.20494.1.S1\_at \| \| Bt.23418.1.S1\_at \| 280794 \| 1/3 \| XM\_001252030  XM\_864390  XM\_873485  XM\_873679  XM\_873775  XM\_873966  XM\_874054  XM\_874147 \| XM\_864390 \|  \| 3780752  6218503  6304699  10972305  12658628  12933807  16168584 \| FN1 \| Bt.23418.1.S1\_at \| \| Bt.15267.1.A1\_at \| 510322 \| 1/1 \| NM\_001046051 \|  \|  \|  \| PRPF3 \| Bt.15267.1.A1\_at \| \| Bt.20316.1.S1\_at \|  \|  \|  \| XM\_001249467 \|  \|  \| ANKRD23 \| Bt.20316.1.S1\_at \| \| Bt.7540.2.S1\_a\_at \| 327703 \| 1/2 \| NM\_175819 \| NM\_175819 \| GO:0005245  GO:0005509  GO:0006811  GO:0006816 \| 10684870  11282978 \| CACNB1 \| Bt.7540.2.S1\_a\_at \| \| Bt.25038.1.S1\_at \| 520974 \| 1/1 \| XM\_599229 \| XM\_599229 \|  \|  \| MED12 \| Bt.25038.1.S1\_at \| \| Bt.4503.1.S1\_at \| 337927 \| 1/1 \| NM\_176634 \|  \| GO:0005488  GO:0005739  GO:0006810  GO:0016020  GO:0016021 \| 11282978  16305752 \| MTCH2 \| Bt.4503.1.S1\_at \| \| Bt.63.1.S1\_at \| 327670 \| 1/1 \| NM\_175791 \| NM\_175791 \| GO:0003954  GO:0005739  GO:0006979  GO:0008137  GO:0016491  GO:0031966 \| 1518044  12477932  12857734 \| NDUFA6 \| Bt.63.1.S1\_at \| \| Bt.15643.1.A1\_at \|  \|  \|  \|  \|  \|  \| RC3H1 \| Bt.15643.1.A1\_at \| \| Bt.5047.1.S1\_at \| 533227 \| 1/1 \| NM\_001075985 \| NM\_001075985 \|  \|  \|  \| Bt.5047.1.S1\_at \| \| Bt.24211.1.A1\_at \| 507990 \| 2/2 \| NM\_001034309 \|  \|  \| 12477932 \| ASPN \| Bt.24211.1.A1\_at \| \| Bt.61.1.S1\_at \| 338073 \| 1/1 \| NM\_176667 \|  \| GO:0003954  GO:0005739  GO:0005743  GO:0006118  GO:0008137  GO:0016491 \| 1518044 \| NDUFB3 \| Bt.61.1.S1\_at \| \| Bt.16314.1.S1\_at \| 505123 \| 1/2 \| XM\_581363 \|  \|  \|  \| CHD1 \| Bt.16314.1.S1\_at \| \| Bt.18352.1.A1\_at \| 504718 \| 1/1 \| XM\_580882 \|  \|  \|  \| GADD45GIP1 \| Bt.18352.1.A1\_at \| \| Bt.7636.1.S1\_at \|  \|  \|  \|  \|  \|  \| ZNF436 \| Bt.7636.1.S1\_at \| \| Bt.28081.1.S1\_at \| 535254 \| 1/4 \| XM\_870012  XM\_883122 \| XM\_883122 \| GO:0003677  GO:0005622  GO:0005634  GO:0008270  GO:0045449 \|  \| POGZ \| Bt.28081.1.S1\_at \| \| Bt.22369.2.S1\_at \|  \|  \|  \|  \|  \|  \|  \| Bt.22369.2.S1\_at \| \| Bt.14624.1.A1\_at \| 535683 \| 2/2 \| NM\_001076067 \|  \| GO:0005829  GO:0043234 \|  \| PSMD14 \| Bt.14624.1.A1\_at \| \| Bt.2873.1.S1\_at \| 613802 \| 1/1 \| NM\_001035437 \| NM\_001035437 \| GO:0016020  GO:0016021 \| 12477932 \| MGC126931 \| Bt.2873.1.S1\_at \| \| Bt.13349.1.S1\_at \| 613774 \| 1/1 \| XM\_864869 \| XM\_864869 \|  \|  \|  \| Bt.13349.1.S1\_at \| \| Bt.16329.1.A1\_at \| 510050 \| 1/4 \| XM\_587139 \|  \|  \| 12477932 \| TGIF \| Bt.16329.1.A1\_at \| \| Bt.13610.1.A1\_at \| 534185 \| 1/1 \| NM\_001076015 \|  \| GO:0005739  GO:0030529 \| 11279123 \| MRPS31 \| Bt.13610.1.A1\_at \| \| Bt.1366.1.S1\_at \| 515783 \| 1/1 \| NM\_001046212 \| NM\_001046212 \| GO:0001501  GO:0005529 \|  \| CLEC3B \| Bt.1366.1.S1\_at \| \| Bt.400.1.S1\_at \| 327674 \| 1/1 \| NM\_175795 \| NM\_175795 \| GO:0004129  GO:0005739  GO:0005746  GO:0006118  GO:0016020  GO:0016021  GO:0016491 \| 2550462  2844245  8638158  10089392  10771420 \| COX7B \| Bt.400.1.S1\_at \| \| Bt.7655.1.S1\_at \| 533297 \| 1/1 \| XM\_612661 \| XM\_612661 \| GO:0003677  GO:0006355 \|  \| CSDA \| Bt.7655.1.S1\_at \| \| Bt.1667.1.S1\_at \| 616156 \| 1/1 \| XM\_868121 \| XM\_868121 \| GO:0006464  GO:0006512  GO:0019787 \|  \| CDC34 \| Bt.1667.1.S1\_at \| \| Bt.1552.1.S1\_at \| 281476 \| 1/1 \| NM\_174175 \|  \|  \| 12477932  15213404 \| SARS \| Bt.1552.1.S1\_at \| \| Bt.21151.1.S1\_at \| 532203 \| 1/2 \| NM\_001034576 \| NM\_001034576 \| GO:0003735  GO:0005622  GO:0005739  GO:0005840  GO:0006412 \| 12477932  16510155 \| MRPL24 \| Bt.21151.1.S1\_at \| \| Bt.15714.1.S1\_at \| 540194 \| 2/2 \| NM\_001024571 \| NM\_001024571 \|  \| 11282978  16305752 \| FLJ20436 \| Bt.15714.1.S1\_at \| \| Bt.10111.1.S1\_at \|  \|  \|  \|  \|  \|  \| C1orf24 \| Bt.10111.1.S1\_at \| \| Bt.13128.1.S1\_at \| 286840 \| 1/1 \| NM\_001040483 \|  \| GO:0005739  GO:0005743  GO:0006118  GO:0009055  GO:0016020  GO:0051536 \| 16751257 \| SDHB \| Bt.13128.1.S1\_at \| \| Bt.11240.1.A1\_at \| 538481 \| 1/2 \| XR\_028158 \|  \|  \|  \| FLJ20032 \| Bt.11240.1.A1\_at \| \| Bt.9966.1.S1\_at \| 538590 \| 2/2 \| XR\_028495 \| XM\_001251070 \|  \|  \| COL5A2 \| Bt.9966.1.S1\_at \| \| Bt.21877.1.S1\_at \| 521244 \| 3/3 \|  \|  \|  \|  \|  \| Bt.21877.1.S1\_at \| \| Bt.12916.1.S1\_at \| 281210 \| 1/1 \| NM\_174077 \|  \| GO:0004602  GO:0005615  GO:0006982  GO:0008134  GO:0008430  GO:0016491  GO:0042744  GO:0051289 \| 8262911 \| GPX3 \| Bt.12916.1.S1\_at \| \| Bt.11446.1.A1\_at \| 617976 \| 1/1 \| NM\_001046611 \| NM\_001046611 \|  \|  \| LDB2 \| Bt.11446.1.A1\_at \| \| Bt.7595.1.S1\_at \| 360006 \| 1/1 \| NM\_001040491 \| NM\_001040491 \| GO:0006457  GO:0031072  GO:0051082 \| 11282978  14555765 \| DNAJA2 \| Bt.7595.1.S1\_at \| \| Bt.5153.1.S1\_at \| 507537 \| 1/1 \| NM\_001034302 \| NM\_001034302 \|  \| 12477932 \| SPARCL1 \| Bt.5153.1.S1\_at \| \| Bt.5176.2.A1\_at \| 538533 \| 2/5 \| NM\_001076109 \| NM\_001076109 \| GO:0000166  GO:0003676 \|  \| TIA1 \| Bt.5176.2.A1\_at \| \| Bt.27192.1.S1\_at \| 541077 \| 1/1 \| NM\_001076254 \| NM\_001076254 \| GO:0005515  GO:0005737  GO:0006415  GO:0016149 \|  \| ETF1 \| Bt.27192.1.S1\_at \| \| Bt.12217.1.S1\_at \|  \|  \|  \|  \|  \|  \| NTRK2 \| Bt.12217.1.S1\_at \| \| Bt.24550.1.S1\_at \| 514949 \| 1/2 \| XR\_027975 \| XR\_027975 \|  \|  \| COMTD1 \| Bt.24550.1.S1\_at \| \| Bt.20064.1.S1\_at \|  \|  \|  \| XM\_594245 \|  \|  \| SDCCAG33 \| Bt.20064.1.S1\_at \| \| Bt.13483.1.A1\_at \| 505430 \| 1/1 \| XM\_001252143  XM\_581717 \|  \|  \|  \| ATPAF1 \| Bt.13483.1.A1\_at \| \| Bt.5223.1.S1\_at \| 281117 \| 1/2 \| NM\_174037 \| NM\_174037 \|  \| 10543989  12568810  14668210 \| DLK1 \| Bt.5223.1.S1\_at \| \| Bt.1311.1.S1\_at \| 511090 \| 1/1 \| NM\_001075493 \|  \| GO:0005524  GO:0008152  GO:0016874 \|  \| SUCLA2 \| Bt.1311.1.S1\_at \| \| Bt.27121.1.S1\_at \| 509192 \| 1/1 \| NM\_001046026 \|  \|  \|  \| DULLARD \| Bt.27121.1.S1\_at \| \| Bt.26718.1.S1\_a\_at \| 506250 \| 1/1 \| XM\_582675 \| XM\_582675 \|  \|  \| HCCS \| Bt.26718.1.S1\_a\_at \| \| Bt.1670.1.A1\_at \|  \|  \|  \| XR\_028756 \|  \|  \|  \| Bt.1670.1.A1\_at \| \| Bt.3260.1.S1\_at \| 614633 \| 1/1 \| NM\_001035453 \|  \| GO:0005739  GO:0006605  GO:0015031  GO:0016020  GO:0016021 \| 12477932 \| C18orf55 \| Bt.3260.1.S1\_at \| \| Bt.8829.1.S1\_a\_at \| 536731 \| 1/2 \| XM\_875299 \|  \| GO:0008270 \| 12140684  16305752 \| WDR10 \| Bt.8829.1.S1\_a\_at \| \| Bt.6247.1.S1\_at \|  \|  \|  \|  \|  \|  \| IGF1R \| Bt.6247.1.S1\_at \| \| Bt.1197.1.A1\_at \|  \|  \|  \|  \|  \|  \| S100PBPR \| Bt.1197.1.A1\_at \| \| Bt.15724.1.S1\_at \| 506149 \| 1/1 \| NM\_001045937 \|  \|  \|  \|  \| Bt.15724.1.S1\_at \| \| Bt.11216.1.S1\_at \| 280695 \| 1/1 \| NM\_173881 \| NM\_173881 \| GO:0001666  GO:0005344  GO:0005506  GO:0006810  GO:0015671  GO:0019825  GO:0020037  GO:0043353  GO:0046872 \| 2659586  5477290  12477932  14966674 \| MB \| Bt.11216.1.S1\_at \| \| Bt.4550.1.A1\_at \| 507801 \| 1/2 \| XM\_864599 \| XM\_864599 \|  \|  \| SFRS12 \| Bt.4550.1.A1\_at \| \| Bt.15857.1.S1\_at \| 534286 \| 1/1 \| XM\_866156 \| XM\_866156 \|  \|  \| ALAS1 \| Bt.15857.1.S1\_at \| \| Bt.14566.1.A1\_at \|  \|  \|  \| XM\_582713 \|  \|  \| ICK \| Bt.14566.1.A1\_at \| \| Bt.6473.1.S1\_at \| 505050 \| 1/1 \| NM\_001034233 \| NM\_001034233 \|  \| 12477932 \| PSMA7 \| Bt.6473.1.S1\_at \| \| Bt.5605.1.S1\_at \|  \|  \|  \|  \|  \|  \| MARCKS \| Bt.5605.1.S1\_at \| \| Bt.4819.1.S1\_at \| 327704 \| 1/2 \| NM\_175820 \|  \| GO:0003954  GO:0005739  GO:0005743  GO:0008137  GO:0016491 \| 1518044  12477932 \| NDUFA4 \| Bt.4819.1.S1\_at \| \| Bt.4563.1.S1\_at \| 514455 \| 1/2 \| XR\_028518 \|  \|  \|  \| TM9SF2 \| Bt.4563.1.S1\_at \| \| Bt.24616.1.S1\_at \|  \|  \|  \|  \|  \|  \| ABI2 \| Bt.24616.1.S1\_at \| \| Bt.4506.1.S1\_at \| 281935 \| 1/1 \| NM\_174394 \| NM\_174394 \| GO:0000166  GO:0003774  GO:0003779  GO:0005524  GO:0005856  GO:0007165  GO:0016459 \| 10984435  15705568  15961399  16030012 \| MYO10 \| Bt.4506.1.S1\_at \| \| Bt.15726.1.S1\_at \| 541239 \| 1/2 \| XM\_615238 \|  \|  \|  \| C10orf6 \| Bt.15726.1.S1\_at \| \| Bt.2524.1.S1\_at \| 281479 \| 1/1 \|  \|  \|  \| 10556424  10984454 \| CXCL12 \| Bt.2524.1.S1\_at \| \| Bt.5546.1.S1\_at \| 281790 \| 1/1 \| NM\_174324 \|  \| GO:0000166  GO:0004871  GO:0005515  GO:0005525  GO:0007165  GO:0007186 \| 2419165  3094012  10343125 \| GNAI1 \| Bt.5546.1.S1\_at \| \| Bt.3218.1.S1\_at \| 515093 \| 1/2 \|  \|  \|  \|  \| KIAA0323 \| Bt.3218.1.S1\_at \| \| Bt.9708.2.S1\_at \| 535065 \| 1/1 \| XM\_001251030  XM\_615049 \|  \|  \|  \| CUTC \| Bt.9708.2.S1\_at \| \| Bt.8679.3.S1\_at \| 539637 \| 1/1 \|  \| XM\_590291 \|  \|  \| DLAT \| Bt.8679.3.S1\_at \| \| Bt.10096.1.S1\_at \|  \|  \|  \|  \|  \|  \| NCALD \| Bt.10096.1.S1\_at \| \| Bt.28651.1.S1\_at \| 538697 \| 1/1 \| XM\_582091 \| XM\_582091 \| GO:0005622  GO:0007165 \|  \| STARD13 \| Bt.28651.1.S1\_at \| \| Bt.1844.1.S1\_at \| 613349 \| 4/4 \| XM\_864015 \|  \|  \|  \| TCTE1L \| Bt.1844.1.S1\_at \| \| Bt.1237.1.S1\_at \| 540801 \| 1/2 \|  \|  \|  \|  \|  \| Bt.1237.1.S1\_at \| \| Bt.9797.1.S1\_at \| 534546 \| 1/2 \| NM\_001034613 \| XM\_001252378 \| GO:0004240  GO:0005739  GO:0006508  GO:0008270  GO:0046872 \| 12477932 \| PMPCB \| Bt.9797.1.S1\_at \| \| Bt.5605.2.S1\_at \|  \|  \|  \|  \|  \|  \| MARCKS \| Bt.5605.2.S1\_at \| \| Bt.24039.1.A1\_at \| 522998 \| 1/1 \| XR\_028333 \|  \|  \|  \| CCDC6 \| Bt.24039.1.A1\_at \| \| Bt.26878.1.S1\_at \| 539344 \| 1/1 \| XM\_586545 \| XM\_586545 \| GO:0007242 \|  \| ASB12 \| Bt.26878.1.S1\_at \| \| Bt.2527.1.S1\_at \| 539203 \| 1/2 \| XR\_027865 \|  \|  \|  \|  \| Bt.2527.1.S1\_at \| \| Bt.21471.1.S1\_at \| 538604 \| 1/1 \| XM\_581418 \|  \|  \| 11282978  16305752 \| ABI3BP \| Bt.21471.1.S1\_at \| \| Bt.8351.1.S1\_at \| 504867 \| 1/2 \| NM\_001034226 \| NM\_001034226 \|  \| 12477932 \| HSPC138 \| Bt.8351.1.S1\_at \| \| Bt.19218.1.A1\_at \|  \|  \|  \|  \|  \|  \| CNOT6 \| Bt.19218.1.A1\_at \| \| Bt.66.1.S1\_at \| 338064 \| 1/1 \| NM\_176659 \| NM\_176659 \| GO:0003954  GO:0005739  GO:0008137  GO:0016020  GO:0016021  GO:0016491 \| 1518044  7958365  12477932 \| NDUFA3 \| Bt.66.1.S1\_at \| \| Bt.4985.1.S1\_a\_at \| 508825 \| 2/3 \| NM\_001077884  XM\_864930 \|  \|  \|  \|  \| Bt.4985.1.S1\_a\_at \| \| Bt.4224.1.S1\_at \| 280715 \| 1/1 \| NM\_001013582 \|  \| GO:0000166  GO:0004017  GO:0005524  GO:0005737  GO:0005739  GO:0005886  GO:0006139  GO:0007050  GO:0016301  GO:0016740  GO:0046034 \| 6089869  11282978  16305752 \| AK1 \| Bt.4224.1.S1\_at \| \| Bt.26577.1.A1\_at \|  \|  \|  \|  \|  \|  \| G3BP2 \| Bt.26577.1.A1\_at \| \| Bt.2077.1.S1\_at \|  \|  \|  \| XM\_001256472 \|  \|  \| SCRIB \| Bt.2077.1.S1\_at \| \| Bt.543.1.S1\_at \| 327684 \| 1/1 \| NM\_175804 \|  \| GO:0001764  GO:0003700  GO:0003707  GO:0005634  GO:0006350  GO:0006355  GO:0008270  GO:0030900  GO:0043565  GO:0046872 \|  \| NR2F1 \| Bt.543.1.S1\_at \| \| Bt.5078.1.S1\_at \| 514372 \| 1/1 \| NM\_001075665 \| NM\_001075665 \|  \|  \| MAGED2 \| Bt.5078.1.S1\_at \| \| Bt.22054.2.S1\_a\_at \| 522230 \| 2/2 \| XM\_600505 \| XM\_600505 \|  \|  \| C20orf7 \| Bt.22054.2.S1\_a\_at \| \| Bt.21020.1.S1\_at \|  \|  \|  \| XM\_611981 \|  \|  \| C1orf96 \| Bt.21020.1.S1\_at \| \| Bt.8046.1.A1\_at \| 613835 \| 1/2 \| NM\_001035438 \|  \| GO:0005739  GO:0005763  GO:0030529 \| 11279123  12477932 \| MRPS36 \| Bt.8046.1.A1\_at \| \| Bt.24413.1.S1\_at \|  \|  \|  \|  \|  \|  \| DKFZp762A217 \| Bt.24413.1.S1\_at \| \| Bt.26130.1.A1\_at \|  \|  \|  \|  \|  \|  \| USP2 \| Bt.26130.1.A1\_at \| \| Bt.1332.1.S1\_a\_at \| 511440 \| 1/2 \| XM\_864678 \|  \| GO:0004659  GO:0006783  GO:0008495  GO:0016021 \|  \| COX10 \| Bt.1332.1.S1\_a\_at \| \| Bt.4875.2.S1\_at \| 615352 \| 1/1 \| XM\_867138 \| XM\_867138 \| GO:0005488 \|  \| FANCG \| Bt.4875.2.S1\_at \| \| Bt.9956.1.S1\_at \| 404159 \| 1/1 \| XM\_593976 \| XM\_593976 \| GO:0045454 \|  \| TXNL5 \| Bt.9956.1.S1\_at \| \| Bt.20046.1.S1\_a\_at \| 539785 \| 1/2 \| XM\_589446 \| XM\_589446 \| GO:0000166  GO:0003676 \|  \| SAFB \| Bt.20046.1.S1\_a\_at \| \| Bt.1517.1.S1\_at \| 617809 \| 1/1 \| NM\_001078082  XM\_001255292 \| NM\_001078082 \|  \|  \| BCL2L13 \| Bt.1517.1.S1\_at \| \| Bt.23509.1.S1\_at \| 617707 \| 1/1 \| NM\_001076473 \|  \|  \|  \| PPAP2B \| Bt.23509.1.S1\_at \| \| Bt.24305.1.A1\_at \|  \|  \|  \|  \|  \|  \| PCGF5 \| Bt.24305.1.A1\_at \| \| Bt.4705.1.S1\_at \| 327953 \| 1/1 \| NM\_175832 \|  \| GO:0008083  GO:0008201 \|  \| HDGF \| Bt.4705.1.S1\_at \| \| Bt.5418.1.S1\_at \| 541101 \| 1/1 \| NM\_001076255 \|  \| GO:0003924  GO:0005525  GO:0005874  GO:0007017  GO:0043234  GO:0051258 \|  \|  \| Bt.5418.1.S1\_at \| \| Bt.746.1.S1\_at \| 399683 \| 1/1 \| NM\_203363 \| NM\_203363 \| GO:0016020  GO:0016021 \| 12477932 \| PLP2 \| Bt.746.1.S1\_at \| \| Bt.11242.1.S1\_at \| 767977 \| 1/1 \| NM\_001077042 \|  \|  \|  \| BRP44L \| Bt.11242.1.S1\_at \| \| Bt.5108.1.S1\_at \| 515902 \| 1/1 \| NM\_001038548 \|  \| GO:0004658  GO:0005739  GO:0016874 \|  \| PCCB \| Bt.5108.1.S1\_at \| \| Bt.58.1.S1\_at \| 327680 \| 1/1 \| NM\_175800 \|  \| GO:0003954  GO:0005739  GO:0005747  GO:0006118  GO:0006800  GO:0007420  GO:0008137  GO:0008137  GO:0019933  GO:0032981  GO:0045333  GO:0048146  GO:0051591 \| 1518044  8603710  12477932 \| NDUFS4 \| Bt.58.1.S1\_at \| \| Bt.4205.1.S1\_at \| 286809 \| 1/1 \| NM\_174770 \| NM\_174770 \| GO:0004602  GO:0005739  GO:0006979  GO:0007275  GO:0008430  GO:0016491  GO:0047066 \| 12477932  12658628  15746255 \| GPX4 \| Bt.4205.1.S1\_at \| \| Bt.25390.1.A1\_at \| 613877 \| 1/1 \| XM\_876237 \|  \|  \|  \| MEIS1 \| Bt.25390.1.A1\_at \| \| Bt.4606.2.S1\_at \| 505884 \| 3/5 \| NM\_001035271 \| NM\_001035271 \|  \| 12477932 \| KLF6 \| Bt.4606.2.S1\_at \| \| Bt.24892.1.A1\_at \|  \|  \|  \| XM\_613117 \|  \|  \| RIT1 \| Bt.24892.1.A1\_at \| \| Bt.8486.1.S1\_at \| 539627 \| 2/3 \| XM\_588438 \| XM\_588438 \|  \|  \| TNIK \| Bt.8486.1.S1\_at \| \| Bt.5848.1.S1\_at \| 518117 \| 2/2 \| NM\_001075767 \|  \| GO:0000166  GO:0000287  GO:0005388  GO:0005509  GO:0005515  GO:0005524  GO:0006812  GO:0006816  GO:0008152  GO:0015992  GO:0016020  GO:0016021  GO:0016529  GO:0016787  GO:0016820  GO:0031448 \|  \| ATP2A1 \| Bt.5848.1.S1\_at \| \| Bt.25338.1.S1\_at \|  \|  \|  \|  \|  \|  \| BTBD7 \| Bt.25338.1.S1\_at \| \| Bt.3989.1.S1\_at \|  \|  \|  \|  \|  \|  \| EMP2 \| Bt.3989.1.S1\_at \| \| Bt.25072.1.S1\_at \| 615964 \| 1/1 \| NM\_001046566 \| NM\_001046566 \| GO:0005506  GO:0016226  GO:0051536 \|  \| HIRIP5 \| Bt.25072.1.S1\_at \| \| Bt.9169.1.A1\_at \|  \|  \|  \| XM\_867242 \|  \|  \| NOTCH2 \| Bt.9169.1.A1\_at \| \| Bt.24848.1.A1\_at \|  \|  \|  \|  \|  \|  \| PTPRD \| Bt.24848.1.A1\_at \| \| Bt.8001.1.S1\_at \| 506676 \| 1/1 \| NM\_001037588 \| NM\_001037588 \|  \|  \|  \| Bt.8001.1.S1\_at \| \| Bt.1426.1.S1\_at \| 338081 \| 1/1 \| NM\_176670 \| NM\_176670 \| GO:0005739  GO:0005740  GO:0005743  GO:0006811  GO:0015986  GO:0015992  GO:0016469  GO:0016787  GO:0045261  GO:0046872  GO:0046933  GO:0046961 \| 1827992  2138455  2864455  8065448 \| ATP5D \| Bt.1426.1.S1\_at \| \| Bt.13170.1.S1\_at \| 327696 \| 1/1 \| NM\_175814 \| NM\_175814 \| GO:0000104  GO:0005506  GO:0005739  GO:0006099  GO:0006118  GO:0006810  GO:0016020  GO:0016021  GO:0046872 \| 1447196  7890754  7947903  11282978  12477932  16305752  16717460 \| SDHC \| Bt.13170.1.S1\_at \| \| Bt.21383.2.S1\_a\_at \| 614973 \| 2/2 \| XM\_878838 \|  \|  \|  \|  \| Bt.21383.2.S1\_a\_at \| \| Bt.24236.1.S1\_at \| 511433 \| 1/1 \| XR\_027491 \| XR\_027491 \|  \|  \| DLC1 \| Bt.24236.1.S1\_at \| \| Bt.21969.1.A1\_at \| 517876 \| 1/4 \| XM\_596055 \| XM\_596055 \| GO:0003677  GO:0005622  GO:0008270 \|  \| ARID2 \| Bt.21969.1.A1\_at \| \| Bt.19097.1.A1\_at \|  \|  \|  \|  \|  \|  \| IGF1 \| Bt.19097.1.A1\_at \| \| Bt.10215.1.A1\_at \|  \|  \|  \|  \|  \|  \| RAB10 \| Bt.10215.1.A1\_at \| \| Bt.2493.1.S1\_at \| 513622 \| 1/1 \| XM\_591331 \|  \| GO:0003735  GO:0005739  GO:0005762  GO:0006412  GO:0030529 \| 11402041  11551941 \| MRPL51 \| Bt.2493.1.S1\_at \| \| Bt.22165.1.S1\_at \| 615436 \| 1/1 \| NM\_001076375 \| NM\_001076375 \|  \|  \| KIAA1914 \| Bt.22165.1.S1\_at \| \| Bt.6549.1.S1\_at \|  \|  \|  \|  \|  \|  \| IRF2 \| Bt.6549.1.S1\_at \| \| Bt.7237.2.S1\_a\_at \| 281810 \| 1/1 \| NM\_174335 \| NM\_174335 \| GO:0003857  GO:0004300  GO:0005739  GO:0006631  GO:0006635  GO:0008152  GO:0016491  GO:0016507  GO:0050662 \| 10417336 \| HADHA \| Bt.7237.2.S1\_a\_at \| \| Bt.4886.1.A1\_at \| 777690 \| 1/1 \| NM\_001078150  XM\_001250633 \| NM\_001078150 \| GO:0005515 \| 12140684  16305752 \| LRRC17 \| Bt.4886.1.A1\_at \| \| Bt.28676.1.A1\_at \| 533077 \| 1/2 \| NM\_001076890 \| NM\_001076890 \| GO:0004872 \|  \| PEX7 \| Bt.28676.1.A1\_at \| \| Bt.1950.1.S1\_at \| 540719 \| 2/2 \| XM\_612814 \| XM\_612814 \|  \|  \| KPNA3 \| Bt.1950.1.S1\_at \| \| Bt.16020.1.A2\_at \|  \|  \|  \|  \|  \|  \| ACVR1 \| Bt.16020.1.A2\_at \| \| Bt.9585.1.S1\_at \| 510679 \| 1/1 \| NM\_001014895 \| NM\_001014895 \| GO:0003824  GO:0004655  GO:0008152  GO:0033014  GO:0046872 \| 11282978 \| ALAD \| Bt.9585.1.S1\_at \| \| Bt.7478.1.S1\_at \| 534551 \| 1/1 \| NM\_001076027 \| NM\_001076027 \| GO:0005515 \|  \| HSPB6 \| Bt.7478.1.S1\_at \| \| Bt.10259.1.A1\_at \| 539449 \| 1/1 \| XM\_587229 \| XM\_587229 \|  \|  \| PLK2 \| Bt.10259.1.A1\_at \| \| Bt.5074.1.S1\_at \|  \|  \|  \|  \|  \|  \| SFPQ \| Bt.5074.1.S1\_at \| \| Bt.14095.1.S1\_at \|  \|  \|  \|  \|  \|  \|  \| Bt.14095.1.S1\_at \| \| Bt.1613.1.S1\_at \| 282326 \| 1/1 \| XM\_612097 \| XM\_612097 \| GO:0004252  GO:0005515  GO:0006508  GO:0008233 \| 9852107 \| HTRA1 \| Bt.1613.1.S1\_at \| \| Bt.1699.3.S1\_a\_at \| 767928 \| 2/2 \| NM\_001077001 \| NM\_001077001 \| GO:0001525  GO:0001837  GO:0003677  GO:0005515  GO:0005634  GO:0006350  GO:0006357  GO:0007219  GO:0007507  GO:0008284  GO:0030097  GO:0030183  GO:0042742 \|  \|  \| Bt.1699.3.S1\_a\_at \| \| Bt.26829.1.S1\_at \| 540985 \| 1/1 \| XM\_613987 \| XM\_613987 \| GO:0004470  GO:0006108  GO:0016616  GO:0051287 \|  \| ME1 \| Bt.26829.1.S1\_at \| \| Bt.3821.2.S1\_at \| 514741 \| 2/2 \| NM\_001038109 \| NM\_001038109 \| GO:0016020  GO:0016021 \|  \| TSPAN6 \| Bt.3821.2.S1\_at \| \| Bt.14853.1.A1\_at \| 533284 \| 1/1 \| NM\_001046347 \|  \|  \|  \| RAB3IP \| Bt.14853.1.A1\_at \| \| Bt.6397.2.S1\_at \| 540444  618297 \| 1/1 \| NM\_001037616  XM\_870627 \| NM\_001037616 \|  \|  \| HMGB2 \| Bt.6397.2.S1\_at \| \| Bt.5378.1.S1\_at \| 282517 \| 1/1 \| NM\_174667 \| NM\_174667 \| GO:0003954  GO:0005739  GO:0006118  GO:0008137  GO:0016491 \| 1518044  12477932 \| NDUFB8 \| Bt.5378.1.S1\_at \| \| Bt.1789.1.S1\_at \|  \|  \|  \|  \|  \|  \| TGFBR2 \| Bt.1789.1.S1\_at \| \| Bt.20431.2.S1\_at \| 614509 \| 2/4 \| NM\_001076331 \| NM\_001076331 \| GO:0005634  GO:0030528  GO:0045449 \|  \| MXI1 \| Bt.20431.2.S1\_at \| \| Bt.1767.1.S1\_at \| 281225 \| 1/1 \| NM\_174080 \| NM\_174080 \| GO:0004842  GO:0006464  GO:0006512  GO:0016874 \| 1322903  1714895  9657692  15723079 \| HIP2 \| Bt.1767.1.S1\_at \| \| Bt.25202.1.A1\_at \| 534295 \| 3/3 \| XM\_614016 \|  \| GO:0004190  GO:0006508 \|  \| KIAA1109 \| Bt.25202.1.A1\_at \| \| Bt.5664.1.S1\_at \|  \|  \|  \|  \|  \|  \|  \| Bt.5664.1.S1\_at \| \| Bt.20.1.S1\_at \| 338063 \| 1/2 \| NM\_176658 \| NM\_176658 \| GO:0003954  GO:0005739  GO:0005739  GO:0005743  GO:0005743  GO:0006120  GO:0008137  GO:0016491  GO:0016491  GO:0042773  GO:0042773 \| 1426273  12644575  12837546 \| NDUFA7 \| Bt.20.1.S1\_at \| \| Bt.23176.2.S1\_a\_at \| 281240 \| 1/4 \| NM\_174087 \| NM\_174087 \| GO:0000074  GO:0005179  GO:0005576  GO:0008083  GO:0008283 \| 1280544  2302223  2388846  3390164  3941093  12112582  12658628  12873229  16120826  16644179 \| IGF2 \| Bt.23176.2.S1\_a\_at \| \| Bt.20228.1.S1\_at \| 359720 \| 1/1 \| NM\_182656 \| NM\_182656 \| GO:0005783 \| 12832288 \| RTN2 \| Bt.20228.1.S1\_at \| \| Bt.5406.1.S1\_at \|  \|  \|  \| XM\_001252217 \|  \|  \| RAB6IP1 \| Bt.5406.1.S1\_at \| \| Bt.23526.1.S1\_at \| 617466 \| 1/1 \| NM\_001046601 \| NM\_001046601 \|  \| 10938081  11402041 \| MRPS24 \| Bt.23526.1.S1\_at \| \| Bt.4203.1.S1\_at \| 506492 \| 1/1 \| BC108208 \|  \|  \| 8011660 \|  \| Bt.4203.1.S1\_at \| \| Bt.12126.1.S1\_at \| 511783 \| 1/1 \| XM\_589182 \| XM\_589182 \| GO:0005516  GO:0005977 \|  \| PHKB \| Bt.12126.1.S1\_at \| \| Bt.23603.1.S1\_a\_at \| 511798 \| 3/3 \| NM\_001034412 \| NM\_001034412 \| GO:0006512 \| 12477932 \| FBXO9 \| Bt.23603.1.S1\_a\_at \| \| Bt.15742.1.S2\_at \|  \|  \|  \|  \|  \|  \| CD47 \| Bt.15742.1.S2\_at \| \| Bt.4792.1.S1\_at \| 509725 \| 1/2 \| NM\_001046036 \| NM\_001046036 \|  \|  \| KIF2 \| Bt.4792.1.S1\_at \| \| Bt.22451.1.S1\_at \| 532337 \| 1/1 \| XM\_610849 \|  \|  \|  \| GSPT1 \| Bt.22451.1.S1\_at \| \| Bt.17526.1.A1\_at \|  \|  \|  \|  \|  \|  \|  \| Bt.17526.1.A1\_at \| \| Bt.11192.1.S1\_at \| 534576 \| 1/1 \| XM\_875610 \|  \|  \|  \| PABPC4 \| Bt.11192.1.S1\_at \| \| Bt.3227.1.S1\_at \| 505503 \| 1/1 \| NM\_001015524 \| NM\_001015524 \|  \| 11282978 \| ASCC2 \| Bt.3227.1.S1\_at \| \| Bt.20854.1.S1\_at \| 512902 \| 2/2 \| NM\_001075587 \|  \| GO:0008415  GO:0016740 \|  \|  \| Bt.20854.1.S1\_at \| \| Bt.23081.1.S1\_a\_at \| 540340 \| 2/3 \| XM\_593330 \|  \|  \|  \|  \| Bt.23081.1.S1\_a\_at \| \| Bt.3867.1.S1\_at \| 282701 \| 1/2 \| NM\_001038501 \| NM\_001038501 \| GO:0005739  GO:0005743  GO:0006811  GO:0015986  GO:0015992  GO:0016469  GO:0045263  GO:0046933  GO:0046961 \| 1827992  2890767 \| ATP5F1 \| Bt.3867.1.S1\_at \| \| Bt.12912.1.S2\_at \| 282191 \| 2/2 \| XM\_580317 \| XM\_580317 \| GO:0005201  GO:0005581  GO:0005604  GO:0005737  GO:0006817 \| 11970952  15299013 \|  \| Bt.12912.1.S2\_at \| \| Bt.869.2.S1\_at \| 613759 \| 1/2 \| XM\_864841 \| XM\_864841 \|  \|  \|  \| Bt.869.2.S1\_at \| \| Bt.4891.1.S1\_at \| 539673 \| 2/2 \| XM\_868578 \| XM\_868578 \|  \| 16305752 \| POLDIP2 \| Bt.4891.1.S1\_at \| \| Bt.26764.1.A1\_at \| 281278 \| 2/2 \| NM\_181013 \|  \| GO:0004872  GO:0004930  GO:0005529  GO:0007165  GO:0007218  GO:0016020  GO:0016021  GO:0016524 \| 9261169  10025961 \| LPHN2 \| Bt.26764.1.A1\_at \| \| Bt.17641.1.A1\_at \| 615298 \| 1/2 \| NM\_001038206 \| NM\_001038206 \|  \|  \| C13orf1 \| Bt.17641.1.A1\_at \| \| Bt.7373.3.S1\_a\_at \| 504463 \| 2/4 \| XM\_580596 \|  \|  \|  \|  \| Bt.7373.3.S1\_a\_at \| \| Bt.12249.2.S1\_at \| 536073 \| 2/2 \| XM\_616193 \| XM\_616193 \|  \|  \| JMJD1A \| Bt.12249.2.S1\_at \| \| Bt.21019.1.S1\_at \| 538483 \| 1/2 \| XR\_028795 \|  \|  \|  \| CIC \| Bt.21019.1.S1\_at \| \| Bt.5748.2.S1\_at \| 780856 \| 1/1 \| NM\_001079793  XM\_001249349  XM\_001249402 \| NM\_001079793 \|  \|  \| YPEL5 \| Bt.5748.2.S1\_at \| \| Bt.16039.1.S1\_at \| 508371 \| 1/1 \| NM\_001035056 \| NM\_001035056 \|  \| 12477932 \| MGC128026 \| Bt.16039.1.S1\_at \| \| Bt.8322.1.S1\_at \| 519943 \| 1/3 \| XM\_872166 \|  \|  \|  \| FAM76B \| Bt.8322.1.S1\_at \| \| Bt.19431.1.S1\_at \| 445421 \| 2/2 \| NM\_001003902 \| NM\_001003902 \| GO:0003735  GO:0005622  GO:0005739  GO:0005840  GO:0006412 \| 10938081  12477932 \| MRPS14 \| Bt.19431.1.S1\_at \| \| Bt.20849.1.S1\_at \| 528986 \| 1/1 \| XM\_607424 \| XM\_607424 \| GO:0003676  GO:0008270  GO:0016779 \|  \| ZCCHC11 \| Bt.20849.1.S1\_at \| \| Bt.3753.1.S1\_at \| 534434 \| 1/5 \| XM\_614200 \| XM\_614200 \| GO:0004842  GO:0005622  GO:0006464  GO:0006512 \|  \| FLJ30092 \| Bt.3753.1.S1\_at \| \| Bt.4478.1.S1\_at \| 515069 \| 1/1 \| NM\_001038112 \| NM\_001038112 \| GO:0003755  GO:0005634  GO:0006457  GO:0016853 \| 1371698 \| FKBP3 \| Bt.4478.1.S1\_at \| \| Bt.21122.1.S1\_at \| 540345 \| 1/1 \| NM\_001076214 \| NM\_001076214 \| GO:0005622  GO:0005694  GO:0043565 \|  \| DRAP1 \| Bt.21122.1.S1\_at \| \| Bt.4271.1.S1\_at \| 509464 \| 1/1 \|  \|  \|  \|  \| CYFIP1 \| Bt.4271.1.S1\_at \| \| Bt.2729.1.S1\_at \| 536359 \| 1/1 \| NM\_001046416 \|  \| GO:0006886  GO:0030662 \|  \| AP3S1 \| Bt.2729.1.S1\_at \| \| Bt.13780.1.A1\_at \| 507095 \| 1/1 \| XM\_001249879  XM\_001249928  XM\_583649 \| XM\_001249879 \|  \|  \| SPRY1 \| Bt.13780.1.A1\_at \| \| Bt.10360.1.S1\_at \|  \|  \|  \|  \|  \|  \| KIAA1546 \| Bt.10360.1.S1\_at \| \| Bt.23854.1.A1\_at \| 616069 \| 1/1 \| NM\_001046574 \| NM\_001046574 \|  \|  \| CG018 \| Bt.23854.1.A1\_at \| \| Bt.2824.1.S1\_at \| 615149 \| 1/1 \| NM\_001076362 \|  \|  \|  \|  \| Bt.2824.1.S1\_at \| \| Bt.6080.1.S1\_at \| 507431 \| 1/2 \| XM\_866888 \| XM\_866888 \|  \| 12477932 \| ECH1 \| Bt.6080.1.S1\_at \| \| Bt.13833.1.S1\_at \|  \|  \|  \|  \|  \|  \| RYBP \| Bt.13833.1.S1\_at \| \| Bt.7596.1.S1\_at \| 616632 \| 1/1 \| XM\_868703 \|  \|  \|  \| MGC3196 \| Bt.7596.1.S1\_at \| \| Bt.7133.1.S1\_at \| 281365 \| 1/2 \| NM\_174130 \|  \|  \| 7774801  10520448 \| ODC1 \| Bt.7133.1.S1\_at \| \| Bt.3211.1.S2\_at \| 338472 \| 2/2 \| NM\_001033616 \| NM\_001033616 \| GO:0005874 \| 12438634  12477932 \| GABARAPL1 \| Bt.3211.1.S2\_at \| \| Bt.16978.1.S1\_at \| 540719 \| 2/2 \| XM\_612814 \| XM\_612814 \|  \|  \| KPNA3 \| Bt.16978.1.S1\_at \| \| Bt.24662.1.S1\_at \| 514859 \| 1/1 \| XM\_869458 \| XM\_869458 \|  \|  \| AKT1S1 \| Bt.24662.1.S1\_at \| \| Bt.3913.1.S1\_at \| 282122 \| 1/1 \| NM\_174488 \| NM\_174488 \| GO:0008083  GO:0016020 \| 10575000 \| VEGFC \| Bt.3913.1.S1\_at \| \| Bt.4492.1.S1\_at \| 504741 \| 1/2 \| XM\_611082 \|  \|  \|  \| COLEC12 \| Bt.4492.1.S1\_at \| \| Bt.21435.1.S1\_at \| 510324 \| 1/1 \| NM\_001038068 \| NM\_001038068 \|  \|  \| STOML2 \| Bt.21435.1.S1\_at \| \| Bt.21603.1.S1\_at \| 536781 \| 1/1 \| NM\_001035416 \| NM\_001035416 \| GO:0005515 \| 12477932 \| LDB3 \| Bt.21603.1.S1\_at \| \| Bt.3604.1.A1\_at \| 516010 \| 1/2 \| NM\_001046218 \| NM\_001046218 \| GO:0005622  GO:0005634  GO:0006350  GO:0006355 \|  \| SMAD2 \| Bt.3604.1.A1\_at \| \| Bt.19519.1.S1\_at \| 617843 \| 1/1 \| XM\_870167 \| XM\_588490 \|  \|  \| SMARCA3 \| Bt.19519.1.S1\_at \| \| Bt.4957.1.S2\_at \| 281937 \| 1/2 \| NM\_174396 \|  \| GO:0000166  GO:0003774  GO:0003779  GO:0005516  GO:0005524  GO:0005737  GO:0005902  GO:0009925  GO:0016328  GO:0016459  GO:0031941 \| 8022785  8313976 \| MYO1C \| Bt.4957.1.S2\_at \| \| Bt.20146.1.S1\_at \| 282146 \| 1/2 \| XM\_001251849  XM\_001251893  XM\_001251926  XM\_001251961  XM\_588082 \| XM\_001251893 \| GO:0003824  GO:0005388  GO:0005509  GO:0005524  GO:0006810  GO:0006812  GO:0006816  GO:0008152  GO:0015662  GO:0015992  GO:0016020  GO:0016820 \| 1531651  2966397  12552084 \|  \| Bt.20146.1.S1\_at \| \| Bt.2374.1.S2\_at \| 281201 \| 1/2 \| NM\_175777 \| NM\_175777 \| GO:0004871  GO:0005834  GO:0007165  GO:0007200  GO:0008283  GO:0050909 \| 2414128  3083416  8001974  8521505  8552196  9739091  12477932  12764189  14595696 \| GNB1 \| Bt.2374.1.S2\_at \| \| Bt.18348.1.S1\_at \|  \|  \|  \|  \|  \|  \| C14orf32 \| Bt.18348.1.S1\_at \| \| Bt.27403.1.S1\_at \| 521683 \| 1/1 \| NM\_001038552 \| XM\_614001 \|  \|  \| C5orf5 \| Bt.27403.1.S1\_at \| \| Bt.23234.1.S1\_at \| 539673 \| 2/2 \| XM\_868578 \| XM\_868578 \|  \| 16305752 \| POLDIP2 \| Bt.23234.1.S1\_at \| \| Bt.27089.1.S1\_at \|  \|  \|  \|  \|  \|  \| NOPE \| Bt.27089.1.S1\_at \| \| Bt.21469.1.S1\_at \| 533643 \| 1/1 \| XM\_613111  XM\_876937 \| XM\_876937 \| GO:0003677  GO:0005622  GO:0005634  GO:0008270 \|  \| ZNF326 \| Bt.21469.1.S1\_at \| \| Bt.3782.1.A1\_at \|  \|  \|  \|  \|  \|  \|  \| Bt.3782.1.A1\_at \| \| Bt.23520.1.S1\_at \| 540089 \| 1/1 \| NM\_001014960 \| NM\_001014960 \|  \| 12140684  16305752 \| C10orf104 \| Bt.23520.1.S1\_at \| \| Bt.11798.1.S1\_at \| 533764 \| 1/3 \| XM\_001251964  XM\_613276  XM\_866370 \|  \| GO:0003824  GO:0004750  GO:0005975  GO:0008152 \| 11282978  16305752 \| FLJ23861 \| Bt.11798.1.S1\_at \| \| Bt.17942.1.S1\_at \|  \|  \|  \| XM\_868111 \|  \|  \| LBH \| Bt.17942.1.S1\_at \| \| Bt.6693.1.A1\_at \| 540212 \| 1/1 \| XM\_592318 \| XM\_592318 \|  \|  \| NAT5 \| Bt.6693.1.A1\_at \| \| Bt.21818.1.S1\_at \| 533087 \| 1/1 \| XM\_867545 \|  \| GO:0003677  GO:0004519  GO:0005622  GO:0005634  GO:0006281 \|  \| EZH1 \| Bt.21818.1.S1\_at \| \| Bt.11154.2.S1\_at \| 613711 \| 1/3 \| XM\_864978 \|  \|  \|  \| C10orf9 \| Bt.11154.2.S1\_at \| \| Bt.16471.1.S1\_at \|  \|  \|  \|  \|  \|  \|  \| Bt.16471.1.S1\_at \| \| Bt.16380.2.A1\_at \| 504205 \| 1/2 \|  \|  \|  \|  \| RGL1 \| Bt.16380.2.A1\_at \| \| Bt.9706.2.S1\_at \| 512377 \| 1/1 \| NM\_001034421 \|  \|  \| 12477932 \| C20orf24 \| Bt.9706.2.S1\_at \| \| Bt.19706.1.S1\_at \|  \|  \|  \|  \|  \|  \| ZFP36L2 \| Bt.19706.1.S1\_at \| \| Bt.2235.1.S1\_at \| 281188 \| 2/2 \| NM\_174064 \| NM\_174064 \|  \| 1904992  2115118  8609986 \| GDI1 \| Bt.2235.1.S1\_at \| \| Bt.19862.2.S1\_at \| 518458 \| 2/2 \| XM\_001249780  XM\_001249831  XM\_001249925  XM\_001249962  XM\_596649 \|  \| GO:0004872  GO:0007275  GO:0016020 \|  \| SEMA6D \| Bt.19862.2.S1\_at \| \| Bt.21745.1.S1\_at \| 511268 \| 1/1 \| NM\_001015572 \|  \| GO:0005515  GO:0005634  GO:0005634  GO:0005737  GO:0005737  GO:0006457  GO:0008344  GO:0042542  GO:0051583 \| 12140684  12477932  16305752 \| PARK7 \| Bt.21745.1.S1\_at \| \| Bt.12661.1.A1\_at \| 513504 \| 1/2 \| XM\_591194 \| XM\_591194 \| GO:0005524  GO:0008152  GO:0009374  GO:0016874 \|  \| MCCC1 \| Bt.12661.1.A1\_at \| \| Bt.16437.1.S1\_at \| 614576 \| 1/1 \| NM\_001046541 \| NM\_001046541 \| GO:0005515  GO:0005737  GO:0030100 \|  \| BIN1 \| Bt.16437.1.S1\_at \| \| Bt.2717.1.A1\_at \| 508882 \| 1/1 \| XM\_585727 \|  \|  \|  \| FAP \| Bt.2717.1.A1\_at \| \| Bt.15779.3.S1\_at \| 509784 \| 1/5 \| XM\_874620 \| XM\_874620 \|  \|  \| PHLDB2 \| Bt.15779.3.S1\_at \| \| Bt.10266.1.S1\_at \| 515503 \| 1/1 \| NM\_001035310 \| NM\_001035310 \| GO:0004298  GO:0005829  GO:0005839  GO:0006511 \| 12477932 \| PSMA1 \| Bt.10266.1.S1\_at \| \| Bt.18278.1.S1\_at \| 509717 \| 2/4 \| NM\_001034358 \| XM\_001251293 \|  \| 12477932 \|  \| Bt.18278.1.S1\_at \| \| Bt.2399.1.S1\_at \|  \|  \|  \| XM\_594710 \|  \|  \| TNS3 \| Bt.2399.1.S1\_at \| \| Bt.814.1.S1\_at \| 511085 \| 1/1 \| NM\_001075490 \| NM\_001075490 \|  \|  \| FLJ20850 \| Bt.814.1.S1\_at \| \| Bt.19581.1.A1\_at \| 535290 \| 1/2 \| NM\_001046401 \| NM\_001046401 \| GO:0003723  GO:0005739  GO:0030529 \| 10938081 \| MRPS28 \| Bt.19581.1.A1\_at \| \| Bt.2989.2.S1\_at \| 539494 \| 2/4 \| XM\_001252000  XM\_877687 \| XM\_001252000 \| GO:0005215  GO:0005488  GO:0005509  GO:0005743  GO:0006810  GO:0016020 \|  \| SLC25A12 \| Bt.2989.2.S1\_at \| \| Bt.13152.1.S1\_s\_at \| 280722 \| 2/2 \| NM\_001076796 \|  \| GO:0005488  GO:0016021 \| 1656157  10594237  14709559 \| APP \| Bt.13152.1.S1\_s\_at \| \| Bt.1154.1.S1\_at \| 533223 \| 1/1 \| NM\_001046346 \| NM\_001046346 \| GO:0003779  GO:0005856 \|  \| WDR1 \| Bt.1154.1.S1\_at \| \| Bt.20683.1.S1\_at \|  \|  \|  \|  \|  \|  \|  \| Bt.20683.1.S1\_at \| \| Bt.9210.1.S1\_at \| 617104 \| 1/1 \| NM\_001046592 \|  \|  \|  \| E2IG5 \| Bt.9210.1.S1\_at \| \| Bt.24310.1.S1\_at \|  \|  \|  \|  \|  \|  \|  \| Bt.24310.1.S1\_at \| \| Bt.25098.1.S1\_at \| 520518 \| 1/1 \| XM\_598765 \| XM\_598765 \|  \|  \| C1orf163 \| Bt.25098.1.S1\_at \| \| Bt.8262.1.A1\_at \|  \|  \|  \|  \|  \|  \| GOPC \| Bt.8262.1.A1\_at \| \| Bt.29910.1.S1\_at \| 407209 \| 2/2 \| NM\_001075145 \|  \| GO:0005509  GO:0005515  GO:0005856  GO:0007156  GO:0016012  GO:0016020  GO:0016021 \| 14668210 \| SGCE \| Bt.29910.1.S1\_at \| \| Bt.9176.2.S1\_at \|  \|  \|  \| XM\_588651 \|  \|  \|  \| Bt.9176.2.S1\_at \| \| Bt.12366.1.S1\_at \| 538796 \| 1/1 \| NM\_001034650 \|  \|  \| 12477932 \| CCNL2 \| Bt.12366.1.S1\_at \| \| Bt.22310.1.S1\_at \| 505320 \| 1/2 \| XM\_868367 \|  \|  \|  \| ARIH2 \| Bt.22310.1.S1\_at \| \| Bt.12910.1.S1\_at \| 534599 \| 1/1 \| NM\_001076030 \| NM\_001076030 \| GO:0004591  GO:0006096  GO:0008152  GO:0030976 \|  \| OGDH \| Bt.12910.1.S1\_at \| \| Bt.6679.1.S1\_at \| 512427 \| 1/3 \| NM\_001038088 \|  \| GO:0001824  GO:0005515  GO:0005634  GO:0006260  GO:0007049  GO:0008283  GO:0015031  GO:0043486  GO:0051879 \|  \| NASP \| Bt.6679.1.S1\_at \| \| Bt.5560.1.S1\_at \| 404059 \| 1/3 \| NM\_205771 \|  \|  \| 12874223 \|  \| Bt.5560.1.S1\_at \| \| Bt.23254.1.S1\_at \| 768311 \| 1/1 \| NM\_001078127  XM\_001252704 \|  \| GO:0006605  GO:0007264  GO:0019904 \|  \| YWHAQ \| Bt.23254.1.S1\_at \| \| Bt.10579.1.S1\_at \|  \|  \|  \| XR\_027461 \|  \|  \| IRF2BP2 \| Bt.10579.1.S1\_at \| \| Bt.22114.1.S1\_at \| 506546 \| 1/2 \| XM\_583007 \|  \|  \|  \| DZIP1 \| Bt.22114.1.S1\_at \| \| Bt.22142.2.S1\_a\_at \| 511624 \| 1/1 \| NM\_001046093 \|  \| GO:0004497  GO:0006118  GO:0006725  GO:0006744  GO:0008152  GO:0015997  GO:0050660 \|  \| COQ6 \| Bt.22142.2.S1\_a\_at \| \| Bt.23158.1.S1\_at \| 505453 \| 1/1 \| XM\_581740 \| XM\_581740 \|  \|  \| CREBBP \| Bt.23158.1.S1\_at \| \| Bt.21981.3.S1\_at \|  \|  \|  \|  \|  \|  \| ANTXR1 \| Bt.21981.3.S1\_at \| \| Bt.5033.1.S1\_at \| 281480 \| 1/3 \| NM\_174178 \| NM\_174178 \|  \| 1375942  8142412  10612251  16324220  16751257 \| SDHA \| Bt.5033.1.S1\_at \| \| Bt.1699.2.S1\_a\_at \| 767928 \| 2/2 \| NM\_001077001 \| NM\_001077001 \| GO:0001525  GO:0001837  GO:0003677  GO:0005515  GO:0005634  GO:0006350  GO:0006357  GO:0007219  GO:0007507  GO:0008284  GO:0030097  GO:0030183  GO:0042742 \|  \|  \| Bt.1699.2.S1\_a\_at \| \| Bt.19415.1.A1\_at \| 407109 \| 3/4 \| XM\_581602 \| XM\_581602 \| GO:0008152  GO:0016624 \|  \| PDHA1 \| Bt.19415.1.A1\_at \| \| Bt.5054.3.S1\_at \|  \|  \|  \| XM\_001253071 \|  \|  \| TGFBR3 \| Bt.5054.3.S1\_at \| \| Bt.19936.1.A1\_at \|  \|  \|  \|  \|  \|  \| ZNF704 \| Bt.19936.1.A1\_at \| \| Bt.22084.1.A1\_at \| 515903 \| 3/5 \| XM\_001249521  XM\_001249570  XM\_878572  XM\_878731  XM\_878792 \| XM\_001249521 \|  \|  \| FOXP1 \| Bt.22084.1.A1\_at \| \| Bt.1430.1.S1\_at \| 282848 \| 1/1 \| NM\_174732 \| NM\_174732 \|  \| 12477932 \| PFDN5 \| Bt.1430.1.S1\_at \| \| Bt.27386.1.A1\_at \| 527956 \| 2/2 \| XM\_606363 \| XM\_606363 \|  \|  \| KIAA1223 \| Bt.27386.1.A1\_at \| \| Bt.22634.1.S1\_at \| 538209 \| 1/4 \| XM\_618406 \| XM\_618406 \|  \|  \| PTGFRN \| Bt.22634.1.S1\_at \| \| Bt.4670.1.S1\_at \| 539596 \| 1/1 \| XR\_028016 \| XR\_028016 \| GO:0005515  GO:0005578  GO:0007155 \| 8557636 \| TGFBI \| Bt.4670.1.S1\_at \| \| Bt.7481.1.S1\_at \| 514665 \| 1/1 \| NM\_001075676 \|  \| GO:0005215  GO:0006810  GO:0008021  GO:0016020 \|  \|  \| Bt.7481.1.S1\_at \| \| Bt.4232.2.S1\_at \|  \|  \|  \| XM\_584740 \|  \|  \| DOCK6 \| Bt.4232.2.S1\_at \| \| Bt.380.1.S1\_at \| 327699 \| 1/1 \| NM\_175816 \|  \| GO:0001937  GO:0005516  GO:0005739  GO:0009268  GO:0009986  GO:0042030  GO:0042030  GO:0042803  GO:0043532  GO:0045980  GO:0051117  GO:0051117  GO:0051289  GO:0051346  GO:0051346 \| 1083159  2894843  6461003  7397110  11327770  16341776 \| ATPIF1 \| Bt.380.1.S1\_at \| \| Bt.4653.1.S2\_at \| 282303 \| 2/3 \| NM\_174571 \|  \| GO:0005515  GO:0007155  GO:0016020  GO:0016021 \| 8558001  15668248 \| PECAM1 \| Bt.4653.1.S2\_at \| \| Bt.24727.1.S1\_a\_at \| 538804 \| 1/1 \| NM\_001038178 \| NM\_001038178 \|  \|  \| C6orf136 \| Bt.24727.1.S1\_a\_at \| \| Bt.7804.1.S1\_at \| 280812 \| 1/1 \| NM\_001045867 \| NM\_001045867 \|  \| 1281472 \| GYG \| Bt.7804.1.S1\_at \| \| Bt.3494.1.A1\_s\_at \| 540195 \| 2/3 \| XM\_592217 \| XM\_592217 \|  \|  \| RNF19 \| Bt.3494.1.A1\_s\_at \| \| Bt.27221.1.S1\_at \|  \|  \|  \| XM\_615986 \|  \|  \| SLC16A2 \| Bt.27221.1.S1\_at \| \| Bt.3484.1.S1\_at \| 407236 \| 1/2 \| NM\_001001158 \|  \| GO:0005529 \|  \| CD302 \| Bt.3484.1.S1\_at \| \| Bt.13588.3.A1\_at \| 533044 \| 2/2 \| XM\_612314 \|  \| GO:0003824  GO:0006564  GO:0008152  GO:0008483  GO:0030170 \|  \| PSAT1 \| Bt.13588.3.A1\_at \| \| Bt.16004.1.A1\_at \| 281339 \| 3/3 \| XM\_865879 \|  \| GO:0003774  GO:0016459 \| 8014155 \| MYH8 \| Bt.16004.1.A1\_at \| \| Bt.28612.1.S1\_at \|  \|  \|  \|  \|  \|  \| SPIN \| Bt.28612.1.S1\_at \| \| Bt.5322.1.S1\_at \| 534640 \| 1/1 \| NM\_001037612 \|  \| GO:0004298  GO:0005829  GO:0005839  GO:0006511 \|  \| PSMB5 \| Bt.5322.1.S1\_at \| \| Bt.21790.1.S1\_at \| 540311 \| 2/3 \| XM\_593096 \| XM\_593096 \|  \| 11282978  16305752 \| HNRPA0 \| Bt.21790.1.S1\_at \| \| Bt.65.1.S1\_at \| 327698 \| 1/1 \| NM\_175815 \| NM\_175815 \| GO:0003954  GO:0005739  GO:0005747  GO:0008137  GO:0016491  GO:0031966  GO:0031966 \| 1518044 \| NDUFA2 \| Bt.65.1.S1\_at \| \| Bt.1399.1.S1\_at \| 282405 \| 1/1 \| NM\_001045869 \| NM\_001045869 \| GO:0006811  GO:0015986  GO:0015992  GO:0016469  GO:0016787  GO:0046872  GO:0046933  GO:0046961 \| 8621738 \| ATP6V1F \| Bt.1399.1.S1\_at \| \| Bt.752.1.S1\_a\_at \| 541216 \| 1/1 \| NM\_001034726 \| NM\_001034726 \| GO:0006464  GO:0006512  GO:0019787 \| 12477932 \| RWDD1 \| Bt.752.1.S1\_a\_at \| \| Bt.20402.1.S1\_at \| 530393 \| 3/3 \| NM\_001046312 \| NM\_001046312 \| GO:0005216  GO:0005509  GO:0005515  GO:0005929  GO:0005932  GO:0006811  GO:0006816  GO:0006874  GO:0007050  GO:0007259  GO:0007368  GO:0015629  GO:0016020  GO:0016021  GO:0050974 \| 15998908  16229973 \| PKD2 \| Bt.20402.1.S1\_at \| \| Bt.29463.1.S1\_at \|  \|  \|  \|  \|  \|  \|  \| Bt.29463.1.S1\_at \| \| Bt.23651.1.A1\_at \| 525385 \| 1/1 \| XM\_603738 \| XM\_617553 \|  \|  \| DDAH1 \| Bt.23651.1.A1\_at \| \| Bt.20236.1.S1\_at \| 507654 \| 1/2 \| XM\_584307 \| XM\_584307 \|  \| 11282978  16305752 \| TSRC1 \| Bt.20236.1.S1\_at \| \| Bt.979.1.A1\_at \| 537282 \| 2/3 \| XM\_617440 \|  \|  \|  \| MYO1B \| Bt.979.1.A1\_at \| \| Bt.9754.1.S1\_at \| 519632 \| 1/1 \| NM\_001034535 \|  \| GO:0006118  GO:0016491  GO:0050662 \| 12477932 \| NQO1 \| Bt.9754.1.S1\_at \| \| Bt.29697.1.S1\_at \| 407147 \| 1/2 \| NM\_001040496 \|  \| GO:0003956  GO:0006471  GO:0016740 \|  \| ART3 \| Bt.29697.1.S1\_at \| \| Bt.2696.2.S1\_at \| 535363 \| 2/2 \| XM\_615431 \|  \| GO:0005509  GO:0007156  GO:0016020 \|  \| CDH11 \| Bt.2696.2.S1\_at \| \| Bt.15715.1.S1\_at \|  \|  \|  \|  \|  \|  \| CDH5 \| Bt.15715.1.S1\_at \| \| Bt.21182.1.S1\_at \|  \|  \|  \|  \|  \|  \| CCDC6 \| Bt.21182.1.S1\_at \| \| Bt.7836.1.S1\_at \| 614921 \| 1/1 \| NM\_001037624 \| NM\_001037624 \| GO:0005515  GO:0005622  GO:0015031 \|  \| NUTF2 \| Bt.7836.1.S1\_at \| \| Bt.3448.1.S1\_at \| 526940 \| 1/1 \|  \|  \|  \|  \|  \| Bt.3448.1.S1\_at \| \| Bt.6586.1.S1\_at \|  \|  \|  \| XR\_028762 \|  \|  \| NOTCH3 \| Bt.6586.1.S1\_at \| \| Bt.15868.1.S1\_at \|  \|  \|  \|  \|  \|  \|  \| Bt.15868.1.S1\_at \| \| Bt.2451.2.A1\_at \| 538594 \| 2/3 \| XM\_876869 \|  \| GO:0003700  GO:0005634  GO:0006355  GO:0043565  GO:0046983 \|  \| ZF \| Bt.2451.2.A1\_at \| \| Bt.8047.1.S1\_at \| 540176 \| 1/1 \| NM\_001034692 \|  \| GO:0005515  GO:0005739  GO:0006811  GO:0008289  GO:0015986  GO:0015992  GO:0016020  GO:0016469  GO:0045263  GO:0046933  GO:0046961 \| 12477932 \| ATP5G3 \| Bt.8047.1.S1\_at \| \| Bt.20605.2.S1\_at \|  \|  \|  \|  \|  \|  \| CLDN1 \| Bt.20605.2.S1\_at \| \| Bt.15853.1.S1\_at \| 532209 \| 3/3 \| XM\_610719 \|  \|  \|  \| EVI1 \| Bt.15853.1.S1\_at \| \| Bt.16067.1.S1\_at \| 338044 \| 2/2 \| XM\_588265 \| NM\_001082424 \|  \|  \| SLC38A2 \| Bt.16067.1.S1\_at \| \| Bt.29053.1.A1\_at \| 537533 \| 1/2 \| XM\_617702 \|  \| GO:0004143  GO:0007205  GO:0007242 \|  \| DGKH \| Bt.29053.1.A1\_at \| \| Bt.11060.1.S1\_at \| 511861 \| 1/1 \| XM\_589279 \| XM\_589279 \|  \|  \| TDE1 \| Bt.11060.1.S1\_at \| \| Bt.11322.1.A1\_at \| 509995 \| 1/1 \| XR\_028400 \| XR\_028400 \|  \|  \| LRPPRC \| Bt.11322.1.A1\_at \| \| Bt.6399.2.S1\_a\_at \| 518738 \| 1/1 \| NM\_001075778 \| NM\_001075778 \| GO:0003676  GO:0005622 \| 11282978  16305752 \| GPATC1 \| Bt.6399.2.S1\_a\_at \| \| Bt.21366.2.S1\_at \| 522459 \| 1/3 \| XM\_600742 \| XM\_600742 \|  \|  \| FLJ13220 \| Bt.21366.2.S1\_at \| \| Bt.16935.1.A1\_at \|  \|  \|  \|  \|  \|  \| SENP2 \| Bt.16935.1.A1\_at \| \| Bt.9252.1.A1\_at \|  \|  \|  \| XM\_001249420 \|  \|  \| JMJD1C \| Bt.9252.1.A1\_at \| \| Bt.8690.2.S1\_at \|  \|  \|  \|  \|  \|  \|  \| Bt.8690.2.S1\_at \| \| Bt.21468.1.S1\_at \|  \|  \|  \| XM\_001251214 \|  \|  \| DEXI \| Bt.21468.1.S1\_at \| \| Bt.8912.1.A1\_at \| 540475 \| 1/4 \| XM\_879584 \|  \|  \|  \| CUGBP1 \| Bt.8912.1.A1\_at \| \| Bt.5541.1.S1\_at \| 404188 \| 1/1 \| NM\_205817 \| NM\_205817 \| GO:0003954  GO:0005739  GO:0005743  GO:0005747  GO:0008137  GO:0016491  GO:0044237  GO:0050662 \| 1832859  11282978  12477932  16305752 \| NDUFA9 \| Bt.5541.1.S1\_at \| \| Bt.3288.1.A1\_at \| 514374 \| 1/1 \| NM\_001038102 \|  \| GO:0005739 \|  \| PIG-Y \| Bt.3288.1.A1\_at \| \| Bt.18332.1.S1\_at \|  \|  \|  \|  \|  \|  \| LAMA2 \| Bt.18332.1.S1\_at \| \| Bt.9705.1.S1\_at \| 513371 \| 1/1 \|  \|  \|  \|  \| NKTR \| Bt.9705.1.S1\_at \| \| Bt.22630.2.A1\_at \| 617564 \| 2/2 \| NM\_001076469 \| NM\_001076469 \|  \|  \| DHDH \| Bt.22630.2.A1\_at \| \| Bt.20129.2.S1\_at \| 523683 \| 3/5 \| XM\_601985 \| XM\_601985 \|  \|  \| CUTL1 \| Bt.20129.2.S1\_at \| \| Bt.27211.1.S1\_at \| 616048 \| 1/1 \| XM\_868011 \|  \| GO:0006470  GO:0008138  GO:0016787  GO:0016791 \|  \| DUSP13 \| Bt.27211.1.S1\_at \| \| Bt.18057.1.A1\_at \| 529115 \| 1/2 \| XM\_607555 \| XM\_607555 \| GO:0003676  GO:0005622  GO:0008270 \|  \| HIVEP1 \| Bt.18057.1.A1\_at \| \| Bt.21288.1.A1\_at \|  \|  \|  \|  \|  \|  \|  \| Bt.21288.1.A1\_at \| \| Bt.13063.1.S1\_at \| 282107 \| 1/2 \| AF419316 \|  \| GO:0006464  GO:0006512  GO:0016874  GO:0019787 \|  \| UBE2A \| Bt.13063.1.S1\_at \| \| Bt.1505.1.S1\_at \| 615692 \| 1/1 \| NM\_001035467 \|  \| GO:0005515  GO:0006350  GO:0006355 \| 12477932 \| SAP18 \| Bt.1505.1.S1\_at \| \| Bt.2815.1.S1\_at \| 513309 \| 1/3 \| XM\_864958 \|  \|  \|  \| LXN \| Bt.2815.1.S1\_at \| \| Bt.22736.1.S1\_at \|  \|  \|  \|  \|  \|  \| ARHGAP12 \| Bt.22736.1.S1\_at \| \| Bt.19105.1.A1\_at \| 529124 \| 1/1 \| NM\_001046298 \| NM\_001046298 \| GO:0003700  GO:0005634  GO:0006355  GO:0043565 \|  \| FLI1 \| Bt.19105.1.A1\_at \| \| Bt.9233.3.S1\_at \| 519729 \| 1/2 \| XM\_866050 \| NM\_001080282 \|  \| 12140684  16305752 \|  \| Bt.9233.3.S1\_at \| \| Bt.25202.3.S1\_at \| 534295 \| 3/3 \| XM\_614016 \|  \| GO:0004190  GO:0006508 \|  \| KIAA1109 \| Bt.25202.3.S1\_at \| \| Bt.11409.1.A1\_a\_at \|  \|  \|  \|  \|  \|  \| MAP4K4 \| Bt.11409.1.A1\_a\_at \| \| Bt.23606.1.S1\_at \| 317697 \| 1/2 \| NM\_174841 \| NM\_174841 \| GO:0004872  GO:0005216  GO:0005262  GO:0005509  GO:0005515  GO:0005783  GO:0006811  GO:0006816  GO:0016020  GO:0016021 \| 2174422  11584008  12714606  14506248 \| ITPR1 \| Bt.23606.1.S1\_at \| \| Bt.3216.1.A1\_at \| 504577 \| 1/1 \| NM\_001075167 \| NM\_001075167 \|  \|  \| ARMCX1 \| Bt.3216.1.A1\_at \| \| Bt.4903.1.S2\_at \| 281945 \| 2/3 \| NM\_174402 \| NM\_174402 \| GO:0003700  GO:0003707  GO:0005634  GO:0006350  GO:0006355  GO:0008270  GO:0043565  GO:0046872 \| 10343125  15171724 \| NR2F2 \| Bt.4903.1.S2\_at \| \| Bt.2870.1.A1\_at \| 540040 \| 1/4 \| NM\_001038567 \| NM\_001038567 \| GO:0000166  GO:0004871  GO:0005525  GO:0005622  GO:0007186  GO:0007264 \|  \| ARL3 \| Bt.2870.1.A1\_at \| \| Bt.9629.2.S1\_at \| 614773 \| 2/3 \| XM\_865938 \| XM\_865938 \|  \|  \| ZFP36L1 \| Bt.9629.2.S1\_at \| \| Bt.404.1.S1\_at \| 282690 \| 1/1 \| NM\_174717 \|  \| GO:0005739  GO:0005743  GO:0006811  GO:0015986  GO:0015992  GO:0016469  GO:0045263  GO:0046933  GO:0046961 \| 1827992  2894843  6149548  12477932 \| ATP5J \| Bt.404.1.S1\_at \| \| Bt.24676.1.S1\_at \| 515999 \| 2/2 \| NM\_001035315 \| NM\_001035315 \| GO:0000166  GO:0000287  GO:0005525  GO:0005622  GO:0005783  GO:0006886  GO:0006888  GO:0007264  GO:0016020 \| 12477932 \| SAR1B \| Bt.24676.1.S1\_at \| \| Bt.22526.1.S1\_at \| 539524 \| 1/1 \| NM\_001014955 \|  \| GO:0005622  GO:0006457  GO:0042802 \| 11282978  12477932  16305752 \| HSPB8 \| Bt.22526.1.S1\_at \| \| Bt.20582.1.S1\_at \| 530971 \| 2/2 \|  \|  \|  \|  \| HDGFRP3 \| Bt.20582.1.S1\_at \| \| Bt.8730.1.S1\_at \| 536673 \| 1/1 \|  \|  \|  \|  \| RAPGEF2 \| Bt.8730.1.S1\_at \| \| Bt.27112.1.S1\_at \| 538533 \| 2/5 \| NM\_001076109 \|  \| GO:0000166  GO:0003676 \|  \|  \| Bt.27112.1.S1\_at \| \| Bt.11182.1.S1\_at \| 514918 \| 2/3 \| NM\_001075689 \|  \| GO:0003700  GO:0005634  GO:0045449 \|  \| C21orf66 \| Bt.11182.1.S1\_at \| \| Bt.26568.2.S1\_a\_at \| 531049 \| 2/2 \| XM\_883226 \| XM\_883226 \| GO:0003677  GO:0005488  GO:0008152  GO:0016491 \|  \| KIAA0664 \| Bt.26568.2.S1\_a\_at \| \| Bt.2347.2.S1\_at \| 506544 \| 2/3 \| NM\_001075268 \| NM\_001075268 \|  \| 8001974 \| PFKM \| Bt.2347.2.S1\_at \| \| Bt.20242.1.S1\_at \| 534213 \| 1/1 \|  \| XM\_582772 \|  \|  \|  \| Bt.20242.1.S1\_at \| \| Bt.16296.1.A1\_at \| 540568 \| 3/4 \| XM\_612129 \|  \| GO:0003824  GO:0005524  GO:0006810  GO:0006812  GO:0008152  GO:0015662  GO:0016020 \|  \| ATP2A2 \| Bt.16296.1.A1\_at \| \| Bt.5510.1.S1\_at \| 338088  617230 \| 1/1 \| XM\_868567  XM\_869452 \|  \|  \|  \| ATP5E \| Bt.5510.1.S1\_at \| \| Bt.21737.1.S1\_at \|  \|  \|  \|  \|  \|  \| C14orf43 \| Bt.21737.1.S1\_at \| \| Bt.5965.1.S1\_at \| 538292 \| 2/3 \| XM\_618492 \| XM\_618492 \| GO:0004674  GO:0005089  GO:0005524  GO:0005622  GO:0006468  GO:0035023 \|  \| TRIO \| Bt.5965.1.S1\_at \| \| Bt.12603.1.S1\_at \| 504657 \| 1/2 \| XR\_027700 \| XR\_027700 \|  \|  \| ARHGAP29 \| Bt.12603.1.S1\_at \| \| Bt.19928.1.S1\_at \| 525652 \| 1/2 \|  \| XM\_589886 \|  \|  \|  \| Bt.19928.1.S1\_at \| \| Bt.4725.1.S1\_at \| 281032 \| 1/1 \| NM\_173999 \|  \|  \| 12477932  15033446 \| BTG1 \| Bt.4725.1.S1\_at \| \| Bt.19826.1.A1\_at \|  \|  \|  \|  \|  \|  \| P2RY1 \| Bt.19826.1.A1\_at \| \| Bt.2989.3.S1\_at \| 539494 \| 2/4 \| XM\_001252000  XM\_877687 \| XM\_877687 \| GO:0005215  GO:0005488  GO:0005509  GO:0005743  GO:0006810  GO:0016020 \|  \| SLC25A12 \| Bt.2989.3.S1\_at \| \| Bt.13765.1.S1\_at \|  \|  \|  \|  \|  \|  \|  \| Bt.13765.1.S1\_at \| \| Bt.16041.1.S1\_at \| 504968 \| 2/2 \| XM\_866358  XM\_878203 \| XM\_866358 \|  \|  \| ABLIM1 \| Bt.16041.1.S1\_at \| \| Bt.11662.1.A1\_at \| 523311 \| 1/1 \| XM\_601607 \|  \|  \|  \| GPX7 \| Bt.11662.1.A1\_at \| \| Bt.26999.1.S1\_at \| 615975 \| 1/1 \| NM\_001037626 \|  \|  \| 12477932 \| SMPX \| Bt.26999.1.S1\_at \| \| Bt.12379.1.S1\_at \| 617775 \| 1/2 \| XM\_870096 \|  \|  \|  \| PTOV1 \| Bt.12379.1.S1\_at \| \| Bt.20528.1.S1\_at \|  \|  \|  \|  \|  \|  \| PHC3 \| Bt.20528.1.S1\_at \| \| Bt.25052.1.S1\_at \|  \|  \|  \| XM\_586655 \|  \|  \| CAPN6 \| Bt.25052.1.S1\_at \| \| Bt.8077.1.S1\_at \| 507476 \| 1/1 \| NM\_001034299 \| NM\_001034299 \|  \| 11282978  12477932  12488440  12658628 \| PGK1 \| Bt.8077.1.S1\_at \| \| Bt.4263.1.S1\_at \| 281801 \| 1/1 \| NM\_174331 \| NM\_174331 \| GO:0000166  GO:0004703  GO:0004871  GO:0005524  GO:0006468  GO:0007165  GO:0016740 \| 8120045 \| GRK5 \| Bt.4263.1.S1\_at \| \| Bt.13366.1.S1\_at \| 539676 \| 1/2 \| XM\_864821 \| XM\_864821 \|  \|  \| DMTF1 \| Bt.13366.1.S1\_at \| \| Bt.16078.1.A1\_s\_at \|  \|  \|  \|  \|  \|  \| CPNE1 \| Bt.16078.1.A1\_s\_at \| \| Bt.7069.1.S1\_at \| 505807 \| 1/1 \| NM\_001024479 \|  \|  \| 11282978  16305752 \| PCMTD2 \| Bt.7069.1.S1\_at \| \| Bt.23174.2.S1\_at \| 515646 \| 1/1 \| NM\_001015608 \|  \| GO:0005247  GO:0005515  GO:0005624  GO:0005625  GO:0005634  GO:0005737  GO:0005903  GO:0006811  GO:0006821  GO:0016020  GO:0031404 \| 12140684  12477932  16305752 \| CLIC1 \| Bt.23174.2.S1\_at \| \| Bt.4867.1.A1\_at \| 281339 \| 3/3 \| XM\_865879 \|  \| GO:0003774  GO:0016459 \| 8014155 \| MYH8 \| Bt.4867.1.A1\_at \| \| Bt.727.2.S1\_at \|  \|  \|  \|  \|  \|  \| NUP37 \| Bt.727.2.S1\_at \| \| Bt.7696.1.S1\_at \| 516619 \| 2/2 \| NM\_001015614 \| NM\_001015614 \| GO:0004663  GO:0005515  GO:0008270  GO:0008318  GO:0016740  GO:0018346  GO:0046872 \| 11282978  16305752 \| RABGGTA \| Bt.7696.1.S1\_at \| \| Bt.29655.1.S1\_a\_at \| 614078 \| 1/1 \| XM\_865417 \| XM\_865417 \|  \|  \|  \| Bt.29655.1.S1\_a\_at \| \| Bt.7079.1.S1\_at \| 526771 \| 1/1 \| XM\_605145 \| XM\_605145 \|  \|  \| TEAD4 \| Bt.7079.1.S1\_at \| \| Bt.14340.1.A1\_at \| 511639 \| 2/2 \| XR\_027604 \|  \|  \|  \| RNF103 \| Bt.14340.1.A1\_at \| \| Bt.5468.1.S1\_at \| 503685 \| 1/1 \| NM\_001012764 \| NM\_001012764 \| GO:0004869 \|  \| CST6 \| Bt.5468.1.S1\_at \| \| Bt.20318.1.S1\_a\_at \| 508566 \| 1/2 \| NM\_001034323 \| NM\_001034323 \|  \| 12477932 \| NQO2 \| Bt.20318.1.S1\_a\_at \| \| Bt.20489.1.S1\_at \|  \|  \|  \|  \|  \|  \| KIAA0460 \| Bt.20489.1.S1\_at \| \| Bt.2359.1.A1\_at \|  \|  \|  \| XM\_001251202 \|  \|  \| FYN \| Bt.2359.1.A1\_at \| \| Bt.13865.1.A1\_at \| 515270 \| 1/1 \| NM\_001038544 \| NM\_001038544 \| GO:0006601  GO:0007283  GO:0008168  GO:0009887  GO:0016740  GO:0030731  GO:0040014 \|  \| GAMT \| Bt.13865.1.A1\_at \| \| Bt.10722.1.S1\_at \| 618835 \| 1/3 \| XM\_883394 \|  \|  \|  \| FLJ21616 \| Bt.10722.1.S1\_at \| \| Bt.29381.1.S1\_at \|  \|  \|  \|  \|  \|  \| VLDLR \| Bt.29381.1.S1\_at \| \| Bt.24210.1.S1\_at \| 537161 \| 1/1 \| NM\_001076085 \|  \| GO:0003824  GO:0008152 \|  \| ACSL1 \| Bt.24210.1.S1\_at \| \| Bt.22166.1.S1\_at \|  \|  \|  \|  \|  \|  \| HCCS \| Bt.22166.1.S1\_at \| \| Bt.19746.1.S1\_at \|  \|  \|  \|  \|  \|  \| ZBTB4 \| Bt.19746.1.S1\_at \| \| Bt.11093.1.S1\_at \|  \|  \|  \|  \|  \|  \| EML4 \| Bt.11093.1.S1\_at \| \| Bt.3940.1.S1\_at \|  \|  \|  \| NM\_001077885 \|  \|  \| TCF12 \| Bt.3940.1.S1\_at \| \| Bt.21191.2.A1\_at \| 508547 \| 2/5 \| XM\_867519 \|  \|  \|  \| PDE4DIP \| Bt.21191.2.A1\_at \| \| Bt.10999.1.A1\_at \| 526430 \| 2/2 \| XM\_604801 \|  \|  \|  \| NCK2 \| Bt.10999.1.A1\_at \| \| Bt.2070.2.A1\_at \| 541175 \| 3/3 \| NM\_001034725 \| NM\_001034725 \| GO:0004725  GO:0005634  GO:0006470  GO:0016787  GO:0017017 \| 11282978 \| DUSP10 \| Bt.2070.2.A1\_at \| \| Bt.17778.1.A1\_at \| 515650 \| 1/1 \| XM\_593707 \| XM\_593707 \|  \|  \|  \| Bt.17778.1.A1\_at \| \| Bt.22630.1.S1\_at \| 617564 \| 2/2 \| NM\_001076469 \|  \|  \|  \| DHDH \| Bt.22630.1.S1\_at \| \| Bt.21036.2.A1\_at \| 615221 \| 2/2 \|  \|  \|  \|  \| FAM73A \| Bt.21036.2.A1\_at \| \| Bt.24916.1.A1\_at \|  \|  \|  \|  \|  \|  \| DOCK7 \| Bt.24916.1.A1\_at \| \| Bt.28623.1.S1\_at \| 508251 \| 1/2 \| XM\_864984 \| XM\_864984 \|  \|  \| FAT \| Bt.28623.1.S1\_at \| \| Bt.4571.1.S1\_at \| 281051 \| 1/1 \| NM\_174009 \| NM\_174009 \|  \| 11642278 \| CD34 \| Bt.4571.1.S1\_at \| \| Bt.23237.1.S1\_at \| 510245 \| 1/1 \| XM\_587369 \| XM\_587369 \|  \| 11282978  16305752 \| PPP5C \| Bt.23237.1.S1\_at \| \| Bt.9772.1.S1\_at \|  \|  \|  \|  \|  \|  \| DKFZP566M1046 \| Bt.9772.1.S1\_at \| \| Bt.13118.1.S1\_at \| 281600 \| 1/3 \| XM\_614306 \| XM\_614306 \| GO:0007229 \| 10429942 \| ADAM9 \| Bt.13118.1.S1\_at \| \| Bt.9633.2.S1\_at \| 518540 \| 1/1 \| XM\_596733 \| XM\_596733 \|  \|  \| MEOX2 \| Bt.9633.2.S1\_at \| \| Bt.5527.1.S1\_at \| 615845 \| 2/2 \| NM\_001046562 \|  \| GO:0006450  GO:0017068 \|  \| 15E1.2 \| Bt.5527.1.S1\_at \| \| Bt.11256.1.S1\_at \| 533968 \| 1/2 \| XM\_613555 \| XM\_613555 \|  \|  \| CNOT1 \| Bt.11256.1.S1\_at \| \| Bt.25087.1.A1\_at \| 521244 \| 3/3 \|  \|  \|  \|  \|  \| Bt.25087.1.A1\_at \| \| Bt.20080.1.S1\_at \| 614851 \| 1/1 \| XM\_880078 \| XM\_880078 \|  \|  \| WSB1 \| Bt.20080.1.S1\_at \| \| Bt.29.1.S1\_at \| 338047 \| 1/1 \| NM\_176643 \| NM\_176643 \| GO:0016042  GO:0016787  GO:0046872 \| 9488669 \| DDHD1 \| Bt.29.1.S1\_at \| \| Bt.22852.1.S1\_at \| 617087 \| 1/1 \| XM\_001253301  XM\_883174 \|  \|  \|  \| MXRA7 \| Bt.22852.1.S1\_at \| \| Bt.10920.1.S1\_at \| 506569 \| 2/4 \| XM\_864201 \|  \|  \|  \| TTC3 \| Bt.10920.1.S1\_at \| \| Bt.23225.1.S1\_at \|  \|  \|  \|  \|  \|  \| AK1 \| Bt.23225.1.S1\_at \| \| Bt.21813.1.S1\_at \|  \|  \|  \|  \|  \|  \|  \| Bt.21813.1.S1\_at \| \| Bt.9958.1.S1\_at \| 404186 \| 1/1 \| NM\_001040495 \|  \| GO:0001558  GO:0005520  GO:0005576 \| 1282670  14966674 \| IGFBP6 \| Bt.9958.1.S1\_at \| \| Bt.70.1.S1\_at \| 327701 \| 1/1 \| NM\_175818 \| NM\_175818 \| GO:0003954  GO:0005739  GO:0005743  GO:0008137  GO:0016491 \| 1518044  12477932 \| NDUFB10 \| Bt.70.1.S1\_at \| \| Bt.3426.1.S1\_at \|  \|  \|  \|  \|  \|  \| KITLG \| Bt.3426.1.S1\_at \| \| Bt.16194.1.A1\_at \| 528138 \| 1/1 \| XM\_871667 \|  \| GO:0005515 \| 11282978  16305752 \| KLHL13 \| Bt.16194.1.A1\_at \| \| Bt.4972.1.S1\_at \| 526027 \| 2/4 \| XM\_588094 \| XM\_588094 \|  \|  \| SPTBN1 \| Bt.4972.1.S1\_at \| \| Bt.19621.1.A1\_at \|  \|  \|  \|  \|  \|  \|  \| Bt.19621.1.A1\_at \| \| Bt.20275.1.S1\_at \| 613421 \| 1/1 \| NM\_001034737 \|  \| GO:0005856  GO:0030833 \| 12140684 \| ARPC5L \| Bt.20275.1.S1\_at \| \| Bt.19100.1.A1\_at \|  \|  \|  \|  \|  \|  \|  \| Bt.19100.1.A1\_at \| \| Bt.18278.2.A1\_at \| 509717 \| 2/4 \| NM\_001034358 \| XM\_001251293 \|  \| 12477932 \|  \| Bt.18278.2.A1\_at \| \| Bt.26805.1.S1\_at \| 528615 \| 1/2 \| XM\_607042 \|  \| GO:0006457  GO:0031072  GO:0051082 \|  \| DNAJA4 \| Bt.26805.1.S1\_at \| \| Bt.25226.1.A1\_at \|  \|  \|  \|  \|  \|  \| KIAA1244 \| Bt.25226.1.A1\_at \| \| Bt.18986.1.S1\_at \| 511682 \| 1/1 \| NM\_001025330 \| NM\_001025330 \| GO:0004289  GO:0006508  GO:0008233 \|  \| MBTPS1 \| Bt.18986.1.S1\_at \| \| Bt.9405.1.S1\_at \| 514443 \| 1/1 \| XM\_592297 \|  \|  \|  \| TAF10 \| Bt.9405.1.S1\_at \| \| Bt.22163.1.S1\_at \| 540083 \| 1/3 \| NM\_001014959 \|  \| GO:0006457  GO:0031072  GO:0051082 \| 11282978 \| DNAJB5 \| Bt.22163.1.S1\_at \| \| Bt.13860.1.S1\_at \| 407170 \| 1/4 \| XM\_611785 \| XM\_611785 \| GO:0004714  GO:0004872  GO:0005021  GO:0005524  GO:0006468  GO:0007169  GO:0016020  GO:0016301 \| 1323289  8626709  15501243 \| KDR \| Bt.13860.1.S1\_at \| \| Bt.20613.2.A1\_at \| 506231 \| 1/2 \| NM\_001024483 \| NM\_001024483 \|  \| 11282978  16305752 \| BCAR3 \| Bt.20613.2.A1\_at \| \| Bt.16053.1.S1\_at \|  \|  \|  \|  \|  \|  \| PLXND1 \| Bt.16053.1.S1\_at \| \| Bt.9983.1.S1\_at \| 613636 \| 1/1 \| NM\_001046511 \|  \| GO:0004596  GO:0005634  GO:0008152  GO:0008415  GO:0016740 \|  \| ARD1A \| Bt.9983.1.S1\_at \| \| Bt.19526.1.S1\_at \| 506428 \| 2/2 \|  \| XR\_027451 \|  \|  \| UTRN \| Bt.19526.1.S1\_at \| \| Bt.9998.1.S1\_at \|  \|  \|  \|  \|  \|  \| MGC14289 \| Bt.9998.1.S1\_at \| \| Bt.7984.1.S1\_at \| 529670 \| 1/2 \| XM\_608123 \|  \| GO:0005102  GO:0005606  GO:0030155  GO:0030334  GO:0045995 \|  \| LAMA4 \| Bt.7984.1.S1\_at \| \| Bt.20402.2.A1\_at \| 530393 \| 3/3 \| NM\_001046312 \| NM\_001046312 \| GO:0005216  GO:0005509  GO:0005515  GO:0005929  GO:0005932  GO:0006811  GO:0006816  GO:0006874  GO:0007050  GO:0007259  GO:0007368  GO:0015629  GO:0016020  GO:0016021  GO:0050974 \| 15998908  16229973 \| PKD2 \| Bt.20402.2.A1\_at \| \| Bt.23667.1.S1\_at \|  \|  \|  \| XM\_001250744 \|  \|  \| P2RY5 \| Bt.23667.1.S1\_at \| \| Bt.2494.2.S1\_a\_at \| 615785 \| 1/2 \| NM\_001034788 \|  \| GO:0008092  GO:0016020  GO:0016021 \| 11282978  16305752 \| SDC2 \| Bt.2494.2.S1\_a\_at \| \| Bt.1217.1.A1\_a\_at \| 507707 \| 2/3 \| NM\_001075328 \|  \|  \|  \| TOMM70A \| Bt.1217.1.A1\_a\_at \| \| Bt.25016.1.S1\_at \| 537712 \| 2/3 \| XM\_617891 \|  \| GO:0003677  GO:0005634  GO:0006355  GO:0030528 \|  \| EBF \| Bt.25016.1.S1\_at \| \| Bt.23081.1.S1\_at \| 540340 \| 2/3 \| XM\_593330 \|  \|  \|  \|  \| Bt.23081.1.S1\_at \| \| Bt.1739.2.S1\_at \| 445416 \| 1/4 \| XM\_869491 \|  \| GO:0004926  GO:0004930  GO:0007166  GO:0016020 \| 17013885 \| FZD4 \| Bt.1739.2.S1\_at \| \| Bt.17194.1.S1\_at \| 618226 \| 1/2 \| XM\_870555 \|  \|  \|  \| SFPQ \| Bt.17194.1.S1\_at \| \| Bt.97.1.S1\_at \| 281759 \| 1/1 \| NM\_174314 \| NM\_174314 \| GO:0006810  GO:0008289 \| 8702709  16879357 \| FABP4 \| Bt.97.1.S1\_at \| \| Bt.28489.1.S1\_at \| 520833 \| 1/1 \| XR\_027272 \| XR\_027272 \|  \|  \| UBE4B \| Bt.28489.1.S1\_at \| \| Bt.12669.1.A1\_at \| 531382 \| 1/2 \| XM\_609874 \|  \|  \|  \| KIAA1627 \| Bt.12669.1.A1\_at \| \| Bt.10880.1.S1\_at \| 505489 \| 1/1 \| NM\_001035024 \|  \|  \| 11282978  12477932  16305752 \| MGC128406 \| Bt.10880.1.S1\_at \| \| Bt.8976.1.S1\_at \| 540481 \| 1/2 \| XM\_611661 \| XM\_611661 \| GO:0003677  GO:0006355 \|  \| TCF7L2 \| Bt.8976.1.S1\_at \| \| Bt.9244.1.A1\_at \| 538571 \| 1/1 \| XM\_581186 \|  \|  \|  \| KIAA1972 \| Bt.9244.1.A1\_at \| \| Bt.2065.1.A1\_at \|  \|  \|  \|  \|  \|  \| WDR68 \| Bt.2065.1.A1\_at \| \| Bt.17985.1.S1\_at \| 508065 \| 1/1 \| XM\_584793 \| XM\_584793 \|  \|  \| FLJ25476 \| Bt.17985.1.S1\_at \| \| Bt.12217.2.S1\_at \| 505824 \| 1/1 \| NM\_001075225 \|  \|  \|  \| NTRK2 \| Bt.12217.2.S1\_at \| \| Bt.4476.1.S1\_at \| 281998 \| 1/1 \| NM\_174432 \|  \| GO:0005739  GO:0016491  GO:0016763  GO:0042802  GO:0051920 \| 8089078  8912927  12477932  12773537 \| PRDX3 \| Bt.4476.1.S1\_at \| \| Bt.10777.1.S1\_at \| 515903 \| 3/5 \| XM\_001249521  XM\_001249570  XM\_878572  XM\_878731  XM\_878792 \|  \|  \|  \| FOXP1 \| Bt.10777.1.S1\_at \| \| Bt.28058.1.S1\_at \|  \|  \|  \|  \|  \|  \| ELK4 \| Bt.28058.1.S1\_at \| \| Bt.19937.2.S1\_at \|  \|  \|  \| XR\_027597 \|  \|  \| CPD \| Bt.19937.2.S1\_at \| \| Bt.12728.3.S1\_at \|  \|  \|  \|  \|  \|  \| ZNF704 \| Bt.12728.3.S1\_at \| \| Bt.6651.1.S1\_at \|  \|  \|  \| XR\_027267 \|  \|  \| TBX3 \| Bt.6651.1.S1\_at \| \| Bt.17989.1.S1\_at \|  \|  \|  \|  \|  \|  \| WDR71 \| Bt.17989.1.S1\_at \| \| Bt.20515.1.S1\_at \| 528175 \| 1/1 \| XM\_868130 \| XM\_868130 \| GO:0005488 \|  \| KIAA0892 \| Bt.20515.1.S1\_at \| \| Bt.23499.1.S1\_at \|  \|  \|  \| XM\_001253337 \|  \|  \| GYS1 \| Bt.23499.1.S1\_at \| \| Bt.25980.1.A1\_at \|  \|  \|  \|  \|  \|  \|  \| Bt.25980.1.A1\_at \| \| Bt.5021.1.S1\_at \| 281154 \| 1/1 \| NM\_174053 \|  \|  \| 7835900  8557636  12560590  12648102  12946356  15612324  15776436 \| FBN1 \| Bt.5021.1.S1\_at \| \| Bt.26622.1.S1\_a\_at \| 504914 \| 1/2 \| XM\_581108 \| XM\_581108 \|  \|  \| UROD \| Bt.26622.1.S1\_a\_at \| \| Bt.15854.1.A1\_at \| 513562 \| 1/4 \| NM\_001076846 \|  \| GO:0003723  GO:0004386 \|  \| FUBP1 \| Bt.15854.1.A1\_at \| \| Bt.9154.1.S1\_at \| 512187 \| 1/1 \| XM\_589646 \|  \|  \|  \| NUMB \| Bt.9154.1.S1\_at \| \| Bt.21792.2.S1\_a\_at \| 614102 \| 1/1 \| XM\_876688 \| XM\_876688 \| GO:0005739  GO:0030529 \| 11279123  11402041 \| MRPS18A \| Bt.21792.2.S1\_a\_at \| \| Bt.5603.2.S1\_at \| 534759 \| 1/2 \| XM\_866439 \| XM\_866439 \|  \|  \|  \| Bt.5603.2.S1\_at \| \| Bt.401.2.S1\_at \| 768330 \| 1/1 \| XM\_864209 \|  \| GO:0000062  GO:0006810  GO:0008289 \| 1518047  1622397  1931985  2881742  3525533  3663196  8358232 \| DBI \| Bt.401.2.S1\_at \| \| Bt.4580.1.A1\_at \|  \|  \|  \|  \|  \|  \| BAZ1B \| Bt.4580.1.A1\_at \| \| Bt.12267.1.S1\_at \| 613830 \| 1/1 \| XM\_864370 \| XM\_864370 \|  \|  \| FLJ23342 \| Bt.12267.1.S1\_at \| \| Bt.19937.1.S1\_at \|  \|  \|  \|  \|  \|  \| CPD \| Bt.19937.1.S1\_at \| \| Bt.25069.1.A1\_at \|  \|  \|  \|  \|  \|  \|  \| Bt.25069.1.A1\_at \| \| Bt.3605.1.S1\_at \| 508853 \| 1/1 \| NM\_001046015 \| NM\_001046015 \|  \|  \| IMMT \| Bt.3605.1.S1\_at \| \| Bt.23315.1.S1\_a\_at \| 280710 \| 1/3 \| NM\_173885 \|  \| GO:0000166  GO:0004713  GO:0005515  GO:0005524  GO:0006468  GO:0016740  GO:0030424  GO:0030425  GO:0030426  GO:0043025 \| 9312079  10085085  11278777  11535592  11799118  15123659 \| TNK2 \| Bt.23315.1.S1\_a\_at \| \| Bt.1606.1.S1\_at \| 404069 \| 1/1 \| NM\_205772 \| NM\_205772 \| GO:0005829  GO:0030140  GO:0042609  GO:0050851 \| 12131876 \| SPG21 \| Bt.1606.1.S1\_at \| \| Bt.2391.1.S1\_at \| 534664 \| 1/2 \| XM\_614507 \| XM\_614507 \|  \|  \| C1orf55 \| Bt.2391.1.S1\_at \| \| Bt.22047.1.S1\_at \| 535981 \| 1/1 \| NM\_001046414 \| NM\_001046414 \| GO:0005739  GO:0006508  GO:0008233  GO:0008462 \|  \| CLPP \| Bt.22047.1.S1\_at \| \| Bt.15698.1.S1\_at \| 540167 \| 1/2 \| NM\_001035428 \| NM\_001035428 \| GO:0016020  GO:0016021 \| 12140684  12477932  16305752 \| GK001 \| Bt.15698.1.S1\_at \| \| Bt.10135.1.A1\_at \|  \|  \|  \|  \|  \|  \| SLC35A5 \| Bt.10135.1.A1\_at \| \| Bt.3272.1.A1\_at \| 535714 \| 1/1 \| XM\_864867  XM\_874530  XM\_874789 \| XM\_864867 \| GO:0005737  GO:0006118  GO:0016491  GO:0045454  GO:0050660 \| 17158896 \| PDCD8 \| Bt.3272.1.A1\_at \| \| Bt.4320.1.S1\_at \|  \|  \|  \| XR\_028381 \|  \|  \| RNF38 \| Bt.4320.1.S1\_at \| \| Bt.20323.1.S1\_at \| 513740 \| 1/2 \| XM\_591483 \|  \|  \|  \| FAM21C \| Bt.20323.1.S1\_at \| \| Bt.20591.2.A1\_at \|  \|  \|  \|  \|  \|  \| FLJ12505 \| Bt.20591.2.A1\_at \| \| Bt.7728.1.A1\_at \|  \|  \|  \|  \|  \|  \| DCBLD2 \| Bt.7728.1.A1\_at \| \| Bt.20282.1.S1\_at \| 520864 \| 1/1 \| XM\_878970 \| XM\_878970 \|  \|  \| HIF1AN \| Bt.20282.1.S1\_at \| \| Bt.26675.1.S1\_at \| 534423 \| 1/4 \| NM\_001076023 \| NM\_001076023 \|  \|  \| FLJ21415 \| Bt.26675.1.S1\_at \| \| Bt.26919.1.S1\_at \| 537455 \| 1/1 \| XM\_617620 \|  \| GO:0005578 \|  \| NTN4 \| Bt.26919.1.S1\_at \| \| Bt.2116.1.S1\_at \| 510069 \| 1/1 \| NM\_001034369 \| NM\_001034369 \|  \| 12477932 \| PSMB6 \| Bt.2116.1.S1\_at \| \| Bt.28150.1.S1\_at \|  \|  \|  \|  \|  \|  \| TRIM7 \| Bt.28150.1.S1\_at \| \| Bt.1742.1.S1\_at \| 520030 \| 1/1 \| XM\_598260 \| XM\_598260 \| GO:0005578  GO:0016491  GO:0046914 \|  \| LAMB1 \| Bt.1742.1.S1\_at \| \| Bt.27846.1.S1\_at \|  \|  \|  \| XM\_001255826 \|  \|  \| SPATA7 \| Bt.27846.1.S1\_at \| \| Bt.10723.1.S1\_at \| 539035 \| 1/1 \| NM\_001076142 \| NM\_001076142 \|  \|  \| RNF13 \| Bt.10723.1.S1\_at \| \| Bt.20278.1.S1\_at \| 522721 \| 1/3 \| XM\_879427 \| XM\_879427 \|  \|  \| ZNF261 \| Bt.20278.1.S1\_at \| \| Bt.4555.1.S1\_at \| 617210 \| 1/1 \| NM\_001038582 \| NM\_001038582 \|  \|  \| ETFB \| Bt.4555.1.S1\_at \| \| Bt.4948.1.S1\_at \| 282705 \| 1/1 \| NM\_174721 \| NM\_174721 \|  \|  \| PSEN1 \| Bt.4948.1.S1\_at \| \| Bt.3990.1.S1\_at \| 540222 \| 1/2 \| XR\_027956 \|  \|  \|  \| KIAA0423 \| Bt.3990.1.S1\_at \| \| Bt.15836.2.S1\_at \| 510376 \| 2/2 \| NM\_001034378 \| NM\_001034378 \|  \| 12477932 \| ANKRD1 \| Bt.15836.2.S1\_at \| \| Bt.21176.1.S1\_at \|  \|  \|  \|  \|  \|  \| PHF13 \| Bt.21176.1.S1\_at \| \| Bt.9362.1.S1\_at \| 514403 \| 1/1 \| NM\_001040527 \|  \| GO:0003735  GO:0005622  GO:0005739  GO:0005840  GO:0006412 \| 11551941 \| MRPL2 \| Bt.9362.1.S1\_at \| \| Bt.12204.1.S1\_at \|  \|  \|  \|  \|  \|  \| KIAA0355 \| Bt.12204.1.S1\_at \| \| Bt.6007.1.S1\_at \|  \|  \|  \|  \|  \|  \|  \| Bt.6007.1.S1\_at \| \| Bt.11262.1.S1\_at \| 444864 \| 3/5 \| NM\_001002887 \| NM\_001002887 \| GO:0003676  GO:0003700  GO:0005622  GO:0005634  GO:0006355  GO:0006915  GO:0008270  GO:0046872 \| 15203203 \| PEG3 \| Bt.11262.1.S1\_at \| \| Bt.10922.1.A1\_at \|  \|  \|  \|  \|  \|  \| IGSF4 \| Bt.10922.1.A1\_at \| \| Bt.28754.1.S1\_at \|  \|  \|  \|  \|  \|  \| PURB \| Bt.28754.1.S1\_at \| \| Bt.3236.2.S1\_at \|  \|  \|  \| XM\_001250879 \|  \|  \| ARHGEF3 \| Bt.3236.2.S1\_at \| \| Bt.12514.1.S1\_at \| 515363 \| 1/1 \| NM\_001046198 \| NM\_001046198 \| GO:0004872  GO:0005634  GO:0005739  GO:0006350  GO:0006355  GO:0016020 \|  \| PHB2 \| Bt.12514.1.S1\_at \| \| Bt.26756.1.S1\_at \|  \|  \|  \|  \|  \|  \| MAP3K7IP1 \| Bt.26756.1.S1\_at \| \| Bt.2070.1.A1\_at \| 541175 \| 3/3 \| NM\_001034725 \|  \| GO:0004725  GO:0005634  GO:0006470  GO:0016787  GO:0017017 \| 11282978 \| DUSP10 \| Bt.2070.1.A1\_at \| \| Bt.6171.1.A1\_at \| 512002 \| 1/1 \| NM\_001046106 \| NM\_001046106 \| GO:0004616  GO:0006098  GO:0006573  GO:0008442  GO:0016491  GO:0051287 \|  \| HIBADH \| Bt.6171.1.A1\_at \| \| Bt.4689.1.S2\_at \| 282110 \| 1/1 \| NM\_174481 \| NM\_174481 \| GO:0004221  GO:0005622  GO:0005829  GO:0006511  GO:0006512  GO:0008234  GO:0043234 \| 12477932  16906146 \| UCHL5 \| Bt.4689.1.S2\_at \| \| Bt.9559.2.S1\_at \| 280722 \| 2/2 \| NM\_001076796 \|  \| GO:0005488  GO:0016021 \| 1656157  10594237  14709559 \| APP \| Bt.9559.2.S1\_at \| \| Bt.2510.1.A1\_at \| 510474 \| 1/1 \| XM\_878051  XM\_878096  XM\_878253 \|  \|  \|  \| SFRS5 \| Bt.2510.1.A1\_at \| \| Bt.4985.2.S1\_a\_at \| 508825 \| 2/3 \| NM\_001077884  XM\_864930 \| NM\_001077884 \|  \|  \|  \| Bt.4985.2.S1\_a\_at \| \| Bt.25689.1.S1\_at \|  \|  \|  \| XM\_001252094 \|  \|  \|  \| Bt.25689.1.S1\_at \| \| Bt.4449.1.S1\_at \| 618607 \| 1/1 \| NM\_001076513 \| NM\_001076513 \|  \|  \| AKR1A1 \| Bt.4449.1.S1\_at \| \| Bt.8807.1.S1\_at \| 510412 \| 1/1 \| XM\_587552 \|  \|  \|  \| GOLGA5 \| Bt.8807.1.S1\_at \| \| Bt.18381.1.S1\_at \| 540910 \| 1/1 \| XM\_613654  XM\_873352 \| XM\_873352 \| GO:0003677  GO:0006355 \|  \| SMARCE1 \| Bt.18381.1.S1\_at \| \| Bt.9623.1.A1\_at \| 539188 \| 1/1 \| XM\_585516 \|  \| GO:0003824  GO:0009058  GO:0016769  GO:0016847  GO:0030170 \| 14667821 \| GPT \| Bt.9623.1.A1\_at \| \| Bt.18755.1.A1\_at \| 534761 \| 1/4 \| XM\_873360 \| XM\_001257234 \| GO:0004672  GO:0005524  GO:0006468 \| 16519507 \| GSK3B \| Bt.18755.1.A1\_at \| \| Bt.20139.1.S1\_at \|  \|  \|  \|  \|  \|  \| DKFZP564J0863 \| Bt.20139.1.S1\_at \| \| Bt.23572.1.S1\_at \| 617512 \| 1/2 \| NM\_001046604 \|  \|  \|  \|  \| Bt.23572.1.S1\_at \| \| Bt.20977.1.S1\_at \| 538508 \| 1/4 \| XM\_580792 \|  \| GO:0004601  GO:0006979 \|  \| CCPG1 \| Bt.20977.1.S1\_at \| \| Bt.4797.1.S1\_at \| 508535 \| 1/1 \| NM\_001034322 \|  \|  \| 1279700  1371107  12140684  12477932  16305752 \| FKBP4 \| Bt.4797.1.S1\_at \| \| Bt.10813.1.S1\_at \| 515410 \| 1/1 \| NM\_001034491 \| NM\_001034491 \| GO:0005515  GO:0008270  GO:0046872 \| 12477932 \| PDLIM4 \| Bt.10813.1.S1\_at \| \| Bt.14473.1.S1\_at \| 535152 \| 1/1 \| XM\_615157 \| XM\_615157 \| GO:0003723  GO:0005524  GO:0005634  GO:0008026  GO:0016818 \|  \| DDX54 \| Bt.14473.1.S1\_at \| \| Bt.20744.1.A1\_at \|  \|  \|  \|  \|  \|  \| PDZK3 \| Bt.20744.1.A1\_at \| \| Bt.22034.1.S1\_at \|  \|  \|  \|  \|  \|  \| C1orf115 \| Bt.22034.1.S1\_at \| \| Bt.22187.1.S1\_at \|  \|  \|  \|  \|  \|  \| RCBTB1 \| Bt.22187.1.S1\_at \| \| Bt.13722.2.A1\_at \| 504716 \| 1/2 \| XM\_580880 \|  \|  \|  \| AHCTF1 \| Bt.13722.2.A1\_at \| \| Bt.13410.1.S1\_at \| 511190 \| 1/1 \| XM\_588472 \| XM\_588472 \| GO:0004674  GO:0004713  GO:0004867  GO:0005524  GO:0006468 \|  \| RPS6KA3 \| Bt.13410.1.S1\_at \| \| Bt.11138.1.S1\_at \| 613788 \| 1/1 \| XM\_864900 \|  \| GO:0003677  GO:0003899  GO:0006351  GO:0030880  GO:0045449 \|  \|  \| Bt.11138.1.S1\_at \| \| Bt.9949.2.S1\_at \| 533851 \| 2/3 \| NM\_001017947 \| NM\_001017947 \| GO:0001503  GO:0005515  GO:0005856  GO:0007275  GO:0008270  GO:0030154  GO:0046872 \| 11282978  16305752 \| PDLIM7 \| Bt.9949.2.S1\_at \| \| Bt.16412.1.S1\_a\_at \| 508724 \| 1/1 \| XM\_585546 \| XM\_585546 \|  \|  \| FAH \| Bt.16412.1.S1\_a\_at \| \| Bt.9246.1.A1\_at \|  \|  \|  \|  \|  \|  \| FRS2 \| Bt.9246.1.A1\_at \| \| Bt.21196.1.S1\_at \| 286850 \| 1/3 \| NM\_174785 \|  \| GO:0004871  GO:0005834  GO:0007165  GO:0007186  GO:0016020 \| 7493986 \| GNG12 \| Bt.21196.1.S1\_at \| \| Bt.18623.1.A1\_at \| 510159 \| 1/2 \| NM\_001034374 \| NM\_001034374 \|  \| 12477932 \| MAPRE2 \| Bt.18623.1.A1\_at \| \| Bt.18113.2.S1\_at \| 511816 \| 1/1 \| XM\_589227 \| XM\_589227 \|  \|  \| SNAPAP \| Bt.18113.2.S1\_at \| \| Bt.7696.2.S1\_a\_at \| 516619 \| 2/2 \| NM\_001015614 \|  \| GO:0004663  GO:0005515  GO:0008270  GO:0008318  GO:0016740  GO:0018346  GO:0046872 \| 11282978  16305752 \| RABGGTA \| Bt.7696.2.S1\_a\_at \| \| Bt.7039.1.S1\_a\_at \| 510434 \| 1/5 \| NM\_001038071 \|  \|  \|  \| MGC134398 \| Bt.7039.1.S1\_a\_at \| \| Bt.4985.1.S1\_at \|  \|  \|  \| XM\_864930 \|  \|  \|  \| Bt.4985.1.S1\_at \| \| Bt.5035.1.S1\_at \| 404132 \| 1/1 \| NM\_205796 \| NM\_205796 \|  \|  \| APOA1BP \| Bt.5035.1.S1\_at \| \| Bt.7342.1.S1\_at \| 509591 \| 1/1 \| XM\_875611 \|  \|  \|  \|  \| Bt.7342.1.S1\_at \| \| Bt.11014.1.S1\_at \| 513889 \| 1/1 \| NM\_001046159 \| NM\_001046159 \| GO:0005739  GO:0006605  GO:0015031 \|  \| CHCHD4 \| Bt.11014.1.S1\_at \| \| Bt.14176.1.S1\_at \|  \|  \|  \|  \|  \|  \| TIMP2 \| Bt.14176.1.S1\_at \| \| Bt.2625.1.S1\_at \|  \|  \|  \|  \|  \|  \| FLJ10154 \| Bt.2625.1.S1\_at \| \| Bt.10600.1.S1\_at \| 513632 \| 1/1 \| XM\_864976 \| XM\_864976 \| GO:0005515 \|  \| RCBTB2 \| Bt.10600.1.S1\_at \| \| Bt.6964.1.S1\_at \| 780784 \| 1/1 \| NM\_001079640  XM\_001252269 \| XM\_868823 \| GO:0003779  GO:0005523  GO:0005856 \|  \| TMOD1 \| Bt.6964.1.S1\_at \| \| Bt.4834.1.S1\_at \| 281161 \| 1/1 \| NM\_174056 \| NM\_174056 \|  \| 2425435  3472745  3556754  3596000  3741423  3809608  3863109  3940857  4081126  6591194  10867016  12441094  12461785  12591230  14561657  15389540  15591023  16307186  16338792  16574787  16769728 \| FGF2 \| Bt.4834.1.S1\_at \| \| Bt.23331.1.S1\_at \| 510341 \| 1/2 \| XM\_587468 \|  \|  \|  \|  \| Bt.23331.1.S1\_at \| \| Bt.9055.2.S1\_a\_at \| 616451 \| 1/2 \| NM\_001038213 \| NM\_001038213 \|  \|  \| TRMU \| Bt.9055.2.S1\_a\_at \| \| Bt.4384.1.S1\_at \| 504482 \| 1/2 \| XM\_865880 \|  \|  \|  \| WASF2 \| Bt.4384.1.S1\_at \| \| Bt.20800.1.S1\_at \| 613349 \| 4/4 \| XM\_864015 \|  \|  \|  \| TCTE1L \| Bt.20800.1.S1\_at \| \| Bt.22054.1.S1\_at \| 522230 \| 2/2 \| XM\_600505 \| XM\_600505 \|  \|  \| C20orf7 \| Bt.22054.1.S1\_at \| \| Bt.5723.1.S1\_at \| 326346 \| 1/2 \| NM\_175718 \| NM\_175718 \| GO:0003954  GO:0005739  GO:0008137  GO:0016020  GO:0016021  GO:0016491 \| 12381726  12477932 \| NDUFA11 \| Bt.5723.1.S1\_at \| \| Bt.10354.1.S1\_at \|  \|  \|  \|  \|  \|  \|  \| Bt.10354.1.S1\_at \| \| Bt.17012.1.A1\_s\_at \| 530254 \| 2/2 \| XM\_001254999  XM\_608719 \| XM\_001254999 \| GO:0005488  GO:0031072 \|  \| DNAJC7 \| Bt.17012.1.A1\_s\_at \| \| Bt.8673.1.S1\_a\_at \|  \|  \|  \|  \|  \|  \|  \| Bt.8673.1.S1\_a\_at \| \| Bt.7183.1.S1\_at \| 505055 \| 1/1 \|  \| NM\_001046247 \|  \|  \|  \| Bt.7183.1.S1\_at \| \| Bt.27952.1.S1\_at \| 616748 \| 2/2 \| NM\_001034796 \| NM\_001034796 \|  \| 12477932 \| MGC127642 \| Bt.27952.1.S1\_at \| \| Bt.21239.1.S1\_at \| 535625 \| 1/4 \| XM\_615733 \|  \| GO:0004674  GO:0005524  GO:0006468 \|  \| MAPKAPK5 \| Bt.21239.1.S1\_at \| \| Bt.28684.1.S1\_at \|  \|  \|  \|  \|  \|  \| KPNA6 \| Bt.28684.1.S1\_at \| \| Bt.3975.1.S1\_at \| 506522 \| 1/2 \| XM\_582979 \| XM\_582979 \|  \|  \| C9orf112 \| Bt.3975.1.S1\_at \| \| Bt.22459.1.S1\_at \|  \|  \|  \| XM\_001250911 \|  \|  \| NCAM1 \| Bt.22459.1.S1\_at \| \| Bt.20852.1.A1\_at \|  \|  \|  \|  \|  \|  \|  \| Bt.20852.1.A1\_at \| \| Bt.18079.1.A1\_at \| 512341 \| 1/1 \| XM\_864349 \|  \|  \|  \| MLYCD \| Bt.18079.1.A1\_at \| \| Bt.28480.1.S1\_at \| 506073 \| 1/1 \| XM\_865033 \|  \|  \|  \| PLAA \| Bt.28480.1.S1\_at \| \| Bt.8674.1.A1\_at \|  \|  \|  \|  \|  \|  \| CREB3L2 \| Bt.8674.1.A1\_at \| \| Bt.28105.1.S1\_at \| 615534 \| 1/2 \| XM\_001249400  XM\_867375 \| XM\_001249400 \| GO:0004896  GO:0016020 \|  \| FNDC3B \| Bt.28105.1.S1\_at \| \| Bt.11023.1.S1\_at \| 518910 \| 1/1 \| NM\_001077937  XM\_001250023 \|  \|  \|  \| ZBTB5 \| Bt.11023.1.S1\_at \| \| Bt.20326.1.S1\_at \| 534386 \| 1/2 \| XM\_001250786  XM\_001250840  XM\_614137 \|  \|  \|  \|  \| Bt.20326.1.S1\_at \| \| Bt.24253.1.S1\_at \| 613828 \| 1/3 \| NM\_001046519 \|  \| GO:0005622  GO:0005634  GO:0043565 \|  \| DR1 \| Bt.24253.1.S1\_at \| \| Bt.23509.2.A1\_at \|  \|  \|  \|  \|  \|  \| PPAP2B \| Bt.23509.2.A1\_at \| \| Bt.7633.1.S1\_at \| 617105 \| 1/1 \| NM\_001040599 \| NM\_001040599 \|  \|  \|  \| Bt.7633.1.S1\_at \| \| Bt.7421.1.S1\_at \| 616223 \| 1/1 \| XM\_869283 \| XM\_869283 \|  \|  \|  \| Bt.7421.1.S1\_at \| \| Bt.9486.1.S1\_at \| 532578 \| 1/2 \| XM\_611700 \| XM\_611700 \|  \|  \| FLJ10707 \| Bt.9486.1.S1\_at \| \| Bt.6701.1.S1\_at \| 510604 \| 1/4 \| XR\_028094 \| XR\_028094 \|  \|  \| PCF11 \| Bt.6701.1.S1\_at \| \| Bt.7373.2.S1\_a\_at \| 504463 \| 2/4 \| XM\_580596 \|  \|  \|  \| SIRT2 \| Bt.7373.2.S1\_a\_at \| \| Bt.4768.1.S2\_at \| 281258 \| 1/2 \| NM\_174095 \| NM\_174095 \| GO:0005923  GO:0016020  GO:0016021 \| 10395639  11282978  12477932  16305752 \| F11R \| Bt.4768.1.S2\_at \| \| Bt.4503.1.S2\_at \|  \|  \|  \|  \|  \|  \| MTCH2 \| Bt.4503.1.S2\_at \| \| Bt.11704.1.S1\_at \|  \|  \|  \|  \|  \|  \| LDLRAD3 \| Bt.11704.1.S1\_at \| \| Bt.24624.2.S1\_at \| 519302 \| 2/3 \| NM\_001035332 \| NM\_001035332 \| GO:0003735  GO:0005622  GO:0005739  GO:0005840  GO:0006412 \| 11282978  11344316  16305752 \| MRPS9 \| Bt.24624.2.S1\_at \| \| Bt.22071.1.S1\_at \| 516039 \| 1/1 \| XM\_594169 \| XM\_594169 \|  \|  \| MAP2K3 \| Bt.22071.1.S1\_at \| \| Bt.5465.2.A1\_a\_at \| 511560 \| 1/1 \| NM\_001079588  XM\_001251855 \| XM\_001251855 \| GO:0005515  GO:0005622  GO:0005634  GO:0005730  GO:0006364  GO:0006412  GO:0019843  GO:0030529 \|  \| IMP3 \| Bt.5465.2.A1\_a\_at \| \| Bt.27123.1.A1\_at \| 532209 \| 3/3 \| XM\_610719 \| XM\_610719 \|  \|  \| EVI1 \| Bt.27123.1.A1\_at \| \| Bt.9012.1.S1\_at \| 508247 \| 1/1 \| XM\_585000 \| XM\_585000 \|  \|  \| KIAA1639 \| Bt.9012.1.S1\_at \| \| Bt.1418.2.S1\_at \|  \|  \|  \|  \|  \|  \| MGC12966 \| Bt.1418.2.S1\_at \| \| Bt.25637.1.A1\_at \|  \|  \|  \|  \|  \|  \| IDH3A \| Bt.25637.1.A1\_at \| \| Bt.14040.1.S1\_at \| 506263 \| 1/1 \| NM\_001075249 \|  \|  \|  \| ZADH1 \| Bt.14040.1.S1\_at \| \| Bt.11717.1.S1\_s\_at \|  \|  \|  \|  \|  \|  \|  \| Bt.11717.1.S1\_s\_at \| \| Bt.7430.1.S1\_at \| 538584 \| 1/1 \| XM\_581232 \| XM\_581232 \| GO:0005515  GO:0005622  GO:0007165 \|  \| ARHGAP21 \| Bt.7430.1.S1\_at \| \| Bt.24945.1.S1\_at \| 539978 \| 1/1 \| NM\_001034686 \| NM\_001034686 \| GO:0006071  GO:0008889  GO:0016020  GO:0016021  GO:0016787  GO:0047395 \| 12477932 \| MIR16 \| Bt.24945.1.S1\_at \| \| Bt.25479.1.S1\_at \| 618186 \| 1/1 \| XM\_870509 \| XM\_870509 \|  \|  \| DERPC \| Bt.25479.1.S1\_at \| \| Bt.9337.1.S1\_at \|  \|  \|  \|  \|  \|  \| FLJ31951 \| Bt.9337.1.S1\_at \| \| Bt.7397.1.S1\_at \| 534482 \| 1/1 \| NM\_001017950 \|  \| GO:0005509 \| 11282978 \| FSTL1 \| Bt.7397.1.S1\_at \| \| Bt.4702.1.S1\_at \|  \|  \|  \|  \|  \|  \| FOXO3A \| Bt.4702.1.S1\_at \| \| Bt.12466.1.A1\_at \|  \|  \|  \|  \|  \|  \| CDGAP \| Bt.12466.1.A1\_at \| \| Bt.9114.1.A1\_at \| 540564 \| 2/3 \| XM\_612101 \| XM\_612101 \|  \|  \|  \| Bt.9114.1.A1\_at \| \| Bt.5089.1.S1\_at \| 617051 \| 3/5 \| XM\_883110 \|  \|  \|  \| H3F3B \| Bt.5089.1.S1\_at \| \| Bt.10.2.S1\_a\_at \| 281698 \| 1/1 \| NM\_174277 \| NM\_174277 \| GO:0005509  GO:0005905  GO:0006886  GO:0030125 \| 2434865  2930486  3821891  12426379  16460735 \| CLTB \| Bt.10.2.S1\_a\_at \| \| Bt.26550.1.S1\_at \| 513061 \| 1/2 \| NM\_001046136 \| NM\_001046136 \| GO:0000166  GO:0005524  GO:0016887 \| 11282978  16305752 \|  \| Bt.26550.1.S1\_at \| \| Bt.20780.2.S1\_a\_at \| 508149 \| 1/1 \| XM\_584888  XM\_875829 \| XM\_875829 \|  \|  \|  \| Bt.20780.2.S1\_a\_at \| \| Bt.2052.1.S1\_a\_at \| 533188 \| 1/4 \| XM\_612512 \|  \|  \| 12477932 \|  \| Bt.2052.1.S1\_a\_at \| \| Bt.19061.1.A1\_at \| 534203 \| 1/2 \| NM\_001035405 \|  \|  \| 12477932 \| KIAA1458 \| Bt.19061.1.A1\_at \| \| Bt.7257.1.S1\_at \|  \|  \|  \| XM\_001255662 \|  \|  \|  \| Bt.7257.1.S1\_at \| \| Bt.10947.1.S1\_at \| 511088 \| 1/1 \| NM\_001075492 \| NM\_001075492 \|  \|  \| C7orf28A \| Bt.10947.1.S1\_at \| \| Bt.997.2.S1\_a\_at \| 516287 \| 2/2 \| NM\_001034518 \|  \| GO:0004128  GO:0006118  GO:0016491  GO:0050660  GO:0051287 \| 12477932 \| CYB5R1 \| Bt.997.2.S1\_a\_at \| \| Bt.21119.1.A1\_at \| 525346 \| 4/4 \| XM\_867432 \| XM\_867432 \|  \|  \| NCOA1 \| Bt.21119.1.A1\_at \| \| Bt.16945.1.S1\_at \| 509150 \| 1/1 \| NM\_001034344 \|  \|  \| 12477932 \| MGC127386 \| Bt.16945.1.S1\_at \| \| Bt.21491.1.S1\_at \| 614915 \| 1/1 \| NM\_001034772 \| NM\_001034772 \| GO:0000785  GO:0003677  GO:0005634 \| 456580  12477932 \| HMGN1 \| Bt.21491.1.S1\_at \| \| Bt.28212.1.A1\_at \|  \|  \|  \|  \|  \|  \| SMAD5 \| Bt.28212.1.A1\_at \| \| Bt.7184.1.S1\_at \| 540421 \| 1/1 \| NM\_001014964 \| NM\_001014964 \| GO:0004674  GO:0005057  GO:0005524  GO:0006468  GO:0007242 \| 11282978 \| ARAF \| Bt.7184.1.S1\_at \| \| Bt.22552.1.S2\_at \| 514447 \| 1/3 \| XM\_592305 \|  \|  \|  \| CLSTN1 \| Bt.22552.1.S2\_at \| \| Bt.1262.1.S1\_at \| 281813 \| 1/1 \| NM\_174338 \| NM\_174338 \|  \|  \| HCBP6 \| Bt.1262.1.S1\_at \| \| Bt.935.1.A1\_at \| 539354 \| 2/2 \| NM\_001079612  XM\_001250904 \| XM\_001250904 \| GO:0000139  GO:0005351  GO:0008643  GO:0016021 \|  \| SLC35A4 \| Bt.935.1.A1\_at \| \| Bt.28462.1.S1\_at \| 507702 \| 1/3 \| NM\_001075326 \| NM\_001075326 \|  \|  \| C6orf93 \| Bt.28462.1.S1\_at \| \| Bt.22553.1.A1\_at \|  \|  \|  \|  \|  \|  \|  \| Bt.22553.1.A1\_at \| \| Bt.23182.1.S1\_at \| 281156 \| 1/1 \| NM\_177497 \| NM\_177497 \|  \| 11773414 \| FDPS \| Bt.23182.1.S1\_at \| \| Bt.4355.1.S1\_at \| 282885 \| 1/1 \| NM\_174749 \|  \| GO:0005739  GO:0005777  GO:0016491  GO:0051920 \| 12477932  14662316 \| PRDX5 \| Bt.4355.1.S1\_at \| \| Bt.15900.1.A1\_at \| 532836 \| 1/3 \| XM\_612032  XM\_876398 \| XM\_612032 \| GO:0004013  GO:0006730 \|  \| KIAA0828 \| Bt.15900.1.A1\_at \| \| Bt.5445.2.A1\_at \| 281555 \| 1/1 \| NM\_001003900 \|  \|  \| 1946367  9428769  11700061  15518544  15959508  16159957  16371510 \| TUBB2 \| Bt.5445.2.A1\_at \| \| Bt.28699.1.S1\_at \|  \|  \|  \|  \|  \|  \| WASL \| Bt.28699.1.S1\_at \| \| Bt.27118.1.A1\_at \| 510651 \| 1/3 \| XM\_587818 \| XM\_587818 \|  \|  \| FLJ10652 \| Bt.27118.1.A1\_at \| \| Bt.16753.1.A1\_at \|  \|  \|  \|  \|  \|  \| ZNF652 \| Bt.16753.1.A1\_at \| \| Bt.13834.1.S1\_at \|  \|  \|  \| XM\_580860 \|  \|  \| TFRC \| Bt.13834.1.S1\_at \| \| Bt.21404.1.S1\_at \|  \|  \|  \|  \|  \|  \| EP400 \| Bt.21404.1.S1\_at \| \| Bt.28071.1.S1\_at \| 511554 \| 1/1 \| XM\_588909 \|  \| GO:0016020  GO:0016021 \|  \| C18orf19 \| Bt.28071.1.S1\_at \| \| Bt.13887.1.S1\_at \| 617375 \| 1/1 \| XM\_001253384 \| XM\_001253384 \|  \|  \|  \| Bt.13887.1.S1\_at \| \| Bt.2001.1.S1\_at \| 613488 \| 1/1 \| XM\_873100 \|  \|  \|  \|  \| Bt.2001.1.S1\_at \| \| Bt.27027.1.S1\_a\_at \| 508928 \| 1/1 \| XM\_585783 \| XM\_585783 \|  \|  \| MRPS15 \| Bt.27027.1.S1\_a\_at \| \| Bt.23649.1.A1\_at \| 534331 \| 1/2 \| XM\_614067 \| XM\_614067 \| GO:0003723  GO:0004386  GO:0005524  GO:0005634 \| 12477932 \|  \| Bt.23649.1.A1\_at \| \| Bt.8961.1.S1\_at \|  \|  \|  \|  \|  \|  \| PIP5K3 \| Bt.8961.1.S1\_at \| \| Bt.2378.1.S1\_at \|  \|  \|  \|  \|  \|  \| IHPK1 \| Bt.2378.1.S1\_at \| \| Bt.22633.1.S1\_at \| 616899 \| 1/1 \| XM\_869036 \| XM\_869036 \|  \|  \|  \| Bt.22633.1.S1\_at \| \| Bt.1719.1.S1\_at \| 520335 \| 1/1 \| NM\_001035335 \| NM\_001035335 \| GO:0005739  GO:0005743  GO:0016020  GO:0016021  GO:0051205 \| 12477932 \| MGC127460 \| Bt.1719.1.S1\_at \| \| Bt.25339.1.S1\_at \|  \|  \|  \| XM\_865894 \|  \|  \| MORC3 \| Bt.25339.1.S1\_at \| \| Bt.3817.1.A1\_at \|  \|  \|  \|  \|  \|  \| SACS \| Bt.3817.1.A1\_at \| \| Bt.13548.1.A1\_at \| 538498 \| 1/3 \| XM\_580692 \| XM\_580692 \|  \|  \| ZZZ3 \| Bt.13548.1.A1\_at \| \| Bt.20463.1.S1\_at \| 534612 \| 1/1 \| XM\_001249935  XM\_864319  XM\_873095 \| XM\_873095 \| GO:0000785  GO:0003677  GO:0003682  GO:0005622  GO:0005634  GO:0006333 \|  \| ARID4B \| Bt.20463.1.S1\_at \| \| Bt.22647.3.A1\_at \| 532952 \| 1/2 \| XM\_001251033  XM\_612177 \| XM\_612177 \| GO:0005515 \|  \| MAGI3 \| Bt.22647.3.A1\_at \| \| Bt.29874.1.S1\_at \| 406231 \| 1/3 \| NM\_001001133 \|  \|  \|  \| EIF2C3 \| Bt.29874.1.S1\_at \| \| Bt.21372.1.S1\_at \| 513054 \| 3/6 \| NM\_001075595 \| NM\_001075595 \|  \|  \| WHSC1L1 \| Bt.21372.1.S1\_at \| \| Bt.22466.1.A1\_at \| 540614 \| 1/2 \| NM\_001076230 \|  \| GO:0000166  GO:0005525  GO:0007049 \|  \| SEPT8 \| Bt.22466.1.A1\_at \| \| Bt.3789.1.S1\_at \| 530616 \| 1/2 \| XM\_609091 \| XM\_609091 \| GO:0005634  GO:0030528  GO:0045449 \|  \| TCF3 \| Bt.3789.1.S1\_at \| \| Bt.28123.1.S1\_at \|  \|  \|  \|  \|  \|  \| UGCG \| Bt.28123.1.S1\_at \| \| Bt.24307.1.A1\_at \| 537711 \| 1/3 \| XM\_001251750  XM\_617890 \|  \|  \|  \| RAPH1 \| Bt.24307.1.A1\_at \| \| Bt.3193.1.A1\_at \| 538625 \| 1/1 \| NM\_001034649 \| NM\_001034649 \| GO:0005829  GO:0043234 \| 12477932 \| PSMD6 \| Bt.3193.1.A1\_at \| \| Bt.7007.1.S1\_at \| 404143 \| 1/3 \| NM\_001034052 \|  \| GO:0016021 \| 12477932  12658628 \| GHITM \| Bt.7007.1.S1\_at \| \| Bt.17608.1.A1\_at \|  \|  \|  \|  \|  \|  \| DNAJB14 \| Bt.17608.1.A1\_at \| \| Bt.25657.1.S1\_at \|  \|  \|  \|  \|  \|  \| GULP1 \| Bt.25657.1.S1\_at \| \| Bt.5682.1.S1\_at \|  \|  \|  \|  \|  \|  \| TCF4 \| Bt.5682.1.S1\_at \| \| Bt.24023.1.A1\_at \|  \|  \|  \|  \|  \|  \| GATA6 \| Bt.24023.1.A1\_at \| \| Bt.21215.1.A1\_at \| 506721 \| 2/3 \| XM\_001251311  XM\_583206  XM\_865524 \| XM\_583206 \|  \|  \| PITX2 \| Bt.21215.1.A1\_at \| \| Bt.10990.1.S1\_at \| 539560 \| 2/2 \| NM\_001076173 \| NM\_001076173 \|  \|  \|  \| Bt.10990.1.S1\_at \| \| Bt.8736.2.S1\_at \|  \|  \|  \| XM\_598835 \|  \|  \| C1orf9 \| Bt.8736.2.S1\_at \| \| Bt.9240.1.A1\_at \| 511058 \| 2/3 \| XM\_588314 \| XM\_588314 \| GO:0016787 \|  \| PPP6C \| Bt.9240.1.A1\_at \| \| Bt.12088.1.S1\_at \| 540353 \| 1/1 \| XM\_593424 \| XM\_593424 \| GO:0005515  GO:0008270 \|  \| RNF111 \| Bt.12088.1.S1\_at \| \| Bt.9629.1.S1\_at \| 614773 \| 2/3 \| XM\_865938 \| XM\_865938 \|  \|  \| ZFP36L1 \| Bt.9629.1.S1\_at \| \| Bt.16525.1.A1\_at \|  \|  \|  \|  \|  \|  \| ZNF609 \| Bt.16525.1.A1\_at \| \| Bt.13925.1.S1\_at \| 504637 \| 1/1 \| NM\_001045894 \| NM\_001045894 \|  \|  \| RFC4 \| Bt.13925.1.S1\_at \| \| Bt.27095.1.S1\_at \| 513265 \| 1/1 \| NM\_001046143 \| NM\_001046143 \|  \|  \| CARF \| Bt.27095.1.S1\_at \| \| Bt.16264.1.A1\_at \|  \|  \|  \| XM\_864068 \|  \|  \| BRAF \| Bt.16264.1.A1\_at \| \| Bt.9197.1.S1\_at \| 532940 \| 1/2 \| XM\_612163 \| XM\_612163 \|  \|  \| CCDC28A \| Bt.9197.1.S1\_at \| \| Bt.21372.2.S1\_at \| 513054 \| 3/6 \| NM\_001075595 \|  \|  \|  \|  \| Bt.21372.2.S1\_at \| \| Bt.1286.1.S1\_a\_at \| 508990 \| 1/1 \| NM\_001046020 \|  \|  \|  \|  \| Bt.1286.1.S1\_a\_at \| \| Bt.4390.1.S1\_at \| 280939 \| 1/1 \| NM\_173964 \| NM\_173964 \|  \| 8415706 \| TEK \| Bt.4390.1.S1\_at \| \| Bt.3843.1.S1\_at \| 280821 \| 1/1 \| NM\_175773 \| NM\_175773 \|  \| 1427820  8286037  12477932 \| IGJ \| Bt.3843.1.S1\_at \| \| Bt.7962.1.S1\_at \|  \|  \|  \| XM\_001254874 \|  \|  \| YPEL3 \| Bt.7962.1.S1\_at \| \| Bt.13254.1.S1\_at \| 529423 \| 1/1 \| XM\_607870 \|  \|  \|  \| FAM40B \| Bt.13254.1.S1\_at \| \| Bt.16782.2.S1\_at \|  \|  \|  \|  \|  \|  \| ADSSL1 \| Bt.16782.2.S1\_at \| \| Bt.21938.1.S1\_at \|  \|  \|  \|  \|  \|  \| PDE7B \| Bt.21938.1.S1\_at \| \| Bt.10251.1.S1\_at \|  \|  \|  \|  \|  \|  \| MLR2 \| Bt.10251.1.S1\_at \| \| Bt.22290.1.S1\_at \| 616926 \| 1/1 \| NM\_001078066  XM\_001249839  XM\_001249888 \| XM\_001249888 \|  \|  \| ESAM \| Bt.22290.1.S1\_at \| \| Bt.9301.1.A1\_at \| 529591 \| 1/3 \| XM\_608043 \|  \| GO:0003676  GO:0005515  GO:0005622  GO:0008270 \|  \| PHF20 \| Bt.9301.1.A1\_at \| \| Bt.27396.1.S1\_at \|  \|  \|  \|  \|  \|  \| ABI1 \| Bt.27396.1.S1\_at \| \| Bt.27129.1.S1\_at \| 539399 \| 1/2 \| XM\_586888  XM\_877675 \| XM\_877675 \| GO:0016020  GO:0030001  GO:0046873 \|  \| SLC39A9 \| Bt.27129.1.S1\_at \| \| Bt.28043.1.S1\_at \| 513934 \| 1/2 \| XM\_591699 \| XM\_591699 \| GO:0005515 \|  \| GCL \| Bt.28043.1.S1\_at \| \| Bt.13065.1.S1\_at \| 282572 \| 1/1 \| NM\_001040482 \| NM\_001040482 \| GO:0006813  GO:0015272  GO:0016020 \| 16305752 \| KCNJ8 \| Bt.13065.1.S1\_at \| \| Bt.10175.2.A1\_a\_at \| 538549 \| 1/1 \| XM\_581058 \|  \|  \|  \|  \| Bt.10175.2.A1\_a\_at \| \| Bt.2606.1.S1\_at \|  \|  \|  \|  \|  \|  \| LAMP2 \| Bt.2606.1.S1\_at \| \| Bt.5434.1.S1\_at \| 614538 \| 1/1 \| NM\_001034761 \| NM\_001034761 \| GO:0004579  GO:0005783  GO:0006915  GO:0016020  GO:0016021  GO:0016740 \| 12140684  12477932  16305752 \| DAD1 \| Bt.5434.1.S1\_at \| \| Bt.22763.2.S1\_a\_at \| 407767 \| 1/2 \| XM\_001252032  XM\_609765 \| XM\_001251609 \| GO:0004421  GO:0006084 \| 12477932  14510667 \| HMGCS1 \| Bt.22763.2.S1\_a\_at \| \| Bt.3945.1.S1\_at \| 529494 \| 1/3 \| XM\_607943 \|  \| GO:0005488 \|  \| DKFZP564D166 \| Bt.3945.1.S1\_at \| \| Bt.5529.2.S1\_a\_at \| 614288 \| 2/2 \| XM\_865734 \| XM\_001252127 \|  \|  \|  \| Bt.5529.2.S1\_a\_at \| \| Bt.4658.1.S1\_a\_at \| 504533 \| 1/1 \| NM\_001034214 \| NM\_001034214 \|  \| 12477932 \| UBL4A \| Bt.4658.1.S1\_a\_at \| \| Bt.26920.2.A1\_a\_at \|  \|  \|  \|  \|  \|  \| XK \| Bt.26920.2.A1\_a\_at \| \| Bt.19522.2.S1\_at \|  \|  \|  \|  \|  \|  \| RAB11A \| Bt.19522.2.S1\_at \| \| Bt.9578.1.S1\_at \| 282318 \| 1/1 \| NM\_174581 \| NM\_174581 \| GO:0004721  GO:0005506  GO:0005975  GO:0005977  GO:0007049  GO:0016787  GO:0030145  GO:0046872  GO:0051301 \| 12065625 \| PPP1CC \| Bt.9578.1.S1\_at \| \| Bt.9643.1.S1\_at \| 512662  617985 \| 1/1 \| NM\_001075576  XM\_001255339  XM\_870317 \| XM\_870317 \|  \|  \| CHP \| Bt.9643.1.S1\_at \| \| Bt.20193.1.S1\_at \| 408010 \| 1/1 \| XM\_614980 \|  \| GO:0004820  GO:0005524  GO:0006426  GO:0016874 \|  \| GARS \| Bt.20193.1.S1\_at \| \| Bt.18634.1.A1\_at \| 540329 \| 4/4 \| NM\_001046474 \|  \| GO:0000287  GO:0005739  GO:0006470  GO:0008287  GO:0015071  GO:0016787 \| 16229973 \| PPM1K \| Bt.18634.1.A1\_at \| \| Bt.20485.1.S1\_at \| 533746 \| 1/3 \| NM\_001076000 \|  \| GO:0005739  GO:0007275  GO:0007399  GO:0016787  GO:0030154  GO:0030424  GO:0030425  GO:0043025 \|  \| DPYSL2 \| Bt.20485.1.S1\_at \| \| Bt.7030.1.S1\_at \| 520505 \| 1/1 \| XR\_028223 \| XR\_028223 \|  \|  \| SIN3B \| Bt.7030.1.S1\_at \| \| Bt.16243.1.S1\_at \| 533671 \| 1/1 \| NM\_001046358 \| NM\_001046358 \| GO:0016020 \|  \| CKLFSF8 \| Bt.16243.1.S1\_at \| \| Bt.6087.2.S1\_a\_at \| 533038 \| 2/2 \| NM\_001075980 \|  \| GO:0016021 \| 12140684  16305752 \| TM4SF1 \| Bt.6087.2.S1\_a\_at \| \| Bt.4480.1.S2\_at \| 281216 \| 1/2 \| NM\_175778 \|  \| GO:0004383  GO:0006182  GO:0007242 \| 1973124  9391039 \| GUCY1A3 \| Bt.4480.1.S2\_at \| \| Bt.26894.1.S1\_at \|  \|  \|  \|  \|  \|  \| FBXL17 \| Bt.26894.1.S1\_at \| \| Bt.1484.1.S1\_at \|  \|  \|  \| XM\_868188 \|  \|  \| GALNT2 \| Bt.1484.1.S1\_at \| \| Bt.27147.1.S1\_at \|  \|  \|  \|  \|  \|  \| YES1 \| Bt.27147.1.S1\_at \| \| Bt.23109.1.S1\_at \| 404126 \| 1/2 \| NM\_001037444 \| NM\_001037444 \| GO:0004407  GO:0005634  GO:0016575 \| 12477932  16941691 \| HDAC1 \| Bt.23109.1.S1\_at \| \| Bt.12510.3.S1\_at \| 509824 \| 4/6 \| XM\_872590 \| XM\_872590 \|  \|  \|  \| Bt.12510.3.S1\_at \| \| Bt.4466.1.A1\_at \| 537728 \| 1/3 \| XM\_617909 \|  \| GO:0008270 \|  \| PHF2 \| Bt.4466.1.A1\_at \| \| Bt.4533.1.S1\_at \| 535038 \| 1/1 \| NM\_001034624 \|  \| GO:0016020  GO:0016021 \| 12477932 \| NIFIE14 \| Bt.4533.1.S1\_at \| \| Bt.6673.1.S1\_at \| 536553 \| 1/1 \| NM\_001076078 \|  \| GO:0005905  GO:0006810  GO:0006886  GO:0030662 \|  \| AP2S1 \| Bt.6673.1.S1\_at \| \| Bt.15938.1.S1\_at \| 515494 \| 1/1 \| XM\_593524 \|  \| GO:0004872 \| 11282978  16305752 \| LRP10 \| Bt.15938.1.S1\_at \| \| Bt.5484.1.S1\_at \| 282123 \| 1/4 \| NM\_174489 \| NM\_174489 \| GO:0004872  GO:0005509  GO:0016020 \| 9799803  10356983  15056566  15095337 \| VLDLR \| Bt.5484.1.S1\_at \| \| Bt.21715.1.S1\_at \| 532986 \| 2/2 \| XM\_001249745  XM\_612229 \|  \| GO:0005515  GO:0008270 \|  \| FALZ \| Bt.21715.1.S1\_at \| \| Bt.25335.1.A1\_at \|  \|  \|  \|  \|  \|  \| GATAD2B \| Bt.25335.1.A1\_at \| \| Bt.7870.1.S1\_at \| 280701 \| 1/1 \| NM\_001075118 \|  \| GO:0000287  GO:0004427  GO:0005737  GO:0006796  GO:0016787 \| 1339444 \| PPA1 \| Bt.7870.1.S1\_at \| \| Bt.12096.1.S1\_at \| 524142 \| 1/1 \| XM\_864454 \|  \|  \|  \|  \| Bt.12096.1.S1\_at \| \| Bt.24832.1.A1\_a\_at \| 538860 \| 1/1 \| NM\_001076133 \| NM\_001076133 \|  \|  \| 2-PDE \| Bt.24832.1.A1\_a\_at \| \| Bt.16628.2.A1\_at \|  \|  \|  \|  \|  \|  \| ADAM12 \| Bt.16628.2.A1\_at \| \| Bt.17958.1.S1\_at \|  \|  \|  \| XR\_028807 \|  \|  \| TAF4 \| Bt.17958.1.S1\_at \| \| Bt.27.1.S1\_at \| 286862 \| 1/1 \| NM\_174793 \| NM\_174793 \| GO:0003779  GO:0005080  GO:0005159  GO:0005737  GO:0006469  GO:0006605  GO:0006605  GO:0007010  GO:0007088  GO:0008426  GO:0009966  GO:0019904  GO:0019904  GO:0043066  GO:0045664  GO:0048167 \| 1671102  7931346 \| YWHAG \| Bt.27.1.S1\_at \| \| Bt.3050.1.S1\_at \| 513958 \| 1/1 \| NM\_001015593 \| NM\_001015593 \| GO:0001541  GO:0003743  GO:0005085  GO:0005515  GO:0005524  GO:0005525  GO:0005737  GO:0005851  GO:0006413  GO:0007417  GO:0014003  GO:0042552  GO:0044237  GO:0051716 \| 12140684  16305752 \| EIF2B2 \| Bt.3050.1.S1\_at \| \| Bt.15819.2.S1\_at \| 615424 \| 1/2 \| XM\_001250938  XM\_867160 \| XM\_867160 \|  \|  \| MTX2 \| Bt.15819.2.S1\_at \| \| Bt.11171.1.S1\_at \| 526652 \| 1/2 \| NM\_001035363 \|  \|  \| 11282978  12477932  16305752 \| FLCN \| Bt.11171.1.S1\_at \| \| Bt.20988.1.S1\_at \| 614801 \| 1/1 \| XM\_001255516  XM\_866432 \|  \|  \|  \|  \| Bt.20988.1.S1\_at \| \| Bt.16639.1.A1\_at \| 529061 \| 1/1 \| XR\_027889 \|  \|  \|  \|  \| Bt.16639.1.A1\_at \| \| Bt.24594.1.A1\_at \| 525813 \| 1/2 \| NM\_001075877 \|  \|  \|  \| ME3 \| Bt.24594.1.A1\_at \| \| Bt.22725.1.S1\_at \| 512841 \| 1/2 \| XM\_001251540  XM\_590426 \| XM\_001251540 \| GO:0007242 \|  \| CRK \| Bt.22725.1.S1\_at \| \| Bt.8606.1.S1\_a\_at \| 506115 \| 2/3 \| NM\_001045935 \|  \|  \|  \| CTDSP2 \| Bt.8606.1.S1\_a\_at \| \| Bt.9041.1.S1\_at \| 513451 \| 1/1 \| NM\_001075621 \|  \| GO:0000166  GO:0003676 \|  \| SFRS11 \| Bt.9041.1.S1\_at \| \| Bt.13028.1.S1\_at \| 281960 \| 1/1 \| NM\_001040479 \|  \| GO:0007155 \| 12761672 \| POSTN \| Bt.13028.1.S1\_at \| \| Bt.20226.1.A1\_at \| 506264 \| 1/4 \| XM\_582692 \|  \|  \|  \| CRIM1 \| Bt.20226.1.A1\_at \| \| Bt.21133.1.S1\_at \|  \|  \|  \|  \|  \|  \| UQCRB \| Bt.21133.1.S1\_at \| \| Bt.27930.1.S1\_at \|  \|  \|  \|  \|  \|  \| PRRG1 \| Bt.27930.1.S1\_at \| \| Bt.16125.2.S1\_at \| 616080 \| 3/4 \| NM\_001076407 \|  \|  \|  \|  \| Bt.16125.2.S1\_at \| \| Bt.23600.1.S1\_at \|  \|  \|  \| XM\_001252557 \|  \|  \| MGC2963 \| Bt.23600.1.S1\_at \| \| Bt.9866.1.S1\_at \| 615979 \| 1/1 \| NM\_001046568 \|  \|  \|  \| C14orf112 \| Bt.9866.1.S1\_at \| \| Bt.7701.1.S1\_at \| 614105 \| 1/1 \| NM\_001034748 \| NM\_001034748 \| GO:0016021 \| 12477932 \| GPSN2 \| Bt.7701.1.S1\_at \| \| Bt.29325.1.S1\_at \| 444858 \| 1/1 \| XM\_585239 \| XM\_585239 \|  \| 15448358 \| EIF4G1 \| Bt.29325.1.S1\_at \| \| Bt.1932.1.S1\_at \| 539220 \| 1/1 \| NM\_001034666 \|  \|  \| 12477932 \| AHSA1 \| Bt.1932.1.S1\_at \| \| Bt.68.1.S1\_at \|  \|  \|  \|  \|  \|  \| NDUFB1 \| Bt.68.1.S1\_at \| \| Bt.23615.1.S1\_at \|  \|  \|  \|  \|  \|  \| TUG1 \| Bt.23615.1.S1\_at \| \| Bt.12957.1.A1\_a\_at \| 528167 \| 1/2 \| XM\_001252363  XM\_001252395  XM\_606579 \|  \| GO:0000166 \|  \| TNRC6B \| Bt.12957.1.A1\_a\_at \| \| Bt.665.1.S1\_s\_at \| 282211 \| 1/2 \| NM\_174529 \|  \| GO:0005506  GO:0005783  GO:0005792  GO:0006118  GO:0016020  GO:0020037  GO:0046872  GO:0050381 \| 1396678 \| CYP2D6 \| Bt.665.1.S1\_s\_at \| \| Bt.8648.1.S1\_at \|  \|  \|  \|  \|  \|  \| DCUN1D4 \| Bt.8648.1.S1\_at \| \| Bt.22615.2.A1\_a\_at \| 613854 \| 2/2 \| NM\_001079617  XM\_001250680  XM\_001250721 \|  \| GO:0004719  GO:0006464  GO:0008168  GO:0016740 \| 1627573  2777770  3167043 \| PCMT1 \| Bt.22615.2.A1\_a\_at \| \| Bt.3895.1.S1\_at \|  \|  \|  \| XM\_868008 \|  \|  \| DOCK9 \| Bt.3895.1.S1\_at \| \| Bt.422.1.S2\_at \| 282261 \| 2/2 \| NM\_174556 \|  \| GO:0001558  GO:0005520  GO:0005576  GO:0008160  GO:0009968  GO:0043065  GO:0045663  GO:0046872 \| 1701128  1711841  9202228  9363609  12912978  14561651  15192040  15927849  17302958 \| IGFBP3 \| Bt.422.1.S2\_at \| \| Bt.668.1.S1\_at \|  \|  \|  \| XM\_001249927 \|  \|  \| TMEPAI \| Bt.668.1.S1\_at \| \| Bt.5237.1.S1\_at \| 282262 \| 1/1 \| NM\_174557 \|  \| GO:0001558  GO:0005520  GO:0005576 \| 1282670  16159699 \| IGFBP4 \| Bt.5237.1.S1\_at \| \| Bt.21584.1.S1\_at \| 513530 \| 1/1 \| NM\_001075624 \| NM\_001075624 \| GO:0003824  GO:0006633 \|  \| KS \| Bt.21584.1.S1\_at \| \| Bt.16111.1.S1\_at \|  \|  \|  \|  \|  \|  \| GMFB \| Bt.16111.1.S1\_at \| \| Bt.19097.2.S1\_at \|  \|  \|  \|  \|  \|  \| IGF1 \| Bt.19097.2.S1\_at \| \| Bt.7788.1.S1\_at \| 515235 \| 1/2 \| XM\_593217 \| XM\_593217 \|  \|  \| URB \| Bt.7788.1.S1\_at \| \| Bt.8163.1.S1\_at \| 529699 \| 1/1 \| NM\_001032302 \| NM\_001032302 \|  \|  \| ZC3H11A \| Bt.8163.1.S1\_at \| \| Bt.16108.1.A1\_at \| 508170 \| 1/1 \| NM\_001045995 \|  \|  \|  \| MCEE \| Bt.16108.1.A1\_at \| \| Bt.1760.1.S1\_at \| 540139 \| 1/1 \| XM\_591769 \| NM\_001081735 \|  \|  \| REXO2 \| Bt.1760.1.S1\_at \| \| Bt.12820.1.S1\_at \| 317706 \| 1/1 \| NM\_001075133 \| NM\_001075133 \| GO:0004872  GO:0005496  GO:0005783  GO:0005792  GO:0008289  GO:0009986  GO:0016020  GO:0016021  GO:0020037  GO:0046914 \|  \| PGRMC1 \| Bt.12820.1.S1\_at \| \| Bt.1325.1.S1\_at \| 615868 \| 1/3 \| NM\_001076399 \|  \| GO:0016301 \|  \| FN3KRP \| Bt.1325.1.S1\_at \| \| Bt.23330.1.S1\_at \|  \|  \|  \|  \|  \|  \| RAB30 \| Bt.23330.1.S1\_at \| \| Bt.24433.1.S1\_at \| 515194 \| 2/3 \| XM\_593171 \| NM\_001080730 \|  \|  \| MRPL39 \| Bt.24433.1.S1\_at \| \| Bt.12290.1.S1\_at \| 282011 \| 1/1 \| XM\_590212 \|  \| GO:0003677  GO:0005634  GO:0006350  GO:0006355 \|  \| PSIP1 \| Bt.12290.1.S1\_at \| \| Bt.12108.1.S1\_at \| 526138 \| 1/2 \| NM\_001075881 \| NM\_001075881 \|  \|  \| MTERFD1 \| Bt.12108.1.S1\_at \| \| Bt.422.1.S1\_at \| 282261 \| 2/2 \| NM\_174556 \| NM\_174556 \| GO:0001558  GO:0005520  GO:0005576  GO:0008160  GO:0009968  GO:0043065  GO:0045663  GO:0046872 \| 1701128  1711841  9202228  9363609  12912978  14561651  15192040  15927849  17302958 \| IGFBP3 \| Bt.422.1.S1\_at \| \| Bt.23971.1.S1\_at \| 505134 \| 1/3 \| XM\_581374 \| XM\_581374 \|  \|  \| ADAR \| Bt.23971.1.S1\_at \| \| Bt.7575.1.A1\_at \|  \|  \|  \| XM\_870732 \|  \|  \| GPT2 \| Bt.7575.1.A1\_at \| \| Bt.21607.1.S1\_at \| 521378 \| 1/1 \| NM\_001075819 \| NM\_001075819 \|  \|  \| CITED2 \| Bt.21607.1.S1\_at \| \| Bt.27457.1.S1\_at \|  \|  \|  \|  \|  \|  \|  \| Bt.27457.1.S1\_at \| \| Bt.5939.1.S1\_a\_at \| 538931 \| 1/1 \| XM\_583738 \| XM\_583738 \|  \|  \| MGC14151 \| Bt.5939.1.S1\_a\_at \| \| Bt.22168.1.A1\_at \| 536558 \| 1/1 \| XM\_878246 \|  \|  \|  \| HSPA4 \| Bt.22168.1.A1\_at \| \| Bt.13913.1.S1\_at \|  \|  \|  \|  \|  \|  \| AGPAT3 \| Bt.13913.1.S1\_at \| \| Bt.561.1.S1\_at \| 286883 \| 1/1 \| NM\_001034045 \| NM\_001034045 \| GO:0000166  GO:0004674  GO:0004713  GO:0005524  GO:0006468  GO:0016740 \| 11282978  16305752 \| MAP2K6 \| Bt.561.1.S1\_at \| \| Bt.16597.1.S1\_at \| 618292 \| 1/2 \| NM\_001078086  XM\_001253586  XM\_001253606 \| XM\_001253586 \|  \| 14667821 \| EXOSC4 \| Bt.16597.1.S1\_at \| \| Bt.13205.2.S1\_at \| 513438 \| 1/1 \| NM\_001075619 \| NM\_001075619 \| GO:0005739  GO:0005763  GO:0030529 \| 11344316 \| MRPS35 \| Bt.13205.2.S1\_at \| \| Bt.29938.1.S1\_at \| 508943 \| 1/3 \| XM\_880929 \| XM\_880929 \|  \|  \| MAP3K3 \| Bt.29938.1.S1\_at \| \| Bt.9544.1.S1\_at \| 539120 \| 2/2 \| XM\_584969 \|  \| GO:0003676  GO:0005622  GO:0008270 \|  \| ZNF161 \| Bt.9544.1.S1\_at \| \| Bt.2765.1.S1\_at \|  \|  \|  \|  \|  \|  \| FMNL2 \| Bt.2765.1.S1\_at \| \| Bt.13998.1.S1\_at \| 510968 \| 1/1 \| NM\_001075482 \| NM\_001075482 \| GO:0017134 \|  \| FIBP \| Bt.13998.1.S1\_at \| \| Bt.59.1.S1\_at \| 327710 \| 1/1 \| NM\_175826 \| NM\_175826 \| GO:0003954  GO:0005739  GO:0005743  GO:0008137  GO:0016491 \| 1830204 \| NDUFA8 \| Bt.59.1.S1\_at \| \| Bt.21622.1.A1\_at \|  \|  \|  \|  \|  \|  \| TRIO \| Bt.21622.1.A1\_at \| \| Bt.5079.1.S1\_at \| 510029 \| 1/1 \| NM\_001035083 \| NM\_001035083 \|  \| 12477932 \| PSMC4 \| Bt.5079.1.S1\_at \| \| Bt.23076.1.S1\_at \|  \|  \|  \| NM\_001040598 \|  \|  \| C1orf93 \| Bt.23076.1.S1\_at \| \| Bt.23662.1.S1\_at \| 506764 \| 2/2 \| NM\_001014858 \| NM\_001014858 \|  \| 11282978  16305752 \| RNF34 \| Bt.23662.1.S1\_at \| \| Bt.3857.1.S1\_at \| 327707 \| 1/1 \| NM\_175823 \|  \| GO:0000287  GO:0003676  GO:0004519  GO:0005739  GO:0016787  GO:0030145 \| 3360771  7688144  7789991  7870594  15066427 \| ENDOG \| Bt.3857.1.S1\_at \| \| Bt.20473.1.A1\_at \| 504811 \| 1/2 \| XM\_580986 \| XM\_580986 \|  \|  \| CAMTA1 \| Bt.20473.1.A1\_at \| \| Bt.4867.2.S1\_at \| 281339 \| 3/3 \| XM\_865879 \|  \| GO:0003774  GO:0016459 \| 8014155 \| MYH8 \| Bt.4867.2.S1\_at \| \| Bt.28138.1.S1\_at \|  \|  \|  \|  \|  \|  \| KIAA2002 \| Bt.28138.1.S1\_at \| \| Bt.22639.2.S1\_at \| 614703 \| 1/1 \| XM\_866291 \|  \|  \|  \| NSUN4 \| Bt.22639.2.S1\_at \| \| Bt.23734.1.A1\_at \|  \|  \|  \| XR\_028511 \|  \|  \| PAWR \| Bt.23734.1.A1\_at \| \| Bt.3236.1.A1\_at \|  \|  \|  \|  \|  \|  \| ARHGEF3 \| Bt.3236.1.A1\_at \| \| Bt.25832.1.S1\_at \|  \|  \|  \|  \|  \|  \| TRPC1 \| Bt.25832.1.S1\_at \| \| Bt.25282.1.A1\_at \| 536187 \| 1/2 \| XM\_871851 \| XM\_871851 \|  \|  \| C1QDC1 \| Bt.25282.1.A1\_at \| \| Bt.15616.1.S1\_at \| 510085 \| 2/3 \| XM\_587181 \| XM\_587181 \|  \|  \| SLC16A3 \| Bt.15616.1.S1\_at \| \| Bt.13321.1.S1\_at \| 524180 \| 1/2 \| XM\_865517 \| XM\_865517 \|  \|  \| CSPP1 \| Bt.13321.1.S1\_at \| \| Bt.4267.1.S1\_at \| 280857 \| 1/1 \| NM\_173936 \| NM\_173936 \|  \| 8621736 \| MIA \| Bt.4267.1.S1\_at \| \| Bt.11246.1.A1\_at \|  \|  \|  \|  \|  \|  \| MECP2 \| Bt.11246.1.A1\_at \| \| Bt.28087.1.S1\_at \|  \|  \|  \| XM\_614418 \|  \|  \| NCOA7 \| Bt.28087.1.S1\_at \| \| Bt.3880.1.S1\_at \| 539555 \| 1/1 \| NM\_001076172 \|  \| GO:0005516 \|  \| MARCKSL1 \| Bt.3880.1.S1\_at \| \| Bt.27793.1.A1\_at \| 521962 \| 1/2 \| XM\_600235 \| XM\_600235 \|  \|  \| MLLT3 \| Bt.27793.1.A1\_at \| \| Bt.22650.1.A1\_at \|  \|  \|  \|  \|  \|  \| MYEF2 \| Bt.22650.1.A1\_at \| \| Bt.8492.1.A1\_at \| 513412 \| 1/2 \| XM\_591084 \| XM\_591084 \|  \|  \| JAM3 \| Bt.8492.1.A1\_at \| \| Bt.2956.1.A1\_at \| 614073 \| 1/1 \| NM\_001040575 \| NM\_001040575 \|  \|  \| CCDC23 \| Bt.2956.1.A1\_at \| \| Bt.27430.1.S1\_at \| 506029 \| 2/2 \| XM\_582418 \|  \|  \|  \| ALS2CR2 \| Bt.27430.1.S1\_at \| \| Bt.25227.1.A1\_at \|  \|  \|  \| XR\_028313 \|  \|  \| HOXB8 \| Bt.25227.1.A1\_at \| \| Bt.20102.1.S1\_at \| 618791 \| 1/1 \| XM\_871114 \|  \|  \|  \| FKSG24 \| Bt.20102.1.S1\_at \| \| Bt.27207.1.A1\_at \|  \|  \|  \|  \|  \|  \| GPC6 \| Bt.27207.1.A1\_at \| \| Bt.13588.2.S1\_at \| 617679 \| 1/1 \|  \|  \|  \|  \| PSAT1 \| Bt.13588.2.S1\_at \| \| Bt.18459.1.A1\_at \| 522509 \| 1/2 \| XM\_001251867  XM\_600794 \|  \|  \|  \| PDE7B \| Bt.18459.1.A1\_at \| \| Bt.22150.1.A1\_at \| 512570 \| 1/2 \| NM\_001034425 \| NM\_001034425 \|  \| 12140684  12477932  16305752 \| LZTFL1 \| Bt.22150.1.A1\_at \| \| Bt.26080.2.S1\_at \| 532872 \| 1/3 \| XM\_612077 \| XM\_612077 \|  \|  \| MYOM3 \| Bt.26080.2.S1\_at \| \| Bt.20038.1.S1\_at \| 511885 \| 1/1 \| NM\_001046103 \|  \|  \|  \| PLEKHB1 \| Bt.20038.1.S1\_at \| \| Bt.303.1.S1\_at \| 281963 \| 1/1 \| NM\_174410 \| NM\_174410 \| GO:0001584  GO:0004872  GO:0007165  GO:0007186  GO:0016020  GO:0016021  GO:0016494  GO:0045028 \| 7626079  7682696  8508924  9848096 \| P2RY1 \| Bt.303.1.S1\_at \| \| Bt.1267.1.S1\_at \|  \|  \|  \|  \|  \|  \| KREMEN1 \| Bt.1267.1.S1\_at \| \| Bt.19862.1.A1\_at \| 518458 \| 2/2 \| XM\_001249780  XM\_001249831  XM\_001249925  XM\_001249962  XM\_596649 \|  \| GO:0004872  GO:0007275  GO:0016020 \|  \| SEMA6D \| Bt.19862.1.A1\_at \| \| Bt.14366.1.S1\_at \| 532209 \| 3/3 \| XM\_610719 \| XM\_610719 \|  \|  \| EVI1 \| Bt.14366.1.S1\_at \| \| Bt.22785.1.S1\_at \|  \|  \|  \| NM\_176788 \|  \|  \| CEBPB \| Bt.22785.1.S1\_at \| \| Bt.20820.1.A1\_at \|  \|  \|  \| XR\_027261 \|  \|  \|  \| Bt.20820.1.A1\_at \| \| Bt.7319.1.S1\_at \|  \|  \|  \| XM\_586668 \|  \|  \| PTPRK \| Bt.7319.1.S1\_at \| \| Bt.26568.1.S1\_a\_at \| 531049 \| 2/2 \| XM\_883226 \|  \| GO:0003677  GO:0005488  GO:0008152  GO:0016491 \|  \| KIAA0664 \| Bt.26568.1.S1\_a\_at \| \| Bt.12875.1.S1\_at \| 535273 \| 1/1 \| NM\_001024562 \| NM\_001024562 \| GO:0016020  GO:0016021  GO:0016049 \| 12140684  16305752 \| EMP3 \| Bt.12875.1.S1\_at \| \| Bt.13845.1.S1\_at \| 613695 \| 1/2 \| XM\_864764  XM\_873995 \|  \|  \|  \| C6orf203 \| Bt.13845.1.S1\_at \| \| Bt.5278.1.A1\_at \| 539713 \| 2/2 \| NM\_001015671 \| NM\_001015671 \| GO:0000166  GO:0005524  GO:0005739  GO:0006457  GO:0016020  GO:0016021  GO:0017111 \| 11282978  12477932 \| BCS1L \| Bt.5278.1.A1\_at \| \| Bt.20328.1.S1\_at \| 508957 \| 1/3 \| XM\_001253743  XM\_585817 \| XM\_001253743 \|  \|  \| PHF1 \| Bt.20328.1.S1\_at \| \| Bt.27196.1.A1\_at \| 511421 \| 1/1 \|  \|  \|  \|  \| CRTAC1 \| Bt.27196.1.A1\_at \| \| Bt.6937.1.S1\_at \| 533303 \| 1/1 \| XR\_027533 \| XR\_027533 \|  \|  \|  \| Bt.6937.1.S1\_at \| \| Bt.1020.1.S1\_at \| 508317 \| 1/2 \|  \| XM\_875205 \|  \|  \| CLK1 \| Bt.1020.1.S1\_at \| \| Bt.3996.1.S1\_at \|  \|  \|  \|  \|  \|  \| MEGF10 \| Bt.3996.1.S1\_at \| \| Bt.25346.1.A1\_at \|  \|  \|  \|  \|  \|  \| EDNRA \| Bt.25346.1.A1\_at \| \| Bt.24624.1.S1\_at \| 519302 \| 2/3 \| NM\_001035332 \|  \| GO:0003735  GO:0005622  GO:0005739  GO:0005840  GO:0006412 \| 11282978  11344316  16305752 \| MRPS9 \| Bt.24624.1.S1\_at \| \| Bt.4748.1.S1\_at \| 281496 \| 1/1 \| NM\_201527 \| NM\_201527 \|  \| 8292376  12140684  12658628  16305752 \| SOD2 \| Bt.4748.1.S1\_at \| \| Bt.5226.1.S1\_at \| 282068 \| 1/1 \| NM\_174460 \| NM\_174460 \| GO:0001756  GO:0007275  GO:0016055  GO:0030154 \| 10381896 \| SFRP1 \| Bt.5226.1.S1\_at \| \| Bt.26609.1.S1\_at \| 541159 \| 1/2 \| XM\_614802 \|  \|  \|  \| MTMR12 \| Bt.26609.1.S1\_at \| \| Bt.1993.1.S1\_a\_at \|  \|  \|  \| XM\_001250677 \|  \|  \| KIAA0433 \| Bt.1993.1.S1\_a\_at \| \| Bt.13850.3.S1\_a\_at \| 539992 \| 1/2 \| NM\_001034688 \| NM\_001034688 \| GO:0006629  GO:0006631  GO:0016787 \| 12477932 \| LYPLA1 \| Bt.13850.3.S1\_a\_at \| \| Bt.6483.1.A1\_at \|  \|  \|  \| XM\_601798 \|  \|  \| MTSS1 \| Bt.6483.1.A1\_at \| \| Bt.21997.2.A1\_at \| 540329 \| 4/4 \| NM\_001046474 \| NM\_001046474 \| GO:0000287  GO:0005739  GO:0006470  GO:0008287  GO:0015071  GO:0016787 \| 16229973 \|  \| Bt.21997.2.A1\_at \| \| Bt.1523.1.S1\_at \| 510219 \| 1/1 \| NM\_001075438 \| NM\_001075438 \|  \|  \| TOMM40 \| Bt.1523.1.S1\_at \| \| Bt.16985.1.S1\_at \| 509821 \| 1/2 \| NM\_001024505 \|  \|  \| 11282978 \| ELMO1 \| Bt.16985.1.S1\_at \| \| Bt.11027.1.A1\_at \| 516232 \| 1/1 \| XM\_594384 \|  \|  \|  \| ZNF653 \| Bt.11027.1.A1\_at \| \| Bt.8809.1.S1\_at \| 538100 \| 1/1 \| NM\_001076912 \|  \| GO:0005216 \|  \| SCN4B \| Bt.8809.1.S1\_at \| \| Bt.6983.1.S1\_at \|  \|  \|  \|  \|  \|  \| SOX9 \| Bt.6983.1.S1\_at \| \| Bt.27937.1.A1\_at \|  \|  \|  \|  \|  \|  \|  \| Bt.27937.1.A1\_at \| \| Bt.17843.1.A1\_at \| 539700 \| 1/2 \| XM\_864732 \|  \| GO:0003677  GO:0005634 \| 12477932 \| AKAP8L \| Bt.17843.1.A1\_at \| \| Bt.1652.1.S1\_at \|  \|  \|  \| XM\_863840 \|  \|  \| KIAA0738 \| Bt.1652.1.S1\_at \| \| Bt.29506.1.S1\_at \|  \|  \|  \|  \|  \|  \| FLJ23518 \| Bt.29506.1.S1\_at \| \| Bt.27262.1.S1\_at \| 513230 \| 1/2 \| XM\_590885 \| XM\_590885 \| GO:0004197  GO:0006508  GO:0031072 \|  \| PITRM1 \| Bt.27262.1.S1\_at \| \| Bt.20091.1.S1\_at \| 523151 \| 1/1 \| XM\_601446 \|  \|  \|  \|  \| Bt.20091.1.S1\_at \| \| Bt.23285.1.S1\_at \| 616719 \| 1/1 \| XM\_868815 \| XM\_868815 \|  \|  \|  \| Bt.23285.1.S1\_at \| \| Bt.4817.1.A1\_at \| 508268 \| 1/2 \| NM\_001035055 \| NM\_001035055 \|  \| 12477932 \| CLDN11 \| Bt.4817.1.A1\_at \| \| Bt.12468.1.S1\_at \| 520979 \| 1/1 \| NM\_001075813 \| NM\_001075813 \|  \|  \|  \| Bt.12468.1.S1\_at \| \| Bt.16092.1.S1\_at \|  \|  \|  \|  \|  \|  \| GGCX \| Bt.16092.1.S1\_at \| \| Bt.17886.1.A1\_at \|  \|  \|  \|  \|  \|  \| SPATA6 \| Bt.17886.1.A1\_at \| \| Bt.19309.3.A1\_at \| 616742 \| 1/4 \| NM\_001076432 \|  \|  \|  \| EFNA5 \| Bt.19309.3.A1\_at \| \| Bt.25712.1.A1\_s\_at \| 615173 \| 3/4 \| NM\_001034777 \|  \|  \| 11282978 \|  \| Bt.25712.1.A1\_s\_at \| \| Bt.25015.1.A1\_at \| 540839 \| 1/2 \| XM\_613382 \|  \| GO:0003677  GO:0003899  GO:0006350 \|  \| POLR3B \| Bt.25015.1.A1\_at \| \| Bt.26135.1.A1\_at \|  \|  \|  \|  \|  \|  \| KCNAB1 \| Bt.26135.1.A1\_at \| \| Bt.2121.1.S1\_at \| 522383 \| 1/2 \| XM\_600663 \| XM\_600663 \|  \|  \| NES \| Bt.2121.1.S1\_at \| \| Bt.16861.1.A1\_at \|  \|  \|  \| XR\_028719 \|  \|  \| DKFZp761N1114 \| Bt.16861.1.A1\_at \| \| Bt.2418.1.S1\_at \|  \|  \|  \|  \|  \|  \| KIAA0934 \| Bt.2418.1.S1\_at \| \| Bt.21824.1.A1\_at \| 513188 \| 1/1 \| NM\_001075603 \| NM\_001075603 \| GO:0004246  GO:0006508  GO:0016020  GO:0016021 \|  \| TMEM27 \| Bt.21824.1.A1\_at \| \| Bt.23505.1.S1\_at \| 507367 \| 3/3 \| XM\_583960 \|  \|  \|  \|  \| Bt.23505.1.S1\_at \| \| Bt.7130.1.S1\_at \| 534774 \| 1/1 \| XM\_001250437  XM\_614655 \| XM\_001250437 \| GO:0004194  GO:0006508  GO:0009049 \|  \| BACE2 \| Bt.7130.1.S1\_at \| \| Bt.25616.1.S1\_at \|  \|  \|  \|  \|  \|  \| GPR27 \| Bt.25616.1.S1\_at \| \| Bt.4653.1.S1\_at \| 282303 \| 2/3 \| NM\_174571 \|  \| GO:0005515  GO:0007155  GO:0016020  GO:0016021 \| 8558001  15668248 \| PECAM1 \| Bt.4653.1.S1\_at \| \| Bt.4868.2.S1\_a\_at \| 613382 \| 1/1 \| XM\_864279  XM\_872589 \|  \| GO:0003824 \|  \| HDDC3 \| Bt.4868.2.S1\_a\_at \| \| Bt.4040.1.S1\_at \| 282515 \| 1/1 \| NM\_174665 \| NM\_174665 \| GO:0003847  GO:0005515  GO:0005515  GO:0005737  GO:0007283  GO:0016042  GO:0016788 \| 8083218  8985254  11522926 \| PAFAH1B3 \| Bt.4040.1.S1\_at \| \| Bt.22538.2.S1\_at \| 616913 \| 1/2 \| XM\_869051 \|  \|  \|  \| FZD8 \| Bt.22538.2.S1\_at \| \| Bt.26792.1.S1\_at \| 512056 \| 1/2 \| XR\_028764 \| XR\_028764 \|  \|  \| RASGRP2 \| Bt.26792.1.S1\_at \| \| Bt.19352.1.A1\_at \|  \|  \|  \|  \|  \|  \|  \| Bt.19352.1.A1\_at \| \| Bt.28305.1.S1\_at \|  \|  \|  \| XM\_593269 \|  \|  \|  \| Bt.28305.1.S1\_at \| \| Bt.20783.1.A1\_at \| 532887 \| 2/3 \| XM\_612103 \| XM\_612103 \|  \|  \| PLEKHA5 \| Bt.20783.1.A1\_at \| \| Bt.24434.1.A1\_at \| 516019 \| 1/3 \| XM\_873210 \| XM\_873210 \|  \|  \| SEMA6A \| Bt.24434.1.A1\_at \| \| Bt.9828.1.S1\_at \| 281817 \| 1/1 \| NM\_174341 \| NM\_174341 \|  \|  \| HINT3 \| Bt.9828.1.S1\_at \| \| Bt.11502.1.A1\_at \| 533660 \| 1/2 \| NM\_001015649 \| NM\_001015649 \| GO:0006457  GO:0016020  GO:0016021  GO:0031072  GO:0051082 \| 11282978  12477932 \| DNAJC18 \| Bt.11502.1.A1\_at \| \| Bt.2587.2.S1\_a\_at \| 520260 \| 2/2 \| NM\_001075803 \| NM\_001075803 \| GO:0003824  GO:0004333  GO:0006106  GO:0045239 \|  \| FH \| Bt.2587.2.S1\_a\_at \| \| Bt.21620.1.A1\_at \|  \|  \|  \|  \|  \|  \| PHF16 \| Bt.21620.1.A1\_at \| \| Bt.5132.1.S1\_at \| 540386 \| 1/1 \| NM\_001034704 \| NM\_001034704 \| GO:0016403  GO:0016787 \| 12477932 \| DDAH2 \| Bt.5132.1.S1\_at \| \| Bt.28472.1.S1\_at \| 505318 \| 1/1 \| NM\_001075197 \| NM\_001075197 \|  \|  \| OLFML3 \| Bt.28472.1.S1\_at \| \| Bt.25085.1.A1\_at \|  \|  \|  \|  \|  \|  \| CNNM3 \| Bt.25085.1.A1\_at \| \| Bt.10398.1.S1\_at \| 541148 \| 1/1 \| NM\_001076259 \| NM\_001076259 \|  \| 16769728 \| PTX3 \| Bt.10398.1.S1\_at \| \| Bt.23486.1.A1\_at \| 521389 \| 2/3 \| XM\_599652 \| XM\_599652 \|  \|  \|  \| Bt.23486.1.A1\_at \| \| Bt.22046.1.S1\_at \| 540376 \| 1/1 \| XM\_593629 \| XM\_593629 \| GO:0003824  GO:0008152 \|  \| AMACR \| Bt.22046.1.S1\_at \| \| Bt.23456.1.A1\_s\_at \|  \|  \|  \|  \|  \|  \|  \| Bt.23456.1.A1\_s\_at \| \| Bt.28568.1.A1\_at \|  \|  \|  \|  \|  \|  \|  \| Bt.28568.1.A1\_at \| \| Bt.2120.2.S1\_at \| 540923 \| 1/2 \| NM\_001076248 \|  \| GO:0004180  GO:0006508  GO:0008233 \|  \| PGCP \| Bt.2120.2.S1\_at \| \| Bt.24766.1.S1\_at \| 520694 \| 1/1 \| XM\_598942 \| XM\_598942 \| GO:0005515 \|  \| LRRN6A \| Bt.24766.1.S1\_at \| \| Bt.13427.1.S1\_at \| 523799 \| 1/1 \| XM\_602105 \|  \|  \|  \| FLJ44216 \| Bt.13427.1.S1\_at \| \| Bt.13334.1.S1\_at \|  \|  \|  \|  \|  \|  \|  \| Bt.13334.1.S1\_at \| \| Bt.23131.1.S1\_s\_at \| 508529 \| 1/2 \| XM\_585317 \| XM\_001255597 \|  \|  \|  \| Bt.23131.1.S1\_s\_at \| \| Bt.20072.1.S1\_at \|  \|  \|  \|  \|  \|  \| FAM80B \| Bt.20072.1.S1\_at \| \| Bt.17943.1.S1\_at \|  \|  \|  \| XM\_001253893 \|  \|  \| FTO \| Bt.17943.1.S1\_at \| \| Bt.3228.1.S1\_at \| 507783 \| 1/1 \| NM\_001075332 \| NM\_001075332 \|  \| 16820722 \| PTHR1 \| Bt.3228.1.S1\_at \| \| Bt.21342.1.S1\_at \| 618250 \| 1/1 \| NM\_001079634  XM\_001253822 \|  \|  \|  \| S100A14 \| Bt.21342.1.S1\_at \| \| Bt.17081.1.A1\_at \| 614876 \| 2/2 \| NM\_001076352 \| NM\_001076352 \| GO:0005515  GO:0005634  GO:0008270  GO:0046872 \|  \| LMO2 \| Bt.17081.1.A1\_at \| \| Bt.9968.1.A1\_at \| 533847 \| 2/2 \| XM\_613386 \| XM\_613386 \| GO:0003676  GO:0005622  GO:0008270 \|  \| ZNF532 \| Bt.9968.1.A1\_at \| \| Bt.6665.1.S1\_at \|  \|  \|  \|  \|  \|  \| BNIP3L \| Bt.6665.1.S1\_at \| \| Bt.23396.2.S1\_a\_at \| 507834 \| 2/4 \| XR\_028758 \|  \|  \| 15772903 \|  \| Bt.23396.2.S1\_a\_at \| \| Bt.29050.1.S1\_at \|  \|  \|  \|  \|  \|  \| PDF \| Bt.29050.1.S1\_at \| \| Bt.3967.1.S1\_a\_at \| 616441 \| 1/4 \| NM\_001076422 \| NM\_001076422 \|  \|  \| THY28 \| Bt.3967.1.S1\_a\_at \| \| Bt.27918.1.S1\_at \| 505059 \| 1/1 \| XM\_581286 \| XM\_581286 \|  \|  \|  \| Bt.27918.1.S1\_at \| \| Bt.23373.1.S1\_at \| 528390 \| 1/1 \| NM\_001075911 \|  \| GO:0003700  GO:0003707  GO:0005634  GO:0006350  GO:0006355  GO:0008270  GO:0043565  GO:0046872 \| 11282978  16305752 \| NR4A1 \| Bt.23373.1.S1\_at \| \| Bt.21166.1.S1\_at \| 540328 \| 1/1 \| NM\_001076211 \| NM\_001076211 \|  \|  \| FBXO25 \| Bt.21166.1.S1\_at \| \| Bt.4685.1.S1\_at \| 538834 \| 1/3 \|  \| XM\_617252 \|  \|  \| WDFY3 \| Bt.4685.1.S1\_at \| \| Bt.13648.1.A1\_at \| 527732 \| 1/2 \| XM\_001249881  XM\_606129 \| XM\_606129 \| GO:0005488 \|  \|  \| Bt.13648.1.A1\_at \| \| Bt.4118.1.A1\_at \| 514472 \| 1/1 \| NM\_001038675 \|  \| GO:0000166  GO:0005524  GO:0005739  GO:0006457  GO:0051082 \|  \| TRAP1 \| Bt.4118.1.A1\_at \| \| Bt.17081.2.S1\_at \| 614876 \| 2/2 \| NM\_001076352 \| NM\_001076352 \| GO:0005515  GO:0005634  GO:0008270  GO:0046872 \|  \| LMO2 \| Bt.17081.2.S1\_at \| \| Bt.11270.2.S1\_at \| 505556 \| 1/3 \| XM\_581858 \|  \|  \|  \| VARS \| Bt.11270.2.S1\_at \| \| Bt.16503.1.A1\_at \|  \|  \|  \|  \|  \|  \| HSPA9B \| Bt.16503.1.A1\_at \| \| Bt.25107.1.A1\_at \|  \|  \|  \| XM\_589244 \|  \|  \| FGD5 \| Bt.25107.1.A1\_at \| \| Bt.10458.2.S1\_at \| 513774 \| 2/2 \| XM\_591518 \|  \| GO:0005515  GO:0006605  GO:0007165  GO:0016491  GO:0046914 \|  \| AKAP12 \| Bt.10458.2.S1\_at \| \| Bt.3814.1.S1\_at \| 509221 \| 1/2 \| XM\_879138 \| XM\_879138 \|  \| 11282978  16305752 \| DAB2 \| Bt.3814.1.S1\_at \| \| Bt.9416.1.S1\_at \| 508306 \| 1/1 \| XM\_864490 \|  \|  \|  \| MICAL1 \| Bt.9416.1.S1\_at \| \| Bt.22672.1.A1\_at \| 512259 \| 2/2 \| NM\_001034419 \| NM\_001034419 \| GO:0004957  GO:0005829  GO:0006693  GO:0007179  GO:0007565  GO:0007567  GO:0008152  GO:0016404  GO:0016491  GO:0019372  GO:0042803  GO:0045786  GO:0051287 \| 12477932 \| HPGD \| Bt.22672.1.A1\_at \| \| Bt.19469.1.A1\_at \| 614509 \| 2/4 \| NM\_001076331 \| XM\_867376 \| GO:0005634  GO:0030528  GO:0045449 \|  \| MXI1 \| Bt.19469.1.A1\_at \| \| Bt.12712.1.A1\_at \| 540313 \| 1/3 \| XM\_865918  XM\_877179 \| XM\_877179 \| GO:0003700  GO:0005634  GO:0006355  GO:0043565 \|  \| ETS1 \| Bt.12712.1.A1\_at \| \| Bt.17332.2.S1\_at \| 538390 \| 1/1 \|  \| XM\_864996 \|  \|  \|  \| Bt.17332.2.S1\_at \| \| Bt.29009.1.A1\_at \| 539899 \| 1/2 \| XM\_590220 \| XM\_590221 \| GO:0005216  GO:0005219  GO:0005509  GO:0006811  GO:0006874  GO:0016020  GO:0016021 \|  \| RYR3 \| Bt.29009.1.A1\_at \| \| Bt.4044.1.S1\_at \| 618849 \| 1/1 \| NM\_001076523 \| NM\_001076523 \|  \|  \| HMGA1 \| Bt.4044.1.S1\_at \| \| Bt.1692.1.S1\_at \| 615423 \| 1/1 \| XM\_868697 \| XM\_868697 \|  \|  \| C15orf24 \| Bt.1692.1.S1\_at \| \| Bt.9324.1.S1\_at \| 509816 \| 1/1 \| NM\_001035078 \|  \|  \| 11279123  12477932 \| MRPS11 \| Bt.9324.1.S1\_at \| \| Bt.3888.1.S1\_a\_at \| 535390 \| 1/1 \| NM\_001076056 \| NM\_001076056 \| GO:0004759  GO:0005515  GO:0006482  GO:0006725  GO:0016787  GO:0016789  GO:0051721  GO:0051722 \| 11282978  16305752 \|  \| Bt.3888.1.S1\_a\_at \| \| Bt.2235.1.S2\_at \| 281188 \| 2/2 \| NM\_174064 \|  \|  \| 1904992  2115118  8609986 \| GDI1 \| Bt.2235.1.S2\_at \| \| Bt.4011.1.S1\_at \| 520173 \| 1/1 \| NM\_001079600  XM\_001252004 \| NM\_001079600 \| GO:0016021 \|  \| TMPIT \| Bt.4011.1.S1\_at \| \| Bt.20300.1.A1\_at \| 509859 \| 1/3 \| XR\_028825 \| XR\_028825 \|  \|  \| KIAA0342 \| Bt.20300.1.A1\_at \| \| Bt.24563.1.S1\_at \| 536504 \| 1/1 \| NM\_001076077 \| XM\_612177 \| GO:0003677 \|  \| MAGI3 \| Bt.24563.1.S1\_at \| \| Bt.23113.1.A1\_at \|  \|  \|  \|  \|  \|  \| MGC16121 \| Bt.23113.1.A1\_at \| \| Bt.20470.2.S1\_a\_at \| 525071 \| 2/2 \| NM\_001075863 \| NM\_001075863 \|  \|  \| MTP18 \| Bt.20470.2.S1\_a\_at \| \| Bt.7056.3.A1\_a\_at \| 511735 \| 3/6 \| NM\_001014902 \|  \| GO:0005344  GO:0005506  GO:0005833  GO:0006810  GO:0015671  GO:0019825  GO:0020037  GO:0046872 \| 5958205  6322113 \| HBG \| Bt.7056.3.A1\_a\_at \| \| Bt.10719.1.S1\_at \| 504201 \| 1/1 \| NM\_001075149 \| NM\_001075149 \| GO:0005509 \|  \|  \| Bt.10719.1.S1\_at \| \| Bt.7772.1.S1\_at \|  \|  \|  \| XM\_586374 \|  \|  \| SMO \| Bt.7772.1.S1\_at \| \| Bt.21528.1.A1\_at \| 280852 \| 1/1 \| NM\_173935 \|  \|  \| 10954861  12477932  14502603 \| MDK \| Bt.21528.1.A1\_at \| \| Bt.20470.1.S1\_a\_at \| 525071 \| 2/2 \| NM\_001075863 \| NM\_001075863 \|  \|  \| MTP18 \| Bt.20470.1.S1\_a\_at \| \| Bt.2138.1.S1\_s\_at \| 404113 \| 1/1 \| NM\_207166 \|  \|  \| 2425311 \| MRPL43 \| Bt.2138.1.S1\_s\_at \| \| Bt.16668.1.A1\_at \| 539091 \| 1/1 \| NM\_001014952 \| NM\_001014952 \| GO:0005515  GO:0005622  GO:0005634  GO:0008270  GO:0046872 \| 11282978  16305752 \| TRIM45 \| Bt.16668.1.A1\_at \| \| Bt.1421.1.S1\_at \| 509824 \| 4/6 \| XM\_872590 \|  \|  \|  \|  \| Bt.1421.1.S1\_at \| \| Bt.20361.2.A1\_at \|  \|  \|  \| NM\_001035096 \|  \|  \| FBXL20 \| Bt.20361.2.A1\_at \| \| Bt.20405.1.S1\_at \|  \|  \|  \|  \|  \|  \| ATXN3 \| Bt.20405.1.S1\_at \| \| Bt.19423.1.S1\_at \| 535379 \| 1/2 \| NM\_001024693 \|  \| GO:0000166  GO:0005524  GO:0016020  GO:0016887 \| 17032780 \| ABCA1 \| Bt.19423.1.S1\_at \| \| Bt.4954.1.S1\_at \|  \|  \|  \|  \|  \|  \| LTBP4 \| Bt.4954.1.S1\_at \| \| Bt.11415.1.A1\_at \|  \|  \|  \|  \|  \|  \|  \| Bt.11415.1.A1\_at \| \| Bt.12721.1.A1\_at \| 768035 \| 1/1 \| NM\_001077094 \|  \| GO:0005086  GO:0005515  GO:0005622  GO:0032012 \|  \| PSCD2 \| Bt.12721.1.A1\_at \| \| Bt.27806.1.S1\_at \| 513586 \| 1/1 \| NM\_001035284 \| NM\_001035284 \| GO:0004476  GO:0005975  GO:0008270  GO:0016853  GO:0046872 \| 12477932 \| MPI \| Bt.27806.1.S1\_at \| \| Bt.26728.1.S1\_at \| 535498 \| 1/1 \| XM\_001250055  XM\_871598 \| XM\_871598 \| GO:0003677  GO:0003723  GO:0005515  GO:0005525  GO:0006614  GO:0008270  GO:0016020 \|  \| BAZ2B \| Bt.26728.1.S1\_at \| \| Bt.10458.1.A1\_at \| 513774 \| 2/2 \| XM\_591518 \|  \| GO:0005515  GO:0006605  GO:0007165  GO:0016491  GO:0046914 \|  \| AKAP12 \| Bt.10458.1.A1\_at \| \| Bt.11622.1.A1\_at \|  \|  \|  \| XM\_001250326 \|  \|  \|  \| Bt.11622.1.A1\_at \| \| Bt.1229.1.S1\_at \| 281631 \| 1/1 \| NM\_174242 \|  \| GO:0005319  GO:0005515  GO:0005576  GO:0006629  GO:0006869  GO:0008202  GO:0008203  GO:0008289  GO:0030300  GO:0042157 \| 1739745  2105728  2117227  8672146  12477932 \| APOA1 \| Bt.1229.1.S1\_at \| \| Bt.4698.1.S1\_at \| 280941 \| 1/1 \| NM\_173965 \|  \|  \| 8415706 \| TIE1 \| Bt.4698.1.S1\_at \| \| Bt.8682.1.S1\_at \|  \|  \|  \|  \|  \|  \| HDAC4 \| Bt.8682.1.S1\_at \| \| Bt.3452.1.S1\_at \| 510718 \| 1/1 \| XM\_587895 \|  \| GO:0003735  GO:0005622  GO:0005840  GO:0006412 \|  \|  \| Bt.3452.1.S1\_at \| \| Bt.8177.1.S1\_at \| 399560 \| 1/1 \| NM\_201606 \| NM\_201606 \| GO:0003735  GO:0005622  GO:0005739  GO:0005840  GO:0006412 \| 11282978  12140684  14757048  16305752  16510155 \| MRPL12 \| Bt.8177.1.S1\_at \| \| Bt.26621.1.S1\_at \| 617080 \| 1/1 \| NM\_001046591 \| NM\_001046591 \|  \| 11282978  16305752 \| LGI1 \| Bt.26621.1.S1\_at \| \| Bt.22735.1.S1\_at \| 614406 \| 1/1 \| XM\_865860 \|  \|  \|  \| SYTL2 \| Bt.22735.1.S1\_at \| \| Bt.24154.1.A1\_at \| 506114 \| 1/1 \| NM\_001075241 \| NM\_001075241 \|  \| 11282978 \| TFDP2 \| Bt.24154.1.A1\_at \| \| Bt.28733.1.S1\_at \|  \|  \|  \| NM\_001075262 \|  \|  \| ZNF397 \| Bt.28733.1.S1\_at \| \| Bt.15840.1.A1\_at \|  \|  \|  \| XR\_027657 \|  \|  \| INPP5F \| Bt.15840.1.A1\_at \| \| Bt.923.1.A1\_at \| 507116 \| 2/2 \| XM\_583674 \|  \|  \|  \| ZCCHC3 \| Bt.923.1.A1\_at \| \| Bt.15666.2.S1\_at \| 508609 \| 1/4 \| NM\_001046008 \|  \|  \|  \| PXMP2 \| Bt.15666.2.S1\_at \| \| Bt.10814.1.S1\_at \| 526585 \| 1/3 \| XM\_604958 \| XM\_604958 \|  \|  \| F2R \| Bt.10814.1.S1\_at \| \| Bt.18420.2.A1\_at \|  \|  \|  \|  \|  \|  \| EPB41L5 \| Bt.18420.2.A1\_at \| \| Bt.5393.2.S1\_a\_at \| 506569 \| 2/4 \| XM\_864201 \|  \|  \|  \| TTC3 \| Bt.5393.2.S1\_a\_at \| \| Bt.10995.1.S1\_at \| 533611 \| 1/1 \| NM\_001038160 \|  \|  \|  \| BBS2 \| Bt.10995.1.S1\_at \| \| Bt.11436.1.A1\_at \|  \|  \|  \|  \|  \|  \| SHANK3 \| Bt.11436.1.A1\_at \| \| Bt.20354.1.S1\_at \| 529126 \| 1/1 \|  \|  \|  \|  \| MAGED4 \| Bt.20354.1.S1\_at \| \| Bt.4872.1.S1\_at \| 522392 \| 1/1 \| NM\_001075830 \| NM\_001075830 \|  \|  \| MXRA8 \| Bt.4872.1.S1\_at \| \| Bt.5805.1.S1\_at \|  \|  \|  \| XM\_001251783 \|  \|  \|  \| Bt.5805.1.S1\_at \| \| Bt.17332.1.S1\_at \| 613833 \| 2/2 \| XM\_864996 \|  \| GO:0003700  GO:0003707  GO:0005634  GO:0006355  GO:0008270  GO:0043565 \|  \| NR3C2 \| Bt.17332.1.S1\_at \| \| Bt.11019.1.S1\_at \|  \|  \|  \| XR\_027984 \|  \|  \| FBXO40 \| Bt.11019.1.S1\_at \| \| Bt.3714.2.S1\_at \|  \|  \|  \|  \|  \|  \| ADRBK2 \| Bt.3714.2.S1\_at \| \| Bt.21997.1.S1\_at \| 540329 \| 4/4 \| NM\_001046474 \|  \| GO:0000287  GO:0005739  GO:0006470  GO:0008287  GO:0015071  GO:0016787 \| 16229973 \| PPM1K \| Bt.21997.1.S1\_at \| \| Bt.24802.1.S1\_at \| 510085 \| 2/3 \| XM\_587181 \|  \|  \|  \| SLC16A3 \| Bt.24802.1.S1\_at \| \| Bt.1112.1.S1\_at \| 618204 \| 1/1 \| NM\_001076496 \| NM\_001076496 \|  \|  \| SCN1B \| Bt.1112.1.S1\_at \| \| Bt.1389.1.S1\_at \| 511052 \| 1/1 \| XM\_867588 \|  \|  \|  \| C1orf60 \| Bt.1389.1.S1\_at \| \| Bt.23232.1.S1\_at \| 535578 \| 1/1 \| XM\_866836 \|  \|  \|  \| EGLN3 \| Bt.23232.1.S1\_at \| \| Bt.4950.1.S1\_at \| 286838 \| 1/1 \| NM\_174777 \|  \| GO:0009987  GO:0015012  GO:0015016  GO:0016020  GO:0016021  GO:0016740  GO:0016787 \| 9712870  12658628 \| NDST2 \| Bt.4950.1.S1\_at \| \| Bt.26462.1.S1\_at \| 513753 \| 1/1 \| XM\_591496 \| XM\_591496 \| GO:0005576  GO:0009405  GO:0019229 \|  \| EDN3 \| Bt.26462.1.S1\_at \| \| Bt.14340.2.S1\_at \| 511639 \| 2/2 \| XR\_027604 \|  \|  \|  \| RNF103 \| Bt.14340.2.S1\_at \| \| Bt.18048.1.A1\_at \| 540312 \| 2/3 \| XM\_865657 \|  \|  \|  \| DDX26B \| Bt.18048.1.A1\_at \| \| Bt.12634.1.A1\_at \| 616371 \| 1/1 \| XM\_868377 \| XM\_868377 \|  \|  \|  \| Bt.12634.1.A1\_at \| \| Bt.13357.1.S1\_at \| 508527 \| 1/1 \| XM\_585315 \|  \|  \|  \|  \| Bt.13357.1.S1\_at \| \| Bt.22198.1.S1\_at \| 533917 \| 1/1 \| NM\_001015651 \| NM\_001015651 \| GO:0004364  GO:0008152  GO:0016740 \| 12140684  16305752 \| GSTA4 \| Bt.22198.1.S1\_at \| \| Bt.16573.1.A1\_at \| 404098 \| 1/2 \| NM\_205784 \|  \| GO:0003676  GO:0004518  GO:0005634  GO:0006350  GO:0006355 \| 11691653  12477932  15627504 \|  \| Bt.16573.1.A1\_at \| \| Bt.2151.2.A1\_at \| 282022 \| 1/2 \| XM\_864482 \| XM\_864482 \| GO:0001516  GO:0004601  GO:0004666  GO:0005506  GO:0005737  GO:0005792  GO:0006633  GO:0008217  GO:0016020  GO:0016491  GO:0016702  GO:0030216  GO:0046872 \| 9348208  12080013 \| PTGS1 \| Bt.2151.2.A1\_at \| \| Bt.22200.1.S1\_at \|  \|  \|  \|  \|  \|  \| IGSF3 \| Bt.22200.1.S1\_at \| \| Bt.18023.1.S1\_at \|  \|  \|  \|  \|  \|  \|  \| Bt.18023.1.S1\_at \| \| Bt.6842.1.S1\_at \|  \|  \|  \| XM\_877619 \|  \|  \| CDC14B \| Bt.6842.1.S1\_at \| \| Bt.16637.1.A1\_at \| 515178 \| 1/1 \| NM\_001046190 \| NM\_001046190 \| GO:0000166  GO:0005524  GO:0006810  GO:0016020  GO:0016021  GO:0016887  GO:0042626 \|  \| ABCD1 \| Bt.16637.1.A1\_at \| \| Bt.27058.1.A1\_at \|  \|  \|  \| XR\_027842 \|  \|  \| ITGA8 \| Bt.27058.1.A1\_at \| \| Bt.2818.2.S1\_a\_at \| 768044 \| 1/1 \| NM\_001077102 \|  \| GO:0004081 \|  \| NUDT2 \| Bt.2818.2.S1\_a\_at \| \| Bt.11496.1.S1\_at \|  \|  \|  \|  \|  \|  \| CBFA2T2 \| Bt.11496.1.S1\_at \| \| Bt.26061.1.A1\_at \|  \|  \|  \|  \|  \|  \|  \| Bt.26061.1.A1\_at \| \| Bt.2656.1.S1\_at \|  \|  \|  \| XM\_001252042 \|  \|  \| TNFRSF25 \| Bt.2656.1.S1\_at \| \| Bt.2853.1.S1\_at \|  \|  \|  \| XM\_001252118 \|  \|  \| CHCHD8 \| Bt.2853.1.S1\_at \| \| Bt.21561.1.S1\_at \|  \|  \|  \|  \|  \|  \| FAM38A \| Bt.21561.1.S1\_at \| \| Bt.5602.1.A1\_at \| 516156 \| 1/1 \| XR\_028735 \|  \|  \|  \|  \| Bt.5602.1.A1\_at \| \| Bt.15907.2.S1\_at \| 513433 \| 1/2 \| NM\_001015588 \| NM\_001015588 \| GO:0031419  GO:0050897 \| 12140684  16305752 \| DKFZp564I122 \| Bt.15907.2.S1\_at \| \| Bt.4631.2.S1\_at \| 507912 \| 1/3 \| NM\_001045984 \|  \|  \|  \|  \| Bt.4631.2.S1\_at \| \| Bt.19261.1.S1\_at \| 519439 \| 1/1 \| NM\_001075789 \| NM\_001075789 \| GO:0003677  GO:0006355 \|  \| SOX18 \| Bt.19261.1.S1\_at \| \| BtAffx.29967.1.S1\_at \|  \|  \|  \| NM\_174318 \|  \|  \| FGFR3 \| BtAffx.29967.1.S1\_at \| \| Bt.23143.1.A1\_at \| 615804 \| 1/2 \| XM\_867711 \|  \| GO:0005525  GO:0007264  GO:0015031 \|  \|  \| Bt.23143.1.A1\_at \| \| Bt.15589.1.A1\_at \| 525806 \| 1/2 \| XM\_604162 \|  \|  \|  \| C13orf23 \| Bt.15589.1.A1\_at \| \| Bt.19332.1.S1\_at \|  \|  \|  \|  \|  \|  \| CLCN5 \| Bt.19332.1.S1\_at \| \| Bt.20591.1.S1\_at \|  \|  \|  \|  \|  \|  \| FLJ12505 \| Bt.20591.1.S1\_at \| \| Bt.7574.1.S1\_at \| 519597 \| 1/1 \| XM\_865606 \|  \|  \|  \| SNX4 \| Bt.7574.1.S1\_at \| \| Bt.621.1.S1\_at \| 505060 \| 1/1 \| NM\_001025319 \| NM\_001025319 \|  \| 15836952  16508140 \| CYP51A1 \| Bt.621.1.S1\_at \| \| Bt.22615.1.S1\_at \| 613854 \| 2/2 \| NM\_001079617  XM\_001250680  XM\_001250721 \| XM\_001250680 \| GO:0004719  GO:0006464  GO:0008168  GO:0016740 \| 1627573  2777770  3167043 \| PCMT1 \| Bt.22615.1.S1\_at \| \| Bt.20151.1.S1\_at \| 514044 \| 1/1 \| NM\_001046162 \|  \| GO:0008152  GO:0016491 \|  \| DHRS7 \| Bt.20151.1.S1\_at \| \| Bt.23496.1.S1\_at \| 767909 \| 1/1 \| XM\_001255704 \| XM\_001255704 \| GO:0005739 \| 2298292 \|  \| Bt.23496.1.S1\_at \| \| Bt.21761.1.S1\_a\_at \| 505645 \| 1/1 \| NM\_001075215 \| NM\_001075215 \|  \|  \| TMOD4 \| Bt.21761.1.S1\_a\_at \| \| Bt.20113.1.S1\_at \| 538409 \| 1/1 \| NM\_001035419 \| NM\_001035419 \| GO:0006508  GO:0006915  GO:0008234  GO:0030693 \| 12477932 \| CASP6 \| Bt.20113.1.S1\_at \| \| Bt.11237.1.S1\_at \| 613858 \| 1/3 \| NM\_001046520 \| NM\_001046520 \|  \| 12477932 \| YT521 \| Bt.11237.1.S1\_at \| \| Bt.10271.1.A1\_at \| 528265 \| 1/1 \| NM\_001079778  XM\_001252144 \| NM\_001079778 \| GO:0005515  GO:0007154  GO:0015031  GO:0035091 \|  \| SNX3 \| Bt.10271.1.A1\_at \| \| Bt.11262.2.S1\_at \| 444864 \| 3/5 \| NM\_001002887 \| NM\_001002887 \| GO:0003676  GO:0003700  GO:0005622  GO:0005634  GO:0006355  GO:0006915  GO:0008270  GO:0046872 \| 15203203 \| ZIM2 \| Bt.11262.2.S1\_at \| \| Bt.1638.2.S1\_at \|  \|  \|  \|  \|  \|  \| CXorf39 \| Bt.1638.2.S1\_at \| \| Bt.2814.1.S1\_at \|  \|  \|  \|  \|  \|  \| YWHAG \| Bt.2814.1.S1\_at \| \| Bt.22444.1.A1\_at \| 541264 \| 1/3 \| XM\_615363 \| XM\_615363 \| GO:0003676  GO:0005622  GO:0008270 \|  \| ZNF292 \| Bt.22444.1.A1\_at \| \| Bt.6279.1.A1\_at \| 535127 \| 1/2 \| XM\_615128 \|  \| GO:0003774  GO:0005524  GO:0016459 \|  \| MYO6 \| Bt.6279.1.A1\_at \| \| Bt.1283.1.S1\_at \| 360196 \| 1/1 \| NM\_182988 \|  \| GO:0003677  GO:0005634  GO:0005694 \| 12140684  12477932  16305752 \| BANF1 \| Bt.1283.1.S1\_at \| \| Bt.282.1.S1\_at \| 282716 \| 1/1 \| NM\_174729 \| NM\_174729 \|  \| 14739283 \| VDAC3 \| Bt.282.1.S1\_at \| \| Bt.25343.1.S1\_at \| 525164 \| 1/1 \| NM\_001079776  XM\_603511 \|  \|  \|  \| P53CSV \| Bt.25343.1.S1\_at \| \| Bt.20895.1.S1\_at \| 767980 \| 1/2 \| NM\_001077044 \| NM\_001077044 \| GO:0005783  GO:0005784  GO:0006613  GO:0016020  GO:0016021  GO:0030176 \|  \| SSR3 \| Bt.20895.1.S1\_at \| \| Bt.16132.2.S1\_a\_at \| 540859 \| 1/1 \| NM\_001046493 \| NM\_001046493 \| GO:0005515  GO:0005634  GO:0006350  GO:0006355  GO:0006512 \|  \| TCEB1 \| Bt.16132.2.S1\_a\_at \| \| Bt.19839.1.A1\_at \| 530827 \| 1/3 \| XM\_609305 \|  \|  \|  \| PPIG \| Bt.19839.1.A1\_at \| \| Bt.26641.1.S1\_at \| 513156 \| 1/1 \| NM\_001075602 \| NM\_001075602 \| GO:0004177  GO:0006508  GO:0008235 \| 11282978  16305752 \| XPNPEP1 \| Bt.26641.1.S1\_at \| \| Bt.10150.1.S1\_at \|  \|  \|  \|  \|  \|  \| CAPN6 \| Bt.10150.1.S1\_at \| \| Bt.3631.1.S1\_at \| 326581 \| 1/1 \| NM\_001040487 \| NM\_001040487 \| GO:0003746  GO:0005853  GO:0006414 \| 11282978  12658628  16305752 \|  \| Bt.3631.1.S1\_at \| \| Bt.1825.1.S1\_at \| 510264 \| 1/1 \| NM\_001035088 \|  \|  \| 12477932 \| MGC4172 \| Bt.1825.1.S1\_at \| \| Bt.23401.1.S1\_at \| 509056 \| 1/1 \| NM\_001034341 \| NM\_001034341 \|  \| 12477932 \| FHL1 \| Bt.23401.1.S1\_at \| \| Bt.28207.1.S1\_at \| 540195 \| 2/3 \| XM\_592217 \| XM\_592217 \|  \|  \| RNF19 \| Bt.28207.1.S1\_at \| \| Bt.13969.1.A1\_at \|  \|  \|  \|  \|  \|  \| SBNO1 \| Bt.13969.1.A1\_at \| \| Bt.7024.1.A1\_at \| 614435 \| 2/2 \| XM\_001254288  XM\_865931 \| XM\_001254288 \|  \|  \| PTP4A2 \| Bt.7024.1.A1\_at \| \| Bt.23607.1.S1\_at \| 532785 \| 1/1 \| NM\_001046334 \| NM\_001046334 \| GO:0006631  GO:0016491  GO:0050662 \|  \|  \| Bt.23607.1.S1\_at \| \| Bt.11193.1.S1\_at \| 509841 \| 1/1 \| XM\_586890 \| XM\_586890 \|  \|  \|  \| Bt.11193.1.S1\_at \| \| Bt.6938.1.S1\_at \| 516091 \| 1/1 \| NM\_001075737 \| NM\_001075737 \| GO:0005634  GO:0006512 \|  \| CUEDC2 \| Bt.6938.1.S1\_at \| \| Bt.14219.1.S1\_at \| 613715 \| 1/1 \| NM\_001046515 \| NM\_001046515 \|  \|  \| C10orf10 \| Bt.14219.1.S1\_at \| \| Bt.17777.1.S1\_at \| 534150 \| 2/3 \| NM\_001034602 \| NM\_001034602 \|  \| 12477932 \| OPTN \| Bt.17777.1.S1\_at \| \| Bt.15531.1.S1\_at \| 614172 \| 1/1 \|  \| XM\_001250306 \|  \|  \| GARNL1 \| Bt.15531.1.S1\_at \| \| Bt.5941.1.S1\_at \|  \|  \|  \|  \|  \|  \| KIAA0494 \| Bt.5941.1.S1\_at \| \| Bt.22563.1.A1\_s\_at \| 513993 \| 2/4 \| XM\_591773 \|  \| GO:0003677  GO:0004601  GO:0006355  GO:0006979 \|  \|  \| Bt.22563.1.A1\_s\_at \| \| Bt.15742.1.S1\_at \|  \|  \|  \|  \|  \|  \| CD47 \| Bt.15742.1.S1\_at \| \| Bt.23551.2.S1\_at \| 517211 \| 1/2 \| NM\_001075750 \|  \| GO:0008080  GO:0008152  GO:0008415  GO:0016740 \|  \| MAK3 \| Bt.23551.2.S1\_at \| \| Bt.231.1.S1\_at \| 280950 \| 1/1 \| NM\_173968 \| NM\_173968 \|  \| 10647825  10727087  12477932 \| TXN \| Bt.231.1.S1\_at \| \| Bt.10195.1.S1\_at \| 533554 \| 1/1 \| XM\_001249428  XM\_612994  XM\_878307 \| XM\_878307 \|  \|  \| FBXO38 \| Bt.10195.1.S1\_at \| \| Bt.13132.1.S1\_at \| 286969 \| 1/1 \| NM\_001040484 \|  \| GO:0001584  GO:0001604  GO:0004872  GO:0007165  GO:0007186  GO:0016020  GO:0016021 \| 7733947  11282978  16305752 \| UTS2R \| Bt.13132.1.S1\_at \| \| Bt.1543.1.A1\_at \| 520472 \| 1/2 \| XM\_878444 \|  \|  \|  \| PBEF1 \| Bt.1543.1.A1\_at \| \| Bt.5582.1.S1\_at \|  \|  \|  \|  \|  \|  \|  \| Bt.5582.1.S1\_at \| \| Bt.28162.2.A1\_at \| 537625 \| 1/1 \| XM\_617798 \|  \|  \|  \| PLN \| Bt.28162.2.A1\_at \| \| Bt.4279.2.S1\_at \| 507196 \| 2/4 \| NM\_001075305 \| NM\_001075305 \|  \|  \| SHC1 \| Bt.4279.2.S1\_at \| \| Bt.23143.2.S1\_at \| 513993 \| 2/4 \| XM\_591773 \| XM\_591773 \| GO:0003677  GO:0004601  GO:0006355  GO:0006979 \|  \| CSDE1 \| Bt.23143.2.S1\_at \| \| Bt.6441.1.S1\_at \| 614893 \| 1/1 \| NM\_001037488 \|  \| GO:0005634  GO:0006350  GO:0006355  GO:0042127 \| 12477932 \| CDCA7 \| Bt.6441.1.S1\_at \| \| Bt.28113.1.S1\_at \| 535227 \| 1/1 \| NM\_001076050 \|  \| GO:0005506  GO:0006118  GO:0016712  GO:0020037 \|  \| CYP2U1 \| Bt.28113.1.S1\_at \| \| Bt.1240.1.S1\_at \| 282218 \| 1/1 \| NM\_001075127 \| NM\_001075127 \| GO:0004519  GO:0004531  GO:0005764  GO:0006308  GO:0006915  GO:0007275  GO:0016787  GO:0030218 \| 9812984  12594037 \| DNASE2 \| Bt.1240.1.S1\_at \| \| Bt.8448.1.S1\_at \| 533847 \| 2/2 \| XM\_613386 \|  \| GO:0003676  GO:0005622  GO:0008270 \|  \| ZNF532 \| Bt.8448.1.S1\_at \| \| Bt.21215.2.S1\_at \| 506721 \| 2/3 \| XM\_001251311  XM\_583206  XM\_865524 \| XM\_583206 \|  \|  \| PITX2 \| Bt.21215.2.S1\_at \| \| Bt.9546.2.S1\_a\_at \| 534630 \| 1/1 \| XM\_864580 \|  \| GO:0000166  GO:0003676 \|  \| RBMX2 \| Bt.9546.2.S1\_a\_at \| \| Bt.19338.1.S1\_at \|  \|  \|  \|  \|  \|  \| PVRL3 \| Bt.19338.1.S1\_at \| \| Bt.605.1.S1\_at \| 281187 \| 1/2 \| NM\_001001525 \| NM\_001001525 \|  \| 9314496  9356471  11451380  15975431  16110474  16424609 \| GDF8 \| Bt.605.1.S1\_at \| \| Bt.21843.1.A1\_at \| 533443 \| 1/1 \| XM\_612851 \|  \|  \|  \| CNOT8 \| Bt.21843.1.A1\_at \| \| Bt.21238.1.S1\_at \| 541004 \| 1/4 \| XM\_614062  XM\_865994  XM\_877502 \| XM\_614062 \| GO:0007155  GO:0016020 \|  \| NRP2 \| Bt.21238.1.S1\_at \| \| Bt.12859.1.S1\_at \| 541202 \| 1/1 \| XM\_876757 \|  \|  \|  \| HNRPH1 \| Bt.12859.1.S1\_at \| \| Bt.12867.3.S1\_at \|  \|  \|  \|  \|  \|  \| ZNF318 \| Bt.12867.3.S1\_at \| \| Bt.14552.1.A1\_at \| 509827 \| 1/1 \| XM\_001250156  XM\_001250303  XM\_865229  XM\_875924 \|  \|  \|  \| ATF2 \| Bt.14552.1.A1\_at \| \| Bt.8260.1.A1\_at \|  \|  \|  \|  \|  \|  \| SDC2 \| Bt.8260.1.A1\_at \| \| Bt.2642.1.S1\_at \| 614279 \| 1/1 \| XM\_865722 \| XM\_865722 \| GO:0003676  GO:0005622  GO:0008270 \| 12477932 \| ZNF706 \| Bt.2642.1.S1\_at \| \| Bt.8333.1.S1\_at \| 507897 \| 1/1 \| NM\_001075337 \| NM\_001075337 \|  \|  \|  \| Bt.8333.1.S1\_at \| \| Bt.10043.1.S1\_at \| 614183 \| 2/2 \| XM\_865921 \|  \|  \|  \|  \| Bt.10043.1.S1\_at \| \| Bt.9749.1.S1\_at \| 511453 \| 1/1 \| NM\_001034404 \| NM\_001034404 \| GO:0005783  GO:0005787  GO:0005792  GO:0006465  GO:0009003  GO:0016020  GO:0016021  GO:0030176 \| 12477932 \| SPCS1 \| Bt.9749.1.S1\_at \| \| Bt.22155.1.A1\_at \|  \|  \|  \|  \|  \|  \| KIF13A \| Bt.22155.1.A1\_at \| \| Bt.27061.1.S1\_at \| 768016 \| 1/1 \| NM\_001077075 \| XM\_001252503 \|  \| 12140684  16305752 \| TOB1 \| Bt.27061.1.S1\_at \| \| Bt.5319.2.S1\_a\_at \| 282438 \| 1/3 \| NM\_174643 \| NM\_174643 \| GO:0000302  GO:0005764  GO:0005829  GO:0016042  GO:0016491  GO:0016787  GO:0051920 \| 2373154  9748299  10409692  12140684  12477932  12810819  15240427  16305752 \| PRDX6 \| Bt.5319.2.S1\_a\_at \| \| Bt.222.1.S1\_at \| 281719 \| 1/1 \| NM\_174290 \| NM\_174290 \| GO:0005212  GO:0005515  GO:0005625  GO:0005626  GO:0005737  GO:0005737  GO:0005886  GO:0006916  GO:0007517  GO:0030018  GO:0032387  GO:0043292 \| 1304359  2599111  3579961  4477528  8114688  12477932  15075233 \| CRYAB \| Bt.222.1.S1\_at \| \| Bt.22584.1.A1\_at \|  \|  \|  \|  \|  \|  \| FCHSD2 \| Bt.22584.1.A1\_at \| \| Bt.18316.2.S1\_at \| 513587 \| 2/2 \| XM\_591287  XM\_876203 \|  \|  \|  \|  \| Bt.18316.2.S1\_at \| \| Bt.2156.2.S1\_a\_at \| 508598 \| 1/2 \| XM\_585394 \| XM\_585394 \|  \|  \| TMEM11 \| Bt.2156.2.S1\_a\_at \| \| Bt.16025.1.S1\_at \| 281090 \| 1/1 \| NM\_001001439 \|  \|  \| 227779  227780  2822541  6093095  7557450  8638158  10089392  10771420  11311561  12140684  12477932  16305752 \| COX4I1 \| Bt.16025.1.S1\_at \| \| Bt.3306.1.S1\_at \| 512397 \| 1/1 \| XM\_001252640  XM\_589910 \| XM\_589910 \| GO:0005737  GO:0016020 \|  \| ProSAPiP1 \| Bt.3306.1.S1\_at \| \| Bt.13440.3.S1\_at \| 506615 \| 1/2 \| XM\_583086 \|  \|  \|  \| LKAP \| Bt.13440.3.S1\_at \| \| Bt.21296.1.S1\_at \| 519558 \| 1/1 \| XM\_001254073  XM\_001254086  XM\_869465  XM\_882582 \| XM\_001254073 \| GO:0003676  GO:0004674  GO:0005083  GO:0005524  GO:0005622  GO:0006468  GO:0008270  GO:0016301 \| 12087176 \| MINK1 \| Bt.21296.1.S1\_at \| \| Bt.8699.1.S1\_at \| 540045 \| 1/2 \| XM\_001251971  XM\_611497  XM\_872514  XM\_872608 \| XM\_872608 \| GO:0003676  GO:0005622  GO:0005634  GO:0008270 \|  \| ZNF385 \| Bt.8699.1.S1\_at \| \| Bt.2816.1.S1\_at \|  \|  \|  \| XM\_001250061 \|  \|  \|  \| Bt.2816.1.S1\_at \| \| Bt.15837.1.S1\_at \| 507213 \| 1/1 \| NM\_001045962 \| NM\_001045962 \|  \|  \| PSMA6 \| Bt.15837.1.S1\_at \| \| Bt.6534.1.S1\_at \|  \|  \|  \|  \|  \|  \| CNNM4 \| Bt.6534.1.S1\_at \| \| Bt.26682.1.S1\_at \|  \|  \|  \|  \|  \|  \|  \| Bt.26682.1.S1\_at \| \| Bt.6686.1.S1\_at \|  \|  \|  \| XM\_001249492 \|  \|  \| CASK \| Bt.6686.1.S1\_at \| \| Bt.26872.1.S1\_at \|  \|  \|  \| XM\_001253708 \|  \|  \|  \| Bt.26872.1.S1\_at \| \| Bt.11908.1.S1\_at \|  \|  \|  \|  \|  \|  \| FNDC3A \| Bt.11908.1.S1\_at \| \| Bt.6778.1.S1\_at \|  \|  \|  \|  \|  \|  \| APLN \| Bt.6778.1.S1\_at \| \| Bt.27956.1.A1\_at \| 540786 \| 1/3 \| XM\_613125 \| XM\_613125 \| GO:0006470  GO:0008138  GO:0016791 \|  \| PTEN \| Bt.27956.1.A1\_at \| \| Bt.4756.1.S1\_at \| 530186 \| 2/2 \| NM\_001015639 \| NM\_001015639 \| GO:0000166  GO:0005524  GO:0005634  GO:0005737  GO:0005829  GO:0016787  GO:0017111  GO:0030163  GO:0043234 \| 12140684  12477932  16305752 \| PSMC2 \| Bt.4756.1.S1\_at \| \| Bt.28393.1.S1\_at \| 518609 \| 1/1 \| NM\_001030303 \|  \|  \|  \| DAF \| Bt.28393.1.S1\_at \| \| Bt.28146.1.A1\_at \| 537932 \| 1/2 \| XR\_028356 \|  \|  \|  \| ZNF650 \| Bt.28146.1.A1\_at \| \| Bt.25887.1.A1\_at \| 514357 \| 1/1 \| NM\_001076850 \|  \| GO:0016740 \|  \| UGCG \| Bt.25887.1.A1\_at \| \| Bt.27149.1.S1\_at \| 614070 \| 1/1 \| NM\_001035442 \|  \|  \| 12477932 \| MGC127958 \| Bt.27149.1.S1\_at \| \| Bt.21113.1.S1\_a\_at \| 509459 \| 1/1 \| NM\_001034349 \| NM\_001034349 \|  \| 11282978  16305752 \| CPT1B \| Bt.21113.1.S1\_a\_at \| \| Bt.2821.1.S1\_at \| 511207 \| 1/1 \| NM\_001038527 \| NM\_001038527 \| GO:0004298  GO:0005829  GO:0005839  GO:0006511 \|  \| PSMB7 \| Bt.2821.1.S1\_at \| \| Bt.9322.1.S1\_at \| 614311 \| 1/1 \| NM\_001046530 \| NM\_001046530 \|  \|  \| FLJ10287 \| Bt.9322.1.S1\_at \| \| Bt.7056.2.A1\_x\_at \| 511735 \| 3/6 \| NM\_001014902 \| XM\_001255618 \| GO:0005344  GO:0005506  GO:0005833  GO:0006810  GO:0015671  GO:0019825  GO:0020037  GO:0046872 \| 5958205  6322113 \| HBG \| Bt.7056.2.A1\_x\_at \| \| Bt.26779.1.S1\_at \| 617851 \| 1/1 \| XM\_870175 \|  \|  \| 11282978  16305752 \| PADI2 \| Bt.26779.1.S1\_at \| \| Bt.9741.1.S1\_at \|  \|  \|  \|  \|  \|  \| MTMR4 \| Bt.9741.1.S1\_at \| \| Bt.868.1.S1\_at \| 616375 \| 2/4 \| NM\_001034791 \| NM\_001034791 \|  \| 12477932 \| MAPBPIP \| Bt.868.1.S1\_at \| \| Bt.24017.1.A1\_s\_at \| 530186 \| 2/2 \| NM\_001015639 \| NM\_001015639 \| GO:0000166  GO:0005524  GO:0005634  GO:0005737  GO:0005829  GO:0016787  GO:0017111  GO:0030163  GO:0043234 \| 12140684  12477932  16305752 \| PSMC2 \| Bt.24017.1.A1\_s\_at \| \| Bt.3174.1.A1\_at \|  \|  \|  \|  \|  \|  \| UBPH \| Bt.3174.1.A1\_at \| \| Bt.15714.2.S1\_a\_at \| 540194 \| 2/2 \| NM\_001024571 \|  \|  \| 11282978  16305752 \| FLJ20436 \| Bt.15714.2.S1\_a\_at \| \| Bt.9176.1.A1\_at \|  \|  \|  \| XM\_588651 \|  \|  \|  \| Bt.9176.1.A1\_at \| \| Bt.22340.1.S1\_at \| 540170 \| 1/1 \| XM\_864456 \|  \|  \|  \| PUM2 \| Bt.22340.1.S1\_at \| \| Bt.20672.1.S1\_at \| 515332 \| 1/2 \| XM\_593335 \|  \| GO:0003677  GO:0005634  GO:0045449 \|  \| KIAA1915 \| Bt.20672.1.S1\_at \| \| Bt.60.1.S1\_at \| 327713 \| 1/1 \| NM\_175828 \| NM\_175828 \| GO:0003954  GO:0005739  GO:0008137  GO:0016491 \| 1518044 \| NDUFB2 \| Bt.60.1.S1\_at \| \| Bt.16041.2.S1\_at \| 504968 \| 2/2 \| XM\_866358  XM\_878203 \| XM\_866358 \|  \|  \| ABLIM1 \| Bt.16041.2.S1\_at \| \| Bt.14054.2.S1\_at \| 541283 \| 1/2 \| NM\_001034729 \|  \| GO:0030154 \| 12477932 \| IFRD1 \| Bt.14054.2.S1\_at \| \| Bt.2501.1.S1\_at \|  \|  \|  \|  \|  \|  \| SOD2 \| Bt.2501.1.S1\_at \| \| Bt.7434.1.S1\_at \| 616333 \| 1/1 \| NM\_001076418 \| NM\_001076418 \|  \| 16305752 \| FAM79A \| Bt.7434.1.S1\_at \| \| Bt.14395.2.S1\_at \| 780785 \| 1/2 \| XM\_001253530 \|  \|  \|  \| ADMP \| Bt.14395.2.S1\_at \| \| Bt.4584.1.S1\_at \|  \|  \|  \|  \|  \|  \| C1orf21 \| Bt.4584.1.S1\_at \| \| Bt.13771.3.S1\_at \|  \|  \|  \|  \|  \|  \| ZNF262 \| Bt.13771.3.S1\_at \| \| Bt.11326.1.S1\_at \| 614292 \| 2/3 \| XM\_001250807  XM\_878516 \|  \|  \|  \| C6orf166 \| Bt.11326.1.S1\_at \| \| Bt.6493.1.S1\_at \| 540311 \| 2/3 \| XM\_593096 \| XM\_593096 \|  \| 11282978  16305752 \| HNRPA0 \| Bt.6493.1.S1\_at \| \| Bt.5527.2.S1\_a\_at \| 615845 \| 2/2 \| NM\_001046562 \| NM\_001046562 \| GO:0006450  GO:0017068 \|  \| 15E1.2 \| Bt.5527.2.S1\_a\_at \| \| Bt.20194.1.S1\_at \| 767923 \| 1/1 \| NM\_001076997 \|  \|  \|  \| HSPC268 \| Bt.20194.1.S1\_at \| \| Bt.7873.1.S1\_at \| 282862 \| 1/1 \| NM\_174741 \|  \|  \| 10908043  12096143 \| LU \| Bt.7873.1.S1\_at \| \| Bt.21351.1.S1\_a\_at \| 512350 \| 2/3 \| NM\_001046117 \|  \| GO:0003743  GO:0003924  GO:0005525  GO:0006413 \| 12140684  16305752 \| EIF2S3 \| Bt.21351.1.S1\_a\_at \| \| Bt.6490.2.S1\_at \| 535054 \| 1/1 \| NM\_001078004  XM\_001252088 \| XM\_001252088 \|  \|  \| PELI1 \| Bt.6490.2.S1\_at \| \| Bt.21453.1.S1\_at \| 507107 \| 1/1 \| NM\_001024488 \| NM\_001024488 \|  \| 11282978  12477932 \| SLC3A2 \| Bt.21453.1.S1\_at \| \| Bt.9288.2.A1\_at \| 510738 \| 2/3 \| XM\_587922 \| XM\_587922 \|  \|  \|  \| Bt.9288.2.A1\_at \| \| Bt.12249.1.S1\_at \| 536073 \| 2/2 \| XM\_616193 \| XM\_616193 \|  \|  \| JMJD1A \| Bt.12249.1.S1\_at \| \| Bt.20422.1.S1\_at \| 533578 \| 1/2 \| NM\_001038680 \|  \|  \|  \| NUDT22 \| Bt.20422.1.S1\_at \| \| Bt.6449.1.S1\_at \| 535185 \| 1/1 \| NM\_001014946 \| NM\_001014946 \| GO:0004888  GO:0005509  GO:0005515  GO:0007155  GO:0007596  GO:0016020 \| 11282978  16305752 \| FBLN5 \| Bt.6449.1.S1\_at \| \| Bt.24817.1.S1\_at \| 767889 \| 1/1 \| XM\_001249877  XM\_001249926 \| XM\_001249926 \| GO:0003998  GO:0016787 \| 3011706 \|  \| Bt.24817.1.S1\_at \| \| Bt.15561.1.S1\_at \|  \|  \|  \| XR\_028711 \|  \|  \| EFHD2 \| Bt.15561.1.S1\_at \| \| Bt.26095.1.A1\_at \|  \|  \|  \|  \|  \|  \| KIAA1462 \| Bt.26095.1.A1\_at \| \| Bt.22770.1.S1\_at \| 511229 \| 1/2 \| XM\_588526 \|  \| GO:0005515  GO:0007165 \|  \|  \| Bt.22770.1.S1\_at \| \| Bt.23673.1.A1\_at \| 534216 \| 2/6 \| NM\_001046374 \|  \| GO:0000166  GO:0003723  GO:0005622  GO:0005634  GO:0007049  GO:0008270  GO:0045786  GO:0046872 \|  \| RBM6 \| Bt.23673.1.A1\_at \| \| Bt.11038.1.S1\_at \|  \|  \|  \|  \|  \|  \| TULP4 \| Bt.11038.1.S1\_at \| \| Bt.28162.1.S1\_at \|  \|  \|  \|  \|  \|  \| PLN \| Bt.28162.1.S1\_at \| \| Bt.24471.1.S1\_a\_at \| 532986 \| 2/2 \| XM\_001249745  XM\_612229 \|  \| GO:0005515  GO:0008270 \|  \| FALZ \| Bt.24471.1.S1\_a\_at \| \| Bt.1415.1.S1\_at \| 515990 \| 1/1 \| NM\_001014932 \|  \| GO:0003735  GO:0005622  GO:0005739  GO:0005762  GO:0005840  GO:0006412 \| 12140684 \| MRPL11 \| Bt.1415.1.S1\_at \| \| Bt.15867.1.S1\_at \| 527852 \| 2/5 \| XM\_877649 \|  \|  \|  \| SERF2 \| Bt.15867.1.S1\_at \| \| Bt.9981.1.S1\_at \| 535320 \| 1/1 \| NM\_001017951 \| NM\_001017951 \| GO:0005515  GO:0005622  GO:0005856  GO:0005874  GO:0005875  GO:0007026  GO:0007275  GO:0008017  GO:0008270  GO:0030154  GO:0046872 \| 11282978  16305752 \| TRIM54 \| Bt.9981.1.S1\_at \| \| Bt.22295.1.S1\_at \| 531770 \| 1/2 \| NM\_001046318 \| NM\_001046318 \|  \|  \|  \| Bt.22295.1.S1\_at \| \| Bt.24474.1.S1\_at \| 515757 \| 2/5 \| NM\_001046211 \| NM\_001046211 \| GO:0000166  GO:0004222  GO:0005524  GO:0005739  GO:0005743  GO:0006508  GO:0008270  GO:0016020  GO:0016021  GO:0017111  GO:0030163  GO:0046872 \|  \|  \| Bt.24474.1.S1\_at \| \| Bt.5135.1.S1\_at \| 540564 \| 2/3 \| XM\_612101 \| XM\_612101 \|  \|  \| RAD23A \| Bt.5135.1.S1\_at \| \| Bt.4972.2.S1\_at \| 526027 \| 2/4 \| XM\_588094 \| XM\_588094 \|  \|  \| SPTBN1 \| Bt.4972.2.S1\_at \| \| Bt.10565.1.A1\_at \| 532927 \| 1/1 \| XR\_028888 \| XM\_615211 \|  \|  \| SYNE1 \| Bt.10565.1.A1\_at \| \| Bt.6440.1.S1\_at \| 281730 \| 1/1 \| NM\_175712 \| NM\_175712 \| GO:0005634  GO:0008152  GO:0016491  GO:0016616  GO:0048037  GO:0051287 \| 11163272 \| CTBP2 \| Bt.6440.1.S1\_at \| \| Bt.24717.2.S1\_a\_at \| 505293 \| 1/1 \| NM\_001034237 \|  \|  \| 12477932 \| MED6 \| Bt.24717.2.S1\_a\_at \| \| Bt.4831.1.S1\_at \| 281942 \| 1/1 \| NM\_174400 \|  \|  \|  \| NDP52 \| Bt.4831.1.S1\_at \| \| Bt.5415.1.S1\_at \| 515022 \| 1/1 \| NM\_001046187 \| NM\_001046187 \| GO:0004785  GO:0006801  GO:0030001  GO:0046872 \|  \| CCS \| Bt.5415.1.S1\_at \| \| Bt.10352.1.S1\_at \| 507410 \| 1/1 \| NM\_001075312 \|  \|  \|  \| POP5 \| Bt.10352.1.S1\_at \| \| Bt.13991.2.S1\_a\_at \| 507736 \| 2/5 \| NM\_001034308 \| NM\_001034308 \|  \| 12477932 \| PNRC1 \| Bt.13991.2.S1\_a\_at \| \| Bt.12684.2.S1\_at \|  \|  \|  \|  \|  \|  \|  \| Bt.12684.2.S1\_at \| \| Bt.27938.1.A1\_at \| 536203 \| 1/2 \| NM\_001037478 \| NM\_001037478 \| GO:0000166  GO:0005524  GO:0016020  GO:0016887 \| 12477932  15998908  17087837 \| ABCG2 \| Bt.27938.1.A1\_at \| \| Bt.27139.1.S1\_at \| 777691 \| 1/1 \| NM\_001078151  XM\_001251480  XM\_001251511 \| NM\_001078151 \|  \|  \| MTCP1 \| Bt.27139.1.S1\_at \| \| Bt.3395.1.S1\_at \| 777769 \| 1/1 \| NM\_001078154  XM\_001249587  XM\_001249638  XM\_001249672 \| NM\_001078154 \| GO:0005634 \|  \| SMARCD3 \| Bt.3395.1.S1\_at \| \| Bt.9223.1.A1\_at \| 515469 \| 1/1 \| XM\_864187 \|  \| GO:0005488  GO:0005856 \|  \| FKSG44 \| Bt.9223.1.A1\_at \| \| Bt.15909.1.S1\_at \| 530070 \| 1/1 \| XM\_608533 \|  \|  \|  \| DKFZP564F0522 \| Bt.15909.1.S1\_at \| \| Bt.20257.1.S1\_at \|  \|  \|  \|  \|  \|  \|  \| Bt.20257.1.S1\_at \| \| Bt.20340.2.S1\_at \| 513675 \| 1/2 \| XR\_028836 \|  \|  \|  \| DMPK \| Bt.20340.2.S1\_at \| \| Bt.18894.1.A1\_at \|  \|  \|  \| XM\_001253244 \|  \|  \| ZBTB10 \| Bt.18894.1.A1\_at \| \| Bt.27116.2.S1\_at \| 618131 \| 1/1 \| NM\_001037629 \| NM\_001037629 \|  \| 12477932 \| TIMM8B \| Bt.27116.2.S1\_at \| \| Bt.20917.2.S1\_a\_at \| 510469 \| 1/1 \| NM\_001046055 \|  \| GO:0006396 \|  \| RTCD1 \| Bt.20917.2.S1\_a\_at \| \| Bt.23326.2.A1\_at \|  \|  \|  \|  \|  \|  \| CALD1 \| Bt.23326.2.A1\_at \| \| Bt.26726.1.S1\_at \|  \|  \|  \|  \|  \|  \| PBX3 \| Bt.26726.1.S1\_at \| \| Bt.12337.2.S1\_a\_at \| 514560 \| 2/2 \| NM\_001037601 \| NM\_001037601 \|  \|  \| MGC134601 \| Bt.12337.2.S1\_a\_at \| \| Bt.20582.2.A1\_at \| 530971 \| 2/2 \|  \|  \|  \|  \| HDGFRP3 \| Bt.20582.2.A1\_at \| \| Bt.23169.1.S1\_at \|  \|  \|  \| XR\_027437 \|  \|  \| PTPNS1 \| Bt.23169.1.S1\_at \| \| Bt.13908.1.S1\_at \| 532044 \| 1/1 \| NM\_001046319 \| NM\_001046319 \| GO:0005739  GO:0030529 \| 10938081 \| MRPS22 \| Bt.13908.1.S1\_at \| \| Bt.18165.1.A1\_at \| 538094 \| 2/3 \| XM\_605323 \| XM\_605323 \| GO:0004674  GO:0004707  GO:0005524  GO:0006468 \|  \| MAPK6 \| Bt.18165.1.A1\_at \| \| Bt.13748.1.S1\_at \|  \|  \|  \| XM\_592833 \|  \|  \| DYRK2 \| Bt.13748.1.S1\_at \| \| Bt.12609.1.A1\_at \|  \|  \|  \|  \|  \|  \| RASGRP3 \| Bt.12609.1.A1\_at \| \| Bt.13183.1.A1\_at \|  \|  \|  \|  \|  \|  \| NRAS \| Bt.13183.1.A1\_at \| \| Bt.11257.2.S1\_at \| 506501 \| 1/2 \| XM\_582955 \| XM\_582955 \|  \|  \| TERF2 \| Bt.11257.2.S1\_at \| \| Bt.21072.1.A1\_at \| 512445 \| 1/1 \| XM\_589962 \|  \| GO:0003676  GO:0003700  GO:0005622  GO:0005634  GO:0006355  GO:0008270 \|  \| ZNF187 \| Bt.21072.1.A1\_at \| \| Bt.3029.1.S1\_at \| 518023 \| 1/2 \| XM\_596205 \| XM\_596205 \|  \|  \| LEMD2 \| Bt.3029.1.S1\_at \| \| Bt.21369.1.S1\_at \|  \|  \|  \|  \|  \|  \| MLLT6 \| Bt.21369.1.S1\_at \| \| Bt.16737.1.A1\_at \|  \|  \|  \|  \|  \|  \| PRDM2 \| Bt.16737.1.A1\_at \| \| Bt.8208.1.A1\_at \| 538749 \| 1/1 \| XM\_582415 \| XM\_582415 \|  \|  \| DAAM1 \| Bt.8208.1.A1\_at \| \| Bt.12906.1.S1\_at \| 535077 \| 1/1 \| NM\_001034627 \|  \| GO:0003779  GO:0005509  GO:0005856  GO:0051016 \| 12477932 \| GSN \| Bt.12906.1.S1\_at \| \| Bt.22854.1.S1\_at \| 280740 \| 1/1 \| NM\_178572 \| NM\_178572 \| GO:0002009  GO:0004089  GO:0005737  GO:0006730  GO:0008270  GO:0015670  GO:0016829  GO:0046872  GO:0046903 \| 19093  826282  12477932  15039588  16213706 \| CA2 \| Bt.22854.1.S1\_at \| \| Bt.4031.1.S1\_at \| 444879 \| 2/2 \| NM\_001002892 \| NM\_001002892 \| GO:0006486  GO:0008373  GO:0030173 \| 12477932  15843597 \| SIAT4B \| Bt.4031.1.S1\_at \| \| Bt.6865.1.S1\_at \|  \|  \|  \|  \|  \|  \| AXL \| Bt.6865.1.S1\_at \| \| Bt.11745.1.S1\_at \|  \|  \|  \|  \|  \|  \| KLHDC5 \| Bt.11745.1.S1\_at \| \| Bt.21272.1.S1\_at \| 533918 \| 1/1 \| NM\_001077993  XM\_001254372 \| NM\_001077993 \| GO:0005515  GO:0005737  GO:0006915  GO:0008289  GO:0008415  GO:0016740 \|  \| SH3GLB1 \| Bt.21272.1.S1\_at \| \| Bt.9889.1.S1\_at \| 613265 \| 1/1 \| NM\_001033121 \| NM\_001033121 \| GO:0003677  GO:0005622  GO:0005634  GO:0006350  GO:0006355  GO:0007276  GO:0008270  GO:0046872 \| 16621236 \|  \| Bt.9889.1.S1\_at \| \| Bt.4844.1.S1\_at \| 280904 \| 1/1 \| NM\_173955 \| NM\_173955 \|  \| 1550956  2229039  2270483  2610682  8484780  12477932 \| PTN \| Bt.4844.1.S1\_at \| \| Bt.21578.1.S1\_at \| 513978 \| 1/2 \| XM\_001254675  XM\_591752 \| XM\_591752 \|  \|  \| DUSP23 \| Bt.21578.1.S1\_at \| \| Bt.8286.1.S1\_at \| 524075 \| 1/1 \| NM\_001075853 \| NM\_001075853 \|  \| 11282978  16305752 \| PDK2 \| Bt.8286.1.S1\_at \| \| Bt.3589.2.A1\_at \| 512888 \| 1/3 \| XM\_865971 \|  \|  \|  \| TLE1 \| Bt.3589.2.A1\_at \| \| Bt.2599.1.S1\_at \| 614413 \| 1/3 \| XR\_027338 \|  \|  \|  \| CUGBP2 \| Bt.2599.1.S1\_at \| \| Bt.5879.1.S1\_at \|  \|  \|  \|  \|  \|  \|  \| Bt.5879.1.S1\_at \| \| Bt.250.1.S1\_at \| 281351 \| 1/1 \| NM\_177504 \| NM\_177504 \| GO:0004379  GO:0006499  GO:0008415  GO:0016740 \| 8452528  9390183  15041955 \| NMT1 \| Bt.250.1.S1\_at \| \| Bt.10122.1.S1\_at \|  \|  \|  \|  \|  \|  \| SPTLC2 \| Bt.10122.1.S1\_at \| \| Bt.22555.1.S1\_at \| 514045 \| 1/2 \| XM\_591833 \| XM\_591833 \|  \|  \|  \| Bt.22555.1.S1\_at \| \| Bt.841.2.A1\_at \|  \|  \|  \|  \|  \|  \|  \| Bt.841.2.A1\_at \| \| Bt.17332.3.A1\_at \| 613833 \| 2/2 \| XM\_864996 \| XM\_864996 \| GO:0003700  GO:0003707  GO:0005634  GO:0006355  GO:0008270  GO:0043565 \|  \| NR3C2 \| Bt.17332.3.A1\_at \| \| Bt.28208.1.S1\_at \| 514066 \| 1/1 \| NM\_001046164 \| NM\_001046164 \| GO:0005975  GO:0006094  GO:0016787  GO:0042132  GO:0042578 \|  \|  \| Bt.28208.1.S1\_at \| \| Bt.23668.1.A1\_at \|  \|  \|  \|  \|  \|  \| MITF \| Bt.23668.1.A1\_at \| \| Bt.7177.1.S1\_at \| 280719 \| 1/1 \| NM\_177485 \| NM\_177485 \| GO:0004047  GO:0005737  GO:0005739  GO:0005739  GO:0005739  GO:0005743  GO:0006546  GO:0006546  GO:0008483  GO:0016740  GO:0031405 \| 2002038  2211640 \| AMT \| Bt.7177.1.S1\_at \| \| Bt.7041.1.S1\_at \| 533187 \| 1/4 \| XM\_001250349  XM\_612509 \|  \| GO:0003677  GO:0006355 \|  \| BBX \| Bt.7041.1.S1\_at \| \| Bt.10962.1.S1\_at \| 539643 \| 1/2 \| XM\_588521 \| XM\_588521 \|  \|  \| VCPIP1 \| Bt.10962.1.S1\_at \| \| Bt.23647.1.A1\_a\_at \|  \|  \|  \|  \|  \|  \|  \| Bt.23647.1.A1\_a\_at \| \| Bt.20965.1.S1\_at \| 530409 \| 1/1 \| NM\_001034572 \| NM\_001034572 \| GO:0000074  GO:0005515  GO:0005654  GO:0005739  GO:0005743  GO:0005887  GO:0006260  GO:0016020  GO:0016481  GO:0016563  GO:0016564  GO:0016575 \| 12477932 \| PHB \| Bt.20965.1.S1\_at \| \| Bt.7619.2.S1\_at \|  \|  \|  \|  \|  \|  \| CAMK2A \| Bt.7619.2.S1\_at \| \| Bt.21191.3.S1\_at \| 508547 \| 2/5 \| XM\_867519 \| XM\_867519 \|  \|  \| PDE4DIP \| Bt.21191.3.S1\_at \| \| Bt.14644.1.A1\_at \|  \|  \|  \|  \|  \|  \| CTNNBIP1 \| Bt.14644.1.A1\_at \| \| Bt.23268.1.S1\_at \| 280815 \| 1/1 \| NM\_173918 \|  \|  \| 9030770  12140684  12477932  12591954  16305752 \| NPC2 \| Bt.23268.1.S1\_at \| \| Bt.9190.1.S1\_at \|  \|  \|  \|  \|  \|  \| UBE2G1 \| Bt.9190.1.S1\_at \| \| Bt.21485.1.S1\_at \| 614042 \| 1/1 \| NM\_001035112 \|  \|  \| 11282978  12477932 \| CDC42SE1 \| Bt.21485.1.S1\_at \| \| Bt.16106.1.A1\_at \| 539095 \| 1/3 \| XM\_882402 \|  \| GO:0003700  GO:0005634  GO:0006355  GO:0043565 \|  \| HOXB6 \| Bt.16106.1.A1\_at \| \| Bt.20955.1.A1\_at \|  \|  \|  \|  \|  \|  \| HLF \| Bt.20955.1.A1\_at \| \| Bt.10797.2.S1\_a\_at \| 615093 \| 1/1 \| XM\_866800 \| XM\_866800 \|  \|  \|  \| Bt.10797.2.S1\_a\_at \| \| Bt.17253.1.A1\_at \| 618291 \| 2/3 \|  \|  \|  \|  \| SUV420H1 \| Bt.17253.1.A1\_at \| \| Bt.13966.1.S1\_at \| 614731 \| 1/2 \| XM\_867601  XM\_880561 \| XM\_867601 \|  \|  \|  \| Bt.13966.1.S1\_at \| \| Bt.3821.1.A1\_at \| 514741 \| 2/2 \| NM\_001038109 \|  \| GO:0016020  GO:0016021 \|  \| TSPAN6 \| Bt.3821.1.A1\_at \| \| Bt.2394.1.S1\_at \| 513461 \| 1/1 \| NM\_001046148 \| NM\_001046148 \| GO:0005829  GO:0043234 \| 12140684  16305752 \| PSMD11 \| Bt.2394.1.S1\_at \| \| Bt.20692.1.A1\_at \|  \|  \|  \| XM\_876130 \|  \|  \| BZW1 \| Bt.20692.1.A1\_at \| \| Bt.28210.1.S1\_at \|  \|  \|  \|  \|  \|  \| NFIA \| Bt.28210.1.S1\_at \| \| Bt.4279.3.S1\_a\_at \| 507196 \| 2/4 \| NM\_001075305 \| NM\_001075305 \|  \|  \|  \| Bt.4279.3.S1\_a\_at \| \| Bt.3541.1.S1\_at \| 526135 \| 1/1 \| NM\_001035361 \|  \|  \| 12477932 \| RBMS1 \| Bt.3541.1.S1\_at \| \| Bt.5351.1.S1\_a\_at \| 280733 \| 1/2 \| NM\_178318 \|  \| GO:0005515  GO:0005578  GO:0030133 \| 2656687  2914936  8485158  8673009  11282978  11723132  12354766  14600270  15313168  16305752  16547006 \| BGN \| Bt.5351.1.S1\_a\_at \| \| Bt.19330.1.A1\_s\_at \| 540617 \| 2/2 \|  \| XR\_027877 \|  \|  \| FNBP1L \| Bt.19330.1.A1\_s\_at \| \| Bt.27431.1.A1\_at \|  \|  \|  \|  \|  \|  \| FCHO2 \| Bt.27431.1.A1\_at \| \| Bt.11966.1.A1\_at \| 540090 \| 2/2 \| XM\_591423 \| XM\_591423 \| GO:0005515  GO:0008270 \|  \| RNF2 \| Bt.11966.1.A1\_at \| \| Bt.22355.1.S1\_at \| 540265 \| 1/1 \| XM\_865387 \| XM\_865387 \|  \|  \| TRIM41 \| Bt.22355.1.S1\_at \| \| Bt.13248.1.A1\_at \|  \|  \|  \|  \|  \|  \| SEC63 \| Bt.13248.1.A1\_at \| \| Bt.22125.2.A1\_at \|  \|  \|  \|  \|  \|  \|  \| Bt.22125.2.A1\_at \| \| Bt.5112.1.A1\_a\_at \| 512060 \| 1/1 \| XR\_028325 \|  \|  \|  \| SEPHS2 \| Bt.5112.1.A1\_a\_at \| \| Bt.23392.1.S1\_at \| 538959 \| 1/2 \| XR\_027542 \| XR\_027542 \|  \|  \| EPB41L2 \| Bt.23392.1.S1\_at \| \| Bt.8015.3.A1\_at \|  \|  \|  \|  \|  \|  \| ACSL4 \| Bt.8015.3.A1\_at \| \| Bt.8213.1.S1\_at \| 517688 \| 2/2 \| XM\_595862 \| XM\_595862 \|  \|  \| FLJ14466 \| Bt.8213.1.S1\_at \| \| Bt.20355.1.S1\_at \| 505840 \| 1/1 \| NM\_001075226 \| NM\_001075226 \|  \|  \| FLJ39378 \| Bt.20355.1.S1\_at \| \| Bt.23941.1.A1\_at \|  \|  \|  \|  \|  \|  \| ZFP161 \| Bt.23941.1.A1\_at \| \| Bt.9767.1.S1\_a\_at \|  \|  \|  \| XM\_604908 \|  \|  \| SQLE \| Bt.9767.1.S1\_a\_at \| \| Bt.14624.2.S1\_at \| 535683 \| 2/2 \| NM\_001076067 \| NM\_001076067 \| GO:0005829  GO:0043234 \|  \| PSMD14 \| Bt.14624.2.S1\_at \| \| Bt.7384.1.A1\_at \| 534852 \| 1/6 \| NM\_001034617 \|  \| GO:0000209  GO:0000723  GO:0005515  GO:0006511  GO:0006512  GO:0031398 \| 12477932 \|  \| Bt.7384.1.A1\_at \| \| Bt.7727.1.S1\_at \| 767586 \| 1/1 \| NM\_001076531 \| NM\_001076531 \|  \|  \| ANKRD25 \| Bt.7727.1.S1\_at \| \| Bt.18402.1.S1\_at \|  \|  \|  \|  \|  \|  \| SAR1B \| Bt.18402.1.S1\_at \| \| Bt.2092.1.S1\_at \| 506953 \| 1/4 \| XM\_867721 \| XM\_583479 \|  \|  \| ITGA7 \| Bt.2092.1.S1\_at \| \| Bt.28059.1.S1\_at \|  \|  \|  \|  \|  \|  \| RSN \| Bt.28059.1.S1\_at \| \| Bt.2109.1.S1\_at \| 508285  614177 \| 1/1 \| NM\_001046526  XM\_585042 \| NM\_001046526 \|  \|  \| MAT2B \| Bt.2109.1.S1\_at \| \| Bt.3098.1.S1\_at \| 509775 \| 1/1 \| NM\_001035076 \| NM\_001035076 \|  \| 12477932 \| TMEM59 \| Bt.3098.1.S1\_at \| \| Bt.25092.1.S1\_at \| 505511 \| 1/1 \| XM\_581806 \|  \|  \|  \| AHCYL1 \| Bt.25092.1.S1\_at \| \| Bt.28390.1.S1\_at \|  \|  \|  \|  \|  \|  \| TSC22D3 \| Bt.28390.1.S1\_at \| \| Bt.22125.1.S1\_at \|  \|  \|  \|  \|  \|  \| ATRX \| Bt.22125.1.S1\_at \| \| Bt.27800.1.A1\_at \| 504474 \| 1/2 \| XM\_580609 \|  \|  \|  \| SIMP \| Bt.27800.1.A1\_at \| \| Bt.27160.1.A1\_at \|  \|  \|  \|  \|  \|  \| SSBP3 \| Bt.27160.1.A1\_at \| \| Bt.6789.1.S1\_at \| 614425 \| 1/1 \| NM\_001046536 \| NM\_001046536 \|  \|  \| C19orf27 \| Bt.6789.1.S1\_at \| \| Bt.22534.1.S1\_at \| 534497 \| 1/1 \| XM\_001255352  XM\_614282 \| XM\_001255352 \| GO:0007049  GO:0007050  GO:0016020  GO:0016021 \| 7568893  10899964 \| PMP22 \| Bt.22534.1.S1\_at \| \| Bt.18039.1.S1\_at \| 617635 \| 2/2 \| NM\_001034802 \|  \|  \| 12477932 \|  \| Bt.18039.1.S1\_at \| \| Bt.28057.1.S1\_at \| 508731 \| 1/1 \| XM\_864386 \| XM\_864386 \|  \|  \| ZNF198 \| Bt.28057.1.S1\_at \| \| Bt.7425.1.S1\_at \| 613811 \| 1/1 \| XM\_001250631  XM\_864886  XM\_874568 \|  \| GO:0005576  GO:0006955  GO:0008009 \|  \| CXCL12 \| Bt.7425.1.S1\_at \| \| Bt.3427.1.S1\_at \| 618441 \| 1/1 \| NM\_001034810 \| NM\_001034810 \|  \| 12477932 \| MGC128294 \| Bt.3427.1.S1\_at \| \| Bt.16188.1.S1\_at \| 539799 \| 1/1 \|  \|  \|  \|  \| CYYR1 \| Bt.16188.1.S1\_at \| \| Bt.13645.1.S1\_at \| 513649 \| 1/2 \| XM\_591366 \| XM\_591366 \|  \|  \| FLJ31795 \| Bt.13645.1.S1\_at \| \| Bt.9803.1.S1\_at \|  \|  \|  \|  \|  \|  \| ETF1 \| Bt.9803.1.S1\_at \| \| Bt.9391.2.S1\_at \| 514386 \| 1/2 \| NM\_001035293 \| NM\_001035293 \| GO:0005515  GO:0005622  GO:0006916  GO:0008270  GO:0042981 \| 12477932 \|  \| Bt.9391.2.S1\_at \| \| Bt.17425.1.A1\_at \| 511058 \| 2/3 \| XM\_588314 \| XM\_588314 \| GO:0016787 \|  \| PPP6C \| Bt.17425.1.A1\_at \| \| Bt.5412.1.S1\_at \| 282150 \| 1/1 \| NM\_174507 \| NM\_174507 \| GO:0003826  GO:0003863  GO:0005739 \| 1730724  2322554 \| BCKDHB \| Bt.5412.1.S1\_at \| \| Bt.26684.1.S1\_at \| 613751 \| 1/2 \| NM\_001076287 \| NM\_001076287 \|  \|  \| GPBP1 \| Bt.26684.1.S1\_at \| \| Bt.18945.1.A1\_at \| 525772 \| 1/1 \| XM\_604128 \|  \|  \|  \|  \| Bt.18945.1.A1\_at \| \| Bt.13874.1.A1\_at \|  \|  \|  \| XM\_618434 \|  \|  \|  \| Bt.13874.1.A1\_at \| \| Bt.3414.1.S1\_at \| 536149 \| 1/3 \| NM\_001038171 \|  \| GO:0003677  GO:0005634  GO:0006810  GO:0007275  GO:0007283  GO:0008270  GO:0030154  GO:0043087  GO:0046872 \|  \| HRB \| Bt.3414.1.S1\_at \| \| Bt.18748.1.A1\_at \| 505696 \| 1/2 \| XM\_582025 \| XM\_582025 \|  \|  \| PTPRB \| Bt.18748.1.A1\_at \| \| Bt.5376.1.S1\_at \| 506336 \| 1/1 \| NM\_001034269 \|  \|  \| 12477932 \| YIF1A \| Bt.5376.1.S1\_at \| \| Bt.9877.1.S1\_at \| 539950 \| 1/1 \| NM\_001076920 \| NM\_001076920 \|  \|  \| HNRPU \| Bt.9877.1.S1\_at \| \| Bt.6111.1.S1\_at \| 510314 \| 1/2 \| NM\_001034376 \| NM\_001034376 \|  \| 12477932 \| MGC127942 \| Bt.6111.1.S1\_at \| \| Bt.22084.2.S1\_at \| 515903 \| 3/5 \| XM\_001249521  XM\_001249570  XM\_878572  XM\_878731  XM\_878792 \| XM\_001249521 \|  \|  \| FOXP1 \| Bt.22084.2.S1\_at \| \| Bt.24137.1.A1\_at \| 539761 \| 1/1 \| XR\_027726 \| XR\_027726 \|  \|  \| PLAGL1 \| Bt.24137.1.A1\_at \| \| Bt.21774.2.S1\_a\_at \| 520111 \| 2/2 \| NM\_001075799 \| NM\_001075799 \| GO:0005515  GO:0005622  GO:0008270 \|  \| RFP \| Bt.21774.2.S1\_a\_at \| \| Bt.20720.1.S1\_at \| 614585 \| 1/2 \| XM\_866139 \| XM\_866139 \|  \|  \| CGI-128 \| Bt.20720.1.S1\_at \| \| Bt.4662.1.S1\_at \| 287024 \| 1/1 \| NM\_181037 \|  \| GO:0004517  GO:0005506  GO:0005509  GO:0005516  GO:0006118  GO:0006809  GO:0007596  GO:0008270  GO:0010181  GO:0016491 \| 1378626  1379225  1385480  7682550  8524847  9875848  11051558  11331003  11331290  11590164  11695891  12381415  12488320  12501214  12511559  12521608  12591925  12676772  12757411  12767233  12799359  12869534  12952971  12969888  14529282  14551684  14568906  14581470  14583337  14715665  14769821  14983058  15004019  15242742  15383526  15607726  15637154  15665327  15713835  15743275  15774480  15922337  16123043  16172287  16257964  16458937  16601232  16614401  16679377  16737962  16921253  16998480  17071725  17170139 \| NOS3 \| Bt.4662.1.S1\_at \| \| Bt.21351.1.S1\_at \| 512350 \| 2/3 \| NM\_001046117 \|  \| GO:0003743  GO:0003924  GO:0005525  GO:0006413 \| 12140684  16305752 \| EIF2S3 \| Bt.21351.1.S1\_at \| \| Bt.20846.1.A1\_a\_at \| 538425 \| 1/2 \| XM\_867393 \|  \| GO:0016791 \|  \| CTDSPL \| Bt.20846.1.A1\_a\_at \| \| Bt.6019.1.S1\_at \| 506235 \| 1/1 \| NM\_001076809 \|  \|  \| 12077321  14667821 \|  \| Bt.6019.1.S1\_at \| \| Bt.22543.1.S1\_at \|  \|  \|  \|  \|  \|  \| KPNA6 \| Bt.22543.1.S1\_at \| \| Bt.2230.1.S2\_at \| 281040 \| 3/3 \| NM\_174004 \| NM\_174004 \|  \| 12477932  12917688  15292187  15471980  15637154  16325778  16894547 \| CAV1 \| Bt.2230.1.S2\_at \| \| Bt.6020.1.S1\_at \| 508137 \| 1/1 \| NM\_001045993 \| NM\_001045993 \|  \|  \| DNAJC11 \| Bt.6020.1.S1\_at \| \| Bt.3500.1.S1\_at \| 515169 \| 1/1 \| NM\_001046189 \| NM\_001046189 \| GO:0000287  GO:0004749  GO:0009116  GO:0009156  GO:0009165  GO:0016301  GO:0016740  GO:0016978 \|  \| PRPS1 \| Bt.3500.1.S1\_at \| \| Bt.20872.1.A1\_at \|  \|  \|  \| XM\_001251679 \|  \|  \| PIGO \| Bt.20872.1.A1\_at \| \| Bt.27371.1.S1\_at \|  \|  \|  \|  \|  \|  \|  \| Bt.27371.1.S1\_at \| \| Bt.4743.1.A1\_at \| 507581 \| 1/2 \| NM\_001076813 \|  \|  \|  \|  \| Bt.4743.1.A1\_at \| \| Bt.22516.1.A1\_at \| 505949 \| 1/1 \| NM\_001075233 \| NM\_001075233 \|  \|  \| RDH14 \| Bt.22516.1.A1\_at \| \| Bt.27018.1.S1\_at \| 521237 \| 1/1 \| NM\_001046255 \| NM\_001046255 \| GO:0004672  GO:0005524  GO:0006468 \|  \| STK16 \| Bt.27018.1.S1\_at \| \| Bt.7266.1.S1\_at \| 515109 \| 1/1 \| NM\_001034480 \| NM\_001034480 \| GO:0005739  GO:0006457  GO:0008270  GO:0015031  GO:0016020  GO:0042719  GO:0045039  GO:0046872 \| 12477932 \| TIMM8A \| Bt.7266.1.S1\_at \| \| Bt.23218.1.S1\_at \| 513038 \| 1/1 \| NM\_001034435 \| NM\_001034435 \| GO:0004216  GO:0005764  GO:0006508 \| 12140684  16305752 \| CTSK \| Bt.23218.1.S1\_at \| \| Bt.1171.1.S1\_at \| 532143 \| 1/1 \| NM\_001046320 \| NM\_001046320 \|  \|  \| FLJ42461 \| Bt.1171.1.S1\_at \| \| Bt.3898.1.S1\_at \| 282446 \| 1/1 \| NM\_174644 \| NM\_174644 \| GO:0004449  GO:0005739  GO:0005739  GO:0006099  GO:0008152  GO:0016491  GO:0016616 \| 215197  7654189 \| IDH3A \| Bt.3898.1.S1\_at \| \| Bt.20445.1.S1\_at \| 618689 \| 1/1 \| XR\_027943 \| XR\_027943 \|  \| 12477932 \| SS18L2 \| Bt.20445.1.S1\_at \| \| Bt.21015.1.S1\_at \| 512902 \| 2/2 \| NM\_001075587 \| NM\_001075587 \| GO:0008415  GO:0016740 \|  \| CRAT \| Bt.21015.1.S1\_at \| \| Bt.3388.1.S1\_at \| 540602 \| 1/1 \| NM\_001046482 \| NM\_001046482 \| GO:0003824  GO:0008152  GO:0016051 \|  \| NANS \| Bt.3388.1.S1\_at \| \| Bt.5494.1.S1\_at \| 281057 \| 1/1 \| NM\_174013 \| NM\_174013 \|  \| 1922105  12496181  15846044 \| CD44 \| Bt.5494.1.S1\_at \| \| Bt.10608.2.S1\_a\_at \| 505736 \| 1/2 \| NM\_001034256 \|  \|  \| 11282978  12477932  16305752 \| FLJ10803 \| Bt.10608.2.S1\_a\_at \| \| Bt.9242.1.S1\_at \| 506521 \| 1/1 \| XM\_582978 \| XM\_582978 \|  \|  \| MRPL41 \| Bt.9242.1.S1\_at \| \| Bt.23216.1.S1\_at \| 532663 \| 1/2 \| XM\_611807 \| NM\_001080293 \| GO:0003676  GO:0004519  GO:0006935  GO:0008152  GO:0009117  GO:0016042  GO:0016787  GO:0046872  GO:0047391 \| 12119361 \| ENPP2 \| Bt.23216.1.S1\_at \| \| Bt.12862.1.S1\_at \|  \|  \|  \|  \|  \|  \| TSPYL5 \| Bt.12862.1.S1\_at \| \| Bt.23605.2.S1\_at \| 532621 \| 1/1 \| NM\_001046329 \| NM\_001046329 \| GO:0003700  GO:0003707  GO:0004887  GO:0005634  GO:0006355  GO:0008270  GO:0043565 \|  \| THRA \| Bt.23605.2.S1\_at \| \| Bt.16094.1.S1\_at \| 509106 \| 1/1 \| XM\_585997  XM\_865659 \| XM\_585997 \|  \|  \| EZH2 \| Bt.16094.1.S1\_at \| \| Bt.21440.1.S1\_at \| 514323 \| 1/2 \| XM\_880706 \|  \| GO:0003676  GO:0005524  GO:0008026 \|  \| DDX17 \| Bt.21440.1.S1\_at \| \| Bt.4071.1.S1\_at \| 616916 \| 1/2 \| XM\_869056 \|  \|  \|  \| C2orf23 \| Bt.4071.1.S1\_at \| \| Bt.4520.1.S1\_at \| 282346 \| 1/1 \| NM\_174597 \|  \| GO:0000299  GO:0004872  GO:0005515  GO:0005887  GO:0007155  GO:0016020  GO:0016599 \| 9406850 \| SCARB1 \| Bt.4520.1.S1\_at \| \| Bt.12333.1.A1\_at \|  \|  \|  \|  \|  \|  \| ZNF265 \| Bt.12333.1.A1\_at \| \| Bt.24350.1.A1\_at \|  \|  \|  \| XM\_869255 \|  \|  \| MGA \| Bt.24350.1.A1\_at \| \| Bt.3137.1.S1\_at \| 616317 \| 1/1 \| NM\_001034790 \| NM\_001034790 \|  \| 12477932 \| STMN1 \| Bt.3137.1.S1\_at \| \| Bt.16206.2.S1\_at \| 514264 \| 1/2 \|  \| XM\_870563 \|  \|  \|  \| Bt.16206.2.S1\_at \| \| Bt.13537.1.A1\_at \| 505775 \| 1/1 \| NM\_001037319 \| NM\_001037319 \|  \| 12477932  15894023  16122954  16857719 \| SLC16A1 \| Bt.13537.1.A1\_at \| \| Bt.22333.1.S1\_at \| 510194 \| 1/1 \| NM\_001075436 \| NM\_001075436 \|  \|  \| HP1BP3 \| Bt.22333.1.S1\_at \| \| Bt.13249.1.A1\_at \| 617419 \| 1/1 \| NM\_001076945 \| NM\_001076945 \|  \|  \|  \| Bt.13249.1.A1\_at \| \| Bt.26122.2.S1\_at \| 525680 \| 1/1 \| NM\_001037471 \| NM\_001037471 \|  \| 12477932 \| UXT \| Bt.26122.2.S1\_at \| \| Bt.27696.1.A1\_at \| 614007 \| 1/2 \| NM\_001035440 \| NM\_001035440 \|  \| 12140684  12477932 \| SH3BGRL \| Bt.27696.1.A1\_at \| \| Bt.22170.2.A1\_at \| 530414 \| 3/5 \| NM\_001075932 \|  \| GO:0008152  GO:0008415  GO:0016740 \|  \| AGPAT5 \| Bt.22170.2.A1\_at \| \| Bt.16142.2.S1\_at \|  \|  \|  \|  \|  \|  \| YWHAG \| Bt.16142.2.S1\_at \| \| Bt.13391.1.S1\_at \| 493728 \| 1/1 \| NM\_001012998 \|  \| GO:0003779  GO:0008290  GO:0030036  GO:0051016 \| 12140684  12477932  12917688  16305752 \| CAPZA2 \| Bt.13391.1.S1\_at \| \| Bt.8486.2.S1\_at \| 539627 \| 2/3 \| XM\_588438 \| XM\_588438 \|  \|  \| TNIK \| Bt.8486.2.S1\_at \| \| Bt.15972.1.S1\_at \| 281193 \| 1/2 \| NM\_174068 \|  \|  \| 1695901  11824477  15123628  15319221  16310041  16552723 \| GJA1 \| Bt.15972.1.S1\_at \| \| Bt.20481.1.S1\_at \|  \|  \|  \|  \|  \|  \| PIAS1 \| Bt.20481.1.S1\_at \| \| Bt.3211.1.S1\_at \| 338472 \| 2/2 \| NM\_001033616 \| NM\_001033616 \| GO:0005874 \| 12438634  12477932 \| GABARAPL1 \| Bt.3211.1.S1\_at \| \| Bt.4370.2.S1\_at \| 337889 \| 1/3 \| NM\_001045873 \| NM\_001045873 \| GO:0005515 \| 12658628 \| LRRC41 \| Bt.4370.2.S1\_at \| \| Bt.27199.1.S1\_at \| 616383 \| 1/1 \|  \|  \|  \|  \| MKLN1 \| Bt.27199.1.S1\_at \| \| Bt.21051.1.S1\_at \| 514753 \| 1/1 \| NM\_001046180 \| NM\_001046180 \| GO:0005739  GO:0030529 \| 11551941 \| MRPL50 \| Bt.21051.1.S1\_at \| \| Bt.5542.2.S1\_at \| 540136 \| 1/2 \| XM\_872313 \|  \|  \|  \|  \| Bt.5542.2.S1\_at \| \| Bt.11052.1.S1\_at \| 614611 \| 1/1 \| NM\_001076338 \|  \|  \|  \| MOBK1B \| Bt.11052.1.S1\_at \| \| Bt.5088.1.S1\_at \| 617609 \| 1/3 \| XM\_869904 \| XM\_868350 \|  \|  \| KIAA0515 \| Bt.5088.1.S1\_at \| \| Bt.15836.1.S1\_at \| 510376 \| 2/2 \| NM\_001034378 \|  \|  \| 12477932 \| ANKRD1 \| Bt.15836.1.S1\_at \| \| Bt.27008.1.A1\_at \| 407102 \| 1/3 \| XM\_582218 \|  \| GO:0005515  GO:0005923 \| 16269664 \| TJP1 \| Bt.27008.1.A1\_at \| \| Bt.8798.1.S1\_at \|  \|  \|  \| XM\_583647 \|  \|  \| ALDH1A3 \| Bt.8798.1.S1\_at \| \| Bt.1731.1.S1\_at \| 282215 \| 1/1 \| NM\_174532 \| NM\_174532 \| GO:0031072 \| 11896048 \| DNAJB6 \| Bt.1731.1.S1\_at \| \| Bt.2537.2.S1\_at \|  \|  \|  \| NM\_174719 \|  \|  \| TIMP3 \| Bt.2537.2.S1\_at \| \| Bt.27100.1.S1\_at \| 533430 \| 1/1 \| NM\_001046351 \| NM\_001046351 \| GO:0004872  GO:0005634  GO:0008270  GO:0030528  GO:0045449 \|  \|  \| Bt.27100.1.S1\_at \| \| Bt.7991.1.S1\_a\_at \| 541229 \| 1/2 \| XM\_001254088  XM\_867666  XM\_880840 \|  \|  \|  \| SF1 \| Bt.7991.1.S1\_a\_at \| \| Bt.9674.1.S1\_at \|  \|  \|  \|  \|  \|  \| FKBP9 \| Bt.9674.1.S1\_at \| \| Bt.19807.1.S1\_at \| 616545 \| 1/1 \| NM\_001038214 \| NM\_001038214 \|  \|  \| SCAP2 \| Bt.19807.1.S1\_at \| \| Bt.28162.3.S1\_at \|  \|  \|  \|  \|  \|  \| PLN \| Bt.28162.3.S1\_at \| \| Bt.5038.5.S1\_at \| 617635 \| 2/2 \| NM\_001034802 \| NM\_001034802 \|  \| 12477932 \| RAB24 \| Bt.5038.5.S1\_at \| \| Bt.24433.2.A1\_at \| 515194 \| 2/3 \| XM\_593171 \| NM\_001080730 \|  \|  \| MRPL39 \| Bt.24433.2.A1\_at \| \| Bt.25342.1.A1\_at \| 535248 \| 1/3 \| XM\_615280 \| XM\_615280 \|  \|  \| FLJ20097 \| Bt.25342.1.A1\_at \| \| Bt.3060.1.S1\_a\_at \| 615190 \| 1/4 \| NM\_001078048  XM\_001249875 \|  \|  \|  \|  \| Bt.3060.1.S1\_a\_at \| \| Bt.13440.1.S1\_at \|  \|  \|  \|  \|  \|  \| LKAP \| Bt.13440.1.S1\_at \| \| Bt.22092.1.S1\_at \|  \|  \|  \|  \|  \|  \| TAF9 \| Bt.22092.1.S1\_at \| \| Bt.21456.1.S1\_at \|  \|  \|  \|  \|  \|  \| LHFP \| Bt.21456.1.S1\_at \| \| Bt.10359.1.S1\_at \| 510177 \| 2/4 \| XM\_865029 \| XM\_865029 \|  \|  \| SF3B1 \| Bt.10359.1.S1\_at \| \| Bt.23265.1.S1\_at \| 519882 \| 1/1 \| XM\_598113 \| XM\_598113 \|  \|  \| SPRED1 \| Bt.23265.1.S1\_at \| \| Bt.27717.1.A1\_at \|  \|  \|  \|  \|  \|  \| ANK3 \| Bt.27717.1.A1\_at \| \| Bt.13237.1.S1\_at \| 510423 \| 1/1 \| NM\_001034381 \|  \|  \| 12477932 \| PSMA4 \| Bt.13237.1.S1\_at \| \| Bt.22122.1.S1\_at \|  \|  \|  \|  \|  \|  \| ABLIM1 \| Bt.22122.1.S1\_at \| \| Bt.12912.1.S1\_at \| 282191 \| 2/2 \| XM\_580317 \|  \| GO:0005201  GO:0005581  GO:0005604  GO:0005737  GO:0006817 \| 11970952  15299013 \| COL4A1 \| Bt.12912.1.S1\_at \| \| Bt.22170.1.S1\_at \| 530414 \| 3/5 \| NM\_001075932 \| NM\_001075932 \| GO:0008152  GO:0008415  GO:0016740 \|  \| AGPAT5 \| Bt.22170.1.S1\_at \| \| Bt.28728.1.A1\_at \| 517688 \| 2/2 \| XM\_595862 \| XM\_595862 \|  \|  \| FLJ14466 \| Bt.28728.1.A1\_at \| \| Bt.29915.1.S1\_at \| 506632 \| 1/2 \| NM\_001076811 \| NM\_001076811 \|  \|  \| KLHL21 \| Bt.29915.1.S1\_at \| \| Bt.6950.1.S1\_at \|  \|  \|  \|  \|  \|  \| C20orf35 \| Bt.6950.1.S1\_at \| \| Bt.15668.1.S1\_at \| 540966 \| 1/1 \| NM\_001034717 \| NM\_001034717 \| GO:0000228  GO:0005515  GO:0005634  GO:0006281  GO:0006915  GO:0007001  GO:0007049  GO:0007059  GO:0007067  GO:0051301 \| 12477932 \| RAD21 \| Bt.15668.1.S1\_at \| \| Bt.413.1.S1\_at \| 281780 \| 1/1 \| NM\_177511 \| NM\_177511 \| GO:0005975  GO:0016020  GO:0016021  GO:0016758  GO:0030145  GO:0046872  GO:0047276 \| 2503516  11179209  11592969  12011052 \| GGTA1 \| Bt.413.1.S1\_at \| \| Bt.17837.1.A1\_at \| 506047 \| 1/1 \| XM\_582439 \|  \|  \|  \| PIK3C2B \| Bt.17837.1.A1\_at \| \| Bt.6989.1.S1\_at \| 404134 \| 1/1 \| NM\_205798 \| XM\_001257081 \|  \| 11282978  16305752 \| TMBIM1 \| Bt.6989.1.S1\_at \| \| Bt.1217.2.S1\_at \| 507707 \| 2/3 \| NM\_001075328 \|  \|  \|  \| TOMM70A \| Bt.1217.2.S1\_at \| \| Bt.5466.4.S1\_at \| 286798 \| 1/3 \| NM\_174766 \|  \| GO:0003779  GO:0005856  GO:0007242 \| 11023826 \| TNS1 \| Bt.5466.4.S1\_at \| \| Bt.5241.1.S1\_at \| 513405 \| 1/1 \| NM\_001075617 \|  \| GO:0005515  GO:0005634  GO:0005730  GO:0006364  GO:0030529 \|  \| IMP4 \| Bt.5241.1.S1\_at \| \| Bt.17932.1.S1\_at \| 514613 \| 1/2 \| XM\_875093 \| XM\_875093 \|  \|  \| CCNL1 \| Bt.17932.1.S1\_at \| \| Bt.11629.1.A1\_at \| 540861 \| 1/2 \| XM\_613474 \| XM\_613474 \|  \|  \| RW1 \| Bt.11629.1.A1\_at \| \| Bt.3610.1.S1\_at \| 613899 \| 1/1 \| NM\_001034745 \|  \| GO:0005739  GO:0005746  GO:0006122  GO:0006810  GO:0008121  GO:0016020  GO:0016491 \| 3003044  6126477  9204897  9651245  12477932 \| MGC127994 \| Bt.3610.1.S1\_at \| \| Bt.15799.1.S1\_at \| 516462 \| 1/3 \| XM\_594616  XM\_875185 \| XM\_600524 \| GO:0003677  GO:0003725  GO:0005622 \| 15777723 \| SON \| Bt.15799.1.S1\_at \| \| Bt.27430.2.S1\_at \| 506029 \| 2/2 \| XM\_582418 \| XM\_582418 \|  \|  \| ALS2CR2 \| Bt.27430.2.S1\_at \| \| Bt.13345.1.A1\_at \| 533388 \| 1/2 \| XM\_612781 \|  \| GO:0004221  GO:0006511 \|  \| USP54 \| Bt.13345.1.A1\_at \| \| Bt.2518.1.S1\_at \|  \|  \|  \|  \|  \|  \| EGFR \| Bt.2518.1.S1\_at \| \| Bt.2230.1.S1\_at \| 281040 \| 3/3 \| NM\_174004 \| NM\_174004 \|  \| 12477932  12917688  15292187  15471980  15637154  16325778  16894547 \| CAV1 \| Bt.2230.1.S1\_at \| \| Bt.903.1.S1\_at \| 538887 \| 1/1 \| XM\_001251974  XM\_583463 \|  \|  \|  \| SLC44A1 \| Bt.903.1.S1\_at \| \| Bt.18270.1.A1\_at \| 541118 \| 1/7 \| XM\_614640 \|  \| GO:0000166  GO:0003676 \|  \| TNRC6A \| Bt.18270.1.A1\_at \| \| Bt.20243.2.S1\_at \| 614713 \| 1/3 \| XR\_027547 \|  \|  \|  \| SLC25A26 \| Bt.20243.2.S1\_at \| \| Bt.28758.1.A1\_at \| 531877 \| 1/2 \| XM\_610380 \|  \| GO:0004725  GO:0006470  GO:0016791 \|  \| PTPN12 \| Bt.28758.1.A1\_at \| \| Bt.8106.1.S1\_at \| 534319 \| 1/1 \| XM\_614049 \| XM\_614049 \| GO:0008218  GO:0018298 \| 1745013  15296935 \| NID1 \| Bt.8106.1.S1\_at \| \| Bt.1390.1.A1\_at \|  \|  \|  \|  \|  \|  \| KBTBD8 \| Bt.1390.1.A1\_at \| \| Bt.26995.2.S1\_at \| 616365 \| 1/2 \| XM\_868370 \|  \|  \|  \|  \| Bt.26995.2.S1\_at \| \| Bt.5288.1.S1\_at \| 281557 \| 1/1 \| NM\_174208 \|  \|  \| 9363753 \| TXN2 \| Bt.5288.1.S1\_at \| \| Bt.23227.1.S1\_at \|  \|  \|  \|  \|  \|  \| MXD1 \| Bt.23227.1.S1\_at \| \| Bt.1062.1.S1\_at \| 529318 \| 1/3 \| XM\_607763 \| XM\_607763 \| GO:0003723  GO:0005515  GO:0008270  GO:0046872 \|  \| RKHD2 \| Bt.1062.1.S1\_at \| \| Bt.23106.1.S1\_at \| 613381 \| 1/1 \| NM\_001034734 \|  \| GO:0005634 \| 12477932 \| HN1 \| Bt.23106.1.S1\_at \| \| Bt.26835.1.S1\_at \| 512506 \| 1/1 \| NM\_001037460 \|  \|  \| 12477932 \| RANGNRF \| Bt.26835.1.S1\_at \| \| Bt.25957.1.S1\_at \| 618508 \| 1/2 \| NM\_001046620 \| NM\_001046620 \|  \|  \| KIAA1271 \| Bt.25957.1.S1\_at \| \| Bt.18914.1.S1\_at \|  \|  \|  \|  \|  \|  \| PODXL \| Bt.18914.1.S1\_at \| \| Bt.20619.1.S1\_at \|  \|  \|  \|  \|  \|  \| MSI2 \| Bt.20619.1.S1\_at \| \| Bt.24319.1.A1\_at \|  \|  \|  \|  \|  \|  \| BMPR2 \| Bt.24319.1.A1\_at \| \| Bt.26751.1.S1\_at \| 508388 \| 1/1 \| NM\_001075357 \|  \|  \| 11282978  16305752 \| WSB2 \| Bt.26751.1.S1\_at \| \| Bt.23603.2.S1\_a\_at \| 511798 \| 3/3 \| NM\_001034412 \|  \| GO:0006512 \| 12477932 \| FBXO9 \| Bt.23603.2.S1\_a\_at \| \| Bt.28566.1.S1\_at \|  \|  \|  \|  \|  \|  \| NR3C2 \| Bt.28566.1.S1\_at \| \| Bt.16139.1.A1\_at \| 538623 \| 1/1 \|  \|  \|  \|  \| PCYOX1 \| Bt.16139.1.A1\_at \| \| Bt.3791.1.S1\_at \| 508716 \| 1/1 \| NM\_001075371 \| NM\_001075371 \|  \| 12140684  16305752 \|  \| Bt.3791.1.S1\_at \| \| Bt.6768.1.S1\_at \| 282077 \| 1/1 \| NM\_174464 \|  \| GO:0005507  GO:0005509  GO:0005604 \| 2535844  2834720  2835093  2982834  3012473  3421938  12140684  12477932  16305752 \| SPARC \| Bt.6768.1.S1\_at \| \| Bt.21031.1.S1\_at \| 507524 \| 1/5 \| XM\_001250438  XM\_001250487  XM\_865076 \|  \|  \|  \| LEO1 \| Bt.21031.1.S1\_at \| \| Bt.25202.2.S1\_at \| 534295 \| 3/3 \| XM\_614016 \|  \| GO:0004190  GO:0006508 \|  \| KIAA1109 \| Bt.25202.2.S1\_at \| \| Bt.18115.1.A1\_at \|  \|  \|  \| NM\_001007817 \|  \|  \| QKI \| Bt.18115.1.A1\_at \| \| Bt.21580.1.S1\_at \| 407210 \| 1/2 \| XM\_602256 \| XM\_602256 \| GO:0004872  GO:0007165 \| 14668210 \| GRB10 \| Bt.21580.1.S1\_at \| \| Bt.2513.2.S1\_at \| 538086 \| 2/4 \| XM\_865366 \| XM\_865366 \|  \|  \| CAND1 \| Bt.2513.2.S1\_at \| \| Bt.16137.1.S1\_at \| 537539 \| 1/2 \| NM\_001046423 \|  \| GO:0004029  GO:0005829  GO:0006081  GO:0006118  GO:0008152  GO:0009437  GO:0016491  GO:0019145  GO:0042136  GO:0047105 \|  \| ALDH9A1 \| Bt.16137.1.S1\_at \| \| Bt.11230.1.S1\_at \| 519739 \| 1/1 \| XM\_597964 \|  \| GO:0008152  GO:0008415 \|  \| MGC11324 \| Bt.11230.1.S1\_at \| \| Bt.8437.1.A1\_at \| 539762 \| 1/1 \| XM\_874567 \| XM\_874567 \| GO:0003677  GO:0005622  GO:0008270 \| 11282978  16305752 \| ZFHX4 \| Bt.8437.1.A1\_at \| \| Bt.8543.1.S1\_at \| 282091 \| 1/2 \| NM\_174824 \| NM\_174824 \| GO:0005886  GO:0007165  GO:0019903 \| 12055102 \| PPP1R16B \| Bt.8543.1.S1\_at \| \| Bt.23405.1.S1\_at \| 505031 \| 1/1 \| XM\_581254 \| XM\_581254 \|  \|  \| C6orf49 \| Bt.23405.1.S1\_at \| \| Bt.5278.2.S1\_at \| 539713 \| 2/2 \| NM\_001015671 \| NM\_001015671 \| GO:0000166  GO:0005524  GO:0005739  GO:0006457  GO:0016020  GO:0016021  GO:0017111 \| 11282978  12477932 \| BCS1L \| Bt.5278.2.S1\_at \| \| Bt.12429.1.S1\_at \| 515380 \| 1/1 \| NM\_001035307 \| NM\_001035307 \| GO:0005516  GO:0005634  GO:0006350  GO:0006355  GO:0007275  GO:0030154  GO:0043565 \| 12477932 \| EDF1 \| Bt.12429.1.S1\_at \| \| Bt.29459.1.A1\_at \| 539432 \| 1/1 \| XM\_587119 \|  \|  \|  \| EPC2 \| Bt.29459.1.A1\_at \| \| Bt.19429.2.S1\_a\_at \| 614004 \| 1/2 \|  \|  \|  \|  \|  \| Bt.19429.2.S1\_a\_at \| \| Bt.16499.1.A1\_at \| 505325 \| 1/3 \| XM\_581598 \|  \|  \|  \| LRCH1 \| Bt.16499.1.A1\_at \| \| Bt.21589.1.S1\_at \| 512019 \| 1/1 \| XM\_864781 \| XM\_864781 \|  \|  \| ANGPTL2 \| Bt.21589.1.S1\_at \| \| Bt.6116.1.S1\_a\_at \|  \|  \|  \| NM\_001077956 \|  \|  \| HTRA2 \| Bt.6116.1.S1\_a\_at \| \| Bt.9438.1.S1\_at \| 511763 \| 1/1 \| XM\_589161 \| XM\_589161 \| GO:0000166  GO:0003676 \|  \| SYNCRIP \| Bt.9438.1.S1\_at \| \| Bt.15839.1.S1\_at \|  \|  \|  \|  \|  \|  \| CFL2 \| Bt.15839.1.S1\_at \| \| Bt.11339.1.S1\_at \| 514740 \| 1/1 \| NM\_001038108 \|  \| GO:0005739  GO:0030529 \| 11344316  12477932 \| MRPS27 \| Bt.11339.1.S1\_at \| \| Bt.22170.1.S1\_a\_at \| 530414 \| 3/5 \| NM\_001075932 \| NM\_001075932 \| GO:0008152  GO:0008415  GO:0016740 \|  \| AGPAT5 \| Bt.22170.1.S1\_a\_at \| \| Bt.1998.1.S1\_at \| 516056 \| 1/1 \| NM\_001075735 \| XM\_001254742 \|  \|  \| NGFRAP1 \| Bt.1998.1.S1\_at \| \| Bt.15607.1.S1\_at \|  \|  \|  \| XM\_584067 \|  \|  \| SNX16 \| Bt.15607.1.S1\_at \| \| Bt.22651.1.A1\_at \|  \|  \|  \|  \|  \|  \|  \| Bt.22651.1.A1\_at \| \| Bt.4311.1.S1\_at \| 281791 \| 1/1 \| XM\_589440 \| XM\_589440 \| GO:0004871  GO:0005525  GO:0007165  GO:0007186 \|  \| GNAI2 \| Bt.4311.1.S1\_at \| \| Bt.24395.1.S1\_at \|  \|  \|  \|  \|  \|  \| KCNIP2 \| Bt.24395.1.S1\_at \| \| Bt.11301.1.S1\_at \| 613794 \| 1/5 \| XM\_865922 \| XM\_865922 \| GO:0003779  GO:0007010  GO:0008270 \|  \| ABLIM3 \| Bt.11301.1.S1\_at \| \| Bt.22325.2.A1\_at \|  \|  \|  \|  \|  \|  \|  \| Bt.22325.2.A1\_at \| \| Bt.2214.1.S1\_at \| 616368 \| 1/1 \| XM\_868373 \| XM\_868373 \|  \|  \| IGFBP7 \| Bt.2214.1.S1\_at \| \| Bt.4970.1.S1\_at \| 327688 \| 1/1 \| NM\_175807 \|  \| GO:0004129  GO:0005739  GO:0005746  GO:0006118  GO:0009055  GO:0016020 \| 1717471  2549516  2844245  8307562 \| COX7A2 \| Bt.4970.1.S1\_at \| \| Bt.20414.1.S1\_at \|  \|  \|  \|  \|  \|  \| ROCK1 \| Bt.20414.1.S1\_at \| \| Bt.10303.1.S1\_at \| 511245 \| 1/1 \| XM\_588542 \|  \|  \|  \| D4ST1 \| Bt.10303.1.S1\_at \| \| Bt.1386.1.S1\_at \| 507869 \| 1/3 \| XM\_584555 \| XM\_584555 \|  \|  \| TPR \| Bt.1386.1.S1\_at \| \| Bt.10956.1.S1\_at \|  \|  \|  \|  \|  \|  \|  \| Bt.10956.1.S1\_at \| \| Bt.888.1.S1\_at \| 534615 \| 1/1 \| NM\_001034614 \| NM\_001034614 \| GO:0005515  GO:0005634  GO:0005739  GO:0005740  GO:0006915  GO:0006917  GO:0008634  GO:0016020  GO:0016021  GO:0051607 \| 12140684  12477932  16305752 \| BNIP3L \| Bt.888.1.S1\_at \| \| Bt.21433.2.S1\_at \| 517812 \| 1/2 \| NM\_001046234 \| NM\_001046234 \| GO:0000166  GO:0003677  GO:0003678  GO:0003697  GO:0005524  GO:0005634  GO:0006260  GO:0006268  GO:0006270  GO:0006350  GO:0006355  GO:0007049  GO:0008094  GO:0042802 \|  \| MCM6 \| Bt.21433.2.S1\_at \| \| Bt.16224.3.A1\_at \| 533745 \| 1/3 \|  \|  \|  \|  \| FLJ12975 \| Bt.16224.3.A1\_at \| \| Bt.21718.1.S1\_at \| 509684 \| 1/1 \| NM\_001075415 \| NM\_001075415 \|  \|  \| CGI-69 \| Bt.21718.1.S1\_at \| \| Bt.13419.1.S1\_at \| 541040 \| 1/2 \| XM\_614253 \|  \|  \|  \| SEC63 \| Bt.13419.1.S1\_at \| \| Bt.13950.1.A1\_at \| 507032 \| 1/1 \| XM\_583577 \|  \|  \|  \| MGC13096 \| Bt.13950.1.A1\_at \| \| Bt.14122.1.A1\_at \| 521961 \| 1/2 \| XM\_615898 \|  \|  \|  \| VAV3 \| Bt.14122.1.A1\_at \| \| Bt.25081.1.S1\_at \| 538606 \| 1/2 \| XM\_865032 \|  \|  \|  \| PPP1R12A \| Bt.25081.1.S1\_at \| \| Bt.27106.1.S1\_at \| 615905 \| 1/1 \| XM\_001253123  XM\_867433 \| XM\_867433 \| GO:0003676  GO:0005622  GO:0005634  GO:0008270  GO:0046872 \| 14667821 \| ZNF34 \| Bt.27106.1.S1\_at \| \| Bt.13953.1.A1\_at \| 533581 \| 1/3 \| XM\_613030 \|  \|  \|  \| HOMER1 \| Bt.13953.1.A1\_at \| \| Bt.6827.1.S1\_at \| 505176 \| 1/1 \| NM\_001034235 \| NM\_001034235 \|  \| 12140684  16305752 \| PSMA3 \| Bt.6827.1.S1\_at \| \| Bt.7500.1.S1\_at \| 614242 \| 1/2 \| NM\_001076317 \| NM\_001076317 \| GO:0000166  GO:0005525  GO:0007264  GO:0015031  GO:0016020 \|  \| RAB8B \| Bt.7500.1.S1\_at \| \| Bt.4732.1.S1\_at \| 281615 \| 1/1 \| NM\_174239 \| NM\_174239 \| GO:0001758  GO:0008152  GO:0016491 \| 7786847  12477932 \| ALDH1A1 \| Bt.4732.1.S1\_at \| \| Bt.26928.1.S1\_at \| 618291 \| 2/3 \|  \|  \|  \|  \| SUV420H1 \| Bt.26928.1.S1\_at \| \| Bt.20331.1.A1\_at \| 508162 \| 2/3 \| XM\_584905 \|  \|  \|  \| NCOA2 \| Bt.20331.1.A1\_at \| \| Bt.721.1.A1\_at \| 407215 \| 1/4 \| XM\_613744 \| XM\_613744 \| GO:0008080  GO:0008152  GO:0030001  GO:0046872 \|  \| PCAF \| Bt.721.1.A1\_at \| \| Bt.4726.1.S1\_at \|  \|  \|  \|  \|  \|  \|  \| Bt.4726.1.S1\_at \| \| Bt.20261.1.S1\_at \| 511010 \| 1/3 \| XM\_588254 \| XM\_588254 \| GO:0004725  GO:0005737  GO:0005856  GO:0006470  GO:0008092  GO:0016020  GO:0016791 \|  \| PTPN3 \| Bt.20261.1.S1\_at \| \| Bt.27617.1.S1\_at \| 527935 \| 1/1 \| XM\_606342 \|  \| GO:0003676  GO:0005622  GO:0008270 \|  \| GLIS2 \| Bt.27617.1.S1\_at \| \| Bt.24976.1.S1\_at \| 530254 \| 2/2 \| XM\_001254999  XM\_608719 \| XM\_001254999 \| GO:0005488  GO:0031072 \|  \| DNAJC7 \| Bt.24976.1.S1\_at \| \| Bt.13439.1.S1\_at \| 768074 \| 1/1 \| NM\_001077130 \| NM\_001077130 \|  \|  \| ETFDH \| Bt.13439.1.S1\_at \| \| Bt.27141.1.A1\_at \| 531673 \| 1/4 \| XM\_610173 \| XM\_001254563 \|  \|  \|  \| Bt.27141.1.A1\_at \| \| Bt.24517.2.A1\_at \| 516334 \| 1/2 \| NM\_001075741 \| NM\_001075741 \| GO:0004872  GO:0008067  GO:0016020 \| 11282978  16305752 \| GPRC5B \| Bt.24517.2.A1\_at \| \| Bt.20465.1.S1\_at \| 613629 \| 1/2 \| XM\_864818 \|  \|  \|  \| SIRT1 \| Bt.20465.1.S1\_at \| \| Bt.23318.1.S1\_at \| 510833 \| 2/2 \| NM\_001076831 \|  \| GO:0005198  GO:0005201  GO:0005578  GO:0005581  GO:0005737  GO:0006817 \| 488906  488907  488908  488909  488910  488911 \| COL3A1 \| Bt.23318.1.S1\_at \| \| Bt.8706.1.S1\_at \| 615215 \| 1/1 \| NM\_001034779 \| NM\_001034779 \| GO:0000166  GO:0004674  GO:0005524  GO:0005634  GO:0006468  GO:0016740 \| 11282978  12477932  16305752 \| MAPKAPK3 \| Bt.8706.1.S1\_at \| \| Bt.24896.1.A1\_at \| 518402 \| 1/1 \| NM\_001035329 \| NM\_001035329 \| GO:0005488  GO:0005871  GO:0019894 \| 12477932 \| KIFAP3 \| Bt.24896.1.A1\_at \| \| Bt.19949.1.A1\_at \| 534839 \| 1/2 \| XM\_879429 \| XM\_879429 \|  \|  \| SIPA1L1 \| Bt.19949.1.A1\_at \| \| Bt.13204.1.S1\_at \| 508090 \| 1/1 \| NM\_001034313 \|  \|  \| 12477932 \| FGL1 \| Bt.13204.1.S1\_at \| \| Bt.11588.1.A1\_at \| 533393 \| 1/1 \| XM\_001254303  XM\_001254328  XM\_863927 \| XM\_863927 \| GO:0004221  GO:0006464  GO:0006511 \|  \| USP48 \| Bt.11588.1.A1\_at \| \| Bt.23091.1.S1\_at \| 511803 \| 1/1 \| XM\_867844 \|  \| GO:0000074  GO:0005488 \|  \| PSMD1 \| Bt.23091.1.S1\_at \| \| Bt.22356.1.S1\_at \| 540181 \| 1/2 \| XM\_001252068  XM\_592084 \|  \| GO:0003677  GO:0005622 \|  \| ARID1A \| Bt.22356.1.S1\_at \| \| Bt.12337.1.S1\_a\_at \| 514560 \| 2/2 \| NM\_001037601 \| NM\_001037601 \|  \|  \| MGC134601 \| Bt.12337.1.S1\_a\_at \| \| Bt.4119.1.S1\_at \| 509231 \| 1/3 \| NM\_001075396 \| NM\_001075396 \|  \|  \| PIAS1 \| Bt.4119.1.S1\_at \| \| Bt.11182.2.S1\_a\_at \| 514918 \| 2/3 \| NM\_001075689 \| NM\_001075689 \| GO:0003700  GO:0005634  GO:0045449 \|  \| C21orf66 \| Bt.11182.2.S1\_a\_at \| \| Bt.9785.1.S1\_at \| 538831 \| 1/1 \| XM\_583052 \|  \|  \|  \| KIAA0831 \| Bt.9785.1.S1\_at \| \| Bt.5103.1.S1\_at \| 613855 \| 1/2 \| NM\_001076291 \| NM\_001076291 \| GO:0004872  GO:0005515  GO:0006464  GO:0006915 \|  \|  \| Bt.5103.1.S1\_at \| \| Bt.26808.1.S1\_at \| 514187 \| 1/1 \| XR\_027914 \|  \|  \|  \| THADA \| Bt.26808.1.S1\_at \| \| Bt.20458.1.S1\_at \| 512106 \| 1/1 \| XM\_589552 \|  \|  \|  \| RPRC1 \| Bt.20458.1.S1\_at \| \| Bt.16125.3.S1\_at \| 616080 \| 3/4 \| NM\_001076407 \| NM\_001076407 \|  \|  \|  \| Bt.16125.3.S1\_at \| \| Bt.28535.1.S1\_at \|  \|  \|  \|  \|  \|  \|  \| Bt.28535.1.S1\_at \| \| Bt.5326.1.S1\_at \| 532673 \| 1/1 \| XR\_028724 \| XR\_028724 \|  \|  \| CHD3 \| Bt.5326.1.S1\_at \| \| Bt.26629.1.S1\_a\_at \| 540932 \| 1/1 \| NM\_001076249 \| NM\_001076249 \|  \|  \| FBXW7 \| Bt.26629.1.S1\_a\_at \| \| Bt.19209.2.S1\_at \|  \|  \|  \|  \|  \|  \| SLC35A3 \| Bt.19209.2.S1\_at \| \| Bt.4031.2.S1\_at \| 444879 \| 2/2 \| NM\_001002892 \| NM\_001002892 \| GO:0006486  GO:0008373  GO:0030173 \| 12477932  15843597 \| SIAT4B \| Bt.4031.2.S1\_at \| \| Bt.13478.1.A1\_at \| 618089 \| 1/1 \| XM\_870417 \|  \|  \|  \| LSM10 \| Bt.13478.1.A1\_at \| \| Bt.886.1.S1\_at \| 510155 \| 1/1 \| NM\_001015566 \|  \|  \| 12140684  12477932  16305752 \| PSMA5 \| Bt.886.1.S1\_at \| \| Bt.17777.2.S1\_at \| 534150 \| 2/3 \| NM\_001034602 \|  \|  \| 12477932 \| OPTN \| Bt.17777.2.S1\_at \| \| Bt.5075.1.S1\_at \| 507484 \| 1/1 \| NM\_001045971 \|  \|  \|  \| SPINT2 \| Bt.5075.1.S1\_at \| \| Bt.13881.1.S1\_at \|  \|  \|  \| XM\_590268 \|  \|  \| C10orf74 \| Bt.13881.1.S1\_at \| \| Bt.364.1.S2\_at \| 280961 \| 1/1 \| NM\_177490 \| NM\_177490 \|  \| 8410797  9457673  11734898  12356942 \| ZFX \| Bt.364.1.S2\_at \| \| Bt.20675.1.S1\_at \| 504586 \| 1/1 \| XM\_580733 \|  \|  \|  \| ASNA1 \| Bt.20675.1.S1\_at \| \| Bt.13370.1.A1\_at \|  \|  \|  \| XM\_605851 \|  \|  \| AVO3 \| Bt.13370.1.A1\_at \| \| Bt.13229.1.S1\_at \| 504219 \| 1/1 \| XM\_580300 \| XM\_580300 \|  \|  \| PRKAG2 \| Bt.13229.1.S1\_at \| \| Bt.1131.1.S1\_at \| 281419 \| 1/1 \| NM\_174152 \|  \|  \| 8010972  8013656  12140684  12477932  12658628  16305752 \| PPIB \| Bt.1131.1.S1\_at \| \| Bt.23648.1.A1\_at \|  \|  \|  \|  \|  \|  \| ROD1 \| Bt.23648.1.A1\_at \| \| Bt.1987.1.S1\_at \| 514852 \| 1/1 \| NM\_001034474 \| NM\_001034474 \| GO:0005515 \| 12477932 \| TAX1BP3 \| Bt.1987.1.S1\_at \| \| Bt.23236.1.A1\_at \| 505129 \| 2/5 \| XM\_001252366  XM\_001252396  XM\_001252429  XM\_581371 \|  \|  \|  \|  \| Bt.23236.1.A1\_at \| \| Bt.22536.2.S1\_at \| 613522 \| 1/2 \| NM\_001076275 \|  \| GO:0003676  GO:0005216  GO:0005622  GO:0008270 \|  \| KCMF1 \| Bt.22536.2.S1\_at \| \| Bt.17764.1.A1\_at \|  \|  \|  \|  \|  \|  \| C4orf8 \| Bt.17764.1.A1\_at \| \| Bt.13637.1.A1\_at \| 533264 \| 1/2 \| XM\_863846 \|  \|  \|  \| NCOA3 \| Bt.13637.1.A1\_at \| \| Bt.23209.1.S1\_a\_at \| 281278 \| 2/2 \| NM\_181013 \| NM\_181013 \| GO:0004872  GO:0004930  GO:0005529  GO:0007165  GO:0007218  GO:0016020  GO:0016021  GO:0016524 \| 9261169  10025961 \| LPHN2 \| Bt.23209.1.S1\_a\_at \| \| Bt.12217.3.A1\_at \|  \|  \|  \|  \|  \|  \| NTRK2 \| Bt.12217.3.A1\_at \| \| Bt.15687.2.S1\_at \|  \|  \|  \|  \|  \|  \| HERC4 \| Bt.15687.2.S1\_at \| \| Bt.868.1.S1\_a\_at \| 616375 \| 2/4 \| NM\_001034791 \|  \|  \| 12477932 \| MAPBPIP \| Bt.868.1.S1\_a\_at \| \| Bt.6313.1.S1\_at \|  \|  \|  \|  \|  \|  \| RNF44 \| Bt.6313.1.S1\_at \| \| Bt.26992.2.S1\_at \| 535192 \| 2/3 \| NM\_001035410 \|  \| GO:0004806  GO:0005319  GO:0008201  GO:0016042  GO:0016787 \| 12477932 \| ADAM10 \| Bt.26992.2.S1\_at \| \| Bt.3210.1.S1\_at \| 526848 \| 1/1 \| NM\_001035366 \| NM\_001035366 \|  \| 12477932 \| CTNNA1 \| Bt.3210.1.S1\_at \| \| Bt.1750.1.A1\_at \| 533517 \| 1/1 \|  \|  \|  \|  \| BRD1 \| Bt.1750.1.A1\_at \| \| Bt.26150.1.A1\_at \| 514230 \| 1/3 \| XM\_864101 \|  \|  \|  \| L2HGDH \| Bt.26150.1.A1\_at \| \| Bt.5536.1.S1\_at \| 282564 \| 1/1 \| NM\_174679 \| NM\_174679 \|  \| 12551988  14510667 \| ITGB5 \| Bt.5536.1.S1\_at \| \| Bt.12299.1.A1\_at \| 281338 \| 1/1 \| XM\_866281 \|  \| GO:0003774  GO:0016459 \|  \| MYH3 \| Bt.12299.1.A1\_at \| \| Bt.5106.1.S1\_a\_at \| 281793 \| 2/5 \| NM\_181021 \|  \| GO:0000166  GO:0004871  GO:0005525  GO:0007165  GO:0007186  GO:0016020 \| 3015900  3080331  3081893  9111083  9395396  9417641  10427002  10729789  12477932  12574119  15457345  15546818 \| GNAS1 \| Bt.5106.1.S1\_a\_at \| \| Bt.4900.1.A1\_at \| 512469 \| 1/1 \| NM\_001046123 \| NM\_001046123 \| GO:0003723  GO:0003735  GO:0005622  GO:0005739  GO:0005840  GO:0006412 \| 11344316 \| MRPS5 \| Bt.4900.1.A1\_at \| \| Bt.9425.1.S1\_at \| 535156 \| 1/3 \| XM\_615162 \| XM\_615162 \|  \|  \| SPPL3 \| Bt.9425.1.S1\_at \| \| Bt.26668.1.S1\_at \| 539275 \| 1/2 \| XM\_001252092  XM\_586145 \| XM\_001252092 \| GO:0000785  GO:0003677  GO:0003682  GO:0004386  GO:0005524  GO:0005634  GO:0006333 \|  \| CHD9 \| Bt.26668.1.S1\_at \| \| Bt.7903.1.A1\_at \| 510937 \| 1/1 \| NM\_001046066 \| NM\_001046066 \| GO:0000166  GO:0004821  GO:0005524  GO:0006418  GO:0016874 \| 12477932 \| HARS \| Bt.7903.1.A1\_at \| \| Bt.20007.1.S1\_a\_at \|  \|  \|  \| XM\_001251920 \|  \|  \| ABCC5 \| Bt.20007.1.S1\_a\_at \| \| Bt.27832.1.S1\_at \| 616561 \| 1/1 \| NM\_001046583 \| NM\_001046583 \|  \|  \|  \| Bt.27832.1.S1\_at \| \| Bt.2110.1.S1\_at \| 510200 \| 1/1 \| NM\_001024513 \| NM\_001024513 \|  \| 11282978  12477932 \| DPP3 \| Bt.2110.1.S1\_at \| \| Bt.3321.1.S1\_at \|  \|  \|  \|  \|  \|  \| AKAP11 \| Bt.3321.1.S1\_at \| \| Bt.24276.1.A1\_at \|  \|  \|  \|  \|  \|  \| THAP1 \| Bt.24276.1.A1\_at \| \| Bt.3583.1.S1\_at \| 281574 \| 1/1 \| NM\_174217 \|  \| GO:0001931  GO:0005198  GO:0005737  GO:0005884  GO:0005932  GO:0008092  GO:0008360  GO:0016020  GO:0016324  GO:0019898  GO:0035088  GO:0043621  GO:0051015  GO:0051017 \| 8660651  12477932 \| VIL2 \| Bt.3583.1.S1\_at \| \| Bt.4402.1.S1\_at \| 538054 \| 1/1 \| NM\_001034641 \|  \| GO:0003735  GO:0005622  GO:0005739  GO:0005762  GO:0005840  GO:0006412 \| 12477932 \| MRPL16 \| Bt.4402.1.S1\_at \| \| Bt.23129.2.S1\_at \| 532572 \| 2/7 \| XM\_611689 \|  \| GO:0007155  GO:0031012 \|  \| LAMC1 \| Bt.23129.2.S1\_at \| \| Bt.6069.1.S1\_at \|  \|  \|  \|  \|  \|  \| PALM2-AKAP2 \| Bt.6069.1.S1\_at \| \| Bt.27046.1.S1\_at \|  \|  \|  \| XM\_001254443 \|  \|  \| FLJ00193 \| Bt.27046.1.S1\_at \| \| Bt.20362.2.S1\_at \| 516567 \| 1/4 \| XM\_879722 \|  \| GO:0003723  GO:0005622  GO:0006396 \|  \| SFRS14 \| Bt.20362.2.S1\_at \| \| Bt.5866.1.S1\_at \|  \|  \|  \|  \|  \|  \| MBD3 \| Bt.5866.1.S1\_at \| \| Bt.27339.2.S1\_at \| 536741 \| 1/2 \| XM\_616885 \| XM\_616885 \|  \|  \| MME \| Bt.27339.2.S1\_at \| \| Bt.25268.1.A1\_at \|  \|  \|  \|  \|  \|  \| CENTB2 \| Bt.25268.1.A1\_at \| \| Bt.1819.1.S1\_at \| 616109 \| 1/1 \| XM\_868076 \| XM\_868076 \| GO:0005739  GO:0005740  GO:0005746  GO:0006122  GO:0006810  GO:0008121  GO:0016020  GO:0016491 \| 6305819  9204897  9651245  12477932 \| UCRC \| Bt.1819.1.S1\_at \| \| Bt.10999.2.S1\_at \| 526430 \| 2/2 \| XM\_604801 \| XM\_604801 \|  \|  \|  \| Bt.10999.2.S1\_at \| \| Bt.27023.1.S1\_at \|  \|  \|  \|  \|  \|  \| RNPC3 \| Bt.27023.1.S1\_at \| \| Bt.3422.1.S1\_at \|  \|  \|  \| XM\_864533 \|  \|  \| C9orf88 \| Bt.3422.1.S1\_at \| \| Bt.7955.1.S1\_at \| 504471 \| 2/2 \| NM\_001045888 \| NM\_001045888 \|  \|  \| PCOLCE \| Bt.7955.1.S1\_at \| \| Bt.23985.1.A1\_at \| 514473 \| 1/1 \| XM\_592333 \| XM\_592333 \| GO:0003677  GO:0004386  GO:0005515  GO:0005524  GO:0008270 \|  \| ATRX \| Bt.23985.1.A1\_at \| \| Bt.9490.1.S1\_at \| 514371 \| 1/2 \| XM\_879421 \| XM\_879421 \| GO:0005515  GO:0005524 \| 12477932 \| ACTR1A \| Bt.9490.1.S1\_at \| \| Bt.20314.2.S1\_at \| 538005 \| 1/2 \| NM\_001076098 \| XM\_001255869 \|  \|  \|  \| Bt.20314.2.S1\_at \| \| Bt.9973.1.S2\_at \| 281876 \| 1/1 \| NM\_174368 \|  \| GO:0000074  GO:0000082  GO:0001701  GO:0004872  GO:0005178  GO:0005515  GO:0005886  GO:0007155  GO:0007160  GO:0007229  GO:0007275  GO:0008284  GO:0008305  GO:0010002  GO:0016021  GO:0019717  GO:0042685  GO:0045214  GO:0045596 \| 7545439  9657852  12551988  15471980 \| ITGB1 \| Bt.9973.1.S2\_at \| \| Bt.2513.1.S1\_at \| 538086 \| 2/4 \| XM\_865366 \|  \|  \|  \| CAND1 \| Bt.2513.1.S1\_at \| \| Bt.9278.1.S1\_at \| 540141 \| 1/2 \| XM\_591785 \|  \| GO:0003677  GO:0005515  GO:0006118  GO:0008270  GO:0009055 \|  \| FBXL11 \| Bt.9278.1.S1\_at \| \| Bt.1310.1.S1\_at \| 532775 \| 1/1 \| XM\_611947 \| XM\_611947 \|  \|  \| TMEM38A \| Bt.1310.1.S1\_at \| \| Bt.9413.1.S1\_at \| 505585 \| 1/3 \| NM\_001045920 \|  \|  \|  \| NUCKS1 \| Bt.9413.1.S1\_at \| \| Bt.4394.1.S1\_at \| 282433 \| 1/1 \| NM\_174641 \| NM\_174641 \| GO:0004383  GO:0005506  GO:0006182  GO:0006811  GO:0006826  GO:0007242  GO:0020037  GO:0020037  GO:0046872 \| 2903071  7908439  9391039  9521770 \| GUCY1B3 \| Bt.4394.1.S1\_at \| \| Bt.27655.1.S1\_at \|  \|  \|  \| XM\_868663 \|  \|  \|  \| Bt.27655.1.S1\_at \| \| Bt.1678.1.S1\_at \| 616187 \| 1/1 \| XR\_027814 \| XR\_027814 \|  \|  \| RNF5 \| Bt.1678.1.S1\_at \| \| Bt.21630.1.S1\_at \|  \|  \|  \|  \|  \|  \| DNAJB5 \| Bt.21630.1.S1\_at \| \| Bt.1595.1.S1\_at \| 539955 \| 1/3 \| NM\_001034684 \| NM\_001034684 \| GO:0003677  GO:0005634 \| 12477932 \| CROP \| Bt.1595.1.S1\_at \| \| Bt.5300.1.S1\_at \| 613766 \| 1/2 \| XM\_865520 \| XM\_865520 \|  \|  \|  \| Bt.5300.1.S1\_at \| \| Bt.16636.2.A1\_at \| 533126 \| 1/2 \| XM\_863840 \| XM\_863840 \|  \|  \| KIAA0738 \| Bt.16636.2.A1\_at \| \| Bt.12345.1.S1\_at \| 617227 \| 1/1 \|  \| XM\_583217 \|  \|  \| MACF1 \| Bt.12345.1.S1\_at \| \| Bt.18019.1.A1\_at \|  \|  \|  \|  \|  \|  \| ZFHX1B \| Bt.18019.1.A1\_at \| \| Bt.654.1.A1\_at \| 506325 \| 1/1 \| NM\_001045940 \| NM\_001045940 \|  \|  \| ZMYND11 \| Bt.654.1.A1\_at \| \| Bt.13730.1.A1\_at \| 408003 \| 2/2 \| NM\_001001171 \| NM\_001001171 \| GO:0006512  GO:0006914  GO:0008234  GO:0015031 \| 15140988 \|  \| Bt.13730.1.A1\_at \| \| Bt.3633.1.A1\_at \|  \|  \|  \|  \|  \|  \| PSEN1 \| Bt.3633.1.A1\_at \| \| Bt.9695.1.S1\_at \| 540947 \| 1/1 \| XM\_613810 \|  \|  \|  \| SS18 \| Bt.9695.1.S1\_at \| \| Bt.28855.1.A1\_at \|  \|  \|  \|  \|  \|  \| HEG \| Bt.28855.1.A1\_at \| \| Bt.1101.1.S1\_at \| 539802 \| 1/1 \| XM\_611424 \|  \|  \|  \| CGGBP1 \| Bt.1101.1.S1\_at \| \| Bt.4161.1.S1\_at \| 529978 \| 1/1 \|  \|  \|  \|  \| SOS2 \| Bt.4161.1.S1\_at \| \| Bt.9639.1.S1\_at \| 615677 \| 1/2 \| NM\_001076940 \| NM\_001076940 \|  \|  \| CPNE1 \| Bt.9639.1.S1\_at \| \| Bt.22441.1.S1\_at \| 540904 \| 1/1 \| XM\_613634 \| XM\_001253543 \| GO:0003677  GO:0004386  GO:0005524 \|  \| SMARCA2 \| Bt.22441.1.S1\_at \| \| Bt.19431.1.S1\_s\_at \| 445421 \| 2/2 \| NM\_001003902 \| NM\_001003902 \| GO:0003735  GO:0005622  GO:0005739  GO:0005840  GO:0006412 \| 10938081  12477932 \| MRPS14 \| Bt.19431.1.S1\_s\_at \| \| Bt.20041.1.S1\_at \| 506060 \| 1/1 \| XM\_582453 \| XM\_582453 \|  \|  \| CSNK1G2 \| Bt.20041.1.S1\_at \| \| Bt.7143.1.S1\_at \| 510899 \| 1/1 \| NM\_001075474 \|  \| GO:0003735  GO:0005622  GO:0005739  GO:0005840  GO:0006412 \| 11279123 \|  \| Bt.7143.1.S1\_at \| \| Bt.1322.1.S1\_at \| 509037 \| 1/1 \| NM\_001075385 \| NM\_001075385 \|  \| 12140684  16305752 \| TXNDC \| Bt.1322.1.S1\_at \| \| Bt.22197.1.S1\_at \| 532389 \| 1/1 \| NM\_001046321 \|  \| GO:0003723  GO:0004519  GO:0004525  GO:0005739  GO:0006396  GO:0016787  GO:0030529 \|  \| MRPL44 \| Bt.22197.1.S1\_at \| \| Bt.13562.1.S1\_at \|  \|  \|  \|  \|  \|  \| SH3MD1 \| Bt.13562.1.S1\_at \| \| Bt.26774.1.S1\_at \| 537487 \| 1/1 \| XM\_617655 \|  \| GO:0005515 \|  \| IGSF10 \| Bt.26774.1.S1\_at \| \| Bt.12966.1.S1\_at \| 520891 \| 1/1 \| NM\_001015626 \| NM\_001015626 \| GO:0003723  GO:0005634  GO:0006364  GO:0030529 \| 12140684  12477932  16305752 \| NOLA2 \| Bt.12966.1.S1\_at \| \| Bt.948.1.S1\_at \| 538948 \| 1/1 \| XM\_583852 \| NM\_001080308 \|  \| 11282978  16305752 \| USP11 \| Bt.948.1.S1\_at \| \| Bt.7139.1.S1\_at \| 512673 \| 1/1 \| XM\_865272 \| XM\_001249751 \| GO:0005742  GO:0006605 \|  \| TOMM20 \| Bt.7139.1.S1\_at \| \| Bt.3163.1.A1\_at \|  \|  \|  \| XM\_592279 \|  \|  \| MAP3K7IP2 \| Bt.3163.1.A1\_at \| \| Bt.4314.1.S1\_at \| 282689 \| 1/2 \| NM\_174716 \| NM\_174716 \| GO:0001525  GO:0004859  GO:0005509  GO:0005544  GO:0005737  GO:0005769  GO:0008092  GO:0030199  GO:0042383  GO:0042730 \| 2942542  2945590  12140684  12477932  14522961  14668336  14991775  16305752 \| ANXA2 \| Bt.4314.1.S1\_at \| \| Bt.13969.2.S1\_at \|  \|  \|  \|  \|  \|  \| SBNO1 \| Bt.13969.2.S1\_at \| \| Bt.10417.1.S1\_at \| 506270 \| 1/3 \| NM\_001075250 \|  \|  \| 16510472 \| PTK2 \| Bt.10417.1.S1\_at \| \| Bt.22283.1.S1\_at \|  \|  \|  \|  \|  \|  \| PLEKHA2 \| Bt.22283.1.S1\_at \| \| Bt.6289.1.S1\_at \| 614165 \| 1/1 \| NM\_001034749 \| NM\_001034749 \| GO:0004758  GO:0005783  GO:0008415  GO:0009058  GO:0016020  GO:0016021  GO:0016769  GO:0030170 \| 12477932 \| SPTLC1 \| Bt.6289.1.S1\_at \| \| Bt.20029.1.S1\_at \| 527938 \| 1/1 \| XM\_606345 \| XM\_606345 \|  \|  \| ARS2 \| Bt.20029.1.S1\_at \| \| Bt.13593.1.A1\_at \|  \|  \|  \|  \|  \|  \| ZFYVE20 \| Bt.13593.1.A1\_at \| \| Bt.5038.4.A1\_at \| 615175 \| 1/1 \| NM\_001046554 \| NM\_001046554 \| GO:0000166  GO:0003676 \|  \| NONO \| Bt.5038.4.A1\_at \| \| Bt.1165.1.S1\_at \| 281603 \| 1/1 \| NM\_174230 \| NM\_174230 \| GO:0000287  GO:0004016  GO:0006171  GO:0007242  GO:0016020  GO:0016021 \| 8557110 \| ADCY7 \| Bt.1165.1.S1\_at \| \| Bt.26643.1.S1\_at \| 511168 \| 1/4 \| XM\_588451 \|  \| GO:0005622 \|  \| MDC1 \| Bt.26643.1.S1\_at \| \| Bt.21534.3.S1\_a\_at \| 533826 \| 1/1 \| XR\_027534 \| XR\_027534 \|  \|  \| TIP120B \| Bt.21534.3.S1\_a\_at \| \| Bt.676.1.S1\_at \| 613618 \| 1/2 \| NM\_001038197 \| NM\_001038197 \| GO:0000786  GO:0003677  GO:0005634  GO:0005694  GO:0006334  GO:0007001 \|  \| H2AFV \| Bt.676.1.S1\_at \| \| Bt.9948.1.S1\_at \|  \|  \|  \|  \|  \|  \| NOTCH1 \| Bt.9948.1.S1\_at \| \| Bt.13768.1.S1\_at \| 613349 \| 4/4 \| XM\_864015 \|  \|  \|  \| TCTE1L \| Bt.13768.1.S1\_at \| \| Bt.2262.1.S1\_at \| 539381 \| 1/1 \| NM\_001038183 \|  \| GO:0005634  GO:0007275  GO:0008270  GO:0030154  GO:0046872 \|  \| CSRP2 \| Bt.2262.1.S1\_at \| \| Bt.23094.6.A1\_at \| 617917 \| 1/1 \| NM\_001038584 \| NM\_001038584 \|  \|  \|  \| Bt.23094.6.A1\_at \| \| Bt.21036.1.S1\_at \| 615221 \| 2/2 \|  \|  \|  \|  \| FAM73A \| Bt.21036.1.S1\_at \| \| Bt.1785.1.A1\_at \| 514105 \| 1/2 \|  \| XR\_027597 \|  \|  \| CPD \| Bt.1785.1.A1\_at \| \| Bt.17380.1.S1\_at \| 513699 \| 1/1 \| NM\_001037599 \|  \|  \|  \| UNQ501 \| Bt.17380.1.S1\_at \| \| Bt.11258.2.A1\_a\_at \| 509856 \| 1/1 \| XM\_586911 \|  \|  \|  \| RNF31 \| Bt.11258.2.A1\_a\_at \| \| Bt.23178.1.S1\_at \| 280760 \| 1/2 \| NM\_173906 \| NM\_173906 \|  \| 2914936  3435485  3936484  6654908  8907183  9895299  10737760  11322944  11723132  12097842  12140684  12477932  12601001  12658628  14600270  14615389  15313168  15501918  15811857  16263082  16293388  16305752 \| DCN \| Bt.23178.1.S1\_at \| \| Bt.28280.1.A1\_at \| 528207 \| 1/2 \| XM\_606619 \| XM\_606619 \|  \|  \| KIAA0971 \| Bt.28280.1.A1\_at \| \| Bt.22737.1.S1\_at \| 512866 \| 1/3 \| XM\_001252422  XM\_865008 \| XM\_865008 \| GO:0005515 \|  \| ERBB2IP \| Bt.22737.1.S1\_at \| \| Bt.16230.1.S1\_at \| 616227 \| 1/1 \| XM\_868201 \|  \|  \|  \| CAMK2N1 \| Bt.16230.1.S1\_at \| \| Bt.25436.1.S1\_a\_at \| 540090 \| 2/2 \| XM\_591423 \| XM\_591423 \| GO:0005515  GO:0008270 \|  \| RNF2 \| Bt.25436.1.S1\_a\_at \| \| Bt.10257.1.A1\_at \| 535231 \| 1/2 \| XM\_866386 \| XM\_866386 \| GO:0003700  GO:0005634  GO:0006355  GO:0043565 \|  \| ERG \| Bt.10257.1.A1\_at \| \| Bt.12583.1.A1\_at \|  \|  \|  \|  \|  \|  \| BHLHB3 \| Bt.12583.1.A1\_at \| \| Bt.26313.1.A1\_at \| 533862 \| 1/2 \| NM\_001046361 \|  \|  \| 11282978  16305752 \| PAPD4 \| Bt.26313.1.A1\_at \| \| Bt.5459.1.S1\_at \| 534352 \| 1/1 \| NM\_001034608 \| NM\_001034608 \| GO:0003735  GO:0005622  GO:0005739  GO:0005840  GO:0006412 \| 12477932 \| MRPL18 \| Bt.5459.1.S1\_at \| \| Bt.21217.1.S1\_at \| 515765 \| 1/1 \| NM\_001037466 \| NM\_001037466 \| GO:0003697  GO:0006260 \| 12477932 \| MGC133588 \| Bt.21217.1.S1\_at \| \| Bt.20456.1.S1\_at \| 539560 \| 2/2 \| NM\_001076173 \| NM\_001076173 \|  \|  \|  \| Bt.20456.1.S1\_at \| \| Bt.1697.1.S1\_at \| 780787 \| 1/1 \| NM\_001079642 \| NM\_001079642 \| GO:0004872 \|  \| SRA1 \| Bt.1697.1.S1\_at \| \| Bt.20129.1.S1\_at \| 523683 \| 3/5 \| XM\_601985 \|  \|  \|  \| CUTL1 \| Bt.20129.1.S1\_at \| \| Bt.27057.1.A1\_at \| 513324 \| 1/1 \| NM\_001075614 \| NM\_001075614 \| GO:0005509  GO:0016021 \|  \| LETM1 \| Bt.27057.1.A1\_at \| \| Bt.24051.1.S1\_at \|  \|  \|  \|  \|  \|  \| PURB \| Bt.24051.1.S1\_at \| \| Bt.23519.1.S1\_at \| 539244 \| 1/1 \| NM\_001034669 \|  \| GO:0006512  GO:0007242 \| 12477932 \| ASB2 \| Bt.23519.1.S1\_at \| \| Bt.13900.1.S1\_at \| 616748 \| 2/2 \| NM\_001034796 \|  \|  \| 12477932 \| TRAPPC2 \| Bt.13900.1.S1\_at \| \| Bt.19938.1.A1\_at \| 540766 \| 1/1 \| XM\_613036 \| XM\_613036 \| GO:0003676  GO:0005622  GO:0008270 \|  \| SP3 \| Bt.19938.1.A1\_at \| \| Bt.18890.1.A1\_at \|  \|  \|  \|  \|  \|  \| E2F3 \| Bt.18890.1.A1\_at \| \| Bt.4544.1.S1\_at \| 506342 \| 1/1 \|  \|  \|  \|  \| ITGA6 \| Bt.4544.1.S1\_at \| \| Bt.21410.1.S1\_at \| 518169 \| 1/1 \| NM\_001035326 \| NM\_001035326 \| GO:0004186  GO:0005764  GO:0006508  GO:0008233 \| 12477932 \| PPGB \| Bt.21410.1.S1\_at \| \| Bt.2195.1.A1\_at \| 613930 \| 1/2 \| XM\_864823 \|  \| GO:0004871  GO:0005834  GO:0007186 \| 12477932 \|  \| Bt.2195.1.A1\_at \| \| Bt.2825.1.S1\_at \| 617901 \| 1/1 \| NM\_001034805 \|  \|  \| 12477932 \| MRPL13 \| Bt.2825.1.S1\_at \| \| Bt.13946.2.A1\_at \| 511965 \| 1/2 \| NM\_001038531 \| NM\_001038531 \| GO:0003677  GO:0005622  GO:0005634  GO:0006350  GO:0006355  GO:0008270  GO:0046872 \|  \| MIZF \| Bt.13946.2.A1\_at \| \| Bt.1729.1.A1\_at \| 286861 \| 1/1 \| NM\_174792 \| NM\_174792 \| GO:0005488  GO:0006826  GO:0006879  GO:0008199  GO:0016491  GO:0046914 \| 12477932  12658628 \| FTL \| Bt.1729.1.A1\_at \| \| Bt.26393.1.A1\_at \|  \|  \|  \|  \|  \|  \| ZXDB \| Bt.26393.1.A1\_at \| \| Bt.8117.1.S1\_at \|  \|  \|  \|  \|  \|  \| ULK2 \| Bt.8117.1.S1\_at \| \| Bt.11932.1.S1\_at \|  \|  \|  \|  \|  \|  \| SAR1A \| Bt.11932.1.S1\_at \| \| Bt.24895.1.A1\_at \| 532856 \| 1/4 \| XM\_612059 \| XM\_612059 \|  \|  \| ANKRD11 \| Bt.24895.1.A1\_at \| \| Bt.9833.1.S1\_a\_at \| 616237 \| 1/2 \| XM\_868211 \|  \|  \|  \|  \| Bt.9833.1.S1\_a\_at \| \| Bt.4339.1.S1\_at \| 515589 \| 1/1 \| NM\_001046205 \|  \|  \|  \| C20orf27 \| Bt.4339.1.S1\_at \| \| Bt.10260.1.S1\_at \|  \|  \|  \|  \|  \|  \|  \| Bt.10260.1.S1\_at \| \| Bt.7869.1.A1\_at \| 535699 \| 1/7 \| XM\_615809 \| XM\_615809 \| GO:0003735  GO:0005515  GO:0005622  GO:0005840  GO:0006412  GO:0042981 \|  \|  \| Bt.7869.1.A1\_at \| \| Bt.21159.1.S1\_at \| 514094 \| 1/1 \| XM\_591893 \| XM\_591893 \| GO:0008270 \|  \| NCOR1 \| Bt.21159.1.S1\_at \| \| Bt.5302.1.S1\_a\_at \| 281918 \| 1/1 \| NM\_174392  NM\_194465 \| NM\_174392 \| GO:0004872  GO:0016301 \| 10724174  16166082 \| MRVI1 \| Bt.5302.1.S1\_a\_at \| \| Bt.5507.2.S1\_at \| 506218 \| 1/2 \| XM\_582637 \|  \|  \|  \| SNX9 \| Bt.5507.2.S1\_at \| \| Bt.10312.1.A1\_at \| 514220 \| 1/2 \| XM\_592033 \|  \|  \|  \| C22orf5 \| Bt.10312.1.A1\_at \| \| Bt.13755.1.S1\_at \|  \|  \|  \|  \|  \|  \| MGAT4A \| Bt.13755.1.S1\_at \| \| Bt.29948.1.S1\_at \| 539120 \| 2/2 \| XM\_584969 \| XM\_584969 \| GO:0003676  GO:0005622  GO:0008270 \|  \| ZNF161 \| Bt.29948.1.S1\_at \| \| Bt.8243.1.A1\_at \| 529006 \| 1/1 \| XM\_607444 \| XM\_607444 \| GO:0003676  GO:0005622  GO:0008270 \|  \| ZFP91 \| Bt.8243.1.A1\_at \| \| Bt.19692.1.S1\_at \| 540298 \| 1/1 \| NM\_001076210 \| NM\_001076210 \| GO:0004395  GO:0005739  GO:0006744  GO:0008152  GO:0008168  GO:0008425  GO:0016740 \|  \| COQ3 \| Bt.19692.1.S1\_at \| \| Bt.15684.1.S1\_at \| 510494 \| 1/2 \| XM\_881783 \| XM\_881783 \|  \|  \| ST13 \| Bt.15684.1.S1\_at \| \| Bt.22306.1.S1\_at \| 509696 \| 1/2 \| XM\_586717 \| XM\_586717 \|  \|  \| TPRT \| Bt.22306.1.S1\_at \| \| Bt.5104.1.S1\_at \| 540984 \| 1/1 \| NM\_001034719 \| NM\_001034719 \|  \| 12477932 \| OTUB1 \| Bt.5104.1.S1\_at \| \| Bt.1617.2.S1\_a\_at \| 281078 \| 1/2 \| NM\_174022 \| NM\_174022 \|  \| 2434865  3821891  12426379 \| CLTA \| Bt.1617.2.S1\_a\_at \| \| Bt.2451.1.S1\_at \| 538594 \| 2/3 \| XM\_876869 \|  \| GO:0003700  GO:0005634  GO:0006355  GO:0043565  GO:0046983 \|  \| ZF \| Bt.2451.1.S1\_at \| \| Bt.4054.1.S1\_at \|  \|  \|  \|  \|  \|  \| RBM8A \| Bt.4054.1.S1\_at \| \| Bt.16040.1.S1\_at \| 614672 \| 1/1 \| NM\_001034763 \| NM\_001034763 \|  \| 12477932 \| SH3BGRL3 \| Bt.16040.1.S1\_at \| \| Bt.27887.1.S1\_at \|  \|  \|  \| XM\_001251637 \|  \|  \| YPEL2 \| Bt.27887.1.S1\_at \| \| Bt.24109.1.S1\_at \| 519720 \| 1/1 \|  \| XM\_602479 \|  \|  \|  \| Bt.24109.1.S1\_at \| \| Bt.25093.1.A1\_at \| 515827 \| 1/1 \|  \|  \|  \|  \|  \| Bt.25093.1.A1\_at \| \| Bt.24767.1.S1\_at \| 535035 \| 1/2 \| XM\_615012 \| XM\_615012 \|  \|  \| ESCO1 \| Bt.24767.1.S1\_at \| \| Bt.4221.1.S1\_at \| 511030 \| 1/3 \| NM\_001038525 \|  \| GO:0000139  GO:0006886  GO:0006888  GO:0016020  GO:0016021 \|  \|  \| Bt.4221.1.S1\_at \| \| Bt.11013.1.S1\_at \|  \|  \|  \|  \|  \|  \|  \| Bt.11013.1.S1\_at \| \| Bt.12567.1.A1\_at \|  \|  \|  \| XM\_001252993 \|  \|  \| HRASLS \| Bt.12567.1.A1\_at \| \| Bt.26803.1.A1\_at \|  \|  \|  \| XM\_001256048 \|  \|  \| TES \| Bt.26803.1.A1\_at \| \| Bt.850.1.S1\_at \|  \|  \|  \| XM\_001251422 \|  \|  \| INPPL1 \| Bt.850.1.S1\_at \| \| Bt.15575.1.A1\_at \| 527852 \| 2/5 \| XM\_877649 \|  \|  \|  \|  \| Bt.15575.1.A1\_at \| \| Bt.22064.1.A1\_at \| 532701 \| 1/3 \| XM\_611857 \|  \|  \|  \| FLJ11021 \| Bt.22064.1.A1\_at \| \| Bt.7980.1.S1\_at \| 532851 \| 1/1 \| NM\_001035394 \| XM\_001255031 \| GO:0003779  GO:0005856  GO:0006811  GO:0008270  GO:0046872 \| 12477932 \| LASP1 \| Bt.7980.1.S1\_at \| \| Bt.6455.1.S1\_at \| 617935 \| 1/2 \| XM\_870269 \| XM\_870269 \|  \|  \| CUL4A \| Bt.6455.1.S1\_at \| \| Bt.14007.3.A1\_at \| 504409 \| 2/5 \| XM\_001250100  XM\_580523 \| XM\_580523 \| GO:0000166  GO:0003676  GO:0008270 \|  \| C13orf10 \| Bt.14007.3.A1\_at \| \| Bt.26594.1.S1\_at \|  \|  \|  \|  \|  \|  \| PRKG1 \| Bt.26594.1.S1\_at \| \| Bt.20308.2.S1\_at \|  \|  \|  \|  \|  \|  \| BACH2 \| Bt.20308.2.S1\_at \| \| Bt.9847.1.S1\_at \| 510095 \| 1/4 \| XM\_587195  XM\_865126 \| XM\_587195 \|  \|  \| RAP140 \| Bt.9847.1.S1\_at \| \| Bt.27140.1.S1\_at \|  \|  \|  \| XR\_027912 \|  \|  \|  \| Bt.27140.1.S1\_at \| \| Bt.20331.2.S1\_at \| 508162 \| 2/3 \| XM\_584905 \|  \|  \|  \| NCOA2 \| Bt.20331.2.S1\_at \| \| Bt.25104.1.S1\_at \|  \|  \|  \| XM\_865825 \|  \|  \|  \| Bt.25104.1.S1\_at \| \| Bt.11035.1.S1\_at \| 616452 \| 1/1 \| XM\_868479 \|  \|  \|  \| MESDC1 \| Bt.11035.1.S1\_at \| \| Bt.8024.1.A1\_at \| 541155 \| 1/2 \| XM\_614787 \| XM\_614787 \| GO:0004437  GO:0046839 \|  \| MTMR1 \| Bt.8024.1.A1\_at \| \| Bt.26613.1.A1\_at \| 615530 \| 1/1 \| XM\_867371 \| XM\_867371 \|  \|  \| FLJ22471 \| Bt.26613.1.A1\_at \| \| Bt.10609.2.A1\_at \|  \|  \|  \| NM\_001015644 \|  \|  \| CYP20A1 \| Bt.10609.2.A1\_at \| \| Bt.18002.2.S1\_at \| 444866 \| 2/2 \| XM\_614169 \| XM\_614169 \| GO:0003676  GO:0005622  GO:0005634  GO:0006355  GO:0008270  GO:0046872 \| 15302581 \| ZNF470 \| Bt.18002.2.S1\_at \| \| Bt.28836.1.S1\_at \|  \|  \|  \| XM\_582415 \|  \|  \|  \| Bt.28836.1.S1\_at \| \| Bt.23770.1.A1\_at \|  \|  \|  \|  \|  \|  \| ALCAM \| Bt.23770.1.A1\_at \| \| Bt.28569.1.S1\_at \|  \|  \|  \|  \|  \|  \| PDE5A \| Bt.28569.1.S1\_at \| \| Bt.9965.1.S1\_at \| 533910 \| 1/3 \| XM\_865718 \| XM\_865718 \| GO:0004148  GO:0005737  GO:0006118  GO:0016491  GO:0045454  GO:0050660 \|  \| DLD \| Bt.9965.1.S1\_at \| \| Bt.18519.1.A1\_at \| 508231 \| 1/2 \| XM\_867943 \|  \|  \|  \| RSBN1 \| Bt.18519.1.A1\_at \| \| Bt.6157.1.S1\_at \| 507365 \| 1/1 \| NM\_001034296 \|  \|  \| 12477932 \| NET1 \| Bt.6157.1.S1\_at \| \| Bt.13741.1.A1\_at \|  \|  \|  \| XR\_027555 \|  \|  \| Cep192 \| Bt.13741.1.A1\_at \| \| Bt.981.2.S1\_at \|  \|  \|  \|  \|  \|  \| CLIC4 \| Bt.981.2.S1\_at \| \| Bt.25097.1.S1\_at \| 511200 \| 1/1 \| XM\_588486 \| XM\_588486 \| GO:0003824  GO:0003922  GO:0005524  GO:0006164  GO:0006177  GO:0006541  GO:0009058 \|  \| GMPS \| Bt.25097.1.S1\_at \| \| Bt.12745.1.A1\_at \| 510080 \| 4/5 \| NM\_001076826 \|  \|  \|  \| ANTXR2 \| Bt.12745.1.A1\_at \| \| Bt.1255.1.A1\_at \| 513970 \| 1/1 \| XM\_591741 \|  \|  \|  \| JTB \| Bt.1255.1.A1\_at \| \| Bt.23129.3.S1\_a\_at \| 532572 \| 2/7 \| XM\_611689 \|  \| GO:0007155  GO:0031012 \|  \| LAMC1 \| Bt.23129.3.S1\_a\_at \| \| Bt.6585.1.S1\_at \|  \|  \|  \|  \|  \|  \| SMARCC1 \| Bt.6585.1.S1\_at \| \| Bt.13489.1.S1\_at \|  \|  \|  \|  \|  \|  \| RAI17 \| Bt.13489.1.S1\_at \| \| Bt.8641.1.A1\_at \| 538724 \| 1/1 \| XM\_582247 \|  \|  \|  \| GSPT2 \| Bt.8641.1.A1\_at \| \| Bt.2439.1.S1\_at \| 513091 \| 1/1 \| XM\_590721 \| XM\_590721 \|  \| 11420620 \| NUMA1 \| Bt.2439.1.S1\_at \| \| Bt.21774.1.S1\_at \| 520111 \| 2/2 \| NM\_001075799 \|  \| GO:0005515  GO:0005622  GO:0008270 \|  \| RFP \| Bt.21774.1.S1\_at \| \| Bt.21827.2.S1\_at \| 510889 \| 1/2 \| XM\_001256079 \| XM\_001256079 \| GO:0004222  GO:0005515  GO:0006508  GO:0006518  GO:0007243  GO:0008270  GO:0046872 \| 11282978  14726412  16305752 \| THOP1 \| Bt.21827.2.S1\_at \| \| Bt.3447.2.S1\_at \| 509885 \| 1/2 \| XM\_866343 \| XM\_866343 \|  \|  \| IARS \| Bt.3447.2.S1\_at \| \| Bt.12888.1.S1\_at \| 613305 \| 1/1 \| NM\_001034732 \|  \| GO:0003723 \| 12477932 \| AD023 \| Bt.12888.1.S1\_at \| \| Bt.23468.1.A1\_at \| 532555 \| 1/3 \| XM\_001255086  XM\_611669 \| XM\_611669 \|  \|  \| GOLPH3L \| Bt.23468.1.A1\_at \| \| Bt.2453.1.S1\_at \| 505599 \| 1/1 \| XM\_581913 \| XM\_581913 \|  \|  \| POLR2H \| Bt.2453.1.S1\_at \| \| Bt.13847.1.S1\_at \| 617441 \| 1/1 \| NM\_001076459 \|  \|  \| 11282978  16305752 \| C14orf153 \| Bt.13847.1.S1\_at \| \| Bt.2187.1.S1\_at \| 532422 \| 1/1 \| NM\_001046324 \| NM\_001046324 \| GO:0008152  GO:0016491 \|  \| HSD17B8 \| Bt.2187.1.S1\_at \| \| Bt.4786.1.S2\_at \| 281080 \| 1/2 \| NM\_174023 \| NM\_174023 \|  \| 7585943  12505986 \| CLTC \| Bt.4786.1.S2\_at \| \| Bt.12348.1.A1\_at \| 508722 \| 1/1 \| XM\_868289  XM\_881624 \|  \|  \|  \| RNPC2 \| Bt.12348.1.A1\_at \| \| Bt.12382.1.S1\_at \|  \|  \|  \|  \|  \|  \| PCDHGC3 \| Bt.12382.1.S1\_at \| \| Bt.6733.1.S1\_at \| 615333 \| 1/2 \| XM\_868513 \|  \|  \|  \| DKFZP564G2022 \| Bt.6733.1.S1\_at \| \| Bt.24676.2.A1\_at \| 515999 \| 2/2 \| NM\_001035315 \| NM\_001035315 \| GO:0000166  GO:0000287  GO:0005525  GO:0005622  GO:0005783  GO:0006886  GO:0006888  GO:0007264  GO:0016020 \| 12477932 \| SAR1B \| Bt.24676.2.A1\_at \| \| Bt.23399.1.S1\_at \| 512571 \| 1/2 \| XM\_590109 \| XM\_590109 \| GO:0000287  GO:0004743  GO:0006096  GO:0016740  GO:0030955 \| 12140684  12477932  16305752 \|  \| Bt.23399.1.S1\_at \| \| Bt.21835.1.S1\_at \| 404169 \| 1/1 \| NM\_205812 \| NM\_205812 \| GO:0004862  GO:0006469 \|  \| PKIG \| Bt.21835.1.S1\_at \| \| Bt.19332.2.A1\_at \|  \|  \|  \|  \|  \|  \| CLCN5 \| Bt.19332.2.A1\_at \| \| Bt.5439.1.S1\_at \| 509796 \| 1/1 \| XM\_001253680  XM\_586836 \| XM\_001253680 \|  \|  \| DPYSL3 \| Bt.5439.1.S1\_at \| \| Bt.21487.2.S1\_at \|  \|  \|  \|  \|  \|  \|  \| Bt.21487.2.S1\_at \| \| Bt.8164.1.S1\_at \| 513071 \| 1/1 \| XM\_590695 \| XM\_590695 \|  \|  \| GNB1L \| Bt.8164.1.S1\_at \| \| Bt.20247.1.S1\_at \| 509931 \| 1/1 \| XM\_586995 \| XM\_586995 \|  \|  \|  \| Bt.20247.1.S1\_at \| \| Bt.167.1.S1\_at \| 327672 \| 1/1 \| NM\_175793 \| NM\_175793 \| GO:0000166  GO:0004674  GO:0004707  GO:0005524  GO:0006468  GO:0007049  GO:0016740 \| 15078886  15304367 \| MAPK1 \| Bt.167.1.S1\_at \| \| Bt.4621.1.S1\_a\_at \| 528013 \| 1/1 \| NM\_001076879 \| NM\_001076879 \|  \|  \| SMTN \| Bt.4621.1.S1\_a\_at \| \| Bt.12261.1.A1\_at \| 532606 \| 1/3 \| NM\_001034577 \|  \| GO:0004067  GO:0006516 \| 11282978 \| TASP1 \| Bt.12261.1.A1\_at \| \| Bt.4280.1.A1\_at \| 506991 \| 1/1 \| NM\_001015536 \| NM\_001015536 \|  \| 12140684  12477932  16305752 \| TXNDC12 \| Bt.4280.1.A1\_at \| \| Bt.566.1.S1\_at \| 282200 \| 1/1 \| NM\_174522 \| NM\_174522 \| GO:0004129  GO:0005739  GO:0005740  GO:0006118  GO:0016020  GO:0016021  GO:0016491 \| 1647214  2994692  7687470  8638158  10089392  10771420  12477932 \| COX6A2 \| Bt.566.1.S1\_at \| \| Bt.20377.1.S1\_at \| 508608 \| 1/1 \| XM\_001250792  XM\_585405 \| XM\_001250792 \|  \|  \| MGC5352 \| Bt.20377.1.S1\_at \| \| Bt.27934.1.S1\_at \| 532989 \| 1/2 \| XM\_001250005  XM\_001250048  XM\_612233 \| XM\_612233 \| GO:0004674  GO:0005524  GO:0006468 \|  \| DYRK1A \| Bt.27934.1.S1\_at \| \| Bt.26445.1.A1\_at \|  \|  \|  \| XM\_612028 \|  \|  \| KIT \| Bt.26445.1.A1\_at \| \| Bt.1244.1.S1\_at \| 514939 \| 1/2 \| XR\_027433 \|  \| GO:0004616  GO:0006098  GO:0016491  GO:0050661 \| 12477932  12658628 \| PGD \| Bt.1244.1.S1\_at \| \| Bt.23224.1.S1\_at \|  \|  \|  \|  \|  \|  \| HIPK1 \| Bt.23224.1.S1\_at \| \| Bt.13912.1.S1\_at \| 540357 \| 1/6 \| XM\_872345 \|  \|  \|  \| CHST7 \| Bt.13912.1.S1\_at \| \| Bt.26837.1.S1\_at \| 539347 \| 1/2 \| NM\_001034673 \| NM\_001034673 \| GO:0016020  GO:0016021 \| 12477932 \| MGC17943 \| Bt.26837.1.S1\_at \| \| Bt.27626.1.A1\_at \|  \|  \|  \|  \|  \|  \| HIC1 \| Bt.27626.1.A1\_at \| \| Bt.13199.1.S1\_at \|  \|  \|  \|  \|  \|  \| PPP1CB \| Bt.13199.1.S1\_at \| \| Bt.4020.1.S1\_at \| 520757 \| 1/1 \| NM\_001076869 \| NM\_001076869 \| GO:0005515  GO:0008270  GO:0046872 \| 12658628 \|  \| Bt.4020.1.S1\_at \| \| Bt.12370.1.S1\_at \| 513843 \| 1/1 \| NM\_001034456 \| NM\_001034456 \|  \| 12477932 \| MLF2 \| Bt.12370.1.S1\_at \| \| Bt.23002.1.S1\_s\_at \| 282199 \| 2/3 \| NM\_001077831  XM\_001250845  XM\_001250892 \| NM\_001077831 \| GO:0004129  GO:0005739  GO:0005740  GO:0006118  GO:0016020  GO:0016491 \| 1720401  2844245  10984454 \| COX6A1 \| Bt.23002.1.S1\_s\_at \| \| Bt.13768.2.S1\_at \| 613349 \| 4/4 \| XM\_864015 \| XM\_864015 \|  \|  \| TCTE1L \| Bt.13768.2.S1\_at \| \| Bt.5888.1.S1\_at \|  \|  \|  \| XM\_001251557 \|  \|  \|  \| Bt.5888.1.S1\_at \| \| Bt.26595.1.S1\_at \| 504317 \| 1/1 \| NM\_001077843  XM\_001255262 \| XM\_001255262 \| GO:0004872  GO:0005509  GO:0016020 \| 17319939 \| LRP4 \| Bt.26595.1.S1\_at \| \| Bt.18634.2.S1\_at \| 540329 \| 4/4 \| NM\_001046474 \| NM\_001046474 \| GO:0000287  GO:0005739  GO:0006470  GO:0008287  GO:0015071  GO:0016787 \| 16229973 \| PPM1K \| Bt.18634.2.S1\_at \| \| Bt.69.1.S1\_at \| 327673 \| 1/1 \| NM\_175794 \|  \| GO:0003954  GO:0005739  GO:0008137  GO:0016491  GO:0031966 \| 1518044  9224902  12477932  15056672 \| NDUFA1 \| Bt.69.1.S1\_at \| \| Bt.22040.1.S1\_at \| 614288 \| 2/2 \| XM\_865734 \|  \|  \|  \| MKKS \| Bt.22040.1.S1\_at \| \| Bt.26549.1.A1\_at \| 616487 \| 1/1 \| XM\_001249705  XM\_001249799  XM\_868518 \| XM\_001249705 \|  \|  \| STAG2 \| Bt.26549.1.A1\_at \| \| Bt.22362.1.S1\_at \| 510489 \| 1/2 \| XM\_587629 \| XM\_587629 \|  \|  \| SH3KBP1 \| Bt.22362.1.S1\_at \| \| Bt.16125.1.A1\_s\_at \| 616080 \| 3/4 \| NM\_001076407 \| NM\_001076407 \|  \|  \|  \| Bt.16125.1.A1\_s\_at \| \| Bt.21085.1.S1\_at \| 510879 \| 1/2 \| XM\_001249464  XM\_001249559  XM\_875828 \|  \| GO:0005509 \|  \| ITSN1 \| Bt.21085.1.S1\_at \| \| Bt.11925.1.A1\_at \| 512588 \| 1/2 \| NM\_001040518 \|  \|  \|  \|  \| Bt.11925.1.A1\_at \| \| Bt.24474.1.S1\_a\_at \| 515757 \| 2/5 \| NM\_001046211 \|  \| GO:0000166  GO:0004222  GO:0005524  GO:0005739  GO:0005743  GO:0006508  GO:0008270  GO:0016020  GO:0016021  GO:0017111  GO:0030163  GO:0046872 \|  \| AFG3L2 \| Bt.24474.1.S1\_a\_at \| \| Bt.8012.1.S1\_at \| 540396 \| 1/2 \| XM\_593747 \|  \| GO:0003676  GO:0005622  GO:0008270 \|  \| HIVEP2 \| Bt.8012.1.S1\_at \| \| Bt.13198.1.S1\_at \|  \|  \|  \| XR\_027437 \|  \|  \| PTPNS1 \| Bt.13198.1.S1\_at \| \| Bt.5099.1.S1\_at \| 538975 \| 1/1 \| NM\_001038179 \|  \| GO:0003779  GO:0003779  GO:0005516  GO:0008017  GO:0031032 \|  \| CNN3 \| Bt.5099.1.S1\_at \| \| Bt.19482.1.A1\_at \| 504698 \| 1/1 \| XM\_580860 \|  \|  \|  \| TFRC \| Bt.19482.1.A1\_at \| \| Bt.7814.1.S1\_at \|  \|  \|  \|  \|  \|  \| ARF4 \| Bt.7814.1.S1\_at \| \| Bt.27927.1.S1\_at \| 532674 \| 1/2 \| XM\_878913 \| XM\_878913 \|  \|  \| GPR116 \| Bt.27927.1.S1\_at \| \| Bt.5542.1.A1\_at \| 613882 \| 1/1 \| XM\_864286  XM\_872834 \| XM\_872834 \| GO:0005634  GO:0006334 \|  \| NAP1L1 \| Bt.5542.1.A1\_at \| \| Bt.13986.1.S1\_at \| 404110 \| 1/1 \| XM\_875877  XM\_876095 \|  \| GO:0008270 \| 7713329 \| ZA20D2 \| Bt.13986.1.S1\_at \| \| Bt.11390.1.A1\_at \|  \|  \|  \|  \|  \|  \|  \| Bt.11390.1.A1\_at \| \| Bt.24234.1.S1\_at \| 532597 \| 1/2 \| XM\_611722 \|  \|  \|  \| WAC \| Bt.24234.1.S1\_at \| \| Bt.6075.1.S1\_at \|  \|  \|  \|  \|  \|  \| RBM9 \| Bt.6075.1.S1\_at \| \| Bt.1318.1.S1\_at \| 507011  513685 \| 1/1 \| XM\_583550  XM\_591413 \| XM\_591413 \|  \|  \| FLJ23436 \| Bt.1318.1.S1\_at \| \| Bt.22443.1.A1\_at \|  \|  \|  \|  \|  \|  \| PITPNC1 \| Bt.22443.1.A1\_at \| \| Bt.23784.1.A1\_at \| 533945 \| 1/1 \| XR\_028162 \|  \|  \|  \| CNOT2 \| Bt.23784.1.A1\_at \| \| Bt.2106.1.S1\_at \|  \|  \|  \| XM\_001249562 \|  \|  \| UPF2 \| Bt.2106.1.S1\_at \| \| Bt.26143.1.A1\_at \| 532635 \| 1/1 \| XM\_611771 \| XM\_611771 \|  \|  \| RCOR3 \| Bt.26143.1.A1\_at \| \| Bt.2046.1.S1\_at \| 337918 \| 1/1 \| NM\_001040489 \| NM\_001040489 \| GO:0007155  GO:0016020 \| 14550394 \|  \| Bt.2046.1.S1\_at \| \| Bt.21981.1.A1\_at \|  \|  \|  \|  \|  \|  \| ANTXR1 \| Bt.21981.1.A1\_at \| \| Bt.16831.1.A1\_at \|  \|  \|  \|  \|  \|  \|  \| Bt.16831.1.A1\_at \| \| Bt.24643.1.S1\_at \| 509619 \| 1/1 \| NM\_001034355 \|  \|  \| 12477932 \| APOBEC2 \| Bt.24643.1.S1\_at \| \| Bt.17767.1.A1\_at \| 527058 \| 1/1 \| XM\_605445 \| XM\_605445 \|  \|  \| RLF \| Bt.17767.1.A1\_at \| \| Bt.5313.1.S1\_at \| 282872 \| 1/1 \| NM\_174745 \|  \|  \| 11204721  12102173  12236590  12586316  12606436  14967948  15026147  15219830  15374620  15570615  15792356 \| MMP2 \| Bt.5313.1.S1\_at \| \| Bt.712.1.S2\_at \| 510780 \| 1/1 \| XM\_587978 \| XM\_587978 \|  \|  \| TOMM22 \| Bt.712.1.S2\_at \| \| Bt.6390.1.S1\_at \| 508143 \| 1/1 \| NM\_001024500 \|  \|  \| 11279123 \| MRPS17 \| Bt.6390.1.S1\_at \| \| Bt.26615.1.S1\_at \| 510778 \| 1/1 \| NM\_001038523 \|  \|  \|  \|  \| Bt.26615.1.S1\_at \| \| Bt.4056.1.S1\_at \| 780821 \| 1/1 \| NM\_001079649 \| NM\_001079649 \| GO:0008270  GO:0046872 \|  \| CRIP2 \| Bt.4056.1.S1\_at \| \| Bt.9966.2.S1\_a\_at \| 538590 \| 2/2 \| XR\_028495 \| XM\_001251070 \|  \|  \| COL5A2 \| Bt.9966.2.S1\_a\_at \| \| Bt.16105.1.S1\_at \|  \|  \|  \|  \|  \|  \|  \| Bt.16105.1.S1\_at \| \| Bt.17672.1.S1\_at \| 505940 \| 2/3 \| XM\_001251347  XM\_582310 \|  \|  \|  \| ARHGEF2 \| Bt.17672.1.S1\_at \| \| Bt.27892.1.S1\_at \| 523325 \| 1/1 \| XM\_001252162  XM\_001252190  XM\_601621 \| XM\_001252190 \|  \|  \| DVL1 \| Bt.27892.1.S1\_at \| \| Bt.12505.1.S1\_at \| 540108 \| 1/1 \| XM\_866848  XM\_879380 \| XM\_879380 \| GO:0005509 \|  \| SPTAN1 \| Bt.12505.1.S1\_at \| \| Bt.21060.1.S1\_at \| 513722 \| 1/1 \| XR\_028402 \| XR\_028402 \|  \|  \| CENTD3 \| Bt.21060.1.S1\_at \| \| Bt.6125.1.S1\_at \|  \|  \|  \| XM\_001251494 \|  \|  \| JAG1 \| Bt.6125.1.S1\_at \| \| Bt.4599.1.S1\_at \| 281092 \| 1/1 \| NM\_177494 \| NM\_177494 \|  \| 9288928 \| CROT \| Bt.4599.1.S1\_at \| \| Bt.7955.2.S1\_at \| 504471 \| 2/2 \| NM\_001045888 \|  \|  \|  \| PCOLCE \| Bt.7955.2.S1\_at \| \| Bt.16733.1.A1\_s\_at \| 618340 \| 1/2 \| XM\_870671 \| XM\_870671 \|  \|  \| NARF \| Bt.16733.1.A1\_s\_at \| \| Bt.2007.1.S1\_at \| 525750 \| 1/2 \| XM\_864309 \|  \|  \|  \|  \| Bt.2007.1.S1\_at \| \| Bt.23307.1.S1\_at \| 281301 \| 1/2 \| XM\_586044 \|  \|  \|  \| MCAM \| Bt.23307.1.S1\_at \| \| Bt.656.1.S1\_at \| 515798 \| 1/3 \| XM\_867749 \|  \|  \|  \| CORO1C \| Bt.656.1.S1\_at \| \| Bt.23002.1.S1\_at \| 282199 \| 2/3 \| NM\_001077831  XM\_001250845  XM\_001250892 \|  \| GO:0004129  GO:0005739  GO:0005740  GO:0006118  GO:0016020  GO:0016491 \| 1720401  2844245  10984454 \| COX6A1 \| Bt.23002.1.S1\_at \| \| Bt.17008.1.S1\_at \|  \|  \|  \|  \|  \|  \| SH3GLB1 \| Bt.17008.1.S1\_at \| \| Bt.8920.1.S1\_at \|  \|  \|  \|  \|  \|  \| SLC25A36 \| Bt.8920.1.S1\_at \| \| Bt.9238.1.S1\_at \| 338044 \| 2/2 \| XM\_588265 \| NM\_001082424 \|  \|  \| SLC38A2 \| Bt.9238.1.S1\_at \| \| Bt.20023.1.S1\_at \| 507114 \| 1/1 \| NM\_001075298 \|  \|  \| 11282978  16305752 \| KIAA1967 \| Bt.20023.1.S1\_at \| \| Bt.1464.1.A1\_at \| 338408 \| 1/1 \|  \|  \|  \| 10984454 \| ZNF236 \| Bt.1464.1.A1\_at \| \| Bt.2017.1.S1\_at \| 360007 \| 1/1 \| NM\_182788 \| NM\_182788 \| GO:0004867  GO:0005576  GO:0007596 \| 12477932  12921785 \| TFPI2 \| Bt.2017.1.S1\_at \| \| Bt.19676.2.S1\_a\_at \| 516874 \| 1/2 \| NM\_001038127 \|  \| GO:0000154  GO:0000179  GO:0005739  GO:0006350  GO:0006355  GO:0006364  GO:0008168  GO:0008649  GO:0016740 \|  \| TFB2M \| Bt.19676.2.S1\_a\_at \| \| Bt.5006.1.S1\_at \| 536619 \| 1/1 \| NM\_001034636 \| NM\_001034636 \| GO:0005515  GO:0005737  GO:0006417  GO:0030371  GO:0045947 \| 12477932 \| PAIP2 \| Bt.5006.1.S1\_at \| \| Bt.4261.1.S1\_at \| 282024 \| 1/3 \| XM\_591897 \| XM\_591897 \| GO:0003779  GO:0004872  GO:0005622 \|  \| PTK9L \| Bt.4261.1.S1\_at \| \| Bt.18843.1.A1\_at \|  \|  \|  \|  \|  \|  \| WNT5A \| Bt.18843.1.A1\_at \| \| Bt.22442.3.S1\_a\_at \| 512943 \| 1/2 \| XM\_869517  XM\_882708 \| XM\_869517 \| GO:0004674  GO:0004707  GO:0005524  GO:0006468 \|  \| MAPK12 \| Bt.22442.3.S1\_a\_at \| \| Bt.15860.1.S1\_at \|  \|  \|  \| NM\_001078016 \|  \|  \| ZNF238 \| Bt.15860.1.S1\_at \| \| Bt.5748.3.S1\_at \|  \|  \|  \|  \|  \|  \| YPEL5 \| Bt.5748.3.S1\_at \| \| Bt.14305.1.A1\_at \| 512267 \| 1/1 \|  \|  \|  \|  \|  \| Bt.14305.1.A1\_at \| \| Bt.1734.1.S1\_at \| 616278 \| 1/2 \| NM\_001046576 \|  \|  \|  \| SPFH2 \| Bt.1734.1.S1\_at \| \| Bt.25228.1.A1\_at \|  \|  \|  \|  \|  \|  \| SMURF2 \| Bt.25228.1.A1\_at \| \| Bt.13991.2.S1\_at \| 507736 \| 2/5 \| NM\_001034308 \| NM\_001034308 \|  \| 12477932 \| PNRC1 \| Bt.13991.2.S1\_at \| \| Bt.18408.1.A1\_at \|  \|  \|  \| XR\_027701 \|  \|  \| FLJ33814 \| Bt.18408.1.A1\_at \| \| Bt.19382.1.S1\_at \|  \|  \|  \|  \|  \|  \| PAG1 \| Bt.19382.1.S1\_at \| \| Bt.13730.2.S1\_at \| 408003 \| 2/2 \| NM\_001001171 \| NM\_001001171 \| GO:0006512  GO:0006914  GO:0008234  GO:0015031 \| 15140988 \| ATG4A \| Bt.13730.2.S1\_at \| \| Bt.3158.1.S1\_at \| 539896 \| 1/2 \| XM\_590199 \|  \|  \|  \| BRD8 \| Bt.3158.1.S1\_at \| \| Bt.1125.1.S1\_at \| 539003 \| 1/1 \| NM\_001076141 \| NM\_001076141 \| GO:0005198  GO:0005515  GO:0005634  GO:0005856  GO:0006350  GO:0006355  GO:0007155  GO:0016055 \|  \| CTNNB1 \| Bt.1125.1.S1\_at \| \| Bt.23380.2.S1\_at \| 505093 \| 1/1 \| NM\_001045911 \|  \|  \| 2994041  3032834  3941105 \| HNRPA1 \| Bt.23380.2.S1\_at \| \| Bt.22335.1.S1\_a\_at \|  \|  \|  \|  \|  \|  \| ZCCHC14 \| Bt.22335.1.S1\_a\_at \| \| Bt.5039.1.S1\_at \| 515652 \| 1/1 \| NM\_001034504 \|  \| GO:0000785  GO:0003677  GO:0003682  GO:0005634 \| 12477932 \| HMGN3 \| Bt.5039.1.S1\_at \| \| Bt.18513.1.S1\_at \| 518258 \| 1/2 \| XM\_001252368  XM\_596444 \|  \|  \|  \| FUNDC1 \| Bt.18513.1.S1\_at \| \| Bt.11389.1.S1\_at \| 504847 \| 1/3 \| XM\_872917 \|  \|  \|  \| CCNI \| Bt.11389.1.S1\_at \| \| Bt.2005.1.S1\_at \| 535447 \| 1/1 \| NM\_001017952 \| XR\_027786 \| GO:0003723  GO:0005515  GO:0005634  GO:0006397  GO:0008380  GO:0030529 \| 11282978  16305752 \| LSM1 \| Bt.2005.1.S1\_at \| \| Bt.9448.1.S2\_at \| 497019  512435  540799 \| 1/1 \| NM\_001011674  XM\_589950  XM\_869727 \| XM\_589950 \|  \|  \|  \| Bt.9448.1.S2\_at \| \| Bt.22011.1.S1\_at \| 532053 \| 1/3 \| XM\_610562 \| XM\_610562 \|  \|  \| OGT \| Bt.22011.1.S1\_at \| \| Bt.9186.1.A1\_at \| 540712 \| 1/1 \| XM\_612754 \|  \|  \|  \| FBXL3 \| Bt.9186.1.A1\_at \| \| Bt.935.2.S1\_at \| 539354 \| 2/2 \| NM\_001079612  XM\_001250904 \|  \| GO:0000139  GO:0005351  GO:0008643  GO:0016021 \|  \| SLC35A4 \| Bt.935.2.S1\_at \| \| Bt.23522.1.S1\_at \| 510575 \| 1/1 \| NM\_001035093 \|  \| GO:0005515  GO:0005524  GO:0006915  GO:0006917  GO:0016020  GO:0016021 \| 12477932 \| ITM2B \| Bt.23522.1.S1\_at \| \| Bt.1967.1.S1\_at \| 618855 \| 1/1 \| XM\_871173 \|  \|  \|  \| NUDT3 \| Bt.1967.1.S1\_at \| \| Bt.17812.1.S1\_at \| 614694 \| 1/1 \| XM\_001250351  XM\_866275 \|  \| GO:0006118  GO:0016491 \|  \| NUPL1 \| Bt.17812.1.S1\_at \| \| Bt.13529.1.S1\_at \| 510177 \| 2/4 \| XM\_865029 \|  \|  \|  \| SF3B1 \| Bt.13529.1.S1\_at \| \| Bt.2070.3.S1\_at \| 541175 \| 3/3 \| NM\_001034725 \| NM\_001034725 \| GO:0004725  GO:0005634  GO:0006470  GO:0016787  GO:0017017 \| 11282978 \| DUSP10 \| Bt.2070.3.S1\_at \| \| Bt.5872.1.S1\_at \|  \|  \|  \|  \|  \|  \|  \| Bt.5872.1.S1\_at \| \| Bt.16975.1.S1\_at \| 538487 \| 1/3 \| XM\_863921 \|  \|  \|  \| PRO0149 \| Bt.16975.1.S1\_at \| \| Bt.11149.1.S1\_at \| 280955 \| 1/2 \| NM\_173969 \| NM\_173969 \|  \| 8144034  12658628  16876394 \| VIM \| Bt.11149.1.S1\_at \| \| Bt.4458.1.A1\_at \| 515984 \| 1/1 \|  \|  \|  \|  \|  \| Bt.4458.1.A1\_at \| \| Bt.24397.1.S1\_at \| 506046 \| 1/4 \| XM\_582437 \| XM\_582437 \|  \|  \| MDS025 \| Bt.24397.1.S1\_at \| \| Bt.14037.1.S1\_at \|  \|  \|  \|  \|  \|  \| SAP30L \| Bt.14037.1.S1\_at \| \| Bt.5598.1.S1\_at \| 617047 \| 1/1 \| NM\_001035490 \| NM\_001035490 \|  \| 12140684  12477932  16305752 \| C8orf4 \| Bt.5598.1.S1\_at \| \| Bt.14193.1.A1\_at \|  \|  \|  \| XM\_001251178 \|  \|  \| GRSF1 \| Bt.14193.1.A1\_at \| \| Bt.3820.2.A1\_at \| 326578 \| 1/2 \| NM\_001077834  XM\_001252075 \|  \| GO:0003723  GO:0004872  GO:0005525  GO:0005634  GO:0005786  GO:0006614 \| 12658628 \| SNW1 \| Bt.3820.2.A1\_at \| \| Bt.8966.1.S2\_at \| 282090 \| 1/2 \| NM\_174470 \|  \| GO:0004016  GO:0006091  GO:0006171  GO:0006772  GO:0016311  GO:0016787  GO:0050333 \| 11827967 \| THTPA \| Bt.8966.1.S2\_at \| \| Bt.5388.1.S1\_at \| 326601 \| 2/3 \| NM\_001014389 \|  \| GO:0000786  GO:0003677  GO:0005634  GO:0005694  GO:0006334  GO:0007001 \| 10464286  11282978  12140684  12477932  12658628  16305752 \| H3F3A \| Bt.5388.1.S1\_at \| \| Bt.13871.1.S1\_at \| 538856 \| 1/2 \|  \|  \|  \|  \| BCL6B \| Bt.13871.1.S1\_at \| \| Bt.618.1.S1\_at \| 616275 \| 1/1 \| XM\_869340  XM\_882563 \| XM\_882563 \|  \| 12477932 \| ANAPC11 \| Bt.618.1.S1\_at \| \| Bt.23318.1.S2\_at \| 510833 \| 2/2 \| NM\_001076831 \|  \| GO:0005198  GO:0005201  GO:0005578  GO:0005581  GO:0005737  GO:0006817 \| 488906  488907  488908  488909  488910  488911 \| COL3A1 \| Bt.23318.1.S2\_at \| \| Bt.7467.1.S1\_at \| 616416 \| 1/1 \| XM\_868438 \|  \|  \|  \| KCTD12 \| Bt.7467.1.S1\_at \| \| Bt.24873.1.S1\_at \| 519864 \| 1/3 \| XM\_598095 \|  \|  \|  \| MLLT10 \| Bt.24873.1.S1\_at \| \| Bt.9078.2.S1\_a\_at \| 505916 \| 1/1 \| XM\_582280 \| XM\_582280 \|  \|  \| SRM \| Bt.9078.2.S1\_a\_at \| \| Bt.22433.1.A1\_at \| 541266 \| 1/2 \| NM\_001046502 \| NM\_001046502 \| GO:0006811  GO:0015075  GO:0016020  GO:0016021 \| 11282978  16305752 \| SLC22A5 \| Bt.22433.1.A1\_at \| \| Bt.3573.1.S1\_at \| 282220 \| 1/5 \| NM\_174535 \| XM\_001249987 \| GO:0000166  GO:0003746  GO:0003924  GO:0005515  GO:0005525 \| 12078752  12658628 \| EEF1A1 \| Bt.3573.1.S1\_at \| \| Bt.3478.1.A1\_s\_at \| 281040 \| 3/3 \| NM\_174004 \| NM\_174004 \|  \| 12477932  12917688  15292187  15471980  15637154  16325778  16894547 \| CAV1 \| Bt.3478.1.A1\_s\_at \| \| Bt.4859.1.S1\_at \| 286807 \| 1/1 \| NM\_174769 \|  \| GO:0005634  GO:0006464  GO:0006512 \| 9833880  12140684  12477932  16305752 \| SMT3H2 \| Bt.4859.1.S1\_at \| \| Bt.5467.1.S2\_at \|  \|  \|  \|  \|  \|  \| PSAP \| Bt.5467.1.S2\_at \| \| Bt.26524.1.S1\_at \|  \|  \|  \| XM\_869323 \|  \|  \|  \| Bt.26524.1.S1\_at \| \| Bt.10056.1.S1\_at \|  \|  \|  \| XM\_615934 \|  \|  \|  \| Bt.10056.1.S1\_at \| \| Bt.4552.1.S1\_a\_at \| 281985 \| 1/3 \| NM\_174424 \|  \| GO:0004435  GO:0004871  GO:0005509  GO:0007242  GO:0016042  GO:0016787 \| 8327481  8389372 \| PLCB4 \| Bt.4552.1.S1\_a\_at \| \| Bt.9144.1.A1\_at \| 617074 \| 1/2 \| XM\_001252424  XM\_869258 \| XM\_869258 \|  \|  \| SPFH1 \| Bt.9144.1.A1\_at \| \| Bt.8028.1.S1\_at \| 525572 \| 1/2 \| XM\_603925 \|  \|  \|  \| GPS1 \| Bt.8028.1.S1\_at \| \| Bt.21920.1.S1\_at \|  \|  \|  \|  \|  \|  \|  \| Bt.21920.1.S1\_at \| \| Bt.57.1.S1\_at \| 338057 \| 1/1 \| NM\_176652 \| NM\_176652 \| GO:0003954  GO:0005739  GO:0008137  GO:0016491 \| 1518044  12477932 \| NDUFS5 \| Bt.57.1.S1\_at \| \| Bt.8566.1.S1\_at \| 538427 \| 1/2 \| XM\_612559 \| XM\_612559 \| GO:0005515  GO:0005634  GO:0005737  GO:0005783  GO:0006913  GO:0007242  GO:0016363  GO:0048471 \| 7937870  8679524  15895553 \| ANP32A \| Bt.8566.1.S1\_at \| \| Bt.4057.1.S1\_at \| 317655 \| 1/4 \| NM\_174834 \| NM\_174834 \| GO:0000166  GO:0001725  GO:0001764  GO:0001778  GO:0003774  GO:0003779  GO:0005516  GO:0005524  GO:0005737  GO:0006887  GO:0006930  GO:0008360  GO:0016459 \| 7782316 \| MYH10 \| Bt.4057.1.S1\_at \| \| Bt.7785.1.S1\_at \| 513312 \| 1/1 \| NM\_001014916 \|  \| GO:0003677  GO:0005634  GO:0006350  GO:0015031 \| 8047162  10982809  12140684  12477932  16305752 \| NACA \| Bt.7785.1.S1\_at \| \| Bt.24087.1.A1\_at \|  \|  \|  \|  \|  \|  \|  \| Bt.24087.1.A1\_at \| \| Bt.17079.2.A1\_at \|  \|  \|  \|  \|  \|  \| VGLL4 \| Bt.17079.2.A1\_at \| \| Bt.22525.1.A1\_at \| 540254 \| 1/2 \| XM\_876103 \| XM\_876103 \|  \|  \| DGCR8 \| Bt.22525.1.A1\_at \| \| Bt.4294.1.S1\_at \| 407240 \| 1/1 \| NM\_001001162 \|  \| GO:0005634  GO:0030528  GO:0045449 \| 14660559 \| USF2 \| Bt.4294.1.S1\_at \| \| Bt.2798.1.S1\_a\_at \| 282385 \| 1/1 \| NM\_174623 \|  \| GO:0003779  GO:0005737  GO:0005856  GO:0007010 \| 3818171  6952223  9108730  11895978 \| TMSB10 \| Bt.2798.1.S1\_a\_at \| \| Bt.11058.1.A1\_at \| 280891 \| 1/2 \| NM\_173949 \|  \|  \| 6288374  8396421  11842080  15897476 \| PPM2C \| Bt.11058.1.A1\_at \| \| Bt.4241.1.S1\_at \| 282050 \| 1/1 \| NM\_001077830  XM\_001250860 \|  \| GO:0003735  GO:0005622  GO:0005840  GO:0006412 \| 11683713  12658628 \| RPS14 \| Bt.4241.1.S1\_at \| \| Bt.22981.1.S1\_at \| 359716 \| 1/3 \| XM\_001252215  XM\_867643 \| XM\_001252215 \| GO:0000791  GO:0003677  GO:0003677  GO:0003886  GO:0005634  GO:0005737  GO:0006306  GO:0006306  GO:0006349  GO:0008168  GO:0008270  GO:0016363  GO:0016740 \|  \| DNMT3A \| Bt.22981.1.S1\_at \| \| Bt.11448.1.S1\_at \| 615626 \| 1/1 \| NM\_001039953 \|  \|  \|  \| PTMA \| Bt.11448.1.S1\_at \| \| Bt.5106.2.S1\_at \| 281793 \| 2/5 \| NM\_181021 \|  \| GO:0000166  GO:0004871  GO:0005525  GO:0007165  GO:0007186  GO:0016020 \| 3015900  3080331  3081893  9111083  9395396  9417641  10427002  10729789  12477932  12574119  15457345  15546818 \| GNAS \| Bt.5106.2.S1\_at \| \| Bt.5737.1.S1\_at \| 510574 \| 1/3 \| NM\_001075455 \| NM\_001075455 \| GO:0007034  GO:0015031  GO:0016020  GO:0030904 \| 12140684  16305752 \| VPS26 \| Bt.5737.1.S1\_at \| \| Bt.22243.1.S1\_at \| 506295 \| 1/1 \| NM\_001075252 \|  \|  \|  \| MYADM \| Bt.22243.1.S1\_at \| \| Bt.23229.2.S1\_at \| 510763 \| 1/3 \| XR\_027587 \| XR\_027587 \|  \|  \| MYBPC1 \| Bt.23229.2.S1\_at \| \| Bt.19601.1.S1\_at \| 532953 \| 1/2 \| NM\_001075976 \|  \| GO:0005509 \|  \| CETN3 \| Bt.19601.1.S1\_at \| \| Bt.12498.1.A1\_at \| 530657 \| 1/1 \| XM\_609132 \| XM\_609132 \| GO:0004867  GO:0005737  GO:0006817 \|  \| COL6A3 \| Bt.12498.1.A1\_at \| \| Bt.9207.1.S1\_at \| 540426 \| 1/1 \| NM\_001046477 \|  \| GO:0001931  GO:0005102  GO:0005198  GO:0005488  GO:0005737  GO:0005856  GO:0008092  GO:0016020  GO:0016323  GO:0016324 \|  \| MSN \| Bt.9207.1.S1\_at \| \| Bt.5388.1.S1\_s\_at \| 326601 \| 2/3 \| NM\_001014389 \| NM\_001014389 \| GO:0000786  GO:0003677  GO:0005634  GO:0005694  GO:0006334  GO:0007001 \| 10464286  11282978  12140684  12477932  12658628  16305752 \| H3F3A \| Bt.5388.1.S1\_s\_at \| \| Bt.23326.1.S1\_at \| 404122 \| 1/1 \| NM\_001033618 \| NM\_001033618 \| GO:0000166  GO:0005198  GO:0005515  GO:0005524  GO:0005856 \| 213279  6195151  12477932  16324220 \| ACTG1 \| Bt.23326.1.S1\_at \| \| Bt.26817.1.S1\_at \|  \|  \|  \|  \|  \|  \|  \| Bt.26817.1.S1\_at \| \| Bt.15357.1.A1\_at \| 614194 \| 1/2 \| NM\_001035444 \|  \|  \| 12477932 \|  \| Bt.15357.1.A1\_at \| \| Bt.8122.1.S1\_at \| 282386 \| 1/2 \| NM\_001002885 \|  \| GO:0003779  GO:0005737  GO:0007010 \| 16677835 \| TMSB4X \| Bt.8122.1.S1\_at \| \| Bt.3599.1.S1\_at \| 507211  614028 \| 1/1 \| NM\_001035441  XM\_583781 \| XM\_001249312 \|  \|  \|  \| Bt.3599.1.S1\_at \| \| Bt.10822.1.S1\_at \|  \|  \|  \|  \|  \|  \|  \| Bt.10822.1.S1\_at \| \| Bt.12558.1.S1\_at \|  \|  \|  \| XM\_870226 \|  \|  \| KIAA0152 \| Bt.12558.1.S1\_at \| \| Bt.16841.1.A1\_at \|  \|  \|  \| XR\_028008 \|  \|  \| SNRK \| Bt.16841.1.A1\_at \| \| Bt.4877.1.S1\_at \| 281986 \| 1/1 \| XM\_593661 \|  \|  \| 3390863 \| PLCD1 \| Bt.4877.1.S1\_at \| \| Bt.11067.1.S1\_at \| 522965 \| 1/1 \| XM\_601255 \|  \|  \|  \| CLIPR-59 \| Bt.11067.1.S1\_at \| \| Bt.6675.1.A1\_at \|  \|  \|  \|  \|  \|  \|  \| Bt.6675.1.A1\_at \| \| Bt.17789.1.A1\_at \| 407768 \| 1/1 \| NM\_001001163 \|  \| GO:0003828  GO:0003828  GO:0006486  GO:0016020  GO:0016021  GO:0030173 \| 15843597 \| ST8SIA4 \| Bt.17789.1.A1\_at \| \| Bt.28817.1.A1\_at \|  \|  \|  \| XM\_001250794 \|  \|  \| GLS \| Bt.28817.1.A1\_at \| \| Bt.2905.1.S1\_at \| 515063 \| 1/1 \| NM\_001035304 \| NM\_001035304 \| GO:0007275  GO:0007399  GO:0030154 \| 12477932 \| NDRG2 \| Bt.2905.1.S1\_at \| \| Bt.25165.1.S1\_at \| 533953 \| 1/2 \| NM\_001040555 \|  \| GO:0000166  GO:0000287  GO:0004674  GO:0005515  GO:0005524  GO:0006468  GO:0007165  GO:0016740 \| 12477932  16621030 \|  \| Bt.25165.1.S1\_at \| \| Bt.1069.1.A1\_at \| 536337 \| 1/1 \| NM\_001015662 \|  \| GO:0004725  GO:0004872  GO:0006470  GO:0016787  GO:0016791 \| 11282978 \| PTPRR \| Bt.1069.1.A1\_at \| \| Bt.21427.1.S1\_at \|  \|  \|  \|  \|  \|  \|  \| Bt.21427.1.S1\_at \| \| Bt.24260.2.S1\_at \|  \|  \|  \|  \|  \|  \| CENTB2 \| Bt.24260.2.S1\_at \| \| Bt.19774.2.S1\_at \| 532571 \| 1/3 \| XM\_611687 \|  \| GO:0003677  GO:0005634 \|  \| BOMB \| Bt.19774.2.S1\_at \| \| Bt.15864.1.S1\_at \|  \|  \|  \|  \|  \|  \| FGF2 \| Bt.15864.1.S1\_at \| \| Bt.12769.1.S1\_at \| 282856 \| 1/1 \| XM\_583200 \|  \|  \| 12388798  12477932 \| PCK2 \| Bt.12769.1.S1\_at \| \| Bt.5379.1.S1\_at \|  \|  \|  \| XM\_001250802 \|  \|  \| PTPRF \| Bt.5379.1.S1\_at \| \| Bt.23869.1.A1\_at \| 510080 \| 4/5 \| NM\_001076826 \|  \|  \|  \|  \| Bt.23869.1.A1\_at \| \| Bt.5027.1.A1\_at \| 444864 \| 3/5 \| NM\_001002887 \| NM\_001002887 \| GO:0003676  GO:0003700  GO:0005622  GO:0005634  GO:0006355  GO:0006915  GO:0008270  GO:0046872 \| 15203203 \| ZIM2 \| Bt.5027.1.A1\_at \| \| Bt.20402.3.S1\_at \| 530393 \| 3/3 \| NM\_001046312 \| NM\_001046312 \| GO:0005216  GO:0005509  GO:0005515  GO:0005929  GO:0005932  GO:0006811  GO:0006816  GO:0006874  GO:0007050  GO:0007259  GO:0007368  GO:0015629  GO:0016020  GO:0016021  GO:0050974 \| 15998908  16229973 \| PKD2 \| Bt.20402.3.S1\_at \| \| Bt.23240.1.S1\_at \| 616464 \| 1/1 \|  \|  \|  \|  \|  \| Bt.23240.1.S1\_at \| \| Bt.9221.1.A1\_at \|  \|  \|  \| XM\_001254700 \|  \|  \| DKFZp762C186 \| Bt.9221.1.A1\_at \| \| Bt.997.1.S1\_at \| 516287 \| 2/2 \| NM\_001034518 \|  \| GO:0004128  GO:0006118  GO:0016491  GO:0050660  GO:0051287 \| 12477932 \| CYB5R1 \| Bt.997.1.S1\_at \| \| BtAffx.29968.1.S1\_at \|  \|  \|  \|  \|  \|  \|  \| BtAffx.29968.1.S1\_at \| \| Bt.24536.1.S1\_at \| 540617 \| 2/2 \|  \|  \|  \|  \| FNBP1L \| Bt.24536.1.S1\_at \| \| Bt.5788.1.S1\_at \|  \|  \|  \|  \|  \|  \| ELK3 \| Bt.5788.1.S1\_at \| \| Bt.26094.1.A1\_at \|  \|  \|  \|  \|  \|  \| MAP3K2 \| Bt.26094.1.A1\_at \| \| Bt.8802.1.S1\_at \| 508425 \| 1/1 \| XM\_585198 \| NM\_001081516 \|  \|  \| FAM57A \| Bt.8802.1.S1\_at \| \| Bt.15735.1.S1\_at \| 525795 \| 1/2 \| XM\_604151 \| XM\_604151 \|  \|  \| AGRN \| Bt.15735.1.S1\_at \| \| Bt.9059.1.S1\_at \|  \|  \|  \| XR\_028492 \|  \|  \| PPP1R9B \| Bt.9059.1.S1\_at \| \| Bt.25654.1.A1\_at \| 511699 \| 1/1 \| NM\_001031757 \| NM\_001031757 \| GO:0005247  GO:0006821  GO:0016020 \|  \| CLCN4 \| Bt.25654.1.A1\_at \| \| Bt.5341.1.S1\_at \| 280884 \| 1/1 \| NM\_173946 \| NM\_173946 \|  \| 2180954  2372374  2584240  9346963  10373482  12140684  12477932  16305752 \| OGN \| Bt.5341.1.S1\_at \| \| Bt.24321.1.S1\_at \|  \|  \|  \|  \|  \|  \| EPM2AIP1 \| Bt.24321.1.S1\_at \| \| Bt.29853.1.A1\_at \| 407121 \| 1/2 \| XM\_613028 \|  \|  \|  \|  \| Bt.29853.1.A1\_at \| \| Bt.20487.1.S1\_at \| 508216 \| 1/1 \| NM\_001045996 \| NM\_001045996 \|  \|  \| MRPL28 \| Bt.20487.1.S1\_at \| \| Bt.14007.1.S1\_at \| 504409 \| 2/5 \| XM\_001250100  XM\_580523 \|  \| GO:0000166  GO:0003676  GO:0008270 \|  \| C13orf10 \| Bt.14007.1.S1\_at \| \| Bt.3014.1.A1\_at \|  \|  \|  \|  \|  \|  \| PDGFC \| Bt.3014.1.A1\_at \| \| Bt.29472.1.A1\_at \|  \|  \|  \|  \|  \|  \| TIA1 \| Bt.29472.1.A1\_at \| \| Bt.19637.1.A1\_at \|  \|  \|  \|  \|  \|  \| STRN \| Bt.19637.1.A1\_at \| \| Bt.11194.1.S1\_at \| 281360 \| 1/1 \| NM\_174822 \| NM\_174822 \| GO:0004090  GO:0005739  GO:0005777  GO:0008152  GO:0016491 \| 15487511 \| NDRD \| Bt.11194.1.S1\_at \| \| Bt.21365.1.S1\_a\_at \| 513829 \| 1/6 \| XR\_028194 \| XR\_028194 \|  \|  \| EIF2AK4 \| Bt.21365.1.S1\_a\_at \| \| Bt.10324.1.A1\_at \| 327714 \| 2/2 \| NM\_175829 \|  \| GO:0003954  GO:0005739  GO:0005743  GO:0006118  GO:0008137 \| 1518044  1907966  12477932 \|  \| Bt.10324.1.A1\_at \| \| Bt.4744.1.S1\_at \| 281206 \| 1/2 \| NM\_177502 \| NM\_177502 \| GO:0004069  GO:0006520  GO:0009058  GO:0030170 \| 8224363  11282978  16305752 \| GOT1 \| Bt.4744.1.S1\_at \| \| Bt.10734.1.A1\_at \|  \|  \|  \|  \|  \|  \|  \| Bt.10734.1.A1\_at \| \| Bt.2997.1.A1\_at \|  \|  \|  \|  \|  \|  \| PTGIS \| Bt.2997.1.A1\_at \| \| Bt.8671.1.S1\_at \|  \|  \|  \|  \|  \|  \|  \| Bt.8671.1.S1\_at \| \| Bt.14304.2.S1\_at \| 539922 \| 1/2 \| XM\_590319 \| NM\_001081611 \|  \|  \| FBXO34 \| Bt.14304.2.S1\_at \| \| Bt.26604.1.S1\_at \|  \|  \|  \|  \|  \|  \| AGTRL1 \| Bt.26604.1.S1\_at \| \| Bt.8785.1.S1\_at \|  \|  \|  \|  \|  \|  \| DRCTNNB1A \| Bt.8785.1.S1\_at \| \| Bt.2667.1.S1\_a\_at \| 510729 \| 1/2 \| XM\_001256148 \| XM\_001256148 \| GO:0006388  GO:0016772 \| 12477932  16305752 \| TRPT1 \| Bt.2667.1.S1\_a\_at \| \| Bt.9412.2.S1\_at \|  \|  \|  \|  \|  \|  \| COL14A1 \| Bt.9412.2.S1\_at \| \| Bt.14572.1.A1\_at \| 613449 \| 2/11 \| XM\_864237 \|  \| GO:0004674  GO:0005524  GO:0006468 \|  \| DCAMKL1 \| Bt.14572.1.A1\_at \| \| Bt.11126.3.S1\_a\_at \| 510960 \| 2/2 \| NM\_001046069 \| NM\_001046069 \| GO:0004607  GO:0006629  GO:0008415  GO:0016740 \|  \| LCAT \| Bt.11126.3.S1\_a\_at \| \| Bt.1963.1.S1\_at \| 504889 \| 1/1 \| XM\_865942 \| XM\_865942 \|  \|  \| GMPPA \| Bt.1963.1.S1\_at \| \| Bt.24396.1.S1\_at \| 539201 \| 1/2 \|  \|  \|  \|  \| NUDT10 \| Bt.24396.1.S1\_at \| \| Bt.19898.1.A1\_at \|  \|  \|  \| XM\_592731 \|  \|  \|  \| Bt.19898.1.A1\_at \| \| Bt.24381.1.S1\_at \|  \|  \|  \|  \|  \|  \| DCTD \| Bt.24381.1.S1\_at \| \| Bt.7240.1.S1\_at \| 281271 \| 1/1 \| BC105385 \|  \|  \| 8369298  15466433 \| LAP3 \| Bt.7240.1.S1\_at \| \| Bt.21053.2.S1\_at \| 534995 \| 1/2 \| NM\_001034623 \| NM\_001034623 \| GO:0016020 \| 12477932 \| SYNGR1 \| Bt.21053.2.S1\_at \| \| Bt.9288.1.S1\_at \| 510738 \| 2/3 \| XM\_587922 \| XM\_587922 \|  \|  \|  \| Bt.9288.1.S1\_at \| \| Bt.18434.1.A1\_at \|  \|  \|  \|  \|  \|  \| HNRPR \| Bt.18434.1.A1\_at \| \| Bt.5328.1.S1\_at \| 529878 \| 1/1 \| XM\_608339 \| XM\_608339 \|  \|  \| DKFZP434C171 \| Bt.5328.1.S1\_at \| \| Bt.2331.1.A1\_at \| 617017 \| 1/2 \| XM\_869191 \| XM\_869191 \|  \|  \| RAMP1 \| Bt.2331.1.A1\_at \| \| Bt.13205.1.A1\_at \|  \|  \|  \|  \|  \|  \| MRPS35 \| Bt.13205.1.A1\_at \| \| Bt.14207.1.S1\_at \| 319141 \| 1/1 \| NM\_001075134 \|  \| GO:0005739  GO:0008415  GO:0008890  GO:0009058  GO:0016769  GO:0030170 \| 8307963  10712613 \| GCAT \| Bt.14207.1.S1\_at \| \| Bt.12445.2.S1\_at \| 507342 \| 1/2 \| NM\_001035045 \|  \|  \| 12477932 \| FSCN1 \| Bt.12445.2.S1\_at \| \| Bt.3215.1.S1\_at \| 507319 \| 1/1 \| NM\_001034292 \| NM\_001034292 \|  \| 12477932 \| EFNA1 \| Bt.3215.1.S1\_at \| \| Bt.9930.1.S1\_at \|  \|  \|  \|  \|  \|  \| GABARAPL1 \| Bt.9930.1.S1\_at \| \| Bt.3092.1.S1\_at \| 281912 \| 1/1 \| NM\_174388 \|  \| GO:0001527  GO:0005578 \| 2019589  8286390  11284693  11723132 \| MFAP2 \| Bt.3092.1.S1\_at \| \| Bt.28193.1.S1\_at \|  \|  \|  \|  \|  \|  \| RICS \| Bt.28193.1.S1\_at \| \| Bt.9953.1.S1\_at \| 510483 \| 1/1 \| NM\_001034383 \| NM\_001034383 \| GO:0006506  GO:0016021  GO:0016757  GO:0017176 \| 12477932 \| PIGC \| Bt.9953.1.S1\_at \| \| Bt.7872.1.S1\_at \| 515946 \| 1/1 \| NM\_001034513  XM\_001249829 \| XM\_001249829 \| GO:0004090  GO:0008152  GO:0016491 \| 12477932 \| CBR1 \| Bt.7872.1.S1\_at \| \| Bt.16259.1.A1\_at \|  \|  \|  \|  \|  \|  \| SLK \| Bt.16259.1.A1\_at \| \| Bt.11165.1.S1\_at \| 533980 \| 1/1 \| XM\_613573 \| XM\_613573 \|  \|  \| CKAP5 \| Bt.11165.1.S1\_at \| \| Bt.8970.1.S1\_at \| 510462 \| 1/1 \| NM\_001035090 \|  \|  \| 12140684 \| MGC15763 \| Bt.8970.1.S1\_at \| \| Bt.23669.1.A1\_at \|  \|  \|  \|  \|  \|  \| TAL1 \| Bt.23669.1.A1\_at \| \| Bt.19957.1.A1\_at \|  \|  \|  \|  \|  \|  \|  \| Bt.19957.1.A1\_at \| \| Bt.6577.1.S1\_at \| 505862 \| 1/1 \| NM\_001037447 \|  \|  \| 11282978  12477932  16305752 \| FLJ10922 \| Bt.6577.1.S1\_at \| \| Bt.25016.2.S1\_at \| 537712 \| 2/3 \| XM\_617891 \|  \| GO:0003677  GO:0005634  GO:0006355  GO:0030528 \|  \| EBF \| Bt.25016.2.S1\_at \| \| Bt.28188.1.S1\_at \| 539790 \| 1/3 \| XM\_611420 \|  \| GO:0003677  GO:0005622  GO:0005634  GO:0006355 \|  \| POGK \| Bt.28188.1.S1\_at \| \| Bt.26316.1.A1\_at \|  \|  \|  \|  \|  \|  \| ARL7 \| Bt.26316.1.A1\_at \| \| Bt.7716.1.S1\_at \| 532120 \| 1/1 \| NM\_001075956 \|  \| GO:0000242  GO:0003777  GO:0005488  GO:0005856  GO:0051297 \|  \| BBS4 \| Bt.7716.1.S1\_at \| \| Bt.17188.1.A1\_at \|  \|  \|  \|  \|  \|  \|  \| Bt.17188.1.A1\_at \| \| Bt.20544.1.S1\_at \|  \|  \|  \|  \|  \|  \| SMUG1 \| Bt.20544.1.S1\_at \| \| Bt.11599.1.S1\_at \|  \|  \|  \|  \|  \|  \| SEMA6A \| Bt.11599.1.S1\_at \| \| Bt.11725.1.A1\_at \|  \|  \|  \|  \|  \|  \| NEDD4L \| Bt.11725.1.A1\_at \| \| Bt.8957.1.S1\_at \| 281736 \| 1/1 \| NM\_174301 \|  \| GO:0001569  GO:0001584  GO:0001667  GO:0001764  GO:0003779  GO:0004872  GO:0005515  GO:0007165  GO:0007186  GO:0007281  GO:0007420  GO:0008045  GO:0008354  GO:0016020  GO:0016021  GO:0016493  GO:0016494  GO:0030334  GO:0030426  GO:0031252  GO:0032027  GO:0042098 \| 1661837  8234909  12477932 \| CXCR4 \| Bt.8957.1.S1\_at \| \| Bt.22181.1.A1\_at \| 513707 \| 1/2 \| XM\_591440 \|  \|  \|  \| IPO7 \| Bt.22181.1.A1\_at \| \| Bt.26915.2.S1\_at \|  \|  \|  \|  \|  \|  \| HIP1 \| Bt.26915.2.S1\_at \| \| Bt.9036.1.A1\_at \| 540922 \| 1/2 \| XM\_864178 \| XM\_864178 \|  \|  \| ZC3H7A \| Bt.9036.1.A1\_at \| \| Bt.11088.1.S1\_a\_at \| 338066 \| 1/1 \| NM\_176661 \| NM\_176661 \| GO:0004872  GO:0004930  GO:0005509  GO:0005515  GO:0007155  GO:0007165  GO:0007218  GO:0016020  GO:0016021 \| 15234554 \| CD97 \| Bt.11088.1.S1\_a\_at \| \| Bt.6017.1.A1\_at \|  \|  \|  \| XM\_001255353 \|  \|  \|  \| Bt.6017.1.A1\_at \| \| Bt.13530.1.S1\_at \| 535174 \| 1/1 \| NM\_001046396 \| NM\_001046396 \| GO:0008152  GO:0016853 \|  \| DCI \| Bt.13530.1.S1\_at \| \| Bt.22414.2.S1\_at \|  \|  \|  \| NM\_175789 \|  \|  \| NSUN6 \| Bt.22414.2.S1\_at \| \| Bt.25522.1.S1\_at \|  \|  \|  \|  \|  \|  \| KIAA1274 \| Bt.25522.1.S1\_at \| \| Bt.12297.1.S1\_at \| 281904 \| 1/2 \| NM\_174384 \|  \| GO:0004720  GO:0005044  GO:0005507  GO:0005576  GO:0016020  GO:0016491  GO:0046872 \|  \| LOXL4 \| Bt.12297.1.S1\_at \| \| Bt.1175.1.S1\_at \| 521073 \| 1/1 \| NM\_001046254 \| NM\_001046254 \| GO:0005739  GO:0006457  GO:0008270  GO:0015031  GO:0016020  GO:0042719  GO:0045039  GO:0046872 \|  \| TIMM9 \| Bt.1175.1.S1\_at \| \| Bt.11530.1.A1\_at \|  \|  \|  \|  \|  \|  \|  \| Bt.11530.1.A1\_at \| \| Bt.17456.1.S1\_at \| 535680 \| 1/1 \| NM\_001076066 \| NM\_001076066 \| GO:0004001  GO:0006166  GO:0016301 \|  \| ADK \| Bt.17456.1.S1\_at \| \| Bt.12867.2.S1\_at \|  \|  \|  \|  \|  \|  \| ZNF318 \| Bt.12867.2.S1\_at \| \| Bt.26848.1.S1\_at \| 510313 \| 1/1 \| XM\_587442 \| XM\_587442 \|  \|  \| MGC13125 \| Bt.26848.1.S1\_at \| \| Bt.8766.1.S1\_at \| 510620 \| 1/1 \| XM\_587784 \| XM\_587784 \|  \|  \| ASAH1 \| Bt.8766.1.S1\_at \| \| Bt.9936.1.S1\_at \|  \|  \|  \| XM\_581038 \|  \|  \| MLLT4 \| Bt.9936.1.S1\_at \| \| Bt.819.1.S1\_at \| 515086 \| 1/4 \| NM\_001038543 \| XR\_028650 \| GO:0003676 \|  \| CAMTA2 \| Bt.819.1.S1\_at \| \| Bt.28150.2.S1\_at \|  \|  \|  \| XM\_001252990 \|  \|  \| TRIM7 \| Bt.28150.2.S1\_at \| \| Bt.10657.1.A1\_at \| 515233 \| 1/1 \| NM\_001037464 \|  \| GO:0000166  GO:0003746  GO:0003924  GO:0005525  GO:0005634  GO:0005737  GO:0005853  GO:0006414  GO:0006916 \| 12477932 \| MGC128473 \| Bt.10657.1.A1\_at \| \| Bt.3698.1.S1\_at \|  \|  \|  \|  \|  \|  \| BEXL1 \| Bt.3698.1.S1\_at \| \| Bt.12861.1.S1\_at \| 768014 \| 1/1 \| NM\_001077073 \|  \| GO:0000166  GO:0003777  GO:0005524  GO:0005874  GO:0005875  GO:0007018 \|  \| KIF2 \| Bt.12861.1.S1\_at \| \| Bt.6567.1.S1\_at \| 513606 \| 1/2 \| NM\_001034450 \|  \| GO:0006281 \| 12477932 \| POLH \| Bt.6567.1.S1\_at \| \| Bt.9993.1.S1\_at \|  \|  \|  \| NM\_001076378 \|  \|  \| AQP7 \| Bt.9993.1.S1\_at \| \| Bt.15922.1.S1\_at \| 535099 \| 1/1 \| XM\_866127 \| NM\_001081605 \|  \|  \| MTMR3 \| Bt.15922.1.S1\_at \| \| Bt.22176.1.A1\_at \| 515897 \| 1/2 \|  \|  \|  \|  \| IGSF1 \| Bt.22176.1.A1\_at \| \| Bt.22320.3.S1\_at \| 505819 \| 1/5 \| XM\_582170 \|  \|  \|  \|  \| Bt.22320.3.S1\_at \| \| Bt.755.1.S1\_at \| 615788 \| 1/1 \| XM\_867686 \|  \| GO:0005515  GO:0005622  GO:0006508  GO:0030693  GO:0042981 \|  \| CRADD \| Bt.755.1.S1\_at \| \| Bt.4437.1.S1\_at \| 282182 \| 1/2 \| NM\_174518 \| NM\_174518 \| GO:0005634  GO:0030528  GO:0045449 \| 12477932 \| CITED1 \| Bt.4437.1.S1\_at \| \| Bt.5732.2.A1\_at \|  \|  \|  \|  \|  \|  \|  \| Bt.5732.2.A1\_at \| \| Bt.8608.1.S1\_at \|  \|  \|  \|  \|  \|  \| ZNF605 \| Bt.8608.1.S1\_at \| \| Bt.26430.1.S1\_at \| 515674 \| 1/1 \| XM\_593739 \|  \| GO:0003676  GO:0005622  GO:0005634  GO:0006355  GO:0008270 \|  \| ZNF184 \| Bt.26430.1.S1\_at \| \| Bt.21177.1.A1\_s\_at \|  \|  \|  \|  \|  \|  \|  \| Bt.21177.1.A1\_s\_at \| \| Bt.27428.1.A1\_at \|  \|  \|  \|  \|  \|  \| GPM6A \| Bt.27428.1.A1\_at \| \| Bt.13350.1.S1\_at \| 539675 \| 1/1 \|  \|  \|  \|  \|  \| Bt.13350.1.S1\_at \| \| Bt.10129.1.A1\_at \|  \|  \|  \|  \|  \|  \| VTI1A \| Bt.10129.1.A1\_at \| \| Bt.25419.1.A1\_at \|  \|  \|  \|  \|  \|  \| PACRG \| Bt.25419.1.A1\_at \| \| Bt.16495.2.A1\_at \| 511602 \| 1/2 \| XM\_588970 \|  \| GO:0005201  GO:0005581  GO:0005737  GO:0006817 \|  \| COL4A5 \| Bt.16495.2.A1\_at \| \| Bt.27481.1.A1\_at \| 535424 \| 1/2 \| NM\_001034631 \| NM\_001034631 \| GO:0000166  GO:0003924  GO:0005525  GO:0006955 \| 11282978  12477932  16305752 \| SPG3A \| Bt.27481.1.A1\_at \| \| Bt.25836.2.A1\_at \| 531068 \| 1/2 \| NM\_001035385 \|  \|  \| 11282978 \|  \| Bt.25836.2.A1\_at \| \| Bt.15887.2.S1\_a\_at \|  \|  \|  \| XM\_585964 \|  \|  \| ZNRF1 \| Bt.15887.2.S1\_a\_at \| \| Bt.8479.1.A1\_at \| 282876 \| 1/4 \| NM\_001076799 \| NM\_001076799 \|  \| 7536776  7558036  12016516  16207333  16461547  16966449 \| NOS2A \| Bt.8479.1.A1\_at \| \| Bt.18002.1.A1\_at \| 444866 \| 2/2 \| XM\_614169 \| XM\_614169 \| GO:0003676  GO:0005622  GO:0005634  GO:0006355  GO:0008270  GO:0046872 \| 15302581 \| ZNF470 \| Bt.18002.1.A1\_at \| \| Bt.18340.1.A1\_at \| 504650 \| 1/1 \| XM\_868988  XM\_882164 \|  \|  \|  \| CEPT1 \| Bt.18340.1.A1\_at \| \| Bt.5129.2.A1\_at \|  \|  \|  \|  \|  \|  \| NNAT \| Bt.5129.2.A1\_at \| \| Bt.11711.1.A1\_at \| 532409 \| 1/1 \| XR\_027998 \| XR\_027998 \|  \|  \| HMGB3 \| Bt.11711.1.A1\_at \| \| Bt.13581.1.S1\_at \|  \|  \|  \| XM\_001251636 \|  \|  \| SBBI54 \| Bt.13581.1.S1\_at \| \| Bt.25044.1.S1\_at \| 535090 \| 1/2 \| XR\_027688 \| XR\_027688 \|  \|  \| KPNA4 \| Bt.25044.1.S1\_at \| \| Bt.1151.1.S1\_at \| 493998 \| 1/1 \| NM\_001008670 \|  \| GO:0001972  GO:0006810  GO:0008289  GO:0016918  GO:0035115  GO:0042573 \|  \| CRABP2 \| Bt.1151.1.S1\_at \| \| Bt.12688.2.A1\_at \|  \|  \|  \|  \|  \|  \|  \| Bt.12688.2.A1\_at \| \| Bt.20815.1.A1\_at \|  \|  \|  \|  \|  \|  \| TMEFF1 \| Bt.20815.1.A1\_at \| \| Bt.20230.1.S1\_at \| 445543 \| 1/1 \| NM\_001004024 \| NM\_001004024 \| GO:0005198  GO:0005624  GO:0005737  GO:0005829  GO:0005856  GO:0005913  GO:0007155  GO:0008092  GO:0030057 \|  \| JUP \| Bt.20230.1.S1\_at \| \| Bt.5170.1.S1\_at \| 504764 \| 1/1 \| XM\_580934 \| XM\_580934 \|  \|  \| GRHPR \| Bt.5170.1.S1\_at \| \| Bt.27413.1.A1\_at \| 538292 \| 2/3 \| XM\_618492 \| XM\_618492 \| GO:0004674  GO:0005089  GO:0005524  GO:0005622  GO:0006468  GO:0035023 \|  \| TRIO \| Bt.27413.1.A1\_at \| \| Bt.16836.2.A1\_at \| 537282 \| 2/3 \| XM\_617440 \| XM\_617440 \|  \|  \| MYO1B \| Bt.16836.2.A1\_at \| \| Bt.27400.1.S1\_at \| 523226 \| 1/1 \| XR\_028510 \| XR\_028510 \|  \|  \| PCDHAC1 \| Bt.27400.1.S1\_at \| \| Bt.27872.1.S1\_at \| 615173 \| 3/4 \| NM\_001034777 \|  \|  \| 11282978 \| C6orf134 \| Bt.27872.1.S1\_at \| \| Bt.10329.1.A1\_at \| 510498 \| 1/1 \| XR\_027901 \|  \|  \|  \| SBF2 \| Bt.10329.1.A1\_at \| \| Bt.876.1.S1\_at \| 507002 \| 1/2 \| XM\_583541 \| XM\_583541 \|  \|  \| OLFM1 \| Bt.876.1.S1\_at \| \| Bt.20106.1.S1\_at \| 505945 \| 1/2 \| XM\_590213 \|  \|  \|  \|  \| Bt.20106.1.S1\_at \| \| Bt.18126.2.S1\_at \|  \|  \|  \| XR\_028039 \|  \|  \| PRICKLE1 \| Bt.18126.2.S1\_at \| \| Bt.25966.1.A1\_at \| 513830 \| 1/2 \| XM\_868118 \|  \| GO:0000785  GO:0003682  GO:0005634  GO:0006333 \|  \| CBX6 \| Bt.25966.1.A1\_at \| \| Bt.17061.1.A1\_at \|  \|  \|  \|  \|  \|  \|  \| Bt.17061.1.A1\_at \| \| Bt.22405.1.A1\_at \| 538695 \| 1/1 \| XM\_582079 \| XM\_582079 \|  \|  \| KLHL11 \| Bt.22405.1.A1\_at \| \| Bt.23230.1.S1\_at \|  \|  \|  \|  \|  \|  \| KIAA1553 \| Bt.23230.1.S1\_at \| \| Bt.23486.3.S1\_at \| 521389 \| 2/3 \| XM\_599652 \| XM\_599652 \|  \|  \| MYO9A \| Bt.23486.3.S1\_at \| \| Bt.11408.1.A1\_at \|  \|  \|  \|  \|  \|  \| ODZ3 \| Bt.11408.1.A1\_at \| \| Bt.18727.1.A1\_at \| 505006 \| 1/2 \| XM\_581225 \| XM\_581225 \|  \|  \| C21orf6 \| Bt.18727.1.A1\_at \| \| Bt.24269.1.S1\_at \|  \|  \|  \|  \|  \|  \| KIAA1394 \| Bt.24269.1.S1\_at \| \| Bt.5626.2.S1\_at \| 508999 \| 1/4 \| XM\_877555 \|  \|  \|  \| KNS2 \| Bt.5626.2.S1\_at \| \| Bt.26993.1.S1\_at \| 514446 \| 3/4 \| XM\_592304 \|  \| GO:0003779  GO:0005515 \|  \| FLII \| Bt.26993.1.S1\_at \| \| Bt.19281.2.S1\_at \| 515490 \| 1/2 \| XM\_593520 \|  \| GO:0005622  GO:0007165 \|  \| CDGAP \| Bt.19281.2.S1\_at \| \| Bt.29679.1.S1\_s\_at \|  \|  \|  \|  \|  \|  \| POFUT2 \| Bt.29679.1.S1\_s\_at \| \| Bt.18316.1.A1\_at \| 513587 \| 2/2 \| XM\_591287  XM\_876203 \| XM\_876203 \|  \|  \|  \| Bt.18316.1.A1\_at \| \| Bt.7612.1.S1\_at \| 509259 \| 1/2 \| NM\_001038064 \| NM\_001038064 \|  \|  \| GTF2F2 \| Bt.7612.1.S1\_at \| \| Bt.10810.1.A1\_at \|  \|  \|  \|  \|  \|  \| NRK \| Bt.10810.1.A1\_at \| \| Bt.12745.3.S1\_at \| 510080 \| 4/5 \| NM\_001076826 \|  \|  \|  \| ANTXR2 \| Bt.12745.3.S1\_at \| \| Bt.177.1.S1\_at \| 282032 \| 1/1 \| NM\_174449 \| NM\_174449 \| GO:0000910  GO:0001570  GO:0001726  GO:0001953  GO:0005099  GO:0005102  GO:0005102  GO:0005622  GO:0005737  GO:0005886  GO:0007155  GO:0007162  GO:0007242  GO:0008360  GO:0009790  GO:0019870  GO:0030036  GO:0030833  GO:0043524  GO:0045768  GO:0051020  GO:0051020  GO:0051056  GO:0051252 \| 2842690 \| RASA1 \| Bt.177.1.S1\_at \| \| Bt.23246.1.S1\_at \| 414735 \| 1/1 \| NM\_001001601 \| NM\_001001601 \| GO:0005509  GO:0005515  GO:0007155  GO:0007156  GO:0016020  GO:0016021 \| 12876221  14672921  15075376  15579907  16446427  17060906 \| CDH5 \| Bt.23246.1.S1\_at \| \| Bt.29900.1.S1\_at \|  \|  \|  \| XR\_028470 \|  \|  \| HS2ST1 \| Bt.29900.1.S1\_at \| \| Bt.27401.1.A1\_at \| 535869 \| 1/2 \| NM\_001076071 \|  \| GO:0004842  GO:0005515  GO:0005770  GO:0005783  GO:0005794  GO:0006512  GO:0008270  GO:0016020  GO:0016021  GO:0016874  GO:0042036  GO:0046872 \|  \|  \| Bt.27401.1.A1\_at \| \| Bt.4964.1.S1\_at \| 509111 \| 1/2 \| XM\_865493 \| XM\_865493 \|  \|  \| TP53BP1 \| Bt.4964.1.S1\_at \| \| Bt.4726.2.S1\_at \| 535307 \| 1/2 \| XM\_864937 \|  \|  \|  \| NEO1 \| Bt.4726.2.S1\_at \| \| Bt.6028.1.S1\_at \| 514160 \| 1/3 \| XM\_866978 \| XM\_866978 \|  \|  \|  \| Bt.6028.1.S1\_at \| \| Bt.9660.1.A1\_s\_at \|  \|  \|  \| XR\_027729 \|  \|  \| TRIM8 \| Bt.9660.1.A1\_s\_at \| \| Bt.22094.1.A1\_at \| 507054 \| 1/2 \| NM\_001075297 \|  \|  \|  \| C9orf26 \| Bt.22094.1.A1\_at \| \| Bt.26572.1.S1\_at \|  \|  \|  \|  \|  \|  \| LEPREL1 \| Bt.26572.1.S1\_at \| \| Bt.26750.1.S1\_at \| 513822 \| 2/2 \| NM\_001014922 \|  \|  \| 11282978 \|  \| Bt.26750.1.S1\_at \| \| Bt.9306.2.S1\_a\_at \| 512531 \| 2/2 \| NM\_001038091 \| NM\_001038091 \| GO:0000049  GO:0000166  GO:0004825  GO:0005524  GO:0006431  GO:0016874 \|  \| MARS \| Bt.9306.2.S1\_a\_at \| \| Bt.18702.1.A1\_at \| 523683 \| 3/5 \| XM\_601985 \| XM\_601985 \|  \|  \| CUTL1 \| Bt.18702.1.A1\_at \| \| Bt.4008.1.S1\_at \| 282389 \| 1/2 \| NM\_174626 \| NM\_174626 \| GO:0000305  GO:0004791  GO:0005515  GO:0005737  GO:0005739  GO:0006118  GO:0007507  GO:0008430  GO:0016491  GO:0016654  GO:0030097  GO:0045454  GO:0050660  GO:0050661 \| 10447675 \| TXNRD2 \| Bt.4008.1.S1\_at \| \| Bt.18048.2.S1\_at \| 540312 \| 2/3 \| XM\_865657 \| XM\_865657 \|  \|  \| DDX26B \| Bt.18048.2.S1\_at \| \| Bt.20862.1.A1\_at \| 517539 \| 1/3 \| NM\_001075755 \|  \| GO:0009396  GO:0016787 \|  \| MTHFD2 \| Bt.20862.1.A1\_at \| \| Bt.11770.1.S1\_at \|  \|  \|  \| NM\_001077936 \|  \|  \|  \| Bt.11770.1.S1\_at \| \| Bt.22230.1.S1\_at \| 517451 \| 1/1 \| XM\_001251507  XM\_595621 \|  \| GO:0008270 \|  \| ZDHHC14 \| Bt.22230.1.S1\_at \| \| Bt.21277.1.A1\_at \| 512656 \| 1/1 \| XR\_027363 \|  \|  \|  \| VPS13B \| Bt.21277.1.A1\_at \| \| Bt.27627.1.A1\_at \| 527488 \| 1/1 \| NM\_001075898 \| NM\_001075898 \| GO:0005515 \|  \| SNTA1 \| Bt.27627.1.A1\_at \| \| Bt.16259.2.S1\_at \|  \|  \|  \|  \|  \|  \| SLK \| Bt.16259.2.S1\_at \| \| Bt.10092.1.S1\_at \|  \|  \|  \|  \|  \|  \| TNFRSF19 \| Bt.10092.1.S1\_at \| \| Bt.26364.1.A1\_at \| 539008 \| 1/1 \| XM\_584193 \| XM\_001256703 \|  \|  \| KIAA1107 \| Bt.26364.1.A1\_at \| \| Bt.3372.2.S1\_a\_at \| 511614 \| 1/2 \| XM\_866018 \| XM\_866018 \|  \|  \| AGPAT6 \| Bt.3372.2.S1\_a\_at \| \| Bt.17005.1.S1\_at \| 519845 \| 1/2 \| XM\_598075 \| XM\_598075 \| GO:0000785  GO:0003677  GO:0005634  GO:0006355 \|  \| C20orf100 \| Bt.17005.1.S1\_at \| \| Bt.4719.1.S1\_at \| 281749 \| 1/2 \| NM\_174308 \|  \| GO:0001569  GO:0001584  GO:0001666  GO:0001701  GO:0004872  GO:0004962  GO:0005515  GO:0007165  GO:0007186  GO:0007507  GO:0007585  GO:0014032  GO:0015758  GO:0016020  GO:0016021  GO:0030818  GO:0042310 \| 2175396  12788101 \| EDNRA \| Bt.4719.1.S1\_at \| \| Bt.10317.1.A1\_at \| 513301 \| 1/4 \| XM\_590963 \|  \|  \|  \| C14orf118 \| Bt.10317.1.A1\_at \| \| Bt.20372.1.S1\_at \| 510953 \| 1/1 \| NM\_001075479 \| NM\_001075479 \| GO:0004064 \|  \| PON3 \| Bt.20372.1.S1\_at \| \| Bt.20629.3.A1\_at \| 617337 \| 1/1 \|  \|  \|  \|  \|  \| Bt.20629.3.A1\_at \| \| Bt.7120.1.S1\_a\_at \| 534249 \| 1/1 \| NM\_001035406 \| NM\_001035406 \| GO:0005634  GO:0006281  GO:0006310  GO:0008270  GO:0046872 \| 12477932 \| NSMCE1 \| Bt.7120.1.S1\_a\_at \| \| Bt.8109.2.A1\_at \| 514742 \| 1/2 \| NM\_001014926  XM\_001249732 \|  \|  \| 11282978  16305752 \| SRPX2 \| Bt.8109.2.A1\_at \| \| Bt.10930.1.S1\_at \|  \|  \|  \|  \|  \|  \| C16orf28 \| Bt.10930.1.S1\_at \| \| Bt.26402.1.A1\_at \|  \|  \|  \|  \|  \|  \| PAWR \| Bt.26402.1.A1\_at \| \| Bt.8909.1.S1\_at \| 525869 \| 1/1 \| XM\_604226 \|  \|  \|  \| RAI14 \| Bt.8909.1.S1\_at \| \| Bt.984.1.S1\_at \|  \|  \|  \|  \|  \|  \|  \| Bt.984.1.S1\_at \| \| Bt.3394.1.S1\_at \| 615173 \| 3/4 \| NM\_001034777 \|  \|  \| 11282978 \| C6orf134 \| Bt.3394.1.S1\_at \| \| Bt.28455.1.S1\_at \| 536307 \| 1/1 \| XM\_616433 \|  \| GO:0003676  GO:0005622  GO:0006355 \|  \|  \| Bt.28455.1.S1\_at \| \| Bt.10044.1.A1\_at \| 511062 \| 1/3 \| NM\_001046073 \|  \| GO:0005097  GO:0005622  GO:0032313 \|  \|  \| Bt.10044.1.A1\_at \| \| Bt.15561.2.A1\_s\_at \| 505991 \| 1/2 \| XM\_582369 \|  \|  \|  \|  \| Bt.15561.2.A1\_s\_at \| \| Bt.26710.1.S1\_at \|  \|  \|  \| XM\_605041 \|  \|  \|  \| Bt.26710.1.S1\_at \| \| Bt.4800.1.S1\_at \|  \|  \|  \|  \|  \|  \| FLJ13710 \| Bt.4800.1.S1\_at \| \| Bt.19199.1.A1\_at \| 613449 \| 2/11 \| XM\_864237 \|  \| GO:0004674  GO:0005524  GO:0006468 \|  \| DCAMKL1 \| Bt.19199.1.A1\_at \| \| Bt.16510.1.A1\_at \| 522707 \| 1/1 \| XM\_863890 \|  \|  \|  \| RAP2A \| Bt.16510.1.A1\_at \| \| Bt.11040.1.S1\_at \|  \|  \|  \| XM\_001250911 \|  \|  \| NCAM1 \| Bt.11040.1.S1\_at \| \| Bt.29625.1.S1\_at \|  \|  \|  \|  \|  \|  \| EIF5A2 \| Bt.29625.1.S1\_at \| \| Bt.26447.1.A1\_at \|  \|  \|  \|  \|  \|  \| KIAA1005 \| Bt.26447.1.A1\_at \| \| Bt.9070.1.S1\_at \| 618954 \| 1/1 \| NM\_001076525 \| NM\_001076525 \|  \|  \| C7orf20 \| Bt.9070.1.S1\_at \| \| Bt.26423.1.A1\_at \|  \|  \|  \|  \|  \|  \| ZC3H6 \| Bt.26423.1.A1\_at \| \| Bt.9321.1.S1\_at \| 615359 \| 1/2 \| XM\_867149 \| XM\_867149 \| GO:0003950  GO:0005622  GO:0005634  GO:0006471  GO:0015986  GO:0016469  GO:0046933  GO:0046961 \|  \|  \| Bt.9321.1.S1\_at \| \| Bt.7168.2.A1\_at \|  \|  \|  \| XM\_602219 \|  \|  \|  \| Bt.7168.2.A1\_at \| \| Bt.13994.1.S1\_at \|  \|  \|  \|  \|  \|  \| TCP11L1 \| Bt.13994.1.S1\_at \| \| Bt.13965.1.S1\_at \|  \|  \|  \|  \|  \|  \| CNTNAP1 \| Bt.13965.1.S1\_at \| \| Bt.17447.1.A1\_at \| 537850 \| 1/1 \|  \| XM\_614661 \|  \|  \| C6orf60 \| Bt.17447.1.A1\_at \| \| Bt.8275.1.S1\_at \| 616247 \| 1/2 \| XM\_868222 \|  \|  \|  \|  \| Bt.8275.1.S1\_at \| \| Bt.3323.1.S1\_at \| 508175 \| 1/2 \| XM\_584919 \| XM\_584919 \|  \|  \| PLXNB2 \| Bt.3323.1.S1\_at \| \| Bt.22002.1.S1\_at \|  \|  \|  \|  \|  \|  \| FABP4 \| Bt.22002.1.S1\_at \| \| Bt.26871.1.S1\_at \| 539431 \| 1/2 \| NM\_001076161 \|  \| GO:0005634 \|  \| MEN1 \| Bt.26871.1.S1\_at \| \| Bt.1000.1.S1\_at \| 504558 \| 1/3 \| XM\_580700 \| XM\_580700 \|  \|  \|  \| Bt.1000.1.S1\_at \| \| Bt.23094.3.S1\_x\_at \| 282138 \| 2/8 \| NM\_181027 \| XM\_001249710 \| GO:0001516  GO:0006633  GO:0016491  GO:0047017 \| 1339268  2829166  9867836  12551929 \|  \| Bt.23094.3.S1\_x\_at \| \| Bt.25997.1.S1\_at \| 614297 \| 1/1 \|  \|  \|  \|  \| CABLES1 \| Bt.25997.1.S1\_at \| \| Bt.923.2.S1\_at \| 507116 \| 2/2 \| XM\_583674 \| NM\_001080234 \|  \|  \| ZCCHC3 \| Bt.923.2.S1\_at \| \| Bt.26750.2.A1\_at \| 513822 \| 2/2 \| NM\_001014922 \| NM\_001014922 \|  \| 11282978 \|  \| Bt.26750.2.A1\_at \| \| Bt.1768.1.A1\_at \| 537706 \| 1/1 \| NM\_001035417 \|  \| GO:0000166  GO:0003676 \| 12477932 \| THOC4 \| Bt.1768.1.A1\_at \| \| Bt.12105.1.A1\_at \| 539567 \| 1/1 \| XR\_028176 \|  \|  \|  \| PPM1J \| Bt.12105.1.A1\_at \| \| Bt.2556.1.S1\_at \| 512259 \| 2/2 \| NM\_001034419 \| NM\_001034419 \| GO:0004957  GO:0005829  GO:0006693  GO:0007179  GO:0007565  GO:0007567  GO:0008152  GO:0016404  GO:0016491  GO:0019372  GO:0042803  GO:0045786  GO:0051287 \| 12477932 \| HPGD \| Bt.2556.1.S1\_at \| \| Bt.6570.2.S1\_at \| 525110 \| 1/2 \| XM\_603456 \|  \|  \|  \| FLJ21742 \| Bt.6570.2.S1\_at \| \| Bt.11476.1.S1\_at \| 516209 \| 1/1 \| NM\_001046222 \| NM\_001046222 \| GO:0007275  GO:0016020  GO:0016021  GO:0016757 \|  \| LFNG \| Bt.11476.1.S1\_at \| \| Bt.3139.1.A1\_at \|  \|  \|  \|  \|  \|  \| SLITRK6 \| Bt.3139.1.A1\_at \| \| Bt.5674.1.S1\_at \| 281769 \| 1/7 \| NM\_174318 \|  \| GO:0004713  GO:0004872  GO:0005524  GO:0006468 \| 12354143 \| LETM1 \| Bt.5674.1.S1\_at \| \| Bt.1793.3.S1\_at \| 512666 \| 2/2 \| XM\_590221 \| XM\_590221 \|  \|  \| AVEN \| Bt.1793.3.S1\_at \| \| Bt.28431.1.S1\_at \|  \|  \|  \|  \|  \|  \|  \| Bt.28431.1.S1\_at \| \| Bt.467.1.S1\_at \| 281296 \| 1/3 \| NM\_174106 \| NM\_174106 \| GO:0005515  GO:0005856  GO:0005874  GO:0007026 \| 2498649  2498650  8910513  11264299  16519507  16781671 \| MAPT \| Bt.467.1.S1\_at \| \| Bt.27386.2.A1\_at \| 527956 \| 2/2 \| XM\_606363 \| XM\_606363 \|  \|  \| KIAA1223 \| Bt.27386.2.A1\_at \| \| Bt.8617.1.S1\_at \| 539715 \| 1/1 \| NM\_001076183 \| NM\_001076183 \| GO:0005515 \|  \| C2orf32 \| Bt.8617.1.S1\_at \| \| Bt.20601.2.S1\_at \| 407101 \| 2/4 \| XM\_873993 \| XM\_873993 \| GO:0005515 \|  \| TJP2 \| Bt.20601.2.S1\_at \| \| Bt.25099.2.S1\_at \| 533630 \| 2/2 \| NM\_001046355 \| NM\_001046355 \| GO:0000287  GO:0004647  GO:0006564  GO:0008152  GO:0008652  GO:0016787 \|  \|  \| Bt.25099.2.S1\_at \| \| Bt.27781.1.S1\_at \| 515330 \| 1/1 \| XM\_593333 \|  \|  \|  \| MYO9A \| Bt.27781.1.S1\_at \| \| Bt.11599.2.S1\_at \|  \|  \|  \|  \|  \|  \| SEMA6A \| Bt.11599.2.S1\_at \| \| Bt.11016.1.S1\_at \|  \|  \|  \|  \|  \|  \| KIAA1754L \| Bt.11016.1.S1\_at \| \| Bt.13408.2.S1\_at \| 532923 \| 1/7 \| XR\_027990 \| XR\_027990 \|  \|  \| GOLGB1 \| Bt.13408.2.S1\_at \| \| Bt.6926.1.A1\_a\_at \| 281945 \| 2/3 \| NM\_174402 \|  \| GO:0003700  GO:0003707  GO:0005634  GO:0006350  GO:0006355  GO:0008270  GO:0043565  GO:0046872 \| 10343125  15171724 \|  \| Bt.6926.1.A1\_a\_at \| \| Bt.11325.1.S1\_at \| 533757 \| 1/2 \| XM\_613264 \| XM\_613264 \| GO:0003676  GO:0003700  GO:0005622  GO:0005634  GO:0006355  GO:0008270  GO:0043565 \|  \| ADNP \| Bt.11325.1.S1\_at \| \| Bt.11326.3.S1\_at \| 614292 \| 2/3 \| XM\_001250807  XM\_878516 \| XM\_878516 \|  \|  \| C6orf166 \| Bt.11326.3.S1\_at \| \| Bt.26513.1.S1\_at \| 515133 \| 1/1 \| NM\_001038113 \| NM\_001038113 \|  \|  \| ECRG4 \| Bt.26513.1.S1\_at \| \| Bt.4764.1.S1\_at \|  \|  \|  \| XM\_001256191 \|  \|  \| HDLBP \| Bt.4764.1.S1\_at \| \| Bt.211.1.S1\_at \| 286770 \| 1/1 \| NM\_174756 \|  \| GO:0006457  GO:0006986  GO:0031072 \| 7511204 \| DNAJC3 \| Bt.211.1.S1\_at \| \| Bt.9306.3.S1\_at \| 512531 \| 2/2 \| NM\_001038091 \| NM\_001038091 \| GO:0000049  GO:0000166  GO:0004825  GO:0005524  GO:0006431  GO:0016874 \|  \| MARS \| Bt.9306.3.S1\_at \| \| Bt.17460.1.A1\_at \| 540117 \| 1/1 \| XM\_591589 \|  \| GO:0005515 \|  \| SLITRK2 \| Bt.17460.1.A1\_at \| \| Bt.22564.3.S1\_at \|  \|  \|  \|  \|  \|  \|  \| Bt.22564.3.S1\_at \| \| Bt.21641.1.S1\_at \|  \|  \|  \| NM\_001078067 \|  \|  \| ENC1 \| Bt.21641.1.S1\_at \| \| Bt.19811.1.A1\_at \|  \|  \|  \|  \|  \|  \| TTLL7 \| Bt.19811.1.A1\_at \| \| Bt.22502.1.S1\_at \|  \|  \|  \| XM\_001252275 \|  \|  \| ROBO4 \| Bt.22502.1.S1\_at \| \| Bt.13972.1.A1\_at \| 615967 \| 1/1 \| NM\_001046567 \|  \| GO:0005515  GO:0006350  GO:0006355  GO:0008270  GO:0046872 \|  \| PCGF3 \| Bt.13972.1.A1\_at \| \| Bt.1681.1.S1\_at \| 510109 \| 1/1 \| NM\_001034372 \|  \|  \| 12477932 \| ABHD11 \| Bt.1681.1.S1\_at \| \| Bt.12030.1.S1\_at \| 522269 \| 1/4 \| XM\_001252980  XM\_001253010  XM\_001253060  XM\_001253079  XM\_872287  XM\_872491 \|  \|  \|  \|  \| Bt.12030.1.S1\_at \| \| Bt.28821.1.S1\_at \| 514738 \| 1/1 \| XM\_592634 \| XM\_592634 \|  \|  \| OSGEPL1 \| Bt.28821.1.S1\_at \| \| Bt.27565.1.S1\_at \| 509657 \| 1/1 \| XM\_586668 \| XM\_586668 \|  \|  \| PTPRK \| Bt.27565.1.S1\_at \| \| Bt.8392.1.S1\_at \|  \|  \|  \|  \|  \|  \|  \| Bt.8392.1.S1\_at \| \| Bt.9944.1.S1\_at \|  \|  \|  \|  \|  \|  \| DPYSL5 \| Bt.9944.1.S1\_at \| \| Bt.1807.1.A1\_at \|  \|  \|  \|  \|  \|  \| PAK3 \| Bt.1807.1.A1\_at \| \| Bt.15915.1.S1\_at \|  \|  \|  \|  \|  \|  \| SLRN \| Bt.15915.1.S1\_at \| \| Bt.24470.1.S1\_a\_at \| 617078 \| 1/3 \| XM\_869261 \|  \|  \|  \|  \| Bt.24470.1.S1\_a\_at \| \| Bt.25921.1.A1\_at \|  \|  \|  \|  \|  \|  \| PCDH19 \| Bt.25921.1.A1\_at \| \| Bt.13287.1.S1\_at \| 515186 \| 1/2 \| NM\_001015605 \|  \| GO:0008483  GO:0016740  GO:0030170 \| 11282978 \| AGXT2L1 \| Bt.13287.1.S1\_at \| \| Bt.1705.1.S1\_at \|  \|  \|  \|  \|  \|  \| MGC16121 \| Bt.1705.1.S1\_at \| \| Bt.20020.1.S1\_at \| 514807 \| 1/2 \| XM\_592712 \| NM\_001081523 \|  \|  \| HABP4 \| Bt.20020.1.S1\_at \| \| Bt.4652.1.S1\_at \| 506657 \| 1/1 \| NM\_001075276 \|  \|  \| 11551941 \|  \| Bt.4652.1.S1\_at \| \| Bt.18414.1.S1\_at \| 505987 \| 1/1 \| NM\_001075236 \| NM\_001075236 \|  \|  \| GK \| Bt.18414.1.S1\_at \| \| Bt.22349.1.A1\_at \|  \|  \|  \|  \|  \|  \| GNG2 \| Bt.22349.1.A1\_at \| \| Bt.3726.1.A1\_at \|  \|  \|  \| XM\_870342 \|  \|  \| DDEF2 \| Bt.3726.1.A1\_at \| \| Bt.2123.1.S1\_at \| 510084 \| 1/5 \| XM\_587180 \|  \|  \|  \| ANKRD10 \| Bt.2123.1.S1\_at \| \| Bt.26502.1.A1\_at \| 507983 \| 1/2 \| XM\_584693 \| XM\_584693 \|  \|  \| TANC \| Bt.26502.1.A1\_at \| \| Bt.13784.1.S1\_at \| 512818 \| 1/1 \| NM\_001046132 \| NM\_001046132 \| GO:0005739  GO:0006457  GO:0008270  GO:0015031  GO:0016020  GO:0042719  GO:0045039  GO:0046872 \|  \|  \| Bt.13784.1.S1\_at \| \| Bt.24359.1.S1\_at \| 506969 \| 1/3 \| XR\_028441 \| XR\_028441 \|  \|  \| ALS2CL \| Bt.24359.1.S1\_at \| \| Bt.9778.1.S1\_at \|  \|  \|  \|  \|  \|  \| ABLIM1 \| Bt.9778.1.S1\_at \| \| Bt.26674.1.S1\_at \| 517405 \| 1/1 \| XM\_595574 \| XM\_595574 \| GO:0005509 \|  \| JAG2 \| Bt.26674.1.S1\_at \| \| Bt.18043.1.S1\_at \|  \|  \|  \|  \|  \|  \|  \| Bt.18043.1.S1\_at \| \| Bt.11126.1.S1\_at \| 510960 \| 2/2 \| NM\_001046069 \| NM\_001046069 \| GO:0004607  GO:0006629  GO:0008415  GO:0016740 \|  \| LCAT \| Bt.11126.1.S1\_at \| \| Bt.16659.1.A1\_at \|  \|  \|  \|  \|  \|  \|  \| Bt.16659.1.A1\_at \| \| Bt.9612.1.S1\_at \|  \|  \|  \|  \|  \|  \|  \| Bt.9612.1.S1\_at \| \| Bt.2850.1.A1\_at \|  \|  \|  \|  \|  \|  \| ZNF397 \| Bt.2850.1.A1\_at \| \| Bt.9533.1.S1\_at \|  \|  \|  \|  \|  \|  \| JMJD3 \| Bt.9533.1.S1\_at \| \| Bt.15796.1.S1\_at \| 508226 \| 1/1 \| XR\_028604 \|  \|  \|  \| CDC42BPB \| Bt.15796.1.S1\_at \| \| Bt.3391.1.S1\_at \|  \|  \|  \|  \|  \|  \| MARVELD1 \| Bt.3391.1.S1\_at \| \| Bt.6087.1.S1\_at \| 533038 \| 2/2 \| NM\_001075980 \|  \| GO:0016021 \| 12140684  16305752 \| TM4SF1 \| Bt.6087.1.S1\_at \| \| Bt.2304.1.A1\_at \|  \|  \|  \|  \|  \|  \|  \| Bt.2304.1.A1\_at \| \| Bt.20817.1.A1\_at \|  \|  \|  \|  \|  \|  \| POLA \| Bt.20817.1.A1\_at \| \| Bt.17701.1.A1\_at \| 767896 \| 1/1 \| BT025426 \|  \|  \| 11282978  16305752 \| ZFP2 \| Bt.17701.1.A1\_at \| \| Bt.23094.2.A1\_x\_at \| 282138 \| 2/8 \| NM\_181027 \| XM\_001250814 \| GO:0001516  GO:0006633  GO:0016491  GO:0047017 \| 1339268  2829166  9867836  12551929 \|  \| Bt.23094.2.A1\_x\_at \| \| Bt.23355.1.S1\_at \| 615191 \| 2/2 \| XM\_866930 \| XM\_866930 \| GO:0005515  GO:0005524  GO:0016311  GO:0016791  GO:0044267 \|  \| PTPLA \| Bt.23355.1.S1\_at \| |
