# Supplementary material for: A gene network switch enhances the oxidative capacity of ovine skeletal muscle during late fetal development
Source: BMC Genomics. 2010 Jun 15;11:378. doi: 10.1186/1471-2164-11-378 (PMC2894804; doi:10.1186/1471-2164-11-378)
Supplement: Additional File 1 — Gene expression data. This file contains links to the gene expression data. The genes that are significantly differentially expressed between adjacent development times are listed as Experiment 1 (80 d vs 100 d), Experiment 2 (100 d vs 120 d), Experiment 3 (120 d vs 150 d) and Experiment 4 (150 d vs 230 d) in the section entitled Open microarray analysis report for developmental expression. Each experiment contains an MA plot, Volcano plot and Heatmap for each of the three microarray processing programs MAS5, RMA and GCRMA. For each of these processing programs there is a list of significantly differentially expressed probe sets, and for each of these there is an FDR corrected probability, fold change, percentage of microarrays with a MAS5 Present call, and a convergence percentage for all three microarray processing programs. The file also contains annotation of the probe sets. In a second section entitled Time series analysis of developmental expression, there is a list of genes in each gene expression cluster and diagrams showing MAS5 gene expression values for these genes. [file 1471-2164-11-378-S1.ZIP › Report/AnnotationExp4.html]

## Annotation of differentially expressed genes for experiment 4.

The links connect to external resources. The first column is the probe ID and links to the Affymetrix annotation site. The second column links to Entrez. The third column shows how many times the Entrez gene ID was detected as significantly differentially expressed and how many probes target the gene on the chip - this provides an additional indication of reliability of differential expression. The fourth column is the RefSeq annotation (there can be more than one link). The fifth column is the RefSeq annotation provided by SheepGenomics (it should be more reliable than the original Affymetrix annotation). The sixth column is the gene ontology annotation. The seventh column links to Pubmed. The eighth column links to the Gene Symbol in Entrez. The last column links to SheepGenomics. Note: the GO and Pubmed annotation were derived from the EntrezGene ID, the gene symbols are based on the human annotation.

|  |  |  |  |  |  |  |  |  |  |  |  |  |  |  |  |  |  |  |  |  |  |  |  |  |  |  |  |  |  |  |  |  |  |  |  |  |  |  |  |  |  |  |  |  |  |  |  |  |  |  |  |  |  |  |  |  |  |  |  |  |  |  |  |  |  |  |  |  |  |  |  |  |  |  |  |  |  |  |  |  |  |  |  |  |  |  |  |  |  |  |  |  |  |  |  |  |  |  |  |  |  |  |  |  |  |  |  |  |  |  |  |  |  |  |  |  |  |  |  |  |  |  |  |  |  |  |  |  |  |  |  |  |  |  |  |  |  |  |  |  |  |  |  |  |  |  |  |  |  |  |  |  |  |  |  |  |  |  |  |  |  |  |  |  |  |  |  |  |  |  |  |  |  |  |  |  |  |  |  |  |  |  |  |  |  |  |  |  |  |  |  |  |  |  |  |  |  |  |  |  |  |  |  |  |  |  |  |  |  |  |  |  |  |  |  |  |  |  |  |  |  |  |  |  |  |  |  |  |  |  |  |  |  |  |  |  |  |  |  |  |  |  |  |  |  |  |  |  |  |  |  |  |  |  |  |  |  |  |  |  |  |  |  |  |  |  |  |  |  |  |  |  |  |  |  |  |  |  |  |  |  |  |  |  |  |  |  |  |  |  |  |  |  |  |  |  |  |  |  |  |  |  |  |  |  |  |  |  |  |  |  |  |  |  |  |  |  |  |  |  |  |  |  |  |  |  |  |  |  |  |  |  |  |  |  |  |  |  |  |  |  |  |  |  |  |  |  |  |  |  |  |  |  |  |  |  |  |  |  |  |  |  |  |  |  |  |  |  |  |  |  |  |  |  |  |  |  |  |  |  |  |  |  |  |  |  |  |  |  |  |  |  |  |  |  |  |  |  |  |  |  |  |  |  |  |  |  |  |  |  |  |  |  |  |  |  |  |  |  |  |  |  |  |  |  |  |  |  |  |  |  |  |  |  |  |  |  |  |  |  |  |  |  |  |  |  |  |  |  |  |  |  |  |  |  |  |  |  |  |  |  |  |  |  |  |  |  |  |  |  |  |  |  |  |  |  |  |  |  |  |  |  |  |  |  |  |  |  |  |  |  |  |  |  |  |  |  |  |  |  |  |  |  |  |  |  |  |  |  |  |  |  |  |  |  |  |  |  |  |  |  |  |  |  |  |  |  |  |  |  |  |  |  |  |  |  |  |  |  |  |  |  |  |  |  |  |  |  |  |  |  |  |  |  |  |  |  |  |  |  |  |  |  |  |  |  |  |  |  |  |  |  |  |  |  |  |  |  |  |  |  |  |  |  |  |  |  |  |  |  |  |  |  |  |  |  |  |  |  |  |  |  |  |  |  |  |  |  |  |  |  |  |  |  |  |  |  |  |  |  |  |  |  |  |  |  |  |  |  |  |  |  |  |  |  |  |  |  |  |  |  |  |  |  |  |  |  |  |  |  |  |  |  |  |  |  |  |  |  |  |  |  |  |  |  |  |  |  |  |  |  |  |  |  |  |  |  |  |  |  |  |  |  |  |  |  |  |  |  |  |  |  |  |  |  |  |  |  |  |  |  |  |  |  |  |  |  |  |  |  |  |  |  |  |  |  |  |  |  |  |  |  |  |  |  |  |  |  |  |  |  |  |  |  |  |  |  |  |  |  |  |  |  |  |  |  |  |  |  |  |  |  |  |  |  |  |  |  |  |  |  |  |  |  |  |  |  |  |  |  |  |  |  |  |  |  |  |  |  |  |  |  |  |  |  |  |  |  |  |  |  |  |  |  |  |  |  |  |  |  |  |  |  |  |  |  |  |  |  |  |  |  |  |  |  |  |  |  |  |  |  |  |  |  |  |  |  |  |  |  |  |  |  |  |  |  |  |  |  |  |  |  |  |  |  |  |  |  |  |  |  |  |  |  |  |  |  |  |  |  |  |  |  |  |  |  |  |  |  |  |  |  |  |  |  |  |  |  |  |  |  |  |  |  |  |  |  |  |  |  |  |  |  |  |  |  |  |  |  |  |  |  |  |  |  |  |  |  |  |  |  |  |  |  |  |  |  |  |  |  |  |  |  |  |  |  |  |  |  |  |  |  |  |  |  |  |  |  |  |  |  |  |  |  |  |  |  |  |  |  |  |  |  |  |  |  |  |  |  |  |  |  |  |  |  |  |  |  |  |  |  |  |  |  |  |  |  |  |  |  |  |  |  |  |  |  |  |  |  |  |  |  |  |  |  |  |  |  |  |  |  |  |  |  |  |  |  |  |  |  |  |  |  |  |  |  |  |  |  |  |  |  |  |  |  |  |  |  |  |  |  |  |  |  |  |  |  |  |  |  |  |  |  |  |  |  |  |  |  |  |  |  |  |  |  |  |  |  |  |  |  |  |  |  |  |  |  |  |  |  |  |  |  |  |  |  |  |  |  |  |  |  |  |  |  |  |  |  |  |  |  |  |  |  |  |  |  |  |  |  |  |  |  |  |  |  |  |  |  |  |  |  |  |  |  |  |  |  |  |  |  |  |  |  |  |  |  |  |  |  |  |  |  |  |  |  |  |  |  |  |  |  |  |  |  |  |  |  |  |  |  |  |  |  |  |  |  |  |  |  |  |  |  |  |  |  |  |  |  |  |  |  |  |  |  |  |  |  |  |  |  |  |  |  |  |  |  |  |  |  |  |  |  |  |  |  |  |  |  |  |  |  |  |  |  |  |  |  |  |  |  |  |  |  |  |  |  |  |  |  |  |  |  |  |  |  |  |  |  |  |  |  |  |  |  |  |  |  |  |  |  |  |  |  |  |  |  |  |  |  |  |  |  |  |  |  |  |  |  |  |  |  |  |  |  |  |  |  |  |  |  |  |  |  |  |  |  |  |  |  |  |  |  |  |  |  |  |  |  |  |  |  |  |  |  |  |  |  |  |  |  |  |  |  |  |  |  |  |  |  |  |  |  |  |  |  |  |  |  |  |  |  |  |  |  |  |  |  |  |  |  |  |  |  |  |  |  |  |  |  |  |  |  |  |  |  |  |  |  |  |  |  |  |  |  |  |  |  |  |  |  |  |  |  |  |  |  |  |  |  |  |  |  |  |  |  |  |  |  |  |  |  |  |  |  |  |  |  |  |  |  |  |  |  |  |  |  |  |  |  |  |  |  |  |  |  |  |  |  |  |  |  |  |  |  |  |  |  |  |  |  |  |  |  |  |  |  |  |  |  |  |  |  |  |  |  |  |  |  |  |  |  |  |  |  |  |  |  |  |  |  |  |  |  |  |  |  |  |  |  |  |  |  |  |  |  |  |  |  |  |  |  |  |  |  |  |  |  |  |  |  |  |  |  |  |  |  |  |  |  |  |  |  |  |  |  |  |  |  |  |  |  |  |  |  |  |  |  |  |  |  |  |  |  |  |  |  |  |  |  |  |  |  |  |  |  |  |  |  |  |  |  |  |  |  |  |  |  |  |  |  |  |  |  |  |  |  |  |  |  |  |  |  |  |  |  |  |  |  |  |  |  |  |  |  |  |  |  |  |  |  |  |  |  |  |  |  |  |  |  |  |  |  |  |  |  |  |  |  |  |  |  |  |  |  |  |  |  |  |  |  |  |  |  |  |  |  |  |  |  |  |  |  |  |  |  |  |  |  |  |  |  |  |  |  |  |  |  |  |  |  |  |  |  |  |  |  |  |  |  |  |  |  |  |  |  |  |  |  |  |  |  |  |  |  |  |  |  |  |  |  |  |  |  |  |  |  |  |  |  |  |  |  |  |  |  |  |  |  |  |  |  |  |  |  |  |  |  |  |  |  |  |  |  |  |  |  |  |  |  |  |  |  |  |  |  |  |  |  |  |  |  |  |  |  |  |  |  |  |  |  |  |  |  |  |  |  |  |  |  |  |  |  |  |  |  |  |  |  |  |  |  |  |  |  |  |  |  |  |  |  |  |  |  |  |  |  |  |  |  |  |  |  |  |  |  |  |  |  |  |  |  |  |  |  |  |  |  |  |  |  |  |  |  |  |  |  |  |  |  |  |  |  |  |  |  |  |  |  |  |  |  |  |  |  |  |  |  |  |  |  |  |  |  |  |  |  |  |  |  |  |  |  |  |  |  |  |  |  |  |  |  |  |  |  |  |  |  |  |  |  |  |  |  |  |  |  |  |  |  |  |  |  |  |  |  |  |  |  |  |  |  |  |  |  |  |  |  |  |  |  |  |  |  |  |  |  |  |  |  |  |  |  |  |  |  |  |  |  |  |  |  |  |  |  |  |  |  |  |  |  |  |  |  |  |  |  |  |  |  |  |  |  |  |  |  |  |  |  |  |  |  |  |  |  |  |  |  |  |  |  |  |  |  |  |  |  |  |  |  |  |  |  |  |  |  |  |  |  |  |  |  |  |  |  |  |  |  |  |  |  |  |  |  |  |  |  |  |  |  |  |  |  |  |  |  |  |  |  |  |  |  |  |  |  |  |  |  |  |  |  |  |  |  |  |  |  |  |  |  |  |  |  |  |  |  |  |  |  |  |  |  |  |  |  |  |  |  |  |  |  |  |  |  |  |  |  |  |  |  |  |  |  |  |  |  |  |  |  |  |  |  |  |  |  |  |  |  |  |  |  |  |  |  |  |  |  |  |  |  |  |  |  |  |  |  |  |  |  |  |  |  |  |  |  |  |  |  |  |  |  |  |  |  |  |  |  |  |  |  |  |  |  |  |  |  |  |  |  |  |  |  |  |  |  |  |  |  |  |  |  |  |  |  |  |  |  |  |  |  |  |  |  |  |  |  |  |  |  |  |  |  |  |  |  |  |  |  |  |  |  |  |  |  |  |  |  |  |  |  |  |  |  |  |  |  |  |  |  |  |  |  |  |  |  |  |  |  |  |  |  |  |  |  |  |  |  |  |  |  |  |  |  |  |  |  |  |  |  |  |  |  |  |  |  |  |  |  |  |  |  |  |  |  |  |  |  |  |  |  |  |  |  |  |  |  |  |  |  |  |  |  |  |  |  |  |  |  |  |  |  |  |  |  |  |  |  |  |  |  |  |  |  |  |  |  |  |  |  |  |  |  |  |  |  |  |  |  |  |  |  |  |  |  |  |  |  |  |  |  |  |  |  |  |  |  |  |  |  |  |  |  |  |  |  |  |  |  |  |  |  |  |  |  |  |  |  |  |  |  |  |  |  |  |  |  |  |  |  |  |  |  |  |  |  |  |  |  |  |  |  |  |  |  |  |  |  |  |  |  |  |  |  |  |  |  |  |  |  |  |  |  |  |  |  |  |  |  |  |  |  |  |  |  |  |  |  |  |  |  |  |  |  |  |  |  |  |  |  |  |  |  |  |  |  |  |  |  |  |  |  |  |  |  |  |  |  |  |  |  |  |  |  |  |  |  |  |  |  |  |  |  |  |  |  |  |  |  |  |  |  |  |  |  |  |  |  |  |  |  |  |  |  |  |  |  |  |  |  |  |  |  |  |  |  |  |  |  |  |  |  |  |  |  |  |  |  |  |  |  |  |  |  |  |  |  |  |  |  |  |  |  |  |  |  |  |  |  |  |  |  |  |  |  |  |  |  |  |  |  |  |  |  |  |  |  |  |  |  |  |  |  |  |  |  |  |  |  |  |  |  |  |  |  |  |  |  |  |  |  |  |  |  |  |  |  |  |  |  |  |  |  |  |  |  |  |  |  |  |  |  |  |  |  |  |  |  |  |  |  |  |  |  |  |  |  |  |  |  |  |  |  |  |  |  |  |  |  |  |  |  |  |  |  |  |  |  |  |  |  |  |  |  |  |  |  |  |  |  |  |  |  |  |  |  |  |  |  |  |  |  |  |  |  |  |  |  |  |  |  |  |  |  |  |  |  |  |  |  |  |  |  |  |  |  |  |  |  |  |  |  |  |  |  |  |  |  |  |  |  |  |  |  |  |  |  |  |  |  |  |  |  |  |  |  |  |  |  |  |  |  |  |  |  |  |  |  |  |  |  |  |  |  |  |  |  |  |  |  |  |  |  |  |  |  |  |  |  |  |  |  |  |  |  |  |  |  |  |  |  |  |  |  |  |  |  |  |  |  |  |  |  |  |  |  |  |  |  |  |  |  |  |  |  |  |  |  |  |  |  |  |  |  |  |  |  |  |  |  |  |  |  |  |  |  |  |  |  |  |  |  |  |  |  |  |  |  |  |  |  |  |  |  |  |  |  |  |  |  |  |  |  |  |  |  |  |  |  |  |  |  |  |  |  |  |  |  |  |  |  |  |  |  |  |  |  |  |  |  |  |  |  |  |  |  |  |  |  |  |  |  |  |  |  |  |  |  |  |  |  |  |  |  |  |  |  |  |  |  |  |  |  |  |  |  |  |  |  |  |  |  |  |  |  |  |  |  |  |  |  |  |  |  |  |  |  |  |  |  |  |  |  |  |  |  |  |  |  |  |  |  |  |  |  |  |  |  |  |  |  |  |  |  |  |  |  |  |  |  |  |  |  |  |  |  |  |  |  |  |  |  |  |  |  |  |  |  |  |  |  |  |  |  |  |  |  |  |  |  |  |  |  |  |  |  |  |  |  |  |  |  |  |  |  |  |  |  |  |  |  |  |  |  |  |  |  |  |  |  |  |  |  |  |  |  |  |  |  |  |  |  |  |  |  |  |  |  |  |  |  |  |  |  |  |  |  |  |  |  |  |  |  |  |  |  |  |  |  |  |  |  |  |  |  |  |  |  |  |  |  |  |  |  |  |  |  |  |  |  |  |  |  |  |  |  |  |  |  |  |  |  |  |  |  |  |  |  |  |  |  |  |  |  |  |  |  |  |  |  |  |  |  |  |  |  |  |  |  |  |  |  |  |  |  |  |  |  |  |  |  |  |  |  |  |  |  |  |  |  |  |  |  |  |  |  |  |  |  |  |  |  |  |  |  |  |  |  |  |  |  |  |  |  |  |  |  |  |  |  |  |  |  |  |  |  |  |  |  |  |  |  |  |  |  |  |  |  |  |  |  |  |  |  |  |  |  |  |  |  |  |  |  |  |  |  |  |  |  |  |  |  |  |  |  |  |  |  |  |  |  |  |  |  |  |  |  |  |  |  |  |  |  |  |  |  |  |  |  |  |  |  |  |  |  |  |  |  |  |  |  |  |  |  |  |  |  |  |  |  |  |  |  |  |  |  |  |  |  |  |  |  |  |  |  |  |  |  |  |  |  |  |  |  |  |  |  |  |  |  |  |  |  |  |  |  |  |  |  |  |  |  |  |  |  |  |  |  |  |  |  |  |  |  |  |  |  |  |  |  |  |  |  |  |  |  |  |  |  |  |  |  |  |  |  |  |  |  |  |  |  |  |  |  |  |  |  |  |  |  |  |  |  |  |  |  |  |  |  |  |  |  |  |  |  |  |  |  |  |  |  |  |  |  |  |  |  |  |  |  |  |  |  |  |  |  |  |  |  |  |  |  |  |  |  |  |  |  |  |  |  |  |  |  |  |  |  |  |  |  |  |  |  |  |  |  |  |  |  |  |  |  |  |  |  |  |  |  |  |  |  |  |  |  |  |  |  |  |  |  |  |  |  |  |  |  |  |  |  |  |  |  |  |  |  |  |  |  |  |  |  |  |  |  |  |  |  |  |  |  |  |  |  |  |  |  |  |  |  |  |  |  |  |  |  |  |  |  |  |  |  |  |  |  |  |  |  |  |  |  |  |  |  |  |  |  |  |  |  |  |  |  |  |  |  |  |  |  |  |  |  |  |  |  |  |  |  |  |  |  |  |  |  |  |  |  |  |  |  |  |  |  |  |  |  |  |  |  |  |  |  |  |  |  |  |  |  |  |  |  |  |  |  |  |  |  |  |  |  |  |  |  |  |  |  |  |  |  |  |  |  |  |  |  |  |  |  |  |  |  |  |  |  |  |  |  |  |  |  |  |  |  |  |  |  |  |  |  |  |  |  |  |  |  |  |  |  |  |  |  |  |  |  |  |  |  |  |  |  |  |  |  |  |  |  |  |  |  |  |  |  |  |  |  |  |  |  |  |  |  |  |  |  |  |  |  |  |  |  |  |  |  |  |  |  |  |  |  |  |  |  |  |  |  |  |  |  |  |  |  |  |  |  |  |  |  |  |  |  |  |  |  |  |  |  |  |  |  |  |  |  |  |  |  |  |  |  |  |  |  |  |  |  |  |  |  |  |  |  |  |  |  |  |  |  |  |  |  |  |  |  |  |  |  |  |  |  |  |  |  |  |  |  |  |  |  |  |  |  |  |  |  |  |  |  |  |  |  |  |  |  |  |  |  |  |  |  |  |  |  |  |  |  |  |  |  |  |  |  |  |  |  |  |  |  |  |  |  |  |  |  |  |  |  |  |  |  |  |  |  |  |  |  |  |  |  |  |  |  |  |  |  |  |  |  |  |  |  |  |  |  |  |  |  |  |  |  |  |  |  |  |  |  |  |  |  |  |  |  |  |  |  |  |  |  |  |  |  |  |  |  |  |  |  |  |  |  |  |  |  |  |  |  |  |  |  |  |  |  |  |  |  |  |  |  |  |  |  |  |  |  |  |  |  |  |  |  |  |  |  |  |  |  |  |  |  |  |  |  |  |  |  |  |  |  |  |  |  |  |  |  |  |  |  |  |  |  |  |  |  |  |  |  |  |  |  |  |  |  |  |  |  |  |  |  |  |  |  |  |  |  |  |  |  |  |  |  |  |  |  |  |  |  |  |  |  |  |  |  |  |  |  |  |  |  |  |  |  |  |  |  |  |  |  |  |  |  |  |  |  |  |  |  |  |  |  |  |  |  |  |  |  |  |  |  |  |  |  |  |  |  |  |  |  |  |  |  |  |  |  |  |  |  |  |  |  |  |  |  |  |  |  |  |  |  |  |  |  |  |  |  |  |  |  |  |  |  |  |  |  |  |  |  |  |  |  |  |  |  |  |  |  |  |  |  |  |  |  |  |  |  |  |  |  |  |  |  |  |  |  |  |  |  |  |  |  |  |  |  |  |  |  |  |  |  |  |  |  |  |  |  |  |  |  |  |  |  |  |  |  |  |  |  |  |  |  |  |  |  |  |  |  |  |  |  |  |  |  |  |  |  |  |  |  |  |  |  |  |  |  |  |  |  |  |  |  |  |  |  |  |  |  |  |  |  |  |  |  |  |  |  |  |  |  |  |  |  |  |  |  |  |  |  |  |  |  |  |  |  |  |  |  |  |  |  |  |  |  |  |  |  |  |  |  |  |  |  |  |  |  |  |  |  |  |  |  |  |  |  |  |  |  |  |  |  |  |  |  |  |  |  |  |  |  |  |  |  |  |  |  |  |  |  |  |  |  |  |  |  |  |  |  |  |  |  |  |  |  |  |  |  |  |  |  |  |  |  |  |  |  |  |  |  |  |  |  |  |  |  |  |  |  |  |  |  |  |  |  |  |  |  |  |  |  |  |  |  |  |  |  |  |  |  |  |  |  |  |  |  |  |  |  |  |  |  |  |  |  |  |  |  |  |  |  |  |  |  |  |  |  |  |  |  |  |  |  |  |  |  |  |  |  |  |  |  |  |  |  |  |  |  |  |  |  |  |  |  |  |  |  |  |  |  |  |  |  |  |  |  |  |  |  |  |  |  |  |  |  |  |  |  |  |  |  |  |  |  |  |  |  |  |  |  |  |  |  |  |  |  |  |  |  |  |  |  |  |  |  |  |  |  |  |  |  |  |  |  |  |  |  |  |  |  |  |  |  |  |  |  |  |  |  |  |  |  |  |  |  |  |  |  |  |  |  |  |  |  |  |  |  |  |  |  |  |  |  |  |  |  |  |  |  |  |  |  |  |  |  |  |  |  |  |  |  |  |  |  |  |  |  |  |  |  |  |  |  |  |  |  |  |  |  |  |  |  |  |  |  |  |  |  |  |  |  |  |  |  |  |  |  |  |  |  |  |  |  |  |  |  |  |  |  |  |  |  |  |  |  |  |  |  |  |  |  |  |  |  |  |  |  |  |  |  |  |  |  |  |  |  |  |  |  |  |  |  |  |  |  |  |  |  |  |  |  |  |  |  |  |  |  |  |  |  |  |  |  |  |  |  |  |  |  |  |  |  |  |  |  |  |  |  |  |  |  |  |  |  |  |  |  |  |  |  |  |  |  |  |  |  |  |  |  |  |  |  |  |  |  |  |  |  |  |  |  |  |  |  |  |  |  |  |  |  |  |  |  |  |  |  |  |  |  |  |  |  |  |  |  |  |  |  |  |  |  |  |  |  |  |  |  |  |  |  |  |  |  |  |  |  |  |  |  |  |  |  |  |  |  |  |  |  |  |  |  |  |  |  |  |  |  |  |  |  |  |  |  |  |  |  |  |  |  |  |  |  |  |  |  |  |  |  |  |  |  |  |  |  |  |  |  |  |  |  |  |  |  |  |  |  |  |  |  |  |  |  |  |  |  |  |  |  |  |  |  |  |  |  |  |  |  |  |  |  |  |  |  |  |  |  |  |  |  |  |  |  |  |  |  |  |  |  |  |  |  |  |  |  |  |  |  |  |  |  |  |  |  |  |  |  |  |  |  |  |  |  |  |  |  |  |  |  |  |  |  |  |  |  |  |  |  |  |  |  |  |  |  |  |  |  |  |  |  |  |  |  |  |  |  |  |  |  |  |  |  |  |  |  |  |  |  |  |  |  |  |  |  |  |  |  |  |  |  |  |  |  |  |  |  |  |  |  |  |  |  |  |  |  |  |  |  |  |  |  |  |  |  |  |  |  |  |  |  |  |  |  |  |  |  |  |  |  |  |  |  |  |  |  |  |  |  |  |  |  |  |  |  |  |  |  |  |  |  |  |  |  |  |  |  |  |  |  |  |  |  |  |  |  |  |  |  |  |  |  |  |  |  |  |  |  |  |  |  |  |  |  |  |  |  |  |  |  |  |  |  |  |  |  |  |  |  |  |  |  |  |  |  |  |  |  |  |  |  |  |  |  |  |  |  |  |  |  |  |  |  |  |  |  |  |  |  |  |  |  |  |  |  |  |  |  |  |  |  |  |  |  |  |  |  |  |  |  |  |  |  |  |  |  |  |  |  |  |  |  |  |  |  |  |  |  |  |  |  |  |  |  |  |  |  |  |  |  |  |  |  |  |  |  |  |  |  |  |  |  |  |  |  |  |  |  |  |  |  |  |  |  |  |  |  |  |  |  |  |  |  |  |  |  |  |  |  |  |  |  |  |  |  |  |  |  |  |  |  |  |  |  |  |  |  |  |  |  |  |  |  |  |  |  |  |  |  |  |  |  |  |  |  |  |  |  |  |  |  |  |  |  |  |  |  |  |  |  |  |  |  |  |  |  |  |  |  |  |  |  |  |  |  |  |  |  |  |  |  |  |  |  |  |  |  |  |  |  |  |  |  |  |  |  |  |  |  |  |  |  |  |  |  |  |  |  |  |  |  |  |  |  |  |  |  |  |  |  |  |  |  |  |  |  |  |  |  |  |  |  |  |  |  |  |  |  |  |  |  |  |  |  |  |  |  |  |  |  |  |  |  |  |  |  |  |  |  |  |  |  |  |  |  |  |  |  |  |  |  |  |  |  |  |  |  |  |  |  |  |  |  |  |  |  |  |  |  |  |  |  |  |  |  |  |  |  |  |  |  |  |  |  |  |  |  |  |  |  |  |  |  |  |  |  |  |  |  |  |  |  |  |  |  |  |  |  |  |  |  |  |  |  |  |  |  |  |  |  |  |  |  |  |  |  |  |  |  |  |  |  |  |  |  |  |  |  |  |  |  |  |  |  |  |  |  |  |  |  |  |  |  |  |  |  |  |  |  |  |  |  |  |  |  |  |  |  |  |  |  |  |  |  |  |  |  |  |  |  |  |  |  |  |  |  |  |  |  |  |  |  |  |  |  |  |  |  |  |  |  |  |  |  |  |  |  |  |  |  |  |  |  |  |  |  |  |  |  |  |  |  |  |  |  |  |  |  |  |  |  |  |  |  |  |  |  |  |  |  |  |  |  |  |  |  |  |  |  |  |  |  |  |  |  |  |  |  |  |  |  |  |  |  |  |  |  |  |  |  |  |  |  |  |  |  |  |  |  |  |  |  |  |  |  |  |  |  |  |  |  |  |  |  |  |  |  |  |  |  |  |  |  |  |  |  |  |  |  |  |  |  |  |  |  |  |  |  |  |  |  |  |  |  |  |  |  |  |  |  |  |  |  |  |  |  |  |  |  |  |  |  |  |  |  |  |  |  |  |  |  |  |  |  |  |  |  |  |  |  |  |  |  |  |  |  |  |  |  |  |  |  |  |  |  |  |  |  |  |  |  |  |  |  |  |  |  |  |  |  |  |  |  |  |  |  |  |  |  |  |  |  |  |  |  |  |  |  |  |  |  |  |  |  |  |  |  |  |  |  |  |  |  |  |  |  |  |  |  |  |  |  |  |  |  |  |  |  |  |  |  |  |  |  |  |  |  |  |  |  |  |  |  |  |  |  |  |  |  |  |  |  |  |  |  |  |  |  |  |  |  |  |  |  |  |  |  |  |  |  |  |  |  |  |  |  |  |  |  |  |  |  |  |  |  |  |  |  |  |  |  |  |  |  |  |  |  |  |  |  |  |  |  |  |  |  |  |  |  |  |  |  |  |  |  |  |  |  |  |  |  |  |  |  |  |  |  |  |  |  |  |  |  |  |  |  |  |  |  |  |  |  |  |  |  |  |  |  |  |  |  |  |  |  |  |  |  |  |  |  |  |  |  |  |  |  |  |  |  |  |  |  |  |  |  |  |  |  |  |  |  |  |  |  |  |  |  |  |  |  |  |  |  |  |  |  |  |  |  |  |  |  |  |  |  |  |  |  |  |  |  |  |  |  |  |  |  |  |  |  |  |  |  |  |  |  |  |  |  |  |  |  |  |  |  |  |  |  |  |  |  |  |  |  |  |  |  |  |  |  |  |  |  |  |  |  |  |  |  |  |  |  |  |  |  |  |  |  |  |  |  |  |  |  |  |  |  |  |  |  |  |  |  |  |  |  |  |  |  |  |  |  |  |  |  |  |  |  |  |  |  |  |  |  |  |  |  |  |  |  |  |  |  |  |  |  |  |  |  |  |  |  |
| --- | --- | --- | --- | --- | --- | --- | --- | --- | --- | --- | --- | --- | --- | --- | --- | --- | --- | --- | --- | --- | --- | --- | --- | --- | --- | --- | --- | --- | --- | --- | --- | --- | --- | --- | --- | --- | --- | --- | --- | --- | --- | --- | --- | --- | --- | --- | --- | --- | --- | --- | --- | --- | --- | --- | --- | --- | --- | --- | --- | --- | --- | --- | --- | --- | --- | --- | --- | --- | --- | --- | --- | --- | --- | --- | --- | --- | --- | --- | --- | --- | --- | --- | --- | --- | --- | --- | --- | --- | --- | --- | --- | --- | --- | --- | --- | --- | --- | --- | --- | --- | --- | --- | --- | --- | --- | --- | --- | --- | --- | --- | --- | --- | --- | --- | --- | --- | --- | --- | --- | --- | --- | --- | --- | --- | --- | --- | --- | --- | --- | --- | --- | --- | --- | --- | --- | --- | --- | --- | --- | --- | --- | --- | --- | --- | --- | --- | --- | --- | --- | --- | --- | --- | --- | --- | --- | --- | --- | --- | --- | --- | --- | --- | --- | --- | --- | --- | --- | --- | --- | --- | --- | --- | --- | --- | --- | --- | --- | --- | --- | --- | --- | --- | --- | --- | --- | --- | --- | --- | --- | --- | --- | --- | --- | --- | --- | --- | --- | --- | --- | --- | --- | --- | --- | --- | --- | --- | --- | --- | --- | --- | --- | --- | --- | --- | --- | --- | --- | --- | --- | --- | --- | --- | --- | --- | --- | --- | --- | --- | --- | --- | --- | --- | --- | --- | --- | --- | --- | --- | --- | --- | --- | --- | --- | --- | --- | --- | --- | --- | --- | --- | --- | --- | --- | --- | --- | --- | --- | --- | --- | --- | --- | --- | --- | --- | --- | --- | --- | --- | --- | --- | --- | --- | --- | --- | --- | --- | --- | --- | --- | --- | --- | --- | --- | --- | --- | --- | --- | --- | --- | --- | --- | --- | --- | --- | --- | --- | --- | --- | --- | --- | --- | --- | --- | --- | --- | --- | --- | --- | --- | --- | --- | --- | --- | --- | --- | --- | --- | --- | --- | --- | --- | --- | --- | --- | --- | --- | --- | --- | --- | --- | --- | --- | --- | --- | --- | --- | --- | --- | --- | --- | --- | --- | --- | --- | --- | --- | --- | --- | --- | --- | --- | --- | --- | --- | --- | --- | --- | --- | --- | --- | --- | --- | --- | --- | --- | --- | --- | --- | --- | --- | --- | --- | --- | --- | --- | --- | --- | --- | --- | --- | --- | --- | --- | --- | --- | --- | --- | --- | --- | --- | --- | --- | --- | --- | --- | --- | --- | --- | --- | --- | --- | --- | --- | --- | --- | --- | --- | --- | --- | --- | --- | --- | --- | --- | --- | --- | --- | --- | --- | --- | --- | --- | --- | --- | --- | --- | --- | --- | --- | --- | --- | --- | --- | --- | --- | --- | --- | --- | --- | --- | --- | --- | --- | --- | --- | --- | --- | --- | --- | --- | --- | --- | --- | --- | --- | --- | --- | --- | --- | --- | --- | --- | --- | --- | --- | --- | --- | --- | --- | --- | --- | --- | --- | --- | --- | --- | --- | --- | --- | --- | --- | --- | --- | --- | --- | --- | --- | --- | --- | --- | --- | --- | --- | --- | --- | --- | --- | --- | --- | --- | --- | --- | --- | --- | --- | --- | --- | --- | --- | --- | --- | --- | --- | --- | --- | --- | --- | --- | --- | --- | --- | --- | --- | --- | --- | --- | --- | --- | --- | --- | --- | --- | --- | --- | --- | --- | --- | --- | --- | --- | --- | --- | --- | --- | --- | --- | --- | --- | --- | --- | --- | --- | --- | --- | --- | --- | --- | --- | --- | --- | --- | --- | --- | --- | --- | --- | --- | --- | --- | --- | --- | --- | --- | --- | --- | --- | --- | --- | --- | --- | --- | --- | --- | --- | --- | --- | --- | --- | --- | --- | --- | --- | --- | --- | --- | --- | --- | --- | --- | --- | --- | --- | --- | --- | --- | --- | --- | --- | --- | --- | --- | --- | --- | --- | --- | --- | --- | --- | --- | --- | --- | --- | --- | --- | --- | --- | --- | --- | --- | --- | --- | --- | --- | --- | --- | --- | --- | --- | --- | --- | --- | --- | --- | --- | --- | --- | --- | --- | --- | --- | --- | --- | --- | --- | --- | --- | --- | --- | --- | --- | --- | --- | --- | --- | --- | --- | --- | --- | --- | --- | --- | --- | --- | --- | --- | --- | --- | --- | --- | --- | --- | --- | --- | --- | --- | --- | --- | --- | --- | --- | --- | --- | --- | --- | --- | --- | --- | --- | --- | --- | --- | --- | --- | --- | --- | --- | --- | --- | --- | --- | --- | --- | --- | --- | --- | --- | --- | --- | --- | --- | --- | --- | --- | --- | --- | --- | --- | --- | --- | --- | --- | --- | --- | --- | --- | --- | --- | --- | --- | --- | --- | --- | --- | --- | --- | --- | --- | --- | --- | --- | --- | --- | --- | --- | --- | --- | --- | --- | --- | --- | --- | --- | --- | --- | --- | --- | --- | --- | --- | --- | --- | --- | --- | --- | --- | --- | --- | --- | --- | --- | --- | --- | --- | --- | --- | --- | --- | --- | --- | --- | --- | --- | --- | --- | --- | --- | --- | --- | --- | --- | --- | --- | --- | --- | --- | --- | --- | --- | --- | --- | --- | --- | --- | --- | --- | --- | --- | --- | --- | --- | --- | --- | --- | --- | --- | --- | --- | --- | --- | --- | --- | --- | --- | --- | --- | --- | --- | --- | --- | --- | --- | --- | --- | --- | --- | --- | --- | --- | --- | --- | --- | --- | --- | --- | --- | --- | --- | --- | --- | --- | --- | --- | --- | --- | --- | --- | --- | --- | --- | --- | --- | --- | --- | --- | --- | --- | --- | --- | --- | --- | --- | --- | --- | --- | --- | --- | --- | --- | --- | --- | --- | --- | --- | --- | --- | --- | --- | --- | --- | --- | --- | --- | --- | --- | --- | --- | --- | --- | --- | --- | --- | --- | --- | --- | --- | --- | --- | --- | --- | --- | --- | --- | --- | --- | --- | --- | --- | --- | --- | --- | --- | --- | --- | --- | --- | --- | --- | --- | --- | --- | --- | --- | --- | --- | --- | --- | --- | --- | --- | --- | --- | --- | --- | --- | --- | --- | --- | --- | --- | --- | --- | --- | --- | --- | --- | --- | --- | --- | --- | --- | --- | --- | --- | --- | --- | --- | --- | --- | --- | --- | --- | --- | --- | --- | --- | --- | --- | --- | --- | --- | --- | --- | --- | --- | --- | --- | --- | --- | --- | --- | --- | --- | --- | --- | --- | --- | --- | --- | --- | --- | --- | --- | --- | --- | --- | --- | --- | --- | --- | --- | --- | --- | --- | --- | --- | --- | --- | --- | --- | --- | --- | --- | --- | --- | --- | --- | --- | --- | --- | --- | --- | --- | --- | --- | --- | --- | --- | --- | --- | --- | --- | --- | --- | --- | --- | --- | --- | --- | --- | --- | --- | --- | --- | --- | --- | --- | --- | --- | --- | --- | --- | --- | --- | --- | --- | --- | --- | --- | --- | --- | --- | --- | --- | --- | --- | --- | --- | --- | --- | --- | --- | --- | --- | --- | --- | --- | --- | --- | --- | --- | --- | --- | --- | --- | --- | --- | --- | --- | --- | --- | --- | --- | --- | --- | --- | --- | --- | --- | --- | --- | --- | --- | --- | --- | --- | --- | --- | --- | --- | --- | --- | --- | --- | --- | --- | --- | --- | --- | --- | --- | --- | --- | --- | --- | --- | --- | --- | --- | --- | --- | --- | --- | --- | --- | --- | --- | --- | --- | --- | --- | --- | --- | --- | --- | --- | --- | --- | --- | --- | --- | --- | --- | --- | --- | --- | --- | --- | --- | --- | --- | --- | --- | --- | --- | --- | --- | --- | --- | --- | --- | --- | --- | --- | --- | --- | --- | --- | --- | --- | --- | --- | --- | --- | --- | --- | --- | --- | --- | --- | --- | --- | --- | --- | --- | --- | --- | --- | --- | --- | --- | --- | --- | --- | --- | --- | --- | --- | --- | --- | --- | --- | --- | --- | --- | --- | --- | --- | --- | --- | --- | --- | --- | --- | --- | --- | --- | --- | --- | --- | --- | --- | --- | --- | --- | --- | --- | --- | --- | --- | --- | --- | --- | --- | --- | --- | --- | --- | --- | --- | --- | --- | --- | --- | --- | --- | --- | --- | --- | --- | --- | --- | --- | --- | --- | --- | --- | --- | --- | --- | --- | --- | --- | --- | --- | --- | --- | --- | --- | --- | --- | --- | --- | --- | --- | --- | --- | --- | --- | --- | --- | --- | --- | --- | --- | --- | --- | --- | --- | --- | --- | --- | --- | --- | --- | --- | --- | --- | --- | --- | --- | --- | --- | --- | --- | --- | --- | --- | --- | --- | --- | --- | --- | --- | --- | --- | --- | --- | --- | --- | --- | --- | --- | --- | --- | --- | --- | --- | --- | --- | --- | --- | --- | --- | --- | --- | --- | --- | --- | --- | --- | --- | --- | --- | --- | --- | --- | --- | --- | --- | --- | --- | --- | --- | --- | --- | --- | --- | --- | --- | --- | --- | --- | --- | --- | --- | --- | --- | --- | --- | --- | --- | --- | --- | --- | --- | --- | --- | --- | --- | --- | --- | --- | --- | --- | --- | --- | --- | --- | --- | --- | --- | --- | --- | --- | --- | --- | --- | --- | --- | --- | --- | --- | --- | --- | --- | --- | --- | --- | --- | --- | --- | --- | --- | --- | --- | --- | --- | --- | --- | --- | --- | --- | --- | --- | --- | --- | --- | --- | --- | --- | --- | --- | --- | --- | --- | --- | --- | --- | --- | --- | --- | --- | --- | --- | --- | --- | --- | --- | --- | --- | --- | --- | --- | --- | --- | --- | --- | --- | --- | --- | --- | --- | --- | --- | --- | --- | --- | --- | --- | --- | --- | --- | --- | --- | --- | --- | --- | --- | --- | --- | --- | --- | --- | --- | --- | --- | --- | --- | --- | --- | --- | --- | --- | --- | --- | --- | --- | --- | --- | --- | --- | --- | --- | --- | --- | --- | --- | --- | --- | --- | --- | --- | --- | --- | --- | --- | --- | --- | --- | --- | --- | --- | --- | --- | --- | --- | --- | --- | --- | --- | --- | --- | --- | --- | --- | --- | --- | --- | --- | --- | --- | --- | --- | --- | --- | --- | --- | --- | --- | --- | --- | --- | --- | --- | --- | --- | --- | --- | --- | --- | --- | --- | --- | --- | --- | --- | --- | --- | --- | --- | --- | --- | --- | --- | --- | --- | --- | --- | --- | --- | --- | --- | --- | --- | --- | --- | --- | --- | --- | --- | --- | --- | --- | --- | --- | --- | --- | --- | --- | --- | --- | --- | --- | --- | --- | --- | --- | --- | --- | --- | --- | --- | --- | --- | --- | --- | --- | --- | --- | --- | --- | --- | --- | --- | --- | --- | --- | --- | --- | --- | --- | --- | --- | --- | --- | --- | --- | --- | --- | --- | --- | --- | --- | --- | --- | --- | --- | --- | --- | --- | --- | --- | --- | --- | --- | --- | --- | --- | --- | --- | --- | --- | --- | --- | --- | --- | --- | --- | --- | --- | --- | --- | --- | --- | --- | --- | --- | --- | --- | --- | --- | --- | --- | --- | --- | --- | --- | --- | --- | --- | --- | --- | --- | --- | --- | --- | --- | --- | --- | --- | --- | --- | --- | --- | --- | --- | --- | --- | --- | --- | --- | --- | --- | --- | --- | --- | --- | --- | --- | --- | --- | --- | --- | --- | --- | --- | --- | --- | --- | --- | --- | --- | --- | --- | --- | --- | --- | --- | --- | --- | --- | --- | --- | --- | --- | --- | --- | --- | --- | --- | --- | --- | --- | --- | --- | --- | --- | --- | --- | --- | --- | --- | --- | --- | --- | --- | --- | --- | --- | --- | --- | --- | --- | --- | --- | --- | --- | --- | --- | --- | --- | --- | --- | --- | --- | --- | --- | --- | --- | --- | --- | --- | --- | --- | --- | --- | --- | --- | --- | --- | --- | --- | --- | --- | --- | --- | --- | --- | --- | --- | --- | --- | --- | --- | --- | --- | --- | --- | --- | --- | --- | --- | --- | --- | --- | --- | --- | --- | --- | --- | --- | --- | --- | --- | --- | --- | --- | --- | --- | --- | --- | --- | --- | --- | --- | --- | --- | --- | --- | --- | --- | --- | --- | --- | --- | --- | --- | --- | --- | --- | --- | --- | --- | --- | --- | --- | --- | --- | --- | --- | --- | --- | --- | --- | --- | --- | --- | --- | --- | --- | --- | --- | --- | --- | --- | --- | --- | --- | --- | --- | --- | --- | --- | --- | --- | --- | --- | --- | --- | --- | --- | --- | --- | --- | --- | --- | --- | --- | --- | --- | --- | --- | --- | --- | --- | --- | --- | --- | --- | --- | --- | --- | --- | --- | --- | --- | --- | --- | --- | --- | --- | --- | --- | --- | --- | --- | --- | --- | --- | --- | --- | --- | --- | --- | --- | --- | --- | --- | --- | --- | --- | --- | --- | --- | --- | --- | --- | --- | --- | --- | --- | --- | --- | --- | --- | --- | --- | --- | --- | --- | --- | --- | --- | --- | --- | --- | --- | --- | --- | --- | --- | --- | --- | --- | --- | --- | --- | --- | --- | --- | --- | --- | --- | --- | --- | --- | --- | --- | --- | --- | --- | --- | --- | --- | --- | --- | --- | --- | --- | --- | --- | --- | --- | --- | --- | --- | --- | --- | --- | --- | --- | --- | --- | --- | --- | --- | --- | --- | --- | --- | --- | --- | --- | --- | --- | --- | --- | --- | --- | --- | --- | --- | --- | --- | --- | --- | --- | --- | --- | --- | --- | --- | --- | --- | --- | --- | --- | --- | --- | --- | --- | --- | --- | --- | --- | --- | --- | --- | --- | --- | --- | --- | --- | --- | --- | --- | --- | --- | --- | --- | --- | --- | --- | --- | --- | --- | --- | --- | --- | --- | --- | --- | --- | --- | --- | --- | --- | --- | --- | --- | --- | --- | --- | --- | --- | --- | --- | --- | --- | --- | --- | --- | --- | --- | --- | --- | --- | --- | --- | --- | --- | --- | --- | --- | --- | --- | --- | --- | --- | --- | --- | --- | --- | --- | --- | --- | --- | --- | --- | --- | --- | --- | --- | --- | --- | --- | --- | --- | --- | --- | --- | --- | --- | --- | --- | --- | --- | --- | --- | --- | --- | --- | --- | --- | --- | --- | --- | --- | --- | --- | --- | --- | --- | --- | --- | --- | --- | --- | --- | --- | --- | --- | --- | --- | --- | --- | --- | --- | --- | --- | --- | --- | --- | --- | --- | --- | --- | --- | --- | --- | --- | --- | --- | --- | --- | --- | --- | --- | --- | --- | --- | --- | --- | --- | --- | --- | --- | --- | --- | --- | --- | --- | --- | --- | --- | --- | --- | --- | --- | --- | --- | --- | --- | --- | --- | --- | --- | --- | --- | --- | --- | --- | --- | --- | --- | --- | --- | --- | --- | --- | --- | --- | --- | --- | --- | --- | --- | --- | --- | --- | --- | --- | --- | --- | --- | --- | --- | --- | --- | --- | --- | --- | --- | --- | --- | --- | --- | --- | --- | --- | --- | --- | --- | --- | --- | --- | --- | --- | --- | --- | --- | --- | --- | --- | --- | --- | --- | --- | --- | --- | --- | --- | --- | --- | --- | --- | --- | --- | --- | --- | --- | --- | --- | --- | --- | --- | --- | --- | --- | --- | --- | --- | --- | --- | --- | --- | --- | --- | --- | --- | --- | --- | --- | --- | --- | --- | --- | --- | --- | --- | --- | --- | --- | --- | --- | --- | --- | --- | --- | --- | --- | --- | --- | --- | --- | --- | --- | --- | --- | --- | --- | --- | --- | --- | --- | --- | --- | --- | --- | --- | --- | --- | --- | --- | --- | --- | --- | --- | --- | --- | --- | --- | --- | --- | --- | --- | --- | --- | --- | --- | --- | --- | --- | --- | --- | --- | --- | --- | --- | --- | --- | --- | --- | --- | --- | --- | --- | --- | --- | --- | --- | --- | --- | --- | --- | --- | --- | --- | --- | --- | --- | --- | --- | --- | --- | --- | --- | --- | --- | --- | --- | --- | --- | --- | --- | --- | --- | --- | --- | --- | --- | --- | --- | --- | --- | --- | --- | --- | --- | --- | --- | --- | --- | --- | --- | --- | --- | --- | --- | --- | --- | --- | --- | --- | --- | --- | --- | --- | --- | --- | --- | --- | --- | --- | --- | --- | --- | --- | --- | --- | --- | --- | --- | --- | --- | --- | --- | --- | --- | --- | --- | --- | --- | --- | --- | --- | --- | --- | --- | --- | --- | --- | --- | --- | --- | --- | --- | --- | --- | --- | --- | --- | --- | --- | --- | --- | --- | --- | --- | --- | --- | --- | --- | --- | --- | --- | --- | --- | --- | --- | --- | --- | --- | --- | --- | --- | --- | --- | --- | --- | --- | --- | --- | --- | --- | --- | --- | --- | --- | --- | --- | --- | --- | --- | --- | --- | --- | --- | --- | --- | --- | --- | --- | --- | --- | --- | --- | --- | --- | --- | --- | --- | --- | --- | --- | --- | --- | --- | --- | --- | --- | --- | --- | --- | --- | --- | --- | --- | --- | --- | --- | --- | --- | --- | --- | --- | --- | --- | --- | --- | --- | --- | --- | --- | --- | --- | --- | --- | --- | --- | --- | --- | --- | --- | --- | --- | --- | --- | --- | --- | --- | --- | --- | --- | --- | --- | --- | --- | --- | --- | --- | --- | --- | --- | --- | --- | --- | --- | --- | --- | --- | --- | --- | --- | --- | --- | --- | --- | --- | --- | --- | --- | --- | --- | --- | --- | --- | --- | --- | --- | --- | --- | --- | --- | --- | --- | --- | --- | --- | --- | --- | --- | --- | --- | --- | --- | --- | --- | --- | --- | --- | --- | --- | --- | --- | --- | --- | --- | --- | --- | --- | --- | --- | --- | --- | --- | --- | --- | --- | --- | --- | --- | --- | --- | --- | --- | --- | --- | --- | --- | --- | --- | --- | --- | --- | --- | --- | --- | --- | --- | --- | --- | --- | --- | --- | --- | --- | --- | --- | --- | --- | --- | --- | --- | --- | --- | --- | --- | --- | --- | --- | --- | --- | --- | --- | --- | --- | --- | --- | --- | --- | --- | --- | --- | --- | --- | --- | --- | --- | --- | --- | --- | --- | --- | --- | --- | --- | --- | --- | --- | --- | --- | --- | --- | --- | --- | --- | --- | --- | --- | --- | --- | --- | --- | --- | --- | --- | --- | --- | --- | --- | --- | --- | --- | --- | --- | --- | --- | --- | --- | --- | --- | --- | --- | --- | --- | --- | --- | --- | --- | --- | --- | --- | --- | --- | --- | --- | --- | --- | --- | --- | --- | --- | --- | --- | --- | --- | --- | --- | --- | --- | --- | --- | --- | --- | --- | --- | --- | --- | --- | --- | --- | --- | --- | --- | --- | --- | --- | --- | --- | --- | --- | --- | --- | --- | --- | --- | --- | --- | --- | --- | --- | --- | --- | --- | --- | --- | --- | --- | --- | --- | --- | --- | --- | --- | --- | --- | --- | --- | --- | --- | --- | --- | --- | --- | --- | --- | --- | --- | --- | --- | --- | --- | --- | --- | --- | --- | --- | --- | --- | --- | --- | --- | --- | --- | --- | --- | --- | --- | --- | --- | --- | --- | --- | --- | --- | --- | --- | --- | --- | --- | --- | --- | --- | --- | --- | --- | --- | --- | --- | --- | --- | --- | --- | --- | --- | --- | --- | --- | --- | --- | --- | --- | --- | --- | --- | --- | --- | --- | --- | --- | --- | --- | --- | --- | --- | --- | --- | --- | --- | --- | --- | --- | --- | --- | --- | --- | --- | --- | --- | --- | --- | --- | --- | --- | --- | --- | --- | --- | --- | --- | --- | --- | --- | --- | --- | --- | --- | --- | --- | --- | --- | --- | --- | --- | --- | --- | --- | --- | --- | --- | --- | --- | --- | --- | --- | --- | --- | --- | --- | --- | --- | --- | --- | --- | --- | --- | --- | --- | --- | --- | --- | --- | --- | --- | --- | --- | --- | --- | --- | --- | --- | --- | --- | --- | --- | --- | --- | --- | --- | --- | --- | --- | --- | --- | --- | --- | --- | --- | --- | --- | --- | --- | --- | --- | --- | --- | --- | --- | --- | --- | --- | --- | --- | --- | --- | --- | --- | --- | --- | --- | --- | --- | --- | --- | --- | --- | --- | --- | --- | --- | --- | --- | --- | --- | --- | --- | --- | --- | --- | --- | --- | --- | --- | --- | --- | --- | --- | --- | --- | --- | --- | --- | --- | --- | --- | --- | --- | --- | --- | --- | --- | --- | --- | --- | --- | --- | --- | --- | --- | --- | --- | --- | --- | --- | --- | --- | --- | --- | --- | --- | --- | --- | --- | --- | --- | --- | --- | --- | --- | --- | --- | --- | --- | --- | --- | --- | --- | --- | --- | --- | --- | --- | --- | --- | --- | --- | --- | --- | --- | --- | --- | --- | --- | --- | --- | --- | --- | --- | --- | --- | --- | --- | --- | --- | --- | --- | --- | --- | --- | --- | --- | --- | --- | --- | --- | --- | --- | --- | --- | --- | --- | --- | --- | --- | --- | --- | --- | --- | --- | --- | --- | --- | --- | --- | --- | --- | --- | --- | --- | --- | --- | --- | --- | --- | --- | --- | --- | --- | --- | --- | --- | --- | --- | --- | --- | --- | --- | --- | --- | --- | --- | --- | --- | --- | --- | --- | --- | --- | --- | --- | --- | --- | --- | --- | --- | --- | --- | --- | --- | --- | --- | --- | --- | --- | --- | --- | --- | --- | --- | --- | --- | --- | --- | --- | --- | --- | --- | --- | --- | --- | --- | --- | --- | --- | --- | --- | --- | --- | --- | --- | --- | --- | --- | --- | --- | --- | --- | --- | --- | --- | --- | --- | --- | --- | --- | --- | --- | --- | --- | --- | --- | --- | --- | --- | --- | --- | --- | --- | --- | --- | --- | --- | --- | --- | --- | --- | --- | --- | --- | --- | --- | --- | --- | --- | --- | --- | --- | --- | --- | --- | --- | --- | --- | --- | --- | --- | --- | --- | --- | --- | --- | --- | --- | --- | --- | --- | --- | --- | --- | --- | --- | --- | --- | --- | --- | --- | --- | --- | --- | --- | --- | --- | --- | --- | --- | --- | --- | --- | --- | --- | --- | --- | --- | --- | --- | --- | --- | --- | --- | --- | --- | --- | --- | --- | --- | --- | --- | --- | --- | --- | --- | --- | --- | --- | --- | --- | --- | --- | --- | --- | --- | --- | --- | --- | --- | --- | --- | --- | --- | --- | --- | --- | --- | --- | --- | --- | --- | --- | --- | --- | --- | --- | --- | --- | --- | --- | --- | --- | --- | --- | --- | --- | --- | --- | --- | --- | --- | --- | --- | --- | --- | --- | --- | --- | --- | --- | --- | --- | --- | --- | --- | --- | --- | --- | --- | --- | --- | --- | --- | --- | --- | --- | --- | --- | --- | --- | --- | --- | --- | --- | --- | --- | --- | --- | --- | --- | --- | --- | --- | --- | --- | --- | --- | --- | --- | --- | --- | --- | --- | --- | --- | --- | --- | --- | --- | --- | --- | --- | --- | --- | --- | --- | --- | --- | --- | --- | --- | --- | --- | --- | --- | --- | --- | --- | --- | --- | --- | --- | --- | --- | --- | --- | --- | --- | --- | --- | --- | --- | --- | --- | --- | --- | --- | --- | --- | --- | --- | --- | --- | --- | --- | --- | --- | --- | --- | --- | --- | --- | --- | --- | --- | --- | --- | --- | --- | --- | --- | --- | --- | --- | --- | --- | --- | --- | --- | --- | --- | --- | --- | --- | --- | --- | --- | --- | --- | --- | --- | --- | --- | --- | --- | --- | --- | --- | --- | --- | --- | --- | --- | --- | --- | --- | --- | --- | --- | --- | --- | --- | --- | --- | --- | --- | --- | --- | --- | --- | --- | --- | --- | --- | --- | --- | --- | --- | --- | --- | --- | --- | --- | --- | --- | --- | --- | --- | --- | --- | --- | --- | --- | --- | --- | --- | --- | --- | --- | --- | --- | --- | --- | --- | --- | --- | --- | --- | --- | --- | --- | --- | --- | --- | --- | --- | --- | --- | --- | --- | --- | --- | --- | --- | --- | --- | --- | --- | --- | --- | --- | --- | --- | --- | --- | --- | --- | --- | --- | --- | --- | --- | --- | --- | --- | --- | --- | --- | --- | --- | --- | --- | --- | --- | --- | --- | --- | --- | --- | --- | --- | --- | --- | --- | --- | --- | --- | --- | --- | --- | --- | --- | --- | --- | --- | --- | --- | --- | --- | --- | --- | --- | --- | --- | --- | --- | --- | --- | --- | --- | --- | --- | --- | --- | --- | --- | --- | --- | --- | --- | --- | --- | --- | --- | --- | --- | --- | --- | --- | --- | --- | --- | --- | --- | --- | --- | --- | --- | --- | --- | --- | --- | --- | --- | --- | --- | --- | --- | --- | --- | --- | --- | --- | --- | --- | --- | --- | --- | --- | --- | --- | --- | --- | --- | --- | --- | --- | --- | --- | --- | --- | --- | --- | --- | --- | --- | --- | --- | --- | --- | --- | --- | --- | --- | --- | --- | --- | --- | --- | --- | --- | --- | --- | --- | --- | --- | --- | --- | --- | --- | --- | --- | --- | --- | --- | --- | --- | --- | --- | --- | --- | --- | --- | --- | --- | --- | --- | --- | --- | --- | --- | --- | --- | --- | --- | --- | --- | --- | --- | --- | --- | --- | --- | --- | --- | --- | --- | --- | --- | --- | --- | --- | --- | --- | --- | --- | --- | --- | --- | --- | --- | --- | --- | --- | --- | --- | --- | --- | --- | --- | --- | --- | --- | --- | --- | --- | --- | --- | --- | --- | --- | --- | --- | --- | --- | --- | --- | --- | --- | --- | --- | --- | --- | --- | --- | --- | --- | --- | --- | --- | --- | --- | --- | --- | --- | --- | --- | --- | --- | --- | --- | --- | --- | --- | --- | --- | --- | --- | --- | --- | --- | --- | --- | --- | --- | --- | --- | --- | --- | --- | --- | --- | --- | --- | --- | --- | --- | --- | --- | --- | --- | --- | --- | --- | --- | --- | --- | --- | --- | --- | --- | --- | --- | --- | --- | --- | --- | --- | --- | --- | --- | --- | --- | --- | --- | --- | --- | --- | --- | --- | --- | --- | --- | --- | --- | --- | --- | --- | --- | --- | --- | --- | --- | --- | --- | --- | --- | --- | --- | --- | --- | --- | --- | --- | --- | --- | --- | --- | --- | --- | --- | --- | --- | --- | --- | --- | --- | --- | --- | --- | --- | --- | --- | --- | --- | --- | --- | --- | --- | --- | --- | --- | --- | --- | --- | --- | --- | --- | --- | --- | --- | --- | --- | --- | --- | --- | --- | --- | --- | --- | --- | --- | --- | --- | --- | --- | --- | --- | --- | --- | --- | --- | --- | --- | --- | --- | --- | --- | --- | --- | --- | --- | --- | --- | --- | --- | --- | --- | --- | --- | --- | --- | --- | --- | --- | --- | --- | --- | --- | --- | --- | --- | --- | --- | --- | --- | --- | --- | --- | --- | --- | --- | --- | --- | --- | --- | --- | --- | --- | --- | --- | --- | --- | --- | --- | --- | --- | --- | --- | --- | --- | --- | --- | --- | --- | --- | --- | --- | --- | --- | --- | --- | --- | --- | --- | --- | --- | --- | --- | --- | --- | --- | --- | --- | --- | --- | --- | --- | --- | --- | --- | --- | --- | --- | --- | --- | --- | --- | --- | --- | --- | --- | --- | --- | --- | --- | --- | --- | --- | --- | --- | --- | --- | --- | --- | --- | --- | --- | --- | --- | --- | --- | --- | --- | --- | --- | --- | --- | --- | --- | --- | --- | --- | --- | --- | --- | --- | --- | --- | --- | --- | --- | --- | --- | --- | --- | --- | --- | --- | --- | --- | --- | --- | --- | --- | --- | --- | --- | --- | --- | --- | --- | --- | --- | --- | --- | --- | --- | --- | --- | --- | --- | --- | --- | --- | --- | --- | --- | --- | --- | --- | --- | --- | --- | --- | --- | --- | --- | --- | --- | --- | --- | --- | --- | --- | --- | --- | --- | --- | --- | --- | --- | --- | --- | --- | --- | --- | --- | --- | --- | --- | --- | --- | --- | --- | --- | --- | --- | --- | --- | --- | --- | --- | --- | --- | --- | --- | --- | --- | --- | --- | --- | --- | --- | --- | --- | --- | --- | --- | --- | --- | --- | --- | --- | --- | --- | --- | --- | --- | --- | --- | --- | --- | --- | --- | --- | --- | --- | --- | --- | --- | --- | --- | --- | --- | --- | --- | --- | --- | --- | --- | --- | --- | --- | --- | --- | --- | --- | --- | --- | --- | --- | --- | --- | --- | --- | --- | --- | --- | --- | --- | --- | --- | --- | --- | --- | --- | --- | --- | --- | --- | --- | --- | --- | --- | --- | --- | --- | --- | --- | --- | --- | --- | --- | --- | --- | --- | --- | --- | --- | --- | --- | --- | --- | --- | --- | --- | --- | --- | --- | --- | --- | --- | --- | --- | --- | --- | --- | --- | --- | --- | --- | --- | --- | --- | --- | --- | --- | --- | --- | --- | --- | --- | --- | --- | --- | --- | --- | --- | --- | --- | --- | --- | --- | --- | --- | --- | --- | --- | --- | --- | --- | --- | --- | --- | --- | --- | --- | --- | --- | --- | --- | --- | --- | --- | --- | --- | --- | --- | --- | --- | --- | --- | --- | --- | --- | --- | --- | --- | --- | --- | --- | --- | --- | --- | --- | --- | --- | --- | --- | --- | --- | --- | --- | --- | --- | --- | --- | --- | --- | --- | --- | --- | --- | --- | --- | --- | --- | --- | --- | --- | --- | --- | --- | --- | --- | --- | --- | --- | --- | --- | --- | --- | --- | --- | --- | --- | --- | --- | --- | --- | --- | --- | --- | --- | --- | --- | --- | --- | --- | --- | --- | --- | --- | --- | --- | --- | --- | --- | --- | --- | --- | --- | --- | --- | --- | --- | --- | --- | --- | --- | --- | --- | --- | --- | --- | --- | --- | --- | --- | --- | --- | --- | --- | --- | --- | --- | --- | --- | --- | --- | --- | --- | --- | --- | --- | --- | --- | --- | --- | --- | --- | --- | --- | --- | --- | --- | --- | --- | --- | --- | --- | --- | --- | --- | --- | --- | --- | --- | --- | --- | --- | --- | --- | --- | --- | --- | --- | --- | --- | --- | --- | --- | --- | --- | --- | --- | --- | --- | --- | --- | --- | --- | --- | --- | --- | --- | --- | --- | --- | --- | --- | --- | --- | --- | --- | --- | --- | --- | --- | --- | --- | --- | --- | --- | --- | --- | --- | --- | --- | --- | --- | --- | --- | --- | --- | --- | --- | --- | --- | --- | --- | --- | --- | --- | --- | --- | --- | --- | --- | --- | --- | --- | --- | --- | --- | --- | --- | --- | --- | --- | --- | --- | --- | --- | --- | --- | --- | --- | --- | --- | --- | --- | --- | --- | --- | --- | --- | --- | --- | --- | --- | --- | --- | --- | --- | --- | --- | --- | --- | --- | --- | --- | --- | --- | --- | --- | --- | --- | --- | --- | --- | --- | --- | --- | --- | --- | --- | --- | --- | --- | --- | --- | --- | --- | --- | --- | --- | --- | --- | --- | --- | --- | --- | --- | --- | --- | --- | --- | --- | --- | --- | --- | --- | --- | --- | --- | --- | --- | --- | --- | --- | --- | --- | --- | --- | --- | --- | --- | --- | --- | --- | --- | --- | --- | --- | --- | --- | --- | --- | --- | --- | --- | --- | --- | --- | --- | --- | --- | --- | --- | --- | --- | --- | --- | --- | --- | --- | --- | --- | --- | --- | --- | --- | --- | --- | --- | --- | --- | --- | --- | --- | --- | --- | --- | --- | --- | --- | --- | --- | --- | --- | --- | --- | --- | --- | --- | --- | --- | --- | --- | --- | --- | --- | --- | --- | --- | --- | --- | --- | --- | --- | --- | --- | --- | --- | --- | --- | --- | --- | --- | --- | --- | --- | --- | --- | --- | --- | --- | --- | --- | --- | --- | --- | --- | --- | --- | --- | --- | --- | --- | --- | --- | --- | --- | --- | --- | --- | --- | --- | --- | --- | --- | --- | --- | --- | --- | --- | --- | --- | --- | --- | --- | --- | --- | --- | --- | --- | --- | --- | --- | --- | --- | --- | --- | --- | --- | --- | --- | --- | --- | --- | --- | --- | --- | --- | --- | --- | --- | --- | --- | --- | --- | --- | --- | --- | --- | --- | --- | --- | --- | --- | --- | --- | --- | --- | --- | --- | --- | --- | --- | --- | --- | --- | --- | --- | --- | --- | --- | --- | --- | --- | --- | --- | --- | --- | --- | --- | --- | --- | --- | --- | --- | --- | --- | --- | --- | --- | --- | --- | --- | --- | --- | --- | --- | --- | --- | --- | --- | --- | --- | --- | --- | --- | --- | --- | --- | --- | --- | --- | --- | --- | --- | --- | --- | --- | --- | --- | --- | --- | --- | --- | --- | --- | --- | --- | --- | --- | --- | --- | --- | --- | --- | --- | --- | --- | --- | --- | --- | --- | --- | --- | --- | --- | --- | --- | --- | --- | --- | --- | --- | --- | --- | --- | --- | --- | --- | --- | --- | --- | --- | --- | --- | --- | --- | --- | --- | --- | --- | --- | --- | --- | --- | --- | --- | --- | --- | --- | --- | --- | --- | --- | --- | --- | --- | --- | --- | --- | --- | --- | --- | --- | --- | --- | --- | --- | --- | --- | --- | --- | --- | --- | --- | --- | --- | --- | --- | --- | --- | --- | --- | --- | --- | --- | --- | --- | --- | --- | --- | --- | --- | --- | --- | --- | --- | --- | --- | --- | --- | --- | --- | --- | --- | --- | --- | --- | --- | --- | --- | --- | --- | --- | --- | --- | --- | --- | --- | --- | --- | --- | --- | --- | --- | --- | --- | --- | --- | --- | --- | --- | --- | --- | --- | --- | --- | --- | --- | --- | --- | --- | --- | --- | --- | --- | --- | --- | --- | --- | --- | --- | --- | --- | --- | --- | --- | --- | --- | --- | --- | --- | --- | --- | --- | --- | --- | --- | --- | --- | --- | --- | --- | --- | --- | --- | --- | --- | --- | --- | --- | --- | --- | --- | --- | --- | --- | --- | --- | --- | --- | --- | --- | --- | --- | --- | --- | --- | --- | --- | --- | --- | --- | --- | --- | --- | --- | --- | --- | --- | --- | --- | --- | --- | --- | --- | --- | --- | --- | --- | --- | --- | --- | --- | --- | --- | --- | --- | --- | --- | --- | --- | --- | --- | --- | --- | --- | --- | --- | --- | --- | --- | --- | --- | --- | --- | --- | --- | --- | --- | --- | --- | --- | --- | --- | --- | --- | --- | --- | --- | --- | --- | --- | --- | --- | --- | --- | --- | --- | --- | --- | --- | --- | --- | --- | --- | --- | --- | --- | --- | --- | --- | --- | --- | --- | --- | --- | --- | --- | --- | --- | --- | --- | --- | --- | --- | --- | --- | --- | --- | --- | --- | --- | --- | --- | --- | --- | --- | --- | --- | --- | --- | --- | --- | --- | --- | --- | --- | --- | --- | --- | --- | --- | --- | --- | --- | --- | --- | --- | --- | --- | --- | --- | --- | --- | --- | --- | --- | --- | --- | --- | --- | --- | --- | --- | --- | --- | --- | --- | --- | --- | --- | --- | --- | --- | --- | --- | --- | --- | --- | --- | --- | --- | --- | --- | --- | --- | --- | --- | --- | --- | --- | --- | --- | --- | --- | --- | --- | --- | --- | --- | --- | --- | --- | --- | --- | --- | --- | --- | --- | --- | --- | --- | --- | --- | --- | --- | --- | --- | --- | --- | --- | --- | --- | --- | --- | --- | --- | --- | --- | --- | --- | --- | --- | --- | --- | --- | --- | --- | --- | --- | --- | --- | --- | --- | --- | --- | --- | --- | --- | --- | --- | --- | --- | --- | --- | --- | --- | --- | --- | --- | --- | --- | --- | --- | --- | --- | --- | --- | --- | --- | --- | --- | --- | --- | --- | --- | --- | --- | --- | --- | --- | --- | --- | --- | --- | --- | --- | --- | --- | --- | --- | --- | --- | --- | --- | --- | --- | --- | --- | --- | --- | --- | --- | --- | --- | --- | --- | --- | --- | --- | --- | --- | --- | --- | --- | --- | --- | --- | --- | --- | --- | --- | --- | --- | --- | --- | --- | --- | --- | --- | --- | --- | --- | --- | --- | --- | --- | --- | --- | --- | --- | --- | --- | --- | --- | --- | --- | --- | --- | --- | --- | --- | --- | --- | --- | --- | --- | --- | --- | --- | --- | --- | --- | --- | --- | --- | --- | --- | --- | --- | --- | --- | --- | --- | --- | --- | --- | --- | --- | --- | --- | --- | --- | --- | --- | --- | --- | --- | --- | --- | --- | --- | --- | --- | --- | --- | --- | --- | --- | --- | --- | --- | --- | --- | --- | --- | --- | --- | --- | --- | --- | --- | --- | --- | --- | --- | --- | --- | --- | --- | --- | --- | --- | --- | --- | --- | --- | --- | --- | --- | --- | --- | --- | --- | --- | --- | --- | --- | --- | --- | --- | --- | --- | --- | --- | --- | --- | --- | --- | --- | --- | --- | --- | --- | --- | --- | --- | --- | --- | --- | --- | --- | --- | --- | --- | --- | --- | --- | --- | --- | --- | --- | --- | --- | --- | --- | --- | --- | --- | --- | --- | --- | --- | --- | --- | --- | --- | --- | --- | --- | --- | --- | --- | --- | --- | --- | --- | --- | --- | --- | --- | --- | --- | --- | --- | --- | --- | --- | --- | --- | --- | --- | --- | --- | --- | --- | --- | --- | --- | --- | --- | --- | --- | --- | --- | --- | --- | --- | --- | --- | --- | --- | --- | --- | --- | --- | --- | --- | --- | --- | --- | --- | --- | --- | --- | --- | --- | --- | --- | --- | --- | --- | --- | --- | --- | --- | --- | --- | --- | --- | --- | --- | --- | --- | --- | --- | --- | --- | --- | --- | --- | --- | --- | --- | --- | --- | --- | --- | --- | --- | --- | --- | --- | --- | --- | --- | --- | --- | --- | --- | --- | --- | --- | --- | --- | --- | --- | --- | --- | --- | --- | --- | --- | --- | --- | --- | --- | --- | --- | --- | --- | --- | --- | --- | --- | --- |
| AFFY Probe | Entrez Gene | Dif. Expressed | RefSeq | SG RefSeq | Gene Ontology | Pubmed | Gene Symbol | SheepGenomics\|  \|  \|  \|  \|  \|  \|  \|  \|  \| \| --- \| --- \| --- \| --- \| --- \| --- \| --- \| --- \| --- \| \| Bt.13986.1.S1\_at \| 404110 \| 1/1 \| XM\_875877  XM\_876095 \|  \| GO:0008270 \| 7713329 \| ZA20D2 \| Bt.13986.1.S1\_at \| \| Bt.13289.1.S1\_at \|  \|  \|  \|  \|  \|  \| ARID5B \| Bt.13289.1.S1\_at \| \| Bt.4818.1.S1\_at \| 768028 \| 1/1 \| NM\_001077087 \| NM\_001077087 \|  \|  \|  \| Bt.4818.1.S1\_at \| \| Bt.8972.1.S1\_at \| 505524 \| 1/1 \| XM\_864458 \| XM\_864458 \|  \|  \| DNTTIP1 \| Bt.8972.1.S1\_at \| \| Bt.7034.1.S1\_at \| 780788 \| 1/1 \| NM\_001079643 \|  \| GO:0004849  GO:0005524  GO:0007229 \|  \| ITGB1BP3 \| Bt.7034.1.S1\_at \| \| Bt.9556.1.S1\_at \| 404180 \| 2/3 \| XM\_591933 \|  \| GO:0003824  GO:0016020  GO:0016021 \| 14668210 \| MEST \| Bt.9556.1.S1\_at \| \| Bt.20501.1.S1\_at \| 513776 \| 3/3 \| NM\_001046155 \|  \| GO:0005515  GO:0006512 \|  \| FBXO32 \| Bt.20501.1.S1\_at \| \| Bt.12299.1.A1\_at \| 281338 \| 1/1 \| XM\_866281 \|  \| GO:0003774  GO:0016459 \|  \| MYH3 \| Bt.12299.1.A1\_at \| \| Bt.6642.2.S1\_at \| 537224 \| 3/4 \| XM\_865119 \|  \|  \|  \| LPIN1 \| Bt.6642.2.S1\_at \| \| Bt.13289.2.S1\_at \|  \|  \|  \|  \|  \|  \| ARID5B \| Bt.13289.2.S1\_at \| \| Bt.7394.1.A1\_at \| 615191 \| 2/2 \| XM\_866930 \|  \| GO:0005515  GO:0005524  GO:0016311  GO:0016791  GO:0044267 \|  \| PTPLA \| Bt.7394.1.A1\_at \| \| Bt.25052.1.S1\_at \|  \|  \|  \| XM\_586655 \|  \|  \| CAPN6 \| Bt.25052.1.S1\_at \| \| Bt.3071.1.S1\_at \| 515784 \| 1/1 \| XM\_868540 \| XM\_868540 \|  \|  \| CKAP4 \| Bt.3071.1.S1\_at \| \| Bt.5070.1.S1\_x\_at \| 444874 \| 2/2 \| XM\_874550 \| XM\_874550 \| GO:0006464 \| 1846490 \| UBC \| Bt.5070.1.S1\_x\_at \| \| Bt.6991.1.S1\_at \| 516550 \| 1/1 \| NM\_001046226 \| NM\_001046226 \|  \|  \| FEM1A \| Bt.6991.1.S1\_at \| \| Bt.7133.1.S1\_at \| 281365 \| 1/2 \| NM\_174130 \|  \|  \| 7774801  10520448 \| ODC1 \| Bt.7133.1.S1\_at \| \| Bt.13588.1.A1\_at \| 533044 \| 2/2 \| XM\_612314 \| XM\_612314 \| GO:0003824  GO:0006564  GO:0008152  GO:0008483  GO:0030170 \|  \|  \| Bt.13588.1.A1\_at \| \| Bt.15682.1.A1\_at \|  \|  \|  \|  \|  \|  \| BRI3BP \| Bt.15682.1.A1\_at \| \| Bt.1035.1.S1\_a\_at \| 338062 \| 1/1 \| NM\_176657 \|  \| GO:0002474  GO:0004872  GO:0006955  GO:0016020  GO:0019882  GO:0042612 \| 10657638  12477932  12514749  16095719 \| FCGRT \| Bt.1035.1.S1\_a\_at \| \| Bt.22803.1.S1\_at \| 615087 \| 1/1 \| NM\_001046549 \| NM\_001046549 \| GO:0003924  GO:0005198  GO:0005525  GO:0005874  GO:0007018  GO:0043234  GO:0051258 \| 12140684  16305752 \| TUBB \| Bt.22803.1.S1\_at \| \| Bt.25319.2.S1\_at \| 517422 \| 2/2 \| XM\_001251272  XM\_865801 \| XM\_865801 \| GO:0000166  GO:0003677  GO:0003887  GO:0005634  GO:0006139  GO:0006260  GO:0008152  GO:0016740 \|  \| REV3L \| Bt.25319.2.S1\_at \| \| Bt.5340.1.S1\_s\_at \| 404189 \| 1/1 \| NM\_205818 \| NM\_205818 \| GO:0000166  GO:0000287  GO:0004550  GO:0005524  GO:0005625  GO:0005634  GO:0005792  GO:0006183  GO:0006228  GO:0006241  GO:0007595  GO:0009117  GO:0016301  GO:0016740  GO:0030879 \| 9760230  10225434 \| NME1 \| Bt.5340.1.S1\_s\_at \| \| Bt.6774.1.S1\_at \| 408001 \| 2/2 \| NM\_001001169 \|  \| GO:0000045  GO:0005737  GO:0005776  GO:0005874  GO:0006512  GO:0006914  GO:0031090 \| 7908909  9557703  12477932  15140988  15325588 \|  \| Bt.6774.1.S1\_at \| \| Bt.4184.1.S2\_at \| 281814 \| 1/1 \| NM\_174339 \|  \| GO:0001525  GO:0001666  GO:0001755  GO:0001947  GO:0003700  GO:0004871  GO:0005515  GO:0005634  GO:0006355  GO:0007165  GO:0009434  GO:0030154  GO:0035035  GO:0042981  GO:0045941  GO:0045944  GO:0046982 \| 10320777  15548892 \| HIF1A \| Bt.4184.1.S2\_at \| \| Bt.8213.1.S1\_at \| 517688 \| 2/2 \| XM\_595862 \| XM\_595862 \|  \|  \| FLJ14466 \| Bt.8213.1.S1\_at \| \| Bt.5348.1.S1\_at \| 511905 \| 1/1 \| XM\_611412 \| XM\_611412 \|  \|  \|  \| Bt.5348.1.S1\_at \| \| Bt.10330.1.S1\_at \| 515320 \| 2/2 \| NM\_001046196 \|  \| GO:0003677  GO:0005634  GO:0006350  GO:0006355  GO:0016055 \|  \| HBP1 \| Bt.10330.1.S1\_at \| \| Bt.2153.1.S1\_at \| 505455 \| 1/1 \| NM\_001075202 \| NM\_001075202 \|  \|  \| IER3 \| Bt.2153.1.S1\_at \| \| Bt.19706.1.S1\_at \|  \|  \|  \|  \|  \|  \| ZFP36L2 \| Bt.19706.1.S1\_at \| \| Bt.4822.1.A1\_at \|  \|  \|  \| NM\_001077116 \|  \|  \|  \| Bt.4822.1.A1\_at \| \| Bt.26808.1.S1\_at \| 514187 \| 1/1 \| XR\_027914 \|  \|  \|  \| THADA \| Bt.26808.1.S1\_at \| \| Bt.23883.1.A1\_at \|  \|  \|  \|  \|  \|  \| GLCE \| Bt.23883.1.A1\_at \| \| Bt.1582.1.S1\_at \| 615447 \| 1/1 \| NM\_001076376 \|  \| GO:0000166  GO:0000287  GO:0004550  GO:0005524  GO:0006183  GO:0006228  GO:0006241  GO:0009117  GO:0016020  GO:0016301  GO:0016740 \|  \|  \| Bt.1582.1.S1\_at \| \| Bt.23176.2.S1\_a\_at \| 281240 \| 1/4 \| NM\_174087 \| NM\_174087 \| GO:0000074  GO:0005179  GO:0005576  GO:0008083  GO:0008283 \| 1280544  2302223  2388846  3390164  3941093  12112582  12658628  12873229  16120826  16644179 \| IGF2 \| Bt.23176.2.S1\_a\_at \| \| Bt.11659.1.S1\_at \| 615666 \| 1/2 \| NM\_001034787 \|  \| GO:0003735  GO:0005739  GO:0005761  GO:0006412  GO:0030529 \| 11402041  12477932 \| MRP63 \| Bt.11659.1.S1\_at \| \| Bt.25806.2.A1\_at \| 539945 \| 2/2 \| XM\_590441 \| XM\_590441 \|  \|  \| ITGB1BP2 \| Bt.25806.2.A1\_at \| \| Bt.24310.1.S1\_at \|  \|  \|  \|  \|  \|  \|  \| Bt.24310.1.S1\_at \| \| Bt.9012.1.S1\_at \| 508247 \| 1/1 \| XM\_585000 \| XM\_585000 \|  \|  \| KIAA1639 \| Bt.9012.1.S1\_at \| \| Bt.12412.1.S1\_at \| 515320 \| 2/2 \| NM\_001046196 \|  \| GO:0003677  GO:0005634  GO:0006350  GO:0006355  GO:0016055 \|  \| HBP1 \| Bt.12412.1.S1\_at \| \| Bt.20104.1.S1\_at \| 538226 \| 1/3 \| XM\_618424  XM\_865104 \|  \|  \|  \| SESN1 \| Bt.20104.1.S1\_at \| \| Bt.4702.1.S1\_at \|  \|  \|  \|  \|  \|  \| FOXO3A \| Bt.4702.1.S1\_at \| \| Bt.10150.1.S1\_at \|  \|  \|  \|  \|  \|  \| CAPN6 \| Bt.10150.1.S1\_at \| \| Bt.17963.1.A1\_at \| 517345 \| 1/1 \| NM\_001046229 \|  \| GO:0000166  GO:0005525  GO:0005622  GO:0005794  GO:0007264 \|  \| ARL1 \| Bt.17963.1.A1\_at \| \| Bt.1169.1.S1\_at \|  \|  \|  \|  \|  \|  \| ADD3 \| Bt.1169.1.S1\_at \| \| Bt.23521.1.S1\_at \| 533896 \| 1/2 \| NM\_001076004 \|  \| GO:0006470  GO:0008138  GO:0016791 \|  \| DUSP26 \| Bt.23521.1.S1\_at \| \| Bt.9810.1.S1\_at \| 613730 \| 1/1 \| XM\_865378 \|  \| GO:0005622  GO:0043565 \|  \| POLE4 \| Bt.9810.1.S1\_at \| \| Bt.28312.1.S1\_at \|  \|  \|  \|  \|  \|  \| TFDP2 \| Bt.28312.1.S1\_at \| \| Bt.3306.1.S1\_at \| 512397 \| 1/1 \| XM\_001252640  XM\_589910 \| XM\_589910 \| GO:0005737  GO:0016020 \|  \| ProSAPiP1 \| Bt.3306.1.S1\_at \| \| Bt.6642.1.S1\_a\_at \| 537224 \| 3/4 \| XM\_865119 \|  \|  \|  \| LPIN1 \| Bt.6642.1.S1\_a\_at \| \| Bt.25099.1.A1\_at \| 533630 \| 2/2 \| NM\_001046355 \| NM\_001046355 \| GO:0000287  GO:0004647  GO:0006564  GO:0008152  GO:0008652  GO:0016787 \|  \| PSPH \| Bt.25099.1.A1\_at \| \| Bt.22510.1.S1\_at \| 511492 \| 1/1 \| XM\_588837 \| XM\_588837 \|  \|  \| C2orf7 \| Bt.22510.1.S1\_at \| \| Bt.20145.1.S1\_at \| 523609 \| 1/1 \| NM\_001038138 \| NM\_001038138 \|  \|  \| PX19 \| Bt.20145.1.S1\_at \| \| Bt.7908.1.S1\_at \| 508628 \| 1/1 \| NM\_001015548 \|  \|  \| 12140684  12477932  16305752  16816403 \| RPS6 \| Bt.7908.1.S1\_at \| \| Bt.15643.1.A1\_at \|  \|  \|  \|  \|  \|  \| RC3H1 \| Bt.15643.1.A1\_at \| \| Bt.21551.2.S1\_a\_at \| 534046 \| 2/2 \| XM\_865607 \| XM\_865607 \|  \|  \| UCKL1 \| Bt.21551.2.S1\_a\_at \| \| Bt.2347.2.S1\_at \| 506544 \| 1/3 \| NM\_001075268 \| NM\_001075268 \|  \| 8001974 \| PFKM \| Bt.2347.2.S1\_at \| \| Bt.21385.1.S1\_at \| 510850 \| 1/1 \| NM\_001046063 \| NM\_001046063 \| GO:0004867  GO:0005783  GO:0005793  GO:0006457  GO:0051082 \|  \| SERPINH1 \| Bt.21385.1.S1\_at \| \| Bt.2091.1.S1\_at \| 529235 \| 1/1 \| NM\_001075922 \| NM\_001075922 \|  \|  \| DDIT4 \| Bt.2091.1.S1\_at \| \| Bt.3388.1.S1\_at \| 540602 \| 1/1 \| NM\_001046482 \| NM\_001046482 \| GO:0003824  GO:0008152  GO:0016051 \|  \| NANS \| Bt.3388.1.S1\_at \| \| Bt.16639.1.A1\_at \| 529061 \| 1/1 \| XR\_027889 \|  \|  \|  \|  \| Bt.16639.1.A1\_at \| \| Bt.22568.1.S1\_at \| 507270 \| 1/1 \| NM\_001014862 \| NM\_001014862 \|  \| 12140684  12477932  16305752 \| RPL29 \| Bt.22568.1.S1\_at \| \| Bt.6774.2.S1\_at \| 408001 \| 2/2 \| NM\_001001169 \| NM\_001001169 \| GO:0000045  GO:0005737  GO:0005776  GO:0005874  GO:0006512  GO:0006914  GO:0031090 \| 7908909  9557703  12477932  15140988  15325588 \|  \| Bt.6774.2.S1\_at \| \| Bt.9180.1.S1\_a\_at \| 506537 \| 1/2 \| NM\_001077863  XM\_001250632 \|  \|  \|  \| ALG14 \| Bt.9180.1.S1\_a\_at \| \| Bt.2070.1.A1\_at \| 541175 \| 3/3 \| NM\_001034725 \|  \| GO:0004725  GO:0005634  GO:0006470  GO:0016787  GO:0017017 \| 11282978 \| DUSP10 \| Bt.2070.1.A1\_at \| \| Bt.21142.1.S1\_at \| 535392 \| 1/1 \| XM\_615468 \|  \|  \|  \| KIAA0408 \| Bt.21142.1.S1\_at \| \| Bt.16127.1.S1\_at \|  \|  \|  \|  \|  \|  \| HECA \| Bt.16127.1.S1\_at \| \| Bt.7962.1.S1\_at \|  \|  \|  \| XM\_001254874 \|  \|  \| YPEL3 \| Bt.7962.1.S1\_at \| \| Bt.14204.1.A1\_at \| 538880 \| 1/2 \| XM\_001250951  XM\_583432 \|  \| GO:0000166  GO:0003676 \|  \| CPEB2 \| Bt.14204.1.A1\_at \| \| Bt.23519.1.S1\_at \| 539244 \| 1/1 \| NM\_001034669 \|  \| GO:0006512  GO:0007242 \| 12477932 \| ASB2 \| Bt.23519.1.S1\_at \| \| Bt.14023.1.S1\_at \| 507997 \| 1/2 \| XM\_584709 \|  \|  \|  \| C9orf10 \| Bt.14023.1.S1\_at \| \| Bt.20340.2.S1\_at \| 513675 \| 1/2 \| XR\_028836 \|  \|  \|  \| DMPK \| Bt.20340.2.S1\_at \| \| Bt.13988.1.S1\_at \|  \|  \|  \|  \|  \|  \| DUSP3 \| Bt.13988.1.S1\_at \| \| Bt.29029.1.S1\_at \| 533768 \| 1/2 \| XM\_613281 \| XM\_613281 \|  \|  \| TACC2 \| Bt.29029.1.S1\_at \| \| Bt.24524.1.S1\_at \|  \|  \|  \|  \|  \|  \| ARL1 \| Bt.24524.1.S1\_at \| \| Bt.5123.2.S1\_at \| 514446 \| 2/4 \| XM\_592304 \| XM\_592304 \| GO:0003779  GO:0005515 \|  \| FLII \| Bt.5123.2.S1\_at \| \| Bt.9021.1.S1\_at \| 534742 \| 1/1 \| XM\_614616 \| XM\_614616 \|  \|  \| SLC25A24 \| Bt.9021.1.S1\_at \| \| Bt.6802.1.S1\_at \|  \|  \|  \|  \|  \|  \| RGS5 \| Bt.6802.1.S1\_at \| \| Bt.4725.1.S1\_at \| 281032 \| 1/1 \| NM\_173999 \|  \|  \| 12477932  15033446 \| BTG1 \| Bt.4725.1.S1\_at \| \| Bt.13975.1.S1\_a\_at \| 613972 \| 1/1 \| NM\_001076301 \|  \| GO:0005215  GO:0005501  GO:0006810  GO:0008289 \|  \|  \| Bt.13975.1.S1\_a\_at \| \| Bt.4353.1.S1\_at \| 282711 \| 1/2 \| NM\_174725 \| NM\_174725 \|  \| 10320777 \| EPAS1 \| Bt.4353.1.S1\_at \| \| Bt.25973.1.S1\_at \| 505802 \| 1/2 \| XM\_582150 \|  \|  \|  \|  \| Bt.25973.1.S1\_at \| \| Bt.7537.1.S1\_at \|  \|  \|  \|  \|  \|  \| AUTS2 \| Bt.7537.1.S1\_at \| \| Bt.7670.1.A1\_at \| 539020 \| 1/1 \| XM\_001250887  XM\_584289  XM\_865691 \| XM\_865691 \| GO:0003676  GO:0005622  GO:0008270 \|  \| BCL6 \| Bt.7670.1.A1\_at \| \| Bt.12314.1.S1\_at \| 407183 \| 1/2 \| NM\_001077837  XM\_001252652 \| NM\_001077837 \| GO:0000166  GO:0003824  GO:0003873  GO:0004331  GO:0005524  GO:0006003  GO:0008152  GO:0016301  GO:0016740  GO:0016787 \| 7733968 \| PFKFB3 \| Bt.12314.1.S1\_at \| \| Bt.20420.1.S1\_at \| 505691 \| 1/1 \| XM\_582018 \|  \|  \|  \| CDKN2C \| Bt.20420.1.S1\_at \| \| Bt.24137.1.A1\_at \| 539761 \| 1/1 \| XR\_027726 \| XR\_027726 \|  \|  \| PLAGL1 \| Bt.24137.1.A1\_at \| \| Bt.21432.2.A1\_at \|  \|  \|  \|  \|  \|  \|  \| Bt.21432.2.A1\_at \| \| Bt.5070.1.S1\_at \| 444874 \| 2/2 \| XM\_874550 \| XM\_874550 \| GO:0006464 \| 1846490 \| UBC \| Bt.5070.1.S1\_at \| \| Bt.4659.1.S1\_at \| 508629 \| 1/1 \| NM\_001075367 \|  \|  \|  \| ALDH2 \| Bt.4659.1.S1\_at \| \| Bt.13330.1.S1\_at \| 507367 \| 2/3 \| XM\_583960 \| XM\_583960 \|  \|  \| PDK4 \| Bt.13330.1.S1\_at \| \| Bt.23668.1.A1\_at \|  \|  \|  \|  \|  \|  \| MITF \| Bt.23668.1.A1\_at \| \| Bt.28090.1.S1\_at \| 509896 \| 1/1 \| NM\_001034368 \| NM\_001034368 \|  \| 11282978  16305752 \| ABHD4 \| Bt.28090.1.S1\_at \| \| Bt.13324.1.S1\_a\_at \| 281235 \| 2/2 \| NM\_181012 \| NM\_181012 \| GO:0000287  GO:0004450  GO:0004450  GO:0005737  GO:0005829  GO:0006097  GO:0006099  GO:0006749  GO:0006979  GO:0008152  GO:0016491  GO:0016616  GO:0030145 \| 10358094  10677231  12477932  16636309  16751257 \| IDH1 \| Bt.13324.1.S1\_a\_at \| \| Bt.3616.1.S1\_at \| 282688 \| 1/2 \| NM\_174715 \| NM\_174715 \| GO:0003735  GO:0005622  GO:0005840  GO:0006412 \| 7948030  10684968  12140684  12477932  12658628  16305752 \| Rpl3 \| Bt.3616.1.S1\_at \| \| Bt.5123.3.S1\_a\_at \| 514446 \| 2/4 \| XM\_592304 \| XM\_592304 \| GO:0003779  GO:0005515 \|  \| FLII \| Bt.5123.3.S1\_a\_at \| \| Bt.7069.1.S1\_at \| 505807 \| 1/1 \| NM\_001024479 \|  \|  \| 11282978  16305752 \| PCMTD2 \| Bt.7069.1.S1\_at \| \| Bt.3780.1.S1\_at \|  \|  \|  \|  \|  \|  \| C5orf13 \| Bt.3780.1.S1\_at \| \| Bt.12491.1.A1\_at \| 533219 \| 1/1 \| NM\_001034585 \| NM\_001034585 \| GO:0000166  GO:0005198  GO:0005515  GO:0005524  GO:0005856 \| 12477932  16980359 \| ACTC \| Bt.12491.1.A1\_at \| \| Bt.1998.1.S1\_at \| 516056 \| 1/1 \| NM\_001075735 \| XM\_001254742 \|  \|  \| NGFRAP1 \| Bt.1998.1.S1\_at \| \| Bt.26688.2.S1\_a\_at \|  \|  \|  \|  \|  \|  \| FRMD3 \| Bt.26688.2.S1\_a\_at \| \| Bt.911.1.S1\_at \|  \|  \|  \|  \|  \|  \| FLJ13910 \| Bt.911.1.S1\_at \| \| Bt.22783.1.S1\_at \| 281141 \| 1/1 \| NM\_174049 \| NM\_174049 \|  \| 7499243 \| ENO1 \| Bt.22783.1.S1\_at \| \| Bt.7540.2.S1\_a\_at \| 327703 \| 1/2 \| NM\_175819 \| NM\_175819 \| GO:0005245  GO:0005509  GO:0006811  GO:0006816 \| 10684870  11282978 \| CACNB1 \| Bt.7540.2.S1\_a\_at \| \| Bt.18755.1.A1\_at \| 534761 \| 2/4 \| XM\_873360 \| XM\_001257234 \| GO:0004672  GO:0005524  GO:0006468 \| 16519507 \| GSK3B \| Bt.18755.1.A1\_at \| \| Bt.9804.1.S1\_at \| 616539 \| 1/1 \| NM\_001046581 \| NM\_001046581 \|  \|  \| CHURC1 \| Bt.9804.1.S1\_at \| \| Bt.7288.1.S1\_at \| 504438 \| 1/1 \| NM\_001038503 \|  \| GO:0005783  GO:0016020  GO:0016021 \|  \| SSR4 \| Bt.7288.1.S1\_at \| \| Bt.23603.3.S1\_at \| 511798 \| 2/3 \| NM\_001034412 \| NM\_001034412 \| GO:0006512 \| 12477932 \| FBXO9 \| Bt.23603.3.S1\_at \| \| Bt.5304.1.S1\_at \| 615507 \| 1/1 \| NM\_001046560 \| NM\_001046560 \| GO:0004364  GO:0008152  GO:0016740 \|  \| GSTM3 \| Bt.5304.1.S1\_at \| \| Bt.7481.1.S1\_at \| 514665 \| 1/1 \| NM\_001075676 \|  \| GO:0005215  GO:0006810  GO:0008021  GO:0016020 \|  \|  \| Bt.7481.1.S1\_at \| \| Bt.9956.1.S1\_at \| 404159 \| 1/1 \| XM\_593976 \| XM\_593976 \| GO:0045454 \|  \| TXNL5 \| Bt.9956.1.S1\_at \| \| Bt.3863.1.S1\_at \| 282127 \| 1/1 \| NM\_174493 \| NM\_174493 \| GO:0003676  GO:0008270 \| 7559666  7768935 \| ZFP36 \| Bt.3863.1.S1\_at \| \| Bt.1268.1.A1\_at \|  \|  \|  \|  \|  \|  \| SET7 \| Bt.1268.1.A1\_at \| \| Bt.11492.1.A1\_at \| 538561 \| 1/2 \| XR\_027849 \| XR\_027849 \|  \|  \| MGEA5 \| Bt.11492.1.A1\_at \| \| Bt.21724.2.S1\_a\_at \| 281767 \| 1/2 \| NM\_001013004 \|  \| GO:0004310  GO:0008610  GO:0016021  GO:0016740 \|  \| FDFT1 \| Bt.21724.2.S1\_a\_at \| \| Bt.18623.1.A1\_at \| 510159 \| 1/2 \| NM\_001034374 \| NM\_001034374 \|  \| 12477932 \| MAPRE2 \| Bt.18623.1.A1\_at \| \| Bt.25319.1.A1\_at \| 517422 \| 2/2 \| XM\_001251272  XM\_865801 \| XM\_865801 \| GO:0000166  GO:0003677  GO:0003887  GO:0005634  GO:0006139  GO:0006260  GO:0008152  GO:0016740 \|  \| REV3L \| Bt.25319.1.A1\_at \| \| Bt.9643.1.S1\_at \| 512662  617985 \| 1/1 \| NM\_001075576  XM\_001255339  XM\_870317 \| XM\_870317 \|  \|  \| CHP \| Bt.9643.1.S1\_at \| \| Bt.22651.1.A1\_at \|  \|  \|  \|  \|  \|  \|  \| Bt.22651.1.A1\_at \| \| Bt.20141.1.S1\_at \| 509274 \| 1/1 \| NM\_001035274 \| NM\_001035274 \|  \| 12477932 \| HAGH \| Bt.20141.1.S1\_at \| \| Bt.1330.1.S1\_at \| 317748 \| 1/1 \| NM\_001012519 \|  \| GO:0016491 \| 2105951  2515032  8431420  12140684  12551929  12658628 \| AKR1B1 \| Bt.1330.1.S1\_at \| \| Bt.5472.1.S1\_at \| 326598 \| 1/1 \| NM\_175782 \| NM\_175782 \| GO:0004871  GO:0005529  GO:0005622  GO:0043123 \| 1587821  1900835  2470348  3569527  7773775  8108426  12477932  12658628 \| LGALS1 \| Bt.5472.1.S1\_at \| \| Bt.26805.1.S1\_at \| 528615 \| 1/2 \| XM\_607042 \|  \| GO:0006457  GO:0031072  GO:0051082 \|  \| DNAJA4 \| Bt.26805.1.S1\_at \| \| Bt.2696.1.A1\_at \| 535363 \| 2/2 \| XM\_615431 \| XM\_001256656 \| GO:0005509  GO:0007156  GO:0016020 \|  \| CDH11 \| Bt.2696.1.A1\_at \| \| Bt.9190.1.S1\_at \|  \|  \|  \|  \|  \|  \| UBE2G1 \| Bt.9190.1.S1\_at \| \| Bt.2537.2.S1\_at \|  \|  \|  \| NM\_174719 \|  \|  \| TIMP3 \| Bt.2537.2.S1\_at \| \| Bt.25806.1.S1\_at \| 539945 \| 2/2 \| XM\_590441 \| XM\_590441 \|  \|  \| ITGB1BP2 \| Bt.25806.1.S1\_at \| \| Bt.4624.1.S1\_at \| 282376 \| 1/1 \| NM\_174617 \| NM\_174617 \| GO:0001553  GO:0001553  GO:0001779  GO:0001779  GO:0003700  GO:0004871  GO:0004871  GO:0005509  GO:0005515  GO:0005515  GO:0005634  GO:0005634  GO:0005737  GO:0005737  GO:0006350  GO:0006355  GO:0007165  GO:0007242  GO:0007259  GO:0007259  GO:0007565  GO:0007565  GO:0007595  GO:0007595  GO:0008284  GO:0008284  GO:0019218  GO:0019218  GO:0019221  GO:0019221  GO:0019915  GO:0019915  GO:0030155  GO:0030155  GO:0030856  GO:0030856  GO:0040018  GO:0040018  GO:0042104  GO:0042104  GO:0042448  GO:0042448  GO:0043029  GO:0043029  GO:0043066  GO:0043066  GO:0045086  GO:0045086  GO:0045579  GO:0045579  GO:0045647  GO:0045647  GO:0045885  GO:0045885  GO:0045931  GO:0045931  GO:0045944  GO:0045944  GO:0046543  GO:0046543  GO:0046544  GO:0046544  GO:0050729  GO:0050729 \| 10050298  10835485 \| STAT5B \| Bt.4624.1.S1\_at \| \| Bt.659.2.S1\_at \| 534928 \| 2/3 \| XM\_614870  XM\_881407  XM\_881423 \|  \| GO:0003824  GO:0005524  GO:0006003  GO:0008152 \|  \| PFKFB4 \| Bt.659.2.S1\_at \| \| Bt.21133.1.S1\_at \|  \|  \|  \|  \|  \|  \| UQCRB \| Bt.21133.1.S1\_at \| \| Bt.3312.1.S1\_at \|  \|  \|  \|  \|  \|  \| DUSP7 \| Bt.3312.1.S1\_at \| \| Bt.20270.1.S1\_at \| 531673 \| 1/4 \| XM\_610173 \|  \|  \|  \|  \| Bt.20270.1.S1\_at \| \| Bt.15157.1.S1\_at \| 613575 \| 1/3 \| NM\_001034739 \|  \| GO:0007242  GO:0016020  GO:0016021 \| 12140684  16305752 \| CNIH \| Bt.15157.1.S1\_at \| \| Bt.22785.1.S1\_at \|  \|  \|  \| NM\_176788 \|  \|  \| CEBPB \| Bt.22785.1.S1\_at \| \| Bt.22216.1.S1\_at \| 541180 \| 2/3 \| XM\_614920 \| XM\_614920 \|  \|  \| FEM1C \| Bt.22216.1.S1\_at \| \| Bt.23992.1.A1\_at \|  \|  \|  \|  \|  \|  \| AK3L1 \| Bt.23992.1.A1\_at \| \| Bt.6156.1.S1\_at \| 616771 \| 1/1 \|  \|  \|  \|  \| C2orf28 \| Bt.6156.1.S1\_at \| \| Bt.5252.1.S1\_at \| 404148 \| 1/1 \| NM\_001034054 \| NM\_001034054 \| GO:0003824  GO:0004082  GO:0004083  GO:0004619  GO:0006096  GO:0008152  GO:0016787  GO:0016853  GO:0016868 \| 12477932  12658628 \| PGAM1 \| Bt.5252.1.S1\_at \| \| Bt.9915.1.S1\_at \| 540892 \| 1/1 \| NM\_001046494 \| NM\_001046494 \| GO:0000166  GO:0003924  GO:0005525  GO:0016020  GO:0016787 \|  \| DNM1L \| Bt.9915.1.S1\_at \| \| Bt.11034.1.S1\_at \| 615572 \| 1/1 \| NM\_001076383 \|  \|  \|  \| MID1IP1 \| Bt.11034.1.S1\_at \| \| Bt.4410.1.S1\_at \| 506986 \| 1/2 \| NM\_001075292 \|  \|  \|  \|  \| Bt.4410.1.S1\_at \| \| Bt.1131.1.S1\_at \| 281419 \| 1/1 \| NM\_174152 \|  \|  \| 8010972  8013656  12140684  12477932  12658628  16305752 \| PPIB \| Bt.1131.1.S1\_at \| \| Bt.4353.2.S1\_at \|  \|  \|  \|  \|  \|  \| EPAS1 \| Bt.4353.2.S1\_at \| \| Bt.1418.2.S1\_at \|  \|  \|  \|  \|  \|  \| MGC12966 \| Bt.1418.2.S1\_at \| \| Bt.4704.2.S1\_at \| 514355 \| 1/1 \| NM\_001046171 \| NM\_001046171 \| GO:0000166  GO:0005524  GO:0005832  GO:0006457  GO:0042802  GO:0051082 \|  \| CCT7 \| Bt.4704.2.S1\_at \| \| Bt.3414.1.S1\_at \| 536149 \| 1/3 \| NM\_001038171 \|  \| GO:0003677  GO:0005634  GO:0006810  GO:0007275  GO:0007283  GO:0008270  GO:0030154  GO:0043087  GO:0046872 \|  \| HRB \| Bt.3414.1.S1\_at \| \| Bt.9033.2.S1\_at \| 281546 \| 1/2 \| NM\_001040476 \|  \|  \|  \| TRAM1 \| Bt.9033.2.S1\_at \| \| Bt.22763.2.S1\_a\_at \| 407767 \| 1/2 \| XM\_001252032  XM\_609765 \| XM\_001251609 \| GO:0004421  GO:0006084 \| 12477932  14510667 \| HMGCS1 \| Bt.22763.2.S1\_a\_at \| \| Bt.21116.1.S1\_at \| 510154 \| 1/1 \| NM\_001046048 \|  \|  \|  \| SELENBP1 \| Bt.21116.1.S1\_at \| \| Bt.6680.1.S1\_a\_at \| 538998 \| 1/2 \| NM\_001037481 \| NM\_001037481 \| GO:0003755  GO:0005783  GO:0006457  GO:0016853 \| 12477932 \| FKBP2 \| Bt.6680.1.S1\_a\_at \| \| Bt.7776.1.S2\_at \| 326599 \| 1/4 \| NM\_001014388 \|  \| GO:0005509  GO:0005615  GO:0005737  GO:0005771  GO:0045298 \| 12140684  12477932  12658628  16305752 \| TPT1 \| Bt.7776.1.S2\_at \| \| Bt.24538.1.S1\_at \|  \|  \|  \| XM\_001249769 \|  \|  \| NEK7 \| Bt.24538.1.S1\_at \| \| Bt.28390.1.S1\_at \|  \|  \|  \|  \|  \|  \| TSC22D3 \| Bt.28390.1.S1\_at \| \| Bt.28567.1.A1\_at \|  \|  \|  \|  \|  \|  \| NFE2L1 \| Bt.28567.1.A1\_at \| \| Bt.4970.1.S1\_at \| 327688 \| 1/1 \| NM\_175807 \|  \| GO:0004129  GO:0005739  GO:0005746  GO:0006118  GO:0009055  GO:0016020 \| 1717471  2549516  2844245  8307562 \| COX7A2 \| Bt.4970.1.S1\_at \| \| Bt.18637.1.A1\_at \| 506860 \| 1/1 \| XM\_583363 \|  \|  \|  \| PTS \| Bt.18637.1.A1\_at \| \| Bt.24766.1.S1\_at \| 520694 \| 1/1 \| XM\_598942 \| XM\_598942 \| GO:0005515 \|  \| LRRN6A \| Bt.24766.1.S1\_at \| \| Bt.24727.1.S1\_a\_at \| 538804 \| 1/1 \| NM\_001038178 \| NM\_001038178 \|  \|  \| C6orf136 \| Bt.24727.1.S1\_a\_at \| \| Bt.18639.1.A1\_at \| 513776 \| 3/3 \| NM\_001046155 \| NM\_001046155 \| GO:0005515  GO:0006512 \|  \| FBXO32 \| Bt.18639.1.A1\_at \| \| Bt.24614.1.S1\_at \|  \|  \|  \|  \|  \|  \| INSIG1 \| Bt.24614.1.S1\_at \| \| Bt.29724.1.S1\_at \|  \|  \|  \| NM\_001077837 \|  \|  \| PFKFB3 \| Bt.29724.1.S1\_at \| \| Bt.16563.1.A1\_at \|  \|  \|  \|  \|  \|  \| PTPLA \| Bt.16563.1.A1\_at \| \| Bt.22167.1.S1\_at \| 541113 \| 2/2 \| XM\_614609 \|  \| GO:0005515 \|  \| LRRC2 \| Bt.22167.1.S1\_at \| \| Bt.28732.1.S1\_s\_at \| 404120  404176  407199 \| 1/2 \| D90419  XM\_603355  XM\_867018 \| XM\_603355 \|  \|  \|  \| Bt.28732.1.S1\_s\_at \| \| Bt.2070.2.A1\_at \| 541175 \| 3/3 \| NM\_001034725 \| NM\_001034725 \| GO:0004725  GO:0005634  GO:0006470  GO:0016787  GO:0017017 \| 11282978 \| DUSP10 \| Bt.2070.2.A1\_at \| \| Bt.29873.1.S1\_at \| 404180 \| 2/3 \| XM\_591933 \|  \| GO:0003824  GO:0016020  GO:0016021 \| 14668210 \| MEST \| Bt.29873.1.S1\_at \| \| Bt.24439.1.S1\_at \| 540056 \| 1/1 \| XM\_591217 \| XM\_591217 \|  \|  \| C10orf26 \| Bt.24439.1.S1\_at \| \| Bt.17102.3.S1\_at \| 532726 \| 2/5 \| NM\_001035392 \|  \| GO:0004864  GO:0005975  GO:0005977  GO:0009966 \| 12477932 \| MGC127677 \| Bt.17102.3.S1\_at \| \| Bt.20227.1.S1\_at \| 532560 \| 1/2 \| NM\_001046326 \|  \| GO:0005215  GO:0005488  GO:0005743  GO:0006810  GO:0016020 \|  \| SLC25A15 \| Bt.20227.1.S1\_at \| \| Bt.5468.1.S1\_at \| 503685 \| 1/1 \| NM\_001012764 \| NM\_001012764 \| GO:0004869 \|  \| CST6 \| Bt.5468.1.S1\_at \| \| Bt.5356.1.S1\_s\_at \| 282530 \| 1/2 \| NM\_001012680 \| NM\_001012680 \| GO:0002504  GO:0006955  GO:0016020  GO:0016021  GO:0019884  GO:0019886  GO:0042613 \| 1559718  1952283  2134154  7590975  7733993  8258704  8572364  9110933  11261936  11282978  12140684  12648094  12722639  12859595  15102783  15182329  15363847  15487587  16029434  16305752 \| BoLA-DRB3 \| Bt.5356.1.S1\_s\_at \| \| Bt.8189.2.A1\_at \|  \|  \|  \|  \|  \|  \| RANBP1 \| Bt.8189.2.A1\_at \| \| Bt.9571.2.S1\_a\_at \| 509771 \| 1/3 \| NM\_001034363 \| NM\_001034363 \|  \| 12477932 \| RBM3 \| Bt.9571.2.S1\_a\_at \| \| Bt.23250.2.S1\_at \| 414732 \| 1/1 \| NM\_001045878 \| NM\_001045878 \| GO:0016740 \|  \| GATM \| Bt.23250.2.S1\_at \| \| Bt.13765.1.S1\_at \|  \|  \|  \|  \|  \|  \|  \| Bt.13765.1.S1\_at \| \| Bt.2874.1.S1\_at \|  \|  \|  \| XM\_001253965 \|  \|  \|  \| Bt.2874.1.S1\_at \| \| Bt.20143.2.S1\_at \|  \|  \|  \|  \|  \|  \| STAT5B \| Bt.20143.2.S1\_at \| \| Bt.13315.1.S1\_at \| 768237 \| 1/1 \| NM\_001078112 \|  \| GO:0003824  GO:0008152 \|  \| FLJ20920 \| Bt.13315.1.S1\_at \| \| Bt.12559.1.S1\_at \| 511135 \| 1/1 \| NM\_001037457 \|  \| GO:0000166  GO:0000287  GO:0003878  GO:0005515  GO:0005524  GO:0008152  GO:0008610  GO:0016740 \| 12477932 \| ACLY \| Bt.12559.1.S1\_at \| \| Bt.8699.1.S1\_at \| 540045 \| 1/2 \| XM\_001251971  XM\_611497  XM\_872514  XM\_872608 \| XM\_872608 \| GO:0003676  GO:0005622  GO:0005634  GO:0008270 \|  \| ZNF385 \| Bt.8699.1.S1\_at \| \| Bt.7651.1.S1\_at \| 511852 \| 1/1 \| NM\_001034413 \|  \| GO:0007242 \| 12477932 \| ASB11 \| Bt.7651.1.S1\_at \| \| Bt.13588.3.A1\_at \| 533044 \| 2/2 \| XM\_612314 \|  \| GO:0003824  GO:0006564  GO:0008152  GO:0008483  GO:0030170 \|  \| PSAT1 \| Bt.13588.3.A1\_at \| \| Bt.9767.1.S1\_a\_at \|  \|  \|  \| XM\_604908 \|  \|  \| SQLE \| Bt.9767.1.S1\_a\_at \| \| Bt.19023.1.S1\_at \| 507234 \| 1/2 \| XM\_583804 \|  \|  \|  \| TRAF5 \| Bt.19023.1.S1\_at \| \| Bt.24517.2.A1\_at \| 516334 \| 1/2 \| NM\_001075741 \| NM\_001075741 \| GO:0004872  GO:0008067  GO:0016020 \| 11282978  16305752 \| GPRC5B \| Bt.24517.2.A1\_at \| \| Bt.24804.1.S1\_at \| 533378 \| 1/1 \| XM\_612769 \| XM\_612769 \|  \|  \| MYLK2 \| Bt.24804.1.S1\_at \| \| Bt.18639.2.S1\_at \| 513776 \| 3/3 \| NM\_001046155 \| NM\_001046155 \| GO:0005515  GO:0006512 \|  \| FBXO32 \| Bt.18639.2.S1\_at \| \| Bt.15675.1.S1\_at \| 286806 \| 1/1 \| NM\_181667 \|  \| GO:0004222  GO:0005578  GO:0006508  GO:0008270  GO:0046872 \| 10356395  10403768  10625599  14715656 \| ADAMTS4 \| Bt.15675.1.S1\_at \| \| Bt.4631.2.S1\_at \| 507912 \| 1/3 \| NM\_001045984 \|  \|  \|  \|  \| Bt.4631.2.S1\_at \| \| Bt.4888.1.S1\_at \| 616435 \| 1/1 \| XM\_868459 \|  \|  \|  \| KIAA0657 \| Bt.4888.1.S1\_at \| \| Bt.27196.1.A1\_at \| 511421 \| 1/1 \|  \|  \|  \|  \| CRTAC1 \| Bt.27196.1.A1\_at \| \| Bt.16389.1.S1\_at \| 404121 \| 1/2 \| NM\_205789 \| NM\_205789 \| GO:0005783  GO:0006986  GO:0015031  GO:0016020  GO:0016021 \| 11282978  16305752 \| DERL1 \| Bt.16389.1.S1\_at \| \| Bt.16597.1.S1\_at \| 618292 \| 1/2 \| NM\_001078086  XM\_001253586  XM\_001253606 \| XM\_001253586 \|  \| 14667821 \| EXOSC4 \| Bt.16597.1.S1\_at \| \| Bt.13588.2.S1\_at \| 617679 \| 1/1 \|  \|  \|  \|  \| PSAT1 \| Bt.13588.2.S1\_at \| \| Bt.20470.1.S1\_a\_at \| 525071 \| 2/2 \| NM\_001075863 \| NM\_001075863 \|  \|  \| MTP18 \| Bt.20470.1.S1\_a\_at \| \| Bt.19508.1.A1\_at \| 540179 \| 1/1 \| XM\_592079 \| XM\_592079 \|  \|  \| GPC4 \| Bt.19508.1.A1\_at \| \| Bt.19352.1.A1\_at \|  \|  \|  \|  \|  \|  \|  \| Bt.19352.1.A1\_at \| \| Bt.17883.1.S1\_at \|  \|  \|  \|  \|  \|  \| PYGO1 \| Bt.17883.1.S1\_at \| \| Bt.21039.1.A1\_at \| 507449 \| 1/1 \| XM\_869115 \| XM\_869115 \|  \| 12477932 \| RASD1 \| Bt.21039.1.A1\_at \| \| Bt.8552.1.S1\_at \| 506214 \| 1/1 \| NM\_001012677 \| NM\_001012677 \|  \| 2298463  7945359  8082897  10449907  12140684  12477932  15182329 \| HLA-DRA \| Bt.8552.1.S1\_at \| \| Bt.22480.1.A1\_at \|  \|  \|  \|  \|  \|  \| SLC35E3 \| Bt.22480.1.A1\_at \| \| Bt.20431.2.S1\_at \| 614509 \| 2/4 \| NM\_001076331 \| NM\_001076331 \| GO:0005634  GO:0030528  GO:0045449 \|  \| MXI1 \| Bt.20431.2.S1\_at \| \| Bt.10579.1.S1\_at \|  \|  \|  \| XR\_027461 \|  \|  \| IRF2BP2 \| Bt.10579.1.S1\_at \| \| Bt.21818.1.S1\_at \| 533087 \| 1/1 \| XM\_867545 \|  \| GO:0003677  GO:0004519  GO:0005622  GO:0005634  GO:0006281 \|  \| EZH1 \| Bt.21818.1.S1\_at \| \| Bt.12472.1.S1\_at \| 281199 \| 1/1 \| NM\_001040474 \|  \| GO:0004356  GO:0006542  GO:0006807  GO:0016874 \| 2893372  9321487 \| GLUL \| Bt.12472.1.S1\_at \| \| Bt.621.1.S1\_at \| 505060 \| 1/1 \| NM\_001025319 \| NM\_001025319 \|  \| 15836952  16508140 \| CYP51A1 \| Bt.621.1.S1\_at \| \| Bt.25390.1.A1\_at \| 613877 \| 1/1 \| XM\_876237 \|  \|  \|  \| MEIS1 \| Bt.25390.1.A1\_at \| \| Bt.2392.1.S1\_at \| 404124 \| 1/1 \| NM\_205791 \| NM\_205791 \| GO:0006486  GO:0008373  GO:0030173  GO:0047290 \| 15843597 \| ST6GALNAC4 \| Bt.2392.1.S1\_at \| \| Bt.18316.2.S1\_at \| 513587 \| 2/2 \| XM\_591287  XM\_876203 \|  \|  \|  \|  \| Bt.18316.2.S1\_at \| \| Bt.11014.1.S1\_at \| 513889 \| 1/1 \| NM\_001046159 \| NM\_001046159 \| GO:0005739  GO:0006605  GO:0015031 \|  \| CHCHD4 \| Bt.11014.1.S1\_at \| \| Bt.27926.1.S1\_at \| 520170 \| 1/1 \| NM\_001040541 \| NM\_001040541 \| GO:0004221  GO:0005622  GO:0006511  GO:0006512  GO:0008234 \|  \| UCHL3 \| Bt.27926.1.S1\_at \| \| Bt.13235.1.S1\_at \| 614348 \| 1/2 \| XM\_865827 \| XM\_865827 \|  \|  \| RGC32 \| Bt.13235.1.S1\_at \| \| Bt.8250.1.S1\_at \|  \|  \|  \|  \|  \|  \| C10orf56 \| Bt.8250.1.S1\_at \| \| Bt.28728.1.A1\_at \| 517688 \| 2/2 \| XM\_595862 \| XM\_595862 \|  \|  \| FLJ14466 \| Bt.28728.1.A1\_at \| \| Bt.11794.1.S1\_at \| 768057 \| 1/1 \| NM\_001077114 \|  \|  \|  \|  \| Bt.11794.1.S1\_at \| \| Bt.25718.1.A1\_at \| 524624 \| 1/1 \| XM\_602953 \| XM\_602953 \|  \|  \| PDE4C \| Bt.25718.1.A1\_at \| \| Bt.12685.1.S1\_at \| 530050 \| 1/1 \| XM\_608513 \| XM\_608513 \|  \|  \| MYH11 \| Bt.12685.1.S1\_at \| \| Bt.15931.1.S1\_at \| 539373 \| 1/1 \| NM\_001034674 \| NM\_001034674 \| GO:0003735  GO:0005622  GO:0005840  GO:0006412 \| 12477932 \| RPL14 \| Bt.15931.1.S1\_at \| \| Bt.15783.1.S1\_at \| 506715 \| 1/1 \| XM\_001250457  XM\_583196 \| XM\_001250457 \|  \|  \|  \| Bt.15783.1.S1\_at \| \| Bt.9959.3.S1\_a\_at \| 506118 \| 1/2 \| XM\_866286 \| XM\_866286 \|  \|  \| GLT28D1 \| Bt.9959.3.S1\_a\_at \| \| Bt.3314.1.S1\_at \| 515552 \| 1/2 \| XM\_593587 \| XM\_593587 \| GO:0005515  GO:0005524  GO:0008152  GO:0016491  GO:0044267 \|  \| PLEKHM2 \| Bt.3314.1.S1\_at \| \| Bt.23109.1.S1\_at \| 404126 \| 1/2 \| NM\_001037444 \| NM\_001037444 \| GO:0004407  GO:0005634  GO:0016575 \| 12477932  16941691 \| HDAC1 \| Bt.23109.1.S1\_at \| \| Bt.2515.1.S1\_at \| 287016 \| 1/1 \| NM\_174809 \| NM\_174809 \| GO:0000786  GO:0003677  GO:0005634  GO:0005694  GO:0006334  GO:0007001 \| 2713375  3344202  6306766  6832364 \| H2AFZ \| Bt.2515.1.S1\_at \| \| Bt.19332.2.A1\_at \|  \|  \|  \|  \|  \|  \| CLCN5 \| Bt.19332.2.A1\_at \| \| Bt.9811.1.S1\_at \| 533993 \| 1/1 \| NM\_001046365 \|  \|  \|  \| TNFSF5IP1 \| Bt.9811.1.S1\_at \| \| Bt.16150.1.A1\_at \| 512254 \| 1/2 \| NM\_001046113 \| NM\_001046113 \| GO:0003700  GO:0005634  GO:0006355  GO:0043565 \|  \| MEF2C \| Bt.16150.1.A1\_at \| \| Bt.12613.1.S1\_at \| 505884 \| 1/5 \| NM\_001035271 \|  \|  \| 12477932 \| KLF6 \| Bt.12613.1.S1\_at \| \| Bt.7266.1.S1\_at \| 515109 \| 1/1 \| NM\_001034480 \| NM\_001034480 \| GO:0005739  GO:0006457  GO:0008270  GO:0015031  GO:0016020  GO:0042719  GO:0045039  GO:0046872 \| 12477932 \| TIMM8A \| Bt.7266.1.S1\_at \| \| Bt.15784.1.S1\_at \| 521424 \| 1/1 \| XM\_599688 \|  \|  \|  \| EMP1 \| Bt.15784.1.S1\_at \| \| Bt.21705.1.S1\_at \| 513449 \| 1/1 \| XM\_869766 \|  \|  \|  \| SEC14L1 \| Bt.21705.1.S1\_at \| \| Bt.8124.1.S2\_at \| 282188 \| 2/2 \| NM\_174520 \|  \| GO:0005201  GO:0005581  GO:0005737  GO:0006817 \| 173531  4412529  4435743  4609475  9628255 \| COL1A2 \| Bt.8124.1.S2\_at \| \| Bt.5176.1.S1\_at \| 282480 \| 1/1 \| NM\_174660 \| NM\_174660 \| GO:0005215  GO:0005488  GO:0005739  GO:0005743  GO:0006810  GO:0006839  GO:0006915  GO:0016020  GO:0016021 \| 2540808  12140684  15595696  16305752 \| SLC25A6 \| Bt.5176.1.S1\_at \| \| Bt.18748.1.A1\_at \| 505696 \| 1/2 \| XM\_582025 \| XM\_582025 \|  \|  \| PTPRB \| Bt.18748.1.A1\_at \| \| Bt.10277.2.S1\_a\_at \| 404165 \| 1/1 \| NM\_001033622 \| NM\_001033622 \| GO:0005634 \| 12477932 \| NOL5A \| Bt.10277.2.S1\_a\_at \| \| Bt.665.1.S1\_s\_at \| 282211 \| 1/2 \| NM\_174529 \|  \| GO:0005506  GO:0005783  GO:0005792  GO:0006118  GO:0016020  GO:0020037  GO:0046872  GO:0050381 \| 1396678 \| CYP2D6 \| Bt.665.1.S1\_s\_at \| \| Bt.5605.1.S1\_at \|  \|  \|  \|  \|  \|  \| MARCKS \| Bt.5605.1.S1\_at \| \| Bt.4357.1.S1\_at \| 615596 \| 1/1 \| NM\_001076384 \|  \| GO:0016021 \|  \| TSPAN7 \| Bt.4357.1.S1\_at \| \| Bt.25255.1.A1\_at \|  \|  \|  \|  \|  \|  \| SNTB2 \| Bt.25255.1.A1\_at \| \| Bt.15722.1.S1\_at \| 538548 \| 1/1 \| XM\_581057 \| XM\_581057 \| GO:0003723  GO:0005634  GO:0006364 \|  \| FBL \| Bt.15722.1.S1\_at \| \| Bt.21978.1.S1\_at \| 515039 \| 1/1 \| XM\_592983 \|  \| GO:0004106  GO:0009073 \|  \| KIAA1509 \| Bt.21978.1.S1\_at \| \| Bt.23002.1.S1\_s\_at \| 282199 \| 2/3 \| NM\_001077831  XM\_001250845  XM\_001250892 \| NM\_001077831 \| GO:0004129  GO:0005739  GO:0005740  GO:0006118  GO:0016020  GO:0016491 \| 1720401  2844245  10984454 \| COX6A1 \| Bt.23002.1.S1\_s\_at \| \| Bt.5306.1.S1\_at \| 513257 \| 2/2 \| NM\_001014915 \| NM\_001014915 \| GO:0005515 \| 12477932 \| TCAP \| Bt.5306.1.S1\_at \| \| Bt.20977.1.S1\_at \| 538508 \| 1/4 \| XM\_580792 \|  \| GO:0004601  GO:0006979 \|  \| CCPG1 \| Bt.20977.1.S1\_at \| \| Bt.5269.2.S1\_a\_at \| 280729 \| 2/3 \| NM\_173893 \| NM\_173893 \| GO:0002474  GO:0006955  GO:0042612 \| 3889925  6174509  8319984  12658628  15014972 \| B2M \| Bt.5269.2.S1\_a\_at \| \| Bt.7856.1.S1\_at \| 505005 \| 1/1 \| NM\_001045906 \| NM\_001045906 \|  \|  \| BCKDK \| Bt.7856.1.S1\_at \| \| Bt.27331.1.S1\_at \|  \|  \|  \|  \|  \|  \| C5orf13 \| Bt.27331.1.S1\_at \| \| Bt.13330.2.A1\_at \| 507367 \| 2/3 \| XM\_583960 \|  \|  \|  \| PDK4 \| Bt.13330.2.A1\_at \| \| Bt.23317.1.S1\_at \| 508221 \| 1/1 \| NM\_001015543 \|  \|  \| 12477932 \| RPL13 \| Bt.23317.1.S1\_at \| \| Bt.23354.1.S1\_at \| 535293 \| 1/2 \| NM\_001034629 \|  \| GO:0004301  GO:0005792  GO:0009636  GO:0016020  GO:0016787 \| 12477932 \| EPHX1 \| Bt.23354.1.S1\_at \| \| Bt.1767.1.S1\_at \| 281225 \| 1/1 \| NM\_174080 \| NM\_174080 \| GO:0004842  GO:0006464  GO:0006512  GO:0016874 \| 1322903  1714895  9657692  15723079 \| HIP2 \| Bt.1767.1.S1\_at \| \| Bt.1310.1.S1\_at \| 532775 \| 1/1 \| XM\_611947 \| XM\_611947 \|  \|  \| TMEM38A \| Bt.1310.1.S1\_at \| \| Bt.20505.1.S1\_at \| 532427 \| 1/1 \| XM\_610945 \|  \|  \|  \| TIGD2 \| Bt.20505.1.S1\_at \| \| Bt.9242.1.S1\_at \| 506521 \| 1/1 \| XM\_582978 \| XM\_582978 \|  \|  \| MRPL41 \| Bt.9242.1.S1\_at \| \| Bt.23603.1.S1\_a\_at \| 511798 \| 2/3 \| NM\_001034412 \| NM\_001034412 \| GO:0006512 \| 12477932 \| FBXO9 \| Bt.23603.1.S1\_a\_at \| \| Bt.8234.1.S1\_at \| 510316 \| 1/1 \| XM\_866189 \| XM\_866189 \|  \|  \| WDR42A \| Bt.8234.1.S1\_at \| \| Bt.16784.1.S1\_at \| 527394 \| 1/1 \| XM\_605785 \| XM\_605785 \| GO:0005515  GO:0008270 \|  \| PYGO1 \| Bt.16784.1.S1\_at \| \| Bt.20470.2.S1\_a\_at \| 525071 \| 2/2 \| NM\_001075863 \| NM\_001075863 \|  \|  \| MTP18 \| Bt.20470.2.S1\_a\_at \| \| Bt.26872.1.S1\_at \|  \|  \|  \| XM\_001253708 \|  \|  \|  \| Bt.26872.1.S1\_at \| \| Bt.23227.1.S1\_at \|  \|  \|  \|  \|  \|  \| MXD1 \| Bt.23227.1.S1\_at \| \| Bt.23173.1.S1\_at \| 506990 \| 1/1 \| NM\_001075294 \| NM\_001075294 \|  \|  \|  \| Bt.23173.1.S1\_at \| \| Bt.5267.1.S1\_at \| 327685 \| 1/1 \| XM\_585815 \|  \| GO:0005509  GO:0005544  GO:0006816  GO:0006937  GO:0048471 \| 9748523 \| ANXA6 \| Bt.5267.1.S1\_at \| \| Bt.7955.2.S1\_at \| 504471 \| 2/2 \| NM\_001045888 \|  \|  \|  \| PCOLCE \| Bt.7955.2.S1\_at \| \| Bt.20194.1.S1\_at \| 767923 \| 1/1 \| NM\_001076997 \|  \|  \|  \| HSPC268 \| Bt.20194.1.S1\_at \| \| Bt.2249.1.S1\_at \| 531753 \| 1/1 \| NM\_001040551 \|  \|  \|  \|  \| Bt.2249.1.S1\_at \| \| Bt.5596.1.A1\_at \| 414274 \| 1/5 \| XM\_868368 \| XM\_868368 \|  \|  \| SMARCA4 \| Bt.5596.1.A1\_at \| \| Bt.4292.1.S1\_at \| 281597 \| 1/1 \| NM\_174226 \|  \| GO:0000166  GO:0005198  GO:0005515  GO:0005524  GO:0005856 \| 1530599  11721045  12176354  14757065  15117967  15505213  15592479  16253999  17251352 \| ACTR3 \| Bt.4292.1.S1\_at \| \| Bt.21670.1.A1\_at \| 767932 \| 1/1 \| NM\_001077005 \| NM\_001077005 \|  \|  \| AZI2 \| Bt.21670.1.A1\_at \| \| Bt.11196.1.S1\_at \| 281816 \| 1/1 \| NM\_174340 \|  \| GO:0005739  GO:0016787 \|  \| HINT2 \| Bt.11196.1.S1\_at \| \| Bt.26942.1.S1\_at \| 530164 \| 1/1 \| XM\_608629 \| XM\_608629 \|  \|  \| SLC17A5 \| Bt.26942.1.S1\_at \| \| Bt.4917.1.S1\_at \| 539339 \| 1/1 \| NM\_001033628 \|  \| GO:0005524  GO:0005525  GO:0005622  GO:0006355  GO:0007264  GO:0008134  GO:0015031 \| 11282978  12477932  16305752 \| RAB1A \| Bt.4917.1.S1\_at \| \| Bt.20955.1.A1\_at \|  \|  \|  \|  \|  \|  \| HLF \| Bt.20955.1.A1\_at \| \| Bt.18755.2.S1\_at \| 534761 \| 2/4 \| XM\_873360 \| XM\_873360 \| GO:0004672  GO:0005524  GO:0006468 \| 16519507 \| GSK3B \| Bt.18755.2.S1\_at \| \| Bt.28583.1.S1\_s\_at \| 282187  404102 \| 1/1 \| AB098904  NM\_001034039 \|  \|  \|  \| COL1A1 \| Bt.28583.1.S1\_s\_at \| \| Bt.2889.1.S1\_at \| 445424 \| 1/1 \| NM\_001003905 \| NM\_001003905 \|  \|  \| GTL3 \| Bt.2889.1.S1\_at \| \| Bt.2262.1.S1\_at \| 539381 \| 1/1 \| NM\_001038183 \|  \| GO:0005634  GO:0007275  GO:0008270  GO:0030154  GO:0046872 \|  \| CSRP2 \| Bt.2262.1.S1\_at \| \| Bt.21721.1.A1\_at \| 522980 \| 1/1 \| NM\_001046263 \| NM\_001046263 \|  \|  \| USP2 \| Bt.21721.1.A1\_at \| \| Bt.23094.3.S1\_x\_at \| 282138 \| 3/8 \| NM\_181027 \| XM\_001249710 \| GO:0001516  GO:0006633  GO:0016491  GO:0047017 \| 1339268  2829166  9867836  12551929 \|  \| Bt.23094.3.S1\_x\_at \| \| Bt.9092.1.S1\_at \| 526320 \| 1/1 \| NM\_001015633 \| NM\_001015633 \|  \| 12140684  12477932  16305752 \| POLR2G \| Bt.9092.1.S1\_at \| \| Bt.4959.1.A1\_at \|  \|  \|  \|  \|  \|  \| FADS1 \| Bt.4959.1.A1\_at \| \| Bt.8618.1.S1\_at \| 505129 \| 1/5 \| XM\_001252366  XM\_001252396  XM\_001252429  XM\_581371 \| NM\_001080921 \|  \|  \|  \| Bt.8618.1.S1\_at \| \| Bt.3843.1.S1\_at \| 280821 \| 1/1 \| NM\_175773 \| NM\_175773 \|  \| 1427820  8286037  12477932 \| IGJ \| Bt.3843.1.S1\_at \| \| Bt.26483.1.A1\_at \| 540513 \| 1/1 \| XM\_868353 \|  \|  \|  \| RHOBTB1 \| Bt.26483.1.A1\_at \| \| Bt.1696.1.S1\_at \| 618777 \| 1/1 \| NM\_001046623 \| NM\_001046623 \|  \| 11282978  16305752 \| SAMM50 \| Bt.1696.1.S1\_at \| \| Bt.16123.1.S1\_at \| 515854 \| 1/3 \| XM\_864644 \| XM\_864644 \|  \|  \| SGK \| Bt.16123.1.S1\_at \| \| Bt.26629.1.S1\_a\_at \| 540932 \| 1/1 \| NM\_001076249 \| NM\_001076249 \|  \|  \| FBXW7 \| Bt.26629.1.S1\_a\_at \| \| Bt.14340.1.A1\_at \| 511639 \| 2/2 \| XR\_027604 \|  \|  \|  \| RNF103 \| Bt.14340.1.A1\_at \| \| Bt.1269.1.S1\_a\_at \| 326582 \| 1/3 \| NM\_001014387 \| NM\_001014387 \| GO:0003735  GO:0005622  GO:0005840  GO:0006412 \| 12477932  12658628 \| RPS12 \| Bt.1269.1.S1\_a\_at \| \| Bt.21031.1.S1\_at \| 507524 \| 1/5 \| XM\_001250438  XM\_001250487  XM\_865076 \|  \|  \|  \| LEO1 \| Bt.21031.1.S1\_at \| \| Bt.21681.1.A1\_at \| 535452 \| 1/2 \| NM\_001076059 \|  \| GO:0005634  GO:0006512 \|  \| FBXL4 \| Bt.21681.1.A1\_at \| \| Bt.3567.1.S1\_at \| 281565 \| 1/2 \| NM\_174212 \|  \|  \| 8407878 \| UGP2 \| Bt.3567.1.S1\_at \| \| Bt.27194.1.S1\_at \| 534958 \| 1/2 \| XM\_001252536  XM\_614905 \|  \| GO:0004182  GO:0006508 \|  \| AGTPBP1 \| Bt.27194.1.S1\_at \| \| Bt.4175.2.S1\_at \| 512534 \| 1/3 \| NM\_001046124 \|  \| GO:0008717  GO:0016021 \|  \|  \| Bt.4175.2.S1\_at \| \| Bt.8549.1.S2\_at \|  \|  \|  \|  \|  \|  \| COL1A1 \| Bt.8549.1.S2\_at \| \| Bt.9588.1.S1\_at \| 513128 \| 1/1 \| NM\_001040520 \| NM\_001040520 \| GO:0003735  GO:0005622  GO:0005840  GO:0006412  GO:0042254 \| 11683713  12140684  16305752 \|  \| Bt.9588.1.S1\_at \| \| Bt.13886.2.S1\_at \| 534067 \| 1/3 \| XM\_613697 \| XM\_613697 \| GO:0004221  GO:0005097  GO:0005622  GO:0006511  GO:0006810  GO:0032313 \|  \| USP34 \| Bt.13886.2.S1\_at \| \| Bt.9405.1.S1\_at \| 514443 \| 1/1 \| XM\_592297 \|  \|  \|  \| TAF10 \| Bt.9405.1.S1\_at \| \| Bt.4831.1.S1\_at \| 281942 \| 1/1 \| NM\_174400 \|  \|  \|  \| NDP52 \| Bt.4831.1.S1\_at \| \| Bt.13610.1.A1\_at \| 534185 \| 1/1 \| NM\_001076015 \|  \| GO:0005739  GO:0030529 \| 11279123 \| MRPS31 \| Bt.13610.1.A1\_at \| \| Bt.3427.1.S1\_at \| 618441 \| 1/1 \| NM\_001034810 \| NM\_001034810 \|  \| 12477932 \| MGC128294 \| Bt.3427.1.S1\_at \| \| Bt.10730.1.S1\_at \| 540637 \| 1/1 \| XM\_612416 \|  \|  \|  \| EPLIN \| Bt.10730.1.S1\_at \| \| Bt.689.1.S1\_at \| 615565 \| 1/2 \| NM\_001034784 \| NM\_001034784 \| GO:0005488  GO:0005739  GO:0006915  GO:0016020  GO:0016021 \| 12477932 \| TTC11 \| Bt.689.1.S1\_at \| \| Bt.23094.6.A1\_at \| 617917 \| 1/1 \| NM\_001038584 \| NM\_001038584 \|  \|  \|  \| Bt.23094.6.A1\_at \| \| Bt.7307.2.A1\_a\_at \|  \|  \|  \|  \|  \|  \|  \| Bt.7307.2.A1\_a\_at \| \| Bt.7008.1.S1\_at \| 528253 \| 1/3 \| XM\_606667 \| XM\_606667 \| GO:0003677  GO:0005622  GO:0005634 \|  \| JARID2 \| Bt.7008.1.S1\_at \| \| Bt.10515.1.S1\_at \| 531367 \| 1/1 \| XM\_609859 \| XM\_609859 \| GO:0005506  GO:0006118  GO:0009055  GO:0016209  GO:0020037  GO:0045454 \|  \| NXN \| Bt.10515.1.S1\_at \| \| Bt.15717.1.A1\_at \| 512918 \| 1/2 \| XM\_595995 \| XM\_595995 \| GO:0005515  GO:0007154  GO:0035091 \|  \|  \| Bt.15717.1.A1\_at \| \| Bt.20559.2.A1\_at \| 522692 \| 1/2 \| XM\_600977 \|  \|  \|  \| AFURS1 \| Bt.20559.2.A1\_at \| \| Bt.11662.1.A1\_at \| 523311 \| 1/1 \| XM\_601607 \|  \|  \|  \| GPX7 \| Bt.11662.1.A1\_at \| \| Bt.27563.1.A1\_at \| 538576 \| 1/1 \| XM\_865427 \| XM\_865427 \|  \|  \| OSBPL11 \| Bt.27563.1.A1\_at \| \| Bt.9238.1.S1\_at \| 338044 \| 1/2 \| XM\_588265 \| NM\_001082424 \|  \|  \| SLC38A2 \| Bt.9238.1.S1\_at \| \| Bt.7700.1.S1\_at \| 524770 \| 1/3 \| NM\_001035351 \| NM\_001035351 \|  \| 153745  12477932 \| ACTN1 \| Bt.7700.1.S1\_at \| \| Bt.13991.2.S1\_at \| 507736 \| 1/5 \| NM\_001034308 \| NM\_001034308 \|  \| 12477932 \| PNRC1 \| Bt.13991.2.S1\_at \| \| Bt.1821.1.S1\_at \|  \|  \|  \|  \|  \|  \|  \| Bt.1821.1.S1\_at \| \| Bt.3437.1.S1\_at \| 533700 \| 1/2 \| XM\_613184 \|  \| GO:0000166  GO:0003676  GO:0005524  GO:0008026  GO:0016787 \| 12477932 \| DDX5 \| Bt.3437.1.S1\_at \| \| Bt.5676.1.S1\_at \| 533208 \| 1/2 \| NM\_001075984 \| NM\_001075984 \| GO:0005739  GO:0006744  GO:0008168  GO:0016740 \|  \|  \| Bt.5676.1.S1\_at \| \| Bt.2934.1.S1\_at \| 509426 \| 1/1 \| NM\_001015561 \| XM\_001251505 \|  \|  \|  \| Bt.2934.1.S1\_at \| \| Bt.5466.2.S1\_a\_at \| 286873  614241 \| 1/1 \| NM\_001035445  XM\_591678 \|  \|  \|  \| RPS4Y1 \| Bt.5466.2.S1\_a\_at \| \| Bt.15298.1.A1\_at \|  \|  \|  \|  \|  \|  \| FLJ14054 \| Bt.15298.1.A1\_at \| \| Bt.11855.1.S1\_at \| 510727 \| 1/1 \| NM\_001038074 \| NM\_001038074 \|  \|  \| COX4NB \| Bt.11855.1.S1\_at \| \| Bt.24395.1.S1\_at \|  \|  \|  \|  \|  \|  \| KCNIP2 \| Bt.24395.1.S1\_at \| \| Bt.11178.1.S1\_at \| 615239 \| 1/1 \| NM\_001035463 \| NM\_001035463 \| GO:0005578 \| 12477932 \| GPC3 \| Bt.11178.1.S1\_at \| \| Bt.5631.1.S1\_at \| 613998 \| 1/1 \| XM\_877831 \| XM\_877831 \| GO:0030001  GO:0046872 \| 12477932 \| ATOX1 \| Bt.5631.1.S1\_at \| \| Bt.17573.1.A1\_at \| 534401 \| 1/2 \| NM\_001037476 \|  \| GO:0003676  GO:0005515  GO:0005622  GO:0006350  GO:0006355  GO:0008270  GO:0046872 \| 11282978  12477932  16305752 \| ZBTB16 \| Bt.17573.1.A1\_at \| \| Bt.8876.1.S1\_at \|  \|  \|  \|  \|  \|  \| FAM20B \| Bt.8876.1.S1\_at \| \| Bt.4937.1.S1\_at \| 505941 \| 1/3 \| XR\_028249 \|  \|  \|  \| RRBP1 \| Bt.4937.1.S1\_at \| \| Bt.4671.1.S1\_at \| 281994 \| 1/1 \| NM\_174429 \| NM\_174429 \| GO:0000287  GO:0006470  GO:0008287  GO:0015071  GO:0016787  GO:0030145 \| 9486768 \| PPM1A \| Bt.4671.1.S1\_at \| \| Bt.25109.2.A1\_at \|  \|  \|  \|  \|  \|  \| PLOD2 \| Bt.25109.2.A1\_at \| \| Bt.5203.1.S1\_at \| 281999 \| 1/1 \| NM\_174433 \| NM\_174433 \| GO:0016491  GO:0051920 \| 14662316 \| PRDX4 \| Bt.5203.1.S1\_at \| \| Bt.6950.1.S1\_at \|  \|  \|  \|  \|  \|  \| C20orf35 \| Bt.6950.1.S1\_at \| \| Bt.5306.2.S1\_a\_at \| 513257 \| 2/2 \| NM\_001014915 \| NM\_001014915 \| GO:0005515 \| 12477932 \| TCAP \| Bt.5306.2.S1\_a\_at \| \| Bt.27237.1.S1\_a\_at \|  \|  \|  \| NM\_001045943 \|  \|  \| ALG5 \| Bt.27237.1.S1\_a\_at \| \| Bt.7187.1.S1\_at \| 505507 \| 1/3 \| XM\_581800 \| XM\_581800 \|  \|  \|  \| Bt.7187.1.S1\_at \| \| Bt.19721.1.S1\_at \| 535414 \| 1/1 \| NM\_001046406 \| NM\_001046406 \| GO:0005829  GO:0043234 \|  \| PSMD10 \| Bt.19721.1.S1\_at \| \| Bt.9424.1.S1\_at \| 514186 \| 1/1 \| XM\_870453 \| XM\_870453 \|  \|  \| DMN \| Bt.9424.1.S1\_at \| \| Bt.9086.1.S1\_at \| 616331 \| 1/2 \| NM\_001040591 \| NM\_001040591 \|  \|  \| AP1S2 \| Bt.9086.1.S1\_at \| \| Bt.20229.1.S1\_at \| 767855 \| 1/1 \| NM\_001076967 \| NM\_001076967 \| GO:0004672  GO:0005524  GO:0006915 \|  \| TBRG4 \| Bt.20229.1.S1\_at \| \| Bt.23552.1.S1\_at \| 509905 \| 1/1 \| XM\_586968 \|  \|  \|  \| SFRS9 \| Bt.23552.1.S1\_at \| \| Bt.20852.1.A1\_at \|  \|  \|  \|  \|  \|  \|  \| Bt.20852.1.A1\_at \| \| Bt.8643.1.A1\_at \|  \|  \|  \| NM\_001075579 \|  \|  \| RCN2 \| Bt.8643.1.A1\_at \| \| Bt.26692.1.S1\_a\_at \| 615342 \| 1/2 \| NM\_001076366 \| NM\_001076366 \| GO:0005635  GO:0005739  GO:0006309  GO:0006338  GO:0006800  GO:0006915  GO:0006917  GO:0008219  GO:0008634  GO:0016020  GO:0016021  GO:0031307  GO:0031966  GO:0042803  GO:0045837  GO:0046902  GO:0046982  GO:0051607 \|  \| BNIP3 \| Bt.26692.1.S1\_a\_at \| \| Bt.26685.1.S1\_at \| 616007 \| 1/1 \| NM\_001046570 \|  \|  \|  \| HSPB3 \| Bt.26685.1.S1\_at \| \| Bt.3767.2.S1\_at \| 532791 \| 1/3 \| XM\_881853 \| XM\_881853 \| GO:0005515 \| 11282978  16305752 \| KEAP1 \| Bt.3767.2.S1\_at \| \| Bt.7968.2.S1\_at \| 532726 \| 2/5 \| NM\_001035392 \|  \| GO:0004864  GO:0005975  GO:0005977  GO:0009966 \| 12477932 \|  \| Bt.7968.2.S1\_at \| \| Bt.10869.1.S1\_at \|  \|  \|  \| XR\_028406 \|  \|  \| DIAPH1 \| Bt.10869.1.S1\_at \| \| Bt.3541.1.S1\_at \| 526135 \| 1/1 \| NM\_001035361 \|  \|  \| 12477932 \| RBMS1 \| Bt.3541.1.S1\_at \| \| Bt.23148.1.S1\_at \|  \|  \|  \|  \|  \|  \| COL5A1 \| Bt.23148.1.S1\_at \| \| Bt.5519.2.S1\_at \| 512562 \| 1/1 \| NM\_001046125 \| NM\_001046125 \|  \|  \| MAGED1 \| Bt.5519.2.S1\_at \| \| Bt.25098.1.S1\_at \| 520518 \| 1/1 \| XM\_598765 \| XM\_598765 \|  \|  \| C1orf163 \| Bt.25098.1.S1\_at \| \| Bt.1268.2.S1\_at \|  \|  \|  \|  \|  \|  \| SET7 \| Bt.1268.2.S1\_at \| \| Bt.8279.3.S1\_a\_at \| 511968 \| 1/3 \| NM\_001046105 \|  \| GO:0005634 \|  \| DKFZP564J0123 \| Bt.8279.3.S1\_a\_at \| \| Bt.22106.2.S1\_at \| 533729 \| 1/4 \| NM\_001076898 \|  \| GO:0000166  GO:0004674  GO:0005524  GO:0006468  GO:0016740 \|  \| PAK1 \| Bt.22106.2.S1\_at \| \| Bt.27101.1.S1\_at \| 540901 \| 1/1 \| XM\_866850 \|  \|  \|  \| PRRX1 \| Bt.27101.1.S1\_at \| \| Bt.5466.4.S1\_at \| 286798 \| 1/3 \| NM\_174766 \|  \| GO:0003779  GO:0005856  GO:0007242 \| 11023826 \| TNS1 \| Bt.5466.4.S1\_at \| \| Bt.22472.1.S1\_at \| 540605 \| 1/5 \| NM\_001034710 \| NM\_001034710 \| GO:0003700  GO:0005634  GO:0006350  GO:0006355  GO:0043565  GO:0046983 \| 12477932 \| CREM \| Bt.22472.1.S1\_at \| \| Bt.15672.2.S1\_at \|  \|  \|  \| XM\_866255 \|  \|  \| CBFB \| Bt.15672.2.S1\_at \| \| Bt.22803.1.S2\_at \| 533307 \| 1/1 \| XM\_612678 \| XM\_612678 \|  \|  \|  \| Bt.22803.1.S2\_at \| \| Bt.2439.1.S1\_at \| 513091 \| 1/1 \| XM\_590721 \| XM\_590721 \|  \| 11420620 \| NUMA1 \| Bt.2439.1.S1\_at \| \| Bt.4825.1.A1\_at \| 507607 \| 1/1 \| NM\_001034305 \| NM\_001034305 \|  \| 12477932 \| CCDC28B \| Bt.4825.1.A1\_at \| \| Bt.20897.2.S1\_a\_at \| 505225 \| 1/3 \| NM\_001075194 \|  \|  \|  \| ADD3 \| Bt.20897.2.S1\_a\_at \| \| Bt.9662.1.S1\_at \| 282331 \| 1/1 \| NM\_174589 \| NM\_174589 \| GO:0001584  GO:0004872  GO:0004957  GO:0007165  GO:0007186  GO:0016020  GO:0016021 \| 11282978  12810564  16305752 \| PTGER4 \| Bt.9662.1.S1\_at \| \| Bt.3975.1.S1\_at \| 506522 \| 1/2 \| XM\_582979 \| XM\_582979 \|  \|  \| C9orf112 \| Bt.3975.1.S1\_at \| \| Bt.27267.1.S1\_at \| 506534 \| 1/1 \| NM\_001075267 \| NM\_001075267 \|  \|  \| TMEM4 \| Bt.27267.1.S1\_at \| \| Bt.28263.1.S1\_at \| 509965 \| 1/1 \| NM\_001046044 \| NM\_001046044 \|  \|  \| CMPK \| Bt.28263.1.S1\_at \| \| Bt.26711.2.S1\_at \|  \|  \|  \|  \|  \|  \| LRRC20 \| Bt.26711.2.S1\_at \| \| Bt.13218.2.S1\_at \| 528100 \| 1/4 \| XM\_606511 \| XM\_606511 \| GO:0005783  GO:0006605  GO:0015031  GO:0016020  GO:0016021 \| 12477932 \| SERP1 \| Bt.13218.2.S1\_at \| \| Bt.21151.1.S1\_at \| 532203 \| 1/2 \| NM\_001034576 \| NM\_001034576 \| GO:0003735  GO:0005622  GO:0005739  GO:0005840  GO:0006412 \| 12477932  16510155 \| MRPL24 \| Bt.21151.1.S1\_at \| \| Bt.27617.1.S1\_at \| 527935 \| 1/1 \| XM\_606342 \|  \| GO:0003676  GO:0005622  GO:0008270 \|  \| GLIS2 \| Bt.27617.1.S1\_at \| \| Bt.14037.1.S1\_at \|  \|  \|  \|  \|  \|  \| SAP30L \| Bt.14037.1.S1\_at \| \| Bt.1552.1.S1\_at \| 281476 \| 1/1 \| NM\_174175 \|  \|  \| 12477932  15213404 \| SARS \| Bt.1552.1.S1\_at \| \| Bt.8073.1.S1\_at \| 514547 \| 1/1 \| NM\_001046175 \|  \|  \|  \| MAP1LC3A \| Bt.8073.1.S1\_at \| \| Bt.1537.1.S1\_at \| 504499 \| 1/1 \| NM\_001035009 \| NM\_001035009 \|  \| 12477932 \| NDRG1 \| Bt.1537.1.S1\_at \| \| Bt.5650.1.S1\_at \| 541054 \| 1/1 \| NM\_001034722 \| NM\_001034722 \|  \| 12477932 \| BTG3 \| Bt.5650.1.S1\_at \| \| Bt.12327.1.S1\_at \| 506790 \| 1/1 \| XM\_583286 \| XM\_583286 \|  \|  \| TXNIP \| Bt.12327.1.S1\_at \| \| Bt.12885.2.S1\_at \| 534582 \| 1/1 \| XR\_027954 \|  \|  \|  \| NFE2L1 \| Bt.12885.2.S1\_at \| \| Bt.27130.1.S1\_at \| 507289 \| 1/1 \| NM\_001034288 \| NM\_001034288 \|  \| 12477932 \| SCCPDH \| Bt.27130.1.S1\_at \| \| Bt.946.1.S1\_at \| 280716 \| 1/3 \| NM\_173889 \| NM\_173889 \| GO:0000166  GO:0004017  GO:0005524  GO:0005739  GO:0005739  GO:0005743  GO:0005743  GO:0006139  GO:0016301  GO:0016740 \| 2227435  3002789  3040716  8868479  11282978  16305752 \| AK2 \| Bt.946.1.S1\_at \| \| Bt.15397.1.S1\_a\_at \| 282884 \| 1/2 \| NM\_001024469 \|  \| GO:0003735  GO:0005622  GO:0005840  GO:0006412 \| 12477932  12658628 \| RPL9 \| Bt.15397.1.S1\_a\_at \| \| Bt.4790.1.S1\_at \| 540207 \| 1/1 \| NM\_001034693 \| NM\_001034693 \| GO:0000166  GO:0004674  GO:0004713  GO:0005524  GO:0006468  GO:0016740 \| 12477932  15316070  16723384 \| ILK \| Bt.4790.1.S1\_at \| \| Bt.2856.1.S1\_at \| 511545 \| 1/1 \| NM\_001075525 \| NM\_001075525 \|  \|  \| GRCC10 \| Bt.2856.1.S1\_at \| \| Bt.2001.1.S1\_at \| 613488 \| 1/1 \| XM\_873100 \|  \|  \|  \|  \| Bt.2001.1.S1\_at \| \| Bt.3921.1.S1\_at \| 540965 \| 1/1 \| NM\_001034716 \| NM\_001034716 \| GO:0030529 \| 12477932  12658628 \| MGC127433 \| Bt.3921.1.S1\_at \| \| Bt.23182.1.S1\_at \| 281156 \| 1/1 \| NM\_177497 \| NM\_177497 \|  \| 11773414 \| FDPS \| Bt.23182.1.S1\_at \| \| Bt.21032.1.S1\_at \| 532693 \| 1/1 \| NM\_001040553 \| NM\_001040553 \|  \|  \| NUDCD2 \| Bt.21032.1.S1\_at \| \| Bt.26358.1.S1\_at \|  \|  \|  \|  \|  \|  \| STAM \| Bt.26358.1.S1\_at \| \| Bt.7633.1.S1\_at \| 617105 \| 1/1 \| NM\_001040599 \| NM\_001040599 \|  \|  \|  \| Bt.7633.1.S1\_at \| \| Bt.5605.2.S1\_at \|  \|  \|  \|  \|  \|  \| MARCKS \| Bt.5605.2.S1\_at \| \| Bt.9055.2.S1\_a\_at \| 616451 \| 1/2 \| NM\_001038213 \| NM\_001038213 \|  \|  \| TRMU \| Bt.9055.2.S1\_a\_at \| \| Bt.10442.2.S1\_a\_at \| 359712 \| 1/2 \| XM\_611630 \| XM\_611630 \| GO:0005198  GO:0005578  GO:0005737  GO:0006817  GO:0007155 \| 15157235 \| COL12A1 \| Bt.10442.2.S1\_a\_at \| \| Bt.2519.1.S1\_at \| 281408 \| 1/1 \| NM\_174147 \| NM\_174147 \|  \| 8385052  11204721  15128599 \| PLAU \| Bt.2519.1.S1\_at \| \| Bt.8641.1.A1\_at \| 538724 \| 1/1 \| XM\_582247 \|  \|  \|  \| GSPT2 \| Bt.8641.1.A1\_at \| \| Bt.4986.1.S1\_at \| 515878 \| 1/1 \| NM\_001014931 \| NM\_001014931 \| GO:0005783  GO:0016020  GO:0016021 \| 12140684  16305752 \| ORMDL2 \| Bt.4986.1.S1\_at \| \| Bt.23273.1.S1\_at \| 507120 \| 1/1 \| NM\_001034278 \| NM\_001034278 \|  \| 12477932 \| CIRBP \| Bt.23273.1.S1\_at \| \| Bt.2841.1.S1\_at \| 281576 \| 1/3 \| NM\_174218 \| XM\_001256626 \| GO:0000166  GO:0004830  GO:0005524  GO:0006436  GO:0016874 \| 1907847  2631684  12477932  15579907 \| WARS \| Bt.2841.1.S1\_at \| \| Bt.3573.1.S2\_at \| 282220 \| 1/5 \| NM\_174535 \|  \| GO:0000166  GO:0003746  GO:0003924  GO:0005515  GO:0005525 \| 12078752  12658628 \|  \| Bt.3573.1.S2\_at \| \| Bt.9053.1.S1\_a\_at \| 613568 \| 1/2 \| NM\_001046509 \| NM\_001046509 \|  \|  \|  \| Bt.9053.1.S1\_a\_at \| \| Bt.2575.1.S1\_at \| 510041 \| 1/1 \| NM\_001024509 \|  \|  \| 7989312 \| PSME1 \| Bt.2575.1.S1\_at \| \| Bt.7192.1.S1\_at \| 506988 \| 1/1 \| NM\_001034274 \| NM\_001034274 \|  \| 12477932 \| MRPL17 \| Bt.7192.1.S1\_at \| \| Bt.7648.1.S1\_at \| 516063 \| 1/1 \| NM\_001037467 \| NM\_001037467 \| GO:0003735  GO:0005515  GO:0005622  GO:0005730  GO:0005840  GO:0005843  GO:0006412  GO:0030218 \| 12477932 \| MGC133566 \| Bt.7648.1.S1\_at \| \| Bt.13237.1.S1\_at \| 510423 \| 1/1 \| NM\_001034381 \|  \|  \| 12477932 \| PSMA4 \| Bt.13237.1.S1\_at \| \| Bt.20193.1.S1\_at \| 408010 \| 1/1 \| XM\_614980 \|  \| GO:0004820  GO:0005524  GO:0006426  GO:0016874 \|  \| GARS \| Bt.20193.1.S1\_at \| \| Bt.22167.2.A1\_at \| 541113 \| 2/2 \| XM\_614609 \| NM\_001080311 \| GO:0005515 \|  \| LRRC2 \| Bt.22167.2.A1\_at \| \| Bt.16329.1.A1\_at \| 510050 \| 1/4 \| XM\_587139 \|  \|  \| 12477932 \| TGIF \| Bt.16329.1.A1\_at \| \| Bt.12429.1.S1\_at \| 515380 \| 1/1 \| NM\_001035307 \| NM\_001035307 \| GO:0005516  GO:0005634  GO:0006350  GO:0006355  GO:0007275  GO:0030154  GO:0043565 \| 12477932 \| EDF1 \| Bt.12429.1.S1\_at \| \| Bt.15882.2.S1\_a\_at \| 508319 \| 1/3 \| NM\_001075350 \|  \|  \|  \| SRI \| Bt.15882.2.S1\_a\_at \| \| Bt.2847.1.S1\_a\_at \| 281341 \| 1/1 \| NM\_175780 \| NM\_175780 \| GO:0003774  GO:0005509  GO:0008307  GO:0016461  GO:0030898 \| 2263497  2971667  12477932 \| MYL6 \| Bt.2847.1.S1\_a\_at \| \| Bt.22556.1.S1\_at \|  \|  \|  \|  \|  \|  \| RPP14 \| Bt.22556.1.S1\_at \| \| Bt.3296.1.A1\_at \| 518288 \| 1/1 \| NM\_001075770 \| NM\_001075770 \| GO:0005488  GO:0016702  GO:0016706  GO:0019538 \|  \| P4HA1 \| Bt.3296.1.A1\_at \| \| Bt.3240.1.S1\_at \| 616999 \| 1/1 \| XR\_027593 \| XR\_027593 \|  \|  \| SELT \| Bt.3240.1.S1\_at \| \| Bt.24200.1.S1\_at \|  \|  \|  \|  \|  \|  \|  \| Bt.24200.1.S1\_at \| \| Bt.3162.1.S1\_at \| 281373 \| 1/1 \| NM\_174135 \| NM\_174135 \|  \| 2351674  3619939 \| P4HB \| Bt.3162.1.S1\_at \| \| Bt.9664.1.A1\_at \| 538743 \| 1/2 \| NM\_001076126 \|  \| GO:0004221  GO:0006511  GO:0008233 \|  \| USP8 \| Bt.9664.1.A1\_at \| \| Bt.4263.1.S1\_at \| 281801 \| 1/1 \| NM\_174331 \| NM\_174331 \| GO:0000166  GO:0004703  GO:0004871  GO:0005524  GO:0006468  GO:0007165  GO:0016740 \| 8120045 \| GRK5 \| Bt.4263.1.S1\_at \| \| Bt.27018.1.S1\_at \| 521237 \| 1/1 \| NM\_001046255 \| NM\_001046255 \| GO:0004672  GO:0005524  GO:0006468 \|  \| STK16 \| Bt.27018.1.S1\_at \| \| Bt.2822.1.S1\_at \| 535056 \| 1/1 \| NM\_001034625 \|  \| GO:0003735  GO:0005622  GO:0005840  GO:0006412  GO:0019843 \| 12477932 \| RPL8 \| Bt.2822.1.S1\_at \| \| Bt.27904.1.A1\_at \|  \|  \|  \|  \|  \|  \| GSK3B \| Bt.27904.1.A1\_at \| \| Bt.9027.1.S1\_at \| 282291 \| 1/1 \| NM\_001045868 \| NM\_001045868 \|  \| 12459277  12711254  15310755 \| NFKBIA \| Bt.9027.1.S1\_at \| \| Bt.27927.1.S1\_at \| 532674 \| 1/2 \| XM\_878913 \| XM\_878913 \|  \|  \| GPR116 \| Bt.27927.1.S1\_at \| \| Bt.22329.1.S1\_at \| 614795 \| 1/1 \| NM\_001035456 \|  \| GO:0003755  GO:0006457  GO:0016853 \| 1701173  1706222  1710319  1710782  1718307  1868545  1915353  2041572  2253615  2477715  7543369  12140684  12477932  16305752 \| FKBP1A \| Bt.22329.1.S1\_at \| \| Bt.8124.1.S1\_at \| 282188 \| 2/2 \| NM\_174520 \|  \| GO:0005201  GO:0005581  GO:0005737  GO:0006817 \| 173531  4412529  4435743  4609475  9628255 \| COL1A2 \| Bt.8124.1.S1\_at \| \| Bt.24039.1.A1\_at \| 522998 \| 1/1 \| XR\_028333 \|  \|  \|  \| CCDC6 \| Bt.24039.1.A1\_at \| \| Bt.10916.2.S1\_at \| 507430 \| 1/5 \| NM\_001034298 \| NM\_001034298 \|  \| 12477932 \| NIPA2 \| Bt.10916.2.S1\_at \| \| Bt.13440.1.S1\_at \|  \|  \|  \|  \|  \|  \| LKAP \| Bt.13440.1.S1\_at \| \| Bt.25689.1.S1\_at \|  \|  \|  \| XM\_001252094 \|  \|  \|  \| Bt.25689.1.S1\_at \| \| Bt.3560.1.S1\_at \|  \|  \|  \| XM\_001254241 \|  \|  \|  \| Bt.3560.1.S1\_at \| \| Bt.15878.1.S1\_at \| 540559 \| 1/1 \| NM\_001076227 \|  \|  \|  \| LANCL1 \| Bt.15878.1.S1\_at \| \| Bt.21376.1.S1\_at \| 507815 \| 1/1 \| NM\_001045980 \|  \|  \|  \| ITM1 \| Bt.21376.1.S1\_at \| \| Bt.21981.3.S1\_at \|  \|  \|  \|  \|  \|  \| ANTXR1 \| Bt.21981.3.S1\_at \| \| Bt.12510.1.S1\_a\_at \| 509824 \| 2/6 \| XM\_872590 \| XM\_872590 \|  \|  \|  \| Bt.12510.1.S1\_a\_at \| \| Bt.18352.1.A1\_at \| 504718 \| 1/1 \| XM\_580882 \|  \|  \|  \| GADD45GIP1 \| Bt.18352.1.A1\_at \| \| Bt.15870.1.S1\_at \| 528165 \| 1/1 \| XM\_606577 \| XM\_606577 \| GO:0005744  GO:0006886  GO:0008152  GO:0015450  GO:0016740 \|  \|  \| Bt.15870.1.S1\_at \| \| Bt.5246.1.S1\_at \| 510949 \| 1/2 \| XM\_868352  XM\_881618 \| XM\_868352 \| GO:0003824  GO:0004018  GO:0009152 \|  \| ADSL \| Bt.5246.1.S1\_at \| \| Bt.20025.1.S1\_at \| 618609 \| 1/1 \| NM\_001076514 \|  \|  \| 11551941 \| MRPL21 \| Bt.20025.1.S1\_at \| \| Bt.5377.1.S1\_at \| 282028 \| 1/1 \| NM\_001033612 \|  \| GO:0000166  GO:0005525  GO:0007264  GO:0016020 \| 12477932 \| RAB34 \| Bt.5377.1.S1\_at \| \| Bt.24421.1.A1\_at \| 537248 \| 1/1 \| XR\_028059 \| XR\_028059 \|  \|  \| ACPL2 \| Bt.24421.1.A1\_at \| \| Bt.11194.1.S1\_at \| 281360 \| 1/1 \| NM\_174822 \| NM\_174822 \| GO:0004090  GO:0005739  GO:0005777  GO:0008152  GO:0016491 \| 15487511 \| NDRD \| Bt.11194.1.S1\_at \| \| Bt.4773.4.S1\_at \| 282147 \| 1/4 \| NM\_174504 \| NM\_174504 \| GO:0000166  GO:0005524  GO:0005739  GO:0006811  GO:0015986  GO:0015992  GO:0016469  GO:0016787  GO:0046872  GO:0046933  GO:0046961 \| 1386191  1722207  1835703  12477932  12708314 \| ATP6V1A \| Bt.4773.4.S1\_at \| \| Bt.8356.1.S1\_at \| 529761 \| 1/1 \| NM\_001040549 \| NM\_001040549 \| GO:0006888  GO:0015031  GO:0016020  GO:0016021 \| 12140684  16305752 \| TMED10 \| Bt.8356.1.S1\_at \| \| Bt.3455.1.S1\_at \| 515573 \| 1/1 \| XM\_001252935  XM\_593614 \| XM\_001252935 \| GO:0006464  GO:0006512  GO:0019787 \| 12354154 \| UBE2I \| Bt.3455.1.S1\_at \| \| Bt.2564.1.S1\_at \| 338049 \| 1/1 \| NM\_176645 \| NM\_176645 \| GO:0000166  GO:0000287  GO:0003924  GO:0004871  GO:0005525  GO:0005622  GO:0005856  GO:0007264  GO:0007266  GO:0016020  GO:0030036  GO:0042346  GO:0043123 \| 1905729  2174426  2498316  2506852  3141419  12477932  15075376  15371459  15937065  16627366 \| RHOA \| Bt.2564.1.S1\_at \| \| Bt.19332.1.S1\_at \|  \|  \|  \|  \|  \|  \| CLCN5 \| Bt.19332.1.S1\_at \| \| Bt.5334.1.S1\_at \| 281898 \| 1/1 \| NM\_174379 \|  \| GO:0003735  GO:0005622  GO:0005719  GO:0006412  GO:0007507  GO:0015935 \| 1849001  12477932  12658628 \| RPSA \| Bt.5334.1.S1\_at \| \| Bt.5243.1.S1\_at \| 516473 \| 1/2 \| XM\_594628 \| XM\_594628 \|  \| 12140684  16305752 \| EEF1D \| Bt.5243.1.S1\_at \| \| Bt.6815.1.A1\_at \| 506056 \| 1/1 \| XM\_582448 \| XM\_582448 \|  \|  \| CRI1 \| Bt.6815.1.A1\_at \| \| Bt.8051.1.S1\_at \|  \|  \|  \|  \|  \|  \| KRAS \| Bt.8051.1.S1\_at \| \| Bt.7701.1.S1\_at \| 614105 \| 1/1 \| NM\_001034748 \| NM\_001034748 \| GO:0016021 \| 12477932 \| GPSN2 \| Bt.7701.1.S1\_at \| \| Bt.7814.1.S1\_at \|  \|  \|  \|  \|  \|  \| ARF4 \| Bt.7814.1.S1\_at \| \| Bt.1154.1.S1\_at \| 533223 \| 1/1 \| NM\_001046346 \| NM\_001046346 \| GO:0003779  GO:0005856 \|  \| WDR1 \| Bt.1154.1.S1\_at \| \| Bt.5376.1.S1\_at \| 506336 \| 1/1 \| NM\_001034269 \|  \|  \| 12477932 \| YIF1A \| Bt.5376.1.S1\_at \| \| Bt.7903.1.A1\_at \| 510937 \| 1/1 \| NM\_001046066 \| NM\_001046066 \| GO:0000166  GO:0004821  GO:0005524  GO:0006418  GO:0016874 \| 12477932 \| HARS \| Bt.7903.1.A1\_at \| \| Bt.2898.2.S1\_a\_at \| 507679 \| 1/2 \| NM\_001034307 \| NM\_001034307 \|  \| 12477932 \| GNL3L \| Bt.2898.2.S1\_a\_at \| \| Bt.5038.4.A1\_at \| 615175 \| 1/1 \| NM\_001046554 \| NM\_001046554 \| GO:0000166  GO:0003676 \|  \| NONO \| Bt.5038.4.A1\_at \| \| Bt.23218.1.S1\_at \| 513038 \| 1/1 \| NM\_001034435 \| NM\_001034435 \| GO:0004216  GO:0005764  GO:0006508 \| 12140684  16305752 \| CTSK \| Bt.23218.1.S1\_at \| \| Bt.16980.1.S1\_at \| 515602 \| 1/1 \| NM\_001038547 \|  \| GO:0000166  GO:0003677  GO:0005515  GO:0005634  GO:0006260  GO:0017111 \|  \| RFC3 \| Bt.16980.1.S1\_at \| \| Bt.20008.1.S1\_at \| 535312 \| 1/1 \| XM\_865050 \|  \| GO:0003676  GO:0008270 \|  \| PIAS3 \| Bt.20008.1.S1\_at \| \| Bt.29546.1.A1\_s\_at \| 511583 \| 1/5 \| XM\_588946 \|  \|  \|  \|  \| Bt.29546.1.A1\_s\_at \| \| Bt.7322.1.S1\_at \|  \|  \|  \|  \|  \|  \| BRWD3 \| Bt.7322.1.S1\_at \| \| Bt.13324.4.S1\_at \| 281235 \| 2/2 \| NM\_181012 \| NM\_181012 \| GO:0000287  GO:0004450  GO:0004450  GO:0005737  GO:0005829  GO:0006097  GO:0006099  GO:0006749  GO:0006979  GO:0008152  GO:0016491  GO:0016616  GO:0030145 \| 10358094  10677231  12477932  16636309  16751257 \| IDH1 \| Bt.13324.4.S1\_at \| \| Bt.6685.2.A1\_at \|  \|  \|  \|  \|  \|  \| MTHFD2 \| Bt.6685.2.A1\_at \| \| Bt.28194.1.S1\_at \| 505642 \| 1/1 \| NM\_001075214 \| NM\_001075214 \|  \|  \| GSTO1 \| Bt.28194.1.S1\_at \| \| Bt.19575.1.S1\_at \| 534751 \| 1/4 \| NM\_001046388 \| NM\_001046388 \| GO:0000166  GO:0005524 \|  \| HSPA14 \| Bt.19575.1.S1\_at \| \| Bt.8122.1.S1\_at \| 282386 \| 1/2 \| NM\_001002885 \|  \| GO:0003779  GO:0005737  GO:0007010 \| 16677835 \| TMSB4X \| Bt.8122.1.S1\_at \| \| Bt.11163.1.S1\_at \|  \|  \|  \|  \|  \|  \|  \| Bt.11163.1.S1\_at \| \| Bt.20988.1.S1\_at \| 614801 \| 1/1 \| XM\_001255516  XM\_866432 \|  \|  \|  \|  \| Bt.20988.1.S1\_at \| \| Bt.7437.1.S1\_at \| 515570 \| 1/1 \| XM\_001251029  XM\_593609 \| XM\_593609 \| GO:0030246 \|  \|  \| Bt.7437.1.S1\_at \| \| Bt.7139.1.S1\_at \| 512673 \| 1/1 \| XM\_865272 \| XM\_001249751 \| GO:0005742  GO:0006605 \|  \| TOMM20 \| Bt.7139.1.S1\_at \| \| Bt.4637.1.A1\_a\_at \| 281091 \| 1/2 \| NM\_174024 \| NM\_174024 \|  \| 1689292  2176624  2844245  6327490  7766994  8638158  10089392  10771420 \| COX8A \| Bt.4637.1.A1\_a\_at \| \| Bt.24875.1.S1\_at \| 617922 \| 1/1 \| NM\_001076948 \|  \|  \|  \|  \| Bt.24875.1.S1\_at \| \| Bt.26999.1.S1\_at \| 615975 \| 1/1 \| NM\_001037626 \|  \|  \| 12477932 \| SMPX \| Bt.26999.1.S1\_at \| \| Bt.18278.1.S1\_at \| 509717 \| 1/4 \| NM\_001034358 \| XM\_001251293 \|  \| 12477932 \|  \| Bt.18278.1.S1\_at \| \| Bt.27226.1.S1\_at \|  \|  \|  \| XM\_001255517 \|  \|  \| ZFP106 \| Bt.27226.1.S1\_at \| \| Bt.26885.1.S1\_at \| 509422 \| 1/1 \| XM\_586370  XM\_866765 \|  \|  \|  \| FKBP7 \| Bt.26885.1.S1\_at \| \| Bt.21551.1.S1\_at \| 534046 \| 2/2 \| XM\_865607 \| XM\_865607 \|  \|  \| UCKL1 \| Bt.21551.1.S1\_at \| \| Bt.19208.1.A1\_at \| 520387 \| 1/2 \| XR\_027771 \| XR\_027771 \|  \|  \| KIAA0528 \| Bt.19208.1.A1\_at \| \| Bt.10608.2.S1\_a\_at \| 505736 \| 1/2 \| NM\_001034256 \|  \|  \| 11282978  12477932  16305752 \| FLJ10803 \| Bt.10608.2.S1\_a\_at \| \| Bt.997.2.S1\_a\_at \| 516287 \| 1/2 \| NM\_001034518 \|  \| GO:0004128  GO:0006118  GO:0016491  GO:0050660  GO:0051287 \| 12477932 \| CYB5R1 \| Bt.997.2.S1\_a\_at \| \| Bt.23254.1.S1\_at \| 768311 \| 1/1 \| NM\_001078127  XM\_001252704 \|  \| GO:0006605  GO:0007264  GO:0019904 \|  \| YWHAQ \| Bt.23254.1.S1\_at \| \| Bt.7540.3.S1\_at \| 534092 \| 1/1 \| NM\_001076012 \| NM\_001076012 \| GO:0004714  GO:0004872  GO:0005524  GO:0006468  GO:0007155  GO:0007169  GO:0016020 \| 11282978  16305752 \| DDR1 \| Bt.7540.3.S1\_at \| \| Bt.3715.1.S1\_at \|  \|  \|  \|  \|  \|  \|  \| Bt.3715.1.S1\_at \| \| Bt.22721.1.A1\_a\_at \| 509181 \| 1/2 \| XM\_586084 \| NM\_001080247 \|  \| 11282978  16305752 \| C14orf100 \| Bt.22721.1.A1\_a\_at \| \| Bt.25135.1.A1\_at \|  \|  \|  \|  \|  \|  \| UBE2D3 \| Bt.25135.1.A1\_at \| \| Bt.21027.1.S1\_at \| 519030 \| 1/2 \| XM\_597238 \|  \| GO:0008270 \|  \| SPIRE1 \| Bt.21027.1.S1\_at \| \| Bt.2825.1.S1\_at \| 617901 \| 1/1 \| NM\_001034805 \|  \|  \| 12477932 \| MRPL13 \| Bt.2825.1.S1\_at \| \| Bt.9799.1.S1\_at \| 508491 \| 1/1 \| XR\_027312 \| XR\_027876 \|  \|  \| CSTF3 \| Bt.9799.1.S1\_at \| \| Bt.1214.1.S1\_at \| 529312 \| 2/4 \| XM\_607757 \|  \| GO:0000175  GO:0003723  GO:0006396 \|  \| SSR1 \| Bt.1214.1.S1\_at \| \| Bt.17704.1.S1\_at \|  \|  \|  \|  \|  \|  \| CHSY1 \| Bt.17704.1.S1\_at \| \| Bt.11448.1.S1\_at \| 615626 \| 1/1 \| NM\_001039953 \|  \|  \|  \| PTMA \| Bt.11448.1.S1\_at \| \| Bt.15819.2.S1\_at \| 615424 \| 1/2 \| XM\_001250938  XM\_867160 \| XM\_867160 \|  \|  \| MTX2 \| Bt.15819.2.S1\_at \| \| Bt.7870.1.S1\_at \| 280701 \| 1/1 \| NM\_001075118 \|  \| GO:0000287  GO:0004427  GO:0005737  GO:0006796  GO:0016787 \| 1339444 \| PPA1 \| Bt.7870.1.S1\_at \| \| Bt.18890.1.A1\_at \|  \|  \|  \|  \|  \|  \| E2F3 \| Bt.18890.1.A1\_at \| \| Bt.23002.1.S1\_at \| 282199 \| 2/3 \| NM\_001077831  XM\_001250845  XM\_001250892 \|  \| GO:0004129  GO:0005739  GO:0005740  GO:0006118  GO:0016020  GO:0016491 \| 1720401  2844245  10984454 \| COX6A1 \| Bt.23002.1.S1\_at \| \| Bt.2428.1.S1\_at \| 538357 \| 1/1 \| XR\_027475 \| XR\_027475 \|  \|  \| EPRS \| Bt.2428.1.S1\_at \| \| Bt.23308.1.S1\_at \| 535746 \| 1/5 \| XM\_615857 \|  \|  \|  \| SEPT11 \| Bt.23308.1.S1\_at \| \| Bt.4844.1.S1\_at \| 280904 \| 1/1 \| NM\_173955 \| NM\_173955 \|  \| 1550956  2229039  2270483  2610682  8484780  12477932 \| PTN \| Bt.4844.1.S1\_at \| \| Bt.15845.1.S1\_at \| 510230 \| 1/2 \| XM\_587353 \|  \|  \|  \|  \| Bt.15845.1.S1\_at \| \| Bt.5598.1.S1\_at \| 617047 \| 1/1 \| NM\_001035490 \| NM\_001035490 \|  \| 12140684  12477932  16305752 \| C8orf4 \| Bt.5598.1.S1\_at \| \| Bt.9741.1.S1\_at \|  \|  \|  \|  \|  \|  \| MTMR4 \| Bt.9741.1.S1\_at \| \| Bt.1405.1.S1\_at \| 507914 \| 1/1 \| NM\_001045985 \| NM\_001045985 \|  \|  \| SORBS3 \| Bt.1405.1.S1\_at \| \| Bt.422.1.S1\_at \| 282261 \| 1/2 \| NM\_174556 \| NM\_174556 \| GO:0001558  GO:0005520  GO:0005576  GO:0008160  GO:0009968  GO:0043065  GO:0045663  GO:0046872 \| 1701128  1711841  9202228  9363609  12912978  14561651  15192040  15927849  17302958 \| IGFBP3 \| Bt.422.1.S1\_at \| \| Bt.24960.1.S1\_at \| 530962 \| 1/3 \| XM\_609444 \|  \|  \|  \| MPHOSPH9 \| Bt.24960.1.S1\_at \| \| Bt.21896.1.S1\_at \| 533456 \| 1/1 \| XM\_612868 \|  \| GO:0005737  GO:0006817 \|  \| COL21A1 \| Bt.21896.1.S1\_at \| \| Bt.3854.1.S1\_at \|  \|  \|  \|  \|  \|  \| SH3MD1 \| Bt.3854.1.S1\_at \| \| Bt.21396.1.A1\_s\_at \| 359721 \| 1/2 \| NM\_182657 \| NM\_182657 \| GO:0005783  GO:0006810  GO:0006888  GO:0006915  GO:0016020  GO:0016021 \| 12140684  12832288  16305752 \| RTN3 \| Bt.21396.1.A1\_s\_at \| \| Bt.9057.1.A1\_at \| 505171 \| 1/2 \| XM\_864736 \| XM\_864736 \|  \| 12477932 \| TFG \| Bt.9057.1.A1\_at \| \| Bt.22306.1.S1\_at \| 509696 \| 1/2 \| XM\_586717 \| XM\_586717 \|  \|  \| TPRT \| Bt.22306.1.S1\_at \| \| Bt.3015.1.A1\_at \|  \|  \|  \|  \|  \|  \| LHFPL2 \| Bt.3015.1.A1\_at \| \| Bt.13834.1.S1\_at \|  \|  \|  \| XM\_580860 \|  \|  \| TFRC \| Bt.13834.1.S1\_at \| \| Bt.27552.1.A1\_at \| 517520 \| 1/1 \| XM\_595692 \|  \| GO:0004289  GO:0006508 \|  \| CCDC21 \| Bt.27552.1.A1\_at \| \| Bt.19519.1.S1\_at \| 617843 \| 1/1 \| XM\_870167 \| XM\_588490 \|  \|  \| SMARCA3 \| Bt.19519.1.S1\_at \| \| Bt.5131.1.S1\_at \| 504781 \| 1/1 \| NM\_001015515 \| NM\_001015515 \|  \| 11282978  16305752 \| MGC3234 \| Bt.5131.1.S1\_at \| \| Bt.11687.2.S1\_at \|  \|  \|  \| XM\_591672 \|  \|  \| SRL \| Bt.11687.2.S1\_at \| \| Bt.9589.1.S1\_at \| 535277 \| 1/1 \| XM\_868523 \| XM\_868523 \|  \|  \| TPM4 \| Bt.9589.1.S1\_at \| \| Bt.21731.1.S1\_at \| 505406 \| 1/2 \| NM\_001045917 \|  \|  \|  \| DBN1 \| Bt.21731.1.S1\_at \| \| Bt.23589.2.S1\_at \| 529312 \| 2/4 \| XM\_607757 \| XM\_607757 \| GO:0000175  GO:0003723  GO:0006396 \|  \| SSR1 \| Bt.23589.2.S1\_at \| \| Bt.1244.1.S1\_at \| 514939 \| 1/2 \| XR\_027433 \|  \| GO:0004616  GO:0006098  GO:0016491  GO:0050661 \| 12477932  12658628 \| PGD \| Bt.1244.1.S1\_at \| \| Bt.13604.1.S1\_at \|  \|  \|  \|  \|  \|  \| ADAM19 \| Bt.13604.1.S1\_at \| \| Bt.4442.1.S1\_at \| 327687 \| 1/1 \| NM\_175806 \| NM\_175806 \| GO:0000166  GO:0005104  GO:0005524  GO:0005634  GO:0005773  GO:0006811  GO:0008083  GO:0008201  GO:0015986  GO:0015992  GO:0016020  GO:0016469  GO:0016787  GO:0046872  GO:0046933  GO:0046961  GO:0050677 \| 7929063  12477932 \| ATP6IP1 \| Bt.4442.1.S1\_at \| \| Bt.5096.1.S1\_at \| 504735 \| 1/1 \| NM\_001017934 \| NM\_001017934 \|  \| 11282978  12477932 \| CCT3 \| Bt.5096.1.S1\_at \| \| Bt.22466.1.A1\_at \| 540614 \| 1/2 \| NM\_001076230 \|  \| GO:0000166  GO:0005525  GO:0007049 \|  \| SEPT8 \| Bt.22466.1.A1\_at \| \| Bt.21313.1.S1\_at \| 616503 \| 1/1 \| NM\_001038688 \|  \|  \|  \| RRAS \| Bt.21313.1.S1\_at \| \| Bt.26811.1.S1\_at \| 508169 \| 1/2 \| XM\_584911 \| XM\_584911 \|  \|  \| SLC30A5 \| Bt.26811.1.S1\_at \| \| Bt.26817.1.S1\_at \|  \|  \|  \|  \|  \|  \|  \| Bt.26817.1.S1\_at \| \| Bt.18228.1.A1\_at \| 614128 \| 1/4 \| NM\_001046524 \| NM\_001046524 \| GO:0005783  GO:0006605  GO:0009306  GO:0015031  GO:0015450  GO:0016020  GO:0016021 \|  \| SEC61A2 \| Bt.18228.1.A1\_at \| \| Bt.12888.1.S1\_at \| 613305 \| 1/1 \| NM\_001034732 \|  \| GO:0003723 \| 12477932 \| AD023 \| Bt.12888.1.S1\_at \| \| Bt.27283.1.S1\_x\_at \| 515712 \| 1/12 \| NM\_001040532 \| NM\_001038518 \| GO:0002474  GO:0006955  GO:0016020  GO:0019882  GO:0042612 \| 10630296  16451353  17206412 \|  \| Bt.27283.1.S1\_x\_at \| \| Bt.4864.2.S1\_at \| 532483 \| 1/3 \| NM\_001035389 \|  \| GO:0005515  GO:0005634  GO:0007049  GO:0008270  GO:0045786  GO:0046872 \| 12477932 \| MGC127324 \| Bt.4864.2.S1\_at \| \| Bt.4868.2.S1\_a\_at \| 613382 \| 1/1 \| XM\_864279  XM\_872589 \|  \| GO:0003824 \|  \| HDDC3 \| Bt.4868.2.S1\_a\_at \| \| Bt.23094.2.A1\_at \| 282138 \| 3/8 \| NM\_181027 \| XM\_001250814 \| GO:0001516  GO:0006633  GO:0016491  GO:0047017 \| 1339268  2829166  9867836  12551929 \|  \| Bt.23094.2.A1\_at \| \| Bt.1187.1.S1\_at \| 514688 \| 1/1 \| NM\_001046178 \| NM\_001046178 \|  \|  \| PPP1R15A \| Bt.1187.1.S1\_at \| \| Bt.20095.2.S1\_at \| 511573 \| 1/2 \| XM\_872705 \|  \| GO:0003676  GO:0005622  GO:0008270 \|  \| ZNF142 \| Bt.20095.2.S1\_at \| \| Bt.23734.1.A1\_at \|  \|  \|  \| XR\_028511 \|  \|  \| PAWR \| Bt.23734.1.A1\_at \| \| Bt.16004.1.A1\_at \| 281339 \| 1/3 \| XM\_865879 \|  \| GO:0003774  GO:0016459 \| 8014155 \| MYH8 \| Bt.16004.1.A1\_at \| \| Bt.17512.1.S1\_at \|  \|  \|  \|  \|  \|  \| FYCO1 \| Bt.17512.1.S1\_at \| \| Bt.8212.1.S1\_at \| 511451 \| 1/2 \| NM\_001046087 \| NM\_001046087 \|  \|  \| CAB39 \| Bt.8212.1.S1\_at \| \| Bt.1370.1.S1\_at \| 280726 \| 1/1 \| NM\_173892 \| NM\_173892 \| GO:0000050  GO:0000166  GO:0004055  GO:0005524  GO:0006526  GO:0008652  GO:0016874 \| 2813370  12477932  14970240  15851478 \| ASS \| Bt.1370.1.S1\_at \| \| Bt.21468.1.S1\_at \|  \|  \|  \| XM\_001251214 \|  \|  \| DEXI \| Bt.21468.1.S1\_at \| \| Bt.12510.1.S1\_at \| 509824 \| 2/6 \| XM\_872590 \|  \|  \|  \| GTL2 \| Bt.12510.1.S1\_at \| \| Bt.5038.1.S2\_at \| 281160 \| 1/9 \| NM\_174055 \|  \|  \| 1280126  1702556  2425435  2714282  2849564  3205724  3532107  3768327  4065099  4071057  12477932  16415522 \| HIST2H2BE \| Bt.5038.1.S2\_at \| \| Bt.12862.1.S1\_at \|  \|  \|  \|  \|  \|  \| TSPYL5 \| Bt.12862.1.S1\_at \| \| Bt.27552.2.S1\_at \|  \|  \|  \| XM\_595692 \|  \|  \| CCDC21 \| Bt.27552.2.S1\_at \| \| Bt.15925.1.S1\_at \| 511716 \| 1/2 \| NM\_001075534 \|  \| GO:0006725  GO:0008152  GO:0016787 \|  \| EPHX2 \| Bt.15925.1.S1\_at \| \| Bt.4798.1.S2\_at \| 280924 \| 1/1 \| NM\_173959 \|  \|  \| 10945276  15834635 \| SCD \| Bt.4798.1.S2\_at \| \| Bt.10153.1.S1\_at \| 528046 \| 1/1 \| XM\_606455 \|  \|  \|  \| EIF2C4 \| Bt.10153.1.S1\_at \| \| Bt.6483.1.A1\_at \|  \|  \|  \| XM\_601798 \|  \|  \| MTSS1 \| Bt.6483.1.A1\_at \| \| Bt.9870.2.A1\_at \| 533317 \| 1/2 \| XM\_612690 \|  \|  \|  \| FLVCR \| Bt.9870.2.A1\_at \| \| Bt.17148.2.S1\_at \| 541180 \| 2/3 \| XM\_614920 \| XM\_614920 \|  \|  \|  \| Bt.17148.2.S1\_at \| \| Bt.12508.1.S1\_at \| 614103 \| 1/1 \| NM\_001038202 \| NM\_001038202 \|  \|  \| XTP3TPA \| Bt.12508.1.S1\_at \| \| Bt.12411.1.S1\_at \| 404144 \| 1/2 \| NM\_001034053 \| NM\_001034053 \| GO:0005198  GO:0005882 \| 12477932  12658628 \| LMNA \| Bt.12411.1.S1\_at \| \| Bt.23094.2.A1\_x\_at \| 282138 \| 3/8 \| NM\_181027 \| XM\_001250814 \| GO:0001516  GO:0006633  GO:0016491  GO:0047017 \| 1339268  2829166  9867836  12551929 \|  \| Bt.23094.2.A1\_x\_at \| \| Bt.17883.2.A1\_at \|  \|  \|  \|  \|  \|  \| PYGO1 \| Bt.17883.2.A1\_at \| \| Bt.29928.1.S1\_at \|  \|  \|  \|  \|  \|  \| SSH2 \| Bt.29928.1.S1\_at \| \| Bt.5269.1.S1\_at \| 280729 \| 2/3 \| NM\_173893 \| NM\_173893 \| GO:0002474  GO:0006955  GO:0042612 \| 3889925  6174509  8319984  12658628  15014972 \|  \| Bt.5269.1.S1\_at \| \| Bt.2696.2.S1\_at \| 535363 \| 2/2 \| XM\_615431 \|  \| GO:0005509  GO:0007156  GO:0016020 \|  \| CDH11 \| Bt.2696.2.S1\_at \| \| Bt.23605.1.S1\_at \| 768225 \| 1/1 \| NM\_001078100 \|  \| GO:0003700  GO:0003707  GO:0005634  GO:0006355  GO:0008270  GO:0043565 \| 17075855 \| NR1D1 \| Bt.23605.1.S1\_at \| \| Bt.350.1.S1\_s\_at \| 539241 \| 1/6 \| NM\_001034668 \| NM\_001034668 \| GO:0002504  GO:0006955  GO:0016020  GO:0016021  GO:0042613 \| 1503262  1537611  1776708  9226127  11132144  15771733  16220347 \| HLA-DQB1 \| Bt.350.1.S1\_s\_at \| \| Bt.5333.1.S1\_at \| 504854 \| 1/2 \| NM\_001014851 \| NM\_001014851 \|  \| 11282978  16305752 \| CRELD1 \| Bt.5333.1.S1\_at \| \| Bt.28623.1.S1\_at \| 508251 \| 1/2 \| XM\_864984 \| XM\_864984 \|  \|  \| FAT \| Bt.28623.1.S1\_at \| \| Bt.16567.1.A1\_at \| 282875 \| 1/3 \| NM\_001045870 \| NM\_001045870 \|  \| 12440939 \| PRKRA \| Bt.16567.1.A1\_at \| \| Bt.5530.1.S1\_at \| 281482 \| 1/1 \| NM\_174180 \| NM\_174180 \|  \| 9705317  12477932 \| DHRS3 \| Bt.5530.1.S1\_at \| \| Bt.22325.2.A1\_at \|  \|  \|  \|  \|  \|  \|  \| Bt.22325.2.A1\_at \| \| Bt.18689.2.A1\_at \|  \|  \|  \| XM\_618606 \|  \|  \| ARSB \| Bt.18689.2.A1\_at \| \| Bt.20479.1.S1\_at \| 533608 \| 1/2 \| NM\_001038159 \| NM\_001038159 \| GO:0006118  GO:0016491  GO:0020037  GO:0046914 \|  \| CYB5R4 \| Bt.20479.1.S1\_at \| \| Bt.11239.3.S1\_at \| 515269 \| 1/3 \| XM\_593253 \|  \| GO:0000166  GO:0004222  GO:0005524  GO:0006508  GO:0008270  GO:0016020  GO:0016021  GO:0017111  GO:0030163 \|  \| SPG7 \| Bt.11239.3.S1\_at \| \| Bt.1318.1.S1\_at \| 507011  513685 \| 1/1 \| XM\_583550  XM\_591413 \| XM\_591413 \|  \|  \| FLJ23436 \| Bt.1318.1.S1\_at \| \| Bt.3562.1.S1\_at \| 281276 \| 1/2 \| XM\_868927 \| XM\_868927 \| GO:0004872  GO:0005319  GO:0005509  GO:0005905  GO:0006629  GO:0006869  GO:0006897  GO:0008202  GO:0008203  GO:0016020  GO:0016021 \| 2999774  3755212  6143315  6327078  15056566  15095337 \| LDLR \| Bt.3562.1.S1\_at \| \| Bt.1178.1.S1\_a\_at \| 528735 \| 1/2 \| XM\_001253608  XM\_607166 \|  \|  \|  \| S100A1 \| Bt.1178.1.S1\_a\_at \| \| Bt.16362.2.S1\_at \| 512324 \| 1/2 \| XM\_589822 \| XM\_589822 \| GO:0005634 \| 11282978  16305752 \| C14orf154 \| Bt.16362.2.S1\_at \| \| Bt.26122.2.S1\_at \| 525680 \| 1/1 \| NM\_001037471 \| NM\_001037471 \|  \| 12477932 \| UXT \| Bt.26122.2.S1\_at \| \| Bt.16628.2.A1\_at \|  \|  \|  \|  \|  \|  \| ADAM12 \| Bt.16628.2.A1\_at \| \| Bt.10990.1.S1\_at \| 539560 \| 1/2 \| NM\_001076173 \| NM\_001076173 \|  \|  \|  \| Bt.10990.1.S1\_at \| \| Bt.2846.1.A1\_at \|  \|  \|  \|  \|  \|  \| RHOU \| Bt.2846.1.A1\_at \| \| Bt.4339.1.S1\_at \| 515589 \| 1/1 \| NM\_001046205 \|  \|  \|  \| C20orf27 \| Bt.4339.1.S1\_at \| \| Bt.26524.1.S1\_at \|  \|  \|  \| XM\_869323 \|  \|  \|  \| Bt.26524.1.S1\_at \| \| Bt.15540.1.A1\_at \|  \|  \|  \|  \|  \|  \| MBNL1 \| Bt.15540.1.A1\_at \| \| Bt.11013.1.S1\_at \|  \|  \|  \|  \|  \|  \|  \| Bt.11013.1.S1\_at \| \| Bt.15753.1.S1\_at \| 538662 \| 1/1 \| XM\_581859 \| XM\_581859 \| GO:0016071  GO:0030529 \|  \| LSM2 \| Bt.15753.1.S1\_at \| \| Bt.1388.1.S1\_at \| 506085 \| 1/1 \| NM\_001024482 \| NM\_001024482 \|  \| 11282978  16305752 \| ZDHHC16 \| Bt.1388.1.S1\_at \| \| Bt.16105.1.S1\_at \|  \|  \|  \|  \|  \|  \|  \| Bt.16105.1.S1\_at \| \| Bt.19469.1.A1\_at \| 614509 \| 2/4 \| NM\_001076331 \| XM\_867376 \| GO:0005634  GO:0030528  GO:0045449 \|  \| MXI1 \| Bt.19469.1.A1\_at \| \| Bt.4670.1.S1\_at \| 539596 \| 1/1 \| XR\_028016 \| XR\_028016 \| GO:0005515  GO:0005578  GO:0007155 \| 8557636 \| TGFBI \| Bt.4670.1.S1\_at \| \| Bt.22234.1.S1\_at \|  \|  \|  \| XM\_582266 \|  \|  \| PRKCQ \| Bt.22234.1.S1\_at \| \| Bt.5465.2.A1\_a\_at \| 511560 \| 1/1 \| NM\_001079588  XM\_001251855 \| XM\_001251855 \| GO:0005515  GO:0005622  GO:0005634  GO:0005730  GO:0006364  GO:0006412  GO:0019843  GO:0030529 \|  \| IMP3 \| Bt.5465.2.A1\_a\_at \| \| Bt.3251.1.S1\_at \| 515749 \| 1/2 \| XM\_001251315  XM\_872743 \| XM\_872743 \| GO:0005198  GO:0007155  GO:0015629  GO:0045296 \|  \| CTNNAL1 \| Bt.3251.1.S1\_at \| \| Bt.2749.1.S1\_at \| 513497 \| 1/1 \| XM\_591188 \| XM\_591188 \| GO:0004861  GO:0005634  GO:0007050 \|  \| CDKN1A \| Bt.2749.1.S1\_at \| \| Bt.17579.1.A1\_at \| 539619 \| 1/1 \| XM\_588383 \| NM\_001080738 \| GO:0005515  GO:0016020  GO:0016021 \|  \| LRRN1 \| Bt.17579.1.A1\_at \| \| Bt.21409.1.A1\_at \| 287013 \| 1/7 \| XM\_001253222  XM\_001253240  XM\_001253287  XM\_587946 \|  \| GO:0005198  GO:0005201  GO:0005578  GO:0005581  GO:0005737  GO:0006817  GO:0007155 \| 1744123  7642541  8486632 \|  \| Bt.21409.1.A1\_at \| \| Bt.4751.1.S1\_a\_at \| 282535 \| 2/3 \| NM\_001012681 \|  \| GO:0002504  GO:0006955  GO:0016020  GO:0016021  GO:0042613 \| 7611993  9089108  12140684  12658628 \|  \| Bt.4751.1.S1\_a\_at \| \| Bt.10810.1.A1\_at \|  \|  \|  \|  \|  \|  \| NRK \| Bt.10810.1.A1\_at \| \| Bt.4751.2.S1\_a\_at \| 282535 \| 2/3 \| NM\_001012681 \| NM\_001012681 \| GO:0002504  GO:0006955  GO:0016020  GO:0016021  GO:0042613 \| 7611993  9089108  12140684  12658628 \| BOLA-DQA2 \| Bt.4751.2.S1\_a\_at \| \| Bt.13306.2.S1\_at \|  \|  \|  \|  \|  \|  \| SORBS1 \| Bt.13306.2.S1\_at \| \| Bt.21578.1.S1\_at \| 513978 \| 1/2 \| XM\_001254675  XM\_591752 \| XM\_591752 \|  \|  \| DUSP23 \| Bt.21578.1.S1\_at \| \| Bt.4680.1.S1\_at \| 281259 \| 1/1 \| NM\_174096 \| NM\_174096 \| GO:0006457  GO:0031072  GO:0051082 \| 10760603 \| DNAJC12 \| Bt.4680.1.S1\_at \| \| Bt.25099.2.S1\_at \| 533630 \| 2/2 \| NM\_001046355 \| NM\_001046355 \| GO:0000287  GO:0004647  GO:0006564  GO:0008152  GO:0008652  GO:0016787 \|  \|  \| Bt.25099.2.S1\_at \| \| Bt.28524.1.S1\_at \| 514520 \| 1/1 \| XM\_865857 \|  \|  \|  \| C21orf5 \| Bt.28524.1.S1\_at \| \| Bt.21054.1.A1\_at \| 506787 \| 1/1 \| XM\_583282 \| XM\_583282 \|  \|  \| PDE8A \| Bt.21054.1.A1\_at \| \| Bt.7642.1.S1\_at \| 616274 \| 1/1 \|  \|  \|  \|  \|  \| Bt.7642.1.S1\_at \| \| Bt.21800.1.S1\_at \| 504422 \| 1/2 \| XM\_580541 \| XM\_580541 \| GO:0005515  GO:0006886  GO:0006888  GO:0008270  GO:0030127 \|  \| SEC24D \| Bt.21800.1.S1\_at \| \| Bt.24269.1.S1\_at \|  \|  \|  \|  \|  \|  \| KIAA1394 \| Bt.24269.1.S1\_at \| \| Bt.876.1.S1\_at \| 507002 \| 1/2 \| XM\_583541 \| XM\_583541 \|  \|  \| OLFM1 \| Bt.876.1.S1\_at \| \| Bt.2070.3.S1\_at \| 541175 \| 3/3 \| NM\_001034725 \| NM\_001034725 \| GO:0004725  GO:0005634  GO:0006470  GO:0016787  GO:0017017 \| 11282978 \| DUSP10 \| Bt.2070.3.S1\_at \| \| Bt.4274.1.S1\_at \| 507845 \| 1/1 \| NM\_001075334 \| NM\_001075334 \|  \| 10984454 \| MAPRE1 \| Bt.4274.1.S1\_at \| \| Bt.562.1.S1\_at \| 282304 \| 1/1 \| NM\_174572 \|  \| GO:0000166  GO:0003873  GO:0004331  GO:0005524  GO:0006003  GO:0006094  GO:0006096  GO:0008152  GO:0016301  GO:0016740  GO:0016787  GO:0043540 \| 1654864 \| PFKFB1 \| Bt.562.1.S1\_at \| \| Bt.4803.1.S1\_at \| 286764 \| 1/1 \| NM\_174751 \| NM\_174751 \| GO:0000723  GO:0003677  GO:0003950  GO:0005622  GO:0005634  GO:0005635  GO:0005730  GO:0006284  GO:0006471  GO:0008270  GO:0016757  GO:0040009  GO:0042802  GO:0046872  GO:0051287 \| 2119324  2450019  15361875  15389540 \| PARP1 \| Bt.4803.1.S1\_at \| \| Bt.2110.1.S1\_at \| 510200 \| 1/1 \| NM\_001024513 \| NM\_001024513 \|  \| 11282978  12477932 \| DPP3 \| Bt.2110.1.S1\_at \| \| Bt.23212.1.S1\_at \| 504481 \| 1/1 \| XM\_001250395  XM\_580615  XM\_878782 \|  \|  \|  \| SC4MOL \| Bt.23212.1.S1\_at \| \| Bt.15847.1.S1\_at \| 534647 \| 1/1 \| NM\_001046383 \|  \|  \|  \| CDR2 \| Bt.15847.1.S1\_at \| \| Bt.89.1.S1\_at \| 281687 \| 1/1 \| NM\_174271 \|  \| GO:0004889  GO:0005216  GO:0005230  GO:0005794  GO:0005886  GO:0006811  GO:0016021  GO:0030594  GO:0045211 \| 3838938 \| CHRND \| Bt.89.1.S1\_at \| \| Bt.18101.1.A1\_at \|  \|  \|  \|  \|  \|  \| OLFM1 \| Bt.18101.1.A1\_at \| \| Bt.24856.2.S1\_at \| 538915 \| 1/3 \| XM\_583609 \|  \|  \|  \| KIAA0007 \| Bt.24856.2.S1\_at \| \| Bt.21724.1.S1\_at \|  \|  \|  \|  \|  \|  \| FDFT1 \| Bt.21724.1.S1\_at \| \| Bt.6840.1.S1\_a\_at \| 506043 \| 1/1 \| NM\_001045932 \| NM\_001045932 \|  \|  \| FKBP11 \| Bt.6840.1.S1\_a\_at \| \| Bt.7056.1.S1\_at \| 280813 \| 1/1 \| NM\_173917 \| NM\_173917 \|  \| 4561255  6048711  6322113  8411160  10449916  11369847 \| HBB \| Bt.7056.1.S1\_at \| \| Bt.25186.1.S1\_at \|  \|  \|  \|  \|  \|  \| BCAT1 \| Bt.25186.1.S1\_at \| \| Bt.7431.1.S1\_a\_at \|  \|  \|  \| XR\_028576 \|  \|  \| FHOD1 \| Bt.7431.1.S1\_a\_at \| \| Bt.25398.1.A1\_at \|  \|  \|  \|  \|  \|  \| CCNJ \| Bt.25398.1.A1\_at \| \| Bt.6642.1.S1\_at \| 537224 \| 3/4 \| XM\_865119 \|  \|  \|  \| LPIN1 \| Bt.6642.1.S1\_at \| \| Bt.7955.1.S1\_at \| 504471 \| 2/2 \| NM\_001045888 \| NM\_001045888 \|  \|  \| PCOLCE \| Bt.7955.1.S1\_at \| \| Bt.20862.1.A1\_at \| 517539 \| 1/3 \| NM\_001075755 \|  \| GO:0009396  GO:0016787 \|  \| MTHFD2 \| Bt.20862.1.A1\_at \| \| Bt.26135.1.A1\_at \|  \|  \|  \|  \|  \|  \| KCNAB1 \| Bt.26135.1.A1\_at \| \| Bt.8319.3.S1\_a\_at \| 515016 \| 1/4 \| NM\_001075692 \|  \|  \|  \| MTUS1 \| Bt.8319.3.S1\_a\_at \| \| Bt.26760.2.S1\_a\_at \| 523285 \| 1/2 \| XM\_881458 \| NM\_001081592 \|  \|  \| ARRDC2 \| Bt.26760.2.S1\_a\_at \| \| Bt.2786.1.S1\_at \| 509055 \| 1/1 \| NM\_001075388 \|  \|  \|  \| C11orf24 \| Bt.2786.1.S1\_at \| \| Bt.1720.1.S1\_at \|  \|  \|  \| XR\_028689 \|  \|  \| SC65 \| Bt.1720.1.S1\_at \| \| Bt.3980.1.S1\_at \| 281478 \| 1/1 \| NM\_174177 \|  \|  \| 1651929  1847925  2153078  7891673  8119933  8780652  15686479 \| SCIN \| Bt.3980.1.S1\_at \| \| Bt.14340.2.S1\_at \| 511639 \| 2/2 \| XR\_027604 \|  \|  \|  \| RNF103 \| Bt.14340.2.S1\_at \| \| Bt.10092.1.S1\_at \|  \|  \|  \|  \|  \|  \| TNFRSF19 \| Bt.10092.1.S1\_at \| \| Bt.659.1.S1\_at \| 534928 \| 2/3 \| XM\_614870  XM\_881407  XM\_881423 \|  \| GO:0003824  GO:0005524  GO:0006003  GO:0008152 \|  \| PFKFB4 \| Bt.659.1.S1\_at \| \| Bt.13334.1.S1\_at \|  \|  \|  \|  \|  \|  \|  \| Bt.13334.1.S1\_at \| \| Bt.26711.1.S1\_at \|  \|  \|  \|  \|  \|  \| LRRC20 \| Bt.26711.1.S1\_at \| \| Bt.16979.3.S1\_at \|  \|  \|  \|  \|  \|  \| NAPE-PLD \| Bt.16979.3.S1\_at \| \| Bt.18316.1.A1\_at \| 513587 \| 2/2 \| XM\_591287  XM\_876203 \| XM\_876203 \|  \|  \|  \| Bt.18316.1.A1\_at \| \| Bt.13435.1.A1\_at \| 539445 \| 1/2 \| XM\_587191 \|  \| GO:0004674  GO:0005524  GO:0006468 \|  \| MASK \| Bt.13435.1.A1\_at \| \| Bt.4523.1.S1\_at \| 506441 \| 1/1 \| NM\_001024484 \| NM\_001024484 \|  \| 11282978  12477932 \| DCTN5 \| Bt.4523.1.S1\_at \| \| Bt.13445.2.S1\_a\_at \| 506370 \| 1/4 \| XM\_582806 \| XM\_582806 \|  \|  \|  \| Bt.13445.2.S1\_a\_at \| \| Bt.20570.1.S1\_at \| 615827 \| 1/1 \| XM\_868710 \| XM\_868710 \| GO:0007049  GO:0016538  GO:0051301 \|  \|  \| Bt.20570.1.S1\_at \| \| Bt.22576.3.S1\_at \|  \|  \|  \|  \|  \|  \| PPP1R3D \| Bt.22576.3.S1\_at \| \| Bt.16087.1.S1\_at \| 521854 \| 1/1 \| XM\_600125 \|  \|  \|  \| NID2 \| Bt.16087.1.S1\_at \| \| Bt.21942.1.S1\_at \|  \|  \|  \| XM\_001252116 \|  \|  \|  \| Bt.21942.1.S1\_at \| \| Bt.27301.1.S1\_at \| 512204 \| 1/3 \|  \|  \|  \|  \| SYNCRIP \| Bt.27301.1.S1\_at \| \| Bt.5170.1.S1\_at \| 504764 \| 1/1 \| XM\_580934 \| XM\_580934 \|  \|  \| GRHPR \| Bt.5170.1.S1\_at \| \| Bt.16821.1.S1\_at \| 507280 \| 1/1 \| NM\_001034287 \|  \|  \| 12477932 \| TM4SF18 \| Bt.16821.1.S1\_at \| \| Bt.8963.1.S1\_at \| 281903 \| 1/1 \| NM\_174383 \| NM\_174383 \|  \| 11684696 \| LOXL1 \| Bt.8963.1.S1\_at \| \| Bt.14223.3.S1\_at \| 507781 \| 1/2 \| XM\_584456  XM\_878522 \|  \|  \|  \| NEDD4 \| Bt.14223.3.S1\_at \| \| Bt.4816.1.S1\_at \| 509963 \| 1/1 \| NM\_001046043 \| NM\_001046043 \|  \|  \| ANGPTL4 \| Bt.4816.1.S1\_at \| \| Bt.22002.1.S1\_at \|  \|  \|  \|  \|  \|  \| FABP4 \| Bt.22002.1.S1\_at \| \| Bt.21537.1.A1\_at \| 535704 \| 1/1 \| XM\_615814 \|  \| GO:0006457 \|  \| FKBP5 \| Bt.21537.1.A1\_at \| \| Bt.9828.1.S2\_at \|  \|  \|  \|  \|  \|  \| HINT3 \| Bt.9828.1.S2\_at \| \| Bt.23355.1.S1\_at \| 615191 \| 2/2 \| XM\_866930 \| XM\_866930 \| GO:0005515  GO:0005524  GO:0016311  GO:0016791  GO:0044267 \|  \| PTPLA \| Bt.23355.1.S1\_at \| |
