# Supplementary material for: A gene network switch enhances the oxidative capacity of ovine skeletal muscle during late fetal development
Source: BMC Genomics. 2010 Jun 15;11:378. doi: 10.1186/1471-2164-11-378 (PMC2894804; doi:10.1186/1471-2164-11-378)
Supplement: Additional File 1 — Gene expression data. This file contains links to the gene expression data. The genes that are significantly differentially expressed between adjacent development times are listed as Experiment 1 (80 d vs 100 d), Experiment 2 (100 d vs 120 d), Experiment 3 (120 d vs 150 d) and Experiment 4 (150 d vs 230 d) in the section entitled Open microarray analysis report for developmental expression. Each experiment contains an MA plot, Volcano plot and Heatmap for each of the three microarray processing programs MAS5, RMA and GCRMA. For each of these processing programs there is a list of significantly differentially expressed probe sets, and for each of these there is an FDR corrected probability, fold change, percentage of microarrays with a MAS5 Present call, and a convergence percentage for all three microarray processing programs. The file also contains annotation of the probe sets. In a second section entitled Time series analysis of developmental expression, there is a list of genes in each gene expression cluster and diagrams showing MAS5 gene expression values for these genes. [file 1471-2164-11-378-S1.ZIP › Report/Experiment2.html]

## Experiment 2

### Experimental Question

Which genes are differentially expressed between day 100 and day 120?

### Arrays

Array reference names used in the experiment and their corresponding original CEL file names.

| Reference | CEL File |
| --- | --- |
| d100\_1 | af1c1228-2-204-161007.CEL |
| d100\_2 | af1c1228-3-205-161007.CEL |
| d100\_3 | af1c1228-9-288-161007.CEL |
| d120\_1 | af1c1228-1-196-161007.CEL |
| d120\_2 | af1c1228-4-215-161007.CEL |
| d120\_3 | af1c1228-8-261-161007.CEL |

### Probe Summary

Total number of probe sets on chip: 24128 .

Total number of Affymetrix control probe sets on chip: 112 (removed from analysis).

Total number of probe sets detected as absent or marginal on all slides: 8374 (removed from analysis).

Total number of probe sets used in analysis: 15642 .

### Annotation

Annotation of differentially expressed genes for experiment 2.

### Expression Plots

MA plots for MAS5, RMA and GCRMA for contrast 1.

Volcano plots for MAS5, RMA and GCRMA for contrast 1.

Heatmap plots for MAS5, RMA and GCRMA.

### Functional Profile Analyses

Gene Ontology analyses.

### Summary gene list with 43 differentially expressed genes

Briefly - the best starting point for significantly expressed genes is for the genes with a value of 100 in the convergence column and 3 stars (\*\*\*) in the FDR\_Corrected column.

Differential expression was tested on log2 expression intensities obtained using MAS, RMA and GCRMA summarization methods. P-values for each method are shown for the moderated F-statistic which stabilizes the gene-specific variance using an empirical Bayes method to shrink the probe-wise sample variances towards a common value and to augment the degrees of freedom for the individual variances (Smyth,2004). Significance after correction for false discovery rates (FDR) using the method BH for a targeted p-value of 0.01 is also shown for each summarization set (ns - not significant, \* - significant; this means that if the gene has three stars it is significant for all three summarizations, and a very reliable indication that the gene is truly differentially expressed). Log2 fold changes are also shown in the table (remember that a log fold change of one equals a fold change of two). The fold changes are based on the raw data and not the fitted intensities. See the MA and volcano plots for a visual representation. If the gene was detected as present or not in each array is shown in the table in percentage (Presence\_Calls column). The last column shows the convergence in percentage of the gene across the three methods. A value of 100 indicates that the gene was detected as significantly differentially expressed for all three summarizations. The table was filtered for genes with a convergence greater than 50% and called as present in at least half of the samples. Note that the log fold change used is simply the average of the differences of the contrasts, if there is more than one contrast. It does not necessarily mean that the fold change is above the threshold for each individual contrast.

MAS parameters: background correction - for both PM and MM, the lowest 2% of probe intensities are used as a background value for each of 16 grids. Each probe is then adjusted based upon a weighted (distance between the location of the probe and the centroid) average of the background of each grid; normalization - scale normalization, all arrays scaled to the same mean value (200); summarization - log2 scale average using 1-step Tukey biweight.

RMA parameters: background correction - convolution; normalization - quantile; summarization - median polish. Note: MM intensities are not used, which does not account for non-specific binding on the different probes (can underestimate the background).

GCRMA parameters: background correction - an affinity measure model based on probe sequences and MM intensities; normalization - quantile; summarization - median polish.

|  |  |  |  |  |  |  |  |  |  |
| --- | --- | --- | --- | --- | --- | --- | --- | --- | --- |
| **AFFY** | **MAS\_ModFtest** | **RMA\_ModFtest** | **GCRMA\_ModFtest** | **FDR\_Corrected** | **MAS\_FoldChange** | **RMA\_FoldChange** | **GCRMA\_FoldChange** | **Presence\_Calls** | **Convergence** |
| Bt.29854.1.S1\_x\_at | 3.96e-07 | 1.52e-08 | 1.31e-06 | \* \* \* | -3.24 | -3.44 | -4.06 | 100 | 100 |
| Bt.15968.1.S1\_at | 5.68e-07 | 9.94e-09 | 6.08e-09 | \* \* \* | -1.75 | -1.73 | -2.29 | 100 | 100 |
| Bt.11622.1.A1\_at | 5.96e-07 | 1.02e-09 | 7.38e-10 | \* \* \* | 3.04 | 2.02 | 2.89 | 100 | 100 |
| Bt.12300.1.S1\_at | 1.28e-06 | 3.57e-07 | 8.15e-06 | \* \* \* | -2.06 | -2.08 | -2.67 | 100 | 100 |
| Bt.13980.2.A1\_a\_at | 3.85e-06 | 6.22e-10 | 9.87e-08 | \* \* \* | -1.98 | -2.13 | -2.5 | 100 | 100 |
| Bt.22379.1.S1\_at | 3.87e-06 | 2.06e-10 | 4.16e-07 | \* \* \* | -1.61 | -1.71 | -2.54 | 100 | 100 |
| Bt.29854.1.S1\_at | 3.66e-07 | 1.53e-08 | 2.73e-06 | \* \* \* | -3.33 | -3.51 | -4.19 | 83.3 | 100 |
| Bt.5848.2.S1\_at | 8.09e-06 | 9.59e-06 | 5.78e-07 | ns \* \* | -1.17 | -1.05 | -1.24 | 100 | 66.7 |
| Bt.19517.1.S1\_at | 8.17e-06 | 5.09e-09 | 8.56e-06 | ns \* \* | 1.71 | 1.72 | 2.15 | 100 | 66.7 |
| Bt.76.1.S1\_at | 1.13e-05 | 1.47e-06 | 6.93e-06 | ns \* \* | -1.4 | -1.07 | -1.22 | 100 | 66.7 |
| Bt.29854.1.A1\_at | 1.55e-05 | 2.99e-08 | 5.86e-08 | ns \* \* | -2.06 | -2.08 | -4.28 | 100 | 66.7 |
| Bt.29697.1.S1\_at | 1.75e-05 | 1.99e-06 | 8.46e-06 | ns \* \* | -1.69 | -1.48 | -1.82 | 100 | 66.7 |
| Bt.7394.1.A1\_at | 2.89e-05 | 8.91e-06 | 5.82e-06 | ns \* \* | -1.23 | -1.13 | -1.3 | 100 | 66.7 |
| Bt.2129.2.S1\_at | 2.91e-05 | 1.4e-06 | 6.03e-06 | ns \* \* | 1.62 | 1.7 | 2 | 100 | 66.7 |
| Bt.20316.1.S1\_at | 3.11e-05 | 2.78e-07 | 4.8e-07 | ns \* \* | -1.77 | -1.83 | -2.15 | 100 | 66.7 |
| Bt.25689.1.S1\_at | 7.91e-05 | 4.04e-08 | 8.98e-08 | ns \* \* | -1.37 | -1.31 | -1.74 | 100 | 66.7 |
| Bt.13980.1.A1\_at | 8.09e-05 | 9.36e-09 | 5.06e-07 | ns \* \* | -1.74 | -1.55 | -2.02 | 100 | 66.7 |
| Bt.4584.1.S1\_at | 9.2e-05 | 1.03e-08 | 1.6e-06 | ns \* \* | -1.34 | -1.37 | -1.64 | 100 | 66.7 |
| Bt.25282.1.A1\_at | 0.000115 | 4.47e-05 | 1.44e-06 | ns \* \* | 0.925 | 0.794 | 0.983 | 100 | 66.7 |
| Bt.25658.1.A1\_at | 0.000127 | 3.42e-05 | 1.67e-05 | ns \* \* | -1.14 | -1.16 | -1.53 | 100 | 66.7 |
| Bt.6993.1.S1\_at | 0.000136 | 9.87e-06 | 1.46e-05 | ns \* \* | -1.1 | -1.04 | -1.43 | 100 | 66.7 |
| Bt.10278.1.S1\_at | 0.000285 | 1.46e-05 | 2.85e-05 | ns \* \* | -0.851 | -0.9 | -1.05 | 100 | 66.7 |
| Bt.4138.2.S1\_at | 0.000287 | 3.19e-05 | 3.51e-06 | ns \* \* | -1.11 | -0.931 | -1.14 | 100 | 66.7 |
| Bt.17613.1.A1\_at | 0.000309 | 4.56e-05 | 2.69e-05 | ns \* \* | -1.1 | -0.73 | -1.31 | 100 | 66.7 |
| Bt.28162.1.S1\_at | 0.000314 | 1.45e-06 | 4.77e-06 | ns \* \* | -1.58 | -1.5 | -1.77 | 100 | 66.7 |
| Bt.5078.1.S1\_at | 0.000372 | 3.65e-06 | 2.84e-06 | ns \* \* | 1.01 | 0.881 | 1.06 | 100 | 66.7 |
| Bt.25086.1.S1\_at | 0.000753 | 1.6e-05 | 6.93e-06 | ns \* \* | -0.908 | -0.742 | -0.934 | 100 | 66.7 |
| Bt.25052.1.S1\_at | 0.00129 | 2.6e-05 | 4.19e-05 | ns \* \* | 0.651 | 0.851 | 1.14 | 100 | 66.7 |
| Bt.2660.1.S1\_at | 0.00215 | 1.22e-05 | 3.62e-05 | ns \* \* | 0.789 | 0.821 | 0.888 | 100 | 66.7 |
| Bt.22169.1.S1\_at | 0.00229 | 2.22e-05 | 2.71e-05 | ns \* \* | -0.802 | -0.839 | -0.981 | 100 | 66.7 |
| Bt.13128.1.S1\_at | 0.00237 | 3.68e-05 | 1.93e-06 | ns \* \* | -0.8 | -0.649 | -0.837 | 100 | 66.7 |
| Bt.16843.1.A1\_at | 0.00397 | 1.65e-05 | 8.97e-06 | ns \* \* | -0.588 | -0.703 | -0.859 | 100 | 66.7 |
| Bt.21731.1.S1\_at | 0.00614 | 3.08e-06 | 1.45e-05 | ns \* \* | 0.832 | 0.811 | 1.11 | 100 | 66.7 |
| Bt.28208.1.S1\_at | 0.013 | 1.33e-08 | 7.68e-07 | ns \* \* | -1.76 | -1.61 | -1.93 | 100 | 66.7 |
| Bt.22174.1.S1\_at | 0.0184 | 5.47e-06 | 5.46e-07 | ns \* \* | 1.69 | 1.35 | 3.15 | 100 | 66.7 |
| Bt.2129.1.S1\_at | 0.000117 | 3.2e-06 | 1.34e-06 | ns \* \* | 1.73 | 1.56 | 2.47 | 83.3 | 66.7 |
| Bt.5002.1.S1\_at | 0.000177 | 4.6e-08 | 2.57e-06 | ns \* \* | -1.26 | -1.26 | -1.85 | 83.3 | 66.7 |
| Bt.8319.3.S1\_at | 0.000311 | 1.68e-05 | 2.71e-05 | ns \* \* | -1.2 | -0.883 | -1.77 | 83.3 | 66.7 |
| Bt.2892.1.S1\_at | 0.117 | 4.5e-05 | 5.36e-05 | ns \* \* | 1.05 | 0.946 | 1.48 | 83.3 | 66.7 |
| Bt.17278.3.S1\_a\_at | 0.142 | 1.17e-05 | 4.1e-06 | ns \* \* | 1.7 | 1.27 | 2.18 | 83.3 | 66.7 |
| Bt.5129.2.A1\_at | 3.55e-06 | 2.46e-06 | 0.00116 | \* \* ns | 1.96 | 1.31 | 2.02 | 66.7 | 66.7 |
| Bt.27202.1.A1\_at | 0.0069 | 1.51e-07 | 2.18e-05 | ns \* \* | 3.26 | 1.95 | 2.8 | 66.7 | 66.7 |
| Bt.5129.1.S1\_a\_at | 0.000542 | 5.38e-07 | 5.24e-06 | ns \* \* | 2.57 | 2.12 | 3.18 | 50 | 66.7 |

Differentially expressed genes for MAS: 10

| AFFY | p-value | FoldChange |
| --- | --- | --- |
| Bt.29854.1.S1\_at | 3.661e-07 | -3.334 |
| Bt.29854.1.S1\_x\_at | 3.959e-07 | -3.244 |
| Bt.15968.1.S1\_at | 5.677e-07 | -1.749 |
| Bt.11622.1.A1\_at | 5.956e-07 | 3.039 |
| Bt.12300.1.S1\_at | 1.276e-06 | -2.06 |
| Bt.13922.1.A1\_at | 1.762e-06 | 3.436 |
| Bt.5129.2.A1\_at | 3.546e-06 | 1.955 |
| Bt.13980.2.A1\_a\_at | 3.846e-06 | -1.983 |
| Bt.22379.1.S1\_at | 3.873e-06 | -1.615 |
| Bt.17113.1.A1\_at | 5.175e-06 | -4.4 |

Differentially expressed genes for RMA: 72

| AFFY | p-value | FoldChange |
| --- | --- | --- |
| Bt.22379.1.S1\_at | 2.057e-10 | -1.711 |
| Bt.13980.2.A1\_a\_at | 6.218e-10 | -2.135 |
| Bt.11622.1.A1\_at | 1.022e-09 | 2.023 |
| Bt.19517.1.S1\_at | 5.086e-09 | 1.722 |
| Bt.13980.1.A1\_at | 9.36e-09 | -1.551 |
| Bt.15968.1.S1\_at | 9.939e-09 | -1.73 |
| Bt.4584.1.S1\_at | 1.026e-08 | -1.373 |
| Bt.28208.1.S1\_at | 1.334e-08 | -1.612 |
| Bt.29854.1.S1\_x\_at | 1.521e-08 | -3.443 |
| Bt.29854.1.S1\_at | 1.531e-08 | -3.51 |
| Bt.29854.1.A1\_at | 2.991e-08 | -2.085 |
| Bt.25689.1.S1\_at | 4.036e-08 | -1.306 |
| Bt.5002.1.S1\_at | 4.596e-08 | -1.257 |
| Bt.27202.1.A1\_at | 1.507e-07 | 1.954 |
| Bt.20316.1.S1\_at | 2.776e-07 | -1.828 |
| Bt.12300.1.S1\_at | 3.567e-07 | -2.08 |
| Bt.5129.1.S1\_a\_at | 5.381e-07 | 2.123 |
| Bt.12555.1.S1\_at | 6.379e-07 | -1.255 |
| Bt.13287.1.S1\_at | 1.184e-06 | -1.158 |
| Bt.2129.2.S1\_at | 1.405e-06 | 1.696 |
| Bt.28162.1.S1\_at | 1.451e-06 | -1.497 |
| Bt.76.1.S1\_at | 1.468e-06 | -1.072 |
| Bt.29697.1.S1\_at | 1.994e-06 | -1.479 |
| Bt.27323.1.S1\_at | 2.1e-06 | -1.102 |
| Bt.3782.1.A1\_at | 2.341e-06 | 1.21 |
| Bt.5129.2.A1\_at | 2.456e-06 | 1.313 |
| Bt.8199.1.S1\_at | 2.797e-06 | -1.847 |
| Bt.21731.1.S1\_at | 3.081e-06 | 0.8105 |
| Bt.2129.1.S1\_at | 3.203e-06 | 1.557 |
| Bt.5078.1.S1\_at | 3.652e-06 | 0.8814 |
| Bt.17810.1.S1\_a\_at | 4.631e-06 | 1.002 |
| Bt.22174.1.S1\_at | 5.474e-06 | 1.354 |
| Bt.26905.2.S1\_at | 6.358e-06 | -0.979 |
| Bt.9767.1.S1\_a\_at | 8.381e-06 | 1.227 |
| Bt.8964.2.S1\_a\_at | 8.824e-06 | -1.254 |
| Bt.7394.1.A1\_at | 8.911e-06 | -1.125 |
| Bt.5848.2.S1\_at | 9.585e-06 | -1.047 |
| Bt.6993.1.S1\_at | 9.875e-06 | -1.042 |
| Bt.25707.1.A1\_at | 1.112e-05 | 1.066 |
| Bt.17278.3.S1\_a\_at | 1.17e-05 | 1.268 |
| Bt.1074.1.A1\_a\_at | 1.203e-05 | 1.082 |
| Bt.2660.1.S1\_at | 1.221e-05 | 0.8214 |
| Bt.10278.1.S1\_at | 1.457e-05 | -0.9004 |
| Bt.25086.1.S1\_at | 1.601e-05 | -0.7423 |
| Bt.16843.1.A1\_at | 1.653e-05 | -0.7026 |
| Bt.8319.3.S1\_at | 1.677e-05 | -0.8827 |
| Bt.22533.1.S1\_at | 1.946e-05 | -0.7961 |
| Bt.24200.1.S1\_at | 1.952e-05 | 1.193 |
| Bt.5395.1.S1\_a\_at | 1.995e-05 | 1.284 |
| Bt.8679.3.S1\_at | 2.109e-05 | -1.163 |
| Bt.14398.1.S1\_at | 2.189e-05 | -1.383 |
| Bt.10931.1.S1\_at | 2.193e-05 | 0.8644 |
| Bt.22169.1.S1\_at | 2.224e-05 | -0.8393 |
| Bt.28162.3.S1\_at | 2.4e-05 | -1.422 |
| Bt.5353.1.S1\_a\_at | 2.464e-05 | 0.7272 |
| Bt.14572.1.A1\_at | 2.488e-05 | 1.108 |
| Bt.25052.1.S1\_at | 2.597e-05 | 0.8514 |
| Bt.9556.1.S1\_at | 2.693e-05 | 0.8341 |
| Bt.25855.2.S1\_at | 2.843e-05 | -1.125 |
| Bt.23651.1.A1\_at | 2.955e-05 | 0.685 |
| Bt.29381.1.S1\_at | 2.972e-05 | -0.9892 |
| Bt.4138.2.S1\_at | 3.193e-05 | -0.9311 |
| Bt.25658.1.A1\_at | 3.424e-05 | -1.156 |
| Bt.13128.1.S1\_at | 3.683e-05 | -0.6486 |
| Bt.9944.1.S1\_at | 3.786e-05 | 1.296 |
| Bt.24926.1.S1\_at | 3.817e-05 | -1.507 |
| Bt.8384.1.S1\_at | 3.933e-05 | -0.6969 |
| Bt.9202.1.S1\_at | 4.343e-05 | -1.543 |
| Bt.25282.1.A1\_at | 4.471e-05 | 0.7944 |
| Bt.2892.1.S1\_at | 4.504e-05 | 0.9456 |
| Bt.25186.1.S1\_at | 4.512e-05 | 1.943 |
| Bt.17613.1.A1\_at | 4.556e-05 | -0.7297 |

Differentially expressed genes for GCRMA: 85

| AFFY | p-value | FoldChange |
| --- | --- | --- |
| Bt.11622.1.A1\_at | 7.384e-10 | 2.886 |
| Bt.15968.1.S1\_at | 6.078e-09 | -2.292 |
| Bt.29854.1.A1\_at | 5.861e-08 | -4.278 |
| Bt.25689.1.S1\_at | 8.984e-08 | -1.742 |
| Bt.13980.2.A1\_a\_at | 9.866e-08 | -2.505 |
| Bt.22379.1.S1\_at | 4.156e-07 | -2.537 |
| Bt.20316.1.S1\_at | 4.796e-07 | -2.149 |
| Bt.13980.1.A1\_at | 5.059e-07 | -2.016 |
| Bt.22174.1.S1\_at | 5.463e-07 | 3.149 |
| Bt.5848.2.S1\_at | 5.776e-07 | -1.24 |
| Bt.28208.1.S1\_at | 7.676e-07 | -1.932 |
| Bt.25524.1.S1\_at | 8.519e-07 | 0.7326 |
| Bt.29854.1.S1\_x\_at | 1.308e-06 | -4.062 |
| Bt.2129.1.S1\_at | 1.337e-06 | 2.473 |
| Bt.25282.1.A1\_at | 1.435e-06 | 0.983 |
| Bt.4584.1.S1\_at | 1.599e-06 | -1.638 |
| Bt.13128.1.S1\_at | 1.933e-06 | -0.837 |
| Bt.5002.1.S1\_at | 2.569e-06 | -1.854 |
| Bt.29854.1.S1\_at | 2.73e-06 | -4.192 |
| Bt.5078.1.S1\_at | 2.843e-06 | 1.057 |
| Bt.23148.1.S1\_at | 3.031e-06 | 0.7016 |
| Bt.11178.1.S1\_at | 3.209e-06 | 0.8632 |
| Bt.848.1.S1\_at | 3.506e-06 | -0.6346 |
| Bt.4138.2.S1\_at | 3.515e-06 | -1.142 |
| Bt.298.1.S1\_at | 3.544e-06 | -0.7179 |
| Bt.14570.1.A1\_at | 4.051e-06 | 1.059 |
| Bt.17278.3.S1\_a\_at | 4.097e-06 | 2.177 |
| Bt.28162.1.S1\_at | 4.771e-06 | -1.765 |
| Bt.13287.1.S1\_at | 5.134e-06 | -1.149 |
| Bt.5129.1.S1\_a\_at | 5.24e-06 | 3.182 |
| Bt.7394.1.A1\_at | 5.822e-06 | -1.296 |
| Bt.2129.2.S1\_at | 6.033e-06 | 2.001 |
| Bt.18890.1.A1\_at | 6.193e-06 | 0.7867 |
| Bt.22634.1.S1\_at | 6.469e-06 | 0.9179 |
| Bt.76.1.S1\_at | 6.928e-06 | -1.217 |
| Bt.25086.1.S1\_at | 6.934e-06 | -0.9343 |
| Bt.6705.1.S1\_at | 7.054e-06 | 1.19 |
| Bt.21176.1.S1\_at | 7.773e-06 | 0.6419 |
| Bt.12300.1.S1\_at | 8.147e-06 | -2.672 |
| Bt.16003.1.S1\_at | 8.285e-06 | -0.9414 |
| Bt.8650.1.A1\_at | 8.35e-06 | 0.5324 |
| Bt.29697.1.S1\_at | 8.464e-06 | -1.82 |
| Bt.19517.1.S1\_at | 8.559e-06 | 2.148 |
| Bt.16843.1.A1\_at | 8.965e-06 | -0.8588 |
| Bt.24396.3.A1\_at | 1.096e-05 | 0.8166 |
| Bt.21180.1.S1\_at | 1.188e-05 | 1.203 |
| Bt.5519.2.S1\_at | 1.242e-05 | 0.7294 |
| Bt.20228.1.S1\_at | 1.328e-05 | -0.4866 |
| Bt.21731.1.S1\_at | 1.447e-05 | 1.108 |
| Bt.6993.1.S1\_at | 1.46e-05 | -1.425 |
| Bt.3960.2.S1\_at | 1.557e-05 | -0.4734 |
| Bt.23230.1.S1\_at | 1.616e-05 | 0.5087 |
| Bt.25658.1.A1\_at | 1.667e-05 | -1.531 |
| Bt.100.1.S1\_at | 1.784e-05 | -1.694 |
| Bt.396.2.S1\_a\_at | 1.839e-05 | 1.433 |
| Bt.1696.1.S1\_at | 1.973e-05 | -0.5217 |
| Bt.26905.1.A1\_at | 2.157e-05 | -1.003 |
| Bt.27202.1.A1\_at | 2.179e-05 | 2.795 |
| Bt.20591.2.A1\_at | 2.224e-05 | 1.084 |
| Bt.3940.1.S1\_at | 2.412e-05 | 0.5152 |
| Bt.9773.1.A1\_at | 2.528e-05 | -0.5552 |
| Bt.59.1.S1\_at | 2.673e-05 | -0.7538 |
| Bt.17613.1.A1\_at | 2.692e-05 | -1.308 |
| Bt.8319.3.S1\_at | 2.705e-05 | -1.769 |
| Bt.22169.1.S1\_at | 2.707e-05 | -0.9809 |
| Bt.22519.1.S1\_at | 2.718e-05 | -0.9459 |
| Bt.15751.1.S1\_at | 2.757e-05 | 0.6988 |
| Bt.10278.1.S1\_at | 2.847e-05 | -1.048 |
| Bt.8319.1.S1\_at | 2.921e-05 | -0.8771 |
| Bt.27816.3.S1\_a\_at | 2.99e-05 | -0.8101 |
| Bt.13605.1.A1\_at | 3.255e-05 | -1.002 |
| Bt.4473.1.S1\_at | 3.265e-05 | -0.7842 |
| Bt.5848.1.S1\_at | 3.36e-05 | -0.9126 |
| Bt.2660.1.S1\_at | 3.621e-05 | 0.8879 |
| Bt.1059.3.S1\_a\_at | 3.65e-05 | -0.6397 |
| Bt.3809.1.S1\_at | 3.758e-05 | -0.928 |
| Bt.7523.1.S1\_at | 3.858e-05 | 0.6082 |
| Bt.3452.1.S1\_at | 3.905e-05 | 1.077 |
| Bt.25052.1.S1\_at | 4.19e-05 | 1.141 |
| Bt.12327.1.S1\_at | 4.243e-05 | -0.8456 |
| Bt.7215.1.S1\_at | 4.641e-05 | -1.232 |
| Bt.923.1.A1\_at | 5.11e-05 | 0.9265 |
| Bt.5456.1.S2\_at | 5.188e-05 | -0.8751 |
| Bt.5515.1.S1\_at | 5.321e-05 | -0.4229 |
| Bt.2892.1.S1\_at | 5.362e-05 | 1.485 |

*Venn diagram of differential expression for the three different methods.*
