# Supplementary material for: A gene network switch enhances the oxidative capacity of ovine skeletal muscle during late fetal development
Source: BMC Genomics. 2010 Jun 15;11:378. doi: 10.1186/1471-2164-11-378 (PMC2894804; doi:10.1186/1471-2164-11-378)
Supplement: Additional File 1 — Gene expression data. This file contains links to the gene expression data. The genes that are significantly differentially expressed between adjacent development times are listed as Experiment 1 (80 d vs 100 d), Experiment 2 (100 d vs 120 d), Experiment 3 (120 d vs 150 d) and Experiment 4 (150 d vs 230 d) in the section entitled Open microarray analysis report for developmental expression. Each experiment contains an MA plot, Volcano plot and Heatmap for each of the three microarray processing programs MAS5, RMA and GCRMA. For each of these processing programs there is a list of significantly differentially expressed probe sets, and for each of these there is an FDR corrected probability, fold change, percentage of microarrays with a MAS5 Present call, and a convergence percentage for all three microarray processing programs. The file also contains annotation of the probe sets. In a second section entitled Time series analysis of developmental expression, there is a list of genes in each gene expression cluster and diagrams showing MAS5 gene expression values for these genes. [file 1471-2164-11-378-S1.ZIP › Report/Experiment3.html]

## Experiment 3

### Experimental Question

Which genes are differentially expressed between day 120 and day 150?

### Arrays

Array reference names used in the experiment and their corresponding original CEL file names.

| Reference | CEL File |
| --- | --- |
| d120\_1 | af1c1228-1-196-161007.CEL |
| d120\_2 | af1c1228-4-215-161007.CEL |
| d120\_3 | af1c1228-8-261-161007.CEL |
| d150\_1 | af1c1228-12-4555-161007.CEL |
| d150\_2 | af1c1228-14-4576-161007.CEL |
| d150\_3 | af1c1228-15-4580-161007.CEL |

### Probe Summary

Total number of probe sets on chip: 24128 .

Total number of Affymetrix control probe sets on chip: 112 (removed from analysis).

Total number of probe sets detected as absent or marginal on all slides: 8374 (removed from analysis).

Total number of probe sets used in analysis: 15642 .

### Annotation

Annotation of differentially expressed genes for experiment 3.

### Expression Plots

MA plots for MAS5, RMA and GCRMA for contrast 1.

Volcano plots for MAS5, RMA and GCRMA for contrast 1.

Heatmap plots for MAS5, RMA and GCRMA.

### Functional Profile Analyses

Gene Ontology analyses.

### Summary gene list with 2471 differentially expressed genes

Briefly - the best starting point for significantly expressed genes is for the genes with a value of 100 in the convergence column and 3 stars (\*\*\*) in the FDR\_Corrected column.

Differential expression was tested on log2 expression intensities obtained using MAS, RMA and GCRMA summarization methods. P-values for each method are shown for the moderated F-statistic which stabilizes the gene-specific variance using an empirical Bayes method to shrink the probe-wise sample variances towards a common value and to augment the degrees of freedom for the individual variances (Smyth,2004). Significance after correction for false discovery rates (FDR) using the method BH for a targeted p-value of 0.01 is also shown for each summarization set (ns - not significant, \* - significant; this means that if the gene has three stars it is significant for all three summarizations, and a very reliable indication that the gene is truly differentially expressed). Log2 fold changes are also shown in the table (remember that a log fold change of one equals a fold change of two). The fold changes are based on the raw data and not the fitted intensities. See the MA and volcano plots for a visual representation. If the gene was detected as present or not in each array is shown in the table in percentage (Presence\_Calls column). The last column shows the convergence in percentage of the gene across the three methods. A value of 100 indicates that the gene was detected as significantly differentially expressed for all three summarizations. The table was filtered for genes with a convergence greater than 50% and called as present in at least half of the samples. Note that the log fold change used is simply the average of the differences of the contrasts, if there is more than one contrast. It does not necessarily mean that the fold change is above the threshold for each individual contrast.

MAS parameters: background correction - for both PM and MM, the lowest 2% of probe intensities are used as a background value for each of 16 grids. Each probe is then adjusted based upon a weighted (distance between the location of the probe and the centroid) average of the background of each grid; normalization - scale normalization, all arrays scaled to the same mean value (200); summarization - log2 scale average using 1-step Tukey biweight.

RMA parameters: background correction - convolution; normalization - quantile; summarization - median polish. Note: MM intensities are not used, which does not account for non-specific binding on the different probes (can underestimate the background).

GCRMA parameters: background correction - an affinity measure model based on probe sequences and MM intensities; normalization - quantile; summarization - median polish.

|  |  |  |  |  |  |  |  |  |  |
| --- | --- | --- | --- | --- | --- | --- | --- | --- | --- |
| **AFFY** | **MAS\_ModFtest** | **RMA\_ModFtest** | **GCRMA\_ModFtest** | **FDR\_Corrected** | **MAS\_FoldChange** | **RMA\_FoldChange** | **GCRMA\_FoldChange** | **Presence\_Calls** | **Convergence** |
| Bt.13980.1.A1\_at | 1.89e-09 | 2.76e-13 | 7.37e-10 | \* \* \* | -4.67 | -3.04 | -3.7 | 100 | 100 |
| Bt.3960.1.S1\_a\_at | 5.69e-09 | 1.9e-09 | 7.87e-10 | \* \* \* | -2.04 | -1.75 | -2.12 | 100 | 100 |
| Bt.13980.2.A1\_a\_at | 6.56e-09 | 6.72e-12 | 2.08e-09 | \* \* \* | -3.52 | -2.86 | -3.58 | 100 | 100 |
| Bt.1316.1.S1\_at | 6.65e-09 | 2.24e-11 | 1.95e-08 | \* \* \* | -2.85 | -2.56 | -3.27 | 100 | 100 |
| Bt.23502.1.S1\_at | 1.01e-08 | 1.34e-08 | 4.41e-08 | \* \* \* | 1.9 | 1.23 | 1.65 | 100 | 100 |
| Bt.23662.2.S1\_at | 1.11e-08 | 1.53e-09 | 1.13e-07 | \* \* \* | -2.29 | -1.96 | -2.9 | 100 | 100 |
| Bt.22664.1.A1\_at | 1.47e-08 | 1.01e-09 | 2.14e-09 | \* \* \* | 2.96 | 2.28 | 2.79 | 100 | 100 |
| Bt.24931.1.A1\_at | 1.58e-08 | 1.03e-09 | 1.54e-09 | \* \* \* | -1.97 | -1.61 | -2.05 | 100 | 100 |
| Bt.7034.1.S1\_at | 1.72e-08 | 1.41e-10 | 8.32e-08 | \* \* \* | -3.93 | -2.9 | -3.56 | 100 | 100 |
| Bt.2894.1.S1\_at | 2.51e-08 | 6.54e-12 | 1.79e-08 | \* \* \* | -2.35 | -2.33 | -3.12 | 100 | 100 |
| Bt.16843.1.A1\_at | 2.67e-08 | 2.66e-09 | 2.15e-09 | \* \* \* | -2.05 | -1.34 | -1.91 | 100 | 100 |
| Bt.11178.1.S1\_at | 2.88e-08 | 8.62e-11 | 1.01e-11 | \* \* \* | 2.16 | 1.99 | 2.8 | 100 | 100 |
| Bt.27381.1.S1\_at | 2.91e-08 | 7.08e-12 | 2.12e-08 | \* \* \* | -3.05 | -2.93 | -4.08 | 100 | 100 |
| Bt.1153.1.S1\_at | 3.12e-08 | 6.86e-09 | 1.02e-08 | \* \* \* | -1.87 | -1.52 | -2 | 100 | 100 |
| Bt.27421.1.S1\_at | 3.44e-08 | 1.57e-07 | 1.68e-07 | \* \* \* | 2.06 | 1.63 | 2.05 | 100 | 100 |
| Bt.3865.3.S1\_a\_at | 3.63e-08 | 5.06e-12 | 1.04e-08 | \* \* \* | -2.79 | -2.49 | -3.34 | 100 | 100 |
| Bt.21766.3.S1\_at | 4.33e-08 | 2.48e-08 | 1.16e-07 | \* \* \* | -2.3 | -2.02 | -2.55 | 100 | 100 |
| Bt.22519.1.S1\_at | 5.33e-08 | 1.03e-09 | 1.27e-09 | \* \* \* | -2.26 | -1.87 | -2.48 | 100 | 100 |
| Bt.848.1.S1\_at | 5.55e-08 | 1.63e-07 | 4.24e-10 | \* \* \* | -1.8 | -1.17 | -1.48 | 100 | 100 |
| Bt.7056.5.A1\_x\_at | 5.69e-08 | 1.68e-08 | 1.73e-08 | \* \* \* | 5.71 | 3.82 | 7.27 | 100 | 100 |
| Bt.11236.1.S1\_at | 5.95e-08 | 5.55e-11 | 1.88e-08 | \* \* \* | -4.63 | -3.21 | -4.56 | 100 | 100 |
| Bt.12510.1.S1\_a\_at | 7.64e-08 | 1.12e-10 | 3.97e-08 | \* \* \* | 2.63 | 2.77 | 4.29 | 100 | 100 |
| Bt.2696.1.A1\_at | 7.96e-08 | 5.19e-08 | 6.41e-07 | \* \* \* | 2.1 | 2.14 | 2.4 | 100 | 100 |
| Bt.11770.1.S1\_a\_at | 8.01e-08 | 3.69e-11 | 3.34e-10 | \* \* \* | -2.76 | -2.23 | -2.58 | 100 | 100 |
| Bt.5002.1.S1\_at | 8.1e-08 | 1.89e-12 | 6.59e-09 | \* \* \* | -2.66 | -2.44 | -3.27 | 100 | 100 |
| Bt.17613.1.A1\_at | 8.17e-08 | 1.56e-11 | 9.9e-09 | \* \* \* | -2.47 | -2.13 | -2.84 | 100 | 100 |
| Bt.11794.1.S1\_at | 8.81e-08 | 5.41e-09 | 2.38e-07 | \* \* \* | -2.45 | -1.95 | -2.42 | 100 | 100 |
| Bt.4770.1.S1\_at | 9.14e-08 | 2.76e-09 | 1.9e-09 | \* \* \* | 1.97 | 1.66 | 2.03 | 100 | 100 |
| Bt.6576.1.S1\_at | 9.37e-08 | 4.88e-07 | 1.74e-07 | \* \* \* | 1.56 | 1.03 | 1.11 | 100 | 100 |
| Bt.4777.1.S1\_at | 9.73e-08 | 1.93e-08 | 1.27e-07 | \* \* \* | -2.12 | -1.74 | -2.17 | 100 | 100 |
| Bt.9222.1.S1\_at | 9.81e-08 | 1.4e-08 | 2.22e-07 | \* \* \* | -1.92 | -1.45 | -1.9 | 100 | 100 |
| Bt.20229.1.S1\_at | 1.11e-07 | 7.03e-10 | 1.33e-09 | \* \* \* | -2.3 | -1.75 | -2.34 | 100 | 100 |
| Bt.4704.1.S1\_at | 1.12e-07 | 8.89e-08 | 3.21e-07 | \* \* \* | -2.3 | -1.65 | -2 | 100 | 100 |
| Bt.21896.1.S1\_at | 1.18e-07 | 3.57e-10 | 2.22e-08 | \* \* \* | 2.61 | 2.64 | 3.3 | 100 | 100 |
| Bt.4072.1.S1\_at | 1.26e-07 | 2.07e-08 | 1.47e-07 | \* \* \* | -1.98 | -1.5 | -2.01 | 100 | 100 |
| Bt.1316.1.S2\_at | 1.27e-07 | 7.13e-11 | 2.99e-09 | \* \* \* | -3.12 | -2.18 | -3 | 100 | 100 |
| Bt.3117.1.S1\_at | 1.32e-07 | 6.52e-09 | 6.33e-09 | \* \* \* | -2.13 | -1.54 | -1.86 | 100 | 100 |
| Bt.27814.1.S1\_at | 1.35e-07 | 1.15e-07 | 2.54e-06 | \* \* \* | 1.97 | 2 | 2.48 | 100 | 100 |
| Bt.836.1.S1\_at | 1.36e-07 | 2.5e-09 | 4.11e-08 | \* \* \* | -2.28 | -1.69 | -2.12 | 100 | 100 |
| Bt.4758.1.S1\_at | 1.5e-07 | 2.14e-08 | 6.21e-06 | \* \* \* | -2.59 | -2.13 | -2.41 | 100 | 100 |
| Bt.18753.1.A1\_at | 1.74e-07 | 3.7e-10 | 1.04e-07 | \* \* \* | 2.55 | 1.94 | 2.54 | 100 | 100 |
| Bt.5120.1.S1\_at | 1.99e-07 | 2.05e-09 | 1.46e-07 | \* \* \* | -2.31 | -1.9 | -2.31 | 100 | 100 |
| Bt.17963.1.A1\_at | 2.16e-07 | 6.83e-07 | 1.15e-06 | \* \* \* | -1.7 | -1.34 | -1.79 | 100 | 100 |
| Bt.13330.2.A1\_at | 2.23e-07 | 1.26e-09 | 2.14e-07 | \* \* \* | -4.19 | -3.67 | -4.73 | 100 | 100 |
| Bt.3247.1.S1\_at | 2.29e-07 | 1.02e-08 | 3.83e-08 | \* \* \* | 2.76 | 2.31 | 2.79 | 100 | 100 |
| Bt.3960.2.S1\_at | 2.31e-07 | 9.16e-07 | 8.54e-11 | \* \* \* | -1.66 | -1.07 | -1.49 | 100 | 100 |
| Bt.1793.1.A1\_at | 2.31e-07 | 1.48e-06 | 8.82e-06 | \* \* \* | -2.16 | -1.54 | -2.09 | 100 | 100 |
| Bt.18572.1.A1\_at | 2.39e-07 | 4.75e-10 | 1.2e-08 | \* \* \* | 2.53 | 2.3 | 3.4 | 100 | 100 |
| Bt.3873.1.S1\_at | 2.6e-07 | 3.03e-10 | 1.09e-08 | \* \* \* | -1.77 | -1.42 | -1.85 | 100 | 100 |
| Bt.6391.1.A1\_at | 2.65e-07 | 1.16e-08 | 1.21e-05 | \* \* \* | -2.74 | -2.15 | -3.17 | 100 | 100 |
| Bt.18752.1.S1\_at | 2.72e-07 | 2.6e-06 | 7.24e-06 | \* \* \* | -1.64 | -1.52 | -2.17 | 100 | 100 |
| Bt.12510.1.S1\_at | 2.82e-07 | 8.83e-11 | 1.06e-07 | \* \* \* | 3.48 | 3.33 | 4.04 | 100 | 100 |
| Bt.19508.1.A1\_at | 2.88e-07 | 2.85e-08 | 1.38e-07 | \* \* \* | 2.22 | 1.76 | 2.35 | 100 | 100 |
| Bt.4473.1.S1\_at | 2.95e-07 | 4.11e-09 | 2.96e-09 | \* \* \* | -1.7 | -1.65 | -1.94 | 100 | 100 |
| Bt.946.2.A1\_a\_at | 3.02e-07 | 2.1e-08 | 1.5e-07 | \* \* \* | -1.74 | -1.44 | -2.2 | 100 | 100 |
| Bt.11516.1.S1\_at | 3.05e-07 | 1.77e-09 | 2.56e-07 | \* \* \* | -2.13 | -1.46 | -1.83 | 100 | 100 |
| Bt.5395.1.S1\_a\_at | 3.05e-07 | 4.26e-07 | 9.32e-07 | \* \* \* | 2.1 | 1.75 | 2.93 | 100 | 100 |
| Bt.19517.1.S1\_at | 3.07e-07 | 1.55e-11 | 1.5e-08 | \* \* \* | 2.33 | 2.52 | 3.97 | 100 | 100 |
| Bt.22082.1.S1\_at | 3.28e-07 | 6.91e-08 | 1.71e-07 | \* \* \* | -2.35 | -1.76 | -2.18 | 100 | 100 |
| Bt.11449.1.S1\_at | 3.46e-07 | 1.35e-07 | 1.52e-07 | \* \* \* | -1.79 | -1.29 | -1.84 | 100 | 100 |
| Bt.1403.1.S1\_at | 3.54e-07 | 1.09e-09 | 2.37e-10 | \* \* \* | -1.96 | -1.81 | -2.23 | 100 | 100 |
| Bt.16082.1.S1\_at | 3.54e-07 | 4.75e-08 | 2.38e-07 | \* \* \* | -2.23 | -1.64 | -2.15 | 100 | 100 |
| Bt.13313.1.S1\_at | 3.58e-07 | 6.84e-07 | 6.27e-07 | \* \* \* | 1.53 | 1.34 | 1.83 | 100 | 100 |
| Bt.21372.3.A1\_at | 4.11e-07 | 5.3e-06 | 1.71e-07 | \* \* \* | 2.12 | 1.2 | 1.79 | 100 | 100 |
| Bt.27926.1.S1\_at | 4.16e-07 | 4.74e-07 | 1.58e-07 | \* \* \* | -1.82 | -1.5 | -1.85 | 100 | 100 |
| Bt.21.1.S1\_at | 4.51e-07 | 1.74e-05 | 1.27e-07 | \* \* \* | -1.6 | -0.786 | -1.04 | 100 | 100 |
| Bt.7215.1.S1\_at | 4.54e-07 | 1.02e-09 | 6.4e-08 | \* \* \* | -2.52 | -1.8 | -2.39 | 100 | 100 |
| Bt.12694.1.S1\_at | 4.82e-07 | 1.24e-08 | 5.08e-08 | \* \* \* | 1.69 | 1.45 | 2.06 | 100 | 100 |
| Bt.5732.1.S1\_at | 4.94e-07 | 1.1e-07 | 1.72e-07 | \* \* \* | 2.02 | 1.98 | 2.66 | 100 | 100 |
| Bt.5519.2.S1\_at | 5.11e-07 | 6.43e-08 | 1.32e-08 | \* \* \* | 1.47 | 1.27 | 1.42 | 100 | 100 |
| Bt.18479.1.A1\_at | 5.4e-07 | 1.01e-10 | 6.62e-07 | \* \* \* | 2.19 | 2 | 2.65 | 100 | 100 |
| Bt.5483.1.S1\_at | 5.46e-07 | 1.79e-08 | 7.89e-09 | \* \* \* | -1.71 | -1.31 | -1.74 | 100 | 100 |
| Bt.99.1.S1\_at | 5.67e-07 | 8.25e-07 | 1.29e-06 | \* \* \* | -1.68 | -1.27 | -1.58 | 100 | 100 |
| Bt.378.1.S1\_at | 5.83e-07 | 1.08e-05 | 4.9e-07 | \* \* \* | -1.42 | -0.785 | -1.07 | 100 | 100 |
| Bt.9556.1.S1\_at | 6.02e-07 | 3.58e-09 | 8.14e-07 | \* \* \* | 1.81 | 1.63 | 1.87 | 100 | 100 |
| Bt.4262.1.S1\_a\_at | 6.09e-07 | 5.46e-07 | 1.38e-05 | \* \* \* | -2.61 | -1.42 | -1.87 | 100 | 100 |
| Bt.27215.1.S1\_at | 6.12e-07 | 1.51e-07 | 9.92e-07 | \* \* \* | -1.83 | -1.43 | -1.74 | 100 | 100 |
| Bt.5210.1.S1\_at | 6.31e-07 | 1.43e-06 | 9.72e-07 | \* \* \* | -1.58 | -1.11 | -1.28 | 100 | 100 |
| Bt.27193.1.A1\_at | 6.41e-07 | 3.08e-08 | 2.65e-08 | \* \* \* | 1.68 | 1.47 | 1.9 | 100 | 100 |
| Bt.1758.1.S1\_at | 6.6e-07 | 1.23e-07 | 9.38e-08 | \* \* \* | 1.4 | 1.41 | 1.69 | 100 | 100 |
| Bt.27318.1.S1\_at | 6.72e-07 | 2.84e-08 | 3.9e-07 | \* \* \* | 1.81 | 1.92 | 2.19 | 100 | 100 |
| Bt.3448.2.S1\_at | 6.91e-07 | 3.69e-08 | 1.78e-07 | \* \* \* | -1.77 | -1.55 | -1.87 | 100 | 100 |
| Bt.10013.1.S1\_at | 7.06e-07 | 2.35e-08 | 8.63e-08 | \* \* \* | 1.92 | 1.81 | 2.47 | 100 | 100 |
| Bt.298.1.S1\_at | 7.17e-07 | 1.79e-05 | 4.62e-08 | \* \* \* | -1.42 | -0.899 | -1.09 | 100 | 100 |
| Bt.23164.1.S1\_at | 7.34e-07 | 1.35e-09 | 1.83e-07 | \* \* \* | -2.16 | -1.65 | -2.15 | 100 | 100 |
| Bt.20899.1.A1\_at | 7.44e-07 | 2.06e-10 | 2.71e-07 | \* \* \* | -2.32 | -2.07 | -2.6 | 100 | 100 |
| Bt.13313.2.S1\_at | 7.53e-07 | 5.5e-08 | 2.38e-08 | \* \* \* | 1.53 | 1.48 | 1.71 | 100 | 100 |
| Bt.216.1.S1\_at | 7.58e-07 | 5.26e-06 | 9.82e-06 | \* \* \* | 1.53 | 1.31 | 1.7 | 100 | 100 |
| Bt.28704.1.S1\_at | 7.61e-07 | 1.03e-05 | 4.85e-06 | \* \* \* | 2.3 | 2.17 | 2.86 | 100 | 100 |
| Bt.28975.1.S1\_at | 7.66e-07 | 7e-07 | 2.38e-07 | \* \* \* | 1.84 | 1.41 | 1.94 | 100 | 100 |
| Bt.20141.1.S1\_at | 7.8e-07 | 4.41e-09 | 3.12e-07 | \* \* \* | -1.92 | -1.51 | -2.16 | 100 | 100 |
| Bt.3487.1.S1\_at | 7.85e-07 | 6.24e-06 | 5.32e-06 | \* \* \* | -1.77 | -1.08 | -1.41 | 100 | 100 |
| Bt.1242.1.S1\_at | 8.04e-07 | 3.47e-08 | 1.3e-06 | \* \* \* | -2.12 | -1.58 | -2.02 | 100 | 100 |
| Bt.27039.1.A1\_at | 8.11e-07 | 1.04e-07 | 1.29e-06 | \* \* \* | -1.86 | -2.2 | -2.65 | 100 | 100 |
| Bt.20505.1.S1\_at | 8.79e-07 | 2.64e-09 | 5.48e-09 | \* \* \* | -1.79 | -1.68 | -2.11 | 100 | 100 |
| Bt.9811.1.S1\_at | 9.16e-07 | 1.71e-06 | 2.64e-06 | \* \* \* | -1.34 | -0.925 | -1.12 | 100 | 100 |
| Bt.22379.1.S1\_at | 9.39e-07 | 1.21e-09 | 7.4e-06 | \* \* \* | -1.85 | -1.52 | -1.9 | 100 | 100 |
| Bt.23098.1.S1\_at | 9.51e-07 | 3.22e-07 | 7.85e-06 | \* \* \* | 2.22 | 1.03 | 1.25 | 100 | 100 |
| Bt.1035.1.S1\_a\_at | 9.65e-07 | 8.21e-07 | 4.14e-08 | \* \* \* | -1.37 | -0.902 | -1.22 | 100 | 100 |
| Bt.24211.2.S1\_at | 1.03e-06 | 1.4e-06 | 2.3e-06 | \* \* \* | 2.07 | 2 | 2.53 | 100 | 100 |
| Bt.27091.2.S1\_at | 1.07e-06 | 7.39e-07 | 5.07e-06 | \* \* \* | 2.55 | 1.62 | 2.19 | 100 | 100 |
| Bt.5571.1.S1\_at | 1.09e-06 | 1.42e-06 | 2.13e-06 | \* \* \* | -1.55 | -1.05 | -1.37 | 100 | 100 |
| Bt.22510.1.S1\_at | 1.11e-06 | 8.61e-07 | 2.1e-07 | \* \* \* | -1.64 | -1.21 | -1.85 | 100 | 100 |
| Bt.20984.1.S1\_at | 1.13e-06 | 2.84e-06 | 5.79e-06 | \* \* \* | -1.9 | -1.28 | -1.53 | 100 | 100 |
| Bt.10278.1.S1\_at | 1.22e-06 | 7.98e-08 | 4.42e-07 | \* \* \* | -1.49 | -1.35 | -1.6 | 100 | 100 |
| Bt.5922.1.S1\_at | 1.27e-06 | 1.34e-07 | 1.87e-06 | \* \* \* | -2.66 | -1.83 | -2.42 | 100 | 100 |
| Bt.136.1.S1\_at | 1.32e-06 | 3.19e-07 | 1.41e-05 | \* \* \* | -3 | -2.6 | -3.51 | 100 | 100 |
| Bt.19266.1.A1\_at | 1.36e-06 | 0.00037 | 0.00187 | \* \* \* | 1.9 | 0.808 | 1.17 | 100 | 100 |
| Bt.1408.1.S1\_at | 1.37e-06 | 1.99e-06 | 8.15e-06 | \* \* \* | 1.35 | 1.29 | 1.5 | 100 | 100 |
| Bt.76.1.S1\_at | 1.4e-06 | 1.45e-06 | 2.26e-07 | \* \* \* | -1.71 | -1.07 | -1.71 | 100 | 100 |
| Bt.1808.1.S1\_at | 1.42e-06 | 6.94e-08 | 3.85e-06 | \* \* \* | -1.88 | -1.13 | -1.54 | 100 | 100 |
| Bt.1129.2.S1\_at | 1.42e-06 | 4.26e-08 | 1.78e-08 | \* \* \* | -1.88 | -1.47 | -2.01 | 100 | 100 |
| Bt.75.1.S1\_at | 1.44e-06 | 1.03e-06 | 3.49e-06 | \* \* \* | -1.52 | -0.949 | -1.26 | 100 | 100 |
| Bt.13324.2.S1\_a\_at | 1.46e-06 | 3.59e-09 | 9.25e-07 | \* \* \* | -1.83 | -1.48 | -1.79 | 100 | 100 |
| Bt.20512.2.S1\_at | 1.49e-06 | 8.54e-08 | 9.46e-07 | \* \* \* | 2 | 1.68 | 2.11 | 100 | 100 |
| Bt.1280.2.S1\_a\_at | 1.64e-06 | 4.27e-07 | 6.8e-07 | \* \* \* | -1.86 | -1.29 | -1.76 | 100 | 100 |
| Bt.12358.1.S1\_at | 1.65e-06 | 2.28e-07 | 1.22e-08 | \* \* \* | 1.43 | 1.15 | 1.77 | 100 | 100 |
| Bt.12453.1.A1\_a\_at | 1.68e-06 | 3.49e-09 | 7.97e-07 | \* \* \* | 2.44 | 2 | 2.43 | 100 | 100 |
| Bt.6494.1.S1\_at | 1.72e-06 | 4.72e-07 | 1.24e-08 | \* \* \* | -1.25 | -0.951 | -1.14 | 100 | 100 |
| Bt.1582.1.S1\_at | 1.73e-06 | 1.44e-06 | 2.9e-06 | \* \* \* | -1.53 | -1.2 | -1.54 | 100 | 100 |
| Bt.20295.1.A1\_at | 1.74e-06 | 4.07e-08 | 5.42e-07 | \* \* \* | 1.89 | 1.91 | 2.4 | 100 | 100 |
| Bt.5123.3.S1\_a\_at | 1.77e-06 | 4.14e-06 | 1.41e-06 | \* \* \* | -1.3 | -0.992 | -1.28 | 100 | 100 |
| Bt.4475.1.S1\_at | 1.82e-06 | 1.06e-07 | 9.37e-08 | \* \* \* | -1.78 | -1.34 | -1.69 | 100 | 100 |
| Bt.21383.1.S1\_at | 1.84e-06 | 2.05e-06 | 5.37e-05 | \* \* \* | 1.88 | 1.32 | 1.66 | 100 | 100 |
| Bt.8549.2.S1\_at | 1.88e-06 | 1.76e-08 | 1.68e-08 | \* \* \* | 1.71 | 1.45 | 1.94 | 100 | 100 |
| Bt.321.1.S1\_at | 2.02e-06 | 1.1e-06 | 9.18e-06 | \* \* \* | -1.79 | -1.29 | -1.58 | 100 | 100 |
| Bt.1861.1.S1\_at | 2.11e-06 | 5.54e-08 | 5.73e-08 | \* \* \* | 1.66 | 1.56 | 2.01 | 100 | 100 |
| Bt.893.1.S1\_at | 2.14e-06 | 9.23e-05 | 6.92e-05 | \* \* \* | -1.5 | -0.974 | -1.11 | 100 | 100 |
| Bt.15941.1.S1\_at | 2.19e-06 | 7.62e-07 | 3.33e-07 | \* \* \* | -1.54 | -1.27 | -1.58 | 100 | 100 |
| Bt.1059.2.S1\_at | 2.27e-06 | 9.75e-05 | 9.74e-07 | \* \* \* | -1.38 | -0.717 | -0.781 | 100 | 100 |
| Bt.20308.1.S1\_at | 2.32e-06 | 2.12e-07 | 1.23e-05 | \* \* \* | 2.62 | 2.05 | 2.35 | 100 | 100 |
| Bt.23497.1.S1\_at | 2.35e-06 | 6.55e-08 | 6e-06 | \* \* \* | -4.43 | -3.04 | -3.86 | 100 | 100 |
| Bt.8964.2.S1\_a\_at | 2.41e-06 | 9.5e-09 | 1.1e-06 | \* \* \* | -2.58 | -2.09 | -2.51 | 100 | 100 |
| Bt.25555.1.S1\_at | 2.44e-06 | 1.02e-08 | 2.54e-08 | \* \* \* | -2.06 | -1.42 | -2.12 | 100 | 100 |
| Bt.21218.1.A1\_at | 2.5e-06 | 5.4e-09 | 4.98e-07 | \* \* \* | -2.13 | -2.01 | -2.7 | 100 | 100 |
| Bt.26992.1.A1\_at | 2.51e-06 | 1.71e-06 | 2.16e-06 | \* \* \* | 1.19 | 1.22 | 1.37 | 100 | 100 |
| Bt.13505.1.S1\_at | 2.55e-06 | 8.29e-06 | 0.000129 | \* \* \* | -1.85 | -1.18 | -1.49 | 100 | 100 |
| Bt.7856.1.S1\_at | 2.69e-06 | 4.42e-08 | 3.31e-07 | \* \* \* | -1.8 | -1.57 | -2.08 | 100 | 100 |
| Bt.24824.1.S1\_at | 2.72e-06 | 8.79e-06 | 7.85e-08 | \* \* \* | 1.66 | 1.13 | 1.91 | 100 | 100 |
| Bt.8062.1.S1\_at | 2.75e-06 | 3.29e-06 | 4.52e-07 | \* \* \* | -1.2 | -1.01 | -1.21 | 100 | 100 |
| Bt.315.1.S1\_at | 2.79e-06 | 4.67e-08 | 2.84e-06 | \* \* \* | -1.87 | -1.43 | -1.87 | 100 | 100 |
| Bt.1129.1.S1\_at | 2.87e-06 | 1.63e-08 | 3.77e-06 | \* \* \* | -2.04 | -1.87 | -2.41 | 100 | 100 |
| Bt.27908.1.S1\_at | 2.93e-06 | 3.7e-07 | 7.21e-07 | \* \* \* | -1.35 | -1.11 | -1.49 | 100 | 100 |
| Bt.13240.1.S1\_at | 3.01e-06 | 5.65e-06 | 5.56e-07 | \* \* \* | 1.26 | 1.15 | 1.47 | 100 | 100 |
| Bt.16003.1.S1\_at | 3.03e-06 | 2.69e-07 | 4.75e-08 | \* \* \* | -1.84 | -1.31 | -1.56 | 100 | 100 |
| Bt.6683.1.S1\_at | 3.07e-06 | 7.39e-07 | 5.16e-06 | \* \* \* | -1.85 | -1.54 | -1.85 | 100 | 100 |
| Bt.5344.1.S1\_at | 3.08e-06 | 2.81e-07 | 6.8e-07 | \* \* \* | -1.84 | -1.28 | -1.86 | 100 | 100 |
| Bt.27543.1.A1\_at | 3.1e-06 | 2.13e-07 | 5.59e-06 | \* \* \* | 1.75 | 1.59 | 2.14 | 100 | 100 |
| Bt.17079.1.S1\_a\_at | 3.17e-06 | 1.64e-06 | 1.36e-07 | \* \* \* | 1.52 | 1.34 | 1.78 | 100 | 100 |
| Bt.1696.1.S1\_at | 3.19e-06 | 7.82e-09 | 6.38e-11 | \* \* \* | -1.57 | -1.19 | -1.73 | 100 | 100 |
| Bt.371.1.S1\_at | 3.21e-06 | 1.82e-06 | 3.27e-06 | \* \* \* | -1.76 | -1.04 | -1.25 | 100 | 100 |
| Bt.5520.1.S1\_at | 3.24e-06 | 4.62e-08 | 2.75e-07 | \* \* \* | -2.19 | -1.59 | -1.98 | 100 | 100 |
| Bt.25087.3.S1\_at | 3.31e-06 | 1.29e-06 | 1.02e-07 | \* \* \* | -1.33 | -1.15 | -1.46 | 100 | 100 |
| Bt.4606.1.S1\_at | 3.32e-06 | 2.82e-07 | 3.64e-06 | \* \* \* | 1.87 | 1.69 | 1.94 | 100 | 100 |
| Bt.11791.1.A1\_at | 3.32e-06 | 3.35e-07 | 1.3e-05 | \* \* \* | -1.93 | -1.76 | -1.92 | 100 | 100 |
| Bt.4804.2.A1\_at | 3.37e-06 | 1.02e-10 | 1.51e-09 | \* \* \* | 1.79 | 1.84 | 2.19 | 100 | 100 |
| Bt.22518.1.S1\_a\_at | 3.48e-06 | 3.75e-08 | 3.45e-08 | \* \* \* | -1.75 | -1.47 | -1.82 | 100 | 100 |
| Bt.13330.1.S1\_at | 3.49e-06 | 7.08e-06 | 1.22e-06 | \* \* \* | -2.67 | -2.42 | -3.68 | 100 | 100 |
| Bt.23127.1.S1\_at | 3.6e-06 | 5.37e-07 | 1.96e-05 | \* \* \* | 1.59 | 1.33 | 1.63 | 100 | 100 |
| Bt.7915.1.S1\_at | 3.61e-06 | 6.73e-06 | 1.74e-05 | \* \* \* | -1.59 | -0.817 | -0.888 | 100 | 100 |
| Bt.13943.1.A1\_at | 3.61e-06 | 6.19e-07 | 1.92e-09 | \* \* \* | 1.33 | 1.08 | 1.44 | 100 | 100 |
| Bt.22843.1.S1\_at | 3.63e-06 | 6.18e-07 | 2.57e-06 | \* \* \* | -1.45 | -1.01 | -1.22 | 100 | 100 |
| Bt.23603.3.S1\_at | 3.64e-06 | 7.67e-07 | 3.74e-08 | \* \* \* | -1.89 | -0.969 | -1.33 | 100 | 100 |
| Bt.7394.1.A1\_at | 3.66e-06 | 2.56e-05 | 7.04e-06 | \* \* \* | -1.51 | -1.03 | -1.27 | 100 | 100 |
| Bt.26702.1.S1\_a\_at | 3.71e-06 | 3.46e-09 | 3.38e-07 | \* \* \* | -1.8 | -1.47 | -2.39 | 100 | 100 |
| Bt.9949.3.S1\_a\_at | 3.72e-06 | 3.97e-06 | 1.64e-05 | \* \* \* | -1.73 | -1.15 | -1.54 | 100 | 100 |
| Bt.18990.1.S1\_at | 3.74e-06 | 0.000597 | 0.000771 | \* \* \* | 1.38 | 0.804 | 0.95 | 100 | 100 |
| Bt.29931.1.S1\_at | 3.77e-06 | 3.36e-08 | 6.4e-08 | \* \* \* | -1.62 | -1.27 | -2.08 | 100 | 100 |
| Bt.5042.1.S1\_at | 3.86e-06 | 2.76e-06 | 5.93e-07 | \* \* \* | -1.18 | -0.964 | -1.23 | 100 | 100 |
| Bt.19355.1.A1\_at | 3.9e-06 | 0.00116 | 0.000525 | \* \* \* | 2.41 | 0.669 | 1.94 | 100 | 100 |
| Bt.5537.1.S1\_at | 4.01e-06 | 2.98e-08 | 1.12e-07 | \* \* \* | -1.74 | -1.56 | -2.06 | 100 | 100 |
| Bt.5246.1.S1\_at | 4.06e-06 | 3.14e-08 | 3.62e-08 | \* \* \* | -2.1 | -1.46 | -1.75 | 100 | 100 |
| Bt.5848.2.S1\_at | 4.07e-06 | 0.00047 | 1.42e-05 | \* \* \* | -1.25 | -0.726 | -0.898 | 100 | 100 |
| Bt.27152.1.A1\_at | 4.07e-06 | 1.07e-08 | 2.9e-06 | \* \* \* | 2.75 | 2.54 | 3.11 | 100 | 100 |
| Bt.28133.1.S1\_at | 4.13e-06 | 4.12e-06 | 4.07e-07 | \* \* \* | 1.25 | 1.13 | 1.34 | 100 | 100 |
| Bt.17968.1.S1\_at | 4.16e-06 | 2.4e-05 | 5.28e-05 | \* \* \* | -1.77 | -1.4 | -1.75 | 100 | 100 |
| Bt.16815.1.S1\_at | 4.26e-06 | 6.72e-08 | 7.83e-09 | \* \* \* | -1.26 | -1.16 | -1.73 | 100 | 100 |
| Bt.9730.1.S1\_at | 4.39e-06 | 0.000179 | 1.56e-06 | \* \* \* | -1.06 | -0.661 | -0.917 | 100 | 100 |
| Bt.26975.1.A1\_a\_at | 4.43e-06 | 3.19e-06 | 2.7e-05 | \* \* \* | 1.62 | 1.01 | 2.02 | 100 | 100 |
| Bt.5123.2.S1\_at | 4.45e-06 | 2.33e-07 | 4.05e-08 | \* \* \* | -1.27 | -1.01 | -1.29 | 100 | 100 |
| Bt.2049.1.S1\_at | 4.46e-06 | 1.75e-09 | 5.03e-07 | \* \* \* | -1.6 | -1.76 | -2.47 | 100 | 100 |
| Bt.5089.1.S2\_at | 4.49e-06 | 1.29e-07 | 4.42e-08 | \* \* \* | 1.36 | 1.46 | 1.7 | 100 | 100 |
| Bt.3856.1.S1\_at | 4.49e-06 | 1.26e-07 | 4.04e-06 | \* \* \* | -1.57 | -1.45 | -2.09 | 100 | 100 |
| Bt.16900.1.A1\_at | 4.54e-06 | 1.55e-05 | 3e-07 | \* \* \* | 1.16 | 1.17 | 1.37 | 100 | 100 |
| Bt.265.1.S1\_at | 4.57e-06 | 2.5e-06 | 5.52e-06 | \* \* \* | -1.54 | -0.962 | -1.21 | 100 | 100 |
| Bt.11492.1.A1\_at | 4.62e-06 | 1.87e-07 | 4.39e-06 | \* \* \* | 1.54 | 1.53 | 2.01 | 100 | 100 |
| Bt.20601.1.A1\_at | 4.65e-06 | 2.81e-08 | 2.65e-07 | \* \* \* | 1.54 | 1.48 | 1.57 | 100 | 100 |
| Bt.5447.1.S1\_at | 4.72e-06 | 1.79e-06 | 0.000148 | \* \* \* | 1.24 | 0.953 | 1.15 | 100 | 100 |
| Bt.22122.2.A1\_at | 4.75e-06 | 8.61e-08 | 1.17e-07 | \* \* \* | 1.55 | 1.34 | 1.56 | 100 | 100 |
| Bt.1568.1.S1\_at | 4.76e-06 | 2.13e-06 | 1.02e-05 | \* \* \* | -1.5 | -0.846 | -1.13 | 100 | 100 |
| Bt.11659.1.S1\_at | 4.77e-06 | 8.92e-08 | 8.5e-08 | \* \* \* | -1.42 | -1.23 | -1.72 | 100 | 100 |
| Bt.23361.1.S1\_at | 4.79e-06 | 0.000357 | 0.000193 | \* \* \* | -1.12 | -0.541 | -0.616 | 100 | 100 |
| Bt.28187.1.S1\_at | 4.89e-06 | 8.93e-05 | 0.000172 | \* \* \* | 1.27 | 0.941 | 1.09 | 100 | 100 |
| Bt.21533.1.S1\_at | 4.99e-06 | 1.09e-06 | 3.57e-06 | \* \* \* | 1.2 | 1.31 | 1.82 | 100 | 100 |
| Bt.19167.1.A1\_at | 4.99e-06 | 2.53e-07 | 8.18e-07 | \* \* \* | 2.02 | 1.41 | 2 | 100 | 100 |
| Bt.9826.1.S1\_at | 5.04e-06 | 7.26e-07 | 1.33e-07 | \* \* \* | -1.45 | -0.891 | -1.14 | 100 | 100 |
| Bt.4864.2.S1\_at | 5.04e-06 | 6.51e-07 | 3.86e-07 | \* \* \* | 1.38 | 0.866 | 1.26 | 100 | 100 |
| Bt.2726.1.S1\_at | 5.11e-06 | 2.38e-06 | 2.86e-06 | \* \* \* | 1.67 | 1.46 | 2.28 | 100 | 100 |
| Bt.12303.1.S1\_at | 5.12e-06 | 1.67e-08 | 1.35e-08 | \* \* \* | 1.72 | 1.28 | 1.79 | 100 | 100 |
| Bt.3015.1.A1\_at | 5.13e-06 | 6.94e-08 | 2.05e-10 | \* \* \* | 1.51 | 1.53 | 2.02 | 100 | 100 |
| Bt.9047.1.S1\_at | 5.13e-06 | 2.09e-06 | 6.52e-06 | \* \* \* | -1.92 | -1.18 | -2.29 | 100 | 100 |
| Bt.8950.1.S1\_at | 5.13e-06 | 1.2e-06 | 3.19e-06 | \* \* \* | -1.34 | -0.978 | -1.23 | 100 | 100 |
| Bt.11855.1.S1\_at | 5.27e-06 | 1.59e-07 | 3.19e-06 | \* \* \* | -1.29 | -1.02 | -1.26 | 100 | 100 |
| Bt.2973.2.S1\_a\_at | 5.31e-06 | 9.7e-06 | 2.36e-06 | \* \* \* | -1.38 | -0.865 | -1.08 | 100 | 100 |
| Bt.2129.2.S1\_at | 5.35e-06 | 3.86e-07 | 1.46e-06 | \* \* \* | 1.92 | 1.87 | 2.31 | 100 | 100 |
| Bt.5290.1.S1\_at | 5.4e-06 | 3.59e-05 | 2.48e-06 | \* \* \* | 1.2 | 0.746 | 0.999 | 100 | 100 |
| Bt.25099.1.A1\_at | 5.44e-06 | 4.48e-07 | 3.25e-06 | \* \* \* | -3.6 | -2.97 | -3.76 | 100 | 100 |
| Bt.5183.1.S1\_at | 5.46e-06 | 7.09e-08 | 4.28e-06 | \* \* \* | -2.49 | -1.8 | -2.19 | 100 | 100 |
| Bt.7415.1.S1\_at | 5.49e-06 | 3.19e-06 | 1.89e-06 | \* \* \* | 1.39 | 0.915 | 1.17 | 100 | 100 |
| Bt.3101.1.A1\_at | 5.53e-06 | 9.64e-06 | 4.27e-05 | \* \* \* | -2.09 | -1.53 | -1.87 | 100 | 100 |
| Bt.10154.1.S1\_at | 5.54e-06 | 0.000166 | 7.18e-05 | \* \* \* | -1.36 | -0.839 | -1.03 | 100 | 100 |
| Bt.9825.1.S1\_at | 5.62e-06 | 2.15e-06 | 2.01e-06 | \* \* \* | -1.87 | -1.23 | -1.51 | 100 | 100 |
| Bt.20307.1.S1\_at | 5.65e-06 | 2.94e-07 | 8.15e-08 | \* \* \* | -1.75 | -1.18 | -1.73 | 100 | 100 |
| Bt.9434.1.S1\_at | 5.66e-06 | 3.46e-06 | 1.57e-07 | \* \* \* | -1.48 | -1.07 | -1.38 | 100 | 100 |
| Bt.11791.2.S1\_at | 5.7e-06 | 1.12e-06 | 6.4e-06 | \* \* \* | -1.86 | -1.44 | -1.86 | 100 | 100 |
| Bt.1294.1.S1\_at | 5.71e-06 | 3e-06 | 4.56e-06 | \* \* \* | -1.82 | -1.45 | -2.11 | 100 | 100 |
| Bt.22842.2.S1\_at | 5.73e-06 | 2.51e-08 | 2.64e-06 | \* \* \* | 1.42 | 1.25 | 1.81 | 100 | 100 |
| Bt.14143.1.S1\_at | 5.8e-06 | 4.64e-05 | 0.00145 | \* \* \* | 2.03 | 0.876 | 1.66 | 100 | 100 |
| Bt.25051.1.A1\_at | 5.87e-06 | 2.5e-05 | 1.38e-05 | \* \* \* | 1.78 | 1.48 | 1.78 | 100 | 100 |
| Bt.5750.1.S1\_at | 5.94e-06 | 3.46e-05 | 3.82e-06 | \* \* \* | -1.15 | -0.928 | -1.25 | 100 | 100 |
| Bt.6063.1.S1\_at | 6.07e-06 | 1.64e-05 | 2.02e-05 | \* \* \* | -1.49 | -1.16 | -1.52 | 100 | 100 |
| Bt.9586.1.A1\_at | 6.21e-06 | 6.25e-07 | 2.56e-08 | \* \* \* | 1.17 | 1.18 | 1.47 | 100 | 100 |
| Bt.12211.1.S1\_at | 6.22e-06 | 1.98e-07 | 8.82e-07 | \* \* \* | -1.51 | -1.29 | -2.14 | 100 | 100 |
| Bt.24265.1.S1\_a\_at | 6.31e-06 | 3.1e-07 | 1.59e-08 | \* \* \* | -1.28 | -1 | -1.59 | 100 | 100 |
| Bt.27091.1.S1\_at | 6.47e-06 | 1.24e-07 | 1.47e-06 | \* \* \* | 1.66 | 1.63 | 2.02 | 100 | 100 |
| Bt.20281.2.S1\_a\_at | 6.52e-06 | 0.000221 | 0.000316 | \* \* \* | -1.33 | -0.834 | -1.15 | 100 | 100 |
| Bt.28559.1.S1\_a\_at | 6.59e-06 | 4.51e-06 | 3.25e-05 | \* \* \* | -1.16 | -1.08 | -1.36 | 100 | 100 |
| Bt.23569.1.S1\_at | 6.69e-06 | 0.000411 | 3.24e-06 | \* \* \* | -1.21 | -0.723 | -1.04 | 100 | 100 |
| Bt.23396.1.S1\_at | 6.69e-06 | 2.53e-08 | 5.09e-08 | \* \* \* | -2.15 | -2.01 | -2.7 | 100 | 100 |
| Bt.4651.1.S1\_at | 6.82e-06 | 3.86e-07 | 1.48e-06 | \* \* \* | 1.13 | 1.46 | 1.74 | 100 | 100 |
| Bt.8504.1.A1\_at | 6.88e-06 | 6.35e-07 | 1.11e-06 | \* \* \* | 1.33 | 1.45 | 1.71 | 100 | 100 |
| Bt.22528.1.A1\_at | 6.89e-06 | 8.25e-05 | 0.000127 | \* \* \* | -1.11 | -0.896 | -1.27 | 100 | 100 |
| Bt.12423.1.S1\_at | 6.9e-06 | 1.68e-06 | 5.98e-08 | \* \* \* | 1.15 | 0.988 | 1.39 | 100 | 100 |
| Bt.27939.2.A1\_a\_at | 7.32e-06 | 3.67e-07 | 1.05e-08 | \* \* \* | 1.24 | 1.03 | 1.47 | 100 | 100 |
| Bt.8679.1.A1\_at | 7.43e-06 | 5.79e-08 | 1.25e-07 | \* \* \* | -1.87 | -1.31 | -1.78 | 100 | 100 |
| Bt.884.1.S1\_at | 7.54e-06 | 8.38e-05 | 0.000229 | \* \* \* | 2.05 | 1.04 | 1.14 | 100 | 100 |
| Bt.14229.1.S1\_at | 7.58e-06 | 2.46e-07 | 5.32e-07 | \* \* \* | -1.18 | -0.971 | -1.22 | 100 | 100 |
| Bt.4099.1.S1\_at | 7.63e-06 | 0.000342 | 3.33e-05 | \* \* \* | -1.22 | -0.628 | -0.768 | 100 | 100 |
| Bt.9878.1.A1\_at | 7.66e-06 | 4.01e-07 | 2.1e-05 | \* \* \* | 1.43 | 1.27 | 1.42 | 100 | 100 |
| Bt.1059.3.S1\_a\_at | 7.74e-06 | 3.06e-05 | 6.44e-07 | \* \* \* | -1.17 | -0.718 | -0.967 | 100 | 100 |
| Bt.5676.1.S1\_at | 7.86e-06 | 6.9e-08 | 1.55e-06 | \* \* \* | -1.9 | -1.69 | -2.2 | 100 | 100 |
| Bt.20025.1.S1\_at | 7.97e-06 | 2.14e-08 | 4.32e-07 | \* \* \* | -1.66 | -1.49 | -1.89 | 100 | 100 |
| Bt.5966.1.A1\_at | 7.98e-06 | 2.04e-08 | 6.25e-08 | \* \* \* | -2.27 | -1.69 | -2.11 | 100 | 100 |
| Bt.7175.1.S1\_at | 7.98e-06 | 1.57e-09 | 3.5e-08 | \* \* \* | -1.63 | -1.42 | -2.19 | 100 | 100 |
| Bt.5029.1.S1\_at | 8.04e-06 | 9.25e-06 | 1.2e-06 | \* \* \* | -1.3 | -0.838 | -1.05 | 100 | 100 |
| Bt.24404.1.A1\_at | 8.31e-06 | 3.26e-05 | 6.58e-07 | \* \* \* | 1.54 | 1.21 | 2.22 | 100 | 100 |
| Bt.10942.1.S1\_at | 8.32e-06 | 2.57e-06 | 2.06e-05 | \* \* \* | 1.45 | 0.846 | 1 | 100 | 100 |
| Bt.27412.1.A1\_at | 8.53e-06 | 3.54e-07 | 1.24e-05 | \* \* \* | 1.7 | 1.41 | 1.68 | 100 | 100 |
| Bt.20984.1.S1\_a\_at | 8.67e-06 | 4.87e-06 | 2.82e-07 | \* \* \* | -2.2 | -0.969 | -1.31 | 100 | 100 |
| Bt.21766.2.S1\_at | 9.02e-06 | 4.62e-09 | 1.08e-07 | \* \* \* | -1.9 | -1.84 | -2.15 | 100 | 100 |
| Bt.26453.1.S1\_at | 9.14e-06 | 0.000549 | 3.57e-07 | \* \* \* | 1.41 | 0.828 | 1.85 | 100 | 100 |
| Bt.8650.1.A1\_at | 9.24e-06 | 2.9e-06 | 2.71e-08 | \* \* \* | 1.34 | 0.854 | 0.93 | 100 | 100 |
| Bt.21721.1.A1\_at | 9.29e-06 | 6.25e-08 | 5.45e-06 | \* \* \* | -2.21 | -1.96 | -2.57 | 100 | 100 |
| Bt.4977.1.S2\_at | 9.45e-06 | 2.53e-08 | 7.88e-10 | \* \* \* | 1.17 | 1.29 | 1.49 | 100 | 100 |
| Bt.21698.1.S1\_a\_at | 9.45e-06 | 0.00127 | 0.00117 | \* \* \* | -1.18 | -0.779 | -0.875 | 100 | 100 |
| Bt.56.1.S1\_at | 9.6e-06 | 1.29e-06 | 5.31e-06 | \* \* \* | -1.53 | -1.22 | -1.34 | 100 | 100 |
| Bt.28269.1.S1\_at | 9.63e-06 | 2.49e-07 | 1.36e-07 | \* \* \* | 1.37 | 1.39 | 1.59 | 100 | 100 |
| Bt.29746.1.S1\_at | 9.65e-06 | 1.34e-08 | 1.12e-07 | \* \* \* | -1.7 | -1.41 | -2.52 | 100 | 100 |
| Bt.615.1.S1\_at | 9.77e-06 | 1.85e-05 | 1.02e-05 | \* \* \* | 1.53 | 0.981 | 1.17 | 100 | 100 |
| Bt.5495.1.S1\_at | 9.82e-06 | 0.000219 | 5.64e-05 | \* \* \* | -1.58 | -1.05 | -1.62 | 100 | 100 |
| Bt.23438.1.S1\_at | 9.91e-06 | 2.6e-08 | 5.13e-08 | \* \* \* | 1.64 | 1.25 | 1.78 | 100 | 100 |
| Bt.3922.1.S1\_a\_at | 1.01e-05 | 4.29e-08 | 1.52e-07 | \* \* \* | -1.54 | -1.53 | -2.46 | 100 | 100 |
| Bt.5183.2.S1\_at | 1.01e-05 | 2.16e-08 | 1.62e-06 | \* \* \* | -2.44 | -2.18 | -2.69 | 100 | 100 |
| Bt.3149.1.S1\_at | 1.01e-05 | 1.05e-06 | 8.41e-06 | \* \* \* | 1.14 | 1.08 | 1.54 | 100 | 100 |
| Bt.20411.1.S1\_at | 1.02e-05 | 1.31e-06 | 2.42e-07 | \* \* \* | -2.09 | -1.54 | -1.75 | 100 | 100 |
| Bt.405.1.S1\_at | 1.03e-05 | 2.04e-06 | 6.21e-05 | \* \* \* | 2.03 | 1.44 | 1.83 | 100 | 100 |
| Bt.6465.1.S1\_at | 1.03e-05 | 1.05e-08 | 1.81e-05 | \* \* \* | -3.52 | -2.63 | -3.89 | 100 | 100 |
| Bt.24875.1.S1\_at | 1.03e-05 | 8.76e-08 | 1.48e-09 | \* \* \* | -1.49 | -1.19 | -1.73 | 100 | 100 |
| Bt.4565.1.S1\_at | 1.04e-05 | 7.24e-06 | 0.00185 | \* \* \* | 1.31 | 1.05 | 1.41 | 100 | 100 |
| Bt.10189.1.S1\_at | 1.04e-05 | 6.43e-05 | 2.04e-06 | \* \* \* | -1.09 | -0.915 | -1.1 | 100 | 100 |
| Bt.2587.1.S1\_at | 1.05e-05 | 5.52e-06 | 4.62e-07 | \* \* \* | -1.69 | -0.996 | -1.3 | 100 | 100 |
| Bt.547.1.S1\_at | 1.07e-05 | 2.75e-05 | 8.49e-06 | \* \* \* | -2.37 | -1.05 | -1.78 | 100 | 100 |
| Bt.24096.1.A1\_at | 1.11e-05 | 2.83e-06 | 1.21e-05 | \* \* \* | 1.33 | 1.24 | 1.35 | 100 | 100 |
| Bt.8679.2.S1\_at | 1.11e-05 | 5.1e-07 | 9.77e-07 | \* \* \* | -2.01 | -1.45 | -1.63 | 100 | 100 |
| Bt.4694.1.S1\_at | 1.12e-05 | 5.85e-05 | 2.62e-06 | \* \* \* | -0.991 | -0.737 | -1.06 | 100 | 100 |
| Bt.20321.1.S1\_at | 1.12e-05 | 1.37e-07 | 6.16e-07 | \* \* \* | 1.16 | 1.13 | 1.35 | 100 | 100 |
| Bt.12728.1.A1\_a\_at | 1.12e-05 | 5.33e-07 | 2.46e-06 | \* \* \* | 1.98 | 1.72 | 2.12 | 100 | 100 |
| Bt.8618.1.S1\_at | 1.14e-05 | 1.7e-09 | 4.51e-09 | \* \* \* | -2.38 | -1.57 | -2.35 | 100 | 100 |
| Bt.28226.3.A1\_s\_at | 1.15e-05 | 5.96e-07 | 1.39e-07 | \* \* \* | 1.18 | 1.24 | 1.45 | 100 | 100 |
| Bt.4880.1.S1\_at | 1.16e-05 | 1.17e-06 | 1.31e-05 | \* \* \* | -1.44 | -0.95 | -1.16 | 100 | 100 |
| Bt.3428.1.A1\_s\_at | 1.16e-05 | 6.71e-06 | 9.06e-05 | \* \* \* | 1.4 | 1.14 | 1.2 | 100 | 100 |
| Bt.15664.1.S1\_at | 1.18e-05 | 6.03e-05 | 4.35e-06 | \* \* \* | -1.19 | -0.919 | -1.06 | 100 | 100 |
| Bt.23576.1.A1\_at | 1.18e-05 | 1.69e-06 | 7.68e-07 | \* \* \* | 1.17 | 1.27 | 1.43 | 100 | 100 |
| Bt.28238.1.A1\_at | 1.21e-05 | 1.1e-07 | 8.96e-06 | \* \* \* | 2.17 | 1.63 | 2.07 | 100 | 100 |
| Bt.22270.1.S1\_at | 1.24e-05 | 1.3e-09 | 7.73e-07 | \* \* \* | 1.57 | 1.57 | 2.28 | 100 | 100 |
| Bt.20064.2.S1\_at | 1.25e-05 | 2.13e-05 | 3.87e-05 | \* \* \* | 1.3 | 0.962 | 1.33 | 100 | 100 |
| Bt.23308.1.S1\_at | 1.26e-05 | 4.03e-06 | 1.75e-07 | \* \* \* | 1.3 | 0.974 | 1.34 | 100 | 100 |
| Bt.10965.1.S1\_at | 1.26e-05 | 2.29e-06 | 2.66e-06 | \* \* \* | -1.41 | -0.934 | -1.11 | 100 | 100 |
| Bt.20937.1.A1\_at | 1.3e-05 | 2.09e-06 | 8.77e-06 | \* \* \* | 2.19 | 1.99 | 2.32 | 100 | 100 |
| Bt.8279.3.S1\_a\_at | 1.3e-05 | 8.33e-07 | 1.26e-06 | \* \* \* | -1.22 | -0.966 | -1.31 | 100 | 100 |
| Bt.7459.1.S1\_at | 1.31e-05 | 4.55e-07 | 8.98e-08 | \* \* \* | 1.48 | 1.27 | 1.67 | 100 | 100 |
| Bt.2138.1.S1\_at | 1.31e-05 | 5.42e-07 | 0.000209 | \* \* \* | -1.56 | -1.05 | -1.15 | 100 | 100 |
| Bt.19934.1.S1\_at | 1.32e-05 | 2.25e-05 | 1.87e-05 | \* \* \* | -1.49 | -1.17 | -1.33 | 100 | 100 |
| Bt.15870.1.S1\_at | 1.32e-05 | 3.38e-07 | 4.91e-06 | \* \* \* | -1.34 | -1.16 | -1.82 | 100 | 100 |
| Bt.25185.1.S1\_at | 1.32e-05 | 5.55e-06 | 2.13e-05 | \* \* \* | 1.6 | 1.38 | 1.74 | 100 | 100 |
| Bt.9756.1.S1\_at | 1.34e-05 | 1.8e-05 | 1.57e-05 | \* \* \* | -1.65 | -1.13 | -1.27 | 100 | 100 |
| Bt.23357.1.S1\_at | 1.35e-05 | 1.11e-08 | 1.72e-06 | \* \* \* | -1.83 | -1.41 | -2.06 | 100 | 100 |
| Bt.64.1.S1\_at | 1.36e-05 | 1.03e-07 | 4.09e-06 | \* \* \* | -1.54 | -1.15 | -1.45 | 100 | 100 |
| Bt.19409.1.A1\_s\_at | 1.36e-05 | 2.29e-08 | 9.82e-09 | \* \* \* | 1.51 | 1.59 | 1.93 | 100 | 100 |
| Bt.21731.1.S1\_at | 1.38e-05 | 3.51e-09 | 1.26e-07 | \* \* \* | 1.75 | 1.32 | 1.77 | 100 | 100 |
| Bt.2347.1.S1\_at | 1.39e-05 | 8.96e-05 | 2.09e-05 | \* \* \* | -1.17 | -0.852 | -1.06 | 100 | 100 |
| Bt.2711.1.S1\_at | 1.42e-05 | 1.75e-05 | 1.78e-07 | \* \* \* | -1.12 | -0.917 | -1.18 | 100 | 100 |
| Bt.6546.1.A1\_at | 1.43e-05 | 8.99e-05 | 1.21e-05 | \* \* \* | 1.09 | 0.977 | 1.32 | 100 | 100 |
| Bt.77.1.S1\_at | 1.46e-05 | 0.000159 | 1.27e-05 | \* \* \* | -1.29 | -0.875 | -1.03 | 100 | 100 |
| Bt.5164.1.S1\_at | 1.46e-05 | 5.56e-06 | 8.38e-05 | \* \* \* | -2.66 | -1.81 | -2.58 | 100 | 100 |
| Bt.29873.1.S1\_at | 1.47e-05 | 1.13e-07 | 1.27e-07 | \* \* \* | 1.81 | 1.72 | 2.35 | 100 | 100 |
| Bt.12722.1.A1\_at | 1.48e-05 | 0.000303 | 0.000676 | \* \* \* | 1.77 | 1.01 | 1 | 100 | 100 |
| Bt.12872.1.S1\_at | 1.49e-05 | 1.28e-06 | 3.36e-06 | \* \* \* | 1.89 | 1.06 | 1.54 | 100 | 100 |
| Bt.22461.1.S1\_at | 1.5e-05 | 0.000462 | 1.99e-05 | \* \* \* | 0.936 | 0.865 | 1.06 | 100 | 100 |
| Bt.20426.1.S1\_at | 1.51e-05 | 0.000129 | 1.92e-05 | \* \* \* | 1.08 | 0.782 | 0.819 | 100 | 100 |
| Bt.23335.1.S1\_at | 1.51e-05 | 1.11e-05 | 2.65e-05 | \* \* \* | 1.31 | 1.3 | 1.46 | 100 | 100 |
| Bt.26628.1.S1\_at | 1.51e-05 | 8.65e-06 | 1.73e-05 | \* \* \* | 1.25 | 1.09 | 1.26 | 100 | 100 |
| Bt.13289.2.S1\_at | 1.53e-05 | 1.31e-05 | 5.96e-05 | \* \* \* | 2.79 | 1.98 | 2.39 | 100 | 100 |
| Bt.7395.1.S1\_at | 1.54e-05 | 1.97e-05 | 4.42e-06 | \* \* \* | 2.19 | 0.789 | 1.07 | 100 | 100 |
| Bt.5040.1.S1\_at | 1.56e-05 | 6.58e-05 | 4.36e-06 | \* \* \* | -1.43 | -0.633 | -1.03 | 100 | 100 |
| Bt.11059.1.S1\_at | 1.57e-05 | 3.36e-07 | 7.55e-07 | \* \* \* | 1.32 | 1.4 | 1.71 | 100 | 100 |
| Bt.13289.1.S1\_at | 1.59e-05 | 3.47e-06 | 2.19e-05 | \* \* \* | 1.86 | 2.02 | 2.59 | 100 | 100 |
| Bt.12412.1.S1\_at | 1.62e-05 | 0.000419 | 0.000184 | \* \* \* | 1.52 | 0.865 | 1.01 | 100 | 100 |
| Bt.11055.1.S1\_at | 1.63e-05 | 6.9e-07 | 1.12e-06 | \* \* \* | 1.15 | 1.35 | 1.65 | 100 | 100 |
| Bt.3126.1.A1\_at | 1.65e-05 | 1.26e-07 | 1.03e-06 | \* \* \* | 1.18 | 1.42 | 1.61 | 100 | 100 |
| Bt.4431.1.S1\_a\_at | 1.69e-05 | 0.000688 | 9.85e-06 | \* \* \* | -1.17 | -0.624 | -0.752 | 100 | 100 |
| Bt.6832.1.S1\_at | 1.7e-05 | 2.23e-05 | 4.19e-06 | \* \* \* | 1.18 | 1.06 | 1.33 | 100 | 100 |
| Bt.10969.1.S1\_at | 1.7e-05 | 9.72e-07 | 3.67e-05 | \* \* \* | 1.58 | 1.21 | 1.46 | 100 | 100 |
| Bt.13588.1.A1\_at | 1.72e-05 | 1.55e-06 | 1.04e-05 | \* \* \* | -4.21 | -3.45 | -4.77 | 100 | 100 |
| Bt.24376.1.S1\_at | 1.72e-05 | 1.19e-05 | 4.77e-05 | \* \* \* | 1.5 | 1.32 | 1.81 | 100 | 100 |
| Bt.12613.1.S1\_at | 1.72e-05 | 4.18e-08 | 6.68e-07 | \* \* \* | 1.59 | 1.76 | 2.08 | 100 | 100 |
| Bt.12453.1.A1\_at | 1.73e-05 | 1.18e-08 | 8.99e-07 | \* \* \* | 2.09 | 2.22 | 2.59 | 100 | 100 |
| Bt.28143.1.S1\_at | 1.74e-05 | 5.57e-07 | 3.13e-06 | \* \* \* | 1.58 | 1.5 | 1.71 | 100 | 100 |
| Bt.28618.1.S1\_at | 1.74e-05 | 1.17e-05 | 1.08e-05 | \* \* \* | 1.24 | 1.3 | 1.47 | 100 | 100 |
| Bt.10543.1.S1\_at | 1.76e-05 | 1.26e-07 | 4.85e-06 | \* \* \* | 1.86 | 1.35 | 1.89 | 100 | 100 |
| Bt.27901.1.S1\_at | 1.76e-05 | 3.74e-06 | 1.75e-07 | \* \* \* | 1.36 | 1.27 | 1.76 | 100 | 100 |
| Bt.11990.1.S1\_at | 1.77e-05 | 2.79e-05 | 0.00121 | \* \* \* | 1.25 | 1.11 | 1.61 | 100 | 100 |
| Bt.4540.1.S1\_at | 1.85e-05 | 3.05e-05 | 6.1e-05 | \* \* \* | -1.11 | -0.63 | -0.78 | 100 | 100 |
| Bt.67.1.S1\_at | 1.85e-05 | 6.14e-07 | 1.56e-06 | \* \* \* | -1.72 | -1.28 | -1.72 | 100 | 100 |
| Bt.4864.3.S1\_a\_at | 1.85e-05 | 2.6e-05 | 2.66e-06 | \* \* \* | 1.45 | 0.979 | 1.3 | 100 | 100 |
| Bt.832.1.S1\_at | 1.86e-05 | 1.92e-08 | 3.87e-10 | \* \* \* | -2.34 | -1.51 | -2.05 | 100 | 100 |
| Bt.3854.1.S1\_at | 1.87e-05 | 2.1e-07 | 3.07e-07 | \* \* \* | 1.49 | 1.47 | 1.85 | 100 | 100 |
| Bt.14095.2.S1\_at | 1.9e-05 | 5.31e-05 | 8.65e-05 | \* \* \* | 1.59 | 1.32 | 1.48 | 100 | 100 |
| Bt.21766.1.A1\_at | 1.93e-05 | 0.000161 | 0.000473 | \* \* \* | -2.37 | -1.59 | -1.7 | 100 | 100 |
| Bt.9857.1.S1\_at | 1.97e-05 | 7.63e-07 | 2.78e-06 | \* \* \* | -1.76 | -1.3 | -1.75 | 100 | 100 |
| Bt.12377.1.S1\_at | 2.01e-05 | 6.92e-05 | 0.000301 | \* \* \* | 0.927 | 0.768 | 0.785 | 100 | 100 |
| Bt.2988.1.S1\_at | 2.03e-05 | 3.26e-07 | 1.43e-08 | \* \* \* | 1.18 | 1.18 | 1.58 | 100 | 100 |
| Bt.20512.1.S1\_at | 2.04e-05 | 5.18e-08 | 4.37e-07 | \* \* \* | 1.59 | 1.53 | 1.65 | 100 | 100 |
| Bt.22130.1.S1\_at | 2.05e-05 | 1.84e-06 | 1.78e-08 | \* \* \* | 1.19 | 1.11 | 1.4 | 100 | 100 |
| Bt.21009.1.S1\_at | 2.08e-05 | 1.58e-06 | 2.13e-05 | \* \* \* | 1.59 | 1.59 | 2.06 | 100 | 100 |
| Bt.23148.1.S1\_at | 2.08e-05 | 3.88e-08 | 2.05e-09 | \* \* \* | 1.15 | 1.17 | 1.4 | 100 | 100 |
| Bt.4898.1.S1\_at | 2.09e-05 | 3.88e-06 | 0.00019 | \* \* \* | 1.48 | 1.53 | 2.22 | 100 | 100 |
| Bt.9708.1.S1\_at | 2.09e-05 | 1.7e-06 | 3.98e-06 | \* \* \* | -1.46 | -1.37 | -1.78 | 100 | 100 |
| Bt.14116.1.A1\_at | 2.12e-05 | 6.41e-09 | 2.12e-07 | \* \* \* | 1.31 | 1.33 | 1.54 | 100 | 100 |
| Bt.4775.1.S1\_at | 2.12e-05 | 3.61e-05 | 6.42e-05 | \* \* \* | 1.56 | 0.997 | 1.3 | 100 | 100 |
| Bt.5345.1.S1\_at | 2.12e-05 | 0.000372 | 5.24e-05 | \* \* \* | -1.32 | -0.741 | -0.966 | 100 | 100 |
| Bt.15968.1.S1\_at | 2.15e-05 | 3.52e-05 | 3.2e-06 | \* \* \* | -1.23 | -0.926 | -1.26 | 100 | 100 |
| Bt.11196.1.S1\_at | 2.16e-05 | 2e-07 | 2.03e-07 | \* \* \* | -1.57 | -1.33 | -1.73 | 100 | 100 |
| Bt.9731.1.S1\_at | 2.17e-05 | 9.26e-07 | 8.5e-06 | \* \* \* | -1.96 | -1.62 | -2.2 | 100 | 100 |
| Bt.27657.1.A1\_at | 2.17e-05 | 7.33e-09 | 8.35e-06 | \* \* \* | -2.33 | -1.78 | -2.47 | 100 | 100 |
| Bt.20494.1.S1\_at | 2.21e-05 | 1.49e-05 | 1.2e-06 | \* \* \* | 1.29 | 0.964 | 1.21 | 100 | 100 |
| Bt.23418.1.S1\_at | 2.23e-05 | 1.13e-07 | 1.66e-07 | \* \* \* | 1.64 | 1.22 | 1.44 | 100 | 100 |
| Bt.15267.1.A1\_at | 2.29e-05 | 1.63e-06 | 1.98e-05 | \* \* \* | 1.57 | 1.02 | 1.38 | 100 | 100 |
| Bt.20316.1.S1\_at | 2.31e-05 | 4.51e-05 | 3.29e-06 | \* \* \* | -1.82 | -1.21 | -1.78 | 100 | 100 |
| Bt.7540.2.S1\_a\_at | 2.34e-05 | 7.37e-05 | 1.32e-05 | \* \* \* | -1.14 | -0.932 | -1.13 | 100 | 100 |
| Bt.25038.1.S1\_at | 2.37e-05 | 4.15e-06 | 1.84e-08 | \* \* \* | 1.06 | 0.944 | 1.14 | 100 | 100 |
| Bt.4503.1.S1\_at | 2.42e-05 | 5.34e-06 | 4.74e-06 | \* \* \* | -1.14 | -1.09 | -1.29 | 100 | 100 |
| Bt.63.1.S1\_at | 2.42e-05 | 5.19e-05 | 0.000129 | \* \* \* | -1.49 | -1.16 | -1.43 | 100 | 100 |
| Bt.15643.1.A1\_at | 2.43e-05 | 9.16e-06 | 1.03e-05 | \* \* \* | 1.36 | 0.953 | 1.29 | 100 | 100 |
| Bt.5047.1.S1\_at | 2.47e-05 | 4.53e-05 | 6.09e-05 | \* \* \* | 1.43 | 0.941 | 1.5 | 100 | 100 |
| Bt.24211.1.A1\_at | 2.51e-05 | 2.63e-06 | 5.97e-06 | \* \* \* | 2.11 | 1.68 | 1.93 | 100 | 100 |
| Bt.61.1.S1\_at | 2.51e-05 | 6e-04 | 3.49e-05 | \* \* \* | -1.12 | -0.57 | -0.759 | 100 | 100 |
| Bt.16314.1.S1\_at | 2.51e-05 | 2.62e-05 | 3.4e-05 | \* \* \* | 1.16 | 0.971 | 1.13 | 100 | 100 |
| Bt.18352.1.A1\_at | 2.53e-05 | 4.02e-08 | 2.58e-07 | \* \* \* | -1.38 | -1.28 | -2.36 | 100 | 100 |
| Bt.7636.1.S1\_at | 2.53e-05 | 1.79e-06 | 1.49e-05 | \* \* \* | 1.33 | 1.24 | 1.52 | 100 | 100 |
| Bt.28081.1.S1\_at | 2.56e-05 | 0.000923 | 0.00138 | \* \* \* | 1.09 | 0.582 | 0.89 | 100 | 100 |
| Bt.22369.2.S1\_at | 2.6e-05 | 6.1e-08 | 1.29e-08 | \* \* \* | 1.76 | 1.27 | 1.93 | 100 | 100 |
| Bt.14624.1.A1\_at | 2.61e-05 | 4.87e-05 | 0.000111 | \* \* \* | -1.23 | -0.951 | -1.1 | 100 | 100 |
| Bt.2873.1.S1\_at | 2.61e-05 | 0.000435 | 0.000576 | \* \* \* | -1.23 | -0.808 | -0.93 | 100 | 100 |
| Bt.13349.1.S1\_at | 2.64e-05 | 1.44e-06 | 5.23e-07 | \* \* \* | -1.61 | -1.01 | -1.15 | 100 | 100 |
| Bt.16329.1.A1\_at | 2.65e-05 | 1.71e-08 | 2.57e-09 | \* \* \* | 1.23 | 1.69 | 1.93 | 100 | 100 |
| Bt.13610.1.A1\_at | 2.65e-05 | 1.84e-06 | 2.22e-06 | \* \* \* | -1.67 | -1.31 | -1.76 | 100 | 100 |
| Bt.1366.1.S1\_at | 2.65e-05 | 3.93e-07 | 5.88e-06 | \* \* \* | 1.39 | 1.36 | 1.54 | 100 | 100 |
| Bt.400.1.S1\_at | 2.67e-05 | 8.36e-06 | 7.67e-07 | \* \* \* | -1.4 | -0.781 | -1.02 | 100 | 100 |
| Bt.7655.1.S1\_at | 2.71e-05 | 6.96e-06 | 5e-06 | \* \* \* | -1.67 | -0.916 | -1.26 | 100 | 100 |
| Bt.1667.1.S1\_at | 2.75e-05 | 1.8e-06 | 4.82e-06 | \* \* \* | -1.33 | -1.16 | -1.48 | 100 | 100 |
| Bt.1552.1.S1\_at | 2.78e-05 | 1.38e-07 | 8.92e-07 | \* \* \* | -3.14 | -1.81 | -2.5 | 100 | 100 |
| Bt.21151.1.S1\_at | 2.81e-05 | 5.36e-05 | 2.3e-05 | \* \* \* | -0.94 | -0.7 | -0.874 | 100 | 100 |
| Bt.15714.1.S1\_at | 2.83e-05 | 2.73e-05 | 2.38e-06 | \* \* \* | 1.23 | 0.88 | 1.36 | 100 | 100 |
| Bt.10111.1.S1\_at | 2.85e-05 | 7.79e-08 | 1.12e-06 | \* \* \* | 1.6 | 1.7 | 1.99 | 100 | 100 |
| Bt.13128.1.S1\_at | 2.87e-05 | 1.01e-06 | 1.81e-07 | \* \* \* | -1.35 | -0.872 | -1.05 | 100 | 100 |
| Bt.11240.1.A1\_at | 2.9e-05 | 4.52e-05 | 2.36e-05 | \* \* \* | 1.37 | 1.36 | 1.56 | 100 | 100 |
| Bt.9966.1.S1\_at | 2.9e-05 | 4.44e-08 | 2.53e-07 | \* \* \* | 1.36 | 1.56 | 1.88 | 100 | 100 |
| Bt.21877.1.S1\_at | 2.93e-05 | 1.15e-05 | 1.57e-07 | \* \* \* | -1.46 | -0.976 | -1.12 | 100 | 100 |
| Bt.12916.1.S1\_at | 2.94e-05 | 5.61e-06 | 6.26e-05 | \* \* \* | -1.92 | -1.86 | -2.77 | 100 | 100 |
| Bt.11446.1.A1\_at | 3.02e-05 | 1e-05 | 5.66e-06 | \* \* \* | 1.17 | 1.02 | 1.17 | 100 | 100 |
| Bt.7595.1.S1\_at | 3.03e-05 | 1.87e-05 | 0.000199 | \* \* \* | -1.08 | -0.797 | -1.04 | 100 | 100 |
| Bt.5153.1.S1\_at | 3.05e-05 | 5.12e-07 | 7.75e-07 | \* \* \* | 1.35 | 1.37 | 1.5 | 100 | 100 |
| Bt.5176.2.A1\_at | 3.1e-05 | 2.07e-06 | 1.31e-06 | \* \* \* | 1.14 | 1.11 | 1.4 | 100 | 100 |
| Bt.27192.1.S1\_at | 3.11e-05 | 1.07e-07 | 1.99e-07 | \* \* \* | -1.23 | -1.55 | -2.08 | 100 | 100 |
| Bt.12217.1.S1\_at | 3.12e-05 | 0.000195 | 0.000651 | \* \* \* | 1.6 | 1.24 | 1.39 | 100 | 100 |
| Bt.24550.1.S1\_at | 3.13e-05 | 1.06e-07 | 2.66e-06 | \* \* \* | -1.43 | -1.29 | -2.74 | 100 | 100 |
| Bt.20064.1.S1\_at | 3.14e-05 | 8.56e-06 | 1.41e-06 | \* \* \* | 1.18 | 0.913 | 1.09 | 100 | 100 |
| Bt.13483.1.A1\_at | 3.16e-05 | 1.36e-06 | 5.94e-07 | \* \* \* | -1.53 | -1.01 | -1.4 | 100 | 100 |
| Bt.5223.1.S1\_at | 3.18e-05 | 1.76e-10 | 1.27e-07 | \* \* \* | 1.74 | 1.89 | 2.15 | 100 | 100 |
| Bt.1311.1.S1\_at | 3.2e-05 | 5.29e-06 | 5.37e-05 | \* \* \* | -2.6 | -1.75 | -1.91 | 100 | 100 |
| Bt.27121.1.S1\_at | 3.21e-05 | 8.92e-05 | 3.01e-06 | \* \* \* | -1.11 | -0.69 | -0.986 | 100 | 100 |
| Bt.26718.1.S1\_a\_at | 3.21e-05 | 1.3e-09 | 2.28e-08 | \* \* \* | -1.69 | -1.68 | -2.48 | 100 | 100 |
| Bt.1670.1.A1\_at | 3.24e-05 | 0.000243 | 2.87e-05 | \* \* \* | -1.14 | -0.794 | -1.06 | 100 | 100 |
| Bt.3260.1.S1\_at | 3.25e-05 | 2.34e-05 | 0.000428 | \* \* \* | -1.32 | -0.683 | -0.868 | 100 | 100 |
| Bt.8829.1.S1\_a\_at | 3.28e-05 | 4.67e-08 | 3.8e-07 | \* \* \* | 1.69 | 2.08 | 2.76 | 100 | 100 |
| Bt.6247.1.S1\_at | 3.3e-05 | 1.03e-07 | 9.57e-07 | \* \* \* | 1.89 | 1.95 | 2.51 | 100 | 100 |
| Bt.1197.1.A1\_at | 3.32e-05 | 2.28e-08 | 5.62e-06 | \* \* \* | 1.61 | 1.15 | 1.28 | 100 | 100 |
| Bt.15724.1.S1\_at | 3.32e-05 | 9.92e-06 | 5.25e-06 | \* \* \* | -1.11 | -0.909 | -1.19 | 100 | 100 |
| Bt.11216.1.S1\_at | 3.33e-05 | 1.85e-08 | 1.13e-06 | \* \* \* | -2.46 | -2.39 | -3.17 | 100 | 100 |
| Bt.4550.1.A1\_at | 3.39e-05 | 0.00084 | 0.00151 | \* \* \* | 1.37 | 0.727 | 0.979 | 100 | 100 |
| Bt.15857.1.S1\_at | 3.42e-05 | 3.25e-07 | 2.96e-06 | \* \* \* | -2.03 | -1.41 | -1.95 | 100 | 100 |
| Bt.14566.1.A1\_at | 3.43e-05 | 5.33e-05 | 3.71e-05 | \* \* \* | 1.52 | 1.3 | 1.64 | 100 | 100 |
| Bt.6473.1.S1\_at | 3.49e-05 | 0.00179 | 0.000182 | \* \* \* | -1.09 | -0.57 | -0.756 | 100 | 100 |
| Bt.5605.1.S1\_at | 3.59e-05 | 7.17e-07 | 8.45e-08 | \* \* \* | 0.984 | 1.2 | 1.34 | 100 | 100 |
| Bt.4819.1.S1\_at | 3.63e-05 | 7.27e-06 | 3.5e-06 | \* \* \* | -1.26 | -0.725 | -0.895 | 100 | 100 |
| Bt.4563.1.S1\_at | 3.65e-05 | 9.02e-05 | 3.93e-07 | \* \* \* | 0.892 | 0.652 | 0.766 | 100 | 100 |
| Bt.24616.1.S1\_at | 3.65e-05 | 7.51e-06 | 8.36e-05 | \* \* \* | 1.7 | 1.36 | 2 | 100 | 100 |
| Bt.4506.1.S1\_at | 3.65e-05 | 8.25e-07 | 2.77e-06 | \* \* \* | 1.02 | 1.1 | 1.35 | 100 | 100 |
| Bt.15726.1.S1\_at | 3.73e-05 | 0.000627 | 1.65e-06 | \* \* \* | 1.42 | 0.699 | 1.49 | 100 | 100 |
| Bt.2524.1.S1\_at | 3.78e-05 | 4.21e-06 | 1.11e-08 | \* \* \* | 1.02 | 0.936 | 1.76 | 100 | 100 |
| Bt.5546.1.S1\_at | 3.83e-05 | 4.07e-05 | 2.26e-06 | \* \* \* | 1.18 | 1.05 | 1.27 | 100 | 100 |
| Bt.3218.1.S1\_at | 3.88e-05 | 9.9e-08 | 3.52e-07 | \* \* \* | 1.51 | 1.17 | 1.96 | 100 | 100 |
| Bt.9708.2.S1\_at | 3.89e-05 | 4.55e-07 | 6e-07 | \* \* \* | -1.79 | -1.5 | -1.97 | 100 | 100 |
| Bt.8679.3.S1\_at | 3.96e-05 | 2e-07 | 1.38e-06 | \* \* \* | -1.72 | -1.68 | -2.32 | 100 | 100 |
| Bt.10096.1.S1\_at | 3.96e-05 | 5.94e-06 | 0.000142 | \* \* \* | 1.43 | 0.935 | 1.61 | 100 | 100 |
| Bt.28651.1.S1\_at | 3.98e-05 | 9.09e-08 | 6.69e-06 | \* \* \* | 1.42 | 1.25 | 1.72 | 100 | 100 |
| Bt.1844.1.S1\_at | 3.98e-05 | 6.31e-08 | 6.56e-07 | \* \* \* | 1.17 | 1.14 | 1.21 | 100 | 100 |
| Bt.1237.1.S1\_at | 3.99e-05 | 0.000298 | 3.68e-05 | \* \* \* | 1 | 0.852 | 1.02 | 100 | 100 |
| Bt.9797.1.S1\_at | 4e-05 | 1.96e-06 | 2.7e-06 | \* \* \* | -1.33 | -1.13 | -1.35 | 100 | 100 |
| Bt.5605.2.S1\_at | 4e-05 | 3.68e-06 | 1.28e-06 | \* \* \* | 1.16 | 1.13 | 1.46 | 100 | 100 |
| Bt.24039.1.A1\_at | 4.02e-05 | 1.59e-07 | 2.32e-09 | \* \* \* | -1.3 | -1.04 | -1.6 | 100 | 100 |
| Bt.26878.1.S1\_at | 4.06e-05 | 3.14e-06 | 4.94e-05 | \* \* \* | -1.65 | -1.39 | -1.83 | 100 | 100 |
| Bt.2527.1.S1\_at | 4.09e-05 | 0.000119 | 6.38e-05 | \* \* \* | 1.01 | 0.923 | 1.09 | 100 | 100 |
| Bt.21471.1.S1\_at | 4.15e-05 | 3.02e-07 | 0.00055 | \* \* \* | 1.39 | 1.64 | 1.64 | 100 | 100 |
| Bt.8351.1.S1\_at | 4.26e-05 | 2.28e-06 | 3.44e-08 | \* \* \* | -1.37 | -0.86 | -1.18 | 100 | 100 |
| Bt.19218.1.A1\_at | 4.27e-05 | 5.21e-05 | 4.77e-06 | \* \* \* | 0.854 | 0.809 | 0.889 | 100 | 100 |
| Bt.66.1.S1\_at | 4.28e-05 | 2.3e-08 | 6.63e-08 | \* \* \* | -1.37 | -1.11 | -1.5 | 100 | 100 |
| Bt.4985.1.S1\_a\_at | 4.29e-05 | 3.47e-06 | 5.85e-07 | \* \* \* | -1.04 | -0.766 | -1.05 | 100 | 100 |
| Bt.4224.1.S1\_at | 4.29e-05 | 0.000113 | 2.29e-05 | \* \* \* | -1.28 | -0.789 | -0.948 | 100 | 100 |
| Bt.26577.1.A1\_at | 4.31e-05 | 0.00193 | 0.000135 | \* \* \* | -1.01 | -0.607 | -0.774 | 100 | 100 |
| Bt.2077.1.S1\_at | 4.35e-05 | 0.000389 | 5.24e-05 | \* \* \* | 1.19 | 0.686 | 1.23 | 100 | 100 |
| Bt.543.1.S1\_at | 4.36e-05 | 1.52e-05 | 1.39e-05 | \* \* \* | 1.49 | 1.26 | 1.77 | 100 | 100 |
| Bt.5078.1.S1\_at | 4.38e-05 | 2.7e-08 | 6.78e-08 | \* \* \* | 1.28 | 1.27 | 1.52 | 100 | 100 |
| Bt.22054.2.S1\_a\_at | 4.39e-05 | 4.22e-07 | 5.37e-05 | \* \* \* | -2.1 | -2.34 | -2.82 | 100 | 100 |
| Bt.21020.1.S1\_at | 4.46e-05 | 1.6e-05 | 0.000642 | \* \* \* | 1.48 | 1.01 | 1.18 | 100 | 100 |
| Bt.8046.1.A1\_at | 4.47e-05 | 1.45e-06 | 4.48e-06 | \* \* \* | -1.86 | -1.46 | -1.96 | 100 | 100 |
| Bt.24413.1.S1\_at | 4.51e-05 | 2.27e-10 | 5.53e-08 | \* \* \* | 1.55 | 1.69 | 2.17 | 100 | 100 |
| Bt.26130.1.A1\_at | 4.54e-05 | 2.08e-06 | 2.3e-05 | \* \* \* | -2.02 | -1.28 | -1.77 | 100 | 100 |
| Bt.1332.1.S1\_a\_at | 4.58e-05 | 5.66e-07 | 2.52e-08 | \* \* \* | -1.3 | -1.19 | -1.77 | 100 | 100 |
| Bt.4875.2.S1\_at | 4.58e-05 | 2.2e-07 | 0.000212 | \* \* \* | 1.34 | 1.09 | 1.56 | 100 | 100 |
| Bt.9956.1.S1\_at | 4.58e-05 | 1.8e-05 | 8e-07 | \* \* \* | -1.2 | -0.891 | -1.32 | 100 | 100 |
| Bt.20046.1.S1\_a\_at | 4.63e-05 | 1.29e-06 | 3.64e-07 | \* \* \* | 0.871 | 0.933 | 1.09 | 100 | 100 |
| Bt.1517.1.S1\_at | 4.66e-05 | 2.69e-06 | 5.96e-08 | \* \* \* | -1.06 | -0.804 | -1.25 | 100 | 100 |
| Bt.23509.1.S1\_at | 4.67e-05 | 1.82e-06 | 3.46e-06 | \* \* \* | 1.23 | 0.956 | 1.19 | 100 | 100 |
| Bt.24305.1.A1\_at | 4.67e-05 | 1.29e-05 | 4.99e-07 | \* \* \* | 1.54 | 0.925 | 1.14 | 100 | 100 |
| Bt.4705.1.S1\_at | 4.67e-05 | 0.000275 | 0.00066 | \* \* \* | -1.23 | -0.65 | -1.03 | 100 | 100 |
| Bt.5418.1.S1\_at | 4.71e-05 | 0.000129 | 0.00013 | \* \* \* | -1.32 | -0.959 | -1.32 | 100 | 100 |
| Bt.746.1.S1\_at | 4.71e-05 | 1.93e-05 | 3.78e-06 | \* \* \* | 1.11 | 0.915 | 1.26 | 100 | 100 |
| Bt.11242.1.S1\_at | 4.72e-05 | 2.13e-05 | 9.94e-06 | \* \* \* | -1.59 | -0.836 | -1.1 | 100 | 100 |
| Bt.5108.1.S1\_at | 4.73e-05 | 4.56e-07 | 2.48e-06 | \* \* \* | -1.41 | -1.17 | -1.49 | 100 | 100 |
| Bt.58.1.S1\_at | 4.78e-05 | 0.000316 | 1.29e-06 | \* \* \* | -0.852 | -0.542 | -0.799 | 100 | 100 |
| Bt.4205.1.S1\_at | 4.8e-05 | 1.88e-05 | 3.86e-06 | \* \* \* | -1.09 | -0.731 | -1.09 | 100 | 100 |
| Bt.25390.1.A1\_at | 4.8e-05 | 0.000139 | 0.00016 | \* \* \* | 1.16 | 0.922 | 1.14 | 100 | 100 |
| Bt.4606.2.S1\_at | 4.84e-05 | 1.39e-05 | 0.000138 | \* \* \* | 2.3 | 1.81 | 2.39 | 100 | 100 |
| Bt.24892.1.A1\_at | 4.85e-05 | 5.52e-06 | 0.000159 | \* \* \* | 1.62 | 1.5 | 1.88 | 100 | 100 |
| Bt.8486.1.S1\_at | 4.86e-05 | 2.03e-05 | 0.000316 | \* \* \* | 1.67 | 1.28 | 1.73 | 100 | 100 |
| Bt.5848.1.S1\_at | 4.86e-05 | 0.000411 | 6.41e-05 | \* \* \* | -1.18 | -0.718 | -0.85 | 100 | 100 |
| Bt.25338.1.S1\_at | 4.89e-05 | 4.06e-05 | 2.11e-06 | \* \* \* | 1.21 | 0.858 | 0.839 | 100 | 100 |
| Bt.3989.1.S1\_at | 4.93e-05 | 2.94e-06 | 5.91e-06 | \* \* \* | 1.22 | 1.32 | 1.67 | 100 | 100 |
| Bt.25072.1.S1\_at | 4.94e-05 | 1.93e-05 | 4.06e-06 | \* \* \* | -1.16 | -1.01 | -1.42 | 100 | 100 |
| Bt.9169.1.A1\_at | 4.94e-05 | 1.36e-05 | 4.36e-06 | \* \* \* | 1.17 | 1.31 | 1.56 | 100 | 100 |
| Bt.24848.1.A1\_at | 4.95e-05 | 3.74e-08 | 8.76e-08 | \* \* \* | 1.88 | 1.86 | 3.4 | 100 | 100 |
| Bt.8001.1.S1\_at | 4.97e-05 | 2.52e-06 | 1.99e-06 | \* \* \* | -1.29 | -0.943 | -1.36 | 100 | 100 |
| Bt.1426.1.S1\_at | 4.97e-05 | 0.000234 | 5.87e-05 | \* \* \* | -1.14 | -0.733 | -0.903 | 100 | 100 |
| Bt.13170.1.S1\_at | 5e-05 | 3.2e-06 | 5.74e-06 | \* \* \* | -1.47 | -1.06 | -1.33 | 100 | 100 |
| Bt.21383.2.S1\_a\_at | 5.03e-05 | 1.96e-06 | 1.94e-07 | \* \* \* | 1.47 | 1.06 | 1.92 | 100 | 100 |
| Bt.24236.1.S1\_at | 5.04e-05 | 3.26e-06 | 2.54e-06 | \* \* \* | 1.11 | 1.21 | 1.44 | 100 | 100 |
| Bt.21969.1.A1\_at | 5.07e-05 | 1e-05 | 4.74e-07 | \* \* \* | 0.844 | 0.867 | 0.922 | 100 | 100 |
| Bt.19097.1.A1\_at | 5.19e-05 | 3.46e-08 | 3.32e-06 | \* \* \* | 1.34 | 1.29 | 1.44 | 100 | 100 |
| Bt.10215.1.A1\_at | 5.28e-05 | 0.00084 | 1.43e-05 | \* \* \* | -0.914 | -0.611 | -0.805 | 100 | 100 |
| Bt.2493.1.S1\_at | 5.29e-05 | 2.97e-05 | 2.77e-05 | \* \* \* | -1.31 | -1.28 | -1.57 | 100 | 100 |
| Bt.22165.1.S1\_at | 5.32e-05 | 0.00188 | 0.000223 | \* \* \* | 1.7 | 1.08 | 1.62 | 100 | 100 |
| Bt.6549.1.S1\_at | 5.36e-05 | 2.67e-06 | 9.06e-06 | \* \* \* | 1.39 | 1.58 | 1.78 | 100 | 100 |
| Bt.7237.2.S1\_a\_at | 5.49e-05 | 1.42e-07 | 1.26e-06 | \* \* \* | -1.27 | -1.37 | -1.96 | 100 | 100 |
| Bt.4886.1.A1\_at | 5.51e-05 | 6.13e-06 | 3.59e-05 | \* \* \* | 2.37 | 2.17 | 2.89 | 100 | 100 |
| Bt.28676.1.A1\_at | 5.52e-05 | 5.02e-06 | 5.7e-05 | \* \* \* | -1.09 | -0.812 | -1.06 | 100 | 100 |
| Bt.1950.1.S1\_at | 5.52e-05 | 0.000389 | 0.000626 | \* \* \* | -1.03 | -0.718 | -0.849 | 100 | 100 |
| Bt.16020.1.A2\_at | 5.53e-05 | 3.33e-05 | 3.16e-05 | \* \* \* | 1.21 | 1.08 | 1.22 | 100 | 100 |
| Bt.9585.1.S1\_at | 5.67e-05 | 5.92e-05 | 1.98e-05 | \* \* \* | -1.46 | -1.05 | -1.66 | 100 | 100 |
| Bt.7478.1.S1\_at | 5.67e-05 | 2.34e-06 | 0.000107 | \* \* \* | -2.02 | -1.6 | -1.95 | 100 | 100 |
| Bt.10259.1.A1\_at | 5.76e-05 | 2.27e-05 | 0.000114 | \* \* \* | 1.39 | 1.38 | 1.67 | 100 | 100 |
| Bt.5074.1.S1\_at | 5.79e-05 | 2.22e-05 | 8.45e-05 | \* \* \* | 1.46 | 1.22 | 1.5 | 100 | 100 |
| Bt.14095.1.S1\_at | 5.84e-05 | 1.41e-06 | 7.04e-07 | \* \* \* | 1.11 | 1.08 | 1.33 | 100 | 100 |
| Bt.1613.1.S1\_at | 5.85e-05 | 4.08e-07 | 8.69e-06 | \* \* \* | 1.58 | 1.5 | 1.79 | 100 | 100 |
| Bt.1699.3.S1\_a\_at | 5.86e-05 | 3.86e-06 | 3.02e-05 | \* \* \* | 1.07 | 1.2 | 1.44 | 100 | 100 |
| Bt.26829.1.S1\_at | 5.89e-05 | 6.47e-07 | 4.19e-06 | \* \* \* | -1.91 | -1.49 | -2.7 | 100 | 100 |
| Bt.3821.2.S1\_at | 5.99e-05 | 1.46e-06 | 1.09e-05 | \* \* \* | 1.18 | 1.13 | 1.35 | 100 | 100 |
| Bt.14853.1.A1\_at | 6.09e-05 | 3.89e-05 | 4.33e-05 | \* \* \* | 1.37 | 1.05 | 1.35 | 100 | 100 |
| Bt.6397.2.S1\_at | 6.15e-05 | 2.86e-05 | 0.000336 | \* \* \* | 1.49 | 1.17 | 1.47 | 100 | 100 |
| Bt.5378.1.S1\_at | 6.18e-05 | 2e-04 | 3.9e-05 | \* \* \* | -1.21 | -0.755 | -0.921 | 100 | 100 |
| Bt.1789.1.S1\_at | 6.2e-05 | 0.000148 | 8.25e-06 | \* \* \* | 1.81 | 0.78 | 1.12 | 100 | 100 |
| Bt.20431.2.S1\_at | 6.21e-05 | 0.000212 | 0.00163 | \* \* \* | 1.25 | 1.13 | 1.45 | 100 | 100 |
| Bt.1767.1.S1\_at | 6.26e-05 | 1.93e-05 | 1.64e-05 | \* \* \* | -1.06 | -0.834 | -0.916 | 100 | 100 |
| Bt.25202.1.A1\_at | 6.34e-05 | 2.57e-06 | 1.6e-05 | \* \* \* | 1.67 | 1.68 | 1.86 | 100 | 100 |
| Bt.5664.1.S1\_at | 6.39e-05 | 2.95e-05 | 0.000139 | \* \* \* | 1.18 | 0.724 | 1.25 | 100 | 100 |
| Bt.20.1.S1\_at | 6.45e-05 | 2.25e-09 | 3.12e-07 | \* \* \* | -1.72 | -1.45 | -2.01 | 100 | 100 |
| Bt.23176.2.S1\_a\_at | 6.49e-05 | 2.94e-09 | 1.82e-07 | \* \* \* | 1.76 | 2.23 | 2.57 | 100 | 100 |
| Bt.20228.1.S1\_at | 6.71e-05 | 0.00134 | 8.91e-07 | \* \* \* | -0.788 | -0.458 | -0.64 | 100 | 100 |
| Bt.5406.1.S1\_at | 6.73e-05 | 3.82e-06 | 1.56e-07 | \* \* \* | 1.2 | 1.04 | 1.3 | 100 | 100 |
| Bt.23526.1.S1\_at | 6.76e-05 | 9.79e-08 | 3.66e-09 | \* \* \* | -0.998 | -0.986 | -1.26 | 100 | 100 |
| Bt.4203.1.S1\_at | 6.76e-05 | 0.00173 | 0.000207 | \* \* \* | -1.15 | -0.748 | -0.927 | 100 | 100 |
| Bt.12126.1.S1\_at | 6.83e-05 | 1.34e-07 | 1.13e-07 | \* \* \* | -1.56 | -1.24 | -1.71 | 100 | 100 |
| Bt.23603.1.S1\_a\_at | 6.86e-05 | 1.4e-05 | 8.5e-07 | \* \* \* | -1.38 | -1.11 | -1.47 | 100 | 100 |
| Bt.15742.1.S2\_at | 6.87e-05 | 2.12e-06 | 2.6e-05 | \* \* \* | 0.929 | 1.06 | 1.08 | 100 | 100 |
| Bt.4792.1.S1\_at | 6.9e-05 | 1.85e-06 | 7.57e-07 | \* \* \* | 1.42 | 1.3 | 1.56 | 100 | 100 |
| Bt.22451.1.S1\_at | 6.93e-05 | 2.67e-05 | 1.84e-05 | \* \* \* | -1.17 | -0.827 | -1.13 | 100 | 100 |
| Bt.17526.1.A1\_at | 6.94e-05 | 0.000426 | 2.28e-05 | \* \* \* | 1.46 | 0.77 | 1.11 | 100 | 100 |
| Bt.11192.1.S1\_at | 6.97e-05 | 0.00206 | 0.000151 | \* \* \* | -1 | -0.478 | -0.64 | 100 | 100 |
| Bt.3227.1.S1\_at | 7.07e-05 | 2.72e-07 | 1.16e-08 | \* \* \* | -1.12 | -1.2 | -1.66 | 100 | 100 |
| Bt.20854.1.S1\_at | 7.08e-05 | 2.62e-09 | 2.17e-09 | \* \* \* | -1.47 | -1.39 | -1.86 | 100 | 100 |
| Bt.23081.1.S1\_a\_at | 7.18e-05 | 1.88e-06 | 1.25e-05 | \* \* \* | -1.98 | -1.86 | -2.28 | 100 | 100 |
| Bt.3867.1.S1\_at | 7.19e-05 | 0.000764 | 7.52e-06 | \* \* \* | -1.12 | -0.543 | -0.638 | 100 | 100 |
| Bt.12912.1.S2\_at | 7.26e-05 | 9e-08 | 4.36e-06 | \* \* \* | 1.22 | 1.22 | 1.3 | 100 | 100 |
| Bt.869.2.S1\_at | 7.26e-05 | 7.95e-08 | 8.19e-07 | \* \* \* | 1.17 | 1.17 | 1.43 | 100 | 100 |
| Bt.4891.1.S1\_at | 7.33e-05 | 9.28e-10 | 1.96e-09 | \* \* \* | -1.75 | -1.31 | -1.57 | 100 | 100 |
| Bt.26764.1.A1\_at | 7.34e-05 | 8.13e-06 | 9.07e-07 | \* \* \* | 0.94 | 1.03 | 1.17 | 100 | 100 |
| Bt.17641.1.A1\_at | 7.36e-05 | 8.51e-05 | 0.000471 | \* \* \* | -1.1 | -1.1 | -1.32 | 100 | 100 |
| Bt.7373.3.S1\_a\_at | 7.37e-05 | 1.55e-05 | 4.9e-05 | \* \* \* | -1.12 | -0.894 | -1.52 | 100 | 100 |
| Bt.12249.2.S1\_at | 7.39e-05 | 4.7e-05 | 1.12e-05 | \* \* \* | 1.15 | 0.833 | 1.21 | 100 | 100 |
| Bt.21019.1.S1\_at | 7.48e-05 | 7.89e-06 | 2.41e-08 | \* \* \* | 0.878 | 0.855 | 1.27 | 100 | 100 |
| Bt.5748.2.S1\_at | 7.5e-05 | 6.04e-06 | 1.55e-06 | \* \* \* | 0.932 | 1.06 | 1.29 | 100 | 100 |
| Bt.16039.1.S1\_at | 7.59e-05 | 6.82e-07 | 5.67e-06 | \* \* \* | -1.1 | -0.963 | -1.5 | 100 | 100 |
| Bt.8322.1.S1\_at | 7.63e-05 | 1.64e-06 | 2.02e-06 | \* \* \* | 1.24 | 1.34 | 1.53 | 100 | 100 |
| Bt.19431.1.S1\_at | 7.66e-05 | 0.000176 | 4.5e-06 | \* \* \* | -1.19 | -0.634 | -0.894 | 100 | 100 |
| Bt.20849.1.S1\_at | 7.82e-05 | 3.11e-05 | 1.18e-05 | \* \* \* | 0.97 | 1.09 | 1.26 | 100 | 100 |
| Bt.3753.1.S1\_at | 7.89e-05 | 4.03e-06 | 9.26e-05 | \* \* \* | 1.29 | 1.12 | 1.76 | 100 | 100 |
| Bt.4478.1.S1\_at | 7.92e-05 | 0.000234 | 4.52e-05 | \* \* \* | -1.79 | -0.889 | -1.09 | 100 | 100 |
| Bt.21122.1.S1\_at | 7.96e-05 | 0.000211 | 0.000287 | \* \* \* | -0.945 | -0.822 | -0.921 | 100 | 100 |
| Bt.4271.1.S1\_at | 7.97e-05 | 2.68e-06 | 1.12e-07 | \* \* \* | 1.06 | 0.952 | 1.03 | 100 | 100 |
| Bt.2729.1.S1\_at | 8.02e-05 | 3.51e-06 | 4.9e-06 | \* \* \* | 1.16 | 0.856 | 0.937 | 100 | 100 |
| Bt.13780.1.A1\_at | 8.07e-05 | 7.7e-06 | 8.58e-06 | \* \* \* | 1.59 | 1.52 | 1.73 | 100 | 100 |
| Bt.10360.1.S1\_at | 8.08e-05 | 0.000314 | 0.000172 | \* \* \* | 1.03 | 0.835 | 1.2 | 100 | 100 |
| Bt.23854.1.A1\_at | 8.09e-05 | 9.85e-07 | 3.59e-06 | \* \* \* | 1.62 | 1.64 | 2.27 | 100 | 100 |
| Bt.2824.1.S1\_at | 8.1e-05 | 5.11e-06 | 1.04e-05 | \* \* \* | -1.23 | -0.9 | -1.23 | 100 | 100 |
| Bt.6080.1.S1\_at | 8.15e-05 | 1.09e-07 | 1.66e-06 | \* \* \* | -1.26 | -1.1 | -1.43 | 100 | 100 |
| Bt.13833.1.S1\_at | 8.17e-05 | 9.24e-07 | 1.18e-07 | \* \* \* | 1.13 | 1.09 | 1.29 | 100 | 100 |
| Bt.7596.1.S1\_at | 8.21e-05 | 9.43e-05 | 2.96e-05 | \* \* \* | -1.02 | -0.632 | -0.996 | 100 | 100 |
| Bt.7133.1.S1\_at | 8.33e-05 | 0.000209 | 0.000461 | \* \* \* | -1.02 | -0.768 | -1.01 | 100 | 100 |
| Bt.3211.1.S2\_at | 8.42e-05 | 7.38e-05 | 4.24e-05 | \* \* \* | 1.19 | 0.822 | 0.961 | 100 | 100 |
| Bt.16978.1.S1\_at | 8.51e-05 | 1.65e-06 | 1.11e-06 | \* \* \* | -1.1 | -1.16 | -1.58 | 100 | 100 |
| Bt.24662.1.S1\_at | 8.53e-05 | 1.04e-05 | 0.00109 | \* \* \* | -1.22 | -1.01 | -1.47 | 100 | 100 |
| Bt.3913.1.S1\_at | 8.53e-05 | 2.78e-05 | 8.07e-05 | \* \* \* | 1.06 | 1.13 | 1.21 | 100 | 100 |
| Bt.4492.1.S1\_at | 8.55e-05 | 8.29e-05 | 2.03e-05 | \* \* \* | 1.27 | 1.03 | 1.58 | 100 | 100 |
| Bt.21435.1.S1\_at | 8.57e-05 | 0.000117 | 2.75e-05 | \* \* \* | -0.906 | -0.607 | -0.831 | 100 | 100 |
| Bt.21603.1.S1\_at | 8.65e-05 | 2.02e-06 | 2.34e-06 | \* \* \* | -1.58 | -1.05 | -1.64 | 100 | 100 |
| Bt.3604.1.A1\_at | 8.66e-05 | 0.000281 | 1.81e-05 | \* \* \* | 1.05 | 0.801 | 1 | 100 | 100 |
| Bt.19519.1.S1\_at | 8.73e-05 | 0.00168 | 1.76e-05 | \* \* \* | 1.83 | 0.562 | 0.778 | 100 | 100 |
| Bt.4957.1.S2\_at | 8.73e-05 | 4.61e-06 | 2.58e-05 | \* \* \* | 1.31 | 0.906 | 1.13 | 100 | 100 |
| Bt.20146.1.S1\_at | 8.81e-05 | 3.14e-07 | 1.53e-06 | \* \* \* | 0.995 | 1.54 | 1.89 | 100 | 100 |
| Bt.2374.1.S2\_at | 8.83e-05 | 2.87e-05 | 0.000665 | \* \* \* | 1.02 | 0.611 | 0.505 | 100 | 100 |
| Bt.18348.1.S1\_at | 8.83e-05 | 1.82e-05 | 1.18e-05 | \* \* \* | 1.2 | 1.08 | 1.44 | 100 | 100 |
| Bt.27403.1.S1\_at | 8.86e-05 | 1.81e-07 | 1.4e-06 | \* \* \* | 1.43 | 1.37 | 1.64 | 100 | 100 |
| Bt.23234.1.S1\_at | 8.88e-05 | 1.3e-06 | 2.14e-06 | \* \* \* | -1.28 | -1.13 | -1.66 | 100 | 100 |
| Bt.27089.1.S1\_at | 8.96e-05 | 7.42e-07 | 3.28e-06 | \* \* \* | 1.23 | 1.26 | 1.45 | 100 | 100 |
| Bt.21469.1.S1\_at | 9.05e-05 | 1.75e-05 | 2.36e-05 | \* \* \* | 0.94 | 1.02 | 1.11 | 100 | 100 |
| Bt.3782.1.A1\_at | 9.07e-05 | 1.66e-08 | 9.77e-08 | \* \* \* | 2.26 | 1.74 | 3.29 | 100 | 100 |
| Bt.23520.1.S1\_at | 9.08e-05 | 1.81e-05 | 7.71e-08 | \* \* \* | 0.938 | 0.858 | 1.01 | 100 | 100 |
| Bt.11798.1.S1\_at | 9.12e-05 | 2.79e-05 | 0.000109 | \* \* \* | 1.44 | 1.37 | 1.65 | 100 | 100 |
| Bt.17942.1.S1\_at | 9.13e-05 | 1.91e-09 | 2.25e-06 | \* \* \* | 1.57 | 1.38 | 1.55 | 100 | 100 |
| Bt.6693.1.A1\_at | 9.15e-05 | 8.8e-06 | 4.22e-05 | \* \* \* | -1.71 | -1.18 | -1.41 | 100 | 100 |
| Bt.21818.1.S1\_at | 9.21e-05 | 8.57e-06 | 5.86e-05 | \* \* \* | 1.21 | 1.06 | 1.45 | 100 | 100 |
| Bt.11154.2.S1\_at | 9.26e-05 | 2.72e-05 | 2.42e-05 | \* \* \* | -1.08 | -0.957 | -1.29 | 100 | 100 |
| Bt.16471.1.S1\_at | 9.29e-05 | 8.26e-06 | 0.000301 | \* \* \* | 1.38 | 1.23 | 1.48 | 100 | 100 |
| Bt.16380.2.A1\_at | 9.31e-05 | 0.00151 | 0.000529 | \* \* \* | 1.18 | 0.654 | 1.03 | 100 | 100 |
| Bt.9706.2.S1\_at | 9.33e-05 | 0.000126 | 2.31e-06 | \* \* \* | -0.931 | -0.589 | -0.887 | 100 | 100 |
| Bt.19706.1.S1\_at | 9.34e-05 | 1.29e-05 | 2.93e-05 | \* \* \* | 1.24 | 1.28 | 1.5 | 100 | 100 |
| Bt.2235.1.S1\_at | 9.4e-05 | 7.39e-05 | 0.000107 | \* \* \* | 0.927 | 0.937 | 1.23 | 100 | 100 |
| Bt.19862.2.S1\_at | 9.43e-05 | 2.62e-08 | 1.51e-05 | \* \* \* | 1.47 | 1.5 | 2.61 | 100 | 100 |
| Bt.21745.1.S1\_at | 9.45e-05 | 0.000101 | 1.89e-05 | \* \* \* | -1 | -0.864 | -1.18 | 100 | 100 |
| Bt.12661.1.A1\_at | 9.5e-05 | 0.00047 | 1.7e-07 | \* \* \* | 1.36 | 0.894 | 1.45 | 100 | 100 |
| Bt.16437.1.S1\_at | 9.57e-05 | 0.00184 | 0.000461 | \* \* \* | -0.96 | -0.442 | -0.679 | 100 | 100 |
| Bt.2717.1.A1\_at | 9.57e-05 | 1.05e-06 | 0.000479 | \* \* \* | 2.01 | 1.67 | 2.05 | 100 | 100 |
| Bt.15779.3.S1\_at | 9.57e-05 | 0.000645 | 0.00109 | \* \* \* | 1.71 | 0.881 | 1.7 | 100 | 100 |
| Bt.10266.1.S1\_at | 9.6e-05 | 0.000216 | 0.000125 | \* \* \* | -1.37 | -0.869 | -1.17 | 100 | 100 |
| Bt.18278.1.S1\_at | 9.63e-05 | 3.9e-10 | 5.77e-09 | \* \* \* | -1.9 | -1.84 | -2.27 | 100 | 100 |
| Bt.2399.1.S1\_at | 9.66e-05 | 3.33e-05 | 5.04e-06 | \* \* \* | 1.47 | 0.949 | 1.27 | 100 | 100 |
| Bt.814.1.S1\_at | 9.68e-05 | 1.17e-06 | 7.83e-08 | \* \* \* | -0.997 | -0.755 | -1.33 | 100 | 100 |
| Bt.19581.1.A1\_at | 9.77e-05 | 9.44e-06 | 8.58e-05 | \* \* \* | -1.18 | -0.95 | -1.41 | 100 | 100 |
| Bt.2989.2.S1\_at | 9.79e-05 | 3.53e-06 | 1.53e-05 | \* \* \* | -1.38 | -1.16 | -1.4 | 100 | 100 |
| Bt.13152.1.S1\_s\_at | 9.8e-05 | 2.26e-05 | 0.000204 | \* \* \* | 1.74 | 1.41 | 1.94 | 100 | 100 |
| Bt.1154.1.S1\_at | 9.94e-05 | 2.66e-08 | 3.16e-07 | \* \* \* | -1.69 | -1.51 | -1.95 | 100 | 100 |
| Bt.20683.1.S1\_at | 9.96e-05 | 1.5e-06 | 1.07e-05 | \* \* \* | -1.69 | -1.64 | -3.08 | 100 | 100 |
| Bt.9210.1.S1\_at | 1e-04 | 8.44e-05 | 0.000193 | \* \* \* | -0.923 | -0.754 | -0.775 | 100 | 100 |
| Bt.24310.1.S1\_at | 1e-04 | 1.54e-06 | 8.9e-06 | \* \* \* | -1.57 | -1.1 | -1.65 | 100 | 100 |
| Bt.25098.1.S1\_at | 0.000101 | 7.63e-06 | 6.67e-05 | \* \* \* | -1.34 | -1.17 | -1.87 | 100 | 100 |
| Bt.8262.1.A1\_at | 0.000101 | 0.000131 | 6.73e-05 | \* \* \* | 1.03 | 0.766 | 0.773 | 100 | 100 |
| Bt.29910.1.S1\_at | 0.000102 | 1.92e-07 | 2.02e-06 | \* \* \* | 1.43 | 1.3 | 1.56 | 100 | 100 |
| Bt.9176.2.S1\_at | 0.000102 | 3.95e-05 | 2.31e-05 | \* \* \* | 1.16 | 0.903 | 0.875 | 100 | 100 |
| Bt.12366.1.S1\_at | 0.000102 | 9.75e-06 | 0.00029 | \* \* \* | 0.926 | 0.774 | 0.665 | 100 | 100 |
| Bt.22310.1.S1\_at | 0.000102 | 4.03e-06 | 4.17e-06 | \* \* \* | -1.3 | -0.958 | -1.48 | 100 | 100 |
| Bt.12910.1.S1\_at | 0.000103 | 5.92e-06 | 1.28e-06 | \* \* \* | -1.51 | -0.774 | -1.15 | 100 | 100 |
| Bt.6679.1.S1\_at | 0.000104 | 0.000124 | 0.000401 | \* \* \* | 0.993 | 0.843 | 0.984 | 100 | 100 |
| Bt.5560.1.S1\_at | 0.000104 | 0.000243 | 5.71e-05 | \* \* \* | 1.06 | 0.968 | 1.23 | 100 | 100 |
| Bt.23254.1.S1\_at | 0.000104 | 6.3e-07 | 2.2e-06 | \* \* \* | 1.09 | 0.928 | 1.05 | 100 | 100 |
| Bt.10579.1.S1\_at | 0.000105 | 4.58e-05 | 1.82e-05 | \* \* \* | 1.07 | 1.13 | 1.41 | 100 | 100 |
| Bt.22114.1.S1\_at | 0.000105 | 0.000864 | 3.91e-06 | \* \* \* | 1.08 | 0.669 | 1.13 | 100 | 100 |
| Bt.22142.2.S1\_a\_at | 0.000105 | 2.13e-06 | 1.11e-05 | \* \* \* | -1.44 | -1.51 | -2.54 | 100 | 100 |
| Bt.23158.1.S1\_at | 0.000106 | 0.000111 | 1.51e-06 | \* \* \* | 1 | 1.05 | 1.58 | 100 | 100 |
| Bt.21981.3.S1\_at | 0.000106 | 4.89e-08 | 6.47e-07 | \* \* \* | 1.33 | 1.38 | 1.66 | 100 | 100 |
| Bt.5033.1.S1\_at | 0.000106 | 1.09e-07 | 1.27e-06 | \* \* \* | -1.47 | -1.07 | -1.44 | 100 | 100 |
| Bt.1699.2.S1\_a\_at | 0.000107 | 0.000584 | 0.00179 | \* \* \* | 1.24 | 1.11 | 1.33 | 100 | 100 |
| Bt.19415.1.A1\_at | 0.000107 | 1.43e-07 | 1.39e-05 | \* \* \* | -2.35 | -2.09 | -2.51 | 100 | 100 |
| Bt.5054.3.S1\_at | 0.000107 | 1.26e-05 | 0.000144 | \* \* \* | 1.46 | 1.25 | 1.5 | 100 | 100 |
| Bt.19936.1.A1\_at | 0.000109 | 2.02e-08 | 5.93e-06 | \* \* \* | 1.81 | 1.66 | 2.3 | 100 | 100 |
| Bt.22084.1.A1\_at | 0.000109 | 1.61e-07 | 1.85e-08 | \* \* \* | 1.25 | 1.35 | 1.63 | 100 | 100 |
| Bt.1430.1.S1\_at | 0.00011 | 4.52e-05 | 2.97e-06 | \* \* \* | 0.868 | 0.77 | 0.868 | 100 | 100 |
| Bt.27386.1.A1\_at | 0.000111 | 7.38e-05 | 1.75e-05 | \* \* \* | 1.27 | 1.18 | 1.64 | 100 | 100 |
| Bt.22634.1.S1\_at | 0.000112 | 3.49e-09 | 8.66e-09 | \* \* \* | 1.32 | 1.51 | 1.74 | 100 | 100 |
| Bt.4670.1.S1\_at | 0.000112 | 1.36e-08 | 4.83e-07 | \* \* \* | 1.29 | 1.58 | 2.01 | 100 | 100 |
| Bt.7481.1.S1\_at | 0.000113 | 5.26e-06 | 0.00012 | \* \* \* | -1.84 | -1.39 | -1.81 | 100 | 100 |
| Bt.4232.2.S1\_at | 0.000113 | 1.76e-06 | 1.01e-07 | \* \* \* | 1.29 | 1.01 | 1.53 | 100 | 100 |
| Bt.380.1.S1\_at | 0.000113 | 2.93e-05 | 3.37e-05 | \* \* \* | -1.39 | -0.868 | -1.13 | 100 | 100 |
| Bt.4653.1.S2\_at | 0.000114 | 8.58e-07 | 2.36e-05 | \* \* \* | 1.28 | 1.28 | 1.33 | 100 | 100 |
| Bt.24727.1.S1\_a\_at | 0.000117 | 0.000385 | 4.81e-05 | \* \* \* | -1.18 | -0.776 | -1.14 | 100 | 100 |
| Bt.7804.1.S1\_at | 0.000117 | 1.24e-08 | 6.43e-10 | \* \* \* | -1.4 | -1.43 | -2 | 100 | 100 |
| Bt.3494.1.A1\_s\_at | 0.000118 | 0.000223 | 0.000187 | \* \* \* | 0.964 | 0.854 | 1.03 | 100 | 100 |
| Bt.27221.1.S1\_at | 0.00012 | 1.17e-05 | 0.000871 | \* \* \* | 1.56 | 1.04 | 2.12 | 100 | 100 |
| Bt.3484.1.S1\_at | 0.00012 | 0.000211 | 3.6e-05 | \* \* \* | 1 | 0.876 | 0.946 | 100 | 100 |
| Bt.13588.3.A1\_at | 0.000121 | 0.000225 | 0.000803 | \* \* \* | -3.2 | -2.31 | -2.9 | 100 | 100 |
| Bt.16004.1.A1\_at | 0.000121 | 3.12e-07 | 2.24e-05 | \* \* \* | 3.66 | 4.04 | 4.72 | 100 | 100 |
| Bt.28612.1.S1\_at | 0.000121 | 2.24e-09 | 2.22e-09 | \* \* \* | 0.994 | 1.35 | 1.47 | 100 | 100 |
| Bt.5322.1.S1\_at | 0.000121 | 2.77e-06 | 3.23e-05 | \* \* \* | -1.32 | -0.89 | -1.16 | 100 | 100 |
| Bt.21790.1.S1\_at | 0.000122 | 3.29e-06 | 1.55e-07 | \* \* \* | 1.03 | 1.04 | 1.27 | 100 | 100 |
| Bt.65.1.S1\_at | 0.000123 | 0.00157 | 0.000404 | \* \* \* | -0.919 | -0.586 | -0.759 | 100 | 100 |
| Bt.1399.1.S1\_at | 0.000123 | 1.24e-08 | 4.62e-07 | \* \* \* | -1.23 | -1.21 | -1.52 | 100 | 100 |
| Bt.752.1.S1\_a\_at | 0.000123 | 0.00174 | 7.71e-05 | \* \* \* | -0.934 | -0.525 | -0.605 | 100 | 100 |
| Bt.20402.1.S1\_at | 0.000124 | 1.45e-06 | 8.02e-07 | \* \* \* | 1.96 | 1.36 | 1.82 | 100 | 100 |
| Bt.29463.1.S1\_at | 0.000125 | 0.000841 | 0.0011 | \* \* \* | 1.33 | 0.786 | 1.06 | 100 | 100 |
| Bt.23651.1.A1\_at | 0.000125 | 6.98e-07 | 3.84e-06 | \* \* \* | 1.39 | 0.929 | 1.83 | 100 | 100 |
| Bt.20236.1.S1\_at | 0.000126 | 2.67e-07 | 4.88e-05 | \* \* \* | 1.17 | 1.03 | 1.31 | 100 | 100 |
| Bt.979.1.A1\_at | 0.000127 | 4.3e-07 | 6.35e-07 | \* \* \* | 1.69 | 1.53 | 1.84 | 100 | 100 |
| Bt.9754.1.S1\_at | 0.000127 | 4.81e-08 | 1.5e-06 | \* \* \* | -2.28 | -1.88 | -2.31 | 100 | 100 |
| Bt.29697.1.S1\_at | 0.000127 | 6.73e-05 | 4.89e-05 | \* \* \* | -1.37 | -1.1 | -1.51 | 100 | 100 |
| Bt.2696.2.S1\_at | 0.000128 | 1.04e-05 | 0.000236 | \* \* \* | 1.18 | 1.21 | 1.57 | 100 | 100 |
| Bt.15715.1.S1\_at | 0.000128 | 8.77e-07 | 2.75e-05 | \* \* \* | 1.47 | 1.16 | 1.45 | 100 | 100 |
| Bt.21182.1.S1\_at | 0.000128 | 9.95e-06 | 1.32e-05 | \* \* \* | -0.924 | -0.716 | -0.846 | 100 | 100 |
| Bt.7836.1.S1\_at | 0.00013 | 1.1e-05 | 3.48e-05 | \* \* \* | -1.1 | -0.867 | -1.03 | 100 | 100 |
| Bt.3448.1.S1\_at | 0.00013 | 2e-04 | 0.000401 | \* \* \* | -1.63 | -1.28 | -1.59 | 100 | 100 |
| Bt.6586.1.S1\_at | 0.00013 | 1.19e-05 | 3.28e-06 | \* \* \* | 1.27 | 1.07 | 1.62 | 100 | 100 |
| Bt.15868.1.S1\_at | 0.00013 | 4.24e-06 | 5.85e-06 | \* \* \* | 1.14 | 1.05 | 1.34 | 100 | 100 |
| Bt.2451.2.A1\_at | 0.000131 | 0.000165 | 0.000248 | \* \* \* | 1.29 | 1.15 | 1.24 | 100 | 100 |
| Bt.8047.1.S1\_at | 0.000131 | 3.9e-05 | 1.8e-05 | \* \* \* | -1.16 | -0.732 | -0.897 | 100 | 100 |
| Bt.20605.2.S1\_at | 0.000132 | 0.00013 | 0.000331 | \* \* \* | 1.37 | 0.735 | 0.904 | 100 | 100 |
| Bt.15853.1.S1\_at | 0.000134 | 0.000145 | 1.1e-06 | \* \* \* | 1.24 | 0.869 | 1.41 | 100 | 100 |
| Bt.16067.1.S1\_at | 0.000134 | 0.000154 | 0.000384 | \* \* \* | -1.41 | -1.02 | -1.54 | 100 | 100 |
| Bt.29053.1.A1\_at | 0.000134 | 1.8e-08 | 2.11e-07 | \* \* \* | 1.73 | 1.37 | 1.7 | 100 | 100 |
| Bt.11060.1.S1\_at | 0.000134 | 1.85e-06 | 5.76e-07 | \* \* \* | 1.78 | 1.02 | 1.25 | 100 | 100 |
| Bt.11322.1.A1\_at | 0.000135 | 5.14e-06 | 1.07e-05 | \* \* \* | -1.18 | -0.824 | -1.16 | 100 | 100 |
| Bt.6399.2.S1\_a\_at | 0.000136 | 0.000187 | 0.000563 | \* \* \* | 1.57 | 0.824 | 1.33 | 100 | 100 |
| Bt.21366.2.S1\_at | 0.000136 | 2e-06 | 4.94e-06 | \* \* \* | -1.37 | -1.22 | -1.71 | 100 | 100 |
| Bt.16935.1.A1\_at | 0.000137 | 7.19e-05 | 1.77e-05 | \* \* \* | 0.874 | 0.765 | 0.87 | 100 | 100 |
| Bt.9252.1.A1\_at | 0.000138 | 2.34e-05 | 4.28e-05 | \* \* \* | 1.33 | 1.39 | 1.67 | 100 | 100 |
| Bt.8690.2.S1\_at | 0.000138 | 0.000283 | 0.000253 | \* \* \* | -1.14 | -0.694 | -0.982 | 100 | 100 |
| Bt.21468.1.S1\_at | 0.00014 | 7.07e-06 | 1.17e-05 | \* \* \* | -1.28 | -1.17 | -1.71 | 100 | 100 |
| Bt.8912.1.A1\_at | 0.000141 | 3.81e-05 | 0.000277 | \* \* \* | -1.3 | -1.03 | -1.68 | 100 | 100 |
| Bt.5541.1.S1\_at | 0.000142 | 1.7e-06 | 8.38e-07 | \* \* \* | -1.41 | -0.926 | -1.34 | 100 | 100 |
| Bt.3288.1.A1\_at | 0.000143 | 0.000127 | 6.37e-05 | \* \* \* | -0.934 | -0.737 | -0.807 | 100 | 100 |
| Bt.18332.1.S1\_at | 0.000144 | 3.35e-06 | 4.96e-07 | \* \* \* | 0.964 | 1.15 | 1.42 | 100 | 100 |
| Bt.9705.1.S1\_at | 0.000144 | 5.47e-05 | 7.05e-05 | \* \* \* | 0.993 | 0.934 | 1.36 | 100 | 100 |
| Bt.22630.2.A1\_at | 0.000145 | 1.11e-06 | 1.78e-05 | \* \* \* | -1.18 | -1.09 | -1.57 | 100 | 100 |
| Bt.20129.2.S1\_at | 0.000145 | 1.73e-07 | 1.24e-05 | \* \* \* | -1.08 | -0.979 | -1.32 | 100 | 100 |
| Bt.27211.1.S1\_at | 0.000145 | 0.000146 | 0.000906 | \* \* \* | -1.41 | -0.978 | -1.27 | 100 | 100 |
| Bt.18057.1.A1\_at | 0.000147 | 1.14e-05 | 0.000314 | \* \* \* | 1.63 | 1.07 | 1.35 | 100 | 100 |
| Bt.21288.1.A1\_at | 0.000148 | 1.42e-05 | 0.000384 | \* \* \* | 1.3 | 0.831 | 0.626 | 100 | 100 |
| Bt.13063.1.S1\_at | 0.000149 | 1.11e-05 | 1.59e-06 | \* \* \* | -0.978 | -0.969 | -1.19 | 100 | 100 |
| Bt.1505.1.S1\_at | 0.00015 | 1.86e-06 | 1.54e-07 | \* \* \* | -1.39 | -0.929 | -1.41 | 100 | 100 |
| Bt.2815.1.S1\_at | 0.00015 | 7.52e-06 | 2.14e-05 | \* \* \* | 1.58 | 0.998 | 1.09 | 100 | 100 |
| Bt.22736.1.S1\_at | 0.00015 | 5.98e-05 | 0.000725 | \* \* \* | 1.09 | 1 | 1.21 | 100 | 100 |
| Bt.19105.1.A1\_at | 0.00015 | 5.73e-05 | 8.79e-05 | \* \* \* | 1.16 | 0.879 | 0.927 | 100 | 100 |
| Bt.9233.3.S1\_at | 0.000151 | 0.000156 | 0.000348 | \* \* \* | -1.28 | -0.873 | -1.21 | 100 | 100 |
| Bt.25202.3.S1\_at | 0.000154 | 7.45e-06 | 6.02e-05 | \* \* \* | 1.34 | 1.43 | 1.92 | 100 | 100 |
| Bt.11409.1.A1\_a\_at | 0.000154 | 1.71e-07 | 8.71e-05 | \* \* \* | 0.827 | 1 | 1.29 | 100 | 100 |
| Bt.23606.1.S1\_at | 0.000154 | 8.01e-07 | 1.9e-06 | \* \* \* | 1.34 | 1.37 | 1.49 | 100 | 100 |
| Bt.3216.1.A1\_at | 0.00016 | 1.84e-06 | 2.92e-05 | \* \* \* | 1.22 | 1.23 | 1.37 | 100 | 100 |
| Bt.4903.1.S2\_at | 0.00016 | 6.6e-05 | 1.34e-05 | \* \* \* | 0.947 | 0.99 | 1.14 | 100 | 100 |
| Bt.2870.1.A1\_at | 0.000162 | 1.1e-05 | 0.000287 | \* \* \* | 1.34 | 1.15 | 1.3 | 100 | 100 |
| Bt.9629.2.S1\_at | 0.000162 | 2.14e-05 | 6.72e-06 | \* \* \* | 0.902 | 1.22 | 1.4 | 100 | 100 |
| Bt.404.1.S1\_at | 0.000162 | 2.97e-06 | 3.7e-06 | \* \* \* | -1.23 | -0.889 | -1.2 | 100 | 100 |
| Bt.24676.1.S1\_at | 0.000162 | 1.07e-05 | 0.000139 | \* \* \* | -1.48 | -1.07 | -1.75 | 100 | 100 |
| Bt.22526.1.S1\_at | 0.000163 | 0.000331 | 0.000354 | \* \* \* | -1.1 | -0.673 | -0.934 | 100 | 100 |
| Bt.20582.1.S1\_at | 0.000163 | 0.00131 | 4e-04 | \* \* \* | 1.29 | 0.994 | 1.35 | 100 | 100 |
| Bt.8730.1.S1\_at | 0.000164 | 1.79e-08 | 1.25e-09 | \* \* \* | 0.914 | 1.16 | 1.32 | 100 | 100 |
| Bt.27112.1.S1\_at | 0.000165 | 2.01e-05 | 7.55e-05 | \* \* \* | 1.57 | 1.17 | 1.49 | 100 | 100 |
| Bt.11182.1.S1\_at | 0.000167 | 0.000767 | 0.000286 | \* \* \* | 0.829 | 0.803 | 0.92 | 100 | 100 |
| Bt.26568.2.S1\_a\_at | 0.000168 | 2.74e-06 | 2.94e-07 | \* \* \* | -1.99 | -0.817 | -1.93 | 100 | 100 |
| Bt.2347.2.S1\_at | 0.000168 | 0.00109 | 0.000234 | \* \* \* | -0.921 | -0.712 | -0.923 | 100 | 100 |
| Bt.20242.1.S1\_at | 0.00017 | 2.02e-05 | 0.000328 | \* \* \* | 1.19 | 1.29 | 1.58 | 100 | 100 |
| Bt.16296.1.A1\_at | 0.00017 | 1.36e-05 | 0.000102 | \* \* \* | -1.67 | -1.21 | -1.61 | 100 | 100 |
| Bt.5510.1.S1\_at | 0.000177 | 5.83e-05 | 3.13e-05 | \* \* \* | -1.23 | -0.618 | -0.565 | 100 | 100 |
| Bt.21737.1.S1\_at | 0.000179 | 1.08e-07 | 9.62e-05 | \* \* \* | 1.21 | 1.11 | 1.42 | 100 | 100 |
| Bt.5965.1.S1\_at | 0.00018 | 6.81e-07 | 8.52e-06 | \* \* \* | 1.48 | 1.07 | 1.33 | 100 | 100 |
| Bt.12603.1.S1\_at | 0.00018 | 0.000314 | 0.000407 | \* \* \* | 1.23 | 0.819 | 0.835 | 100 | 100 |
| Bt.19928.1.S1\_at | 0.000181 | 1.97e-06 | 6.48e-06 | \* \* \* | 1.27 | 1.03 | 0.998 | 100 | 100 |
| Bt.4725.1.S1\_at | 0.000181 | 1.74e-08 | 1.17e-06 | \* \* \* | 1.07 | 1.29 | 1.45 | 100 | 100 |
| Bt.19826.1.A1\_at | 0.000183 | 2.78e-06 | 0.000718 | \* \* \* | -1.38 | -1.36 | -1.5 | 100 | 100 |
| Bt.2989.3.S1\_at | 0.000183 | 5.09e-05 | 0.000928 | \* \* \* | -1.39 | -1.08 | -1.37 | 100 | 100 |
| Bt.13765.1.S1\_at | 0.000183 | 5.61e-06 | 0.00017 | \* \* \* | -1.58 | -1.35 | -1.63 | 100 | 100 |
| Bt.16041.1.S1\_at | 0.000183 | 0.000305 | 0.000132 | \* \* \* | 1.08 | 0.928 | 1.24 | 100 | 100 |
| Bt.11662.1.A1\_at | 0.000183 | 3.25e-05 | 5.87e-05 | \* \* \* | 1.42 | 0.994 | 1.3 | 100 | 100 |
| Bt.26999.1.S1\_at | 0.000188 | 0.000322 | 0.000239 | \* \* \* | -1.08 | -0.615 | -0.737 | 100 | 100 |
| Bt.12379.1.S1\_at | 0.000188 | 0.000239 | 0.000945 | \* \* \* | 1.33 | 0.773 | 0.964 | 100 | 100 |
| Bt.20528.1.S1\_at | 0.000189 | 0.000245 | 7.49e-05 | \* \* \* | 1.21 | 0.806 | 0.881 | 100 | 100 |
| Bt.25052.1.S1\_at | 0.000192 | 0.000197 | 0.000467 | \* \* \* | 0.822 | 0.704 | 0.86 | 100 | 100 |
| Bt.8077.1.S1\_at | 0.000193 | 1.8e-10 | 5.61e-10 | \* \* \* | -2.59 | -2.09 | -2.69 | 100 | 100 |
| Bt.4263.1.S1\_at | 0.000193 | 1.7e-06 | 7.88e-08 | \* \* \* | 1.08 | 1.09 | 1.62 | 100 | 100 |
| Bt.13366.1.S1\_at | 0.000193 | 2.36e-05 | 0.000208 | \* \* \* | 1.22 | 1.18 | 1.7 | 100 | 100 |
| Bt.16078.1.A1\_s\_at | 0.000193 | 4.82e-06 | 2.58e-05 | \* \* \* | 1.1 | 0.975 | 1.1 | 100 | 100 |
| Bt.7069.1.S1\_at | 0.000194 | 0.000108 | 0.00122 | \* \* \* | 1.26 | 0.882 | 0.9 | 100 | 100 |
| Bt.23174.2.S1\_at | 0.000195 | 1.25e-06 | 2.28e-05 | \* \* \* | 1.07 | 0.879 | 1.1 | 100 | 100 |
| Bt.4867.1.A1\_at | 0.000195 | 7.09e-07 | 4.86e-05 | \* \* \* | 3.92 | 3.27 | 3.57 | 100 | 100 |
| Bt.727.2.S1\_at | 0.000195 | 0.00145 | 6.29e-05 | \* \* \* | -0.756 | -0.583 | -0.827 | 100 | 100 |
| Bt.7696.1.S1\_at | 0.000195 | 1.57e-07 | 3.04e-07 | \* \* \* | -1.66 | -1.19 | -1.84 | 100 | 100 |
| Bt.29655.1.S1\_a\_at | 0.000196 | 9.92e-06 | 3.99e-06 | \* \* \* | -0.878 | -0.829 | -1.18 | 100 | 100 |
| Bt.7079.1.S1\_at | 0.000196 | 7.7e-09 | 2.85e-07 | \* \* \* | -1.74 | -1.81 | -3.27 | 100 | 100 |
| Bt.14340.1.A1\_at | 0.000197 | 6.44e-07 | 3.18e-07 | \* \* \* | 1.11 | 1.13 | 1.42 | 100 | 100 |
| Bt.5468.1.S1\_at | 0.000201 | 1.27e-06 | 5.88e-05 | \* \* \* | -2.41 | -1.92 | -2.58 | 100 | 100 |
| Bt.20318.1.S1\_a\_at | 0.000202 | 0.000563 | 0.000204 | \* \* \* | -0.909 | -0.683 | -1.09 | 100 | 100 |
| Bt.20489.1.S1\_at | 0.000203 | 1.46e-05 | 8.15e-06 | \* \* \* | 1.22 | 0.838 | 0.994 | 100 | 100 |
| Bt.2359.1.A1\_at | 0.000204 | 1.6e-06 | 1.47e-06 | \* \* \* | 0.938 | 0.981 | 1.18 | 100 | 100 |
| Bt.13865.1.A1\_at | 0.000206 | 4.36e-06 | 7.14e-05 | \* \* \* | -1.37 | -1.13 | -1.5 | 100 | 100 |
| Bt.10722.1.S1\_at | 0.000207 | 7.3e-08 | 1.84e-06 | \* \* \* | 1.21 | 1.08 | 1.06 | 100 | 100 |
| Bt.29381.1.S1\_at | 0.000207 | 4.7e-07 | 3.31e-06 | \* \* \* | -1.55 | -1.38 | -1.81 | 100 | 100 |
| Bt.24210.1.S1\_at | 0.000208 | 5.07e-05 | 4.15e-05 | \* \* \* | -1.78 | -0.922 | -1.13 | 100 | 100 |
| Bt.22166.1.S1\_at | 0.000209 | 9.42e-06 | 5.24e-05 | \* \* \* | -1.06 | -1.16 | -1.83 | 100 | 100 |
| Bt.19746.1.S1\_at | 0.000209 | 0.000953 | 0.000108 | \* \* \* | 0.958 | 0.746 | 1.07 | 100 | 100 |
| Bt.11093.1.S1\_at | 0.000212 | 1.53e-07 | 1.96e-07 | \* \* \* | 1.27 | 1.11 | 1.81 | 100 | 100 |
| Bt.3940.1.S1\_at | 0.000212 | 3.64e-07 | 5.76e-09 | \* \* \* | 0.881 | 1.03 | 1.16 | 100 | 100 |
| Bt.21191.2.A1\_at | 0.000215 | 0.000123 | 0.00186 | \* \* \* | -2.26 | -1.69 | -2.36 | 100 | 100 |
| Bt.10999.1.A1\_at | 0.000216 | 6.49e-06 | 1.34e-05 | \* \* \* | 1.32 | 1.02 | 1.31 | 100 | 100 |
| Bt.2070.2.A1\_at | 0.000217 | 3.47e-05 | 0.000747 | \* \* \* | -1.35 | -1.08 | -1.29 | 100 | 100 |
| Bt.17778.1.A1\_at | 0.000218 | 1.06e-08 | 2.21e-07 | \* \* \* | -1.24 | -1.56 | -1.76 | 100 | 100 |
| Bt.22630.1.S1\_at | 0.000219 | 4.08e-06 | 0.000662 | \* \* \* | -1.23 | -1.16 | -1.62 | 100 | 100 |
| Bt.21036.2.A1\_at | 0.00022 | 4.36e-05 | 0.000158 | \* \* \* | 0.832 | 0.922 | 0.981 | 100 | 100 |
| Bt.24916.1.A1\_at | 0.000221 | 5.79e-05 | 4.29e-05 | \* \* \* | 1.22 | 0.957 | 1.12 | 100 | 100 |
| Bt.28623.1.S1\_at | 0.000226 | 6e-08 | 1.25e-06 | \* \* \* | 1.07 | 1.21 | 1.85 | 100 | 100 |
| Bt.4571.1.S1\_at | 0.000226 | 1.98e-06 | 2.13e-05 | \* \* \* | 1.28 | 1.17 | 1.75 | 100 | 100 |
| Bt.23237.1.S1\_at | 0.000227 | 6.85e-05 | 0.00011 | \* \* \* | -0.998 | -0.884 | -1.63 | 100 | 100 |
| Bt.9772.1.S1\_at | 0.000228 | 2.32e-05 | 1.05e-05 | \* \* \* | 1.43 | 1.21 | 1.5 | 100 | 100 |
| Bt.13118.1.S1\_at | 0.000229 | 2.48e-05 | 0.000334 | \* \* \* | 1.54 | 0.972 | 1 | 100 | 100 |
| Bt.9633.2.S1\_at | 0.000229 | 0.000236 | 0.000838 | \* \* \* | 1.19 | 1.2 | 1.26 | 100 | 100 |
| Bt.5527.1.S1\_at | 0.000229 | 1.16e-06 | 4.33e-06 | \* \* \* | -1.09 | -0.972 | -1.17 | 100 | 100 |
| Bt.11256.1.S1\_at | 0.00023 | 0.0012 | 6.44e-06 | \* \* \* | 0.771 | 0.54 | 0.636 | 100 | 100 |
| Bt.25087.1.A1\_at | 0.00023 | 5.43e-05 | 3.15e-05 | \* \* \* | -0.903 | -0.848 | -1.08 | 100 | 100 |
| Bt.20080.1.S1\_at | 0.000231 | 7.63e-05 | 9.36e-05 | \* \* \* | 1.68 | 1.27 | 1.42 | 100 | 100 |
| Bt.29.1.S1\_at | 0.000232 | 6.06e-05 | 0.000341 | \* \* \* | 1.32 | 0.992 | 1.76 | 100 | 100 |
| Bt.22852.1.S1\_at | 0.000232 | 1.63e-06 | 8.81e-05 | \* \* \* | 1.37 | 1.23 | 1.41 | 100 | 100 |
| Bt.10920.1.S1\_at | 0.000234 | 5.96e-06 | 6.33e-06 | \* \* \* | 1.65 | 1.26 | 1.6 | 100 | 100 |
| Bt.23225.1.S1\_at | 0.000234 | 2.53e-05 | 3.85e-05 | \* \* \* | -1.69 | -0.957 | -1.18 | 100 | 100 |
| Bt.21813.1.S1\_at | 0.000235 | 5.52e-07 | 0.000126 | \* \* \* | 1.71 | 1.63 | 2.34 | 100 | 100 |
| Bt.9958.1.S1\_at | 0.000235 | 1.45e-05 | 2.32e-05 | \* \* \* | 1.17 | 1.13 | 1.38 | 100 | 100 |
| Bt.70.1.S1\_at | 0.000237 | 1.01e-06 | 1.13e-05 | \* \* \* | -1.96 | -0.904 | -1.3 | 100 | 100 |
| Bt.3426.1.S1\_at | 0.000238 | 0.000196 | 9.2e-05 | \* \* \* | 1.04 | 0.742 | 0.788 | 100 | 100 |
| Bt.16194.1.A1\_at | 0.000238 | 7.95e-06 | 1.16e-05 | \* \* \* | 1.38 | 1.4 | 2.4 | 100 | 100 |
| Bt.4972.1.S1\_at | 0.000238 | 1.03e-07 | 1.42e-07 | \* \* \* | 0.917 | 1.25 | 1.42 | 100 | 100 |
| Bt.19621.1.A1\_at | 0.000244 | 3.86e-07 | 1.56e-06 | \* \* \* | 1.11 | 1.18 | 1.18 | 100 | 100 |
| Bt.20275.1.S1\_at | 0.000244 | 4.56e-05 | 8.15e-05 | \* \* \* | -1.15 | -0.81 | -0.939 | 100 | 100 |
| Bt.19100.1.A1\_at | 0.000245 | 1.85e-06 | 2.88e-06 | \* \* \* | 1.17 | 1.53 | 2.19 | 100 | 100 |
| Bt.18278.2.A1\_at | 0.000246 | 3e-08 | 1.9e-05 | \* \* \* | -2.33 | -1.7 | -2.23 | 100 | 100 |
| Bt.26805.1.S1\_at | 0.000246 | 8.56e-06 | 0.000117 | \* \* \* | -1.56 | -1.46 | -2.18 | 100 | 100 |
| Bt.25226.1.A1\_at | 0.000246 | 0.000352 | 4e-05 | \* \* \* | 1.24 | 1.16 | 1.88 | 100 | 100 |
| Bt.18986.1.S1\_at | 0.000248 | 2.44e-07 | 7.24e-08 | \* \* \* | 1.1 | 1.12 | 1.59 | 100 | 100 |
| Bt.9405.1.S1\_at | 0.000249 | 0.000156 | 9e-06 | \* \* \* | -0.788 | -0.505 | -0.776 | 100 | 100 |
| Bt.22163.1.S1\_at | 0.00025 | 1.32e-05 | 0.000522 | \* \* \* | -2.23 | -1.86 | -2.32 | 100 | 100 |
| Bt.13860.1.S1\_at | 0.000251 | 6.74e-06 | 4.24e-05 | \* \* \* | 1.18 | 1.09 | 1.21 | 100 | 100 |
| Bt.20613.2.A1\_at | 0.000252 | 2.38e-06 | 6.42e-06 | \* \* \* | 1.64 | 1.18 | 1.44 | 100 | 100 |
| Bt.16053.1.S1\_at | 0.000253 | 3.58e-07 | 2.34e-06 | \* \* \* | 1.64 | 1.65 | 2.01 | 100 | 100 |
| Bt.9983.1.S1\_at | 0.000255 | 2.41e-06 | 2.34e-08 | \* \* \* | -0.931 | -0.858 | -1.33 | 100 | 100 |
| Bt.19526.1.S1\_at | 0.000258 | 5.25e-07 | 1.1e-05 | \* \* \* | 1.23 | 0.979 | 1.15 | 100 | 100 |
| Bt.9998.1.S1\_at | 0.000262 | 1.07e-05 | 0.000214 | \* \* \* | 1.34 | 1.31 | 1.65 | 100 | 100 |
| Bt.7984.1.S1\_at | 0.000264 | 1.71e-08 | 4.78e-08 | \* \* \* | 1.33 | 1.35 | 1.59 | 100 | 100 |
| Bt.20402.2.A1\_at | 0.000265 | 2.83e-06 | 1.69e-05 | \* \* \* | 1.26 | 1.17 | 1.19 | 100 | 100 |
| Bt.23667.1.S1\_at | 0.000265 | 4.32e-06 | 1.19e-05 | \* \* \* | 1.36 | 1.45 | 1.86 | 100 | 100 |
| Bt.2494.2.S1\_a\_at | 0.000266 | 5.66e-05 | 5.56e-07 | \* \* \* | 1.03 | 1.05 | 1.34 | 100 | 100 |
| Bt.1217.1.A1\_a\_at | 0.000266 | 0.00135 | 0.000239 | \* \* \* | -0.96 | -0.601 | -0.868 | 100 | 100 |
| Bt.25016.1.S1\_at | 0.000269 | 2.48e-05 | 3.36e-05 | \* \* \* | 1.39 | 1.24 | 1.58 | 100 | 100 |
| Bt.23081.1.S1\_at | 0.00027 | 7.57e-06 | 2.99e-05 | \* \* \* | -2.17 | -1.5 | -1.96 | 100 | 100 |
| Bt.1739.2.S1\_at | 0.000272 | 4.15e-05 | 3.48e-06 | \* \* \* | 1.18 | 0.807 | 1.06 | 100 | 100 |
| Bt.17194.1.S1\_at | 0.000272 | 6.83e-05 | 7.98e-05 | \* \* \* | 1.31 | 1.04 | 2.52 | 100 | 100 |
| Bt.97.1.S1\_at | 0.000272 | 7.32e-06 | 0.000288 | \* \* \* | -2.69 | -2.81 | -3.68 | 100 | 100 |
| Bt.28489.1.S1\_at | 0.000272 | 0.00133 | 0.00126 | \* \* \* | 0.863 | 0.706 | 0.875 | 100 | 100 |
| Bt.12669.1.A1\_at | 0.000273 | 1.03e-05 | 2.08e-06 | \* \* \* | 1.29 | 0.905 | 1.41 | 100 | 100 |
| Bt.10880.1.S1\_at | 0.000274 | 9.85e-06 | 0.000152 | \* \* \* | -1.11 | -0.999 | -1.27 | 100 | 100 |
| Bt.8976.1.S1\_at | 0.000275 | 0.00026 | 0.000205 | \* \* \* | 1.28 | 1.09 | 1.42 | 100 | 100 |
| Bt.9244.1.A1\_at | 0.000276 | 6.95e-06 | 6.82e-05 | \* \* \* | 1.1 | 0.945 | 1.23 | 100 | 100 |
| Bt.2065.1.A1\_at | 0.000276 | 0.000293 | 5.8e-07 | \* \* \* | 0.859 | 0.537 | 0.798 | 100 | 100 |
| Bt.17985.1.S1\_at | 0.000279 | 1.54e-05 | 0.00017 | \* \* \* | 1.04 | 1.09 | 1.54 | 100 | 100 |
| Bt.12217.2.S1\_at | 0.00028 | 2.24e-05 | 0.000272 | \* \* \* | 2.06 | 1.51 | 1.86 | 100 | 100 |
| Bt.4476.1.S1\_at | 0.000281 | 1.69e-07 | 8.48e-07 | \* \* \* | -1.46 | -1.4 | -1.85 | 100 | 100 |
| Bt.10777.1.S1\_at | 0.000283 | 3.58e-05 | 1.04e-05 | \* \* \* | 1.39 | 1.09 | 1.7 | 100 | 100 |
| Bt.28058.1.S1\_at | 0.000283 | 8.5e-06 | 2.16e-06 | \* \* \* | 1.13 | 0.894 | 1.03 | 100 | 100 |
| Bt.19937.2.S1\_at | 0.000283 | 9.52e-08 | 6.99e-07 | \* \* \* | 0.972 | 1.06 | 1.36 | 100 | 100 |
| Bt.12728.3.S1\_at | 0.000284 | 4.64e-05 | 3.42e-05 | \* \* \* | 0.838 | 0.715 | 1.23 | 100 | 100 |
| Bt.6651.1.S1\_at | 0.000287 | 0.000153 | 0.000869 | \* \* \* | 1.35 | 1.3 | 1.44 | 100 | 100 |
| Bt.17989.1.S1\_at | 0.000287 | 0.000653 | 8.66e-05 | \* \* \* | 1.21 | 0.598 | 0.967 | 100 | 100 |
| Bt.20515.1.S1\_at | 0.000288 | 6.72e-07 | 5.65e-08 | \* \* \* | 1.26 | 1.32 | 1.64 | 100 | 100 |
| Bt.23499.1.S1\_at | 0.000289 | 7.18e-06 | 2.54e-05 | \* \* \* | -1.08 | -1.02 | -1.74 | 100 | 100 |
| Bt.25980.1.A1\_at | 0.00029 | 1.72e-05 | 0.000136 | \* \* \* | -1.56 | -1.11 | -1.54 | 100 | 100 |
| Bt.5021.1.S1\_at | 0.00029 | 1.86e-05 | 5.83e-05 | \* \* \* | 1.02 | 1.13 | 1.39 | 100 | 100 |
| Bt.26622.1.S1\_a\_at | 0.000292 | 4.38e-05 | 3.52e-05 | \* \* \* | -0.902 | -0.774 | -1.24 | 100 | 100 |
| Bt.15854.1.A1\_at | 0.000292 | 0.000541 | 0.000315 | \* \* \* | 1.34 | 1.26 | 1.38 | 100 | 100 |
| Bt.9154.1.S1\_at | 0.000294 | 5.93e-06 | 1.79e-06 | \* \* \* | 1.01 | 1.09 | 1.33 | 100 | 100 |
| Bt.21792.2.S1\_a\_at | 0.000294 | 3.16e-06 | 6.75e-05 | \* \* \* | -1.01 | -0.985 | -1.41 | 100 | 100 |
| Bt.5603.2.S1\_at | 0.000294 | 0.000105 | 6.65e-05 | \* \* \* | 1.22 | 0.812 | 1.13 | 100 | 100 |
| Bt.401.2.S1\_at | 0.000295 | 5.23e-06 | 1.3e-05 | \* \* \* | -1.13 | -1.01 | -1.4 | 100 | 100 |
| Bt.4580.1.A1\_at | 0.000295 | 0.00028 | 4.7e-05 | \* \* \* | 0.746 | 0.7 | 0.861 | 100 | 100 |
| Bt.12267.1.S1\_at | 0.000297 | 8.28e-05 | 6.31e-05 | \* \* \* | 1.14 | 0.784 | 0.991 | 100 | 100 |
| Bt.19937.1.S1\_at | 0.000297 | 7.14e-06 | 7.83e-06 | \* \* \* | 0.891 | 0.925 | 1.07 | 100 | 100 |
| Bt.25069.1.A1\_at | 0.000302 | 0.000183 | 0.000421 | \* \* \* | -0.932 | -0.551 | -0.613 | 100 | 100 |
| Bt.3605.1.S1\_at | 0.000303 | 2.82e-08 | 2.45e-05 | \* \* \* | -2.24 | -1.74 | -2.36 | 100 | 100 |
| Bt.23315.1.S1\_a\_at | 0.000303 | 3.52e-05 | 0.0018 | \* \* \* | 1.11 | 0.637 | 0.972 | 100 | 100 |
| Bt.1606.1.S1\_at | 0.000305 | 1.9e-05 | 0.000806 | \* \* \* | 1.51 | 1.27 | 1.49 | 100 | 100 |
| Bt.2391.1.S1\_at | 0.000307 | 0.000916 | 0.000516 | \* \* \* | 1.13 | 0.622 | 0.623 | 100 | 100 |
| Bt.22047.1.S1\_at | 0.000309 | 8.81e-07 | 6.45e-07 | \* \* \* | -1.2 | -0.999 | -1.33 | 100 | 100 |
| Bt.15698.1.S1\_at | 0.000311 | 1.09e-05 | 9.06e-05 | \* \* \* | -1.41 | -1.09 | -1.31 | 100 | 100 |
| Bt.10135.1.A1\_at | 0.000312 | 0.000399 | 0.000696 | \* \* \* | 1.22 | 1.12 | 1.16 | 100 | 100 |
| Bt.3272.1.A1\_at | 0.000312 | 2.1e-07 | 4.39e-06 | \* \* \* | -0.952 | -1.43 | -1.83 | 100 | 100 |
| Bt.4320.1.S1\_at | 0.000312 | 1.13e-06 | 1.51e-05 | \* \* \* | 1.19 | 1.36 | 1.59 | 100 | 100 |
| Bt.20323.1.S1\_at | 0.000313 | 0.000102 | 0.000664 | \* \* \* | 1.17 | 1.1 | 1.4 | 100 | 100 |
| Bt.20591.2.A1\_at | 0.000314 | 3.94e-08 | 9.46e-08 | \* \* \* | 1.92 | 1.64 | 1.87 | 100 | 100 |
| Bt.7728.1.A1\_at | 0.000318 | 7.55e-06 | 2.4e-06 | \* \* \* | 1.34 | 1.09 | 1.23 | 100 | 100 |
| Bt.20282.1.S1\_at | 0.000321 | 6.12e-07 | 6.69e-07 | \* \* \* | -1.43 | -1.12 | -1.48 | 100 | 100 |
| Bt.26675.1.S1\_at | 0.000321 | 6.84e-05 | 0.000284 | \* \* \* | -1.05 | -0.881 | -1.08 | 100 | 100 |
| Bt.26919.1.S1\_at | 0.000322 | 4.48e-07 | 9.01e-05 | \* \* \* | 1.71 | 1.6 | 2.29 | 100 | 100 |
| Bt.2116.1.S1\_at | 0.000324 | 4.35e-05 | 0.000276 | \* \* \* | -1.02 | -0.642 | -0.78 | 100 | 100 |
| Bt.28150.1.S1\_at | 0.000325 | 1.53e-06 | 1.41e-05 | \* \* \* | -1.72 | -1.5 | -2.3 | 100 | 100 |
| Bt.1742.1.S1\_at | 0.000327 | 2.85e-05 | 2.09e-05 | \* \* \* | 1.15 | 1.26 | 1.48 | 100 | 100 |
| Bt.27846.1.S1\_at | 0.000327 | 3.21e-06 | 3.12e-05 | \* \* \* | 1.44 | 1.37 | 1.64 | 100 | 100 |
| Bt.10723.1.S1\_at | 0.000328 | 0.000607 | 0.000393 | \* \* \* | 0.959 | 0.699 | 0.777 | 100 | 100 |
| Bt.20278.1.S1\_at | 0.000331 | 6.94e-06 | 0.000208 | \* \* \* | 1.19 | 0.78 | 1.48 | 100 | 100 |
| Bt.4555.1.S1\_at | 0.000332 | 3.92e-05 | 1.35e-05 | \* \* \* | -1.29 | -1.05 | -1.38 | 100 | 100 |
| Bt.4948.1.S1\_at | 0.000332 | 4.67e-05 | 6.91e-06 | \* \* \* | 1.1 | 0.716 | 1.16 | 100 | 100 |
| Bt.3990.1.S1\_at | 0.000332 | 0.000186 | 0.00022 | \* \* \* | 0.822 | 0.792 | 0.901 | 100 | 100 |
| Bt.15836.2.S1\_at | 0.000335 | 5.53e-05 | 0.000552 | \* \* \* | -3.81 | -3.33 | -3.98 | 100 | 100 |
| Bt.21176.1.S1\_at | 0.000336 | 2.34e-06 | 1.37e-08 | \* \* \* | 0.981 | 1 | 1.19 | 100 | 100 |
| Bt.9362.1.S1\_at | 0.000337 | 2.23e-07 | 5.55e-08 | \* \* \* | -1.17 | -1.1 | -1.41 | 100 | 100 |
| Bt.12204.1.S1\_at | 0.000341 | 8.42e-06 | 6.53e-06 | \* \* \* | 1.14 | 0.972 | 1.27 | 100 | 100 |
| Bt.6007.1.S1\_at | 0.000342 | 0.000847 | 0.000108 | \* \* \* | 0.809 | 0.607 | 1.4 | 100 | 100 |
| Bt.11262.1.S1\_at | 0.000343 | 1.1e-07 | 6.2e-06 | \* \* \* | 1.21 | 1.49 | 2.11 | 100 | 100 |
| Bt.10922.1.A1\_at | 0.000344 | 3.67e-05 | 0.000451 | \* \* \* | 1.28 | 1.42 | 1.51 | 100 | 100 |
| Bt.28754.1.S1\_at | 0.000344 | 1.89e-06 | 3.19e-06 | \* \* \* | -1.19 | -0.926 | -1.46 | 100 | 100 |
| Bt.3236.2.S1\_at | 0.000345 | 3.5e-06 | 9.8e-08 | \* \* \* | 1.2 | 1.08 | 1.61 | 100 | 100 |
| Bt.12514.1.S1\_at | 0.000349 | 0.000742 | 8.87e-06 | \* \* \* | -0.946 | -0.642 | -0.857 | 100 | 100 |
| Bt.26756.1.S1\_at | 0.000349 | 0.000304 | 3.4e-05 | \* \* \* | 0.769 | 0.514 | 0.567 | 100 | 100 |
| Bt.2070.1.A1\_at | 0.000351 | 4.63e-06 | 7.85e-07 | \* \* \* | -1.54 | -1.13 | -1.42 | 100 | 100 |
| Bt.6171.1.A1\_at | 0.000354 | 0.00135 | 0.000561 | \* \* \* | -0.927 | -0.763 | -0.996 | 100 | 100 |
| Bt.4689.1.S2\_at | 0.000359 | 0.00172 | 0.000124 | \* \* \* | -0.863 | -0.47 | -0.68 | 100 | 100 |
| Bt.9559.2.S1\_at | 0.000364 | 1.95e-06 | 0.000135 | \* \* \* | 1.64 | 1.67 | 2.04 | 100 | 100 |
| Bt.2510.1.A1\_at | 0.000364 | 0.000392 | 0.000114 | \* \* \* | 1.08 | 0.882 | 1.14 | 100 | 100 |
| Bt.4985.2.S1\_a\_at | 0.000369 | 0.00011 | 9.22e-05 | \* \* \* | -0.998 | -0.722 | -1.1 | 100 | 100 |
| Bt.25689.1.S1\_at | 0.000372 | 6.28e-05 | 4.36e-05 | \* \* \* | -1.15 | -0.731 | -0.936 | 100 | 100 |
| Bt.4449.1.S1\_at | 0.000381 | 3.28e-05 | 0.000141 | \* \* \* | 0.918 | 0.946 | 1.22 | 100 | 100 |
| Bt.8807.1.S1\_at | 0.000383 | 0.000145 | 0.000508 | \* \* \* | 1.01 | 0.744 | 0.999 | 100 | 100 |
| Bt.18381.1.S1\_at | 0.000384 | 1.44e-06 | 1.45e-07 | \* \* \* | 0.997 | 1.19 | 1.32 | 100 | 100 |
| Bt.9623.1.A1\_at | 0.000385 | 1.37e-05 | 0.000226 | \* \* \* | -1.48 | -1.22 | -1.67 | 100 | 100 |
| Bt.18755.1.A1\_at | 0.000389 | 1.52e-05 | 1.05e-05 | \* \* \* | -0.785 | -0.908 | -1.26 | 100 | 100 |
| Bt.20139.1.S1\_at | 0.00039 | 0.000148 | 6.07e-05 | \* \* \* | 1.1 | 0.852 | 0.926 | 100 | 100 |
| Bt.23572.1.S1\_at | 0.00039 | 0.00126 | 0.00096 | \* \* \* | 0.884 | 0.688 | 1.12 | 100 | 100 |
| Bt.20977.1.S1\_at | 0.00039 | 1.14e-05 | 8.11e-05 | \* \* \* | 1.19 | 1.02 | 1.12 | 100 | 100 |
| Bt.4797.1.S1\_at | 0.000392 | 5.97e-05 | 0.000403 | \* \* \* | -1.11 | -0.806 | -1.23 | 100 | 100 |
| Bt.10813.1.S1\_at | 0.000393 | 8.75e-06 | 5.47e-05 | \* \* \* | 1.4 | 1.27 | 1.81 | 100 | 100 |
| Bt.14473.1.S1\_at | 0.000394 | 0.000776 | 3.86e-05 | \* \* \* | -0.865 | -0.548 | -1.07 | 100 | 100 |
| Bt.20744.1.A1\_at | 0.000402 | 0.000764 | 0.000338 | \* \* \* | 1.3 | 0.821 | 1.1 | 100 | 100 |
| Bt.22034.1.S1\_at | 0.000404 | 1.64e-05 | 1e-04 | \* \* \* | 1.87 | 1.19 | 1.67 | 100 | 100 |
| Bt.22187.1.S1\_at | 0.000405 | 0.000124 | 0.00168 | \* \* \* | 1.18 | 0.905 | 0.9 | 100 | 100 |
| Bt.13722.2.A1\_at | 0.000406 | 0.00048 | 0.00073 | \* \* \* | 1.11 | 0.639 | 0.694 | 100 | 100 |
| Bt.13410.1.S1\_at | 0.000406 | 0.000198 | 9.99e-05 | \* \* \* | -0.781 | -0.72 | -0.932 | 100 | 100 |
| Bt.11138.1.S1\_at | 0.000408 | 4.67e-05 | 9.11e-05 | \* \* \* | -0.932 | -0.755 | -0.93 | 100 | 100 |
| Bt.9949.2.S1\_at | 0.000413 | 0.000578 | 0.000553 | \* \* \* | -1.23 | -0.778 | -1.23 | 100 | 100 |
| Bt.16412.1.S1\_a\_at | 0.000417 | 1.78e-05 | 6.02e-05 | \* \* \* | 1.55 | 0.778 | 1.15 | 100 | 100 |
| Bt.9246.1.A1\_at | 0.000423 | 0.00115 | 0.000179 | \* \* \* | 1.34 | 1.01 | 1.11 | 100 | 100 |
| Bt.21196.1.S1\_at | 0.000424 | 6.18e-05 | 9.97e-05 | \* \* \* | 0.956 | 1.04 | 1.14 | 100 | 100 |
| Bt.18623.1.A1\_at | 0.000424 | 0.000683 | 0.000259 | \* \* \* | -1.14 | -0.918 | -1.64 | 100 | 100 |
| Bt.18113.2.S1\_at | 0.000426 | 1.48e-05 | 9.37e-07 | \* \* \* | 0.665 | 0.634 | 0.694 | 100 | 100 |
| Bt.7696.2.S1\_a\_at | 0.000426 | 1.71e-06 | 1.79e-07 | \* \* \* | -1.23 | -1.25 | -2.14 | 100 | 100 |
| Bt.7039.1.S1\_a\_at | 0.000427 | 4.28e-05 | 9.1e-06 | \* \* \* | -0.934 | -0.78 | -0.998 | 100 | 100 |
| Bt.4985.1.S1\_at | 0.00043 | 2.78e-06 | 2.13e-06 | \* \* \* | -0.857 | -0.894 | -1.04 | 100 | 100 |
| Bt.5035.1.S1\_at | 0.000431 | 9e-05 | 0.000306 | \* \* \* | -0.951 | -0.732 | -1.06 | 100 | 100 |
| Bt.7342.1.S1\_at | 0.000436 | 8.65e-06 | 2.95e-05 | \* \* \* | 1.06 | 0.982 | 1.22 | 100 | 100 |
| Bt.11014.1.S1\_at | 0.000436 | 3.72e-05 | 0.000864 | \* \* \* | -1.35 | -1.01 | -1.81 | 100 | 100 |
| Bt.14176.1.S1\_at | 0.000438 | 9.23e-06 | 0.000163 | \* \* \* | 1.1 | 1.1 | 1.39 | 100 | 100 |
| Bt.2625.1.S1\_at | 0.00044 | 1.52e-06 | 7.02e-06 | \* \* \* | 0.895 | 0.975 | 1.08 | 100 | 100 |
| Bt.10600.1.S1\_at | 0.00044 | 7.94e-08 | 0.000132 | \* \* \* | 1.11 | 1.25 | 1.47 | 100 | 100 |
| Bt.6964.1.S1\_at | 0.000441 | 2.69e-06 | 5.09e-06 | \* \* \* | -1.07 | -0.946 | -1.39 | 100 | 100 |
| Bt.4834.1.S1\_at | 0.000441 | 2.21e-05 | 2.29e-05 | \* \* \* | 1.19 | 1.05 | 1.25 | 100 | 100 |
| Bt.23331.1.S1\_at | 0.000445 | 1.59e-05 | 9.04e-06 | \* \* \* | 0.899 | 1.07 | 1.18 | 100 | 100 |
| Bt.9055.2.S1\_a\_at | 0.000446 | 6.91e-07 | 8.44e-06 | \* \* \* | -1.34 | -1.21 | -1.61 | 100 | 100 |
| Bt.4384.1.S1\_at | 0.000447 | 6.4e-05 | 0.000743 | \* \* \* | 0.981 | 0.76 | 0.837 | 100 | 100 |
| Bt.20800.1.S1\_at | 0.00045 | 0.000237 | 0.000203 | \* \* \* | 0.932 | 0.867 | 1.13 | 100 | 100 |
| Bt.22054.1.S1\_at | 0.000453 | 0.000263 | 0.000338 | \* \* \* | -1.5 | -0.713 | -1.14 | 100 | 100 |
| Bt.5723.1.S1\_at | 0.000455 | 1.43e-05 | 8.63e-06 | \* \* \* | -1.14 | -1.01 | -1.41 | 100 | 100 |
| Bt.10354.1.S1\_at | 0.000456 | 3.25e-06 | 4.37e-05 | \* \* \* | 1.27 | 0.943 | 1.1 | 100 | 100 |
| Bt.17012.1.A1\_s\_at | 0.000457 | 5.25e-05 | 0.000369 | \* \* \* | -1.18 | -0.918 | -1.25 | 100 | 100 |
| Bt.8673.1.S1\_a\_at | 0.000457 | 9.96e-07 | 1.45e-05 | \* \* \* | 1.16 | 1.33 | 1.79 | 100 | 100 |
| Bt.7183.1.S1\_at | 0.000459 | 2.87e-06 | 0.000122 | \* \* \* | 1.04 | 0.952 | 1.04 | 100 | 100 |
| Bt.27952.1.S1\_at | 0.000462 | 6.85e-05 | 6.24e-05 | \* \* \* | -0.915 | -1 | -1.36 | 100 | 100 |
| Bt.21239.1.S1\_at | 0.000471 | 1.71e-08 | 4.96e-09 | \* \* \* | -0.965 | -1.31 | -1.79 | 100 | 100 |
| Bt.28684.1.S1\_at | 0.000473 | 0.000401 | 0.00126 | \* \* \* | -1.08 | -0.647 | -0.92 | 100 | 100 |
| Bt.3975.1.S1\_at | 0.000473 | 0.000573 | 0.000407 | \* \* \* | -0.758 | -0.516 | -0.755 | 100 | 100 |
| Bt.22459.1.S1\_at | 0.000478 | 1.99e-06 | 2.39e-06 | \* \* \* | 1.26 | 1.05 | 1.94 | 100 | 100 |
| Bt.20852.1.A1\_at | 0.000479 | 0.000184 | 0.000303 | \* \* \* | 1.15 | 0.92 | 1.31 | 100 | 100 |
| Bt.18079.1.A1\_at | 0.00048 | 9.74e-06 | 0.000668 | \* \* \* | -1.49 | -1.04 | -0.714 | 100 | 100 |
| Bt.28480.1.S1\_at | 0.000486 | 2.94e-06 | 8.27e-07 | \* \* \* | -1.23 | -0.943 | -1.1 | 100 | 100 |
| Bt.8674.1.A1\_at | 0.000487 | 1.69e-06 | 7.59e-07 | \* \* \* | 1.02 | 1.06 | 1.23 | 100 | 100 |
| Bt.28105.1.S1\_at | 0.000489 | 0.000172 | 1.34e-05 | \* \* \* | 0.779 | 0.759 | 0.962 | 100 | 100 |
| Bt.11023.1.S1\_at | 0.000497 | 7.59e-07 | 3.46e-05 | \* \* \* | 0.801 | 0.875 | 1.27 | 100 | 100 |
| Bt.20326.1.S1\_at | 0.000499 | 9.62e-06 | 9.96e-05 | \* \* \* | 0.912 | 0.974 | 1.19 | 100 | 100 |
| Bt.24253.1.S1\_at | 5e-04 | 0.000401 | 3.15e-05 | \* \* \* | 0.784 | 0.832 | 1.03 | 100 | 100 |
| Bt.23509.2.A1\_at | 0.000503 | 3.72e-06 | 1.49e-06 | \* \* \* | 0.818 | 0.953 | 1.04 | 100 | 100 |
| Bt.7633.1.S1\_at | 0.000503 | 2.52e-06 | 0.000114 | \* \* \* | -1.12 | -1.21 | -1.53 | 100 | 100 |
| Bt.7421.1.S1\_at | 0.000504 | 0.000694 | 0.00135 | \* \* \* | -1.28 | -0.983 | -1.31 | 100 | 100 |
| Bt.9486.1.S1\_at | 0.000509 | 4.24e-06 | 9.27e-06 | \* \* \* | 1.13 | 1.04 | 1.52 | 100 | 100 |
| Bt.6701.1.S1\_at | 0.000515 | 0.000218 | 1.16e-05 | \* \* \* | 1.16 | 0.691 | 1.16 | 100 | 100 |
| Bt.7373.2.S1\_a\_at | 0.000515 | 4.09e-05 | 2.29e-06 | \* \* \* | -0.81 | -0.626 | -0.841 | 100 | 100 |
| Bt.4768.1.S2\_at | 0.000515 | 1.4e-05 | 3.35e-05 | \* \* \* | 1.22 | 0.776 | 1.14 | 100 | 100 |
| Bt.4503.1.S2\_at | 0.000519 | 2.18e-05 | 8.53e-06 | \* \* \* | -1.08 | -0.908 | -1.1 | 100 | 100 |
| Bt.11704.1.S1\_at | 0.00052 | 5.38e-07 | 8.15e-05 | \* \* \* | 1.52 | 1.51 | 1.65 | 100 | 100 |
| Bt.24624.2.S1\_at | 0.00052 | 0.000822 | 0.000261 | \* \* \* | -1.15 | -0.798 | -1.25 | 100 | 100 |
| Bt.22071.1.S1\_at | 0.000522 | 1.25e-05 | 1.11e-05 | \* \* \* | -1.01 | -0.838 | -1.18 | 100 | 100 |
| Bt.5465.2.A1\_a\_at | 0.000523 | 0.00165 | 0.000886 | \* \* \* | -1.02 | -0.723 | -0.957 | 100 | 100 |
| Bt.27123.1.A1\_at | 0.000524 | 0.000113 | 0.00109 | \* \* \* | 0.808 | 0.817 | 1 | 100 | 100 |
| Bt.9012.1.S1\_at | 0.000525 | 0.000143 | 0.000207 | \* \* \* | 1.26 | 0.796 | 1.08 | 100 | 100 |
| Bt.1418.2.S1\_at | 0.000527 | 4.41e-06 | 6.3e-06 | \* \* \* | -1.1 | -0.977 | -1.45 | 100 | 100 |
| Bt.25637.1.A1\_at | 0.000529 | 1.48e-05 | 4.78e-06 | \* \* \* | -1.41 | -1.46 | -2.17 | 100 | 100 |
| Bt.14040.1.S1\_at | 0.000531 | 5.01e-05 | 0.000267 | \* \* \* | -1.48 | -1.1 | -1.31 | 100 | 100 |
| Bt.11717.1.S1\_s\_at | 0.000535 | 0.000427 | 1.1e-05 | \* \* \* | -0.713 | -0.581 | -0.857 | 100 | 100 |
| Bt.7430.1.S1\_at | 0.000535 | 0.000154 | 4.11e-05 | \* \* \* | 0.706 | 0.721 | 0.757 | 100 | 100 |
| Bt.24945.1.S1\_at | 0.000538 | 0.000202 | 1.17e-05 | \* \* \* | -0.735 | -0.631 | -0.829 | 100 | 100 |
| Bt.25479.1.S1\_at | 0.000539 | 0.000166 | 0.000126 | \* \* \* | 0.775 | 0.56 | 1.44 | 100 | 100 |
| Bt.9337.1.S1\_at | 0.00054 | 5.1e-05 | 0.000148 | \* \* \* | 1.07 | 1.04 | 1.21 | 100 | 100 |
| Bt.7397.1.S1\_at | 0.000541 | 3.82e-06 | 0.000369 | \* \* \* | 1.48 | 1.07 | 1.21 | 100 | 100 |
| Bt.4702.1.S1\_at | 0.000542 | 3.48e-05 | 1.34e-05 | \* \* \* | 1.1 | 0.846 | 1.12 | 100 | 100 |
| Bt.12466.1.A1\_at | 0.000543 | 1.76e-06 | 0.00049 | \* \* \* | 1.05 | 0.999 | 0.85 | 100 | 100 |
| Bt.9114.1.A1\_at | 0.000543 | 0.00107 | 0.000705 | \* \* \* | -0.865 | -0.662 | -0.812 | 100 | 100 |
| Bt.5089.1.S1\_at | 0.000543 | 4.17e-08 | 8.37e-07 | \* \* \* | 0.88 | 1.29 | 1.41 | 100 | 100 |
| Bt.10.2.S1\_a\_at | 0.000545 | 5.65e-07 | 1.7e-08 | \* \* \* | -0.926 | -0.88 | -1.26 | 100 | 100 |
| Bt.26550.1.S1\_at | 0.000551 | 3.59e-06 | 3.11e-05 | \* \* \* | -1.22 | -1.05 | -1.77 | 100 | 100 |
| Bt.20780.2.S1\_a\_at | 0.000559 | 0.000885 | 0.000449 | \* \* \* | -1.1 | -0.93 | -1.2 | 100 | 100 |
| Bt.2052.1.S1\_a\_at | 0.000559 | 0.000304 | 0.000131 | \* \* \* | 1.4 | 0.696 | 0.701 | 100 | 100 |
| Bt.19061.1.A1\_at | 0.000561 | 0.000104 | 0.000392 | \* \* \* | -0.958 | -0.684 | -0.904 | 100 | 100 |
| Bt.7257.1.S1\_at | 0.000562 | 1.34e-05 | 0.000119 | \* \* \* | -1.01 | -0.81 | -1.1 | 100 | 100 |
| Bt.10947.1.S1\_at | 0.000564 | 0.000114 | 9.28e-05 | \* \* \* | 0.733 | 0.704 | 0.86 | 100 | 100 |
| Bt.997.2.S1\_a\_at | 0.000564 | 3.5e-06 | 2.39e-05 | \* \* \* | -1.13 | -1.19 | -1.86 | 100 | 100 |
| Bt.21119.1.A1\_at | 0.000567 | 0.00128 | 0.00129 | \* \* \* | 1.22 | 0.681 | 1.1 | 100 | 100 |
| Bt.16945.1.S1\_at | 0.000569 | 2.33e-05 | 1.84e-05 | \* \* \* | -0.732 | -0.685 | -0.999 | 100 | 100 |
| Bt.21491.1.S1\_at | 0.000574 | 5.69e-06 | 1.36e-06 | \* \* \* | 0.837 | 0.892 | 1.09 | 100 | 100 |
| Bt.28212.1.A1\_at | 0.00058 | 0.000522 | 0.00125 | \* \* \* | 0.988 | 0.73 | 1.01 | 100 | 100 |
| Bt.7184.1.S1\_at | 0.000582 | 0.000729 | 2.73e-05 | \* \* \* | -0.687 | -0.538 | -0.792 | 100 | 100 |
| Bt.22552.1.S2\_at | 0.000584 | 1.05e-05 | 2.89e-07 | \* \* \* | 0.809 | 0.746 | 0.814 | 100 | 100 |
| Bt.1262.1.S1\_at | 0.000585 | 5.44e-06 | 3.57e-05 | \* \* \* | -0.985 | -0.884 | -1.18 | 100 | 100 |
| Bt.935.1.A1\_at | 0.000585 | 1.93e-05 | 0.000442 | \* \* \* | -1.16 | -0.731 | -1.36 | 100 | 100 |
| Bt.28462.1.S1\_at | 0.000588 | 0.000118 | 0.000747 | \* \* \* | -1.15 | -0.912 | -1.18 | 100 | 100 |
| Bt.22553.1.A1\_at | 0.000588 | 0.000342 | 0.000486 | \* \* \* | 1.28 | 0.752 | 0.85 | 100 | 100 |
| Bt.23182.1.S1\_at | 0.000593 | 2.12e-07 | 5.97e-06 | \* \* \* | -1.12 | -1.36 | -1.72 | 100 | 100 |
| Bt.4355.1.S1\_at | 0.000595 | 3.89e-07 | 1.48e-05 | \* \* \* | -1.12 | -1.06 | -1.18 | 100 | 100 |
| Bt.15900.1.A1\_at | 0.000597 | 5.04e-05 | 0.000202 | \* \* \* | -1.07 | -0.736 | -1.25 | 100 | 100 |
| Bt.5445.2.A1\_at | 0.000599 | 0.000514 | 0.000473 | \* \* \* | 1.27 | 0.71 | 0.805 | 100 | 100 |
| Bt.28699.1.S1\_at | 0.000603 | 0.00146 | 0.000977 | \* \* \* | 0.767 | 0.781 | 1.01 | 100 | 100 |
| Bt.27118.1.A1\_at | 0.000605 | 1.14e-05 | 1.49e-05 | \* \* \* | 1.5 | 1.09 | 1.28 | 100 | 100 |
| Bt.16753.1.A1\_at | 0.000609 | 0.000165 | 5.61e-05 | \* \* \* | 0.826 | 0.826 | 1.04 | 100 | 100 |
| Bt.13834.1.S1\_at | 0.000615 | 7.32e-10 | 4.66e-08 | \* \* \* | -2.97 | -2.19 | -2.63 | 100 | 100 |
| Bt.21404.1.S1\_at | 0.000617 | 0.000351 | 0.00141 | \* \* \* | 0.881 | 0.616 | 0.822 | 100 | 100 |
| Bt.28071.1.S1\_at | 0.000618 | 5.97e-07 | 4.35e-08 | \* \* \* | -0.759 | -0.933 | -1.37 | 100 | 100 |
| Bt.13887.1.S1\_at | 0.00062 | 1.91e-05 | 0.000187 | \* \* \* | -1.3 | -1.02 | -1.39 | 100 | 100 |
| Bt.2001.1.S1\_at | 0.000621 | 4.48e-06 | 2.05e-06 | \* \* \* | -1.2 | -0.824 | -1.27 | 100 | 100 |
| Bt.27027.1.S1\_a\_at | 0.000622 | 5.47e-06 | 4.11e-06 | \* \* \* | -1.24 | -1.05 | -1.26 | 100 | 100 |
| Bt.23649.1.A1\_at | 0.000623 | 1.07e-05 | 0.00048 | \* \* \* | 0.806 | 0.895 | 0.917 | 100 | 100 |
| Bt.8961.1.S1\_at | 0.000626 | 8.22e-05 | 9.04e-06 | \* \* \* | 1.12 | 0.919 | 1.15 | 100 | 100 |
| Bt.2378.1.S1\_at | 0.000628 | 1.2e-05 | 1.28e-06 | \* \* \* | 1.11 | 0.787 | 1.13 | 100 | 100 |
| Bt.22633.1.S1\_at | 0.00063 | 6.87e-06 | 2.34e-05 | \* \* \* | 0.851 | 0.925 | 1.15 | 100 | 100 |
| Bt.1719.1.S1\_at | 0.00063 | 1.36e-05 | 1.83e-05 | \* \* \* | -0.964 | -0.841 | -1.32 | 100 | 100 |
| Bt.25339.1.S1\_at | 0.000636 | 0.000919 | 0.000216 | \* \* \* | 1.04 | 0.7 | 0.617 | 100 | 100 |
| Bt.3817.1.A1\_at | 0.000638 | 0.000113 | 0.000141 | \* \* \* | 0.989 | 0.629 | 1.03 | 100 | 100 |
| Bt.13548.1.A1\_at | 0.000642 | 0.000114 | 1.7e-06 | \* \* \* | 1.1 | 0.752 | 0.812 | 100 | 100 |
| Bt.20463.1.S1\_at | 0.000644 | 7.72e-05 | 0.000139 | \* \* \* | 0.984 | 1.18 | 1.32 | 100 | 100 |
| Bt.22647.3.A1\_at | 0.000646 | 8.67e-05 | 0.000909 | \* \* \* | 1.31 | 1.21 | 2.03 | 100 | 100 |
| Bt.29874.1.S1\_at | 0.000647 | 0.000226 | 0.000109 | \* \* \* | 0.952 | 0.874 | 1.08 | 100 | 100 |
| Bt.21372.1.S1\_at | 0.000648 | 0.000284 | 0.000398 | \* \* \* | 1.07 | 1.02 | 1.24 | 100 | 100 |
| Bt.22466.1.A1\_at | 0.000649 | 2.04e-07 | 7.2e-07 | \* \* \* | 0.918 | 1.07 | 1.48 | 100 | 100 |
| Bt.3789.1.S1\_at | 0.000654 | 3.66e-05 | 2.06e-05 | \* \* \* | 0.757 | 0.785 | 1.05 | 100 | 100 |
| Bt.28123.1.S1\_at | 0.000657 | 0.00054 | 7.97e-05 | \* \* \* | 0.824 | 0.884 | 1.43 | 100 | 100 |
| Bt.24307.1.A1\_at | 0.00066 | 4.95e-05 | 2.1e-05 | \* \* \* | 1.75 | 0.988 | 1.3 | 100 | 100 |
| Bt.3193.1.A1\_at | 0.000663 | 0.000146 | 0.000289 | \* \* \* | -0.856 | -0.765 | -0.893 | 100 | 100 |
| Bt.7007.1.S1\_at | 0.000663 | 4.54e-07 | 4.04e-06 | \* \* \* | -1.32 | -1.02 | -1.44 | 100 | 100 |
| Bt.17608.1.A1\_at | 0.000664 | 0.000538 | 1.53e-05 | \* \* \* | 0.86 | 0.628 | 0.553 | 100 | 100 |
| Bt.25657.1.S1\_at | 0.000665 | 1.65e-06 | 3.11e-05 | \* \* \* | 2.71 | 1.76 | 1.94 | 100 | 100 |
| Bt.5682.1.S1\_at | 0.000666 | 4.57e-06 | 9.49e-05 | \* \* \* | 1.44 | 1.05 | 1.12 | 100 | 100 |
| Bt.24023.1.A1\_at | 0.000677 | 2.77e-05 | 3.29e-05 | \* \* \* | 1.33 | 0.875 | 1.33 | 100 | 100 |
| Bt.21215.1.A1\_at | 0.000681 | 0.00124 | 0.000725 | \* \* \* | 1.15 | 0.92 | 1.06 | 100 | 100 |
| Bt.10990.1.S1\_at | 0.000684 | 6.08e-06 | 2.31e-05 | \* \* \* | -1.56 | -1.02 | -1.19 | 100 | 100 |
| Bt.8736.2.S1\_at | 0.000686 | 0.00111 | 0.000489 | \* \* \* | 0.853 | 0.49 | 0.729 | 100 | 100 |
| Bt.9240.1.A1\_at | 0.000693 | 0.000372 | 1.21e-05 | \* \* \* | -0.819 | -0.607 | -0.858 | 100 | 100 |
| Bt.12088.1.S1\_at | 0.000697 | 0.000467 | 0.000379 | \* \* \* | 0.842 | 0.848 | 0.993 | 100 | 100 |
| Bt.9629.1.S1\_at | 7e-04 | 1.22e-07 | 1.4e-06 | \* \* \* | 0.797 | 1.09 | 1.15 | 100 | 100 |
| Bt.16525.1.A1\_at | 0.000702 | 6.6e-06 | 5.07e-06 | \* \* \* | 0.884 | 0.78 | 1.08 | 100 | 100 |
| Bt.13925.1.S1\_at | 0.000704 | 0.000267 | 7.34e-05 | \* \* \* | 0.698 | 0.668 | 0.986 | 100 | 100 |
| Bt.27095.1.S1\_at | 0.000706 | 4.39e-05 | 5.65e-05 | \* \* \* | 0.845 | 0.909 | 0.815 | 100 | 100 |
| Bt.16264.1.A1\_at | 0.000711 | 0.000125 | 5.81e-05 | \* \* \* | 1.42 | 0.648 | 0.939 | 100 | 100 |
| Bt.9197.1.S1\_at | 0.000726 | 0.000326 | 0.00152 | \* \* \* | 0.971 | 0.891 | 1.07 | 100 | 100 |
| Bt.21372.2.S1\_at | 0.000729 | 0.000312 | 7.89e-05 | \* \* \* | 1.41 | 1.02 | 1.4 | 100 | 100 |
| Bt.1286.1.S1\_a\_at | 0.000731 | 1.02e-06 | 8.56e-08 | \* \* \* | 1.33 | 1.15 | 1.4 | 100 | 100 |
| Bt.4390.1.S1\_at | 0.000732 | 7.8e-06 | 9.61e-06 | \* \* \* | 1.38 | 0.932 | 1.19 | 100 | 100 |
| Bt.3843.1.S1\_at | 0.000739 | 9.26e-07 | 1.6e-06 | \* \* \* | -1.03 | -1.15 | -1.24 | 100 | 100 |
| Bt.7962.1.S1\_at | 0.000741 | 6.77e-05 | 0.000503 | \* \* \* | 1.69 | 1.58 | 2.19 | 100 | 100 |
| Bt.13254.1.S1\_at | 0.000744 | 8e-05 | 0.000598 | \* \* \* | 1.34 | 1.25 | 1.32 | 100 | 100 |
| Bt.16782.2.S1\_at | 0.000752 | 1.29e-05 | 0.00013 | \* \* \* | -1.68 | -1.04 | -1.39 | 100 | 100 |
| Bt.21938.1.S1\_at | 0.000763 | 1.63e-05 | 4.3e-05 | \* \* \* | 1.09 | 1.25 | 1.56 | 100 | 100 |
| Bt.10251.1.S1\_at | 0.000765 | 2.08e-05 | 4.44e-05 | \* \* \* | 0.937 | 1.13 | 1.27 | 100 | 100 |
| Bt.22290.1.S1\_at | 0.000765 | 2.75e-05 | 0.000114 | \* \* \* | 1.03 | 0.888 | 0.964 | 100 | 100 |
| Bt.9301.1.A1\_at | 0.000765 | 1.41e-05 | 5.89e-05 | \* \* \* | 0.996 | 0.9 | 1.37 | 100 | 100 |
| Bt.27396.1.S1\_at | 0.000768 | 0.000235 | 2.9e-05 | \* \* \* | 1.06 | 0.769 | 1 | 100 | 100 |
| Bt.27129.1.S1\_at | 0.000774 | 0.00155 | 0.00151 | \* \* \* | 0.883 | 0.547 | 0.47 | 100 | 100 |
| Bt.28043.1.S1\_at | 0.000775 | 6.3e-05 | 0.000118 | \* \* \* | 1.32 | 0.918 | 1.46 | 100 | 100 |
| Bt.13065.1.S1\_at | 0.000778 | 4.44e-05 | 0.00172 | \* \* \* | 1.23 | 1.28 | 1.5 | 100 | 100 |
| Bt.10175.2.A1\_a\_at | 0.000782 | 4.81e-07 | 9.82e-07 | \* \* \* | 0.751 | 0.991 | 1.05 | 100 | 100 |
| Bt.2606.1.S1\_at | 0.000785 | 0.000127 | 2.38e-05 | \* \* \* | 1.27 | 1.11 | 1.65 | 100 | 100 |
| Bt.5434.1.S1\_at | 0.000789 | 0.000616 | 0.000676 | \* \* \* | -0.788 | -0.599 | -0.733 | 100 | 100 |
| Bt.22763.2.S1\_a\_at | 0.00079 | 1.96e-06 | 1.47e-05 | \* \* \* | -0.957 | -1.11 | -1.39 | 100 | 100 |
| Bt.3945.1.S1\_at | 0.00079 | 3.21e-06 | 2.23e-06 | \* \* \* | 0.986 | 1.21 | 1.63 | 100 | 100 |
| Bt.5529.2.S1\_a\_at | 0.000799 | 0.000912 | 4.19e-05 | \* \* \* | -0.88 | -0.662 | -0.834 | 100 | 100 |
| Bt.4658.1.S1\_a\_at | 0.000802 | 8.15e-06 | 2.43e-07 | \* \* \* | -1.01 | -0.664 | -1.01 | 100 | 100 |
| Bt.26920.2.A1\_a\_at | 0.000805 | 2.16e-05 | 2.21e-05 | \* \* \* | -1.3 | -0.976 | -1.3 | 100 | 100 |
| Bt.19522.2.S1\_at | 0.000806 | 0.000187 | 0.00159 | \* \* \* | 0.893 | 0.667 | 0.665 | 100 | 100 |
| Bt.9578.1.S1\_at | 0.000808 | 0.000177 | 0.000654 | \* \* \* | -0.846 | -0.739 | -0.806 | 100 | 100 |
| Bt.9643.1.S1\_at | 0.000808 | 0.0015 | 0.000224 | \* \* \* | -0.943 | -0.678 | -0.917 | 100 | 100 |
| Bt.20193.1.S1\_at | 0.000814 | 7.78e-05 | 0.000109 | \* \* \* | -1.44 | -1.57 | -2.27 | 100 | 100 |
| Bt.18634.1.A1\_at | 0.000815 | 1.95e-07 | 1.71e-05 | \* \* \* | 1.33 | 1.8 | 1.97 | 100 | 100 |
| Bt.20485.1.S1\_at | 0.000815 | 2.79e-06 | 5.87e-08 | \* \* \* | 0.708 | 0.903 | 1.06 | 100 | 100 |
| Bt.7030.1.S1\_at | 0.000816 | 6.22e-05 | 6.33e-05 | \* \* \* | 0.887 | 0.766 | 0.919 | 100 | 100 |
| Bt.16243.1.S1\_at | 0.000817 | 0.000232 | 0.00122 | \* \* \* | 1.1 | 0.908 | 1.4 | 100 | 100 |
| Bt.6087.2.S1\_a\_at | 0.000822 | 0.000136 | 4.39e-06 | \* \* \* | 0.947 | 0.808 | 1.12 | 100 | 100 |
| Bt.4480.1.S2\_at | 0.000824 | 2.46e-05 | 0.000596 | \* \* \* | 1.19 | 1.06 | 1.2 | 100 | 100 |
| Bt.26894.1.S1\_at | 0.000825 | 1.27e-06 | 0.000162 | \* \* \* | -0.914 | -0.976 | -1.14 | 100 | 100 |
| Bt.1484.1.S1\_at | 0.000825 | 2.52e-05 | 4.21e-05 | \* \* \* | 0.844 | 0.742 | 0.867 | 100 | 100 |
| Bt.27147.1.S1\_at | 0.000827 | 1.27e-05 | 2.66e-05 | \* \* \* | 1.23 | 1.04 | 1.59 | 100 | 100 |
| Bt.23109.1.S1\_at | 0.000827 | 0.00139 | 6.19e-05 | \* \* \* | -1.02 | -0.516 | -1.02 | 100 | 100 |
| Bt.12510.3.S1\_at | 0.000829 | 6.87e-08 | 3.22e-07 | \* \* \* | 1.24 | 1.7 | 2.78 | 100 | 100 |
| Bt.4466.1.A1\_at | 0.000835 | 5.08e-05 | 8.39e-05 | \* \* \* | 0.74 | 0.705 | 0.875 | 100 | 100 |
| Bt.4533.1.S1\_at | 0.00084 | 9.31e-05 | 1.55e-05 | \* \* \* | -0.906 | -0.606 | -0.847 | 100 | 100 |
| Bt.6673.1.S1\_at | 0.000842 | 0.00181 | 0.000401 | \* \* \* | -0.74 | -0.44 | -0.603 | 100 | 100 |
| Bt.15938.1.S1\_at | 0.000843 | 0.000137 | 1.57e-05 | \* \* \* | 0.916 | 0.797 | 1.32 | 100 | 100 |
| Bt.5484.1.S1\_at | 0.000846 | 0.000234 | 0.000187 | \* \* \* | -0.812 | -0.77 | -0.958 | 100 | 100 |
| Bt.21715.1.S1\_at | 0.000849 | 3.8e-05 | 0.000708 | \* \* \* | 1.17 | 1.03 | 1.07 | 100 | 100 |
| Bt.25335.1.A1\_at | 0.00085 | 0.000149 | 1.09e-05 | \* \* \* | 1.09 | 0.746 | 0.735 | 100 | 100 |
| Bt.7870.1.S1\_at | 0.000857 | 2.14e-06 | 1.46e-06 | \* \* \* | -1.83 | -1.56 | -2.26 | 100 | 100 |
| Bt.12096.1.S1\_at | 0.000858 | 9.27e-06 | 2.24e-05 | \* \* \* | -1.34 | -1.07 | -1.24 | 100 | 100 |
| Bt.24832.1.A1\_a\_at | 0.000859 | 0.000124 | 0.00122 | \* \* \* | -1.39 | -1.16 | -1.63 | 100 | 100 |
| Bt.16628.2.A1\_at | 0.00086 | 0.000204 | 0.000815 | \* \* \* | 1.84 | 1.17 | 1.31 | 100 | 100 |
| Bt.17958.1.S1\_at | 0.000869 | 0.00095 | 0.000198 | \* \* \* | 0.753 | 0.52 | 0.725 | 100 | 100 |
| Bt.27.1.S1\_at | 0.00087 | 0.000168 | 0.00125 | \* \* \* | -0.924 | -0.889 | -1.24 | 100 | 100 |
| Bt.3050.1.S1\_at | 0.000872 | 0.00133 | 0.00094 | \* \* \* | -0.677 | -0.507 | -0.695 | 100 | 100 |
| Bt.15819.2.S1\_at | 0.000879 | 0.000172 | 0.00035 | \* \* \* | -0.767 | -0.802 | -0.957 | 100 | 100 |
| Bt.11171.1.S1\_at | 0.000884 | 0.000718 | 0.000609 | \* \* \* | 0.899 | 0.674 | 0.915 | 100 | 100 |
| Bt.20988.1.S1\_at | 0.000885 | 2.91e-05 | 4.88e-05 | \* \* \* | -0.989 | -0.918 | -1.26 | 100 | 100 |
| Bt.16639.1.A1\_at | 0.000885 | 4.5e-05 | 0.000112 | \* \* \* | 0.854 | 0.712 | 0.902 | 100 | 100 |
| Bt.24594.1.A1\_at | 0.000888 | 0.000358 | 0.000651 | \* \* \* | -1.31 | -1.42 | -2.28 | 100 | 100 |
| Bt.22725.1.S1\_at | 0.000888 | 0.000546 | 0.00172 | \* \* \* | 0.876 | 0.579 | 0.645 | 100 | 100 |
| Bt.8606.1.S1\_a\_at | 0.000892 | 9.93e-08 | 7.71e-06 | \* \* \* | 0.974 | 1.13 | 1.57 | 100 | 100 |
| Bt.9041.1.S1\_at | 0.000893 | 2.44e-05 | 1.34e-05 | \* \* \* | 0.676 | 0.835 | 0.961 | 100 | 100 |
| Bt.13028.1.S1\_at | 0.000893 | 3.18e-05 | 0.000429 | \* \* \* | 2.85 | 2.44 | 2.97 | 100 | 100 |
| Bt.20226.1.A1\_at | 9e-04 | 1.07e-05 | 6.53e-05 | \* \* \* | 1.56 | 1.44 | 1.59 | 100 | 100 |
| Bt.21133.1.S1\_at | 0.000901 | 0.000197 | 0.00126 | \* \* \* | 1.43 | 1.62 | 2.06 | 100 | 100 |
| Bt.27930.1.S1\_at | 0.00091 | 2.13e-06 | 2.47e-05 | \* \* \* | 0.779 | 0.896 | 1.14 | 100 | 100 |
| Bt.16125.2.S1\_at | 0.00091 | 0.000317 | 5.78e-05 | \* \* \* | -0.984 | -1.04 | -1.38 | 100 | 100 |
| Bt.23600.1.S1\_at | 0.00091 | 6.43e-05 | 2.87e-06 | \* \* \* | -0.739 | -0.685 | -0.985 | 100 | 100 |
| Bt.9866.1.S1\_at | 0.000916 | 0.000292 | 0.000726 | \* \* \* | -0.973 | -0.911 | -1.21 | 100 | 100 |
| Bt.7701.1.S1\_at | 0.000921 | 1.72e-07 | 3.13e-06 | \* \* \* | -0.949 | -1.13 | -1.5 | 100 | 100 |
| Bt.29325.1.S1\_at | 0.000921 | 9.25e-05 | 0.000174 | \* \* \* | -0.893 | -0.813 | -1.06 | 100 | 100 |
| Bt.1932.1.S1\_at | 0.000921 | 0.00107 | 0.00154 | \* \* \* | -0.909 | -0.755 | -0.875 | 100 | 100 |
| Bt.68.1.S1\_at | 0.000924 | 9.46e-07 | 4.37e-05 | \* \* \* | -1.46 | -1.12 | -1.41 | 100 | 100 |
| Bt.23615.1.S1\_at | 0.000925 | 4.04e-05 | 2.98e-06 | \* \* \* | 0.71 | 0.665 | 0.825 | 100 | 100 |
| Bt.12957.1.A1\_a\_at | 0.000926 | 4.97e-05 | 9.45e-05 | \* \* \* | 0.893 | 0.72 | 0.702 | 100 | 100 |
| Bt.665.1.S1\_s\_at | 0.000934 | 5.54e-06 | 5.79e-05 | \* \* \* | -1.07 | -0.776 | -1.19 | 100 | 100 |
| Bt.8648.1.S1\_at | 0.000935 | 5.75e-05 | 9.69e-05 | \* \* \* | 0.751 | 0.853 | 1.15 | 100 | 100 |
| Bt.22615.2.A1\_a\_at | 0.00094 | 0.000491 | 0.000301 | \* \* \* | -0.965 | -0.727 | -0.879 | 100 | 100 |
| Bt.3895.1.S1\_at | 0.000942 | 1.09e-06 | 1.71e-06 | \* \* \* | 1.28 | 1.02 | 0.951 | 100 | 100 |
| Bt.422.1.S2\_at | 0.000944 | 5.42e-07 | 7.2e-06 | \* \* \* | 0.864 | 1.18 | 1.62 | 100 | 100 |
| Bt.668.1.S1\_at | 0.000949 | 1.01e-05 | 0.000498 | \* \* \* | 0.931 | 0.757 | 0.856 | 100 | 100 |
| Bt.5237.1.S1\_at | 0.000963 | 0.000479 | 0.000229 | \* \* \* | 0.974 | 0.725 | 1.05 | 100 | 100 |
| Bt.21584.1.S1\_at | 0.000964 | 5.92e-07 | 5.35e-06 | \* \* \* | -1.09 | -1.5 | -1.86 | 100 | 100 |
| Bt.16111.1.S1\_at | 0.000972 | 2.72e-05 | 8.68e-08 | \* \* \* | 0.725 | 0.768 | 0.965 | 100 | 100 |
| Bt.19097.2.S1\_at | 0.000974 | 1.62e-05 | 2.06e-05 | \* \* \* | 0.972 | 1.12 | 1.51 | 100 | 100 |
| Bt.7788.1.S1\_at | 0.00098 | 2.05e-05 | 0.000505 | \* \* \* | 1.06 | 1.04 | 1.21 | 100 | 100 |
| Bt.8163.1.S1\_at | 0.000982 | 6.19e-05 | 1.25e-05 | \* \* \* | 0.76 | 0.814 | 0.913 | 100 | 100 |
| Bt.16108.1.A1\_at | 0.000985 | 6.37e-05 | 0.000167 | \* \* \* | -1.55 | -1.22 | -1.89 | 100 | 100 |
| Bt.1760.1.S1\_at | 0.000985 | 1.14e-05 | 3.52e-05 | \* \* \* | -0.79 | -0.819 | -1.18 | 100 | 100 |
| Bt.12820.1.S1\_at | 0.000985 | 2.47e-05 | 6.25e-05 | \* \* \* | 0.943 | 0.782 | 0.697 | 100 | 100 |
| Bt.1325.1.S1\_at | 0.000987 | 0.000852 | 1.17e-05 | \* \* \* | 1.2 | 0.674 | 1.49 | 100 | 100 |
| Bt.23330.1.S1\_at | 0.000994 | 2.61e-06 | 1.41e-06 | \* \* \* | 0.897 | 1.1 | 1.37 | 100 | 100 |
| Bt.24433.1.S1\_at | 0.000995 | 0.000817 | 0.000225 | \* \* \* | -0.784 | -0.584 | -0.794 | 100 | 100 |
| Bt.12290.1.S1\_at | 0.000996 | 3.2e-07 | 8.07e-07 | \* \* \* | 1.43 | 1.42 | 1.57 | 100 | 100 |
| Bt.12108.1.S1\_at | 0.00102 | 2.41e-05 | 0.000697 | \* \* \* | -1.21 | -0.91 | -1 | 100 | 100 |
| Bt.422.1.S1\_at | 0.00102 | 9.17e-05 | 9.91e-05 | \* \* \* | 0.869 | 1 | 1.03 | 100 | 100 |
| Bt.23971.1.S1\_at | 0.00102 | 1.61e-05 | 8.68e-06 | \* \* \* | 1.02 | 0.861 | 1.19 | 100 | 100 |
| Bt.7575.1.A1\_at | 0.00102 | 9.3e-05 | 0.000706 | \* \* \* | -1.05 | -1.01 | -1.31 | 100 | 100 |
| Bt.21607.1.S1\_at | 0.00103 | 7.11e-05 | 0.000255 | \* \* \* | 1.35 | 1.47 | 1.72 | 100 | 100 |
| Bt.27457.1.S1\_at | 0.00103 | 3.51e-08 | 8.58e-10 | \* \* \* | 1.54 | 1.58 | 2.19 | 100 | 100 |
| Bt.5939.1.S1\_a\_at | 0.00103 | 0.000412 | 3.64e-05 | \* \* \* | -0.639 | -0.514 | -0.784 | 100 | 100 |
| Bt.22168.1.A1\_at | 0.00105 | 0.000344 | 0.00189 | \* \* \* | -1.1 | -1.04 | -1.18 | 100 | 100 |
| Bt.13913.1.S1\_at | 0.00106 | 0.000902 | 5.43e-05 | \* \* \* | -0.764 | -0.542 | -1.14 | 100 | 100 |
| Bt.561.1.S1\_at | 0.00107 | 0.000106 | 0.00079 | \* \* \* | -1.52 | -1.3 | -1.65 | 100 | 100 |
| Bt.16597.1.S1\_at | 0.00107 | 1.91e-06 | 8.89e-06 | \* \* \* | -1.33 | -1.05 | -1.63 | 100 | 100 |
| Bt.13205.2.S1\_at | 0.00107 | 0.000403 | 2.45e-05 | \* \* \* | -1.03 | -0.819 | -1.32 | 100 | 100 |
| Bt.29938.1.S1\_at | 0.00108 | 0.000996 | 0.000914 | \* \* \* | 0.989 | 0.656 | 0.949 | 100 | 100 |
| Bt.9544.1.S1\_at | 0.00108 | 0.000317 | 2.06e-05 | \* \* \* | 0.699 | 0.699 | 0.854 | 100 | 100 |
| Bt.2765.1.S1\_at | 0.00108 | 5e-04 | 0.000362 | \* \* \* | 0.948 | 0.732 | 0.948 | 100 | 100 |
| Bt.13998.1.S1\_at | 0.00109 | 9.96e-05 | 3.58e-05 | \* \* \* | -0.924 | -0.63 | -0.939 | 100 | 100 |
| Bt.59.1.S1\_at | 0.0011 | 3.7e-06 | 4.42e-07 | \* \* \* | -0.888 | -0.825 | -1.14 | 100 | 100 |
| Bt.21622.1.A1\_at | 0.0011 | 1.25e-06 | 5.52e-07 | \* \* \* | 1.32 | 1.3 | 1.52 | 100 | 100 |
| Bt.5079.1.S1\_at | 0.00111 | 6.02e-05 | 0.000212 | \* \* \* | -1.05 | -1.01 | -1.21 | 100 | 100 |
| Bt.23076.1.S1\_at | 1.4e-09 | 6.54e-11 | 5.47e-09 | \* \* \* | 2.82 | 2.42 | 3.75 | 83.3 | 100 |
| Bt.23662.1.S1\_at | 7.22e-08 | 1.52e-10 | 1.14e-09 | \* \* \* | -2.15 | -1.82 | -2.18 | 83.3 | 100 |
| Bt.3857.1.S1\_at | 8.14e-08 | 7e-11 | 6.61e-08 | \* \* \* | -2.62 | -2.66 | -3.56 | 83.3 | 100 |
| Bt.20473.1.A1\_at | 1.69e-07 | 1.76e-09 | 8.89e-10 | \* \* \* | 2.4 | 2.32 | 3.89 | 83.3 | 100 |
| Bt.4867.2.S1\_at | 5.34e-07 | 3.24e-09 | 5.79e-06 | \* \* \* | 4.59 | 4.62 | 6.4 | 83.3 | 100 |
| Bt.28138.1.S1\_at | 6.72e-07 | 3.74e-08 | 1.04e-06 | \* \* \* | 1.86 | 1.16 | 1.8 | 83.3 | 100 |
| Bt.22639.2.S1\_at | 1.82e-06 | 5.18e-05 | 0.00117 | \* \* \* | -1.64 | -1.19 | -1.46 | 83.3 | 100 |
| Bt.23734.1.A1\_at | 2.62e-06 | 1.31e-06 | 9.17e-06 | \* \* \* | 2.77 | 1.72 | 3.08 | 83.3 | 100 |
| Bt.3236.1.A1\_at | 4.52e-06 | 1.02e-06 | 3.99e-05 | \* \* \* | 1.52 | 1.31 | 1.37 | 83.3 | 100 |
| Bt.25832.1.S1\_at | 5.66e-06 | 2.32e-07 | 3.37e-06 | \* \* \* | 1.5 | 1.31 | 1.98 | 83.3 | 100 |
| Bt.25282.1.A1\_at | 7.44e-06 | 9.26e-07 | 8.67e-09 | \* \* \* | 1.23 | 1.09 | 1.6 | 83.3 | 100 |
| Bt.15616.1.S1\_at | 1.26e-05 | 5.69e-05 | 7.56e-05 | \* \* \* | -1.48 | -0.945 | -1.58 | 83.3 | 100 |
| Bt.13321.1.S1\_at | 1.37e-05 | 1.36e-08 | 1.12e-07 | \* \* \* | 1.65 | 1.62 | 2.07 | 83.3 | 100 |
| Bt.4267.1.S1\_at | 2.08e-05 | 0.00044 | 0.000106 | \* \* \* | 1.6 | 0.564 | 1.38 | 83.3 | 100 |
| Bt.11246.1.A1\_at | 2.25e-05 | 1.88e-06 | 3.99e-05 | \* \* \* | 1.46 | 0.98 | 1.12 | 83.3 | 100 |
| Bt.28087.1.S1\_at | 2.6e-05 | 0.000737 | 5.51e-07 | \* \* \* | 1.16 | 0.805 | 1.24 | 83.3 | 100 |
| Bt.3880.1.S1\_at | 2.63e-05 | 6.11e-08 | 5.26e-07 | \* \* \* | 2.33 | 1.92 | 2.95 | 83.3 | 100 |
| Bt.27793.1.A1\_at | 2.97e-05 | 4.19e-07 | 8.17e-05 | \* \* \* | 1.47 | 1.72 | 2.89 | 83.3 | 100 |
| Bt.22650.1.A1\_at | 4.46e-05 | 2.28e-05 | 6.63e-05 | \* \* \* | 1.67 | 1.26 | 1.27 | 83.3 | 100 |
| Bt.8492.1.A1\_at | 5.14e-05 | 3.84e-05 | 0.000214 | \* \* \* | 1.57 | 1.02 | 1.37 | 83.3 | 100 |
| Bt.2956.1.A1\_at | 5.25e-05 | 1.85e-05 | 1.15e-06 | \* \* \* | 1.36 | 1.01 | 1.22 | 83.3 | 100 |
| Bt.27430.1.S1\_at | 6.19e-05 | 2.33e-05 | 5.79e-05 | \* \* \* | -1.18 | -0.857 | -0.941 | 83.3 | 100 |
| Bt.25227.1.A1\_at | 7.18e-05 | 7.32e-09 | 4.67e-07 | \* \* \* | 1.87 | 1.52 | 2.05 | 83.3 | 100 |
| Bt.20102.1.S1\_at | 7.19e-05 | 9.19e-05 | 0.00141 | \* \* \* | -1.78 | -1.06 | -2.1 | 83.3 | 100 |
| Bt.27207.1.A1\_at | 7.2e-05 | 5.74e-06 | 9.21e-06 | \* \* \* | 1.81 | 1.04 | 1.99 | 83.3 | 100 |
| Bt.13588.2.S1\_at | 7.68e-05 | 4.41e-06 | 4.18e-05 | \* \* \* | -4.22 | -2.67 | -4.76 | 83.3 | 100 |
| Bt.18459.1.A1\_at | 8.51e-05 | 0.000198 | 5.26e-05 | \* \* \* | 1.24 | 0.731 | 0.733 | 83.3 | 100 |
| Bt.22150.1.A1\_at | 9.46e-05 | 1.07e-05 | 1.93e-05 | \* \* \* | 1.2 | 0.85 | 0.831 | 83.3 | 100 |
| Bt.26080.2.S1\_at | 9.9e-05 | 8.53e-06 | 0.000636 | \* \* \* | 1.56 | 1.03 | 2.17 | 83.3 | 100 |
| Bt.20038.1.S1\_at | 0.000107 | 2.44e-05 | 1.82e-05 | \* \* \* | -1.32 | -0.83 | -2.48 | 83.3 | 100 |
| Bt.303.1.S1\_at | 0.000116 | 5.39e-07 | 7.8e-05 | \* \* \* | -1.94 | -1.3 | -2.42 | 83.3 | 100 |
| Bt.1267.1.S1\_at | 0.000133 | 4.2e-07 | 4.89e-06 | \* \* \* | 1.4 | 1.37 | 2.16 | 83.3 | 100 |
| Bt.19862.1.A1\_at | 0.000151 | 1.96e-05 | 9.3e-06 | \* \* \* | 1.82 | 1.2 | 1.42 | 83.3 | 100 |
| Bt.14366.1.S1\_at | 0.000154 | 1.12e-05 | 0.000542 | \* \* \* | 1 | 1.01 | 1.07 | 83.3 | 100 |
| Bt.22785.1.S1\_at | 0.000161 | 5.31e-05 | 6.71e-06 | \* \* \* | -1.64 | -1.24 | -2.04 | 83.3 | 100 |
| Bt.20820.1.A1\_at | 0.000188 | 0.000645 | 5.1e-05 | \* \* \* | 1.06 | 0.798 | 1.96 | 83.3 | 100 |
| Bt.7319.1.S1\_at | 0.000198 | 1.39e-06 | 1.22e-05 | \* \* \* | 1.65 | 1.84 | 2.35 | 83.3 | 100 |
| Bt.26568.1.S1\_a\_at | 0.000216 | 3.42e-07 | 2.94e-06 | \* \* \* | -1.42 | -1.46 | -2.34 | 83.3 | 100 |
| Bt.12875.1.S1\_at | 0.000222 | 2.17e-07 | 4.17e-08 | \* \* \* | 1.58 | 1.18 | 1.91 | 83.3 | 100 |
| Bt.13845.1.S1\_at | 0.000262 | 1.88e-06 | 8.85e-05 | \* \* \* | -1.59 | -1.07 | -1.51 | 83.3 | 100 |
| Bt.5278.1.A1\_at | 0.000272 | 8.21e-06 | 8.65e-07 | \* \* \* | -0.966 | -0.952 | -1.7 | 83.3 | 100 |
| Bt.20328.1.S1\_at | 0.000274 | 0.000261 | 0.000162 | \* \* \* | 0.897 | 0.507 | 0.807 | 83.3 | 100 |
| Bt.27196.1.A1\_at | 0.000291 | 3.39e-06 | 6.02e-06 | \* \* \* | -2.58 | -1.71 | -3.65 | 83.3 | 100 |
| Bt.6937.1.S1\_at | 0.000306 | 9.46e-05 | 9.83e-06 | \* \* \* | -1.5 | -0.797 | -0.882 | 83.3 | 100 |
| Bt.1020.1.S1\_at | 0.000322 | 9.3e-07 | 3.41e-06 | \* \* \* | 1.17 | 1.32 | 1.49 | 83.3 | 100 |
| Bt.3996.1.S1\_at | 0.000325 | 1.21e-06 | 1.73e-05 | \* \* \* | 2.63 | 1.96 | 2.27 | 83.3 | 100 |
| Bt.25346.1.A1\_at | 0.00033 | 1.25e-07 | 2.11e-05 | \* \* \* | 1.3 | 1.08 | 0.723 | 83.3 | 100 |
| Bt.24624.1.S1\_at | 0.000343 | 2.17e-06 | 1.37e-05 | \* \* \* | -1.27 | -0.983 | -1.62 | 83.3 | 100 |
| Bt.4748.1.S1\_at | 0.000384 | 9.63e-06 | 0.000955 | \* \* \* | -1.38 | -0.997 | -1.09 | 83.3 | 100 |
| Bt.5226.1.S1\_at | 0.00041 | 1.68e-07 | 0.000101 | \* \* \* | 1.15 | 0.993 | 1.5 | 83.3 | 100 |
| Bt.26609.1.S1\_at | 0.00042 | 0.000318 | 0.000148 | \* \* \* | 1.2 | 0.746 | 0.813 | 83.3 | 100 |
| Bt.1993.1.S1\_a\_at | 0.000421 | 0.00123 | 7e-04 | \* \* \* | 1.48 | 0.782 | 1.51 | 83.3 | 100 |
| Bt.13850.3.S1\_a\_at | 0.000475 | 0.000134 | 0.000659 | \* \* \* | -1.04 | -0.83 | -0.749 | 83.3 | 100 |
| Bt.6483.1.A1\_at | 0.000478 | 2.2e-06 | 2.31e-06 | \* \* \* | 0.89 | 0.925 | 1.16 | 83.3 | 100 |
| Bt.21997.2.A1\_at | 0.000533 | 0.00116 | 0.000555 | \* \* \* | 1.56 | 0.968 | 2.23 | 83.3 | 100 |
| Bt.1523.1.S1\_at | 0.000543 | 4.39e-05 | 2.97e-05 | \* \* \* | -1.22 | -0.813 | -1.34 | 83.3 | 100 |
| Bt.16985.1.S1\_at | 0.00056 | 9.43e-06 | 5.02e-05 | \* \* \* | 0.986 | 1.04 | 1.16 | 83.3 | 100 |
| Bt.11027.1.A1\_at | 0.000579 | 0.000606 | 0.00042 | \* \* \* | 1.37 | 0.6 | 1.36 | 83.3 | 100 |
| Bt.8809.1.S1\_at | 0.000605 | 0.000104 | 0.00081 | \* \* \* | -2.25 | -1.41 | -2.68 | 83.3 | 100 |
| Bt.6983.1.S1\_at | 0.000624 | 1.12e-05 | 8.69e-05 | \* \* \* | 1.75 | 1.56 | 2.33 | 83.3 | 100 |
| Bt.27937.1.A1\_at | 0.000644 | 2.97e-06 | 8.92e-06 | \* \* \* | 1.38 | 1.32 | 1.59 | 83.3 | 100 |
| Bt.17843.1.A1\_at | 0.000666 | 0.000761 | 4.9e-06 | \* \* \* | 0.883 | 0.605 | 1.21 | 83.3 | 100 |
| Bt.1652.1.S1\_at | 0.000674 | 2.39e-07 | 7.76e-07 | \* \* \* | 1.98 | 1.47 | 1.64 | 83.3 | 100 |
| Bt.29506.1.S1\_at | 0.000711 | 2.06e-05 | 1.7e-05 | \* \* \* | 1.98 | 1.27 | 2.06 | 83.3 | 100 |
| Bt.27262.1.S1\_at | 0.000733 | 0.00186 | 0.000594 | \* \* \* | -1.15 | -0.579 | -0.951 | 83.3 | 100 |
| Bt.20091.1.S1\_at | 0.00076 | 0.000389 | 0.000268 | \* \* \* | 1.39 | 0.719 | 0.777 | 83.3 | 100 |
| Bt.23285.1.S1\_at | 0.000824 | 2.51e-05 | 0.000105 | \* \* \* | -0.785 | -0.754 | -1.2 | 83.3 | 100 |
| Bt.4817.1.A1\_at | 0.000828 | 0.000512 | 0.00117 | \* \* \* | 1.75 | 1.17 | 1.44 | 83.3 | 100 |
| Bt.12468.1.S1\_at | 0.000829 | 2.94e-06 | 1.9e-06 | \* \* \* | -1.69 | -1.23 | -1.94 | 83.3 | 100 |
| Bt.16092.1.S1\_at | 0.000837 | 1.92e-07 | 6.49e-05 | \* \* \* | 0.964 | 0.978 | 2.23 | 83.3 | 100 |
| Bt.17886.1.A1\_at | 0.000869 | 1.65e-05 | 3.18e-06 | \* \* \* | 1.86 | 1.15 | 2.44 | 83.3 | 100 |
| Bt.19309.3.A1\_at | 0.000881 | 0.000119 | 0.000576 | \* \* \* | 1.36 | 0.875 | 1.28 | 83.3 | 100 |
| Bt.25712.1.A1\_s\_at | 0.000917 | 1.79e-05 | 1.34e-06 | \* \* \* | 1.78 | 0.821 | 1.58 | 83.3 | 100 |
| Bt.25015.1.A1\_at | 0.000936 | 0.00168 | 0.000692 | \* \* \* | 1 | 0.605 | 1 | 83.3 | 100 |
| Bt.26135.1.A1\_at | 0.000996 | 9.5e-11 | 6.2e-10 | \* \* \* | -2.24 | -2.34 | -3.71 | 83.3 | 100 |
| Bt.2121.1.S1\_at | 0.000997 | 3.45e-07 | 0.000259 | \* \* \* | 2.1 | 1.56 | 2.36 | 83.3 | 100 |
| Bt.16861.1.A1\_at | 0.000999 | 0.000251 | 5.73e-05 | \* \* \* | 1.14 | 0.753 | 1.59 | 83.3 | 100 |
| Bt.2418.1.S1\_at | 0.00101 | 0.000197 | 8.59e-05 | \* \* \* | 0.873 | 0.72 | 0.971 | 83.3 | 100 |
| Bt.21824.1.A1\_at | 0.00105 | 3.85e-05 | 0.000271 | \* \* \* | 1.29 | 1.26 | 1.64 | 83.3 | 100 |
| Bt.23505.1.S1\_at | 0.0011 | 9.66e-07 | 1.81e-05 | \* \* \* | -2.27 | -2.21 | -3.43 | 83.3 | 100 |
| Bt.7130.1.S1\_at | 0.00111 | 0.000143 | 8.56e-05 | \* \* \* | 0.977 | 0.77 | 0.989 | 83.3 | 100 |
| Bt.25616.1.S1\_at | 1.17e-06 | 8.21e-07 | 5.75e-07 | \* \* \* | 1.5 | 1.09 | 1.57 | 66.7 | 100 |
| Bt.4653.1.S1\_at | 1.47e-06 | 5.87e-06 | 1.31e-06 | \* \* \* | 1.94 | 1.11 | 1.88 | 66.7 | 100 |
| Bt.4868.2.S1\_a\_at | 2.14e-06 | 2.87e-06 | 7.73e-08 | \* \* \* | -1.41 | -0.821 | -1.34 | 66.7 | 100 |
| Bt.4040.1.S1\_at | 4.05e-06 | 1.54e-05 | 3.63e-08 | \* \* \* | 1.56 | 1.05 | 2.18 | 66.7 | 100 |
| Bt.22538.2.S1\_at | 8.17e-06 | 2.7e-07 | 7.82e-06 | \* \* \* | 1.58 | 1.59 | 2.48 | 66.7 | 100 |
| Bt.26792.1.S1\_at | 9.45e-06 | 3.75e-08 | 1.07e-07 | \* \* \* | 2.31 | 2.06 | 3.78 | 66.7 | 100 |
| Bt.19352.1.A1\_at | 1.03e-05 | 5.03e-06 | 1.32e-07 | \* \* \* | -1.99 | -1.56 | -1.41 | 66.7 | 100 |
| Bt.28305.1.S1\_at | 1.2e-05 | 2.38e-08 | 4.11e-07 | \* \* \* | 1.79 | 1.84 | 2.41 | 66.7 | 100 |
| Bt.20783.1.A1\_at | 1.3e-05 | 2.52e-07 | 1.44e-06 | \* \* \* | 1.59 | 1.51 | 2.62 | 66.7 | 100 |
| Bt.24434.1.A1\_at | 1.33e-05 | 1.11e-06 | 2.07e-05 | \* \* \* | 2.11 | 1.03 | 1.84 | 66.7 | 100 |
| Bt.9828.1.S1\_at | 1.34e-05 | 4.92e-06 | 0.000435 | \* \* \* | -1.37 | -1.15 | -1.38 | 66.7 | 100 |
| Bt.11502.1.A1\_at | 1.41e-05 | 1.6e-05 | 5.61e-05 | \* \* \* | 1.38 | 0.988 | 1.08 | 66.7 | 100 |
| Bt.2587.2.S1\_a\_at | 1.5e-05 | 6.36e-06 | 8.4e-05 | \* \* \* | -1.24 | -1.1 | -1.15 | 66.7 | 100 |
| Bt.21620.1.A1\_at | 1.58e-05 | 1.34e-06 | 2.09e-06 | \* \* \* | 1.33 | 1.26 | 2.09 | 66.7 | 100 |
| Bt.5132.1.S1\_at | 1.59e-05 | 8.04e-07 | 3.11e-06 | \* \* \* | 1.93 | 1.4 | 1.87 | 66.7 | 100 |
| Bt.28472.1.S1\_at | 1.87e-05 | 5.46e-05 | 7.22e-05 | \* \* \* | 2.31 | 0.938 | 1.19 | 66.7 | 100 |
| Bt.25085.1.A1\_at | 2.03e-05 | 1.38e-06 | 2.01e-05 | \* \* \* | 1.86 | 1.46 | 2.18 | 66.7 | 100 |
| Bt.10398.1.S1\_at | 2.27e-05 | 1.74e-06 | 1.08e-05 | \* \* \* | 1.94 | 1.24 | 1.83 | 66.7 | 100 |
| Bt.23486.1.A1\_at | 2.37e-05 | 8.66e-06 | 1.09e-07 | \* \* \* | 1.87 | 1 | 1.89 | 66.7 | 100 |
| Bt.22046.1.S1\_at | 3.14e-05 | 1.22e-05 | 9.73e-05 | \* \* \* | -1.75 | -0.739 | -1.11 | 66.7 | 100 |
| Bt.23456.1.A1\_s\_at | 3.34e-05 | 2.99e-05 | 0.00143 | \* \* \* | 1.71 | 0.678 | 0.58 | 66.7 | 100 |
| Bt.28568.1.A1\_at | 4.3e-05 | 4.74e-08 | 2.28e-07 | \* \* \* | 3.04 | 2.16 | 4.37 | 66.7 | 100 |
| Bt.2120.2.S1\_at | 5e-05 | 4.95e-05 | 0.000794 | \* \* \* | 2.37 | 1.7 | 2.91 | 66.7 | 100 |
| Bt.24766.1.S1\_at | 6.13e-05 | 3.15e-06 | 7.52e-05 | \* \* \* | -1.62 | -1.53 | -2.28 | 66.7 | 100 |
| Bt.13427.1.S1\_at | 6.72e-05 | 2.42e-06 | 1.19e-06 | \* \* \* | 1.72 | 0.987 | 2.66 | 66.7 | 100 |
| Bt.13334.1.S1\_at | 6.92e-05 | 4.56e-07 | 1.03e-06 | \* \* \* | 1.34 | 1.12 | 1.52 | 66.7 | 100 |
| Bt.23131.1.S1\_s\_at | 7.23e-05 | 1.08e-05 | 2.42e-07 | \* \* \* | 1.49 | 1.12 | 2.3 | 66.7 | 100 |
| Bt.20072.1.S1\_at | 7.6e-05 | 1.52e-09 | 1.94e-06 | \* \* \* | 1.99 | 1.96 | 2.9 | 66.7 | 100 |
| Bt.17943.1.S1\_at | 7.63e-05 | 0.00196 | 0.000941 | \* \* \* | 1.08 | 0.555 | 0.918 | 66.7 | 100 |
| Bt.3228.1.S1\_at | 7.98e-05 | 0.000329 | 8.78e-05 | \* \* \* | 1.19 | 0.707 | 0.591 | 66.7 | 100 |
| Bt.21342.1.S1\_at | 8.02e-05 | 1.29e-05 | 3.66e-05 | \* \* \* | -1.12 | -0.908 | -1.2 | 66.7 | 100 |
| Bt.17081.1.A1\_at | 8.43e-05 | 1.46e-07 | 2.05e-06 | \* \* \* | 2.05 | 1.62 | 2.29 | 66.7 | 100 |
| Bt.9968.1.A1\_at | 9.24e-05 | 2.44e-07 | 0.000116 | \* \* \* | 1.28 | 1.21 | 1.32 | 66.7 | 100 |
| Bt.6665.1.S1\_at | 0.000101 | 7.12e-05 | 3.81e-05 | \* \* \* | 1.6 | 1.24 | 1.49 | 66.7 | 100 |
| Bt.23396.2.S1\_a\_at | 0.00012 | 4.3e-07 | 4.07e-05 | \* \* \* | -2.85 | -1.29 | -2.56 | 66.7 | 100 |
| Bt.29050.1.S1\_at | 0.000126 | 4.63e-06 | 2.4e-05 | \* \* \* | -1.9 | -1.1 | -2.31 | 66.7 | 100 |
| Bt.3967.1.S1\_a\_at | 0.000162 | 2.31e-06 | 6.06e-06 | \* \* \* | -1.53 | -1.12 | -1.73 | 66.7 | 100 |
| Bt.27918.1.S1\_at | 0.000195 | 2.69e-05 | 0.00069 | \* \* \* | 1.23 | 0.949 | 1.13 | 66.7 | 100 |
| Bt.23373.1.S1\_at | 0.000234 | 7.24e-05 | 5.03e-05 | \* \* \* | -1.56 | -1.01 | -1.74 | 66.7 | 100 |
| Bt.21166.1.S1\_at | 0.000235 | 4.54e-07 | 9.8e-06 | \* \* \* | 1.63 | 1.18 | 1.8 | 66.7 | 100 |
| Bt.4685.1.S1\_at | 0.000253 | 0.000685 | 0.000111 | \* \* \* | 1.02 | 0.85 | 1.27 | 66.7 | 100 |
| Bt.13648.1.A1\_at | 0.000264 | 3.84e-05 | 0.000108 | \* \* \* | 1.18 | 0.932 | 1.02 | 66.7 | 100 |
| Bt.4118.1.A1\_at | 0.000264 | 1.34e-05 | 6.53e-05 | \* \* \* | -1.04 | -1.06 | -1.27 | 66.7 | 100 |
| Bt.17081.2.S1\_at | 0.000268 | 6.74e-08 | 0.000144 | \* \* \* | 2.25 | 1.54 | 2.28 | 66.7 | 100 |
| Bt.11270.2.S1\_at | 0.000332 | 0.000343 | 0.00138 | \* \* \* | -2.21 | -0.82 | -1.19 | 66.7 | 100 |
| Bt.16503.1.A1\_at | 0.000343 | 0.000107 | 0.00012 | \* \* \* | -1.97 | -0.668 | -0.435 | 66.7 | 100 |
| Bt.25107.1.A1\_at | 0.000366 | 0.000134 | 9.61e-07 | \* \* \* | 1.51 | 0.711 | 1.08 | 66.7 | 100 |
| Bt.10458.2.S1\_at | 0.000371 | 0.000308 | 5.41e-05 | \* \* \* | 1.25 | 1.06 | 1.94 | 66.7 | 100 |
| Bt.3814.1.S1\_at | 0.000436 | 9.76e-05 | 0.000484 | \* \* \* | 1.27 | 1.1 | 1.3 | 66.7 | 100 |
| Bt.9416.1.S1\_at | 0.00049 | 0.000251 | 0.000152 | \* \* \* | 1.56 | 0.832 | 1.68 | 66.7 | 100 |
| Bt.22672.1.A1\_at | 0.000545 | 0.000174 | 0.000361 | \* \* \* | 1.51 | 1.55 | 1.97 | 66.7 | 100 |
| Bt.19469.1.A1\_at | 0.000655 | 0.000201 | 3.57e-05 | \* \* \* | 1.14 | 0.564 | 0.6 | 66.7 | 100 |
| Bt.12712.1.A1\_at | 0.000689 | 0.000265 | 0.000915 | \* \* \* | 1.75 | 0.794 | 0.786 | 66.7 | 100 |
| Bt.17332.2.S1\_at | 0.000734 | 6.25e-06 | 2.1e-06 | \* \* \* | 0.956 | 1.05 | 1.66 | 66.7 | 100 |
| Bt.29009.1.A1\_at | 0.000768 | 5.88e-06 | 2.53e-05 | \* \* \* | 2.15 | 2.07 | 3.17 | 66.7 | 100 |
| Bt.4044.1.S1\_at | 0.000773 | 4.76e-07 | 0.000142 | \* \* \* | -1.17 | -1.17 | -2.23 | 66.7 | 100 |
| Bt.1692.1.S1\_at | 0.000796 | 0.000449 | 9.07e-06 | \* \* \* | -1.01 | -0.691 | -0.798 | 66.7 | 100 |
| Bt.9324.1.S1\_at | 0.000834 | 2.42e-07 | 2.36e-06 | \* \* \* | -1.36 | -1.01 | -1.68 | 66.7 | 100 |
| Bt.3888.1.S1\_a\_at | 0.000876 | 1.56e-05 | 0.000142 | \* \* \* | -1.15 | -1.35 | -1.6 | 66.7 | 100 |
| Bt.2235.1.S2\_at | 0.000977 | 0.000279 | 9.48e-05 | \* \* \* | 1.01 | 0.863 | 1.05 | 66.7 | 100 |
| Bt.4011.1.S1\_at | 0.00103 | 7.51e-08 | 8.82e-07 | \* \* \* | -1.61 | -1.17 | -2.27 | 66.7 | 100 |
| Bt.20300.1.A1\_at | 0.00104 | 5.34e-05 | 1.7e-06 | \* \* \* | 0.818 | 0.657 | 1.3 | 66.7 | 100 |
| Bt.24563.1.S1\_at | 0.00111 | 1.36e-05 | 2.93e-05 | \* \* \* | 1.08 | 1.1 | 1.5 | 66.7 | 100 |
| Bt.23113.1.A1\_at | 1.32e-08 | 7.02e-07 | 1.1e-07 | \* \* \* | 3.09 | 2.04 | 3.21 | 50 | 100 |
| Bt.20470.2.S1\_a\_at | 2.96e-08 | 1.06e-08 | 2.78e-09 | \* \* \* | -2.76 | -1.78 | -4.69 | 50 | 100 |
| Bt.7056.3.A1\_a\_at | 3.88e-08 | 9.25e-14 | 7.5e-12 | \* \* \* | 8.64 | 6.25 | 8.44 | 50 | 100 |
| Bt.10719.1.S1\_at | 1.58e-07 | 2.79e-12 | 4.38e-08 | \* \* \* | 6.66 | 5.23 | 7.03 | 50 | 100 |
| Bt.7772.1.S1\_at | 2.37e-07 | 6.92e-06 | 4.82e-05 | \* \* \* | 2.19 | 1.2 | 1.74 | 50 | 100 |
| Bt.21528.1.A1\_at | 5.19e-07 | 7.14e-11 | 1.53e-05 | \* \* \* | 5.42 | 2.26 | 3.65 | 50 | 100 |
| Bt.20470.1.S1\_a\_at | 5.42e-07 | 8.19e-10 | 1.44e-05 | \* \* \* | -5.42 | -2.37 | -3.93 | 50 | 100 |
| Bt.2138.1.S1\_s\_at | 6.79e-07 | 4.07e-08 | 2.82e-07 | \* \* \* | -1.61 | -1.36 | -2.23 | 50 | 100 |
| Bt.16668.1.A1\_at | 7.9e-07 | 4.41e-08 | 1.01e-06 | \* \* \* | 1.71 | 1.53 | 1.94 | 50 | 100 |
| Bt.1421.1.S1\_at | 8.8e-07 | 8.88e-10 | 2.01e-06 | \* \* \* | 2.92 | 2.7 | 3.78 | 50 | 100 |
| Bt.20361.2.A1\_at | 9.49e-07 | 1.75e-07 | 1.57e-06 | \* \* \* | 1.76 | 1.83 | 2.24 | 50 | 100 |
| Bt.20405.1.S1\_at | 9.72e-07 | 7.6e-06 | 3.92e-05 | \* \* \* | 1.41 | 1.33 | 1.96 | 50 | 100 |
| Bt.19423.1.S1\_at | 1.07e-06 | 5.29e-06 | 0.000124 | \* \* \* | 2.05 | 1.24 | 1.71 | 50 | 100 |
| Bt.4954.1.S1\_at | 1.16e-06 | 4.16e-06 | 7.69e-06 | \* \* \* | 1.83 | 1.14 | 2.28 | 50 | 100 |
| Bt.11415.1.A1\_at | 2.4e-06 | 7.33e-08 | 5.73e-07 | \* \* \* | 2 | 2.32 | 3.11 | 50 | 100 |
| Bt.12721.1.A1\_at | 2.45e-06 | 4.68e-06 | 9.98e-08 | \* \* \* | 1.31 | 0.884 | 1.28 | 50 | 100 |
| Bt.27806.1.S1\_at | 3.3e-06 | 9.98e-08 | 7.7e-10 | \* \* \* | -2.07 | -1.25 | -2.14 | 50 | 100 |
| Bt.26728.1.S1\_at | 4.1e-06 | 1.45e-06 | 4.21e-07 | \* \* \* | 1.29 | 1.16 | 1.81 | 50 | 100 |
| Bt.10458.1.A1\_at | 5.87e-06 | 4.6e-07 | 1.1e-06 | \* \* \* | 1.31 | 1.07 | 1.82 | 50 | 100 |
| Bt.11622.1.A1\_at | 7.05e-06 | 0.000167 | 3.26e-07 | \* \* \* | 2.41 | 0.802 | 1.64 | 50 | 100 |
| Bt.1229.1.S1\_at | 7.4e-06 | 1.23e-06 | 1.71e-06 | \* \* \* | 2.52 | 1.28 | 2.66 | 50 | 100 |
| Bt.4698.1.S1\_at | 8.5e-06 | 5.64e-07 | 2.81e-06 | \* \* \* | 1.76 | 1.42 | 2.12 | 50 | 100 |
| Bt.8682.1.S1\_at | 1.03e-05 | 0.00132 | 0.000365 | \* \* \* | 1.28 | 0.508 | 1.11 | 50 | 100 |
| Bt.3452.1.S1\_at | 1.08e-05 | 6.07e-05 | 2.43e-05 | \* \* \* | 1.63 | 0.794 | 1.13 | 50 | 100 |
| Bt.8177.1.S1\_at | 1.31e-05 | 7.74e-07 | 1.37e-05 | \* \* \* | -1.84 | -1.17 | -1.57 | 50 | 100 |
| Bt.26621.1.S1\_at | 1.46e-05 | 3.09e-05 | 9.42e-05 | \* \* \* | 1.8 | 0.972 | 1.41 | 50 | 100 |
| Bt.22735.1.S1\_at | 1.98e-05 | 4.87e-06 | 9.19e-06 | \* \* \* | 1.63 | 1.59 | 2.35 | 50 | 100 |
| Bt.24154.1.A1\_at | 2.03e-05 | 1.42e-05 | 8.07e-08 | \* \* \* | -1.59 | -0.926 | -1.75 | 50 | 100 |
| Bt.28733.1.S1\_at | 3.46e-05 | 0.000192 | 0.00122 | \* \* \* | 1.01 | 0.646 | 0.823 | 50 | 100 |
| Bt.15840.1.A1\_at | 3.62e-05 | 1.73e-06 | 1.47e-07 | \* \* \* | 1.45 | 1.1 | 1.55 | 50 | 100 |
| Bt.923.1.A1\_at | 3.68e-05 | 7.98e-05 | 8.82e-06 | \* \* \* | 1.23 | 0.773 | 1.12 | 50 | 100 |
| Bt.15666.2.S1\_at | 4.62e-05 | 7.72e-07 | 3.87e-07 | \* \* \* | 1.3 | 1.15 | 2.35 | 50 | 100 |
| Bt.10814.1.S1\_at | 4.99e-05 | 0.000106 | 7.97e-05 | \* \* \* | 1.57 | 0.894 | 1.05 | 50 | 100 |
| Bt.18420.2.A1\_at | 6.19e-05 | 1.23e-06 | 1.77e-07 | \* \* \* | 1.59 | 1.28 | 2.41 | 50 | 100 |
| Bt.5393.2.S1\_a\_at | 6.38e-05 | 0.000353 | 2.54e-05 | \* \* \* | 1.64 | 0.668 | 1.29 | 50 | 100 |
| Bt.10995.1.S1\_at | 6.49e-05 | 0.000937 | 2.89e-07 | \* \* \* | 2.04 | 0.729 | 1.75 | 50 | 100 |
| Bt.11436.1.A1\_at | 7.37e-05 | 6.38e-06 | 1.1e-08 | \* \* \* | 1.01 | 0.937 | 1.43 | 50 | 100 |
| Bt.20354.1.S1\_at | 7.59e-05 | 4.3e-12 | 6.94e-08 | \* \* \* | 1.91 | 1.91 | 2.42 | 50 | 100 |
| Bt.4872.1.S1\_at | 7.69e-05 | 1.16e-05 | 4.59e-06 | \* \* \* | 1.15 | 0.927 | 1.38 | 50 | 100 |
| Bt.5805.1.S1\_at | 8.69e-05 | 4.85e-07 | 8.03e-09 | \* \* \* | -1.73 | -1.02 | -1.99 | 50 | 100 |
| Bt.17332.1.S1\_at | 0.000108 | 4.88e-05 | 7.94e-05 | \* \* \* | 1.72 | 0.882 | 1.77 | 50 | 100 |
| Bt.11019.1.S1\_at | 0.000109 | 6.96e-08 | 0.000235 | \* \* \* | -4.39 | -1.4 | -2.07 | 50 | 100 |
| Bt.3714.2.S1\_at | 0.000132 | 2.96e-05 | 4.23e-06 | \* \* \* | 1.21 | 1.06 | 2.55 | 50 | 100 |
| Bt.21997.1.S1\_at | 0.000135 | 1.53e-06 | 0.000102 | \* \* \* | 2.59 | 1.27 | 2.5 | 50 | 100 |
| Bt.24802.1.S1\_at | 0.000159 | 2.45e-07 | 0.0013 | \* \* \* | -1.67 | -1.43 | -2.24 | 50 | 100 |
| Bt.1112.1.S1\_at | 0.000169 | 1.41e-09 | 3.64e-10 | \* \* \* | -2.75 | -2.33 | -4.87 | 50 | 100 |
| Bt.1389.1.S1\_at | 0.000172 | 0.00072 | 0.000178 | \* \* \* | 0.902 | 0.528 | 1.17 | 50 | 100 |
| Bt.23232.1.S1\_at | 0.000173 | 2.53e-06 | 9.38e-06 | \* \* \* | -1.7 | -1.52 | -2.39 | 50 | 100 |
| Bt.4950.1.S1\_at | 0.000248 | 2.19e-05 | 0.00159 | \* \* \* | 1.34 | 0.752 | 1.38 | 50 | 100 |
| Bt.26462.1.S1\_at | 0.000257 | 2.72e-06 | 1.45e-08 | \* \* \* | 2.24 | 1.18 | 2.18 | 50 | 100 |
| Bt.14340.2.S1\_at | 0.000264 | 1.27e-07 | 2.37e-07 | \* \* \* | 1.55 | 1.44 | 2.64 | 50 | 100 |
| Bt.18048.1.A1\_at | 0.000279 | 4.76e-07 | 6.23e-06 | \* \* \* | 1.23 | 1.22 | 1.42 | 50 | 100 |
| Bt.12634.1.A1\_at | 0.000288 | 4.78e-08 | 4.42e-05 | \* \* \* | 1.78 | 1.25 | 2.09 | 50 | 100 |
| Bt.13357.1.S1\_at | 0.000334 | 0.000166 | 0.00143 | \* \* \* | 0.96 | 0.668 | 0.785 | 50 | 100 |
| Bt.22198.1.S1\_at | 0.000364 | 0.000252 | 0.000694 | \* \* \* | 1.04 | 0.854 | 1.12 | 50 | 100 |
| Bt.16573.1.A1\_at | 0.000396 | 0.00114 | 0.000331 | \* \* \* | 1.11 | 0.63 | 0.949 | 50 | 100 |
| Bt.2151.2.A1\_at | 0.00044 | 5.83e-05 | 0.00144 | \* \* \* | 2.72 | 0.83 | 1.27 | 50 | 100 |
| Bt.22200.1.S1\_at | 0.000442 | 7.55e-06 | 5.34e-07 | \* \* \* | 1.27 | 1.03 | 2.83 | 50 | 100 |
| Bt.18023.1.S1\_at | 0.000464 | 4.52e-06 | 1.43e-05 | \* \* \* | 1.31 | 0.912 | 1.23 | 50 | 100 |
| Bt.6842.1.S1\_at | 0.000478 | 2.57e-06 | 3.23e-06 | \* \* \* | 1.66 | 1.26 | 2.21 | 50 | 100 |
| Bt.16637.1.A1\_at | 0.000562 | 4.23e-07 | 0.000126 | \* \* \* | -1.31 | -1.18 | -1.99 | 50 | 100 |
| Bt.27058.1.A1\_at | 0.000605 | 2.55e-07 | 0.000328 | \* \* \* | 1.16 | 1.56 | 2.13 | 50 | 100 |
| Bt.2818.2.S1\_a\_at | 0.000701 | 1.18e-05 | 2.33e-05 | \* \* \* | 1.21 | 0.684 | 1.42 | 50 | 100 |
| Bt.11496.1.S1\_at | 0.000728 | 7.76e-05 | 1.05e-05 | \* \* \* | 1.43 | 1.24 | 2.1 | 50 | 100 |
| Bt.26061.1.A1\_at | 0.00075 | 0.000121 | 7.7e-05 | \* \* \* | 1.02 | 0.777 | 1.34 | 50 | 100 |
| Bt.2656.1.S1\_at | 0.000768 | 7.9e-05 | 2.36e-05 | \* \* \* | 1.13 | 1.05 | 2.2 | 50 | 100 |
| Bt.2853.1.S1\_at | 0.000775 | 0.00031 | 0.000801 | \* \* \* | -0.89 | -0.802 | -1.09 | 50 | 100 |
| Bt.21561.1.S1\_at | 0.00087 | 4.78e-07 | 1.09e-07 | \* \* \* | 0.942 | 0.997 | 1.59 | 50 | 100 |
| Bt.5602.1.A1\_at | 0.000946 | 0.000129 | 0.0016 | \* \* \* | 0.965 | 0.942 | 1.36 | 50 | 100 |
| Bt.15907.2.S1\_at | 0.000986 | 2.25e-06 | 6.75e-05 | \* \* \* | -2.06 | -1.47 | -1.98 | 50 | 100 |
| Bt.4631.2.S1\_at | 0.00107 | 2.89e-05 | 5.31e-05 | \* \* \* | 1.02 | 0.853 | 1.31 | 50 | 100 |
| Bt.19261.1.S1\_at | 0.00111 | 8.44e-05 | 0.0019 | \* \* \* | 1.53 | 0.677 | 1.1 | 50 | 100 |
| BtAffx.29967.1.S1\_at | 1.5e-06 | 0.00115 | 0.00273 | \* \* ns | -2.76 | -0.781 | -0.772 | 100 | 66.7 |
| Bt.23143.1.A1\_at | 6.62e-06 | 0.00311 | 8.05e-05 | \* ns \* | -1.16 | -0.501 | -0.679 | 100 | 66.7 |
| Bt.15589.1.A1\_at | 3.56e-05 | 6.61e-05 | 0.00195 | \* \* ns | 1.3 | 1.24 | 1.39 | 100 | 66.7 |
| Bt.19332.1.S1\_at | 4.88e-05 | 0.000319 | 0.00487 | \* \* ns | 1.27 | 1.09 | 1.13 | 100 | 66.7 |
| Bt.20591.1.S1\_at | 5.67e-05 | 0.00609 | 1.21e-05 | \* ns \* | 1.55 | 0.684 | 1.22 | 100 | 66.7 |
| Bt.7574.1.S1\_at | 5.78e-05 | 0.00655 | 0.0016 | \* ns \* | 1.49 | 0.511 | 0.629 | 100 | 66.7 |
| Bt.621.1.S1\_at | 5.8e-05 | 0.000189 | 0.00503 | \* \* ns | -1.64 | -0.915 | -1.38 | 100 | 66.7 |
| Bt.22615.1.S1\_at | 6.24e-05 | 0.00503 | 0.000635 | \* ns \* | -1.03 | -0.588 | -0.743 | 100 | 66.7 |
| Bt.20151.1.S1\_at | 6.48e-05 | 0.000238 | 0.00574 | \* \* ns | 1.66 | 1.36 | 1.57 | 100 | 66.7 |
| Bt.23496.1.S1\_at | 7.07e-05 | 0.00482 | 0.000351 | \* ns \* | -1.13 | -0.464 | -0.651 | 100 | 66.7 |
| Bt.21761.1.S1\_a\_at | 7.11e-05 | 0.000485 | 0.00273 | \* \* ns | -1.5 | -0.821 | -1.02 | 100 | 66.7 |
| Bt.20113.1.S1\_at | 8.22e-05 | 0.000581 | 0.00393 | \* \* ns | 1.49 | 0.564 | 0.707 | 100 | 66.7 |
| Bt.11237.1.S1\_at | 9.42e-05 | 0.000719 | 0.0154 | \* \* ns | 1.53 | 0.924 | 1.02 | 100 | 66.7 |
| Bt.10271.1.A1\_at | 0.000107 | 0.017 | 0.000104 | \* ns \* | -1.31 | -0.331 | -0.494 | 100 | 66.7 |
| Bt.11262.2.S1\_at | 0.000108 | 0.000351 | 0.00243 | \* \* ns | 1.82 | 1.12 | 1.39 | 100 | 66.7 |
| Bt.1638.2.S1\_at | 0.000123 | 0.00135 | 0.00325 | \* \* ns | 1.4 | 0.862 | 1.09 | 100 | 66.7 |
| Bt.2814.1.S1\_at | 0.000135 | 0.0268 | 0.00106 | \* ns \* | -0.989 | -0.39 | -0.537 | 100 | 66.7 |
| Bt.22444.1.A1\_at | 0.000146 | 0.00032 | 0.00318 | \* \* ns | 1.45 | 1.09 | 1.24 | 100 | 66.7 |
| Bt.6279.1.A1\_at | 0.000159 | 0.000329 | 0.00362 | \* \* ns | 1.14 | 0.731 | 0.607 | 100 | 66.7 |
| Bt.1283.1.S1\_at | 0.000188 | 0.0254 | 0.00127 | \* ns \* | -0.92 | -0.358 | -0.571 | 100 | 66.7 |
| Bt.282.1.S1\_at | 0.000203 | 0.014 | 0.00019 | \* ns \* | -0.926 | -0.329 | -0.524 | 100 | 66.7 |
| Bt.25343.1.S1\_at | 0.000216 | 0.000168 | 0.00531 | \* \* ns | -1.71 | -0.723 | -0.891 | 100 | 66.7 |
| Bt.20895.1.S1\_at | 0.000239 | 0.00429 | 0.000156 | \* ns \* | -0.805 | -0.502 | -0.622 | 100 | 66.7 |
| Bt.16132.2.S1\_a\_at | 0.000239 | 0.0101 | 0.00174 | \* ns \* | -0.783 | -0.563 | -0.615 | 100 | 66.7 |
| Bt.19839.1.A1\_at | 0.000243 | 0.00141 | 0.00539 | \* \* ns | 1.36 | 1.07 | 1.08 | 100 | 66.7 |
| Bt.26641.1.S1\_at | 0.000249 | 0.00134 | 0.00456 | \* \* ns | -1.04 | -0.673 | -0.887 | 100 | 66.7 |
| Bt.10150.1.S1\_at | 0.000258 | 0.00234 | 0.000611 | \* ns \* | 1.04 | 0.64 | 0.843 | 100 | 66.7 |
| Bt.3631.1.S1\_at | 0.000264 | 0.00755 | 0.000235 | \* ns \* | -0.857 | -0.307 | -0.362 | 100 | 66.7 |
| Bt.1825.1.S1\_at | 0.00027 | 0.00376 | 6.82e-05 | \* ns \* | -0.907 | -0.372 | -0.865 | 100 | 66.7 |
| Bt.23401.1.S1\_at | 0.000273 | 0.00052 | 0.00376 | \* \* ns | -1.4 | -0.726 | -0.835 | 100 | 66.7 |
| Bt.28207.1.S1\_at | 0.000274 | 0.000695 | 0.00925 | \* \* ns | 1.14 | 0.838 | 0.843 | 100 | 66.7 |
| Bt.13969.1.A1\_at | 0.000281 | 0.00038 | 0.00261 | \* \* ns | 1.18 | 0.708 | 0.701 | 100 | 66.7 |
| Bt.7024.1.A1\_at | 0.000291 | 0.00295 | 0.00116 | \* ns \* | -0.929 | -0.608 | -0.684 | 100 | 66.7 |
| Bt.23607.1.S1\_at | 0.000301 | 0.00518 | 0.000147 | \* ns \* | -1.06 | -0.432 | -0.686 | 100 | 66.7 |
| Bt.11193.1.S1\_at | 0.000308 | 2.77e-06 | 0.0391 | \* \* ns | -1.66 | -1.47 | -0.421 | 100 | 66.7 |
| Bt.6938.1.S1\_at | 0.000309 | 0.00333 | 0.0015 | \* ns \* | -0.764 | -0.626 | -0.814 | 100 | 66.7 |
| Bt.14219.1.S1\_at | 0.000312 | 7.51e-06 | 0.00293 | \* \* ns | 1.42 | 1.13 | 1.2 | 100 | 66.7 |
| Bt.17777.1.S1\_at | 0.000328 | 0.000658 | 0.002 | \* \* ns | -1.03 | -0.699 | -0.862 | 100 | 66.7 |
| Bt.15531.1.S1\_at | 0.000337 | 0.00219 | 0.000272 | \* ns \* | 0.713 | 0.607 | 0.781 | 100 | 66.7 |
| Bt.5941.1.S1\_at | 0.000337 | 0.00141 | 0.00592 | \* \* ns | 0.931 | 0.531 | 0.56 | 100 | 66.7 |
| Bt.22563.1.A1\_s\_at | 0.00035 | 0.00581 | 0.000345 | \* ns \* | -0.77 | -0.542 | -0.638 | 100 | 66.7 |
| Bt.15742.1.S1\_at | 0.000361 | 6.98e-05 | 0.00923 | \* \* ns | 1.2 | 1.15 | 0.621 | 100 | 66.7 |
| Bt.23551.2.S1\_at | 0.000364 | 0.000233 | 0.00246 | \* \* ns | -0.981 | -0.849 | -1.01 | 100 | 66.7 |
| Bt.231.1.S1\_at | 0.000372 | 0.00968 | 0.000655 | \* ns \* | -0.924 | -0.488 | -0.817 | 100 | 66.7 |
| Bt.10195.1.S1\_at | 0.000381 | 0.00323 | 0.00023 | \* ns \* | 0.801 | 0.582 | 0.812 | 100 | 66.7 |
| Bt.13132.1.S1\_at | 0.000398 | 0.00102 | 0.0785 | \* \* ns | -0.86 | -0.673 | -0.668 | 100 | 66.7 |
| Bt.1543.1.A1\_at | 0.000409 | 0.00266 | 0.000197 | \* ns \* | -1.21 | -0.775 | -1.22 | 100 | 66.7 |
| Bt.5582.1.S1\_at | 0.000416 | 0.00144 | 0.00198 | \* \* ns | -1.32 | -0.801 | -1.15 | 100 | 66.7 |
| Bt.28162.2.A1\_at | 0.000458 | 0.000546 | 0.00284 | \* \* ns | -1.52 | -1.22 | -1.26 | 100 | 66.7 |
| Bt.4279.2.S1\_at | 0.000462 | 0.00992 | 0.000305 | \* ns \* | 1.04 | 0.471 | 1.11 | 100 | 66.7 |
| Bt.23143.2.S1\_at | 0.000487 | 0.00842 | 0.000546 | \* ns \* | -0.942 | -0.604 | -0.817 | 100 | 66.7 |
| Bt.6441.1.S1\_at | 0.000491 | 0.000741 | 0.0527 | \* \* ns | 1.43 | 0.819 | 0.667 | 100 | 66.7 |
| Bt.28113.1.S1\_at | 0.000516 | 0.000659 | 0.00439 | \* \* ns | 1.23 | 0.942 | 0.838 | 100 | 66.7 |
| Bt.1240.1.S1\_at | 0.000524 | 3.21e-05 | 0.00514 | \* \* ns | 1.08 | 0.743 | 0.937 | 100 | 66.7 |
| Bt.8448.1.S1\_at | 0.000526 | 8.78e-05 | 0.0195 | \* \* ns | 1.24 | 0.922 | 1.31 | 100 | 66.7 |
| Bt.21215.2.S1\_at | 0.000527 | 0.000738 | 0.00696 | \* \* ns | 0.821 | 0.708 | 0.872 | 100 | 66.7 |
| Bt.9546.2.S1\_a\_at | 0.000536 | 0.00242 | 0.00134 | \* ns \* | 0.82 | 0.712 | 0.916 | 100 | 66.7 |
| Bt.19338.1.S1\_at | 0.00054 | 0.000516 | 0.00213 | \* \* ns | 1.39 | 0.96 | 1.03 | 100 | 66.7 |
| Bt.605.1.S1\_at | 0.000546 | 0.000293 | 0.00411 | \* \* ns | 1.78 | 1.45 | 1.93 | 100 | 66.7 |
| Bt.21843.1.A1\_at | 0.000581 | 0.000275 | 0.0153 | \* \* ns | 1.51 | 1.1 | 0.981 | 100 | 66.7 |
| Bt.21238.1.S1\_at | 0.000598 | 4.99e-05 | 0.0248 | \* \* ns | 1.03 | 0.873 | 1 | 100 | 66.7 |
| Bt.12859.1.S1\_at | 0.000598 | 0.00493 | 0.000686 | \* ns \* | 1.23 | 0.591 | 0.772 | 100 | 66.7 |
| Bt.12867.3.S1\_at | 0.000602 | 0.00591 | 0.000872 | \* ns \* | 0.957 | 0.429 | 0.809 | 100 | 66.7 |
| Bt.14552.1.A1\_at | 0.000642 | 0.00104 | 0.00324 | \* \* ns | 0.791 | 0.734 | 0.934 | 100 | 66.7 |
| Bt.8260.1.A1\_at | 0.000649 | 0.00368 | 0.000381 | \* ns \* | 1.65 | 0.67 | 0.908 | 100 | 66.7 |
| Bt.2642.1.S1\_at | 0.000659 | 0.00259 | 5.9e-05 | \* ns \* | -0.768 | -0.546 | -0.655 | 100 | 66.7 |
| Bt.8333.1.S1\_at | 0.000674 | 0.00322 | 0.000584 | \* ns \* | -0.832 | -0.649 | -0.82 | 100 | 66.7 |
| Bt.10043.1.S1\_at | 0.000676 | 0.000421 | 0.0023 | \* \* ns | -0.903 | -0.525 | -0.612 | 100 | 66.7 |
| Bt.9749.1.S1\_at | 0.000683 | 0.0181 | 0.000165 | \* ns \* | -0.659 | -0.398 | -0.55 | 100 | 66.7 |
| Bt.22155.1.A1\_at | 7e-04 | 0.00338 | 0.000125 | \* ns \* | 0.722 | 0.489 | 0.63 | 100 | 66.7 |
| Bt.27061.1.S1\_at | 0.000708 | 0.0271 | 0.00181 | \* ns \* | -0.765 | -0.48 | -0.642 | 100 | 66.7 |
| Bt.5319.2.S1\_a\_at | 0.000713 | 0.0131 | 0.000449 | \* ns \* | -0.787 | -0.459 | -0.594 | 100 | 66.7 |
| Bt.222.1.S1\_at | 0.000753 | 0.000534 | 0.00267 | \* \* ns | -2.14 | -1.55 | -1.96 | 100 | 66.7 |
| Bt.22584.1.A1\_at | 0.000762 | 0.000426 | 0.00307 | \* \* ns | 1.25 | 0.757 | 1.05 | 100 | 66.7 |
| Bt.18316.2.S1\_at | 0.00079 | 0.000288 | 0.00483 | \* \* ns | 1.29 | 1.1 | 1.06 | 100 | 66.7 |
| Bt.2156.2.S1\_a\_at | 0.00079 | 0.00327 | 9.88e-05 | \* ns \* | -0.706 | -0.501 | -0.659 | 100 | 66.7 |
| Bt.16025.1.S1\_at | 0.000794 | 0.032 | 0.00146 | \* ns \* | -0.852 | -0.26 | -0.328 | 100 | 66.7 |
| Bt.3306.1.S1\_at | 0.000804 | 0.000509 | 0.00281 | \* \* ns | 0.98 | 0.587 | 0.767 | 100 | 66.7 |
| Bt.13440.3.S1\_at | 0.000805 | 0.002 | 0.00588 | \* \* ns | 1.04 | 0.79 | 1.01 | 100 | 66.7 |
| Bt.21296.1.S1\_at | 0.000811 | 0.000161 | 0.0086 | \* \* ns | 0.775 | 0.571 | 0.775 | 100 | 66.7 |
| Bt.8699.1.S1\_at | 0.000847 | 0.000113 | 0.00258 | \* \* ns | -1.06 | -1.21 | -1.48 | 100 | 66.7 |
| Bt.2816.1.S1\_at | 0.000847 | 0.00313 | 0.000749 | \* ns \* | -0.854 | -0.376 | -0.512 | 100 | 66.7 |
| Bt.15837.1.S1\_at | 0.000863 | 0.0847 | 0.000516 | \* ns \* | -0.685 | -0.267 | -0.454 | 100 | 66.7 |
| Bt.6534.1.S1\_at | 0.000896 | 0.00086 | 0.0484 | \* \* ns | 1.18 | 0.596 | 0.64 | 100 | 66.7 |
| Bt.26682.1.S1\_at | 0.000901 | 0.00187 | 0.00594 | \* \* ns | 0.988 | 0.868 | 1.3 | 100 | 66.7 |
| Bt.6686.1.S1\_at | 0.000913 | 0.000184 | 0.00383 | \* \* ns | 0.878 | 0.892 | 1.02 | 100 | 66.7 |
| Bt.26872.1.S1\_at | 0.000916 | 0.000943 | 0.0148 | \* \* ns | 1.01 | 0.946 | 1.18 | 100 | 66.7 |
| Bt.11908.1.S1\_at | 0.000924 | 0.00274 | 2.69e-06 | \* ns \* | 0.912 | 0.528 | 0.811 | 100 | 66.7 |
| Bt.6778.1.S1\_at | 0.000932 | 0.000495 | 0.00746 | \* \* ns | 1.69 | 1.13 | 1.72 | 100 | 66.7 |
| Bt.27956.1.A1\_at | 0.000939 | 0.000642 | 0.00267 | \* \* ns | 0.8 | 0.631 | 0.673 | 100 | 66.7 |
| Bt.4756.1.S1\_at | 0.000948 | 0.00889 | 0.00118 | \* ns \* | -0.662 | -0.367 | -0.418 | 100 | 66.7 |
| Bt.28393.1.S1\_at | 0.000958 | 0.000819 | 0.00218 | \* \* ns | 1.4 | 0.831 | 0.924 | 100 | 66.7 |
| Bt.28146.1.A1\_at | 0.00101 | 0.000636 | 0.00432 | \* \* ns | -1.12 | -0.886 | -1.1 | 100 | 66.7 |
| Bt.25887.1.A1\_at | 0.00104 | 0.00268 | 0.000721 | \* ns \* | 1.26 | 0.932 | 1.35 | 100 | 66.7 |
| Bt.27149.1.S1\_at | 0.00107 | 0.00587 | 0.0011 | \* ns \* | -0.845 | -0.625 | -0.744 | 100 | 66.7 |
| Bt.21113.1.S1\_a\_at | 0.00108 | 0.000137 | 0.00193 | \* \* ns | -1.19 | -0.983 | -1.31 | 100 | 66.7 |
| Bt.2821.1.S1\_at | 0.0011 | 0.00317 | 0.00164 | \* ns \* | -0.787 | -0.415 | -0.558 | 100 | 66.7 |
| Bt.9322.1.S1\_at | 0.0011 | 0.000429 | 0.00264 | \* \* ns | 1.38 | 1.05 | 1.32 | 100 | 66.7 |
| Bt.7056.2.A1\_x\_at | 0.00111 | 2.16e-05 | 0.00205 | \* \* ns | 1.71 | 1.58 | 1.26 | 100 | 66.7 |
| Bt.26779.1.S1\_at | 0.00112 | 3.05e-05 | 0.00142 | ns \* \* | -1.43 | -1.08 | -1.92 | 100 | 66.7 |
| Bt.9741.1.S1\_at | 0.00112 | 0.000267 | 0.00156 | ns \* \* | 0.965 | 0.722 | 0.834 | 100 | 66.7 |
| Bt.868.1.S1\_at | 0.00113 | 4.85e-05 | 3.25e-05 | ns \* \* | -0.755 | -0.624 | -0.854 | 100 | 66.7 |
| Bt.24017.1.A1\_s\_at | 0.00113 | 0.000632 | 5.46e-05 | ns \* \* | -0.921 | -0.571 | -0.601 | 100 | 66.7 |
| Bt.3174.1.A1\_at | 0.00113 | 0.000105 | 3.06e-05 | ns \* \* | 0.794 | 0.762 | 0.856 | 100 | 66.7 |
| Bt.15714.2.S1\_a\_at | 0.00114 | 6.3e-06 | 0.000299 | ns \* \* | 0.94 | 1.03 | 1.43 | 100 | 66.7 |
| Bt.9176.1.A1\_at | 0.00114 | 5.09e-06 | 0.00144 | ns \* \* | 0.941 | 0.952 | 1.08 | 100 | 66.7 |
| Bt.22340.1.S1\_at | 0.00114 | 2.26e-05 | 0.000225 | ns \* \* | 0.804 | 0.682 | 0.629 | 100 | 66.7 |
| Bt.20672.1.S1\_at | 0.00116 | 5.28e-05 | 0.000487 | ns \* \* | 1.04 | 0.882 | 1.12 | 100 | 66.7 |
| Bt.60.1.S1\_at | 0.00116 | 0.000168 | 0.000313 | ns \* \* | -0.82 | -0.57 | -0.816 | 100 | 66.7 |
| Bt.16041.2.S1\_at | 0.00117 | 1.41e-05 | 0.000703 | ns \* \* | 0.986 | 0.939 | 1.74 | 100 | 66.7 |
| Bt.14054.2.S1\_at | 0.00117 | 0.000451 | 0.00149 | ns \* \* | -1.62 | -1.21 | -1.64 | 100 | 66.7 |
| Bt.2501.1.S1\_at | 0.00117 | 3.99e-07 | 1.81e-06 | ns \* \* | -1.1 | -1.38 | -1.8 | 100 | 66.7 |
| Bt.7434.1.S1\_at | 0.00117 | 0.00144 | 0.000348 | ns \* \* | 0.683 | 0.588 | 0.577 | 100 | 66.7 |
| Bt.14395.2.S1\_at | 0.00118 | 2.49e-05 | 0.00136 | ns \* \* | 0.9 | 0.739 | 1.67 | 100 | 66.7 |
| Bt.4584.1.S1\_at | 0.00119 | 3.7e-06 | 7.01e-05 | ns \* \* | -0.99 | -0.891 | -1.1 | 100 | 66.7 |
| Bt.13771.3.S1\_at | 0.0012 | 0.00119 | 9.11e-05 | ns \* \* | 1.59 | 0.592 | 0.761 | 100 | 66.7 |
| Bt.11326.1.S1\_at | 0.00121 | 0.00138 | 1.57e-05 | ns \* \* | -0.605 | -0.508 | -0.706 | 100 | 66.7 |
| Bt.6493.1.S1\_at | 0.00123 | 0.000288 | 0.000161 | ns \* \* | 0.667 | 0.574 | 0.684 | 100 | 66.7 |
| Bt.5527.2.S1\_a\_at | 0.00123 | 5.14e-07 | 1.39e-06 | ns \* \* | -1.5 | -1.15 | -1.51 | 100 | 66.7 |
| Bt.20194.1.S1\_at | 0.00124 | 0.000624 | 2.92e-05 | ns \* \* | -0.714 | -0.58 | -0.83 | 100 | 66.7 |
| Bt.7873.1.S1\_at | 0.00125 | 8.12e-05 | 5.52e-05 | ns \* \* | 0.912 | 0.671 | 0.998 | 100 | 66.7 |
| Bt.21351.1.S1\_a\_at | 0.00125 | 0.000175 | 8.23e-05 | ns \* \* | 0.872 | 0.834 | 1.04 | 100 | 66.7 |
| Bt.6490.2.S1\_at | 0.00126 | 4.37e-06 | 1.07e-05 | ns \* \* | 1.43 | 1.29 | 1.28 | 100 | 66.7 |
| Bt.21453.1.S1\_at | 0.00127 | 9.28e-06 | 7.57e-05 | ns \* \* | -1.39 | -1.15 | -1.77 | 100 | 66.7 |
| Bt.9288.2.A1\_at | 0.00127 | 7.84e-05 | 0.000233 | ns \* \* | 1.23 | 0.689 | 0.857 | 100 | 66.7 |
| Bt.12249.1.S1\_at | 0.00127 | 0.00041 | 0.000725 | ns \* \* | 1.76 | 0.577 | 0.837 | 100 | 66.7 |
| Bt.20422.1.S1\_at | 0.00127 | 2e-05 | 8.23e-05 | ns \* \* | -0.965 | -0.916 | -1.78 | 100 | 66.7 |
| Bt.6449.1.S1\_at | 0.00128 | 3.11e-06 | 1.92e-05 | ns \* \* | 1.07 | 1.2 | 1.47 | 100 | 66.7 |
| Bt.24817.1.S1\_at | 0.00128 | 5.93e-07 | 6.03e-07 | ns \* \* | -2.14 | -1.28 | -1.73 | 100 | 66.7 |
| Bt.15561.1.S1\_at | 0.00129 | 1.37e-05 | 1.92e-05 | ns \* \* | 1.02 | 0.869 | 1.16 | 100 | 66.7 |
| Bt.26095.1.A1\_at | 0.0013 | 3.73e-06 | 0.000477 | ns \* \* | 1.51 | 1.3 | 1.77 | 100 | 66.7 |
| Bt.22770.1.S1\_at | 0.0013 | 0.000258 | 4.31e-06 | ns \* \* | 1.38 | 0.94 | 1.6 | 100 | 66.7 |
| Bt.23673.1.A1\_at | 0.00131 | 4.06e-05 | 0.000347 | ns \* \* | 0.976 | 1.07 | 1.31 | 100 | 66.7 |
| Bt.11038.1.S1\_at | 0.00131 | 8.35e-06 | 1.71e-05 | ns \* \* | 1.5 | 1.24 | 1.45 | 100 | 66.7 |
| Bt.28162.1.S1\_at | 0.00132 | 4.51e-06 | 2.6e-05 | ns \* \* | -1.32 | -1.37 | -1.48 | 100 | 66.7 |
| Bt.24471.1.S1\_a\_at | 0.00133 | 0.00093 | 0.000579 | ns \* \* | 1.09 | 1.05 | 1.33 | 100 | 66.7 |
| Bt.1415.1.S1\_at | 0.00134 | 0.000178 | 0.000144 | ns \* \* | -0.934 | -0.863 | -1.28 | 100 | 66.7 |
| Bt.15867.1.S1\_at | 0.00135 | 0.000554 | 0.00139 | ns \* \* | 0.686 | 0.734 | 1.17 | 100 | 66.7 |
| Bt.9981.1.S1\_at | 0.00135 | 0.000737 | 0.00104 | ns \* \* | -0.941 | -0.711 | -0.989 | 100 | 66.7 |
| Bt.22295.1.S1\_at | 0.00135 | 0.000352 | 6.03e-07 | ns \* \* | -0.641 | -0.622 | -0.908 | 100 | 66.7 |
| Bt.24474.1.S1\_at | 0.00135 | 1.02e-05 | 2.15e-05 | ns \* \* | -1.01 | -0.785 | -1.13 | 100 | 66.7 |
| Bt.5135.1.S1\_at | 0.00136 | 4.64e-06 | 4.96e-05 | ns \* \* | -0.939 | -1.06 | -1.39 | 100 | 66.7 |
| Bt.4972.2.S1\_at | 0.00136 | 0.00183 | 0.00167 | ns \* \* | 0.882 | 0.842 | 1.1 | 100 | 66.7 |
| Bt.10565.1.A1\_at | 0.00138 | 1.14e-06 | 1.75e-06 | ns \* \* | 0.79 | 1.03 | 1.3 | 100 | 66.7 |
| Bt.6440.1.S1\_at | 0.00138 | 0.000216 | 4.95e-06 | ns \* \* | 0.67 | 0.816 | 0.929 | 100 | 66.7 |
| Bt.24717.2.S1\_a\_at | 0.00138 | 0.000859 | 0.000153 | ns \* \* | 0.878 | 0.667 | 0.814 | 100 | 66.7 |
| Bt.4831.1.S1\_at | 0.00138 | 0.000123 | 2.83e-05 | ns \* \* | -0.799 | -0.597 | -0.782 | 100 | 66.7 |
| Bt.5415.1.S1\_at | 0.00139 | 0.000604 | 0.00132 | ns \* \* | 0.793 | 0.582 | 0.966 | 100 | 66.7 |
| Bt.10352.1.S1\_at | 0.00141 | 4.34e-05 | 0.000295 | ns \* \* | -1.05 | -0.779 | -1.12 | 100 | 66.7 |
| Bt.13991.2.S1\_a\_at | 0.00141 | 0.000718 | 0.000141 | ns \* \* | 0.891 | 0.844 | 1.15 | 100 | 66.7 |
| Bt.12684.2.S1\_at | 0.00141 | 6.46e-05 | 1.57e-06 | ns \* \* | 0.744 | 0.704 | 1.1 | 100 | 66.7 |
| Bt.27938.1.A1\_at | 0.00142 | 1.17e-05 | 0.000217 | ns \* \* | 1.16 | 1.04 | 1.44 | 100 | 66.7 |
| Bt.27139.1.S1\_at | 0.00143 | 0.000177 | 0.000631 | ns \* \* | -0.901 | -0.648 | -0.856 | 100 | 66.7 |
| Bt.3395.1.S1\_at | 0.00143 | 0.000773 | 1.42e-05 | ns \* \* | -0.676 | -0.607 | -0.82 | 100 | 66.7 |
| Bt.9223.1.A1\_at | 0.00144 | 5.56e-06 | 2.39e-05 | ns \* \* | 0.987 | 0.966 | 1.29 | 100 | 66.7 |
| Bt.15909.1.S1\_at | 0.00144 | 0.000414 | 0.000379 | ns \* \* | -0.887 | -0.923 | -1.13 | 100 | 66.7 |
| Bt.20257.1.S1\_at | 0.00144 | 3.67e-06 | 9.14e-09 | ns \* \* | 0.89 | 0.865 | 1.13 | 100 | 66.7 |
| Bt.20340.2.S1\_at | 0.00145 | 2e-04 | 0.000616 | ns \* \* | 0.82 | 1.05 | 1.32 | 100 | 66.7 |
| Bt.18894.1.A1\_at | 0.00145 | 0.000101 | 0.000104 | ns \* \* | 0.883 | 0.758 | 1.42 | 100 | 66.7 |
| Bt.27116.2.S1\_at | 0.00145 | 3.43e-06 | 1.84e-06 | ns \* \* | -1.02 | -0.817 | -1.08 | 100 | 66.7 |
| Bt.20917.2.S1\_a\_at | 0.00145 | 0.000205 | 0.000319 | ns \* \* | -0.929 | -0.899 | -1.22 | 100 | 66.7 |
| Bt.23326.2.A1\_at | 0.00146 | 5.49e-05 | 0.000106 | ns \* \* | 0.885 | 0.802 | 0.889 | 100 | 66.7 |
| Bt.26726.1.S1\_at | 0.00146 | 7.63e-05 | 0.000142 | ns \* \* | 1.11 | 1.32 | 1.64 | 100 | 66.7 |
| Bt.12337.2.S1\_a\_at | 0.00146 | 0.000138 | 2.75e-06 | ns \* \* | -0.934 | -0.574 | -0.928 | 100 | 66.7 |
| Bt.20582.2.A1\_at | 0.00147 | 1.89e-06 | 2.78e-06 | ns \* \* | 1.1 | 1.46 | 1.6 | 100 | 66.7 |
| Bt.23169.1.S1\_at | 0.00148 | 0.000188 | 0.000351 | ns \* \* | 0.862 | 0.784 | 0.919 | 100 | 66.7 |
| Bt.13908.1.S1\_at | 0.00148 | 5.77e-05 | 6.97e-05 | ns \* \* | -1.25 | -0.814 | -1.3 | 100 | 66.7 |
| Bt.18165.1.A1\_at | 0.00149 | 3.67e-05 | 0.00175 | ns \* \* | -2.71 | -2.48 | -3.03 | 100 | 66.7 |
| Bt.13748.1.S1\_at | 0.0015 | 4.08e-06 | 0.000216 | ns \* \* | 1.01 | 1.07 | 1.17 | 100 | 66.7 |
| Bt.12609.1.A1\_at | 0.0015 | 0.00116 | 0.00128 | ns \* \* | 1.01 | 0.721 | 0.929 | 100 | 66.7 |
| Bt.13183.1.A1\_at | 0.00151 | 0.000121 | 5.42e-05 | ns \* \* | 1.05 | 0.975 | 1.12 | 100 | 66.7 |
| Bt.11257.2.S1\_at | 0.00151 | 0.00129 | 6.6e-05 | ns \* \* | 0.598 | 0.555 | 0.813 | 100 | 66.7 |
| Bt.21072.1.A1\_at | 0.00151 | 3.06e-05 | 0.000297 | ns \* \* | 1.15 | 0.954 | 1.45 | 100 | 66.7 |
| Bt.3029.1.S1\_at | 0.00151 | 0.00012 | 0.000211 | ns \* \* | 0.735 | 0.708 | 0.889 | 100 | 66.7 |
| Bt.21369.1.S1\_at | 0.00151 | 0.000834 | 0.000328 | ns \* \* | 0.784 | 0.616 | 0.828 | 100 | 66.7 |
| Bt.16737.1.A1\_at | 0.00152 | 0.000279 | 0.000731 | ns \* \* | 0.861 | 0.915 | 0.932 | 100 | 66.7 |
| Bt.8208.1.A1\_at | 0.00152 | 0.000129 | 0.000293 | ns \* \* | 1.27 | 1.17 | 1.38 | 100 | 66.7 |
| Bt.12906.1.S1\_at | 0.00153 | 0.000814 | 0.000928 | ns \* \* | 1.11 | 1.01 | 1.25 | 100 | 66.7 |
| Bt.22854.1.S1\_at | 0.00153 | 2.63e-05 | 0.000662 | ns \* \* | -1.22 | -1.46 | -2.12 | 100 | 66.7 |
| Bt.4031.1.S1\_at | 0.00154 | 1.22e-06 | 2.42e-06 | ns \* \* | 0.825 | 0.975 | 1.03 | 100 | 66.7 |
| Bt.6865.1.S1\_at | 0.00154 | 0.00115 | 0.00103 | ns \* \* | 1.57 | 1.11 | 1.39 | 100 | 66.7 |
| Bt.11745.1.S1\_at | 0.00155 | 6.36e-05 | 2.5e-06 | ns \* \* | 1.06 | 1 | 1.68 | 100 | 66.7 |
| Bt.21272.1.S1\_at | 0.00157 | 0.000213 | 0.000158 | ns \* \* | -0.599 | -0.69 | -0.75 | 100 | 66.7 |
| Bt.9889.1.S1\_at | 0.00158 | 7.92e-05 | 1.38e-05 | ns \* \* | 0.752 | 0.82 | 0.977 | 100 | 66.7 |
| Bt.4844.1.S1\_at | 0.00158 | 1.26e-05 | 0.00014 | ns \* \* | 1.86 | 1.65 | 1.71 | 100 | 66.7 |
| Bt.21578.1.S1\_at | 0.00158 | 4.09e-06 | 1.2e-05 | ns \* \* | -1.19 | -0.925 | -1.5 | 100 | 66.7 |
| Bt.8286.1.S1\_at | 0.00159 | 0.00019 | 0.000478 | ns \* \* | -0.683 | -0.761 | -1.16 | 100 | 66.7 |
| Bt.3589.2.A1\_at | 0.00159 | 0.000258 | 0.00107 | ns \* \* | 1 | 1.11 | 1.33 | 100 | 66.7 |
| Bt.2599.1.S1\_at | 0.00159 | 6.92e-06 | 0.000816 | ns \* \* | 1.62 | 1.08 | 1.97 | 100 | 66.7 |
| Bt.5879.1.S1\_at | 0.00159 | 0.000867 | 4.47e-06 | ns \* \* | 0.75 | 0.62 | 1.09 | 100 | 66.7 |
| Bt.250.1.S1\_at | 0.00162 | 0.000301 | 4.51e-07 | ns \* \* | -0.788 | -0.633 | -0.74 | 100 | 66.7 |
| Bt.10122.1.S1\_at | 0.00164 | 0.000113 | 0.000108 | ns \* \* | 1.13 | 0.742 | 0.937 | 100 | 66.7 |
| Bt.22555.1.S1\_at | 0.00164 | 3.94e-06 | 7.66e-05 | ns \* \* | -0.981 | -1.14 | -1.56 | 100 | 66.7 |
| Bt.841.2.A1\_at | 0.00165 | 1e-05 | 3.17e-05 | ns \* \* | 1.23 | 1.11 | 1.57 | 100 | 66.7 |
| Bt.17332.3.A1\_at | 0.00166 | 0.000103 | 0.000269 | ns \* \* | 0.766 | 0.845 | 1.18 | 100 | 66.7 |
| Bt.28208.1.S1\_at | 0.00167 | 7.86e-10 | 1.01e-07 | ns \* \* | -2.43 | -1.95 | -2.35 | 100 | 66.7 |
| Bt.23668.1.A1\_at | 0.00167 | 0.00173 | 0.000396 | ns \* \* | -0.673 | -0.593 | -0.777 | 100 | 66.7 |
| Bt.7177.1.S1\_at | 0.00167 | 0.000139 | 0.000619 | ns \* \* | 0.799 | 0.676 | 0.876 | 100 | 66.7 |
| Bt.7041.1.S1\_at | 0.00168 | 0.000643 | 0.000499 | ns \* \* | 0.661 | 0.672 | 0.755 | 100 | 66.7 |
| Bt.10962.1.S1\_at | 0.00168 | 0.00134 | 2.91e-05 | ns \* \* | 0.585 | 0.488 | 0.575 | 100 | 66.7 |
| Bt.23647.1.A1\_a\_at | 0.00168 | 0.000133 | 2.31e-05 | ns \* \* | 1.41 | 1.02 | 1.21 | 100 | 66.7 |
| Bt.20965.1.S1\_at | 0.00169 | 0.000105 | 0.000403 | ns \* \* | -0.802 | -0.77 | -0.957 | 100 | 66.7 |
| Bt.7619.2.S1\_at | 0.0017 | 7.24e-06 | 4.7e-05 | ns \* \* | -1.06 | -1.06 | -1.59 | 100 | 66.7 |
| Bt.21191.3.S1\_at | 0.0017 | 0.000769 | 0.0017 | ns \* \* | -1.27 | -1.32 | -1.91 | 100 | 66.7 |
| Bt.14644.1.A1\_at | 0.00171 | 0.00102 | 0.00175 | ns \* \* | 0.855 | 0.647 | 0.826 | 100 | 66.7 |
| Bt.23268.1.S1\_at | 0.00172 | 6.62e-05 | 0.00025 | ns \* \* | 1.04 | 0.933 | 1.14 | 100 | 66.7 |
| Bt.9190.1.S1\_at | 0.00174 | 0.000656 | 7.56e-05 | ns \* \* | -0.75 | -0.647 | -0.909 | 100 | 66.7 |
| Bt.21485.1.S1\_at | 0.00174 | 0.00198 | 1e-04 | ns \* \* | 0.614 | 0.563 | 0.781 | 100 | 66.7 |
| Bt.16106.1.A1\_at | 0.00175 | 1.61e-05 | 1.3e-05 | ns \* \* | 1.31 | 0.989 | 1.45 | 100 | 66.7 |
| Bt.20955.1.A1\_at | 0.00175 | 0.00151 | 0.000543 | ns \* \* | -1.19 | -0.915 | -1.25 | 100 | 66.7 |
| Bt.10797.2.S1\_a\_at | 0.00177 | 1.22e-05 | 5.85e-07 | ns \* \* | -0.962 | -0.829 | -1.12 | 100 | 66.7 |
| Bt.17253.1.A1\_at | 0.00179 | 0.00082 | 0.000957 | ns \* \* | 1.48 | 0.711 | 0.753 | 100 | 66.7 |
| Bt.13966.1.S1\_at | 0.00181 | 0.000859 | 3.09e-05 | ns \* \* | -0.664 | -0.531 | -0.812 | 100 | 66.7 |
| Bt.3821.1.A1\_at | 0.00181 | 4.2e-05 | 0.000331 | ns \* \* | 0.87 | 0.879 | 0.944 | 100 | 66.7 |
| Bt.2394.1.S1\_at | 0.00182 | 0.000684 | 1.92e-05 | ns \* \* | -0.731 | -0.63 | -0.859 | 100 | 66.7 |
| Bt.20692.1.A1\_at | 0.00183 | 0.000827 | 0.000545 | ns \* \* | 0.856 | 0.586 | 0.728 | 100 | 66.7 |
| Bt.28210.1.S1\_at | 0.00183 | 1.37e-05 | 3.07e-06 | ns \* \* | 0.551 | 0.904 | 0.963 | 100 | 66.7 |
| Bt.4279.3.S1\_a\_at | 0.00183 | 1.5e-05 | 1.8e-06 | ns \* \* | 0.761 | 0.902 | 1.05 | 100 | 66.7 |
| Bt.3541.1.S1\_at | 0.00184 | 4.44e-06 | 5.05e-08 | ns \* \* | 0.657 | 0.905 | 0.99 | 100 | 66.7 |
| Bt.5351.1.S1\_a\_at | 0.00189 | 0.000179 | 0.00141 | ns \* \* | 0.97 | 0.745 | 1.23 | 100 | 66.7 |
| Bt.19330.1.A1\_s\_at | 0.00189 | 3.61e-06 | 3.44e-06 | ns \* \* | 1.37 | 1.2 | 1.49 | 100 | 66.7 |
| Bt.27431.1.A1\_at | 0.00189 | 2.99e-05 | 2.31e-05 | ns \* \* | 0.974 | 0.994 | 1.14 | 100 | 66.7 |
| Bt.11966.1.A1\_at | 0.0019 | 0.000545 | 0.000401 | ns \* \* | 1.69 | 0.944 | 1.06 | 100 | 66.7 |
| Bt.22355.1.S1\_at | 0.00191 | 6.21e-05 | 0.000455 | ns \* \* | 0.685 | 0.683 | 0.955 | 100 | 66.7 |
| Bt.13248.1.A1\_at | 0.00191 | 0.000243 | 0.000323 | ns \* \* | 1.01 | 1.04 | 1.21 | 100 | 66.7 |
| Bt.22125.2.A1\_at | 0.00191 | 6e-04 | 0.00116 | ns \* \* | 0.752 | 0.903 | 0.916 | 100 | 66.7 |
| Bt.5112.1.A1\_a\_at | 0.00192 | 3.25e-05 | 0.000972 | ns \* \* | -0.961 | -1.14 | -1.3 | 100 | 66.7 |
| Bt.23392.1.S1\_at | 0.00194 | 2.02e-06 | 3.77e-09 | ns \* \* | 0.831 | 1.04 | 1.22 | 100 | 66.7 |
| Bt.8015.3.A1\_at | 0.00196 | 3.23e-05 | 7.09e-05 | ns \* \* | -1.25 | -1.17 | -1.45 | 100 | 66.7 |
| Bt.8213.1.S1\_at | 0.00197 | 4.33e-05 | 7.76e-05 | ns \* \* | -0.736 | -0.796 | -1.13 | 100 | 66.7 |
| Bt.20355.1.S1\_at | 0.00197 | 1.13e-05 | 0.00011 | ns \* \* | -1.04 | -0.858 | -1.12 | 100 | 66.7 |
| Bt.23941.1.A1\_at | 0.00197 | 5.69e-06 | 1.09e-05 | ns \* \* | 0.722 | 0.884 | 1.03 | 100 | 66.7 |
| Bt.9767.1.S1\_a\_at | 0.00198 | 3.6e-05 | 0.000336 | ns \* \* | -1.22 | -1.08 | -1.37 | 100 | 66.7 |
| Bt.14624.2.S1\_at | 0.00198 | 0.000872 | 0.00175 | ns \* \* | -1.03 | -0.853 | -1.03 | 100 | 66.7 |
| Bt.7384.1.A1\_at | 0.00199 | 1.17e-06 | 1.4e-06 | ns \* \* | 0.675 | 1.03 | 1.47 | 100 | 66.7 |
| Bt.7727.1.S1\_at | 0.002 | 8.26e-05 | 6.08e-06 | ns \* \* | 0.778 | 0.663 | 1.12 | 100 | 66.7 |
| Bt.18402.1.S1\_at | 0.00202 | 1.04e-07 | 1.43e-07 | ns \* \* | -1.15 | -1.27 | -1.64 | 100 | 66.7 |
| Bt.2092.1.S1\_at | 0.00203 | 0.000527 | 0.00164 | ns \* \* | 1.07 | 0.731 | 0.842 | 100 | 66.7 |
| Bt.28059.1.S1\_at | 0.00203 | 0.000541 | 0.000148 | ns \* \* | -1 | -0.825 | -1.24 | 100 | 66.7 |
| Bt.2109.1.S1\_at | 0.00204 | 4.27e-05 | 0.000127 | ns \* \* | 0.81 | 0.835 | 1.1 | 100 | 66.7 |
| Bt.3098.1.S1\_at | 0.00204 | 0.00105 | 0.000303 | ns \* \* | 0.737 | 0.68 | 0.91 | 100 | 66.7 |
| Bt.25092.1.S1\_at | 0.00204 | 0.000196 | 0.000145 | ns \* \* | -0.83 | -0.824 | -1.12 | 100 | 66.7 |
| Bt.28390.1.S1\_at | 0.00204 | 0.000267 | 0.000175 | ns \* \* | -0.854 | -0.768 | -1.01 | 100 | 66.7 |
| Bt.22125.1.S1\_at | 0.00204 | 0.000328 | 0.000707 | ns \* \* | 0.842 | 0.699 | 1.24 | 100 | 66.7 |
| Bt.27800.1.A1\_at | 0.00207 | 0.00148 | 0.000405 | ns \* \* | -0.757 | -0.677 | -0.975 | 100 | 66.7 |
| Bt.27160.1.A1\_at | 0.00208 | 0.000313 | 0.00162 | ns \* \* | 0.864 | 0.713 | 1.23 | 100 | 66.7 |
| Bt.6789.1.S1\_at | 0.00208 | 0.00135 | 0.000384 | ns \* \* | -0.753 | -0.437 | -0.769 | 100 | 66.7 |
| Bt.22534.1.S1\_at | 0.00209 | 8e-05 | 0.000677 | ns \* \* | 0.919 | 0.849 | 1.01 | 100 | 66.7 |
| Bt.18039.1.S1\_at | 0.0021 | 0.000484 | 0.000447 | ns \* \* | 0.907 | 0.608 | 0.829 | 100 | 66.7 |
| Bt.28057.1.S1\_at | 0.00211 | 0.000332 | 0.000131 | ns \* \* | 0.667 | 0.645 | 0.874 | 100 | 66.7 |
| Bt.7425.1.S1\_at | 0.00211 | 5.37e-05 | 2.32e-05 | ns \* \* | 0.901 | 1.31 | 1.87 | 100 | 66.7 |
| Bt.3427.1.S1\_at | 0.00211 | 0.000893 | 0.000209 | ns \* \* | -0.875 | -0.582 | -1.26 | 100 | 66.7 |
| Bt.16188.1.S1\_at | 0.00215 | 0.000141 | 2.08e-06 | ns \* \* | 1.05 | 0.689 | 0.933 | 100 | 66.7 |
| Bt.13645.1.S1\_at | 0.00215 | 0.000526 | 0.00063 | ns \* \* | -1.72 | -1.19 | -1.69 | 100 | 66.7 |
| Bt.9803.1.S1\_at | 0.00215 | 2.19e-05 | 2.33e-05 | ns \* \* | -1 | -1.04 | -1.31 | 100 | 66.7 |
| Bt.9391.2.S1\_at | 0.00219 | 0.000936 | 0.00126 | ns \* \* | 0.892 | 0.679 | 0.91 | 100 | 66.7 |
| Bt.17425.1.A1\_at | 0.00219 | 2.41e-05 | 0.000278 | ns \* \* | -1.09 | -1.05 | -1.18 | 100 | 66.7 |
| Bt.5412.1.S1\_at | 0.00219 | 2.36e-05 | 0.000173 | ns \* \* | -1.07 | -0.887 | -1.06 | 100 | 66.7 |
| Bt.26684.1.S1\_at | 0.00219 | 0.000366 | 5.15e-06 | ns \* \* | 0.796 | 0.608 | 0.744 | 100 | 66.7 |
| Bt.18945.1.A1\_at | 0.00223 | 8.12e-06 | 3.74e-06 | ns \* \* | 0.968 | 0.897 | 0.916 | 100 | 66.7 |
| Bt.13874.1.A1\_at | 0.00223 | 0.000159 | 8.71e-06 | ns \* \* | 0.69 | 0.588 | 0.662 | 100 | 66.7 |
| Bt.3414.1.S1\_at | 0.00224 | 0.000873 | 0.000262 | ns \* \* | -0.88 | -0.66 | -0.852 | 100 | 66.7 |
| Bt.18748.1.A1\_at | 0.00224 | 5.5e-05 | 1.65e-05 | ns \* \* | 0.812 | 0.878 | 1.21 | 100 | 66.7 |
| Bt.5376.1.S1\_at | 0.00225 | 0.000335 | 1.03e-05 | ns \* \* | -0.732 | -0.522 | -0.73 | 100 | 66.7 |
| Bt.9877.1.S1\_at | 0.00226 | 3.35e-05 | 7.26e-08 | ns \* \* | 0.551 | 0.834 | 0.907 | 100 | 66.7 |
| Bt.6111.1.S1\_at | 0.00227 | 0.000569 | 5.97e-05 | ns \* \* | -0.759 | -0.676 | -0.893 | 100 | 66.7 |
| Bt.22084.2.S1\_at | 0.00232 | 0.00026 | 0.000525 | ns \* \* | 0.884 | 1.02 | 1.55 | 100 | 66.7 |
| Bt.24137.1.A1\_at | 0.00232 | 6.95e-05 | 5.56e-05 | ns \* \* | 0.964 | 1.3 | 1.55 | 100 | 66.7 |
| Bt.21774.2.S1\_a\_at | 0.00235 | 0.000303 | 0.0018 | ns \* \* | 0.64 | 0.561 | 0.81 | 100 | 66.7 |
| Bt.20720.1.S1\_at | 0.00237 | 2.38e-05 | 7.66e-05 | ns \* \* | -0.951 | -0.979 | -1.49 | 100 | 66.7 |
| Bt.4662.1.S1\_at | 0.00237 | 3.06e-05 | 1.02e-07 | ns \* \* | 0.619 | 0.817 | 1.49 | 100 | 66.7 |
| Bt.21351.1.S1\_at | 0.00238 | 0.000175 | 0.00144 | ns \* \* | 1.22 | 0.984 | 1.08 | 100 | 66.7 |
| Bt.20846.1.A1\_a\_at | 0.00239 | 7.6e-05 | 8.79e-05 | ns \* \* | 1.05 | 1.17 | 1.78 | 100 | 66.7 |
| Bt.6019.1.S1\_at | 0.00241 | 0.000495 | 0.000484 | ns \* \* | -0.72 | -0.515 | -0.714 | 100 | 66.7 |
| Bt.22543.1.S1\_at | 0.00244 | 9.84e-05 | 3.5e-05 | ns \* \* | -1.31 | -0.671 | -1.02 | 100 | 66.7 |
| Bt.2230.1.S2\_at | 0.00244 | 2.33e-06 | 2.25e-06 | ns \* \* | 0.837 | 1.1 | 1.27 | 100 | 66.7 |
| Bt.6020.1.S1\_at | 0.00247 | 5.42e-05 | 1.82e-05 | ns \* \* | -0.771 | -0.764 | -1.18 | 100 | 66.7 |
| Bt.3500.1.S1\_at | 0.00249 | 0.000941 | 0.000488 | ns \* \* | -0.839 | -0.545 | -0.604 | 100 | 66.7 |
| Bt.20872.1.A1\_at | 0.0025 | 0.000195 | 0.000421 | ns \* \* | 0.714 | 0.749 | 0.803 | 100 | 66.7 |
| Bt.27371.1.S1\_at | 0.00251 | 9.84e-05 | 3.67e-05 | ns \* \* | 1.03 | 0.806 | 1.47 | 100 | 66.7 |
| Bt.4743.1.A1\_at | 0.00252 | 6.02e-05 | 7e-06 | ns \* \* | 0.88 | 0.593 | 1.11 | 100 | 66.7 |
| Bt.22516.1.A1\_at | 0.00252 | 3.67e-05 | 1.61e-05 | ns \* \* | -0.934 | -0.777 | -0.946 | 100 | 66.7 |
| Bt.27018.1.S1\_at | 0.00253 | 0.000142 | 2.42e-05 | ns \* \* | -0.899 | -0.737 | -1.19 | 100 | 66.7 |
| Bt.7266.1.S1\_at | 0.00253 | 0.000289 | 0.000797 | ns \* \* | -0.843 | -1.02 | -1.37 | 100 | 66.7 |
| Bt.23218.1.S1\_at | 0.00256 | 3.6e-07 | 5.58e-07 | ns \* \* | 0.979 | 1.07 | 1.07 | 100 | 66.7 |
| Bt.1171.1.S1\_at | 0.00259 | 0.00172 | 0.00133 | ns \* \* | -1.03 | -0.8 | -1.06 | 100 | 66.7 |
| Bt.3898.1.S1\_at | 0.00259 | 1.34e-05 | 9.76e-05 | ns \* \* | -1.44 | -1.27 | -1.53 | 100 | 66.7 |
| Bt.20445.1.S1\_at | 0.00259 | 0.000598 | 1.48e-06 | ns \* \* | -0.792 | -0.63 | -0.76 | 100 | 66.7 |
| Bt.21015.1.S1\_at | 0.00261 | 2.91e-08 | 0.000178 | ns \* \* | -1.35 | -1.4 | -2.36 | 100 | 66.7 |
| Bt.3388.1.S1\_at | 0.00264 | 0.00127 | 0.00139 | ns \* \* | -0.693 | -0.605 | -0.731 | 100 | 66.7 |
| Bt.5494.1.S1\_at | 0.00266 | 0.000683 | 0.00114 | ns \* \* | 1.11 | 0.824 | 1.11 | 100 | 66.7 |
| Bt.10608.2.S1\_a\_at | 0.00267 | 6.69e-07 | 3.71e-06 | ns \* \* | -1.1 | -0.984 | -1.36 | 100 | 66.7 |
| Bt.9242.1.S1\_at | 0.00268 | 0.000641 | 0.000931 | ns \* \* | -0.761 | -0.653 | -0.917 | 100 | 66.7 |
| Bt.23216.1.S1\_at | 0.00268 | 5.45e-06 | 1.45e-05 | ns \* \* | 0.694 | 1.19 | 1.54 | 100 | 66.7 |
| Bt.12862.1.S1\_at | 0.00269 | 4.45e-05 | 0.00025 | ns \* \* | 1.2 | 1.09 | 1.26 | 100 | 66.7 |
| Bt.23605.2.S1\_at | 0.00269 | 0.000582 | 0.000312 | ns \* \* | 0.684 | 0.805 | 1.11 | 100 | 66.7 |
| Bt.16094.1.S1\_at | 0.00271 | 5.69e-05 | 0.000407 | ns \* \* | 1.19 | 1.33 | 1.66 | 100 | 66.7 |
| Bt.21440.1.S1\_at | 0.00272 | 3.46e-07 | 4.14e-05 | ns \* \* | 0.812 | 1.16 | 1.21 | 100 | 66.7 |
| Bt.4071.1.S1\_at | 0.00272 | 0.000367 | 0.00016 | ns \* \* | -0.625 | -0.541 | -0.772 | 100 | 66.7 |
| Bt.4520.1.S1\_at | 0.00272 | 0.000268 | 0.000529 | ns \* \* | 0.829 | 0.601 | 0.448 | 100 | 66.7 |
| Bt.12333.1.A1\_at | 0.00274 | 0.00166 | 5.04e-05 | ns \* \* | 0.661 | 0.747 | 0.885 | 100 | 66.7 |
| Bt.24350.1.A1\_at | 0.00274 | 0.00163 | 0.00037 | ns \* \* | 0.836 | 0.696 | 0.826 | 100 | 66.7 |
| Bt.3137.1.S1\_at | 0.00275 | 4.68e-07 | 8.6e-05 | ns \* \* | 1.3 | 1.85 | 2.14 | 100 | 66.7 |
| Bt.16206.2.S1\_at | 0.00275 | 6.2e-05 | 0.000577 | ns \* \* | 0.985 | 0.923 | 1.29 | 100 | 66.7 |
| Bt.13537.1.A1\_at | 0.00279 | 0.00158 | 0.000981 | ns \* \* | -0.971 | -0.849 | -1.17 | 100 | 66.7 |
| Bt.22333.1.S1\_at | 0.0028 | 5.63e-05 | 3.37e-05 | ns \* \* | 0.745 | 0.76 | 1.01 | 100 | 66.7 |
| Bt.13249.1.A1\_at | 0.00281 | 1.26e-05 | 5.9e-05 | ns \* \* | 1.61 | 1.11 | 1.31 | 100 | 66.7 |
| Bt.26122.2.S1\_at | 0.00282 | 0.00017 | 4.76e-06 | ns \* \* | -1.21 | -0.711 | -1.08 | 100 | 66.7 |
| Bt.27696.1.A1\_at | 0.00284 | 9.38e-06 | 8.01e-05 | ns \* \* | 0.94 | 1.15 | 1.29 | 100 | 66.7 |
| Bt.22170.2.A1\_at | 0.00285 | 0.00023 | 0.000151 | ns \* \* | -0.86 | -0.562 | -0.606 | 100 | 66.7 |
| Bt.16142.2.S1\_at | 0.00286 | 0.000243 | 0.000309 | ns \* \* | -0.816 | -0.858 | -1.15 | 100 | 66.7 |
| Bt.13391.1.S1\_at | 0.00287 | 0.000535 | 0.000107 | ns \* \* | -0.635 | -0.634 | -0.632 | 100 | 66.7 |
| Bt.8486.2.S1\_at | 0.00287 | 0.000326 | 0.000476 | ns \* \* | 0.663 | 0.515 | 0.797 | 100 | 66.7 |
| Bt.15972.1.S1\_at | 0.00287 | 0.00194 | 0.000434 | ns \* \* | 0.652 | 0.65 | 0.693 | 100 | 66.7 |
| Bt.20481.1.S1\_at | 0.00288 | 0.000459 | 0.000536 | ns \* \* | 0.887 | 1.05 | 1.26 | 100 | 66.7 |
| Bt.3211.1.S1\_at | 0.0029 | 3.89e-05 | 9.78e-05 | ns \* \* | 0.549 | 0.843 | 0.924 | 100 | 66.7 |
| Bt.4370.2.S1\_at | 0.0029 | 0.000152 | 5.62e-05 | ns \* \* | 0.615 | 0.595 | 0.774 | 100 | 66.7 |
| Bt.27199.1.S1\_at | 0.0029 | 0.000123 | 3.02e-05 | ns \* \* | -0.795 | -0.798 | -1.09 | 100 | 66.7 |
| Bt.21051.1.S1\_at | 0.00292 | 2.54e-05 | 3.47e-06 | ns \* \* | -0.95 | -0.922 | -1.1 | 100 | 66.7 |
| Bt.5542.2.S1\_at | 0.00293 | 0.00038 | 3.15e-05 | ns \* \* | 0.673 | 0.806 | 0.989 | 100 | 66.7 |
| Bt.11052.1.S1\_at | 0.00295 | 0.000418 | 3.9e-06 | ns \* \* | 0.765 | 0.646 | 0.791 | 100 | 66.7 |
| Bt.5088.1.S1\_at | 0.00295 | 1.41e-05 | 3.56e-05 | ns \* \* | 1.07 | 0.972 | 1.31 | 100 | 66.7 |
| Bt.15836.1.S1\_at | 0.00296 | 7.29e-05 | 0.000681 | ns \* \* | -2.93 | -2.65 | -3.42 | 100 | 66.7 |
| Bt.27008.1.A1\_at | 0.00299 | 4.68e-06 | 1.54e-06 | ns \* \* | 1.06 | 1.21 | 1.38 | 100 | 66.7 |
| Bt.8798.1.S1\_at | 0.00299 | 2.67e-05 | 2.65e-05 | ns \* \* | 1.03 | 1.26 | 1.7 | 100 | 66.7 |
| Bt.1731.1.S1\_at | 0.003 | 0.00083 | 0.00104 | ns \* \* | 0.805 | 0.837 | 1.24 | 100 | 66.7 |
| Bt.2537.2.S1\_at | 0.003 | 2.92e-05 | 8.14e-06 | ns \* \* | 0.673 | 0.846 | 1.03 | 100 | 66.7 |
| Bt.27100.1.S1\_at | 0.003 | 0.000314 | 0.000279 | ns \* \* | 0.577 | 0.583 | 0.723 | 100 | 66.7 |
| Bt.7991.1.S1\_a\_at | 0.00304 | 0.00045 | 2.68e-05 | ns \* \* | 0.893 | 0.572 | 0.797 | 100 | 66.7 |
| Bt.9674.1.S1\_at | 0.00305 | 1.1e-06 | 2.18e-05 | ns \* \* | 0.922 | 1.1 | 1.43 | 100 | 66.7 |
| Bt.19807.1.S1\_at | 0.00305 | 0.000419 | 1.35e-05 | ns \* \* | 0.836 | 0.49 | 0.512 | 100 | 66.7 |
| Bt.28162.3.S1\_at | 0.00306 | 1.06e-05 | 0.000218 | ns \* \* | -1.53 | -1.53 | -1.81 | 100 | 66.7 |
| Bt.5038.5.S1\_at | 0.00309 | 0.000192 | 8.17e-06 | ns \* \* | 0.744 | 0.736 | 0.865 | 100 | 66.7 |
| Bt.24433.2.A1\_at | 0.00309 | 0.000429 | 1.98e-05 | ns \* \* | -0.682 | -0.513 | -0.875 | 100 | 66.7 |
| Bt.25342.1.A1\_at | 0.00309 | 5.89e-05 | 2.14e-05 | ns \* \* | 1.02 | 0.862 | 1.74 | 100 | 66.7 |
| Bt.3060.1.S1\_a\_at | 0.0031 | 0.00161 | 0.000635 | ns \* \* | 0.97 | 0.646 | 1.01 | 100 | 66.7 |
| Bt.13440.1.S1\_at | 0.00311 | 0.000119 | 0.000102 | ns \* \* | 0.913 | 0.626 | 0.859 | 100 | 66.7 |
| Bt.22092.1.S1\_at | 0.00311 | 1.06e-05 | 0.000106 | ns \* \* | -1.02 | -1.36 | -1.77 | 100 | 66.7 |
| Bt.21456.1.S1\_at | 0.00316 | 1.57e-06 | 3.25e-05 | ns \* \* | 1.1 | 1.19 | 1.36 | 100 | 66.7 |
| Bt.10359.1.S1\_at | 0.00317 | 0.000216 | 0.000769 | ns \* \* | 0.918 | 0.91 | 1.06 | 100 | 66.7 |
| Bt.23265.1.S1\_at | 0.00321 | 0.000227 | 0.000349 | ns \* \* | 0.889 | 0.881 | 1.22 | 100 | 66.7 |
| Bt.27717.1.A1\_at | 0.00323 | 8.58e-06 | 0.000189 | ns \* \* | 0.66 | 1.16 | 1.29 | 100 | 66.7 |
| Bt.13237.1.S1\_at | 0.00323 | 4.53e-05 | 3.16e-07 | ns \* \* | -0.892 | -0.776 | -0.783 | 100 | 66.7 |
| Bt.22122.1.S1\_at | 0.00324 | 0.000234 | 0.000986 | ns \* \* | 0.643 | 0.604 | 0.836 | 100 | 66.7 |
| Bt.12912.1.S1\_at | 0.00329 | 0.000123 | 0.00138 | ns \* \* | 0.995 | 1.06 | 1.21 | 100 | 66.7 |
| Bt.22170.1.S1\_at | 0.0033 | 6.55e-05 | 0.000692 | ns \* \* | -0.887 | -0.867 | -1.13 | 100 | 66.7 |
| Bt.28728.1.A1\_at | 0.00332 | 0.000447 | 0.00102 | ns \* \* | -0.974 | -0.855 | -1.27 | 100 | 66.7 |
| Bt.29915.1.S1\_at | 0.00333 | 0.00123 | 0.000126 | ns \* \* | -0.681 | -0.55 | -0.74 | 100 | 66.7 |
| Bt.6950.1.S1\_at | 0.00336 | 0.000141 | 0.000403 | ns \* \* | -0.871 | -0.579 | -0.841 | 100 | 66.7 |
| Bt.15668.1.S1\_at | 0.00339 | 2.89e-05 | 2.43e-06 | ns \* \* | 0.593 | 1.01 | 1.09 | 100 | 66.7 |
| Bt.413.1.S1\_at | 0.0034 | 0.00186 | 0.000211 | ns \* \* | 0.902 | 0.688 | 0.894 | 100 | 66.7 |
| Bt.17837.1.A1\_at | 0.00341 | 3.11e-06 | 7.47e-06 | ns \* \* | 1.09 | 0.821 | 1.03 | 100 | 66.7 |
| Bt.6989.1.S1\_at | 0.00341 | 6.6e-06 | 6.63e-05 | ns \* \* | 0.819 | 0.903 | 1.04 | 100 | 66.7 |
| Bt.1217.2.S1\_at | 0.00347 | 3.85e-05 | 2.96e-05 | ns \* \* | -1.07 | -0.827 | -1.16 | 100 | 66.7 |
| Bt.5466.4.S1\_at | 0.00349 | 1.95e-06 | 1.3e-05 | ns \* \* | 0.852 | 0.893 | 1.07 | 100 | 66.7 |
| Bt.5241.1.S1\_at | 0.00349 | 0.000609 | 0.00116 | ns \* \* | -0.778 | -0.575 | -0.862 | 100 | 66.7 |
| Bt.17932.1.S1\_at | 0.00351 | 0.000268 | 0.00177 | ns \* \* | 0.741 | 0.87 | 1.44 | 100 | 66.7 |
| Bt.11629.1.A1\_at | 0.00351 | 8.91e-05 | 1.06e-05 | ns \* \* | 0.788 | 0.874 | 1.03 | 100 | 66.7 |
| Bt.3610.1.S1\_at | 0.00351 | 0.000138 | 0.000613 | ns \* \* | -0.843 | -0.596 | -0.768 | 100 | 66.7 |
| Bt.15799.1.S1\_at | 0.00353 | 0.000204 | 0.000306 | ns \* \* | 0.616 | 0.808 | 0.927 | 100 | 66.7 |
| Bt.27430.2.S1\_at | 0.00356 | 0.000162 | 0.000272 | ns \* \* | -0.797 | -0.943 | -1.13 | 100 | 66.7 |
| Bt.13345.1.A1\_at | 0.00357 | 0.000449 | 9.25e-05 | ns \* \* | 0.815 | 0.598 | 1.11 | 100 | 66.7 |
| Bt.2518.1.S1\_at | 0.00357 | 9.32e-05 | 8.28e-05 | ns \* \* | 0.752 | 0.851 | 1.19 | 100 | 66.7 |
| Bt.2230.1.S1\_at | 0.00361 | 0.000324 | 0.000434 | ns \* \* | 1.06 | 1.15 | 1.36 | 100 | 66.7 |
| Bt.903.1.S1\_at | 0.00362 | 1.87e-06 | 2.05e-06 | ns \* \* | 0.83 | 1.06 | 1.4 | 100 | 66.7 |
| Bt.18270.1.A1\_at | 0.00367 | 0.00051 | 2.42e-05 | ns \* \* | 0.614 | 0.789 | 0.995 | 100 | 66.7 |
| Bt.20243.2.S1\_at | 0.0037 | 2.79e-05 | 1.71e-05 | ns \* \* | -0.709 | -0.661 | -1.04 | 100 | 66.7 |
| Bt.28758.1.A1\_at | 0.00372 | 0.000792 | 0.0014 | ns \* \* | 0.816 | 0.829 | 0.896 | 100 | 66.7 |
| Bt.8106.1.S1\_at | 0.00372 | 0.00179 | 0.000397 | ns \* \* | 0.861 | 0.599 | 0.773 | 100 | 66.7 |
| Bt.1390.1.A1\_at | 0.00374 | 0.00103 | 0.000372 | ns \* \* | -0.762 | -0.599 | -0.918 | 100 | 66.7 |
| Bt.26995.2.S1\_at | 0.0038 | 7.41e-05 | 0.00129 | ns \* \* | 0.644 | 0.731 | 0.914 | 100 | 66.7 |
| Bt.5288.1.S1\_at | 0.00382 | 7.06e-06 | 9.04e-06 | ns \* \* | -0.947 | -0.9 | -1.22 | 100 | 66.7 |
| Bt.23227.1.S1\_at | 0.00387 | 4.88e-05 | 2e-04 | ns \* \* | 1.02 | 1.13 | 1.4 | 100 | 66.7 |
| Bt.1062.1.S1\_at | 0.00388 | 0.000699 | 0.000249 | ns \* \* | 0.671 | 0.831 | 0.981 | 100 | 66.7 |
| Bt.23106.1.S1\_at | 0.00389 | 1.98e-05 | 3.84e-05 | ns \* \* | -1.02 | -0.862 | -1.21 | 100 | 66.7 |
| Bt.26835.1.S1\_at | 0.00389 | 4.58e-05 | 0.000157 | ns \* \* | -0.636 | -0.822 | -1.3 | 100 | 66.7 |
| Bt.25957.1.S1\_at | 0.0039 | 0.000177 | 5.16e-07 | ns \* \* | -0.743 | -0.605 | -1.33 | 100 | 66.7 |
| Bt.18914.1.S1\_at | 0.00391 | 0.00015 | 0.000581 | ns \* \* | 1.04 | 0.75 | 0.792 | 100 | 66.7 |
| Bt.20619.1.S1\_at | 0.00395 | 5.73e-05 | 4.6e-05 | ns \* \* | -0.616 | -0.746 | -1.04 | 100 | 66.7 |
| Bt.24319.1.A1\_at | 0.00399 | 0.000435 | 7.61e-05 | ns \* \* | 0.74 | 0.632 | 0.843 | 100 | 66.7 |
| Bt.26751.1.S1\_at | 0.00401 | 0.000279 | 0.00115 | ns \* \* | -0.851 | -0.522 | -0.751 | 100 | 66.7 |
| Bt.23603.2.S1\_a\_at | 0.00403 | 7.25e-06 | 0.00014 | ns \* \* | -1.23 | -0.799 | -0.971 | 100 | 66.7 |
| Bt.28566.1.S1\_at | 0.00405 | 1.44e-06 | 3.21e-07 | ns \* \* | 1.07 | 0.928 | 0.929 | 100 | 66.7 |
| Bt.16139.1.A1\_at | 0.00408 | 0.000279 | 0.00105 | ns \* \* | -0.799 | -0.664 | -0.884 | 100 | 66.7 |
| Bt.3791.1.S1\_at | 0.00408 | 3.22e-05 | 0.00015 | ns \* \* | -1.5 | -0.822 | -1.04 | 100 | 66.7 |
| Bt.6768.1.S1\_at | 0.00409 | 7.63e-05 | 0.000881 | ns \* \* | 0.746 | 0.86 | 0.991 | 100 | 66.7 |
| Bt.21031.1.S1\_at | 0.00411 | 2.09e-05 | 7.71e-06 | ns \* \* | -0.876 | -0.958 | -1.31 | 100 | 66.7 |
| Bt.25202.2.S1\_at | 0.00411 | 8.37e-05 | 0.000588 | ns \* \* | 1.04 | 1.31 | 1.73 | 100 | 66.7 |
| Bt.18115.1.A1\_at | 0.00416 | 0.000728 | 0.00181 | ns \* \* | -1.02 | -0.903 | -1.23 | 100 | 66.7 |
| Bt.21580.1.S1\_at | 0.00419 | 2e-05 | 0.00119 | ns \* \* | 1.17 | 1.22 | 1.02 | 100 | 66.7 |
| Bt.2513.2.S1\_at | 0.00422 | 0.00112 | 0.00119 | ns \* \* | 0.89 | 0.672 | 0.957 | 100 | 66.7 |
| Bt.16137.1.S1\_at | 0.00425 | 9.21e-06 | 9.26e-06 | ns \* \* | 0.692 | 1.05 | 1.69 | 100 | 66.7 |
| Bt.11230.1.S1\_at | 0.00425 | 1.37e-05 | 0.000331 | ns \* \* | -1 | -1.07 | -1.37 | 100 | 66.7 |
| Bt.8437.1.A1\_at | 0.00427 | 4.38e-05 | 0.000169 | ns \* \* | 1.13 | 1.25 | 1.45 | 100 | 66.7 |
| Bt.8543.1.S1\_at | 0.0043 | 3.22e-06 | 4.45e-05 | ns \* \* | 1.4 | 1.04 | 1.27 | 100 | 66.7 |
| Bt.23405.1.S1\_at | 0.00432 | 4.48e-05 | 9.8e-05 | ns \* \* | -0.981 | -0.903 | -1.28 | 100 | 66.7 |
| Bt.5278.2.S1\_at | 0.00432 | 2.84e-05 | 0.000109 | ns \* \* | -0.932 | -0.86 | -1.17 | 100 | 66.7 |
| Bt.12429.1.S1\_at | 0.00435 | 0.000219 | 0.000396 | ns \* \* | -0.68 | -0.704 | -0.783 | 100 | 66.7 |
| Bt.29459.1.A1\_at | 0.00436 | 5.02e-05 | 0.000252 | ns \* \* | 0.681 | 0.87 | 1.26 | 100 | 66.7 |
| Bt.19429.2.S1\_a\_at | 0.00449 | 0.000951 | 7.06e-05 | ns \* \* | 0.713 | 0.653 | 0.812 | 100 | 66.7 |
| Bt.16499.1.A1\_at | 0.00453 | 9.35e-06 | 5.59e-05 | ns \* \* | 0.971 | 1.11 | 1.58 | 100 | 66.7 |
| Bt.21589.1.S1\_at | 0.00456 | 2.86e-05 | 3.3e-05 | ns \* \* | 0.807 | 0.896 | 1.19 | 100 | 66.7 |
| Bt.6116.1.S1\_a\_at | 0.00457 | 0.000129 | 0.000447 | ns \* \* | 0.813 | 0.772 | 0.845 | 100 | 66.7 |
| Bt.9438.1.S1\_at | 0.0046 | 1.67e-05 | 0.000647 | ns \* \* | -0.898 | -0.869 | -0.87 | 100 | 66.7 |
| Bt.15839.1.S1\_at | 0.00463 | 0.000168 | 0.000425 | ns \* \* | -1.31 | -1.08 | -1.43 | 100 | 66.7 |
| Bt.11339.1.S1\_at | 0.00467 | 0.000647 | 3.64e-05 | ns \* \* | -0.821 | -0.602 | -0.951 | 100 | 66.7 |
| Bt.22170.1.S1\_a\_at | 0.00468 | 0.00089 | 0.000654 | ns \* \* | -0.884 | -0.765 | -0.968 | 100 | 66.7 |
| Bt.1998.1.S1\_at | 0.00468 | 7.96e-05 | 5.98e-06 | ns \* \* | 0.669 | 0.833 | 1.05 | 100 | 66.7 |
| Bt.15607.1.S1\_at | 0.00469 | 0.000505 | 0.000519 | ns \* \* | 0.78 | 0.826 | 1.16 | 100 | 66.7 |
| Bt.22651.1.A1\_at | 0.0048 | 2.54e-06 | 7.92e-08 | ns \* \* | 0.719 | 0.983 | 1.15 | 100 | 66.7 |
| Bt.4311.1.S1\_at | 0.00488 | 3.24e-06 | 0.000144 | ns \* \* | 0.668 | 0.785 | 0.866 | 100 | 66.7 |
| Bt.24395.1.S1\_at | 0.00488 | 1.3e-05 | 5.81e-06 | ns \* \* | -0.705 | -1.01 | -1.48 | 100 | 66.7 |
| Bt.11301.1.S1\_at | 0.0049 | 2.22e-05 | 8.31e-05 | ns \* \* | 0.663 | 0.783 | 1.29 | 100 | 66.7 |
| Bt.22325.2.A1\_at | 0.00494 | 0.000184 | 0.000443 | ns \* \* | 0.765 | 0.751 | 0.792 | 100 | 66.7 |
| Bt.2214.1.S1\_at | 0.00501 | 1.8e-06 | 3.49e-05 | ns \* \* | 0.958 | 1.17 | 1.26 | 100 | 66.7 |
| Bt.4970.1.S1\_at | 0.00502 | 0.00133 | 0.0016 | ns \* \* | -0.905 | -0.707 | -0.891 | 100 | 66.7 |
| Bt.20414.1.S1\_at | 0.00508 | 0.000121 | 4.26e-05 | ns \* \* | 0.921 | 0.833 | 0.954 | 100 | 66.7 |
| Bt.10303.1.S1\_at | 0.00509 | 1.37e-06 | 2.93e-06 | ns \* \* | 0.884 | 1 | 1.42 | 100 | 66.7 |
| Bt.1386.1.S1\_at | 0.00514 | 0.000701 | 1.58e-05 | ns \* \* | 0.665 | 0.571 | 0.705 | 100 | 66.7 |
| Bt.10956.1.S1\_at | 0.00517 | 2.4e-05 | 5.72e-08 | ns \* \* | 0.501 | 0.835 | 0.979 | 100 | 66.7 |
| Bt.888.1.S1\_at | 0.00519 | 8.8e-07 | 1.18e-06 | ns \* \* | 0.971 | 1.3 | 1.61 | 100 | 66.7 |
| Bt.21433.2.S1\_at | 0.00522 | 0.000117 | 0.000525 | ns \* \* | 0.966 | 1.04 | 1.65 | 100 | 66.7 |
| Bt.16224.3.A1\_at | 0.00526 | 1.36e-05 | 0.000331 | ns \* \* | 0.949 | 0.929 | 1.38 | 100 | 66.7 |
| Bt.21718.1.S1\_at | 0.0053 | 2.63e-06 | 2.82e-05 | ns \* \* | -1.05 | -1.27 | -1.74 | 100 | 66.7 |
| Bt.13419.1.S1\_at | 0.0053 | 0.000266 | 5.25e-05 | ns \* \* | 0.774 | 0.745 | 0.879 | 100 | 66.7 |
| Bt.13950.1.A1\_at | 0.00531 | 0.000402 | 0.000562 | ns \* \* | -0.854 | -0.598 | -0.882 | 100 | 66.7 |
| Bt.14122.1.A1\_at | 0.00532 | 0.00103 | 0.000786 | ns \* \* | 1.26 | 0.93 | 1.39 | 100 | 66.7 |
| Bt.25081.1.S1\_at | 0.00541 | 0.000962 | 0.00161 | ns \* \* | 0.711 | 0.874 | 1.01 | 100 | 66.7 |
| Bt.27106.1.S1\_at | 0.00543 | 5.22e-05 | 1.8e-05 | ns \* \* | 0.804 | 0.918 | 1.45 | 100 | 66.7 |
| Bt.13953.1.A1\_at | 0.00548 | 0.00163 | 0.0016 | ns \* \* | -1 | -1.14 | -1.35 | 100 | 66.7 |
| Bt.6827.1.S1\_at | 0.0055 | 0.000299 | 0.000631 | ns \* \* | -0.66 | -0.687 | -0.736 | 100 | 66.7 |
| Bt.7500.1.S1\_at | 0.00554 | 4.32e-06 | 0.000348 | ns \* \* | 0.678 | 1.06 | 1.18 | 100 | 66.7 |
| Bt.4732.1.S1\_at | 0.00554 | 7.35e-06 | 0.00068 | ns \* \* | -1.18 | -1.3 | -1.77 | 100 | 66.7 |
| Bt.26928.1.S1\_at | 0.00556 | 1.02e-05 | 3.4e-06 | ns \* \* | 0.951 | 0.985 | 1.41 | 100 | 66.7 |
| Bt.20331.1.A1\_at | 0.00557 | 2.79e-06 | 3.35e-06 | ns \* \* | 0.989 | 1.29 | 1.43 | 100 | 66.7 |
| Bt.721.1.A1\_at | 0.00561 | 0.00129 | 0.000354 | ns \* \* | 0.861 | 0.724 | 0.884 | 100 | 66.7 |
| Bt.4726.1.S1\_at | 0.00563 | 1.65e-05 | 0.000132 | ns \* \* | 1.14 | 1.02 | 0.993 | 100 | 66.7 |
| Bt.20261.1.S1\_at | 0.00565 | 5.98e-05 | 7.34e-06 | ns \* \* | 0.634 | 0.955 | 1.39 | 100 | 66.7 |
| Bt.27617.1.S1\_at | 0.00567 | 0.000284 | 0.00138 | ns \* \* | 0.672 | 0.856 | 1.06 | 100 | 66.7 |
| Bt.24976.1.S1\_at | 0.00571 | 0.000532 | 0.00123 | ns \* \* | -1.16 | -0.982 | -1.15 | 100 | 66.7 |
| Bt.13439.1.S1\_at | 0.0058 | 2.9e-07 | 4.17e-07 | ns \* \* | -1.62 | -1.44 | -2.02 | 100 | 66.7 |
| Bt.27141.1.A1\_at | 0.00581 | 0.000107 | 1.38e-05 | ns \* \* | 0.935 | 0.875 | 1.09 | 100 | 66.7 |
| Bt.24517.2.A1\_at | 0.00584 | 0.000544 | 0.00017 | ns \* \* | -0.609 | -0.66 | -0.821 | 100 | 66.7 |
| Bt.20465.1.S1\_at | 0.00586 | 0.000224 | 9.26e-06 | ns \* \* | 1.47 | 0.603 | 0.679 | 100 | 66.7 |
| Bt.23318.1.S1\_at | 0.0059 | 1.22e-07 | 5.09e-07 | ns \* \* | 0.75 | 1.22 | 1.4 | 100 | 66.7 |
| Bt.8706.1.S1\_at | 0.00594 | 0.000965 | 0.00182 | ns \* \* | -0.651 | -0.622 | -0.848 | 100 | 66.7 |
| Bt.24896.1.A1\_at | 0.00597 | 0.000234 | 0.000411 | ns \* \* | 0.735 | 0.652 | 0.781 | 100 | 66.7 |
| Bt.19949.1.A1\_at | 0.006 | 0.000137 | 8.52e-05 | ns \* \* | 0.771 | 0.703 | 0.91 | 100 | 66.7 |
| Bt.13204.1.S1\_at | 0.00601 | 1.11e-06 | 0.000191 | ns \* \* | 1.06 | 1.37 | 1.33 | 100 | 66.7 |
| Bt.11588.1.A1\_at | 0.00608 | 0.000292 | 0.000423 | ns \* \* | 0.944 | 0.827 | 0.879 | 100 | 66.7 |
| Bt.23091.1.S1\_at | 0.0061 | 0.00097 | 0.00157 | ns \* \* | -0.931 | -0.776 | -1.22 | 100 | 66.7 |
| Bt.22356.1.S1\_at | 0.00613 | 0.000527 | 0.000162 | ns \* \* | 0.534 | 0.722 | 0.833 | 100 | 66.7 |
| Bt.12337.1.S1\_a\_at | 0.00613 | 4.56e-06 | 7.73e-07 | ns \* \* | -0.7 | -0.714 | -1.05 | 100 | 66.7 |
| Bt.4119.1.S1\_at | 0.00615 | 0.000308 | 6.36e-06 | ns \* \* | 0.843 | 0.728 | 1.03 | 100 | 66.7 |
| Bt.11182.2.S1\_a\_at | 0.00617 | 0.000365 | 0.000678 | ns \* \* | 0.659 | 0.839 | 0.985 | 100 | 66.7 |
| Bt.9785.1.S1\_at | 0.00619 | 0.000125 | 3.82e-05 | ns \* \* | 1.42 | 1.05 | 1.4 | 100 | 66.7 |
| Bt.5103.1.S1\_at | 0.00633 | 0.00208 | 0.0013 | ns \* \* | -0.77 | -0.647 | -0.926 | 100 | 66.7 |
| Bt.26808.1.S1\_at | 0.00635 | 0.000603 | 0.00102 | ns \* \* | -0.77 | -0.641 | -0.905 | 100 | 66.7 |
| Bt.20458.1.S1\_at | 0.00641 | 0.000723 | 0.00113 | ns \* \* | -0.975 | -0.574 | -0.854 | 100 | 66.7 |
| Bt.16125.3.S1\_at | 0.00647 | 6.16e-05 | 0.000313 | ns \* \* | -1.02 | -1.08 | -1.18 | 100 | 66.7 |
| Bt.28535.1.S1\_at | 0.00648 | 0.000836 | 0.000217 | ns \* \* | -0.514 | -0.566 | -1.01 | 100 | 66.7 |
| Bt.5326.1.S1\_at | 0.00651 | 2.28e-05 | 1.81e-06 | ns \* \* | 0.674 | 0.656 | 1.55 | 100 | 66.7 |
| Bt.26629.1.S1\_a\_at | 0.00653 | 0.00103 | 0.000961 | ns \* \* | -0.929 | -0.865 | -1.09 | 100 | 66.7 |
| Bt.19209.2.S1\_at | 0.00654 | 0.000524 | 1.68e-07 | ns \* \* | 0.734 | 0.582 | 0.636 | 100 | 66.7 |
| Bt.4031.2.S1\_at | 0.00656 | 0.000206 | 1.22e-05 | ns \* \* | 0.907 | 0.651 | 0.582 | 100 | 66.7 |
| Bt.13478.1.A1\_at | 0.00658 | 0.000164 | 0.000223 | ns \* \* | -0.881 | -0.689 | -0.923 | 100 | 66.7 |
| Bt.886.1.S1\_at | 0.00665 | 0.000115 | 6.03e-05 | ns \* \* | -0.76 | -0.761 | -1.09 | 100 | 66.7 |
| Bt.17777.2.S1\_at | 0.0068 | 2.61e-05 | 0.000201 | ns \* \* | -0.998 | -0.852 | -1.14 | 100 | 66.7 |
| Bt.5075.1.S1\_at | 0.00681 | 4.79e-07 | 8.29e-06 | ns \* \* | 1.49 | 1.28 | 1.7 | 100 | 66.7 |
| Bt.13881.1.S1\_at | 0.00681 | 0.000262 | 0.000394 | ns \* \* | 0.928 | 0.827 | 0.894 | 100 | 66.7 |
| Bt.364.1.S2\_at | 0.00694 | 0.00109 | 0.00175 | ns \* \* | 0.977 | 0.928 | 0.992 | 100 | 66.7 |
| Bt.20675.1.S1\_at | 0.00694 | 0.002 | 6.64e-05 | ns \* \* | -0.599 | -0.539 | -0.769 | 100 | 66.7 |
| Bt.13370.1.A1\_at | 0.00701 | 0.00161 | 0.00114 | ns \* \* | 0.948 | 0.845 | 1.03 | 100 | 66.7 |
| Bt.13229.1.S1\_at | 0.00707 | 9.51e-06 | 4.52e-05 | ns \* \* | 1.38 | 1.26 | 1.42 | 100 | 66.7 |
| Bt.1131.1.S1\_at | 0.0071 | 5.87e-05 | 2.06e-05 | ns \* \* | 0.607 | 0.702 | 0.782 | 100 | 66.7 |
| Bt.23648.1.A1\_at | 0.00711 | 8.15e-06 | 1.11e-05 | ns \* \* | 0.77 | 0.941 | 1.12 | 100 | 66.7 |
| Bt.1987.1.S1\_at | 0.00714 | 0.000395 | 1.19e-05 | ns \* \* | 0.549 | 0.494 | 0.588 | 100 | 66.7 |
| Bt.23236.1.A1\_at | 0.00717 | 7.44e-06 | 1.12e-08 | ns \* \* | 0.508 | 0.913 | 1.04 | 100 | 66.7 |
| Bt.22536.2.S1\_at | 0.00723 | 0.000606 | 4.08e-05 | ns \* \* | -0.695 | -0.622 | -0.839 | 100 | 66.7 |
| Bt.17764.1.A1\_at | 0.00729 | 0.00156 | 2.86e-05 | ns \* \* | 0.537 | 0.482 | 0.701 | 100 | 66.7 |
| Bt.13637.1.A1\_at | 0.0073 | 6.26e-06 | 6.02e-08 | ns \* \* | 0.66 | 0.826 | 0.995 | 100 | 66.7 |
| Bt.23209.1.S1\_a\_at | 0.00733 | 0.000144 | 0.000333 | ns \* \* | 0.717 | 0.909 | 1.08 | 100 | 66.7 |
| Bt.12217.3.A1\_at | 0.00733 | 0.000692 | 0.000649 | ns \* \* | 1.18 | 0.955 | 1.5 | 100 | 66.7 |
| Bt.15687.2.S1\_at | 0.00735 | 0.00211 | 0.000847 | ns \* \* | 0.679 | 0.631 | 0.847 | 100 | 66.7 |
| Bt.868.1.S1\_a\_at | 0.00736 | 0.000201 | 0.000229 | ns \* \* | -0.6 | -0.6 | -0.805 | 100 | 66.7 |
| Bt.6313.1.S1\_at | 0.00737 | 1.01e-05 | 9.19e-05 | ns \* \* | 0.84 | 0.786 | 0.988 | 100 | 66.7 |
| Bt.26992.2.S1\_at | 0.00738 | 1.76e-05 | 0.000767 | ns \* \* | 1.02 | 1.09 | 1.21 | 100 | 66.7 |
| Bt.3210.1.S1\_at | 0.00738 | 0.000107 | 5.3e-05 | ns \* \* | 0.723 | 0.734 | 0.908 | 100 | 66.7 |
| Bt.1750.1.A1\_at | 0.00739 | 0.000864 | 0.00111 | ns \* \* | 1.03 | 0.729 | 1.02 | 100 | 66.7 |
| Bt.26150.1.A1\_at | 0.00741 | 6.84e-07 | 5.63e-07 | ns \* \* | -1.61 | -1.49 | -2.07 | 100 | 66.7 |
| Bt.5536.1.S1\_at | 0.00757 | 0.00131 | 0.00041 | ns \* \* | 0.785 | 0.649 | 1.13 | 100 | 66.7 |
| Bt.12299.1.A1\_at | 0.0076 | 0.000148 | 0.0015 | ns \* \* | 1.66 | 2.03 | 2.36 | 100 | 66.7 |
| Bt.5106.1.S1\_a\_at | 0.00763 | 1.82e-05 | 1.41e-06 | ns \* \* | 0.52 | 0.822 | 1.05 | 100 | 66.7 |
| Bt.4900.1.A1\_at | 0.00765 | 5.64e-06 | 0.000289 | ns \* \* | -0.879 | -0.853 | -1 | 100 | 66.7 |
| Bt.9425.1.S1\_at | 0.00773 | 9.28e-05 | 0.000227 | ns \* \* | 1.19 | 0.759 | 1.22 | 100 | 66.7 |
| Bt.26668.1.S1\_at | 0.0078 | 0.000552 | 5.85e-05 | ns \* \* | 0.686 | 0.717 | 0.848 | 100 | 66.7 |
| Bt.7903.1.A1\_at | 0.00782 | 7.56e-05 | 0.000113 | ns \* \* | -0.632 | -0.771 | -1.12 | 100 | 66.7 |
| Bt.20007.1.S1\_a\_at | 0.00782 | 3.79e-05 | 8.04e-06 | ns \* \* | 0.59 | 0.781 | 0.774 | 100 | 66.7 |
| Bt.27832.1.S1\_at | 0.00783 | 0.000312 | 0.00101 | ns \* \* | -0.489 | -0.511 | -0.706 | 100 | 66.7 |
| Bt.2110.1.S1\_at | 0.00787 | 1.14e-05 | 9.21e-05 | ns \* \* | -0.861 | -1.3 | -2.14 | 100 | 66.7 |
| Bt.3321.1.S1\_at | 0.00793 | 0.000642 | 4.43e-07 | ns \* \* | 0.736 | 0.752 | 1.27 | 100 | 66.7 |
| Bt.24276.1.A1\_at | 0.00793 | 0.00144 | 0.00144 | ns \* \* | 0.902 | 0.51 | 0.564 | 100 | 66.7 |
| Bt.3583.1.S1\_at | 0.00795 | 0.00101 | 0.000836 | ns \* \* | 1.1 | 0.585 | 0.772 | 100 | 66.7 |
| Bt.4402.1.S1\_at | 0.008 | 0.000102 | 9.21e-05 | ns \* \* | -0.922 | -0.687 | -0.915 | 100 | 66.7 |
| Bt.23129.2.S1\_at | 0.00805 | 0.000574 | 0.000112 | ns \* \* | 0.705 | 0.751 | 0.913 | 100 | 66.7 |
| Bt.6069.1.S1\_at | 0.00806 | 0.00112 | 9.88e-06 | ns \* \* | 0.553 | 0.622 | 0.774 | 100 | 66.7 |
| Bt.27046.1.S1\_at | 0.00806 | 9.21e-05 | 8.69e-07 | ns \* \* | 0.643 | 0.614 | 0.82 | 100 | 66.7 |
| Bt.20362.2.S1\_at | 0.00807 | 8.53e-06 | 0.00164 | ns \* \* | 1.11 | 1.26 | 1.3 | 100 | 66.7 |
| Bt.5866.1.S1\_at | 0.00809 | 0.000997 | 0.000608 | ns \* \* | -0.863 | -0.543 | -1.36 | 100 | 66.7 |
| Bt.27339.2.S1\_at | 0.00813 | 0.00139 | 0.00124 | ns \* \* | 0.746 | 0.826 | 1.03 | 100 | 66.7 |
| Bt.25268.1.A1\_at | 0.00819 | 6.32e-05 | 6.21e-05 | ns \* \* | 0.791 | 0.842 | 1.22 | 100 | 66.7 |
| Bt.1819.1.S1\_at | 0.00826 | 0.00208 | 0.000625 | ns \* \* | -1.01 | -0.661 | -0.763 | 100 | 66.7 |
| Bt.10999.2.S1\_at | 0.00828 | 3.22e-06 | 5.87e-05 | ns \* \* | 0.82 | 0.947 | 1.35 | 100 | 66.7 |
| Bt.27023.1.S1\_at | 0.0083 | 0.000136 | 4.85e-05 | ns \* \* | 0.936 | 1.03 | 1.23 | 100 | 66.7 |
| Bt.3422.1.S1\_at | 0.00831 | 0.000696 | 9.81e-05 | ns \* \* | 0.844 | 0.64 | 0.807 | 100 | 66.7 |
| Bt.7955.1.S1\_at | 0.00832 | 0.000252 | 0.000261 | ns \* \* | 0.637 | 0.699 | 0.794 | 100 | 66.7 |
| Bt.23985.1.A1\_at | 0.00839 | 0.000916 | 0.00128 | ns \* \* | 0.62 | 0.728 | 0.972 | 100 | 66.7 |
| Bt.9490.1.S1\_at | 0.00844 | 0.00177 | 0.000128 | ns \* \* | -0.885 | -0.577 | -0.929 | 100 | 66.7 |
| Bt.20314.2.S1\_at | 0.00845 | 0.000406 | 0.000723 | ns \* \* | -0.623 | -0.74 | -0.909 | 100 | 66.7 |
| Bt.9973.1.S2\_at | 0.00855 | 0.000395 | 9.38e-05 | ns \* \* | 0.453 | 0.563 | 0.532 | 100 | 66.7 |
| Bt.2513.1.S1\_at | 0.00861 | 0.000425 | 2.52e-05 | ns \* \* | 0.775 | 0.562 | 0.821 | 100 | 66.7 |
| Bt.9278.1.S1\_at | 0.00865 | 3.55e-05 | 0.000964 | ns \* \* | 0.743 | 0.796 | 0.79 | 100 | 66.7 |
| Bt.1310.1.S1\_at | 0.00871 | 0.00205 | 0.000555 | ns \* \* | -0.646 | -0.456 | -0.597 | 100 | 66.7 |
| Bt.9413.1.S1\_at | 0.00874 | 2.7e-05 | 2.57e-05 | ns \* \* | 0.901 | 0.802 | 0.828 | 100 | 66.7 |
| Bt.4394.1.S1\_at | 0.00889 | 0.0011 | 0.000849 | ns \* \* | 0.577 | 0.507 | 0.558 | 100 | 66.7 |
| Bt.27655.1.S1\_at | 0.00892 | 0.000193 | 0.0019 | ns \* \* | -1.14 | -1.17 | -1.52 | 100 | 66.7 |
| Bt.1678.1.S1\_at | 0.00906 | 0.00109 | 0.00124 | ns \* \* | -0.659 | -0.521 | -0.793 | 100 | 66.7 |
| Bt.21630.1.S1\_at | 0.00907 | 2.35e-06 | 0.000172 | ns \* \* | -1.27 | -1.59 | -2.31 | 100 | 66.7 |
| Bt.1595.1.S1\_at | 0.00913 | 0.00035 | 0.000302 | ns \* \* | 0.576 | 0.768 | 0.951 | 100 | 66.7 |
| Bt.5300.1.S1\_at | 0.00917 | 0.000113 | 2.72e-06 | ns \* \* | -1.73 | -0.757 | -0.595 | 100 | 66.7 |
| Bt.16636.2.A1\_at | 0.00918 | 0.000132 | 0.000315 | ns \* \* | 1.4 | 0.878 | 1.15 | 100 | 66.7 |
| Bt.12345.1.S1\_at | 0.00925 | 5.15e-05 | 2.71e-06 | ns \* \* | 0.619 | 0.737 | 0.857 | 100 | 66.7 |
| Bt.18019.1.A1\_at | 0.00934 | 3.23e-06 | 1.86e-05 | ns \* \* | 0.96 | 1.17 | 1.33 | 100 | 66.7 |
| Bt.654.1.A1\_at | 0.00956 | 0.000527 | 0.000536 | ns \* \* | 0.524 | 0.519 | 0.475 | 100 | 66.7 |
| Bt.13730.1.A1\_at | 0.00964 | 5.93e-06 | 6.56e-06 | ns \* \* | -0.925 | -1.07 | -1.45 | 100 | 66.7 |
| Bt.3633.1.A1\_at | 0.00968 | 3.4e-06 | 0.000126 | ns \* \* | 0.755 | 0.842 | 0.8 | 100 | 66.7 |
| Bt.9695.1.S1\_at | 0.00969 | 7.79e-05 | 0.000137 | ns \* \* | 0.866 | 0.829 | 0.858 | 100 | 66.7 |
| Bt.28855.1.A1\_at | 0.00971 | 0.000125 | 6.83e-06 | ns \* \* | 0.885 | 0.677 | 0.684 | 100 | 66.7 |
| Bt.1101.1.S1\_at | 0.00979 | 3.27e-05 | 2.22e-08 | ns \* \* | 0.601 | 0.7 | 0.954 | 100 | 66.7 |
| Bt.4161.1.S1\_at | 0.00985 | 0.000724 | 0.000291 | ns \* \* | 0.566 | 0.703 | 0.783 | 100 | 66.7 |
| Bt.9639.1.S1\_at | 0.00986 | 0.000364 | 0.00113 | ns \* \* | 0.836 | 0.79 | 1.28 | 100 | 66.7 |
| Bt.22441.1.S1\_at | 0.00998 | 0.000275 | 1.05e-05 | ns \* \* | 0.537 | 0.573 | 0.729 | 100 | 66.7 |
| Bt.19431.1.S1\_s\_at | 0.01 | 0.000755 | 8.19e-05 | ns \* \* | -0.638 | -0.551 | -0.888 | 100 | 66.7 |
| Bt.20041.1.S1\_at | 0.0101 | 0.000714 | 9.31e-05 | ns \* \* | -0.712 | -0.534 | -0.993 | 100 | 66.7 |
| Bt.7143.1.S1\_at | 0.0101 | 0.000137 | 0.000195 | ns \* \* | -0.605 | -0.557 | -0.784 | 100 | 66.7 |
| Bt.1322.1.S1\_at | 0.0102 | 3.32e-05 | 2.85e-05 | ns \* \* | 0.732 | 0.89 | 1.04 | 100 | 66.7 |
| Bt.22197.1.S1\_at | 0.0102 | 7.67e-06 | 1.47e-05 | ns \* \* | -1.12 | -1.02 | -1.5 | 100 | 66.7 |
| Bt.13562.1.S1\_at | 0.0102 | 3.3e-05 | 1.01e-05 | ns \* \* | 0.725 | 0.905 | 1.55 | 100 | 66.7 |
| Bt.26774.1.S1\_at | 0.0102 | 8.51e-05 | 0.000258 | ns \* \* | 1.06 | 1.46 | 2.02 | 100 | 66.7 |
| Bt.12966.1.S1\_at | 0.0103 | 4.31e-06 | 0.000246 | ns \* \* | -1.49 | -1.25 | -1.59 | 100 | 66.7 |
| Bt.948.1.S1\_at | 0.0103 | 0.0013 | 0.000133 | ns \* \* | 0.71 | 0.669 | 0.96 | 100 | 66.7 |
| Bt.7139.1.S1\_at | 0.0103 | 0.000456 | 6.28e-06 | ns \* \* | -0.817 | -0.672 | -0.807 | 100 | 66.7 |
| Bt.3163.1.A1\_at | 0.0103 | 0.000405 | 1.36e-05 | ns \* \* | 0.614 | 0.587 | 0.717 | 100 | 66.7 |
| Bt.4314.1.S1\_at | 0.0103 | 2e-05 | 0.000107 | ns \* \* | 0.695 | 0.778 | 0.936 | 100 | 66.7 |
| Bt.13969.2.S1\_at | 0.0104 | 0.000825 | 0.000194 | ns \* \* | 0.689 | 0.605 | 1.09 | 100 | 66.7 |
| Bt.10417.1.S1\_at | 0.0105 | 3.12e-05 | 0.000165 | ns \* \* | 0.99 | 0.643 | 0.696 | 100 | 66.7 |
| Bt.22283.1.S1\_at | 0.0105 | 4.11e-07 | 9.98e-05 | ns \* \* | 1.07 | 1.16 | 1.46 | 100 | 66.7 |
| Bt.6289.1.S1\_at | 0.0106 | 0.000229 | 8.49e-06 | ns \* \* | 1.03 | 0.755 | 0.828 | 100 | 66.7 |
| Bt.20029.1.S1\_at | 0.0106 | 0.000325 | 4.46e-06 | ns \* \* | 0.464 | 0.541 | 0.799 | 100 | 66.7 |
| Bt.13593.1.A1\_at | 0.0107 | 0.000411 | 0.000133 | ns \* \* | 0.738 | 0.628 | 0.849 | 100 | 66.7 |
| Bt.5038.4.A1\_at | 0.0107 | 8.93e-07 | 1.03e-06 | ns \* \* | 0.514 | 0.838 | 0.963 | 100 | 66.7 |
| Bt.1165.1.S1\_at | 0.0107 | 4.72e-06 | 0.000229 | ns \* \* | 0.752 | 0.939 | 0.994 | 100 | 66.7 |
| Bt.26643.1.S1\_at | 0.0108 | 0.000445 | 0.000289 | ns \* \* | 1.03 | 0.615 | 0.886 | 100 | 66.7 |
| Bt.21534.3.S1\_a\_at | 0.0109 | 0.000684 | 0.00141 | ns \* \* | -0.892 | -0.713 | -0.817 | 100 | 66.7 |
| Bt.676.1.S1\_at | 0.0109 | 0.000177 | 5.46e-07 | ns \* \* | 0.44 | 0.657 | 0.831 | 100 | 66.7 |
| Bt.9948.1.S1\_at | 0.0109 | 0.000176 | 1.75e-05 | ns \* \* | 0.582 | 0.581 | 1.32 | 100 | 66.7 |
| Bt.13768.1.S1\_at | 0.0111 | 2.48e-05 | 5.35e-06 | ns \* \* | 1.12 | 0.972 | 1.17 | 100 | 66.7 |
| Bt.2262.1.S1\_at | 0.0113 | 3.96e-05 | 0.000386 | ns \* \* | -0.848 | -0.779 | -0.943 | 100 | 66.7 |
| Bt.23094.6.A1\_at | 0.0113 | 0.000175 | 0.00043 | ns \* \* | -1.1 | -1.12 | -1.68 | 100 | 66.7 |
| Bt.21036.1.S1\_at | 0.0116 | 0.000145 | 0.000186 | ns \* \* | 0.798 | 0.754 | 1.19 | 100 | 66.7 |
| Bt.1785.1.A1\_at | 0.0117 | 0.000107 | 0.00101 | ns \* \* | 0.594 | 0.806 | 1.11 | 100 | 66.7 |
| Bt.17380.1.S1\_at | 0.012 | 2.86e-05 | 1.24e-05 | ns \* \* | -0.676 | -0.668 | -1.39 | 100 | 66.7 |
| Bt.11258.2.A1\_a\_at | 0.0122 | 0.000371 | 0.00113 | ns \* \* | -0.669 | -0.495 | -0.43 | 100 | 66.7 |
| Bt.23178.1.S1\_at | 0.0122 | 1.81e-07 | 4.31e-07 | ns \* \* | 0.725 | 1.19 | 1.33 | 100 | 66.7 |
| Bt.28280.1.A1\_at | 0.0122 | 0.000986 | 2.32e-05 | ns \* \* | -0.56 | -0.55 | -0.844 | 100 | 66.7 |
| Bt.22737.1.S1\_at | 0.0122 | 0.001 | 0.00011 | ns \* \* | 0.659 | 0.693 | 0.729 | 100 | 66.7 |
| Bt.16230.1.S1\_at | 0.0125 | 0.000101 | 0.00138 | ns \* \* | 0.696 | 0.76 | 0.851 | 100 | 66.7 |
| Bt.25436.1.S1\_a\_at | 0.0125 | 0.000923 | 0.00132 | ns \* \* | 0.787 | 0.93 | 1.17 | 100 | 66.7 |
| Bt.10257.1.A1\_at | 0.0125 | 1.16e-06 | 3.51e-05 | ns \* \* | 0.908 | 1.18 | 1.5 | 100 | 66.7 |
| Bt.12583.1.A1\_at | 0.0125 | 0.000102 | 0.000609 | ns \* \* | -0.796 | -0.965 | -1.28 | 100 | 66.7 |
| Bt.26313.1.A1\_at | 0.0125 | 0.000577 | 1.07e-05 | ns \* \* | 0.542 | 0.662 | 0.777 | 100 | 66.7 |
| Bt.5459.1.S1\_at | 0.0126 | 3.13e-05 | 0.000466 | ns \* \* | -0.905 | -0.879 | -1.02 | 100 | 66.7 |
| Bt.21217.1.S1\_at | 0.0128 | 0.00024 | 1.94e-05 | ns \* \* | -0.481 | -0.62 | -0.706 | 100 | 66.7 |
| Bt.20456.1.S1\_at | 0.013 | 0.000171 | 0.000488 | ns \* \* | -0.636 | -0.941 | -1.61 | 100 | 66.7 |
| Bt.1697.1.S1\_at | 0.013 | 0.00105 | 0.000305 | ns \* \* | -0.533 | -0.485 | -0.39 | 100 | 66.7 |
| Bt.20129.1.S1\_at | 0.013 | 3.06e-05 | 8.37e-06 | ns \* \* | -0.869 | -0.721 | -0.957 | 100 | 66.7 |
| Bt.27057.1.A1\_at | 0.0131 | 0.000128 | 0.0011 | ns \* \* | -0.847 | -0.906 | -1.36 | 100 | 66.7 |
| Bt.24051.1.S1\_at | 0.0133 | 0.00124 | 0.000646 | ns \* \* | 0.778 | 0.779 | 0.931 | 100 | 66.7 |
| Bt.23519.1.S1\_at | 0.0135 | 0.000345 | 8.55e-05 | ns \* \* | -0.783 | -0.723 | -1.15 | 100 | 66.7 |
| Bt.13900.1.S1\_at | 0.0138 | 6.31e-05 | 0.00117 | ns \* \* | -0.764 | -0.885 | -1.27 | 100 | 66.7 |
| Bt.19938.1.A1\_at | 0.0138 | 0.0015 | 7.15e-05 | ns \* \* | 0.469 | 0.693 | 0.803 | 100 | 66.7 |
| Bt.18890.1.A1\_at | 0.0139 | 4.47e-08 | 6.12e-09 | ns \* \* | -0.656 | -1.11 | -1.53 | 100 | 66.7 |
| Bt.4544.1.S1\_at | 0.0141 | 1.86e-05 | 0.000865 | ns \* \* | 0.822 | 0.864 | 0.896 | 100 | 66.7 |
| Bt.21410.1.S1\_at | 0.0141 | 0.000183 | 0.000558 | ns \* \* | -0.667 | -0.628 | -0.812 | 100 | 66.7 |
| Bt.2195.1.A1\_at | 0.0145 | 0.00179 | 0.000261 | ns \* \* | -0.817 | -0.537 | -0.688 | 100 | 66.7 |
| Bt.2825.1.S1\_at | 0.0146 | 1.57e-06 | 1.13e-06 | ns \* \* | -1.27 | -1.11 | -1.7 | 100 | 66.7 |
| Bt.13946.2.A1\_at | 0.0146 | 0.000129 | 6.3e-06 | ns \* \* | 0.736 | 0.634 | 1.06 | 100 | 66.7 |
| Bt.1729.1.A1\_at | 0.0146 | 0.00036 | 0.000439 | ns \* \* | 0.594 | 0.683 | 0.839 | 100 | 66.7 |
| Bt.26393.1.A1\_at | 0.0147 | 0.000199 | 0.00017 | ns \* \* | 0.837 | 0.988 | 1.09 | 100 | 66.7 |
| Bt.8117.1.S1\_at | 0.0147 | 0.00011 | 0.000686 | ns \* \* | 0.888 | 0.845 | 1.01 | 100 | 66.7 |
| Bt.11932.1.S1\_at | 0.0147 | 0.000419 | 7.03e-06 | ns \* \* | 0.733 | 0.59 | 0.786 | 100 | 66.7 |
| Bt.24895.1.A1\_at | 0.0147 | 6.29e-05 | 1.74e-06 | ns \* \* | 0.537 | 0.703 | 0.784 | 100 | 66.7 |
| Bt.9833.1.S1\_a\_at | 0.0147 | 0.00036 | 0.00108 | ns \* \* | -0.993 | -0.985 | -0.815 | 100 | 66.7 |
| Bt.4339.1.S1\_at | 0.0148 | 1.56e-06 | 3.01e-05 | ns \* \* | -0.892 | -0.939 | -1.14 | 100 | 66.7 |
| Bt.10260.1.S1\_at | 0.0149 | 0.00118 | 0.00164 | ns \* \* | 0.887 | 0.595 | 1.12 | 100 | 66.7 |
| Bt.7869.1.A1\_at | 0.015 | 1.1e-06 | 2.93e-06 | ns \* \* | 0.749 | 1.2 | 1.71 | 100 | 66.7 |
| Bt.21159.1.S1\_at | 0.0151 | 0.000136 | 8.39e-05 | ns \* \* | 0.814 | 1.15 | 2.06 | 100 | 66.7 |
| Bt.5302.1.S1\_a\_at | 0.0151 | 2.36e-05 | 0.000402 | ns \* \* | 0.627 | 0.677 | 0.906 | 100 | 66.7 |
| Bt.5507.2.S1\_at | 0.0152 | 0.00033 | 0.00033 | ns \* \* | 0.749 | 0.681 | 0.983 | 100 | 66.7 |
| Bt.10312.1.A1\_at | 0.0153 | 0.000244 | 0.000158 | ns \* \* | 0.459 | 0.556 | 0.572 | 100 | 66.7 |
| Bt.13755.1.S1\_at | 0.0153 | 8.29e-08 | 1.66e-07 | ns \* \* | 0.902 | 0.955 | 1.18 | 100 | 66.7 |
| Bt.29948.1.S1\_at | 0.0153 | 0.000239 | 8.52e-05 | ns \* \* | 0.578 | 0.697 | 0.737 | 100 | 66.7 |
| Bt.8243.1.A1\_at | 0.0154 | 5.92e-05 | 8.39e-05 | ns \* \* | -1.08 | -1.08 | -1.42 | 100 | 66.7 |
| Bt.19692.1.S1\_at | 0.0154 | 0.000176 | 5.75e-05 | ns \* \* | -0.582 | -0.635 | -0.862 | 100 | 66.7 |
| Bt.15684.1.S1\_at | 0.0154 | 3.14e-05 | 6.94e-06 | ns \* \* | 0.558 | 0.694 | 0.818 | 100 | 66.7 |
| Bt.22306.1.S1\_at | 0.0155 | 1.69e-07 | 7.55e-07 | ns \* \* | -0.884 | -1.56 | -2.11 | 100 | 66.7 |
| Bt.5104.1.S1\_at | 0.0156 | 0.00201 | 0.00115 | ns \* \* | -0.836 | -0.668 | -0.789 | 100 | 66.7 |
| Bt.1617.2.S1\_a\_at | 0.0157 | 0.000363 | 0.00015 | ns \* \* | 0.473 | 0.65 | 0.709 | 100 | 66.7 |
| Bt.2451.1.S1\_at | 0.0159 | 0.000325 | 0.000179 | ns \* \* | 0.61 | 0.924 | 1.2 | 100 | 66.7 |
| Bt.4054.1.S1\_at | 0.0161 | 4.17e-05 | 2.51e-05 | ns \* \* | 0.574 | 0.962 | 1.11 | 100 | 66.7 |
| Bt.16040.1.S1\_at | 0.0161 | 0.000431 | 0.00148 | ns \* \* | 0.505 | 0.556 | 0.655 | 100 | 66.7 |
| Bt.27887.1.S1\_at | 0.0165 | 0.000326 | 0.000225 | ns \* \* | 0.736 | 0.99 | 1.45 | 100 | 66.7 |
| Bt.24109.1.S1\_at | 0.0166 | 8.26e-05 | 0.00151 | ns \* \* | -1.18 | -1.52 | -2.08 | 100 | 66.7 |
| Bt.25093.1.A1\_at | 0.0169 | 0.000567 | 0.000278 | ns \* \* | 0.602 | 0.462 | 0.719 | 100 | 66.7 |
| Bt.24767.1.S1\_at | 0.017 | 7.09e-05 | 3.16e-05 | ns \* \* | 0.547 | 0.601 | 0.705 | 100 | 66.7 |
| Bt.4221.1.S1\_at | 0.017 | 8.52e-05 | 0.000882 | ns \* \* | 0.575 | 0.719 | 1.07 | 100 | 66.7 |
| Bt.11013.1.S1\_at | 0.0171 | 1.09e-05 | 0.000147 | ns \* \* | 0.678 | 0.883 | 0.777 | 100 | 66.7 |
| Bt.12567.1.A1\_at | 0.0172 | 0.000283 | 0.000588 | ns \* \* | -0.592 | -0.817 | -0.934 | 100 | 66.7 |
| Bt.26803.1.A1\_at | 0.0172 | 0.000271 | 0.000312 | ns \* \* | 0.583 | 0.663 | 0.932 | 100 | 66.7 |
| Bt.850.1.S1\_at | 0.0174 | 0.000146 | 0.000734 | ns \* \* | 0.711 | 0.666 | 0.807 | 100 | 66.7 |
| Bt.15575.1.A1\_at | 0.018 | 0.000216 | 0.0013 | ns \* \* | -0.595 | -0.695 | -1.01 | 100 | 66.7 |
| Bt.22064.1.A1\_at | 0.0182 | 0.00151 | 0.00123 | ns \* \* | 0.494 | 0.555 | 0.597 | 100 | 66.7 |
| Bt.7980.1.S1\_at | 0.0184 | 2.36e-05 | 0.000425 | ns \* \* | 1.34 | 0.891 | 0.962 | 100 | 66.7 |
| Bt.6455.1.S1\_at | 0.0186 | 4.73e-05 | 2.32e-06 | ns \* \* | -0.799 | -0.752 | -0.961 | 100 | 66.7 |
| Bt.14007.3.A1\_at | 0.0189 | 0.000817 | 0.000212 | ns \* \* | 0.546 | 0.831 | 1.02 | 100 | 66.7 |
| Bt.26594.1.S1\_at | 0.019 | 0.00039 | 0.00105 | ns \* \* | -0.673 | -0.63 | -0.874 | 100 | 66.7 |
| Bt.20308.2.S1\_at | 0.0194 | 0.000167 | 0.000365 | ns \* \* | 1.04 | 0.861 | 1.2 | 100 | 66.7 |
| Bt.9847.1.S1\_at | 0.0195 | 0.00126 | 0.00091 | ns \* \* | 0.573 | 0.787 | 0.915 | 100 | 66.7 |
| Bt.27140.1.S1\_at | 0.0195 | 3.95e-05 | 1.19e-05 | ns \* \* | 0.852 | 0.909 | 0.959 | 100 | 66.7 |
| Bt.20331.2.S1\_at | 0.0198 | 0.00072 | 0.000368 | ns \* \* | 0.727 | 0.795 | 1.13 | 100 | 66.7 |
| Bt.25104.1.S1\_at | 0.0198 | 0.000874 | 0.00019 | ns \* \* | 0.415 | 0.641 | 0.738 | 100 | 66.7 |
| Bt.11035.1.S1\_at | 0.0198 | 9.82e-06 | 5.27e-06 | ns \* \* | 0.952 | 0.759 | 0.973 | 100 | 66.7 |
| Bt.8024.1.A1\_at | 0.0199 | 7.8e-05 | 2.69e-05 | ns \* \* | -0.785 | -0.725 | -1.03 | 100 | 66.7 |
| Bt.26613.1.A1\_at | 0.0199 | 0.00036 | 0.00166 | ns \* \* | -0.514 | -0.787 | -0.961 | 100 | 66.7 |
| Bt.10609.2.A1\_at | 0.0199 | 9.72e-05 | 3.96e-06 | ns \* \* | 1.1 | 0.707 | 0.951 | 100 | 66.7 |
| Bt.18002.2.S1\_at | 0.0199 | 0.00023 | 0.00138 | ns \* \* | 1.05 | 1.04 | 1.26 | 100 | 66.7 |
| Bt.28836.1.S1\_at | 0.02 | 0.000229 | 0.000566 | ns \* \* | 0.974 | 0.866 | 1.06 | 100 | 66.7 |
| Bt.23770.1.A1\_at | 0.02 | 0.00139 | 0.000526 | ns \* \* | 0.739 | 0.612 | 1.41 | 100 | 66.7 |
| Bt.28569.1.S1\_at | 0.0202 | 9.62e-05 | 0.000113 | ns \* \* | 0.743 | 0.907 | 1.01 | 100 | 66.7 |
| Bt.9965.1.S1\_at | 0.0213 | 8.86e-08 | 2.75e-06 | ns \* \* | -1.04 | -1.36 | -1.64 | 100 | 66.7 |
| Bt.18519.1.A1\_at | 0.0214 | 7.43e-05 | 0.00016 | ns \* \* | 1.18 | 0.8 | 0.717 | 100 | 66.7 |
| Bt.6157.1.S1\_at | 0.0216 | 0.000302 | 0.000115 | ns \* \* | 0.591 | 0.721 | 0.876 | 100 | 66.7 |
| Bt.13741.1.A1\_at | 0.022 | 3.8e-07 | 0.000199 | ns \* \* | 1.45 | 1.24 | 0.946 | 100 | 66.7 |
| Bt.981.2.S1\_at | 0.022 | 0.00164 | 0.000315 | ns \* \* | 0.747 | 0.803 | 0.99 | 100 | 66.7 |
| Bt.25097.1.S1\_at | 0.022 | 0.000837 | 7.65e-05 | ns \* \* | -0.587 | -0.564 | -0.876 | 100 | 66.7 |
| Bt.12745.1.A1\_at | 0.0225 | 9.19e-06 | 2.56e-05 | ns \* \* | 0.775 | 0.849 | 1.45 | 100 | 66.7 |
| Bt.1255.1.A1\_at | 0.0225 | 0.000768 | 4.88e-05 | ns \* \* | -0.453 | -0.492 | -0.663 | 100 | 66.7 |
| Bt.23129.3.S1\_a\_at | 0.0234 | 1.18e-05 | 1.94e-07 | ns \* \* | 0.472 | 0.905 | 1.11 | 100 | 66.7 |
| Bt.6585.1.S1\_at | 0.0234 | 0.000126 | 1.7e-05 | ns \* \* | 0.466 | 0.684 | 0.854 | 100 | 66.7 |
| Bt.13489.1.S1\_at | 0.0237 | 0.00184 | 0.00154 | ns \* \* | 0.54 | 0.571 | 0.72 | 100 | 66.7 |
| Bt.8641.1.A1\_at | 0.024 | 0.000322 | 0.000877 | ns \* \* | 0.922 | 0.748 | 1.05 | 100 | 66.7 |
| Bt.2439.1.S1\_at | 0.0242 | 6.05e-06 | 2.12e-05 | ns \* \* | 0.762 | 0.809 | 1.07 | 100 | 66.7 |
| Bt.21774.1.S1\_at | 0.0246 | 2.27e-05 | 1.18e-05 | ns \* \* | 0.574 | 0.813 | 1.06 | 100 | 66.7 |
| Bt.21827.2.S1\_at | 0.0247 | 4.99e-05 | 4.25e-05 | ns \* \* | -0.601 | -0.701 | -1.36 | 100 | 66.7 |
| Bt.3447.2.S1\_at | 0.025 | 0.000539 | 0.0012 | ns \* \* | -1.08 | -0.733 | -1.15 | 100 | 66.7 |
| Bt.12888.1.S1\_at | 0.0253 | 0.000244 | 3.08e-06 | ns \* \* | -0.849 | -0.882 | -1.39 | 100 | 66.7 |
| Bt.23468.1.A1\_at | 0.0253 | 0.00028 | 0.00103 | ns \* \* | 0.397 | 0.628 | 0.392 | 100 | 66.7 |
| Bt.2453.1.S1\_at | 0.0254 | 0.000454 | 2.07e-05 | ns \* \* | 0.469 | 0.488 | 0.675 | 100 | 66.7 |
| Bt.13847.1.S1\_at | 0.0259 | 4.42e-05 | 0.000598 | ns \* \* | -1 | -1.06 | -1.28 | 100 | 66.7 |
| Bt.2187.1.S1\_at | 0.0261 | 6.42e-05 | 3.92e-06 | ns \* \* | -0.521 | -0.663 | -0.925 | 100 | 66.7 |
| Bt.4786.1.S2\_at | 0.0262 | 0.000525 | 0.00117 | ns \* \* | 0.376 | 0.513 | 0.519 | 100 | 66.7 |
| Bt.12348.1.A1\_at | 0.0265 | 7.38e-05 | 2.47e-05 | ns \* \* | 0.876 | 1.15 | 1.44 | 100 | 66.7 |
| Bt.12382.1.S1\_at | 0.0275 | 0.000168 | 3.14e-05 | ns \* \* | 0.854 | 0.641 | 1.31 | 100 | 66.7 |
| Bt.6733.1.S1\_at | 0.0276 | 0.000171 | 4.77e-05 | ns \* \* | 0.636 | 0.721 | 1.02 | 100 | 66.7 |
| Bt.24676.2.A1\_at | 0.0278 | 8.44e-06 | 3.47e-07 | ns \* \* | -1.26 | -0.881 | -1.51 | 100 | 66.7 |
| Bt.23399.1.S1\_at | 0.028 | 2.28e-08 | 5.22e-07 | ns \* \* | -1.31 | -1.77 | -2.11 | 100 | 66.7 |
| Bt.21835.1.S1\_at | 0.0292 | 0.000472 | 4.62e-05 | ns \* \* | 0.719 | 0.668 | 1.3 | 100 | 66.7 |
| Bt.19332.2.A1\_at | 0.0293 | 0.000983 | 0.0011 | ns \* \* | 0.875 | 1.1 | 1.26 | 100 | 66.7 |
| Bt.5439.1.S1\_at | 0.0294 | 0.0013 | 1.82e-05 | ns \* \* | 0.43 | 0.546 | 0.946 | 100 | 66.7 |
| Bt.21487.2.S1\_at | 0.0295 | 0.00139 | 0.000479 | ns \* \* | -0.539 | -0.504 | -0.842 | 100 | 66.7 |
| Bt.8164.1.S1\_at | 0.0295 | 0.000371 | 5.35e-05 | ns \* \* | -0.681 | -0.676 | -0.665 | 100 | 66.7 |
| Bt.20247.1.S1\_at | 0.0296 | 3.72e-06 | 5.73e-05 | ns \* \* | 0.676 | 1.11 | 1.52 | 100 | 66.7 |
| Bt.167.1.S1\_at | 0.0298 | 0.000137 | 0.000128 | ns \* \* | -0.683 | -0.686 | -1.01 | 100 | 66.7 |
| Bt.4621.1.S1\_a\_at | 0.0302 | 0.000742 | 0.000664 | ns \* \* | 0.596 | 0.534 | 0.755 | 100 | 66.7 |
| Bt.12261.1.A1\_at | 0.0303 | 0.000493 | 2.84e-05 | ns \* \* | 0.435 | 0.561 | 0.644 | 100 | 66.7 |
| Bt.4280.1.A1\_at | 0.0305 | 0.000429 | 3.69e-07 | ns \* \* | 0.441 | 0.54 | 0.795 | 100 | 66.7 |
| Bt.566.1.S1\_at | 0.0307 | 0.00073 | 0.0013 | ns \* \* | -0.99 | -0.652 | -1 | 100 | 66.7 |
| Bt.20377.1.S1\_at | 0.0314 | 9.53e-05 | 0.000107 | ns \* \* | -0.806 | -0.962 | -1.22 | 100 | 66.7 |
| Bt.27934.1.S1\_at | 0.0314 | 0.00024 | 6.88e-06 | ns \* \* | 0.377 | 0.61 | 0.659 | 100 | 66.7 |
| Bt.26445.1.A1\_at | 0.0315 | 0.00037 | 1.36e-05 | ns \* \* | 0.72 | 0.896 | 1.23 | 100 | 66.7 |
| Bt.1244.1.S1\_at | 0.0316 | 0.00177 | 0.000148 | ns \* \* | 0.618 | 0.454 | 0.616 | 100 | 66.7 |
| Bt.23224.1.S1\_at | 0.032 | 0.00059 | 5.92e-05 | ns \* \* | 0.78 | 0.645 | 0.905 | 100 | 66.7 |
| Bt.13912.1.S1\_at | 0.0325 | 0.000411 | 2.21e-05 | ns \* \* | 0.8 | 0.654 | 1.12 | 100 | 66.7 |
| Bt.26837.1.S1\_at | 0.0326 | 0.00107 | 0.000846 | ns \* \* | 0.589 | 0.764 | 0.753 | 100 | 66.7 |
| Bt.27626.1.A1\_at | 0.0329 | 1.68e-05 | 0.00175 | ns \* \* | 0.538 | 0.765 | 1 | 100 | 66.7 |
| Bt.13199.1.S1\_at | 0.033 | 2.79e-05 | 0.000354 | ns \* \* | -0.693 | -0.9 | -1.01 | 100 | 66.7 |
| Bt.4020.1.S1\_at | 0.0335 | 0.000684 | 0.000153 | ns \* \* | -0.706 | -0.59 | -0.525 | 100 | 66.7 |
| Bt.12370.1.S1\_at | 0.0338 | 0.000182 | 6.98e-05 | ns \* \* | -0.551 | -0.577 | -0.744 | 100 | 66.7 |
| Bt.23002.1.S1\_s\_at | 0.0341 | 0.000248 | 0.000168 | ns \* \* | 0.425 | 0.575 | 0.574 | 100 | 66.7 |
| Bt.13768.2.S1\_at | 0.0345 | 0.00016 | 3.15e-06 | ns \* \* | 0.69 | 0.793 | 0.904 | 100 | 66.7 |
| Bt.5888.1.S1\_at | 0.0351 | 1.28e-06 | 2.09e-05 | ns \* \* | -0.704 | -1.06 | -1.65 | 100 | 66.7 |
| Bt.26595.1.S1\_at | 0.0351 | 0.000857 | 4.73e-07 | ns \* \* | 0.505 | 0.705 | 1.22 | 100 | 66.7 |
| Bt.18634.2.S1\_at | 0.0352 | 0.000203 | 0.000499 | ns \* \* | 1.51 | 0.867 | 1.09 | 100 | 66.7 |
| Bt.69.1.S1\_at | 0.0365 | 0.000136 | 7.49e-05 | ns \* \* | -0.746 | -0.66 | -0.939 | 100 | 66.7 |
| Bt.22040.1.S1\_at | 0.0365 | 2.04e-06 | 1.03e-06 | ns \* \* | 0.516 | 0.982 | 1.71 | 100 | 66.7 |
| Bt.26549.1.A1\_at | 0.0365 | 0.00169 | 0.000182 | ns \* \* | 0.363 | 0.607 | 0.632 | 100 | 66.7 |
| Bt.22362.1.S1\_at | 0.0367 | 8.41e-05 | 0.00058 | ns \* \* | -0.64 | -0.754 | -1.08 | 100 | 66.7 |
| Bt.16125.1.A1\_s\_at | 0.0368 | 0.00126 | 0.000687 | ns \* \* | -0.627 | -0.915 | -1.06 | 100 | 66.7 |
| Bt.21085.1.S1\_at | 0.0372 | 7.66e-05 | 1.22e-05 | ns \* \* | 0.591 | 1.02 | 1.41 | 100 | 66.7 |
| Bt.11925.1.A1\_at | 0.0379 | 0.000496 | 0.000405 | ns \* \* | 0.557 | 0.614 | 0.634 | 100 | 66.7 |
| Bt.24474.1.S1\_a\_at | 0.038 | 1.65e-05 | 0.00119 | ns \* \* | -0.642 | -0.817 | -1.05 | 100 | 66.7 |
| Bt.8012.1.S1\_at | 0.0381 | 0.00035 | 8.39e-05 | ns \* \* | 0.445 | 0.567 | 0.894 | 100 | 66.7 |
| Bt.13198.1.S1\_at | 0.0383 | 6.43e-07 | 7.25e-05 | ns \* \* | 0.763 | 1.21 | 1.57 | 100 | 66.7 |
| Bt.5099.1.S1\_at | 0.039 | 4.29e-05 | 0.000279 | ns \* \* | 0.759 | 1.07 | 1.05 | 100 | 66.7 |
| Bt.19482.1.A1\_at | 0.0399 | 0.000117 | 0.000429 | ns \* \* | -1.12 | -1.13 | -1.93 | 100 | 66.7 |
| Bt.7814.1.S1\_at | 0.0402 | 0.000124 | 1.84e-05 | ns \* \* | 0.398 | 0.581 | 0.588 | 100 | 66.7 |
| Bt.27927.1.S1\_at | 0.0406 | 1.15e-05 | 1.52e-05 | ns \* \* | 0.764 | 0.876 | 1.03 | 100 | 66.7 |
| Bt.5542.1.A1\_at | 0.0417 | 0.000159 | 3.26e-05 | ns \* \* | 0.423 | 0.896 | 1.06 | 100 | 66.7 |
| Bt.13986.1.S1\_at | 0.0423 | 0.000789 | 2.1e-05 | ns \* \* | 0.337 | 0.574 | 0.716 | 100 | 66.7 |
| Bt.11390.1.A1\_at | 0.0424 | 0.000125 | 0.00111 | ns \* \* | -1.19 | -1.42 | -1.95 | 100 | 66.7 |
| Bt.24234.1.S1\_at | 0.043 | 0.00123 | 9.64e-06 | ns \* \* | 0.354 | 0.51 | 0.598 | 100 | 66.7 |
| Bt.6075.1.S1\_at | 0.0433 | 0.00109 | 0.000244 | ns \* \* | 0.784 | 0.532 | 0.816 | 100 | 66.7 |
| Bt.1318.1.S1\_at | 0.0434 | 0.00177 | 0.0018 | ns \* \* | -0.679 | -0.448 | -0.922 | 100 | 66.7 |
| Bt.22443.1.A1\_at | 0.0436 | 4.47e-05 | 0.000229 | ns \* \* | 0.541 | 0.849 | 0.825 | 100 | 66.7 |
| Bt.23784.1.A1\_at | 0.045 | 0.0011 | 0.00056 | ns \* \* | 0.402 | 0.466 | 0.536 | 100 | 66.7 |
| Bt.2106.1.S1\_at | 0.0456 | 0.00133 | 0.00106 | ns \* \* | 0.487 | 0.635 | 0.704 | 100 | 66.7 |
| Bt.26143.1.A1\_at | 0.046 | 0.000741 | 0.000315 | ns \* \* | 0.82 | 0.652 | 0.757 | 100 | 66.7 |
| Bt.2046.1.S1\_at | 0.047 | 2.91e-05 | 0.00101 | ns \* \* | 0.765 | 0.925 | 1.6 | 100 | 66.7 |
| Bt.21981.1.A1\_at | 0.047 | 1.61e-05 | 7.27e-05 | ns \* \* | 0.561 | 1.13 | 1.33 | 100 | 66.7 |
| Bt.16831.1.A1\_at | 0.0477 | 5.84e-05 | 0.000421 | ns \* \* | 1.02 | 1.19 | 1.24 | 100 | 66.7 |
| Bt.24643.1.S1\_at | 0.0494 | 4.95e-05 | 3.74e-05 | ns \* \* | 0.819 | 0.836 | 1.01 | 100 | 66.7 |
| Bt.17767.1.A1\_at | 0.0509 | 0.0015 | 7.99e-05 | ns \* \* | 0.462 | 0.567 | 0.588 | 100 | 66.7 |
| Bt.5313.1.S1\_at | 0.0514 | 0.00015 | 0.000408 | ns \* \* | 0.52 | 0.787 | 0.99 | 100 | 66.7 |
| Bt.712.1.S2\_at | 0.0526 | 0.00149 | 0.000972 | ns \* \* | -0.411 | -0.509 | -0.989 | 100 | 66.7 |
| Bt.6390.1.S1\_at | 0.0533 | 0.00101 | 0.000689 | ns \* \* | -0.613 | -0.656 | -0.797 | 100 | 66.7 |
| Bt.26615.1.S1\_at | 0.0546 | 0.000719 | 1.07e-05 | ns \* \* | -0.672 | -0.43 | -0.744 | 100 | 66.7 |
| Bt.4056.1.S1\_at | 0.055 | 4.24e-05 | 4.45e-05 | ns \* \* | 0.522 | 0.619 | 0.851 | 100 | 66.7 |
| Bt.9966.2.S1\_a\_at | 0.0556 | 0.000758 | 0.000349 | ns \* \* | 0.907 | 0.953 | 1.24 | 100 | 66.7 |
| Bt.16105.1.S1\_at | 0.0556 | 2.37e-05 | 0.000266 | ns \* \* | 0.502 | 1.3 | 1.49 | 100 | 66.7 |
| Bt.17672.1.S1\_at | 0.0567 | 1.12e-05 | 0.000415 | ns \* \* | 0.526 | 0.772 | 1.14 | 100 | 66.7 |
| Bt.27892.1.S1\_at | 0.0576 | 4.27e-05 | 8.56e-06 | ns \* \* | -0.531 | -0.736 | -0.937 | 100 | 66.7 |
| Bt.12505.1.S1\_at | 0.0586 | 5.65e-05 | 1.83e-06 | ns \* \* | 0.442 | 0.623 | 0.816 | 100 | 66.7 |
| Bt.21060.1.S1\_at | 0.0591 | 0.00101 | 8.06e-06 | ns \* \* | 0.447 | 0.561 | 1.03 | 100 | 66.7 |
| Bt.6125.1.S1\_at | 0.0596 | 0.000598 | 0.000598 | ns \* \* | 0.573 | 0.599 | 1.02 | 100 | 66.7 |
| Bt.4599.1.S1\_at | 0.0602 | 0.00176 | 0.000954 | ns \* \* | -0.518 | -0.6 | -0.909 | 100 | 66.7 |
| Bt.7955.2.S1\_at | 0.0602 | 0.000922 | 5.98e-05 | ns \* \* | 0.584 | 0.747 | 0.94 | 100 | 66.7 |
| Bt.16733.1.A1\_s\_at | 0.0606 | 0.000253 | 0.00134 | ns \* \* | 0.746 | 0.851 | 0.867 | 100 | 66.7 |
| Bt.2007.1.S1\_at | 0.0622 | 0.00054 | 0.000703 | ns \* \* | -0.593 | -0.731 | -1.18 | 100 | 66.7 |
| Bt.23307.1.S1\_at | 0.063 | 0.000544 | 0.00126 | ns \* \* | 0.835 | 0.573 | 0.911 | 100 | 66.7 |
| Bt.656.1.S1\_at | 0.0642 | 0.00187 | 4.39e-05 | ns \* \* | 0.499 | 0.51 | 0.641 | 100 | 66.7 |
| Bt.23002.1.S1\_at | 0.0643 | 1.94e-05 | 2.42e-05 | ns \* \* | -1.01 | -1.05 | -1.63 | 100 | 66.7 |
| Bt.17008.1.S1\_at | 0.0658 | 0.000967 | 1.96e-05 | ns \* \* | 0.306 | 0.554 | 0.689 | 100 | 66.7 |
| Bt.8920.1.S1\_at | 0.0658 | 0.000412 | 0.000663 | ns \* \* | 0.551 | 0.837 | 1.11 | 100 | 66.7 |
| Bt.9238.1.S1\_at | 0.0669 | 0.000196 | 0.000336 | ns \* \* | -0.577 | -0.977 | -1.03 | 100 | 66.7 |
| Bt.20023.1.S1\_at | 0.07 | 0.000499 | 0.000543 | ns \* \* | 0.379 | 0.601 | 0.846 | 100 | 66.7 |
| Bt.1464.1.A1\_at | 0.0703 | 2.2e-06 | 1e-05 | ns \* \* | 0.562 | 1.1 | 1.57 | 100 | 66.7 |
| Bt.2017.1.S1\_at | 0.0712 | 0.000476 | 0.00102 | ns \* \* | 0.558 | 0.664 | 0.903 | 100 | 66.7 |
| Bt.19676.2.S1\_a\_at | 0.0717 | 2.24e-06 | 4.1e-05 | ns \* \* | -0.9 | -1.14 | -1.54 | 100 | 66.7 |
| Bt.5006.1.S1\_at | 0.0718 | 0.000973 | 2.42e-05 | ns \* \* | 0.38 | 0.596 | 0.689 | 100 | 66.7 |
| Bt.4261.1.S1\_at | 0.073 | 6.95e-05 | 0.000271 | ns \* \* | -0.719 | -0.869 | -1.3 | 100 | 66.7 |
| Bt.18843.1.A1\_at | 0.0731 | 1.53e-05 | 9.71e-06 | ns \* \* | 0.603 | 0.788 | 1.15 | 100 | 66.7 |
| Bt.22442.3.S1\_a\_at | 0.0742 | 0.00129 | 0.000979 | ns \* \* | -0.493 | -0.634 | -0.825 | 100 | 66.7 |
| Bt.15860.1.S1\_at | 0.0747 | 0.00164 | 0.000256 | ns \* \* | 0.327 | 0.576 | 0.644 | 100 | 66.7 |
| Bt.5748.3.S1\_at | 0.0749 | 0.000462 | 0.000366 | ns \* \* | 0.792 | 0.843 | 1.14 | 100 | 66.7 |
| Bt.14305.1.A1\_at | 0.0752 | 0.000652 | 9.55e-05 | ns \* \* | -0.396 | -0.557 | -0.799 | 100 | 66.7 |
| Bt.1734.1.S1\_at | 0.076 | 7.37e-06 | 4.88e-06 | ns \* \* | 0.525 | 1.05 | 1.54 | 100 | 66.7 |
| Bt.25228.1.A1\_at | 0.0764 | 2.76e-05 | 0.000123 | ns \* \* | 0.584 | 1.17 | 1.28 | 100 | 66.7 |
| Bt.13991.2.S1\_at | 0.0781 | 0.00013 | 0.00023 | ns \* \* | 0.454 | 0.783 | 1.01 | 100 | 66.7 |
| Bt.18408.1.A1\_at | 0.0792 | 0.000211 | 0.000346 | ns \* \* | 0.478 | 1.1 | 1.32 | 100 | 66.7 |
| Bt.19382.1.S1\_at | 0.0812 | 0.000157 | 0.00127 | ns \* \* | 0.609 | 0.813 | 0.938 | 100 | 66.7 |
| Bt.13730.2.S1\_at | 0.0817 | 2.8e-06 | 3.83e-06 | ns \* \* | -0.488 | -1.25 | -1.5 | 100 | 66.7 |
| Bt.3158.1.S1\_at | 0.0831 | 0.00012 | 1.23e-05 | ns \* \* | 0.521 | 0.664 | 0.794 | 100 | 66.7 |
| Bt.1125.1.S1\_at | 0.0835 | 0.000967 | 7.3e-06 | ns \* \* | 0.287 | 0.57 | 0.679 | 100 | 66.7 |
| Bt.23380.2.S1\_at | 0.0837 | 1.07e-05 | 5.25e-06 | ns \* \* | 0.42 | 0.899 | 1.06 | 100 | 66.7 |
| Bt.22335.1.S1\_a\_at | 0.086 | 0.000854 | 3.6e-06 | ns \* \* | 0.486 | 0.66 | 0.741 | 100 | 66.7 |
| Bt.5039.1.S1\_at | 0.0881 | 0.0018 | 0.000789 | ns \* \* | 0.363 | 0.573 | 0.573 | 100 | 66.7 |
| Bt.18513.1.S1\_at | 0.0895 | 0.0011 | 0.00163 | ns \* \* | -0.488 | -0.471 | -0.512 | 100 | 66.7 |
| Bt.11389.1.S1\_at | 0.0899 | 0.00015 | 0.00109 | ns \* \* | 0.61 | 0.875 | 1.03 | 100 | 66.7 |
| Bt.2005.1.S1\_at | 0.0928 | 0.000249 | 0.000362 | ns \* \* | -0.631 | -0.532 | -0.745 | 100 | 66.7 |
| Bt.9448.1.S2\_at | 0.093 | 2.5e-05 | 0.000245 | ns \* \* | 0.304 | 0.7 | 0.657 | 100 | 66.7 |
| Bt.22011.1.S1\_at | 0.0932 | 0.000786 | 0.00164 | ns \* \* | 0.557 | 0.77 | 0.869 | 100 | 66.7 |
| Bt.9186.1.A1\_at | 0.0939 | 0.00101 | 0.00104 | ns \* \* | 0.668 | 1.04 | 1.23 | 100 | 66.7 |
| Bt.935.2.S1\_at | 0.0949 | 0.000131 | 0.000533 | ns \* \* | -0.427 | -0.767 | -0.983 | 100 | 66.7 |
| Bt.23522.1.S1\_at | 0.102 | 6.22e-06 | 1.14e-05 | ns \* \* | 0.284 | 0.777 | 0.778 | 100 | 66.7 |
| Bt.1967.1.S1\_at | 0.103 | 0.000108 | 0.000123 | ns \* \* | -0.496 | -0.703 | -0.842 | 100 | 66.7 |
| Bt.17812.1.S1\_at | 0.105 | 0.00132 | 9.36e-05 | ns \* \* | 0.377 | 0.535 | 0.763 | 100 | 66.7 |
| Bt.13529.1.S1\_at | 0.105 | 0.000325 | 2.28e-05 | ns \* \* | 0.34 | 0.753 | 0.928 | 100 | 66.7 |
| Bt.2070.3.S1\_at | 0.112 | 2.87e-05 | 0.00019 | ns \* \* | -0.717 | -1.01 | -1.8 | 100 | 66.7 |
| Bt.5872.1.S1\_at | 0.116 | 0.000446 | 0.000859 | ns \* \* | 0.492 | 0.482 | 0.47 | 100 | 66.7 |
| Bt.16975.1.S1\_at | 0.121 | 4.48e-05 | 6.64e-05 | ns \* \* | 0.409 | 0.711 | 0.801 | 100 | 66.7 |
| Bt.11149.1.S1\_at | 0.122 | 5.48e-06 | 8.89e-05 | ns \* \* | 0.351 | 1.06 | 1.1 | 100 | 66.7 |
| Bt.4458.1.A1\_at | 0.127 | 0.000144 | 2.92e-06 | ns \* \* | 0.457 | 0.606 | 1 | 100 | 66.7 |
| Bt.24397.1.S1\_at | 0.13 | 0.000418 | 0.000165 | ns \* \* | 0.325 | 0.532 | 0.549 | 100 | 66.7 |
| Bt.14037.1.S1\_at | 0.138 | 0.000873 | 0.000381 | ns \* \* | 0.293 | 0.489 | 0.687 | 100 | 66.7 |
| Bt.5598.1.S1\_at | 0.142 | 0.000907 | 6.36e-05 | ns \* \* | 0.756 | 0.918 | 1.34 | 100 | 66.7 |
| Bt.14193.1.A1\_at | 0.143 | 0.000281 | 2.1e-07 | ns \* \* | -0.512 | -0.571 | -0.563 | 100 | 66.7 |
| Bt.3820.2.A1\_at | 0.144 | 2.6e-05 | 2.92e-05 | ns \* \* | 0.272 | 0.646 | 0.825 | 100 | 66.7 |
| Bt.8966.1.S2\_at | 0.145 | 0.000297 | 0.000681 | ns \* \* | -0.362 | -0.588 | -0.762 | 100 | 66.7 |
| Bt.5388.1.S1\_at | 0.145 | 1.56e-06 | 3.95e-05 | ns \* \* | 0.383 | 0.785 | 0.852 | 100 | 66.7 |
| Bt.13871.1.S1\_at | 0.15 | 0.000997 | 0.000662 | ns \* \* | 0.436 | 0.625 | 0.854 | 100 | 66.7 |
| Bt.618.1.S1\_at | 0.155 | 0.000814 | 0.00132 | ns \* \* | -0.515 | -0.523 | -0.705 | 100 | 66.7 |
| Bt.23318.1.S2\_at | 0.156 | 2.04e-07 | 1.45e-06 | ns \* \* | 0.348 | 1.05 | 1.07 | 100 | 66.7 |
| Bt.7467.1.S1\_at | 0.158 | 2.8e-05 | 0.000384 | ns \* \* | 0.545 | 0.87 | 1.09 | 100 | 66.7 |
| Bt.24873.1.S1\_at | 0.159 | 1.04e-05 | 2.85e-05 | ns \* \* | 0.427 | 1.01 | 1 | 100 | 66.7 |
| Bt.9078.2.S1\_a\_at | 0.165 | 1.01e-05 | 2.66e-05 | ns \* \* | -0.598 | -0.982 | -1.65 | 100 | 66.7 |
| Bt.22433.1.A1\_at | 0.165 | 1.87e-06 | 5.75e-06 | ns \* \* | -0.884 | -1.04 | -1.76 | 100 | 66.7 |
| Bt.3573.1.S1\_at | 0.167 | 0.000179 | 0.00039 | ns \* \* | -0.327 | 0.497 | 0.482 | 100 | 66.7 |
| Bt.3478.1.A1\_s\_at | 0.169 | 0.000669 | 0.000119 | ns \* \* | 0.3 | 0.857 | 1.08 | 100 | 66.7 |
| Bt.4859.1.S1\_at | 0.169 | 0.000452 | 2.7e-06 | ns \* \* | 0.228 | 0.563 | 0.536 | 100 | 66.7 |
| Bt.5467.1.S2\_at | 0.175 | 0.000784 | 0.00077 | ns \* \* | 0.324 | 0.526 | 0.464 | 100 | 66.7 |
| Bt.26524.1.S1\_at | 0.182 | 5e-05 | 4.92e-05 | ns \* \* | -0.441 | -0.702 | -0.773 | 100 | 66.7 |
| Bt.10056.1.S1\_at | 0.19 | 0.00143 | 0.0019 | ns \* \* | 0.437 | 0.628 | 0.864 | 100 | 66.7 |
| Bt.4552.1.S1\_a\_at | 0.206 | 0.000165 | 2.17e-05 | ns \* \* | 0.353 | 0.737 | 0.834 | 100 | 66.7 |
| Bt.9144.1.A1\_at | 0.206 | 0.000773 | 8.23e-05 | ns \* \* | 0.25 | 0.572 | 0.703 | 100 | 66.7 |
| Bt.8028.1.S1\_at | 0.211 | 0.000156 | 0.000772 | ns \* \* | -0.339 | -0.586 | -0.707 | 100 | 66.7 |
| Bt.21920.1.S1\_at | 0.214 | 0.0011 | 3.98e-06 | ns \* \* | 0.247 | 0.487 | 0.683 | 100 | 66.7 |
| Bt.57.1.S1\_at | 0.251 | 0.000606 | 6.86e-05 | ns \* \* | -0.537 | -0.529 | -0.63 | 100 | 66.7 |
| Bt.8566.1.S1\_at | 0.251 | 0.000732 | 0.000292 | ns \* \* | 0.265 | 0.612 | 0.785 | 100 | 66.7 |
| Bt.4057.1.S1\_at | 0.266 | 1.59e-06 | 0.000109 | ns \* \* | 0.949 | 0.941 | 1.41 | 100 | 66.7 |
| Bt.7785.1.S1\_at | 0.268 | 0.000654 | 0.000142 | ns \* \* | 0.248 | 0.744 | 0.832 | 100 | 66.7 |
| Bt.24087.1.A1\_at | 0.301 | 0.00128 | 0.000549 | ns \* \* | 0.215 | 0.56 | 0.591 | 100 | 66.7 |
| Bt.17079.2.A1\_at | 0.323 | 6.16e-06 | 0.000514 | ns \* \* | 0.417 | 0.869 | 0.895 | 100 | 66.7 |
| Bt.22525.1.A1\_at | 0.341 | 0.00166 | 4.31e-05 | ns \* \* | 0.262 | 0.474 | 1.02 | 100 | 66.7 |
| Bt.4294.1.S1\_at | 0.353 | 0.00031 | 0.000438 | ns \* \* | -0.281 | -0.574 | -0.67 | 100 | 66.7 |
| Bt.2798.1.S1\_a\_at | 0.385 | 7.31e-05 | 0.000537 | ns \* \* | -0.222 | 0.614 | 0.632 | 100 | 66.7 |
| Bt.11058.1.A1\_at | 0.398 | 0.000798 | 0.00115 | ns \* \* | -0.265 | -0.563 | -0.533 | 100 | 66.7 |
| Bt.4241.1.S1\_at | 0.403 | 0.000402 | 0.000312 | ns \* \* | -0.158 | 0.449 | 0.399 | 100 | 66.7 |
| Bt.22981.1.S1\_at | 0.487 | 0.00075 | 0.00102 | ns \* \* | -0.198 | -0.595 | -1.05 | 100 | 66.7 |
| Bt.11448.1.S1\_at | 0.5 | 9.37e-06 | 1.91e-06 | ns \* \* | 0.152 | 0.761 | 0.868 | 100 | 66.7 |
| Bt.5106.2.S1\_at | 0.517 | 0.000182 | 0.000383 | ns \* \* | -0.124 | 0.538 | 0.562 | 100 | 66.7 |
| Bt.5737.1.S1\_at | 0.588 | 0.00183 | 0.00139 | ns \* \* | 0.222 | 0.532 | 0.478 | 100 | 66.7 |
| Bt.22243.1.S1\_at | 0.598 | 2.1e-05 | 0.000135 | ns \* \* | -0.126 | 0.843 | 1.03 | 100 | 66.7 |
| Bt.23229.2.S1\_at | 0.614 | 0.000175 | 0.000666 | ns \* \* | 0.108 | 0.739 | 0.812 | 100 | 66.7 |
| Bt.19601.1.S1\_at | 0.622 | 0.000484 | 0.000229 | ns \* \* | 0.205 | 0.944 | 1.18 | 100 | 66.7 |
| Bt.12498.1.A1\_at | 0.654 | 0.000416 | 0.000553 | ns \* \* | -0.103 | 0.629 | 0.656 | 100 | 66.7 |
| Bt.9207.1.S1\_at | 0.66 | 3.64e-05 | 3.71e-05 | ns \* \* | 0.0805 | 0.741 | 0.83 | 100 | 66.7 |
| Bt.5388.1.S1\_s\_at | 0.728 | 4.42e-06 | 3.11e-05 | ns \* \* | 0.0628 | 0.709 | 0.769 | 100 | 66.7 |
| Bt.23326.1.S1\_at | 0.783 | 0.000621 | 0.00165 | ns \* \* | -0.0593 | 0.49 | 0.475 | 100 | 66.7 |
| Bt.26817.1.S1\_at | 0.79 | 0.0011 | 0.0013 | ns \* \* | 0.0961 | -0.598 | -0.342 | 100 | 66.7 |
| Bt.15357.1.A1\_at | 0.793 | 3.97e-05 | 1.43e-05 | ns \* \* | -0.085 | -0.729 | -1.62 | 100 | 66.7 |
| Bt.8122.1.S1\_at | 0.891 | 4.28e-06 | 4.6e-06 | ns \* \* | 0.0277 | 0.707 | 0.679 | 100 | 66.7 |
| Bt.3599.1.S1\_at | 0.902 | 0.000235 | 1.72e-05 | ns \* \* | -0.0208 | 0.582 | 0.643 | 100 | 66.7 |
| Bt.10822.1.S1\_at | 0.912 | 0.000603 | 0.00191 | ns \* \* | 0.0242 | 0.555 | 0.652 | 100 | 66.7 |
| Bt.12558.1.S1\_at | 0.935 | 0.000506 | 0.00143 | ns \* \* | 0.0173 | 0.684 | 0.975 | 100 | 66.7 |
| Bt.16841.1.A1\_at | 0.956 | 7e-05 | 0.000121 | ns \* \* | -0.0178 | 0.79 | 0.87 | 100 | 66.7 |
| Bt.4877.1.S1\_at | 2.99e-06 | 0.00151 | 0.055 | \* \* ns | 2.25 | 0.608 | 0.447 | 83.3 | 66.7 |
| Bt.11067.1.S1\_at | 5.32e-05 | 0.0065 | 0.00183 | \* ns \* | 1.34 | 0.54 | 0.871 | 83.3 | 66.7 |
| Bt.6675.1.A1\_at | 7.57e-05 | 9.64e-06 | 0.00528 | \* \* ns | -1.2 | -1.04 | -1.29 | 83.3 | 66.7 |
| Bt.17789.1.A1\_at | 7.96e-05 | 1.91e-05 | 0.00362 | \* \* ns | 2.05 | 1.65 | 2.48 | 83.3 | 66.7 |
| Bt.28817.1.A1\_at | 8.8e-05 | 0.000147 | 0.0177 | \* \* ns | 1.56 | 0.842 | 0.511 | 83.3 | 66.7 |
| Bt.2905.1.S1\_at | 9.22e-05 | 3.12e-06 | 0.00292 | \* \* ns | 1.43 | 1 | 1.11 | 83.3 | 66.7 |
| Bt.25165.1.S1\_at | 9.37e-05 | 0.000371 | 0.00199 | \* \* ns | -1.46 | -1.1 | -0.182 | 83.3 | 66.7 |
| Bt.1069.1.A1\_at | 0.000119 | 0.000135 | 0.00252 | \* \* ns | 1.9 | 0.74 | 1.26 | 83.3 | 66.7 |
| Bt.21427.1.S1\_at | 0.000152 | 7.01e-05 | 0.00466 | \* \* ns | 1.47 | 0.75 | 0.777 | 83.3 | 66.7 |
| Bt.24260.2.S1\_at | 0.000207 | 0.00133 | 0.0582 | \* \* ns | 1.7 | 1.05 | 0.951 | 83.3 | 66.7 |
| Bt.19774.2.S1\_at | 0.000266 | 7.09e-05 | 0.0258 | \* \* ns | 1.14 | 0.891 | 0.555 | 83.3 | 66.7 |
| Bt.15864.1.S1\_at | 0.000385 | 2.77e-05 | 0.00692 | \* \* ns | 1.71 | 1.14 | 0.987 | 83.3 | 66.7 |
| Bt.12769.1.S1\_at | 0.000397 | 0.000514 | 0.0124 | \* \* ns | -1.41 | -1.09 | -0.66 | 83.3 | 66.7 |
| Bt.5379.1.S1\_at | 0.000421 | 1.05e-05 | 0.02 | \* \* ns | 1.76 | 1.14 | 0.862 | 83.3 | 66.7 |
| Bt.23869.1.A1\_at | 0.000515 | 7.83e-05 | 0.247 | \* \* ns | 1.28 | 0.82 | 0.337 | 83.3 | 66.7 |
| Bt.5027.1.A1\_at | 0.000555 | 0.000239 | 0.00253 | \* \* ns | 1.16 | 1.13 | 0.876 | 83.3 | 66.7 |
| Bt.20402.3.S1\_at | 0.000991 | 0.00192 | 0.00222 | \* \* ns | 1.7 | 0.82 | 0.863 | 83.3 | 66.7 |
| Bt.23240.1.S1\_at | 0.00104 | 0.000955 | 0.00551 | \* \* ns | 1.24 | 0.716 | 1.08 | 83.3 | 66.7 |
| Bt.9221.1.A1\_at | 0.00114 | 0.000684 | 0.00127 | ns \* \* | -0.945 | -0.756 | -0.932 | 83.3 | 66.7 |
| Bt.997.1.S1\_at | 0.00114 | 5.14e-06 | 3.65e-06 | ns \* \* | -1.04 | -0.929 | -1.42 | 83.3 | 66.7 |
| BtAffx.29968.1.S1\_at | 0.00117 | 5.55e-05 | 0.00124 | ns \* \* | -3.04 | -2.81 | -3.77 | 83.3 | 66.7 |
| Bt.24536.1.S1\_at | 0.00119 | 0.000364 | 0.00126 | ns \* \* | 1.18 | 1.11 | 1.45 | 83.3 | 66.7 |
| Bt.5788.1.S1\_at | 0.00119 | 0.000197 | 0.000872 | ns \* \* | 1.57 | 0.77 | 0.774 | 83.3 | 66.7 |
| Bt.26094.1.A1\_at | 0.00127 | 0.00152 | 0.000319 | ns \* \* | 0.884 | 0.686 | 0.815 | 83.3 | 66.7 |
| Bt.8802.1.S1\_at | 0.00127 | 6.95e-06 | 0.000189 | ns \* \* | 0.964 | 0.748 | 1.03 | 83.3 | 66.7 |
| Bt.15735.1.S1\_at | 0.0013 | 1.39e-07 | 2.63e-06 | ns \* \* | 1.51 | 1.81 | 2.33 | 83.3 | 66.7 |
| Bt.9059.1.S1\_at | 0.00142 | 9.82e-06 | 8.51e-06 | ns \* \* | 1.18 | 0.773 | 0.719 | 83.3 | 66.7 |
| Bt.25654.1.A1\_at | 0.00149 | 3.09e-05 | 0.000243 | ns \* \* | -1.06 | -0.812 | -1.09 | 83.3 | 66.7 |
| Bt.5341.1.S1\_at | 0.00155 | 6.15e-06 | 4.26e-06 | ns \* \* | 1.28 | 1.13 | 1.44 | 83.3 | 66.7 |
| Bt.24321.1.S1\_at | 0.0016 | 0.00032 | 0.000214 | ns \* \* | 1.52 | 0.66 | 1 | 83.3 | 66.7 |
| Bt.29853.1.A1\_at | 0.00163 | 0.000152 | 0.00132 | ns \* \* | -1.26 | -0.973 | -1.67 | 83.3 | 66.7 |
| Bt.20487.1.S1\_at | 0.00169 | 2.8e-05 | 0.000273 | ns \* \* | -1.04 | -0.964 | -1.43 | 83.3 | 66.7 |
| Bt.14007.1.S1\_at | 0.00171 | 0.000493 | 9.35e-05 | ns \* \* | 0.602 | 0.666 | 0.774 | 83.3 | 66.7 |
| Bt.3014.1.A1\_at | 0.00173 | 0.000425 | 0.00139 | ns \* \* | 1.14 | 0.95 | 1.34 | 83.3 | 66.7 |
| Bt.29472.1.A1\_at | 0.00176 | 5e-06 | 0.000234 | ns \* \* | 1.79 | 1.71 | 2.08 | 83.3 | 66.7 |
| Bt.19637.1.A1\_at | 0.00179 | 5.26e-05 | 2.76e-05 | ns \* \* | 0.809 | 1.14 | 1.33 | 83.3 | 66.7 |
| Bt.11194.1.S1\_at | 0.00195 | 1.24e-06 | 5.05e-05 | ns \* \* | -1.82 | -1.32 | -2.05 | 83.3 | 66.7 |
| Bt.21365.1.S1\_a\_at | 0.00232 | 5.45e-05 | 0.00107 | ns \* \* | 1.66 | 1.17 | 1.4 | 83.3 | 66.7 |
| Bt.10324.1.A1\_at | 0.00233 | 0.00052 | 0.000884 | ns \* \* | -1.37 | -0.795 | -1.33 | 83.3 | 66.7 |
| Bt.4744.1.S1\_at | 0.00275 | 2e-06 | 7.4e-05 | ns \* \* | -1.7 | -1.46 | -2.14 | 83.3 | 66.7 |
| Bt.10734.1.A1\_at | 0.00292 | 5.89e-07 | 0.000285 | ns \* \* | 1.52 | 1.16 | 1.38 | 83.3 | 66.7 |
| Bt.2997.1.A1\_at | 0.00302 | 0.000112 | 6.92e-07 | ns \* \* | 1.01 | 0.836 | 1.85 | 83.3 | 66.7 |
| Bt.8671.1.S1\_at | 0.00316 | 9.3e-05 | 0.000236 | ns \* \* | -1 | -0.799 | -1.29 | 83.3 | 66.7 |
| Bt.14304.2.S1\_at | 0.00334 | 0.000926 | 1.38e-05 | ns \* \* | 0.708 | 0.588 | 0.884 | 83.3 | 66.7 |
| Bt.26604.1.S1\_at | 0.00347 | 2.63e-06 | 8.21e-05 | ns \* \* | 2.47 | 1.28 | 1.83 | 83.3 | 66.7 |
| Bt.8785.1.S1\_at | 0.00367 | 0.000391 | 0.0013 | ns \* \* | 0.805 | 0.963 | 1.06 | 83.3 | 66.7 |
| Bt.2667.1.S1\_a\_at | 0.0038 | 6.39e-06 | 0.000286 | ns \* \* | -1.43 | -1.12 | -1.57 | 83.3 | 66.7 |
| Bt.9412.2.S1\_at | 0.00394 | 0.000793 | 0.000542 | ns \* \* | 1.15 | 0.727 | 1.29 | 83.3 | 66.7 |
| Bt.14572.1.A1\_at | 0.00409 | 3.79e-06 | 3.49e-05 | ns \* \* | 0.909 | 1.3 | 1.79 | 83.3 | 66.7 |
| Bt.11126.3.S1\_a\_at | 0.00422 | 0.000176 | 4.23e-05 | ns \* \* | 0.921 | 0.749 | 1.32 | 83.3 | 66.7 |
| Bt.1963.1.S1\_at | 0.00425 | 0.000684 | 7.84e-05 | ns \* \* | -0.65 | -0.514 | -0.656 | 83.3 | 66.7 |
| Bt.24396.1.S1\_at | 0.00447 | 0.000485 | 0.000672 | ns \* \* | -0.774 | -0.503 | -0.679 | 83.3 | 66.7 |
| Bt.19898.1.A1\_at | 0.00461 | 0.00143 | 0.00129 | ns \* \* | 0.899 | 0.636 | 0.938 | 83.3 | 66.7 |
| Bt.24381.1.S1\_at | 0.00473 | 2.71e-05 | 0.000413 | ns \* \* | 1.1 | 1.15 | 2 | 83.3 | 66.7 |
| Bt.7240.1.S1\_at | 0.00529 | 6.45e-05 | 4.36e-05 | ns \* \* | -0.904 | -0.92 | -1.01 | 83.3 | 66.7 |
| Bt.21053.2.S1\_at | 0.00579 | 4.13e-05 | 0.000166 | ns \* \* | -1.14 | -0.793 | -1.21 | 83.3 | 66.7 |
| Bt.9288.1.S1\_at | 0.0059 | 2.34e-05 | 6.63e-05 | ns \* \* | 0.913 | 1.29 | 1.65 | 83.3 | 66.7 |
| Bt.18434.1.A1\_at | 0.00593 | 0.000163 | 6.64e-05 | ns \* \* | 0.97 | 1.15 | 1.31 | 83.3 | 66.7 |
| Bt.5328.1.S1\_at | 0.00613 | 7.7e-05 | 0.000457 | ns \* \* | -1.27 | -1.18 | -1.33 | 83.3 | 66.7 |
| Bt.2331.1.A1\_at | 0.00689 | 2.2e-05 | 0.00057 | ns \* \* | 1.34 | 1.24 | 1.78 | 83.3 | 66.7 |
| Bt.13205.1.A1\_at | 0.00708 | 1.05e-05 | 6.41e-06 | ns \* \* | -0.932 | -0.919 | -1.5 | 83.3 | 66.7 |
| Bt.14207.1.S1\_at | 0.0082 | 1.61e-05 | 0.000977 | ns \* \* | -0.88 | -0.744 | -1.59 | 83.3 | 66.7 |
| Bt.12445.2.S1\_at | 0.00867 | 0.000798 | 0.00188 | ns \* \* | 1.97 | 0.754 | 1.26 | 83.3 | 66.7 |
| Bt.3215.1.S1\_at | 0.00921 | 5.79e-06 | 3.91e-07 | ns \* \* | 0.703 | 0.849 | 1.08 | 83.3 | 66.7 |
| Bt.9930.1.S1\_at | 0.0106 | 1.13e-05 | 0.000397 | ns \* \* | 0.667 | 0.948 | 1.02 | 83.3 | 66.7 |
| Bt.3092.1.S1\_at | 0.0114 | 7.34e-06 | 9.11e-05 | ns \* \* | 0.593 | 1.12 | 1.11 | 83.3 | 66.7 |
| Bt.28193.1.S1\_at | 0.0118 | 2.24e-06 | 1e-06 | ns \* \* | 1.95 | 1.54 | 2.24 | 83.3 | 66.7 |
| Bt.9953.1.S1\_at | 0.012 | 5.3e-05 | 8.2e-07 | ns \* \* | -0.957 | -0.684 | -0.985 | 83.3 | 66.7 |
| Bt.7872.1.S1\_at | 0.013 | 1.34e-05 | 0.000641 | ns \* \* | -1.01 | -0.97 | -1.33 | 83.3 | 66.7 |
| Bt.16259.1.A1\_at | 0.0141 | 4.56e-06 | 0.000211 | ns \* \* | 0.789 | 0.781 | 0.646 | 83.3 | 66.7 |
| Bt.11165.1.S1\_at | 0.0143 | 9.29e-05 | 8.67e-07 | ns \* \* | 0.689 | 0.653 | 0.876 | 83.3 | 66.7 |
| Bt.8970.1.S1\_at | 0.0145 | 0.000978 | 4.79e-06 | ns \* \* | -0.779 | -0.575 | -0.564 | 83.3 | 66.7 |
| Bt.23669.1.A1\_at | 0.0154 | 6.87e-07 | 2.02e-07 | ns \* \* | 0.564 | 1.19 | 2.02 | 83.3 | 66.7 |
| Bt.19957.1.A1\_at | 0.0155 | 2.86e-06 | 3.05e-05 | ns \* \* | 1.32 | 1.17 | 1.63 | 83.3 | 66.7 |
| Bt.6577.1.S1\_at | 0.0156 | 1.09e-05 | 0.000145 | ns \* \* | -0.763 | -0.908 | -1.3 | 83.3 | 66.7 |
| Bt.25016.2.S1\_at | 0.0157 | 0.000145 | 0.000344 | ns \* \* | 0.71 | 0.738 | 1.27 | 83.3 | 66.7 |
| Bt.28188.1.S1\_at | 0.0159 | 2.92e-05 | 0.000314 | ns \* \* | 0.954 | 0.798 | 1.22 | 83.3 | 66.7 |
| Bt.26316.1.A1\_at | 0.0184 | 0.000122 | 0.00111 | ns \* \* | 1.27 | 1.1 | 1.51 | 83.3 | 66.7 |
| Bt.7716.1.S1\_at | 0.019 | 0.000238 | 0.000816 | ns \* \* | 0.901 | 0.777 | 0.961 | 83.3 | 66.7 |
| Bt.17188.1.A1\_at | 0.019 | 6.16e-05 | 2.58e-05 | ns \* \* | 0.689 | 1.09 | 1.68 | 83.3 | 66.7 |
| Bt.20544.1.S1\_at | 0.0203 | 3.13e-05 | 3.34e-05 | ns \* \* | 0.849 | 0.824 | 0.779 | 83.3 | 66.7 |
| Bt.11599.1.S1\_at | 0.0235 | 1.84e-05 | 6.75e-06 | ns \* \* | 0.904 | 1.11 | 1.7 | 83.3 | 66.7 |
| Bt.11725.1.A1\_at | 0.0239 | 0.000134 | 7.48e-05 | ns \* \* | 0.71 | 0.844 | 1.52 | 83.3 | 66.7 |
| Bt.8957.1.S1\_at | 0.025 | 0.00129 | 0.000164 | ns \* \* | 1.17 | 0.745 | 1.42 | 83.3 | 66.7 |
| Bt.22181.1.A1\_at | 0.0266 | 1.12e-05 | 9.93e-05 | ns \* \* | -1.31 | -1.09 | -1.35 | 83.3 | 66.7 |
| Bt.26915.2.S1\_at | 0.0303 | 0.000398 | 0.000792 | ns \* \* | 0.471 | 0.788 | 1.38 | 83.3 | 66.7 |
| Bt.9036.1.A1\_at | 0.0306 | 0.0017 | 0.000157 | ns \* \* | 0.399 | 0.471 | 0.614 | 83.3 | 66.7 |
| Bt.11088.1.S1\_a\_at | 0.031 | 0.00046 | 0.00116 | ns \* \* | 0.647 | 0.636 | 0.991 | 83.3 | 66.7 |
| Bt.6017.1.A1\_at | 0.035 | 0.000204 | 3.73e-05 | ns \* \* | -0.709 | -0.852 | -0.685 | 83.3 | 66.7 |
| Bt.13530.1.S1\_at | 0.0352 | 3.93e-07 | 8.21e-05 | ns \* \* | -1.21 | -1.21 | -1.96 | 83.3 | 66.7 |
| Bt.22414.2.S1\_at | 0.0383 | 0.00023 | 0.00148 | ns \* \* | 1.31 | 1.52 | 2.78 | 83.3 | 66.7 |
| Bt.25522.1.S1\_at | 0.0473 | 9.46e-05 | 2.28e-05 | ns \* \* | 0.619 | 0.627 | 1.28 | 83.3 | 66.7 |
| Bt.12297.1.S1\_at | 0.0501 | 9.27e-05 | 0.000927 | ns \* \* | -0.798 | -0.827 | -1.58 | 83.3 | 66.7 |
| Bt.1175.1.S1\_at | 0.0519 | 2.8e-05 | 0.000422 | ns \* \* | -0.78 | -0.843 | -1.41 | 83.3 | 66.7 |
| Bt.11530.1.A1\_at | 0.0539 | 0.000407 | 0.00182 | ns \* \* | 0.628 | 0.682 | 0.733 | 83.3 | 66.7 |
| Bt.17456.1.S1\_at | 0.0548 | 2.98e-05 | 0.000671 | ns \* \* | -0.91 | -0.838 | -1.25 | 83.3 | 66.7 |
| Bt.12867.2.S1\_at | 0.0594 | 0.000108 | 0.000915 | ns \* \* | 0.506 | 0.546 | 0.656 | 83.3 | 66.7 |
| Bt.26848.1.S1\_at | 0.0656 | 0.000738 | 0.000358 | ns \* \* | 0.538 | 0.748 | 1.14 | 83.3 | 66.7 |
| Bt.8766.1.S1\_at | 0.0685 | 0.000249 | 0.00103 | ns \* \* | 0.854 | 0.666 | 0.739 | 83.3 | 66.7 |
| Bt.9936.1.S1\_at | 0.0779 | 3.45e-06 | 0.000208 | ns \* \* | 0.55 | 0.874 | 0.525 | 83.3 | 66.7 |
| Bt.819.1.S1\_at | 0.0816 | 9.76e-05 | 0.00175 | ns \* \* | -0.512 | -0.605 | -0.792 | 83.3 | 66.7 |
| Bt.28150.2.S1\_at | 0.083 | 1.72e-05 | 9.29e-06 | ns \* \* | -1.11 | -1.05 | -2.3 | 83.3 | 66.7 |
| Bt.10657.1.A1\_at | 0.0861 | 7.17e-06 | 4.7e-05 | ns \* \* | -1.37 | -1.05 | -2.35 | 83.3 | 66.7 |
| Bt.3698.1.S1\_at | 0.0868 | 0.000996 | 0.000242 | ns \* \* | 0.73 | 0.756 | 1.11 | 83.3 | 66.7 |
| Bt.12861.1.S1\_at | 0.117 | 0.000608 | 8.4e-05 | ns \* \* | 0.594 | 0.578 | 1.16 | 83.3 | 66.7 |
| Bt.6567.1.S1\_at | 0.133 | 0.000489 | 6.2e-05 | ns \* \* | 0.656 | 0.653 | 1.25 | 83.3 | 66.7 |
| Bt.9993.1.S1\_at | 0.162 | 4.83e-06 | 1.08e-05 | ns \* \* | -0.961 | -0.942 | -1.52 | 83.3 | 66.7 |
| Bt.15922.1.S1\_at | 0.183 | 0.000389 | 0.000375 | ns \* \* | 0.428 | 0.547 | 0.536 | 83.3 | 66.7 |
| Bt.22176.1.A1\_at | 0.213 | 0.000189 | 7.2e-05 | ns \* \* | 0.873 | 1.06 | 2.05 | 83.3 | 66.7 |
| Bt.22320.3.S1\_at | 0.254 | 5.45e-05 | 0.00179 | ns \* \* | 0.505 | 1 | 0.746 | 83.3 | 66.7 |
| Bt.755.1.S1\_at | 0.323 | 0.00121 | 1.88e-06 | ns \* \* | 0.26 | -0.632 | -0.953 | 83.3 | 66.7 |
| Bt.4437.1.S1\_at | 0.913 | 0.00137 | 1.83e-05 | ns \* \* | 0.0543 | 0.735 | 1.24 | 83.3 | 66.7 |
| Bt.5732.2.A1\_at | 2.76e-06 | 0.00126 | 0.00578 | \* \* ns | 1.86 | 0.915 | 1.22 | 66.7 | 66.7 |
| Bt.8608.1.S1\_at | 0.000129 | 0.000484 | 0.00206 | \* \* ns | 1.93 | 0.687 | 0.661 | 66.7 | 66.7 |
| Bt.26430.1.S1\_at | 0.00021 | 0.000134 | 0.00976 | \* \* ns | 1.16 | 0.842 | 0.742 | 66.7 | 66.7 |
| Bt.21177.1.A1\_s\_at | 0.000352 | 4.19e-06 | 0.0161 | \* \* ns | 1.34 | 1.47 | 1.15 | 66.7 | 66.7 |
| Bt.27428.1.A1\_at | 0.000358 | 0.002 | 0.0024 | \* \* ns | 1.65 | 0.878 | 1.4 | 66.7 | 66.7 |
| Bt.13350.1.S1\_at | 0.000399 | 0.00205 | 0.0546 | \* \* ns | 0.965 | 0.884 | 0.651 | 66.7 | 66.7 |
| Bt.10129.1.A1\_at | 0.000587 | 0.000924 | 0.381 | \* \* ns | 1.26 | 0.581 | 0.0738 | 66.7 | 66.7 |
| Bt.25419.1.A1\_at | 0.000613 | 4e-04 | 0.0149 | \* \* ns | -2.42 | -1.32 | -2.02 | 66.7 | 66.7 |
| Bt.16495.2.A1\_at | 0.000661 | 0.00259 | 0.00119 | \* ns \* | 1.28 | 0.529 | 0.998 | 66.7 | 66.7 |
| Bt.27481.1.A1\_at | 0.000662 | 0.000268 | 0.199 | \* \* ns | 1.78 | 1.02 | 0.503 | 66.7 | 66.7 |
| Bt.25836.2.A1\_at | 0.000724 | 0.0107 | 0.000172 | \* ns \* | 1.2 | 0.599 | 1.78 | 66.7 | 66.7 |
| Bt.15887.2.S1\_a\_at | 0.000782 | 0.00173 | 0.00312 | \* \* ns | -0.818 | -0.537 | -0.565 | 66.7 | 66.7 |
| Bt.8479.1.A1\_at | 0.000808 | 0.000111 | 0.00409 | \* \* ns | 1.6 | 1.04 | 1.72 | 66.7 | 66.7 |
| Bt.18002.1.A1\_at | 0.000874 | 1.8e-05 | 0.00913 | \* \* ns | 2.37 | 0.861 | 0.672 | 66.7 | 66.7 |
| Bt.18340.1.A1\_at | 0.000984 | 0.00523 | 0.000628 | \* ns \* | 0.85 | 0.538 | 0.605 | 66.7 | 66.7 |
| Bt.5129.2.A1\_at | 0.0011 | 0.000937 | 0.0157 | \* \* ns | -1.05 | -0.754 | -1.33 | 66.7 | 66.7 |
| Bt.11711.1.A1\_at | 0.00151 | 5.88e-06 | 5e-04 | ns \* \* | 2.27 | 2.13 | 3.05 | 66.7 | 66.7 |
| Bt.13581.1.S1\_at | 0.00195 | 0.000611 | 0.000401 | ns \* \* | 1.71 | 1.19 | 1.49 | 66.7 | 66.7 |
| Bt.25044.1.S1\_at | 0.00211 | 0.000156 | 0.000891 | ns \* \* | -1.49 | -0.917 | -1.45 | 66.7 | 66.7 |
| Bt.1151.1.S1\_at | 0.00222 | 0.000572 | 0.000704 | ns \* \* | -2.22 | -1.8 | -2.76 | 66.7 | 66.7 |
| Bt.12688.2.A1\_at | 0.00231 | 5.95e-05 | 0.00116 | ns \* \* | 2.67 | 0.724 | 0.875 | 66.7 | 66.7 |
| Bt.20815.1.A1\_at | 0.00249 | 1.84e-05 | 0.000547 | ns \* \* | 2.35 | 1.49 | 2.3 | 66.7 | 66.7 |
| Bt.20230.1.S1\_at | 0.00251 | 5.33e-05 | 0.000534 | ns \* \* | 1.1 | 0.762 | 1.04 | 66.7 | 66.7 |
| Bt.5170.1.S1\_at | 0.00253 | 2.81e-05 | 6.29e-05 | ns \* \* | -1.03 | -0.845 | -1.31 | 66.7 | 66.7 |
| Bt.27413.1.A1\_at | 0.00254 | 3.93e-06 | 0.000112 | ns \* \* | 1.62 | 1.51 | 2.26 | 66.7 | 66.7 |
| Bt.16836.2.A1\_at | 0.00275 | 3.3e-05 | 0.0011 | ns \* \* | 1.51 | 1.06 | 1.81 | 66.7 | 66.7 |
| Bt.27400.1.S1\_at | 0.00323 | 0.000262 | 0.00117 | ns \* \* | 0.989 | 1.09 | 1.88 | 66.7 | 66.7 |
| Bt.27872.1.S1\_at | 0.00342 | 0.000877 | 0.000247 | ns \* \* | 1.13 | 0.573 | 1.4 | 66.7 | 66.7 |
| Bt.10329.1.A1\_at | 0.00349 | 1.48e-05 | 1.86e-05 | ns \* \* | 0.86 | 0.999 | 1.33 | 66.7 | 66.7 |
| Bt.876.1.S1\_at | 0.00362 | 8.32e-06 | 0.000355 | ns \* \* | -1.91 | -1.54 | -2.23 | 66.7 | 66.7 |
| Bt.20106.1.S1\_at | 0.00382 | 8.95e-05 | 1.41e-05 | ns \* \* | 0.868 | 0.739 | 1.14 | 66.7 | 66.7 |
| Bt.18126.2.S1\_at | 0.00428 | 0.00203 | 6.49e-05 | ns \* \* | 1.71 | 0.592 | 1.25 | 66.7 | 66.7 |
| Bt.25966.1.A1\_at | 0.005 | 8.89e-05 | 0.000504 | ns \* \* | 1.38 | 0.697 | 0.625 | 66.7 | 66.7 |
| Bt.17061.1.A1\_at | 0.00506 | 0.000161 | 0.000274 | ns \* \* | 2.1 | 1.12 | 1.82 | 66.7 | 66.7 |
| Bt.22405.1.A1\_at | 0.00527 | 1.89e-06 | 1.55e-05 | ns \* \* | 1.03 | 1.18 | 2.08 | 66.7 | 66.7 |
| Bt.23230.1.S1\_at | 0.00541 | 0.000966 | 2.15e-08 | ns \* \* | 1.06 | 0.621 | 0.973 | 66.7 | 66.7 |
| Bt.23486.3.S1\_at | 0.00553 | 5.71e-05 | 0.000922 | ns \* \* | 2.24 | 1.43 | 2.16 | 66.7 | 66.7 |
| Bt.11408.1.A1\_at | 0.00603 | 0.00187 | 0.00099 | ns \* \* | 1.28 | 0.971 | 1.61 | 66.7 | 66.7 |
| Bt.18727.1.A1\_at | 0.00628 | 0.00105 | 0.0012 | ns \* \* | -0.824 | -0.603 | -0.949 | 66.7 | 66.7 |
| Bt.24269.1.S1\_at | 0.00693 | 2e-07 | 5.01e-05 | ns \* \* | -1.54 | -1.3 | -1.66 | 66.7 | 66.7 |
| Bt.5626.2.S1\_at | 0.00706 | 0.000363 | 1.42e-05 | ns \* \* | -1.39 | -0.72 | -2.11 | 66.7 | 66.7 |
| Bt.26993.1.S1\_at | 0.0071 | 2.72e-05 | 3.16e-05 | ns \* \* | -0.769 | -0.806 | -1.81 | 66.7 | 66.7 |
| Bt.19281.2.S1\_at | 0.00718 | 1.46e-05 | 3.95e-05 | ns \* \* | 0.849 | 0.828 | 0.982 | 66.7 | 66.7 |
| Bt.29679.1.S1\_s\_at | 0.00736 | 0.0011 | 0.000168 | ns \* \* | 1.27 | 0.612 | 1.45 | 66.7 | 66.7 |
| Bt.18316.1.A1\_at | 0.00915 | 2.74e-05 | 0.000153 | ns \* \* | 2.56 | 1.43 | 1.49 | 66.7 | 66.7 |
| Bt.7612.1.S1\_at | 0.00921 | 0.000474 | 0.000477 | ns \* \* | -0.69 | -0.522 | -0.72 | 66.7 | 66.7 |
| Bt.10810.1.A1\_at | 0.00931 | 0.000175 | 0.000232 | ns \* \* | 1.1 | 0.951 | 0.971 | 66.7 | 66.7 |
| Bt.12745.3.S1\_at | 0.00954 | 0.00135 | 0.00181 | ns \* \* | 1.16 | 0.917 | 1.46 | 66.7 | 66.7 |
| Bt.177.1.S1\_at | 0.00962 | 6.37e-05 | 2.77e-06 | ns \* \* | 0.637 | 1.06 | 1.22 | 66.7 | 66.7 |
| Bt.23246.1.S1\_at | 0.00965 | 3.02e-06 | 0.000238 | ns \* \* | 1.08 | 1.15 | 1.38 | 66.7 | 66.7 |
| Bt.29900.1.S1\_at | 0.011 | 8.11e-06 | 0.000496 | ns \* \* | 1.57 | 1.25 | 1.67 | 66.7 | 66.7 |
| Bt.27401.1.A1\_at | 0.0114 | 6.21e-06 | 1e-04 | ns \* \* | -0.87 | -0.776 | -0.773 | 66.7 | 66.7 |
| Bt.4964.1.S1\_at | 0.0116 | 9.23e-06 | 0.000492 | ns \* \* | 0.623 | 0.979 | 1.4 | 66.7 | 66.7 |
| Bt.4726.2.S1\_at | 0.012 | 8.18e-05 | 1.05e-05 | ns \* \* | 1.07 | 0.835 | 1.34 | 66.7 | 66.7 |
| Bt.6028.1.S1\_at | 0.0136 | 0.0014 | 0.000273 | ns \* \* | -0.78 | -0.627 | -0.998 | 66.7 | 66.7 |
| Bt.9660.1.A1\_s\_at | 0.0138 | 0.000436 | 0.00154 | ns \* \* | 0.468 | 0.571 | 0.6 | 66.7 | 66.7 |
| Bt.22094.1.A1\_at | 0.0141 | 3.86e-06 | 0.00106 | ns \* \* | 1.16 | 1.07 | 0.976 | 66.7 | 66.7 |
| Bt.26572.1.S1\_at | 0.0155 | 0.000131 | 4.63e-06 | ns \* \* | 1.37 | 0.85 | 1.8 | 66.7 | 66.7 |
| Bt.26750.1.S1\_at | 0.0155 | 3.76e-05 | 0.000467 | ns \* \* | -2.39 | -1.57 | -2.39 | 66.7 | 66.7 |
| Bt.9306.2.S1\_a\_at | 0.0162 | 8.05e-05 | 8.35e-05 | ns \* \* | -1.22 | -0.868 | -1.31 | 66.7 | 66.7 |
| Bt.18702.1.A1\_at | 0.0179 | 0.000264 | 4.63e-05 | ns \* \* | -0.902 | -0.733 | -1.25 | 66.7 | 66.7 |
| Bt.4008.1.S1\_at | 0.0196 | 0.000203 | 8.85e-05 | ns \* \* | -0.612 | -0.706 | -0.985 | 66.7 | 66.7 |
| Bt.18048.2.S1\_at | 0.0197 | 3.42e-05 | 0.000251 | ns \* \* | 1.04 | 1.05 | 1.42 | 66.7 | 66.7 |
| Bt.20862.1.A1\_at | 0.0204 | 1.38e-05 | 2.88e-06 | ns \* \* | -0.594 | -0.852 | -1.46 | 66.7 | 66.7 |
| Bt.11770.1.S1\_at | 0.0206 | 1.82e-07 | 9.79e-05 | ns \* \* | -1.04 | -1.19 | -2.41 | 66.7 | 66.7 |
| Bt.22230.1.S1\_at | 0.0213 | 9.76e-05 | 0.000337 | ns \* \* | 0.932 | 0.936 | 1.35 | 66.7 | 66.7 |
| Bt.21277.1.A1\_at | 0.0285 | 8.79e-06 | 6.01e-05 | ns \* \* | 1.55 | 0.738 | 0.859 | 66.7 | 66.7 |
| Bt.27627.1.A1\_at | 0.0356 | 0.000256 | 0.000111 | ns \* \* | -1.05 | -0.574 | -1.2 | 66.7 | 66.7 |
| Bt.16259.2.S1\_at | 0.0366 | 0.000141 | 7.79e-06 | ns \* \* | 0.617 | 0.59 | 0.968 | 66.7 | 66.7 |
| Bt.10092.1.S1\_at | 0.038 | 2.36e-05 | 0.000346 | ns \* \* | 0.656 | 1.04 | 1.21 | 66.7 | 66.7 |
| Bt.26364.1.A1\_at | 0.0468 | 0.000126 | 0.000538 | ns \* \* | 1.11 | 1.13 | 1.49 | 66.7 | 66.7 |
| Bt.3372.2.S1\_a\_at | 0.0498 | 0.000918 | 0.000523 | ns \* \* | -0.832 | -0.668 | -1.07 | 66.7 | 66.7 |
| Bt.17005.1.S1\_at | 0.0825 | 0.00205 | 0.000368 | ns \* \* | -0.605 | -0.532 | -1.34 | 66.7 | 66.7 |
| Bt.4719.1.S1\_at | 0.104 | 0.00012 | 7.12e-06 | ns \* \* | 1.02 | 0.828 | 1.63 | 66.7 | 66.7 |
| Bt.10317.1.A1\_at | 0.108 | 5.42e-05 | 0.000182 | ns \* \* | 0.554 | 0.74 | 0.935 | 66.7 | 66.7 |
| Bt.20372.1.S1\_at | 0.119 | 1.05e-07 | 0.000225 | ns \* \* | -1.56 | -1.84 | -2.46 | 66.7 | 66.7 |
| Bt.20629.3.A1\_at | 0.264 | 0.000101 | 0.00134 | ns \* \* | -0.513 | -0.896 | -0.86 | 66.7 | 66.7 |
| Bt.7120.1.S1\_a\_at | 0.269 | 1.97e-05 | 0.000147 | ns \* \* | 0.239 | 0.771 | 0.611 | 66.7 | 66.7 |
| Bt.8109.2.A1\_at | 0.387 | 0.00182 | 8.49e-05 | ns \* \* | 0.625 | 0.641 | 1.54 | 66.7 | 66.7 |
| Bt.10930.1.S1\_at | 5.28e-06 | 9.16e-06 | 0.0291 | \* \* ns | 1.48 | 0.898 | 0.622 | 50 | 66.7 |
| Bt.26402.1.A1\_at | 5.97e-06 | 9.93e-05 | 0.0331 | \* \* ns | 4.06 | 0.745 | 0.33 | 50 | 66.7 |
| Bt.8909.1.S1\_at | 2.33e-05 | 0.000515 | 0.0202 | \* \* ns | 1.29 | 0.811 | 0.922 | 50 | 66.7 |
| Bt.984.1.S1\_at | 3.3e-05 | 0.00513 | 4.26e-06 | \* ns \* | 1.83 | 0.603 | 1.43 | 50 | 66.7 |
| Bt.3394.1.S1\_at | 5.53e-05 | 0.000292 | 0.145 | \* \* ns | 2.17 | 0.755 | 0.962 | 50 | 66.7 |
| Bt.28455.1.S1\_at | 8.01e-05 | 1.66e-05 | 0.0195 | \* \* ns | 1.06 | 0.888 | 0.832 | 50 | 66.7 |
| Bt.10044.1.A1\_at | 0.000113 | 0.00111 | 0.304 | \* \* ns | 1.63 | 0.712 | 0.448 | 50 | 66.7 |
| Bt.15561.2.A1\_s\_at | 0.000132 | 0.000446 | 0.00581 | \* \* ns | -5.17 | -1.1 | -2.46 | 50 | 66.7 |
| Bt.26710.1.S1\_at | 0.000149 | 0.0301 | 0.000912 | \* ns \* | 2.82 | 0.482 | 1.29 | 50 | 66.7 |
| Bt.4800.1.S1\_at | 0.000255 | 0.000827 | 0.112 | \* \* ns | 1.58 | 0.753 | 0.839 | 50 | 66.7 |
| Bt.19199.1.A1\_at | 0.000282 | 0.000423 | 0.00249 | \* \* ns | 1.76 | 1.31 | 2.03 | 50 | 66.7 |
| Bt.16510.1.A1\_at | 0.000291 | 7.78e-05 | 0.0846 | \* \* ns | 2.67 | 0.68 | 0.959 | 50 | 66.7 |
| Bt.11040.1.S1\_at | 0.000356 | 0.00284 | 0.000186 | \* ns \* | 1.2 | 0.554 | 1.58 | 50 | 66.7 |
| Bt.29625.1.S1\_at | 0.000374 | 0.000524 | 0.0279 | \* \* ns | 3.01 | 0.854 | 1.15 | 50 | 66.7 |
| Bt.26447.1.A1\_at | 0.000519 | 0.000217 | 0.205 | \* \* ns | 1.86 | 0.831 | 0.3 | 50 | 66.7 |
| Bt.9070.1.S1\_at | 0.000544 | 0.00159 | 0.0541 | \* \* ns | -0.875 | -0.716 | -0.576 | 50 | 66.7 |
| Bt.26423.1.A1\_at | 0.000558 | 2.05e-06 | 0.00871 | \* \* ns | 1.64 | 1.26 | 1.04 | 50 | 66.7 |
| Bt.9321.1.S1\_at | 0.000628 | 6.45e-05 | 0.28 | \* \* ns | 1.61 | 0.685 | 0.247 | 50 | 66.7 |
| Bt.7168.2.A1\_at | 0.000661 | 3.72e-05 | 0.161 | \* \* ns | 2.48 | 0.994 | 0.499 | 50 | 66.7 |
| Bt.13994.1.S1\_at | 0.000759 | 0.000289 | 0.00641 | \* \* ns | 1.05 | 0.86 | 1.87 | 50 | 66.7 |
| Bt.13965.1.S1\_at | 0.000892 | 0.00378 | 0.000331 | \* ns \* | 1.05 | 0.657 | 1.5 | 50 | 66.7 |
| Bt.17447.1.A1\_at | 0.00106 | 0.00119 | 0.0706 | \* \* ns | 1.83 | 1.03 | 0.649 | 50 | 66.7 |
| Bt.8275.1.S1\_at | 0.00109 | 0.000332 | 0.00531 | \* \* ns | 1.1 | 1.03 | 1.27 | 50 | 66.7 |
| Bt.3323.1.S1\_at | 0.00124 | 0.000357 | 3.33e-05 | ns \* \* | 0.717 | 0.595 | 0.981 | 50 | 66.7 |
| Bt.22002.1.S1\_at | 0.00126 | 3.78e-09 | 1.92e-05 | ns \* \* | -5.07 | -2.28 | -3.3 | 50 | 66.7 |
| Bt.26871.1.S1\_at | 0.00131 | 6e-05 | 2.87e-05 | ns \* \* | 1.06 | 0.604 | 0.98 | 50 | 66.7 |
| Bt.1000.1.S1\_at | 0.00149 | 0.000272 | 1.01e-05 | ns \* \* | 0.954 | 0.708 | 1.92 | 50 | 66.7 |
| Bt.23094.3.S1\_x\_at | 0.00161 | 0.000425 | 0.000141 | ns \* \* | -1.44 | -0.958 | -1.77 | 50 | 66.7 |
| Bt.25997.1.S1\_at | 0.00178 | 5.41e-08 | 2.25e-06 | ns \* \* | 2.33 | 1.36 | 1.9 | 50 | 66.7 |
| Bt.923.2.S1\_at | 0.00203 | 2.25e-05 | 1.31e-05 | ns \* \* | 0.993 | 0.838 | 1.27 | 50 | 66.7 |
| Bt.26750.2.A1\_at | 0.00212 | 4.17e-06 | 3.86e-05 | ns \* \* | -2.8 | -1.58 | -2.77 | 50 | 66.7 |
| Bt.1768.1.A1\_at | 0.00215 | 2.94e-05 | 0.000651 | ns \* \* | -0.904 | -0.705 | -0.833 | 50 | 66.7 |
| Bt.12105.1.A1\_at | 0.00245 | 1.53e-05 | 0.000103 | ns \* \* | -1.28 | -0.805 | -1.5 | 50 | 66.7 |
| Bt.2556.1.S1\_at | 0.0027 | 7.11e-05 | 0.000179 | ns \* \* | 2.65 | 1.29 | 2.08 | 50 | 66.7 |
| Bt.6570.2.S1\_at | 0.0027 | 7.94e-05 | 0.000437 | ns \* \* | 1.28 | 0.855 | 1.43 | 50 | 66.7 |
| Bt.11476.1.S1\_at | 0.00283 | 4.54e-06 | 5.29e-07 | ns \* \* | 1.69 | 0.901 | 1.68 | 50 | 66.7 |
| Bt.3139.1.A1\_at | 0.00302 | 9.79e-07 | 4.81e-05 | ns \* \* | 3.1 | 1.9 | 2.15 | 50 | 66.7 |
| Bt.5674.1.S1\_at | 0.00309 | 0.000173 | 0.000606 | ns \* \* | -1 | -0.63 | -1.22 | 50 | 66.7 |
| Bt.1793.3.S1\_at | 0.00324 | 4.15e-05 | 9.18e-05 | ns \* \* | -1.27 | -1.35 | -1.94 | 50 | 66.7 |
| Bt.28431.1.S1\_at | 0.00357 | 2.87e-06 | 2.12e-07 | ns \* \* | 1.17 | 1.03 | 1.38 | 50 | 66.7 |
| Bt.467.1.S1\_at | 0.00365 | 1.98e-07 | 7.8e-07 | ns \* \* | -2.58 | -1.68 | -3.43 | 50 | 66.7 |
| Bt.27386.2.A1\_at | 0.00366 | 0.000508 | 0.000573 | ns \* \* | 1.8 | 1.06 | 1.97 | 50 | 66.7 |
| Bt.8617.1.S1\_at | 0.00375 | 5.21e-05 | 0.00123 | ns \* \* | 0.857 | 0.981 | 1.22 | 50 | 66.7 |
| Bt.20601.2.S1\_at | 0.00377 | 2.36e-05 | 1.36e-05 | ns \* \* | 1.02 | 0.763 | 1.29 | 50 | 66.7 |
| Bt.25099.2.S1\_at | 0.0039 | 2.47e-07 | 0.000234 | ns \* \* | -2.48 | -2.34 | -3.37 | 50 | 66.7 |
| Bt.27781.1.S1\_at | 0.00396 | 3.78e-05 | 4.71e-05 | ns \* \* | 1.15 | 1.29 | 1.65 | 50 | 66.7 |
| Bt.11599.2.S1\_at | 0.00512 | 3.01e-05 | 0.00162 | ns \* \* | 2.37 | 0.916 | 2.45 | 50 | 66.7 |
| Bt.11016.1.S1\_at | 0.00517 | 0.000521 | 9.72e-06 | ns \* \* | 1.11 | 0.554 | 1.1 | 50 | 66.7 |
| Bt.13408.2.S1\_at | 0.00542 | 0.000338 | 0.000732 | ns \* \* | 1.2 | 0.824 | 0.99 | 50 | 66.7 |
| Bt.6926.1.A1\_a\_at | 0.00542 | 2.79e-05 | 0.000688 | ns \* \* | 1.46 | 1.14 | 1.37 | 50 | 66.7 |
| Bt.11325.1.S1\_at | 0.00707 | 0.000892 | 1.91e-05 | ns \* \* | 0.908 | 0.712 | 0.964 | 50 | 66.7 |
| Bt.11326.3.S1\_at | 0.00722 | 0.00174 | 9.07e-06 | ns \* \* | -0.691 | -0.512 | -0.551 | 50 | 66.7 |
| Bt.26513.1.S1\_at | 0.00729 | 2.82e-05 | 0.000878 | ns \* \* | 1.48 | 1.66 | 2.25 | 50 | 66.7 |
| Bt.4764.1.S1\_at | 0.00784 | 0.00056 | 9.66e-06 | ns \* \* | -0.816 | -0.456 | -0.781 | 50 | 66.7 |
| Bt.211.1.S1\_at | 0.00832 | 0.000227 | 0.000568 | ns \* \* | -0.91 | -1.01 | -1.92 | 50 | 66.7 |
| Bt.9306.3.S1\_at | 0.0087 | 0.000135 | 6.21e-05 | ns \* \* | -1.19 | -0.795 | -1.44 | 50 | 66.7 |
| Bt.17460.1.A1\_at | 0.0108 | 0.000288 | 0.00121 | ns \* \* | 1.5 | 1.24 | 1.04 | 50 | 66.7 |
| Bt.22564.3.S1\_at | 0.0113 | 0.00197 | 0.00012 | ns \* \* | 0.96 | 0.571 | 0.493 | 50 | 66.7 |
| Bt.21641.1.S1\_at | 0.0121 | 3.32e-07 | 0.000111 | ns \* \* | 1.7 | 1.44 | 2.04 | 50 | 66.7 |
| Bt.19811.1.A1\_at | 0.0122 | 3.78e-05 | 0.00108 | ns \* \* | -1.29 | -1.01 | -1.2 | 50 | 66.7 |
| Bt.22502.1.S1\_at | 0.0123 | 0.000336 | 2.71e-06 | ns \* \* | 0.826 | 0.661 | 1.11 | 50 | 66.7 |
| Bt.13972.1.A1\_at | 0.0133 | 4.66e-05 | 1.5e-05 | ns \* \* | 2.03 | 0.687 | 1.81 | 50 | 66.7 |
| Bt.1681.1.S1\_at | 0.0147 | 1.52e-05 | 0.000122 | ns \* \* | -1.43 | -0.778 | -1.63 | 50 | 66.7 |
| Bt.12030.1.S1\_at | 0.0149 | 0.000113 | 9.14e-05 | ns \* \* | 0.629 | 0.666 | 0.873 | 50 | 66.7 |
| Bt.28821.1.S1\_at | 0.0153 | 7.35e-05 | 0.000982 | ns \* \* | -1.01 | -0.988 | -0.746 | 50 | 66.7 |
| Bt.27565.1.S1\_at | 0.0159 | 0.000328 | 0.000557 | ns \* \* | 0.655 | 0.543 | 0.472 | 50 | 66.7 |
| Bt.8392.1.S1\_at | 0.0164 | 0.000237 | 0.000461 | ns \* \* | 0.654 | 0.687 | 1.12 | 50 | 66.7 |
| Bt.9944.1.S1\_at | 0.0196 | 2.89e-05 | 0.000835 | ns \* \* | 1.67 | 1.33 | 2.05 | 50 | 66.7 |
| Bt.1807.1.A1\_at | 0.0197 | 4.96e-08 | 5.88e-07 | ns \* \* | 1.71 | 1.41 | 2.49 | 50 | 66.7 |
| Bt.15915.1.S1\_at | 0.0223 | 0.000123 | 0.000454 | ns \* \* | 0.804 | 0.83 | 0.972 | 50 | 66.7 |
| Bt.24470.1.S1\_a\_at | 0.0231 | 0.00016 | 0.000527 | ns \* \* | 1.56 | 0.843 | 1.07 | 50 | 66.7 |
| Bt.25921.1.A1\_at | 0.0256 | 0.000259 | 0.000314 | ns \* \* | 1.81 | 0.646 | 0.908 | 50 | 66.7 |
| Bt.13287.1.S1\_at | 0.0258 | 1.09e-08 | 2.71e-09 | ns \* \* | -2.13 | -1.63 | -2.36 | 50 | 66.7 |
| Bt.1705.1.S1\_at | 0.0272 | 7.04e-05 | 2.77e-06 | ns \* \* | 1.03 | 1.26 | 2.44 | 50 | 66.7 |
| Bt.20020.1.S1\_at | 0.0278 | 0.000228 | 1.54e-05 | ns \* \* | 0.896 | 0.81 | 1.32 | 50 | 66.7 |
| Bt.4652.1.S1\_at | 0.029 | 0.00039 | 0.000368 | ns \* \* | -0.778 | -0.706 | -0.967 | 50 | 66.7 |
| Bt.18414.1.S1\_at | 0.0293 | 0.00039 | 0.00134 | ns \* \* | -1.3 | -1.06 | -2.49 | 50 | 66.7 |
| Bt.22349.1.A1\_at | 0.0355 | 4.79e-05 | 3.67e-05 | ns \* \* | 1.14 | 0.793 | 1.22 | 50 | 66.7 |
| Bt.3726.1.A1\_at | 0.0356 | 6.56e-05 | 0.000222 | ns \* \* | 0.639 | 0.924 | 1.08 | 50 | 66.7 |
| Bt.2123.1.S1\_at | 0.0387 | 2.75e-05 | 0.00063 | ns \* \* | -1.67 | -0.889 | -1.2 | 50 | 66.7 |
| Bt.26502.1.A1\_at | 0.042 | 3.88e-06 | 5.17e-06 | ns \* \* | 1.5 | 1.01 | 1.04 | 50 | 66.7 |
| Bt.13784.1.S1\_at | 0.0433 | 8.76e-05 | 0.000163 | ns \* \* | -0.541 | -0.698 | -0.816 | 50 | 66.7 |
| Bt.24359.1.S1\_at | 0.0449 | 0.00135 | 0.000551 | ns \* \* | 0.633 | 0.607 | 0.868 | 50 | 66.7 |
| Bt.9778.1.S1\_at | 0.0486 | 8.49e-05 | 0.000121 | ns \* \* | 0.69 | 0.848 | 1 | 50 | 66.7 |
| Bt.26674.1.S1\_at | 0.0572 | 0.000439 | 4.6e-05 | ns \* \* | 0.655 | 0.637 | 1.09 | 50 | 66.7 |
| Bt.18043.1.S1\_at | 0.0645 | 0.00121 | 0.000103 | ns \* \* | 0.554 | 0.515 | 1.18 | 50 | 66.7 |
| Bt.11126.1.S1\_at | 0.0703 | 0.000961 | 0.000907 | ns \* \* | 1.16 | 0.894 | 1.36 | 50 | 66.7 |
| Bt.16659.1.A1\_at | 0.0791 | 0.000732 | 2.4e-06 | ns \* \* | -2.07 | -0.54 | -0.662 | 50 | 66.7 |
| Bt.9612.1.S1\_at | 0.08 | 2.74e-06 | 0.0014 | ns \* \* | -1.22 | -1.26 | -1.49 | 50 | 66.7 |
| Bt.2850.1.A1\_at | 0.148 | 0.000849 | 0.00143 | ns \* \* | 1.02 | 0.992 | 1.43 | 50 | 66.7 |
| Bt.9533.1.S1\_at | 0.168 | 0.000978 | 0.00132 | ns \* \* | 0.67 | 0.572 | 0.921 | 50 | 66.7 |
| Bt.15796.1.S1\_at | 0.174 | 3.11e-05 | 3.13e-05 | ns \* \* | 1.12 | 0.729 | 1.05 | 50 | 66.7 |
| Bt.3391.1.S1\_at | 0.18 | 0.000869 | 0.000209 | ns \* \* | 1.08 | 0.732 | 0.863 | 50 | 66.7 |
| Bt.6087.1.S1\_at | 0.191 | 0.00176 | 0.000529 | ns \* \* | 0.738 | 0.437 | 0.427 | 50 | 66.7 |
| Bt.2304.1.A1\_at | 0.192 | 0.000764 | 0.000117 | ns \* \* | 0.577 | 0.605 | 0.956 | 50 | 66.7 |
| Bt.20817.1.A1\_at | 0.207 | 0.000456 | 4.06e-05 | ns \* \* | 0.388 | 0.65 | 1.06 | 50 | 66.7 |
| Bt.17701.1.A1\_at | 0.441 | 0.00156 | 0.000103 | ns \* \* | 0.454 | 0.491 | 1.01 | 50 | 66.7 |
| Bt.23094.2.A1\_x\_at | 0.479 | 5.82e-05 | 0.000177 | ns \* \* | -0.361 | -1.04 | -1.38 | 50 | 66.7 |
| Bt.23355.1.S1\_at | 0.517 | 0.000541 | 1.56e-05 | ns \* \* | -0.189 | -0.726 | -1.26 | 50 | 66.7 |

Differentially expressed genes for MAS: 1743

| AFFY | p-value | FoldChange |
| --- | --- | --- |
| Bt.4683.1.S1\_at | 4.577e-11 | 5.017 |
| Bt.23076.1.S1\_at | 1.4e-09 | 2.825 |
| Bt.13980.1.A1\_at | 1.891e-09 | -4.665 |
| Bt.3960.1.S1\_a\_at | 5.688e-09 | -2.041 |
| Bt.13980.2.A1\_a\_at | 6.563e-09 | -3.515 |
| Bt.1316.1.S1\_at | 6.649e-09 | -2.848 |
| Bt.23502.1.S1\_at | 1.012e-08 | 1.897 |
| Bt.23662.2.S1\_at | 1.107e-08 | -2.287 |
| Bt.23113.1.A1\_at | 1.316e-08 | 3.088 |
| Bt.22664.1.A1\_at | 1.472e-08 | 2.959 |
| Bt.24931.1.A1\_at | 1.577e-08 | -1.974 |
| Bt.7034.1.S1\_at | 1.721e-08 | -3.932 |
| Bt.2894.1.S1\_at | 2.511e-08 | -2.347 |
| Bt.16843.1.A1\_at | 2.67e-08 | -2.05 |
| Bt.11178.1.S1\_at | 2.881e-08 | 2.156 |
| Bt.27381.1.S1\_at | 2.914e-08 | -3.053 |
| Bt.20470.2.S1\_a\_at | 2.963e-08 | -2.757 |
| Bt.1153.1.S1\_at | 3.116e-08 | -1.871 |
| Bt.27421.1.S1\_at | 3.435e-08 | 2.057 |
| Bt.3865.3.S1\_a\_at | 3.633e-08 | -2.792 |
| Bt.7056.3.A1\_a\_at | 3.876e-08 | 8.644 |
| Bt.21766.3.S1\_at | 4.325e-08 | -2.296 |
| Bt.22519.1.S1\_at | 5.332e-08 | -2.26 |
| Bt.848.1.S1\_at | 5.549e-08 | -1.796 |
| Bt.7056.5.A1\_x\_at | 5.69e-08 | 5.708 |
| Bt.11236.1.S1\_at | 5.945e-08 | -4.635 |
| Bt.23662.1.S1\_at | 7.219e-08 | -2.153 |
| Bt.12510.1.S1\_a\_at | 7.635e-08 | 2.634 |
| Bt.2696.1.A1\_at | 7.958e-08 | 2.099 |
| Bt.11770.1.S1\_a\_at | 8.013e-08 | -2.763 |
| Bt.5002.1.S1\_at | 8.096e-08 | -2.66 |
| Bt.3857.1.S1\_at | 8.144e-08 | -2.623 |
| Bt.17613.1.A1\_at | 8.168e-08 | -2.471 |
| Bt.11794.1.S1\_at | 8.811e-08 | -2.455 |
| Bt.4770.1.S1\_at | 9.141e-08 | 1.97 |
| Bt.6576.1.S1\_at | 9.375e-08 | 1.559 |
| Bt.4777.1.S1\_at | 9.727e-08 | -2.121 |
| Bt.9222.1.S1\_at | 9.807e-08 | -1.923 |
| Bt.20229.1.S1\_at | 1.111e-07 | -2.297 |
| Bt.4704.1.S1\_at | 1.118e-07 | -2.296 |
| Bt.21896.1.S1\_at | 1.182e-07 | 2.608 |
| Bt.4072.1.S1\_at | 1.259e-07 | -1.976 |
| Bt.1316.1.S2\_at | 1.266e-07 | -3.123 |
| Bt.3117.1.S1\_at | 1.319e-07 | -2.134 |
| Bt.27814.1.S1\_at | 1.346e-07 | 1.974 |
| Bt.836.1.S1\_at | 1.362e-07 | -2.281 |
| Bt.4758.1.S1\_at | 1.5e-07 | -2.594 |
| Bt.18126.1.A1\_at | 1.557e-07 | 3.48 |
| Bt.10719.1.S1\_at | 1.578e-07 | 6.661 |
| Bt.20473.1.A1\_at | 1.693e-07 | 2.397 |
| Bt.18753.1.A1\_at | 1.745e-07 | 2.549 |
| Bt.5120.1.S1\_at | 1.99e-07 | -2.314 |
| Bt.4697.1.S1\_at | 2.034e-07 | 3.973 |
| Bt.17963.1.A1\_at | 2.161e-07 | -1.7 |
| Bt.13330.2.A1\_at | 2.235e-07 | -4.192 |
| Bt.3247.1.S1\_at | 2.289e-07 | 2.764 |
| Bt.3960.2.S1\_at | 2.31e-07 | -1.658 |
| Bt.1793.1.A1\_at | 2.313e-07 | -2.16 |
| Bt.7772.1.S1\_at | 2.374e-07 | 2.186 |
| Bt.18572.1.A1\_at | 2.395e-07 | 2.527 |
| Bt.3873.1.S1\_at | 2.604e-07 | -1.767 |
| Bt.6391.1.A1\_at | 2.652e-07 | -2.74 |
| Bt.18752.1.S1\_at | 2.716e-07 | -1.645 |
| Bt.12510.1.S1\_at | 2.824e-07 | 3.478 |
| Bt.19508.1.A1\_at | 2.882e-07 | 2.222 |
| Bt.4473.1.S1\_at | 2.947e-07 | -1.699 |
| Bt.946.2.A1\_a\_at | 3.024e-07 | -1.737 |
| Bt.11516.1.S1\_at | 3.047e-07 | -2.134 |
| Bt.5395.1.S1\_a\_at | 3.052e-07 | 2.103 |
| Bt.19517.1.S1\_at | 3.072e-07 | 2.332 |
| Bt.396.2.S1\_a\_at | 3.152e-07 | 3.66 |
| Bt.22082.1.S1\_at | 3.277e-07 | -2.349 |
| Bt.11449.1.S1\_at | 3.464e-07 | -1.79 |
| Bt.1403.1.S1\_at | 3.536e-07 | -1.962 |
| Bt.16082.1.S1\_at | 3.537e-07 | -2.233 |
| Bt.13313.1.S1\_at | 3.585e-07 | 1.531 |
| Bt.21372.3.A1\_at | 4.106e-07 | 2.12 |
| Bt.27926.1.S1\_at | 4.16e-07 | -1.819 |
| Bt.21.1.S1\_at | 4.512e-07 | -1.597 |
| Bt.7215.1.S1\_at | 4.537e-07 | -2.518 |
| Bt.12694.1.S1\_at | 4.816e-07 | 1.686 |
| Bt.5732.1.S1\_at | 4.943e-07 | 2.018 |
| Bt.5519.2.S1\_at | 5.114e-07 | 1.475 |
| Bt.21528.1.A1\_at | 5.187e-07 | 5.422 |
| Bt.4867.2.S1\_at | 5.337e-07 | 4.59 |
| Bt.18479.1.A1\_at | 5.396e-07 | 2.193 |
| Bt.20470.1.S1\_a\_at | 5.419e-07 | -5.422 |
| Bt.5483.1.S1\_at | 5.464e-07 | -1.707 |
| Bt.99.1.S1\_at | 5.675e-07 | -1.683 |
| Bt.378.1.S1\_at | 5.828e-07 | -1.419 |
| Bt.9556.1.S1\_at | 6.025e-07 | 1.809 |
| Bt.4262.1.S1\_a\_at | 6.094e-07 | -2.608 |
| Bt.27215.1.S1\_at | 6.119e-07 | -1.828 |
| Bt.5210.1.S1\_at | 6.306e-07 | -1.575 |
| Bt.27193.1.A1\_at | 6.406e-07 | 1.677 |
| Bt.1758.1.S1\_at | 6.604e-07 | 1.397 |
| Bt.28138.1.S1\_at | 6.722e-07 | 1.864 |
| Bt.27318.1.S1\_at | 6.724e-07 | 1.814 |
| Bt.2138.1.S1\_s\_at | 6.786e-07 | -1.607 |
| Bt.3448.2.S1\_at | 6.914e-07 | -1.768 |
| Bt.10013.1.S1\_at | 7.062e-07 | 1.923 |
| Bt.298.1.S1\_at | 7.17e-07 | -1.415 |
| Bt.23164.1.S1\_at | 7.338e-07 | -2.156 |
| Bt.20899.1.A1\_at | 7.44e-07 | -2.324 |
| Bt.13313.2.S1\_at | 7.532e-07 | 1.528 |
| Bt.216.1.S1\_at | 7.578e-07 | 1.534 |
| Bt.28704.1.S1\_at | 7.609e-07 | 2.3 |
| Bt.28975.1.S1\_at | 7.664e-07 | 1.84 |
| Bt.20141.1.S1\_at | 7.802e-07 | -1.922 |
| Bt.3487.1.S1\_at | 7.847e-07 | -1.773 |
| Bt.16668.1.A1\_at | 7.905e-07 | 1.712 |
| Bt.1242.1.S1\_at | 8.036e-07 | -2.121 |
| Bt.27039.1.A1\_at | 8.115e-07 | -1.859 |
| Bt.20505.1.S1\_at | 8.791e-07 | -1.794 |
| Bt.1421.1.S1\_at | 8.804e-07 | 2.923 |
| Bt.9811.1.S1\_at | 9.159e-07 | -1.336 |
| Bt.22379.1.S1\_at | 9.394e-07 | -1.845 |
| Bt.20361.2.A1\_at | 9.488e-07 | 1.758 |
| Bt.23098.1.S1\_at | 9.507e-07 | 2.219 |
| Bt.1035.1.S1\_a\_at | 9.649e-07 | -1.372 |
| Bt.20405.1.S1\_at | 9.718e-07 | 1.411 |
| Bt.24211.2.S1\_at | 1.034e-06 | 2.068 |
| Bt.27091.2.S1\_at | 1.068e-06 | 2.552 |
| Bt.19423.1.S1\_at | 1.073e-06 | 2.052 |
| Bt.5571.1.S1\_at | 1.088e-06 | -1.553 |
| Bt.22510.1.S1\_at | 1.107e-06 | -1.638 |
| Bt.20984.1.S1\_at | 1.128e-06 | -1.9 |
| Bt.4954.1.S1\_at | 1.162e-06 | 1.827 |
| Bt.25616.1.S1\_at | 1.173e-06 | 1.499 |
| Bt.10278.1.S1\_at | 1.223e-06 | -1.487 |
| Bt.5922.1.S1\_at | 1.273e-06 | -2.659 |
| Bt.136.1.S1\_at | 1.321e-06 | -3.004 |
| Bt.19266.1.A1\_at | 1.359e-06 | 1.899 |
| Bt.1408.1.S1\_at | 1.366e-06 | 1.346 |
| Bt.76.1.S1\_at | 1.405e-06 | -1.708 |
| Bt.1808.1.S1\_at | 1.418e-06 | -1.881 |
| Bt.1129.2.S1\_at | 1.421e-06 | -1.881 |
| Bt.75.1.S1\_at | 1.437e-06 | -1.517 |
| Bt.13324.2.S1\_a\_at | 1.465e-06 | -1.833 |
| Bt.4653.1.S1\_at | 1.469e-06 | 1.939 |
| Bt.20512.2.S1\_at | 1.488e-06 | 2.002 |
| BtAffx.29967.1.S1\_at | 1.498e-06 | -2.761 |
| Bt.1280.2.S1\_a\_at | 1.644e-06 | -1.864 |
| Bt.12358.1.S1\_at | 1.646e-06 | 1.431 |
| Bt.12453.1.A1\_a\_at | 1.684e-06 | 2.441 |
| Bt.6494.1.S1\_at | 1.725e-06 | -1.251 |
| Bt.1582.1.S1\_at | 1.726e-06 | -1.533 |
| Bt.20295.1.A1\_at | 1.738e-06 | 1.886 |
| Bt.5123.3.S1\_a\_at | 1.769e-06 | -1.304 |
| Bt.4475.1.S1\_at | 1.815e-06 | -1.779 |
| Bt.22639.2.S1\_at | 1.817e-06 | -1.636 |
| Bt.21383.1.S1\_at | 1.837e-06 | 1.884 |
| Bt.8549.2.S1\_at | 1.876e-06 | 1.708 |
| Bt.321.1.S1\_at | 2.016e-06 | -1.792 |
| Bt.1861.1.S1\_at | 2.108e-06 | 1.656 |
| Bt.4868.2.S1\_a\_at | 2.138e-06 | -1.407 |
| Bt.893.1.S1\_at | 2.143e-06 | -1.498 |
| Bt.15941.1.S1\_at | 2.194e-06 | -1.544 |
| Bt.1059.2.S1\_at | 2.27e-06 | -1.378 |
| Bt.20308.1.S1\_at | 2.323e-06 | 2.615 |
| Bt.23497.1.S1\_at | 2.346e-06 | -4.426 |
| Bt.11415.1.A1\_at | 2.395e-06 | 1.995 |
| Bt.8964.2.S1\_a\_at | 2.413e-06 | -2.582 |
| Bt.25555.1.S1\_at | 2.445e-06 | -2.06 |
| Bt.12721.1.A1\_at | 2.449e-06 | 1.307 |
| Bt.21218.1.A1\_at | 2.502e-06 | -2.127 |
| Bt.26992.1.A1\_at | 2.507e-06 | 1.194 |
| Bt.13505.1.S1\_at | 2.547e-06 | -1.845 |
| Bt.23734.1.A1\_at | 2.624e-06 | 2.773 |
| Bt.7856.1.S1\_at | 2.687e-06 | -1.796 |
| Bt.24824.1.S1\_at | 2.722e-06 | 1.659 |
| Bt.8062.1.S1\_at | 2.746e-06 | -1.202 |
| Bt.5732.2.A1\_at | 2.764e-06 | 1.86 |
| Bt.315.1.S1\_at | 2.789e-06 | -1.871 |
| Bt.1129.1.S1\_at | 2.873e-06 | -2.04 |
| Bt.27908.1.S1\_at | 2.933e-06 | -1.348 |
| Bt.4877.1.S1\_at | 2.987e-06 | 2.25 |
| Bt.13240.1.S1\_at | 3.005e-06 | 1.256 |
| Bt.16003.1.S1\_at | 3.026e-06 | -1.844 |
| Bt.6683.1.S1\_at | 3.07e-06 | -1.848 |
| Bt.9049.1.S1\_at | 3.079e-06 | 2.184 |
| Bt.5344.1.S1\_at | 3.084e-06 | -1.837 |
| Bt.27543.1.A1\_at | 3.098e-06 | 1.751 |
| Bt.17079.1.S1\_a\_at | 3.169e-06 | 1.523 |
| Bt.1696.1.S1\_at | 3.187e-06 | -1.572 |
| Bt.371.1.S1\_at | 3.206e-06 | -1.756 |
| Bt.5520.1.S1\_at | 3.24e-06 | -2.193 |
| Bt.27806.1.S1\_at | 3.301e-06 | -2.073 |
| Bt.25087.3.S1\_at | 3.307e-06 | -1.326 |
| Bt.4606.1.S1\_at | 3.317e-06 | 1.867 |
| Bt.11791.1.A1\_at | 3.321e-06 | -1.928 |
| Bt.4804.2.A1\_at | 3.373e-06 | 1.792 |
| Bt.22518.1.S1\_a\_at | 3.481e-06 | -1.755 |
| Bt.13330.1.S1\_at | 3.493e-06 | -2.667 |
| Bt.23127.1.S1\_at | 3.599e-06 | 1.594 |
| Bt.7915.1.S1\_at | 3.609e-06 | -1.592 |
| Bt.13943.1.A1\_at | 3.612e-06 | 1.33 |
| Bt.22843.1.S1\_at | 3.631e-06 | -1.452 |
| Bt.23603.3.S1\_at | 3.645e-06 | -1.887 |
| Bt.7394.1.A1\_at | 3.659e-06 | -1.515 |
| Bt.26702.1.S1\_a\_at | 3.714e-06 | -1.801 |
| Bt.9949.3.S1\_a\_at | 3.72e-06 | -1.734 |
| Bt.18990.1.S1\_at | 3.744e-06 | 1.379 |
| Bt.29931.1.S1\_at | 3.768e-06 | -1.615 |
| Bt.5042.1.S1\_at | 3.859e-06 | -1.179 |
| Bt.19355.1.A1\_at | 3.904e-06 | 2.41 |
| Bt.27204.1.S1\_at | 3.94e-06 | -1.897 |
| Bt.5537.1.S1\_at | 4.007e-06 | -1.739 |
| Bt.4040.1.S1\_at | 4.052e-06 | 1.558 |
| Bt.5246.1.S1\_at | 4.065e-06 | -2.1 |
| Bt.5848.2.S1\_at | 4.073e-06 | -1.25 |
| Bt.27152.1.A1\_at | 4.074e-06 | 2.753 |
| Bt.26728.1.S1\_at | 4.1e-06 | 1.285 |
| Bt.28133.1.S1\_at | 4.131e-06 | 1.251 |
| Bt.17968.1.S1\_at | 4.164e-06 | -1.769 |
| Bt.22626.2.S1\_at | 4.223e-06 | 2.941 |
| Bt.16815.1.S1\_at | 4.258e-06 | -1.257 |
| Bt.6969.1.S1\_at | 4.361e-06 | 1.499 |
| Bt.9730.1.S1\_at | 4.388e-06 | -1.056 |
| Bt.26975.1.A1\_a\_at | 4.426e-06 | 1.618 |
| Bt.5123.2.S1\_at | 4.447e-06 | -1.272 |
| Bt.2049.1.S1\_at | 4.46e-06 | -1.601 |
| Bt.5089.1.S2\_at | 4.486e-06 | 1.361 |
| Bt.3856.1.S1\_at | 4.495e-06 | -1.568 |
| Bt.3236.1.A1\_at | 4.515e-06 | 1.523 |
| Bt.16900.1.A1\_at | 4.54e-06 | 1.162 |
| Bt.6009.1.S1\_at | 4.555e-06 | 1.423 |
| Bt.265.1.S1\_at | 4.57e-06 | -1.54 |
| Bt.11492.1.A1\_at | 4.624e-06 | 1.536 |
| Bt.20601.1.A1\_at | 4.652e-06 | 1.536 |
| Bt.5447.1.S1\_at | 4.717e-06 | 1.235 |
| Bt.22122.2.A1\_at | 4.75e-06 | 1.551 |
| Bt.1568.1.S1\_at | 4.764e-06 | -1.497 |
| Bt.11659.1.S1\_at | 4.771e-06 | -1.424 |
| Bt.23361.1.S1\_at | 4.786e-06 | -1.117 |
| Bt.28187.1.S1\_at | 4.888e-06 | 1.269 |
| Bt.21533.1.S1\_at | 4.986e-06 | 1.202 |
| Bt.19167.1.A1\_at | 4.99e-06 | 2.017 |
| Bt.9826.1.S1\_at | 5.037e-06 | -1.448 |
| Bt.4864.2.S1\_at | 5.042e-06 | 1.38 |
| Bt.2726.1.S1\_at | 5.106e-06 | 1.67 |
| Bt.12303.1.S1\_at | 5.116e-06 | 1.715 |
| Bt.3015.1.A1\_at | 5.13e-06 | 1.509 |
| Bt.9047.1.S1\_at | 5.131e-06 | -1.916 |
| Bt.8950.1.S1\_at | 5.132e-06 | -1.342 |
| Bt.11855.1.S1\_at | 5.267e-06 | -1.294 |
| Bt.10930.1.S1\_at | 5.276e-06 | 1.477 |
| Bt.2973.2.S1\_a\_at | 5.312e-06 | -1.377 |
| Bt.2129.2.S1\_at | 5.351e-06 | 1.923 |
| Bt.5290.1.S1\_at | 5.399e-06 | 1.204 |
| Bt.25099.1.A1\_at | 5.442e-06 | -3.601 |
| Bt.5183.1.S1\_at | 5.463e-06 | -2.486 |
| Bt.7415.1.S1\_at | 5.495e-06 | 1.394 |
| Bt.3101.1.A1\_at | 5.532e-06 | -2.086 |
| Bt.10154.1.S1\_at | 5.536e-06 | -1.36 |
| Bt.9825.1.S1\_at | 5.617e-06 | -1.875 |
| Bt.20307.1.S1\_at | 5.649e-06 | -1.75 |
| Bt.9434.1.S1\_at | 5.657e-06 | -1.483 |
| Bt.25832.1.S1\_at | 5.659e-06 | 1.505 |
| Bt.11791.2.S1\_at | 5.696e-06 | -1.856 |
| Bt.1294.1.S1\_at | 5.711e-06 | -1.824 |
| Bt.22842.2.S1\_at | 5.726e-06 | 1.425 |
| Bt.14143.1.S1\_at | 5.801e-06 | 2.03 |
| Bt.25051.1.A1\_at | 5.865e-06 | 1.779 |
| Bt.10458.1.A1\_at | 5.869e-06 | 1.308 |
| Bt.5750.1.S1\_at | 5.94e-06 | -1.154 |
| Bt.26402.1.A1\_at | 5.974e-06 | 4.062 |
| Bt.6063.1.S1\_at | 6.066e-06 | -1.486 |
| Bt.9586.1.A1\_at | 6.207e-06 | 1.17 |
| Bt.12211.1.S1\_at | 6.218e-06 | -1.513 |
| Bt.24265.1.S1\_a\_at | 6.312e-06 | -1.283 |
| Bt.27091.1.S1\_at | 6.475e-06 | 1.657 |
| Bt.20281.2.S1\_a\_at | 6.524e-06 | -1.329 |
| Bt.28559.1.S1\_a\_at | 6.59e-06 | -1.164 |
| Bt.23143.1.A1\_at | 6.616e-06 | -1.164 |
| Bt.23569.1.S1\_at | 6.686e-06 | -1.207 |
| Bt.23396.1.S1\_at | 6.689e-06 | -2.15 |
| Bt.4651.1.S1\_at | 6.824e-06 | 1.126 |
| Bt.8504.1.A1\_at | 6.882e-06 | 1.325 |
| Bt.22528.1.A1\_at | 6.889e-06 | -1.11 |
| Bt.12423.1.S1\_at | 6.905e-06 | 1.149 |
| Bt.11622.1.A1\_at | 7.046e-06 | 2.407 |
| Bt.27939.2.A1\_a\_at | 7.322e-06 | 1.237 |
| Bt.1229.1.S1\_at | 7.404e-06 | 2.525 |
| Bt.8679.1.A1\_at | 7.435e-06 | -1.866 |
| Bt.25282.1.A1\_at | 7.438e-06 | 1.229 |
| Bt.884.1.S1\_at | 7.542e-06 | 2.05 |
| Bt.14229.1.S1\_at | 7.577e-06 | -1.179 |
| Bt.4099.1.S1\_at | 7.63e-06 | -1.224 |
| Bt.9878.1.A1\_at | 7.662e-06 | 1.426 |
| Bt.5055.1.S1\_at | 7.716e-06 | 1.436 |
| Bt.1059.3.S1\_a\_at | 7.741e-06 | -1.168 |
| Bt.24396.3.A1\_at | 7.833e-06 | 2.965 |
| Bt.5676.1.S1\_at | 7.858e-06 | -1.9 |
| Bt.20025.1.S1\_at | 7.966e-06 | -1.663 |
| Bt.5966.1.A1\_at | 7.977e-06 | -2.274 |
| Bt.7175.1.S1\_at | 7.98e-06 | -1.635 |
| Bt.5029.1.S1\_at | 8.037e-06 | -1.301 |
| Bt.22538.2.S1\_at | 8.171e-06 | 1.58 |
| Bt.24404.1.A1\_at | 8.311e-06 | 1.544 |
| Bt.10942.1.S1\_at | 8.315e-06 | 1.45 |
| Bt.4698.1.S1\_at | 8.503e-06 | 1.761 |
| Bt.27412.1.A1\_at | 8.534e-06 | 1.697 |
| Bt.20984.1.S1\_a\_at | 8.667e-06 | -2.202 |
| Bt.21766.2.S1\_at | 9.019e-06 | -1.902 |
| Bt.26453.1.S1\_at | 9.138e-06 | 1.408 |
| Bt.8650.1.A1\_at | 9.237e-06 | 1.345 |
| Bt.21721.1.A1\_at | 9.289e-06 | -2.208 |
| Bt.4977.1.S2\_at | 9.448e-06 | 1.174 |
| Bt.26792.1.S1\_at | 9.451e-06 | 2.312 |
| Bt.21698.1.S1\_a\_at | 9.453e-06 | -1.181 |
| Bt.56.1.S1\_at | 9.604e-06 | -1.531 |
| Bt.28269.1.S1\_at | 9.626e-06 | 1.37 |
| Bt.29746.1.S1\_at | 9.646e-06 | -1.697 |
| Bt.615.1.S1\_at | 9.768e-06 | 1.531 |
| Bt.5495.1.S1\_at | 9.818e-06 | -1.584 |
| Bt.23438.1.S1\_at | 9.906e-06 | 1.641 |
| Bt.3922.1.S1\_a\_at | 1.008e-05 | -1.544 |
| Bt.5183.2.S1\_at | 1.009e-05 | -2.435 |
| Bt.3149.1.S1\_at | 1.011e-05 | 1.144 |
| Bt.20411.1.S1\_at | 1.018e-05 | -2.091 |
| Bt.19352.1.A1\_at | 1.026e-05 | -1.993 |
| Bt.8682.1.S1\_at | 1.027e-05 | 1.275 |
| Bt.405.1.S1\_at | 1.028e-05 | 2.03 |
| Bt.6465.1.S1\_at | 1.029e-05 | -3.519 |
| Bt.24875.1.S1\_at | 1.033e-05 | -1.486 |
| Bt.4565.1.S1\_at | 1.037e-05 | 1.31 |
| Bt.10189.1.S1\_at | 1.042e-05 | -1.093 |
| Bt.2587.1.S1\_at | 1.047e-05 | -1.691 |
| Bt.11140.1.S1\_at | 1.052e-05 | 1.973 |
| Bt.24849.1.A1\_at | 1.053e-05 | -1.514 |
| Bt.547.1.S1\_at | 1.068e-05 | -2.367 |
| Bt.3452.1.S1\_at | 1.079e-05 | 1.631 |
| Bt.24096.1.A1\_at | 1.108e-05 | 1.327 |
| Bt.8679.2.S1\_at | 1.111e-05 | -2.014 |
| Bt.4694.1.S1\_at | 1.118e-05 | -0.9906 |
| Bt.20321.1.S1\_at | 1.119e-05 | 1.155 |
| Bt.12728.1.A1\_a\_at | 1.12e-05 | 1.979 |
| Bt.8618.1.S1\_at | 1.141e-05 | -2.382 |
| Bt.28226.3.A1\_s\_at | 1.148e-05 | 1.181 |
| Bt.4880.1.S1\_at | 1.164e-05 | -1.435 |
| Bt.3428.1.A1\_s\_at | 1.164e-05 | 1.403 |
| Bt.15664.1.S1\_at | 1.18e-05 | -1.188 |
| Bt.23576.1.A1\_at | 1.182e-05 | 1.169 |
| Bt.28305.1.S1\_at | 1.196e-05 | 1.791 |
| Bt.28238.1.A1\_at | 1.209e-05 | 2.168 |
| Bt.22270.1.S1\_at | 1.243e-05 | 1.57 |
| Bt.20064.2.S1\_at | 1.252e-05 | 1.295 |
| Bt.23308.1.S1\_at | 1.255e-05 | 1.297 |
| Bt.10965.1.S1\_at | 1.256e-05 | -1.411 |
| Bt.15616.1.S1\_at | 1.259e-05 | -1.478 |
| Bt.20937.1.A1\_at | 1.296e-05 | 2.188 |
| Bt.20783.1.A1\_at | 1.299e-05 | 1.594 |
| Bt.8279.3.S1\_a\_at | 1.301e-05 | -1.215 |
| Bt.7459.1.S1\_at | 1.306e-05 | 1.477 |
| Bt.2138.1.S1\_at | 1.308e-05 | -1.557 |
| Bt.8177.1.S1\_at | 1.311e-05 | -1.838 |
| Bt.19934.1.S1\_at | 1.319e-05 | -1.493 |
| Bt.15870.1.S1\_at | 1.319e-05 | -1.337 |
| Bt.25185.1.S1\_at | 1.32e-05 | 1.6 |
| Bt.24434.1.A1\_at | 1.33e-05 | 2.11 |
| Bt.9828.1.S1\_at | 1.335e-05 | -1.371 |
| Bt.9756.1.S1\_at | 1.343e-05 | -1.653 |
| Bt.23357.1.S1\_at | 1.349e-05 | -1.833 |
| Bt.64.1.S1\_at | 1.361e-05 | -1.536 |
| Bt.19409.1.A1\_s\_at | 1.363e-05 | 1.513 |
| Bt.13321.1.S1\_at | 1.366e-05 | 1.653 |
| Bt.21731.1.S1\_at | 1.375e-05 | 1.747 |
| Bt.2347.1.S1\_at | 1.392e-05 | -1.168 |
| Bt.11502.1.A1\_at | 1.408e-05 | 1.375 |
| Bt.2711.1.S1\_at | 1.423e-05 | -1.121 |
| Bt.6546.1.A1\_at | 1.43e-05 | 1.088 |
| Bt.77.1.S1\_at | 1.457e-05 | -1.286 |
| Bt.26621.1.S1\_at | 1.461e-05 | 1.797 |
| Bt.5164.1.S1\_at | 1.465e-05 | -2.662 |
| Bt.29873.1.S1\_at | 1.467e-05 | 1.805 |
| Bt.20882.1.S1\_at | 1.472e-05 | 2.166 |
| Bt.12722.1.A1\_at | 1.483e-05 | 1.765 |
| Bt.12872.1.S1\_at | 1.49e-05 | 1.888 |
| Bt.2587.2.S1\_a\_at | 1.498e-05 | -1.242 |
| Bt.22461.1.S1\_at | 1.504e-05 | 0.9356 |
| Bt.20426.1.S1\_at | 1.509e-05 | 1.08 |
| Bt.24713.1.S1\_at | 1.511e-05 | 1.404 |
| Bt.23335.1.S1\_at | 1.513e-05 | 1.31 |
| Bt.26628.1.S1\_at | 1.513e-05 | 1.252 |
| Bt.13289.2.S1\_at | 1.526e-05 | 2.79 |
| Bt.7395.1.S1\_at | 1.537e-05 | 2.194 |
| Bt.5040.1.S1\_at | 1.558e-05 | -1.434 |
| Bt.11059.1.S1\_at | 1.567e-05 | 1.315 |
| Bt.21620.1.A1\_at | 1.582e-05 | 1.329 |
| Bt.5132.1.S1\_at | 1.585e-05 | 1.931 |
| Bt.13289.1.S1\_at | 1.587e-05 | 1.855 |
| Bt.12412.1.S1\_at | 1.619e-05 | 1.522 |
| Bt.11055.1.S1\_at | 1.627e-05 | 1.15 |
| Bt.3126.1.A1\_at | 1.647e-05 | 1.178 |
| Bt.797.1.S1\_at | 1.678e-05 | 1.909 |
| Bt.4431.1.S1\_a\_at | 1.688e-05 | -1.168 |
| Bt.6832.1.S1\_at | 1.698e-05 | 1.176 |
| Bt.10969.1.S1\_at | 1.705e-05 | 1.583 |
| Bt.13588.1.A1\_at | 1.718e-05 | -4.205 |
| Bt.24376.1.S1\_at | 1.723e-05 | 1.496 |
| Bt.12613.1.S1\_at | 1.724e-05 | 1.586 |
| Bt.12453.1.A1\_at | 1.729e-05 | 2.094 |
| Bt.28143.1.S1\_at | 1.737e-05 | 1.582 |
| Bt.28618.1.S1\_at | 1.738e-05 | 1.242 |
| Bt.10543.1.S1\_at | 1.757e-05 | 1.858 |
| Bt.27901.1.S1\_at | 1.762e-05 | 1.363 |
| Bt.11990.1.S1\_at | 1.767e-05 | 1.248 |
| Bt.4540.1.S1\_at | 1.846e-05 | -1.108 |
| Bt.67.1.S1\_at | 1.848e-05 | -1.725 |
| Bt.4864.3.S1\_a\_at | 1.853e-05 | 1.452 |
| Bt.832.1.S1\_at | 1.862e-05 | -2.342 |
| Bt.28472.1.S1\_at | 1.865e-05 | 2.313 |
| Bt.3854.1.S1\_at | 1.874e-05 | 1.489 |
| Bt.14095.2.S1\_at | 1.897e-05 | 1.588 |
| Bt.21766.1.A1\_at | 1.929e-05 | -2.374 |
| Bt.9857.1.S1\_at | 1.967e-05 | -1.758 |
| Bt.22735.1.S1\_at | 1.977e-05 | 1.627 |
| Bt.12377.1.S1\_at | 2.014e-05 | 0.9274 |
| Bt.9950.1.S1\_at | 2.017e-05 | 1.869 |
| Bt.29931.2.S1\_at | 2.024e-05 | -2.086 |
| Bt.24154.1.A1\_at | 2.025e-05 | -1.589 |
| Bt.2988.1.S1\_at | 2.027e-05 | 1.185 |
| Bt.25085.1.A1\_at | 2.034e-05 | 1.862 |
| Bt.20512.1.S1\_at | 2.036e-05 | 1.595 |
| Bt.22130.1.S1\_at | 2.05e-05 | 1.189 |
| Bt.9954.1.S1\_at | 2.071e-05 | -0.9736 |
| Bt.21009.1.S1\_at | 2.08e-05 | 1.594 |
| Bt.23148.1.S1\_at | 2.083e-05 | 1.153 |
| Bt.4267.1.S1\_at | 2.085e-05 | 1.598 |
| Bt.4898.1.S1\_at | 2.091e-05 | 1.482 |
| Bt.9708.1.S1\_at | 2.094e-05 | -1.458 |
| Bt.14116.1.A1\_at | 2.121e-05 | 1.314 |
| Bt.4775.1.S1\_at | 2.122e-05 | 1.56 |
| Bt.5345.1.S1\_at | 2.124e-05 | -1.324 |
| Bt.15968.1.S1\_at | 2.151e-05 | -1.234 |
| Bt.11196.1.S1\_at | 2.157e-05 | -1.569 |
| Bt.9731.1.S1\_at | 2.165e-05 | -1.961 |
| Bt.27657.1.A1\_at | 2.175e-05 | -2.326 |
| Bt.20494.1.S1\_at | 2.21e-05 | 1.288 |
| Bt.23418.1.S1\_at | 2.231e-05 | 1.645 |
| Bt.11246.1.A1\_at | 2.252e-05 | 1.464 |
| Bt.24381.2.S1\_at | 2.259e-05 | 2.191 |
| Bt.10398.1.S1\_at | 2.266e-05 | 1.942 |
| Bt.15267.1.A1\_at | 2.29e-05 | 1.575 |
| Bt.20316.1.S1\_at | 2.311e-05 | -1.824 |
| Bt.8909.1.S1\_at | 2.334e-05 | 1.292 |
| Bt.7540.2.S1\_a\_at | 2.338e-05 | -1.14 |
| Bt.25038.1.S1\_at | 2.368e-05 | 1.057 |
| Bt.23486.1.A1\_at | 2.371e-05 | 1.875 |
| Bt.4503.1.S1\_at | 2.42e-05 | -1.136 |
| Bt.63.1.S1\_at | 2.423e-05 | -1.486 |
| Bt.15643.1.A1\_at | 2.428e-05 | 1.363 |
| Bt.4984.3.S1\_at | 2.445e-05 | 2.34 |
| Bt.5047.1.S1\_at | 2.467e-05 | 1.433 |
| Bt.26415.1.A1\_at | 2.494e-05 | 1.547 |
| Bt.15789.1.S1\_at | 2.505e-05 | -1.474 |
| Bt.24211.1.A1\_at | 2.506e-05 | 2.106 |
| Bt.61.1.S1\_at | 2.51e-05 | -1.124 |
| Bt.16314.1.S1\_at | 2.512e-05 | 1.164 |
| Bt.18352.1.A1\_at | 2.529e-05 | -1.378 |
| Bt.7636.1.S1\_at | 2.533e-05 | 1.331 |
| Bt.28081.1.S1\_at | 2.565e-05 | 1.092 |
| Bt.28087.1.S1\_at | 2.598e-05 | 1.164 |
| Bt.22369.2.S1\_at | 2.602e-05 | 1.763 |
| Bt.14624.1.A1\_at | 2.605e-05 | -1.233 |
| Bt.2873.1.S1\_at | 2.607e-05 | -1.229 |
| Bt.3880.1.S1\_at | 2.629e-05 | 2.331 |
| Bt.13349.1.S1\_at | 2.64e-05 | -1.614 |
| Bt.16329.1.A1\_at | 2.646e-05 | 1.232 |
| Bt.13610.1.A1\_at | 2.651e-05 | -1.667 |
| Bt.1366.1.S1\_at | 2.654e-05 | 1.392 |
| Bt.400.1.S1\_at | 2.669e-05 | -1.403 |
| Bt.7655.1.S1\_at | 2.715e-05 | -1.672 |
| Bt.1667.1.S1\_at | 2.747e-05 | -1.331 |
| Bt.1552.1.S1\_at | 2.779e-05 | -3.138 |
| Bt.21151.1.S1\_at | 2.807e-05 | -0.9395 |
| Bt.15714.1.S1\_at | 2.834e-05 | 1.229 |
| Bt.10111.1.S1\_at | 2.846e-05 | 1.6 |
| Bt.13128.1.S1\_at | 2.867e-05 | -1.35 |
| Bt.11240.1.A1\_at | 2.898e-05 | 1.366 |
| Bt.9966.1.S1\_at | 2.901e-05 | 1.362 |
| Bt.21877.1.S1\_at | 2.932e-05 | -1.463 |
| Bt.12916.1.S1\_at | 2.939e-05 | -1.923 |
| Bt.27793.1.A1\_at | 2.972e-05 | 1.465 |
| Bt.8743.1.S1\_at | 2.985e-05 | 3.85 |
| Bt.11446.1.A1\_at | 3.015e-05 | 1.171 |
| Bt.7595.1.S1\_at | 3.025e-05 | -1.082 |
| Bt.1476.1.S1\_at | 3.042e-05 | 1.66 |
| Bt.5153.1.S1\_at | 3.05e-05 | 1.351 |
| Bt.5176.2.A1\_at | 3.101e-05 | 1.143 |
| Bt.27192.1.S1\_at | 3.114e-05 | -1.234 |
| Bt.12217.1.S1\_at | 3.123e-05 | 1.598 |
| Bt.24550.1.S1\_at | 3.128e-05 | -1.427 |
| Bt.12364.1.S1\_at | 3.136e-05 | -1.436 |
| Bt.22046.1.S1\_at | 3.138e-05 | -1.749 |
| Bt.20064.1.S1\_at | 3.139e-05 | 1.185 |
| Bt.13483.1.A1\_at | 3.158e-05 | -1.527 |
| Bt.5223.1.S1\_at | 3.181e-05 | 1.743 |
| Bt.1311.1.S1\_at | 3.199e-05 | -2.6 |
| Bt.27121.1.S1\_at | 3.205e-05 | -1.109 |
| Bt.26718.1.S1\_a\_at | 3.212e-05 | -1.688 |
| Bt.1670.1.A1\_at | 3.239e-05 | -1.136 |
| Bt.3260.1.S1\_at | 3.252e-05 | -1.322 |
| Bt.8829.1.S1\_a\_at | 3.28e-05 | 1.692 |
| Bt.6247.1.S1\_at | 3.297e-05 | 1.885 |
| Bt.984.1.S1\_at | 3.303e-05 | 1.831 |
| Bt.1197.1.A1\_at | 3.315e-05 | 1.61 |
| Bt.15724.1.S1\_at | 3.322e-05 | -1.111 |
| Bt.11216.1.S1\_at | 3.328e-05 | -2.462 |
| Bt.23456.1.A1\_s\_at | 3.338e-05 | 1.707 |
| Bt.4550.1.A1\_at | 3.385e-05 | 1.37 |
| Bt.15857.1.S1\_at | 3.419e-05 | -2.032 |
| Bt.7888.1.S1\_at | 3.427e-05 | 2.961 |
| Bt.14566.1.A1\_at | 3.432e-05 | 1.523 |
| Bt.28733.1.S1\_at | 3.459e-05 | 1.006 |
| Bt.19116.1.A1\_at | 3.488e-05 | 1.947 |
| Bt.6473.1.S1\_at | 3.491e-05 | -1.088 |
| Bt.15589.1.A1\_at | 3.556e-05 | 1.303 |
| Bt.5605.1.S1\_at | 3.587e-05 | 0.9838 |
| Bt.15840.1.A1\_at | 3.617e-05 | 1.45 |
| Bt.4819.1.S1\_at | 3.632e-05 | -1.257 |
| Bt.4563.1.S1\_at | 3.649e-05 | 0.8918 |
| Bt.24616.1.S1\_at | 3.653e-05 | 1.696 |
| Bt.4506.1.S1\_at | 3.654e-05 | 1.021 |
| Bt.11696.1.A1\_at | 3.677e-05 | 2.886 |
| Bt.923.1.A1\_at | 3.683e-05 | 1.226 |
| Bt.15726.1.S1\_at | 3.73e-05 | 1.418 |
| Bt.2524.1.S1\_at | 3.784e-05 | 1.016 |
| Bt.5546.1.S1\_at | 3.83e-05 | 1.182 |
| Bt.3218.1.S1\_at | 3.884e-05 | 1.508 |
| Bt.9855.1.S1\_at | 3.894e-05 | -1.234 |
| Bt.9708.2.S1\_at | 3.895e-05 | -1.786 |
| Bt.8679.3.S1\_at | 3.957e-05 | -1.721 |
| Bt.10096.1.S1\_at | 3.963e-05 | 1.434 |
| Bt.28651.1.S1\_at | 3.981e-05 | 1.425 |
| Bt.1844.1.S1\_at | 3.982e-05 | 1.166 |
| Bt.1237.1.S1\_at | 3.985e-05 | 1.005 |
| Bt.9797.1.S1\_at | 3.996e-05 | -1.332 |
| Bt.5605.2.S1\_at | 4.003e-05 | 1.163 |
| Bt.24039.1.A1\_at | 4.024e-05 | -1.298 |
| Bt.26878.1.S1\_at | 4.064e-05 | -1.654 |
| Bt.2527.1.S1\_at | 4.087e-05 | 1.006 |
| Bt.21471.1.S1\_at | 4.155e-05 | 1.39 |
| Bt.7484.1.S1\_at | 4.189e-05 | 2.218 |
| Bt.8351.1.S1\_at | 4.258e-05 | -1.372 |
| Bt.19218.1.A1\_at | 4.265e-05 | 0.8544 |
| Bt.66.1.S1\_at | 4.277e-05 | -1.372 |
| Bt.4985.1.S1\_a\_at | 4.292e-05 | -1.044 |
| Bt.4224.1.S1\_at | 4.292e-05 | -1.276 |
| Bt.28568.1.A1\_at | 4.302e-05 | 3.045 |
| Bt.26577.1.A1\_at | 4.314e-05 | -1.014 |
| Bt.2077.1.S1\_at | 4.347e-05 | 1.194 |
| Bt.543.1.S1\_at | 4.363e-05 | 1.489 |
| Bt.5078.1.S1\_at | 4.376e-05 | 1.279 |
| Bt.22054.2.S1\_a\_at | 4.391e-05 | -2.101 |
| Bt.22650.1.A1\_at | 4.456e-05 | 1.67 |
| Bt.21020.1.S1\_at | 4.457e-05 | 1.484 |
| Bt.8046.1.A1\_at | 4.469e-05 | -1.859 |
| Bt.24413.1.S1\_at | 4.507e-05 | 1.551 |
| Bt.26130.1.A1\_at | 4.544e-05 | -2.019 |
| Bt.1332.1.S1\_a\_at | 4.577e-05 | -1.301 |
| Bt.4875.2.S1\_at | 4.581e-05 | 1.338 |
| Bt.9956.1.S1\_at | 4.585e-05 | -1.199 |
| Bt.22663.1.A1\_at | 4.589e-05 | -1.718 |
| Bt.15666.2.S1\_at | 4.618e-05 | 1.298 |
| Bt.20046.1.S1\_a\_at | 4.63e-05 | 0.871 |
| Bt.19813.1.A1\_at | 4.631e-05 | 3.477 |
| Bt.1517.1.S1\_at | 4.658e-05 | -1.06 |
| Bt.23509.1.S1\_at | 4.665e-05 | 1.229 |
| Bt.24305.1.A1\_at | 4.668e-05 | 1.541 |
| Bt.4705.1.S1\_at | 4.672e-05 | -1.232 |
| Bt.5418.1.S1\_at | 4.706e-05 | -1.322 |
| Bt.746.1.S1\_at | 4.711e-05 | 1.111 |
| Bt.11242.1.S1\_at | 4.716e-05 | -1.586 |
| Bt.5108.1.S1\_at | 4.729e-05 | -1.409 |
| Bt.58.1.S1\_at | 4.782e-05 | -0.8516 |
| Bt.4205.1.S1\_at | 4.797e-05 | -1.087 |
| Bt.25390.1.A1\_at | 4.804e-05 | 1.156 |
| Bt.4606.2.S1\_at | 4.835e-05 | 2.297 |
| Bt.24892.1.A1\_at | 4.849e-05 | 1.621 |
| Bt.8486.1.S1\_at | 4.859e-05 | 1.675 |
| Bt.5848.1.S1\_at | 4.864e-05 | -1.185 |
| Bt.19332.1.S1\_at | 4.88e-05 | 1.272 |
| Bt.25338.1.S1\_at | 4.89e-05 | 1.213 |
| Bt.3989.1.S1\_at | 4.93e-05 | 1.222 |
| Bt.25072.1.S1\_at | 4.935e-05 | -1.161 |
| Bt.9169.1.A1\_at | 4.945e-05 | 1.167 |
| Bt.24848.1.A1\_at | 4.951e-05 | 1.875 |
| Bt.8001.1.S1\_at | 4.97e-05 | -1.292 |
| Bt.1426.1.S1\_at | 4.971e-05 | -1.139 |
| Bt.10814.1.S1\_at | 4.985e-05 | 1.571 |
| Bt.2120.2.S1\_at | 5.001e-05 | 2.367 |
| Bt.13170.1.S1\_at | 5.002e-05 | -1.468 |
| Bt.21383.2.S1\_a\_at | 5.033e-05 | 1.472 |
| Bt.24236.1.S1\_at | 5.035e-05 | 1.108 |
| Bt.21969.1.A1\_at | 5.07e-05 | 0.8444 |
| Bt.8492.1.A1\_at | 5.137e-05 | 1.565 |
| Bt.20322.2.S1\_at | 5.16e-05 | -1.44 |
| Bt.19097.1.A1\_at | 5.186e-05 | 1.34 |
| Bt.2956.1.A1\_at | 5.252e-05 | 1.358 |
| Bt.10215.1.A1\_at | 5.28e-05 | -0.9137 |
| Bt.2493.1.S1\_at | 5.286e-05 | -1.308 |
| Bt.11067.1.S1\_at | 5.316e-05 | 1.343 |
| Bt.22165.1.S1\_at | 5.324e-05 | 1.697 |
| Bt.6549.1.S1\_at | 5.359e-05 | 1.392 |
| Bt.7237.2.S1\_a\_at | 5.495e-05 | -1.269 |
| Bt.4886.1.A1\_at | 5.515e-05 | 2.367 |
| Bt.28676.1.A1\_at | 5.52e-05 | -1.094 |
| Bt.1950.1.S1\_at | 5.525e-05 | -1.03 |
| Bt.16020.1.A2\_at | 5.526e-05 | 1.213 |
| Bt.3394.1.S1\_at | 5.532e-05 | 2.17 |
| Bt.26467.1.A1\_at | 5.657e-05 | 3.104 |
| Bt.20591.1.S1\_at | 5.667e-05 | 1.55 |
| Bt.7619.1.S1\_at | 5.669e-05 | -3.682 |
| Bt.9585.1.S1\_at | 5.67e-05 | -1.463 |
| Bt.7478.1.S1\_at | 5.67e-05 | -2.022 |
| Bt.12619.1.S1\_at | 5.692e-05 | 1.397 |
| Bt.10259.1.A1\_at | 5.756e-05 | 1.393 |
| Bt.7574.1.S1\_at | 5.783e-05 | 1.493 |
| Bt.5074.1.S1\_at | 5.789e-05 | 1.458 |
| Bt.621.1.S1\_at | 5.796e-05 | -1.641 |
| Bt.14095.1.S1\_at | 5.839e-05 | 1.108 |
| Bt.1613.1.S1\_at | 5.846e-05 | 1.579 |
| Bt.1699.3.S1\_a\_at | 5.862e-05 | 1.068 |
| Bt.26829.1.S1\_at | 5.89e-05 | -1.907 |
| Bt.3230.1.A1\_at | 5.947e-05 | 3.935 |
| Bt.3821.2.S1\_at | 5.992e-05 | 1.175 |
| Bt.20481.2.S1\_at | 6.02e-05 | 1.803 |
| Bt.14853.1.A1\_at | 6.09e-05 | 1.374 |
| Bt.24766.1.S1\_at | 6.128e-05 | -1.616 |
| Bt.6397.2.S1\_at | 6.154e-05 | 1.49 |
| Bt.5378.1.S1\_at | 6.183e-05 | -1.207 |
| Bt.18420.2.A1\_at | 6.187e-05 | 1.589 |
| Bt.27430.1.S1\_at | 6.192e-05 | -1.18 |
| Bt.1789.1.S1\_at | 6.202e-05 | 1.811 |
| Bt.20431.2.S1\_at | 6.214e-05 | 1.251 |
| Bt.4977.1.S1\_at | 6.218e-05 | 1.044 |
| Bt.19247.2.S1\_at | 6.226e-05 | 2.176 |
| Bt.22615.1.S1\_at | 6.24e-05 | -1.033 |
| Bt.1767.1.S1\_at | 6.265e-05 | -1.057 |
| Bt.25202.1.A1\_at | 6.344e-05 | 1.668 |
| Bt.21809.1.S1\_at | 6.35e-05 | 2.274 |
| Bt.5393.2.S1\_a\_at | 6.376e-05 | 1.639 |
| Bt.5664.1.S1\_at | 6.392e-05 | 1.184 |
| Bt.20.1.S1\_at | 6.453e-05 | -1.725 |
| Bt.20151.1.S1\_at | 6.478e-05 | 1.662 |
| Bt.23176.2.S1\_a\_at | 6.491e-05 | 1.762 |
| Bt.10995.1.S1\_at | 6.494e-05 | 2.037 |
| Bt.5400.1.S1\_at | 6.511e-05 | -1.366 |
| Bt.2566.2.S1\_at | 6.68e-05 | 1.695 |
| Bt.20228.1.S1\_at | 6.71e-05 | -0.7884 |
| Bt.13427.1.S1\_at | 6.725e-05 | 1.716 |
| Bt.5406.1.S1\_at | 6.726e-05 | 1.2 |
| Bt.23526.1.S1\_at | 6.76e-05 | -0.9983 |
| Bt.4203.1.S1\_at | 6.763e-05 | -1.15 |
| Bt.12126.1.S1\_at | 6.828e-05 | -1.557 |
| Bt.23603.1.S1\_a\_at | 6.858e-05 | -1.378 |
| Bt.15742.1.S2\_at | 6.872e-05 | 0.9293 |
| Bt.4792.1.S1\_at | 6.902e-05 | 1.417 |
| Bt.13334.1.S1\_at | 6.918e-05 | 1.343 |
| Bt.22451.1.S1\_at | 6.932e-05 | -1.169 |
| Bt.25478.1.A1\_at | 6.936e-05 | 1.28 |
| Bt.17526.1.A1\_at | 6.937e-05 | 1.46 |
| Bt.3847.1.S1\_at | 6.955e-05 | 1.677 |
| Bt.11192.1.S1\_at | 6.967e-05 | -1.002 |
| Bt.23496.1.S1\_at | 7.066e-05 | -1.133 |
| Bt.3227.1.S1\_at | 7.073e-05 | -1.122 |
| Bt.20854.1.S1\_at | 7.082e-05 | -1.467 |
| Bt.23718.1.S1\_at | 7.097e-05 | -1.302 |
| Bt.21761.1.S1\_a\_at | 7.109e-05 | -1.503 |
| Bt.27327.1.A1\_at | 7.113e-05 | 2.981 |
| Bt.25227.1.A1\_at | 7.178e-05 | 1.867 |
| Bt.23081.1.S1\_a\_at | 7.179e-05 | -1.981 |
| Bt.20102.1.S1\_at | 7.189e-05 | -1.783 |
| Bt.3867.1.S1\_at | 7.192e-05 | -1.115 |
| Bt.27207.1.A1\_at | 7.203e-05 | 1.806 |
| Bt.23131.1.S1\_s\_at | 7.226e-05 | 1.489 |
| Bt.12912.1.S2\_at | 7.256e-05 | 1.223 |
| Bt.869.2.S1\_at | 7.262e-05 | 1.166 |
| Bt.12039.1.S1\_at | 7.32e-05 | 1.75 |
| Bt.4891.1.S1\_at | 7.326e-05 | -1.75 |
| Bt.26764.1.A1\_at | 7.341e-05 | 0.9401 |
| Bt.17641.1.A1\_at | 7.355e-05 | -1.102 |
| Bt.7373.3.S1\_a\_at | 7.367e-05 | -1.117 |
| Bt.11436.1.A1\_at | 7.37e-05 | 1.011 |
| Bt.12249.2.S1\_at | 7.392e-05 | 1.152 |
| Bt.21019.1.S1\_at | 7.479e-05 | 0.8779 |
| Bt.5748.2.S1\_at | 7.502e-05 | 0.9325 |
| Bt.6675.1.A1\_at | 7.565e-05 | -1.197 |
| Bt.20354.1.S1\_at | 7.587e-05 | 1.906 |
| Bt.16039.1.S1\_at | 7.587e-05 | -1.103 |
| Bt.20072.1.S1\_at | 7.597e-05 | 1.989 |
| Bt.17943.1.S1\_at | 7.629e-05 | 1.081 |
| Bt.8322.1.S1\_at | 7.63e-05 | 1.237 |
| Bt.19431.1.S1\_at | 7.656e-05 | -1.187 |
| Bt.13588.2.S1\_at | 7.675e-05 | -4.223 |
| Bt.4872.1.S1\_at | 7.694e-05 | 1.146 |
| Bt.20849.1.S1\_at | 7.817e-05 | 0.9695 |
| Bt.6386.2.S1\_at | 7.849e-05 | 2.339 |
| Bt.3753.1.S1\_at | 7.894e-05 | 1.291 |
| Bt.4478.1.S1\_at | 7.92e-05 | -1.787 |
| Bt.17789.1.A1\_at | 7.955e-05 | 2.054 |
| Bt.21122.1.S1\_at | 7.96e-05 | -0.9448 |
| Bt.4271.1.S1\_at | 7.966e-05 | 1.06 |
| Bt.3228.1.S1\_at | 7.983e-05 | 1.191 |
| Bt.20529.1.A1\_at | 7.983e-05 | 2.695 |
| Bt.28455.1.S1\_at | 8.01e-05 | 1.064 |
| Bt.10853.1.S1\_at | 8.012e-05 | 1.651 |
| Bt.2729.1.S1\_at | 8.016e-05 | 1.155 |
| Bt.21342.1.S1\_at | 8.023e-05 | -1.124 |
| Bt.13780.1.A1\_at | 8.072e-05 | 1.594 |
| Bt.10360.1.S1\_at | 8.085e-05 | 1.035 |
| Bt.23854.1.A1\_at | 8.092e-05 | 1.625 |
| Bt.2824.1.S1\_at | 8.097e-05 | -1.234 |
| Bt.6080.1.S1\_at | 8.147e-05 | -1.263 |
| Bt.5460.1.A1\_at | 8.149e-05 | 0.9678 |
| Bt.13833.1.S1\_at | 8.174e-05 | 1.13 |
| Bt.7596.1.S1\_at | 8.205e-05 | -1.016 |
| Bt.20113.1.S1\_at | 8.215e-05 | 1.49 |
| Bt.7133.1.S1\_at | 8.331e-05 | -1.018 |
| Bt.25404.2.S1\_at | 8.354e-05 | 2.956 |
| Bt.3211.1.S2\_at | 8.417e-05 | 1.192 |
| Bt.17081.1.A1\_at | 8.432e-05 | 2.046 |
| Bt.16978.1.S1\_at | 8.508e-05 | -1.104 |
| Bt.18459.1.A1\_at | 8.512e-05 | 1.245 |
| Bt.24662.1.S1\_at | 8.533e-05 | -1.217 |
| Bt.3913.1.S1\_at | 8.533e-05 | 1.06 |
| Bt.4492.1.S1\_at | 8.548e-05 | 1.272 |
| Bt.21435.1.S1\_at | 8.566e-05 | -0.9055 |
| Bt.21603.1.S1\_at | 8.655e-05 | -1.58 |
| Bt.3604.1.A1\_at | 8.655e-05 | 1.049 |
| Bt.5805.1.S1\_at | 8.692e-05 | -1.734 |
| Bt.19519.1.S1\_at | 8.728e-05 | 1.834 |
| Bt.4957.1.S2\_at | 8.729e-05 | 1.31 |
| Bt.28817.1.A1\_at | 8.797e-05 | 1.56 |
| Bt.20146.1.S1\_at | 8.81e-05 | 0.9951 |
| Bt.2374.1.S2\_at | 8.828e-05 | 1.021 |
| Bt.18348.1.S1\_at | 8.83e-05 | 1.201 |
| Bt.27403.1.S1\_at | 8.864e-05 | 1.431 |
| Bt.23234.1.S1\_at | 8.882e-05 | -1.276 |
| Bt.27089.1.S1\_at | 8.964e-05 | 1.231 |
| Bt.25031.1.S1\_at | 8.996e-05 | 1.502 |
| Bt.25886.1.A1\_at | 9.009e-05 | 3.181 |
| Bt.21469.1.S1\_at | 9.049e-05 | 0.9402 |
| Bt.3782.1.A1\_at | 9.073e-05 | 2.26 |
| Bt.23520.1.S1\_at | 9.078e-05 | 0.9376 |
| Bt.11798.1.S1\_at | 9.116e-05 | 1.442 |
| Bt.17942.1.S1\_at | 9.132e-05 | 1.574 |
| Bt.6693.1.A1\_at | 9.153e-05 | -1.705 |
| Bt.21818.1.S1\_at | 9.212e-05 | 1.21 |
| Bt.2905.1.S1\_at | 9.215e-05 | 1.433 |
| Bt.9968.1.A1\_at | 9.236e-05 | 1.282 |
| Bt.11154.2.S1\_at | 9.263e-05 | -1.085 |
| Bt.16471.1.S1\_at | 9.285e-05 | 1.379 |
| Bt.16380.2.A1\_at | 9.313e-05 | 1.181 |
| Bt.9706.2.S1\_at | 9.332e-05 | -0.9307 |
| Bt.19706.1.S1\_at | 9.34e-05 | 1.241 |
| Bt.8103.1.S1\_a\_at | 9.363e-05 | -1.242 |
| Bt.25165.1.S1\_at | 9.37e-05 | -1.464 |
| Bt.13195.1.A1\_at | 9.384e-05 | 2.541 |
| Bt.2235.1.S1\_at | 9.395e-05 | 0.9267 |
| Bt.11237.1.S1\_at | 9.416e-05 | 1.532 |
| Bt.19862.2.S1\_at | 9.429e-05 | 1.471 |
| Bt.21745.1.S1\_at | 9.446e-05 | -1.004 |
| Bt.22150.1.A1\_at | 9.458e-05 | 1.2 |
| Bt.2347.3.S1\_a\_at | 9.475e-05 | -1.087 |
| Bt.12661.1.A1\_at | 9.496e-05 | 1.359 |
| Bt.16437.1.S1\_at | 9.568e-05 | -0.9595 |
| Bt.2717.1.A1\_at | 9.574e-05 | 2.007 |
| Bt.15779.3.S1\_at | 9.575e-05 | 1.708 |
| Bt.20503.1.A1\_at | 9.6e-05 | 1.712 |
| Bt.10266.1.S1\_at | 9.604e-05 | -1.366 |
| Bt.771.1.S1\_at | 9.625e-05 | 2.044 |
| Bt.18278.1.S1\_at | 9.627e-05 | -1.896 |
| Bt.2399.1.S1\_at | 9.662e-05 | 1.472 |
| Bt.814.1.S1\_at | 9.678e-05 | -0.9967 |
| Bt.19581.1.A1\_at | 9.775e-05 | -1.184 |
| Bt.2989.2.S1\_at | 9.789e-05 | -1.377 |
| Bt.13152.1.S1\_s\_at | 9.804e-05 | 1.736 |
| Bt.26080.2.S1\_at | 9.9e-05 | 1.559 |
| Bt.1154.1.S1\_at | 9.936e-05 | -1.691 |
| Bt.20683.1.S1\_at | 9.959e-05 | -1.687 |
| Bt.27111.1.A1\_at | 9.985e-05 | 2.149 |
| Bt.9210.1.S1\_at | 0.0001001 | -0.9225 |
| Bt.24310.1.S1\_at | 0.0001003 | -1.573 |
| Bt.25098.1.S1\_at | 0.0001009 | -1.337 |
| Bt.6665.1.S1\_at | 0.000101 | 1.596 |
| Bt.8262.1.A1\_at | 0.000101 | 1.03 |
| Bt.29910.1.S1\_at | 0.0001016 | 1.428 |
| Bt.9176.2.S1\_at | 0.000102 | 1.16 |
| Bt.12366.1.S1\_at | 0.0001023 | 0.9259 |
| Bt.22310.1.S1\_at | 0.0001025 | -1.303 |
| Bt.2289.3.S1\_at | 0.000103 | 1.816 |
| Bt.12910.1.S1\_at | 0.0001031 | -1.505 |
| Bt.6679.1.S1\_at | 0.0001037 | 0.9935 |
| Bt.5560.1.S1\_at | 0.0001041 | 1.065 |
| Bt.23254.1.S1\_at | 0.0001044 | 1.087 |
| Bt.29194.1.S1\_at | 0.0001047 | -1.419 |
| Bt.10579.1.S1\_at | 0.0001048 | 1.07 |
| Bt.22114.1.S1\_at | 0.0001048 | 1.077 |
| Bt.22142.2.S1\_a\_at | 0.0001052 | -1.444 |
| Bt.20768.1.S1\_at | 0.0001057 | 1.201 |
| Bt.23158.1.S1\_at | 0.0001058 | 1.001 |
| Bt.21981.3.S1\_at | 0.0001058 | 1.334 |
| Bt.5033.1.S1\_at | 0.0001061 | -1.471 |
| Bt.1699.2.S1\_a\_at | 0.0001066 | 1.236 |
| Bt.10631.2.S1\_at | 0.0001067 | 1.833 |
| Bt.10271.1.A1\_at | 0.0001068 | -1.315 |
| Bt.20038.1.S1\_at | 0.000107 | -1.319 |
| Bt.19415.1.A1\_at | 0.0001073 | -2.353 |
| Bt.5054.3.S1\_at | 0.0001075 | 1.462 |
| Bt.17332.1.S1\_at | 0.0001075 | 1.722 |
| Bt.11262.2.S1\_at | 0.000108 | 1.82 |
| Bt.27471.1.S1\_at | 0.0001083 | 0.961 |
| Bt.19936.1.A1\_at | 0.0001087 | 1.812 |
| Bt.22084.1.A1\_at | 0.0001094 | 1.254 |
| Bt.11019.1.S1\_at | 0.0001095 | -4.392 |
| Bt.1430.1.S1\_at | 0.0001103 | 0.868 |
| Bt.27386.1.A1\_at | 0.000111 | 1.265 |
| Bt.2919.1.S1\_at | 0.0001113 | 1.067 |
| Bt.15895.2.S1\_at | 0.0001114 | 2.043 |
| Bt.22634.1.S1\_at | 0.0001117 | 1.322 |
| Bt.4670.1.S1\_at | 0.0001119 | 1.286 |
| Bt.26047.1.A1\_at | 0.0001124 | 2.126 |
| Bt.22466.2.S1\_at | 0.0001125 | 1.077 |
| Bt.7481.1.S1\_at | 0.000113 | -1.845 |
| Bt.4232.2.S1\_at | 0.0001132 | 1.293 |
| Bt.380.1.S1\_at | 0.0001132 | -1.39 |
| Bt.10044.1.A1\_at | 0.0001134 | 1.63 |
| Bt.4653.1.S2\_at | 0.0001138 | 1.276 |
| Bt.303.1.S1\_at | 0.0001157 | -1.944 |
| Bt.24727.1.S1\_a\_at | 0.0001168 | -1.178 |
| Bt.7804.1.S1\_at | 0.0001174 | -1.395 |
| Bt.3494.1.A1\_s\_at | 0.0001183 | 0.9643 |
| Bt.1069.1.A1\_at | 0.0001186 | 1.897 |
| Bt.27221.1.S1\_at | 0.0001196 | 1.556 |
| Bt.27664.1.A1\_at | 0.00012 | 1.813 |
| Bt.23396.2.S1\_a\_at | 0.0001202 | -2.845 |
| Bt.3484.1.S1\_at | 0.0001204 | 1.001 |
| Bt.13588.3.A1\_at | 0.0001208 | -3.196 |
| Bt.16004.1.A1\_at | 0.0001209 | 3.662 |
| Bt.28612.1.S1\_at | 0.0001211 | 0.9944 |
| Bt.5322.1.S1\_at | 0.0001212 | -1.322 |
| Bt.21790.1.S1\_at | 0.0001216 | 1.033 |
| Bt.65.1.S1\_at | 0.0001228 | -0.9194 |
| Bt.1399.1.S1\_at | 0.0001228 | -1.23 |
| Bt.1638.2.S1\_at | 0.0001229 | 1.396 |
| Bt.20406.1.S1\_at | 0.0001233 | 1.257 |
| Bt.752.1.S1\_a\_at | 0.0001234 | -0.9336 |
| Bt.20402.1.S1\_at | 0.0001243 | 1.957 |
| Bt.29463.1.S1\_at | 0.0001248 | 1.333 |
| Bt.23651.1.A1\_at | 0.0001251 | 1.385 |
| Bt.29050.1.S1\_at | 0.0001261 | -1.895 |
| Bt.20236.1.S1\_at | 0.0001261 | 1.173 |
| Bt.979.1.A1\_at | 0.0001268 | 1.689 |
| Bt.9754.1.S1\_at | 0.0001272 | -2.285 |
| Bt.29697.1.S1\_at | 0.0001274 | -1.369 |
| Bt.2696.2.S1\_at | 0.0001277 | 1.184 |
| Bt.15715.1.S1\_at | 0.0001278 | 1.468 |
| Bt.21182.1.S1\_at | 0.0001285 | -0.9241 |
| Bt.8608.1.S1\_at | 0.0001293 | 1.931 |
| Bt.7836.1.S1\_at | 0.0001296 | -1.098 |
| Bt.3448.1.S1\_at | 0.0001297 | -1.634 |
| Bt.6586.1.S1\_at | 0.00013 | 1.266 |
| Bt.15868.1.S1\_at | 0.00013 | 1.144 |
| Bt.2451.2.A1\_at | 0.0001306 | 1.288 |
| Bt.8047.1.S1\_at | 0.0001309 | -1.159 |
| Bt.3714.2.S1\_at | 0.0001316 | 1.206 |
| Bt.15561.2.A1\_s\_at | 0.0001317 | -5.172 |
| Bt.27065.1.A1\_at | 0.0001323 | 4.576 |
| Bt.20605.2.S1\_at | 0.0001324 | 1.371 |
| Bt.1267.1.S1\_at | 0.000133 | 1.402 |
| Bt.15853.1.S1\_at | 0.0001337 | 1.24 |
| Bt.16067.1.S1\_at | 0.0001337 | -1.415 |
| Bt.29053.1.A1\_at | 0.0001343 | 1.733 |
| Bt.11060.1.S1\_at | 0.0001343 | 1.785 |
| Bt.21794.1.S1\_at | 0.0001349 | 3.242 |
| Bt.2814.1.S1\_at | 0.000135 | -0.9886 |
| Bt.11322.1.A1\_at | 0.000135 | -1.176 |
| Bt.21997.1.S1\_at | 0.0001351 | 2.588 |
| Bt.6399.2.S1\_a\_at | 0.0001358 | 1.569 |
| Bt.9377.1.S1\_at | 0.0001361 | 2.362 |
| Bt.21366.2.S1\_at | 0.0001364 | -1.369 |
| Bt.16935.1.A1\_at | 0.0001368 | 0.8737 |
| Bt.9252.1.A1\_at | 0.0001376 | 1.329 |
| Bt.12562.1.S1\_at | 0.0001377 | 0.9986 |
| Bt.8690.2.S1\_at | 0.000138 | -1.14 |
| Bt.854.1.S1\_at | 0.00014 | 1.071 |
| Bt.21468.1.S1\_at | 0.0001404 | -1.275 |
| Bt.8912.1.A1\_at | 0.000141 | -1.3 |
| Bt.5541.1.S1\_at | 0.0001423 | -1.414 |
| Bt.3288.1.A1\_at | 0.0001427 | -0.9336 |
| Bt.18332.1.S1\_at | 0.0001437 | 0.9641 |
| Bt.9705.1.S1\_at | 0.000144 | 0.9925 |
| Bt.22630.2.A1\_at | 0.0001446 | -1.179 |
| Bt.20129.2.S1\_at | 0.0001447 | -1.08 |
| Bt.27211.1.S1\_at | 0.0001454 | -1.406 |
| Bt.22444.1.A1\_at | 0.0001456 | 1.455 |
| Bt.18057.1.A1\_at | 0.0001467 | 1.625 |
| Bt.21288.1.A1\_at | 0.0001479 | 1.305 |
| Bt.24508.1.A1\_at | 0.0001488 | 2.56 |
| Bt.13063.1.S1\_at | 0.000149 | -0.9778 |
| Bt.26710.1.S1\_at | 0.0001491 | 2.816 |
| Bt.1505.1.S1\_at | 0.0001495 | -1.393 |
| Bt.2815.1.S1\_at | 0.0001495 | 1.576 |
| Bt.11264.1.S1\_at | 0.0001499 | 1.251 |
| Bt.22736.1.S1\_at | 0.0001501 | 1.086 |
| Bt.19105.1.A1\_at | 0.0001502 | 1.155 |
| Bt.12434.1.S1\_at | 0.0001506 | -1.377 |
| Bt.19862.1.A1\_at | 0.0001506 | 1.821 |
| Bt.9233.3.S1\_at | 0.0001511 | -1.276 |
| Bt.16384.1.A1\_at | 0.0001512 | -3.257 |
| Bt.21427.1.S1\_at | 0.0001521 | 1.466 |
| Bt.25202.3.S1\_at | 0.0001539 | 1.344 |
| Bt.14366.1.S1\_at | 0.0001539 | 1.004 |
| Bt.11409.1.A1\_a\_at | 0.000154 | 0.8265 |
| Bt.23606.1.S1\_at | 0.0001545 | 1.34 |
| Bt.6279.1.A1\_at | 0.0001592 | 1.143 |
| Bt.24802.1.S1\_at | 0.0001594 | -1.672 |
| Bt.3216.1.A1\_at | 0.00016 | 1.222 |
| Bt.4903.1.S2\_at | 0.0001604 | 0.9467 |
| Bt.22785.1.S1\_at | 0.0001613 | -1.642 |
| Bt.3967.1.S1\_a\_at | 0.0001616 | -1.535 |
| Bt.21275.1.S1\_at | 0.0001616 | 2.024 |
| Bt.2870.1.A1\_at | 0.0001616 | 1.342 |
| Bt.9629.2.S1\_at | 0.0001617 | 0.9023 |
| Bt.404.1.S1\_at | 0.0001618 | -1.23 |
| Bt.24676.1.S1\_at | 0.0001619 | -1.477 |
| Bt.18206.1.A1\_at | 0.0001622 | 2.263 |
| Bt.22526.1.S1\_at | 0.000163 | -1.104 |
| Bt.3809.1.S1\_at | 0.0001633 | -1.071 |
| Bt.20582.1.S1\_at | 0.0001634 | 1.291 |
| Bt.26579.2.A1\_a\_at | 0.0001635 | 1.546 |
| Bt.8730.1.S1\_at | 0.0001636 | 0.9139 |
| Bt.27112.1.S1\_at | 0.0001653 | 1.571 |
| Bt.11182.1.S1\_at | 0.0001666 | 0.8292 |
| Bt.26568.2.S1\_a\_at | 0.0001683 | -1.989 |
| Bt.2347.2.S1\_at | 0.0001684 | -0.9207 |
| Bt.1112.1.S1\_at | 0.0001694 | -2.751 |
| Bt.20242.1.S1\_at | 0.0001701 | 1.187 |
| Bt.16296.1.A1\_at | 0.0001702 | -1.669 |
| Bt.1389.1.S1\_at | 0.0001718 | 0.9019 |
| Bt.23232.1.S1\_at | 0.000173 | -1.703 |
| Bt.1065.1.S1\_at | 0.0001733 | -0.9149 |
| Bt.9353.1.S1\_at | 0.0001754 | 1.968 |
| Bt.5510.1.S1\_at | 0.0001773 | -1.231 |
| Bt.20784.1.A1\_at | 0.0001788 | 3.295 |
| Bt.21737.1.S1\_at | 0.0001794 | 1.214 |
| Bt.5965.1.S1\_at | 0.0001803 | 1.477 |
| Bt.12603.1.S1\_at | 0.0001804 | 1.234 |
| Bt.19928.1.S1\_at | 0.0001806 | 1.269 |
| Bt.4725.1.S1\_at | 0.0001809 | 1.072 |
| Bt.20511.1.S1\_at | 0.0001816 | 1.993 |
| Bt.19826.1.A1\_at | 0.0001826 | -1.376 |
| Bt.2989.3.S1\_at | 0.000183 | -1.389 |
| Bt.13765.1.S1\_at | 0.000183 | -1.577 |
| Bt.16041.1.S1\_at | 0.0001832 | 1.077 |
| Bt.11662.1.A1\_at | 0.0001834 | 1.423 |
| Bt.18224.1.S1\_at | 0.0001867 | 0.9366 |
| Bt.26999.1.S1\_at | 0.0001879 | -1.08 |
| Bt.22320.2.S1\_at | 0.000188 | 1.422 |
| Bt.12379.1.S1\_at | 0.000188 | 1.329 |
| Bt.20820.1.A1\_at | 0.0001881 | 1.057 |
| Bt.19786.1.A1\_at | 0.0001882 | 1.844 |
| Bt.1283.1.S1\_at | 0.0001884 | -0.9201 |
| Bt.20528.1.S1\_at | 0.0001888 | 1.208 |
| Bt.25052.1.S1\_at | 0.000192 | 0.8223 |
| Bt.8077.1.S1\_at | 0.0001926 | -2.592 |
| Bt.4263.1.S1\_at | 0.000193 | 1.076 |
| Bt.13366.1.S1\_at | 0.0001932 | 1.222 |
| Bt.16078.1.A1\_s\_at | 0.0001933 | 1.098 |
| Bt.7069.1.S1\_at | 0.0001942 | 1.262 |
| Bt.23174.2.S1\_at | 0.0001949 | 1.075 |
| Bt.4867.1.A1\_at | 0.000195 | 3.92 |
| Bt.727.2.S1\_at | 0.0001953 | -0.7556 |
| Bt.7696.1.S1\_at | 0.0001954 | -1.661 |
| Bt.27918.1.S1\_at | 0.0001954 | 1.225 |
| Bt.29655.1.S1\_a\_at | 0.0001958 | -0.8784 |
| Bt.7079.1.S1\_at | 0.000196 | -1.737 |
| Bt.14340.1.A1\_at | 0.0001973 | 1.111 |
| Bt.7319.1.S1\_at | 0.0001984 | 1.651 |
| Bt.5468.1.S1\_at | 0.0002013 | -2.415 |
| Bt.20318.1.S1\_a\_at | 0.0002018 | -0.909 |
| Bt.20489.1.S1\_at | 0.0002028 | 1.22 |
| Bt.282.1.S1\_at | 0.0002033 | -0.9257 |
| Bt.2359.1.A1\_at | 0.000204 | 0.9384 |
| Bt.13865.1.A1\_at | 0.0002064 | -1.369 |
| Bt.10722.1.S1\_at | 0.0002068 | 1.209 |
| Bt.24260.2.S1\_at | 0.0002072 | 1.697 |
| Bt.29381.1.S1\_at | 0.0002072 | -1.548 |
| Bt.24210.1.S1\_at | 0.0002081 | -1.779 |
| Bt.22166.1.S1\_at | 0.0002085 | -1.063 |
| Bt.19746.1.S1\_at | 0.0002093 | 0.9579 |
| Bt.27807.1.A1\_at | 0.0002097 | 1.328 |
| Bt.26430.1.S1\_at | 0.0002098 | 1.16 |
| Bt.11093.1.S1\_at | 0.000212 | 1.271 |
| Bt.3940.1.S1\_at | 0.0002122 | 0.8808 |
| Bt.21191.2.A1\_at | 0.0002146 | -2.256 |
| Bt.21058.2.S1\_at | 0.0002153 | -1.497 |
| Bt.25343.1.S1\_at | 0.0002156 | -1.711 |
| Bt.26568.1.S1\_a\_at | 0.0002158 | -1.418 |
| Bt.10999.1.A1\_at | 0.0002161 | 1.323 |
| Bt.2070.2.A1\_at | 0.0002172 | -1.351 |
| Bt.25093.2.S1\_at | 0.0002175 | 2.09 |
| Bt.17778.1.A1\_at | 0.0002183 | -1.237 |
| Bt.22630.1.S1\_at | 0.0002194 | -1.229 |
| Bt.21036.2.A1\_at | 0.0002197 | 0.832 |
| Bt.15708.1.S1\_at | 0.0002197 | -1.399 |
| Bt.22465.1.S1\_at | 0.0002209 | 2.599 |
| Bt.24916.1.A1\_at | 0.0002214 | 1.221 |
| Bt.12875.1.S1\_at | 0.0002219 | 1.576 |
| Bt.6537.1.S1\_at | 0.0002242 | 2.378 |
| Bt.28623.1.S1\_at | 0.0002256 | 1.071 |
| Bt.4571.1.S1\_at | 0.0002264 | 1.277 |
| Bt.23237.1.S1\_at | 0.0002272 | -0.9976 |
| Bt.9772.1.S1\_at | 0.0002282 | 1.427 |
| Bt.4702.2.S1\_at | 0.0002286 | 1.177 |
| Bt.13118.1.S1\_at | 0.0002289 | 1.541 |
| Bt.9633.2.S1\_at | 0.000229 | 1.189 |
| Bt.5527.1.S1\_at | 0.0002295 | -1.09 |
| Bt.11256.1.S1\_at | 0.0002295 | 0.771 |
| Bt.25087.1.A1\_at | 0.0002303 | -0.9029 |
| Bt.20080.1.S1\_at | 0.0002315 | 1.678 |
| Bt.29.1.S1\_at | 0.0002321 | 1.321 |
| Bt.22852.1.S1\_at | 0.0002321 | 1.374 |
| Bt.23373.1.S1\_at | 0.0002339 | -1.557 |
| Bt.10920.1.S1\_at | 0.0002343 | 1.651 |
| Bt.23225.1.S1\_at | 0.0002343 | -1.694 |
| Bt.21813.1.S1\_at | 0.0002348 | 1.714 |
| Bt.9958.1.S1\_at | 0.0002353 | 1.167 |
| Bt.21166.1.S1\_at | 0.0002353 | 1.626 |
| Bt.4194.1.S1\_at | 0.0002368 | 2.73 |
| Bt.70.1.S1\_at | 0.0002373 | -1.958 |
| Bt.3426.1.S1\_at | 0.0002376 | 1.04 |
| Bt.16194.1.A1\_at | 0.0002376 | 1.38 |
| Bt.4972.1.S1\_at | 0.0002383 | 0.9171 |
| Bt.20895.1.S1\_at | 0.0002385 | -0.8053 |
| Bt.16132.2.S1\_a\_at | 0.0002392 | -0.7831 |
| Bt.4677.1.S1\_at | 0.0002397 | -0.8133 |
| Bt.8290.1.S1\_at | 0.0002425 | 1.255 |
| Bt.19839.1.A1\_at | 0.0002425 | 1.36 |
| Bt.19621.1.A1\_at | 0.0002441 | 1.107 |
| Bt.20275.1.S1\_at | 0.0002444 | -1.15 |
| Bt.16939.1.A1\_at | 0.0002446 | 2.711 |
| Bt.19100.1.A1\_at | 0.0002451 | 1.17 |
| Bt.18278.2.A1\_at | 0.0002459 | -2.331 |
| Bt.26805.1.S1\_at | 0.0002464 | -1.564 |
| Bt.25226.1.A1\_at | 0.0002465 | 1.238 |
| Bt.4950.1.S1\_at | 0.0002476 | 1.338 |
| Bt.18986.1.S1\_at | 0.0002476 | 1.105 |
| Bt.26641.1.S1\_at | 0.0002492 | -1.045 |
| Bt.9405.1.S1\_at | 0.0002493 | -0.7879 |
| Bt.19360.1.A1\_at | 0.0002498 | 2.501 |
| Bt.22163.1.S1\_at | 0.00025 | -2.23 |
| Bt.13860.1.S1\_at | 0.0002508 | 1.179 |
| Bt.20613.2.A1\_at | 0.0002521 | 1.64 |
| Bt.16053.1.S1\_at | 0.0002525 | 1.639 |
| Bt.4685.1.S1\_at | 0.0002533 | 1.02 |
| Bt.9983.1.S1\_at | 0.0002553 | -0.9309 |
| Bt.4800.1.S1\_at | 0.0002554 | 1.581 |
| Bt.26462.1.S1\_at | 0.0002568 | 2.242 |
| Bt.10150.1.S1\_at | 0.0002579 | 1.044 |
| Bt.19526.1.S1\_at | 0.0002583 | 1.23 |
| Bt.9998.1.S1\_at | 0.0002616 | 1.338 |
| Bt.13845.1.S1\_at | 0.0002622 | -1.588 |
| Bt.7984.1.S1\_at | 0.0002636 | 1.331 |
| Bt.14340.2.S1\_at | 0.0002637 | 1.548 |
| Bt.3631.1.S1\_at | 0.000264 | -0.8574 |
| Bt.13648.1.A1\_at | 0.0002641 | 1.175 |
| Bt.4118.1.A1\_at | 0.0002641 | -1.044 |
| Bt.20402.2.A1\_at | 0.0002648 | 1.26 |
| Bt.23667.1.S1\_at | 0.0002653 | 1.365 |
| Bt.2494.2.S1\_a\_at | 0.0002658 | 1.031 |
| Bt.19774.2.S1\_at | 0.000266 | 1.143 |
| Bt.1217.1.A1\_a\_at | 0.0002665 | -0.96 |
| Bt.26647.1.A1\_at | 0.0002676 | 3.427 |
| Bt.17081.2.S1\_at | 0.0002681 | 2.252 |
| Bt.25016.1.S1\_at | 0.0002692 | 1.39 |
| Bt.1825.1.S1\_at | 0.0002696 | -0.9069 |
| Bt.23081.1.S1\_at | 0.0002703 | -2.166 |
| Bt.26515.1.S1\_at | 0.0002704 | 0.9072 |
| Bt.5278.1.A1\_at | 0.0002716 | -0.9662 |
| Bt.8131.1.S1\_at | 0.0002719 | 1.565 |
| Bt.1739.2.S1\_at | 0.000272 | 1.178 |
| Bt.17194.1.S1\_at | 0.000272 | 1.312 |
| Bt.97.1.S1\_at | 0.0002723 | -2.69 |
| Bt.28489.1.S1\_at | 0.0002723 | 0.8627 |
| Bt.23401.1.S1\_at | 0.0002727 | -1.4 |
| Bt.12669.1.A1\_at | 0.0002729 | 1.286 |
| Bt.19267.2.S1\_at | 0.0002732 | -1.055 |
| Bt.28207.1.S1\_at | 0.0002735 | 1.14 |
| Bt.10880.1.S1\_at | 0.0002736 | -1.106 |
| Bt.20328.1.S1\_at | 0.0002743 | 0.8966 |
| Bt.8976.1.S1\_at | 0.0002752 | 1.278 |
| Bt.9244.1.A1\_at | 0.0002755 | 1.1 |
| Bt.2065.1.A1\_at | 0.0002759 | 0.8587 |
| Bt.18048.1.A1\_at | 0.0002791 | 1.231 |
| Bt.17985.1.S1\_at | 0.0002793 | 1.038 |
| Bt.12217.2.S1\_at | 0.0002802 | 2.062 |
| Bt.4476.1.S1\_at | 0.0002812 | -1.464 |
| Bt.13969.1.A1\_at | 0.0002813 | 1.181 |
| Bt.19199.1.A1\_at | 0.0002816 | 1.76 |
| Bt.29197.1.S1\_a\_at | 0.0002821 | 3.58 |
| Bt.10777.1.S1\_at | 0.000283 | 1.39 |
| Bt.28058.1.S1\_at | 0.000283 | 1.134 |
| Bt.19937.2.S1\_at | 0.0002831 | 0.9722 |
| Bt.12728.3.S1\_at | 0.0002838 | 0.838 |
| Bt.12864.1.S1\_at | 0.0002859 | -0.8936 |
| Bt.18335.1.A1\_at | 0.0002869 | 1.133 |
| Bt.6651.1.S1\_at | 0.000287 | 1.351 |
| Bt.17989.1.S1\_at | 0.0002871 | 1.209 |
| Bt.20515.1.S1\_at | 0.0002883 | 1.26 |
| Bt.12634.1.A1\_at | 0.0002884 | 1.783 |
| Bt.23499.1.S1\_at | 0.0002891 | -1.079 |
| Bt.25980.1.A1\_at | 0.0002899 | -1.557 |
| Bt.5021.1.S1\_at | 0.00029 | 1.017 |
| Bt.27196.1.A1\_at | 0.0002908 | -2.575 |
| Bt.16510.1.A1\_at | 0.000291 | 2.673 |
| Bt.7024.1.A1\_at | 0.0002914 | -0.9287 |
| Bt.26622.1.S1\_a\_at | 0.0002918 | -0.9019 |
| Bt.15854.1.A1\_at | 0.0002924 | 1.34 |
| Bt.9154.1.S1\_at | 0.000294 | 1.009 |
| Bt.21792.2.S1\_a\_at | 0.0002941 | -1.011 |
| Bt.5603.2.S1\_at | 0.0002942 | 1.217 |
| Bt.401.2.S1\_at | 0.0002953 | -1.134 |
| Bt.4580.1.A1\_at | 0.0002955 | 0.7457 |
| Bt.12267.1.S1\_at | 0.0002965 | 1.144 |
| Bt.19937.1.S1\_at | 0.0002971 | 0.8913 |
| Bt.28670.1.A1\_at | 0.0002999 | -1.078 |
| Bt.23607.1.S1\_at | 0.0003005 | -1.058 |
| Bt.2634.1.S1\_at | 0.0003008 | 2.037 |
| Bt.25069.1.A1\_at | 0.0003016 | -0.9318 |
| Bt.3605.1.S1\_at | 0.0003029 | -2.237 |
| Bt.10121.1.A1\_at | 0.000303 | -0.7522 |
| Bt.23315.1.S1\_a\_at | 0.0003034 | 1.107 |
| Bt.1606.1.S1\_at | 0.0003046 | 1.514 |
| Bt.6937.1.S1\_at | 0.0003061 | -1.504 |
| Bt.2391.1.S1\_at | 0.0003066 | 1.13 |
| Bt.11193.1.S1\_at | 0.0003077 | -1.656 |
| Bt.6938.1.S1\_at | 0.0003086 | -0.7641 |
| Bt.22047.1.S1\_at | 0.0003093 | -1.205 |
| Bt.20052.1.S1\_at | 0.0003101 | 1.261 |
| Bt.15698.1.S1\_at | 0.0003111 | -1.407 |
| Bt.10135.1.A1\_at | 0.0003115 | 1.22 |
| Bt.3272.1.A1\_at | 0.000312 | -0.9525 |
| Bt.14219.1.S1\_at | 0.0003121 | 1.416 |
| Bt.4320.1.S1\_at | 0.0003122 | 1.187 |
| Bt.20323.1.S1\_at | 0.0003125 | 1.173 |
| Bt.20591.2.A1\_at | 0.000314 | 1.915 |
| Bt.29257.1.A1\_at | 0.0003145 | 2.55 |
| Bt.25417.1.A1\_at | 0.0003153 | 1.146 |
| Bt.7728.1.A1\_at | 0.000318 | 1.339 |
| Bt.20282.1.S1\_at | 0.0003206 | -1.426 |
| Bt.26675.1.S1\_at | 0.0003215 | -1.047 |
| Bt.1020.1.S1\_at | 0.0003216 | 1.168 |
| Bt.26919.1.S1\_at | 0.000322 | 1.713 |
| Bt.2116.1.S1\_at | 0.0003237 | -1.025 |
| Bt.4218.2.S1\_a\_at | 0.0003246 | 2.254 |
| Bt.3996.1.S1\_at | 0.000325 | 2.63 |
| Bt.28150.1.S1\_at | 0.0003251 | -1.721 |
| Bt.1742.1.S1\_at | 0.0003272 | 1.148 |
| Bt.27846.1.S1\_at | 0.0003273 | 1.44 |
| Bt.10723.1.S1\_at | 0.0003276 | 0.9592 |
| Bt.17777.1.S1\_at | 0.0003278 | -1.028 |
| Bt.25346.1.A1\_at | 0.0003301 | 1.298 |
| Bt.20278.1.S1\_at | 0.0003312 | 1.194 |
| Bt.4555.1.S1\_at | 0.0003319 | -1.287 |
| Bt.11270.2.S1\_at | 0.000332 | -2.205 |
| Bt.4948.1.S1\_at | 0.0003324 | 1.104 |
| Bt.3990.1.S1\_at | 0.0003325 | 0.8225 |
| Bt.13357.1.S1\_at | 0.0003342 | 0.9604 |
| Bt.9715.1.S1\_at | 0.0003345 | 0.806 |
| Bt.15836.2.S1\_at | 0.0003352 | -3.809 |
| Bt.21176.1.S1\_at | 0.0003361 | 0.9807 |
| Bt.9362.1.S1\_at | 0.0003368 | -1.165 |
| Bt.15531.1.S1\_at | 0.0003371 | 0.7135 |
| Bt.5941.1.S1\_at | 0.0003374 | 0.9306 |
| Bt.12204.1.S1\_at | 0.000341 | 1.142 |
| Bt.6007.1.S1\_at | 0.0003418 | 0.8092 |
| Bt.11262.1.S1\_at | 0.0003425 | 1.209 |
| Bt.24624.1.S1\_at | 0.0003427 | -1.271 |
| Bt.16503.1.A1\_at | 0.0003432 | -1.967 |
| Bt.10922.1.A1\_at | 0.0003438 | 1.276 |
| Bt.28754.1.S1\_at | 0.000344 | -1.189 |
| Bt.3236.2.S1\_at | 0.0003451 | 1.204 |
| Bt.10781.1.S1\_at | 0.0003485 | 1.364 |
| Bt.12514.1.S1\_at | 0.0003485 | -0.9465 |
| Bt.26756.1.S1\_at | 0.0003493 | 0.7691 |
| Bt.22563.1.A1\_s\_at | 0.0003504 | -0.7696 |
| Bt.562.1.S1\_at | 0.0003507 | -4.451 |
| Bt.2070.1.A1\_at | 0.0003511 | -1.542 |
| Bt.21177.1.A1\_s\_at | 0.0003517 | 1.342 |
| Bt.6171.1.A1\_at | 0.0003539 | -0.9266 |
| Bt.11040.1.S1\_at | 0.0003559 | 1.197 |
| Bt.27428.1.A1\_at | 0.0003584 | 1.649 |
| Bt.4689.1.S2\_at | 0.0003589 | -0.8631 |
| Bt.15742.1.S1\_at | 0.0003607 | 1.196 |
| Bt.7452.1.S1\_at | 0.0003617 | -0.7276 |
| Bt.22198.1.S1\_at | 0.0003637 | 1.035 |
| Bt.9559.2.S1\_at | 0.0003639 | 1.641 |
| Bt.2510.1.A1\_at | 0.000364 | 1.084 |
| Bt.23551.2.S1\_at | 0.0003643 | -0.9809 |
| Bt.22363.1.S1\_at | 0.0003643 | 0.9547 |
| Bt.25107.1.A1\_at | 0.0003663 | 1.514 |
| Bt.4985.2.S1\_a\_at | 0.0003686 | -0.9981 |
| Bt.10458.2.S1\_at | 0.0003711 | 1.247 |
| Bt.231.1.S1\_at | 0.0003716 | -0.9236 |
| Bt.25689.1.S1\_at | 0.0003722 | -1.147 |
| Bt.29625.1.S1\_at | 0.0003739 | 3.006 |
| Bt.25426.1.A1\_at | 0.0003772 | -3.239 |
| Bt.4449.1.S1\_at | 0.0003807 | 0.9183 |
| Bt.10195.1.S1\_at | 0.0003809 | 0.8008 |
| Bt.8807.1.S1\_at | 0.0003827 | 1.015 |
| Bt.4748.1.S1\_at | 0.0003838 | -1.375 |
| Bt.18381.1.S1\_at | 0.000384 | 0.9968 |
| Bt.15864.1.S1\_at | 0.0003849 | 1.711 |
| Bt.9623.1.A1\_at | 0.0003851 | -1.479 |
| Bt.23558.1.S1\_at | 0.0003873 | 1.129 |
| Bt.18755.1.A1\_at | 0.0003885 | -0.7852 |
| Bt.21249.1.S1\_at | 0.0003889 | 2.718 |
| Bt.20139.1.S1\_at | 0.0003898 | 1.102 |
| Bt.23572.1.S1\_at | 0.00039 | 0.8836 |
| Bt.20977.1.S1\_at | 0.0003901 | 1.188 |
| Bt.13312.1.S1\_at | 0.0003909 | 3.526 |
| Bt.4797.1.S1\_at | 0.0003919 | -1.107 |
| Bt.10813.1.S1\_at | 0.0003933 | 1.403 |
| Bt.14473.1.S1\_at | 0.0003937 | -0.8654 |
| Bt.11303.1.A1\_at | 0.000395 | 1.252 |
| Bt.16573.1.A1\_at | 0.0003961 | 1.105 |
| Bt.12769.1.S1\_at | 0.0003974 | -1.407 |
| Bt.7676.3.S1\_at | 0.0003982 | 1.854 |
| Bt.13132.1.S1\_at | 0.0003985 | -0.8601 |
| Bt.13350.1.S1\_at | 0.0003992 | 0.965 |
| Bt.20744.1.A1\_at | 0.0004018 | 1.296 |
| Bt.22034.1.S1\_at | 0.0004039 | 1.871 |
| Bt.8094.1.S1\_at | 0.0004045 | 0.8372 |
| Bt.22187.1.S1\_at | 0.0004048 | 1.184 |
| Bt.13722.2.A1\_at | 0.0004062 | 1.106 |
| Bt.13410.1.S1\_at | 0.0004062 | -0.7805 |
| Bt.11138.1.S1\_at | 0.0004076 | -0.9321 |
| Bt.10818.1.A1\_at | 0.0004084 | 2.903 |
| Bt.1543.1.A1\_at | 0.000409 | -1.205 |
| Bt.5226.1.S1\_at | 0.00041 | 1.15 |
| Bt.26913.1.S1\_at | 0.0004105 | -0.7407 |
| Bt.9949.2.S1\_at | 0.0004125 | -1.232 |
| Bt.5582.1.S1\_at | 0.0004157 | -1.319 |
| Bt.16412.1.S1\_a\_at | 0.0004165 | 1.554 |
| Bt.9576.1.S1\_a\_at | 0.000419 | 0.964 |
| Bt.26609.1.S1\_at | 0.0004199 | 1.197 |
| Bt.1993.1.S1\_a\_at | 0.0004214 | 1.478 |
| Bt.5379.1.S1\_at | 0.0004215 | 1.759 |
| Bt.9246.1.A1\_at | 0.0004232 | 1.342 |
| Bt.21196.1.S1\_at | 0.0004237 | 0.9563 |
| Bt.18623.1.A1\_at | 0.0004238 | -1.135 |
| Bt.18113.2.S1\_at | 0.0004256 | 0.665 |
| Bt.17773.1.S1\_at | 0.0004256 | 1.4 |
| Bt.7696.2.S1\_a\_at | 0.000426 | -1.235 |
| Bt.7039.1.S1\_a\_at | 0.0004266 | -0.934 |
| Bt.4985.1.S1\_at | 0.0004302 | -0.8569 |
| Bt.5035.1.S1\_at | 0.0004315 | -0.9515 |
| Bt.7342.1.S1\_at | 0.0004355 | 1.056 |
| Bt.11014.1.S1\_at | 0.0004362 | -1.348 |
| Bt.3814.1.S1\_at | 0.0004363 | 1.268 |
| Bt.7968.2.S1\_at | 0.0004372 | -0.9378 |
| Bt.14176.1.S1\_at | 0.0004377 | 1.099 |
| Bt.2625.1.S1\_at | 0.0004395 | 0.895 |
| Bt.7883.1.S1\_at | 0.0004397 | 0.7676 |
| Bt.2151.2.A1\_at | 0.0004401 | 2.72 |
| Bt.10600.1.S1\_at | 0.0004404 | 1.11 |
| Bt.6964.1.S1\_at | 0.0004407 | -1.074 |
| Bt.22128.1.S1\_at | 0.0004413 | 1.57 |
| Bt.4834.1.S1\_at | 0.0004414 | 1.191 |
| Bt.22200.1.S1\_at | 0.0004425 | 1.274 |
| Bt.6993.1.S1\_at | 0.0004432 | -0.9575 |
| Bt.18526.1.A1\_at | 0.0004445 | 1.96 |
| Bt.23331.1.S1\_at | 0.0004452 | 0.8989 |
| Bt.9055.2.S1\_a\_at | 0.0004463 | -1.345 |
| Bt.4384.1.S1\_at | 0.0004466 | 0.9813 |
| Bt.6899.1.S1\_at | 0.0004479 | -1.503 |
| Bt.20800.1.S1\_at | 0.00045 | 0.9322 |
| Bt.22054.1.S1\_at | 0.000453 | -1.496 |
| Bt.9139.1.A1\_at | 0.0004539 | -1.784 |
| Bt.5723.1.S1\_at | 0.000455 | -1.142 |
| Bt.10354.1.S1\_at | 0.0004555 | 1.266 |
| Bt.17012.1.A1\_s\_at | 0.0004572 | -1.178 |
| Bt.8673.1.S1\_a\_at | 0.0004574 | 1.159 |
| Bt.28162.2.A1\_at | 0.0004576 | -1.522 |
| Bt.659.1.S1\_at | 0.0004577 | -2.672 |
| Bt.7183.1.S1\_at | 0.0004588 | 1.038 |
| Bt.4279.2.S1\_at | 0.0004618 | 1.039 |
| Bt.27952.1.S1\_at | 0.0004622 | -0.9152 |
| Bt.18023.1.S1\_at | 0.0004637 | 1.312 |
| Bt.21239.1.S1\_at | 0.0004709 | -0.9648 |
| Bt.28684.1.S1\_at | 0.000473 | -1.083 |
| Bt.3975.1.S1\_at | 0.0004733 | -0.7578 |
| Bt.196.1.S1\_at | 0.0004733 | 1.673 |
| Bt.13850.3.S1\_a\_at | 0.0004754 | -1.035 |
| Bt.6483.1.A1\_at | 0.0004775 | 0.8897 |
| Bt.6842.1.S1\_at | 0.0004777 | 1.658 |
| Bt.22459.1.S1\_at | 0.0004784 | 1.265 |
| Bt.504.1.S1\_at | 0.0004792 | -0.9479 |
| Bt.20852.1.A1\_at | 0.0004793 | 1.147 |
| Bt.18079.1.A1\_at | 0.0004796 | -1.486 |
| Bt.9162.1.A1\_at | 0.0004811 | -1.718 |
| Bt.28480.1.S1\_at | 0.0004857 | -1.23 |
| Bt.8674.1.A1\_at | 0.0004867 | 1.017 |
| Bt.23143.2.S1\_at | 0.0004872 | -0.9421 |
| Bt.28105.1.S1\_at | 0.0004886 | 0.7791 |
| Bt.9416.1.S1\_at | 0.0004903 | 1.56 |
| Bt.6441.1.S1\_at | 0.0004905 | 1.43 |
| Bt.14559.1.S1\_at | 0.0004939 | 0.7611 |
| Bt.11023.1.S1\_at | 0.000497 | 0.8011 |
| Bt.20326.1.S1\_at | 0.0004989 | 0.9121 |
| Bt.8545.1.S1\_at | 0.0004994 | -1.304 |
| Bt.24253.1.S1\_at | 5e-04 | 0.784 |
| Bt.23509.2.A1\_at | 0.0005027 | 0.8184 |
| Bt.7633.1.S1\_at | 0.0005027 | -1.119 |
| Bt.20738.1.S1\_at | 0.0005029 | 1.283 |
| Bt.7421.1.S1\_at | 0.0005043 | -1.283 |
| Bt.9486.1.S1\_at | 0.0005089 | 1.127 |
| Bt.28257.1.S1\_at | 0.0005106 | 0.8969 |
| Bt.4495.1.S1\_at | 0.0005138 | 1.325 |
| Bt.6701.1.S1\_at | 0.0005149 | 1.156 |
| Bt.7373.2.S1\_a\_at | 0.0005154 | -0.8104 |
| Bt.4768.1.S2\_at | 0.0005154 | 1.221 |
| Bt.23869.1.A1\_at | 0.0005154 | 1.277 |
| Bt.28113.1.S1\_at | 0.0005159 | 1.23 |
| Bt.9312.1.A1\_at | 0.000518 | 1.911 |
| Bt.4503.1.S2\_at | 0.000519 | -1.08 |
| Bt.26447.1.A1\_at | 0.0005192 | 1.86 |
| Bt.11704.1.S1\_at | 0.0005196 | 1.517 |
| Bt.24624.2.S1\_at | 0.0005199 | -1.146 |
| Bt.22071.1.S1\_at | 0.0005219 | -1.015 |
| Bt.29864.1.A1\_at | 0.0005221 | 2.712 |
| Bt.5465.2.A1\_a\_at | 0.0005231 | -1.024 |
| Bt.8279.2.S1\_at | 0.0005238 | -1.687 |
| Bt.27123.1.A1\_at | 0.0005239 | 0.8076 |
| Bt.1240.1.S1\_at | 0.0005244 | 1.082 |
| Bt.23396.2.S1\_at | 0.0005245 | -1.957 |
| Bt.9012.1.S1\_at | 0.0005252 | 1.256 |
| Bt.8448.1.S1\_at | 0.0005264 | 1.238 |
| Bt.1418.2.S1\_at | 0.000527 | -1.103 |
| Bt.21215.2.S1\_at | 0.0005273 | 0.8208 |
| Bt.25637.1.A1\_at | 0.0005287 | -1.411 |
| Bt.14040.1.S1\_at | 0.0005311 | -1.484 |
| Bt.21997.2.A1\_at | 0.0005332 | 1.559 |
| Bt.11717.1.S1\_s\_at | 0.0005346 | -0.7132 |
| Bt.7430.1.S1\_at | 0.0005347 | 0.7062 |
| Bt.9546.2.S1\_a\_at | 0.0005359 | 0.8199 |
| Bt.24945.1.S1\_at | 0.0005378 | -0.7353 |
| Bt.25479.1.S1\_at | 0.0005386 | 0.7746 |
| Bt.26796.1.S1\_at | 0.0005389 | 2.74 |
| Bt.9337.1.S1\_at | 0.0005398 | 1.066 |
| Bt.19338.1.S1\_at | 0.0005403 | 1.392 |
| Bt.7397.1.S1\_at | 0.0005411 | 1.483 |
| Bt.28323.1.S1\_at | 0.0005423 | 1.918 |
| Bt.4702.1.S1\_at | 0.0005425 | 1.102 |
| Bt.12466.1.A1\_at | 0.0005425 | 1.05 |
| Bt.8739.1.S1\_at | 0.0005426 | 1.481 |
| Bt.9114.1.A1\_at | 0.0005428 | -0.8646 |
| Bt.1523.1.S1\_at | 0.0005432 | -1.224 |
| Bt.5089.1.S1\_at | 0.0005435 | 0.8796 |
| Bt.1193.1.S1\_at | 0.0005435 | -0.7713 |
| Bt.9070.1.S1\_at | 0.0005436 | -0.8752 |
| Bt.10.2.S1\_a\_at | 0.0005451 | -0.9265 |
| Bt.22672.1.A1\_at | 0.0005452 | 1.505 |
| Bt.605.1.S1\_at | 0.0005462 | 1.776 |
| Bt.1140.1.S1\_at | 0.000549 | 1.249 |
| Bt.26550.1.S1\_at | 0.0005506 | -1.225 |
| Bt.5027.1.A1\_at | 0.0005554 | 1.16 |
| Bt.4574.1.S1\_at | 0.0005557 | 0.9431 |
| Bt.20397.1.S1\_at | 0.0005573 | 1.567 |
| Bt.26423.1.A1\_at | 0.0005575 | 1.639 |
| Bt.20780.2.S1\_a\_at | 0.0005585 | -1.095 |
| Bt.2052.1.S1\_a\_at | 0.0005591 | 1.396 |
| Bt.16985.1.S1\_at | 0.0005595 | 0.9857 |
| Bt.18835.1.S1\_at | 0.0005603 | -2.692 |
| Bt.19061.1.A1\_at | 0.000561 | -0.9579 |
| Bt.7257.1.S1\_at | 0.0005617 | -1.013 |
| Bt.16637.1.A1\_at | 0.0005621 | -1.313 |
| Bt.18229.1.A1\_at | 0.0005627 | 1.332 |
| Bt.10947.1.S1\_at | 0.0005636 | 0.7331 |
| Bt.997.2.S1\_a\_at | 0.0005645 | -1.128 |
| Bt.21119.1.A1\_at | 0.0005668 | 1.215 |
| Bt.16945.1.S1\_at | 0.0005687 | -0.7322 |
| Bt.709.1.S1\_at | 0.0005714 | 2.162 |
| Bt.21491.1.S1\_at | 0.0005737 | 0.8369 |
| Bt.11027.1.A1\_at | 0.0005785 | 1.371 |
| Bt.28212.1.A1\_at | 0.0005805 | 0.9881 |
| Bt.21843.1.A1\_at | 0.0005809 | 1.507 |
| Bt.5951.1.S1\_at | 0.000582 | 0.9014 |
| Bt.7184.1.S1\_at | 0.0005821 | -0.6874 |
| Bt.22552.1.S2\_at | 0.0005845 | 0.8086 |
| Bt.1262.1.S1\_at | 0.0005849 | -0.9849 |
| Bt.935.1.A1\_at | 0.0005855 | -1.156 |
| Bt.26747.1.A1\_at | 0.0005856 | 1.203 |
| Bt.13411.1.S1\_at | 0.0005864 | 0.9572 |
| Bt.10129.1.A1\_at | 0.0005868 | 1.262 |
| Bt.5332.1.S1\_at | 0.0005881 | -0.7 |
| Bt.28462.1.S1\_at | 0.0005883 | -1.15 |
| Bt.22553.1.A1\_at | 0.0005884 | 1.277 |
| Bt.14304.1.S1\_at | 0.0005889 | 0.991 |
| Bt.16380.1.S1\_at | 0.0005899 | 1.063 |
| Bt.25057.1.A1\_at | 0.0005914 | 1.043 |
| Bt.23182.1.S1\_at | 0.000593 | -1.119 |
| Bt.4355.1.S1\_at | 0.0005953 | -1.122 |
| Bt.15900.1.A1\_at | 0.0005971 | -1.074 |
| Bt.21238.1.S1\_at | 0.0005979 | 1.029 |
| Bt.12859.1.S1\_at | 0.000598 | 1.232 |
| Bt.24359.1.S1\_a\_at | 0.0005983 | 1.434 |
| Bt.5445.2.A1\_at | 0.0005993 | 1.272 |
| Bt.12867.3.S1\_at | 0.000602 | 0.957 |
| Bt.28699.1.S1\_at | 0.0006026 | 0.7673 |
| Bt.27118.1.A1\_at | 0.0006048 | 1.498 |
| Bt.8809.1.S1\_at | 0.0006048 | -2.251 |
| Bt.27058.1.A1\_at | 0.0006051 | 1.159 |
| Bt.9415.1.S1\_at | 0.000608 | 1.057 |
| Bt.16753.1.A1\_at | 0.0006092 | 0.8256 |
| Bt.25419.1.A1\_at | 0.0006134 | -2.423 |
| Bt.11208.1.S1\_at | 0.0006153 | -1.115 |
| Bt.13834.1.S1\_at | 0.0006154 | -2.967 |
| Bt.21404.1.S1\_at | 0.0006171 | 0.8814 |
| Bt.28071.1.S1\_at | 0.0006176 | -0.7593 |
| Bt.13887.1.S1\_at | 0.00062 | -1.301 |
| Bt.2001.1.S1\_at | 0.0006209 | -1.197 |
| Bt.27027.1.S1\_a\_at | 0.0006215 | -1.242 |
| Bt.23649.1.A1\_at | 0.0006235 | 0.8064 |
| Bt.6983.1.S1\_at | 0.000624 | 1.754 |
| Bt.19599.1.A1\_at | 0.0006256 | 1.563 |
| Bt.8961.1.S1\_at | 0.0006257 | 1.118 |
| Bt.15977.1.S1\_at | 0.0006267 | -1.398 |
| Bt.9321.1.S1\_at | 0.0006276 | 1.611 |
| Bt.2378.1.S1\_at | 0.0006281 | 1.113 |
| Bt.22633.1.S1\_at | 0.0006296 | 0.8508 |
| Bt.1719.1.S1\_at | 0.0006299 | -0.9641 |
| Bt.18203.2.S1\_at | 0.0006319 | 1.207 |
| Bt.22446.1.S1\_at | 0.0006353 | 0.9368 |
| Bt.25339.1.S1\_at | 0.0006356 | 1.043 |
| Bt.3817.1.A1\_at | 0.0006376 | 0.9888 |
| Bt.14552.1.A1\_at | 0.0006418 | 0.7906 |
| Bt.13548.1.A1\_at | 0.0006419 | 1.105 |
| Bt.20463.1.S1\_at | 0.0006436 | 0.9842 |
| Bt.27937.1.A1\_at | 0.0006438 | 1.377 |
| Bt.22647.3.A1\_at | 0.0006456 | 1.309 |
| Bt.8324.1.S1\_at | 0.000646 | -0.9889 |
| Bt.9457.1.S1\_at | 0.000646 | -0.7965 |
| Bt.6850.2.S1\_at | 0.0006461 | 2.131 |
| Bt.29874.1.S1\_at | 0.0006473 | 0.9516 |
| Bt.21372.1.S1\_at | 0.0006481 | 1.066 |
| Bt.19100.2.S1\_at | 0.0006484 | 1.634 |
| Bt.8260.1.A1\_at | 0.0006486 | 1.649 |
| Bt.22466.1.A1\_at | 0.0006492 | 0.9177 |
| Bt.3789.1.S1\_at | 0.0006536 | 0.7569 |
| Bt.19469.1.A1\_at | 0.0006552 | 1.135 |
| Bt.28123.1.S1\_at | 0.0006567 | 0.8242 |
| Bt.6728.1.A1\_at | 0.0006575 | 0.741 |
| Bt.2642.1.S1\_at | 0.0006593 | -0.7678 |
| Bt.24307.1.A1\_at | 0.0006604 | 1.754 |
| Bt.7168.2.A1\_at | 0.000661 | 2.482 |
| Bt.16495.2.A1\_at | 0.0006615 | 1.282 |
| Bt.27481.1.A1\_at | 0.0006621 | 1.784 |
| Bt.3193.1.A1\_at | 0.0006631 | -0.8557 |
| Bt.7007.1.S1\_at | 0.0006632 | -1.318 |
| Bt.17608.1.A1\_at | 0.0006642 | 0.8605 |
| Bt.25657.1.S1\_at | 0.0006648 | 2.713 |
| Bt.4505.1.S1\_at | 0.0006659 | -0.6718 |
| Bt.17843.1.A1\_at | 0.000666 | 0.8828 |
| Bt.5682.1.S1\_at | 0.0006663 | 1.44 |
| Bt.1652.1.S1\_at | 0.0006737 | 1.983 |
| Bt.8333.1.S1\_at | 0.0006745 | -0.832 |
| Bt.10043.1.S1\_at | 0.0006763 | -0.9028 |
| Bt.24023.1.A1\_at | 0.0006774 | 1.325 |
| Bt.24630.2.S1\_at | 0.000678 | 1.516 |
| Bt.21215.1.A1\_at | 0.0006808 | 1.153 |
| Bt.9749.1.S1\_at | 0.0006829 | -0.6588 |
| Bt.10990.1.S1\_at | 0.0006838 | -1.559 |
| Bt.27652.1.A1\_at | 0.000684 | 2.576 |
| Bt.6581.1.S1\_at | 0.0006849 | 1.25 |
| Bt.8736.2.S1\_at | 0.0006862 | 0.8529 |
| Bt.12712.1.A1\_at | 0.0006888 | 1.75 |
| Bt.9868.3.S1\_a\_at | 0.0006924 | 1.124 |
| Bt.9240.1.A1\_at | 0.0006925 | -0.8188 |
| Bt.12088.1.S1\_at | 0.0006972 | 0.8419 |
| Bt.22155.1.A1\_at | 0.0006996 | 0.7217 |
| Bt.9629.1.S1\_at | 0.0007003 | 0.7973 |
| Bt.2818.2.S1\_a\_at | 0.0007008 | 1.207 |
| Bt.16525.1.A1\_at | 0.000702 | 0.8844 |
| Bt.13925.1.S1\_at | 0.0007044 | 0.698 |
| Bt.27095.1.S1\_at | 0.0007056 | 0.8452 |
| Bt.15957.1.S1\_at | 0.0007077 | 0.8209 |
| Bt.27061.1.S1\_at | 0.0007078 | -0.7652 |
| Bt.25077.1.S1\_at | 0.0007082 | 0.8546 |
| Bt.16264.1.A1\_at | 0.0007113 | 1.425 |
| Bt.29506.1.S1\_at | 0.0007114 | 1.982 |
| Bt.5319.2.S1\_a\_at | 0.0007128 | -0.7869 |
| Bt.2848.1.S1\_at | 0.0007188 | -0.8607 |
| Bt.25836.2.A1\_at | 0.0007241 | 1.198 |
| Bt.9197.1.S1\_at | 0.0007263 | 0.9708 |
| Bt.21321.1.S1\_at | 0.0007265 | 1.341 |
| Bt.11496.1.S1\_at | 0.0007275 | 1.427 |
| Bt.21372.2.S1\_at | 0.0007293 | 1.409 |
| Bt.1286.1.S1\_a\_at | 0.0007307 | 1.326 |
| Bt.4390.1.S1\_at | 0.0007316 | 1.377 |
| Bt.11601.1.A1\_at | 0.0007318 | -1.227 |
| Bt.27262.1.S1\_at | 0.0007331 | -1.155 |
| Bt.17332.2.S1\_at | 0.0007342 | 0.956 |
| Bt.16020.1.A1\_at | 0.0007344 | 1.062 |
| Bt.27708.1.A1\_at | 0.0007346 | 1.287 |
| Bt.3843.1.S1\_at | 0.0007388 | -1.033 |
| Bt.28909.1.A1\_at | 0.0007392 | -1.589 |
| Bt.7962.1.S1\_at | 0.0007407 | 1.688 |
| Bt.28312.1.S1\_at | 0.0007422 | 0.9979 |
| Bt.13254.1.S1\_at | 0.0007438 | 1.337 |
| Bt.21314.1.S1\_at | 0.0007449 | 0.6814 |
| Bt.3860.1.S1\_at | 0.0007465 | 1.06 |
| Bt.26061.1.A1\_at | 0.0007502 | 1.019 |
| Bt.16782.2.S1\_at | 0.0007515 | -1.683 |
| Bt.222.1.S1\_at | 0.0007527 | -2.141 |
| Bt.20431.1.A1\_at | 0.0007541 | 0.9331 |
| Bt.26431.1.S1\_at | 0.0007592 | 1.443 |
| Bt.13994.1.S1\_at | 0.0007593 | 1.051 |
| Bt.20091.1.S1\_at | 0.0007599 | 1.391 |
| Bt.22584.1.A1\_at | 0.0007618 | 1.251 |
| Bt.21938.1.S1\_at | 0.0007635 | 1.089 |
| Bt.10251.1.S1\_at | 0.0007651 | 0.9374 |
| Bt.22290.1.S1\_at | 0.0007651 | 1.03 |
| Bt.9301.1.A1\_at | 0.0007652 | 0.9965 |
| Bt.24894.1.S1\_at | 0.0007675 | 1.114 |
| Bt.27396.1.S1\_at | 0.0007675 | 1.061 |
| Bt.29009.1.A1\_at | 0.0007682 | 2.153 |
| Bt.2656.1.S1\_at | 0.0007683 | 1.126 |
| Bt.20290.1.A1\_at | 0.0007687 | -0.9369 |
| Bt.4044.1.S1\_at | 0.0007731 | -1.167 |
| Bt.20414.3.S1\_at | 0.0007732 | 0.9054 |
| Bt.27129.1.S1\_at | 0.0007736 | 0.8833 |
| Bt.28043.1.S1\_at | 0.000775 | 1.323 |
| Bt.2853.1.S1\_at | 0.0007754 | -0.8898 |
| Bt.13065.1.S1\_at | 0.0007779 | 1.23 |
| Bt.10175.2.A1\_a\_at | 0.0007824 | 0.7507 |
| Bt.15887.2.S1\_a\_at | 0.0007825 | -0.8184 |
| Bt.22475.1.S1\_at | 0.0007842 | 0.9979 |
| Bt.2606.1.S1\_at | 0.0007846 | 1.274 |
| Bt.5434.1.S1\_at | 0.0007889 | -0.7877 |
| Bt.22763.2.S1\_a\_at | 0.0007895 | -0.9568 |
| Bt.18316.2.S1\_at | 0.0007898 | 1.291 |
| Bt.3945.1.S1\_at | 0.0007901 | 0.9865 |
| Bt.2156.2.S1\_a\_at | 0.0007902 | -0.7057 |
| Bt.16025.1.S1\_at | 0.0007943 | -0.8519 |
| Bt.1692.1.S1\_at | 0.0007964 | -1.005 |
| Bt.5529.2.S1\_a\_at | 0.0007992 | -0.8796 |
| Bt.4658.1.S1\_a\_at | 0.0008018 | -1.011 |
| Bt.5534.1.S1\_at | 0.0008025 | -1.336 |
| Bt.3306.1.S1\_at | 0.0008037 | 0.9797 |
| Bt.26920.2.A1\_a\_at | 0.0008048 | -1.304 |
| Bt.13440.3.S1\_at | 0.000805 | 1.039 |
| Bt.19522.2.S1\_at | 0.0008057 | 0.8925 |
| Bt.9578.1.S1\_at | 0.000808 | -0.8465 |
| Bt.9643.1.S1\_at | 0.0008081 | -0.9433 |
| Bt.8479.1.A1\_at | 0.0008084 | 1.599 |
| Bt.3319.1.A1\_at | 0.0008094 | 2.547 |
| Bt.21296.1.S1\_at | 0.0008109 | 0.7747 |
| Bt.20304.1.S1\_at | 0.0008125 | 0.6639 |
| Bt.20193.1.S1\_at | 0.0008135 | -1.443 |
| Bt.18634.1.A1\_at | 0.0008146 | 1.332 |
| Bt.20485.1.S1\_at | 0.0008154 | 0.7079 |
| Bt.7030.1.S1\_at | 0.0008161 | 0.8873 |
| Bt.16243.1.S1\_at | 0.0008169 | 1.101 |
| Bt.28298.1.S1\_at | 0.0008184 | 0.8133 |
| Bt.7043.2.S1\_a\_at | 0.0008185 | 1.177 |
| Bt.6382.1.S1\_at | 0.0008198 | 1.32 |
| Bt.22745.2.S1\_at | 0.0008213 | 0.967 |
| Bt.6087.2.S1\_a\_at | 0.0008224 | 0.9466 |
| Bt.4480.1.S2\_at | 0.0008238 | 1.194 |
| Bt.23285.1.S1\_at | 0.0008244 | -0.785 |
| Bt.26894.1.S1\_at | 0.0008248 | -0.9136 |
| Bt.1484.1.S1\_at | 0.0008253 | 0.8436 |
| Bt.27147.1.S1\_at | 0.0008269 | 1.227 |
| Bt.23109.1.S1\_at | 0.0008272 | -1.022 |
| Bt.4817.1.A1\_at | 0.0008284 | 1.746 |
| Bt.12468.1.S1\_at | 0.0008287 | -1.688 |
| Bt.12510.3.S1\_at | 0.0008291 | 1.243 |
| Bt.9324.1.S1\_at | 0.0008338 | -1.363 |
| Bt.28695.2.A1\_at | 0.0008349 | 0.9008 |
| Bt.4466.1.A1\_at | 0.0008355 | 0.7398 |
| Bt.19098.1.A1\_s\_at | 0.0008366 | 1.021 |
| Bt.16092.1.S1\_at | 0.0008374 | 0.9642 |
| Bt.7221.1.S1\_at | 0.0008398 | -0.9873 |
| Bt.4533.1.S1\_at | 0.0008398 | -0.906 |
| Bt.9403.1.S1\_at | 0.0008408 | 1.019 |
| Bt.6673.1.S1\_at | 0.000842 | -0.7401 |
| Bt.15938.1.S1\_at | 0.0008431 | 0.9156 |
| Bt.5484.1.S1\_at | 0.0008465 | -0.8118 |
| Bt.8699.1.S1\_at | 0.0008471 | -1.062 |
| Bt.2816.1.S1\_at | 0.0008473 | -0.8538 |
| Bt.3026.1.A1\_at | 0.0008474 | 0.9799 |
| Bt.21715.1.S1\_at | 0.0008492 | 1.168 |
| Bt.25335.1.A1\_at | 0.0008497 | 1.094 |
| Bt.22294.1.S1\_at | 0.0008537 | 0.673 |
| Bt.16836.1.S1\_at | 0.0008544 | 1.414 |
| Bt.7870.1.S1\_at | 0.0008566 | -1.833 |
| Bt.12096.1.S1\_at | 0.0008578 | -1.339 |
| Bt.24832.1.A1\_a\_at | 0.0008586 | -1.389 |
| Bt.16628.2.A1\_at | 0.0008599 | 1.837 |
| Bt.15837.1.S1\_at | 0.0008633 | -0.6846 |
| Bt.21027.2.S1\_at | 0.000866 | 1.553 |
| Bt.17958.1.S1\_at | 0.0008687 | 0.7534 |
| Bt.17886.1.A1\_at | 0.0008693 | 1.86 |
| Bt.27.1.S1\_at | 0.00087 | -0.9239 |
| Bt.21561.1.S1\_at | 0.0008703 | 0.9419 |
| Bt.3050.1.S1\_at | 0.0008716 | -0.6772 |
| Bt.11623.1.A1\_a\_at | 0.0008734 | 2.821 |
| Bt.18002.1.A1\_at | 0.0008738 | 2.375 |
| Bt.3090.1.S1\_at | 0.0008762 | 0.7561 |
| Bt.3888.1.S1\_a\_at | 0.0008765 | -1.153 |
| Bt.15819.2.S1\_at | 0.0008787 | -0.7674 |
| Bt.19309.3.A1\_at | 0.0008812 | 1.36 |
| Bt.11171.1.S1\_at | 0.000884 | 0.8986 |
| Bt.20988.1.S1\_at | 0.0008852 | -0.9889 |
| Bt.16639.1.A1\_at | 0.0008852 | 0.8542 |
| Bt.1117.1.S1\_at | 0.0008857 | 2.532 |
| Bt.24594.1.A1\_at | 0.0008877 | -1.311 |
| Bt.22725.1.S1\_at | 0.0008883 | 0.876 |
| Bt.13965.1.S1\_at | 0.0008915 | 1.053 |
| Bt.8606.1.S1\_a\_at | 0.0008918 | 0.9736 |
| Bt.9041.1.S1\_at | 0.0008925 | 0.6756 |
| Bt.13028.1.S1\_at | 0.0008935 | 2.85 |
| Bt.6534.1.S1\_at | 0.0008963 | 1.181 |
| Bt.20226.1.A1\_at | 0.0009001 | 1.556 |
| Bt.21133.1.S1\_at | 0.0009008 | 1.427 |
| Bt.26682.1.S1\_at | 0.0009013 | 0.9875 |
| Bt.22505.1.S1\_at | 0.0009019 | 2.771 |
| Bt.27930.1.S1\_at | 0.0009097 | 0.7786 |
| Bt.16125.2.S1\_at | 0.0009098 | -0.9844 |
| Bt.23600.1.S1\_at | 0.00091 | -0.7392 |
| Bt.1573.2.S1\_a\_at | 0.000911 | -1.177 |
| Bt.6686.1.S1\_at | 0.0009126 | 0.8778 |
| Bt.26872.1.S1\_at | 0.0009159 | 1.014 |
| Bt.9866.1.S1\_at | 0.0009164 | -0.9727 |
| Bt.25712.1.A1\_s\_at | 0.000917 | 1.78 |
| Bt.7701.1.S1\_at | 0.0009207 | -0.9492 |
| Bt.29325.1.S1\_at | 0.0009208 | -0.8932 |
| Bt.1932.1.S1\_at | 0.0009211 | -0.9087 |
| Bt.19643.2.S1\_at | 0.0009231 | 1.141 |
| Bt.11908.1.S1\_at | 0.0009238 | 0.9116 |
| Bt.68.1.S1\_at | 0.000924 | -1.459 |
| Bt.23615.1.S1\_at | 0.000925 | 0.7096 |
| Bt.12957.1.A1\_a\_at | 0.0009261 | 0.8927 |
| Bt.6778.1.S1\_at | 0.0009324 | 1.694 |
| Bt.665.1.S1\_s\_at | 0.0009339 | -1.066 |
| Bt.8648.1.S1\_at | 0.0009354 | 0.7513 |
| Bt.25015.1.A1\_at | 0.0009356 | 1.001 |
| Bt.27956.1.A1\_at | 0.0009388 | 0.7999 |
| Bt.22615.2.A1\_a\_at | 0.0009396 | -0.965 |
| Bt.3895.1.S1\_at | 0.0009417 | 1.279 |
| Bt.422.1.S2\_at | 0.0009443 | 0.8644 |
| Bt.5602.1.A1\_at | 0.0009461 | 0.9647 |
| Bt.4756.1.S1\_at | 0.0009478 | -0.6622 |
| Bt.668.1.S1\_at | 0.0009494 | 0.9306 |
| Bt.28393.1.S1\_at | 0.0009576 | 1.404 |
| Bt.5237.1.S1\_at | 0.0009634 | 0.9743 |
| Bt.21584.1.S1\_at | 0.000964 | -1.094 |
| Bt.20947.1.S1\_at | 0.0009656 | 1.23 |
| Bt.16111.1.S1\_at | 0.0009716 | 0.7246 |
| Bt.11180.1.A1\_a\_at | 0.0009737 | 1.023 |
| Bt.19097.2.S1\_at | 0.0009738 | 0.9722 |
| Bt.2235.1.S2\_at | 0.0009772 | 1.015 |
| Bt.10892.1.S1\_at | 0.0009803 | -2.842 |
| Bt.7788.1.S1\_at | 0.0009804 | 1.062 |
| Bt.8163.1.S1\_at | 0.0009824 | 0.7603 |
| Bt.21911.1.S1\_at | 0.000984 | 1.559 |
| Bt.18340.1.A1\_at | 0.000984 | 0.8498 |
| Bt.16108.1.A1\_at | 0.0009853 | -1.554 |
| Bt.1760.1.S1\_at | 0.0009853 | -0.7903 |
| Bt.12820.1.S1\_at | 0.0009853 | 0.9431 |
| Bt.15907.2.S1\_at | 0.0009858 | -2.057 |
| Bt.1325.1.S1\_at | 0.0009875 | 1.196 |
| Bt.7641.2.S1\_a\_at | 0.0009875 | -3.441 |
| Bt.20402.3.S1\_at | 0.0009907 | 1.702 |
| Bt.23330.1.S1\_at | 0.0009943 | 0.8967 |
| Bt.22339.2.S1\_at | 0.0009953 | -1.399 |
| Bt.24433.1.S1\_at | 0.0009953 | -0.7844 |
| Bt.12290.1.S1\_at | 0.0009958 | 1.435 |
| Bt.26135.1.A1\_at | 0.0009961 | -2.24 |
| Bt.2121.1.S1\_at | 0.0009969 | 2.102 |
| Bt.16473.1.A1\_at | 0.000999 | 1.611 |
| Bt.16861.1.A1\_at | 0.0009992 | 1.137 |
| Bt.25075.1.A1\_at | 0.001002 | 1.302 |
| Bt.2418.1.S1\_at | 0.001007 | 0.8726 |
| Bt.28146.1.A1\_at | 0.001014 | -1.122 |
| Bt.23542.1.S1\_at | 0.001015 | -1.296 |
| Bt.12108.1.S1\_at | 0.001017 | -1.213 |
| Bt.422.1.S1\_at | 0.001018 | 0.8692 |
| Bt.23971.1.S1\_at | 0.001023 | 1.019 |
| Bt.7575.1.A1\_at | 0.001023 | -1.051 |
| Bt.19100.3.A1\_at | 0.001024 | 2.372 |
| Bt.24396.2.S1\_at | 0.001027 | 2.272 |
| Bt.4011.1.S1\_at | 0.001028 | -1.606 |
| Bt.21607.1.S1\_at | 0.001029 | 1.351 |
| Bt.27457.1.S1\_at | 0.001029 | 1.542 |
| Bt.5939.1.S1\_a\_at | 0.001033 | -0.6389 |
| Bt.9170.1.A1\_at | 0.001035 | 1.586 |
| Bt.23240.1.S1\_at | 0.001041 | 1.239 |
| Bt.20300.1.A1\_at | 0.001043 | 0.8175 |
| Bt.25887.1.A1\_at | 0.001043 | 1.259 |
| Bt.23655.1.S1\_at | 0.001044 | 1.511 |
| Bt.22168.1.A1\_at | 0.001045 | -1.105 |
| Bt.11315.1.A1\_at | 0.001052 | 0.9935 |
| Bt.21824.1.A1\_at | 0.001053 | 1.286 |
| Bt.8180.2.S1\_at | 0.001055 | 1.5 |
| Bt.16487.1.S1\_at | 0.001056 | 0.9082 |
| Bt.17447.1.A1\_at | 0.00106 | 1.829 |
| Bt.13913.1.S1\_at | 0.001062 | -0.7644 |
| Bt.561.1.S1\_at | 0.001066 | -1.516 |
| Bt.27149.1.S1\_at | 0.001068 | -0.8448 |
| Bt.4631.2.S1\_at | 0.001068 | 1.02 |
| Bt.28649.1.A1\_a\_at | 0.001069 | -1.431 |
| Bt.16597.1.S1\_at | 0.001071 | -1.327 |
| Bt.25461.1.A1\_at | 0.001071 | -1.169 |
| Bt.13205.2.S1\_at | 0.001074 | -1.034 |
| Bt.29938.1.S1\_at | 0.001075 | 0.9888 |
| Bt.3777.1.S1\_at | 0.001077 | -0.6822 |
| Bt.27982.1.S1\_at | 0.001081 | -0.8487 |
| Bt.21113.1.S1\_a\_at | 0.001081 | -1.186 |
| Bt.9544.1.S1\_at | 0.001082 | 0.6991 |
| Bt.2765.1.S1\_at | 0.001084 | 0.948 |
| Bt.13998.1.S1\_at | 0.001086 | -0.9235 |
| Bt.21219.1.S1\_at | 0.00109 | 0.9374 |
| Bt.15674.1.S1\_at | 0.001091 | 1.414 |
| Bt.8275.1.S1\_at | 0.001093 | 1.097 |
| Bt.59.1.S1\_at | 0.001095 | -0.8883 |
| Bt.7058.1.S1\_at | 0.001096 | 1.699 |
| Bt.2821.1.S1\_at | 0.001096 | -0.787 |
| Bt.5129.2.A1\_at | 0.001097 | -1.046 |
| Bt.9322.1.S1\_at | 0.001097 | 1.383 |
| Bt.21622.1.A1\_at | 0.001102 | 1.321 |
| Bt.6667.1.S1\_at | 0.001104 | 0.9834 |
| Bt.23505.1.S1\_at | 0.001105 | -2.271 |
| Bt.7056.2.A1\_x\_at | 0.001109 | 1.707 |
| Bt.24563.1.S1\_at | 0.001109 | 1.077 |
| Bt.7130.1.S1\_at | 0.001109 | 0.9769 |
| Bt.5079.1.S1\_at | 0.001112 | -1.054 |
| Bt.19261.1.S1\_at | 0.001112 | 1.526 |

Differentially expressed genes for RMA: 3326

| AFFY | p-value | FoldChange |
| --- | --- | --- |
| Bt.7056.3.A1\_a\_at | 9.253e-14 | 6.252 |
| Bt.13980.1.A1\_at | 2.764e-13 | -3.042 |
| Bt.5002.1.S1\_at | 1.892e-12 | -2.442 |
| Bt.10719.1.S1\_at | 2.787e-12 | 5.228 |
| Bt.20354.1.S1\_at | 4.296e-12 | 1.914 |
| Bt.3865.3.S1\_a\_at | 5.056e-12 | -2.495 |
| Bt.2894.1.S1\_at | 6.543e-12 | -2.331 |
| Bt.13980.2.A1\_a\_at | 6.725e-12 | -2.858 |
| Bt.27381.1.S1\_at | 7.084e-12 | -2.925 |
| Bt.19517.1.S1\_at | 1.553e-11 | 2.516 |
| Bt.17613.1.A1\_at | 1.559e-11 | -2.133 |
| Bt.1316.1.S1\_at | 2.236e-11 | -2.561 |
| Bt.11770.1.S1\_a\_at | 3.692e-11 | -2.233 |
| Bt.11236.1.S1\_at | 5.55e-11 | -3.212 |
| Bt.23076.1.S1\_at | 6.539e-11 | 2.416 |
| Bt.3857.1.S1\_at | 6.998e-11 | -2.656 |
| Bt.1316.1.S2\_at | 7.127e-11 | -2.184 |
| Bt.21528.1.A1\_at | 7.139e-11 | 2.26 |
| Bt.11178.1.S1\_at | 8.623e-11 | 1.988 |
| Bt.12510.1.S1\_at | 8.827e-11 | 3.329 |
| Bt.26135.1.A1\_at | 9.496e-11 | -2.337 |
| Bt.18479.1.A1\_at | 1.01e-10 | 2.003 |
| Bt.4804.2.A1\_at | 1.018e-10 | 1.837 |
| Bt.12510.1.S1\_a\_at | 1.123e-10 | 2.765 |
| Bt.7034.1.S1\_at | 1.407e-10 | -2.897 |
| Bt.23662.1.S1\_at | 1.52e-10 | -1.821 |
| Bt.5223.1.S1\_at | 1.758e-10 | 1.894 |
| Bt.8077.1.S1\_at | 1.803e-10 | -2.086 |
| Bt.20899.1.A1\_at | 2.059e-10 | -2.071 |
| Bt.24413.1.S1\_at | 2.266e-10 | 1.688 |
| Bt.3873.1.S1\_at | 3.031e-10 | -1.419 |
| Bt.21896.1.S1\_at | 3.572e-10 | 2.64 |
| Bt.18753.1.A1\_at | 3.703e-10 | 1.936 |
| Bt.18278.1.S1\_at | 3.904e-10 | -1.842 |
| Bt.18572.1.A1\_at | 4.747e-10 | 2.299 |
| Bt.20229.1.S1\_at | 7.029e-10 | -1.751 |
| Bt.13834.1.S1\_at | 7.317e-10 | -2.192 |
| Bt.28208.1.S1\_at | 7.856e-10 | -1.951 |
| Bt.20470.1.S1\_a\_at | 8.193e-10 | -2.369 |
| Bt.1421.1.S1\_at | 8.884e-10 | 2.7 |
| Bt.4891.1.S1\_at | 9.279e-10 | -1.307 |
| Bt.22664.1.A1\_at | 1.006e-09 | 2.277 |
| Bt.7215.1.S1\_at | 1.018e-09 | -1.804 |
| Bt.22519.1.S1\_at | 1.032e-09 | -1.872 |
| Bt.24931.1.A1\_at | 1.035e-09 | -1.61 |
| Bt.1403.1.S1\_at | 1.091e-09 | -1.812 |
| Bt.22379.1.S1\_at | 1.212e-09 | -1.523 |
| Bt.13330.2.A1\_at | 1.264e-09 | -3.668 |
| Bt.26718.1.S1\_a\_at | 1.302e-09 | -1.678 |
| Bt.22270.1.S1\_at | 1.305e-09 | 1.571 |
| Bt.23164.1.S1\_at | 1.348e-09 | -1.646 |
| Bt.1112.1.S1\_at | 1.406e-09 | -2.329 |
| Bt.20072.1.S1\_at | 1.516e-09 | 1.961 |
| Bt.23662.2.S1\_at | 1.533e-09 | -1.958 |
| Bt.7175.1.S1\_at | 1.574e-09 | -1.422 |
| Bt.8618.1.S1\_at | 1.698e-09 | -1.574 |
| Bt.2049.1.S1\_at | 1.749e-09 | -1.756 |
| Bt.20473.1.A1\_at | 1.765e-09 | 2.319 |
| Bt.11516.1.S1\_at | 1.77e-09 | -1.464 |
| Bt.3960.1.S1\_a\_at | 1.897e-09 | -1.746 |
| Bt.17942.1.S1\_at | 1.908e-09 | 1.377 |
| Bt.5120.1.S1\_at | 2.054e-09 | -1.905 |
| Bt.28612.1.S1\_at | 2.237e-09 | 1.348 |
| Bt.20.1.S1\_at | 2.248e-09 | -1.454 |
| Bt.836.1.S1\_at | 2.5e-09 | -1.693 |
| Bt.20854.1.S1\_at | 2.621e-09 | -1.393 |
| Bt.20505.1.S1\_at | 2.642e-09 | -1.681 |
| Bt.16843.1.A1\_at | 2.658e-09 | -1.344 |
| Bt.4770.1.S1\_at | 2.764e-09 | 1.663 |
| Bt.23176.2.S1\_a\_at | 2.942e-09 | 2.228 |
| Bt.562.1.S1\_at | 2.945e-09 | -1.832 |
| Bt.4867.2.S1\_at | 3.245e-09 | 4.622 |
| Bt.26702.1.S1\_a\_at | 3.462e-09 | -1.467 |
| Bt.22634.1.S1\_at | 3.49e-09 | 1.507 |
| Bt.12453.1.A1\_a\_at | 3.491e-09 | 2.004 |
| Bt.21731.1.S1\_at | 3.512e-09 | 1.324 |
| Bt.9556.1.S1\_at | 3.577e-09 | 1.631 |
| Bt.13324.2.S1\_a\_at | 3.591e-09 | -1.479 |
| Bt.22002.1.S1\_at | 3.782e-09 | -2.281 |
| Bt.4473.1.S1\_at | 4.105e-09 | -1.646 |
| Bt.20141.1.S1\_at | 4.414e-09 | -1.514 |
| Bt.21766.2.S1\_at | 4.616e-09 | -1.841 |
| Bt.21218.1.A1\_at | 5.397e-09 | -2.014 |
| Bt.11794.1.S1\_at | 5.414e-09 | -1.953 |
| Bt.14116.1.A1\_at | 6.406e-09 | 1.327 |
| Bt.3117.1.S1\_at | 6.518e-09 | -1.538 |
| Bt.1153.1.S1\_at | 6.862e-09 | -1.517 |
| Bt.25227.1.A1\_at | 7.324e-09 | 1.518 |
| Bt.27657.1.A1\_at | 7.327e-09 | -1.78 |
| Bt.7079.1.S1\_at | 7.7e-09 | -1.812 |
| Bt.1696.1.S1\_at | 7.822e-09 | -1.194 |
| Bt.8964.2.S1\_a\_at | 9.504e-09 | -2.086 |
| Bt.25555.1.S1\_at | 1.016e-08 | -1.417 |
| Bt.3247.1.S1\_at | 1.023e-08 | 2.31 |
| Bt.6465.1.S1\_at | 1.054e-08 | -2.631 |
| Bt.17778.1.A1\_at | 1.059e-08 | -1.556 |
| Bt.20470.2.S1\_a\_at | 1.064e-08 | -1.784 |
| Bt.27152.1.A1\_at | 1.067e-08 | 2.544 |
| Bt.13287.1.S1\_at | 1.085e-08 | -1.625 |
| Bt.23357.1.S1\_at | 1.11e-08 | -1.414 |
| Bt.6391.1.A1\_at | 1.157e-08 | -2.15 |
| Bt.12453.1.A1\_at | 1.185e-08 | 2.223 |
| Bt.7804.1.S1\_at | 1.236e-08 | -1.428 |
| Bt.1399.1.S1\_at | 1.237e-08 | -1.212 |
| Bt.12694.1.S1\_at | 1.238e-08 | 1.446 |
| Bt.23502.1.S1\_at | 1.339e-08 | 1.234 |
| Bt.29746.1.S1\_at | 1.344e-08 | -1.411 |
| Bt.13321.1.S1\_at | 1.359e-08 | 1.624 |
| Bt.4670.1.S1\_at | 1.363e-08 | 1.58 |
| Bt.9222.1.S1\_at | 1.395e-08 | -1.445 |
| Bt.1129.1.S1\_at | 1.628e-08 | -1.868 |
| Bt.3782.1.A1\_at | 1.664e-08 | 1.739 |
| Bt.12303.1.S1\_at | 1.668e-08 | 1.275 |
| Bt.7056.5.A1\_x\_at | 1.684e-08 | 3.82 |
| Bt.16329.1.A1\_at | 1.707e-08 | 1.691 |
| Bt.7984.1.S1\_at | 1.707e-08 | 1.345 |
| Bt.21239.1.S1\_at | 1.711e-08 | -1.313 |
| Bt.4725.1.S1\_at | 1.744e-08 | 1.286 |
| Bt.8549.2.S1\_at | 1.762e-08 | 1.454 |
| Bt.5483.1.S1\_at | 1.787e-08 | -1.309 |
| Bt.8730.1.S1\_at | 1.789e-08 | 1.159 |
| Bt.29053.1.A1\_at | 1.802e-08 | 1.365 |
| Bt.11216.1.S1\_at | 1.85e-08 | -2.387 |
| Bt.832.1.S1\_at | 1.919e-08 | -1.507 |
| Bt.4777.1.S1\_at | 1.934e-08 | -1.744 |
| Bt.19936.1.A1\_at | 2.017e-08 | 1.664 |
| Bt.5966.1.A1\_at | 2.038e-08 | -1.685 |
| Bt.4072.1.S1\_at | 2.071e-08 | -1.502 |
| Bt.946.2.A1\_a\_at | 2.101e-08 | -1.438 |
| Bt.4758.1.S1\_at | 2.144e-08 | -2.128 |
| Bt.20025.1.S1\_at | 2.145e-08 | -1.487 |
| Bt.5183.2.S1\_at | 2.155e-08 | -2.178 |
| Bt.1197.1.A1\_at | 2.278e-08 | 1.151 |
| Bt.23399.1.S1\_at | 2.279e-08 | -1.766 |
| Bt.19409.1.A1\_s\_at | 2.286e-08 | 1.595 |
| Bt.66.1.S1\_at | 2.298e-08 | -1.111 |
| Bt.10013.1.S1\_at | 2.348e-08 | 1.811 |
| Bt.28305.1.S1\_at | 2.378e-08 | 1.836 |
| Bt.21766.3.S1\_at | 2.479e-08 | -2.02 |
| Bt.22842.2.S1\_at | 2.505e-08 | 1.248 |
| Bt.23396.1.S1\_at | 2.527e-08 | -2.013 |
| Bt.4977.1.S2\_at | 2.532e-08 | 1.287 |
| Bt.23438.1.S1\_at | 2.598e-08 | 1.251 |
| Bt.19862.2.S1\_at | 2.615e-08 | 1.496 |
| Bt.1154.1.S1\_at | 2.665e-08 | -1.508 |
| Bt.5078.1.S1\_at | 2.699e-08 | 1.269 |
| Bt.20601.1.A1\_at | 2.806e-08 | 1.484 |
| Bt.24802.2.S1\_a\_at | 2.815e-08 | -1.597 |
| Bt.3605.1.S1\_at | 2.817e-08 | -1.742 |
| Bt.27318.1.S1\_at | 2.841e-08 | 1.919 |
| Bt.19508.1.A1\_at | 2.849e-08 | 1.757 |
| Bt.21015.1.S1\_at | 2.907e-08 | -1.401 |
| Bt.5537.1.S1\_at | 2.98e-08 | -1.56 |
| Bt.18278.2.A1\_at | 2.998e-08 | -1.701 |
| Bt.27193.1.A1\_at | 3.083e-08 | 1.472 |
| Bt.7576.1.S1\_at | 3.09e-08 | 1.37 |
| Bt.5246.1.S1\_at | 3.14e-08 | -1.456 |
| Bt.29931.1.S1\_at | 3.357e-08 | -1.274 |
| Bt.19097.1.A1\_at | 3.462e-08 | 1.285 |
| Bt.1242.1.S1\_at | 3.469e-08 | -1.577 |
| Bt.11208.1.S1\_at | 3.483e-08 | -1.218 |
| Bt.27457.1.S1\_at | 3.513e-08 | 1.578 |
| Bt.3448.2.S1\_at | 3.694e-08 | -1.546 |
| Bt.28138.1.S1\_at | 3.736e-08 | 1.165 |
| Bt.24848.1.A1\_at | 3.745e-08 | 1.855 |
| Bt.26792.1.S1\_at | 3.746e-08 | 2.062 |
| Bt.22518.1.S1\_a\_at | 3.755e-08 | -1.472 |
| Bt.23148.1.S1\_at | 3.883e-08 | 1.168 |
| Bt.20591.2.A1\_at | 3.937e-08 | 1.637 |
| Bt.18352.1.A1\_at | 4.018e-08 | -1.276 |
| Bt.2138.1.S1\_s\_at | 4.073e-08 | -1.356 |
| Bt.20295.1.A1\_at | 4.074e-08 | 1.911 |
| Bt.5089.1.S1\_at | 4.168e-08 | 1.289 |
| Bt.12613.1.S1\_at | 4.184e-08 | 1.763 |
| Bt.1129.2.S1\_at | 4.257e-08 | -1.469 |
| Bt.3922.1.S1\_a\_at | 4.293e-08 | -1.529 |
| Bt.16668.1.A1\_at | 4.407e-08 | 1.53 |
| Bt.7856.1.S1\_at | 4.424e-08 | -1.568 |
| Bt.9966.1.S1\_at | 4.437e-08 | 1.564 |
| Bt.18890.1.A1\_at | 4.469e-08 | -1.112 |
| Bt.5520.1.S1\_at | 4.623e-08 | -1.585 |
| Bt.8829.1.S1\_a\_at | 4.671e-08 | 2.076 |
| Bt.315.1.S1\_at | 4.674e-08 | -1.426 |
| Bt.28568.1.A1\_at | 4.744e-08 | 2.164 |
| Bt.16082.1.S1\_at | 4.75e-08 | -1.639 |
| Bt.12634.1.A1\_at | 4.783e-08 | 1.254 |
| Bt.9754.1.S1\_at | 4.813e-08 | -1.884 |
| Bt.21981.3.S1\_at | 4.886e-08 | 1.384 |
| Bt.1807.1.A1\_at | 4.957e-08 | 1.407 |
| Bt.20512.1.S1\_at | 5.183e-08 | 1.528 |
| Bt.2696.1.A1\_at | 5.194e-08 | 2.145 |
| Bt.25997.1.S1\_at | 5.414e-08 | 1.365 |
| Bt.13313.2.S1\_at | 5.501e-08 | 1.484 |
| Bt.1861.1.S1\_at | 5.543e-08 | 1.56 |
| Bt.8679.1.A1\_at | 5.792e-08 | -1.315 |
| Bt.2726.2.S1\_at | 5.973e-08 | 1.782 |
| Bt.28623.1.S1\_at | 5.999e-08 | 1.208 |
| Bt.22369.2.S1\_at | 6.1e-08 | 1.266 |
| Bt.3880.1.S1\_at | 6.106e-08 | 1.917 |
| Bt.21721.1.A1\_at | 6.253e-08 | -1.962 |
| Bt.1844.1.S1\_at | 6.308e-08 | 1.138 |
| Bt.5519.2.S1\_at | 6.431e-08 | 1.269 |
| Bt.23497.1.S1\_at | 6.55e-08 | -3.041 |
| Bt.16815.1.S1\_at | 6.717e-08 | -1.158 |
| Bt.17081.2.S1\_at | 6.737e-08 | 1.536 |
| Bt.12510.3.S1\_at | 6.871e-08 | 1.697 |
| Bt.5676.1.S1\_at | 6.896e-08 | -1.691 |
| Bt.22082.1.S1\_at | 6.911e-08 | -1.764 |
| Bt.3015.1.A1\_at | 6.939e-08 | 1.526 |
| Bt.1808.1.S1\_at | 6.94e-08 | -1.13 |
| Bt.11019.1.S1\_at | 6.964e-08 | -1.399 |
| Bt.5183.1.S1\_at | 7.09e-08 | -1.796 |
| Bt.10722.1.S1\_at | 7.297e-08 | 1.084 |
| Bt.11415.1.A1\_at | 7.328e-08 | 2.322 |
| Bt.4011.1.S1\_at | 7.513e-08 | -1.171 |
| Bt.10111.1.S1\_at | 7.792e-08 | 1.697 |
| Bt.10600.1.S1\_at | 7.943e-08 | 1.249 |
| Bt.869.2.S1\_at | 7.948e-08 | 1.167 |
| Bt.10278.1.S1\_at | 7.98e-08 | -1.347 |
| Bt.13755.1.S1\_at | 8.292e-08 | 0.9555 |
| Bt.20512.2.S1\_at | 8.538e-08 | 1.681 |
| Bt.22122.2.A1\_at | 8.61e-08 | 1.339 |
| Bt.24875.1.S1\_at | 8.762e-08 | -1.193 |
| Bt.9965.1.S1\_at | 8.857e-08 | -1.359 |
| Bt.4704.1.S1\_at | 8.886e-08 | -1.649 |
| Bt.11659.1.S1\_at | 8.925e-08 | -1.231 |
| Bt.12912.1.S2\_at | 9.002e-08 | 1.221 |
| Bt.28651.1.S1\_at | 9.09e-08 | 1.245 |
| Bt.19937.2.S1\_at | 9.521e-08 | 1.061 |
| Bt.23526.1.S1\_at | 9.79e-08 | -0.9858 |
| Bt.3218.1.S1\_at | 9.901e-08 | 1.17 |
| Bt.8606.1.S1\_a\_at | 9.931e-08 | 1.133 |
| Bt.27806.1.S1\_at | 9.98e-08 | -1.246 |
| Bt.64.1.S1\_at | 1.028e-07 | -1.146 |
| Bt.6247.1.S1\_at | 1.031e-07 | 1.949 |
| Bt.4972.1.S1\_at | 1.031e-07 | 1.247 |
| Bt.27039.1.A1\_at | 1.036e-07 | -2.201 |
| Bt.18402.1.S1\_at | 1.045e-07 | -1.272 |
| Bt.20372.1.S1\_at | 1.049e-07 | -1.84 |
| Bt.4475.1.S1\_at | 1.059e-07 | -1.339 |
| Bt.24550.1.S1\_at | 1.06e-07 | -1.286 |
| Bt.27192.1.S1\_at | 1.071e-07 | -1.551 |
| Bt.21737.1.S1\_at | 1.084e-07 | 1.11 |
| Bt.6080.1.S1\_at | 1.089e-07 | -1.104 |
| Bt.5033.1.S1\_at | 1.089e-07 | -1.066 |
| Bt.11262.1.S1\_at | 1.096e-07 | 1.489 |
| Bt.28238.1.A1\_at | 1.103e-07 | 1.633 |
| Bt.5732.1.S1\_at | 1.104e-07 | 1.976 |
| Bt.23418.1.S1\_at | 1.127e-07 | 1.225 |
| Bt.29873.1.S1\_at | 1.133e-07 | 1.724 |
| Bt.27814.1.S1\_at | 1.146e-07 | 1.996 |
| Bt.9629.1.S1\_at | 1.217e-07 | 1.086 |
| Bt.23318.1.S1\_at | 1.221e-07 | 1.217 |
| Bt.1758.1.S1\_at | 1.227e-07 | 1.41 |
| Bt.27091.1.S1\_at | 1.235e-07 | 1.633 |
| Bt.25346.1.A1\_at | 1.249e-07 | 1.078 |
| Bt.3126.1.A1\_at | 1.26e-07 | 1.416 |
| Bt.10543.1.S1\_at | 1.261e-07 | 1.347 |
| Bt.3856.1.S1\_at | 1.263e-07 | -1.452 |
| Bt.14340.2.S1\_at | 1.273e-07 | 1.441 |
| Bt.5089.1.S2\_at | 1.295e-07 | 1.455 |
| Bt.5922.1.S1\_at | 1.336e-07 | -1.833 |
| Bt.12126.1.S1\_at | 1.337e-07 | -1.237 |
| Bt.11449.1.S1\_at | 1.35e-07 | -1.287 |
| Bt.20321.1.S1\_at | 1.372e-07 | 1.128 |
| Bt.1552.1.S1\_at | 1.376e-07 | -1.808 |
| Bt.15735.1.S1\_at | 1.388e-07 | 1.806 |
| Bt.7237.2.S1\_a\_at | 1.415e-07 | -1.369 |
| Bt.19415.1.A1\_at | 1.428e-07 | -2.093 |
| Bt.17081.1.A1\_at | 1.458e-07 | 1.617 |
| Bt.27215.1.S1\_at | 1.508e-07 | -1.432 |
| Bt.11093.1.S1\_at | 1.533e-07 | 1.107 |
| Bt.27421.1.S1\_at | 1.569e-07 | 1.628 |
| Bt.7696.1.S1\_at | 1.575e-07 | -1.186 |
| Bt.11855.1.S1\_at | 1.594e-07 | -1.023 |
| Bt.24039.1.A1\_at | 1.595e-07 | -1.036 |
| Bt.22084.1.A1\_at | 1.612e-07 | 1.346 |
| Bt.848.1.S1\_at | 1.632e-07 | -1.173 |
| Bt.5226.1.S1\_at | 1.677e-07 | 0.9927 |
| Bt.4476.1.S1\_at | 1.693e-07 | -1.404 |
| Bt.22306.1.S1\_at | 1.694e-07 | -1.565 |
| Bt.11409.1.A1\_a\_at | 1.706e-07 | 1.002 |
| Bt.7701.1.S1\_at | 1.721e-07 | -1.134 |
| Bt.20129.2.S1\_at | 1.734e-07 | -0.9792 |
| Bt.20361.2.A1\_at | 1.751e-07 | 1.826 |
| Bt.23178.1.S1\_at | 1.805e-07 | 1.191 |
| Bt.27403.1.S1\_at | 1.809e-07 | 1.365 |
| Bt.11770.1.S1\_at | 1.817e-07 | -1.192 |
| Bt.24849.1.A1\_at | 1.848e-07 | -1.421 |
| Bt.11492.1.A1\_at | 1.869e-07 | 1.534 |
| Bt.29910.1.S1\_at | 1.915e-07 | 1.297 |
| Bt.16092.1.S1\_at | 1.917e-07 | 0.9781 |
| Bt.18634.1.A1\_at | 1.947e-07 | 1.798 |
| Bt.12211.1.S1\_at | 1.982e-07 | -1.287 |
| Bt.467.1.S1\_at | 1.984e-07 | -1.678 |
| Bt.8679.3.S1\_at | 1.996e-07 | -1.681 |
| Bt.24269.1.S1\_at | 1.996e-07 | -1.298 |
| Bt.11196.1.S1\_at | 1.999e-07 | -1.332 |
| Bt.22466.1.A1\_at | 2.04e-07 | 1.071 |
| Bt.23318.1.S2\_at | 2.043e-07 | 1.045 |
| Bt.3272.1.A1\_at | 2.101e-07 | -1.431 |
| Bt.3854.1.S1\_at | 2.105e-07 | 1.467 |
| Bt.20308.1.S1\_at | 2.122e-07 | 2.048 |
| Bt.23182.1.S1\_at | 2.122e-07 | -1.363 |
| Bt.27543.1.A1\_at | 2.13e-07 | 1.59 |
| Bt.12875.1.S1\_at | 2.166e-07 | 1.185 |
| Bt.4875.2.S1\_at | 2.197e-07 | 1.087 |
| Bt.9362.1.S1\_at | 2.233e-07 | -1.098 |
| Bt.12358.1.S1\_at | 2.281e-07 | 1.152 |
| Bt.25832.1.S1\_at | 2.324e-07 | 1.309 |
| Bt.5123.2.S1\_at | 2.328e-07 | -1.008 |
| Bt.1652.1.S1\_at | 2.395e-07 | 1.466 |
| Bt.9324.1.S1\_at | 2.421e-07 | -1.013 |
| Bt.18986.1.S1\_at | 2.438e-07 | 1.121 |
| Bt.9968.1.A1\_at | 2.44e-07 | 1.206 |
| Bt.24802.1.S1\_at | 2.447e-07 | -1.426 |
| Bt.14229.1.S1\_at | 2.46e-07 | -0.9709 |
| Bt.25099.2.S1\_at | 2.469e-07 | -2.341 |
| Bt.28269.1.S1\_at | 2.489e-07 | 1.389 |
| Bt.20783.1.A1\_at | 2.522e-07 | 1.513 |
| Bt.19167.1.A1\_at | 2.527e-07 | 1.406 |
| Bt.27058.1.A1\_at | 2.554e-07 | 1.556 |
| Bt.20236.1.S1\_at | 2.668e-07 | 1.035 |
| Bt.16003.1.S1\_at | 2.694e-07 | -1.307 |
| Bt.22538.2.S1\_at | 2.695e-07 | 1.594 |
| Bt.3227.1.S1\_at | 2.715e-07 | -1.197 |
| Bt.5344.1.S1\_at | 2.813e-07 | -1.284 |
| Bt.4606.1.S1\_at | 2.824e-07 | 1.687 |
| Bt.13439.1.S1\_at | 2.9e-07 | -1.443 |
| Bt.20307.1.S1\_at | 2.939e-07 | -1.178 |
| Bt.7676.3.S1\_at | 2.944e-07 | 1.405 |
| Bt.21471.1.S1\_at | 3.025e-07 | 1.638 |
| Bt.24265.1.S1\_a\_at | 3.1e-07 | -1.001 |
| Bt.16004.1.A1\_at | 3.118e-07 | 4.041 |
| Bt.20146.1.S1\_at | 3.139e-07 | 1.536 |
| Bt.136.1.S1\_at | 3.191e-07 | -2.599 |
| Bt.12290.1.S1\_at | 3.199e-07 | 1.419 |
| Bt.23098.1.S1\_at | 3.216e-07 | 1.03 |
| Bt.15857.1.S1\_at | 3.253e-07 | -1.409 |
| Bt.2988.1.S1\_at | 3.26e-07 | 1.179 |
| Bt.21641.1.S1\_at | 3.319e-07 | 1.444 |
| Bt.11791.1.A1\_at | 3.354e-07 | -1.765 |
| Bt.11059.1.S1\_at | 3.356e-07 | 1.397 |
| Bt.15870.1.S1\_at | 3.375e-07 | -1.159 |
| Bt.26568.1.S1\_a\_at | 3.416e-07 | -1.456 |
| Bt.2121.1.S1\_at | 3.451e-07 | 1.563 |
| Bt.21440.1.S1\_at | 3.461e-07 | 1.16 |
| Bt.2421.1.A1\_at | 3.467e-07 | 1.253 |
| Bt.27412.1.A1\_at | 3.54e-07 | 1.407 |
| Bt.16053.1.S1\_at | 3.576e-07 | 1.646 |
| Bt.23218.1.S1\_at | 3.603e-07 | 1.069 |
| Bt.3940.1.S1\_at | 3.643e-07 | 1.032 |
| Bt.7641.2.S1\_a\_at | 3.656e-07 | -1.847 |
| Bt.27939.2.A1\_a\_at | 3.666e-07 | 1.035 |
| Bt.27908.1.S1\_at | 3.702e-07 | -1.105 |
| Bt.13741.1.A1\_at | 3.798e-07 | 1.238 |
| Bt.7056.3.A1\_x\_at | 3.856e-07 | 2.413 |
| Bt.2129.2.S1\_at | 3.857e-07 | 1.871 |
| Bt.4651.1.S1\_at | 3.861e-07 | 1.464 |
| Bt.19621.1.A1\_at | 3.862e-07 | 1.184 |
| Bt.4355.1.S1\_at | 3.886e-07 | -1.062 |
| Bt.13530.1.S1\_at | 3.929e-07 | -1.207 |
| Bt.1366.1.S1\_at | 3.93e-07 | 1.36 |
| Bt.2501.1.S1\_at | 3.988e-07 | -1.38 |
| Bt.9878.1.A1\_at | 4.013e-07 | 1.273 |
| Bt.1613.1.S1\_at | 4.078e-07 | 1.5 |
| Bt.22283.1.S1\_at | 4.109e-07 | 1.156 |
| Bt.27793.1.A1\_at | 4.193e-07 | 1.72 |
| Bt.1267.1.S1\_at | 4.204e-07 | 1.373 |
| Bt.22054.2.S1\_a\_at | 4.221e-07 | -2.336 |
| Bt.16637.1.A1\_at | 4.228e-07 | -1.183 |
| Bt.5395.1.S1\_a\_at | 4.257e-07 | 1.747 |
| Bt.1280.2.S1\_a\_at | 4.272e-07 | -1.293 |
| Bt.979.1.A1\_at | 4.297e-07 | 1.532 |
| Bt.23396.2.S1\_a\_at | 4.302e-07 | -1.286 |
| Bt.25099.1.A1\_at | 4.479e-07 | -2.973 |
| Bt.26919.1.S1\_at | 4.483e-07 | 1.605 |
| Bt.21166.1.S1\_at | 4.536e-07 | 1.179 |
| Bt.7007.1.S1\_at | 4.536e-07 | -1.02 |
| Bt.9708.2.S1\_at | 4.55e-07 | -1.502 |
| Bt.7459.1.S1\_at | 4.552e-07 | 1.267 |
| Bt.5108.1.S1\_at | 4.559e-07 | -1.174 |
| Bt.13334.1.S1\_at | 4.564e-07 | 1.123 |
| Bt.10458.1.A1\_at | 4.596e-07 | 1.066 |
| Bt.27204.1.S1\_at | 4.618e-07 | -1.817 |
| Bt.3137.1.S1\_at | 4.68e-07 | 1.851 |
| Bt.29381.1.S1\_at | 4.699e-07 | -1.383 |
| Bt.6494.1.S1\_at | 4.716e-07 | -0.9513 |
| Bt.27926.1.S1\_at | 4.736e-07 | -1.502 |
| Bt.4044.1.S1\_at | 4.757e-07 | -1.168 |
| Bt.18048.1.A1\_at | 4.762e-07 | 1.221 |
| Bt.21561.1.S1\_at | 4.776e-07 | 0.9967 |
| Bt.5075.1.S1\_at | 4.793e-07 | 1.277 |
| Bt.10175.2.A1\_a\_at | 4.814e-07 | 0.9914 |
| Bt.5805.1.S1\_at | 4.854e-07 | -1.017 |
| Bt.6576.1.S1\_at | 4.876e-07 | 1.026 |
| Bt.7619.1.S1\_at | 5.023e-07 | -1.665 |
| Bt.8679.2.S1\_at | 5.098e-07 | -1.446 |
| Bt.5153.1.S1\_at | 5.119e-07 | 1.371 |
| Bt.22350.1.A1\_at | 5.134e-07 | 1.427 |
| Bt.5527.2.S1\_a\_at | 5.139e-07 | -1.151 |
| Bt.19526.1.S1\_at | 5.246e-07 | 0.9789 |
| Bt.23396.2.S1\_at | 5.327e-07 | -1.072 |
| Bt.12728.1.A1\_a\_at | 5.332e-07 | 1.719 |
| Bt.23127.1.S1\_at | 5.371e-07 | 1.327 |
| Bt.11704.1.S1\_at | 5.378e-07 | 1.512 |
| Bt.303.1.S1\_at | 5.391e-07 | -1.304 |
| Bt.422.1.S2\_at | 5.421e-07 | 1.18 |
| Bt.2138.1.S1\_at | 5.422e-07 | -1.051 |
| Bt.4262.1.S1\_a\_at | 5.46e-07 | -1.415 |
| Bt.21813.1.S1\_at | 5.517e-07 | 1.633 |
| Bt.28143.1.S1\_at | 5.566e-07 | 1.501 |
| Bt.4698.1.S1\_at | 5.637e-07 | 1.424 |
| Bt.10.2.S1\_a\_at | 5.654e-07 | -0.88 |
| Bt.1332.1.S1\_a\_at | 5.656e-07 | -1.186 |
| Bt.6057.1.S1\_at | 5.79e-07 | 1.064 |
| Bt.10734.1.A1\_at | 5.894e-07 | 1.161 |
| Bt.21584.1.S1\_at | 5.925e-07 | -1.5 |
| Bt.24817.1.S1\_at | 5.935e-07 | -1.282 |
| Bt.28226.3.A1\_s\_at | 5.955e-07 | 1.242 |
| Bt.28071.1.S1\_at | 5.968e-07 | -0.9329 |
| Bt.20282.1.S1\_at | 6.12e-07 | -1.12 |
| Bt.67.1.S1\_at | 6.139e-07 | -1.282 |
| Bt.22843.1.S1\_at | 6.179e-07 | -1.013 |
| Bt.13943.1.A1\_at | 6.192e-07 | 1.083 |
| Bt.9586.1.A1\_at | 6.249e-07 | 1.177 |
| Bt.23254.1.S1\_at | 6.304e-07 | 0.9281 |
| Bt.8504.1.A1\_at | 6.353e-07 | 1.446 |
| Bt.13198.1.S1\_at | 6.43e-07 | 1.212 |
| Bt.14340.1.A1\_at | 6.438e-07 | 1.133 |
| Bt.26829.1.S1\_at | 6.475e-07 | -1.486 |
| Bt.4864.2.S1\_at | 6.507e-07 | 0.866 |
| Bt.10608.2.S1\_a\_at | 6.693e-07 | -0.9837 |
| Bt.20515.1.S1\_at | 6.724e-07 | 1.316 |
| Bt.5965.1.S1\_at | 6.809e-07 | 1.072 |
| Bt.16039.1.S1\_at | 6.817e-07 | -0.963 |
| Bt.17963.1.A1\_at | 6.829e-07 | -1.342 |
| Bt.13313.1.S1\_at | 6.841e-07 | 1.34 |
| Bt.26150.1.A1\_at | 6.844e-07 | -1.491 |
| Bt.23669.1.A1\_at | 6.87e-07 | 1.186 |
| Bt.11055.1.S1\_at | 6.896e-07 | 1.347 |
| Bt.9055.2.S1\_a\_at | 6.91e-07 | -1.208 |
| Bt.23651.1.A1\_at | 6.978e-07 | 0.9292 |
| Bt.28975.1.S1\_at | 7.003e-07 | 1.41 |
| Bt.23113.1.A1\_at | 7.021e-07 | 2.044 |
| Bt.4867.1.A1\_at | 7.086e-07 | 3.27 |
| Bt.18327.1.A1\_at | 7.128e-07 | -1.463 |
| Bt.5605.1.S1\_at | 7.166e-07 | 1.199 |
| Bt.9826.1.S1\_at | 7.256e-07 | -0.8909 |
| Bt.6683.1.S1\_at | 7.394e-07 | -1.541 |
| Bt.27091.2.S1\_at | 7.394e-07 | 1.618 |
| Bt.27089.1.S1\_at | 7.42e-07 | 1.264 |
| Bt.11023.1.S1\_at | 7.594e-07 | 0.8752 |
| Bt.15941.1.S1\_at | 7.625e-07 | -1.271 |
| Bt.9857.1.S1\_at | 7.634e-07 | -1.304 |
| Bt.23603.3.S1\_at | 7.672e-07 | -0.9692 |
| Bt.15666.2.S1\_at | 7.725e-07 | 1.15 |
| Bt.8177.1.S1\_at | 7.745e-07 | -1.17 |
| Bt.23606.1.S1\_at | 8.009e-07 | 1.371 |
| Bt.5132.1.S1\_at | 8.039e-07 | 1.396 |
| Bt.1035.1.S1\_a\_at | 8.208e-07 | -0.9021 |
| Bt.25616.1.S1\_at | 8.211e-07 | 1.093 |
| Bt.4506.1.S1\_at | 8.246e-07 | 1.1 |
| Bt.99.1.S1\_at | 8.252e-07 | -1.27 |
| Bt.8279.3.S1\_a\_at | 8.329e-07 | -0.9662 |
| Bt.4653.1.S2\_at | 8.58e-07 | 1.282 |
| Bt.22510.1.S1\_at | 8.607e-07 | -1.208 |
| Bt.15715.1.S1\_at | 8.773e-07 | 1.165 |
| Bt.888.1.S1\_at | 8.803e-07 | 1.296 |
| Bt.22047.1.S1\_at | 8.811e-07 | -0.9995 |
| Bt.5038.4.A1\_at | 8.933e-07 | 0.8378 |
| Bt.3960.2.S1\_at | 9.157e-07 | -1.066 |
| Bt.8545.1.S1\_at | 9.203e-07 | -1.074 |
| Bt.13833.1.S1\_at | 9.242e-07 | 1.093 |
| Bt.9731.1.S1\_at | 9.259e-07 | -1.616 |
| Bt.3843.1.S1\_at | 9.261e-07 | -1.154 |
| Bt.25282.1.A1\_at | 9.263e-07 | 1.094 |
| Bt.1020.1.S1\_at | 9.296e-07 | 1.318 |
| Bt.68.1.S1\_at | 9.46e-07 | -1.116 |
| Bt.23505.1.S1\_at | 9.661e-07 | -2.207 |
| Bt.10969.1.S1\_at | 9.715e-07 | 1.212 |
| Bt.3139.1.A1\_at | 9.788e-07 | 1.904 |
| Bt.23854.1.A1\_at | 9.847e-07 | 1.638 |
| Bt.8673.1.S1\_a\_at | 9.959e-07 | 1.334 |
| Bt.11180.1.A1\_a\_at | 1.002e-06 | 1.19 |
| Bt.70.1.S1\_at | 1.011e-06 | -0.9036 |
| Bt.13128.1.S1\_at | 1.013e-06 | -0.8721 |
| Bt.3236.1.A1\_at | 1.017e-06 | 1.307 |
| Bt.1286.1.S1\_a\_at | 1.023e-06 | 1.153 |
| Bt.75.1.S1\_at | 1.033e-06 | -0.9493 |
| Bt.3149.1.S1\_at | 1.051e-06 | 1.082 |
| Bt.2717.1.A1\_at | 1.055e-06 | 1.666 |
| Bt.21533.1.S1\_at | 1.087e-06 | 1.315 |
| Bt.3895.1.S1\_at | 1.089e-06 | 1.021 |
| Bt.7869.1.A1\_at | 1.1e-06 | 1.203 |
| Bt.9674.1.S1\_at | 1.102e-06 | 1.101 |
| Bt.321.1.S1\_at | 1.103e-06 | -1.287 |
| Bt.24434.1.A1\_at | 1.106e-06 | 1.034 |
| Bt.13204.1.S1\_at | 1.106e-06 | 1.373 |
| Bt.22630.2.A1\_at | 1.115e-06 | -1.085 |
| Bt.1299.1.S1\_a\_at | 1.12e-06 | 2.084 |
| Bt.11791.2.S1\_at | 1.122e-06 | -1.436 |
| Bt.4320.1.S1\_at | 1.127e-06 | 1.357 |
| Bt.10565.1.A1\_at | 1.143e-06 | 1.03 |
| Bt.10257.1.A1\_at | 1.161e-06 | 1.177 |
| Bt.5527.1.S1\_at | 1.164e-06 | -0.9716 |
| Bt.814.1.S1\_at | 1.167e-06 | -0.7554 |
| Bt.4880.1.S1\_at | 1.167e-06 | -0.9501 |
| Bt.7384.1.A1\_at | 1.17e-06 | 1.032 |
| Bt.8950.1.S1\_at | 1.201e-06 | -0.9776 |
| Bt.3996.1.S1\_at | 1.207e-06 | 1.958 |
| Bt.4031.1.S1\_at | 1.218e-06 | 0.9749 |
| Bt.18420.2.A1\_at | 1.23e-06 | 1.278 |
| Bt.1229.1.S1\_at | 1.232e-06 | 1.283 |
| Bt.11194.1.S1\_at | 1.236e-06 | -1.316 |
| Bt.23174.2.S1\_at | 1.245e-06 | 0.8791 |
| Bt.21622.1.A1\_at | 1.251e-06 | 1.296 |
| Bt.26894.1.S1\_at | 1.268e-06 | -0.9756 |
| Bt.5468.1.S1\_at | 1.271e-06 | -1.922 |
| Bt.12872.1.S1\_at | 1.275e-06 | 1.056 |
| Bt.5888.1.S1\_at | 1.281e-06 | -1.063 |
| Bt.56.1.S1\_at | 1.287e-06 | -1.217 |
| Bt.20046.1.S1\_a\_at | 1.289e-06 | 0.9332 |
| Bt.25087.3.S1\_at | 1.293e-06 | -1.148 |
| Bt.8279.2.S1\_at | 1.297e-06 | -0.9027 |
| Bt.23234.1.S1\_at | 1.304e-06 | -1.132 |
| Bt.20411.1.S1\_at | 1.31e-06 | -1.542 |
| Bt.23734.1.A1\_at | 1.313e-06 | 1.715 |
| Bt.21620.1.A1\_at | 1.335e-06 | 1.265 |
| Bt.13483.1.A1\_at | 1.361e-06 | -1.008 |
| Bt.10303.1.S1\_at | 1.368e-06 | 1 |
| Bt.25085.1.A1\_at | 1.376e-06 | 1.465 |
| Bt.7319.1.S1\_at | 1.392e-06 | 1.839 |
| Bt.24211.2.S1\_at | 1.396e-06 | 2.003 |
| Bt.4510.1.S1\_at | 1.401e-06 | 1.083 |
| Bt.14095.1.S1\_at | 1.41e-06 | 1.076 |
| Bt.5571.1.S1\_at | 1.421e-06 | -1.055 |
| Bt.5210.1.S1\_at | 1.427e-06 | -1.112 |
| Bt.28566.1.S1\_at | 1.437e-06 | 0.9281 |
| Bt.13349.1.S1\_at | 1.44e-06 | -1.011 |
| Bt.18381.1.S1\_at | 1.442e-06 | 1.189 |
| Bt.1582.1.S1\_at | 1.445e-06 | -1.199 |
| Bt.26728.1.S1\_at | 1.447e-06 | 1.16 |
| Bt.76.1.S1\_at | 1.447e-06 | -1.073 |
| Bt.8046.1.A1\_at | 1.449e-06 | -1.461 |
| Bt.20402.1.S1\_at | 1.454e-06 | 1.364 |
| Bt.3821.2.S1\_at | 1.459e-06 | 1.133 |
| Bt.1793.1.A1\_at | 1.479e-06 | -1.538 |
| Bt.20683.1.S1\_at | 1.5e-06 | -1.643 |
| Bt.2625.1.S1\_at | 1.522e-06 | 0.975 |
| Bt.21997.1.S1\_at | 1.525e-06 | 1.272 |
| Bt.28150.1.S1\_at | 1.528e-06 | -1.495 |
| Bt.24310.1.S1\_at | 1.541e-06 | -1.098 |
| Bt.13588.1.A1\_at | 1.546e-06 | -3.451 |
| Bt.4339.1.S1\_at | 1.555e-06 | -0.9391 |
| Bt.5388.1.S1\_at | 1.562e-06 | 0.785 |
| Bt.21456.1.S1\_at | 1.571e-06 | 1.194 |
| Bt.2825.1.S1\_at | 1.573e-06 | -1.105 |
| Bt.21009.1.S1\_at | 1.582e-06 | 1.587 |
| Bt.4057.1.S1\_at | 1.587e-06 | 0.9415 |
| Bt.2359.1.A1\_at | 1.604e-06 | 0.9808 |
| Bt.15267.1.A1\_at | 1.629e-06 | 1.018 |
| Bt.22852.1.S1\_at | 1.631e-06 | 1.229 |
| Bt.17079.1.S1\_a\_at | 1.641e-06 | 1.341 |
| Bt.8322.1.S1\_at | 1.642e-06 | 1.343 |
| Bt.16978.1.S1\_at | 1.645e-06 | -1.159 |
| Bt.25657.1.S1\_at | 1.648e-06 | 1.762 |
| Bt.12423.1.S1\_at | 1.675e-06 | 0.9877 |
| Bt.8674.1.A1\_at | 1.692e-06 | 1.058 |
| Bt.23576.1.A1\_at | 1.692e-06 | 1.265 |
| Bt.5541.1.S1\_at | 1.698e-06 | -0.9258 |
| Bt.4263.1.S1\_at | 1.701e-06 | 1.094 |
| Bt.9708.1.S1\_at | 1.705e-06 | -1.368 |
| Bt.9811.1.S1\_at | 1.706e-06 | -0.9246 |
| Bt.26992.1.A1\_at | 1.712e-06 | 1.216 |
| Bt.7696.2.S1\_a\_at | 1.712e-06 | -1.253 |
| Bt.15840.1.A1\_at | 1.732e-06 | 1.097 |
| Bt.10398.1.S1\_at | 1.744e-06 | 1.239 |
| Bt.12466.1.A1\_at | 1.756e-06 | 0.9986 |
| Bt.4232.2.S1\_at | 1.763e-06 | 1.007 |
| Bt.5447.1.S1\_at | 1.788e-06 | 0.9533 |
| Bt.7636.1.S1\_at | 1.789e-06 | 1.242 |
| Bt.2214.1.S1\_at | 1.802e-06 | 1.168 |
| Bt.1667.1.S1\_at | 1.802e-06 | -1.162 |
| Bt.371.1.S1\_at | 1.815e-06 | -1.042 |
| Bt.23509.1.S1\_at | 1.818e-06 | 0.9564 |
| Bt.22130.1.S1\_at | 1.836e-06 | 1.112 |
| Bt.3216.1.A1\_at | 1.837e-06 | 1.226 |
| Bt.13610.1.A1\_at | 1.84e-06 | -1.309 |
| Bt.11060.1.S1\_at | 1.845e-06 | 1.019 |
| Bt.19100.1.A1\_at | 1.853e-06 | 1.533 |
| Bt.4792.1.S1\_at | 1.853e-06 | 1.301 |
| Bt.1505.1.S1\_at | 1.864e-06 | -0.9294 |
| Bt.903.1.S1\_at | 1.869e-06 | 1.057 |
| Bt.22433.1.A1\_at | 1.87e-06 | -1.041 |
| Bt.11246.1.A1\_at | 1.877e-06 | 0.98 |
| Bt.23081.1.S1\_a\_at | 1.88e-06 | -1.861 |
| Bt.13845.1.S1\_at | 1.88e-06 | -1.07 |
| Bt.20582.2.A1\_at | 1.886e-06 | 1.462 |
| Bt.22405.1.A1\_at | 1.893e-06 | 1.178 |
| Bt.28754.1.S1\_at | 1.894e-06 | -0.9262 |
| Bt.16597.1.S1\_at | 1.915e-06 | -1.048 |
| Bt.9559.2.S1\_at | 1.953e-06 | 1.674 |
| Bt.5466.4.S1\_at | 1.954e-06 | 0.8927 |
| Bt.21383.2.S1\_a\_at | 1.959e-06 | 1.061 |
| Bt.9797.1.S1\_at | 1.961e-06 | -1.127 |
| Bt.22763.2.S1\_a\_at | 1.964e-06 | -1.107 |
| Bt.19928.1.S1\_at | 1.966e-06 | 1.033 |
| Bt.4571.1.S1\_at | 1.98e-06 | 1.167 |
| Bt.22459.1.S1\_at | 1.989e-06 | 1.045 |
| Bt.1408.1.S1\_at | 1.99e-06 | 1.289 |
| Bt.21366.2.S1\_at | 1.996e-06 | -1.219 |
| Bt.4744.1.S1\_at | 1.999e-06 | -1.458 |
| Bt.21603.1.S1\_at | 2.024e-06 | -1.052 |
| Bt.23392.1.S1\_at | 2.024e-06 | 1.038 |
| Bt.405.1.S1\_at | 2.035e-06 | 1.44 |
| Bt.22040.1.S1\_at | 2.036e-06 | 0.9823 |
| Bt.26423.1.A1\_at | 2.046e-06 | 1.262 |
| Bt.21383.1.S1\_at | 2.053e-06 | 1.323 |
| Bt.5176.2.A1\_at | 2.067e-06 | 1.105 |
| Bt.26130.1.A1\_at | 2.08e-06 | -1.283 |
| Bt.9047.1.S1\_at | 2.088e-06 | -1.179 |
| Bt.20937.1.A1\_at | 2.092e-06 | 1.988 |
| Bt.1674.2.S1\_at | 2.1e-06 | -0.9725 |
| Bt.15742.1.S2\_at | 2.12e-06 | 1.056 |
| Bt.27930.1.S1\_at | 2.132e-06 | 0.896 |
| Bt.1568.1.S1\_at | 2.134e-06 | -0.8465 |
| Bt.22142.2.S1\_a\_at | 2.134e-06 | -1.514 |
| Bt.7870.1.S1\_at | 2.135e-06 | -1.558 |
| Bt.9825.1.S1\_at | 2.15e-06 | -1.229 |
| Bt.24624.1.S1\_at | 2.166e-06 | -0.9826 |
| Bt.1464.1.A1\_at | 2.2e-06 | 1.1 |
| Bt.6483.1.A1\_at | 2.202e-06 | 0.9251 |
| Bt.19676.2.S1\_a\_at | 2.238e-06 | -1.14 |
| Bt.28193.1.S1\_at | 2.241e-06 | 1.537 |
| Bt.15907.2.S1\_at | 2.251e-06 | -1.474 |
| Bt.8351.1.S1\_at | 2.282e-06 | -0.8598 |
| Bt.10965.1.S1\_at | 2.292e-06 | -0.9342 |
| Bt.3967.1.S1\_a\_at | 2.307e-06 | -1.124 |
| Bt.2230.1.S2\_at | 2.331e-06 | 1.104 |
| Bt.21176.1.S1\_at | 2.339e-06 | 1.003 |
| Bt.7478.1.S1\_at | 2.339e-06 | -1.596 |
| Bt.21630.1.S1\_at | 2.345e-06 | -1.593 |
| Bt.2726.1.S1\_at | 2.376e-06 | 1.459 |
| Bt.20613.2.A1\_at | 2.377e-06 | 1.18 |
| Bt.9983.1.S1\_at | 2.406e-06 | -0.8583 |
| Bt.13427.1.S1\_at | 2.423e-06 | 0.9867 |
| Bt.265.1.S1\_at | 2.501e-06 | -0.9616 |
| Bt.8001.1.S1\_at | 2.52e-06 | -0.9434 |
| Bt.7633.1.S1\_at | 2.523e-06 | -1.209 |
| Bt.23232.1.S1\_at | 2.528e-06 | -1.52 |
| Bt.22651.1.A1\_at | 2.539e-06 | 0.983 |
| Bt.25202.1.A1\_at | 2.567e-06 | 1.679 |
| Bt.6842.1.S1\_at | 2.571e-06 | 1.258 |
| Bt.10942.1.S1\_at | 2.572e-06 | 0.8456 |
| Bt.18752.1.S1\_at | 2.599e-06 | -1.52 |
| Bt.23330.1.S1\_at | 2.61e-06 | 1.1 |
| Bt.26604.1.S1\_at | 2.628e-06 | 1.276 |
| Bt.21718.1.S1\_at | 2.631e-06 | -1.267 |
| Bt.24211.1.A1\_at | 2.632e-06 | 1.675 |
| Bt.6549.1.S1\_at | 2.674e-06 | 1.579 |
| Bt.4271.1.S1\_at | 2.676e-06 | 0.9524 |
| Bt.6964.1.S1\_at | 2.692e-06 | -0.9459 |
| Bt.1517.1.S1\_at | 2.694e-06 | -0.804 |
| Bt.26462.1.S1\_at | 2.718e-06 | 1.175 |
| Bt.29931.2.S1\_at | 2.726e-06 | -1.123 |
| Bt.9612.1.S1\_at | 2.742e-06 | -1.255 |
| Bt.26568.2.S1\_a\_at | 2.744e-06 | -0.8167 |
| Bt.5042.1.S1\_at | 2.762e-06 | -0.9643 |
| Bt.5322.1.S1\_at | 2.768e-06 | -0.8905 |
| Bt.11193.1.S1\_at | 2.773e-06 | -1.468 |
| Bt.19826.1.A1\_at | 2.78e-06 | -1.358 |
| Bt.4985.1.S1\_at | 2.782e-06 | -0.8936 |
| Bt.20331.1.A1\_at | 2.789e-06 | 1.292 |
| Bt.20485.1.S1\_at | 2.793e-06 | 0.9026 |
| Bt.13730.2.S1\_at | 2.8e-06 | -1.254 |
| Bt.26415.1.A1\_at | 2.804e-06 | 0.7925 |
| Bt.24096.1.A1\_at | 2.831e-06 | 1.237 |
| Bt.20402.2.A1\_at | 2.834e-06 | 1.172 |
| Bt.20984.1.S1\_at | 2.842e-06 | -1.284 |
| Bt.19957.1.A1\_at | 2.856e-06 | 1.165 |
| Bt.28431.1.S1\_at | 2.869e-06 | 1.027 |
| Bt.7183.1.S1\_at | 2.872e-06 | 0.9522 |
| Bt.4868.2.S1\_a\_at | 2.872e-06 | -0.8214 |
| Bt.8650.1.A1\_at | 2.904e-06 | 0.8538 |
| Bt.3989.1.S1\_at | 2.936e-06 | 1.32 |
| Bt.28480.1.S1\_at | 2.939e-06 | -0.9431 |
| Bt.12468.1.S1\_at | 2.94e-06 | -1.225 |
| Bt.4009.3.S1\_at | 2.964e-06 | 0.7849 |
| Bt.404.1.S1\_at | 2.969e-06 | -0.8887 |
| Bt.27937.1.A1\_at | 2.97e-06 | 1.317 |
| Bt.1294.1.S1\_at | 3.002e-06 | -1.454 |
| Bt.23246.1.S1\_at | 3.023e-06 | 1.152 |
| Bt.17837.1.A1\_at | 3.107e-06 | 0.8214 |
| Bt.6449.1.S1\_at | 3.11e-06 | 1.197 |
| Bt.2905.1.S1\_at | 3.117e-06 | 1.004 |
| Bt.26878.1.S1\_at | 3.136e-06 | -1.387 |
| Bt.12364.1.S1\_at | 3.141e-06 | -1.327 |
| Bt.24766.1.S1\_at | 3.151e-06 | -1.531 |
| Bt.21792.2.S1\_a\_at | 3.164e-06 | -0.9849 |
| Bt.7415.1.S1\_at | 3.19e-06 | 0.9147 |
| Bt.26975.1.A1\_a\_at | 3.191e-06 | 1.013 |
| Bt.13170.1.S1\_at | 3.198e-06 | -1.062 |
| Bt.3945.1.S1\_at | 3.206e-06 | 1.214 |
| Bt.27846.1.S1\_at | 3.206e-06 | 1.368 |
| Bt.8543.1.S1\_at | 3.22e-06 | 1.044 |
| Bt.10999.2.S1\_at | 3.222e-06 | 0.9469 |
| Bt.18019.1.A1\_at | 3.228e-06 | 1.171 |
| Bt.4311.1.S1\_at | 3.243e-06 | 0.7852 |
| Bt.10354.1.S1\_at | 3.251e-06 | 0.9425 |
| Bt.24236.1.S1\_at | 3.257e-06 | 1.207 |
| Bt.8062.1.S1\_at | 3.285e-06 | -1.013 |
| Bt.21790.1.S1\_at | 3.289e-06 | 1.037 |
| Bt.18332.1.S1\_at | 3.349e-06 | 1.155 |
| Bt.27196.1.A1\_at | 3.385e-06 | -1.715 |
| Bt.10144.1.A1\_s\_at | 3.399e-06 | 0.9053 |
| Bt.3633.1.A1\_at | 3.4e-06 | 0.8424 |
| Bt.27116.2.S1\_at | 3.434e-06 | -0.8173 |
| Bt.9936.1.S1\_at | 3.452e-06 | 0.8736 |
| Bt.3015.2.S1\_at | 3.457e-06 | 1.264 |
| Bt.12843.1.S1\_at | 3.459e-06 | 0.8577 |
| Bt.9434.1.S1\_at | 3.46e-06 | -1.068 |
| Bt.4985.1.S1\_a\_at | 3.466e-06 | -0.7661 |
| Bt.13289.1.S1\_at | 3.473e-06 | 2.021 |
| Bt.997.2.S1\_a\_at | 3.495e-06 | -1.193 |
| Bt.3236.2.S1\_at | 3.5e-06 | 1.078 |
| Bt.2729.1.S1\_at | 3.506e-06 | 0.8557 |
| Bt.2989.2.S1\_at | 3.534e-06 | -1.161 |
| Bt.22369.1.S1\_at | 3.587e-06 | 0.9486 |
| Bt.26550.1.S1\_at | 3.591e-06 | -1.046 |
| Bt.12703.2.S1\_at | 3.602e-06 | -0.8702 |
| Bt.19330.1.A1\_s\_at | 3.614e-06 | 1.198 |
| Bt.20257.1.S1\_at | 3.672e-06 | 0.8646 |
| Bt.5605.2.S1\_at | 3.677e-06 | 1.128 |
| Bt.59.1.S1\_at | 3.695e-06 | -0.8247 |
| Bt.4584.1.S1\_at | 3.699e-06 | -0.8911 |
| Bt.20247.1.S1\_at | 3.717e-06 | 1.107 |
| Bt.23509.2.A1\_at | 3.718e-06 | 0.9535 |
| Bt.26095.1.A1\_at | 3.732e-06 | 1.295 |
| Bt.27901.1.S1\_at | 3.744e-06 | 1.267 |
| Bt.14572.1.A1\_at | 3.791e-06 | 1.298 |
| Bt.7397.1.S1\_at | 3.818e-06 | 1.069 |
| Bt.5406.1.S1\_at | 3.823e-06 | 1.037 |
| Bt.1699.3.S1\_a\_at | 3.858e-06 | 1.202 |
| Bt.22094.1.A1\_at | 3.863e-06 | 1.065 |
| Bt.4898.1.S1\_at | 3.877e-06 | 1.532 |
| Bt.26502.1.A1\_at | 3.88e-06 | 1.012 |
| Bt.27413.1.A1\_at | 3.929e-06 | 1.506 |
| Bt.22555.1.S1\_at | 3.94e-06 | -1.143 |
| Bt.9949.3.S1\_a\_at | 3.974e-06 | -1.154 |
| Bt.3753.1.S1\_at | 4.025e-06 | 1.122 |
| Bt.22310.1.S1\_at | 4.03e-06 | -0.9578 |
| Bt.23308.1.S1\_at | 4.034e-06 | 0.9739 |
| Bt.13748.1.S1\_at | 4.078e-06 | 1.067 |
| Bt.22630.1.S1\_at | 4.082e-06 | -1.156 |
| Bt.21578.1.S1\_at | 4.088e-06 | -0.9253 |
| Bt.28133.1.S1\_at | 4.117e-06 | 1.131 |
| Bt.5123.3.S1\_a\_at | 4.136e-06 | -0.9921 |
| Bt.25038.1.S1\_at | 4.153e-06 | 0.944 |
| Bt.4954.1.S1\_at | 4.161e-06 | 1.138 |
| Bt.26750.2.A1\_at | 4.172e-06 | -1.577 |
| Bt.21177.1.A1\_s\_at | 4.188e-06 | 1.471 |
| Bt.2524.1.S1\_at | 4.214e-06 | 0.9359 |
| Bt.15868.1.S1\_at | 4.24e-06 | 1.046 |
| Bt.9486.1.S1\_at | 4.241e-06 | 1.037 |
| Bt.8122.1.S1\_at | 4.283e-06 | 0.7068 |
| Bt.12966.1.S1\_at | 4.311e-06 | -1.248 |
| Bt.23667.1.S1\_at | 4.316e-06 | 1.449 |
| Bt.7500.1.S1\_at | 4.324e-06 | 1.058 |
| Bt.4798.1.S2\_at | 4.329e-06 | -1.429 |
| Bt.13865.1.A1\_at | 4.359e-06 | -1.134 |
| Bt.6490.2.S1\_at | 4.367e-06 | 1.293 |
| Bt.18461.1.A1\_at | 4.368e-06 | 0.8962 |
| Bt.24487.1.S1\_at | 4.376e-06 | -0.9715 |
| Bt.13588.2.S1\_at | 4.408e-06 | -2.671 |
| Bt.1418.2.S1\_at | 4.415e-06 | -0.9773 |
| Bt.5388.1.S1\_s\_at | 4.415e-06 | 0.7091 |
| Bt.3541.1.S1\_at | 4.441e-06 | 0.9054 |
| Bt.2001.1.S1\_at | 4.476e-06 | -0.8237 |
| Bt.28162.1.S1\_at | 4.514e-06 | -1.369 |
| Bt.28559.1.S1\_a\_at | 4.515e-06 | -1.084 |
| Bt.18023.1.S1\_at | 4.519e-06 | 0.912 |
| Bt.11476.1.S1\_at | 4.542e-06 | 0.9011 |
| Bt.12337.1.S1\_a\_at | 4.557e-06 | -0.7143 |
| Bt.16259.1.A1\_at | 4.562e-06 | 0.7814 |
| Bt.5682.1.S1\_at | 4.573e-06 | 1.048 |
| Bt.4957.1.S2\_at | 4.607e-06 | 0.9059 |
| Bt.2070.1.A1\_at | 4.627e-06 | -1.131 |
| Bt.29050.1.S1\_at | 4.63e-06 | -1.097 |
| Bt.5135.1.S1\_at | 4.639e-06 | -1.061 |
| Bt.12721.1.A1\_at | 4.677e-06 | 0.8844 |
| Bt.27008.1.A1\_at | 4.681e-06 | 1.207 |
| Bt.1165.1.S1\_at | 4.717e-06 | 0.9387 |
| Bt.16078.1.A1\_s\_at | 4.816e-06 | 0.9745 |
| Bt.9993.1.S1\_at | 4.828e-06 | -0.9421 |
| Bt.22634.3.S1\_at | 4.829e-06 | 0.8715 |
| Bt.20984.1.S1\_a\_at | 4.87e-06 | -0.9685 |
| Bt.22735.1.S1\_at | 4.871e-06 | 1.593 |
| Bt.9828.1.S1\_at | 4.92e-06 | -1.152 |
| Bt.29472.1.A1\_at | 5.001e-06 | 1.711 |
| Bt.28676.1.A1\_at | 5.016e-06 | -0.8117 |
| Bt.278.1.S1\_at | 5.023e-06 | -1.066 |
| Bt.19352.1.A1\_at | 5.03e-06 | -1.557 |
| Bt.9176.1.A1\_at | 5.088e-06 | 0.9516 |
| Bt.2824.1.S1\_at | 5.114e-06 | -0.8999 |
| Bt.5293.2.A1\_at | 5.139e-06 | 1.012 |
| Bt.11322.1.A1\_at | 5.141e-06 | -0.8243 |
| Bt.997.1.S1\_at | 5.144e-06 | -0.9289 |
| Bt.401.2.S1\_at | 5.232e-06 | -1.009 |
| Bt.7481.1.S1\_at | 5.258e-06 | -1.394 |
| Bt.216.1.S1\_at | 5.258e-06 | 1.306 |
| Bt.1311.1.S1\_at | 5.289e-06 | -1.747 |
| Bt.19423.1.S1\_at | 5.291e-06 | 1.236 |
| Bt.2871.2.A1\_at | 5.299e-06 | 0.7674 |
| Bt.21372.3.A1\_at | 5.3e-06 | 1.205 |
| Bt.11869.1.S1\_at | 5.318e-06 | -0.8987 |
| Bt.4503.1.S1\_at | 5.341e-06 | -1.091 |
| Bt.1262.1.S1\_at | 5.437e-06 | -0.8838 |
| Bt.23216.1.S1\_at | 5.454e-06 | 1.193 |
| Bt.27027.1.S1\_a\_at | 5.466e-06 | -1.054 |
| Bt.11149.1.S1\_at | 5.477e-06 | 1.062 |
| Bt.22759.1.S1\_at | 5.479e-06 | 0.8529 |
| Bt.24892.1.A1\_at | 5.518e-06 | 1.503 |
| Bt.2587.1.S1\_at | 5.518e-06 | -0.9964 |
| Bt.665.1.S1\_s\_at | 5.544e-06 | -0.7758 |
| Bt.25185.1.S1\_at | 5.553e-06 | 1.376 |
| Bt.10892.1.S1\_at | 5.555e-06 | -0.8844 |
| Bt.9223.1.A1\_at | 5.557e-06 | 0.9656 |
| Bt.5164.1.S1\_at | 5.561e-06 | -1.807 |
| Bt.13765.1.S1\_at | 5.61e-06 | -1.347 |
| Bt.12916.1.S1\_at | 5.614e-06 | -1.858 |
| Bt.4900.1.A1\_at | 5.645e-06 | -0.8531 |
| Bt.13240.1.S1\_at | 5.645e-06 | 1.154 |
| Bt.21491.1.S1\_at | 5.693e-06 | 0.8922 |
| Bt.23941.1.A1\_at | 5.694e-06 | 0.8845 |
| Bt.11264.1.S1\_at | 5.737e-06 | 0.8735 |
| Bt.27207.1.A1\_at | 5.738e-06 | 1.044 |
| Bt.3215.1.S1\_at | 5.786e-06 | 0.8495 |
| Bt.10341.1.S1\_at | 5.821e-06 | 0.8339 |
| Bt.4653.1.S1\_at | 5.866e-06 | 1.107 |
| Bt.11711.1.A1\_at | 5.877e-06 | 2.133 |
| Bt.29009.1.A1\_at | 5.878e-06 | 2.07 |
| Bt.12910.1.S1\_at | 5.923e-06 | -0.774 |
| Bt.9154.1.S1\_at | 5.929e-06 | 1.088 |
| Bt.13730.1.A1\_at | 5.93e-06 | -1.073 |
| Bt.10096.1.S1\_at | 5.944e-06 | 0.9349 |
| Bt.10920.1.S1\_at | 5.956e-06 | 1.264 |
| Bt.4733.1.S1\_at | 5.996e-06 | -1.765 |
| Bt.5748.2.S1\_at | 6.037e-06 | 1.056 |
| Bt.2439.1.S1\_at | 6.045e-06 | 0.8087 |
| Bt.10990.1.S1\_at | 6.078e-06 | -1.021 |
| Bt.4886.1.A1\_at | 6.126e-06 | 2.173 |
| Bt.5341.1.S1\_at | 6.145e-06 | 1.126 |
| Bt.17079.2.A1\_at | 6.159e-06 | 0.8687 |
| Bt.27401.1.A1\_at | 6.21e-06 | -0.7757 |
| Bt.23522.1.S1\_at | 6.219e-06 | 0.7767 |
| Bt.3487.1.S1\_at | 6.242e-06 | -1.082 |
| Bt.17332.2.S1\_at | 6.253e-06 | 1.046 |
| Bt.13637.1.A1\_at | 6.258e-06 | 0.826 |
| Bt.11140.1.S1\_at | 6.261e-06 | 1.147 |
| Bt.347.1.S1\_at | 6.278e-06 | -1.149 |
| Bt.15714.2.S1\_a\_at | 6.304e-06 | 1.032 |
| Bt.1185.2.S1\_at | 6.319e-06 | -0.7105 |
| Bt.2587.2.S1\_a\_at | 6.357e-06 | -1.098 |
| Bt.11436.1.A1\_at | 6.385e-06 | 0.937 |
| Bt.2667.1.S1\_a\_at | 6.394e-06 | -1.116 |
| Bt.10999.1.A1\_at | 6.486e-06 | 1.021 |
| Bt.6989.1.S1\_at | 6.601e-06 | 0.9028 |
| Bt.16525.1.A1\_at | 6.605e-06 | 0.7803 |
| Bt.6999.1.S1\_at | 6.657e-06 | 0.6745 |
| Bt.3428.1.A1\_s\_at | 6.712e-06 | 1.139 |
| Bt.7915.1.S1\_at | 6.733e-06 | -0.8172 |
| Bt.13860.1.S1\_at | 6.738e-06 | 1.086 |
| Bt.22633.1.S1\_at | 6.866e-06 | 0.9247 |
| Bt.5277.2.S1\_a\_at | 6.899e-06 | -0.8464 |
| Bt.7772.1.S1\_at | 6.92e-06 | 1.201 |
| Bt.2599.1.S1\_at | 6.923e-06 | 1.083 |
| Bt.20278.1.S1\_at | 6.942e-06 | 0.7801 |
| Bt.9244.1.A1\_at | 6.953e-06 | 0.9448 |
| Bt.8802.1.S1\_at | 6.953e-06 | 0.7475 |
| Bt.7655.1.S1\_at | 6.963e-06 | -0.916 |
| Bt.5288.1.S1\_at | 7.061e-06 | -0.8995 |
| Bt.21468.1.S1\_at | 7.069e-06 | -1.17 |
| Bt.13330.1.S1\_at | 7.08e-06 | -2.425 |
| Bt.24713.1.S1\_at | 7.139e-06 | 0.9333 |
| Bt.19937.1.S1\_at | 7.141e-06 | 0.9249 |
| Bt.10657.1.A1\_at | 7.169e-06 | -1.048 |
| Bt.23499.1.S1\_at | 7.184e-06 | -1.015 |
| Bt.4565.1.S1\_at | 7.237e-06 | 1.048 |
| Bt.7619.2.S1\_at | 7.238e-06 | -1.056 |
| Bt.23603.2.S1\_a\_at | 7.248e-06 | -0.7994 |
| Bt.4819.1.S1\_at | 7.265e-06 | -0.7252 |
| Bt.97.1.S1\_at | 7.318e-06 | -2.815 |
| Bt.3092.1.S1\_at | 7.345e-06 | 1.12 |
| Bt.4732.1.S1\_at | 7.354e-06 | -1.298 |
| Bt.1734.1.S1\_at | 7.365e-06 | 1.052 |
| Bt.10683.1.S1\_at | 7.37e-06 | 0.8207 |
| Bt.23236.1.A1\_at | 7.444e-06 | 0.9132 |
| Bt.25202.3.S1\_at | 7.446e-06 | 1.427 |
| Bt.24616.1.S1\_at | 7.507e-06 | 1.355 |
| Bt.14219.1.S1\_at | 7.509e-06 | 1.125 |
| Bt.2815.1.S1\_at | 7.521e-06 | 0.9978 |
| Bt.9868.2.S1\_a\_at | 7.524e-06 | 0.744 |
| Bt.12039.1.S1\_at | 7.531e-06 | 0.9468 |
| Bt.7728.1.A1\_at | 7.549e-06 | 1.086 |
| Bt.22200.1.S1\_at | 7.554e-06 | 1.034 |
| Bt.23081.1.S1\_at | 7.574e-06 | -1.504 |
| Bt.15435.1.A1\_at | 7.601e-06 | 1.185 |
| Bt.20405.1.S1\_at | 7.604e-06 | 1.329 |
| Bt.25098.1.S1\_at | 7.626e-06 | -1.166 |
| Bt.22197.1.S1\_at | 7.674e-06 | -1.017 |
| Bt.13780.1.A1\_at | 7.7e-06 | 1.515 |
| Bt.21071.2.A1\_at | 7.761e-06 | 1.078 |
| Bt.4390.1.S1\_at | 7.804e-06 | 0.9319 |
| Bt.21019.1.S1\_at | 7.894e-06 | 0.8552 |
| Bt.18835.1.S1\_at | 7.923e-06 | -1.022 |
| Bt.16194.1.A1\_at | 7.954e-06 | 1.395 |
| Bt.29900.1.S1\_at | 8.11e-06 | 1.252 |
| Bt.18945.1.A1\_at | 8.122e-06 | 0.8973 |
| Bt.26764.1.A1\_at | 8.13e-06 | 1.03 |
| Bt.4658.1.S1\_a\_at | 8.146e-06 | -0.6644 |
| Bt.23648.1.A1\_at | 8.151e-06 | 0.9414 |
| Bt.5278.1.A1\_at | 8.211e-06 | -0.9522 |
| Bt.16471.1.S1\_at | 8.258e-06 | 1.228 |
| Bt.20361.1.S1\_at | 8.263e-06 | 0.9166 |
| Bt.24149.1.A1\_at | 8.282e-06 | -0.8635 |
| Bt.13505.1.S1\_at | 8.291e-06 | -1.184 |
| Bt.876.1.S1\_at | 8.316e-06 | -1.537 |
| Bt.27252.1.A1\_at | 8.317e-06 | -1.199 |
| Bt.11038.1.S1\_at | 8.352e-06 | 1.238 |
| Bt.400.1.S1\_at | 8.36e-06 | -0.781 |
| Bt.12204.1.S1\_at | 8.418e-06 | 0.9722 |
| Bt.24676.2.A1\_at | 8.44e-06 | -0.8811 |
| Bt.27248.1.A1\_at | 8.486e-06 | 0.8958 |
| Bt.28058.1.S1\_at | 8.503e-06 | 0.8938 |
| Bt.20362.2.S1\_at | 8.531e-06 | 1.261 |
| Bt.26080.2.S1\_at | 8.532e-06 | 1.033 |
| Bt.26805.1.S1\_at | 8.559e-06 | -1.464 |
| Bt.20064.1.S1\_at | 8.564e-06 | 0.9134 |
| Bt.21818.1.S1\_at | 8.572e-06 | 1.056 |
| Bt.27717.1.A1\_at | 8.581e-06 | 1.159 |
| Bt.26550.2.S1\_at | 8.616e-06 | -1.2 |
| Bt.26628.1.S1\_at | 8.65e-06 | 1.086 |
| Bt.7342.1.S1\_at | 8.654e-06 | 0.9818 |
| Bt.23486.1.A1\_at | 8.661e-06 | 1.004 |
| Bt.10813.1.S1\_at | 8.75e-06 | 1.265 |
| Bt.876.2.S1\_at | 8.75e-06 | -1.24 |
| Bt.24824.1.S1\_at | 8.793e-06 | 1.134 |
| Bt.21277.1.A1\_at | 8.794e-06 | 0.7377 |
| Bt.6693.1.A1\_at | 8.799e-06 | -1.177 |
| Bt.14176.2.S1\_at | 9.027e-06 | 0.92 |
| Bt.1185.1.A1\_at | 9.069e-06 | -0.7833 |
| Bt.10930.1.S1\_at | 9.162e-06 | 0.8978 |
| Bt.15643.1.A1\_at | 9.162e-06 | 0.9531 |
| Bt.7033.2.S1\_a\_at | 9.178e-06 | -1.994 |
| Bt.12745.1.A1\_at | 9.186e-06 | 0.8495 |
| Bt.16137.1.S1\_at | 9.214e-06 | 1.054 |
| Bt.14176.1.S1\_at | 9.226e-06 | 1.103 |
| Bt.4964.1.S1\_at | 9.228e-06 | 0.9789 |
| Bt.4194.1.S1\_at | 9.251e-06 | 0.7304 |
| Bt.28101.1.S1\_at | 9.252e-06 | 0.8487 |
| Bt.5029.1.S1\_at | 9.255e-06 | -0.8384 |
| Bt.12096.1.S1\_at | 9.271e-06 | -1.069 |
| Bt.21453.1.S1\_at | 9.278e-06 | -1.147 |
| Bt.19510.1.S1\_at | 9.317e-06 | 1.262 |
| Bt.16499.1.A1\_at | 9.345e-06 | 1.109 |
| Bt.11448.1.S1\_at | 9.374e-06 | 0.7607 |
| Bt.27696.1.A1\_at | 9.383e-06 | 1.153 |
| Bt.22166.1.S1\_at | 9.42e-06 | -1.157 |
| Bt.16985.1.S1\_at | 9.434e-06 | 1.042 |
| Bt.19581.1.A1\_at | 9.444e-06 | -0.9503 |
| Bt.13229.1.S1\_at | 9.513e-06 | 1.265 |
| Bt.20326.1.S1\_at | 9.623e-06 | 0.9742 |
| Bt.4748.1.S1\_at | 9.629e-06 | -0.9974 |
| Bt.3101.1.A1\_at | 9.635e-06 | -1.527 |
| Bt.6675.1.A1\_at | 9.638e-06 | -1.04 |
| Bt.2973.2.S1\_a\_at | 9.701e-06 | -0.8653 |
| Bt.18079.1.A1\_at | 9.744e-06 | -1.036 |
| Bt.12366.1.S1\_at | 9.749e-06 | 0.7742 |
| Bt.9059.1.S1\_at | 9.817e-06 | 0.7734 |
| Bt.11035.1.S1\_at | 9.82e-06 | 0.7593 |
| Bt.5055.1.S1\_at | 9.821e-06 | 0.9007 |
| Bt.10880.1.S1\_at | 9.849e-06 | -0.9993 |
| Bt.15724.1.S1\_at | 9.92e-06 | -0.9095 |
| Bt.29655.1.S1\_a\_at | 9.922e-06 | -0.8294 |
| Bt.21182.1.S1\_at | 9.953e-06 | -0.716 |
| Bt.21969.1.A1\_at | 1.001e-05 | 0.8665 |
| Bt.11446.1.A1\_at | 1.001e-05 | 1.019 |
| Bt.841.2.A1\_at | 1.001e-05 | 1.114 |
| Bt.9078.2.S1\_a\_at | 1.01e-05 | -0.9818 |
| Bt.16122.1.S1\_at | 1.013e-05 | -1.047 |
| Bt.668.1.S1\_at | 1.013e-05 | 0.7571 |
| Bt.6313.1.S1\_at | 1.013e-05 | 0.7862 |
| Bt.26928.1.S1\_at | 1.021e-05 | 0.9848 |
| Bt.24474.1.S1\_at | 1.023e-05 | -0.7852 |
| Bt.28704.1.S1\_at | 1.027e-05 | 2.171 |
| Bt.12669.1.A1\_at | 1.029e-05 | 0.9052 |
| Bt.24873.1.S1\_at | 1.036e-05 | 1.013 |
| Bt.24662.1.S1\_at | 1.042e-05 | -1.014 |
| Bt.2696.2.S1\_at | 1.044e-05 | 1.207 |
| Bt.22552.1.S2\_at | 1.045e-05 | 0.7458 |
| Bt.5379.1.S1\_at | 1.049e-05 | 1.144 |
| Bt.13205.1.A1\_at | 1.054e-05 | -0.9194 |
| Bt.28162.3.S1\_at | 1.058e-05 | -1.525 |
| Bt.22092.1.S1\_at | 1.064e-05 | -1.361 |
| Bt.9998.1.S1\_at | 1.066e-05 | 1.306 |
| Bt.22150.1.A1\_at | 1.067e-05 | 0.8498 |
| Bt.20226.1.A1\_at | 1.068e-05 | 1.436 |
| Bt.23380.2.S1\_at | 1.071e-05 | 0.899 |
| Bt.24676.1.S1\_at | 1.074e-05 | -1.072 |
| Bt.23649.1.A1\_at | 1.075e-05 | 0.8949 |
| Bt.23131.1.S1\_s\_at | 1.084e-05 | 1.118 |
| Bt.378.1.S1\_at | 1.084e-05 | -0.7855 |
| Bt.6577.1.S1\_at | 1.087e-05 | -0.9079 |
| Bt.11013.1.S1\_at | 1.088e-05 | 0.8833 |
| Bt.15698.1.S1\_at | 1.095e-05 | -1.093 |
| Bt.24552.1.S1\_at | 1.099e-05 | 0.7642 |
| Bt.7836.1.S1\_at | 1.1e-05 | -0.8669 |
| Bt.2870.1.A1\_at | 1.101e-05 | 1.151 |
| Bt.23335.1.S1\_at | 1.111e-05 | 1.303 |
| Bt.13063.1.S1\_at | 1.113e-05 | -0.9692 |
| Bt.17672.1.S1\_at | 1.116e-05 | 0.7718 |
| Bt.22181.1.A1\_at | 1.119e-05 | -1.089 |
| Bt.14366.1.S1\_at | 1.122e-05 | 1.011 |
| Bt.6983.1.S1\_at | 1.123e-05 | 1.558 |
| Bt.20355.1.S1\_at | 1.127e-05 | -0.8578 |
| Bt.9930.1.S1\_at | 1.129e-05 | 0.9475 |
| Bt.1760.1.S1\_at | 1.138e-05 | -0.8195 |
| Bt.2110.1.S1\_at | 1.138e-05 | -1.301 |
| Bt.18057.1.A1\_at | 1.139e-05 | 1.068 |
| Bt.20977.1.S1\_at | 1.141e-05 | 1.02 |
| Bt.17107.1.S1\_at | 1.142e-05 | -0.9631 |
| Bt.27118.1.A1\_at | 1.143e-05 | 1.094 |
| Bt.27927.1.S1\_at | 1.146e-05 | 0.8758 |
| Bt.21877.1.S1\_at | 1.151e-05 | -0.9755 |
| Bt.4872.1.S1\_at | 1.161e-05 | 0.9265 |
| Bt.28618.1.S1\_at | 1.165e-05 | 1.299 |
| Bt.27221.1.S1\_at | 1.167e-05 | 1.041 |
| Bt.27938.1.A1\_at | 1.172e-05 | 1.045 |
| Bt.2818.2.S1\_a\_at | 1.175e-05 | 0.684 |
| Bt.23129.3.S1\_a\_at | 1.182e-05 | 0.9048 |
| Bt.24376.1.S1\_at | 1.188e-05 | 1.318 |
| Bt.6586.1.S1\_at | 1.192e-05 | 1.073 |
| Bt.2378.1.S1\_at | 1.203e-05 | 0.7866 |
| Bt.10797.2.S1\_a\_at | 1.22e-05 | -0.8292 |
| Bt.22046.1.S1\_at | 1.224e-05 | -0.7392 |
| Bt.27870.1.S1\_at | 1.227e-05 | -1.046 |
| Bt.22071.1.S1\_at | 1.253e-05 | -0.838 |
| Bt.12054.1.S1\_at | 1.255e-05 | 0.8277 |
| Bt.4844.1.S1\_at | 1.258e-05 | 1.646 |
| Bt.13249.1.A1\_at | 1.261e-05 | 1.113 |
| Bt.5054.3.S1\_at | 1.264e-05 | 1.246 |
| Bt.25021.1.A1\_at | 1.27e-05 | 0.9822 |
| Bt.27147.1.S1\_at | 1.274e-05 | 1.041 |
| Bt.18851.1.S1\_at | 1.277e-05 | -0.7223 |
| Bt.23807.1.A1\_at | 1.285e-05 | 1.369 |
| Bt.19706.1.S1\_at | 1.286e-05 | 1.281 |
| Bt.24305.1.A1\_at | 1.289e-05 | 0.9255 |
| Bt.21342.1.S1\_at | 1.289e-05 | -0.9085 |
| Bt.16782.2.S1\_at | 1.292e-05 | -1.044 |
| Bt.24395.1.S1\_at | 1.297e-05 | -1.013 |
| Bt.13289.2.S1\_at | 1.306e-05 | 1.977 |
| Bt.22163.1.S1\_at | 1.324e-05 | -1.857 |
| Bt.27935.1.A1\_at | 1.334e-05 | 0.909 |
| Bt.7257.1.S1\_at | 1.335e-05 | -0.8096 |
| Bt.3898.1.S1\_at | 1.336e-05 | -1.267 |
| Bt.7872.1.S1\_at | 1.339e-05 | -0.9703 |
| Bt.4118.1.A1\_at | 1.34e-05 | -1.056 |
| Bt.9807.1.S1\_at | 1.353e-05 | 1.705 |
| Bt.1719.1.S1\_at | 1.356e-05 | -0.8411 |
| Bt.24563.1.S1\_at | 1.356e-05 | 1.099 |
| Bt.16296.1.A1\_at | 1.356e-05 | -1.214 |
| Bt.16224.3.A1\_at | 1.359e-05 | 0.9291 |
| Bt.9169.1.A1\_at | 1.365e-05 | 1.311 |
| Bt.9623.1.A1\_at | 1.366e-05 | -1.217 |
| Bt.11230.1.S1\_at | 1.37e-05 | -1.068 |
| Bt.15561.1.S1\_at | 1.374e-05 | 0.8686 |
| Bt.28210.1.S1\_at | 1.374e-05 | 0.9044 |
| Bt.20862.1.A1\_at | 1.379e-05 | -0.8516 |
| Bt.4606.2.S1\_at | 1.391e-05 | 1.805 |
| Bt.23603.1.S1\_a\_at | 1.402e-05 | -1.11 |
| Bt.4768.1.S2\_at | 1.402e-05 | 0.7764 |
| Bt.16041.2.S1\_at | 1.41e-05 | 0.9386 |
| Bt.9301.1.A1\_at | 1.411e-05 | 0.8997 |
| Bt.5088.1.S1\_at | 1.414e-05 | 0.9717 |
| Bt.21288.1.A1\_at | 1.42e-05 | 0.8314 |
| Bt.24154.1.A1\_at | 1.424e-05 | -0.9262 |
| Bt.5723.1.S1\_at | 1.426e-05 | -1.01 |
| Bt.9958.1.S1\_at | 1.453e-05 | 1.134 |
| Bt.20489.1.S1\_at | 1.456e-05 | 0.8377 |
| Bt.19281.2.S1\_at | 1.463e-05 | 0.8277 |
| Bt.25637.1.A1\_at | 1.476e-05 | -1.456 |
| Bt.10329.1.A1\_at | 1.481e-05 | 0.9989 |
| Bt.18113.2.S1\_at | 1.482e-05 | 0.6335 |
| Bt.20494.1.S1\_at | 1.485e-05 | 0.9639 |
| Bt.4279.3.S1\_a\_at | 1.501e-05 | 0.9018 |
| Bt.18755.1.A1\_at | 1.516e-05 | -0.9078 |
| Bt.543.1.S1\_at | 1.52e-05 | 1.261 |
| Bt.1681.1.S1\_at | 1.52e-05 | -0.7777 |
| Bt.18843.1.A1\_at | 1.528e-05 | 0.7875 |
| Bt.26723.1.S1\_at | 1.529e-05 | -0.8379 |
| Bt.12105.1.A1\_at | 1.53e-05 | -0.8051 |
| Bt.8131.1.S1\_at | 1.534e-05 | 1.091 |
| Bt.4040.1.S1\_at | 1.537e-05 | 1.046 |
| Bt.17985.1.S1\_at | 1.543e-05 | 1.087 |
| Bt.7373.3.S1\_a\_at | 1.55e-05 | -0.8942 |
| Bt.16900.1.A1\_at | 1.554e-05 | 1.167 |
| Bt.3888.1.S1\_a\_at | 1.563e-05 | -1.348 |
| Bt.23331.1.S1\_at | 1.594e-05 | 1.07 |
| Bt.21020.1.S1\_at | 1.601e-05 | 1.014 |
| Bt.11502.1.A1\_at | 1.604e-05 | 0.9877 |
| Bt.21981.1.A1\_at | 1.607e-05 | 1.13 |
| Bt.14207.1.S1\_at | 1.609e-05 | -0.7437 |
| Bt.23971.1.S1\_at | 1.609e-05 | 0.8606 |
| Bt.16106.1.A1\_at | 1.614e-05 | 0.989 |
| Bt.19097.2.S1\_at | 1.618e-05 | 1.121 |
| Bt.21938.1.S1\_at | 1.63e-05 | 1.253 |
| Bt.22034.1.S1\_at | 1.64e-05 | 1.191 |
| Bt.6063.1.S1\_at | 1.641e-05 | -1.163 |
| Bt.29921.1.S1\_at | 1.642e-05 | 1.125 |
| Bt.24474.1.S1\_a\_at | 1.646e-05 | -0.8165 |
| Bt.4726.1.S1\_at | 1.648e-05 | 1.02 |
| Bt.17886.1.A1\_at | 1.654e-05 | 1.155 |
| Bt.28455.1.S1\_at | 1.662e-05 | 0.8884 |
| Bt.9438.1.S1\_at | 1.675e-05 | -0.8688 |
| Bt.27626.1.A1\_at | 1.68e-05 | 0.7646 |
| Bt.22339.1.A1\_at | 1.715e-05 | -0.9132 |
| Bt.28150.2.S1\_at | 1.72e-05 | -1.049 |
| Bt.25980.1.A1\_at | 1.723e-05 | -1.113 |
| Bt.21.1.S1\_at | 1.738e-05 | -0.786 |
| Bt.21469.1.S1\_at | 1.747e-05 | 1.02 |
| Bt.2711.1.S1\_at | 1.752e-05 | -0.9169 |
| Bt.26992.2.S1\_at | 1.763e-05 | 1.091 |
| Bt.16412.1.S1\_a\_at | 1.78e-05 | 0.7779 |
| Bt.25712.1.A1\_s\_at | 1.79e-05 | 0.8215 |
| Bt.298.1.S1\_at | 1.793e-05 | -0.8985 |
| Bt.9756.1.S1\_at | 1.798e-05 | -1.13 |
| Bt.18002.1.A1\_at | 1.8e-05 | 0.8609 |
| Bt.9956.1.S1\_at | 1.802e-05 | -0.8911 |
| Bt.23520.1.S1\_at | 1.808e-05 | 0.8583 |
| Bt.18348.1.S1\_at | 1.818e-05 | 1.075 |
| Bt.5106.1.S1\_a\_at | 1.822e-05 | 0.8223 |
| Bt.8103.1.S1\_a\_at | 1.828e-05 | -1.045 |
| Bt.11599.1.S1\_at | 1.842e-05 | 1.111 |
| Bt.20815.1.A1\_at | 1.842e-05 | 1.486 |
| Bt.615.1.S1\_at | 1.845e-05 | 0.9809 |
| Bt.2956.1.A1\_at | 1.852e-05 | 1.014 |
| Bt.4544.1.S1\_at | 1.858e-05 | 0.8644 |
| Bt.5021.1.S1\_at | 1.861e-05 | 1.132 |
| Bt.7595.1.S1\_at | 1.867e-05 | -0.7968 |
| Bt.4205.1.S1\_at | 1.881e-05 | -0.7314 |
| Bt.1606.1.S1\_at | 1.899e-05 | 1.272 |
| Bt.12443.1.S1\_at | 1.904e-05 | 0.6549 |
| Bt.17789.1.A1\_at | 1.907e-05 | 1.647 |
| Bt.13887.1.S1\_at | 1.909e-05 | -1.023 |
| Bt.746.1.S1\_at | 1.926e-05 | 0.9154 |
| Bt.935.1.A1\_at | 1.929e-05 | -0.7311 |
| Bt.1767.1.S1\_at | 1.933e-05 | -0.8337 |
| Bt.25072.1.S1\_at | 1.934e-05 | -1.013 |
| Bt.6004.1.S1\_at | 1.937e-05 | 0.6439 |
| Bt.23002.1.S1\_at | 1.943e-05 | -1.054 |
| Bt.29324.1.S1\_at | 1.948e-05 | 0.6784 |
| Bt.19862.1.A1\_at | 1.958e-05 | 1.198 |
| Bt.7395.1.S1\_at | 1.967e-05 | 0.7888 |
| Bt.10066.1.S1\_at | 1.972e-05 | 1.07 |
| Bt.7120.1.S1\_a\_at | 1.974e-05 | 0.7707 |
| Bt.23106.1.S1\_at | 1.984e-05 | -0.8621 |
| Bt.9398.1.S1\_at | 1.988e-05 | 0.6722 |
| Bt.4314.1.S1\_at | 1.997e-05 | 0.7784 |
| Bt.21580.1.S1\_at | 1.997e-05 | 1.222 |
| Bt.20422.1.S1\_at | 1.998e-05 | -0.9155 |
| Bt.27112.1.S1\_at | 2.011e-05 | 1.167 |
| Bt.20242.1.S1\_at | 2.021e-05 | 1.292 |
| Bt.8486.1.S1\_at | 2.033e-05 | 1.284 |
| Bt.1284.1.S1\_at | 2.034e-05 | -1.055 |
| Bt.7788.1.S1\_at | 2.047e-05 | 1.042 |
| Bt.14566.2.S1\_at | 2.049e-05 | 1.226 |
| Bt.29506.1.S1\_at | 2.06e-05 | 1.275 |
| Bt.10251.1.S1\_at | 2.082e-05 | 1.128 |
| Bt.21031.1.S1\_at | 2.091e-05 | -0.9585 |
| Bt.27843.1.S1\_at | 2.092e-05 | -0.8675 |
| Bt.22243.1.S1\_at | 2.095e-05 | 0.8428 |
| Bt.11242.1.S1\_at | 2.132e-05 | -0.836 |
| Bt.20064.2.S1\_at | 2.133e-05 | 0.9622 |
| Bt.9629.2.S1\_at | 2.138e-05 | 1.215 |
| Bt.12628.1.S1\_at | 2.156e-05 | 0.9962 |
| Bt.7056.2.A1\_x\_at | 2.161e-05 | 1.578 |
| Bt.26920.2.A1\_a\_at | 2.163e-05 | -0.9757 |
| Bt.23394.1.S1\_at | 2.168e-05 | -0.9464 |
| Bt.4503.1.S2\_at | 2.178e-05 | -0.9081 |
| Bt.4950.1.S1\_at | 2.186e-05 | 0.752 |
| Bt.9803.1.S1\_at | 2.187e-05 | -1.04 |
| Bt.2331.1.A1\_at | 2.197e-05 | 1.24 |
| Bt.4834.1.S1\_at | 2.21e-05 | 1.051 |
| Bt.11301.1.S1\_at | 2.221e-05 | 0.7829 |
| Bt.5074.1.S1\_at | 2.223e-05 | 1.222 |
| Bt.6832.1.S1\_at | 2.231e-05 | 1.056 |
| Bt.7363.1.S1\_at | 2.236e-05 | 0.8174 |
| Bt.12217.2.S1\_at | 2.241e-05 | 1.513 |
| Bt.923.2.S1\_at | 2.25e-05 | 0.8375 |
| Bt.19934.1.S1\_at | 2.255e-05 | -1.171 |
| Bt.13152.1.S1\_s\_at | 2.258e-05 | 1.413 |
| Bt.22340.1.S1\_at | 2.261e-05 | 0.6817 |
| Bt.10259.1.A1\_at | 2.266e-05 | 1.382 |
| Bt.10931.1.S1\_at | 2.268e-05 | 0.8619 |
| Bt.21774.1.S1\_at | 2.274e-05 | 0.8133 |
| Bt.22650.1.A1\_at | 2.28e-05 | 1.262 |
| Bt.5326.1.S1\_at | 2.284e-05 | 0.6559 |
| Bt.9772.1.S1\_at | 2.323e-05 | 1.205 |
| Bt.16945.1.S1\_at | 2.331e-05 | -0.6854 |
| Bt.27430.1.S1\_at | 2.333e-05 | -0.8571 |
| Bt.9252.1.A1\_at | 2.335e-05 | 1.392 |
| Bt.3260.1.S1\_at | 2.336e-05 | -0.6827 |
| Bt.9288.1.S1\_at | 2.342e-05 | 1.293 |
| Bt.24623.1.A1\_at | 2.347e-05 | 0.6452 |
| Bt.7980.1.S1\_at | 2.356e-05 | 0.8909 |
| Bt.20601.2.S1\_at | 2.357e-05 | 0.7634 |
| Bt.10092.1.S1\_at | 2.359e-05 | 1.041 |
| Bt.5412.1.S1\_at | 2.359e-05 | -0.8872 |
| Bt.5302.1.S1\_a\_at | 2.362e-05 | 0.6774 |
| Bt.14214.1.S1\_at | 2.363e-05 | 1.046 |
| Bt.13366.1.S1\_at | 2.365e-05 | 1.184 |
| Bt.16105.1.S1\_at | 2.368e-05 | 1.299 |
| Bt.11751.1.A1\_at | 2.369e-05 | -0.7728 |
| Bt.20720.1.S1\_at | 2.376e-05 | -0.979 |
| Bt.10956.1.S1\_at | 2.399e-05 | 0.8349 |
| Bt.17968.1.S1\_at | 2.4e-05 | -1.402 |
| Bt.17425.1.A1\_at | 2.409e-05 | -1.054 |
| Bt.12108.1.S1\_at | 2.41e-05 | -0.9096 |
| Bt.1418.1.S1\_at | 2.422e-05 | -0.9674 |
| Bt.20038.1.S1\_at | 2.436e-05 | -0.8299 |
| Bt.9041.1.S1\_at | 2.438e-05 | 0.8355 |
| Bt.24783.2.S1\_at | 2.446e-05 | 1.03 |
| Bt.9868.3.S1\_a\_at | 2.449e-05 | 0.7527 |
| Bt.13747.1.A1\_at | 2.459e-05 | 1.046 |
| Bt.4480.1.S2\_at | 2.459e-05 | 1.057 |
| Bt.12820.1.S1\_at | 2.466e-05 | 0.782 |
| Bt.25016.1.S1\_at | 2.477e-05 | 1.238 |
| Bt.13118.1.S1\_at | 2.481e-05 | 0.9717 |
| Bt.13768.1.S1\_at | 2.484e-05 | 0.9723 |
| Bt.14395.2.S1\_at | 2.49e-05 | 0.7386 |
| Bt.25051.1.A1\_at | 2.495e-05 | 1.477 |
| Bt.21284.1.A1\_at | 2.496e-05 | 1.378 |
| Bt.9448.1.S2\_at | 2.503e-05 | 0.7 |
| Bt.23285.1.S1\_at | 2.513e-05 | -0.7542 |
| Bt.1484.1.S1\_at | 2.519e-05 | 0.742 |
| Bt.23225.1.S1\_at | 2.527e-05 | -0.9566 |
| Bt.21051.1.S1\_at | 2.54e-05 | -0.9218 |
| Bt.7394.1.A1\_at | 2.562e-05 | -1.028 |
| Bt.12867.1.A1\_at | 2.579e-05 | 0.7543 |
| Bt.4864.3.S1\_a\_at | 2.599e-05 | 0.9795 |
| Bt.3820.2.A1\_at | 2.605e-05 | 0.646 |
| Bt.17777.2.S1\_at | 2.607e-05 | -0.8524 |
| Bt.16314.1.S1\_at | 2.621e-05 | 0.9712 |
| Bt.22854.1.S1\_at | 2.629e-05 | -1.463 |
| Bt.8798.1.S1\_at | 2.666e-05 | 1.262 |
| Bt.22451.1.S1\_at | 2.675e-05 | -0.8268 |
| Bt.27918.1.S1\_at | 2.69e-05 | 0.9491 |
| Bt.9413.1.S1\_at | 2.696e-05 | 0.8024 |
| Bt.24381.1.S1\_at | 2.71e-05 | 1.151 |
| Bt.11154.2.S1\_at | 2.716e-05 | -0.957 |
| Bt.26993.1.S1\_at | 2.72e-05 | -0.8063 |
| Bt.16111.1.S1\_at | 2.722e-05 | 0.7682 |
| Bt.15714.1.S1\_at | 2.732e-05 | 0.8799 |
| Bt.18316.1.A1\_at | 2.737e-05 | 1.43 |
| Bt.2123.1.S1\_at | 2.747e-05 | -0.889 |
| Bt.547.1.S1\_at | 2.75e-05 | -1.054 |
| Bt.22290.1.S1\_at | 2.754e-05 | 0.8882 |
| Bt.25228.1.A1\_at | 2.76e-05 | 1.168 |
| Bt.24023.1.A1\_at | 2.769e-05 | 0.8748 |
| Bt.15864.1.S1\_at | 2.772e-05 | 1.144 |
| Bt.3913.1.S1\_at | 2.781e-05 | 1.13 |
| Bt.11798.1.S1\_at | 2.787e-05 | 1.366 |
| Bt.13327.1.S1\_at | 2.788e-05 | -0.7309 |
| Bt.6926.1.A1\_a\_at | 2.789e-05 | 1.141 |
| Bt.20243.2.S1\_at | 2.79e-05 | -0.6605 |
| Bt.11990.1.S1\_at | 2.793e-05 | 1.112 |
| Bt.13199.1.S1\_at | 2.793e-05 | -0.8995 |
| Bt.7467.1.S1\_at | 2.798e-05 | 0.8704 |
| Bt.1175.1.S1\_at | 2.801e-05 | -0.8431 |
| Bt.20487.1.S1\_at | 2.803e-05 | -0.9639 |
| Bt.5170.1.S1\_at | 2.808e-05 | -0.8451 |
| Bt.26513.1.S1\_at | 2.817e-05 | 1.658 |
| Bt.5278.2.S1\_at | 2.835e-05 | -0.8597 |
| Bt.1742.1.S1\_at | 2.845e-05 | 1.264 |
| Bt.6397.2.S1\_at | 2.856e-05 | 1.166 |
| Bt.17380.1.S1\_at | 2.858e-05 | -0.6682 |
| Bt.21589.1.S1\_at | 2.858e-05 | 0.8959 |
| Bt.13214.1.S1\_at | 2.863e-05 | 0.8709 |
| Bt.2374.1.S2\_at | 2.869e-05 | 0.6108 |
| Bt.2070.3.S1\_at | 2.87e-05 | -1.007 |
| Bt.9944.1.S1\_at | 2.886e-05 | 1.328 |
| Bt.15668.1.S1\_at | 2.887e-05 | 1.012 |
| Bt.4631.2.S1\_at | 2.887e-05 | 0.8527 |
| Bt.20988.1.S1\_at | 2.905e-05 | -0.918 |
| Bt.2046.1.S1\_at | 2.908e-05 | 0.9254 |
| Bt.2537.2.S1\_at | 2.919e-05 | 0.8461 |
| Bt.28188.1.S1\_at | 2.924e-05 | 0.7975 |
| Bt.380.1.S1\_at | 2.933e-05 | -0.8685 |
| Bt.1768.1.A1\_at | 2.943e-05 | -0.7047 |
| Bt.11857.1.A1\_at | 2.943e-05 | -0.8212 |
| Bt.5664.1.S1\_at | 2.945e-05 | 0.7243 |
| Bt.3714.2.S1\_at | 2.957e-05 | 1.062 |
| Bt.2493.1.S1\_at | 2.967e-05 | -1.282 |
| Bt.17456.1.S1\_at | 2.976e-05 | -0.8384 |
| Bt.12927.1.S1\_at | 2.981e-05 | 1.115 |
| Bt.27431.1.A1\_at | 2.986e-05 | 0.9942 |
| Bt.23456.1.A1\_s\_at | 2.986e-05 | 0.6776 |
| Bt.12589.1.S1\_at | 2.995e-05 | 0.7856 |
| Bt.3138.1.S1\_at | 3.004e-05 | 0.9805 |
| Bt.11599.2.S1\_at | 3.01e-05 | 0.9158 |
| Bt.4540.1.S1\_at | 3.052e-05 | -0.6304 |
| Bt.26779.1.S1\_at | 3.055e-05 | -1.078 |
| Bt.20129.1.S1\_at | 3.056e-05 | -0.7206 |
| Bt.4662.1.S1\_at | 3.057e-05 | 0.817 |
| Bt.1059.3.S1\_a\_at | 3.061e-05 | -0.7179 |
| Bt.21072.1.A1\_at | 3.062e-05 | 0.9544 |
| Bt.25654.1.A1\_at | 3.086e-05 | -0.8121 |
| Bt.26621.1.S1\_at | 3.09e-05 | 0.972 |
| Bt.8180.3.A1\_a\_at | 3.093e-05 | 0.8367 |
| Bt.20849.1.S1\_at | 3.112e-05 | 1.087 |
| Bt.15796.1.S1\_at | 3.113e-05 | 0.7292 |
| Bt.10417.1.S1\_at | 3.115e-05 | 0.6426 |
| Bt.5459.1.S1\_at | 3.127e-05 | -0.8789 |
| Bt.27114.1.A1\_at | 3.127e-05 | 0.7862 |
| Bt.20544.1.S1\_at | 3.127e-05 | 0.8235 |
| Bt.15684.1.S1\_at | 3.14e-05 | 0.6941 |
| Bt.26080.3.S1\_at | 3.159e-05 | 1.014 |
| Bt.9426.1.S1\_at | 3.163e-05 | 0.9135 |
| Bt.5016.1.S1\_at | 3.169e-05 | -0.6532 |
| Bt.10953.2.S1\_at | 3.176e-05 | 1.135 |
| Bt.13028.1.S1\_at | 3.178e-05 | 2.438 |
| Bt.1240.1.S1\_at | 3.205e-05 | 0.7429 |
| Bt.29952.1.S1\_at | 3.214e-05 | -0.9425 |
| Bt.17006.1.A1\_at | 3.219e-05 | 0.7561 |
| Bt.3791.1.S1\_at | 3.221e-05 | -0.8218 |
| Bt.8015.3.A1\_at | 3.229e-05 | -1.166 |
| Bt.5112.1.A1\_a\_at | 3.247e-05 | -1.136 |
| Bt.11662.1.A1\_at | 3.254e-05 | 0.9935 |
| Bt.24404.1.A1\_at | 3.26e-05 | 1.209 |
| Bt.1101.1.S1\_at | 3.274e-05 | 0.7004 |
| Bt.4449.1.S1\_at | 3.283e-05 | 0.9463 |
| Bt.16836.2.A1\_at | 3.299e-05 | 1.057 |
| Bt.13562.1.S1\_at | 3.3e-05 | 0.9047 |
| Bt.21053.1.A1\_at | 3.32e-05 | -0.9487 |
| Bt.1322.1.S1\_at | 3.322e-05 | 0.8904 |
| Bt.2399.1.S1\_at | 3.328e-05 | 0.9492 |
| Bt.16020.1.A2\_at | 3.33e-05 | 1.085 |
| Bt.9877.1.S1\_at | 3.345e-05 | 0.8341 |
| Bt.29194.1.S1\_at | 3.353e-05 | -1.064 |
| Bt.4697.1.S1\_at | 3.394e-05 | 0.7611 |
| Bt.18048.2.S1\_at | 3.422e-05 | 1.049 |
| Bt.5750.1.S1\_at | 3.461e-05 | -0.9282 |
| Bt.2070.2.A1\_at | 3.466e-05 | -1.076 |
| Bt.4702.1.S1\_at | 3.48e-05 | 0.8456 |
| Bt.659.1.S1\_at | 3.497e-05 | -0.9371 |
| Bt.15968.1.S1\_at | 3.518e-05 | -0.9259 |
| Bt.23315.1.S1\_a\_at | 3.521e-05 | 0.6371 |
| Bt.9278.1.S1\_at | 3.55e-05 | 0.7961 |
| Bt.25105.2.A1\_at | 3.573e-05 | 0.8209 |
| Bt.10777.1.S1\_at | 3.579e-05 | 1.087 |
| Bt.5290.1.S1\_at | 3.59e-05 | 0.7458 |
| Bt.9767.1.S1\_a\_at | 3.598e-05 | -1.082 |
| Bt.4775.1.S1\_at | 3.61e-05 | 0.997 |
| Bt.9207.1.S1\_at | 3.638e-05 | 0.7406 |
| Bt.24348.1.A1\_at | 3.643e-05 | 1.111 |
| Bt.3789.1.S1\_at | 3.659e-05 | 0.7855 |
| Bt.10922.1.A1\_at | 3.671e-05 | 1.418 |
| Bt.22516.1.A1\_at | 3.673e-05 | -0.7768 |
| Bt.18165.1.A1\_at | 3.674e-05 | -2.481 |
| Bt.11014.1.S1\_at | 3.723e-05 | -1.005 |
| Bt.7168.2.A1\_at | 3.724e-05 | 0.9937 |
| Bt.28829.1.S1\_at | 3.737e-05 | -0.8076 |
| Bt.26750.1.S1\_at | 3.764e-05 | -1.571 |
| Bt.28477.1.S1\_at | 3.77e-05 | 0.6484 |
| Bt.27781.1.S1\_at | 3.779e-05 | 1.29 |
| Bt.19811.1.A1\_at | 3.782e-05 | -1.007 |
| Bt.20007.1.S1\_a\_at | 3.793e-05 | 0.7813 |
| Bt.21715.1.S1\_at | 3.803e-05 | 1.027 |
| Bt.8912.1.A1\_at | 3.815e-05 | -1.035 |
| Bt.8492.1.A1\_at | 3.837e-05 | 1.024 |
| Bt.13648.1.A1\_at | 3.843e-05 | 0.9319 |
| Bt.21824.1.A1\_at | 3.848e-05 | 1.263 |
| Bt.1217.2.S1\_at | 3.848e-05 | -0.8267 |
| Bt.14853.1.A1\_at | 3.889e-05 | 1.048 |
| Bt.3211.1.S1\_at | 3.892e-05 | 0.8428 |
| Bt.8047.1.S1\_at | 3.897e-05 | -0.7318 |
| Bt.4555.1.S1\_at | 3.92e-05 | -1.05 |
| Bt.28323.1.S1\_at | 3.937e-05 | 1.552 |
| Bt.9176.2.S1\_at | 3.948e-05 | 0.9033 |
| Bt.27140.1.S1\_at | 3.95e-05 | 0.909 |
| Bt.20422.2.S1\_a\_at | 3.951e-05 | -0.721 |
| Bt.2262.1.S1\_at | 3.964e-05 | -0.779 |
| Bt.15357.1.A1\_at | 3.97e-05 | -0.7289 |
| Bt.23615.1.S1\_at | 4.041e-05 | 0.6649 |
| Bt.23673.1.A1\_at | 4.06e-05 | 1.072 |
| Bt.25338.1.S1\_at | 4.06e-05 | 0.8579 |
| Bt.5546.1.S1\_at | 4.073e-05 | 1.05 |
| Bt.15896.1.S1\_at | 4.079e-05 | 0.7962 |
| Bt.7373.2.S1\_a\_at | 4.094e-05 | -0.6255 |
| Bt.24510.1.S1\_at | 4.095e-05 | 0.8125 |
| Bt.21053.2.S1\_at | 4.134e-05 | -0.7931 |
| Bt.1793.3.S1\_at | 4.145e-05 | -1.348 |
| Bt.1739.2.S1\_at | 4.152e-05 | 0.8067 |
| Bt.4054.1.S1\_at | 4.169e-05 | 0.9621 |
| Bt.3821.1.A1\_at | 4.197e-05 | 0.8787 |
| Bt.4056.1.S1\_at | 4.238e-05 | 0.6194 |
| Bt.27892.1.S1\_at | 4.266e-05 | -0.7361 |
| Bt.2109.1.S1\_at | 4.273e-05 | 0.8352 |
| Bt.7039.1.S1\_a\_at | 4.284e-05 | -0.7799 |
| Bt.5099.1.S1\_at | 4.286e-05 | 1.067 |
| Bt.21597.1.S1\_at | 4.31e-05 | -0.715 |
| Bt.8213.1.S1\_at | 4.328e-05 | -0.7957 |
| Bt.10352.1.S1\_at | 4.34e-05 | -0.779 |
| Bt.2116.1.S1\_at | 4.348e-05 | -0.6415 |
| Bt.21036.2.A1\_at | 4.359e-05 | 0.9215 |
| Bt.17630.1.S1\_at | 4.376e-05 | 0.8151 |
| Bt.26622.1.S1\_a\_at | 4.379e-05 | -0.7742 |
| Bt.8437.1.A1\_at | 4.383e-05 | 1.246 |
| Bt.27095.1.S1\_at | 4.387e-05 | 0.9092 |
| Bt.1523.1.S1\_at | 4.387e-05 | -0.8128 |
| Bt.13847.1.S1\_at | 4.416e-05 | -1.057 |
| Bt.10631.2.S1\_at | 4.435e-05 | 1.161 |
| Bt.13065.1.S1\_at | 4.438e-05 | 1.282 |
| Bt.23094.4.S1\_at | 4.448e-05 | -1.064 |
| Bt.12862.1.S1\_at | 4.45e-05 | 1.091 |
| Bt.22443.1.A1\_at | 4.473e-05 | 0.8493 |
| Bt.16975.1.S1\_at | 4.482e-05 | 0.711 |
| Bt.23405.1.S1\_at | 4.483e-05 | -0.9029 |
| Bt.16639.1.A1\_at | 4.495e-05 | 0.712 |
| Bt.20316.1.S1\_at | 4.505e-05 | -1.211 |
| Bt.11240.1.A1\_at | 4.516e-05 | 1.36 |
| Bt.1430.1.S1\_at | 4.52e-05 | 0.7697 |
| Bt.6537.1.S1\_at | 4.525e-05 | 1.03 |
| Bt.13237.1.S1\_at | 4.527e-05 | -0.7757 |
| Bt.5047.1.S1\_at | 4.529e-05 | 0.9409 |
| Bt.20275.1.S1\_at | 4.558e-05 | -0.8099 |
| Bt.10579.1.S1\_at | 4.58e-05 | 1.135 |
| Bt.26835.1.S1\_at | 4.582e-05 | -0.8221 |
| Bt.28700.1.S1\_at | 4.612e-05 | 0.6998 |
| Bt.12728.3.S1\_at | 4.636e-05 | 0.7153 |
| Bt.14143.1.S1\_at | 4.639e-05 | 0.8762 |
| Bt.13972.1.A1\_at | 4.662e-05 | 0.6865 |
| Bt.11138.1.S1\_at | 4.667e-05 | -0.7553 |
| Bt.4948.1.S1\_at | 4.674e-05 | 0.7164 |
| Bt.25850.1.S1\_at | 4.688e-05 | 0.7846 |
| Bt.12249.2.S1\_at | 4.695e-05 | 0.8334 |
| Bt.6455.1.S1\_at | 4.726e-05 | -0.7522 |
| Bt.22349.1.A1\_at | 4.786e-05 | 0.7931 |
| Bt.868.1.S1\_at | 4.849e-05 | -0.6244 |
| Bt.14624.1.A1\_at | 4.869e-05 | -0.9512 |
| Bt.15880.1.S1\_at | 4.872e-05 | 0.8666 |
| Bt.23227.1.S1\_at | 4.876e-05 | 1.129 |
| Bt.17332.1.S1\_at | 4.881e-05 | 0.8821 |
| Bt.23055.1.A1\_s\_at | 4.91e-05 | 0.9868 |
| Bt.27859.1.S1\_at | 4.936e-05 | 0.8461 |
| Bt.24307.1.A1\_at | 4.948e-05 | 0.9875 |
| Bt.2120.2.S1\_at | 4.95e-05 | 1.699 |
| Bt.24643.1.S1\_at | 4.952e-05 | 0.8361 |
| Bt.12957.1.A1\_a\_at | 4.968e-05 | 0.7198 |
| Bt.21238.1.S1\_at | 4.991e-05 | 0.8725 |
| Bt.21827.2.S1\_at | 4.994e-05 | -0.7013 |
| Bt.26524.1.S1\_at | 5e-05 | -0.7016 |
| Bt.14040.1.S1\_at | 5.006e-05 | -1.099 |
| Bt.29459.1.A1\_at | 5.023e-05 | 0.8699 |
| Bt.15900.1.A1\_at | 5.037e-05 | -0.736 |
| Bt.24210.1.S1\_at | 5.072e-05 | -0.9217 |
| Bt.4466.1.A1\_at | 5.081e-05 | 0.7054 |
| Bt.2989.3.S1\_at | 5.092e-05 | -1.076 |
| Bt.9337.1.S1\_at | 5.099e-05 | 1.044 |
| Bt.12345.1.S1\_at | 5.145e-05 | 0.7372 |
| Bt.22639.2.S1\_at | 5.178e-05 | -1.191 |
| Bt.63.1.S1\_at | 5.186e-05 | -1.161 |
| Bt.19218.1.A1\_at | 5.214e-05 | 0.8092 |
| Bt.8617.1.S1\_at | 5.215e-05 | 0.9814 |
| Bt.27106.1.S1\_at | 5.221e-05 | 0.9179 |
| Bt.17012.1.A1\_s\_at | 5.251e-05 | -0.9179 |
| Bt.19637.1.A1\_at | 5.257e-05 | 1.145 |
| Bt.19100.3.A1\_at | 5.28e-05 | 0.8865 |
| Bt.20672.1.S1\_at | 5.284e-05 | 0.8825 |
| Bt.19542.1.S1\_at | 5.287e-05 | 0.7143 |
| Bt.9953.1.S1\_at | 5.296e-05 | -0.6836 |
| Bt.14095.2.S1\_at | 5.305e-05 | 1.321 |
| Bt.28625.1.S1\_at | 5.308e-05 | 0.626 |
| Bt.22785.1.S1\_at | 5.314e-05 | -1.235 |
| Bt.17080.1.A1\_at | 5.317e-05 | 0.7026 |
| Bt.17063.1.S1\_at | 5.318e-05 | 0.6909 |
| Bt.20230.1.S1\_at | 5.327e-05 | 0.7617 |
| Bt.14566.1.A1\_at | 5.334e-05 | 1.295 |
| Bt.20300.1.A1\_at | 5.338e-05 | 0.6569 |
| Bt.21736.1.A1\_at | 5.353e-05 | 0.768 |
| Bt.21151.1.S1\_at | 5.359e-05 | -0.6999 |
| Bt.7425.1.S1\_at | 5.37e-05 | 1.305 |
| Bt.6020.1.S1\_at | 5.419e-05 | -0.7644 |
| Bt.10317.1.A1\_at | 5.423e-05 | 0.7397 |
| Bt.25087.1.A1\_at | 5.434e-05 | -0.8485 |
| Bt.22320.3.S1\_at | 5.449e-05 | 1.001 |
| Bt.21365.1.S1\_a\_at | 5.454e-05 | 1.168 |
| Bt.28472.1.S1\_at | 5.464e-05 | 0.9381 |
| Bt.9705.1.S1\_at | 5.466e-05 | 0.9342 |
| Bt.23326.2.A1\_at | 5.494e-05 | 0.802 |
| Bt.18748.1.A1\_at | 5.497e-05 | 0.8779 |
| Bt.8775.2.S1\_a\_at | 5.517e-05 | -0.7796 |
| Bt.15836.2.S1\_at | 5.535e-05 | -3.332 |
| BtAffx.29968.1.S1\_at | 5.551e-05 | -2.815 |
| Bt.28314.1.S1\_at | 5.624e-05 | 0.8924 |
| Bt.10159.1.S1\_at | 5.628e-05 | -0.8393 |
| Bt.22333.1.S1\_at | 5.629e-05 | 0.7598 |
| Bt.12505.1.S1\_at | 5.646e-05 | 0.6228 |
| Bt.2494.2.S1\_a\_at | 5.658e-05 | 1.053 |
| Bt.15616.1.S1\_at | 5.687e-05 | -0.9449 |
| Bt.16094.1.S1\_at | 5.694e-05 | 1.326 |
| Bt.23486.3.S1\_at | 5.712e-05 | 1.435 |
| Bt.21929.1.S1\_at | 5.715e-05 | 0.7603 |
| Bt.19105.1.A1\_at | 5.732e-05 | 0.8789 |
| Bt.20619.1.S1\_at | 5.734e-05 | -0.7461 |
| Bt.8648.1.S1\_at | 5.75e-05 | 0.8527 |
| Bt.13908.1.S1\_at | 5.775e-05 | -0.8136 |
| Bt.24916.1.A1\_at | 5.791e-05 | 0.957 |
| Bt.23094.2.A1\_x\_at | 5.824e-05 | -1.039 |
| Bt.2151.2.A1\_at | 5.827e-05 | 0.8296 |
| Bt.5510.1.S1\_at | 5.828e-05 | -0.6183 |
| Bt.16831.1.A1\_at | 5.835e-05 | 1.188 |
| Bt.4694.1.S1\_at | 5.846e-05 | -0.7366 |
| Bt.1131.1.S1\_at | 5.872e-05 | 0.7019 |
| Bt.25342.1.A1\_at | 5.893e-05 | 0.8622 |
| Bt.8243.1.A1\_at | 5.917e-05 | -1.085 |
| Bt.9585.1.S1\_at | 5.92e-05 | -1.045 |
| Bt.12688.2.A1\_at | 5.949e-05 | 0.7244 |
| Bt.4797.1.S1\_at | 5.97e-05 | -0.8056 |
| Bt.20261.1.S1\_at | 5.976e-05 | 0.955 |
| Bt.22736.1.S1\_at | 5.982e-05 | 1.005 |
| Bt.26871.1.S1\_at | 6.001e-05 | 0.6039 |
| Bt.4743.1.A1\_at | 6.016e-05 | 0.5932 |
| Bt.5079.1.S1\_at | 6.025e-05 | -1.008 |
| Bt.15664.1.S1\_at | 6.027e-05 | -0.9193 |
| Bt.29.1.S1\_at | 6.063e-05 | 0.9916 |
| Bt.3452.1.S1\_at | 6.07e-05 | 0.794 |
| Bt.16125.3.S1\_at | 6.159e-05 | -1.077 |
| Bt.17188.1.A1\_at | 6.161e-05 | 1.088 |
| Bt.4270.1.S1\_at | 6.165e-05 | 0.9266 |
| Bt.21196.1.S1\_at | 6.176e-05 | 1.045 |
| Bt.8163.1.S1\_at | 6.192e-05 | 0.8142 |
| Bt.16206.2.S1\_at | 6.199e-05 | 0.9232 |
| Bt.8640.1.S1\_at | 6.202e-05 | 1.176 |
| Bt.22355.1.S1\_at | 6.211e-05 | 0.6827 |
| Bt.7030.1.S1\_at | 6.218e-05 | 0.7656 |
| Bt.2577.1.S1\_at | 6.25e-05 | 1.008 |
| Bt.25689.1.S1\_at | 6.277e-05 | -0.7307 |
| Bt.24895.1.A1\_at | 6.289e-05 | 0.7026 |
| Bt.28043.1.S1\_at | 6.303e-05 | 0.9177 |
| Bt.13900.1.S1\_at | 6.311e-05 | -0.8849 |
| Bt.25268.1.A1\_at | 6.324e-05 | 0.8421 |
| Bt.11745.1.S1\_at | 6.356e-05 | 1.005 |
| Bt.177.1.S1\_at | 6.365e-05 | 1.057 |
| Bt.16108.1.A1\_at | 6.373e-05 | -1.221 |
| Bt.4384.1.S1\_at | 6.403e-05 | 0.7604 |
| Bt.2187.1.S1\_at | 6.42e-05 | -0.6631 |
| Bt.10189.1.S1\_at | 6.429e-05 | -0.9151 |
| Bt.23600.1.S1\_at | 6.432e-05 | -0.6852 |
| Bt.18105.1.A1\_at | 6.439e-05 | 1.022 |
| Bt.9321.1.S1\_at | 6.448e-05 | 0.6847 |
| Bt.7240.1.S1\_at | 6.451e-05 | -0.9196 |
| Bt.12684.2.S1\_at | 6.462e-05 | 0.7042 |
| Bt.22170.1.S1\_at | 6.55e-05 | -0.8673 |
| Bt.3726.1.A1\_at | 6.56e-05 | 0.9241 |
| Bt.6172.1.A1\_at | 6.564e-05 | 0.9995 |
| Bt.5040.1.S1\_at | 6.577e-05 | -0.6327 |
| Bt.4903.1.S2\_at | 6.598e-05 | 0.9902 |
| Bt.15589.1.A1\_at | 6.611e-05 | 1.244 |
| Bt.23268.1.S1\_at | 6.618e-05 | 0.933 |
| Bt.13722.1.S1\_at | 6.677e-05 | 0.6279 |
| Bt.29697.1.S1\_at | 6.728e-05 | -1.097 |
| Bt.4998.1.S1\_at | 6.756e-05 | 0.7173 |
| Bt.3192.1.S1\_at | 6.758e-05 | 0.7945 |
| Bt.7962.1.S1\_at | 6.769e-05 | 1.578 |
| Bt.17194.1.S1\_at | 6.83e-05 | 1.044 |
| Bt.26675.1.S1\_at | 6.839e-05 | -0.8811 |
| Bt.27952.1.S1\_at | 6.845e-05 | -1.001 |
| Bt.23237.1.S1\_at | 6.851e-05 | -0.8843 |
| Bt.4588.1.S1\_at | 6.859e-05 | 0.905 |
| Bt.12377.1.S1\_at | 6.922e-05 | 0.7682 |
| Bt.24137.1.A1\_at | 6.951e-05 | 1.3 |
| Bt.4261.1.S1\_at | 6.952e-05 | -0.8687 |
| Bt.10025.1.A1\_at | 6.964e-05 | -0.7508 |
| Bt.15742.1.S1\_at | 6.98e-05 | 1.148 |
| Bt.16841.1.A1\_at | 6.997e-05 | 0.7903 |
| Bt.21427.1.S1\_at | 7.009e-05 | 0.7499 |
| Bt.1705.1.S1\_at | 7.041e-05 | 1.264 |
| Bt.21170.1.A1\_at | 7.058e-05 | 0.8446 |
| Bt.2331.2.S1\_at | 7.059e-05 | 0.9989 |
| Bt.19774.2.S1\_at | 7.087e-05 | 0.8908 |
| Bt.24767.1.S1\_at | 7.094e-05 | 0.6011 |
| Bt.28506.1.A1\_at | 7.097e-05 | -1.072 |
| Bt.21607.1.S1\_at | 7.109e-05 | 1.467 |
| Bt.2556.1.S1\_at | 7.11e-05 | 1.29 |
| Bt.6665.1.S1\_at | 7.12e-05 | 1.242 |
| Bt.16935.1.A1\_at | 7.187e-05 | 0.7653 |
| Bt.23373.1.S1\_at | 7.238e-05 | -1.008 |
| Bt.15836.1.S1\_at | 7.288e-05 | -2.648 |
| Bt.2798.1.S1\_a\_at | 7.311e-05 | 0.6143 |
| Bt.28821.1.S1\_at | 7.35e-05 | -0.9878 |
| Bt.7540.2.S1\_a\_at | 7.371e-05 | -0.9322 |
| Bt.3211.1.S2\_at | 7.38e-05 | 0.822 |
| Bt.12348.1.A1\_at | 7.382e-05 | 1.153 |
| Bt.27386.1.A1\_at | 7.383e-05 | 1.183 |
| Bt.2235.1.S1\_at | 7.392e-05 | 0.9371 |
| Bt.26995.2.S1\_at | 7.415e-05 | 0.7314 |
| Bt.18519.1.A1\_at | 7.434e-05 | 0.8 |
| Bt.7903.1.A1\_at | 7.559e-05 | -0.771 |
| Bt.20846.1.A1\_a\_at | 7.599e-05 | 1.172 |
| Bt.23633.3.S1\_at | 7.614e-05 | -0.8317 |
| Bt.26726.1.S1\_at | 7.627e-05 | 1.322 |
| Bt.20080.1.S1\_at | 7.63e-05 | 1.271 |
| Bt.6768.1.S1\_at | 7.634e-05 | 0.8604 |
| Bt.12403.1.S1\_at | 7.634e-05 | 0.8774 |
| Bt.21085.1.S1\_at | 7.661e-05 | 1.024 |
| Bt.3546.1.S1\_at | 7.666e-05 | 1.055 |
| Bt.7170.1.S1\_at | 7.684e-05 | 0.7527 |
| Bt.5328.1.S1\_at | 7.703e-05 | -1.175 |
| Bt.20463.1.S1\_at | 7.724e-05 | 1.179 |
| Bt.11496.1.S1\_at | 7.758e-05 | 1.236 |
| Bt.26505.1.S1\_at | 7.764e-05 | 0.8488 |
| Bt.20193.1.S1\_at | 7.781e-05 | -1.57 |
| Bt.16510.1.A1\_at | 7.784e-05 | 0.68 |
| Bt.9695.1.S1\_at | 7.785e-05 | 0.8292 |
| Bt.17109.1.A1\_at | 7.791e-05 | -1.029 |
| Bt.8024.1.A1\_at | 7.798e-05 | -0.7254 |
| Bt.2131.1.S1\_at | 7.815e-05 | 0.7228 |
| Bt.23869.1.A1\_at | 7.832e-05 | 0.8204 |
| Bt.9288.2.A1\_at | 7.836e-05 | 0.6894 |
| Bt.2656.1.S1\_at | 7.895e-05 | 1.053 |
| Bt.9889.1.S1\_at | 7.924e-05 | 0.8202 |
| Bt.6570.2.S1\_at | 7.942e-05 | 0.8547 |
| Bt.1998.1.S1\_at | 7.964e-05 | 0.8331 |
| Bt.923.1.A1\_at | 7.982e-05 | 0.7729 |
| Bt.13254.1.S1\_at | 7.995e-05 | 1.247 |
| Bt.22534.1.S1\_at | 7.998e-05 | 0.8488 |
| Bt.9306.2.S1\_a\_at | 8.052e-05 | -0.8676 |
| Bt.7873.1.S1\_at | 8.124e-05 | 0.6706 |
| Bt.387.1.S1\_at | 8.137e-05 | -1.103 |
| Bt.4726.2.S1\_at | 8.183e-05 | 0.8352 |
| Bt.8961.1.S1\_at | 8.219e-05 | 0.919 |
| Bt.22528.1.A1\_at | 8.246e-05 | -0.8962 |
| Bt.7727.1.S1\_at | 8.258e-05 | 0.6625 |
| Bt.24109.1.S1\_at | 8.261e-05 | -1.521 |
| Bt.12267.1.S1\_at | 8.281e-05 | 0.7837 |
| Bt.4492.1.S1\_at | 8.293e-05 | 1.029 |
| Bt.25056.1.A1\_at | 8.354e-05 | 1.238 |
| Bt.9712.1.S1\_at | 8.357e-05 | 0.7959 |
| Bt.25202.2.S1\_at | 8.367e-05 | 1.306 |
| Bt.884.1.S1\_at | 8.384e-05 | 1.041 |
| Bt.22362.1.S1\_at | 8.409e-05 | -0.7541 |
| Bt.19261.1.S1\_at | 8.437e-05 | 0.6767 |
| Bt.9210.1.S1\_at | 8.443e-05 | -0.754 |
| Bt.9778.1.S1\_at | 8.489e-05 | 0.8484 |
| Bt.17641.1.A1\_at | 8.507e-05 | -1.102 |
| Bt.26774.1.S1\_at | 8.508e-05 | 1.459 |
| Bt.4221.1.S1\_at | 8.522e-05 | 0.719 |
| Bt.22647.3.A1\_at | 8.671e-05 | 1.213 |
| Bt.20817.2.S1\_at | 8.686e-05 | 0.579 |
| Bt.13784.1.S1\_at | 8.757e-05 | -0.6978 |
| Bt.8448.1.S1\_at | 8.781e-05 | 0.9224 |
| Bt.25966.1.A1\_at | 8.89e-05 | 0.6965 |
| Bt.11629.1.A1\_at | 8.906e-05 | 0.8742 |
| Bt.27121.1.S1\_at | 8.918e-05 | -0.6903 |
| Bt.28187.1.S1\_at | 8.934e-05 | 0.9415 |
| Bt.20106.1.S1\_at | 8.947e-05 | 0.7387 |
| Bt.2347.1.S1\_at | 8.962e-05 | -0.852 |
| Bt.6546.1.A1\_at | 8.988e-05 | 0.9767 |
| Bt.5035.1.S1\_at | 9.004e-05 | -0.7315 |
| Bt.4563.1.S1\_at | 9.019e-05 | 0.6519 |
| Bt.4247.1.S1\_at | 9.048e-05 | 0.785 |
| Bt.24222.1.S1\_at | 9.101e-05 | 0.7276 |
| Bt.20652.2.S1\_at | 9.137e-05 | -1.032 |
| Bt.422.1.S1\_at | 9.166e-05 | 1.001 |
| Bt.26579.1.S1\_at | 9.186e-05 | 0.6627 |
| Bt.20102.1.S1\_at | 9.19e-05 | -1.06 |
| Bt.15967.1.S1\_at | 9.202e-05 | 0.5659 |
| Bt.27046.1.S1\_at | 9.214e-05 | 0.6141 |
| Bt.893.1.S1\_at | 9.231e-05 | -0.9738 |
| Bt.8331.1.S1\_at | 9.248e-05 | -0.9989 |
| Bt.29325.1.S1\_at | 9.254e-05 | -0.8128 |
| Bt.12297.1.S1\_at | 9.273e-05 | -0.8273 |
| Bt.9425.1.S1\_at | 9.28e-05 | 0.7591 |
| Bt.11165.1.S1\_at | 9.294e-05 | 0.6528 |
| Bt.8671.1.S1\_at | 9.304e-05 | -0.7992 |
| Bt.7575.1.A1\_at | 9.305e-05 | -1.008 |
| Bt.4533.1.S1\_at | 9.307e-05 | -0.6058 |
| Bt.2518.1.S1\_at | 9.315e-05 | 0.8505 |
| Bt.7596.1.S1\_at | 9.431e-05 | -0.6318 |
| Bt.2266.3.S1\_at | 9.436e-05 | -0.9255 |
| Bt.6937.1.S1\_at | 9.458e-05 | -0.7968 |
| Bt.25522.1.S1\_at | 9.46e-05 | 0.6273 |
| Bt.20377.1.S1\_at | 9.532e-05 | -0.9619 |
| Bt.28569.1.S1\_at | 9.619e-05 | 0.9073 |
| Bt.12138.1.S1\_at | 9.709e-05 | -0.7065 |
| Bt.10609.2.A1\_at | 9.722e-05 | 0.7068 |
| Bt.1059.2.S1\_at | 9.745e-05 | -0.717 |
| Bt.819.1.S1\_at | 9.759e-05 | -0.605 |
| Bt.3814.1.S1\_at | 9.76e-05 | 1.1 |
| Bt.22230.1.S1\_at | 9.764e-05 | 0.9363 |
| Bt.27371.1.S1\_at | 9.836e-05 | 0.8055 |
| Bt.22543.1.S1\_at | 9.836e-05 | -0.6713 |
| Bt.23548.2.S1\_at | 9.845e-05 | 0.9703 |
| Bt.13097.1.S1\_at | 9.911e-05 | -0.6042 |
| Bt.26402.1.A1\_at | 9.933e-05 | 0.7449 |
| Bt.18126.1.A1\_at | 9.952e-05 | 1.17 |
| Bt.13998.1.S1\_at | 9.961e-05 | -0.6296 |
| Bt.9312.1.A1\_at | 9.997e-05 | 0.8883 |
| Bt.20629.3.A1\_at | 0.0001011 | -0.8962 |
| Bt.16230.1.S1\_at | 0.0001014 | 0.7603 |
| Bt.16046.1.A1\_at | 0.0001014 | 0.9272 |
| Bt.18894.1.A1\_at | 0.0001014 | 0.7575 |
| Bt.21745.1.S1\_at | 0.0001014 | -0.8639 |
| Bt.4402.1.S1\_at | 0.0001019 | -0.6871 |
| Bt.12583.1.A1\_at | 0.0001023 | -0.9651 |
| Bt.20323.1.S1\_at | 0.0001024 | 1.095 |
| Bt.17332.3.A1\_at | 0.0001025 | 0.8452 |
| Bt.6169.2.S1\_at | 0.0001028 | 0.7691 |
| Bt.29836.1.S1\_at | 0.000104 | 0.7298 |
| Bt.8809.1.S1\_at | 0.0001042 | -1.411 |
| Bt.19061.1.A1\_at | 0.0001044 | -0.6842 |
| Bt.5603.2.S1\_at | 0.0001045 | 0.812 |
| Bt.3174.1.A1\_at | 0.0001048 | 0.7618 |
| Bt.7327.2.S1\_a\_at | 0.0001049 | 0.7145 |
| Bt.20965.1.S1\_at | 0.0001052 | -0.7701 |
| Bt.561.1.S1\_at | 0.0001058 | -1.304 |
| Bt.10814.1.S1\_at | 0.0001062 | 0.8938 |
| Bt.27141.1.A1\_at | 0.0001071 | 0.8751 |
| Bt.3210.1.S1\_at | 0.0001072 | 0.734 |
| Bt.16503.1.A1\_at | 0.0001073 | -0.668 |
| Bt.1785.1.A1\_at | 0.0001073 | 0.8056 |
| Bt.16149.1.A1\_at | 0.0001075 | -0.8299 |
| Bt.29213.1.S1\_at | 0.0001079 | 0.8556 |
| Bt.12867.2.S1\_at | 0.0001081 | 0.5455 |
| Bt.7069.1.S1\_at | 0.0001082 | 0.8825 |
| Bt.1967.1.S1\_at | 0.0001082 | -0.7026 |
| Bt.28659.1.A1\_at | 0.0001091 | 0.7037 |
| Bt.11926.1.A1\_at | 0.0001098 | -0.7195 |
| Bt.4985.2.S1\_a\_at | 0.0001102 | -0.7221 |
| Bt.8117.1.S1\_at | 0.0001105 | 0.845 |
| Bt.5022.1.A1\_at | 0.0001111 | 0.762 |
| Bt.8479.1.A1\_at | 0.0001111 | 1.037 |
| Bt.23158.1.S1\_at | 0.0001112 | 1.051 |
| Bt.2997.1.A1\_at | 0.0001116 | 0.8359 |
| Bt.4474.2.S1\_a\_at | 0.0001119 | -0.9202 |
| Bt.11054.1.S1\_at | 0.000112 | 0.5887 |
| Bt.3817.1.A1\_at | 0.0001127 | 0.6288 |
| Bt.5300.1.S1\_at | 0.0001128 | -0.7568 |
| Bt.8699.1.S1\_at | 0.0001128 | -1.205 |
| Bt.27123.1.A1\_at | 0.000113 | 0.8174 |
| Bt.10122.1.S1\_at | 0.0001131 | 0.7417 |
| Bt.4224.1.S1\_at | 0.0001134 | -0.7888 |
| Bt.12030.1.S1\_at | 0.0001134 | 0.6662 |
| Bt.13548.1.A1\_at | 0.0001136 | 0.7519 |
| Bt.10947.1.S1\_at | 0.0001139 | 0.7043 |
| Bt.886.1.S1\_at | 0.0001155 | -0.761 |
| Bt.21435.1.S1\_at | 0.0001171 | -0.6065 |
| Bt.21433.2.S1\_at | 0.0001173 | 1.037 |
| Bt.19482.1.A1\_at | 0.0001174 | -1.131 |
| Bt.10027.1.S2\_at | 0.0001176 | 0.9013 |
| Bt.28462.1.S1\_at | 0.0001182 | -0.9119 |
| Bt.19309.3.A1\_at | 0.0001187 | 0.8749 |
| Bt.2527.1.S1\_at | 0.0001188 | 0.9226 |
| Bt.13440.1.S1\_at | 0.0001195 | 0.6263 |
| Bt.3158.1.S1\_at | 0.0001198 | 0.6639 |
| Bt.27116.3.S1\_at | 0.00012 | 0.7422 |
| Bt.3029.1.S1\_at | 0.00012 | 0.708 |
| Bt.4719.1.S1\_at | 0.0001202 | 0.828 |
| Bt.13183.1.A1\_at | 0.0001206 | 0.9746 |
| Bt.26061.1.A1\_at | 0.0001206 | 0.7773 |
| Bt.20414.1.S1\_at | 0.000121 | 0.8329 |
| Bt.9139.1.A1\_at | 0.0001219 | -1.153 |
| Bt.26316.1.A1\_at | 0.0001221 | 1.1 |
| Bt.28631.1.S1\_at | 0.0001222 | 0.6695 |
| Bt.9049.1.S1\_at | 0.0001224 | 0.9886 |
| Bt.12912.1.S1\_at | 0.0001226 | 1.061 |
| Bt.4831.1.S1\_at | 0.0001232 | -0.597 |
| Bt.21191.2.A1\_at | 0.0001233 | -1.695 |
| Bt.27199.1.S1\_at | 0.0001234 | -0.7975 |
| Bt.15915.1.S1\_at | 0.0001234 | 0.8299 |
| Bt.22187.1.S1\_at | 0.0001236 | 0.9052 |
| Bt.6679.1.S1\_at | 0.0001237 | 0.843 |
| Bt.9918.1.S1\_at | 0.0001239 | 0.8707 |
| Bt.24832.1.A1\_a\_at | 0.000124 | -1.159 |
| Bt.7814.1.S1\_at | 0.0001242 | 0.5805 |
| Bt.9191.1.S1\_at | 0.0001244 | 0.8583 |
| Bt.21482.1.A1\_at | 0.0001244 | 0.8408 |
| Bt.16264.1.A1\_at | 0.0001248 | 0.6483 |
| Bt.11390.1.A1\_at | 0.000125 | -1.415 |
| Bt.28855.1.A1\_at | 0.000125 | 0.6774 |
| Bt.9785.1.S1\_at | 0.0001253 | 1.05 |
| Bt.6585.1.S1\_at | 0.000126 | 0.6836 |
| Bt.26364.1.A1\_at | 0.0001261 | 1.127 |
| Bt.9706.2.S1\_at | 0.0001261 | -0.589 |
| Bt.22435.1.S1\_at | 0.0001267 | 0.8617 |
| Bt.2606.1.S1\_at | 0.0001268 | 1.109 |
| Bt.3288.1.A1\_at | 0.000127 | -0.7365 |
| Bt.20660.1.A1\_at | 0.0001272 | -0.6605 |
| Bt.1097.1.S1\_at | 0.0001275 | 0.6262 |
| Bt.27057.1.A1\_at | 0.0001278 | -0.9059 |
| Bt.13314.1.S1\_at | 0.0001279 | 0.5805 |
| Bt.13946.2.A1\_at | 0.0001287 | 0.6336 |
| Bt.5602.1.A1\_at | 0.0001287 | 0.9415 |
| Bt.8208.1.A1\_at | 0.000129 | 1.173 |
| Bt.4317.1.S1\_at | 0.0001292 | -1.217 |
| Bt.6116.1.S1\_a\_at | 0.0001293 | 0.772 |
| Bt.19301.1.A1\_at | 0.0001293 | 0.9344 |
| Bt.20426.1.S1\_at | 0.0001294 | 0.7823 |
| Bt.5418.1.S1\_at | 0.0001295 | -0.9594 |
| Bt.13991.2.S1\_at | 0.0001296 | 0.7828 |
| Bt.27097.2.S1\_at | 0.0001297 | 0.7892 |
| Bt.9185.1.A1\_at | 0.0001299 | 1.147 |
| Bt.20605.2.S1\_at | 0.0001299 | 0.7346 |
| Bt.12332.1.A1\_at | 0.00013 | 0.6324 |
| Bt.12164.1.S1\_at | 0.0001301 | 0.6948 |
| Bt.1573.2.S1\_a\_at | 0.0001305 | -0.7145 |
| Bt.21027.2.S1\_at | 0.0001305 | 0.5654 |
| Bt.935.2.S1\_at | 0.0001308 | -0.7666 |
| Bt.26572.1.S1\_at | 0.0001309 | 0.8496 |
| Bt.8262.1.A1\_at | 0.0001309 | 0.7664 |
| Bt.1770.2.A1\_at | 0.0001317 | 0.7517 |
| Bt.16636.2.A1\_at | 0.0001318 | 0.8784 |
| Bt.23647.1.A1\_a\_at | 0.0001326 | 1.016 |
| Bt.24272.1.S1\_at | 0.0001327 | 0.6863 |
| Bt.11725.1.A1\_at | 0.0001337 | 0.8437 |
| Bt.13850.3.S1\_a\_at | 0.0001339 | -0.8298 |
| Bt.25107.1.A1\_at | 0.0001339 | 0.7108 |
| Bt.26430.1.S1\_at | 0.0001343 | 0.8417 |
| Bt.9306.3.S1\_at | 0.000135 | -0.7947 |
| Bt.20226.2.A1\_at | 0.0001352 | 0.9188 |
| Bt.1069.1.A1\_at | 0.0001354 | 0.7401 |
| Bt.6542.1.S1\_at | 0.0001357 | -0.6376 |
| Bt.6087.2.S1\_a\_at | 0.0001359 | 0.8079 |
| Bt.27023.1.S1\_at | 0.0001362 | 1.03 |
| Bt.69.1.S1\_at | 0.0001363 | -0.6603 |
| Bt.21159.1.S1\_at | 0.0001363 | 1.15 |
| Bt.7143.1.S1\_at | 0.0001366 | -0.5567 |
| Bt.15938.1.S1\_at | 0.0001368 | 0.7971 |
| Bt.167.1.S1\_at | 0.0001369 | -0.6864 |
| Bt.19949.1.A1\_at | 0.000137 | 0.7031 |
| Bt.3023.1.S1\_at | 0.0001371 | -0.7505 |
| Bt.21113.1.S1\_a\_at | 0.0001373 | -0.9827 |
| Bt.20322.2.S1\_at | 0.0001373 | -0.5899 |
| Bt.3610.1.S1\_at | 0.0001377 | -0.5957 |
| Bt.12337.2.S1\_a\_at | 0.0001377 | -0.5736 |
| Bt.12205.1.S1\_at | 0.0001382 | -0.7721 |
| Bt.25390.1.A1\_at | 0.0001386 | 0.9218 |
| Bt.7177.1.S1\_at | 0.0001391 | 0.6755 |
| Bt.22308.1.A1\_at | 0.0001395 | 0.5947 |
| Bt.16920.1.A1\_s\_at | 0.0001398 | 1.011 |
| Bt.6950.1.S1\_at | 0.0001406 | -0.5786 |
| Bt.16188.1.S1\_at | 0.0001409 | 0.6892 |
| Bt.16259.2.S1\_at | 0.000141 | 0.5899 |
| Bt.13741.2.S1\_at | 0.0001422 | 0.8525 |
| Bt.27018.1.S1\_at | 0.0001423 | -0.7371 |
| Bt.7130.1.S1\_at | 0.0001425 | 0.7702 |
| Bt.9012.1.S1\_at | 0.0001426 | 0.7956 |
| Bt.22380.1.S1\_at | 0.0001429 | 1.175 |
| Bt.23209.1.S1\_a\_at | 0.0001439 | 0.9092 |
| Bt.4458.1.A1\_at | 0.000144 | 0.6057 |
| Bt.15853.1.S1\_at | 0.0001447 | 0.8691 |
| Bt.25016.2.S1\_at | 0.0001447 | 0.7383 |
| Bt.21036.1.S1\_at | 0.0001448 | 0.7537 |
| Bt.8807.1.S1\_at | 0.0001448 | 0.7444 |
| Bt.19559.1.S1\_at | 0.0001451 | 0.9156 |
| Bt.26978.1.A1\_at | 0.0001456 | 0.6425 |
| Bt.850.1.S1\_at | 0.0001458 | 0.6657 |
| Bt.22848.1.S1\_at | 0.0001461 | -0.5511 |
| Bt.27211.1.S1\_at | 0.0001464 | -0.9782 |
| Bt.3193.1.A1\_at | 0.0001464 | -0.7654 |
| Bt.28817.1.A1\_at | 0.0001473 | 0.8423 |
| Bt.20139.1.S1\_at | 0.0001479 | 0.8521 |
| Bt.12299.1.A1\_at | 0.000148 | 2.03 |
| Bt.1789.1.S1\_at | 0.0001484 | 0.7799 |
| Bt.25335.1.A1\_at | 0.0001494 | 0.7456 |
| Bt.18914.1.S1\_at | 0.0001499 | 0.7503 |
| Bt.5313.1.S1\_at | 0.0001499 | 0.7866 |
| Bt.7888.1.S1\_at | 0.0001502 | 0.5571 |
| Bt.11389.1.S1\_at | 0.0001505 | 0.8753 |
| Bt.10884.1.S1\_at | 0.0001507 | 0.5916 |
| Bt.29853.1.A1\_at | 0.0001523 | -0.9731 |
| Bt.4370.2.S1\_at | 0.0001524 | 0.5949 |
| Bt.6651.1.S1\_at | 0.0001525 | 1.304 |
| Bt.7430.1.S1\_at | 0.0001535 | 0.7215 |
| Bt.16067.1.S1\_at | 0.0001537 | -1.023 |
| Bt.27450.1.A1\_at | 0.000154 | -0.9336 |
| Bt.4038.1.S1\_at | 0.0001551 | 0.6492 |
| Bt.27826.1.S1\_at | 0.0001551 | 1.127 |
| Bt.13845.2.S1\_at | 0.0001551 | -0.6975 |
| Bt.9405.1.S1\_at | 0.0001555 | -0.5045 |
| Bt.9233.3.S1\_at | 0.0001558 | -0.8725 |
| Bt.8028.1.S1\_at | 0.0001562 | -0.586 |
| Bt.25044.1.S1\_at | 0.0001563 | -0.9169 |
| Bt.3811.1.S1\_at | 0.0001566 | -0.7533 |
| Bt.19382.1.S1\_at | 0.000157 | 0.813 |
| Bt.5542.1.A1\_at | 0.0001588 | 0.8959 |
| Bt.13874.1.A1\_at | 0.0001594 | 0.5883 |
| Bt.77.1.S1\_at | 0.0001594 | -0.8749 |
| Bt.18835.2.S1\_at | 0.0001599 | -1.093 |
| Bt.20549.1.S1\_at | 0.0001602 | 0.6634 |
| Bt.13768.2.S1\_at | 0.0001603 | 0.7929 |
| Bt.24470.1.S1\_a\_at | 0.0001604 | 0.8428 |
| Bt.21296.1.S1\_at | 0.0001609 | 0.5711 |
| Bt.21766.1.A1\_at | 0.0001611 | -1.585 |
| Bt.27071.1.S1\_at | 0.0001611 | 0.6253 |
| Bt.18804.1.A1\_at | 0.0001613 | 0.7343 |
| Bt.17061.1.A1\_at | 0.0001615 | 1.117 |
| Bt.27430.2.S1\_at | 0.0001616 | -0.9426 |
| Bt.27654.1.S1\_at | 0.0001626 | 0.8144 |
| Bt.18434.1.A1\_at | 0.0001631 | 1.147 |
| Bt.13478.1.A1\_at | 0.000164 | -0.6887 |
| Bt.18569.1.A1\_at | 0.0001646 | 1.03 |
| Bt.16753.1.A1\_at | 0.0001647 | 0.8256 |
| Bt.2451.2.A1\_at | 0.0001648 | 1.153 |
| Bt.4552.1.S1\_a\_at | 0.0001654 | 0.7372 |
| Bt.25479.1.S1\_at | 0.0001655 | 0.5597 |
| Bt.27323.1.S1\_at | 0.0001658 | -0.7523 |
| Bt.10154.1.S1\_at | 0.0001664 | -0.839 |
| Bt.13357.1.S1\_at | 0.0001665 | 0.668 |
| Bt.11622.1.A1\_at | 0.0001666 | 0.8025 |
| Bt.20308.2.S1\_at | 0.0001674 | 0.8607 |
| Bt.1487.1.S1\_at | 0.0001675 | -0.7583 |
| Bt.27.1.S1\_at | 0.0001678 | -0.8891 |
| Bt.15839.1.S1\_at | 0.0001679 | -1.084 |
| Bt.25343.1.S1\_at | 0.0001679 | -0.7233 |
| Bt.60.1.S1\_at | 0.0001682 | -0.5701 |
| Bt.12382.1.S1\_at | 0.0001682 | 0.641 |
| Bt.22769.1.S1\_at | 0.0001693 | 1.068 |
| Bt.20551.1.S1\_at | 0.0001695 | 0.6329 |
| Bt.26122.2.S1\_at | 0.0001699 | -0.7109 |
| Bt.6733.1.S1\_at | 0.0001713 | 0.7206 |
| Bt.20456.1.S1\_at | 0.0001713 | -0.941 |
| Bt.15819.2.S1\_at | 0.000172 | -0.8015 |
| Bt.28105.1.S1\_at | 0.0001723 | 0.7591 |
| Bt.2225.1.S1\_at | 0.0001723 | 0.7648 |
| Bt.19581.2.S1\_at | 0.000173 | -1.055 |
| Bt.5674.1.S1\_at | 0.0001733 | -0.6301 |
| Bt.21433.1.S1\_at | 0.0001734 | 0.9426 |
| Bt.22672.1.A1\_at | 0.0001737 | 1.55 |
| Bt.26302.1.A1\_at | 0.0001741 | 0.6296 |
| Bt.23094.6.A1\_at | 0.0001746 | -1.123 |
| Bt.16384.1.A1\_at | 0.0001748 | -0.8841 |
| Bt.4063.1.S1\_at | 0.0001749 | -1.373 |
| Bt.23229.2.S1\_at | 0.000175 | 0.7394 |
| Bt.21351.1.S1\_a\_at | 0.0001751 | 0.8336 |
| Bt.10810.1.A1\_at | 0.0001752 | 0.9508 |
| Bt.21351.1.S1\_at | 0.0001752 | 0.9842 |
| Bt.11126.3.S1\_a\_at | 0.0001758 | 0.7487 |
| Bt.19431.1.S1\_at | 0.0001758 | -0.6344 |
| Bt.11797.1.S1\_at | 0.0001761 | 0.8933 |
| Bt.9948.1.S1\_at | 0.0001761 | 0.5811 |
| Bt.19692.1.S1\_at | 0.0001762 | -0.6353 |
| Bt.25957.1.S1\_at | 0.0001769 | -0.6051 |
| Bt.27139.1.S1\_at | 0.0001772 | -0.6477 |
| Bt.676.1.S1\_at | 0.0001772 | 0.6566 |
| Bt.9578.1.S1\_at | 0.0001773 | -0.7393 |
| Bt.1415.1.S1\_at | 0.0001784 | -0.8627 |
| Bt.9730.1.S1\_at | 0.0001786 | -0.6611 |
| Bt.5351.1.S1\_a\_at | 0.000179 | 0.7447 |
| Bt.29638.1.A1\_at | 0.0001791 | -1.035 |
| Bt.24899.1.A1\_at | 0.0001793 | 0.902 |
| Bt.3573.1.S1\_at | 0.0001795 | 0.4968 |
| Bt.5106.2.S1\_at | 0.0001818 | 0.538 |
| Bt.12370.1.S1\_at | 0.0001819 | -0.5765 |
| Bt.25069.1.A1\_at | 0.0001827 | -0.5511 |
| Bt.21410.1.S1\_at | 0.0001829 | -0.6276 |
| Bt.4683.1.S1\_at | 0.0001833 | 1.151 |
| Bt.22325.2.A1\_at | 0.0001835 | 0.7507 |
| Bt.20852.1.A1\_at | 0.0001835 | 0.9203 |
| Bt.25312.1.A1\_at | 0.000184 | 0.7818 |
| Bt.6686.1.S1\_at | 0.0001845 | 0.8917 |
| Bt.22453.2.S1\_at | 0.0001847 | 0.671 |
| Bt.3990.1.S1\_at | 0.0001859 | 0.7921 |
| Bt.6399.2.S1\_a\_at | 0.0001866 | 0.8235 |
| Bt.19522.2.S1\_at | 0.0001867 | 0.6667 |
| Bt.28244.1.S1\_at | 0.0001873 | -0.751 |
| Bt.3342.1.S1\_at | 0.000188 | -0.7938 |
| Bt.23169.1.S1\_at | 0.0001883 | 0.7842 |
| Bt.621.1.S1\_at | 0.0001891 | -0.9152 |
| Bt.22176.1.A1\_at | 0.0001891 | 1.057 |
| Bt.2120.1.S1\_at | 0.0001891 | 1.155 |
| Bt.8286.1.S1\_at | 0.0001895 | -0.7607 |
| Bt.25871.1.A1\_at | 0.0001898 | 0.7335 |
| Bt.5038.5.S1\_at | 0.0001917 | 0.7363 |
| Bt.28733.1.S1\_at | 0.0001918 | 0.6457 |
| Bt.12054.2.S1\_at | 0.0001923 | 0.7452 |
| Bt.27655.1.S1\_at | 0.0001932 | -1.173 |
| Bt.2362.1.S1\_at | 0.0001933 | -0.7524 |
| Bt.28596.1.S1\_at | 0.0001944 | 0.6918 |
| Bt.20872.1.A1\_at | 0.0001952 | 0.7493 |
| Bt.12217.1.S1\_at | 0.0001953 | 1.24 |
| Bt.9238.1.S1\_at | 0.0001955 | -0.9773 |
| Bt.25092.1.S1\_at | 0.0001958 | -0.8245 |
| Bt.3426.1.S1\_at | 0.0001958 | 0.7421 |
| Bt.10218.1.S1\_s\_at | 0.0001961 | 0.6805 |
| Bt.5788.1.S1\_at | 0.0001971 | 0.7703 |
| Bt.2418.1.S1\_at | 0.0001971 | 0.7198 |
| Bt.21133.1.S1\_at | 0.0001972 | 1.622 |
| Bt.25052.1.S1\_at | 0.0001973 | 0.7039 |
| Bt.18459.1.A1\_at | 0.0001976 | 0.7315 |
| Bt.13410.1.S1\_at | 0.0001979 | -0.7196 |
| Bt.23622.1.A1\_at | 0.0001981 | 0.6885 |
| Bt.28562.1.S1\_at | 0.000199 | 0.6176 |
| Bt.26393.1.A1\_at | 0.000199 | 0.9877 |
| Bt.24881.1.S1\_at | 0.0001994 | 0.8007 |
| Bt.20340.2.S1\_at | 2e-04 | 1.055 |
| Bt.5378.1.S1\_at | 0.0002001 | -0.7555 |
| Bt.3448.1.S1\_at | 0.0002003 | -1.275 |
| Bt.19469.1.A1\_at | 0.0002013 | 0.564 |
| Bt.868.1.S1\_a\_at | 0.0002014 | -0.5999 |
| Bt.24945.1.S1\_at | 0.0002015 | -0.6305 |
| Bt.4008.1.S1\_at | 0.0002029 | -0.7061 |
| Bt.2082.1.S1\_at | 0.0002033 | 0.7676 |
| Bt.18634.2.S1\_at | 0.0002034 | 0.8672 |
| Bt.16628.2.A1\_at | 0.0002036 | 1.166 |
| Bt.15799.1.S1\_at | 0.0002039 | 0.8077 |
| Bt.2064.1.S1\_at | 0.0002039 | -0.5795 |
| Bt.6017.1.A1\_at | 0.0002044 | -0.8523 |
| Bt.20917.2.S1\_a\_at | 0.0002046 | -0.899 |
| Bt.4031.2.S1\_at | 0.000206 | 0.6506 |
| Bt.26726.2.S1\_at | 0.0002061 | 0.7328 |
| Bt.25404.2.S1\_at | 0.0002072 | 0.8995 |
| Bt.26771.1.S1\_at | 0.0002075 | 0.9523 |
| Bt.7133.1.S1\_at | 0.000209 | -0.7681 |
| Bt.2297.1.S1\_at | 0.0002095 | -0.5605 |
| Bt.18408.1.A1\_at | 0.0002109 | 1.1 |
| Bt.3484.1.S1\_at | 0.0002113 | 0.8765 |
| Bt.21122.1.S1\_at | 0.0002114 | -0.822 |
| Bt.20431.2.S1\_at | 0.0002123 | 1.126 |
| Bt.21272.1.S1\_at | 0.0002131 | -0.6901 |
| Bt.9710.1.S1\_at | 0.0002138 | 0.6514 |
| Bt.29572.1.A1\_at | 0.0002151 | 0.6974 |
| Bt.149.1.S1\_at | 0.0002153 | 0.8282 |
| Bt.10266.1.S1\_at | 0.0002156 | -0.8692 |
| Bt.10359.1.S1\_at | 0.0002157 | 0.91 |
| Bt.6440.1.S1\_at | 0.0002157 | 0.8158 |
| Bt.26306.1.A1\_at | 0.0002158 | 0.7279 |
| Bt.15575.1.A1\_at | 0.000216 | -0.6954 |
| Bt.26447.1.A1\_at | 0.0002175 | 0.8312 |
| Bt.6701.1.S1\_at | 0.0002182 | 0.6909 |
| Bt.5495.1.S1\_at | 0.0002188 | -1.05 |
| Bt.12429.1.S1\_at | 0.0002194 | -0.7035 |
| Bt.396.2.S1\_a\_at | 0.0002202 | 0.7659 |
| Bt.9026.1.S1\_at | 0.0002209 | 0.7943 |
| Bt.20281.2.S1\_a\_at | 0.0002213 | -0.8345 |
| Bt.3494.1.A1\_s\_at | 0.0002226 | 0.854 |
| Bt.17810.1.S1\_a\_at | 0.0002233 | 0.7074 |
| Bt.20465.1.S1\_at | 0.0002242 | 0.6035 |
| Bt.7457.1.S1\_at | 0.0002243 | -0.6298 |
| Bt.13588.3.A1\_at | 0.0002249 | -2.311 |
| Bt.3213.1.A1\_at | 0.000225 | 1.45 |
| Bt.29874.1.S1\_at | 0.0002255 | 0.8744 |
| Bt.19676.1.S1\_at | 0.0002256 | -0.6818 |
| Bt.211.1.S1\_at | 0.0002266 | -1.012 |
| Bt.23265.1.S1\_at | 0.0002267 | 0.8814 |
| Bt.20020.1.S1\_at | 0.0002283 | 0.8105 |
| Bt.19295.1.S1\_at | 0.0002286 | -0.7359 |
| Bt.6289.1.S1\_at | 0.0002291 | 0.7546 |
| Bt.28836.1.S1\_at | 0.0002292 | 0.8662 |
| Bt.22170.2.A1\_at | 0.0002299 | -0.5622 |
| Bt.22414.2.S1\_at | 0.0002301 | 1.52 |
| Bt.9162.1.A1\_at | 0.0002303 | -0.9534 |
| Bt.18002.2.S1\_at | 0.0002304 | 1.041 |
| Bt.9184.1.S1\_at | 0.0002309 | 0.506 |
| Bt.28729.1.A1\_at | 0.0002317 | 0.9509 |
| Bt.16243.1.S1\_at | 0.0002325 | 0.9083 |
| Bt.23551.2.S1\_at | 0.0002329 | -0.8491 |
| Bt.12768.1.S1\_at | 0.0002335 | -0.6745 |
| Bt.22122.1.S1\_at | 0.0002336 | 0.6041 |
| Bt.17029.1.A1\_at | 0.0002337 | -0.6431 |
| Bt.5484.1.S1\_at | 0.0002339 | -0.7697 |
| Bt.24896.1.A1\_at | 0.0002342 | 0.6524 |
| Bt.4478.1.S1\_at | 0.0002343 | -0.8886 |
| Bt.1426.1.S1\_at | 0.0002343 | -0.733 |
| Bt.24584.1.S1\_at | 0.0002351 | 0.5978 |
| Bt.27396.1.S1\_at | 0.0002351 | 0.7691 |
| Bt.3599.1.S1\_at | 0.0002353 | 0.5817 |
| Bt.9633.2.S1\_at | 0.0002361 | 1.196 |
| Bt.8392.1.S1\_at | 0.0002372 | 0.6866 |
| Bt.20800.1.S1\_at | 0.0002372 | 0.8668 |
| Bt.7716.1.S1\_at | 0.0002378 | 0.7774 |
| Bt.20151.1.S1\_at | 0.0002385 | 1.356 |
| Bt.12379.1.S1\_at | 0.0002385 | 0.7726 |
| Bt.27187.1.S1\_at | 0.0002388 | -0.7209 |
| Bt.29948.1.S1\_at | 0.0002389 | 0.6968 |
| Bt.5027.1.A1\_at | 0.0002389 | 1.132 |
| Bt.20604.1.S1\_at | 0.0002394 | -0.6903 |
| Bt.21217.1.S1\_at | 0.0002396 | -0.6196 |
| Bt.27934.1.S1\_at | 0.0002402 | 0.6099 |
| Bt.20652.1.A1\_at | 0.0002411 | -0.7104 |
| Bt.16392.2.A1\_at | 0.0002416 | -0.5479 |
| Bt.6259.1.S1\_at | 0.0002418 | 0.8922 |
| Bt.2871.1.S1\_at | 0.0002423 | 0.6904 |
| Bt.13248.1.A1\_at | 0.0002426 | 1.038 |
| Bt.1670.1.A1\_at | 0.000243 | -0.7942 |
| Bt.5560.1.S1\_at | 0.0002433 | 0.9677 |
| Bt.16142.2.S1\_at | 0.0002434 | -0.858 |
| Bt.12888.1.S1\_at | 0.0002435 | -0.8819 |
| Bt.10312.1.A1\_at | 0.0002442 | 0.5563 |
| Bt.20528.1.S1\_at | 0.0002452 | 0.8056 |
| Bt.3823.1.S1\_at | 0.0002463 | -0.7344 |
| Bt.25198.1.A1\_at | 0.000247 | 0.6689 |
| Bt.23002.1.S1\_s\_at | 0.0002484 | 0.5753 |
| Bt.8766.1.S1\_at | 0.0002487 | 0.6663 |
| Bt.2005.1.S1\_at | 0.0002488 | -0.5318 |
| Bt.28185.1.S1\_at | 0.0002504 | 1.408 |
| Bt.16861.1.A1\_at | 0.0002511 | 0.7526 |
| Bt.9416.1.S1\_at | 0.0002512 | 0.8317 |
| Bt.22198.1.S1\_at | 0.0002516 | 0.8543 |
| Bt.7955.1.S1\_at | 0.0002521 | 0.6989 |
| Bt.16733.1.A1\_s\_at | 0.000253 | 0.8506 |
| Bt.12315.1.S1\_at | 0.0002543 | 0.7495 |
| Bt.24963.1.A1\_at | 0.000255 | -0.7339 |
| Bt.10610.1.S1\_at | 0.0002552 | 0.8176 |
| Bt.27627.1.A1\_at | 0.0002556 | -0.5739 |
| Bt.29926.2.S1\_at | 0.0002556 | 0.777 |
| Bt.22770.1.S1\_at | 0.0002578 | 0.9398 |
| Bt.3589.2.A1\_at | 0.0002579 | 1.109 |
| Bt.25921.1.A1\_at | 0.0002595 | 0.6461 |
| Bt.8976.1.S1\_at | 0.0002596 | 1.093 |
| Bt.22084.2.S1\_at | 0.0002599 | 1.016 |
| Bt.20328.1.S1\_at | 0.0002605 | 0.5073 |
| Bt.25137.1.A1\_at | 0.0002614 | 0.703 |
| Bt.13881.1.S1\_at | 0.0002616 | 0.827 |
| Bt.27400.1.S1\_at | 0.0002621 | 1.091 |
| Bt.22054.1.S1\_at | 0.0002631 | -0.7126 |
| Bt.18702.1.A1\_at | 0.0002644 | -0.7335 |
| Bt.12712.1.A1\_at | 0.0002651 | 0.7941 |
| Bt.28050.1.A1\_at | 0.0002662 | 0.5207 |
| Bt.13419.1.S1\_at | 0.0002663 | 0.7448 |
| Bt.13925.1.S1\_at | 0.0002667 | 0.6677 |
| Bt.27223.2.S1\_a\_at | 0.0002671 | -0.8067 |
| Bt.9741.1.S1\_at | 0.0002672 | 0.7215 |
| Bt.28390.1.S1\_at | 0.0002674 | -0.768 |
| Bt.27481.1.A1\_at | 0.0002675 | 1.02 |
| Bt.4520.1.S1\_at | 0.0002676 | 0.6008 |
| Bt.17932.1.S1\_at | 0.0002681 | 0.8696 |
| Bt.26803.1.A1\_at | 0.0002711 | 0.6627 |
| Bt.1000.1.S1\_at | 0.0002717 | 0.7075 |
| Bt.1560.1.S1\_at | 0.0002721 | 0.9513 |
| Bt.17594.1.S1\_at | 0.0002733 | -0.5243 |
| Bt.21843.1.A1\_at | 0.0002746 | 1.104 |
| Bt.4705.1.S1\_at | 0.0002754 | -0.6497 |
| Bt.22441.1.S1\_at | 0.0002754 | 0.5728 |
| Bt.12111.1.S1\_at | 0.0002759 | -0.6005 |
| Bt.17488.1.A1\_at | 0.0002761 | 0.7675 |
| Bt.20936.1.S1\_at | 0.0002777 | 1.079 |
| Bt.17105.1.A1\_at | 0.0002783 | 0.64 |
| Bt.16737.1.A1\_at | 0.0002786 | 0.915 |
| Bt.26751.1.S1\_at | 0.0002793 | -0.522 |
| Bt.2235.1.S2\_at | 0.0002793 | 0.8628 |
| Bt.16139.1.A1\_at | 0.0002795 | -0.6642 |
| Bt.4580.1.A1\_at | 0.0002797 | 0.6997 |
| Bt.23468.1.A1\_at | 0.0002804 | 0.6285 |
| Bt.14193.1.A1\_at | 0.0002806 | -0.5706 |
| Bt.27301.1.S1\_at | 0.0002811 | -1.021 |
| Bt.3604.1.A1\_at | 0.0002812 | 0.8009 |
| Bt.25725.1.A1\_at | 0.0002821 | -0.8152 |
| Bt.1463.1.S1\_at | 0.0002829 | 0.5646 |
| Bt.8690.2.S1\_at | 0.0002829 | -0.694 |
| Bt.11411.1.S1\_at | 0.000283 | -0.747 |
| Bt.21741.2.A1\_at | 0.0002831 | 1.062 |
| Bt.12567.1.A1\_at | 0.0002835 | -0.8175 |
| Bt.27617.1.S1\_at | 0.0002836 | 0.8562 |
| Bt.23830.1.A1\_at | 0.0002837 | 0.7794 |
| Bt.21372.1.S1\_at | 0.0002841 | 1.022 |
| Bt.2975.1.S1\_at | 0.0002857 | 0.7602 |
| Bt.6493.1.S1\_at | 0.0002877 | 0.5744 |
| Bt.3059.1.S1\_at | 0.0002877 | -0.6316 |
| Bt.17460.1.A1\_at | 0.0002877 | 1.238 |
| Bt.18316.2.S1\_at | 0.0002881 | 1.102 |
| Bt.24611.1.S1\_at | 0.0002884 | 0.6905 |
| Bt.7266.1.S1\_at | 0.0002889 | -1.021 |
| Bt.13994.1.S1\_at | 0.000289 | 0.8601 |
| Bt.9866.1.S1\_at | 0.0002918 | -0.9111 |
| Bt.3394.1.S1\_at | 0.0002921 | 0.755 |
| Bt.11588.1.A1\_at | 0.0002924 | 0.8267 |
| Bt.2065.1.A1\_at | 0.0002927 | 0.5368 |
| Bt.605.1.S1\_at | 0.0002931 | 1.446 |
| Bt.21284.2.S1\_at | 0.0002948 | 1.076 |
| Bt.16382.1.A1\_at | 0.0002956 | 0.6213 |
| Bt.28486.1.S1\_at | 0.0002966 | -0.486 |
| Bt.8966.1.S2\_at | 0.0002974 | -0.588 |
| Bt.1237.1.S1\_at | 0.0002979 | 0.8517 |
| Bt.6827.1.S1\_at | 0.0002988 | -0.6871 |
| Bt.20182.1.S1\_at | 0.0002989 | 0.6889 |
| Bt.20677.1.S1\_at | 0.0003005 | 0.789 |
| Bt.10101.2.S1\_at | 0.0003005 | 0.6382 |
| Bt.250.1.S1\_at | 0.0003007 | -0.6333 |
| Bt.22768.1.A1\_at | 0.000301 | 0.7178 |
| Bt.13259.1.S1\_at | 0.0003015 | -0.7684 |
| Bt.6157.1.S1\_at | 0.0003021 | 0.7212 |
| Bt.12722.1.A1\_at | 0.0003029 | 1.01 |
| Bt.21774.2.S1\_a\_at | 0.0003035 | 0.5606 |
| Bt.2052.1.S1\_a\_at | 0.0003038 | 0.6959 |
| Bt.26756.1.S1\_at | 0.0003043 | 0.5136 |
| Bt.16041.1.S1\_at | 0.0003046 | 0.9281 |
| Bt.12698.1.A1\_at | 0.0003065 | 0.8325 |
| Bt.4977.1.S1\_at | 0.0003075 | 0.7016 |
| Bt.4119.1.S1\_at | 0.0003076 | 0.7277 |
| Bt.10458.2.S1\_at | 0.0003078 | 1.062 |
| Bt.21876.1.A1\_at | 0.0003081 | 0.7817 |
| Bt.18135.1.S1\_at | 0.0003088 | 0.9216 |
| Bt.2853.1.S1\_at | 0.0003097 | -0.8018 |
| Bt.16606.1.A1\_at | 0.0003098 | 0.794 |
| Bt.4294.1.S1\_at | 0.0003102 | -0.5738 |
| Bt.21372.2.S1\_at | 0.0003121 | 1.018 |
| Bt.27832.1.S1\_at | 0.0003123 | -0.5109 |
| Bt.27160.1.A1\_at | 0.0003126 | 0.7129 |
| Bt.12603.1.S1\_at | 0.0003139 | 0.819 |
| Bt.10360.1.S1\_at | 0.000314 | 0.8349 |
| Bt.27100.1.S1\_at | 0.000314 | 0.5831 |
| Bt.23654.1.S1\_at | 0.000315 | 0.9058 |
| Bt.20214.2.S1\_at | 0.0003154 | -1.012 |
| Bt.58.1.S1\_at | 0.000316 | -0.5424 |
| Bt.16125.2.S1\_at | 0.000317 | -1.04 |
| Bt.18709.1.A1\_s\_at | 0.0003171 | 0.7151 |
| Bt.9544.1.S1\_at | 0.0003174 | 0.6987 |
| Bt.26609.1.S1\_at | 0.0003176 | 0.746 |
| Bt.12191.1.S1\_at | 0.0003179 | 0.6809 |
| Bt.19332.1.S1\_at | 0.0003192 | 1.093 |
| Bt.22444.1.A1\_at | 0.0003197 | 1.088 |
| Bt.24321.1.S1\_at | 0.0003198 | 0.6604 |
| Bt.28978.1.A1\_at | 0.0003219 | -0.5998 |
| Bt.26999.1.S1\_at | 0.000322 | -0.6154 |
| Bt.8641.1.A1\_at | 0.0003221 | 0.7477 |
| Bt.10381.1.S1\_at | 0.0003223 | 0.6393 |
| Bt.20322.3.S1\_a\_at | 0.0003236 | -0.6606 |
| Bt.2230.1.S1\_at | 0.0003244 | 1.147 |
| Bt.20029.1.S1\_at | 0.0003246 | 0.5406 |
| Bt.2451.1.S1\_at | 0.0003251 | 0.9243 |
| Bt.13529.1.S1\_at | 0.0003254 | 0.7525 |
| Bt.9197.1.S1\_at | 0.0003255 | 0.8911 |
| Bt.20238.1.S1\_at | 0.0003255 | 0.807 |
| Bt.8486.2.S1\_at | 0.0003258 | 0.5152 |
| Bt.27887.1.S1\_at | 0.0003261 | 0.9903 |
| Bt.27565.1.S1\_at | 0.000328 | 0.5429 |
| Bt.22125.1.S1\_at | 0.000328 | 0.6992 |
| Bt.3228.1.S1\_at | 0.0003286 | 0.7071 |
| Bt.6279.1.A1\_at | 0.000329 | 0.7312 |
| Bt.5507.2.S1\_at | 0.0003303 | 0.6807 |
| Bt.22526.1.S1\_at | 0.0003307 | -0.6729 |
| Bt.8275.1.S1\_at | 0.0003318 | 1.025 |
| Bt.3879.1.S1\_at | 0.0003319 | 1.011 |
| Bt.28057.1.S1\_at | 0.0003323 | 0.6446 |
| Bt.5376.1.S1\_at | 0.0003351 | -0.5222 |
| Bt.22502.1.S1\_at | 0.0003362 | 0.6612 |
| Bt.532.1.S1\_at | 0.0003366 | -0.7205 |
| Bt.21787.1.S1\_at | 0.000337 | 0.6018 |
| Bt.13408.2.S1\_at | 0.0003376 | 0.8236 |
| Bt.13291.1.S1\_at | 0.0003389 | 0.8456 |
| Bt.22553.1.A1\_at | 0.0003416 | 0.7521 |
| Bt.4099.1.S1\_at | 0.0003423 | -0.6281 |
| Bt.26881.3.S1\_at | 0.0003424 | -0.6676 |
| Bt.22639.1.A1\_at | 0.000343 | -0.9327 |
| Bt.11270.2.S1\_at | 0.0003433 | -0.8201 |
| Bt.22168.1.A1\_at | 0.0003445 | -1.045 |
| Bt.23519.1.S1\_at | 0.000345 | -0.7228 |
| Bt.13124.1.S1\_at | 0.0003455 | -0.8466 |
| Bt.4697.1.S2\_at | 0.0003478 | 0.8645 |
| Bt.19295.2.S1\_at | 0.0003487 | -0.7176 |
| Bt.21288.2.S1\_at | 0.0003493 | 0.5746 |
| Bt.20170.1.S1\_at | 0.00035 | 0.7006 |
| Bt.8012.1.S1\_at | 0.0003501 | 0.5672 |
| Bt.1595.1.S1\_at | 0.0003502 | 0.7676 |
| Bt.11262.2.S1\_at | 0.0003507 | 1.116 |
| Bt.21404.1.S1\_at | 0.0003509 | 0.6157 |
| Bt.19425.1.S1\_at | 0.0003509 | -0.6775 |
| Bt.28783.1.A1\_at | 0.0003517 | -0.7105 |
| Bt.25226.1.A1\_at | 0.0003517 | 1.161 |
| Bt.22295.1.S1\_at | 0.0003523 | -0.622 |
| Bt.5393.2.S1\_a\_at | 0.000353 | 0.6677 |
| Bt.26812.1.S1\_at | 0.0003536 | -0.664 |
| Bt.3323.1.S1\_at | 0.0003568 | 0.5947 |
| Bt.23361.1.S1\_at | 0.0003569 | -0.541 |
| Bt.24594.1.A1\_at | 0.0003578 | -1.417 |
| Bt.16061.1.S1\_at | 0.0003582 | 0.534 |
| Bt.236.1.S1\_at | 0.0003589 | 1.305 |
| Bt.1788.1.A1\_at | 0.0003594 | 0.7035 |
| Bt.14137.1.A1\_at | 0.0003596 | 0.8213 |
| Bt.9833.1.S1\_a\_at | 0.0003597 | -0.9854 |
| Bt.26613.1.A1\_at | 0.00036 | -0.7866 |
| Bt.1729.1.A1\_at | 0.0003603 | 0.6832 |
| Bt.1920.1.S1\_at | 0.0003609 | -0.5995 |
| Bt.26575.1.S1\_at | 0.000361 | 0.8482 |
| Bt.6685.1.S1\_at | 0.0003624 | -0.7306 |
| Bt.5626.2.S1\_at | 0.0003628 | -0.7205 |
| Bt.1617.2.S1\_a\_at | 0.0003629 | 0.6496 |
| Bt.24536.1.S1\_at | 0.0003637 | 1.114 |
| Bt.9639.1.S1\_at | 0.0003639 | 0.7899 |
| Bt.11182.2.S1\_a\_at | 0.0003653 | 0.8389 |
| Bt.26684.1.S1\_at | 0.0003656 | 0.6082 |
| Bt.4071.1.S1\_at | 0.0003671 | -0.5413 |
| Bt.10358.1.S1\_at | 0.0003675 | 0.702 |
| Bt.6688.1.S1\_at | 0.0003686 | -0.7191 |
| Bt.19266.1.A1\_at | 0.0003696 | 0.8075 |
| Bt.26445.1.A1\_at | 0.0003697 | 0.8964 |
| Bt.25165.1.S1\_at | 0.0003708 | -1.098 |
| Bt.11258.2.A1\_a\_at | 0.0003713 | -0.4953 |
| Bt.8164.1.S1\_at | 0.0003714 | -0.6757 |
| Bt.5345.1.S1\_at | 0.0003719 | -0.7413 |
| Bt.9240.1.A1\_at | 0.000372 | -0.6071 |
| Bt.22538.1.A1\_at | 0.0003742 | 1.1 |
| Bt.26080.1.S1\_at | 0.0003754 | 0.7838 |
| Bt.21365.2.S1\_at | 0.0003766 | 0.5467 |
| Bt.28442.1.S1\_at | 0.0003786 | -0.6453 |
| Bt.9459.1.S1\_at | 0.0003787 | -0.7583 |
| Bt.13969.1.A1\_at | 0.0003804 | 0.7084 |
| Bt.5542.2.S1\_at | 0.0003805 | 0.8063 |
| Bt.759.1.S1\_at | 0.0003846 | 0.5681 |
| Bt.22663.1.A1\_at | 0.0003852 | -0.5238 |
| Bt.24727.1.S1\_a\_at | 0.0003853 | -0.7757 |
| Bt.22349.2.S1\_at | 0.0003884 | 1.193 |
| Bt.15922.1.S1\_at | 0.0003887 | 0.547 |
| Bt.20091.1.S1\_at | 0.000389 | 0.7189 |
| Bt.17758.1.S1\_at | 0.0003892 | 0.7026 |
| Bt.1950.1.S1\_at | 0.0003892 | -0.7177 |
| Bt.2077.1.S1\_at | 0.0003893 | 0.6862 |
| Bt.18414.1.S1\_at | 0.0003896 | -1.063 |
| Bt.4652.1.S1\_at | 0.0003898 | -0.7059 |
| Bt.9089.1.S1\_at | 0.0003902 | -0.5396 |
| Bt.26594.1.S1\_at | 0.0003903 | -0.6301 |
| Bt.8785.1.S1\_at | 0.0003909 | 0.9627 |
| Bt.2510.1.A1\_at | 0.0003921 | 0.8816 |
| Bt.21308.1.A1\_at | 0.0003926 | 0.5318 |
| Bt.27269.2.S1\_at | 0.0003936 | 0.5661 |
| Bt.10349.1.S1\_at | 0.0003938 | 0.9919 |
| Bt.8003.1.A1\_at | 0.0003947 | -0.6819 |
| Bt.9973.1.S2\_at | 0.000395 | 0.5633 |
| Bt.1987.1.S1\_at | 0.0003953 | 0.4938 |
| Bt.27044.1.A1\_at | 0.0003956 | -0.9025 |
| Bt.15906.1.S1\_at | 0.0003963 | 0.8138 |
| Bt.25412.1.A1\_at | 0.0003965 | 1.797 |
| Bt.5828.1.S1\_at | 0.0003966 | -0.7641 |
| Bt.26915.2.S1\_at | 0.0003975 | 0.788 |
| Bt.10135.1.A1\_at | 0.0003988 | 1.122 |
| Bt.25419.1.A1\_at | 0.0003997 | -1.322 |
| Bt.28684.1.S1\_at | 0.0004008 | -0.647 |
| Bt.24253.1.S1\_at | 0.0004015 | 0.8317 |
| Bt.13950.1.A1\_at | 0.0004018 | -0.5983 |
| Bt.4241.1.S1\_at | 0.0004023 | 0.4493 |
| Bt.3516.1.S1\_at | 0.0004029 | -0.5695 |
| Bt.1747.1.S1\_at | 0.0004031 | -1.066 |
| Bt.13205.2.S1\_at | 0.0004034 | -0.8191 |
| Bt.3163.1.A1\_at | 0.0004047 | 0.5875 |
| Bt.18532.1.A1\_at | 0.000405 | 0.9125 |
| Bt.14351.1.A1\_at | 0.0004061 | -0.857 |
| Bt.20314.2.S1\_at | 0.0004062 | -0.7398 |
| Bt.5174.1.S1\_at | 0.0004072 | 0.6774 |
| Bt.11530.1.A1\_at | 0.0004075 | 0.6822 |
| Bt.12249.1.S1\_at | 0.0004099 | 0.5769 |
| Bt.23569.1.S1\_at | 0.0004107 | -0.7234 |
| Bt.5848.1.S1\_at | 0.000411 | -0.7178 |
| Bt.13912.1.S1\_at | 0.0004112 | 0.654 |
| Bt.13593.1.A1\_at | 0.0004114 | 0.6283 |
| Bt.5939.1.S1\_a\_at | 0.0004117 | -0.514 |
| Bt.8920.1.S1\_at | 0.0004119 | 0.8368 |
| Bt.15774.1.S1\_at | 0.0004126 | 0.8168 |
| Bt.15909.1.S1\_at | 0.0004135 | -0.9232 |
| Bt.12498.1.A1\_at | 0.0004158 | 0.6287 |
| Bt.11052.1.S1\_at | 0.0004176 | 0.6457 |
| Bt.24397.1.S1\_at | 0.0004183 | 0.5319 |
| Bt.11932.1.S1\_at | 0.0004186 | 0.5897 |
| Bt.12412.1.S1\_at | 0.0004186 | 0.8649 |
| Bt.19807.1.S1\_at | 0.0004187 | 0.4898 |
| Bt.3363.1.S1\_at | 0.0004199 | 0.8714 |
| Bt.10043.1.S1\_at | 0.0004212 | -0.5249 |
| Bt.1057.1.S1\_at | 0.0004219 | 0.929 |
| Bt.19199.1.A1\_at | 0.0004231 | 1.315 |
| Bt.11315.1.A1\_at | 0.0004236 | 0.7727 |
| Bt.2513.1.S1\_at | 0.0004245 | 0.5617 |
| Bt.23094.3.S1\_x\_at | 0.0004247 | -0.9583 |
| Bt.3014.1.A1\_at | 0.0004254 | 0.9496 |
| Bt.28669.1.A1\_at | 0.0004254 | -0.796 |
| Bt.17526.1.A1\_at | 0.0004256 | 0.7699 |
| Bt.22584.1.A1\_at | 0.0004258 | 0.7567 |
| Bt.11717.1.S1\_s\_at | 0.0004267 | -0.5812 |
| Bt.9156.1.A1\_at | 0.0004276 | 1.2 |
| Bt.24433.2.A1\_at | 0.0004288 | -0.5133 |
| Bt.9322.1.S1\_at | 0.0004291 | 1.05 |
| Bt.4280.1.A1\_at | 0.0004294 | 0.5395 |
| Bt.16040.1.S1\_at | 0.0004306 | 0.5561 |
| Bt.16932.1.A1\_at | 0.0004329 | -0.6984 |
| Bt.1537.1.S1\_at | 0.0004329 | 0.8014 |
| Bt.28945.1.A1\_at | 0.0004336 | 0.7164 |
| Bt.27841.1.A1\_at | 0.0004336 | 1.106 |
| Bt.4140.1.S1\_at | 0.0004342 | 0.6022 |
| Bt.24319.1.A1\_at | 0.0004348 | 0.632 |
| Bt.2873.1.S1\_at | 0.000435 | -0.8078 |
| Bt.9660.1.A1\_s\_at | 0.0004362 | 0.5714 |
| Bt.26674.1.S1\_at | 0.0004388 | 0.637 |
| Bt.4267.1.S1\_at | 0.0004396 | 0.5636 |
| Bt.20210.1.S1\_at | 0.0004412 | 0.5996 |
| Bt.26643.1.S1\_at | 0.0004452 | 0.6153 |
| Bt.19324.1.A1\_at | 0.0004457 | 0.6561 |
| Bt.5872.1.S1\_at | 0.0004461 | 0.4824 |
| Bt.15561.2.A1\_s\_at | 0.0004463 | -1.102 |
| Bt.4091.3.S1\_a\_at | 0.0004465 | -0.7601 |
| Bt.11125.1.A1\_at | 0.0004466 | -0.8283 |
| Bt.28728.1.A1\_at | 0.0004474 | -0.8545 |
| Bt.7384.2.S1\_at | 0.0004476 | 0.7856 |
| Bt.13345.1.A1\_at | 0.0004488 | 0.5983 |
| Bt.1692.1.S1\_at | 0.000449 | -0.6909 |
| Bt.7991.1.S1\_a\_at | 0.0004499 | 0.5722 |
| Bt.14054.2.S1\_at | 0.000451 | -1.214 |
| Bt.28914.1.A1\_at | 0.0004521 | 1.074 |
| Bt.4859.1.S1\_at | 0.0004525 | 0.5632 |
| Bt.2453.1.S1\_at | 0.0004544 | 0.4876 |
| Bt.8392.2.S1\_at | 0.0004547 | 0.7515 |
| Bt.12811.1.S1\_at | 0.0004555 | 0.5774 |
| Bt.7139.1.S1\_at | 0.000456 | -0.6721 |
| Bt.20817.1.A1\_at | 0.0004562 | 0.65 |
| Bt.27232.1.S1\_at | 0.000458 | 0.4769 |
| Bt.20481.1.S1\_at | 0.0004587 | 1.049 |
| Bt.11088.1.S1\_a\_at | 0.0004603 | 0.6362 |
| Bt.5748.3.S1\_at | 0.0004617 | 0.8433 |
| Bt.22461.1.S1\_at | 0.0004623 | 0.8655 |
| Bt.10387.1.S1\_at | 0.0004635 | -0.7273 |
| Bt.12088.1.S1\_at | 0.0004671 | 0.848 |
| Bt.24259.3.S1\_at | 0.0004686 | -0.5081 |
| Bt.12661.1.A1\_at | 0.0004704 | 0.8939 |
| Bt.5848.2.S1\_at | 0.0004704 | -0.7257 |
| Bt.12220.1.S1\_a\_at | 0.0004714 | 0.7875 |
| Bt.21835.1.S1\_at | 0.0004725 | 0.6679 |
| Bt.7612.1.S1\_at | 0.0004743 | -0.5224 |
| Bt.19687.2.S1\_at | 0.0004747 | -0.7975 |
| Bt.13930.2.S1\_at | 0.0004749 | 0.6214 |
| Bt.2017.1.S1\_at | 0.0004761 | 0.664 |
| Bt.21064.1.S1\_at | 0.0004763 | -0.5104 |
| Bt.24296.1.A1\_at | 0.0004785 | 0.7868 |
| Bt.5237.1.S1\_at | 0.0004789 | 0.7252 |
| Bt.13722.2.A1\_at | 0.0004801 | 0.6394 |
| Bt.4395.1.S1\_at | 0.0004805 | 0.4891 |
| Bt.18039.1.S1\_at | 0.0004835 | 0.608 |
| Bt.8608.1.S1\_at | 0.0004836 | 0.6875 |
| Bt.19601.1.S1\_at | 0.000484 | 0.9439 |
| Bt.21761.1.S1\_a\_at | 0.0004848 | -0.8214 |
| Bt.24396.1.S1\_at | 0.000485 | -0.5034 |
| Bt.6567.1.S1\_at | 0.0004895 | 0.6532 |
| Bt.12535.1.S1\_at | 0.0004907 | -0.5742 |
| Bt.22615.2.A1\_a\_at | 0.000491 | -0.7269 |
| Bt.9412.1.S1\_at | 0.0004925 | 1.061 |
| Bt.14007.1.S1\_at | 0.0004932 | 0.666 |
| Bt.12261.1.A1\_at | 0.0004932 | 0.5607 |
| Bt.805.1.S1\_at | 0.0004946 | -0.8198 |
| Bt.6019.1.S1\_at | 0.0004949 | -0.5149 |
| Bt.6778.1.S1\_at | 0.0004954 | 1.129 |
| Bt.11925.1.A1\_at | 0.0004955 | 0.6137 |
| Bt.12024.1.S1\_at | 0.0004972 | 0.7466 |
| Bt.27208.2.S1\_at | 0.0004981 | -0.5551 |
| Bt.29226.1.A1\_at | 0.0004981 | -0.7027 |
| Bt.20023.1.S1\_at | 0.0004994 | 0.6007 |
| Bt.2765.1.S1\_at | 0.0004996 | 0.7325 |
| Bt.5458.1.S1\_at | 0.0005016 | 0.6317 |
| Bt.11078.2.S1\_at | 0.0005025 | -0.5457 |
| Bt.15607.1.S1\_at | 0.0005051 | 0.8256 |
| Bt.27097.1.A1\_at | 0.0005052 | 0.615 |
| Bt.12558.1.S1\_at | 0.000506 | 0.6835 |
| Bt.27386.2.A1\_at | 0.0005081 | 1.06 |
| Bt.3306.1.S1\_at | 0.0005089 | 0.5874 |
| Bt.18270.1.A1\_at | 0.0005105 | 0.789 |
| Bt.4817.1.A1\_at | 0.0005122 | 1.17 |
| Bt.4875.1.S1\_s\_at | 0.0005123 | 0.5749 |
| Bt.5736.1.S1\_at | 0.000513 | 0.7112 |
| Bt.29026.1.A1\_at | 0.0005138 | 0.5747 |
| Bt.12769.1.S1\_at | 0.0005139 | -1.092 |
| Bt.5445.2.A1\_at | 0.0005141 | 0.7097 |
| Bt.8909.1.S1\_at | 0.000515 | 0.8106 |
| Bt.19338.1.S1\_at | 0.0005157 | 0.9605 |
| Bt.20542.1.S1\_at | 0.0005188 | -0.5579 |
| Bt.20929.1.S1\_at | 0.000519 | 0.543 |
| Bt.10324.1.A1\_at | 0.0005198 | -0.7948 |
| Bt.2147.1.S1\_at | 0.0005199 | 0.5618 |
| Bt.23401.1.S1\_at | 0.0005203 | -0.7256 |
| Bt.11016.1.S1\_at | 0.0005211 | 0.554 |
| Bt.28212.1.A1\_at | 0.0005224 | 0.7302 |
| Bt.29625.1.S1\_at | 0.0005235 | 0.8539 |
| Bt.7728.2.S1\_at | 0.0005237 | 1.166 |
| Bt.19209.2.S1\_at | 0.000524 | 0.582 |
| Bt.4786.1.S2\_at | 0.0005247 | 0.5133 |
| Bt.13645.1.S1\_at | 0.000526 | -1.193 |
| Bt.2092.1.S1\_at | 0.0005265 | 0.7315 |
| Bt.654.1.A1\_at | 0.0005267 | 0.5187 |
| Bt.22356.1.S1\_at | 0.0005268 | 0.7222 |
| Bt.12257.1.S1\_at | 0.0005287 | 0.7072 |
| Bt.6453.1.S1\_at | 0.0005302 | 0.5049 |
| Bt.24625.1.S1\_at | 0.0005308 | 0.631 |
| Bt.24976.1.S1\_at | 0.0005315 | -0.9818 |
| Bt.17883.2.A1\_at | 0.0005324 | 0.5727 |
| Bt.222.1.S1\_at | 0.0005344 | -1.553 |
| Bt.13391.1.S1\_at | 0.0005348 | -0.6335 |
| Bt.6798.1.A1\_at | 0.0005367 | 0.8244 |
| Bt.8592.1.S1\_a\_at | 0.0005374 | -0.6057 |
| Bt.3385.2.S1\_at | 0.0005378 | 0.6294 |
| Bt.17608.1.A1\_at | 0.0005384 | 0.628 |
| Bt.615.2.S1\_a\_at | 0.0005386 | 0.9534 |
| Bt.3447.2.S1\_at | 0.0005393 | -0.7333 |
| Bt.28123.1.S1\_at | 0.0005397 | 0.8836 |
| Bt.2007.1.S1\_at | 0.0005398 | -0.7309 |
| Bt.15854.1.A1\_at | 0.0005406 | 1.265 |
| Bt.23355.1.S1\_at | 0.000541 | -0.7258 |
| Bt.28059.1.S1\_at | 0.0005414 | -0.8249 |
| Bt.4816.1.S1\_at | 0.000544 | -0.7417 |
| Bt.23307.1.S1\_at | 0.0005442 | 0.5732 |
| Bt.24517.2.A1\_at | 0.0005442 | -0.66 |
| Bt.11966.1.A1\_at | 0.0005453 | 0.9438 |
| Bt.28162.2.A1\_at | 0.000546 | -1.216 |
| Bt.22725.1.S1\_at | 0.0005463 | 0.5792 |
| Bt.10360.2.S1\_a\_at | 0.0005466 | 1.167 |
| Bt.20001.1.S1\_at | 0.0005476 | -0.5733 |
| Bt.1533.1.S1\_at | 0.0005483 | 0.5401 |
| Bt.26453.1.S1\_at | 0.0005492 | 0.8281 |
| Bt.1303.1.S1\_at | 0.0005493 | 0.8063 |
| Bt.26668.1.S1\_at | 0.0005522 | 0.7175 |
| Bt.26966.1.S1\_at | 0.0005527 | -0.8485 |
| Bt.13248.2.S1\_at | 0.000553 | 0.6364 |
| Bt.15867.1.S1\_at | 0.0005544 | 0.734 |
| Bt.8382.2.S1\_at | 0.000555 | 0.8967 |
| Bt.4388.1.S1\_at | 0.0005564 | 0.5352 |
| Bt.20240.1.S1\_at | 0.0005574 | 0.6974 |
| Bt.4764.1.S1\_at | 0.0005599 | -0.4558 |
| Bt.21071.1.S1\_at | 0.0005609 | 0.64 |
| Bt.20318.1.S1\_a\_at | 0.0005631 | -0.6828 |
| Bt.25161.1.A1\_at | 0.0005658 | 0.5252 |
| Bt.25093.1.A1\_at | 0.000567 | 0.4618 |
| Bt.22626.2.S1\_at | 0.0005674 | 0.7277 |
| Bt.29854.1.A1\_at | 0.000568 | -0.915 |
| Bt.6111.1.S1\_at | 0.0005688 | -0.676 |
| Bt.12640.1.S1\_at | 0.0005707 | -0.5838 |
| Bt.1151.1.S1\_at | 0.0005722 | -1.805 |
| Bt.3975.1.S1\_at | 0.0005731 | -0.516 |
| Bt.23129.2.S1\_at | 0.0005737 | 0.7509 |
| Bt.5202.1.S1\_at | 0.0005759 | 0.613 |
| Bt.26313.1.A1\_at | 0.0005772 | 0.6624 |
| Bt.18734.1.S1\_at | 0.0005774 | -0.6188 |
| Bt.9949.2.S1\_at | 0.0005783 | -0.778 |
| Bt.21537.1.A1\_at | 0.0005803 | -0.7097 |
| Bt.20113.1.S1\_at | 0.0005814 | 0.5643 |
| Bt.23605.2.S1\_at | 0.000582 | 0.805 |
| Bt.21777.1.S1\_at | 0.0005821 | 1.222 |
| Bt.25417.1.A1\_at | 0.0005835 | 0.6626 |
| Bt.1699.2.S1\_a\_at | 0.0005837 | 1.11 |
| Bt.22188.1.S1\_at | 0.0005897 | 0.716 |
| Bt.23224.1.S1\_at | 0.0005899 | 0.6445 |
| Bt.18990.1.S1\_at | 0.0005973 | 0.8039 |
| Bt.5366.1.S1\_at | 0.0005976 | 0.8123 |
| Bt.20445.1.S1\_at | 0.0005982 | -0.6296 |
| Bt.6125.1.S1\_at | 0.0005983 | 0.5992 |
| Bt.22125.2.A1\_at | 0.0006001 | 0.9031 |
| Bt.61.1.S1\_at | 0.0006003 | -0.57 |
| Bt.10822.1.S1\_at | 0.0006026 | 0.5549 |
| Bt.24559.1.A1\_at | 0.000603 | 0.6605 |
| Bt.26808.1.S1\_at | 0.0006032 | -0.6405 |
| Bt.19038.1.A1\_at | 0.0006034 | 0.8866 |
| Bt.5393.1.S1\_at | 0.0006043 | 0.7181 |
| Bt.5415.1.S1\_at | 0.0006043 | 0.5819 |
| Bt.11027.1.A1\_at | 0.0006058 | 0.5999 |
| Bt.22536.2.S1\_at | 0.0006059 | -0.622 |
| Bt.57.1.S1\_at | 0.000606 | -0.5291 |
| Bt.10723.1.S1\_at | 0.0006067 | 0.6992 |
| Bt.26685.1.S1\_at | 0.000607 | -0.7498 |
| Bt.1706.1.S1\_at | 0.000607 | 0.7275 |
| Bt.28662.1.A1\_at | 0.000608 | 1.084 |
| Bt.12861.1.S1\_at | 0.0006082 | 0.5778 |
| Bt.5241.1.S1\_at | 0.0006094 | -0.5748 |
| Bt.13581.1.S1\_at | 0.0006108 | 1.185 |
| Bt.8322.2.S1\_at | 0.0006115 | 0.9614 |
| Bt.5434.1.S1\_at | 0.0006161 | -0.5989 |
| Bt.22231.1.S1\_at | 0.0006168 | -0.8072 |
| Bt.16473.1.A1\_at | 0.0006169 | 0.7319 |
| Bt.23326.1.S1\_at | 0.0006208 | 0.4897 |
| Bt.9759.1.S1\_at | 0.0006217 | 0.6968 |
| Bt.14464.1.A1\_at | 0.0006235 | -0.5678 |
| Bt.20194.1.S1\_at | 0.0006245 | -0.5797 |
| Bt.15726.1.S1\_at | 0.0006272 | 0.6991 |
| Bt.19578.1.S1\_at | 0.0006315 | -0.5203 |
| Bt.24017.1.A1\_s\_at | 0.0006316 | -0.571 |
| Bt.5351.2.A1\_x\_at | 0.0006344 | 0.5427 |
| Bt.28146.1.A1\_at | 0.0006356 | -0.8859 |
| Bt.16673.1.S1\_at | 0.0006362 | -0.7418 |
| Bt.9242.1.S1\_at | 0.0006413 | -0.6534 |
| Bt.25014.1.A1\_at | 0.0006417 | -0.7036 |
| Bt.3321.1.S1\_at | 0.000642 | 0.7522 |
| Bt.27956.1.A1\_at | 0.000642 | 0.6314 |
| Bt.26606.1.S1\_at | 0.0006421 | 0.5035 |
| Bt.7041.1.S1\_at | 0.000643 | 0.6716 |
| Bt.20820.1.A1\_at | 0.0006447 | 0.7985 |
| Bt.15779.3.S1\_at | 0.0006453 | 0.8809 |
| Bt.11339.1.S1\_at | 0.0006473 | -0.602 |
| Bt.2129.1.S1\_at | 0.000648 | 0.9525 |
| Bt.18045.1.S1\_at | 0.0006494 | -0.5547 |
| Bt.19649.1.A1\_at | 0.0006503 | 0.5899 |
| Bt.14305.1.A1\_at | 0.0006518 | -0.5568 |
| Bt.17989.1.S1\_at | 0.0006528 | 0.5985 |
| Bt.7785.1.S1\_at | 0.0006544 | 0.7444 |
| Bt.9190.1.S1\_at | 0.0006563 | -0.6472 |
| Bt.20319.1.S1\_at | 0.0006565 | -0.6781 |
| Bt.22564.1.A1\_at | 0.0006569 | 0.7821 |
| Bt.20471.2.S1\_a\_at | 0.0006578 | -0.544 |
| Bt.17777.1.S1\_at | 0.0006583 | -0.6992 |
| Bt.12580.1.S1\_at | 0.0006589 | -0.6739 |
| Bt.28113.1.S1\_at | 0.000659 | 0.9423 |
| Bt.368.1.S1\_at | 0.0006607 | -0.6013 |
| Bt.12885.1.S1\_at | 0.0006658 | -0.5585 |
| Bt.2881.1.S1\_at | 0.0006671 | 0.9983 |
| Bt.11039.2.S1\_a\_at | 0.0006692 | -0.5902 |
| Bt.3478.1.A1\_s\_at | 0.0006694 | 0.8573 |
| Bt.12434.1.S1\_at | 0.0006702 | -0.6569 |
| Bt.1638.1.S1\_at | 0.0006718 | 0.7389 |
| Bt.6499.1.S1\_at | 0.0006736 | 0.7067 |
| Bt.4863.1.S1\_at | 0.0006749 | 0.741 |
| Bt.12508.1.S1\_at | 0.0006796 | -0.7437 |
| Bt.18623.1.A1\_at | 0.0006826 | -0.9183 |
| Bt.5494.1.S1\_at | 0.0006831 | 0.8236 |
| Bt.21534.3.S1\_a\_at | 0.0006837 | -0.7125 |
| Bt.9221.1.A1\_at | 0.000684 | -0.7557 |
| Bt.2394.1.S1\_at | 0.0006841 | -0.6297 |
| Bt.1963.1.S1\_at | 0.0006842 | -0.5137 |
| Bt.4020.1.S1\_at | 0.0006843 | -0.5896 |
| Bt.4685.1.S1\_at | 0.0006845 | 0.8501 |
| Bt.28209.1.S1\_at | 0.0006859 | 0.6346 |
| Bt.4431.1.S1\_a\_at | 0.0006876 | -0.6241 |
| Bt.26976.1.S1\_at | 0.0006892 | -0.7667 |
| Bt.12217.3.A1\_at | 0.0006917 | 0.9548 |
| Bt.22865.1.S2\_at | 0.0006931 | 0.6213 |
| Bt.7421.1.S1\_at | 0.0006937 | -0.9827 |
| Bt.16124.2.S1\_at | 0.0006939 | 0.8412 |
| Bt.28207.1.S1\_at | 0.0006953 | 0.8384 |
| Bt.3422.1.S1\_at | 0.0006955 | 0.64 |
| Bt.22483.1.S1\_at | 0.0006978 | 0.6338 |
| Bt.21380.2.S1\_at | 0.0006985 | -0.7169 |
| Bt.1062.1.S1\_at | 0.000699 | 0.8308 |
| Bt.1386.1.S1\_at | 0.0007009 | 0.571 |
| Bt.14123.1.S1\_at | 0.0007023 | -1.086 |
| Bt.28140.1.S1\_at | 0.0007025 | -0.6458 |
| Bt.26573.2.S1\_at | 0.0007077 | -0.8648 |
| Bt.24333.1.S1\_at | 0.0007083 | 0.5918 |
| Bt.20132.1.S1\_at | 0.0007083 | 0.6691 |
| Bt.25285.1.A1\_at | 0.0007085 | 0.6354 |
| Bt.18571.3.A1\_at | 0.000709 | -0.8145 |
| Bt.20041.1.S1\_at | 0.000714 | -0.5345 |
| Bt.11171.1.S1\_at | 0.0007178 | 0.6737 |
| Bt.13464.1.S1\_at | 0.0007179 | -0.618 |
| Bt.13991.2.S1\_a\_at | 0.0007181 | 0.8441 |
| Bt.26615.1.S1\_at | 0.0007189 | -0.4299 |
| Bt.11237.1.S1\_at | 0.0007191 | 0.9243 |
| Bt.20331.2.S1\_at | 0.00072 | 0.7952 |
| Bt.1389.1.S1\_at | 0.00072 | 0.5278 |
| Bt.16360.1.A1\_at | 0.0007203 | 0.5075 |
| Bt.26851.1.S1\_at | 0.0007212 | 0.6127 |
| Bt.11485.1.A1\_at | 0.0007217 | -0.7781 |
| Bt.20458.1.S1\_at | 0.0007229 | -0.5743 |
| Bt.4161.1.S1\_at | 0.0007239 | 0.7035 |
| Bt.18115.1.A1\_at | 0.0007283 | -0.9034 |
| Bt.7184.1.S1\_at | 0.0007287 | -0.538 |
| Bt.566.1.S1\_at | 0.0007299 | -0.6518 |
| Bt.8566.1.S1\_at | 0.0007316 | 0.6116 |
| Bt.16659.1.A1\_at | 0.0007316 | -0.5402 |
| Bt.6154.1.S1\_at | 0.0007336 | -0.6073 |
| Bt.9981.1.S1\_at | 0.0007368 | -0.7108 |
| Bt.28087.1.S1\_at | 0.000737 | 0.8051 |
| Bt.26848.1.S1\_at | 0.0007377 | 0.7476 |
| Bt.21215.2.S1\_at | 0.0007378 | 0.708 |
| Bt.26143.1.A1\_at | 0.0007414 | 0.6516 |
| Bt.6441.1.S1\_at | 0.0007415 | 0.819 |
| Bt.4621.1.S1\_a\_at | 0.0007419 | 0.5339 |
| Bt.12514.1.S1\_at | 0.0007425 | -0.6416 |
| Bt.23123.1.S1\_at | 0.00075 | 0.9738 |
| Bt.22981.1.S1\_at | 0.0007504 | -0.5946 |
| Bt.22665.1.S1\_at | 0.0007518 | 0.5419 |
| Bt.7619.3.S1\_at | 0.0007528 | -0.7037 |
| Bt.7300.1.S1\_at | 0.0007547 | -0.6329 |
| Bt.19431.1.S1\_s\_at | 0.0007555 | -0.551 |
| Bt.17092.1.A1\_at | 0.000757 | -0.5866 |
| Bt.9966.2.S1\_a\_at | 0.0007584 | 0.9529 |
| Bt.20598.1.S1\_at | 0.0007607 | -0.6959 |
| Bt.17843.1.A1\_at | 0.0007607 | 0.6054 |
| Bt.2304.1.A1\_at | 0.0007635 | 0.6049 |
| Bt.3867.1.S1\_at | 0.0007639 | -0.5432 |
| Bt.20744.1.A1\_at | 0.0007642 | 0.8214 |
| Bt.11182.1.S1\_at | 0.0007667 | 0.8026 |
| Bt.24417.1.A1\_at | 0.0007677 | 0.5093 |
| Bt.1255.1.A1\_at | 0.0007678 | -0.4921 |
| Bt.20154.1.S1\_at | 0.0007683 | 0.5205 |
| Bt.21191.3.S1\_at | 0.0007687 | -1.322 |
| Bt.3395.1.S1\_at | 0.0007726 | -0.6067 |
| Bt.9144.1.A1\_at | 0.0007727 | 0.572 |
| Bt.14473.1.S1\_at | 0.0007765 | -0.5475 |
| Bt.9037.1.S1\_at | 0.0007783 | 0.6314 |
| Bt.26788.1.S1\_at | 0.0007813 | 0.9058 |
| Bt.5467.1.S2\_at | 0.0007844 | 0.5264 |
| Bt.9050.1.S1\_a\_at | 0.0007854 | -0.4599 |
| Bt.10180.1.A1\_at | 0.0007856 | 0.6149 |
| Bt.22011.1.S1\_at | 0.0007864 | 0.7705 |
| Bt.6017.2.S1\_at | 0.0007875 | -0.7278 |
| Bt.13986.1.S1\_at | 0.0007894 | 0.574 |
| Bt.28758.1.A1\_at | 0.0007917 | 0.8294 |
| Bt.9412.2.S1\_at | 0.0007933 | 0.7274 |
| Bt.8335.1.S1\_at | 0.0007943 | 0.6312 |
| Bt.7543.1.S1\_at | 0.0007973 | 0.5799 |
| Bt.11058.1.A1\_at | 0.0007975 | -0.5634 |
| Bt.21058.2.S1\_at | 0.0007982 | -0.7058 |
| Bt.10017.1.S1\_at | 0.0007984 | 0.7524 |
| Bt.12445.2.S1\_at | 0.0007985 | 0.7542 |
| Bt.2568.1.S1\_at | 0.0008022 | 0.59 |
| Bt.20449.1.S1\_at | 0.0008024 | -0.741 |
| Bt.16177.1.S1\_at | 0.0008025 | 0.6263 |
| Bt.12445.1.A1\_at | 0.0008033 | 0.6903 |
| Bt.15440.1.A1\_at | 0.0008071 | -0.6234 |
| Bt.618.1.S1\_at | 0.0008136 | -0.523 |
| Bt.12906.1.S1\_at | 0.0008138 | 1.007 |
| Bt.14007.3.A1\_at | 0.0008167 | 0.831 |
| Bt.29958.1.S1\_at | 0.000817 | 0.5672 |
| Bt.24433.1.S1\_at | 0.0008171 | -0.5839 |
| Bt.2425.2.S1\_at | 0.0008184 | -0.5228 |
| Bt.28393.1.S1\_at | 0.0008188 | 0.8305 |
| Bt.17253.1.A1\_at | 0.0008197 | 0.7111 |
| Bt.24624.2.S1\_at | 0.0008216 | -0.7982 |
| Bt.8114.1.A1\_at | 0.0008226 | 0.6514 |
| Bt.18315.3.A1\_at | 0.0008237 | 0.6633 |
| Bt.13969.2.S1\_at | 0.0008251 | 0.6047 |
| Bt.24819.1.S1\_at | 0.0008252 | -0.9127 |
| Bt.18958.1.S1\_at | 0.0008252 | -0.5459 |
| Bt.4800.1.S1\_at | 0.0008273 | 0.7532 |
| Bt.20692.1.A1\_at | 0.0008275 | 0.5859 |
| Bt.15846.1.S1\_at | 0.0008281 | 0.8858 |
| Bt.1731.1.S1\_at | 0.0008302 | 0.8368 |
| Bt.1815.1.S1\_at | 0.0008326 | -0.5954 |
| Bt.21369.1.S1\_at | 0.0008344 | 0.616 |
| Bt.13959.1.S1\_at | 0.0008354 | 0.5075 |
| Bt.28535.1.S1\_at | 0.000836 | -0.5657 |
| Bt.25097.1.S1\_at | 0.0008373 | -0.5639 |
| Bt.12504.1.S1\_at | 0.0008385 | 0.7299 |
| Bt.10215.1.A1\_at | 0.0008395 | -0.6109 |
| Bt.4550.1.A1\_at | 0.0008403 | 0.7273 |
| Bt.29463.1.S1\_at | 0.0008413 | 0.786 |
| Bt.13312.1.S1\_at | 0.0008443 | 0.5461 |
| Bt.13546.1.A1\_at | 0.000846 | 1.06 |
| Bt.19502.1.A1\_at | 0.000847 | 0.6722 |
| Bt.6007.1.S1\_at | 0.0008471 | 0.6067 |
| Bt.2850.1.A1\_at | 0.000849 | 0.9924 |
| Bt.3278.1.A1\_at | 0.0008512 | 0.9363 |
| Bt.1325.1.S1\_at | 0.0008516 | 0.6741 |
| Bt.14007.2.S1\_at | 0.0008534 | 0.8786 |
| Bt.22335.1.S1\_a\_at | 0.0008538 | 0.6596 |
| Bt.26595.1.S1\_at | 0.0008569 | 0.7046 |
| Bt.3240.1.S1\_at | 0.0008575 | -0.8282 |
| Bt.3778.1.S1\_at | 0.0008583 | 0.6546 |
| Bt.13966.1.S1\_at | 0.0008589 | -0.5314 |
| Bt.24717.2.S1\_a\_at | 0.0008592 | 0.6667 |
| Bt.6534.1.S1\_at | 0.0008602 | 0.5957 |
| Bt.1750.1.A1\_at | 0.0008643 | 0.7285 |
| Bt.22114.1.S1\_at | 0.0008645 | 0.669 |
| Bt.1938.2.S1\_at | 0.0008661 | 0.5867 |
| Bt.5879.1.S1\_at | 0.0008675 | 0.6203 |
| Bt.3391.1.S1\_at | 0.0008691 | 0.7317 |
| Bt.14624.2.S1\_at | 0.0008721 | -0.8534 |
| Bt.3414.1.S1\_at | 0.0008726 | -0.6603 |
| Bt.14037.1.S1\_at | 0.0008735 | 0.4892 |
| Bt.25104.1.S1\_at | 0.0008744 | 0.6413 |
| Bt.10900.1.S1\_at | 0.0008747 | -0.5361 |
| Bt.8610.1.S1\_at | 0.0008755 | 0.5694 |
| Bt.8555.2.S1\_at | 0.0008758 | -0.5047 |
| Bt.27872.1.S1\_at | 0.0008769 | 0.5734 |
| Bt.20322.1.S1\_at | 0.0008772 | -0.6427 |
| Bt.9027.1.S1\_at | 0.0008792 | 0.5494 |
| Bt.1602.1.S1\_at | 0.0008839 | 0.457 |
| Bt.20780.2.S1\_a\_at | 0.0008849 | -0.9302 |
| Bt.194.1.S1\_at | 0.0008857 | -0.9088 |
| Bt.25936.1.A1\_at | 0.0008877 | -0.665 |
| Bt.3385.1.A1\_at | 0.0008882 | 0.8364 |
| Bt.22170.1.S1\_a\_at | 0.0008903 | -0.7649 |
| Bt.26533.1.S1\_at | 0.0008904 | -0.7729 |
| Bt.10851.1.S1\_at | 0.0008913 | -0.5216 |
| Bt.8308.2.S1\_at | 0.0008913 | 0.6347 |
| Bt.11325.1.S1\_at | 0.0008917 | 0.7119 |
| Bt.3427.1.S1\_at | 0.0008932 | -0.582 |
| Bt.2966.1.A1\_at | 0.0008974 | 0.8285 |
| Bt.15400.1.A1\_at | 0.0009014 | 0.4924 |
| Bt.13913.1.S1\_at | 0.0009017 | -0.5424 |
| Bt.4815.1.S1\_at | 0.0009067 | -0.7462 |
| Bt.5598.1.S1\_at | 0.0009074 | 0.9177 |
| Bt.5529.2.S1\_a\_at | 0.0009122 | -0.6624 |
| Bt.28772.1.A1\_at | 0.0009131 | -0.5197 |
| Bt.19032.1.S1\_at | 0.0009153 | 0.6232 |
| Bt.23985.1.A1\_at | 0.0009161 | 0.728 |
| Bt.2391.1.S1\_at | 0.0009165 | 0.6217 |
| Bt.29918.1.S1\_at | 0.0009173 | -0.52 |
| Bt.3372.2.S1\_a\_at | 0.000918 | -0.6682 |
| Bt.1539.1.S1\_at | 0.0009188 | 0.4949 |
| Bt.25339.1.S1\_at | 0.0009189 | 0.7001 |
| Bt.7955.2.S1\_at | 0.0009221 | 0.7473 |
| Bt.28081.1.S1\_at | 0.0009225 | 0.5825 |
| Bt.25436.1.S1\_a\_at | 0.0009235 | 0.9296 |
| Bt.10129.1.A1\_at | 0.0009241 | 0.5807 |
| Bt.14304.2.S1\_at | 0.0009263 | 0.5878 |
| Bt.9.2.S1\_a\_at | 0.000928 | -0.6457 |
| Bt.24471.1.S1\_a\_at | 0.0009298 | 1.052 |
| Bt.24295.1.S1\_at | 0.0009329 | -0.6708 |
| Bt.9391.2.S1\_at | 0.0009356 | 0.6793 |
| Bt.13898.2.S1\_at | 0.0009358 | 1.393 |
| Bt.4551.2.S1\_at | 0.0009363 | -0.7363 |
| Bt.3485.1.S1\_at | 0.0009365 | 0.6165 |
| Bt.10995.1.S1\_at | 0.0009371 | 0.7292 |
| Bt.5129.2.A1\_at | 0.0009372 | -0.7542 |
| Bt.28220.1.S1\_at | 0.0009408 | 1.021 |
| Bt.3500.1.S1\_at | 0.0009414 | -0.545 |
| Bt.26872.1.S1\_at | 0.000943 | 0.9458 |
| Bt.10385.1.A1\_at | 0.0009444 | 0.5805 |
| Bt.9118.1.S1\_at | 0.0009477 | 0.5622 |
| Bt.17958.1.S1\_at | 0.0009498 | 0.5204 |
| Bt.13431.1.S1\_a\_at | 0.000951 | 0.5242 |
| Bt.19429.2.S1\_a\_at | 0.000951 | 0.6529 |
| Bt.6698.1.S1\_at | 0.0009521 | -0.7387 |
| Bt.19746.1.S1\_at | 0.0009531 | 0.7463 |
| Bt.23240.1.S1\_at | 0.0009548 | 0.7161 |
| Bt.1953.1.S1\_at | 0.0009549 | 0.8034 |
| Bt.25895.2.A1\_at | 0.000958 | -0.6583 |
| Bt.21428.1.S1\_at | 0.0009581 | 0.5073 |
| Bt.7318.1.S1\_at | 0.0009582 | 0.7055 |
| Bt.29868.1.S1\_at | 0.0009583 | -0.7236 |
| Bt.8292.1.A1\_at | 0.0009594 | 0.5563 |
| Bt.25855.2.S1\_at | 0.0009608 | 0.7901 |
| Bt.11126.1.S1\_at | 0.000961 | 0.894 |
| Bt.25081.1.S1\_at | 0.0009617 | 0.8737 |
| Bt.7614.1.S1\_at | 0.0009621 | -0.5083 |
| Bt.8706.1.S1\_at | 0.0009652 | -0.6216 |
| Bt.23230.1.S1\_at | 0.0009659 | 0.6214 |
| Bt.1125.1.S1\_at | 0.0009667 | 0.5697 |
| Bt.17008.1.S1\_at | 0.0009674 | 0.5542 |
| Bt.23091.1.S1\_at | 0.0009701 | -0.7755 |
| Bt.24370.1.A1\_at | 0.0009721 | 0.8925 |
| Bt.12705.1.S1\_at | 0.0009728 | -0.5732 |
| Bt.5006.1.S1\_at | 0.0009733 | 0.5961 |
| Bt.9533.1.S1\_at | 0.0009778 | 0.5722 |
| Bt.8970.1.S1\_at | 0.0009783 | -0.5751 |
| Bt.28844.1.S1\_at | 0.0009799 | -0.5132 |
| Bt.27243.1.A1\_s\_at | 0.0009801 | 0.6601 |
| Bt.27741.1.A1\_at | 0.0009817 | 0.5392 |
| Bt.19332.2.A1\_at | 0.0009827 | 1.098 |
| Bt.12650.1.S1\_at | 0.0009842 | 0.845 |
| Bt.28280.1.A1\_at | 0.0009857 | -0.5495 |
| Bt.29079.1.A1\_at | 0.0009934 | -0.763 |
| Bt.3457.1.S1\_at | 0.0009942 | -0.4985 |
| Bt.29938.1.S1\_at | 0.0009957 | 0.6557 |
| Bt.3698.1.S1\_at | 0.000996 | 0.7565 |
| Bt.5866.1.S1\_at | 0.0009969 | -0.5432 |
| Bt.13871.1.S1\_at | 0.000997 | 0.6251 |
| Bt.23672.1.A1\_at | 0.0009979 | 1.271 |
| Bt.22737.1.S1\_at | 0.0009998 | 0.6931 |
| Bt.21060.1.S1\_at | 0.001006 | 0.5611 |
| Bt.7594.1.S1\_at | 0.001007 | 0.92 |
| Bt.19496.2.A1\_at | 0.001008 | 0.7678 |
| Bt.5365.1.S1\_at | 0.001008 | 0.7055 |
| Bt.6390.1.S1\_at | 0.001013 | -0.6559 |
| Bt.9186.1.A1\_at | 0.001013 | 1.035 |
| Bt.3583.1.S1\_at | 0.001015 | 0.5848 |
| Bt.13132.1.S1\_at | 0.001019 | -0.6734 |
| Bt.10299.1.S1\_at | 0.001019 | 0.6238 |
| Bt.14644.1.A1\_at | 0.001019 | 0.647 |
| Bt.5589.1.S1\_at | 0.001025 | -0.5417 |
| Bt.14122.1.A1\_at | 0.001026 | 0.93 |
| Bt.16508.1.A1\_at | 0.001028 | 0.7322 |
| Bt.1390.1.A1\_at | 0.001029 | -0.5989 |
| Bt.28583.1.S1\_s\_at | 0.00103 | 0.8551 |
| Bt.16380.1.S1\_at | 0.00103 | 0.52 |
| Bt.26629.1.S1\_a\_at | 0.001033 | -0.8649 |
| Bt.14552.1.A1\_at | 0.001037 | 0.7344 |
| Bt.21385.1.S1\_at | 0.001042 | 0.5212 |
| Bt.27011.1.A1\_at | 0.001044 | 0.9983 |
| Bt.18727.1.A1\_at | 0.001048 | -0.6028 |
| Bt.3098.1.S1\_at | 0.001049 | 0.6803 |
| Bt.1697.1.S1\_at | 0.00105 | -0.4846 |
| Bt.361.1.S1\_at | 0.001053 | -0.6312 |
| Bt.9194.1.S1\_at | 0.001055 | 0.4264 |
| Bt.24522.3.S1\_a\_at | 0.001057 | 1.215 |
| Bt.12221.1.S1\_at | 0.001059 | 0.7101 |
| Bt.20391.1.A1\_at | 0.001064 | 0.9607 |
| Bt.3767.1.A1\_at | 0.001064 | -0.5841 |
| Bt.20205.1.S1\_at | 0.001066 | -0.5579 |
| Bt.17440.1.A1\_at | 0.001068 | 0.5571 |
| Bt.9114.1.A1\_at | 0.001069 | -0.6622 |
| Bt.22332.1.A1\_at | 0.00107 | 0.7808 |
| Bt.5165.1.S1\_at | 0.001071 | -0.6001 |
| Bt.26837.1.S1\_at | 0.001073 | 0.7636 |
| Bt.1932.1.S1\_at | 0.001074 | -0.7546 |
| Bt.13307.1.S1\_at | 0.001077 | 0.6332 |
| Bt.26473.1.A1\_at | 0.001078 | 0.9562 |
| Bt.11617.1.A1\_at | 0.001083 | 0.5684 |
| Bt.26866.1.S1\_at | 0.001086 | -0.4849 |
| Bt.6075.1.S1\_at | 0.001088 | 0.5321 |
| Bt.3448.3.S1\_at | 0.001088 | -1.847 |
| Bt.1678.1.S1\_at | 0.001091 | -0.5215 |
| Bt.20437.1.S1\_at | 0.001091 | -0.5627 |
| Bt.2347.2.S1\_at | 0.001094 | -0.7119 |
| Bt.364.1.S2\_at | 0.001094 | 0.928 |
| Bt.21920.1.S1\_at | 0.001096 | 0.4871 |
| Bt.25402.1.S1\_at | 0.001096 | -0.5329 |
| Bt.12496.1.S1\_at | 0.001096 | 0.5348 |
| Bt.19770.1.S1\_at | 0.001097 | -0.5252 |
| Bt.4394.1.S1\_at | 0.001099 | 0.507 |
| Bt.29679.1.S1\_s\_at | 0.0011 | 0.6123 |
| Bt.18513.1.S1\_at | 0.001101 | -0.471 |
| Bt.26817.1.S1\_at | 0.001103 | -0.5983 |
| Bt.23853.1.S1\_at | 0.001104 | 0.5814 |
| Bt.23784.1.A1\_at | 0.001105 | 0.4664 |
| Bt.8736.2.S1\_at | 0.001106 | 0.4898 |
| Bt.5998.1.S1\_at | 0.001107 | 0.8885 |
| Bt.25364.1.A1\_at | 0.001108 | -0.6214 |
| Bt.12035.1.S1\_at | 0.001111 | -0.8328 |
| Bt.10044.1.A1\_at | 0.001114 | 0.7119 |
| Bt.29733.1.S1\_at | 0.001114 | -0.5332 |
| Bt.3593.1.S1\_at | 0.001115 | -0.6779 |
| Bt.21160.1.A1\_at | 0.001116 | 0.7858 |
| Bt.2513.2.S1\_at | 0.001117 | 0.6722 |
| Bt.6069.1.S1\_at | 0.001124 | 0.6221 |
| Bt.22035.2.S1\_at | 0.001125 | -0.4685 |
| Bt.16052.2.A1\_at | 0.001131 | 0.813 |
| Bt.27907.1.S1\_at | 0.001133 | 0.7511 |
| Bt.27041.1.A1\_at | 0.001134 | 0.825 |
| Bt.22304.1.S1\_at | 0.001136 | -0.6542 |
| Bt.3387.1.S1\_at | 0.001137 | 0.5102 |
| Bt.16573.1.A1\_at | 0.001141 | 0.6297 |
| Bt.28264.2.S1\_at | 0.001144 | 0.7955 |
| Bt.3300.1.S1\_at | 0.001144 | 0.5376 |
| Bt.7264.1.S1\_at | 0.001144 | 0.7727 |
| Bt.9246.1.A1\_at | 0.001145 | 1.01 |
| BtAffx.29967.1.S1\_at | 0.001147 | -0.7807 |
| Bt.16248.1.A1\_at | 0.001148 | 0.6218 |
| Bt.6865.1.S1\_at | 0.001152 | 1.107 |
| Bt.26901.2.S1\_a\_at | 0.001154 | -0.5733 |
| Bt.2178.1.S1\_at | 0.001155 | 0.4934 |
| Bt.14083.1.A1\_at | 0.001156 | -0.7278 |
| Bt.19355.1.A1\_at | 0.001156 | 0.6693 |
| Bt.21997.2.A1\_at | 0.001156 | 0.9682 |
| Bt.11580.1.S1\_at | 0.001157 | 0.706 |
| Bt.12609.1.A1\_at | 0.001162 | 0.7208 |
| Bt.18116.3.A1\_s\_at | 0.001175 | 0.4635 |
| Bt.26259.3.A1\_a\_at | 0.001176 | -0.4812 |
| Bt.3374.1.A1\_at | 0.00118 | 0.5981 |
| Bt.10260.1.S1\_at | 0.00118 | 0.5948 |
| Bt.26828.1.S1\_at | 0.001181 | 0.8592 |
| Bt.5369.1.S1\_at | 0.001183 | 0.5248 |
| Bt.10397.1.A1\_at | 0.001183 | 0.6355 |
| Bt.12142.1.S1\_at | 0.001184 | 0.7301 |
| Bt.24486.1.A1\_at | 0.001185 | -0.6028 |
| Bt.17447.1.A1\_at | 0.001186 | 1.028 |
| Bt.17233.1.A1\_at | 0.001189 | -0.5063 |
| Bt.3887.1.S1\_at | 0.00119 | 0.6012 |
| Bt.13771.3.S1\_at | 0.00119 | 0.5924 |
| Bt.2220.2.A1\_a\_at | 0.001201 | 0.5728 |
| Bt.11256.1.S1\_at | 0.001202 | 0.5398 |
| Bt.755.1.S1\_at | 0.001205 | -0.6319 |
| Bt.26703.1.S1\_at | 0.001206 | 0.504 |
| Bt.28110.1.S1\_at | 0.00121 | 0.6523 |
| Bt.18043.1.S1\_at | 0.00121 | 0.5148 |
| Bt.16450.2.A1\_at | 0.00121 | 0.6179 |
| Bt.16619.1.A1\_at | 0.001212 | 1.031 |
| Bt.6169.1.A1\_at | 0.001212 | 0.6369 |
| Bt.20314.1.S1\_at | 0.001214 | -0.63 |
| Bt.23276.2.S1\_at | 0.001214 | 0.7613 |
| Bt.16538.1.S1\_at | 0.001216 | 0.8097 |
| Bt.1313.1.S1\_at | 0.001221 | 0.4933 |
| Bt.1993.1.S1\_a\_at | 0.001228 | 0.7821 |
| Bt.24234.1.S1\_at | 0.00123 | 0.51 |
| Bt.29915.1.S1\_at | 0.001233 | -0.5501 |
| Bt.4989.1.S1\_at | 0.001233 | 0.6703 |
| Bt.21215.1.A1\_at | 0.00124 | 0.9198 |
| Bt.24680.1.A1\_at | 0.001241 | 0.6825 |
| Bt.24051.1.S1\_at | 0.001244 | 0.7786 |
| Bt.1580.1.S1\_at | 0.001248 | 1.016 |
| Bt.18203.1.A1\_at | 0.001253 | 0.5055 |
| Bt.23572.1.S1\_at | 0.001255 | 0.6882 |
| Bt.9847.1.S1\_at | 0.001255 | 0.7872 |
| Bt.5732.2.A1\_at | 0.001258 | 0.9146 |
| Bt.16125.1.A1\_s\_at | 0.001264 | -0.9154 |
| Bt.5513.1.A1\_at | 0.001265 | 0.5565 |
| Bt.7994.2.S1\_at | 0.001268 | -0.6459 |
| Bt.21515.1.S1\_at | 0.001272 | -0.5918 |
| Bt.3388.1.S1\_at | 0.001272 | -0.6047 |
| Bt.8265.1.A1\_at | 0.001273 | 0.7631 |
| Bt.21698.1.S1\_a\_at | 0.001273 | -0.779 |
| Bt.11826.1.S1\_at | 0.001275 | 0.6657 |
| Bt.23708.1.A1\_at | 0.001275 | -0.4685 |
| Bt.15819.1.A1\_a\_at | 0.001276 | -0.5928 |
| Bt.18477.1.A1\_at | 0.001279 | -0.6593 |
| Bt.26051.1.A1\_at | 0.00128 | 0.8418 |
| Bt.1257.1.S1\_at | 0.001281 | 0.652 |
| Bt.24087.1.A1\_at | 0.001283 | 0.5602 |
| Bt.24511.1.A1\_at | 0.001283 | 0.6962 |
| Bt.2452.1.S1\_at | 0.001284 | 0.684 |
| Bt.21119.1.A1\_at | 0.001284 | 0.6807 |
| Bt.721.1.A1\_at | 0.001288 | 0.7241 |
| Bt.20840.1.A1\_at | 0.001289 | 0.7018 |
| Bt.7826.1.S1\_at | 0.001289 | 0.5718 |
| Bt.8957.1.S1\_at | 0.001289 | 0.7451 |
| Bt.349.1.S1\_at | 0.001291 | 0.5352 |
| Bt.11257.2.S1\_at | 0.001292 | 0.5548 |
| Bt.22442.3.S1\_a\_at | 0.001292 | -0.6344 |
| Bt.16617.1.A1\_at | 0.001294 | 0.7103 |
| Bt.21833.1.S1\_at | 0.001297 | -0.5639 |
| Bt.948.1.S1\_at | 0.001297 | 0.6691 |
| Bt.4737.1.S2\_s\_at | 0.001299 | 0.5174 |
| Bt.5439.1.S1\_at | 0.0013 | 0.5459 |
| Bt.20342.2.S1\_a\_at | 0.001302 | -0.5323 |
| Bt.20582.1.S1\_at | 0.001307 | 0.9939 |
| Bt.5536.1.S1\_at | 0.001308 | 0.6485 |
| Bt.22440.1.S1\_at | 0.001312 | 0.5244 |
| Bt.27856.1.S1\_at | 0.001315 | 0.8983 |
| Bt.17812.1.S1\_at | 0.001316 | 0.5352 |
| Bt.25094.2.S1\_a\_at | 0.001317 | -0.6593 |
| Bt.18.1.S1\_at | 0.001317 | -0.6861 |
| Bt.8682.1.S1\_at | 0.00132 | 0.5083 |
| Bt.7514.1.S1\_at | 0.00132 | 0.604 |
| Bt.26612.2.S1\_at | 0.00132 | 0.6274 |
| Bt.15688.1.S1\_at | 0.001322 | -0.7802 |
| Bt.27675.1.A1\_at | 0.001323 | 0.4874 |
| Bt.4970.1.S1\_at | 0.001327 | -0.7069 |
| Bt.28489.1.S1\_at | 0.001327 | 0.7063 |
| Bt.3050.1.S1\_at | 0.001328 | -0.5071 |
| Bt.27778.1.A1\_at | 0.001329 | 0.639 |
| Bt.2106.1.S1\_at | 0.00133 | 0.6351 |
| Bt.24260.2.S1\_at | 0.001331 | 1.051 |
| Bt.21765.1.S1\_at | 0.001332 | -1.239 |
| Bt.6556.1.S1\_at | 0.001332 | -0.5733 |
| Bt.13425.2.S1\_at | 0.001335 | 0.6248 |
| Bt.26783.1.A1\_at | 0.001335 | 0.6224 |
| Bt.26641.1.S1\_at | 0.001335 | -0.673 |
| Bt.10962.1.S1\_at | 0.001337 | 0.4884 |
| Bt.12510.2.S1\_at | 0.001338 | 1.06 |
| Bt.20228.1.S1\_at | 0.001341 | -0.4584 |
| Bt.1455.1.S1\_at | 0.001342 | 0.9193 |
| Bt.23825.1.S1\_at | 0.001343 | 0.4933 |
| Bt.10515.1.S1\_at | 0.001346 | -0.5348 |
| Bt.12745.3.S1\_at | 0.001347 | 0.9167 |
| Bt.6171.1.A1\_at | 0.001348 | -0.763 |
| Bt.1638.2.S1\_at | 0.001348 | 0.8621 |
| Bt.24359.1.S1\_at | 0.001348 | 0.6065 |
| Bt.25213.1.A1\_at | 0.00135 | 0.8282 |
| Bt.6789.1.S1\_at | 0.00135 | -0.4372 |
| Bt.27955.1.A1\_at | 0.00135 | 0.9734 |
| Bt.1217.1.A1\_a\_at | 0.001352 | -0.601 |
| Bt.24214.1.S1\_at | 0.001354 | -0.7293 |
| Bt.8090.1.A1\_at | 0.001356 | -0.5489 |
| Bt.13907.1.S1\_at | 0.001366 | 0.5495 |
| Bt.26470.1.S1\_at | 0.001368 | -0.635 |
| Bt.8823.3.S1\_a\_at | 0.001369 | -0.5871 |
| Bt.28695.1.S1\_at | 0.001372 | 0.6711 |
| Bt.4437.1.S1\_at | 0.001374 | 0.7348 |
| Bt.25054.1.A1\_at | 0.001375 | 0.5479 |
| Bt.11326.1.S1\_at | 0.001376 | -0.5082 |
| Bt.9933.1.S1\_at | 0.001378 | 0.6736 |
| Bt.22999.1.S1\_at | 0.001383 | 0.4638 |
| Bt.27339.2.S1\_at | 0.001385 | 0.826 |
| Bt.15854.2.S1\_at | 0.001386 | 1.076 |
| Bt.24783.1.A1\_at | 0.001386 | 0.7402 |
| Bt.19681.1.A1\_at | 0.001386 | 0.5766 |
| Bt.20177.1.S1\_at | 0.001388 | 0.5076 |
| Bt.23770.1.A1\_at | 0.001388 | 0.6124 |
| Bt.6581.1.S1\_at | 0.001389 | 0.53 |
| Bt.23109.1.S1\_at | 0.001389 | -0.5156 |
| Bt.21487.2.S1\_at | 0.001392 | -0.5045 |
| Bt.10878.3.S1\_a\_at | 0.001395 | -0.5221 |
| Bt.5657.2.S1\_at | 0.001396 | 0.8487 |
| Bt.13127.1.S1\_at | 0.0014 | -0.5957 |
| Bt.14054.1.A1\_at | 0.0014 | -1.121 |
| Bt.24385.1.A1\_at | 0.001401 | 0.5426 |
| Bt.6028.1.S1\_at | 0.001402 | -0.6269 |
| Bt.19839.1.A1\_at | 0.001407 | 1.072 |
| Bt.18197.1.A1\_s\_at | 0.001408 | 0.5783 |
| Bt.11739.2.A1\_a\_at | 0.00141 | -0.4611 |
| Bt.8794.2.S1\_at | 0.001412 | 0.8136 |
| Bt.12606.1.S1\_a\_at | 0.001412 | 0.834 |
| Bt.5941.1.S1\_at | 0.001414 | 0.5312 |
| Bt.21603.2.S1\_at | 0.001416 | -0.5213 |
| Bt.1853.1.S1\_at | 0.001416 | -0.5448 |
| Bt.6899.2.S1\_at | 0.001417 | -0.768 |
| Bt.4767.1.S1\_at | 0.001422 | 0.7388 |
| Bt.215.1.S1\_at | 0.001424 | -0.4768 |
| Bt.3786.1.S1\_a\_at | 0.001427 | -0.6233 |
| Bt.10056.1.S1\_at | 0.001428 | 0.6283 |
| Bt.19898.1.A1\_at | 0.001431 | 0.6364 |
| Bt.24276.1.A1\_at | 0.001436 | 0.5101 |
| Bt.19408.1.A1\_at | 0.00144 | 1.15 |
| Bt.5151.1.S1\_at | 0.00144 | 0.6237 |
| Bt.7434.1.S1\_at | 0.001441 | 0.5882 |
| Bt.24604.2.A1\_at | 0.001441 | 0.5595 |
| Bt.5582.1.S1\_at | 0.001441 | -0.8007 |
| Bt.25076.1.A1\_at | 0.001445 | 0.7545 |
| Bt.727.2.S1\_at | 0.001455 | -0.583 |
| Bt.21994.1.S1\_at | 0.001459 | 0.4473 |
| Bt.28699.1.S1\_at | 0.001462 | 0.7814 |
| Bt.9739.1.S1\_at | 0.001462 | 0.6553 |
| Bt.21593.2.S1\_at | 0.001464 | -0.6433 |
| Bt.2128.1.S1\_at | 0.001464 | 0.6901 |
| Bt.1319.1.S1\_at | 0.00147 | -0.6599 |
| Bt.23663.1.S1\_at | 0.00147 | 0.5416 |
| Bt.17265.1.A1\_a\_at | 0.001483 | -0.535 |
| Bt.27800.1.A1\_at | 0.001484 | -0.6766 |
| Bt.13665.1.A1\_at | 0.001484 | 0.4663 |
| Bt.23872.1.A1\_at | 0.001487 | -0.555 |
| Bt.26545.1.A1\_at | 0.001489 | 0.5623 |
| Bt.712.1.S2\_at | 0.001493 | -0.5089 |
| Bt.9643.1.S1\_at | 0.001497 | -0.6779 |
| Bt.11765.1.A1\_at | 0.001499 | -0.647 |
| Bt.19938.1.A1\_at | 0.001501 | 0.6933 |
| Bt.17767.1.A1\_at | 0.001503 | 0.5672 |
| Bt.20955.1.A1\_at | 0.001505 | -0.9151 |
| Bt.16016.1.S1\_at | 0.001506 | -0.9055 |
| Bt.4877.1.S1\_at | 0.001508 | 0.6085 |
| Bt.10231.1.S1\_at | 0.001508 | 0.6325 |
| Bt.16380.2.A1\_at | 0.001509 | 0.6541 |
| Bt.22064.1.A1\_at | 0.001513 | 0.5545 |
| Bt.26094.1.A1\_at | 0.001516 | 0.6863 |
| Bt.11631.3.S1\_at | 0.001536 | -0.6557 |
| Bt.22377.1.S1\_a\_at | 0.001547 | 0.5477 |
| Bt.27129.1.S1\_at | 0.001548 | 0.5467 |
| Bt.22053.1.S1\_at | 0.00155 | 0.6314 |
| Bt.1993.3.A1\_a\_at | 0.001553 | 0.9406 |
| Bt.17701.1.A1\_at | 0.001557 | 0.4915 |
| Bt.17764.1.A1\_at | 0.00156 | 0.4823 |
| Bt.24315.1.A1\_at | 0.001561 | 0.7353 |
| Bt.13194.1.A1\_at | 0.001562 | 0.4664 |
| Bt.20929.2.A1\_at | 0.001564 | 0.9939 |
| Bt.65.1.S1\_at | 0.001566 | -0.5858 |
| Bt.9354.1.S1\_at | 0.001568 | -0.4666 |
| Bt.1308.1.S1\_at | 0.001571 | 0.7517 |
| Bt.22174.1.S1\_at | 0.001576 | 0.7797 |
| Bt.13537.1.A1\_at | 0.001579 | -0.8491 |
| Bt.2860.1.S1\_at | 0.001581 | 0.4058 |
| Bt.26611.1.S1\_at | 0.001589 | 0.6832 |
| Bt.9070.1.S1\_at | 0.001591 | -0.7164 |
| Bt.29582.1.A1\_at | 0.001593 | 0.7038 |
| Bt.20768.1.S1\_at | 0.0016 | 0.4626 |
| Bt.22148.1.S1\_at | 0.001602 | 0.6459 |
| Bt.21210.1.A1\_at | 0.001602 | 0.4851 |
| Bt.16611.1.A1\_at | 0.001606 | 0.6566 |
| Bt.11078.1.S1\_a\_at | 0.001606 | -0.5863 |
| Bt.13370.1.A1\_at | 0.001606 | 0.8451 |
| Bt.3060.1.S1\_a\_at | 0.001613 | 0.6456 |
| Bt.3980.1.S1\_at | 0.00162 | 0.8869 |
| Bt.13401.1.S1\_at | 0.00162 | 0.5728 |
| Bt.5398.1.S1\_at | 0.001622 | 0.6583 |
| Bt.9071.2.S1\_a\_at | 0.001623 | -0.534 |
| Bt.460.1.S1\_s\_at | 0.001625 | -0.4781 |
| Bt.24350.1.A1\_at | 0.001628 | 0.6963 |
| Bt.13953.1.A1\_at | 0.00163 | -1.14 |
| Bt.2898.3.S1\_at | 0.001634 | -0.5785 |
| Bt.29823.1.S1\_at | 0.001636 | -0.8894 |
| Bt.17452.1.S1\_at | 0.001637 | 0.665 |
| Bt.981.2.S1\_at | 0.00164 | 0.8026 |
| Bt.15860.1.S1\_at | 0.00164 | 0.5758 |
| Bt.18689.2.A1\_at | 0.00164 | 0.683 |
| Bt.8166.3.A1\_s\_at | 0.001644 | 0.8223 |
| Bt.5465.2.A1\_a\_at | 0.001646 | -0.7234 |
| Bt.21286.1.S1\_at | 0.001647 | 0.794 |
| Bt.22525.1.A1\_at | 0.001661 | 0.4735 |
| Bt.18230.1.S1\_a\_at | 0.001661 | 0.5686 |
| Bt.21476.1.S1\_at | 0.001662 | -0.6248 |
| Bt.12333.1.A1\_at | 0.001662 | 0.7467 |
| Bt.22339.2.S1\_at | 0.001664 | -0.492 |
| Bt.8769.1.A1\_at | 0.001669 | -0.9044 |
| Bt.3722.1.S1\_at | 0.001669 | 0.6195 |
| Bt.1797.1.S1\_at | 0.001669 | 0.5387 |
| Bt.25331.1.A1\_at | 0.001679 | 0.542 |
| Bt.19519.1.S1\_at | 0.001681 | 0.5619 |
| Bt.25015.1.A1\_at | 0.001683 | 0.6051 |
| Bt.4431.2.A1\_at | 0.001687 | -0.5936 |
| Bt.27345.2.S1\_at | 0.001687 | -0.4557 |
| Bt.26549.1.A1\_at | 0.001688 | 0.6068 |
| Bt.20938.1.S1\_at | 0.001689 | 0.5292 |
| Bt.4796.1.S1\_at | 0.001692 | -0.64 |
| Bt.5200.1.S1\_at | 0.001692 | -0.8003 |
| Bt.9654.1.S1\_at | 0.001693 | -0.7302 |
| Bt.9036.1.A1\_at | 0.001697 | 0.4711 |
| Bt.29197.1.S1\_a\_at | 0.001699 | 0.6997 |
| Bt.8039.2.S1\_a\_at | 0.001704 | 0.4201 |
| Bt.21858.1.S1\_at | 0.001707 | 0.6609 |
| Bt.22740.1.A1\_at | 0.001709 | 0.5436 |
| Bt.1387.1.A1\_at | 0.001717 | 0.4885 |
| Bt.4689.1.S2\_at | 0.00172 | -0.4701 |
| Bt.17928.1.A1\_at | 0.00172 | 0.5396 |
| Bt.1171.1.S1\_at | 0.001724 | -0.8 |
| Bt.612.1.S1\_at | 0.001726 | -0.5716 |
| Bt.4203.1.S1\_at | 0.001731 | -0.7476 |
| Bt.15887.2.S1\_a\_at | 0.001732 | -0.5371 |
| Bt.27899.1.A1\_at | 0.001733 | -0.6125 |
| Bt.23668.1.A1\_at | 0.001734 | -0.5933 |
| Bt.752.1.S1\_a\_at | 0.001739 | -0.525 |
| Bt.11326.3.S1\_at | 0.001745 | -0.512 |
| Bt.9164.1.A1\_at | 0.001749 | 0.4508 |
| Bt.2317.1.S1\_at | 0.00175 | -0.605 |
| Bt.24396.2.S1\_at | 0.001751 | 0.6949 |
| Bt.6087.1.S1\_at | 0.001759 | 0.437 |
| Bt.3035.2.S1\_at | 0.001759 | 0.5638 |
| Bt.4599.1.S1\_at | 0.001764 | -0.5999 |
| Bt.596.1.S1\_at | 0.001765 | -0.7518 |
| Bt.24381.2.S1\_at | 0.001772 | 0.6298 |
| Bt.1318.1.S1\_at | 0.001772 | -0.4482 |
| Bt.26953.1.A1\_at | 0.001773 | -0.6076 |
| Bt.9490.1.S1\_at | 0.001773 | -0.5771 |
| Bt.1244.1.S1\_at | 0.001775 | 0.4539 |
| Bt.29084.1.S1\_at | 0.00178 | 0.6751 |
| Bt.16901.1.A1\_at | 0.001781 | 0.5737 |
| Bt.227.1.A1\_at | 0.001784 | -0.8606 |
| Bt.11257.1.S1\_at | 0.001784 | 0.4841 |
| Bt.2195.1.A1\_at | 0.001785 | -0.5374 |
| Bt.3070.2.S1\_at | 0.001786 | -0.7132 |
| Bt.471.1.S1\_at | 0.001786 | 0.6676 |
| Bt.6473.1.S1\_at | 0.001789 | -0.5704 |
| Bt.21246.1.A1\_at | 0.001793 | 0.7747 |
| Bt.8106.1.S1\_at | 0.001793 | 0.5991 |
| Bt.15717.2.S1\_at | 0.001799 | 0.7256 |
| Bt.5039.1.S1\_at | 0.0018 | 0.5728 |
| Bt.29853.1.S1\_at | 0.001812 | -1.875 |
| Bt.5534.1.S1\_at | 0.001812 | -0.5012 |
| Bt.9622.1.A1\_at | 0.001812 | -0.5263 |
| Bt.6673.1.S1\_at | 0.001814 | -0.4404 |
| Bt.5208.1.S1\_at | 0.001818 | -0.7957 |
| Bt.8109.2.A1\_at | 0.001825 | 0.6406 |
| Bt.4972.2.S1\_at | 0.001825 | 0.8421 |
| Bt.5737.1.S1\_at | 0.001829 | 0.5324 |
| Bt.13549.2.S1\_at | 0.00183 | 0.83 |
| Bt.4358.1.S1\_at | 0.001831 | -0.4893 |
| Bt.25089.2.A1\_at | 0.001843 | 0.9616 |
| Bt.4466.2.S1\_at | 0.001844 | 0.5853 |
| Bt.13489.1.S1\_at | 0.001845 | 0.571 |
| Bt.16437.1.S1\_at | 0.001845 | -0.4424 |
| Bt.10934.1.A1\_at | 0.001845 | 0.4463 |
| Bt.8690.1.S1\_at | 0.001845 | -0.6376 |
| Bt.5184.2.S1\_at | 0.001857 | -0.4472 |
| Bt.413.1.S1\_at | 0.001862 | 0.6885 |
| Bt.27262.1.S1\_at | 0.001863 | -0.5793 |
| Bt.23940.1.A1\_at | 0.001863 | -0.4994 |
| Bt.2837.1.A1\_at | 0.001865 | -0.4456 |
| Bt.656.1.S1\_at | 0.001866 | 0.5097 |
| Bt.20262.1.S1\_at | 0.001866 | 0.9005 |
| Bt.11408.1.A1\_at | 0.001871 | 0.9708 |
| Bt.24779.2.S1\_at | 0.001872 | -0.5953 |
| Bt.27202.1.A1\_at | 0.001872 | 0.8363 |
| Bt.18229.1.A1\_at | 0.001873 | 0.7669 |
| Bt.26682.1.S1\_at | 0.001874 | 0.8682 |
| Bt.22165.1.S1\_at | 0.001877 | 1.078 |
| Bt.12888.2.S1\_at | 0.001879 | -0.7918 |
| Bt.6043.1.S1\_at | 0.001881 | 0.4886 |
| Bt.16138.1.S1\_at | 0.001891 | 0.4641 |
| Bt.3735.1.A1\_at | 0.001897 | -1.293 |
| Bt.28909.1.A1\_at | 0.001898 | -0.5101 |
| Bt.27254.1.A1\_at | 0.001904 | 0.5745 |
| Bt.7760.1.S1\_at | 0.001905 | -0.5711 |
| Bt.25882.1.A1\_at | 0.001905 | -0.5841 |
| Bt.20561.1.S1\_at | 0.001912 | -0.5352 |
| Bt.29365.1.A1\_at | 0.001914 | -0.4886 |
| Bt.6117.1.S1\_at | 0.001916 | 0.5914 |
| Bt.23542.1.S1\_at | 0.001918 | -0.5775 |
| Bt.24508.1.A1\_at | 0.001919 | 0.5759 |
| Bt.20402.3.S1\_at | 0.001923 | 0.8202 |
| Bt.771.1.S1\_at | 0.001926 | 0.4078 |
| Bt.23658.1.A1\_at | 0.001929 | -0.8106 |
| Bt.26577.1.A1\_at | 0.00193 | -0.6071 |
| Bt.5605.3.S1\_at | 0.001932 | 0.7764 |
| Bt.24273.2.S1\_a\_at | 0.001938 | -0.6194 |
| Bt.15972.1.S1\_at | 0.001943 | 0.6497 |
| Bt.19546.1.A1\_at | 0.001953 | -0.7682 |
| Bt.7168.1.S1\_at | 0.001954 | 0.6602 |
| Bt.24614.1.S1\_at | 0.001956 | -0.8394 |
| Bt.3874.1.S1\_at | 0.001958 | 0.5139 |
| Bt.17943.1.S1\_at | 0.001961 | 0.5549 |
| Bt.24241.1.A1\_at | 0.001962 | 0.6763 |
| Bt.1985.1.S1\_at | 0.001969 | -0.6178 |
| Bt.22564.3.S1\_at | 0.001974 | 0.571 |
| Bt.9940.1.S1\_at | 0.001982 | -0.8565 |
| Bt.21485.1.S1\_at | 0.001984 | 0.5626 |
| Bt.14520.1.A1\_at | 0.001986 | 0.5798 |
| Bt.20116.1.S1\_at | 0.001988 | 0.5401 |
| Bt.8024.2.S1\_at | 0.001996 | -0.6523 |
| Bt.27428.1.A1\_at | 0.001999 | 0.8779 |
| Bt.20675.1.S1\_at | 0.002 | -0.5385 |
| Bt.2073.1.S1\_at | 0.002001 | -0.8433 |
| Bt.13440.3.S1\_at | 0.002001 | 0.79 |
| Bt.13989.3.S1\_at | 0.002003 | 0.9715 |
| Bt.6206.1.S1\_at | 0.002004 | -0.4519 |
| Bt.2749.1.S1\_at | 0.002007 | -1.149 |
| Bt.28147.1.S1\_at | 0.00201 | 0.5798 |
| Bt.5104.1.S1\_at | 0.00201 | -0.6679 |
| Bt.11085.1.S1\_at | 0.002013 | 0.4126 |
| Bt.6284.1.S1\_at | 0.002017 | 0.5677 |
| Bt.20341.1.S1\_at | 0.00202 | 0.4368 |
| Bt.15907.1.A1\_at | 0.002023 | -0.6973 |
| Bt.7671.1.S1\_at | 0.002027 | 0.5458 |
| Bt.13702.1.S1\_at | 0.002027 | 0.462 |
| Bt.18126.2.S1\_at | 0.002028 | 0.5916 |
| Bt.3704.1.S1\_at | 0.002037 | 0.5187 |
| Bt.14626.1.S1\_at | 0.002038 | -1.167 |
| Bt.1310.1.S1\_at | 0.002048 | -0.4564 |
| Bt.13350.1.S1\_at | 0.002053 | 0.8835 |
| Bt.17005.1.S1\_at | 0.002055 | -0.5321 |
| Bt.677.1.S1\_at | 0.002057 | 0.5315 |
| Bt.18822.1.A1\_at | 0.00206 | 0.5446 |
| Bt.11192.1.S1\_at | 0.002061 | -0.4777 |
| Bt.10609.1.S1\_at | 0.002064 | 0.6402 |
| Bt.14310.1.S1\_a\_at | 0.002067 | -0.6461 |
| Bt.13323.1.S1\_at | 0.002068 | 0.6737 |
| Bt.10003.1.S1\_at | 0.002073 | 0.5605 |
| Bt.1655.1.A1\_at | 0.002074 | 0.7097 |
| Bt.21827.1.A1\_s\_at | 0.002077 | -0.5063 |
| Bt.5103.1.S1\_at | 0.002078 | -0.6475 |
| Bt.13990.3.A1\_at | 0.00208 | 0.628 |
| Bt.1819.1.S1\_at | 0.002082 | -0.6612 |
| Bt.22126.1.S1\_at | 0.002085 | 0.5596 |
| Bt.18660.1.A1\_at | 0.002085 | -0.4156 |
| Bt.22602.1.A1\_at | 0.002088 | 0.5637 |
| Bt.28523.1.S1\_at | 0.002089 | 0.4894 |
| Bt.22723.2.S1\_at | 0.002091 | -0.4573 |
| Bt.5282.1.S1\_at | 0.002094 | 0.3778 |
| Bt.3393.1.S1\_at | 0.002105 | 0.4492 |
| Bt.16566.1.A1\_at | 0.002106 | 0.6251 |
| Bt.28166.1.A1\_at | 0.002109 | -0.5072 |
| Bt.15687.2.S1\_at | 0.00211 | 0.6308 |
| Bt.13867.1.S1\_at | 0.00211 | -0.6317 |
| Bt.24856.2.S1\_at | 0.00211 | -0.6843 |
| Bt.16142.1.S1\_at | 0.002111 | -0.7259 |
| Bt.22094.2.S1\_at | 0.002119 | 0.6899 |
| Bt.1579.1.S1\_at | 0.002123 | 0.5267 |

Differentially expressed genes for GCRMA: 2994

| AFFY | p-value | FoldChange |
| --- | --- | --- |
| Bt.7056.3.A1\_a\_at | 7.502e-12 | 8.442 |
| Bt.11178.1.S1\_at | 1.012e-11 | 2.796 |
| Bt.1696.1.S1\_at | 6.375e-11 | -1.73 |
| Bt.3960.2.S1\_at | 8.537e-11 | -1.492 |
| Bt.3015.1.A1\_at | 2.048e-10 | 2.022 |
| Bt.1403.1.S1\_at | 2.366e-10 | -2.228 |
| Bt.11770.1.S1\_a\_at | 3.339e-10 | -2.578 |
| Bt.1112.1.S1\_at | 3.638e-10 | -4.874 |
| Bt.832.1.S1\_at | 3.869e-10 | -2.051 |
| Bt.848.1.S1\_at | 4.239e-10 | -1.484 |
| Bt.8077.1.S1\_at | 5.605e-10 | -2.686 |
| Bt.26135.1.A1\_at | 6.201e-10 | -3.712 |
| Bt.7804.1.S1\_at | 6.432e-10 | -2 |
| Bt.13980.1.A1\_at | 7.368e-10 | -3.703 |
| Bt.27806.1.S1\_at | 7.703e-10 | -2.137 |
| Bt.3960.1.S1\_a\_at | 7.871e-10 | -2.12 |
| Bt.4977.1.S2\_at | 7.879e-10 | 1.488 |
| Bt.27457.1.S1\_at | 8.585e-10 | 2.186 |
| Bt.20473.1.A1\_at | 8.887e-10 | 3.893 |
| Bt.23662.1.S1\_at | 1.143e-09 | -2.175 |
| Bt.8730.1.S1\_at | 1.249e-09 | 1.323 |
| Bt.22519.1.S1\_at | 1.273e-09 | -2.478 |
| Bt.20229.1.S1\_at | 1.333e-09 | -2.341 |
| Bt.24875.1.S1\_at | 1.477e-09 | -1.735 |
| Bt.4804.2.A1\_at | 1.513e-09 | 2.19 |
| Bt.24931.1.A1\_at | 1.538e-09 | -2.051 |
| Bt.4770.1.S1\_at | 1.901e-09 | 2.027 |
| Bt.13943.1.A1\_at | 1.923e-09 | 1.44 |
| Bt.4891.1.S1\_at | 1.961e-09 | -1.569 |
| Bt.23148.1.S1\_at | 2.047e-09 | 1.401 |
| Bt.13980.2.A1\_a\_at | 2.084e-09 | -3.58 |
| Bt.22664.1.A1\_at | 2.141e-09 | 2.786 |
| Bt.16843.1.A1\_at | 2.147e-09 | -1.908 |
| Bt.20854.1.S1\_at | 2.171e-09 | -1.858 |
| Bt.28612.1.S1\_at | 2.223e-09 | 1.473 |
| Bt.24039.1.A1\_at | 2.315e-09 | -1.605 |
| Bt.16329.1.A1\_at | 2.57e-09 | 1.931 |
| Bt.13287.1.S1\_at | 2.714e-09 | -2.359 |
| Bt.20470.2.S1\_a\_at | 2.784e-09 | -4.689 |
| Bt.4473.1.S1\_at | 2.958e-09 | -1.941 |
| Bt.1316.1.S2\_at | 2.99e-09 | -3.001 |
| Bt.23526.1.S1\_at | 3.663e-09 | -1.261 |
| Bt.23392.1.S1\_at | 3.773e-09 | 1.219 |
| Bt.8618.1.S1\_at | 4.508e-09 | -2.351 |
| Bt.21239.1.S1\_at | 4.959e-09 | -1.787 |
| Bt.23076.1.S1\_at | 5.473e-09 | 3.748 |
| Bt.20505.1.S1\_at | 5.476e-09 | -2.112 |
| Bt.3940.1.S1\_at | 5.759e-09 | 1.161 |
| Bt.18278.1.S1\_at | 5.769e-09 | -2.27 |
| Bt.18890.1.A1\_at | 6.121e-09 | -1.529 |
| Bt.3117.1.S1\_at | 6.331e-09 | -1.862 |
| Bt.5002.1.S1\_at | 6.594e-09 | -3.272 |
| Bt.16815.1.S1\_at | 7.83e-09 | -1.728 |
| Bt.5483.1.S1\_at | 7.891e-09 | -1.739 |
| Bt.5805.1.S1\_at | 8.029e-09 | -1.991 |
| Bt.22634.1.S1\_at | 8.663e-09 | 1.735 |
| Bt.25282.1.A1\_at | 8.672e-09 | 1.597 |
| Bt.20257.1.S1\_at | 9.137e-09 | 1.135 |
| Bt.19409.1.A1\_s\_at | 9.816e-09 | 1.926 |
| Bt.17613.1.A1\_at | 9.903e-09 | -2.838 |
| Bt.1153.1.S1\_at | 1.023e-08 | -1.995 |
| Bt.3865.3.S1\_a\_at | 1.043e-08 | -3.343 |
| Bt.27939.2.A1\_a\_at | 1.052e-08 | 1.473 |
| Bt.3873.1.S1\_at | 1.095e-08 | -1.851 |
| Bt.11436.1.A1\_at | 1.103e-08 | 1.429 |
| Bt.2524.1.S1\_at | 1.112e-08 | 1.759 |
| Bt.23236.1.A1\_at | 1.117e-08 | 1.042 |
| Bt.3227.1.S1\_at | 1.155e-08 | -1.657 |
| Bt.18572.1.A1\_at | 1.195e-08 | 3.403 |
| Bt.12358.1.S1\_at | 1.218e-08 | 1.77 |
| Bt.6494.1.S1\_at | 1.238e-08 | -1.139 |
| Bt.22369.2.S1\_at | 1.291e-08 | 1.926 |
| Bt.5519.2.S1\_at | 1.316e-08 | 1.42 |
| Bt.12303.1.S1\_at | 1.35e-08 | 1.786 |
| Bt.21176.1.S1\_at | 1.374e-08 | 1.185 |
| Bt.2988.1.S1\_at | 1.427e-08 | 1.577 |
| Bt.26462.1.S1\_at | 1.446e-08 | 2.179 |
| Bt.19517.1.S1\_at | 1.497e-08 | 3.973 |
| Bt.24265.1.S1\_a\_at | 1.59e-08 | -1.588 |
| Bt.8549.2.S1\_at | 1.675e-08 | 1.944 |
| Bt.10.2.S1\_a\_at | 1.704e-08 | -1.263 |
| Bt.7056.5.A1\_x\_at | 1.726e-08 | 7.265 |
| Bt.1129.2.S1\_at | 1.778e-08 | -2.005 |
| Bt.22130.1.S1\_at | 1.781e-08 | 1.4 |
| Bt.2894.1.S1\_at | 1.787e-08 | -3.12 |
| Bt.25038.1.S1\_at | 1.838e-08 | 1.136 |
| Bt.22084.1.A1\_at | 1.848e-08 | 1.632 |
| Bt.11236.1.S1\_at | 1.877e-08 | -4.556 |
| Bt.1316.1.S1\_at | 1.947e-08 | -3.27 |
| Bt.27381.1.S1\_at | 2.12e-08 | -4.08 |
| Bt.23230.1.S1\_at | 2.152e-08 | 0.9727 |
| Bt.21896.1.S1\_at | 2.218e-08 | 3.296 |
| Bt.1101.1.S1\_at | 2.222e-08 | 0.9535 |
| Bt.26718.1.S1\_a\_at | 2.284e-08 | -2.483 |
| Bt.9983.1.S1\_at | 2.336e-08 | -1.329 |
| Bt.13313.2.S1\_at | 2.376e-08 | 1.714 |
| Bt.21019.1.S1\_at | 2.407e-08 | 1.272 |
| Bt.1332.1.S1\_a\_at | 2.518e-08 | -1.768 |
| Bt.25555.1.S1\_at | 2.537e-08 | -2.117 |
| Bt.9586.1.A1\_at | 2.56e-08 | 1.474 |
| Bt.3015.2.S1\_at | 2.624e-08 | 1.863 |
| Bt.27193.1.A1\_at | 2.648e-08 | 1.9 |
| Bt.8650.1.A1\_at | 2.713e-08 | 0.9298 |
| Bt.8351.1.S1\_at | 3.439e-08 | -1.179 |
| Bt.22518.1.S1\_a\_at | 3.451e-08 | -1.816 |
| Bt.7175.1.S1\_at | 3.501e-08 | -2.193 |
| Bt.5246.1.S1\_at | 3.623e-08 | -1.748 |
| Bt.4040.1.S1\_at | 3.635e-08 | 2.176 |
| Bt.23603.3.S1\_at | 3.743e-08 | -1.332 |
| Bt.3247.1.S1\_at | 3.827e-08 | 2.788 |
| Bt.12510.1.S1\_a\_at | 3.974e-08 | 4.291 |
| Bt.5123.2.S1\_at | 4.051e-08 | -1.287 |
| Bt.836.1.S1\_at | 4.11e-08 | -2.125 |
| Bt.1035.1.S1\_a\_at | 4.14e-08 | -1.22 |
| Bt.12875.1.S1\_at | 4.172e-08 | 1.907 |
| Bt.28071.1.S1\_at | 4.349e-08 | -1.37 |
| Bt.10719.1.S1\_at | 4.379e-08 | 7.027 |
| Bt.23502.1.S1\_at | 4.412e-08 | 1.648 |
| Bt.5089.1.S2\_at | 4.416e-08 | 1.7 |
| Bt.298.1.S1\_at | 4.621e-08 | -1.093 |
| Bt.13834.1.S1\_at | 4.656e-08 | -2.628 |
| Bt.16003.1.S1\_at | 4.746e-08 | -1.559 |
| Bt.7984.1.S1\_at | 4.775e-08 | 1.588 |
| Bt.12434.1.S1\_at | 5.039e-08 | -1.411 |
| Bt.3541.1.S1\_at | 5.05e-08 | 0.9904 |
| Bt.12694.1.S1\_at | 5.075e-08 | 2.056 |
| Bt.23396.1.S1\_at | 5.094e-08 | -2.704 |
| Bt.23438.1.S1\_at | 5.131e-08 | 1.777 |
| Bt.1674.2.S1\_at | 5.306e-08 | -1.695 |
| Bt.24413.1.S1\_at | 5.528e-08 | 2.173 |
| Bt.9362.1.S1\_at | 5.549e-08 | -1.412 |
| Bt.20515.1.S1\_at | 5.654e-08 | 1.638 |
| Bt.10956.1.S1\_at | 5.717e-08 | 0.9789 |
| Bt.1861.1.S1\_at | 5.73e-08 | 2.014 |
| Bt.20485.1.S1\_at | 5.867e-08 | 1.065 |
| Bt.1517.1.S1\_at | 5.963e-08 | -1.249 |
| Bt.12423.1.S1\_at | 5.985e-08 | 1.391 |
| Bt.13637.1.A1\_at | 6.024e-08 | 0.9952 |
| Bt.5966.1.A1\_at | 6.248e-08 | -2.113 |
| Bt.7215.1.S1\_at | 6.398e-08 | -2.385 |
| Bt.29931.1.S1\_at | 6.402e-08 | -2.075 |
| Bt.3857.1.S1\_at | 6.607e-08 | -3.555 |
| Bt.66.1.S1\_at | 6.627e-08 | -1.496 |
| Bt.5078.1.S1\_at | 6.775e-08 | 1.518 |
| Bt.20354.1.S1\_at | 6.943e-08 | 2.423 |
| Bt.18986.1.S1\_at | 7.236e-08 | 1.589 |
| Bt.9877.1.S1\_at | 7.261e-08 | 0.9066 |
| Bt.23520.1.S1\_at | 7.715e-08 | 1.006 |
| Bt.4868.2.S1\_a\_at | 7.734e-08 | -1.335 |
| Bt.814.1.S1\_at | 7.833e-08 | -1.335 |
| Bt.24824.1.S1\_at | 7.85e-08 | 1.906 |
| Bt.4263.1.S1\_at | 7.883e-08 | 1.619 |
| Bt.22651.1.A1\_at | 7.921e-08 | 1.153 |
| Bt.24154.1.A1\_at | 8.065e-08 | -1.746 |
| Bt.20307.1.S1\_at | 8.146e-08 | -1.732 |
| Bt.7034.1.S1\_at | 8.324e-08 | -3.563 |
| Bt.5605.1.S1\_at | 8.45e-08 | 1.342 |
| Bt.11659.1.S1\_at | 8.503e-08 | -1.721 |
| Bt.1286.1.S1\_a\_at | 8.559e-08 | 1.401 |
| Bt.10013.1.S1\_at | 8.631e-08 | 2.469 |
| Bt.16111.1.S1\_at | 8.679e-08 | 0.965 |
| Bt.24848.1.A1\_at | 8.762e-08 | 3.405 |
| Bt.7459.1.S1\_at | 8.98e-08 | 1.668 |
| Bt.11180.1.A1\_a\_at | 9.165e-08 | 1.942 |
| Bt.4475.1.S1\_at | 9.365e-08 | -1.694 |
| Bt.1758.1.S1\_at | 9.381e-08 | 1.689 |
| Bt.20591.2.A1\_at | 9.462e-08 | 1.866 |
| Bt.3782.1.A1\_at | 9.773e-08 | 3.289 |
| Bt.3236.2.S1\_at | 9.795e-08 | 1.608 |
| Bt.12721.1.A1\_at | 9.981e-08 | 1.284 |
| Bt.28208.1.S1\_at | 1.006e-07 | -2.348 |
| Bt.4232.2.S1\_at | 1.007e-07 | 1.528 |
| Bt.25087.3.S1\_at | 1.022e-07 | -1.464 |
| Bt.4662.1.S1\_at | 1.023e-07 | 1.49 |
| Bt.18753.1.A1\_at | 1.035e-07 | 2.537 |
| Bt.12510.1.S1\_at | 1.063e-07 | 4.04 |
| Bt.26792.1.S1\_at | 1.069e-07 | 3.775 |
| Bt.21766.2.S1\_at | 1.083e-07 | -2.149 |
| Bt.21561.1.S1\_at | 1.087e-07 | 1.591 |
| Bt.23486.1.A1\_at | 1.093e-07 | 1.887 |
| Bt.23113.1.A1\_at | 1.103e-07 | 3.215 |
| Bt.4271.1.S1\_at | 1.116e-07 | 1.026 |
| Bt.5537.1.S1\_at | 1.117e-07 | -2.06 |
| Bt.13321.1.S1\_at | 1.123e-07 | 2.071 |
| Bt.29746.1.S1\_at | 1.125e-07 | -2.516 |
| Bt.23662.2.S1\_at | 1.129e-07 | -2.895 |
| Bt.12126.1.S1\_at | 1.134e-07 | -1.711 |
| Bt.21766.3.S1\_at | 1.164e-07 | -2.552 |
| Bt.22122.2.A1\_at | 1.173e-07 | 1.557 |
| Bt.13833.1.S1\_at | 1.18e-07 | 1.29 |
| Bt.8679.1.A1\_at | 1.249e-07 | -1.785 |
| Bt.21731.1.S1\_at | 1.261e-07 | 1.774 |
| Bt.5223.1.S1\_at | 1.271e-07 | 2.151 |
| Bt.4777.1.S1\_at | 1.271e-07 | -2.172 |
| Bt.29873.1.S1\_at | 1.273e-07 | 2.345 |
| Bt.21.1.S1\_at | 1.275e-07 | -1.036 |
| Bt.19352.1.A1\_at | 1.325e-07 | -1.411 |
| Bt.9826.1.S1\_at | 1.334e-07 | -1.137 |
| Bt.28269.1.S1\_at | 1.359e-07 | 1.59 |
| Bt.17079.1.S1\_a\_at | 1.359e-07 | 1.777 |
| Bt.19508.1.A1\_at | 1.379e-07 | 2.346 |
| Bt.28226.3.A1\_s\_at | 1.392e-07 | 1.447 |
| Bt.4972.1.S1\_at | 1.422e-07 | 1.416 |
| Bt.18402.1.S1\_at | 1.431e-07 | -1.643 |
| Bt.18381.1.S1\_at | 1.448e-07 | 1.323 |
| Bt.5120.1.S1\_at | 1.465e-07 | -2.311 |
| Bt.15840.1.A1\_at | 1.466e-07 | 1.55 |
| Bt.4072.1.S1\_at | 1.467e-07 | -2.008 |
| Bt.946.2.A1\_a\_at | 1.499e-07 | -2.197 |
| Bt.11449.1.S1\_at | 1.519e-07 | -1.841 |
| Bt.3922.1.S1\_a\_at | 1.522e-07 | -2.465 |
| Bt.1505.1.S1\_at | 1.545e-07 | -1.409 |
| Bt.21790.1.S1\_at | 1.548e-07 | 1.274 |
| Bt.5406.1.S1\_at | 1.565e-07 | 1.305 |
| Bt.21877.1.S1\_at | 1.569e-07 | -1.119 |
| Bt.9434.1.S1\_at | 1.575e-07 | -1.38 |
| Bt.27926.1.S1\_at | 1.584e-07 | -1.849 |
| Bt.23418.1.S1\_at | 1.662e-07 | 1.444 |
| Bt.13755.1.S1\_at | 1.663e-07 | 1.181 |
| Bt.27421.1.S1\_at | 1.677e-07 | 2.052 |
| Bt.19209.2.S1\_at | 1.682e-07 | 0.6361 |
| Bt.12661.1.A1\_at | 1.698e-07 | 1.453 |
| Bt.22082.1.S1\_at | 1.707e-07 | -2.184 |
| Bt.21372.3.A1\_at | 1.71e-07 | 1.79 |
| Bt.5732.1.S1\_at | 1.717e-07 | 2.66 |
| Bt.6576.1.S1\_at | 1.741e-07 | 1.112 |
| Bt.23308.1.S1\_at | 1.745e-07 | 1.342 |
| Bt.27901.1.S1\_at | 1.746e-07 | 1.764 |
| Bt.18420.2.A1\_at | 1.768e-07 | 2.413 |
| Bt.2711.1.S1\_at | 1.775e-07 | -1.177 |
| Bt.3448.2.S1\_at | 1.783e-07 | -1.865 |
| Bt.7696.2.S1\_a\_at | 1.791e-07 | -2.141 |
| Bt.24849.1.A1\_at | 1.794e-07 | -1.928 |
| Bt.13128.1.S1\_at | 1.809e-07 | -1.054 |
| Bt.23176.2.S1\_a\_at | 1.822e-07 | 2.566 |
| Bt.23164.1.S1\_at | 1.831e-07 | -2.15 |
| Bt.11208.1.S1\_at | 1.871e-07 | -1.733 |
| Bt.21383.2.S1\_a\_at | 1.935e-07 | 1.92 |
| Bt.23129.3.S1\_a\_at | 1.941e-07 | 1.107 |
| Bt.11093.1.S1\_at | 1.958e-07 | 1.808 |
| Bt.27192.1.S1\_at | 1.99e-07 | -2.084 |
| Bt.23669.1.A1\_at | 2.021e-07 | 2.023 |
| Bt.11196.1.S1\_at | 2.029e-07 | -1.731 |
| Bt.14193.1.A1\_at | 2.096e-07 | -0.5632 |
| Bt.22510.1.S1\_at | 2.099e-07 | -1.845 |
| Bt.29053.1.A1\_at | 2.108e-07 | 1.7 |
| Bt.28431.1.S1\_at | 2.116e-07 | 1.38 |
| Bt.14116.1.A1\_at | 2.12e-07 | 1.538 |
| Bt.13330.2.A1\_at | 2.143e-07 | -4.727 |
| Bt.17778.1.A1\_at | 2.215e-07 | -1.764 |
| Bt.9222.1.S1\_at | 2.221e-07 | -1.9 |
| Bt.76.1.S1\_at | 2.257e-07 | -1.707 |
| Bt.28568.1.A1\_at | 2.281e-07 | 4.367 |
| Bt.14340.2.S1\_at | 2.366e-07 | 2.637 |
| Bt.28975.1.S1\_at | 2.376e-07 | 1.936 |
| Bt.11794.1.S1\_at | 2.38e-07 | -2.422 |
| Bt.16082.1.S1\_at | 2.384e-07 | -2.147 |
| Bt.23131.1.S1\_s\_at | 2.417e-07 | 2.295 |
| Bt.20411.1.S1\_at | 2.425e-07 | -1.749 |
| Bt.4658.1.S1\_a\_at | 2.429e-07 | -1.006 |
| Bt.9966.1.S1\_at | 2.527e-07 | 1.879 |
| Bt.11516.1.S1\_at | 2.561e-07 | -1.827 |
| Bt.18352.1.A1\_at | 2.583e-07 | -2.361 |
| Bt.20601.1.A1\_at | 2.646e-07 | 1.568 |
| Bt.20899.1.A1\_at | 2.711e-07 | -2.599 |
| Bt.5520.1.S1\_at | 2.751e-07 | -1.981 |
| Bt.22350.1.A1\_at | 2.784e-07 | 1.748 |
| Bt.2138.1.S1\_s\_at | 2.821e-07 | -2.23 |
| Bt.20984.1.S1\_a\_at | 2.822e-07 | -1.309 |
| Bt.7079.1.S1\_at | 2.848e-07 | -3.268 |
| Bt.10995.1.S1\_at | 2.886e-07 | 1.753 |
| Bt.22552.1.S2\_at | 2.887e-07 | 0.8144 |
| Bt.26568.2.S1\_a\_at | 2.938e-07 | -1.927 |
| Bt.18851.1.S1\_at | 2.971e-07 | -1.315 |
| Bt.16900.1.A1\_at | 3.004e-07 | 1.366 |
| Bt.7696.1.S1\_at | 3.045e-07 | -1.838 |
| Bt.3854.1.S1\_at | 3.07e-07 | 1.855 |
| Bt.20.1.S1\_at | 3.115e-07 | -2.01 |
| Bt.20141.1.S1\_at | 3.123e-07 | -2.16 |
| Bt.13237.1.S1\_at | 3.156e-07 | -0.7833 |
| Bt.1154.1.S1\_at | 3.158e-07 | -1.947 |
| Bt.14340.1.A1\_at | 3.18e-07 | 1.417 |
| Bt.28566.1.S1\_at | 3.208e-07 | 0.9286 |
| Bt.4704.1.S1\_at | 3.215e-07 | -1.998 |
| Bt.12510.3.S1\_at | 3.216e-07 | 2.784 |
| Bt.11622.1.A1\_at | 3.263e-07 | 1.64 |
| Bt.7856.1.S1\_at | 3.312e-07 | -2.077 |
| Bt.15941.1.S1\_at | 3.329e-07 | -1.584 |
| Bt.26702.1.S1\_a\_at | 3.381e-07 | -2.389 |
| Bt.24676.2.A1\_at | 3.474e-07 | -1.509 |
| Bt.3218.1.S1\_at | 3.518e-07 | 1.956 |
| Bt.26453.1.S1\_at | 3.574e-07 | 1.854 |
| Bt.20046.1.S1\_a\_at | 3.638e-07 | 1.086 |
| Bt.21251.1.S1\_at | 3.682e-07 | 0.5822 |
| Bt.4280.1.A1\_at | 3.689e-07 | 0.7951 |
| Bt.8829.1.S1\_a\_at | 3.8e-07 | 2.759 |
| Bt.4864.2.S1\_at | 3.856e-07 | 1.257 |
| Bt.15666.2.S1\_at | 3.872e-07 | 2.348 |
| Bt.27318.1.S1\_at | 3.897e-07 | 2.19 |
| Bt.3215.1.S1\_at | 3.913e-07 | 1.081 |
| Bt.4563.1.S1\_at | 3.926e-07 | 0.7661 |
| Bt.28133.1.S1\_at | 4.066e-07 | 1.34 |
| Bt.28305.1.S1\_at | 4.105e-07 | 2.412 |
| Bt.13439.1.S1\_at | 4.169e-07 | -2.023 |
| Bt.26728.1.S1\_at | 4.21e-07 | 1.806 |
| Bt.23178.1.S1\_at | 4.305e-07 | 1.326 |
| Bt.20025.1.S1\_at | 4.316e-07 | -1.891 |
| Bt.20512.1.S1\_at | 4.366e-07 | 1.648 |
| Bt.59.1.S1\_at | 4.419e-07 | -1.143 |
| Bt.10278.1.S1\_at | 4.419e-07 | -1.6 |
| Bt.3321.1.S1\_at | 4.426e-07 | 1.267 |
| Bt.250.1.S1\_at | 4.513e-07 | -0.74 |
| Bt.8062.1.S1\_at | 4.515e-07 | -1.211 |
| Bt.1399.1.S1\_at | 4.615e-07 | -1.518 |
| Bt.2587.1.S1\_at | 4.62e-07 | -1.304 |
| Bt.25227.1.A1\_at | 4.673e-07 | 2.047 |
| Bt.26595.1.S1\_at | 4.729e-07 | 1.215 |
| Bt.21969.1.A1\_at | 4.737e-07 | 0.9219 |
| Bt.4670.1.S1\_at | 4.827e-07 | 2.012 |
| Bt.378.1.S1\_at | 4.896e-07 | -1.074 |
| Bt.18332.1.S1\_at | 4.965e-07 | 1.42 |
| Bt.21218.1.A1\_at | 4.983e-07 | -2.696 |
| Bt.24305.1.A1\_at | 4.992e-07 | 1.143 |
| Bt.26415.1.A1\_at | 5.003e-07 | 1.049 |
| Bt.2049.1.S1\_at | 5.034e-07 | -2.469 |
| Bt.23318.1.S1\_at | 5.094e-07 | 1.402 |
| Bt.25957.1.S1\_at | 5.156e-07 | -1.327 |
| Bt.23399.1.S1\_at | 5.215e-07 | -2.109 |
| Bt.13349.1.S1\_at | 5.225e-07 | -1.154 |
| Bt.3880.1.S1\_at | 5.264e-07 | 2.949 |
| Bt.11476.1.S1\_at | 5.289e-07 | 1.68 |
| Bt.14229.1.S1\_at | 5.325e-07 | -1.218 |
| Bt.22200.1.S1\_at | 5.336e-07 | 2.83 |
| Bt.20295.1.A1\_at | 5.423e-07 | 2.403 |
| Bt.676.1.S1\_at | 5.462e-07 | 0.8311 |
| Bt.28087.1.S1\_at | 5.505e-07 | 1.239 |
| Bt.21622.1.A1\_at | 5.523e-07 | 1.522 |
| Bt.2494.2.S1\_a\_at | 5.558e-07 | 1.343 |
| Bt.13240.1.S1\_at | 5.56e-07 | 1.468 |
| Bt.23218.1.S1\_at | 5.58e-07 | 1.066 |
| Bt.26150.1.A1\_at | 5.627e-07 | -2.067 |
| Bt.13327.1.S1\_at | 5.688e-07 | -0.9374 |
| Bt.11415.1.A1\_at | 5.735e-07 | 3.111 |
| Bt.25616.1.S1\_at | 5.747e-07 | 1.575 |
| Bt.11060.1.S1\_at | 5.758e-07 | 1.252 |
| Bt.2065.1.A1\_at | 5.795e-07 | 0.7981 |
| Bt.4985.1.S1\_a\_at | 5.845e-07 | -1.053 |
| Bt.10797.2.S1\_a\_at | 5.852e-07 | -1.123 |
| Bt.1807.1.A1\_at | 5.88e-07 | 2.493 |
| Bt.5042.1.S1\_at | 5.927e-07 | -1.23 |
| Bt.13483.1.A1\_at | 5.941e-07 | -1.404 |
| Bt.9708.2.S1\_at | 5.995e-07 | -1.968 |
| Bt.24817.1.S1\_at | 6.027e-07 | -1.732 |
| Bt.22295.1.S1\_at | 6.033e-07 | -0.9084 |
| Bt.20321.1.S1\_at | 6.165e-07 | 1.347 |
| Bt.13313.1.S1\_at | 6.268e-07 | 1.827 |
| Bt.979.1.A1\_at | 6.352e-07 | 1.843 |
| Bt.2696.1.A1\_at | 6.409e-07 | 2.401 |
| Bt.1059.3.S1\_a\_at | 6.436e-07 | -0.9673 |
| Bt.22047.1.S1\_at | 6.453e-07 | -1.33 |
| Bt.21981.3.S1\_at | 6.465e-07 | 1.661 |
| Bt.1844.1.S1\_at | 6.557e-07 | 1.205 |
| Bt.24404.1.A1\_at | 6.577e-07 | 2.22 |
| Bt.18479.1.A1\_at | 6.62e-07 | 2.648 |
| Bt.12613.1.S1\_at | 6.68e-07 | 2.084 |
| Bt.20282.1.S1\_at | 6.692e-07 | -1.476 |
| Bt.5344.1.S1\_at | 6.796e-07 | -1.861 |
| Bt.1280.2.S1\_a\_at | 6.798e-07 | -1.761 |
| Bt.2997.1.A1\_at | 6.923e-07 | 1.853 |
| Bt.19937.2.S1\_at | 6.988e-07 | 1.357 |
| Bt.14095.1.S1\_at | 7.036e-07 | 1.331 |
| Bt.22466.1.A1\_at | 7.198e-07 | 1.484 |
| Bt.27908.1.S1\_at | 7.214e-07 | -1.489 |
| Bt.22306.1.S1\_at | 7.552e-07 | -2.111 |
| Bt.11059.1.S1\_at | 7.554e-07 | 1.711 |
| Bt.4792.1.S1\_at | 7.573e-07 | 1.559 |
| Bt.8674.1.A1\_at | 7.587e-07 | 1.226 |
| Bt.400.1.S1\_at | 7.667e-07 | -1.024 |
| Bt.23576.1.A1\_at | 7.677e-07 | 1.427 |
| Bt.22270.1.S1\_at | 7.731e-07 | 2.284 |
| Bt.12337.1.S1\_a\_at | 7.735e-07 | -1.054 |
| Bt.5153.1.S1\_at | 7.746e-07 | 1.505 |
| Bt.1652.1.S1\_at | 7.765e-07 | 1.637 |
| Bt.467.1.S1\_at | 7.8e-07 | -3.431 |
| Bt.2070.1.A1\_at | 7.852e-07 | -1.423 |
| Bt.12453.1.A1\_a\_at | 7.969e-07 | 2.428 |
| Bt.9956.1.S1\_at | 8.002e-07 | -1.323 |
| Bt.20402.1.S1\_at | 8.02e-07 | 1.817 |
| Bt.12290.1.S1\_at | 8.07e-07 | 1.573 |
| Bt.9556.1.S1\_at | 8.138e-07 | 1.866 |
| Bt.19167.1.A1\_at | 8.184e-07 | 2 |
| Bt.869.2.S1\_at | 8.194e-07 | 1.427 |
| Bt.9953.1.S1\_at | 8.2e-07 | -0.9854 |
| Bt.28480.1.S1\_at | 8.265e-07 | -1.095 |
| Bt.5089.1.S1\_at | 8.37e-07 | 1.409 |
| Bt.5541.1.S1\_at | 8.382e-07 | -1.336 |
| Bt.1284.1.S1\_at | 8.422e-07 | -2.251 |
| Bt.4476.1.S1\_at | 8.479e-07 | -1.85 |
| Bt.23603.1.S1\_a\_at | 8.502e-07 | -1.467 |
| Bt.5278.1.A1\_at | 8.65e-07 | -1.7 |
| Bt.11165.1.S1\_at | 8.669e-07 | 0.8759 |
| Bt.27046.1.S1\_at | 8.692e-07 | 0.8203 |
| Bt.12211.1.S1\_at | 8.819e-07 | -2.144 |
| Bt.4011.1.S1\_at | 8.822e-07 | -2.266 |
| Bt.20228.1.S1\_at | 8.913e-07 | -0.6396 |
| Bt.1552.1.S1\_at | 8.922e-07 | -2.502 |
| Bt.12453.1.A1\_at | 8.988e-07 | 2.587 |
| Bt.1258.1.S1\_at | 8.992e-07 | 0.9009 |
| Bt.26764.1.A1\_at | 9.07e-07 | 1.175 |
| Bt.13324.2.S1\_a\_at | 9.253e-07 | -1.789 |
| Bt.5395.1.S1\_a\_at | 9.316e-07 | 2.931 |
| Bt.18113.2.S1\_at | 9.365e-07 | 0.6939 |
| Bt.20512.2.S1\_at | 9.463e-07 | 2.108 |
| Bt.6247.1.S1\_at | 9.572e-07 | 2.511 |
| Bt.25107.1.A1\_at | 9.609e-07 | 1.081 |
| Bt.5210.1.S1\_at | 9.724e-07 | -1.284 |
| Bt.1059.2.S1\_at | 9.744e-07 | -0.7813 |
| Bt.8679.2.S1\_at | 9.767e-07 | -1.627 |
| Bt.10175.2.A1\_a\_at | 9.818e-07 | 1.052 |
| Bt.27215.1.S1\_at | 9.921e-07 | -1.743 |
| Bt.28193.1.S1\_at | 1.004e-06 | 2.242 |
| Bt.16668.1.A1\_at | 1.013e-06 | 1.94 |
| Bt.5038.4.A1\_at | 1.025e-06 | 0.9627 |
| Bt.13334.1.S1\_at | 1.026e-06 | 1.523 |
| Bt.3126.1.A1\_at | 1.029e-06 | 1.606 |
| Bt.22040.1.S1\_at | 1.033e-06 | 1.712 |
| Bt.28138.1.S1\_at | 1.042e-06 | 1.803 |
| Bt.10458.1.A1\_at | 1.097e-06 | 1.822 |
| Bt.15853.1.S1\_at | 1.097e-06 | 1.411 |
| Bt.8964.2.S1\_a\_at | 1.1e-06 | -2.507 |
| Bt.8504.1.A1\_at | 1.106e-06 | 1.708 |
| Bt.16978.1.S1\_at | 1.115e-06 | -1.583 |
| Bt.11055.1.S1\_at | 1.119e-06 | 1.651 |
| Bt.10111.1.S1\_at | 1.119e-06 | 1.987 |
| Bt.2825.1.S1\_at | 1.128e-06 | -1.704 |
| Bt.11216.1.S1\_at | 1.129e-06 | -3.173 |
| Bt.2956.1.A1\_at | 1.148e-06 | 1.225 |
| Bt.17963.1.A1\_at | 1.154e-06 | -1.793 |
| Bt.4725.1.S1\_at | 1.166e-06 | 1.445 |
| Bt.888.1.S1\_at | 1.178e-06 | 1.609 |
| Bt.13427.1.S1\_at | 1.194e-06 | 2.655 |
| Bt.5029.1.S1\_at | 1.195e-06 | -1.047 |
| Bt.20494.1.S1\_at | 1.202e-06 | 1.213 |
| Bt.13330.1.S1\_at | 1.22e-06 | -3.682 |
| Bt.28623.1.S1\_at | 1.246e-06 | 1.846 |
| Bt.7237.2.S1\_a\_at | 1.255e-06 | -1.963 |
| Bt.8279.3.S1\_a\_at | 1.258e-06 | -1.311 |
| Bt.2919.1.S1\_a\_at | 1.259e-06 | 1.411 |
| Bt.5033.1.S1\_at | 1.273e-06 | -1.438 |
| Bt.5605.2.S1\_at | 1.276e-06 | 1.457 |
| Bt.12910.1.S1\_at | 1.276e-06 | -1.154 |
| Bt.2378.1.S1\_at | 1.282e-06 | 1.135 |
| Bt.58.1.S1\_at | 1.288e-06 | -0.7993 |
| Bt.99.1.S1\_at | 1.288e-06 | -1.58 |
| Bt.27039.1.A1\_at | 1.289e-06 | -2.647 |
| Bt.1242.1.S1\_at | 1.301e-06 | -2.021 |
| Bt.5176.2.A1\_at | 1.306e-06 | 1.397 |
| Bt.4653.1.S1\_at | 1.31e-06 | 1.876 |
| Bt.25712.1.A1\_s\_at | 1.344e-06 | 1.582 |
| Bt.21491.1.S1\_at | 1.364e-06 | 1.092 |
| Bt.8679.3.S1\_at | 1.377e-06 | -2.319 |
| Bt.5527.2.S1\_a\_at | 1.389e-06 | -1.508 |
| Bt.11184.1.S1\_at | 1.39e-06 | -0.7046 |
| Bt.9629.1.S1\_at | 1.396e-06 | 1.147 |
| Bt.7384.1.A1\_at | 1.396e-06 | 1.469 |
| Bt.27403.1.S1\_at | 1.404e-06 | 1.64 |
| Bt.23330.1.S1\_at | 1.407e-06 | 1.368 |
| Bt.20064.1.S1\_at | 1.409e-06 | 1.09 |
| Bt.5106.1.S1\_a\_at | 1.41e-06 | 1.054 |
| Bt.5123.3.S1\_a\_at | 1.411e-06 | -1.284 |
| Bt.20783.1.A1\_at | 1.442e-06 | 2.619 |
| Bt.23318.1.S2\_at | 1.446e-06 | 1.067 |
| Bt.7870.1.S1\_at | 1.458e-06 | -2.261 |
| Bt.2129.2.S1\_at | 1.458e-06 | 2.309 |
| Bt.2359.1.A1\_at | 1.47e-06 | 1.181 |
| Bt.27091.1.S1\_at | 1.475e-06 | 2.02 |
| Bt.4651.1.S1\_at | 1.477e-06 | 1.737 |
| Bt.20445.1.S1\_at | 1.477e-06 | -0.7604 |
| Bt.23509.2.A1\_at | 1.492e-06 | 1.039 |
| Bt.9754.1.S1\_at | 1.504e-06 | -2.312 |
| Bt.23158.1.S1\_at | 1.509e-06 | 1.575 |
| Bt.20146.1.S1\_at | 1.531e-06 | 1.893 |
| Bt.27008.1.A1\_at | 1.54e-06 | 1.378 |
| Bt.5676.1.S1\_at | 1.545e-06 | -2.202 |
| Bt.5748.2.S1\_at | 1.548e-06 | 1.29 |
| Bt.19621.1.A1\_at | 1.557e-06 | 1.181 |
| Bt.9730.1.S1\_at | 1.557e-06 | -0.9174 |
| Bt.67.1.S1\_at | 1.56e-06 | -1.722 |
| Bt.12684.2.S1\_at | 1.568e-06 | 1.099 |
| Bt.20361.2.A1\_at | 1.568e-06 | 2.244 |
| Bt.13063.1.S1\_at | 1.59e-06 | -1.192 |
| Bt.3843.1.S1\_at | 1.603e-06 | -1.245 |
| Bt.5183.2.S1\_at | 1.624e-06 | -2.688 |
| Bt.15726.1.S1\_at | 1.645e-06 | 1.486 |
| Bt.6080.1.S1\_at | 1.661e-06 | -1.432 |
| Bt.20300.1.A1\_at | 1.701e-06 | 1.304 |
| Bt.13548.1.A1\_at | 1.702e-06 | 0.8122 |
| Bt.1229.1.S1\_at | 1.707e-06 | 2.66 |
| Bt.3895.1.S1\_at | 1.709e-06 | 0.9508 |
| Bt.23357.1.S1\_at | 1.722e-06 | -2.062 |
| Bt.24895.1.A1\_at | 1.74e-06 | 0.7838 |
| Bt.10565.1.A1\_at | 1.747e-06 | 1.305 |
| Bt.9154.1.S1\_at | 1.79e-06 | 1.326 |
| Bt.4279.3.S1\_a\_at | 1.804e-06 | 1.048 |
| Bt.5326.1.S1\_at | 1.806e-06 | 1.554 |
| Bt.2501.1.S1\_at | 1.808e-06 | -1.796 |
| Bt.12505.1.S1\_at | 1.826e-06 | 0.8158 |
| Bt.10722.1.S1\_at | 1.838e-06 | 1.059 |
| Bt.27116.2.S1\_at | 1.841e-06 | -1.075 |
| Bt.5922.1.S1\_at | 1.872e-06 | -2.416 |
| Bt.755.1.S1\_at | 1.881e-06 | -0.9532 |
| Bt.7415.1.S1\_at | 1.886e-06 | 1.174 |
| Bt.23606.1.S1\_at | 1.901e-06 | 1.49 |
| Bt.12468.1.S1\_at | 1.903e-06 | -1.945 |
| Bt.11448.1.S1\_at | 1.907e-06 | 0.8681 |
| Bt.20072.1.S1\_at | 1.941e-06 | 2.895 |
| Bt.8001.1.S1\_at | 1.989e-06 | -1.36 |
| Bt.9825.1.S1\_at | 2.007e-06 | -1.507 |
| Bt.1421.1.S1\_at | 2.008e-06 | 3.78 |
| Bt.29910.1.S1\_at | 2.017e-06 | 1.557 |
| Bt.8322.1.S1\_at | 2.019e-06 | 1.528 |
| Bt.10189.1.S1\_at | 2.038e-06 | -1.096 |
| Bt.2001.1.S1\_at | 2.046e-06 | -1.274 |
| Bt.17081.1.A1\_at | 2.053e-06 | 2.291 |
| Bt.903.1.S1\_at | 2.054e-06 | 1.397 |
| Bt.16188.1.S1\_at | 2.08e-06 | 0.9326 |
| Bt.12669.1.A1\_at | 2.085e-06 | 1.411 |
| Bt.21620.1.A1\_at | 2.087e-06 | 2.087 |
| Bt.17332.2.S1\_at | 2.102e-06 | 1.663 |
| Bt.25338.1.S1\_at | 2.106e-06 | 0.8388 |
| Bt.5571.1.S1\_at | 2.131e-06 | -1.372 |
| Bt.4985.1.S1\_at | 2.133e-06 | -1.04 |
| Bt.23234.1.S1\_at | 2.143e-06 | -1.66 |
| Bt.28058.1.S1\_at | 2.157e-06 | 1.035 |
| Bt.26992.1.A1\_at | 2.158e-06 | 1.369 |
| Bt.23254.1.S1\_at | 2.198e-06 | 1.055 |
| Bt.13610.1.A1\_at | 2.225e-06 | -1.762 |
| Bt.3945.1.S1\_at | 2.234e-06 | 1.632 |
| Bt.25997.1.S1\_at | 2.249e-06 | 1.903 |
| Bt.2230.1.S2\_at | 2.251e-06 | 1.269 |
| Bt.17942.1.S1\_at | 2.253e-06 | 1.548 |
| Bt.5546.1.S1\_at | 2.26e-06 | 1.275 |
| Bt.21396.1.A1\_s\_at | 2.262e-06 | 0.513 |
| Bt.7373.2.S1\_a\_at | 2.285e-06 | -0.8406 |
| Bt.24211.2.S1\_at | 2.302e-06 | 2.525 |
| Bt.6483.1.A1\_at | 2.309e-06 | 1.165 |
| Bt.9706.2.S1\_at | 2.31e-06 | -0.8871 |
| Bt.6455.1.S1\_at | 2.32e-06 | -0.961 |
| Bt.21603.1.S1\_at | 2.345e-06 | -1.644 |
| Bt.16053.1.S1\_at | 2.345e-06 | 2.008 |
| Bt.2973.2.S1\_a\_at | 2.362e-06 | -1.078 |
| Bt.9324.1.S1\_at | 2.364e-06 | -1.675 |
| Bt.27204.1.S1\_at | 2.372e-06 | -2.661 |
| Bt.15714.1.S1\_at | 2.38e-06 | 1.36 |
| Bt.22459.1.S1\_at | 2.393e-06 | 1.942 |
| Bt.16659.1.A1\_at | 2.398e-06 | -0.6616 |
| Bt.7728.1.A1\_at | 2.405e-06 | 1.232 |
| Bt.4031.1.S1\_at | 2.42e-06 | 1.032 |
| Bt.15668.1.S1\_at | 2.431e-06 | 1.09 |
| Bt.12728.1.A1\_a\_at | 2.459e-06 | 2.116 |
| Bt.5108.1.S1\_at | 2.478e-06 | -1.489 |
| Bt.5290.1.S1\_at | 2.481e-06 | 0.9992 |
| Bt.11745.1.S1\_at | 2.502e-06 | 1.68 |
| Bt.27814.1.S1\_at | 2.542e-06 | 2.484 |
| Bt.24236.1.S1\_at | 2.543e-06 | 1.444 |
| Bt.22843.1.S1\_at | 2.567e-06 | -1.22 |
| Bt.4694.1.S1\_at | 2.618e-06 | -1.062 |
| Bt.15735.1.S1\_at | 2.626e-06 | 2.326 |
| Bt.22842.2.S1\_at | 2.635e-06 | 1.808 |
| Bt.9811.1.S1\_at | 2.639e-06 | -1.122 |
| Bt.24550.1.S1\_at | 2.656e-06 | -2.742 |
| Bt.10965.1.S1\_at | 2.659e-06 | -1.114 |
| Bt.4864.3.S1\_a\_at | 2.663e-06 | 1.302 |
| Bt.11908.1.S1\_at | 2.695e-06 | 0.8109 |
| Bt.9797.1.S1\_at | 2.7e-06 | -1.346 |
| Bt.4859.1.S1\_at | 2.704e-06 | 0.5357 |
| Bt.22502.1.S1\_at | 2.71e-06 | 1.112 |
| Bt.12345.1.S1\_at | 2.714e-06 | 0.8573 |
| Bt.22507.1.S1\_at | 2.719e-06 | 1.246 |
| Bt.5300.1.S1\_at | 2.72e-06 | -0.5951 |
| Bt.12337.2.S1\_a\_at | 2.747e-06 | -0.9278 |
| Bt.9965.1.S1\_at | 2.749e-06 | -1.636 |
| Bt.5534.1.S1\_at | 2.752e-06 | -0.6416 |
| Bt.1705.1.S1\_at | 2.765e-06 | 2.436 |
| Bt.177.1.S1\_at | 2.767e-06 | 1.222 |
| Bt.4506.1.S1\_at | 2.77e-06 | 1.351 |
| Bt.9857.1.S1\_at | 2.781e-06 | -1.751 |
| Bt.20582.2.A1\_at | 2.784e-06 | 1.596 |
| Bt.15916.1.S1\_at | 2.787e-06 | 0.5327 |
| Bt.4698.1.S1\_at | 2.81e-06 | 2.124 |
| Bt.315.1.S1\_at | 2.837e-06 | -1.87 |
| Bt.2726.1.S1\_at | 2.855e-06 | 2.285 |
| Bt.23600.1.S1\_at | 2.866e-06 | -0.9847 |
| Bt.19100.1.A1\_at | 2.882e-06 | 2.191 |
| Bt.20862.1.A1\_at | 2.883e-06 | -1.463 |
| Bt.27152.1.A1\_at | 2.903e-06 | 3.114 |
| Bt.1582.1.S1\_at | 2.903e-06 | -1.535 |
| Bt.4458.1.A1\_at | 2.915e-06 | 1.005 |
| Bt.7869.1.A1\_at | 2.928e-06 | 1.713 |
| Bt.10303.1.S1\_at | 2.934e-06 | 1.422 |
| Bt.26568.1.S1\_a\_at | 2.942e-06 | -2.341 |
| Bt.15857.1.S1\_at | 2.956e-06 | -1.945 |
| Bt.1430.1.S1\_at | 2.975e-06 | 0.8684 |
| Bt.23615.1.S1\_at | 2.983e-06 | 0.8251 |
| Bt.27121.1.S1\_at | 3.013e-06 | -0.9861 |
| Bt.28210.1.S1\_at | 3.067e-06 | 0.9629 |
| Bt.12888.1.S1\_at | 3.081e-06 | -1.394 |
| Bt.8279.2.S1\_at | 3.09e-06 | -1.512 |
| Bt.5132.1.S1\_at | 3.109e-06 | 1.868 |
| Bt.8545.1.S1\_at | 3.109e-06 | -1.47 |
| Bt.7701.1.S1\_at | 3.13e-06 | -1.502 |
| Bt.28143.1.S1\_at | 3.132e-06 | 1.71 |
| Bt.13768.2.S1\_at | 3.146e-06 | 0.904 |
| Bt.17886.1.A1\_at | 3.183e-06 | 2.439 |
| Bt.28754.1.S1\_at | 3.187e-06 | -1.465 |
| Bt.8950.1.S1\_at | 3.188e-06 | -1.232 |
| Bt.11855.1.S1\_at | 3.195e-06 | -1.264 |
| Bt.15968.1.S1\_at | 3.2e-06 | -1.26 |
| Bt.6842.1.S1\_at | 3.229e-06 | 2.207 |
| Bt.23569.1.S1\_at | 3.236e-06 | -1.041 |
| Bt.25099.1.A1\_at | 3.249e-06 | -3.761 |
| Bt.371.1.S1\_at | 3.269e-06 | -1.248 |
| Bt.27089.1.S1\_at | 3.278e-06 | 1.451 |
| Bt.6586.1.S1\_at | 3.282e-06 | 1.617 |
| Bt.20316.1.S1\_at | 3.287e-06 | -1.777 |
| Bt.29381.1.S1\_at | 3.313e-06 | -1.813 |
| Bt.19097.1.A1\_at | 3.324e-06 | 1.44 |
| Bt.12443.1.S1\_at | 3.342e-06 | 0.8665 |
| Bt.20331.1.A1\_at | 3.346e-06 | 1.426 |
| Bt.12872.1.S1\_at | 3.36e-06 | 1.541 |
| Bt.25832.1.S1\_at | 3.366e-06 | 1.984 |
| Bt.19606.1.A1\_at | 3.369e-06 | -0.4869 |
| Bt.20422.2.S1\_a\_at | 3.372e-06 | -1.012 |
| Bt.26928.1.S1\_at | 3.403e-06 | 1.408 |
| Bt.1020.1.S1\_at | 3.411e-06 | 1.493 |
| Bt.19330.1.A1\_s\_at | 3.443e-06 | 1.493 |
| Bt.23509.1.S1\_at | 3.464e-06 | 1.188 |
| Bt.21051.1.S1\_at | 3.468e-06 | -1.099 |
| Bt.1739.2.S1\_at | 3.476e-06 | 1.064 |
| Bt.75.1.S1\_at | 3.491e-06 | -1.261 |
| Bt.4819.1.S1\_at | 3.495e-06 | -0.8948 |
| Bt.21533.1.S1\_at | 3.573e-06 | 1.82 |
| Bt.23854.1.A1\_at | 3.589e-06 | 2.273 |
| Bt.22335.1.S1\_a\_at | 3.601e-06 | 0.7409 |
| Bt.4606.1.S1\_at | 3.636e-06 | 1.939 |
| Bt.997.1.S1\_at | 3.65e-06 | -1.42 |
| Bt.404.1.S1\_at | 3.698e-06 | -1.196 |
| Bt.10608.2.S1\_a\_at | 3.708e-06 | -1.355 |
| Bt.18945.1.A1\_at | 3.744e-06 | 0.9156 |
| Bt.1129.1.S1\_at | 3.773e-06 | -2.414 |
| Bt.746.1.S1\_at | 3.777e-06 | 1.264 |
| Bt.5750.1.S1\_at | 3.825e-06 | -1.253 |
| Bt.13730.2.S1\_at | 3.829e-06 | -1.498 |
| Bt.23651.1.A1\_at | 3.844e-06 | 1.833 |
| Bt.1808.1.S1\_at | 3.848e-06 | -1.542 |
| Bt.11140.1.S1\_at | 3.855e-06 | 1.568 |
| Bt.4205.1.S1\_at | 3.858e-06 | -1.091 |
| Bt.11052.1.S1\_at | 3.9e-06 | 0.791 |
| Bt.22114.1.S1\_at | 3.906e-06 | 1.133 |
| Bt.2187.1.S1\_at | 3.92e-06 | -0.9255 |
| Bt.4477.2.S1\_a\_at | 3.941e-06 | 0.5279 |
| Bt.10609.2.A1\_at | 3.957e-06 | 0.9507 |
| Bt.21920.1.S1\_at | 3.978e-06 | 0.6828 |
| Bt.9708.1.S1\_at | 3.979e-06 | -1.783 |
| Bt.29655.1.S1\_a\_at | 3.992e-06 | -1.185 |
| Bt.3856.1.S1\_at | 4.038e-06 | -2.089 |
| Bt.7007.1.S1\_at | 4.042e-06 | -1.437 |
| Bt.25072.1.S1\_at | 4.058e-06 | -1.423 |
| Bt.64.1.S1\_at | 4.087e-06 | -1.454 |
| Bt.27027.1.S1\_a\_at | 4.115e-06 | -1.261 |
| Bt.22310.1.S1\_at | 4.166e-06 | -1.481 |
| Bt.6832.1.S1\_at | 4.185e-06 | 1.33 |
| Bt.26829.1.S1\_at | 4.187e-06 | -2.697 |
| Bt.3714.2.S1\_at | 4.225e-06 | 2.553 |
| Bt.5341.1.S1\_at | 4.256e-06 | 1.441 |
| Bt.984.1.S1\_at | 4.261e-06 | 1.426 |
| Bt.5183.1.S1\_at | 4.279e-06 | -2.185 |
| Bt.22770.1.S1\_at | 4.309e-06 | 1.6 |
| Bt.5527.1.S1\_at | 4.328e-06 | -1.168 |
| Bt.15664.1.S1\_at | 4.346e-06 | -1.065 |
| Bt.5040.1.S1\_at | 4.358e-06 | -1.035 |
| Bt.9169.1.A1\_at | 4.359e-06 | 1.565 |
| Bt.12912.1.S2\_at | 4.36e-06 | 1.302 |
| Bt.11492.1.A1\_at | 4.39e-06 | 2.01 |
| Bt.6087.2.S1\_a\_at | 4.391e-06 | 1.115 |
| Bt.3272.1.A1\_at | 4.394e-06 | -1.826 |
| Bt.7395.1.S1\_at | 4.424e-06 | 1.073 |
| Bt.20029.1.S1\_at | 4.462e-06 | 0.7988 |
| Bt.5879.1.S1\_at | 4.473e-06 | 1.087 |
| Bt.8046.1.A1\_at | 4.478e-06 | -1.961 |
| Bt.19431.1.S1\_at | 4.497e-06 | -0.8938 |
| Bt.1294.1.S1\_at | 4.563e-06 | -2.112 |
| Bt.4872.1.S1\_at | 4.587e-06 | 1.378 |
| Bt.8122.1.S1\_at | 4.605e-06 | 0.6795 |
| Bt.26572.1.S1\_at | 4.63e-06 | 1.798 |
| Bt.24625.1.S1\_at | 4.704e-06 | 1.269 |
| Bt.4503.1.S1\_at | 4.737e-06 | -1.292 |
| Bt.26122.2.S1\_at | 4.765e-06 | -1.077 |
| Bt.19218.1.A1\_at | 4.766e-06 | 0.8894 |
| Bt.25637.1.A1\_at | 4.779e-06 | -2.17 |
| Bt.8970.1.S1\_at | 4.794e-06 | -0.5639 |
| Bt.1667.1.S1\_at | 4.816e-06 | -1.484 |
| Bt.28704.1.S1\_at | 4.846e-06 | 2.865 |
| Bt.10543.1.S1\_at | 4.849e-06 | 1.887 |
| Bt.1734.1.S1\_at | 4.877e-06 | 1.54 |
| Bt.1267.1.S1\_at | 4.887e-06 | 2.165 |
| Bt.4037.1.S1\_at | 4.893e-06 | 0.8083 |
| Bt.2729.1.S1\_at | 4.899e-06 | 0.9369 |
| Bt.17843.1.A1\_at | 4.9e-06 | 1.21 |
| Bt.15870.1.S1\_at | 4.905e-06 | -1.816 |
| Bt.21366.2.S1\_at | 4.936e-06 | -1.713 |
| Bt.6440.1.S1\_at | 4.953e-06 | 0.9287 |
| Bt.7655.1.S1\_at | 5.001e-06 | -1.257 |
| Bt.2399.1.S1\_at | 5.044e-06 | 1.266 |
| Bt.16525.1.A1\_at | 5.07e-06 | 1.076 |
| Bt.27091.2.S1\_at | 5.073e-06 | 2.185 |
| Bt.6964.1.S1\_at | 5.093e-06 | -1.394 |
| Bt.26684.1.S1\_at | 5.15e-06 | 0.7443 |
| Bt.6683.1.S1\_at | 5.164e-06 | -1.852 |
| Bt.26502.1.A1\_at | 5.172e-06 | 1.041 |
| Bt.15724.1.S1\_at | 5.247e-06 | -1.195 |
| Bt.23380.2.S1\_at | 5.247e-06 | 1.059 |
| Bt.11035.1.S1\_at | 5.272e-06 | 0.9727 |
| Bt.56.1.S1\_at | 5.308e-06 | -1.336 |
| Bt.3487.1.S1\_at | 5.321e-06 | -1.408 |
| Bt.13768.1.S1\_at | 5.348e-06 | 1.174 |
| Bt.21584.1.S1\_at | 5.35e-06 | -1.862 |
| Bt.7457.1.S1\_at | 5.359e-06 | -1.66 |
| Bt.21721.1.A1\_at | 5.454e-06 | -2.572 |
| Bt.265.1.S1\_at | 5.521e-06 | -1.211 |
| Bt.27543.1.A1\_at | 5.588e-06 | 2.141 |
| Bt.1197.1.A1\_at | 5.621e-06 | 1.283 |
| Bt.11446.1.A1\_at | 5.661e-06 | 1.173 |
| Bt.16039.1.S1\_at | 5.665e-06 | -1.503 |
| Bt.13170.1.S1\_at | 5.738e-06 | -1.332 |
| Bt.22433.1.A1\_at | 5.746e-06 | -1.758 |
| Bt.659.1.S1\_at | 5.781e-06 | -2.266 |
| Bt.4867.2.S1\_at | 5.789e-06 | 6.396 |
| Bt.20984.1.S1\_at | 5.792e-06 | -1.53 |
| Bt.24395.1.S1\_at | 5.813e-06 | -1.475 |
| Bt.15868.1.S1\_at | 5.855e-06 | 1.342 |
| Bt.1366.1.S1\_at | 5.878e-06 | 1.545 |
| Bt.3989.1.S1\_at | 5.911e-06 | 1.672 |
| Bt.19936.1.A1\_at | 5.935e-06 | 2.304 |
| Bt.24211.1.A1\_at | 5.966e-06 | 1.933 |
| Bt.23182.1.S1\_at | 5.972e-06 | -1.721 |
| Bt.1998.1.S1\_at | 5.975e-06 | 1.048 |
| Bt.23497.1.S1\_at | 6.003e-06 | -3.863 |
| Bt.27196.1.A1\_at | 6.015e-06 | -3.648 |
| Bt.3967.1.S1\_a\_at | 6.055e-06 | -1.731 |
| Bt.7727.1.S1\_at | 6.077e-06 | 1.118 |
| Bt.11262.1.S1\_at | 6.196e-06 | 2.106 |
| Bt.4758.1.S1\_at | 6.208e-06 | -2.415 |
| Bt.18048.1.A1\_at | 6.226e-06 | 1.416 |
| Bt.12364.1.S1\_at | 6.27e-06 | -1.685 |
| Bt.7139.1.S1\_at | 6.275e-06 | -0.8072 |
| Bt.1418.2.S1\_at | 6.298e-06 | -1.446 |
| Bt.13946.2.A1\_at | 6.303e-06 | 1.064 |
| Bt.10920.1.S1\_at | 6.328e-06 | 1.599 |
| Bt.4119.1.S1\_at | 6.362e-06 | 1.031 |
| Bt.1418.1.S1\_at | 6.378e-06 | -1.405 |
| Bt.11791.2.S1\_at | 6.403e-06 | -1.861 |
| Bt.13205.1.A1\_at | 6.41e-06 | -1.495 |
| Bt.20613.2.A1\_at | 6.422e-06 | 1.436 |
| Bt.11256.1.S1\_at | 6.441e-06 | 0.6359 |
| Bt.19928.1.S1\_at | 6.478e-06 | 0.9981 |
| Bt.29834.1.S1\_at | 6.484e-06 | -0.8212 |
| Bt.9047.1.S1\_at | 6.518e-06 | -2.288 |
| Bt.12204.1.S1\_at | 6.534e-06 | 1.265 |
| Bt.13730.1.A1\_at | 6.562e-06 | -1.453 |
| Bt.5166.1.S1\_at | 6.62e-06 | 2.245 |
| Bt.28651.1.S1\_at | 6.694e-06 | 1.721 |
| Bt.22785.1.S1\_at | 6.715e-06 | -2.043 |
| Bt.9629.2.S1\_at | 6.715e-06 | 1.398 |
| Bt.11599.1.S1\_at | 6.75e-06 | 1.698 |
| Bt.28855.1.A1\_at | 6.829e-06 | 0.6838 |
| Bt.27934.1.S1\_at | 6.88e-06 | 0.659 |
| Bt.4948.1.S1\_at | 6.914e-06 | 1.157 |
| Bt.15684.1.S1\_at | 6.939e-06 | 0.8182 |
| Bt.4743.1.A1\_at | 6.995e-06 | 1.11 |
| Bt.2625.1.S1\_at | 7.016e-06 | 1.078 |
| Bt.11932.1.S1\_at | 7.026e-06 | 0.7864 |
| Bt.7394.1.A1\_at | 7.039e-06 | -1.271 |
| Bt.4719.1.S1\_at | 7.118e-06 | 1.626 |
| Bt.27054.2.S1\_a\_at | 7.138e-06 | 1.107 |
| Bt.422.1.S2\_at | 7.199e-06 | 1.623 |
| Bt.18752.1.S1\_at | 7.242e-06 | -2.171 |
| Bt.1125.1.S1\_at | 7.301e-06 | 0.6794 |
| Bt.20261.1.S1\_at | 7.344e-06 | 1.393 |
| Bt.17972.1.A1\_at | 7.387e-06 | 0.7587 |
| Bt.22379.1.S1\_at | 7.398e-06 | -1.904 |
| Bt.17837.1.A1\_at | 7.474e-06 | 1.033 |
| Bt.3867.1.S1\_at | 7.515e-06 | -0.6384 |
| Bt.4954.1.S1\_at | 7.688e-06 | 2.284 |
| Bt.8606.1.S1\_a\_at | 7.707e-06 | 1.571 |
| Bt.21031.1.S1\_at | 7.713e-06 | -1.307 |
| Bt.16259.2.S1\_at | 7.785e-06 | 0.9681 |
| Bt.22538.2.S1\_at | 7.821e-06 | 2.477 |
| Bt.19937.1.S1\_at | 7.835e-06 | 1.074 |
| Bt.18075.1.A1\_at | 7.838e-06 | -1.383 |
| Bt.23098.1.S1\_at | 7.846e-06 | 1.252 |
| Bt.18492.1.A1\_at | 7.973e-06 | -0.4917 |
| Bt.20007.1.S1\_a\_at | 8.039e-06 | 0.7742 |
| Bt.21060.1.S1\_at | 8.061e-06 | 1.028 |
| Bt.2537.2.S1\_at | 8.14e-06 | 1.026 |
| Bt.1408.1.S1\_at | 8.146e-06 | 1.502 |
| Bt.20489.1.S1\_at | 8.15e-06 | 0.9939 |
| Bt.5038.5.S1\_at | 8.174e-06 | 0.8646 |
| Bt.23396.2.S1\_at | 8.219e-06 | -1.747 |
| Bt.1789.1.S1\_at | 8.251e-06 | 1.121 |
| Bt.5075.1.S1\_at | 8.291e-06 | 1.697 |
| Bt.27657.1.A1\_at | 8.347e-06 | -2.465 |
| Bt.20129.1.S1\_at | 8.365e-06 | -0.9575 |
| Bt.3149.1.S1\_at | 8.41e-06 | 1.537 |
| Bt.9055.2.S1\_a\_at | 8.438e-06 | -1.609 |
| Bt.547.1.S1\_at | 8.486e-06 | -1.781 |
| Bt.6289.1.S1\_at | 8.487e-06 | 0.8284 |
| Bt.9731.1.S1\_at | 8.501e-06 | -2.2 |
| Bt.9059.1.S1\_at | 8.513e-06 | 0.7187 |
| Bt.5965.1.S1\_at | 8.518e-06 | 1.331 |
| Bt.4503.1.S2\_at | 8.528e-06 | -1.099 |
| Bt.27892.1.S1\_at | 8.557e-06 | -0.9366 |
| Bt.13780.1.A1\_at | 8.583e-06 | 1.725 |
| Bt.5723.1.S1\_at | 8.635e-06 | -1.409 |
| Bt.10727.1.S1\_at | 8.675e-06 | -0.7836 |
| Bt.23971.1.S1\_at | 8.682e-06 | 1.185 |
| Bt.1613.1.S1\_at | 8.686e-06 | 1.792 |
| Bt.13874.1.A1\_at | 8.707e-06 | 0.6622 |
| Bt.20937.1.A1\_at | 8.766e-06 | 2.323 |
| Bt.1793.1.A1\_at | 8.817e-06 | -2.086 |
| Bt.923.1.A1\_at | 8.822e-06 | 1.118 |
| Bt.12514.1.S1\_at | 8.865e-06 | -0.8574 |
| Bt.16597.1.S1\_at | 8.889e-06 | -1.629 |
| Bt.24310.1.S1\_at | 8.899e-06 | -1.645 |
| Bt.27937.1.A1\_at | 8.923e-06 | 1.592 |
| Bt.28238.1.A1\_at | 8.955e-06 | 2.068 |
| Bt.9405.1.S1\_at | 9.002e-06 | -0.7764 |
| Bt.5288.1.S1\_at | 9.035e-06 | -1.222 |
| Bt.8961.1.S1\_at | 9.037e-06 | 1.152 |
| Bt.23331.1.S1\_at | 9.043e-06 | 1.185 |
| Bt.6549.1.S1\_at | 9.059e-06 | 1.782 |
| Bt.1692.1.S1\_at | 9.065e-06 | -0.7977 |
| Bt.11326.3.S1\_at | 9.067e-06 | -0.5507 |
| Bt.7039.1.S1\_a\_at | 9.098e-06 | -0.9977 |
| Bt.23734.1.A1\_at | 9.171e-06 | 3.079 |
| Bt.321.1.S1\_at | 9.177e-06 | -1.584 |
| Bt.22735.1.S1\_at | 9.19e-06 | 2.352 |
| Bt.27207.1.A1\_at | 9.206e-06 | 1.988 |
| Bt.16137.1.S1\_at | 9.257e-06 | 1.695 |
| Bt.20465.1.S1\_at | 9.259e-06 | 0.6786 |
| Bt.9486.1.S1\_at | 9.273e-06 | 1.516 |
| Bt.28150.2.S1\_at | 9.292e-06 | -2.302 |
| Bt.19862.1.A1\_at | 9.299e-06 | 1.419 |
| Bt.23232.1.S1\_at | 9.382e-06 | -2.387 |
| Bt.4390.1.S1\_at | 9.614e-06 | 1.19 |
| Bt.24234.1.S1\_at | 9.635e-06 | 0.5982 |
| Bt.4764.1.S1\_at | 9.656e-06 | -0.7805 |
| Bt.18843.1.A1\_at | 9.71e-06 | 1.153 |
| Bt.11016.1.S1\_at | 9.724e-06 | 1.1 |
| Bt.21166.1.S1\_at | 9.796e-06 | 1.801 |
| Bt.216.1.S1\_at | 9.817e-06 | 1.697 |
| Bt.6937.1.S1\_at | 9.827e-06 | -0.8819 |
| Bt.4431.1.S1\_a\_at | 9.852e-06 | -0.7524 |
| Bt.6069.1.S1\_at | 9.884e-06 | 0.7744 |
| Bt.11242.1.S1\_at | 9.941e-06 | -1.099 |
| Bt.1464.1.A1\_at | 1.001e-05 | 1.567 |
| Bt.1000.1.S1\_at | 1.009e-05 | 1.922 |
| Bt.13562.1.S1\_at | 1.01e-05 | 1.549 |
| Bt.615.1.S1\_at | 1.019e-05 | 1.169 |
| Bt.1568.1.S1\_at | 1.019e-05 | -1.129 |
| Bt.15643.1.A1\_at | 1.027e-05 | 1.289 |
| Bt.5376.1.S1\_at | 1.033e-05 | -0.7303 |
| Bt.13588.1.A1\_at | 1.036e-05 | -4.767 |
| Bt.10777.1.S1\_at | 1.038e-05 | 1.705 |
| Bt.2824.1.S1\_at | 1.042e-05 | -1.226 |
| Bt.22441.1.S1\_at | 1.046e-05 | 0.7294 |
| Bt.11496.1.S1\_at | 1.048e-05 | 2.098 |
| Bt.4726.2.S1\_at | 1.052e-05 | 1.342 |
| Bt.9772.1.S1\_at | 1.052e-05 | 1.501 |
| Bt.18755.1.A1\_at | 1.054e-05 | -1.262 |
| Bt.11629.1.A1\_at | 1.061e-05 | 1.031 |
| Bt.11322.1.A1\_at | 1.066e-05 | -1.159 |
| Bt.6490.2.S1\_at | 1.071e-05 | 1.285 |
| Bt.26615.1.S1\_at | 1.072e-05 | -0.744 |
| Bt.26313.1.A1\_at | 1.074e-05 | 0.7774 |
| Bt.20683.1.S1\_at | 1.074e-05 | -3.077 |
| Bt.10398.1.S1\_at | 1.077e-05 | 1.832 |
| Bt.9993.1.S1\_at | 1.084e-05 | -1.517 |
| Bt.28618.1.S1\_at | 1.085e-05 | 1.469 |
| Bt.25335.1.A1\_at | 1.085e-05 | 0.7349 |
| Bt.23941.1.A1\_at | 1.086e-05 | 1.033 |
| Bt.3821.2.S1\_at | 1.092e-05 | 1.348 |
| Bt.11717.1.S1\_s\_at | 1.098e-05 | -0.8571 |
| Bt.19526.1.S1\_at | 1.104e-05 | 1.154 |
| Bt.23648.1.A1\_at | 1.106e-05 | 1.122 |
| Bt.22071.1.S1\_at | 1.106e-05 | -1.179 |
| Bt.22142.2.S1\_a\_at | 1.11e-05 | -2.542 |
| Bt.12249.2.S1\_at | 1.116e-05 | 1.21 |
| Bt.70.1.S1\_at | 1.132e-05 | -1.305 |
| Bt.23522.1.S1\_at | 1.136e-05 | 0.7776 |
| Bt.562.1.S1\_at | 1.138e-05 | -2.08 |
| Bt.4733.1.S1\_at | 1.146e-05 | -2.584 |
| Bt.16194.1.A1\_at | 1.155e-05 | 2.4 |
| Bt.6701.1.S1\_at | 1.161e-05 | 1.16 |
| Bt.1325.1.S1\_at | 1.171e-05 | 1.493 |
| Bt.24945.1.S1\_at | 1.171e-05 | -0.8286 |
| Bt.21468.1.S1\_at | 1.172e-05 | -1.706 |
| Bt.21774.1.S1\_at | 1.176e-05 | 1.063 |
| Bt.20849.1.S1\_at | 1.179e-05 | 1.263 |
| Bt.18348.1.S1\_at | 1.184e-05 | 1.44 |
| Bt.27140.1.S1\_at | 1.187e-05 | 0.9586 |
| Bt.1987.1.S1\_at | 1.188e-05 | 0.5883 |
| Bt.23667.1.S1\_at | 1.19e-05 | 1.856 |
| Bt.21578.1.S1\_at | 1.202e-05 | -1.5 |
| Bt.20591.1.S1\_at | 1.208e-05 | 1.22 |
| Bt.6546.1.A1\_at | 1.208e-05 | 1.319 |
| Bt.6391.1.A1\_at | 1.21e-05 | -3.174 |
| Bt.9240.1.A1\_at | 1.212e-05 | -0.8576 |
| Bt.24096.1.A1\_at | 1.214e-05 | 1.346 |
| Bt.4031.2.S1\_at | 1.22e-05 | 0.5824 |
| Bt.7319.1.S1\_at | 1.221e-05 | 2.35 |
| Bt.21085.1.S1\_at | 1.224e-05 | 1.411 |
| Bt.20308.1.S1\_at | 1.227e-05 | 2.35 |
| Bt.3158.1.S1\_at | 1.228e-05 | 0.7939 |
| Bt.20129.2.S1\_at | 1.237e-05 | -1.32 |
| Bt.27412.1.A1\_at | 1.238e-05 | 1.683 |
| Bt.17380.1.S1\_at | 1.241e-05 | -1.389 |
| Bt.23081.1.S1\_a\_at | 1.25e-05 | -2.275 |
| Bt.8163.1.S1\_at | 1.253e-05 | 0.9133 |
| Bt.77.1.S1\_at | 1.269e-05 | -1.026 |
| Bt.16106.1.A1\_at | 1.297e-05 | 1.447 |
| Bt.401.2.S1\_at | 1.298e-05 | -1.403 |
| Bt.11791.1.A1\_at | 1.298e-05 | -1.919 |
| Bt.5466.4.S1\_at | 1.302e-05 | 1.074 |
| Bt.923.2.S1\_at | 1.309e-05 | 1.267 |
| Bt.4880.1.S1\_at | 1.309e-05 | -1.163 |
| Bt.7619.1.S1\_at | 1.312e-05 | -3.155 |
| Bt.21182.1.S1\_at | 1.315e-05 | -0.8462 |
| Bt.10683.1.S1\_at | 1.316e-05 | 1.387 |
| Bt.7540.2.S1\_a\_at | 1.316e-05 | -1.133 |
| Bt.10999.1.A1\_at | 1.336e-05 | 1.312 |
| Bt.4903.1.S2\_at | 1.338e-05 | 1.137 |
| Bt.4702.1.S1\_at | 1.338e-05 | 1.122 |
| Bt.28105.1.S1\_at | 1.343e-05 | 0.9623 |
| Bt.9041.1.S1\_at | 1.344e-05 | 0.9606 |
| Bt.4555.1.S1\_at | 1.348e-05 | -1.376 |
| Bt.19807.1.S1\_at | 1.354e-05 | 0.5117 |
| Bt.26445.1.A1\_at | 1.356e-05 | 1.233 |
| Bt.3163.1.A1\_at | 1.356e-05 | 0.7167 |
| Bt.20601.2.S1\_at | 1.356e-05 | 1.289 |
| Bt.8177.1.S1\_at | 1.366e-05 | -1.574 |
| Bt.24604.2.A1\_at | 1.37e-05 | 0.9386 |
| Bt.24624.1.S1\_at | 1.374e-05 | -1.619 |
| Bt.26788.1.S1\_at | 1.375e-05 | 2.465 |
| Bt.25051.1.A1\_at | 1.38e-05 | 1.781 |
| Bt.9889.1.S1\_at | 1.381e-05 | 0.9766 |
| Bt.27141.1.A1\_at | 1.383e-05 | 1.089 |
| Bt.14304.2.S1\_at | 1.384e-05 | 0.8841 |
| Bt.4262.1.S1\_a\_at | 1.385e-05 | -1.871 |
| Bt.19415.1.A1\_at | 1.389e-05 | -2.512 |
| Bt.543.1.S1\_at | 1.392e-05 | 1.771 |
| Bt.28150.1.S1\_at | 1.405e-05 | -2.299 |
| Bt.20106.1.S1\_at | 1.408e-05 | 1.138 |
| Bt.136.1.S1\_at | 1.414e-05 | -3.513 |
| Bt.5848.2.S1\_at | 1.419e-05 | -0.898 |
| Bt.3395.1.S1\_at | 1.42e-05 | -0.8196 |
| Bt.1167.1.S1\_at | 1.424e-05 | 0.5717 |
| Bt.5626.2.S1\_at | 1.424e-05 | -2.105 |
| Bt.15357.1.A1\_at | 1.426e-05 | -1.622 |
| Bt.18023.1.S1\_at | 1.433e-05 | 1.227 |
| Bt.10215.1.A1\_at | 1.434e-05 | -0.8052 |
| Bt.20470.1.S1\_a\_at | 1.437e-05 | -3.933 |
| Bt.8673.1.S1\_a\_at | 1.451e-05 | 1.791 |
| Bt.23216.1.S1\_at | 1.453e-05 | 1.54 |
| Bt.22197.1.S1\_at | 1.472e-05 | -1.498 |
| Bt.22763.2.S1\_a\_at | 1.474e-05 | -1.388 |
| Bt.5505.1.S1\_at | 1.477e-05 | 1.212 |
| Bt.4355.1.S1\_at | 1.483e-05 | -1.18 |
| Bt.20955.2.S1\_at | 1.484e-05 | -0.7988 |
| Bt.27118.1.A1\_at | 1.487e-05 | 1.285 |
| Bt.7636.1.S1\_at | 1.495e-05 | 1.523 |
| Bt.13972.1.A1\_at | 1.5e-05 | 1.807 |
| Bt.4320.1.S1\_at | 1.509e-05 | 1.588 |
| Bt.19862.2.S1\_at | 1.51e-05 | 2.608 |
| Bt.27927.1.S1\_at | 1.525e-05 | 1.034 |
| Bt.21528.1.A1\_at | 1.53e-05 | 3.648 |
| Bt.17608.1.A1\_at | 1.532e-05 | 0.5534 |
| Bt.2989.2.S1\_at | 1.535e-05 | -1.401 |
| Bt.20020.1.S1\_at | 1.536e-05 | 1.322 |
| Bt.22405.1.A1\_at | 1.55e-05 | 2.078 |
| Bt.4533.1.S1\_at | 1.555e-05 | -0.8468 |
| Bt.23355.1.S1\_at | 1.559e-05 | -1.263 |
| Bt.20652.2.S1\_at | 1.561e-05 | -1.254 |
| Bt.9756.1.S1\_at | 1.568e-05 | -1.274 |
| Bt.15938.1.S1\_at | 1.572e-05 | 1.316 |
| Bt.11326.1.S1\_at | 1.573e-05 | -0.7062 |
| Bt.1386.1.S1\_at | 1.579e-05 | 0.7047 |
| Bt.1065.1.S1\_at | 1.59e-05 | -1.391 |
| Bt.25202.1.A1\_at | 1.601e-05 | 1.863 |
| Bt.22516.1.A1\_at | 1.611e-05 | -0.9461 |
| Bt.9949.3.S1\_a\_at | 1.636e-05 | -1.537 |
| Bt.1767.1.S1\_at | 1.64e-05 | -0.9164 |
| Bt.18748.1.A1\_at | 1.649e-05 | 1.21 |
| Bt.12885.1.S1\_at | 1.692e-05 | -1.089 |
| Bt.20402.2.A1\_at | 1.694e-05 | 1.187 |
| Bt.29506.1.S1\_at | 1.701e-05 | 2.06 |
| Bt.6585.1.S1\_at | 1.705e-05 | 0.8544 |
| Bt.11038.1.S1\_at | 1.707e-05 | 1.454 |
| Bt.20243.2.S1\_at | 1.707e-05 | -1.044 |
| Bt.18634.1.A1\_at | 1.708e-05 | 1.97 |
| Bt.3599.1.S1\_at | 1.715e-05 | 0.6432 |
| Bt.26628.1.S1\_at | 1.725e-05 | 1.261 |
| Bt.3996.1.S1\_at | 1.73e-05 | 2.269 |
| Bt.3538.1.S1\_at | 1.737e-05 | 0.6733 |
| Bt.7915.1.S1\_at | 1.74e-05 | -0.8884 |
| Bt.9948.1.S1\_at | 1.753e-05 | 1.319 |
| Bt.27386.1.A1\_at | 1.753e-05 | 1.638 |
| Bt.149.1.S1\_at | 1.758e-05 | 1.908 |
| Bt.19519.1.S1\_at | 1.763e-05 | 0.7782 |
| Bt.6316.1.S1\_at | 1.766e-05 | -0.6523 |
| Bt.16935.1.A1\_at | 1.769e-05 | 0.8703 |
| Bt.22630.2.A1\_at | 1.78e-05 | -1.569 |
| Bt.8047.1.S1\_at | 1.795e-05 | -0.8966 |
| Bt.27106.1.S1\_at | 1.796e-05 | 1.45 |
| Bt.23505.1.S1\_at | 1.806e-05 | -3.433 |
| Bt.3604.1.A1\_at | 1.808e-05 | 1.004 |
| Bt.6465.1.S1\_at | 1.81e-05 | -3.888 |
| Bt.20038.1.S1\_at | 1.816e-05 | -2.484 |
| Bt.10579.1.S1\_at | 1.819e-05 | 1.406 |
| Bt.6020.1.S1\_at | 1.822e-05 | -1.18 |
| Bt.5439.1.S1\_at | 1.822e-05 | 0.9457 |
| Bt.4437.1.S1\_at | 1.828e-05 | 1.242 |
| Bt.1719.1.S1\_at | 1.834e-05 | -1.317 |
| Bt.16945.1.S1\_at | 1.836e-05 | -0.999 |
| Bt.7814.1.S1\_at | 1.838e-05 | 0.5877 |
| Bt.22451.1.S1\_at | 1.842e-05 | -1.133 |
| Bt.24543.1.A1\_at | 1.854e-05 | 1.501 |
| Bt.10329.1.A1\_at | 1.858e-05 | 1.334 |
| Bt.18019.1.A1\_at | 1.863e-05 | 1.326 |
| Bt.19934.1.S1\_at | 1.865e-05 | -1.33 |
| Bt.21745.1.S1\_at | 1.887e-05 | -1.181 |
| Bt.18278.2.A1\_at | 1.901e-05 | -2.23 |
| Bt.11325.1.S1\_at | 1.912e-05 | 0.9636 |
| Bt.20426.1.S1\_at | 1.915e-05 | 0.8186 |
| Bt.22002.1.S1\_at | 1.918e-05 | -3.298 |
| Bt.2394.1.S1\_at | 1.919e-05 | -0.8592 |
| Bt.6449.1.S1\_at | 1.921e-05 | 1.47 |
| Bt.15561.1.S1\_at | 1.922e-05 | 1.165 |
| Bt.22150.1.A1\_at | 1.93e-05 | 0.8306 |
| Bt.21217.1.S1\_at | 1.943e-05 | -0.7063 |
| Bt.23127.1.S1\_at | 1.961e-05 | 1.628 |
| Bt.17008.1.S1\_at | 1.961e-05 | 0.6891 |
| Bt.15267.1.A1\_at | 1.979e-05 | 1.381 |
| Bt.9585.1.S1\_at | 1.981e-05 | -1.662 |
| Bt.24433.2.A1\_at | 1.984e-05 | -0.875 |
| Bt.22461.1.S1\_at | 1.989e-05 | 1.062 |
| Bt.25085.1.A1\_at | 2.013e-05 | 2.182 |
| Bt.6063.1.S1\_at | 2.023e-05 | -1.523 |
| Bt.4492.1.S1\_at | 2.028e-05 | 1.581 |
| Bt.19097.2.S1\_at | 2.058e-05 | 1.512 |
| Bt.3789.1.S1\_at | 2.059e-05 | 1.047 |
| Bt.9544.1.S1\_at | 2.059e-05 | 0.8537 |
| Bt.1131.1.S1\_at | 2.062e-05 | 0.7816 |
| Bt.10942.1.S1\_at | 2.064e-05 | 1.001 |
| Bt.2453.1.S1\_at | 2.072e-05 | 0.6753 |
| Bt.24434.1.A1\_at | 2.073e-05 | 1.841 |
| Bt.1742.1.S1\_at | 2.085e-05 | 1.484 |
| Bt.5888.1.S1\_at | 2.087e-05 | -1.649 |
| Bt.2347.1.S1\_at | 2.088e-05 | -1.061 |
| Bt.13986.1.S1\_at | 2.098e-05 | 0.7163 |
| Bt.24307.1.A1\_at | 2.1e-05 | 1.3 |
| Bt.9878.1.A1\_at | 2.104e-05 | 1.416 |
| Bt.25346.1.A1\_at | 2.11e-05 | 0.7233 |
| Bt.2439.1.S1\_at | 2.123e-05 | 1.074 |
| Bt.25185.1.S1\_at | 2.126e-05 | 1.74 |
| Bt.21009.1.S1\_at | 2.126e-05 | 2.058 |
| Bt.4571.1.S1\_at | 2.127e-05 | 1.749 |
| Bt.2815.1.S1\_at | 2.138e-05 | 1.088 |
| Bt.25342.1.A1\_at | 2.144e-05 | 1.737 |
| Bt.10218.1.S1\_s\_at | 2.148e-05 | 1.131 |
| Bt.24474.1.S1\_at | 2.151e-05 | -1.129 |
| Bt.17237.1.A1\_at | 2.165e-05 | -0.3724 |
| Bt.4552.1.S1\_a\_at | 2.172e-05 | 0.8336 |
| Bt.9674.1.S1\_at | 2.181e-05 | 1.426 |
| Bt.13289.1.S1\_at | 2.191e-05 | 2.595 |
| Bt.13912.1.S1\_at | 2.207e-05 | 1.123 |
| Bt.26920.2.A1\_a\_at | 2.208e-05 | -1.301 |
| Bt.12096.1.S1\_at | 2.238e-05 | -1.238 |
| Bt.16004.1.A1\_at | 2.243e-05 | 4.722 |
| Bt.25522.1.S1\_at | 2.277e-05 | 1.277 |
| Bt.17526.1.A1\_at | 2.279e-05 | 1.11 |
| Bt.13529.1.S1\_at | 2.28e-05 | 0.9283 |
| Bt.23174.2.S1\_at | 2.281e-05 | 1.103 |
| Bt.4834.1.S1\_at | 2.292e-05 | 1.255 |
| Bt.4224.1.S1\_at | 2.293e-05 | -0.9479 |
| Bt.26130.1.A1\_at | 2.298e-05 | -1.767 |
| Bt.21151.1.S1\_at | 2.3e-05 | -0.8742 |
| Bt.23647.1.A1\_a\_at | 2.308e-05 | 1.212 |
| Bt.9176.2.S1\_at | 2.313e-05 | 0.875 |
| Bt.10990.1.S1\_at | 2.314e-05 | -1.187 |
| Bt.27431.1.A1\_at | 2.314e-05 | 1.136 |
| Bt.7425.1.S1\_at | 2.316e-05 | 1.872 |
| Bt.28280.1.A1\_at | 2.316e-05 | -0.8437 |
| Bt.9958.1.S1\_at | 2.322e-05 | 1.383 |
| Bt.9803.1.S1\_at | 2.329e-05 | -1.314 |
| Bt.2818.2.S1\_a\_at | 2.335e-05 | 1.425 |
| Bt.22633.1.S1\_at | 2.343e-05 | 1.151 |
| Bt.11240.1.A1\_at | 2.355e-05 | 1.565 |
| Bt.2656.1.S1\_at | 2.356e-05 | 2.197 |
| Bt.21469.1.S1\_at | 2.361e-05 | 1.11 |
| Bt.4653.1.S2\_at | 2.363e-05 | 1.33 |
| Bt.2606.1.S1\_at | 2.377e-05 | 1.645 |
| Bt.9223.1.A1\_at | 2.389e-05 | 1.294 |
| Bt.997.2.S1\_a\_at | 2.394e-05 | -1.864 |
| Bt.29050.1.S1\_at | 2.401e-05 | -2.315 |
| Bt.23002.1.S1\_at | 2.416e-05 | -1.634 |
| Bt.27018.1.S1\_at | 2.417e-05 | -1.187 |
| Bt.11154.2.S1\_at | 2.418e-05 | -1.285 |
| Bt.18270.1.A1\_at | 2.422e-05 | 0.9946 |
| Bt.5006.1.S1\_at | 2.425e-05 | 0.6894 |
| Bt.3452.1.S1\_at | 2.432e-05 | 1.134 |
| Bt.3605.1.S1\_at | 2.452e-05 | -2.356 |
| Bt.13205.2.S1\_at | 2.454e-05 | -1.32 |
| Bt.27930.1.S1\_at | 2.47e-05 | 1.138 |
| Bt.12348.1.A1\_at | 2.473e-05 | 1.444 |
| Bt.22567.1.S1\_at | 2.486e-05 | -0.7385 |
| Bt.4054.1.S1\_at | 2.507e-05 | 1.109 |
| Bt.2513.1.S1\_at | 2.517e-05 | 0.8214 |
| Bt.29009.1.A1\_at | 2.527e-05 | 3.169 |
| Bt.5393.2.S1\_a\_at | 2.539e-05 | 1.291 |
| Bt.23499.1.S1\_at | 2.544e-05 | -1.736 |
| Bt.22663.1.A1\_at | 2.557e-05 | -0.9034 |
| Bt.12745.1.A1\_at | 2.559e-05 | 1.45 |
| Bt.9413.1.S1\_at | 2.569e-05 | 0.8282 |
| Bt.17188.1.A1\_at | 2.576e-05 | 1.685 |
| Bt.16078.1.A1\_s\_at | 2.577e-05 | 1.096 |
| Bt.4957.1.S2\_at | 2.584e-05 | 1.128 |
| Bt.28162.1.S1\_at | 2.598e-05 | -1.479 |
| Bt.15742.1.S2\_at | 2.598e-05 | 1.078 |
| Bt.3418.1.S1\_at | 2.601e-05 | 0.5375 |
| Bt.8798.1.S1\_at | 2.648e-05 | 1.702 |
| Bt.23335.1.S1\_at | 2.65e-05 | 1.455 |
| Bt.27147.1.S1\_at | 2.658e-05 | 1.591 |
| Bt.9078.2.S1\_a\_at | 2.66e-05 | -1.647 |
| Bt.7991.1.S1\_a\_at | 2.675e-05 | 0.7969 |
| Bt.8024.1.A1\_at | 2.693e-05 | -1.026 |
| Bt.26975.1.A1\_a\_at | 2.699e-05 | 2.022 |
| Bt.7184.1.S1\_at | 2.732e-05 | -0.792 |
| Bt.21435.1.S1\_at | 2.751e-05 | -0.8315 |
| Bt.15715.1.S1\_at | 2.752e-05 | 1.45 |
| Bt.19637.1.A1\_at | 2.763e-05 | 1.329 |
| Bt.2493.1.S1\_at | 2.766e-05 | -1.569 |
| Bt.21718.1.S1\_at | 2.822e-05 | -1.744 |
| Bt.4831.1.S1\_at | 2.831e-05 | -0.7823 |
| Bt.12261.1.A1\_at | 2.842e-05 | 0.6437 |
| Bt.1322.1.S1\_at | 2.845e-05 | 1.036 |
| Bt.24873.1.S1\_at | 2.855e-05 | 1.001 |
| Bt.17764.1.A1\_at | 2.857e-05 | 0.7011 |
| Bt.26871.1.S1\_at | 2.868e-05 | 0.9799 |
| Bt.1670.1.A1\_at | 2.871e-05 | -1.062 |
| Bt.27396.1.S1\_at | 2.897e-05 | 1.002 |
| Bt.6501.1.S1\_at | 2.904e-05 | 0.7669 |
| Bt.10962.1.S1\_at | 2.914e-05 | 0.5754 |
| Bt.3216.1.A1\_at | 2.918e-05 | 1.365 |
| Bt.20194.1.S1\_at | 2.919e-05 | -0.8297 |
| Bt.3820.2.A1\_at | 2.924e-05 | 0.8248 |
| Bt.19706.1.S1\_at | 2.926e-05 | 1.501 |
| Bt.24563.1.S1\_at | 2.935e-05 | 1.495 |
| Bt.7342.1.S1\_at | 2.946e-05 | 1.223 |
| Bt.1217.2.S1\_at | 2.962e-05 | -1.156 |
| Bt.7596.1.S1\_at | 2.964e-05 | -0.9958 |
| Bt.1523.1.S1\_at | 2.965e-05 | -1.342 |
| Bt.23081.1.S1\_at | 2.992e-05 | -1.961 |
| Bt.4339.1.S1\_at | 3.011e-05 | -1.14 |
| Bt.27199.1.S1\_at | 3.017e-05 | -1.092 |
| Bt.11169.1.S1\_at | 3.017e-05 | 2.163 |
| Bt.1699.3.S1\_a\_at | 3.024e-05 | 1.445 |
| Bt.19957.1.A1\_at | 3.052e-05 | 1.635 |
| Bt.3174.1.A1\_at | 3.064e-05 | 0.8555 |
| Bt.13966.1.S1\_at | 3.088e-05 | -0.8116 |
| Bt.26550.1.S1\_at | 3.105e-05 | -1.768 |
| Bt.5388.1.S1\_s\_at | 3.106e-05 | 0.7687 |
| Bt.25657.1.S1\_at | 3.113e-05 | 1.937 |
| Bt.27846.1.S1\_at | 3.122e-05 | 1.643 |
| Bt.5510.1.S1\_at | 3.132e-05 | -0.5654 |
| Bt.15796.1.S1\_at | 3.133e-05 | 1.047 |
| Bt.12382.1.S1\_at | 3.142e-05 | 1.312 |
| Bt.5542.2.S1\_at | 3.15e-05 | 0.9895 |
| Bt.25087.1.A1\_at | 3.15e-05 | -1.079 |
| Bt.24253.1.S1\_at | 3.15e-05 | 1.032 |
| Bt.26993.1.S1\_at | 3.156e-05 | -1.808 |
| Bt.24767.1.S1\_at | 3.158e-05 | 0.7052 |
| Bt.16020.1.A2\_at | 3.161e-05 | 1.223 |
| Bt.841.2.A1\_at | 3.171e-05 | 1.566 |
| Bt.15754.1.S1\_at | 3.208e-05 | 0.5565 |
| Bt.5322.1.S1\_at | 3.227e-05 | -1.164 |
| Bt.868.1.S1\_at | 3.249e-05 | -0.8542 |
| Bt.28559.1.S1\_a\_at | 3.251e-05 | -1.365 |
| Bt.21456.1.S1\_at | 3.253e-05 | 1.36 |
| Bt.19054.1.A1\_at | 3.253e-05 | -0.3113 |
| Bt.5542.1.A1\_at | 3.259e-05 | 1.06 |
| Bt.14201.1.S1\_at | 3.265e-05 | -0.5146 |
| Bt.7631.1.S1\_at | 3.288e-05 | -1.135 |
| Bt.24023.1.A1\_at | 3.289e-05 | 1.328 |
| Bt.21589.1.S1\_at | 3.299e-05 | 1.185 |
| Bt.3323.1.S1\_at | 3.328e-05 | 0.9811 |
| Bt.4099.1.S1\_at | 3.332e-05 | -0.7681 |
| Bt.20544.1.S1\_at | 3.338e-05 | 0.779 |
| Bt.6961.1.S1\_at | 3.34e-05 | 2.469 |
| Bt.4768.1.S2\_at | 3.352e-05 | 1.142 |
| Bt.25016.1.S1\_at | 3.358e-05 | 1.581 |
| Bt.380.1.S1\_at | 3.365e-05 | -1.131 |
| Bt.29540.1.S1\_at | 3.37e-05 | -0.7114 |
| Bt.22333.1.S1\_at | 3.374e-05 | 1.012 |
| Bt.26756.1.S1\_at | 3.401e-05 | 0.567 |
| Bt.16314.1.S1\_at | 3.403e-05 | 1.126 |
| Bt.12728.3.S1\_at | 3.415e-05 | 1.228 |
| Bt.11023.1.S1\_at | 3.455e-05 | 1.268 |
| Bt.7836.1.S1\_at | 3.485e-05 | -1.034 |
| Bt.14572.1.A1\_at | 3.487e-05 | 1.788 |
| Bt.61.1.S1\_at | 3.489e-05 | -0.7586 |
| Bt.2214.1.S1\_at | 3.495e-05 | 1.262 |
| Bt.26987.2.S1\_at | 3.495e-05 | -1.263 |
| Bt.22543.1.S1\_at | 3.504e-05 | -1.023 |
| Bt.10257.1.A1\_at | 3.509e-05 | 1.497 |
| Bt.26622.1.S1\_a\_at | 3.516e-05 | -1.237 |
| Bt.1760.1.S1\_at | 3.525e-05 | -1.181 |
| Bt.5088.1.S1\_at | 3.557e-05 | 1.306 |
| Bt.19469.1.A1\_at | 3.572e-05 | 0.5996 |
| Bt.1262.1.S1\_at | 3.574e-05 | -1.179 |
| Bt.13998.1.S1\_at | 3.578e-05 | -0.9385 |
| Bt.4886.1.A1\_at | 3.592e-05 | 2.886 |
| Bt.3484.1.S1\_at | 3.597e-05 | 0.9464 |
| Bt.6152.1.S1\_at | 3.62e-05 | 0.717 |
| Bt.11339.1.S1\_at | 3.636e-05 | -0.9513 |
| Bt.5939.1.S1\_a\_at | 3.645e-05 | -0.7838 |
| Bt.21342.1.S1\_at | 3.663e-05 | -1.201 |
| Bt.22349.1.A1\_at | 3.667e-05 | 1.222 |
| Bt.27371.1.S1\_at | 3.672e-05 | 1.475 |
| Bt.10969.1.S1\_at | 3.674e-05 | 1.459 |
| Bt.1237.1.S1\_at | 3.685e-05 | 1.016 |
| Bt.9207.1.S1\_at | 3.71e-05 | 0.8298 |
| Bt.14566.1.A1\_at | 3.71e-05 | 1.64 |
| Bt.6017.1.A1\_at | 3.73e-05 | -0.6852 |
| Bt.24643.1.S1\_at | 3.744e-05 | 1.012 |
| Bt.25138.1.A1\_at | 3.79e-05 | -0.4665 |
| Bt.6665.1.S1\_at | 3.807e-05 | 1.486 |
| Bt.9785.1.S1\_at | 3.816e-05 | 1.403 |
| Bt.23106.1.S1\_at | 3.838e-05 | -1.205 |
| Bt.23225.1.S1\_at | 3.85e-05 | -1.178 |
| Bt.26750.2.A1\_at | 3.858e-05 | -2.766 |
| Bt.14473.1.S1\_at | 3.865e-05 | -1.071 |
| Bt.20064.2.S1\_at | 3.867e-05 | 1.329 |
| Bt.5378.1.S1\_at | 3.899e-05 | -0.9214 |
| Bt.20405.1.S1\_at | 3.922e-05 | 1.955 |
| Bt.19281.2.S1\_at | 3.949e-05 | 0.982 |
| Bt.10421.1.S1\_at | 3.953e-05 | 1.312 |
| Bt.5388.1.S1\_at | 3.955e-05 | 0.8523 |
| Bt.3236.1.A1\_at | 3.989e-05 | 1.37 |
| Bt.11246.1.A1\_at | 3.991e-05 | 1.118 |
| Bt.25226.1.A1\_at | 4.003e-05 | 1.876 |
| Bt.20817.1.A1\_at | 4.062e-05 | 1.059 |
| Bt.23396.2.S1\_a\_at | 4.068e-05 | -2.561 |
| Bt.22692.1.A1\_at | 4.077e-05 | -0.8018 |
| Bt.22536.2.S1\_at | 4.078e-05 | -0.839 |
| Bt.19676.2.S1\_a\_at | 4.097e-05 | -1.537 |
| Bt.26960.1.S1\_at | 4.105e-05 | -0.6221 |
| Bt.7430.1.S1\_at | 4.112e-05 | 0.7569 |
| Bt.21440.1.S1\_at | 4.144e-05 | 1.213 |
| Bt.24210.1.S1\_at | 4.149e-05 | -1.133 |
| Bt.18738.1.A1\_at | 4.163e-05 | -0.3334 |
| Bt.13588.2.S1\_at | 4.176e-05 | -4.761 |
| Bt.5529.2.S1\_a\_at | 4.188e-05 | -0.8336 |
| Bt.1484.1.S1\_at | 4.209e-05 | 0.8675 |
| Bt.18122.1.A1\_at | 4.218e-05 | -0.3719 |
| Bt.2914.1.S1\_at | 4.219e-05 | 0.7988 |
| Bt.6693.1.A1\_at | 4.225e-05 | -1.412 |
| Bt.11126.3.S1\_a\_at | 4.227e-05 | 1.322 |
| Bt.3211.1.S2\_at | 4.241e-05 | 0.9614 |
| Bt.13860.1.S1\_at | 4.245e-05 | 1.212 |
| Bt.21827.2.S1\_at | 4.247e-05 | -1.361 |
| Bt.12963.1.A1\_at | 4.257e-05 | 0.606 |
| Bt.20414.1.S1\_at | 4.258e-05 | 0.9537 |
| Bt.3101.1.A1\_at | 4.272e-05 | -1.866 |
| Bt.9252.1.A1\_at | 4.283e-05 | 1.67 |
| Bt.24916.1.A1\_at | 4.288e-05 | 1.119 |
| Bt.21938.1.S1\_at | 4.297e-05 | 1.563 |
| Bt.22525.1.A1\_at | 4.308e-05 | 1.023 |
| Bt.14853.1.A1\_at | 4.325e-05 | 1.355 |
| Bt.25689.1.S1\_at | 4.356e-05 | -0.936 |
| Bt.7240.1.S1\_at | 4.362e-05 | -1.008 |
| Bt.10354.1.S1\_at | 4.366e-05 | 1.099 |
| Bt.68.1.S1\_at | 4.368e-05 | -1.409 |
| Bt.656.1.S1\_at | 4.39e-05 | 0.6408 |
| Bt.12634.1.A1\_at | 4.42e-05 | 2.094 |
| Bt.10251.1.S1\_at | 4.444e-05 | 1.274 |
| Bt.8543.1.S1\_at | 4.45e-05 | 1.275 |
| Bt.4056.1.S1\_at | 4.453e-05 | 0.8514 |
| Bt.21222.1.S1\_at | 4.514e-05 | 0.8064 |
| Bt.13229.1.S1\_at | 4.516e-05 | 1.42 |
| Bt.4478.1.S1\_at | 4.519e-05 | -1.087 |
| Bt.5515.1.S1\_at | 4.54e-05 | 0.4304 |
| Bt.26674.1.S1\_at | 4.602e-05 | 1.094 |
| Bt.20619.1.S1\_at | 4.605e-05 | -1.042 |
| Bt.21835.1.S1\_at | 4.624e-05 | 1.302 |
| Bt.18702.1.A1\_at | 4.629e-05 | -1.247 |
| Bt.7619.2.S1\_at | 4.698e-05 | -1.595 |
| Bt.4580.1.A1\_at | 4.699e-05 | 0.8609 |
| Bt.10657.1.A1\_at | 4.704e-05 | -2.355 |
| Bt.27781.1.S1\_at | 4.708e-05 | 1.654 |
| Bt.26812.1.S1\_at | 4.717e-05 | -0.9168 |
| Bt.6733.1.S1\_at | 4.765e-05 | 1.02 |
| Bt.24376.1.S1\_at | 4.772e-05 | 1.806 |
| Bt.24727.1.S1\_a\_at | 4.81e-05 | -1.141 |
| Bt.3139.1.A1\_at | 4.811e-05 | 2.155 |
| Bt.7772.1.S1\_at | 4.815e-05 | 1.745 |
| Bt.27023.1.S1\_at | 4.848e-05 | 1.226 |
| Bt.4867.1.A1\_at | 4.859e-05 | 3.569 |
| Bt.24819.1.S1\_at | 4.872e-05 | -1.259 |
| Bt.20988.1.S1\_at | 4.875e-05 | -1.263 |
| Bt.20236.1.S1\_at | 4.876e-05 | 1.309 |
| Bt.1255.1.A1\_at | 4.883e-05 | -0.6626 |
| Bt.1207.1.S1\_at | 4.885e-05 | 1.491 |
| Bt.29697.1.S1\_at | 4.895e-05 | -1.508 |
| Bt.7373.3.S1\_a\_at | 4.898e-05 | -1.521 |
| Bt.5423.1.S1\_at | 4.916e-05 | -0.566 |
| Bt.26524.1.S1\_at | 4.924e-05 | -0.7729 |
| Bt.26878.1.S1\_at | 4.941e-05 | -1.834 |
| Bt.27475.1.A1\_at | 4.95e-05 | -0.6409 |
| Bt.5135.1.S1\_at | 4.959e-05 | -1.392 |
| Bt.24269.1.S1\_at | 5.006e-05 | -1.658 |
| Bt.16985.1.S1\_at | 5.016e-05 | 1.163 |
| Bt.23373.1.S1\_at | 5.03e-05 | -1.738 |
| Bt.12333.1.A1\_at | 5.043e-05 | 0.8849 |
| Bt.11194.1.S1\_at | 5.055e-05 | -2.054 |
| Bt.21064.1.S1\_at | 5.058e-05 | -0.8416 |
| Bt.20820.1.A1\_at | 5.103e-05 | 1.958 |
| Bt.27929.1.S1\_at | 5.198e-05 | -0.659 |
| Bt.2077.1.S1\_at | 5.236e-05 | 1.232 |
| Bt.22166.1.S1\_at | 5.242e-05 | -1.835 |
| Bt.5345.1.S1\_at | 5.245e-05 | -0.966 |
| Bt.13419.1.S1\_at | 5.252e-05 | 0.8788 |
| Bt.18459.1.A1\_at | 5.262e-05 | 0.7327 |
| Bt.17968.1.S1\_at | 5.28e-05 | -1.755 |
| Bt.3210.1.S1\_at | 5.299e-05 | 0.9076 |
| Bt.4631.2.S1\_at | 5.305e-05 | 1.311 |
| Bt.8164.1.S1\_at | 5.348e-05 | -0.6648 |
| Bt.1311.1.S1\_at | 5.367e-05 | -1.909 |
| Bt.21383.1.S1\_at | 5.367e-05 | 1.662 |
| Bt.22054.2.S1\_a\_at | 5.374e-05 | -2.825 |
| Bt.10458.2.S1\_at | 5.411e-05 | 1.942 |
| Bt.13183.1.A1\_at | 5.418e-05 | 1.122 |
| Bt.13913.1.S1\_at | 5.433e-05 | -1.144 |
| Bt.13345.2.S1\_at | 5.443e-05 | 0.7131 |
| Bt.24017.1.A1\_s\_at | 5.462e-05 | -0.6011 |
| Bt.10813.1.S1\_at | 5.475e-05 | 1.808 |
| Bt.6830.1.S1\_at | 5.495e-05 | 0.4264 |
| Bt.7873.1.S1\_at | 5.523e-05 | 0.9982 |
| Bt.24137.1.A1\_at | 5.559e-05 | 1.546 |
| Bt.16499.1.A1\_at | 5.591e-05 | 1.581 |
| Bt.11502.1.A1\_at | 5.609e-05 | 1.081 |
| Bt.16753.1.A1\_at | 5.615e-05 | 1.041 |
| Bt.4370.2.S1\_at | 5.624e-05 | 0.7738 |
| Bt.5495.1.S1\_at | 5.64e-05 | -1.622 |
| Bt.27095.1.S1\_at | 5.649e-05 | 0.815 |
| Bt.6648.1.A1\_at | 5.675e-05 | -0.5186 |
| Bt.28676.1.A1\_at | 5.696e-05 | -1.063 |
| Bt.5560.1.S1\_at | 5.707e-05 | 1.233 |
| Bt.20247.1.S1\_at | 5.727e-05 | 1.521 |
| Bt.16861.1.A1\_at | 5.734e-05 | 1.589 |
| Bt.19692.1.S1\_at | 5.748e-05 | -0.8625 |
| Bt.16125.2.S1\_at | 5.778e-05 | -1.377 |
| Bt.27430.1.S1\_at | 5.79e-05 | -0.9405 |
| Bt.665.1.S1\_s\_at | 5.794e-05 | -1.194 |
| Bt.16264.1.A1\_at | 5.812e-05 | 0.9395 |
| Bt.5021.1.S1\_at | 5.825e-05 | 1.394 |
| Bt.26668.1.S1\_at | 5.853e-05 | 0.8477 |
| Bt.21818.1.S1\_at | 5.862e-05 | 1.454 |
| Bt.10999.2.S1\_at | 5.866e-05 | 1.347 |
| Bt.11662.1.A1\_at | 5.866e-05 | 1.299 |
| Bt.1426.1.S1\_at | 5.87e-05 | -0.9034 |
| Bt.5468.1.S1\_at | 5.877e-05 | -2.58 |
| Bt.9301.1.A1\_at | 5.886e-05 | 1.368 |
| Bt.2642.1.S1\_at | 5.9e-05 | -0.6549 |
| Bt.13249.1.A1\_at | 5.903e-05 | 1.308 |
| Bt.23224.1.S1\_at | 5.92e-05 | 0.9054 |
| Bt.13289.2.S1\_at | 5.961e-05 | 2.386 |
| Bt.6111.1.S1\_at | 5.969e-05 | -0.8931 |
| Bt.7955.2.S1\_at | 5.981e-05 | 0.9399 |
| Bt.21277.1.A1\_at | 6.011e-05 | 0.8589 |
| Bt.25202.3.S1\_at | 6.016e-05 | 1.916 |
| Bt.16412.1.S1\_a\_at | 6.021e-05 | 1.148 |
| Bt.886.1.S1\_at | 6.035e-05 | -1.086 |
| Bt.6359.1.S1\_at | 6.069e-05 | -0.4859 |
| Bt.20139.1.S1\_at | 6.073e-05 | 0.926 |
| Bt.5047.1.S1\_at | 6.088e-05 | 1.497 |
| Bt.4540.1.S1\_at | 6.104e-05 | -0.7798 |
| Bt.13003.12.S1\_at | 6.148e-05 | -0.4366 |
| Bt.23109.1.S1\_at | 6.194e-05 | -1.018 |
| Bt.6567.1.S1\_at | 6.198e-05 | 1.25 |
| Bt.405.1.S1\_at | 6.208e-05 | 1.828 |
| Bt.25268.1.A1\_at | 6.211e-05 | 1.224 |
| Bt.9306.3.S1\_at | 6.214e-05 | -1.438 |
| Bt.17871.1.S1\_a\_at | 6.237e-05 | 0.8392 |
| Bt.27952.1.S1\_at | 6.241e-05 | -1.359 |
| Bt.12820.1.S1\_at | 6.254e-05 | 0.697 |
| Bt.11527.1.A1\_at | 6.257e-05 | 0.5135 |
| Bt.12916.1.S1\_at | 6.261e-05 | -2.774 |
| Bt.727.2.S1\_at | 6.285e-05 | -0.8269 |
| Bt.5170.1.S1\_at | 6.286e-05 | -1.311 |
| Bt.12267.1.S1\_at | 6.314e-05 | 0.9906 |
| Bt.28567.1.A1\_at | 6.323e-05 | 0.6646 |
| Bt.7030.1.S1\_at | 6.326e-05 | 0.919 |
| Bt.5598.1.S1\_at | 6.358e-05 | 1.339 |
| Bt.3288.1.A1\_at | 6.367e-05 | -0.8074 |
| Bt.21308.1.A1\_at | 6.383e-05 | 0.7406 |
| Bt.2527.1.S1\_at | 6.384e-05 | 1.091 |
| Bt.5848.1.S1\_at | 6.406e-05 | -0.8498 |
| Bt.4775.1.S1\_at | 6.416e-05 | 1.3 |
| Bt.18126.2.S1\_at | 6.49e-05 | 1.247 |
| Bt.16092.1.S1\_at | 6.493e-05 | 2.225 |
| Bt.19502.1.A1\_at | 6.511e-05 | 1.008 |
| Bt.4118.1.A1\_at | 6.53e-05 | -1.269 |
| Bt.20226.1.A1\_at | 6.532e-05 | 1.593 |
| Bt.28279.1.S1\_at | 6.571e-05 | 1.014 |
| Bt.11257.2.S1\_at | 6.602e-05 | 0.813 |
| Bt.22650.1.A1\_at | 6.625e-05 | 1.274 |
| Bt.6989.1.S1\_at | 6.628e-05 | 1.038 |
| Bt.9288.1.S1\_at | 6.628e-05 | 1.651 |
| Bt.20675.1.S1\_at | 6.639e-05 | -0.7687 |
| Bt.18434.1.A1\_at | 6.64e-05 | 1.307 |
| Bt.16975.1.S1\_at | 6.643e-05 | 0.8015 |
| Bt.4697.1.S1\_at | 6.647e-05 | 1.424 |
| Bt.5603.2.S1\_at | 6.649e-05 | 1.131 |
| Bt.25098.1.S1\_at | 6.667e-05 | -1.873 |
| Bt.7033.2.S1\_a\_at | 6.678e-05 | -3.305 |
| Bt.8262.1.A1\_at | 6.726e-05 | 0.7735 |
| Bt.21792.2.S1\_a\_at | 6.754e-05 | -1.408 |
| Bt.15907.2.S1\_at | 6.754e-05 | -1.981 |
| Bt.4848.1.S1\_at | 6.775e-05 | 0.8105 |
| Bt.1825.1.S1\_at | 6.817e-05 | -0.8648 |
| Bt.9244.1.A1\_at | 6.821e-05 | 1.226 |
| Bt.57.1.S1\_at | 6.864e-05 | -0.6305 |
| Bt.24963.1.A1\_at | 6.868e-05 | -1.222 |
| Bt.893.1.S1\_at | 6.918e-05 | -1.115 |
| Bt.13908.1.S1\_at | 6.97e-05 | -1.296 |
| Bt.12370.1.S1\_at | 6.977e-05 | -0.7437 |
| Bt.28205.2.S1\_at | 6.999e-05 | 1.614 |
| Bt.4584.1.S1\_at | 7.015e-05 | -1.102 |
| Bt.9705.1.S1\_at | 7.053e-05 | 1.361 |
| Bt.19429.2.S1\_a\_at | 7.06e-05 | 0.8122 |
| Bt.12464.1.S1\_at | 7.062e-05 | 1.16 |
| Bt.8015.3.A1\_at | 7.093e-05 | -1.45 |
| Bt.13865.1.A1\_at | 7.141e-05 | -1.504 |
| Bt.19938.1.A1\_at | 7.147e-05 | 0.8025 |
| Bt.26737.1.S1\_at | 7.154e-05 | 0.8721 |
| Bt.8676.1.S1\_at | 7.169e-05 | 0.9153 |
| Bt.10154.1.S1\_at | 7.181e-05 | -1.027 |
| Bt.22176.1.A1\_at | 7.202e-05 | 2.047 |
| Bt.28472.1.S1\_at | 7.225e-05 | 1.189 |
| Bt.28829.1.S1\_at | 7.242e-05 | -0.9549 |
| Bt.13198.1.S1\_at | 7.249e-05 | 1.568 |
| Bt.21981.1.A1\_at | 7.274e-05 | 1.333 |
| Bt.13925.1.S1\_at | 7.337e-05 | 0.9865 |
| Bt.11233.1.S1\_at | 7.384e-05 | 0.5041 |
| Bt.4744.1.S1\_at | 7.401e-05 | -2.142 |
| Bt.13408.1.S1\_at | 7.437e-05 | 0.8695 |
| Bt.20471.2.S1\_a\_at | 7.453e-05 | -1.019 |
| Bt.17845.1.A1\_at | 7.464e-05 | -0.3496 |
| Bt.11725.1.A1\_at | 7.48e-05 | 1.521 |
| Bt.20528.1.S1\_at | 7.487e-05 | 0.881 |
| Bt.69.1.S1\_at | 7.493e-05 | -0.9387 |
| Bt.24766.1.S1\_at | 7.516e-05 | -2.281 |
| Bt.27112.1.S1\_at | 7.552e-05 | 1.485 |
| Bt.9190.1.S1\_at | 7.558e-05 | -0.909 |
| Bt.15616.1.S1\_at | 7.561e-05 | -1.585 |
| Bt.21453.1.S1\_at | 7.574e-05 | -1.767 |
| Bt.24319.1.A1\_at | 7.613e-05 | 0.8432 |
| Bt.28909.1.A1\_at | 7.616e-05 | -0.7274 |
| Bt.5423.2.A1\_at | 7.641e-05 | 0.7803 |
| Bt.25097.1.S1\_at | 7.649e-05 | -0.8764 |
| Bt.22555.1.S1\_at | 7.66e-05 | -1.559 |
| Bt.20720.1.S1\_at | 7.663e-05 | -1.492 |
| Bt.26909.1.S1\_at | 7.681e-05 | -0.3441 |
| Bt.26061.1.A1\_at | 7.704e-05 | 1.34 |
| Bt.752.1.S1\_a\_at | 7.706e-05 | -0.6049 |
| Bt.8213.1.S1\_at | 7.76e-05 | -1.133 |
| Bt.630.1.S1\_at | 7.796e-05 | 0.6305 |
| Bt.303.1.S1\_at | 7.801e-05 | -2.42 |
| Bt.11264.1.S1\_at | 7.835e-05 | 1.003 |
| Bt.1963.1.S1\_at | 7.845e-05 | -0.6559 |
| Bt.21372.2.S1\_at | 7.891e-05 | 1.403 |
| Bt.3400.1.A1\_at | 7.93e-05 | 0.4766 |
| Bt.17332.1.S1\_at | 7.938e-05 | 1.775 |
| Bt.28123.1.S1\_at | 7.966e-05 | 1.427 |
| Bt.10814.1.S1\_at | 7.972e-05 | 1.049 |
| Bt.17194.1.S1\_at | 7.975e-05 | 2.523 |
| Bt.17767.1.A1\_at | 7.987e-05 | 0.5876 |
| Bt.13151.1.S1\_at | 8.007e-05 | -2.009 |
| Bt.27696.1.A1\_at | 8.01e-05 | 1.29 |
| Bt.23143.1.A1\_at | 8.049e-05 | -0.6791 |
| Bt.3913.1.S1\_at | 8.075e-05 | 1.207 |
| Bt.20977.1.S1\_at | 8.114e-05 | 1.117 |
| Bt.18380.1.A1\_at | 8.129e-05 | -0.4149 |
| Bt.9090.1.S1\_at | 8.144e-05 | 0.6735 |
| Bt.20275.1.S1\_at | 8.15e-05 | -0.9394 |
| Bt.11704.1.S1\_at | 8.152e-05 | 1.65 |
| Bt.27793.1.A1\_at | 8.166e-05 | 2.894 |
| Bt.4832.1.S1\_at | 8.182e-05 | 0.2834 |
| Bt.19431.1.S1\_s\_at | 8.191e-05 | -0.8881 |
| Bt.13530.1.S1\_at | 8.205e-05 | -1.955 |
| Bt.26604.1.S1\_at | 8.206e-05 | 1.826 |
| Bt.20422.1.S1\_at | 8.227e-05 | -1.779 |
| Bt.21351.1.S1\_a\_at | 8.228e-05 | 1.043 |
| Bt.9144.1.A1\_at | 8.232e-05 | 0.7031 |
| Bt.2518.1.S1\_at | 8.281e-05 | 1.194 |
| Bt.11301.1.S1\_at | 8.309e-05 | 1.294 |
| Bt.9306.2.S1\_a\_at | 8.354e-05 | -1.307 |
| Bt.24616.1.S1\_at | 8.357e-05 | 2 |
| Bt.5164.1.S1\_at | 8.375e-05 | -2.584 |
| Bt.21870.1.S1\_at | 8.382e-05 | -0.6825 |
| Bt.4466.1.A1\_at | 8.386e-05 | 0.875 |
| Bt.8243.1.A1\_at | 8.39e-05 | -1.423 |
| Bt.8012.1.S1\_at | 8.392e-05 | 0.8943 |
| Bt.21159.1.S1\_at | 8.394e-05 | 2.056 |
| Bt.2587.2.S1\_a\_at | 8.395e-05 | -1.149 |
| Bt.12861.1.S1\_at | 8.396e-05 | 1.156 |
| Bt.5074.1.S1\_at | 8.445e-05 | 1.496 |
| Bt.8109.2.A1\_at | 8.491e-05 | 1.544 |
| Bt.20551.1.S1\_at | 8.492e-05 | 0.9342 |
| Bt.29948.1.S1\_at | 8.517e-05 | 0.7366 |
| Bt.19949.1.A1\_at | 8.524e-05 | 0.9098 |
| Bt.23519.1.S1\_at | 8.553e-05 | -1.148 |
| Bt.7130.1.S1\_at | 8.557e-05 | 0.9886 |
| Bt.19581.1.A1\_at | 8.576e-05 | -1.41 |
| Bt.17323.1.A1\_at | 8.587e-05 | -0.397 |
| Bt.2418.1.S1\_at | 8.591e-05 | 0.9711 |
| Bt.3137.1.S1\_at | 8.599e-05 | 2.138 |
| Bt.14095.2.S1\_at | 8.646e-05 | 1.482 |
| Bt.17989.1.S1\_at | 8.661e-05 | 0.967 |
| Bt.6983.1.S1\_at | 8.695e-05 | 2.33 |
| Bt.11409.1.A1\_a\_at | 8.711e-05 | 1.286 |
| Bt.17488.1.A1\_at | 8.758e-05 | 1.008 |
| Bt.3228.1.S1\_at | 8.781e-05 | 0.5908 |
| Bt.20846.1.A1\_a\_at | 8.786e-05 | 1.784 |
| Bt.19105.1.A1\_at | 8.787e-05 | 0.9267 |
| Bt.16122.1.S1\_at | 8.792e-05 | -1.616 |
| Bt.22852.1.S1\_at | 8.812e-05 | 1.414 |
| Bt.4008.1.S1\_at | 8.849e-05 | -0.9854 |
| Bt.13845.1.S1\_at | 8.85e-05 | -1.506 |
| Bt.8131.1.S1\_at | 8.851e-05 | 1.228 |
| Bt.11149.1.S1\_at | 8.895e-05 | 1.105 |
| Bt.9582.1.A1\_at | 8.96e-05 | -0.4311 |
| Bt.9060.1.S1\_at | 8.966e-05 | 0.8168 |
| Bt.7619.3.S1\_at | 8.989e-05 | -1.605 |
| Bt.26919.1.S1\_at | 9.014e-05 | 2.291 |
| Bt.3428.1.A1\_s\_at | 9.057e-05 | 1.2 |
| Bt.15698.1.S1\_at | 9.058e-05 | -1.311 |
| Bt.13771.3.S1\_at | 9.11e-05 | 0.7614 |
| Bt.11138.1.S1\_at | 9.111e-05 | -0.9302 |
| Bt.3092.1.S1\_at | 9.113e-05 | 1.109 |
| Bt.6156.1.S1\_at | 9.129e-05 | -0.5197 |
| Bt.12030.1.S1\_at | 9.14e-05 | 0.8727 |
| Bt.1793.3.S1\_at | 9.18e-05 | -1.945 |
| Bt.6313.1.S1\_at | 9.191e-05 | 0.9876 |
| Bt.3426.1.S1\_at | 9.196e-05 | 0.7878 |
| Bt.2110.1.S1\_at | 9.205e-05 | -2.135 |
| Bt.4402.1.S1\_at | 9.214e-05 | -0.9146 |
| Bt.4985.2.S1\_a\_at | 9.222e-05 | -1.101 |
| Bt.13345.1.A1\_at | 9.253e-05 | 1.113 |
| Bt.3753.1.S1\_at | 9.258e-05 | 1.763 |
| Bt.10947.1.S1\_at | 9.279e-05 | 0.8596 |
| Bt.20041.1.S1\_at | 9.308e-05 | -0.9929 |
| Bt.14007.1.S1\_at | 9.346e-05 | 0.7738 |
| Bt.20080.1.S1\_at | 9.355e-05 | 1.42 |
| Bt.17812.1.S1\_at | 9.363e-05 | 0.7633 |
| Bt.9973.1.S2\_at | 9.377e-05 | 0.532 |
| Bt.26621.1.S1\_at | 9.424e-05 | 1.411 |
| Bt.13003.14.A1\_x\_at | 9.441e-05 | -0.3928 |
| Bt.12957.1.A1\_a\_at | 9.447e-05 | 0.7019 |
| Bt.2235.1.S2\_at | 9.484e-05 | 1.046 |
| Bt.5682.1.S1\_at | 9.485e-05 | 1.119 |
| Bt.8800.1.S1\_at | 9.487e-05 | -0.7206 |
| Bt.14305.1.A1\_at | 9.549e-05 | -0.7993 |
| Bt.21737.1.S1\_at | 9.616e-05 | 1.422 |
| Bt.8648.1.S1\_at | 9.692e-05 | 1.155 |
| Bt.24713.1.S1\_at | 9.712e-05 | 0.6939 |
| Bt.22046.1.S1\_at | 9.734e-05 | -1.113 |
| Bt.3898.1.S1\_at | 9.76e-05 | -1.532 |
| Bt.3211.1.S1\_at | 9.777e-05 | 0.9239 |
| Bt.28490.1.S1\_at | 9.777e-05 | -0.5316 |
| Bt.11770.1.S1\_at | 9.793e-05 | -2.415 |
| Bt.23405.1.S1\_at | 9.796e-05 | -1.28 |
| Bt.12138.1.S1\_at | 9.801e-05 | -0.9437 |
| Bt.3422.1.S1\_at | 9.808e-05 | 0.8069 |
| Bt.2156.2.S1\_a\_at | 9.877e-05 | -0.6595 |
| Bt.422.1.S1\_at | 9.905e-05 | 1.033 |
| Bt.22181.1.A1\_at | 9.929e-05 | -1.347 |
| Bt.20326.1.S1\_at | 9.963e-05 | 1.192 |
| Bt.21196.1.S1\_at | 9.971e-05 | 1.145 |
| Bt.22283.1.S1\_at | 9.98e-05 | 1.462 |
| Bt.13410.1.S1\_at | 9.992e-05 | -0.9315 |
| Bt.27401.1.A1\_at | 0.0001001 | -0.7733 |
| Bt.22034.1.S1\_at | 0.0001005 | 1.674 |
| Bt.21485.1.S1\_at | 0.0001005 | 0.7811 |
| Bt.5226.1.S1\_at | 0.0001008 | 1.495 |
| Bt.13440.1.S1\_at | 0.000102 | 0.8589 |
| Bt.21997.1.S1\_at | 0.0001021 | 2.497 |
| Bt.16296.1.A1\_at | 0.0001022 | -1.611 |
| Bt.20510.1.S1\_at | 0.0001023 | -0.5325 |
| Bt.17701.1.A1\_at | 0.0001026 | 1.013 |
| Bt.18043.1.S1\_at | 0.0001028 | 1.182 |
| Bt.442.1.S1\_at | 0.0001029 | -0.537 |
| Bt.12105.1.A1\_at | 0.0001031 | -1.501 |
| Bt.10271.1.A1\_at | 0.0001036 | -0.4938 |
| Bt.18894.1.A1\_at | 0.0001036 | 1.416 |
| Bt.9051.1.S1\_at | 0.0001043 | -0.7467 |
| Bt.12821.1.S1\_at | 0.0001046 | 1.388 |
| Bt.23285.1.S1\_at | 0.0001051 | -1.204 |
| Bt.22092.1.S1\_at | 0.0001055 | -1.774 |
| Bt.4267.1.S1\_at | 0.0001061 | 1.376 |
| Bt.23326.2.A1\_at | 0.0001063 | 0.8893 |
| Bt.4314.1.S1\_at | 0.0001066 | 0.9356 |
| Bt.15977.1.S1\_at | 0.0001066 | -0.3895 |
| Bt.20377.1.S1\_at | 0.000107 | -1.218 |
| Bt.7478.1.S1\_at | 0.0001073 | -1.952 |
| Bt.13391.1.S1\_at | 0.0001073 | -0.6321 |
| Bt.2235.1.S1\_at | 0.0001074 | 1.233 |
| Bt.19746.1.S1\_at | 0.0001076 | 1.068 |
| Bt.13648.1.A1\_at | 0.0001079 | 1.024 |
| Bt.6007.1.S1\_at | 0.0001079 | 1.396 |
| Bt.10122.1.S1\_at | 0.0001081 | 0.9371 |
| Bt.20193.1.S1\_at | 0.0001086 | -2.267 |
| Bt.666.1.S1\_at | 0.0001087 | -0.6244 |
| Bt.5278.2.S1\_at | 0.0001087 | -1.168 |
| Bt.25108.1.S1\_at | 0.0001089 | -0.3095 |
| Bt.4057.1.S1\_at | 0.0001091 | 1.413 |
| Bt.29874.1.S1\_at | 0.0001093 | 1.083 |
| Bt.11798.1.S1\_at | 0.0001094 | 1.653 |
| Bt.6488.1.S1\_at | 0.0001098 | -0.9454 |
| Bt.22737.1.S1\_at | 0.0001099 | 0.7293 |
| Bt.20355.1.S1\_at | 0.00011 | -1.12 |
| Bt.23237.1.S1\_at | 0.0001101 | -1.631 |
| Bt.14624.1.A1\_at | 0.0001105 | -1.096 |
| Bt.21641.1.S1\_at | 0.0001106 | 2.044 |
| Bt.27627.1.A1\_at | 0.0001106 | -1.205 |
| Bt.4685.1.S1\_at | 0.0001109 | 1.268 |
| Bt.15925.1.S1\_at | 0.0001116 | -1.156 |
| Bt.16639.1.A1\_at | 0.0001118 | 0.9017 |
| Bt.20788.1.A1\_at | 0.0001118 | -0.5687 |
| Bt.27413.1.A1\_at | 0.000112 | 2.262 |
| Bt.23129.2.S1\_at | 0.0001121 | 0.9128 |
| Bt.7903.1.A1\_at | 0.0001133 | -1.123 |
| Bt.28569.1.S1\_at | 0.0001133 | 1.005 |
| Bt.12469.1.S1\_at | 0.0001136 | 0.3542 |
| Bt.2510.1.A1\_at | 0.0001137 | 1.142 |
| Bt.22290.1.S1\_at | 0.0001138 | 0.9643 |
| Bt.10259.1.A1\_at | 0.0001139 | 1.671 |
| Bt.7633.1.S1\_at | 0.0001139 | -1.535 |
| Bt.6157.1.S1\_at | 0.0001151 | 0.8762 |
| Bt.9968.1.A1\_at | 0.0001158 | 1.318 |
| Bt.25402.1.S1\_at | 0.0001163 | -0.8079 |
| Bt.26805.1.S1\_at | 0.0001166 | -2.183 |
| Bt.2304.1.A1\_at | 0.0001166 | 0.9563 |
| Bt.28043.1.S1\_at | 0.0001175 | 1.462 |
| Bt.7257.1.S1\_at | 0.0001191 | -1.104 |
| Bt.3478.1.A1\_s\_at | 0.0001194 | 1.084 |
| Bt.16503.1.A1\_at | 0.0001197 | -0.4351 |
| Bt.22564.3.S1\_at | 0.0001201 | 0.4931 |
| Bt.7481.1.S1\_at | 0.0001205 | -1.808 |
| Bt.9778.1.S1\_at | 0.000121 | 1.001 |
| Bt.16841.1.A1\_at | 0.000121 | 0.8702 |
| Bt.1681.1.S1\_at | 0.0001217 | -1.63 |
| Bt.7183.1.S1\_at | 0.0001224 | 1.038 |
| Bt.25850.1.S1\_at | 0.0001225 | 0.8009 |
| Bt.1967.1.S1\_at | 0.0001228 | -0.8418 |
| Bt.25228.1.A1\_at | 0.0001228 | 1.281 |
| Bt.4689.1.S2\_at | 0.0001238 | -0.6796 |
| Bt.9398.1.S1\_at | 0.000124 | 0.728 |
| Bt.19423.1.S1\_at | 0.0001241 | 1.707 |
| Bt.10266.1.S1\_at | 0.0001248 | -1.17 |
| Bt.4870.1.S1\_at | 0.000125 | 0.4759 |
| Bt.22155.1.A1\_at | 0.000125 | 0.6299 |
| Bt.16637.1.A1\_at | 0.0001255 | -1.99 |
| Bt.25479.1.S1\_at | 0.0001255 | 1.438 |
| Bt.29915.1.S1\_at | 0.0001257 | -0.7403 |
| Bt.3633.1.A1\_at | 0.0001257 | 0.8002 |
| Bt.21813.1.S1\_at | 0.0001264 | 2.341 |
| Bt.20306.1.S1\_at | 0.0001267 | -0.5888 |
| Bt.2109.1.S1\_at | 0.0001268 | 1.095 |
| Bt.22528.1.A1\_at | 0.0001268 | -1.275 |
| Bt.9490.1.S1\_at | 0.0001275 | -0.9287 |
| Bt.18812.1.A1\_at | 0.0001279 | -0.3078 |
| Bt.167.1.S1\_at | 0.000128 | -1.008 |
| Bt.63.1.S1\_at | 0.0001288 | -1.426 |
| Bt.13505.1.S1\_at | 0.0001289 | -1.487 |
| Bt.16782.2.S1\_at | 0.0001297 | -1.386 |
| Bt.5418.1.S1\_at | 0.0001303 | -1.324 |
| Bt.2052.1.S1\_a\_at | 0.0001309 | 0.7008 |
| Bt.28057.1.S1\_at | 0.0001311 | 0.8741 |
| Bt.4726.1.S1\_at | 0.0001319 | 0.9931 |
| Bt.16041.1.S1\_at | 0.0001322 | 1.239 |
| Bt.29409.1.S1\_at | 0.0001324 | -0.6597 |
| Bt.24381.2.S1\_at | 0.0001324 | 1.818 |
| Bt.10600.1.S1\_at | 0.0001325 | 1.471 |
| Bt.948.1.S1\_at | 0.0001327 | 0.9597 |
| Bt.13593.1.A1\_at | 0.0001331 | 0.8495 |
| Bt.23654.1.S1\_at | 0.0001344 | 1.042 |
| Bt.26577.1.A1\_at | 0.0001347 | -0.7744 |
| Bt.22243.1.S1\_at | 0.0001351 | 1.028 |
| Bt.9559.2.S1\_at | 0.0001352 | 2.037 |
| Bt.25980.1.A1\_at | 0.0001363 | -1.539 |
| Bt.9695.1.S1\_at | 0.0001374 | 0.858 |
| Bt.4606.2.S1\_at | 0.0001378 | 2.387 |
| Bt.12408.1.S1\_at | 0.0001386 | -0.5993 |
| Bt.20463.1.S1\_at | 0.0001391 | 1.317 |
| Bt.24676.1.S1\_at | 0.0001392 | -1.749 |
| Bt.5664.1.S1\_at | 0.0001392 | 1.253 |
| Bt.23603.2.S1\_a\_at | 0.0001396 | -0.9709 |
| Bt.4844.1.S1\_at | 0.0001399 | 1.711 |
| Bt.2325.1.S1\_at | 0.0001404 | -0.4491 |
| Bt.13991.2.S1\_a\_at | 0.0001405 | 1.154 |
| Bt.23094.3.S1\_x\_at | 0.0001408 | -1.772 |
| Bt.4449.1.S1\_at | 0.0001409 | 1.219 |
| Bt.3817.1.A1\_at | 0.0001412 | 1.032 |
| Bt.18126.1.A1\_at | 0.0001414 | 1.694 |
| Bt.26726.1.S1\_at | 0.0001418 | 1.641 |
| Bt.10096.1.S1\_at | 0.000142 | 1.609 |
| Bt.3888.1.S1\_a\_at | 0.0001422 | -1.604 |
| Bt.7785.1.S1\_at | 0.0001424 | 0.832 |
| Bt.4044.1.S1\_at | 0.0001424 | -2.229 |
| Bt.4145.1.S1\_at | 0.0001429 | -0.6753 |
| Bt.5054.3.S1\_at | 0.0001436 | 1.497 |
| Bt.1415.1.S1\_at | 0.0001436 | -1.283 |
| Bt.17081.2.S1\_at | 0.0001436 | 2.28 |
| Bt.9312.1.A1\_at | 0.0001436 | 1.447 |
| Bt.4311.1.S1\_at | 0.0001438 | 0.8664 |
| Bt.6577.1.S1\_at | 0.0001449 | -1.301 |
| Bt.25092.1.S1\_at | 0.0001453 | -1.115 |
| Bt.8234.1.S1\_at | 0.0001464 | -0.4441 |
| Bt.7120.1.S1\_a\_at | 0.0001465 | 0.6106 |
| Bt.11013.1.S1\_at | 0.000147 | 0.7769 |
| Bt.23607.1.S1\_at | 0.0001474 | -0.6857 |
| Bt.5447.1.S1\_at | 0.000148 | 1.147 |
| Bt.1244.1.S1\_at | 0.000148 | 0.616 |
| Bt.26609.1.S1\_at | 0.000148 | 0.8128 |
| Bt.28059.1.S1\_at | 0.0001481 | -1.243 |
| Bt.8180.3.A1\_a\_at | 0.0001482 | 1.32 |
| Bt.9337.1.S1\_at | 0.0001483 | 1.214 |
| Bt.4543.1.S1\_at | 0.0001496 | 0.5924 |
| Bt.3791.1.S1\_at | 0.0001504 | -1.039 |
| Bt.1617.2.S1\_a\_at | 0.0001504 | 0.7091 |
| Bt.22170.2.A1\_at | 0.0001507 | -0.6059 |
| Bt.11192.1.S1\_at | 0.0001511 | -0.6402 |
| Bt.9416.1.S1\_at | 0.0001515 | 1.68 |
| Bt.10880.1.S1\_at | 0.000152 | -1.265 |
| Bt.4020.1.S1\_at | 0.0001527 | -0.5252 |
| Bt.18316.1.A1\_at | 0.0001528 | 1.488 |
| Bt.24717.2.S1\_a\_at | 0.0001529 | 0.8143 |
| Bt.2266.3.S1\_at | 0.0001531 | -1.346 |
| Bt.23073.1.S1\_at | 0.0001548 | 0.5327 |
| Bt.5857.1.S1\_at | 0.0001549 | -0.2629 |
| Bt.20895.1.S1\_at | 0.0001564 | -0.6224 |
| Bt.26835.1.S1\_at | 0.0001569 | -1.297 |
| Bt.9036.1.A1\_at | 0.0001572 | 0.6142 |
| Bt.21272.1.S1\_at | 0.0001578 | -0.7505 |
| Bt.10312.1.A1\_at | 0.0001579 | 0.5716 |
| Bt.21036.2.A1\_at | 0.0001583 | 0.9806 |
| Bt.24892.1.A1\_at | 0.0001588 | 1.877 |
| Bt.3011.2.S1\_at | 0.0001594 | 1.259 |
| Bt.4071.1.S1\_at | 0.0001597 | -0.772 |
| Bt.25390.1.A1\_at | 0.0001599 | 1.144 |
| Bt.18519.1.A1\_at | 0.0001602 | 0.7165 |
| Bt.1990.1.S1\_at | 0.0001604 | 0.4153 |
| Bt.6493.1.S1\_at | 0.0001605 | 0.6845 |
| Bt.15781.1.A1\_at | 0.0001613 | -0.5488 |
| Bt.22356.1.S1\_at | 0.0001615 | 0.8334 |
| Bt.20328.1.S1\_at | 0.0001619 | 0.807 |
| Bt.26894.1.S1\_at | 0.0001619 | -1.14 |
| Bt.13784.1.S1\_at | 0.0001632 | -0.8156 |
| Bt.14176.1.S1\_at | 0.0001634 | 1.389 |
| Bt.22369.1.S1\_at | 0.000164 | 1.979 |
| Bt.24802.2.S1\_a\_at | 0.000164 | -2.607 |
| Bt.8957.1.S1\_at | 0.0001644 | 1.424 |
| Bt.10417.1.S1\_at | 0.0001651 | 0.6959 |
| Bt.24397.1.S1\_at | 0.0001653 | 0.5493 |
| Bt.9749.1.S1\_at | 0.0001654 | -0.5501 |
| Bt.21053.2.S1\_at | 0.0001659 | -1.214 |
| Bt.16108.1.A1\_at | 0.000167 | -1.889 |
| Bt.29679.1.S1\_s\_at | 0.0001681 | 1.446 |
| Bt.23002.1.S1\_s\_at | 0.0001683 | 0.5737 |
| Bt.8437.1.A1\_at | 0.0001687 | 1.45 |
| Bt.24517.2.A1\_at | 0.0001698 | -0.8205 |
| Bt.13765.1.S1\_at | 0.0001698 | -1.627 |
| Bt.17985.1.S1\_at | 0.0001699 | 1.542 |
| Bt.26393.1.A1\_at | 0.0001701 | 1.094 |
| Bt.25836.2.A1\_at | 0.0001715 | 1.783 |
| Bt.10360.1.S1\_at | 0.0001722 | 1.197 |
| Bt.28187.1.S1\_at | 0.0001724 | 1.088 |
| Bt.21630.1.S1\_at | 0.0001725 | -2.307 |
| Bt.5412.1.S1\_at | 0.0001728 | -1.058 |
| Bt.29325.1.S1\_at | 0.0001739 | -1.063 |
| Bt.1690.1.S1\_at | 0.0001746 | 0.5349 |
| Bt.28390.1.S1\_at | 0.0001751 | -1.013 |
| Bt.22532.3.S1\_at | 0.0001752 | -0.6137 |
| Bt.23094.2.A1\_x\_at | 0.0001771 | -1.385 |
| Bt.1389.1.S1\_at | 0.0001779 | 1.168 |
| Bt.18755.2.S1\_at | 0.0001779 | -0.6439 |
| Bt.21015.1.S1\_at | 0.0001783 | -2.358 |
| Bt.2451.1.S1\_at | 0.000179 | 1.201 |
| Bt.9246.1.A1\_at | 0.0001791 | 1.115 |
| Bt.2556.1.S1\_at | 0.0001794 | 2.081 |
| Bt.10349.1.S1\_at | 0.0001803 | 1.158 |
| Bt.10317.1.A1\_at | 0.0001819 | 0.9351 |
| Bt.910.3.S1\_at | 0.000182 | -0.2406 |
| Bt.6473.1.S1\_at | 0.000182 | -0.7558 |
| Bt.27889.1.S1\_at | 0.0001821 | -0.9472 |
| Bt.26549.1.A1\_at | 0.0001822 | 0.6321 |
| Bt.1097.1.S1\_at | 0.0001833 | 1.087 |
| Bt.7888.1.S1\_at | 0.0001835 | 1.046 |
| Bt.12412.1.S1\_at | 0.0001837 | 1.013 |
| Bt.1806.1.S1\_at | 0.0001844 | -0.897 |
| Bt.9658.1.S1\_at | 0.0001853 | -0.6651 |
| Bt.21036.1.S1\_at | 0.0001858 | 1.193 |
| Bt.11040.1.S1\_at | 0.0001862 | 1.581 |
| Bt.3494.1.A1\_s\_at | 0.0001868 | 1.03 |
| Bt.4489.1.S1\_at | 0.0001869 | -0.565 |
| Bt.5484.1.S1\_at | 0.0001872 | -0.9584 |
| Bt.13887.1.S1\_at | 0.0001874 | -1.39 |
| Bt.27717.1.A1\_at | 0.0001885 | 1.292 |
| Bt.8802.1.S1\_at | 0.000189 | 1.033 |
| Bt.25104.1.S1\_at | 0.0001896 | 0.7377 |
| Bt.4898.1.S1\_at | 0.0001899 | 2.218 |
| Bt.282.1.S1\_at | 0.0001901 | -0.5241 |
| Bt.2070.3.S1\_at | 0.0001901 | -1.8 |
| Bt.23589.2.S1\_at | 0.0001907 | 0.5818 |
| Bt.13204.1.S1\_at | 0.0001911 | 1.328 |
| Bt.1541.1.S1\_at | 0.0001916 | 1.156 |
| Bt.2972.1.A1\_at | 0.0001924 | 1.383 |
| Bt.27037.1.A1\_at | 0.0001926 | 0.4897 |
| Bt.9210.1.S1\_at | 0.0001929 | -0.7751 |
| Bt.23361.1.S1\_at | 0.0001931 | -0.6159 |
| Bt.13969.2.S1\_at | 0.0001936 | 1.089 |
| Bt.7143.1.S1\_at | 0.0001949 | -0.7844 |
| Bt.11028.1.S1\_at | 0.0001958 | -0.6694 |
| Bt.28464.1.S1\_at | 0.0001972 | 0.7013 |
| Bt.100.1.S1\_at | 0.0001972 | -1.294 |
| Bt.1543.1.A1\_at | 0.0001974 | -1.22 |
| Bt.17958.1.S1\_at | 0.0001984 | 0.7252 |
| Bt.7595.1.S1\_at | 0.0001988 | -1.039 |
| Bt.13741.1.A1\_at | 0.0001991 | 0.9456 |
| Bt.22336.1.A1\_at | 0.0001992 | -0.488 |
| Bt.23227.1.S1\_at | 0.0002002 | 1.404 |
| Bt.23342.1.S1\_at | 0.0002005 | 0.4423 |
| Bt.17777.2.S1\_at | 0.0002007 | -1.138 |
| Bt.15900.1.A1\_at | 0.0002024 | -1.247 |
| Bt.20800.1.S1\_at | 0.000203 | 1.125 |
| Bt.13152.1.S1\_s\_at | 0.0002038 | 1.935 |
| Bt.20318.1.S1\_a\_at | 0.0002045 | -1.094 |
| Bt.8976.1.S1\_at | 0.0002053 | 1.421 |
| Bt.7154.1.S2\_at | 0.0002064 | 0.5012 |
| Bt.24359.1.S1\_a\_at | 0.0002073 | 1.56 |
| Bt.9012.1.S1\_at | 0.0002074 | 1.08 |
| Bt.4203.1.S1\_at | 0.0002074 | -0.9266 |
| Bt.13366.1.S1\_at | 0.0002075 | 1.704 |
| Bt.9936.1.S1\_at | 0.0002076 | 0.5252 |
| Bt.20278.1.S1\_at | 0.0002076 | 1.482 |
| Bt.17701.2.A1\_at | 0.0002087 | 0.4174 |
| Bt.2138.1.S1\_at | 0.0002089 | -1.153 |
| Bt.3427.1.S1\_at | 0.000209 | -1.264 |
| Bt.3391.1.S1\_at | 0.0002094 | 0.8633 |
| Bt.19100.3.A1\_at | 0.0002103 | 1.457 |
| Bt.413.1.S1\_at | 0.0002108 | 0.8937 |
| Bt.16259.1.A1\_at | 0.000211 | 0.646 |
| Bt.3029.1.S1\_at | 0.0002113 | 0.8889 |
| Bt.4875.2.S1\_at | 0.0002119 | 1.556 |
| Bt.7676.3.S1\_at | 0.000212 | 1.567 |
| Bt.14007.3.A1\_at | 0.0002122 | 1.016 |
| Bt.5079.1.S1\_at | 0.0002123 | -1.211 |
| Bt.9998.1.S1\_at | 0.0002136 | 1.654 |
| Bt.27807.1.A1\_at | 0.0002136 | 1.334 |
| Bt.8492.1.A1\_at | 0.0002141 | 1.374 |
| Bt.24321.1.S1\_at | 0.0002143 | 1.003 |
| Bt.2007.2.S1\_at | 0.0002144 | -0.5075 |
| Bt.16602.1.A1\_at | 0.0002151 | -0.2998 |
| Bt.25339.1.S1\_at | 0.0002159 | 0.6171 |
| Bt.13748.1.S1\_at | 0.0002164 | 1.168 |
| Bt.28535.1.S1\_at | 0.0002167 | -1.012 |
| Bt.2219.1.S1\_at | 0.0002169 | -0.7657 |
| Bt.27938.1.A1\_at | 0.0002174 | 1.44 |
| Bt.28162.3.S1\_at | 0.000218 | -1.809 |
| Bt.19916.1.S1\_at | 0.0002197 | 0.568 |
| Bt.3990.1.S1\_at | 0.00022 | 0.9012 |
| Bt.3726.1.A1\_at | 0.0002224 | 1.082 |
| Bt.22165.1.S1\_at | 0.0002229 | 1.615 |
| Bt.13478.1.A1\_at | 0.0002229 | -0.923 |
| Bt.9643.1.S1\_at | 0.000224 | -0.917 |
| Bt.27887.1.S1\_at | 0.0002245 | 1.448 |
| Bt.20372.1.S1\_at | 0.0002246 | -2.463 |
| Bt.22340.1.S1\_at | 0.0002246 | 0.6292 |
| Bt.24433.1.S1\_at | 0.0002248 | -0.7943 |
| Bt.1753.1.S1\_at | 0.0002257 | -0.4552 |
| Bt.9623.1.A1\_at | 0.0002264 | -1.674 |
| Bt.9425.1.S1\_at | 0.0002269 | 1.222 |
| Bt.19601.1.S1\_at | 0.0002285 | 1.18 |
| Bt.1165.1.S1\_at | 0.0002286 | 0.9945 |
| Bt.22443.1.A1\_at | 0.0002288 | 0.8248 |
| Bt.1978.14.S1\_at | 0.000229 | -0.3594 |
| Bt.5237.1.S1\_at | 0.000229 | 1.052 |
| Bt.884.1.S1\_at | 0.0002294 | 1.143 |
| Bt.868.1.S1\_a\_at | 0.0002294 | -0.8046 |
| Bt.10195.1.S1\_at | 0.0002302 | 0.8117 |
| Bt.13991.2.S1\_at | 0.0002303 | 1.005 |
| Bt.10810.1.A1\_at | 0.000232 | 0.9706 |
| Bt.10056.2.S1\_at | 0.0002332 | 1.16 |
| Bt.9288.2.A1\_at | 0.0002334 | 0.8572 |
| Bt.2347.2.S1\_at | 0.0002336 | -0.9235 |
| Bt.25099.2.S1\_at | 0.0002338 | -3.37 |
| Bt.18928.1.A1\_at | 0.0002339 | 0.5215 |
| Bt.29472.1.A1\_at | 0.0002342 | 2.078 |
| Bt.11019.1.S1\_at | 0.0002349 | -2.075 |
| Bt.3631.1.S1\_at | 0.0002352 | -0.3623 |
| Bt.8671.1.S1\_at | 0.0002363 | -1.294 |
| Bt.2696.2.S1\_at | 0.0002363 | 1.566 |
| Bt.23246.1.S1\_at | 0.0002375 | 1.38 |
| Bt.3404.1.S1\_at | 0.0002382 | 0.5027 |
| Bt.26999.1.S1\_at | 0.0002387 | -0.7373 |
| Bt.1217.1.A1\_a\_at | 0.0002388 | -0.8681 |
| Bt.11687.2.S1\_at | 0.0002396 | -0.5861 |
| Bt.4467.2.S1\_a\_at | 0.0002402 | -0.6056 |
| Bt.21365.2.S1\_at | 0.0002422 | 0.7552 |
| Bt.3698.1.S1\_at | 0.0002424 | 1.113 |
| Bt.26652.1.S1\_at | 0.0002427 | -0.7627 |
| Bt.25654.1.A1\_at | 0.0002429 | -1.086 |
| Bt.4638.1.S1\_at | 0.0002431 | -0.8972 |
| Bt.6075.1.S1\_at | 0.0002439 | 0.8158 |
| Bt.9448.1.S2\_at | 0.0002448 | 0.6572 |
| Bt.8691.1.S1\_at | 0.000245 | 1.392 |
| Bt.2156.1.S1\_at | 0.0002455 | -0.4716 |
| Bt.12966.1.S1\_at | 0.0002463 | -1.592 |
| Bt.27872.1.S1\_at | 0.0002472 | 1.4 |
| Bt.2451.2.A1\_at | 0.0002476 | 1.236 |
| Bt.1062.1.S1\_at | 0.0002489 | 0.9812 |
| Bt.23268.1.S1\_at | 0.0002495 | 1.139 |
| Bt.12862.1.S1\_at | 0.0002503 | 1.262 |
| Bt.18048.2.S1\_at | 0.0002509 | 1.422 |
| Bt.29459.1.A1\_at | 0.0002525 | 1.265 |
| Bt.1185.1.A1\_at | 0.0002529 | -1.087 |
| Bt.8690.2.S1\_at | 0.0002531 | -0.9822 |
| Bt.21607.1.S1\_at | 0.0002549 | 1.723 |
| Bt.24494.1.A1\_at | 0.0002555 | -0.3393 |
| Bt.15860.1.S1\_at | 0.0002555 | 0.6442 |
| Bt.5092.1.S1\_at | 0.0002563 | -0.5591 |
| Bt.26774.1.S1\_at | 0.0002578 | 2.021 |
| Bt.2121.1.S1\_at | 0.0002586 | 2.358 |
| Bt.18623.1.A1\_at | 0.0002588 | -1.636 |
| Bt.3593.1.S1\_at | 0.0002609 | -1.142 |
| Bt.17490.1.S1\_a\_at | 0.0002609 | -0.6057 |
| Bt.2195.1.A1\_at | 0.000261 | -0.6883 |
| Bt.24624.2.S1\_at | 0.000261 | -1.254 |
| Bt.7955.1.S1\_at | 0.0002615 | 0.7943 |
| Bt.3414.1.S1\_at | 0.0002615 | -0.8518 |
| Bt.28669.1.A1\_at | 0.0002622 | -0.6993 |
| Bt.26550.2.S1\_at | 0.0002635 | -1.75 |
| Bt.26883.1.S1\_at | 0.0002655 | -0.8843 |
| Bt.16105.1.S1\_at | 0.000266 | 1.494 |
| Bt.14040.1.S1\_at | 0.0002671 | -1.309 |
| Bt.20091.1.S1\_at | 0.0002684 | 0.7772 |
| Bt.17332.3.A1\_at | 0.000269 | 1.18 |
| Bt.4261.1.S1\_at | 0.0002705 | -1.296 |
| Bt.17585.1.S2\_at | 0.0002707 | 1.06 |
| Bt.21824.1.A1\_at | 0.0002712 | 1.644 |
| Bt.15531.1.S1\_at | 0.0002717 | 0.7814 |
| Bt.18152.1.S1\_at | 0.0002717 | 0.5794 |
| Bt.12217.2.S1\_at | 0.0002723 | 1.863 |
| Bt.27430.2.S1\_at | 0.0002723 | -1.13 |
| Bt.6028.1.S1\_at | 0.0002727 | -0.9983 |
| Bt.20487.1.S1\_at | 0.0002729 | -1.43 |
| Bt.2889.1.S1\_at | 0.0002729 | 0.5576 |
| Bt.17061.1.A1\_at | 0.0002737 | 1.819 |
| Bt.9773.1.A1\_at | 0.0002751 | 0.423 |
| Bt.17999.3.A1\_a\_at | 0.0002756 | 0.5443 |
| Bt.2116.1.S1\_at | 0.0002765 | -0.7796 |
| Bt.8912.1.A1\_at | 0.0002771 | -1.676 |
| Bt.3472.1.A1\_at | 0.0002772 | -0.5245 |
| Bt.25093.1.A1\_at | 0.0002776 | 0.7194 |
| Bt.17425.1.A1\_at | 0.000278 | -1.181 |
| Bt.5099.1.S1\_at | 0.0002786 | 1.054 |
| Bt.27100.1.S1\_at | 0.0002792 | 0.7228 |
| Bt.1334.1.S1\_at | 0.0002806 | -0.6967 |
| Bt.347.1.S1\_at | 0.0002817 | -2.176 |
| Bt.20245.1.S1\_at | 0.0002834 | 1.396 |
| Bt.26675.1.S1\_at | 0.0002842 | -1.075 |
| Bt.10734.1.A1\_at | 0.0002853 | 1.381 |
| Bt.2667.1.S1\_a\_at | 0.0002858 | -1.575 |
| Bt.11182.1.S1\_at | 0.0002861 | 0.9195 |
| Bt.2870.1.A1\_at | 0.0002866 | 1.302 |
| Bt.21122.1.S1\_at | 0.0002871 | -0.9215 |
| Bt.11832.2.A1\_at | 0.0002872 | 0.9215 |
| Bt.29803.1.S1\_at | 0.0002873 | -0.5056 |
| Bt.97.1.S1\_at | 0.0002878 | -3.684 |
| Bt.26643.1.S1\_at | 0.0002889 | 0.8861 |
| Bt.3193.1.A1\_at | 0.0002889 | -0.8928 |
| Bt.4900.1.A1\_at | 0.0002892 | -1.001 |
| Bt.18358.1.A1\_at | 0.0002896 | -0.3427 |
| Bt.12366.1.S1\_at | 0.0002902 | 0.6654 |
| Bt.4161.1.S1\_at | 0.0002909 | 0.7832 |
| Bt.8566.1.S1\_at | 0.0002916 | 0.7849 |
| Bt.8208.1.A1\_at | 0.0002931 | 1.376 |
| Bt.2999.1.A1\_at | 0.0002945 | -0.2817 |
| Bt.10352.1.S1\_at | 0.0002946 | -1.117 |
| Bt.4862.1.S1\_at | 0.000295 | -0.5343 |
| Bt.21072.1.A1\_at | 0.0002974 | 1.453 |
| Bt.15714.2.S1\_a\_at | 0.0002992 | 1.427 |
| Bt.12377.1.S1\_at | 0.0003008 | 0.7848 |
| Bt.16471.1.S1\_at | 0.0003011 | 1.482 |
| Bt.22615.2.A1\_a\_at | 0.0003013 | -0.8787 |
| Bt.1595.1.S1\_at | 0.0003015 | 0.9509 |
| Bt.29928.1.S1\_at | 0.0003022 | 0.5577 |
| Bt.20852.1.A1\_at | 0.0003027 | 1.313 |
| Bt.3098.1.S1\_at | 0.0003032 | 0.9104 |
| Bt.3359.1.S1\_at | 0.0003034 | -0.463 |
| Bt.18970.1.A1\_at | 0.0003039 | -0.276 |
| Bt.17146.1.A1\_at | 0.0003048 | -0.502 |
| Bt.1697.1.S1\_at | 0.0003049 | -0.3899 |
| Bt.4279.2.S1\_at | 0.0003054 | 1.11 |
| Bt.5035.1.S1\_at | 0.0003063 | -1.056 |
| Bt.15799.1.S1\_at | 0.0003063 | 0.9273 |
| Bt.9377.1.S1\_at | 0.0003075 | 1.393 |
| Bt.6661.1.S1\_at | 0.0003075 | -0.4418 |
| Bt.16884.1.A1\_at | 0.0003079 | -0.4726 |
| Bt.16142.2.S1\_at | 0.0003092 | -1.147 |
| Bt.24377.2.S1\_at | 0.0003093 | -0.8393 |
| Bt.26803.1.A1\_at | 0.0003117 | 0.9321 |
| Bt.23815.1.S1\_at | 0.0003118 | -0.4221 |
| Bt.4241.1.S1\_at | 0.000312 | 0.399 |
| Bt.23605.2.S1\_at | 0.0003122 | 1.109 |
| Bt.16125.3.S1\_at | 0.0003126 | -1.184 |
| Bt.12691.1.A1\_at | 0.0003128 | 0.5788 |
| Bt.7040.1.S1\_at | 0.0003133 | -0.5602 |
| Bt.60.1.S1\_at | 0.0003133 | -0.8161 |
| Bt.28188.1.S1\_at | 0.0003137 | 1.223 |
| Bt.18057.1.A1\_at | 0.0003143 | 1.349 |
| Bt.25921.1.A1\_at | 0.0003143 | 0.9082 |
| Bt.16636.2.A1\_at | 0.0003146 | 1.152 |
| Bt.981.2.S1\_at | 0.0003147 | 0.9898 |
| Bt.15854.1.A1\_at | 0.0003153 | 1.377 |
| Bt.26143.1.A1\_at | 0.0003154 | 0.7572 |
| Bt.8486.1.S1\_at | 0.000316 | 1.729 |
| Bt.20281.2.S1\_a\_at | 0.0003165 | -1.146 |
| Bt.26094.1.A1\_at | 0.0003188 | 0.815 |
| Bt.20917.2.S1\_a\_at | 0.000319 | -1.223 |
| Bt.13248.1.A1\_at | 0.0003226 | 1.209 |
| Bt.16700.1.A1\_at | 0.000323 | -0.2738 |
| Bt.15287.1.A1\_at | 0.0003254 | -0.2074 |
| Bt.21369.1.S1\_at | 0.0003276 | 0.828 |
| Bt.20242.1.S1\_at | 0.000328 | 1.584 |
| Bt.27058.1.A1\_at | 0.0003284 | 2.128 |
| Bt.28491.1.S1\_at | 0.0003295 | 0.675 |
| Bt.5507.2.S1\_at | 0.0003304 | 0.9831 |
| Bt.13965.1.S1\_at | 0.0003306 | 1.501 |
| Bt.16224.3.A1\_at | 0.0003307 | 1.381 |
| Bt.3821.1.A1\_at | 0.0003309 | 0.9445 |
| Bt.16573.1.A1\_at | 0.0003313 | 0.9488 |
| Bt.20605.2.S1\_at | 0.0003315 | 0.9043 |
| Bt.11230.1.S1\_at | 0.0003315 | -1.373 |
| Bt.23209.1.S1\_a\_at | 0.000333 | 1.08 |
| Bt.1429.3.A1\_s\_at | 0.0003338 | 0.5103 |
| Bt.13118.1.S1\_at | 0.0003338 | 1 |
| Bt.9238.1.S1\_at | 0.0003357 | -1.031 |
| Bt.9767.1.S1\_a\_at | 0.0003358 | -1.368 |
| Bt.6397.2.S1\_at | 0.0003362 | 1.469 |
| Bt.22230.1.S1\_at | 0.0003365 | 1.347 |
| Bt.22054.1.S1\_at | 0.0003376 | -1.139 |
| Bt.20744.1.A1\_at | 0.000338 | 1.097 |
| Bt.29.1.S1\_at | 0.0003406 | 1.761 |
| Bt.25016.2.S1\_at | 0.0003445 | 1.271 |
| Bt.22563.1.A1\_s\_at | 0.000345 | -0.6383 |
| Bt.10092.1.S1\_at | 0.0003458 | 1.208 |
| Bt.18408.1.A1\_at | 0.000346 | 1.318 |
| Bt.13355.1.S1\_at | 0.0003465 | 0.59 |
| Bt.23673.1.A1\_at | 0.0003472 | 1.307 |
| Bt.7434.1.S1\_at | 0.0003475 | 0.5767 |
| Bt.21378.1.S1\_at | 0.0003477 | 0.7602 |
| Bt.9233.3.S1\_at | 0.0003482 | -1.213 |
| Bt.7500.1.S1\_at | 0.0003484 | 1.179 |
| Bt.23265.1.S1\_at | 0.0003488 | 1.225 |
| Bt.9966.2.S1\_a\_at | 0.0003488 | 1.239 |
| Bt.15819.2.S1\_at | 0.0003501 | -0.9573 |
| Bt.23169.1.S1\_at | 0.0003505 | 0.919 |
| Bt.12639.1.S1\_at | 0.0003508 | 0.5886 |
| Bt.23496.1.S1\_at | 0.0003515 | -0.6512 |
| Bt.721.1.A1\_at | 0.0003542 | 0.8836 |
| Bt.13199.1.S1\_at | 0.0003542 | -1.01 |
| Bt.22526.1.S1\_at | 0.0003543 | -0.9339 |
| Bt.11189.1.S1\_at | 0.0003547 | -0.7195 |
| Bt.876.1.S1\_at | 0.0003549 | -2.231 |
| Bt.26848.1.S1\_at | 0.0003577 | 1.143 |
| Bt.22672.1.A1\_at | 0.0003607 | 1.965 |
| Bt.2005.1.S1\_at | 0.0003618 | -0.7447 |
| Bt.2765.1.S1\_at | 0.0003622 | 0.9481 |
| Bt.26921.1.A1\_at | 0.0003625 | -0.4647 |
| Bt.22486.1.S1\_at | 0.0003638 | 0.5372 |
| Bt.8682.1.S1\_at | 0.000365 | 1.115 |
| Bt.20308.2.S1\_at | 0.0003652 | 1.203 |
| Bt.4270.1.S1\_at | 0.0003654 | 1.775 |
| Bt.5748.3.S1\_at | 0.0003661 | 1.143 |
| Bt.4652.1.S1\_at | 0.0003678 | -0.9667 |
| Bt.20331.2.S1\_at | 0.0003682 | 1.132 |
| Bt.17005.1.S1\_at | 0.0003683 | -1.34 |
| Bt.17012.1.A1\_s\_at | 0.0003685 | -1.252 |
| Bt.7397.1.S1\_at | 0.0003695 | 1.212 |
| Bt.24350.1.A1\_at | 0.0003702 | 0.8257 |
| Bt.16387.2.A1\_at | 0.0003715 | -0.6394 |
| Bt.7263.2.A1\_at | 0.0003718 | -0.264 |
| Bt.1390.1.A1\_at | 0.000372 | -0.918 |
| Bt.1299.1.S1\_a\_at | 0.0003733 | 2.325 |
| Bt.15922.1.S1\_at | 0.0003751 | 0.5365 |
| Bt.12088.1.S1\_at | 0.0003791 | 0.9926 |
| Bt.15909.1.S1\_at | 0.0003793 | -1.125 |
| Bt.8260.1.A1\_at | 0.0003807 | 0.9078 |
| Bt.14037.1.S1\_at | 0.0003809 | 0.6865 |
| Bt.27218.1.A1\_at | 0.0003812 | -0.8905 |
| Bt.5106.2.S1\_at | 0.0003831 | 0.5625 |
| Bt.7467.1.S1\_at | 0.0003839 | 1.085 |
| Bt.21288.1.A1\_at | 0.0003841 | 0.6263 |
| Bt.16067.1.S1\_at | 0.0003842 | -1.544 |
| Bt.6789.1.S1\_at | 0.0003842 | -0.7685 |
| Bt.4030.1.S1\_at | 0.0003842 | -0.6986 |
| Bt.5906.1.S1\_at | 0.000385 | -0.6383 |
| Bt.27071.1.S1\_at | 0.0003857 | 1.171 |
| Bt.2262.1.S1\_at | 0.0003862 | -0.9426 |
| Bt.16932.1.A1\_at | 0.0003887 | -0.8587 |
| Bt.28772.1.A1\_at | 0.0003891 | -0.8111 |
| Bt.1461.1.S1\_at | 0.00039 | -0.3913 |
| Bt.3573.1.S1\_at | 0.0003902 | 0.4823 |
| Bt.19649.1.A1\_at | 0.0003913 | 0.7977 |
| Bt.19061.1.A1\_at | 0.000392 | -0.9039 |
| Bt.13464.1.S1\_at | 0.0003927 | -0.8961 |
| Bt.10723.1.S1\_at | 0.0003929 | 0.7767 |
| Bt.13881.1.S1\_at | 0.0003944 | 0.8941 |
| Bt.12429.1.S1\_at | 0.0003956 | -0.7834 |
| Bt.16209.1.A1\_at | 0.0003958 | -0.3985 |
| Bt.23668.1.A1\_at | 0.0003959 | -0.7766 |
| Bt.9930.1.S1\_at | 0.0003967 | 1.02 |
| Bt.8106.1.S1\_at | 0.0003968 | 0.7728 |
| Bt.21372.1.S1\_at | 0.0003983 | 1.238 |
| Bt.12039.1.S1\_at | 0.0003998 | 1.428 |
| Bt.20582.1.S1\_at | 0.0003999 | 1.353 |
| Bt.6679.1.S1\_at | 0.0004005 | 0.9842 |
| Bt.3448.1.S1\_at | 0.0004007 | -1.594 |
| Bt.13581.1.S1\_at | 0.0004008 | 1.489 |
| Bt.11966.1.A1\_at | 0.000401 | 1.056 |
| Bt.6673.1.S1\_at | 0.0004015 | -0.6026 |
| Bt.5302.1.S1\_a\_at | 0.0004024 | 0.9062 |
| Bt.20965.1.S1\_at | 0.0004029 | -0.9569 |
| Bt.6950.1.S1\_at | 0.0004029 | -0.8413 |
| Bt.4797.1.S1\_at | 0.0004031 | -1.228 |
| Bt.65.1.S1\_at | 0.0004036 | -0.7592 |
| Bt.11168.1.S1\_at | 0.0004039 | 0.8693 |
| Bt.11925.1.A1\_at | 0.0004054 | 0.6343 |
| Bt.3998.1.S1\_at | 0.0004054 | 0.4539 |
| Bt.27800.1.A1\_at | 0.0004055 | -0.9747 |
| Bt.3975.1.S1\_at | 0.0004067 | -0.7554 |
| Bt.12603.1.S1\_at | 0.000407 | 0.8354 |
| Bt.16094.1.S1\_at | 0.0004074 | 1.664 |
| Bt.25759.1.A1\_at | 0.0004079 | 0.8335 |
| Bt.5313.1.S1\_at | 0.0004085 | 0.9901 |
| Bt.5536.1.S1\_at | 0.0004097 | 1.129 |
| Bt.20821.1.A1\_at | 0.0004099 | -0.2895 |
| Bt.18739.1.A1\_at | 0.0004111 | -0.2781 |
| Bt.24896.1.A1\_at | 0.0004113 | 0.7806 |
| Bt.24381.1.S1\_at | 0.0004132 | 2 |
| Bt.17672.1.S1\_at | 0.0004148 | 1.144 |
| Bt.3036.1.S1\_at | 0.0004199 | 0.4349 |
| Bt.11027.1.A1\_at | 0.0004204 | 1.36 |
| Bt.25069.1.A1\_at | 0.0004205 | -0.6134 |
| Bt.20872.1.A1\_at | 0.0004207 | 0.8034 |
| Bt.16831.1.A1\_at | 0.0004207 | 1.245 |
| Bt.1175.1.S1\_at | 0.0004216 | -1.412 |
| Bt.11588.1.A1\_at | 0.0004234 | 0.8788 |
| Bt.19574.1.A1\_at | 0.0004242 | -0.302 |
| Bt.15839.1.S1\_at | 0.0004249 | -1.426 |
| Bt.7980.1.S1\_at | 0.0004252 | 0.962 |
| Bt.3260.1.S1\_at | 0.0004277 | -0.8683 |
| Bt.28783.1.A1\_at | 0.0004285 | -1.305 |
| Bt.9136.1.S1\_at | 0.0004288 | 0.5766 |
| Bt.19482.1.A1\_at | 0.0004289 | -1.929 |
| Bt.13028.1.S1\_at | 0.0004293 | 2.97 |
| Bt.5277.2.S1\_a\_at | 0.0004297 | -1.517 |
| Bt.23094.6.A1\_at | 0.0004298 | -1.679 |
| Bt.19485.1.A1\_at | 0.0004324 | -1.33 |
| Bt.6089.1.S1\_at | 0.0004333 | -0.5671 |
| Bt.15972.1.S1\_at | 0.0004336 | 0.6935 |
| Bt.2230.1.S1\_at | 0.000434 | 1.36 |
| Bt.9828.1.S1\_at | 0.0004347 | -1.381 |
| Bt.4007.1.S1\_at | 0.0004354 | -0.7724 |
| Bt.6570.2.S1\_at | 0.0004372 | 1.427 |
| Bt.4294.1.S1\_at | 0.0004379 | -0.6701 |
| Bt.1729.1.A1\_at | 0.0004392 | 0.8386 |
| Bt.2483.1.A1\_at | 0.0004404 | -0.2678 |
| Bt.21876.1.A1\_at | 0.0004413 | 1.067 |
| Bt.12870.1.S1\_at | 0.0004421 | -0.6048 |
| Bt.935.1.A1\_at | 0.0004424 | -1.356 |
| Bt.22325.2.A1\_at | 0.0004428 | 0.7918 |
| Bt.6043.1.S1\_at | 0.0004431 | 1.114 |
| Bt.10321.1.S1\_at | 0.0004453 | 0.7441 |
| Bt.23994.1.A1\_at | 0.0004457 | 0.5346 |
| Bt.6116.1.S1\_a\_at | 0.0004472 | 0.8447 |
| Bt.18039.1.S1\_at | 0.0004472 | 0.8293 |
| Bt.19958.1.A1\_at | 0.0004475 | -0.2242 |
| Bt.20780.2.S1\_a\_at | 0.0004492 | -1.198 |
| Bt.5319.2.S1\_a\_at | 0.0004495 | -0.5942 |
| Bt.10922.1.A1\_at | 0.000451 | 1.507 |
| Bt.15790.2.S1\_at | 0.0004521 | -0.7202 |
| Bt.15915.1.S1\_at | 0.0004542 | 0.9722 |
| Bt.22355.1.S1\_at | 0.0004551 | 0.9548 |
| Bt.5328.1.S1\_at | 0.0004567 | -1.327 |
| Bt.1553.2.S1\_a\_at | 0.0004571 | -0.62 |
| Bt.25291.1.A1\_at | 0.0004601 | -0.1861 |
| Bt.7133.1.S1\_at | 0.0004606 | -1.007 |
| Bt.8392.1.S1\_at | 0.0004609 | 1.115 |
| Bt.16437.1.S1\_at | 0.0004611 | -0.6791 |
| Bt.4194.1.S1\_at | 0.0004615 | 0.7702 |
| Bt.5459.1.S1\_at | 0.0004662 | -1.02 |
| Bt.25002.1.S1\_at | 0.0004665 | -0.9838 |
| Bt.25052.1.S1\_at | 0.0004667 | 0.8601 |
| Bt.19569.1.A1\_at | 0.000467 | -0.4653 |
| Bt.26750.1.S1\_at | 0.0004673 | -2.393 |
| Bt.5599.1.S1\_at | 0.0004678 | 0.6724 |
| Bt.28755.1.S1\_at | 0.0004707 | 0.5384 |
| Bt.17641.1.A1\_at | 0.0004715 | -1.32 |
| Bt.27315.1.S1\_at | 0.0004719 | -0.4686 |
| Bt.21766.1.A1\_at | 0.0004729 | -1.696 |
| Bt.5445.2.A1\_at | 0.0004731 | 0.8053 |
| Bt.19669.1.A1\_at | 0.000475 | -0.4863 |
| Bt.8486.2.S1\_at | 0.0004761 | 0.797 |
| Bt.26095.1.A1\_at | 0.0004765 | 1.768 |
| Bt.7612.1.S1\_at | 0.0004774 | -0.7199 |
| Bt.8286.1.S1\_at | 0.0004784 | -1.158 |
| Bt.2717.1.A1\_at | 0.0004785 | 2.045 |
| Bt.21487.2.S1\_at | 0.0004786 | -0.8416 |
| Bt.17092.1.A1\_at | 0.0004799 | -0.8594 |
| Bt.23649.1.A1\_at | 0.0004802 | 0.917 |
| Bt.28194.1.S1\_at | 0.0004812 | 0.6491 |
| Bt.22264.2.A1\_a\_at | 0.0004833 | 1.425 |
| Bt.3814.1.S1\_at | 0.0004837 | 1.301 |
| Bt.6019.1.S1\_at | 0.0004838 | -0.7142 |
| Bt.3302.1.S1\_at | 0.0004846 | 0.589 |
| Bt.7959.1.A1\_at | 0.0004846 | -0.2273 |
| Bt.13351.1.S1\_at | 0.0004853 | 0.9087 |
| Bt.22553.1.A1\_at | 0.0004862 | 0.8504 |
| Bt.13496.2.S1\_a\_at | 0.0004864 | 0.7615 |
| Bt.20672.1.S1\_at | 0.000487 | 1.119 |
| Bt.3500.1.S1\_at | 0.0004878 | -0.6038 |
| Bt.17343.1.S1\_at | 0.000488 | 1.019 |
| Bt.20456.1.S1\_at | 0.0004883 | -1.611 |
| Bt.8736.2.S1\_at | 0.0004888 | 0.7287 |
| Bt.12466.1.A1\_at | 0.0004905 | 0.8498 |
| Bt.4964.1.S1\_at | 0.000492 | 1.395 |
| Bt.29900.1.S1\_at | 0.0004959 | 1.667 |
| Bt.668.1.S1\_at | 0.0004982 | 0.8562 |
| Bt.18634.2.S1\_at | 0.0004988 | 1.088 |
| Bt.7041.1.S1\_at | 0.000499 | 0.7554 |
| Bt.4406.1.S1\_at | 0.0004995 | 0.3751 |
| Bt.11711.1.A1\_at | 5e-04 | 3.048 |
| Bt.7962.1.S1\_at | 0.0005035 | 2.187 |
| Bt.25966.1.A1\_at | 0.0005041 | 0.6251 |
| Bt.7788.1.S1\_at | 0.0005048 | 1.206 |
| Bt.8807.1.S1\_at | 0.0005084 | 0.9992 |
| Bt.5008.1.S1\_at | 0.0005105 | 0.6504 |
| Bt.27187.1.S1\_at | 0.0005112 | -0.9451 |
| Bt.19483.1.A1\_at | 0.0005113 | -0.3055 |
| Bt.17079.2.A1\_at | 0.0005136 | 0.8948 |
| Bt.2391.1.S1\_at | 0.0005161 | 0.623 |
| Bt.15837.1.S1\_at | 0.0005164 | -0.454 |
| Bt.10333.1.S1\_at | 0.0005179 | 0.5403 |
| Bt.15607.1.S1\_at | 0.0005185 | 1.158 |
| Bt.2098.1.S1\_at | 0.0005219 | 0.6631 |
| Bt.22163.1.S1\_at | 0.000522 | -2.32 |
| Bt.3372.2.S1\_a\_at | 0.000523 | -1.075 |
| Bt.22084.2.S1\_at | 0.0005246 | 1.554 |
| Bt.21433.2.S1\_at | 0.0005247 | 1.647 |
| Bt.19355.1.A1\_at | 0.0005255 | 1.94 |
| Bt.23770.1.A1\_at | 0.0005258 | 1.41 |
| Bt.24470.1.S1\_a\_at | 0.0005267 | 1.072 |
| Bt.29216.1.S1\_at | 0.0005268 | -0.2356 |
| Bt.24921.1.A1\_at | 0.0005271 | -0.2139 |
| Bt.16380.2.A1\_at | 0.0005292 | 1.028 |
| Bt.6087.1.S1\_at | 0.0005294 | 0.4268 |
| Bt.4520.1.S1\_at | 0.0005294 | 0.4475 |
| Bt.18930.1.A1\_at | 0.0005331 | -0.2316 |
| Bt.935.2.S1\_at | 0.0005334 | -0.9829 |
| Bt.20230.1.S1\_at | 0.0005342 | 1.044 |
| Bt.28012.1.A1\_at | 0.0005345 | -0.3293 |
| Bt.20481.1.S1\_at | 0.0005358 | 1.256 |
| Bt.654.1.A1\_at | 0.0005364 | 0.475 |
| Bt.2798.1.S1\_a\_at | 0.0005373 | 0.6316 |
| Bt.26364.1.A1\_at | 0.0005384 | 1.493 |
| Bt.10159.1.S1\_at | 0.0005415 | -1.06 |
| Bt.9412.2.S1\_at | 0.0005416 | 1.295 |
| Bt.14366.1.S1\_at | 0.0005416 | 1.066 |
| Bt.26815.1.S1\_at | 0.0005427 | 0.7753 |
| Bt.20023.1.S1\_at | 0.000543 | 0.8463 |
| Bt.2466.1.S1\_at | 0.0005432 | -0.5733 |
| Bt.20955.1.A1\_at | 0.0005433 | -1.254 |
| Bt.20692.1.A1\_at | 0.000545 | 0.7283 |
| Bt.20221.1.S1\_at | 0.0005451 | 0.5807 |
| Bt.23143.2.S1\_at | 0.0005457 | -0.817 |
| Bt.20815.1.A1\_at | 0.0005471 | 2.303 |
| Bt.24087.1.A1\_at | 0.0005494 | 0.5914 |
| Bt.21471.1.S1\_at | 0.00055 | 1.635 |
| Bt.20846.1.A1\_at | 0.0005503 | 1.532 |
| Bt.20913.1.S1\_at | 0.0005508 | 0.7551 |
| Bt.24359.1.S1\_at | 0.0005508 | 0.868 |
| Bt.15836.2.S1\_at | 0.000552 | -3.985 |
| Bt.9949.2.S1\_at | 0.0005526 | -1.228 |
| Bt.12498.1.A1\_at | 0.0005534 | 0.6558 |
| Bt.1310.1.S1\_at | 0.0005546 | -0.5973 |
| Bt.4816.1.S1\_at | 0.0005547 | -0.9365 |
| Bt.21997.2.A1\_at | 0.0005552 | 2.229 |
| Bt.27565.1.S1\_at | 0.0005569 | 0.4721 |
| Bt.21410.1.S1\_at | 0.000558 | -0.8121 |
| Bt.23784.1.A1\_at | 0.0005602 | 0.5355 |
| Bt.278.1.S1\_at | 0.0005604 | -2.086 |
| Bt.6171.1.A1\_at | 0.0005615 | -0.9958 |
| Bt.13950.1.A1\_at | 0.000562 | -0.8818 |
| Bt.6399.2.S1\_a\_at | 0.0005627 | 1.334 |
| Bt.2040.1.S1\_at | 0.0005652 | 0.5503 |
| Bt.28836.1.S1\_at | 0.0005659 | 1.058 |
| Bt.7715.1.S1\_at | 0.0005663 | 1.207 |
| Bt.211.1.S1\_at | 0.000568 | -1.922 |
| Bt.18270.2.S1\_at | 0.0005683 | 0.5241 |
| Bt.19412.1.A1\_at | 0.0005704 | -0.2734 |
| Bt.2331.1.A1\_at | 0.0005705 | 1.78 |
| Bt.2179.1.S1\_a\_at | 0.000572 | 0.9311 |
| Bt.27386.2.A1\_at | 0.0005735 | 1.971 |
| Bt.22095.1.S1\_at | 0.0005741 | -0.2976 |
| Bt.1404.1.S1\_at | 0.0005742 | 0.9477 |
| Bt.12371.1.S1\_at | 0.0005757 | -0.5912 |
| Bt.2873.1.S1\_at | 0.0005758 | -0.9301 |
| Bt.19309.3.A1\_at | 0.000576 | 1.277 |
| Bt.16206.2.S1\_at | 0.0005767 | 1.292 |
| Bt.24471.1.S1\_a\_at | 0.0005793 | 1.327 |
| Bt.22362.1.S1\_at | 0.0005797 | -1.08 |
| Bt.18914.1.S1\_at | 0.0005811 | 0.7915 |
| Bt.8333.1.S1\_at | 0.0005838 | -0.8204 |
| Bt.12567.1.A1\_at | 0.0005878 | -0.934 |
| Bt.25202.2.S1\_at | 0.0005882 | 1.731 |
| Bt.27262.1.S1\_at | 0.000594 | -0.9508 |
| Bt.1580.1.S1\_at | 0.0005954 | 1.681 |
| Bt.4480.1.S2\_at | 0.0005963 | 1.204 |
| Bt.11475.1.A1\_at | 0.0005974 | -0.5357 |
| Bt.13847.1.S1\_at | 0.0005978 | -1.285 |
| Bt.6125.1.S1\_at | 0.000598 | 1.021 |
| Bt.13254.1.S1\_at | 0.0005984 | 1.316 |
| Bt.10913.1.S1\_at | 0.0006001 | -0.6818 |
| Bt.4140.1.S1\_at | 0.0006014 | 1.423 |
| Bt.5674.1.S1\_at | 0.0006063 | -1.223 |
| Bt.5866.1.S1\_at | 0.0006083 | -1.363 |
| Bt.12583.1.A1\_at | 0.0006091 | -1.284 |
| Bt.11171.1.S1\_at | 0.0006092 | 0.9147 |
| Bt.16761.1.S1\_a\_at | 0.0006095 | -0.6351 |
| Bt.12961.1.A1\_at | 0.0006096 | -0.2257 |
| Bt.10150.1.S1\_at | 0.0006108 | 0.843 |
| Bt.20974.1.S1\_at | 0.0006122 | 0.7955 |
| Bt.18853.1.A1\_at | 0.0006125 | -0.5978 |
| Bt.3610.1.S1\_at | 0.0006134 | -0.7677 |
| Bt.20340.2.S1\_at | 0.0006163 | 1.317 |
| Bt.7177.1.S1\_at | 0.0006186 | 0.8761 |
| Bt.29733.1.S1\_at | 0.0006214 | -0.5163 |
| Bt.5070.1.S1\_at | 0.0006217 | 0.5011 |
| Bt.4033.1.S1\_at | 0.0006244 | 1.063 |
| Bt.1819.1.S1\_at | 0.0006249 | -0.7628 |
| Bt.2660.1.S1\_at | 0.0006249 | -0.6348 |
| Bt.1950.1.S1\_at | 0.0006261 | -0.8488 |
| Bt.18340.1.A1\_at | 0.0006275 | 0.6045 |
| Bt.13645.1.S1\_at | 0.00063 | -1.687 |
| Bt.2123.1.S1\_at | 0.0006303 | -1.204 |
| Bt.6827.1.S1\_at | 0.0006309 | -0.7362 |
| Bt.11751.1.A1\_at | 0.0006309 | -0.8843 |
| Bt.28786.1.A1\_at | 0.0006309 | -0.7081 |
| Bt.27139.1.S1\_at | 0.000631 | -0.8556 |
| Bt.22615.1.S1\_at | 0.0006349 | -0.7433 |
| Bt.3060.1.S1\_a\_at | 0.0006354 | 1.005 |
| Bt.26080.2.S1\_at | 0.0006364 | 2.168 |
| Bt.24439.1.S1\_at | 0.0006368 | 0.884 |
| Bt.23508.1.A1\_at | 0.0006372 | 0.4389 |
| Bt.7872.1.S1\_at | 0.0006408 | -1.335 |
| Bt.21020.1.S1\_at | 0.0006418 | 1.179 |
| Bt.21231.1.S1\_a\_at | 0.0006444 | 0.3635 |
| Bt.24051.1.S1\_at | 0.0006455 | 0.9312 |
| Bt.9438.1.S1\_at | 0.0006475 | -0.8696 |
| Bt.12217.3.A1\_at | 0.0006487 | 1.501 |
| Bt.12217.1.S1\_at | 0.0006507 | 1.392 |
| Bt.24594.1.A1\_at | 0.0006509 | -2.279 |
| Bt.1768.1.A1\_at | 0.0006509 | -0.8331 |
| Bt.9578.1.S1\_at | 0.0006544 | -0.806 |
| Bt.22170.1.S1\_a\_at | 0.0006545 | -0.9681 |
| Bt.231.1.S1\_at | 0.0006546 | -0.8174 |
| Bt.9698.1.S1\_at | 0.0006551 | -0.7465 |
| Bt.13850.3.S1\_a\_at | 0.0006589 | -0.7486 |
| Bt.4705.1.S1\_at | 0.0006604 | -1.031 |
| Bt.13871.1.S1\_at | 0.0006617 | 0.8542 |
| Bt.22854.1.S1\_at | 0.0006618 | -2.117 |
| Bt.22630.1.S1\_at | 0.0006621 | -1.623 |
| Bt.8920.1.S1\_at | 0.0006628 | 1.11 |
| Bt.20323.1.S1\_at | 0.0006642 | 1.403 |
| Bt.4621.1.S1\_a\_at | 0.0006642 | 0.7549 |
| Bt.2374.1.S2\_at | 0.0006655 | 0.5047 |
| Bt.23229.2.S1\_at | 0.000666 | 0.812 |
| Bt.18079.1.A1\_at | 0.0006681 | -0.714 |
| Bt.17456.1.S1\_at | 0.0006711 | -1.25 |
| Bt.20506.1.S1\_at | 0.0006715 | 0.7904 |
| Bt.24396.1.S1\_at | 0.0006716 | -0.679 |
| Bt.12722.1.A1\_at | 0.0006762 | 1.002 |
| Bt.5434.1.S1\_at | 0.0006762 | -0.7333 |
| Bt.22534.1.S1\_at | 0.0006769 | 1.005 |
| Bt.11182.2.S1\_a\_at | 0.0006778 | 0.9846 |
| Bt.4732.1.S1\_at | 0.0006803 | -1.774 |
| Bt.8966.1.S2\_at | 0.0006805 | -0.7622 |
| Bt.15836.1.S1\_at | 0.0006806 | -3.42 |
| Bt.22511.1.S1\_at | 0.0006808 | -0.8579 |
| Bt.17096.2.A1\_at | 0.0006816 | -0.2671 |
| Bt.25783.1.A1\_at | 0.0006839 | 0.5196 |
| Bt.29175.1.A1\_at | 0.0006841 | 0.6071 |
| Bt.12859.1.S1\_at | 0.0006857 | 0.7722 |
| Bt.8117.1.S1\_at | 0.0006859 | 1.006 |
| Bt.16125.1.A1\_s\_at | 0.0006872 | -1.059 |
| Bt.6926.1.A1\_a\_at | 0.0006879 | 1.374 |
| Bt.6390.1.S1\_at | 0.0006887 | -0.7974 |
| Bt.9089.1.S1\_a\_at | 0.0006899 | -0.7186 |
| Bt.27918.1.S1\_at | 0.0006903 | 1.134 |
| Bt.4783.1.S1\_at | 0.0006913 | 0.3765 |
| Bt.22170.1.S1\_at | 0.0006924 | -1.126 |
| Bt.25015.1.A1\_at | 0.0006925 | 1.001 |
| Bt.22198.1.S1\_at | 0.0006944 | 1.115 |
| Bt.10135.1.A1\_at | 0.0006964 | 1.163 |
| Bt.17213.1.A1\_at | 0.0006971 | -0.2464 |
| Bt.12108.1.S1\_at | 0.0006972 | -1 |
| Bt.19079.1.A1\_s\_at | 0.0006972 | -0.3871 |
| Bt.1993.1.S1\_a\_at | 0.0006998 | 1.508 |
| Bt.22594.3.S1\_at | 0.0007003 | -0.2497 |
| Bt.24903.1.A1\_at | 0.0007014 | -0.3185 |
| Bt.2007.1.S1\_at | 0.0007025 | -1.176 |
| Bt.16041.2.S1\_at | 0.0007027 | 1.736 |
| Bt.1151.1.S1\_at | 0.0007039 | -2.762 |
| Bt.18284.1.A1\_at | 0.0007044 | -0.2717 |
| Bt.9114.1.A1\_at | 0.0007048 | -0.8121 |
| Bt.7575.1.A1\_at | 0.0007062 | -1.314 |
| Bt.22125.1.S1\_at | 0.000707 | 1.238 |
| Bt.2225.1.S1\_at | 0.000708 | 2.143 |
| Bt.21715.1.S1\_at | 0.0007082 | 1.073 |
| Bt.2099.1.S1\_at | 0.0007101 | -0.3738 |
| Bt.4138.2.S1\_at | 0.0007129 | 0.6289 |
| Bt.19826.1.A1\_at | 0.0007184 | -1.499 |
| Bt.25887.1.A1\_at | 0.0007207 | 1.354 |
| Bt.20314.2.S1\_at | 0.000723 | -0.9088 |
| Bt.21215.1.A1\_at | 0.0007246 | 1.062 |
| Bt.22736.1.S1\_at | 0.0007246 | 1.21 |
| Bt.12249.1.S1\_at | 0.0007247 | 0.8375 |
| Bt.9866.1.S1\_at | 0.0007259 | -1.214 |
| Bt.13722.2.A1\_at | 0.0007304 | 0.6943 |
| Bt.16737.1.A1\_at | 0.000731 | 0.9321 |
| Bt.13408.2.S1\_at | 0.000732 | 0.9903 |
| Bt.850.1.S1\_at | 0.0007336 | 0.8072 |
| Bt.12263.1.S1\_at | 0.0007358 | 0.8455 |
| Bt.18423.1.A1\_at | 0.0007392 | -0.3935 |
| Bt.4384.1.S1\_at | 0.0007434 | 0.8366 |
| Bt.25229.1.A1\_at | 0.0007437 | -0.2407 |
| Bt.23315.1.S1\_at | 0.0007437 | 0.8837 |
| Bt.12959.1.S1\_at | 0.0007459 | 0.6136 |
| Bt.2070.2.A1\_at | 0.0007465 | -1.295 |
| Bt.28462.1.S1\_at | 0.0007469 | -1.177 |
| Bt.4942.1.S1\_at | 0.0007471 | -0.6629 |
| Bt.2816.1.S1\_at | 0.0007492 | -0.5116 |
| Bt.616.1.S1\_at | 0.0007493 | 0.432 |
| Bt.3516.1.S1\_at | 0.0007509 | -0.7171 |
| Bt.17731.1.A1\_at | 0.000752 | 0.4733 |
| Bt.16011.1.S1\_at | 0.0007589 | -0.3391 |
| Bt.19598.1.A1\_at | 0.000763 | -0.4967 |
| Bt.26992.2.S1\_at | 0.0007674 | 1.207 |
| Bt.10359.1.S1\_at | 0.0007686 | 1.059 |
| Bt.5467.1.S2\_at | 0.0007703 | 0.4638 |
| Bt.532.1.S1\_at | 0.0007705 | -0.696 |
| Bt.901.2.S1\_a\_at | 0.0007706 | -0.578 |
| Bt.18990.1.S1\_at | 0.000771 | 0.9504 |
| Bt.8028.1.S1\_at | 0.0007717 | -0.7069 |
| Bt.12291.1.S1\_at | 0.0007766 | -1.196 |
| Bt.13706.1.A1\_at | 0.0007783 | -0.2746 |
| Bt.15440.1.A1\_at | 0.0007799 | -0.3541 |
| Bt.23746.1.A1\_at | 0.0007853 | -0.2132 |
| Bt.14122.1.A1\_at | 0.0007862 | 1.388 |
| Bt.18177.2.S1\_at | 0.0007863 | 0.2577 |
| Bt.21480.1.A1\_s\_at | 0.0007881 | 0.6917 |
| Bt.5039.1.S1\_at | 0.0007885 | 0.5725 |
| Bt.22245.1.S1\_at | 0.0007897 | 0.4078 |
| Bt.561.1.S1\_at | 0.0007903 | -1.652 |
| Bt.26915.2.S1\_at | 0.0007915 | 1.38 |
| Bt.2120.2.S1\_at | 0.0007943 | 2.911 |
| Bt.7266.1.S1\_at | 0.0007973 | -1.374 |
| Bt.22074.1.S1\_at | 0.0008001 | -0.7969 |
| Bt.2853.1.S1\_at | 0.0008013 | -1.088 |
| Bt.13588.3.A1\_at | 0.0008026 | -2.9 |
| Bt.3284.2.A1\_at | 0.0008065 | -0.3684 |
| Bt.1606.1.S1\_at | 0.0008065 | 1.486 |
| Bt.8809.1.S1\_at | 0.0008097 | -2.684 |
| Bt.16628.2.A1\_at | 0.0008151 | 1.309 |
| Bt.7716.1.S1\_at | 0.0008158 | 0.961 |
| Bt.2599.1.S1\_at | 0.0008163 | 1.97 |
| Bt.7849.1.S1\_at | 0.0008194 | 0.3217 |
| Bt.20210.1.S1\_at | 0.0008211 | 0.838 |
| Bt.6816.1.S1\_at | 0.0008241 | 0.7969 |
| Bt.26019.1.A1\_at | 0.0008245 | -0.3295 |
| Bt.16125.1.A1\_at | 0.0008306 | -0.6213 |
| Bt.19522.1.S1\_at | 0.0008348 | -0.5331 |
| Bt.9944.1.S1\_at | 0.0008354 | 2.052 |
| Bt.3583.1.S1\_at | 0.0008358 | 0.7716 |
| Bt.10421.2.S1\_at | 0.0008364 | 0.8357 |
| Bt.9633.2.S1\_at | 0.0008377 | 1.26 |
| Bt.18798.1.A1\_at | 0.0008399 | -0.1865 |
| Bt.18481.1.S1\_at | 0.0008444 | 0.5108 |
| Bt.26837.1.S1\_at | 0.0008456 | 0.7533 |
| Bt.15687.2.S1\_at | 0.0008467 | 0.8475 |
| Bt.18570.2.A1\_s\_at | 0.0008485 | -0.5862 |
| Bt.4394.1.S1\_at | 0.000849 | 0.5577 |
| Bt.9828.1.S2\_at | 0.0008528 | -0.6269 |
| Bt.11315.1.A1\_at | 0.0008537 | 1.327 |
| Bt.5872.1.S1\_at | 0.0008588 | 0.4703 |
| Bt.17987.1.A1\_at | 0.0008627 | -0.3309 |
| Bt.11014.1.S1\_at | 0.0008635 | -1.808 |
| Bt.4544.1.S1\_at | 0.0008649 | 0.8962 |
| Bt.6651.1.S1\_at | 0.0008686 | 1.437 |
| Bt.8122.1.S1\_a\_at | 0.0008687 | 0.7498 |
| Bt.27221.1.S1\_at | 0.0008711 | 2.119 |
| Bt.19593.1.A1\_at | 0.0008719 | -0.3536 |
| Bt.5788.1.S1\_at | 0.0008721 | 0.7742 |
| Bt.12867.3.S1\_at | 0.0008725 | 0.8087 |
| Bt.7641.2.S1\_a\_at | 0.0008766 | -2.23 |
| Bt.8641.1.A1\_at | 0.0008767 | 1.048 |
| Bt.26513.1.S1\_at | 0.0008779 | 2.249 |
| Bt.6768.1.S1\_at | 0.0008811 | 0.9908 |
| Bt.4221.1.S1\_at | 0.0008819 | 1.07 |
| Bt.10324.1.A1\_at | 0.0008844 | -1.328 |
| Bt.5465.2.A1\_a\_at | 0.0008862 | -0.9572 |
| Bt.25044.1.S1\_at | 0.0008911 | -1.446 |
| Bt.19759.1.S1\_at | 0.0009047 | 1.088 |
| Bt.24348.1.A1\_at | 0.0009059 | 1.412 |
| Bt.27211.1.S1\_at | 0.0009063 | -1.273 |
| Bt.11126.1.S1\_at | 0.0009074 | 1.363 |
| Bt.22647.3.A1\_at | 0.0009091 | 2.026 |
| Bt.9847.1.S1\_at | 0.0009105 | 0.915 |
| Bt.8439.1.S1\_at | 0.0009107 | 0.3764 |
| Bt.19907.1.A1\_at | 0.0009107 | -0.1925 |
| Bt.26710.1.S1\_at | 0.0009119 | 1.291 |
| Bt.29938.1.S1\_at | 0.0009144 | 0.949 |
| Bt.12867.2.S1\_at | 0.0009148 | 0.6562 |
| Bt.12712.1.A1\_at | 0.0009154 | 0.786 |
| Bt.20071.1.A1\_at | 0.0009155 | 0.9852 |
| Bt.9959.3.S1\_a\_at | 0.0009165 | -0.9017 |
| Bt.20888.1.S1\_at | 0.0009168 | -0.3512 |
| Bt.24219.1.A1\_at | 0.0009168 | 0.7154 |
| Bt.23486.3.S1\_at | 0.0009222 | 2.163 |
| Bt.12297.1.S1\_at | 0.0009273 | -1.581 |
| Bt.12906.1.S1\_at | 0.000928 | 1.246 |
| Bt.2989.3.S1\_at | 0.0009281 | -1.366 |
| Bt.742.1.S2\_at | 0.0009295 | 0.3658 |
| Bt.9242.1.S1\_at | 0.0009305 | -0.9166 |
| Bt.3050.1.S1\_at | 0.0009405 | -0.6951 |
| Bt.17943.1.S1\_at | 0.0009407 | 0.918 |
| Bt.22005.1.S1\_at | 0.0009443 | 0.8245 |
| Bt.12379.1.S1\_at | 0.0009446 | 0.964 |
| Bt.13216.3.S1\_at | 0.0009484 | -0.7039 |
| Bt.8078.1.S1\_at | 0.0009489 | -0.6905 |
| Bt.26403.1.A1\_at | 0.0009493 | -0.5548 |
| Bt.8927.1.S1\_at | 0.0009538 | -0.2434 |
| Bt.4599.1.S1\_at | 0.0009544 | -0.9091 |
| Bt.4748.1.S1\_at | 0.0009547 | -1.095 |
| Bt.16072.1.S1\_at | 0.0009563 | 0.4181 |
| Bt.22069.1.A1\_at | 0.0009567 | 0.74 |
| Bt.17253.1.A1\_at | 0.000957 | 0.7534 |
| Bt.21371.1.A1\_at | 0.0009589 | 0.5802 |
| Bt.20322.2.S1\_at | 0.0009596 | -0.8843 |
| Bt.23572.1.S1\_at | 0.0009604 | 1.118 |
| Bt.26629.1.S1\_a\_at | 0.0009611 | -1.086 |
| Bt.9278.1.S1\_at | 0.0009645 | 0.7897 |
| Bt.10793.1.A1\_at | 0.0009664 | -0.1897 |
| Bt.18710.2.A1\_at | 0.0009682 | -0.4002 |
| Bt.20172.1.S1\_at | 0.0009706 | 1.003 |
| Bt.712.1.S2\_at | 0.0009717 | -0.9887 |
| Bt.5112.1.A1\_a\_at | 0.0009719 | -1.304 |
| Bt.16750.2.A1\_at | 0.0009723 | -0.5279 |
| Bt.9076.1.S1\_at | 0.0009724 | -0.6066 |
| Bt.27380.1.A1\_at | 0.0009734 | -0.2347 |
| Bt.23274.1.S1\_at | 0.0009737 | 0.5008 |
| Bt.1899.1.A1\_at | 0.0009747 | 1.315 |
| Bt.8667.1.S1\_at | 0.000975 | -0.2606 |
| Bt.3773.1.S1\_at | 0.0009758 | 0.3182 |
| Bt.18356.1.S1\_at | 0.0009759 | 0.56 |
| Bt.28699.1.S1\_at | 0.0009767 | 1.01 |
| Bt.14207.1.S1\_at | 0.0009771 | -1.594 |
| Bt.17299.2.S1\_at | 0.0009777 | -0.3023 |
| Bt.22442.3.S1\_a\_at | 0.0009786 | -0.8245 |
| Bt.13537.1.A1\_at | 0.000981 | -1.173 |
| Bt.28821.1.S1\_at | 0.0009817 | -0.7458 |
| Bt.22122.1.S1\_at | 0.0009863 | 0.8361 |
| Bt.11408.1.A1\_at | 0.0009896 | 1.611 |
| Bt.20435.1.S1\_at | 0.001002 | -0.6575 |
| Bt.9594.1.S1\_at | 0.001007 | 0.6367 |
| Bt.1785.1.A1\_at | 0.001008 | 1.111 |
| Bt.15707.1.S1\_at | 0.00101 | 1.122 |
| Bt.27832.1.S1\_at | 0.001014 | -0.7057 |
| Bt.2046.1.S1\_at | 0.001014 | 1.604 |
| Bt.28728.1.A1\_at | 0.001017 | -1.271 |
| Bt.2017.1.S1\_at | 0.00102 | 0.9028 |
| Bt.26808.1.S1\_at | 0.00102 | -0.9045 |
| Bt.22981.1.S1\_at | 0.001023 | -1.05 |
| Bt.12187.1.S1\_at | 0.001026 | -0.7245 |
| Bt.6865.1.S1\_at | 0.001029 | 1.393 |
| Bt.23468.1.A1\_at | 0.001031 | 0.3918 |
| Bt.28280.2.S1\_at | 0.001032 | -0.6828 |
| Bt.8766.1.S1\_at | 0.001034 | 0.7389 |
| Bt.25296.1.A1\_at | 0.001034 | -0.7458 |
| Bt.1731.1.S1\_at | 0.001035 | 1.239 |
| Bt.4184.1.S2\_at | 0.001036 | 0.4145 |
| Bt.9186.1.A1\_at | 0.001039 | 1.233 |
| Bt.9981.1.S1\_at | 0.001044 | -0.9889 |
| Bt.23599.1.S1\_at | 0.001044 | 0.5286 |
| Bt.236.1.S1\_at | 0.001044 | 1.62 |
| Bt.16139.1.A1\_at | 0.00105 | -0.8837 |
| Bt.26594.1.S1\_at | 0.00105 | -0.8741 |
| Bt.21913.1.S1\_at | 0.001051 | -0.2978 |
| Bt.11585.1.A1\_at | 0.001052 | 0.5634 |
| Bt.2814.1.S1\_at | 0.001056 | -0.5375 |
| Bt.2106.1.S1\_at | 0.001057 | 0.7039 |
| Bt.8715.1.S1\_at | 0.00106 | 0.4794 |
| Bt.22094.1.A1\_at | 0.001061 | 0.9756 |
| Bt.18566.1.A1\_at | 0.001061 | -1.479 |
| Bt.19002.1.A1\_at | 0.001066 | -0.2161 |
| Bt.3890.1.S1\_at | 0.001066 | 0.4948 |
| Bt.21365.1.S1\_a\_at | 0.001069 | 1.398 |
| Bt.3589.2.A1\_at | 0.00107 | 1.332 |
| Bt.12843.1.S1\_at | 0.001074 | 1.031 |
| Bt.6698.1.S1\_at | 0.00108 | -1.297 |
| Bt.19811.1.A1\_at | 0.001081 | -1.199 |
| Bt.9833.1.S1\_a\_at | 0.001082 | -0.8152 |
| Bt.12662.1.S1\_at | 0.001087 | 0.857 |
| Bt.27123.1.A1\_at | 0.00109 | 1.001 |
| Bt.15779.3.S1\_at | 0.001091 | 1.701 |
| Bt.24662.1.S1\_at | 0.001093 | -1.465 |
| Bt.11389.1.S1\_at | 0.001095 | 1.029 |
| Bt.19332.2.A1\_at | 0.001096 | 1.263 |
| Bt.27057.1.A1\_at | 0.001096 | -1.36 |
| Bt.16836.2.A1\_at | 0.0011 | 1.808 |
| Bt.29463.1.S1\_at | 0.001102 | 1.06 |
| Bt.27149.1.S1\_at | 0.001103 | -0.7438 |
| Bt.11390.1.A1\_at | 0.00111 | -1.952 |
| Bt.19269.1.A1\_at | 0.00111 | -0.2792 |
| Bt.26316.1.A1\_at | 0.001113 | 1.515 |
| Bt.7178.1.S1\_at | 0.001114 | -1.314 |
| Bt.1750.1.A1\_at | 0.001114 | 1.02 |
| Bt.28494.2.A1\_x\_at | 0.001123 | -0.1944 |
| Bt.25461.1.A1\_at | 0.001124 | -0.322 |
| Bt.19161.1.S1\_at | 0.001125 | -0.7678 |
| Bt.20458.1.S1\_at | 0.001125 | -0.8544 |
| Bt.11258.2.A1\_a\_at | 0.001125 | -0.4296 |
| Bt.9639.1.S1\_at | 0.001133 | 1.276 |
| Bt.5494.1.S1\_at | 0.00114 | 1.112 |
| Bt.13370.1.A1\_at | 0.001144 | 1.032 |
| Bt.28682.1.A1\_at | 0.001148 | 1.625 |
| Bt.26751.1.S1\_at | 0.001148 | -0.751 |
| Bt.5104.1.S1\_at | 0.00115 | -0.7887 |
| Bt.11058.1.A1\_at | 0.001153 | -0.5334 |
| Bt.12688.2.A1\_at | 0.001155 | 0.8746 |
| Bt.22125.2.A1\_at | 0.001156 | 0.9158 |
| Bt.7770.1.S1\_at | 0.001159 | -0.4554 |
| Bt.5241.1.S1\_at | 0.001159 | -0.8623 |
| Bt.7024.1.A1\_at | 0.001161 | -0.684 |
| Bt.11088.1.S1\_a\_at | 0.001165 | 0.9914 |
| Bt.21698.1.S1\_a\_at | 0.001166 | -0.8755 |
| Bt.4817.1.A1\_at | 0.001166 | 1.445 |
| Bt.4786.1.S2\_at | 0.001168 | 0.5189 |
| Bt.9459.1.S1\_at | 0.001169 | -0.9243 |
| Bt.13900.1.S1\_at | 0.001169 | -1.267 |
| Bt.22639.2.S1\_at | 0.00117 | -1.461 |
| Bt.27400.1.S1\_at | 0.001173 | 1.883 |
| Bt.4756.1.S1\_at | 0.001177 | -0.418 |
| Bt.16064.1.S1\_a\_at | 0.001178 | 0.3522 |
| Bt.3444.1.A1\_at | 0.001181 | -0.7849 |
| Bt.27837.1.S1\_at | 0.001182 | 0.4499 |
| Bt.2513.2.S1\_at | 0.001185 | 0.9573 |
| Bt.12032.1.S1\_at | 0.001187 | 0.6018 |
| Bt.16495.2.A1\_at | 0.001191 | 0.9975 |
| Bt.7924.1.S1\_at | 0.001191 | -0.5225 |
| Bt.21580.1.S1\_at | 0.001193 | 1.024 |
| Bt.20661.1.A1\_at | 0.001194 | -0.4315 |
| Bt.24474.1.S1\_a\_at | 0.001194 | -1.055 |
| Bt.6105.1.S1\_at | 0.001195 | 0.5909 |
| Bt.3447.2.S1\_at | 0.001196 | -1.148 |
| Bt.8869.1.S1\_at | 0.001198 | 0.6645 |
| Bt.18727.1.A1\_at | 0.001201 | -0.9491 |
| Bt.11990.1.S1\_at | 0.001206 | 1.611 |
| Bt.10348.1.A1\_at | 0.001207 | -0.5923 |
| Bt.17460.1.A1\_at | 0.00121 | 1.043 |
| Bt.4618.1.S1\_at | 0.001212 | 0.3492 |
| Bt.13314.1.S1\_at | 0.001217 | 0.7525 |
| Bt.6057.1.S1\_at | 0.001217 | 1.307 |
| Bt.9171.1.A1\_at | 0.001218 | -0.8627 |
| Bt.28733.1.S1\_at | 0.001219 | 0.8233 |
| Bt.16243.1.S1\_at | 0.001219 | 1.398 |
| Bt.20817.2.S1\_at | 0.001221 | 0.7207 |
| Bt.24832.1.A1\_a\_at | 0.001224 | -1.63 |
| Bt.7069.1.S1\_at | 0.001225 | 0.9005 |
| Bt.28178.1.A1\_at | 0.001225 | 0.7653 |
| Bt.22064.1.A1\_at | 0.001226 | 0.5973 |
| Bt.8617.1.S1\_at | 0.00123 | 1.219 |
| Bt.24976.1.S1\_at | 0.001231 | -1.152 |
| Bt.27339.2.S1\_at | 0.001236 | 1.035 |
| Bt.28064.2.S1\_at | 0.001237 | -0.7221 |
| Bt.1678.1.S1\_at | 0.00124 | -0.7928 |
| Bt.4913.3.A1\_at | 0.001242 | -0.4532 |
| BtAffx.29968.1.S1\_at | 0.001242 | -3.767 |
| Bt.17758.1.S1\_at | 0.001243 | 0.6864 |
| Bt.8735.2.S1\_a\_at | 0.001246 | -0.4284 |
| Bt.28212.1.A1\_at | 0.001246 | 1.012 |
| Bt.27.1.S1\_at | 0.001249 | -1.24 |
| Bt.5636.3.S1\_at | 0.001249 | -0.2242 |
| Bt.10101.1.S1\_at | 0.001253 | 0.5946 |
| Bt.23307.1.S1\_at | 0.001256 | 0.9112 |
| Bt.28489.1.S1\_at | 0.001257 | 0.8753 |
| Bt.21133.1.S1\_at | 0.001258 | 2.058 |
| Bt.19712.1.A1\_at | 0.001258 | -0.1936 |
| Bt.28684.1.S1\_at | 0.00126 | -0.9197 |
| Bt.9391.2.S1\_at | 0.00126 | 0.91 |
| Bt.24536.1.S1\_at | 0.00126 | 1.448 |
| Bt.22654.1.A1\_at | 0.001263 | 0.5554 |
| Bt.19382.1.S1\_at | 0.001268 | 0.9381 |
| Bt.19754.1.A1\_at | 0.001271 | -0.4178 |
| Bt.1283.1.S1\_at | 0.001273 | -0.5708 |
| Bt.9221.1.A1\_at | 0.001274 | -0.9316 |
| Bt.12609.1.A1\_at | 0.001277 | 0.9293 |
| Bt.23985.1.A1\_at | 0.001285 | 0.9717 |
| Bt.3741.1.S1\_at | 0.001285 | 0.774 |
| Bt.19898.1.A1\_at | 0.001286 | 0.9378 |
| Bt.8051.1.S1\_at | 0.001288 | -0.4445 |
| Bt.6712.1.S1\_at | 0.00129 | 0.8874 |
| Bt.22375.1.A1\_at | 0.001291 | 0.513 |
| Bt.26995.2.S1\_at | 0.001292 | 0.9144 |
| Bt.8451.1.S1\_a\_at | 0.001292 | -0.6277 |
| Bt.21119.1.A1\_at | 0.001294 | 1.102 |
| Bt.26817.1.S1\_at | 0.001296 | -0.3416 |
| Bt.566.1.S1\_at | 0.001299 | -1.004 |
| Bt.5103.1.S1\_at | 0.001301 | -0.9262 |
| Bt.24802.1.S1\_at | 0.001301 | -2.236 |
| Bt.11857.1.A1\_at | 0.001303 | -0.7535 |
| Bt.8785.1.S1\_at | 0.001303 | 1.065 |
| Bt.15575.1.A1\_at | 0.001303 | -1.012 |
| Bt.8987.1.S1\_at | 0.001307 | -0.1997 |
| Bt.2532.1.S1\_at | 0.001308 | -0.3527 |
| Bt.5377.1.S1\_at | 0.00131 | 0.8264 |
| Bt.5096.1.S1\_at | 0.001312 | -0.7025 |
| Bt.20251.1.S1\_at | 0.001315 | 0.3232 |
| Bt.618.1.S1\_at | 0.001316 | -0.7047 |
| Bt.4967.1.S1\_at | 0.001317 | 0.2481 |
| Bt.5415.1.S1\_at | 0.001318 | 0.9659 |
| Bt.9533.1.S1\_at | 0.001318 | 0.9214 |
| Bt.29853.1.A1\_at | 0.00132 | -1.667 |
| Bt.25436.1.S1\_a\_at | 0.001322 | 1.175 |
| Bt.16931.1.A1\_at | 0.001328 | -0.3281 |
| Bt.6016.1.S1\_at | 0.001328 | -0.5423 |
| Bt.1171.1.S1\_at | 0.00133 | -1.058 |
| Bt.18414.1.S1\_at | 0.001337 | -2.492 |
| Bt.21214.1.S1\_at | 0.001341 | -0.5997 |
| Bt.20290.1.A1\_at | 0.001341 | -0.776 |
| Bt.20629.3.A1\_at | 0.001341 | -0.8603 |
| Bt.9546.2.S1\_a\_at | 0.001343 | 0.9156 |
| Bt.16733.1.A1\_s\_at | 0.001345 | 0.8674 |
| Bt.7421.1.S1\_at | 0.001349 | -1.308 |
| Bt.14395.2.S1\_at | 0.001357 | 1.669 |
| Bt.24524.1.S1\_at | 0.001367 | -0.703 |
| Bt.13669.1.A1\_at | 0.00137 | -0.7193 |
| Bt.26905.1.A1\_at | 0.001371 | 0.6103 |
| Bt.12823.1.S1\_at | 0.001371 | 1.367 |
| Bt.21976.1.S1\_at | 0.001372 | 0.6093 |
| Bt.28419.1.S1\_at | 0.001373 | -0.2591 |
| Bt.28081.1.S1\_at | 0.001375 | 0.8898 |
| Bt.16230.1.S1\_at | 0.001378 | 0.8507 |
| Bt.11270.2.S1\_at | 0.001378 | -1.186 |
| Bt.18002.2.S1\_at | 0.001379 | 1.263 |
| Bt.17555.1.A1\_at | 0.001379 | -0.2114 |
| Bt.27617.1.S1\_at | 0.001381 | 1.057 |
| Bt.12419.1.S1\_at | 0.001382 | 0.8422 |
| Bt.3866.1.S1\_at | 0.001382 | 0.4458 |
| Bt.12912.1.S1\_at | 0.001385 | 1.211 |
| Bt.15867.1.S1\_at | 0.001389 | 1.171 |
| Bt.3388.1.S1\_at | 0.00139 | -0.7313 |
| Bt.23611.1.A1\_at | 0.001391 | -0.7475 |
| Bt.5737.1.S1\_at | 0.001393 | 0.4778 |
| Bt.3014.1.A1\_at | 0.001394 | 1.336 |
| Bt.17041.1.A1\_at | 0.001394 | -0.4082 |
| Bt.28758.1.A1\_at | 0.001401 | 0.8959 |
| Bt.9612.1.S1\_at | 0.001401 | -1.493 |
| Bt.11029.1.S1\_at | 0.001404 | 0.5289 |
| Bt.29739.1.S1\_at | 0.001406 | -0.2232 |
| Bt.5351.1.S1\_a\_at | 0.001407 | 1.234 |
| Bt.20102.1.S1\_at | 0.001408 | -2.105 |
| Bt.2129.1.S1\_at | 0.001408 | 1.128 |
| Bt.21534.3.S1\_a\_at | 0.001411 | -0.817 |
| Bt.21404.1.S1\_at | 0.001414 | 0.8222 |
| Bt.24700.1.A1\_at | 0.001414 | -0.2446 |
| Bt.20198.1.S1\_at | 0.001415 | -0.2621 |
| Bt.19933.1.S1\_at | 0.001419 | 0.8138 |
| Bt.26779.1.S1\_at | 0.00142 | -1.918 |
| Bt.16449.1.A1\_at | 0.001425 | -0.2062 |
| Bt.12558.1.S1\_at | 0.001426 | 0.975 |
| Bt.9220.1.S1\_at | 0.001427 | 0.7088 |
| Bt.13357.1.S1\_at | 0.001429 | 0.7855 |
| Bt.2850.1.A1\_at | 0.00143 | 1.431 |
| Bt.23456.1.A1\_s\_at | 0.00143 | 0.5799 |
| Bt.4070.1.S1\_at | 0.001432 | -0.8569 |
| Bt.2311.1.S1\_at | 0.001434 | 0.7677 |
| Bt.1404.2.S1\_at | 0.001434 | 0.464 |
| Bt.17045.1.A1\_at | 0.001435 | -0.2101 |
| Bt.21351.1.S1\_at | 0.001439 | 1.082 |
| Bt.9176.1.A1\_at | 0.00144 | 1.08 |
| Bt.24276.1.A1\_at | 0.001443 | 0.5645 |
| Bt.2151.2.A1\_at | 0.001444 | 1.273 |
| Bt.8609.1.S1\_s\_at | 0.001448 | -0.5581 |
| Bt.14143.1.S1\_at | 0.001455 | 1.662 |
| Bt.16025.1.S1\_at | 0.001457 | -0.3277 |
| Bt.23531.1.S1\_at | 0.001457 | -0.41 |
| Bt.5587.1.S1\_at | 0.001463 | 0.9209 |
| Bt.7572.1.S1\_at | 0.001471 | 0.7845 |
| Bt.27099.1.A1\_at | 0.001473 | 0.7592 |
| Bt.22527.1.A1\_at | 0.001473 | 0.5942 |
| Bt.22414.2.S1\_at | 0.001482 | 2.776 |
| Bt.16040.1.S1\_at | 0.001483 | 0.6554 |
| Bt.28326.1.S1\_at | 0.001483 | 0.4585 |
| Bt.27223.2.S1\_a\_at | 0.001484 | -1.467 |
| Bt.20189.1.S1\_at | 0.001485 | 0.6188 |
| Bt.14054.2.S1\_at | 0.001486 | -1.64 |
| Bt.194.1.S1\_at | 0.001489 | -2.533 |
| Bt.9069.1.S1\_at | 0.001489 | -0.3891 |
| Bt.4049.1.S1\_at | 0.001498 | 0.812 |
| Bt.12299.1.A1\_at | 0.001499 | 2.363 |
| Bt.6938.1.S1\_at | 0.001503 | -0.8144 |
| Bt.18999.1.S1\_at | 0.001507 | -0.7647 |
| Bt.4550.1.A1\_at | 0.001508 | 0.9794 |
| Bt.24109.1.S1\_at | 0.001508 | -2.078 |
| Bt.15809.2.S1\_a\_at | 0.001511 | -0.5247 |
| Bt.18229.1.A1\_at | 0.001513 | 0.7023 |
| Bt.27129.1.S1\_at | 0.001514 | 0.4705 |
| Bt.17890.1.A1\_at | 0.001518 | -0.2527 |
| Bt.9197.1.S1\_at | 0.001523 | 1.071 |
| Bt.19258.1.A1\_at | 0.001525 | 1.192 |
| Bt.11100.1.S1\_at | 0.001529 | -0.7971 |
| Bt.20562.1.S1\_at | 0.001534 | -0.3563 |
| Bt.3387.1.S1\_at | 0.001534 | 0.7234 |
| Bt.8090.2.S1\_at | 0.001537 | -1.025 |
| Bt.9660.1.A1\_s\_at | 0.001539 | 0.6002 |
| Bt.1932.1.S1\_at | 0.001541 | -0.8748 |
| Bt.13489.1.S1\_at | 0.001544 | 0.7199 |
| Bt.22380.1.S1\_at | 0.001548 | 1.838 |
| Bt.14058.1.S1\_at | 0.001551 | -0.5449 |
| Bt.9611.1.A1\_at | 0.00156 | 0.9577 |
| Bt.9741.1.S1\_at | 0.001562 | 0.8335 |
| Bt.9303.1.S1\_at | 0.001568 | 0.6053 |
| Bt.13741.2.S1\_at | 0.001571 | 1.816 |
| Bt.23091.1.S1\_at | 0.001574 | -1.217 |
| Bt.21634.1.S1\_at | 0.001575 | -0.5911 |
| Bt.19153.1.A1\_at | 0.001576 | -0.2934 |
| Bt.17777.3.S1\_at | 0.001577 | -0.9766 |
| Bt.3382.1.S1\_at | 0.001581 | 0.3819 |
| Bt.17807.1.A1\_at | 0.001581 | -0.1985 |
| Bt.4950.1.S1\_at | 0.001585 | 1.378 |
| Bt.7714.1.S1\_at | 0.001587 | -0.613 |
| Bt.26747.1.A1\_at | 0.001589 | 1.029 |
| Bt.19522.2.S1\_at | 0.001592 | 0.6654 |
| Bt.4970.1.S1\_at | 0.001599 | -0.8911 |
| Bt.20061.1.S1\_at | 0.001601 | -0.927 |
| Bt.5602.1.A1\_at | 0.001601 | 1.357 |
| Bt.7574.1.S1\_at | 0.001601 | 0.6294 |
| Bt.13953.1.A1\_at | 0.001603 | -1.348 |
| Bt.28982.1.A1\_at | 0.001606 | -0.1833 |
| Bt.24627.2.A1\_a\_at | 0.001608 | -0.8187 |
| Bt.20698.1.A1\_at | 0.00161 | -0.229 |
| Bt.21226.2.S1\_at | 0.001612 | 1.099 |
| Bt.25081.1.S1\_at | 0.001613 | 1.007 |
| Bt.10631.2.S1\_at | 0.001615 | 1.87 |
| Bt.11599.2.S1\_at | 0.001615 | 2.455 |
| Bt.19864.1.A1\_at | 0.001617 | -0.2198 |
| Bt.27160.1.A1\_at | 0.001618 | 1.225 |
| Bt.19624.1.A1\_at | 0.001619 | -0.2745 |
| Bt.23179.1.S1\_at | 0.001621 | -0.844 |
| Bt.7968.1.S1\_at | 0.001623 | -0.5255 |
| Bt.24528.1.A1\_at | 0.001623 | -0.3246 |
| Bt.20431.2.S1\_at | 0.001626 | 1.453 |
| Bt.18513.1.S1\_at | 0.001626 | -0.5116 |
| Bt.3183.3.A1\_at | 0.001628 | 0.3909 |
| Bt.22011.1.S1\_at | 0.001637 | 0.8686 |
| Bt.12661.2.S1\_at | 0.001638 | 0.8588 |
| Bt.20362.2.S1\_at | 0.00164 | 1.296 |
| Bt.2821.1.S1\_at | 0.001642 | -0.5581 |
| Bt.10260.1.S1\_at | 0.001644 | 1.116 |
| Bt.2092.1.S1\_at | 0.001644 | 0.8415 |
| Bt.23551.3.S1\_at | 0.001646 | -0.7043 |
| Bt.23326.1.S1\_at | 0.001652 | 0.4751 |
| Bt.26613.1.A1\_at | 0.001661 | -0.9606 |
| Bt.21275.1.S1\_at | 0.001666 | 0.9547 |
| Bt.16546.1.S1\_at | 0.001669 | 0.9112 |
| Bt.23861.1.A1\_at | 0.001673 | -0.2083 |
| Bt.1507.1.S1\_at | 0.001673 | 0.5388 |
| Bt.4972.2.S1\_at | 0.001674 | 1.101 |
| Bt.22187.1.S1\_at | 0.001679 | 0.8995 |
| Bt.19859.1.S1\_at | 0.001684 | -0.9037 |
| Bt.5748.1.S1\_at | 0.001688 | 1.158 |
| Bt.15763.1.S1\_at | 0.001688 | 0.425 |
| Bt.10116.1.A1\_at | 0.0017 | -0.8113 |
| Bt.2349.1.S1\_at | 0.001701 | -0.2857 |
| Bt.21191.3.S1\_at | 0.001703 | -1.914 |
| Bt.10025.1.A1\_at | 0.001706 | -1.127 |
| Bt.15723.1.S1\_at | 0.001706 | 0.8621 |
| Bt.11931.1.A1\_at | 0.001707 | -0.692 |
| Bt.21380.2.S1\_at | 0.00171 | -0.6746 |
| Bt.23528.1.S1\_at | 0.00171 | -0.7591 |
| Bt.4642.1.A1\_at | 0.001712 | -0.8132 |
| Bt.8024.2.S1\_at | 0.001714 | -0.3994 |
| Bt.13065.1.S1\_at | 0.001716 | 1.505 |
| Bt.16951.1.A1\_at | 0.001718 | 0.9376 |
| Bt.3024.1.S1\_at | 0.001719 | -1.095 |
| Bt.22725.1.S1\_at | 0.001725 | 0.6451 |
| Bt.1927.1.S1\_at | 0.001729 | -0.6195 |
| Bt.20977.2.S1\_at | 0.00173 | 0.7945 |
| Bt.16265.1.S1\_at | 0.001733 | -0.5394 |
| Bt.3849.1.S1\_at | 0.001735 | 0.5433 |
| Bt.16132.2.S1\_a\_at | 0.001743 | -0.6154 |
| Bt.2214.2.A1\_at | 0.001745 | 0.3504 |
| Bt.819.1.S1\_at | 0.00175 | -0.7918 |
| Bt.27626.1.A1\_at | 0.00175 | 0.9997 |
| Bt.14624.2.S1\_at | 0.001751 | -1.033 |
| Bt.18165.1.A1\_at | 0.001752 | -3.031 |
| Bt.14644.1.A1\_at | 0.001753 | 0.8262 |
| Bt.364.1.S2\_at | 0.001753 | 0.9918 |
| Bt.18926.1.A1\_s\_at | 0.001759 | -0.8941 |
| Bt.20288.1.S1\_at | 0.001763 | 0.6348 |
| Bt.26831.1.S1\_at | 0.001764 | 0.8616 |
| Bt.18744.1.A1\_at | 0.001765 | -0.2626 |
| Bt.17932.1.S1\_at | 0.001767 | 1.443 |
| Bt.5350.1.S1\_at | 0.001783 | -0.7037 |
| Bt.22320.3.S1\_at | 0.001786 | 0.7464 |
| Bt.1699.2.S1\_a\_at | 0.001787 | 1.327 |
| Bt.21838.1.A1\_at | 0.001787 | -0.6921 |
| Bt.26520.2.A1\_at | 0.001789 | 0.3418 |
| Bt.23315.1.S1\_a\_at | 0.001798 | 0.9723 |
| Bt.21774.2.S1\_a\_at | 0.001799 | 0.8096 |
| Bt.1318.1.S1\_at | 0.001802 | -0.9224 |
| Bt.24477.1.S1\_a\_at | 0.001807 | 0.6061 |
| Bt.18115.1.A1\_at | 0.001807 | -1.233 |
| Bt.27061.1.S1\_at | 0.001808 | -0.6424 |
| Bt.12745.3.S1\_at | 0.001808 | 1.458 |
| Bt.10262.1.A1\_at | 0.001813 | 0.4994 |
| Bt.11530.1.A1\_at | 0.001815 | 0.7326 |
| Bt.8706.1.S1\_at | 0.001821 | -0.8482 |
| Bt.22702.1.A1\_at | 0.001821 | -0.3247 |
| Bt.11067.1.S1\_at | 0.00183 | 0.8715 |
| Bt.20087.1.S1\_at | 0.001834 | -0.3323 |
| Bt.26286.1.A1\_at | 0.001838 | 1.047 |
| Bt.1953.1.S1\_at | 0.001838 | 1.569 |
| Bt.2612.1.S1\_at | 0.001841 | 0.5038 |
| Bt.27102.1.S1\_at | 0.001843 | -0.5087 |
| Bt.2984.1.A1\_at | 0.001844 | -0.5031 |
| Bt.19735.1.A1\_at | 0.001845 | -0.2149 |
| Bt.9139.1.A1\_at | 0.001847 | -1.974 |
| Bt.7194.1.S1\_at | 0.001848 | -0.6156 |
| Bt.7363.1.S1\_at | 0.001851 | 0.9725 |
| Bt.4565.1.S1\_at | 0.001852 | 1.411 |
| Bt.13390.2.S1\_a\_at | 0.001854 | -0.5928 |
| Bt.21191.2.A1\_at | 0.001856 | -2.362 |
| Bt.27108.1.A1\_at | 0.00186 | 0.5648 |
| Bt.16817.1.A1\_at | 0.001863 | -0.1938 |
| Bt.19266.1.A1\_at | 0.001872 | 1.168 |
| Bt.5037.1.S1\_at | 0.001877 | 0.581 |
| Bt.8054.2.S1\_a\_at | 0.001881 | -0.5369 |
| Bt.12445.2.S1\_at | 0.001882 | 1.259 |
| Bt.10900.1.S1\_at | 0.001882 | -0.8822 |
| Bt.24486.1.A1\_at | 0.001892 | -0.7302 |
| Bt.22168.1.A1\_at | 0.001893 | -1.179 |
| Bt.10056.1.S1\_at | 0.001898 | 0.8635 |
| Bt.24508.1.A1\_at | 0.0019 | 1.502 |
| Bt.27655.1.S1\_at | 0.001903 | -1.521 |
| Bt.19261.1.S1\_at | 0.001904 | 1.104 |
| Bt.12452.2.S1\_at | 0.001904 | -0.6629 |
| Bt.10822.1.S1\_at | 0.001905 | 0.6515 |
| Bt.9609.1.S1\_a\_at | 0.001907 | -0.299 |

*Venn diagram of differential expression for the three different methods.*
