# Supplementary material for: A gene network switch enhances the oxidative capacity of ovine skeletal muscle during late fetal development
Source: BMC Genomics. 2010 Jun 15;11:378. doi: 10.1186/1471-2164-11-378 (PMC2894804; doi:10.1186/1471-2164-11-378)
Supplement: Additional File 1 — Gene expression data. This file contains links to the gene expression data. The genes that are significantly differentially expressed between adjacent development times are listed as Experiment 1 (80 d vs 100 d), Experiment 2 (100 d vs 120 d), Experiment 3 (120 d vs 150 d) and Experiment 4 (150 d vs 230 d) in the section entitled Open microarray analysis report for developmental expression. Each experiment contains an MA plot, Volcano plot and Heatmap for each of the three microarray processing programs MAS5, RMA and GCRMA. For each of these processing programs there is a list of significantly differentially expressed probe sets, and for each of these there is an FDR corrected probability, fold change, percentage of microarrays with a MAS5 Present call, and a convergence percentage for all three microarray processing programs. The file also contains annotation of the probe sets. In a second section entitled Time series analysis of developmental expression, there is a list of genes in each gene expression cluster and diagrams showing MAS5 gene expression values for these genes. [file 1471-2164-11-378-S1.ZIP › Report/Experiment4.html]

## Experiment 4

### Experimental Question

Which genes are differentially expressed between day 150 and day 230?

### Arrays

Array reference names used in the experiment and their corresponding original CEL file names.

| Reference | CEL File |
| --- | --- |
| d150\_1 | af1c1228-12-4555-161007.CEL |
| d150\_2 | af1c1228-14-4576-161007.CEL |
| d150\_3 | af1c1228-15-4580-161007.CEL |
| d230\_1 | af1c1228-10-4531-161007.CEL |
| d230\_2 | af1c1228-11-4544-161007.CEL |
| d230\_3 | af1c1228-13-4572-161007.CEL |

### Probe Summary

Total number of probe sets on chip: 24128 .

Total number of Affymetrix control probe sets on chip: 112 (removed from analysis).

Total number of probe sets detected as absent or marginal on all slides: 8374 (removed from analysis).

Total number of probe sets used in analysis: 15642 .

### Annotation

Annotation of differentially expressed genes for experiment 4.

### Expression Plots

MA plots for MAS5, RMA and GCRMA for contrast 1.

Volcano plots for MAS5, RMA and GCRMA for contrast 1.

Heatmap plots for MAS5, RMA and GCRMA.

### Functional Profile Analyses

Gene Ontology analyses.

### Summary gene list with 646 differentially expressed genes

Briefly - the best starting point for significantly expressed genes is for the genes with a value of 100 in the convergence column and 3 stars (\*\*\*) in the FDR\_Corrected column.

Differential expression was tested on log2 expression intensities obtained using MAS, RMA and GCRMA summarization methods. P-values for each method are shown for the moderated F-statistic which stabilizes the gene-specific variance using an empirical Bayes method to shrink the probe-wise sample variances towards a common value and to augment the degrees of freedom for the individual variances (Smyth,2004). Significance after correction for false discovery rates (FDR) using the method BH for a targeted p-value of 0.01 is also shown for each summarization set (ns - not significant, \* - significant; this means that if the gene has three stars it is significant for all three summarizations, and a very reliable indication that the gene is truly differentially expressed). Log2 fold changes are also shown in the table (remember that a log fold change of one equals a fold change of two). The fold changes are based on the raw data and not the fitted intensities. See the MA and volcano plots for a visual representation. If the gene was detected as present or not in each array is shown in the table in percentage (Presence\_Calls column). The last column shows the convergence in percentage of the gene across the three methods. A value of 100 indicates that the gene was detected as significantly differentially expressed for all three summarizations. The table was filtered for genes with a convergence greater than 50% and called as present in at least half of the samples. Note that the log fold change used is simply the average of the differences of the contrasts, if there is more than one contrast. It does not necessarily mean that the fold change is above the threshold for each individual contrast.

MAS parameters: background correction - for both PM and MM, the lowest 2% of probe intensities are used as a background value for each of 16 grids. Each probe is then adjusted based upon a weighted (distance between the location of the probe and the centroid) average of the background of each grid; normalization - scale normalization, all arrays scaled to the same mean value (200); summarization - log2 scale average using 1-step Tukey biweight.

RMA parameters: background correction - convolution; normalization - quantile; summarization - median polish. Note: MM intensities are not used, which does not account for non-specific binding on the different probes (can underestimate the background).

GCRMA parameters: background correction - an affinity measure model based on probe sequences and MM intensities; normalization - quantile; summarization - median polish.

|  |  |  |  |  |  |  |  |  |  |
| --- | --- | --- | --- | --- | --- | --- | --- | --- | --- |
| **AFFY** | **MAS\_ModFtest** | **RMA\_ModFtest** | **GCRMA\_ModFtest** | **FDR\_Corrected** | **MAS\_FoldChange** | **RMA\_FoldChange** | **GCRMA\_FoldChange** | **Presence\_Calls** | **Convergence** |
| Bt.13986.1.S1\_at | 8.15e-08 | 2.45e-08 | 2.36e-09 | \* \* \* | -1.66 | -1.38 | -1.73 | 100 | 100 |
| Bt.13289.1.S1\_at | 1.16e-07 | 8.03e-08 | 7.5e-07 | \* \* \* | -2.94 | -2.68 | -3.66 | 100 | 100 |
| Bt.4818.1.S1\_at | 1.23e-07 | 2.51e-10 | 8.91e-08 | \* \* \* | 1.62 | 1.76 | 1.88 | 100 | 100 |
| Bt.8972.1.S1\_at | 1.35e-07 | 7.05e-10 | 4.53e-09 | \* \* \* | -2.13 | -1.65 | -2.15 | 100 | 100 |
| Bt.7034.1.S1\_at | 1.4e-07 | 1.92e-09 | 1.18e-06 | \* \* \* | 3.27 | 2.44 | 2.76 | 100 | 100 |
| Bt.9556.1.S1\_at | 3.22e-07 | 3.61e-11 | 1.43e-08 | \* \* \* | 1.92 | 2.2 | 2.74 | 100 | 100 |
| Bt.20501.1.S1\_at | 3.34e-07 | 1.14e-08 | 1.97e-06 | \* \* \* | -4.62 | -4.01 | -5.12 | 100 | 100 |
| Bt.12299.1.A1\_at | 3.96e-07 | 2.29e-08 | 1.27e-06 | \* \* \* | 5.04 | 4.1 | 5.25 | 100 | 100 |
| Bt.6642.2.S1\_at | 4.85e-07 | 1.76e-09 | 1.36e-09 | \* \* \* | -2.73 | -2.29 | -3.92 | 100 | 100 |
| Bt.13289.2.S1\_at | 5.22e-07 | 3.9e-07 | 3.08e-06 | \* \* \* | -3.85 | -2.61 | -3.26 | 100 | 100 |
| Bt.7394.1.A1\_at | 6.39e-07 | 4.52e-07 | 1.39e-06 | \* \* \* | 1.78 | 1.42 | 1.5 | 100 | 100 |
| Bt.25052.1.S1\_at | 8.87e-07 | 2.1e-07 | 1.62e-06 | \* \* \* | 1.42 | 1.25 | 1.6 | 100 | 100 |
| Bt.3071.1.S1\_at | 1.02e-06 | 1.11e-09 | 1.23e-09 | \* \* \* | 1.66 | 1.69 | 2.48 | 100 | 100 |
| Bt.5070.1.S1\_x\_at | 1.12e-06 | 1.46e-05 | 1.99e-05 | \* \* \* | -1.53 | -1.09 | -1.27 | 100 | 100 |
| Bt.6991.1.S1\_at | 1.2e-06 | 1.16e-06 | 2.36e-06 | \* \* \* | -1.58 | -1.05 | -1.45 | 100 | 100 |
| Bt.7133.1.S1\_at | 1.28e-06 | 9.84e-07 | 1.3e-05 | \* \* \* | 1.54 | 1.22 | 1.51 | 100 | 100 |
| Bt.13588.1.A1\_at | 1.31e-06 | 1.83e-07 | 4.44e-06 | \* \* \* | 5.39 | 4.05 | 5.2 | 100 | 100 |
| Bt.15682.1.A1\_at | 2.12e-06 | 4.97e-08 | 1.75e-07 | \* \* \* | 1.96 | 2.43 | 3.14 | 100 | 100 |
| Bt.1035.1.S1\_a\_at | 2.31e-06 | 3.85e-08 | 2.36e-08 | \* \* \* | 1.26 | 1.13 | 1.29 | 100 | 100 |
| Bt.22803.1.S1\_at | 2.53e-06 | 6.01e-08 | 1.03e-06 | \* \* \* | 1.61 | 1.33 | 1.58 | 100 | 100 |
| Bt.25319.2.S1\_at | 2.81e-06 | 1.99e-05 | 5.03e-06 | \* \* \* | -1.28 | -1.03 | -1.51 | 100 | 100 |
| Bt.5340.1.S1\_s\_at | 2.87e-06 | 5.99e-08 | 1.15e-07 | \* \* \* | 1.38 | 1.33 | 1.6 | 100 | 100 |
| Bt.6774.1.S1\_at | 2.98e-06 | 6.69e-06 | 1.98e-07 | \* \* \* | -1.51 | -1.21 | -1.55 | 100 | 100 |
| Bt.4184.1.S2\_at | 3.23e-06 | 5.65e-07 | 3.38e-08 | \* \* \* | 1.12 | 1.17 | 1.24 | 100 | 100 |
| Bt.8213.1.S1\_at | 4.31e-06 | 7.73e-07 | 9.71e-06 | \* \* \* | 1.46 | 1.11 | 1.42 | 100 | 100 |
| Bt.5348.1.S1\_at | 4.37e-06 | 1.01e-07 | 8.32e-06 | \* \* \* | 2.11 | 1.46 | 1.6 | 100 | 100 |
| Bt.10330.1.S1\_at | 5.62e-06 | 8.91e-06 | 0.000143 | \* \* \* | -1.46 | -1.24 | -1.67 | 100 | 100 |
| Bt.2153.1.S1\_at | 6.52e-06 | 1.65e-06 | 1.03e-05 | \* \* \* | 1.94 | 1.37 | 1.81 | 100 | 100 |
| Bt.19706.1.S1\_at | 7.07e-06 | 1.9e-05 | 1.09e-05 | \* \* \* | -1.62 | -1.24 | -1.67 | 100 | 100 |
| Bt.4822.1.A1\_at | 8.17e-06 | 1.59e-06 | 8.49e-07 | \* \* \* | 1.2 | 1.07 | 1.1 | 100 | 100 |
| Bt.26808.1.S1\_at | 8.47e-06 | 1.79e-05 | 7.88e-06 | \* \* \* | -1.7 | -0.9 | -1.59 | 100 | 100 |
| Bt.23883.1.A1\_at | 8.96e-06 | 3.75e-05 | 8.05e-06 | \* \* \* | 1.23 | 0.863 | 1.31 | 100 | 100 |
| Bt.1582.1.S1\_at | 9.11e-06 | 1.73e-06 | 7.35e-06 | \* \* \* | 1.31 | 1.18 | 1.4 | 100 | 100 |
| Bt.23176.2.S1\_a\_at | 9.78e-06 | 1.61e-08 | 4.88e-07 | \* \* \* | 2.14 | 1.98 | 2.33 | 100 | 100 |
| Bt.11659.1.S1\_at | 9.84e-06 | 1.65e-07 | 3.05e-07 | \* \* \* | 1.33 | 1.18 | 1.52 | 100 | 100 |
| Bt.25806.2.A1\_at | 9.96e-06 | 2.25e-07 | 6.2e-06 | \* \* \* | 1.53 | 1.39 | 1.59 | 100 | 100 |
| Bt.24310.1.S1\_at | 1e-05 | 1.62e-09 | 6.05e-08 | \* \* \* | -2 | -1.79 | -2.68 | 100 | 100 |
| Bt.9012.1.S1\_at | 1.17e-05 | 2.22e-06 | 2.07e-06 | \* \* \* | -1.9 | -1.14 | -1.77 | 100 | 100 |
| Bt.12412.1.S1\_at | 1.34e-05 | 5.25e-05 | 1.52e-05 | \* \* \* | -1.55 | -1.06 | -1.34 | 100 | 100 |
| Bt.20104.1.S1\_at | 1.34e-05 | 3.37e-07 | 6.03e-07 | \* \* \* | -2.02 | -1.59 | -2.18 | 100 | 100 |
| Bt.4702.1.S1\_at | 1.39e-05 | 4.96e-07 | 3.12e-07 | \* \* \* | -1.65 | -1.19 | -1.63 | 100 | 100 |
| Bt.10150.1.S1\_at | 1.45e-05 | 8.01e-07 | 7.7e-06 | \* \* \* | 1.42 | 1.36 | 1.39 | 100 | 100 |
| Bt.17963.1.A1\_at | 1.48e-05 | 1.25e-05 | 4.36e-05 | \* \* \* | 1.14 | 1.06 | 1.23 | 100 | 100 |
| Bt.1169.1.S1\_at | 1.51e-05 | 5.8e-05 | 1.08e-05 | \* \* \* | 1.27 | 0.872 | 0.904 | 100 | 100 |
| Bt.23521.1.S1\_at | 1.54e-05 | 2.21e-05 | 3.16e-05 | \* \* \* | -1.54 | -1.15 | -2.08 | 100 | 100 |
| Bt.9810.1.S1\_at | 1.6e-05 | 3.52e-10 | 1.28e-08 | \* \* \* | 1.57 | 1.75 | 2.04 | 100 | 100 |
| Bt.28312.1.S1\_at | 1.69e-05 | 0.000156 | 1.65e-05 | \* \* \* | -1.52 | -1.04 | -1.35 | 100 | 100 |
| Bt.3306.1.S1\_at | 1.72e-05 | 2.05e-06 | 8.46e-06 | \* \* \* | -1.51 | -0.968 | -1.54 | 100 | 100 |
| Bt.6642.1.S1\_a\_at | 1.83e-05 | 2.01e-08 | 6.39e-06 | \* \* \* | -2.56 | -2.05 | -2.33 | 100 | 100 |
| Bt.25099.1.A1\_at | 1.91e-05 | 5.6e-07 | 9.9e-06 | \* \* \* | 3.18 | 2.92 | 3.36 | 100 | 100 |
| Bt.22510.1.S1\_at | 1.97e-05 | 1.2e-05 | 2.23e-06 | \* \* \* | 1.24 | 0.979 | 1.47 | 100 | 100 |
| Bt.20145.1.S1\_at | 2.06e-05 | 5.74e-06 | 1.94e-05 | \* \* \* | 1.6 | 1.37 | 1.91 | 100 | 100 |
| Bt.7908.1.S1\_at | 2.15e-05 | 1.82e-07 | 1.87e-07 | \* \* \* | 1.03 | 0.949 | 0.943 | 100 | 100 |
| Bt.15643.1.A1\_at | 2.37e-05 | 3.17e-05 | 3.86e-06 | \* \* \* | -1.37 | -0.857 | -1.43 | 100 | 100 |
| Bt.21551.2.S1\_a\_at | 2.45e-05 | 0.000302 | 1.08e-05 | \* \* \* | -1.09 | -0.84 | -0.929 | 100 | 100 |
| Bt.2347.2.S1\_at | 2.5e-05 | 0.000186 | 4.57e-05 | \* \* \* | -1.13 | -0.863 | -1.11 | 100 | 100 |
| Bt.21385.1.S1\_at | 2.56e-05 | 3.84e-08 | 8.78e-07 | \* \* \* | 1.39 | 1.25 | 1.58 | 100 | 100 |
| Bt.2091.1.S1\_at | 2.58e-05 | 1.65e-06 | 7.5e-06 | \* \* \* | -2.11 | -1.72 | -2.15 | 100 | 100 |
| Bt.3388.1.S1\_at | 2.63e-05 | 1.06e-06 | 8.74e-06 | \* \* \* | 1.2 | 1.16 | 1.32 | 100 | 100 |
| Bt.16639.1.A1\_at | 2.71e-05 | 7.43e-06 | 5.26e-06 | \* \* \* | -1.27 | -0.832 | -1.25 | 100 | 100 |
| Bt.22568.1.S1\_at | 2.93e-05 | 5.71e-05 | 6.61e-05 | \* \* \* | 1.12 | 1.1 | 1.19 | 100 | 100 |
| Bt.6774.2.S1\_at | 2.96e-05 | 2.51e-06 | 1.57e-06 | \* \* \* | -1.17 | -0.924 | -1.21 | 100 | 100 |
| Bt.9180.1.S1\_a\_at | 2.99e-05 | 4.67e-07 | 2.92e-06 | \* \* \* | 1.32 | 1.46 | 1.79 | 100 | 100 |
| Bt.2070.1.A1\_at | 3.07e-05 | 2.38e-07 | 1.57e-07 | \* \* \* | 2.02 | 1.42 | 1.66 | 100 | 100 |
| Bt.21142.1.S1\_at | 3.21e-05 | 1.48e-06 | 2.99e-05 | \* \* \* | -2.26 | -1.85 | -2.81 | 100 | 100 |
| Bt.16127.1.S1\_at | 3.28e-05 | 8.62e-05 | 2.61e-05 | \* \* \* | -0.968 | -0.719 | -0.757 | 100 | 100 |
| Bt.7962.1.S1\_at | 3.45e-05 | 3.77e-06 | 3.72e-05 | \* \* \* | -2.39 | -2.02 | -2.97 | 100 | 100 |
| Bt.14204.1.A1\_at | 3.62e-05 | 0.000171 | 0.000153 | \* \* \* | -1.16 | -0.905 | -1.21 | 100 | 100 |
| Bt.23519.1.S1\_at | 3.99e-05 | 9.91e-08 | 9.86e-08 | \* \* \* | -1.69 | -1.43 | -2.28 | 100 | 100 |
| Bt.14023.1.S1\_at | 4e-05 | 8.32e-06 | 1.14e-05 | \* \* \* | 0.932 | 0.889 | 0.851 | 100 | 100 |
| Bt.20340.2.S1\_at | 4.18e-05 | 6.95e-05 | 3.77e-05 | \* \* \* | 1.24 | 1.17 | 1.83 | 100 | 100 |
| Bt.13988.1.S1\_at | 4.31e-05 | 1.12e-05 | 1.99e-06 | \* \* \* | -1.26 | -0.86 | -1.32 | 100 | 100 |
| Bt.29029.1.S1\_at | 4.32e-05 | 4.93e-05 | 4.35e-07 | \* \* \* | -1.18 | -0.693 | -1.09 | 100 | 100 |
| Bt.24524.1.S1\_at | 4.39e-05 | 8.92e-08 | 3.35e-07 | \* \* \* | 1.55 | 1.41 | 1.76 | 100 | 100 |
| Bt.5123.2.S1\_at | 4.54e-05 | 6.28e-07 | 5.63e-07 | \* \* \* | 1.01 | 0.936 | 1 | 100 | 100 |
| Bt.9021.1.S1\_at | 4.57e-05 | 1.19e-06 | 1.82e-05 | \* \* \* | 1.63 | 1.88 | 2.15 | 100 | 100 |
| Bt.6802.1.S1\_at | 5.24e-05 | 0.000197 | 0.00019 | \* \* \* | -1.66 | -0.937 | -1.06 | 100 | 100 |
| Bt.4725.1.S1\_at | 5.29e-05 | 6.85e-07 | 4.71e-06 | \* \* \* | -1.23 | -0.987 | -1.26 | 100 | 100 |
| Bt.13975.1.S1\_a\_at | 5.48e-05 | 2.35e-06 | 5.27e-06 | \* \* \* | -1.39 | -1.26 | -1.61 | 100 | 100 |
| Bt.4353.1.S1\_at | 5.49e-05 | 5.93e-05 | 5.27e-05 | \* \* \* | -1.25 | -0.844 | -1.57 | 100 | 100 |
| Bt.25973.1.S1\_at | 5.61e-05 | 0.000495 | 0.000139 | \* \* \* | -1.43 | -0.804 | -1.69 | 100 | 100 |
| Bt.7537.1.S1\_at | 5.72e-05 | 3.43e-06 | 2.99e-06 | \* \* \* | 1.06 | 1.13 | 1.26 | 100 | 100 |
| Bt.7670.1.A1\_at | 6.09e-05 | 3.3e-05 | 4.76e-06 | \* \* \* | -1.33 | -1.1 | -1.44 | 100 | 100 |
| Bt.12314.1.S1\_at | 6.38e-05 | 1.11e-05 | 0.000103 | \* \* \* | -2.98 | -2.53 | -3.07 | 100 | 100 |
| Bt.20420.1.S1\_at | 6.45e-05 | 5.66e-07 | 3.45e-07 | \* \* \* | -1.72 | -1.33 | -1.61 | 100 | 100 |
| Bt.24137.1.A1\_at | 6.72e-05 | 6.78e-06 | 3.46e-05 | \* \* \* | 1.48 | 1.59 | 1.63 | 100 | 100 |
| Bt.21432.2.A1\_at | 7.74e-05 | 1.57e-06 | 3.54e-07 | \* \* \* | 1.13 | 1.46 | 2.44 | 100 | 100 |
| Bt.5070.1.S1\_at | 7.8e-05 | 2.65e-05 | 1.74e-07 | \* \* \* | -1.26 | -1.13 | -1.2 | 100 | 100 |
| Bt.4659.1.S1\_at | 7.95e-05 | 2.73e-06 | 0.000131 | \* \* \* | -2.05 | -1.63 | -2.06 | 100 | 100 |
| Bt.13330.1.S1\_at | 7.96e-05 | 4.69e-05 | 0.000401 | \* \* \* | -1.94 | -2.06 | -1.96 | 100 | 100 |
| Bt.23668.1.A1\_at | 8.42e-05 | 0.000244 | 1.91e-05 | \* \* \* | -0.965 | -0.739 | -1.1 | 100 | 100 |
| Bt.28090.1.S1\_at | 8.49e-05 | 2.05e-06 | 3.84e-05 | \* \* \* | -1.63 | -1.19 | -2.1 | 100 | 100 |
| Bt.13324.1.S1\_a\_at | 8.58e-05 | 5.78e-06 | 0.000117 | \* \* \* | 1.43 | 1.24 | 1.42 | 100 | 100 |
| Bt.3616.1.S1\_at | 9.22e-05 | 7.57e-06 | 0.00012 | \* \* \* | 0.955 | 0.953 | 0.982 | 100 | 100 |
| Bt.5123.3.S1\_a\_at | 9.37e-05 | 5.08e-05 | 4.76e-05 | \* \* \* | 0.875 | 0.8 | 0.89 | 100 | 100 |
| Bt.7069.1.S1\_at | 9.54e-05 | 7.2e-06 | 7.44e-05 | \* \* \* | -1.37 | -1.12 | -1.27 | 100 | 100 |
| Bt.3780.1.S1\_at | 9.76e-05 | 4.81e-06 | 2.01e-05 | \* \* \* | 1.27 | 1.09 | 1.15 | 100 | 100 |
| Bt.12491.1.A1\_at | 9.79e-05 | 1.08e-06 | 0.000207 | \* \* \* | 2.13 | 2.09 | 2.41 | 100 | 100 |
| Bt.1998.1.S1\_at | 9.79e-05 | 9.06e-07 | 1.92e-07 | \* \* \* | 1.1 | 1.21 | 1.47 | 100 | 100 |
| Bt.26688.2.S1\_a\_at | 9.83e-05 | 1.08e-05 | 2.41e-05 | \* \* \* | -1.42 | -1.27 | -2 | 100 | 100 |
| Bt.911.1.S1\_at | 9.88e-05 | 5.9e-06 | 1.37e-06 | \* \* \* | -1.15 | -0.94 | -1.29 | 100 | 100 |
| Bt.22783.1.S1\_at | 9.99e-05 | 9.78e-07 | 3.4e-06 | \* \* \* | 1.2 | 1.41 | 1.87 | 100 | 100 |
| Bt.7540.2.S1\_a\_at | 0.000101 | 2.71e-05 | 3.1e-05 | \* \* \* | 0.976 | 1.02 | 1.03 | 100 | 100 |
| Bt.18755.1.A1\_at | 0.000101 | 1.3e-06 | 7.19e-06 | \* \* \* | 0.916 | 1.11 | 1.31 | 100 | 100 |
| Bt.9804.1.S1\_at | 0.000102 | 6.71e-05 | 0.000326 | \* \* \* | -1.01 | -0.79 | -0.894 | 100 | 100 |
| Bt.7288.1.S1\_at | 0.000104 | 8.1e-07 | 1.94e-06 | \* \* \* | 1.11 | 0.974 | 1.05 | 100 | 100 |
| Bt.23603.3.S1\_at | 0.000107 | 2.77e-06 | 6.67e-07 | \* \* \* | 1.34 | 0.877 | 1.01 | 100 | 100 |
| Bt.5304.1.S1\_at | 0.000109 | 4e-05 | 0.000147 | \* \* \* | -1.83 | -1.41 | -1.86 | 100 | 100 |
| Bt.7481.1.S1\_at | 0.000109 | 2.6e-06 | 0.000177 | \* \* \* | 1.85 | 1.48 | 1.73 | 100 | 100 |
| Bt.9956.1.S1\_at | 0.000114 | 4.3e-05 | 4.67e-06 | \* \* \* | 1.09 | 0.825 | 1.11 | 100 | 100 |
| Bt.3863.1.S1\_at | 0.000114 | 3.12e-07 | 2.96e-07 | \* \* \* | -1.74 | -1.35 | -2.3 | 100 | 100 |
| Bt.1268.1.A1\_at | 0.000116 | 0.00017 | 0.000104 | \* \* \* | -1.3 | -0.988 | -1.22 | 100 | 100 |
| Bt.11492.1.A1\_at | 0.00012 | 5.04e-05 | 0.000133 | \* \* \* | -1.1 | -0.977 | -1.39 | 100 | 100 |
| Bt.21724.2.S1\_a\_at | 0.000122 | 5.37e-05 | 0.000166 | \* \* \* | -1.51 | -1.31 | -1.85 | 100 | 100 |
| Bt.18623.1.A1\_at | 0.000126 | 0.000204 | 0.000197 | \* \* \* | -1.31 | -1.04 | -1.69 | 100 | 100 |
| Bt.25319.1.A1\_at | 0.000127 | 8.52e-05 | 7.71e-06 | \* \* \* | -1.11 | -0.888 | -1.11 | 100 | 100 |
| Bt.9643.1.S1\_at | 0.00013 | 4.25e-07 | 7.28e-07 | \* \* \* | 1.17 | 1.43 | 1.69 | 100 | 100 |
| Bt.22651.1.A1\_at | 0.000131 | 2.83e-05 | 4.02e-06 | \* \* \* | 1.14 | 0.804 | 0.787 | 100 | 100 |
| Bt.20141.1.S1\_at | 0.000134 | 7e-06 | 0.000177 | \* \* \* | 1.15 | 0.88 | 1.11 | 100 | 100 |
| Bt.1330.1.S1\_at | 0.000134 | 2.16e-06 | 3.14e-05 | \* \* \* | 1.16 | 0.999 | 1.04 | 100 | 100 |
| Bt.5472.1.S1\_at | 0.000136 | 1.82e-06 | 1.68e-05 | \* \* \* | 1.4 | 1.1 | 1.2 | 100 | 100 |
| Bt.26805.1.S1\_at | 0.000136 | 5.09e-08 | 2.46e-05 | \* \* \* | 1.67 | 2.16 | 2.6 | 100 | 100 |
| Bt.2696.1.A1\_at | 0.000137 | 0.000275 | 0.000215 | \* \* \* | 1.02 | 1.06 | 1.3 | 100 | 100 |
| Bt.9190.1.S1\_at | 0.000138 | 5.23e-05 | 7.27e-06 | \* \* \* | -1.02 | -0.835 | -1.17 | 100 | 100 |
| Bt.2537.2.S1\_at | 0.000138 | 9.53e-05 | 1.42e-05 | \* \* \* | -0.992 | -0.759 | -0.967 | 100 | 100 |
| Bt.25806.1.S1\_at | 0.000138 | 9.75e-07 | 2.65e-05 | \* \* \* | 1.59 | 1.46 | 1.66 | 100 | 100 |
| Bt.4624.1.S1\_at | 0.000139 | 1.81e-06 | 2.31e-06 | \* \* \* | -1.31 | -1.19 | -1.67 | 100 | 100 |
| Bt.659.2.S1\_at | 0.00014 | 8.61e-07 | 7.3e-08 | \* \* \* | -1.53 | -1.63 | -3.34 | 100 | 100 |
| Bt.21133.1.S1\_at | 0.000141 | 0.00016 | 0.000722 | \* \* \* | -1.78 | -1.66 | -2.22 | 100 | 100 |
| Bt.3312.1.S1\_at | 0.000144 | 6.46e-10 | 2.49e-13 | \* \* \* | -1.28 | -1.4 | -2.1 | 100 | 100 |
| Bt.20270.1.S1\_at | 0.000145 | 9.37e-07 | 3.97e-08 | \* \* \* | 1.01 | 1.18 | 1.54 | 100 | 100 |
| Bt.15157.1.S1\_at | 0.000145 | 0.000363 | 8.65e-06 | \* \* \* | 1.1 | 0.797 | 0.857 | 100 | 100 |
| Bt.22785.1.S1\_at | 0.000145 | 2.06e-05 | 1.32e-05 | \* \* \* | -1.66 | -1.34 | -1.9 | 100 | 100 |
| Bt.22216.1.S1\_at | 0.000147 | 1.06e-06 | 1.78e-05 | \* \* \* | 1.18 | 1.69 | 2.13 | 100 | 100 |
| Bt.23992.1.A1\_at | 0.000153 | 4.41e-05 | 0.000291 | \* \* \* | -1.23 | -0.941 | -1.18 | 100 | 100 |
| Bt.6156.1.S1\_at | 0.000154 | 4.64e-06 | 7.14e-06 | \* \* \* | 0.805 | 0.764 | 0.684 | 100 | 100 |
| Bt.5252.1.S1\_at | 0.000157 | 6.44e-05 | 0.000458 | \* \* \* | 1.64 | 1.35 | 1.47 | 100 | 100 |
| Bt.9915.1.S1\_at | 0.000161 | 0.000315 | 3.35e-05 | \* \* \* | 1.07 | 0.803 | 1.66 | 100 | 100 |
| Bt.11034.1.S1\_at | 0.000161 | 2.11e-06 | 2.77e-05 | \* \* \* | -1.81 | -1.34 | -1.74 | 100 | 100 |
| Bt.4410.1.S1\_at | 0.000163 | 2.71e-06 | 2.67e-06 | \* \* \* | 1.06 | 0.934 | 0.986 | 100 | 100 |
| Bt.1131.1.S1\_at | 0.000167 | 3.59e-06 | 2.93e-06 | \* \* \* | 1 | 0.892 | 0.956 | 100 | 100 |
| Bt.4353.2.S1\_at | 0.000169 | 3.93e-05 | 4.34e-05 | \* \* \* | -0.988 | -0.81 | -1.07 | 100 | 100 |
| Bt.1418.2.S1\_at | 0.00017 | 1.69e-05 | 1.06e-05 | \* \* \* | -1.26 | -0.874 | -1.37 | 100 | 100 |
| Bt.4704.2.S1\_at | 0.000171 | 0.00011 | 0.000245 | \* \* \* | 1.22 | 0.946 | 0.951 | 100 | 100 |
| Bt.3414.1.S1\_at | 0.000171 | 1.31e-06 | 1.12e-05 | \* \* \* | 1.21 | 1.2 | 1.21 | 100 | 100 |
| Bt.9033.2.S1\_at | 0.000173 | 4.33e-05 | 9.08e-07 | \* \* \* | 0.812 | 0.71 | 0.757 | 100 | 100 |
| Bt.22763.2.S1\_a\_at | 0.000177 | 3.29e-05 | 0.000407 | \* \* \* | 1.14 | 0.875 | 0.952 | 100 | 100 |
| Bt.21116.1.S1\_at | 0.00018 | 0.000584 | 0.000146 | \* \* \* | -1.09 | -0.736 | -1.25 | 100 | 100 |
| Bt.6680.1.S1\_a\_at | 0.00018 | 5.17e-05 | 0.000157 | \* \* \* | 1.12 | 0.966 | 0.991 | 100 | 100 |
| Bt.7776.1.S2\_at | 0.000182 | 3.08e-05 | 3.96e-05 | \* \* \* | -1.46 | -0.8 | -1.12 | 100 | 100 |
| Bt.24538.1.S1\_at | 0.000182 | 0.000622 | 9.01e-05 | \* \* \* | -1.42 | -0.928 | -1.35 | 100 | 100 |
| Bt.28390.1.S1\_at | 0.000183 | 5.81e-05 | 3.67e-05 | \* \* \* | -1.15 | -0.891 | -1.21 | 100 | 100 |
| Bt.28567.1.A1\_at | 0.000185 | 3.47e-05 | 2.09e-08 | \* \* \* | -1.01 | -0.752 | -1.48 | 100 | 100 |
| Bt.4970.1.S1\_at | 0.000193 | 1.6e-08 | 1.4e-06 | \* \* \* | 1.39 | 1.86 | 1.98 | 100 | 100 |
| Bt.18637.1.A1\_at | 0.000195 | 7.75e-05 | 0.000507 | \* \* \* | 1.1 | 0.809 | 1.25 | 100 | 100 |
| Bt.24766.1.S1\_at | 0.000195 | 0.000129 | 0.000641 | \* \* \* | -1.42 | -1.11 | -1.76 | 100 | 100 |
| Bt.24727.1.S1\_a\_at | 0.000196 | 0.000283 | 7.25e-05 | \* \* \* | 1.11 | 0.801 | 1.09 | 100 | 100 |
| Bt.18639.1.A1\_at | 1.73e-07 | 8.91e-10 | 9.21e-08 | \* \* \* | -4.75 | -3.61 | -4.74 | 83.3 | 100 |
| Bt.24614.1.S1\_at | 2.81e-07 | 2.53e-06 | 1.99e-05 | \* \* \* | 1.94 | 1.59 | 1.87 | 83.3 | 100 |
| Bt.29724.1.S1\_at | 7.25e-06 | 1.89e-06 | 5.25e-05 | \* \* \* | -3.18 | -2.05 | -3.33 | 83.3 | 100 |
| Bt.16563.1.A1\_at | 7.26e-06 | 7.19e-08 | 6.2e-06 | \* \* \* | 2.26 | 1.84 | 2.2 | 83.3 | 100 |
| Bt.22167.1.S1\_at | 9.12e-06 | 3.04e-09 | 7.3e-10 | \* \* \* | -2.94 | -2.11 | -4.16 | 83.3 | 100 |
| Bt.28732.1.S1\_s\_at | 1.67e-05 | 2.33e-06 | 0.000404 | \* \* \* | -2.31 | -1.34 | -2.69 | 83.3 | 100 |
| Bt.2070.2.A1\_at | 2.52e-05 | 1.26e-06 | 0.000143 | \* \* \* | 1.71 | 1.41 | 1.59 | 83.3 | 100 |
| Bt.29873.1.S1\_at | 4.82e-05 | 5.74e-08 | 6.43e-09 | \* \* \* | 1.6 | 1.81 | 3.1 | 83.3 | 100 |
| Bt.24439.1.S1\_at | 6.01e-05 | 0.000257 | 2.94e-05 | \* \* \* | -1.4 | -0.816 | -1.27 | 83.3 | 100 |
| Bt.17102.3.S1\_at | 7.82e-05 | 5.53e-05 | 1.31e-05 | \* \* \* | -1.45 | -0.812 | -1.61 | 83.3 | 100 |
| Bt.20227.1.S1\_at | 9.81e-05 | 2.4e-06 | 5.79e-05 | \* \* \* | 1.97 | 1.51 | 1.91 | 83.3 | 100 |
| Bt.5468.1.S1\_at | 9.85e-05 | 2.84e-07 | 1.99e-05 | \* \* \* | 2.62 | 2.15 | 2.9 | 83.3 | 100 |
| Bt.5356.1.S1\_s\_at | 0.000102 | 3.68e-07 | 1.81e-05 | \* \* \* | -2.45 | -1.96 | -3.6 | 83.3 | 100 |
| Bt.8189.2.A1\_at | 0.000149 | 3.22e-07 | 3.68e-06 | \* \* \* | 1.28 | 1.52 | 1.73 | 83.3 | 100 |
| Bt.9571.2.S1\_a\_at | 0.00016 | 2.77e-05 | 0.000164 | \* \* \* | 1.53 | 1.37 | 1.87 | 83.3 | 100 |
| Bt.23250.2.S1\_at | 3.06e-06 | 1.52e-09 | 2.57e-07 | \* \* \* | 3.32 | 3.46 | 3.67 | 66.7 | 100 |
| Bt.13765.1.S1\_at | 7.96e-06 | 2.39e-07 | 2.83e-05 | \* \* \* | 2.19 | 1.72 | 1.99 | 66.7 | 100 |
| Bt.2874.1.S1\_at | 1.56e-05 | 5.24e-08 | 1.02e-06 | \* \* \* | 1.86 | 1.22 | 1.11 | 66.7 | 100 |
| Bt.20143.2.S1\_at | 2.62e-05 | 5.18e-07 | 4.55e-06 | \* \* \* | -1.25 | -0.879 | -1.12 | 66.7 | 100 |
| Bt.13315.1.S1\_at | 8.4e-05 | 1.31e-06 | 5.99e-05 | \* \* \* | 1.53 | 0.999 | 1.29 | 66.7 | 100 |
| Bt.12559.1.S1\_at | 0.00018 | 3.86e-05 | 1.93e-05 | \* \* \* | 1.31 | 0.924 | 1.65 | 66.7 | 100 |
| Bt.8699.1.S1\_at | 2.95e-07 | 1.01e-05 | 2.9e-05 | \* \* \* | 2.42 | 1.5 | 2.57 | 50 | 100 |
| Bt.7651.1.S1\_at | 1.03e-06 | 2.7e-09 | 9.82e-09 | \* \* \* | -2.19 | -1.64 | -2.91 | 50 | 100 |
| Bt.13588.3.A1\_at | 3.81e-06 | 4.29e-05 | 0.000396 | \* \* \* | 4.55 | 2.71 | 3.17 | 50 | 100 |
| Bt.9767.1.S1\_a\_at | 4.31e-06 | 1.39e-11 | 1.35e-08 | \* \* \* | 2.42 | 3.12 | 3.85 | 50 | 100 |
| Bt.19023.1.S1\_at | 5.96e-06 | 3.8e-06 | 0.000109 | \* \* \* | 1.91 | 1.57 | 2.44 | 50 | 100 |
| Bt.24517.2.A1\_at | 1.02e-05 | 2.24e-08 | 9.57e-08 | \* \* \* | 1.31 | 1.53 | 1.77 | 50 | 100 |
| Bt.24804.1.S1\_at | 3.74e-05 | 0.000532 | 7.45e-05 | \* \* \* | -1.9 | -0.74 | -1.6 | 50 | 100 |
| Bt.18639.2.S1\_at | 4.04e-05 | 2.79e-10 | 2.81e-07 | \* \* \* | -6.42 | -3.77 | -6.18 | 50 | 100 |
| Bt.15675.1.S1\_at | 4.18e-05 | 9.67e-06 | 8.22e-06 | \* \* \* | 2.94 | 1.02 | 2.57 | 50 | 100 |
| Bt.4631.2.S1\_at | 4.83e-05 | 1.36e-06 | 3.4e-06 | \* \* \* | -1.46 | -1.1 | -1.75 | 50 | 100 |
| Bt.4888.1.S1\_at | 4.99e-05 | 1.61e-06 | 5.12e-06 | \* \* \* | 1.54 | 0.965 | 2.16 | 50 | 100 |
| Bt.27196.1.A1\_at | 5.68e-05 | 1.3e-05 | 6.66e-06 | \* \* \* | 3.09 | 1.53 | 3.61 | 50 | 100 |
| Bt.16389.1.S1\_at | 6.09e-05 | 1.88e-05 | 0.000249 | \* \* \* | -1.48 | -0.76 | -0.903 | 50 | 100 |
| Bt.16597.1.S1\_at | 6.45e-05 | 1.37e-06 | 6.87e-06 | \* \* \* | 1.85 | 1.08 | 1.67 | 50 | 100 |
| Bt.13588.2.S1\_at | 6.48e-05 | 2.66e-06 | 2.34e-05 | \* \* \* | 4.3 | 2.78 | 5.07 | 50 | 100 |
| Bt.20470.1.S1\_a\_at | 6.49e-05 | 1.92e-07 | 0.000288 | \* \* \* | 3.39 | 1.63 | 2.81 | 50 | 100 |
| Bt.19508.1.A1\_at | 6.75e-05 | 0.000135 | 5.84e-05 | \* \* \* | 1.31 | 0.892 | 1.27 | 50 | 100 |
| Bt.19352.1.A1\_at | 8.93e-05 | 5.99e-05 | 8.41e-07 | \* \* \* | 1.59 | 1.26 | 1.18 | 50 | 100 |
| Bt.17883.1.S1\_at | 0.000109 | 3.12e-06 | 6.27e-05 | \* \* \* | -1.73 | -1.17 | -1.48 | 50 | 100 |
| Bt.21039.1.A1\_at | 0.000154 | 9.1e-07 | 2.74e-05 | \* \* \* | -3.17 | -3.15 | -4.95 | 50 | 100 |
| Bt.8552.1.S1\_at | 0.000156 | 2.9e-06 | 0.000124 | \* \* \* | -2.74 | -2.03 | -3.1 | 50 | 100 |
| Bt.22480.1.A1\_at | 0.000196 | 9.53e-06 | 3.28e-05 | \* \* \* | 1.61 | 1.31 | 2.2 | 50 | 100 |
| Bt.20431.2.S1\_at | 1.44e-05 | 0.000273 | 0.00088 | \* \* ns | -1.46 | -1.1 | -1.58 | 100 | 66.7 |
| Bt.10579.1.S1\_at | 8.9e-05 | 0.000989 | 0.000125 | \* ns \* | -1.09 | -0.829 | -1.14 | 100 | 66.7 |
| Bt.21818.1.S1\_at | 9.64e-05 | 0.000119 | 0.000766 | \* \* ns | -1.2 | -0.835 | -1.07 | 100 | 66.7 |
| Bt.12472.1.S1\_at | 0.000114 | 0.00102 | 5e-04 | \* ns \* | -1.34 | -0.769 | -1.09 | 100 | 66.7 |
| Bt.621.1.S1\_at | 0.000117 | 8.92e-06 | 0.000877 | \* \* ns | 1.52 | 1.21 | 1.78 | 100 | 66.7 |
| Bt.25390.1.A1\_at | 0.000124 | 0.0146 | 0.000157 | \* ns \* | -1.04 | -0.511 | -1.15 | 100 | 66.7 |
| Bt.2392.1.S1\_at | 0.000152 | 0.00214 | 1.84e-06 | \* ns \* | 0.913 | 0.594 | 1.35 | 100 | 66.7 |
| Bt.18316.2.S1\_at | 0.000156 | 0.000187 | 0.00142 | \* \* ns | -1.57 | -1.15 | -1.27 | 100 | 66.7 |
| Bt.11014.1.S1\_at | 0.000191 | 4.1e-05 | 0.000917 | \* \* ns | 1.49 | 0.997 | 1.79 | 100 | 66.7 |
| Bt.27926.1.S1\_at | 0.000206 | 0.000126 | 5.85e-05 | ns \* \* | 0.978 | 0.94 | 1.01 | 100 | 66.7 |
| Bt.13235.1.S1\_at | 0.000213 | 0.000527 | 0.00017 | ns \* \* | -1.31 | -1.12 | -1.7 | 100 | 66.7 |
| Bt.8250.1.S1\_at | 0.000227 | 0.000589 | 0.000176 | ns \* \* | -0.948 | -0.549 | -0.817 | 100 | 66.7 |
| Bt.28728.1.A1\_at | 0.000228 | 3.81e-06 | 1.31e-05 | ns \* \* | 1.38 | 1.32 | 2.11 | 100 | 66.7 |
| Bt.11794.1.S1\_at | 0.000234 | 1.25e-05 | 0.000301 | ns \* \* | 1.13 | 1.1 | 1.14 | 100 | 66.7 |
| Bt.25718.1.A1\_at | 0.000248 | 9.97e-05 | 3.92e-05 | ns \* \* | -1.76 | -0.738 | -1.31 | 100 | 66.7 |
| Bt.12685.1.S1\_at | 0.000248 | 0.000111 | 7.67e-05 | ns \* \* | -1.65 | -0.755 | -1.19 | 100 | 66.7 |
| Bt.15931.1.S1\_at | 0.000254 | 4.36e-05 | 1.32e-05 | ns \* \* | 0.819 | 0.631 | 0.631 | 100 | 66.7 |
| Bt.15783.1.S1\_at | 0.000261 | 3.97e-05 | 9.85e-05 | ns \* \* | 0.777 | 0.929 | 0.81 | 100 | 66.7 |
| Bt.9959.3.S1\_a\_at | 0.000264 | 3.79e-05 | 7.69e-05 | ns \* \* | 1.06 | 1.15 | 1.22 | 100 | 66.7 |
| Bt.3314.1.S1\_at | 0.00029 | 5.85e-05 | 0.000224 | ns \* \* | -0.907 | -0.627 | -0.987 | 100 | 66.7 |
| Bt.23109.1.S1\_at | 0.000297 | 7.02e-06 | 8.67e-06 | ns \* \* | 1.16 | 0.864 | 1.26 | 100 | 66.7 |
| Bt.2515.1.S1\_at | 0.000301 | 0.000401 | 2.54e-05 | ns \* \* | 0.703 | 0.709 | 0.691 | 100 | 66.7 |
| Bt.19332.2.A1\_at | 0.000303 | 8.73e-06 | 3.36e-05 | ns \* \* | 1.76 | 1.73 | 1.92 | 100 | 66.7 |
| Bt.9811.1.S1\_at | 0.000305 | 5.36e-05 | 0.000432 | ns \* \* | 0.738 | 0.692 | 0.639 | 100 | 66.7 |
| Bt.16150.1.A1\_at | 0.000308 | 0.000157 | 0.00011 | ns \* \* | 1.75 | 1.14 | 1.65 | 100 | 66.7 |
| Bt.12613.1.S1\_at | 0.000313 | 0.000182 | 8.83e-05 | ns \* \* | -1.16 | -0.893 | -1.25 | 100 | 66.7 |
| Bt.7266.1.S1\_at | 0.000315 | 3.22e-05 | 0.000394 | ns \* \* | 1.1 | 1.26 | 1.5 | 100 | 66.7 |
| Bt.15784.1.S1\_at | 0.000318 | 1.05e-05 | 5.02e-05 | ns \* \* | 1.22 | 0.979 | 1.15 | 100 | 66.7 |
| Bt.21705.1.S1\_at | 0.00034 | 5.62e-06 | 1.88e-05 | ns \* \* | 1.36 | 1.21 | 1.41 | 100 | 66.7 |
| Bt.8124.1.S2\_at | 0.000343 | 2.7e-05 | 0.000413 | ns \* \* | 1.68 | 1.04 | 0.968 | 100 | 66.7 |
| Bt.5176.1.S1\_at | 0.000353 | 0.000553 | 0.000447 | ns \* \* | 1.07 | 0.714 | 0.937 | 100 | 66.7 |
| Bt.18748.1.A1\_at | 0.000353 | 0.000283 | 3.49e-05 | ns \* \* | -1.03 | -0.748 | -1.12 | 100 | 66.7 |
| Bt.10277.2.S1\_a\_at | 0.000363 | 8.42e-05 | 0.000157 | ns \* \* | 1.22 | 1.1 | 1.46 | 100 | 66.7 |
| Bt.665.1.S1\_s\_at | 0.000365 | 2.78e-05 | 0.000111 | ns \* \* | 1.2 | 0.677 | 1.11 | 100 | 66.7 |
| Bt.5605.1.S1\_at | 0.000368 | 8.2e-05 | 1.18e-05 | ns \* \* | 0.76 | 0.807 | 0.825 | 100 | 66.7 |
| Bt.4357.1.S1\_at | 0.000374 | 2.42e-05 | 4.05e-06 | ns \* \* | 0.695 | 0.809 | 0.818 | 100 | 66.7 |
| Bt.25255.1.A1\_at | 0.000374 | 3.62e-06 | 4.23e-06 | ns \* \* | 0.942 | 1.04 | 1 | 100 | 66.7 |
| Bt.15722.1.S1\_at | 0.000379 | 9.75e-05 | 0.000246 | ns \* \* | 1.39 | 0.88 | 0.96 | 100 | 66.7 |
| Bt.21978.1.S1\_at | 0.000382 | 0.000138 | 1.42e-05 | ns \* \* | -0.954 | -0.738 | -1.24 | 100 | 66.7 |
| Bt.23002.1.S1\_s\_at | 0.000385 | 1.41e-05 | 1.34e-05 | ns \* \* | 0.86 | 0.751 | 0.759 | 100 | 66.7 |
| Bt.5306.1.S1\_at | 0.000387 | 4.63e-05 | 4.54e-05 | ns \* \* | -1.43 | -0.841 | -1.18 | 100 | 66.7 |
| Bt.20977.1.S1\_at | 0.000389 | 0.000141 | 7.19e-05 | ns \* \* | -1.19 | -0.812 | -1.13 | 100 | 66.7 |
| Bt.5269.2.S1\_a\_at | 0.000395 | 0.000337 | 0.000487 | ns \* \* | -1.28 | -0.916 | -1.18 | 100 | 66.7 |
| Bt.7856.1.S1\_at | 0.000397 | 9.53e-06 | 1.7e-05 | ns \* \* | 1.06 | 1.04 | 1.4 | 100 | 66.7 |
| Bt.27331.1.S1\_at | 0.000397 | 3.31e-06 | 6.51e-05 | ns \* \* | 1.41 | 1.44 | 1.55 | 100 | 66.7 |
| Bt.13330.2.A1\_at | 0.000408 | 1.33e-05 | 0.00046 | ns \* \* | -1.97 | -1.86 | -2.09 | 100 | 66.7 |
| Bt.23317.1.S1\_at | 0.000432 | 1.46e-05 | 4.78e-06 | ns \* \* | 0.779 | 0.728 | 0.762 | 100 | 66.7 |
| Bt.23354.1.S1\_at | 0.000437 | 5.62e-05 | 0.000313 | ns \* \* | -1.46 | -1.12 | -1.68 | 100 | 66.7 |
| Bt.1767.1.S1\_at | 0.000448 | 3.69e-05 | 0.000209 | ns \* \* | 0.846 | 0.787 | 0.689 | 100 | 66.7 |
| Bt.1310.1.S1\_at | 0.000451 | 2.2e-05 | 0.000164 | ns \* \* | 0.982 | 0.729 | 0.693 | 100 | 66.7 |
| Bt.20505.1.S1\_at | 0.000451 | 6.98e-07 | 9.36e-06 | ns \* \* | 0.942 | 1.13 | 1.03 | 100 | 66.7 |
| Bt.9242.1.S1\_at | 0.000458 | 6.81e-06 | 7.84e-05 | ns \* \* | 0.961 | 1 | 1.24 | 100 | 66.7 |
| Bt.23603.1.S1\_a\_at | 0.000461 | 0.000121 | 3.9e-05 | ns \* \* | 1.11 | 0.913 | 0.99 | 100 | 66.7 |
| Bt.8234.1.S1\_at | 0.000499 | 0.000483 | 1.71e-07 | ns \* \* | -0.724 | -0.618 | -0.892 | 100 | 66.7 |
| Bt.16784.1.S1\_at | 0.000502 | 0.000621 | 8.92e-05 | ns \* \* | -0.991 | -0.811 | -1.06 | 100 | 66.7 |
| Bt.20470.2.S1\_a\_at | 0.000516 | 4.55e-05 | 8.51e-07 | ns \* \* | 1.05 | 0.937 | 2.74 | 100 | 66.7 |
| Bt.26872.1.S1\_at | 0.000521 | 4.02e-06 | 9.97e-05 | ns \* \* | 1.09 | 1.58 | 2.4 | 100 | 66.7 |
| Bt.23227.1.S1\_at | 0.000522 | 1.77e-05 | 1.88e-05 | ns \* \* | -1.34 | -1.24 | -1.83 | 100 | 66.7 |
| Bt.23173.1.S1\_at | 0.000535 | 9.36e-05 | 7.58e-05 | ns \* \* | -1.17 | -0.937 | -1.24 | 100 | 66.7 |
| Bt.5267.1.S1\_at | 0.000549 | 5.55e-05 | 0.000109 | ns \* \* | 0.763 | 0.912 | 1.02 | 100 | 66.7 |
| Bt.7955.2.S1\_at | 0.000554 | 1.38e-05 | 1.58e-06 | ns \* \* | 1.3 | 1.13 | 1.37 | 100 | 66.7 |
| Bt.20194.1.S1\_at | 0.000596 | 5.81e-06 | 4.29e-06 | ns \* \* | 0.784 | 0.899 | 1.01 | 100 | 66.7 |
| Bt.2249.1.S1\_at | 0.000596 | 7.22e-06 | 1.04e-05 | ns \* \* | 0.86 | 0.92 | 1.27 | 100 | 66.7 |
| Bt.5596.1.A1\_at | 0.000596 | 8.79e-06 | 2.71e-05 | ns \* \* | 1.07 | 0.911 | 1.06 | 100 | 66.7 |
| Bt.4292.1.S1\_at | 0.000617 | 0.000136 | 1.34e-05 | ns \* \* | 0.832 | 0.694 | 0.897 | 100 | 66.7 |
| Bt.21670.1.A1\_at | 0.000619 | 5.98e-06 | 2.4e-07 | ns \* \* | 1.23 | 0.893 | 1.37 | 100 | 66.7 |
| Bt.11196.1.S1\_at | 0.000622 | 3.32e-05 | 3.63e-05 | ns \* \* | 1.08 | 0.886 | 1.03 | 100 | 66.7 |
| Bt.26942.1.S1\_at | 0.000624 | 0.000151 | 1.84e-05 | ns \* \* | 0.867 | 0.752 | 0.975 | 100 | 66.7 |
| Bt.4917.1.S1\_at | 0.00063 | 1.26e-05 | 7.93e-07 | ns \* \* | 0.68 | 0.691 | 0.729 | 100 | 66.7 |
| Bt.20955.1.A1\_at | 0.00063 | 0.000296 | 5e-04 | ns \* \* | 1.36 | 1.1 | 1.27 | 100 | 66.7 |
| Bt.18755.2.S1\_at | 0.000637 | 4.25e-05 | 2.64e-06 | ns \* \* | 0.87 | 0.693 | 1.01 | 100 | 66.7 |
| Bt.28583.1.S1\_s\_at | 0.000639 | 3.67e-05 | 4e-04 | ns \* \* | 1.36 | 1.2 | 1.42 | 100 | 66.7 |
| Bt.2889.1.S1\_at | 0.000649 | 0.000126 | 6.93e-06 | ns \* \* | 0.817 | 0.637 | 0.838 | 100 | 66.7 |
| Bt.2262.1.S1\_at | 0.000669 | 4.62e-08 | 7.96e-06 | ns \* \* | 1.28 | 1.32 | 1.46 | 100 | 66.7 |
| Bt.21721.1.A1\_at | 0.000698 | 3.56e-05 | 0.000683 | ns \* \* | 1.37 | 1.19 | 1.49 | 100 | 66.7 |
| Bt.23094.3.S1\_x\_at | 0.000713 | 3.08e-06 | 4.7e-06 | ns \* \* | -1.6 | -1.5 | -2.56 | 100 | 66.7 |
| Bt.9092.1.S1\_at | 0.000732 | 0.000116 | 0.00012 | ns \* \* | 1.28 | 0.811 | 1.12 | 100 | 66.7 |
| Bt.4959.1.A1\_at | 0.000748 | 4.43e-07 | 8.48e-06 | ns \* \* | 1.35 | 1.34 | 1.74 | 100 | 66.7 |
| Bt.8618.1.S1\_at | 0.000752 | 7.13e-05 | 3.55e-05 | ns \* \* | 1.5 | 0.701 | 0.978 | 100 | 66.7 |
| Bt.3843.1.S1\_at | 0.000753 | 3.43e-07 | 1.98e-06 | ns \* \* | 1.03 | 1.24 | 1.22 | 100 | 66.7 |
| Bt.26483.1.A1\_at | 0.000761 | 0.000151 | 0.000283 | ns \* \* | -1.48 | -1 | -1.39 | 100 | 66.7 |
| Bt.1696.1.S1\_at | 0.000762 | 7.87e-06 | 2.96e-08 | ns \* \* | 0.873 | 0.715 | 0.989 | 100 | 66.7 |
| Bt.16123.1.S1\_at | 0.000771 | 6.52e-05 | 0.000478 | ns \* \* | 1.4 | 1.2 | 1.44 | 100 | 66.7 |
| Bt.26629.1.S1\_a\_at | 0.000776 | 5.32e-06 | 5.74e-05 | ns \* \* | 1.26 | 1.43 | 1.53 | 100 | 66.7 |
| Bt.14340.1.A1\_at | 0.000803 | 0.000184 | 1.2e-05 | ns \* \* | -0.938 | -0.699 | -0.986 | 100 | 66.7 |
| Bt.1269.1.S1\_a\_at | 0.000861 | 0.000603 | 0.000237 | ns \* \* | 0.667 | 0.533 | 0.578 | 100 | 66.7 |
| Bt.21031.1.S1\_at | 0.000882 | 0.000463 | 7.72e-06 | ns \* \* | 1.09 | 0.711 | 1.31 | 100 | 66.7 |
| Bt.21681.1.A1\_at | 0.000903 | 0.000214 | 0.000107 | ns \* \* | -1.76 | -1.25 | -2.1 | 100 | 66.7 |
| Bt.3567.1.S1\_at | 0.000944 | 0.000289 | 5.99e-05 | ns \* \* | -0.779 | -0.641 | -0.793 | 100 | 66.7 |
| Bt.27194.1.S1\_at | 0.000948 | 5.57e-05 | 0.000141 | ns \* \* | -1.38 | -1 | -1.46 | 100 | 66.7 |
| Bt.4175.2.S1\_at | 0.000962 | 6.88e-06 | 2.5e-05 | ns \* \* | 0.658 | 0.758 | 1.08 | 100 | 66.7 |
| Bt.8549.1.S2\_at | 0.000962 | 3.85e-05 | 0.000225 | ns \* \* | 1.88 | 1.5 | 1.73 | 100 | 66.7 |
| Bt.9588.1.S1\_at | 0.000965 | 0.000262 | 0.000598 | ns \* \* | 0.86 | 0.654 | 0.675 | 100 | 66.7 |
| Bt.13886.2.S1\_at | 0.000976 | 4.24e-05 | 2.17e-06 | ns \* \* | -0.966 | -0.741 | -1.02 | 100 | 66.7 |
| Bt.9405.1.S1\_at | 0.000991 | 1.69e-05 | 4.67e-05 | ns \* \* | 0.665 | 0.619 | 0.651 | 100 | 66.7 |
| Bt.4831.1.S1\_at | 0.000998 | 0.000229 | 1.17e-05 | ns \* \* | -0.834 | -0.561 | -0.86 | 100 | 66.7 |
| Bt.13610.1.A1\_at | 0.001 | 0.000105 | 0.000105 | ns \* \* | 1.1 | 0.925 | 1.17 | 100 | 66.7 |
| Bt.3427.1.S1\_at | 0.00101 | 1.28e-05 | 4.36e-05 | ns \* \* | 0.967 | 0.88 | 1.51 | 100 | 66.7 |
| Bt.10730.1.S1\_at | 0.00103 | 8.4e-05 | 0.000241 | ns \* \* | 1.19 | 1.2 | 1.2 | 100 | 66.7 |
| Bt.689.1.S1\_at | 0.00109 | 0.00062 | 1.05e-05 | ns \* \* | -0.685 | -0.526 | -0.874 | 100 | 66.7 |
| Bt.23094.6.A1\_at | 0.00113 | 0.000129 | 3.15e-05 | ns \* \* | -1.55 | -1.16 | -2.27 | 100 | 66.7 |
| Bt.7307.2.A1\_a\_at | 0.00114 | 1.91e-07 | 1.47e-06 | ns \* \* | -1.11 | -0.991 | -1.23 | 100 | 66.7 |
| Bt.7008.1.S1\_at | 0.00116 | 3.82e-07 | 1.95e-05 | ns \* \* | 1.4 | 1.42 | 1.47 | 100 | 66.7 |
| Bt.10515.1.S1\_at | 0.00119 | 6.13e-05 | 0.000228 | ns \* \* | -1.06 | -0.739 | -1.17 | 100 | 66.7 |
| Bt.15717.1.A1\_at | 0.00123 | 0.000285 | 0.000259 | ns \* \* | 1.52 | 1.24 | 1.49 | 100 | 66.7 |
| Bt.20559.2.A1\_at | 0.00128 | 4.85e-05 | 0.000364 | ns \* \* | 1.13 | 1.11 | 1.29 | 100 | 66.7 |
| Bt.11662.1.A1\_at | 0.0013 | 8.36e-07 | 1e-05 | ns \* \* | 1.12 | 1.34 | 1.57 | 100 | 66.7 |
| Bt.27563.1.A1\_at | 0.00131 | 0.000555 | 2.22e-06 | ns \* \* | -0.901 | -0.886 | -1.1 | 100 | 66.7 |
| Bt.9238.1.S1\_at | 0.00135 | 2.81e-05 | 0.000101 | ns \* \* | 1.18 | 1.17 | 1.19 | 100 | 66.7 |
| Bt.7700.1.S1\_at | 0.00138 | 2e-04 | 9.83e-05 | ns \* \* | 0.845 | 0.838 | 1.15 | 100 | 66.7 |
| Bt.13991.2.S1\_at | 0.00138 | 0.000157 | 5.62e-05 | ns \* \* | -0.969 | -0.768 | -1.18 | 100 | 66.7 |
| Bt.1821.1.S1\_at | 0.00139 | 4.09e-05 | 0.000269 | ns \* \* | 0.91 | 1.12 | 1.06 | 100 | 66.7 |
| Bt.3437.1.S1\_at | 0.0014 | 0.000245 | 0.000731 | ns \* \* | 0.918 | 0.981 | 0.941 | 100 | 66.7 |
| Bt.5676.1.S1\_at | 0.00143 | 1.94e-05 | 0.000357 | ns \* \* | 1.06 | 1.09 | 1.22 | 100 | 66.7 |
| Bt.2934.1.S1\_at | 0.00144 | 1.06e-05 | 0.000143 | ns \* \* | 0.885 | 0.719 | 0.708 | 100 | 66.7 |
| Bt.5466.2.S1\_a\_at | 0.00151 | 3.62e-05 | 3.4e-05 | ns \* \* | 0.68 | 0.732 | 0.728 | 100 | 66.7 |
| Bt.15298.1.A1\_at | 0.00156 | 1.96e-05 | 6.36e-05 | ns \* \* | 1.76 | 2.42 | 2.85 | 100 | 66.7 |
| Bt.11855.1.S1\_at | 0.00158 | 6.61e-05 | 0.000733 | ns \* \* | 0.685 | 0.628 | 0.687 | 100 | 66.7 |
| Bt.24395.1.S1\_at | 0.00159 | 1.99e-05 | 3.13e-05 | ns \* \* | 0.83 | 0.976 | 1.24 | 100 | 66.7 |
| Bt.11178.1.S1\_at | 0.00165 | 4.76e-06 | 3.26e-06 | ns \* \* | 0.706 | 0.92 | 0.862 | 100 | 66.7 |
| Bt.5631.1.S1\_at | 0.00171 | 9.38e-06 | 0.000122 | ns \* \* | 0.805 | 0.735 | 0.808 | 100 | 66.7 |
| Bt.17573.1.A1\_at | 0.00173 | 2.66e-06 | 1.74e-05 | ns \* \* | -1.79 | -1.82 | -2.79 | 100 | 66.7 |
| Bt.8876.1.S1\_at | 0.00181 | 6.9e-07 | 3.73e-06 | ns \* \* | 0.848 | 0.947 | 1.03 | 100 | 66.7 |
| Bt.4937.1.S1\_at | 0.00183 | 2.63e-07 | 1.71e-06 | ns \* \* | 0.819 | 1.25 | 1.18 | 100 | 66.7 |
| Bt.4671.1.S1\_at | 0.00186 | 6.66e-05 | 6.04e-05 | ns \* \* | -0.821 | -0.704 | -0.96 | 100 | 66.7 |
| Bt.25109.2.A1\_at | 0.00187 | 0.000183 | 0.000736 | ns \* \* | 1.1 | 1.02 | 1.6 | 100 | 66.7 |
| Bt.5203.1.S1\_at | 0.00189 | 5.66e-05 | 5.21e-05 | ns \* \* | 0.656 | 0.776 | 0.887 | 100 | 66.7 |
| Bt.6950.1.S1\_at | 0.00207 | 2.54e-05 | 0.000169 | ns \* \* | -0.934 | -0.678 | -0.935 | 100 | 66.7 |
| Bt.5306.2.S1\_a\_at | 0.00209 | 0.000141 | 0.000206 | ns \* \* | -1.35 | -1 | -1.37 | 100 | 66.7 |
| Bt.27237.1.S1\_a\_at | 0.00211 | 3.89e-06 | 8.41e-06 | ns \* \* | 0.812 | 1.18 | 1.4 | 100 | 66.7 |
| Bt.7187.1.S1\_at | 0.00216 | 2.68e-05 | 3.04e-06 | ns \* \* | 0.68 | 0.632 | 0.631 | 100 | 66.7 |
| Bt.19721.1.S1\_at | 0.00218 | 4.41e-07 | 5.96e-05 | ns \* \* | 1.37 | 1.68 | 1.82 | 100 | 66.7 |
| Bt.9424.1.S1\_at | 0.00218 | 6.48e-05 | 0.000201 | ns \* \* | -1.35 | -0.995 | -1.36 | 100 | 66.7 |
| Bt.9086.1.S1\_at | 0.00221 | 6.16e-05 | 0.000383 | ns \* \* | 0.776 | 0.872 | 0.987 | 100 | 66.7 |
| Bt.20229.1.S1\_at | 0.00234 | 5.26e-05 | 2.12e-05 | ns \* \* | 0.807 | 0.756 | 0.921 | 100 | 66.7 |
| Bt.23552.1.S1\_at | 0.00236 | 0.00043 | 3.01e-05 | ns \* \* | 0.555 | 0.611 | 0.64 | 100 | 66.7 |
| Bt.20852.1.A1\_at | 0.00237 | 3.84e-06 | 6.9e-05 | ns \* \* | 0.93 | 1.3 | 1.56 | 100 | 66.7 |
| Bt.8643.1.A1\_at | 0.00243 | 3.31e-06 | 8.05e-05 | ns \* \* | 1.05 | 1.22 | 1.42 | 100 | 66.7 |
| Bt.26692.1.S1\_a\_at | 0.0025 | 0.000116 | 5.32e-05 | ns \* \* | -1.08 | -0.941 | -1.33 | 100 | 66.7 |
| Bt.26685.1.S1\_at | 0.00257 | 8.94e-07 | 1.78e-05 | ns \* \* | 1.02 | 1.34 | 1.49 | 100 | 66.7 |
| Bt.3767.2.S1\_at | 0.00268 | 0.000262 | 6.66e-05 | ns \* \* | -0.798 | -0.648 | -1.08 | 100 | 66.7 |
| Bt.7968.2.S1\_at | 0.00269 | 0.000119 | 1.53e-06 | ns \* \* | -0.738 | -0.644 | -0.991 | 100 | 66.7 |
| Bt.10869.1.S1\_at | 0.00274 | 1.32e-06 | 2.46e-05 | ns \* \* | -1.33 | -1.16 | -1.93 | 100 | 66.7 |
| Bt.3541.1.S1\_at | 0.00277 | 0.000274 | 2.35e-06 | ns \* \* | 0.62 | 0.624 | 0.684 | 100 | 66.7 |
| Bt.23148.1.S1\_at | 0.0028 | 3.48e-05 | 3.78e-07 | ns \* \* | 0.646 | 0.688 | 0.861 | 100 | 66.7 |
| Bt.5519.2.S1\_at | 0.00288 | 2.07e-06 | 2.17e-08 | ns \* \* | 0.577 | 0.98 | 1.36 | 100 | 66.7 |
| Bt.25098.1.S1\_at | 0.00295 | 0.000357 | 0.000539 | ns \* \* | 0.878 | 0.817 | 1.46 | 100 | 66.7 |
| Bt.1268.2.S1\_at | 0.0031 | 5.73e-05 | 0.000152 | ns \* \* | -0.968 | -0.819 | -1.22 | 100 | 66.7 |
| Bt.8279.3.S1\_a\_at | 0.00313 | 0.000197 | 0.000316 | ns \* \* | 0.639 | 0.604 | 0.722 | 100 | 66.7 |
| Bt.22106.2.S1\_at | 0.00322 | 6.25e-05 | 0.000759 | ns \* \* | 0.8 | 1.08 | 1.36 | 100 | 66.7 |
| Bt.27101.1.S1\_at | 0.00323 | 1.41e-05 | 3.28e-05 | ns \* \* | 0.997 | 1.18 | 1.45 | 100 | 66.7 |
| Bt.5466.4.S1\_at | 0.00334 | 0.000278 | 0.000279 | ns \* \* | -0.857 | -0.575 | -0.761 | 100 | 66.7 |
| Bt.22472.1.S1\_at | 0.00335 | 9.55e-05 | 0.000378 | ns \* \* | -1.27 | -0.936 | -1.31 | 100 | 66.7 |
| Bt.15672.2.S1\_at | 0.00336 | 0.000108 | 0.000151 | ns \* \* | 0.659 | 0.799 | 0.822 | 100 | 66.7 |
| Bt.22803.1.S2\_at | 0.00344 | 1.1e-07 | 9.25e-05 | ns \* \* | 1.05 | 1.27 | 1.72 | 100 | 66.7 |
| Bt.2439.1.S1\_at | 0.00349 | 0.000514 | 0.000205 | ns \* \* | -1.07 | -0.534 | -0.832 | 100 | 66.7 |
| Bt.4825.1.A1\_at | 0.00352 | 5.03e-05 | 1.29e-05 | ns \* \* | 1.24 | 1.12 | 2.17 | 100 | 66.7 |
| Bt.20897.2.S1\_a\_at | 0.00355 | 0.000378 | 9.34e-05 | ns \* \* | 0.856 | 0.901 | 1.48 | 100 | 66.7 |
| Bt.9662.1.S1\_at | 0.00372 | 4.58e-05 | 0.000226 | ns \* \* | -1.06 | -1.01 | -1.38 | 100 | 66.7 |
| Bt.3975.1.S1\_at | 0.00388 | 0.000213 | 0.000418 | ns \* \* | 0.571 | 0.573 | 0.753 | 100 | 66.7 |
| Bt.27267.1.S1\_at | 0.0039 | 0.000489 | 0.000241 | ns \* \* | 0.63 | 0.697 | 0.761 | 100 | 66.7 |
| Bt.28263.1.S1\_at | 0.00394 | 0.000179 | 0.000281 | ns \* \* | 0.889 | 0.971 | 0.929 | 100 | 66.7 |
| Bt.26711.2.S1\_at | 0.00398 | 5.79e-05 | 0.000754 | ns \* \* | -1.41 | -0.928 | -1.96 | 100 | 66.7 |
| Bt.13218.2.S1\_at | 0.00414 | 4.16e-05 | 0.000119 | ns \* \* | 0.864 | 1.13 | 1.2 | 100 | 66.7 |
| Bt.21151.1.S1\_at | 0.00417 | 0.000229 | 0.000393 | ns \* \* | 0.511 | 0.608 | 0.631 | 100 | 66.7 |
| Bt.27617.1.S1\_at | 0.00423 | 3.65e-05 | 0.000249 | ns \* \* | 0.703 | 1.04 | 1.32 | 100 | 66.7 |
| Bt.14037.1.S1\_at | 0.00429 | 0.000241 | 7.64e-05 | ns \* \* | -0.645 | -0.563 | -0.83 | 100 | 66.7 |
| Bt.1552.1.S1\_at | 0.00439 | 0.000106 | 0.000226 | ns \* \* | 1.69 | 1.05 | 1.39 | 100 | 66.7 |
| Bt.8073.1.S1\_at | 0.00444 | 4.4e-05 | 3.31e-05 | ns \* \* | -0.92 | -0.892 | -1.36 | 100 | 66.7 |
| Bt.1537.1.S1\_at | 0.00445 | 3.91e-05 | 0.000713 | ns \* \* | -1.04 | -1.01 | -1.18 | 100 | 66.7 |
| Bt.5650.1.S1\_at | 0.00448 | 0.00013 | 0.000161 | ns \* \* | 0.785 | 0.976 | 1.17 | 100 | 66.7 |
| Bt.12327.1.S1\_at | 0.00449 | 8.02e-06 | 3.44e-06 | ns \* \* | -1.93 | -0.925 | -1.1 | 100 | 66.7 |
| Bt.12885.2.S1\_at | 0.00464 | 8.35e-05 | 1.21e-05 | ns \* \* | -0.926 | -0.694 | -0.977 | 100 | 66.7 |
| Bt.27130.1.S1\_at | 0.00501 | 5.11e-05 | 6.78e-06 | ns \* \* | 0.623 | 0.765 | 0.899 | 100 | 66.7 |
| Bt.946.1.S1\_at | 0.00514 | 2.8e-06 | 0.000149 | ns \* \* | 0.636 | 0.988 | 1.3 | 100 | 66.7 |
| Bt.15397.1.S1\_a\_at | 0.00524 | 0.000121 | 2.4e-06 | ns \* \* | 0.554 | 0.567 | 0.571 | 100 | 66.7 |
| Bt.4790.1.S1\_at | 0.00525 | 0.00015 | 0.000231 | ns \* \* | 0.551 | 0.61 | 0.634 | 100 | 66.7 |
| Bt.2856.1.S1\_at | 0.00542 | 2.84e-05 | 0.000211 | ns \* \* | 0.871 | 1.01 | 1.04 | 100 | 66.7 |
| Bt.2001.1.S1\_at | 0.00556 | 3.39e-06 | 6.43e-06 | ns \* \* | 0.882 | 0.842 | 1.14 | 100 | 66.7 |
| Bt.3921.1.S1\_at | 0.00569 | 4.74e-05 | 4.2e-08 | ns \* \* | 0.582 | 0.628 | 0.64 | 100 | 66.7 |
| Bt.23182.1.S1\_at | 0.00613 | 4.34e-07 | 7.84e-06 | ns \* \* | 0.807 | 1.29 | 1.67 | 100 | 66.7 |
| Bt.21032.1.S1\_at | 0.00633 | 1.41e-05 | 0.000483 | ns \* \* | 0.674 | 0.792 | 0.968 | 100 | 66.7 |
| Bt.26358.1.S1\_at | 0.00642 | 3.82e-06 | 2.82e-05 | ns \* \* | 0.882 | 1.47 | 1.81 | 100 | 66.7 |
| Bt.7633.1.S1\_at | 0.00695 | 3.85e-05 | 0.000689 | ns \* \* | 0.775 | 0.961 | 1.23 | 100 | 66.7 |
| Bt.5605.2.S1\_at | 0.00697 | 0.000259 | 0.000634 | ns \* \* | 0.606 | 0.77 | 0.736 | 100 | 66.7 |
| Bt.9055.2.S1\_a\_at | 0.00703 | 3.47e-05 | 2.37e-05 | ns \* \* | 0.916 | 0.877 | 1.44 | 100 | 66.7 |
| Bt.10442.2.S1\_a\_at | 0.00728 | 3.55e-05 | 0.000261 | ns \* \* | 0.876 | 1.3 | 1.6 | 100 | 66.7 |
| Bt.2519.1.S1\_at | 0.00786 | 0.000282 | 6.05e-05 | ns \* \* | 0.727 | 0.626 | 0.872 | 100 | 66.7 |
| Bt.8641.1.A1\_at | 0.00789 | 1.71e-05 | 0.000115 | ns \* \* | 1.14 | 0.986 | 1.35 | 100 | 66.7 |
| Bt.4986.1.S1\_at | 0.00807 | 0.000348 | 2.32e-05 | ns \* \* | 0.442 | 0.674 | 1.04 | 100 | 66.7 |
| Bt.23273.1.S1\_at | 0.00857 | 0.000321 | 0.000171 | ns \* \* | 0.575 | 0.708 | 0.967 | 100 | 66.7 |
| Bt.2841.1.S1\_at | 0.00885 | 0.000502 | 0.000377 | ns \* \* | 0.824 | 0.706 | 0.661 | 100 | 66.7 |
| Bt.3573.1.S2\_at | 0.00888 | 5.02e-05 | 2.58e-05 | ns \* \* | 0.651 | 1.13 | 1.55 | 100 | 66.7 |
| Bt.9053.1.S1\_a\_at | 0.00889 | 0.000211 | 0.000306 | ns \* \* | -0.908 | -0.744 | -0.903 | 100 | 66.7 |
| Bt.2575.1.S1\_at | 0.00893 | 4.31e-05 | 2.02e-07 | ns \* \* | 0.514 | 0.758 | 0.79 | 100 | 66.7 |
| Bt.7192.1.S1\_at | 0.00907 | 0.000309 | 0.000657 | ns \* \* | 0.706 | 0.799 | 0.926 | 100 | 66.7 |
| Bt.7648.1.S1\_at | 0.00907 | 2.46e-05 | 1.21e-05 | ns \* \* | 0.559 | 0.672 | 0.725 | 100 | 66.7 |
| Bt.13237.1.S1\_at | 0.00918 | 0.000184 | 3.17e-05 | ns \* \* | 0.755 | 0.679 | 0.491 | 100 | 66.7 |
| Bt.20193.1.S1\_at | 0.00921 | 0.000181 | 0.00026 | ns \* \* | 1.01 | 1.45 | 2.05 | 100 | 66.7 |
| Bt.22167.2.A1\_at | 0.00942 | 2.88e-07 | 8.24e-05 | ns \* \* | -1.69 | -1.94 | -2.32 | 100 | 66.7 |
| Bt.16329.1.A1\_at | 0.0101 | 2.67e-05 | 2.56e-05 | ns \* \* | 0.577 | 0.963 | 0.791 | 100 | 66.7 |
| Bt.12429.1.S1\_at | 0.0101 | 0.000202 | 0.000725 | ns \* \* | 0.592 | 0.709 | 0.725 | 100 | 66.7 |
| Bt.15882.2.S1\_a\_at | 0.0103 | 7.78e-05 | 0.000442 | ns \* \* | 0.682 | 0.781 | 0.757 | 100 | 66.7 |
| Bt.2847.1.S1\_a\_at | 0.0104 | 0.000396 | 0.000539 | ns \* \* | 0.514 | 0.473 | 0.505 | 100 | 66.7 |
| Bt.22556.1.S1\_at | 0.0106 | 1.82e-05 | 1.19e-08 | ns \* \* | -0.975 | -0.642 | -0.784 | 100 | 66.7 |
| Bt.3296.1.A1\_at | 0.0107 | 9.48e-05 | 1.89e-05 | ns \* \* | 0.882 | 0.805 | 0.987 | 100 | 66.7 |
| Bt.3240.1.S1\_at | 0.0109 | 8.04e-06 | 0.00041 | ns \* \* | 0.933 | 1.3 | 1.17 | 100 | 66.7 |
| Bt.24200.1.S1\_at | 0.011 | 0.000143 | 0.000459 | ns \* \* | 0.878 | 0.993 | 1.16 | 100 | 66.7 |
| Bt.3162.1.S1\_at | 0.011 | 2.7e-05 | 1.05e-05 | ns \* \* | 0.596 | 0.673 | 0.858 | 100 | 66.7 |
| Bt.9664.1.A1\_at | 0.0113 | 3.63e-05 | 3.9e-06 | ns \* \* | 0.656 | 0.877 | 1.06 | 100 | 66.7 |
| Bt.4263.1.S1\_at | 0.0115 | 0.000286 | 1.28e-06 | ns \* \* | -0.61 | -0.696 | -1.24 | 100 | 66.7 |
| Bt.27018.1.S1\_at | 0.012 | 4.3e-05 | 1.22e-06 | ns \* \* | 0.701 | 0.825 | 1.61 | 100 | 66.7 |
| Bt.2822.1.S1\_at | 0.0124 | 8.47e-07 | 2.63e-07 | ns \* \* | 0.657 | 0.804 | 0.876 | 100 | 66.7 |
| Bt.27904.1.A1\_at | 0.0125 | 0.000191 | 0.000437 | ns \* \* | 0.619 | 0.727 | 0.985 | 100 | 66.7 |
| Bt.9027.1.S1\_at | 0.0126 | 8.22e-05 | 0.000691 | ns \* \* | -1.09 | -0.703 | -1.01 | 100 | 66.7 |
| Bt.27927.1.S1\_at | 0.0127 | 0.000179 | 0.000173 | ns \* \* | -0.973 | -0.681 | -0.789 | 100 | 66.7 |
| Bt.22329.1.S1\_at | 0.0128 | 9.73e-06 | 6.5e-06 | ns \* \* | 0.699 | 0.772 | 0.886 | 100 | 66.7 |
| Bt.8124.1.S1\_at | 0.0131 | 1.94e-05 | 0.00024 | ns \* \* | 0.675 | 0.975 | 1.01 | 100 | 66.7 |
| Bt.24039.1.A1\_at | 0.0136 | 8.57e-05 | 4.66e-06 | ns \* \* | 0.602 | 0.621 | 0.778 | 100 | 66.7 |
| Bt.10916.2.S1\_at | 0.0143 | 6.9e-05 | 3.59e-05 | ns \* \* | 0.491 | 0.758 | 1.08 | 100 | 66.7 |
| Bt.13440.1.S1\_at | 0.0146 | 9.5e-05 | 9.43e-06 | ns \* \* | -0.707 | -0.64 | -1.11 | 100 | 66.7 |
| Bt.25689.1.S1\_at | 0.015 | 2.23e-05 | 4.72e-05 | ns \* \* | 0.669 | 0.802 | 0.928 | 100 | 66.7 |
| Bt.3560.1.S1\_at | 0.0151 | 1.02e-05 | 0.000257 | ns \* \* | -1.39 | -1.35 | -2.03 | 100 | 66.7 |
| Bt.15878.1.S1\_at | 0.0152 | 0.000449 | 0.000376 | ns \* \* | 0.558 | 0.727 | 0.944 | 100 | 66.7 |
| Bt.21376.1.S1\_at | 0.0155 | 2.86e-05 | 8.46e-06 | ns \* \* | 0.552 | 0.755 | 0.793 | 100 | 66.7 |
| Bt.21981.3.S1\_at | 0.0155 | 8.77e-06 | 2.48e-05 | ns \* \* | 0.669 | 0.933 | 1.15 | 100 | 66.7 |
| Bt.12510.1.S1\_a\_at | 0.0156 | 0.000587 | 1.65e-06 | ns \* \* | 0.657 | 0.832 | 3 | 100 | 66.7 |
| Bt.18352.1.A1\_at | 0.0157 | 0.00053 | 0.00028 | ns \* \* | 0.592 | 0.576 | 1.13 | 100 | 66.7 |
| Bt.15870.1.S1\_at | 0.0158 | 0.000438 | 0.000597 | ns \* \* | 0.537 | 0.623 | 1.06 | 100 | 66.7 |
| Bt.5246.1.S1\_at | 0.0162 | 0.000509 | 0.000501 | ns \* \* | 0.749 | 0.649 | 0.647 | 100 | 66.7 |
| Bt.20025.1.S1\_at | 0.0162 | 2.33e-05 | 0.000439 | ns \* \* | 0.633 | 0.87 | 0.901 | 100 | 66.7 |
| Bt.5377.1.S1\_at | 0.0168 | 2.69e-05 | 3.47e-05 | ns \* \* | 0.393 | 0.975 | 1.28 | 100 | 66.7 |
| Bt.24421.1.A1\_at | 0.0169 | 7.16e-05 | 0.000114 | ns \* \* | 0.601 | 0.833 | 1.39 | 100 | 66.7 |
| Bt.11194.1.S1\_at | 0.018 | 0.000354 | 4e-04 | ns \* \* | 1.26 | 0.798 | 1.61 | 100 | 66.7 |
| Bt.4773.4.S1\_at | 0.018 | 0.000155 | 8.24e-05 | ns \* \* | 0.661 | 0.638 | 0.491 | 100 | 66.7 |
| Bt.8356.1.S1\_at | 0.0198 | 0.000119 | 0.000304 | ns \* \* | 0.581 | 0.692 | 0.727 | 100 | 66.7 |
| Bt.3455.1.S1\_at | 0.0204 | 0.000207 | 0.000407 | ns \* \* | 0.626 | 0.714 | 1.04 | 100 | 66.7 |
| Bt.2564.1.S1\_at | 0.0206 | 0.000109 | 2.4e-05 | ns \* \* | 0.434 | 0.544 | 0.55 | 100 | 66.7 |
| Bt.19332.1.S1\_at | 0.0211 | 0.000203 | 0.000706 | ns \* \* | 0.553 | 1.15 | 1.49 | 100 | 66.7 |
| Bt.5334.1.S1\_at | 0.0221 | 0.000301 | 1.2e-06 | ns \* \* | 0.421 | 0.486 | 0.482 | 100 | 66.7 |
| Bt.5243.1.S1\_at | 0.0224 | 9.94e-05 | 1.13e-05 | ns \* \* | 0.505 | 0.619 | 0.685 | 100 | 66.7 |
| Bt.6815.1.A1\_at | 0.0231 | 0.000329 | 0.000239 | ns \* \* | 0.628 | 0.605 | 0.879 | 100 | 66.7 |
| Bt.8051.1.S1\_at | 0.0232 | 0.000181 | 6.24e-05 | ns \* \* | 0.509 | 0.678 | 0.645 | 100 | 66.7 |
| Bt.7701.1.S1\_at | 0.0251 | 5.88e-06 | 0.000286 | ns \* \* | 0.559 | 0.865 | 0.916 | 100 | 66.7 |
| Bt.7814.1.S1\_at | 0.0253 | 0.000159 | 7.78e-05 | ns \* \* | 0.442 | 0.567 | 0.502 | 100 | 66.7 |
| Bt.1154.1.S1\_at | 0.0255 | 3.26e-06 | 3.31e-05 | ns \* \* | 0.762 | 1.06 | 1.21 | 100 | 66.7 |
| Bt.5376.1.S1\_at | 0.0261 | 5.26e-05 | 3.22e-05 | ns \* \* | 0.482 | 0.626 | 0.647 | 100 | 66.7 |
| Bt.7903.1.A1\_at | 0.0274 | 8.66e-05 | 8.13e-05 | ns \* \* | 0.499 | 0.761 | 1.17 | 100 | 66.7 |
| Bt.2898.2.S1\_a\_at | 0.0284 | 0.000137 | 9.49e-05 | ns \* \* | 0.412 | 0.693 | 0.809 | 100 | 66.7 |
| Bt.5038.4.A1\_at | 0.0286 | 0.000261 | 0.000381 | ns \* \* | 0.424 | 0.512 | 0.508 | 100 | 66.7 |
| Bt.23218.1.S1\_at | 0.0288 | 0.000402 | 1.96e-05 | ns \* \* | 0.643 | 0.583 | 0.743 | 100 | 66.7 |
| Bt.16980.1.S1\_at | 0.0298 | 1.86e-06 | 9.26e-05 | ns \* \* | 0.607 | 0.948 | 1.25 | 100 | 66.7 |
| Bt.20008.1.S1\_at | 0.0306 | 0.000221 | 3.46e-06 | ns \* \* | 0.551 | 0.645 | 0.726 | 100 | 66.7 |
| Bt.29546.1.A1\_s\_at | 0.0353 | 4.63e-05 | 0.000239 | ns \* \* | 0.578 | 1.07 | 1.25 | 100 | 66.7 |
| Bt.7322.1.S1\_at | 0.0359 | 4.07e-05 | 1.55e-05 | ns \* \* | 0.964 | 1.12 | 1.93 | 100 | 66.7 |
| Bt.13324.4.S1\_at | 0.0372 | 0.000174 | 3.36e-05 | ns \* \* | 0.733 | 0.914 | 1.64 | 100 | 66.7 |
| Bt.6685.2.A1\_at | 0.0378 | 2.19e-05 | 0.000282 | ns \* \* | 0.944 | 1.07 | 1.16 | 100 | 66.7 |
| Bt.28194.1.S1\_at | 0.038 | 6e-04 | 0.000562 | ns \* \* | 0.618 | 0.554 | 0.636 | 100 | 66.7 |
| Bt.19575.1.S1\_at | 0.0411 | 0.000101 | 0.000124 | ns \* \* | 0.515 | 0.96 | 1.1 | 100 | 66.7 |
| Bt.8122.1.S1\_at | 0.0429 | 0.000558 | 0.000142 | ns \* \* | -0.449 | -0.45 | -0.469 | 100 | 66.7 |
| Bt.11163.1.S1\_at | 0.0439 | 0.000273 | 2.21e-06 | ns \* \* | -0.692 | -0.569 | -0.762 | 100 | 66.7 |
| Bt.20988.1.S1\_at | 0.0445 | 3e-04 | 0.000512 | ns \* \* | 0.508 | 0.734 | 0.957 | 100 | 66.7 |
| Bt.7437.1.S1\_at | 0.0448 | 0.000187 | 4.82e-06 | ns \* \* | 0.461 | 0.62 | 0.961 | 100 | 66.7 |
| Bt.7139.1.S1\_at | 0.0451 | 0.000381 | 2.25e-05 | ns \* \* | 0.603 | 0.685 | 0.706 | 100 | 66.7 |
| Bt.4637.1.A1\_a\_at | 0.0465 | 6.56e-06 | 3.29e-05 | ns \* \* | 0.699 | 1.07 | 1.14 | 100 | 66.7 |
| Bt.24875.1.S1\_at | 0.0476 | 0.000577 | 2.33e-05 | ns \* \* | 0.459 | 0.564 | 0.682 | 100 | 66.7 |
| Bt.26999.1.S1\_at | 0.0498 | 0.00032 | 0.000664 | ns \* \* | 0.448 | 0.616 | 0.649 | 100 | 66.7 |
| Bt.18278.1.S1\_at | 0.0501 | 4.68e-07 | 1.47e-05 | ns \* \* | 0.729 | 1.13 | 1.06 | 100 | 66.7 |
| Bt.27226.1.S1\_at | 0.0518 | 3.79e-05 | 0.000501 | ns \* \* | 0.623 | 0.917 | 1.06 | 100 | 66.7 |
| Bt.26885.1.S1\_at | 0.0541 | 0.000235 | 4.35e-05 | ns \* \* | 0.729 | 0.987 | 1.02 | 100 | 66.7 |
| Bt.21551.1.S1\_at | 0.0569 | 1e-05 | 7.8e-05 | ns \* \* | -0.799 | -0.864 | -0.982 | 100 | 66.7 |
| Bt.19208.1.A1\_at | 0.0581 | 0.000124 | 0.000199 | ns \* \* | 0.389 | 0.839 | 0.944 | 100 | 66.7 |
| Bt.10608.2.S1\_a\_at | 0.0666 | 1.81e-06 | 1.8e-05 | ns \* \* | 0.59 | 0.911 | 1.15 | 100 | 66.7 |
| Bt.997.2.S1\_a\_at | 0.067 | 0.000189 | 0.000494 | ns \* \* | 0.492 | 0.838 | 1.31 | 100 | 66.7 |
| Bt.23254.1.S1\_at | 0.069 | 0.000366 | 0.000731 | ns \* \* | 0.387 | 0.533 | 0.552 | 100 | 66.7 |
| Bt.7540.3.S1\_at | 0.0699 | 2.04e-06 | 6.94e-07 | ns \* \* | 0.538 | 1.22 | 1.92 | 100 | 66.7 |
| Bt.3715.1.S1\_at | 0.0716 | 0.00027 | 4.53e-05 | ns \* \* | 0.498 | 0.66 | 0.715 | 100 | 66.7 |
| Bt.22721.1.A1\_a\_at | 0.075 | 0.000437 | 0.000129 | ns \* \* | 0.509 | 0.891 | 1.04 | 100 | 66.7 |
| Bt.25135.1.A1\_at | 0.076 | 0.00013 | 0.000636 | ns \* \* | 0.483 | 0.771 | 1.05 | 100 | 66.7 |
| Bt.21027.1.S1\_at | 0.0793 | 5.01e-05 | 0.000324 | ns \* \* | 0.511 | 1.05 | 1.27 | 100 | 66.7 |
| Bt.2825.1.S1\_at | 0.0811 | 9.92e-05 | 3.45e-05 | ns \* \* | 0.848 | 0.776 | 1.2 | 100 | 66.7 |
| Bt.9799.1.S1\_at | 0.0844 | 2.28e-05 | 3.54e-05 | ns \* \* | 0.408 | 0.901 | 1.38 | 100 | 66.7 |
| Bt.1214.1.S1\_at | 0.0856 | 0.000108 | 7.46e-06 | ns \* \* | 0.262 | 0.817 | 1.39 | 100 | 66.7 |
| Bt.17704.1.S1\_at | 0.0856 | 0.000282 | 6.61e-05 | ns \* \* | 0.563 | 0.785 | 0.897 | 100 | 66.7 |
| Bt.11448.1.S1\_at | 0.0857 | 0.000556 | 0.000428 | ns \* \* | 0.407 | 0.517 | 0.48 | 100 | 66.7 |
| Bt.15819.2.S1\_at | 0.0873 | 2.66e-05 | 0.00027 | ns \* \* | 0.327 | 0.954 | 0.988 | 100 | 66.7 |
| Bt.7870.1.S1\_at | 0.0891 | 0.000256 | 0.000709 | ns \* \* | 0.774 | 1.02 | 1.14 | 100 | 66.7 |
| Bt.18890.1.A1\_at | 0.092 | 1.44e-07 | 1.51e-06 | ns \* \* | 0.419 | 1.02 | 0.907 | 100 | 66.7 |
| Bt.23002.1.S1\_at | 0.0954 | 9.6e-05 | 0.000479 | ns \* \* | -0.895 | -0.911 | -1.16 | 100 | 66.7 |
| Bt.2428.1.S1\_at | 0.0994 | 0.000579 | 0.000751 | ns \* \* | 0.836 | 0.915 | 1.02 | 100 | 66.7 |
| Bt.23308.1.S1\_at | 0.104 | 0.000255 | 5.98e-05 | ns \* \* | 0.326 | 0.671 | 0.742 | 100 | 66.7 |
| Bt.4844.1.S1\_at | 0.112 | 9.16e-05 | 0.000459 | ns \* \* | 0.79 | 1.38 | 1.48 | 100 | 66.7 |
| Bt.15845.1.S1\_at | 0.119 | 0.000325 | 2.91e-05 | ns \* \* | 0.299 | 0.654 | 0.895 | 100 | 66.7 |
| Bt.5598.1.S1\_at | 0.14 | 0.000437 | 4.54e-05 | ns \* \* | -0.76 | -0.996 | -1.39 | 100 | 66.7 |
| Bt.9741.1.S1\_at | 0.15 | 0.000281 | 0.000273 | ns \* \* | 0.351 | 0.718 | 1.05 | 100 | 66.7 |
| Bt.1405.1.S1\_at | 0.152 | 0.000117 | 0.000439 | ns \* \* | -0.684 | -0.629 | -0.858 | 100 | 66.7 |
| Bt.422.1.S1\_at | 0.184 | 4.23e-05 | 1.1e-05 | ns \* \* | 0.286 | 1.08 | 1.31 | 100 | 66.7 |
| Bt.24960.1.S1\_at | 0.189 | 5.6e-05 | 0.000283 | ns \* \* | 0.324 | 0.932 | 1.08 | 100 | 66.7 |
| Bt.21896.1.S1\_at | 0.201 | 0.000136 | 0.000544 | ns \* \* | 0.326 | 0.997 | 1.15 | 100 | 66.7 |
| Bt.3854.1.S1\_at | 0.235 | 0.000475 | 0.000579 | ns \* \* | 0.277 | 0.755 | 0.825 | 100 | 66.7 |
| Bt.21396.1.A1\_s\_at | 0.241 | 8.12e-05 | 3.02e-07 | ns \* \* | 0.467 | 0.766 | 0.625 | 100 | 66.7 |
| Bt.9057.1.A1\_at | 0.256 | 0.000129 | 1.84e-05 | ns \* \* | 0.405 | 0.573 | 0.554 | 100 | 66.7 |
| Bt.22306.1.S1\_at | 0.269 | 3.6e-07 | 1.9e-06 | ns \* \* | 0.365 | 1.48 | 1.93 | 100 | 66.7 |
| Bt.3015.1.A1\_at | 0.285 | 0.000113 | 3.75e-07 | ns \* \* | 0.221 | 0.839 | 1.01 | 100 | 66.7 |
| Bt.13834.1.S1\_at | 0.297 | 3.66e-05 | 0.000229 | ns \* \* | 0.709 | 0.981 | 1.1 | 100 | 66.7 |
| Bt.27552.1.A1\_at | 0.303 | 0.000552 | 0.000551 | ns \* \* | -0.28 | -0.726 | -1.12 | 100 | 66.7 |
| Bt.19519.1.S1\_at | 0.304 | 0.000393 | 1.26e-05 | ns \* \* | 0.344 | 0.664 | 0.807 | 100 | 66.7 |
| Bt.5131.1.S1\_at | 0.305 | 0.00015 | 9.6e-06 | ns \* \* | 0.27 | 0.746 | 1.05 | 100 | 66.7 |
| Bt.11687.2.S1\_at | 0.322 | 0.000511 | 0.000155 | ns \* \* | 0.366 | 0.532 | 0.617 | 100 | 66.7 |
| Bt.9589.1.S1\_at | 0.395 | 0.000301 | 5.62e-05 | ns \* \* | 0.217 | 0.773 | 0.771 | 100 | 66.7 |
| Bt.21731.1.S1\_at | 0.396 | 6.68e-05 | 4.06e-06 | ns \* \* | 0.222 | 0.623 | 1.26 | 100 | 66.7 |
| Bt.23589.2.S1\_at | 0.413 | 0.000459 | 1.23e-05 | ns \* \* | 0.192 | 0.582 | 0.789 | 100 | 66.7 |
| Bt.1244.1.S1\_at | 0.415 | 0.000123 | 0.000598 | ns \* \* | 0.215 | 0.608 | 0.519 | 100 | 66.7 |
| Bt.13604.1.S1\_at | 0.509 | 2.15e-05 | 0.000105 | ns \* \* | 0.214 | 0.752 | 0.767 | 100 | 66.7 |
| Bt.4442.1.S1\_at | 0.558 | 0.000275 | 0.000153 | ns \* \* | 0.108 | 0.629 | 0.766 | 100 | 66.7 |
| Bt.5096.1.S1\_at | 0.579 | 0.000382 | 0.000282 | ns \* \* | 0.156 | 0.62 | 0.857 | 100 | 66.7 |
| Bt.22466.1.A1\_at | 0.588 | 3.17e-05 | 0.000636 | ns \* \* | 0.113 | 0.716 | 0.709 | 100 | 66.7 |
| Bt.21313.1.S1\_at | 0.727 | 0.000444 | 1.56e-05 | ns \* \* | 0.0954 | 0.554 | 0.64 | 100 | 66.7 |
| Bt.26811.1.S1\_at | 0.821 | 9.26e-05 | 0.000305 | ns \* \* | 0.0505 | 1 | 1.11 | 100 | 66.7 |
| Bt.26817.1.S1\_at | 0.872 | 2.93e-05 | 0.000376 | ns \* \* | 0.0579 | 0.863 | 0.401 | 100 | 66.7 |
| Bt.18228.1.A1\_at | 0.997 | 0.000436 | 0.000361 | ns \* \* | 0.000811 | 0.589 | 0.778 | 100 | 66.7 |
| Bt.12888.1.S1\_at | 0.999 | 0.000332 | 4.36e-05 | ns \* \* | -0.00023 | 0.854 | 1.06 | 100 | 66.7 |
| Bt.27283.1.S1\_x\_at | 1.33e-05 | 0.0011 | 0.000103 | \* ns \* | -3.5 | -0.605 | -0.77 | 83.3 | 66.7 |
| Bt.4864.2.S1\_at | 4.73e-05 | 0.00197 | 0.000655 | \* ns \* | -1.1 | -0.41 | -0.563 | 83.3 | 66.7 |
| Bt.4868.2.S1\_a\_at | 5.53e-05 | 0.00662 | 3.01e-05 | \* ns \* | 1.02 | 0.369 | 0.736 | 83.3 | 66.7 |
| Bt.23094.2.A1\_at | 5.54e-05 | 0.00021 | 0.00434 | \* \* ns | -1.28 | -0.718 | -1.24 | 83.3 | 66.7 |
| Bt.1187.1.S1\_at | 8.82e-05 | 0.00427 | 0.000401 | \* ns \* | -1.5 | -0.498 | -0.838 | 83.3 | 66.7 |
| Bt.20095.2.S1\_at | 0.000114 | 0.00814 | 3.4e-05 | \* ns \* | -0.981 | -0.353 | -0.744 | 83.3 | 66.7 |
| Bt.23734.1.A1\_at | 0.000186 | 0.048 | 0.000434 | \* ns \* | -1.79 | -0.499 | -1.99 | 83.3 | 66.7 |
| Bt.16004.1.A1\_at | 0.000262 | 2.91e-05 | 0.000343 | ns \* \* | 3.35 | 2.81 | 3.46 | 83.3 | 66.7 |
| Bt.17512.1.S1\_at | 0.000268 | 3.78e-06 | 6.4e-06 | ns \* \* | -1.65 | -1.05 | -0.945 | 83.3 | 66.7 |
| Bt.8212.1.S1\_at | 0.000285 | 2.68e-05 | 2.16e-06 | ns \* \* | -1.28 | -0.755 | -1.02 | 83.3 | 66.7 |
| Bt.1370.1.S1\_at | 0.000453 | 0.000195 | 8.54e-05 | ns \* \* | -1.81 | -1.13 | -1.5 | 83.3 | 66.7 |
| Bt.21468.1.S1\_at | 0.000458 | 0.000178 | 0.000411 | ns \* \* | 1.11 | 0.875 | 1.14 | 83.3 | 66.7 |
| Bt.12510.1.S1\_at | 0.000476 | 2.99e-08 | 1.88e-06 | ns \* \* | 1.64 | 2.26 | 3.06 | 83.3 | 66.7 |
| Bt.5038.1.S2\_at | 0.000519 | 5.68e-05 | 0.00069 | ns \* \* | -0.939 | -0.871 | -1.26 | 83.3 | 66.7 |
| Bt.12862.1.S1\_at | 0.000932 | 0.000252 | 0.000111 | ns \* \* | 1.38 | 0.923 | 1.39 | 83.3 | 66.7 |
| Bt.27552.2.S1\_at | 0.000932 | 1.12e-05 | 0.000234 | ns \* \* | -1.45 | -1.07 | -1.97 | 83.3 | 66.7 |
| Bt.15925.1.S1\_at | 0.000999 | 0.000129 | 7.68e-06 | ns \* \* | 1.32 | 0.834 | 1.55 | 83.3 | 66.7 |
| Bt.4798.1.S2\_at | 0.00114 | 1.02e-06 | 0.000133 | ns \* \* | 1.52 | 1.6 | 3.41 | 83.3 | 66.7 |
| Bt.10153.1.S1\_at | 0.00128 | 1.9e-06 | 1.6e-06 | ns \* \* | -1.33 | -1.33 | -1.75 | 83.3 | 66.7 |
| Bt.6483.1.A1\_at | 0.00132 | 1.69e-05 | 8.86e-05 | ns \* \* | 0.781 | 0.783 | 0.792 | 83.3 | 66.7 |
| Bt.9870.2.A1\_at | 0.00166 | 2.21e-05 | 0.000258 | ns \* \* | 0.845 | 1.07 | 1.44 | 83.3 | 66.7 |
| Bt.17148.2.S1\_at | 0.00186 | 6.96e-07 | 6.22e-05 | ns \* \* | 1.09 | 1.41 | 1.35 | 83.3 | 66.7 |
| Bt.12508.1.S1\_at | 0.00221 | 8.77e-06 | 6.33e-05 | ns \* \* | 1.16 | 1.13 | 1.72 | 83.3 | 66.7 |
| Bt.12411.1.S1\_at | 0.00304 | 2.98e-06 | 5.48e-05 | ns \* \* | 1.13 | 0.993 | 1.21 | 83.3 | 66.7 |
| Bt.23094.2.A1\_x\_at | 0.00344 | 1.42e-05 | 5.79e-08 | ns \* \* | -1.78 | -1.18 | -3.15 | 83.3 | 66.7 |
| Bt.17883.2.A1\_at | 0.00349 | 9.28e-07 | 2.39e-05 | ns \* \* | -1.48 | -1.01 | -1.61 | 83.3 | 66.7 |
| Bt.29928.1.S1\_at | 0.00507 | 1.25e-05 | 1.18e-07 | ns \* \* | -0.95 | -0.802 | -1.27 | 83.3 | 66.7 |
| Bt.5269.1.S1\_at | 0.0059 | 4.53e-05 | 1.02e-06 | ns \* \* | -0.912 | -0.878 | -1.08 | 83.3 | 66.7 |
| Bt.2696.2.S1\_at | 0.00697 | 1.69e-06 | 0.000395 | ns \* \* | 0.699 | 1.4 | 1.47 | 83.3 | 66.7 |
| Bt.23605.1.S1\_at | 0.00826 | 0.000106 | 4.82e-06 | ns \* \* | -1.04 | -0.809 | -1.33 | 83.3 | 66.7 |
| Bt.350.1.S1\_s\_at | 0.00858 | 3.35e-06 | 6.62e-09 | ns \* \* | -3.13 | -1.44 | -3.82 | 83.3 | 66.7 |
| Bt.5333.1.S1\_at | 0.0108 | 0.000345 | 2.33e-05 | ns \* \* | 0.694 | 0.696 | 1.65 | 83.3 | 66.7 |
| Bt.28623.1.S1\_at | 0.0146 | 2.33e-06 | 2.51e-05 | ns \* \* | 0.592 | 0.92 | 1.36 | 83.3 | 66.7 |
| Bt.16567.1.A1\_at | 0.0147 | 4.8e-06 | 2.07e-05 | ns \* \* | 1.35 | 0.944 | 1.71 | 83.3 | 66.7 |
| Bt.5530.1.S1\_at | 0.0236 | 4.47e-06 | 3.44e-06 | ns \* \* | -0.926 | -1 | -1.46 | 83.3 | 66.7 |
| Bt.22325.2.A1\_at | 0.0257 | 1.28e-05 | 1.88e-06 | ns \* \* | 0.567 | 0.958 | 1.44 | 83.3 | 66.7 |
| Bt.18689.2.A1\_at | 0.0347 | 2.43e-05 | 0.000628 | ns \* \* | 0.617 | 1.05 | 1.03 | 83.3 | 66.7 |
| Bt.20479.1.S1\_at | 0.0383 | 0.000425 | 0.000645 | ns \* \* | 1.06 | 0.973 | 1.1 | 83.3 | 66.7 |
| Bt.11239.3.S1\_at | 0.0409 | 4.91e-05 | 0.000125 | ns \* \* | -0.758 | -0.814 | -1.19 | 83.3 | 66.7 |
| Bt.1318.1.S1\_at | 0.054 | 0.000559 | 0.000131 | ns \* \* | 0.642 | 0.513 | 1.29 | 83.3 | 66.7 |
| Bt.3562.1.S1\_at | 0.0683 | 1.68e-06 | 3.59e-06 | ns \* \* | 0.648 | 1.51 | 2.32 | 83.3 | 66.7 |
| Bt.1178.1.S1\_a\_at | 0.0753 | 8.08e-06 | 3.93e-07 | ns \* \* | -1.42 | -1.06 | -2.1 | 83.3 | 66.7 |
| Bt.16362.2.S1\_at | 0.0942 | 0.000215 | 0.000669 | ns \* \* | 0.477 | 0.641 | 0.495 | 83.3 | 66.7 |
| Bt.26122.2.S1\_at | 0.108 | 0.00014 | 4.75e-06 | ns \* \* | 0.564 | 0.725 | 1.08 | 83.3 | 66.7 |
| Bt.16628.2.A1\_at | 0.211 | 9.63e-05 | 0.000349 | ns \* \* | 0.554 | 1.25 | 1.46 | 83.3 | 66.7 |
| Bt.10990.1.S1\_at | 0.222 | 0.000188 | 5.46e-05 | ns \* \* | 0.446 | 0.75 | 1.08 | 83.3 | 66.7 |
| Bt.2846.1.A1\_at | 0.25 | 0.000222 | 0.000332 | ns \* \* | 0.452 | 0.753 | 1.14 | 83.3 | 66.7 |
| Bt.4339.1.S1\_at | 0.272 | 3.5e-05 | 0.000314 | ns \* \* | 0.362 | 0.725 | 0.871 | 83.3 | 66.7 |
| Bt.26524.1.S1\_at | 0.402 | 3.61e-05 | 0.000574 | ns \* \* | 0.27 | 0.722 | 0.578 | 83.3 | 66.7 |
| Bt.15540.1.A1\_at | 0.424 | 2.83e-06 | 7.59e-05 | ns \* \* | -0.23 | -0.857 | -1.38 | 83.3 | 66.7 |
| Bt.11013.1.S1\_at | 0.473 | 0.000568 | 3.6e-05 | ns \* \* | 0.182 | 0.606 | 0.911 | 83.3 | 66.7 |
| Bt.15753.1.S1\_at | 0.916 | 5.25e-05 | 3.34e-05 | ns \* \* | -0.0332 | 0.665 | 0.765 | 83.3 | 66.7 |
| Bt.1388.1.S1\_at | 0.942 | 0.000248 | 0.000184 | ns \* \* | -0.0158 | 0.537 | 1.09 | 83.3 | 66.7 |
| Bt.16105.1.S1\_at | 5.62e-07 | 0.003 | 9.72e-05 | \* ns \* | 2.23 | 0.779 | 1.68 | 66.7 | 66.7 |
| Bt.19469.1.A1\_at | 0.000122 | 0.0171 | 5.66e-05 | \* ns \* | -1.38 | -0.316 | -0.57 | 66.7 | 66.7 |
| Bt.4670.1.S1\_at | 0.000226 | 6.13e-06 | 1.49e-05 | ns \* \* | 1.19 | 1 | 1.42 | 66.7 | 66.7 |
| Bt.22234.1.S1\_at | 0.00043 | 2.8e-06 | 0.000416 | ns \* \* | -2.97 | -1.76 | -2.55 | 66.7 | 66.7 |
| Bt.5465.2.A1\_a\_at | 0.00044 | 3.82e-05 | 2.58e-05 | ns \* \* | 1.05 | 1.07 | 1.45 | 66.7 | 66.7 |
| Bt.3251.1.S1\_at | 0.000451 | 0.000367 | 1.09e-06 | ns \* \* | -0.96 | -0.651 | -1.24 | 66.7 | 66.7 |
| Bt.2749.1.S1\_at | 0.00055 | 7.49e-06 | 0.000178 | ns \* \* | 2.15 | 2 | 3.2 | 66.7 | 66.7 |
| Bt.17579.1.A1\_at | 0.000805 | 0.000108 | 9.82e-05 | ns \* \* | 1.43 | 1.53 | 2.64 | 66.7 | 66.7 |
| Bt.21409.1.A1\_at | 0.00115 | 0.000159 | 0.000752 | ns \* \* | 3.11 | 2.53 | 3.61 | 66.7 | 66.7 |
| Bt.4751.1.S1\_a\_at | 0.00177 | 1.48e-05 | 0.000155 | ns \* \* | -1.84 | -1.48 | -2.73 | 66.7 | 66.7 |
| Bt.10810.1.A1\_at | 0.00205 | 5.34e-08 | 1.04e-07 | ns \* \* | 1.39 | 1.84 | 2.16 | 66.7 | 66.7 |
| Bt.4751.2.S1\_a\_at | 0.00353 | 1.49e-05 | 7.7e-05 | ns \* \* | -3.62 | -1.5 | -3.09 | 66.7 | 66.7 |
| Bt.13306.2.S1\_at | 0.00388 | 5.33e-06 | 4.66e-05 | ns \* \* | -1.58 | -1.05 | -1.73 | 66.7 | 66.7 |
| Bt.21578.1.S1\_at | 0.00467 | 1.96e-06 | 6.06e-06 | ns \* \* | 1.01 | 0.981 | 1.61 | 66.7 | 66.7 |
| Bt.4680.1.S1\_at | 0.00501 | 1.02e-05 | 8.7e-05 | ns \* \* | 0.877 | 1.1 | 1.66 | 66.7 | 66.7 |
| Bt.25099.2.S1\_at | 0.00536 | 1.95e-06 | 0.000638 | ns \* \* | 2.36 | 2 | 2.97 | 66.7 | 66.7 |
| Bt.28524.1.S1\_at | 0.00592 | 4.97e-07 | 5.79e-05 | ns \* \* | 0.727 | 1.05 | 1.29 | 66.7 | 66.7 |
| Bt.21054.1.A1\_at | 0.00775 | 1.42e-05 | 5.4e-05 | ns \* \* | -1.34 | -1.36 | -2.19 | 66.7 | 66.7 |
| Bt.7642.1.S1\_at | 0.0125 | 3.94e-05 | 1.28e-05 | ns \* \* | 0.97 | 0.9 | 1.95 | 66.7 | 66.7 |
| Bt.21800.1.S1\_at | 0.013 | 7.31e-05 | 0.00032 | ns \* \* | 1.15 | 1.13 | 1.28 | 66.7 | 66.7 |
| Bt.24269.1.S1\_at | 0.0164 | 7.07e-06 | 0.000252 | ns \* \* | 1.32 | 0.985 | 1.38 | 66.7 | 66.7 |
| Bt.876.1.S1\_at | 0.0513 | 9.91e-06 | 0.000462 | ns \* \* | 1.15 | 1.51 | 2.16 | 66.7 | 66.7 |
| Bt.2070.3.S1\_at | 0.117 | 6.48e-06 | 0.000253 | ns \* \* | 0.705 | 1.14 | 1.74 | 66.7 | 66.7 |
| Bt.4274.1.S1\_at | 0.129 | 5.13e-05 | 3.05e-07 | ns \* \* | 0.338 | 0.688 | 1.37 | 66.7 | 66.7 |
| Bt.562.1.S1\_at | 0.133 | 2.4e-06 | 1.63e-06 | ns \* \* | -1.47 | -1.13 | -2.53 | 66.7 | 66.7 |
| Bt.4803.1.S1\_at | 0.146 | 0.000544 | 2.41e-05 | ns \* \* | 0.302 | 0.508 | 0.56 | 66.7 | 66.7 |
| Bt.2110.1.S1\_at | 0.157 | 2.86e-05 | 0.000323 | ns \* \* | 0.41 | 1.2 | 1.84 | 66.7 | 66.7 |
| Bt.23212.1.S1\_at | 0.778 | 1.03e-07 | 1.58e-05 | ns \* \* | 0.0898 | 1.27 | 1.45 | 66.7 | 66.7 |
| Bt.15847.1.S1\_at | 0.791 | 0.000267 | 1.29e-05 | ns \* \* | 0.0644 | 0.633 | 1.24 | 66.7 | 66.7 |
| Bt.89.1.S1\_at | 3.73e-05 | 0.00357 | 9.96e-05 | \* ns \* | 1.9 | 0.51 | 1.62 | 50 | 66.7 |
| Bt.18101.1.A1\_at | 4.69e-05 | 0.000528 | 0.00681 | \* \* ns | 3.52 | 1.16 | 1.32 | 50 | 66.7 |
| Bt.24856.2.S1\_at | 5.88e-05 | 0.00038 | 0.00145 | \* \* ns | 1.38 | 0.835 | 1.23 | 50 | 66.7 |
| Bt.21724.1.S1\_at | 0.000224 | 1.38e-06 | 4.26e-07 | ns \* \* | -1.45 | -0.972 | -1.24 | 50 | 66.7 |
| Bt.6840.1.S1\_a\_at | 0.000233 | 1.31e-05 | 8.4e-05 | ns \* \* | 2.06 | 1.18 | 2.07 | 50 | 66.7 |
| Bt.7056.1.S1\_at | 0.00024 | 1.07e-07 | 1.03e-08 | ns \* \* | 3.49 | 2.55 | 3.24 | 50 | 66.7 |
| Bt.25186.1.S1\_at | 0.000242 | 1.97e-05 | 0.000488 | ns \* \* | 5.06 | 2.09 | 2.38 | 50 | 66.7 |
| Bt.7431.1.S1\_a\_at | 0.000283 | 0.000128 | 2.33e-05 | ns \* \* | -1.1 | -0.705 | -1.78 | 50 | 66.7 |
| Bt.25398.1.A1\_at | 0.000343 | 7.07e-07 | 1.91e-05 | ns \* \* | 1.48 | 1.39 | 1.81 | 50 | 66.7 |
| Bt.6642.1.S1\_at | 0.000522 | 9.38e-09 | 1.44e-10 | ns \* \* | -1.72 | -1.71 | -3.14 | 50 | 66.7 |
| Bt.7955.1.S1\_at | 0.000619 | 1.45e-05 | 9.91e-06 | ns \* \* | 0.925 | 0.912 | 1.14 | 50 | 66.7 |
| Bt.20862.1.A1\_at | 0.000769 | 1.42e-07 | 6.49e-09 | ns \* \* | 0.99 | 1.22 | 2.62 | 50 | 66.7 |
| Bt.26135.1.A1\_at | 0.000988 | 9.1e-11 | 3.8e-10 | ns \* \* | 2.24 | 2.34 | 3.88 | 50 | 66.7 |
| Bt.8319.3.S1\_a\_at | 0.00111 | 5.76e-05 | 0.000564 | ns \* \* | -0.989 | -0.827 | -1.22 | 50 | 66.7 |
| Bt.26760.2.S1\_a\_at | 0.00145 | 1.1e-10 | 1.05e-10 | ns \* \* | -3.89 | -1.98 | -5.25 | 50 | 66.7 |
| Bt.2786.1.S1\_at | 0.00156 | 1.55e-05 | 2.05e-06 | ns \* \* | 0.815 | 0.941 | 1.92 | 50 | 66.7 |
| Bt.1720.1.S1\_at | 0.00231 | 0.000188 | 7.69e-05 | ns \* \* | 0.877 | 0.725 | 1.63 | 50 | 66.7 |
| Bt.3980.1.S1\_at | 0.00262 | 6.17e-05 | 0.000167 | ns \* \* | 1.1 | 1.25 | 1.66 | 50 | 66.7 |
| Bt.14340.2.S1\_at | 0.0034 | 4.28e-05 | 5.71e-06 | ns \* \* | -1.11 | -0.906 | -1.93 | 50 | 66.7 |
| Bt.10092.1.S1\_at | 0.00419 | 0.000289 | 0.000614 | ns \* \* | -0.988 | -0.821 | -1.12 | 50 | 66.7 |
| Bt.659.1.S1\_at | 0.005 | 8.4e-08 | 1.79e-06 | ns \* \* | -1.93 | -1.51 | -2.55 | 50 | 66.7 |
| Bt.13334.1.S1\_at | 0.00513 | 0.00042 | 0.000173 | ns \* \* | -0.779 | -0.62 | -0.884 | 50 | 66.7 |
| Bt.26711.1.S1\_at | 0.006 | 0.000444 | 1.89e-06 | ns \* \* | -1.49 | -0.811 | -1.78 | 50 | 66.7 |
| Bt.16979.3.S1\_at | 0.00687 | 0.000115 | 3.04e-06 | ns \* \* | -0.962 | -0.771 | -0.669 | 50 | 66.7 |
| Bt.18316.1.A1\_at | 0.00835 | 0.000206 | 8.53e-05 | ns \* \* | -2.6 | -1.18 | -1.59 | 50 | 66.7 |
| Bt.13435.1.A1\_at | 0.00855 | 5.89e-08 | 1.88e-06 | ns \* \* | 2.04 | 2.11 | 2.44 | 50 | 66.7 |
| Bt.4523.1.S1\_at | 0.00859 | 0.000287 | 0.000477 | ns \* \* | 1.8 | 0.649 | 0.818 | 50 | 66.7 |
| Bt.13445.2.S1\_a\_at | 0.00991 | 8.44e-05 | 0.000305 | ns \* \* | 0.729 | 0.96 | 1.35 | 50 | 66.7 |
| Bt.20570.1.S1\_at | 0.0117 | 8.63e-05 | 8.2e-05 | ns \* \* | 1.2 | 0.827 | 1.27 | 50 | 66.7 |
| Bt.22576.3.S1\_at | 0.0123 | 0.000253 | 3.72e-06 | ns \* \* | -1.46 | -0.652 | -1.27 | 50 | 66.7 |
| Bt.16087.1.S1\_at | 0.0132 | 0.000117 | 2.97e-05 | ns \* \* | 1.09 | 1.02 | 1.26 | 50 | 66.7 |
| Bt.21942.1.S1\_at | 0.0136 | 0.000231 | 0.000566 | ns \* \* | 1.38 | 0.94 | 1.08 | 50 | 66.7 |
| Bt.27301.1.S1\_at | 0.0153 | 4.63e-06 | 1.15e-05 | ns \* \* | 0.954 | 1.48 | 2.63 | 50 | 66.7 |
| Bt.5170.1.S1\_at | 0.0179 | 0.000458 | 0.000414 | ns \* \* | 0.747 | 0.644 | 1.05 | 50 | 66.7 |
| Bt.16821.1.S1\_at | 0.0198 | 0.000181 | 0.000416 | ns \* \* | 0.917 | 1.1 | 1.77 | 50 | 66.7 |
| Bt.8963.1.S1\_at | 0.0238 | 5.31e-06 | 2.03e-06 | ns \* \* | 0.925 | 1.23 | 1.99 | 50 | 66.7 |
| Bt.14223.3.S1\_at | 0.029 | 4.64e-05 | 0.00051 | ns \* \* | -2.76 | -0.726 | -1.17 | 50 | 66.7 |
| Bt.4816.1.S1\_at | 0.0617 | 2.82e-05 | 3.71e-07 | ns \* \* | -1.21 | -0.991 | -2.06 | 50 | 66.7 |
| Bt.22002.1.S1\_at | 0.0654 | 7.57e-08 | 0.000108 | ns \* \* | 2.47 | 1.85 | 2.73 | 50 | 66.7 |
| Bt.21537.1.A1\_at | 0.0868 | 1.41e-06 | 0.00014 | ns \* \* | -1.3 | -1.22 | -1.95 | 50 | 66.7 |
| Bt.9828.1.S2\_at | 0.575 | 0.000415 | 6.44e-07 | ns \* \* | -0.601 | -0.705 | -1.38 | 50 | 66.7 |
| Bt.23355.1.S1\_at | 0.831 | 0.000399 | 1.22e-05 | ns \* \* | 0.0618 | 0.75 | 1.3 | 50 | 66.7 |

Differentially expressed genes for MAS: 317

| AFFY | p-value | FoldChange |
| --- | --- | --- |
| Bt.7471.1.S1\_at | 1.766e-08 | 2.965 |
| Bt.24478.1.S1\_at | 2.949e-08 | 3.232 |
| Bt.23113.1.A1\_at | 7.198e-08 | 2.662 |
| Bt.13986.1.S1\_at | 8.15e-08 | -1.663 |
| Bt.8121.1.S1\_x\_at | 8.24e-08 | -4.773 |
| Bt.13289.1.S1\_at | 1.164e-07 | -2.942 |
| Bt.4818.1.S1\_at | 1.225e-07 | 1.623 |
| Bt.8972.1.S1\_at | 1.354e-07 | -2.133 |
| Bt.7034.1.S1\_at | 1.4e-07 | 3.271 |
| Bt.18639.1.A1\_at | 1.726e-07 | -4.75 |
| Bt.24614.1.S1\_at | 2.81e-07 | 1.935 |
| Bt.8699.1.S1\_at | 2.955e-07 | 2.416 |
| Bt.9556.1.S1\_at | 3.224e-07 | 1.915 |
| Bt.20501.1.S1\_at | 3.341e-07 | -4.619 |
| Bt.12299.1.A1\_at | 3.964e-07 | 5.04 |
| Bt.4867.2.S1\_at | 4.084e-07 | 4.703 |
| Bt.5129.2.A1\_at | 4.727e-07 | 2.359 |
| Bt.6642.2.S1\_at | 4.847e-07 | -2.734 |
| Bt.13289.2.S1\_at | 5.223e-07 | -3.849 |
| Bt.16105.1.S1\_at | 5.622e-07 | 2.229 |
| Bt.7394.1.A1\_at | 6.388e-07 | 1.783 |
| Bt.13195.1.A1\_at | 7.177e-07 | -4.118 |
| Bt.19423.1.S1\_at | 8.071e-07 | -2.107 |
| Bt.25052.1.S1\_at | 8.874e-07 | 1.415 |
| Bt.27878.1.S1\_at | 9.511e-07 | 3.931 |
| Bt.2522.1.S1\_at | 9.914e-07 | 3.182 |
| Bt.3071.1.S1\_at | 1.025e-06 | 1.655 |
| Bt.7651.1.S1\_at | 1.027e-06 | -2.188 |
| Bt.5070.1.S1\_x\_at | 1.122e-06 | -1.526 |
| Bt.6991.1.S1\_at | 1.2e-06 | -1.578 |
| Bt.7133.1.S1\_at | 1.284e-06 | 1.543 |
| Bt.13588.1.A1\_at | 1.311e-06 | 5.393 |
| Bt.13021.1.S1\_at | 1.571e-06 | 3.525 |
| Bt.5717.1.S1\_at | 2.08e-06 | 2.493 |
| Bt.15682.1.A1\_at | 2.118e-06 | 1.965 |
| Bt.1035.1.S1\_a\_at | 2.307e-06 | 1.265 |
| Bt.1029.1.S1\_at | 2.43e-06 | 3.501 |
| Bt.22803.1.S1\_at | 2.527e-06 | 1.609 |
| Bt.793.1.S1\_at | 2.758e-06 | 2.314 |
| Bt.25319.2.S1\_at | 2.81e-06 | -1.284 |
| Bt.5340.1.S1\_s\_at | 2.873e-06 | 1.383 |
| Bt.6774.1.S1\_at | 2.977e-06 | -1.514 |
| Bt.23250.2.S1\_at | 3.06e-06 | 3.316 |
| Bt.27664.1.A1\_at | 3.216e-06 | 2.623 |
| Bt.4184.1.S2\_at | 3.226e-06 | 1.116 |
| Bt.26621.1.S1\_at | 3.379e-06 | -2.074 |
| Bt.9559.1.S1\_at | 3.77e-06 | 2.54 |
| Bt.13588.3.A1\_at | 3.812e-06 | 4.553 |
| Bt.15895.2.S1\_at | 3.898e-06 | -2.877 |
| Bt.13581.1.S1\_at | 4.1e-06 | 3.401 |
| Bt.8213.1.S1\_at | 4.306e-06 | 1.461 |
| Bt.9767.1.S1\_a\_at | 4.311e-06 | 2.422 |
| Bt.5348.1.S1\_at | 4.372e-06 | 2.108 |
| Bt.4702.2.S1\_at | 5.431e-06 | -1.741 |
| Bt.11580.1.S1\_at | 5.581e-06 | 3.925 |
| Bt.10330.1.S1\_at | 5.624e-06 | -1.455 |
| Bt.19023.1.S1\_at | 5.958e-06 | 1.909 |
| Bt.2153.1.S1\_at | 6.52e-06 | 1.944 |
| Bt.11696.1.A1\_at | 6.788e-06 | -3.421 |
| Bt.2096.1.S1\_at | 6.952e-06 | 1.88 |
| Bt.19706.1.S1\_at | 7.072e-06 | -1.619 |
| Bt.29724.1.S1\_at | 7.251e-06 | -3.178 |
| Bt.16563.1.A1\_at | 7.258e-06 | 2.256 |
| Bt.8682.1.S1\_at | 7.377e-06 | -1.317 |
| Bt.13765.1.S1\_at | 7.964e-06 | 2.191 |
| Bt.4822.1.A1\_at | 8.174e-06 | 1.196 |
| Bt.26808.1.S1\_at | 8.469e-06 | -1.705 |
| Bt.23883.1.A1\_at | 8.955e-06 | 1.23 |
| Bt.1582.1.S1\_at | 9.11e-06 | 1.307 |
| Bt.22167.1.S1\_at | 9.125e-06 | -2.944 |
| Bt.12223.1.S1\_at | 9.169e-06 | 2.226 |
| Bt.23176.2.S1\_a\_at | 9.775e-06 | 2.141 |
| Bt.11659.1.S1\_at | 9.844e-06 | 1.327 |
| Bt.25806.2.A1\_at | 9.955e-06 | 1.534 |
| Bt.7179.1.S1\_a\_at | 9.996e-06 | 2.047 |
| Bt.24310.1.S1\_at | 1.002e-05 | -1.998 |
| Bt.24517.2.A1\_at | 1.015e-05 | 1.307 |
| Bt.3532.1.A1\_at | 1.153e-05 | 2.133 |
| Bt.9012.1.S1\_at | 1.169e-05 | -1.903 |
| Bt.19623.1.S1\_at | 1.314e-05 | -2.457 |
| Bt.27283.1.S1\_x\_at | 1.325e-05 | -3.502 |
| Bt.12412.1.S1\_at | 1.337e-05 | -1.552 |
| Bt.20104.1.S1\_at | 1.342e-05 | -2.019 |
| Bt.22626.2.S1\_at | 1.346e-05 | -2.625 |
| Bt.4702.1.S1\_at | 1.389e-05 | -1.648 |
| Bt.20431.2.S1\_at | 1.444e-05 | -1.455 |
| Bt.10150.1.S1\_at | 1.447e-05 | 1.422 |
| Bt.17963.1.A1\_at | 1.478e-05 | 1.141 |
| Bt.24396.3.A1\_at | 1.481e-05 | -2.784 |
| Bt.1169.1.S1\_at | 1.506e-05 | 1.273 |
| Bt.23521.1.S1\_at | 1.545e-05 | -1.543 |
| Bt.2874.1.S1\_at | 1.556e-05 | 1.862 |
| Bt.6850.2.S1\_at | 1.579e-05 | -3.215 |
| Bt.9810.1.S1\_at | 1.598e-05 | 1.572 |
| Bt.28011.1.S1\_at | 1.603e-05 | -2.069 |
| Bt.18206.1.A1\_at | 1.633e-05 | -2.888 |
| Bt.28732.1.S1\_s\_at | 1.667e-05 | -2.306 |
| Bt.18526.1.A1\_at | 1.684e-05 | -2.805 |
| Bt.28312.1.S1\_at | 1.691e-05 | -1.521 |
| Bt.3306.1.S1\_at | 1.716e-05 | -1.506 |
| Bt.23829.1.A1\_at | 1.783e-05 | -1.581 |
| Bt.6642.1.S1\_a\_at | 1.827e-05 | -2.56 |
| Bt.25099.1.A1\_at | 1.914e-05 | 3.179 |
| Bt.2159.1.S1\_at | 1.959e-05 | 1.243 |
| Bt.4381.1.A1\_at | 1.972e-05 | 1.293 |
| Bt.22510.1.S1\_at | 1.973e-05 | 1.24 |
| Bt.19247.2.S1\_at | 2.048e-05 | -2.443 |
| Bt.20145.1.S1\_at | 2.058e-05 | 1.598 |
| Bt.3883.1.S1\_at | 2.081e-05 | -1.37 |
| Bt.7908.1.S1\_at | 2.147e-05 | 1.031 |
| Bt.16668.1.A1\_at | 2.187e-05 | -1.243 |
| Bt.25668.2.S1\_at | 2.237e-05 | 1.224 |
| Bt.15623.1.S1\_at | 2.242e-05 | 2.944 |
| Bt.17444.1.S1\_at | 2.363e-05 | 2.545 |
| Bt.15643.1.A1\_at | 2.373e-05 | -1.366 |
| Bt.21551.2.S1\_a\_at | 2.451e-05 | -1.092 |
| Bt.24933.1.S1\_at | 2.461e-05 | 3.675 |
| Bt.2347.2.S1\_at | 2.503e-05 | -1.13 |
| Bt.2070.2.A1\_at | 2.517e-05 | 1.706 |
| Bt.21385.1.S1\_at | 2.558e-05 | 1.392 |
| Bt.2091.1.S1\_at | 2.577e-05 | -2.108 |
| Bt.20143.2.S1\_at | 2.623e-05 | -1.25 |
| Bt.3388.1.S1\_at | 2.634e-05 | 1.198 |
| Bt.16639.1.A1\_at | 2.708e-05 | -1.269 |
| Bt.22568.1.S1\_at | 2.928e-05 | 1.117 |
| Bt.6774.2.S1\_at | 2.962e-05 | -1.171 |
| Bt.9180.1.S1\_a\_at | 2.991e-05 | 1.316 |
| Bt.19030.1.A1\_at | 3.044e-05 | -3.736 |
| Bt.2070.1.A1\_at | 3.069e-05 | 2.017 |
| Bt.12619.1.S1\_at | 3.16e-05 | -1.485 |
| Bt.21142.1.S1\_at | 3.212e-05 | -2.26 |
| Bt.16127.1.S1\_at | 3.277e-05 | -0.9683 |
| Bt.149.1.S1\_at | 3.344e-05 | 2.679 |
| Bt.7962.1.S1\_at | 3.447e-05 | -2.391 |
| Bt.16168.1.A1\_at | 3.584e-05 | 3.234 |
| Bt.14204.1.A1\_at | 3.624e-05 | -1.163 |
| Bt.4954.1.S1\_at | 3.702e-05 | -1.302 |
| Bt.89.1.S1\_at | 3.727e-05 | 1.902 |
| Bt.24804.1.S1\_at | 3.736e-05 | -1.897 |
| Bt.20431.1.A1\_at | 3.97e-05 | -1.306 |
| Bt.23519.1.S1\_at | 3.989e-05 | -1.689 |
| Bt.14023.1.S1\_at | 3.997e-05 | 0.9316 |
| Bt.18639.2.S1\_at | 4.044e-05 | -6.417 |
| Bt.21661.1.S1\_at | 4.11e-05 | -2.042 |
| Bt.15675.1.S1\_at | 4.177e-05 | 2.94 |
| Bt.20340.2.S1\_at | 4.183e-05 | 1.243 |
| Bt.13988.1.S1\_at | 4.306e-05 | -1.26 |
| Bt.29029.1.S1\_at | 4.325e-05 | -1.177 |
| Bt.24524.1.S1\_at | 4.389e-05 | 1.555 |
| Bt.13247.1.S1\_at | 4.5e-05 | 2.093 |
| Bt.5123.2.S1\_at | 4.539e-05 | 1.007 |
| Bt.9021.1.S1\_at | 4.572e-05 | 1.632 |
| Bt.18101.1.A1\_at | 4.689e-05 | 3.524 |
| Bt.4864.2.S1\_at | 4.732e-05 | -1.101 |
| Bt.29873.1.S1\_at | 4.822e-05 | 1.597 |
| Bt.4631.2.S1\_at | 4.832e-05 | -1.462 |
| Bt.19502.1.A1\_at | 4.945e-05 | 2.307 |
| Bt.4888.1.S1\_at | 4.985e-05 | 1.537 |
| Bt.6802.1.S1\_at | 5.242e-05 | -1.663 |
| Bt.4725.1.S1\_at | 5.291e-05 | -1.226 |
| Bt.13975.1.S1\_a\_at | 5.477e-05 | -1.39 |
| Bt.4353.1.S1\_at | 5.486e-05 | -1.253 |
| Bt.4868.2.S1\_a\_at | 5.527e-05 | 1.018 |
| Bt.23094.2.A1\_at | 5.539e-05 | -1.282 |
| Bt.25973.1.S1\_at | 5.61e-05 | -1.433 |
| Bt.27196.1.A1\_at | 5.682e-05 | 3.089 |
| Bt.7537.1.S1\_at | 5.717e-05 | 1.063 |
| Bt.4688.1.S1\_a\_at | 5.821e-05 | 1.561 |
| Bt.24856.2.S1\_at | 5.88e-05 | 1.378 |
| Bt.20361.2.A1\_at | 5.94e-05 | -1.17 |
| Bt.13084.1.S1\_at | 5.971e-05 | -0.9861 |
| Bt.24439.1.S1\_at | 6.01e-05 | -1.403 |
| Bt.7670.1.A1\_at | 6.092e-05 | -1.329 |
| Bt.16389.1.S1\_at | 6.093e-05 | -1.477 |
| Bt.4638.1.S1\_at | 6.317e-05 | 1.109 |
| Bt.12314.1.S1\_at | 6.38e-05 | -2.979 |
| Bt.16597.1.S1\_at | 6.449e-05 | 1.845 |
| Bt.20420.1.S1\_at | 6.451e-05 | -1.718 |
| Bt.13588.2.S1\_at | 6.478e-05 | 4.301 |
| Bt.20470.1.S1\_a\_at | 6.486e-05 | 3.394 |
| Bt.21994.1.S1\_at | 6.69e-05 | 1.351 |
| Bt.24137.1.A1\_at | 6.718e-05 | 1.483 |
| Bt.16326.1.S1\_at | 6.729e-05 | -2.013 |
| Bt.19508.1.A1\_at | 6.75e-05 | 1.308 |
| Bt.27652.1.A1\_at | 6.975e-05 | -3.354 |
| Bt.12885.1.S1\_at | 7.26e-05 | 1.86 |
| Bt.19643.1.S1\_at | 7.336e-05 | -1.884 |
| Bt.14369.1.A1\_at | 7.418e-05 | 2.283 |
| Bt.21432.2.A1\_at | 7.743e-05 | 1.132 |
| Bt.5070.1.S1\_at | 7.801e-05 | -1.259 |
| Bt.17102.3.S1\_at | 7.818e-05 | -1.451 |
| Bt.4659.1.S1\_at | 7.952e-05 | -2.054 |
| Bt.13330.1.S1\_at | 7.962e-05 | -1.943 |
| Bt.22176.1.A1\_at | 8.077e-05 | 3.828 |
| Bt.26402.1.A1\_at | 8.208e-05 | -3.107 |
| Bt.14387.1.S1\_at | 8.35e-05 | 2.615 |
| Bt.13315.1.S1\_at | 8.4e-05 | 1.533 |
| Bt.23668.1.A1\_at | 8.423e-05 | -0.9651 |
| Bt.28090.1.S1\_at | 8.486e-05 | -1.63 |
| Bt.13324.1.S1\_a\_at | 8.575e-05 | 1.428 |
| Bt.1187.1.S1\_at | 8.82e-05 | -1.497 |
| Bt.10579.1.S1\_at | 8.9e-05 | -1.089 |
| Bt.19352.1.A1\_at | 8.926e-05 | 1.593 |
| Bt.27079.1.S1\_at | 9.061e-05 | 2.23 |
| Bt.3616.1.S1\_at | 9.219e-05 | 0.9545 |
| Bt.26745.1.A1\_at | 9.261e-05 | 2.58 |
| Bt.5123.3.S1\_a\_at | 9.367e-05 | 0.8754 |
| Bt.7069.1.S1\_at | 9.544e-05 | -1.366 |
| Bt.23456.1.A1\_s\_at | 9.636e-05 | -1.525 |
| Bt.21818.1.S1\_at | 9.64e-05 | -1.204 |
| Bt.3780.1.S1\_at | 9.757e-05 | 1.271 |
| Bt.12491.1.A1\_at | 9.788e-05 | 2.126 |
| Bt.1998.1.S1\_at | 9.789e-05 | 1.096 |
| Bt.20227.1.S1\_at | 9.807e-05 | 1.972 |
| Bt.26688.2.S1\_a\_at | 9.827e-05 | -1.42 |
| Bt.5468.1.S1\_at | 9.846e-05 | 2.616 |
| Bt.911.1.S1\_at | 9.882e-05 | -1.148 |
| Bt.15545.1.S1\_at | 9.93e-05 | 1.353 |
| Bt.22783.1.S1\_at | 9.987e-05 | 1.198 |
| Bt.7540.2.S1\_a\_at | 0.0001008 | 0.9763 |
| Bt.18755.1.A1\_at | 0.0001011 | 0.9161 |
| Bt.9804.1.S1\_at | 0.0001018 | -1.011 |
| Bt.5356.1.S1\_s\_at | 0.0001019 | -2.455 |
| Bt.17124.1.A1\_s\_at | 0.0001019 | 2.117 |
| Bt.7288.1.S1\_at | 0.0001043 | 1.109 |
| Bt.25487.1.A1\_at | 0.000105 | 1.569 |
| Bt.18603.1.A1\_at | 0.0001054 | -3.657 |
| Bt.23603.3.S1\_at | 0.0001073 | 1.336 |
| Bt.1440.1.S1\_at | 0.0001076 | 1.585 |
| Bt.5304.1.S1\_at | 0.0001086 | -1.831 |
| Bt.7481.1.S1\_at | 0.000109 | 1.852 |
| Bt.17883.1.S1\_at | 0.0001092 | -1.732 |
| Bt.20095.2.S1\_at | 0.0001135 | -0.9811 |
| Bt.18752.1.S1\_at | 0.0001138 | -0.9098 |
| Bt.9956.1.S1\_at | 0.0001139 | 1.087 |
| Bt.12472.1.S1\_at | 0.000114 | -1.341 |
| Bt.3863.1.S1\_at | 0.0001142 | -1.745 |
| Bt.1268.1.A1\_at | 0.0001164 | -1.302 |
| Bt.621.1.S1\_at | 0.0001173 | 1.52 |
| Bt.11492.1.A1\_at | 0.0001198 | -1.1 |
| Bt.19469.1.A1\_at | 0.0001217 | -1.383 |
| Bt.21724.2.S1\_a\_at | 0.0001219 | -1.512 |
| Bt.25390.1.A1\_at | 0.0001236 | -1.044 |
| Bt.18623.1.A1\_at | 0.0001257 | -1.306 |
| Bt.25319.1.A1\_at | 0.0001273 | -1.105 |
| Bt.9643.1.S1\_at | 0.0001295 | 1.172 |
| Bt.26796.1.S1\_at | 0.0001296 | -3.236 |
| Bt.21658.1.S1\_at | 0.0001303 | -0.8792 |
| Bt.22651.1.A1\_at | 0.0001311 | 1.145 |
| Bt.20141.1.S1\_at | 0.0001338 | 1.149 |
| Bt.1330.1.S1\_at | 0.0001341 | 1.161 |
| Bt.2726.2.S1\_at | 0.0001357 | 2.167 |
| Bt.771.1.S1\_at | 0.0001357 | -1.967 |
| Bt.5472.1.S1\_at | 0.0001358 | 1.401 |
| Bt.26805.1.S1\_at | 0.0001358 | 1.674 |
| Bt.13886.3.S1\_a\_at | 0.0001362 | -0.7701 |
| Bt.2696.1.A1\_at | 0.0001365 | 1.02 |
| Bt.9190.1.S1\_at | 0.000138 | -1.024 |
| Bt.2537.2.S1\_at | 0.0001382 | -0.9924 |
| Bt.25806.1.S1\_at | 0.0001383 | 1.593 |
| Bt.4624.1.S1\_at | 0.0001393 | -1.311 |
| Bt.659.2.S1\_at | 0.0001401 | -1.53 |
| Bt.21133.1.S1\_at | 0.0001406 | -1.782 |
| Bt.3312.1.S1\_at | 0.0001437 | -1.278 |
| Bt.20281.2.S1\_a\_at | 0.0001446 | -0.9636 |
| Bt.20270.1.S1\_at | 0.0001447 | 1.014 |
| Bt.5125.1.S1\_at | 0.0001448 | -3.663 |
| Bt.15157.1.S1\_at | 0.000145 | 1.101 |
| Bt.22785.1.S1\_at | 0.0001455 | -1.661 |
| Bt.28362.1.S1\_at | 0.0001463 | 1.194 |
| Bt.22216.1.S1\_at | 0.0001473 | 1.182 |
| Bt.28340.1.S1\_at | 0.0001477 | 2.292 |
| Bt.8189.2.A1\_at | 0.0001495 | 1.284 |
| Bt.2392.1.S1\_at | 0.0001519 | 0.9128 |
| Bt.23992.1.A1\_at | 0.0001532 | -1.227 |
| Bt.6156.1.S1\_at | 0.0001536 | 0.8048 |
| Bt.21039.1.A1\_at | 0.0001543 | -3.168 |
| Bt.18316.2.S1\_at | 0.000156 | -1.567 |
| Bt.8552.1.S1\_at | 0.0001564 | -2.738 |
| Bt.18835.1.S1\_at | 0.0001567 | 3.127 |
| Bt.5252.1.S1\_at | 0.0001568 | 1.637 |
| Bt.9571.2.S1\_a\_at | 0.0001598 | 1.532 |
| Bt.5141.1.S2\_at | 0.0001602 | 1.254 |
| Bt.9915.1.S1\_at | 0.0001606 | 1.067 |
| Bt.28506.1.A1\_at | 0.000161 | 2.03 |
| Bt.26641.1.S1\_at | 0.0001611 | 1.098 |
| Bt.11034.1.S1\_at | 0.0001614 | -1.808 |
| Bt.4410.1.S1\_at | 0.0001633 | 1.059 |
| Bt.1131.1.S1\_at | 0.0001669 | 1.002 |
| Bt.4353.2.S1\_at | 0.0001694 | -0.9883 |
| Bt.1418.2.S1\_at | 0.0001698 | -1.26 |
| Bt.26682.1.S1\_at | 0.0001699 | -1.207 |
| Bt.4704.2.S1\_at | 0.0001711 | 1.22 |
| Bt.3414.1.S1\_at | 0.0001711 | 1.215 |
| Bt.9033.2.S1\_at | 0.0001734 | 0.8116 |
| Bt.3090.1.S1\_at | 0.0001766 | -0.9167 |
| Bt.22763.2.S1\_a\_at | 0.0001769 | 1.145 |
| Bt.27175.2.S1\_at | 0.0001788 | -1.044 |
| Bt.21116.1.S1\_at | 0.0001797 | -1.092 |
| Bt.6680.1.S1\_a\_at | 0.00018 | 1.117 |
| Bt.12559.1.S1\_at | 0.0001803 | 1.315 |
| Bt.7776.1.S2\_at | 0.0001815 | -1.457 |
| Bt.24538.1.S1\_at | 0.0001817 | -1.422 |
| Bt.20601.1.A1\_at | 0.0001819 | -1.05 |
| Bt.28390.1.S1\_at | 0.0001831 | -1.155 |
| Bt.28567.1.A1\_at | 0.0001848 | -1.014 |
| Bt.23734.1.A1\_at | 0.0001862 | -1.789 |
| Bt.23187.2.S1\_a\_at | 0.0001866 | 2.625 |
| Bt.1681.1.S1\_at | 0.0001897 | 2.637 |
| Bt.11014.1.S1\_at | 0.0001907 | 1.485 |
| Bt.4970.1.S1\_at | 0.0001932 | 1.388 |
| Bt.18637.1.A1\_at | 0.0001951 | 1.104 |
| Bt.24766.1.S1\_at | 0.0001955 | -1.422 |
| Bt.24727.1.S1\_a\_at | 0.0001959 | 1.111 |
| Bt.9049.1.S1\_at | 0.0001959 | -1.422 |
| Bt.22480.1.A1\_at | 0.000196 | 1.606 |
| Bt.24057.1.A1\_at | 0.0001997 | -1.943 |

Differentially expressed genes for RMA: 984

| AFFY | p-value | FoldChange |
| --- | --- | --- |
| Bt.9767.1.S1\_a\_at | 1.394e-11 | 3.119 |
| Bt.9556.1.S1\_at | 3.612e-11 | 2.205 |
| Bt.26135.1.A1\_at | 9.102e-11 | 2.343 |
| Bt.26760.2.S1\_a\_at | 1.099e-10 | -1.981 |
| Bt.4818.1.S1\_at | 2.513e-10 | 1.755 |
| Bt.18639.2.S1\_at | 2.793e-10 | -3.768 |
| Bt.9810.1.S1\_at | 3.52e-10 | 1.755 |
| Bt.3312.1.S1\_at | 6.465e-10 | -1.405 |
| Bt.8972.1.S1\_at | 7.049e-10 | -1.652 |
| Bt.18639.1.A1\_at | 8.914e-10 | -3.613 |
| Bt.3071.1.S1\_at | 1.112e-09 | 1.688 |
| Bt.23250.2.S1\_at | 1.523e-09 | 3.455 |
| Bt.24310.1.S1\_at | 1.615e-09 | -1.79 |
| Bt.6642.2.S1\_at | 1.757e-09 | -2.287 |
| Bt.7034.1.S1\_at | 1.918e-09 | 2.439 |
| Bt.7651.1.S1\_at | 2.701e-09 | -1.642 |
| Bt.22167.1.S1\_at | 3.037e-09 | -2.112 |
| Bt.6642.1.S1\_at | 9.379e-09 | -1.705 |
| Bt.20501.1.S1\_at | 1.137e-08 | -4.014 |
| Bt.4970.1.S1\_at | 1.597e-08 | 1.855 |
| Bt.23176.2.S1\_a\_at | 1.613e-08 | 1.984 |
| Bt.6642.1.S1\_a\_at | 2.008e-08 | -2.049 |
| Bt.24517.2.A1\_at | 2.236e-08 | 1.527 |
| Bt.12299.1.A1\_at | 2.288e-08 | 4.097 |
| Bt.13986.1.S1\_at | 2.45e-08 | -1.376 |
| Bt.12510.1.S1\_at | 2.987e-08 | 2.255 |
| Bt.27856.1.S1\_at | 3.593e-08 | 2.226 |
| Bt.21385.1.S1\_at | 3.838e-08 | 1.25 |
| Bt.1035.1.S1\_a\_at | 3.854e-08 | 1.128 |
| Bt.2262.1.S1\_at | 4.621e-08 | 1.322 |
| Bt.15682.1.A1\_at | 4.973e-08 | 2.425 |
| Bt.26805.1.S1\_at | 5.086e-08 | 2.161 |
| Bt.2874.1.S1\_at | 5.245e-08 | 1.221 |
| Bt.10810.1.A1\_at | 5.338e-08 | 1.839 |
| Bt.29873.1.S1\_at | 5.736e-08 | 1.81 |
| Bt.13435.1.A1\_at | 5.889e-08 | 2.107 |
| Bt.5340.1.S1\_s\_at | 5.987e-08 | 1.328 |
| Bt.22803.1.S1\_at | 6.012e-08 | 1.328 |
| Bt.16563.1.A1\_at | 7.194e-08 | 1.836 |
| Bt.22002.1.S1\_at | 7.573e-08 | 1.854 |
| Bt.13289.1.S1\_at | 8.031e-08 | -2.684 |
| Bt.659.1.S1\_at | 8.403e-08 | -1.507 |
| Bt.24524.1.S1\_at | 8.924e-08 | 1.41 |
| Bt.23519.1.S1\_at | 9.907e-08 | -1.434 |
| Bt.5348.1.S1\_at | 1.008e-07 | 1.459 |
| Bt.23212.1.S1\_at | 1.025e-07 | 1.272 |
| Bt.7056.1.S1\_at | 1.068e-07 | 2.55 |
| Bt.22803.1.S2\_at | 1.099e-07 | 1.266 |
| Bt.20862.1.A1\_at | 1.418e-07 | 1.216 |
| Bt.18890.1.A1\_at | 1.44e-07 | 1.023 |
| Bt.11659.1.S1\_at | 1.651e-07 | 1.178 |
| Bt.7908.1.S1\_at | 1.818e-07 | 0.9492 |
| Bt.13588.1.A1\_at | 1.833e-07 | 4.05 |
| Bt.7307.2.A1\_a\_at | 1.907e-07 | -0.9911 |
| Bt.20470.1.S1\_a\_at | 1.916e-07 | 1.626 |
| Bt.25052.1.S1\_at | 2.102e-07 | 1.248 |
| Bt.25806.2.A1\_at | 2.246e-07 | 1.395 |
| Bt.2070.1.A1\_at | 2.381e-07 | 1.42 |
| Bt.13765.1.S1\_at | 2.391e-07 | 1.718 |
| Bt.4937.1.S1\_at | 2.631e-07 | 1.246 |
| Bt.5468.1.S1\_at | 2.84e-07 | 2.152 |
| Bt.22167.2.A1\_at | 2.882e-07 | -1.945 |
| Bt.3863.1.S1\_at | 3.117e-07 | -1.352 |
| Bt.8189.2.A1\_at | 3.218e-07 | 1.523 |
| Bt.20104.1.S1\_at | 3.369e-07 | -1.588 |
| Bt.3843.1.S1\_at | 3.43e-07 | 1.244 |
| Bt.22306.1.S1\_at | 3.596e-07 | 1.48 |
| Bt.5356.1.S1\_s\_at | 3.677e-07 | -1.964 |
| Bt.7008.1.S1\_at | 3.818e-07 | 1.422 |
| Bt.13289.2.S1\_at | 3.903e-07 | -2.609 |
| Bt.23521.2.S1\_a\_at | 4.082e-07 | -1.236 |
| Bt.9643.1.S1\_at | 4.245e-07 | 1.428 |
| Bt.23182.1.S1\_at | 4.341e-07 | 1.293 |
| Bt.19721.1.S1\_at | 4.414e-07 | 1.682 |
| Bt.4959.1.A1\_at | 4.43e-07 | 1.338 |
| Bt.7394.1.A1\_at | 4.518e-07 | 1.423 |
| Bt.9180.1.S1\_a\_at | 4.666e-07 | 1.46 |
| Bt.18278.1.S1\_at | 4.683e-07 | 1.127 |
| Bt.4702.1.S1\_at | 4.955e-07 | -1.194 |
| Bt.28524.1.S1\_at | 4.966e-07 | 1.053 |
| Bt.20143.2.S1\_at | 5.176e-07 | -0.8792 |
| Bt.25099.1.A1\_at | 5.598e-07 | 2.923 |
| Bt.4184.1.S2\_at | 5.649e-07 | 1.171 |
| Bt.20420.1.S1\_at | 5.659e-07 | -1.331 |
| Bt.5123.2.S1\_at | 6.278e-07 | 0.936 |
| Bt.26286.1.A1\_at | 6.714e-07 | 0.8701 |
| Bt.4725.1.S1\_at | 6.847e-07 | -0.9872 |
| Bt.8876.1.S1\_at | 6.9e-07 | 0.9467 |
| Bt.17148.2.S1\_at | 6.962e-07 | 1.407 |
| Bt.20505.1.S1\_at | 6.979e-07 | 1.133 |
| Bt.7676.3.S1\_at | 7.039e-07 | 1.316 |
| Bt.25398.1.A1\_at | 7.073e-07 | 1.395 |
| Bt.28477.1.S1\_at | 7.567e-07 | -0.8932 |
| Bt.8213.1.S1\_at | 7.728e-07 | 1.108 |
| Bt.10150.1.S1\_at | 8.009e-07 | 1.36 |
| Bt.7288.1.S1\_at | 8.105e-07 | 0.9739 |
| Bt.7056.5.A1\_x\_at | 8.138e-07 | 2.886 |
| Bt.1175.1.S1\_at | 8.269e-07 | 1.124 |
| Bt.11662.1.A1\_at | 8.365e-07 | 1.341 |
| Bt.2822.1.S1\_at | 8.472e-07 | 0.8036 |
| Bt.659.2.S1\_at | 8.613e-07 | -1.629 |
| Bt.26685.1.S1\_at | 8.944e-07 | 1.344 |
| Bt.1998.1.S1\_at | 9.061e-07 | 1.213 |
| Bt.21039.1.A1\_at | 9.096e-07 | -3.153 |
| Bt.17883.2.A1\_at | 9.276e-07 | -1.009 |
| Bt.20270.1.S1\_at | 9.373e-07 | 1.178 |
| Bt.25806.1.S1\_at | 9.751e-07 | 1.46 |
| Bt.22783.1.S1\_at | 9.783e-07 | 1.408 |
| Bt.7133.1.S1\_at | 9.843e-07 | 1.221 |
| Bt.4798.1.S2\_at | 1.018e-06 | 1.601 |
| Bt.24478.1.S1\_at | 1.025e-06 | 0.9996 |
| Bt.22216.1.S1\_at | 1.06e-06 | 1.687 |
| Bt.3388.1.S1\_at | 1.061e-06 | 1.165 |
| Bt.12491.1.A1\_at | 1.084e-06 | 2.087 |
| Bt.6991.1.S1\_at | 1.157e-06 | -1.051 |
| Bt.9021.1.S1\_at | 1.187e-06 | 1.883 |
| Bt.2070.2.A1\_at | 1.261e-06 | 1.414 |
| Bt.18755.1.A1\_at | 1.301e-06 | 1.108 |
| Bt.2159.1.S1\_at | 1.301e-06 | 1.057 |
| Bt.13315.1.S1\_at | 1.307e-06 | 0.9986 |
| Bt.3414.1.S1\_at | 1.315e-06 | 1.197 |
| Bt.10869.1.S1\_at | 1.315e-06 | -1.16 |
| Bt.4631.2.S1\_at | 1.361e-06 | -1.097 |
| Bt.16597.1.S1\_at | 1.372e-06 | 1.076 |
| Bt.21724.1.S1\_at | 1.382e-06 | -0.9718 |
| Bt.21537.1.A1\_at | 1.405e-06 | -1.222 |
| Bt.16520.1.A1\_at | 1.466e-06 | -1.9 |
| Bt.21142.1.S1\_at | 1.476e-06 | -1.849 |
| Bt.21432.2.A1\_at | 1.565e-06 | 1.465 |
| Bt.4822.1.A1\_at | 1.59e-06 | 1.065 |
| Bt.4888.1.S1\_at | 1.612e-06 | 0.9649 |
| Bt.2091.1.S1\_at | 1.646e-06 | -1.723 |
| Bt.2153.1.S1\_at | 1.648e-06 | 1.37 |
| Bt.3562.1.S1\_at | 1.679e-06 | 1.507 |
| Bt.2696.2.S1\_at | 1.695e-06 | 1.398 |
| Bt.1582.1.S1\_at | 1.733e-06 | 1.182 |
| Bt.4624.1.S1\_at | 1.805e-06 | -1.192 |
| Bt.10608.2.S1\_a\_at | 1.811e-06 | 0.9113 |
| Bt.5472.1.S1\_at | 1.817e-06 | 1.103 |
| Bt.1920.1.S1\_at | 1.839e-06 | -0.9604 |
| Bt.16980.1.S1\_at | 1.855e-06 | 0.9478 |
| Bt.29724.1.S1\_at | 1.893e-06 | -2.05 |
| Bt.10153.1.S1\_at | 1.898e-06 | -1.33 |
| Bt.25099.2.S1\_at | 1.948e-06 | 2.002 |
| Bt.21578.1.S1\_at | 1.956e-06 | 0.9811 |
| Bt.7540.3.S1\_at | 2.044e-06 | 1.222 |
| Bt.28090.1.S1\_at | 2.047e-06 | -1.193 |
| Bt.3306.1.S1\_at | 2.055e-06 | -0.968 |
| Bt.20354.1.S1\_at | 2.056e-06 | 0.7826 |
| Bt.5519.2.S1\_at | 2.071e-06 | 0.9797 |
| Bt.11034.1.S1\_at | 2.107e-06 | -1.344 |
| Bt.1681.1.S1\_at | 2.109e-06 | 0.9142 |
| Bt.1330.1.S1\_at | 2.157e-06 | 0.9989 |
| Bt.9012.1.S1\_at | 2.217e-06 | -1.143 |
| Bt.28732.1.S1\_s\_at | 2.326e-06 | -1.344 |
| Bt.28623.1.S1\_at | 2.329e-06 | 0.9196 |
| Bt.13975.1.S1\_a\_at | 2.347e-06 | -1.262 |
| Bt.562.1.S1\_at | 2.401e-06 | -1.131 |
| Bt.20227.1.S1\_at | 2.405e-06 | 1.513 |
| Bt.26444.1.A1\_at | 2.5e-06 | -1.466 |
| Bt.6774.2.S1\_at | 2.508e-06 | -0.9241 |
| Bt.24614.1.S1\_at | 2.535e-06 | 1.593 |
| Bt.7481.1.S1\_at | 2.595e-06 | 1.476 |
| Bt.2592.1.A1\_x\_at | 2.64e-06 | -1.003 |
| Bt.17573.1.A1\_at | 2.66e-06 | -1.822 |
| Bt.13588.2.S1\_at | 2.664e-06 | 2.781 |
| Bt.4410.1.S1\_at | 2.714e-06 | 0.9336 |
| Bt.4659.1.S1\_at | 2.727e-06 | -1.633 |
| Bt.23603.3.S1\_at | 2.768e-06 | 0.8774 |
| Bt.5897.1.S1\_at | 2.772e-06 | -0.8451 |
| Bt.946.1.S1\_at | 2.798e-06 | 0.9877 |
| Bt.22234.1.S1\_at | 2.804e-06 | -1.761 |
| Bt.15540.1.A1\_at | 2.827e-06 | -0.857 |
| Bt.8552.1.S1\_at | 2.895e-06 | -2.033 |
| Bt.16951.1.A1\_at | 2.921e-06 | 1.14 |
| Bt.12411.1.S1\_at | 2.981e-06 | 0.9935 |
| Bt.23094.3.S1\_x\_at | 3.081e-06 | -1.5 |
| Bt.17883.1.S1\_at | 3.123e-06 | -1.169 |
| Bt.1154.1.S1\_at | 3.26e-06 | 1.056 |
| Bt.8643.1.A1\_at | 3.308e-06 | 1.217 |
| Bt.27331.1.S1\_at | 3.313e-06 | 1.438 |
| Bt.350.1.S1\_s\_at | 3.353e-06 | -1.438 |
| Bt.2001.1.S1\_at | 3.389e-06 | 0.8423 |
| Bt.7537.1.S1\_at | 3.426e-06 | 1.127 |
| Bt.12443.1.S1\_at | 3.481e-06 | -0.7543 |
| Bt.23505.1.S1\_at | 3.49e-06 | -1.996 |
| Bt.1131.1.S1\_at | 3.589e-06 | 0.8918 |
| Bt.25255.1.A1\_at | 3.621e-06 | 1.036 |
| Bt.7962.1.S1\_at | 3.77e-06 | -2.023 |
| Bt.17512.1.S1\_at | 3.782e-06 | -1.052 |
| Bt.19023.1.S1\_at | 3.805e-06 | 1.573 |
| Bt.28728.1.A1\_at | 3.814e-06 | 1.322 |
| Bt.26358.1.S1\_at | 3.819e-06 | 1.467 |
| Bt.20852.1.A1\_at | 3.839e-06 | 1.297 |
| Bt.27237.1.S1\_a\_at | 3.887e-06 | 1.183 |
| Bt.26872.1.S1\_at | 4.022e-06 | 1.584 |
| Bt.5530.1.S1\_at | 4.47e-06 | -1.001 |
| Bt.4733.1.S1\_at | 4.589e-06 | 1.804 |
| Bt.27301.1.S1\_at | 4.63e-06 | 1.481 |
| Bt.6156.1.S1\_at | 4.637e-06 | 0.7637 |
| Bt.7033.2.S1\_a\_at | 4.687e-06 | 2.108 |
| Bt.28506.1.A1\_at | 4.721e-06 | 1.356 |
| Bt.11178.1.S1\_at | 4.758e-06 | 0.9204 |
| Bt.16567.1.A1\_at | 4.803e-06 | 0.9443 |
| Bt.3780.1.S1\_at | 4.813e-06 | 1.087 |
| Bt.16917.1.S1\_at | 4.97e-06 | -1.019 |
| Bt.8963.1.S1\_at | 5.309e-06 | 1.23 |
| Bt.26629.1.S1\_a\_at | 5.318e-06 | 1.431 |
| Bt.13306.2.S1\_at | 5.328e-06 | -1.046 |
| Bt.16122.1.S1\_at | 5.498e-06 | 1.101 |
| Bt.21705.1.S1\_at | 5.623e-06 | 1.207 |
| Bt.20145.1.S1\_at | 5.739e-06 | 1.368 |
| Bt.7179.1.S1\_a\_at | 5.75e-06 | 1.178 |
| Bt.13324.1.S1\_a\_at | 5.782e-06 | 1.244 |
| Bt.20194.1.S1\_at | 5.812e-06 | 0.8991 |
| Bt.7701.1.S1\_at | 5.884e-06 | 0.8648 |
| Bt.911.1.S1\_at | 5.9e-06 | -0.9404 |
| Bt.21670.1.A1\_at | 5.981e-06 | 0.8932 |
| Bt.4670.1.S1\_at | 6.13e-06 | 1.004 |
| Bt.25997.1.S1\_at | 6.383e-06 | -0.9513 |
| Bt.2070.3.S1\_at | 6.484e-06 | 1.143 |
| Bt.4637.1.A1\_a\_at | 6.563e-06 | 1.067 |
| Bt.4688.1.S1\_a\_at | 6.585e-06 | 0.9491 |
| Bt.25018.1.A1\_at | 6.676e-06 | -0.958 |
| Bt.6774.1.S1\_at | 6.688e-06 | -1.212 |
| Bt.28829.1.S1\_at | 6.781e-06 | 0.9351 |
| Bt.24137.1.A1\_at | 6.784e-06 | 1.593 |
| Bt.9242.1.S1\_at | 6.805e-06 | 1.003 |
| Bt.4175.2.S1\_at | 6.879e-06 | 0.7583 |
| Bt.20141.1.S1\_at | 7.005e-06 | 0.8804 |
| Bt.23109.1.S1\_at | 7.022e-06 | 0.864 |
| Bt.24269.1.S1\_at | 7.071e-06 | 0.985 |
| Bt.9996.1.S1\_at | 7.082e-06 | 0.8702 |
| Bt.7069.1.S1\_at | 7.198e-06 | -1.122 |
| Bt.2249.1.S1\_at | 7.222e-06 | 0.9201 |
| Bt.5106.1.S1\_at | 7.392e-06 | 1.054 |
| Bt.16639.1.A1\_at | 7.432e-06 | -0.832 |
| Bt.2749.1.S1\_at | 7.494e-06 | 2.004 |
| Bt.3616.1.S1\_at | 7.567e-06 | 0.9527 |
| Bt.1696.1.S1\_at | 7.871e-06 | 0.7149 |
| Bt.12327.1.S1\_at | 8.023e-06 | -0.9247 |
| Bt.3240.1.S1\_at | 8.042e-06 | 1.296 |
| Bt.1178.1.S1\_a\_at | 8.085e-06 | -1.055 |
| Bt.14023.1.S1\_at | 8.317e-06 | 0.8886 |
| Bt.19423.1.S1\_at | 8.324e-06 | -1.191 |
| Bt.19332.2.A1\_at | 8.733e-06 | 1.735 |
| Bt.12508.1.S1\_at | 8.767e-06 | 1.126 |
| Bt.21981.3.S1\_at | 8.772e-06 | 0.933 |
| Bt.5596.1.A1\_at | 8.79e-06 | 0.9114 |
| Bt.27851.1.S1\_at | 8.876e-06 | 1.197 |
| Bt.10330.1.S1\_at | 8.915e-06 | -1.242 |
| Bt.621.1.S1\_at | 8.915e-06 | 1.208 |
| Bt.21661.1.S1\_at | 9.161e-06 | -1.695 |
| Bt.15545.1.S1\_at | 9.302e-06 | 1.112 |
| Bt.5631.1.S1\_at | 9.377e-06 | 0.7354 |
| Bt.17672.1.S1\_at | 9.526e-06 | 0.7822 |
| Bt.22480.1.A1\_at | 9.529e-06 | 1.31 |
| Bt.7856.1.S1\_at | 9.529e-06 | 1.042 |
| Bt.24625.1.S1\_at | 9.602e-06 | -0.9221 |
| Bt.15675.1.S1\_at | 9.672e-06 | 1.019 |
| Bt.22329.1.S1\_at | 9.734e-06 | 0.7717 |
| Bt.876.1.S1\_at | 9.915e-06 | 1.515 |
| Bt.21551.1.S1\_at | 1.005e-05 | -0.8644 |
| Bt.8699.1.S1\_at | 1.01e-05 | 1.496 |
| Bt.4680.1.S1\_at | 1.017e-05 | 1.098 |
| Bt.3560.1.S1\_at | 1.023e-05 | -1.345 |
| Bt.1726.3.S1\_at | 1.027e-05 | -0.7871 |
| Bt.15784.1.S1\_at | 1.052e-05 | 0.9794 |
| Bt.2934.1.S1\_at | 1.058e-05 | 0.7194 |
| Bt.26688.2.S1\_a\_at | 1.076e-05 | -1.267 |
| Bt.12314.1.S1\_at | 1.112e-05 | -2.526 |
| Bt.27552.2.S1\_at | 1.118e-05 | -1.075 |
| Bt.13988.1.S1\_at | 1.122e-05 | -0.8604 |
| Bt.13464.1.S1\_at | 1.163e-05 | 0.919 |
| Bt.21238.1.S1\_at | 1.171e-05 | 0.9909 |
| Bt.14114.1.A1\_at | 1.174e-05 | -1.024 |
| Bt.19102.1.S1\_at | 1.181e-05 | 0.8685 |
| Bt.22510.1.S1\_at | 1.198e-05 | 0.9788 |
| Bt.11794.1.S1\_at | 1.248e-05 | 1.097 |
| Bt.29928.1.S1\_at | 1.249e-05 | -0.8018 |
| Bt.17963.1.A1\_at | 1.252e-05 | 1.064 |
| Bt.4917.1.S1\_at | 1.259e-05 | 0.6913 |
| Bt.3427.1.S1\_at | 1.283e-05 | 0.8801 |
| Bt.22325.2.A1\_at | 1.284e-05 | 0.958 |
| Bt.27196.1.A1\_at | 1.303e-05 | 1.535 |
| Bt.6840.1.S1\_a\_at | 1.309e-05 | 1.182 |
| Bt.24449.1.S1\_at | 1.31e-05 | 0.7998 |
| Bt.5024.1.S1\_at | 1.313e-05 | 0.8387 |
| Bt.13330.2.A1\_at | 1.329e-05 | -1.859 |
| Bt.23180.1.S1\_at | 1.37e-05 | 0.7858 |
| Bt.7955.2.S1\_at | 1.378e-05 | 1.127 |
| Bt.21032.1.S1\_at | 1.408e-05 | 0.7921 |
| Bt.23002.1.S1\_s\_at | 1.409e-05 | 0.751 |
| Bt.27101.1.S1\_at | 1.413e-05 | 1.182 |
| Bt.23094.2.A1\_x\_at | 1.416e-05 | -1.178 |
| Bt.21054.1.A1\_at | 1.416e-05 | -1.363 |
| Bt.7955.1.S1\_at | 1.448e-05 | 0.9116 |
| Bt.23317.1.S1\_at | 1.458e-05 | 0.728 |
| Bt.5070.1.S1\_x\_at | 1.459e-05 | -1.094 |
| Bt.4381.1.A1\_at | 1.472e-05 | 0.8813 |
| Bt.4751.1.S1\_a\_at | 1.484e-05 | -1.477 |
| Bt.21464.3.S1\_a\_at | 1.488e-05 | 0.8531 |
| Bt.4751.2.S1\_a\_at | 1.489e-05 | -1.505 |
| Bt.2304.1.A1\_at | 1.491e-05 | 0.8869 |
| Bt.2786.1.S1\_at | 1.553e-05 | 0.941 |
| Bt.5125.1.S1\_at | 1.567e-05 | -1.185 |
| Bt.6483.1.A1\_at | 1.685e-05 | 0.7828 |
| Bt.1418.2.S1\_at | 1.687e-05 | -0.874 |
| Bt.9405.1.S1\_at | 1.689e-05 | 0.6186 |
| Bt.19293.1.A1\_at | 1.704e-05 | 1.345 |
| Bt.8641.1.A1\_at | 1.715e-05 | 0.986 |
| Bt.23227.1.S1\_at | 1.766e-05 | -1.236 |
| Bt.6406.1.S2\_at | 1.768e-05 | -1.996 |
| Bt.26905.1.A1\_at | 1.778e-05 | 0.783 |
| Bt.26808.1.S1\_at | 1.794e-05 | -0.9002 |
| Bt.18278.2.A1\_at | 1.813e-05 | 1.041 |
| Bt.22556.1.S1\_at | 1.817e-05 | -0.6418 |
| Bt.16326.1.S1\_at | 1.821e-05 | -0.8911 |
| Bt.20449.1.S1\_at | 1.88e-05 | 1.071 |
| Bt.16389.1.S1\_at | 1.884e-05 | -0.7602 |
| Bt.29152.2.A1\_at | 1.892e-05 | 1.153 |
| Bt.16668.1.A1\_at | 1.892e-05 | -0.9591 |
| Bt.19706.1.S1\_at | 1.899e-05 | -1.239 |
| Bt.8124.1.S1\_at | 1.938e-05 | 0.9747 |
| Bt.5676.1.S1\_at | 1.942e-05 | 1.091 |
| Bt.15298.1.A1\_at | 1.96e-05 | 2.424 |
| Bt.25186.1.S1\_at | 1.966e-05 | 2.091 |
| Bt.25319.2.S1\_at | 1.99e-05 | -1.029 |
| Bt.24395.1.S1\_at | 1.993e-05 | 0.9759 |
| Bt.25616.1.S1\_at | 2.018e-05 | 0.8435 |
| Bt.22785.1.S1\_at | 2.058e-05 | -1.345 |
| Bt.13604.1.S1\_at | 2.147e-05 | 0.7515 |
| Bt.9998.1.S1\_at | 2.177e-05 | 1.229 |
| Bt.6685.2.A1\_at | 2.191e-05 | 1.068 |
| Bt.1310.1.S1\_at | 2.198e-05 | 0.7286 |
| Bt.9870.2.A1\_at | 2.206e-05 | 1.065 |
| Bt.23521.1.S1\_at | 2.21e-05 | -1.15 |
| Bt.16720.1.A1\_at | 2.22e-05 | -1.057 |
| Bt.25689.1.S1\_at | 2.235e-05 | 0.8018 |
| Bt.15666.2.S1\_at | 2.267e-05 | -0.8749 |
| Bt.9799.1.S1\_at | 2.284e-05 | 0.901 |
| Bt.20025.1.S1\_at | 2.33e-05 | 0.8702 |
| Bt.9438.1.S1\_at | 2.356e-05 | 0.8435 |
| Bt.4357.1.S1\_at | 2.417e-05 | 0.8091 |
| Bt.18689.2.A1\_at | 2.431e-05 | 1.051 |
| Bt.7648.1.S1\_at | 2.46e-05 | 0.6721 |
| Bt.24487.1.S1\_at | 2.511e-05 | 0.8387 |
| Bt.6950.1.S1\_at | 2.544e-05 | -0.6779 |
| Bt.2818.2.S1\_a\_at | 2.56e-05 | 0.6396 |
| Bt.5070.1.S1\_at | 2.646e-05 | -1.134 |
| Bt.15819.2.S1\_at | 2.66e-05 | 0.9542 |
| Bt.16329.1.A1\_at | 2.669e-05 | 0.9627 |
| Bt.7187.1.S1\_at | 2.681e-05 | 0.6322 |
| Bt.8212.1.S1\_at | 2.681e-05 | -0.7549 |
| Bt.5377.1.S1\_at | 2.69e-05 | 0.9754 |
| Bt.8124.1.S2\_at | 2.697e-05 | 1.042 |
| Bt.3162.1.S1\_at | 2.697e-05 | 0.673 |
| Bt.11216.1.S1\_at | 2.708e-05 | -1.365 |
| Bt.7540.2.S1\_a\_at | 2.714e-05 | 1.021 |
| Bt.11058.1.A1\_at | 2.718e-05 | 0.788 |
| Bt.9571.2.S1\_a\_at | 2.767e-05 | 1.368 |
| Bt.665.1.S1\_s\_at | 2.781e-05 | 0.6766 |
| Bt.9238.1.S1\_at | 2.812e-05 | 1.173 |
| Bt.4816.1.S1\_at | 2.821e-05 | -0.9906 |
| Bt.2562.1.S1\_at | 2.828e-05 | 0.7746 |
| Bt.22651.1.A1\_at | 2.835e-05 | 0.8039 |
| Bt.2856.1.S1\_at | 2.841e-05 | 1.012 |
| Bt.21376.1.S1\_at | 2.859e-05 | 0.755 |
| Bt.2110.1.S1\_at | 2.865e-05 | 1.202 |
| Bt.16004.1.A1\_at | 2.908e-05 | 2.809 |
| Bt.28035.1.S1\_at | 2.913e-05 | 0.9767 |
| Bt.26817.1.S1\_at | 2.93e-05 | 0.8632 |
| Bt.7776.1.S2\_at | 3.083e-05 | -0.7995 |
| Bt.15643.1.A1\_at | 3.167e-05 | -0.8566 |
| Bt.22466.1.A1\_at | 3.169e-05 | 0.7162 |
| Bt.9559.1.S1\_at | 3.206e-05 | 1.272 |
| Bt.7266.1.S1\_at | 3.218e-05 | 1.26 |
| Bt.12343.1.S1\_at | 3.232e-05 | -0.7294 |
| Bt.22763.2.S1\_a\_at | 3.292e-05 | 0.8753 |
| Bt.7670.1.A1\_at | 3.301e-05 | -1.096 |
| Bt.11196.1.S1\_at | 3.316e-05 | 0.8858 |
| Bt.24356.1.S1\_at | 3.349e-05 | -1.164 |
| Bt.28567.1.A1\_at | 3.466e-05 | -0.7523 |
| Bt.9055.2.S1\_a\_at | 3.466e-05 | 0.8774 |
| Bt.23148.1.S1\_at | 3.477e-05 | 0.6877 |
| Bt.4339.1.S1\_at | 3.503e-05 | 0.7252 |
| Bt.10442.2.S1\_a\_at | 3.548e-05 | 1.301 |
| Bt.21721.1.A1\_at | 3.559e-05 | 1.192 |
| Bt.26524.1.S1\_at | 3.613e-05 | 0.7225 |
| Bt.5466.2.S1\_a\_at | 3.619e-05 | 0.7321 |
| Bt.6114.1.S1\_at | 3.63e-05 | 1.01 |
| Bt.9664.1.A1\_at | 3.631e-05 | 0.8772 |
| Bt.27617.1.S1\_at | 3.65e-05 | 1.043 |
| Bt.15819.1.A1\_a\_at | 3.659e-05 | 0.8531 |
| Bt.13834.1.S1\_at | 3.66e-05 | 0.981 |
| Bt.28583.1.S1\_s\_at | 3.669e-05 | 1.2 |
| Bt.1767.1.S1\_at | 3.69e-05 | 0.7875 |
| Bt.15744.1.S1\_at | 3.7e-05 | 0.8405 |
| Bt.23883.1.A1\_at | 3.749e-05 | 0.8631 |
| Bt.27226.1.S1\_at | 3.786e-05 | 0.9174 |
| Bt.9959.3.S1\_a\_at | 3.789e-05 | 1.146 |
| Bt.5465.2.A1\_a\_at | 3.82e-05 | 1.07 |
| Bt.8549.1.S2\_at | 3.846e-05 | 1.497 |
| Bt.7633.1.S1\_at | 3.851e-05 | 0.9612 |
| Bt.12559.1.S1\_at | 3.857e-05 | 0.9239 |
| Bt.11428.1.A1\_at | 3.866e-05 | 1.682 |
| Bt.1537.1.S1\_at | 3.912e-05 | -1.014 |
| Bt.4353.2.S1\_at | 3.931e-05 | -0.8102 |
| Bt.7642.1.S1\_at | 3.943e-05 | 0.9005 |
| Bt.15783.1.S1\_at | 3.972e-05 | 0.9294 |
| Bt.26659.1.S1\_at | 3.996e-05 | 0.8103 |
| Bt.5304.1.S1\_at | 4.003e-05 | -1.411 |
| Bt.7322.1.S1\_at | 4.072e-05 | 1.123 |
| Bt.1821.1.S1\_at | 4.09e-05 | 1.119 |
| Bt.11014.1.S1\_at | 4.105e-05 | 0.9966 |
| Bt.13218.2.S1\_at | 4.158e-05 | 1.135 |
| Bt.2513.1.S1\_at | 4.204e-05 | 0.7047 |
| Bt.422.1.S1\_at | 4.227e-05 | 1.075 |
| Bt.13886.2.S1\_at | 4.239e-05 | -0.7409 |
| Bt.18755.2.S1\_at | 4.25e-05 | 0.6931 |
| Bt.14340.2.S1\_at | 4.282e-05 | -0.9064 |
| Bt.13588.3.A1\_at | 4.287e-05 | 2.709 |
| Bt.9956.1.S1\_at | 4.3e-05 | 0.8252 |
| Bt.27018.1.S1\_at | 4.302e-05 | 0.825 |
| Bt.2575.1.S1\_at | 4.31e-05 | 0.7581 |
| Bt.5605.3.S1\_at | 4.314e-05 | 1.159 |
| Bt.9033.2.S1\_at | 4.329e-05 | 0.7102 |
| Bt.3605.1.S1\_at | 4.329e-05 | 0.9835 |
| Bt.15931.1.S1\_at | 4.356e-05 | 0.6313 |
| Bt.8073.1.S1\_at | 4.402e-05 | -0.8924 |
| Bt.23992.1.A1\_at | 4.415e-05 | -0.9407 |
| Bt.6685.1.S1\_at | 4.497e-05 | 0.8958 |
| Bt.6516.1.A1\_at | 4.5e-05 | 0.7523 |
| Bt.5269.1.S1\_at | 4.531e-05 | -0.8777 |
| Bt.20470.2.S1\_a\_at | 4.555e-05 | 0.9371 |
| Bt.10236.1.S1\_at | 4.564e-05 | 1.592 |
| Bt.9662.1.S1\_at | 4.584e-05 | -1.011 |
| Bt.5306.1.S1\_at | 4.632e-05 | -0.8406 |
| Bt.29546.1.A1\_s\_at | 4.635e-05 | 1.073 |
| Bt.14223.3.S1\_at | 4.644e-05 | -0.7265 |
| Bt.13330.1.S1\_at | 4.692e-05 | -2.059 |
| Bt.3921.1.S1\_at | 4.739e-05 | 0.6278 |
| Bt.28163.1.S1\_at | 4.753e-05 | 1.41 |
| Bt.20559.2.A1\_at | 4.848e-05 | 1.111 |
| Bt.4167.1.S1\_at | 4.863e-05 | 1.064 |
| Bt.7641.2.S1\_a\_at | 4.89e-05 | 1.24 |
| Bt.11239.3.S1\_at | 4.912e-05 | -0.8136 |
| Bt.29029.1.S1\_at | 4.931e-05 | -0.693 |
| Bt.12364.1.S1\_at | 4.943e-05 | 1.05 |
| Bt.11617.1.A1\_at | 4.978e-05 | 0.7802 |
| Bt.21027.1.S1\_at | 5.009e-05 | 1.055 |
| Bt.3573.1.S2\_at | 5.021e-05 | 1.133 |
| Bt.876.2.S1\_at | 5.026e-05 | 1.065 |
| Bt.4825.1.A1\_at | 5.027e-05 | 1.119 |
| Bt.3077.1.S1\_at | 5.029e-05 | 1.411 |
| Bt.11492.1.A1\_at | 5.036e-05 | -0.9775 |
| Bt.5123.3.S1\_a\_at | 5.079e-05 | 0.8004 |
| Bt.27130.1.S1\_at | 5.113e-05 | 0.7648 |
| Bt.4274.1.S1\_at | 5.128e-05 | 0.688 |
| Bt.6680.1.S1\_a\_at | 5.17e-05 | 0.9662 |
| Bt.9190.1.S1\_at | 5.23e-05 | -0.835 |
| Bt.15753.1.S1\_at | 5.25e-05 | 0.6651 |
| Bt.12412.1.S1\_at | 5.253e-05 | -1.062 |
| Bt.5376.1.S1\_at | 5.258e-05 | 0.626 |
| Bt.20229.1.S1\_at | 5.264e-05 | 0.7561 |
| Bt.24919.1.S1\_at | 5.32e-05 | 1.175 |
| Bt.9811.1.S1\_at | 5.359e-05 | 0.6919 |
| Bt.21724.2.S1\_a\_at | 5.372e-05 | -1.314 |
| Bt.17102.3.S1\_at | 5.53e-05 | -0.8121 |
| Bt.5267.1.S1\_at | 5.546e-05 | 0.9115 |
| Bt.1546.1.S1\_at | 5.551e-05 | 1.207 |
| Bt.27194.1.S1\_at | 5.569e-05 | -1.005 |
| Bt.24960.1.S1\_at | 5.604e-05 | 0.9316 |
| Bt.23354.1.S1\_at | 5.625e-05 | -1.116 |
| Bt.5203.1.S1\_at | 5.659e-05 | 0.7758 |
| Bt.26905.2.S1\_at | 5.672e-05 | 0.8097 |
| Bt.5038.1.S2\_at | 5.68e-05 | -0.8706 |
| Bt.22568.1.S1\_at | 5.706e-05 | 1.097 |
| Bt.15534.1.S1\_a\_at | 5.713e-05 | 0.7728 |
| Bt.13991.3.S1\_at | 5.715e-05 | -0.6652 |
| Bt.1268.2.S1\_at | 5.729e-05 | -0.8188 |
| Bt.8319.3.S1\_a\_at | 5.761e-05 | -0.8266 |
| Bt.26711.2.S1\_at | 5.789e-05 | -0.9283 |
| Bt.1169.1.S1\_at | 5.803e-05 | 0.872 |
| Bt.28390.1.S1\_at | 5.809e-05 | -0.8911 |
| Bt.3314.1.S1\_at | 5.849e-05 | -0.6275 |
| Bt.4353.1.S1\_at | 5.926e-05 | -0.8437 |
| Bt.19352.1.A1\_at | 5.994e-05 | 1.257 |
| Bt.10515.1.S1\_at | 6.128e-05 | -0.7391 |
| Bt.9086.1.S1\_at | 6.155e-05 | 0.8716 |
| Bt.27204.1.S1\_at | 6.167e-05 | 1.215 |
| Bt.3980.1.S1\_at | 6.169e-05 | 1.253 |
| Bt.22106.2.S1\_at | 6.253e-05 | 1.079 |
| Bt.7614.1.S1\_at | 6.277e-05 | 0.6735 |
| Bt.347.1.S1\_at | 6.301e-05 | 0.9399 |
| Bt.26736.2.S1\_at | 6.441e-05 | 1.091 |
| Bt.5252.1.S1\_at | 6.445e-05 | 1.347 |
| Bt.9424.1.S1\_at | 6.477e-05 | -0.9946 |
| Bt.16123.1.S1\_at | 6.52e-05 | 1.203 |
| Bt.11855.1.S1\_at | 6.612e-05 | 0.6281 |
| Bt.5361.2.S1\_a\_at | 6.616e-05 | 1.289 |
| Bt.21164.1.S1\_at | 6.623e-05 | 0.8447 |
| Bt.4671.1.S1\_at | 6.655e-05 | -0.704 |
| Bt.18220.1.A1\_at | 6.677e-05 | 1.234 |
| Bt.21731.1.S1\_at | 6.678e-05 | 0.6227 |
| Bt.20439.1.S1\_at | 6.705e-05 | 0.8186 |
| Bt.9804.1.S1\_at | 6.714e-05 | -0.7902 |
| Bt.15937.1.A1\_at | 6.865e-05 | 0.9982 |
| Bt.10916.2.S1\_at | 6.902e-05 | 0.7583 |
| Bt.20340.2.S1\_at | 6.952e-05 | 1.168 |
| Bt.892.1.S1\_at | 6.996e-05 | 0.6015 |
| Bt.8618.1.S1\_at | 7.128e-05 | 0.7007 |
| Bt.14036.1.S1\_at | 7.14e-05 | -0.6138 |
| Bt.24421.1.A1\_at | 7.163e-05 | 0.8331 |
| Bt.21800.1.S1\_at | 7.308e-05 | 1.134 |
| Bt.24827.1.A1\_at | 7.446e-05 | -0.8289 |
| Bt.4044.1.S1\_at | 7.487e-05 | 0.7684 |
| Bt.11580.1.S1\_at | 7.697e-05 | 0.938 |
| Bt.27323.1.S1\_at | 7.698e-05 | 0.8102 |
| Bt.18637.1.A1\_at | 7.752e-05 | 0.8093 |
| Bt.15882.2.S1\_a\_at | 7.783e-05 | 0.7806 |
| Bt.23658.1.A1\_at | 7.784e-05 | 1.145 |
| Bt.7358.1.S1\_at | 7.921e-05 | 0.9486 |
| Bt.20593.2.S1\_at | 7.949e-05 | 1.217 |
| Bt.12024.1.S1\_at | 8.046e-05 | -0.8974 |
| Bt.21396.1.A1\_s\_at | 8.124e-05 | 0.7662 |
| Bt.5605.1.S1\_at | 8.197e-05 | 0.8071 |
| Bt.9027.1.S1\_at | 8.216e-05 | -0.7026 |
| Bt.12885.2.S1\_at | 8.346e-05 | -0.6941 |
| Bt.10730.1.S1\_at | 8.398e-05 | 1.199 |
| Bt.10277.2.S1\_a\_at | 8.422e-05 | 1.102 |
| Bt.13445.2.S1\_a\_at | 8.441e-05 | 0.9602 |
| Bt.25319.1.A1\_at | 8.522e-05 | -0.888 |
| Bt.24039.1.A1\_at | 8.571e-05 | 0.6211 |
| Bt.18362.1.A1\_at | 8.585e-05 | 0.9108 |
| Bt.16127.1.S1\_at | 8.618e-05 | -0.7193 |
| Bt.20570.1.S1\_at | 8.628e-05 | 0.8274 |
| Bt.7903.1.A1\_at | 8.663e-05 | 0.7612 |
| Bt.24779.2.S1\_at | 8.665e-05 | -0.8295 |
| Bt.24555.1.S1\_at | 8.674e-05 | 0.7254 |
| Bt.26596.1.S1\_at | 8.807e-05 | 0.6368 |
| Bt.27400.1.S1\_at | 8.934e-05 | -1.213 |
| Bt.1801.2.S1\_a\_at | 9.087e-05 | 0.8588 |
| Bt.4844.1.S1\_at | 9.156e-05 | 1.378 |
| Bt.26811.1.S1\_at | 9.258e-05 | 1.004 |
| Bt.23173.1.S1\_at | 9.357e-05 | -0.9372 |
| Bt.3764.1.S1\_at | 9.392e-05 | 1.109 |
| Bt.5432.1.S1\_at | 9.436e-05 | -0.5926 |
| Bt.3296.1.A1\_at | 9.481e-05 | 0.8047 |
| Bt.13440.1.S1\_at | 9.497e-05 | -0.6403 |
| Bt.2537.2.S1\_at | 9.531e-05 | -0.759 |
| Bt.22472.1.S1\_at | 9.545e-05 | -0.9359 |
| Bt.1768.1.A1\_at | 9.574e-05 | 0.6324 |
| Bt.23002.1.S1\_at | 9.605e-05 | -0.9114 |
| Bt.17659.1.S1\_at | 9.608e-05 | 0.802 |
| Bt.28166.1.A1\_at | 9.611e-05 | 0.7106 |
| Bt.16628.2.A1\_at | 9.634e-05 | 1.255 |
| Bt.13570.1.S1\_at | 9.675e-05 | 0.7626 |
| Bt.4287.1.A1\_at | 9.716e-05 | 0.7586 |
| Bt.15722.1.S1\_at | 9.753e-05 | 0.8803 |
| Bt.2825.1.S1\_at | 9.92e-05 | 0.7758 |
| Bt.5243.1.S1\_at | 9.943e-05 | 0.6186 |
| Bt.25718.1.A1\_at | 9.974e-05 | -0.7381 |
| Bt.23829.1.A1\_at | 0.0001004 | -0.8002 |
| Bt.19575.1.S1\_at | 0.0001011 | 0.9596 |
| Bt.18660.1.A1\_at | 0.0001015 | 0.5783 |
| Bt.471.1.S1\_at | 0.0001036 | -0.9093 |
| Bt.28271.1.A1\_at | 0.0001045 | -0.933 |
| Bt.13610.1.A1\_at | 0.0001052 | 0.9253 |
| Bt.1552.1.S1\_at | 0.000106 | 1.051 |
| Bt.7193.1.S1\_at | 0.0001064 | 0.6846 |
| Bt.23605.1.S1\_at | 0.0001064 | -0.8087 |
| Bt.22085.1.S1\_at | 0.0001076 | 0.8741 |
| Bt.1214.1.S1\_at | 0.0001078 | 0.8175 |
| Bt.8686.1.S1\_at | 0.000108 | 0.9344 |
| Bt.5108.1.S1\_at | 0.0001081 | 0.7436 |
| Bt.15672.2.S1\_at | 0.0001082 | 0.7991 |
| Bt.17579.1.A1\_at | 0.0001083 | 1.527 |
| Bt.2564.1.S1\_at | 0.0001085 | 0.5441 |
| Bt.29324.1.S1\_at | 0.000109 | -0.5799 |
| Bt.4704.2.S1\_at | 0.0001102 | 0.9459 |
| Bt.12685.1.S1\_at | 0.0001115 | -0.7551 |
| Bt.3015.1.A1\_at | 0.000113 | 0.8392 |
| Bt.21641.1.S1\_at | 0.0001131 | 0.8898 |
| Bt.16979.3.S1\_at | 0.0001154 | -0.7712 |
| Bt.26692.1.S1\_a\_at | 0.000116 | -0.9412 |
| Bt.9092.1.S1\_at | 0.0001165 | 0.8114 |
| Bt.16087.1.S1\_at | 0.0001165 | 1.016 |
| Bt.1405.1.S1\_at | 0.0001168 | -0.6292 |
| Bt.8356.1.S1\_at | 0.0001186 | 0.6918 |
| Bt.7968.2.S1\_at | 0.0001187 | -0.644 |
| Bt.21818.1.S1\_at | 0.0001188 | -0.835 |
| Bt.15397.1.S1\_a\_at | 0.000121 | 0.567 |
| Bt.23603.1.S1\_a\_at | 0.0001211 | 0.9131 |
| Bt.1244.1.S1\_at | 0.0001227 | 0.6077 |
| Bt.19208.1.A1\_at | 0.0001239 | 0.8391 |
| Bt.25346.1.A1\_at | 0.000124 | -0.6127 |
| Bt.28695.2.A1\_at | 0.0001242 | 0.6696 |
| Bt.7151.1.S1\_at | 0.0001253 | 0.6008 |
| Bt.2889.1.S1\_at | 0.0001259 | 0.6373 |
| Bt.27926.1.S1\_at | 0.0001261 | 0.9403 |
| Bt.7431.1.S1\_a\_at | 0.0001277 | -0.7054 |
| Bt.23094.6.A1\_at | 0.0001285 | -1.157 |
| Bt.24766.1.S1\_at | 0.0001291 | -1.107 |
| Bt.22101.1.A1\_at | 0.0001293 | 1.001 |
| Bt.9057.1.A1\_at | 0.0001293 | 0.5727 |
| Bt.15925.1.S1\_at | 0.0001294 | 0.8345 |
| Bt.5650.1.S1\_at | 0.0001301 | 0.9759 |
| Bt.25135.1.A1\_at | 0.0001304 | 0.7713 |
| Bt.9306.2.S1\_a\_at | 0.0001309 | 0.8281 |
| Bt.17299.1.A1\_at | 0.0001313 | 0.7091 |
| Bt.3835.1.S1\_at | 0.0001322 | 0.7545 |
| Bt.97.1.S1\_at | 0.0001323 | 2.174 |
| Bt.3447.1.S1\_at | 0.0001328 | 0.879 |
| Bt.28150.1.S1\_at | 0.0001332 | 1.018 |
| Bt.13273.1.S1\_at | 0.0001334 | 1.107 |
| Bt.1439.1.S1\_at | 0.0001341 | 1.035 |
| Bt.29952.1.S1\_at | 0.000135 | 0.8245 |
| Bt.19508.1.A1\_at | 0.0001353 | 0.8916 |
| Bt.21896.1.S1\_at | 0.0001356 | 0.997 |
| Bt.4292.1.S1\_at | 0.0001364 | 0.6938 |
| Bt.2898.2.S1\_a\_at | 0.0001369 | 0.6931 |
| Bt.16821.2.S1\_at | 0.000137 | 1.165 |
| Bt.21978.1.S1\_at | 0.0001384 | -0.7378 |
| Bt.21049.1.S1\_at | 0.00014 | 1.092 |
| Bt.26122.2.S1\_at | 0.0001401 | 0.7246 |
| Bt.25574.1.A1\_at | 0.0001404 | -0.9222 |
| Bt.5306.2.S1\_a\_at | 0.0001408 | -1.002 |
| Bt.20977.1.S1\_at | 0.0001414 | -0.8122 |
| Bt.24200.1.S1\_at | 0.0001427 | 0.9933 |
| Bt.3791.1.S1\_at | 0.0001432 | 0.715 |
| Bt.23662.1.S1\_at | 0.0001432 | 0.647 |
| Bt.9571.1.S1\_at | 0.0001433 | 1.061 |
| Bt.24932.1.A1\_at | 0.0001441 | -0.639 |
| Bt.28225.1.A1\_at | 0.000148 | -0.5447 |
| Bt.14209.1.A1\_at | 0.0001481 | 0.7697 |
| Bt.17939.1.S1\_at | 0.0001492 | 1.302 |
| Bt.5131.1.S1\_at | 0.0001497 | 0.7462 |
| Bt.4790.1.S1\_at | 0.0001502 | 0.6102 |
| Bt.19717.1.S1\_at | 0.0001508 | 0.5909 |
| Bt.26483.1.A1\_at | 0.0001508 | -1.005 |
| Bt.26942.1.S1\_at | 0.000151 | 0.7516 |
| Bt.8682.1.S1\_at | 0.0001516 | -0.6428 |
| Bt.19826.1.A1\_at | 0.0001554 | 0.9543 |
| Bt.4773.4.S1\_at | 0.0001554 | 0.638 |
| Bt.28312.1.S1\_at | 0.0001563 | -1.041 |
| Bt.13991.2.S1\_at | 0.0001566 | -0.7684 |
| Bt.16150.1.A1\_at | 0.0001568 | 1.136 |
| Bt.6391.1.A1\_at | 0.0001571 | 1.01 |
| Bt.9612.1.S1\_at | 0.0001574 | 0.8802 |
| Bt.7814.1.S1\_at | 0.0001589 | 0.5667 |
| Bt.21409.1.A1\_at | 0.0001591 | 2.528 |
| Bt.13975.2.A1\_at | 0.0001592 | -0.8838 |
| Bt.21133.1.S1\_at | 0.0001596 | -1.657 |
| Bt.12063.1.S1\_at | 0.0001602 | 1.025 |
| Bt.5368.1.S1\_at | 0.0001611 | -0.7105 |
| Bt.783.1.S1\_at | 0.0001612 | -0.734 |
| Bt.12158.1.S1\_at | 0.0001613 | -0.6004 |
| Bt.26621.1.S1\_at | 0.0001627 | -0.832 |
| Bt.21681.2.S1\_at | 0.0001657 | -1.159 |
| Bt.13688.1.S1\_at | 0.0001668 | 1.076 |
| Bt.1268.1.A1\_at | 0.0001701 | -0.9882 |
| Bt.14204.1.A1\_at | 0.0001712 | -0.9048 |
| Bt.5141.1.S2\_at | 0.000173 | 0.8213 |
| Bt.13324.4.S1\_at | 0.0001735 | 0.9138 |
| Bt.20404.1.S1\_at | 0.0001752 | 1.258 |
| Bt.6460.1.S1\_at | 0.0001753 | 0.9501 |
| Bt.24860.1.A1\_at | 0.0001756 | -0.9437 |
| Bt.6434.2.S1\_at | 0.0001759 | -0.6305 |
| Bt.14624.1.A1\_at | 0.0001774 | 0.8409 |
| Bt.21468.1.S1\_at | 0.0001776 | 0.8754 |
| Bt.10880.1.S1\_at | 0.0001784 | 0.768 |
| Bt.28263.1.S1\_at | 0.0001791 | 0.9709 |
| Bt.27927.1.S1\_at | 0.0001795 | -0.6813 |
| Bt.4867.2.S1\_at | 0.0001805 | 1.965 |
| Bt.20193.1.S1\_at | 0.0001808 | 1.447 |
| Bt.8051.1.S1\_at | 0.000181 | 0.6785 |
| Bt.1300.1.S1\_at | 0.0001811 | -0.8263 |
| Bt.22574.1.S1\_at | 0.0001811 | 1.033 |
| Bt.16821.1.S1\_at | 0.0001812 | 1.104 |
| Bt.12613.1.S1\_at | 0.0001818 | -0.893 |
| Bt.3272.1.A1\_at | 0.0001824 | 0.8135 |
| Bt.2121.1.S1\_at | 0.0001825 | 0.9214 |
| Bt.25109.2.A1\_at | 0.0001832 | 1.018 |
| Bt.14340.1.A1\_at | 0.0001836 | -0.6995 |
| Bt.13237.1.S1\_at | 0.0001839 | 0.6788 |
| Bt.13331.2.S1\_a\_at | 0.0001842 | 0.7511 |
| Bt.2347.2.S1\_at | 0.0001857 | -0.8627 |
| Bt.7437.1.S1\_at | 0.0001868 | 0.6201 |
| Bt.18316.2.S1\_at | 0.0001873 | -1.151 |
| Bt.1720.1.S1\_at | 0.0001877 | 0.7251 |
| Bt.10990.1.S1\_at | 0.0001882 | 0.7497 |
| Bt.997.2.S1\_a\_at | 0.0001888 | 0.8378 |
| Bt.11942.1.S1\_at | 0.0001895 | 0.7948 |
| Bt.27596.2.A1\_a\_at | 0.0001904 | 0.6677 |
| Bt.27904.1.A1\_at | 0.0001911 | 0.727 |
| Bt.13757.1.S1\_at | 0.0001936 | -0.7983 |
| Bt.8775.1.S1\_at | 0.0001939 | 0.5977 |
| Bt.12769.1.S1\_at | 0.0001952 | 1.209 |
| Bt.1370.1.S1\_at | 0.0001954 | -1.129 |
| Bt.6802.1.S1\_at | 0.0001966 | -0.9373 |
| Bt.8279.3.S1\_a\_at | 0.0001969 | 0.6039 |
| Bt.3735.1.A1\_at | 0.0001979 | 1.665 |
| Bt.23396.1.S1\_at | 0.0001987 | 0.9752 |
| Bt.1560.1.S1\_at | 0.0001995 | 0.9818 |
| Bt.7700.1.S1\_at | 0.0002002 | 0.8382 |
| Bt.3171.1.A1\_at | 0.0002006 | 0.7135 |
| Bt.12429.1.S1\_at | 0.0002021 | 0.7094 |
| Bt.19332.1.S1\_at | 0.0002026 | 1.145 |
| Bt.24609.1.S1\_at | 0.0002028 | 0.8069 |
| Bt.13708.2.S1\_at | 0.0002037 | -0.5393 |
| Bt.18623.1.A1\_at | 0.0002041 | -1.044 |
| Bt.23135.1.S1\_at | 0.0002043 | 0.5688 |
| Bt.9304.1.S1\_at | 0.0002045 | -0.6924 |
| Bt.9180.3.S1\_a\_at | 0.0002057 | 0.7342 |
| Bt.18316.1.A1\_at | 0.0002063 | -1.183 |
| Bt.3455.1.S1\_at | 0.0002073 | 0.7141 |
| Bt.29689.1.S1\_s\_at | 0.000209 | -1.273 |
| Bt.8518.1.S1\_at | 0.0002097 | 0.8068 |
| Bt.23094.2.A1\_at | 0.0002098 | -0.7185 |
| Bt.26600.1.S1\_at | 0.0002103 | -0.6295 |
| Bt.9053.1.S1\_a\_at | 0.0002112 | -0.7443 |
| Bt.3975.1.S1\_at | 0.0002125 | 0.573 |
| Bt.19161.1.S1\_at | 0.0002128 | -0.6687 |
| Bt.21681.1.A1\_at | 0.0002145 | -1.254 |
| Bt.16362.2.S1\_at | 0.0002148 | 0.6407 |
| Bt.401.2.S1\_at | 0.0002178 | 0.7215 |
| Bt.29882.1.S1\_at | 0.0002193 | -0.6748 |
| Bt.20008.1.S1\_at | 0.0002212 | 0.6454 |
| Bt.2846.1.A1\_at | 0.0002225 | 0.753 |
| Bt.4551.2.S1\_at | 0.0002244 | 0.8596 |
| Bt.5520.1.S1\_at | 0.0002245 | 0.7914 |
| Bt.4740.1.S1\_at | 0.0002273 | 0.5895 |
| Bt.3525.1.S1\_at | 0.0002277 | 0.6082 |
| Bt.20231.1.S1\_at | 0.0002285 | 0.6337 |
| Bt.4831.1.S1\_at | 0.0002286 | -0.5614 |
| Bt.21151.1.S1\_at | 0.0002291 | 0.6084 |
| Bt.21580.1.S1\_at | 0.0002306 | -0.9716 |
| Bt.21942.1.S1\_at | 0.0002309 | 0.9396 |
| Bt.26885.1.S1\_at | 0.0002348 | 0.9872 |
| Bt.12619.1.S1\_at | 0.0002351 | -0.6283 |
| Bt.26415.1.A1\_at | 0.0002361 | -0.5344 |
| Bt.14037.1.S1\_at | 0.0002405 | -0.5626 |
| Bt.3005.1.S1\_at | 0.0002408 | 0.7877 |
| Bt.9221.1.A1\_at | 0.0002426 | 0.8444 |
| Bt.14054.2.S1\_at | 0.0002432 | 1.296 |
| Bt.23668.1.A1\_at | 0.0002444 | -0.7394 |
| Bt.3437.1.S1\_at | 0.0002448 | 0.9811 |
| Bt.13256.1.A1\_at | 0.0002472 | 0.595 |
| Bt.1388.1.S1\_at | 0.0002477 | 0.5372 |
| Bt.11278.1.S1\_at | 0.0002489 | 1.651 |
| Bt.26246.1.A1\_at | 0.0002493 | 0.6279 |
| Bt.20176.1.S1\_at | 0.0002505 | 0.8916 |
| Bt.22460.1.S1\_at | 0.0002513 | -1.012 |
| Bt.12862.1.S1\_at | 0.0002515 | 0.9234 |
| Bt.3744.1.S1\_at | 0.0002521 | 0.7992 |
| Bt.22576.3.S1\_at | 0.0002534 | -0.6517 |
| Bt.1807.1.A1\_at | 0.0002535 | 0.697 |
| Bt.23308.1.S1\_at | 0.0002553 | 0.6708 |
| Bt.7870.1.S1\_at | 0.0002558 | 1.02 |
| Bt.24439.1.S1\_at | 0.0002574 | -0.8158 |
| Bt.8482.1.A1\_at | 0.000259 | 0.5872 |
| Bt.5605.2.S1\_at | 0.0002592 | 0.7702 |
| Bt.7181.1.S1\_at | 0.0002601 | 0.5434 |
| Bt.7439.1.S1\_at | 0.000261 | 0.7045 |
| Bt.5038.4.A1\_at | 0.0002612 | 0.5115 |
| Bt.3767.2.S1\_at | 0.0002616 | -0.6478 |
| Bt.20422.1.S1\_at | 0.0002619 | 0.7188 |
| Bt.9588.1.S1\_at | 0.0002619 | 0.6539 |
| Bt.7014.1.S1\_at | 0.000262 | 0.685 |
| Bt.29385.1.A1\_at | 0.0002621 | -0.5995 |
| Bt.15847.1.S1\_at | 0.0002666 | 0.6332 |
| Bt.10159.1.S1\_at | 0.0002673 | 0.7213 |
| Bt.23494.1.S1\_at | 0.0002689 | 1.047 |
| Bt.3715.1.S1\_at | 0.0002699 | 0.6598 |
| Bt.28593.1.S1\_at | 0.0002724 | -0.8604 |
| Bt.11163.1.S1\_at | 0.0002733 | -0.5694 |
| Bt.20431.2.S1\_at | 0.0002735 | -1.097 |
| Bt.3541.1.S1\_at | 0.0002743 | 0.6238 |
| Bt.4442.1.S1\_at | 0.0002747 | 0.6287 |
| Bt.2696.1.A1\_at | 0.0002749 | 1.057 |
| Bt.5466.4.S1\_at | 0.0002777 | -0.5754 |
| Bt.23676.1.S1\_at | 0.0002779 | -0.94 |
| Bt.14055.1.S1\_at | 0.0002798 | -0.6375 |
| Bt.9741.1.S1\_at | 0.0002814 | 0.7177 |
| Bt.17704.1.S1\_at | 0.0002819 | 0.7853 |
| Bt.2519.1.S1\_at | 0.000282 | 0.6255 |
| Bt.18748.1.A1\_at | 0.000283 | -0.7484 |
| Bt.24727.1.S1\_a\_at | 0.0002834 | 0.8011 |
| Bt.3277.1.S1\_at | 0.0002845 | 0.7276 |
| Bt.15717.1.A1\_at | 0.0002847 | 1.245 |
| Bt.4263.1.S1\_at | 0.0002856 | -0.6956 |
| Bt.15857.1.S1\_at | 0.0002858 | 0.7903 |
| Bt.23244.1.S1\_at | 0.0002859 | 0.6172 |
| Bt.4523.1.S1\_at | 0.0002866 | 0.649 |
| Bt.24780.2.A1\_at | 0.0002873 | -1.676 |
| Bt.17219.1.A1\_at | 0.0002887 | 0.5966 |
| Bt.10092.1.S1\_at | 0.0002893 | -0.8209 |
| Bt.3567.1.S1\_at | 0.0002893 | -0.6412 |
| Bt.28866.1.A1\_at | 0.0002899 | 0.6079 |
| Bt.14163.2.S1\_at | 0.0002932 | -0.7312 |
| Bt.20955.1.A1\_at | 0.0002961 | 1.099 |
| Bt.4314.1.S1\_at | 0.0002969 | 0.6032 |
| Bt.20988.1.S1\_at | 0.0002998 | 0.7345 |
| Bt.5334.1.S1\_at | 0.0003009 | 0.4861 |
| Bt.9589.1.S1\_at | 0.0003009 | 0.7727 |
| Bt.21551.2.S1\_a\_at | 0.000302 | -0.8398 |
| Bt.16265.1.S1\_at | 0.0003032 | 0.6877 |
| Bt.28429.1.S1\_at | 0.0003039 | 0.9928 |
| Bt.7192.1.S1\_at | 0.0003089 | 0.7986 |
| Bt.22197.1.S1\_at | 0.0003105 | 0.7238 |
| Bt.2098.1.S1\_at | 0.0003105 | 0.6103 |
| Bt.17148.1.S1\_at | 0.0003108 | 1.126 |
| Bt.13480.1.S1\_at | 0.0003133 | 0.7592 |
| Bt.9915.1.S1\_at | 0.0003153 | 0.803 |
| Bt.3912.1.S1\_at | 0.0003157 | -0.6294 |
| Bt.21433.1.S1\_at | 0.0003164 | 0.8865 |
| Bt.26999.1.S1\_at | 0.00032 | 0.6158 |
| Bt.21981.1.A1\_at | 0.0003211 | 0.8526 |
| Bt.23273.1.S1\_at | 0.0003212 | 0.7084 |
| Bt.12924.1.S1\_at | 0.0003241 | 1.378 |
| Bt.15845.1.S1\_at | 0.000325 | 0.6539 |
| Bt.22463.1.A1\_at | 0.0003292 | 0.6097 |
| Bt.6815.1.A1\_at | 0.0003294 | 0.6049 |
| Bt.9218.1.A1\_at | 0.0003295 | -0.701 |
| Bt.4786.1.S2\_at | 0.0003296 | 0.5395 |
| Bt.22522.1.S1\_at | 0.0003299 | 0.5598 |
| Bt.12888.1.S1\_at | 0.0003322 | 0.854 |
| Bt.8102.1.S1\_at | 0.0003372 | 0.7725 |
| Bt.5269.2.S1\_a\_at | 0.0003373 | -0.9157 |
| Bt.11157.1.S1\_a\_at | 0.0003399 | 0.551 |
| Bt.20314.2.S1\_at | 0.0003399 | 0.7539 |
| Bt.12404.2.S1\_a\_at | 0.0003418 | 0.5857 |
| Bt.27035.1.A1\_at | 0.0003445 | -0.5109 |
| Bt.5333.1.S1\_at | 0.000345 | 0.6962 |
| Bt.4986.1.S1\_at | 0.0003482 | 0.6745 |
| Bt.8549.1.S1\_at | 0.0003492 | 1.209 |
| Bt.21286.1.S1\_at | 0.00035 | -0.9473 |
| Bt.23155.1.S1\_at | 0.0003519 | -0.5826 |
| Bt.22869.1.S2\_at | 0.000353 | -0.912 |
| Bt.14054.1.A1\_at | 0.0003535 | 1.31 |
| Bt.11194.1.S1\_at | 0.0003538 | 0.7982 |
| Bt.4142.1.S1\_at | 0.0003544 | 0.6926 |
| Bt.26718.1.S1\_a\_at | 0.0003549 | 0.6259 |
| Bt.1800.1.S1\_at | 0.0003563 | 0.65 |
| Bt.25098.1.S1\_at | 0.0003568 | 0.8172 |
| Bt.13972.1.A1\_at | 0.0003602 | -0.5621 |
| Bt.15157.1.S1\_at | 0.0003625 | 0.7968 |
| Bt.19852.1.A1\_at | 0.0003634 | -0.5525 |
| Bt.7786.1.S1\_at | 0.0003657 | -0.676 |
| Bt.23254.1.S1\_at | 0.0003664 | 0.5327 |
| Bt.3251.1.S1\_at | 0.0003667 | -0.651 |
| Bt.2263.1.A1\_at | 0.0003694 | 0.6522 |
| Bt.27657.1.A1\_at | 0.0003703 | 0.7426 |
| Bt.4355.1.S1\_at | 0.0003709 | 0.5869 |
| Bt.1929.1.S1\_at | 0.0003745 | 1.819 |
| Bt.24109.1.S1\_at | 0.0003758 | 1.308 |
| Bt.28379.1.S1\_at | 0.0003766 | 1.409 |
| Bt.20897.2.S1\_a\_at | 0.0003776 | 0.9013 |
| Bt.4863.1.S1\_at | 0.0003789 | 0.7893 |
| Bt.24856.2.S1\_at | 0.0003802 | 0.8349 |
| Bt.7139.1.S1\_at | 0.0003805 | 0.6853 |
| Bt.11133.2.S1\_a\_at | 0.0003808 | 0.6415 |
| Bt.5096.1.S1\_at | 0.0003825 | 0.6196 |
| Bt.15867.2.S1\_at | 0.0003843 | -0.5316 |
| Bt.18835.1.S1\_at | 0.0003844 | 0.7124 |
| Bt.771.1.S1\_at | 0.0003915 | -0.4903 |
| Bt.5916.1.S1\_at | 0.0003928 | 0.6278 |
| Bt.5630.1.S1\_at | 0.0003929 | 0.7239 |
| Bt.19519.1.S1\_at | 0.000393 | 0.6638 |
| Bt.2847.1.S1\_a\_at | 0.0003957 | 0.4726 |
| Bt.20383.1.S1\_at | 0.0003969 | 1.135 |
| Bt.23355.1.S1\_at | 0.0003992 | 0.7501 |
| Bt.2515.1.S1\_at | 0.0004012 | 0.7087 |
| Bt.23218.1.S1\_at | 0.0004017 | 0.5826 |
| Bt.29621.1.S1\_at | 0.0004036 | -1.159 |
| Bt.17456.1.S1\_at | 0.0004064 | 0.6509 |
| Bt.3856.1.S1\_at | 0.0004077 | 0.7319 |
| Bt.4702.2.S1\_at | 0.0004093 | -0.6612 |
| Bt.11394.1.S1\_at | 0.0004147 | 0.717 |
| Bt.23456.1.A1\_s\_at | 0.000415 | -0.5251 |
| Bt.9828.1.S2\_at | 0.0004151 | -0.7051 |
| Bt.13537.1.A1\_at | 0.0004158 | 0.9894 |
| Bt.13334.1.S1\_at | 0.0004204 | -0.62 |
| Bt.3137.1.S1\_at | 0.0004242 | 1.023 |
| Bt.20479.1.S1\_at | 0.0004248 | 0.9726 |
| Bt.20917.2.S1\_a\_at | 0.0004248 | 0.8332 |
| Bt.5112.1.A1\_a\_at | 0.0004261 | 0.884 |
| Bt.3750.1.S1\_at | 0.0004286 | 0.781 |
| Bt.23552.1.S1\_at | 0.0004295 | 0.6114 |
| Bt.3821.2.S1\_at | 0.0004297 | 0.6818 |
| Bt.4273.1.S1\_at | 0.0004313 | 0.891 |
| Bt.18228.1.A1\_at | 0.0004362 | 0.5888 |
| Bt.5598.1.S1\_at | 0.0004366 | -0.9957 |
| Bt.22721.1.A1\_a\_at | 0.0004374 | 0.8908 |
| Bt.15870.1.S1\_at | 0.0004385 | 0.6228 |
| Bt.28695.1.S1\_at | 0.0004399 | 0.764 |
| Bt.13950.1.A1\_at | 0.0004403 | 0.5924 |
| Bt.16230.1.S1\_at | 0.0004419 | 0.6549 |
| Bt.22082.1.S1\_at | 0.000442 | 0.8442 |
| Bt.26711.1.S1\_at | 0.0004437 | -0.8107 |
| Bt.11628.1.A1\_at | 0.000444 | -0.6383 |
| Bt.21313.1.S1\_at | 0.0004442 | 0.5541 |
| Bt.17445.1.A1\_at | 0.0004447 | 0.7913 |
| Bt.15878.1.S1\_at | 0.0004487 | 0.7274 |
| Bt.5170.1.S1\_at | 0.0004581 | 0.6444 |
| Bt.23589.2.S1\_at | 0.0004591 | 0.5819 |
| Bt.8319.3.S1\_at | 0.000461 | -0.6431 |
| Bt.20040.1.S1\_at | 0.0004624 | 0.5625 |
| Bt.21031.1.S1\_at | 0.0004628 | 0.7114 |
| Bt.18540.1.A1\_at | 0.0004641 | -0.7229 |
| Bt.28183.1.S1\_at | 0.0004662 | 0.6616 |
| Bt.26911.1.A1\_at | 0.0004717 | -0.6766 |
| Bt.3854.1.S1\_at | 0.0004747 | 0.7551 |
| Bt.26784.1.S1\_at | 0.0004752 | -0.5275 |
| Bt.12144.2.S1\_a\_at | 0.0004754 | 0.7487 |
| Bt.28481.1.A1\_at | 0.0004791 | 0.6396 |
| Bt.28671.1.S1\_at | 0.0004791 | -0.5753 |
| Bt.12916.1.S1\_at | 0.0004796 | -1.23 |
| Bt.7728.2.S1\_at | 0.0004796 | -1.178 |
| Bt.864.2.S1\_at | 0.0004815 | 1.118 |
| Bt.8234.1.S1\_at | 0.000483 | -0.618 |
| Bt.27371.1.S1\_at | 0.000483 | 0.6852 |
| Bt.5596.2.S1\_at | 0.0004871 | 0.9642 |
| Bt.27267.1.S1\_at | 0.0004891 | 0.6967 |
| Bt.25402.1.S1\_at | 0.0004905 | 0.5837 |
| Bt.25973.1.S1\_at | 0.0004954 | -0.8036 |
| Bt.20172.1.S1\_at | 0.0004958 | 0.7314 |
| Bt.8593.2.A1\_at | 0.0004976 | -0.6625 |
| Bt.2841.1.S1\_at | 0.0005017 | 0.7056 |
| Bt.63.1.S1\_at | 0.0005044 | 0.9257 |
| Bt.5246.1.S1\_at | 0.0005092 | 0.6489 |
| Bt.11687.2.S1\_at | 0.0005111 | 0.5321 |
| Bt.2439.1.S1\_at | 0.0005141 | -0.5345 |
| Bt.3888.1.S1\_a\_at | 0.0005186 | 0.9635 |
| Bt.16695.2.A1\_at | 0.0005246 | 0.7712 |
| Bt.13235.1.S1\_at | 0.0005268 | -1.124 |
| Bt.18101.1.A1\_at | 0.0005281 | 1.155 |
| Bt.22168.1.A1\_at | 0.0005283 | 0.9978 |
| Bt.18352.1.A1\_at | 0.0005298 | 0.5761 |
| Bt.12216.1.S1\_at | 0.0005305 | 0.5539 |
| Bt.15996.1.S2\_at | 0.0005315 | -0.6238 |
| Bt.24804.1.S1\_at | 0.0005318 | -0.7397 |
| Bt.3216.1.A1\_at | 0.0005372 | 0.733 |
| Bt.4803.1.S1\_at | 0.0005439 | 0.5084 |
| Bt.28238.1.A1\_at | 0.0005446 | 0.7898 |
| Bt.976.1.A1\_at | 0.0005447 | 0.8595 |
| Bt.22865.1.S2\_at | 0.0005494 | -0.6376 |
| Bt.27552.1.A1\_at | 0.0005523 | -0.7256 |
| Bt.5176.1.S1\_at | 0.0005533 | 0.7137 |
| Bt.27563.1.A1\_at | 0.0005553 | -0.8861 |
| Bt.11448.1.S1\_at | 0.0005561 | 0.5168 |
| Bt.8122.1.S1\_at | 0.0005583 | -0.4501 |
| Bt.1318.1.S1\_at | 0.0005588 | 0.5131 |
| Bt.18212.1.S1\_at | 0.0005589 | 0.5902 |
| Bt.22577.2.S1\_at | 0.0005592 | -1.145 |
| Bt.15724.1.S1\_at | 0.0005639 | 0.6199 |
| Bt.11013.1.S1\_at | 0.0005679 | 0.6063 |
| Bt.4078.2.S1\_a\_at | 0.0005763 | 0.6902 |
| Bt.24875.1.S1\_at | 0.0005773 | 0.5641 |
| Bt.2428.1.S1\_at | 0.0005791 | 0.9149 |
| Bt.7863.1.S1\_at | 0.0005807 | 0.5411 |
| Bt.4869.1.S1\_at | 0.0005815 | -0.5923 |
| Bt.3414.3.A1\_a\_at | 0.0005825 | 0.7242 |
| Bt.20361.2.A1\_at | 0.0005825 | -0.9063 |
| Bt.22637.1.S1\_at | 0.0005832 | -0.6392 |
| Bt.19927.1.A1\_at | 0.0005833 | 0.6625 |
| Bt.3857.1.S1\_at | 0.0005842 | 0.7755 |
| Bt.21116.1.S1\_at | 0.0005843 | -0.7362 |
| Bt.12510.1.S1\_a\_at | 0.0005873 | 0.832 |
| Bt.8250.1.S1\_at | 0.0005894 | -0.5492 |
| Bt.1344.1.S1\_at | 0.0005963 | 1.007 |
| Bt.28194.1.S1\_at | 6e-04 | 0.5536 |
| Bt.23646.1.S1\_at | 0.0006022 | 0.73 |
| Bt.3070.2.S1\_a\_at | 0.0006022 | 0.882 |
| Bt.1269.1.S1\_a\_at | 0.0006028 | 0.5333 |
| Bt.26130.1.A1\_at | 0.0006049 | 0.7643 |
| Bt.18731.1.A1\_at | 0.0006112 | 0.5453 |
| Bt.4661.1.S1\_at | 0.0006133 | 0.5984 |
| Bt.9959.2.S1\_a\_at | 0.0006203 | 0.888 |
| Bt.689.1.S1\_at | 0.0006204 | -0.5255 |
| Bt.16784.1.S1\_at | 0.000621 | -0.8106 |
| Bt.24538.1.S1\_at | 0.000622 | -0.9276 |
| Bt.14211.1.A1\_at | 0.0006253 | 0.6086 |

Differentially expressed genes for GCRMA: 1189

| AFFY | p-value | FoldChange |
| --- | --- | --- |
| Bt.3312.1.S1\_at | 2.487e-13 | -2.1 |
| Bt.26760.2.S1\_a\_at | 1.045e-10 | -5.253 |
| Bt.6642.1.S1\_at | 1.442e-10 | -3.145 |
| Bt.26135.1.A1\_at | 3.804e-10 | 3.879 |
| Bt.22167.1.S1\_at | 7.299e-10 | -4.158 |
| Bt.3071.1.S1\_at | 1.226e-09 | 2.482 |
| Bt.6642.2.S1\_at | 1.359e-09 | -3.917 |
| Bt.13986.1.S1\_at | 2.356e-09 | -1.726 |
| Bt.9599.1.A1\_at | 4.142e-09 | 1.505 |
| Bt.8972.1.S1\_at | 4.529e-09 | -2.148 |
| Bt.29873.1.S1\_at | 6.426e-09 | 3.098 |
| Bt.20862.1.A1\_at | 6.492e-09 | 2.617 |
| Bt.350.1.S1\_s\_at | 6.623e-09 | -3.825 |
| Bt.7651.1.S1\_at | 9.817e-09 | -2.908 |
| Bt.7056.1.S1\_at | 1.031e-08 | 3.236 |
| Bt.22556.1.S1\_at | 1.194e-08 | -0.7838 |
| Bt.9810.1.S1\_at | 1.279e-08 | 2.036 |
| Bt.9767.1.S1\_a\_at | 1.349e-08 | 3.852 |
| Bt.9556.1.S1\_at | 1.429e-08 | 2.737 |
| Bt.29882.1.S1\_at | 1.782e-08 | -1.739 |
| Bt.28567.1.A1\_at | 2.09e-08 | -1.478 |
| Bt.5519.2.S1\_at | 2.169e-08 | 1.356 |
| Bt.1035.1.S1\_a\_at | 2.363e-08 | 1.286 |
| Bt.1696.1.S1\_at | 2.963e-08 | 0.989 |
| Bt.4184.1.S2\_at | 3.382e-08 | 1.239 |
| Bt.20270.1.S1\_at | 3.97e-08 | 1.544 |
| Bt.3921.1.S1\_at | 4.197e-08 | 0.6402 |
| Bt.5144.1.S1\_at | 5.705e-08 | 1.779 |
| Bt.23094.2.A1\_x\_at | 5.793e-08 | -3.153 |
| Bt.24310.1.S1\_at | 6.048e-08 | -2.683 |
| Bt.24625.1.S1\_at | 6.658e-08 | -1.921 |
| Bt.659.2.S1\_at | 7.303e-08 | -3.338 |
| Bt.12343.1.S1\_at | 8.312e-08 | -1.611 |
| Bt.4818.1.S1\_at | 8.912e-08 | 1.883 |
| Bt.18639.1.A1\_at | 9.209e-08 | -4.736 |
| Bt.24517.2.A1\_at | 9.57e-08 | 1.773 |
| Bt.23519.1.S1\_at | 9.857e-08 | -2.285 |
| Bt.10810.1.A1\_at | 1.042e-07 | 2.159 |
| Bt.5340.1.S1\_s\_at | 1.154e-07 | 1.602 |
| Bt.29928.1.S1\_at | 1.182e-07 | -1.266 |
| Bt.2159.1.S1\_at | 1.569e-07 | 2.061 |
| Bt.2070.1.A1\_at | 1.572e-07 | 1.662 |
| Bt.8234.1.S1\_at | 1.708e-07 | -0.8924 |
| Bt.5070.1.S1\_at | 1.742e-07 | -1.2 |
| Bt.15682.1.A1\_at | 1.748e-07 | 3.138 |
| Bt.7908.1.S1\_at | 1.87e-07 | 0.9428 |
| Bt.1998.1.S1\_at | 1.924e-07 | 1.47 |
| Bt.6774.1.S1\_at | 1.982e-07 | -1.548 |
| Bt.2575.1.S1\_at | 2.024e-07 | 0.7897 |
| Bt.21670.1.A1\_at | 2.404e-07 | 1.372 |
| Bt.12434.1.S1\_at | 2.451e-07 | 1.215 |
| Bt.23250.2.S1\_at | 2.567e-07 | 3.672 |
| Bt.2822.1.S1\_at | 2.63e-07 | 0.8764 |
| Bt.18639.2.S1\_at | 2.808e-07 | -6.179 |
| Bt.3863.1.S1\_at | 2.961e-07 | -2.302 |
| Bt.21396.1.A1\_s\_at | 3.016e-07 | 0.6248 |
| Bt.11659.1.S1\_at | 3.045e-07 | 1.524 |
| Bt.4274.1.S1\_at | 3.05e-07 | 1.37 |
| Bt.4702.1.S1\_at | 3.123e-07 | -1.634 |
| Bt.24524.1.S1\_at | 3.347e-07 | 1.757 |
| Bt.20420.1.S1\_at | 3.45e-07 | -1.612 |
| Bt.21432.2.A1\_at | 3.543e-07 | 2.436 |
| Bt.4816.1.S1\_at | 3.713e-07 | -2.056 |
| Bt.3015.1.A1\_at | 3.753e-07 | 1.009 |
| Bt.23148.1.S1\_at | 3.78e-07 | 0.8611 |
| Bt.1178.1.S1\_a\_at | 3.929e-07 | -2.102 |
| Bt.21724.1.S1\_at | 4.257e-07 | -1.242 |
| Bt.29029.1.S1\_at | 4.352e-07 | -1.088 |
| Bt.4437.1.S1\_at | 4.566e-07 | 1.802 |
| Bt.23176.2.S1\_a\_at | 4.884e-07 | 2.334 |
| Bt.5123.2.S1\_at | 5.626e-07 | 1.002 |
| Bt.20104.1.S1\_at | 6.028e-07 | -2.179 |
| Bt.9828.1.S2\_at | 6.438e-07 | -1.379 |
| Bt.7676.3.S1\_at | 6.482e-07 | 2.894 |
| Bt.23603.3.S1\_at | 6.669e-07 | 1.013 |
| Bt.7540.3.S1\_at | 6.945e-07 | 1.921 |
| Bt.1920.1.S1\_at | 7.227e-07 | -2.252 |
| Bt.9643.1.S1\_at | 7.276e-07 | 1.686 |
| Bt.13289.1.S1\_at | 7.502e-07 | -3.657 |
| Bt.4917.1.S1\_at | 7.932e-07 | 0.729 |
| Bt.6873.1.A1\_at | 8.107e-07 | -0.3986 |
| Bt.3163.1.A1\_at | 8.399e-07 | -0.9496 |
| Bt.19352.1.A1\_at | 8.405e-07 | 1.181 |
| Bt.4822.1.A1\_at | 8.491e-07 | 1.097 |
| Bt.20470.2.S1\_a\_at | 8.513e-07 | 2.739 |
| Bt.21385.1.S1\_at | 8.78e-07 | 1.579 |
| Bt.9033.2.S1\_at | 9.08e-07 | 0.7574 |
| Bt.27856.1.S1\_at | 9.318e-07 | 2.989 |
| Bt.4715.1.S1\_at | 9.844e-07 | 0.9979 |
| Bt.5269.1.S1\_at | 1.022e-06 | -1.078 |
| Bt.2874.1.S1\_at | 1.022e-06 | 1.107 |
| Bt.22803.1.S1\_at | 1.026e-06 | 1.576 |
| Bt.4381.1.A1\_at | 1.047e-06 | 1.798 |
| Bt.3251.1.S1\_at | 1.092e-06 | -1.235 |
| Bt.7034.1.S1\_at | 1.18e-06 | 2.761 |
| Bt.5334.1.S1\_at | 1.197e-06 | 0.4816 |
| Bt.27018.1.S1\_at | 1.222e-06 | 1.612 |
| Bt.12443.1.S1\_at | 1.263e-06 | -0.9548 |
| Bt.12299.1.A1\_at | 1.271e-06 | 5.253 |
| Bt.4263.1.S1\_at | 1.277e-06 | -1.239 |
| Bt.911.1.S1\_at | 1.37e-06 | -1.288 |
| Bt.7394.1.A1\_at | 1.394e-06 | 1.497 |
| Bt.4970.1.S1\_at | 1.399e-06 | 1.98 |
| Bt.7307.2.A1\_a\_at | 1.468e-06 | -1.232 |
| Bt.18890.1.A1\_at | 1.507e-06 | 0.907 |
| Bt.7968.2.S1\_at | 1.526e-06 | -0.9911 |
| Bt.7179.1.S1\_a\_at | 1.557e-06 | 2.603 |
| Bt.6774.2.S1\_at | 1.575e-06 | -1.214 |
| Bt.7955.2.S1\_at | 1.582e-06 | 1.374 |
| Bt.10153.1.S1\_at | 1.603e-06 | -1.75 |
| Bt.25052.1.S1\_at | 1.617e-06 | 1.599 |
| Bt.562.1.S1\_at | 1.633e-06 | -2.534 |
| Bt.25880.1.A1\_at | 1.647e-06 | -0.7735 |
| Bt.12510.1.S1\_a\_at | 1.648e-06 | 3.001 |
| Bt.17417.1.A1\_at | 1.679e-06 | -0.3962 |
| Bt.4937.1.S1\_at | 1.708e-06 | 1.181 |
| Bt.659.1.S1\_at | 1.788e-06 | -2.551 |
| Bt.12963.1.A1\_at | 1.836e-06 | -0.8406 |
| Bt.2392.1.S1\_at | 1.837e-06 | 1.353 |
| Bt.13435.1.A1\_at | 1.876e-06 | 2.443 |
| Bt.12510.1.S1\_at | 1.88e-06 | 3.059 |
| Bt.22325.2.A1\_at | 1.881e-06 | 1.441 |
| Bt.26711.1.S1\_at | 1.889e-06 | -1.779 |
| Bt.22306.1.S1\_at | 1.905e-06 | 1.927 |
| Bt.7288.1.S1\_at | 1.941e-06 | 1.048 |
| Bt.13991.3.S1\_at | 1.953e-06 | -0.517 |
| Bt.20501.1.S1\_at | 1.973e-06 | -5.116 |
| Bt.3843.1.S1\_at | 1.984e-06 | 1.219 |
| Bt.13988.1.S1\_at | 1.992e-06 | -1.316 |
| Bt.8963.1.S1\_at | 2.027e-06 | 1.991 |
| Bt.2786.1.S1\_at | 2.055e-06 | 1.916 |
| Bt.3241.1.S1\_at | 2.06e-06 | 0.7178 |
| Bt.9012.1.S1\_at | 2.073e-06 | -1.773 |
| Bt.8212.1.S1\_at | 2.16e-06 | -1.019 |
| Bt.13886.2.S1\_at | 2.17e-06 | -1.021 |
| Bt.11163.1.S1\_at | 2.21e-06 | -0.7616 |
| Bt.27563.1.A1\_at | 2.22e-06 | -1.097 |
| Bt.22510.1.S1\_at | 2.232e-06 | 1.465 |
| Bt.4832.1.S1\_at | 2.251e-06 | 0.4144 |
| Bt.24154.1.A1\_at | 2.263e-06 | -1.264 |
| Bt.4624.1.S1\_at | 2.307e-06 | -1.671 |
| Bt.3541.1.S1\_at | 2.348e-06 | 0.6838 |
| Bt.6991.1.S1\_at | 2.365e-06 | -1.448 |
| Bt.15397.1.S1\_a\_at | 2.398e-06 | 0.5712 |
| Bt.16951.1.A1\_at | 2.512e-06 | 1.985 |
| Bt.18755.2.S1\_at | 2.641e-06 | 1.014 |
| Bt.4410.1.S1\_at | 2.669e-06 | 0.9856 |
| Bt.9180.1.S1\_a\_at | 2.92e-06 | 1.79 |
| Bt.4688.1.S1\_a\_at | 2.923e-06 | 1.382 |
| Bt.1131.1.S1\_at | 2.933e-06 | 0.9564 |
| Bt.7537.1.S1\_at | 2.988e-06 | 1.259 |
| Bt.5141.1.S2\_at | 2.996e-06 | 1.699 |
| Bt.16979.3.S1\_at | 3.036e-06 | -0.6686 |
| Bt.7187.1.S1\_at | 3.038e-06 | 0.6305 |
| Bt.13289.2.S1\_at | 3.077e-06 | -3.263 |
| Bt.26415.1.A1\_at | 3.08e-06 | -0.8764 |
| Bt.11178.1.S1\_at | 3.261e-06 | 0.8618 |
| Bt.4631.2.S1\_at | 3.398e-06 | -1.751 |
| Bt.22783.1.S1\_at | 3.405e-06 | 1.869 |
| Bt.15916.1.S1\_at | 3.416e-06 | -0.5219 |
| Bt.12327.1.S1\_at | 3.435e-06 | -1.101 |
| Bt.5530.1.S1\_at | 3.438e-06 | -1.461 |
| Bt.20008.1.S1\_at | 3.458e-06 | 0.7257 |
| Bt.3562.1.S1\_at | 3.593e-06 | 2.321 |
| Bt.8189.2.A1\_at | 3.68e-06 | 1.73 |
| Bt.22576.3.S1\_at | 3.716e-06 | -1.268 |
| Bt.8876.1.S1\_at | 3.731e-06 | 1.03 |
| Bt.15643.1.A1\_at | 3.859e-06 | -1.426 |
| Bt.9664.1.A1\_at | 3.898e-06 | 1.063 |
| Bt.22651.1.A1\_at | 4.023e-06 | 0.7868 |
| Bt.4357.1.S1\_at | 4.047e-06 | 0.8179 |
| Bt.21731.1.S1\_at | 4.062e-06 | 1.264 |
| Bt.21966.1.S1\_at | 4.194e-06 | -0.5705 |
| Bt.25255.1.A1\_at | 4.232e-06 | 1.004 |
| Bt.20194.1.S1\_at | 4.286e-06 | 1.014 |
| Bt.13588.1.A1\_at | 4.44e-06 | 5.201 |
| Bt.23521.2.S1\_a\_at | 4.448e-06 | -2.062 |
| Bt.20143.2.S1\_at | 4.549e-06 | -1.12 |
| Bt.24039.1.A1\_at | 4.655e-06 | 0.7779 |
| Bt.9956.1.S1\_at | 4.675e-06 | 1.109 |
| Bt.23094.3.S1\_x\_at | 4.702e-06 | -2.561 |
| Bt.4725.1.S1\_at | 4.708e-06 | -1.257 |
| Bt.26122.2.S1\_at | 4.751e-06 | 1.077 |
| Bt.7670.1.A1\_at | 4.761e-06 | -1.445 |
| Bt.23317.1.S1\_at | 4.782e-06 | 0.7623 |
| Bt.7437.1.S1\_at | 4.815e-06 | 0.9606 |
| Bt.23605.1.S1\_at | 4.817e-06 | -1.334 |
| Bt.15666.2.S1\_at | 4.849e-06 | -1.828 |
| Bt.25319.2.S1\_at | 5.032e-06 | -1.514 |
| Bt.4888.1.S1\_at | 5.122e-06 | 2.165 |
| Bt.16639.1.A1\_at | 5.258e-06 | -1.254 |
| Bt.13975.1.S1\_a\_at | 5.273e-06 | -1.609 |
| Bt.14340.2.S1\_at | 5.713e-06 | -1.926 |
| Bt.26286.1.A1\_at | 5.826e-06 | 2.055 |
| Bt.21578.1.S1\_at | 6.057e-06 | 1.611 |
| Bt.25806.2.A1\_at | 6.202e-06 | 1.594 |
| Bt.16563.1.A1\_at | 6.205e-06 | 2.204 |
| Bt.18851.1.S1\_at | 6.336e-06 | 0.9711 |
| Bt.23076.1.S1\_at | 6.358e-06 | 1.904 |
| Bt.6642.1.S1\_a\_at | 6.393e-06 | -2.328 |
| Bt.17512.1.S1\_at | 6.403e-06 | -0.9449 |
| Bt.2001.1.S1\_at | 6.431e-06 | 1.135 |
| Bt.7861.1.A1\_at | 6.46e-06 | -0.8861 |
| Bt.22329.1.S1\_at | 6.498e-06 | 0.8863 |
| Bt.27196.1.A1\_at | 6.663e-06 | 3.61 |
| Bt.27130.1.S1\_at | 6.777e-06 | 0.8994 |
| Bt.20733.1.S1\_at | 6.843e-06 | -1.65 |
| Bt.16597.1.S1\_at | 6.866e-06 | 1.673 |
| Bt.755.1.S1\_at | 6.899e-06 | 0.8357 |
| Bt.2889.1.S1\_at | 6.932e-06 | 0.8377 |
| Bt.6156.1.S1\_at | 7.14e-06 | 0.6844 |
| Bt.18755.1.A1\_at | 7.192e-06 | 1.313 |
| Bt.9190.1.S1\_at | 7.267e-06 | -1.169 |
| Bt.1582.1.S1\_at | 7.347e-06 | 1.397 |
| Bt.1214.1.S1\_at | 7.457e-06 | 1.394 |
| Bt.2091.1.S1\_at | 7.503e-06 | -2.154 |
| Bt.15925.1.S1\_at | 7.682e-06 | 1.546 |
| Bt.10150.1.S1\_at | 7.7e-06 | 1.385 |
| Bt.25319.1.A1\_at | 7.713e-06 | -1.105 |
| Bt.21031.1.S1\_at | 7.724e-06 | 1.307 |
| Bt.9180.3.S1\_a\_at | 7.742e-06 | 1.07 |
| Bt.23182.1.S1\_at | 7.835e-06 | 1.673 |
| Bt.26808.1.S1\_at | 7.882e-06 | -1.586 |
| Bt.5471.1.S1\_at | 7.937e-06 | -0.3942 |
| Bt.2262.1.S1\_at | 7.963e-06 | 1.457 |
| Bt.23883.1.A1\_at | 8.051e-06 | 1.307 |
| Bt.15675.1.S1\_at | 8.218e-06 | 2.569 |
| Bt.5348.1.S1\_at | 8.32e-06 | 1.598 |
| Bt.27237.1.S1\_a\_at | 8.411e-06 | 1.396 |
| Bt.21376.1.S1\_at | 8.461e-06 | 0.7934 |
| Bt.3306.1.S1\_at | 8.461e-06 | -1.541 |
| Bt.4959.1.A1\_at | 8.485e-06 | 1.739 |
| Bt.15157.1.S1\_at | 8.649e-06 | 0.8573 |
| Bt.23109.1.S1\_at | 8.668e-06 | 1.257 |
| Bt.3388.1.S1\_at | 8.741e-06 | 1.324 |
| Bt.15545.1.S1\_at | 8.786e-06 | 1.301 |
| Bt.5300.1.S1\_at | 9.203e-06 | 0.5255 |
| Bt.20505.1.S1\_at | 9.364e-06 | 1.031 |
| Bt.13440.1.S1\_at | 9.431e-06 | -1.113 |
| Bt.5131.1.S1\_at | 9.595e-06 | 1.053 |
| Bt.8213.1.S1\_at | 9.708e-06 | 1.419 |
| Bt.18410.1.A1\_at | 9.877e-06 | -0.7096 |
| Bt.25099.1.A1\_at | 9.899e-06 | 3.355 |
| Bt.7955.1.S1\_at | 9.909e-06 | 1.144 |
| Bt.11662.1.A1\_at | 9.997e-06 | 1.572 |
| Bt.2153.1.S1\_at | 1.031e-05 | 1.807 |
| Bt.24779.2.S1\_at | 1.036e-05 | -1.929 |
| Bt.3418.1.S1\_at | 1.036e-05 | 0.5926 |
| Bt.2249.1.S1\_at | 1.043e-05 | 1.27 |
| Bt.689.1.S1\_at | 1.048e-05 | -0.8742 |
| Bt.3162.1.S1\_at | 1.048e-05 | 0.8576 |
| Bt.1418.2.S1\_at | 1.061e-05 | -1.37 |
| Bt.1169.1.S1\_at | 1.079e-05 | 0.9041 |
| Bt.21551.2.S1\_a\_at | 1.084e-05 | -0.9294 |
| Bt.19706.1.S1\_at | 1.09e-05 | -1.667 |
| Bt.13637.1.A1\_at | 1.092e-05 | -0.5971 |
| Bt.422.1.S1\_at | 1.103e-05 | 1.313 |
| Bt.3414.1.S1\_at | 1.12e-05 | 1.211 |
| Bt.5243.1.S1\_at | 1.134e-05 | 0.6853 |
| Bt.14023.1.S1\_at | 1.14e-05 | 0.851 |
| Bt.2524.1.S1\_at | 1.145e-05 | -0.8975 |
| Bt.5432.1.S1\_at | 1.146e-05 | -0.8599 |
| Bt.27301.1.S1\_at | 1.148e-05 | 2.633 |
| Bt.9983.1.S1\_at | 1.154e-05 | 0.7258 |
| Bt.4831.1.S1\_at | 1.169e-05 | -0.8595 |
| Bt.5605.1.S1\_at | 1.181e-05 | 0.8247 |
| Bt.14340.1.A1\_at | 1.201e-05 | -0.9856 |
| Bt.12885.2.S1\_at | 1.207e-05 | -0.977 |
| Bt.7648.1.S1\_at | 1.212e-05 | 0.7245 |
| Bt.23355.1.S1\_at | 1.225e-05 | 1.295 |
| Bt.23589.2.S1\_at | 1.234e-05 | 0.7885 |
| Bt.15751.1.S1\_at | 1.234e-05 | 0.7612 |
| Bt.19519.1.S1\_at | 1.255e-05 | 0.8067 |
| Bt.7642.1.S1\_at | 1.279e-05 | 1.953 |
| Bt.4825.1.A1\_at | 1.294e-05 | 2.173 |
| Bt.15847.1.S1\_at | 1.294e-05 | 1.242 |
| Bt.7133.1.S1\_at | 1.298e-05 | 1.512 |
| Bt.17102.3.S1\_at | 1.308e-05 | -1.608 |
| Bt.28728.1.A1\_at | 1.313e-05 | 2.112 |
| Bt.15931.1.S1\_at | 1.319e-05 | 0.6311 |
| Bt.22785.1.S1\_at | 1.322e-05 | -1.904 |
| Bt.4292.1.S1\_at | 1.337e-05 | 0.8972 |
| Bt.23002.1.S1\_s\_at | 1.342e-05 | 0.7594 |
| Bt.15436.1.S1\_s\_at | 1.382e-05 | 0.4269 |
| Bt.10727.1.S1\_at | 1.396e-05 | 0.7456 |
| Bt.21978.1.S1\_at | 1.417e-05 | -1.242 |
| Bt.2537.2.S1\_at | 1.425e-05 | -0.9674 |
| Bt.18278.1.S1\_at | 1.468e-05 | 1.062 |
| Bt.5897.1.S1\_at | 1.484e-05 | -1.141 |
| Bt.4670.1.S1\_at | 1.491e-05 | 1.424 |
| Bt.2532.1.S1\_at | 1.501e-05 | -0.5983 |
| Bt.24828.1.A1\_at | 1.506e-05 | 1.076 |
| Bt.12412.1.S1\_at | 1.517e-05 | -1.338 |
| Bt.11631.2.S1\_at | 1.532e-05 | -1.469 |
| Bt.1681.1.S1\_at | 1.537e-05 | 2.048 |
| Bt.7322.1.S1\_at | 1.55e-05 | 1.929 |
| Bt.21313.1.S1\_at | 1.556e-05 | 0.6402 |
| Bt.27140.1.S1\_at | 1.568e-05 | 0.9309 |
| Bt.23212.1.S1\_at | 1.581e-05 | 1.454 |
| Bt.28312.1.S1\_at | 1.652e-05 | -1.348 |
| Bt.5472.1.S1\_at | 1.68e-05 | 1.197 |
| Bt.7856.1.S1\_at | 1.698e-05 | 1.399 |
| Bt.4477.2.S1\_a\_at | 1.712e-05 | -0.4533 |
| Bt.17573.1.A1\_at | 1.74e-05 | -2.795 |
| Bt.22216.1.S1\_at | 1.776e-05 | 2.134 |
| Bt.26685.1.S1\_at | 1.776e-05 | 1.491 |
| Bt.10608.2.S1\_a\_at | 1.797e-05 | 1.151 |
| Bt.5356.1.S1\_s\_at | 1.815e-05 | -3.599 |
| Bt.9021.1.S1\_at | 1.817e-05 | 2.147 |
| Bt.23520.1.S1\_at | 1.825e-05 | -0.5852 |
| Bt.26942.1.S1\_at | 1.84e-05 | 0.9748 |
| Bt.9057.1.A1\_at | 1.842e-05 | 0.5544 |
| Bt.21705.1.S1\_at | 1.878e-05 | 1.414 |
| Bt.23227.1.S1\_at | 1.884e-05 | -1.831 |
| Bt.3296.1.A1\_at | 1.893e-05 | 0.9869 |
| Bt.23668.1.A1\_at | 1.906e-05 | -1.099 |
| Bt.25398.1.A1\_at | 1.906e-05 | 1.814 |
| Bt.12559.1.S1\_at | 1.93e-05 | 1.652 |
| Bt.20145.1.S1\_at | 1.937e-05 | 1.909 |
| Bt.7008.1.S1\_at | 1.949e-05 | 1.472 |
| Bt.27430.1.S1\_at | 1.954e-05 | -1.059 |
| Bt.23218.1.S1\_at | 1.961e-05 | 0.7432 |
| Bt.24614.1.S1\_at | 1.994e-05 | 1.867 |
| Bt.5468.1.S1\_at | 1.994e-05 | 2.903 |
| Bt.5070.1.S1\_x\_at | 1.994e-05 | -1.265 |
| Bt.3780.1.S1\_at | 2.014e-05 | 1.155 |
| Bt.16567.1.A1\_at | 2.066e-05 | 1.714 |
| Bt.23542.1.S1\_at | 2.084e-05 | -1.282 |
| Bt.23169.1.S1\_at | 2.113e-05 | 1.267 |
| Bt.10253.1.A1\_at | 2.116e-05 | 2.064 |
| Bt.20229.1.S1\_at | 2.121e-05 | 0.9213 |
| Bt.7052.1.S1\_at | 2.18e-05 | 0.6535 |
| Bt.7139.1.S1\_at | 2.25e-05 | 0.7061 |
| Bt.19364.1.A1\_at | 2.268e-05 | -0.3011 |
| Bt.4733.1.S1\_at | 2.317e-05 | 2.398 |
| Bt.4986.1.S1\_at | 2.318e-05 | 1.036 |
| Bt.24875.1.S1\_at | 2.329e-05 | 0.6824 |
| Bt.21661.1.S1\_at | 2.329e-05 | -2.834 |
| Bt.7431.1.S1\_a\_at | 2.333e-05 | -1.783 |
| Bt.5333.1.S1\_at | 2.335e-05 | 1.65 |
| Bt.13588.2.S1\_at | 2.34e-05 | 5.07 |
| Bt.5402.1.S1\_a\_at | 2.34e-05 | -0.6375 |
| Bt.9055.2.S1\_a\_at | 2.37e-05 | 1.443 |
| Bt.17883.2.A1\_at | 2.395e-05 | -1.611 |
| Bt.2564.1.S1\_at | 2.404e-05 | 0.5495 |
| Bt.4803.1.S1\_at | 2.406e-05 | 0.5597 |
| Bt.26688.2.S1\_a\_at | 2.407e-05 | -1.998 |
| Bt.24478.1.S1\_at | 2.435e-05 | 1.566 |
| Bt.23596.1.S1\_at | 2.439e-05 | 0.4606 |
| Bt.26805.1.S1\_at | 2.457e-05 | 2.596 |
| Bt.10869.1.S1\_at | 2.462e-05 | -1.926 |
| Bt.21981.3.S1\_at | 2.478e-05 | 1.146 |
| Bt.4175.2.S1\_at | 2.505e-05 | 1.082 |
| Bt.28623.1.S1\_at | 2.507e-05 | 1.356 |
| Bt.20007.1.S1\_a\_at | 2.536e-05 | -0.6858 |
| Bt.2515.1.S1\_at | 2.545e-05 | 0.6905 |
| Bt.16329.1.A1\_at | 2.557e-05 | 0.7908 |
| Bt.9014.1.A1\_at | 2.569e-05 | 1.419 |
| Bt.5465.2.A1\_a\_at | 2.583e-05 | 1.454 |
| Bt.3573.1.S2\_at | 2.585e-05 | 1.547 |
| Bt.8319.1.S1\_at | 2.588e-05 | -0.8886 |
| Bt.1400.1.S1\_at | 2.592e-05 | 1.048 |
| Bt.16127.1.S1\_at | 2.613e-05 | -0.7567 |
| Bt.22410.1.S1\_at | 2.644e-05 | 1.113 |
| Bt.25806.1.S1\_at | 2.648e-05 | 1.66 |
| Bt.3835.1.S1\_at | 2.657e-05 | 1.208 |
| Bt.5078.1.S1\_at | 2.666e-05 | 0.8378 |
| Bt.20542.1.S1\_at | 2.668e-05 | -0.7839 |
| Bt.28340.1.S1\_at | 2.699e-05 | 1.43 |
| Bt.5596.1.A1\_at | 2.705e-05 | 1.063 |
| Bt.5085.1.S1\_at | 2.732e-05 | 1.262 |
| Bt.21039.1.A1\_at | 2.737e-05 | -4.947 |
| Bt.19709.1.S1\_at | 2.764e-05 | 0.8688 |
| Bt.24582.1.S1\_at | 2.766e-05 | -0.7638 |
| Bt.11034.1.S1\_at | 2.769e-05 | -1.739 |
| Bt.25273.1.A1\_at | 2.772e-05 | -0.2585 |
| Bt.26358.1.S1\_at | 2.816e-05 | 1.806 |
| Bt.13765.1.S1\_at | 2.829e-05 | 1.992 |
| Bt.12221.1.S1\_at | 2.84e-05 | 1.413 |
| Bt.7968.1.S1\_at | 2.88e-05 | -0.8567 |
| Bt.28806.1.S1\_at | 2.886e-05 | -0.4121 |
| Bt.20465.1.S1\_at | 2.886e-05 | -0.6016 |
| Bt.8699.1.S1\_at | 2.896e-05 | 2.574 |
| Bt.15845.1.S1\_at | 2.905e-05 | 0.8947 |
| Bt.24439.1.S1\_at | 2.936e-05 | -1.268 |
| Bt.13287.1.S1\_at | 2.938e-05 | -0.9564 |
| Bt.16087.1.S1\_at | 2.968e-05 | 1.256 |
| Bt.2494.2.S1\_a\_at | 2.982e-05 | 0.8951 |
| Bt.21142.1.S1\_at | 2.993e-05 | -2.81 |
| Bt.16048.1.S1\_at | 2.999e-05 | -1.014 |
| Bt.3788.1.S1\_at | 3.007e-05 | 0.2947 |
| Bt.23552.1.S1\_at | 3.007e-05 | 0.6405 |
| Bt.4868.2.S1\_a\_at | 3.008e-05 | 0.7364 |
| Bt.20422.2.S1\_a\_at | 3.059e-05 | 0.8036 |
| Bt.7540.2.S1\_a\_at | 3.102e-05 | 1.034 |
| Bt.24395.1.S1\_at | 3.132e-05 | 1.236 |
| Bt.1330.1.S1\_at | 3.14e-05 | 1.041 |
| Bt.23094.6.A1\_at | 3.154e-05 | -2.273 |
| Bt.23521.1.S1\_at | 3.163e-05 | -2.077 |
| Bt.13237.1.S1\_at | 3.173e-05 | 0.491 |
| Bt.5376.1.S1\_at | 3.222e-05 | 0.647 |
| Bt.22480.1.A1\_at | 3.278e-05 | 2.198 |
| Bt.27101.1.S1\_at | 3.282e-05 | 1.448 |
| Bt.4637.1.A1\_a\_at | 3.286e-05 | 1.144 |
| Bt.20955.2.S1\_at | 3.293e-05 | 0.7334 |
| Bt.8073.1.S1\_at | 3.309e-05 | -1.363 |
| Bt.1154.1.S1\_at | 3.312e-05 | 1.214 |
| Bt.5024.1.S1\_at | 3.321e-05 | 0.8522 |
| Bt.5609.1.A1\_a\_at | 3.331e-05 | 2.027 |
| Bt.15753.1.S1\_at | 3.338e-05 | 0.7654 |
| Bt.9915.1.S1\_at | 3.354e-05 | 1.658 |
| Bt.13324.4.S1\_at | 3.359e-05 | 1.641 |
| Bt.19332.2.A1\_at | 3.362e-05 | 1.918 |
| Bt.3907.1.S2\_at | 3.397e-05 | 0.5274 |
| Bt.20095.2.S1\_at | 3.399e-05 | -0.7441 |
| Bt.5466.2.S1\_a\_at | 3.403e-05 | 0.7277 |
| Bt.25335.1.A1\_at | 3.434e-05 | -0.65 |
| Bt.2825.1.S1\_at | 3.454e-05 | 1.198 |
| Bt.24137.1.A1\_at | 3.459e-05 | 1.629 |
| Bt.5377.1.S1\_at | 3.473e-05 | 1.281 |
| Bt.18748.1.A1\_at | 3.486e-05 | -1.116 |
| Bt.9799.1.S1\_at | 3.541e-05 | 1.38 |
| Bt.11133.2.S1\_a\_at | 3.548e-05 | 1.076 |
| Bt.8618.1.S1\_at | 3.552e-05 | 0.9782 |
| Bt.10916.2.S1\_at | 3.586e-05 | 1.084 |
| Bt.11013.1.S1\_at | 3.604e-05 | 0.9105 |
| Bt.20508.1.S1\_at | 3.623e-05 | 1.048 |
| Bt.11196.1.S1\_at | 3.625e-05 | 1.025 |
| Bt.28390.1.S1\_at | 3.673e-05 | -1.209 |
| Bt.7962.1.S1\_at | 3.717e-05 | -2.967 |
| Bt.13587.2.S1\_a\_at | 3.761e-05 | 1.733 |
| Bt.20340.2.S1\_at | 3.77e-05 | 1.831 |
| Bt.28090.1.S1\_at | 3.837e-05 | -2.097 |
| Bt.26412.1.A1\_at | 3.844e-05 | -0.4675 |
| Bt.2815.1.S1\_at | 3.901e-05 | 1.02 |
| Bt.23603.1.S1\_a\_at | 3.903e-05 | 0.9897 |
| Bt.25718.1.A1\_at | 3.918e-05 | -1.312 |
| Bt.7776.1.S2\_at | 3.963e-05 | -1.118 |
| Bt.2106.1.S1\_at | 4.045e-05 | -1.042 |
| Bt.24955.1.S1\_at | 4.126e-05 | 0.8879 |
| Bt.9047.1.S1\_at | 4.204e-05 | 1.877 |
| Bt.16051.1.S1\_at | 4.293e-05 | 1.056 |
| Bt.4353.2.S1\_at | 4.336e-05 | -1.071 |
| Bt.20197.1.S1\_at | 4.343e-05 | 1.054 |
| Bt.26885.1.S1\_at | 4.347e-05 | 1.02 |
| Bt.3427.1.S1\_at | 4.357e-05 | 1.513 |
| Bt.12888.1.S1\_at | 4.359e-05 | 1.056 |
| Bt.17963.1.A1\_at | 4.36e-05 | 1.231 |
| Bt.4638.1.S1\_at | 4.387e-05 | 1.092 |
| Bt.3400.1.A1\_at | 4.411e-05 | -0.5088 |
| Bt.3715.1.S1\_at | 4.533e-05 | 0.7146 |
| Bt.5598.1.S1\_at | 4.537e-05 | -1.39 |
| Bt.5306.1.S1\_at | 4.538e-05 | -1.183 |
| Bt.21104.1.S1\_at | 4.546e-05 | 0.7617 |
| Bt.694.1.A1\_at | 4.552e-05 | 0.8712 |
| Bt.2347.2.S1\_at | 4.569e-05 | -1.114 |
| Bt.11932.1.S1\_at | 4.582e-05 | 0.6442 |
| Bt.13306.2.S1\_at | 4.656e-05 | -1.733 |
| Bt.9637.1.S1\_at | 4.66e-05 | 0.403 |
| Bt.9405.1.S1\_at | 4.674e-05 | 0.651 |
| Bt.16916.1.S1\_at | 4.691e-05 | -1.366 |
| Bt.25689.1.S1\_at | 4.719e-05 | 0.9278 |
| Bt.5123.3.S1\_a\_at | 4.765e-05 | 0.8903 |
| Bt.1527.1.A1\_at | 4.776e-05 | -0.8799 |
| Bt.13605.1.A1\_at | 4.899e-05 | -0.9579 |
| Bt.15784.1.S1\_at | 5.016e-05 | 1.148 |
| Bt.1978.14.S1\_at | 5.16e-05 | -0.4267 |
| Bt.5203.1.S1\_at | 5.208e-05 | 0.887 |
| Bt.29724.1.S1\_at | 5.252e-05 | -3.33 |
| Bt.4353.1.S1\_at | 5.275e-05 | -1.574 |
| Bt.26692.1.S1\_a\_at | 5.322e-05 | -1.332 |
| Bt.7283.1.S1\_at | 5.341e-05 | 0.5061 |
| Bt.21054.1.A1\_at | 5.402e-05 | -2.191 |
| Bt.27046.1.S1\_at | 5.436e-05 | -0.5347 |
| Bt.10990.1.S1\_at | 5.465e-05 | 1.081 |
| Bt.12411.1.S1\_at | 5.48e-05 | 1.206 |
| Bt.13821.1.S1\_at | 5.491e-05 | -0.9324 |
| Bt.16976.3.A1\_at | 5.548e-05 | -0.2752 |
| Bt.13991.2.S1\_at | 5.619e-05 | -1.183 |
| Bt.9589.1.S1\_at | 5.622e-05 | 0.7706 |
| Bt.20114.1.S1\_at | 5.632e-05 | 0.6379 |
| Bt.19469.1.A1\_at | 5.66e-05 | -0.5699 |
| Bt.16111.1.S1\_at | 5.662e-05 | 0.5019 |
| Bt.26629.1.S1\_a\_at | 5.736e-05 | 1.527 |
| Bt.20361.2.A1\_at | 5.74e-05 | -1.541 |
| Bt.20227.1.S1\_at | 5.787e-05 | 1.912 |
| Bt.28524.1.S1\_at | 5.788e-05 | 1.29 |
| Bt.19508.1.A1\_at | 5.839e-05 | 1.27 |
| Bt.27926.1.S1\_at | 5.849e-05 | 1.014 |
| Bt.13189.2.S1\_at | 5.864e-05 | 0.6918 |
| Bt.25524.1.S1\_at | 5.866e-05 | 0.4725 |
| Bt.6899.1.S1\_at | 5.904e-05 | -0.3987 |
| Bt.19721.1.S1\_at | 5.96e-05 | 1.815 |
| Bt.23308.1.S1\_at | 5.975e-05 | 0.7416 |
| Bt.3567.1.S1\_at | 5.987e-05 | -0.7929 |
| Bt.13315.1.S1\_at | 5.994e-05 | 1.286 |
| Bt.4671.1.S1\_at | 6.043e-05 | -0.96 |
| Bt.2519.1.S1\_at | 6.046e-05 | 0.8723 |
| Bt.1674.2.S1\_at | 6.062e-05 | 0.8353 |
| Bt.14223.1.A1\_at | 6.062e-05 | -0.6161 |
| Bt.24404.1.A1\_at | 6.132e-05 | 1.39 |
| Bt.13801.1.S1\_at | 6.148e-05 | -1.162 |
| Bt.7434.1.S1\_at | 6.177e-05 | -0.7061 |
| Bt.17148.2.S1\_at | 6.216e-05 | 1.353 |
| Bt.8051.1.S1\_at | 6.237e-05 | 0.6445 |
| Bt.17883.1.S1\_at | 6.27e-05 | -1.477 |
| Bt.12508.1.S1\_at | 6.326e-05 | 1.719 |
| Bt.21417.1.S1\_at | 6.347e-05 | 0.7209 |
| Bt.15298.1.A1\_at | 6.362e-05 | 2.849 |
| Bt.27331.1.S1\_at | 6.515e-05 | 1.548 |
| Bt.12885.1.S1\_at | 6.522e-05 | 0.9405 |
| Bt.7772.1.S1\_at | 6.545e-05 | 1.686 |
| Bt.22568.1.S1\_at | 6.606e-05 | 1.192 |
| Bt.17704.1.S1\_at | 6.612e-05 | 0.8965 |
| Bt.13327.1.S1\_at | 6.644e-05 | 0.5732 |
| Bt.3767.2.S1\_at | 6.655e-05 | -1.083 |
| Bt.20515.1.S1\_at | 6.732e-05 | -0.8028 |
| Bt.20852.1.A1\_at | 6.897e-05 | 1.561 |
| Bt.25018.1.A1\_at | 6.925e-05 | -1.591 |
| Bt.13408.1.S1\_at | 6.948e-05 | -0.8762 |
| Bt.19054.1.A1\_at | 6.998e-05 | -0.286 |
| Bt.16931.1.A1\_at | 6.999e-05 | -0.4717 |
| Bt.3767.3.S1\_a\_at | 7.021e-05 | -1.229 |
| Bt.14201.1.S1\_at | 7.043e-05 | 0.4726 |
| Bt.7033.2.S1\_a\_at | 7.078e-05 | 3.283 |
| Bt.20977.1.S1\_at | 7.189e-05 | -1.132 |
| Bt.22564.3.S1\_at | 7.204e-05 | -0.5227 |
| Bt.3049.1.A1\_at | 7.239e-05 | 1.107 |
| Bt.24727.1.S1\_a\_at | 7.255e-05 | 1.09 |
| Bt.5155.1.S1\_at | 7.347e-05 | 1.881 |
| Bt.5534.1.S1\_at | 7.353e-05 | 0.4532 |
| Bt.7069.1.S1\_at | 7.444e-05 | -1.271 |
| Bt.24804.1.S1\_at | 7.446e-05 | -1.599 |
| Bt.23173.1.S1\_at | 7.585e-05 | -1.237 |
| Bt.15540.1.A1\_at | 7.592e-05 | -1.375 |
| Bt.2748.1.S1\_at | 7.608e-05 | 1.02 |
| Bt.14037.1.S1\_at | 7.643e-05 | -0.8301 |
| Bt.12685.1.S1\_at | 7.674e-05 | -1.192 |
| Bt.9959.3.S1\_a\_at | 7.689e-05 | 1.22 |
| Bt.1720.1.S1\_at | 7.692e-05 | 1.628 |
| Bt.4751.2.S1\_a\_at | 7.705e-05 | -3.092 |
| Bt.23383.1.S1\_at | 7.767e-05 | 0.4734 |
| Bt.3244.1.A1\_at | 7.768e-05 | 0.6875 |
| Bt.7814.1.S1\_at | 7.777e-05 | 0.5018 |
| Bt.21551.1.S1\_at | 7.803e-05 | -0.9824 |
| Bt.9242.1.S1\_at | 7.842e-05 | 1.24 |
| Bt.23191.1.S1\_at | 7.951e-05 | 0.4346 |
| Bt.8643.1.A1\_at | 8.049e-05 | 1.42 |
| Bt.19423.1.S1\_at | 8.073e-05 | -1.794 |
| Bt.7903.1.A1\_at | 8.13e-05 | 1.167 |
| Bt.20570.1.S1\_at | 8.197e-05 | 1.27 |
| Bt.4773.4.S1\_at | 8.238e-05 | 0.4907 |
| Bt.22167.2.A1\_at | 8.245e-05 | -2.319 |
| Bt.6840.1.S1\_a\_at | 8.398e-05 | 2.066 |
| Bt.2304.1.A1\_at | 8.462e-05 | 0.9921 |
| Bt.24550.1.S1\_at | 8.469e-05 | 1.899 |
| Bt.9795.1.S1\_at | 8.492e-05 | 0.8429 |
| Bt.18316.1.A1\_at | 8.527e-05 | -1.591 |
| Bt.1370.1.S1\_at | 8.543e-05 | -1.503 |
| Bt.1418.1.S1\_at | 8.555e-05 | -1.062 |
| Bt.5348.1.S2\_at | 8.556e-05 | 0.4661 |
| Bt.22705.1.A1\_at | 8.622e-05 | -0.2916 |
| Bt.27204.1.S1\_at | 8.642e-05 | 1.818 |
| Bt.6032.1.S1\_s\_at | 8.694e-05 | 0.43 |
| Bt.4680.1.S1\_at | 8.697e-05 | 1.664 |
| Bt.13441.1.S1\_at | 8.765e-05 | -0.6412 |
| Bt.12364.1.S1\_at | 8.833e-05 | 1.268 |
| Bt.12613.1.S1\_at | 8.833e-05 | -1.254 |
| Bt.6483.1.A1\_at | 8.862e-05 | 0.7916 |
| Bt.16784.1.S1\_at | 8.918e-05 | -1.061 |
| Bt.1175.1.S1\_at | 9.002e-05 | 1.698 |
| Bt.24538.1.S1\_at | 9.014e-05 | -1.347 |
| Bt.9141.2.S1\_a\_at | 9.034e-05 | 1.333 |
| Bt.24819.1.S1\_at | 9.076e-05 | 1.174 |
| Bt.13240.1.S1\_at | 9.181e-05 | 0.8634 |
| Bt.15476.1.A1\_at | 9.233e-05 | -0.395 |
| Bt.22803.1.S2\_at | 9.249e-05 | 1.721 |
| Bt.16980.1.S1\_at | 9.258e-05 | 1.25 |
| Bt.20897.2.S1\_a\_at | 9.338e-05 | 1.482 |
| Bt.2898.2.S1\_a\_at | 9.486e-05 | 0.809 |
| Bt.3694.1.S1\_at | 9.568e-05 | -0.5095 |
| Bt.4014.1.S1\_s\_at | 9.613e-05 | 0.9892 |
| Bt.16105.1.S1\_at | 9.717e-05 | 1.682 |
| Bt.17579.1.A1\_at | 9.816e-05 | 2.639 |
| Bt.1987.1.S1\_at | 9.826e-05 | 0.4671 |
| Bt.7700.1.S1\_at | 9.83e-05 | 1.153 |
| Bt.15783.1.S1\_at | 9.848e-05 | 0.81 |
| Bt.21.1.S1\_at | 9.86e-05 | 0.5247 |
| Bt.1065.1.S1\_at | 9.928e-05 | 1.137 |
| Bt.89.1.S1\_at | 9.955e-05 | 1.617 |
| Bt.26872.1.S1\_at | 9.966e-05 | 2.395 |
| Bt.16122.1.S1\_at | 9.983e-05 | 1.593 |
| Bt.25616.1.S1\_at | 0.0001001 | 0.9203 |
| Bt.8736.2.S1\_at | 0.0001008 | 0.8809 |
| Bt.6588.1.S1\_at | 0.0001009 | 0.9233 |
| Bt.9238.1.S1\_at | 0.000101 | 1.189 |
| Bt.27283.1.S1\_x\_at | 0.0001028 | -0.7698 |
| Bt.12314.1.S1\_at | 0.0001032 | -3.071 |
| Bt.1268.1.A1\_at | 0.0001037 | -1.219 |
| Bt.13610.1.A1\_at | 0.0001046 | 1.171 |
| Bt.13604.1.S1\_at | 0.0001049 | 0.7667 |
| Bt.8486.2.S1\_at | 0.0001061 | 0.9547 |
| Bt.21681.1.A1\_at | 0.0001074 | -2.098 |
| Bt.22002.1.S1\_at | 0.0001077 | 2.726 |
| Bt.1202.1.S1\_at | 0.0001078 | -0.541 |
| Bt.5267.1.S1\_at | 0.0001085 | 1.015 |
| Bt.5748.2.S1\_at | 0.0001086 | -0.8234 |
| Bt.19023.1.S1\_at | 0.0001089 | 2.439 |
| Bt.16150.1.A1\_at | 0.0001103 | 1.648 |
| Bt.9324.1.S1\_at | 0.0001105 | 1.113 |
| Bt.12862.1.S1\_at | 0.0001107 | 1.389 |
| Bt.665.1.S1\_s\_at | 0.0001109 | 1.109 |
| Bt.23305.1.S1\_at | 0.000111 | 1.029 |
| Bt.10303.1.S1\_at | 0.0001124 | 0.9631 |
| Bt.24421.1.A1\_at | 0.0001139 | 1.387 |
| Bt.2698.1.S1\_at | 0.0001151 | 1.03 |
| Bt.8641.1.A1\_at | 0.0001154 | 1.346 |
| Bt.7639.1.S1\_at | 0.0001154 | 0.8432 |
| Bt.11631.1.A1\_at | 0.0001156 | -0.846 |
| Bt.7457.1.S1\_at | 0.0001169 | 1.19 |
| Bt.10046.1.A1\_at | 0.000117 | -0.6013 |
| Bt.13324.1.S1\_a\_at | 0.0001172 | 1.425 |
| Bt.870.1.S1\_at | 0.0001176 | -0.9582 |
| Bt.20198.1.S1\_at | 0.0001177 | -0.3582 |
| Bt.11060.1.S1\_at | 0.0001178 | -0.7183 |
| Bt.13218.2.S1\_at | 0.0001186 | 1.199 |
| Bt.5423.1.S1\_at | 0.0001193 | 0.512 |
| Bt.9092.1.S1\_at | 0.0001201 | 1.124 |
| Bt.3616.1.S1\_at | 0.0001203 | 0.9818 |
| Bt.15968.1.S1\_at | 0.000121 | -0.854 |
| Bt.5631.1.S1\_at | 0.0001223 | 0.8078 |
| Bt.19575.1.S1\_at | 0.0001235 | 1.103 |
| Bt.17807.1.A1\_at | 0.0001236 | -0.2739 |
| Bt.8552.1.S1\_at | 0.0001244 | -3.103 |
| Bt.11239.3.S1\_at | 0.0001245 | -1.194 |
| Bt.13455.1.S1\_at | 0.0001245 | 0.666 |
| Bt.26495.1.S1\_at | 0.0001246 | -0.6173 |
| Bt.27095.1.S1\_at | 0.0001249 | -0.7447 |
| Bt.3211.1.S2\_at | 0.0001252 | -0.8509 |
| Bt.10579.1.S1\_at | 0.0001254 | -1.135 |
| Bt.27889.1.S1\_at | 0.0001255 | -0.9895 |
| Bt.27386.1.A1\_at | 0.0001263 | 1.317 |
| Bt.26621.1.S1\_at | 0.0001264 | -1.364 |
| Bt.3946.1.S1\_at | 0.0001264 | 1.538 |
| Bt.23510.2.A1\_at | 0.0001278 | 0.3244 |
| Bt.8164.1.S1\_at | 0.0001281 | 0.602 |
| Bt.22721.1.A1\_a\_at | 0.0001286 | 1.037 |
| Bt.22190.1.S1\_a\_at | 0.0001305 | 0.5157 |
| Bt.1318.1.S1\_at | 0.0001309 | 1.288 |
| Bt.4659.1.S1\_at | 0.0001313 | -2.057 |
| Bt.4798.1.S2\_at | 0.0001329 | 3.411 |
| Bt.11492.1.A1\_at | 0.0001332 | -1.391 |
| Bt.7776.2.S1\_s\_at | 0.000135 | -0.3558 |
| Bt.13464.1.S1\_at | 0.0001378 | 1.017 |
| Bt.28829.1.S1\_at | 0.000138 | 0.8869 |
| Bt.6641.1.S1\_at | 0.0001386 | 0.8966 |
| Bt.25973.1.S1\_at | 0.0001391 | -1.693 |
| Bt.3528.1.A1\_at | 0.0001395 | -0.5074 |
| Bt.21537.1.A1\_at | 0.0001397 | -1.949 |
| Bt.21965.1.S1\_at | 0.0001404 | -0.7549 |
| Bt.27194.1.S1\_at | 0.0001409 | -1.458 |
| Bt.27008.1.A1\_at | 0.0001419 | -0.8517 |
| Bt.8122.1.S1\_at | 0.0001421 | -0.4688 |
| Bt.2416.1.S2\_at | 0.0001425 | -0.472 |
| Bt.10330.1.S1\_at | 0.0001431 | -1.674 |
| Bt.2070.2.A1\_at | 0.0001431 | 1.587 |
| Bt.2934.1.S1\_at | 0.0001435 | 0.7076 |
| Bt.2686.1.S1\_at | 0.0001435 | 0.5062 |
| Bt.2981.1.S1\_at | 0.0001454 | 0.4228 |
| Bt.21116.1.S1\_at | 0.0001462 | -1.251 |
| Bt.1078.2.S1\_at | 0.0001467 | -0.8463 |
| Bt.5304.1.S1\_at | 0.0001471 | -1.856 |
| Bt.946.1.S1\_at | 0.0001493 | 1.299 |
| Bt.19652.1.A1\_at | 0.0001501 | -0.5035 |
| Bt.21913.1.S1\_at | 0.0001507 | -0.3798 |
| Bt.15672.2.S1\_at | 0.0001513 | 0.8215 |
| Bt.5408.1.A1\_at | 0.0001514 | 1.878 |
| Bt.1268.2.S1\_at | 0.0001523 | -1.224 |
| Bt.14204.1.A1\_at | 0.0001529 | -1.212 |
| Bt.4442.1.S1\_at | 0.0001534 | 0.7659 |
| Bt.11687.2.S1\_at | 0.0001549 | 0.6173 |
| Bt.4751.1.S1\_a\_at | 0.0001551 | -2.727 |
| Bt.24895.1.A1\_at | 0.0001553 | -0.4853 |
| Bt.20977.2.S1\_at | 0.0001568 | -1.08 |
| Bt.25390.1.A1\_at | 0.0001568 | -1.147 |
| Bt.6680.1.S1\_a\_at | 0.000157 | 0.991 |
| Bt.10277.2.S1\_a\_at | 0.0001572 | 1.463 |
| Bt.2606.1.S1\_at | 0.0001572 | -1.331 |
| Bt.4842.1.S1\_at | 0.0001575 | 0.4437 |
| Bt.5650.1.S1\_at | 0.0001608 | 1.167 |
| Bt.10189.1.S1\_at | 0.0001609 | -0.6863 |
| Bt.20259.1.S1\_at | 0.0001631 | 1.374 |
| Bt.9571.2.S1\_a\_at | 0.0001636 | 1.867 |
| Bt.1310.1.S1\_at | 0.0001639 | 0.6935 |
| Bt.680.1.S1\_at | 0.0001652 | 0.5102 |
| Bt.21724.2.S1\_a\_at | 0.0001659 | -1.852 |
| Bt.3980.1.S1\_at | 0.0001667 | 1.662 |
| Bt.4977.1.S2\_at | 0.0001672 | 0.443 |
| Bt.1403.1.S1\_at | 0.0001677 | 0.5952 |
| Bt.16326.1.S1\_at | 0.0001684 | -1.473 |
| Bt.6950.1.S1\_at | 0.0001692 | -0.9346 |
| Bt.13235.1.S1\_at | 0.0001697 | -1.696 |
| Bt.17971.1.A1\_at | 0.00017 | 0.839 |
| Bt.23273.1.S1\_at | 0.0001705 | 0.9668 |
| Bt.7323.1.S1\_at | 0.0001716 | 0.4577 |
| Bt.13334.1.S1\_at | 0.0001728 | -0.8837 |
| Bt.27927.1.S1\_at | 0.0001732 | -0.7892 |
| Bt.12220.1.S1\_a\_at | 0.0001733 | 1.286 |
| Bt.22093.1.S1\_at | 0.0001756 | 0.8503 |
| Bt.8250.1.S1\_at | 0.0001765 | -0.8175 |
| Bt.7481.1.S1\_at | 0.0001768 | 1.729 |
| Bt.8970.1.S1\_at | 0.0001771 | 0.3807 |
| Bt.26770.1.S1\_at | 0.0001772 | 1.009 |
| Bt.28163.1.S1\_at | 0.0001773 | 1.853 |
| Bt.20141.1.S1\_at | 0.0001774 | 1.113 |
| Bt.4573.1.S1\_at | 0.0001778 | -0.6156 |
| Bt.2749.1.S1\_at | 0.0001782 | 3.199 |
| Bt.29332.1.A1\_at | 0.0001782 | -0.3609 |
| Bt.20533.1.S1\_at | 0.0001817 | -0.7675 |
| Bt.13584.1.A1\_at | 0.0001834 | 0.8873 |
| Bt.22155.1.A1\_at | 0.0001836 | -0.6022 |
| Bt.1388.1.S1\_at | 0.0001837 | 1.086 |
| Bt.10333.1.S1\_at | 0.0001847 | -0.6131 |
| Bt.3391.1.S1\_at | 0.0001853 | 0.8759 |
| Bt.3809.1.S1\_at | 0.0001877 | -0.7729 |
| Bt.4031.1.S1\_at | 0.0001896 | -0.6449 |
| Bt.6802.1.S1\_at | 0.0001904 | -1.06 |
| Bt.29670.1.A1\_at | 0.0001923 | -0.2723 |
| Bt.15922.1.S1\_at | 0.0001923 | -0.5818 |
| Bt.5006.1.S1\_at | 0.0001927 | -0.5458 |
| Bt.3767.1.A1\_at | 0.0001931 | -0.9783 |
| Bt.16538.1.S1\_at | 0.0001932 | 1.081 |
| Bt.5935.2.S1\_a\_at | 0.0001935 | 0.4271 |
| Bt.1258.1.S1\_at | 0.000194 | -0.5091 |
| Bt.12430.1.S1\_at | 0.0001944 | 0.5174 |
| Bt.11436.1.A1\_at | 0.0001947 | -0.5321 |
| Bt.3909.1.S1\_at | 0.000195 | 1.007 |
| Bt.18623.1.A1\_at | 0.000197 | -1.691 |
| Bt.19208.1.A1\_at | 0.0001987 | 0.9443 |
| Bt.21464.1.S1\_at | 2e-04 | 2.275 |
| Bt.1601.1.S1\_at | 0.0002009 | 0.3443 |
| Bt.11326.3.S1\_at | 0.0002013 | 0.3909 |
| Bt.9424.1.S1\_at | 0.0002013 | -1.357 |
| Bt.23225.1.S1\_at | 0.0002014 | 0.9757 |
| Bt.20680.1.S1\_at | 0.0002021 | -0.6761 |
| Bt.14305.1.A1\_at | 0.0002023 | 0.7323 |
| Bt.24963.1.A1\_at | 0.0002036 | 1.078 |
| Bt.2439.1.S1\_at | 0.0002051 | -0.8323 |
| Bt.18358.1.A1\_at | 0.0002057 | -0.3572 |
| Bt.29915.1.S1\_at | 0.0002057 | -0.6986 |
| Bt.5306.2.S1\_a\_at | 0.000206 | -1.371 |
| Bt.12491.1.A1\_at | 0.000207 | 2.406 |
| Bt.16520.1.A1\_at | 0.0002075 | -2.745 |
| Bt.1767.1.S1\_at | 0.0002093 | 0.6891 |
| Bt.2856.1.S1\_at | 0.0002105 | 1.044 |
| Bt.23143.1.A1\_at | 0.0002109 | -0.6071 |
| Bt.25997.1.S1\_at | 0.0002114 | -1.166 |
| Bt.19784.1.A1\_at | 0.0002127 | -1.528 |
| Bt.3227.1.S1\_at | 0.0002127 | 0.6131 |
| Bt.24873.1.S1\_at | 0.0002137 | -0.7967 |
| Bt.1927.1.S1\_at | 0.0002151 | -0.8112 |
| Bt.2696.1.A1\_at | 0.0002153 | 1.297 |
| Bt.5467.1.S2\_at | 0.000217 | -0.5434 |
| Bt.4271.1.S1\_at | 0.0002174 | 0.4679 |
| Bt.22822.1.S1\_at | 0.0002181 | 0.8776 |
| Bt.26568.2.S1\_a\_at | 0.0002182 | 0.9635 |
| Bt.8279.2.S1\_at | 0.0002212 | 0.9506 |
| Bt.3314.1.S1\_at | 0.000224 | -0.9872 |
| Bt.8549.1.S2\_at | 0.0002251 | 1.735 |
| Bt.9662.1.S1\_at | 0.0002256 | -1.383 |
| Bt.9051.1.S1\_at | 0.0002258 | 0.682 |
| Bt.1552.1.S1\_at | 0.0002263 | 1.387 |
| Bt.10515.1.S1\_at | 0.0002276 | -1.17 |
| Bt.13834.1.S1\_at | 0.0002285 | 1.097 |
| Bt.26993.1.S1\_at | 0.000229 | 1.442 |
| Bt.12505.1.S1\_at | 0.0002305 | 0.4843 |
| Bt.4790.1.S1\_at | 0.0002306 | 0.6337 |
| Bt.11631.3.S1\_at | 0.0002338 | -1.217 |
| Bt.27552.2.S1\_at | 0.0002339 | -1.969 |
| Bt.26198.1.A1\_at | 0.0002349 | -0.5252 |
| Bt.17701.2.A1\_at | 0.0002359 | -0.4113 |
| Bt.1269.1.S1\_a\_at | 0.0002371 | 0.578 |
| Bt.6815.1.A1\_at | 0.0002386 | 0.8792 |
| Bt.29546.1.A1\_s\_at | 0.000239 | 1.249 |
| Bt.8124.1.S1\_at | 0.0002399 | 1.009 |
| Bt.15664.1.S1\_at | 0.0002405 | 0.6862 |
| Bt.721.1.A1\_at | 0.0002406 | -0.9263 |
| Bt.27267.1.S1\_at | 0.0002407 | 0.7613 |
| Bt.10730.1.S1\_at | 0.0002409 | 1.199 |
| Bt.5699.1.S1\_at | 0.0002421 | 0.4127 |
| Bt.13998.1.S1\_at | 0.0002447 | 0.7532 |
| Bt.4704.2.S1\_at | 0.0002453 | 0.951 |
| Bt.15722.1.S1\_at | 0.0002462 | 0.9597 |
| Bt.2994.1.A1\_at | 0.0002464 | -0.8433 |
| Bt.13434.2.A1\_at | 0.000248 | -0.789 |
| Bt.27617.1.S1\_at | 0.0002492 | 1.318 |
| Bt.16389.1.S1\_at | 0.0002494 | -0.9029 |
| Bt.27193.1.A1\_at | 0.0002504 | 0.7445 |
| Bt.26974.1.S1\_at | 0.0002507 | -0.5469 |
| Bt.8258.1.S1\_at | 0.0002515 | -0.8321 |
| Bt.24269.1.S1\_at | 0.0002515 | 1.376 |
| Bt.11616.1.A1\_at | 0.0002519 | -0.3696 |
| Bt.23158.1.S1\_at | 0.000253 | -0.9072 |
| Bt.2070.3.S1\_at | 0.0002533 | 1.739 |
| Bt.17310.2.A1\_at | 0.0002557 | -0.3553 |
| Bt.13766.2.S1\_at | 0.0002568 | -0.6614 |
| Bt.6064.1.S1\_at | 0.0002569 | -0.7575 |
| Bt.3560.1.S1\_at | 0.000257 | -2.033 |
| Bt.9870.2.A1\_at | 0.0002583 | 1.443 |
| Bt.15717.1.A1\_at | 0.0002594 | 1.485 |
| Bt.20193.1.S1\_at | 0.0002597 | 2.046 |
| Bt.12408.1.S1\_at | 0.000261 | 0.5559 |
| Bt.10442.2.S1\_a\_at | 0.0002611 | 1.597 |
| Bt.12514.1.S1\_at | 0.0002617 | 0.5884 |
| Bt.1333.1.S1\_at | 0.0002617 | -0.6846 |
| Bt.29152.1.S1\_at | 0.0002634 | 1.084 |
| Bt.5423.2.A1\_at | 0.000264 | -0.6751 |
| Bt.26477.1.A1\_at | 0.0002647 | -0.257 |
| Bt.18894.1.A1\_at | 0.0002663 | -1.267 |
| Bt.1821.1.S1\_at | 0.0002685 | 1.06 |
| Bt.15819.2.S1\_at | 0.0002697 | 0.9884 |
| Bt.149.1.S1\_at | 0.0002721 | 1.4 |
| Bt.9741.1.S1\_at | 0.0002728 | 1.046 |
| Bt.16094.1.S1\_at | 0.0002732 | 1.747 |
| Bt.13934.1.S1\_at | 0.0002741 | -0.5541 |
| Bt.15993.1.S1\_s\_at | 0.0002764 | 0.2927 |
| Bt.13966.1.S1\_at | 0.0002778 | 0.6312 |
| Bt.28150.2.S1\_at | 0.0002786 | 1.576 |
| Bt.3036.1.S1\_at | 0.0002792 | 0.4574 |
| Bt.5466.4.S1\_at | 0.0002793 | -0.7612 |
| Bt.18352.1.A1\_at | 0.0002796 | 1.131 |
| Bt.26595.1.S1\_at | 0.0002797 | 0.6173 |
| Bt.28506.1.A1\_at | 0.0002812 | 1.79 |
| Bt.28263.1.S1\_at | 0.0002813 | 0.9293 |
| Bt.6685.2.A1\_at | 0.0002818 | 1.159 |
| Bt.5096.1.S1\_at | 0.0002823 | 0.8566 |
| Bt.3140.1.A1\_at | 0.0002825 | -0.8908 |
| Bt.26483.1.A1\_at | 0.0002829 | -1.386 |
| Bt.24960.1.S1\_at | 0.0002833 | 1.078 |
| Bt.4867.2.S1\_at | 0.0002839 | 4.16 |
| Bt.18122.1.A1\_at | 0.0002847 | -0.2983 |
| Bt.12063.1.S1\_at | 0.0002847 | 1.561 |
| Bt.7701.1.S1\_at | 0.0002861 | 0.9165 |
| Bt.23381.1.S1\_at | 0.0002871 | 0.4623 |
| Bt.20470.1.S1\_a\_at | 0.0002878 | 2.806 |
| Bt.18603.1.A1\_at | 0.0002879 | -0.2441 |
| Bt.27131.1.S1\_at | 0.0002893 | -0.3804 |
| Bt.23992.1.A1\_at | 0.0002914 | -1.182 |
| Bt.13991.2.S1\_a\_at | 0.0002927 | -1.057 |
| Bt.10750.1.A1\_at | 0.0002934 | 0.9802 |
| Bt.24517.1.S1\_at | 0.0002963 | 1.237 |
| Bt.14198.1.S1\_at | 0.0002989 | 0.9518 |
| Bt.23001.1.S1\_a\_at | 0.0002999 | 0.4976 |
| Bt.11794.1.S1\_at | 0.0003012 | 1.141 |
| Bt.28105.1.S1\_at | 0.0003038 | 0.6773 |
| Bt.8356.1.S1\_at | 0.0003043 | 0.7268 |
| Bt.13445.2.S1\_a\_at | 0.0003046 | 1.352 |
| Bt.26811.1.S1\_at | 0.0003049 | 1.11 |
| Bt.9053.1.S1\_a\_at | 0.0003058 | -0.9035 |
| Bt.1429.3.A1\_s\_at | 0.0003066 | 0.5157 |
| Bt.27737.1.A1\_at | 0.0003079 | -0.3948 |
| Bt.668.1.S1\_at | 0.0003093 | 0.9086 |
| Bt.23354.1.S1\_at | 0.0003126 | -1.675 |
| Bt.4339.1.S1\_at | 0.0003144 | 0.8713 |
| Bt.8279.3.S1\_a\_at | 0.0003161 | 0.7217 |
| Bt.21800.1.S1\_at | 0.0003196 | 1.283 |
| Bt.4586.1.S1\_at | 0.0003202 | 0.8677 |
| Bt.27816.3.S1\_a\_at | 0.0003208 | 0.6169 |
| Bt.13491.1.S1\_at | 0.0003211 | -0.3674 |
| Bt.4620.1.S1\_at | 0.0003214 | -0.3878 |
| Bt.25330.1.A1\_at | 0.0003221 | -0.2513 |
| Bt.2110.1.S1\_at | 0.0003225 | 1.841 |
| Bt.21027.1.S1\_at | 0.0003235 | 1.272 |
| Bt.8082.1.S1\_at | 0.0003239 | 0.5589 |
| Bt.14098.1.S1\_at | 0.0003251 | -0.9718 |
| Bt.9804.1.S1\_at | 0.0003262 | -0.8936 |
| Bt.22006.1.S1\_at | 0.0003264 | 0.8104 |
| Bt.11233.1.S1\_at | 0.000327 | -0.4232 |
| Bt.28270.1.S1\_at | 0.0003276 | 0.4615 |
| Bt.25980.1.A1\_at | 0.0003279 | 1.386 |
| Bt.16027.1.S1\_at | 0.0003281 | -0.3199 |
| Bt.4646.1.S1\_at | 0.0003293 | 1.006 |
| Bt.60.2.A1\_at | 0.0003303 | -0.5072 |
| Bt.2846.1.A1\_at | 0.0003319 | 1.138 |
| Bt.29954.1.S1\_at | 0.0003325 | -0.9687 |
| Bt.11605.1.S1\_at | 0.0003332 | 1.214 |
| Bt.18059.1.S1\_at | 0.0003358 | -0.492 |
| Bt.17237.1.A1\_at | 0.0003373 | -0.2722 |
| Bt.2347.1.S1\_at | 0.0003377 | -0.7722 |
| Bt.23366.1.S1\_at | 0.0003394 | 0.7072 |
| Bt.16004.1.A1\_at | 0.0003428 | 3.458 |
| Bt.12510.2.S1\_at | 0.0003451 | 2.652 |
| Bt.13965.1.S1\_at | 0.0003465 | 1.492 |
| Bt.12209.1.S1\_at | 0.000348 | -0.6486 |
| Bt.16628.2.A1\_at | 0.0003486 | 1.459 |
| Bt.6549.1.S1\_at | 0.0003494 | -1.183 |
| Bt.16991.1.S1\_a\_at | 0.0003513 | 0.6242 |
| Bt.5281.2.S1\_a\_at | 0.0003524 | 1.027 |
| Bt.17009.1.A1\_at | 0.0003529 | -0.3561 |
| Bt.5676.1.S1\_at | 0.0003571 | 1.219 |
| Bt.12870.1.S1\_at | 0.0003572 | 0.6211 |
| Bt.19297.2.A1\_a\_at | 0.0003579 | -0.9284 |
| Bt.21217.1.S1\_at | 0.0003584 | 0.5065 |
| Bt.28477.1.S1\_at | 0.00036 | -0.5939 |
| Bt.18228.1.A1\_at | 0.0003611 | 0.7776 |
| Bt.17299.2.S1\_at | 0.0003621 | 0.3435 |
| Bt.8591.1.S1\_at | 0.0003631 | 0.9322 |
| Bt.20559.2.A1\_at | 0.0003642 | 1.293 |
| Bt.7786.1.S1\_at | 0.0003642 | -0.7002 |
| Bt.17923.1.A1\_at | 0.0003696 | -0.363 |
| Bt.2818.2.S1\_a\_at | 0.0003713 | 1.038 |
| Bt.21839.1.A1\_at | 0.0003717 | 0.5595 |
| Bt.9706.2.S1\_at | 0.0003717 | 0.5086 |
| Bt.16978.1.S1\_at | 0.0003729 | -0.844 |
| Bt.20860.2.S1\_at | 0.000374 | -0.563 |
| Bt.4080.1.S1\_at | 0.0003753 | 0.6146 |
| Bt.26817.1.S1\_at | 0.0003756 | 0.4014 |
| Bt.15878.1.S1\_at | 0.000376 | 0.9436 |
| Bt.2841.1.S1\_at | 0.0003768 | 0.6612 |
| Bt.24263.1.S1\_at | 0.0003775 | 0.7101 |
| Bt.22472.1.S1\_at | 0.0003783 | -1.308 |
| Bt.24208.1.S1\_at | 0.0003808 | -0.877 |
| Bt.5038.4.A1\_at | 0.0003812 | 0.5076 |
| Bt.26911.1.A1\_at | 0.0003824 | -0.947 |
| Bt.9086.1.S1\_at | 0.0003829 | 0.9866 |
| Bt.3593.1.S1\_at | 0.0003908 | 1.087 |
| Bt.1034.1.S1\_at | 0.0003922 | 0.4622 |
| Bt.17490.1.S1\_a\_at | 0.0003922 | 0.5761 |
| Bt.21151.1.S1\_at | 0.0003928 | 0.6311 |
| Bt.29739.1.S1\_at | 0.0003936 | -0.2638 |
| Bt.7266.1.S1\_at | 0.0003943 | 1.504 |
| Bt.2696.2.S1\_at | 0.0003954 | 1.47 |
| Bt.28267.1.S1\_at | 0.0003956 | 0.4822 |
| Bt.1174.1.S1\_at | 0.0003962 | -0.6757 |
| Bt.13588.3.A1\_at | 0.0003965 | 3.174 |
| Bt.23372.1.S1\_a\_at | 0.0003976 | 0.4172 |
| Bt.10499.1.S1\_at | 0.0003989 | -0.9829 |
| Bt.28583.1.S1\_s\_at | 0.0003996 | 1.422 |
| Bt.11194.1.S1\_at | 0.0004004 | 1.612 |
| Bt.23331.1.S1\_at | 0.0004008 | -0.7732 |
| Bt.1187.1.S1\_at | 0.0004009 | -0.8379 |
| Bt.13330.1.S1\_at | 0.000401 | -1.963 |
| Bt.29895.1.S1\_at | 0.0004011 | 1.028 |
| Bt.687.1.S1\_at | 0.0004012 | -0.5657 |
| Bt.9413.1.S1\_at | 0.0004027 | -0.6032 |
| Bt.9559.1.S1\_at | 0.0004035 | 1.557 |
| Bt.28732.1.S1\_s\_at | 0.0004038 | -2.692 |
| Bt.22763.2.S1\_a\_at | 0.0004065 | 0.9516 |
| Bt.3455.1.S1\_at | 0.0004072 | 1.041 |
| Bt.19447.1.A1\_at | 0.0004082 | -0.7331 |
| Bt.17607.1.A1\_at | 0.000409 | -0.3153 |
| Bt.3240.1.S1\_at | 0.0004097 | 1.169 |
| Bt.10995.1.S1\_at | 0.0004102 | -0.8098 |
| Bt.19100.1.A1\_at | 0.0004103 | -1.268 |
| Bt.27905.1.S1\_at | 0.0004106 | -0.6124 |
| Bt.20411.1.S1\_at | 0.0004113 | 0.7944 |
| Bt.21468.1.S1\_at | 0.0004114 | 1.14 |
| Bt.8124.1.S2\_at | 0.0004132 | 0.9677 |
| Bt.5170.1.S1\_at | 0.0004141 | 1.05 |
| Bt.22234.1.S1\_at | 0.0004155 | -2.553 |
| Bt.16821.1.S1\_at | 0.0004162 | 1.768 |
| Bt.7804.1.S1\_at | 0.0004167 | -0.5236 |
| Bt.3975.1.S1\_at | 0.0004179 | 0.7528 |
| Bt.28721.1.S1\_at | 0.0004189 | -0.4213 |
| Bt.3729.1.S1\_at | 0.0004202 | -0.3932 |
| Bt.26764.1.A1\_at | 0.0004203 | 0.6046 |
| Bt.13278.1.S1\_at | 0.0004224 | 1.574 |
| Bt.24745.2.S1\_a\_at | 0.0004225 | -0.6929 |
| Bt.11004.1.S1\_at | 0.0004246 | -0.738 |
| Bt.24626.1.S1\_at | 0.0004246 | 0.9989 |
| Bt.11955.1.S1\_at | 0.0004277 | -0.6953 |
| Bt.11448.1.S1\_at | 0.0004281 | 0.4797 |
| Bt.4641.1.S1\_at | 0.0004289 | -0.7945 |
| Bt.746.1.S1\_at | 0.0004292 | 0.748 |
| Bt.9811.1.S1\_at | 0.0004323 | 0.6394 |
| Bt.23734.1.A1\_at | 0.0004339 | -1.993 |
| Bt.27904.1.A1\_at | 0.0004367 | 0.985 |
| Bt.4466.1.A1\_at | 0.0004382 | -0.7187 |
| Bt.20025.1.S1\_at | 0.0004386 | 0.9008 |
| Bt.1405.1.S1\_at | 0.0004394 | -0.8581 |
| Bt.24919.1.S1\_at | 0.0004406 | 1.887 |
| Bt.7176.1.S1\_at | 0.0004407 | 1.672 |
| Bt.3751.1.S1\_at | 0.0004413 | 1.91 |
| Bt.15882.2.S1\_a\_at | 0.0004416 | 0.7569 |
| Bt.26987.2.S1\_at | 0.0004437 | 0.9394 |
| Bt.25002.1.S1\_at | 0.0004444 | 0.9898 |
| Bt.23367.1.S1\_at | 0.0004448 | -0.2494 |
| Bt.10554.2.S1\_at | 0.0004468 | -0.6372 |
| Bt.5176.1.S1\_at | 0.0004469 | 0.9373 |
| Bt.24640.1.S1\_at | 0.0004472 | 0.7851 |
| Bt.22693.1.A1\_at | 0.0004491 | -0.2507 |
| Bt.3940.1.S1\_at | 0.0004497 | 0.3676 |
| Bt.5261.1.S1\_at | 0.0004506 | 0.6461 |
| Bt.21231.1.S1\_a\_at | 0.0004573 | -0.3797 |
| Bt.20228.1.S1\_at | 0.0004574 | -0.3251 |
| Bt.5252.1.S1\_at | 0.0004582 | 1.474 |
| Bt.4844.1.S1\_at | 0.0004587 | 1.481 |
| Bt.9129.1.S1\_at | 0.0004593 | 0.7837 |
| Bt.24200.1.S1\_at | 0.0004594 | 1.165 |
| Bt.13330.2.A1\_at | 0.00046 | -2.092 |
| Bt.16148.1.A1\_at | 0.0004619 | 0.8066 |
| Bt.876.1.S1\_at | 0.0004623 | 2.158 |
| Bt.9658.1.S1\_at | 0.0004624 | -0.5948 |
| Bt.2582.1.S1\_at | 0.0004627 | 0.7071 |
| Bt.7543.1.S1\_at | 0.0004629 | 0.9326 |
| Bt.2867.1.S1\_at | 0.0004633 | -0.5863 |
| Bt.12280.1.S2\_at | 0.0004638 | 0.7814 |
| Bt.26619.1.S1\_at | 0.0004661 | -0.5762 |
| Bt.1020.1.S1\_at | 0.0004675 | -0.8651 |
| Bt.24330.1.S1\_at | 0.000468 | 0.6181 |
| Bt.20251.1.S1\_at | 0.0004692 | -0.3701 |
| Bt.29194.1.S1\_at | 0.0004707 | -1.436 |
| Bt.21614.1.A1\_at | 0.0004749 | 0.6215 |
| Bt.20601.2.S1\_at | 0.0004758 | -0.8586 |
| Bt.16668.1.A1\_at | 0.0004761 | -0.9937 |
| Bt.4523.1.S1\_at | 0.0004769 | 0.8176 |
| Bt.15888.1.A1\_at | 0.000478 | -0.9489 |
| Bt.16123.1.S1\_at | 0.0004784 | 1.441 |
| Bt.23002.1.S1\_at | 0.0004787 | -1.157 |
| Bt.5917.1.S1\_at | 0.0004788 | 0.936 |
| Bt.9078.2.S1\_a\_at | 0.0004812 | 1.177 |
| Bt.15718.1.S1\_at | 0.0004818 | 0.7489 |
| Bt.4870.1.S1\_at | 0.0004819 | -0.4043 |
| Bt.21032.1.S1\_at | 0.0004828 | 0.9684 |
| Bt.10332.1.A1\_at | 0.0004831 | -0.2101 |
| Bt.4606.1.S1\_at | 0.0004871 | -1.125 |
| Bt.5269.2.S1\_a\_at | 0.0004873 | -1.184 |
| Bt.25186.1.S1\_at | 0.0004879 | 2.384 |
| Bt.22441.1.S1\_at | 0.0004905 | -0.4712 |
| Bt.16106.1.A1\_at | 0.0004924 | 0.9554 |
| Bt.997.2.S1\_a\_at | 0.000494 | 1.313 |
| Bt.1670.1.A1\_at | 0.0004949 | 0.7625 |
| Bt.15947.1.S1\_at | 0.0004969 | 0.8541 |
| Bt.7896.1.S1\_at | 0.0004977 | 0.5305 |
| Bt.12472.1.S1\_at | 0.0004999 | -1.088 |
| Bt.20955.1.A1\_at | 0.0005003 | 1.267 |
| Bt.5246.1.S1\_at | 0.0005007 | 0.647 |
| Bt.27226.1.S1\_at | 0.0005011 | 1.063 |
| Bt.7984.1.S1\_at | 0.0005067 | 0.6025 |
| Bt.18637.1.A1\_at | 0.000507 | 1.251 |
| Bt.4503.1.S1\_at | 0.0005076 | 0.766 |
| Bt.14223.3.S1\_at | 0.0005103 | -1.166 |
| Bt.20988.1.S1\_at | 0.0005115 | 0.9573 |
| Bt.1844.1.S1\_at | 0.0005153 | -0.5857 |
| Bt.3609.1.S1\_at | 0.0005156 | -0.3166 |
| Bt.5608.1.A1\_at | 0.0005172 | -0.8365 |
| Bt.9785.1.S1\_at | 0.0005199 | -1.033 |
| Bt.18380.1.A1\_at | 0.0005239 | -0.332 |
| Bt.22692.1.A1\_at | 0.000526 | 0.5937 |
| Bt.22547.1.A1\_a\_at | 0.0005264 | -0.5816 |
| Bt.3433.1.A1\_at | 0.0005267 | -0.3907 |
| Bt.25402.1.S1\_at | 0.0005296 | 0.6726 |
| Bt.3148.1.S1\_at | 0.00053 | 1.582 |
| Bt.10610.1.S1\_at | 0.0005338 | 1.206 |
| Bt.2688.1.S1\_at | 0.0005372 | -0.6382 |
| Bt.27929.1.S1\_at | 0.0005383 | 0.4998 |
| Bt.2847.1.S1\_a\_at | 0.0005388 | 0.5051 |
| Bt.25098.1.S1\_at | 0.0005392 | 1.46 |
| Bt.21896.1.S1\_at | 0.0005439 | 1.154 |
| Bt.2853.1.S1\_at | 0.0005482 | 1.143 |
| Bt.27552.1.A1\_at | 0.0005511 | -1.12 |
| Bt.14304.2.S1\_at | 0.0005514 | -0.5794 |
| Bt.5397.1.S1\_at | 0.0005539 | 0.4203 |
| Bt.12691.1.A1\_at | 0.0005547 | -0.5387 |
| Bt.13581.1.S1\_at | 0.0005573 | 1.429 |
| Bt.24502.1.S1\_at | 0.0005588 | 0.8629 |
| Bt.28701.1.S1\_at | 0.0005591 | -0.9961 |
| Bt.28194.1.S1\_at | 0.0005623 | 0.6364 |
| Bt.19502.1.A1\_at | 0.0005625 | 0.7795 |
| Bt.8319.3.S1\_a\_at | 0.000564 | -1.218 |
| Bt.21942.1.S1\_at | 0.0005661 | 1.078 |
| Bt.2191.2.S1\_at | 0.0005684 | -0.6969 |
| Bt.17845.1.A1\_at | 0.0005692 | -0.2741 |
| Bt.20341.1.S1\_at | 0.0005722 | -0.5308 |
| Bt.18377.1.S1\_at | 0.0005722 | -0.378 |
| Bt.24826.1.A1\_at | 0.0005731 | -0.3796 |
| Bt.26524.1.S1\_at | 0.0005744 | 0.5778 |
| Bt.3854.1.S1\_at | 0.0005785 | 0.8252 |
| Bt.4078.1.S1\_at | 0.0005793 | 1.601 |
| Bt.6493.1.S1\_at | 0.0005811 | 0.5846 |
| Bt.13911.1.S1\_s\_at | 0.0005866 | 0.9842 |
| Bt.8682.1.S1\_at | 0.0005867 | -1.05 |
| Bt.9119.1.A1\_at | 0.0005942 | -0.4681 |
| Bt.15870.1.S1\_at | 0.0005971 | 1.058 |
| Bt.22518.1.S1\_a\_at | 0.0005976 | 0.6545 |
| Bt.1244.1.S1\_at | 0.0005978 | 0.5192 |
| Bt.9588.1.S1\_at | 0.0005981 | 0.6752 |
| Bt.13705.1.S1\_at | 0.0006001 | 0.5744 |
| Bt.7184.1.S1\_at | 0.0006027 | 0.5517 |
| Bt.17731.1.A1\_at | 0.0006038 | -0.487 |
| Bt.468.1.S1\_at | 0.0006046 | -0.2218 |
| Bt.22040.1.S1\_at | 0.0006054 | 0.8518 |
| Bt.28491.1.S1\_at | 0.0006074 | -0.625 |
| Bt.28073.1.S1\_at | 0.0006102 | -0.9175 |
| Bt.10092.1.S1\_at | 0.0006135 | -1.124 |
| Bt.25492.1.A1\_at | 0.0006148 | -0.2846 |
| Bt.4552.1.S1\_a\_at | 0.0006153 | -0.5651 |
| Bt.16887.1.A1\_at | 0.0006159 | 0.4964 |
| Bt.5175.1.S1\_at | 0.0006162 | 0.9244 |
| Bt.5443.1.S1\_at | 0.0006187 | -0.3571 |
| Bt.28417.1.S1\_at | 0.00062 | 0.5645 |
| Bt.26265.2.S1\_at | 0.0006225 | -1.08 |
| Bt.12024.1.S1\_at | 0.0006245 | -0.4808 |
| Bt.18689.2.A1\_at | 0.0006277 | 1.031 |
| Bt.4413.1.S1\_at | 0.0006289 | -0.5779 |
| Bt.11865.1.A1\_at | 0.0006297 | 0.5019 |
| Bt.23829.1.A1\_at | 0.0006318 | -0.6191 |
| Bt.16388.1.A1\_at | 0.0006334 | -0.5528 |
| Bt.4026.1.S1\_at | 0.0006339 | 1.577 |
| Bt.5605.2.S1\_at | 0.0006343 | 0.7361 |
| Bt.25135.1.A1\_at | 0.0006355 | 1.05 |
| Bt.4954.1.S1\_at | 0.0006357 | -1.382 |
| Bt.22466.1.A1\_at | 0.0006364 | 0.7086 |
| Bt.1697.1.S1\_at | 0.0006372 | 0.3554 |
| Bt.25099.2.S1\_at | 0.0006379 | 2.974 |
| Bt.4864.3.S1\_a\_at | 0.000638 | -0.7072 |
| Bt.24766.1.S1\_at | 0.0006414 | -1.762 |
| Bt.4721.1.S1\_at | 0.0006416 | 0.5032 |
| Bt.12158.1.S1\_at | 0.0006431 | -0.5065 |
| Bt.20479.1.S1\_at | 0.0006445 | 1.102 |
| Bt.8891.1.S1\_at | 0.0006458 | -0.7926 |
| Bt.24182.1.A1\_at | 0.0006508 | -0.3845 |
| Bt.6334.1.A1\_at | 0.0006521 | 0.8524 |
| Bt.25069.1.A1\_at | 0.0006541 | -0.5798 |
| Bt.1874.1.S1\_at | 0.0006544 | 0.4247 |
| Bt.4864.2.S1\_at | 0.0006545 | -0.5625 |
| Bt.7192.1.S1\_at | 0.0006569 | 0.926 |
| Bt.3015.2.S1\_at | 0.0006576 | 0.6465 |
| Bt.946.2.A1\_a\_at | 0.0006612 | 0.8973 |
| Bt.26999.1.S1\_at | 0.0006641 | 0.6489 |
| Bt.11028.1.S1\_at | 0.0006643 | 0.5753 |
| Bt.28083.1.S1\_at | 0.0006671 | -0.2267 |
| Bt.16362.2.S1\_at | 0.000669 | 0.4953 |
| Bt.21364.2.S1\_at | 0.0006717 | 1.367 |
| Bt.17485.1.A1\_at | 0.0006722 | -0.2397 |
| Bt.19306.2.S1\_at | 0.0006727 | -0.8895 |
| Bt.11152.1.S1\_at | 0.0006766 | 0.6854 |
| Bt.19632.1.S1\_at | 0.0006786 | 0.6918 |
| Bt.7206.1.S1\_at | 0.0006795 | -0.5247 |
| Bt.16916.2.A1\_at | 0.0006825 | -1.049 |
| Bt.21721.1.A1\_at | 0.0006832 | 1.489 |
| Bt.16209.1.A1\_at | 0.000685 | -0.3716 |
| Bt.24224.1.S1\_at | 0.0006877 | 0.6483 |
| Bt.7633.1.S1\_at | 0.0006889 | 1.232 |
| Bt.5038.1.S2\_at | 0.0006897 | -1.264 |
| Bt.9027.1.S1\_at | 0.0006906 | -1.009 |
| Bt.29558.1.A1\_at | 0.0006916 | -0.229 |
| Bt.6411.1.S1\_at | 0.0006942 | 0.2444 |
| Bt.422.1.S2\_at | 0.0006957 | 0.9641 |
| Bt.27800.1.A1\_at | 0.0006958 | 0.9099 |
| Bt.4682.1.S1\_at | 0.0006963 | -0.6756 |
| Bt.24882.2.S1\_at | 0.0006967 | 0.9929 |
| Bt.22832.1.S1\_at | 0.0007047 | -0.9942 |
| Bt.11409.1.A1\_a\_at | 0.0007058 | 0.9981 |
| Bt.19332.1.S1\_at | 0.0007062 | 1.493 |
| Bt.440.1.S1\_at | 0.0007068 | -0.1912 |
| Bt.7870.1.S1\_at | 0.0007085 | 1.141 |
| Bt.1537.1.S1\_at | 0.0007135 | -1.18 |
| Bt.24220.1.S1\_at | 0.0007179 | 0.5678 |
| Bt.11651.1.A1\_at | 0.0007215 | 0.5253 |
| Bt.21133.1.S1\_at | 0.0007223 | -2.216 |
| Bt.6921.1.S1\_at | 0.0007244 | 0.9944 |
| Bt.12429.1.S1\_at | 0.000725 | 0.7252 |
| Bt.13781.1.S1\_at | 0.0007256 | -0.6923 |
| Bt.22634.1.S1\_at | 0.0007261 | 0.5364 |
| Bt.903.1.S1\_at | 0.0007279 | 0.7268 |
| Bt.8319.2.S1\_at | 0.0007297 | -0.8549 |
| Bt.23254.1.S1\_at | 0.0007307 | 0.5521 |
| Bt.3437.1.S1\_at | 0.0007312 | 0.9415 |
| Bt.11855.1.S1\_at | 0.0007331 | 0.6869 |
| Bt.923.2.S1\_at | 0.0007334 | 0.7958 |
| Bt.17102.2.S1\_at | 0.0007354 | -1.106 |
| Bt.25109.2.A1\_at | 0.000736 | 1.605 |
| Bt.3773.1.S1\_at | 0.0007366 | -0.3302 |
| Bt.7056.3.A1\_x\_at | 0.0007371 | 3.683 |
| Bt.24444.2.S1\_a\_at | 0.0007376 | -1.221 |
| Bt.9695.1.S1\_at | 0.0007415 | 0.6973 |
| Bt.4827.1.S1\_at | 0.0007416 | 0.6682 |
| Bt.997.1.S1\_at | 0.0007466 | 0.7799 |
| Bt.13768.2.S1\_at | 0.0007469 | -0.4891 |
| Bt.25393.1.A1\_at | 0.0007494 | -0.227 |
| Bt.7276.1.S1\_at | 0.0007497 | -0.6045 |
| Bt.2428.1.S1\_at | 0.0007508 | 1.016 |
| Bt.21409.1.A1\_at | 0.0007525 | 3.611 |
| Bt.26711.2.S1\_at | 0.0007537 | -1.961 |
| Bt.28941.1.A1\_at | 0.0007541 | -0.3745 |
| Bt.15790.2.S1\_at | 0.0007552 | -0.6743 |
| Bt.15528.1.S1\_at | 0.0007586 | 1.277 |
| Bt.22106.2.S1\_at | 0.0007594 | 1.356 |

*Venn diagram of differential expression for the three different methods.*
