# Supplementary material for: A gene network switch enhances the oxidative capacity of ovine skeletal muscle during late fetal development
Source: BMC Genomics. 2010 Jun 15;11:378. doi: 10.1186/1471-2164-11-378 (PMC2894804; doi:10.1186/1471-2164-11-378)
Supplement: Additional File 1 — Gene expression data. This file contains links to the gene expression data. The genes that are significantly differentially expressed between adjacent development times are listed as Experiment 1 (80 d vs 100 d), Experiment 2 (100 d vs 120 d), Experiment 3 (120 d vs 150 d) and Experiment 4 (150 d vs 230 d) in the section entitled Open microarray analysis report for developmental expression. Each experiment contains an MA plot, Volcano plot and Heatmap for each of the three microarray processing programs MAS5, RMA and GCRMA. For each of these processing programs there is a list of significantly differentially expressed probe sets, and for each of these there is an FDR corrected probability, fold change, percentage of microarrays with a MAS5 Present call, and a convergence percentage for all three microarray processing programs. The file also contains annotation of the probe sets. In a second section entitled Time series analysis of developmental expression, there is a list of genes in each gene expression cluster and diagrams showing MAS5 gene expression values for these genes. [file 1471-2164-11-378-S1.ZIP › Report/GO1/Report/TopNodesCC.html]

### Top terms for Cellular Component

Top terms sorted by the weight algorithm. The first column is the GO term id and links to GO, the second column is a short description of the term, the third column is the number of genes annotated to the term, the fourth is the number of genes significant for the term and the fifth column is the expected number of genes for the term. The last four columns are the p-values for different tests of significance. The results are ordered by the weight algorithm (last column).

|  |  |  |  |  |  |  |  |  |
| --- | --- | --- | --- | --- | --- | --- | --- | --- |
| **GO** | **Term** | **Annotated** | **Significant** | **Expected** | **classic** | **KS** | **elim** | **weight** |
| GO:0030017 | sarcomere | 27 | 3 | 0.14 | 0.00034 | 0.996 | 0.084 | 0.00034 |
| GO:0000015 | phosphopyruvate hydratase complex | 2 | 1 | 0.01 | 0.01044 | 0.963 | 0.010 | 0.01044 |
| GO:0033017 | sarcoplasmic reticulum membrane | 3 | 1 | 0.02 | 0.01562 | 0.726 | 0.016 | 0.01562 |
| GO:0001725 | stress fiber | 4 | 1 | 0.02 | 0.02078 | 1.000 | 0.021 | 0.02078 |
| GO:0005884 | actin filament | 7 | 1 | 0.04 | 0.03609 | 1.000 | 0.036 | 0.03609 |
| GO:0030027 | lamellipodium | 8 | 1 | 0.04 | 0.04114 | 0.852 | 0.041 | 0.04114 |
| GO:0005912 | adherens junction | 9 | 1 | 0.05 | 0.04616 | 0.227 | 0.046 | 0.04616 |
| GO:0005743 | mitochondrial inner membrane | 188 | 3 | 0.98 | 0.07327 | 0.990 | 0.073 | 0.07327 |
| GO:0005625 | soluble fraction | 20 | 1 | 0.1 | 0.09981 | 0.501 | 0.100 | 0.09981 |
| GO:0005938 | cell cortex | 26 | 1 | 0.14 | 0.12783 | 0.795 | 0.128 | 0.12783 |
| GO:0009986 | cell surface | 39 | 1 | 0.2 | 0.18570 | 0.064 | 0.186 | 0.18570 |
| GO:0045202 | synapse | 41 | 1 | 0.21 | 0.19427 | 0.397 | 0.194 | 0.19427 |
| GO:0005576 | extracellular region | 372 | 3 | 1.95 | 0.30814 | 0.454 | 0.308 | 0.30814 |
| GO:0042995 | cell projection | 80 | 2 | 0.42 | 0.06488 | 1.000 | 0.065 | 0.31031 |
| GO:0005739 | mitochondrion | 504 | 5 | 2.64 | 0.11657 | 0.805 | 0.117 | 0.40568 |
| GO:0016021 | integral to membrane | 1213 | 7 | 6.35 | 0.45682 | 0.697 | 0.457 | 0.45682 |
| GO:0005829 | cytosol | 154 | 2 | 0.81 | 0.19199 | 0.122 | 0.192 | 0.54117 |
| GO:0005840 | ribosome | 186 | 1 | 0.97 | 0.63016 | 0.796 | 0.630 | 0.63016 |
| GO:0031224 | intrinsic to membrane | 1234 | 7 | 6.46 | 0.47705 | 0.679 | 0.477 | 0.68133 |
| GO:0005856 | cytoskeleton | 310 | 4 | 1.62 | 0.07508 | 0.893 | 0.433 | 0.73964 |

### DE genes for top GO terms for Cellular Component

List of DE genes for top GO terms. The first column is the Affymetrix probe ID, the second column is the Entrez ID, the third column is the gene symbol and the fourth column is the gene description. Note that the same gene can appear more than once in the list since different probes can target the same gene (can help detect overepresented genes due to multiple probe targets).

### GO:0030017

|  |  |  |  |
| --- | --- | --- | --- |
| **probe** | **entrez** | **symbol** | **description** |
| Bt.12300.1.S1\_at | 281337 | MYH1 | myosin, heavy chain 1, skeletal muscle, adult |
| Bt.12825.1.S1\_at | 515610 | ACTA2 | actin, alpha 2, smooth muscle, aorta |
| Bt.29854.1.S1\_x\_at | 281337 | MYH1 | myosin, heavy chain 1, skeletal muscle, adult |

### GO:0000015

|  |  |  |  |
| --- | --- | --- | --- |
| **probe** | **entrez** | **symbol** | **description** |
| Bt.22169.1.S1\_at | 540303 | ENO3 | enolase 3 (beta, muscle) |

### GO:0033017

|  |  |  |  |
| --- | --- | --- | --- |
| **probe** | **entrez** | **symbol** | **description** |
| Bt.5848.1.S1\_at | 518117 | ATP2A1 | ATPase, Ca++ transporting, cardiac muscle, fast twitch 1 |

### GO:0001725

|  |  |  |  |
| --- | --- | --- | --- |
| **probe** | **entrez** | **symbol** | **description** |
| Bt.12825.1.S1\_at | 515610 | ACTA2 | actin, alpha 2, smooth muscle, aorta |

### GO:0005884

|  |  |  |  |
| --- | --- | --- | --- |
| **probe** | **entrez** | **symbol** | **description** |
| Bt.12825.1.S1\_at | 515610 | ACTA2 | actin, alpha 2, smooth muscle, aorta |

### GO:0030027

|  |  |  |  |
| --- | --- | --- | --- |
| **probe** | **entrez** | **symbol** | **description** |
| Bt.4417.1.S1\_at | 281062 | CDH2 | cadherin 2, type 1, N-cadherin (neuronal) |

### GO:0005912

|  |  |  |  |
| --- | --- | --- | --- |
| **probe** | **entrez** | **symbol** | **description** |
| Bt.4417.1.S1\_at | 281062 | CDH2 | cadherin 2, type 1, N-cadherin (neuronal) |

### GO:0005743

|  |  |  |  |
| --- | --- | --- | --- |
| **probe** | **entrez** | **symbol** | **description** |
| Bt.13980.1.A1\_at | 538944 | CKMT2 | creatine kinase, mitochondrial 2 (sarcomeric) |
| Bt.13980.2.A1\_a\_at | 538944 | CKMT2 | creatine kinase, mitochondrial 2 (sarcomeric) |
| Bt.76.1.S1\_at | 338086 | COX7A1 | cytochrome c oxidase subunit VIIa polypeptide 1 (muscle) |

### GO:0005625

|  |  |  |  |
| --- | --- | --- | --- |
| **probe** | **entrez** | **symbol** | **description** |
| Bt.12825.1.S1\_at | 515610 | ACTA2 | actin, alpha 2, smooth muscle, aorta |

### GO:0005938

|  |  |  |  |
| --- | --- | --- | --- |
| **probe** | **entrez** | **symbol** | **description** |
| Bt.3980.1.S1\_at | 281478 | SCIN | scinderin |

### GO:0009986

|  |  |  |  |
| --- | --- | --- | --- |
| **probe** | **entrez** | **symbol** | **description** |
| Bt.9202.1.S1\_at | 511711 | FGL2 | fibrinogen-like 2 |

### GO:0045202

|  |  |  |  |
| --- | --- | --- | --- |
| **probe** | **entrez** | **symbol** | **description** |
| Bt.4417.1.S1\_at | 281062 | CDH2 | cadherin 2, type 1, N-cadherin (neuronal) |

### GO:0005576

|  |  |  |  |
| --- | --- | --- | --- |
| **probe** | **entrez** | **symbol** | **description** |
| Bt.3595.1.S1\_at | 282660 | MGP | matrix Gla protein |
| Bt.3804.1.S1\_at | 513618 | PCOLCE2 | procollagen C-endopeptidase enhancer 2 |
| Bt.9202.1.S1\_at | 511711 | FGL2 | fibrinogen-like 2 |

### GO:0042995

|  |  |  |  |
| --- | --- | --- | --- |
| **probe** | **entrez** | **symbol** | **description** |
| Bt.11199.1.S1\_at | 281939 | MYOZ1 | myozenin 1 |
| Bt.4417.1.S1\_at | 281062 | CDH2 | cadherin 2, type 1, N-cadherin (neuronal) |

### GO:0005739

|  |  |  |  |
| --- | --- | --- | --- |
| **probe** | **entrez** | **symbol** | **description** |
| Bt.13980.1.A1\_at | 538944 | CKMT2 | creatine kinase, mitochondrial 2 (sarcomeric) |
| Bt.13980.2.A1\_a\_at | 538944 | CKMT2 | creatine kinase, mitochondrial 2 (sarcomeric) |
| Bt.22379.1.S1\_at | 536925 | CABC1 | chaperone, ABC1 activity of bc1 complex homolog (S. pombe) |
| Bt.5084.1.S1\_at | 516210 | CKB | creatine kinase, brain |
| Bt.76.1.S1\_at | 338086 | COX7A1 | cytochrome c oxidase subunit VIIa polypeptide 1 (muscle) |

### GO:0016021

|  |  |  |  |
| --- | --- | --- | --- |
| **probe** | **entrez** | **symbol** | **description** |
| Bt.10349.1.S1\_at | 532626 | GPR37 | G protein-coupled receptor 37 (endothelin receptor type B-like) |
| Bt.25689.1.S1\_at | 516072 | HHATL | hedgehog acyltransferase-like |
| Bt.28008.1.S1\_s\_at | 404177 | KCNN2 | potassium intermediate/small conductance calcium-activated channel, subfamily N, member 2 |
| Bt.2911.1.A1\_at | 540068 | FAM134B | family with sequence similarity 134, member B |
| Bt.4417.1.S1\_at | 281062 | CDH2 | cadherin 2, type 1, N-cadherin (neuronal) |
| Bt.5848.1.S1\_at | 518117 | ATP2A1 | ATPase, Ca++ transporting, cardiac muscle, fast twitch 1 |
| Bt.76.1.S1\_at | 338086 | COX7A1 | cytochrome c oxidase subunit VIIa polypeptide 1 (muscle) |

### GO:0005829

|  |  |  |  |
| --- | --- | --- | --- |
| **probe** | **entrez** | **symbol** | **description** |
| Bt.12825.1.S1\_at | 515610 | ACTA2 | actin, alpha 2, smooth muscle, aorta |
| Bt.22169.1.S1\_at | 540303 | ENO3 | enolase 3 (beta, muscle) |

### GO:0005840

|  |  |  |  |
| --- | --- | --- | --- |
| **probe** | **entrez** | **symbol** | **description** |
| Bt.25658.1.A1\_at | 618440 | RPL3L | ribosomal protein L3-like |

### GO:0031224

|  |  |  |  |
| --- | --- | --- | --- |
| **probe** | **entrez** | **symbol** | **description** |
| Bt.10349.1.S1\_at | 532626 | GPR37 | G protein-coupled receptor 37 (endothelin receptor type B-like) |
| Bt.25689.1.S1\_at | 516072 | HHATL | hedgehog acyltransferase-like |
| Bt.28008.1.S1\_s\_at | 404177 | KCNN2 | potassium intermediate/small conductance calcium-activated channel, subfamily N, member 2 |
| Bt.2911.1.A1\_at | 540068 | FAM134B | family with sequence similarity 134, member B |
| Bt.4417.1.S1\_at | 281062 | CDH2 | cadherin 2, type 1, N-cadherin (neuronal) |
| Bt.5848.1.S1\_at | 518117 | ATP2A1 | ATPase, Ca++ transporting, cardiac muscle, fast twitch 1 |
| Bt.76.1.S1\_at | 338086 | COX7A1 | cytochrome c oxidase subunit VIIa polypeptide 1 (muscle) |

### GO:0005856

|  |  |  |  |
| --- | --- | --- | --- |
| **probe** | **entrez** | **symbol** | **description** |
| Bt.12300.1.S1\_at | 281337 | MYH1 | myosin, heavy chain 1, skeletal muscle, adult |
| Bt.12825.1.S1\_at | 515610 | ACTA2 | actin, alpha 2, smooth muscle, aorta |
| Bt.21761.1.S1\_a\_at | 505645 | TMOD4 | tropomodulin 4 (muscle) |
| Bt.29854.1.S1\_x\_at | 281337 | MYH1 | myosin, heavy chain 1, skeletal muscle, adult |
