# Supplementary material for: A gene network switch enhances the oxidative capacity of ovine skeletal muscle during late fetal development
Source: BMC Genomics. 2010 Jun 15;11:378. doi: 10.1186/1471-2164-11-378 (PMC2894804; doi:10.1186/1471-2164-11-378)
Supplement: Additional File 1 — Gene expression data. This file contains links to the gene expression data. The genes that are significantly differentially expressed between adjacent development times are listed as Experiment 1 (80 d vs 100 d), Experiment 2 (100 d vs 120 d), Experiment 3 (120 d vs 150 d) and Experiment 4 (150 d vs 230 d) in the section entitled Open microarray analysis report for developmental expression. Each experiment contains an MA plot, Volcano plot and Heatmap for each of the three microarray processing programs MAS5, RMA and GCRMA. For each of these processing programs there is a list of significantly differentially expressed probe sets, and for each of these there is an FDR corrected probability, fold change, percentage of microarrays with a MAS5 Present call, and a convergence percentage for all three microarray processing programs. The file also contains annotation of the probe sets. In a second section entitled Time series analysis of developmental expression, there is a list of genes in each gene expression cluster and diagrams showing MAS5 gene expression values for these genes. [file 1471-2164-11-378-S1.ZIP › Report/GO1/Report/TopNodesMF.html]

### Top terms for Molecular Function

Top terms sorted by the weight algorithm. The first column is the GO term id and links to GO, the second column is a short description of the term, the third column is the number of genes annotated to the term, the fourth is the number of genes significant for the term and the fifth column is the expected number of genes for the term. The last four columns are the p-values for different tests of significance. The results are ordered by the weight algorithm (last column).

|  |  |  |  |  |  |  |  |  |
| --- | --- | --- | --- | --- | --- | --- | --- | --- |
| **GO** | **Term** | **Annotated** | **Significant** | **Expected** | **classic** | **KS** | **elim** | **weight** |
| GO:0004111 | creatine kinase activity | 5 | 3 | 0.03 | 1.1e-06 | 1.00 | 1.1e-06 | 1.1e-06 |
| GO:0003779 | actin binding | 118 | 6 | 0.59 | 2.1e-05 | 0.19 | 2.1e-05 | 2.1e-05 |
| GO:0005516 | calmodulin binding | 42 | 4 | 0.21 | 5.1e-05 | 0.79 | 5.1e-05 | 5.1e-05 |
| GO:0001786 | phosphatidylserine binding | 1 | 1 | 0.01 | 0.005 | 1.00 | 0.005 | 0.005 |
| GO:0005546 | phosphatidylinositol-4,5-bisphosphate bi... | 1 | 1 | 0.01 | 0.005 | 1.00 | 0.005 | 0.005 |
| GO:0016286 | small conductance calcium-activated pota... | 1 | 1 | 0.01 | 0.005 | 1.00 | 0.005 | 0.005 |
| GO:0004082 | bisphosphoglycerate mutase activity | 2 | 1 | 0.01 | 0.010 | 1.00 | 0.010 | 0.010 |
| GO:0004083 | bisphosphoglycerate phosphatase activity | 2 | 1 | 0.01 | 0.010 | 1.00 | 0.010 | 0.010 |
| GO:0004619 | phosphoglycerate mutase activity | 2 | 1 | 0.01 | 0.010 | 1.00 | 0.010 | 0.010 |
| GO:0004634 | phosphopyruvate hydratase activity | 2 | 1 | 0.01 | 0.010 | 0.97 | 0.010 | 0.010 |
| GO:0030567 | prothrombin activator activity | 2 | 1 | 0.01 | 0.010 | 0.53 | 0.010 | 0.010 |
| GO:0042132 | fructose-bisphosphatase activity | 2 | 1 | 0.01 | 0.010 | 0.55 | 0.010 | 0.010 |
| GO:0005524 | ATP binding | 630 | 8 | 3.17 | 0.010 | 0.91 | 0.010 | 0.010 |
| GO:0005509 | calcium ion binding | 292 | 5 | 1.47 | 0.014 | 0.62 | 0.014 | 0.014 |
| GO:0003956 | NAD(P)+-protein-arginine ADP-ribosyltran... | 3 | 1 | 0.02 | 0.015 | 0.99 | 0.015 | 0.015 |
| GO:0004427 | inorganic diphosphatase activity | 4 | 1 | 0.02 | 0.020 | 1.00 | 0.020 | 0.020 |
| GO:0005388 | calcium-transporting ATPase activity | 6 | 1 | 0.03 | 0.030 | 1.00 | 0.030 | 0.030 |
| GO:0005523 | tropomyosin binding | 6 | 1 | 0.03 | 0.030 | 0.74 | 0.030 | 0.030 |
| GO:0000287 | magnesium ion binding | 136 | 3 | 0.68 | 0.030 | 0.29 | 0.030 | 0.030 |
| GO:0003774 | motor activity | 60 | 2 | 0.3 | 0.036 | 0.78 | 0.036 | 0.036 |

### DE genes for top GO terms for Molecular Function

List of DE genes for top GO terms. The first column is the Affymetrix probe ID, the second column is the Entrez ID, the third column is the gene symbol and the fourth column is the gene description. Note that the same gene can appear more than once in the list since different probes can target the same gene (can help detect overepresented genes due to multiple probe targets).

### GO:0004111

|  |  |  |  |
| --- | --- | --- | --- |
| **probe** | **entrez** | **symbol** | **description** |
| Bt.13980.1.A1\_at | 538944 | CKMT2 | creatine kinase, mitochondrial 2 (sarcomeric) |
| Bt.13980.2.A1\_a\_at | 538944 | CKMT2 | creatine kinase, mitochondrial 2 (sarcomeric) |
| Bt.5084.1.S1\_at | 516210 | CKB | creatine kinase, brain |

### GO:0003779

|  |  |  |  |
| --- | --- | --- | --- |
| **probe** | **entrez** | **symbol** | **description** |
| Bt.12300.1.S1\_at | 281337 | MYH1 | myosin, heavy chain 1, skeletal muscle, adult |
| Bt.21761.1.S1\_a\_at | 505645 | TMOD4 | tropomodulin 4 (muscle) |
| Bt.29854.1.S1\_x\_at | 281337 | MYH1 | myosin, heavy chain 1, skeletal muscle, adult |
| Bt.3980.1.S1\_at | 281478 | SCIN | scinderin |
| Bt.6630.1.S1\_at | 534583 | CNN1 | calponin 1, basic, smooth muscle |
| Bt.7084.1.S1\_at | 539375 | ACTN3 | actinin, alpha 3 |

### GO:0005516

|  |  |  |  |
| --- | --- | --- | --- |
| **probe** | **entrez** | **symbol** | **description** |
| Bt.12300.1.S1\_at | 281337 | MYH1 | myosin, heavy chain 1, skeletal muscle, adult |
| Bt.28008.1.S1\_s\_at | 404177 | KCNN2 | potassium intermediate/small conductance calcium-activated channel, subfamily N, member 2 |
| Bt.29854.1.S1\_x\_at | 281337 | MYH1 | myosin, heavy chain 1, skeletal muscle, adult |
| Bt.6630.1.S1\_at | 534583 | CNN1 | calponin 1, basic, smooth muscle |

### GO:0001786

|  |  |  |  |
| --- | --- | --- | --- |
| **probe** | **entrez** | **symbol** | **description** |
| Bt.3980.1.S1\_at | 281478 | SCIN | scinderin |

### GO:0005546

|  |  |  |  |
| --- | --- | --- | --- |
| **probe** | **entrez** | **symbol** | **description** |
| Bt.3980.1.S1\_at | 281478 | SCIN | scinderin |

### GO:0016286

|  |  |  |  |
| --- | --- | --- | --- |
| **probe** | **entrez** | **symbol** | **description** |
| Bt.28008.1.S1\_s\_at | 404177 | KCNN2 | potassium intermediate/small conductance calcium-activated channel, subfamily N, member 2 |

### GO:0004082

|  |  |  |  |
| --- | --- | --- | --- |
| **probe** | **entrez** | **symbol** | **description** |
| Bt.23217.1.S1\_at | 515067 | PGAM2 | phosphoglycerate mutase 2 (muscle) |

### GO:0004083

|  |  |  |  |
| --- | --- | --- | --- |
| **probe** | **entrez** | **symbol** | **description** |
| Bt.23217.1.S1\_at | 515067 | PGAM2 | phosphoglycerate mutase 2 (muscle) |

### GO:0004619

|  |  |  |  |
| --- | --- | --- | --- |
| **probe** | **entrez** | **symbol** | **description** |
| Bt.23217.1.S1\_at | 515067 | PGAM2 | phosphoglycerate mutase 2 (muscle) |

### GO:0004634

|  |  |  |  |
| --- | --- | --- | --- |
| **probe** | **entrez** | **symbol** | **description** |
| Bt.22169.1.S1\_at | 540303 | ENO3 | enolase 3 (beta, muscle) |

### GO:0030567

|  |  |  |  |
| --- | --- | --- | --- |
| **probe** | **entrez** | **symbol** | **description** |
| Bt.9202.1.S1\_at | 511711 | FGL2 | fibrinogen-like 2 |

### GO:0042132

|  |  |  |  |
| --- | --- | --- | --- |
| **probe** | **entrez** | **symbol** | **description** |
| Bt.28208.1.S1\_at | 514066 | FBP2 | fructose-1,6-bisphosphatase 2 |

### GO:0005524

|  |  |  |  |
| --- | --- | --- | --- |
| **probe** | **entrez** | **symbol** | **description** |
| Bt.12300.1.S1\_at | 281337 | MYH1 | myosin, heavy chain 1, skeletal muscle, adult |
| Bt.12825.1.S1\_at | 515610 | ACTA2 | actin, alpha 2, smooth muscle, aorta |
| Bt.13980.1.A1\_at | 538944 | CKMT2 | creatine kinase, mitochondrial 2 (sarcomeric) |
| Bt.13980.2.A1\_a\_at | 538944 | CKMT2 | creatine kinase, mitochondrial 2 (sarcomeric) |
| Bt.22379.1.S1\_at | 536925 | CABC1 | chaperone, ABC1 activity of bc1 complex homolog (S. pombe) |
| Bt.29854.1.S1\_x\_at | 281337 | MYH1 | myosin, heavy chain 1, skeletal muscle, adult |
| Bt.5084.1.S1\_at | 516210 | CKB | creatine kinase, brain |
| Bt.5848.1.S1\_at | 518117 | ATP2A1 | ATPase, Ca++ transporting, cardiac muscle, fast twitch 1 |

### GO:0005509

|  |  |  |  |
| --- | --- | --- | --- |
| **probe** | **entrez** | **symbol** | **description** |
| Bt.3595.1.S1\_at | 282660 | MGP | matrix Gla protein |
| Bt.3980.1.S1\_at | 281478 | SCIN | scinderin |
| Bt.4417.1.S1\_at | 281062 | CDH2 | cadherin 2, type 1, N-cadherin (neuronal) |
| Bt.5848.1.S1\_at | 518117 | ATP2A1 | ATPase, Ca++ transporting, cardiac muscle, fast twitch 1 |
| Bt.7084.1.S1\_at | 539375 | ACTN3 | actinin, alpha 3 |

### GO:0003956

|  |  |  |  |
| --- | --- | --- | --- |
| **probe** | **entrez** | **symbol** | **description** |
| Bt.29697.1.S1\_at | 407147 | ART3 | ADP-ribosyltransferase 3 |

### GO:0004427

|  |  |  |  |
| --- | --- | --- | --- |
| **probe** | **entrez** | **symbol** | **description** |
| Bt.26688.2.S1\_a\_at | 533321 | FRMD3 | FERM domain containing 3 |

### GO:0005388

|  |  |  |  |
| --- | --- | --- | --- |
| **probe** | **entrez** | **symbol** | **description** |
| Bt.5848.1.S1\_at | 518117 | ATP2A1 | ATPase, Ca++ transporting, cardiac muscle, fast twitch 1 |

### GO:0005523

|  |  |  |  |
| --- | --- | --- | --- |
| **probe** | **entrez** | **symbol** | **description** |
| Bt.21761.1.S1\_a\_at | 505645 | TMOD4 | tropomodulin 4 (muscle) |

### GO:0000287

|  |  |  |  |
| --- | --- | --- | --- |
| **probe** | **entrez** | **symbol** | **description** |
| Bt.22169.1.S1\_at | 540303 | ENO3 | enolase 3 (beta, muscle) |
| Bt.26688.2.S1\_a\_at | 533321 | FRMD3 | FERM domain containing 3 |
| Bt.5848.1.S1\_at | 518117 | ATP2A1 | ATPase, Ca++ transporting, cardiac muscle, fast twitch 1 |

### GO:0003774

|  |  |  |  |
| --- | --- | --- | --- |
| **probe** | **entrez** | **symbol** | **description** |
| Bt.12300.1.S1\_at | 281337 | MYH1 | myosin, heavy chain 1, skeletal muscle, adult |
| Bt.29854.1.S1\_x\_at | 281337 | MYH1 | myosin, heavy chain 1, skeletal muscle, adult |
