# Supplementary material for: A gene network switch enhances the oxidative capacity of ovine skeletal muscle during late fetal development
Source: BMC Genomics. 2010 Jun 15;11:378. doi: 10.1186/1471-2164-11-378 (PMC2894804; doi:10.1186/1471-2164-11-378)
Supplement: Additional File 1 — Gene expression data. This file contains links to the gene expression data. The genes that are significantly differentially expressed between adjacent development times are listed as Experiment 1 (80 d vs 100 d), Experiment 2 (100 d vs 120 d), Experiment 3 (120 d vs 150 d) and Experiment 4 (150 d vs 230 d) in the section entitled Open microarray analysis report for developmental expression. Each experiment contains an MA plot, Volcano plot and Heatmap for each of the three microarray processing programs MAS5, RMA and GCRMA. For each of these processing programs there is a list of significantly differentially expressed probe sets, and for each of these there is an FDR corrected probability, fold change, percentage of microarrays with a MAS5 Present call, and a convergence percentage for all three microarray processing programs. The file also contains annotation of the probe sets. In a second section entitled Time series analysis of developmental expression, there is a list of genes in each gene expression cluster and diagrams showing MAS5 gene expression values for these genes. [file 1471-2164-11-378-S1.ZIP › Report/GO2/Start.html]

## Functional profiles based on Gene Ontology for project DevelopmentalExpression

A total of 43 differentially expressed probes were used. Of these 10 are not annotated and were excluded from the analysis (includes duplicated probes that target the same gene). The results are for 33 annotated genes.

Functional profiles at different levels

### Induced tree graph for all DE genes

Biological Process

Cellular Component

Molecular Function

### Induced graphs of top ranking nodes

Biological Process

Cellular Component

Molecular Function

### Top ranking nodes summary

Biological Process

Cellular Component

Molecular Function

### Annotation - Link to DE genes in NCBI and EMBL-EBI

Biological process

Cellular component

Molecular function
