# Supplementary material for: A gene network switch enhances the oxidative capacity of ovine skeletal muscle during late fetal development
Source: BMC Genomics. 2010 Jun 15;11:378. doi: 10.1186/1471-2164-11-378 (PMC2894804; doi:10.1186/1471-2164-11-378)
Supplement: Additional File 1 — Gene expression data. This file contains links to the gene expression data. The genes that are significantly differentially expressed between adjacent development times are listed as Experiment 1 (80 d vs 100 d), Experiment 2 (100 d vs 120 d), Experiment 3 (120 d vs 150 d) and Experiment 4 (150 d vs 230 d) in the section entitled Open microarray analysis report for developmental expression. Each experiment contains an MA plot, Volcano plot and Heatmap for each of the three microarray processing programs MAS5, RMA and GCRMA. For each of these processing programs there is a list of significantly differentially expressed probe sets, and for each of these there is an FDR corrected probability, fold change, percentage of microarrays with a MAS5 Present call, and a convergence percentage for all three microarray processing programs. The file also contains annotation of the probe sets. In a second section entitled Time series analysis of developmental expression, there is a list of genes in each gene expression cluster and diagrams showing MAS5 gene expression values for these genes. [file 1471-2164-11-378-S1.ZIP › Report/GO3/Report/BP.html]

### Biological process

| Entrez | GO ID | Evidence Code |
| --- | --- | --- |
 280682 | GO:0006099 | IEA || 280682 | GO:0006099 | IEA |
| 280695 | GO:0043353 | IEA |
| 280695 | GO:0007507 | IEA |
| 280695 | GO:0006810 | IEA |
| 280695 | GO:0001666 | IEA |
| 280695 | GO:0015671 | IEA |
| 280701 | GO:0006796 | IEA |
| 280715 | GO:0006139 | IEA |
| 280715 | GO:0046034 | IEA |
| 280715 | GO:0046034 | IEA |
| 280715 | GO:0006139 | IEA |
| 280716 | GO:0006139 | IEA |
| 280719 | GO:0006546 | IEA |
| 280740 | GO:0006730 | IEA |
| 280740 | GO:0015670 | IEA |
| 280740 | GO:0046903 | IEA |
| 280740 | GO:0002009 | IEA |
| 280753 | GO:0030070 | IEA |
| 280753 | GO:0006508 | IEA |
| 280808 | GO:0006096 | IEA |
| 280808 | GO:0006094 | IEA |
| 280812 | GO:0016051 | IEA |
| 280815 | GO:0032367 | IEA |
| 280815 | GO:0042632 | IEA |
| 280832 | GO:0019221 | IEA |
| 280832 | GO:0030097 | IEA |
| 280832 | GO:0043473 | IEA |
| 280832 | GO:0018108 | IEA |
| 280832 | GO:0006468 | IEA |
| 280832 | GO:0048066 | IEA |
| 280832 | GO:0043406 | IEA |
| 280832 | GO:0007169 | IEA |
| 280832 | GO:0007243 | IEA |
| 280832 | GO:0008284 | IEA |
| 280832 | GO:0009314 | IEA |
| 280852 | GO:0009611 | IEA |
| 280857 | GO:0030198 | IEA |
| 280857 | GO:0007160 | IEA |
| 280878 | GO:0015992 | IEA |
| 280878 | GO:0006118 | IEA |
| 280878 | GO:0006810 | IEA |
| 280878 | GO:0006118 | IEA |
| 280878 | GO:0006810 | IEA |
| 280878 | GO:0015992 | IEA |
| 280890 | GO:0006518 | IEA |
| 280904 | GO:0008283 | IEA |
| 280904 | GO:0007612 | IEA |
| 280904 | GO:0030282 | IEA |
| 280939 | GO:0016337 | IEA |
| 280939 | GO:0045765 | IEA |
| 280939 | GO:0030334 | IEA |
| 280939 | GO:0007160 | IEA |
| 280939 | GO:0006468 | IEA |
| 280941 | GO:0016525 | IEA |
| 280941 | GO:0045026 | IEA |
| 280941 | GO:0006468 | IEA |
| 280941 | GO:0030336 | IEA |
| 280950 | GO:0006810 | IEA |
| 280950 | GO:0006118 | IEA |
| 280950 | GO:0045454 | IEA |
| 280955 | GO:0008150 | ND |
| 280955 | GO:0045103 | IEA |
| 280961 | GO:0045449 | IEA |
| 280961 | GO:0006355 | IEA |
| 280961 | GO:0006350 | IEA |
| 280962 | GO:0006350 | IEA |
| 280962 | GO:0045449 | IEA |
| 280962 | GO:0006355 | IEA |
| 280976 | GO:0006091 | ISS |
| 280976 | GO:0006099 | IEA |
| 280976 | GO:0006101 | ISS |
| 280976 | GO:0008152 | IEA |
| 281032 | GO:0045603 | IEA |
| 281032 | GO:0030308 | ISS |
| 281032 | GO:0042981 | IEA |
| 281032 | GO:0042981 | ISS |
| 281032 | GO:0045766 | IEA |
| 281032 | GO:0045449 | ISS |
| 281032 | GO:0007286 | ISS |
| 281032 | GO:0045663 | ISS |
| 281032 | GO:0008285 | IEA |
| 281032 | GO:0016477 | ISS |
| 281032 | GO:0043085 | ISS |
| 281032 | GO:0045603 | ISS |
| 281032 | GO:0045766 | ISS |
| 281032 | GO:0008285 | ISS |
| 281040 | GO:0008150 | ND |
| 281040 | GO:0008150 | ND |
| 281040 | GO:0008150 | ND |
| 281051 | GO:0007155 | IEA |
| 281057 | GO:0007155 | IEA |
| 281078 | GO:0006886 | IEA |
| 281092 | GO:0015908 | IEA |
| 281092 | GO:0006629 | IEA |
| 281092 | GO:0006631 | IEA |
| 281092 | GO:0006810 | IEA |
| 281156 | GO:0008299 | IEA |
| 281156 | GO:0006695 | IEA |
| 281156 | GO:0016126 | IEA |
| 281156 | GO:0006694 | IEA |
| 281156 | GO:0008610 | IEA |
| 281161 | GO:0001759 | IEA |
| 281161 | GO:0045892 | ISS |
| 281161 | GO:0017158 | ISS |
| 281161 | GO:0000186 | IEA |
| 281161 | GO:0000189 | ISS |
| 281161 | GO:0001934 | ISS |
| 281161 | GO:0008284 | ISS |
| 281161 | GO:0050679 | IEA |
| 281161 | GO:0030324 | IEA |
| 281161 | GO:0001525 | IEA |
| 281161 | GO:0006700 | ISS |
| 281161 | GO:0045766 | IEA |
| 281161 | GO:0045597 | ISS |
| 281161 | GO:0030308 | ISS |
| 281161 | GO:0001934 | IEA |
| 281161 | GO:0000186 | ISS |
| 281161 | GO:0030324 | ISS |
| 281161 | GO:0021940 | IEA |
| 281161 | GO:0008283 | IEA |
| 281161 | GO:0046668 | ISS |
| 281161 | GO:0006355 | ISS |
| 281161 | GO:0045597 | IEA |
| 281161 | GO:0001759 | ISS |
| 281161 | GO:0010001 | IEA |
| 281161 | GO:0007165 | ISS |
| 281161 | GO:0007243 | ISS |
| 281161 | GO:0007275 | IEA |
| 281161 | GO:0046668 | IEA |
| 281161 | GO:0030154 | IEA |
| 281161 | GO:0045941 | ISS |
| 281161 | GO:0008285 | IEA |
| 281161 | GO:0010001 | ISS |
| 281161 | GO:0007268 | ISS |
| 281187 | GO:0045941 | IEA |
| 281187 | GO:0040007 | IEA |
| 281187 | GO:0007179 | IEA |
| 281188 | GO:0007264 | ISS |
| 281188 | GO:0015031 | IEA |
| 281188 | GO:0043087 | IEA |
| 281188 | GO:0007264 | ISS |
| 281188 | GO:0015031 | IEA |
| 281188 | GO:0043087 | IEA |
| 281190 | GO:0017187 | IEA |
| 281193 | GO:0007154 | IEA |
| 281193 | GO:0035050 | IEA |
| 281193 | GO:0007267 | IEA |
| 281193 | GO:0045844 | IEA |
| 281193 | GO:0008016 | IEA |
| 281193 | GO:0007507 | IEA |
| 281193 | GO:0001701 | IEA |
| 281193 | GO:0001764 | IEA |
| 281193 | GO:0007512 | IEA |
| 281193 | GO:0048514 | IEA |
| 281193 | GO:0043123 | IEA |
| 281193 | GO:0001947 | IEA |
| 281193 | GO:0002070 | IEA |
| 281193 | GO:0043403 | IEA |
| 281201 | GO:0008283 | IEA |
| 281201 | GO:0007165 | IEA |
| 281201 | GO:0050909 | IEA |
| 281201 | GO:0007200 | IEA |
| 281206 | GO:0009058 | IEA |
| 281206 | GO:0006520 | IEA |
| 281210 | GO:0042744 | IEA |
| 281210 | GO:0042744 | ISS |
| 281210 | GO:0051289 | IEA |
| 281210 | GO:0006982 | ISS |
| 281210 | GO:0051289 | ISS |
| 281210 | GO:0006979 | IEA |
| 281216 | GO:0030828 | IEA |
| 281216 | GO:0060087 | IEA |
| 281216 | GO:0006182 | IEA |
| 281216 | GO:0009190 | IEA |
| 281216 | GO:0052565 | IEA |
| 281216 | GO:0008217 | IEA |
| 281216 | GO:0007242 | IEA |
| 281225 | GO:0006464 | IEA |
| 281225 | GO:0006512 | IEA |
| 281240 | GO:0008283 | IEA |
| 281278 | GO:0007218 | IEA |
| 281278 | GO:0007165 | IEA |
| 281278 | GO:0007186 | IEA |
| 281278 | GO:0007186 | IEA |
| 281278 | GO:0007165 | IEA |
| 281278 | GO:0007218 | IEA |
| 281296 | GO:0007026 | IEA |
| 281306 | GO:0006100 | IEA |
| 281306 | GO:0006099 | IEA |
| 281306 | GO:0006096 | IEA |
| 281306 | GO:0006108 | IEA |
| 281351 | GO:0001701 | IEA |
| 281351 | GO:0006499 | IEA |
| 281357 | GO:0009190 | IEA |
| 281357 | GO:0006182 | IEA |
| 281357 | GO:0006468 | IEA |
| 281357 | GO:0007242 | IEA |
| 281360 | GO:0008152 | IEA |
| 281365 | GO:0006596 | IEA |
| 281419 | GO:0006457 | IEA |
| 281433 | GO:0006629 | IEA |
| 281433 | GO:0006665 | IEA |
| 281433 | GO:0007585 | IEA |
| 281476 | GO:0006434 | IEA |
| 281476 | GO:0006418 | IEA |
| 281476 | GO:0006412 | IEA |
| 281543 | GO:0008610 | IEA |
| 281543 | GO:0006094 | IEA |
| 281543 | GO:0006098 | IEA |
| 281543 | GO:0008152 | IEA |
| 281543 | GO:0006633 | IEA |
| 281543 | GO:0006096 | IEA |
| 281543 | GO:0019682 | IEA |
| 281556 | GO:0006412 | IEA |
| 281556 | GO:0006414 | IEA |
| 281557 | GO:0006810 | IEA |
| 281557 | GO:0006662 | IEA |
| 281557 | GO:0045454 | IEA |
| 281557 | GO:0006118 | IEA |
| 281563 | GO:0000303 | IEA |
| 281563 | GO:0006839 | IEA |
| 281563 | GO:0006631 | IEA |
| 281563 | GO:0006810 | IEA |
| 281570 | GO:0006810 | IEA |
| 281570 | GO:0006118 | IEA |
| 281574 | GO:0035088 | IEA |
| 281574 | GO:0035088 | ISS |
| 281574 | GO:0051017 | ISS |
| 281574 | GO:0008360 | IEA |
| 281600 | GO:0007229 | IEA |
| 281603 | GO:0006171 | IEA |
| 281603 | GO:0009190 | IEA |
| 281603 | GO:0007242 | IEA |
| 281615 | GO:0008152 | IEA |
| 281631 | GO:0008202 | IEA |
| 281631 | GO:0051347 | IEA |
| 281631 | GO:0042158 | IEA |
| 281631 | GO:0008203 | IEA |
| 281631 | GO:0006695 | IEA |
| 281631 | GO:0006869 | IEA |
| 281631 | GO:0051346 | IEA |
| 281631 | GO:0001935 | IEA |
| 281631 | GO:0030325 | IEA |
| 281631 | GO:0006810 | IEA |
| 281631 | GO:0008211 | IEA |
| 281631 | GO:0033344 | IEA |
| 281631 | GO:0042157 | IEA |
| 281631 | GO:0001932 | IEA |
| 281631 | GO:0006644 | IEA |
| 281631 | GO:0043534 | IEA |
| 281631 | GO:0030300 | IEA |
| 281631 | GO:0006629 | IEA |
| 281631 | GO:0030301 | IEA |
| 281631 | GO:0019915 | IEA |
| 281660 | GO:0006936 | IEA |
| 281719 | GO:0032387 | IEA |
| 281719 | GO:0006916 | IEA |
| 281729 | GO:0007052 | ISS |
| 281729 | GO:0007165 | ISS |
| 281729 | GO:0006281 | IEA |
| 281729 | GO:0007049 | IEA |
| 281729 | GO:0006974 | IEA |
| 281729 | GO:0007126 | IEA |
| 281729 | GO:0007067 | IEA |
| 281729 | GO:0051301 | IEA |
| 281729 | GO:0007062 | ISS |
| 281729 | GO:0007059 | IEA |
| 281729 | GO:0006259 | IEA |
| 281729 | GO:0051276 | IEA |
| 281730 | GO:0006564 | IEA |
| 281730 | GO:0045892 | IEA |
| 281730 | GO:0008152 | IEA |
| 281736 | GO:0001764 | IEA |
| 281736 | GO:0007281 | IEA |
| 281736 | GO:0042098 | IEA |
| 281736 | GO:0030334 | IEA |
| 281736 | GO:0001667 | IEA |
| 281736 | GO:0008045 | IEA |
| 281736 | GO:0008354 | IEA |
| 281736 | GO:0001666 | IEA |
| 281736 | GO:0007420 | IEA |
| 281736 | GO:0001569 | IEA |
| 281736 | GO:0007165 | IEA |
| 281736 | GO:0007186 | IEA |
| 281742 | GO:0006979 | IEA |
| 281742 | GO:0006118 | IEA |
| 281749 | GO:0007165 | IEA |
| 281749 | GO:0007507 | IEA |
| 281749 | GO:0001569 | IEA |
| 281749 | GO:0007585 | IEA |
| 281749 | GO:0001701 | IEA |
| 281749 | GO:0030818 | IEA |
| 281749 | GO:0014032 | IEA |
| 281749 | GO:0007186 | IEA |
| 281749 | GO:0015758 | IEA |
| 281749 | GO:0001666 | IEA |
| 281749 | GO:0042310 | IEA |
| 281749 | GO:0015758 | IEA |
| 281749 | GO:0001569 | IEA |
| 281749 | GO:0007585 | IEA |
| 281749 | GO:0030818 | IEA |
| 281749 | GO:0001701 | IEA |
| 281749 | GO:0007507 | IEA |
| 281749 | GO:0007165 | IEA |
| 281749 | GO:0014032 | IEA |
| 281749 | GO:0001666 | IEA |
| 281749 | GO:0007186 | IEA |
| 281749 | GO:0042310 | IEA |
| 281758 | GO:0006810 | IEA |
| 281758 | GO:0006656 | IEA |
| 281759 | GO:0006810 | IEA |
| 281759 | GO:0006810 | IEA |
| 281780 | GO:0005975 | IEA |
| 281780 | GO:0005975 | IEA |
| 281790 | GO:0007186 | IEA |
| 281790 | GO:0007165 | IEA |
| 281791 | GO:0007165 | IEA |
| 281791 | GO:0007186 | IEA |
| 281801 | GO:0006468 | IEA |
| 281801 | GO:0007165 | IEA |
| 281810 | GO:0008152 | IEA |
| 281810 | GO:0006629 | IEA |
| 281810 | GO:0006631 | IEA |
| 281810 | GO:0006635 | IEA |
| 281811 | GO:0006629 | IEA |
| 281811 | GO:0006631 | IEA |
| 281849 | GO:0006810 | IEA |
| 281876 | GO:0007229 | IEA |
| 281876 | GO:0007160 | IEA |
| 281876 | GO:0007155 | IEA |
| 281876 | GO:0045214 | IEA |
| 281876 | GO:0007275 | IEA |
| 281876 | GO:0000082 | IEA |
| 281876 | GO:0042685 | IEA |
| 281876 | GO:0010002 | IEA |
| 281876 | GO:0008284 | IEA |
| 281876 | GO:0051726 | IEA |
| 281876 | GO:0001701 | IEA |
| 281876 | GO:0045596 | IEA |
| 281935 | GO:0007165 | IEA |
| 281937 | GO:0006810 | IEA |
| 281945 | GO:0009956 | IEA |
| 281945 | GO:0006350 | IEA |
| 281945 | GO:0007519 | IEA |
| 281945 | GO:0006355 | IEA |
| 281945 | GO:0009952 | IEA |
| 281945 | GO:0048514 | IEA |
| 281945 | GO:0060173 | IEA |
| 281945 | GO:0030900 | IEA |
| 281945 | GO:0001764 | IEA |
| 281963 | GO:0007165 | IEA |
| 281963 | GO:0007186 | IEA |
| 281986 | GO:0007165 | IEA |
| 281986 | GO:0007242 | IEA |
| 281986 | GO:0016042 | IEA |
| 281986 | GO:0006629 | IEA |
| 281987 | GO:0006629 | IEA |
| 281987 | GO:0007242 | IEA |
| 281987 | GO:0016042 | IEA |
| 281987 | GO:0007165 | IEA |
| 281987 | GO:0001701 | IEA |
| 282011 | GO:0006355 | IEA |
| 282011 | GO:0006350 | IEA |
| 282015 | GO:0043161 | ISS |
| 282015 | GO:0043069 | ISS |
| 282015 | GO:0043193 | ISS |
| 282015 | GO:0030163 | IEA |
| 282022 | GO:0006633 | IEA |
| 282022 | GO:0030216 | ISS |
| 282022 | GO:0008217 | IEA |
| 282022 | GO:0008610 | IEA |
| 282022 | GO:0001516 | IEA |
| 282032 | GO:0045768 | ISS |
| 282032 | GO:0051056 | IEA |
| 282032 | GO:0051252 | ISS |
| 282032 | GO:0043524 | ISS |
| 282032 | GO:0007165 | ISS |
| 282032 | GO:0043524 | IEA |
| 282032 | GO:0001953 | ISS |
| 282032 | GO:0007162 | ISS |
| 282032 | GO:0007155 | ISS |
| 282032 | GO:0001570 | ISS |
| 282032 | GO:0009790 | ISS |
| 282032 | GO:0001570 | IEA |
| 282032 | GO:0009790 | IEA |
| 282032 | GO:0000910 | ISS |
| 282032 | GO:0030036 | ISS |
| 282032 | GO:0008360 | ISS |
| 282032 | GO:0030833 | ISS |
| 282032 | GO:0007242 | IEA |
| 282050 | GO:0006412 | IEA |
| 282068 | GO:0007275 | IEA |
| 282068 | GO:0001756 | IEA |
| 282068 | GO:0030154 | IEA |
| 282068 | GO:0009952 | IEA |
| 282068 | GO:0016055 | IEA |
| 282090 | GO:0006171 | IEA |
| 282090 | GO:0006091 | ISS |
| 282090 | GO:0016311 | IEA |
| 282090 | GO:0006772 | IEA |
| 282091 | GO:0007165 | ISS |
| 282100 | GO:0006810 | IEA |
| 282100 | GO:0006816 | IEA |
| 282100 | GO:0006811 | IEA |
| 282110 | GO:0006511 | IEA |
| 282110 | GO:0006512 | IEA |
| 282119 | GO:0006820 | IEA |
| 282119 | GO:0006811 | IEA |
| 282119 | GO:0006810 | IEA |
| 282119 | GO:0006915 | IEA |
| 282119 | GO:0006810 | IEA |
| 282119 | GO:0006820 | IEA |
| 282119 | GO:0006811 | IEA |
| 282119 | GO:0006915 | IEA |
| 282120 | GO:0006820 | IEA |
| 282120 | GO:0006810 | IEA |
| 282120 | GO:0006811 | IEA |
| 282122 | GO:0016331 | IEA |
| 282122 | GO:0048010 | IEA |
| 282122 | GO:0009887 | IEA |
| 282130 | GO:0006629 | IEA |
| 282130 | GO:0006118 | IEA |
| 282130 | GO:0006631 | IEA |
| 282130 | GO:0008152 | IEA |
| 282132 | GO:0007162 | ISS |
| 282132 | GO:0006913 | ISS |
| 282132 | GO:0001701 | IEA |
| 282132 | GO:0007219 | IEA |
| 282132 | GO:0006468 | IEA |
| 282132 | GO:0006509 | ISS |
| 282132 | GO:0007220 | ISS |
| 282132 | GO:0001701 | ISS |
| 282132 | GO:0006508 | IEA |
| 282132 | GO:0006468 | ISS |
| 282132 | GO:0007267 | ISS |
| 282132 | GO:0007162 | ISS |
| 282132 | GO:0007220 | ISS |
| 282132 | GO:0006913 | ISS |
| 282132 | GO:0007267 | ISS |
| 282132 | GO:0006468 | IEA |
| 282132 | GO:0006509 | ISS |
| 282132 | GO:0006508 | IEA |
| 282132 | GO:0001701 | IEA |
| 282132 | GO:0007219 | IEA |
| 282132 | GO:0006468 | ISS |
| 282132 | GO:0001701 | ISS |
| 282200 | GO:0006118 | IEA |
| 282211 | GO:0006118 | IEA |
| 282215 | GO:0006457 | IEA |
| 282218 | GO:0006308 | IEA |
| 282218 | GO:0007275 | IEA |
| 282218 | GO:0006259 | IEA |
| 282218 | GO:0030218 | IEA |
| 282218 | GO:0006915 | IEA |
| 282220 | GO:0006414 | IEA |
| 282220 | GO:0006412 | IEA |
| 282261 | GO:0001558 | IEA |
| 282261 | GO:0045663 | ISS |
| 282261 | GO:0009968 | ISS |
| 282261 | GO:0043065 | ISS |
| 282262 | GO:0001558 | IEA |
| 282290 | GO:0006120 | IEA |
| 282303 | GO:0007155 | IEA |
| 282303 | GO:0007155 | IEA |
| 282318 | GO:0051301 | IEA |
| 282318 | GO:0005975 | IEA |
| 282318 | GO:0005977 | IEA |
| 282318 | GO:0007049 | IEA |
| 282326 | GO:0006508 | IEA |
| 282326 | GO:0030512 | IEA |
| 282326 | GO:0030514 | IEA |
| 282346 | GO:0006707 | IEA |
| 282346 | GO:0030301 | IEA |
| 282346 | GO:0001935 | IEA |
| 282346 | GO:0043534 | IEA |
| 282346 | GO:0007155 | IEA |
| 282385 | GO:0007010 | IEA |
| 282386 | GO:0007010 | IEA |
| 282389 | GO:0000305 | TAS |
| 282389 | GO:0045454 | IEA |
| 282389 | GO:0006118 | IEA |
| 282393 | GO:0006810 | IEA |
| 282393 | GO:0006118 | IEA |
| 282394 | GO:0006508 | IEA |
| 282394 | GO:0006810 | IEA |
| 282394 | GO:0006118 | IEA |
| 282405 | GO:0015992 | IEA |
| 282405 | GO:0006811 | IEA |
| 282405 | GO:0015986 | IEA |
| 282405 | GO:0006754 | IEA |
| 282405 | GO:0006810 | IEA |
| 282420 | GO:0051726 | IEA |
| 282420 | GO:0006468 | IEA |
| 282420 | GO:0016055 | IEA |
| 282433 | GO:0006182 | IEA |
| 282433 | GO:0006810 | IEA |
| 282433 | GO:0006811 | IEA |
| 282433 | GO:0006826 | IEA |
| 282433 | GO:0009190 | IEA |
| 282433 | GO:0007242 | IEA |
| 282438 | GO:0016042 | IEA |
| 282438 | GO:0000302 | ISS |
| 282446 | GO:0006099 | IEA |
| 282446 | GO:0008152 | IEA |
| 282446 | GO:0006099 | IEA |
| 282446 | GO:0008152 | IEA |
| 282477 | GO:0006810 | IEA |
| 282478 | GO:0006839 | IEA |
| 282478 | GO:0006810 | IEA |
| 282515 | GO:0006629 | IEA |
| 282515 | GO:0016042 | IEA |
| 282517 | GO:0006118 | IEA |
| 282523 | GO:0006810 | IEA |
| 282564 | GO:0007229 | IEA |
| 282564 | GO:0007275 | IEA |
| 282564 | GO:0007155 | IEA |
| 282564 | GO:0007160 | IEA |
| 282572 | GO:0043330 | IEA |
| 282572 | GO:0006813 | IEA |
| 282572 | GO:0006811 | IEA |
| 282572 | GO:0051607 | IEA |
| 282572 | GO:0007507 | IEA |
| 282572 | GO:0032496 | IEA |
| 282572 | GO:0006810 | IEA |
| 282689 | GO:0042730 | IEA |
| 282689 | GO:0001525 | IEA |
| 282689 | GO:0030199 | IEA |
| 282690 | GO:0006810 | IEA |
| 282690 | GO:0006811 | IEA |
| 282690 | GO:0015986 | IEA |
| 282690 | GO:0015992 | IEA |
| 282701 | GO:0006810 | IEA |
| 282701 | GO:0015986 | IEA |
| 282701 | GO:0006811 | IEA |
| 282701 | GO:0015992 | IEA |
| 282705 | GO:0007219 | IEA |
| 282705 | GO:0016485 | ISS |
| 282705 | GO:0007155 | IEA |
| 282705 | GO:0006916 | ISS |
| 282705 | GO:0006915 | IEA |
| 282705 | GO:0006509 | ISS |
| 282705 | GO:0007059 | ISS |
| 282705 | GO:0007242 | IEA |
| 282705 | GO:0007220 | ISS |
| 282705 | GO:0043085 | ISS |
| 282705 | GO:0042987 | ISS |
| 282705 | GO:0016485 | ISS |
| 282705 | GO:0007220 | ISS |
| 282705 | GO:0007155 | IEA |
| 282705 | GO:0006915 | IEA |
| 282705 | GO:0007242 | IEA |
| 282705 | GO:0006509 | ISS |
| 282705 | GO:0007219 | IEA |
| 282705 | GO:0006916 | ISS |
| 282705 | GO:0007059 | ISS |
| 282705 | GO:0043085 | ISS |
| 282705 | GO:0042987 | ISS |
| 282710 | GO:0015986 | IEA |
| 282710 | GO:0015992 | IEA |
| 282710 | GO:0006810 | IEA |
| 282710 | GO:0006811 | IEA |
| 282716 | GO:0006810 | IEA |
| 282716 | GO:0006811 | IEA |
| 282716 | GO:0006820 | IEA |
| 282848 | GO:0006457 | IEA |
| 282856 | GO:0006094 | IEA |
| 282862 | GO:0007155 | IEA |
| 282872 | GO:0000270 | IEA |
| 282872 | GO:0030574 | IEA |
| 282872 | GO:0006508 | IEA |
| 282876 | GO:0006809 | IEA |
| 282876 | GO:0001666 | IEA |
| 282876 | GO:0006118 | IEA |
| 282876 | GO:0042742 | IEA |
| 286770 | GO:0006986 | IEA |
| 286770 | GO:0006457 | IEA |
| 286807 | GO:0006512 | IEA |
| 286807 | GO:0006464 | IEA |
| 286809 | GO:0007275 | IEA |
| 286809 | GO:0006979 | IEA |
| 286838 | GO:0006412 | IEA |
| 286838 | GO:0009987 | IEA |
| 286838 | GO:0015012 | IEA |
| 286840 | GO:0006810 | IEA |
| 286840 | GO:0006118 | IEA |
| 286840 | GO:0006099 | IEA |
| 286861 | GO:0006879 | IEA |
| 286861 | GO:0006826 | IEA |
| 286862 | GO:0045664 | IEA |
| 286862 | GO:0006605 | IEA |
| 286862 | GO:0048167 | ISS |
| 286862 | GO:0007088 | ISS |
| 286862 | GO:0048167 | IEA |
| 286862 | GO:0045664 | ISS |
| 286862 | GO:0009966 | ISS |
| 286862 | GO:0006605 | ISS |
| 286862 | GO:0007010 | ISS |
| 286862 | GO:0006469 | ISS |
| 286862 | GO:0043066 | ISS |
| 286862 | GO:0006469 | ISS |
| 286862 | GO:0045664 | ISS |
| 286862 | GO:0006605 | IEA |
| 286862 | GO:0006605 | ISS |
| 286862 | GO:0045664 | IEA |
| 286862 | GO:0007088 | ISS |
| 286862 | GO:0048167 | IEA |
| 286862 | GO:0009966 | ISS |
| 286862 | GO:0043066 | ISS |
| 286862 | GO:0048167 | ISS |
| 286862 | GO:0007010 | ISS |
| 286883 | GO:0006468 | IEA |
| 286885 | GO:0006810 | IEA |
| 286885 | GO:0006118 | IEA |
| 286886 | GO:0045471 | IEA |
| 286886 | GO:0009058 | IEA |
| 286886 | GO:0015908 | IEA |
| 286886 | GO:0006520 | IEA |
| 286886 | GO:0006520 | IEA |
| 286886 | GO:0015908 | IEA |
| 286886 | GO:0045471 | IEA |
| 286886 | GO:0009058 | IEA |
| 286969 | GO:0007186 | IEA |
| 286969 | GO:0007165 | IEA |
| 287012 | GO:0006118 | IEA |
| 287014 | GO:0006120 | IEA |
| 287014 | GO:0006118 | IEA |
| 287020 | GO:0006810 | IEA |
| 287020 | GO:0006118 | IEA |
| 287024 | GO:0043267 | IEA |
| 287024 | GO:0002028 | IEA |
| 287024 | GO:0031663 | IEA |
| 287024 | GO:0030324 | IEA |
| 287024 | GO:0006809 | IEA |
| 287024 | GO:0051926 | IEA |
| 287024 | GO:0051346 | IEA |
| 287024 | GO:0001525 | IEA |
| 287024 | GO:0001542 | IEA |
| 287024 | GO:0006118 | IEA |
| 287024 | GO:0007612 | IEA |
| 287024 | GO:0007596 | IEA |
| 287027 | GO:0006118 | IEA |
| 287027 | GO:0006979 | IEA |
| 287027 | GO:0032981 | IEA |
| 287327 | GO:0030308 | IEA |
| 287327 | GO:0006917 | IEA |
| 287327 | GO:0006800 | IEA |
| 287327 | GO:0006120 | IEA |
| 287327 | GO:0006118 | IEA |
| 288380 | GO:0006120 | IEA |
| 288380 | GO:0045333 | IEA |
| 288380 | GO:0046034 | IEA |
| 288380 | GO:0006915 | IEA |
| 288380 | GO:0006118 | IEA |
| 288380 | GO:0006800 | IEA |
| 288380 | GO:0051881 | IEA |
| 317655 | GO:0001778 | ISS |
| 317655 | GO:0001764 | ISS |
| 317655 | GO:0006930 | ISS |
| 317655 | GO:0006887 | ISS |
| 317655 | GO:0008360 | IEA |
| 317697 | GO:0006816 | IEA |
| 317697 | GO:0006811 | IEA |
| 317697 | GO:0006810 | IEA |
| 319141 | GO:0009058 | IEA |
| 326578 | GO:0006614 | IEA |
| 326579 | GO:0006446 | IEA |
| 326581 | GO:0006412 | IEA |
| 326581 | GO:0006414 | IEA |
| 326601 | GO:0006334 | IEA |
| 326601 | GO:0006334 | IEA |
| 326601 | GO:0006334 | IEA |
| 326601 | GO:0006334 | IEA |
| 326601 | GO:0006334 | IEA |
| 327664 | GO:0005980 | IEA |
| 327664 | GO:0005975 | IEA |
| 327664 | GO:0005977 | IEA |
| 327668 | GO:0006754 | IEA |
| 327668 | GO:0006811 | IEA |
| 327668 | GO:0015992 | IEA |
| 327668 | GO:0006810 | IEA |
| 327668 | GO:0015986 | IEA |
| 327669 | GO:0008152 | IEA |
| 327669 | GO:0006099 | IEA |
| 327669 | GO:0006097 | IEA |
| 327670 | GO:0006979 | IEA |
| 327674 | GO:0006118 | IEA |
| 327680 | GO:0006118 | IEA |
| 327681 | GO:0045596 | IEA |
| 327681 | GO:0043616 | IEA |
| 327681 | GO:0042475 | IEA |
| 327681 | GO:0007276 | IEA |
| 327681 | GO:0030509 | IEA |
| 327681 | GO:0031069 | IEA |
| 327681 | GO:0007389 | IEA |
| 327681 | GO:0008585 | IEA |
| 327681 | GO:0042475 | IEA |
| 327681 | GO:0008585 | IEA |
| 327681 | GO:0007389 | IEA |
| 327681 | GO:0007276 | IEA |
| 327681 | GO:0043616 | IEA |
| 327681 | GO:0045596 | IEA |
| 327681 | GO:0030509 | IEA |
| 327681 | GO:0031069 | IEA |
| 327684 | GO:0001764 | IEA |
| 327684 | GO:0030900 | IEA |
| 327684 | GO:0006355 | IEA |
| 327684 | GO:0006350 | IEA |
| 327688 | GO:0006118 | IEA |
| 327690 | GO:0006118 | IEA |
| 327696 | GO:0006810 | IEA |
| 327696 | GO:0006118 | IEA |
| 327696 | GO:0006099 | IEA |
| 327697 | GO:0006979 | IEA |
| 327697 | GO:0006118 | IEA |
| 327699 | GO:0045980 | IEA |
| 327699 | GO:0001937 | ISS |
| 327699 | GO:0051260 | IDA |
| 327699 | GO:0009268 | IDA |
| 327699 | GO:0051289 | IDA |
| 327699 | GO:0051346 | IDA |
| 327702 | GO:0006120 | TAS |
| 327702 | GO:0006633 | IEA |
| 327702 | GO:0008610 | IEA |
| 327703 | GO:0006816 | IEA |
| 327703 | GO:0006811 | IEA |
| 327703 | GO:0006810 | IEA |
| 327706 | GO:0006118 | IEA |
| 327718 | GO:0006118 | IEA |
| 337916 | GO:0006355 | IEA |
| 337918 | GO:0007155 | IEA |
| 337927 | GO:0006810 | IEA |
| 337927 | GO:0006810 | IEA |
| 338040 | GO:0015986 | IEA |
| 338040 | GO:0006811 | IEA |
| 338040 | GO:0006754 | IEA |
| 338040 | GO:0015992 | IEA |
| 338040 | GO:0006810 | IEA |
| 338044 | GO:0006865 | IEA |
| 338046 | GO:0006120 | IEA |
| 338047 | GO:0016042 | IEA |
| 338053 | GO:0006810 | IEA |
| 338053 | GO:0015986 | IEA |
| 338053 | GO:0015992 | IEA |
| 338053 | GO:0006811 | IEA |
| 338057 | GO:0032981 | IEA |
| 338060 | GO:0006139 | IEA |
| 338062 | GO:0002474 | IEA |
| 338062 | GO:0019882 | IEA |
| 338062 | GO:0006955 | IEA |
| 338063 | GO:0042773 | IEA |
| 338063 | GO:0006120 | IDA |
| 338065 | GO:0006118 | IEA |
| 338066 | GO:0007165 | IEA |
| 338066 | GO:0007186 | IEA |
| 338066 | GO:0007218 | IEA |
| 338066 | GO:0007155 | IEA |
| 338068 | GO:0001569 | IEA |
| 338068 | GO:0043066 | IEA |
| 338068 | GO:0045941 | IEA |
| 338068 | GO:0032926 | IEA |
| 338068 | GO:0000082 | IEA |
| 338068 | GO:0001701 | ISS |
| 338068 | GO:0001702 | IEA |
| 338068 | GO:0002526 | IEA |
| 338068 | GO:0001755 | ISS |
| 338068 | GO:0007369 | IEA |
| 338068 | GO:0001707 | IEA |
| 338068 | GO:0030278 | IEA |
| 338068 | GO:0006468 | IEA |
| 338068 | GO:0001755 | IEA |
| 338068 | GO:0051145 | IEA |
| 338068 | GO:0060037 | IEA |
| 338068 | GO:0007178 | IEA |
| 338068 | GO:0009790 | ISS |
| 338068 | GO:0001655 | ISS |
| 338068 | GO:0007179 | ISS |
| 338068 | GO:0007179 | IEA |
| 338068 | GO:0007498 | IEA |
| 338068 | GO:0007281 | ISS |
| 338068 | GO:0007281 | IEA |
| 338068 | GO:0001701 | IEA |
| 338068 | GO:0007507 | IEA |
| 338068 | GO:0007368 | IEA |
| 338068 | GO:0009790 | IEA |
| 338068 | GO:0007368 | ISS |
| 338068 | GO:0007507 | ISS |
| 338073 | GO:0006118 | IEA |
| 338079 | GO:0006120 | IEA |
| 338081 | GO:0006811 | IEA |
| 338081 | GO:0006754 | IEA |
| 338081 | GO:0006810 | IEA |
| 338081 | GO:0015986 | IEA |
| 338081 | GO:0015992 | IEA |
| 338084 | GO:0008624 | IEA |
| 338084 | GO:0045892 | IEA |
| 338084 | GO:0030262 | IEA |
| 338084 | GO:0006800 | IEA |
| 338084 | GO:0006915 | IEA |
| 338084 | GO:0017148 | IEA |
| 338084 | GO:0045892 | ISS |
| 338084 | GO:0030262 | ISS |
| 338084 | GO:0030308 | ISS |
| 338084 | GO:0006606 | ISS |
| 338084 | GO:0030308 | IEA |
| 338084 | GO:0008624 | ISS |
| 338086 | GO:0006118 | IEA |
| 338087 | GO:0006118 | IEA |
| 338088 | GO:0015992 | IEA |
| 338088 | GO:0015986 | IEA |
| 338088 | GO:0006811 | IEA |
| 338088 | GO:0006810 | IEA |
| 338088 | GO:0006754 | IEA |
| 353217 | GO:0006810 | IEA |
| 359716 | GO:0006306 | IEA |
| 359716 | GO:0006349 | ISS |
| 360006 | GO:0006457 | IEA |
| 360007 | GO:0007596 | IEA |
| 360196 | GO:0015074 | IEA |
| 399560 | GO:0006412 | IEA |
| 404069 | GO:0050851 | ISS |
| 404126 | GO:0016575 | IEA |
| 404159 | GO:0045454 | IEA |
| 404169 | GO:0006469 | IEA |
| 404186 | GO:0001558 | IEA |
| 404188 | GO:0044237 | IEA |
| 407147 | GO:0006471 | IEA |
| 407170 | GO:0045446 | IEA |
| 407170 | GO:0048754 | IEA |
| 407170 | GO:0043129 | IEA |
| 407170 | GO:0001541 | IEA |
| 407170 | GO:0030324 | IEA |
| 407170 | GO:0048010 | IEA |
| 407170 | GO:0045165 | IEA |
| 407170 | GO:0050679 | IEA |
| 407170 | GO:0048469 | IEA |
| 407170 | GO:0007169 | IEA |
| 407170 | GO:0048286 | IEA |
| 407170 | GO:0055074 | IEA |
| 407170 | GO:0001570 | IEA |
| 407170 | GO:0006468 | IEA |
| 407170 | GO:0002053 | IEA |
| 407170 | GO:0016477 | IEA |
| 407170 | GO:0030097 | IEA |
| 407209 | GO:0007156 | IEA |
| 407209 | GO:0007156 | IEA |
| 407240 | GO:0045449 | IEA |
| 407767 | GO:0006084 | IEA |
| 408003 | GO:0006914 | IEA |
| 408003 | GO:0006512 | IEA |
| 408003 | GO:0006810 | IEA |
| 408003 | GO:0015031 | IEA |
| 408003 | GO:0006512 | IEA |
| 408003 | GO:0015031 | IEA |
| 408003 | GO:0006810 | IEA |
| 408003 | GO:0006914 | IEA |
| 408010 | GO:0006418 | IEA |
| 408010 | GO:0006412 | IEA |
| 408010 | GO:0006426 | IEA |
| 414735 | GO:0007155 | IEA |
| 414735 | GO:0007156 | IEA |
| 414735 | GO:0007156 | IEA |
| 414735 | GO:0007155 | IEA |
| 414922 | GO:0016338 | IEA |
| 444864 | GO:0006915 | IEA |
| 444864 | GO:0006355 | IEA |
| 444864 | GO:0006915 | IEA |
| 444864 | GO:0006355 | IEA |
| 444866 | GO:0006355 | IEA |
| 444866 | GO:0006355 | IEA |
| 444878 | GO:0006118 | IEA |
| 444879 | GO:0006486 | IEA |
| 445421 | GO:0006412 | IEA |
| 445421 | GO:0006412 | IEA |
| 445543 | GO:0007155 | IEA |
| 493728 | GO:0051016 | IEA |
| 493728 | GO:0030036 | IEA |
| 493998 | GO:0042573 | IEA |
| 493998 | GO:0035115 | IEA |
| 493998 | GO:0006810 | IEA |
| 503685 | GO:0008544 | IEA |
| 504248 | GO:0009156 | IEA |
| 504248 | GO:0009116 | IEA |
| 504248 | GO:0009165 | IEA |
| 504317 | GO:0008104 | IEA |
| 504317 | GO:0009953 | IEA |
| 504317 | GO:0051124 | IEA |
| 504317 | GO:0030178 | IEA |
| 504317 | GO:0030326 | IEA |
| 504317 | GO:0001942 | IEA |
| 504317 | GO:0048856 | IEA |
| 504317 | GO:0001822 | IEA |
| 504317 | GO:0043113 | IEA |
| 504317 | GO:0009954 | IEA |
| 504317 | GO:0042733 | IEA |
| 504317 | GO:0042475 | IEA |
| 504474 | GO:0018279 | IEA |
| 504533 | GO:0006464 | IEA |
| 504534 | GO:0006814 | IEA |
| 504534 | GO:0015711 | IEA |
| 504599 | GO:0006334 | IEA |
| 504599 | GO:0006334 | IEA |
| 504599 | GO:0006334 | IEA |
| 504599 | GO:0006334 | IEA |
| 504599 | GO:0006334 | IEA |
| 504637 | GO:0006260 | IEA |
| 504650 | GO:0008654 | IEA |
| 504716 | GO:0045449 | IEA |
| 504741 | GO:0006955 | IEA |
| 504867 | GO:0007275 | IEA |
| 504914 | GO:0042168 | IEA |
| 505005 | GO:0018106 | IEA |
| 505005 | GO:0016310 | IEA |
| 505050 | GO:0006511 | IEA |
| 505060 | GO:0006694 | IEA |
| 505060 | GO:0006118 | IEA |
| 505060 | GO:0008610 | IEA |
| 505060 | GO:0006695 | IEA |
| 505060 | GO:0016126 | IEA |
| 505093 | GO:0006397 | IEA |
| 505093 | GO:0008380 | IEA |
| 505093 | GO:0051028 | IEA |
| 505093 | GO:0006810 | IEA |
| 505176 | GO:0006511 | IEA |
| 505219 | GO:0006810 | IEA |
| 505293 | GO:0045449 | IEA |
| 505293 | GO:0045944 | IEA |
| 505320 | GO:0006511 | IEA |
| 505453 | GO:0045893 | IEA |
| 505489 | GO:0006810 | IEA |
| 505489 | GO:0015031 | IEA |
| 505489 | GO:0006605 | IEA |
| 505489 | GO:0007006 | IEA |
| 505489 | GO:0006470 | IEA |
| 505775 | GO:0006810 | IEA |
| 505775 | GO:0015711 | IEA |
| 505807 | GO:0006464 | IEA |
| 505884 | GO:0019221 | IEA |
| 505884 | GO:0019221 | IEA |
| 505884 | GO:0019221 | IEA |
| 505949 | GO:0008152 | IEA |
| 505968 | GO:0009791 | IEA |
| 505968 | GO:0008152 | IEA |
| 505968 | GO:0009409 | IEA |
| 505968 | GO:0055007 | IEA |
| 505968 | GO:0007507 | IEA |
| 505968 | GO:0042594 | IEA |
| 505968 | GO:0006635 | IEA |
| 505968 | GO:0001889 | IEA |
| 505968 | GO:0006118 | IEA |
| 505968 | GO:0006082 | IEA |
| 505968 | GO:0009437 | IEA |
| 505968 | GO:0006635 | ISS |
| 505987 | GO:0005975 | IEA |
| 505987 | GO:0006072 | IEA |
| 506029 | GO:0007254 | IEA |
| 506029 | GO:0000902 | IEA |
| 506029 | GO:0006916 | IEA |
| 506029 | GO:0000902 | IEA |
| 506029 | GO:0007254 | IEA |
| 506029 | GO:0006916 | IEA |
| 506114 | GO:0006355 | IEA |
| 506114 | GO:0007049 | IEA |
| 506114 | GO:0006350 | IEA |
| 506218 | GO:0006886 | IEA |
| 506231 | GO:0007264 | IEA |
| 506231 | GO:0007242 | IEA |
| 506235 | GO:0006457 | IEA |
| 506235 | GO:0006986 | IEA |
| 506235 | GO:0006350 | IEA |
| 506235 | GO:0006355 | IEA |
| 506336 | GO:0015031 | IEA |
| 506336 | GO:0006888 | IEA |
| 506336 | GO:0006810 | IEA |
| 506448 | GO:0006355 | IEA |
| 506448 | GO:0006350 | IEA |
| 506544 | GO:0006096 | IEA |
| 506544 | GO:0006096 | IEA |
| 506730 | GO:0007163 | IEA |
| 506730 | GO:0016055 | IEA |
| 506730 | GO:0001707 | IEA |
| 506730 | GO:0006928 | IEA |
| 506730 | GO:0006620 | IEA |
| 506764 | GO:0006512 | IEA |
| 506764 | GO:0006915 | IEA |
| 506764 | GO:0006915 | IEA |
| 506764 | GO:0006512 | IEA |
| 506882 | GO:0006508 | IEA |
| 506969 | GO:0008104 | IEA |
| 506991 | GO:0045454 | IEA |
| 507093 | GO:0048048 | IEA |
| 507093 | GO:0042573 | IEA |
| 507093 | GO:0021768 | IEA |
| 507093 | GO:0060166 | IEA |
| 507093 | GO:0043065 | IEA |
| 507093 | GO:0002072 | IEA |
| 507095 | GO:0043407 | IEA |
| 507095 | GO:0001656 | IEA |
| 507095 | GO:0001759 | IEA |
| 507095 | GO:0001657 | IEA |
| 507107 | GO:0005975 | IEA |
| 507114 | GO:0006915 | IEA |
| 507179 | GO:0051726 | IEA |
| 507196 | GO:0016337 | IEA |
| 507196 | GO:0007242 | IEA |
| 507196 | GO:0001525 | IEA |
| 507196 | GO:0031532 | IEA |
| 507196 | GO:0007507 | IEA |
| 507196 | GO:0007242 | IEA |
| 507196 | GO:0001525 | IEA |
| 507196 | GO:0007507 | IEA |
| 507196 | GO:0031532 | IEA |
| 507196 | GO:0016337 | IEA |
| 507213 | GO:0006511 | IEA |
| 507216 | GO:0006810 | IEA |
| 507216 | GO:0006820 | IEA |
| 507216 | GO:0006811 | IEA |
| 507319 | GO:0030182 | IEA |
| 507319 | GO:0048013 | IEA |
| 507319 | GO:0000187 | IEA |
| 507319 | GO:0050770 | IEA |
| 507365 | GO:0035023 | IEA |
| 507365 | GO:0007242 | IEA |
| 507410 | GO:0008033 | IEA |
| 507431 | GO:0008152 | IEA |
| 507476 | GO:0006096 | IEA |
| 507632 | GO:0015758 | IEA |
| 507783 | GO:0008284 | IEA |
| 507783 | GO:0008285 | IEA |
| 507783 | GO:0048469 | IEA |
| 507783 | GO:0030282 | IEA |
| 507783 | GO:0045453 | IEA |
| 507783 | GO:0007200 | IEA |
| 507783 | GO:0007186 | IEA |
| 507783 | GO:0002062 | IEA |
| 507783 | GO:0001501 | IEA |
| 507783 | GO:0001503 | IEA |
| 507783 | GO:0007189 | IEA |
| 507783 | GO:0002076 | IEA |
| 507783 | GO:0006874 | IEA |
| 507783 | GO:0007165 | IEA |
| 508090 | GO:0007165 | IEA |
| 508137 | GO:0006457 | IEA |
| 508143 | GO:0006810 | IEA |
| 508143 | GO:0006412 | IEA |
| 508175 | GO:0050772 | IEA |
| 508268 | GO:0007283 | IEA |
| 508268 | GO:0007155 | IEA |
| 508268 | GO:0008366 | IEA |
| 508285 | GO:0045226 | IEA |
| 508388 | GO:0007242 | IEA |
| 508442 | GO:0006333 | IEA |
| 508442 | GO:0006338 | IEA |
| 508465 | GO:0030968 | ISS |
| 508465 | GO:0008284 | ISS |
| 508465 | GO:0030970 | ISS |
| 508465 | GO:0030307 | IEA |
| 508465 | GO:0030307 | ISS |
| 508465 | GO:0030970 | IEA |
| 508465 | GO:0030968 | IEA |
| 508465 | GO:0030433 | ISS |
| 508465 | GO:0030433 | IEA |
| 508465 | GO:0008284 | IEA |
| 508535 | GO:0031503 | IEA |
| 508535 | GO:0006463 | IEA |
| 508535 | GO:0007566 | IEA |
| 508535 | GO:0006457 | IEA |
| 508535 | GO:0046661 | IEA |
| 508566 | GO:0006118 | IEA |
| 508712 | GO:0009058 | IEA |
| 508722 | GO:0006397 | IEA |
| 508724 | GO:0006527 | IEA |
| 508840 | GO:0016337 | IEA |
| 508840 | GO:0009566 | IEA |
| 508840 | GO:0007286 | IEA |
| 508990 | GO:0001523 | IEA |
| 509037 | GO:0045454 | IEA |
| 509037 | GO:0006916 | IEA |
| 509192 | GO:0006998 | IEA |
| 509221 | GO:0006898 | IEA |
| 509221 | GO:0006907 | IEA |
| 509221 | GO:0000904 | IEA |
| 509221 | GO:0001701 | IEA |
| 509221 | GO:0007588 | IEA |
| 509273 | GO:0008152 | IEA |
| 509459 | GO:0006629 | IEA |
| 509459 | GO:0006810 | IEA |
| 509459 | GO:0006631 | IEA |
| 509619 | GO:0016556 | IEA |
| 509619 | GO:0006397 | IEA |
| 509684 | GO:0006810 | IEA |
| 509696 | GO:0006744 | IEA |
| 509696 | GO:0008299 | IEA |
| 509696 | GO:0051290 | IEA |
| 509816 | GO:0006412 | IEA |
| 509816 | GO:0043043 | IC |
| 509983 | GO:0006099 | IEA |
| 509983 | GO:0008152 | IEA |
| 510029 | GO:0030163 | IEA |
| 510050 | GO:0045449 | IEA |
| 510050 | GO:0006355 | IEA |
| 510069 | GO:0006511 | IEA |
| 510109 | GO:0006725 | IEA |
| 510155 | GO:0006511 | IEA |
| 510159 | GO:0007049 | IEA |
| 510159 | GO:0051301 | IEA |
| 510159 | GO:0007067 | IEA |
| 510194 | GO:0006334 | IEA |
| 510200 | GO:0006508 | IEA |
| 510219 | GO:0006820 | IEA |
| 510245 | GO:0006470 | IEA |
| 510264 | GO:0008152 | IEA |
| 510314 | GO:0006955 | IEA |
| 510314 | GO:0006457 | IEA |
| 510322 | GO:0006397 | IEA |
| 510376 | GO:0006357 | IEA |
| 510376 | GO:0006357 | IEA |
| 510423 | GO:0006511 | IEA |
| 510434 | GO:0006468 | IEA |
| 510462 | GO:0006118 | IEA |
| 510469 | GO:0006396 | IEA |
| 510483 | GO:0006506 | IEA |
| 510498 | GO:0051262 | IEA |
| 510568 | GO:0006611 | IEA |
| 510574 | GO:0007034 | IEA |
| 510575 | GO:0006917 | IEA |
| 510575 | GO:0006915 | IEA |
| 510679 | GO:0006783 | IEA |
| 510679 | GO:0008152 | IEA |
| 510679 | GO:0006779 | IEA |
| 510729 | GO:0006388 | IEA |
| 510767 | GO:0006810 | IEA |
| 510767 | GO:0006118 | IEA |
| 510833 | GO:0006817 | IEA |
| 510833 | GO:0006817 | IEA |
| 510889 | GO:0007243 | IEA |
| 510889 | GO:0006508 | IEA |
| 510889 | GO:0006518 | IEA |
| 510899 | GO:0006412 | IEA |
| 510937 | GO:0006427 | IEA |
| 510937 | GO:0006418 | IEA |
| 510937 | GO:0006412 | IEA |
| 510960 | GO:0043691 | IEA |
| 510960 | GO:0006629 | IEA |
| 510960 | GO:0042632 | IEA |
| 510960 | GO:0008203 | IEA |
| 510960 | GO:0006656 | IEA |
| 510960 | GO:0042158 | IEA |
| 510960 | GO:0006656 | IEA |
| 510960 | GO:0008203 | IEA |
| 510960 | GO:0043691 | IEA |
| 510960 | GO:0042632 | IEA |
| 510960 | GO:0042158 | IEA |
| 510960 | GO:0006629 | IEA |
| 511007 | GO:0006512 | IEA |
| 511047 | GO:0005978 | IEA |
| 511047 | GO:0007507 | IEA |
| 511090 | GO:0008152 | IEA |
| 511200 | GO:0006177 | IEA |
| 511200 | GO:0009058 | IEA |
| 511200 | GO:0006164 | IEA |
| 511200 | GO:0006541 | IEA |
| 511245 | GO:0050655 | IEA |
| 511268 | GO:0006457 | IEA |
| 511291 | GO:0043066 | IEA |
| 511291 | GO:0008654 | IEA |
| 511291 | GO:0043154 | IEA |
| 511291 | GO:0006663 | IEA |
| 511433 | GO:0001843 | IEA |
| 511433 | GO:0030900 | IEA |
| 511433 | GO:0048041 | IEA |
| 511433 | GO:0003007 | IEA |
| 511433 | GO:0021575 | IEA |
| 511433 | GO:0030036 | IEA |
| 511440 | GO:0006784 | IEA |
| 511440 | GO:0009060 | IEA |
| 511440 | GO:0000266 | IEA |
| 511440 | GO:0007005 | IEA |
| 511440 | GO:0017004 | IEA |
| 511440 | GO:0008535 | IEA |
| 511453 | GO:0006465 | IEA |
| 511560 | GO:0006412 | IEA |
| 511560 | GO:0006364 | IEA |
| 511614 | GO:0008610 | IEA |
| 511614 | GO:0019432 | IEA |
| 511614 | GO:0046339 | IEA |
| 511614 | GO:0030879 | IEA |
| 511614 | GO:0006631 | IEA |
| 511614 | GO:0006641 | IEA |
| 511614 | GO:0007595 | IEA |
| 511614 | GO:0002071 | IEA |
| 511614 | GO:0040014 | IEA |
| 511624 | GO:0006744 | IEA |
| 511624 | GO:0008152 | IEA |
| 511624 | GO:0006725 | IEA |
| 511624 | GO:0006118 | IEA |
| 511682 | GO:0006629 | IEA |
| 511682 | GO:0042990 | IEA |
| 511699 | GO:0006821 | IEA |
| 511735 | GO:0015671 | IEA |
| 511735 | GO:0006810 | IEA |
| 511735 | GO:0015671 | IEA |
| 511735 | GO:0006810 | IEA |
| 511735 | GO:0015671 | IEA |
| 511735 | GO:0006810 | IEA |
| 511798 | GO:0006512 | IEA |
| 511798 | GO:0006512 | IEA |
| 511798 | GO:0006512 | IEA |
| 511812 | GO:0007186 | IEA |
| 511812 | GO:0007165 | IEA |
| 511861 | GO:0006917 | IEA |
| 512002 | GO:0006573 | IEA |
| 512002 | GO:0006098 | IEA |
| 512136 | GO:0006405 | IEA |
| 512136 | GO:0016973 | IEA |
| 512136 | GO:0006810 | IEA |
| 512259 | GO:0006629 | IEA |
| 512259 | GO:0045786 | ISS |
| 512259 | GO:0006693 | ISS |
| 512259 | GO:0007565 | ISS |
| 512259 | GO:0019372 | ISS |
| 512259 | GO:0006631 | IEA |
| 512259 | GO:0008152 | IEA |
| 512259 | GO:0007179 | ISS |
| 512259 | GO:0007567 | ISS |
| 512259 | GO:0007565 | ISS |
| 512259 | GO:0006693 | ISS |
| 512259 | GO:0007567 | ISS |
| 512259 | GO:0045786 | ISS |
| 512259 | GO:0006631 | IEA |
| 512259 | GO:0007179 | ISS |
| 512259 | GO:0008152 | IEA |
| 512259 | GO:0019372 | ISS |
| 512259 | GO:0006629 | IEA |
| 512350 | GO:0006412 | IEA |
| 512350 | GO:0006413 | ISS |
| 512350 | GO:0006412 | IEA |
| 512350 | GO:0006413 | ISS |
| 512427 | GO:0008283 | ISS |
| 512427 | GO:0001824 | ISS |
| 512427 | GO:0043486 | ISS |
| 512427 | GO:0007049 | IEA |
| 512427 | GO:0006260 | IEA |
| 512427 | GO:0015031 | IEA |
| 512427 | GO:0006810 | IEA |
| 512469 | GO:0006412 | IEA |
| 512500 | GO:0006810 | IEA |
| 512500 | GO:0006118 | IEA |
| 512531 | GO:0006412 | IEA |
| 512531 | GO:0006431 | IEA |
| 512531 | GO:0006418 | IEA |
| 512531 | GO:0006431 | IEA |
| 512531 | GO:0006412 | IEA |
| 512531 | GO:0006418 | IEA |
| 512571 | GO:0006096 | IEA |
| 512666 | GO:0006916 | IEA |
| 512673 | GO:0043681 | IEA |
| 512741 | GO:0006334 | IEA |
| 512741 | GO:0006334 | IEA |
| 512741 | GO:0006334 | IEA |
| 512741 | GO:0006334 | IEA |
| 512741 | GO:0006334 | IEA |
| 512771 | GO:0006526 | IEA |
| 512771 | GO:0008652 | IEA |
| 512771 | GO:0042450 | IEA |
| 512818 | GO:0006810 | IEA |
| 512818 | GO:0045039 | IEA |
| 512818 | GO:0006605 | IEA |
| 512818 | GO:0006457 | IEA |
| 512818 | GO:0006626 | IEA |
| 512818 | GO:0015031 | IEA |
| 512891 | GO:0006508 | IEA |
| 512978 | GO:0006397 | IEA |
| 512978 | GO:0006810 | IEA |
| 512978 | GO:0008380 | IEA |
| 512978 | GO:0051028 | IEA |
| 513038 | GO:0006508 | IEA |
| 513155 | GO:0019835 | IEA |
| 513188 | GO:0006508 | IEA |
| 513265 | GO:0031647 | IEA |
| 513265 | GO:0030308 | IEA |
| 513265 | GO:0009967 | IEA |
| 513530 | GO:0051792 | IEA |
| 513530 | GO:0051790 | IEA |
| 513530 | GO:0006637 | IEA |
| 513530 | GO:0006633 | IEA |
| 513573 | GO:0006457 | IEA |
| 513586 | GO:0005975 | IEA |
| 513699 | GO:0017157 | IEA |
| 513753 | GO:0008284 | IEA |
| 513753 | GO:0001755 | IEA |
| 513753 | GO:0030318 | IEA |
| 513753 | GO:0030182 | IEA |
| 513762 | GO:0009268 | IEA |
| 513762 | GO:0010033 | IEA |
| 513762 | GO:0045454 | IEA |
| 513762 | GO:0042542 | IEA |
| 513762 | GO:0006118 | IEA |
| 513843 | GO:0006952 | IEA |
| 513889 | GO:0015031 | IEA |
| 513889 | GO:0006810 | IEA |
| 513889 | GO:0006605 | IEA |
| 513934 | GO:0045449 | IEA |
| 513958 | GO:0042552 | ISS |
| 513958 | GO:0007417 | ISS |
| 513958 | GO:0014003 | IEA |
| 513958 | GO:0042552 | IEA |
| 513958 | GO:0051716 | ISS |
| 513958 | GO:0044237 | IEA |
| 513958 | GO:0014003 | ISS |
| 513958 | GO:0051716 | IEA |
| 513958 | GO:0006412 | IEA |
| 513958 | GO:0006413 | ISS |
| 513958 | GO:0001541 | ISS |
| 513958 | GO:0001541 | IEA |
| 513958 | GO:0007417 | IEA |
| 514044 | GO:0008152 | IEA |
| 514066 | GO:0005975 | IEA |
| 514066 | GO:0006094 | IEA |
| 514230 | GO:0044267 | IEA |
| 514386 | GO:0042981 | IEA |
| 514386 | GO:0006916 | IEA |
| 514403 | GO:0006412 | IEA |
| 514446 | GO:0007275 | IEA |
| 514446 | GO:0007275 | IEA |
| 514446 | GO:0007275 | IEA |
| 514472 | GO:0006457 | IEA |
| 514473 | GO:0030900 | IEA |
| 514665 | GO:0006810 | IEA |
| 514665 | GO:0006874 | IEA |
| 514852 | GO:0007266 | IEA |
| 514859 | GO:0043526 | IEA |
| 514859 | GO:0045884 | IEA |
| 514918 | GO:0045449 | IEA |
| 514918 | GO:0045449 | IEA |
| 514928 | GO:0006890 | IEA |
| 515022 | GO:0030001 | IEA |
| 515022 | GO:0006801 | IEA |
| 515063 | GO:0007399 | IEA |
| 515063 | GO:0007275 | IEA |
| 515063 | GO:0030154 | IEA |
| 515109 | GO:0006457 | IEA |
| 515109 | GO:0045039 | IEA |
| 515109 | GO:0006810 | IEA |
| 515109 | GO:0006626 | IEA |
| 515109 | GO:0006605 | IEA |
| 515109 | GO:0015031 | IEA |
| 515169 | GO:0009156 | IEA |
| 515169 | GO:0009165 | IEA |
| 515169 | GO:0009116 | IEA |
| 515176 | GO:0006308 | IEA |
| 515178 | GO:0006810 | IEA |
| 515320 | GO:0006355 | IEA |
| 515363 | GO:0006350 | IEA |
| 515363 | GO:0006355 | IEA |
| 515380 | GO:0030154 | IEA |
| 515380 | GO:0007275 | IEA |
| 515380 | GO:0006350 | IEA |
| 515380 | GO:0006355 | IEA |
| 515490 | GO:0007264 | IEA |
| 515494 | GO:0006869 | IEA |
| 515494 | GO:0006629 | IEA |
| 515503 | GO:0006511 | IEA |
| 515614 | GO:0006783 | IEA |
| 515614 | GO:0006779 | IEA |
| 515646 | GO:0006810 | IEA |
| 515646 | GO:0006821 | IEA |
| 515646 | GO:0006811 | IEA |
| 515696 | GO:0015992 | IEA |
| 515696 | GO:0006811 | IEA |
| 515696 | GO:0015986 | IEA |
| 515696 | GO:0006754 | IEA |
| 515696 | GO:0006810 | IEA |
| 515757 | GO:0030163 | IEA |
| 515757 | GO:0006508 | IEA |
| 515757 | GO:0006508 | IEA |
| 515757 | GO:0030163 | IEA |
| 515765 | GO:0006260 | IEA |
| 515783 | GO:0001501 | IEA |
| 515946 | GO:0008152 | IEA |
| 515990 | GO:0006412 | IEA |
| 515999 | GO:0006888 | IEA |
| 515999 | GO:0015031 | IEA |
| 515999 | GO:0006886 | IEA |
| 515999 | GO:0007264 | IEA |
| 515999 | GO:0006810 | IEA |
| 515999 | GO:0006810 | IEA |
| 515999 | GO:0006888 | IEA |
| 515999 | GO:0015031 | IEA |
| 515999 | GO:0007264 | IEA |
| 515999 | GO:0006886 | IEA |
| 516039 | GO:0000165 | IEA |
| 516039 | GO:0060048 | IEA |
| 516039 | GO:0042035 | IEA |
| 516039 | GO:0006954 | IEA |
| 516039 | GO:0000187 | IEA |
| 516287 | GO:0006118 | IEA |
| 516287 | GO:0006118 | IEA |
| 516619 | GO:0018346 | IEA |
| 516619 | GO:0018344 | IEA |
| 516619 | GO:0018346 | IEA |
| 516619 | GO:0018344 | IEA |
| 516874 | GO:0006390 | IEA |
| 516874 | GO:0000154 | IEA |
| 517139 | GO:0006334 | IEA |
| 517139 | GO:0006334 | IEA |
| 517139 | GO:0006334 | IEA |
| 517139 | GO:0006334 | IEA |
| 517139 | GO:0006334 | IEA |
| 517171 | GO:0006888 | IEA |
| 517171 | GO:0015031 | IEA |
| 517171 | GO:0006886 | IEA |
| 517171 | GO:0007264 | IEA |
| 517171 | GO:0016192 | IEA |
| 517171 | GO:0006810 | IEA |
| 517345 | GO:0007264 | IEA |
| 517402 | GO:0008152 | IEA |
| 517405 | GO:0042475 | IEA |
| 517405 | GO:0009912 | IEA |
| 517405 | GO:0016331 | IEA |
| 517405 | GO:0030155 | IEA |
| 517405 | GO:0001501 | IEA |
| 517405 | GO:0042492 | IEA |
| 517405 | GO:0042981 | IEA |
| 517535 | GO:0006986 | IEA |
| 517535 | GO:0006457 | IEA |
| 517535 | GO:0006611 | IEA |
| 517539 | GO:0009396 | IEA |
| 517688 | GO:0051928 | IEA |
| 517688 | GO:0051928 | IEA |
| 517812 | GO:0006260 | IEA |
| 517812 | GO:0006355 | IEA |
| 517812 | GO:0006350 | IEA |
| 517812 | GO:0007049 | IEA |
| 517812 | GO:0006270 | IEA |
| 517812 | GO:0006268 | IEA |
| 518045 | GO:0006396 | IEA |
| 518117 | GO:0015992 | IEA |
| 518117 | GO:0006812 | IEA |
| 518117 | GO:0008152 | IEA |
| 518117 | GO:0006811 | IEA |
| 518117 | GO:0006810 | IEA |
| 518117 | GO:0006816 | IEA |
| 518117 | GO:0008152 | IEA |
| 518117 | GO:0015992 | IEA |
| 518117 | GO:0006810 | IEA |
| 518117 | GO:0006816 | IEA |
| 518117 | GO:0006812 | IEA |
| 518117 | GO:0006811 | IEA |
| 518169 | GO:0006508 | IEA |
| 518318 | GO:0006334 | IEA |
| 518318 | GO:0006334 | IEA |
| 518318 | GO:0006334 | IEA |
| 518318 | GO:0006334 | IEA |
| 518318 | GO:0006334 | IEA |
| 518540 | GO:0001757 | IEA |
| 518601 | GO:0006888 | IEA |
| 518601 | GO:0006810 | IEA |
| 518601 | GO:0015031 | IEA |
| 518601 | GO:0007264 | IEA |
| 518659 | GO:0042384 | IEA |
| 518659 | GO:0043584 | IEA |
| 518659 | GO:0043010 | IEA |
| 518659 | GO:0035116 | IEA |
| 518659 | GO:0042305 | IEA |
| 518659 | GO:0035108 | IEA |
| 518659 | GO:0007382 | IEA |
| 518659 | GO:0007368 | IEA |
| 518659 | GO:0035115 | IEA |
| 518659 | GO:0007420 | IEA |
| 518659 | GO:0008589 | IEA |
| 518659 | GO:0001701 | IEA |
| 518659 | GO:0021532 | IEA |
| 518659 | GO:0021772 | IEA |
| 518659 | GO:0021537 | IEA |
| 518659 | GO:0022038 | IEA |
| 518659 | GO:0001822 | IEA |
| 518659 | GO:0060039 | IEA |
| 518659 | GO:0001889 | IEA |
| 518659 | GO:0021549 | IEA |
| 518659 | GO:0021670 | IEA |
| 518758 | GO:0006810 | IEA |
| 518758 | GO:0006810 | IEA |
| 519213 | GO:0006506 | IEA |
| 519302 | GO:0006412 | IEA |
| 519302 | GO:0006412 | IEA |
| 519439 | GO:0006355 | IEA |
| 519439 | GO:0001570 | IEA |
| 519439 | GO:0001525 | IEA |
| 519632 | GO:0006979 | IEA |
| 519632 | GO:0006118 | IEA |
| 519777 | GO:0016571 | IEA |
| 519958 | GO:0030324 | IEA |
| 519958 | GO:0000185 | IEA |
| 519958 | GO:0007179 | IEA |
| 519958 | GO:0003007 | IEA |
| 519958 | GO:0001701 | IEA |
| 520260 | GO:0006106 | IEA |
| 520260 | GO:0048873 | IEA |
| 520260 | GO:0006106 | IEA |
| 520260 | GO:0048873 | IEA |
| 520335 | GO:0051205 | IEA |
| 520472 | GO:0009435 | IEA |
| 520631 | GO:0007275 | IEA |
| 520631 | GO:0009314 | IEA |
| 520631 | GO:0006355 | IEA |
| 520631 | GO:0006350 | IEA |
| 520694 | GO:0043491 | IEA |
| 520694 | GO:0031175 | IEA |
| 520694 | GO:0048715 | IEA |
| 520694 | GO:0021954 | IEA |
| 520891 | GO:0042254 | IEA |
| 520891 | GO:0006364 | IEA |
| 521073 | GO:0006605 | IEA |
| 521073 | GO:0015031 | IEA |
| 521073 | GO:0006457 | IEA |
| 521073 | GO:0006626 | IEA |
| 521073 | GO:0045039 | IEA |
| 521073 | GO:0006810 | IEA |
| 521237 | GO:0006468 | IEA |
| 521378 | GO:0045449 | IEA |
| 521581 | GO:0006334 | IEA |
| 521581 | GO:0006334 | IEA |
| 521581 | GO:0006334 | IEA |
| 521581 | GO:0006334 | IEA |
| 521581 | GO:0006334 | IEA |
| 521747 | GO:0006412 | IEA |
| 521962 | GO:0009952 | IEA |
| 521962 | GO:0007379 | IEA |
| 522980 | GO:0006511 | IEA |
| 522980 | GO:0006511 | IEA |
| 523039 | GO:0048666 | IEA |
| 523039 | GO:0040014 | IEA |
| 523039 | GO:0007628 | IEA |
| 523039 | GO:0007005 | IEA |
| 523039 | GO:0008629 | IEA |
| 523039 | GO:0008344 | IEA |
| 523039 | GO:0030900 | IEA |
| 523214 | GO:0006334 | IEA |
| 523214 | GO:0006334 | IEA |
| 523214 | GO:0006334 | IEA |
| 523214 | GO:0006334 | IEA |
| 523214 | GO:0006334 | IEA |
| 523294 | GO:0016051 | IEA |
| 523325 | GO:0001505 | IEA |
| 523325 | GO:0007528 | IEA |
| 523325 | GO:0048813 | IEA |
| 523325 | GO:0043113 | IEA |
| 523325 | GO:0050808 | IEA |
| 523325 | GO:0007269 | IEA |
| 523745 | GO:0043087 | IEA |
| 523745 | GO:0007265 | IEA |
| 523752 | GO:0006508 | IEA |
| 524075 | GO:0018106 | IEA |
| 525042 | GO:0005975 | IEA |
| 525042 | GO:0046168 | IEA |
| 525042 | GO:0006072 | IEA |
| 525042 | GO:0006094 | IEA |
| 525164 | GO:0006916 | IEA |
| 525365 | GO:0006631 | IEA |
| 525511 | GO:0006334 | IEA |
| 525511 | GO:0006334 | IEA |
| 525511 | GO:0006334 | IEA |
| 525511 | GO:0006334 | IEA |
| 525511 | GO:0006334 | IEA |
| 525680 | GO:0006457 | IEA |
| 525716 | GO:0006417 | NAS |
| 525716 | GO:0006874 | ISS |
| 525716 | GO:0042035 | ISS |
| 525716 | GO:0048168 | IEA |
| 525716 | GO:0007409 | NAS |
| 525716 | GO:0006917 | ISS |
| 525716 | GO:0048143 | NAS |
| 525716 | GO:0045917 | NAS |
| 525716 | GO:0006112 | ISS |
| 525716 | GO:0048169 | ISS |
| 525716 | GO:0048151 | NAS |
| 525716 | GO:0007611 | ISS |
| 525716 | GO:0007613 | IEA |
| 525795 | GO:0043113 | IEA |
| 525795 | GO:0045213 | IEA |
| 525795 | GO:0007009 | IEA |
| 525795 | GO:0007528 | IEA |
| 525795 | GO:0008582 | IEA |
| 525795 | GO:0045944 | IEA |
| 525813 | GO:0006108 | IEA |
| 526027 | GO:0007182 | IEA |
| 526027 | GO:0007184 | IEA |
| 526027 | GO:0007182 | IEA |
| 526027 | GO:0007184 | IEA |
| 526430 | GO:0016477 | IEA |
| 526430 | GO:0030032 | IEA |
| 526430 | GO:0007015 | IEA |
| 526430 | GO:0030032 | IEA |
| 526430 | GO:0007015 | IEA |
| 526430 | GO:0016477 | IEA |
| 526652 | GO:0045786 | IEA |
| 526652 | GO:0007049 | IEA |
| 527488 | GO:0007528 | IEA |
| 527935 | GO:0045941 | IEA |
| 527935 | GO:0016481 | IEA |
| 528265 | GO:0006810 | IEA |
| 528265 | GO:0007154 | IEA |
| 528265 | GO:0015031 | IEA |
| 528390 | GO:0006355 | IEA |
| 529115 | GO:0045892 | IEA |
| 529124 | GO:0008015 | IEA |
| 529124 | GO:0006350 | IEA |
| 529124 | GO:0006355 | IEA |
| 529124 | GO:0009887 | IEA |
| 529670 | GO:0001568 | IEA |
| 529670 | GO:0030334 | IEA |
| 529670 | GO:0045995 | IEA |
| 529670 | GO:0030155 | IEA |
| 530393 | GO:0006874 | IEA |
| 530393 | GO:0006811 | IEA |
| 530393 | GO:0006816 | IEA |
| 530393 | GO:0006810 | IEA |
| 530393 | GO:0007368 | IEA |
| 530393 | GO:0050982 | IEA |
| 530393 | GO:0007259 | IEA |
| 530393 | GO:0007050 | IEA |
| 530393 | GO:0006810 | IEA |
| 530393 | GO:0007368 | IEA |
| 530393 | GO:0007259 | IEA |
| 530393 | GO:0006811 | IEA |
| 530393 | GO:0007050 | IEA |
| 530393 | GO:0006816 | IEA |
| 530393 | GO:0050982 | IEA |
| 530393 | GO:0006874 | IEA |
| 530393 | GO:0007050 | IEA |
| 530393 | GO:0007368 | IEA |
| 530393 | GO:0006816 | IEA |
| 530393 | GO:0006810 | IEA |
| 530393 | GO:0006811 | IEA |
| 530393 | GO:0007259 | IEA |
| 530393 | GO:0050982 | IEA |
| 530393 | GO:0006874 | IEA |
| 530409 | GO:0006260 | IEA |
| 530616 | GO:0006355 | IEA |
| 530865 | GO:0009105 | IEA |
| 530865 | GO:0009107 | IEA |
| 530865 | GO:0008152 | IEA |
| 531770 | GO:0030155 | IEA |
| 531877 | GO:0006470 | IEA |
| 531990 | GO:0006334 | IEA |
| 531990 | GO:0006334 | IEA |
| 531990 | GO:0006334 | IEA |
| 531990 | GO:0006334 | IEA |
| 531990 | GO:0006334 | IEA |
| 532120 | GO:0030534 | IEA |
| 532120 | GO:0007286 | IEA |
| 532120 | GO:0035058 | IEA |
| 532120 | GO:0045494 | IEA |
| 532120 | GO:0046548 | IEA |
| 532120 | GO:0045724 | IEA |
| 532120 | GO:0019216 | IEA |
| 532120 | GO:0007608 | IEA |
| 532120 | GO:0001843 | IEA |
| 532120 | GO:0016358 | IEA |
| 532120 | GO:0000226 | IEA |
| 532120 | GO:0001895 | IEA |
| 532203 | GO:0006412 | IEA |
| 532231 | GO:0006096 | IEA |
| 532231 | GO:0006006 | IEA |
| 532389 | GO:0006396 | IEA |
| 532483 | GO:0045786 | IEA |
| 532483 | GO:0006355 | IEA |
| 532483 | GO:0007049 | IEA |
| 532483 | GO:0006355 | IEA |
| 532483 | GO:0007049 | IEA |
| 532483 | GO:0045786 | IEA |
| 532572 | GO:0007155 | IEA |
| 532572 | GO:0022617 | IEA |
| 532572 | GO:0016477 | IEA |
| 532572 | GO:0006461 | IEA |
| 532572 | GO:0031581 | IEA |
| 532621 | GO:0016481 | IEA |
| 532621 | GO:0009887 | IEA |
| 532621 | GO:0001503 | IEA |
| 532621 | GO:0006350 | IEA |
| 532621 | GO:0008016 | IEA |
| 532621 | GO:0001502 | IEA |
| 532621 | GO:0006355 | IEA |
| 532785 | GO:0006631 | IEA |
| 532795 | GO:0006414 | IEA |
| 532795 | GO:0006412 | IEA |
| 532945 | GO:0006118 | IEA |
| 533077 | GO:0001764 | IEA |
| 533077 | GO:0006635 | IEA |
| 533077 | GO:0016558 | IEA |
| 533077 | GO:0001958 | IEA |
| 533077 | GO:0006625 | IEA |
| 533336 | GO:0006464 | IEA |
| 533336 | GO:0006512 | IEA |
| 533379 | GO:0002318 | IEA |
| 533379 | GO:0030154 | IEA |
| 533379 | GO:0006350 | IEA |
| 533379 | GO:0007275 | IEA |
| 533379 | GO:0002318 | IEA |
| 533379 | GO:0030154 | IEA |
| 533379 | GO:0006350 | IEA |
| 533379 | GO:0007275 | IEA |
| 533379 | GO:0007275 | IEA |
| 533379 | GO:0006350 | IEA |
| 533379 | GO:0030154 | IEA |
| 533379 | GO:0002318 | IEA |
| 533430 | GO:0045449 | IEA |
| 533611 | GO:0007288 | IEA |
| 533611 | GO:0040015 | IEA |
| 533611 | GO:0008104 | IEA |
| 533611 | GO:0030534 | IEA |
| 533611 | GO:0045494 | IEA |
| 533630 | GO:0008652 | IEA |
| 533630 | GO:0006564 | IEA |
| 533630 | GO:0008152 | IEA |
| 533630 | GO:0008152 | IEA |
| 533630 | GO:0008652 | IEA |
| 533630 | GO:0006564 | IEA |
| 533643 | GO:0045941 | IEA |
| 533660 | GO:0006457 | IEA |
| 533785 | GO:0006096 | IEA |
| 533851 | GO:0030036 | IEA |
| 533851 | GO:0030036 | IEA |
| 533917 | GO:0008152 | IEA |
| 533918 | GO:0008654 | IEA |
| 533918 | GO:0051084 | IEA |
| 533918 | GO:0001844 | IEA |
| 533918 | GO:0006654 | IEA |
| 533918 | GO:0006916 | IEA |
| 533953 | GO:0032496 | IEA |
| 533953 | GO:0019221 | IEA |
| 533953 | GO:0006468 | IEA |
| 533953 | GO:0031663 | IEA |
| 533953 | GO:0007165 | IEA |
| 533993 | GO:0051726 | IEA |
| 533993 | GO:0007094 | IEA |
| 533993 | GO:0042981 | IEA |
| 534182 | GO:0006457 | IEA |
| 534216 | GO:0007049 | IEA |
| 534216 | GO:0045786 | IEA |
| 534232 | GO:0045449 | IEA |
| 534232 | GO:0006351 | IEA |
| 534249 | GO:0006512 | IEA |
| 534249 | GO:0007242 | IEA |
| 534319 | GO:0008218 | IEA |
| 534319 | GO:0018298 | IEA |
| 534319 | GO:0007160 | IEA |
| 534319 | GO:0032836 | IEA |
| 534352 | GO:0006412 | IEA |
| 534391 | GO:0006397 | IEA |
| 534391 | GO:0006810 | IEA |
| 534391 | GO:0051028 | IEA |
| 534391 | GO:0008380 | IEA |
| 534402 | GO:0005975 | IEA |
| 534488 | GO:0008643 | IEA |
| 534546 | GO:0006508 | IEA |
| 534551 | GO:0006986 | IEA |
| 534551 | GO:0006457 | IEA |
| 534599 | GO:0006096 | IEA |
| 534599 | GO:0008152 | IEA |
| 534615 | GO:0008634 | ISS |
| 534615 | GO:0051607 | ISS |
| 534615 | GO:0043065 | IEA |
| 534615 | GO:0006917 | ISS |
| 534615 | GO:0006915 | IEA |
| 534615 | GO:0006915 | IEA |
| 534615 | GO:0008634 | ISS |
| 534615 | GO:0043065 | IEA |
| 534615 | GO:0051607 | ISS |
| 534615 | GO:0006917 | ISS |
| 534640 | GO:0006511 | IEA |
| 534640 | GO:0043161 | IEA |
| 534640 | GO:0006979 | IEA |
| 534708 | GO:0007264 | IEA |
| 534708 | GO:0015031 | IEA |
| 534708 | GO:0006810 | IEA |
| 534927 | GO:0006508 | IEA |
| 535077 | GO:0016192 | IEA |
| 535090 | GO:0006606 | IEA |
| 535174 | GO:0008152 | IEA |
| 535174 | GO:0006635 | IEA |
| 535182 | GO:0006100 | IEA |
| 535182 | GO:0006099 | IEA |
| 535182 | GO:0006108 | IEA |
| 535182 | GO:0006096 | IEA |
| 535185 | GO:0007155 | IEA |
| 535185 | GO:0007596 | IEA |
| 535227 | GO:0006118 | IEA |
| 535273 | GO:0016049 | IEA |
| 535320 | GO:0030154 | IEA |
| 535320 | GO:0007275 | IEA |
| 535390 | GO:0006725 | IEA |
| 535424 | GO:0006955 | IEA |
| 535447 | GO:0008380 | IEA |
| 535447 | GO:0006397 | IEA |
| 535447 | GO:0016071 | IEA |
| 535601 | GO:0005975 | IEA |
| 535680 | GO:0006166 | IEA |
| 535820 | GO:0031018 | IEA |
| 535820 | GO:0001501 | IEA |
| 535820 | GO:0031016 | IEA |
| 535820 | GO:0032092 | IEA |
| 535820 | GO:0051260 | IEA |
| 535820 | GO:0002063 | IEA |
| 535820 | GO:0030282 | IEA |
| 535820 | GO:0019722 | IEA |
| 535820 | GO:0006919 | IEA |
| 535820 | GO:0008632 | IEA |
| 535820 | GO:0030968 | IEA |
| 535820 | GO:0006468 | IEA |
| 535820 | GO:0031642 | IEA |
| 535820 | GO:0030073 | IEA |
| 535820 | GO:0006412 | IEA |
| 535820 | GO:0017148 | IEA |
| 535820 | GO:0046777 | IEA |
| 535820 | GO:0006926 | IEA |
| 535820 | GO:0048009 | IEA |
| 535820 | GO:0007029 | IEA |
| 535820 | GO:0006983 | IEA |
| 535869 | GO:0006508 | IEA |
| 535869 | GO:0006512 | IEA |
| 535869 | GO:0042036 | IEA |
| 535981 | GO:0006508 | IEA |
| 536149 | GO:0007283 | IEA |
| 536149 | GO:0043087 | IEA |
| 536149 | GO:0030154 | IEA |
| 536149 | GO:0006810 | IEA |
| 536149 | GO:0007275 | IEA |
| 536203 | GO:0006810 | IEA |
| 536337 | GO:0006470 | IEA |
| 536359 | GO:0015031 | IEA |
| 536359 | GO:0006810 | IEA |
| 536359 | GO:0006886 | IEA |
| 536553 | GO:0006810 | IEA |
| 536553 | GO:0006886 | IEA |
| 536619 | GO:0006417 | IEA |
| 536619 | GO:0045947 | IEA |
| 537455 | GO:0016322 | IEA |
| 537539 | GO:0009437 | IEA |
| 537539 | GO:0008152 | IEA |
| 537539 | GO:0042136 | IEA |
| 537539 | GO:0006118 | IEA |
| 537539 | GO:0006081 | IEA |
| 537932 | GO:0001701 | IEA |
| 537932 | GO:0042048 | IEA |
| 537932 | GO:0001967 | IEA |
| 538054 | GO:0006412 | IEA |
| 538094 | GO:0006468 | IEA |
| 538094 | GO:0006468 | IEA |
| 538173 | GO:0001501 | IEA |
| 538173 | GO:0042475 | IEA |
| 538173 | GO:0042981 | IEA |
| 538173 | GO:0030155 | IEA |
| 538173 | GO:0008152 | IEA |
| 538173 | GO:0016331 | IEA |
| 538236 | GO:0006610 | IEA |
| 538409 | GO:0006508 | IEA |
| 538427 | GO:0006913 | ISS |
| 538427 | GO:0007242 | ISS |
| 538594 | GO:0006350 | IEA |
| 538594 | GO:0045814 | IEA |
| 538594 | GO:0051090 | IEA |
| 538594 | GO:0009615 | IEA |
| 538594 | GO:0016481 | IEA |
| 538702 | GO:0005975 | IEA |
| 538702 | GO:0006072 | IEA |
| 538829 | GO:0006470 | IEA |
| 538931 | GO:0016071 | IEA |
| 538975 | GO:0031032 | IEA |
| 539003 | GO:0045596 | IEA |
| 539003 | GO:0030154 | IEA |
| 539003 | GO:0045669 | IEA |
| 539003 | GO:0045892 | IEA |
| 539003 | GO:0045667 | IEA |
| 539003 | GO:0007507 | IEA |
| 539003 | GO:0030316 | IEA |
| 539003 | GO:0001569 | IEA |
| 539003 | GO:0060070 | IEA |
| 539003 | GO:0050808 | IEA |
| 539003 | GO:0009950 | IEA |
| 539003 | GO:0045671 | IEA |
| 539003 | GO:0008283 | IEA |
| 539003 | GO:0030097 | IEA |
| 539003 | GO:0001702 | IEA |
| 539003 | GO:0048489 | IEA |
| 539003 | GO:0045941 | IEA |
| 539003 | GO:0045944 | IEA |
| 539003 | GO:0042127 | IEA |
| 539003 | GO:0001706 | IEA |
| 539003 | GO:0006915 | IEA |
| 539003 | GO:0001708 | IEA |
| 539003 | GO:0048593 | IEA |
| 539003 | GO:0007160 | IEA |
| 539003 | GO:0016337 | IEA |
| 539003 | GO:0001709 | IEA |
| 539003 | GO:0042475 | IEA |
| 539003 | GO:0032331 | IEA |
| 539003 | GO:0000904 | IEA |
| 539003 | GO:0030324 | IEA |
| 539003 | GO:0006355 | IEA |
| 539003 | GO:0042733 | IEA |
| 539003 | GO:0030858 | IEA |
| 539003 | GO:0035116 | IEA |
| 539003 | GO:0045893 | IEA |
| 539003 | GO:0035117 | IEA |
| 539003 | GO:0030900 | IEA |
| 539003 | GO:0001501 | IEA |
| 539003 | GO:0000122 | IEA |
| 539003 | GO:0009987 | IEA |
| 539003 | GO:0045453 | IEA |
| 539003 | GO:0045449 | IEA |
| 539003 | GO:0048469 | IEA |
| 539003 | GO:0016331 | IEA |
| 539003 | GO:0009954 | IEA |
| 539003 | GO:0031016 | IEA |
| 539003 | GO:0016055 | IEA |
| 539003 | GO:0001711 | IEA |
| 539003 | GO:0007268 | IEA |
| 539003 | GO:0045595 | IEA |
| 539003 | GO:0009953 | IEA |
| 539003 | GO:0007398 | IEA |
| 539005 | GO:0006412 | IEA |
| 539035 | GO:0006508 | IEA |
| 539091 | GO:0006754 | IEA |
| 539091 | GO:0015986 | IEA |
| 539120 | GO:0001525 | IEA |
| 539120 | GO:0001885 | IEA |
| 539164 | GO:0007224 | IEA |
| 539164 | GO:0009953 | IEA |
| 539164 | GO:0001841 | IEA |
| 539164 | GO:0016485 | IEA |
| 539164 | GO:0007368 | IEA |
| 539188 | GO:0009058 | IEA |
| 539220 | GO:0006457 | IEA |
| 539220 | GO:0006986 | IEA |
| 539244 | GO:0006512 | IEA |
| 539244 | GO:0007242 | IEA |
| 539249 | GO:0006810 | IEA |
| 539308 | GO:0001570 | IEA |
| 539308 | GO:0009953 | IEA |
| 539308 | GO:0007417 | IEA |
| 539308 | GO:0007389 | IEA |
| 539308 | GO:0008284 | IEA |
| 539308 | GO:0001649 | IEA |
| 539308 | GO:0021953 | IEA |
| 539308 | GO:0007224 | IEA |
| 539308 | GO:0002052 | IEA |
| 539308 | GO:0021696 | IEA |
| 539308 | GO:0043066 | IEA |
| 539308 | GO:0042475 | IEA |
| 539308 | GO:0048468 | IEA |
| 539308 | GO:0043392 | IEA |
| 539308 | GO:0003007 | IEA |
| 539308 | GO:0021938 | IEA |
| 539308 | GO:0048589 | IEA |
| 539308 | GO:0007368 | IEA |
| 539308 | GO:0001755 | IEA |
| 539354 | GO:0008643 | IEA |
| 539354 | GO:0008643 | IEA |
| 539379 | GO:0045944 | IEA |
| 539379 | GO:0000060 | IEA |
| 539379 | GO:0051091 | IEA |
| 539379 | GO:0045941 | IEA |
| 539379 | GO:0035025 | IEA |
| 539381 | GO:0030154 | IEA |
| 539381 | GO:0007275 | IEA |
| 539431 | GO:0045941 | IEA |
| 539431 | GO:0016571 | IEA |
| 539449 | GO:0000278 | IEA |
| 539449 | GO:0043123 | IEA |
| 539524 | GO:0006986 | IEA |
| 539524 | GO:0006457 | IEA |
| 539567 | GO:0006470 | IEA |
| 539771 | GO:0006974 | IEA |
| 539771 | GO:0006281 | IEA |
| 539771 | GO:0006284 | ISS |
| 539771 | GO:0008152 | IEA |
| 539771 | GO:0006284 | IEA |
| 539789 | GO:0051272 | IEA |
| 539789 | GO:0007052 | IEA |
| 539789 | GO:0007409 | IEA |
| 539789 | GO:0007242 | IEA |
| 539789 | GO:0031115 | IEA |
| 539789 | GO:0007019 | IEA |
| 539875 | GO:0006334 | IEA |
| 539875 | GO:0006334 | IEA |
| 539875 | GO:0006334 | IEA |
| 539875 | GO:0006334 | IEA |
| 539875 | GO:0006334 | IEA |
| 539978 | GO:0006071 | IEA |
| 539992 | GO:0006631 | IEA |
| 539992 | GO:0006629 | IEA |
| 540040 | GO:0007186 | IEA |
| 540040 | GO:0007264 | IEA |
| 540083 | GO:0006986 | IEA |
| 540083 | GO:0006457 | IEA |
| 540083 | GO:0006457 | IEA |
| 540083 | GO:0006986 | IEA |
| 540085 | GO:0007257 | IEA |
| 540085 | GO:0030334 | IEA |
| 540085 | GO:0007165 | IEA |
| 540085 | GO:0000187 | IEA |
| 540085 | GO:0006979 | IEA |
| 540085 | GO:0006979 | IEA |
| 540085 | GO:0030334 | IEA |
| 540085 | GO:0007257 | IEA |
| 540085 | GO:0000187 | IEA |
| 540085 | GO:0007165 | IEA |
| 540090 | GO:0016574 | IEA |
| 540090 | GO:0009948 | IEA |
| 540090 | GO:0001702 | IEA |
| 540090 | GO:0000278 | IEA |
| 540090 | GO:0000122 | IEA |
| 540090 | GO:0016574 | IEA |
| 540090 | GO:0000278 | IEA |
| 540090 | GO:0000122 | IEA |
| 540090 | GO:0009948 | IEA |
| 540090 | GO:0001702 | IEA |
| 540117 | GO:0007409 | IEA |
| 540139 | GO:0006139 | IEA |
| 540148 | GO:0006334 | IEA |
| 540148 | GO:0006334 | IEA |
| 540148 | GO:0006334 | IEA |
| 540148 | GO:0006334 | IEA |
| 540148 | GO:0006334 | IEA |
| 540176 | GO:0015986 | IEA |
| 540176 | GO:0006810 | IEA |
| 540176 | GO:0015992 | IEA |
| 540176 | GO:0006811 | IEA |
| 540181 | GO:0006325 | IEA |
| 540329 | GO:0006470 | IEA |
| 540329 | GO:0006470 | IEA |
| 540329 | GO:0006470 | IEA |
| 540329 | GO:0006470 | IEA |
| 540329 | GO:0006470 | IEA |
| 540353 | GO:0007389 | IEA |
| 540376 | GO:0008152 | IEA |
| 540421 | GO:0007242 | IEA |
| 540421 | GO:0007165 | IEA |
| 540421 | GO:0006468 | IEA |
| 540444 | GO:0006288 | ISS |
| 540444 | GO:0006357 | ISS |
| 540444 | GO:0006355 | IEA |
| 540444 | GO:0006357 | IEA |
| 540444 | GO:0006288 | IEA |
| 540526 | GO:0009411 | IEA |
| 540526 | GO:0008629 | IEA |
| 540526 | GO:0045910 | IEA |
| 540526 | GO:0006298 | IEA |
| 540526 | GO:0045190 | IEA |
| 540526 | GO:0016447 | IEA |
| 540526 | GO:0008630 | IEA |
| 540526 | GO:0016446 | IEA |
| 540526 | GO:0008340 | IEA |
| 540564 | GO:0006974 | IEA |
| 540564 | GO:0006974 | IEA |
| 540568 | GO:0006812 | IEA |
| 540568 | GO:0008152 | IEA |
| 540568 | GO:0006810 | IEA |
| 540602 | GO:0008152 | IEA |
| 540602 | GO:0016051 | IEA |
| 540614 | GO:0007049 | IEA |
| 540619 | GO:0008360 | IEA |
| 540619 | GO:0007266 | IEA |
| 540619 | GO:0030036 | IEA |
| 540766 | GO:0030217 | IEA |
| 540766 | GO:0030851 | IEA |
| 540766 | GO:0006355 | IEA |
| 540766 | GO:0030224 | IEA |
| 540766 | GO:0001779 | IEA |
| 540766 | GO:0030183 | IEA |
| 540766 | GO:0030218 | IEA |
| 540859 | GO:0006355 | IEA |
| 540859 | GO:0006512 | IEA |
| 540859 | GO:0006350 | IEA |
| 540932 | GO:0007219 | IEA |
| 540966 | GO:0007067 | IEA |
| 540966 | GO:0006915 | IEA |
| 540966 | GO:0051301 | IEA |
| 540966 | GO:0007059 | IEA |
| 540966 | GO:0006281 | IEA |
| 540966 | GO:0007049 | IEA |
| 540966 | GO:0006974 | IEA |
| 540985 | GO:0006108 | IEA |
| 541077 | GO:0006415 | IEA |
| 541077 | GO:0006412 | IEA |
| 541077 | GO:0006412 | IEA |
| 541077 | GO:0006415 | IEA |
| 541101 | GO:0007018 | IEA |
| 541101 | GO:0051258 | IEA |
| 541125 | GO:0006355 | IEA |
| 541125 | GO:0016481 | IEA |
| 541125 | GO:0042060 | IEA |
| 541125 | GO:0001525 | IEA |
| 541148 | GO:0001878 | IEA |
| 541148 | GO:0050766 | IEA |
| 541148 | GO:0045429 | IEA |
| 541148 | GO:0008228 | IEA |
| 541175 | GO:0006470 | IEA |
| 541175 | GO:0006470 | IEA |
| 541175 | GO:0006470 | IEA |
| 541216 | GO:0006464 | IEA |
| 541216 | GO:0006512 | IEA |
| 541266 | GO:0006814 | IEA |
| 541266 | GO:0006811 | IEA |
| 541266 | GO:0006810 | IEA |
| 541283 | GO:0042692 | IEA |
| 541283 | GO:0007527 | IEA |
| 541283 | GO:0030154 | IEA |
| 613338 | GO:0006099 | IEA |
| 613338 | GO:0008152 | IEA |
| 613345 | GO:0006334 | IEA |
| 613345 | GO:0006334 | IEA |
| 613345 | GO:0006334 | IEA |
| 613345 | GO:0006334 | IEA |
| 613345 | GO:0006334 | IEA |
| 613421 | GO:0030833 | IEA |
| 613570 | GO:0006631 | IEA |
| 613610 | GO:0006096 | IEA |
| 613618 | GO:0006334 | IEA |
| 613629 | GO:0016481 | IEA |
| 613629 | GO:0007283 | IEA |
| 613629 | GO:0016575 | IEA |
| 613629 | GO:0001542 | IEA |
| 613729 | GO:0006355 | IEA |
| 613751 | GO:0045893 | IEA |
| 613787 | GO:0008284 | IEA |
| 613787 | GO:0009887 | IEA |
| 613787 | GO:0048008 | IEA |
| 613787 | GO:0050730 | IEA |
| 613787 | GO:0007171 | IEA |
| 613788 | GO:0045449 | IEA |
| 613788 | GO:0006351 | IEA |
| 613854 | GO:0006479 | IEA |
| 613854 | GO:0006464 | IEA |
| 613855 | GO:0006464 | IEA |
| 613855 | GO:0030182 | IEA |
| 613855 | GO:0006915 | IEA |
| 613877 | GO:0006355 | IEA |
| 613899 | GO:0006118 | IEA |
| 613899 | GO:0006122 | IEA |
| 613899 | GO:0006810 | IEA |
| 613907 | GO:0006355 | IEA |
| 613907 | GO:0045449 | IEA |
| 613930 | GO:0007186 | IEA |
| 614043 | GO:0006118 | IEA |
| 614114 | GO:0006118 | IEA |
| 614145 | GO:0008152 | IEA |
| 614145 | GO:0006099 | IEA |
| 614177 | GO:0045226 | IEA |
| 614242 | GO:0007264 | IEA |
| 614242 | GO:0006810 | IEA |
| 614242 | GO:0015031 | IEA |
| 614244 | GO:0006754 | IEA |
| 614244 | GO:0006810 | IEA |
| 614244 | GO:0006811 | IEA |
| 614244 | GO:0015986 | IEA |
| 614244 | GO:0015992 | IEA |
| 614288 | GO:0035058 | IEA |
| 614288 | GO:0050910 | IEA |
| 614288 | GO:0035176 | IEA |
| 614288 | GO:0007286 | IEA |
| 614288 | GO:0007608 | IEA |
| 614288 | GO:0045494 | IEA |
| 614288 | GO:0009296 | IEA |
| 614509 | GO:0045449 | IEA |
| 614538 | GO:0006915 | IEA |
| 614576 | GO:0030100 | IEA |
| 614581 | GO:0007264 | IEA |
| 614581 | GO:0006810 | IEA |
| 614581 | GO:0015031 | IEA |
| 614581 | GO:0006888 | IEA |
| 614630 | GO:0016055 | IEA |
| 614630 | GO:0007275 | IEA |
| 614633 | GO:0015031 | IEA |
| 614633 | GO:0006810 | IEA |
| 614633 | GO:0006605 | IEA |
| 614732 | GO:0006457 | IEA |
| 614773 | GO:0006417 | IEA |
| 614773 | GO:0001570 | IEA |
| 614773 | GO:0000288 | IEA |
| 614773 | GO:0001570 | IEA |
| 614773 | GO:0000288 | IEA |
| 614773 | GO:0006417 | IEA |
| 614893 | GO:0006350 | IEA |
| 614893 | GO:0006355 | IEA |
| 614921 | GO:0006810 | IEA |
| 614921 | GO:0015031 | IEA |
| 614922 | GO:0006397 | IEA |
| 614922 | GO:0008380 | IEA |
| 614922 | GO:0006810 | IEA |
| 614922 | GO:0051028 | IEA |
| 615066 | GO:0006512 | IEA |
| 615215 | GO:0006468 | IEA |
| 615239 | GO:0001658 | IEA |
| 615239 | GO:0009887 | IEA |
| 615239 | GO:0030513 | IEA |
| 615239 | GO:0045926 | IEA |
| 615239 | GO:0008285 | IEA |
| 615352 | GO:0006974 | IEA |
| 615352 | GO:0009314 | IEA |
| 615352 | GO:0001541 | IEA |
| 615352 | GO:0007286 | IEA |
| 615447 | GO:0009117 | IEA |
| 615447 | GO:0006183 | IEA |
| 615447 | GO:0006241 | IEA |
| 615447 | GO:0006228 | IEA |
| 615447 | GO:0009209 | IEA |
| 615498 | GO:0006810 | IEA |
| 615666 | GO:0006412 | IEA |
| 615692 | GO:0006355 | IEA |
| 615692 | GO:0006350 | IEA |
| 615845 | GO:0006450 | IEA |
| 615845 | GO:0006450 | IEA |
| 615978 | GO:0006790 | IEA |
| 615978 | GO:0006044 | IEA |
| 616066 | GO:0006915 | IEA |
| 616109 | GO:0006810 | IEA |
| 616109 | GO:0006122 | IEA |
| 616109 | GO:0006118 | IEA |
| 616148 | GO:0006357 | IEA |
| 616217 | GO:0000122 | IEA |
| 616275 | GO:0006512 | IEA |
| 616317 | GO:0031115 | IEA |
| 616317 | GO:0051272 | IEA |
| 616317 | GO:0007019 | IEA |
| 616317 | GO:0007242 | IEA |
| 616317 | GO:0007052 | IEA |
| 616317 | GO:0007409 | IEA |
| 616375 | GO:0000186 | IEA |
| 616375 | GO:0000186 | IEA |
| 616748 | GO:0006350 | IEA |
| 616748 | GO:0006810 | IEA |
| 616748 | GO:0006888 | IEA |
| 616748 | GO:0006350 | IEA |
| 616748 | GO:0006888 | IEA |
| 616748 | GO:0006810 | IEA |
| 616800 | GO:0006334 | IEA |
| 616800 | GO:0006334 | IEA |
| 616800 | GO:0006334 | IEA |
| 616800 | GO:0006334 | IEA |
| 616800 | GO:0006334 | IEA |
| 616819 | GO:0006334 | IEA |
| 616819 | GO:0006334 | IEA |
| 616819 | GO:0006334 | IEA |
| 616819 | GO:0006334 | IEA |
| 616819 | GO:0006334 | IEA |
| 616871 | GO:0006122 | IEA |
| 616871 | GO:0006118 | IEA |
| 616871 | GO:0006810 | IEA |
| 616926 | GO:0007156 | IEA |
| 616926 | GO:0007155 | IEA |
| 616926 | GO:0016337 | IEA |
| 617051 | GO:0006334 | IEA |
| 617051 | GO:0006334 | IEA |
| 617051 | GO:0006334 | IEA |
| 617051 | GO:0006334 | IEA |
| 617051 | GO:0006334 | IEA |
| 617105 | GO:0043065 | IEA |
| 617105 | GO:0008285 | ISS |
| 617105 | GO:0043065 | ISS |
| 617105 | GO:0009790 | ISS |
| 617105 | GO:0043517 | ISS |
| 617105 | GO:0008285 | IEA |
| 617105 | GO:0043517 | IEA |
| 617105 | GO:0006281 | ISS |
| 617210 | GO:0006118 | IEA |
| 617210 | GO:0006810 | IEA |
| 617230 | GO:0006754 | IEA |
| 617230 | GO:0015986 | IEA |
| 617230 | GO:0006811 | IEA |
| 617230 | GO:0015992 | IEA |
| 617230 | GO:0006810 | IEA |
| 617267 | GO:0006096 | IEA |
| 617375 | GO:0045449 | IEA |
| 617375 | GO:0006350 | IEA |
| 617419 | GO:0006633 | IEA |
| 617419 | GO:0006629 | IEA |
| 617441 | GO:0008629 | IEA |
| 617564 | GO:0008152 | IEA |
| 617564 | GO:0006118 | IEA |
| 617564 | GO:0006118 | IEA |
| 617564 | GO:0008152 | IEA |
| 617635 | GO:0007264 | IEA |
| 617694 | GO:0006118 | IEA |
| 617707 | GO:0030111 | IEA |
| 617707 | GO:0001702 | IEA |
| 617707 | GO:0006644 | IEA |
| 617707 | GO:0001568 | IEA |
| 617707 | GO:0030111 | IEA |
| 617707 | GO:0001568 | IEA |
| 617707 | GO:0006644 | IEA |
| 617707 | GO:0001702 | IEA |
| 617809 | GO:0042981 | IEA |
| 617888 | GO:0006334 | IEA |
| 617888 | GO:0006334 | IEA |
| 617888 | GO:0006334 | IEA |
| 617888 | GO:0006334 | IEA |
| 617888 | GO:0006334 | IEA |
| 617901 | GO:0006412 | IEA |
| 617944 | GO:0006412 | IEA |
| 617944 | GO:0006414 | IEA |
| 617976 | GO:0007275 | IEA |
| 618131 | GO:0006457 | IEA |
| 618131 | GO:0006605 | IEA |
| 618131 | GO:0006626 | IEA |
| 618131 | GO:0045039 | IEA |
| 618131 | GO:0015031 | IEA |
| 618131 | GO:0006810 | IEA |
| 618149 | GO:0006334 | IEA |
| 618149 | GO:0006334 | IEA |
| 618149 | GO:0006334 | IEA |
| 618149 | GO:0006334 | IEA |
| 618149 | GO:0006334 | IEA |
| 618292 | GO:0006396 | IEA |
| 618292 | GO:0006364 | IEA |
| 618297 | GO:0006357 | IEA |
| 618297 | GO:0006355 | IEA |
| 618297 | GO:0006288 | ISS |
| 618297 | GO:0006288 | IEA |
| 618297 | GO:0006357 | ISS |
| 618377 | GO:0009611 | IEA |
| 618441 | GO:0030091 | IEA |
| 618508 | GO:0043123 | IEA |
| 618573 | GO:0045449 | IEA |
| 618607 | GO:0042840 | IEA |
| 618607 | GO:0046185 | IEA |
| 618607 | GO:0019853 | IEA |
| 618609 | GO:0006412 | IEA |
| 618622 | GO:0007368 | IEA |
| 618622 | GO:0030326 | IEA |
| 618849 | GO:0045941 | ISS |
| 618849 | GO:0006355 | IEA |
| 619141 | GO:0006334 | IEA |
| 619141 | GO:0006334 | IEA |
| 619141 | GO:0006334 | IEA |
| 619141 | GO:0006334 | IEA |
| 619141 | GO:0006334 | IEA |
| 654400 | GO:0045944 | IEA |
| 654400 | GO:0006355 | IEA |
| 654400 | GO:0001889 | IEA |
| 767855 | GO:0006915 | IEA |
| 767898 | GO:0000288 | IEA |
| 767898 | GO:0043488 | IEA |
| 767980 | GO:0006613 | IEA |
| 768014 | GO:0007018 | IEA |
| 768016 | GO:0030514 | IEA |
| 768016 | GO:0007184 | IEA |
| 768016 | GO:0045668 | IEA |
| 768035 | GO:0032012 | IEA |
| 768074 | GO:0006118 | IEA |
| 768074 | GO:0006810 | IEA |
| 768330 | GO:0006810 | IEA |
| 777769 | GO:0043393 | IEA |
| 777769 | GO:0045893 | IEA |
| 777769 | GO:0045941 | IEA |
| 777769 | GO:0042692 | IEA |
| 777769 | GO:0003007 | IEA |
| 777775 | GO:0051258 | IEA |
| 777775 | GO:0007018 | IEA |
| 777775 | GO:0007018 | IEA |
| 777775 | GO:0051258 | IEA |
| 780784 | GO:0030239 | IEA |
| 780962 | GO:0008152 | IEA |
| 780978 | GO:0006810 | IEA |
| 780978 | GO:0006886 | IEA |
| 780978 | GO:0015031 | IEA |
| 781014 | GO:0006998 | IEA |
| 781064 | GO:0006979 | IEA |
| 781064 | GO:0006118 | IEA |
| 781070 | GO:0006414 | IEA |
| 781070 | GO:0006412 | IEA |
| 781100 | GO:0006260 | IEA |
| 781111 | GO:0006334 | IEA |
| 781111 | GO:0006334 | IEA |
| 781119 | GO:0007409 | IEA |
| 781197 | GO:0006465 | IEA |
| 781224 | GO:0006334 | IEA |
| 781224 | GO:0006334 | IEA |
| 781224 | GO:0006334 | IEA |
| 781224 | GO:0006334 | IEA |
| 781224 | GO:0006334 | IEA |
| 781227 | GO:0009165 | IEA |
| 781227 | GO:0009116 | IEA |
| 781227 | GO:0009156 | IEA |
| 781334 | GO:0007010 | IEA |
| 781462 | GO:0015031 | IEA |
| 781462 | GO:0006626 | IEA |
| 781462 | GO:0006605 | IEA |
| 781462 | GO:0045039 | IEA |
| 781462 | GO:0006457 | IEA |
| 781462 | GO:0006810 | IEA |
| 781532 | GO:0006457 | IEA |
| 781532 | GO:0006955 | IEA |
| 781542 | GO:0006412 | IEA |
| 781566 | GO:0006334 | IEA |
| 781566 | GO:0006334 | IEA |
| 781566 | GO:0006334 | IEA |
| 781566 | GO:0006334 | IEA |
| 781566 | GO:0006334 | IEA |
| 781575 | GO:0043681 | IEA |
| 781609 | GO:0006118 | IEA |
| 781610 | GO:0006635 | ISS |
| 781610 | GO:0007507 | IEA |
| 781610 | GO:0009791 | IEA |
| 781610 | GO:0009409 | IEA |
| 781610 | GO:0008152 | IEA |
| 781610 | GO:0042594 | IEA |
| 781610 | GO:0006635 | IEA |
| 781610 | GO:0006118 | IEA |
| 781610 | GO:0006082 | IEA |
| 781610 | GO:0055007 | IEA |
| 781610 | GO:0001889 | IEA |
| 781610 | GO:0009437 | IEA |
| 781625 | GO:0001569 | IEA |
| 781648 | GO:0019538 | IEA |
| 781648 | GO:0006508 | IEA |
| 781719 | GO:0006810 | IEA |
| 781719 | GO:0006412 | IEA |
| 781741 | GO:0006464 | IEA |
| 781741 | GO:0006915 | IEA |
| 781741 | GO:0030182 | IEA |
| 781770 | GO:0006464 | IEA |
| 781770 | GO:0006512 | IEA |
| 781813 | GO:0006414 | IEA |
| 781813 | GO:0006412 | IEA |
| 781824 | GO:0006120 | IEA |
| 781969 | GO:0051607 | ISS |
| 781969 | GO:0008634 | ISS |
| 781969 | GO:0006917 | ISS |
| 781969 | GO:0043065 | IEA |
| 781969 | GO:0006915 | IEA |
| 781969 | GO:0008634 | ISS |
| 781969 | GO:0051607 | ISS |
| 781969 | GO:0043065 | IEA |
| 781969 | GO:0006915 | IEA |
| 781969 | GO:0006917 | ISS |
| 782024 | GO:0016071 | IEA |
| 782024 | GO:0006397 | IEA |
| 782024 | GO:0008380 | IEA |
| 782058 | GO:0006457 | IEA |
| 782114 | GO:0006629 | IEA |
| 782114 | GO:0006631 | IEA |
| 782233 | GO:0015992 | IEA |
| 782233 | GO:0006754 | IEA |
| 782233 | GO:0006810 | IEA |
| 782233 | GO:0006811 | IEA |
| 782233 | GO:0015986 | IEA |
| 782259 | GO:0006508 | IEA |
| 782270 | GO:0006810 | IEA |
| 782270 | GO:0006811 | IEA |
| 782270 | GO:0006754 | IEA |
| 782270 | GO:0015986 | IEA |
| 782270 | GO:0015992 | IEA |
| 782344 | GO:0051028 | IEA |
| 782344 | GO:0006810 | IEA |
| 782344 | GO:0006397 | IEA |
| 782344 | GO:0008380 | IEA |
| 782393 | GO:0043065 | IEA |
| 782393 | GO:0008285 | ISS |
| 782393 | GO:0043065 | ISS |
| 782393 | GO:0009790 | ISS |
| 782393 | GO:0008285 | IEA |
| 782393 | GO:0043517 | ISS |
| 782393 | GO:0043517 | IEA |
| 782393 | GO:0006281 | ISS |
| 782416 | GO:0006120 | TAS |
| 782416 | GO:0008610 | IEA |
| 782416 | GO:0006633 | IEA |
| 782525 | GO:0006412 | IEA |
| 782525 | GO:0006414 | IEA |
| 782666 | GO:0015992 | IEA |
| 782666 | GO:0006811 | IEA |
| 782666 | GO:0006810 | IEA |
| 782666 | GO:0015986 | IEA |
| 782666 | GO:0006754 | IEA |
| 782671 | GO:0043123 | IEA |
| 782776 | GO:0006414 | IEA |
| 782776 | GO:0006412 | IEA |
| 782921 | GO:0006826 | IEA |
| 782921 | GO:0006879 | IEA |
| 782954 | GO:0006414 | IEA |
| 782954 | GO:0006412 | IEA |
| 782989 | GO:0006414 | IEA |
| 782989 | GO:0006412 | IEA |
| 783184 | GO:0006084 | IEA |
| 783220 | GO:0006879 | IEA |
| 783220 | GO:0006826 | IEA |
| 783224 | GO:0006879 | IEA |
| 783224 | GO:0006826 | IEA |
| 783294 | GO:0006820 | IEA |
| 783294 | GO:0006811 | IEA |
| 783294 | GO:0006810 | IEA |
| 783301 | GO:0006810 | IEA |
| 783301 | GO:0006397 | IEA |
| 783301 | GO:0051028 | IEA |
| 783301 | GO:0008380 | IEA |
| 783337 | GO:0006412 | IEA |
| 783337 | GO:0006412 | IEA |
| 783421 | GO:0006413 | ISS |
| 783421 | GO:0006412 | IEA |
| 783421 | GO:0006412 | IEA |
| 783421 | GO:0006413 | ISS |
| 783467 | GO:0006979 | IEA |
| 783467 | GO:0006118 | IEA |
| 783502 | GO:0006118 | IEA |
| 783502 | GO:0006810 | IEA |
| 783502 | GO:0006508 | IEA |
| 783523 | GO:0051028 | IEA |
| 783523 | GO:0006810 | IEA |
| 783523 | GO:0006397 | IEA |
| 783523 | GO:0008380 | IEA |
| 783681 | GO:0048839 | IEA |
| 783681 | GO:0002011 | IEA |
| 783681 | GO:0009887 | IEA |
| 783681 | GO:0045747 | IEA |
| 783681 | GO:0045596 | IEA |
| 783681 | GO:0045639 | IEA |
| 783681 | GO:0042491 | IEA |
| 783686 | GO:0006120 | IEA |
| 783687 | GO:0043681 | IEA |
| 783837 | GO:0051289 | IEA |
| 783837 | GO:0045893 | IEA |
| 783837 | GO:0051291 | IEA |
| 783838 | GO:0006414 | IEA |
| 783838 | GO:0006412 | IEA |
| 783928 | GO:0006508 | IEA |
| 783991 | GO:0006754 | IEA |
| 783991 | GO:0006810 | IEA |
| 783991 | GO:0006811 | IEA |
| 783991 | GO:0015986 | IEA |
| 783991 | GO:0015992 | IEA |
| 783993 | GO:0006826 | IEA |
| 783993 | GO:0006879 | IEA |
| 784025 | GO:0006879 | IEA |
| 784025 | GO:0006826 | IEA |
| 784088 | GO:0030155 | IEA |
| 784097 | GO:0006120 | IEA |
| 784131 | GO:0006414 | IEA |
| 784131 | GO:0006412 | IEA |
| 784220 | GO:0008152 | IEA |
| 784289 | GO:0006826 | IEA |
| 784289 | GO:0006879 | IEA |
| 784294 | GO:0006810 | IEA |
| 784294 | GO:0006811 | IEA |
| 784294 | GO:0006820 | IEA |
| 784340 | GO:0006096 | IEA |
| 784442 | GO:0030900 | IEA |
| 784492 | GO:0051607 | ISS |
| 784492 | GO:0043065 | IEA |
| 784492 | GO:0006915 | IEA |
| 784492 | GO:0006917 | ISS |
| 784492 | GO:0008634 | ISS |
| 784492 | GO:0043065 | IEA |
| 784492 | GO:0051607 | ISS |
| 784492 | GO:0006917 | ISS |
| 784492 | GO:0006915 | IEA |
| 784492 | GO:0008634 | ISS |
| 784606 | GO:0006397 | IEA |
| 784606 | GO:0006810 | IEA |
| 784606 | GO:0051028 | IEA |
| 784606 | GO:0008380 | IEA |
| 784615 | GO:0006512 | IEA |
| 784642 | GO:0006879 | IEA |
| 784642 | GO:0006826 | IEA |
| 784643 | GO:0007010 | IEA |
| 784737 | GO:0045668 | IEA |
| 784737 | GO:0030514 | IEA |
| 784737 | GO:0007184 | IEA |
| 784774 | GO:0006879 | IEA |
| 784774 | GO:0006826 | IEA |
| 785087 | GO:0006414 | IEA |
| 785087 | GO:0006412 | IEA |
| 785134 | GO:0006118 | IEA |
| 785214 | GO:0006464 | IEA |
| 785297 | GO:0006879 | IEA |
| 785297 | GO:0006826 | IEA |
| 785317 | GO:0006879 | IEA |
| 785317 | GO:0006826 | IEA |
| 785387 | GO:0006334 | IEA |
| 785387 | GO:0006334 | IEA |
| 785387 | GO:0006334 | IEA |
| 785388 | GO:0006397 | IEA |
| 785388 | GO:0008380 | IEA |
| 785388 | GO:0051028 | IEA |
| 785388 | GO:0006810 | IEA |
| 785395 | GO:0006810 | IEA |
| 785395 | GO:0006118 | IEA |
| 785395 | GO:0006099 | IEA |
| 785455 | GO:0007010 | IEA |
| 785484 | GO:0006334 | IEA |
| 785484 | GO:0006334 | IEA |
| 785484 | GO:0006334 | IEA |
| 785653 | GO:0006508 | IEA |
| 785655 | GO:0014037 | IEA |
| 785655 | GO:0007507 | IEA |
| 785655 | GO:0021545 | IEA |
| 785655 | GO:0007422 | IEA |
| 785655 | GO:0043524 | IEA |
| 785657 | GO:0051726 | IEA |
| 785761 | GO:0006397 | IEA |
| 785761 | GO:0006810 | IEA |
| 785761 | GO:0051028 | IEA |
| 785761 | GO:0008380 | IEA |
| 785763 | GO:0006096 | IEA |
| 785824 | GO:0006118 | IEA |
| 785842 | GO:0006879 | IEA |
| 785842 | GO:0006826 | IEA |
| 785922 | GO:0006412 | IEA |
| 785922 | GO:0006414 | IEA |
| 785958 | GO:0015074 | IEA |
| 785986 | GO:0006355 | IEA |
| 785986 | GO:0007049 | IEA |
| 785986 | GO:0045786 | IEA |
| 785986 | GO:0006355 | IEA |
| 785986 | GO:0045786 | IEA |
| 785986 | GO:0007049 | IEA |
| 785987 | GO:0006879 | IEA |
| 785987 | GO:0006826 | IEA |
| 786048 | GO:0030154 | IEA |
| 786048 | GO:0006350 | IEA |
| 786048 | GO:0006355 | IEA |
| 786048 | GO:0007275 | IEA |
| 786055 | GO:0006414 | IEA |
| 786055 | GO:0006412 | IEA |
| 786108 | GO:0007368 | IEA |
| 786108 | GO:0030326 | IEA |
| 786128 | GO:0007018 | IEA |
| 786128 | GO:0051258 | IEA |
| 786426 | GO:0006811 | IEA |
| 786426 | GO:0006810 | IEA |
| 786426 | GO:0006820 | IEA |
| 786431 | GO:0015986 | IEA |
| 786431 | GO:0006754 | IEA |
| 786431 | GO:0015992 | IEA |
| 786431 | GO:0006810 | IEA |
| 786431 | GO:0006811 | IEA |
| 786533 | GO:0016042 | IEA |
| 786533 | GO:0000302 | ISS |
| 786673 | GO:0006811 | IEA |
| 786673 | GO:0015986 | IEA |
| 786673 | GO:0006810 | IEA |
| 786673 | GO:0015992 | IEA |
| 786733 | GO:0050965 | IEA |
| 787289 | GO:0008380 | IEA |
| 787289 | GO:0006397 | IEA |
| 787289 | GO:0006810 | IEA |
| 787289 | GO:0051028 | IEA |
| 787327 | GO:0045449 | IEA |
| 787486 | GO:0008152 | IEA |
| 787497 | GO:0006120 | TAS |
| 787497 | GO:0008610 | IEA |
| 787497 | GO:0006633 | IEA |
| 787528 | GO:0006334 | IEA |
| 787528 | GO:0006334 | IEA |
| 787528 | GO:0006334 | IEA |
| 787528 | GO:0006334 | IEA |
| 787528 | GO:0006334 | IEA |
| 787530 | GO:0031032 | IEA |
| 787920 | GO:0008152 | IEA |
| 788157 | GO:0006414 | IEA |
| 788157 | GO:0006412 | IEA |
| 788193 | GO:0006334 | IEA |
| 788193 | GO:0006334 | IEA |
| 788193 | GO:0006334 | IEA |
| 788193 | GO:0006334 | IEA |
| 788193 | GO:0006334 | IEA |
| 788250 | GO:0006334 | IEA |
| 788250 | GO:0006334 | IEA |
| 788250 | GO:0006334 | IEA |
| 788250 | GO:0006334 | IEA |
| 788250 | GO:0006334 | IEA |
| 788293 | GO:0006099 | IEA |
| 788293 | GO:0006099 | IEA |
| 788392 | GO:0006412 | IEA |
| 788392 | GO:0006413 | ISS |
| 788392 | GO:0006413 | ISS |
| 788392 | GO:0006412 | IEA |
| 788417 | GO:0045910 | IEA |
| 788417 | GO:0006298 | IEA |
| 788417 | GO:0008630 | IEA |
| 788417 | GO:0008629 | IEA |
| 788417 | GO:0016447 | IEA |
| 788417 | GO:0008340 | IEA |
| 788417 | GO:0045190 | IEA |
| 788417 | GO:0016446 | IEA |
| 788417 | GO:0009411 | IEA |
| 788596 | GO:0007165 | IEA |
| 788596 | GO:0007186 | IEA |
| 788609 | GO:0006414 | IEA |
| 788609 | GO:0006412 | IEA |
| 788610 | GO:0015671 | IEA |
| 788610 | GO:0006810 | IEA |
| 788610 | GO:0015671 | IEA |
| 788610 | GO:0006810 | IEA |
| 788610 | GO:0006810 | IEA |
| 788610 | GO:0015671 | IEA |
| 788668 | GO:0006334 | IEA |
| 788668 | GO:0006334 | IEA |
| 788668 | GO:0006334 | IEA |
| 788672 | GO:0006412 | IEA |
| 788672 | GO:0006414 | IEA |
| 788745 | GO:0006810 | IEA |
| 788745 | GO:0015986 | IEA |
| 788745 | GO:0015992 | IEA |
| 788745 | GO:0006811 | IEA |
| 788801 | GO:0006879 | IEA |
| 788801 | GO:0006826 | IEA |
| 788907 | GO:0006879 | IEA |
| 788907 | GO:0006826 | IEA |
| 789002 | GO:0006464 | IEA |
| 789255 | GO:0006118 | IEA |
| 789369 | GO:0006334 | IEA |
| 789369 | GO:0006334 | IEA |
| 789369 | GO:0006334 | IEA |
| 789369 | GO:0006334 | IEA |
| 789369 | GO:0006334 | IEA |
| 789447 | GO:0006464 | IEA |
| 789447 | GO:0006512 | IEA |
| 789638 | GO:0006096 | IEA |
| 789819 | GO:0006464 | IEA |
| 789819 | GO:0006512 | IEA |
| 789854 | GO:0006357 | IEA |
| 789854 | GO:0001541 | IEA |
| 789867 | GO:0006412 | IEA |
| 789867 | GO:0006414 | IEA |
| 790012 | GO:0016042 | IEA |
| 790012 | GO:0006629 | IEA |
| 790012 | GO:0007165 | IEA |
| 790012 | GO:0007242 | IEA |
| 790040 | GO:0016322 | IEA |
| 790360 | GO:0006810 | IEA |
| 790360 | GO:0006886 | IEA |
| 790402 | GO:0007613 | IEA |
| 790402 | GO:0030198 | IEA |
| 790402 | GO:0042472 | IEA |
| 790402 | GO:0030030 | IEA |
| 790402 | GO:0030511 | IEA |
| 790402 | GO:0001656 | IEA |
| 790454 | GO:0042048 | IEA |
| 790454 | GO:0001967 | IEA |
| 790454 | GO:0001701 | IEA |
| 790539 | GO:0005975 | IEA |
| 790539 | GO:0006072 | IEA |
| 790541 | GO:0005975 | IEA |
| 790541 | GO:0006094 | IEA |
| 790667 | GO:0007275 | IEA |
| 790667 | GO:0002318 | IEA |
| 790667 | GO:0030154 | IEA |
| 790667 | GO:0006350 | IEA |
| 790667 | GO:0007275 | IEA |
| 790667 | GO:0030154 | IEA |
| 790667 | GO:0002318 | IEA |
| 790667 | GO:0006350 | IEA |
| 790667 | GO:0006350 | IEA |
| 790667 | GO:0002318 | IEA |
| 790667 | GO:0030154 | IEA |
| 790667 | GO:0007275 | IEA |
| 790836 | GO:0006351 | IEA |
| 790836 | GO:0045449 | IEA |
| 790868 | GO:0006508 | IEA |
| 790868 | GO:0006810 | IEA |
| 790868 | GO:0006118 | IEA |
|  |  |  |
