# Supplementary material for: A gene network switch enhances the oxidative capacity of ovine skeletal muscle during late fetal development
Source: BMC Genomics. 2010 Jun 15;11:378. doi: 10.1186/1471-2164-11-378 (PMC2894804; doi:10.1186/1471-2164-11-378)
Supplement: Additional File 1 — Gene expression data. This file contains links to the gene expression data. The genes that are significantly differentially expressed between adjacent development times are listed as Experiment 1 (80 d vs 100 d), Experiment 2 (100 d vs 120 d), Experiment 3 (120 d vs 150 d) and Experiment 4 (150 d vs 230 d) in the section entitled Open microarray analysis report for developmental expression. Each experiment contains an MA plot, Volcano plot and Heatmap for each of the three microarray processing programs MAS5, RMA and GCRMA. For each of these processing programs there is a list of significantly differentially expressed probe sets, and for each of these there is an FDR corrected probability, fold change, percentage of microarrays with a MAS5 Present call, and a convergence percentage for all three microarray processing programs. The file also contains annotation of the probe sets. In a second section entitled Time series analysis of developmental expression, there is a list of genes in each gene expression cluster and diagrams showing MAS5 gene expression values for these genes. [file 1471-2164-11-378-S1.ZIP › Report/GO3/Report/CC.html]

### Cellular Component

| Entrez | GO ID | Evidence Code |
| --- | --- | --- |
 280682 | GO:0005739 | IEA || 280682 | GO:0005737 | IEA |
| 280682 | GO:0005737 | IEA |
| 280682 | GO:0005739 | IEA |
| 280701 | GO:0005737 | IEA |
| 280715 | GO:0005737 | IEA |
| 280715 | GO:0005737 | IEA |
| 280719 | GO:0005739 | IEA |
| 280719 | GO:0005743 | TAS |
| 280719 | GO:0005737 | IEA |
| 280733 | GO:0005578 | IEA |
| 280740 | GO:0005737 | IEA |
| 280760 | GO:0005578 | IEA |
| 280815 | GO:0005764 | IEA |
| 280832 | GO:0016021 | IEA |
| 280832 | GO:0009897 | IEA |
| 280832 | GO:0016020 | IEA |
| 280878 | GO:0005739 | IEA |
| 280878 | GO:0016021 | IEA |
| 280878 | GO:0005743 | IEA |
| 280878 | GO:0016020 | IEA |
| 280878 | GO:0016021 | IEA |
| 280878 | GO:0005739 | IEA |
| 280878 | GO:0016020 | IEA |
| 280878 | GO:0005743 | IEA |
| 280884 | GO:0005783 | IEA |
| 280884 | GO:0005578 | IEA |
| 280890 | GO:0016020 | IEA |
| 280890 | GO:0016021 | IEA |
| 280904 | GO:0005783 | IEA |
| 280939 | GO:0016021 | IEA |
| 280939 | GO:0016020 | IEA |
| 280941 | GO:0016021 | IEA |
| 280941 | GO:0016020 | IEA |
| 280955 | GO:0005882 | IEA |
| 280955 | GO:0005737 | IEA |
| 280955 | GO:0005737 | ISS |
| 280961 | GO:0005634 | IEA |
| 280961 | GO:0005622 | IEA |
| 280962 | GO:0005622 | IEA |
| 280962 | GO:0005634 | IEA |
| 280976 | GO:0005739 | IEA |
| 280979 | GO:0035267 | ISS |
| 280979 | GO:0005856 | IEA |
| 281032 | GO:0005634 | ISS |
| 281032 | GO:0005634 | IEA |
| 281032 | GO:0005737 | IEA |
| 281032 | GO:0005737 | ISS |
| 281040 | GO:0016021 | IEA |
| 281040 | GO:0016020 | IEA |
| 281040 | GO:0016021 | IEA |
| 281040 | GO:0016020 | IEA |
| 281040 | GO:0016021 | IEA |
| 281040 | GO:0016020 | IEA |
| 281051 | GO:0016020 | IEA |
| 281057 | GO:0016020 | IEA |
| 281057 | GO:0016021 | IEA |
| 281078 | GO:0030125 | IEA |
| 281078 | GO:0005905 | IEA |
| 281092 | GO:0005777 | IEA |
| 281154 | GO:0005578 | IEA |
| 281161 | GO:0005615 | IEA |
| 281161 | GO:0005615 | ISS |
| 281187 | GO:0005615 | IEA |
| 281188 | GO:0005624 | ISS |
| 281188 | GO:0005737 | ISS |
| 281188 | GO:0005624 | ISS |
| 281188 | GO:0005737 | ISS |
| 281190 | GO:0005783 | IEA |
| 281190 | GO:0016020 | IEA |
| 281190 | GO:0016021 | IEA |
| 281193 | GO:0005922 | IEA |
| 281193 | GO:0005921 | IEA |
| 281193 | GO:0030054 | IEA |
| 281193 | GO:0016020 | IEA |
| 281193 | GO:0016021 | IEA |
| 281201 | GO:0005834 | IEA |
| 281210 | GO:0005615 | IEA |
| 281210 | GO:0005615 | ISS |
| 281239 | GO:0005576 | IEA |
| 281240 | GO:0005576 | IEA |
| 281258 | GO:0016020 | IEA |
| 281258 | GO:0005923 | IEA |
| 281258 | GO:0016021 | IEA |
| 281278 | GO:0016021 | IEA |
| 281278 | GO:0016020 | IEA |
| 281278 | GO:0016021 | IEA |
| 281278 | GO:0016020 | IEA |
| 281296 | GO:0005856 | IEA |
| 281296 | GO:0005874 | IEA |
| 281306 | GO:0005743 | IEA |
| 281306 | GO:0005739 | IEA |
| 281338 | GO:0016459 | IEA |
| 281339 | GO:0016459 | IEA |
| 281339 | GO:0016459 | IEA |
| 281339 | GO:0016459 | IEA |
| 281357 | GO:0016020 | IEA |
| 281357 | GO:0016021 | IEA |
| 281360 | GO:0005739 | ISS |
| 281360 | GO:0005777 | IEA |
| 281419 | GO:0005783 | IEA |
| 281433 | GO:0005764 | IEA |
| 281433 | GO:0005739 | IEA |
| 281556 | GO:0005622 | IEA |
| 281556 | GO:0005739 | IEA |
| 281557 | GO:0005739 | IEA |
| 281563 | GO:0005739 | IEA |
| 281563 | GO:0016020 | IEA |
| 281563 | GO:0016021 | IEA |
| 281563 | GO:0005743 | IEA |
| 281570 | GO:0016020 | IEA |
| 281570 | GO:0016021 | IEA |
| 281570 | GO:0005739 | IEA |
| 281570 | GO:0005743 | IEA |
| 281570 | GO:0005746 | IEA |
| 281574 | GO:0005737 | IEA |
| 281574 | GO:0015629 | ISS |
| 281574 | GO:0001931 | ISS |
| 281574 | GO:0001931 | IEA |
| 281574 | GO:0016324 | IEA |
| 281574 | GO:0005932 | IEA |
| 281574 | GO:0005932 | ISS |
| 281574 | GO:0016324 | ISS |
| 281574 | GO:0016020 | IEA |
| 281574 | GO:0045177 | ISS |
| 281574 | GO:0019898 | ISS |
| 281574 | GO:0005856 | IEA |
| 281574 | GO:0005884 | ISS |
| 281600 | GO:0005737 | IEA |
| 281600 | GO:0005886 | IEA |
| 281603 | GO:0016021 | IEA |
| 281603 | GO:0016020 | IEA |
| 281631 | GO:0005615 | IEA |
| 281631 | GO:0005576 | IEA |
| 281719 | GO:0005737 | IEA |
| 281729 | GO:0016363 | ISS |
| 281729 | GO:0016020 | IEA |
| 281729 | GO:0005634 | IEA |
| 281729 | GO:0005737 | ISS |
| 281729 | GO:0005694 | IEA |
| 281729 | GO:0008278 | ISS |
| 281729 | GO:0000922 | ISS |
| 281730 | GO:0005634 | IEA |
| 281736 | GO:0031252 | IEA |
| 281736 | GO:0030426 | IEA |
| 281736 | GO:0016020 | IEA |
| 281736 | GO:0016021 | IEA |
| 281742 | GO:0005739 | IEA |
| 281742 | GO:0016020 | IEA |
| 281749 | GO:0016021 | IEA |
| 281749 | GO:0016020 | IEA |
| 281749 | GO:0016021 | IEA |
| 281749 | GO:0016020 | IEA |
| 281780 | GO:0016021 | IEA |
| 281780 | GO:0016020 | IEA |
| 281780 | GO:0016021 | IEA |
| 281780 | GO:0016020 | IEA |
| 281810 | GO:0016507 | IEA |
| 281810 | GO:0005739 | IEA |
| 281811 | GO:0005739 | IEA |
| 281811 | GO:0005743 | IEA |
| 281816 | GO:0005739 | IEA |
| 281816 | GO:0005739 | ISS |
| 281849 | GO:0016021 | IEA |
| 281849 | GO:0016020 | IEA |
| 281849 | GO:0005764 | IEA |
| 281876 | GO:0005886 | IEA |
| 281876 | GO:0016021 | IEA |
| 281876 | GO:0008305 | IEA |
| 281876 | GO:0042383 | IEA |
| 281876 | GO:0019717 | IEA |
| 281876 | GO:0016020 | IEA |
| 281876 | GO:0031594 | IEA |
| 281904 | GO:0016020 | IEA |
| 281904 | GO:0005576 | IEA |
| 281912 | GO:0005578 | IEA |
| 281912 | GO:0001527 | IEA |
| 281935 | GO:0005856 | IEA |
| 281935 | GO:0016459 | IEA |
| 281937 | GO:0005903 | IEA |
| 281937 | GO:0005737 | IEA |
| 281937 | GO:0045160 | IEA |
| 281937 | GO:0016459 | IEA |
| 281937 | GO:0016328 | IEA |
| 281937 | GO:0005902 | IEA |
| 281937 | GO:0009925 | IEA |
| 281937 | GO:0032420 | IEA |
| 281937 | GO:0031941 | IEA |
| 281942 | GO:0005737 | IEA |
| 281945 | GO:0005634 | IEA |
| 281963 | GO:0005886 | IEA |
| 281963 | GO:0016021 | IEA |
| 281963 | GO:0016020 | IEA |
| 281985 | GO:0005790 | IEA |
| 281985 | GO:0005792 | IEA |
| 281985 | GO:0005634 | IEA |
| 281985 | GO:0014069 | IEA |
| 281985 | GO:0030425 | IEA |
| 281987 | GO:0005737 | IEA |
| 281998 | GO:0005739 | IEA |
| 282011 | GO:0005634 | IEA |
| 282015 | GO:0000502 | ISS |
| 282015 | GO:0005829 | IEA |
| 282015 | GO:0005634 | IEA |
| 282015 | GO:0043234 | IEA |
| 282015 | GO:0005737 | IEA |
| 282022 | GO:0005737 | IEA |
| 282022 | GO:0005792 | IEA |
| 282022 | GO:0016020 | IEA |
| 282024 | GO:0005622 | IEA |
| 282032 | GO:0005737 | ISS |
| 282032 | GO:0005886 | IEA |
| 282032 | GO:0005622 | IEA |
| 282032 | GO:0001726 | IEA |
| 282050 | GO:0030529 | IEA |
| 282050 | GO:0005622 | IEA |
| 282050 | GO:0005840 | IEA |
| 282077 | GO:0005604 | IEA |
| 282077 | GO:0005578 | IEA |
| 282091 | GO:0016020 | IEA |
| 282091 | GO:0005886 | ISS |
| 282100 | GO:0016020 | IEA |
| 282100 | GO:0016021 | IEA |
| 282110 | GO:0043234 | IEA |
| 282110 | GO:0005829 | IEA |
| 282110 | GO:0005622 | IEA |
| 282119 | GO:0019867 | IEA |
| 282119 | GO:0016020 | IEA |
| 282119 | GO:0005741 | IEA |
| 282119 | GO:0016021 | IEA |
| 282119 | GO:0016020 | IEA |
| 282119 | GO:0005741 | IEA |
| 282119 | GO:0016021 | IEA |
| 282119 | GO:0019867 | IEA |
| 282120 | GO:0005741 | IEA |
| 282120 | GO:0019867 | IEA |
| 282120 | GO:0005739 | IEA |
| 282120 | GO:0005743 | IEA |
| 282120 | GO:0016020 | IEA |
| 282120 | GO:0016021 | IEA |
| 282122 | GO:0005615 | IEA |
| 282122 | GO:0016020 | IEA |
| 282123 | GO:0016020 | IEA |
| 282130 | GO:0005739 | IEA |
| 282132 | GO:0005794 | ISS |
| 282132 | GO:0016020 | IEA |
| 282132 | GO:0005798 | ISS |
| 282132 | GO:0016021 | IEA |
| 282132 | GO:0005737 | IEA |
| 282132 | GO:0009986 | ISS |
| 282132 | GO:0005634 | ISS |
| 282132 | GO:0005634 | IEA |
| 282132 | GO:0005886 | IEA |
| 282132 | GO:0005737 | ISS |
| 282132 | GO:0005794 | ISS |
| 282132 | GO:0005798 | ISS |
| 282132 | GO:0009986 | ISS |
| 282132 | GO:0005737 | ISS |
| 282132 | GO:0005886 | IEA |
| 282132 | GO:0005634 | IEA |
| 282132 | GO:0005634 | ISS |
| 282132 | GO:0016020 | IEA |
| 282132 | GO:0005737 | IEA |
| 282132 | GO:0016021 | IEA |
| 282150 | GO:0005739 | IEA |
| 282191 | GO:0005581 | IEA |
| 282191 | GO:0005578 | IEA |
| 282191 | GO:0005604 | IEA |
| 282191 | GO:0005581 | IEA |
| 282191 | GO:0005578 | IEA |
| 282191 | GO:0005604 | IEA |
| 282200 | GO:0005740 | IEA |
| 282200 | GO:0016021 | IEA |
| 282200 | GO:0005739 | IEA |
| 282200 | GO:0016020 | IEA |
| 282211 | GO:0005783 | IEA |
| 282211 | GO:0005792 | IEA |
| 282211 | GO:0016020 | IEA |
| 282218 | GO:0005764 | IEA |
| 282220 | GO:0005737 | IEA |
| 282261 | GO:0005615 | IEA |
| 282261 | GO:0005576 | IEA |
| 282262 | GO:0005576 | IEA |
| 282262 | GO:0005615 | IEA |
| 282277 | GO:0005762 | IEA |
| 282277 | GO:0030529 | IEA |
| 282277 | GO:0005739 | IEA |
| 282277 | GO:0005739 | IEA |
| 282277 | GO:0030529 | IEA |
| 282277 | GO:0005762 | IEA |
| 282289 | GO:0005739 | IEA |
| 282290 | GO:0005739 | IEA |
| 282303 | GO:0016020 | IEA |
| 282303 | GO:0016021 | IEA |
| 282303 | GO:0016021 | IEA |
| 282303 | GO:0016020 | IEA |
| 282346 | GO:0016021 | IEA |
| 282346 | GO:0016020 | IEA |
| 282346 | GO:0005887 | IEA |
| 282346 | GO:0000299 | IEA |
| 282385 | GO:0005856 | IEA |
| 282385 | GO:0005737 | IEA |
| 282386 | GO:0005737 | IEA |
| 282386 | GO:0005856 | IEA |
| 282389 | GO:0005739 | IEA |
| 282389 | GO:0005737 | IEA |
| 282393 | GO:0005746 | IEA |
| 282393 | GO:0005743 | ISS |
| 282393 | GO:0005739 | IEA |
| 282393 | GO:0016020 | IEA |
| 282393 | GO:0005743 | IEA |
| 282394 | GO:0005746 | IEA |
| 282394 | GO:0005743 | IEA |
| 282394 | GO:0005739 | IEA |
| 282394 | GO:0016020 | IEA |
| 282405 | GO:0016469 | IEA |
| 282438 | GO:0005829 | ISS |
| 282438 | GO:0005764 | IEA |
| 282446 | GO:0005739 | IEA |
| 282446 | GO:0005739 | IEA |
| 282477 | GO:0005739 | IEA |
| 282477 | GO:0005743 | IEA |
| 282477 | GO:0016021 | IEA |
| 282477 | GO:0016020 | IEA |
| 282477 | GO:0005743 | ISS |
| 282478 | GO:0005739 | IEA |
| 282478 | GO:0016020 | IEA |
| 282478 | GO:0016021 | IEA |
| 282478 | GO:0005743 | IEA |
| 282517 | GO:0005739 | IEA |
| 282523 | GO:0005743 | IEA |
| 282523 | GO:0005739 | IEA |
| 282523 | GO:0016021 | IEA |
| 282523 | GO:0016020 | IEA |
| 282564 | GO:0016021 | IEA |
| 282564 | GO:0016020 | IEA |
| 282564 | GO:0008305 | IEA |
| 282572 | GO:0016020 | IEA |
| 282572 | GO:0016021 | IEA |
| 282572 | GO:0030016 | IEA |
| 282689 | GO:0005737 | IEA |
| 282689 | GO:0005769 | IEA |
| 282689 | GO:0042383 | IEA |
| 282690 | GO:0045263 | IEA |
| 282690 | GO:0016469 | IEA |
| 282690 | GO:0005739 | IEA |
| 282690 | GO:0005743 | IEA |
| 282701 | GO:0016469 | IEA |
| 282701 | GO:0005739 | IEA |
| 282701 | GO:0005743 | IEA |
| 282701 | GO:0045263 | IEA |
| 282705 | GO:0005887 | ISS |
| 282705 | GO:0016020 | IEA |
| 282705 | GO:0000776 | ISS |
| 282705 | GO:0016021 | IEA |
| 282705 | GO:0005639 | ISS |
| 282705 | GO:0005739 | ISS |
| 282705 | GO:0000775 | ISS |
| 282705 | GO:0005783 | IEA |
| 282705 | GO:0005624 | ISS |
| 282705 | GO:0005794 | ISS |
| 282705 | GO:0000775 | ISS |
| 282705 | GO:0005739 | ISS |
| 282705 | GO:0005887 | ISS |
| 282705 | GO:0005624 | ISS |
| 282705 | GO:0005639 | ISS |
| 282705 | GO:0000776 | ISS |
| 282705 | GO:0005783 | IEA |
| 282705 | GO:0005794 | ISS |
| 282705 | GO:0016021 | IEA |
| 282705 | GO:0016020 | IEA |
| 282710 | GO:0045263 | IEA |
| 282710 | GO:0005739 | IEA |
| 282710 | GO:0016469 | IEA |
| 282716 | GO:0005739 | IEA |
| 282716 | GO:0005741 | IEA |
| 282716 | GO:0019867 | IEA |
| 282716 | GO:0016021 | IEA |
| 282716 | GO:0016020 | IEA |
| 282848 | GO:0016272 | IEA |
| 282848 | GO:0005737 | IEA |
| 282856 | GO:0005739 | IEA |
| 282862 | GO:0005886 | IEA |
| 282872 | GO:0005578 | IEA |
| 282876 | GO:0005737 | IEA |
| 282876 | GO:0030863 | IEA |
| 282876 | GO:0048471 | IEA |
| 282885 | GO:0005777 | IEA |
| 282885 | GO:0005739 | IEA |
| 286770 | GO:0005737 | IEA |
| 286807 | GO:0005634 | IEA |
| 286809 | GO:0005739 | IEA |
| 286838 | GO:0005622 | IEA |
| 286838 | GO:0016021 | IEA |
| 286838 | GO:0016020 | IEA |
| 286838 | GO:0005840 | IEA |
| 286840 | GO:0016020 | IEA |
| 286840 | GO:0005743 | IEA |
| 286840 | GO:0005739 | IEA |
| 286862 | GO:0005737 | ISS |
| 286862 | GO:0005737 | ISS |
| 286885 | GO:0005739 | IEA |
| 286885 | GO:0005746 | IEA |
| 286885 | GO:0016020 | IEA |
| 286886 | GO:0005739 | IEA |
| 286886 | GO:0005743 | IEA |
| 286886 | GO:0005886 | IEA |
| 286886 | GO:0005739 | ISS |
| 286886 | GO:0009986 | ISS |
| 286886 | GO:0005743 | ISS |
| 286886 | GO:0005743 | IEA |
| 286886 | GO:0005886 | IEA |
| 286886 | GO:0009986 | ISS |
| 286886 | GO:0005739 | IEA |
| 286886 | GO:0005739 | ISS |
| 286886 | GO:0005743 | ISS |
| 286969 | GO:0016021 | IEA |
| 286969 | GO:0016020 | IEA |
| 287012 | GO:0016020 | IEA |
| 287012 | GO:0005739 | IEA |
| 287012 | GO:0005740 | IEA |
| 287014 | GO:0005743 | IEA |
| 287014 | GO:0005739 | IEA |
| 287020 | GO:0016020 | IEA |
| 287020 | GO:0005743 | IEA |
| 287020 | GO:0016021 | IEA |
| 287020 | GO:0005746 | IEA |
| 287020 | GO:0005739 | IEA |
| 287027 | GO:0005739 | IEA |
| 287027 | GO:0005747 | IEA |
| 287327 | GO:0005739 | IEA |
| 287327 | GO:0005743 | IEA |
| 288380 | GO:0005739 | IEA |
| 288380 | GO:0005758 | IEA |
| 317655 | GO:0016459 | IEA |
| 317655 | GO:0001725 | ISS |
| 317655 | GO:0005737 | ISS |
| 317697 | GO:0016021 | IEA |
| 317697 | GO:0005783 | IEA |
| 317697 | GO:0016020 | IEA |
| 317706 | GO:0005783 | IEA |
| 317706 | GO:0005792 | IEA |
| 317706 | GO:0009986 | IEA |
| 317706 | GO:0016020 | IEA |
| 317706 | GO:0016021 | IEA |
| 319141 | GO:0005739 | IEA |
| 319141 | GO:0005743 | IEA |
| 326346 | GO:0016021 | IEA |
| 326346 | GO:0005739 | IEA |
| 326346 | GO:0016020 | IEA |
| 326578 | GO:0005786 | IEA |
| 326578 | GO:0005634 | IEA |
| 326581 | GO:0005853 | IEA |
| 326601 | GO:0005634 | IEA |
| 326601 | GO:0005694 | IEA |
| 326601 | GO:0000786 | IEA |
| 326601 | GO:0005694 | IEA |
| 326601 | GO:0005634 | IEA |
| 326601 | GO:0000786 | IEA |
| 326601 | GO:0000786 | IEA |
| 326601 | GO:0005694 | IEA |
| 326601 | GO:0005634 | IEA |
| 326601 | GO:0005694 | IEA |
| 326601 | GO:0005634 | IEA |
| 326601 | GO:0000786 | IEA |
| 326601 | GO:0005634 | IEA |
| 326601 | GO:0005694 | IEA |
| 326601 | GO:0000786 | IEA |
| 327660 | GO:0005739 | IEA |
| 327660 | GO:0005743 | IEA |
| 327665 | GO:0005739 | IEA |
| 327666 | GO:0016021 | IEA |
| 327666 | GO:0016020 | IEA |
| 327666 | GO:0016020 | IEA |
| 327666 | GO:0016021 | IEA |
| 327668 | GO:0045261 | IEA |
| 327668 | GO:0016469 | IEA |
| 327668 | GO:0005743 | IEA |
| 327668 | GO:0005739 | IEA |
| 327668 | GO:0016020 | IEA |
| 327669 | GO:0005739 | IEA |
| 327670 | GO:0005747 | IEA |
| 327670 | GO:0005739 | IEA |
| 327673 | GO:0005739 | IEA |
| 327674 | GO:0016020 | IEA |
| 327674 | GO:0005746 | IEA |
| 327674 | GO:0016021 | IEA |
| 327674 | GO:0005739 | IEA |
| 327680 | GO:0005739 | IEA |
| 327684 | GO:0005634 | IEA |
| 327688 | GO:0005746 | IEA |
| 327688 | GO:0005739 | IEA |
| 327688 | GO:0016020 | IEA |
| 327690 | GO:0005739 | IEA |
| 327691 | GO:0005739 | IEA |
| 327696 | GO:0005739 | IEA |
| 327696 | GO:0016020 | IEA |
| 327696 | GO:0016021 | IEA |
| 327697 | GO:0005747 | IEA |
| 327697 | GO:0005739 | IEA |
| 327698 | GO:0005739 | IEA |
| 327699 | GO:0005739 | IEA |
| 327699 | GO:0009986 | ISS |
| 327701 | GO:0005743 | IEA |
| 327701 | GO:0005739 | IEA |
| 327702 | GO:0005759 | IDA |
| 327702 | GO:0005739 | IEA |
| 327702 | GO:0031966 | IDA |
| 327704 | GO:0005739 | IEA |
| 327704 | GO:0005743 | IEA |
| 327706 | GO:0005739 | IEA |
| 327706 | GO:0005743 | IEA |
| 327707 | GO:0005739 | IEA |
| 327710 | GO:0005743 | IEA |
| 327710 | GO:0005739 | IEA |
| 327713 | GO:0005739 | IEA |
| 327717 | GO:0005739 | IEA |
| 327718 | GO:0016020 | IEA |
| 327718 | GO:0016021 | IEA |
| 327718 | GO:0005739 | IEA |
| 337916 | GO:0005634 | IEA |
| 337918 | GO:0009986 | IEA |
| 337918 | GO:0016020 | IEA |
| 337927 | GO:0016020 | IEA |
| 337927 | GO:0005743 | IEA |
| 337927 | GO:0005739 | IEA |
| 337927 | GO:0016021 | IEA |
| 337927 | GO:0016021 | IEA |
| 337927 | GO:0005739 | IEA |
| 337927 | GO:0005743 | IEA |
| 337927 | GO:0016020 | IEA |
| 338040 | GO:0016469 | IEA |
| 338040 | GO:0045263 | IEA |
| 338040 | GO:0005739 | IEA |
| 338044 | GO:0005886 | IEA |
| 338046 | GO:0005743 | IEA |
| 338046 | GO:0005739 | IEA |
| 338053 | GO:0016021 | IEA |
| 338053 | GO:0005743 | IEA |
| 338053 | GO:0005739 | IEA |
| 338053 | GO:0045263 | IEA |
| 338053 | GO:0016469 | IEA |
| 338053 | GO:0016020 | IEA |
| 338057 | GO:0005739 | IEA |
| 338060 | GO:0005739 | IEA |
| 338062 | GO:0042612 | IEA |
| 338063 | GO:0005743 | IEA |
| 338063 | GO:0005739 | IEA |
| 338064 | GO:0016021 | IEA |
| 338064 | GO:0016020 | IEA |
| 338064 | GO:0005739 | IEA |
| 338065 | GO:0005743 | IEA |
| 338065 | GO:0005739 | IEA |
| 338066 | GO:0016021 | IEA |
| 338066 | GO:0016020 | IEA |
| 338068 | GO:0016021 | IEA |
| 338068 | GO:0048179 | ISS |
| 338068 | GO:0048179 | IEA |
| 338068 | GO:0005887 | ISS |
| 338068 | GO:0016020 | IEA |
| 338068 | GO:0045177 | IEA |
| 338068 | GO:0005887 | IEA |
| 338073 | GO:0005743 | IEA |
| 338073 | GO:0005739 | IEA |
| 338079 | GO:0005739 | IEA |
| 338079 | GO:0005743 | IEA |
| 338081 | GO:0005739 | IEA |
| 338081 | GO:0016469 | IEA |
| 338081 | GO:0005740 | IDA |
| 338081 | GO:0045259 | IDA |
| 338081 | GO:0045261 | IEA |
| 338081 | GO:0005743 | IEA |
| 338084 | GO:0005737 | IEA |
| 338084 | GO:0005654 | IEA |
| 338084 | GO:0016020 | IEA |
| 338084 | GO:0005747 | IEA |
| 338084 | GO:0005654 | ISS |
| 338084 | GO:0005634 | IEA |
| 338084 | GO:0016021 | IEA |
| 338084 | GO:0005737 | ISS |
| 338084 | GO:0005743 | IEA |
| 338084 | GO:0005746 | IDA |
| 338084 | GO:0005739 | IEA |
| 338086 | GO:0016020 | IEA |
| 338086 | GO:0005739 | IEA |
| 338086 | GO:0016021 | IEA |
| 338086 | GO:0005746 | IEA |
| 338087 | GO:0005739 | IEA |
| 338088 | GO:0045261 | IEA |
| 338088 | GO:0005739 | IEA |
| 338088 | GO:0016469 | IEA |
| 338472 | GO:0005776 | IEA |
| 338472 | GO:0005622 | IEA |
| 353217 | GO:0016021 | IEA |
| 353217 | GO:0005783 | IEA |
| 353217 | GO:0016020 | IEA |
| 353348 | GO:0005635 | IEA |
| 353348 | GO:0019031 | IEA |
| 359716 | GO:0005634 | ISS |
| 359716 | GO:0005737 | ISS |
| 359716 | GO:0000791 | ISS |
| 359716 | GO:0016363 | ISS |
| 359720 | GO:0005783 | IEA |
| 360005 | GO:0005634 | IEA |
| 360007 | GO:0005576 | IEA |
| 360196 | GO:0005634 | IEA |
| 360196 | GO:0005694 | IEA |
| 399560 | GO:0005840 | IEA |
| 399560 | GO:0005739 | IEA |
| 399560 | GO:0030529 | IEA |
| 399560 | GO:0005622 | IEA |
| 399683 | GO:0016020 | IEA |
| 399683 | GO:0016021 | IEA |
| 404069 | GO:0005829 | ISS |
| 404069 | GO:0030140 | ISS |
| 404069 | GO:0005829 | IEA |
| 404122 | GO:0035267 | ISS |
| 404122 | GO:0005856 | IEA |
| 404126 | GO:0005634 | IEA |
| 404186 | GO:0005576 | IEA |
| 404188 | GO:0005743 | IEA |
| 404188 | GO:0005739 | IEA |
| 407170 | GO:0016020 | IEA |
| 407209 | GO:0016020 | IEA |
| 407209 | GO:0016012 | IEA |
| 407209 | GO:0016012 | IEA |
| 407209 | GO:0016020 | IEA |
| 407240 | GO:0005634 | IEA |
| 414735 | GO:0016020 | IEA |
| 414735 | GO:0016021 | IEA |
| 414735 | GO:0016020 | IEA |
| 414735 | GO:0016021 | IEA |
| 414922 | GO:0016020 | IEA |
| 414922 | GO:0016021 | IEA |
| 414922 | GO:0005923 | IEA |
| 444858 | GO:0005737 | IEA |
| 444864 | GO:0005622 | IEA |
| 444864 | GO:0005634 | IEA |
| 444864 | GO:0005634 | IEA |
| 444864 | GO:0005622 | IEA |
| 444866 | GO:0005622 | IEA |
| 444866 | GO:0005634 | IEA |
| 444866 | GO:0005634 | IEA |
| 444866 | GO:0005622 | IEA |
| 444878 | GO:0016020 | IEA |
| 444878 | GO:0005739 | IEA |
| 444878 | GO:0005743 | ISS |
| 444878 | GO:0005743 | IEA |
| 444879 | GO:0030173 | IEA |
| 445421 | GO:0030529 | IEA |
| 445421 | GO:0005739 | IEA |
| 445421 | GO:0005622 | IEA |
| 445421 | GO:0005840 | IEA |
| 445421 | GO:0005739 | IEA |
| 445421 | GO:0030529 | IEA |
| 445421 | GO:0005622 | IEA |
| 445421 | GO:0005840 | IEA |
| 445543 | GO:0005737 | ISS |
| 445543 | GO:0005856 | IEA |
| 445543 | GO:0005624 | ISS |
| 493728 | GO:0008290 | IEA |
| 503685 | GO:0001533 | IEA |
| 504317 | GO:0016020 | IEA |
| 504317 | GO:0005886 | IEA |
| 504474 | GO:0008250 | IEA |
| 504534 | GO:0016020 | IEA |
| 504534 | GO:0016021 | IEA |
| 504599 | GO:0005694 | IEA |
| 504599 | GO:0000786 | IEA |
| 504599 | GO:0005634 | IEA |
| 504599 | GO:0000786 | IEA |
| 504599 | GO:0005694 | IEA |
| 504599 | GO:0005634 | IEA |
| 504599 | GO:0005634 | IEA |
| 504599 | GO:0005694 | IEA |
| 504599 | GO:0000786 | IEA |
| 504599 | GO:0000786 | IEA |
| 504599 | GO:0005694 | IEA |
| 504599 | GO:0005634 | IEA |
| 504599 | GO:0005634 | IEA |
| 504599 | GO:0005694 | IEA |
| 504599 | GO:0000786 | IEA |
| 504637 | GO:0016020 | IEA |
| 504637 | GO:0005663 | IEA |
| 504716 | GO:0005737 | IEA |
| 504716 | GO:0016363 | IEA |
| 504862 | GO:0030018 | IEA |
| 504862 | GO:0005916 | IEA |
| 505005 | GO:0005739 | IEA |
| 505050 | GO:0005829 | IEA |
| 505050 | GO:0043234 | IEA |
| 505050 | GO:0005839 | IEA |
| 505060 | GO:0016020 | IEA |
| 505060 | GO:0016021 | IEA |
| 505093 | GO:0030529 | IEA |
| 505093 | GO:0005681 | IEA |
| 505093 | GO:0005634 | IEA |
| 505176 | GO:0005829 | IEA |
| 505176 | GO:0043234 | IEA |
| 505176 | GO:0005839 | IEA |
| 505219 | GO:0016021 | IEA |
| 505219 | GO:0016020 | IEA |
| 505293 | GO:0005634 | IEA |
| 505293 | GO:0000119 | IEA |
| 505406 | GO:0005622 | IEA |
| 505453 | GO:0000123 | IEA |
| 505453 | GO:0000940 | IEA |
| 505453 | GO:0005634 | IEA |
| 505471 | GO:0000785 | IEA |
| 505471 | GO:0005634 | IEA |
| 505489 | GO:0005739 | IEA |
| 505489 | GO:0016020 | IEA |
| 505489 | GO:0016021 | IEA |
| 505489 | GO:0016607 | IEA |
| 505489 | GO:0005744 | IEA |
| 505645 | GO:0005856 | IEA |
| 505775 | GO:0016020 | IEA |
| 505775 | GO:0016021 | IEA |
| 505884 | GO:0005622 | IEA |
| 505884 | GO:0005634 | IEA |
| 505884 | GO:0005737 | IEA |
| 505884 | GO:0005622 | IEA |
| 505884 | GO:0005634 | IEA |
| 505884 | GO:0005737 | IEA |
| 505884 | GO:0005622 | IEA |
| 505884 | GO:0005737 | IEA |
| 505884 | GO:0005634 | IEA |
| 505968 | GO:0005739 | ISS |
| 505968 | GO:0005759 | IEA |
| 505968 | GO:0005739 | IEA |
| 505968 | GO:0005759 | ISS |
| 506114 | GO:0005634 | IEA |
| 506114 | GO:0005667 | IEA |
| 506218 | GO:0005625 | IEA |
| 506218 | GO:0005737 | IEA |
| 506218 | GO:0005886 | IEA |
| 506218 | GO:0005792 | IEA |
| 506231 | GO:0005622 | IEA |
| 506235 | GO:0005634 | IEA |
| 506250 | GO:0005739 | IEA |
| 506250 | GO:0005739 | IEA |
| 506295 | GO:0016020 | IEA |
| 506336 | GO:0016020 | IEA |
| 506336 | GO:0016021 | IEA |
| 506336 | GO:0005783 | IEA |
| 506448 | GO:0005622 | IEA |
| 506448 | GO:0005634 | IEA |
| 506544 | GO:0005737 | IEA |
| 506544 | GO:0005945 | IEA |
| 506544 | GO:0005737 | IEA |
| 506544 | GO:0005945 | IEA |
| 506730 | GO:0015630 | IEA |
| 506730 | GO:0015629 | IEA |
| 506764 | GO:0016020 | IEA |
| 506764 | GO:0005634 | IEA |
| 506764 | GO:0005634 | IEA |
| 506764 | GO:0016020 | IEA |
| 506882 | GO:0005622 | IEA |
| 506882 | GO:0005773 | IEA |
| 506969 | GO:0016023 | IEA |
| 506991 | GO:0005783 | IEA |
| 507107 | GO:0009986 | IEA |
| 507114 | GO:0005759 | IEA |
| 507114 | GO:0005737 | IEA |
| 507114 | GO:0005634 | IEA |
| 507179 | GO:0008180 | IEA |
| 507211 | GO:0005634 | IEA |
| 507213 | GO:0043234 | IEA |
| 507213 | GO:0005829 | IEA |
| 507213 | GO:0005839 | IEA |
| 507216 | GO:0005739 | IEA |
| 507216 | GO:0019867 | IEA |
| 507216 | GO:0016021 | IEA |
| 507216 | GO:0005741 | IEA |
| 507216 | GO:0016020 | IEA |
| 507319 | GO:0016020 | IEA |
| 507342 | GO:0030175 | IEA |
| 507365 | GO:0005622 | IEA |
| 507367 | GO:0005739 | IEA |
| 507367 | GO:0005743 | IEA |
| 507367 | GO:0005739 | IEA |
| 507367 | GO:0005743 | IEA |
| 507389 | GO:0009986 | IEA |
| 507410 | GO:0005655 | IEA |
| 507431 | GO:0005739 | IEA |
| 507484 | GO:0005737 | IEA |
| 507707 | GO:0005739 | IEA |
| 507707 | GO:0005739 | IEA |
| 507783 | GO:0016021 | IEA |
| 507783 | GO:0016020 | IEA |
| 507869 | GO:0005635 | IEA |
| 507897 | GO:0005634 | IEA |
| 507990 | GO:0016021 | IEA |
| 507990 | GO:0019867 | IEA |
| 507990 | GO:0005578 | IEA |
| 507990 | GO:0005578 | IEA |
| 507990 | GO:0016021 | IEA |
| 507990 | GO:0019867 | IEA |
| 508143 | GO:0005840 | IEA |
| 508143 | GO:0016020 | IEA |
| 508143 | GO:0005622 | IEA |
| 508143 | GO:0030529 | IEA |
| 508143 | GO:0005739 | IEA |
| 508170 | GO:0005739 | IEA |
| 508231 | GO:0005634 | IEA |
| 508268 | GO:0016020 | IEA |
| 508268 | GO:0016021 | IEA |
| 508268 | GO:0005923 | IEA |
| 508285 | GO:0005634 | IEA |
| 508285 | GO:0048269 | IEA |
| 508371 | GO:0016020 | IEA |
| 508371 | GO:0016021 | IEA |
| 508442 | GO:0005634 | IEA |
| 508442 | GO:0005721 | IEA |
| 508442 | GO:0000793 | IEA |
| 508465 | GO:0005783 | IEA |
| 508465 | GO:0030176 | ISS |
| 508465 | GO:0030176 | IEA |
| 508535 | GO:0005634 | IEA |
| 508609 | GO:0005777 | IEA |
| 508609 | GO:0016020 | IEA |
| 508609 | GO:0016021 | IEA |
| 508716 | GO:0016020 | IEA |
| 508722 | GO:0005634 | IEA |
| 508840 | GO:0001669 | IEA |
| 508840 | GO:0031410 | IEA |
| 508840 | GO:0012506 | IEA |
| 508840 | GO:0042598 | IEA |
| 508840 | GO:0005829 | IEA |
| 508882 | GO:0030027 | IEA |
| 509037 | GO:0005624 | IEA |
| 509037 | GO:0005789 | IEA |
| 509111 | GO:0005654 | IEA |
| 509111 | GO:0005737 | IEA |
| 509150 | GO:0005739 | IEA |
| 509192 | GO:0005635 | IEA |
| 509192 | GO:0005789 | IEA |
| 509459 | GO:0019867 | IEA |
| 509459 | GO:0005739 | IEA |
| 509459 | GO:0016020 | IEA |
| 509459 | GO:0016021 | IEA |
| 509684 | GO:0016020 | IEA |
| 509684 | GO:0005739 | IEA |
| 509775 | GO:0016021 | IEA |
| 509816 | GO:0005622 | IEA |
| 509816 | GO:0005739 | IEA |
| 509816 | GO:0030529 | IEA |
| 509816 | GO:0005763 | IDA |
| 509816 | GO:0005840 | IEA |
| 509827 | GO:0005624 | IEA |
| 509856 | GO:0009898 | IEA |
| 509856 | GO:0005829 | IEA |
| 509966 | GO:0005622 | IEA |
| 509983 | GO:0005739 | IEA |
| 509983 | GO:0005743 | IEA |
| 510029 | GO:0043234 | IEA |
| 510029 | GO:0005634 | IEA |
| 510029 | GO:0005829 | IEA |
| 510029 | GO:0005737 | IEA |
| 510050 | GO:0005634 | IEA |
| 510069 | GO:0005839 | IEA |
| 510069 | GO:0043234 | IEA |
| 510069 | GO:0005829 | IEA |
| 510080 | GO:0016021 | IEA |
| 510080 | GO:0016021 | IEA |
| 510080 | GO:0016021 | IEA |
| 510155 | GO:0005839 | IEA |
| 510155 | GO:0005829 | IEA |
| 510155 | GO:0043234 | IEA |
| 510159 | GO:0005874 | IEA |
| 510194 | GO:0000786 | IEA |
| 510194 | GO:0005634 | IEA |
| 510200 | GO:0005737 | IEA |
| 510219 | GO:0005739 | IEA |
| 510219 | GO:0005741 | IEA |
| 510245 | GO:0005829 | IEA |
| 510245 | GO:0005634 | IEA |
| 510245 | GO:0005737 | IEA |
| 510314 | GO:0005634 | IEA |
| 510314 | GO:0043234 | IEA |
| 510314 | GO:0005829 | IEA |
| 510324 | GO:0005856 | IEA |
| 510324 | GO:0005743 | IEA |
| 510324 | GO:0016020 | IEA |
| 510324 | GO:0005739 | IEA |
| 510376 | GO:0031674 | IEA |
| 510376 | GO:0005737 | IEA |
| 510376 | GO:0005634 | IEA |
| 510376 | GO:0005667 | IEA |
| 510376 | GO:0031674 | IEA |
| 510376 | GO:0005634 | IEA |
| 510376 | GO:0005667 | IEA |
| 510376 | GO:0005737 | IEA |
| 510412 | GO:0000139 | IEA |
| 510423 | GO:0043234 | IEA |
| 510423 | GO:0005829 | IEA |
| 510423 | GO:0005839 | IEA |
| 510483 | GO:0016021 | IEA |
| 510498 | GO:0005774 | IEA |
| 510498 | GO:0016020 | IEA |
| 510568 | GO:0005642 | IEA |
| 510574 | GO:0030904 | IEA |
| 510575 | GO:0016021 | IEA |
| 510575 | GO:0016020 | IEA |
| 510727 | GO:0005739 | IEA |
| 510767 | GO:0005739 | IEA |
| 510767 | GO:0005746 | IEA |
| 510811 | GO:0005737 | IEA |
| 510833 | GO:0005737 | IEA |
| 510833 | GO:0005581 | IEA |
| 510833 | GO:0005737 | IEA |
| 510833 | GO:0005581 | IEA |
| 510899 | GO:0005739 | IEA |
| 510899 | GO:0005763 | IEA |
| 510899 | GO:0005840 | IEA |
| 510899 | GO:0005622 | IEA |
| 510937 | GO:0005737 | IEA |
| 510960 | GO:0005615 | IEA |
| 510960 | GO:0005615 | IEA |
| 511090 | GO:0005739 | IEA |
| 511268 | GO:0005737 | IEA |
| 511268 | GO:0005634 | IEA |
| 511268 | GO:0005737 | ISS |
| 511291 | GO:0005737 | IEA |
| 511440 | GO:0005739 | IEA |
| 511453 | GO:0016021 | IEA |
| 511453 | GO:0030176 | IEA |
| 511453 | GO:0016020 | IEA |
| 511453 | GO:0005787 | IEA |
| 511453 | GO:0005783 | IEA |
| 511453 | GO:0005792 | IEA |
| 511492 | GO:0005576 | IEA |
| 511560 | GO:0030529 | IEA |
| 511560 | GO:0005622 | IEA |
| 511560 | GO:0005634 | IEA |
| 511560 | GO:0005840 | IEA |
| 511560 | GO:0005730 | IEA |
| 511614 | GO:0005783 | IEA |
| 511682 | GO:0005795 | IEA |
| 511699 | GO:0016020 | IEA |
| 511735 | GO:0005833 | IEA |
| 511735 | GO:0005833 | IEA |
| 511735 | GO:0005833 | IEA |
| 511763 | GO:0005737 | IEA |
| 511812 | GO:0016020 | IEA |
| 511812 | GO:0005834 | IEA |
| 511861 | GO:0005886 | IEA |
| 511861 | GO:0005794 | IEA |
| 511965 | GO:0005622 | IEA |
| 512002 | GO:0005739 | IEA |
| 512136 | GO:0005643 | IEA |
| 512136 | GO:0005622 | IEA |
| 512136 | GO:0005634 | IEA |
| 512136 | GO:0042405 | IEA |
| 512259 | GO:0005829 | ISS |
| 512259 | GO:0005829 | ISS |
| 512341 | GO:0005739 | IEA |
| 512427 | GO:0005634 | IEA |
| 512435 | GO:0030863 | IEA |
| 512435 | GO:0043005 | IEA |
| 512435 | GO:0005737 | IEA |
| 512435 | GO:0002102 | IEA |
| 512435 | GO:0030426 | IEA |
| 512435 | GO:0032154 | IEA |
| 512435 | GO:0031941 | IEA |
| 512469 | GO:0030529 | IEA |
| 512469 | GO:0005622 | IEA |
| 512469 | GO:0005840 | IEA |
| 512469 | GO:0005739 | IEA |
| 512500 | GO:0005743 | IEA |
| 512500 | GO:0016021 | IEA |
| 512500 | GO:0016020 | IEA |
| 512500 | GO:0005739 | IEA |
| 512500 | GO:0005746 | IEA |
| 512666 | GO:0005624 | IEA |
| 512666 | GO:0005622 | IEA |
| 512673 | GO:0005740 | IEA |
| 512673 | GO:0005739 | IEA |
| 512741 | GO:0005694 | IEA |
| 512741 | GO:0000786 | IEA |
| 512741 | GO:0005634 | IEA |
| 512741 | GO:0005694 | IEA |
| 512741 | GO:0005634 | IEA |
| 512741 | GO:0000786 | IEA |
| 512741 | GO:0005634 | IEA |
| 512741 | GO:0005694 | IEA |
| 512741 | GO:0000786 | IEA |
| 512741 | GO:0005694 | IEA |
| 512741 | GO:0005634 | IEA |
| 512741 | GO:0000786 | IEA |
| 512741 | GO:0005694 | IEA |
| 512741 | GO:0000786 | IEA |
| 512741 | GO:0005634 | IEA |
| 512818 | GO:0005739 | IEA |
| 512818 | GO:0016020 | IEA |
| 512818 | GO:0042719 | IEA |
| 512891 | GO:0005838 | IEA |
| 512891 | GO:0005829 | IEA |
| 512891 | GO:0043234 | IEA |
| 512902 | GO:0005739 | IEA |
| 512902 | GO:0005739 | IEA |
| 512978 | GO:0005681 | IEA |
| 512978 | GO:0030529 | IEA |
| 512978 | GO:0005634 | IEA |
| 513038 | GO:0005764 | IEA |
| 513155 | GO:0016021 | IEA |
| 513188 | GO:0016021 | IEA |
| 513188 | GO:0016020 | IEA |
| 513265 | GO:0001652 | IEA |
| 513265 | GO:0005730 | IEA |
| 513265 | GO:0005654 | IEA |
| 513265 | GO:0005634 | IEA |
| 513324 | GO:0016021 | IEA |
| 513324 | GO:0005739 | IEA |
| 513324 | GO:0005743 | IEA |
| 513324 | GO:0005743 | IEA |
| 513324 | GO:0005739 | IEA |
| 513324 | GO:0016021 | IEA |
| 513397 | GO:0005759 | IEA |
| 513405 | GO:0005730 | IEA |
| 513438 | GO:0030529 | IEA |
| 513438 | GO:0005739 | IEA |
| 513438 | GO:0005763 | IDA |
| 513438 | GO:0005739 | IEA |
| 513438 | GO:0030529 | IEA |
| 513438 | GO:0005763 | IDA |
| 513461 | GO:0005829 | IEA |
| 513461 | GO:0043234 | IEA |
| 513504 | GO:0005743 | IEA |
| 513504 | GO:0005739 | IEA |
| 513530 | GO:0005739 | IEA |
| 513622 | GO:0005761 | IEA |
| 513699 | GO:0042588 | IEA |
| 513762 | GO:0005739 | IEA |
| 513762 | GO:0005783 | IEA |
| 513762 | GO:0005634 | IEA |
| 513847 | GO:0016021 | IEA |
| 513847 | GO:0016020 | IEA |
| 513889 | GO:0005739 | IEA |
| 513934 | GO:0016363 | IEA |
| 513958 | GO:0005851 | IEA |
| 513958 | GO:0005851 | ISS |
| 513958 | GO:0005737 | IEA |
| 513958 | GO:0005737 | ISS |
| 514230 | GO:0016021 | IEA |
| 514230 | GO:0005739 | IEA |
| 514374 | GO:0005739 | IEA |
| 514386 | GO:0005634 | IEA |
| 514386 | GO:0005737 | IEA |
| 514386 | GO:0005622 | IEA |
| 514403 | GO:0005622 | IEA |
| 514403 | GO:0030529 | IEA |
| 514403 | GO:0005739 | IEA |
| 514403 | GO:0005840 | IEA |
| 514443 | GO:0005669 | IEA |
| 514443 | GO:0005737 | IEA |
| 514443 | GO:0005634 | IEA |
| 514443 | GO:0048471 | IEA |
| 514472 | GO:0005739 | IEA |
| 514473 | GO:0000228 | IEA |
| 514473 | GO:0000792 | IEA |
| 514665 | GO:0008021 | IEA |
| 514665 | GO:0016020 | IEA |
| 514740 | GO:0005739 | IEA |
| 514740 | GO:0030529 | IEA |
| 514753 | GO:0005739 | IEA |
| 514753 | GO:0030529 | IEA |
| 514807 | GO:0005737 | IEA |
| 514859 | GO:0044445 | IEA |
| 514918 | GO:0005634 | IEA |
| 514918 | GO:0005634 | IEA |
| 514928 | GO:0005794 | IEA |
| 515109 | GO:0005739 | IEA |
| 515109 | GO:0016020 | IEA |
| 515109 | GO:0042719 | IEA |
| 515176 | GO:0005783 | IEA |
| 515178 | GO:0005778 | IEA |
| 515178 | GO:0016021 | IEA |
| 515178 | GO:0016020 | IEA |
| 515178 | GO:0005777 | IEA |
| 515194 | GO:0005739 | IEA |
| 515194 | GO:0005739 | IEA |
| 515363 | GO:0005739 | IEA |
| 515363 | GO:0005634 | IEA |
| 515363 | GO:0016020 | IEA |
| 515380 | GO:0005634 | IEA |
| 515490 | GO:0030027 | IEA |
| 515494 | GO:0016021 | IEA |
| 515503 | GO:0005839 | IEA |
| 515503 | GO:0043234 | IEA |
| 515503 | GO:0005829 | IEA |
| 515646 | GO:0005737 | ISS |
| 515646 | GO:0005624 | IEA |
| 515646 | GO:0005625 | IEA |
| 515646 | GO:0005625 | ISS |
| 515646 | GO:0005737 | IEA |
| 515646 | GO:0005903 | ISS |
| 515646 | GO:0016020 | IEA |
| 515646 | GO:0005634 | IEA |
| 515646 | GO:0005624 | ISS |
| 515652 | GO:0005634 | IEA |
| 515652 | GO:0000785 | IEA |
| 515696 | GO:0016469 | IEA |
| 515696 | GO:0005739 | IEA |
| 515696 | GO:0045263 | IEA |
| 515757 | GO:0016021 | IEA |
| 515757 | GO:0005743 | IEA |
| 515757 | GO:0016020 | IEA |
| 515757 | GO:0005739 | IEA |
| 515757 | GO:0016020 | IEA |
| 515757 | GO:0016021 | IEA |
| 515757 | GO:0005743 | IEA |
| 515757 | GO:0005739 | IEA |
| 515902 | GO:0005739 | IEA |
| 515990 | GO:0005762 | IEA |
| 515990 | GO:0005840 | IEA |
| 515990 | GO:0005739 | IEA |
| 515990 | GO:0005622 | IEA |
| 515990 | GO:0030529 | IEA |
| 515999 | GO:0005622 | IEA |
| 515999 | GO:0005783 | IEA |
| 515999 | GO:0016020 | IEA |
| 515999 | GO:0005783 | IEA |
| 515999 | GO:0016020 | IEA |
| 515999 | GO:0005622 | IEA |
| 516334 | GO:0016020 | IEA |
| 516874 | GO:0005759 | IEA |
| 517139 | GO:0005634 | IEA |
| 517139 | GO:0000786 | IEA |
| 517139 | GO:0005694 | IEA |
| 517139 | GO:0005694 | IEA |
| 517139 | GO:0000786 | IEA |
| 517139 | GO:0005634 | IEA |
| 517139 | GO:0005634 | IEA |
| 517139 | GO:0000786 | IEA |
| 517139 | GO:0005694 | IEA |
| 517139 | GO:0005694 | IEA |
| 517139 | GO:0000786 | IEA |
| 517139 | GO:0005634 | IEA |
| 517139 | GO:0005694 | IEA |
| 517139 | GO:0005634 | IEA |
| 517139 | GO:0000786 | IEA |
| 517171 | GO:0005622 | IEA |
| 517171 | GO:0016529 | IEA |
| 517171 | GO:0005783 | IEA |
| 517345 | GO:0005794 | IEA |
| 517345 | GO:0005622 | IEA |
| 517402 | GO:0005739 | IEA |
| 517405 | GO:0005624 | IEA |
| 517405 | GO:0005887 | IEA |
| 517535 | GO:0005739 | IEA |
| 517535 | GO:0009986 | IEA |
| 517539 | GO:0005739 | IEA |
| 517688 | GO:0005887 | IEA |
| 517688 | GO:0005887 | IEA |
| 517812 | GO:0005634 | IEA |
| 518045 | GO:0005737 | IEA |
| 518045 | GO:0005634 | IEA |
| 518117 | GO:0016529 | IEA |
| 518117 | GO:0016021 | IEA |
| 518117 | GO:0016020 | IEA |
| 518117 | GO:0005783 | IEA |
| 518117 | GO:0005783 | IEA |
| 518117 | GO:0016020 | IEA |
| 518117 | GO:0016529 | IEA |
| 518117 | GO:0016021 | IEA |
| 518169 | GO:0005739 | IEA |
| 518169 | GO:0005764 | IEA |
| 518318 | GO:0005634 | IEA |
| 518318 | GO:0005694 | IEA |
| 518318 | GO:0000786 | IEA |
| 518318 | GO:0005694 | IEA |
| 518318 | GO:0005634 | IEA |
| 518318 | GO:0000786 | IEA |
| 518318 | GO:0000786 | IEA |
| 518318 | GO:0005634 | IEA |
| 518318 | GO:0005694 | IEA |
| 518318 | GO:0000786 | IEA |
| 518318 | GO:0005694 | IEA |
| 518318 | GO:0005634 | IEA |
| 518318 | GO:0005634 | IEA |
| 518318 | GO:0000786 | IEA |
| 518318 | GO:0005694 | IEA |
| 518321 | GO:0005759 | IEA |
| 518321 | GO:0005739 | IEA |
| 518402 | GO:0005871 | IEA |
| 518601 | GO:0005737 | IEA |
| 518601 | GO:0005886 | IEA |
| 518601 | GO:0005622 | IEA |
| 518659 | GO:0005932 | IEA |
| 518738 | GO:0005622 | IEA |
| 518758 | GO:0016021 | IEA |
| 518758 | GO:0016020 | IEA |
| 518758 | GO:0005743 | IEA |
| 518758 | GO:0005743 | IEA |
| 518758 | GO:0016020 | IEA |
| 518758 | GO:0016021 | IEA |
| 519213 | GO:0005789 | IEA |
| 519302 | GO:0005739 | IEA |
| 519302 | GO:0005622 | IEA |
| 519302 | GO:0030529 | IEA |
| 519302 | GO:0005840 | IEA |
| 519302 | GO:0030529 | IEA |
| 519302 | GO:0005622 | IEA |
| 519302 | GO:0005739 | IEA |
| 519302 | GO:0005840 | IEA |
| 519597 | GO:0005737 | IEA |
| 519777 | GO:0000780 | IEA |
| 520024 | GO:0005624 | IEA |
| 520111 | GO:0005737 | IEA |
| 520111 | GO:0005622 | IEA |
| 520111 | GO:0005737 | IEA |
| 520111 | GO:0005622 | IEA |
| 520260 | GO:0005739 | IEA |
| 520260 | GO:0045239 | IEA |
| 520260 | GO:0045239 | IEA |
| 520260 | GO:0005739 | IEA |
| 520335 | GO:0016021 | IEA |
| 520335 | GO:0005739 | IEA |
| 520335 | GO:0005743 | IEA |
| 520472 | GO:0005737 | IEA |
| 520631 | GO:0005622 | IEA |
| 520631 | GO:0005634 | IEA |
| 520891 | GO:0030529 | IEA |
| 520891 | GO:0005634 | IEA |
| 521073 | GO:0016020 | IEA |
| 521073 | GO:0042719 | IEA |
| 521073 | GO:0005739 | IEA |
| 521378 | GO:0005634 | IEA |
| 521581 | GO:0000786 | IEA |
| 521581 | GO:0005634 | IEA |
| 521581 | GO:0005694 | IEA |
| 521581 | GO:0005634 | IEA |
| 521581 | GO:0005694 | IEA |
| 521581 | GO:0000786 | IEA |
| 521581 | GO:0000786 | IEA |
| 521581 | GO:0005634 | IEA |
| 521581 | GO:0005694 | IEA |
| 521581 | GO:0000786 | IEA |
| 521581 | GO:0005694 | IEA |
| 521581 | GO:0005634 | IEA |
| 521581 | GO:0005694 | IEA |
| 521581 | GO:0005634 | IEA |
| 521581 | GO:0000786 | IEA |
| 522006 | GO:0005743 | IEA |
| 522006 | GO:0005739 | IEA |
| 523039 | GO:0005739 | IEA |
| 523214 | GO:0005694 | IEA |
| 523214 | GO:0000786 | IEA |
| 523214 | GO:0005634 | IEA |
| 523214 | GO:0005634 | IEA |
| 523214 | GO:0005694 | IEA |
| 523214 | GO:0000786 | IEA |
| 523214 | GO:0000786 | IEA |
| 523214 | GO:0005634 | IEA |
| 523214 | GO:0005694 | IEA |
| 523214 | GO:0000786 | IEA |
| 523214 | GO:0005694 | IEA |
| 523214 | GO:0005634 | IEA |
| 523214 | GO:0000786 | IEA |
| 523214 | GO:0005694 | IEA |
| 523214 | GO:0005634 | IEA |
| 523325 | GO:0016023 | IEA |
| 523325 | GO:0045202 | IEA |
| 523325 | GO:0019717 | IEA |
| 523745 | GO:0005737 | IEA |
| 524075 | GO:0005739 | IEA |
| 524142 | GO:0005743 | IEA |
| 524142 | GO:0030425 | IEA |
| 524142 | GO:0005758 | IEA |
| 524142 | GO:0005739 | IEA |
| 525042 | GO:0009331 | IEA |
| 525042 | GO:0005737 | IEA |
| 525164 | GO:0005739 | IEA |
| 525365 | GO:0005634 | IEA |
| 525365 | GO:0031674 | IEA |
| 525511 | GO:0005694 | IEA |
| 525511 | GO:0005634 | IEA |
| 525511 | GO:0000786 | IEA |
| 525511 | GO:0005694 | IEA |
| 525511 | GO:0005634 | IEA |
| 525511 | GO:0000786 | IEA |
| 525511 | GO:0005634 | IEA |
| 525511 | GO:0000786 | IEA |
| 525511 | GO:0005694 | IEA |
| 525511 | GO:0005694 | IEA |
| 525511 | GO:0000786 | IEA |
| 525511 | GO:0005634 | IEA |
| 525511 | GO:0005694 | IEA |
| 525511 | GO:0005634 | IEA |
| 525511 | GO:0000786 | IEA |
| 525572 | GO:0008180 | IEA |
| 525680 | GO:0016272 | IEA |
| 525716 | GO:0005576 | ISS |
| 525716 | GO:0005634 | IEA |
| 525716 | GO:0005737 | IDA |
| 525716 | GO:0043025 | IEA |
| 525795 | GO:0005605 | IEA |
| 525795 | GO:0009986 | IEA |
| 525795 | GO:0005615 | IEA |
| 525869 | GO:0005739 | IEA |
| 526027 | GO:0005886 | IEA |
| 526027 | GO:0005634 | IEA |
| 526027 | GO:0005886 | IEA |
| 526027 | GO:0005634 | IEA |
| 526430 | GO:0012506 | IEA |
| 526430 | GO:0012506 | IEA |
| 527488 | GO:0042383 | IEA |
| 527488 | GO:0045211 | IEA |
| 527935 | GO:0016607 | IEA |
| 528165 | GO:0005743 | IEA |
| 528165 | GO:0005739 | IEA |
| 528390 | GO:0005634 | IEA |
| 529115 | GO:0005634 | IEA |
| 529124 | GO:0005634 | IEA |
| 529670 | GO:0005606 | IEA |
| 529670 | GO:0005604 | IEA |
| 529670 | GO:0005605 | IEA |
| 529762 | GO:0005739 | IEA |
| 530393 | GO:0005929 | IEA |
| 530393 | GO:0005932 | IEA |
| 530393 | GO:0015629 | IEA |
| 530393 | GO:0016020 | IEA |
| 530393 | GO:0016021 | IEA |
| 530393 | GO:0016020 | IEA |
| 530393 | GO:0005932 | IEA |
| 530393 | GO:0015629 | IEA |
| 530393 | GO:0005929 | IEA |
| 530393 | GO:0016021 | IEA |
| 530393 | GO:0005932 | IEA |
| 530393 | GO:0016020 | IEA |
| 530393 | GO:0005929 | IEA |
| 530393 | GO:0015629 | IEA |
| 530393 | GO:0016021 | IEA |
| 530409 | GO:0016020 | IEA |
| 530409 | GO:0005739 | IEA |
| 530414 | GO:0005739 | IEA |
| 530414 | GO:0005739 | IEA |
| 530414 | GO:0005739 | IEA |
| 530506 | GO:0005634 | IEA |
| 530616 | GO:0005667 | IEA |
| 530616 | GO:0005634 | IEA |
| 530865 | GO:0005739 | IEA |
| 531444 | GO:0005739 | IEA |
| 531770 | GO:0005635 | IEA |
| 531770 | GO:0005737 | IEA |
| 531877 | GO:0005829 | IEA |
| 531990 | GO:0005634 | IEA |
| 531990 | GO:0000786 | IEA |
| 531990 | GO:0005694 | IEA |
| 531990 | GO:0000786 | IEA |
| 531990 | GO:0005694 | IEA |
| 531990 | GO:0005634 | IEA |
| 531990 | GO:0005634 | IEA |
| 531990 | GO:0000786 | IEA |
| 531990 | GO:0005694 | IEA |
| 531990 | GO:0005694 | IEA |
| 531990 | GO:0000786 | IEA |
| 531990 | GO:0005634 | IEA |
| 531990 | GO:0005694 | IEA |
| 531990 | GO:0000786 | IEA |
| 531990 | GO:0005634 | IEA |
| 532120 | GO:0005813 | IEA |
| 532203 | GO:0005840 | IEA |
| 532203 | GO:0030529 | IEA |
| 532203 | GO:0005622 | IEA |
| 532231 | GO:0009434 | IEA |
| 532333 | GO:0005829 | IEA |
| 532389 | GO:0005739 | IEA |
| 532389 | GO:0030529 | IEA |
| 532483 | GO:0005634 | IEA |
| 532483 | GO:0005634 | IEA |
| 532572 | GO:0005605 | IEA |
| 532572 | GO:0005604 | IEA |
| 532572 | GO:0043259 | IEA |
| 532621 | GO:0005634 | IEA |
| 532785 | GO:0005737 | IEA |
| 532785 | GO:0005743 | IEA |
| 532785 | GO:0005739 | IEA |
| 532795 | GO:0005737 | IEA |
| 532945 | GO:0016020 | IEA |
| 532953 | GO:0005814 | IEA |
| 533038 | GO:0016021 | IEA |
| 533038 | GO:0005887 | IEA |
| 533038 | GO:0016021 | IEA |
| 533038 | GO:0005887 | IEA |
| 533077 | GO:0005829 | IEA |
| 533223 | GO:0005856 | IEA |
| 533227 | GO:0005737 | IEA |
| 533227 | GO:0005739 | IEA |
| 533227 | GO:0005829 | IEA |
| 533336 | GO:0005622 | IEA |
| 533379 | GO:0005737 | IEA |
| 533379 | GO:0005634 | IEA |
| 533379 | GO:0005737 | IEA |
| 533379 | GO:0005634 | IEA |
| 533379 | GO:0005634 | IEA |
| 533379 | GO:0005737 | IEA |
| 533430 | GO:0005634 | IEA |
| 533430 | GO:0005737 | IEA |
| 533443 | GO:0005622 | IEA |
| 533643 | GO:0005634 | IEA |
| 533643 | GO:0005626 | IEA |
| 533643 | GO:0016363 | IEA |
| 533660 | GO:0016021 | IEA |
| 533660 | GO:0016020 | IEA |
| 533671 | GO:0016020 | IEA |
| 533746 | GO:0030424 | IEA |
| 533746 | GO:0005739 | IEA |
| 533746 | GO:0030425 | IEA |
| 533746 | GO:0043025 | IEA |
| 533851 | GO:0001725 | IEA |
| 533851 | GO:0005737 | IEA |
| 533851 | GO:0001726 | IEA |
| 533851 | GO:0001726 | IEA |
| 533851 | GO:0001725 | IEA |
| 533851 | GO:0005737 | IEA |
| 533910 | GO:0005739 | IEA |
| 533918 | GO:0005737 | IEA |
| 533918 | GO:0016020 | IEA |
| 533918 | GO:0005792 | IEA |
| 533993 | GO:0005634 | IEA |
| 534182 | GO:0005783 | IEA |
| 534216 | GO:0005634 | IEA |
| 534216 | GO:0005622 | IEA |
| 534232 | GO:0030880 | IEA |
| 534293 | GO:0005737 | IEA |
| 534293 | GO:0005634 | IEA |
| 534319 | GO:0005604 | IEA |
| 534319 | GO:0005578 | IEA |
| 534331 | GO:0005634 | IEA |
| 534352 | GO:0005840 | IEA |
| 534352 | GO:0005739 | IEA |
| 534352 | GO:0005622 | IEA |
| 534352 | GO:0030529 | IEA |
| 534391 | GO:0030529 | IEA |
| 534391 | GO:0005681 | IEA |
| 534391 | GO:0005634 | IEA |
| 534488 | GO:0000139 | IEA |
| 534488 | GO:0016021 | IEA |
| 534546 | GO:0005739 | IEA |
| 534599 | GO:0005739 | IEA |
| 534615 | GO:0016021 | IEA |
| 534615 | GO:0016020 | IEA |
| 534615 | GO:0005739 | IEA |
| 534615 | GO:0005634 | IEA |
| 534615 | GO:0005740 | IEA |
| 534615 | GO:0005739 | IEA |
| 534615 | GO:0005740 | IEA |
| 534615 | GO:0016021 | IEA |
| 534615 | GO:0016020 | IEA |
| 534615 | GO:0005634 | IEA |
| 534624 | GO:0005881 | IEA |
| 534640 | GO:0005829 | IEA |
| 534640 | GO:0005839 | IEA |
| 534640 | GO:0043234 | IEA |
| 534708 | GO:0005802 | IEA |
| 534995 | GO:0016020 | IEA |
| 535077 | GO:0030027 | IEA |
| 535166 | GO:0009986 | IEA |
| 535174 | GO:0005743 | IEA |
| 535185 | GO:0016020 | IEA |
| 535273 | GO:0016020 | IEA |
| 535273 | GO:0016021 | IEA |
| 535290 | GO:0005739 | IEA |
| 535290 | GO:0030529 | IEA |
| 535320 | GO:0005856 | IEA |
| 535320 | GO:0005622 | IEA |
| 535320 | GO:0005874 | IEA |
| 535379 | GO:0016020 | IEA |
| 535447 | GO:0005634 | IEA |
| 535447 | GO:0030529 | IEA |
| 535601 | GO:0005743 | IEA |
| 535683 | GO:0005829 | IEA |
| 535683 | GO:0043234 | IEA |
| 535683 | GO:0043234 | IEA |
| 535683 | GO:0005829 | IEA |
| 535699 | GO:0005913 | IEA |
| 535714 | GO:0005634 | IEA |
| 535714 | GO:0005792 | IEA |
| 535714 | GO:0005737 | IEA |
| 535714 | GO:0005739 | IEA |
| 535714 | GO:0005625 | IEA |
| 535820 | GO:0005737 | IEA |
| 535820 | GO:0005789 | IEA |
| 535820 | GO:0005783 | IEA |
| 535869 | GO:0005794 | IEA |
| 535869 | GO:0016021 | IEA |
| 535869 | GO:0005783 | IEA |
| 535869 | GO:0005770 | IEA |
| 535869 | GO:0016020 | IEA |
| 535981 | GO:0005739 | IEA |
| 536051 | GO:0005737 | IEA |
| 536051 | GO:0005886 | IEA |
| 536073 | GO:0005634 | IEA |
| 536073 | GO:0005634 | IEA |
| 536149 | GO:0005634 | IEA |
| 536203 | GO:0016020 | IEA |
| 536203 | GO:0016021 | IEA |
| 536359 | GO:0030662 | IEA |
| 536553 | GO:0030662 | IEA |
| 536619 | GO:0005737 | IEA |
| 536741 | GO:0016020 | IEA |
| 537455 | GO:0005604 | IEA |
| 537539 | GO:0005829 | IEA |
| 538054 | GO:0030529 | IEA |
| 538054 | GO:0005622 | IEA |
| 538054 | GO:0005840 | IEA |
| 538054 | GO:0005762 | IEA |
| 538054 | GO:0005739 | IEA |
| 538094 | GO:0005737 | IEA |
| 538094 | GO:0005737 | IEA |
| 538427 | GO:0005737 | ISS |
| 538427 | GO:0016363 | IEA |
| 538427 | GO:0048471 | ISS |
| 538427 | GO:0005783 | ISS |
| 538427 | GO:0005634 | IEA |
| 538594 | GO:0005634 | IEA |
| 538625 | GO:0043234 | IEA |
| 538625 | GO:0005829 | IEA |
| 538931 | GO:0030529 | IEA |
| 538944 | GO:0005739 | IEA |
| 538944 | GO:0005739 | IEA |
| 539003 | GO:0005667 | IEA |
| 539003 | GO:0005911 | IEA |
| 539003 | GO:0005912 | IEA |
| 539003 | GO:0005916 | IEA |
| 539003 | GO:0005913 | IEA |
| 539003 | GO:0031528 | IEA |
| 539003 | GO:0030027 | IEA |
| 539003 | GO:0005624 | IEA |
| 539003 | GO:0005886 | IEA |
| 539003 | GO:0016328 | IEA |
| 539003 | GO:0016020 | IEA |
| 539003 | GO:0005634 | IEA |
| 539003 | GO:0045177 | IEA |
| 539003 | GO:0030877 | IEA |
| 539003 | GO:0043296 | IEA |
| 539003 | GO:0030018 | IEA |
| 539003 | GO:0016323 | IEA |
| 539003 | GO:0005737 | IEA |
| 539005 | GO:0030529 | IEA |
| 539005 | GO:0005840 | IEA |
| 539005 | GO:0005622 | IEA |
| 539091 | GO:0016469 | IEA |
| 539091 | GO:0005622 | IEA |
| 539091 | GO:0005634 | IEA |
| 539120 | GO:0005634 | IEA |
| 539249 | GO:0005622 | IEA |
| 539308 | GO:0005929 | IEA |
| 539332 | GO:0005634 | IEA |
| 539332 | GO:0005622 | IEA |
| 539332 | GO:0005856 | IEA |
| 539332 | GO:0005856 | IEA |
| 539332 | GO:0005622 | IEA |
| 539332 | GO:0005634 | IEA |
| 539347 | GO:0016021 | IEA |
| 539347 | GO:0016020 | IEA |
| 539354 | GO:0000139 | IEA |
| 539354 | GO:0016021 | IEA |
| 539354 | GO:0000139 | IEA |
| 539354 | GO:0016021 | IEA |
| 539379 | GO:0030016 | IEA |
| 539379 | GO:0015629 | IEA |
| 539379 | GO:0005886 | IEA |
| 539381 | GO:0005634 | IEA |
| 539431 | GO:0005634 | IEA |
| 539431 | GO:0035097 | IEA |
| 539431 | GO:0032154 | IEA |
| 539431 | GO:0005829 | IEA |
| 539494 | GO:0005743 | IEA |
| 539494 | GO:0005743 | IEA |
| 539524 | GO:0005622 | IEA |
| 539639 | GO:0005643 | IEA |
| 539643 | GO:0005737 | IEA |
| 539673 | GO:0005739 | IEA |
| 539673 | GO:0005739 | IEA |
| 539713 | GO:0016020 | IEA |
| 539713 | GO:0016020 | IEA |
| 539761 | GO:0005622 | IEA |
| 539771 | GO:0005634 | IEA |
| 539771 | GO:0005730 | IEA |
| 539771 | GO:0005730 | ISS |
| 539789 | GO:0005737 | IEA |
| 539789 | GO:0005874 | IEA |
| 539789 | GO:0005622 | IEA |
| 539804 | GO:0005634 | IEA |
| 539804 | GO:0005737 | IEA |
| 539804 | GO:0005634 | IEA |
| 539804 | GO:0005737 | IEA |
| 539875 | GO:0005694 | IEA |
| 539875 | GO:0000786 | IEA |
| 539875 | GO:0005634 | IEA |
| 539875 | GO:0000786 | IEA |
| 539875 | GO:0005634 | IEA |
| 539875 | GO:0005694 | IEA |
| 539875 | GO:0000786 | IEA |
| 539875 | GO:0005694 | IEA |
| 539875 | GO:0005634 | IEA |
| 539875 | GO:0005634 | IEA |
| 539875 | GO:0005694 | IEA |
| 539875 | GO:0000786 | IEA |
| 539875 | GO:0005694 | IEA |
| 539875 | GO:0000786 | IEA |
| 539875 | GO:0005634 | IEA |
| 539950 | GO:0030529 | IEA |
| 539955 | GO:0005634 | IEA |
| 539978 | GO:0016021 | IEA |
| 539978 | GO:0016020 | IEA |
| 540040 | GO:0005622 | IEA |
| 540045 | GO:0005634 | IEA |
| 540045 | GO:0005622 | IEA |
| 540085 | GO:0005737 | IEA |
| 540085 | GO:0005737 | IEA |
| 540090 | GO:0016604 | IEA |
| 540090 | GO:0001739 | IEA |
| 540090 | GO:0031519 | IEA |
| 540090 | GO:0005634 | IEA |
| 540090 | GO:0000151 | IEA |
| 540090 | GO:0000151 | IEA |
| 540090 | GO:0016604 | IEA |
| 540090 | GO:0001739 | IEA |
| 540090 | GO:0031519 | IEA |
| 540090 | GO:0005634 | IEA |
| 540096 | GO:0031941 | IEA |
| 540096 | GO:0002102 | IEA |
| 540096 | GO:0005737 | IEA |
| 540096 | GO:0030863 | IEA |
| 540096 | GO:0032154 | IEA |
| 540096 | GO:0043005 | IEA |
| 540096 | GO:0030426 | IEA |
| 540139 | GO:0005739 | IEA |
| 540148 | GO:0005634 | IEA |
| 540148 | GO:0005694 | IEA |
| 540148 | GO:0000786 | IEA |
| 540148 | GO:0005694 | IEA |
| 540148 | GO:0000786 | IEA |
| 540148 | GO:0005634 | IEA |
| 540148 | GO:0005634 | IEA |
| 540148 | GO:0005694 | IEA |
| 540148 | GO:0000786 | IEA |
| 540148 | GO:0000786 | IEA |
| 540148 | GO:0005634 | IEA |
| 540148 | GO:0005694 | IEA |
| 540148 | GO:0005694 | IEA |
| 540148 | GO:0005634 | IEA |
| 540148 | GO:0000786 | IEA |
| 540167 | GO:0016020 | IEA |
| 540167 | GO:0016021 | IEA |
| 540176 | GO:0016020 | IEA |
| 540176 | GO:0016469 | IEA |
| 540176 | GO:0016021 | IEA |
| 540176 | GO:0045263 | IEA |
| 540176 | GO:0005739 | IEA |
| 540181 | GO:0016514 | IEA |
| 540329 | GO:0008287 | IEA |
| 540329 | GO:0008287 | IEA |
| 540329 | GO:0008287 | IEA |
| 540329 | GO:0008287 | IEA |
| 540329 | GO:0008287 | IEA |
| 540345 | GO:0005694 | IEA |
| 540345 | GO:0005622 | IEA |
| 540345 | GO:0005634 | IEA |
| 540353 | GO:0005634 | IEA |
| 540376 | GO:0005739 | IEA |
| 540421 | GO:0005739 | IEA |
| 540426 | GO:0016020 | IEA |
| 540426 | GO:0001931 | IEA |
| 540426 | GO:0005856 | IEA |
| 540426 | GO:0016324 | IEA |
| 540426 | GO:0045177 | IEA |
| 540426 | GO:0005737 | IEA |
| 540426 | GO:0016323 | IEA |
| 540444 | GO:0000793 | ISS |
| 540444 | GO:0005737 | IEA |
| 540444 | GO:0048471 | ISS |
| 540444 | GO:0000793 | IEA |
| 540444 | GO:0005737 | ISS |
| 540444 | GO:0005634 | IEA |
| 540444 | GO:0048471 | IEA |
| 540444 | GO:0000785 | IEA |
| 540444 | GO:0005694 | IEA |
| 540526 | GO:0032301 | IEA |
| 540526 | GO:0000790 | IEA |
| 540568 | GO:0016020 | IEA |
| 540602 | GO:0005829 | IEA |
| 540766 | GO:0005634 | IEA |
| 540799 | GO:0002102 | IEA |
| 540799 | GO:0043005 | IEA |
| 540799 | GO:0005737 | IEA |
| 540799 | GO:0030863 | IEA |
| 540799 | GO:0032154 | IEA |
| 540799 | GO:0031941 | IEA |
| 540799 | GO:0030426 | IEA |
| 540859 | GO:0005634 | IEA |
| 540932 | GO:0005794 | IEA |
| 540932 | GO:0005783 | IEA |
| 540966 | GO:0000228 | IEA |
| 540966 | GO:0005634 | IEA |
| 541077 | GO:0005737 | IEA |
| 541077 | GO:0005737 | IEA |
| 541081 | GO:0005622 | IEA |
| 541101 | GO:0043234 | IEA |
| 541101 | GO:0005874 | IEA |
| 541125 | GO:0005634 | IEA |
| 541229 | GO:0005681 | IEA |
| 541266 | GO:0016020 | IEA |
| 541266 | GO:0016021 | IEA |
| 541283 | GO:0005634 | IEA |
| 613338 | GO:0005739 | IEA |
| 613345 | GO:0000786 | IEA |
| 613345 | GO:0005634 | IEA |
| 613345 | GO:0005694 | IEA |
| 613345 | GO:0000786 | IEA |
| 613345 | GO:0005694 | IEA |
| 613345 | GO:0005634 | IEA |
| 613345 | GO:0005694 | IEA |
| 613345 | GO:0000786 | IEA |
| 613345 | GO:0005634 | IEA |
| 613345 | GO:0005694 | IEA |
| 613345 | GO:0005634 | IEA |
| 613345 | GO:0000786 | IEA |
| 613345 | GO:0005694 | IEA |
| 613345 | GO:0005634 | IEA |
| 613345 | GO:0000786 | IEA |
| 613381 | GO:0005634 | IEA |
| 613416 | GO:0005739 | IEA |
| 613416 | GO:0005829 | IEA |
| 613416 | GO:0005737 | IEA |
| 613421 | GO:0005856 | IEA |
| 613522 | GO:0005622 | IEA |
| 613548 | GO:0005737 | IEA |
| 613548 | GO:0005938 | IEA |
| 613548 | GO:0042585 | IEA |
| 613548 | GO:0005813 | IEA |
| 613548 | GO:0016020 | IEA |
| 613548 | GO:0005737 | IEA |
| 613548 | GO:0005938 | IEA |
| 613548 | GO:0042585 | IEA |
| 613548 | GO:0016020 | IEA |
| 613548 | GO:0005813 | IEA |
| 613551 | GO:0016021 | IEA |
| 613551 | GO:0016020 | IEA |
| 613570 | GO:0005739 | IEA |
| 613570 | GO:0005737 | IEA |
| 613570 | GO:0005743 | IEA |
| 613610 | GO:0005739 | IEA |
| 613618 | GO:0000786 | IEA |
| 613618 | GO:0005694 | IEA |
| 613618 | GO:0005634 | IEA |
| 613629 | GO:0005634 | IEA |
| 613715 | GO:0005739 | IEA |
| 613729 | GO:0000785 | IEA |
| 613729 | GO:0005634 | IEA |
| 613751 | GO:0043234 | IEA |
| 613751 | GO:0005634 | IEA |
| 613787 | GO:0005576 | IEA |
| 613788 | GO:0030880 | IEA |
| 613802 | GO:0016021 | IEA |
| 613802 | GO:0016020 | IEA |
| 613828 | GO:0005634 | IEA |
| 613828 | GO:0005622 | IEA |
| 613828 | GO:0005694 | IEA |
| 613835 | GO:0005763 | IEA |
| 613835 | GO:0030529 | IEA |
| 613835 | GO:0005739 | IEA |
| 613855 | GO:0005829 | IEA |
| 613855 | GO:0005634 | IEA |
| 613877 | GO:0005667 | IEA |
| 613877 | GO:0005634 | IEA |
| 613899 | GO:0016020 | IEA |
| 613899 | GO:0005739 | IEA |
| 613899 | GO:0005743 | IEA |
| 613899 | GO:0005746 | IEA |
| 613907 | GO:0005634 | IEA |
| 613930 | GO:0005834 | IEA |
| 614028 | GO:0005634 | IEA |
| 614043 | GO:0005739 | IEA |
| 614043 | GO:0005743 | IEA |
| 614114 | GO:0005739 | IEA |
| 614145 | GO:0005739 | IEA |
| 614177 | GO:0005634 | IEA |
| 614177 | GO:0048269 | IEA |
| 614242 | GO:0016020 | IEA |
| 614244 | GO:0016469 | IEA |
| 614279 | GO:0005622 | IEA |
| 614288 | GO:0005622 | IEA |
| 614509 | GO:0005634 | IEA |
| 614538 | GO:0005783 | IEA |
| 614538 | GO:0016020 | IEA |
| 614538 | GO:0016021 | IEA |
| 614576 | GO:0005737 | IEA |
| 614581 | GO:0005737 | IEA |
| 614581 | GO:0005622 | IEA |
| 614581 | GO:0005886 | IEA |
| 614630 | GO:0005634 | IEA |
| 614633 | GO:0016021 | IEA |
| 614633 | GO:0005739 | IEA |
| 614633 | GO:0016020 | IEA |
| 614773 | GO:0005634 | IEA |
| 614773 | GO:0005737 | IEA |
| 614773 | GO:0005737 | IEA |
| 614773 | GO:0005634 | IEA |
| 614876 | GO:0005634 | IEA |
| 614876 | GO:0005634 | IEA |
| 614893 | GO:0005634 | IEA |
| 614915 | GO:0000785 | IEA |
| 614915 | GO:0005634 | IEA |
| 614921 | GO:0005622 | IEA |
| 614922 | GO:0005681 | IEA |
| 614922 | GO:0030529 | IEA |
| 614922 | GO:0005634 | IEA |
| 615215 | GO:0005634 | IEA |
| 615229 | GO:0005634 | IEA |
| 615239 | GO:0005578 | IEA |
| 615424 | GO:0005739 | IEA |
| 615447 | GO:0016020 | IEA |
| 615498 | GO:0016021 | IEA |
| 615498 | GO:0016020 | IEA |
| 615498 | GO:0019867 | IEA |
| 615626 | GO:0005634 | IEA |
| 615666 | GO:0005761 | IEA |
| 615666 | GO:0005739 | IEA |
| 615666 | GO:0030529 | IEA |
| 615785 | GO:0016021 | IEA |
| 615785 | GO:0016020 | IEA |
| 615785 | GO:0016020 | IEA |
| 615785 | GO:0016021 | IEA |
| 615846 | GO:0005739 | IEA |
| 615975 | GO:0005737 | IEA |
| 615975 | GO:0005634 | IEA |
| 615978 | GO:0005802 | IEA |
| 616109 | GO:0005746 | IEA |
| 616109 | GO:0005739 | IEA |
| 616109 | GO:0005740 | IEA |
| 616109 | GO:0016020 | IEA |
| 616148 | GO:0005634 | IEA |
| 616180 | GO:0005634 | IEA |
| 616217 | GO:0005654 | IEA |
| 616217 | GO:0005634 | IEA |
| 616227 | GO:0014069 | IEA |
| 616227 | GO:0019717 | IEA |
| 616317 | GO:0005874 | IEA |
| 616317 | GO:0005737 | IEA |
| 616317 | GO:0005622 | IEA |
| 616375 | GO:0005770 | IEA |
| 616375 | GO:0016020 | IEA |
| 616375 | GO:0005764 | IEA |
| 616375 | GO:0005770 | IEA |
| 616375 | GO:0016020 | IEA |
| 616375 | GO:0005764 | IEA |
| 616477 | GO:0005739 | IEA |
| 616477 | GO:0005743 | IEA |
| 616718 | GO:0005739 | IEA |
| 616742 | GO:0016020 | IEA |
| 616748 | GO:0005622 | IEA |
| 616748 | GO:0005783 | IEA |
| 616748 | GO:0005783 | IEA |
| 616748 | GO:0005622 | IEA |
| 616800 | GO:0005634 | IEA |
| 616800 | GO:0005694 | IEA |
| 616800 | GO:0000786 | IEA |
| 616800 | GO:0005634 | IEA |
| 616800 | GO:0000786 | IEA |
| 616800 | GO:0005694 | IEA |
| 616800 | GO:0005694 | IEA |
| 616800 | GO:0005634 | IEA |
| 616800 | GO:0000786 | IEA |
| 616800 | GO:0000786 | IEA |
| 616800 | GO:0005634 | IEA |
| 616800 | GO:0005694 | IEA |
| 616800 | GO:0005694 | IEA |
| 616800 | GO:0000786 | IEA |
| 616800 | GO:0005634 | IEA |
| 616819 | GO:0005634 | IEA |
| 616819 | GO:0000786 | IEA |
| 616819 | GO:0005694 | IEA |
| 616819 | GO:0005634 | IEA |
| 616819 | GO:0000786 | IEA |
| 616819 | GO:0005694 | IEA |
| 616819 | GO:0005634 | IEA |
| 616819 | GO:0000786 | IEA |
| 616819 | GO:0005694 | IEA |
| 616819 | GO:0005694 | IEA |
| 616819 | GO:0005634 | IEA |
| 616819 | GO:0000786 | IEA |
| 616819 | GO:0005694 | IEA |
| 616819 | GO:0005634 | IEA |
| 616819 | GO:0000786 | IEA |
| 616871 | GO:0016020 | IEA |
| 616871 | GO:0005746 | IEA |
| 616871 | GO:0005739 | IEA |
| 616926 | GO:0005923 | IEA |
| 616926 | GO:0005886 | IEA |
| 616926 | GO:0005912 | IEA |
| 617005 | GO:0005739 | IEA |
| 617005 | GO:0030529 | IEA |
| 617051 | GO:0000786 | IEA |
| 617051 | GO:0005694 | IEA |
| 617051 | GO:0005634 | IEA |
| 617051 | GO:0005694 | IEA |
| 617051 | GO:0005634 | IEA |
| 617051 | GO:0000786 | IEA |
| 617051 | GO:0005694 | IEA |
| 617051 | GO:0005634 | IEA |
| 617051 | GO:0000786 | IEA |
| 617051 | GO:0005694 | IEA |
| 617051 | GO:0000786 | IEA |
| 617051 | GO:0005634 | IEA |
| 617051 | GO:0005694 | IEA |
| 617051 | GO:0005634 | IEA |
| 617051 | GO:0000786 | IEA |
| 617074 | GO:0005789 | IEA |
| 617104 | GO:0016020 | IEA |
| 617104 | GO:0016021 | IEA |
| 617105 | GO:0005737 | IEA |
| 617105 | GO:0017101 | ISS |
| 617105 | GO:0005634 | IEA |
| 617105 | GO:0005737 | ISS |
| 617105 | GO:0005634 | ISS |
| 617210 | GO:0005739 | IEA |
| 617230 | GO:0045261 | IEA |
| 617230 | GO:0005739 | IEA |
| 617230 | GO:0016469 | IEA |
| 617326 | GO:0043005 | IEA |
| 617326 | GO:0002102 | IEA |
| 617326 | GO:0031941 | IEA |
| 617326 | GO:0032154 | IEA |
| 617326 | GO:0030863 | IEA |
| 617326 | GO:0005737 | IEA |
| 617326 | GO:0030426 | IEA |
| 617419 | GO:0016020 | IEA |
| 617419 | GO:0005783 | IEA |
| 617419 | GO:0016021 | IEA |
| 617441 | GO:0005739 | IEA |
| 617512 | GO:0005634 | IEA |
| 617635 | GO:0016020 | IEA |
| 617694 | GO:0005739 | IEA |
| 617694 | GO:0005743 | IEA |
| 617888 | GO:0005634 | IEA |
| 617888 | GO:0000786 | IEA |
| 617888 | GO:0005694 | IEA |
| 617888 | GO:0000786 | IEA |
| 617888 | GO:0005634 | IEA |
| 617888 | GO:0005694 | IEA |
| 617888 | GO:0005634 | IEA |
| 617888 | GO:0005694 | IEA |
| 617888 | GO:0000786 | IEA |
| 617888 | GO:0005694 | IEA |
| 617888 | GO:0000786 | IEA |
| 617888 | GO:0005634 | IEA |
| 617888 | GO:0000786 | IEA |
| 617888 | GO:0005694 | IEA |
| 617888 | GO:0005634 | IEA |
| 617901 | GO:0005840 | IEA |
| 617901 | GO:0005762 | IEA |
| 617901 | GO:0005739 | IEA |
| 617901 | GO:0030529 | IEA |
| 617901 | GO:0005622 | IEA |
| 617922 | GO:0016020 | IEA |
| 617944 | GO:0005737 | IEA |
| 617976 | GO:0005634 | IEA |
| 618131 | GO:0042719 | IEA |
| 618131 | GO:0016020 | IEA |
| 618131 | GO:0005739 | IEA |
| 618149 | GO:0005634 | IEA |
| 618149 | GO:0005694 | IEA |
| 618149 | GO:0000786 | IEA |
| 618149 | GO:0000786 | IEA |
| 618149 | GO:0005634 | IEA |
| 618149 | GO:0005694 | IEA |
| 618149 | GO:0005634 | IEA |
| 618149 | GO:0005694 | IEA |
| 618149 | GO:0000786 | IEA |
| 618149 | GO:0005694 | IEA |
| 618149 | GO:0000786 | IEA |
| 618149 | GO:0005634 | IEA |
| 618149 | GO:0005694 | IEA |
| 618149 | GO:0000786 | IEA |
| 618149 | GO:0005634 | IEA |
| 618292 | GO:0000178 | IEA |
| 618292 | GO:0005634 | IEA |
| 618297 | GO:0000793 | IEA |
| 618297 | GO:0005694 | IEA |
| 618297 | GO:0000785 | IEA |
| 618297 | GO:0048471 | ISS |
| 618297 | GO:0048471 | IEA |
| 618297 | GO:0005634 | IEA |
| 618297 | GO:0005737 | IEA |
| 618297 | GO:0000793 | ISS |
| 618297 | GO:0005737 | ISS |
| 618441 | GO:0005634 | IEA |
| 618441 | GO:0005737 | IEA |
| 618550 | GO:0005737 | IEA |
| 618550 | GO:0005829 | IEA |
| 618550 | GO:0005739 | IEA |
| 618573 | GO:0005737 | IEA |
| 618573 | GO:0005634 | IEA |
| 618607 | GO:0016324 | IEA |
| 618607 | GO:0005829 | IEA |
| 618609 | GO:0005622 | IEA |
| 618609 | GO:0005840 | IEA |
| 618849 | GO:0000785 | IEA |
| 618849 | GO:0005634 | IEA |
| 619141 | GO:0005634 | IEA |
| 619141 | GO:0005694 | IEA |
| 619141 | GO:0000786 | IEA |
| 619141 | GO:0000786 | IEA |
| 619141 | GO:0005694 | IEA |
| 619141 | GO:0005634 | IEA |
| 619141 | GO:0005634 | IEA |
| 619141 | GO:0000786 | IEA |
| 619141 | GO:0005694 | IEA |
| 619141 | GO:0000786 | IEA |
| 619141 | GO:0005694 | IEA |
| 619141 | GO:0005634 | IEA |
| 619141 | GO:0005694 | IEA |
| 619141 | GO:0005634 | IEA |
| 619141 | GO:0000786 | IEA |
| 654400 | GO:0005634 | IEA |
| 767828 | GO:0005634 | IEA |
| 767898 | GO:0005737 | IEA |
| 767898 | GO:0005634 | IEA |
| 767909 | GO:0005739 | IEA |
| 767977 | GO:0005743 | IEA |
| 767980 | GO:0030176 | IEA |
| 767980 | GO:0005784 | IEA |
| 768014 | GO:0005874 | IEA |
| 768014 | GO:0005875 | IEA |
| 768016 | GO:0005737 | IEA |
| 768035 | GO:0005622 | IEA |
| 768074 | GO:0005743 | IEA |
| 768074 | GO:0005739 | IEA |
| 768074 | GO:0016021 | IEA |
| 768074 | GO:0016020 | IEA |
| 777691 | GO:0005739 | IEA |
| 777769 | GO:0005634 | IEA |
| 777775 | GO:0043234 | IEA |
| 777775 | GO:0005874 | IEA |
| 777775 | GO:0005874 | IEA |
| 777775 | GO:0043234 | IEA |
| 780784 | GO:0030016 | IEA |
| 780978 | GO:0030662 | IEA |
| 781014 | GO:0005635 | IEA |
| 781014 | GO:0005789 | IEA |
| 781020 | GO:0005739 | IEA |
| 781064 | GO:0005739 | IEA |
| 781064 | GO:0016020 | IEA |
| 781070 | GO:0005737 | IEA |
| 781100 | GO:0005739 | IEA |
| 781100 | GO:0016020 | IEA |
| 781111 | GO:0005694 | IEA |
| 781111 | GO:0005634 | IEA |
| 781111 | GO:0000786 | IEA |
| 781111 | GO:0005634 | IEA |
| 781111 | GO:0005694 | IEA |
| 781111 | GO:0000786 | IEA |
| 781197 | GO:0005783 | IEA |
| 781197 | GO:0005787 | IEA |
| 781197 | GO:0030176 | IEA |
| 781197 | GO:0016021 | IEA |
| 781197 | GO:0005792 | IEA |
| 781197 | GO:0016020 | IEA |
| 781224 | GO:0005634 | IEA |
| 781224 | GO:0000786 | IEA |
| 781224 | GO:0005694 | IEA |
| 781224 | GO:0005694 | IEA |
| 781224 | GO:0005634 | IEA |
| 781224 | GO:0000786 | IEA |
| 781224 | GO:0000786 | IEA |
| 781224 | GO:0005634 | IEA |
| 781224 | GO:0005694 | IEA |
| 781224 | GO:0000786 | IEA |
| 781224 | GO:0005694 | IEA |
| 781224 | GO:0005634 | IEA |
| 781224 | GO:0005694 | IEA |
| 781224 | GO:0000786 | IEA |
| 781224 | GO:0005634 | IEA |
| 781334 | GO:0005737 | IEA |
| 781334 | GO:0005856 | IEA |
| 781462 | GO:0016020 | IEA |
| 781462 | GO:0005739 | IEA |
| 781462 | GO:0042719 | IEA |
| 781477 | GO:0005886 | IEA |
| 781477 | GO:0005798 | IEA |
| 781477 | GO:0005794 | IEA |
| 781532 | GO:0005829 | IEA |
| 781532 | GO:0005634 | IEA |
| 781532 | GO:0043234 | IEA |
| 781542 | GO:0005622 | IEA |
| 781542 | GO:0030529 | IEA |
| 781542 | GO:0005840 | IEA |
| 781566 | GO:0005634 | IEA |
| 781566 | GO:0000786 | IEA |
| 781566 | GO:0005694 | IEA |
| 781566 | GO:0005634 | IEA |
| 781566 | GO:0000786 | IEA |
| 781566 | GO:0005694 | IEA |
| 781566 | GO:0005634 | IEA |
| 781566 | GO:0000786 | IEA |
| 781566 | GO:0005694 | IEA |
| 781566 | GO:0000786 | IEA |
| 781566 | GO:0005634 | IEA |
| 781566 | GO:0005694 | IEA |
| 781566 | GO:0005634 | IEA |
| 781566 | GO:0005694 | IEA |
| 781566 | GO:0000786 | IEA |
| 781575 | GO:0005739 | IEA |
| 781575 | GO:0005740 | IEA |
| 781609 | GO:0005739 | IEA |
| 781609 | GO:0005743 | IEA |
| 781610 | GO:0005739 | IEA |
| 781610 | GO:0005739 | ISS |
| 781610 | GO:0005759 | ISS |
| 781610 | GO:0005759 | IEA |
| 781648 | GO:0005737 | IEA |
| 781648 | GO:0005622 | IEA |
| 781648 | GO:0005739 | IEA |
| 781719 | GO:0016020 | IEA |
| 781719 | GO:0005840 | IEA |
| 781719 | GO:0030529 | IEA |
| 781719 | GO:0005739 | IEA |
| 781719 | GO:0005622 | IEA |
| 781741 | GO:0005829 | IEA |
| 781741 | GO:0005634 | IEA |
| 781813 | GO:0005737 | IEA |
| 781824 | GO:0005739 | IEA |
| 781953 | GO:0005578 | IEA |
| 781969 | GO:0016021 | IEA |
| 781969 | GO:0005739 | IEA |
| 781969 | GO:0016020 | IEA |
| 781969 | GO:0005740 | IEA |
| 781969 | GO:0005634 | IEA |
| 781969 | GO:0005740 | IEA |
| 781969 | GO:0016021 | IEA |
| 781969 | GO:0005739 | IEA |
| 781969 | GO:0005634 | IEA |
| 781969 | GO:0016020 | IEA |
| 782024 | GO:0005634 | IEA |
| 782024 | GO:0030529 | IEA |
| 782233 | GO:0005739 | IEA |
| 782233 | GO:0045263 | IEA |
| 782233 | GO:0016469 | IEA |
| 782259 | GO:0016020 | IEA |
| 782259 | GO:0016021 | IEA |
| 782270 | GO:0045261 | IEA |
| 782270 | GO:0016469 | IEA |
| 782270 | GO:0005739 | IEA |
| 782344 | GO:0005681 | IEA |
| 782344 | GO:0030529 | IEA |
| 782344 | GO:0005634 | IEA |
| 782393 | GO:0005737 | ISS |
| 782393 | GO:0005634 | ISS |
| 782393 | GO:0005737 | IEA |
| 782393 | GO:0005634 | IEA |
| 782393 | GO:0017101 | ISS |
| 782416 | GO:0005759 | IDA |
| 782416 | GO:0005739 | IEA |
| 782416 | GO:0031966 | IDA |
| 782444 | GO:0005634 | IEA |
| 782525 | GO:0005853 | IEA |
| 782666 | GO:0045263 | IEA |
| 782666 | GO:0016469 | IEA |
| 782666 | GO:0005739 | IEA |
| 782776 | GO:0005737 | IEA |
| 782954 | GO:0005737 | IEA |
| 782989 | GO:0005737 | IEA |
| 783153 | GO:0005634 | IEA |
| 783153 | GO:0000785 | IEA |
| 783188 | GO:0043005 | IEA |
| 783188 | GO:0002102 | IEA |
| 783188 | GO:0032154 | IEA |
| 783188 | GO:0031941 | IEA |
| 783188 | GO:0030863 | IEA |
| 783188 | GO:0030426 | IEA |
| 783188 | GO:0005737 | IEA |
| 783286 | GO:0005887 | IEA |
| 783286 | GO:0016021 | IEA |
| 783286 | GO:0005887 | IEA |
| 783286 | GO:0016021 | IEA |
| 783294 | GO:0016021 | IEA |
| 783294 | GO:0019867 | IEA |
| 783294 | GO:0005739 | IEA |
| 783294 | GO:0016020 | IEA |
| 783294 | GO:0005741 | IEA |
| 783301 | GO:0005681 | IEA |
| 783301 | GO:0030529 | IEA |
| 783301 | GO:0005634 | IEA |
| 783337 | GO:0005840 | IEA |
| 783337 | GO:0005739 | IEA |
| 783337 | GO:0030529 | IEA |
| 783337 | GO:0005622 | IEA |
| 783337 | GO:0005840 | IEA |
| 783337 | GO:0005622 | IEA |
| 783337 | GO:0005739 | IEA |
| 783337 | GO:0030529 | IEA |
| 783373 | GO:0005794 | IEA |
| 783467 | GO:0005739 | IEA |
| 783467 | GO:0016020 | IEA |
| 783502 | GO:0005739 | IEA |
| 783502 | GO:0005746 | IEA |
| 783502 | GO:0005743 | IEA |
| 783502 | GO:0016020 | IEA |
| 783523 | GO:0005634 | IEA |
| 783523 | GO:0030529 | IEA |
| 783523 | GO:0005681 | IEA |
| 783681 | GO:0005886 | IEA |
| 783686 | GO:0005739 | IEA |
| 783687 | GO:0005739 | IEA |
| 783687 | GO:0005740 | IEA |
| 783837 | GO:0005634 | IEA |
| 783838 | GO:0005737 | IEA |
| 783928 | GO:0005739 | IEA |
| 783991 | GO:0005743 | IEA |
| 783991 | GO:0045261 | IEA |
| 783991 | GO:0016020 | IEA |
| 783991 | GO:0005739 | IEA |
| 783991 | GO:0016469 | IEA |
| 784010 | GO:0005634 | IEA |
| 784088 | GO:0005635 | IEA |
| 784088 | GO:0005737 | IEA |
| 784097 | GO:0005739 | IEA |
| 784131 | GO:0005737 | IEA |
| 784220 | GO:0005739 | IEA |
| 784223 | GO:0002102 | IEA |
| 784223 | GO:0030426 | IEA |
| 784223 | GO:0043005 | IEA |
| 784223 | GO:0030863 | IEA |
| 784223 | GO:0005737 | IEA |
| 784223 | GO:0031941 | IEA |
| 784223 | GO:0032154 | IEA |
| 784294 | GO:0016021 | IEA |
| 784294 | GO:0019867 | IEA |
| 784294 | GO:0005743 | IEA |
| 784294 | GO:0016020 | IEA |
| 784294 | GO:0005741 | IEA |
| 784294 | GO:0005739 | IEA |
| 784385 | GO:0005634 | IEA |
| 784442 | GO:0000792 | IEA |
| 784442 | GO:0000228 | IEA |
| 784492 | GO:0005739 | IEA |
| 784492 | GO:0005634 | IEA |
| 784492 | GO:0016021 | IEA |
| 784492 | GO:0005740 | IEA |
| 784492 | GO:0016020 | IEA |
| 784492 | GO:0005740 | IEA |
| 784492 | GO:0005739 | IEA |
| 784492 | GO:0016020 | IEA |
| 784492 | GO:0016021 | IEA |
| 784492 | GO:0005634 | IEA |
| 784606 | GO:0005634 | IEA |
| 784606 | GO:0005681 | IEA |
| 784606 | GO:0030529 | IEA |
| 784619 | GO:0000139 | IEA |
| 784643 | GO:0005856 | IEA |
| 784643 | GO:0005737 | IEA |
| 784737 | GO:0005737 | IEA |
| 784757 | GO:0005739 | IEA |
| 784895 | GO:0005737 | IEA |
| 784895 | GO:0005829 | IEA |
| 784895 | GO:0005739 | IEA |
| 784928 | GO:0000139 | IEA |
| 785087 | GO:0005737 | IEA |
| 785134 | GO:0016020 | IEA |
| 785134 | GO:0005739 | IEA |
| 785134 | GO:0005746 | IEA |
| 785381 | GO:0016020 | IEA |
| 785387 | GO:0005634 | IEA |
| 785387 | GO:0000786 | IEA |
| 785387 | GO:0005694 | IEA |
| 785387 | GO:0000786 | IEA |
| 785387 | GO:0005634 | IEA |
| 785387 | GO:0005694 | IEA |
| 785387 | GO:0005634 | IEA |
| 785387 | GO:0005694 | IEA |
| 785387 | GO:0000786 | IEA |
| 785388 | GO:0005634 | IEA |
| 785388 | GO:0005681 | IEA |
| 785388 | GO:0030529 | IEA |
| 785395 | GO:0016021 | IEA |
| 785395 | GO:0005739 | IEA |
| 785395 | GO:0016020 | IEA |
| 785455 | GO:0005737 | IEA |
| 785455 | GO:0005856 | IEA |
| 785484 | GO:0005694 | IEA |
| 785484 | GO:0000786 | IEA |
| 785484 | GO:0005634 | IEA |
| 785484 | GO:0005694 | IEA |
| 785484 | GO:0005634 | IEA |
| 785484 | GO:0000786 | IEA |
| 785484 | GO:0005634 | IEA |
| 785484 | GO:0000786 | IEA |
| 785484 | GO:0005694 | IEA |
| 785629 | GO:0005739 | IEA |
| 785653 | GO:0043234 | IEA |
| 785653 | GO:0005829 | IEA |
| 785653 | GO:0005838 | IEA |
| 785654 | GO:0005739 | IEA |
| 785655 | GO:0016324 | IEA |
| 785655 | GO:0016328 | IEA |
| 785657 | GO:0008180 | IEA |
| 785761 | GO:0030529 | IEA |
| 785761 | GO:0005681 | IEA |
| 785761 | GO:0005634 | IEA |
| 785824 | GO:0016020 | IEA |
| 785922 | GO:0005737 | IEA |
| 785958 | GO:0005634 | IEA |
| 785958 | GO:0005694 | IEA |
| 785986 | GO:0005634 | IEA |
| 785986 | GO:0005634 | IEA |
| 786048 | GO:0005634 | IEA |
| 786055 | GO:0005737 | IEA |
| 786128 | GO:0043234 | IEA |
| 786128 | GO:0005874 | IEA |
| 786336 | GO:0005634 | IEA |
| 786426 | GO:0005741 | IEA |
| 786426 | GO:0005743 | IEA |
| 786426 | GO:0019867 | IEA |
| 786426 | GO:0016021 | IEA |
| 786426 | GO:0005739 | IEA |
| 786426 | GO:0016020 | IEA |
| 786431 | GO:0045263 | IEA |
| 786431 | GO:0005739 | IEA |
| 786431 | GO:0016469 | IEA |
| 786533 | GO:0005829 | ISS |
| 786533 | GO:0005764 | IEA |
| 786551 | GO:0005739 | IEA |
| 786618 | GO:0005739 | IEA |
| 786618 | GO:0005737 | IEA |
| 786618 | GO:0005829 | IEA |
| 786673 | GO:0005739 | IEA |
| 786673 | GO:0045263 | IEA |
| 786673 | GO:0005743 | IEA |
| 786673 | GO:0016469 | IEA |
| 786845 | GO:0005739 | IEA |
| 787289 | GO:0005634 | IEA |
| 787289 | GO:0030529 | IEA |
| 787289 | GO:0005681 | IEA |
| 787327 | GO:0005634 | IEA |
| 787327 | GO:0005737 | IEA |
| 787497 | GO:0031966 | IDA |
| 787497 | GO:0005759 | IDA |
| 787497 | GO:0005739 | IEA |
| 787513 | GO:0005622 | IEA |
| 787528 | GO:0005694 | IEA |
| 787528 | GO:0000786 | IEA |
| 787528 | GO:0005634 | IEA |
| 787528 | GO:0000786 | IEA |
| 787528 | GO:0005634 | IEA |
| 787528 | GO:0005694 | IEA |
| 787528 | GO:0005694 | IEA |
| 787528 | GO:0000786 | IEA |
| 787528 | GO:0005634 | IEA |
| 787528 | GO:0000786 | IEA |
| 787528 | GO:0005634 | IEA |
| 787528 | GO:0005694 | IEA |
| 787528 | GO:0005694 | IEA |
| 787528 | GO:0005634 | IEA |
| 787528 | GO:0000786 | IEA |
| 787580 | GO:0016020 | IEA |
| 787679 | GO:0016021 | IEA |
| 787920 | GO:0005739 | IEA |
| 788012 | GO:0005739 | IEA |
| 788157 | GO:0005737 | IEA |
| 788193 | GO:0005634 | IEA |
| 788193 | GO:0000786 | IEA |
| 788193 | GO:0005694 | IEA |
| 788193 | GO:0005634 | IEA |
| 788193 | GO:0005694 | IEA |
| 788193 | GO:0000786 | IEA |
| 788193 | GO:0005634 | IEA |
| 788193 | GO:0000786 | IEA |
| 788193 | GO:0005694 | IEA |
| 788193 | GO:0000786 | IEA |
| 788193 | GO:0005694 | IEA |
| 788193 | GO:0005634 | IEA |
| 788193 | GO:0005634 | IEA |
| 788193 | GO:0000786 | IEA |
| 788193 | GO:0005694 | IEA |
| 788250 | GO:0005694 | IEA |
| 788250 | GO:0000786 | IEA |
| 788250 | GO:0005634 | IEA |
| 788250 | GO:0005694 | IEA |
| 788250 | GO:0005634 | IEA |
| 788250 | GO:0000786 | IEA |
| 788250 | GO:0005694 | IEA |
| 788250 | GO:0005634 | IEA |
| 788250 | GO:0000786 | IEA |
| 788250 | GO:0000786 | IEA |
| 788250 | GO:0005694 | IEA |
| 788250 | GO:0005634 | IEA |
| 788250 | GO:0005694 | IEA |
| 788250 | GO:0005634 | IEA |
| 788250 | GO:0000786 | IEA |
| 788293 | GO:0005737 | IEA |
| 788293 | GO:0005739 | IEA |
| 788293 | GO:0005737 | IEA |
| 788293 | GO:0005739 | IEA |
| 788417 | GO:0032301 | IEA |
| 788417 | GO:0000790 | IEA |
| 788482 | GO:0005739 | IEA |
| 788482 | GO:0005743 | IEA |
| 788596 | GO:0016020 | IEA |
| 788596 | GO:0005834 | IEA |
| 788609 | GO:0005737 | IEA |
| 788610 | GO:0005833 | IEA |
| 788610 | GO:0005833 | IEA |
| 788610 | GO:0005833 | IEA |
| 788668 | GO:0000786 | IEA |
| 788668 | GO:0005694 | IEA |
| 788668 | GO:0005634 | IEA |
| 788668 | GO:0005694 | IEA |
| 788668 | GO:0000786 | IEA |
| 788668 | GO:0005634 | IEA |
| 788668 | GO:0005634 | IEA |
| 788668 | GO:0005694 | IEA |
| 788668 | GO:0000786 | IEA |
| 788672 | GO:0005737 | IEA |
| 788745 | GO:0005743 | IEA |
| 788745 | GO:0016469 | IEA |
| 788745 | GO:0005739 | IEA |
| 788745 | GO:0045263 | IEA |
| 789255 | GO:0005739 | IEA |
| 789369 | GO:0005694 | IEA |
| 789369 | GO:0005634 | IEA |
| 789369 | GO:0000786 | IEA |
| 789369 | GO:0005694 | IEA |
| 789369 | GO:0005634 | IEA |
| 789369 | GO:0000786 | IEA |
| 789369 | GO:0000786 | IEA |
| 789369 | GO:0005694 | IEA |
| 789369 | GO:0005634 | IEA |
| 789369 | GO:0005694 | IEA |
| 789369 | GO:0000786 | IEA |
| 789369 | GO:0005634 | IEA |
| 789369 | GO:0000786 | IEA |
| 789369 | GO:0005634 | IEA |
| 789369 | GO:0005694 | IEA |
| 789447 | GO:0005634 | IEA |
| 789819 | GO:0005622 | IEA |
| 789867 | GO:0005737 | IEA |
| 790040 | GO:0005604 | IEA |
| 790175 | GO:0005635 | IEA |
| 790175 | GO:0019031 | IEA |
| 790360 | GO:0030662 | IEA |
| 790402 | GO:0045177 | IEA |
| 790465 | GO:0005739 | IEA |
| 790529 | GO:0005871 | IEA |
| 790539 | GO:0005739 | IEA |
| 790667 | GO:0005634 | IEA |
| 790667 | GO:0005737 | IEA |
| 790667 | GO:0005634 | IEA |
| 790667 | GO:0005737 | IEA |
| 790667 | GO:0005737 | IEA |
| 790667 | GO:0005634 | IEA |
| 790786 | GO:0005634 | IEA |
| 790819 | GO:0005635 | IEA |
| 790836 | GO:0030880 | IEA |
| 790868 | GO:0005739 | IEA |
| 790868 | GO:0005746 | IEA |
| 790868 | GO:0005743 | IEA |
| 790868 | GO:0016020 | IEA |
|  |  |  |
