# Supplementary material for: A gene network switch enhances the oxidative capacity of ovine skeletal muscle during late fetal development
Source: BMC Genomics. 2010 Jun 15;11:378. doi: 10.1186/1471-2164-11-378 (PMC2894804; doi:10.1186/1471-2164-11-378)
Supplement: Additional File 1 — Gene expression data. This file contains links to the gene expression data. The genes that are significantly differentially expressed between adjacent development times are listed as Experiment 1 (80 d vs 100 d), Experiment 2 (100 d vs 120 d), Experiment 3 (120 d vs 150 d) and Experiment 4 (150 d vs 230 d) in the section entitled Open microarray analysis report for developmental expression. Each experiment contains an MA plot, Volcano plot and Heatmap for each of the three microarray processing programs MAS5, RMA and GCRMA. For each of these processing programs there is a list of significantly differentially expressed probe sets, and for each of these there is an FDR corrected probability, fold change, percentage of microarrays with a MAS5 Present call, and a convergence percentage for all three microarray processing programs. The file also contains annotation of the probe sets. In a second section entitled Time series analysis of developmental expression, there is a list of genes in each gene expression cluster and diagrams showing MAS5 gene expression values for these genes. [file 1471-2164-11-378-S1.ZIP › Report/GO3/Report/MF.html]

### Molecular function

| Entrez | GO ID | Evidence Code |
| --- | --- | --- |
 280682 | GO:0016740 | IEA || 280682 | GO:0046912 | IEA |
| 280682 | GO:0004108 | IEA |
| 280682 | GO:0016740 | IEA |
| 280682 | GO:0004108 | IEA |
| 280682 | GO:0046912 | IEA |
| 280695 | GO:0020037 | IEA |
| 280695 | GO:0005506 | IEA |
| 280695 | GO:0005488 | IEA |
| 280695 | GO:0019825 | IEA |
| 280695 | GO:0005344 | IEA |
| 280695 | GO:0046872 | IEA |
| 280701 | GO:0046872 | IEA |
| 280701 | GO:0004427 | IEA |
| 280701 | GO:0016787 | IEA |
| 280701 | GO:0000287 | IEA |
| 280715 | GO:0016740 | IEA |
| 280715 | GO:0000166 | IEA |
| 280715 | GO:0019201 | IEA |
| 280715 | GO:0004017 | IEA |
| 280715 | GO:0005524 | IEA |
| 280715 | GO:0016301 | IEA |
| 280715 | GO:0019201 | IEA |
| 280715 | GO:0016740 | IEA |
| 280715 | GO:0016301 | IEA |
| 280715 | GO:0005524 | IEA |
| 280715 | GO:0000166 | IEA |
| 280715 | GO:0004017 | IEA |
| 280716 | GO:0016740 | IEA |
| 280716 | GO:0005524 | IEA |
| 280716 | GO:0019201 | IEA |
| 280716 | GO:0016776 | IEA |
| 280716 | GO:0004017 | IEA |
| 280716 | GO:0000166 | IEA |
| 280716 | GO:0016301 | IEA |
| 280719 | GO:0004047 | IEA |
| 280719 | GO:0016740 | IEA |
| 280719 | GO:0031405 | IDA |
| 280719 | GO:0008483 | IEA |
| 280733 | GO:0005515 | IEA |
| 280740 | GO:0016829 | IEA |
| 280740 | GO:0046872 | IEA |
| 280740 | GO:0008270 | IEA |
| 280740 | GO:0004089 | IEA |
| 280753 | GO:0016787 | IEA |
| 280753 | GO:0004182 | IEA |
| 280753 | GO:0008233 | IEA |
| 280753 | GO:0004183 | IEA |
| 280753 | GO:0008270 | IEA |
| 280753 | GO:0046872 | IEA |
| 280753 | GO:0004180 | IEA |
| 280753 | GO:0008237 | IEA |
| 280760 | GO:0005515 | IEA |
| 280808 | GO:0016853 | IEA |
| 280808 | GO:0004347 | IEA |
| 280812 | GO:0016758 | IEA |
| 280815 | GO:0015485 | IEA |
| 280815 | GO:0019899 | IEA |
| 280832 | GO:0004714 | IEA |
| 280832 | GO:0016740 | IEA |
| 280832 | GO:0004713 | IEA |
| 280832 | GO:0005020 | IEA |
| 280832 | GO:0016301 | IEA |
| 280832 | GO:0004672 | IEA |
| 280832 | GO:0004872 | IEA |
| 280832 | GO:0005515 | IEA |
| 280832 | GO:0000166 | IEA |
| 280832 | GO:0005524 | IEA |
| 280852 | GO:0008083 | IEA |
| 280857 | GO:0008083 | IEA |
| 280878 | GO:0008746 | IEA |
| 280878 | GO:0016491 | IEA |
| 280878 | GO:0005215 | IEA |
| 280878 | GO:0050661 | IEA |
| 280878 | GO:0008750 | IEA |
| 280878 | GO:0016491 | IEA |
| 280878 | GO:0008750 | IEA |
| 280878 | GO:0008746 | IEA |
| 280878 | GO:0050661 | IEA |
| 280878 | GO:0005215 | IEA |
| 280884 | GO:0005515 | IEA |
| 280884 | GO:0008083 | IEA |
| 280890 | GO:0004497 | IEA |
| 280890 | GO:0008270 | IEA |
| 280890 | GO:0003824 | IEA |
| 280890 | GO:0004598 | IEA |
| 280890 | GO:0046872 | IEA |
| 280890 | GO:0016829 | IEA |
| 280890 | GO:0005507 | IEA |
| 280890 | GO:0031418 | IEA |
| 280890 | GO:0004504 | IEA |
| 280890 | GO:0016491 | IEA |
| 280904 | GO:0008083 | IEA |
| 280904 | GO:0008201 | IEA |
| 280939 | GO:0005524 | IEA |
| 280939 | GO:0000166 | IEA |
| 280939 | GO:0016740 | IEA |
| 280939 | GO:0004713 | IEA |
| 280939 | GO:0004872 | IEA |
| 280939 | GO:0016301 | IEA |
| 280939 | GO:0004672 | IEA |
| 280941 | GO:0016740 | IEA |
| 280941 | GO:0005524 | IEA |
| 280941 | GO:0004713 | IEA |
| 280941 | GO:0004872 | IEA |
| 280941 | GO:0016301 | IEA |
| 280941 | GO:0000166 | IEA |
| 280941 | GO:0004672 | IEA |
| 280950 | GO:0015035 | IEA |
| 280950 | GO:0009055 | IEA |
| 280955 | GO:0005515 | ISS |
| 280955 | GO:0005200 | ISS |
| 280955 | GO:0005198 | IEA |
| 280955 | GO:0005515 | IEA |
| 280955 | GO:0005200 | IEA |
| 280961 | GO:0030528 | IEA |
| 280961 | GO:0003677 | IEA |
| 280961 | GO:0046872 | IEA |
| 280961 | GO:0003676 | IEA |
| 280961 | GO:0008270 | IEA |
| 280962 | GO:0008270 | IEA |
| 280962 | GO:0003676 | IEA |
| 280962 | GO:0030528 | IEA |
| 280962 | GO:0046872 | IEA |
| 280962 | GO:0003677 | IEA |
| 280976 | GO:0016829 | IEA |
| 280976 | GO:0005506 | IEA |
| 280976 | GO:0046872 | IEA |
| 280976 | GO:0003994 | IEA |
| 280976 | GO:0016836 | IEA |
| 280976 | GO:0051539 | IEA |
| 280976 | GO:0051536 | IEA |
| 280979 | GO:0005198 | IEA |
| 280979 | GO:0005515 | IEA |
| 280979 | GO:0000166 | IEA |
| 280979 | GO:0005524 | IEA |
| 281032 | GO:0019900 | ISS |
| 281032 | GO:0003712 | ISS |
| 281032 | GO:0019899 | ISS |
| 281032 | GO:0005515 | ISS |
| 281057 | GO:0005540 | IEA |
| 281057 | GO:0004872 | IEA |
| 281057 | GO:0005515 | IEA |
| 281078 | GO:0005509 | IEA |
| 281092 | GO:0008415 | IEA |
| 281092 | GO:0016740 | IEA |
| 281092 | GO:0008458 | IEA |
| 281154 | GO:0005509 | IEA |
| 281154 | GO:0005201 | IEA |
| 281156 | GO:0016740 | IEA |
| 281156 | GO:0004337 | IEA |
| 281156 | GO:0004161 | IEA |
| 281161 | GO:0005515 | IEA |
| 281161 | GO:0008083 | IEA |
| 281161 | GO:0005245 | ISS |
| 281161 | GO:0005515 | ISS |
| 281161 | GO:0008201 | IEA |
| 281187 | GO:0008083 | IEA |
| 281187 | GO:0005125 | IEA |
| 281187 | GO:0005515 | IEA |
| 281187 | GO:0005102 | IEA |
| 281188 | GO:0005515 | ISS |
| 281188 | GO:0005096 | IEA |
| 281188 | GO:0005093 | IEA |
| 281188 | GO:0005093 | IEA |
| 281188 | GO:0005515 | ISS |
| 281188 | GO:0005096 | IEA |
| 281190 | GO:0016874 | IEA |
| 281190 | GO:0008488 | IEA |
| 281193 | GO:0005243 | IEA |
| 281193 | GO:0004871 | IEA |
| 281201 | GO:0004871 | IEA |
| 281206 | GO:0004069 | IEA |
| 281206 | GO:0008483 | IEA |
| 281206 | GO:0016740 | IEA |
| 281206 | GO:0016769 | IEA |
| 281206 | GO:0003824 | IEA |
| 281210 | GO:0008134 | ISS |
| 281210 | GO:0004602 | IEA |
| 281210 | GO:0005515 | ISS |
| 281210 | GO:0008430 | IEA |
| 281210 | GO:0004601 | IEA |
| 281210 | GO:0016491 | IEA |
| 281216 | GO:0016829 | IEA |
| 281216 | GO:0016849 | IEA |
| 281216 | GO:0004383 | IEA |
| 281225 | GO:0016874 | IEA |
| 281225 | GO:0004842 | IEA |
| 281239 | GO:0008083 | IEA |
| 281239 | GO:0005179 | IEA |
| 281239 | GO:0018445 | IEA |
| 281240 | GO:0005179 | IEA |
| 281240 | GO:0008083 | IEA |
| 281278 | GO:0004872 | IEA |
| 281278 | GO:0004930 | IEA |
| 281278 | GO:0004871 | IEA |
| 281278 | GO:0005529 | IEA |
| 281278 | GO:0016524 | IEA |
| 281278 | GO:0004872 | IEA |
| 281278 | GO:0005529 | IEA |
| 281278 | GO:0004930 | IEA |
| 281278 | GO:0004871 | IEA |
| 281278 | GO:0016524 | IEA |
| 281296 | GO:0005515 | IPI |
| 281306 | GO:0030060 | IEA |
| 281306 | GO:0016615 | IEA |
| 281306 | GO:0004459 | IEA |
| 281306 | GO:0016491 | IEA |
| 281338 | GO:0003774 | IEA |
| 281339 | GO:0003774 | IEA |
| 281339 | GO:0003774 | IEA |
| 281339 | GO:0003774 | IEA |
| 281351 | GO:0008415 | IEA |
| 281351 | GO:0016740 | IEA |
| 281351 | GO:0004379 | IEA |
| 281357 | GO:0004672 | IEA |
| 281357 | GO:0008528 | IEA |
| 281357 | GO:0042802 | IEA |
| 281357 | GO:0004872 | IEA |
| 281357 | GO:0016849 | IEA |
| 281357 | GO:0005524 | IEA |
| 281357 | GO:0016829 | IEA |
| 281357 | GO:0004383 | IEA |
| 281357 | GO:0016941 | IEA |
| 281360 | GO:0016491 | IEA |
| 281360 | GO:0004090 | IEA |
| 281365 | GO:0016829 | IEA |
| 281365 | GO:0003824 | IEA |
| 281365 | GO:0004586 | IEA |
| 281365 | GO:0016831 | IEA |
| 281419 | GO:0016853 | IEA |
| 281419 | GO:0042277 | IEA |
| 281419 | GO:0003755 | IEA |
| 281433 | GO:0004565 | IEA |
| 281433 | GO:0004767 | IEA |
| 281433 | GO:0004557 | IEA |
| 281433 | GO:0004348 | IEA |
| 281433 | GO:0004098 | IEA |
| 281433 | GO:0004336 | IEA |
| 281476 | GO:0016874 | IEA |
| 281476 | GO:0004828 | IEA |
| 281476 | GO:0005524 | IEA |
| 281476 | GO:0000166 | IEA |
| 281476 | GO:0004812 | IEA |
| 281543 | GO:0003824 | IEA |
| 281543 | GO:0004807 | IEA |
| 281543 | GO:0016853 | IEA |
| 281556 | GO:0000166 | IEA |
| 281556 | GO:0005525 | IEA |
| 281556 | GO:0005515 | IPI |
| 281556 | GO:0005515 | IEA |
| 281556 | GO:0003746 | IEA |
| 281557 | GO:0015035 | IEA |
| 281557 | GO:0009055 | IEA |
| 281563 | GO:0017077 | IEA |
| 281563 | GO:0005488 | IEA |
| 281563 | GO:0005215 | IEA |
| 281570 | GO:0016491 | IEA |
| 281570 | GO:0008121 | IEA |
| 281574 | GO:0005515 | ISS |
| 281574 | GO:0005488 | IEA |
| 281574 | GO:0005198 | IEA |
| 281574 | GO:0051015 | ISS |
| 281574 | GO:0008092 | IEA |
| 281574 | GO:0043621 | ISS |
| 281603 | GO:0004016 | IEA |
| 281603 | GO:0016849 | IEA |
| 281603 | GO:0000287 | IEA |
| 281603 | GO:0016829 | IEA |
| 281603 | GO:0046872 | IEA |
| 281615 | GO:0001758 | IEA |
| 281615 | GO:0016491 | IEA |
| 281631 | GO:0017127 | IEA |
| 281631 | GO:0008034 | IEA |
| 281631 | GO:0008289 | IEA |
| 281631 | GO:0008035 | IEA |
| 281631 | GO:0005319 | IEA |
| 281631 | GO:0042802 | IEA |
| 281660 | GO:0005516 | IEA |
| 281660 | GO:0017022 | IEA |
| 281660 | GO:0003779 | IEA |
| 281719 | GO:0005212 | IEA |
| 281729 | GO:0000166 | IEA |
| 281729 | GO:0005515 | IEA |
| 281729 | GO:0045502 | ISS |
| 281729 | GO:0005524 | IEA |
| 281729 | GO:0046982 | ISS |
| 281729 | GO:0016887 | IEA |
| 281729 | GO:0003777 | ISS |
| 281730 | GO:0003714 | IEA |
| 281730 | GO:0016616 | IEA |
| 281730 | GO:0051287 | IEA |
| 281730 | GO:0005515 | IEA |
| 281736 | GO:0004871 | IEA |
| 281736 | GO:0001584 | IEA |
| 281736 | GO:0032027 | IEA |
| 281736 | GO:0005515 | IEA |
| 281736 | GO:0003779 | IEA |
| 281736 | GO:0004930 | IEA |
| 281736 | GO:0016493 | IEA |
| 281736 | GO:0004872 | IEA |
| 281736 | GO:0016494 | IEA |
| 281742 | GO:0003954 | IEA |
| 281742 | GO:0016491 | IEA |
| 281742 | GO:0008137 | IEA |
| 281742 | GO:0009055 | IEA |
| 281749 | GO:0004930 | IEA |
| 281749 | GO:0004871 | IEA |
| 281749 | GO:0004872 | IEA |
| 281749 | GO:0004962 | IEA |
| 281749 | GO:0005515 | IEA |
| 281749 | GO:0001584 | IEA |
| 281749 | GO:0001584 | IEA |
| 281749 | GO:0004871 | IEA |
| 281749 | GO:0004930 | IEA |
| 281749 | GO:0004962 | IEA |
| 281749 | GO:0005515 | IEA |
| 281749 | GO:0004872 | IEA |
| 281758 | GO:0008289 | IEA |
| 281758 | GO:0005488 | IEA |
| 281759 | GO:0005488 | IEA |
| 281759 | GO:0008289 | IEA |
| 281759 | GO:0005488 | IEA |
| 281759 | GO:0008289 | IEA |
| 281780 | GO:0016740 | IEA |
| 281780 | GO:0046872 | IEA |
| 281780 | GO:0030145 | IEA |
| 281780 | GO:0016758 | IEA |
| 281780 | GO:0047276 | IEA |
| 281780 | GO:0016757 | IEA |
| 281780 | GO:0030145 | IEA |
| 281780 | GO:0047276 | IEA |
| 281780 | GO:0046872 | IEA |
| 281780 | GO:0016740 | IEA |
| 281780 | GO:0016758 | IEA |
| 281780 | GO:0016757 | IEA |
| 281790 | GO:0005515 | IEA |
| 281790 | GO:0004871 | IEA |
| 281790 | GO:0000166 | IEA |
| 281790 | GO:0019001 | IEA |
| 281790 | GO:0005525 | IEA |
| 281791 | GO:0019001 | IEA |
| 281791 | GO:0004871 | IEA |
| 281791 | GO:0005525 | IEA |
| 281801 | GO:0004871 | IEA |
| 281801 | GO:0000166 | IEA |
| 281801 | GO:0016301 | IEA |
| 281801 | GO:0005524 | IEA |
| 281801 | GO:0004703 | IEA |
| 281801 | GO:0004674 | IEA |
| 281801 | GO:0004672 | IEA |
| 281801 | GO:0016740 | IEA |
| 281810 | GO:0050662 | IEA |
| 281810 | GO:0003857 | IEA |
| 281810 | GO:0004300 | IEA |
| 281810 | GO:0016829 | IEA |
| 281810 | GO:0003824 | IEA |
| 281810 | GO:0016491 | IEA |
| 281811 | GO:0008415 | IEA |
| 281811 | GO:0003988 | IEA |
| 281811 | GO:0016740 | IEA |
| 281816 | GO:0016787 | IEA |
| 281849 | GO:0005215 | IEA |
| 281849 | GO:0005515 | IPI |
| 281849 | GO:0004872 | IEA |
| 281876 | GO:0005178 | IEA |
| 281876 | GO:0004872 | IEA |
| 281876 | GO:0005515 | IEA |
| 281904 | GO:0046872 | IEA |
| 281904 | GO:0005044 | IEA |
| 281904 | GO:0016491 | IEA |
| 281904 | GO:0004720 | IEA |
| 281904 | GO:0005507 | IEA |
| 281935 | GO:0003779 | IEA |
| 281935 | GO:0005488 | IEA |
| 281935 | GO:0005524 | IEA |
| 281935 | GO:0003774 | IEA |
| 281935 | GO:0000166 | IEA |
| 281937 | GO:0005516 | IEA |
| 281937 | GO:0005515 | IEA |
| 281937 | GO:0000166 | IEA |
| 281937 | GO:0005524 | IEA |
| 281937 | GO:0003779 | IEA |
| 281937 | GO:0003774 | IEA |
| 281942 | GO:0005515 | IEA |
| 281945 | GO:0003677 | IEA |
| 281945 | GO:0004872 | IEA |
| 281945 | GO:0008270 | IEA |
| 281945 | GO:0004879 | IEA |
| 281945 | GO:0043565 | IEA |
| 281945 | GO:0003707 | IEA |
| 281945 | GO:0003700 | IEA |
| 281945 | GO:0046872 | IEA |
| 281963 | GO:0004930 | IEA |
| 281963 | GO:0016494 | IEA |
| 281963 | GO:0004871 | IEA |
| 281963 | GO:0001584 | IEA |
| 281963 | GO:0045028 | IEA |
| 281963 | GO:0004872 | IEA |
| 281985 | GO:0005515 | IEA |
| 281986 | GO:0004871 | IEA |
| 281986 | GO:0046872 | IEA |
| 281986 | GO:0004629 | IEA |
| 281986 | GO:0005509 | IEA |
| 281986 | GO:0016787 | IEA |
| 281986 | GO:0004435 | IEA |
| 281987 | GO:0016787 | IEA |
| 281987 | GO:0005515 | IEA |
| 281987 | GO:0035254 | IEA |
| 281987 | GO:0004871 | IEA |
| 281987 | GO:0005509 | IEA |
| 281987 | GO:0004435 | IEA |
| 281987 | GO:0004629 | IEA |
| 281998 | GO:0016209 | IEA |
| 281998 | GO:0016491 | IEA |
| 281998 | GO:0051920 | IEA |
| 281998 | GO:0004601 | IEA |
| 281998 | GO:0042802 | IEA |
| 282011 | GO:0003677 | IEA |
| 282015 | GO:0005515 | ISS |
| 282015 | GO:0000166 | IEA |
| 282015 | GO:0005524 | IEA |
| 282015 | GO:0016787 | IEA |
| 282015 | GO:0016887 | ISS |
| 282015 | GO:0017111 | IEA |
| 282015 | GO:0031531 | ISS |
| 282015 | GO:0008134 | ISS |
| 282022 | GO:0016702 | IEA |
| 282022 | GO:0004666 | IEA |
| 282022 | GO:0046872 | IEA |
| 282022 | GO:0004601 | IEA |
| 282022 | GO:0016491 | IEA |
| 282022 | GO:0005506 | IEA |
| 282024 | GO:0003779 | IEA |
| 282024 | GO:0004872 | IEA |
| 282032 | GO:0005096 | IEA |
| 282032 | GO:0005099 | ISS |
| 282032 | GO:0005515 | IEA |
| 282032 | GO:0019870 | ISS |
| 282050 | GO:0005515 | IEA |
| 282050 | GO:0003735 | IEA |
| 282077 | GO:0005507 | IEA |
| 282077 | GO:0005509 | IEA |
| 282090 | GO:0016787 | IEA |
| 282090 | GO:0004016 | IEA |
| 282090 | GO:0050333 | IEA |
| 282091 | GO:0019903 | ISS |
| 282100 | GO:0005262 | IEA |
| 282100 | GO:0005509 | IEA |
| 282100 | GO:0005216 | IEA |
| 282100 | GO:0004872 | IEA |
| 282110 | GO:0004221 | IEA |
| 282110 | GO:0008233 | IEA |
| 282110 | GO:0008234 | IEA |
| 282110 | GO:0016787 | IEA |
| 282119 | GO:0008308 | IEA |
| 282119 | GO:0005515 | IEA |
| 282119 | GO:0015288 | IEA |
| 282119 | GO:0005515 | IEA |
| 282119 | GO:0008308 | IEA |
| 282119 | GO:0015288 | IEA |
| 282120 | GO:0015288 | IEA |
| 282120 | GO:0008308 | IEA |
| 282122 | GO:0043185 | IEA |
| 282122 | GO:0008083 | IEA |
| 282123 | GO:0004872 | IEA |
| 282123 | GO:0005509 | IEA |
| 282130 | GO:0016627 | IEA |
| 282130 | GO:0003995 | IEA |
| 282130 | GO:0050660 | IEA |
| 282130 | GO:0016491 | IEA |
| 282132 | GO:0008233 | IEA |
| 282132 | GO:0019901 | ISS |
| 282132 | GO:0042803 | IEA |
| 282132 | GO:0005515 | ISS |
| 282132 | GO:0016787 | IEA |
| 282132 | GO:0008237 | IEA |
| 282132 | GO:0042169 | ISS |
| 282132 | GO:0005102 | ISS |
| 282132 | GO:0004222 | IEA |
| 282132 | GO:0042803 | ISS |
| 282132 | GO:0019901 | IEA |
| 282132 | GO:0004175 | IEA |
| 282132 | GO:0046872 | IEA |
| 282132 | GO:0008270 | IEA |
| 282132 | GO:0004175 | ISS |
| 282132 | GO:0046872 | IEA |
| 282132 | GO:0042169 | ISS |
| 282132 | GO:0042803 | ISS |
| 282132 | GO:0008233 | IEA |
| 282132 | GO:0008237 | IEA |
| 282132 | GO:0005102 | ISS |
| 282132 | GO:0042803 | IEA |
| 282132 | GO:0008270 | IEA |
| 282132 | GO:0019901 | IEA |
| 282132 | GO:0004222 | IEA |
| 282132 | GO:0005515 | ISS |
| 282132 | GO:0016787 | IEA |
| 282132 | GO:0019901 | ISS |
| 282132 | GO:0004175 | ISS |
| 282132 | GO:0004175 | IEA |
| 282150 | GO:0003863 | IEA |
| 282150 | GO:0016491 | IEA |
| 282150 | GO:0003826 | IEA |
| 282191 | GO:0005198 | IEA |
| 282191 | GO:0005201 | IEA |
| 282191 | GO:0005201 | IEA |
| 282191 | GO:0005198 | IEA |
| 282200 | GO:0004129 | IEA |
| 282200 | GO:0016491 | IEA |
| 282211 | GO:0046872 | IEA |
| 282211 | GO:0016491 | IEA |
| 282211 | GO:0005506 | IEA |
| 282211 | GO:0004497 | IEA |
| 282211 | GO:0050381 | IEA |
| 282211 | GO:0016712 | IEA |
| 282211 | GO:0020037 | IEA |
| 282215 | GO:0031072 | IEA |
| 282218 | GO:0004518 | IEA |
| 282218 | GO:0004519 | IEA |
| 282218 | GO:0005515 | IEA |
| 282218 | GO:0016787 | IEA |
| 282218 | GO:0004531 | IEA |
| 282220 | GO:0000166 | IEA |
| 282220 | GO:0005525 | IEA |
| 282220 | GO:0003746 | IEA |
| 282261 | GO:0046872 | ISS |
| 282261 | GO:0008160 | ISS |
| 282261 | GO:0005520 | IEA |
| 282261 | GO:0019838 | IEA |
| 282262 | GO:0019838 | IEA |
| 282262 | GO:0005520 | IEA |
| 282289 | GO:0008137 | IEA |
| 282289 | GO:0016491 | IEA |
| 282289 | GO:0003954 | IEA |
| 282290 | GO:0051537 | IEA |
| 282290 | GO:0008137 | IEA |
| 282290 | GO:0005506 | IEA |
| 282290 | GO:0051536 | IEA |
| 282290 | GO:0046872 | IEA |
| 282290 | GO:0016491 | IEA |
| 282290 | GO:0003954 | IEA |
| 282303 | GO:0005515 | IEA |
| 282303 | GO:0005515 | IEA |
| 282318 | GO:0016787 | IEA |
| 282318 | GO:0004721 | IEA |
| 282318 | GO:0030145 | IEA |
| 282318 | GO:0005506 | IEA |
| 282318 | GO:0046872 | IEA |
| 282326 | GO:0008233 | IEA |
| 282326 | GO:0005515 | IEA |
| 282326 | GO:0016787 | IEA |
| 282326 | GO:0004252 | IEA |
| 282346 | GO:0004872 | IEA |
| 282385 | GO:0003779 | IEA |
| 282386 | GO:0003779 | IEA |
| 282389 | GO:0050660 | IEA |
| 282389 | GO:0016491 | IEA |
| 282389 | GO:0050661 | IEA |
| 282389 | GO:0004791 | IEA |
| 282389 | GO:0008430 | IEA |
| 282389 | GO:0015036 | IEA |
| 282389 | GO:0016654 | IEA |
| 282393 | GO:0008121 | IEA |
| 282393 | GO:0005515 | IEA |
| 282393 | GO:0016491 | IEA |
| 282394 | GO:0004222 | IEA |
| 282394 | GO:0005515 | IEA |
| 282394 | GO:0008121 | IEA |
| 282394 | GO:0016491 | IEA |
| 282405 | GO:0015078 | IEA |
| 282405 | GO:0016787 | IEA |
| 282405 | GO:0046961 | IEA |
| 282405 | GO:0046872 | IEA |
| 282405 | GO:0046933 | IEA |
| 282420 | GO:0004674 | IEA |
| 282420 | GO:0047485 | IEA |
| 282420 | GO:0005524 | IEA |
| 282420 | GO:0004682 | IEA |
| 282420 | GO:0000166 | IEA |
| 282420 | GO:0016740 | IEA |
| 282420 | GO:0016301 | IEA |
| 282420 | GO:0004672 | IEA |
| 282433 | GO:0020037 | IEA |
| 282433 | GO:0004383 | IEA |
| 282433 | GO:0016849 | IEA |
| 282433 | GO:0005506 | IEA |
| 282433 | GO:0016829 | IEA |
| 282433 | GO:0046872 | IEA |
| 282438 | GO:0003824 | IEA |
| 282438 | GO:0016209 | IEA |
| 282438 | GO:0004601 | IEA |
| 282438 | GO:0016787 | IEA |
| 282438 | GO:0016491 | IEA |
| 282438 | GO:0051920 | IEA |
| 282446 | GO:0004449 | IEA |
| 282446 | GO:0016652 | IEA |
| 282446 | GO:0016491 | IEA |
| 282446 | GO:0016491 | IEA |
| 282446 | GO:0004449 | IEA |
| 282446 | GO:0016652 | IEA |
| 282477 | GO:0015293 | IEA |
| 282477 | GO:0005488 | IEA |
| 282478 | GO:0005215 | IEA |
| 282478 | GO:0005488 | IEA |
| 282515 | GO:0016788 | IEA |
| 282515 | GO:0016787 | IEA |
| 282515 | GO:0005515 | IEA |
| 282515 | GO:0003847 | IEA |
| 282515 | GO:0005515 | IPI |
| 282517 | GO:0008137 | IEA |
| 282517 | GO:0003954 | IEA |
| 282517 | GO:0016491 | IEA |
| 282523 | GO:0005215 | IEA |
| 282523 | GO:0005488 | IEA |
| 282564 | GO:0005515 | IEA |
| 282564 | GO:0005178 | IEA |
| 282572 | GO:0005242 | IEA |
| 282572 | GO:0005216 | IEA |
| 282572 | GO:0030955 | IEA |
| 282572 | GO:0015272 | IEA |
| 282572 | GO:0005244 | IEA |
| 282689 | GO:0005544 | IEA |
| 282689 | GO:0005509 | IEA |
| 282689 | GO:0004859 | IEA |
| 282689 | GO:0008092 | IEA |
| 282690 | GO:0046961 | IEA |
| 282690 | GO:0046933 | IEA |
| 282690 | GO:0015078 | IEA |
| 282701 | GO:0015078 | IEA |
| 282701 | GO:0046961 | IEA |
| 282701 | GO:0046933 | IEA |
| 282705 | GO:0016787 | IEA |
| 282705 | GO:0005515 | IEA |
| 282705 | GO:0008233 | IEA |
| 282705 | GO:0005515 | IEA |
| 282705 | GO:0008233 | IEA |
| 282705 | GO:0016787 | IEA |
| 282710 | GO:0015078 | IEA |
| 282710 | GO:0046933 | IEA |
| 282710 | GO:0046961 | IEA |
| 282716 | GO:0008308 | IEA |
| 282716 | GO:0015288 | IEA |
| 282848 | GO:0005515 | IEA |
| 282848 | GO:0051082 | IEA |
| 282856 | GO:0016301 | IEA |
| 282856 | GO:0005525 | IEA |
| 282856 | GO:0004611 | IEA |
| 282862 | GO:0005515 | IEA |
| 282862 | GO:0005055 | IEA |
| 282872 | GO:0005509 | IEA |
| 282872 | GO:0008270 | IEA |
| 282872 | GO:0016787 | IEA |
| 282872 | GO:0004228 | IEA |
| 282872 | GO:0008237 | IEA |
| 282872 | GO:0008233 | IEA |
| 282872 | GO:0004222 | IEA |
| 282872 | GO:0046872 | IEA |
| 282876 | GO:0005506 | IEA |
| 282876 | GO:0042803 | IEA |
| 282876 | GO:0004517 | IEA |
| 282876 | GO:0005509 | IEA |
| 282876 | GO:0050661 | IEA |
| 282876 | GO:0005515 | IEA |
| 282876 | GO:0020037 | IEA |
| 282876 | GO:0005516 | IEA |
| 282876 | GO:0046872 | IEA |
| 282876 | GO:0010181 | IEA |
| 282876 | GO:0050660 | IEA |
| 282876 | GO:0009055 | IEA |
| 282876 | GO:0016491 | IEA |
| 282885 | GO:0004601 | IEA |
| 282885 | GO:0016491 | IEA |
| 282885 | GO:0051920 | IEA |
| 282885 | GO:0016209 | IEA |
| 286770 | GO:0031072 | IEA |
| 286770 | GO:0005488 | IEA |
| 286770 | GO:0005515 | IPI |
| 286809 | GO:0008430 | IEA |
| 286809 | GO:0004601 | IEA |
| 286809 | GO:0004602 | IEA |
| 286809 | GO:0047066 | IEA |
| 286809 | GO:0016491 | IEA |
| 286838 | GO:0016740 | IEA |
| 286838 | GO:0003824 | IEA |
| 286838 | GO:0008146 | IEA |
| 286838 | GO:0016787 | IEA |
| 286838 | GO:0003735 | IEA |
| 286838 | GO:0015016 | IEA |
| 286840 | GO:0051538 | IEA |
| 286840 | GO:0051537 | IEA |
| 286840 | GO:0008177 | IEA |
| 286840 | GO:0051539 | IEA |
| 286840 | GO:0016491 | IEA |
| 286840 | GO:0009055 | IEA |
| 286840 | GO:0051536 | IEA |
| 286840 | GO:0046872 | IEA |
| 286840 | GO:0005506 | IEA |
| 286861 | GO:0005506 | IEA |
| 286861 | GO:0005488 | IEA |
| 286861 | GO:0046872 | IEA |
| 286861 | GO:0008199 | IEA |
| 286862 | GO:0019904 | IEA |
| 286862 | GO:0008426 | ISS |
| 286862 | GO:0005159 | ISS |
| 286862 | GO:0005515 | ISS |
| 286862 | GO:0003779 | ISS |
| 286862 | GO:0005159 | IEA |
| 286862 | GO:0005080 | IEA |
| 286862 | GO:0005159 | IEA |
| 286862 | GO:0003779 | ISS |
| 286862 | GO:0005159 | ISS |
| 286862 | GO:0019904 | IEA |
| 286862 | GO:0005515 | ISS |
| 286862 | GO:0005080 | IEA |
| 286862 | GO:0008426 | ISS |
| 286883 | GO:0005524 | IEA |
| 286883 | GO:0016301 | IEA |
| 286883 | GO:0004672 | IEA |
| 286883 | GO:0000166 | IEA |
| 286883 | GO:0016740 | IEA |
| 286883 | GO:0004713 | IEA |
| 286883 | GO:0004674 | IEA |
| 286885 | GO:0008121 | IEA |
| 286885 | GO:0016491 | IEA |
| 286886 | GO:0004069 | IEA |
| 286886 | GO:0004069 | ISS |
| 286886 | GO:0003824 | IEA |
| 286886 | GO:0016740 | IEA |
| 286886 | GO:0008483 | IEA |
| 286886 | GO:0016769 | IEA |
| 286886 | GO:0008483 | IEA |
| 286886 | GO:0004069 | ISS |
| 286886 | GO:0004069 | IEA |
| 286886 | GO:0003824 | IEA |
| 286886 | GO:0016740 | IEA |
| 286886 | GO:0016769 | IEA |
| 286969 | GO:0004872 | IEA |
| 286969 | GO:0004930 | IEA |
| 286969 | GO:0001604 | IEA |
| 286969 | GO:0001584 | IEA |
| 286969 | GO:0004871 | IEA |
| 287012 | GO:0008270 | IEA |
| 287012 | GO:0004129 | IEA |
| 287012 | GO:0016491 | IEA |
| 287012 | GO:0046872 | IEA |
| 287014 | GO:0051287 | IEA |
| 287014 | GO:0046872 | IEA |
| 287014 | GO:0016651 | IEA |
| 287014 | GO:0016491 | IEA |
| 287014 | GO:0010181 | IEA |
| 287014 | GO:0008137 | IEA |
| 287014 | GO:0051539 | IEA |
| 287014 | GO:0005506 | IEA |
| 287014 | GO:0051536 | IEA |
| 287014 | GO:0003954 | IEA |
| 287020 | GO:0008121 | IEA |
| 287020 | GO:0051536 | IEA |
| 287020 | GO:0046872 | IEA |
| 287020 | GO:0016491 | IEA |
| 287020 | GO:0005506 | IEA |
| 287020 | GO:0051537 | IEA |
| 287024 | GO:0050661 | IEA |
| 287024 | GO:0005509 | IEA |
| 287024 | GO:0004517 | IEA |
| 287024 | GO:0050660 | IEA |
| 287024 | GO:0010181 | IEA |
| 287024 | GO:0005506 | IEA |
| 287024 | GO:0046872 | IEA |
| 287024 | GO:0009055 | IEA |
| 287024 | GO:0008270 | IEA |
| 287024 | GO:0005516 | IEA |
| 287024 | GO:0016491 | IEA |
| 287024 | GO:0020037 | IEA |
| 287027 | GO:0016491 | IEA |
| 287027 | GO:0051539 | IEA |
| 287027 | GO:0046872 | IEA |
| 287027 | GO:0008137 | IEA |
| 287027 | GO:0009055 | IEA |
| 287027 | GO:0051536 | IEA |
| 287027 | GO:0016651 | IEA |
| 287027 | GO:0003954 | IEA |
| 287027 | GO:0005506 | IEA |
| 287327 | GO:0003954 | IEA |
| 287327 | GO:0016651 | IEA |
| 287327 | GO:0008137 | IEA |
| 287327 | GO:0016491 | IEA |
| 288380 | GO:0003954 | IEA |
| 288380 | GO:0005515 | IEA |
| 288380 | GO:0016651 | IEA |
| 288380 | GO:0005506 | IEA |
| 288380 | GO:0051536 | IEA |
| 288380 | GO:0008137 | IEA |
| 288380 | GO:0051539 | IEA |
| 288380 | GO:0009055 | IEA |
| 288380 | GO:0016491 | IEA |
| 288380 | GO:0046872 | IEA |
| 288380 | GO:0051537 | IEA |
| 317655 | GO:0005516 | IEA |
| 317655 | GO:0000166 | IEA |
| 317655 | GO:0003774 | IEA |
| 317655 | GO:0003779 | IEA |
| 317655 | GO:0005515 | ISS |
| 317655 | GO:0005524 | IEA |
| 317697 | GO:0004872 | IEA |
| 317697 | GO:0005216 | IEA |
| 317697 | GO:0005509 | IEA |
| 317697 | GO:0005220 | IEA |
| 317697 | GO:0005262 | IEA |
| 317706 | GO:0008289 | IEA |
| 317706 | GO:0005496 | IEA |
| 317706 | GO:0005515 | IEA |
| 317706 | GO:0004872 | IEA |
| 319141 | GO:0008415 | IEA |
| 319141 | GO:0016740 | IEA |
| 319141 | GO:0008890 | IEA |
| 319141 | GO:0016874 | IEA |
| 319141 | GO:0016769 | IEA |
| 326346 | GO:0016491 | IEA |
| 326346 | GO:0008137 | IEA |
| 326346 | GO:0003954 | IEA |
| 326578 | GO:0005525 | IEA |
| 326578 | GO:0003723 | IEA |
| 326579 | GO:0003743 | IEA |
| 326581 | GO:0003746 | IEA |
| 326601 | GO:0003677 | IEA |
| 326601 | GO:0005515 | IPI |
| 326601 | GO:0003677 | IEA |
| 326601 | GO:0005515 | IPI |
| 326601 | GO:0005515 | IPI |
| 326601 | GO:0003677 | IEA |
| 326601 | GO:0003677 | IEA |
| 326601 | GO:0005515 | IPI |
| 326601 | GO:0003677 | IEA |
| 326601 | GO:0005515 | IPI |
| 327660 | GO:0003954 | IEA |
| 327660 | GO:0016491 | IEA |
| 327660 | GO:0008137 | IEA |
| 327664 | GO:0000166 | IEA |
| 327664 | GO:0030170 | IEA |
| 327664 | GO:0016757 | IEA |
| 327664 | GO:0004645 | IEA |
| 327664 | GO:0016740 | IEA |
| 327664 | GO:0003824 | IEA |
| 327664 | GO:0008184 | IEA |
| 327665 | GO:0003954 | IEA |
| 327665 | GO:0008137 | IEA |
| 327665 | GO:0016491 | IEA |
| 327668 | GO:0005515 | IPI |
| 327668 | GO:0046961 | IEA |
| 327668 | GO:0046933 | IEA |
| 327668 | GO:0016787 | IEA |
| 327668 | GO:0046872 | IEA |
| 327668 | GO:0015078 | IEA |
| 327669 | GO:0004450 | IEA |
| 327669 | GO:0016652 | IEA |
| 327669 | GO:0016491 | IEA |
| 327670 | GO:0016491 | IEA |
| 327670 | GO:0008137 | IEA |
| 327670 | GO:0003954 | IEA |
| 327673 | GO:0016491 | IEA |
| 327673 | GO:0003954 | IEA |
| 327673 | GO:0008137 | IEA |
| 327674 | GO:0016491 | IEA |
| 327674 | GO:0004129 | IEA |
| 327680 | GO:0016651 | IEA |
| 327680 | GO:0003954 | IEA |
| 327680 | GO:0008137 | IEA |
| 327680 | GO:0016491 | IEA |
| 327684 | GO:0043565 | IEA |
| 327684 | GO:0003700 | IEA |
| 327684 | GO:0003677 | IEA |
| 327684 | GO:0003707 | IEA |
| 327684 | GO:0046872 | IEA |
| 327684 | GO:0004872 | IEA |
| 327684 | GO:0008270 | IEA |
| 327684 | GO:0004879 | IEA |
| 327688 | GO:0009055 | IEA |
| 327688 | GO:0004129 | IEA |
| 327688 | GO:0016491 | IEA |
| 327690 | GO:0016491 | IEA |
| 327690 | GO:0008137 | IEA |
| 327690 | GO:0003954 | IEA |
| 327691 | GO:0008137 | IEA |
| 327691 | GO:0016491 | IEA |
| 327691 | GO:0003954 | IEA |
| 327696 | GO:0046872 | IEA |
| 327696 | GO:0005506 | IEA |
| 327696 | GO:0000104 | IEA |
| 327697 | GO:0008137 | IEA |
| 327697 | GO:0003954 | IEA |
| 327697 | GO:0016651 | IEA |
| 327697 | GO:0009055 | IEA |
| 327697 | GO:0046872 | IEA |
| 327697 | GO:0005506 | IEA |
| 327697 | GO:0051536 | IEA |
| 327697 | GO:0051287 | IEA |
| 327697 | GO:0051539 | IEA |
| 327697 | GO:0016491 | IEA |
| 327698 | GO:0016491 | IEA |
| 327698 | GO:0003954 | IEA |
| 327698 | GO:0008137 | IEA |
| 327699 | GO:0043532 | ISS |
| 327699 | GO:0005516 | IDA |
| 327699 | GO:0005515 | IPI |
| 327699 | GO:0042030 | IDA |
| 327699 | GO:0051117 | IDA |
| 327699 | GO:0042803 | IDA |
| 327699 | GO:0004857 | IEA |
| 327701 | GO:0003954 | IEA |
| 327701 | GO:0008137 | IEA |
| 327701 | GO:0005515 | IEA |
| 327701 | GO:0016491 | IEA |
| 327702 | GO:0008137 | TAS |
| 327702 | GO:0031177 | IEA |
| 327702 | GO:0016491 | IEA |
| 327702 | GO:0000036 | IEA |
| 327702 | GO:0048037 | IEA |
| 327702 | GO:0005504 | IDA |
| 327703 | GO:0005245 | IEA |
| 327703 | GO:0005216 | IEA |
| 327703 | GO:0005509 | IEA |
| 327703 | GO:0005244 | IEA |
| 327703 | GO:0005262 | IEA |
| 327704 | GO:0008137 | IEA |
| 327704 | GO:0003954 | IEA |
| 327704 | GO:0016491 | IEA |
| 327706 | GO:0016491 | IEA |
| 327706 | GO:0003954 | IEA |
| 327706 | GO:0008137 | IEA |
| 327707 | GO:0004518 | IEA |
| 327707 | GO:0004519 | IEA |
| 327707 | GO:0030145 | IEA |
| 327707 | GO:0003676 | IEA |
| 327707 | GO:0000287 | IEA |
| 327707 | GO:0046872 | IEA |
| 327707 | GO:0016787 | IEA |
| 327710 | GO:0016491 | IEA |
| 327710 | GO:0003954 | IEA |
| 327710 | GO:0008137 | IEA |
| 327713 | GO:0016491 | IEA |
| 327713 | GO:0003954 | IEA |
| 327713 | GO:0008137 | IEA |
| 327717 | GO:0003954 | IEA |
| 327717 | GO:0008137 | IEA |
| 327717 | GO:0016491 | IEA |
| 327718 | GO:0004129 | IEA |
| 327718 | GO:0016491 | IEA |
| 337889 | GO:0005515 | IEA |
| 337916 | GO:0003700 | IEA |
| 337927 | GO:0005488 | IEA |
| 337927 | GO:0005488 | IEA |
| 338040 | GO:0046961 | IEA |
| 338040 | GO:0015078 | IEA |
| 338040 | GO:0046872 | IEA |
| 338040 | GO:0046933 | IEA |
| 338044 | GO:0015171 | IEA |
| 338046 | GO:0016491 | IEA |
| 338046 | GO:0003954 | IEA |
| 338046 | GO:0008137 | IEA |
| 338047 | GO:0016787 | IEA |
| 338047 | GO:0046872 | IEA |
| 338053 | GO:0046933 | IEA |
| 338053 | GO:0015078 | IEA |
| 338053 | GO:0008289 | IEA |
| 338053 | GO:0046961 | IEA |
| 338057 | GO:0003954 | IEA |
| 338057 | GO:0016491 | IEA |
| 338057 | GO:0008137 | IEA |
| 338060 | GO:0003954 | IEA |
| 338060 | GO:0005524 | IEA |
| 338060 | GO:0016491 | IEA |
| 338060 | GO:0008137 | IEA |
| 338060 | GO:0016773 | IEA |
| 338062 | GO:0004872 | IEA |
| 338063 | GO:0003954 | IEA |
| 338063 | GO:0008137 | IEA |
| 338063 | GO:0016491 | IEA |
| 338064 | GO:0016491 | IEA |
| 338064 | GO:0008137 | IEA |
| 338064 | GO:0003954 | IEA |
| 338065 | GO:0008137 | IEA |
| 338065 | GO:0016491 | IEA |
| 338065 | GO:0003954 | IEA |
| 338066 | GO:0004872 | IEA |
| 338066 | GO:0004930 | IEA |
| 338066 | GO:0005515 | IEA |
| 338066 | GO:0004871 | IEA |
| 338066 | GO:0005509 | IEA |
| 338068 | GO:0050431 | IEA |
| 338068 | GO:0000166 | IEA |
| 338068 | GO:0005524 | IEA |
| 338068 | GO:0030145 | IEA |
| 338068 | GO:0004872 | IEA |
| 338068 | GO:0046332 | IEA |
| 338068 | GO:0046872 | IEA |
| 338068 | GO:0050431 | ISS |
| 338068 | GO:0004674 | IEA |
| 338068 | GO:0004675 | IEA |
| 338068 | GO:0016361 | IEA |
| 338068 | GO:0000287 | IEA |
| 338068 | GO:0048185 | IEA |
| 338068 | GO:0016301 | IEA |
| 338068 | GO:0004672 | IEA |
| 338068 | GO:0016361 | ISS |
| 338068 | GO:0004702 | IEA |
| 338068 | GO:0016740 | IEA |
| 338068 | GO:0019838 | IEA |
| 338068 | GO:0005024 | IEA |
| 338068 | GO:0005025 | IEA |
| 338073 | GO:0003954 | IEA |
| 338073 | GO:0016491 | IEA |
| 338073 | GO:0008137 | IEA |
| 338079 | GO:0046872 | IEA |
| 338079 | GO:0051539 | IEA |
| 338079 | GO:0016491 | IEA |
| 338079 | GO:0008137 | IEA |
| 338079 | GO:0051536 | IEA |
| 338079 | GO:0005506 | IEA |
| 338079 | GO:0003954 | IEA |
| 338081 | GO:0046872 | IEA |
| 338081 | GO:0046961 | IEA |
| 338081 | GO:0046933 | IEA |
| 338081 | GO:0015078 | IEA |
| 338081 | GO:0016787 | IEA |
| 338084 | GO:0016491 | IEA |
| 338084 | GO:0005515 | IEA |
| 338084 | GO:0003954 | IEA |
| 338084 | GO:0005524 | ISS |
| 338084 | GO:0005515 | ISS |
| 338084 | GO:0008137 | IEA |
| 338086 | GO:0009055 | IEA |
| 338086 | GO:0016491 | IEA |
| 338086 | GO:0004129 | IEA |
| 338087 | GO:0004129 | IEA |
| 338087 | GO:0016491 | IEA |
| 338088 | GO:0046872 | IEA |
| 338088 | GO:0016787 | IEA |
| 338088 | GO:0046961 | IEA |
| 338088 | GO:0015078 | IEA |
| 338088 | GO:0046933 | IEA |
| 353217 | GO:0005351 | IEA |
| 353348 | GO:0005515 | IEA |
| 359716 | GO:0003677 | IEA |
| 359716 | GO:0008168 | IEA |
| 359716 | GO:0003886 | ISS |
| 359716 | GO:0008270 | IEA |
| 359716 | GO:0016740 | IEA |
| 360006 | GO:0051082 | IEA |
| 360006 | GO:0031072 | IEA |
| 360006 | GO:0008270 | IEA |
| 360006 | GO:0046872 | IEA |
| 360007 | GO:0004866 | IEA |
| 360007 | GO:0030414 | IEA |
| 360007 | GO:0004867 | IEA |
| 360196 | GO:0003677 | IEA |
| 399560 | GO:0003735 | IEA |
| 404069 | GO:0042609 | IEA |
| 404069 | GO:0042609 | ISS |
| 404122 | GO:0005515 | IEA |
| 404122 | GO:0005524 | IEA |
| 404122 | GO:0000166 | IEA |
| 404122 | GO:0005198 | IEA |
| 404126 | GO:0004407 | IEA |
| 404149 | GO:0016301 | IEA |
| 404159 | GO:0009055 | IEA |
| 404159 | GO:0015035 | IEA |
| 404169 | GO:0004862 | IEA |
| 404169 | GO:0005515 | IEA |
| 404169 | GO:0004860 | IEA |
| 404180 | GO:0003824 | IEA |
| 404180 | GO:0003824 | IEA |
| 404186 | GO:0019838 | IEA |
| 404186 | GO:0005520 | IEA |
| 404188 | GO:0003954 | IEA |
| 404188 | GO:0003824 | IEA |
| 404188 | GO:0005515 | IEA |
| 404188 | GO:0050662 | IEA |
| 404188 | GO:0016491 | IEA |
| 404188 | GO:0008137 | IEA |
| 407101 | GO:0005515 | IEA |
| 407101 | GO:0005515 | IEA |
| 407147 | GO:0003956 | IEA |
| 407147 | GO:0016740 | IEA |
| 407170 | GO:0005515 | IEA |
| 407170 | GO:0016301 | IEA |
| 407170 | GO:0005524 | IEA |
| 407170 | GO:0004714 | IEA |
| 407170 | GO:0004872 | IEA |
| 407170 | GO:0019838 | IEA |
| 407170 | GO:0004672 | IEA |
| 407170 | GO:0004713 | IEA |
| 407170 | GO:0005021 | IEA |
| 407209 | GO:0005509 | IEA |
| 407209 | GO:0005515 | IEA |
| 407209 | GO:0005509 | IEA |
| 407209 | GO:0005515 | IEA |
| 407240 | GO:0030528 | IEA |
| 407767 | GO:0004421 | IEA |
| 408003 | GO:0016787 | IEA |
| 408003 | GO:0008233 | IEA |
| 408003 | GO:0008234 | IEA |
| 408003 | GO:0016787 | IEA |
| 408003 | GO:0008234 | IEA |
| 408003 | GO:0008233 | IEA |
| 408010 | GO:0016874 | IEA |
| 408010 | GO:0005524 | IEA |
| 408010 | GO:0004812 | IEA |
| 408010 | GO:0004820 | IEA |
| 414735 | GO:0005515 | IEA |
| 414735 | GO:0005509 | IEA |
| 414735 | GO:0005515 | IEA |
| 414735 | GO:0005509 | IEA |
| 414922 | GO:0005515 | IEA |
| 414922 | GO:0005198 | IEA |
| 414922 | GO:0042802 | IEA |
| 444864 | GO:0003700 | IEA |
| 444864 | GO:0008270 | IEA |
| 444864 | GO:0046872 | IEA |
| 444864 | GO:0003676 | IEA |
| 444864 | GO:0003700 | IEA |
| 444864 | GO:0003676 | IEA |
| 444864 | GO:0008270 | IEA |
| 444864 | GO:0046872 | IEA |
| 444866 | GO:0046872 | IEA |
| 444866 | GO:0008270 | IEA |
| 444866 | GO:0003676 | IEA |
| 444866 | GO:0003676 | IEA |
| 444866 | GO:0008270 | IEA |
| 444866 | GO:0046872 | IEA |
| 444878 | GO:0016491 | IEA |
| 444878 | GO:0005506 | IEA |
| 444878 | GO:0046872 | IEA |
| 444878 | GO:0004129 | IEA |
| 444879 | GO:0016757 | IEA |
| 444879 | GO:0016740 | IEA |
| 444879 | GO:0008373 | IEA |
| 445421 | GO:0003735 | IEA |
| 445421 | GO:0003735 | IEA |
| 445543 | GO:0005488 | IEA |
| 445543 | GO:0005198 | IEA |
| 445543 | GO:0005515 | IEA |
| 445543 | GO:0008092 | ISS |
| 493728 | GO:0003779 | IEA |
| 493998 | GO:0016918 | IEA |
| 493998 | GO:0005488 | IEA |
| 493998 | GO:0008289 | IEA |
| 493998 | GO:0001972 | IEA |
| 503685 | GO:0004869 | IEA |
| 504201 | GO:0005509 | IEA |
| 504248 | GO:0016740 | IEA |
| 504248 | GO:0016301 | IEA |
| 504248 | GO:0000287 | IEA |
| 504248 | GO:0046872 | IEA |
| 504248 | GO:0004749 | IEA |
| 504317 | GO:0004872 | IEA |
| 504317 | GO:0005509 | IEA |
| 504534 | GO:0008508 | IEA |
| 504599 | GO:0005515 | IPI |
| 504599 | GO:0003677 | IEA |
| 504599 | GO:0003677 | IEA |
| 504599 | GO:0005515 | IPI |
| 504599 | GO:0003677 | IEA |
| 504599 | GO:0005515 | IPI |
| 504599 | GO:0003677 | IEA |
| 504599 | GO:0005515 | IPI |
| 504599 | GO:0005515 | IPI |
| 504599 | GO:0003677 | IEA |
| 504637 | GO:0000166 | IEA |
| 504637 | GO:0017111 | IEA |
| 504637 | GO:0005515 | IEA |
| 504637 | GO:0005524 | IEA |
| 504650 | GO:0016740 | IEA |
| 504716 | GO:0003700 | IEA |
| 504718 | GO:0005515 | IEA |
| 504914 | GO:0004853 | IEA |
| 505005 | GO:0016740 | IEA |
| 505005 | GO:0005524 | IEA |
| 505005 | GO:0004673 | IEA |
| 505005 | GO:0004672 | IEA |
| 505005 | GO:0016301 | IEA |
| 505005 | GO:0016772 | IEA |
| 505050 | GO:0016787 | IEA |
| 505050 | GO:0008233 | IEA |
| 505050 | GO:0004298 | IEA |
| 505050 | GO:0004175 | IEA |
| 505060 | GO:0005506 | IEA |
| 505060 | GO:0016491 | IEA |
| 505060 | GO:0046872 | IEA |
| 505060 | GO:0008398 | IEA |
| 505060 | GO:0004497 | IEA |
| 505060 | GO:0020037 | IEA |
| 505093 | GO:0003723 | IEA |
| 505093 | GO:0016853 | IEA |
| 505093 | GO:0003676 | IEA |
| 505093 | GO:0000166 | IEA |
| 505176 | GO:0008233 | IEA |
| 505176 | GO:0016787 | IEA |
| 505176 | GO:0004298 | IEA |
| 505176 | GO:0004175 | IEA |
| 505176 | GO:0005515 | IEA |
| 505219 | GO:0005524 | IEA |
| 505219 | GO:0000166 | IEA |
| 505219 | GO:0017111 | IEA |
| 505219 | GO:0042803 | IEA |
| 505219 | GO:0016887 | IEA |
| 505293 | GO:0003713 | IEA |
| 505293 | GO:0030528 | IEA |
| 505293 | GO:0005515 | IEA |
| 505406 | GO:0003779 | IEA |
| 505453 | GO:0004402 | IEA |
| 505453 | GO:0003677 | IEA |
| 505453 | GO:0016407 | IEA |
| 505453 | GO:0005515 | IEA |
| 505471 | GO:0003677 | IEA |
| 505489 | GO:0005515 | IEA |
| 505489 | GO:0004722 | IEA |
| 505489 | GO:0004725 | IEA |
| 505489 | GO:0043021 | IEA |
| 505645 | GO:0005523 | IEA |
| 505775 | GO:0015355 | IEA |
| 505775 | GO:0005215 | IEA |
| 505775 | GO:0005515 | IEA |
| 505807 | GO:0004719 | IEA |
| 505824 | GO:0016301 | IEA |
| 505824 | GO:0004872 | IEA |
| 505824 | GO:0005515 | IEA |
| 505884 | GO:0008270 | IEA |
| 505884 | GO:0046872 | IEA |
| 505884 | GO:0003676 | IEA |
| 505884 | GO:0046872 | IEA |
| 505884 | GO:0003676 | IEA |
| 505884 | GO:0008270 | IEA |
| 505884 | GO:0003676 | IEA |
| 505884 | GO:0008270 | IEA |
| 505884 | GO:0046872 | IEA |
| 505949 | GO:0016491 | IEA |
| 505968 | GO:0016627 | IEA |
| 505968 | GO:0050660 | IEA |
| 505968 | GO:0003995 | IEA |
| 505968 | GO:0016491 | IEA |
| 505987 | GO:0016301 | IEA |
| 505987 | GO:0016740 | IEA |
| 505987 | GO:0004370 | IEA |
| 506029 | GO:0004672 | IEA |
| 506029 | GO:0004672 | IEA |
| 506114 | GO:0003700 | IEA |
| 506114 | GO:0003677 | IEA |
| 506115 | GO:0016791 | IEA |
| 506231 | GO:0005085 | IEA |
| 506235 | GO:0003677 | IEA |
| 506235 | GO:0043565 | IEA |
| 506235 | GO:0003700 | IEA |
| 506261 | GO:0019904 | IEA |
| 506263 | GO:0008270 | IEA |
| 506263 | GO:0016491 | IEA |
| 506325 | GO:0008270 | IEA |
| 506448 | GO:0003676 | IEA |
| 506448 | GO:0003700 | IEA |
| 506448 | GO:0003677 | IEA |
| 506448 | GO:0008270 | IEA |
| 506448 | GO:0046872 | IEA |
| 506522 | GO:0005515 | IEA |
| 506544 | GO:0008443 | IEA |
| 506544 | GO:0003872 | IEA |
| 506544 | GO:0016301 | IEA |
| 506544 | GO:0016301 | IEA |
| 506544 | GO:0008443 | IEA |
| 506544 | GO:0003872 | IEA |
| 506730 | GO:0003779 | IEA |
| 506730 | GO:0005515 | IEA |
| 506730 | GO:0008017 | IEA |
| 506764 | GO:0005515 | IEA |
| 506764 | GO:0046872 | IEA |
| 506764 | GO:0016874 | IEA |
| 506764 | GO:0008270 | IEA |
| 506764 | GO:0016874 | IEA |
| 506764 | GO:0005515 | IEA |
| 506764 | GO:0046872 | IEA |
| 506764 | GO:0008270 | IEA |
| 506880 | GO:0005515 | IEA |
| 506880 | GO:0000166 | IEA |
| 506880 | GO:0042802 | IEA |
| 506880 | GO:0003676 | IEA |
| 506882 | GO:0004177 | IEA |
| 506882 | GO:0004250 | IEA |
| 506882 | GO:0003725 | IEA |
| 506969 | GO:0005515 | IEA |
| 506969 | GO:0017137 | IEA |
| 506991 | GO:0019153 | IEA |
| 506991 | GO:0016491 | IEA |
| 506991 | GO:0009055 | IEA |
| 506991 | GO:0015035 | IEA |
| 507095 | GO:0005515 | IEA |
| 507107 | GO:0043169 | IEA |
| 507107 | GO:0005515 | IEA |
| 507107 | GO:0003824 | IEA |
| 507114 | GO:0005515 | IEA |
| 507179 | GO:0005515 | IEA |
| 507211 | GO:0003723 | IEA |
| 507211 | GO:0003676 | IEA |
| 507213 | GO:0016787 | IEA |
| 507213 | GO:0005515 | IEA |
| 507213 | GO:0004175 | IEA |
| 507213 | GO:0008233 | IEA |
| 507213 | GO:0004298 | IEA |
| 507216 | GO:0015288 | IEA |
| 507216 | GO:0008308 | IEA |
| 507342 | GO:0030674 | IEA |
| 507342 | GO:0051015 | IEA |
| 507365 | GO:0005085 | IEA |
| 507365 | GO:0005089 | IEA |
| 507410 | GO:0004540 | IEA |
| 507410 | GO:0004526 | IEA |
| 507410 | GO:0005515 | IEA |
| 507431 | GO:0005515 | IEA |
| 507431 | GO:0003824 | IEA |
| 507476 | GO:0016301 | IEA |
| 507476 | GO:0004618 | IEA |
| 507476 | GO:0016740 | IEA |
| 507476 | GO:0000166 | IEA |
| 507476 | GO:0005524 | IEA |
| 507482 | GO:0003676 | IEA |
| 507482 | GO:0003723 | IEA |
| 507484 | GO:0004867 | IEA |
| 507484 | GO:0008233 | IEA |
| 507537 | GO:0005509 | IEA |
| 507632 | GO:0005515 | IEA |
| 507654 | GO:0008233 | IEA |
| 507707 | GO:0004872 | IEA |
| 507707 | GO:0005488 | IEA |
| 507707 | GO:0005515 | IEA |
| 507707 | GO:0005488 | IEA |
| 507707 | GO:0005515 | IEA |
| 507707 | GO:0004872 | IEA |
| 507736 | GO:0004872 | IEA |
| 507736 | GO:0004872 | IEA |
| 507783 | GO:0004872 | IEA |
| 507783 | GO:0004991 | IEA |
| 507783 | GO:0004871 | IEA |
| 507783 | GO:0004930 | IEA |
| 507801 | GO:0005515 | IEA |
| 507834 | GO:0005515 | IEA |
| 507834 | GO:0005515 | IEA |
| 507897 | GO:0031072 | IEA |
| 507897 | GO:0003677 | IEA |
| 507912 | GO:0003676 | IEA |
| 507990 | GO:0005515 | IEA |
| 507990 | GO:0015288 | IEA |
| 507990 | GO:0015288 | IEA |
| 507990 | GO:0005515 | IEA |
| 508090 | GO:0005102 | IEA |
| 508137 | GO:0031072 | IEA |
| 508143 | GO:0005488 | IEA |
| 508143 | GO:0003723 | IEA |
| 508143 | GO:0019843 | IEA |
| 508143 | GO:0003735 | IEA |
| 508170 | GO:0016853 | IEA |
| 508170 | GO:0004493 | IEA |
| 508175 | GO:0005515 | IEA |
| 508231 | GO:0005515 | IEA |
| 508268 | GO:0005515 | IEA |
| 508268 | GO:0005198 | IEA |
| 508285 | GO:0016740 | IEA |
| 508285 | GO:0008831 | IEA |
| 508306 | GO:0004497 | IEA |
| 508442 | GO:0005515 | IEA |
| 508465 | GO:0005515 | ISS |
| 508465 | GO:0005515 | IEA |
| 508535 | GO:0005488 | IEA |
| 508535 | GO:0003755 | IEA |
| 508535 | GO:0005525 | IEA |
| 508535 | GO:0005524 | IEA |
| 508535 | GO:0035259 | IEA |
| 508535 | GO:0051219 | IEA |
| 508535 | GO:0031072 | IEA |
| 508535 | GO:0016853 | IEA |
| 508566 | GO:0003955 | IEA |
| 508609 | GO:0005515 | IEA |
| 508712 | GO:0016847 | IEA |
| 508712 | GO:0016769 | IEA |
| 508712 | GO:0016740 | IEA |
| 508712 | GO:0008483 | IEA |
| 508722 | GO:0003676 | IEA |
| 508722 | GO:0003723 | IEA |
| 508722 | GO:0005515 | IEA |
| 508722 | GO:0000166 | IEA |
| 508724 | GO:0004334 | IEA |
| 508882 | GO:0004274 | IEA |
| 508957 | GO:0005515 | IEA |
| 509037 | GO:0009055 | IEA |
| 509037 | GO:0015035 | IEA |
| 509037 | GO:0030508 | IEA |
| 509056 | GO:0008270 | IEA |
| 509056 | GO:0046872 | IEA |
| 509111 | GO:0005515 | IEA |
| 509192 | GO:0004722 | IEA |
| 509192 | GO:0016791 | IEA |
| 509231 | GO:0003676 | IEA |
| 509231 | GO:0008270 | IEA |
| 509273 | GO:0003824 | IEA |
| 509273 | GO:0016787 | IEA |
| 509274 | GO:0004416 | IEA |
| 509274 | GO:0016787 | IEA |
| 509459 | GO:0016740 | IEA |
| 509459 | GO:0008415 | IEA |
| 509459 | GO:0004095 | IEA |
| 509619 | GO:0008270 | IEA |
| 509619 | GO:0016787 | IEA |
| 509619 | GO:0016814 | IEA |
| 509619 | GO:0046872 | IEA |
| 509684 | GO:0005488 | IEA |
| 509696 | GO:0000010 | IEA |
| 509696 | GO:0050347 | IEA |
| 509696 | GO:0046982 | IEA |
| 509775 | GO:0004867 | IEA |
| 509816 | GO:0003735 | IEA |
| 509827 | GO:0005515 | IEA |
| 509827 | GO:0003677 | IEA |
| 509856 | GO:0005515 | IEA |
| 509966 | GO:0005525 | IEA |
| 509983 | GO:0003824 | IEA |
| 509983 | GO:0016874 | IEA |
| 510029 | GO:0005524 | IEA |
| 510029 | GO:0017111 | IEA |
| 510029 | GO:0000166 | IEA |
| 510029 | GO:0016787 | IEA |
| 510050 | GO:0003700 | IEA |
| 510050 | GO:0043565 | IEA |
| 510050 | GO:0003677 | IEA |
| 510069 | GO:0004298 | IEA |
| 510069 | GO:0016787 | IEA |
| 510069 | GO:0004175 | IEA |
| 510069 | GO:0008233 | IEA |
| 510080 | GO:0004872 | IEA |
| 510080 | GO:0004872 | IEA |
| 510080 | GO:0004872 | IEA |
| 510109 | GO:0016787 | IEA |
| 510109 | GO:0003824 | IEA |
| 510155 | GO:0008233 | IEA |
| 510155 | GO:0004298 | IEA |
| 510155 | GO:0005515 | IEA |
| 510155 | GO:0004175 | IEA |
| 510155 | GO:0016787 | IEA |
| 510159 | GO:0008017 | IEA |
| 510194 | GO:0003677 | IEA |
| 510200 | GO:0017039 | IEA |
| 510219 | GO:0004872 | IEA |
| 510219 | GO:0008308 | IEA |
| 510245 | GO:0004721 | IEA |
| 510245 | GO:0005488 | IEA |
| 510245 | GO:0016787 | IEA |
| 510245 | GO:0005515 | IEA |
| 510264 | GO:0016491 | IEA |
| 510322 | GO:0005515 | IEA |
| 510376 | GO:0005515 | IEA |
| 510376 | GO:0003714 | IEA |
| 510376 | GO:0005515 | IEA |
| 510376 | GO:0003714 | IEA |
| 510412 | GO:0005515 | IEA |
| 510423 | GO:0004298 | IEA |
| 510423 | GO:0042802 | IEA |
| 510423 | GO:0004175 | IEA |
| 510434 | GO:0004672 | IEA |
| 510434 | GO:0000166 | IEA |
| 510434 | GO:0004674 | IEA |
| 510434 | GO:0016301 | IEA |
| 510434 | GO:0016740 | IEA |
| 510434 | GO:0005524 | IEA |
| 510462 | GO:0016491 | IEA |
| 510469 | GO:0003963 | IEA |
| 510469 | GO:0016886 | IEA |
| 510483 | GO:0017176 | IEA |
| 510483 | GO:0016757 | IEA |
| 510483 | GO:0016740 | IEA |
| 510498 | GO:0005515 | IEA |
| 510498 | GO:0035091 | IEA |
| 510498 | GO:0019902 | IEA |
| 510498 | GO:0019208 | IEA |
| 510498 | GO:0042803 | IEA |
| 510568 | GO:0008565 | IEA |
| 510568 | GO:0005515 | IEA |
| 510575 | GO:0005515 | IEA |
| 510575 | GO:0005524 | IEA |
| 510679 | GO:0004655 | IEA |
| 510679 | GO:0016829 | IEA |
| 510679 | GO:0003824 | IEA |
| 510729 | GO:0008665 | IEA |
| 510729 | GO:0016772 | IEA |
| 510767 | GO:0020037 | IEA |
| 510767 | GO:0005506 | IEA |
| 510767 | GO:0046872 | IEA |
| 510767 | GO:0009055 | IEA |
| 510780 | GO:0005515 | IEA |
| 510811 | GO:0005515 | IEA |
| 510811 | GO:0030160 | IEA |
| 510811 | GO:0003779 | IEA |
| 510833 | GO:0005201 | IEA |
| 510833 | GO:0005201 | IEA |
| 510889 | GO:0008270 | IEA |
| 510889 | GO:0004222 | IEA |
| 510889 | GO:0016787 | IEA |
| 510889 | GO:0008237 | IEA |
| 510889 | GO:0046872 | IEA |
| 510889 | GO:0008233 | IEA |
| 510899 | GO:0003735 | IEA |
| 510937 | GO:0004821 | IEA |
| 510937 | GO:0005524 | IEA |
| 510937 | GO:0000166 | IEA |
| 510937 | GO:0004812 | IEA |
| 510937 | GO:0016874 | IEA |
| 510949 | GO:0004018 | IEA |
| 510949 | GO:0016829 | IEA |
| 510953 | GO:0004064 | IEA |
| 510960 | GO:0016740 | IEA |
| 510960 | GO:0005515 | IEA |
| 510960 | GO:0004607 | IEA |
| 510960 | GO:0008415 | IEA |
| 510960 | GO:0005515 | IEA |
| 510960 | GO:0016740 | IEA |
| 510960 | GO:0008415 | IEA |
| 510960 | GO:0004607 | IEA |
| 510968 | GO:0017134 | IEA |
| 511007 | GO:0005515 | IEA |
| 511047 | GO:0004373 | IEA |
| 511047 | GO:0005515 | IEA |
| 511058 | GO:0005506 | IEA |
| 511058 | GO:0016787 | IEA |
| 511090 | GO:0005524 | IEA |
| 511090 | GO:0003824 | IEA |
| 511090 | GO:0016874 | IEA |
| 511190 | GO:0004674 | IEA |
| 511200 | GO:0003824 | IEA |
| 511200 | GO:0005524 | IEA |
| 511200 | GO:0003922 | IEA |
| 511200 | GO:0000166 | IEA |
| 511245 | GO:0005515 | IEA |
| 511245 | GO:0001537 | IEA |
| 511268 | GO:0005515 | IEA |
| 511291 | GO:0016740 | IEA |
| 511291 | GO:0004142 | IEA |
| 511440 | GO:0004129 | IEA |
| 511453 | GO:0008233 | IEA |
| 511453 | GO:0016787 | IEA |
| 511453 | GO:0009003 | IEA |
| 511560 | GO:0003735 | IEA |
| 511560 | GO:0003723 | IEA |
| 511560 | GO:0005515 | IEA |
| 511614 | GO:0016740 | IEA |
| 511614 | GO:0008415 | IEA |
| 511624 | GO:0050660 | IEA |
| 511624 | GO:0004497 | IEA |
| 511624 | GO:0016491 | IEA |
| 511682 | GO:0008233 | IEA |
| 511682 | GO:0004252 | IEA |
| 511682 | GO:0004175 | IEA |
| 511699 | GO:0005247 | IEA |
| 511735 | GO:0005506 | IEA |
| 511735 | GO:0005488 | IEA |
| 511735 | GO:0005344 | IEA |
| 511735 | GO:0046872 | IEA |
| 511735 | GO:0019825 | IEA |
| 511735 | GO:0020037 | IEA |
| 511735 | GO:0005344 | IEA |
| 511735 | GO:0019825 | IEA |
| 511735 | GO:0046872 | IEA |
| 511735 | GO:0020037 | IEA |
| 511735 | GO:0005506 | IEA |
| 511735 | GO:0005488 | IEA |
| 511735 | GO:0020037 | IEA |
| 511735 | GO:0046872 | IEA |
| 511735 | GO:0005488 | IEA |
| 511735 | GO:0005344 | IEA |
| 511735 | GO:0019825 | IEA |
| 511735 | GO:0005506 | IEA |
| 511763 | GO:0005515 | IEA |
| 511763 | GO:0008143 | IEA |
| 511801 | GO:0005515 | IEA |
| 511812 | GO:0004871 | IEA |
| 511861 | GO:0005515 | IEA |
| 511965 | GO:0008270 | IEA |
| 511965 | GO:0003676 | IEA |
| 511965 | GO:0046872 | IEA |
| 512002 | GO:0004616 | IEA |
| 512002 | GO:0051287 | IEA |
| 512002 | GO:0008442 | IEA |
| 512002 | GO:0050662 | IEA |
| 512002 | GO:0016491 | IEA |
| 512136 | GO:0000166 | IEA |
| 512136 | GO:0003723 | IEA |
| 512136 | GO:0005487 | IEA |
| 512136 | GO:0003729 | IEA |
| 512136 | GO:0005515 | IEA |
| 512259 | GO:0051287 | ISS |
| 512259 | GO:0004957 | ISS |
| 512259 | GO:0042803 | ISS |
| 512259 | GO:0016491 | IEA |
| 512259 | GO:0016404 | ISS |
| 512259 | GO:0003824 | ISS |
| 512259 | GO:0016491 | IEA |
| 512259 | GO:0003824 | ISS |
| 512259 | GO:0016404 | ISS |
| 512259 | GO:0004957 | ISS |
| 512259 | GO:0051287 | ISS |
| 512259 | GO:0042803 | ISS |
| 512350 | GO:0003743 | IEA |
| 512350 | GO:0005525 | IEA |
| 512350 | GO:0008135 | ISS |
| 512350 | GO:0008135 | ISS |
| 512350 | GO:0003743 | IEA |
| 512350 | GO:0005525 | IEA |
| 512427 | GO:0005515 | ISS |
| 512427 | GO:0051879 | ISS |
| 512427 | GO:0005488 | IEA |
| 512469 | GO:0003735 | IEA |
| 512469 | GO:0003723 | IEA |
| 512500 | GO:0046872 | IEA |
| 512500 | GO:0020037 | IEA |
| 512500 | GO:0005506 | IEA |
| 512500 | GO:0009055 | IEA |
| 512531 | GO:0004825 | IEA |
| 512531 | GO:0005524 | IEA |
| 512531 | GO:0004812 | IEA |
| 512531 | GO:0003723 | IEA |
| 512531 | GO:0000049 | IEA |
| 512531 | GO:0016874 | IEA |
| 512531 | GO:0000166 | IEA |
| 512531 | GO:0004812 | IEA |
| 512531 | GO:0005524 | IEA |
| 512531 | GO:0000166 | IEA |
| 512531 | GO:0003723 | IEA |
| 512531 | GO:0016874 | IEA |
| 512531 | GO:0000049 | IEA |
| 512531 | GO:0004825 | IEA |
| 512571 | GO:0016301 | IEA |
| 512571 | GO:0004743 | IEA |
| 512571 | GO:0016740 | IEA |
| 512571 | GO:0000287 | IEA |
| 512666 | GO:0005515 | IEA |
| 512673 | GO:0051082 | IEA |
| 512741 | GO:0005515 | IPI |
| 512741 | GO:0003677 | IEA |
| 512741 | GO:0003677 | IEA |
| 512741 | GO:0005515 | IPI |
| 512741 | GO:0005515 | IPI |
| 512741 | GO:0003677 | IEA |
| 512741 | GO:0005515 | IPI |
| 512741 | GO:0003677 | IEA |
| 512741 | GO:0003677 | IEA |
| 512741 | GO:0005515 | IPI |
| 512771 | GO:0004056 | IEA |
| 512771 | GO:0016829 | IEA |
| 512771 | GO:0003824 | IEA |
| 512818 | GO:0008270 | IEA |
| 512818 | GO:0046872 | IEA |
| 512841 | GO:0042169 | IEA |
| 512841 | GO:0045309 | IEA |
| 512841 | GO:0005515 | IEA |
| 512902 | GO:0016740 | IEA |
| 512902 | GO:0008415 | IEA |
| 512902 | GO:0016740 | IEA |
| 512902 | GO:0008415 | IEA |
| 512978 | GO:0016853 | IEA |
| 512978 | GO:0003723 | IEA |
| 512978 | GO:0003676 | IEA |
| 512978 | GO:0000166 | IEA |
| 513038 | GO:0008233 | IEA |
| 513038 | GO:0016787 | IEA |
| 513038 | GO:0004216 | IEA |
| 513038 | GO:0004197 | IEA |
| 513038 | GO:0008234 | IEA |
| 513061 | GO:0005524 | IEA |
| 513061 | GO:0000166 | IEA |
| 513061 | GO:0016887 | IEA |
| 513061 | GO:0017111 | IEA |
| 513188 | GO:0004246 | IEA |
| 513265 | GO:0002039 | IEA |
| 513324 | GO:0005509 | IEA |
| 513324 | GO:0005509 | IEA |
| 513405 | GO:0005515 | IEA |
| 513433 | GO:0031419 | IEA |
| 513433 | GO:0050897 | IEA |
| 513451 | GO:0000166 | IEA |
| 513451 | GO:0003676 | IEA |
| 513461 | GO:0005515 | IEA |
| 513530 | GO:0004315 | IEA |
| 513530 | GO:0003824 | IEA |
| 513573 | GO:0031072 | IEA |
| 513586 | GO:0004476 | IEA |
| 513586 | GO:0008270 | IEA |
| 513586 | GO:0046872 | IEA |
| 513586 | GO:0016853 | IEA |
| 513587 | GO:0005515 | IEA |
| 513699 | GO:0005515 | IEA |
| 513762 | GO:0009055 | IEA |
| 513762 | GO:0015035 | IEA |
| 513843 | GO:0005515 | IEA |
| 513934 | GO:0005515 | IEA |
| 513958 | GO:0003743 | ISS |
| 513958 | GO:0005085 | IEA |
| 513958 | GO:0003743 | IEA |
| 513958 | GO:0005525 | ISS |
| 513958 | GO:0005515 | ISS |
| 513958 | GO:0005525 | IEA |
| 513958 | GO:0005085 | ISS |
| 513958 | GO:0005524 | IEA |
| 513958 | GO:0005524 | ISS |
| 513958 | GO:0005515 | IEA |
| 514044 | GO:0016491 | IEA |
| 514066 | GO:0042578 | IEA |
| 514066 | GO:0016787 | IEA |
| 514066 | GO:0042132 | IEA |
| 514230 | GO:0047545 | IEA |
| 514357 | GO:0016740 | IEA |
| 514371 | GO:0005524 | IEA |
| 514371 | GO:0005515 | IEA |
| 514386 | GO:0004842 | IEA |
| 514386 | GO:0008270 | IEA |
| 514386 | GO:0005515 | IEA |
| 514403 | GO:0003735 | IEA |
| 514443 | GO:0005515 | IEA |
| 514472 | GO:0000166 | IEA |
| 514472 | GO:0005524 | IEA |
| 514472 | GO:0051082 | IEA |
| 514473 | GO:0005515 | IEA |
| 514473 | GO:0003682 | IEA |
| 514665 | GO:0005215 | IEA |
| 514852 | GO:0005515 | IEA |
| 514918 | GO:0003700 | IEA |
| 514918 | GO:0003677 | IEA |
| 514918 | GO:0003677 | IEA |
| 514918 | GO:0003700 | IEA |
| 515022 | GO:0046872 | IEA |
| 515022 | GO:0004785 | IEA |
| 515091 | GO:0005488 | IEA |
| 515109 | GO:0005515 | IEA |
| 515109 | GO:0046872 | IEA |
| 515109 | GO:0008270 | IEA |
| 515169 | GO:0016740 | IEA |
| 515169 | GO:0016301 | IEA |
| 515169 | GO:0046872 | IEA |
| 515169 | GO:0000287 | IEA |
| 515169 | GO:0004749 | IEA |
| 515176 | GO:0004536 | IEA |
| 515176 | GO:0016787 | IEA |
| 515176 | GO:0004518 | IEA |
| 515176 | GO:0004519 | IEA |
| 515178 | GO:0005524 | IEA |
| 515178 | GO:0005515 | IEA |
| 515178 | GO:0000166 | IEA |
| 515178 | GO:0016887 | IEA |
| 515178 | GO:0042626 | IEA |
| 515178 | GO:0017111 | IEA |
| 515186 | GO:0008483 | IEA |
| 515186 | GO:0030170 | IEA |
| 515186 | GO:0016740 | IEA |
| 515270 | GO:0016740 | IEA |
| 515270 | GO:0008168 | IEA |
| 515270 | GO:0030731 | IEA |
| 515320 | GO:0003677 | IEA |
| 515363 | GO:0005515 | IEA |
| 515363 | GO:0004872 | IEA |
| 515363 | GO:0016566 | IEA |
| 515380 | GO:0005516 | IEA |
| 515380 | GO:0043565 | IEA |
| 515380 | GO:0003677 | IEA |
| 515410 | GO:0008270 | IEA |
| 515410 | GO:0046872 | IEA |
| 515410 | GO:0005515 | IEA |
| 515490 | GO:0005096 | IEA |
| 515490 | GO:0005515 | IEA |
| 515490 | GO:0017124 | IEA |
| 515494 | GO:0004872 | IEA |
| 515494 | GO:0005041 | IEA |
| 515503 | GO:0004298 | IEA |
| 515503 | GO:0004175 | IEA |
| 515503 | GO:0016787 | IEA |
| 515503 | GO:0008233 | IEA |
| 515503 | GO:0005515 | IEA |
| 515509 | GO:0005488 | IEA |
| 515614 | GO:0016740 | IEA |
| 515614 | GO:0004418 | IEA |
| 515646 | GO:0031404 | IEA |
| 515646 | GO:0005254 | IEA |
| 515646 | GO:0005244 | IEA |
| 515646 | GO:0005247 | IEA |
| 515646 | GO:0005515 | IEA |
| 515646 | GO:0005216 | IEA |
| 515652 | GO:0003677 | IEA |
| 515696 | GO:0046872 | IEA |
| 515696 | GO:0046933 | IEA |
| 515696 | GO:0005515 | IEA |
| 515696 | GO:0046961 | IEA |
| 515696 | GO:0015078 | IEA |
| 515757 | GO:0004222 | IEA |
| 515757 | GO:0016787 | IEA |
| 515757 | GO:0008237 | IEA |
| 515757 | GO:0017111 | IEA |
| 515757 | GO:0005524 | IEA |
| 515757 | GO:0046872 | IEA |
| 515757 | GO:0008233 | IEA |
| 515757 | GO:0008270 | IEA |
| 515757 | GO:0000166 | IEA |
| 515757 | GO:0004222 | IEA |
| 515757 | GO:0016787 | IEA |
| 515757 | GO:0008237 | IEA |
| 515757 | GO:0000166 | IEA |
| 515757 | GO:0005524 | IEA |
| 515757 | GO:0008270 | IEA |
| 515757 | GO:0017111 | IEA |
| 515757 | GO:0046872 | IEA |
| 515757 | GO:0008233 | IEA |
| 515765 | GO:0003697 | IEA |
| 515783 | GO:0005529 | IEA |
| 515902 | GO:0016874 | IEA |
| 515946 | GO:0016491 | IEA |
| 515990 | GO:0003735 | IEA |
| 515999 | GO:0000166 | IEA |
| 515999 | GO:0046872 | IEA |
| 515999 | GO:0000287 | IEA |
| 515999 | GO:0005525 | IEA |
| 515999 | GO:0046872 | IEA |
| 515999 | GO:0000166 | IEA |
| 515999 | GO:0005525 | IEA |
| 515999 | GO:0000287 | IEA |
| 516039 | GO:0004708 | IEA |
| 516287 | GO:0051287 | IEA |
| 516287 | GO:0004128 | IEA |
| 516287 | GO:0050660 | IEA |
| 516287 | GO:0016491 | IEA |
| 516287 | GO:0004128 | IEA |
| 516287 | GO:0051287 | IEA |
| 516287 | GO:0016491 | IEA |
| 516287 | GO:0050660 | IEA |
| 516334 | GO:0004872 | IEA |
| 516334 | GO:0008067 | IEA |
| 516462 | GO:0005515 | IEA |
| 516619 | GO:0004659 | IEA |
| 516619 | GO:0004663 | IEA |
| 516619 | GO:0016740 | IEA |
| 516619 | GO:0046872 | IEA |
| 516619 | GO:0008270 | IEA |
| 516619 | GO:0005515 | IEA |
| 516619 | GO:0008318 | IEA |
| 516619 | GO:0004659 | IEA |
| 516619 | GO:0016740 | IEA |
| 516619 | GO:0008270 | IEA |
| 516619 | GO:0004663 | IEA |
| 516619 | GO:0046872 | IEA |
| 516619 | GO:0005515 | IEA |
| 516619 | GO:0008318 | IEA |
| 516874 | GO:0000179 | IEA |
| 516874 | GO:0008649 | IEA |
| 516874 | GO:0003712 | IEA |
| 517139 | GO:0005515 | IPI |
| 517139 | GO:0003677 | IEA |
| 517139 | GO:0005515 | IPI |
| 517139 | GO:0003677 | IEA |
| 517139 | GO:0005515 | IPI |
| 517139 | GO:0003677 | IEA |
| 517139 | GO:0003677 | IEA |
| 517139 | GO:0005515 | IPI |
| 517139 | GO:0005515 | IPI |
| 517139 | GO:0003677 | IEA |
| 517171 | GO:0005525 | IEA |
| 517171 | GO:0000166 | IEA |
| 517345 | GO:0005525 | IEA |
| 517345 | GO:0000166 | IEA |
| 517345 | GO:0005515 | IEA |
| 517402 | GO:0008415 | IEA |
| 517402 | GO:0005515 | IEA |
| 517405 | GO:0005112 | IEA |
| 517405 | GO:0005515 | IEA |
| 517535 | GO:0005524 | IEA |
| 517535 | GO:0005515 | IEA |
| 517535 | GO:0051082 | IEA |
| 517539 | GO:0003824 | IEA |
| 517539 | GO:0016787 | IEA |
| 517688 | GO:0015279 | IEA |
| 517688 | GO:0015279 | IEA |
| 517812 | GO:0042802 | IEA |
| 517812 | GO:0008094 | IEA |
| 517812 | GO:0003677 | IEA |
| 517812 | GO:0003678 | IEA |
| 517812 | GO:0005524 | IEA |
| 517812 | GO:0003697 | IEA |
| 517812 | GO:0000166 | IEA |
| 518045 | GO:0003723 | IEA |
| 518045 | GO:0000166 | IEA |
| 518045 | GO:0003676 | IEA |
| 518045 | GO:0003729 | IEA |
| 518117 | GO:0016820 | IEA |
| 518117 | GO:0000287 | IEA |
| 518117 | GO:0016787 | IEA |
| 518117 | GO:0015662 | IEA |
| 518117 | GO:0046872 | IEA |
| 518117 | GO:0003824 | IEA |
| 518117 | GO:0000166 | IEA |
| 518117 | GO:0005509 | IEA |
| 518117 | GO:0005524 | IEA |
| 518117 | GO:0005388 | IEA |
| 518117 | GO:0003824 | IEA |
| 518117 | GO:0000287 | IEA |
| 518117 | GO:0005524 | IEA |
| 518117 | GO:0005388 | IEA |
| 518117 | GO:0015662 | IEA |
| 518117 | GO:0000166 | IEA |
| 518117 | GO:0016820 | IEA |
| 518117 | GO:0046872 | IEA |
| 518117 | GO:0016787 | IEA |
| 518117 | GO:0005509 | IEA |
| 518169 | GO:0008233 | IEA |
| 518169 | GO:0004186 | IEA |
| 518169 | GO:0004185 | IEA |
| 518169 | GO:0016787 | IEA |
| 518169 | GO:0005515 | IEA |
| 518169 | GO:0004180 | IEA |
| 518318 | GO:0003677 | IEA |
| 518318 | GO:0005515 | IPI |
| 518318 | GO:0005515 | IPI |
| 518318 | GO:0003677 | IEA |
| 518318 | GO:0005515 | IPI |
| 518318 | GO:0003677 | IEA |
| 518318 | GO:0005515 | IPI |
| 518318 | GO:0003677 | IEA |
| 518318 | GO:0003677 | IEA |
| 518318 | GO:0005515 | IPI |
| 518321 | GO:0005515 | IEA |
| 518402 | GO:0005488 | IEA |
| 518402 | GO:0019894 | IEA |
| 518601 | GO:0000166 | IEA |
| 518601 | GO:0005525 | IEA |
| 518738 | GO:0003676 | IEA |
| 518758 | GO:0005215 | IEA |
| 518758 | GO:0005488 | IEA |
| 518758 | GO:0005488 | IEA |
| 518758 | GO:0005215 | IEA |
| 519302 | GO:0003735 | IEA |
| 519302 | GO:0003735 | IEA |
| 519439 | GO:0003677 | IEA |
| 519439 | GO:0003700 | IEA |
| 519597 | GO:0005515 | IEA |
| 519632 | GO:0003955 | IEA |
| 519777 | GO:0042799 | IEA |
| 519958 | GO:0019209 | IEA |
| 520111 | GO:0008270 | IEA |
| 520111 | GO:0005515 | IEA |
| 520111 | GO:0008270 | IEA |
| 520111 | GO:0005515 | IEA |
| 520260 | GO:0004333 | IEA |
| 520260 | GO:0003824 | IEA |
| 520260 | GO:0004333 | IEA |
| 520260 | GO:0003824 | IEA |
| 520472 | GO:0047280 | IEA |
| 520631 | GO:0003677 | IEA |
| 520631 | GO:0008270 | IEA |
| 520631 | GO:0003676 | IEA |
| 520631 | GO:0046872 | IEA |
| 520694 | GO:0005154 | IEA |
| 520757 | GO:0008270 | IEA |
| 520757 | GO:0005515 | IEA |
| 520833 | GO:0005515 | IEA |
| 520864 | GO:0005515 | IEA |
| 520891 | GO:0003723 | IEA |
| 520974 | GO:0005515 | IEA |
| 520979 | GO:0005515 | IEA |
| 521073 | GO:0008270 | IEA |
| 521073 | GO:0046872 | IEA |
| 521073 | GO:0005515 | IEA |
| 521237 | GO:0004674 | IEA |
| 521237 | GO:0000166 | IEA |
| 521237 | GO:0005515 | IEA |
| 521237 | GO:0004672 | IEA |
| 521237 | GO:0005524 | IEA |
| 521237 | GO:0016301 | IEA |
| 521237 | GO:0016740 | IEA |
| 521378 | GO:0030528 | IEA |
| 521581 | GO:0003677 | IEA |
| 521581 | GO:0005515 | IPI |
| 521581 | GO:0005515 | IPI |
| 521581 | GO:0003677 | IEA |
| 521581 | GO:0005515 | IPI |
| 521581 | GO:0003677 | IEA |
| 521581 | GO:0005515 | IPI |
| 521581 | GO:0003677 | IEA |
| 521581 | GO:0003677 | IEA |
| 521581 | GO:0005515 | IPI |
| 521747 | GO:0003743 | IEA |
| 521747 | GO:0005515 | IEA |
| 522006 | GO:0008415 | IEA |
| 522006 | GO:0016740 | IEA |
| 522383 | GO:0005198 | IEA |
| 522509 | GO:0004115 | IEA |
| 522980 | GO:0008233 | IEA |
| 522980 | GO:0004221 | IEA |
| 522980 | GO:0004197 | IEA |
| 522980 | GO:0008233 | IEA |
| 522980 | GO:0004221 | IEA |
| 522980 | GO:0004197 | IEA |
| 523214 | GO:0005515 | IPI |
| 523214 | GO:0003677 | IEA |
| 523214 | GO:0005515 | IPI |
| 523214 | GO:0003677 | IEA |
| 523214 | GO:0005515 | IPI |
| 523214 | GO:0003677 | IEA |
| 523214 | GO:0003677 | IEA |
| 523214 | GO:0005515 | IPI |
| 523214 | GO:0003677 | IEA |
| 523214 | GO:0005515 | IPI |
| 523294 | GO:0016740 | IEA |
| 523294 | GO:0016758 | IEA |
| 523325 | GO:0005515 | IEA |
| 523745 | GO:0017016 | IEA |
| 523745 | GO:0005088 | IEA |
| 523745 | GO:0005515 | IEA |
| 523745 | GO:0046582 | IEA |
| 523752 | GO:0004222 | IEA |
| 523752 | GO:0008233 | IEA |
| 524075 | GO:0005524 | IEA |
| 524075 | GO:0016301 | IEA |
| 524075 | GO:0004673 | IEA |
| 524075 | GO:0016740 | IEA |
| 524075 | GO:0005515 | IEA |
| 525042 | GO:0016614 | IEA |
| 525042 | GO:0051287 | IEA |
| 525042 | GO:0050662 | IEA |
| 525042 | GO:0004367 | IEA |
| 525042 | GO:0016616 | IEA |
| 525042 | GO:0016491 | IEA |
| 525164 | GO:0005515 | IEA |
| 525164 | GO:0043027 | IEA |
| 525511 | GO:0005515 | IPI |
| 525511 | GO:0003677 | IEA |
| 525511 | GO:0003677 | IEA |
| 525511 | GO:0005515 | IPI |
| 525511 | GO:0005515 | IPI |
| 525511 | GO:0003677 | IEA |
| 525511 | GO:0003677 | IEA |
| 525511 | GO:0005515 | IPI |
| 525511 | GO:0005515 | IPI |
| 525511 | GO:0003677 | IEA |
| 525572 | GO:0005515 | IEA |
| 525680 | GO:0005515 | IEA |
| 525716 | GO:0042803 | ISS |
| 525716 | GO:0019210 | NAS |
| 525716 | GO:0008270 | IEA |
| 525716 | GO:0046872 | IEA |
| 525716 | GO:0005509 | IEA |
| 525716 | GO:0048154 | ISS |
| 525716 | GO:0048156 | IPI |
| 525795 | GO:0030548 | IEA |
| 525813 | GO:0004470 | IEA |
| 525813 | GO:0051287 | IEA |
| 525813 | GO:0016616 | IEA |
| 526027 | GO:0003779 | IEA |
| 526027 | GO:0005515 | IEA |
| 526027 | GO:0005515 | IEA |
| 526027 | GO:0003779 | IEA |
| 526135 | GO:0003676 | IEA |
| 526135 | GO:0000166 | IEA |
| 526135 | GO:0003723 | IEA |
| 527263 | GO:0016301 | IEA |
| 527263 | GO:0004713 | IEA |
| 527488 | GO:0005515 | IEA |
| 527935 | GO:0003677 | IEA |
| 527935 | GO:0016563 | IEA |
| 528138 | GO:0005515 | IEA |
| 528165 | GO:0005515 | IEA |
| 528265 | GO:0035091 | IEA |
| 528265 | GO:0005515 | IEA |
| 528390 | GO:0003700 | IEA |
| 528390 | GO:0008270 | IEA |
| 528390 | GO:0004879 | IEA |
| 528390 | GO:0004872 | IEA |
| 528390 | GO:0043565 | IEA |
| 528390 | GO:0003707 | IEA |
| 528390 | GO:0003677 | IEA |
| 529006 | GO:0005515 | IEA |
| 529115 | GO:0016564 | IEA |
| 529115 | GO:0003677 | IEA |
| 529115 | GO:0005515 | IEA |
| 529124 | GO:0003700 | IEA |
| 529124 | GO:0043565 | IEA |
| 529124 | GO:0003677 | IEA |
| 529670 | GO:0005102 | IEA |
| 530254 | GO:0005488 | IEA |
| 530254 | GO:0005488 | IEA |
| 530393 | GO:0005509 | IEA |
| 530393 | GO:0005216 | IEA |
| 530393 | GO:0005515 | IEA |
| 530393 | GO:0005509 | IEA |
| 530393 | GO:0005515 | IEA |
| 530393 | GO:0005216 | IEA |
| 530393 | GO:0005509 | IEA |
| 530393 | GO:0005515 | IEA |
| 530393 | GO:0005216 | IEA |
| 530414 | GO:0003841 | IEA |
| 530414 | GO:0003841 | IEA |
| 530414 | GO:0003841 | IEA |
| 530616 | GO:0042803 | IEA |
| 530616 | GO:0046982 | IEA |
| 530865 | GO:0005506 | IEA |
| 530865 | GO:0016992 | IEA |
| 530865 | GO:0003824 | IEA |
| 530865 | GO:0017140 | IEA |
| 531444 | GO:0008270 | IEA |
| 531444 | GO:0046872 | IEA |
| 531877 | GO:0017124 | IEA |
| 531877 | GO:0004721 | IEA |
| 531990 | GO:0003677 | IEA |
| 531990 | GO:0005515 | IPI |
| 531990 | GO:0003677 | IEA |
| 531990 | GO:0005515 | IPI |
| 531990 | GO:0005515 | IPI |
| 531990 | GO:0003677 | IEA |
| 531990 | GO:0003677 | IEA |
| 531990 | GO:0005515 | IPI |
| 531990 | GO:0005515 | IPI |
| 531990 | GO:0003677 | IEA |
| 532120 | GO:0005488 | IEA |
| 532203 | GO:0003723 | IEA |
| 532203 | GO:0003735 | IEA |
| 532203 | GO:0019843 | IEA |
| 532205 | GO:0047115 | IEA |
| 532205 | GO:0016491 | IEA |
| 532231 | GO:0008943 | IEA |
| 532231 | GO:0005515 | IEA |
| 532231 | GO:0004365 | IEA |
| 532231 | GO:0051287 | IEA |
| 532333 | GO:0001786 | IEA |
| 532389 | GO:0005515 | IEA |
| 532389 | GO:0004518 | IEA |
| 532389 | GO:0004519 | IEA |
| 532389 | GO:0016787 | IEA |
| 532389 | GO:0003723 | IEA |
| 532389 | GO:0004525 | IEA |
| 532483 | GO:0005515 | IEA |
| 532483 | GO:0046872 | IEA |
| 532483 | GO:0008270 | IEA |
| 532483 | GO:0005515 | IEA |
| 532483 | GO:0008270 | IEA |
| 532483 | GO:0046872 | IEA |
| 532572 | GO:0005201 | IEA |
| 532572 | GO:0005515 | IEA |
| 532621 | GO:0046872 | IEA |
| 532621 | GO:0004872 | IEA |
| 532621 | GO:0003700 | IEA |
| 532621 | GO:0008270 | IEA |
| 532621 | GO:0016564 | IEA |
| 532621 | GO:0004879 | IEA |
| 532621 | GO:0003677 | IEA |
| 532621 | GO:0043565 | IEA |
| 532621 | GO:0004887 | IEA |
| 532621 | GO:0005515 | IEA |
| 532621 | GO:0003707 | IEA |
| 532635 | GO:0005515 | IEA |
| 532785 | GO:0016491 | IEA |
| 532785 | GO:0050662 | IEA |
| 532795 | GO:0000166 | IEA |
| 532795 | GO:0003746 | IEA |
| 532795 | GO:0005525 | IEA |
| 532910 | GO:0005515 | IEA |
| 532945 | GO:0016491 | IEA |
| 532945 | GO:0004497 | IEA |
| 532945 | GO:0020037 | IEA |
| 532945 | GO:0005506 | IEA |
| 532945 | GO:0046872 | IEA |
| 532953 | GO:0005509 | IEA |
| 533077 | GO:0004872 | IEA |
| 533087 | GO:0005515 | IEA |
| 533208 | GO:0008168 | IEA |
| 533223 | GO:0003779 | IEA |
| 533227 | GO:0020037 | IEA |
| 533336 | GO:0004842 | IEA |
| 533379 | GO:0003677 | IEA |
| 533379 | GO:0005515 | IEA |
| 533379 | GO:0003677 | IEA |
| 533379 | GO:0005515 | IEA |
| 533379 | GO:0005515 | IEA |
| 533379 | GO:0003677 | IEA |
| 533430 | GO:0004872 | IEA |
| 533430 | GO:0016922 | IEA |
| 533430 | GO:0008270 | IEA |
| 533430 | GO:0030528 | IEA |
| 533630 | GO:0003824 | IEA |
| 533630 | GO:0004647 | IEA |
| 533630 | GO:0000287 | IEA |
| 533630 | GO:0016787 | IEA |
| 533630 | GO:0016791 | IEA |
| 533630 | GO:0016791 | IEA |
| 533630 | GO:0003824 | IEA |
| 533630 | GO:0016787 | IEA |
| 533630 | GO:0004647 | IEA |
| 533630 | GO:0000287 | IEA |
| 533643 | GO:0016563 | IEA |
| 533643 | GO:0003677 | IEA |
| 533643 | GO:0008270 | IEA |
| 533660 | GO:0031072 | IEA |
| 533660 | GO:0051082 | IEA |
| 533746 | GO:0016787 | IEA |
| 533746 | GO:0005515 | IEA |
| 533785 | GO:0016787 | IEA |
| 533785 | GO:0016853 | IEA |
| 533785 | GO:0016868 | IEA |
| 533851 | GO:0005515 | IEA |
| 533851 | GO:0005515 | IEA |
| 533917 | GO:0004364 | IEA |
| 533917 | GO:0016740 | IEA |
| 533918 | GO:0005515 | IEA |
| 533918 | GO:0042171 | IEA |
| 533918 | GO:0042803 | IEA |
| 533918 | GO:0005504 | IEA |
| 533953 | GO:0004674 | IEA |
| 533953 | GO:0005524 | IEA |
| 533953 | GO:0005515 | IEA |
| 533953 | GO:0005149 | IEA |
| 533953 | GO:0016301 | IEA |
| 533953 | GO:0004872 | IEA |
| 533953 | GO:0004672 | IEA |
| 533953 | GO:0000166 | IEA |
| 533953 | GO:0016740 | IEA |
| 534182 | GO:0005509 | IEA |
| 534182 | GO:0016853 | IEA |
| 534182 | GO:0003755 | IEA |
| 534216 | GO:0046872 | IEA |
| 534216 | GO:0008270 | IEA |
| 534216 | GO:0000166 | IEA |
| 534216 | GO:0003723 | IEA |
| 534216 | GO:0003676 | IEA |
| 534232 | GO:0003677 | IEA |
| 534232 | GO:0003899 | IEA |
| 534249 | GO:0005515 | IEA |
| 534249 | GO:0046872 | IEA |
| 534249 | GO:0008270 | IEA |
| 534319 | GO:0005515 | IEA |
| 534331 | GO:0005524 | IEA |
| 534331 | GO:0003676 | IEA |
| 534331 | GO:0003723 | IEA |
| 534331 | GO:0004386 | IEA |
| 534352 | GO:0003735 | IEA |
| 534391 | GO:0003723 | IEA |
| 534391 | GO:0003676 | IEA |
| 534391 | GO:0016853 | IEA |
| 534391 | GO:0000166 | IEA |
| 534402 | GO:0016868 | IEA |
| 534482 | GO:0005509 | IEA |
| 534488 | GO:0005351 | IEA |
| 534546 | GO:0046872 | IEA |
| 534546 | GO:0008237 | IEA |
| 534546 | GO:0016787 | IEA |
| 534546 | GO:0004240 | IEA |
| 534546 | GO:0008270 | IEA |
| 534546 | GO:0004222 | IEA |
| 534546 | GO:0008233 | IEA |
| 534551 | GO:0005515 | IEA |
| 534599 | GO:0004591 | IEA |
| 534599 | GO:0030976 | IEA |
| 534599 | GO:0050164 | IEA |
| 534599 | GO:0016624 | IEA |
| 534615 | GO:0005515 | ISS |
| 534615 | GO:0005515 | ISS |
| 534624 | GO:0005515 | IEA |
| 534624 | GO:0051010 | IEA |
| 534640 | GO:0004175 | IEA |
| 534640 | GO:0004263 | IEA |
| 534640 | GO:0008233 | IEA |
| 534640 | GO:0004298 | IEA |
| 534640 | GO:0016787 | IEA |
| 534708 | GO:0000166 | IEA |
| 534708 | GO:0005525 | IEA |
| 534927 | GO:0008236 | IEA |
| 534927 | GO:0004180 | IEA |
| 534965 | GO:0046872 | IEA |
| 534965 | GO:0008270 | IEA |
| 535077 | GO:0003779 | IEA |
| 535090 | GO:0008565 | IEA |
| 535090 | GO:0005515 | IEA |
| 535166 | GO:0004065 | IEA |
| 535174 | GO:0003824 | IEA |
| 535174 | GO:0016853 | IEA |
| 535174 | GO:0004165 | IEA |
| 535182 | GO:0030060 | IEA |
| 535182 | GO:0016615 | IEA |
| 535182 | GO:0004459 | IEA |
| 535182 | GO:0016491 | IEA |
| 535185 | GO:0004888 | IEA |
| 535185 | GO:0005509 | IEA |
| 535185 | GO:0005515 | IEA |
| 535227 | GO:0005506 | IEA |
| 535227 | GO:0020037 | IEA |
| 535227 | GO:0004497 | IEA |
| 535227 | GO:0016712 | IEA |
| 535254 | GO:0005515 | IEA |
| 535290 | GO:0003723 | IEA |
| 535320 | GO:0046872 | IEA |
| 535320 | GO:0005515 | IEA |
| 535320 | GO:0008270 | IEA |
| 535379 | GO:0000166 | IEA |
| 535379 | GO:0016887 | IEA |
| 535379 | GO:0005524 | IEA |
| 535379 | GO:0017111 | IEA |
| 535390 | GO:0003824 | IEA |
| 535390 | GO:0016787 | IEA |
| 535424 | GO:0000166 | IEA |
| 535424 | GO:0003924 | IEA |
| 535424 | GO:0005525 | IEA |
| 535447 | GO:0003723 | IEA |
| 535447 | GO:0005515 | IEA |
| 535578 | GO:0005515 | IEA |
| 535601 | GO:0003824 | IEA |
| 535601 | GO:0043169 | IEA |
| 535625 | GO:0005515 | IEA |
| 535680 | GO:0016301 | IEA |
| 535680 | GO:0004001 | IEA |
| 535820 | GO:0042802 | IEA |
| 535820 | GO:0004672 | IEA |
| 535820 | GO:0004694 | IEA |
| 535820 | GO:0004674 | IEA |
| 535820 | GO:0005515 | IEA |
| 535869 | GO:0008270 | IEA |
| 535869 | GO:0004842 | IEA |
| 535869 | GO:0046872 | IEA |
| 535869 | GO:0005515 | IEA |
| 535869 | GO:0008233 | IEA |
| 535869 | GO:0016874 | IEA |
| 535981 | GO:0008462 | IEA |
| 535981 | GO:0016787 | IEA |
| 535981 | GO:0004252 | IEA |
| 535981 | GO:0008233 | IEA |
| 536051 | GO:0005515 | IEA |
| 536149 | GO:0008270 | IEA |
| 536149 | GO:0046872 | IEA |
| 536149 | GO:0003677 | IEA |
| 536203 | GO:0000166 | IEA |
| 536203 | GO:0005524 | IEA |
| 536203 | GO:0017111 | IEA |
| 536203 | GO:0016887 | IEA |
| 536203 | GO:0042803 | IEA |
| 536337 | GO:0004872 | IEA |
| 536337 | GO:0004721 | IEA |
| 536337 | GO:0004725 | IEA |
| 536337 | GO:0016787 | IEA |
| 536619 | GO:0030371 | IEA |
| 536619 | GO:0005515 | IEA |
| 536741 | GO:0004245 | IEA |
| 536781 | GO:0005515 | IEA |
| 537539 | GO:0004029 | IEA |
| 537539 | GO:0019145 | IEA |
| 537539 | GO:0016620 | IEA |
| 537539 | GO:0047105 | IEA |
| 537539 | GO:0016491 | IEA |
| 537706 | GO:0005515 | IEA |
| 537706 | GO:0003676 | IEA |
| 537706 | GO:0000166 | IEA |
| 537932 | GO:0005515 | IEA |
| 538054 | GO:0003735 | IEA |
| 538060 | GO:0016491 | IEA |
| 538094 | GO:0005524 | IEA |
| 538094 | GO:0005515 | IEA |
| 538094 | GO:0000166 | IEA |
| 538094 | GO:0016301 | IEA |
| 538094 | GO:0016740 | IEA |
| 538094 | GO:0004672 | IEA |
| 538094 | GO:0004674 | IEA |
| 538094 | GO:0004707 | IEA |
| 538094 | GO:0004674 | IEA |
| 538094 | GO:0000166 | IEA |
| 538094 | GO:0004707 | IEA |
| 538094 | GO:0005515 | IEA |
| 538094 | GO:0005524 | IEA |
| 538094 | GO:0016301 | IEA |
| 538094 | GO:0016740 | IEA |
| 538094 | GO:0004672 | IEA |
| 538173 | GO:0016787 | IEA |
| 538173 | GO:0016791 | IEA |
| 538173 | GO:0003824 | IEA |
| 538236 | GO:0005515 | IEA |
| 538236 | GO:0008565 | IEA |
| 538409 | GO:0008233 | IEA |
| 538409 | GO:0030693 | IEA |
| 538409 | GO:0016787 | IEA |
| 538409 | GO:0008234 | IEA |
| 538427 | GO:0005515 | IEA |
| 538483 | GO:0005515 | IEA |
| 538533 | GO:0003676 | IEA |
| 538533 | GO:0000166 | IEA |
| 538594 | GO:0016566 | IEA |
| 538625 | GO:0005515 | IEA |
| 538702 | GO:0016301 | IEA |
| 538702 | GO:0016740 | IEA |
| 538702 | GO:0004370 | IEA |
| 538778 | GO:0005515 | IEA |
| 538829 | GO:0016787 | IEA |
| 538829 | GO:0005506 | IEA |
| 538829 | GO:0005515 | IEA |
| 538944 | GO:0004111 | IEA |
| 538944 | GO:0016301 | IEA |
| 538944 | GO:0016740 | IEA |
| 538944 | GO:0016772 | IEA |
| 538944 | GO:0016740 | IEA |
| 538944 | GO:0016772 | IEA |
| 538944 | GO:0016301 | IEA |
| 538944 | GO:0004111 | IEA |
| 538975 | GO:0003779 | IEA |
| 538975 | GO:0005516 | IEA |
| 538975 | GO:0008017 | ISS |
| 538975 | GO:0005515 | IEA |
| 539003 | GO:0008022 | IEA |
| 539003 | GO:0003700 | IEA |
| 539003 | GO:0003690 | IEA |
| 539003 | GO:0003682 | IEA |
| 539003 | GO:0003677 | IEA |
| 539003 | GO:0016563 | IEA |
| 539003 | GO:0003713 | IEA |
| 539003 | GO:0005488 | IEA |
| 539003 | GO:0008134 | IEA |
| 539003 | GO:0005515 | IEA |
| 539003 | GO:0045296 | IEA |
| 539003 | GO:0045294 | IEA |
| 539005 | GO:0005515 | IEA |
| 539005 | GO:0003735 | IEA |
| 539035 | GO:0005515 | IEA |
| 539035 | GO:0008233 | IEA |
| 539035 | GO:0008270 | IEA |
| 539091 | GO:0046872 | IEA |
| 539091 | GO:0046961 | IEA |
| 539091 | GO:0046933 | IEA |
| 539091 | GO:0005515 | IEA |
| 539091 | GO:0008270 | IEA |
| 539188 | GO:0016847 | IEA |
| 539188 | GO:0016769 | IEA |
| 539203 | GO:0005515 | IEA |
| 539220 | GO:0051087 | IEA |
| 539249 | GO:0000166 | IEA |
| 539249 | GO:0003676 | IEA |
| 539249 | GO:0005515 | IEA |
| 539332 | GO:0003779 | IEA |
| 539332 | GO:0005515 | IEA |
| 539332 | GO:0005515 | IEA |
| 539332 | GO:0003779 | IEA |
| 539354 | GO:0005351 | IEA |
| 539354 | GO:0005351 | IEA |
| 539379 | GO:0003713 | IEA |
| 539379 | GO:0003779 | IEA |
| 539381 | GO:0008270 | IEA |
| 539381 | GO:0046872 | IEA |
| 539431 | GO:0005515 | IEA |
| 539431 | GO:0003677 | IEA |
| 539431 | GO:0030528 | IEA |
| 539449 | GO:0004871 | IEA |
| 539524 | GO:0005515 | IEA |
| 539524 | GO:0042802 | IEA |
| 539567 | GO:0005515 | IEA |
| 539601 | GO:0005515 | IEA |
| 539639 | GO:0008233 | IEA |
| 539639 | GO:0016929 | IEA |
| 539639 | GO:0005515 | IEA |
| 539668 | GO:0005515 | IEA |
| 539668 | GO:0005515 | IEA |
| 539713 | GO:0017111 | IEA |
| 539713 | GO:0005524 | IEA |
| 539713 | GO:0000166 | IEA |
| 539713 | GO:0017111 | IEA |
| 539713 | GO:0000166 | IEA |
| 539713 | GO:0005524 | IEA |
| 539715 | GO:0005515 | IEA |
| 539761 | GO:0008270 | IEA |
| 539761 | GO:0003676 | IEA |
| 539771 | GO:0005515 | IEA |
| 539771 | GO:0017065 | IEA |
| 539771 | GO:0016787 | IEA |
| 539771 | GO:0004844 | ISS |
| 539771 | GO:0016798 | IEA |
| 539771 | GO:0003677 | IEA |
| 539771 | GO:0003697 | ISS |
| 539771 | GO:0019104 | IDA |
| 539771 | GO:0017065 | ISS |
| 539789 | GO:0015631 | IEA |
| 539789 | GO:0005515 | IEA |
| 539875 | GO:0003677 | IEA |
| 539875 | GO:0005515 | IPI |
| 539875 | GO:0005515 | IPI |
| 539875 | GO:0003677 | IEA |
| 539875 | GO:0005515 | IPI |
| 539875 | GO:0003677 | IEA |
| 539875 | GO:0003677 | IEA |
| 539875 | GO:0005515 | IPI |
| 539875 | GO:0005515 | IPI |
| 539875 | GO:0003677 | IEA |
| 539955 | GO:0003677 | IEA |
| 539978 | GO:0047395 | IEA |
| 539978 | GO:0008889 | IEA |
| 539978 | GO:0016787 | IEA |
| 539992 | GO:0016787 | IEA |
| 540040 | GO:0004871 | IEA |
| 540040 | GO:0000166 | IEA |
| 540040 | GO:0005525 | IEA |
| 540040 | GO:0019001 | IEA |
| 540045 | GO:0003676 | IEA |
| 540045 | GO:0008270 | IEA |
| 540083 | GO:0031072 | IEA |
| 540083 | GO:0051082 | IEA |
| 540083 | GO:0031072 | IEA |
| 540083 | GO:0051082 | IEA |
| 540085 | GO:0004871 | IEA |
| 540085 | GO:0005515 | IEA |
| 540085 | GO:0005515 | IEA |
| 540085 | GO:0004871 | IEA |
| 540090 | GO:0004842 | IEA |
| 540090 | GO:0005515 | IEA |
| 540090 | GO:0003682 | IEA |
| 540090 | GO:0016564 | IEA |
| 540090 | GO:0005515 | IEA |
| 540090 | GO:0016564 | IEA |
| 540090 | GO:0004842 | IEA |
| 540090 | GO:0003682 | IEA |
| 540139 | GO:0008408 | IEA |
| 540148 | GO:0003677 | IEA |
| 540148 | GO:0005515 | IPI |
| 540148 | GO:0005515 | IPI |
| 540148 | GO:0003677 | IEA |
| 540148 | GO:0005515 | IPI |
| 540148 | GO:0003677 | IEA |
| 540148 | GO:0005515 | IPI |
| 540148 | GO:0003677 | IEA |
| 540148 | GO:0003677 | IEA |
| 540148 | GO:0005515 | IPI |
| 540167 | GO:0005515 | IEA |
| 540176 | GO:0008289 | IEA |
| 540176 | GO:0046961 | IEA |
| 540176 | GO:0015078 | IEA |
| 540176 | GO:0046933 | IEA |
| 540176 | GO:0005515 | IEA |
| 540187 | GO:0005515 | IEA |
| 540322 | GO:0005515 | IEA |
| 540329 | GO:0003824 | IEA |
| 540329 | GO:0000287 | IEA |
| 540329 | GO:0004721 | IEA |
| 540329 | GO:0046872 | IEA |
| 540329 | GO:0004722 | IEA |
| 540329 | GO:0016787 | IEA |
| 540329 | GO:0016787 | IEA |
| 540329 | GO:0046872 | IEA |
| 540329 | GO:0000287 | IEA |
| 540329 | GO:0004721 | IEA |
| 540329 | GO:0004722 | IEA |
| 540329 | GO:0003824 | IEA |
| 540329 | GO:0004722 | IEA |
| 540329 | GO:0000287 | IEA |
| 540329 | GO:0016787 | IEA |
| 540329 | GO:0004721 | IEA |
| 540329 | GO:0003824 | IEA |
| 540329 | GO:0046872 | IEA |
| 540329 | GO:0004722 | IEA |
| 540329 | GO:0016787 | IEA |
| 540329 | GO:0003824 | IEA |
| 540329 | GO:0004721 | IEA |
| 540329 | GO:0046872 | IEA |
| 540329 | GO:0000287 | IEA |
| 540329 | GO:0004722 | IEA |
| 540329 | GO:0046872 | IEA |
| 540329 | GO:0016787 | IEA |
| 540329 | GO:0000287 | IEA |
| 540329 | GO:0004721 | IEA |
| 540329 | GO:0003824 | IEA |
| 540345 | GO:0003677 | IEA |
| 540345 | GO:0043565 | IEA |
| 540386 | GO:0016787 | IEA |
| 540421 | GO:0004674 | IEA |
| 540421 | GO:0005524 | IEA |
| 540421 | GO:0016301 | IEA |
| 540421 | GO:0004672 | IEA |
| 540421 | GO:0016740 | IEA |
| 540421 | GO:0005057 | IEA |
| 540421 | GO:0019992 | IEA |
| 540421 | GO:0000166 | IEA |
| 540421 | GO:0008270 | IEA |
| 540421 | GO:0046872 | IEA |
| 540421 | GO:0005515 | IEA |
| 540426 | GO:0005488 | IEA |
| 540426 | GO:0008092 | IEA |
| 540426 | GO:0005198 | IEA |
| 540444 | GO:0003677 | IEA |
| 540444 | GO:0008301 | IEA |
| 540444 | GO:0008301 | ISS |
| 540526 | GO:0003677 | IEA |
| 540526 | GO:0003682 | IEA |
| 540526 | GO:0032137 | IEA |
| 540526 | GO:0003684 | IEA |
| 540564 | GO:0005515 | IEA |
| 540564 | GO:0005515 | IEA |
| 540568 | GO:0003824 | IEA |
| 540568 | GO:0015662 | IEA |
| 540568 | GO:0005524 | IEA |
| 540568 | GO:0048155 | IEA |
| 540602 | GO:0003824 | IEA |
| 540602 | GO:0019007 | IEA |
| 540614 | GO:0005525 | IEA |
| 540619 | GO:0005515 | IEA |
| 540712 | GO:0005515 | IEA |
| 540719 | GO:0005515 | IEA |
| 540766 | GO:0005515 | IEA |
| 540766 | GO:0003682 | IEA |
| 540766 | GO:0003690 | IEA |
| 540859 | GO:0005515 | IEA |
| 540932 | GO:0005515 | IEA |
| 540932 | GO:0003700 | IEA |
| 540962 | GO:0005515 | IEA |
| 540966 | GO:0005515 | IEA |
| 540985 | GO:0030145 | IEA |
| 541077 | GO:0016149 | IEA |
| 541077 | GO:0005515 | IEA |
| 541077 | GO:0005515 | IEA |
| 541077 | GO:0016149 | IEA |
| 541081 | GO:0005525 | IEA |
| 541101 | GO:0005525 | IEA |
| 541101 | GO:0005198 | IEA |
| 541101 | GO:0003924 | IEA |
| 541125 | GO:0003714 | IEA |
| 541125 | GO:0003700 | IEA |
| 541125 | GO:0032422 | IEA |
| 541148 | GO:0001872 | IEA |
| 541175 | GO:0008138 | IEA |
| 541175 | GO:0017017 | IEA |
| 541175 | GO:0004721 | IEA |
| 541175 | GO:0004721 | IEA |
| 541175 | GO:0017017 | IEA |
| 541175 | GO:0008138 | IEA |
| 541175 | GO:0004721 | IEA |
| 541175 | GO:0017017 | IEA |
| 541175 | GO:0008138 | IEA |
| 541216 | GO:0004842 | IEA |
| 541266 | GO:0031402 | IEA |
| 541266 | GO:0000166 | IEA |
| 541266 | GO:0015293 | IEA |
| 541266 | GO:0015075 | IEA |
| 541266 | GO:0005215 | IEA |
| 541266 | GO:0005524 | IEA |
| 541276 | GO:0003676 | IEA |
| 541276 | GO:0005515 | IEA |
| 541276 | GO:0042802 | IEA |
| 541276 | GO:0000166 | IEA |
| 613305 | GO:0003723 | IEA |
| 613338 | GO:0016652 | IEA |
| 613338 | GO:0016491 | IEA |
| 613338 | GO:0004449 | IEA |
| 613345 | GO:0003677 | IEA |
| 613345 | GO:0005515 | IPI |
| 613345 | GO:0003677 | IEA |
| 613345 | GO:0005515 | IPI |
| 613345 | GO:0003677 | IEA |
| 613345 | GO:0005515 | IPI |
| 613345 | GO:0003677 | IEA |
| 613345 | GO:0005515 | IPI |
| 613345 | GO:0003677 | IEA |
| 613345 | GO:0005515 | IPI |
| 613416 | GO:0020037 | IEA |
| 613421 | GO:0005515 | IEA |
| 613522 | GO:0005216 | IEA |
| 613522 | GO:0003676 | IEA |
| 613522 | GO:0046872 | IEA |
| 613522 | GO:0008270 | IEA |
| 613548 | GO:0003779 | IEA |
| 613548 | GO:0005516 | IEA |
| 613548 | GO:0005516 | IEA |
| 613548 | GO:0003779 | IEA |
| 613570 | GO:0016491 | IEA |
| 613570 | GO:0050662 | IEA |
| 613610 | GO:0004739 | IEA |
| 613610 | GO:0016491 | IEA |
| 613618 | GO:0003677 | IEA |
| 613629 | GO:0017136 | IEA |
| 613629 | GO:0016564 | IEA |
| 613729 | GO:0003677 | IEA |
| 613751 | GO:0005515 | IEA |
| 613751 | GO:0003700 | IEA |
| 613787 | GO:0005161 | IEA |
| 613788 | GO:0003899 | IEA |
| 613788 | GO:0003677 | IEA |
| 613828 | GO:0043565 | IEA |
| 613828 | GO:0003677 | IEA |
| 613854 | GO:0004719 | IEA |
| 613854 | GO:0008168 | IEA |
| 613854 | GO:0016740 | IEA |
| 613855 | GO:0004872 | IEA |
| 613855 | GO:0005515 | IEA |
| 613877 | GO:0003705 | IEA |
| 613877 | GO:0003677 | IEA |
| 613877 | GO:0005515 | IEA |
| 613899 | GO:0008121 | IEA |
| 613899 | GO:0016491 | IEA |
| 613907 | GO:0030528 | IEA |
| 613907 | GO:0003677 | IEA |
| 613930 | GO:0004871 | IEA |
| 614028 | GO:0003723 | IEA |
| 614028 | GO:0003676 | IEA |
| 614043 | GO:0016491 | IEA |
| 614043 | GO:0003954 | IEA |
| 614043 | GO:0008137 | IEA |
| 614114 | GO:0016491 | IEA |
| 614114 | GO:0004129 | IEA |
| 614145 | GO:0000166 | IEA |
| 614145 | GO:0005524 | IEA |
| 614145 | GO:0004449 | IEA |
| 614145 | GO:0016652 | IEA |
| 614145 | GO:0016491 | IEA |
| 614177 | GO:0008831 | IEA |
| 614177 | GO:0016740 | IEA |
| 614242 | GO:0005525 | IEA |
| 614242 | GO:0000166 | IEA |
| 614244 | GO:0046933 | IEA |
| 614244 | GO:0046872 | IEA |
| 614244 | GO:0046961 | IEA |
| 614244 | GO:0016787 | IEA |
| 614244 | GO:0015078 | IEA |
| 614279 | GO:0003676 | IEA |
| 614279 | GO:0046872 | IEA |
| 614279 | GO:0008270 | IEA |
| 614368 | GO:0000166 | IEA |
| 614368 | GO:0017111 | IEA |
| 614368 | GO:0005524 | IEA |
| 614368 | GO:0016887 | IEA |
| 614509 | GO:0030528 | IEA |
| 614538 | GO:0004579 | IEA |
| 614538 | GO:0016740 | IEA |
| 614576 | GO:0005515 | IEA |
| 614581 | GO:0000166 | IEA |
| 614581 | GO:0005525 | IEA |
| 614585 | GO:0005515 | IEA |
| 614630 | GO:0008013 | IEA |
| 614694 | GO:0005515 | IEA |
| 614732 | GO:0008270 | IEA |
| 614732 | GO:0046872 | IEA |
| 614732 | GO:0051082 | IEA |
| 614732 | GO:0031072 | IEA |
| 614773 | GO:0003729 | IEA |
| 614773 | GO:0005515 | IEA |
| 614773 | GO:0005515 | IEA |
| 614773 | GO:0003729 | IEA |
| 614876 | GO:0046872 | IEA |
| 614876 | GO:0008270 | IEA |
| 614876 | GO:0046872 | IEA |
| 614876 | GO:0008270 | IEA |
| 614915 | GO:0003677 | IEA |
| 614921 | GO:0008565 | IEA |
| 614921 | GO:0005515 | IEA |
| 614922 | GO:0000166 | IEA |
| 614922 | GO:0003676 | IEA |
| 614922 | GO:0003723 | IEA |
| 614922 | GO:0016853 | IEA |
| 615066 | GO:0008270 | IEA |
| 615066 | GO:0046872 | IEA |
| 615066 | GO:0005515 | IEA |
| 615175 | GO:0003676 | IEA |
| 615175 | GO:0000166 | IEA |
| 615175 | GO:0005515 | IEA |
| 615175 | GO:0042802 | IEA |
| 615215 | GO:0004672 | IEA |
| 615215 | GO:0016301 | IEA |
| 615215 | GO:0004674 | IEA |
| 615215 | GO:0005524 | IEA |
| 615215 | GO:0016740 | IEA |
| 615215 | GO:0000166 | IEA |
| 615229 | GO:0005515 | IEA |
| 615229 | GO:0008270 | IEA |
| 615447 | GO:0016740 | IEA |
| 615447 | GO:0016301 | IEA |
| 615447 | GO:0004550 | IEA |
| 615447 | GO:0000166 | IEA |
| 615447 | GO:0046872 | IEA |
| 615447 | GO:0005524 | IEA |
| 615447 | GO:0000287 | IEA |
| 615498 | GO:0015288 | IEA |
| 615498 | GO:0005215 | IEA |
| 615666 | GO:0003735 | IEA |
| 615692 | GO:0005515 | IEA |
| 615785 | GO:0008092 | IEA |
| 615785 | GO:0008092 | IEA |
| 615868 | GO:0016301 | IEA |
| 615964 | GO:0005515 | IEA |
| 615978 | GO:0001517 | IEA |
| 615978 | GO:0008146 | IEA |
| 616045 | GO:0005089 | IEA |
| 616045 | GO:0017048 | IEA |
| 616089 | GO:0004518 | IEA |
| 616089 | GO:0004519 | IEA |
| 616089 | GO:0003676 | IEA |
| 616089 | GO:0004522 | IEA |
| 616089 | GO:0016787 | IEA |
| 616090 | GO:0005515 | IEA |
| 616109 | GO:0016491 | IEA |
| 616109 | GO:0008121 | IEA |
| 616148 | GO:0016563 | IEA |
| 616156 | GO:0005515 | IEA |
| 616217 | GO:0005515 | IEA |
| 616217 | GO:0003677 | IEA |
| 616217 | GO:0003714 | IEA |
| 616227 | GO:0004860 | IEA |
| 616275 | GO:0046872 | IEA |
| 616275 | GO:0005515 | IEA |
| 616275 | GO:0008270 | IEA |
| 616317 | GO:0015631 | IEA |
| 616317 | GO:0005515 | IEA |
| 616375 | GO:0005515 | IEA |
| 616375 | GO:0005515 | IEA |
| 616477 | GO:0005515 | IEA |
| 616742 | GO:0004872 | IEA |
| 616742 | GO:0016301 | IEA |
| 616800 | GO:0003677 | IEA |
| 616800 | GO:0005515 | IPI |
| 616800 | GO:0005515 | IPI |
| 616800 | GO:0003677 | IEA |
| 616800 | GO:0005515 | IPI |
| 616800 | GO:0003677 | IEA |
| 616800 | GO:0003677 | IEA |
| 616800 | GO:0005515 | IPI |
| 616800 | GO:0005515 | IPI |
| 616800 | GO:0003677 | IEA |
| 616819 | GO:0003677 | IEA |
| 616819 | GO:0005515 | IPI |
| 616819 | GO:0003677 | IEA |
| 616819 | GO:0005515 | IPI |
| 616819 | GO:0005515 | IPI |
| 616819 | GO:0003677 | IEA |
| 616819 | GO:0005515 | IPI |
| 616819 | GO:0003677 | IEA |
| 616819 | GO:0003677 | IEA |
| 616819 | GO:0005515 | IPI |
| 616871 | GO:0016491 | IEA |
| 616871 | GO:0008121 | IEA |
| 616913 | GO:0005515 | IEA |
| 616913 | GO:0042813 | IEA |
| 616913 | GO:0017147 | IEA |
| 616926 | GO:0005515 | IEA |
| 616931 | GO:0000287 | IEA |
| 616931 | GO:0030145 | IEA |
| 616931 | GO:0016787 | IEA |
| 616931 | GO:0008486 | IEA |
| 616931 | GO:0046872 | IEA |
| 617051 | GO:0003677 | IEA |
| 617051 | GO:0005515 | IPI |
| 617051 | GO:0003677 | IEA |
| 617051 | GO:0005515 | IPI |
| 617051 | GO:0003677 | IEA |
| 617051 | GO:0005515 | IPI |
| 617051 | GO:0005515 | IPI |
| 617051 | GO:0003677 | IEA |
| 617051 | GO:0005515 | IPI |
| 617051 | GO:0003677 | IEA |
| 617080 | GO:0005515 | IEA |
| 617105 | GO:0005515 | ISS |
| 617105 | GO:0003746 | IEA |
| 617105 | GO:0005515 | IEA |
| 617210 | GO:0009055 | IEA |
| 617230 | GO:0015078 | IEA |
| 617230 | GO:0046961 | IEA |
| 617230 | GO:0046872 | IEA |
| 617230 | GO:0046933 | IEA |
| 617230 | GO:0016787 | IEA |
| 617267 | GO:0016787 | IEA |
| 617267 | GO:0016853 | IEA |
| 617267 | GO:0016868 | IEA |
| 617375 | GO:0003899 | IEA |
| 617375 | GO:0003677 | IEA |
| 617419 | GO:0016717 | IEA |
| 617419 | GO:0004768 | IEA |
| 617419 | GO:0005506 | IEA |
| 617419 | GO:0016491 | IEA |
| 617564 | GO:0016491 | IEA |
| 617564 | GO:0016491 | IEA |
| 617635 | GO:0005525 | IEA |
| 617635 | GO:0000166 | IEA |
| 617694 | GO:0008137 | IEA |
| 617694 | GO:0003954 | IEA |
| 617694 | GO:0016491 | IEA |
| 617707 | GO:0042577 | IEA |
| 617707 | GO:0042577 | IEA |
| 617888 | GO:0005515 | IPI |
| 617888 | GO:0003677 | IEA |
| 617888 | GO:0003677 | IEA |
| 617888 | GO:0005515 | IPI |
| 617888 | GO:0005515 | IPI |
| 617888 | GO:0003677 | IEA |
| 617888 | GO:0005515 | IPI |
| 617888 | GO:0003677 | IEA |
| 617888 | GO:0003677 | IEA |
| 617888 | GO:0005515 | IPI |
| 617901 | GO:0003735 | IEA |
| 617917 | GO:0047115 | IEA |
| 617917 | GO:0016491 | IEA |
| 617944 | GO:0000166 | IEA |
| 617944 | GO:0003746 | IEA |
| 617944 | GO:0005525 | IEA |
| 617976 | GO:0003712 | IEA |
| 617985 | GO:0005509 | IEA |
| 618089 | GO:0005515 | IEA |
| 618131 | GO:0005515 | IEA |
| 618131 | GO:0008270 | IEA |
| 618131 | GO:0046872 | IEA |
| 618149 | GO:0005515 | IPI |
| 618149 | GO:0003677 | IEA |
| 618149 | GO:0003677 | IEA |
| 618149 | GO:0005515 | IPI |
| 618149 | GO:0005515 | IPI |
| 618149 | GO:0003677 | IEA |
| 618149 | GO:0003677 | IEA |
| 618149 | GO:0005515 | IPI |
| 618149 | GO:0005515 | IPI |
| 618149 | GO:0003677 | IEA |
| 618204 | GO:0005216 | IEA |
| 618250 | GO:0005509 | IEA |
| 618292 | GO:0016787 | IEA |
| 618292 | GO:0004527 | IEA |
| 618292 | GO:0003723 | IEA |
| 618292 | GO:0005515 | IEA |
| 618292 | GO:0004518 | IEA |
| 618292 | GO:0000175 | IEA |
| 618297 | GO:0003677 | IEA |
| 618297 | GO:0008301 | ISS |
| 618297 | GO:0008301 | IEA |
| 618377 | GO:0008083 | IEA |
| 618441 | GO:0000318 | IEA |
| 618441 | GO:0008270 | IEA |
| 618441 | GO:0008113 | IEA |
| 618508 | GO:0004871 | IEA |
| 618508 | GO:0005515 | IEA |
| 618550 | GO:0020037 | IEA |
| 618573 | GO:0005515 | IEA |
| 618607 | GO:0047939 | IEA |
| 618607 | GO:0005515 | IEA |
| 618607 | GO:0004032 | IEA |
| 618607 | GO:0016491 | IEA |
| 618609 | GO:0003735 | IEA |
| 618609 | GO:0003723 | IEA |
| 618849 | GO:0005515 | ISS |
| 618849 | GO:0042975 | ISS |
| 618849 | GO:0003700 | ISS |
| 618849 | GO:0042974 | ISS |
| 618849 | GO:0030374 | ISS |
| 618849 | GO:0046965 | ISS |
| 618849 | GO:0003677 | IEA |
| 618849 | GO:0008134 | ISS |
| 618954 | GO:0005515 | IEA |
| 619141 | GO:0005515 | IPI |
| 619141 | GO:0003677 | IEA |
| 619141 | GO:0003677 | IEA |
| 619141 | GO:0005515 | IPI |
| 619141 | GO:0003677 | IEA |
| 619141 | GO:0005515 | IPI |
| 619141 | GO:0003677 | IEA |
| 619141 | GO:0005515 | IPI |
| 619141 | GO:0005515 | IPI |
| 619141 | GO:0003677 | IEA |
| 654400 | GO:0003677 | IEA |
| 654400 | GO:0008270 | IEA |
| 654400 | GO:0003705 | IEA |
| 654400 | GO:0005515 | IEA |
| 654400 | GO:0016563 | IEA |
| 654400 | GO:0003700 | IEA |
| 654400 | GO:0043565 | IEA |
| 654400 | GO:0003682 | IEA |
| 767834 | GO:0005515 | IEA |
| 767855 | GO:0005524 | IEA |
| 767855 | GO:0004672 | IEA |
| 767889 | GO:0003998 | IEA |
| 767889 | GO:0016787 | IEA |
| 767898 | GO:0008270 | IEA |
| 767898 | GO:0003676 | IEA |
| 767949 | GO:0005515 | IEA |
| 767980 | GO:0005515 | IEA |
| 768014 | GO:0003774 | IEA |
| 768014 | GO:0000166 | IEA |
| 768014 | GO:0003777 | IEA |
| 768014 | GO:0005524 | IEA |
| 768016 | GO:0005515 | IEA |
| 768016 | GO:0003714 | IEA |
| 768016 | GO:0046332 | IEA |
| 768035 | GO:0005085 | IEA |
| 768035 | GO:0005086 | IEA |
| 768035 | GO:0005515 | IEA |
| 768044 | GO:0008796 | IEA |
| 768044 | GO:0016787 | IEA |
| 768044 | GO:0004081 | IEA |
| 768074 | GO:0004174 | IEA |
| 768074 | GO:0016491 | IEA |
| 768311 | GO:0019904 | IEA |
| 768330 | GO:0008289 | IEA |
| 768330 | GO:0000062 | IEA |
| 768330 | GO:0005488 | IEA |
| 777690 | GO:0005515 | IEA |
| 777769 | GO:0035257 | IEA |
| 777769 | GO:0030528 | IEA |
| 777769 | GO:0008134 | IEA |
| 777769 | GO:0005102 | IEA |
| 777769 | GO:0005515 | IEA |
| 777775 | GO:0005198 | IEA |
| 777775 | GO:0000166 | IEA |
| 777775 | GO:0003924 | IEA |
| 777775 | GO:0005525 | IEA |
| 777775 | GO:0005198 | IEA |
| 777775 | GO:0003924 | IEA |
| 777775 | GO:0000166 | IEA |
| 777775 | GO:0005525 | IEA |
| 780962 | GO:0016491 | IEA |
| 780984 | GO:0005509 | IEA |
| 781014 | GO:0004722 | IEA |
| 781014 | GO:0016791 | IEA |
| 781020 | GO:0003954 | IEA |
| 781020 | GO:0016491 | IEA |
| 781020 | GO:0008137 | IEA |
| 781064 | GO:0009055 | IEA |
| 781064 | GO:0003954 | IEA |
| 781064 | GO:0008137 | IEA |
| 781064 | GO:0016491 | IEA |
| 781070 | GO:0000166 | IEA |
| 781070 | GO:0005525 | IEA |
| 781070 | GO:0003746 | IEA |
| 781111 | GO:0005515 | IPI |
| 781111 | GO:0003677 | IEA |
| 781111 | GO:0003677 | IEA |
| 781111 | GO:0005515 | IPI |
| 781197 | GO:0008233 | IEA |
| 781197 | GO:0009003 | IEA |
| 781197 | GO:0016787 | IEA |
| 781224 | GO:0005515 | IPI |
| 781224 | GO:0003677 | IEA |
| 781224 | GO:0003677 | IEA |
| 781224 | GO:0005515 | IPI |
| 781224 | GO:0003677 | IEA |
| 781224 | GO:0005515 | IPI |
| 781224 | GO:0003677 | IEA |
| 781224 | GO:0005515 | IPI |
| 781224 | GO:0003677 | IEA |
| 781224 | GO:0005515 | IPI |
| 781227 | GO:0046872 | IEA |
| 781227 | GO:0000287 | IEA |
| 781227 | GO:0004749 | IEA |
| 781227 | GO:0016740 | IEA |
| 781227 | GO:0016301 | IEA |
| 781334 | GO:0003779 | IEA |
| 781462 | GO:0008270 | IEA |
| 781462 | GO:0046872 | IEA |
| 781462 | GO:0005515 | IEA |
| 781542 | GO:0005515 | IEA |
| 781542 | GO:0003735 | IEA |
| 781566 | GO:0005515 | IPI |
| 781566 | GO:0003677 | IEA |
| 781566 | GO:0005515 | IPI |
| 781566 | GO:0003677 | IEA |
| 781566 | GO:0005515 | IPI |
| 781566 | GO:0003677 | IEA |
| 781566 | GO:0005515 | IPI |
| 781566 | GO:0003677 | IEA |
| 781566 | GO:0003677 | IEA |
| 781566 | GO:0005515 | IPI |
| 781575 | GO:0051082 | IEA |
| 781609 | GO:0008137 | IEA |
| 781609 | GO:0003954 | IEA |
| 781609 | GO:0016491 | IEA |
| 781610 | GO:0016627 | IEA |
| 781610 | GO:0003995 | IEA |
| 781610 | GO:0050660 | IEA |
| 781610 | GO:0016491 | IEA |
| 781625 | GO:0005515 | IEA |
| 781625 | GO:0017154 | IEA |
| 781648 | GO:0004177 | IEA |
| 781648 | GO:0008270 | IEA |
| 781648 | GO:0008233 | IEA |
| 781648 | GO:0016787 | IEA |
| 781648 | GO:0030145 | IEA |
| 781648 | GO:0046872 | IEA |
| 781648 | GO:0000287 | IEA |
| 781648 | GO:0016804 | IEA |
| 781648 | GO:0004178 | IEA |
| 781719 | GO:0019843 | IEA |
| 781719 | GO:0003735 | IEA |
| 781719 | GO:0005488 | IEA |
| 781719 | GO:0003723 | IEA |
| 781741 | GO:0005515 | IEA |
| 781741 | GO:0004872 | IEA |
| 781770 | GO:0004842 | IEA |
| 781813 | GO:0003746 | IEA |
| 781813 | GO:0000166 | IEA |
| 781813 | GO:0005525 | IEA |
| 781824 | GO:0008137 | IEA |
| 781824 | GO:0016491 | IEA |
| 781824 | GO:0051537 | IEA |
| 781824 | GO:0003954 | IEA |
| 781824 | GO:0051536 | IEA |
| 781824 | GO:0046872 | IEA |
| 781824 | GO:0005506 | IEA |
| 781969 | GO:0005515 | ISS |
| 781969 | GO:0005515 | ISS |
| 782024 | GO:0003723 | IEA |
| 782024 | GO:0005515 | IEA |
| 782058 | GO:0051082 | IEA |
| 782058 | GO:0031072 | IEA |
| 782058 | GO:0008270 | IEA |
| 782058 | GO:0046872 | IEA |
| 782061 | GO:0016491 | IEA |
| 782114 | GO:0016787 | IEA |
| 782233 | GO:0046961 | IEA |
| 782233 | GO:0046933 | IEA |
| 782233 | GO:0046872 | IEA |
| 782233 | GO:0005515 | IEA |
| 782233 | GO:0015078 | IEA |
| 782259 | GO:0004246 | IEA |
| 782270 | GO:0016787 | IEA |
| 782270 | GO:0015078 | IEA |
| 782270 | GO:0046933 | IEA |
| 782270 | GO:0046872 | IEA |
| 782270 | GO:0046961 | IEA |
| 782344 | GO:0000166 | IEA |
| 782344 | GO:0003676 | IEA |
| 782344 | GO:0003723 | IEA |
| 782344 | GO:0016853 | IEA |
| 782393 | GO:0003746 | IEA |
| 782393 | GO:0005515 | IEA |
| 782393 | GO:0005515 | ISS |
| 782416 | GO:0005504 | IDA |
| 782416 | GO:0000036 | IEA |
| 782416 | GO:0048037 | IEA |
| 782416 | GO:0016491 | IEA |
| 782416 | GO:0031177 | IEA |
| 782416 | GO:0008137 | TAS |
| 782444 | GO:0005515 | IEA |
| 782525 | GO:0003746 | IEA |
| 782666 | GO:0046961 | IEA |
| 782666 | GO:0005515 | IEA |
| 782666 | GO:0046933 | IEA |
| 782666 | GO:0046872 | IEA |
| 782666 | GO:0015078 | IEA |
| 782671 | GO:0004871 | IEA |
| 782671 | GO:0005515 | IEA |
| 782748 | GO:0016491 | IEA |
| 782748 | GO:0008270 | IEA |
| 782776 | GO:0000166 | IEA |
| 782776 | GO:0005525 | IEA |
| 782776 | GO:0003746 | IEA |
| 782921 | GO:0005506 | IEA |
| 782921 | GO:0046872 | IEA |
| 782921 | GO:0005488 | IEA |
| 782921 | GO:0008199 | IEA |
| 782954 | GO:0000166 | IEA |
| 782954 | GO:0003746 | IEA |
| 782954 | GO:0005525 | IEA |
| 782989 | GO:0005525 | IEA |
| 782989 | GO:0000166 | IEA |
| 782989 | GO:0003746 | IEA |
| 783021 | GO:0003676 | IEA |
| 783153 | GO:0003677 | IEA |
| 783184 | GO:0004421 | IEA |
| 783220 | GO:0005488 | IEA |
| 783220 | GO:0005506 | IEA |
| 783220 | GO:0008199 | IEA |
| 783220 | GO:0046872 | IEA |
| 783224 | GO:0005488 | IEA |
| 783224 | GO:0008199 | IEA |
| 783224 | GO:0005506 | IEA |
| 783224 | GO:0046872 | IEA |
| 783294 | GO:0008308 | IEA |
| 783294 | GO:0015288 | IEA |
| 783301 | GO:0003676 | IEA |
| 783301 | GO:0000166 | IEA |
| 783301 | GO:0003723 | IEA |
| 783301 | GO:0016853 | IEA |
| 783337 | GO:0003735 | IEA |
| 783337 | GO:0003735 | IEA |
| 783373 | GO:0005515 | IEA |
| 783421 | GO:0005525 | IEA |
| 783421 | GO:0003743 | IEA |
| 783421 | GO:0008135 | ISS |
| 783421 | GO:0008135 | ISS |
| 783421 | GO:0003743 | IEA |
| 783421 | GO:0005525 | IEA |
| 783467 | GO:0003954 | IEA |
| 783467 | GO:0008137 | IEA |
| 783467 | GO:0009055 | IEA |
| 783467 | GO:0016491 | IEA |
| 783502 | GO:0005515 | IEA |
| 783502 | GO:0016491 | IEA |
| 783502 | GO:0004222 | IEA |
| 783502 | GO:0008121 | IEA |
| 783523 | GO:0003676 | IEA |
| 783523 | GO:0000166 | IEA |
| 783523 | GO:0003723 | IEA |
| 783523 | GO:0016853 | IEA |
| 783681 | GO:0005515 | IEA |
| 783681 | GO:0005112 | IEA |
| 783686 | GO:0008137 | IEA |
| 783686 | GO:0051536 | IEA |
| 783686 | GO:0003954 | IEA |
| 783686 | GO:0046872 | IEA |
| 783686 | GO:0016491 | IEA |
| 783686 | GO:0005506 | IEA |
| 783686 | GO:0051537 | IEA |
| 783687 | GO:0051082 | IEA |
| 783837 | GO:0004505 | IEA |
| 783837 | GO:0005515 | IEA |
| 783838 | GO:0005525 | IEA |
| 783838 | GO:0000166 | IEA |
| 783838 | GO:0003746 | IEA |
| 783928 | GO:0016787 | IEA |
| 783928 | GO:0004240 | IEA |
| 783928 | GO:0008270 | IEA |
| 783928 | GO:0046872 | IEA |
| 783928 | GO:0008233 | IEA |
| 783928 | GO:0004222 | IEA |
| 783928 | GO:0008237 | IEA |
| 783989 | GO:0005515 | IEA |
| 783991 | GO:0046933 | IEA |
| 783991 | GO:0016787 | IEA |
| 783991 | GO:0005515 | IPI |
| 783991 | GO:0046872 | IEA |
| 783991 | GO:0046961 | IEA |
| 783991 | GO:0015078 | IEA |
| 783993 | GO:0008199 | IEA |
| 783993 | GO:0005488 | IEA |
| 783993 | GO:0005506 | IEA |
| 783993 | GO:0046872 | IEA |
| 784010 | GO:0003677 | IEA |
| 784010 | GO:0031072 | IEA |
| 784025 | GO:0046872 | IEA |
| 784025 | GO:0005506 | IEA |
| 784025 | GO:0008199 | IEA |
| 784025 | GO:0005488 | IEA |
| 784097 | GO:0008137 | IEA |
| 784097 | GO:0051536 | IEA |
| 784097 | GO:0051537 | IEA |
| 784097 | GO:0016491 | IEA |
| 784097 | GO:0005506 | IEA |
| 784097 | GO:0046872 | IEA |
| 784097 | GO:0003954 | IEA |
| 784131 | GO:0000166 | IEA |
| 784131 | GO:0003746 | IEA |
| 784131 | GO:0005525 | IEA |
| 784220 | GO:0005524 | IEA |
| 784220 | GO:0003824 | IEA |
| 784220 | GO:0016874 | IEA |
| 784289 | GO:0005506 | IEA |
| 784289 | GO:0046872 | IEA |
| 784289 | GO:0005488 | IEA |
| 784289 | GO:0008199 | IEA |
| 784294 | GO:0008308 | IEA |
| 784294 | GO:0015288 | IEA |
| 784340 | GO:0016853 | IEA |
| 784340 | GO:0016787 | IEA |
| 784340 | GO:0016868 | IEA |
| 784385 | GO:0003723 | IEA |
| 784385 | GO:0003676 | IEA |
| 784442 | GO:0005515 | IEA |
| 784442 | GO:0003682 | IEA |
| 784484 | GO:0017137 | IEA |
| 784484 | GO:0005515 | IEA |
| 784492 | GO:0005515 | ISS |
| 784492 | GO:0005515 | ISS |
| 784606 | GO:0003676 | IEA |
| 784606 | GO:0003723 | IEA |
| 784606 | GO:0000166 | IEA |
| 784606 | GO:0016853 | IEA |
| 784615 | GO:0008270 | IEA |
| 784615 | GO:0046872 | IEA |
| 784615 | GO:0005515 | IEA |
| 784619 | GO:0005515 | IEA |
| 784642 | GO:0005488 | IEA |
| 784642 | GO:0008199 | IEA |
| 784642 | GO:0046872 | IEA |
| 784642 | GO:0005506 | IEA |
| 784643 | GO:0003779 | IEA |
| 784737 | GO:0003714 | IEA |
| 784737 | GO:0005515 | IEA |
| 784737 | GO:0046332 | IEA |
| 784740 | GO:0003676 | IEA |
| 784740 | GO:0042802 | IEA |
| 784740 | GO:0005515 | IEA |
| 784740 | GO:0000166 | IEA |
| 784774 | GO:0005488 | IEA |
| 784774 | GO:0008199 | IEA |
| 784774 | GO:0005506 | IEA |
| 784774 | GO:0046872 | IEA |
| 784869 | GO:0047115 | IEA |
| 784869 | GO:0016491 | IEA |
| 784895 | GO:0020037 | IEA |
| 784928 | GO:0005515 | IEA |
| 785087 | GO:0000166 | IEA |
| 785087 | GO:0005525 | IEA |
| 785087 | GO:0003746 | IEA |
| 785134 | GO:0004129 | IEA |
| 785134 | GO:0016491 | IEA |
| 785134 | GO:0009055 | IEA |
| 785297 | GO:0005488 | IEA |
| 785297 | GO:0008199 | IEA |
| 785297 | GO:0046872 | IEA |
| 785297 | GO:0005506 | IEA |
| 785317 | GO:0005488 | IEA |
| 785317 | GO:0008199 | IEA |
| 785317 | GO:0046872 | IEA |
| 785317 | GO:0005506 | IEA |
| 785381 | GO:0016887 | IEA |
| 785381 | GO:0017111 | IEA |
| 785381 | GO:0000166 | IEA |
| 785381 | GO:0005524 | IEA |
| 785387 | GO:0005515 | IPI |
| 785387 | GO:0003677 | IEA |
| 785387 | GO:0005515 | IPI |
| 785387 | GO:0003677 | IEA |
| 785387 | GO:0005515 | IPI |
| 785387 | GO:0003677 | IEA |
| 785388 | GO:0003723 | IEA |
| 785388 | GO:0003676 | IEA |
| 785388 | GO:0000166 | IEA |
| 785388 | GO:0016853 | IEA |
| 785395 | GO:0005506 | IEA |
| 785395 | GO:0046872 | IEA |
| 785395 | GO:0000104 | IEA |
| 785455 | GO:0003779 | IEA |
| 785484 | GO:0003677 | IEA |
| 785484 | GO:0005515 | IPI |
| 785484 | GO:0005515 | IPI |
| 785484 | GO:0003677 | IEA |
| 785484 | GO:0005515 | IPI |
| 785484 | GO:0003677 | IEA |
| 785611 | GO:0008486 | IEA |
| 785611 | GO:0046872 | IEA |
| 785611 | GO:0016787 | IEA |
| 785611 | GO:0000287 | IEA |
| 785611 | GO:0030145 | IEA |
| 785629 | GO:0008137 | IEA |
| 785629 | GO:0016491 | IEA |
| 785629 | GO:0003954 | IEA |
| 785654 | GO:0008137 | IEA |
| 785654 | GO:0016491 | IEA |
| 785654 | GO:0003954 | IEA |
| 785655 | GO:0005515 | IEA |
| 785657 | GO:0005515 | IEA |
| 785760 | GO:0003676 | IEA |
| 785760 | GO:0000166 | IEA |
| 785760 | GO:0042802 | IEA |
| 785760 | GO:0005515 | IEA |
| 785761 | GO:0003723 | IEA |
| 785761 | GO:0003676 | IEA |
| 785761 | GO:0000166 | IEA |
| 785761 | GO:0016853 | IEA |
| 785762 | GO:0016491 | IEA |
| 785763 | GO:0016868 | IEA |
| 785763 | GO:0016787 | IEA |
| 785763 | GO:0016853 | IEA |
| 785824 | GO:0046872 | IEA |
| 785824 | GO:0005506 | IEA |
| 785824 | GO:0020037 | IEA |
| 785824 | GO:0016712 | IEA |
| 785824 | GO:0004497 | IEA |
| 785824 | GO:0016491 | IEA |
| 785828 | GO:0003676 | IEA |
| 785842 | GO:0046872 | IEA |
| 785842 | GO:0005488 | IEA |
| 785842 | GO:0008199 | IEA |
| 785842 | GO:0005506 | IEA |
| 785922 | GO:0003746 | IEA |
| 785922 | GO:0000166 | IEA |
| 785922 | GO:0005525 | IEA |
| 785958 | GO:0003677 | IEA |
| 785986 | GO:0046872 | IEA |
| 785986 | GO:0008270 | IEA |
| 785986 | GO:0005515 | IEA |
| 785986 | GO:0046872 | IEA |
| 785986 | GO:0005515 | IEA |
| 785986 | GO:0008270 | IEA |
| 785987 | GO:0005488 | IEA |
| 785987 | GO:0046872 | IEA |
| 785987 | GO:0005506 | IEA |
| 785987 | GO:0008199 | IEA |
| 786048 | GO:0003677 | IEA |
| 786048 | GO:0005516 | IEA |
| 786048 | GO:0043565 | IEA |
| 786055 | GO:0003746 | IEA |
| 786055 | GO:0005525 | IEA |
| 786055 | GO:0000166 | IEA |
| 786128 | GO:0005525 | IEA |
| 786128 | GO:0003924 | IEA |
| 786128 | GO:0005198 | IEA |
| 786426 | GO:0015288 | IEA |
| 786426 | GO:0008308 | IEA |
| 786431 | GO:0046872 | IEA |
| 786431 | GO:0046933 | IEA |
| 786431 | GO:0046961 | IEA |
| 786431 | GO:0005515 | IEA |
| 786431 | GO:0015078 | IEA |
| 786487 | GO:0005509 | IEA |
| 786533 | GO:0003824 | IEA |
| 786533 | GO:0016491 | IEA |
| 786533 | GO:0004601 | IEA |
| 786533 | GO:0051920 | IEA |
| 786533 | GO:0016209 | IEA |
| 786533 | GO:0016787 | IEA |
| 786551 | GO:0003954 | IEA |
| 786551 | GO:0016491 | IEA |
| 786551 | GO:0008137 | IEA |
| 786555 | GO:0005515 | IEA |
| 786555 | GO:0042802 | IEA |
| 786555 | GO:0003676 | IEA |
| 786555 | GO:0000166 | IEA |
| 786618 | GO:0020037 | IEA |
| 786673 | GO:0015078 | IEA |
| 786673 | GO:0046961 | IEA |
| 786673 | GO:0046933 | IEA |
| 786733 | GO:0008191 | IEA |
| 786733 | GO:0005515 | IEA |
| 786845 | GO:0008137 | IEA |
| 786845 | GO:0003954 | IEA |
| 786845 | GO:0016491 | IEA |
| 787289 | GO:0003723 | IEA |
| 787289 | GO:0003676 | IEA |
| 787289 | GO:0016853 | IEA |
| 787289 | GO:0000166 | IEA |
| 787327 | GO:0004872 | IEA |
| 787327 | GO:0008270 | IEA |
| 787327 | GO:0016922 | IEA |
| 787327 | GO:0030528 | IEA |
| 787486 | GO:0016787 | IEA |
| 787486 | GO:0003824 | IEA |
| 787497 | GO:0008137 | TAS |
| 787497 | GO:0000036 | IEA |
| 787497 | GO:0005504 | IDA |
| 787497 | GO:0031177 | IEA |
| 787497 | GO:0048037 | IEA |
| 787497 | GO:0016491 | IEA |
| 787513 | GO:0005525 | IEA |
| 787528 | GO:0003677 | IEA |
| 787528 | GO:0005515 | IPI |
| 787528 | GO:0005515 | IPI |
| 787528 | GO:0003677 | IEA |
| 787528 | GO:0005515 | IPI |
| 787528 | GO:0003677 | IEA |
| 787528 | GO:0003677 | IEA |
| 787528 | GO:0005515 | IPI |
| 787528 | GO:0003677 | IEA |
| 787528 | GO:0005515 | IPI |
| 787530 | GO:0005515 | IEA |
| 787530 | GO:0005516 | IEA |
| 787530 | GO:0008017 | ISS |
| 787530 | GO:0003779 | IEA |
| 787580 | GO:0004245 | IEA |
| 787679 | GO:0004867 | IEA |
| 787903 | GO:0000166 | IEA |
| 787903 | GO:0005515 | IEA |
| 787903 | GO:0003676 | IEA |
| 787903 | GO:0042802 | IEA |
| 788012 | GO:0008137 | IEA |
| 788012 | GO:0016491 | IEA |
| 788012 | GO:0003954 | IEA |
| 788157 | GO:0000166 | IEA |
| 788157 | GO:0003746 | IEA |
| 788157 | GO:0005525 | IEA |
| 788193 | GO:0003677 | IEA |
| 788193 | GO:0005515 | IPI |
| 788193 | GO:0003677 | IEA |
| 788193 | GO:0005515 | IPI |
| 788193 | GO:0003677 | IEA |
| 788193 | GO:0005515 | IPI |
| 788193 | GO:0005515 | IPI |
| 788193 | GO:0003677 | IEA |
| 788193 | GO:0005515 | IPI |
| 788193 | GO:0003677 | IEA |
| 788250 | GO:0003677 | IEA |
| 788250 | GO:0005515 | IPI |
| 788250 | GO:0005515 | IPI |
| 788250 | GO:0003677 | IEA |
| 788250 | GO:0005515 | IPI |
| 788250 | GO:0003677 | IEA |
| 788250 | GO:0005515 | IPI |
| 788250 | GO:0003677 | IEA |
| 788250 | GO:0005515 | IPI |
| 788250 | GO:0003677 | IEA |
| 788293 | GO:0004108 | IEA |
| 788293 | GO:0046912 | IEA |
| 788293 | GO:0016740 | IEA |
| 788293 | GO:0016740 | IEA |
| 788293 | GO:0046912 | IEA |
| 788293 | GO:0004108 | IEA |
| 788392 | GO:0003743 | IEA |
| 788392 | GO:0005525 | IEA |
| 788392 | GO:0008135 | ISS |
| 788392 | GO:0008135 | ISS |
| 788392 | GO:0003743 | IEA |
| 788392 | GO:0005525 | IEA |
| 788417 | GO:0003684 | IEA |
| 788417 | GO:0003677 | IEA |
| 788417 | GO:0032137 | IEA |
| 788417 | GO:0003682 | IEA |
| 788482 | GO:0003954 | IEA |
| 788482 | GO:0016491 | IEA |
| 788482 | GO:0008137 | IEA |
| 788596 | GO:0004871 | IEA |
| 788609 | GO:0000166 | IEA |
| 788609 | GO:0003746 | IEA |
| 788609 | GO:0005525 | IEA |
| 788610 | GO:0005488 | IEA |
| 788610 | GO:0005506 | IEA |
| 788610 | GO:0046872 | IEA |
| 788610 | GO:0019825 | IEA |
| 788610 | GO:0020037 | IEA |
| 788610 | GO:0005344 | IEA |
| 788610 | GO:0005488 | IEA |
| 788610 | GO:0020037 | IEA |
| 788610 | GO:0046872 | IEA |
| 788610 | GO:0019825 | IEA |
| 788610 | GO:0005506 | IEA |
| 788610 | GO:0005344 | IEA |
| 788610 | GO:0020037 | IEA |
| 788610 | GO:0005488 | IEA |
| 788610 | GO:0046872 | IEA |
| 788610 | GO:0019825 | IEA |
| 788610 | GO:0005506 | IEA |
| 788610 | GO:0005344 | IEA |
| 788668 | GO:0005515 | IPI |
| 788668 | GO:0003677 | IEA |
| 788668 | GO:0003677 | IEA |
| 788668 | GO:0005515 | IPI |
| 788668 | GO:0005515 | IPI |
| 788668 | GO:0003677 | IEA |
| 788672 | GO:0000166 | IEA |
| 788672 | GO:0005525 | IEA |
| 788672 | GO:0003746 | IEA |
| 788745 | GO:0046961 | IEA |
| 788745 | GO:0046933 | IEA |
| 788745 | GO:0015078 | IEA |
| 788801 | GO:0008199 | IEA |
| 788801 | GO:0046872 | IEA |
| 788801 | GO:0005506 | IEA |
| 788801 | GO:0005488 | IEA |
| 788907 | GO:0046872 | IEA |
| 788907 | GO:0005488 | IEA |
| 788907 | GO:0008199 | IEA |
| 788907 | GO:0005506 | IEA |
| 789002 | GO:0004719 | IEA |
| 789061 | GO:0003676 | IEA |
| 789240 | GO:0046872 | IEA |
| 789240 | GO:0008270 | IEA |
| 789255 | GO:0004129 | IEA |
| 789255 | GO:0016491 | IEA |
| 789369 | GO:0003677 | IEA |
| 789369 | GO:0005515 | IPI |
| 789369 | GO:0003677 | IEA |
| 789369 | GO:0005515 | IPI |
| 789369 | GO:0005515 | IPI |
| 789369 | GO:0003677 | IEA |
| 789369 | GO:0005515 | IPI |
| 789369 | GO:0003677 | IEA |
| 789369 | GO:0003677 | IEA |
| 789369 | GO:0005515 | IPI |
| 789638 | GO:0016853 | IEA |
| 789638 | GO:0016787 | IEA |
| 789638 | GO:0016868 | IEA |
| 789819 | GO:0004842 | IEA |
| 789867 | GO:0000166 | IEA |
| 789867 | GO:0003746 | IEA |
| 789867 | GO:0005525 | IEA |
| 790012 | GO:0005509 | IEA |
| 790012 | GO:0046872 | IEA |
| 790012 | GO:0004871 | IEA |
| 790012 | GO:0016787 | IEA |
| 790012 | GO:0004435 | IEA |
| 790012 | GO:0004629 | IEA |
| 790175 | GO:0005515 | IEA |
| 790454 | GO:0005515 | IEA |
| 790465 | GO:0004493 | IEA |
| 790465 | GO:0016853 | IEA |
| 790529 | GO:0005488 | IEA |
| 790529 | GO:0019894 | IEA |
| 790539 | GO:0004370 | IEA |
| 790539 | GO:0016301 | IEA |
| 790539 | GO:0016740 | IEA |
| 790541 | GO:0042578 | IEA |
| 790541 | GO:0016787 | IEA |
| 790541 | GO:0042132 | IEA |
| 790667 | GO:0005515 | IEA |
| 790667 | GO:0003677 | IEA |
| 790667 | GO:0005515 | IEA |
| 790667 | GO:0003677 | IEA |
| 790667 | GO:0003677 | IEA |
| 790667 | GO:0005515 | IEA |
| 790827 | GO:0005515 | IEA |
| 790827 | GO:0003676 | IEA |
| 790827 | GO:0000166 | IEA |
| 790827 | GO:0042802 | IEA |
| 790836 | GO:0003899 | IEA |
| 790836 | GO:0003677 | IEA |
| 790868 | GO:0004222 | IEA |
| 790868 | GO:0016491 | IEA |
| 790868 | GO:0008121 | IEA |
| 790868 | GO:0005515 | IEA |
|  |  |  |
