# Supplementary material for: A gene network switch enhances the oxidative capacity of ovine skeletal muscle during late fetal development
Source: BMC Genomics. 2010 Jun 15;11:378. doi: 10.1186/1471-2164-11-378 (PMC2894804; doi:10.1186/1471-2164-11-378)
Supplement: Additional File 1 — Gene expression data. This file contains links to the gene expression data. The genes that are significantly differentially expressed between adjacent development times are listed as Experiment 1 (80 d vs 100 d), Experiment 2 (100 d vs 120 d), Experiment 3 (120 d vs 150 d) and Experiment 4 (150 d vs 230 d) in the section entitled Open microarray analysis report for developmental expression. Each experiment contains an MA plot, Volcano plot and Heatmap for each of the three microarray processing programs MAS5, RMA and GCRMA. For each of these processing programs there is a list of significantly differentially expressed probe sets, and for each of these there is an FDR corrected probability, fold change, percentage of microarrays with a MAS5 Present call, and a convergence percentage for all three microarray processing programs. The file also contains annotation of the probe sets. In a second section entitled Time series analysis of developmental expression, there is a list of genes in each gene expression cluster and diagrams showing MAS5 gene expression values for these genes. [file 1471-2164-11-378-S1.ZIP › Report/GO3/Report/TopNodesBP.html]

### Top terms for Biological Process

Top terms sorted by the weight algorithm. The first column is the GO term id and links to GO, the second column is a short description of the term, the third column is the number of genes annotated to the term, the fourth is the number of genes significant for the term and the fifth column is the expected number of genes for the term. The last four columns are the p-values for different tests of significance. The results are ordered by the weight algorithm (last column).

|  |  |  |  |  |  |  |  |  |
| --- | --- | --- | --- | --- | --- | --- | --- | --- |
| **GO** | **Term** | **Annotated** | **Significant** | **Expected** | **classic** | **KS** | **elim** | **weight** |
| GO:0042775 | organelle ATP synthesis coupled electron... | 12 | 11 | 2.22 | 8.1e-08 | 0.96 | 1.00000 | 8.1e-08 |
| GO:0006099 | tricarboxylic acid cycle | 18 | 12 | 3.32 | 9.1e-06 | 0.55 | 9.1e-06 | 9.1e-06 |
| GO:0015986 | ATP synthesis coupled proton transport | 19 | 12 | 3.51 | 2.1e-05 | 0.99 | 2.1e-05 | 2.1e-05 |
| GO:0006100 | tricarboxylic acid cycle intermediate me... | 15 | 10 | 2.77 | 5.4e-05 | 0.69 | 5.4e-05 | 5.4e-05 |
| GO:0006007 | glucose catabolic process | 31 | 14 | 5.72 | 0.00056 | 0.94 | 0.30311 | 0.00056 |
| GO:0043648 | dicarboxylic acid metabolic process | 8 | 6 | 1.48 | 0.00078 | 1.00 | 0.00078 | 0.00078 |
| GO:0045039 | protein import into mitochondrial inner ... | 4 | 4 | 0.74 | 0.00116 | 1.00 | 0.00116 | 0.00116 |
| GO:0050803 | regulation of synapse structure and acti... | 9 | 6 | 1.66 | 0.00197 | 0.55 | 0.00197 | 0.00197 |
| GO:0006470 | protein amino acid dephosphorylation | 54 | 19 | 9.97 | 0.00254 | 0.84 | 0.00254 | 0.00254 |
| GO:0007050 | cell cycle arrest | 19 | 9 | 3.51 | 0.00375 | 0.87 | 0.00375 | 0.00375 |
| GO:0006979 | response to oxidative stress | 35 | 14 | 6.46 | 0.00236 | 0.26 | 0.00236 | 0.00391 |
| GO:0002318 | myeloid progenitor cell differentiation | 3 | 3 | 0.55 | 0.00628 | 0.92 | 0.00628 | 0.00628 |
| GO:0015908 | fatty acid transport | 3 | 3 | 0.55 | 0.00628 | 0.98 | 0.00628 | 0.00628 |
| GO:0032981 | mitochondrial respiratory chain complex ... | 3 | 3 | 0.55 | 0.00628 | 0.99 | 0.00628 | 0.00628 |
| GO:0045766 | positive regulation of angiogenesis | 3 | 3 | 0.55 | 0.00628 | 0.98 | 0.00628 | 0.00628 |
| GO:0006810 | transport | 985 | 219 | 182 | 0.00044 | 0.99 | 0.00928 | 0.00822 |
| GO:0000186 | activation of MAPKK activity | 6 | 4 | 1.11 | 0.01263 | 1.00 | 0.01263 | 0.01263 |
| GO:0007368 | determination of left/right symmetry | 6 | 4 | 1.11 | 0.01263 | 0.81 | 0.01263 | 0.01263 |
| GO:0055070 | copper ion homeostasis | 6 | 4 | 1.11 | 0.01263 | 0.72 | 0.01263 | 0.01263 |
| GO:0006511 | ubiquitin-dependent protein catabolic pr... | 77 | 22 | 14.2 | 0.01926 | 0.93 | 0.01926 | 0.01926 |

### DE genes for top GO terms for Biological Process

List of DE genes for top GO terms. The first column is the Affymetrix probe ID, the second column is the Entrez ID, the third column is the gene symbol and the fourth column is the gene description. Note that the same gene can appear more than once in the list since different probes can target the same gene (can help detect overepresented genes due to multiple probe targets).

### GO:0042775

|  |  |  |  |
| --- | --- | --- | --- |
| **probe** | **entrez** | **symbol** | **description** |
| Bt.1153.1.S1\_at | 287327 | NDUFS3 | NADH dehydrogenase (ubiquinone) Fe-S protein 3, 30kDa (NADH-coenzyme Q reductase) |
| Bt.1819.1.S1\_at | 616109 | UCRC | ubiquinol-cytochrome c reductase complex 7.2 kDa protein |
| Bt.20.1.S1\_at | 338063 | NDUFA7 | NADH dehydrogenase (ubiquinone) 1 alpha subcomplex, 7, 14.5kDa |
| Bt.21.1.S1\_at | 338046 | NDUFC2 | NADH dehydrogenase (ubiquinone) 1, subcomplex unknown, 2, 14.5kDa |
| Bt.23361.1.S1\_at | 616871 | UQCRB | ubiquinol-cytochrome c reductase binding protein |
| Bt.315.1.S1\_at | 338079 | NDUFS7 | NADH dehydrogenase (ubiquinone) Fe-S protein 7, 20kDa (NADH-coenzyme Q reductase) |
| Bt.321.1.S1\_at | 327702 | NDUFAB1 | NADH dehydrogenase (ubiquinone) 1, alpha/beta subcomplex, 1, 8kDa |
| Bt.3610.1.S1\_at | 613899 | UQCRH | ubiquinol-cytochrome c reductase hinge protein |
| Bt.4072.1.S1\_at | 287014 | NDUFV1 | NADH dehydrogenase (ubiquinone) flavoprotein 1, 51kDa |
| Bt.5040.1.S1\_at | 281570 | UQCR | ubiquinol-cytochrome c reductase (6.4kD) subunit |
| Bt.5378.1.S1\_at | 282517 | NDUFB8 | NADH dehydrogenase (ubiquinone) 1 beta subcomplex, 8, 19kDa |

### GO:0006099

|  |  |  |  |
| --- | --- | --- | --- |
| **probe** | **entrez** | **symbol** | **description** |
| Bt.1129.1.S1\_at | 280682 | CS | citrate synthase |
| Bt.1129.2.S1\_at | 280682 | CS | citrate synthase |
| Bt.13170.1.S1\_at | 327696 | SDHC | succinate dehydrogenase complex, subunit C, integral membrane protein, 15kDa |
| Bt.13324.2.S1\_a\_at | 281235 | IDH1 | isocitrate dehydrogenase 1 (NADP+), soluble |
| Bt.23357.1.S1\_at | 614145 | IDH3G | isocitrate dehydrogenase 3 (NAD+) gamma |
| Bt.2711.1.S1\_at | 509983 | SUCLG1 | succinate-CoA ligase, alpha subunit |
| Bt.3898.1.S1\_at | 282446 | IDH3A | isocitrate dehydrogenase 3 (NAD+) alpha |
| Bt.5033.1.S1\_at | 281480 | SDHA | succinate dehydrogenase complex, subunit A, flavoprotein (Fp) |
| Bt.5210.1.S1\_at | 280976 | ACO2 | aconitase 2, mitochondrial |
| Bt.5345.1.S1\_at | 535182 | MDH1 | malate dehydrogenase 1, NAD (soluble) |
| Bt.5520.1.S1\_at | 327669 | IDH2 | isocitrate dehydrogenase 2 (NADP+), mitochondrial |
| Bt.7915.1.S1\_at | 281306 | MDH2 | malate dehydrogenase 2, NAD (mitochondrial) |

### GO:0015986

|  |  |  |  |
| --- | --- | --- | --- |
| **probe** | **entrez** | **symbol** | **description** |
| Bt.1242.1.S1\_at | 338053 | ATP5G1 | ATP synthase, H+ transporting, mitochondrial F0 complex, subunit C1 (subunit 9) |
| Bt.1426.1.S1\_at | 338081 | ATP5D | ATP synthase, H+ transporting, mitochondrial F1 complex, delta subunit |
| Bt.27657.1.A1\_at | 338053 | ATP5G1 | ATP synthase, H+ transporting, mitochondrial F0 complex, subunit C1 (subunit 9) |
| Bt.2816.1.S1\_at | 515696 | ATP5L | ATP synthase, H+ transporting, mitochondrial F0 complex, subunit G |
| Bt.378.1.S1\_at | 338040 | ATP5I | ATP synthase, H+ transporting, mitochondrial F0 complex, subunit e |
| Bt.3867.1.S1\_at | 282701 | ATP5F1 | ATP synthase, H+ transporting, mitochondrial F0 complex, subunit B1 |
| Bt.404.1.S1\_at | 282690 | ATP5J | ATP synthase, H+ transporting, mitochondrial F0 complex, subunit F6 |
| Bt.4431.1.S1\_a\_at | 327675 | ATP5B | ATP synthase, H+ transporting, mitochondrial F1 complex, beta polypeptide |
| Bt.4540.1.S1\_at | 327668 | ATP5C1 | ATP synthase, H+ transporting, mitochondrial F1 complex, gamma polypeptide 1 |
| Bt.5029.1.S1\_at | 282710 | ATP5H | ATP synthase, H+ transporting, mitochondrial F0 complex, subunit d |
| Bt.5510.1.S1\_at | 338088 | ATP5E | ATP synthase, H+ transporting, mitochondrial F1 complex, epsilon subunit |
| Bt.8047.1.S1\_at | 540176 | ATP5G3 | ATP synthase, H+ transporting, mitochondrial F0 complex, subunit C3 (subunit 9) |

### GO:0006100

|  |  |  |  |
| --- | --- | --- | --- |
| **probe** | **entrez** | **symbol** | **description** |
| Bt.13324.2.S1\_a\_at | 281235 | IDH1 | isocitrate dehydrogenase 1 (NADP+), soluble |
| Bt.23357.1.S1\_at | 614145 | IDH3G | isocitrate dehydrogenase 3 (NAD+) gamma |
| Bt.24594.1.A1\_at | 525813 | ME3 | malic enzyme 3, NADP(+)-dependent, mitochondrial |
| Bt.2587.1.S1\_at | 520260 | FH | fumarate hydratase |
| Bt.2587.2.S1\_a\_at | 520260 | FH | fumarate hydratase |
| Bt.26829.1.S1\_at | 540985 | ME1 | malic enzyme 1, NADP(+)-dependent, cytosolic |
| Bt.5210.1.S1\_at | 280976 | ACO2 | aconitase 2, mitochondrial |
| Bt.5345.1.S1\_at | 535182 | MDH1 | malate dehydrogenase 1, NAD (soluble) |
| Bt.5520.1.S1\_at | 327669 | IDH2 | isocitrate dehydrogenase 2 (NADP+), mitochondrial |
| Bt.7915.1.S1\_at | 281306 | MDH2 | malate dehydrogenase 2, NAD (mitochondrial) |

### GO:0006007

|  |  |  |  |
| --- | --- | --- | --- |
| **probe** | **entrez** | **symbol** | **description** |
| Bt.11516.1.S1\_at | 407109 | PDHA1 | pyruvate dehydrogenase (lipoamide) alpha 1 |
| Bt.1244.1.S1\_at | 514939 | LOC514939 | similar to 6-phosphogluconate dehydrogenase (decarboxylating) |
| Bt.12910.1.S1\_at | 534599 | OGDH | oxoglutarate (alpha-ketoglutarate) dehydrogenase (lipoamide) |
| Bt.13505.1.S1\_at | 280808 | GPI | glucose phosphate isomerase |
| Bt.19415.1.A1\_at | 407109 | PDHA1 | pyruvate dehydrogenase (lipoamide) alpha 1 |
| Bt.2347.1.S1\_at | 506544 | PFKM | phosphofructokinase, muscle |
| Bt.2347.2.S1\_at | 506544 | PFKM | phosphofructokinase, muscle |
| Bt.2973.2.S1\_a\_at | 613610 | PDHB | pyruvate dehydrogenase (lipoamide) beta |
| Bt.29746.1.S1\_at | 407109 | PDHA1 | pyruvate dehydrogenase (lipoamide) alpha 1 |
| Bt.3487.1.S1\_at | 281543 | TPI1 | triosephosphate isomerase 1 |
| Bt.5345.1.S1\_at | 535182 | MDH1 | malate dehydrogenase 1, NAD (soluble) |
| Bt.6171.1.A1\_at | 512002 | HIBADH | 3-hydroxyisobutyrate dehydrogenase |
| Bt.7915.1.S1\_at | 281306 | MDH2 | malate dehydrogenase 2, NAD (mitochondrial) |
| Bt.8077.1.S1\_at | 507476 | PGK1 | phosphoglycerate kinase 1 |

### GO:0043648

|  |  |  |  |
| --- | --- | --- | --- |
| **probe** | **entrez** | **symbol** | **description** |
| Bt.24594.1.A1\_at | 525813 | ME3 | malic enzyme 3, NADP(+)-dependent, mitochondrial |
| Bt.2587.1.S1\_at | 520260 | FH | fumarate hydratase |
| Bt.2587.2.S1\_a\_at | 520260 | FH | fumarate hydratase |
| Bt.26829.1.S1\_at | 540985 | ME1 | malic enzyme 1, NADP(+)-dependent, cytosolic |
| Bt.5345.1.S1\_at | 535182 | MDH1 | malate dehydrogenase 1, NAD (soluble) |
| Bt.7915.1.S1\_at | 281306 | MDH2 | malate dehydrogenase 2, NAD (mitochondrial) |

### GO:0045039

|  |  |  |  |
| --- | --- | --- | --- |
| **probe** | **entrez** | **symbol** | **description** |
| Bt.1175.1.S1\_at | 521073 | TIMM9 | translocase of inner mitochondrial membrane 9 homolog (yeast) |
| Bt.13784.1.S1\_at | 512818 | TIMM10 | translocase of inner mitochondrial membrane 10 homolog (yeast) |
| Bt.27116.2.S1\_at | 618131 | TIMM8B | translocase of inner mitochondrial membrane 8 homolog B (yeast) |
| Bt.7266.1.S1\_at | 515109 | TIMM8A | translocase of inner mitochondrial membrane 8 homolog A (yeast) |

### GO:0050803

|  |  |  |  |
| --- | --- | --- | --- |
| **probe** | **entrez** | **symbol** | **description** |
| Bt.13152.1.S1\_s\_at | 280722 | APP | amyloid beta (A4) precursor protein (peptidase nexin-II, Alzheimer disease) |
| Bt.16142.2.S1\_at | 286862 | YWHAG | tyrosine 3-monooxygenase/tryptophan 5-monooxygenase activation protein, gamma polypeptide |
| Bt.27.1.S1\_at | 286862 | YWHAG | tyrosine 3-monooxygenase/tryptophan 5-monooxygenase activation protein, gamma polypeptide |
| Bt.2814.1.S1\_at | 286862 | YWHAG | tyrosine 3-monooxygenase/tryptophan 5-monooxygenase activation protein, gamma polypeptide |
| Bt.4775.1.S1\_at | 525716 | S100B | S100 calcium binding protein B |
| Bt.9559.2.S1\_at | 280722 | APP | amyloid beta (A4) precursor protein (peptidase nexin-II, Alzheimer disease) |

### GO:0006470

|  |  |  |  |
| --- | --- | --- | --- |
| **probe** | **entrez** | **symbol** | **description** |
| Bt.1069.1.A1\_at | 536337 | PTPRR | protein tyrosine phosphatase, receptor type, R |
| Bt.10880.1.S1\_at | 505489 | TIMM50 | translocase of inner mitochondrial membrane 50 homolog (S. cerevisiae) |
| Bt.11058.1.A1\_at | 280891 | PPM2C | protein phosphatase 2C, magnesium-dependent, catalytic subunit |
| Bt.18634.1.A1\_at | 540329 | PPM1K | protein phosphatase 1K (PP2C domain containing) |
| Bt.18634.2.S1\_at | 540329 | PPM1K | protein phosphatase 1K (PP2C domain containing) |
| Bt.18748.1.A1\_at | 505696 | PTPRB | protein tyrosine phosphatase, receptor type, B |
| Bt.20261.1.S1\_at | 511010 | PTPN3 | protein tyrosine phosphatase, non-receptor type 3 |
| Bt.2070.1.A1\_at | 541175 | DUSP10 | dual specificity phosphatase 10 |
| Bt.2070.2.A1\_at | 541175 | DUSP10 | dual specificity phosphatase 10 |
| Bt.2070.3.S1\_at | 541175 | DUSP10 | dual specificity phosphatase 10 |
| Bt.21997.1.S1\_at | 540329 | PPM1K | protein phosphatase 1K (PP2C domain containing) |
| Bt.21997.2.A1\_at | 540329 | PPM1K | protein phosphatase 1K (PP2C domain containing) |
| Bt.23237.1.S1\_at | 510245 | PPP5C | protein phosphatase 5, catalytic subunit |
| Bt.27211.1.S1\_at | 616048 | DUSP13 | dual specificity phosphatase 13 |
| Bt.27565.1.S1\_at | 509657 | PTPRK | protein tyrosine phosphatase, receptor type, K |
| Bt.27956.1.A1\_at | 540786 | PTEN | phosphatase and tensin homolog (mutated in multiple advanced cancers 1) |
| Bt.28758.1.A1\_at | 531877 | PTPN12 | protein tyrosine phosphatase, non-receptor type 12 |
| Bt.6842.1.S1\_at | 520428 | CDC14B | CDC14 cell division cycle 14 homolog B (S. cerevisiae) |
| Bt.7319.1.S1\_at | 509657 | PTPRK | protein tyrosine phosphatase, receptor type, K |

### GO:0007050

|  |  |  |  |
| --- | --- | --- | --- |
| **probe** | **entrez** | **symbol** | **description** |
| Bt.12345.1.S1\_at | 506730 | MACF1 | microtubule-actin crosslinking factor 1 |
| Bt.20402.1.S1\_at | 530393 | PKD2 | polycystic kidney disease 2 (autosomal dominant) |
| Bt.20402.2.A1\_at | 530393 | PKD2 | polycystic kidney disease 2 (autosomal dominant) |
| Bt.20402.3.S1\_at | 530393 | PKD2 | polycystic kidney disease 2 (autosomal dominant) |
| Bt.21766.1.A1\_at | 533379 | MLF1 | myeloid leukemia factor 1 |
| Bt.21766.2.S1\_at | 533379 | MLF1 | myeloid leukemia factor 1 |
| Bt.21766.3.S1\_at | 533379 | MLF1 | myeloid leukemia factor 1 |
| Bt.22534.1.S1\_at | 534497 | PMP22 | peripheral myelin protein 22 |
| Bt.4804.2.A1\_at | 510972 | CDKN1C | cyclin-dependent kinase inhibitor 1C (p57, Kip2) |

### GO:0006979

|  |  |  |  |
| --- | --- | --- | --- |
| **probe** | **entrez** | **symbol** | **description** |
| Bt.11662.1.A1\_at | 523311 | GPX7 | glutathione peroxidase 7 |
| Bt.12916.1.S1\_at | 281210 | GPX3 | glutathione peroxidase 3 (plasma) |
| Bt.13324.2.S1\_a\_at | 281235 | IDH1 | isocitrate dehydrogenase 1 (NADP+), soluble |
| Bt.136.1.S1\_at | 281563 | UCP3 | uncoupling protein 3 (mitochondrial, proton carrier) |
| Bt.2151.2.A1\_at | 282022 | PTGS1 | prostaglandin-endoperoxide synthase 1 (prostaglandin G/H synthase and cyclooxygenase) |
| Bt.298.1.S1\_at | 281742 | NDUFA12 | NADH dehydrogenase (ubiquinone) 1 alpha subcomplex, 12 |
| Bt.4008.1.S1\_at | 282389 | TXNRD2 | thioredoxin reductase 2 |
| Bt.4205.1.S1\_at | 286809 | GPX4 | glutathione peroxidase 4 (phospholipid hydroperoxidase) |
| Bt.4475.1.S1\_at | 327697 | NDUFS2 | NADH dehydrogenase (ubiquinone) Fe-S protein 2, 49kDa (NADH-coenzyme Q reductase) |
| Bt.4748.1.S1\_at | 281496 | SOD2 | superoxide dismutase 2, mitochondrial |
| Bt.5319.2.S1\_a\_at | 282438 | PRDX6 | peroxiredoxin 6 |
| Bt.5483.1.S1\_at | 287027 | NDUFS8 | NADH dehydrogenase (ubiquinone) Fe-S protein 8, 23kDa (NADH-coenzyme Q reductase) |
| Bt.63.1.S1\_at | 327670 | NDUFA6 | NADH dehydrogenase (ubiquinone) 1 alpha subcomplex, 6, 14kDa |
| Bt.99.1.S1\_at | 327706 | NDUFB4 | NADH dehydrogenase (ubiquinone) 1 beta subcomplex, 4, 15kDa |

### GO:0002318

|  |  |  |  |
| --- | --- | --- | --- |
| **probe** | **entrez** | **symbol** | **description** |
| Bt.21766.1.A1\_at | 533379 | MLF1 | myeloid leukemia factor 1 |
| Bt.21766.2.S1\_at | 533379 | MLF1 | myeloid leukemia factor 1 |
| Bt.21766.3.S1\_at | 533379 | MLF1 | myeloid leukemia factor 1 |

### GO:0015908

|  |  |  |  |
| --- | --- | --- | --- |
| **probe** | **entrez** | **symbol** | **description** |
| Bt.1316.1.S1\_at | 286886 | GOT2 | glutamic-oxaloacetic transaminase 2, mitochondrial (aspartate aminotransferase 2) |
| Bt.1316.1.S2\_at | 286886 | GOT2 | glutamic-oxaloacetic transaminase 2, mitochondrial (aspartate aminotransferase 2) |
| Bt.4599.1.S1\_at | 281092 | CROT | carnitine O-octanoyltransferase |

### GO:0032981

|  |  |  |  |
| --- | --- | --- | --- |
| **probe** | **entrez** | **symbol** | **description** |
| Bt.5483.1.S1\_at | 287027 | NDUFS8 | NADH dehydrogenase (ubiquinone) Fe-S protein 8, 23kDa (NADH-coenzyme Q reductase) |
| Bt.57.1.S1\_at | 338057 | NDUFS5 | NADH dehydrogenase (ubiquinone) Fe-S protein 5, 15kDa (NADH-coenzyme Q reductase) |
| Bt.58.1.S1\_at | 327680 | NDUFS4 | NADH dehydrogenase (ubiquinone) Fe-S protein 4, 18kDa (NADH-coenzyme Q reductase) |

### GO:0045766

|  |  |  |  |
| --- | --- | --- | --- |
| **probe** | **entrez** | **symbol** | **description** |
| Bt.15864.1.S1\_at | 281161 | FGF2 | fibroblast growth factor 2 (basic) |
| Bt.4725.1.S1\_at | 281032 | BTG1 | B-cell translocation gene 1, anti-proliferative |
| Bt.4834.1.S1\_at | 281161 | FGF2 | fibroblast growth factor 2 (basic) |

### GO:0006810

|  |  |  |  |
| --- | --- | --- | --- |
| **probe** | **entrez** | **symbol** | **description** |
| Bt.10.2.S1\_a\_at | 281698 | CLTLB | clathrin, light polypeptide B (light chain B) |
| Bt.10135.1.A1\_at | 534488 | SLC35A5 | solute carrier family 35, member A5 |
| Bt.10271.1.A1\_at | 528265 | SNX3 | sorting nexin 3 |
| Bt.10324.1.A1\_at | 327714 | NDUFA5 | NADH dehydrogenase (ubiquinone) 1 alpha subcomplex, 5, 13kDa |
| Bt.10458.2.S1\_at | 513774 | AKAP12 | A kinase (PRKA) anchor protein (gravin) 12 |
| Bt.1059.2.S1\_at | 540568 | ATP2A2 | ATPase, Ca++ transporting, cardiac muscle, slow twitch 2 |
| Bt.1059.3.S1\_a\_at | 540568 | ATP2A2 | ATPase, Ca++ transporting, cardiac muscle, slow twitch 2 |
| Bt.10880.1.S1\_at | 505489 | TIMM50 | translocase of inner mitochondrial membrane 50 homolog (S. cerevisiae) |
| Bt.11014.1.S1\_at | 513889 | CHCHD4 | coiled-coil-helix-coiled-coil-helix domain containing 4 |
| Bt.1112.1.S1\_at | 618204 | SCN1B | sodium channel, voltage-gated, type I, beta |
| Bt.11216.1.S1\_at | 280695 | MB | myoglobin |
| Bt.1151.1.S1\_at | 493998 | CRABP2 | cellular retinoic acid binding protein 2 |
| Bt.1153.1.S1\_at | 287327 | NDUFS3 | NADH dehydrogenase (ubiquinone) Fe-S protein 3, 30kDa (NADH-coenzyme Q reductase) |
| Bt.1175.1.S1\_at | 521073 | TIMM9 | translocase of inner mitochondrial membrane 9 homolog (yeast) |
| Bt.11770.1.S1\_a\_at | 518758 | SLC25A20 | solute carrier family 25 (carnitine/acylcarnitine translocase), member 20 |
| Bt.11770.1.S1\_at | 518758 | SLC25A20 | solute carrier family 25 (carnitine/acylcarnitine translocase), member 20 |
| Bt.1229.1.S1\_at | 281631 | APOA1 | apolipoprotein A-I |
| Bt.1242.1.S1\_at | 338053 | ATP5G1 | ATP synthase, H+ transporting, mitochondrial F0 complex, subunit C1 (subunit 9) |
| Bt.12498.1.A1\_at | 530657 | COL6A3 | collagen, type VI, alpha 3 |
| Bt.1280.2.S1\_a\_at | 282523 | SLC25A11 | solute carrier family 25 (mitochondrial carrier; oxoglutarate carrier), member 11 |
| Bt.12861.1.S1\_at | 768014 | KIF2A | kinesin heavy chain member 2A |
| Bt.12912.1.S1\_at | 282191 | COL4A1 | collagen, type IV, alpha 1 |
| Bt.12912.1.S2\_at | 282191 | COL4A1 | collagen, type IV, alpha 1 |
| Bt.13065.1.S1\_at | 282572 | KCNJ8 | potassium inwardly-rectifying channel, subfamily J, member 8 |
| Bt.1310.1.S1\_at | 532775 | TMEM38A | transmembrane protein 38A |
| Bt.13152.1.S1\_s\_at | 280722 | APP | amyloid beta (A4) precursor protein (peptidase nexin-II, Alzheimer disease) |
| Bt.1316.1.S1\_at | 286886 | GOT2 | glutamic-oxaloacetic transaminase 2, mitochondrial (aspartate aminotransferase 2) |
| Bt.1316.1.S2\_at | 286886 | GOT2 | glutamic-oxaloacetic transaminase 2, mitochondrial (aspartate aminotransferase 2) |
| Bt.13170.1.S1\_at | 327696 | SDHC | succinate dehydrogenase complex, subunit C, integral membrane protein, 15kDa |
| Bt.1322.1.S1\_at | 509037 | TXNDC1 | thioredoxin domain containing 1 |
| Bt.13439.1.S1\_at | 768074 | ETFDH | electron-transferring-flavoprotein dehydrogenase |
| Bt.13537.1.A1\_at | 505775 | SLC16A1 | solute carrier family 16, member 1 (monocarboxylic acid transporter 1) |
| Bt.136.1.S1\_at | 281563 | UCP3 | uncoupling protein 3 (mitochondrial, proton carrier) |
| Bt.13730.1.A1\_at | 408003 | ATG4A | ATG4 autophagy related 4 homolog A (S. cerevisiae) |
| Bt.13730.2.S1\_at | 408003 | ATG4A | ATG4 autophagy related 4 homolog A (S. cerevisiae) |
| Bt.13784.1.S1\_at | 512818 | TIMM10 | translocase of inner mitochondrial membrane 10 homolog (yeast) |
| Bt.1386.1.S1\_at | 507869 | TPR | translocated promoter region (to activated MET oncogene) |
| Bt.13900.1.S1\_at | 616748 | TRAPPC2 | trafficking protein particle complex 2 |
| Bt.1399.1.S1\_at | 282405 | ATP6V1F | ATPase, H+ transporting, lysosomal 14kDa, V1 subunit F |
| Bt.1403.1.S1\_at | 280878 | NNT | nicotinamide nucleotide transhydrogenase |
| Bt.1426.1.S1\_at | 338081 | ATP5D | ATP synthase, H+ transporting, mitochondrial F1 complex, delta subunit |
| Bt.1523.1.S1\_at | 510219 | TOMM40 | translocase of outer mitochondrial membrane 40 homolog (yeast) |
| Bt.15864.1.S1\_at | 281161 | FGF2 | fibroblast growth factor 2 (basic) |
| Bt.15870.1.S1\_at | 528165 | TIMM44 | translocase of inner mitochondrial membrane 44 homolog (yeast) |
| Bt.15972.1.S1\_at | 281193 | GJA1 | gap junction protein, alpha 1, 43kDa |
| Bt.16067.1.S1\_at | 338044 | SLC38A2 | solute carrier family 38, member 2 |
| Bt.16142.2.S1\_at | 286862 | YWHAG | tyrosine 3-monooxygenase/tryptophan 5-monooxygenase activation protein, gamma polypeptide |
| Bt.1617.2.S1\_a\_at | 281078 | CLTA | clathrin, light chain (Lca) |
| Bt.16296.1.A1\_at | 540568 | ATP2A2 | ATPase, Ca++ transporting, cardiac muscle, slow twitch 2 |
| Bt.16437.1.S1\_at | 614576 | BIN1 | bridging integrator 1 |
| Bt.16495.2.A1\_at | 511602 | COL4A5 | collagen, type IV, alpha 5 (Alport syndrome) |
| Bt.16637.1.A1\_at | 515178 | ABCD1 | ATP-binding cassette, sub-family D (ALD), member 1 |
| Bt.16985.1.S1\_at | 509821 | ELMO1 | engulfment and cell motility 1 |
| Bt.1719.1.S1\_at | 520335 | OXA1L | oxidase (cytochrome c) assembly 1-like |
| Bt.1729.1.A1\_at | 286861 | FTL | ferritin, light polypeptide |
| Bt.1808.1.S1\_at | 512500 | CYC1 | cytochrome c-1 |
| Bt.1819.1.S1\_at | 616109 | UCRC | ubiquinol-cytochrome c reductase complex 7.2 kDa protein |
| Bt.18702.1.A1\_at | 523683 | CUX1 | cut-like homeobox 1 |
| Bt.19209.2.S1\_at | 520918 | SLC35A3 | solute carrier family 35 (UDP-N-acetylglucosamine (UDP-GlcNAc) transporter), member A3 |
| Bt.19522.2.S1\_at | 534708 | RAB11A | RAB11A, member RAS oncogene family |
| Bt.20.1.S1\_at | 338063 | NDUFA7 | NADH dehydrogenase (ubiquinone) 1 alpha subcomplex, 7, 14.5kDa |
| Bt.20007.1.S1\_a\_at | 511769 | ABCC5 | ATP-binding cassette, sub-family C (CFTR/MRP), member 5 |
| Bt.20129.1.S1\_at | 523683 | CUX1 | cut-like homeobox 1 |
| Bt.20129.2.S1\_at | 523683 | CUX1 | cut-like homeobox 1 |
| Bt.20146.1.S1\_at | 282146 | ATP2B4 | ATPase, Ca++ transporting, plasma membrane 4 |
| Bt.20243.2.S1\_at | 614713 | SLC25A26 | solute carrier family 25, member 26 |
| Bt.20402.1.S1\_at | 530393 | PKD2 | polycystic kidney disease 2 (autosomal dominant) |
| Bt.20402.2.A1\_at | 530393 | PKD2 | polycystic kidney disease 2 (autosomal dominant) |
| Bt.20402.3.S1\_at | 530393 | PKD2 | polycystic kidney disease 2 (autosomal dominant) |
| Bt.20895.1.S1\_at | 767980 | SSR3 | signal sequence receptor, gamma (translocon-associated protein gamma) |
| Bt.21.1.S1\_at | 338046 | NDUFC2 | NADH dehydrogenase (ubiquinone) 1, subcomplex unknown, 2, 14.5kDa |
| Bt.21113.1.S1\_a\_at | 509459 | CPT1B | carnitine palmitoyltransferase 1B (muscle) |
| Bt.21485.1.S1\_at | 614042 | CDC42SE1 | CDC42 small effector 1 |
| Bt.21718.1.S1\_at | 509684 | SLC25A39 | solute carrier family 25, member 39 |
| Bt.22002.1.S1\_at | 281759 | FABP4 | fatty acid binding protein 4, adipocyte |
| Bt.222.1.S1\_at | 281719 | CRYAB | crystallin, alpha B |
| Bt.2235.1.S1\_at | 281188 | GDI1 | GDP dissociation inhibitor 1 |
| Bt.2235.1.S2\_at | 281188 | GDI1 | GDP dissociation inhibitor 1 |
| Bt.22433.1.A1\_at | 541266 | SLC22A5 | solute carrier family 22 (organic cation/carnitine transporter), member 5 |
| Bt.22651.1.A1\_at | 521004 | SLC39A10 | solute carrier family 39 (zinc transporter), member 10 |
| Bt.22854.1.S1\_at | 280740 | CA2 | carbonic anhydrase II |
| Bt.231.1.S1\_at | 280950 | TXN | thioredoxin |
| Bt.23164.1.S1\_at | 282393 | UQCRC1 | UQCRC1 protein |
| Bt.23174.2.S1\_at | 515646 | CLIC1 | chloride intracellular channel 1 |
| Bt.23268.1.S1\_at | 280815 | NPC2 | Niemann-Pick disease, type C2 |
| Bt.23318.1.S1\_at | 510833 | COL3A1 | collagen, type III, alpha 1 |
| Bt.23318.1.S2\_at | 510833 | COL3A1 | collagen, type III, alpha 1 |
| Bt.23361.1.S1\_at | 616871 | UQCRB | ubiquinol-cytochrome c reductase binding protein |
| Bt.23380.2.S1\_at | 505093 | HNRPA1 | heterogeneous nuclear ribonucleoprotein A1 |
| Bt.23606.1.S1\_at | 317697 | ITPR1 | inositol 1,4,5-triphosphate receptor, type 1 |
| Bt.24676.1.S1\_at | 515999 | SAR1B | SAR1 gene homolog B (S. cerevisiae) |
| Bt.24676.2.A1\_at | 515999 | SAR1B | SAR1 gene homolog B (S. cerevisiae) |
| Bt.25654.1.A1\_at | 511699 | CLCN4 | chloride channel 4 |
| Bt.265.1.S1\_at | 287020 | UQCRFS1 | ubiquinol-cytochrome c reductase, Rieske iron-sulfur polypeptide 1 |
| Bt.26728.1.S1\_at | 535498 | BAZ2B | bromodomain adjacent to zinc finger domain, 2B |
| Bt.26835.1.S1\_at | 512506 | RANGRF | RAN guanine nucleotide release factor |
| Bt.27.1.S1\_at | 286862 | YWHAG | tyrosine 3-monooxygenase/tryptophan 5-monooxygenase activation protein, gamma polypeptide |
| Bt.27116.2.S1\_at | 618131 | TIMM8B | translocase of inner mitochondrial membrane 8 homolog B (yeast) |
| Bt.27129.1.S1\_at | 539399 | SLC39A9 | solute carrier family 39 (zinc transporter), member 9 |
| Bt.27221.1.S1\_at | 535872 | SLC16A2 | solute carrier family 16, member 2 (monocarboxylic acid transporter 8) |
| Bt.2729.1.S1\_at | 536359 | AP3S1 | adaptor-related protein complex 3, sigma 1 subunit |
| Bt.27657.1.A1\_at | 338053 | ATP5G1 | ATP synthase, H+ transporting, mitochondrial F0 complex, subunit C1 (subunit 9) |
| Bt.27908.1.S1\_at | 513847 | APOO | apolipoprotein O |
| Bt.27952.1.S1\_at | 616748 | TRAPPC2 | trafficking protein particle complex 2 |
| Bt.2814.1.S1\_at | 286862 | YWHAG | tyrosine 3-monooxygenase/tryptophan 5-monooxygenase activation protein, gamma polypeptide |
| Bt.2816.1.S1\_at | 515696 | ATP5L | ATP synthase, H+ transporting, mitochondrial F0 complex, subunit G |
| Bt.28676.1.A1\_at | 533077 | PEX7 | peroxisomal biogenesis factor 7 |
| Bt.29009.1.A1\_at | 539899 | RYR3 | ryanodine receptor 3 |
| Bt.298.1.S1\_at | 281742 | NDUFA12 | NADH dehydrogenase (ubiquinone) 1 alpha subcomplex, 12 |
| Bt.29931.1.S1\_at | 514928 | KIF1C | kinesin family member 1C |
| Bt.3117.1.S1\_at | 327660 | NDUFB9 | NADH dehydrogenase (ubiquinone) 1 beta subcomplex, 9, 22kDa |
| Bt.315.1.S1\_at | 338079 | NDUFS7 | NADH dehydrogenase (ubiquinone) Fe-S protein 7, 20kDa (NADH-coenzyme Q reductase) |
| Bt.321.1.S1\_at | 327702 | NDUFAB1 | NADH dehydrogenase (ubiquinone) 1, alpha/beta subcomplex, 1, 8kDa |
| Bt.3260.1.S1\_at | 614633 | C17H18orf55 | chromosome 18 open reading frame 55 ortholog |
| Bt.3414.1.S1\_at | 536149 | HRB | HIV-1 Rev binding protein |
| Bt.3610.1.S1\_at | 613899 | UQCRH | ubiquinol-cytochrome c reductase hinge protein |
| Bt.371.1.S1\_at | 282478 | SLC25A4 | solute carrier family 25 (mitochondrial carrier; adenine nucleotide translocator), member 4 |
| Bt.378.1.S1\_at | 338040 | ATP5I | ATP synthase, H+ transporting, mitochondrial F0 complex, subunit e |
| Bt.3856.1.S1\_at | 353217 | SLC35B1 | solute carrier family 35, member B1 |
| Bt.3867.1.S1\_at | 282701 | ATP5F1 | ATP synthase, H+ transporting, mitochondrial F0 complex, subunit B1 |
| Bt.3960.1.S1\_a\_at | 282119 | VDAC1 | voltage-dependent anion channel 1 |
| Bt.3960.2.S1\_at | 282119 | VDAC1 | voltage-dependent anion channel 1 |
| Bt.401.2.S1\_at | 768330 | DBI | diazepam binding inhibitor (GABA receptor modulator, acyl-Coenzyme A binding protein) |
| Bt.404.1.S1\_at | 282690 | ATP5J | ATP synthase, H+ transporting, mitochondrial F0 complex, subunit F6 |
| Bt.4057.1.S1\_at | 317655 | MYH10 | myosin, heavy chain 10, non-muscle |
| Bt.4072.1.S1\_at | 287014 | NDUFV1 | NADH dehydrogenase (ubiquinone) flavoprotein 1, 51kDa |
| Bt.4099.1.S1\_at | 282120 | VDAC2 | voltage-dependent anion channel 2 |
| Bt.4203.1.S1\_at | 506492 | ATP5J2 | ATP synthase, H+ transporting, mitochondrial F0 complex, subunit F2 |
| Bt.4262.1.S1\_a\_at | 327717 | NDUFV3 | NADH dehydrogenase (ubiquinone) flavoprotein 3, 10kDa |
| Bt.4394.1.S1\_at | 282433 | GUCY1B3 | guanylate cyclase 1, soluble, beta 3 |
| Bt.4431.1.S1\_a\_at | 327675 | ATP5B | ATP synthase, H+ transporting, mitochondrial F1 complex, beta polypeptide |
| Bt.4473.1.S1\_at | 338060 | NDUFA10 | NADH dehydrogenase (ubiquinone) 1 alpha subcomplex, 10, 42kDa |
| Bt.4475.1.S1\_at | 327697 | NDUFS2 | NADH dehydrogenase (ubiquinone) Fe-S protein 2, 49kDa (NADH-coenzyme Q reductase) |
| Bt.4492.1.S1\_at | 504741 | COLEC12 | collectin sub-family member 12 |
| Bt.4503.1.S1\_at | 337927 | MTCH2 | mitochondrial carrier homolog 2 (C. elegans) |
| Bt.4503.1.S2\_at | 337927 | MTCH2 | mitochondrial carrier homolog 2 (C. elegans) |
| Bt.4520.1.S1\_at | 282346 | SCARB1 | scavenger receptor class B, member 1 |
| Bt.4540.1.S1\_at | 327668 | ATP5C1 | ATP synthase, H+ transporting, mitochondrial F1 complex, gamma polypeptide 1 |
| Bt.4555.1.S1\_at | 617210 | ETFB | electron-transfer-flavoprotein, beta polypeptide |
| Bt.4599.1.S1\_at | 281092 | CROT | carnitine O-octanoyltransferase |
| Bt.4704.1.S1\_at | 327714 | NDUFA5 | NADH dehydrogenase (ubiquinone) 1 alpha subcomplex, 5, 13kDa |
| Bt.4719.1.S1\_at | 281749 | EDNRA | endothelin receptor type A |
| Bt.4758.1.S1\_at | 281758 | FABP3 | fatty acid binding protein 3, muscle and heart (mammary-derived growth inhibitor) |
| Bt.4777.1.S1\_at | 288380 | NDUFS1 | NADH dehydrogenase (ubiquinone) Fe-S protein 1, 75kDa (NADH-coenzyme Q reductase) |
| Bt.4786.1.S2\_at | 281080 | CLTC | clathrin, heavy chain (Hc) |
| Bt.4819.1.S1\_at | 327704 | NDUFA4 | NADH dehydrogenase (ubiquinone) 1 alpha subcomplex, 4, 9kDa |
| Bt.4834.1.S1\_at | 281161 | FGF2 | fibroblast growth factor 2 (basic) |
| Bt.4880.1.S1\_at | 282477 | SLC25A3 | solute carrier family 25 (mitochondrial carrier; phosphate carrier), member 3 |
| Bt.4957.1.S2\_at | 281937 | MYO1C | myosin IC |
| Bt.4977.1.S2\_at | 281849 | IGF2R | insulin-like growth factor 2 receptor |
| Bt.5029.1.S1\_at | 282710 | ATP5H | ATP synthase, H+ transporting, mitochondrial F0 complex, subunit d |
| Bt.5033.1.S1\_at | 281480 | SDHA | succinate dehydrogenase complex, subunit A, flavoprotein (Fp) |
| Bt.5040.1.S1\_at | 281570 | UQCR | ubiquinol-cytochrome c reductase (6.4kD) subunit |
| Bt.5042.1.S1\_at | 282394 | UQCRC2 | ubiquinol-cytochrome c reductase core protein II |
| Bt.5120.1.S1\_at | 280878 | NNT | nicotinamide nucleotide transhydrogenase |
| Bt.5183.1.S1\_at | 777775 | TUBA4A | tubulin, alpha 4a |
| Bt.5183.2.S1\_at | 777775 | TUBA4A | tubulin, alpha 4a |
| Bt.5288.1.S1\_at | 281557 | TXN2 | thioredoxin 2 |
| Bt.5376.1.S1\_at | 506336 | YIF1A | Yip1 interacting factor homolog A (S. cerevisiae) |
| Bt.5378.1.S1\_at | 282517 | NDUFB8 | NADH dehydrogenase (ubiquinone) 1 beta subcomplex, 8, 19kDa |
| Bt.5415.1.S1\_at | 515022 | CCS | copper chaperone for superoxide dismutase |
| Bt.5445.2.A1\_at | 281555 | TUBB2A | tubulin, beta 2A |
| Bt.547.1.S1\_at | 286885 | UQCRQ | low molecular mass ubiquinone-binding protein (9.5kD) |
| Bt.5483.1.S1\_at | 287027 | NDUFS8 | NADH dehydrogenase (ubiquinone) Fe-S protein 8, 23kDa (NADH-coenzyme Q reductase) |
| Bt.5510.1.S1\_at | 338088 | ATP5E | ATP synthase, H+ transporting, mitochondrial F1 complex, epsilon subunit |
| Bt.5541.1.S1\_at | 404188 | NDUFA9 | NADH dehydrogenase (ubiquinone) 1 alpha subcomplex, 9, 39kDa |
| Bt.56.1.S1\_at | 327691 | NDUFS6 | NADH dehydrogenase (ubiquinone) Fe-S protein 6, 13kDa (NADH-coenzyme Q reductase) |
| Bt.57.1.S1\_at | 338057 | NDUFS5 | NADH dehydrogenase (ubiquinone) Fe-S protein 5, 15kDa (NADH-coenzyme Q reductase) |
| Bt.5723.1.S1\_at | 326346 | NDUFA11 | NADH dehydrogenase (ubiquinone) 1 alpha subcomplex, 11, 14.7kDa |
| Bt.5737.1.S1\_at | 510574 | VPS26A | vacuolar protein sorting 26 homolog A (S. pombe) |
| Bt.5750.1.S1\_at | 508465 | DERL2 | Der1-like domain family, member 2 |
| Bt.58.1.S1\_at | 327680 | NDUFS4 | NADH dehydrogenase (ubiquinone) Fe-S protein 4, 18kDa (NADH-coenzyme Q reductase) |
| Bt.5848.1.S1\_at | 518117 | ATP2A1 | ATPase, Ca++ transporting, cardiac muscle, fast twitch 1 |
| Bt.5848.2.S1\_at | 518117 | ATP2A1 | ATPase, Ca++ transporting, cardiac muscle, fast twitch 1 |
| Bt.59.1.S1\_at | 327710 | NDUFA8 | NADH dehydrogenase (ubiquinone) 1 alpha subcomplex, 8, 19kDa |
| Bt.60.1.S1\_at | 327713 | NDUFB2 | NADH dehydrogenase (ubiquinone) 1 beta subcomplex, 2, 8kDa |
| Bt.61.1.S1\_at | 338073 | NDUFB3 | NADH dehydrogenase (ubiquinone) 1 beta subcomplex, 3, 12kDa |
| Bt.63.1.S1\_at | 327670 | NDUFA6 | NADH dehydrogenase (ubiquinone) 1 alpha subcomplex, 6, 14kDa |
| Bt.6390.1.S1\_at | 508143 | MRPS17 | mitochondrial ribosomal protein S17 |
| Bt.64.1.S1\_at | 338065 | NDUFB7 | NADH dehydrogenase (ubiquinone) 1 beta subcomplex, 7, 18kDa |
| Bt.6465.1.S1\_at | 510767 | CYCS | cytochrome c, somatic |
| Bt.6494.1.S1\_at | 404161 | ESSS | mitochondrial NADH:ubiquinone oxidoreductase ESSS subunit |
| Bt.65.1.S1\_at | 327698 | NDUFA2 | NADH dehydrogenase (ubiquinone) 1 alpha subcomplex, 2, 8kDa |
| Bt.66.1.S1\_at | 338064 | NDUFA3 | NADH dehydrogenase (ubiquinone) 1 alpha subcomplex, 3, 9kDa |
| Bt.6673.1.S1\_at | 536553 | AP2S1 | adaptor-related protein complex 2, sigma 1 subunit |
| Bt.6679.1.S1\_at | 512427 | NASP | nuclear autoantigenic sperm protein (histone-binding) |
| Bt.67.1.S1\_at | 282289 | NDUFC1 | NADH dehydrogenase (ubiquinone) 1, subcomplex unknown, 1, 6kDa |
| Bt.68.1.S1\_at | 327690 | NDUFB1 | NADH dehydrogenase (ubiquinone) 1 beta subcomplex, 1, 7kDa |
| Bt.69.1.S1\_at | 327673 | NDUFA1 | NADH dehydrogenase (ubiquinone) 1 alpha subcomplex, 1, 7.5kDa |
| Bt.6950.1.S1\_at | 614236 | SYS1 | SYS1 Golgi-localized integral membrane protein homolog (S. cerevisiae) |
| Bt.70.1.S1\_at | 327701 | NDUFB10 | NADH dehydrogenase (ubiquinone) 1 beta subcomplex, 10, 22kDa |
| Bt.7056.2.A1\_x\_at | 280813 | HBB | hemoglobin, beta |
| Bt.7056.3.A1\_a\_at | 511735 | HBG | hemoglobin, gamma |
| Bt.7056.5.A1\_x\_at | 511735 | HBG | hemoglobin, gamma |
| Bt.7139.1.S1\_at | 781575 | TOMM20 | translocase of outer mitochondrial membrane 20 homolog (yeast) |
| Bt.7168.2.A1\_at | 513668 | COL27A1 | collagen, type XXVII, alpha 1 |
| Bt.7266.1.S1\_at | 515109 | TIMM8A | translocase of inner mitochondrial membrane 8 homolog A (yeast) |
| Bt.7500.1.S1\_at | 614242 | RAB8B | RAB8B, member RAS oncogene family |
| Bt.7540.2.S1\_a\_at | 327703 | CACNB1 | calcium channel, voltage-dependent, beta 1 subunit |
| Bt.7574.1.S1\_at | 519597 | SNX4 | sorting nexin 4 |
| Bt.7785.1.S1\_at | 513312 | NACA | nascent polypeptide-associated complex alpha subunit |
| Bt.7814.1.S1\_at | 518601 | ARF4 | ADP-ribosylation factor 4 |
| Bt.7836.1.S1\_at | 614921 | NUTF2 | nuclear transport factor 2 |
| Bt.7980.1.S1\_at | 532851 | LASP1 | LIM and SH3 protein 1 |
| Bt.8047.1.S1\_at | 540176 | ATP5G3 | ATP synthase, H+ transporting, mitochondrial F0 complex, subunit C3 (subunit 9) |
| Bt.848.1.S1\_at | 282290 | NDUFV2 | NADH dehydrogenase (ubiquinone) flavoprotein 2, 24kDa |
| Bt.8566.1.S1\_at | 538427 | ANP32A | acidic (leucine-rich) nuclear phosphoprotein 32 family, member A |
| Bt.8809.1.S1\_at | 538100 | SCN4B | sodium channel, voltage-gated, type IV, beta |
| Bt.893.1.S1\_at | 327665 | NDUFB6 | NADH dehydrogenase (ubiquinone) 1 beta subcomplex, 6, 17kDa |
| Bt.8950.1.S1\_at | 338084 | NDUFA13 | NADH dehydrogenase (ubiquinone) 1 alpha subcomplex, 13 |
| Bt.9238.1.S1\_at | 338044 | SLC38A2 | solute carrier family 38, member 2 |
| Bt.935.1.A1\_at | 539354 | SLC35A4 | solute carrier family 35, member A4 |
| Bt.935.2.S1\_at | 539354 | SLC35A4 | solute carrier family 35, member A4 |
| Bt.9559.2.S1\_at | 280722 | APP | amyloid beta (A4) precursor protein (peptidase nexin-II, Alzheimer disease) |
| Bt.97.1.S1\_at | 281759 | FABP4 | fatty acid binding protein 4, adipocyte |
| Bt.9731.1.S1\_at | 513762 | GLRX2 | glutaredoxin 2 |
| Bt.981.2.S1\_at | 286823 | CLIC4 | chloride intracellular channel 4 |
| Bt.99.1.S1\_at | 327706 | NDUFB4 | NADH dehydrogenase (ubiquinone) 1 beta subcomplex, 4, 15kDa |
| Bt.9993.1.S1\_at | 615498 | AQP7 | aquaporin 7 |

### GO:0000186

|  |  |  |  |
| --- | --- | --- | --- |
| **probe** | **entrez** | **symbol** | **description** |
| Bt.15864.1.S1\_at | 281161 | FGF2 | fibroblast growth factor 2 (basic) |
| Bt.4834.1.S1\_at | 281161 | FGF2 | fibroblast growth factor 2 (basic) |
| Bt.868.1.S1\_a\_at | 616375 | MAPBPIP | mitogen-activated protein-binding protein-interacting protein |
| Bt.868.1.S1\_at | 616375 | MAPBPIP | mitogen-activated protein-binding protein-interacting protein |

### GO:0007368

|  |  |  |  |
| --- | --- | --- | --- |
| **probe** | **entrez** | **symbol** | **description** |
| Bt.16020.1.A2\_at | 338068 | ACVR1 | activin A receptor, type I |
| Bt.20402.1.S1\_at | 530393 | PKD2 | polycystic kidney disease 2 (autosomal dominant) |
| Bt.20402.2.A1\_at | 530393 | PKD2 | polycystic kidney disease 2 (autosomal dominant) |
| Bt.20402.3.S1\_at | 530393 | PKD2 | polycystic kidney disease 2 (autosomal dominant) |

### GO:0055070

|  |  |  |  |
| --- | --- | --- | --- |
| **probe** | **entrez** | **symbol** | **description** |
| Bt.13152.1.S1\_s\_at | 280722 | APP | amyloid beta (A4) precursor protein (peptidase nexin-II, Alzheimer disease) |
| Bt.9559.2.S1\_at | 280722 | APP | amyloid beta (A4) precursor protein (peptidase nexin-II, Alzheimer disease) |
| Bt.9708.1.S1\_at | 535065 | CUTC | cutC copper transporter homolog (E. coli) |
| Bt.9708.2.S1\_at | 535065 | CUTC | cutC copper transporter homolog (E. coli) |

### GO:0006511

|  |  |  |  |
| --- | --- | --- | --- |
| **probe** | **entrez** | **symbol** | **description** |
| Bt.1000.1.S1\_at | 504558 | CUL7 | cullin 7 |
| Bt.10266.1.S1\_at | 515503 | PSMA1 | proteasome (prosome, macropain) subunit, alpha type, 1 |
| Bt.11588.1.A1\_at | 533393 | USP48 | ubiquitin specific peptidase 48 |
| Bt.13237.1.S1\_at | 510423 | PSMA4 | proteasome (prosome, macropain) subunit, alpha type, 4 |
| Bt.15837.1.S1\_at | 507213 | PSMA6 | proteasome (prosome, macropain) subunit, alpha type, 6 |
| Bt.16132.2.S1\_a\_at | 540859 | TCEB1 | transcription elongation factor B (SIII), polypeptide 1 (15kDa, elongin C) |
| Bt.2116.1.S1\_at | 510069 | PSMB6 | proteasome (prosome, macropain) subunit, beta type, 6 |
| Bt.21721.1.A1\_at | 522980 | USP2 | ubiquitin specific peptidase 2 |
| Bt.24017.1.A1\_s\_at | 530186 | PSMC2 | proteasome (prosome, macropain) 26S subunit, ATPase, 2 |
| Bt.27926.1.S1\_at | 520170 | UCHL3 | ubiquitin carboxyl-terminal esterase L3 (ubiquitin thiolesterase) |
| Bt.2821.1.S1\_at | 511207 | PSMB7 | proteasome (prosome, macropain) subunit, beta type, 7 |
| Bt.4689.1.S2\_at | 282110 | UCHL5 | ubiquitin carboxyl-terminal hydrolase L5 |
| Bt.4756.1.S1\_at | 530186 | PSMC2 | proteasome (prosome, macropain) 26S subunit, ATPase, 2 |
| Bt.5135.1.S1\_at | 540564 | RAD23A | RAD23 homolog A (S. cerevisiae) |
| Bt.5322.1.S1\_at | 534640 | PSMB5 | proteasome (prosome, macropain) subunit, beta type, 5 |
| Bt.5750.1.S1\_at | 508465 | DERL2 | Der1-like domain family, member 2 |
| Bt.6455.1.S1\_at | 617935 | CUL4A | cullin 4A |
| Bt.6473.1.S1\_at | 505050 | PSMA7 | proteasome (prosome, macropain) subunit, alpha type, 7 |
| Bt.6827.1.S1\_at | 505176 | PSMA3 | proteasome (prosome, macropain) subunit, alpha type, 3 |
| Bt.8062.1.S1\_at | 282015 | PSMC5 | proteasome (prosome, macropain) 26S subunit, ATPase, 5 |
| Bt.886.1.S1\_at | 510155 | PSMA5 | proteasome (prosome, macropain) subunit, alpha type, 5 |
| Bt.9114.1.A1\_at | 540564 | RAD23A | RAD23 homolog A (S. cerevisiae) |
