# Supplementary material for: A gene network switch enhances the oxidative capacity of ovine skeletal muscle during late fetal development
Source: BMC Genomics. 2010 Jun 15;11:378. doi: 10.1186/1471-2164-11-378 (PMC2894804; doi:10.1186/1471-2164-11-378)
Supplement: Additional File 1 — Gene expression data. This file contains links to the gene expression data. The genes that are significantly differentially expressed between adjacent development times are listed as Experiment 1 (80 d vs 100 d), Experiment 2 (100 d vs 120 d), Experiment 3 (120 d vs 150 d) and Experiment 4 (150 d vs 230 d) in the section entitled Open microarray analysis report for developmental expression. Each experiment contains an MA plot, Volcano plot and Heatmap for each of the three microarray processing programs MAS5, RMA and GCRMA. For each of these processing programs there is a list of significantly differentially expressed probe sets, and for each of these there is an FDR corrected probability, fold change, percentage of microarrays with a MAS5 Present call, and a convergence percentage for all three microarray processing programs. The file also contains annotation of the probe sets. In a second section entitled Time series analysis of developmental expression, there is a list of genes in each gene expression cluster and diagrams showing MAS5 gene expression values for these genes. [file 1471-2164-11-378-S1.ZIP › Report/GO3/Report/TopNodesCC.html]

### Top terms for Cellular Component

Top terms sorted by the weight algorithm. The first column is the GO term id and links to GO, the second column is a short description of the term, the third column is the number of genes annotated to the term, the fourth is the number of genes significant for the term and the fifth column is the expected number of genes for the term. The last four columns are the p-values for different tests of significance. The results are ordered by the weight algorithm (last column).

|  |  |  |  |  |  |  |  |  |
| --- | --- | --- | --- | --- | --- | --- | --- | --- |
| **GO** | **Term** | **Annotated** | **Significant** | **Expected** | **classic** | **KS** | **elim** | **weight** |
| GO:0005743 | mitochondrial inner membrane | 188 | 115 | 36.2 | < 1e-30 | 1.00 | 2.4e-15 | < 1e-30 |
| GO:0005759 | mitochondrial matrix | 94 | 44 | 18.1 | 8.6e-10 | 1.00 | 1.9e-06 | 1.4e-09 |
| GO:0005739 | mitochondrion | 504 | 225 | 97 | < 1e-30 | 0.99 | 6.3e-08 | 1.0e-06 |
| GO:0045259 | proton-transporting ATP synthase complex | 18 | 12 | 3.46 | 1.4e-05 | 0.99 | 0.0512 | 1.4e-05 |
| GO:0005758 | mitochondrial intermembrane space | 16 | 9 | 3.08 | 0.0011 | 0.98 | 0.0619 | 0.0011 |
| GO:0005932 | basal body | 4 | 4 | 0.77 | 0.0014 | 0.96 | 0.0014 | 0.0014 |
| GO:0000502 | proteasome complex | 32 | 14 | 6.16 | 0.0013 | 0.99 | 0.1116 | 0.0021 |
| GO:0016529 | sarcoplasmic reticulum | 5 | 4 | 0.96 | 0.0058 | 0.99 | 0.3471 | 0.0058 |
| GO:0045239 | tricarboxylic acid cycle enzyme complex | 4 | 3 | 0.77 | 0.0243 | 1.00 | 0.0243 | 0.0370 |
| GO:0001931 | uropod | 2 | 2 | 0.38 | 0.0370 | 1.00 | 0.0370 | 0.0370 |
| GO:0035253 | ciliary rootlet | 2 | 2 | 0.38 | 0.0370 | 1.00 | 0.0370 | 0.0370 |
| GO:0030132 | clathrin coat of coated pit | 5 | 3 | 0.96 | 0.0522 | 0.97 | 0.0522 | 0.0522 |
| GO:0008287 | protein serine/threonine phosphatase com... | 13 | 5 | 2.5 | 0.0863 | 0.68 | 0.0863 | 0.0863 |
| GO:0005737 | cytoplasm | 2306 | 550 | 444 | 1.2e-14 | 1.00 | 0.0320 | 0.1021 |
| GO:0005741 | mitochondrial outer membrane | 26 | 8 | 5 | 0.1099 | 0.96 | 0.1099 | 0.1099 |
| GO:0005833 | hemoglobin complex | 7 | 3 | 1.35 | 0.1351 | 0.99 | 0.1351 | 0.1351 |
| GO:0030130 | clathrin coat of trans-Golgi network ves... | 7 | 3 | 1.35 | 0.1351 | 0.96 | 0.1351 | 0.1351 |
| GO:0031594 | neuromuscular junction | 7 | 3 | 1.35 | 0.1351 | 0.76 | 0.1351 | 0.1351 |
| GO:0016363 | nuclear matrix | 11 | 4 | 2.12 | 0.1446 | 0.73 | 0.1446 | 0.1446 |
| GO:0030173 | integral to Golgi membrane | 15 | 5 | 2.89 | 0.1450 | 0.87 | 0.1450 | 0.1450 |

### DE genes for top GO terms for Cellular Component

List of DE genes for top GO terms. The first column is the Affymetrix probe ID, the second column is the Entrez ID, the third column is the gene symbol and the fourth column is the gene description. Note that the same gene can appear more than once in the list since different probes can target the same gene (can help detect overepresented genes due to multiple probe targets).

### GO:0005743

|  |  |  |  |
| --- | --- | --- | --- |
| **probe** | **entrez** | **symbol** | **description** |
| Bt.10324.1.A1\_at | 327714 | NDUFA5 | NADH dehydrogenase (ubiquinone) 1 alpha subcomplex, 5, 13kDa |
| Bt.10880.1.S1\_at | 505489 | TIMM50 | translocase of inner mitochondrial membrane 50 homolog (S. cerevisiae) |
| Bt.1153.1.S1\_at | 287327 | NDUFS3 | NADH dehydrogenase (ubiquinone) Fe-S protein 3, 30kDa (NADH-coenzyme Q reductase) |
| Bt.1175.1.S1\_at | 521073 | TIMM9 | translocase of inner mitochondrial membrane 9 homolog (yeast) |
| Bt.11770.1.S1\_a\_at | 518758 | SLC25A20 | solute carrier family 25 (carnitine/acylcarnitine translocase), member 20 |
| Bt.11770.1.S1\_at | 518758 | SLC25A20 | solute carrier family 25 (carnitine/acylcarnitine translocase), member 20 |
| Bt.1242.1.S1\_at | 338053 | ATP5G1 | ATP synthase, H+ transporting, mitochondrial F0 complex, subunit C1 (subunit 9) |
| Bt.12514.1.S1\_at | 515363 | PHB2 | prohibitin 2 |
| Bt.1280.2.S1\_a\_at | 282523 | SLC25A11 | solute carrier family 25 (mitochondrial carrier; oxoglutarate carrier), member 11 |
| Bt.13128.1.S1\_at | 286840 | SDHB | succinate dehydrogenase complex, subunit B, iron sulfur (Ip) |
| Bt.1316.1.S1\_at | 286886 | GOT2 | glutamic-oxaloacetic transaminase 2, mitochondrial (aspartate aminotransferase 2) |
| Bt.1316.1.S2\_at | 286886 | GOT2 | glutamic-oxaloacetic transaminase 2, mitochondrial (aspartate aminotransferase 2) |
| Bt.13170.1.S1\_at | 327696 | SDHC | succinate dehydrogenase complex, subunit C, integral membrane protein, 15kDa |
| Bt.13439.1.S1\_at | 768074 | ETFDH | electron-transferring-flavoprotein dehydrogenase |
| Bt.136.1.S1\_at | 281563 | UCP3 | uncoupling protein 3 (mitochondrial, proton carrier) |
| Bt.13784.1.S1\_at | 512818 | TIMM10 | translocase of inner mitochondrial membrane 10 homolog (yeast) |
| Bt.13980.1.A1\_at | 538944 | CKMT2 | creatine kinase, mitochondrial 2 (sarcomeric) |
| Bt.13980.2.A1\_a\_at | 538944 | CKMT2 | creatine kinase, mitochondrial 2 (sarcomeric) |
| Bt.1403.1.S1\_at | 280878 | NNT | nicotinamide nucleotide transhydrogenase |
| Bt.14207.1.S1\_at | 319141 | GCAT | glycine C-acetyltransferase (2-amino-3-ketobutyrate coenzyme A ligase) |
| Bt.1426.1.S1\_at | 338081 | ATP5D | ATP synthase, H+ transporting, mitochondrial F1 complex, delta subunit |
| Bt.1568.1.S1\_at | 287012 | COX5B | cytochrome c oxidase subunit Vb |
| Bt.15870.1.S1\_at | 528165 | TIMM44 | translocase of inner mitochondrial membrane 44 homolog (yeast) |
| Bt.16025.1.S1\_at | 281090 | COX4 | cytochrome c oxydase subunit 4 |
| Bt.1719.1.S1\_at | 520335 | OXA1L | oxidase (cytochrome c) assembly 1-like |
| Bt.1808.1.S1\_at | 512500 | CYC1 | cytochrome c-1 |
| Bt.1819.1.S1\_at | 616109 | UCRC | ubiquinol-cytochrome c reductase complex 7.2 kDa protein |
| Bt.20.1.S1\_at | 338063 | NDUFA7 | NADH dehydrogenase (ubiquinone) 1 alpha subcomplex, 7, 14.5kDa |
| Bt.20243.2.S1\_at | 614713 | SLC25A26 | solute carrier family 25, member 26 |
| Bt.20965.1.S1\_at | 530409 | PHB | prohibitin |
| Bt.21.1.S1\_at | 338046 | NDUFC2 | NADH dehydrogenase (ubiquinone) 1, subcomplex unknown, 2, 14.5kDa |
| Bt.21435.1.S1\_at | 510324 | STOML2 | stomatin (EPB72)-like 2 |
| Bt.21718.1.S1\_at | 509684 | SLC25A39 | solute carrier family 25, member 39 |
| Bt.23002.1.S1\_at | 282199 | COX6A1 | cytochrome c oxidase subunit VIa polypeptide 1 |
| Bt.23002.1.S1\_s\_at | 282199 | COX6A1 | cytochrome c oxidase subunit VIa polypeptide 1 |
| Bt.23164.1.S1\_at | 282393 | UQCRC1 | UQCRC1 protein |
| Bt.23361.1.S1\_at | 616871 | UQCRB | ubiquinol-cytochrome c reductase binding protein |
| Bt.23569.1.S1\_at | 522006 | ACAA2 | acetyl-Coenzyme A acyltransferase 2 |
| Bt.24474.1.S1\_a\_at | 515757 | AFG3L2 | AFG3 ATPase family gene 3-like 2 (yeast) |
| Bt.24474.1.S1\_at | 515757 | AFG3L2 | AFG3 ATPase family gene 3-like 2 (yeast) |
| Bt.265.1.S1\_at | 287020 | UQCRFS1 | ubiquinol-cytochrome c reductase, Rieske iron-sulfur polypeptide 1 |
| Bt.26718.1.S1\_a\_at | 506250 | HCCS | holocytochrome c synthase |
| Bt.2711.1.S1\_at | 509983 | SUCLG1 | succinate-CoA ligase, alpha subunit |
| Bt.27116.2.S1\_at | 618131 | TIMM8B | translocase of inner mitochondrial membrane 8 homolog B (yeast) |
| Bt.27657.1.A1\_at | 338053 | ATP5G1 | ATP synthase, H+ transporting, mitochondrial F0 complex, subunit C1 (subunit 9) |
| Bt.2816.1.S1\_at | 515696 | ATP5L | ATP synthase, H+ transporting, mitochondrial F0 complex, subunit G |
| Bt.298.1.S1\_at | 281742 | NDUFA12 | NADH dehydrogenase (ubiquinone) 1 alpha subcomplex, 12 |
| Bt.3117.1.S1\_at | 327660 | NDUFB9 | NADH dehydrogenase (ubiquinone) 1 beta subcomplex, 9, 22kDa |
| Bt.315.1.S1\_at | 338079 | NDUFS7 | NADH dehydrogenase (ubiquinone) Fe-S protein 7, 20kDa (NADH-coenzyme Q reductase) |
| Bt.321.1.S1\_at | 327702 | NDUFAB1 | NADH dehydrogenase (ubiquinone) 1, alpha/beta subcomplex, 1, 8kDa |
| Bt.3610.1.S1\_at | 613899 | UQCRH | ubiquinol-cytochrome c reductase hinge protein |
| Bt.371.1.S1\_at | 282478 | SLC25A4 | solute carrier family 25 (mitochondrial carrier; adenine nucleotide translocator), member 4 |
| Bt.378.1.S1\_at | 338040 | ATP5I | ATP synthase, H+ transporting, mitochondrial F0 complex, subunit e |
| Bt.3867.1.S1\_at | 282701 | ATP5F1 | ATP synthase, H+ transporting, mitochondrial F0 complex, subunit B1 |
| Bt.400.1.S1\_at | 327674 | COX7B | cytochrome c oxidase subunit VIIb |
| Bt.404.1.S1\_at | 282690 | ATP5J | ATP synthase, H+ transporting, mitochondrial F0 complex, subunit F6 |
| Bt.4072.1.S1\_at | 287014 | NDUFV1 | NADH dehydrogenase (ubiquinone) flavoprotein 1, 51kDa |
| Bt.4099.1.S1\_at | 282120 | VDAC2 | voltage-dependent anion channel 2 |
| Bt.4203.1.S1\_at | 506492 | ATP5J2 | ATP synthase, H+ transporting, mitochondrial F0 complex, subunit F2 |
| Bt.4262.1.S1\_a\_at | 327717 | NDUFV3 | NADH dehydrogenase (ubiquinone) flavoprotein 3, 10kDa |
| Bt.4431.1.S1\_a\_at | 327675 | ATP5B | ATP synthase, H+ transporting, mitochondrial F1 complex, beta polypeptide |
| Bt.4473.1.S1\_at | 338060 | NDUFA10 | NADH dehydrogenase (ubiquinone) 1 alpha subcomplex, 10, 42kDa |
| Bt.4475.1.S1\_at | 327697 | NDUFS2 | NADH dehydrogenase (ubiquinone) Fe-S protein 2, 49kDa (NADH-coenzyme Q reductase) |
| Bt.4503.1.S1\_at | 337927 | MTCH2 | mitochondrial carrier homolog 2 (C. elegans) |
| Bt.4503.1.S2\_at | 337927 | MTCH2 | mitochondrial carrier homolog 2 (C. elegans) |
| Bt.4540.1.S1\_at | 327668 | ATP5C1 | ATP synthase, H+ transporting, mitochondrial F1 complex, gamma polypeptide 1 |
| Bt.4704.1.S1\_at | 327714 | NDUFA5 | NADH dehydrogenase (ubiquinone) 1 alpha subcomplex, 5, 13kDa |
| Bt.4777.1.S1\_at | 288380 | NDUFS1 | NADH dehydrogenase (ubiquinone) Fe-S protein 1, 75kDa (NADH-coenzyme Q reductase) |
| Bt.4819.1.S1\_at | 327704 | NDUFA4 | NADH dehydrogenase (ubiquinone) 1 alpha subcomplex, 4, 9kDa |
| Bt.4880.1.S1\_at | 282477 | SLC25A3 | solute carrier family 25 (mitochondrial carrier; phosphate carrier), member 3 |
| Bt.4970.1.S1\_at | 327688 | COX7A2 | cytochrome c oxidase subunit VIIa polypeptide 2 (liver) |
| Bt.5029.1.S1\_at | 282710 | ATP5H | ATP synthase, H+ transporting, mitochondrial F0 complex, subunit d |
| Bt.5033.1.S1\_at | 281480 | SDHA | succinate dehydrogenase complex, subunit A, flavoprotein (Fp) |
| Bt.5040.1.S1\_at | 281570 | UQCR | ubiquinol-cytochrome c reductase (6.4kD) subunit |
| Bt.5042.1.S1\_at | 282394 | UQCRC2 | ubiquinol-cytochrome c reductase core protein II |
| Bt.5120.1.S1\_at | 280878 | NNT | nicotinamide nucleotide transhydrogenase |
| Bt.5278.1.A1\_at | 539713 | BCS1L | BCS1-like (yeast) |
| Bt.5278.2.S1\_at | 539713 | BCS1L | BCS1-like (yeast) |
| Bt.5344.1.S1\_at | 281556 | TUFM | Tu translation elongation factor, mitochondrial |
| Bt.5378.1.S1\_at | 282517 | NDUFB8 | NADH dehydrogenase (ubiquinone) 1 beta subcomplex, 8, 19kDa |
| Bt.547.1.S1\_at | 286885 | UQCRQ | low molecular mass ubiquinone-binding protein (9.5kD) |
| Bt.5483.1.S1\_at | 287027 | NDUFS8 | NADH dehydrogenase (ubiquinone) Fe-S protein 8, 23kDa (NADH-coenzyme Q reductase) |
| Bt.5520.1.S1\_at | 327669 | IDH2 | isocitrate dehydrogenase 2 (NADP+), mitochondrial |
| Bt.5541.1.S1\_at | 404188 | NDUFA9 | NADH dehydrogenase (ubiquinone) 1 alpha subcomplex, 9, 39kDa |
| Bt.56.1.S1\_at | 327691 | NDUFS6 | NADH dehydrogenase (ubiquinone) Fe-S protein 6, 13kDa (NADH-coenzyme Q reductase) |
| Bt.566.1.S1\_at | 282200 | COX6A2 | cytochrome c oxidase subunit VIa polypeptide 2 |
| Bt.57.1.S1\_at | 338057 | NDUFS5 | NADH dehydrogenase (ubiquinone) Fe-S protein 5, 15kDa (NADH-coenzyme Q reductase) |
| Bt.5723.1.S1\_at | 326346 | NDUFA11 | NADH dehydrogenase (ubiquinone) 1 alpha subcomplex, 11, 14.7kDa |
| Bt.58.1.S1\_at | 327680 | NDUFS4 | NADH dehydrogenase (ubiquinone) Fe-S protein 4, 18kDa (NADH-coenzyme Q reductase) |
| Bt.59.1.S1\_at | 327710 | NDUFA8 | NADH dehydrogenase (ubiquinone) 1 alpha subcomplex, 8, 19kDa |
| Bt.60.1.S1\_at | 327713 | NDUFB2 | NADH dehydrogenase (ubiquinone) 1 beta subcomplex, 2, 8kDa |
| Bt.61.1.S1\_at | 338073 | NDUFB3 | NADH dehydrogenase (ubiquinone) 1 beta subcomplex, 3, 12kDa |
| Bt.63.1.S1\_at | 327670 | NDUFA6 | NADH dehydrogenase (ubiquinone) 1 alpha subcomplex, 6, 14kDa |
| Bt.64.1.S1\_at | 338065 | NDUFB7 | NADH dehydrogenase (ubiquinone) 1 beta subcomplex, 7, 18kDa |
| Bt.6465.1.S1\_at | 510767 | CYCS | cytochrome c, somatic |
| Bt.6494.1.S1\_at | 404161 | ESSS | mitochondrial NADH:ubiquinone oxidoreductase ESSS subunit |
| Bt.65.1.S1\_at | 327698 | NDUFA2 | NADH dehydrogenase (ubiquinone) 1 alpha subcomplex, 2, 8kDa |
| Bt.66.1.S1\_at | 338064 | NDUFA3 | NADH dehydrogenase (ubiquinone) 1 alpha subcomplex, 3, 9kDa |
| Bt.67.1.S1\_at | 282289 | NDUFC1 | NADH dehydrogenase (ubiquinone) 1, subcomplex unknown, 1, 6kDa |
| Bt.68.1.S1\_at | 327690 | NDUFB1 | NADH dehydrogenase (ubiquinone) 1 beta subcomplex, 1, 7kDa |
| Bt.69.1.S1\_at | 327673 | NDUFA1 | NADH dehydrogenase (ubiquinone) 1 alpha subcomplex, 1, 7.5kDa |
| Bt.70.1.S1\_at | 327701 | NDUFB10 | NADH dehydrogenase (ubiquinone) 1 beta subcomplex, 10, 22kDa |
| Bt.7177.1.S1\_at | 280719 | AMT | aminomethyltransferase |
| Bt.7215.1.S1\_at | 282130 | ACADVL | acyl-coenzyme A dehydrogenase, very long chain |
| Bt.7266.1.S1\_at | 515109 | TIMM8A | translocase of inner mitochondrial membrane 8 homolog A (yeast) |
| Bt.75.1.S1\_at | 338087 | COX6B | cytochrome c oxidase subunit VIb |
| Bt.76.1.S1\_at | 338086 | COX7A1 | cytochrome c oxidase subunit VIIa polypeptide 1 (muscle) |
| Bt.77.1.S1\_at | 327718 | COX7C | cytochrome c oxidase subunit VIIc |
| Bt.848.1.S1\_at | 282290 | NDUFV2 | NADH dehydrogenase (ubiquinone) flavoprotein 2, 24kDa |
| Bt.893.1.S1\_at | 327665 | NDUFB6 | NADH dehydrogenase (ubiquinone) 1 beta subcomplex, 6, 17kDa |
| Bt.8950.1.S1\_at | 338084 | NDUFA13 | NADH dehydrogenase (ubiquinone) 1 alpha subcomplex, 13 |
| Bt.946.2.A1\_a\_at | 280716 | AK2 | adenylate kinase 2 |
| Bt.9826.1.S1\_at | 444878 | COX5A | COX5A protein |
| Bt.9857.1.S1\_at | 616477 | CHCHD3 | coiled-coil-helix-coiled-coil-helix domain containing 3 |
| Bt.99.1.S1\_at | 327706 | NDUFB4 | NADH dehydrogenase (ubiquinone) 1 beta subcomplex, 4, 15kDa |

### GO:0005759

|  |  |  |  |
| --- | --- | --- | --- |
| **probe** | **entrez** | **symbol** | **description** |
| Bt.11058.1.A1\_at | 280891 | PPM2C | protein phosphatase 2C, magnesium-dependent, catalytic subunit |
| Bt.1129.1.S1\_at | 280682 | CS | citrate synthase |
| Bt.1129.2.S1\_at | 280682 | CS | citrate synthase |
| Bt.11516.1.S1\_at | 407109 | PDHA1 | pyruvate dehydrogenase (lipoamide) alpha 1 |
| Bt.11659.1.S1\_at | 615666 | MRP63 | mitochondrial ribosomal protein 63 |
| Bt.12910.1.S1\_at | 534599 | OGDH | oxoglutarate (alpha-ketoglutarate) dehydrogenase (lipoamide) |
| Bt.1316.1.S1\_at | 286886 | GOT2 | glutamic-oxaloacetic transaminase 2, mitochondrial (aspartate aminotransferase 2) |
| Bt.1316.1.S2\_at | 286886 | GOT2 | glutamic-oxaloacetic transaminase 2, mitochondrial (aspartate aminotransferase 2) |
| Bt.13205.1.A1\_at | 513438 | MRPS35 | mitochondrial ribosomal protein S35 |
| Bt.13205.2.S1\_at | 513438 | MRPS35 | mitochondrial ribosomal protein S35 |
| Bt.1415.1.S1\_at | 515990 | MRPL11 | mitochondrial ribosomal protein L11 |
| Bt.16082.1.S1\_at | 518321 | C1QBP | complement component 1, q subcomponent binding protein |
| Bt.16945.1.S1\_at | 509150 | ETHE1 | ethylmalonic encephalopathy 1 |
| Bt.18634.1.A1\_at | 540329 | PPM1K | protein phosphatase 1K (PP2C domain containing) |
| Bt.18634.2.S1\_at | 540329 | PPM1K | protein phosphatase 1K (PP2C domain containing) |
| Bt.19415.1.A1\_at | 407109 | PDHA1 | pyruvate dehydrogenase (lipoamide) alpha 1 |
| Bt.19692.1.S1\_at | 540298 | COQ3 | coenzyme Q3 homolog, methyltransferase (S. cerevisiae) |
| Bt.2138.1.S1\_at | 282277 | MRPL43 | mitochondrial ribosomal protein L43 |
| Bt.2138.1.S1\_s\_at | 282277 | MRPL43 | mitochondrial ribosomal protein L43 |
| Bt.21997.1.S1\_at | 540329 | PPM1K | protein phosphatase 1K (PP2C domain containing) |
| Bt.21997.2.A1\_at | 540329 | PPM1K | protein phosphatase 1K (PP2C domain containing) |
| Bt.23526.1.S1\_at | 617466 | MRPS24 | mitochondrial ribosomal protein S24 |
| Bt.2493.1.S1\_at | 513622 | MRPL51 | mitochondrial ribosomal protein L51 |
| Bt.2825.1.S1\_at | 617901 | MRPL13 | mitochondrial ribosomal protein L13 |
| Bt.2973.2.S1\_a\_at | 613610 | PDHB | pyruvate dehydrogenase (lipoamide) beta |
| Bt.29746.1.S1\_at | 407109 | PDHA1 | pyruvate dehydrogenase (lipoamide) alpha 1 |
| Bt.321.1.S1\_at | 327702 | NDUFAB1 | NADH dehydrogenase (ubiquinone) 1, alpha/beta subcomplex, 1, 8kDa |
| Bt.4402.1.S1\_at | 538054 | MRPL16 | mitochondrial ribosomal protein L16 |
| Bt.4473.1.S1\_at | 338060 | NDUFA10 | NADH dehydrogenase (ubiquinone) 1 alpha subcomplex, 10, 42kDa |
| Bt.4555.1.S1\_at | 617210 | ETFB | electron-transfer-flavoprotein, beta polypeptide |
| Bt.4652.1.S1\_at | 506657 | MRPL55 | mitochondrial ribosomal protein L55 |
| Bt.4748.1.S1\_at | 281496 | SOD2 | superoxide dismutase 2, mitochondrial |
| Bt.4758.1.S1\_at | 281758 | FABP3 | fatty acid binding protein 3, muscle and heart (mammary-derived growth inhibitor) |
| Bt.5108.1.S1\_at | 515902 | PCCB | propionyl Coenzyme A carboxylase, beta polypeptide |
| Bt.5412.1.S1\_at | 282150 | BCKDHB | branched chain keto acid dehydrogenase E1, beta polypeptide (maple syrup urine disease) |
| Bt.5541.1.S1\_at | 404188 | NDUFA9 | NADH dehydrogenase (ubiquinone) 1 alpha subcomplex, 9, 39kDa |
| Bt.5966.1.A1\_at | 505968 | ACADM | acyl-coenzyme A dehydrogenase, C-4 to C-12 straight chain |
| Bt.6465.1.S1\_at | 510767 | CYCS | cytochrome c, somatic |
| Bt.7237.2.S1\_a\_at | 281810 | HADHA | hydroxyacyl-Coenzyme A dehydrogenase/3-ketoacyl-Coenzyme A thiolase/enoyl-Coenzyme A hydratase (trifunctional protein), alpha subunit |
| Bt.7856.1.S1\_at | 505005 | BCKDK | branched chain ketoacid dehydrogenase kinase |
| Bt.7915.1.S1\_at | 281306 | MDH2 | malate dehydrogenase 2, NAD (mitochondrial) |
| Bt.8046.1.A1\_at | 613835 | MRPS36 | mitochondrial ribosomal protein S36 |
| Bt.9324.1.S1\_at | 509816 | MRPS11 | mitochondrial ribosomal protein S11 |
| Bt.9797.1.S1\_at | 534546 | PMPCB | peptidase (mitochondrial processing) beta |

### GO:0005739

|  |  |  |  |
| --- | --- | --- | --- |
| **probe** | **entrez** | **symbol** | **description** |
| Bt.10154.1.S1\_at | 531444 | CISD1 | CDGSH iron sulfur domain 1 |
| Bt.10324.1.A1\_at | 327714 | NDUFA5 | NADH dehydrogenase (ubiquinone) 1 alpha subcomplex, 5, 13kDa |
| Bt.10880.1.S1\_at | 505489 | TIMM50 | translocase of inner mitochondrial membrane 50 homolog (S. cerevisiae) |
| Bt.11014.1.S1\_at | 513889 | CHCHD4 | coiled-coil-helix-coiled-coil-helix domain containing 4 |
| Bt.11058.1.A1\_at | 280891 | PPM2C | protein phosphatase 2C, magnesium-dependent, catalytic subunit |
| Bt.11194.1.S1\_at | 281360 | DHRS4 | dehydrogenase/reductase (SDR family) member 4 |
| Bt.11196.1.S1\_at | 281816 | HINT2 | histidine triad nucleotide binding protein 2 |
| Bt.1129.1.S1\_at | 280682 | CS | citrate synthase |
| Bt.1129.2.S1\_at | 280682 | CS | citrate synthase |
| Bt.11339.1.S1\_at | 514740 | MRPS27 | mitochondrial ribosomal protein S27 |
| Bt.11516.1.S1\_at | 407109 | PDHA1 | pyruvate dehydrogenase (lipoamide) alpha 1 |
| Bt.1153.1.S1\_at | 287327 | NDUFS3 | NADH dehydrogenase (ubiquinone) Fe-S protein 3, 30kDa (NADH-coenzyme Q reductase) |
| Bt.11659.1.S1\_at | 615666 | MRP63 | mitochondrial ribosomal protein 63 |
| Bt.1175.1.S1\_at | 521073 | TIMM9 | translocase of inner mitochondrial membrane 9 homolog (yeast) |
| Bt.11770.1.S1\_a\_at | 518758 | SLC25A20 | solute carrier family 25 (carnitine/acylcarnitine translocase), member 20 |
| Bt.11770.1.S1\_at | 518758 | SLC25A20 | solute carrier family 25 (carnitine/acylcarnitine translocase), member 20 |
| Bt.1242.1.S1\_at | 338053 | ATP5G1 | ATP synthase, H+ transporting, mitochondrial F0 complex, subunit C1 (subunit 9) |
| Bt.12514.1.S1\_at | 515363 | PHB2 | prohibitin 2 |
| Bt.1280.2.S1\_a\_at | 282523 | SLC25A11 | solute carrier family 25 (mitochondrial carrier; oxoglutarate carrier), member 11 |
| Bt.12910.1.S1\_at | 534599 | OGDH | oxoglutarate (alpha-ketoglutarate) dehydrogenase (lipoamide) |
| Bt.13128.1.S1\_at | 286840 | SDHB | succinate dehydrogenase complex, subunit B, iron sulfur (Ip) |
| Bt.1316.1.S1\_at | 286886 | GOT2 | glutamic-oxaloacetic transaminase 2, mitochondrial (aspartate aminotransferase 2) |
| Bt.1316.1.S2\_at | 286886 | GOT2 | glutamic-oxaloacetic transaminase 2, mitochondrial (aspartate aminotransferase 2) |
| Bt.13170.1.S1\_at | 327696 | SDHC | succinate dehydrogenase complex, subunit C, integral membrane protein, 15kDa |
| Bt.13205.1.A1\_at | 513438 | MRPS35 | mitochondrial ribosomal protein S35 |
| Bt.13205.2.S1\_at | 513438 | MRPS35 | mitochondrial ribosomal protein S35 |
| Bt.13287.1.S1\_at | 515186 | AGXT2L1 | alanine-glyoxylate aminotransferase 2-like 1 |
| Bt.13439.1.S1\_at | 768074 | ETFDH | electron-transferring-flavoprotein dehydrogenase |
| Bt.13483.1.A1\_at | 505430 | ATPAF1 | ATP synthase mitochondrial F1 complex assembly factor 1 |
| Bt.136.1.S1\_at | 281563 | UCP3 | uncoupling protein 3 (mitochondrial, proton carrier) |
| Bt.13610.1.A1\_at | 534185 | MRPS31 | mitochondrial ribosomal protein S31 |
| Bt.13784.1.S1\_at | 512818 | TIMM10 | translocase of inner mitochondrial membrane 10 homolog (yeast) |
| Bt.13908.1.S1\_at | 532044 | MRPS22 | mitochondrial ribosomal protein S22 |
| Bt.13980.1.A1\_at | 538944 | CKMT2 | creatine kinase, mitochondrial 2 (sarcomeric) |
| Bt.13980.2.A1\_a\_at | 538944 | CKMT2 | creatine kinase, mitochondrial 2 (sarcomeric) |
| Bt.1403.1.S1\_at | 280878 | NNT | nicotinamide nucleotide transhydrogenase |
| Bt.1415.1.S1\_at | 515990 | MRPL11 | mitochondrial ribosomal protein L11 |
| Bt.14207.1.S1\_at | 319141 | GCAT | glycine C-acetyltransferase (2-amino-3-ketobutyrate coenzyme A ligase) |
| Bt.1426.1.S1\_at | 338081 | ATP5D | ATP synthase, H+ transporting, mitochondrial F1 complex, delta subunit |
| Bt.1523.1.S1\_at | 510219 | TOMM40 | translocase of outer mitochondrial membrane 40 homolog (yeast) |
| Bt.1568.1.S1\_at | 287012 | COX5B | cytochrome c oxidase subunit Vb |
| Bt.15724.1.S1\_at | 506149 | C5H22orf32 | chromosome 22 open reading frame 32 ortholog |
| Bt.15870.1.S1\_at | 528165 | TIMM44 | translocase of inner mitochondrial membrane 44 homolog (yeast) |
| Bt.16025.1.S1\_at | 281090 | COX4 | cytochrome c oxydase subunit 4 |
| Bt.16082.1.S1\_at | 518321 | C1QBP | complement component 1, q subcomponent binding protein |
| Bt.16108.1.A1\_at | 508170 | MCEE | methylmalonyl CoA epimerase |
| Bt.16945.1.S1\_at | 509150 | ETHE1 | ethylmalonic encephalopathy 1 |
| Bt.1719.1.S1\_at | 520335 | OXA1L | oxidase (cytochrome c) assembly 1-like |
| Bt.1760.1.S1\_at | 540139 | REXO2 | REX2, RNA exonuclease 2 homolog (S. cerevisiae) |
| Bt.1808.1.S1\_at | 512500 | CYC1 | cytochrome c-1 |
| Bt.1819.1.S1\_at | 616109 | UCRC | ubiquinol-cytochrome c reductase complex 7.2 kDa protein |
| Bt.18634.1.A1\_at | 540329 | PPM1K | protein phosphatase 1K (PP2C domain containing) |
| Bt.18634.2.S1\_at | 540329 | PPM1K | protein phosphatase 1K (PP2C domain containing) |
| Bt.19415.1.A1\_at | 407109 | PDHA1 | pyruvate dehydrogenase (lipoamide) alpha 1 |
| Bt.19431.1.S1\_at | 445421 | MRPS14 | mitochondrial ribosomal protein S14 |
| Bt.19431.1.S1\_s\_at | 445421 | MRPS14 | mitochondrial ribosomal protein S14 |
| Bt.19581.1.A1\_at | 535290 | MRPS28 | mitochondrial ribosomal protein S28 |
| Bt.19676.2.S1\_a\_at | 516874 | TFB2M | transcription factor B2, mitochondrial |
| Bt.19692.1.S1\_at | 540298 | COQ3 | coenzyme Q3 homolog, methyltransferase (S. cerevisiae) |
| Bt.20.1.S1\_at | 338063 | NDUFA7 | NADH dehydrogenase (ubiquinone) 1 alpha subcomplex, 7, 14.5kDa |
| Bt.20025.1.S1\_at | 618609 | MRPL21 | mitochondrial ribosomal protein L21 |
| Bt.20243.2.S1\_at | 614713 | SLC25A26 | solute carrier family 25, member 26 |
| Bt.20485.1.S1\_at | 533746 | DPYSL2 | dihydropyrimidinase-like 2 |
| Bt.20487.1.S1\_at | 508216 | MRPL28 | mitochondrial ribosomal protein L28 |
| Bt.20862.1.A1\_at | 517539 | MTHFD2 | methylenetetrahydrofolate dehydrogenase (NADP+ dependent) 2, methenyltetrahydrofolate cyclohydrolase |
| Bt.20965.1.S1\_at | 530409 | PHB | prohibitin |
| Bt.21.1.S1\_at | 338046 | NDUFC2 | NADH dehydrogenase (ubiquinone) 1, subcomplex unknown, 2, 14.5kDa |
| Bt.21051.1.S1\_at | 514753 | MRPL50 | mitochondrial ribosomal protein L50 |
| Bt.21113.1.S1\_a\_at | 509459 | CPT1B | carnitine palmitoyltransferase 1B (muscle) |
| Bt.21151.1.S1\_at | 532203 | MRPL24 | mitochondrial ribosomal protein L24 |
| Bt.21272.1.S1\_at | 533918 | SH3GLB1 | SH3-domain GRB2-like endophilin B1 |
| Bt.2138.1.S1\_at | 282277 | MRPL43 | mitochondrial ribosomal protein L43 |
| Bt.2138.1.S1\_s\_at | 282277 | MRPL43 | mitochondrial ribosomal protein L43 |
| Bt.21435.1.S1\_at | 510324 | STOML2 | stomatin (EPB72)-like 2 |
| Bt.21584.1.S1\_at | 513530 | OXSM | 3-oxoacyl-ACP synthase, mitochondrial |
| Bt.21718.1.S1\_at | 509684 | SLC25A39 | solute carrier family 25, member 39 |
| Bt.21792.2.S1\_a\_at | 614102 | MRPS18A | mitochondrial ribosomal protein S18A |
| Bt.21997.1.S1\_at | 540329 | PPM1K | protein phosphatase 1K (PP2C domain containing) |
| Bt.21997.2.A1\_at | 540329 | PPM1K | protein phosphatase 1K (PP2C domain containing) |
| Bt.22047.1.S1\_at | 535981 | CLPP | ClpP caseinolytic peptidase, ATP-dependent, proteolytic subunit homolog (E. coli) |
| Bt.22197.1.S1\_at | 532389 | MRPL44 | mitochondrial ribosomal protein L44 |
| Bt.22379.1.S1\_at | 536925 | CABC1 | chaperone, ABC1 activity of bc1 complex homolog (S. pombe) |
| Bt.23002.1.S1\_at | 282199 | COX6A1 | cytochrome c oxidase subunit VIa polypeptide 1 |
| Bt.23002.1.S1\_s\_at | 282199 | COX6A1 | cytochrome c oxidase subunit VIa polypeptide 1 |
| Bt.23164.1.S1\_at | 282393 | UQCRC1 | UQCRC1 protein |
| Bt.23357.1.S1\_at | 614145 | IDH3G | isocitrate dehydrogenase 3 (NAD+) gamma |
| Bt.23361.1.S1\_at | 616871 | UQCRB | ubiquinol-cytochrome c reductase binding protein |
| Bt.23496.1.S1\_at | 767909 | LOC767909 | 6.8 kDa mitochondrial proteolipid |
| Bt.23526.1.S1\_at | 617466 | MRPS24 | mitochondrial ribosomal protein S24 |
| Bt.23569.1.S1\_at | 522006 | ACAA2 | acetyl-Coenzyme A acyltransferase 2 |
| Bt.24474.1.S1\_a\_at | 515757 | AFG3L2 | AFG3 ATPase family gene 3-like 2 (yeast) |
| Bt.24474.1.S1\_at | 515757 | AFG3L2 | AFG3 ATPase family gene 3-like 2 (yeast) |
| Bt.24624.1.S1\_at | 519302 | MRPS9 | mitochondrial ribosomal protein S9 |
| Bt.24624.2.S1\_at | 519302 | MRPS9 | mitochondrial ribosomal protein S9 |
| Bt.2493.1.S1\_at | 513622 | MRPL51 | mitochondrial ribosomal protein L51 |
| Bt.24931.1.A1\_at | 529762 | TMEM126A | transmembrane protein 126A |
| Bt.26150.1.A1\_at | 514230 | L2HGDH | L-2-hydroxyglutarate dehydrogenase |
| Bt.265.1.S1\_at | 287020 | UQCRFS1 | ubiquinol-cytochrome c reductase, Rieske iron-sulfur polypeptide 1 |
| Bt.26718.1.S1\_a\_at | 506250 | HCCS | holocytochrome c synthase |
| Bt.2711.1.S1\_at | 509983 | SUCLG1 | succinate-CoA ligase, alpha subunit |
| Bt.27116.2.S1\_at | 618131 | TIMM8B | translocase of inner mitochondrial membrane 8 homolog B (yeast) |
| Bt.27657.1.A1\_at | 338053 | ATP5G1 | ATP synthase, H+ transporting, mitochondrial F0 complex, subunit C1 (subunit 9) |
| Bt.2816.1.S1\_at | 515696 | ATP5L | ATP synthase, H+ transporting, mitochondrial F0 complex, subunit G |
| Bt.2825.1.S1\_at | 617901 | MRPL13 | mitochondrial ribosomal protein L13 |
| Bt.2894.1.S1\_at | 507245 | ECSIT | ECSIT homolog (Drosophila) |
| Bt.2973.2.S1\_a\_at | 613610 | PDHB | pyruvate dehydrogenase (lipoamide) beta |
| Bt.29746.1.S1\_at | 407109 | PDHA1 | pyruvate dehydrogenase (lipoamide) alpha 1 |
| Bt.298.1.S1\_at | 281742 | NDUFA12 | NADH dehydrogenase (ubiquinone) 1 alpha subcomplex, 12 |
| Bt.3117.1.S1\_at | 327660 | NDUFB9 | NADH dehydrogenase (ubiquinone) 1 beta subcomplex, 9, 22kDa |
| Bt.315.1.S1\_at | 338079 | NDUFS7 | NADH dehydrogenase (ubiquinone) Fe-S protein 7, 20kDa (NADH-coenzyme Q reductase) |
| Bt.321.1.S1\_at | 327702 | NDUFAB1 | NADH dehydrogenase (ubiquinone) 1, alpha/beta subcomplex, 1, 8kDa |
| Bt.3260.1.S1\_at | 614633 | C17H18orf55 | chromosome 18 open reading frame 55 ortholog |
| Bt.3288.1.A1\_at | 514374 | PIGY | phosphatidylinositol glycan anchor biosynthesis, class Y |
| Bt.3610.1.S1\_at | 613899 | UQCRH | ubiquinol-cytochrome c reductase hinge protein |
| Bt.371.1.S1\_at | 282478 | SLC25A4 | solute carrier family 25 (mitochondrial carrier; adenine nucleotide translocator), member 4 |
| Bt.378.1.S1\_at | 338040 | ATP5I | ATP synthase, H+ transporting, mitochondrial F0 complex, subunit e |
| Bt.380.1.S1\_at | 327699 | ATPIF1 | ATPase inhibitory factor 1 |
| Bt.3857.1.S1\_at | 327707 | ENDOG | endonuclease G |
| Bt.3867.1.S1\_at | 282701 | ATP5F1 | ATP synthase, H+ transporting, mitochondrial F0 complex, subunit B1 |
| Bt.3898.1.S1\_at | 282446 | IDH3A | isocitrate dehydrogenase 3 (NAD+) alpha |
| Bt.3922.1.S1\_a\_at | 617005 | MRPL38 | mitochondrial ribosomal protein L38 |
| Bt.3960.1.S1\_a\_at | 282119 | VDAC1 | voltage-dependent anion channel 1 |
| Bt.3960.2.S1\_at | 282119 | VDAC1 | voltage-dependent anion channel 1 |
| Bt.400.1.S1\_at | 327674 | COX7B | cytochrome c oxidase subunit VIIb |
| Bt.4008.1.S1\_at | 282389 | TXNRD2 | thioredoxin reductase 2 |
| Bt.404.1.S1\_at | 282690 | ATP5J | ATP synthase, H+ transporting, mitochondrial F0 complex, subunit F6 |
| Bt.4072.1.S1\_at | 287014 | NDUFV1 | NADH dehydrogenase (ubiquinone) flavoprotein 1, 51kDa |
| Bt.4099.1.S1\_at | 282120 | VDAC2 | voltage-dependent anion channel 2 |
| Bt.4118.1.A1\_at | 514472 | TRAP1 | TNF receptor-associated protein 1 |
| Bt.4203.1.S1\_at | 506492 | ATP5J2 | ATP synthase, H+ transporting, mitochondrial F0 complex, subunit F2 |
| Bt.4205.1.S1\_at | 286809 | GPX4 | glutathione peroxidase 4 (phospholipid hydroperoxidase) |
| Bt.4262.1.S1\_a\_at | 327717 | NDUFV3 | NADH dehydrogenase (ubiquinone) flavoprotein 3, 10kDa |
| Bt.4355.1.S1\_at | 282885 | PRDX5 | peroxiredoxin 5 |
| Bt.4402.1.S1\_at | 538054 | MRPL16 | mitochondrial ribosomal protein L16 |
| Bt.4431.1.S1\_a\_at | 327675 | ATP5B | ATP synthase, H+ transporting, mitochondrial F1 complex, beta polypeptide |
| Bt.4473.1.S1\_at | 338060 | NDUFA10 | NADH dehydrogenase (ubiquinone) 1 alpha subcomplex, 10, 42kDa |
| Bt.4475.1.S1\_at | 327697 | NDUFS2 | NADH dehydrogenase (ubiquinone) Fe-S protein 2, 49kDa (NADH-coenzyme Q reductase) |
| Bt.4476.1.S1\_at | 281998 | PRDX3 | peroxiredoxin 3 |
| Bt.4503.1.S1\_at | 337927 | MTCH2 | mitochondrial carrier homolog 2 (C. elegans) |
| Bt.4503.1.S2\_at | 337927 | MTCH2 | mitochondrial carrier homolog 2 (C. elegans) |
| Bt.4540.1.S1\_at | 327668 | ATP5C1 | ATP synthase, H+ transporting, mitochondrial F1 complex, gamma polypeptide 1 |
| Bt.4555.1.S1\_at | 617210 | ETFB | electron-transfer-flavoprotein, beta polypeptide |
| Bt.4652.1.S1\_at | 506657 | MRPL55 | mitochondrial ribosomal protein L55 |
| Bt.4704.1.S1\_at | 327714 | NDUFA5 | NADH dehydrogenase (ubiquinone) 1 alpha subcomplex, 5, 13kDa |
| Bt.4748.1.S1\_at | 281496 | SOD2 | superoxide dismutase 2, mitochondrial |
| Bt.4758.1.S1\_at | 281758 | FABP3 | fatty acid binding protein 3, muscle and heart (mammary-derived growth inhibitor) |
| Bt.4777.1.S1\_at | 288380 | NDUFS1 | NADH dehydrogenase (ubiquinone) Fe-S protein 1, 75kDa (NADH-coenzyme Q reductase) |
| Bt.4786.1.S2\_at | 281080 | CLTC | clathrin, heavy chain (Hc) |
| Bt.4819.1.S1\_at | 327704 | NDUFA4 | NADH dehydrogenase (ubiquinone) 1 alpha subcomplex, 4, 9kDa |
| Bt.4880.1.S1\_at | 282477 | SLC25A3 | solute carrier family 25 (mitochondrial carrier; phosphate carrier), member 3 |
| Bt.4900.1.A1\_at | 512469 | MRPS5 | mitochondrial ribosomal protein S5 |
| Bt.4948.1.S1\_at | 282705 | PSEN1 | presenilin 1 (Alzheimer disease 3) |
| Bt.4970.1.S1\_at | 327688 | COX7A2 | cytochrome c oxidase subunit VIIa polypeptide 2 (liver) |
| Bt.5029.1.S1\_at | 282710 | ATP5H | ATP synthase, H+ transporting, mitochondrial F0 complex, subunit d |
| Bt.5033.1.S1\_at | 281480 | SDHA | succinate dehydrogenase complex, subunit A, flavoprotein (Fp) |
| Bt.5040.1.S1\_at | 281570 | UQCR | ubiquinol-cytochrome c reductase (6.4kD) subunit |
| Bt.5042.1.S1\_at | 282394 | UQCRC2 | ubiquinol-cytochrome c reductase core protein II |
| Bt.5108.1.S1\_at | 515902 | PCCB | propionyl Coenzyme A carboxylase, beta polypeptide |
| Bt.5120.1.S1\_at | 280878 | NNT | nicotinamide nucleotide transhydrogenase |
| Bt.5210.1.S1\_at | 280976 | ACO2 | aconitase 2, mitochondrial |
| Bt.5278.1.A1\_at | 539713 | BCS1L | BCS1-like (yeast) |
| Bt.5278.2.S1\_at | 539713 | BCS1L | BCS1-like (yeast) |
| Bt.5288.1.S1\_at | 281557 | TXN2 | thioredoxin 2 |
| Bt.5344.1.S1\_at | 281556 | TUFM | Tu translation elongation factor, mitochondrial |
| Bt.5378.1.S1\_at | 282517 | NDUFB8 | NADH dehydrogenase (ubiquinone) 1 beta subcomplex, 8, 19kDa |
| Bt.5412.1.S1\_at | 282150 | BCKDHB | branched chain keto acid dehydrogenase E1, beta polypeptide (maple syrup urine disease) |
| Bt.5459.1.S1\_at | 534352 | MRPL18 | mitochondrial ribosomal protein L18 |
| Bt.5467.1.S2\_at | 281433 | PSAP | prosaposin (variant Gaucher disease and variant metachromatic leukodystrophy) |
| Bt.547.1.S1\_at | 286885 | UQCRQ | low molecular mass ubiquinone-binding protein (9.5kD) |
| Bt.5483.1.S1\_at | 287027 | NDUFS8 | NADH dehydrogenase (ubiquinone) Fe-S protein 8, 23kDa (NADH-coenzyme Q reductase) |
| Bt.5510.1.S1\_at | 338088 | ATP5E | ATP synthase, H+ transporting, mitochondrial F1 complex, epsilon subunit |
| Bt.5520.1.S1\_at | 327669 | IDH2 | isocitrate dehydrogenase 2 (NADP+), mitochondrial |
| Bt.5541.1.S1\_at | 404188 | NDUFA9 | NADH dehydrogenase (ubiquinone) 1 alpha subcomplex, 9, 39kDa |
| Bt.56.1.S1\_at | 327691 | NDUFS6 | NADH dehydrogenase (ubiquinone) Fe-S protein 6, 13kDa (NADH-coenzyme Q reductase) |
| Bt.566.1.S1\_at | 282200 | COX6A2 | cytochrome c oxidase subunit VIa polypeptide 2 |
| Bt.5676.1.S1\_at | 533208 | COQ5 | coenzyme Q5 homolog, methyltransferase (S. cerevisiae) |
| Bt.57.1.S1\_at | 338057 | NDUFS5 | NADH dehydrogenase (ubiquinone) Fe-S protein 5, 15kDa (NADH-coenzyme Q reductase) |
| Bt.5723.1.S1\_at | 326346 | NDUFA11 | NADH dehydrogenase (ubiquinone) 1 alpha subcomplex, 11, 14.7kDa |
| Bt.58.1.S1\_at | 327680 | NDUFS4 | NADH dehydrogenase (ubiquinone) Fe-S protein 4, 18kDa (NADH-coenzyme Q reductase) |
| Bt.59.1.S1\_at | 327710 | NDUFA8 | NADH dehydrogenase (ubiquinone) 1 alpha subcomplex, 8, 19kDa |
| Bt.5966.1.A1\_at | 505968 | ACADM | acyl-coenzyme A dehydrogenase, C-4 to C-12 straight chain |
| Bt.60.1.S1\_at | 327713 | NDUFB2 | NADH dehydrogenase (ubiquinone) 1 beta subcomplex, 2, 8kDa |
| Bt.61.1.S1\_at | 338073 | NDUFB3 | NADH dehydrogenase (ubiquinone) 1 beta subcomplex, 3, 12kDa |
| Bt.6116.1.S1\_a\_at | 523039 | HTRA2 | HtrA serine peptidase 2 |
| Bt.6171.1.A1\_at | 512002 | HIBADH | 3-hydroxyisobutyrate dehydrogenase |
| Bt.63.1.S1\_at | 327670 | NDUFA6 | NADH dehydrogenase (ubiquinone) 1 alpha subcomplex, 6, 14kDa |
| Bt.6390.1.S1\_at | 508143 | MRPS17 | mitochondrial ribosomal protein S17 |
| Bt.64.1.S1\_at | 338065 | NDUFB7 | NADH dehydrogenase (ubiquinone) 1 beta subcomplex, 7, 18kDa |
| Bt.6465.1.S1\_at | 510767 | CYCS | cytochrome c, somatic |
| Bt.6494.1.S1\_at | 404161 | ESSS | mitochondrial NADH:ubiquinone oxidoreductase ESSS subunit |
| Bt.65.1.S1\_at | 327698 | NDUFA2 | NADH dehydrogenase (ubiquinone) 1 alpha subcomplex, 2, 8kDa |
| Bt.66.1.S1\_at | 338064 | NDUFA3 | NADH dehydrogenase (ubiquinone) 1 alpha subcomplex, 3, 9kDa |
| Bt.67.1.S1\_at | 282289 | NDUFC1 | NADH dehydrogenase (ubiquinone) 1, subcomplex unknown, 1, 6kDa |
| Bt.68.1.S1\_at | 327690 | NDUFB1 | NADH dehydrogenase (ubiquinone) 1 beta subcomplex, 1, 7kDa |
| Bt.69.1.S1\_at | 327673 | NDUFA1 | NADH dehydrogenase (ubiquinone) 1 alpha subcomplex, 1, 7.5kDa |
| Bt.70.1.S1\_at | 327701 | NDUFB10 | NADH dehydrogenase (ubiquinone) 1 beta subcomplex, 10, 22kDa |
| Bt.7139.1.S1\_at | 781575 | TOMM20 | translocase of outer mitochondrial membrane 20 homolog (yeast) |
| Bt.7143.1.S1\_at | 510899 | MRPS16 | mitochondrial ribosomal protein S16 |
| Bt.7177.1.S1\_at | 280719 | AMT | aminomethyltransferase |
| Bt.7215.1.S1\_at | 282130 | ACADVL | acyl-coenzyme A dehydrogenase, very long chain |
| Bt.7237.2.S1\_a\_at | 281810 | HADHA | hydroxyacyl-Coenzyme A dehydrogenase/3-ketoacyl-Coenzyme A thiolase/enoyl-Coenzyme A hydratase (trifunctional protein), alpha subunit |
| Bt.7240.1.S1\_at | 781648 | LAP3 | leucine aminopeptidase 3 |
| Bt.7266.1.S1\_at | 515109 | TIMM8A | translocase of inner mitochondrial membrane 8 homolog A (yeast) |
| Bt.75.1.S1\_at | 338087 | COX6B | cytochrome c oxidase subunit VIb |
| Bt.76.1.S1\_at | 338086 | COX7A1 | cytochrome c oxidase subunit VIIa polypeptide 1 (muscle) |
| Bt.77.1.S1\_at | 327718 | COX7C | cytochrome c oxidase subunit VIIc |
| Bt.7856.1.S1\_at | 505005 | BCKDK | branched chain ketoacid dehydrogenase kinase |
| Bt.7915.1.S1\_at | 281306 | MDH2 | malate dehydrogenase 2, NAD (mitochondrial) |
| Bt.8046.1.A1\_at | 613835 | MRPS36 | mitochondrial ribosomal protein S36 |
| Bt.8047.1.S1\_at | 540176 | ATP5G3 | ATP synthase, H+ transporting, mitochondrial F0 complex, subunit C3 (subunit 9) |
| Bt.848.1.S1\_at | 282290 | NDUFV2 | NADH dehydrogenase (ubiquinone) flavoprotein 2, 24kDa |
| Bt.888.1.S1\_at | 534615 | BNIP3L | BCL2/adenovirus E1B 19kDa interacting protein 3-like |
| Bt.893.1.S1\_at | 327665 | NDUFB6 | NADH dehydrogenase (ubiquinone) 1 beta subcomplex, 6, 17kDa |
| Bt.8950.1.S1\_at | 338084 | NDUFA13 | NADH dehydrogenase (ubiquinone) 1 alpha subcomplex, 13 |
| Bt.9324.1.S1\_at | 509816 | MRPS11 | mitochondrial ribosomal protein S11 |
| Bt.9362.1.S1\_at | 514403 | MRPL2 | mitochondrial ribosomal protein L2 |
| Bt.946.2.A1\_a\_at | 280716 | AK2 | adenylate kinase 2 |
| Bt.9731.1.S1\_at | 513762 | GLRX2 | glutaredoxin 2 |
| Bt.9756.1.S1\_at | 530865 | LIAS | lipoic acid synthetase |
| Bt.9797.1.S1\_at | 534546 | PMPCB | peptidase (mitochondrial processing) beta |
| Bt.981.2.S1\_at | 286823 | CLIC4 | chloride intracellular channel 4 |
| Bt.9825.1.S1\_at | 540278 | MRPL35 | mitochondrial ribosomal protein L35 |
| Bt.9826.1.S1\_at | 444878 | COX5A | COX5A protein |
| Bt.9857.1.S1\_at | 616477 | CHCHD3 | coiled-coil-helix-coiled-coil-helix domain containing 3 |
| Bt.99.1.S1\_at | 327706 | NDUFB4 | NADH dehydrogenase (ubiquinone) 1 beta subcomplex, 4, 15kDa |

### GO:0045259

|  |  |  |  |
| --- | --- | --- | --- |
| **probe** | **entrez** | **symbol** | **description** |
| Bt.1242.1.S1\_at | 338053 | ATP5G1 | ATP synthase, H+ transporting, mitochondrial F0 complex, subunit C1 (subunit 9) |
| Bt.1426.1.S1\_at | 338081 | ATP5D | ATP synthase, H+ transporting, mitochondrial F1 complex, delta subunit |
| Bt.27657.1.A1\_at | 338053 | ATP5G1 | ATP synthase, H+ transporting, mitochondrial F0 complex, subunit C1 (subunit 9) |
| Bt.2816.1.S1\_at | 515696 | ATP5L | ATP synthase, H+ transporting, mitochondrial F0 complex, subunit G |
| Bt.378.1.S1\_at | 338040 | ATP5I | ATP synthase, H+ transporting, mitochondrial F0 complex, subunit e |
| Bt.3867.1.S1\_at | 282701 | ATP5F1 | ATP synthase, H+ transporting, mitochondrial F0 complex, subunit B1 |
| Bt.404.1.S1\_at | 282690 | ATP5J | ATP synthase, H+ transporting, mitochondrial F0 complex, subunit F6 |
| Bt.4203.1.S1\_at | 506492 | ATP5J2 | ATP synthase, H+ transporting, mitochondrial F0 complex, subunit F2 |
| Bt.4431.1.S1\_a\_at | 327675 | ATP5B | ATP synthase, H+ transporting, mitochondrial F1 complex, beta polypeptide |
| Bt.4540.1.S1\_at | 327668 | ATP5C1 | ATP synthase, H+ transporting, mitochondrial F1 complex, gamma polypeptide 1 |
| Bt.5029.1.S1\_at | 282710 | ATP5H | ATP synthase, H+ transporting, mitochondrial F0 complex, subunit d |
| Bt.8047.1.S1\_at | 540176 | ATP5G3 | ATP synthase, H+ transporting, mitochondrial F0 complex, subunit C3 (subunit 9) |

### GO:0005758

|  |  |  |  |
| --- | --- | --- | --- |
| **probe** | **entrez** | **symbol** | **description** |
| Bt.11014.1.S1\_at | 513889 | CHCHD4 | coiled-coil-helix-coiled-coil-helix domain containing 4 |
| Bt.1175.1.S1\_at | 521073 | TIMM9 | translocase of inner mitochondrial membrane 9 homolog (yeast) |
| Bt.13784.1.S1\_at | 512818 | TIMM10 | translocase of inner mitochondrial membrane 10 homolog (yeast) |
| Bt.27116.2.S1\_at | 618131 | TIMM8B | translocase of inner mitochondrial membrane 8 homolog B (yeast) |
| Bt.4777.1.S1\_at | 288380 | NDUFS1 | NADH dehydrogenase (ubiquinone) Fe-S protein 1, 75kDa (NADH-coenzyme Q reductase) |
| Bt.6116.1.S1\_a\_at | 523039 | HTRA2 | HtrA serine peptidase 2 |
| Bt.7266.1.S1\_at | 515109 | TIMM8A | translocase of inner mitochondrial membrane 8 homolog A (yeast) |
| Bt.75.1.S1\_at | 338087 | COX6B | cytochrome c oxidase subunit VIb |
| Bt.946.2.A1\_a\_at | 280716 | AK2 | adenylate kinase 2 |

### GO:0005932

|  |  |  |  |
| --- | --- | --- | --- |
| **probe** | **entrez** | **symbol** | **description** |
| Bt.20402.1.S1\_at | 530393 | PKD2 | polycystic kidney disease 2 (autosomal dominant) |
| Bt.20402.2.A1\_at | 530393 | PKD2 | polycystic kidney disease 2 (autosomal dominant) |
| Bt.20402.3.S1\_at | 530393 | PKD2 | polycystic kidney disease 2 (autosomal dominant) |
| Bt.3583.1.S1\_at | 281574 | EZR | ezrin |

### GO:0000502

|  |  |  |  |
| --- | --- | --- | --- |
| **probe** | **entrez** | **symbol** | **description** |
| Bt.10266.1.S1\_at | 515503 | PSMA1 | proteasome (prosome, macropain) subunit, alpha type, 1 |
| Bt.13237.1.S1\_at | 510423 | PSMA4 | proteasome (prosome, macropain) subunit, alpha type, 4 |
| Bt.15837.1.S1\_at | 507213 | PSMA6 | proteasome (prosome, macropain) subunit, alpha type, 6 |
| Bt.17968.1.S1\_at | 512891 | PSMD8 | proteasome (prosome, macropain) 26S subunit, non-ATPase, 8 |
| Bt.2116.1.S1\_at | 510069 | PSMB6 | proteasome (prosome, macropain) subunit, beta type, 6 |
| Bt.24017.1.A1\_s\_at | 530186 | PSMC2 | proteasome (prosome, macropain) 26S subunit, ATPase, 2 |
| Bt.2821.1.S1\_at | 511207 | PSMB7 | proteasome (prosome, macropain) subunit, beta type, 7 |
| Bt.4756.1.S1\_at | 530186 | PSMC2 | proteasome (prosome, macropain) 26S subunit, ATPase, 2 |
| Bt.5322.1.S1\_at | 534640 | PSMB5 | proteasome (prosome, macropain) subunit, beta type, 5 |
| Bt.6473.1.S1\_at | 505050 | PSMA7 | proteasome (prosome, macropain) subunit, alpha type, 7 |
| Bt.6827.1.S1\_at | 505176 | PSMA3 | proteasome (prosome, macropain) subunit, alpha type, 3 |
| Bt.8062.1.S1\_at | 282015 | PSMC5 | proteasome (prosome, macropain) 26S subunit, ATPase, 5 |
| Bt.886.1.S1\_at | 510155 | PSMA5 | proteasome (prosome, macropain) subunit, alpha type, 5 |
| Bt.9233.3.S1\_at | 519729 | ADRM1 | adhesion regulating molecule 1 |

### GO:0016529

|  |  |  |  |
| --- | --- | --- | --- |
| **probe** | **entrez** | **symbol** | **description** |
| Bt.1310.1.S1\_at | 532775 | TMEM38A | transmembrane protein 38A |
| Bt.5302.1.S1\_a\_at | 281918 | MRVI1 | murine retrovirus integration site 1 homolog |
| Bt.5848.1.S1\_at | 518117 | ATP2A1 | ATPase, Ca++ transporting, cardiac muscle, fast twitch 1 |
| Bt.5848.2.S1\_at | 518117 | ATP2A1 | ATPase, Ca++ transporting, cardiac muscle, fast twitch 1 |

### GO:0045239

|  |  |  |  |
| --- | --- | --- | --- |
| **probe** | **entrez** | **symbol** | **description** |
| Bt.2587.1.S1\_at | 520260 | FH | fumarate hydratase |
| Bt.2587.2.S1\_a\_at | 520260 | FH | fumarate hydratase |
| Bt.7856.1.S1\_at | 505005 | BCKDK | branched chain ketoacid dehydrogenase kinase |

### GO:0001931

|  |  |  |  |
| --- | --- | --- | --- |
| **probe** | **entrez** | **symbol** | **description** |
| Bt.3583.1.S1\_at | 281574 | EZR | ezrin |
| Bt.9207.1.S1\_at | 540426 | MSN | moesin |

### GO:0035253

|  |  |  |  |
| --- | --- | --- | --- |
| **probe** | **entrez** | **symbol** | **description** |
| Bt.13152.1.S1\_s\_at | 280722 | APP | amyloid beta (A4) precursor protein (peptidase nexin-II, Alzheimer disease) |
| Bt.9559.2.S1\_at | 280722 | APP | amyloid beta (A4) precursor protein (peptidase nexin-II, Alzheimer disease) |

### GO:0030132

|  |  |  |  |
| --- | --- | --- | --- |
| **probe** | **entrez** | **symbol** | **description** |
| Bt.10.2.S1\_a\_at | 281698 | CLTLB | clathrin, light polypeptide B (light chain B) |
| Bt.1617.2.S1\_a\_at | 281078 | CLTA | clathrin, light chain (Lca) |
| Bt.4786.1.S2\_at | 281080 | CLTC | clathrin, heavy chain (Hc) |

### GO:0008287

|  |  |  |  |
| --- | --- | --- | --- |
| **probe** | **entrez** | **symbol** | **description** |
| Bt.11058.1.A1\_at | 280891 | PPM2C | protein phosphatase 2C, magnesium-dependent, catalytic subunit |
| Bt.18634.1.A1\_at | 540329 | PPM1K | protein phosphatase 1K (PP2C domain containing) |
| Bt.18634.2.S1\_at | 540329 | PPM1K | protein phosphatase 1K (PP2C domain containing) |
| Bt.21997.1.S1\_at | 540329 | PPM1K | protein phosphatase 1K (PP2C domain containing) |
| Bt.21997.2.A1\_at | 540329 | PPM1K | protein phosphatase 1K (PP2C domain containing) |

### GO:0005737

|  |  |  |  |
| --- | --- | --- | --- |
| **probe** | **entrez** | **symbol** | **description** |
| Bt.10.2.S1\_a\_at | 281698 | CLTLB | clathrin, light polypeptide B (light chain B) |
| Bt.10135.1.A1\_at | 534488 | SLC35A5 | solute carrier family 35, member A5 |
| Bt.10154.1.S1\_at | 531444 | CISD1 | CDGSH iron sulfur domain 1 |
| Bt.10266.1.S1\_at | 515503 | PSMA1 | proteasome (prosome, macropain) subunit, alpha type, 1 |
| Bt.10324.1.A1\_at | 327714 | NDUFA5 | NADH dehydrogenase (ubiquinone) 1 alpha subcomplex, 5, 13kDa |
| Bt.10657.1.A1\_at | 515233 | EEF1A2 | eukaryotic translation elongation factor 1 alpha 2 |
| Bt.10880.1.S1\_at | 505489 | TIMM50 | translocase of inner mitochondrial membrane 50 homolog (S. cerevisiae) |
| Bt.11014.1.S1\_at | 513889 | CHCHD4 | coiled-coil-helix-coiled-coil-helix domain containing 4 |
| Bt.11058.1.A1\_at | 280891 | PPM2C | protein phosphatase 2C, magnesium-dependent, catalytic subunit |
| Bt.11149.1.S1\_at | 280955 | VIM | vimentin |
| Bt.11171.1.S1\_at | 526652 | FLCN | folliculin |
| Bt.11194.1.S1\_at | 281360 | DHRS4 | dehydrogenase/reductase (SDR family) member 4 |
| Bt.11196.1.S1\_at | 281816 | HINT2 | histidine triad nucleotide binding protein 2 |
| Bt.1125.1.S1\_at | 539003 | CTNNB1 | catenin (cadherin-associated protein), beta 1, 88kDa |
| Bt.11262.1.S1\_at | 444864 | PEG3 | paternally expressed 3 |
| Bt.11262.2.S1\_at | 444864 | PEG3 | paternally expressed 3 |
| Bt.1129.1.S1\_at | 280682 | CS | citrate synthase |
| Bt.1129.2.S1\_at | 280682 | CS | citrate synthase |
| Bt.1131.1.S1\_at | 281419 | PPIB | peptidylprolyl isomerase B (cyclophilin B) |
| Bt.11339.1.S1\_at | 514740 | MRPS27 | mitochondrial ribosomal protein S27 |
| Bt.11476.1.S1\_at | 516209 | LFNG | LFNG O-fucosylpeptide 3-beta-N-acetylglucosaminyltransferase |
| Bt.1151.1.S1\_at | 493998 | CRABP2 | cellular retinoic acid binding protein 2 |
| Bt.11516.1.S1\_at | 407109 | PDHA1 | pyruvate dehydrogenase (lipoamide) alpha 1 |
| Bt.1153.1.S1\_at | 287327 | NDUFS3 | NADH dehydrogenase (ubiquinone) Fe-S protein 3, 30kDa (NADH-coenzyme Q reductase) |
| Bt.1154.1.S1\_at | 533223 | WDR1 | WD repeat domain 1 |
| Bt.11659.1.S1\_at | 615666 | MRP63 | mitochondrial ribosomal protein 63 |
| Bt.1175.1.S1\_at | 521073 | TIMM9 | translocase of inner mitochondrial membrane 9 homolog (yeast) |
| Bt.11770.1.S1\_a\_at | 518758 | SLC25A20 | solute carrier family 25 (carnitine/acylcarnitine translocase), member 20 |
| Bt.11770.1.S1\_at | 518758 | SLC25A20 | solute carrier family 25 (carnitine/acylcarnitine translocase), member 20 |
| Bt.12303.1.S1\_at | 280890 | PAM | peptidylglycine alpha-amidating monooxygenase |
| Bt.12379.1.S1\_at | 617775 | MGC139265 | similar to Prostate tumor over expressed gene 1 |
| Bt.1240.1.S1\_at | 282218 | DNASE2 | deoxyribonuclease II, lysosomal |
| Bt.1242.1.S1\_at | 338053 | ATP5G1 | ATP synthase, H+ transporting, mitochondrial F0 complex, subunit C1 (subunit 9) |
| Bt.12429.1.S1\_at | 515380 | EDF1 | endothelial differentiation-related factor 1 |
| Bt.12498.1.A1\_at | 530657 | COL6A3 | collagen, type VI, alpha 3 |
| Bt.12514.1.S1\_at | 515363 | PHB2 | prohibitin 2 |
| Bt.1280.2.S1\_a\_at | 282523 | SLC25A11 | solute carrier family 25 (mitochondrial carrier; oxoglutarate carrier), member 11 |
| Bt.12820.1.S1\_at | 317706 | PGRMC1 | progesterone receptor membrane component 1 |
| Bt.1283.1.S1\_at | 360196 | BANF1 | barrier to autointegration factor 1 |
| Bt.12888.1.S1\_at | 613305 | MIF4GD | MIF4G domain containing |
| Bt.12906.1.S1\_at | 535077 | GSN | gelsolin (amyloidosis, Finnish type) |
| Bt.12910.1.S1\_at | 534599 | OGDH | oxoglutarate (alpha-ketoglutarate) dehydrogenase (lipoamide) |
| Bt.12912.1.S1\_at | 282191 | COL4A1 | collagen, type IV, alpha 1 |
| Bt.12912.1.S2\_at | 282191 | COL4A1 | collagen, type IV, alpha 1 |
| Bt.1310.1.S1\_at | 532775 | TMEM38A | transmembrane protein 38A |
| Bt.13128.1.S1\_at | 286840 | SDHB | succinate dehydrogenase complex, subunit B, iron sulfur (Ip) |
| Bt.13152.1.S1\_s\_at | 280722 | APP | amyloid beta (A4) precursor protein (peptidase nexin-II, Alzheimer disease) |
| Bt.1316.1.S1\_at | 286886 | GOT2 | glutamic-oxaloacetic transaminase 2, mitochondrial (aspartate aminotransferase 2) |
| Bt.1316.1.S2\_at | 286886 | GOT2 | glutamic-oxaloacetic transaminase 2, mitochondrial (aspartate aminotransferase 2) |
| Bt.13170.1.S1\_at | 327696 | SDHC | succinate dehydrogenase complex, subunit C, integral membrane protein, 15kDa |
| Bt.13199.1.S1\_at | 538829 | PPP1CB | protein phosphatase 1, catalytic subunit, beta isoform |
| Bt.13205.1.A1\_at | 513438 | MRPS35 | mitochondrial ribosomal protein S35 |
| Bt.13205.2.S1\_at | 513438 | MRPS35 | mitochondrial ribosomal protein S35 |
| Bt.1322.1.S1\_at | 509037 | TXNDC1 | thioredoxin domain containing 1 |
| Bt.13237.1.S1\_at | 510423 | PSMA4 | proteasome (prosome, macropain) subunit, alpha type, 4 |
| Bt.13249.1.A1\_at | 617419 | SCD5 | stearoyl-CoA desaturase 5 |
| Bt.13287.1.S1\_at | 515186 | AGXT2L1 | alanine-glyoxylate aminotransferase 2-like 1 |
| Bt.13324.2.S1\_a\_at | 281235 | IDH1 | isocitrate dehydrogenase 1 (NADP+), soluble |
| Bt.13439.1.S1\_at | 768074 | ETFDH | electron-transferring-flavoprotein dehydrogenase |
| Bt.13483.1.A1\_at | 505430 | ATPAF1 | ATP synthase mitochondrial F1 complex assembly factor 1 |
| Bt.13505.1.S1\_at | 280808 | GPI | glucose phosphate isomerase |
| Bt.136.1.S1\_at | 281563 | UCP3 | uncoupling protein 3 (mitochondrial, proton carrier) |
| Bt.13610.1.A1\_at | 534185 | MRPS31 | mitochondrial ribosomal protein S31 |
| Bt.13730.1.A1\_at | 408003 | ATG4A | ATG4 autophagy related 4 homolog A (S. cerevisiae) |
| Bt.13730.2.S1\_at | 408003 | ATG4A | ATG4 autophagy related 4 homolog A (S. cerevisiae) |
| Bt.13780.1.A1\_at | 507095 | SPRY1 | sprouty homolog 1, antagonist of FGF signaling (Drosophila) |
| Bt.13784.1.S1\_at | 512818 | TIMM10 | translocase of inner mitochondrial membrane 10 homolog (yeast) |
| Bt.13850.3.S1\_a\_at | 539992 | LYPLA1 | lysophospholipase I |
| Bt.1386.1.S1\_at | 507869 | TPR | translocated promoter region (to activated MET oncogene) |
| Bt.13900.1.S1\_at | 616748 | TRAPPC2 | trafficking protein particle complex 2 |
| Bt.13908.1.S1\_at | 532044 | MRPS22 | mitochondrial ribosomal protein S22 |
| Bt.13980.1.A1\_at | 538944 | CKMT2 | creatine kinase, mitochondrial 2 (sarcomeric) |
| Bt.13980.2.A1\_a\_at | 538944 | CKMT2 | creatine kinase, mitochondrial 2 (sarcomeric) |
| Bt.1403.1.S1\_at | 280878 | NNT | nicotinamide nucleotide transhydrogenase |
| Bt.14040.1.S1\_at | 506263 | PTGR2 | prostaglandin reductase 2 |
| Bt.1415.1.S1\_at | 515990 | MRPL11 | mitochondrial ribosomal protein L11 |
| Bt.14207.1.S1\_at | 319141 | GCAT | glycine C-acetyltransferase (2-amino-3-ketobutyrate coenzyme A ligase) |
| Bt.1426.1.S1\_at | 338081 | ATP5D | ATP synthase, H+ transporting, mitochondrial F1 complex, delta subunit |
| Bt.1430.1.S1\_at | 282848 | PFDN5 | prefoldin subunit 5 |
| Bt.14624.1.A1\_at | 535683 | PSMD14 | proteasome (prosome, macropain) 26S subunit, non-ATPase, 14 |
| Bt.14624.2.S1\_at | 535683 | PSMD14 | proteasome (prosome, macropain) 26S subunit, non-ATPase, 14 |
| Bt.14644.1.A1\_at | 614630 | CTNNBIP1 | catenin, beta interacting protein 1 |
| Bt.14853.1.A1\_at | 533284 | RAB3IP | RAB3A interacting protein (rabin3) |
| Bt.1523.1.S1\_at | 510219 | TOMM40 | translocase of outer mitochondrial membrane 40 homolog (yeast) |
| Bt.1552.1.S1\_at | 281476 | SARS | seryl-tRNA synthetase |
| Bt.15666.2.S1\_at | 508609 | PXMP2 | peroxisomal membrane protein 2, 22kDa |
| Bt.1568.1.S1\_at | 287012 | COX5B | cytochrome c oxidase subunit Vb |
| Bt.15724.1.S1\_at | 506149 | C5H22orf32 | chromosome 22 open reading frame 32 ortholog |
| Bt.15735.1.S1\_at | 525795 | AGRN | agrin |
| Bt.1582.1.S1\_at | 615447 | NME2 | non-metastatic cells 2, protein (NM23B) expressed in |
| Bt.15836.1.S1\_at | 510376 | ANKRD1 | ankyrin repeat domain 1 (cardiac muscle) |
| Bt.15836.2.S1\_at | 510376 | ANKRD1 | ankyrin repeat domain 1 (cardiac muscle) |
| Bt.15837.1.S1\_at | 507213 | PSMA6 | proteasome (prosome, macropain) subunit, alpha type, 6 |
| Bt.15870.1.S1\_at | 528165 | TIMM44 | translocase of inner mitochondrial membrane 44 homolog (yeast) |
| Bt.15972.1.S1\_at | 281193 | GJA1 | gap junction protein, alpha 1, 43kDa |
| Bt.16004.1.A1\_at | 281339 | MYH8 | myosin, heavy chain 8, skeletal muscle, perinatal |
| Bt.16025.1.S1\_at | 281090 | COX4 | cytochrome c oxydase subunit 4 |
| Bt.16040.1.S1\_at | 614672 | SH3BGRL3 | SH3 domain binding glutamic acid-rich protein like 3 |
| Bt.1606.1.S1\_at | 404069 | SPG21 | spastic paraplegia 21 (autosomal recessive, Mast syndrome) |
| Bt.16082.1.S1\_at | 518321 | C1QBP | complement component 1, q subcomponent binding protein |
| Bt.16092.1.S1\_at | 281190 | GGCX | gamma-glutamyl carboxylase |
| Bt.16108.1.A1\_at | 508170 | MCEE | methylmalonyl CoA epimerase |
| Bt.16137.1.S1\_at | 537539 | ALDH9A1 | aldehyde dehydrogenase 9 family, member A1 |
| Bt.16142.2.S1\_at | 286862 | YWHAG | tyrosine 3-monooxygenase/tryptophan 5-monooxygenase activation protein, gamma polypeptide |
| Bt.1617.2.S1\_a\_at | 281078 | CLTA | clathrin, light chain (Lca) |
| Bt.16437.1.S1\_at | 614576 | BIN1 | bridging integrator 1 |
| Bt.16495.2.A1\_at | 511602 | COL4A5 | collagen, type IV, alpha 5 (Alport syndrome) |
| Bt.16597.1.S1\_at | 618292 | EXOSC4 | exosome component 4 |
| Bt.16668.1.A1\_at | 539091 | TRIM45 | tripartite motif-containing 45 |
| Bt.16782.2.S1\_at | 784089 | ADSSL1 | adenylosuccinate synthase like 1 |
| Bt.16945.1.S1\_at | 509150 | ETHE1 | ethylmalonic encephalopathy 1 |
| Bt.1719.1.S1\_at | 520335 | OXA1L | oxidase (cytochrome c) assembly 1-like |
| Bt.1731.1.S1\_at | 282215 | DNAJB6 | DnaJ (Hsp40) homolog, subfamily B, member 6 |
| Bt.1734.1.S1\_at | 616278 | ERLIN2 | ER lipid raft associated 2 |
| Bt.1758.1.S1\_at | 512161 | HMGN4 | high mobility group nucleosomal binding domain 4 |
| Bt.1760.1.S1\_at | 540139 | REXO2 | REX2, RNA exonuclease 2 homolog (S. cerevisiae) |
| Bt.1767.1.S1\_at | 281225 | UBE2K | ubiquitin-conjugating enzyme E2K (UBC1 homolog, yeast) |
| Bt.177.1.S1\_at | 282032 | RASA1 | RAS p21 protein activator (GTPase activating protein) 1 |
| Bt.17942.1.S1\_at | 616148 | LBH | limb bud and heart development homolog (mouse) |
| Bt.17963.1.A1\_at | 517345 | ARL1 | ADP-ribosylation factor-like 1 |
| Bt.17968.1.S1\_at | 512891 | PSMD8 | proteasome (prosome, macropain) 26S subunit, non-ATPase, 8 |
| Bt.17989.1.S1\_at | 533348 | PAAF1 | proteasomal ATPase-associated factor 1 |
| Bt.1808.1.S1\_at | 512500 | CYC1 | cytochrome c-1 |
| Bt.1819.1.S1\_at | 616109 | UCRC | ubiquinol-cytochrome c reductase complex 7.2 kDa protein |
| Bt.18623.1.A1\_at | 510159 | MAPRE2 | microtubule-associated protein, RP/EB family, member 2 |
| Bt.18634.1.A1\_at | 540329 | PPM1K | protein phosphatase 1K (PP2C domain containing) |
| Bt.18634.2.S1\_at | 540329 | PPM1K | protein phosphatase 1K (PP2C domain containing) |
| Bt.18702.1.A1\_at | 523683 | CUX1 | cut-like homeobox 1 |
| Bt.19209.2.S1\_at | 520918 | SLC35A3 | solute carrier family 35 (UDP-N-acetylglucosamine (UDP-GlcNAc) transporter), member A3 |
| Bt.1932.1.S1\_at | 539220 | AHSA1 | AHA1, activator of heat shock 90kDa protein ATPase homolog 1 (yeast) |
| Bt.19415.1.A1\_at | 407109 | PDHA1 | pyruvate dehydrogenase (lipoamide) alpha 1 |
| Bt.19431.1.S1\_at | 445421 | MRPS14 | mitochondrial ribosomal protein S14 |
| Bt.19431.1.S1\_s\_at | 445421 | MRPS14 | mitochondrial ribosomal protein S14 |
| Bt.19522.2.S1\_at | 534708 | RAB11A | RAB11A, member RAS oncogene family |
| Bt.19581.1.A1\_at | 535290 | MRPS28 | mitochondrial ribosomal protein S28 |
| Bt.1967.1.S1\_at | 618855 | NUDT3 | nudix (nucleoside diphosphate linked moiety X)-type motif 3 |
| Bt.19676.2.S1\_a\_at | 516874 | TFB2M | transcription factor B2, mitochondrial |
| Bt.19692.1.S1\_at | 540298 | COQ3 | coenzyme Q3 homolog, methyltransferase (S. cerevisiae) |
| Bt.19807.1.S1\_at | 616545 | SKAP2 | src kinase associated phosphoprotein 2 |
| Bt.1998.1.S1\_at | 100034674 | NGFRAP1 | nerve growth factor receptor (TNFRSF16) associated protein 1 |
| Bt.20.1.S1\_at | 338063 | NDUFA7 | NADH dehydrogenase (ubiquinone) 1 alpha subcomplex, 7, 14.5kDa |
| Bt.20025.1.S1\_at | 618609 | MRPL21 | mitochondrial ribosomal protein L21 |
| Bt.20113.1.S1\_at | 538409 | CASP6 | caspase 6, apoptosis-related cysteine peptidase |
| Bt.20129.1.S1\_at | 523683 | CUX1 | cut-like homeobox 1 |
| Bt.20129.2.S1\_at | 523683 | CUX1 | cut-like homeobox 1 |
| Bt.20228.1.S1\_at | 359720 | RTN2 | reticulon 2 |
| Bt.20230.1.S1\_at | 445543 | JUP | junction plakoglobin |
| Bt.20243.2.S1\_at | 614713 | SLC25A26 | solute carrier family 25, member 26 |
| Bt.20261.1.S1\_at | 511010 | PTPN3 | protein tyrosine phosphatase, non-receptor type 3 |
| Bt.20275.1.S1\_at | 613421 | ARPC5L | actin related protein 2/3 complex, subunit 5-like |
| Bt.20307.1.S1\_at | 506882 | DNPEP | aspartyl aminopeptidase |
| Bt.20355.1.S1\_at | 505840 | RILPL1 | Rab interacting lysosomal protein-like 1 |
| Bt.20361.2.A1\_at | 511007 | FBXL20 | F-box and leucine-rich repeat protein 20 |
| Bt.20402.1.S1\_at | 530393 | PKD2 | polycystic kidney disease 2 (autosomal dominant) |
| Bt.20402.2.A1\_at | 530393 | PKD2 | polycystic kidney disease 2 (autosomal dominant) |
| Bt.20402.3.S1\_at | 530393 | PKD2 | polycystic kidney disease 2 (autosomal dominant) |
| Bt.20411.1.S1\_at | 539332 | CFL2 | cofilin 2 (muscle) |
| Bt.20485.1.S1\_at | 533746 | DPYSL2 | dihydropyrimidinase-like 2 |
| Bt.20487.1.S1\_at | 508216 | MRPL28 | mitochondrial ribosomal protein L28 |
| Bt.2070.1.A1\_at | 541175 | DUSP10 | dual specificity phosphatase 10 |
| Bt.2070.2.A1\_at | 541175 | DUSP10 | dual specificity phosphatase 10 |
| Bt.2070.3.S1\_at | 541175 | DUSP10 | dual specificity phosphatase 10 |
| Bt.20862.1.A1\_at | 517539 | MTHFD2 | methylenetetrahydrofolate dehydrogenase (NADP+ dependent) 2, methenyltetrahydrofolate cyclohydrolase |
| Bt.20895.1.S1\_at | 767980 | SSR3 | signal sequence receptor, gamma (translocon-associated protein gamma) |
| Bt.20965.1.S1\_at | 530409 | PHB | prohibitin |
| Bt.21.1.S1\_at | 338046 | NDUFC2 | NADH dehydrogenase (ubiquinone) 1, subcomplex unknown, 2, 14.5kDa |
| Bt.21051.1.S1\_at | 514753 | MRPL50 | mitochondrial ribosomal protein L50 |
| Bt.211.1.S1\_at | 286770 | DNAJC3 | DnaJ (Hsp40) homolog, subfamily C, member 3 |
| Bt.2110.1.S1\_at | 510200 | DPP3 | dipeptidyl-peptidase 3 |
| Bt.21113.1.S1\_a\_at | 509459 | CPT1B | carnitine palmitoyltransferase 1B (muscle) |
| Bt.21151.1.S1\_at | 532203 | MRPL24 | mitochondrial ribosomal protein L24 |
| Bt.2116.1.S1\_at | 510069 | PSMB6 | proteasome (prosome, macropain) subunit, beta type, 6 |
| Bt.21272.1.S1\_at | 533918 | SH3GLB1 | SH3-domain GRB2-like endophilin B1 |
| Bt.21342.1.S1\_at | 618250 | S100A14 | S100 calcium binding protein A14 |
| Bt.2138.1.S1\_at | 282277 | MRPL43 | mitochondrial ribosomal protein L43 |
| Bt.2138.1.S1\_s\_at | 282277 | MRPL43 | mitochondrial ribosomal protein L43 |
| Bt.21410.1.S1\_at | 518169 | CTSA | cathepsin A |
| Bt.21435.1.S1\_at | 510324 | STOML2 | stomatin (EPB72)-like 2 |
| Bt.21485.1.S1\_at | 614042 | CDC42SE1 | CDC42 small effector 1 |
| Bt.2151.2.A1\_at | 282022 | PTGS1 | prostaglandin-endoperoxide synthase 1 (prostaglandin G/H synthase and cyclooxygenase) |
| Bt.21584.1.S1\_at | 513530 | OXSM | 3-oxoacyl-ACP synthase, mitochondrial |
| Bt.216.1.S1\_at | 286825 | WDR44 | WD repeat domain 44 |
| Bt.21718.1.S1\_at | 509684 | SLC25A39 | solute carrier family 25, member 39 |
| Bt.21745.1.S1\_at | 511268 | PARK7 | Parkinson disease (autosomal recessive, early onset) 7 |
| Bt.21761.1.S1\_a\_at | 505645 | TMOD4 | tropomodulin 4 (muscle) |
| Bt.21766.1.A1\_at | 533379 | MLF1 | myeloid leukemia factor 1 |
| Bt.21766.2.S1\_at | 533379 | MLF1 | myeloid leukemia factor 1 |
| Bt.21766.3.S1\_at | 533379 | MLF1 | myeloid leukemia factor 1 |
| Bt.21792.2.S1\_a\_at | 614102 | MRPS18A | mitochondrial ribosomal protein S18A |
| Bt.21827.2.S1\_at | 510889 | THOP1 | thimet oligopeptidase 1 |
| Bt.21997.1.S1\_at | 540329 | PPM1K | protein phosphatase 1K (PP2C domain containing) |
| Bt.21997.2.A1\_at | 540329 | PPM1K | protein phosphatase 1K (PP2C domain containing) |
| Bt.22002.1.S1\_at | 281759 | FABP4 | fatty acid binding protein 4, adipocyte |
| Bt.22047.1.S1\_at | 535981 | CLPP | ClpP caseinolytic peptidase, ATP-dependent, proteolytic subunit homolog (E. coli) |
| Bt.22082.1.S1\_at | 511291 | CHPT1 | choline phosphotransferase 1 |
| Bt.22197.1.S1\_at | 532389 | MRPL44 | mitochondrial ribosomal protein L44 |
| Bt.22198.1.S1\_at | 533917 | GSTA4 | glutathione S-transferase A4 |
| Bt.222.1.S1\_at | 281719 | CRYAB | crystallin, alpha B |
| Bt.2230.1.S1\_at | 281040 | CAV1 | caveolin 1, caveolae protein, 22kDa |
| Bt.2230.1.S2\_at | 281040 | CAV1 | caveolin 1, caveolae protein, 22kDa |
| Bt.2235.1.S1\_at | 281188 | GDI1 | GDP dissociation inhibitor 1 |
| Bt.2235.1.S2\_at | 281188 | GDI1 | GDP dissociation inhibitor 1 |
| Bt.22379.1.S1\_at | 536925 | CABC1 | chaperone, ABC1 activity of bc1 complex homolog (S. pombe) |
| Bt.22615.1.S1\_at | 613854 | PCMT1 | protein-L-isoaspartate (D-aspartate) O-methyltransferase |
| Bt.22615.2.A1\_a\_at | 613854 | PCMT1 | protein-L-isoaspartate (D-aspartate) O-methyltransferase |
| Bt.22854.1.S1\_at | 280740 | CA2 | carbonic anhydrase II |
| Bt.22981.1.S1\_at | 359716 | DNMT3A | DNA (cytosine-5-)-methyltransferase 3 alpha |
| Bt.23002.1.S1\_at | 282199 | COX6A1 | cytochrome c oxidase subunit VIa polypeptide 1 |
| Bt.23002.1.S1\_s\_at | 282199 | COX6A1 | cytochrome c oxidase subunit VIa polypeptide 1 |
| Bt.231.1.S1\_at | 280950 | TXN | thioredoxin |
| Bt.23127.1.S1\_at | 534927 | PRCP | prolylcarboxypeptidase (angiotensinase C) |
| Bt.23164.1.S1\_at | 282393 | UQCRC1 | UQCRC1 protein |
| Bt.23174.2.S1\_at | 515646 | CLIC1 | chloride intracellular channel 1 |
| Bt.23182.1.S1\_at | 281156 | FDPS | farnesyl diphosphate synthase (farnesyl pyrophosphate synthetase, dimethylallyltranstransferase, geranyltranstransferase) |
| Bt.23218.1.S1\_at | 513038 | CTSK | cathepsin K |
| Bt.23237.1.S1\_at | 510245 | PPP5C | protein phosphatase 5, catalytic subunit |
| Bt.23240.1.S1\_at | 768317 | RPL37 | ribosomal protein L37 |
| Bt.23254.1.S1\_at | 768311 | YWHAQ | tyrosine 3-monooxygenase/tryptophan 5-monooxygenase activation protein, theta polypeptide |
| Bt.23268.1.S1\_at | 280815 | NPC2 | Niemann-Pick disease, type C2 |
| Bt.23308.1.S1\_at | 535746 | SEPT11 | septin 11 |
| Bt.23318.1.S1\_at | 510833 | COL3A1 | collagen, type III, alpha 1 |
| Bt.23318.1.S2\_at | 510833 | COL3A1 | collagen, type III, alpha 1 |
| Bt.23326.1.S1\_at | 404122 | ACTG1 | actin, gamma 1 |
| Bt.23357.1.S1\_at | 614145 | IDH3G | isocitrate dehydrogenase 3 (NAD+) gamma |
| Bt.23361.1.S1\_at | 616871 | UQCRB | ubiquinol-cytochrome c reductase binding protein |
| Bt.23380.2.S1\_at | 505093 | HNRPA1 | heterogeneous nuclear ribonucleoprotein A1 |
| Bt.23468.1.A1\_at | 532555 | GOLPH3L | golgi phosphoprotein 3-like |
| Bt.2347.1.S1\_at | 506544 | PFKM | phosphofructokinase, muscle |
| Bt.2347.2.S1\_at | 506544 | PFKM | phosphofructokinase, muscle |
| Bt.23496.1.S1\_at | 767909 | LOC767909 | 6.8 kDa mitochondrial proteolipid |
| Bt.23509.1.S1\_at | 617707 | PPAP2B | phosphatidic acid phosphatase type 2B |
| Bt.23522.1.S1\_at | 510575 | ITM2B | integral membrane protein 2B |
| Bt.23526.1.S1\_at | 617466 | MRPS24 | mitochondrial ribosomal protein S24 |
| Bt.23551.2.S1\_at | 517211 | NAT13 | N-acetyltransferase 13 |
| Bt.23569.1.S1\_at | 522006 | ACAA2 | acetyl-Coenzyme A acyltransferase 2 |
| Bt.23572.1.S1\_at | 617512 | CCNDBP1 | cyclin D-type binding-protein 1 |
| Bt.23606.1.S1\_at | 317697 | ITPR1 | inositol 1,4,5-triphosphate receptor, type 1 |
| Bt.23651.1.A1\_at | 537391 | DDAH1 | dimethylarginine dimethylaminohydrolase 1 |
| Bt.2394.1.S1\_at | 513461 | PSMD11 | proteasome (prosome, macropain) 26S subunit, non-ATPase, 11 |
| Bt.24017.1.A1\_s\_at | 530186 | PSMC2 | proteasome (prosome, macropain) 26S subunit, ATPase, 2 |
| Bt.24359.1.S1\_at | 506969 | ALS2CL | ALS2 C-terminal like |
| Bt.24396.1.S1\_at | 616931 | NUDT10 | nudix (nucleoside diphosphate linked moiety X)-type motif 10 |
| Bt.24404.1.A1\_at | 532616 | EPB41L3 | erythrocyte membrane protein band 4.1-like 3 |
| Bt.24474.1.S1\_a\_at | 515757 | AFG3L2 | AFG3 ATPase family gene 3-like 2 (yeast) |
| Bt.24474.1.S1\_at | 515757 | AFG3L2 | AFG3 ATPase family gene 3-like 2 (yeast) |
| Bt.24624.1.S1\_at | 519302 | MRPS9 | mitochondrial ribosomal protein S9 |
| Bt.24624.2.S1\_at | 519302 | MRPS9 | mitochondrial ribosomal protein S9 |
| Bt.24676.1.S1\_at | 515999 | SAR1B | SAR1 gene homolog B (S. cerevisiae) |
| Bt.24676.2.A1\_at | 515999 | SAR1B | SAR1 gene homolog B (S. cerevisiae) |
| Bt.2493.1.S1\_at | 513622 | MRPL51 | mitochondrial ribosomal protein L51 |
| Bt.24931.1.A1\_at | 529762 | TMEM126A | transmembrane protein 126A |
| Bt.24945.1.S1\_at | 539978 | GDE1 | glycerophosphodiester phosphodiesterase 1 |
| Bt.250.1.S1\_at | 281351 | NMT1 | N-myristoyltransferase 1 |
| Bt.2587.1.S1\_at | 520260 | FH | fumarate hydratase |
| Bt.2587.2.S1\_a\_at | 520260 | FH | fumarate hydratase |
| Bt.26122.2.S1\_at | 525680 | UXT | ubiquitously-expressed transcript |
| Bt.26150.1.A1\_at | 514230 | L2HGDH | L-2-hydroxyglutarate dehydrogenase |
| Bt.265.1.S1\_at | 287020 | UQCRFS1 | ubiquinol-cytochrome c reductase, Rieske iron-sulfur polypeptide 1 |
| Bt.26641.1.S1\_at | 513156 | XPNPEP1 | X-prolyl aminopeptidase (aminopeptidase P) 1, soluble |
| Bt.26718.1.S1\_a\_at | 506250 | HCCS | holocytochrome c synthase |
| Bt.26792.1.S1\_at | 512056 | RASGRP2 | RAS guanyl releasing protein 2 (calcium and DAG-regulated) |
| Bt.26835.1.S1\_at | 512506 | RANGRF | RAN guanine nucleotide release factor |
| Bt.26992.1.A1\_at | 282132 | ADAM10 | ADAM metallopeptidase domain 10 |
| Bt.26992.2.S1\_at | 282132 | ADAM10 | ADAM metallopeptidase domain 10 |
| Bt.27.1.S1\_at | 286862 | YWHAG | tyrosine 3-monooxygenase/tryptophan 5-monooxygenase activation protein, gamma polypeptide |
| Bt.27027.1.S1\_a\_at | 508928 | MRPS15 | mitochondrial ribosomal protein S15 |
| Bt.2711.1.S1\_at | 509983 | SUCLG1 | succinate-CoA ligase, alpha subunit |
| Bt.27116.2.S1\_at | 618131 | TIMM8B | translocase of inner mitochondrial membrane 8 homolog B (yeast) |
| Bt.27121.1.S1\_at | 509192 | DULLARD | dullard homolog (Xenopus laevis) |
| Bt.27192.1.S1\_at | 541077 | ETF1 | eukaryotic translation termination factor 1 |
| Bt.2729.1.S1\_at | 536359 | AP3S1 | adaptor-related protein complex 3, sigma 1 subunit |
| Bt.27401.1.A1\_at | 535869 | RNF128 | ring finger protein 128 |
| Bt.27481.1.A1\_at | 535424 | SPG3A | spastic paraplegia 3A (autosomal dominant) |
| Bt.27627.1.A1\_at | 527488 | SNTA1 | syntrophin, alpha 1 (dystrophin-associated protein A1, 59kDa, acidic component) |
| Bt.27657.1.A1\_at | 338053 | ATP5G1 | ATP synthase, H+ transporting, mitochondrial F0 complex, subunit C1 (subunit 9) |
| Bt.27806.1.S1\_at | 513586 | MPI | mannose phosphate isomerase |
| Bt.27926.1.S1\_at | 520170 | UCHL3 | ubiquitin carboxyl-terminal esterase L3 (ubiquitin thiolesterase) |
| Bt.27952.1.S1\_at | 616748 | TRAPPC2 | trafficking protein particle complex 2 |
| Bt.2798.1.S1\_a\_at | 282385 | TMSB10 | thymosin beta 10 |
| Bt.28113.1.S1\_at | 535227 | CYP2U1 | cytochrome P450, family 2, subfamily U, polypeptide 1 |
| Bt.2814.1.S1\_at | 286862 | YWHAG | tyrosine 3-monooxygenase/tryptophan 5-monooxygenase activation protein, gamma polypeptide |
| Bt.2816.1.S1\_at | 515696 | ATP5L | ATP synthase, H+ transporting, mitochondrial F0 complex, subunit G |
| Bt.2821.1.S1\_at | 511207 | PSMB7 | proteasome (prosome, macropain) subunit, beta type, 7 |
| Bt.2825.1.S1\_at | 617901 | MRPL13 | mitochondrial ribosomal protein L13 |
| Bt.28559.1.S1\_a\_at | 515176 | DNASE1L1 | deoxyribonuclease I-like 1 |
| Bt.28699.1.S1\_at | 281577 | WASL | Wiskott-Aldrich syndrome-like |
| Bt.2894.1.S1\_at | 507245 | ECSIT | ECSIT homolog (Drosophila) |
| Bt.29.1.S1\_at | 338047 | DDHD1 | DDHD domain containing 1 |
| Bt.2905.1.S1\_at | 515063 | NDRG2 | NDRG family member 2 |
| Bt.2973.2.S1\_a\_at | 613610 | PDHB | pyruvate dehydrogenase (lipoamide) beta |
| Bt.29746.1.S1\_at | 407109 | PDHA1 | pyruvate dehydrogenase (lipoamide) alpha 1 |
| Bt.298.1.S1\_at | 281742 | NDUFA12 | NADH dehydrogenase (ubiquinone) 1 alpha subcomplex, 12 |
| Bt.29910.1.S1\_at | 407209 | Sgce | sarcoglycan, epsilon |
| Bt.3050.1.S1\_at | 513958 | EIF2B2 | eukaryotic translation initiation factor 2B, subunit 2 beta, 39kDa |
| Bt.3117.1.S1\_at | 327660 | NDUFB9 | NADH dehydrogenase (ubiquinone) 1 beta subcomplex, 9, 22kDa |
| Bt.3126.1.A1\_at | 407209 | Sgce | sarcoglycan, epsilon |
| Bt.3137.1.S1\_at | 616317 | STMN1 | stathmin 1/oncoprotein 18 |
| Bt.315.1.S1\_at | 338079 | NDUFS7 | NADH dehydrogenase (ubiquinone) Fe-S protein 7, 20kDa (NADH-coenzyme Q reductase) |
| Bt.3193.1.A1\_at | 538625 | PSMD6 | proteasome (prosome, macropain) 26S subunit, non-ATPase, 6 |
| Bt.321.1.S1\_at | 327702 | NDUFAB1 | NADH dehydrogenase (ubiquinone) 1, alpha/beta subcomplex, 1, 8kDa |
| Bt.3210.1.S1\_at | 526848 | CTNNA1 | catenin (cadherin-associated protein), alpha 1, 102kDa |
| Bt.3211.1.S1\_at | 338472 | GABARAPL1 | GABA(A) receptor-associated protein like 1 |
| Bt.3211.1.S2\_at | 338472 | GABARAPL1 | GABA(A) receptor-associated protein like 1 |
| Bt.3260.1.S1\_at | 614633 | C17H18orf55 | chromosome 18 open reading frame 55 ortholog |
| Bt.3272.1.A1\_at | 535714 | AIFM1 | apoptosis-inducing factor, mitochondrion-associated, 1 |
| Bt.3288.1.A1\_at | 514374 | PIGY | phosphatidylinositol glycan anchor biosynthesis, class Y |
| Bt.3306.1.S1\_at | 512397 | LOC512397 | similar to ProSAPiP1 protein |
| Bt.3414.1.S1\_at | 536149 | HRB | HIV-1 Rev binding protein |
| Bt.3427.1.S1\_at | 618441 | SEPX1 | selenoprotein X, 1 |
| Bt.3478.1.A1\_s\_at | 281040 | CAV1 | caveolin 1, caveolae protein, 22kDa |
| Bt.3583.1.S1\_at | 281574 | EZR | ezrin |
| Bt.3604.1.A1\_at | 516010 | SMAD2 | SMAD family member 2 |
| Bt.3610.1.S1\_at | 613899 | UQCRH | ubiquinol-cytochrome c reductase hinge protein |
| Bt.3631.1.S1\_at | 326581 | EEF1G | eukaryotic translation elongation factor 1 gamma |
| Bt.371.1.S1\_at | 282478 | SLC25A4 | solute carrier family 25 (mitochondrial carrier; adenine nucleotide translocator), member 4 |
| Bt.378.1.S1\_at | 338040 | ATP5I | ATP synthase, H+ transporting, mitochondrial F0 complex, subunit e |
| Bt.3791.1.S1\_at | 347698 | BSG | basigin |
| Bt.380.1.S1\_at | 327699 | ATPIF1 | ATPase inhibitory factor 1 |
| Bt.3856.1.S1\_at | 353217 | SLC35B1 | solute carrier family 35, member B1 |
| Bt.3857.1.S1\_at | 327707 | ENDOG | endonuclease G |
| Bt.3867.1.S1\_at | 282701 | ATP5F1 | ATP synthase, H+ transporting, mitochondrial F0 complex, subunit B1 |
| Bt.3898.1.S1\_at | 282446 | IDH3A | isocitrate dehydrogenase 3 (NAD+) alpha |
| Bt.3922.1.S1\_a\_at | 617005 | MRPL38 | mitochondrial ribosomal protein L38 |
| Bt.3960.1.S1\_a\_at | 282119 | VDAC1 | voltage-dependent anion channel 1 |
| Bt.3960.2.S1\_at | 282119 | VDAC1 | voltage-dependent anion channel 1 |
| Bt.400.1.S1\_at | 327674 | COX7B | cytochrome c oxidase subunit VIIb |
| Bt.4008.1.S1\_at | 282389 | TXNRD2 | thioredoxin reductase 2 |
| Bt.4020.1.S1\_at | 520757 | RNF10 | ring finger protein 10 |
| Bt.4031.1.S1\_at | 444879 | ST3GAL2 | ST3 beta-galactoside alpha-2,3-sialyltransferase 2 |
| Bt.4031.2.S1\_at | 444879 | ST3GAL2 | ST3 beta-galactoside alpha-2,3-sialyltransferase 2 |
| Bt.404.1.S1\_at | 282690 | ATP5J | ATP synthase, H+ transporting, mitochondrial F0 complex, subunit F6 |
| Bt.4040.1.S1\_at | 282515 | PAFAH1B3 | platelet-activating factor acetylhydrolase, isoform Ib, gamma subunit 29kDa |
| Bt.4057.1.S1\_at | 317655 | MYH10 | myosin, heavy chain 10, non-muscle |
| Bt.4072.1.S1\_at | 287014 | NDUFV1 | NADH dehydrogenase (ubiquinone) flavoprotein 1, 51kDa |
| Bt.4099.1.S1\_at | 282120 | VDAC2 | voltage-dependent anion channel 2 |
| Bt.4118.1.A1\_at | 514472 | TRAP1 | TNF receptor-associated protein 1 |
| Bt.413.1.S1\_at | 281780 | GGTA1 | alpha-galactosyltransferase 1 (glycoprotein) |
| Bt.4203.1.S1\_at | 506492 | ATP5J2 | ATP synthase, H+ transporting, mitochondrial F0 complex, subunit F2 |
| Bt.4205.1.S1\_at | 286809 | GPX4 | glutathione peroxidase 4 (phospholipid hydroperoxidase) |
| Bt.4224.1.S1\_at | 280715 | AK1 | adenylate kinase 1 |
| Bt.4241.1.S1\_at | 282050 | RPS14 | ribosomal protein S14 |
| Bt.4262.1.S1\_a\_at | 327717 | NDUFV3 | NADH dehydrogenase (ubiquinone) flavoprotein 3, 10kDa |
| Bt.4279.2.S1\_at | 507196 | SHC1 | SHC (Src homology 2 domain containing) transforming protein 1 |
| Bt.4279.3.S1\_a\_at | 507196 | SHC1 | SHC (Src homology 2 domain containing) transforming protein 1 |
| Bt.4280.1.A1\_at | 506991 | TXNDC12 | thioredoxin domain containing 12 (endoplasmic reticulum) |
| Bt.4314.1.S1\_at | 282689 | ANXA2 | annexin A2 |
| Bt.4355.1.S1\_at | 282885 | PRDX5 | peroxiredoxin 5 |
| Bt.4384.1.S1\_at | 504482 | WASF2 | WAS protein family, member 2 |
| Bt.4394.1.S1\_at | 282433 | GUCY1B3 | guanylate cyclase 1, soluble, beta 3 |
| Bt.4402.1.S1\_at | 538054 | MRPL16 | mitochondrial ribosomal protein L16 |
| Bt.4431.1.S1\_a\_at | 327675 | ATP5B | ATP synthase, H+ transporting, mitochondrial F1 complex, beta polypeptide |
| Bt.4473.1.S1\_at | 338060 | NDUFA10 | NADH dehydrogenase (ubiquinone) 1 alpha subcomplex, 10, 42kDa |
| Bt.4475.1.S1\_at | 327697 | NDUFS2 | NADH dehydrogenase (ubiquinone) Fe-S protein 2, 49kDa (NADH-coenzyme Q reductase) |
| Bt.4476.1.S1\_at | 281998 | PRDX3 | peroxiredoxin 3 |
| Bt.4480.1.S2\_at | 281216 | GUCY1A3 | guanylate cyclase 1, soluble, alpha 3 |
| Bt.4492.1.S1\_at | 504741 | COLEC12 | collectin sub-family member 12 |
| Bt.4503.1.S1\_at | 337927 | MTCH2 | mitochondrial carrier homolog 2 (C. elegans) |
| Bt.4503.1.S2\_at | 337927 | MTCH2 | mitochondrial carrier homolog 2 (C. elegans) |
| Bt.4540.1.S1\_at | 327668 | ATP5C1 | ATP synthase, H+ transporting, mitochondrial F1 complex, gamma polypeptide 1 |
| Bt.4555.1.S1\_at | 617210 | ETFB | electron-transfer-flavoprotein, beta polypeptide |
| Bt.4599.1.S1\_at | 281092 | CROT | carnitine O-octanoyltransferase |
| Bt.4652.1.S1\_at | 506657 | MRPL55 | mitochondrial ribosomal protein L55 |
| Bt.4662.1.S1\_at | 287024 | NOS3 | nitric oxide synthase 3 (endothelial cell) |
| Bt.467.1.S1\_at | 281296 | MAPT | microtubule-associated protein tau |
| Bt.4689.1.S2\_at | 282110 | UCHL5 | ubiquitin carboxyl-terminal hydrolase L5 |
| Bt.4704.1.S1\_at | 327714 | NDUFA5 | NADH dehydrogenase (ubiquinone) 1 alpha subcomplex, 5, 13kDa |
| Bt.4705.1.S1\_at | 327953 | HDGF | hepatoma-derived growth factor (high-mobility group protein 1-like) |
| Bt.4725.1.S1\_at | 281032 | BTG1 | B-cell translocation gene 1, anti-proliferative |
| Bt.4732.1.S1\_at | 281615 | ALDH1A1 | aldehyde dehydrogenase 1 family, member A1 |
| Bt.4744.1.S1\_at | 281206 | GOT1 | glutamic-oxaloacetic transaminase 1, soluble (aspartate aminotransferase 1) |
| Bt.4748.1.S1\_at | 281496 | SOD2 | superoxide dismutase 2, mitochondrial |
| Bt.4756.1.S1\_at | 530186 | PSMC2 | proteasome (prosome, macropain) 26S subunit, ATPase, 2 |
| Bt.4758.1.S1\_at | 281758 | FABP3 | fatty acid binding protein 3, muscle and heart (mammary-derived growth inhibitor) |
| Bt.4775.1.S1\_at | 525716 | S100B | S100 calcium binding protein B |
| Bt.4777.1.S1\_at | 288380 | NDUFS1 | NADH dehydrogenase (ubiquinone) Fe-S protein 1, 75kDa (NADH-coenzyme Q reductase) |
| Bt.4786.1.S2\_at | 281080 | CLTC | clathrin, heavy chain (Hc) |
| Bt.4797.1.S1\_at | 508535 | FKBP4 | FK506 binding protein 4, 59kDa |
| Bt.4819.1.S1\_at | 327704 | NDUFA4 | NADH dehydrogenase (ubiquinone) 1 alpha subcomplex, 4, 9kDa |
| Bt.4831.1.S1\_at | 281942 | CALCOCO2 | calcium binding and coiled-coil domain 2 |
| Bt.4844.1.S1\_at | 280904 | PTN | pleiotrophin (heparin binding growth factor 8, neurite growth-promoting factor 1) |
| Bt.4867.1.A1\_at | 281339 | MYH8 | myosin, heavy chain 8, skeletal muscle, perinatal |
| Bt.4867.2.S1\_at | 281339 | MYH8 | myosin, heavy chain 8, skeletal muscle, perinatal |
| Bt.4880.1.S1\_at | 282477 | SLC25A3 | solute carrier family 25 (mitochondrial carrier; phosphate carrier), member 3 |
| Bt.4900.1.A1\_at | 512469 | MRPS5 | mitochondrial ribosomal protein S5 |
| Bt.4948.1.S1\_at | 282705 | PSEN1 | presenilin 1 (Alzheimer disease 3) |
| Bt.4950.1.S1\_at | 286838 | NDST2 | N-deacetylase/N-sulfotransferase (heparan glucosaminyl) 2 |
| Bt.4957.1.S2\_at | 281937 | MYO1C | myosin IC |
| Bt.4970.1.S1\_at | 327688 | COX7A2 | cytochrome c oxidase subunit VIIa polypeptide 2 (liver) |
| Bt.4977.1.S2\_at | 281849 | IGF2R | insulin-like growth factor 2 receptor |
| Bt.4985.1.S1\_a\_at | 508825 | MRPL23 | mitochondrial ribosomal protein L23 |
| Bt.4985.1.S1\_at | 508825 | MRPL23 | mitochondrial ribosomal protein L23 |
| Bt.4985.2.S1\_a\_at | 508825 | MRPL23 | mitochondrial ribosomal protein L23 |
| Bt.5002.1.S1\_at | 525042 | GPD1 | glycerol-3-phosphate dehydrogenase 1 (soluble) |
| Bt.5006.1.S1\_at | 536619 | PAIP2 | poly(A) binding protein interacting protein 2 |
| Bt.5027.1.A1\_at | 444864 | PEG3 | paternally expressed 3 |
| Bt.5029.1.S1\_at | 282710 | ATP5H | ATP synthase, H+ transporting, mitochondrial F0 complex, subunit d |
| Bt.5033.1.S1\_at | 281480 | SDHA | succinate dehydrogenase complex, subunit A, flavoprotein (Fp) |
| Bt.5040.1.S1\_at | 281570 | UQCR | ubiquinol-cytochrome c reductase (6.4kD) subunit |
| Bt.5042.1.S1\_at | 282394 | UQCRC2 | ubiquinol-cytochrome c reductase core protein II |
| Bt.5047.1.S1\_at | 533227 | HEBP1 | heme binding protein 1 |
| Bt.5079.1.S1\_at | 510029 | PSMC4 | proteasome (prosome, macropain) 26S subunit, ATPase, 4 |
| Bt.5108.1.S1\_at | 515902 | PCCB | propionyl Coenzyme A carboxylase, beta polypeptide |
| Bt.5120.1.S1\_at | 280878 | NNT | nicotinamide nucleotide transhydrogenase |
| Bt.5132.1.S1\_at | 540386 | DDAH2 | dimethylarginine dimethylaminohydrolase 2 |
| Bt.5210.1.S1\_at | 280976 | ACO2 | aconitase 2, mitochondrial |
| Bt.5278.1.A1\_at | 539713 | BCS1L | BCS1-like (yeast) |
| Bt.5278.2.S1\_at | 539713 | BCS1L | BCS1-like (yeast) |
| Bt.5288.1.S1\_at | 281557 | TXN2 | thioredoxin 2 |
| Bt.5290.1.S1\_at | 281729 | SMC3 | structural maintenance of chromosomes 3 |
| Bt.5302.1.S1\_a\_at | 281918 | MRVI1 | murine retrovirus integration site 1 homolog |
| Bt.5319.2.S1\_a\_at | 282438 | PRDX6 | peroxiredoxin 6 |
| Bt.5322.1.S1\_at | 534640 | PSMB5 | proteasome (prosome, macropain) subunit, beta type, 5 |
| Bt.5341.1.S1\_at | 280884 | OGN | osteoglycin |
| Bt.5344.1.S1\_at | 281556 | TUFM | Tu translation elongation factor, mitochondrial |
| Bt.5345.1.S1\_at | 535182 | MDH1 | malate dehydrogenase 1, NAD (soluble) |
| Bt.5351.1.S1\_a\_at | 280733 | BGN | biglycan |
| Bt.5376.1.S1\_at | 506336 | YIF1A | Yip1 interacting factor homolog A (S. cerevisiae) |
| Bt.5378.1.S1\_at | 282517 | NDUFB8 | NADH dehydrogenase (ubiquinone) 1 beta subcomplex, 8, 19kDa |
| Bt.5412.1.S1\_at | 282150 | BCKDHB | branched chain keto acid dehydrogenase E1, beta polypeptide (maple syrup urine disease) |
| Bt.5418.1.S1\_at | 541101 | TUBG1 | tubulin, gamma 1 |
| Bt.5434.1.S1\_at | 614538 | DAD1 | defender against cell death 1 |
| Bt.5459.1.S1\_at | 534352 | MRPL18 | mitochondrial ribosomal protein L18 |
| Bt.5467.1.S2\_at | 281433 | PSAP | prosaposin (variant Gaucher disease and variant metachromatic leukodystrophy) |
| Bt.547.1.S1\_at | 286885 | UQCRQ | low molecular mass ubiquinone-binding protein (9.5kD) |
| Bt.5483.1.S1\_at | 287027 | NDUFS8 | NADH dehydrogenase (ubiquinone) Fe-S protein 8, 23kDa (NADH-coenzyme Q reductase) |
| Bt.5510.1.S1\_at | 338088 | ATP5E | ATP synthase, H+ transporting, mitochondrial F1 complex, epsilon subunit |
| Bt.5520.1.S1\_at | 327669 | IDH2 | isocitrate dehydrogenase 2 (NADP+), mitochondrial |
| Bt.5541.1.S1\_at | 404188 | NDUFA9 | NADH dehydrogenase (ubiquinone) 1 alpha subcomplex, 9, 39kDa |
| Bt.56.1.S1\_at | 327691 | NDUFS6 | NADH dehydrogenase (ubiquinone) Fe-S protein 6, 13kDa (NADH-coenzyme Q reductase) |
| Bt.5605.1.S1\_at | 613548 | MARCKS | myristoylated alanine-rich protein kinase C substrate |
| Bt.5605.2.S1\_at | 613548 | MARCKS | myristoylated alanine-rich protein kinase C substrate |
| Bt.566.1.S1\_at | 282200 | COX6A2 | cytochrome c oxidase subunit VIa polypeptide 2 |
| Bt.5676.1.S1\_at | 533208 | COQ5 | coenzyme Q5 homolog, methyltransferase (S. cerevisiae) |
| Bt.57.1.S1\_at | 338057 | NDUFS5 | NADH dehydrogenase (ubiquinone) Fe-S protein 5, 15kDa (NADH-coenzyme Q reductase) |
| Bt.5723.1.S1\_at | 326346 | NDUFA11 | NADH dehydrogenase (ubiquinone) 1 alpha subcomplex, 11, 14.7kDa |
| Bt.5737.1.S1\_at | 510574 | VPS26A | vacuolar protein sorting 26 homolog A (S. pombe) |
| Bt.5750.1.S1\_at | 508465 | DERL2 | Der1-like domain family, member 2 |
| Bt.58.1.S1\_at | 327680 | NDUFS4 | NADH dehydrogenase (ubiquinone) Fe-S protein 4, 18kDa (NADH-coenzyme Q reductase) |
| Bt.5848.1.S1\_at | 518117 | ATP2A1 | ATPase, Ca++ transporting, cardiac muscle, fast twitch 1 |
| Bt.5848.2.S1\_at | 518117 | ATP2A1 | ATPase, Ca++ transporting, cardiac muscle, fast twitch 1 |
| Bt.59.1.S1\_at | 327710 | NDUFA8 | NADH dehydrogenase (ubiquinone) 1 alpha subcomplex, 8, 19kDa |
| Bt.5922.1.S1\_at | 510811 | HOMER2 | homer homolog 2 (Drosophila) |
| Bt.5966.1.A1\_at | 505968 | ACADM | acyl-coenzyme A dehydrogenase, C-4 to C-12 straight chain |
| Bt.60.1.S1\_at | 327713 | NDUFB2 | NADH dehydrogenase (ubiquinone) 1 beta subcomplex, 2, 8kDa |
| Bt.6019.1.S1\_at | 506235 | HSF1 | heat shock transcription factor 1 |
| Bt.61.1.S1\_at | 338073 | NDUFB3 | NADH dehydrogenase (ubiquinone) 1 beta subcomplex, 3, 12kDa |
| Bt.6111.1.S1\_at | 510314 | POMP | proteasome maturation protein |
| Bt.6116.1.S1\_a\_at | 523039 | HTRA2 | HtrA serine peptidase 2 |
| Bt.6171.1.A1\_at | 512002 | HIBADH | 3-hydroxyisobutyrate dehydrogenase |
| Bt.618.1.S1\_at | 616275 | ANAPC11 | APC11 anaphase promoting complex subunit 11 homolog (yeast) |
| Bt.621.1.S1\_at | 505060 | CYP51 | cytochrome P450, family 51, subfamily A, polypeptide 1 |
| Bt.6289.1.S1\_at | 614165 | SPTLC1 | serine palmitoyltransferase, long chain base subunit 1 |
| Bt.63.1.S1\_at | 327670 | NDUFA6 | NADH dehydrogenase (ubiquinone) 1 alpha subcomplex, 6, 14kDa |
| Bt.6390.1.S1\_at | 508143 | MRPS17 | mitochondrial ribosomal protein S17 |
| Bt.6397.2.S1\_at | 540444 | HMGB2 | high-mobility group box 2 |
| Bt.64.1.S1\_at | 338065 | NDUFB7 | NADH dehydrogenase (ubiquinone) 1 beta subcomplex, 7, 18kDa |
| Bt.6465.1.S1\_at | 510767 | CYCS | cytochrome c, somatic |
| Bt.6473.1.S1\_at | 505050 | PSMA7 | proteasome (prosome, macropain) subunit, alpha type, 7 |
| Bt.6494.1.S1\_at | 404161 | ESSS | mitochondrial NADH:ubiquinone oxidoreductase ESSS subunit |
| Bt.65.1.S1\_at | 327698 | NDUFA2 | NADH dehydrogenase (ubiquinone) 1 alpha subcomplex, 2, 8kDa |
| Bt.66.1.S1\_at | 338064 | NDUFA3 | NADH dehydrogenase (ubiquinone) 1 alpha subcomplex, 3, 9kDa |
| Bt.665.1.S1\_s\_at | 282211 | CYP2D6 | cytochrome P450, family 2, subfamily D, polypeptide 6 |
| Bt.6673.1.S1\_at | 536553 | AP2S1 | adaptor-related protein complex 2, sigma 1 subunit |
| Bt.6679.1.S1\_at | 512427 | NASP | nuclear autoantigenic sperm protein (histone-binding) |
| Bt.67.1.S1\_at | 282289 | NDUFC1 | NADH dehydrogenase (ubiquinone) 1, subcomplex unknown, 1, 6kDa |
| Bt.68.1.S1\_at | 327690 | NDUFB1 | NADH dehydrogenase (ubiquinone) 1 beta subcomplex, 1, 7kDa |
| Bt.6827.1.S1\_at | 505176 | PSMA3 | proteasome (prosome, macropain) subunit, alpha type, 3 |
| Bt.69.1.S1\_at | 327673 | NDUFA1 | NADH dehydrogenase (ubiquinone) 1 alpha subcomplex, 1, 7.5kDa |
| Bt.6938.1.S1\_at | 516091 | CUEDC2 | CUE domain containing 2 |
| Bt.6950.1.S1\_at | 614236 | SYS1 | SYS1 Golgi-localized integral membrane protein homolog (S. cerevisiae) |
| Bt.6964.1.S1\_at | 780784 | TMOD1 | tropomodulin 1 |
| Bt.70.1.S1\_at | 327701 | NDUFB10 | NADH dehydrogenase (ubiquinone) 1 beta subcomplex, 10, 22kDa |
| Bt.7056.2.A1\_x\_at | 280813 | HBB | hemoglobin, beta |
| Bt.7056.3.A1\_a\_at | 511735 | HBG | hemoglobin, gamma |
| Bt.7056.5.A1\_x\_at | 511735 | HBG | hemoglobin, gamma |
| Bt.7069.1.S1\_at | 505807 | PCMTD2 | protein-L-isoaspartate (D-aspartate) O-methyltransferase domain containing 2 |
| Bt.7139.1.S1\_at | 781575 | TOMM20 | translocase of outer mitochondrial membrane 20 homolog (yeast) |
| Bt.7143.1.S1\_at | 510899 | MRPS16 | mitochondrial ribosomal protein S16 |
| Bt.7168.2.A1\_at | 513668 | COL27A1 | collagen, type XXVII, alpha 1 |
| Bt.7177.1.S1\_at | 280719 | AMT | aminomethyltransferase |
| Bt.7215.1.S1\_at | 282130 | ACADVL | acyl-coenzyme A dehydrogenase, very long chain |
| Bt.7237.2.S1\_a\_at | 281810 | HADHA | hydroxyacyl-Coenzyme A dehydrogenase/3-ketoacyl-Coenzyme A thiolase/enoyl-Coenzyme A hydratase (trifunctional protein), alpha subunit |
| Bt.7240.1.S1\_at | 781648 | LAP3 | leucine aminopeptidase 3 |
| Bt.7266.1.S1\_at | 515109 | TIMM8A | translocase of inner mitochondrial membrane 8 homolog A (yeast) |
| Bt.7415.1.S1\_at | 506485 | PBXIP1 | pre-B-cell leukemia homeobox interacting protein 1 |
| Bt.75.1.S1\_at | 338087 | COX6B | cytochrome c oxidase subunit VIb |
| Bt.76.1.S1\_at | 338086 | COX7A1 | cytochrome c oxidase subunit VIIa polypeptide 1 (muscle) |
| Bt.7633.1.S1\_at | 617105 | EEF1E1 | eukaryotic translation elongation factor 1 epsilon 1 |
| Bt.77.1.S1\_at | 327718 | COX7C | cytochrome c oxidase subunit VIIc |
| Bt.7701.1.S1\_at | 614105 | GPSN2 | glycoprotein, synaptic 2 |
| Bt.7716.1.S1\_at | 532120 | BBS4 | Bardet-Biedl syndrome 4 |
| Bt.7785.1.S1\_at | 513312 | NACA | nascent polypeptide-associated complex alpha subunit |
| Bt.7814.1.S1\_at | 518601 | ARF4 | ADP-ribosylation factor 4 |
| Bt.7836.1.S1\_at | 614921 | NUTF2 | nuclear transport factor 2 |
| Bt.7856.1.S1\_at | 505005 | BCKDK | branched chain ketoacid dehydrogenase kinase |
| Bt.7870.1.S1\_at | 280701 | PPA1 | pyrophosphatase (inorganic) 1 |
| Bt.7872.1.S1\_at | 515946 | CBR1 | carbonyl reductase 1 |
| Bt.7903.1.A1\_at | 510937 | HARS | histidyl-tRNA synthetase |
| Bt.7915.1.S1\_at | 281306 | MDH2 | malate dehydrogenase 2, NAD (mitochondrial) |
| Bt.7980.1.S1\_at | 532851 | LASP1 | LIM and SH3 protein 1 |
| Bt.8001.1.S1\_at | 506676 | SAKS1 | SAPK substrate protein 1 |
| Bt.8046.1.A1\_at | 613835 | MRPS36 | mitochondrial ribosomal protein S36 |
| Bt.8047.1.S1\_at | 540176 | ATP5G3 | ATP synthase, H+ transporting, mitochondrial F0 complex, subunit C3 (subunit 9) |
| Bt.8062.1.S1\_at | 282015 | PSMC5 | proteasome (prosome, macropain) 26S subunit, ATPase, 5 |
| Bt.8122.1.S1\_at | 282386 | TMSB4X | thymosin beta 4, X-linked |
| Bt.8351.1.S1\_at | 504867 | C29H11orf73 | chromosome 11 open reading frame 73 ortholog |
| Bt.8479.1.A1\_at | 282876 | NOS2A | nitric oxide synthase 2A (inducible, hepatocytes) |
| Bt.848.1.S1\_at | 282290 | NDUFV2 | NADH dehydrogenase (ubiquinone) flavoprotein 2, 24kDa |
| Bt.8566.1.S1\_at | 538427 | ANP32A | acidic (leucine-rich) nuclear phosphoprotein 32 family, member A |
| Bt.868.1.S1\_a\_at | 616375 | MAPBPIP | mitogen-activated protein-binding protein-interacting protein |
| Bt.868.1.S1\_at | 616375 | MAPBPIP | mitogen-activated protein-binding protein-interacting protein |
| Bt.8706.1.S1\_at | 615215 | MAPKAPK3 | mitogen-activated protein kinase-activated protein kinase 3 |
| Bt.886.1.S1\_at | 510155 | PSMA5 | proteasome (prosome, macropain) subunit, alpha type, 5 |
| Bt.888.1.S1\_at | 534615 | BNIP3L | BCL2/adenovirus E1B 19kDa interacting protein 3-like |
| Bt.893.1.S1\_at | 327665 | NDUFB6 | NADH dehydrogenase (ubiquinone) 1 beta subcomplex, 6, 17kDa |
| Bt.8950.1.S1\_at | 338084 | NDUFA13 | NADH dehydrogenase (ubiquinone) 1 alpha subcomplex, 13 |
| Bt.8966.1.S2\_at | 282090 | THTPA | thiamine triphosphatase |
| Bt.9047.1.S1\_at | 615999 | DDT | D-dopachrome tautomerase |
| Bt.9055.2.S1\_a\_at | 616451 | TRMU | tRNA 5-methylaminomethyl-2-thiouridylate methyltransferase |
| Bt.9207.1.S1\_at | 540426 | MSN | moesin |
| Bt.9233.3.S1\_at | 519729 | ADRM1 | adhesion regulating molecule 1 |
| Bt.9306.2.S1\_a\_at | 512531 | MARS | methionyl-tRNA synthetase |
| Bt.9306.3.S1\_at | 512531 | MARS | methionyl-tRNA synthetase |
| Bt.9324.1.S1\_at | 509816 | MRPS11 | mitochondrial ribosomal protein S11 |
| Bt.935.1.A1\_at | 539354 | SLC35A4 | solute carrier family 35, member A4 |
| Bt.935.2.S1\_at | 539354 | SLC35A4 | solute carrier family 35, member A4 |
| Bt.9362.1.S1\_at | 514403 | MRPL2 | mitochondrial ribosomal protein L2 |
| Bt.946.2.A1\_a\_at | 280716 | AK2 | adenylate kinase 2 |
| Bt.9490.1.S1\_at | 514371 | ACTR1A | ARP1 actin-related protein 1 homolog A, centractin alpha (yeast) |
| Bt.9559.2.S1\_at | 280722 | APP | amyloid beta (A4) precursor protein (peptidase nexin-II, Alzheimer disease) |
| Bt.9578.1.S1\_at | 282318 | PPP1CC | protein phosphatase 1, catalytic subunit, gamma isoform |
| Bt.9623.1.A1\_at | 539188 | GPT | glutamic-pyruvate transaminase (alanine aminotransferase) |
| Bt.9643.1.S1\_at | 512662 | CHP | calcium binding protein P22 |
| Bt.97.1.S1\_at | 281759 | FABP4 | fatty acid binding protein 4, adipocyte |
| Bt.9731.1.S1\_at | 513762 | GLRX2 | glutaredoxin 2 |
| Bt.9749.1.S1\_at | 511453 | SPCS1 | signal peptidase complex subunit 1 homolog (S. cerevisiae) |
| Bt.9756.1.S1\_at | 530865 | LIAS | lipoic acid synthetase |
| Bt.9797.1.S1\_at | 534546 | PMPCB | peptidase (mitochondrial processing) beta |
| Bt.981.2.S1\_at | 286823 | CLIC4 | chloride intracellular channel 4 |
| Bt.9825.1.S1\_at | 540278 | MRPL35 | mitochondrial ribosomal protein L35 |
| Bt.9826.1.S1\_at | 444878 | COX5A | COX5A protein |
| Bt.9828.1.S1\_at | 281817 | HINT3 | histidine triad nucleotide binding protein 3 |
| Bt.9857.1.S1\_at | 616477 | CHCHD3 | coiled-coil-helix-coiled-coil-helix domain containing 3 |
| Bt.99.1.S1\_at | 327706 | NDUFB4 | NADH dehydrogenase (ubiquinone) 1 beta subcomplex, 4, 15kDa |
| Bt.9949.2.S1\_at | 533851 | PDLIM7 | PDZ and LIM domain 7 (enigma) |
| Bt.9949.3.S1\_a\_at | 533851 | PDLIM7 | PDZ and LIM domain 7 (enigma) |
| Bt.9965.1.S1\_at | 533910 | DLD | dihydrolipoamide dehydrogenase |
| Bt.9973.1.S2\_at | 281876 | ITGB1 | integrin, beta 1 (fibronectin receptor, beta polypeptide, antigen CD29 includes MDF2, MSK12) |
| Bt.9981.1.S1\_at | 535320 | TRIM54 | tripartite motif-containing 54 |
| Bt.9983.1.S1\_at | 613636 | ARD1A | ARD1 homolog A, N-acetyltransferase |

### GO:0005741

|  |  |  |  |
| --- | --- | --- | --- |
| **probe** | **entrez** | **symbol** | **description** |
| Bt.10154.1.S1\_at | 531444 | CISD1 | CDGSH iron sulfur domain 1 |
| Bt.1523.1.S1\_at | 510219 | TOMM40 | translocase of outer mitochondrial membrane 40 homolog (yeast) |
| Bt.21113.1.S1\_a\_at | 509459 | CPT1B | carnitine palmitoyltransferase 1B (muscle) |
| Bt.21272.1.S1\_at | 533918 | SH3GLB1 | SH3-domain GRB2-like endophilin B1 |
| Bt.3960.1.S1\_a\_at | 282119 | VDAC1 | voltage-dependent anion channel 1 |
| Bt.3960.2.S1\_at | 282119 | VDAC1 | voltage-dependent anion channel 1 |
| Bt.4099.1.S1\_at | 282120 | VDAC2 | voltage-dependent anion channel 2 |
| Bt.7139.1.S1\_at | 781575 | TOMM20 | translocase of outer mitochondrial membrane 20 homolog (yeast) |

### GO:0005833

|  |  |  |  |
| --- | --- | --- | --- |
| **probe** | **entrez** | **symbol** | **description** |
| Bt.7056.2.A1\_x\_at | 280813 | HBB | hemoglobin, beta |
| Bt.7056.3.A1\_a\_at | 511735 | HBG | hemoglobin, gamma |
| Bt.7056.5.A1\_x\_at | 511735 | HBG | hemoglobin, gamma |

### GO:0030130

|  |  |  |  |
| --- | --- | --- | --- |
| **probe** | **entrez** | **symbol** | **description** |
| Bt.10.2.S1\_a\_at | 281698 | CLTLB | clathrin, light polypeptide B (light chain B) |
| Bt.1617.2.S1\_a\_at | 281078 | CLTA | clathrin, light chain (Lca) |
| Bt.4786.1.S2\_at | 281080 | CLTC | clathrin, heavy chain (Hc) |

### GO:0031594

|  |  |  |  |
| --- | --- | --- | --- |
| **probe** | **entrez** | **symbol** | **description** |
| Bt.13152.1.S1\_s\_at | 280722 | APP | amyloid beta (A4) precursor protein (peptidase nexin-II, Alzheimer disease) |
| Bt.9559.2.S1\_at | 280722 | APP | amyloid beta (A4) precursor protein (peptidase nexin-II, Alzheimer disease) |
| Bt.9973.1.S2\_at | 281876 | ITGB1 | integrin, beta 1 (fibronectin receptor, beta polypeptide, antigen CD29 includes MDF2, MSK12) |

### GO:0016363

|  |  |  |  |
| --- | --- | --- | --- |
| **probe** | **entrez** | **symbol** | **description** |
| Bt.20411.1.S1\_at | 539332 | CFL2 | cofilin 2 (muscle) |
| Bt.22981.1.S1\_at | 359716 | DNMT3A | DNA (cytosine-5-)-methyltransferase 3 alpha |
| Bt.5290.1.S1\_at | 281729 | SMC3 | structural maintenance of chromosomes 3 |
| Bt.8566.1.S1\_at | 538427 | ANP32A | acidic (leucine-rich) nuclear phosphoprotein 32 family, member A |

### GO:0030173

|  |  |  |  |
| --- | --- | --- | --- |
| **probe** | **entrez** | **symbol** | **description** |
| Bt.18702.1.A1\_at | 523683 | CUX1 | cut-like homeobox 1 |
| Bt.20129.1.S1\_at | 523683 | CUX1 | cut-like homeobox 1 |
| Bt.20129.2.S1\_at | 523683 | CUX1 | cut-like homeobox 1 |
| Bt.4031.1.S1\_at | 444879 | ST3GAL2 | ST3 beta-galactoside alpha-2,3-sialyltransferase 2 |
| Bt.4031.2.S1\_at | 444879 | ST3GAL2 | ST3 beta-galactoside alpha-2,3-sialyltransferase 2 |
