# Supplementary material for: A gene network switch enhances the oxidative capacity of ovine skeletal muscle during late fetal development
Source: BMC Genomics. 2010 Jun 15;11:378. doi: 10.1186/1471-2164-11-378 (PMC2894804; doi:10.1186/1471-2164-11-378)
Supplement: Additional File 1 — Gene expression data. This file contains links to the gene expression data. The genes that are significantly differentially expressed between adjacent development times are listed as Experiment 1 (80 d vs 100 d), Experiment 2 (100 d vs 120 d), Experiment 3 (120 d vs 150 d) and Experiment 4 (150 d vs 230 d) in the section entitled Open microarray analysis report for developmental expression. Each experiment contains an MA plot, Volcano plot and Heatmap for each of the three microarray processing programs MAS5, RMA and GCRMA. For each of these processing programs there is a list of significantly differentially expressed probe sets, and for each of these there is an FDR corrected probability, fold change, percentage of microarrays with a MAS5 Present call, and a convergence percentage for all three microarray processing programs. The file also contains annotation of the probe sets. In a second section entitled Time series analysis of developmental expression, there is a list of genes in each gene expression cluster and diagrams showing MAS5 gene expression values for these genes. [file 1471-2164-11-378-S1.ZIP › Report/GO3/Report/TopNodesMF.html]

### Top terms for Molecular Function

Top terms sorted by the weight algorithm. The first column is the GO term id and links to GO, the second column is a short description of the term, the third column is the number of genes annotated to the term, the fourth is the number of genes significant for the term and the fifth column is the expected number of genes for the term. The last four columns are the p-values for different tests of significance. The results are ordered by the weight algorithm (last column).

|  |  |  |  |  |  |  |  |  |
| --- | --- | --- | --- | --- | --- | --- | --- | --- |
| **GO** | **Term** | **Annotated** | **Significant** | **Expected** | **classic** | **KS** | **elim** | **weight** |
| GO:0008137 | NADH dehydrogenase (ubiquinone) activity | 18 | 17 | 3.43 | 7.6e-12 | 1.00 | 7.6e-12 | 7.6e-12 |
| GO:0015078 | hydrogen ion transmembrane transporter a... | 65 | 31 | 12.4 | 1.3e-07 | 1.00 | 0.76558 | 1.3e-07 |
| GO:0009055 | electron carrier activity | 116 | 54 | 22.1 | 9.2e-12 | 1.00 | 7.4e-06 | 7.4e-06 |
| GO:0048037 | cofactor binding | 122 | 41 | 23.2 | 8.3e-05 | 0.98 | 0.12271 | 8.3e-05 |
| GO:0016616 | oxidoreductase activity, acting on the C... | 49 | 21 | 9.33 | 0.00010 | 0.97 | 0.00010 | 0.00024 |
| GO:0016624 | oxidoreductase activity, acting on the a... | 7 | 6 | 1.33 | 0.00028 | 0.98 | 0.09438 | 0.00028 |
| GO:0008092 | cytoskeletal protein binding | 148 | 45 | 28.2 | 0.00051 | 0.47 | 0.18745 | 0.00051 |
| GO:0005506 | iron ion binding | 124 | 38 | 23.6 | 0.00116 | 0.98 | 0.00116 | 0.00116 |
| GO:0019829 | cation-transporting ATPase activity | 41 | 16 | 7.81 | 0.00226 | 0.98 | 0.08606 | 0.00226 |
| GO:0004298 | threonine endopeptidase activity | 18 | 9 | 3.43 | 0.00295 | 1.00 | 0.00295 | 0.00295 |
| GO:0051539 | 4 iron, 4 sulfur cluster binding | 15 | 8 | 2.86 | 0.00303 | 0.97 | 0.00303 | 0.00303 |
| GO:0035257 | nuclear hormone receptor binding | 10 | 7 | 1.9 | 0.00062 | 0.95 | 0.00062 | 0.00368 |
| GO:0030145 | manganese ion binding | 58 | 20 | 11 | 0.00382 | 0.92 | 0.00382 | 0.00382 |
| GO:0016769 | transferase activity, transferring nitro... | 19 | 9 | 3.62 | 0.00467 | 0.99 | 0.06589 | 0.00467 |
| GO:0008308 | voltage-gated anion channel activity | 13 | 7 | 2.48 | 0.00521 | 1.00 | 0.00521 | 0.00521 |
| GO:0016709 | oxidoreductase activity, acting on paire... | 5 | 4 | 0.95 | 0.00555 | 0.89 | 0.00555 | 0.00555 |
| GO:0046912 | transferase activity, transferring acyl ... | 5 | 4 | 0.95 | 0.00555 | 0.82 | 0.00555 | 0.00555 |
| GO:0004721 | phosphoprotein phosphatase activity | 74 | 25 | 14.1 | 0.00179 | 0.95 | 0.02585 | 0.00705 |
| GO:0003712 | transcription cofactor activity | 29 | 11 | 5.52 | 0.01359 | 0.86 | 0.01359 | 0.01359 |
| GO:0004707 | MAP kinase activity | 6 | 4 | 1.14 | 0.01413 | 0.95 | 0.01413 | 0.01413 |

### DE genes for top GO terms for Molecular Function

List of DE genes for top GO terms. The first column is the Affymetrix probe ID, the second column is the Entrez ID, the third column is the gene symbol and the fourth column is the gene description. Note that the same gene can appear more than once in the list since different probes can target the same gene (can help detect overepresented genes due to multiple probe targets).

### GO:0008137

|  |  |  |  |
| --- | --- | --- | --- |
| **probe** | **entrez** | **symbol** | **description** |
| Bt.1153.1.S1\_at | 287327 | NDUFS3 | NADH dehydrogenase (ubiquinone) Fe-S protein 3, 30kDa (NADH-coenzyme Q reductase) |
| Bt.20.1.S1\_at | 338063 | NDUFA7 | NADH dehydrogenase (ubiquinone) 1 alpha subcomplex, 7, 14.5kDa |
| Bt.21.1.S1\_at | 338046 | NDUFC2 | NADH dehydrogenase (ubiquinone) 1, subcomplex unknown, 2, 14.5kDa |
| Bt.298.1.S1\_at | 281742 | NDUFA12 | NADH dehydrogenase (ubiquinone) 1 alpha subcomplex, 12 |
| Bt.315.1.S1\_at | 338079 | NDUFS7 | NADH dehydrogenase (ubiquinone) Fe-S protein 7, 20kDa (NADH-coenzyme Q reductase) |
| Bt.321.1.S1\_at | 327702 | NDUFAB1 | NADH dehydrogenase (ubiquinone) 1, alpha/beta subcomplex, 1, 8kDa |
| Bt.4072.1.S1\_at | 287014 | NDUFV1 | NADH dehydrogenase (ubiquinone) flavoprotein 1, 51kDa |
| Bt.4475.1.S1\_at | 327697 | NDUFS2 | NADH dehydrogenase (ubiquinone) Fe-S protein 2, 49kDa (NADH-coenzyme Q reductase) |
| Bt.4777.1.S1\_at | 288380 | NDUFS1 | NADH dehydrogenase (ubiquinone) Fe-S protein 1, 75kDa (NADH-coenzyme Q reductase) |
| Bt.5378.1.S1\_at | 282517 | NDUFB8 | NADH dehydrogenase (ubiquinone) 1 beta subcomplex, 8, 19kDa |
| Bt.5483.1.S1\_at | 287027 | NDUFS8 | NADH dehydrogenase (ubiquinone) Fe-S protein 8, 23kDa (NADH-coenzyme Q reductase) |
| Bt.58.1.S1\_at | 327680 | NDUFS4 | NADH dehydrogenase (ubiquinone) Fe-S protein 4, 18kDa (NADH-coenzyme Q reductase) |
| Bt.61.1.S1\_at | 338073 | NDUFB3 | NADH dehydrogenase (ubiquinone) 1 beta subcomplex, 3, 12kDa |
| Bt.64.1.S1\_at | 338065 | NDUFB7 | NADH dehydrogenase (ubiquinone) 1 beta subcomplex, 7, 18kDa |
| Bt.848.1.S1\_at | 282290 | NDUFV2 | NADH dehydrogenase (ubiquinone) flavoprotein 2, 24kDa |
| Bt.8950.1.S1\_at | 338084 | NDUFA13 | NADH dehydrogenase (ubiquinone) 1 alpha subcomplex, 13 |
| Bt.99.1.S1\_at | 327706 | NDUFB4 | NADH dehydrogenase (ubiquinone) 1 beta subcomplex, 4, 15kDa |

### GO:0015078

|  |  |  |  |
| --- | --- | --- | --- |
| **probe** | **entrez** | **symbol** | **description** |
| Bt.1242.1.S1\_at | 338053 | ATP5G1 | ATP synthase, H+ transporting, mitochondrial F0 complex, subunit C1 (subunit 9) |
| Bt.1399.1.S1\_at | 282405 | ATP6V1F | ATPase, H+ transporting, lysosomal 14kDa, V1 subunit F |
| Bt.1426.1.S1\_at | 338081 | ATP5D | ATP synthase, H+ transporting, mitochondrial F1 complex, delta subunit |
| Bt.1568.1.S1\_at | 287012 | COX5B | cytochrome c oxidase subunit Vb |
| Bt.16025.1.S1\_at | 281090 | COX4 | cytochrome c oxydase subunit 4 |
| Bt.1819.1.S1\_at | 616109 | UCRC | ubiquinol-cytochrome c reductase complex 7.2 kDa protein |
| Bt.23002.1.S1\_at | 282199 | COX6A1 | cytochrome c oxidase subunit VIa polypeptide 1 |
| Bt.23002.1.S1\_s\_at | 282199 | COX6A1 | cytochrome c oxidase subunit VIa polypeptide 1 |
| Bt.23361.1.S1\_at | 616871 | UQCRB | ubiquinol-cytochrome c reductase binding protein |
| Bt.265.1.S1\_at | 287020 | UQCRFS1 | ubiquinol-cytochrome c reductase, Rieske iron-sulfur polypeptide 1 |
| Bt.27657.1.A1\_at | 338053 | ATP5G1 | ATP synthase, H+ transporting, mitochondrial F0 complex, subunit C1 (subunit 9) |
| Bt.2816.1.S1\_at | 515696 | ATP5L | ATP synthase, H+ transporting, mitochondrial F0 complex, subunit G |
| Bt.3610.1.S1\_at | 613899 | UQCRH | ubiquinol-cytochrome c reductase hinge protein |
| Bt.378.1.S1\_at | 338040 | ATP5I | ATP synthase, H+ transporting, mitochondrial F0 complex, subunit e |
| Bt.3867.1.S1\_at | 282701 | ATP5F1 | ATP synthase, H+ transporting, mitochondrial F0 complex, subunit B1 |
| Bt.400.1.S1\_at | 327674 | COX7B | cytochrome c oxidase subunit VIIb |
| Bt.404.1.S1\_at | 282690 | ATP5J | ATP synthase, H+ transporting, mitochondrial F0 complex, subunit F6 |
| Bt.4203.1.S1\_at | 506492 | ATP5J2 | ATP synthase, H+ transporting, mitochondrial F0 complex, subunit F2 |
| Bt.4431.1.S1\_a\_at | 327675 | ATP5B | ATP synthase, H+ transporting, mitochondrial F1 complex, beta polypeptide |
| Bt.4540.1.S1\_at | 327668 | ATP5C1 | ATP synthase, H+ transporting, mitochondrial F1 complex, gamma polypeptide 1 |
| Bt.4970.1.S1\_at | 327688 | COX7A2 | cytochrome c oxidase subunit VIIa polypeptide 2 (liver) |
| Bt.5029.1.S1\_at | 282710 | ATP5H | ATP synthase, H+ transporting, mitochondrial F0 complex, subunit d |
| Bt.5040.1.S1\_at | 281570 | UQCR | ubiquinol-cytochrome c reductase (6.4kD) subunit |
| Bt.547.1.S1\_at | 286885 | UQCRQ | low molecular mass ubiquinone-binding protein (9.5kD) |
| Bt.5510.1.S1\_at | 338088 | ATP5E | ATP synthase, H+ transporting, mitochondrial F1 complex, epsilon subunit |
| Bt.566.1.S1\_at | 282200 | COX6A2 | cytochrome c oxidase subunit VIa polypeptide 2 |
| Bt.75.1.S1\_at | 338087 | COX6B | cytochrome c oxidase subunit VIb |
| Bt.76.1.S1\_at | 338086 | COX7A1 | cytochrome c oxidase subunit VIIa polypeptide 1 (muscle) |
| Bt.77.1.S1\_at | 327718 | COX7C | cytochrome c oxidase subunit VIIc |
| Bt.8047.1.S1\_at | 540176 | ATP5G3 | ATP synthase, H+ transporting, mitochondrial F0 complex, subunit C3 (subunit 9) |
| Bt.9826.1.S1\_at | 444878 | COX5A | COX5A protein |

### GO:0009055

|  |  |  |  |
| --- | --- | --- | --- |
| **probe** | **entrez** | **symbol** | **description** |
| Bt.10609.2.A1\_at | 532945 | CYP20A1 | cytochrome P450, family 20, subfamily A, polypeptide 1 |
| Bt.1153.1.S1\_at | 287327 | NDUFS3 | NADH dehydrogenase (ubiquinone) Fe-S protein 3, 30kDa (NADH-coenzyme Q reductase) |
| Bt.13128.1.S1\_at | 286840 | SDHB | succinate dehydrogenase complex, subunit B, iron sulfur (Ip) |
| Bt.13170.1.S1\_at | 327696 | SDHC | succinate dehydrogenase complex, subunit C, integral membrane protein, 15kDa |
| Bt.13439.1.S1\_at | 768074 | ETFDH | electron-transferring-flavoprotein dehydrogenase |
| Bt.1403.1.S1\_at | 280878 | NNT | nicotinamide nucleotide transhydrogenase |
| Bt.1808.1.S1\_at | 512500 | CYC1 | cytochrome c-1 |
| Bt.20.1.S1\_at | 338063 | NDUFA7 | NADH dehydrogenase (ubiquinone) 1 alpha subcomplex, 7, 14.5kDa |
| Bt.20318.1.S1\_a\_at | 508566 | NQO2 | NAD(P)H dehydrogenase, quinone 2 |
| Bt.21.1.S1\_at | 338046 | NDUFC2 | NADH dehydrogenase (ubiquinone) 1, subcomplex unknown, 2, 14.5kDa |
| Bt.22630.1.S1\_at | 617564 | DHDH | dihydrodiol dehydrogenase (dimeric) |
| Bt.22630.2.A1\_at | 617564 | DHDH | dihydrodiol dehydrogenase (dimeric) |
| Bt.23002.1.S1\_at | 282199 | COX6A1 | cytochrome c oxidase subunit VIa polypeptide 1 |
| Bt.23002.1.S1\_s\_at | 282199 | COX6A1 | cytochrome c oxidase subunit VIa polypeptide 1 |
| Bt.265.1.S1\_at | 287020 | UQCRFS1 | ubiquinol-cytochrome c reductase, Rieske iron-sulfur polypeptide 1 |
| Bt.28113.1.S1\_at | 535227 | CYP2U1 | cytochrome P450, family 2, subfamily U, polypeptide 1 |
| Bt.298.1.S1\_at | 281742 | NDUFA12 | NADH dehydrogenase (ubiquinone) 1 alpha subcomplex, 12 |
| Bt.315.1.S1\_at | 338079 | NDUFS7 | NADH dehydrogenase (ubiquinone) Fe-S protein 7, 20kDa (NADH-coenzyme Q reductase) |
| Bt.321.1.S1\_at | 327702 | NDUFAB1 | NADH dehydrogenase (ubiquinone) 1, alpha/beta subcomplex, 1, 8kDa |
| Bt.3272.1.A1\_at | 535714 | AIFM1 | apoptosis-inducing factor, mitochondrion-associated, 1 |
| Bt.4008.1.S1\_at | 282389 | TXNRD2 | thioredoxin reductase 2 |
| Bt.4072.1.S1\_at | 287014 | NDUFV1 | NADH dehydrogenase (ubiquinone) flavoprotein 1, 51kDa |
| Bt.4475.1.S1\_at | 327697 | NDUFS2 | NADH dehydrogenase (ubiquinone) Fe-S protein 2, 49kDa (NADH-coenzyme Q reductase) |
| Bt.4555.1.S1\_at | 617210 | ETFB | electron-transfer-flavoprotein, beta polypeptide |
| Bt.4662.1.S1\_at | 287024 | NOS3 | nitric oxide synthase 3 (endothelial cell) |
| Bt.4777.1.S1\_at | 288380 | NDUFS1 | NADH dehydrogenase (ubiquinone) Fe-S protein 1, 75kDa (NADH-coenzyme Q reductase) |
| Bt.4970.1.S1\_at | 327688 | COX7A2 | cytochrome c oxidase subunit VIIa polypeptide 2 (liver) |
| Bt.5040.1.S1\_at | 281570 | UQCR | ubiquinol-cytochrome c reductase (6.4kD) subunit |
| Bt.5120.1.S1\_at | 280878 | NNT | nicotinamide nucleotide transhydrogenase |
| Bt.5288.1.S1\_at | 281557 | TXN2 | thioredoxin 2 |
| Bt.5378.1.S1\_at | 282517 | NDUFB8 | NADH dehydrogenase (ubiquinone) 1 beta subcomplex, 8, 19kDa |
| Bt.5483.1.S1\_at | 287027 | NDUFS8 | NADH dehydrogenase (ubiquinone) Fe-S protein 8, 23kDa (NADH-coenzyme Q reductase) |
| Bt.566.1.S1\_at | 282200 | COX6A2 | cytochrome c oxidase subunit VIa polypeptide 2 |
| Bt.58.1.S1\_at | 327680 | NDUFS4 | NADH dehydrogenase (ubiquinone) Fe-S protein 4, 18kDa (NADH-coenzyme Q reductase) |
| Bt.5966.1.A1\_at | 505968 | ACADM | acyl-coenzyme A dehydrogenase, C-4 to C-12 straight chain |
| Bt.61.1.S1\_at | 338073 | NDUFB3 | NADH dehydrogenase (ubiquinone) 1 beta subcomplex, 3, 12kDa |
| Bt.621.1.S1\_at | 505060 | CYP51 | cytochrome P450, family 51, subfamily A, polypeptide 1 |
| Bt.64.1.S1\_at | 338065 | NDUFB7 | NADH dehydrogenase (ubiquinone) 1 beta subcomplex, 7, 18kDa |
| Bt.6465.1.S1\_at | 510767 | CYCS | cytochrome c, somatic |
| Bt.665.1.S1\_s\_at | 282211 | CYP2D6 | cytochrome P450, family 2, subfamily D, polypeptide 6 |
| Bt.68.1.S1\_at | 327690 | NDUFB1 | NADH dehydrogenase (ubiquinone) 1 beta subcomplex, 1, 7kDa |
| Bt.7215.1.S1\_at | 282130 | ACADVL | acyl-coenzyme A dehydrogenase, very long chain |
| Bt.76.1.S1\_at | 338086 | COX7A1 | cytochrome c oxidase subunit VIIa polypeptide 1 (muscle) |
| Bt.8479.1.A1\_at | 282876 | NOS2A | nitric oxide synthase 2A (inducible, hepatocytes) |
| Bt.848.1.S1\_at | 282290 | NDUFV2 | NADH dehydrogenase (ubiquinone) flavoprotein 2, 24kDa |
| Bt.8950.1.S1\_at | 338084 | NDUFA13 | NADH dehydrogenase (ubiquinone) 1 alpha subcomplex, 13 |
| Bt.8970.1.S1\_at | 510462 | OXNAD1 | oxidoreductase NAD-binding domain containing 1 |
| Bt.9278.1.S1\_at | 540141 | FBXL11 | F-box and leucine-rich repeat protein 11 |
| Bt.9731.1.S1\_at | 513762 | GLRX2 | glutaredoxin 2 |
| Bt.9754.1.S1\_at | 519632 | NQO1 | NAD(P)H dehydrogenase, quinone 1 |
| Bt.99.1.S1\_at | 327706 | NDUFB4 | NADH dehydrogenase (ubiquinone) 1 beta subcomplex, 4, 15kDa |
| Bt.9965.1.S1\_at | 533910 | DLD | dihydrolipoamide dehydrogenase |
| Bt.997.1.S1\_at | 516287 | CYB5R1 | cytochrome b5 reductase 1 |
| Bt.997.2.S1\_a\_at | 516287 | CYB5R1 | cytochrome b5 reductase 1 |

### GO:0048037

|  |  |  |  |
| --- | --- | --- | --- |
| **probe** | **entrez** | **symbol** | **description** |
| Bt.1244.1.S1\_at | 514939 | LOC514939 | similar to 6-phosphogluconate dehydrogenase (decarboxylating) |
| Bt.12910.1.S1\_at | 534599 | OGDH | oxoglutarate (alpha-ketoglutarate) dehydrogenase (lipoamide) |
| Bt.1316.1.S1\_at | 286886 | GOT2 | glutamic-oxaloacetic transaminase 2, mitochondrial (aspartate aminotransferase 2) |
| Bt.1316.1.S2\_at | 286886 | GOT2 | glutamic-oxaloacetic transaminase 2, mitochondrial (aspartate aminotransferase 2) |
| Bt.13287.1.S1\_at | 515186 | AGXT2L1 | alanine-glyoxylate aminotransferase 2-like 1 |
| Bt.1403.1.S1\_at | 280878 | NNT | nicotinamide nucleotide transhydrogenase |
| Bt.14207.1.S1\_at | 319141 | GCAT | glycine C-acetyltransferase (2-amino-3-ketobutyrate coenzyme A ligase) |
| Bt.16003.1.S1\_at | 327664 | PYGM | phosphorylase, glycogen; muscle (McArdle syndrome, glycogen storage disease type V) |
| Bt.20318.1.S1\_a\_at | 508566 | NQO2 | NAD(P)H dehydrogenase, quinone 2 |
| Bt.22142.2.S1\_a\_at | 511624 | COQ6 | coenzyme Q6 homolog, monooxygenase (S. cerevisiae) |
| Bt.22672.1.A1\_at | 512259 | HPGD | hydroxyprostaglandin dehydrogenase 15-(NAD) |
| Bt.23607.1.S1\_at | 532785 | HADH | hydroxyacyl-Coenzyme A dehydrogenase |
| Bt.24265.1.S1\_a\_at | 508712 | CCBL2 | cysteine conjugate-beta lyase 2 |
| Bt.24594.1.A1\_at | 525813 | ME3 | malic enzyme 3, NADP(+)-dependent, mitochondrial |
| Bt.2556.1.S1\_at | 512259 | HPGD | hydroxyprostaglandin dehydrogenase 15-(NAD) |
| Bt.26829.1.S1\_at | 540985 | ME1 | malic enzyme 1, NADP(+)-dependent, cytosolic |
| Bt.321.1.S1\_at | 327702 | NDUFAB1 | NADH dehydrogenase (ubiquinone) 1, alpha/beta subcomplex, 1, 8kDa |
| Bt.3272.1.A1\_at | 535714 | AIFM1 | apoptosis-inducing factor, mitochondrion-associated, 1 |
| Bt.4008.1.S1\_at | 282389 | TXNRD2 | thioredoxin reductase 2 |
| Bt.401.2.S1\_at | 768330 | DBI | diazepam binding inhibitor (GABA receptor modulator, acyl-Coenzyme A binding protein) |
| Bt.4072.1.S1\_at | 287014 | NDUFV1 | NADH dehydrogenase (ubiquinone) flavoprotein 1, 51kDa |
| Bt.4475.1.S1\_at | 327697 | NDUFS2 | NADH dehydrogenase (ubiquinone) Fe-S protein 2, 49kDa (NADH-coenzyme Q reductase) |
| Bt.4662.1.S1\_at | 287024 | NOS3 | nitric oxide synthase 3 (endothelial cell) |
| Bt.4744.1.S1\_at | 281206 | GOT1 | glutamic-oxaloacetic transaminase 1, soluble (aspartate aminotransferase 1) |
| Bt.5002.1.S1\_at | 525042 | GPD1 | glycerol-3-phosphate dehydrogenase 1 (soluble) |
| Bt.5033.1.S1\_at | 281480 | SDHA | succinate dehydrogenase complex, subunit A, flavoprotein (Fp) |
| Bt.5120.1.S1\_at | 280878 | NNT | nicotinamide nucleotide transhydrogenase |
| Bt.5541.1.S1\_at | 404188 | NDUFA9 | NADH dehydrogenase (ubiquinone) 1 alpha subcomplex, 9, 39kDa |
| Bt.5966.1.A1\_at | 505968 | ACADM | acyl-coenzyme A dehydrogenase, C-4 to C-12 straight chain |
| Bt.6171.1.A1\_at | 512002 | HIBADH | 3-hydroxyisobutyrate dehydrogenase |
| Bt.6289.1.S1\_at | 614165 | SPTLC1 | serine palmitoyltransferase, long chain base subunit 1 |
| Bt.6440.1.S1\_at | 281730 | CTBP2 | C-terminal binding protein 2 |
| Bt.6683.1.S1\_at | 517402 | PDHX | pyruvate dehydrogenase complex, component X |
| Bt.7177.1.S1\_at | 280719 | AMT | aminomethyltransferase |
| Bt.7215.1.S1\_at | 282130 | ACADVL | acyl-coenzyme A dehydrogenase, very long chain |
| Bt.7237.2.S1\_a\_at | 281810 | HADHA | hydroxyacyl-Coenzyme A dehydrogenase/3-ketoacyl-Coenzyme A thiolase/enoyl-Coenzyme A hydratase (trifunctional protein), alpha subunit |
| Bt.8479.1.A1\_at | 282876 | NOS2A | nitric oxide synthase 2A (inducible, hepatocytes) |
| Bt.848.1.S1\_at | 282290 | NDUFV2 | NADH dehydrogenase (ubiquinone) flavoprotein 2, 24kDa |
| Bt.9623.1.A1\_at | 539188 | GPT | glutamic-pyruvate transaminase (alanine aminotransferase) |
| Bt.9754.1.S1\_at | 519632 | NQO1 | NAD(P)H dehydrogenase, quinone 1 |
| Bt.9965.1.S1\_at | 533910 | DLD | dihydrolipoamide dehydrogenase |

### GO:0016616

|  |  |  |  |
| --- | --- | --- | --- |
| **probe** | **entrez** | **symbol** | **description** |
| Bt.11194.1.S1\_at | 281360 | DHRS4 | dehydrogenase/reductase (SDR family) member 4 |
| Bt.1244.1.S1\_at | 514939 | LOC514939 | similar to 6-phosphogluconate dehydrogenase (decarboxylating) |
| Bt.13324.2.S1\_a\_at | 281235 | IDH1 | isocitrate dehydrogenase 1 (NADP+), soluble |
| Bt.2109.1.S1\_at | 508285 | MAT2B | methionine adenosyltransferase II, beta |
| Bt.22630.1.S1\_at | 617564 | DHDH | dihydrodiol dehydrogenase (dimeric) |
| Bt.22630.2.A1\_at | 617564 | DHDH | dihydrodiol dehydrogenase (dimeric) |
| Bt.22672.1.A1\_at | 512259 | HPGD | hydroxyprostaglandin dehydrogenase 15-(NAD) |
| Bt.23357.1.S1\_at | 614145 | IDH3G | isocitrate dehydrogenase 3 (NAD+) gamma |
| Bt.24594.1.A1\_at | 525813 | ME3 | malic enzyme 3, NADP(+)-dependent, mitochondrial |
| Bt.2556.1.S1\_at | 512259 | HPGD | hydroxyprostaglandin dehydrogenase 15-(NAD) |
| Bt.26829.1.S1\_at | 540985 | ME1 | malic enzyme 1, NADP(+)-dependent, cytosolic |
| Bt.3898.1.S1\_at | 282446 | IDH3A | isocitrate dehydrogenase 3 (NAD+) alpha |
| Bt.4449.1.S1\_at | 618607 | AKR1A1 | aldo-keto reductase family 1, member A1 (aldehyde reductase) |
| Bt.5002.1.S1\_at | 525042 | GPD1 | glycerol-3-phosphate dehydrogenase 1 (soluble) |
| Bt.5345.1.S1\_at | 535182 | MDH1 | malate dehydrogenase 1, NAD (soluble) |
| Bt.5520.1.S1\_at | 327669 | IDH2 | isocitrate dehydrogenase 2 (NADP+), mitochondrial |
| Bt.6171.1.A1\_at | 512002 | HIBADH | 3-hydroxyisobutyrate dehydrogenase |
| Bt.6440.1.S1\_at | 281730 | CTBP2 | C-terminal binding protein 2 |
| Bt.7237.2.S1\_a\_at | 281810 | HADHA | hydroxyacyl-Coenzyme A dehydrogenase/3-ketoacyl-Coenzyme A thiolase/enoyl-Coenzyme A hydratase (trifunctional protein), alpha subunit |
| Bt.7872.1.S1\_at | 515946 | CBR1 | carbonyl reductase 1 |
| Bt.7915.1.S1\_at | 281306 | MDH2 | malate dehydrogenase 2, NAD (mitochondrial) |

### GO:0016624

|  |  |  |  |
| --- | --- | --- | --- |
| **probe** | **entrez** | **symbol** | **description** |
| Bt.11516.1.S1\_at | 407109 | PDHA1 | pyruvate dehydrogenase (lipoamide) alpha 1 |
| Bt.12910.1.S1\_at | 534599 | OGDH | oxoglutarate (alpha-ketoglutarate) dehydrogenase (lipoamide) |
| Bt.19415.1.A1\_at | 407109 | PDHA1 | pyruvate dehydrogenase (lipoamide) alpha 1 |
| Bt.2973.2.S1\_a\_at | 613610 | PDHB | pyruvate dehydrogenase (lipoamide) beta |
| Bt.29746.1.S1\_at | 407109 | PDHA1 | pyruvate dehydrogenase (lipoamide) alpha 1 |
| Bt.5412.1.S1\_at | 282150 | BCKDHB | branched chain keto acid dehydrogenase E1, beta polypeptide (maple syrup urine disease) |

### GO:0008092

|  |  |  |  |
| --- | --- | --- | --- |
| **probe** | **entrez** | **symbol** | **description** |
| Bt.1154.1.S1\_at | 533223 | WDR1 | WD repeat domain 1 |
| Bt.12030.1.S1\_at | 522269 | ACTN4 | actinin, alpha 4 |
| Bt.12345.1.S1\_at | 506730 | MACF1 | microtubule-actin crosslinking factor 1 |
| Bt.12445.2.S1\_at | 507342 | FSCN1 | fascin homolog 1, actin-bundling protein (Strongylocentrotus purpuratus) |
| Bt.12906.1.S1\_at | 535077 | GSN | gelsolin (amyloidosis, Finnish type) |
| Bt.13391.1.S1\_at | 493728 | CAPZA2 | capping protein (actin filament) muscle Z-line, alpha 2 |
| Bt.16111.1.S1\_at | 615255 | GMFB | glia maturation factor, beta |
| Bt.16142.2.S1\_at | 286862 | YWHAG | tyrosine 3-monooxygenase/tryptophan 5-monooxygenase activation protein, gamma polypeptide |
| Bt.18623.1.A1\_at | 510159 | MAPRE2 | microtubule-associated protein, RP/EB family, member 2 |
| Bt.20261.1.S1\_at | 511010 | PTPN3 | protein tyrosine phosphatase, non-receptor type 3 |
| Bt.20275.1.S1\_at | 613421 | ARPC5L | actin related protein 2/3 complex, subunit 5-like |
| Bt.20411.1.S1\_at | 539332 | CFL2 | cofilin 2 (muscle) |
| Bt.21731.1.S1\_at | 505406 | DBN1 | drebrin 1 |
| Bt.21761.1.S1\_a\_at | 505645 | TMOD4 | tropomodulin 4 (muscle) |
| Bt.23326.2.A1\_at | 281660 | CALD1 | caldesmon 1 |
| Bt.24404.1.A1\_at | 532616 | EPB41L3 | erythrocyte membrane protein band 4.1-like 3 |
| Bt.24896.1.A1\_at | 518402 | KIFAP3 | kinesin-associated protein 3 |
| Bt.2494.2.S1\_a\_at | 615785 | SDC2 | syndecan 2 |
| Bt.26993.1.S1\_at | 514446 | FLII | flightless I homolog (Drosophila) |
| Bt.27.1.S1\_at | 286862 | YWHAG | tyrosine 3-monooxygenase/tryptophan 5-monooxygenase activation protein, gamma polypeptide |
| Bt.27627.1.A1\_at | 527488 | SNTA1 | syntrophin, alpha 1 (dystrophin-associated protein A1, 59kDa, acidic component) |
| Bt.2798.1.S1\_a\_at | 282385 | TMSB10 | thymosin beta 10 |
| Bt.2814.1.S1\_at | 286862 | YWHAG | tyrosine 3-monooxygenase/tryptophan 5-monooxygenase activation protein, gamma polypeptide |
| Bt.28699.1.S1\_at | 281577 | WASL | Wiskott-Aldrich syndrome-like |
| Bt.3137.1.S1\_at | 616317 | STMN1 | stathmin 1/oncoprotein 18 |
| Bt.3583.1.S1\_at | 281574 | EZR | ezrin |
| Bt.4057.1.S1\_at | 317655 | MYH10 | myosin, heavy chain 10, non-muscle |
| Bt.4314.1.S1\_at | 282689 | ANXA2 | annexin A2 |
| Bt.4384.1.S1\_at | 504482 | WASF2 | WAS protein family, member 2 |
| Bt.4506.1.S1\_at | 281935 | MYO10 | myosin X |
| Bt.4775.1.S1\_at | 525716 | S100B | S100 calcium binding protein B |
| Bt.4957.1.S2\_at | 281937 | MYO1C | myosin IC |
| Bt.4972.1.S1\_at | 526027 | SPTBN1 | spectrin, beta, non-erythrocytic 1 |
| Bt.4972.2.S1\_at | 526027 | SPTBN1 | spectrin, beta, non-erythrocytic 1 |
| Bt.5099.1.S1\_at | 538975 | CNN3 | calponin 3, acidic |
| Bt.5123.2.S1\_at | 514446 | FLII | flightless I homolog (Drosophila) |
| Bt.5123.3.S1\_a\_at | 514446 | FLII | flightless I homolog (Drosophila) |
| Bt.5605.1.S1\_at | 613548 | MARCKS | myristoylated alanine-rich protein kinase C substrate |
| Bt.5605.2.S1\_at | 613548 | MARCKS | myristoylated alanine-rich protein kinase C substrate |
| Bt.5922.1.S1\_at | 510811 | HOMER2 | homer homolog 2 (Drosophila) |
| Bt.6964.1.S1\_at | 780784 | TMOD1 | tropomodulin 1 |
| Bt.7980.1.S1\_at | 532851 | LASP1 | LIM and SH3 protein 1 |
| Bt.8122.1.S1\_at | 282386 | TMSB4X | thymosin beta 4, X-linked |
| Bt.8957.1.S1\_at | 281736 | CXCR4 | chemokine (C-X-C motif) receptor 4 |
| Bt.9207.1.S1\_at | 540426 | MSN | moesin |

### GO:0005506

|  |  |  |  |
| --- | --- | --- | --- |
| **probe** | **entrez** | **symbol** | **description** |
| Bt.10154.1.S1\_at | 531444 | CISD1 | CDGSH iron sulfur domain 1 |
| Bt.10609.2.A1\_at | 532945 | CYP20A1 | cytochrome P450, family 20, subfamily A, polypeptide 1 |
| Bt.11216.1.S1\_at | 280695 | MB | myoglobin |
| Bt.13170.1.S1\_at | 327696 | SDHC | succinate dehydrogenase complex, subunit C, integral membrane protein, 15kDa |
| Bt.13199.1.S1\_at | 538829 | PPP1CB | protein phosphatase 1, catalytic subunit, beta isoform |
| Bt.13249.1.A1\_at | 617419 | SCD5 | stearoyl-CoA desaturase 5 |
| Bt.13439.1.S1\_at | 768074 | ETFDH | electron-transferring-flavoprotein dehydrogenase |
| Bt.1729.1.A1\_at | 286861 | FTL | ferritin, light polypeptide |
| Bt.17425.1.A1\_at | 511058 | PPP6C | protein phosphatase 6, catalytic subunit |
| Bt.1808.1.S1\_at | 512500 | CYC1 | cytochrome c-1 |
| Bt.2151.2.A1\_at | 282022 | PTGS1 | prostaglandin-endoperoxide synthase 1 (prostaglandin G/H synthase and cyclooxygenase) |
| Bt.23237.1.S1\_at | 510245 | PPP5C | protein phosphatase 5, catalytic subunit |
| Bt.25072.1.S1\_at | 615964 | NFU1 | NFU1 iron-sulfur cluster scaffold homolog (S. cerevisiae) |
| Bt.265.1.S1\_at | 287020 | UQCRFS1 | ubiquinol-cytochrome c reductase, Rieske iron-sulfur polypeptide 1 |
| Bt.26718.1.S1\_a\_at | 506250 | HCCS | holocytochrome c synthase |
| Bt.28113.1.S1\_at | 535227 | CYP2U1 | cytochrome P450, family 2, subfamily U, polypeptide 1 |
| Bt.29050.1.S1\_at | 782980 | LOC782980 | similar to Peptide deformylase (mitochondrial) |
| Bt.315.1.S1\_at | 338079 | NDUFS7 | NADH dehydrogenase (ubiquinone) Fe-S protein 7, 20kDa (NADH-coenzyme Q reductase) |
| Bt.4072.1.S1\_at | 287014 | NDUFV1 | NADH dehydrogenase (ubiquinone) flavoprotein 1, 51kDa |
| Bt.4394.1.S1\_at | 282433 | GUCY1B3 | guanylate cyclase 1, soluble, beta 3 |
| Bt.4475.1.S1\_at | 327697 | NDUFS2 | NADH dehydrogenase (ubiquinone) Fe-S protein 2, 49kDa (NADH-coenzyme Q reductase) |
| Bt.4662.1.S1\_at | 287024 | NOS3 | nitric oxide synthase 3 (endothelial cell) |
| Bt.4777.1.S1\_at | 288380 | NDUFS1 | NADH dehydrogenase (ubiquinone) Fe-S protein 1, 75kDa (NADH-coenzyme Q reductase) |
| Bt.5210.1.S1\_at | 280976 | ACO2 | aconitase 2, mitochondrial |
| Bt.5483.1.S1\_at | 287027 | NDUFS8 | NADH dehydrogenase (ubiquinone) Fe-S protein 8, 23kDa (NADH-coenzyme Q reductase) |
| Bt.621.1.S1\_at | 505060 | CYP51 | cytochrome P450, family 51, subfamily A, polypeptide 1 |
| Bt.6465.1.S1\_at | 510767 | CYCS | cytochrome c, somatic |
| Bt.665.1.S1\_s\_at | 282211 | CYP2D6 | cytochrome P450, family 2, subfamily D, polypeptide 6 |
| Bt.7056.2.A1\_x\_at | 280813 | HBB | hemoglobin, beta |
| Bt.7056.3.A1\_a\_at | 511735 | HBG | hemoglobin, gamma |
| Bt.7056.5.A1\_x\_at | 511735 | HBG | hemoglobin, gamma |
| Bt.8479.1.A1\_at | 282876 | NOS2A | nitric oxide synthase 2A (inducible, hepatocytes) |
| Bt.848.1.S1\_at | 282290 | NDUFV2 | NADH dehydrogenase (ubiquinone) flavoprotein 2, 24kDa |
| Bt.9240.1.A1\_at | 511058 | PPP6C | protein phosphatase 6, catalytic subunit |
| Bt.9578.1.S1\_at | 282318 | PPP1CC | protein phosphatase 1, catalytic subunit, gamma isoform |
| Bt.9731.1.S1\_at | 513762 | GLRX2 | glutaredoxin 2 |
| Bt.9756.1.S1\_at | 530865 | LIAS | lipoic acid synthetase |
| Bt.9826.1.S1\_at | 444878 | COX5A | COX5A protein |

### GO:0019829

|  |  |  |  |
| --- | --- | --- | --- |
| **probe** | **entrez** | **symbol** | **description** |
| Bt.1242.1.S1\_at | 338053 | ATP5G1 | ATP synthase, H+ transporting, mitochondrial F0 complex, subunit C1 (subunit 9) |
| Bt.1399.1.S1\_at | 282405 | ATP6V1F | ATPase, H+ transporting, lysosomal 14kDa, V1 subunit F |
| Bt.1426.1.S1\_at | 338081 | ATP5D | ATP synthase, H+ transporting, mitochondrial F1 complex, delta subunit |
| Bt.20146.1.S1\_at | 282146 | ATP2B4 | ATPase, Ca++ transporting, plasma membrane 4 |
| Bt.27657.1.A1\_at | 338053 | ATP5G1 | ATP synthase, H+ transporting, mitochondrial F0 complex, subunit C1 (subunit 9) |
| Bt.2816.1.S1\_at | 515696 | ATP5L | ATP synthase, H+ transporting, mitochondrial F0 complex, subunit G |
| Bt.378.1.S1\_at | 338040 | ATP5I | ATP synthase, H+ transporting, mitochondrial F0 complex, subunit e |
| Bt.3867.1.S1\_at | 282701 | ATP5F1 | ATP synthase, H+ transporting, mitochondrial F0 complex, subunit B1 |
| Bt.404.1.S1\_at | 282690 | ATP5J | ATP synthase, H+ transporting, mitochondrial F0 complex, subunit F6 |
| Bt.4431.1.S1\_a\_at | 327675 | ATP5B | ATP synthase, H+ transporting, mitochondrial F1 complex, beta polypeptide |
| Bt.4540.1.S1\_at | 327668 | ATP5C1 | ATP synthase, H+ transporting, mitochondrial F1 complex, gamma polypeptide 1 |
| Bt.5029.1.S1\_at | 282710 | ATP5H | ATP synthase, H+ transporting, mitochondrial F0 complex, subunit d |
| Bt.5510.1.S1\_at | 338088 | ATP5E | ATP synthase, H+ transporting, mitochondrial F1 complex, epsilon subunit |
| Bt.5848.1.S1\_at | 518117 | ATP2A1 | ATPase, Ca++ transporting, cardiac muscle, fast twitch 1 |
| Bt.5848.2.S1\_at | 518117 | ATP2A1 | ATPase, Ca++ transporting, cardiac muscle, fast twitch 1 |
| Bt.8047.1.S1\_at | 540176 | ATP5G3 | ATP synthase, H+ transporting, mitochondrial F0 complex, subunit C3 (subunit 9) |

### GO:0004298

|  |  |  |  |
| --- | --- | --- | --- |
| **probe** | **entrez** | **symbol** | **description** |
| Bt.10266.1.S1\_at | 515503 | PSMA1 | proteasome (prosome, macropain) subunit, alpha type, 1 |
| Bt.13237.1.S1\_at | 510423 | PSMA4 | proteasome (prosome, macropain) subunit, alpha type, 4 |
| Bt.15837.1.S1\_at | 507213 | PSMA6 | proteasome (prosome, macropain) subunit, alpha type, 6 |
| Bt.2116.1.S1\_at | 510069 | PSMB6 | proteasome (prosome, macropain) subunit, beta type, 6 |
| Bt.2821.1.S1\_at | 511207 | PSMB7 | proteasome (prosome, macropain) subunit, beta type, 7 |
| Bt.5322.1.S1\_at | 534640 | PSMB5 | proteasome (prosome, macropain) subunit, beta type, 5 |
| Bt.6473.1.S1\_at | 505050 | PSMA7 | proteasome (prosome, macropain) subunit, alpha type, 7 |
| Bt.6827.1.S1\_at | 505176 | PSMA3 | proteasome (prosome, macropain) subunit, alpha type, 3 |
| Bt.886.1.S1\_at | 510155 | PSMA5 | proteasome (prosome, macropain) subunit, alpha type, 5 |

### GO:0051539

|  |  |  |  |
| --- | --- | --- | --- |
| **probe** | **entrez** | **symbol** | **description** |
| Bt.13439.1.S1\_at | 768074 | ETFDH | electron-transferring-flavoprotein dehydrogenase |
| Bt.315.1.S1\_at | 338079 | NDUFS7 | NADH dehydrogenase (ubiquinone) Fe-S protein 7, 20kDa (NADH-coenzyme Q reductase) |
| Bt.4072.1.S1\_at | 287014 | NDUFV1 | NADH dehydrogenase (ubiquinone) flavoprotein 1, 51kDa |
| Bt.4475.1.S1\_at | 327697 | NDUFS2 | NADH dehydrogenase (ubiquinone) Fe-S protein 2, 49kDa (NADH-coenzyme Q reductase) |
| Bt.4777.1.S1\_at | 288380 | NDUFS1 | NADH dehydrogenase (ubiquinone) Fe-S protein 1, 75kDa (NADH-coenzyme Q reductase) |
| Bt.5210.1.S1\_at | 280976 | ACO2 | aconitase 2, mitochondrial |
| Bt.5483.1.S1\_at | 287027 | NDUFS8 | NADH dehydrogenase (ubiquinone) Fe-S protein 8, 23kDa (NADH-coenzyme Q reductase) |
| Bt.9756.1.S1\_at | 530865 | LIAS | lipoic acid synthetase |

### GO:0035257

|  |  |  |  |
| --- | --- | --- | --- |
| **probe** | **entrez** | **symbol** | **description** |
| Bt.13313.1.S1\_at | 525346 | NCOA1 | nuclear receptor coactivator 1 |
| Bt.13313.2.S1\_at | 525346 | NCOA1 | nuclear receptor coactivator 1 |
| Bt.13637.1.A1\_at | 523707 | NCOA3 | nuclear receptor coactivator 3 |
| Bt.21119.1.A1\_at | 525346 | NCOA1 | nuclear receptor coactivator 1 |
| Bt.27939.2.A1\_a\_at | 525346 | NCOA1 | nuclear receptor coactivator 1 |
| Bt.4044.1.S1\_at | 618849 | HMGA1 | high mobility group AT-hook 1 |
| Bt.4797.1.S1\_at | 508535 | FKBP4 | FK506 binding protein 4, 59kDa |

### GO:0030145

|  |  |  |  |
| --- | --- | --- | --- |
| **probe** | **entrez** | **symbol** | **description** |
| Bt.13199.1.S1\_at | 538829 | PPP1CB | protein phosphatase 1, catalytic subunit, beta isoform |
| Bt.13324.2.S1\_a\_at | 281235 | IDH1 | isocitrate dehydrogenase 1 (NADP+), soluble |
| Bt.16020.1.A2\_at | 338068 | ACVR1 | activin A receptor, type I |
| Bt.1760.1.S1\_at | 540139 | REXO2 | REX2, RNA exonuclease 2 homolog (S. cerevisiae) |
| Bt.18634.1.A1\_at | 540329 | PPM1K | protein phosphatase 1K (PP2C domain containing) |
| Bt.18634.2.S1\_at | 540329 | PPM1K | protein phosphatase 1K (PP2C domain containing) |
| Bt.21997.1.S1\_at | 540329 | PPM1K | protein phosphatase 1K (PP2C domain containing) |
| Bt.21997.2.A1\_at | 540329 | PPM1K | protein phosphatase 1K (PP2C domain containing) |
| Bt.22082.1.S1\_at | 511291 | CHPT1 | choline phosphotransferase 1 |
| Bt.23357.1.S1\_at | 614145 | IDH3G | isocitrate dehydrogenase 3 (NAD+) gamma |
| Bt.2359.1.A1\_at | 527263 | FYN | FYN oncogene related to SRC, FGR, YES |
| Bt.24396.1.S1\_at | 616931 | NUDT10 | nudix (nucleoside diphosphate linked moiety X)-type motif 10 |
| Bt.26641.1.S1\_at | 513156 | XPNPEP1 | X-prolyl aminopeptidase (aminopeptidase P) 1, soluble |
| Bt.3857.1.S1\_at | 327707 | ENDOG | endonuclease G |
| Bt.3898.1.S1\_at | 282446 | IDH3A | isocitrate dehydrogenase 3 (NAD+) alpha |
| Bt.413.1.S1\_at | 281780 | GGTA1 | alpha-galactosyltransferase 1 (glycoprotein) |
| Bt.4748.1.S1\_at | 281496 | SOD2 | superoxide dismutase 2, mitochondrial |
| Bt.5520.1.S1\_at | 327669 | IDH2 | isocitrate dehydrogenase 2 (NADP+), mitochondrial |
| Bt.7240.1.S1\_at | 781648 | LAP3 | leucine aminopeptidase 3 |
| Bt.9578.1.S1\_at | 282318 | PPP1CC | protein phosphatase 1, catalytic subunit, gamma isoform |

### GO:0016769

|  |  |  |  |
| --- | --- | --- | --- |
| **probe** | **entrez** | **symbol** | **description** |
| Bt.1316.1.S1\_at | 286886 | GOT2 | glutamic-oxaloacetic transaminase 2, mitochondrial (aspartate aminotransferase 2) |
| Bt.1316.1.S2\_at | 286886 | GOT2 | glutamic-oxaloacetic transaminase 2, mitochondrial (aspartate aminotransferase 2) |
| Bt.13287.1.S1\_at | 515186 | AGXT2L1 | alanine-glyoxylate aminotransferase 2-like 1 |
| Bt.14207.1.S1\_at | 319141 | GCAT | glycine C-acetyltransferase (2-amino-3-ketobutyrate coenzyme A ligase) |
| Bt.24265.1.S1\_a\_at | 508712 | CCBL2 | cysteine conjugate-beta lyase 2 |
| Bt.4744.1.S1\_at | 281206 | GOT1 | glutamic-oxaloacetic transaminase 1, soluble (aspartate aminotransferase 1) |
| Bt.6289.1.S1\_at | 614165 | SPTLC1 | serine palmitoyltransferase, long chain base subunit 1 |
| Bt.7177.1.S1\_at | 280719 | AMT | aminomethyltransferase |
| Bt.9623.1.A1\_at | 539188 | GPT | glutamic-pyruvate transaminase (alanine aminotransferase) |

### GO:0008308

|  |  |  |  |
| --- | --- | --- | --- |
| **probe** | **entrez** | **symbol** | **description** |
| Bt.1523.1.S1\_at | 510219 | TOMM40 | translocase of outer mitochondrial membrane 40 homolog (yeast) |
| Bt.23174.2.S1\_at | 515646 | CLIC1 | chloride intracellular channel 1 |
| Bt.25654.1.A1\_at | 511699 | CLCN4 | chloride channel 4 |
| Bt.3960.1.S1\_a\_at | 282119 | VDAC1 | voltage-dependent anion channel 1 |
| Bt.3960.2.S1\_at | 282119 | VDAC1 | voltage-dependent anion channel 1 |
| Bt.4099.1.S1\_at | 282120 | VDAC2 | voltage-dependent anion channel 2 |
| Bt.981.2.S1\_at | 286823 | CLIC4 | chloride intracellular channel 4 |

### GO:0016709

|  |  |  |  |
| --- | --- | --- | --- |
| **probe** | **entrez** | **symbol** | **description** |
| Bt.22142.2.S1\_a\_at | 511624 | COQ6 | coenzyme Q6 homolog, monooxygenase (S. cerevisiae) |
| Bt.4662.1.S1\_at | 287024 | NOS3 | nitric oxide synthase 3 (endothelial cell) |
| Bt.621.1.S1\_at | 505060 | CYP51 | cytochrome P450, family 51, subfamily A, polypeptide 1 |
| Bt.8479.1.A1\_at | 282876 | NOS2A | nitric oxide synthase 2A (inducible, hepatocytes) |

### GO:0046912

|  |  |  |  |
| --- | --- | --- | --- |
| **probe** | **entrez** | **symbol** | **description** |
| Bt.1129.1.S1\_at | 280682 | CS | citrate synthase |
| Bt.1129.2.S1\_at | 280682 | CS | citrate synthase |
| Bt.22763.2.S1\_a\_at | 407767 | HMGCS1 | 3-hydroxy-3-methylglutaryl-coenzyme A synthase 1 |
| Bt.2711.1.S1\_at | 509983 | SUCLG1 | succinate-CoA ligase, alpha subunit |

### GO:0004721

|  |  |  |  |
| --- | --- | --- | --- |
| **probe** | **entrez** | **symbol** | **description** |
| Bt.1069.1.A1\_at | 536337 | PTPRR | protein tyrosine phosphatase, receptor type, R |
| Bt.10880.1.S1\_at | 505489 | TIMM50 | translocase of inner mitochondrial membrane 50 homolog (S. cerevisiae) |
| Bt.11058.1.A1\_at | 280891 | PPM2C | protein phosphatase 2C, magnesium-dependent, catalytic subunit |
| Bt.13199.1.S1\_at | 538829 | PPP1CB | protein phosphatase 1, catalytic subunit, beta isoform |
| Bt.17425.1.A1\_at | 511058 | PPP6C | protein phosphatase 6, catalytic subunit |
| Bt.18634.1.A1\_at | 540329 | PPM1K | protein phosphatase 1K (PP2C domain containing) |
| Bt.18634.2.S1\_at | 540329 | PPM1K | protein phosphatase 1K (PP2C domain containing) |
| Bt.18748.1.A1\_at | 505696 | PTPRB | protein tyrosine phosphatase, receptor type, B |
| Bt.20261.1.S1\_at | 511010 | PTPN3 | protein tyrosine phosphatase, non-receptor type 3 |
| Bt.2070.1.A1\_at | 541175 | DUSP10 | dual specificity phosphatase 10 |
| Bt.2070.2.A1\_at | 541175 | DUSP10 | dual specificity phosphatase 10 |
| Bt.2070.3.S1\_at | 541175 | DUSP10 | dual specificity phosphatase 10 |
| Bt.21997.1.S1\_at | 540329 | PPM1K | protein phosphatase 1K (PP2C domain containing) |
| Bt.21997.2.A1\_at | 540329 | PPM1K | protein phosphatase 1K (PP2C domain containing) |
| Bt.23237.1.S1\_at | 510245 | PPP5C | protein phosphatase 5, catalytic subunit |
| Bt.27121.1.S1\_at | 509192 | DULLARD | dullard homolog (Xenopus laevis) |
| Bt.27211.1.S1\_at | 616048 | DUSP13 | dual specificity phosphatase 13 |
| Bt.27565.1.S1\_at | 509657 | PTPRK | protein tyrosine phosphatase, receptor type, K |
| Bt.27956.1.A1\_at | 540786 | PTEN | phosphatase and tensin homolog (mutated in multiple advanced cancers 1) |
| Bt.28758.1.A1\_at | 531877 | PTPN12 | protein tyrosine phosphatase, non-receptor type 12 |
| Bt.6842.1.S1\_at | 520428 | CDC14B | CDC14 cell division cycle 14 homolog B (S. cerevisiae) |
| Bt.7319.1.S1\_at | 509657 | PTPRK | protein tyrosine phosphatase, receptor type, K |
| Bt.8024.1.A1\_at | 541155 | MTMR1 | myotubularin related protein 1 |
| Bt.9240.1.A1\_at | 511058 | PPP6C | protein phosphatase 6, catalytic subunit |
| Bt.9578.1.S1\_at | 282318 | PPP1CC | protein phosphatase 1, catalytic subunit, gamma isoform |

### GO:0003712

|  |  |  |  |
| --- | --- | --- | --- |
| **probe** | **entrez** | **symbol** | **description** |
| Bt.11446.1.A1\_at | 617976 | LDB2 | LIM domain binding 2 |
| Bt.13313.1.S1\_at | 525346 | NCOA1 | nuclear receptor coactivator 1 |
| Bt.13313.2.S1\_at | 525346 | NCOA1 | nuclear receptor coactivator 1 |
| Bt.13637.1.A1\_at | 523707 | NCOA3 | nuclear receptor coactivator 3 |
| Bt.15836.1.S1\_at | 510376 | ANKRD1 | ankyrin repeat domain 1 (cardiac muscle) |
| Bt.15836.2.S1\_at | 510376 | ANKRD1 | ankyrin repeat domain 1 (cardiac muscle) |
| Bt.21119.1.A1\_at | 525346 | NCOA1 | nuclear receptor coactivator 1 |
| Bt.23158.1.S1\_at | 505453 | CREBBP | CREB binding protein (Rubinstein-Taybi syndrome) |
| Bt.27939.2.A1\_a\_at | 525346 | NCOA1 | nuclear receptor coactivator 1 |
| Bt.4044.1.S1\_at | 618849 | HMGA1 | high mobility group AT-hook 1 |
| Bt.7785.1.S1\_at | 513312 | NACA | nascent polypeptide-associated complex alpha subunit |

### GO:0004707

|  |  |  |  |
| --- | --- | --- | --- |
| **probe** | **entrez** | **symbol** | **description** |
| Bt.167.1.S1\_at | 327672 | MAPK1 | mitogen-activated protein kinase 1 |
| Bt.18165.1.A1\_at | 538094 | MAPK6 | mitogen-activated protein kinase 6 |
| Bt.3448.1.S1\_at | 538094 | MAPK6 | mitogen-activated protein kinase 6 |
| Bt.3448.2.S1\_at | 538094 | MAPK6 | mitogen-activated protein kinase 6 |
