# Supplementary material for: A gene network switch enhances the oxidative capacity of ovine skeletal muscle during late fetal development
Source: BMC Genomics. 2010 Jun 15;11:378. doi: 10.1186/1471-2164-11-378 (PMC2894804; doi:10.1186/1471-2164-11-378)
Supplement: Additional File 1 — Gene expression data. This file contains links to the gene expression data. The genes that are significantly differentially expressed between adjacent development times are listed as Experiment 1 (80 d vs 100 d), Experiment 2 (100 d vs 120 d), Experiment 3 (120 d vs 150 d) and Experiment 4 (150 d vs 230 d) in the section entitled Open microarray analysis report for developmental expression. Each experiment contains an MA plot, Volcano plot and Heatmap for each of the three microarray processing programs MAS5, RMA and GCRMA. For each of these processing programs there is a list of significantly differentially expressed probe sets, and for each of these there is an FDR corrected probability, fold change, percentage of microarrays with a MAS5 Present call, and a convergence percentage for all three microarray processing programs. The file also contains annotation of the probe sets. In a second section entitled Time series analysis of developmental expression, there is a list of genes in each gene expression cluster and diagrams showing MAS5 gene expression values for these genes. [file 1471-2164-11-378-S1.ZIP › Report/GO4/Report/BP.html]

### Biological process

| Entrez | GO ID | Evidence Code |
| --- | --- | --- |
 280701 | GO:0006796 | IEA || 280716 | GO:0006139 | IEA |
| 280726 | GO:0008652 | IEA |
| 280726 | GO:0006526 | IEA |
| 280726 | GO:0000050 | IEA |
| 280813 | GO:0006810 | IEA |
| 280813 | GO:0015671 | IEA |
| 280904 | GO:0008283 | IEA |
| 280904 | GO:0030282 | IEA |
| 280904 | GO:0007612 | IEA |
| 281032 | GO:0045766 | ISS |
| 281032 | GO:0045603 | IEA |
| 281032 | GO:0045603 | ISS |
| 281032 | GO:0045663 | ISS |
| 281032 | GO:0045449 | ISS |
| 281032 | GO:0030308 | ISS |
| 281032 | GO:0045766 | IEA |
| 281032 | GO:0016477 | ISS |
| 281032 | GO:0008285 | ISS |
| 281032 | GO:0007286 | ISS |
| 281032 | GO:0008285 | IEA |
| 281032 | GO:0042981 | IEA |
| 281032 | GO:0042981 | ISS |
| 281032 | GO:0043085 | ISS |
| 281091 | GO:0006118 | IEA |
| 281141 | GO:0006096 | IEA |
| 281156 | GO:0016126 | IEA |
| 281156 | GO:0008299 | IEA |
| 281156 | GO:0006694 | IEA |
| 281156 | GO:0006695 | IEA |
| 281156 | GO:0008610 | IEA |
| 281195 | GO:0015012 | IDA |
| 281195 | GO:0015012 | IEA |
| 281195 | GO:0030210 | IDA |
| 281195 | GO:0006024 | IEA |
| 281195 | GO:0009252 | IEA |
| 281195 | GO:0008360 | IEA |
| 281195 | GO:0030210 | IEA |
| 281199 | GO:0006807 | IEA |
| 281199 | GO:0006542 | IEA |
| 281225 | GO:0006464 | IEA |
| 281225 | GO:0006512 | IEA |
| 281235 | GO:0006099 | IEA |
| 281235 | GO:0006979 | ISS |
| 281235 | GO:0008152 | IEA |
| 281235 | GO:0006097 | IEA |
| 281235 | GO:0006749 | ISS |
| 281235 | GO:0006099 | IEA |
| 281235 | GO:0006979 | ISS |
| 281235 | GO:0008152 | IEA |
| 281235 | GO:0006749 | ISS |
| 281235 | GO:0006097 | IEA |
| 281240 | GO:0008283 | IEA |
| 281259 | GO:0006457 | IEA |
| 281360 | GO:0008152 | IEA |
| 281365 | GO:0006596 | IEA |
| 281370 | GO:0045941 | IEA |
| 281370 | GO:0006464 | IEA |
| 281370 | GO:0016567 | IEA |
| 281370 | GO:0016567 | IEA |
| 281370 | GO:0045941 | IEA |
| 281370 | GO:0006464 | IEA |
| 281373 | GO:0006457 | IEA |
| 281373 | GO:0018401 | IEA |
| 281373 | GO:0018401 | ISS |
| 281373 | GO:0045454 | IEA |
| 281408 | GO:0006508 | IEA |
| 281419 | GO:0006457 | IEA |
| 281476 | GO:0006434 | IEA |
| 281476 | GO:0006412 | IEA |
| 281476 | GO:0006418 | IEA |
| 281478 | GO:0051016 | IEA |
| 281478 | GO:0045654 | ISS |
| 281478 | GO:0043065 | ISS |
| 281478 | GO:0045010 | IDA |
| 281478 | GO:0051014 | IDA |
| 281478 | GO:0051047 | IDA |
| 281478 | GO:0008285 | ISS |
| 281478 | GO:0017156 | IDA |
| 281482 | GO:0008152 | IEA |
| 281546 | GO:0015031 | IEA |
| 281546 | GO:0006810 | IEA |
| 281546 | GO:0006605 | IEA |
| 281565 | GO:0008152 | IEA |
| 281576 | GO:0006436 | IEA |
| 281576 | GO:0006412 | IEA |
| 281576 | GO:0006418 | IEA |
| 281687 | GO:0042391 | IEA |
| 281687 | GO:0006811 | IEA |
| 281687 | GO:0006810 | IEA |
| 281759 | GO:0006810 | IEA |
| 281767 | GO:0009058 | IEA |
| 281767 | GO:0008610 | IEA |
| 281767 | GO:0009058 | IEA |
| 281767 | GO:0008610 | IEA |
| 281801 | GO:0006468 | IEA |
| 281801 | GO:0007165 | IEA |
| 281814 | GO:0006879 | IEA |
| 281814 | GO:0035162 | IEA |
| 281814 | GO:0045648 | IEA |
| 281814 | GO:0006089 | IEA |
| 281814 | GO:0007165 | IEA |
| 281814 | GO:0042593 | IEA |
| 281814 | GO:0045449 | IEA |
| 281814 | GO:0006350 | IEA |
| 281814 | GO:0001525 | IEA |
| 281814 | GO:0001947 | IEA |
| 281814 | GO:0006355 | IEA |
| 281814 | GO:0045941 | ISS |
| 281814 | GO:0030949 | IEA |
| 281814 | GO:0051216 | IEA |
| 281814 | GO:0001755 | IEA |
| 281814 | GO:0042981 | IEA |
| 281814 | GO:0046716 | IEA |
| 281814 | GO:0045944 | IEA |
| 281814 | GO:0014850 | IEA |
| 281814 | GO:0030154 | IEA |
| 281814 | GO:0001892 | IEA |
| 281814 | GO:0042541 | IEA |
| 281814 | GO:0043619 | IEA |
| 281814 | GO:0045926 | IEA |
| 281814 | GO:0001666 | ISS |
| 281814 | GO:0050790 | IEA |
| 281898 | GO:0007507 | ISS |
| 281898 | GO:0006412 | IEA |
| 281994 | GO:0006470 | IEA |
| 282127 | GO:0050728 | IEA |
| 282127 | GO:0000289 | IEA |
| 282127 | GO:0045638 | IEA |
| 282127 | GO:0050779 | IEA |
| 282127 | GO:0006402 | IEA |
| 282127 | GO:0007243 | IEA |
| 282127 | GO:0043488 | IEA |
| 282127 | GO:0000288 | IEA |
| 282187 | GO:0006817 | IEA |
| 282187 | GO:0006817 | IEA |
| 282188 | GO:0006817 | IEA |
| 282188 | GO:0006817 | IEA |
| 282211 | GO:0006118 | IEA |
| 282261 | GO:0043065 | ISS |
| 282261 | GO:0045663 | ISS |
| 282261 | GO:0001558 | IEA |
| 282261 | GO:0009968 | ISS |
| 282291 | GO:0045746 | IEA |
| 282291 | GO:0032495 | IEA |
| 282291 | GO:0045638 | IEA |
| 282291 | GO:0042127 | IEA |
| 282291 | GO:0032496 | IEA |
| 282291 | GO:0043330 | IEA |
| 282291 | GO:0031663 | IEA |
| 282291 | GO:0000060 | IEA |
| 282304 | GO:0006094 | ISS |
| 282304 | GO:0008152 | IEA |
| 282304 | GO:0006003 | IEA |
| 282304 | GO:0006096 | ISS |
| 282331 | GO:0007186 | IEA |
| 282331 | GO:0007165 | IEA |
| 282331 | GO:0030278 | IEA |
| 282376 | GO:0019915 | IEA |
| 282376 | GO:0050729 | IEA |
| 282376 | GO:0042104 | IEA |
| 282376 | GO:0007259 | IEA |
| 282376 | GO:0007595 | IEA |
| 282376 | GO:0006355 | IEA |
| 282376 | GO:0007242 | IEA |
| 282376 | GO:0045086 | IEA |
| 282376 | GO:0019221 | IEA |
| 282376 | GO:0046544 | IEA |
| 282376 | GO:0008284 | IEA |
| 282376 | GO:0045931 | IEA |
| 282376 | GO:0006350 | IEA |
| 282376 | GO:0030856 | IEA |
| 282376 | GO:0045885 | IEA |
| 282376 | GO:0045944 | IEA |
| 282376 | GO:0019218 | IEA |
| 282376 | GO:0045579 | IEA |
| 282376 | GO:0043066 | IEA |
| 282376 | GO:0042448 | IEA |
| 282376 | GO:0001553 | IEA |
| 282376 | GO:0043029 | IEA |
| 282376 | GO:0040018 | IEA |
| 282376 | GO:0045647 | IEA |
| 282376 | GO:0007165 | IEA |
| 282376 | GO:0001779 | IEA |
| 282376 | GO:0046543 | IEA |
| 282376 | GO:0007565 | IEA |
| 282376 | GO:0030155 | IEA |
| 282376 | GO:0045931 | IEA |
| 282376 | GO:0042104 | IEA |
| 282376 | GO:0045944 | IEA |
| 282376 | GO:0019218 | IEA |
| 282376 | GO:0042448 | IEA |
| 282376 | GO:0050729 | IEA |
| 282376 | GO:0007165 | IEA |
| 282376 | GO:0045647 | IEA |
| 282376 | GO:0019221 | IEA |
| 282376 | GO:0006355 | IEA |
| 282376 | GO:0019915 | IEA |
| 282376 | GO:0045885 | IEA |
| 282376 | GO:0040018 | IEA |
| 282376 | GO:0043029 | IEA |
| 282376 | GO:0007259 | IEA |
| 282376 | GO:0046544 | IEA |
| 282376 | GO:0043066 | IEA |
| 282376 | GO:0030155 | IEA |
| 282376 | GO:0001779 | IEA |
| 282376 | GO:0007595 | IEA |
| 282376 | GO:0001553 | IEA |
| 282376 | GO:0006350 | IEA |
| 282376 | GO:0045579 | IEA |
| 282376 | GO:0030856 | IEA |
| 282376 | GO:0045086 | IEA |
| 282376 | GO:0007565 | IEA |
| 282376 | GO:0008284 | IEA |
| 282376 | GO:0007242 | IEA |
| 282376 | GO:0046543 | IEA |
| 282386 | GO:0007010 | IEA |
| 282688 | GO:0006412 | IEA |
| 282711 | GO:0001666 | IEA |
| 282711 | GO:0045449 | IEA |
| 282711 | GO:0006355 | IEA |
| 282711 | GO:0007165 | IEA |
| 282711 | GO:0045944 | IEA |
| 282711 | GO:0001666 | IEA |
| 282711 | GO:0045449 | IEA |
| 282711 | GO:0045944 | IEA |
| 282711 | GO:0007165 | IEA |
| 282711 | GO:0006355 | IEA |
| 282875 | GO:0006468 | IEA |
| 282875 | GO:0006950 | IEA |
| 282875 | GO:0042473 | IEA |
| 282875 | GO:0006917 | IEA |
| 282875 | GO:0048705 | IEA |
| 282875 | GO:0042474 | IEA |
| 282884 | GO:0006412 | IEA |
| 286764 | GO:0040009 | IEA |
| 286764 | GO:0006974 | IEA |
| 286764 | GO:0006471 | IEA |
| 286764 | GO:0000723 | IEA |
| 286764 | GO:0006284 | IEA |
| 286764 | GO:0006281 | IEA |
| 286764 | GO:0006259 | IEA |
| 286806 | GO:0001501 | ISS |
| 286806 | GO:0006508 | ISS |
| 286806 | GO:0006516 | ISS |
| 286806 | GO:0006508 | IEA |
| 286873 | GO:0006412 | IEA |
| 287016 | GO:0006334 | IEA |
| 326582 | GO:0006412 | IEA |
| 327685 | GO:0006937 | ISS |
| 327685 | GO:0006816 | ISS |
| 327687 | GO:0006811 | IEA |
| 327687 | GO:0006810 | IEA |
| 327687 | GO:0015986 | IEA |
| 327687 | GO:0006754 | IEA |
| 327687 | GO:0015992 | IEA |
| 327688 | GO:0006118 | IEA |
| 327703 | GO:0006816 | IEA |
| 327703 | GO:0006810 | IEA |
| 327703 | GO:0006811 | IEA |
| 338049 | GO:0043123 | IEA |
| 338049 | GO:0043123 | ISS |
| 338049 | GO:0007266 | ISS |
| 338049 | GO:0042346 | ISS |
| 338049 | GO:0030036 | ISS |
| 338049 | GO:0007264 | IEA |
| 338062 | GO:0002474 | IEA |
| 338062 | GO:0006955 | IEA |
| 338062 | GO:0019882 | IEA |
| 404079 | GO:0009209 | IEA |
| 404079 | GO:0006241 | IEA |
| 404079 | GO:0009117 | IEA |
| 404079 | GO:0007595 | IEA |
| 404079 | GO:0030879 | IEA |
| 404079 | GO:0006183 | IEA |
| 404079 | GO:0006228 | IEA |
| 404121 | GO:0006810 | IEA |
| 404121 | GO:0015031 | IEA |
| 404121 | GO:0006986 | IEA |
| 404126 | GO:0016575 | IEA |
| 404148 | GO:0008152 | IEA |
| 404148 | GO:0006096 | IEA |
| 404159 | GO:0045454 | IEA |
| 404189 | GO:0006183 | IEA |
| 404189 | GO:0006241 | IEA |
| 404189 | GO:0007595 | IEA |
| 404189 | GO:0009209 | IEA |
| 404189 | GO:0030879 | IEA |
| 404189 | GO:0009117 | IEA |
| 404189 | GO:0006228 | IEA |
| 407767 | GO:0006084 | IEA |
| 408001 | GO:0006914 | IEA |
| 408001 | GO:0006512 | IEA |
| 408001 | GO:0000045 | ISS |
| 408001 | GO:0006512 | IEA |
| 408001 | GO:0000045 | ISS |
| 408001 | GO:0006914 | IEA |
| 408010 | GO:0006418 | IEA |
| 408010 | GO:0006426 | IEA |
| 408010 | GO:0006412 | IEA |
| 414274 | GO:0030902 | IEA |
| 414274 | GO:0001832 | IEA |
| 414274 | GO:0007403 | IEA |
| 414274 | GO:0001835 | IEA |
| 414274 | GO:0030900 | IEA |
| 414274 | GO:0000122 | IEA |
| 414274 | GO:0006346 | IEA |
| 444874 | GO:0045941 | IEA |
| 444874 | GO:0016567 | IEA |
| 444874 | GO:0006464 | IEA |
| 444874 | GO:0006464 | IEA |
| 444874 | GO:0045941 | IEA |
| 444874 | GO:0016567 | IEA |
| 503685 | GO:0008544 | IEA |
| 504499 | GO:0030154 | IEA |
| 504499 | GO:0045576 | IEA |
| 504735 | GO:0006457 | IEA |
| 504735 | GO:0044267 | IEA |
| 504781 | GO:0005975 | IEA |
| 504781 | GO:0006032 | IEA |
| 505005 | GO:0016310 | IEA |
| 505005 | GO:0018106 | IEA |
| 505060 | GO:0006118 | IEA |
| 505060 | GO:0008610 | IEA |
| 505060 | GO:0006695 | IEA |
| 505060 | GO:0006694 | IEA |
| 505060 | GO:0016126 | IEA |
| 505171 | GO:0043123 | IEA |
| 505199 | GO:0006412 | IEA |
| 505642 | GO:0008152 | IEA |
| 505691 | GO:0000079 | IEA |
| 505691 | GO:0007050 | IEA |
| 505691 | GO:0008285 | IEA |
| 505691 | GO:0000082 | IEA |
| 505691 | GO:0042326 | IEA |
| 505691 | GO:0006917 | IEA |
| 505691 | GO:0030308 | IEA |
| 505807 | GO:0006464 | IEA |
| 505884 | GO:0019221 | IEA |
| 505901 | GO:0006468 | IEA |
| 505901 | GO:0050870 | IEA |
| 505901 | GO:0042102 | IEA |
| 505901 | GO:0007242 | IEA |
| 505901 | GO:0045086 | IEA |
| 505901 | GO:0051092 | IEA |
| 506043 | GO:0006457 | IEA |
| 506085 | GO:0006915 | IEA |
| 506214 | GO:0050778 | IEA |
| 506214 | GO:0016064 | IEA |
| 506214 | GO:0002504 | IEA |
| 506214 | GO:0006955 | IEA |
| 506214 | GO:0019886 | IEA |
| 506214 | GO:0006952 | IEA |
| 506336 | GO:0015031 | IEA |
| 506336 | GO:0006888 | IEA |
| 506336 | GO:0006810 | IEA |
| 506370 | GO:0006810 | IEA |
| 506544 | GO:0006096 | IEA |
| 506566 | GO:0007368 | IEA |
| 506715 | GO:0008104 | IEA |
| 506715 | GO:0031122 | IEA |
| 506715 | GO:0001764 | IEA |
| 506790 | GO:0006979 | IEA |
| 506790 | GO:0030216 | IEA |
| 506988 | GO:0006412 | IEA |
| 506989 | GO:0006412 | IEA |
| 507234 | GO:0007165 | IEA |
| 507234 | GO:0043123 | IEA |
| 507234 | GO:0008284 | IEA |
| 507270 | GO:0006412 | IEA |
| 507449 | GO:0007264 | IEA |
| 507784 | GO:0006412 | IEA |
| 507815 | GO:0018279 | IEA |
| 507815 | GO:0006486 | IEA |
| 507845 | GO:0031115 | ISS |
| 507845 | GO:0007049 | IEA |
| 507845 | GO:0051301 | IEA |
| 507845 | GO:0007067 | IEA |
| 508221 | GO:0006412 | IEA |
| 508319 | GO:0006816 | IEA |
| 508628 | GO:0006412 | IEA |
| 508629 | GO:0008152 | IEA |
| 509426 | GO:0006412 | IEA |
| 509597 | GO:0006955 | IEA |
| 509597 | GO:0002474 | IEA |
| 509597 | GO:0019882 | IEA |
| 509696 | GO:0008299 | IEA |
| 509696 | GO:0051290 | IEA |
| 509696 | GO:0006744 | IEA |
| 509771 | GO:0006412 | IEA |
| 509771 | GO:0035196 | IEA |
| 509771 | GO:0009409 | IEA |
| 509896 | GO:0016042 | IEA |
| 509896 | GO:0006508 | IEA |
| 509896 | GO:0006725 | IEA |
| 509963 | GO:0051005 | IEA |
| 509963 | GO:0007165 | IEA |
| 509963 | GO:0030154 | IEA |
| 509963 | GO:0001525 | IEA |
| 509963 | GO:0007275 | IEA |
| 509963 | GO:0043066 | IEA |
| 509965 | GO:0006139 | IEA |
| 509965 | GO:0006221 | IEA |
| 510050 | GO:0045449 | IEA |
| 510050 | GO:0006355 | IEA |
| 510159 | GO:0007067 | IEA |
| 510159 | GO:0007049 | IEA |
| 510159 | GO:0051301 | IEA |
| 510200 | GO:0006508 | IEA |
| 510423 | GO:0006511 | IEA |
| 510850 | GO:0006457 | IEA |
| 510937 | GO:0006418 | IEA |
| 510937 | GO:0006427 | IEA |
| 510937 | GO:0006412 | IEA |
| 511135 | GO:0008152 | IEA |
| 511560 | GO:0006412 | IEA |
| 511560 | GO:0006364 | IEA |
| 511716 | GO:0006725 | IEA |
| 511716 | GO:0008152 | IEA |
| 511798 | GO:0006512 | IEA |
| 511798 | GO:0006512 | IEA |
| 511852 | GO:0007242 | IEA |
| 511899 | GO:0006991 | IEA |
| 512254 | GO:0006350 | IEA |
| 512254 | GO:0006355 | IEA |
| 512673 | GO:0043681 | IEA |
| 512918 | GO:0007154 | IEA |
| 513038 | GO:0006508 | IEA |
| 513125 | GO:0000122 | IEA |
| 513125 | GO:0009404 | IEA |
| 513128 | GO:0006412 | IEA |
| 513128 | GO:0042254 | IEA |
| 513497 | GO:0030890 | IEA |
| 513497 | GO:0042326 | IEA |
| 513497 | GO:0008285 | IEA |
| 513497 | GO:0031668 | IEA |
| 513497 | GO:0007050 | IEA |
| 513497 | GO:0043066 | IEA |
| 513497 | GO:0030308 | IEA |
| 513497 | GO:0006974 | IEA |
| 513497 | GO:0048146 | IEA |
| 513497 | GO:0043071 | IEA |
| 513497 | GO:0051726 | IEA |
| 513497 | GO:0000082 | IEA |
| 513497 | GO:0009411 | IEA |
| 513497 | GO:0000086 | IEA |
| 513497 | GO:0045736 | IEA |
| 513776 | GO:0006512 | IEA |
| 513776 | GO:0006512 | IEA |
| 513889 | GO:0015031 | IEA |
| 513889 | GO:0006605 | IEA |
| 513889 | GO:0006810 | IEA |
| 514355 | GO:0044267 | IEA |
| 514355 | GO:0006457 | IEA |
| 514446 | GO:0007275 | IEA |
| 514446 | GO:0007275 | IEA |
| 514665 | GO:0006874 | IEA |
| 514665 | GO:0006810 | IEA |
| 515109 | GO:0006605 | IEA |
| 515109 | GO:0006457 | IEA |
| 515109 | GO:0006810 | IEA |
| 515109 | GO:0015031 | IEA |
| 515109 | GO:0045039 | IEA |
| 515109 | GO:0006626 | IEA |
| 515269 | GO:0008089 | IEA |
| 515269 | GO:0007005 | IEA |
| 515320 | GO:0006355 | IEA |
| 515320 | GO:0006355 | IEA |
| 515380 | GO:0006350 | IEA |
| 515380 | GO:0007275 | IEA |
| 515380 | GO:0030154 | IEA |
| 515380 | GO:0006355 | IEA |
| 515573 | GO:0006464 | IEA |
| 515573 | GO:0006512 | IEA |
| 515602 | GO:0006260 | IEA |
| 515712 | GO:0006955 | IEA |
| 515712 | GO:0002474 | IEA |
| 515712 | GO:0019882 | IEA |
| 515854 | GO:0006974 | IEA |
| 515854 | GO:0006468 | IEA |
| 516063 | GO:0030218 | IEA |
| 516063 | GO:0006412 | IEA |
| 516063 | GO:0015669 | IEA |
| 516063 | GO:0048856 | IEA |
| 516287 | GO:0006118 | IEA |
| 517345 | GO:0007264 | IEA |
| 517345 | GO:0007264 | IEA |
| 517422 | GO:0006281 | IEA |
| 517422 | GO:0006281 | IEA |
| 517539 | GO:0009396 | IEA |
| 517539 | GO:0009396 | IEA |
| 517688 | GO:0051928 | IEA |
| 517688 | GO:0051928 | IEA |
| 517688 | GO:0051928 | IEA |
| 518601 | GO:0015031 | IEA |
| 518601 | GO:0007264 | IEA |
| 518601 | GO:0006888 | IEA |
| 518601 | GO:0006810 | IEA |
| 520694 | GO:0031175 | IEA |
| 520694 | GO:0021954 | IEA |
| 520694 | GO:0043491 | IEA |
| 520694 | GO:0048715 | IEA |
| 521237 | GO:0006468 | IEA |
| 522980 | GO:0006511 | IEA |
| 523671 | GO:0006412 | IEA |
| 524507 | GO:0015669 | IEA |
| 524507 | GO:0030218 | IEA |
| 524507 | GO:0048856 | IEA |
| 524507 | GO:0006412 | IEA |
| 524770 | GO:0048041 | IEA |
| 524770 | GO:0051017 | IEA |
| 524770 | GO:0043462 | IDA |
| 524770 | GO:0051271 | IEA |
| 524770 | GO:0046928 | NAS |
| 524770 | GO:0051234 | NAS |
| 525680 | GO:0006457 | IEA |
| 526320 | GO:0006350 | IEA |
| 527394 | GO:0009791 | IEA |
| 527394 | GO:0016055 | IEA |
| 527394 | GO:0001822 | IEA |
| 527935 | GO:0016481 | IEA |
| 527935 | GO:0045941 | IEA |
| 528253 | GO:0008285 | IEA |
| 528253 | GO:0048536 | IEA |
| 528253 | GO:0042127 | IEA |
| 528253 | GO:0045892 | IEA |
| 528253 | GO:0000122 | IEA |
| 528253 | GO:0001889 | IEA |
| 528253 | GO:0048538 | IEA |
| 529761 | GO:0006810 | IEA |
| 529761 | GO:0015031 | IEA |
| 529761 | GO:0045055 | IEA |
| 529761 | GO:0006888 | IEA |
| 529761 | GO:0048199 | IEA |
| 532203 | GO:0006412 | IEA |
| 532483 | GO:0006355 | IEA |
| 532483 | GO:0007049 | IEA |
| 532483 | GO:0045786 | IEA |
| 532560 | GO:0006810 | IEA |
| 532726 | GO:0009966 | IEA |
| 532726 | GO:0009966 | IEA |
| 532791 | GO:0045604 | IEA |
| 532791 | GO:0045449 | IEA |
| 532791 | GO:0001701 | IEA |
| 533251 | GO:0046907 | IEA |
| 533251 | GO:0007051 | IEA |
| 533251 | GO:0046604 | IEA |
| 533307 | GO:0051258 | IEA |
| 533307 | GO:0007018 | IEA |
| 533307 | GO:0007018 | IEA |
| 533307 | GO:0051258 | IEA |
| 533378 | GO:0007274 | IEA |
| 533608 | GO:0006118 | IEA |
| 533630 | GO:0008652 | IEA |
| 533630 | GO:0006564 | IEA |
| 533630 | GO:0008152 | IEA |
| 533630 | GO:0008152 | IEA |
| 533630 | GO:0008652 | IEA |
| 533630 | GO:0006564 | IEA |
| 533642 | GO:0001666 | IEA |
| 533700 | GO:0045941 | IEA |
| 533729 | GO:0016358 | IEA |
| 533729 | GO:0006468 | IEA |
| 533729 | GO:0043113 | IEA |
| 533729 | GO:0007528 | IEA |
| 533785 | GO:0006096 | IEA |
| 533896 | GO:0006470 | IEA |
| 533993 | GO:0051726 | IEA |
| 533993 | GO:0042981 | IEA |
| 533993 | GO:0007094 | IEA |
| 534092 | GO:0007155 | IEA |
| 534092 | GO:0006468 | IEA |
| 534092 | GO:0007169 | IEA |
| 534751 | GO:0006986 | IEA |
| 534751 | GO:0006457 | IEA |
| 535056 | GO:0006412 | IEA |
| 535293 | GO:0009636 | IEA |
| 535312 | GO:0016925 | IEA |
| 536149 | GO:0007283 | IEA |
| 536149 | GO:0007275 | IEA |
| 536149 | GO:0006810 | IEA |
| 536149 | GO:0043087 | IEA |
| 536149 | GO:0030154 | IEA |
| 537224 | GO:0032869 | IEA |
| 537224 | GO:0045598 | IEA |
| 537224 | GO:0031532 | IEA |
| 537224 | GO:0031529 | IEA |
| 537224 | GO:0006629 | IEA |
| 538548 | GO:0006364 | IEA |
| 538548 | GO:0016074 | IEA |
| 538743 | GO:0006511 | IEA |
| 538998 | GO:0006457 | IEA |
| 539020 | GO:0042127 | IEA |
| 539020 | GO:0030183 | IEA |
| 539020 | GO:0043380 | IEA |
| 539020 | GO:0043065 | IEA |
| 539020 | GO:0035024 | IEA |
| 539020 | GO:0051272 | IEA |
| 539020 | GO:0048294 | IEA |
| 539020 | GO:0007266 | IEA |
| 539020 | GO:0032764 | IEA |
| 539020 | GO:0042092 | IEA |
| 539020 | GO:0000060 | IEA |
| 539020 | GO:0030890 | IEA |
| 539020 | GO:0045629 | IEA |
| 539020 | GO:0050727 | IEA |
| 539020 | GO:0032319 | IEA |
| 539020 | GO:0000122 | IEA |
| 539020 | GO:0006974 | IEA |
| 539020 | GO:0007283 | IEA |
| 539020 | GO:0030036 | IEA |
| 539020 | GO:0030308 | IEA |
| 539020 | GO:0000902 | IEA |
| 539020 | GO:0008285 | IEA |
| 539020 | GO:0043066 | IEA |
| 539020 | GO:0045596 | IEA |
| 539020 | GO:0048821 | IEA |
| 539020 | GO:0002467 | IEA |
| 539020 | GO:0002829 | IEA |
| 539020 | GO:0008104 | IEA |
| 539020 | GO:0001953 | IEA |
| 539241 | GO:0002504 | IEA |
| 539241 | GO:0006955 | IEA |
| 539244 | GO:0006512 | IEA |
| 539244 | GO:0007242 | IEA |
| 539339 | GO:0006810 | IEA |
| 539339 | GO:0007264 | IEA |
| 539339 | GO:0006355 | IEA |
| 539339 | GO:0015031 | IEA |
| 539373 | GO:0006412 | IEA |
| 539381 | GO:0007275 | IEA |
| 539381 | GO:0030154 | IEA |
| 539445 | GO:0006468 | IEA |
| 539546 | GO:0006457 | IEA |
| 540207 | GO:0006468 | IEA |
| 540602 | GO:0016051 | IEA |
| 540602 | GO:0008152 | IEA |
| 540614 | GO:0007049 | IEA |
| 540637 | GO:0030835 | IEA |
| 540637 | GO:0031529 | IEA |
| 540637 | GO:0051017 | IEA |
| 540932 | GO:0007219 | IEA |
| 541054 | GO:0045930 | IEA |
| 541175 | GO:0006470 | IEA |
| 541175 | GO:0006470 | IEA |
| 541175 | GO:0006470 | IEA |
| 613519 | GO:0006412 | IEA |
| 613534 | GO:0006412 | IEA |
| 613534 | GO:0042254 | IEA |
| 613575 | GO:0007242 | IEA |
| 613877 | GO:0006355 | IEA |
| 613972 | GO:0006810 | IEA |
| 613998 | GO:0030001 | IEA |
| 614128 | GO:0006810 | IEA |
| 614128 | GO:0006605 | IEA |
| 614128 | GO:0009306 | IEA |
| 614128 | GO:0015031 | IEA |
| 614241 | GO:0006412 | IEA |
| 614366 | GO:0006412 | IEA |
| 614393 | GO:0043123 | IEA |
| 614423 | GO:0008152 | IEA |
| 614423 | GO:0006096 | IEA |
| 614449 | GO:0006412 | IEA |
| 614509 | GO:0045449 | IEA |
| 614581 | GO:0015031 | IEA |
| 614581 | GO:0006888 | IEA |
| 614581 | GO:0006810 | IEA |
| 614581 | GO:0007264 | IEA |
| 614795 | GO:0006457 | IEA |
| 614926 | GO:0030218 | IEA |
| 614926 | GO:0015669 | IEA |
| 614926 | GO:0048856 | IEA |
| 614926 | GO:0006412 | IEA |
| 615087 | GO:0051258 | IEA |
| 615087 | GO:0007018 | IEA |
| 615087 | GO:0051258 | IEA |
| 615087 | GO:0007018 | IEA |
| 615239 | GO:0045926 | IEA |
| 615239 | GO:0030513 | IEA |
| 615239 | GO:0008285 | IEA |
| 615239 | GO:0009887 | IEA |
| 615239 | GO:0001658 | IEA |
| 615342 | GO:0051607 | IEA |
| 615342 | GO:0006915 | IEA |
| 615342 | GO:0046902 | IEA |
| 615342 | GO:0006917 | IEA |
| 615342 | GO:0008634 | IEA |
| 615342 | GO:0008219 | IEA |
| 615342 | GO:0006338 | IEA |
| 615342 | GO:0045837 | IEA |
| 615342 | GO:0006800 | IEA |
| 615342 | GO:0006309 | IEA |
| 615342 | GO:0043065 | IEA |
| 615401 | GO:0006412 | IEA |
| 615447 | GO:0006241 | IEA |
| 615447 | GO:0006183 | IEA |
| 615447 | GO:0009209 | IEA |
| 615447 | GO:0009117 | IEA |
| 615447 | GO:0006228 | IEA |
| 615518 | GO:0006749 | ISS |
| 615518 | GO:0006099 | IEA |
| 615518 | GO:0006097 | IEA |
| 615518 | GO:0006979 | ISS |
| 615518 | GO:0008152 | IEA |
| 615518 | GO:0006979 | ISS |
| 615518 | GO:0006097 | IEA |
| 615518 | GO:0008152 | IEA |
| 615518 | GO:0006749 | ISS |
| 615518 | GO:0006099 | IEA |
| 615565 | GO:0016559 | IEA |
| 615565 | GO:0006915 | IEA |
| 615565 | GO:0000266 | IEA |
| 615572 | GO:0007026 | IEA |
| 615610 | GO:0006096 | IEA |
| 615610 | GO:0008152 | IEA |
| 615666 | GO:0006412 | IEA |
| 615827 | GO:0051301 | IEA |
| 615827 | GO:0007049 | IEA |
| 616007 | GO:0006457 | IEA |
| 616007 | GO:0006986 | IEA |
| 616295 | GO:0006512 | IEA |
| 616295 | GO:0006464 | IEA |
| 616331 | GO:0006810 | IEA |
| 616331 | GO:0006886 | IEA |
| 616503 | GO:0030336 | IEA |
| 616503 | GO:0007264 | IEA |
| 616539 | GO:0007275 | IEA |
| 616539 | GO:0045941 | IEA |
| 617105 | GO:0008285 | IEA |
| 617105 | GO:0043517 | IEA |
| 617105 | GO:0043065 | IEA |
| 617105 | GO:0043065 | ISS |
| 617105 | GO:0009790 | ISS |
| 617105 | GO:0006281 | ISS |
| 617105 | GO:0008285 | ISS |
| 617105 | GO:0043517 | ISS |
| 617267 | GO:0006096 | IEA |
| 617901 | GO:0006412 | IEA |
| 618292 | GO:0006396 | IEA |
| 618292 | GO:0006364 | IEA |
| 618441 | GO:0030091 | IEA |
| 618609 | GO:0006412 | IEA |
| 619061 | GO:0006412 | IEA |
| 767855 | GO:0006915 | IEA |
| 767898 | GO:0000288 | IEA |
| 767898 | GO:0043488 | IEA |
| 767932 | GO:0007249 | IEA |
| 768059 | GO:0006355 | IEA |
| 768059 | GO:0006350 | IEA |
| 781044 | GO:0016601 | IEA |
| 781044 | GO:0030036 | IEA |
| 781044 | GO:0000082 | IEA |
| 781044 | GO:0008360 | IEA |
| 781158 | GO:0006412 | IEA |
| 781300 | GO:0006412 | IEA |
| 781334 | GO:0007010 | IEA |
| 781358 | GO:0006412 | IEA |
| 781412 | GO:0051258 | IEA |
| 781412 | GO:0007018 | IEA |
| 781412 | GO:0051258 | IEA |
| 781412 | GO:0007018 | IEA |
| 781462 | GO:0015031 | IEA |
| 781462 | GO:0006605 | IEA |
| 781462 | GO:0006457 | IEA |
| 781462 | GO:0006810 | IEA |
| 781462 | GO:0045039 | IEA |
| 781462 | GO:0006626 | IEA |
| 781565 | GO:0006412 | IEA |
| 781575 | GO:0043681 | IEA |
| 781579 | GO:0006412 | IEA |
| 781580 | GO:0006412 | IEA |
| 781851 | GO:0006412 | IEA |
| 781894 | GO:0006412 | IEA |
| 781943 | GO:0006412 | IEA |
| 782038 | GO:0006470 | IEA |
| 782393 | GO:0043065 | ISS |
| 782393 | GO:0043065 | IEA |
| 782393 | GO:0008285 | IEA |
| 782393 | GO:0008285 | ISS |
| 782393 | GO:0009790 | ISS |
| 782393 | GO:0043517 | IEA |
| 782393 | GO:0006281 | ISS |
| 782393 | GO:0043517 | ISS |
| 782631 | GO:0006412 | IEA |
| 782738 | GO:0006412 | IEA |
| 782740 | GO:0006412 | IEA |
| 782855 | GO:0006412 | IEA |
| 783065 | GO:0006457 | IEA |
| 783067 | GO:0006412 | IEA |
| 783144 | GO:0006412 | IEA |
| 783148 | GO:0006412 | IEA |
| 783184 | GO:0006084 | IEA |
| 783185 | GO:0006512 | IEA |
| 783185 | GO:0006914 | IEA |
| 783185 | GO:0000045 | ISS |
| 783185 | GO:0000045 | ISS |
| 783185 | GO:0006512 | IEA |
| 783185 | GO:0006914 | IEA |
| 783202 | GO:0006118 | IEA |
| 783215 | GO:0006412 | IEA |
| 783372 | GO:0006412 | IEA |
| 783462 | GO:0006412 | IEA |
| 783463 | GO:0006412 | IEA |
| 783687 | GO:0043681 | IEA |
| 783718 | GO:0006464 | IEA |
| 783718 | GO:0016567 | IEA |
| 783718 | GO:0045941 | IEA |
| 783718 | GO:0006464 | IEA |
| 783718 | GO:0016567 | IEA |
| 783718 | GO:0045941 | IEA |
| 783880 | GO:0042254 | IEA |
| 783880 | GO:0006412 | IEA |
| 783942 | GO:0006412 | IEA |
| 784052 | GO:0006412 | IEA |
| 784054 | GO:0006412 | IEA |
| 784208 | GO:0006412 | IEA |
| 784340 | GO:0006096 | IEA |
| 784521 | GO:0006412 | IEA |
| 784643 | GO:0007010 | IEA |
| 784731 | GO:0006412 | IEA |
| 784738 | GO:0006412 | IEA |
| 785008 | GO:0006412 | IEA |
| 785061 | GO:0006412 | IEA |
| 785134 | GO:0006118 | IEA |
| 785455 | GO:0007010 | IEA |
| 785508 | GO:0006725 | IEA |
| 785508 | GO:0008152 | IEA |
| 785763 | GO:0006096 | IEA |
| 785824 | GO:0006118 | IEA |
| 785986 | GO:0007049 | IEA |
| 785986 | GO:0006355 | IEA |
| 785986 | GO:0045786 | IEA |
| 786048 | GO:0007275 | IEA |
| 786048 | GO:0006355 | IEA |
| 786048 | GO:0006350 | IEA |
| 786048 | GO:0030154 | IEA |
| 786207 | GO:0006412 | IEA |
| 789002 | GO:0006464 | IEA |
| 789264 | GO:0006412 | IEA |
| 789606 | GO:0006412 | IEA |
| 789607 | GO:0006412 | IEA |
| 789638 | GO:0006096 | IEA |
| 790797 | GO:0006355 | IEA |
| 790797 | GO:0007623 | IEA |
|  |  |  |
