# Supplementary material for: A gene network switch enhances the oxidative capacity of ovine skeletal muscle during late fetal development
Source: BMC Genomics. 2010 Jun 15;11:378. doi: 10.1186/1471-2164-11-378 (PMC2894804; doi:10.1186/1471-2164-11-378)
Supplement: Additional File 1 — Gene expression data. This file contains links to the gene expression data. The genes that are significantly differentially expressed between adjacent development times are listed as Experiment 1 (80 d vs 100 d), Experiment 2 (100 d vs 120 d), Experiment 3 (120 d vs 150 d) and Experiment 4 (150 d vs 230 d) in the section entitled Open microarray analysis report for developmental expression. Each experiment contains an MA plot, Volcano plot and Heatmap for each of the three microarray processing programs MAS5, RMA and GCRMA. For each of these processing programs there is a list of significantly differentially expressed probe sets, and for each of these there is an FDR corrected probability, fold change, percentage of microarrays with a MAS5 Present call, and a convergence percentage for all three microarray processing programs. The file also contains annotation of the probe sets. In a second section entitled Time series analysis of developmental expression, there is a list of genes in each gene expression cluster and diagrams showing MAS5 gene expression values for these genes. [file 1471-2164-11-378-S1.ZIP › Report/GO4/Report/CC.html]

### Cellular Component

| Entrez | GO ID | Evidence Code |
| --- | --- | --- |
 280701 | GO:0005737 | IEA || 280813 | GO:0005833 | IEA |
| 280904 | GO:0005783 | IEA |
| 281032 | GO:0005737 | ISS |
| 281032 | GO:0005634 | ISS |
| 281032 | GO:0005634 | IEA |
| 281032 | GO:0005737 | IEA |
| 281091 | GO:0016020 | IEA |
| 281091 | GO:0016021 | IEA |
| 281091 | GO:0005739 | IEA |
| 281141 | GO:0000015 | IEA |
| 281141 | GO:0005737 | ISS |
| 281195 | GO:0005794 | IEA |
| 281195 | GO:0005794 | ISS |
| 281195 | GO:0016021 | IEA |
| 281195 | GO:0016020 | IEA |
| 281235 | GO:0005829 | ISS |
| 281235 | GO:0005737 | ISS |
| 281235 | GO:0005829 | ISS |
| 281235 | GO:0005737 | ISS |
| 281240 | GO:0005576 | IEA |
| 281338 | GO:0016459 | IEA |
| 281339 | GO:0016459 | IEA |
| 281341 | GO:0016459 | IEA |
| 281341 | GO:0016461 | IEA |
| 281360 | GO:0005739 | ISS |
| 281360 | GO:0005777 | IEA |
| 281370 | GO:0005634 | IEA |
| 281370 | GO:0005634 | IEA |
| 281373 | GO:0009986 | IEA |
| 281373 | GO:0005793 | IEA |
| 281373 | GO:0005792 | IEA |
| 281373 | GO:0016020 | IEA |
| 281373 | GO:0005792 | ISS |
| 281373 | GO:0005783 | IEA |
| 281419 | GO:0005783 | IEA |
| 281478 | GO:0005938 | IDA |
| 281478 | GO:0005737 | IDA |
| 281482 | GO:0016021 | IEA |
| 281482 | GO:0016020 | IEA |
| 281546 | GO:0016020 | IEA |
| 281546 | GO:0016021 | IEA |
| 281546 | GO:0005783 | IEA |
| 281597 | GO:0030027 | IEA |
| 281687 | GO:0016021 | IEA |
| 281687 | GO:0016020 | IEA |
| 281687 | GO:0045211 | IEA |
| 281687 | GO:0005892 | IEA |
| 281687 | GO:0005886 | IEA |
| 281767 | GO:0016021 | IEA |
| 281767 | GO:0016021 | IEA |
| 281814 | GO:0005634 | IEA |
| 281814 | GO:0009434 | IEA |
| 281816 | GO:0005739 | IEA |
| 281816 | GO:0005739 | ISS |
| 281898 | GO:0005719 | ISS |
| 281898 | GO:0005840 | IEA |
| 281898 | GO:0005622 | IEA |
| 281898 | GO:0015935 | IEA |
| 281898 | GO:0030529 | IEA |
| 281942 | GO:0005737 | IEA |
| 281994 | GO:0008287 | IEA |
| 281999 | GO:0005739 | IEA |
| 282127 | GO:0005634 | IEA |
| 282127 | GO:0005737 | IEA |
| 282127 | GO:0005829 | IEA |
| 282187 | GO:0005737 | IEA |
| 282187 | GO:0005578 | IEA |
| 282187 | GO:0005581 | IEA |
| 282187 | GO:0005578 | IEA |
| 282187 | GO:0005737 | IEA |
| 282187 | GO:0005581 | IEA |
| 282188 | GO:0005737 | IEA |
| 282188 | GO:0005578 | IEA |
| 282188 | GO:0005581 | IEA |
| 282188 | GO:0005737 | IEA |
| 282188 | GO:0005578 | IEA |
| 282188 | GO:0005581 | IEA |
| 282211 | GO:0005783 | IEA |
| 282211 | GO:0005792 | IEA |
| 282211 | GO:0016020 | IEA |
| 282261 | GO:0005615 | IEA |
| 282261 | GO:0005576 | IEA |
| 282291 | GO:0005829 | IEA |
| 282291 | GO:0005634 | IEA |
| 282291 | GO:0005737 | IEA |
| 282304 | GO:0043540 | ISS |
| 282331 | GO:0016020 | IEA |
| 282331 | GO:0016021 | IEA |
| 282376 | GO:0005634 | IEA |
| 282376 | GO:0005737 | IEA |
| 282376 | GO:0005737 | IEA |
| 282376 | GO:0005634 | IEA |
| 282386 | GO:0005737 | IEA |
| 282386 | GO:0005856 | IEA |
| 282688 | GO:0005840 | IEA |
| 282688 | GO:0005622 | IEA |
| 282688 | GO:0030529 | IEA |
| 282711 | GO:0005634 | IEA |
| 282711 | GO:0005667 | IEA |
| 282711 | GO:0005667 | IEA |
| 282711 | GO:0005634 | IEA |
| 282875 | GO:0005622 | IEA |
| 282875 | GO:0005737 | IEA |
| 282884 | GO:0005840 | IEA |
| 282884 | GO:0030529 | IEA |
| 282884 | GO:0005622 | IEA |
| 286764 | GO:0005635 | ISS |
| 286764 | GO:0005634 | IEA |
| 286764 | GO:0005730 | ISS |
| 286764 | GO:0005622 | IEA |
| 286806 | GO:0005578 | ISS |
| 286806 | GO:0031012 | IEA |
| 286873 | GO:0005840 | IEA |
| 286873 | GO:0030529 | IEA |
| 286873 | GO:0005622 | IEA |
| 287016 | GO:0005694 | IEA |
| 287016 | GO:0000786 | IEA |
| 287016 | GO:0005634 | IEA |
| 326582 | GO:0030529 | IEA |
| 326582 | GO:0005622 | IEA |
| 326582 | GO:0005840 | IEA |
| 326599 | GO:0005737 | IEA |
| 326599 | GO:0005771 | IEA |
| 326599 | GO:0005615 | IEA |
| 326599 | GO:0045298 | IEA |
| 327685 | GO:0048471 | ISS |
| 327687 | GO:0016021 | IEA |
| 327687 | GO:0016469 | IEA |
| 327687 | GO:0005773 | IEA |
| 327687 | GO:0016020 | IEA |
| 327688 | GO:0005739 | IEA |
| 327688 | GO:0005746 | IEA |
| 327688 | GO:0016020 | IEA |
| 338049 | GO:0005622 | IEA |
| 338049 | GO:0005856 | IEA |
| 338049 | GO:0016020 | IEA |
| 338062 | GO:0042612 | IEA |
| 359721 | GO:0005783 | IEA |
| 404079 | GO:0005634 | IEA |
| 404079 | GO:0005792 | IEA |
| 404079 | GO:0005625 | IEA |
| 404121 | GO:0005783 | IEA |
| 404121 | GO:0016021 | IEA |
| 404121 | GO:0016020 | IEA |
| 404126 | GO:0005634 | IEA |
| 404144 | GO:0005882 | IEA |
| 404165 | GO:0005634 | IEA |
| 404189 | GO:0005792 | IEA |
| 404189 | GO:0005634 | IEA |
| 404189 | GO:0005625 | IEA |
| 408001 | GO:0016020 | IEA |
| 408001 | GO:0031090 | ISS |
| 408001 | GO:0005737 | ISS |
| 408001 | GO:0005776 | ISS |
| 408001 | GO:0005773 | IEA |
| 408001 | GO:0005874 | IEA |
| 408001 | GO:0005737 | ISS |
| 408001 | GO:0005773 | IEA |
| 408001 | GO:0005776 | ISS |
| 408001 | GO:0016020 | IEA |
| 408001 | GO:0005874 | IEA |
| 408001 | GO:0031090 | ISS |
| 414274 | GO:0000792 | IEA |
| 414274 | GO:0005634 | IEA |
| 414732 | GO:0005739 | IEA |
| 444874 | GO:0005634 | IEA |
| 444874 | GO:0005634 | IEA |
| 503685 | GO:0001533 | IEA |
| 504438 | GO:0005783 | IEA |
| 504438 | GO:0016021 | IEA |
| 504854 | GO:0016021 | IEA |
| 504854 | GO:0016020 | IEA |
| 505005 | GO:0005739 | IEA |
| 505060 | GO:0016020 | IEA |
| 505060 | GO:0016021 | IEA |
| 505199 | GO:0030529 | IEA |
| 505199 | GO:0005840 | IEA |
| 505199 | GO:0005622 | IEA |
| 505406 | GO:0005622 | IEA |
| 505507 | GO:0005730 | IEA |
| 505507 | GO:0005840 | IEA |
| 505642 | GO:0005737 | IEA |
| 505691 | GO:0005737 | IEA |
| 505691 | GO:0005634 | IEA |
| 505884 | GO:0005622 | IEA |
| 505884 | GO:0005737 | IEA |
| 505884 | GO:0005634 | IEA |
| 505901 | GO:0005886 | IEA |
| 505901 | GO:0001772 | IEA |
| 505901 | GO:0005737 | IEA |
| 506085 | GO:0016020 | IEA |
| 506085 | GO:0005783 | IEA |
| 506085 | GO:0016021 | IEA |
| 506214 | GO:0016020 | IEA |
| 506214 | GO:0009897 | IEA |
| 506214 | GO:0042613 | IEA |
| 506214 | GO:0016021 | IEA |
| 506336 | GO:0016021 | IEA |
| 506336 | GO:0005783 | IEA |
| 506336 | GO:0016020 | IEA |
| 506370 | GO:0016020 | IEA |
| 506370 | GO:0005743 | IEA |
| 506534 | GO:0016021 | IEA |
| 506544 | GO:0005737 | IEA |
| 506544 | GO:0005945 | IEA |
| 506715 | GO:0005886 | IEA |
| 506715 | GO:0005634 | IEA |
| 506715 | GO:0030424 | IEA |
| 506715 | GO:0005813 | IEA |
| 506715 | GO:0005811 | IEA |
| 506790 | GO:0005737 | IEA |
| 506860 | GO:0005739 | IEA |
| 506860 | GO:0005737 | IEA |
| 506988 | GO:0005739 | IEA |
| 506988 | GO:0005743 | IEA |
| 506988 | GO:0005840 | IEA |
| 506988 | GO:0005622 | IEA |
| 506988 | GO:0030529 | IEA |
| 506989 | GO:0005622 | IEA |
| 506989 | GO:0005739 | IEA |
| 506989 | GO:0005743 | IEA |
| 506989 | GO:0030529 | IEA |
| 506989 | GO:0005840 | IEA |
| 507270 | GO:0030529 | IEA |
| 507270 | GO:0005622 | IEA |
| 507270 | GO:0005840 | IEA |
| 507280 | GO:0016021 | IEA |
| 507367 | GO:0005739 | IEA |
| 507367 | GO:0005743 | IEA |
| 507449 | GO:0016020 | IEA |
| 507449 | GO:0005622 | IEA |
| 507679 | GO:0005622 | IEA |
| 507781 | GO:0000151 | IEA |
| 507781 | GO:0005829 | IEA |
| 507784 | GO:0030529 | IEA |
| 507784 | GO:0005622 | IEA |
| 507784 | GO:0005840 | IEA |
| 507815 | GO:0016020 | IEA |
| 507815 | GO:0008250 | IEA |
| 507815 | GO:0005624 | IEA |
| 507845 | GO:0005819 | ISS |
| 507845 | GO:0005874 | IEA |
| 507845 | GO:0030981 | ISS |
| 508221 | GO:0005622 | IEA |
| 508221 | GO:0030529 | IEA |
| 508221 | GO:0005840 | IEA |
| 508319 | GO:0031982 | IEA |
| 508319 | GO:0005622 | IEA |
| 508319 | GO:0005886 | IEA |
| 508319 | GO:0030018 | IEA |
| 508628 | GO:0005840 | IEA |
| 508628 | GO:0030529 | IEA |
| 508628 | GO:0005622 | IEA |
| 508629 | GO:0005739 | IEA |
| 509422 | GO:0005783 | IEA |
| 509426 | GO:0030529 | IEA |
| 509426 | GO:0005622 | IEA |
| 509426 | GO:0005840 | IEA |
| 509597 | GO:0042612 | IEA |
| 509963 | GO:0005576 | IEA |
| 509963 | GO:0005615 | IEA |
| 510041 | GO:0008537 | IEA |
| 510041 | GO:0043234 | IEA |
| 510041 | GO:0005829 | IEA |
| 510050 | GO:0005634 | IEA |
| 510159 | GO:0005874 | IEA |
| 510200 | GO:0005737 | IEA |
| 510423 | GO:0043234 | IEA |
| 510423 | GO:0005839 | IEA |
| 510423 | GO:0005829 | IEA |
| 510727 | GO:0005739 | IEA |
| 510850 | GO:0005793 | IEA |
| 510850 | GO:0005783 | IEA |
| 510937 | GO:0005737 | IEA |
| 511492 | GO:0005576 | IEA |
| 511560 | GO:0005622 | IEA |
| 511560 | GO:0005730 | IEA |
| 511560 | GO:0030529 | IEA |
| 511560 | GO:0005634 | IEA |
| 511560 | GO:0005840 | IEA |
| 512254 | GO:0005634 | IEA |
| 512324 | GO:0005634 | IEA |
| 512534 | GO:0016021 | IEA |
| 512534 | GO:0005783 | IEA |
| 512534 | GO:0005789 | IEA |
| 512673 | GO:0005740 | IEA |
| 512673 | GO:0005739 | IEA |
| 513038 | GO:0005764 | IEA |
| 513125 | GO:0005737 | IEA |
| 513128 | GO:0005840 | IEA |
| 513128 | GO:0030529 | IEA |
| 513128 | GO:0005622 | IEA |
| 513497 | GO:0000307 | IEA |
| 513497 | GO:0005634 | IEA |
| 513497 | GO:0005829 | IEA |
| 513889 | GO:0005739 | IEA |
| 514355 | GO:0005832 | IEA |
| 514443 | GO:0005634 | IEA |
| 514443 | GO:0005669 | IEA |
| 514443 | GO:0048471 | IEA |
| 514443 | GO:0005737 | IEA |
| 514547 | GO:0005776 | IEA |
| 514547 | GO:0031090 | IEA |
| 514665 | GO:0016020 | IEA |
| 514665 | GO:0008021 | IEA |
| 515109 | GO:0016020 | IEA |
| 515109 | GO:0042719 | IEA |
| 515109 | GO:0005739 | IEA |
| 515269 | GO:0005739 | IEA |
| 515380 | GO:0005634 | IEA |
| 515573 | GO:0016605 | IEA |
| 515602 | GO:0005634 | IEA |
| 515712 | GO:0042612 | IEA |
| 515784 | GO:0005783 | IEA |
| 515878 | GO:0016021 | IEA |
| 515878 | GO:0016020 | IEA |
| 515878 | GO:0005783 | IEA |
| 516063 | GO:0030529 | IEA |
| 516063 | GO:0005840 | IEA |
| 516063 | GO:0005730 | IEA |
| 516063 | GO:0005622 | IEA |
| 516334 | GO:0016020 | IEA |
| 517345 | GO:0005794 | IEA |
| 517345 | GO:0005622 | IEA |
| 517345 | GO:0005622 | IEA |
| 517345 | GO:0005794 | IEA |
| 517539 | GO:0005739 | IEA |
| 517539 | GO:0005739 | IEA |
| 517688 | GO:0005887 | IEA |
| 517688 | GO:0005887 | IEA |
| 517688 | GO:0005887 | IEA |
| 518601 | GO:0005886 | IEA |
| 518601 | GO:0005737 | IEA |
| 518601 | GO:0005622 | IEA |
| 523609 | GO:0005739 | IEA |
| 523671 | GO:0005840 | IEA |
| 523671 | GO:0005622 | IEA |
| 523671 | GO:0030529 | IEA |
| 524507 | GO:0030529 | IEA |
| 524507 | GO:0005730 | IEA |
| 524507 | GO:0005622 | IEA |
| 524507 | GO:0005840 | IEA |
| 524770 | GO:0005925 | IEA |
| 524770 | GO:0012506 | NAS |
| 524770 | GO:0030018 | IEA |
| 524770 | GO:0005856 | IEA |
| 525680 | GO:0016272 | IEA |
| 526320 | GO:0005634 | IEA |
| 527394 | GO:0005634 | IEA |
| 527935 | GO:0016607 | IEA |
| 528165 | GO:0005739 | IEA |
| 528165 | GO:0005743 | IEA |
| 528253 | GO:0005634 | IEA |
| 529235 | GO:0005737 | IEA |
| 529235 | GO:0005622 | IEA |
| 529761 | GO:0005793 | IEA |
| 529761 | GO:0016020 | IEA |
| 529761 | GO:0016021 | IEA |
| 530164 | GO:0005886 | IEA |
| 530164 | GO:0016023 | IEA |
| 531367 | GO:0005634 | IEA |
| 532203 | GO:0030529 | IEA |
| 532203 | GO:0005840 | IEA |
| 532203 | GO:0005622 | IEA |
| 532483 | GO:0005634 | IEA |
| 532560 | GO:0016021 | IEA |
| 532560 | GO:0016020 | IEA |
| 532560 | GO:0005743 | IEA |
| 532693 | GO:0005622 | IEA |
| 532791 | GO:0005783 | IEA |
| 532791 | GO:0005737 | IEA |
| 533219 | GO:0005856 | IEA |
| 533223 | GO:0005856 | IEA |
| 533251 | GO:0005813 | IEA |
| 533251 | GO:0005737 | IEA |
| 533307 | GO:0043234 | IEA |
| 533307 | GO:0005874 | IEA |
| 533307 | GO:0005874 | IEA |
| 533307 | GO:0043234 | IEA |
| 533729 | GO:0005634 | IEA |
| 533729 | GO:0030426 | IEA |
| 533729 | GO:0005737 | IEA |
| 533729 | GO:0030424 | IEA |
| 533993 | GO:0005634 | IEA |
| 534092 | GO:0016020 | IEA |
| 534647 | GO:0005737 | IEA |
| 535056 | GO:0030529 | IEA |
| 535056 | GO:0005840 | IEA |
| 535056 | GO:0005622 | IEA |
| 535293 | GO:0005792 | IEA |
| 535293 | GO:0016020 | IEA |
| 535312 | GO:0005634 | IEA |
| 535414 | GO:0005829 | IEA |
| 535414 | GO:0043234 | IEA |
| 536149 | GO:0005634 | IEA |
| 537224 | GO:0005634 | IEA |
| 538548 | GO:0015030 | IEA |
| 538548 | GO:0005730 | IEA |
| 538548 | GO:0005634 | IEA |
| 539020 | GO:0005634 | IEA |
| 539020 | GO:0005657 | IEA |
| 539241 | GO:0016021 | IEA |
| 539241 | GO:0042613 | IEA |
| 539241 | GO:0016020 | IEA |
| 539339 | GO:0016020 | IEA |
| 539339 | GO:0005622 | IEA |
| 539373 | GO:0030529 | IEA |
| 539373 | GO:0005622 | IEA |
| 539373 | GO:0005840 | IEA |
| 539381 | GO:0005634 | IEA |
| 539761 | GO:0005622 | IEA |
| 539945 | GO:0030018 | IEA |
| 539945 | GO:0030018 | IEA |
| 540045 | GO:0005634 | IEA |
| 540045 | GO:0005622 | IEA |
| 540602 | GO:0005829 | IEA |
| 540637 | GO:0005925 | IEA |
| 540637 | GO:0001725 | IEA |
| 540637 | GO:0015629 | IEA |
| 540932 | GO:0005783 | IEA |
| 540932 | GO:0005794 | IEA |
| 540965 | GO:0030529 | IEA |
| 541054 | GO:0005737 | IEA |
| 613519 | GO:0005840 | IEA |
| 613519 | GO:0005622 | IEA |
| 613519 | GO:0030529 | IEA |
| 613534 | GO:0030529 | IEA |
| 613534 | GO:0005622 | IEA |
| 613534 | GO:0005840 | IEA |
| 613548 | GO:0005813 | IEA |
| 613548 | GO:0005938 | IEA |
| 613548 | GO:0005737 | IEA |
| 613548 | GO:0042585 | IEA |
| 613548 | GO:0016020 | IEA |
| 613548 | GO:0005737 | IEA |
| 613548 | GO:0005813 | IEA |
| 613548 | GO:0042585 | IEA |
| 613548 | GO:0005938 | IEA |
| 613548 | GO:0016020 | IEA |
| 613568 | GO:0005739 | IEA |
| 613575 | GO:0016021 | IEA |
| 613575 | GO:0016020 | IEA |
| 613877 | GO:0005634 | IEA |
| 613877 | GO:0005667 | IEA |
| 614128 | GO:0005783 | IEA |
| 614128 | GO:0016020 | IEA |
| 614128 | GO:0016021 | IEA |
| 614241 | GO:0005840 | IEA |
| 614241 | GO:0030529 | IEA |
| 614241 | GO:0005622 | IEA |
| 614366 | GO:0005840 | IEA |
| 614366 | GO:0030529 | IEA |
| 614366 | GO:0005622 | IEA |
| 614449 | GO:0030529 | IEA |
| 614449 | GO:0005840 | IEA |
| 614449 | GO:0005622 | IEA |
| 614509 | GO:0005634 | IEA |
| 614581 | GO:0005886 | IEA |
| 614581 | GO:0005737 | IEA |
| 614581 | GO:0005622 | IEA |
| 614926 | GO:0030529 | IEA |
| 614926 | GO:0005730 | IEA |
| 614926 | GO:0005840 | IEA |
| 614926 | GO:0005622 | IEA |
| 615087 | GO:0005874 | IEA |
| 615087 | GO:0043234 | IEA |
| 615087 | GO:0005874 | IEA |
| 615087 | GO:0043234 | IEA |
| 615239 | GO:0005578 | IEA |
| 615342 | GO:0005740 | IEA |
| 615342 | GO:0005739 | IEA |
| 615342 | GO:0031307 | IEA |
| 615342 | GO:0005635 | IEA |
| 615342 | GO:0016020 | IEA |
| 615342 | GO:0016021 | IEA |
| 615342 | GO:0031966 | IEA |
| 615401 | GO:0005840 | IEA |
| 615401 | GO:0005622 | IEA |
| 615401 | GO:0030529 | IEA |
| 615424 | GO:0005739 | IEA |
| 615447 | GO:0016020 | IEA |
| 615518 | GO:0005737 | ISS |
| 615518 | GO:0005829 | ISS |
| 615518 | GO:0005737 | ISS |
| 615518 | GO:0005829 | ISS |
| 615565 | GO:0016021 | IEA |
| 615565 | GO:0005779 | IEA |
| 615565 | GO:0031307 | IEA |
| 615565 | GO:0005739 | IEA |
| 615565 | GO:0016020 | IEA |
| 615572 | GO:0005737 | IEA |
| 615572 | GO:0005634 | IEA |
| 615572 | GO:0015630 | IEA |
| 615596 | GO:0016021 | IEA |
| 615626 | GO:0005634 | IEA |
| 615666 | GO:0005739 | IEA |
| 615666 | GO:0030529 | IEA |
| 615666 | GO:0005761 | IEA |
| 615975 | GO:0005634 | IEA |
| 615975 | GO:0005737 | IEA |
| 616295 | GO:0016605 | IEA |
| 616331 | GO:0030662 | IEA |
| 616503 | GO:0005622 | IEA |
| 616503 | GO:0016020 | IEA |
| 617105 | GO:0005737 | ISS |
| 617105 | GO:0005737 | IEA |
| 617105 | GO:0005634 | ISS |
| 617105 | GO:0005634 | IEA |
| 617105 | GO:0017101 | ISS |
| 617901 | GO:0005739 | IEA |
| 617901 | GO:0005762 | IEA |
| 617901 | GO:0005622 | IEA |
| 617901 | GO:0005840 | IEA |
| 617901 | GO:0030529 | IEA |
| 617922 | GO:0016020 | IEA |
| 618292 | GO:0000178 | IEA |
| 618292 | GO:0005634 | IEA |
| 618441 | GO:0005737 | IEA |
| 618441 | GO:0005634 | IEA |
| 618609 | GO:0005840 | IEA |
| 618609 | GO:0005622 | IEA |
| 619061 | GO:0005622 | IEA |
| 619061 | GO:0005840 | IEA |
| 619061 | GO:0030529 | IEA |
| 767898 | GO:0005634 | IEA |
| 767898 | GO:0005737 | IEA |
| 767932 | GO:0005737 | IEA |
| 768059 | GO:0005634 | IEA |
| 781158 | GO:0005840 | IEA |
| 781158 | GO:0005622 | IEA |
| 781158 | GO:0030529 | IEA |
| 781300 | GO:0030529 | IEA |
| 781300 | GO:0005622 | IEA |
| 781300 | GO:0005840 | IEA |
| 781334 | GO:0005856 | IEA |
| 781334 | GO:0005737 | IEA |
| 781358 | GO:0005840 | IEA |
| 781358 | GO:0030529 | IEA |
| 781358 | GO:0005622 | IEA |
| 781412 | GO:0043234 | IEA |
| 781412 | GO:0005874 | IEA |
| 781412 | GO:0005874 | IEA |
| 781412 | GO:0043234 | IEA |
| 781462 | GO:0042719 | IEA |
| 781462 | GO:0016020 | IEA |
| 781462 | GO:0005739 | IEA |
| 781565 | GO:0005622 | IEA |
| 781565 | GO:0005840 | IEA |
| 781565 | GO:0030529 | IEA |
| 781575 | GO:0005739 | IEA |
| 781575 | GO:0005740 | IEA |
| 781579 | GO:0030529 | IEA |
| 781579 | GO:0005840 | IEA |
| 781579 | GO:0005622 | IEA |
| 781580 | GO:0005622 | IEA |
| 781580 | GO:0030529 | IEA |
| 781580 | GO:0005840 | IEA |
| 781851 | GO:0005622 | IEA |
| 781851 | GO:0030529 | IEA |
| 781851 | GO:0005840 | IEA |
| 781894 | GO:0005840 | IEA |
| 781894 | GO:0005622 | IEA |
| 781894 | GO:0030529 | IEA |
| 781943 | GO:0030529 | IEA |
| 781943 | GO:0005840 | IEA |
| 781943 | GO:0005622 | IEA |
| 782038 | GO:0008287 | IEA |
| 782393 | GO:0005737 | ISS |
| 782393 | GO:0005634 | IEA |
| 782393 | GO:0005634 | ISS |
| 782393 | GO:0017101 | ISS |
| 782393 | GO:0005737 | IEA |
| 782631 | GO:0005622 | IEA |
| 782631 | GO:0005840 | IEA |
| 782631 | GO:0030529 | IEA |
| 782738 | GO:0030529 | IEA |
| 782738 | GO:0005622 | IEA |
| 782738 | GO:0005840 | IEA |
| 782740 | GO:0005622 | IEA |
| 782740 | GO:0005840 | IEA |
| 782740 | GO:0030529 | IEA |
| 782855 | GO:0005622 | IEA |
| 782855 | GO:0005840 | IEA |
| 782855 | GO:0030529 | IEA |
| 783067 | GO:0005622 | IEA |
| 783067 | GO:0005840 | IEA |
| 783067 | GO:0030529 | IEA |
| 783144 | GO:0005840 | IEA |
| 783144 | GO:0005622 | IEA |
| 783144 | GO:0030529 | IEA |
| 783148 | GO:0030529 | IEA |
| 783148 | GO:0005840 | IEA |
| 783148 | GO:0005622 | IEA |
| 783185 | GO:0005776 | ISS |
| 783185 | GO:0005737 | ISS |
| 783185 | GO:0005773 | IEA |
| 783185 | GO:0016020 | IEA |
| 783185 | GO:0005874 | IEA |
| 783185 | GO:0031090 | ISS |
| 783185 | GO:0005773 | IEA |
| 783185 | GO:0005776 | ISS |
| 783185 | GO:0005874 | IEA |
| 783185 | GO:0031090 | ISS |
| 783185 | GO:0016020 | IEA |
| 783185 | GO:0005737 | ISS |
| 783202 | GO:0016020 | IEA |
| 783202 | GO:0016021 | IEA |
| 783202 | GO:0005739 | IEA |
| 783215 | GO:0030529 | IEA |
| 783215 | GO:0005840 | IEA |
| 783215 | GO:0005622 | IEA |
| 783372 | GO:0030529 | IEA |
| 783372 | GO:0005840 | IEA |
| 783372 | GO:0005622 | IEA |
| 783462 | GO:0005622 | IEA |
| 783462 | GO:0030529 | IEA |
| 783462 | GO:0005840 | IEA |
| 783463 | GO:0030529 | IEA |
| 783463 | GO:0005840 | IEA |
| 783463 | GO:0005622 | IEA |
| 783687 | GO:0005740 | IEA |
| 783687 | GO:0005739 | IEA |
| 783718 | GO:0005634 | IEA |
| 783718 | GO:0005634 | IEA |
| 783880 | GO:0005840 | IEA |
| 783880 | GO:0005622 | IEA |
| 783880 | GO:0030529 | IEA |
| 783942 | GO:0005840 | IEA |
| 783942 | GO:0030529 | IEA |
| 783942 | GO:0005622 | IEA |
| 784052 | GO:0030529 | IEA |
| 784052 | GO:0005622 | IEA |
| 784052 | GO:0005840 | IEA |
| 784054 | GO:0005840 | IEA |
| 784054 | GO:0030529 | IEA |
| 784054 | GO:0005622 | IEA |
| 784208 | GO:0030529 | IEA |
| 784208 | GO:0005840 | IEA |
| 784208 | GO:0005622 | IEA |
| 784521 | GO:0030529 | IEA |
| 784521 | GO:0005840 | IEA |
| 784521 | GO:0005622 | IEA |
| 784643 | GO:0005737 | IEA |
| 784643 | GO:0005856 | IEA |
| 784731 | GO:0030529 | IEA |
| 784731 | GO:0005840 | IEA |
| 784731 | GO:0005622 | IEA |
| 784738 | GO:0030529 | IEA |
| 784738 | GO:0005622 | IEA |
| 784738 | GO:0005840 | IEA |
| 785008 | GO:0005840 | IEA |
| 785008 | GO:0005622 | IEA |
| 785008 | GO:0030529 | IEA |
| 785061 | GO:0030529 | IEA |
| 785061 | GO:0005840 | IEA |
| 785061 | GO:0005622 | IEA |
| 785134 | GO:0005739 | IEA |
| 785134 | GO:0005746 | IEA |
| 785134 | GO:0016020 | IEA |
| 785455 | GO:0005856 | IEA |
| 785455 | GO:0005737 | IEA |
| 785824 | GO:0016020 | IEA |
| 785954 | GO:0030529 | IEA |
| 785986 | GO:0005634 | IEA |
| 786048 | GO:0005634 | IEA |
| 786207 | GO:0005840 | IEA |
| 786207 | GO:0030529 | IEA |
| 786207 | GO:0005622 | IEA |
| 786336 | GO:0005634 | IEA |
| 786705 | GO:0005634 | IEA |
| 787193 | GO:0005737 | IEA |
| 787193 | GO:0005739 | IEA |
| 788457 | GO:0005771 | IEA |
| 788457 | GO:0005737 | IEA |
| 788457 | GO:0005615 | IEA |
| 788457 | GO:0045298 | IEA |
| 789264 | GO:0005840 | IEA |
| 789264 | GO:0030529 | IEA |
| 789264 | GO:0005622 | IEA |
| 789606 | GO:0005622 | IEA |
| 789606 | GO:0030529 | IEA |
| 789606 | GO:0005840 | IEA |
| 789607 | GO:0030529 | IEA |
| 789607 | GO:0005840 | IEA |
| 789607 | GO:0005622 | IEA |
| 790047 | GO:0005829 | IEA |
| 790047 | GO:0000151 | IEA |
| 790786 | GO:0005634 | IEA |
| 790797 | GO:0005634 | IEA |
|  |  |  |
