# Supplementary material for: A gene network switch enhances the oxidative capacity of ovine skeletal muscle during late fetal development
Source: BMC Genomics. 2010 Jun 15;11:378. doi: 10.1186/1471-2164-11-378 (PMC2894804; doi:10.1186/1471-2164-11-378)
Supplement: Additional File 1 — Gene expression data. This file contains links to the gene expression data. The genes that are significantly differentially expressed between adjacent development times are listed as Experiment 1 (80 d vs 100 d), Experiment 2 (100 d vs 120 d), Experiment 3 (120 d vs 150 d) and Experiment 4 (150 d vs 230 d) in the section entitled Open microarray analysis report for developmental expression. Each experiment contains an MA plot, Volcano plot and Heatmap for each of the three microarray processing programs MAS5, RMA and GCRMA. For each of these processing programs there is a list of significantly differentially expressed probe sets, and for each of these there is an FDR corrected probability, fold change, percentage of microarrays with a MAS5 Present call, and a convergence percentage for all three microarray processing programs. The file also contains annotation of the probe sets. In a second section entitled Time series analysis of developmental expression, there is a list of genes in each gene expression cluster and diagrams showing MAS5 gene expression values for these genes. [file 1471-2164-11-378-S1.ZIP › Report/GO4/Report/MF.html]

### Molecular function

| Entrez | GO ID | Evidence Code |
| --- | --- | --- |
 280701 | GO:0000287 | IEA || 280701 | GO:0016787 | IEA |
| 280701 | GO:0004427 | IEA |
| 280701 | GO:0046872 | IEA |
| 280716 | GO:0005524 | IEA |
| 280716 | GO:0000166 | IEA |
| 280716 | GO:0004017 | IEA |
| 280716 | GO:0016740 | IEA |
| 280716 | GO:0016776 | IEA |
| 280716 | GO:0019201 | IEA |
| 280716 | GO:0016301 | IEA |
| 280726 | GO:0004055 | IEA |
| 280726 | GO:0000166 | IEA |
| 280726 | GO:0016874 | IEA |
| 280726 | GO:0005524 | IEA |
| 280813 | GO:0046872 | IEA |
| 280813 | GO:0020037 | IEA |
| 280813 | GO:0019825 | IEA |
| 280813 | GO:0005488 | IEA |
| 280813 | GO:0005506 | IEA |
| 280813 | GO:0005344 | IEA |
| 280904 | GO:0008201 | IEA |
| 280904 | GO:0008083 | IEA |
| 281032 | GO:0019899 | ISS |
| 281032 | GO:0019900 | ISS |
| 281032 | GO:0003712 | ISS |
| 281032 | GO:0005515 | ISS |
| 281091 | GO:0016491 | IEA |
| 281091 | GO:0004129 | IEA |
| 281141 | GO:0046872 | IEA |
| 281141 | GO:0000287 | IEA |
| 281141 | GO:0016829 | IEA |
| 281141 | GO:0008243 | IEA |
| 281141 | GO:0004634 | IEA |
| 281156 | GO:0004337 | IEA |
| 281156 | GO:0016740 | IEA |
| 281156 | GO:0004161 | IEA |
| 281195 | GO:0050379 | IEA |
| 281195 | GO:0016853 | IEA |
| 281195 | GO:0050379 | ISS |
| 281195 | GO:0016857 | IEA |
| 281199 | GO:0016301 | IEA |
| 281199 | GO:0016772 | IEA |
| 281199 | GO:0004356 | IEA |
| 281199 | GO:0016874 | IEA |
| 281225 | GO:0016874 | IEA |
| 281225 | GO:0004842 | IEA |
| 281235 | GO:0004450 | IEA |
| 281235 | GO:0016491 | IEA |
| 281235 | GO:0016652 | IEA |
| 281235 | GO:0016652 | IEA |
| 281235 | GO:0016491 | IEA |
| 281235 | GO:0004450 | IEA |
| 281240 | GO:0005179 | IEA |
| 281240 | GO:0008083 | IEA |
| 281259 | GO:0031072 | IEA |
| 281259 | GO:0051082 | IEA |
| 281338 | GO:0003774 | IEA |
| 281339 | GO:0003774 | IEA |
| 281341 | GO:0003774 | IEA |
| 281341 | GO:0008307 | IEA |
| 281341 | GO:0005509 | IEA |
| 281360 | GO:0016491 | IEA |
| 281360 | GO:0004090 | IEA |
| 281365 | GO:0016829 | IEA |
| 281365 | GO:0004586 | IEA |
| 281365 | GO:0016831 | IEA |
| 281365 | GO:0003824 | IEA |
| 281370 | GO:0030528 | IEA |
| 281370 | GO:0005515 | IPI |
| 281370 | GO:0005515 | IEA |
| 281370 | GO:0005515 | IPI |
| 281370 | GO:0005515 | IEA |
| 281370 | GO:0030528 | IEA |
| 281373 | GO:0016853 | IEA |
| 281373 | GO:0004656 | ISS |
| 281373 | GO:0015035 | IEA |
| 281373 | GO:0009055 | IEA |
| 281373 | GO:0004656 | IEA |
| 281373 | GO:0003756 | IEA |
| 281373 | GO:0005515 | IEA |
| 281408 | GO:0008243 | IEA |
| 281408 | GO:0004252 | IEA |
| 281408 | GO:0016301 | IEA |
| 281408 | GO:0016787 | IEA |
| 281408 | GO:0008233 | IEA |
| 281419 | GO:0016853 | IEA |
| 281419 | GO:0003755 | IEA |
| 281419 | GO:0042277 | IEA |
| 281476 | GO:0016874 | IEA |
| 281476 | GO:0005524 | IEA |
| 281476 | GO:0004812 | IEA |
| 281476 | GO:0004828 | IEA |
| 281476 | GO:0000166 | IEA |
| 281478 | GO:0005546 | IDA |
| 281478 | GO:0051015 | IDA |
| 281478 | GO:0001786 | IDA |
| 281478 | GO:0005509 | IEA |
| 281478 | GO:0003779 | IEA |
| 281482 | GO:0016491 | IEA |
| 281565 | GO:0016779 | IEA |
| 281565 | GO:0003983 | IEA |
| 281565 | GO:0016740 | IEA |
| 281576 | GO:0016874 | IEA |
| 281576 | GO:0004830 | IEA |
| 281576 | GO:0005524 | IEA |
| 281576 | GO:0000166 | IEA |
| 281576 | GO:0004812 | IEA |
| 281597 | GO:0005515 | IEA |
| 281687 | GO:0042166 | IEA |
| 281687 | GO:0004872 | IEA |
| 281687 | GO:0030594 | IEA |
| 281687 | GO:0005515 | IEA |
| 281687 | GO:0005230 | IEA |
| 281687 | GO:0005216 | IEA |
| 281687 | GO:0004889 | IEA |
| 281759 | GO:0005488 | IEA |
| 281759 | GO:0008289 | IEA |
| 281767 | GO:0016740 | IEA |
| 281767 | GO:0004310 | IEA |
| 281767 | GO:0005515 | IEA |
| 281767 | GO:0005515 | IEA |
| 281767 | GO:0004310 | IEA |
| 281767 | GO:0016740 | IEA |
| 281801 | GO:0016740 | IEA |
| 281801 | GO:0005524 | IEA |
| 281801 | GO:0016301 | IEA |
| 281801 | GO:0004871 | IEA |
| 281801 | GO:0004674 | IEA |
| 281801 | GO:0000166 | IEA |
| 281801 | GO:0004703 | IEA |
| 281801 | GO:0004672 | IEA |
| 281814 | GO:0030528 | IEA |
| 281814 | GO:0046982 | ISS |
| 281814 | GO:0005515 | ISS |
| 281814 | GO:0004871 | IEA |
| 281814 | GO:0035035 | ISS |
| 281814 | GO:0003700 | IEA |
| 281814 | GO:0003677 | IEA |
| 281816 | GO:0016787 | IEA |
| 281898 | GO:0003735 | IEA |
| 281898 | GO:0004872 | IEA |
| 281903 | GO:0016491 | IEA |
| 281903 | GO:0046872 | IEA |
| 281903 | GO:0005515 | IEA |
| 281903 | GO:0005507 | IEA |
| 281903 | GO:0004720 | IEA |
| 281942 | GO:0005515 | IEA |
| 281994 | GO:0046872 | IEA |
| 281994 | GO:0016787 | IEA |
| 281994 | GO:0004722 | IEA |
| 281994 | GO:0003824 | IEA |
| 281994 | GO:0030145 | IEA |
| 281994 | GO:0004721 | IEA |
| 281994 | GO:0000287 | IEA |
| 281999 | GO:0016491 | IEA |
| 281999 | GO:0051920 | IEA |
| 281999 | GO:0016209 | IEA |
| 281999 | GO:0004601 | IEA |
| 282127 | GO:0008270 | IEA |
| 282127 | GO:0003676 | IEA |
| 282127 | GO:0017091 | IEA |
| 282127 | GO:0046872 | IEA |
| 282127 | GO:0005515 | IEA |
| 282127 | GO:0003677 | IEA |
| 282127 | GO:0003729 | IEA |
| 282187 | GO:0005198 | IEA |
| 282187 | GO:0005201 | IEA |
| 282187 | GO:0005201 | IEA |
| 282187 | GO:0005198 | IEA |
| 282188 | GO:0005198 | IEA |
| 282188 | GO:0005201 | IEA |
| 282188 | GO:0005201 | IEA |
| 282188 | GO:0005198 | IEA |
| 282211 | GO:0050381 | IEA |
| 282211 | GO:0020037 | IEA |
| 282211 | GO:0046872 | IEA |
| 282211 | GO:0005506 | IEA |
| 282211 | GO:0004497 | IEA |
| 282211 | GO:0016712 | IEA |
| 282211 | GO:0016491 | IEA |
| 282261 | GO:0019838 | IEA |
| 282261 | GO:0005520 | IEA |
| 282261 | GO:0008160 | ISS |
| 282261 | GO:0046872 | ISS |
| 282291 | GO:0008139 | IEA |
| 282291 | GO:0005515 | IEA |
| 282291 | GO:0042802 | IEA |
| 282291 | GO:0031625 | IEA |
| 282291 | GO:0051059 | IEA |
| 282304 | GO:0016740 | IEA |
| 282304 | GO:0016301 | IEA |
| 282304 | GO:0016787 | IEA |
| 282304 | GO:0000166 | IEA |
| 282304 | GO:0005524 | IEA |
| 282304 | GO:0003873 | IEA |
| 282304 | GO:0003824 | IEA |
| 282304 | GO:0004331 | IEA |
| 282331 | GO:0001584 | IEA |
| 282331 | GO:0004872 | IEA |
| 282331 | GO:0004871 | IEA |
| 282331 | GO:0004960 | IEA |
| 282331 | GO:0004955 | IEA |
| 282331 | GO:0004930 | IEA |
| 282331 | GO:0004957 | IEA |
| 282376 | GO:0005509 | IEA |
| 282376 | GO:0005515 | IEA |
| 282376 | GO:0003700 | IEA |
| 282376 | GO:0004871 | IEA |
| 282376 | GO:0003677 | IEA |
| 282376 | GO:0005509 | IEA |
| 282376 | GO:0005515 | IEA |
| 282376 | GO:0003700 | IEA |
| 282376 | GO:0004871 | IEA |
| 282376 | GO:0003677 | IEA |
| 282386 | GO:0003779 | IEA |
| 282688 | GO:0003735 | IEA |
| 282711 | GO:0005515 | IEA |
| 282711 | GO:0004871 | IEA |
| 282711 | GO:0003700 | IEA |
| 282711 | GO:0035035 | IEA |
| 282711 | GO:0030528 | IEA |
| 282711 | GO:0005515 | IEA |
| 282711 | GO:0003700 | IEA |
| 282711 | GO:0030528 | IEA |
| 282711 | GO:0004871 | IEA |
| 282711 | GO:0035035 | IEA |
| 282875 | GO:0003723 | IEA |
| 282875 | GO:0005515 | IEA |
| 282875 | GO:0016301 | IEA |
| 282875 | GO:0003725 | IEA |
| 282884 | GO:0003735 | IEA |
| 286764 | GO:0016740 | IEA |
| 286764 | GO:0003677 | IEA |
| 286764 | GO:0008270 | IEA |
| 286764 | GO:0005515 | ISS |
| 286764 | GO:0003950 | IEA |
| 286764 | GO:0051287 | IEA |
| 286764 | GO:0016757 | IEA |
| 286764 | GO:0042802 | ISS |
| 286764 | GO:0046872 | IEA |
| 286764 | GO:0005515 | IEA |
| 286806 | GO:0016787 | IEA |
| 286806 | GO:0008270 | IEA |
| 286806 | GO:0004222 | IEA |
| 286806 | GO:0008233 | ISS |
| 286806 | GO:0008237 | ISS |
| 286806 | GO:0046872 | IEA |
| 286873 | GO:0003723 | IEA |
| 286873 | GO:0003735 | IEA |
| 286873 | GO:0019843 | IEA |
| 287016 | GO:0003677 | IEA |
| 317748 | GO:0004032 | IEA |
| 317748 | GO:0016491 | IEA |
| 317748 | GO:0047006 | IEA |
| 326582 | GO:0003735 | IEA |
| 326599 | GO:0005509 | IEA |
| 327685 | GO:0005509 | IEA |
| 327685 | GO:0005544 | IEA |
| 327687 | GO:0046961 | IEA |
| 327687 | GO:0015078 | IEA |
| 327687 | GO:0000166 | IEA |
| 327687 | GO:0005524 | IEA |
| 327687 | GO:0016787 | IEA |
| 327687 | GO:0046933 | IEA |
| 327687 | GO:0046872 | IEA |
| 327688 | GO:0004129 | IEA |
| 327688 | GO:0016491 | IEA |
| 327688 | GO:0009055 | IEA |
| 327703 | GO:0005509 | IEA |
| 327703 | GO:0005245 | IEA |
| 327703 | GO:0005262 | IEA |
| 327703 | GO:0005244 | IEA |
| 327703 | GO:0005216 | IEA |
| 338049 | GO:0005515 | IEA |
| 338049 | GO:0000166 | IEA |
| 338049 | GO:0004871 | IEA |
| 338049 | GO:0003924 | ISS |
| 338049 | GO:0005525 | IEA |
| 338049 | GO:0000287 | IEA |
| 338049 | GO:0004871 | ISS |
| 338062 | GO:0004872 | IEA |
| 404079 | GO:0016740 | IEA |
| 404079 | GO:0016301 | IEA |
| 404079 | GO:0000287 | IEA |
| 404079 | GO:0005524 | IEA |
| 404079 | GO:0046872 | IEA |
| 404079 | GO:0004550 | IEA |
| 404079 | GO:0000166 | IEA |
| 404126 | GO:0004407 | IEA |
| 404144 | GO:0005198 | IEA |
| 404148 | GO:0004083 | IEA |
| 404148 | GO:0016868 | IEA |
| 404148 | GO:0016787 | IEA |
| 404148 | GO:0016853 | IEA |
| 404148 | GO:0003824 | IEA |
| 404148 | GO:0004619 | IEA |
| 404148 | GO:0004082 | IEA |
| 404159 | GO:0015035 | IEA |
| 404159 | GO:0009055 | IEA |
| 404180 | GO:0003824 | IEA |
| 404180 | GO:0003824 | IEA |
| 404189 | GO:0000287 | IEA |
| 404189 | GO:0000166 | IEA |
| 404189 | GO:0004550 | IEA |
| 404189 | GO:0016740 | IEA |
| 404189 | GO:0005524 | IEA |
| 404189 | GO:0046872 | IEA |
| 404189 | GO:0016301 | IEA |
| 407767 | GO:0004421 | IEA |
| 408001 | GO:0005515 | IEA |
| 408001 | GO:0005515 | IEA |
| 408010 | GO:0016874 | IEA |
| 408010 | GO:0005524 | IEA |
| 408010 | GO:0004820 | IEA |
| 408010 | GO:0004812 | IEA |
| 414274 | GO:0008134 | IEA |
| 414274 | GO:0047485 | IEA |
| 414274 | GO:0042802 | IEA |
| 414274 | GO:0003682 | IEA |
| 414732 | GO:0016740 | IEA |
| 444874 | GO:0005515 | IPI |
| 444874 | GO:0030528 | IEA |
| 444874 | GO:0005515 | IEA |
| 444874 | GO:0030528 | IEA |
| 444874 | GO:0005515 | IPI |
| 444874 | GO:0005515 | IEA |
| 503685 | GO:0004869 | IEA |
| 504438 | GO:0004872 | IEA |
| 504718 | GO:0005515 | IEA |
| 504735 | GO:0051082 | IEA |
| 504735 | GO:0000166 | IEA |
| 504735 | GO:0005515 | IEA |
| 504735 | GO:0005524 | IEA |
| 504781 | GO:0004568 | IEA |
| 504781 | GO:0004553 | IEA |
| 504781 | GO:0003824 | IEA |
| 504781 | GO:0043169 | IEA |
| 504854 | GO:0005509 | IEA |
| 505005 | GO:0016740 | IEA |
| 505005 | GO:0004672 | IEA |
| 505005 | GO:0004673 | IEA |
| 505005 | GO:0005524 | IEA |
| 505005 | GO:0016772 | IEA |
| 505005 | GO:0016301 | IEA |
| 505060 | GO:0004497 | IEA |
| 505060 | GO:0008398 | IEA |
| 505060 | GO:0005506 | IEA |
| 505060 | GO:0016491 | IEA |
| 505060 | GO:0046872 | IEA |
| 505060 | GO:0020037 | IEA |
| 505171 | GO:0005515 | IEA |
| 505171 | GO:0042802 | IEA |
| 505171 | GO:0004871 | IEA |
| 505199 | GO:0003735 | IEA |
| 505406 | GO:0003779 | IEA |
| 505507 | GO:0005515 | IEA |
| 505507 | GO:0003735 | IEA |
| 505642 | GO:0004364 | IEA |
| 505642 | GO:0016740 | IEA |
| 505691 | GO:0004861 | IEA |
| 505691 | GO:0005515 | IEA |
| 505691 | GO:0019901 | IEA |
| 505807 | GO:0004719 | IEA |
| 505884 | GO:0003676 | IEA |
| 505884 | GO:0008270 | IEA |
| 505884 | GO:0046872 | IEA |
| 505901 | GO:0004697 | IEA |
| 505901 | GO:0005515 | IEA |
| 506043 | GO:0003755 | IEA |
| 506043 | GO:0016853 | IEA |
| 506085 | GO:0016740 | IEA |
| 506085 | GO:0046872 | IEA |
| 506085 | GO:0008415 | IEA |
| 506085 | GO:0008270 | IEA |
| 506261 | GO:0019904 | IEA |
| 506370 | GO:0005215 | IEA |
| 506441 | GO:0008415 | IEA |
| 506522 | GO:0005515 | IEA |
| 506534 | GO:0005515 | IEA |
| 506544 | GO:0003872 | IEA |
| 506544 | GO:0016301 | IEA |
| 506544 | GO:0008443 | IEA |
| 506566 | GO:0016740 | IEA |
| 506715 | GO:0005515 | IEA |
| 506715 | GO:0045502 | IEA |
| 506715 | GO:0016887 | IEA |
| 506787 | GO:0004115 | IEA |
| 506790 | GO:0005515 | IEA |
| 506790 | GO:0004857 | IEA |
| 506860 | GO:0005515 | IEA |
| 506860 | GO:0042802 | IEA |
| 506880 | GO:0042802 | IEA |
| 506880 | GO:0005515 | IEA |
| 506880 | GO:0000166 | IEA |
| 506880 | GO:0003676 | IEA |
| 506880 | GO:0003676 | IEA |
| 506880 | GO:0000166 | IEA |
| 506880 | GO:0042802 | IEA |
| 506880 | GO:0005515 | IEA |
| 506988 | GO:0003735 | IEA |
| 506989 | GO:0003735 | IEA |
| 507120 | GO:0005515 | IEA |
| 507120 | GO:0003676 | IEA |
| 507120 | GO:0000166 | IEA |
| 507234 | GO:0005515 | IEA |
| 507234 | GO:0004871 | IEA |
| 507270 | GO:0003735 | IEA |
| 507430 | GO:0005515 | IEA |
| 507449 | GO:0000166 | IEA |
| 507449 | GO:0005525 | IEA |
| 507679 | GO:0005525 | IEA |
| 507736 | GO:0004872 | IEA |
| 507781 | GO:0004842 | IEA |
| 507784 | GO:0003735 | IEA |
| 507815 | GO:0016740 | IEA |
| 507815 | GO:0004576 | IEA |
| 507845 | GO:0008017 | IEA |
| 507845 | GO:0005515 | ISS |
| 507845 | GO:0051010 | ISS |
| 507912 | GO:0003676 | IEA |
| 508221 | GO:0003735 | IEA |
| 508319 | GO:0005515 | IEA |
| 508319 | GO:0005509 | IEA |
| 508628 | GO:0003735 | IEA |
| 508629 | GO:0016491 | IEA |
| 508629 | GO:0004029 | IEA |
| 509274 | GO:0016787 | IEA |
| 509274 | GO:0004416 | IEA |
| 509422 | GO:0005509 | IEA |
| 509422 | GO:0005515 | IEA |
| 509426 | GO:0003735 | IEA |
| 509696 | GO:0050347 | IEA |
| 509696 | GO:0046982 | IEA |
| 509696 | GO:0000010 | IEA |
| 509771 | GO:0000166 | IEA |
| 509771 | GO:0003676 | IEA |
| 509771 | GO:0043023 | IEA |
| 509896 | GO:0016787 | IEA |
| 509896 | GO:0004177 | IEA |
| 509963 | GO:0004857 | IEA |
| 509963 | GO:0005102 | IEA |
| 509965 | GO:0019201 | IEA |
| 509965 | GO:0000166 | IEA |
| 509965 | GO:0016776 | IEA |
| 509965 | GO:0016740 | IEA |
| 509965 | GO:0005524 | IEA |
| 509965 | GO:0016301 | IEA |
| 510041 | GO:0008538 | IEA |
| 510050 | GO:0003677 | IEA |
| 510050 | GO:0043565 | IEA |
| 510050 | GO:0003700 | IEA |
| 510154 | GO:0008430 | IEA |
| 510159 | GO:0008017 | IEA |
| 510200 | GO:0017039 | IEA |
| 510423 | GO:0004175 | IEA |
| 510423 | GO:0004298 | IEA |
| 510423 | GO:0042802 | IEA |
| 510850 | GO:0051082 | IEA |
| 510850 | GO:0005515 | IEA |
| 510850 | GO:0004867 | IEA |
| 510937 | GO:0016874 | IEA |
| 510937 | GO:0000166 | IEA |
| 510937 | GO:0004812 | IEA |
| 510937 | GO:0005524 | IEA |
| 510937 | GO:0004821 | IEA |
| 510949 | GO:0016829 | IEA |
| 510949 | GO:0004018 | IEA |
| 511135 | GO:0003824 | IEA |
| 511135 | GO:0016829 | IEA |
| 511135 | GO:0005524 | IEA |
| 511560 | GO:0005515 | IEA |
| 511560 | GO:0003735 | IEA |
| 511560 | GO:0003723 | IEA |
| 511716 | GO:0016787 | IEA |
| 511716 | GO:0004301 | IEA |
| 511716 | GO:0003824 | IEA |
| 511899 | GO:0005515 | IEA |
| 512254 | GO:0003700 | IEA |
| 512254 | GO:0003677 | IEA |
| 512254 | GO:0043565 | IEA |
| 512534 | GO:0008717 | IEA |
| 512673 | GO:0051082 | IEA |
| 512717 | GO:0005509 | IEA |
| 512918 | GO:0005515 | IEA |
| 512918 | GO:0035091 | IEA |
| 513038 | GO:0016787 | IEA |
| 513038 | GO:0008233 | IEA |
| 513038 | GO:0008234 | IEA |
| 513038 | GO:0004216 | IEA |
| 513038 | GO:0004197 | IEA |
| 513125 | GO:0003714 | IEA |
| 513125 | GO:0050827 | IEA |
| 513125 | GO:0005515 | IEA |
| 513128 | GO:0003735 | IEA |
| 513257 | GO:0005515 | IEA |
| 513257 | GO:0005515 | IEA |
| 513497 | GO:0030332 | IEA |
| 513497 | GO:0005515 | IEA |
| 513497 | GO:0004861 | IEA |
| 513587 | GO:0005515 | IEA |
| 513776 | GO:0005515 | IEA |
| 513776 | GO:0005515 | IEA |
| 514355 | GO:0000166 | IEA |
| 514355 | GO:0042802 | IEA |
| 514355 | GO:0005515 | IEA |
| 514355 | GO:0005524 | IEA |
| 514355 | GO:0051082 | IEA |
| 514443 | GO:0005515 | IEA |
| 514547 | GO:0005515 | IEA |
| 514665 | GO:0005215 | IEA |
| 515109 | GO:0008270 | IEA |
| 515109 | GO:0046872 | IEA |
| 515109 | GO:0005515 | IEA |
| 515208 | GO:0042802 | IEA |
| 515320 | GO:0003677 | IEA |
| 515320 | GO:0003677 | IEA |
| 515380 | GO:0043565 | IEA |
| 515380 | GO:0005516 | IEA |
| 515380 | GO:0003677 | IEA |
| 515573 | GO:0005515 | IEA |
| 515573 | GO:0043398 | IEA |
| 515573 | GO:0004842 | IEA |
| 515602 | GO:0003677 | IEA |
| 515602 | GO:0000166 | IEA |
| 515602 | GO:0017111 | IEA |
| 515854 | GO:0004672 | IEA |
| 515854 | GO:0005515 | IEA |
| 516063 | GO:0005515 | IEA |
| 516063 | GO:0003735 | IEA |
| 516287 | GO:0051287 | IEA |
| 516287 | GO:0004128 | IEA |
| 516287 | GO:0016491 | IEA |
| 516287 | GO:0050660 | IEA |
| 516334 | GO:0008067 | IEA |
| 516334 | GO:0004872 | IEA |
| 517345 | GO:0005515 | IEA |
| 517345 | GO:0005525 | IEA |
| 517345 | GO:0000166 | IEA |
| 517345 | GO:0005525 | IEA |
| 517345 | GO:0005515 | IEA |
| 517345 | GO:0000166 | IEA |
| 517539 | GO:0016787 | IEA |
| 517539 | GO:0003824 | IEA |
| 517539 | GO:0016787 | IEA |
| 517539 | GO:0003824 | IEA |
| 517688 | GO:0015279 | IEA |
| 517688 | GO:0015279 | IEA |
| 517688 | GO:0015279 | IEA |
| 518601 | GO:0005525 | IEA |
| 518601 | GO:0000166 | IEA |
| 520694 | GO:0005154 | IEA |
| 521237 | GO:0004674 | IEA |
| 521237 | GO:0016740 | IEA |
| 521237 | GO:0005515 | IEA |
| 521237 | GO:0004672 | IEA |
| 521237 | GO:0000166 | IEA |
| 521237 | GO:0005524 | IEA |
| 521237 | GO:0016301 | IEA |
| 522980 | GO:0008233 | IEA |
| 522980 | GO:0004197 | IEA |
| 522980 | GO:0004221 | IEA |
| 523671 | GO:0003735 | IEA |
| 524507 | GO:0003735 | IEA |
| 524507 | GO:0005515 | IEA |
| 524770 | GO:0017166 | IEA |
| 524770 | GO:0051015 | IEA |
| 524770 | GO:0005515 | IEA |
| 524770 | GO:0005523 | IDA |
| 524770 | GO:0003779 | IEA |
| 524770 | GO:0043495 | NAS |
| 524770 | GO:0005178 | IEA |
| 524770 | GO:0051015 | IDA |
| 524770 | GO:0005509 | IEA |
| 525680 | GO:0005515 | IEA |
| 526135 | GO:0003723 | IEA |
| 526135 | GO:0000166 | IEA |
| 526135 | GO:0003676 | IEA |
| 526320 | GO:0003723 | IEA |
| 526320 | GO:0005515 | IEA |
| 526320 | GO:0016740 | IEA |
| 526320 | GO:0016779 | IEA |
| 526320 | GO:0003899 | IEA |
| 527935 | GO:0003677 | IEA |
| 527935 | GO:0016563 | IEA |
| 528165 | GO:0005515 | IEA |
| 528253 | GO:0016564 | IEA |
| 528253 | GO:0005515 | IEA |
| 528253 | GO:0003677 | IEA |
| 528735 | GO:0008270 | IEA |
| 528735 | GO:0005509 | IEA |
| 528735 | GO:0046872 | IEA |
| 529761 | GO:0005515 | IEA |
| 531367 | GO:0004791 | IEA |
| 532203 | GO:0019843 | IEA |
| 532203 | GO:0003723 | IEA |
| 532203 | GO:0003735 | IEA |
| 532205 | GO:0016491 | IEA |
| 532205 | GO:0047115 | IEA |
| 532483 | GO:0046872 | IEA |
| 532483 | GO:0008270 | IEA |
| 532483 | GO:0005515 | IEA |
| 532560 | GO:0005488 | IEA |
| 532560 | GO:0005215 | IEA |
| 532726 | GO:0019212 | IEA |
| 532726 | GO:0004864 | IEA |
| 532726 | GO:0019212 | IEA |
| 532726 | GO:0004864 | IEA |
| 532791 | GO:0030528 | IEA |
| 532791 | GO:0005515 | IEA |
| 533087 | GO:0005515 | IEA |
| 533208 | GO:0008168 | IEA |
| 533219 | GO:0005524 | IEA |
| 533219 | GO:0005515 | IEA |
| 533219 | GO:0005198 | IEA |
| 533219 | GO:0000166 | IEA |
| 533223 | GO:0003779 | IEA |
| 533251 | GO:0005515 | IEA |
| 533307 | GO:0000166 | IEA |
| 533307 | GO:0005525 | IEA |
| 533307 | GO:0005198 | IEA |
| 533307 | GO:0003924 | IEA |
| 533307 | GO:0000166 | IEA |
| 533307 | GO:0005198 | IEA |
| 533307 | GO:0005525 | IEA |
| 533307 | GO:0003924 | IEA |
| 533608 | GO:0016491 | IEA |
| 533630 | GO:0004647 | IEA |
| 533630 | GO:0016787 | IEA |
| 533630 | GO:0000287 | IEA |
| 533630 | GO:0003824 | IEA |
| 533630 | GO:0016791 | IEA |
| 533630 | GO:0000287 | IEA |
| 533630 | GO:0016787 | IEA |
| 533630 | GO:0003824 | IEA |
| 533630 | GO:0004647 | IEA |
| 533630 | GO:0016791 | IEA |
| 533642 | GO:0005515 | IEA |
| 533700 | GO:0008026 | IEA |
| 533700 | GO:0005515 | IEA |
| 533700 | GO:0000166 | IEA |
| 533700 | GO:0003676 | IEA |
| 533700 | GO:0016787 | IEA |
| 533700 | GO:0005524 | IEA |
| 533700 | GO:0003712 | IEA |
| 533700 | GO:0004386 | IEA |
| 533729 | GO:0004672 | IEA |
| 533729 | GO:0005515 | IEA |
| 533729 | GO:0016301 | IEA |
| 533729 | GO:0004674 | IEA |
| 533729 | GO:0005524 | IEA |
| 533785 | GO:0016868 | IEA |
| 533785 | GO:0016787 | IEA |
| 533785 | GO:0016853 | IEA |
| 533896 | GO:0008138 | IEA |
| 533896 | GO:0004721 | IEA |
| 534092 | GO:0004872 | IEA |
| 534092 | GO:0004672 | IEA |
| 534092 | GO:0005524 | IEA |
| 534092 | GO:0004714 | IEA |
| 534092 | GO:0004713 | IEA |
| 534647 | GO:0005515 | IEA |
| 534751 | GO:0005524 | IEA |
| 534751 | GO:0000166 | IEA |
| 535056 | GO:0003735 | IEA |
| 535056 | GO:0019843 | IEA |
| 535056 | GO:0003723 | IEA |
| 535293 | GO:0016787 | IEA |
| 535293 | GO:0004301 | IEA |
| 535293 | GO:0003824 | IEA |
| 535312 | GO:0019789 | IEA |
| 535312 | GO:0051059 | IEA |
| 536149 | GO:0008270 | IEA |
| 536149 | GO:0046872 | IEA |
| 536149 | GO:0003677 | IEA |
| 537224 | GO:0008195 | IEA |
| 538060 | GO:0016491 | IEA |
| 538357 | GO:0005515 | IEA |
| 538548 | GO:0003723 | IEA |
| 538548 | GO:0005515 | IEA |
| 538662 | GO:0005515 | IEA |
| 538662 | GO:0019901 | IEA |
| 538743 | GO:0004221 | IEA |
| 538743 | GO:0016787 | IEA |
| 538743 | GO:0008233 | IEA |
| 538743 | GO:0004197 | IEA |
| 538998 | GO:0003755 | IEA |
| 538998 | GO:0016853 | IEA |
| 538998 | GO:0005515 | IEA |
| 539020 | GO:0003682 | IEA |
| 539020 | GO:0043565 | IEA |
| 539020 | GO:0005515 | IEA |
| 539020 | GO:0016564 | IEA |
| 539020 | GO:0003677 | IEA |
| 539339 | GO:0005525 | IEA |
| 539339 | GO:0005524 | IEA |
| 539339 | GO:0008134 | IEA |
| 539339 | GO:0000166 | IEA |
| 539373 | GO:0003735 | IEA |
| 539381 | GO:0046872 | IEA |
| 539381 | GO:0008270 | IEA |
| 539445 | GO:0004674 | IEA |
| 539445 | GO:0004672 | IEA |
| 539445 | GO:0005524 | IEA |
| 539546 | GO:0016853 | IEA |
| 539546 | GO:0003755 | IEA |
| 539761 | GO:0008270 | IEA |
| 539761 | GO:0003676 | IEA |
| 539945 | GO:0008270 | IEA |
| 539945 | GO:0005509 | IEA |
| 539945 | GO:0005509 | IEA |
| 539945 | GO:0008270 | IEA |
| 540045 | GO:0003676 | IEA |
| 540045 | GO:0008270 | IEA |
| 540207 | GO:0005524 | IEA |
| 540207 | GO:0004674 | IEA |
| 540207 | GO:0004672 | IEA |
| 540207 | GO:0016740 | IEA |
| 540207 | GO:0016301 | IEA |
| 540207 | GO:0004713 | IEA |
| 540207 | GO:0000166 | IEA |
| 540559 | GO:0050750 | IEA |
| 540602 | GO:0003824 | IEA |
| 540602 | GO:0019007 | IEA |
| 540614 | GO:0005525 | IEA |
| 540637 | GO:0003785 | IEA |
| 540637 | GO:0051015 | IEA |
| 540892 | GO:0000166 | IEA |
| 540892 | GO:0003924 | IEA |
| 540892 | GO:0005525 | IEA |
| 540932 | GO:0003700 | IEA |
| 540932 | GO:0005515 | IEA |
| 541113 | GO:0005515 | IEA |
| 541113 | GO:0005515 | IEA |
| 541175 | GO:0008138 | IEA |
| 541175 | GO:0017017 | IEA |
| 541175 | GO:0004721 | IEA |
| 541175 | GO:0004721 | IEA |
| 541175 | GO:0008138 | IEA |
| 541175 | GO:0017017 | IEA |
| 541175 | GO:0008138 | IEA |
| 541175 | GO:0017017 | IEA |
| 541175 | GO:0004721 | IEA |
| 541276 | GO:0042802 | IEA |
| 541276 | GO:0003676 | IEA |
| 541276 | GO:0000166 | IEA |
| 541276 | GO:0005515 | IEA |
| 541276 | GO:0005515 | IEA |
| 541276 | GO:0003676 | IEA |
| 541276 | GO:0042802 | IEA |
| 541276 | GO:0000166 | IEA |
| 613305 | GO:0003723 | IEA |
| 613519 | GO:0003735 | IEA |
| 613534 | GO:0003735 | IEA |
| 613548 | GO:0003779 | IEA |
| 613548 | GO:0005516 | IEA |
| 613548 | GO:0005516 | IEA |
| 613548 | GO:0003779 | IEA |
| 613877 | GO:0003705 | IEA |
| 613877 | GO:0005515 | IEA |
| 613877 | GO:0003677 | IEA |
| 613972 | GO:0005515 | IEA |
| 613972 | GO:0005488 | IEA |
| 613972 | GO:0008289 | IEA |
| 613972 | GO:0005501 | IEA |
| 613972 | GO:0005215 | IEA |
| 613998 | GO:0032767 | IEA |
| 613998 | GO:0016531 | IEA |
| 613998 | GO:0046872 | IEA |
| 614128 | GO:0015450 | IEA |
| 614241 | GO:0003735 | IEA |
| 614241 | GO:0003723 | IEA |
| 614241 | GO:0019843 | IEA |
| 614348 | GO:0005515 | IEA |
| 614366 | GO:0003723 | IEA |
| 614366 | GO:0003735 | IEA |
| 614366 | GO:0019843 | IEA |
| 614393 | GO:0005515 | IEA |
| 614393 | GO:0004871 | IEA |
| 614393 | GO:0042802 | IEA |
| 614423 | GO:0004619 | IEA |
| 614423 | GO:0016787 | IEA |
| 614423 | GO:0016868 | IEA |
| 614423 | GO:0016853 | IEA |
| 614423 | GO:0004082 | IEA |
| 614423 | GO:0004083 | IEA |
| 614423 | GO:0003824 | IEA |
| 614449 | GO:0003735 | IEA |
| 614449 | GO:0003723 | IEA |
| 614449 | GO:0019843 | IEA |
| 614509 | GO:0030528 | IEA |
| 614581 | GO:0000166 | IEA |
| 614581 | GO:0005525 | IEA |
| 614678 | GO:0005515 | IEA |
| 614795 | GO:0016853 | IEA |
| 614795 | GO:0003755 | IEA |
| 614926 | GO:0005515 | IEA |
| 614926 | GO:0003735 | IEA |
| 615087 | GO:0000166 | IEA |
| 615087 | GO:0005198 | IEA |
| 615087 | GO:0005525 | IEA |
| 615087 | GO:0003924 | IEA |
| 615087 | GO:0005525 | IEA |
| 615087 | GO:0000166 | IEA |
| 615087 | GO:0003924 | IEA |
| 615087 | GO:0005198 | IEA |
| 615175 | GO:0005515 | IEA |
| 615175 | GO:0000166 | IEA |
| 615175 | GO:0042802 | IEA |
| 615175 | GO:0003676 | IEA |
| 615175 | GO:0003676 | IEA |
| 615175 | GO:0000166 | IEA |
| 615175 | GO:0005515 | IEA |
| 615175 | GO:0042802 | IEA |
| 615342 | GO:0046982 | IEA |
| 615342 | GO:0042803 | IEA |
| 615401 | GO:0003735 | IEA |
| 615447 | GO:0016740 | IEA |
| 615447 | GO:0004550 | IEA |
| 615447 | GO:0016301 | IEA |
| 615447 | GO:0005524 | IEA |
| 615447 | GO:0000287 | IEA |
| 615447 | GO:0000166 | IEA |
| 615447 | GO:0046872 | IEA |
| 615518 | GO:0004450 | IEA |
| 615518 | GO:0016491 | IEA |
| 615518 | GO:0016652 | IEA |
| 615518 | GO:0004450 | IEA |
| 615518 | GO:0016491 | IEA |
| 615518 | GO:0016652 | IEA |
| 615565 | GO:0005515 | IEA |
| 615565 | GO:0005488 | IEA |
| 615572 | GO:0008022 | IEA |
| 615572 | GO:0005515 | IEA |
| 615610 | GO:0016868 | IEA |
| 615610 | GO:0016787 | IEA |
| 615610 | GO:0004083 | IEA |
| 615610 | GO:0004082 | IEA |
| 615610 | GO:0003824 | IEA |
| 615610 | GO:0016853 | IEA |
| 615610 | GO:0004619 | IEA |
| 615666 | GO:0003735 | IEA |
| 615827 | GO:0016538 | IEA |
| 616295 | GO:0005515 | IEA |
| 616295 | GO:0043398 | IEA |
| 616295 | GO:0004842 | IEA |
| 616503 | GO:0005525 | IEA |
| 616503 | GO:0000166 | IEA |
| 616539 | GO:0016563 | IEA |
| 616539 | GO:0008270 | IEA |
| 617105 | GO:0005515 | ISS |
| 617105 | GO:0003746 | IEA |
| 617105 | GO:0005515 | IEA |
| 617267 | GO:0016787 | IEA |
| 617267 | GO:0016853 | IEA |
| 617267 | GO:0016868 | IEA |
| 617901 | GO:0003735 | IEA |
| 617917 | GO:0016491 | IEA |
| 617917 | GO:0047115 | IEA |
| 617985 | GO:0005509 | IEA |
| 618292 | GO:0000175 | IEA |
| 618292 | GO:0004527 | IEA |
| 618292 | GO:0016787 | IEA |
| 618292 | GO:0005515 | IEA |
| 618292 | GO:0003723 | IEA |
| 618292 | GO:0004518 | IEA |
| 618441 | GO:0000318 | IEA |
| 618441 | GO:0008113 | IEA |
| 618441 | GO:0008270 | IEA |
| 618609 | GO:0003735 | IEA |
| 618609 | GO:0003723 | IEA |
| 619061 | GO:0003735 | IEA |
| 767855 | GO:0004672 | IEA |
| 767855 | GO:0005524 | IEA |
| 767898 | GO:0008270 | IEA |
| 767898 | GO:0003676 | IEA |
| 768311 | GO:0019904 | IEA |
| 780984 | GO:0005509 | IEA |
| 781044 | GO:0003924 | IEA |
| 781158 | GO:0003735 | IEA |
| 781300 | GO:0003735 | IEA |
| 781334 | GO:0003779 | IEA |
| 781358 | GO:0003735 | IEA |
| 781412 | GO:0003924 | IEA |
| 781412 | GO:0005198 | IEA |
| 781412 | GO:0000166 | IEA |
| 781412 | GO:0005525 | IEA |
| 781412 | GO:0000166 | IEA |
| 781412 | GO:0005525 | IEA |
| 781412 | GO:0005198 | IEA |
| 781412 | GO:0003924 | IEA |
| 781462 | GO:0005515 | IEA |
| 781462 | GO:0046872 | IEA |
| 781462 | GO:0008270 | IEA |
| 781565 | GO:0003735 | IEA |
| 781575 | GO:0051082 | IEA |
| 781579 | GO:0003735 | IEA |
| 781580 | GO:0003735 | IEA |
| 781580 | GO:0003723 | IEA |
| 781580 | GO:0019843 | IEA |
| 781851 | GO:0003735 | IEA |
| 781894 | GO:0003723 | IEA |
| 781894 | GO:0003735 | IEA |
| 781894 | GO:0019843 | IEA |
| 781943 | GO:0003735 | IEA |
| 782038 | GO:0016787 | IEA |
| 782038 | GO:0004722 | IEA |
| 782038 | GO:0030145 | IEA |
| 782038 | GO:0004721 | IEA |
| 782038 | GO:0000287 | IEA |
| 782038 | GO:0046872 | IEA |
| 782038 | GO:0003824 | IEA |
| 782061 | GO:0016491 | IEA |
| 782393 | GO:0005515 | ISS |
| 782393 | GO:0003746 | IEA |
| 782393 | GO:0005515 | IEA |
| 782631 | GO:0003735 | IEA |
| 782738 | GO:0003735 | IEA |
| 782740 | GO:0003735 | IEA |
| 782855 | GO:0003735 | IEA |
| 783021 | GO:0003676 | IEA |
| 783065 | GO:0003755 | IEA |
| 783065 | GO:0016853 | IEA |
| 783065 | GO:0005515 | IEA |
| 783067 | GO:0003735 | IEA |
| 783144 | GO:0003735 | IEA |
| 783148 | GO:0003735 | IEA |
| 783184 | GO:0004421 | IEA |
| 783185 | GO:0005515 | IEA |
| 783185 | GO:0005515 | IEA |
| 783202 | GO:0004129 | IEA |
| 783202 | GO:0016491 | IEA |
| 783215 | GO:0003735 | IEA |
| 783372 | GO:0003723 | IEA |
| 783372 | GO:0003735 | IEA |
| 783372 | GO:0019843 | IEA |
| 783462 | GO:0003735 | IEA |
| 783463 | GO:0003735 | IEA |
| 783463 | GO:0003723 | IEA |
| 783463 | GO:0019843 | IEA |
| 783687 | GO:0051082 | IEA |
| 783718 | GO:0005515 | IPI |
| 783718 | GO:0030528 | IEA |
| 783718 | GO:0005515 | IEA |
| 783718 | GO:0030528 | IEA |
| 783718 | GO:0005515 | IPI |
| 783718 | GO:0005515 | IEA |
| 783880 | GO:0003735 | IEA |
| 783942 | GO:0003735 | IEA |
| 784052 | GO:0003735 | IEA |
| 784054 | GO:0003735 | IEA |
| 784208 | GO:0003723 | IEA |
| 784208 | GO:0003735 | IEA |
| 784208 | GO:0019843 | IEA |
| 784340 | GO:0016868 | IEA |
| 784340 | GO:0016853 | IEA |
| 784340 | GO:0016787 | IEA |
| 784521 | GO:0003735 | IEA |
| 784643 | GO:0003779 | IEA |
| 784731 | GO:0003735 | IEA |
| 784731 | GO:0003723 | IEA |
| 784731 | GO:0019843 | IEA |
| 784738 | GO:0003735 | IEA |
| 784740 | GO:0042802 | IEA |
| 784740 | GO:0005515 | IEA |
| 784740 | GO:0000166 | IEA |
| 784740 | GO:0003676 | IEA |
| 784740 | GO:0000166 | IEA |
| 784740 | GO:0042802 | IEA |
| 784740 | GO:0003676 | IEA |
| 784740 | GO:0005515 | IEA |
| 784869 | GO:0047115 | IEA |
| 784869 | GO:0016491 | IEA |
| 785008 | GO:0003735 | IEA |
| 785016 | GO:0005515 | IEA |
| 785061 | GO:0003735 | IEA |
| 785061 | GO:0019843 | IEA |
| 785061 | GO:0003723 | IEA |
| 785134 | GO:0004129 | IEA |
| 785134 | GO:0009055 | IEA |
| 785134 | GO:0016491 | IEA |
| 785455 | GO:0003779 | IEA |
| 785508 | GO:0004301 | IEA |
| 785508 | GO:0003824 | IEA |
| 785508 | GO:0016787 | IEA |
| 785760 | GO:0005515 | IEA |
| 785760 | GO:0042802 | IEA |
| 785760 | GO:0003676 | IEA |
| 785760 | GO:0000166 | IEA |
| 785760 | GO:0003676 | IEA |
| 785760 | GO:0000166 | IEA |
| 785760 | GO:0042802 | IEA |
| 785760 | GO:0005515 | IEA |
| 785762 | GO:0016491 | IEA |
| 785762 | GO:0016491 | IEA |
| 785763 | GO:0016787 | IEA |
| 785763 | GO:0016868 | IEA |
| 785763 | GO:0016853 | IEA |
| 785824 | GO:0004497 | IEA |
| 785824 | GO:0046872 | IEA |
| 785824 | GO:0016712 | IEA |
| 785824 | GO:0020037 | IEA |
| 785824 | GO:0005506 | IEA |
| 785824 | GO:0016491 | IEA |
| 785828 | GO:0003676 | IEA |
| 785986 | GO:0005515 | IEA |
| 785986 | GO:0046872 | IEA |
| 785986 | GO:0008270 | IEA |
| 786048 | GO:0003677 | IEA |
| 786048 | GO:0005516 | IEA |
| 786048 | GO:0043565 | IEA |
| 786100 | GO:0008270 | IEA |
| 786100 | GO:0046872 | IEA |
| 786100 | GO:0005509 | IEA |
| 786207 | GO:0003735 | IEA |
| 786207 | GO:0003723 | IEA |
| 786207 | GO:0019843 | IEA |
| 786555 | GO:0005515 | IEA |
| 786555 | GO:0042802 | IEA |
| 786555 | GO:0003676 | IEA |
| 786555 | GO:0000166 | IEA |
| 786555 | GO:0003676 | IEA |
| 786555 | GO:0042802 | IEA |
| 786555 | GO:0000166 | IEA |
| 786555 | GO:0005515 | IEA |
| 787193 | GO:0005515 | IEA |
| 787193 | GO:0042802 | IEA |
| 787903 | GO:0005515 | IEA |
| 787903 | GO:0003676 | IEA |
| 787903 | GO:0042802 | IEA |
| 787903 | GO:0000166 | IEA |
| 787903 | GO:0005515 | IEA |
| 787903 | GO:0003676 | IEA |
| 787903 | GO:0000166 | IEA |
| 787903 | GO:0042802 | IEA |
| 788457 | GO:0005509 | IEA |
| 789002 | GO:0004719 | IEA |
| 789061 | GO:0003676 | IEA |
| 789264 | GO:0003735 | IEA |
| 789264 | GO:0003723 | IEA |
| 789264 | GO:0019843 | IEA |
| 789606 | GO:0003735 | IEA |
| 789607 | GO:0003735 | IEA |
| 789638 | GO:0016853 | IEA |
| 789638 | GO:0016868 | IEA |
| 789638 | GO:0016787 | IEA |
| 790047 | GO:0004842 | IEA |
| 790574 | GO:0042802 | IEA |
| 790797 | GO:0003700 | IEA |
| 790797 | GO:0003707 | IEA |
| 790797 | GO:0004879 | IEA |
| 790797 | GO:0003677 | IEA |
| 790797 | GO:0043565 | IEA |
| 790797 | GO:0008270 | IEA |
| 790827 | GO:0003676 | IEA |
| 790827 | GO:0005515 | IEA |
| 790827 | GO:0042802 | IEA |
| 790827 | GO:0000166 | IEA |
| 790827 | GO:0003676 | IEA |
| 790827 | GO:0005515 | IEA |
| 790827 | GO:0000166 | IEA |
| 790827 | GO:0042802 | IEA |
|  |  |  |
