# Supplementary material for: A gene network switch enhances the oxidative capacity of ovine skeletal muscle during late fetal development
Source: BMC Genomics. 2010 Jun 15;11:378. doi: 10.1186/1471-2164-11-378 (PMC2894804; doi:10.1186/1471-2164-11-378)
Supplement: Additional File 1 — Gene expression data. This file contains links to the gene expression data. The genes that are significantly differentially expressed between adjacent development times are listed as Experiment 1 (80 d vs 100 d), Experiment 2 (100 d vs 120 d), Experiment 3 (120 d vs 150 d) and Experiment 4 (150 d vs 230 d) in the section entitled Open microarray analysis report for developmental expression. Each experiment contains an MA plot, Volcano plot and Heatmap for each of the three microarray processing programs MAS5, RMA and GCRMA. For each of these processing programs there is a list of significantly differentially expressed probe sets, and for each of these there is an FDR corrected probability, fold change, percentage of microarrays with a MAS5 Present call, and a convergence percentage for all three microarray processing programs. The file also contains annotation of the probe sets. In a second section entitled Time series analysis of developmental expression, there is a list of genes in each gene expression cluster and diagrams showing MAS5 gene expression values for these genes. [file 1471-2164-11-378-S1.ZIP › Report/GO4/Report/TopNodesBP.html]

### Top terms for Biological Process

Top terms sorted by the weight algorithm. The first column is the GO term id and links to GO, the second column is a short description of the term, the third column is the number of genes annotated to the term, the fourth is the number of genes significant for the term and the fifth column is the expected number of genes for the term. The last four columns are the p-values for different tests of significance. The results are ordered by the weight algorithm (last column).

|  |  |  |  |  |  |  |  |  |
| --- | --- | --- | --- | --- | --- | --- | --- | --- |
| **GO** | **Term** | **Annotated** | **Significant** | **Expected** | **classic** | **KS** | **elim** | **weight** |
| GO:0006003 | fructose 2,6-bisphosphate metabolic proc... | 6 | 5 | 0.35 | 3.9e-06 | 1.00 | 3.9e-06 | 3.9e-06 |
| GO:0016125 | sterol metabolic process | 24 | 7 | 1.41 | 0.00033 | 0.80 | 0.0288 | 0.00033 |
| GO:0019882 | antigen processing and presentation | 34 | 9 | 2 | 0.00010 | 1.00 | 0.0207 | 0.00038 |
| GO:0006694 | steroid biosynthetic process | 20 | 6 | 1.18 | 0.00075 | 0.95 | 0.0746 | 0.00075 |
| GO:0008299 | isoprenoid biosynthetic process | 11 | 4 | 0.65 | 0.00277 | 0.97 | 0.0028 | 0.00277 |
| GO:0015976 | carbon utilization | 2 | 2 | 0.12 | 0.00344 | 0.99 | 0.0034 | 0.00344 |
| GO:0006564 | L-serine biosynthetic process | 3 | 2 | 0.18 | 0.00993 | 0.99 | 0.0099 | 0.00993 |
| GO:0006512 | ubiquitin cycle | 160 | 17 | 9.41 | 0.01205 | 0.45 | 0.0121 | 0.01205 |
| GO:0045637 | regulation of myeloid cell differentiati... | 10 | 4 | 0.59 | 0.00185 | 0.74 | 0.0019 | 0.01283 |
| GO:0000045 | autophagic vacuole formation | 4 | 2 | 0.24 | 0.01909 | 0.94 | 0.0191 | 0.01909 |
| GO:0007595 | lactation | 4 | 2 | 0.24 | 0.01909 | 1.00 | 0.0191 | 0.01909 |
| GO:0009967 | positive regulation of signal transducti... | 28 | 5 | 1.65 | 0.02181 | 0.68 | 0.0218 | 0.02181 |
| GO:0006097 | glyoxylate cycle | 5 | 2 | 0.29 | 0.03059 | 0.42 | 0.0306 | 0.03059 |
| GO:0043666 | regulation of phosphoprotein phosphatase... | 5 | 2 | 0.29 | 0.03059 | 0.67 | 0.0306 | 0.03059 |
| GO:0006470 | protein amino acid dephosphorylation | 54 | 7 | 3.17 | 0.03715 | 0.52 | 0.0371 | 0.03715 |
| GO:0006457 | protein folding | 78 | 9 | 4.59 | 0.03798 | 0.97 | 0.0380 | 0.03798 |
| GO:0006102 | isocitrate metabolic process | 6 | 2 | 0.35 | 0.04413 | 0.54 | 0.0441 | 0.04413 |
| GO:0009084 | glutamine family amino acid biosynthetic... | 6 | 2 | 0.35 | 0.04413 | 0.72 | 0.0441 | 0.04413 |
| GO:0006817 | phosphate transport | 45 | 6 | 2.65 | 0.04617 | 0.32 | 0.0462 | 0.04617 |
| GO:0065002 | intracellular protein transport across a... | 35 | 5 | 2.06 | 0.05170 | 0.55 | 0.0517 | 0.05170 |

### DE genes for top GO terms for Biological Process

List of DE genes for top GO terms. The first column is the Affymetrix probe ID, the second column is the Entrez ID, the third column is the gene symbol and the fourth column is the gene description. Note that the same gene can appear more than once in the list since different probes can target the same gene (can help detect overepresented genes due to multiple probe targets).

### GO:0006003

|  |  |  |  |
| --- | --- | --- | --- |
| **probe** | **entrez** | **symbol** | **description** |
| Bt.12314.1.S1\_at | 407183 | PFKFB3 | 6-phosphofructo-2-kinase/fructose-2,6-biphosphatase 3 |
| Bt.29724.1.S1\_at | 407183 | PFKFB3 | 6-phosphofructo-2-kinase/fructose-2,6-biphosphatase 3 |
| Bt.562.1.S1\_at | 282304 | PFKFB1 | 6-phosphofructo-2-kinase/fructose-2,6-biphosphatase 1 |
| Bt.659.1.S1\_at | 534928 | PFKFB4 | 6-phosphofructo-2-kinase/fructose-2,6-biphosphatase 4 |
| Bt.659.2.S1\_at | 534928 | PFKFB4 | 6-phosphofructo-2-kinase/fructose-2,6-biphosphatase 4 |

### GO:0016125

|  |  |  |  |
| --- | --- | --- | --- |
| **probe** | **entrez** | **symbol** | **description** |
| Bt.21724.1.S1\_at | 281767 | FDFT1 | farnesyl-diphosphate farnesyltransferase 1 |
| Bt.21724.2.S1\_a\_at | 281767 | FDFT1 | farnesyl-diphosphate farnesyltransferase 1 |
| Bt.23182.1.S1\_at | 281156 | FDPS | farnesyl diphosphate synthase (farnesyl pyrophosphate synthetase, dimethylallyltranstransferase, geranyltranstransferase) |
| Bt.24614.1.S1\_at | 511899 | INSIG1 | insulin induced gene 1 |
| Bt.3562.1.S1\_at | 281276 | LDLR | low density lipoprotein receptor (familial hypercholesterolemia) |
| Bt.621.1.S1\_at | 505060 | CYP51 | cytochrome P450, family 51, subfamily A, polypeptide 1 |
| Bt.997.2.S1\_a\_at | 516287 | CYB5R1 | cytochrome b5 reductase 1 |

### GO:0019882

|  |  |  |  |
| --- | --- | --- | --- |
| **probe** | **entrez** | **symbol** | **description** |
| Bt.1035.1.S1\_a\_at | 338062 | FCGRT | Fc fragment of IgG, receptor, transporter, alpha |
| Bt.27283.1.S1\_x\_at | 515712 | BOLA | MHC class I heavy chain |
| Bt.350.1.S1\_s\_at | 539241 | BLA-DQB | MHC class II antigen |
| Bt.4751.1.S1\_a\_at | 282535 | BOLA-DQA2 | major histocompatibility complex, class II, DQ alpha 2 |
| Bt.4751.2.S1\_a\_at | 282535 | BOLA-DQA2 | major histocompatibility complex, class II, DQ alpha 2 |
| Bt.5269.1.S1\_at | 280729 | B2M | beta-2-microglobulin |
| Bt.5269.2.S1\_a\_at | 280729 | B2M | beta-2-microglobulin |
| Bt.5356.1.S1\_s\_at | 282530 | BoLA-DRB3 | major histocompatibility complex, class II, DRB3 |
| Bt.8552.1.S1\_at | 506214 | BOLA-DRA | major histocompatibility complex, class II, DR alpha |

### GO:0006694

|  |  |  |  |
| --- | --- | --- | --- |
| **probe** | **entrez** | **symbol** | **description** |
| Bt.21724.1.S1\_at | 281767 | FDFT1 | farnesyl-diphosphate farnesyltransferase 1 |
| Bt.21724.2.S1\_a\_at | 281767 | FDFT1 | farnesyl-diphosphate farnesyltransferase 1 |
| Bt.23182.1.S1\_at | 281156 | FDPS | farnesyl diphosphate synthase (farnesyl pyrophosphate synthetase, dimethylallyltranstransferase, geranyltranstransferase) |
| Bt.621.1.S1\_at | 505060 | CYP51 | cytochrome P450, family 51, subfamily A, polypeptide 1 |
| Bt.7701.1.S1\_at | 614105 | GPSN2 | glycoprotein, synaptic 2 |
| Bt.997.2.S1\_a\_at | 516287 | CYB5R1 | cytochrome b5 reductase 1 |

### GO:0008299

|  |  |  |  |
| --- | --- | --- | --- |
| **probe** | **entrez** | **symbol** | **description** |
| Bt.21724.1.S1\_at | 281767 | FDFT1 | farnesyl-diphosphate farnesyltransferase 1 |
| Bt.21724.2.S1\_a\_at | 281767 | FDFT1 | farnesyl-diphosphate farnesyltransferase 1 |
| Bt.22763.2.S1\_a\_at | 407767 | HMGCS1 | 3-hydroxy-3-methylglutaryl-coenzyme A synthase 1 |
| Bt.23182.1.S1\_at | 281156 | FDPS | farnesyl diphosphate synthase (farnesyl pyrophosphate synthetase, dimethylallyltranstransferase, geranyltranstransferase) |

### GO:0015976

|  |  |  |  |
| --- | --- | --- | --- |
| **probe** | **entrez** | **symbol** | **description** |
| Bt.20470.1.S1\_a\_at | 525071 | MTP18 | mitochondrial protein 18 kDa |
| Bt.20470.2.S1\_a\_at | 525071 | MTP18 | mitochondrial protein 18 kDa |

### GO:0006564

|  |  |  |  |
| --- | --- | --- | --- |
| **probe** | **entrez** | **symbol** | **description** |
| Bt.25099.1.A1\_at | 533630 | PSPH | phosphoserine phosphatase |
| Bt.25099.2.S1\_at | 533630 | PSPH | phosphoserine phosphatase |

### GO:0006512

|  |  |  |  |
| --- | --- | --- | --- |
| **probe** | **entrez** | **symbol** | **description** |
| Bt.14223.3.S1\_at | 507781 | NEDD4 | neural precursor cell expressed, developmentally down-regulated 4 |
| Bt.17148.2.S1\_at | 541180 | FEM1C | fem-1 homolog c (C. elegans) |
| Bt.1767.1.S1\_at | 281225 | UBE2K | ubiquitin-conjugating enzyme E2K (UBC1 homolog, yeast) |
| Bt.18639.1.A1\_at | 513776 | FBXO32 | F-box protein 32 |
| Bt.18639.2.S1\_at | 513776 | FBXO32 | F-box protein 32 |
| Bt.21681.1.A1\_at | 535452 | FBXL4 | F-box and leucine-rich repeat protein 4 |
| Bt.21721.1.A1\_at | 522980 | USP2 | ubiquitin specific peptidase 2 |
| Bt.22216.1.S1\_at | 541180 | FEM1C | fem-1 homolog c (C. elegans) |
| Bt.23519.1.S1\_at | 539244 | ASB2 | ankyrin repeat and SOCS box-containing 2 |
| Bt.23603.1.S1\_a\_at | 511798 | FBXO9 | F-box protein 9 |
| Bt.23603.3.S1\_at | 511798 | FBXO9 | F-box protein 9 |
| Bt.27926.1.S1\_at | 520170 | UCHL3 | ubiquitin carboxyl-terminal esterase L3 (ubiquitin thiolesterase) |
| Bt.6774.1.S1\_at | 408001 | MAP1LC3B | microtubule-associated protein 1 light chain 3 beta |
| Bt.6774.2.S1\_at | 408001 | MAP1LC3B | microtubule-associated protein 1 light chain 3 beta |
| Bt.6991.1.S1\_at | 516550 | FEM1A | fem-1 homolog a (C. elegans) |
| Bt.7651.1.S1\_at | 511852 | ASB11 | ankyrin repeat and SOCS box-containing 11 |
| Bt.8073.1.S1\_at | 514547 | MAP1LC3A | microtubule-associated protein 1 light chain 3 alpha |

### GO:0045637

|  |  |  |  |
| --- | --- | --- | --- |
| **probe** | **entrez** | **symbol** | **description** |
| Bt.3980.1.S1\_at | 281478 | SCIN | scinderin |
| Bt.4184.1.S2\_at | 281814 | HIF1A | hypoxia-inducible factor 1, alpha subunit (basic helix-loop-helix transcription factor) |
| Bt.4624.1.S1\_at | 282376 | STAT5B | signal transducer and activator of transcription 5B |
| Bt.9027.1.S1\_at | 282291 | NFKBIA | nuclear factor of kappa light polypeptide gene enhancer in B-cells inhibitor, alpha |

### GO:0000045

|  |  |  |  |
| --- | --- | --- | --- |
| **probe** | **entrez** | **symbol** | **description** |
| Bt.6774.1.S1\_at | 408001 | MAP1LC3B | microtubule-associated protein 1 light chain 3 beta |
| Bt.6774.2.S1\_at | 408001 | MAP1LC3B | microtubule-associated protein 1 light chain 3 beta |

### GO:0007595

|  |  |  |  |
| --- | --- | --- | --- |
| **probe** | **entrez** | **symbol** | **description** |
| Bt.4624.1.S1\_at | 282376 | STAT5B | signal transducer and activator of transcription 5B |
| Bt.5340.1.S1\_s\_at | 404189 | NME1 | non-metastatic cells 1, protein (NM23A) expressed in |

### GO:0009967

|  |  |  |  |
| --- | --- | --- | --- |
| **probe** | **entrez** | **symbol** | **description** |
| Bt.2564.1.S1\_at | 338049 | RHOA | ras homolog gene family, member A |
| Bt.4184.1.S2\_at | 281814 | HIF1A | hypoxia-inducible factor 1, alpha subunit (basic helix-loop-helix transcription factor) |
| Bt.4671.1.S1\_at | 281994 | PPM1A | protein phosphatase 1A (formerly 2C), magnesium-dependent, alpha isoform |
| Bt.5472.1.S1\_at | 326598 | LGALS1 | lectin, galactoside-binding, soluble, 1 (galectin 1) |
| Bt.7633.1.S1\_at | 617105 | EEF1E1 | eukaryotic translation elongation factor 1 epsilon 1 |

### GO:0006097

|  |  |  |  |
| --- | --- | --- | --- |
| **probe** | **entrez** | **symbol** | **description** |
| Bt.13324.1.S1\_a\_at | 281235 | IDH1 | isocitrate dehydrogenase 1 (NADP+), soluble |
| Bt.13324.4.S1\_at | 281235 | IDH1 | isocitrate dehydrogenase 1 (NADP+), soluble |

### GO:0043666

|  |  |  |  |
| --- | --- | --- | --- |
| **probe** | **entrez** | **symbol** | **description** |
| Bt.17102.3.S1\_at | 532726 | PPP1R2 | protein phosphatase 1, regulatory (inhibitor) subunit 2 |
| Bt.7968.2.S1\_at | 532726 | PPP1R2 | protein phosphatase 1, regulatory (inhibitor) subunit 2 |

### GO:0006470

|  |  |  |  |
| --- | --- | --- | --- |
| **probe** | **entrez** | **symbol** | **description** |
| Bt.13988.1.S1\_at | 615432 | DUSP3 | dual specificity phosphatase 3 |
| Bt.18748.1.A1\_at | 505696 | PTPRB | protein tyrosine phosphatase, receptor type, B |
| Bt.2070.1.A1\_at | 541175 | DUSP10 | dual specificity phosphatase 10 |
| Bt.2070.2.A1\_at | 541175 | DUSP10 | dual specificity phosphatase 10 |
| Bt.2070.3.S1\_at | 541175 | DUSP10 | dual specificity phosphatase 10 |
| Bt.23521.1.S1\_at | 533896 | DUSP26 | dual specificity phosphatase 26 (putative) |
| Bt.4671.1.S1\_at | 281994 | PPM1A | protein phosphatase 1A (formerly 2C), magnesium-dependent, alpha isoform |

### GO:0006457

|  |  |  |  |
| --- | --- | --- | --- |
| **probe** | **entrez** | **symbol** | **description** |
| Bt.1131.1.S1\_at | 281419 | PPIB | peptidylprolyl isomerase B (cyclophilin B) |
| Bt.21537.1.A1\_at | 535704 | FKBP5 | FK506 binding protein 5 |
| Bt.22329.1.S1\_at | 526524 | LOC526524 | FK506 binding protein 1A, 12kDa-like |
| Bt.26122.2.S1\_at | 525680 | UXT | ubiquitously-expressed transcript |
| Bt.4680.1.S1\_at | 281259 | DNAJC12 | DnaJ (Hsp40) homolog, subfamily C, member 12 |
| Bt.4704.2.S1\_at | 514355 | CCT7 | chaperonin containing TCP1, subunit 7 (eta) |
| Bt.5096.1.S1\_at | 504735 | CCT3 | chaperonin containing TCP1, subunit 3 (gamma) |
| Bt.6680.1.S1\_a\_at | 538998 | FKBP2 | FK506 binding protein 2, 13kDa |
| Bt.6840.1.S1\_a\_at | 506043 | FKBP11 | FK506 binding protein 11, 19 kDa |

### GO:0006102

|  |  |  |  |
| --- | --- | --- | --- |
| **probe** | **entrez** | **symbol** | **description** |
| Bt.13324.1.S1\_a\_at | 281235 | IDH1 | isocitrate dehydrogenase 1 (NADP+), soluble |
| Bt.13324.4.S1\_at | 281235 | IDH1 | isocitrate dehydrogenase 1 (NADP+), soluble |

### GO:0009084

|  |  |  |  |
| --- | --- | --- | --- |
| **probe** | **entrez** | **symbol** | **description** |
| Bt.12472.1.S1\_at | 281199 | GLUL | glutamate-ammonia ligase (glutamine synthetase) |
| Bt.1370.1.S1\_at | 280726 | ASS1 | argininosuccinate synthetase 1 |

### GO:0006817

|  |  |  |  |
| --- | --- | --- | --- |
| **probe** | **entrez** | **symbol** | **description** |
| Bt.10442.2.S1\_a\_at | 359712 | COL12A1 | collagen, type XII, alpha 1 |
| Bt.21409.1.A1\_at | 287013 | COL11A1 | collagen, type XI, alpha 1 |
| Bt.28583.1.S1\_s\_at | 282187 | COL1A1 | collagen, type I, alpha 1 |
| Bt.8124.1.S1\_at | 282188 | COL1A2 | collagen, type I, alpha 2 |
| Bt.8124.1.S2\_at | 282188 | COL1A2 | collagen, type I, alpha 2 |
| Bt.8549.1.S2\_at | 282187 | COL1A1 | collagen, type I, alpha 1 |

### GO:0065002

|  |  |  |  |
| --- | --- | --- | --- |
| **probe** | **entrez** | **symbol** | **description** |
| Bt.11014.1.S1\_at | 513889 | CHCHD4 | coiled-coil-helix-coiled-coil-helix domain containing 4 |
| Bt.13218.2.S1\_at | 528100 | SERP1 | stress-associated endoplasmic reticulum protein 1 |
| Bt.18228.1.A1\_at | 614128 | SEC61A2 | Sec61 alpha 2 subunit (S. cerevisiae) |
| Bt.7266.1.S1\_at | 515109 | TIMM8A | translocase of inner mitochondrial membrane 8 homolog A (yeast) |
| Bt.9033.2.S1\_at | 281546 | TRAM1 | translocation associated membrane protein 1 |
