# Supplementary material for: A gene network switch enhances the oxidative capacity of ovine skeletal muscle during late fetal development
Source: BMC Genomics. 2010 Jun 15;11:378. doi: 10.1186/1471-2164-11-378 (PMC2894804; doi:10.1186/1471-2164-11-378)
Supplement: Additional File 1 — Gene expression data. This file contains links to the gene expression data. The genes that are significantly differentially expressed between adjacent development times are listed as Experiment 1 (80 d vs 100 d), Experiment 2 (100 d vs 120 d), Experiment 3 (120 d vs 150 d) and Experiment 4 (150 d vs 230 d) in the section entitled Open microarray analysis report for developmental expression. Each experiment contains an MA plot, Volcano plot and Heatmap for each of the three microarray processing programs MAS5, RMA and GCRMA. For each of these processing programs there is a list of significantly differentially expressed probe sets, and for each of these there is an FDR corrected probability, fold change, percentage of microarrays with a MAS5 Present call, and a convergence percentage for all three microarray processing programs. The file also contains annotation of the probe sets. In a second section entitled Time series analysis of developmental expression, there is a list of genes in each gene expression cluster and diagrams showing MAS5 gene expression values for these genes. [file 1471-2164-11-378-S1.ZIP › Report/GO4/Report/TopNodesCC.html]

### Top terms for Cellular Component

Top terms sorted by the weight algorithm. The first column is the GO term id and links to GO, the second column is a short description of the term, the third column is the number of genes annotated to the term, the fourth is the number of genes significant for the term and the fifth column is the expected number of genes for the term. The last four columns are the p-values for different tests of significance. The results are ordered by the weight algorithm (last column).

|  |  |  |  |  |  |  |  |  |
| --- | --- | --- | --- | --- | --- | --- | --- | --- |
| **GO** | **Term** | **Annotated** | **Significant** | **Expected** | **classic** | **KS** | **elim** | **weight** |
| GO:0005737 | cytoplasm | 2306 | 181 | 129 | 5.4e-11 | 1.00 | 3.3e-06 | 6.0e-06 |
| GO:0044432 | endoplasmic reticulum part | 183 | 26 | 10.2 | 7.3e-06 | 0.99 | 0.02267 | 7.3e-06 |
| GO:0042611 | MHC protein complex | 29 | 9 | 1.62 | 1.7e-05 | 1.00 | 0.02137 | 1.7e-05 |
| GO:0005776 | autophagic vacuole | 4 | 3 | 0.22 | 0.00066 | 1.00 | 0.00066 | 0.00066 |
| GO:0005840 | ribosome | 186 | 20 | 10.4 | 0.00327 | 1.00 | 0.00327 | 0.00392 |
| GO:0005758 | mitochondrial intermembrane space | 16 | 4 | 0.89 | 0.01010 | 0.83 | 0.01010 | 0.01010 |
| GO:0005793 | ER-Golgi intermediate compartment | 10 | 3 | 0.56 | 0.01535 | 0.98 | 0.01535 | 0.01535 |
| GO:0031307 | integral to mitochondrial outer membrane | 4 | 2 | 0.22 | 0.01725 | 1.00 | 0.01725 | 0.01725 |
| GO:0005581 | collagen | 28 | 5 | 1.56 | 0.01774 | 1.00 | 0.01774 | 0.01774 |
| GO:0042585 | germinal vesicle | 5 | 2 | 0.28 | 0.02769 | 1.00 | 0.02769 | 0.02769 |
| GO:0044445 | cytosolic part | 61 | 8 | 3.4 | 0.01894 | 0.31 | 0.01894 | 0.03416 |
| GO:0005792 | microsome | 33 | 5 | 1.84 | 0.03419 | 0.59 | 0.03419 | 0.03419 |
| GO:0005938 | cell cortex | 26 | 5 | 1.45 | 0.01300 | 0.76 | 0.01300 | 0.05527 |
| GO:0005719 | nuclear euchromatin | 1 | 1 | 0.06 | 0.05576 | 1.00 | 0.05576 | 0.05576 |
| GO:0005742 | mitochondrial outer membrane translocase... | 1 | 1 | 0.06 | 0.05576 | 1.00 | 0.05576 | 0.05576 |
| GO:0005779 | integral to peroxisomal membrane | 1 | 1 | 0.06 | 0.05576 | 1.00 | 0.05576 | 0.05576 |
| GO:0030981 | cortical microtubule cytoskeleton | 1 | 1 | 0.06 | 0.05576 | 1.00 | 0.05576 | 0.05576 |
| GO:0005874 | microtubule | 66 | 7 | 3.68 | 0.07291 | 0.98 | 0.07291 | 0.07291 |
| GO:0005813 | centrosome | 31 | 4 | 1.73 | 0.09120 | 0.97 | 0.09120 | 0.09120 |
| GO:0005665 | DNA-directed RNA polymerase II, core com... | 2 | 1 | 0.11 | 0.10842 | 0.99 | 0.10842 | 0.10842 |

### DE genes for top GO terms for Cellular Component

List of DE genes for top GO terms. The first column is the Affymetrix probe ID, the second column is the Entrez ID, the third column is the gene symbol and the fourth column is the gene description. Note that the same gene can appear more than once in the list since different probes can target the same gene (can help detect overepresented genes due to multiple probe targets).

### GO:0005737

|  |  |  |  |
| --- | --- | --- | --- |
| **probe** | **entrez** | **symbol** | **description** |
| Bt.10442.2.S1\_a\_at | 359712 | COL12A1 | collagen, type XII, alpha 1 |
| Bt.10515.1.S1\_at | 531367 | NXN | nucleoredoxin |
| Bt.11014.1.S1\_at | 513889 | CHCHD4 | coiled-coil-helix-coiled-coil-helix domain containing 4 |
| Bt.11194.1.S1\_at | 281360 | DHRS4 | dehydrogenase/reductase (SDR family) member 4 |
| Bt.11196.1.S1\_at | 281816 | HINT2 | histidine triad nucleotide binding protein 2 |
| Bt.1131.1.S1\_at | 281419 | PPIB | peptidylprolyl isomerase B (cyclophilin B) |
| Bt.1154.1.S1\_at | 533223 | WDR1 | WD repeat domain 1 |
| Bt.11659.1.S1\_at | 615666 | MRP63 | mitochondrial ribosomal protein 63 |
| Bt.1187.1.S1\_at | 514688 | PPP1R15A | protein phosphatase 1, regulatory (inhibitor) subunit 15A |
| Bt.1214.1.S1\_at | 529312 | SSR1 | signal sequence receptor, alpha (translocon-associated protein alpha) |
| Bt.12429.1.S1\_at | 515380 | EDF1 | endothelial differentiation-related factor 1 |
| Bt.12472.1.S1\_at | 281199 | GLUL | glutamate-ammonia ligase (glutamine synthetase) |
| Bt.12491.1.A1\_at | 533219 | ACTC1 | actin, alpha, cardiac muscle 1 |
| Bt.12559.1.S1\_at | 511135 | ACLY | ATP citrate lyase |
| Bt.1269.1.S1\_a\_at | 326582 | RPS12 | ribosomal protein S12 |
| Bt.12888.1.S1\_at | 613305 | MIF4GD | MIF4G domain containing |
| Bt.1310.1.S1\_at | 532775 | TMEM38A | transmembrane protein 38A |
| Bt.13218.2.S1\_at | 528100 | SERP1 | stress-associated endoplasmic reticulum protein 1 |
| Bt.13237.1.S1\_at | 510423 | PSMA4 | proteasome (prosome, macropain) subunit, alpha type, 4 |
| Bt.13315.1.S1\_at | 768237 | LOC768237 | hypothetical protein LOC768237 |
| Bt.13324.1.S1\_a\_at | 281235 | IDH1 | isocitrate dehydrogenase 1 (NADP+), soluble |
| Bt.13324.4.S1\_at | 281235 | IDH1 | isocitrate dehydrogenase 1 (NADP+), soluble |
| Bt.13610.1.A1\_at | 534185 | MRPS31 | mitochondrial ribosomal protein S31 |
| Bt.1388.1.S1\_at | 506085 | ZDHHC16 | zinc finger, DHHC-type containing 16 |
| Bt.15157.1.S1\_at | 613575 | CNIH | cornichon homolog (Drosophila) |
| Bt.1537.1.S1\_at | 504499 | NDRG1 | N-myc downstream regulated gene 1 |
| Bt.15397.1.S1\_a\_at | 282884 | RPL9 | ribosomal protein L9 |
| Bt.1552.1.S1\_at | 281476 | SARS | seryl-tRNA synthetase |
| Bt.1582.1.S1\_at | 615447 | NME2 | non-metastatic cells 2, protein (NM23B) expressed in |
| Bt.15870.1.S1\_at | 528165 | TIMM44 | translocase of inner mitochondrial membrane 44 homolog (yeast) |
| Bt.15931.1.S1\_at | 539373 | RPL14 | ribosomal protein L14 |
| Bt.16004.1.A1\_at | 281339 | MYH8 | myosin, heavy chain 8, skeletal muscle, perinatal |
| Bt.16389.1.S1\_at | 404121 | DERL1 | Der1-like domain family, member 1 |
| Bt.16567.1.A1\_at | 282875 | PRKRA | protein kinase, interferon-inducible double stranded RNA dependent activator |
| Bt.16597.1.S1\_at | 618292 | EXOSC4 | exosome component 4 |
| Bt.17148.2.S1\_at | 541180 | FEM1C | fem-1 homolog c (C. elegans) |
| Bt.1767.1.S1\_at | 281225 | UBE2K | ubiquitin-conjugating enzyme E2K (UBC1 homolog, yeast) |
| Bt.17963.1.A1\_at | 517345 | ARL1 | ADP-ribosylation factor-like 1 |
| Bt.18228.1.A1\_at | 614128 | SEC61A2 | Sec61 alpha 2 subunit (S. cerevisiae) |
| Bt.18623.1.A1\_at | 510159 | MAPRE2 | microtubule-associated protein, RP/EB family, member 2 |
| Bt.19721.1.S1\_at | 535414 | PSMD10 | proteasome (prosome, macropain) 26S subunit, non-ATPase, 10 |
| Bt.1998.1.S1\_at | 100034674 | NGFRAP1 | nerve growth factor receptor (TNFRSF16) associated protein 1 |
| Bt.20025.1.S1\_at | 618609 | MRPL21 | mitochondrial ribosomal protein L21 |
| Bt.20145.1.S1\_at | 523609 | PRELID1 | PRELI domain containing 1 |
| Bt.20479.1.S1\_at | 533608 | CYB5R4 | cytochrome b5 reductase 4 |
| Bt.2070.1.A1\_at | 541175 | DUSP10 | dual specificity phosphatase 10 |
| Bt.2070.2.A1\_at | 541175 | DUSP10 | dual specificity phosphatase 10 |
| Bt.2070.3.S1\_at | 541175 | DUSP10 | dual specificity phosphatase 10 |
| Bt.20862.1.A1\_at | 517539 | MTHFD2 | methylenetetrahydrofolate dehydrogenase (NADP+ dependent) 2, methenyltetrahydrofolate cyclohydrolase |
| Bt.2091.1.S1\_at | 529235 | DDIT4 | DNA-damage-inducible transcript 4 |
| Bt.2110.1.S1\_at | 510200 | DPP3 | dipeptidyl-peptidase 3 |
| Bt.21116.1.S1\_at | 510154 | SELENBP1 | selenium binding protein 1 |
| Bt.21151.1.S1\_at | 532203 | MRPL24 | mitochondrial ribosomal protein L24 |
| Bt.21376.1.S1\_at | 507815 | STT3A | STT3, subunit of the oligosaccharyltransferase complex, homolog A (S. cerevisiae) |
| Bt.21385.1.S1\_at | 510850 | SERPINH1 | serpin peptidase inhibitor, clade H (heat shock protein 47), member 1, (collagen binding protein 1) |
| Bt.21396.1.A1\_s\_at | 359721 | RTN3 | reticulon 3 |
| Bt.21409.1.A1\_at | 287013 | COL11A1 | collagen, type XI, alpha 1 |
| Bt.21670.1.A1\_at | 767932 | AZI2 | 5-azacytidine induced 2 |
| Bt.21681.1.A1\_at | 535452 | FBXL4 | F-box and leucine-rich repeat protein 4 |
| Bt.21724.1.S1\_at | 281767 | FDFT1 | farnesyl-diphosphate farnesyltransferase 1 |
| Bt.21724.2.S1\_a\_at | 281767 | FDFT1 | farnesyl-diphosphate farnesyltransferase 1 |
| Bt.21800.1.S1\_at | 504422 | SEC24D | SEC24 related gene family, member D (S. cerevisiae) |
| Bt.22002.1.S1\_at | 281759 | FABP4 | fatty acid binding protein 4, adipocyte |
| Bt.22216.1.S1\_at | 541180 | FEM1C | fem-1 homolog c (C. elegans) |
| Bt.2249.1.S1\_at | 781731 | EIF4E2 | eukaryotic translation initiation factor 4E family member 2 |
| Bt.22568.1.S1\_at | 507270 | RPL29 | ribosomal protein L29 |
| Bt.22783.1.S1\_at | 281141 | ENO1 | enolase 1, (alpha) |
| Bt.23002.1.S1\_at | 282199 | COX6A1 | cytochrome c oxidase subunit VIa polypeptide 1 |
| Bt.23002.1.S1\_s\_at | 282199 | COX6A1 | cytochrome c oxidase subunit VIa polypeptide 1 |
| Bt.23182.1.S1\_at | 281156 | FDPS | farnesyl diphosphate synthase (farnesyl pyrophosphate synthetase, dimethylallyltranstransferase, geranyltranstransferase) |
| Bt.23218.1.S1\_at | 513038 | CTSK | cathepsin K |
| Bt.23250.2.S1\_at | 414732 | GATM | glycine amidinotransferase (L-arginine:glycine amidinotransferase) |
| Bt.23254.1.S1\_at | 768311 | YWHAQ | tyrosine 3-monooxygenase/tryptophan 5-monooxygenase activation protein, theta polypeptide |
| Bt.23308.1.S1\_at | 535746 | SEPT11 | septin 11 |
| Bt.23317.1.S1\_at | 508221 | RPL13 | ribosomal protein L13 |
| Bt.2347.2.S1\_at | 506544 | PFKM | phosphofructokinase, muscle |
| Bt.23521.1.S1\_at | 533896 | DUSP26 | dual specificity phosphatase 26 (putative) |
| Bt.23589.2.S1\_at | 529312 | SSR1 | signal sequence receptor, alpha (translocon-associated protein alpha) |
| Bt.2392.1.S1\_at | 404124 | SIAT7B | beta-galactosamide alpha-2,6-sialyltransferase |
| Bt.24614.1.S1\_at | 511899 | INSIG1 | insulin induced gene 1 |
| Bt.24804.1.S1\_at | 533378 | MYLK2 | myosin light chain kinase 2 |
| Bt.2564.1.S1\_at | 338049 | RHOA | ras homolog gene family, member A |
| Bt.2575.1.S1\_at | 510041 | PSME1 | proteasome (prosome, macropain) activator subunit 1 (PA28 alpha) |
| Bt.26122.2.S1\_at | 525680 | UXT | ubiquitously-expressed transcript |
| Bt.26688.2.S1\_a\_at | 533321 | FRMD3 | FERM domain containing 3 |
| Bt.26692.1.S1\_a\_at | 615342 | BNIP3 | BCL2/adenovirus E1B 19kDa interacting protein 3 |
| Bt.27267.1.S1\_at | 506534 | CNPY2 | canopy 2 homolog (zebrafish) |
| Bt.27926.1.S1\_at | 520170 | UCHL3 | ubiquitin carboxyl-terminal esterase L3 (ubiquitin thiolesterase) |
| Bt.28194.1.S1\_at | 505642 | GSTO1 | glutathione S-transferase omega 1 |
| Bt.2822.1.S1\_at | 535056 | RPL8 | ribosomal protein L8 |
| Bt.2825.1.S1\_at | 617901 | MRPL13 | mitochondrial ribosomal protein L13 |
| Bt.28263.1.S1\_at | 509965 | CMPK1 | cytidine monophosphate (UMP-CMP) kinase 1, cytosolic |
| Bt.2841.1.S1\_at | 281576 | WARS | tryptophanyl-tRNA synthetase |
| Bt.2846.1.A1\_at | 781044 | RHOU | ras homolog gene family, member U |
| Bt.28583.1.S1\_s\_at | 282187 | COL1A1 | collagen, type I, alpha 1 |
| Bt.2934.1.S1\_at | 509426 | RPS26 | ribosomal protein S26 |
| Bt.3162.1.S1\_at | 281373 | P4HB | procollagen-proline, 2-oxoglutarate 4-dioxygenase (proline 4-hydroxylase), beta polypeptide |
| Bt.3296.1.A1\_at | 518288 | P4HA1 | procollagen-proline, 2-oxoglutarate 4-dioxygenase (proline 4-hydroxylase), alpha polypeptide I |
| Bt.3306.1.S1\_at | 512397 | LOC512397 | similar to ProSAPiP1 protein |
| Bt.3414.1.S1\_at | 536149 | HRB | HIV-1 Rev binding protein |
| Bt.3427.1.S1\_at | 618441 | SEPX1 | selenoprotein X, 1 |
| Bt.3567.1.S1\_at | 281565 | UGP2 | UDP-glucose pyrophosphorylase 2 |
| Bt.3616.1.S1\_at | 282688 | Rpl3 | ribosomal protein L3 |
| Bt.3921.1.S1\_at | 540965 | RPS10 | ribosomal protein S10 |
| Bt.3980.1.S1\_at | 281478 | SCIN | scinderin |
| Bt.4184.1.S2\_at | 281814 | HIF1A | hypoxia-inducible factor 1, alpha subunit (basic helix-loop-helix transcription factor) |
| Bt.4274.1.S1\_at | 507845 | MAPRE1 | microtubule-associated protein, RP/EB family, member 1 |
| Bt.4292.1.S1\_at | 281597 | ACTR3 | ARP3 actin-related protein 3 homolog (yeast) |
| Bt.4442.1.S1\_at | 327687 | ATP6AP1 | ATPase, H+ transporting, lysosomal accessory protein 1 |
| Bt.4624.1.S1\_at | 282376 | STAT5B | signal transducer and activator of transcription 5B |
| Bt.4637.1.A1\_a\_at | 281091 | COX8 | cytochrome c oxidase subunit VIII, heart |
| Bt.4659.1.S1\_at | 508629 | ALDH2 | aldehyde dehydrogenase 2 family (mitochondrial) |
| Bt.4704.2.S1\_at | 514355 | CCT7 | chaperonin containing TCP1, subunit 7 (eta) |
| Bt.4725.1.S1\_at | 281032 | BTG1 | B-cell translocation gene 1, anti-proliferative |
| Bt.4773.4.S1\_at | 282147 | ATP6V1A | ATPase, H+ transporting, lysosomal 70kDa, V1 subunit A |
| Bt.4790.1.S1\_at | 540207 | ILK | integrin-linked kinase |
| Bt.4798.1.S2\_at | 280924 | SCD | stearoyl-CoA desaturase (delta-9-desaturase) |
| Bt.4831.1.S1\_at | 281942 | CALCOCO2 | calcium binding and coiled-coil domain 2 |
| Bt.4844.1.S1\_at | 280904 | PTN | pleiotrophin (heparin binding growth factor 8, neurite growth-promoting factor 1) |
| Bt.4970.1.S1\_at | 327688 | COX7A2 | cytochrome c oxidase subunit VIIa polypeptide 2 (liver) |
| Bt.4986.1.S1\_at | 515878 | ORMDL2 | ORM1-like 2 (S. cerevisiae) |
| Bt.5096.1.S1\_at | 504735 | CCT3 | chaperonin containing TCP1, subunit 3 (gamma) |
| Bt.5131.1.S1\_at | 504781 | CHID1 | chitinase domain containing 1 |
| Bt.5176.1.S1\_at | 282480 | SLC25A6 | solute carrier family 25 (mitochondrial carrier; adenine nucleotide translocator), member 6 |
| Bt.5203.1.S1\_at | 281999 | PRDX4 | peroxiredoxin 4 |
| Bt.5243.1.S1\_at | 516473 | EEF1D | eukaryotic translation elongation factor 1 delta (guanine nucleotide exchange protein) |
| Bt.5267.1.S1\_at | 327685 | ANXA6 | annexin A6 |
| Bt.5306.1.S1\_at | 513257 | TCAP | titin-cap (telethonin) |
| Bt.5306.2.S1\_a\_at | 513257 | TCAP | titin-cap (telethonin) |
| Bt.5334.1.S1\_at | 281898 | RPSA | ribosomal protein SA |
| Bt.5340.1.S1\_s\_at | 404189 | NME1 | non-metastatic cells 1, protein (NM23A) expressed in |
| Bt.5376.1.S1\_at | 506336 | YIF1A | Yip1 interacting factor homolog A (S. cerevisiae) |
| Bt.5466.2.S1\_a\_at | 286873 | RPS4Y1 | ribosomal protein S4, Y-linked 1 |
| Bt.5605.1.S1\_at | 613548 | MARCKS | myristoylated alanine-rich protein kinase C substrate |
| Bt.5605.2.S1\_at | 613548 | MARCKS | myristoylated alanine-rich protein kinase C substrate |
| Bt.562.1.S1\_at | 282304 | PFKFB1 | 6-phosphofructo-2-kinase/fructose-2,6-biphosphatase 1 |
| Bt.5676.1.S1\_at | 533208 | COQ5 | coenzyme Q5 homolog, methyltransferase (S. cerevisiae) |
| Bt.621.1.S1\_at | 505060 | CYP51 | cytochrome P450, family 51, subfamily A, polypeptide 1 |
| Bt.665.1.S1\_s\_at | 282211 | CYP2D6 | cytochrome P450, family 2, subfamily D, polypeptide 6 |
| Bt.6680.1.S1\_a\_at | 538998 | FKBP2 | FK506 binding protein 2, 13kDa |
| Bt.6685.2.A1\_at | 517539 | MTHFD2 | methylenetetrahydrofolate dehydrogenase (NADP+ dependent) 2, methenyltetrahydrofolate cyclohydrolase |
| Bt.6774.1.S1\_at | 408001 | MAP1LC3B | microtubule-associated protein 1 light chain 3 beta |
| Bt.6774.2.S1\_at | 408001 | MAP1LC3B | microtubule-associated protein 1 light chain 3 beta |
| Bt.689.1.S1\_at | 615565 | FIS1 | fission 1 (mitochondrial outer membrane) homolog (S. cerevisiae) |
| Bt.6950.1.S1\_at | 614236 | SYS1 | SYS1 Golgi-localized integral membrane protein homolog (S. cerevisiae) |
| Bt.6991.1.S1\_at | 516550 | FEM1A | fem-1 homolog a (C. elegans) |
| Bt.7056.1.S1\_at | 280813 | HBB | hemoglobin, beta |
| Bt.7069.1.S1\_at | 505807 | PCMTD2 | protein-L-isoaspartate (D-aspartate) O-methyltransferase domain containing 2 |
| Bt.7139.1.S1\_at | 781575 | TOMM20 | translocase of outer mitochondrial membrane 20 homolog (yeast) |
| Bt.7192.1.S1\_at | 506988 | MRPL17 | mitochondrial ribosomal protein L17 |
| Bt.7266.1.S1\_at | 515109 | TIMM8A | translocase of inner mitochondrial membrane 8 homolog A (yeast) |
| Bt.7288.1.S1\_at | 504438 | SSR4 | signal sequence receptor, delta (translocon-associated protein delta) |
| Bt.7633.1.S1\_at | 617105 | EEF1E1 | eukaryotic translation elongation factor 1 epsilon 1 |
| Bt.7648.1.S1\_at | 516063 | RPS19 | ribosomal protein S19 |
| Bt.7700.1.S1\_at | 524770 | ACTN1 | actinin, alpha 1 |
| Bt.7701.1.S1\_at | 614105 | GPSN2 | glycoprotein, synaptic 2 |
| Bt.7776.1.S2\_at | 326599 | TPT1 | tumor protein, translationally-controlled 1 |
| Bt.7814.1.S1\_at | 518601 | ARF4 | ADP-ribosylation factor 4 |
| Bt.7856.1.S1\_at | 505005 | BCKDK | branched chain ketoacid dehydrogenase kinase |
| Bt.7870.1.S1\_at | 280701 | PPA1 | pyrophosphatase (inorganic) 1 |
| Bt.7903.1.A1\_at | 510937 | HARS | histidyl-tRNA synthetase |
| Bt.7908.1.S1\_at | 508628 | RPS6 | ribosomal protein S6 |
| Bt.8073.1.S1\_at | 514547 | MAP1LC3A | microtubule-associated protein 1 light chain 3 alpha |
| Bt.8122.1.S1\_at | 282386 | TMSB4X | thymosin beta 4, X-linked |
| Bt.8124.1.S1\_at | 282188 | COL1A2 | collagen, type I, alpha 2 |
| Bt.8124.1.S2\_at | 282188 | COL1A2 | collagen, type I, alpha 2 |
| Bt.8212.1.S1\_at | 511451 | CAB39 | calcium binding protein 39 |
| Bt.8319.3.S1\_a\_at | 515016 | MTUS1 | mitochondrial tumor suppressor 1 |
| Bt.8356.1.S1\_at | 529761 | TMED10 | transmembrane emp24-like trafficking protein 10 (yeast) |
| Bt.8549.1.S2\_at | 282187 | COL1A1 | collagen, type I, alpha 1 |
| Bt.8643.1.A1\_at | 512717 | RCN2 | reticulocalbin 2, EF-hand calcium binding domain |
| Bt.9021.1.S1\_at | 534742 | SLC25A24 | solute carrier family 25 (mitochondrial carrier; phosphate carrier), member 24 |
| Bt.9027.1.S1\_at | 282291 | NFKBIA | nuclear factor of kappa light polypeptide gene enhancer in B-cells inhibitor, alpha |
| Bt.9033.2.S1\_at | 281546 | TRAM1 | translocation associated membrane protein 1 |
| Bt.9055.2.S1\_a\_at | 616451 | TRMU | tRNA 5-methylaminomethyl-2-thiouridylate methyltransferase |
| Bt.9086.1.S1\_at | 616331 | AP1S1 | adaptor-related protein complex 1, sigma 1 subunit |
| Bt.946.1.S1\_at | 280716 | AK2 | adenylate kinase 2 |
| Bt.9588.1.S1\_at | 513128 | RPL7A | ribosomal protein L7a |
| Bt.9643.1.S1\_at | 512662 | CHP | calcium binding protein P22 |
| Bt.9828.1.S2\_at | 281817 | HINT3 | histidine triad nucleotide binding protein 3 |
| Bt.9915.1.S1\_at | 540892 | DNM1L | dynamin 1-like |

### GO:0044432

|  |  |  |  |
| --- | --- | --- | --- |
| **probe** | **entrez** | **symbol** | **description** |
| Bt.1131.1.S1\_at | 281419 | PPIB | peptidylprolyl isomerase B (cyclophilin B) |
| Bt.1214.1.S1\_at | 529312 | SSR1 | signal sequence receptor, alpha (translocon-associated protein alpha) |
| Bt.1310.1.S1\_at | 532775 | TMEM38A | transmembrane protein 38A |
| Bt.13218.2.S1\_at | 528100 | SERP1 | stress-associated endoplasmic reticulum protein 1 |
| Bt.1388.1.S1\_at | 506085 | ZDHHC16 | zinc finger, DHHC-type containing 16 |
| Bt.15157.1.S1\_at | 613575 | CNIH | cornichon homolog (Drosophila) |
| Bt.16389.1.S1\_at | 404121 | DERL1 | Der1-like domain family, member 1 |
| Bt.18228.1.A1\_at | 614128 | SEC61A2 | Sec61 alpha 2 subunit (S. cerevisiae) |
| Bt.21376.1.S1\_at | 507815 | STT3A | STT3, subunit of the oligosaccharyltransferase complex, homolog A (S. cerevisiae) |
| Bt.21385.1.S1\_at | 510850 | SERPINH1 | serpin peptidase inhibitor, clade H (heat shock protein 47), member 1, (collagen binding protein 1) |
| Bt.21396.1.A1\_s\_at | 359721 | RTN3 | reticulon 3 |
| Bt.21724.1.S1\_at | 281767 | FDFT1 | farnesyl-diphosphate farnesyltransferase 1 |
| Bt.21724.2.S1\_a\_at | 281767 | FDFT1 | farnesyl-diphosphate farnesyltransferase 1 |
| Bt.23589.2.S1\_at | 529312 | SSR1 | signal sequence receptor, alpha (translocon-associated protein alpha) |
| Bt.24614.1.S1\_at | 511899 | INSIG1 | insulin induced gene 1 |
| Bt.3162.1.S1\_at | 281373 | P4HB | procollagen-proline, 2-oxoglutarate 4-dioxygenase (proline 4-hydroxylase), beta polypeptide |
| Bt.3296.1.A1\_at | 518288 | P4HA1 | procollagen-proline, 2-oxoglutarate 4-dioxygenase (proline 4-hydroxylase), alpha polypeptide I |
| Bt.4798.1.S2\_at | 280924 | SCD | stearoyl-CoA desaturase (delta-9-desaturase) |
| Bt.4986.1.S1\_at | 515878 | ORMDL2 | ORM1-like 2 (S. cerevisiae) |
| Bt.5376.1.S1\_at | 506336 | YIF1A | Yip1 interacting factor homolog A (S. cerevisiae) |
| Bt.621.1.S1\_at | 505060 | CYP51 | cytochrome P450, family 51, subfamily A, polypeptide 1 |
| Bt.665.1.S1\_s\_at | 282211 | CYP2D6 | cytochrome P450, family 2, subfamily D, polypeptide 6 |
| Bt.6680.1.S1\_a\_at | 538998 | FKBP2 | FK506 binding protein 2, 13kDa |
| Bt.7288.1.S1\_at | 504438 | SSR4 | signal sequence receptor, delta (translocon-associated protein delta) |
| Bt.7701.1.S1\_at | 614105 | GPSN2 | glycoprotein, synaptic 2 |
| Bt.9033.2.S1\_at | 281546 | TRAM1 | translocation associated membrane protein 1 |

### GO:0042611

|  |  |  |  |
| --- | --- | --- | --- |
| **probe** | **entrez** | **symbol** | **description** |
| Bt.1035.1.S1\_a\_at | 338062 | FCGRT | Fc fragment of IgG, receptor, transporter, alpha |
| Bt.27283.1.S1\_x\_at | 515712 | BOLA | MHC class I heavy chain |
| Bt.350.1.S1\_s\_at | 539241 | BLA-DQB | MHC class II antigen |
| Bt.4751.1.S1\_a\_at | 282535 | BOLA-DQA2 | major histocompatibility complex, class II, DQ alpha 2 |
| Bt.4751.2.S1\_a\_at | 282535 | BOLA-DQA2 | major histocompatibility complex, class II, DQ alpha 2 |
| Bt.5269.1.S1\_at | 280729 | B2M | beta-2-microglobulin |
| Bt.5269.2.S1\_a\_at | 280729 | B2M | beta-2-microglobulin |
| Bt.5356.1.S1\_s\_at | 282530 | BoLA-DRB3 | major histocompatibility complex, class II, DRB3 |
| Bt.8552.1.S1\_at | 506214 | BOLA-DRA | major histocompatibility complex, class II, DR alpha |

### GO:0005776

|  |  |  |  |
| --- | --- | --- | --- |
| **probe** | **entrez** | **symbol** | **description** |
| Bt.6774.1.S1\_at | 408001 | MAP1LC3B | microtubule-associated protein 1 light chain 3 beta |
| Bt.6774.2.S1\_at | 408001 | MAP1LC3B | microtubule-associated protein 1 light chain 3 beta |
| Bt.8073.1.S1\_at | 514547 | MAP1LC3A | microtubule-associated protein 1 light chain 3 alpha |

### GO:0005840

|  |  |  |  |
| --- | --- | --- | --- |
| **probe** | **entrez** | **symbol** | **description** |
| Bt.11659.1.S1\_at | 615666 | MRP63 | mitochondrial ribosomal protein 63 |
| Bt.1269.1.S1\_a\_at | 326582 | RPS12 | ribosomal protein S12 |
| Bt.13610.1.A1\_at | 534185 | MRPS31 | mitochondrial ribosomal protein S31 |
| Bt.15397.1.S1\_a\_at | 282884 | RPL9 | ribosomal protein L9 |
| Bt.15931.1.S1\_at | 539373 | RPL14 | ribosomal protein L14 |
| Bt.20025.1.S1\_at | 618609 | MRPL21 | mitochondrial ribosomal protein L21 |
| Bt.21151.1.S1\_at | 532203 | MRPL24 | mitochondrial ribosomal protein L24 |
| Bt.22568.1.S1\_at | 507270 | RPL29 | ribosomal protein L29 |
| Bt.23317.1.S1\_at | 508221 | RPL13 | ribosomal protein L13 |
| Bt.2822.1.S1\_at | 535056 | RPL8 | ribosomal protein L8 |
| Bt.2825.1.S1\_at | 617901 | MRPL13 | mitochondrial ribosomal protein L13 |
| Bt.2934.1.S1\_at | 509426 | RPS26 | ribosomal protein S26 |
| Bt.3616.1.S1\_at | 282688 | Rpl3 | ribosomal protein L3 |
| Bt.3921.1.S1\_at | 540965 | RPS10 | ribosomal protein S10 |
| Bt.5334.1.S1\_at | 281898 | RPSA | ribosomal protein SA |
| Bt.5466.2.S1\_a\_at | 286873 | RPS4Y1 | ribosomal protein S4, Y-linked 1 |
| Bt.7192.1.S1\_at | 506988 | MRPL17 | mitochondrial ribosomal protein L17 |
| Bt.7648.1.S1\_at | 516063 | RPS19 | ribosomal protein S19 |
| Bt.7908.1.S1\_at | 508628 | RPS6 | ribosomal protein S6 |
| Bt.9588.1.S1\_at | 513128 | RPL7A | ribosomal protein L7a |

### GO:0005758

|  |  |  |  |
| --- | --- | --- | --- |
| **probe** | **entrez** | **symbol** | **description** |
| Bt.11014.1.S1\_at | 513889 | CHCHD4 | coiled-coil-helix-coiled-coil-helix domain containing 4 |
| Bt.23250.2.S1\_at | 414732 | GATM | glycine amidinotransferase (L-arginine:glycine amidinotransferase) |
| Bt.7266.1.S1\_at | 515109 | TIMM8A | translocase of inner mitochondrial membrane 8 homolog A (yeast) |
| Bt.946.1.S1\_at | 280716 | AK2 | adenylate kinase 2 |

### GO:0005793

|  |  |  |  |
| --- | --- | --- | --- |
| **probe** | **entrez** | **symbol** | **description** |
| Bt.21385.1.S1\_at | 510850 | SERPINH1 | serpin peptidase inhibitor, clade H (heat shock protein 47), member 1, (collagen binding protein 1) |
| Bt.3162.1.S1\_at | 281373 | P4HB | procollagen-proline, 2-oxoglutarate 4-dioxygenase (proline 4-hydroxylase), beta polypeptide |
| Bt.8356.1.S1\_at | 529761 | TMED10 | transmembrane emp24-like trafficking protein 10 (yeast) |

### GO:0031307

|  |  |  |  |
| --- | --- | --- | --- |
| **probe** | **entrez** | **symbol** | **description** |
| Bt.26692.1.S1\_a\_at | 615342 | BNIP3 | BCL2/adenovirus E1B 19kDa interacting protein 3 |
| Bt.689.1.S1\_at | 615565 | FIS1 | fission 1 (mitochondrial outer membrane) homolog (S. cerevisiae) |

### GO:0005581

|  |  |  |  |
| --- | --- | --- | --- |
| **probe** | **entrez** | **symbol** | **description** |
| Bt.21409.1.A1\_at | 287013 | COL11A1 | collagen, type XI, alpha 1 |
| Bt.28583.1.S1\_s\_at | 282187 | COL1A1 | collagen, type I, alpha 1 |
| Bt.8124.1.S1\_at | 282188 | COL1A2 | collagen, type I, alpha 2 |
| Bt.8124.1.S2\_at | 282188 | COL1A2 | collagen, type I, alpha 2 |
| Bt.8549.1.S2\_at | 282187 | COL1A1 | collagen, type I, alpha 1 |

### GO:0042585

|  |  |  |  |
| --- | --- | --- | --- |
| **probe** | **entrez** | **symbol** | **description** |
| Bt.5605.1.S1\_at | 613548 | MARCKS | myristoylated alanine-rich protein kinase C substrate |
| Bt.5605.2.S1\_at | 613548 | MARCKS | myristoylated alanine-rich protein kinase C substrate |

### GO:0044445

|  |  |  |  |
| --- | --- | --- | --- |
| **probe** | **entrez** | **symbol** | **description** |
| Bt.13237.1.S1\_at | 510423 | PSMA4 | proteasome (prosome, macropain) subunit, alpha type, 4 |
| Bt.22783.1.S1\_at | 281141 | ENO1 | enolase 1, (alpha) |
| Bt.2347.2.S1\_at | 506544 | PFKM | phosphofructokinase, muscle |
| Bt.26122.2.S1\_at | 525680 | UXT | ubiquitously-expressed transcript |
| Bt.4704.2.S1\_at | 514355 | CCT7 | chaperonin containing TCP1, subunit 7 (eta) |
| Bt.562.1.S1\_at | 282304 | PFKFB1 | 6-phosphofructo-2-kinase/fructose-2,6-biphosphatase 1 |
| Bt.7056.1.S1\_at | 280813 | HBB | hemoglobin, beta |
| Bt.7908.1.S1\_at | 508628 | RPS6 | ribosomal protein S6 |

### GO:0005792

|  |  |  |  |
| --- | --- | --- | --- |
| **probe** | **entrez** | **symbol** | **description** |
| Bt.23354.1.S1\_at | 535293 | EPHX1 | epoxide hydrolase 1, microsomal (xenobiotic) |
| Bt.3162.1.S1\_at | 281373 | P4HB | procollagen-proline, 2-oxoglutarate 4-dioxygenase (proline 4-hydroxylase), beta polypeptide |
| Bt.5340.1.S1\_s\_at | 404189 | NME1 | non-metastatic cells 1, protein (NM23A) expressed in |
| Bt.621.1.S1\_at | 505060 | CYP51 | cytochrome P450, family 51, subfamily A, polypeptide 1 |
| Bt.665.1.S1\_s\_at | 282211 | CYP2D6 | cytochrome P450, family 2, subfamily D, polypeptide 6 |

### GO:0005938

|  |  |  |  |
| --- | --- | --- | --- |
| **probe** | **entrez** | **symbol** | **description** |
| Bt.23308.1.S1\_at | 535746 | SEPT11 | septin 11 |
| Bt.3980.1.S1\_at | 281478 | SCIN | scinderin |
| Bt.4274.1.S1\_at | 507845 | MAPRE1 | microtubule-associated protein, RP/EB family, member 1 |
| Bt.5605.1.S1\_at | 613548 | MARCKS | myristoylated alanine-rich protein kinase C substrate |
| Bt.5605.2.S1\_at | 613548 | MARCKS | myristoylated alanine-rich protein kinase C substrate |

### GO:0005719

|  |  |  |  |
| --- | --- | --- | --- |
| **probe** | **entrez** | **symbol** | **description** |
| Bt.5334.1.S1\_at | 281898 | RPSA | ribosomal protein SA |

### GO:0005742

|  |  |  |  |
| --- | --- | --- | --- |
| **probe** | **entrez** | **symbol** | **description** |
| Bt.7139.1.S1\_at | 781575 | TOMM20 | translocase of outer mitochondrial membrane 20 homolog (yeast) |

### GO:0005779

|  |  |  |  |
| --- | --- | --- | --- |
| **probe** | **entrez** | **symbol** | **description** |
| Bt.689.1.S1\_at | 615565 | FIS1 | fission 1 (mitochondrial outer membrane) homolog (S. cerevisiae) |

### GO:0030981

|  |  |  |  |
| --- | --- | --- | --- |
| **probe** | **entrez** | **symbol** | **description** |
| Bt.4274.1.S1\_at | 507845 | MAPRE1 | microtubule-associated protein, RP/EB family, member 1 |

### GO:0005874

|  |  |  |  |
| --- | --- | --- | --- |
| **probe** | **entrez** | **symbol** | **description** |
| Bt.18623.1.A1\_at | 510159 | MAPRE2 | microtubule-associated protein, RP/EB family, member 2 |
| Bt.22803.1.S1\_at | 615087 | TUBB | tubulin, beta |
| Bt.4274.1.S1\_at | 507845 | MAPRE1 | microtubule-associated protein, RP/EB family, member 1 |
| Bt.6774.1.S1\_at | 408001 | MAP1LC3B | microtubule-associated protein 1 light chain 3 beta |
| Bt.6774.2.S1\_at | 408001 | MAP1LC3B | microtubule-associated protein 1 light chain 3 beta |
| Bt.7776.1.S2\_at | 326599 | TPT1 | tumor protein, translationally-controlled 1 |
| Bt.8073.1.S1\_at | 514547 | MAP1LC3A | microtubule-associated protein 1 light chain 3 alpha |

### GO:0005813

|  |  |  |  |
| --- | --- | --- | --- |
| **probe** | **entrez** | **symbol** | **description** |
| Bt.26122.2.S1\_at | 525680 | UXT | ubiquitously-expressed transcript |
| Bt.4274.1.S1\_at | 507845 | MAPRE1 | microtubule-associated protein, RP/EB family, member 1 |
| Bt.5605.1.S1\_at | 613548 | MARCKS | myristoylated alanine-rich protein kinase C substrate |
| Bt.5605.2.S1\_at | 613548 | MARCKS | myristoylated alanine-rich protein kinase C substrate |

### GO:0005665

|  |  |  |  |
| --- | --- | --- | --- |
| **probe** | **entrez** | **symbol** | **description** |
| Bt.1318.1.S1\_at | 507011 | LOC507011 | similar to Zinc finger protein 768 |
