# Supplementary material for: A gene network switch enhances the oxidative capacity of ovine skeletal muscle during late fetal development
Source: BMC Genomics. 2010 Jun 15;11:378. doi: 10.1186/1471-2164-11-378 (PMC2894804; doi:10.1186/1471-2164-11-378)
Supplement: Additional File 1 — Gene expression data. This file contains links to the gene expression data. The genes that are significantly differentially expressed between adjacent development times are listed as Experiment 1 (80 d vs 100 d), Experiment 2 (100 d vs 120 d), Experiment 3 (120 d vs 150 d) and Experiment 4 (150 d vs 230 d) in the section entitled Open microarray analysis report for developmental expression. Each experiment contains an MA plot, Volcano plot and Heatmap for each of the three microarray processing programs MAS5, RMA and GCRMA. For each of these processing programs there is a list of significantly differentially expressed probe sets, and for each of these there is an FDR corrected probability, fold change, percentage of microarrays with a MAS5 Present call, and a convergence percentage for all three microarray processing programs. The file also contains annotation of the probe sets. In a second section entitled Time series analysis of developmental expression, there is a list of genes in each gene expression cluster and diagrams showing MAS5 gene expression values for these genes. [file 1471-2164-11-378-S1.ZIP › Report/GO4/Report/TopNodesMF.html]

### Top terms for Molecular Function

Top terms sorted by the weight algorithm. The first column is the GO term id and links to GO, the second column is a short description of the term, the third column is the number of genes annotated to the term, the fourth is the number of genes significant for the term and the fifth column is the expected number of genes for the term. The last four columns are the p-values for different tests of significance. The results are ordered by the weight algorithm (last column).

|  |  |  |  |  |  |  |  |  |
| --- | --- | --- | --- | --- | --- | --- | --- | --- |
| **GO** | **Term** | **Annotated** | **Significant** | **Expected** | **classic** | **KS** | **elim** | **weight** |
| GO:0003873 | 6-phosphofructo-2-kinase activity | 6 | 5 | 0.33 | 2.7e-06 | 1.00 | 2.7e-06 | 2.7e-06 |
| GO:0000287 | magnesium ion binding | 136 | 18 | 7.43 | 0.00039 | 0.93 | 0.00039 | 0.00039 |
| GO:0005198 | structural molecule activity | 292 | 30 | 15.9 | 0.00053 | 0.72 | 0.06871 | 0.00053 |
| GO:0004331 | fructose-2,6-bisphosphate 2-phosphatase ... | 4 | 3 | 0.22 | 0.00062 | 1.00 | 0.00062 | 0.00062 |
| GO:0017017 | MAP kinase tyrosine/serine/threonine pho... | 5 | 3 | 0.27 | 0.00149 | 0.65 | 0.00149 | 0.00149 |
| GO:0003755 | peptidyl-prolyl cis-trans isomerase acti... | 22 | 5 | 1.2 | 0.00574 | 1.00 | 0.00574 | 0.00574 |
| GO:0004310 | farnesyl-diphosphate farnesyltransferase... | 3 | 2 | 0.16 | 0.00860 | 0.99 | 0.00860 | 0.00860 |
| GO:0004477 | methenyltetrahydrofolate cyclohydrolase ... | 3 | 2 | 0.16 | 0.00860 | 1.00 | 0.00860 | 0.00860 |
| GO:0004487 | methylenetetrahydrofolate dehydrogenase ... | 3 | 2 | 0.16 | 0.00860 | 1.00 | 0.00860 | 0.00860 |
| GO:0004647 | phosphoserine phosphatase activity | 3 | 2 | 0.16 | 0.00860 | 0.99 | 0.00860 | 0.00860 |
| GO:0004656 | procollagen-proline 4-dioxygenase activi... | 3 | 2 | 0.16 | 0.00860 | 0.60 | 0.00860 | 0.00860 |
| GO:0008243 | plasminogen activator activity | 3 | 2 | 0.16 | 0.00860 | 0.64 | 0.00860 | 0.00860 |
| GO:0004129 | cytochrome-c oxidase activity | 16 | 4 | 0.87 | 0.00942 | 0.99 | 0.00942 | 0.00942 |
| GO:0004128 | cytochrome-b5 reductase activity | 4 | 2 | 0.22 | 0.01658 | 1.00 | 0.01658 | 0.01658 |
| GO:0004427 | inorganic diphosphatase activity | 4 | 2 | 0.22 | 0.01658 | 1.00 | 0.01658 | 0.01658 |
| GO:0016776 | phosphotransferase activity, phosphate g... | 21 | 4 | 1.15 | 0.02499 | 0.84 | 0.02499 | 0.02499 |
| GO:0004725 | protein tyrosine phosphatase activity | 42 | 6 | 2.29 | 0.02519 | 0.61 | 0.02519 | 0.02519 |
| GO:0004450 | isocitrate dehydrogenase (NADP+) activit... | 5 | 2 | 0.27 | 0.02664 | 0.42 | 0.02664 | 0.02664 |
| GO:0046912 | transferase activity, transferring acyl ... | 5 | 2 | 0.27 | 0.02664 | 0.26 | 0.02664 | 0.02664 |
| GO:0003887 | DNA-directed DNA polymerase activity | 15 | 3 | 0.82 | 0.04504 | 0.50 | 0.04504 | 0.04504 |

### DE genes for top GO terms for Molecular Function

List of DE genes for top GO terms. The first column is the Affymetrix probe ID, the second column is the Entrez ID, the third column is the gene symbol and the fourth column is the gene description. Note that the same gene can appear more than once in the list since different probes can target the same gene (can help detect overepresented genes due to multiple probe targets).

### GO:0003873

|  |  |  |  |
| --- | --- | --- | --- |
| **probe** | **entrez** | **symbol** | **description** |
| Bt.12314.1.S1\_at | 407183 | PFKFB3 | 6-phosphofructo-2-kinase/fructose-2,6-biphosphatase 3 |
| Bt.29724.1.S1\_at | 407183 | PFKFB3 | 6-phosphofructo-2-kinase/fructose-2,6-biphosphatase 3 |
| Bt.562.1.S1\_at | 282304 | PFKFB1 | 6-phosphofructo-2-kinase/fructose-2,6-biphosphatase 1 |
| Bt.659.1.S1\_at | 534928 | PFKFB4 | 6-phosphofructo-2-kinase/fructose-2,6-biphosphatase 4 |
| Bt.659.2.S1\_at | 534928 | PFKFB4 | 6-phosphofructo-2-kinase/fructose-2,6-biphosphatase 4 |

### GO:0000287

|  |  |  |  |
| --- | --- | --- | --- |
| **probe** | **entrez** | **symbol** | **description** |
| Bt.12559.1.S1\_at | 511135 | ACLY | ATP citrate lyase |
| Bt.13324.1.S1\_a\_at | 281235 | IDH1 | isocitrate dehydrogenase 1 (NADP+), soluble |
| Bt.13324.4.S1\_at | 281235 | IDH1 | isocitrate dehydrogenase 1 (NADP+), soluble |
| Bt.1582.1.S1\_at | 615447 | NME2 | non-metastatic cells 2, protein (NM23B) expressed in |
| Bt.20862.1.A1\_at | 517539 | MTHFD2 | methylenetetrahydrofolate dehydrogenase (NADP+ dependent) 2, methenyltetrahydrofolate cyclohydrolase |
| Bt.21724.1.S1\_at | 281767 | FDFT1 | farnesyl-diphosphate farnesyltransferase 1 |
| Bt.21724.2.S1\_a\_at | 281767 | FDFT1 | farnesyl-diphosphate farnesyltransferase 1 |
| Bt.22783.1.S1\_at | 281141 | ENO1 | enolase 1, (alpha) |
| Bt.2347.2.S1\_at | 506544 | PFKM | phosphofructokinase, muscle |
| Bt.25099.1.A1\_at | 533630 | PSPH | phosphoserine phosphatase |
| Bt.25099.2.S1\_at | 533630 | PSPH | phosphoserine phosphatase |
| Bt.2564.1.S1\_at | 338049 | RHOA | ras homolog gene family, member A |
| Bt.26688.2.S1\_a\_at | 533321 | FRMD3 | FERM domain containing 3 |
| Bt.2846.1.A1\_at | 781044 | RHOU | ras homolog gene family, member U |
| Bt.4671.1.S1\_at | 281994 | PPM1A | protein phosphatase 1A (formerly 2C), magnesium-dependent, alpha isoform |
| Bt.5340.1.S1\_s\_at | 404189 | NME1 | non-metastatic cells 1, protein (NM23A) expressed in |
| Bt.6685.2.A1\_at | 517539 | MTHFD2 | methylenetetrahydrofolate dehydrogenase (NADP+ dependent) 2, methenyltetrahydrofolate cyclohydrolase |
| Bt.7870.1.S1\_at | 280701 | PPA1 | pyrophosphatase (inorganic) 1 |

### GO:0005198

|  |  |  |  |
| --- | --- | --- | --- |
| **probe** | **entrez** | **symbol** | **description** |
| Bt.10442.2.S1\_a\_at | 359712 | COL12A1 | collagen, type XII, alpha 1 |
| Bt.11659.1.S1\_at | 615666 | MRP63 | mitochondrial ribosomal protein 63 |
| Bt.12411.1.S1\_at | 404144 | LMNA | lamin A/C |
| Bt.12491.1.A1\_at | 533219 | ACTC1 | actin, alpha, cardiac muscle 1 |
| Bt.1269.1.S1\_a\_at | 326582 | RPS12 | ribosomal protein S12 |
| Bt.15397.1.S1\_a\_at | 282884 | RPL9 | ribosomal protein L9 |
| Bt.15931.1.S1\_at | 539373 | RPL14 | ribosomal protein L14 |
| Bt.20025.1.S1\_at | 618609 | MRPL21 | mitochondrial ribosomal protein L21 |
| Bt.21151.1.S1\_at | 532203 | MRPL24 | mitochondrial ribosomal protein L24 |
| Bt.21409.1.A1\_at | 287013 | COL11A1 | collagen, type XI, alpha 1 |
| Bt.22568.1.S1\_at | 507270 | RPL29 | ribosomal protein L29 |
| Bt.22803.1.S1\_at | 615087 | TUBB | tubulin, beta |
| Bt.23317.1.S1\_at | 508221 | RPL13 | ribosomal protein L13 |
| Bt.2822.1.S1\_at | 535056 | RPL8 | ribosomal protein L8 |
| Bt.2825.1.S1\_at | 617901 | MRPL13 | mitochondrial ribosomal protein L13 |
| Bt.2847.1.S1\_a\_at | 281341 | MYL6 | myosin, light chain 6, alkali, smooth muscle and non-muscle |
| Bt.28583.1.S1\_s\_at | 282187 | COL1A1 | collagen, type I, alpha 1 |
| Bt.2934.1.S1\_at | 509426 | RPS26 | ribosomal protein S26 |
| Bt.3251.1.S1\_at | 515749 | CTNNAL1 | catenin (cadherin-associated protein), alpha-like 1 |
| Bt.3560.1.S1\_at | 786606 | WIPF3 | WAS/WASL interacting protein family, member 3 |
| Bt.3616.1.S1\_at | 282688 | Rpl3 | ribosomal protein L3 |
| Bt.5334.1.S1\_at | 281898 | RPSA | ribosomal protein SA |
| Bt.5466.2.S1\_a\_at | 286873 | RPS4Y1 | ribosomal protein S4, Y-linked 1 |
| Bt.7192.1.S1\_at | 506988 | MRPL17 | mitochondrial ribosomal protein L17 |
| Bt.7648.1.S1\_at | 516063 | RPS19 | ribosomal protein S19 |
| Bt.7908.1.S1\_at | 508628 | RPS6 | ribosomal protein S6 |
| Bt.8124.1.S1\_at | 282188 | COL1A2 | collagen, type I, alpha 2 |
| Bt.8124.1.S2\_at | 282188 | COL1A2 | collagen, type I, alpha 2 |
| Bt.8549.1.S2\_at | 282187 | COL1A1 | collagen, type I, alpha 1 |
| Bt.9588.1.S1\_at | 513128 | RPL7A | ribosomal protein L7a |

### GO:0004331

|  |  |  |  |
| --- | --- | --- | --- |
| **probe** | **entrez** | **symbol** | **description** |
| Bt.12314.1.S1\_at | 407183 | PFKFB3 | 6-phosphofructo-2-kinase/fructose-2,6-biphosphatase 3 |
| Bt.29724.1.S1\_at | 407183 | PFKFB3 | 6-phosphofructo-2-kinase/fructose-2,6-biphosphatase 3 |
| Bt.562.1.S1\_at | 282304 | PFKFB1 | 6-phosphofructo-2-kinase/fructose-2,6-biphosphatase 1 |

### GO:0017017

|  |  |  |  |
| --- | --- | --- | --- |
| **probe** | **entrez** | **symbol** | **description** |
| Bt.2070.1.A1\_at | 541175 | DUSP10 | dual specificity phosphatase 10 |
| Bt.2070.2.A1\_at | 541175 | DUSP10 | dual specificity phosphatase 10 |
| Bt.2070.3.S1\_at | 541175 | DUSP10 | dual specificity phosphatase 10 |

### GO:0003755

|  |  |  |  |
| --- | --- | --- | --- |
| **probe** | **entrez** | **symbol** | **description** |
| Bt.1131.1.S1\_at | 281419 | PPIB | peptidylprolyl isomerase B (cyclophilin B) |
| Bt.21537.1.A1\_at | 535704 | FKBP5 | FK506 binding protein 5 |
| Bt.22329.1.S1\_at | 526524 | LOC526524 | FK506 binding protein 1A, 12kDa-like |
| Bt.6680.1.S1\_a\_at | 538998 | FKBP2 | FK506 binding protein 2, 13kDa |
| Bt.6840.1.S1\_a\_at | 506043 | FKBP11 | FK506 binding protein 11, 19 kDa |

### GO:0004310

|  |  |  |  |
| --- | --- | --- | --- |
| **probe** | **entrez** | **symbol** | **description** |
| Bt.21724.1.S1\_at | 281767 | FDFT1 | farnesyl-diphosphate farnesyltransferase 1 |
| Bt.21724.2.S1\_a\_at | 281767 | FDFT1 | farnesyl-diphosphate farnesyltransferase 1 |

### GO:0004477

|  |  |  |  |
| --- | --- | --- | --- |
| **probe** | **entrez** | **symbol** | **description** |
| Bt.20862.1.A1\_at | 517539 | MTHFD2 | methylenetetrahydrofolate dehydrogenase (NADP+ dependent) 2, methenyltetrahydrofolate cyclohydrolase |
| Bt.6685.2.A1\_at | 517539 | MTHFD2 | methylenetetrahydrofolate dehydrogenase (NADP+ dependent) 2, methenyltetrahydrofolate cyclohydrolase |

### GO:0004487

|  |  |  |  |
| --- | --- | --- | --- |
| **probe** | **entrez** | **symbol** | **description** |
| Bt.20862.1.A1\_at | 517539 | MTHFD2 | methylenetetrahydrofolate dehydrogenase (NADP+ dependent) 2, methenyltetrahydrofolate cyclohydrolase |
| Bt.6685.2.A1\_at | 517539 | MTHFD2 | methylenetetrahydrofolate dehydrogenase (NADP+ dependent) 2, methenyltetrahydrofolate cyclohydrolase |

### GO:0004647

|  |  |  |  |
| --- | --- | --- | --- |
| **probe** | **entrez** | **symbol** | **description** |
| Bt.25099.1.A1\_at | 533630 | PSPH | phosphoserine phosphatase |
| Bt.25099.2.S1\_at | 533630 | PSPH | phosphoserine phosphatase |

### GO:0004656

|  |  |  |  |
| --- | --- | --- | --- |
| **probe** | **entrez** | **symbol** | **description** |
| Bt.3162.1.S1\_at | 281373 | P4HB | procollagen-proline, 2-oxoglutarate 4-dioxygenase (proline 4-hydroxylase), beta polypeptide |
| Bt.3296.1.A1\_at | 518288 | P4HA1 | procollagen-proline, 2-oxoglutarate 4-dioxygenase (proline 4-hydroxylase), alpha polypeptide I |

### GO:0008243

|  |  |  |  |
| --- | --- | --- | --- |
| **probe** | **entrez** | **symbol** | **description** |
| Bt.22783.1.S1\_at | 281141 | ENO1 | enolase 1, (alpha) |
| Bt.2519.1.S1\_at | 281408 | PLAU | plasminogen activator, urokinase |

### GO:0004129

|  |  |  |  |
| --- | --- | --- | --- |
| **probe** | **entrez** | **symbol** | **description** |
| Bt.23002.1.S1\_at | 282199 | COX6A1 | cytochrome c oxidase subunit VIa polypeptide 1 |
| Bt.23002.1.S1\_s\_at | 282199 | COX6A1 | cytochrome c oxidase subunit VIa polypeptide 1 |
| Bt.4637.1.A1\_a\_at | 281091 | COX8 | cytochrome c oxidase subunit VIII, heart |
| Bt.4970.1.S1\_at | 327688 | COX7A2 | cytochrome c oxidase subunit VIIa polypeptide 2 (liver) |

### GO:0004128

|  |  |  |  |
| --- | --- | --- | --- |
| **probe** | **entrez** | **symbol** | **description** |
| Bt.20479.1.S1\_at | 533608 | CYB5R4 | cytochrome b5 reductase 4 |
| Bt.997.2.S1\_a\_at | 516287 | CYB5R1 | cytochrome b5 reductase 1 |

### GO:0004427

|  |  |  |  |
| --- | --- | --- | --- |
| **probe** | **entrez** | **symbol** | **description** |
| Bt.26688.2.S1\_a\_at | 533321 | FRMD3 | FERM domain containing 3 |
| Bt.7870.1.S1\_at | 280701 | PPA1 | pyrophosphatase (inorganic) 1 |

### GO:0016776

|  |  |  |  |
| --- | --- | --- | --- |
| **probe** | **entrez** | **symbol** | **description** |
| Bt.1582.1.S1\_at | 615447 | NME2 | non-metastatic cells 2, protein (NM23B) expressed in |
| Bt.28263.1.S1\_at | 509965 | CMPK1 | cytidine monophosphate (UMP-CMP) kinase 1, cytosolic |
| Bt.5340.1.S1\_s\_at | 404189 | NME1 | non-metastatic cells 1, protein (NM23A) expressed in |
| Bt.946.1.S1\_at | 280716 | AK2 | adenylate kinase 2 |

### GO:0004725

|  |  |  |  |
| --- | --- | --- | --- |
| **probe** | **entrez** | **symbol** | **description** |
| Bt.13988.1.S1\_at | 615432 | DUSP3 | dual specificity phosphatase 3 |
| Bt.18748.1.A1\_at | 505696 | PTPRB | protein tyrosine phosphatase, receptor type, B |
| Bt.2070.1.A1\_at | 541175 | DUSP10 | dual specificity phosphatase 10 |
| Bt.2070.2.A1\_at | 541175 | DUSP10 | dual specificity phosphatase 10 |
| Bt.2070.3.S1\_at | 541175 | DUSP10 | dual specificity phosphatase 10 |
| Bt.23521.1.S1\_at | 533896 | DUSP26 | dual specificity phosphatase 26 (putative) |

### GO:0004450

|  |  |  |  |
| --- | --- | --- | --- |
| **probe** | **entrez** | **symbol** | **description** |
| Bt.13324.1.S1\_a\_at | 281235 | IDH1 | isocitrate dehydrogenase 1 (NADP+), soluble |
| Bt.13324.4.S1\_at | 281235 | IDH1 | isocitrate dehydrogenase 1 (NADP+), soluble |

### GO:0046912

|  |  |  |  |
| --- | --- | --- | --- |
| **probe** | **entrez** | **symbol** | **description** |
| Bt.12559.1.S1\_at | 511135 | ACLY | ATP citrate lyase |
| Bt.22763.2.S1\_a\_at | 407767 | HMGCS1 | 3-hydroxy-3-methylglutaryl-coenzyme A synthase 1 |

### GO:0003887

|  |  |  |  |
| --- | --- | --- | --- |
| **probe** | **entrez** | **symbol** | **description** |
| Bt.25319.1.A1\_at | 517422 | REV3L | REV3-like, catalytic subunit of DNA polymerase zeta (yeast) |
| Bt.25319.2.S1\_at | 517422 | REV3L | REV3-like, catalytic subunit of DNA polymerase zeta (yeast) |
| Bt.9810.1.S1\_at | 613730 | POLE4 | polymerase (DNA-directed), epsilon 4 (p12 subunit) |
