# Supplementary material for: A gene network switch enhances the oxidative capacity of ovine skeletal muscle during late fetal development
Source: BMC Genomics. 2010 Jun 15;11:378. doi: 10.1186/1471-2164-11-378 (PMC2894804; doi:10.1186/1471-2164-11-378)
Supplement: Additional File 1 — Gene expression data. This file contains links to the gene expression data. The genes that are significantly differentially expressed between adjacent development times are listed as Experiment 1 (80 d vs 100 d), Experiment 2 (100 d vs 120 d), Experiment 3 (120 d vs 150 d) and Experiment 4 (150 d vs 230 d) in the section entitled Open microarray analysis report for developmental expression. Each experiment contains an MA plot, Volcano plot and Heatmap for each of the three microarray processing programs MAS5, RMA and GCRMA. For each of these processing programs there is a list of significantly differentially expressed probe sets, and for each of these there is an FDR corrected probability, fold change, percentage of microarrays with a MAS5 Present call, and a convergence percentage for all three microarray processing programs. The file also contains annotation of the probe sets. In a second section entitled Time series analysis of developmental expression, there is a list of genes in each gene expression cluster and diagrams showing MAS5 gene expression values for these genes. [file 1471-2164-11-378-S1.ZIP › Report/PLMimage8.html]

*d120\_2(af1c1228-4-215-161007.CEL). Four pseudo-images of the chip based on PLM (probe level model) fit, to aid in the identification of spatial effects. Top-left: log transformed raw probe intensities. Top-right: weights used to down-weight outliers, light green shows areas of high weights and dark green low weights (outliers). Bottom-left: residuals. Negative residuals in blue and positive residuals in red. The intensity of the coloring reflects the value of the residuals, residuals close to zero are shown in white. Bottom-right: sign of residuals. Residuals with no intensity scaling - negative in blue and positive in red.*
