# Supplementary material for: A gene network switch enhances the oxidative capacity of ovine skeletal muscle during late fetal development
Source: BMC Genomics. 2010 Jun 15;11:378. doi: 10.1186/1471-2164-11-378 (PMC2894804; doi:10.1186/1471-2164-11-378)
Supplement: Additional File 1 — Gene expression data. This file contains links to the gene expression data. The genes that are significantly differentially expressed between adjacent development times are listed as Experiment 1 (80 d vs 100 d), Experiment 2 (100 d vs 120 d), Experiment 3 (120 d vs 150 d) and Experiment 4 (150 d vs 230 d) in the section entitled Open microarray analysis report for developmental expression. Each experiment contains an MA plot, Volcano plot and Heatmap for each of the three microarray processing programs MAS5, RMA and GCRMA. For each of these processing programs there is a list of significantly differentially expressed probe sets, and for each of these there is an FDR corrected probability, fold change, percentage of microarrays with a MAS5 Present call, and a convergence percentage for all three microarray processing programs. The file also contains annotation of the probe sets. In a second section entitled Time series analysis of developmental expression, there is a list of genes in each gene expression cluster and diagrams showing MAS5 gene expression values for these genes. [file 1471-2164-11-378-S1.ZIP › Start.html]

### Open microarray analysis report for project DevelopmentalExpression

### Time series analysis for project DevelopmentalExpression

Report date: Tue Jul 15 10:10:00 2008

---

### Comments:

This report encompasses the analyses for the Ovine Muscle Developmental Time Course. The slides in general are of good quality, some minor artifacts in slides d100\_1, d100\_3 and d120\_2. Four experimental questions are addressed - namely the DE genes between each time point (80X100, 100X120, 120X150 and 150X230). There is a very large change in expression between day 120x150. A time course analysis for the experiment is also included, with a general summary of expression profiles and main focus on the genes that change profiles between days 120 and 150, and then return to the original level (or show a tendency to do so). Other profiles can be included if biologically interesting.     
     
 For doubts, clarifications and further analyses contact Cedric Gondro at cgondro2@une.edu.au (02 6773 3978). TIGB - The Institute for Genetics and Bioinformatics, University of New England. Armidale, NSW - 2351.     
     
  

*Experimental summary for project DevelopmentalExpression*

|
|  |

**ExpNum** |

**DE\_Genes** |

**Description** |

|
|  |

1 |

65 |

Which genes are differentially expressed between day 80 and day 100? |

|
|  |

2 |

43 |

Which genes are differentially expressed between day 100 and day 120? |

|
|  |

3 |

2471 |

Which genes are differentially expressed between day 120 and day 150? |

|
|  |

4 |

646 |

Which genes are differentially expressed between day 150 and day 230? |
